# Data set from the proteomic analysis of *Bithynia siamensis goniomphalos* snails upon infection with the carcinogenic liver fluke *Opisthorchis viverrini*

**DOI:** 10.1016/j.dib.2014.09.005

**Published:** 2014-11-06

**Authors:** Sattrachai Prasopdee, Smarn Tesana, Cinzia Cantacessi, Thewarach Laha, Jason Mulvenna, Rudi Grams, Alex Loukas, Javier Sotillo

**Affiliations:** a Food-borne Parasite Research Group, Department of Parasitology, Faculty of Medicine, Khon Kaen University, Khon Kaen 40002, Thailand; b Centre for Biodiscovery and Molecular Development of Therapeutics, Australian Institute of Tropical Health and Medicine, James Cook University, Cairns, QLD, Australia; c Chulabhorn International College of Medicine, Thammasat University, Klongluang, Pathum Thani 12120, Thailand; d Department of Veterinary Medicine, University of Cambridge, Cambridge, UK; e QIMR-Berghofer Medical Research Institute, Brisbane, QLD, Australia; f Graduate Program in Biomedical Sciences, Faculty of Allied Health Sciences, Thammasat University, Klongluang, Pathum Thani 12120, Thailand

## Abstract

The snail *Bithynia siamensis goniomphalos* acts as the first intermediate host for the human liver fluke *Opisthorchis viverrini*, the major cause of cholangiocarcinoma (CCA) in Northeast Thailand.

This data article contains the results obtained from the analysis of the proteins differentially expressed in the snail *B. siamensis goniomphalos* upon infection with *O. viverrini*. It contains the data generated from iQuantitator software including a pdf of each sample with a protein׳s relative expression summary and a per-protein detailed analysis of all time points studied and an excel file for each sample containing the raw data from iQuantitator analysis, including ID, mean, standard deviation, credible interval, log_2_ and description for every protein identified in each of the samples.

**Specifications Table**

**Table:** 

Subject area	Biology

More specific subject area	Parasite proteomics
Type of data	Excel tables
How data was acquired	Mass spectrometry, data acquired using a QSTAR Elite instrument (Applied Biosystems)
Data format	Analyzed
Experimental factors	Snail tissues were ground in 600 µl of lysis buffer containing 5 M urea, 2 M thiourea, 0.1% SDS, 1% Triton X-100 and 40 mM Tris (pH 7.4) with a TissueLyser II (QIAGEN) followed by incubation on ice for 30 min, and centrifugation at 12,000*g*, at 4 °C for 20 min. Protein was precipitated with cold methanol and dried protein pellet was re-dissolved in buffer solution containing 0.5 M triethylammonium bicarbonate (TEAB) and 0.05% SDS, centrifuged at 12,000*g* for 10 min at 4 °C and protein content was determined by Bradford assay using BSA as a standard.
Experimental features	After protein precipitation, the proteins were digested and labeled with iTRAQ. Peptides were analyzed by LC–MS/MS using a QSTAR Elite instrument (Applied Biosystems).
Data source location	James Cook University, Cairns, Australia
Data accessibility	Data is supplied with this article and is related to [1]

**Value of the data**

• This is the first in-depth quantitative proteomic analysis of experimentally infected *B. siamensis goniomphalos*.
• A total of 30,545 and 36,179 MS/MS spectra were acquired in body and headfoot samples, respectively, over all iTRAQ runs.
• iTRAQ analysis was performed using iQuantitator.
• A total of 108 and 43 significantly differentially expressed proteins were found in the body and headfoot samples respectively.

## 1 Data, experimental design, materials and methods

A protein report summarizing the analysis of protein expression was generated in the body and headfoot of infected *B. siamensis goniomphalos* (Supplementary file 1 and 2 respectively). This file contains the experiment design, model description, statistical model and data summary as well as a detailed summary of each protein including peptide relative expression estimates in addition to protein level estimates.

Furthermore, a detailed spreadsheet containing the raw data from the iQuantitator analysis including ID, mean, standard deviation, credible interval, log_2_ and description for every protein identified in the body and headfoot samples (Supplementary file 3 and 4 respectively) was generated for each timepoint studied.

### 1.1 Sample preparation and protein extraction

The snail preparation and experimental infections are described in detail in [1]. Two biological replicates from each studied time point with two headfoot and body samples from two male and two female snails were pooled and placed in a 2 ml microcentrifuge tube with 600 μl of lysis buffer containing 5 M urea, 2 M thiourea, 0.1% SDS, 1% Triton X-100 and 40 mM Tris (pH 7.4). Each sample was ground with a TissueLyser II (QIAGEN) using a 5 mm stainless bead at 4 °C for 10 min followed by incubation on ice for 30 min, and centrifugation at 12,000*g*, at 4 C for 20 min. The pellet was discarded and protein supernatant was subsequently precipitated with 10 volumes of cold methanol at −20 C overnight, centrifuged at 8000*g* for 10 min at 4 C, and air-dried for 5–10 min. Dried protein pellet was re-dissolved in buffer solution containing 0.5 M triethylammonium bicarbonate (TEAB) and 0.05% SDS, centrifuged at 12,000*g* for 10 min at 4 °C and protein content was determined by Bradford assay using BSA as a standard. One hundred (100) μg of protein was dried under vacuum before trypsin digestion. Protein extraction from the body portion was performed similarly. Headfoot and body samples from uninfected snails were used as controls and compared with experimentally infected tissues.

### 1.2 Protein digestion and iTRAQ labeling

Dried protein samples were re-suspended in 20 μl of dissolution buffer (0.5 TEAB) prior to reduction, alkylation, digestion and iTRAQ labeling according to the manufacturer׳s protocol (AB Sciex). Briefly, each protein sample was denatured with 2% SDS, reduced with 50 mM Tris-(2-carboxyethyl)-phosphine (TCEP) at 60 °C for 1 h, and cysteine residues were alkylated with 10 mM methyl methanethiosulfate (MMTS) solution at RT for 10 min followed by tryptic digestion using 2 μg of trypsin (Sigma-Aldrich) at 37 °C for 16 h. Digested peptide solutions were individually labeled with one vial of iTRAQ reagent at RT for 2 h. Each sample was labeled with different iTRAQ reagents having distinct isotopic compositions and all samples were subsequently combined into one tube for OFFGEL fractionation and LC-MS/MS analysis.

### 1.3 Peptide OFFGEL fractionation

A 3100 OFFGEL Fractionator (Agilent Technologies) with a 24 well setup was used for peptide separation based on pI. Prior to electrofocusing, desalting of samples was performed using a HiTrap SP HP column (GE Healthcare) and a Sep-Pak C18 cartridge (Waters) was used to remove excess of iTRAQ labeling according to the manufacturer׳s instructions. A total of 3.6 ml of OFFGEL peptide sample solution was used to dissolve the samples. The 24 cm long, 3–10 linear pH range IPG gel strips (GE Healthcare) were rehydrated with IPG Strip Rehydration Solution for 15 min, and 150 μl of dissolved sample was loaded in each well. The samples were focused with a maximum current of 50 μA until 50 kVh was reached. Every peptide fraction was harvested and each well rinsed with 150 μl of a solution of water/methanol/formic acid (49%/50%/1%). After 15 min, rinsing solutions were pooled with their corresponding peptide fraction and all fractions were evaporated using a vacuum concentrator. Prior to LC–MS/MS analysis, peptide fractions were desalted using ZipTip (Millipore) according to manufacturer׳s protocol followed by centrifugation under vacuum.

### 1.4 Reverse-phase (RP) LC–MS/MS analysis

Each dried fraction was reconstituted in 12 μl of 5% formic acid and 3 µl of the resulting suspension was injected into a trap column (LC Packings, PepMap C18 pre-column; 5 mm 300 m i.d.; LC Packings) using an Ultimate 3000 HPLC (Dionex Corporation, Sunnyvalle, CA) via an isocratic flow of 0.1% formic acid in water at a rate of 20 µl/min for 3 min. Peptides were then eluted onto the PepMap C18 analytical column (15 cm 75 µm i.d.; LC Packings) at a flow rate of 300 nl/min and separated using a linear gradient of 4–80% solvent B over 120 min. The mobile phase consisted of solvent A (0.1% formic acid (aqueous)) and solvent B (0.1% formic acid (aqueous) in 90% acetonitrile). The column eluates were subsequently ionized using the NanoSpray II of a QSTAR Elite instrument (Applied Biosystems) operated in information-dependent acquisition mode, in which a 1-s TOF MS scan from 300 to 2000 *m*/*z* was performed, followed by 2-s product ion scans from 100 to 2000 *m*/*z* on the three most intense doubly or triply charged ions. Analyst 2.0 software was used for data acquisition and analysis.

### 1.5 Database searching and bioinformatics analysis

A predicted protein database containing transcriptome data for *B. siamensis goniomphalos* described previously [2] was used for amino acid sequence comparison. The database search was performed using Protein Pilot v4.0.8085 (Applied Biosystems) using the default parameters. Only proteins with a ProteinPilot unused scored above 1.3, which is equivalent to a protein confidence threshold greater than 95%, and for which there was at least one unique peptide match with a confidence >95% were selected. Under these conditions the calculated false discovery rate (FDR) using a reverse decoy database was <1%. The iQuantitator software was used to analyze the differentially expressed proteins in all replicates [3]. This software infers sample-dependent changes in protein expression using Markov Chain Monte Carlo and Bayesian statistical methods. Using iQuantitator, median and 95% confidence intervals were generated for each component peptide and integrating data across replicates. As described previously [3–5], for proteins whose iTRAQ ratios were downregulated in infected snails, the extent of downregulation was considered further if the null value of 1 was above the upper limit of credible interval. Conversely, for proteins whose iTRAQ ratios were upregulated in infected snails, the extent of upregulation was considered further if the lower limit of the credible interval had a value >1. The width of these credible intervals depends on the data available for a given protein. Since the number of peptides observed and the number of spectra used to quantify the change in expression for a given protein are taken into consideration, it is possible to detect small but significant changes in up- or downregulation when many peptides are available. For each protein and each peptide associated with a given protein, the mean, median, and 95% credible intervals were computed for each of the protein and peptide level treatment effects [4,5]. In addition, only proteins with a fold change of at least 1.5 (log_2_=0.6) were considered for further analysis [6].

## 2 Ethics statement

The protocols used for animal experimentation were approved by the Animal Ethics Committee of Khon Kaen University, as described in [1].

## Supplementary materials

*Supplementary file 1*: Document generated by iQuantitator summarizing the analysis of relative protein expression using iTRAQ in the body of infected *Bithynia siamensis goniomphalos*. The reporter ion peak area measurements supplied by the ProteinPilot software are used to estimate treatment-dependent peptide and protein relative expression. The document includes a protein relative expression summary and a per-protein detailed analysis of all time points studied.

*Supplementary file 2*: Document generated by iQuantitator summarizing the analysis of relative protein expression using iTRAQ in the headfoot of infected *Bithynia siamensis goniomphalos*. The reporter ion peak area measurements supplied by the ProteinPilot software are used to estimate treatment-dependent peptide and protein relative expression. The document includes a protein relative expression summary and a per-protein detailed analysis of all time points studied.

*Supplementary file 3*: Raw data from iQuantitator analysis including ID, mean, standard deviation, credible interval, log_2_ and description for every protein identified in the body samples.

*Supplementary file 4*: Raw data from iQuantitator analysis including ID, mean, standard deviation, credible interval, log_2_ and description for every protein identified in the headfoot samples.
