# Supplementary material for: Data set from the proteomic analysis of Bithynia siamensis goniomphalos snails upon infection with the carcinogenic liver fluke Opisthorchis viverrini
Source: Data Brief. 2014 Nov 6;2:16–20. doi: 10.1016/j.dib.2014.09.005 (PMC4459563; doi:10.1016/j.dib.2014.09.005)
Supplement: Supplementary file 1 — Document generated by iQuantitator summarizing the analysis of relative protein expression using iTRAQ in the body of infected Bithynia siamensis goniomphalos. The reporter ion peak area measurements supplied by the ProteinPilot software are used to estimate treatment-dependent peptide and protein relative expression. The document includes a protein relative expression summary and a per-protein detailed analysis of all time points studied. [file mmc1.pdf]

iTRAQ Data Analysis Report

Javier, Body 1d versus control

April 7, 2014

1 Introduction

This document summarizes an analysis of relative protein expression using iTRAQ. The reporter ion peak area measurements supplied by the ABI software are used to estimate treatment-dependent peptide and protein relative expression. Estimation is accomplished using a Bayesian approach with the model given below. The document includes a protein relative expression summary and a per-protein detailed analysis. The document is internally hyperlinked and linked externally to NCBI.

2 Experiment and Model Description

2.1 Experiment Design

The report summarizes data from one or more iTRAQ experiments addressing a common comparison. The experiment design, used in this analysis, is given in the table below.

	Experiment	Treatment	Channel	Sample
1	A	control	113	s1
2	A	test	114	s1
3	B	control	113	s2
4	B	test	114	s2

2.2 Input Files

Data for this analysis was extracted from the following tandem mass spectra (MSMS) summary files.

Experiment	MSMS Summary File
A	body1.txt
B	body2.txt

2.3 Statistical Model

The following statistical model was used to estimate the treatment-dependent effects.

LogIntensity ˜ Channel + Spectrum + Protein + Peptide + Protein:Treatment + Peptide:Treatment

3 Data Summary

The data supplied in the MSMS summary is filtered to remove unidentified proteins, contaminants, and peptides containing selected modifications. The following table sumarizes the data provided and used in the analysis.

	A	B	Combined
Supplied Spectra	11560	18985	30545
Unidentified Spectra	6008	8178	14186
Disallowed Modifications	41	206	247
Spectra from Contaminants	0	0	0
Missing Data	1661	2842	4503
Low Confidence Spectra	6008	8178	14186
Degenerate Peptides	1475	2454	3929
Remaining Spectra			7680
Unique Proteins			814
Unique Peptides			2859
Model R^2			0.807

4 Protein Summary

Each protein identified in one or more of the MSMS summaries is listed below in decreasing order of expression change magnitude. The median and estimated credible interval for each protein is given to the left in the table. Proteins identified by a single peptide are listed in a separate table.

4.1 Identified Proteins

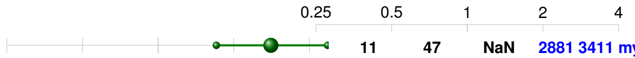

Peptides	Spectra	Coverage	Protein
11	47	NaN	2881 3411 myosin heavy chain [Todarodes pacificus]; 2915 3445 myosin heavy chain [Todarodes pacificus]

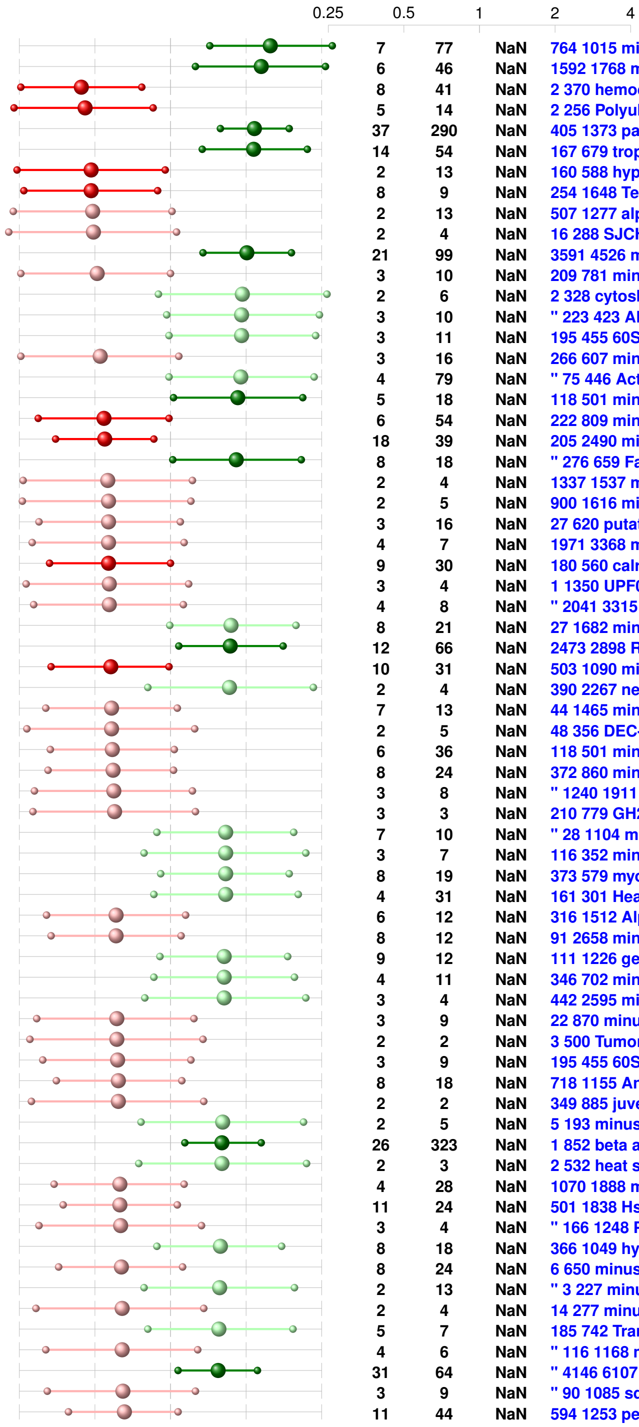

Peptides	Spectra	Coverage	Protein
7	77	NaN	764 1015 minus strand heat shock protein beta-1 [Trichinella spiralis] gi
6	46	NaN	1592 1768 minus strand beta-actin [Diabolocatantops pinguis]
8	41	NaN	2 370 hemocyanin alphaD-subunit [Helix lucorum]
5	14	NaN	2 256 Polyubiquitin (Fragment) OS=Xenopus laevis PE=1 SV=2; 20 388 large subunit ribosomal protein L40e [Clonor
37	290	NaN	405 1373 paramyosin [Haliotis discus discus]
14	54	NaN	167 679 tropomyosin [Haliotis discus discus]
2	13	NaN	160 588 hypothetical protein CGI_10001640 [Crassostrea gigas]
8	9	NaN	254 1648 Tektin-3 [Crassostrea gigas]; 254 1648 Tektin-3 [Crassostrea gigas]
2	13	NaN	507 1277 alpha tubulin [Notothenia coriiceps]
2	4	NaN	16 288 SJCHGC01950 protein [Schistosoma japonicum] gi
21	99	NaN	3591 4526 minus strand myosin heavy chain cardiac muscle specific isoform 1 [Argopecten irradians]
3	10	NaN	209 781 minus strand high mobility group protein 1 [Biomphalaria glabrata]
2	6	NaN	2 328 cytoskeletal actin 2 [Molgula oculata]
3	10	NaN	" 223 423 ADPATP carrier protein; K05863 solute carrier family 25 (mitochondrial adenine nucleotide translocator), m
3	11	NaN	195 455 60S ribosomal protein L23a OS=Rattus norvegicus GN=Rpl23a PE=2 SV=1; 195 512 60S ribosomal protein L
3	16	NaN	266 607 minus strand H2 [Haliotis tuberculata]
4	79	NaN	" 75 446 Actin, cytoplasmic type 8 OS=Xenopus laevis PE=3 SV=1"
5	18	NaN	118 501 minus strand histone 2A.Z [Aplysia californica]; 15 428 PREDICTED: histone H2A.V-like [Apis florea]
6	54	NaN	222 809 minus strand PREDICTED: 15-hydroxyprostaglandin dehydrogenase [NAD+] [Gallus gallus]
18	39	NaN	205 2490 minus strand glucose-regulated protein 94 [Crassostrea gigas]
8	18	NaN	" 276 659 Fatty acid-binding protein, adipocyte [Crassostrea gigas]; 426 809 Fatty acid-binding protein, adipocyte [C
2	4	NaN	1337 1537 minus strand hemocyanin [Haliotis tuberculata]
2	5	NaN	900 1616 minus strand hemocyanin isoform 1 [Haliotis diversicolor]
3	16	NaN	27 620 putative mitochondrial ATP synthase [Haliotis discus discus]
4	7	NaN	1971 3368 minus strand Kyphoscoliosis peptidase [Crassostrea gigas]; 1767 3158 minus strand hillarin [Hirudo med
9	30	NaN	180 560 calreticulin precursor [Aplysia californica] gi; 180 545 calreticulin precursor [Aplysia californica] gi
3	4	NaN	1 1350 UPF0663 transmembrane protein C17orf28 [Crassostrea gigas]; 163 1542 UPF0663 transmembrane protein C
4	8	NaN	" 2041 3315 minus strand Myosin-10 OS=Mus musculus GN=Myh10 PE=1 SV=2; 2041 3345 minus strand Myosin hea
8	21	NaN	27 1682 minus strand PREDICTED: nucleolar protein 56 [Oreochromis niloticus]
12	66	NaN	2473 2898 RecName: Full=Paramyosin gi|397603|emb|CAA79849.1| paramyosin [Echinococcus granulosus]
10	31	NaN	503 1090 minus strand myosin regulatory light chain [Fasciola hepatica]; 278 865 minus strand myosin regulatory lig
2	4	NaN	390 2267 neurofilament protein NF70 [Helix aspersa]; 318 2195 neurofilament protein NF70 [Helix aspersa]
7	13	NaN	44 1465 minus strand Adenylyl cyclase-associated protein 1 [Crassostrea gigas]; 52 1473 minus strand Adenylyl cyc
2	5	NaN	48 356 DEC-3 [Lymnaea stagnalis]
6	36	NaN	118 501 minus strand histone 2A.Z [Aplysia californica]
8	24	NaN	372 860 minus strand small heat shock protein [Tegillarca granosa]
3	8	NaN	" 1240 1911 PREDICTED: ATP synthase subunit alpha, mitochondrial-like [Anolis carolinensis]"
3	3	NaN	210 779 GH20056 [Drosophila grimshawi] gi; 216 785 minus strand GH20056 [Drosophila grimshawi] gi; 210 779 GH2
7	10	NaN	" 28 1104 minus strand Pyruvate dehydrogenase E1 component subunit beta, mitochondrial [Crassostrea gigas]"
3	7	NaN	116 352 minus strand troponin I 4 [Clonorchis sinensis]
8	19	NaN	373 579 myosin heavy chain [Argopecten irradians] gi
4	31	NaN	161 301 Heat shock protein beta-6 OS=Mus musculus GN=Hspb6 PE=1 SV=1
6	12	NaN	316 1512 Alpha-N-acetylgalactosaminidase [Crassostrea gigas]; 282 1478 Alpha-N-acetylgalactosaminidase [Crasso
8	12	NaN	91 2658 minus strand puromycin-sensitive aminopeptidase [Saccoglossus kowalevskii]
9	12	NaN	111 1226 gelsolin [Aplysia californica] gi; 42 1139 minus strand gelsolin [Aplysia californica] gi
4	11	NaN	346 702 minus strand PREDICTED: galectin-4-like [Anolis carolinensis]; 367 669 minus strand PREDICTED: galectin
3	4	NaN	442 2595 minus strand Amiloride-sensitive amine oxidase [copper-containing] [Crassostrea gigas]
3	9	NaN	22 870 minus strand PREDICTED: alcohol dehydrogenase class-3 [Sarcophilus harrisii]
2	2	NaN	3 500 Tumor protein D54 [Crassostrea gigas]; 3 464 Tumor protein D54 [Crassostrea gigas]; 3 464 Tumor protein D54
3	9	NaN	195 455 60S ribosomal protein L23a OS=Rattus norvegicus GN=Rpl23a PE=2 SV=1; 16 465 60S ribosomal protein L2
8	18	NaN	718 1155 Annexin A7 [Crassostrea gigas]
2	2	NaN	349 885 juvenile hormone diol kinase [Danaus plexippus]; 349 885 juvenile hormone diol kinase [Danaus plexippus]
2	5	NaN	5 193 minus strand PREDICTED: 40S ribosomal protein S28-like [Strongylocentrotus purpuratus]; 3 122 PREDICTED
26	323	NaN	1 852 beta actin-like 1 protein [Macrostomum lignano]
2	3	NaN	2 532 heat shock protein 70 [Echinostoma caproni]
4	28	NaN	1070 1888 minus strand galectin [Pinctada fucata]; 1371 2189 minus strand galectin [Pinctada fucata]
11	24	NaN	501 1838 Hsp90A [Haliotis asinina]
3	4	NaN	" 166 1248 PREDICTED: probable pyruvate dehydrogenase E1 component subunit alpha, mitochondrial-like [Metase
8	18	NaN	366 1049 hypothetical protein CGI_10004855 [Crassostrea gigas]
8	24	NaN	6 650 minus strand glutathione S-transferase mu class protein [Crassostrea ariakensis]; 117 761 glutathione S-trans
2	13	NaN	" 3 227 minus strand alpha tubulin, putative [Ixodes scapularis] gi"
2	4	NaN	14 277 minus strand Histidine triad nucleotide-binding protein 1 OS=Oryctolagus cuniculus GN=HINT1 PE=1 SV=2
5	7	NaN	185 742 Transmembrane protein Tmp21 [Crassostrea gigas]
4	6	NaN	" 116 1168 minus strand hypothetical protein CGI_10014553, partial [Crassostrea gigas]"
31	64	NaN	" 4146 6107 minus strand nonmuscle myosin II, partial [Aplysia californica]"
3	9	NaN	" 90 1085 squid, variant A [Blattella germanica]"
11	44	NaN	594 1253 peroxiredoxin 6 [Eriocheir sinensis]

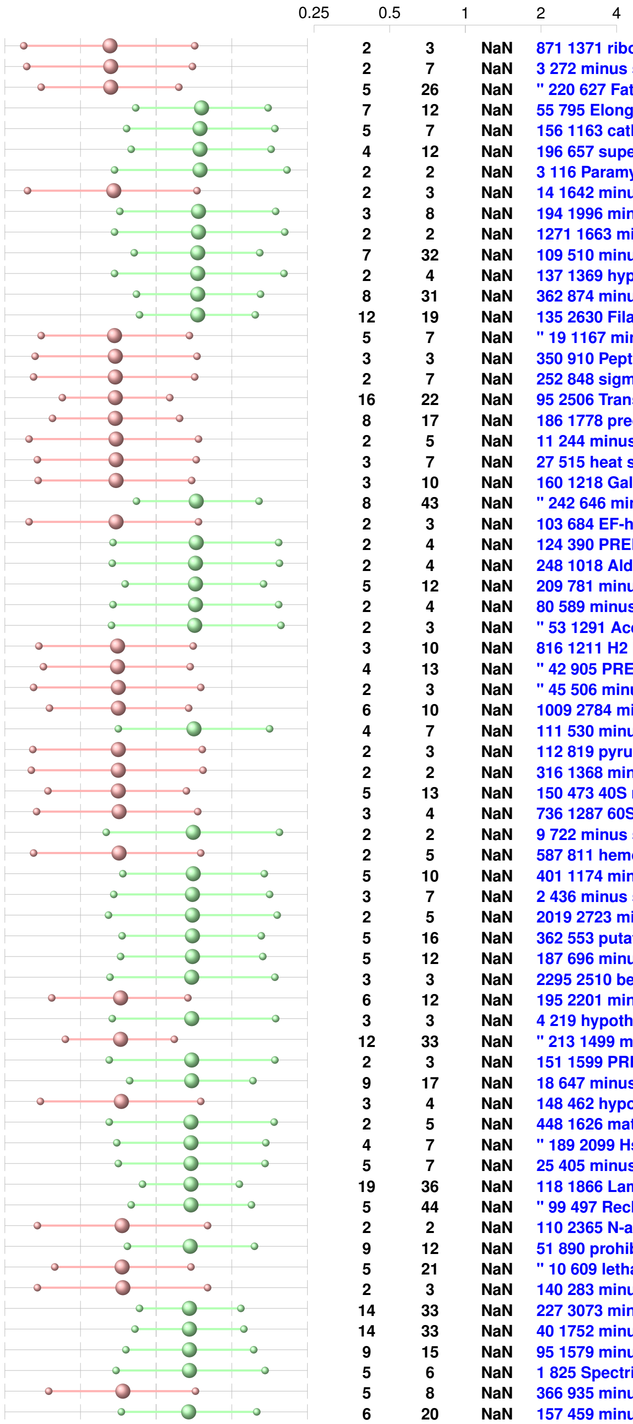

Peptides	Spectra	Coverage	Protein
2	3	NaN	871 1371 ribosomal protein L11 [Triatoma infestans]; 343 843 minus strand ribosomal protein L11 [Triatoma infestan
2	7	NaN	3 272 minus strand ribosomal protein L28 [Haliotis asinina]
5	26	NaN	" 220 627 Fatty acid-binding protein, brain [Crassostrea gigas]"
7	12	NaN	55 795 Elongation factor 1-delta OS=Bos taurus GN=EEF1D PE=2 SV=2; 49 747 PREDICTED: elongation factor 1-del
5	7	NaN	156 1163 cathepsin B [Crassostrea ariakensis]
4	12	NaN	196 657 superoxide dismutase [Aplysia californica] gi; 690 1151 superoxide dismutase [Aplysia californica] gi
2	2	NaN	3 116 Paramyosin [Crassostrea gigas]; 3 107 minus strand Paramyosin [Crassostrea gigas]
2	3	NaN	14 1642 minus strand chaperonin containing tcp1 [Haliotis discus discus]; 15 1619 chaperonin containing tcp1 [Hali
3	8	NaN	194 1996 minus strand Neutral and basic amino acid transport protein rBAT [Crassostrea gigas]; 265 2067 minus str
2	2	NaN	1271 1663 minus strand PREDICTED: similar to AGAP007393-PB [Tribolium castaneum] gi
7	32	NaN	109 510 minus strand hypothetical protein DAPPUDRAFT_231281 [Daphnia pulex]
2	4	NaN	137 1369 hypothetical protein TcasGA2_TC002962 [Tribolium castaneum]
8	31	NaN	362 874 minus strand Transgelin-2 [Crassostrea gigas]
12	19	NaN	135 2630 Filamin-A [Crassostrea gigas]; 118 2613 Filamin-A [Crassostrea gigas]; 157 2652 Filamin-A [Crassostrea gi
5	7	NaN	" 19 1167 minus strand ureidopropionase, beta [Xenopus (Silurana) tropicalis] gi"
3	3	NaN	350 910 Peptidyl-prolyl cis-trans isomerase B [Crassostrea gigas]; 488 1039 Peptidyl-prolyl cis-trans isomerase B [C
2	7	NaN	252 848 sigma class glutathione-s-transferase 2 [Haliotis discus discus]
16	22	NaN	95 2506 Transitional endoplasmic reticulum ATPase [Crassostrea gigas]
8	17	NaN	186 1778 predicted protein [Nematostella vectensis] gi
2	5	NaN	11 244 minus strand Tubulin alpha chain (Fragment) OS=Lytechinus pictus PE=3 SV=1
3	7	NaN	27 515 heat shock protein 70 [Capsaspora owczarzaki ATCC 30864]; 30 464 minus strand hypothetical protein [Trich
3	10	NaN	160 1218 Galectin-9 [Crassostrea gigas]
8	43	NaN	" 242 646 minus strand Fatty acid-binding protein, heart [Crassostrea gigas]; 227 631 minus strand Fatty acid-bindir
2	3	NaN	103 684 EF-hand domain-containing protein D2 [Crassostrea gigas]
2	4	NaN	124 390 PREDICTED: 60S ribosomal protein L23a-like [Callithrix jacchus]
2	4	NaN	248 1018 Aldo-keto reductase family 1 member B10 [Crassostrea gigas]; 44 730 minus strand Aldo-keto reductase fa
5	12	NaN	209 781 minus strand high mobility group protein 1 [Biomphalaria glabrata]; 277 771 high mobility group protein 1 [
2	4	NaN	80 589 minus strand PREDICTED: adenine phosphoribosyltransferase-like [Strongylocentrotus purpuratus]
2	3	NaN	" 53 1291 Acetyl-CoA acetyltransferase B, mitochondrial [Crassostrea gigas]"
3	10	NaN	816 1211 H2 [Haliotis tuberculata]
4	13	NaN	" 42 905 PREDICTED: enoyl-CoA hydratase, mitochondrial-like isoform 1 [Oreochromis niloticus]"
2	3	NaN	" 45 506 minus strand PREDICTED: dihydrolipoyl dehydrogenase, mitochondrial [Monodelphis domestica]"
6	10	NaN	1009 2784 minus strand Collagen alpha-3(VI) chain [Crassostrea gigas]; 1009 2787 minus strand Collagen alpha-3(V
4	7	NaN	111 530 minus strand Nucleoside diphosphate kinase B OS=Homo sapiens GN=NME2 PE=1 SV=1
2	3	NaN	112 819 pyruvate kinase [Crassostrea gigas]; 132 932 pyruvate kinase [Crassostrea gigas]
2	2	NaN	316 1368 minus strand hypothetical protein CGI_10006559 [Crassostrea gigas]
5	13	NaN	150 473 40S ribosomal protein S16 OS=Heteropneustes fossilis GN=rps16 PE=2 SV=1
3	4	NaN	736 1287 60S ribosomal protein L3 OS=Rattus norvegicus GN=Rpl3 PE=1 SV=3
2	2	NaN	9 722 minus strand actin related protein [Lumbricus rubellus]
2	5	NaN	587 811 hemocyanin 2 [Megathura crenulata]
5	10	NaN	401 1174 minus strand Mechanosensory protein 2 [Crassostrea gigas]
3	7	NaN	2 436 minus strand PREDICTED: stress-induced-phosphoprotein 1 [Sarcophilus harrisii]
2	5	NaN	2019 2723 minus strand Kyphoscoliosis peptidase [Crassostrea gigas]; 2060 2764 minus strand Kyphoscoliosis pep
5	16	NaN	362 553 putative RNA-binding protein [Haliotis diversicolor]
5	12	NaN	187 696 minus strand putative ribosomal protein S10 [Sipunculus nudus]
3	3	NaN	2295 2510 beta-thymosin domain repeat protein CSP24KDa_v1 [Hermissenda crassicornis] gi
6	12	NaN	195 2201 minus strand hypothetical protein DAPPUDRAFT_315656 [Daphnia pulex]
3	3	NaN	4 219 hypothetical protein BRAFLDRAFT_66351 [Branchiostoma floridae] gi; 3 239 hypothetical protein BRAFLDRA
12	33	NaN	" 213 1499 minus strand Citrate synthase, mitochondrial [Crassostrea gigas]"
2	3	NaN	151 1599 PREDICTED: galactosamine (N-acetyl)-6-sulfate sulfatase-like [Saccoglossus kowalevskii]
9	17	NaN	18 647 minus strand ATP synthase-like protein [Biomphalaria glabrata]
3	4	NaN	148 462 hypothetical protein DAPPUDRAFT_306954 [Daphnia pulex]; 148 462 hypothetical protein DAPPUDRAFT_30
2	5	NaN	448 1626 matrilin [Biomphalaria glabrata]
4	7	NaN	" 189 2099 Hsp70, putative [Ixodes scapularis] gi"
5	7	NaN	25 405 minus strand Elongation factor 1-delta [Anoplopoma fimbria]
19	36	NaN	118 1866 Lamin Dm0 [Crassostrea gigas]
5	44	NaN	" 99 497 RecName: Full=Histone H2B, gonadal"
2	2	NaN	110 2365 N-acetylated-alpha-linked acidic dipeptidase 2 [Crassostrea gigas]
9	12	NaN	51 890 prohibitin [Octopus tankahkeei]
5	21	NaN	" 10 609 lethal(2)essential for life protein, l2efl [Aedes aegypti] gi"
2	3	NaN	140 283 minus strand elongation factor 1 alpha [Angiostrongylus cantonensis]
14	33	NaN	227 3073 minus strand Lysosomal alpha-mannosidase [Crassostrea gigas]
14	33	NaN	40 1752 minus strand heat shock protein 60 [Biomphalaria glabrata]; 40 1752 minus strand heat shock protein 60 [B
9	15	NaN	95 1579 minus strand hypothetical protein BRAFLDRAFT_113974 [Branchiostoma floridae] gi
5	6	NaN	1 825 Spectrin alpha chain [Crassostrea gigas]
5	8	NaN	366 935 minus strand 40S ribosomal protein S2 OS=Urechis caupo GN=RPS2 PE=2 SV=1
6	20	NaN	157 459 minus strand Synaptojanin-2-binding protein [Crassostrea gigas]

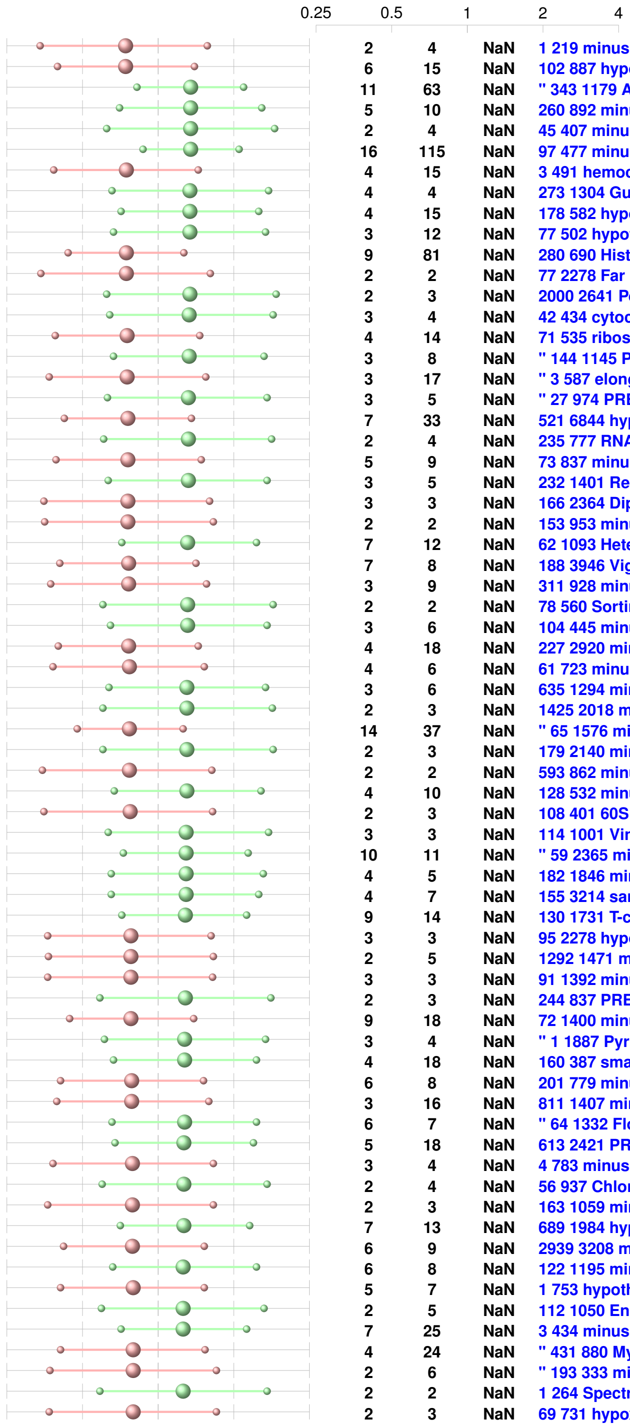

Peptides	Spectra	Coverage	Protein
2	4	NaN	1 219 minus strand PREDICTED: nucleoside diphosphate kinase 3-like [Pan troglodytes]
6	15	NaN	102 887 hypothetical protein DAPPUDRAFT_304712 [Daphnia pulex]
11	63	NaN	" 343 1179 ATP synthase subunit alpha, mitochondrial OS=Pongo abelii GN=ATP5A1 PE=2 SV=1"
5	10	NaN	260 892 minus strand calponin [Haliotis diversicolor]
2	4	NaN	45 407 minus strand 60S ribosomal protein L31 OS=Xenopus laevis GN=rpl31 PE=2 SV=1
16	115	NaN	97 477 minus strand PREDICTED: complement C1q tumor necrosis factor-related protein 3-like [Oreochromis niloti
4	15	NaN	3 491 hemocyanin [Haliotis tuberculata]
4	4	NaN	273 1304 Guanine nucleotide-binding protein subunit beta [Crassostrea gigas]
4	15	NaN	178 582 hypothetical protein CGI_10001640 [Crassostrea gigas]
3	12	NaN	77 502 hypothetical protein CGI_10008263 [Crassostrea gigas]
9	81	NaN	280 690 Histone H3.3 [Harpegnathos saltator]
2	2	NaN	77 2278 Far upstream element-binding protein 3 [Crassostrea gigas]
2	3	NaN	2000 2641 Polyadenylate-binding protein 4 [Crassostrea gigas]
3	4	NaN	42 434 cytochrome b5 [Haliotis discus discus]; 42 434 cytochrome b5 [Haliotis discus discus]
4	14	NaN	71 535 ribosomal protein rpl14 [Arenicola marina]
3	8	NaN	" 144 1145 PREDICTED: RNA-binding protein squid-like isoform 1 [Megachile rotundata]; 90 1085 squid, variant A [B
3	17	NaN	" 3 587 elongation factor 1 alpha, partial [Phorbas sp. nov. SB-2012]"
3	5	NaN	" 27 974 PREDICTED: succinyl-CoA ligase [GDP-forming] subunit alpha, mitochondrial-like [Oreochromis niloticus];
7	33	NaN	521 6844 hypothetical protein CGI_10014469 [Crassostrea gigas]
2	4	NaN	235 777 RNA-binding protein 4.1 [Clonorchis sinensis]
5	9	NaN	73 837 minus strand Electron transfer flavoprotein subunit beta [Crassostrea gigas]; 73 837 minus strand Electron t
3	5	NaN	232 1401 RecName: Full=Guanine nucleotide-binding protein G(s) subunit alpha; AltName: Full=Adenylate cyclase-
3	3	NaN	166 2364 Dipeptidyl-peptidase 3 [Crassostrea gigas]
2	2	NaN	153 953 minus strand ribosomal protein rpl7 [Arenicola marina]
7	12	NaN	62 1093 Heterogeneous nuclear ribonucleoprotein A2-like protein 1 [Crassostrea gigas]
7	8	NaN	188 3946 Vigilin [Crassostrea gigas]; 188 4135 Vigilin [Crassostrea gigas]
3	9	NaN	311 928 minus strand peroxiredoxin 3 [Clonorchis sinensis] gi; 288 905 minus strand peroxiredoxin 3 [Clonorchis si
2	2	NaN	78 560 Sorting nexin-12 [Camponotus floridanus]; 78 578 Sorting nexin-12 [Camponotus floridanus]; 132 605 Sortin
3	6	NaN	104 445 minus strand macrophage migration inhibitory factor [Ascaris suum]
4	18	NaN	227 2920 minus strand Lysosomal alpha-mannosidase [Crassostrea gigas]; 227 3073 minus strand Lysosomal alpha
4	6	NaN	61 723 minus strand 40S ribosomal protein S3-A OS=Xenopus laevis GN=rps3-a PE=2 SV=2
3	6	NaN	635 1294 minus strand Kyphoscoliosis peptidase [Crassostrea gigas]; 643 1302 minus strand Kyphoscoliosis pepti
2	3	NaN	1425 2018 minus strand Ankyrin-2 [Crassostrea gigas]; 1425 2018 minus strand Ankyrin-2 [Crassostrea gigas]
14	37	NaN	" 65 1576 minus strand Aldehyde dehydrogenase, mitochondrial [Crassostrea gigas]"
2	3	NaN	179 2140 minus strand PREDICTED: hypothetical protein [Saccoglossus kowalevskii]
2	2	NaN	593 862 minus strand mitochondrial ATP synthase coupling factor 6 precursor [Ochlerotatus taeniorhynchus]
4	10	NaN	128 532 minus strand mitochondrial ATPase inhibitor [Ornithodoros parkeri]
2	3	NaN	108 401 60S ribosomal L7a-like protein [Phragmatopoma lapidosa]; 108 401 60S ribosomal L7a-like protein [Phragm
3	3	NaN	114 1001 Vinculin [Crassostrea gigas]
10	11	NaN	" 59 2365 minus strand Aconitate hydratase, mitochondrial [Crassostrea gigas]"
4	5	NaN	182 1846 minus strand hypothetical protein DAPPUDRAFT_302108 [Daphnia pulex]; 110 1774 minus strand hypothe
4	7	NaN	155 3214 sarco/endoplasmic reticulum calcium ATPase isoform B [Pinctada fucata]; 155 3214 minus strand sarco/er
9	14	NaN	130 1731 T-complex protein 1 subunit epsilon [Crassostrea gigas]; 130 1731 T-complex protein 1 subunit epsilon [Cr
3	3	NaN	95 2278 hypothetical protein CGI_10017740 [Crassostrea gigas]
2	5	NaN	1292 1471 minus strand hemocyanin isoform 1 [Haliotis diversicolor]
3	3	NaN	91 1392 minus strand hypothetical protein AND_03103 [Anopheles darlingi]
2	3	NaN	244 837 PREDICTED: ras-like GTP-binding protein Rho1-like [Megachile rotundata]
9	18	NaN	72 1400 minus strand hypothetical protein RCJMB04_22i13 [Gallus gallus]
3	4	NaN	" 1 1887 Pyruvate carboxylase, mitochondrial [Crassostrea gigas]"
4	18	NaN	160 387 small nuclear ribonucleoprotein polypeptide G protein [Crassostrea ariakensis]; 180 407 small nuclear ribo
6	8	NaN	201 779 minus strand sigma class glutathione-s-transferase 2 [Haliotis discus discus]
3	16	NaN	811 1407 minus strand mitochondrial malate dehydrogenase 2 [Eospalax baileyi]
6	7	NaN	" 64 1332 Flotillin-1, putative [Pediculus humanus corporis] gi"
5	18	NaN	613 2421 PREDICTED: lysosomal alpha-glucosidase [Danio rerio]
3	4	NaN	4 783 minus strand hemocyanin isoform 2 [Nucula nucleus]
2	4	NaN	56 937 Chloride intracellular channel exc-4 [Crassostrea gigas]
2	3	NaN	163 1059 minus strand Heterogeneous nuclear ribonucleoprotein A2-like protein 1 [Crassostrea gigas]; 99 995 minu
7	13	NaN	689 1984 hypothetical protein BRAFLDRAFT_78257 [Branchiostoma floridae] gi
6	9	NaN	2939 3208 minus strand beta-thymosin domain repeat protein CSP24KDa_v1 [Hermissenda crassicornis] gi
6	8	NaN	122 1195 minus strand Severin [Crassostrea gigas]
5	7	NaN	1 753 hypothetical protein BRAFLDRAFT_131251 [Branchiostoma floridae] gi
2	5	NaN	112 1050 Enolase-phosphatase E1 [Crassostrea gigas]; 112 1050 Enolase-phosphatase E1 [Crassostrea gigas]
7	25	NaN	3 434 minus strand myosin essential light chain [Haliotis discus discus]
4	24	NaN	" 431 880 Myosin essential light chain, striated adductor muscle OS=Aequipecten irradians PE=1 SV=2"
2	6	NaN	" 193 333 minus strand RecName: Full=Globin; AltName: Full=Myoglobin gi|299274|gb|AAB25743.1| myoglobin [Na
2	2	NaN	1 264 Spectrin beta chain [Crassostrea gigas]
2	3	NaN	69 731 hypoxanthine phosphoribosyltransferase 1 [Haliotis discus discus]

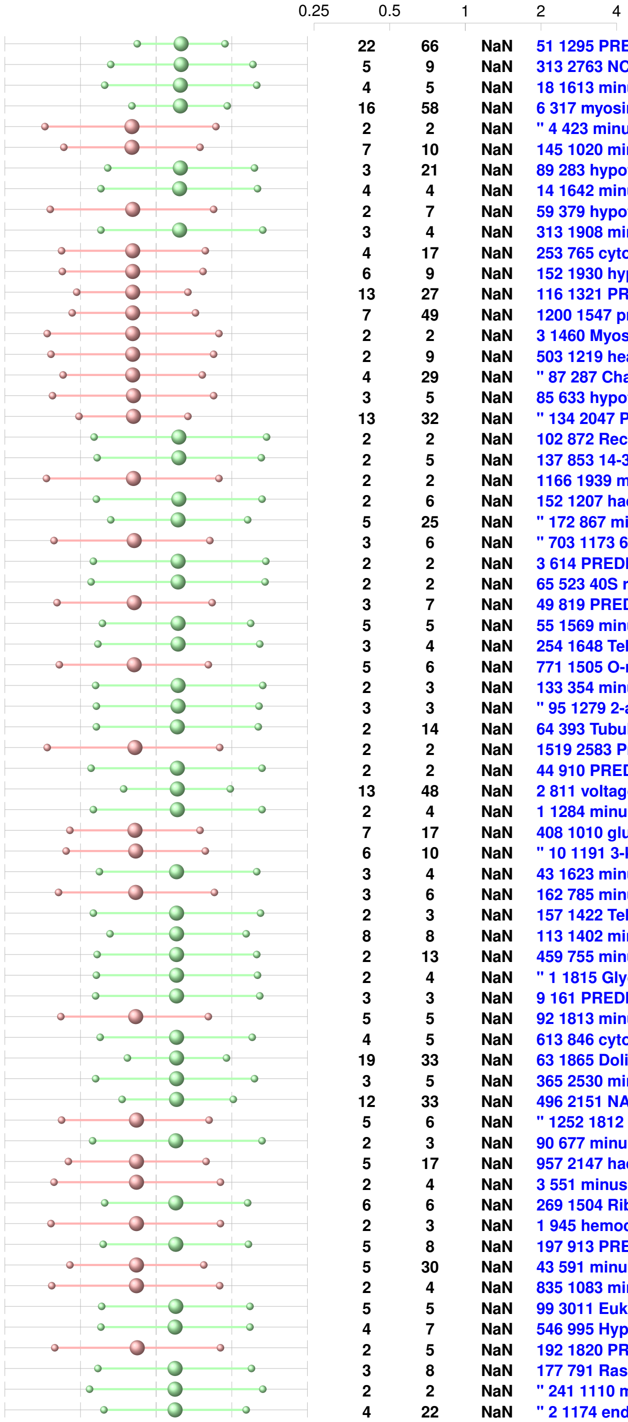

Peptides	Spectra	Coverage	Protein
22	66	NaN	51 1295 PREDICTED: phosphoglycerate kinase-like [Nasonia vitripennis]; 97 1341 PREDICTED: phosphoglycerate k
5	9	NaN	313 2763 NCAM-related cell adhesion molecule precursor [Aplysia californica] gi
4	5	NaN	18 1613 minus strand chaperonin containing T-complex polypeptide subunit zeta [Haliotis discus hannai]; 527 2089
16	58	NaN	6 317 myosin heavy chain cardiac muscle specific isoform 2 [Argopecten irradians]
2	2	NaN	" 4 423 minus strand PREDICTED: similar to NADH dehydrogenase, putative [Tribolium castaneum] gi"
7	10	NaN	145 1020 minus strand PREDICTED: inorganic pyrophosphatase-like [Saccoglossus kowalevskii]
3	21	NaN	89 283 hypothetical protein CGI_10017178 [Crassostrea gigas]
4	4	NaN	14 1642 minus strand chaperonin containing tcp1 [Haliotis discus discus]; 14 1462 minus strand chaperonin contai
2	7	NaN	59 379 hypothetical protein DAPPUDRAFT_230756 [Daphnia pulex]; 117 443 minus strand hypothetical protein DAPP
3	4	NaN	313 1908 minus strand Heterogeneous nuclear ribonucleoprotein L [Crassostrea gigas]
4	17	NaN	253 765 cytochrome c oxidase subunit IV [Urechis caupo]
6	9	NaN	152 1930 hypothetical protein BRAFLDRAFT_223365 [Branchiostoma floridae] gi
13	27	NaN	116 1321 PREDICTED: similar to aspartate aminotransferase [Tribolium castaneum] gi
7	49	NaN	1200 1547 protein disulfide isomerase [Conus eburneus]
2	2	NaN	3 1460 Myosin-VI [Crassostrea gigas]
2	9	NaN	503 1219 heat shock cognate 71 [Mytilus galloprovincialis]
4	29	NaN	" 87 287 Chain A, Structure Of Deoxygenated Hemocyanin From Rapana Thomasiana gi|31615505|pdb|1LNL|B Chai
3	5	NaN	85 633 hypothetical protein [Amblyomma maculatum]
13	32	NaN	" 134 2047 PREDICTED: succinate dehydrogenase [ubiquinone] flavoprotein subunit, mitochondrial-like isoform 3, p
2	2	NaN	102 872 RecName: Full=Gelsolin-like protein 1; AltName: Full=Actin-modulator; Short=EWAM; Short=EWAM-P1 gi|1
2	5	NaN	137 853 14-3-3 protein [Heterodera glycines]
2	2	NaN	1166 1939 minus strand Calcium-binding mitochondrial carrier protein Aralar2 OS=Homo sapiens GN=SLC25A13 PE
2	6	NaN	152 1207 haemocyanin precursor [Nautilus pompilius]
5	25	NaN	" 172 867 minus strand complement C1q-like protein 4 precursor, partial [Oryzias melastigma]"
3	6	NaN	" 703 1173 60S acidic ribosomal protein P0, putative [Ixodes scapularis] gi"
2	2	NaN	3 614 PREDICTED: 60S ribosomal protein L8-like [Papio anubis]
2	2	NaN	65 523 40S ribosomal protein S9 [Crassostrea gigas]; 65 523 40S ribosomal protein S9 [Crassostrea gigas]
3	7	NaN	49 819 PREDICTED: elongation factor 1-delta-like isoform 2 [Strongylocentrotus purpuratus]
5	5	NaN	55 1569 minus strand Putative aminopeptidase W07G4.4 [Crassostrea gigas]; 228 1742 Putative aminopeptidase W0
3	4	NaN	254 1648 Tektin-3 [Crassostrea gigas]
5	6	NaN	771 1505 O-methyltransferase family 3 [Dicentrarchus labrax]
2	3	NaN	133 354 minus strand 60S ribosomal protein L7a OS=Rattus norvegicus GN=Rpl7a PE=1 SV=2
3	3	NaN	" 95 1279 2-amino-3-ketobutyrate coenzyme A ligase, mitochondrial, partial [Crassostrea gigas]"
2	14	NaN	64 393 Tubulin beta chain OS=Paracentrotus lividus PE=2 SV=1; 109 438 Tubulin beta chain OS=Paracentrotus lividu
2	2	NaN	1519 2583 Proliferation-associated protein 2G4 [Crassostrea gigas]; 565 1629 Proliferation-associated protein 2G4 [
2	2	NaN	44 910 PREDICTED: probable ATP-dependent RNA helicase DDX10 [Cricetulus griseus] gi
13	48	NaN	2 811 voltage-dependent anion channel 2-like protein [Haliotis diversicolor]; 2 811 voltage-dependent anion channel
2	4	NaN	1 1284 minus strand Thioredoxin reductase 3 [Crassostrea gigas]
7	17	NaN	408 1010 glutathione S-transferase sigma 2 [Mytilus galloprovincialis]; 166 768 glutathione S-transferase sigma 2 [M
6	10	NaN	" 10 1191 3-ketoacyl-CoA thiolase, mitochondrial [Crassostrea gigas]"
3	4	NaN	43 1623 minus strand MGC84105 protein precursor [Xenopus laevis] gi; 43 1506 minus strand MGC84105 protein pre
3	6	NaN	162 785 minus strand Ropporin-1-like protein OS=Xenopus laevis GN=ropn1l PE=2 SV=1
2	3	NaN	157 1422 Tektin-2 [Crassostrea gigas]
8	8	NaN	113 1402 minus strand S-adenosylhomocysteine hydrolase [Crassostrea ariakensis]; 113 1402 minus strand S-aden
2	13	NaN	459 755 minus strand heat shock protein beta-1 [Trichinella spiralis] gi; 464 763 minus strand heat shock protein bet
2	4	NaN	" 1 1815 Glycogen phosphorylase, muscle form [Crassostrea gigas]"
3	3	NaN	9 161 PREDICTED: tubulin alpha-1C chain-like [Acyrthosiphon pisum]; 9 161 Tubulin alpha-3 chain (Fragments) OS=
5	5	NaN	92 1813 minus strand Dihydropyrimidinase [Crassostrea gigas]
4	5	NaN	613 846 cytochrome c oxidase subunit Va [Antheraea yamamai] gi
19	33	NaN	63 1865 Dolichyl-diphosphooligosaccharide–protein glycosyltransferase subunit 1 [Crassostrea gigas]; 63 1865 Dol
3	5	NaN	365 2530 minus strand calpain B [Gecarcinus lateralis]
12	33	NaN	496 2151 NADP-dependent malic enzyme [Crassostrea gigas]
5	6	NaN	" 1252 1812 minus strand Alanine–glyoxylate aminotransferase 2, mitochondrial [Crassostrea gigas]"
2	3	NaN	90 677 minus strand PREDICTED: 15-hydroxyprostaglandin dehydrogenase [NAD+] [Gallus gallus]; 90 683 minus str
5	17	NaN	957 2147 haemocyanin [Aplysia californica]
2	4	NaN	3 551 minus strand Spectrin beta chain [Crassostrea gigas]
6	6	NaN	269 1504 Ribosome-binding protein 1 [Crassostrea gigas]; 265 1500 Ribosome-binding protein 1 [Crassostrea gigas
2	3	NaN	1 945 hemocyanin isoform 1 [Haliotis diversicolor]
5	8	NaN	197 913 PREDICTED: l-xylulose reductase-like [Monodelphis domestica]; 197 913 PREDICTED: l-xylulose reductase-
5	30	NaN	43 591 minus strand cyclophilin type peptidyl-prolyl cis-trans isomerase [Glossina morsitans morsitans]
2	4	NaN	835 1083 minus strand Calmodulin [Crassostrea gigas]
5	5	NaN	99 3011 Eukaryotic translation initiation factor 3 subunit A [Crassostrea gigas]
4	7	NaN	546 995 Hypothetical protein CBG12002 [Caenorhabditis briggsae]; 543 995 minus strand PREDICTED: tubulin polyr
2	5	NaN	192 1820 PREDICTED: copine IV-like [Saccoglossus kowalevskii]; 125 1753 PREDICTED: copine IV-like [Saccoglossu
3	8	NaN	177 791 Ras-related protein Rab-1A [Haliotis discus discus]
2	2	NaN	" 241 1110 minus strand N(G),N(G)-dimethylarginine dimethylaminohydrolase 1 [Crassostrea gigas]; 241 1110 minus
4	22	NaN	" 2 1174 endo-1,3-beta-D-glucanase [Littorina sitkana]"

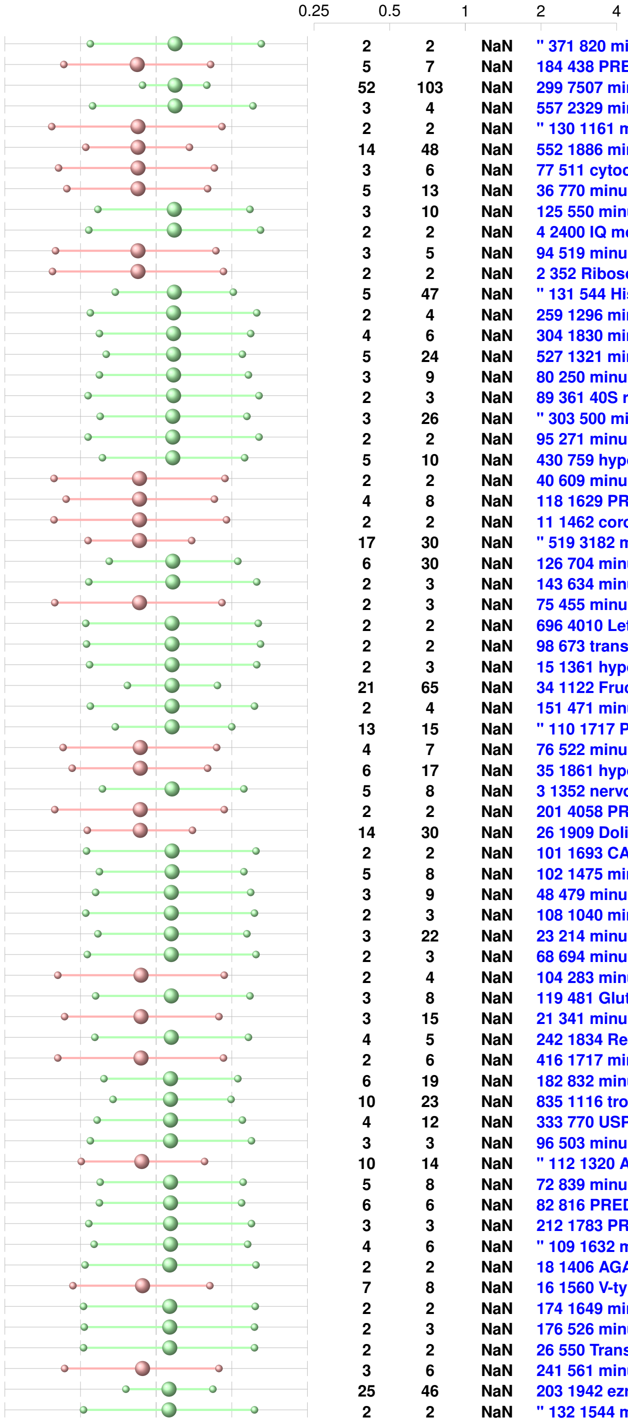

Peptides	Spectra	Coverage	Protein
2	2	NaN	" 371 820 minus strand Peroxiredoxin-5, mitochondrial [Crassostrea gigas]; 91 528 minus strand Peroxiredoxin-5, m
5	7	NaN	184 438 PREDICTED: similar to CG14235 CG14235-PA [Tribolium castaneum] gi
52	103	NaN	299 7507 minus strand filamin [Clonorchis sinensis]
3	4	NaN	557 2329 minus strand Collagen alpha-3(VI) chain [Crassostrea gigas]
2	2	NaN	" 130 1161 minus strand Isocitrate dehydrogenase [NAD] subunit alpha, mitochondrial [Crassostrea gigas]; 198 122
14	48	NaN	552 1886 minus strand intermediate filament protein [Biomphalaria glabrata]
3	6	NaN	77 511 cytochrome c oxidase subunit VIIa polypeptide 2 like [Xenopus (Silurana) tropicalis]; 77 511 cytochrome c ox
5	13	NaN	36 770 minus strand Endoplasmic reticulum protein ERp29 [Crassostrea gigas]
3	10	NaN	125 550 minus strand ribosomal protein S20 [Chlamys farreri]; 125 550 minus strand ribosomal protein S20 [Chlamy
2	2	NaN	4 2400 IQ motif containing GTPase activating protein 2 [Xenopus laevis] gi; 4 2169 IQ motif containing GTPase activ
3	5	NaN	94 519 minus strand hypothetical protein BRAFLDRAFT_115029 [Branchiostoma floridae] gi
2	2	NaN	2 352 Ribosome-binding protein 1 [Crassostrea gigas]
5	47	NaN	" 131 544 Histone H2B, gonadal OS=Patella granatina PE=1 SV=2"
2	4	NaN	259 1296 minus strand GD20916 [Drosophila simulans] gi
4	6	NaN	304 1830 minus strand Methenyltetrahydrofolate synthetase domain-containing protein [Crassostrea gigas]
5	24	NaN	527 1321 minus strand troponin T [Mizuhopecten yessoensis]; 305 1099 minus strand troponin T [Mizuhopecten yes
3	9	NaN	80 250 minus strand 40S ribosomal protein S29 [Ornithodoros parkeri]
2	3	NaN	89 361 40S ribosomal protein S18 OS=Rattus norvegicus GN=Rps18 PE=1 SV=3
3	26	NaN	" 303 500 minus strand protein lethal, putative [Pediculus humanus corporis] gi"
2	2	NaN	95 271 minus strand hypothetical protein EAI_01530 [Harpegnathos saltator]
5	10	NaN	430 759 hypothetical protein [Amblyomma maculatum]; 336 665 hypothetical protein [Amblyomma maculatum]
2	2	NaN	40 609 minus strand ependymin related protein-1 precursor [Haliotis discus discus]
4	8	NaN	118 1629 PREDICTED: similar to tektin 3 [Ciona intestinalis]
2	2	NaN	11 1462 coronin [Schistosoma mansoni]
17	30	NaN	" 519 3182 minus strand Alpha-actinin, sarcomeric [Crassostrea gigas]"
6	30	NaN	126 704 minus strand ribosomal protein L12 [Ixodes scapularis]
2	3	NaN	143 634 minus strand ribosomal protein S14 [Solea senegalensis]
2	3	NaN	75 455 minus strand PREDICTED: calnexin [Strongylocentrotus purpuratus]
2	2	NaN	696 4010 Lethal(2) giant larvae-like protein 1 [Crassostrea gigas]; 657 3971 minus strand Lethal(2) giant larvae-like p
2	2	NaN	98 673 transforming protein Rho-like [Acyrthosiphon pisum]
2	3	NaN	15 1361 hypothetical protein BRAFLDRAFT_231908 [Branchiostoma floridae] gi
21	65	NaN	34 1122 Fructose-bisphosphate aldolase [Crassostrea gigas]
2	4	NaN	151 471 minus strand ribosomal protein rps25 [Arenicola marina]
13	15	NaN	" 110 1717 PREDICTED: glutamate dehydrogenase, mitochondrial-like [Oreochromis niloticus]; 110 1717 PREDICTE
4	7	NaN	76 522 minus strand Troponin C [Crassostrea gigas]; 173 619 minus strand Troponin C [Crassostrea gigas]; 173 619
6	17	NaN	35 1861 hypothetical protein DAPPUDRAFT_309082 [Daphnia pulex]
5	8	NaN	3 1352 nervous system adducin [Aplysia californica] gi; 3 1418 Protein hu-li tai shao [Crassostrea gigas]; 3 1214 ner
2	2	NaN	201 4058 PREDICTED: xanthine dehydrogenase/oxidase-like [Amphimedon queenslandica]
14	30	NaN	26 1909 Dolichyl-diphosphooligosaccharide–protein glycosyltransferase subunit 2 [Crassostrea gigas]
2	2	NaN	101 1693 CALNUC [Spodoptera frugiperda]; 141 1325 Nucleobindin-2 [Crassostrea gigas]; 141 1289 Nucleobindin-2
5	8	NaN	102 1475 minus strand Cytosolic non-specific dipeptidase [Crassostrea gigas]
3	9	NaN	48 479 minus strand 40S ribosomal protein S24 OS=Macaca fascicularis GN=RPS24 PE=2 SV=1
2	3	NaN	108 1040 minus strand PREDICTED: Na(+)/H(+) exchange regulatory cofactor NHE-RF3-like [Metaseiulus occidentali
3	22	NaN	23 214 minus strand hemocyanin isoform 1 [Nucula nucleus]
2	3	NaN	68 694 minus strand hypothetical protein BRAFLDRAFT_272416 [Branchiostoma floridae] gi; 68 667 minus strand h
2	4	NaN	104 283 minus strand RecName: Full=Hemocyanin type 2 unit a; AltName: Full=Hemocyanin RHSS2 subunit; AltNan
3	8	NaN	119 481 Glutamate receptor U1 [Crassostrea gigas]
3	15	NaN	21 341 minus strand hemocyanin isoform 1 [Nucula nucleus]
4	5	NaN	242 1834 Regulator of differentiation 1 [Crassostrea gigas]; 458 2050 minus strand Regulator of differentiation 1 [Cr
2	6	NaN	416 1717 minus strand T-complex protein 1 subunit delta OS=Aedes triseriatus PE=2 SV=1
6	19	NaN	182 832 minus strand qm-like protein [Crassostrea ariakensis]
10	23	NaN	835 1116 tropomyosin [Ilyanassa obsoleta]
4	12	NaN	333 770 USP-like protein isoform 2 [Crassostrea ariakensis]
3	3	NaN	96 503 minus strand hypothetical protein DAPPUDRAFT_230378 [Daphnia pulex]
10	14	NaN	" 112 1320 Aspartate aminotransferase, cytoplasmic [Crassostrea gigas]"
5	8	NaN	72 839 minus strand 60S ribosomal protein L8 OS=Spodoptera frugiperda GN=RpL8 PE=2 SV=1
6	6	NaN	82 816 PREDICTED: triosephosphate isomerase B [Oreochromis niloticus]
3	3	NaN	212 1783 PREDICTED: d-3-phosphoglycerate dehydrogenase-like [Strongylocentrotus purpuratus]
4	6	NaN	" 109 1632 minus strand Seryl-tRNA synthetase, cytoplasmic [Crassostrea gigas]; 109 1614 minus strand Seryl-tRN
2	2	NaN	18 1406 AGAP006996-PA [Anopheles gambiae str. PEST] gi
7	8	NaN	16 1560 V-type proton ATPase subunit B [Crassostrea gigas]
2	2	NaN	174 1649 minus strand Collagen alpha-1(XII) chain [Crassostrea gigas]; 123 1490 minus strand Collagen alpha-1(XII)
2	3	NaN	176 526 minus strand Translocon-associated protein subunit delta [Crassostrea gigas]
2	2	NaN	26 550 Translocon-associated protein subunit gamma [Crassostrea gigas]
3	6	NaN	241 561 minus strand thioredoxin [Crassostrea ariakensis]
25	46	NaN	203 1942 ezrin/radixin/moesin [Aplysia californica] gi; 243 1982 minus strand ezrin/radixin/moesin [Aplysia californic
2	2	NaN	" 132 1544 minus strand Serine hydroxymethyltransferase, cytosolic [Crassostrea gigas]"

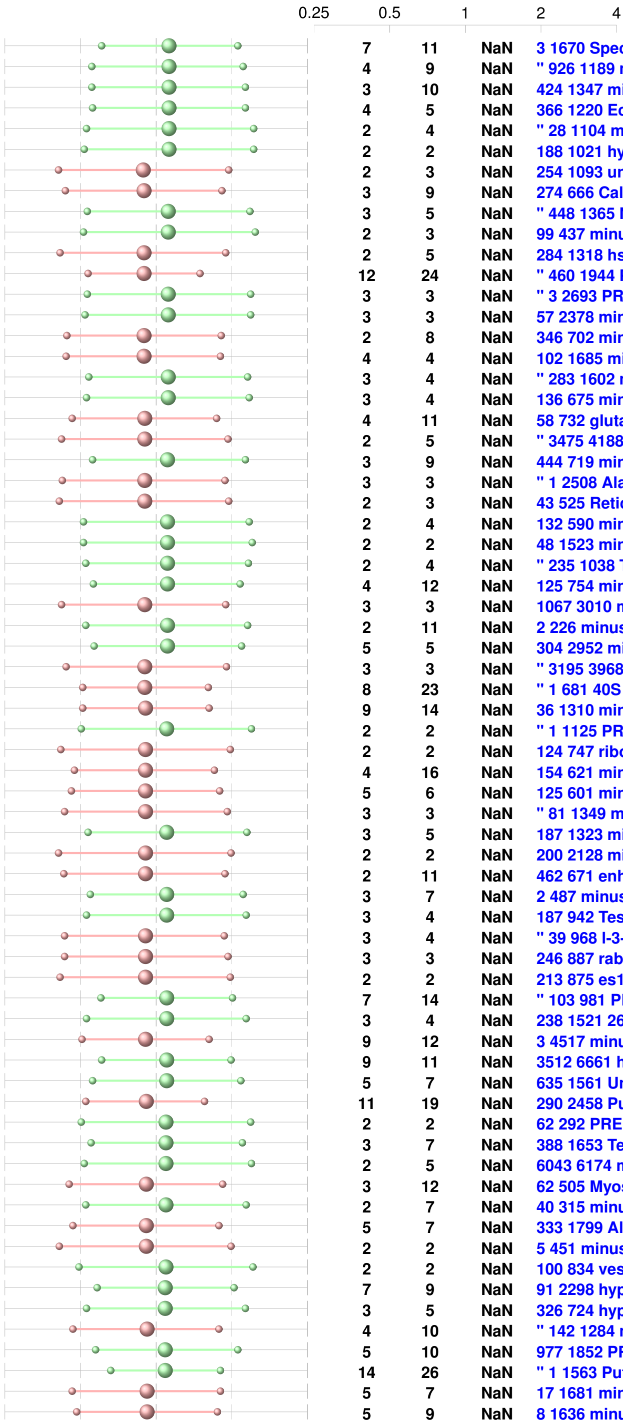

Peptides	Spectra	Coverage	Protein
7	11	NaN	3 1670 Spectrin alpha chain [Crassostrea gigas]
4	9	NaN	" 926 1189 minus strand Myosin heavy chain, striated muscle [Crassostrea gigas]"
3	10	NaN	424 1347 minus strand Tubulin alpha chain [Ascaris suum]
4	5	NaN	366 1220 Ectoine hydroxylase [Crassostrea gigas]
2	4	NaN	" 28 1104 minus strand Pyruvate dehydrogenase E1 component subunit beta, mitochondrial [Crassostrea gigas]; 10
2	2	NaN	188 1021 hypothetical protein BRAFLDRAFT_214346 [Branchiostoma floridae] gi
2	3	NaN	254 1093 uncharacterized protein LOC432187 precursor [Xenopus laevis] gi
3	9	NaN	274 666 Calmodulin-4 [Heterocephalus glaber]
3	5	NaN	" 448 1365 Nitrile-specifier protein 5, partial [Crassostrea gigas]"
2	3	NaN	99 437 minus strand V-type proton ATPase subunit G [Crassostrea gigas]
2	5	NaN	284 1318 hsc70-interacting protein [Gallus gallus] gi; 88 1137 hsc70-interacting protein [Gallus gallus] gi
12	24	NaN	" 460 1944 PREDICTED: succinyl-CoA:3-ketoacid-coenzyme A transferase 1, mitochondrial [Canis lupus familiaris]"
3	3	NaN	" 3 2693 PREDICTED: similar to coatomer protein complex, subunit beta 2 (beta prime) [Taeniopygia guttata]"
3	3	NaN	57 2378 minus strand Heterogeneous nuclear ribonucleoprotein U-like protein 1 [Crassostrea gigas]
2	8	NaN	346 702 minus strand PREDICTED: galectin-4-like [Anolis carolinensis]
4	4	NaN	102 1685 minus strand predicted protein [Nematostella vectensis] gi
3	4	NaN	" 283 1602 minus strand 4-aminobutyrate aminotransferase, mitochondrial [Crassostrea gigas]"
3	4	NaN	136 675 minus strand predicted protein [Nematostella vectensis] gi
4	11	NaN	58 732 glutathione-s-transferase [Haliotis discus discus]
2	5	NaN	" 3475 4188 minus strand Heat shock 70 kDa protein cognate, putative [Pediculus humanus corporis] gi"
3	9	NaN	444 719 minus strand 60S ribosomal protein L9 [Laodelphax striatella]
3	3	NaN	" 1 2508 Alanyl-tRNA synthetase, cytoplasmic [Crassostrea gigas]; 1 2508 Alanyl-tRNA synthetase, cytoplasmic [Cra
2	3	NaN	43 525 Reticulon-4 [Crassostrea gigas]; 143 757 Reticulon-4 [Crassostrea gigas]; 43 564 Reticulon-4 [Crassostrea gi
2	4	NaN	132 590 minus strand Non-neuronal cytoplasmic intermediate filament protein OS=Helix aspersa PE=3 SV=2
2	2	NaN	48 1523 minus strand Spermatogenesis-associated protein 18-like protein [Crassostrea gigas]
2	4	NaN	" 235 1038 Translocon-associated protein subunit alpha [Crassostrea gigas]; 309 725 hypothetical protein [Amblyom
4	12	NaN	125 754 minus strand peptidyl prolyl cis-trans isomerase B [Conus novaehollandiae]
3	3	NaN	1067 3010 minus strand PREDICTED: nucleolin-like isoform 2 [Oreochromis niloticus]
2	11	NaN	2 226 minus strand hypothetical protein CGI_10015501 [Crassostrea gigas]
5	5	NaN	304 2952 minus strand hypothetical protein CGI_10026535 [Crassostrea gigas]
3	3	NaN	" 3195 3968 Spectrin alpha chain, putative [Pediculus humanus corporis] gi"
8	23	NaN	" 1 681 40S ribosomal protein S4, X isoform [Crassostrea gigas]"
9	14	NaN	36 1310 minus strand elongation factor-1 gamma [Diguetia canities]
2	2	NaN	" 1 1125 PREDICTED: trifunctional enzyme subunit beta, mitochondrial-like [Oreochromis niloticus]"
2	2	NaN	124 747 ribosomal protein S10 [Crassostrea gigas] gi
4	16	NaN	154 621 minus strand Stress response protein nhaX [Crassostrea gigas]
5	6	NaN	125 601 minus strand omega class glutathione-s-transferase [Chlamys farreri]; 113 808 minus strand omega class g
3	3	NaN	" 81 1349 minus strand PREDICTED: LOW QUALITY PROTEIN: dolichyl-diphosphooligosaccharide–protein glycosyl
3	5	NaN	187 1323 minus strand gelsolin [Aplysia californica] gi
2	2	NaN	200 2128 minus strand heat shock protein 70 [Cristaria plicata]
2	11	NaN	462 671 enhancer protein [Homo sapiens]
3	7	NaN	2 487 minus strand PREDICTED: hypothetical protein LOC100699750 [Oreochromis niloticus]; 41 535 minus strand
3	4	NaN	187 942 Testis-specific gene 10 protein OS=Mus musculus GN=Tsga10 PE=1 SV=1; 212 967 Testis-specific gene 10
3	4	NaN	" 39 968 l-3-hydroxyacyl-coenzyme a dehydrogenase, short chain [Haliotis discus discus]; 39 968 l-3-hydroxyacyl-co
3	3	NaN	246 887 rab11-2 [Limulus polyphemus]
2	2	NaN	213 875 es1 protein [Haliotis discus discus]
7	14	NaN	" 103 981 PREDICTED: ATP synthase, H+ transporting, mitochondrial F1 complex, gamma subunit isoform H (heart)
3	4	NaN	238 1521 26S protease regulatory subunit 6A [Crassostrea gigas]
9	12	NaN	3 4517 minus strand Clathrin heavy chain 1 [Crassostrea gigas]
9	11	NaN	3512 6661 hypothetical protein BRAFLDRAFT_255626 [Branchiostoma floridae] gi
5	7	NaN	635 1561 Uncharacterized protein yfeX [Crassostrea gigas]
11	19	NaN	290 2458 Putative beta-D-xylosidase 5 [Crassostrea gigas]
2	2	NaN	62 292 PREDICTED: heterogeneous nuclear ribonucleoprotein F-like [Otolemur garnettii]
3	7	NaN	388 1653 Tektin-3 [Crassostrea gigas]
2	5	NaN	6043 6174 myosin heavy chain [Mytilus galloprovincialis]; 5869 6000 myosin heavy chain [Mytilus galloprovincialis]
3	12	NaN	62 505 Myosin-2 essential light chain [Crassostrea gigas]
2	7	NaN	40 315 minus strand 60S ribosomal protein L18a OS=Homo sapiens GN=RPL18A PE=1 SV=2
5	7	NaN	333 1799 Alba-like protein C9orf23-like protein [Crassostrea gigas]
2	2	NaN	5 451 minus strand Spectrin beta chain [Crassostrea gigas]
2	2	NaN	100 834 vesicle-associated membrane protein/synaptobrevin-binding protein [Aplysia californica] gi
7	9	NaN	91 2298 hypothetical protein BRAFLDRAFT_267717 [Branchiostoma floridae] gi; 84 2291 hypothetical protein BRAFL
3	5	NaN	326 724 hypothetical protein [Amblyomma maculatum]
4	10	NaN	" 142 1284 minus strand Chain O, Crsytal Structure Of Human Liver Gapdh gi|67464044|pdb|1ZNQ|P Chain P, Crsyta
5	10	NaN	977 1852 PREDICTED: similar to prohibitin [Tribolium castaneum] gi
14	26	NaN	" 1 1563 Putative methylmalonate-semialdehyde dehydrogenase [acylating], mitochondrial [Crassostrea gigas]"
5	7	NaN	17 1681 minus strand Glucose-6-phosphate isomerase [Crassostrea gigas]
5	9	NaN	8 1636 minus strand V-type proton ATPase catalytic subunit A [Crassostrea gigas]; 8 1636 minus strand V-type proto

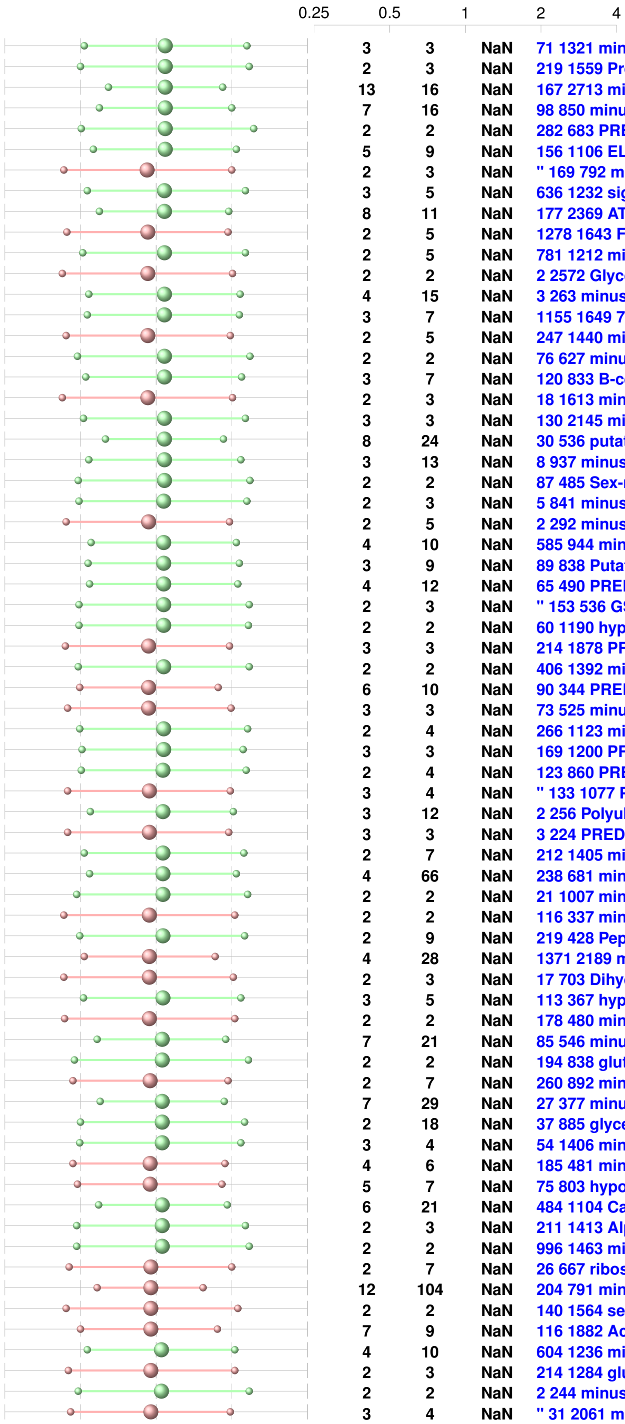

Peptides	Spectra	Coverage	Protein
3	3	NaN	71 1321 minus strand Vinculin [Crassostrea gigas]
2	3	NaN	219 1559 Programmed cell death protein 4 [Crassostrea gigas]
13	16	NaN	167 2713 minus strand Major vault protein [Crassostrea gigas]
7	16	NaN	98 850 minus strand 14-3-3 protein epsilon [Schistocerca gregaria]
2	2	NaN	282 683 PREDICTED: ribonuclease UK114-like [Oreochromis niloticus]
5	9	NaN	156 1106 ELAV 2-like protein [Aplysia californica] gi
2	3	NaN	" 169 792 minus strand rho GDP dissociation inhibitor, putative [Ixodes scapularis] gi"
3	5	NaN	636 1232 sigma class glutathione-s-transferase 3 [Haliotis discus discus]; 963 1559 sigma class glutathione-s-transf
8	11	NaN	177 2369 ATPase family AAA domain-containing protein 2B [Crassostrea gigas]; 177 2369 ATPase family AAA doma
2	5	NaN	1278 1643 Fatty acid-binding-like protein 5 [Crassostrea gigas]
2	5	NaN	781 1212 minus strand cathepsin L2 cysteine protease [Pinctada fucata]
2	2	NaN	2 2572 Glycogen debranching enzyme [Crassostrea gigas]
4	15	NaN	3 263 minus strand beta-actin [Urechis unicinctus]
3	7	NaN	1155 1649 78 kDa glucose-regulated protein OS=Aplysia californica PE=1 SV=1
2	5	NaN	247 1440 minus strand Septin-2 [Crassostrea gigas]
2	2	NaN	76 627 minus strand Heme-binding protein 2 [Crassostrea gigas]; 296 847 minus strand Heme-binding protein 2 [Cra
3	7	NaN	120 833 B-cell receptor-associated protein 31 [Crassostrea gigas]; 238 951 B-cell receptor-associated protein 31 [Cr
2	3	NaN	18 1613 minus strand chaperonin containing T-complex polypeptide subunit zeta [Haliotis discus hannai]; 527 2089
3	3	NaN	130 2145 minus strand Glucosamine–fructose-6-phosphate aminotransferase [isomerizing] 1 [Crassostrea gigas]; 1
8	24	NaN	30 536 putative mitochondrial ATP synthase [Haliotis discus discus]; 27 620 putative mitochondrial ATP synthase [H
3	13	NaN	8 937 minus strand galectin [Crassostrea virginica]
2	2	NaN	87 485 Sex-regulated protein janus-A [Crassostrea gigas]
2	3	NaN	5 841 minus strand Spectrin alpha chain [Crassostrea gigas]
2	5	NaN	2 292 minus strand H2 [Haliotis tuberculata]
4	10	NaN	585 944 minus strand Protein disulfide-isomerase A6 [Crassostrea gigas]
3	9	NaN	89 838 Putative phosphoglycerate mutase [Crassostrea gigas]; 89 838 Putative phosphoglycerate mutase [Crassost
4	12	NaN	65 490 PREDICTED: cofilin-2 [Otolemur garnettii]
2	3	NaN	" 153 536 GST omega, partial [Reishia clavigera]"
2	2	NaN	60 1190 hypothetical protein BRAFLDRAFT_109688 [Branchiostoma floridae] gi; 51 1184 hypothetical protein BRAFI
3	3	NaN	214 1878 PREDICTED: lysyl-tRNA synthetase-like [Oreochromis niloticus]
2	2	NaN	406 1392 minus strand hypothetical protein CGI_10007007 [Crassostrea gigas]
6	10	NaN	90 344 PREDICTED: diazepam binding inhibitor-like [Saccoglossus kowalevskii]
3	3	NaN	73 525 minus strand Actin-related protein 2/3 complex subunit 5-like protein [Salmo salar] gi; 73 525 Actin-related pr
2	4	NaN	266 1123 minus strand mitochondrial malate dehydrogenase precursor [Nucella lapillus]
3	3	NaN	169 1200 PREDICTED: nidogen-2-like isoform 1 [Strongylocentrotus purpuratus]
2	4	NaN	123 860 PREDICTED: proteasome subunit alpha type-6-like [Cavia porcellus]; 1164 1901 PREDICTED: proteasome su
3	4	NaN	" 133 1077 PREDICTED: electron transfer flavoprotein, alpha polypeptide-like isoform 2 [Saccoglossus kowalevskii]"
3	12	NaN	2 256 Polyubiquitin (Fragment) OS=Xenopus laevis PE=1 SV=2
3	3	NaN	3 224 PREDICTED: 40S ribosomal protein S14-like [Sarcophilus harrisii]
2	7	NaN	212 1405 minus strand unnamed protein product [Tetraodon nigroviridis]
4	66	NaN	238 681 minus strand Globin OS=Cerithidea rhizophorarum PE=1 SV=1
2	2	NaN	21 1007 minus strand cathepsin L2 cysteine protease [Pinctada fucata]; 21 1007 minus strand cathepsin L2 cysteine
2	2	NaN	116 337 minus strand Golgi-associated plant pathogenesis-related protein 1 [Crassostrea gigas]; 538 759 minus stra
2	9	NaN	219 428 Peptidyl-prolyl cis-trans isomerase OS=Hemicentrotus pulcherrimus PE=2 SV=1
4	28	NaN	1371 2189 minus strand galectin [Pinctada fucata]
2	3	NaN	17 703 Dihydropteridine reductase [Crassostrea gigas]
3	5	NaN	113 367 hypothetical protein DAPPUDRAFT_329128 [Daphnia pulex]; 113 367 hypothetical protein DAPPUDRAFT_32
2	2	NaN	178 480 minus strand PREDICTED: lipoxygenase homology domain-containing protein 1-like [Danio rerio]
7	21	NaN	85 546 minus strand RecName: Full=Eukaryotic translation initiation factor 5A; Short=eIF-5A gi|51702279|sp|P6292
2	2	NaN	194 838 glutathione S-transferase class mu [Cyphoma gibbosum]
2	7	NaN	260 892 minus strand calponin [Haliotis diversicolor]
7	29	NaN	27 377 minus strand ribosomal protein LP2 [Papilio xuthus] gi; 27 377 minus strand ribosomal protein LP2 [Papilio x
2	18	NaN	37 885 glyceraldehyde 3-phosphate dehydrogenase [Dugesia japonica]
3	4	NaN	54 1406 minus strand Protein henna [Crassostrea gigas]; 54 1406 minus strand Protein henna [Crassostrea gigas]
4	6	NaN	185 481 minus strand Zgc:56530 [Danio rerio]
5	7	NaN	75 803 hypothetical protein DAPPUDRAFT_231350 [Daphnia pulex]
6	21	NaN	484 1104 Calponin-2 [Crassostrea gigas]; 309 938 Calponin-2 [Crassostrea gigas]
2	3	NaN	211 1413 Alpha-N-acetylgalactosaminidase [Crassostrea gigas]
2	2	NaN	996 1463 minus strand catalase [Cristaria plicata] gi
2	7	NaN	26 667 ribosomal protein L15 [Acipenser sinensis] gi; 26 667 ribosomal protein L15 [Acipenser sinensis] gi; 26 667 r
12	104	NaN	204 791 minus strand PREDICTED: 15-hydroxyprostaglandin dehydrogenase [NAD+] [Gallus gallus]
2	2	NaN	140 1564 selenium-binding protein [Haliotis discus hannai]
7	9	NaN	116 1882 Actin-interacting protein 1 [Crassostrea gigas]
4	10	NaN	604 1236 minus strand selenium-dependent glutathione peroxidase [Crassostrea gigas]; 138 770 minus strand seler
2	3	NaN	214 1284 glutamine synthetase [Tegillarca granosa]
2	2	NaN	2 244 minus strand hypothetical protein KGM_08771 [Danaus plexippus]
3	4	NaN	" 31 2061 minus strand 1,4-alpha-glucan-branching enzyme [Crassostrea gigas]"

0.25 0.5 1 2 4

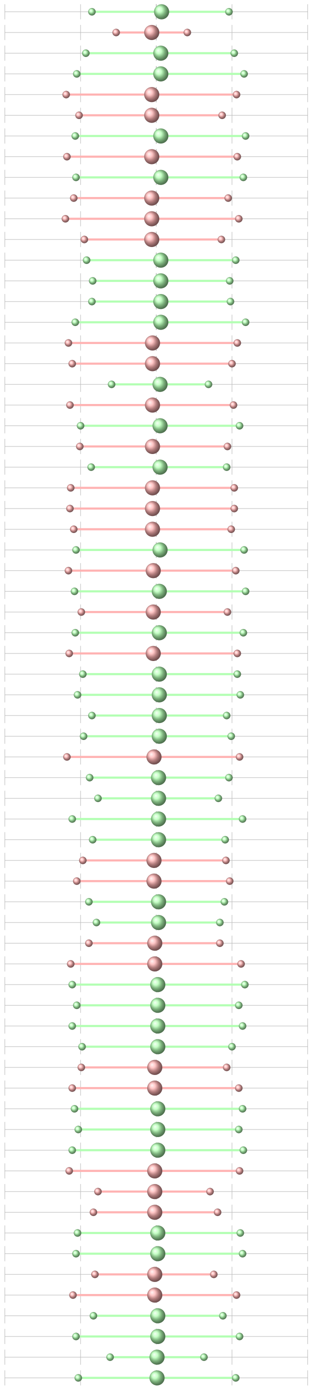

Peptides	Spectra	Coverage	Protein
5	17	NaN	3 218 beta-tubulin [Fasciola hepatica] gi
34	122	NaN	30 1088 arginine kinase [Conus novaehollandiae]
3	15	NaN	49 219 haemocyanin precursor [Nautilus pompilius]
2	2	NaN	158 1318 hypothetical protein BRAFLDRAFT_98992 [Branchiostoma floridae] gi
2	2	NaN	" 2057 2497 Phosphate carrier protein, mitochondrial [Crassostrea gigas]"
5	12	NaN	158 862 X-box binding protein [Haliotis discus discus]
2	3	NaN	325 642 minus strand Protein phosphatase 1B [Crassostrea gigas]
2	2	NaN	" 492 3458 PREDICTED: 2-oxoglutarate dehydrogenase, mitochondrial isoform 1 [Sarcophilus harrisii]"
2	3	NaN	131 541 minus strand nervous system adducin [Aplysia californica] gi
3	6	NaN	113 445 ribosomal protein rps19 [Lineus viridis]
2	2	NaN	230 1189 hypothetical protein [Amblyomma maculatum]
6	10	NaN	915 1505 minus strand Collagen alpha-5(VI) chain [Crassostrea gigas]
4	7	NaN	151 1449 rab GDP-dissociation inhibitor [Branchiostoma belcheri]
6	20	NaN	76 822 14-3-3 protein [Heterodera glycines]; 76 822 minus strand 14-3-3 protein [Heterodera glycines]; 76 822 minus
6	10	NaN	" 143 451 minus strand PREDICTED: 10 kDa heat shock protein, mitochondrial isoform 1 [Canis lupus familiaris]"
2	3	NaN	100 2307 minus strand Xanthine dehydrogenase/oxidase [Crassostrea gigas]
2	4	NaN	35 2953 hypothetical protein CGI_10020867 [Crassostrea gigas]; 97 3015 minus strand hypothetical protein CGI_100
3	5	NaN	" 599 907 minus strand Tryptophanyl-tRNA synthetase, cytoplasmic [Heterocephalus glaber]"
15	47	NaN	52 1599 minus strand ATP synthase beta subunit [Pinctada fucata]
3	3	NaN	4 399 Spectrin beta chain [Crassostrea gigas]
3	4	NaN	4 675 Laminin subunit gamma-1 [Crassostrea gigas]
5	7	NaN	2008 2715 Protein disulfide-isomerase A5 [Crassostrea gigas]
6	11	NaN	260 1597 isocitrate dehydrogenase [Mytilus galloprovincialis]; 260 1597 isocitrate dehydrogenase [Mytilus galloprov
3	5	NaN	28 1455 minus strand PREDICTED: probable serine carboxypeptidase CPVL-like [Anolis carolinensis]
2	6	NaN	243 2165 minus strand Elongation factor 2 OS=Drosophila melanogaster GN=Ef2b PE=2 SV=4
4	4	NaN	93 2876 Hypoxia up-regulated protein 1 [Crassostrea gigas]
2	5	NaN	274 1242 minus strand cathepsin D [Pinctada maxima]; 290 1387 cathepsin D [Pinctada maxima]; 290 1402 minus st
2	3	NaN	479 1732 minus strand Multifunctional protein ADE2 [Crassostrea gigas]; 239 1501 minus strand Multifunctional pro
2	2	NaN	150 716 calponin/transgelin [Papilio xuthus]
5	7	NaN	275 1372 Cystathionine gamma-lyase OS=Bos taurus GN=CTH PE=2 SV=1
2	4	NaN	105 503 Transgelin-2 [Crassostrea gigas]
2	4	NaN	6 581 PREDICTED: mammalian ependymin-related protein 1 [Gallus gallus]
3	11	NaN	712 831 haemocyanin [Aplysia californica]
3	3	NaN	26 250 minus strand fructose-bisphosphate aldolase [Haliotis discus discus]
5	24	NaN	145 459 fk506-binding protein 2 [Papilio xuthus]; 237 551 fk506-binding protein 2 [Papilio xuthus]
5	5	NaN	" 134 1567 minus strand 6-phosphogluconate dehydrogenase, decarboxylating [Crassostrea gigas]; 134 1573 minus
2	2	NaN	369 746 stanniocalcin [Haliotis diversicolor supertexta] gi
6	8	NaN	87 1997 Transketolase-like protein 2 [Crassostrea gigas]; 87 1997 Transketolase-like protein 2 [Crassostrea gigas]; 8
7	55	NaN	" 353 781 alpha-B-crystallin, putative [Ixodes scapularis] gi"
2	2	NaN	172 612 minus strand putative polyadenylate-binding protein 1 [Haliotis diversicolor]; 172 612 putative polyadenylate
5	34	NaN	176 670 hypothetical protein BRAFLDRAFT_122621 [Branchiostoma floridae] gi
5	10	NaN	" 34 1656 Chaperonin containing TCP1, subunit 7 (eta) [Danio rerio] gi; 34 1656 Chaperonin containing TCP1, subun
3	9	NaN	247 726 Annexin A7 [Crassostrea gigas]; 286 675 Annexin A7 [Crassostrea gigas]
6	18	NaN	284 700 Ganglioside GM2 activator [Crassostrea gigas]
10	15	NaN	40 1182 4-hydroxyphenylpyruvate dioxygenase [Crassostrea gigas]
7	16	NaN	1 987 minus strand RecName: Full=Tubulin beta chain; AltName: Full=Beta-tubulin
2	3	NaN	103 1356 minus strand PREDICTED: actin-related protein 3-like [Oreochromis niloticus]
2	2	NaN	91 3270 Valyl-tRNA synthetase [Crassostrea gigas]
3	5	NaN	206 316 minus strand ribosomal protein S17 [Branchiostoma belcheri]
2	2	NaN	266 934 minus strand Kyphoscoliosis peptidase [Crassostrea gigas]
4	7	NaN	293 1795 Amidase [Crassostrea gigas]
5	8	NaN	227 1201 PREDICTED: quinone oxidoreductase-like isoform 1 [Ornithorhynchus anatinus]; 109 1083 PREDICTED: qu
3	3	NaN	54 638 minus strand ubiquitin carboxyl-terminal hydrolase [Aplysia californica] gi
2	4	NaN	154 261 ribosomal protein S18 [Stenella coeruleoalba]
3	3	NaN	440 1645 Tektin-1 [Crassostrea gigas]
2	2	NaN	96 497 AAEL009772-PB [Aedes aegypti]
2	3	NaN	128 1519 minus strand V-type proton ATPase subunit H [Crassostrea gigas]; 128 1519 minus strand V-type proton A
14	21	NaN	424 3507 minus strand putative Na+/K+-ATPase alpha subunit [Paroctopus digueti]; 424 3507 minus strand putative
9	16	NaN	" 3341 4240 minus strand nonmuscle myosin II, partial [Aplysia californica]"
2	8	NaN	32 193 minus strand Ubiquinol-cytochrome C reductase complex [Xenopus laevis] gi; 32 193 minus strand Ubiquino
2	3	NaN	242 1510 minus strand hypothetical protein BRAFLDRAFT_276673 [Branchiostoma floridae] gi
9	26	NaN	288 1487 minus strand putative procollagen type VI alpha 4 [Haliotis diversicolor]
3	3	NaN	70 705 RecName: Full=Ras-related protein Rab-2 gi|288938|emb|CAA51234.1| RAB2 [Lymnaea stagnalis]; 70 705 Re
6	25	NaN	49 1038 Glyceraldehyde-3-phosphate dehydrogenase [Crotalus adamanteus]
3	3	NaN	2 478 Glyoxylate reductase/hydroxypyruvate reductase [Crassostrea gigas]
19	48	NaN	77 1561 retinal dehydrogenase [Lymnaea stagnalis]; 69 1553 retinal dehydrogenase [Lymnaea stagnalis]
4	4	NaN	" 1482 3320 minus strand Phosphoenolpyruvate carboxykinase, cytosolic [GTP] [Crassostrea gigas]; 1326 3164 min

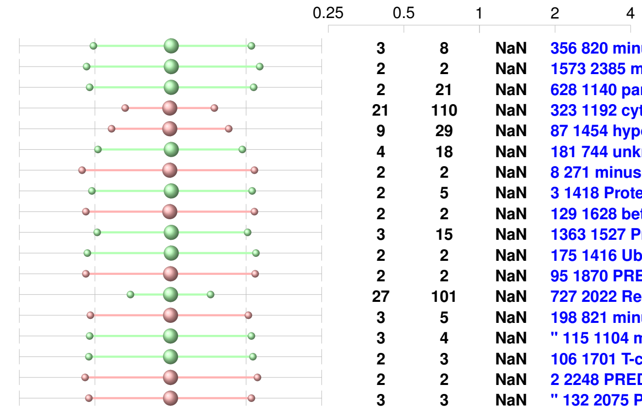

Peptides	Spectra	Coverage	Protein
3	8	NaN	356 820 minus strand calmodulin-1 [Trichinella spiralis] gi
2	2	NaN	1573 2385 minus strand Spectrin beta chain [Crassostrea gigas]
2	21	NaN	628 1140 paramyosin [Haliotis discus discus]
21	110	NaN	323 1192 cytosolic malate dehydrogenase [Lottia scabra]; 323 1192 cytosolic malate dehydrogenase [Lottia scabra];
9	29	NaN	87 1454 hypothetical protein BRAFLDRAFT_287788 [Branchiostoma floridae] gi; 117 1469 hypothetical protein BRAI
4	18	NaN	181 744 unknown [Dendroctonus ponderosae]
2	2	NaN	8 271 minus strand Laminin subunit gamma-1 [Crassostrea gigas]
2	5	NaN	3 1418 Protein hu-li tai shao [Crassostrea gigas]; 3 1523 minus strand Protein hu-li tai shao [Crassostrea gigas]; 3 1
2	2	NaN	129 1628 beta-hexosaminidase subunit beta precursor [Danio rerio] gi
3	15	NaN	1363 1527 Protein disulfide-isomerase A3 [Crassostrea gigas]
2	2	NaN	175 1416 Ubiquinone biosynthesis monooxygenase COQ6 [Crassostrea gigas]
2	2	NaN	95 1870 PREDICTED: acetylcholinesterase-like [Equus caballus]; 99 1874 PREDICTED: acetylcholinesterase-like [Eq
27	101	NaN	727 2022 RecName: Full=Enolase; AltName: Full=2-phospho-D-glycerate hydro-lyase; AltName: Full=2-phosphoglyc
3	5	NaN	198 821 minus strand sigma class glutathione-s-transferase 2 [Haliotis discus discus]
3	4	NaN	" 115 1104 minus strand PREDICTED: KH domain-containing, RNA-binding, signal transduction-associated protein 3
2	3	NaN	106 1701 T-complex protein 1 subunit beta [Crassostrea gigas]
2	2	NaN	2 2248 PREDICTED: heat shock 70 kDa protein 4-like [Oreochromis niloticus]; 2 2227 PREDICTED: heat shock 70 kD
3	3	NaN	" 132 2075 PREDICTED: arginyl-tRNA synthetase, cytoplasmic [Ornithorhynchus anatinus]; 132 2090 minus strand F

4.2 Proteins Identified by a Single Peptide

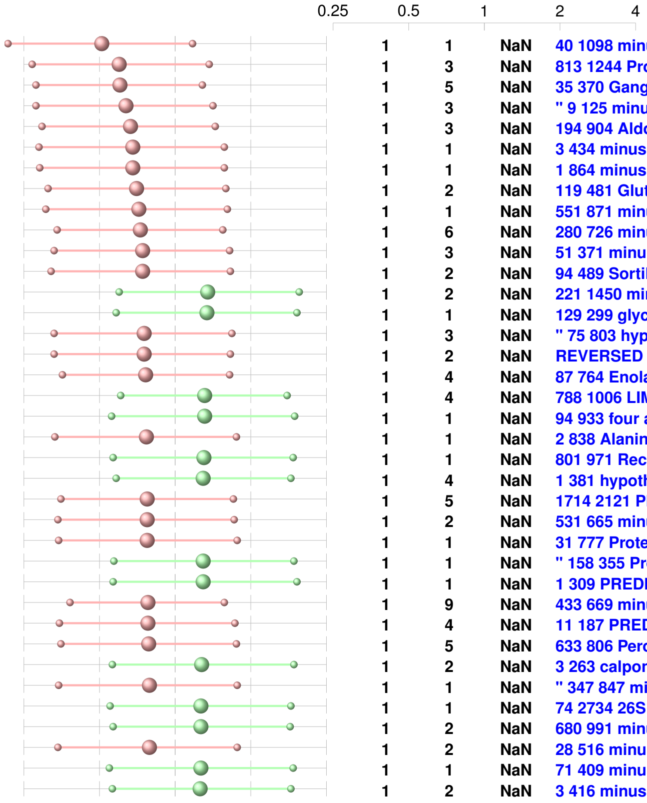

Peptides	Spectra	Coverage	Protein
1	1	NaN	40 1098 minus strand major egg antigen [Clonorchis sinensis]
1	3	NaN	813 1244 Probable protein disulfide-isomerase A6 OS=Caenorhabditis elegans GN=tag-320 PE=2 SV=1
1	5	NaN	35 370 Ganglioside GM2 activator [Crassostrea gigas]; 2 487 minus strand PREDICTED: hypothetical protein LOC10
1	3	NaN	" 9 125 minus strand Hemocyanin, beta-C chain unit D (Fragment) OS=Helix pomatia PE=1 SV=2"
1	3	NaN	194 904 Aldo-keto reductase family 1 member B10 [Crassostrea gigas]
1	1	NaN	3 434 minus strand hypothetical protein CGI_10015501 [Crassostrea gigas]
1	1	NaN	1 864 minus strand Pyruvate kinase isozymes M1/M2 [Crassostrea gigas]; 3 959 minus strand Pyruvate kinase isozy
1	2	NaN	119 481 Glutamate receptor U1 [Crassostrea gigas]; 113 328 glutamate receptor subunit protein GluR2 precursor [Ap
1	1	NaN	551 871 minus strand Protein disulfide-isomerase A4 [Crassostrea gigas]
1	6	NaN	280 726 minus strand RecName: Full=Calmodulin; Short=CaM gi|14588595|dbj|BAB61794.1| calmodulin [Metridium
1	3	NaN	51 371 minus strand putative mitochondrial ATP synthase F chain [Haliotis discus discus]; 51 371 minus strand puta
1	2	NaN	94 489 Sortilin-related receptor [Crassostrea gigas]
1	2	NaN	221 1450 minus strand PREDICTED: dihydrolipoamide branched chain transacylase E2 [Taeniopygia guttata]; 221 16
1	1	NaN	129 299 glycogen phosphorylase [Crassostrea gigas]
1	3	NaN	" 75 803 hypothetical protein DAPPUDRAFT_231350 [Daphnia pulex]; 82 612 ATP synthase B chain, putative [Ixodes
1	2	NaN	REVERSED 216 2639 minus strand polycystin 2 [Gallus gallus]
1	4	NaN	87 764 Enolase-phosphatase E1 [Crassostrea gigas]; 87 1058 Enolase-phosphatase E1 [Crassostrea gigas]; 87 764 I
1	4	NaN	788 1006 LIM protein [Haliotis discus discus]; 768 980 muscle LIM protein isoform 1 [Danaus plexippus]
1	1	NaN	94 933 four and A half lim domains [Schistosoma mansoni]
1	1	NaN	2 838 Alanine aminotransferase 2 [Crassostrea gigas]
1	1	NaN	801 971 RecName: Full=Tropomyosin-2; AltName: Full=Tropomyosin II; Short=BgTMII; Short=TMII gi|155940|gb|AAA
1	4	NaN	1 381 hypothetical protein [Eimeria tenella]
1	5	NaN	1714 2121 Phosphoenolpyruvate carboxykinase [GTP] [Crassostrea gigas]
1	2	NaN	531 665 minus strand Proteasome subunit beta type-6 [Crassostrea gigas]
1	1	NaN	31 777 Proteasome subunit alpha type-7-like protein [Crassostrea gigas]
1	1	NaN	" 158 355 Protein NAS-37, isoform b [Caenorhabditis elegans] gi; 15 227 Hemicentin-1 [Crassostrea gigas]"
1	1	NaN	1 309 PREDICTED: intersectin-1-like [Sus scrofa]
1	9	NaN	433 669 minus strand Superoxide dismutase [Cu-Zn] OS=Prionace glauca GN=sod1 PE=1 SV=1
1	4	NaN	11 187 PREDICTED: 40S ribosomal protein S28-like [Strongylocentrotus purpuratus]
1	5	NaN	633 806 Peroxiredoxin-1 [Crassostrea gigas]; 648 821 Peroxiredoxin-1 [Crassostrea gigas]; 666 839 Peroxiredoxin-1 [
1	2	NaN	3 263 calponin-like protein [Taenia asiatica]
1	1	NaN	" 347 847 minus strand ADP,ATP carrier protein 1 OS=Anopheles gambiae GN=AGAP006782 PE=2 SV=2"
1	1	NaN	74 2734 26S proteasome non-ATPase regulatory subunit 2 [Crassostrea gigas]
1	2	NaN	680 991 minus strand PDZ and LIM domain protein 1 [Crassostrea gigas]; 553 864 minus strand PDZ and LIM domai
1	2	NaN	28 516 minus strand Signal peptidase complex subunit 3 [Crassostrea gigas]; 30 554 minus strand Signal peptidase
1	1	NaN	71 409 minus strand heat shock protein 108 [Alligator mississippiensis]
1	2	NaN	3 416 minus strand myosin essential light chain [Crassostrea gigas]

0.25 0.5 1 2 4

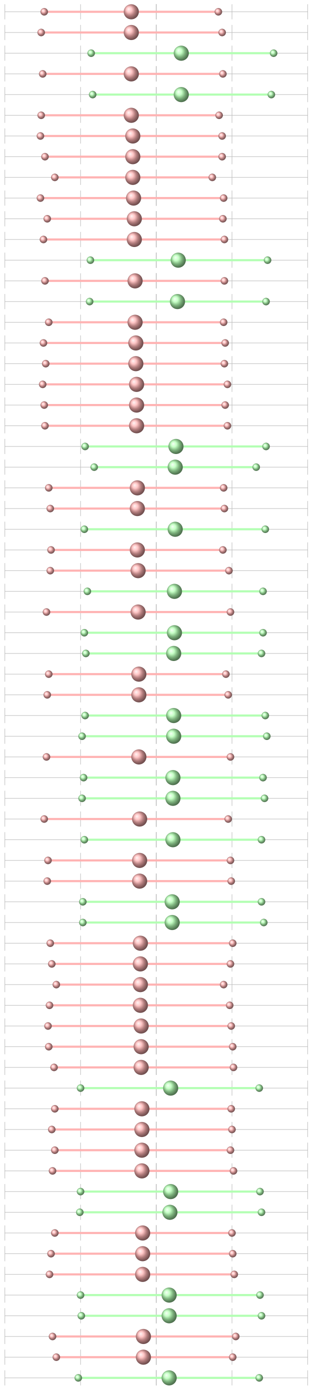

Peptides	Spectra	Coverage	Protein
1	9	NaN	397 510 PREDICTED: actin-5C-like [Megachile rotundata]
1	1	NaN	238 687 60S ribosomal protein L6 [Crassostrea gigas]; 142 789 60S ribosomal protein L6 [Crassostrea gigas]
1	1	NaN	31 375 minus strand 40S ribosomal protein S19 OS=Mya arenaria GN=RPS19 PE=2 SV=1
1	2	NaN	360 764 minus strand Peptidyl-prolyl cis-trans isomerase A OS=Dictyostelium discoideum GN=ppiA PE=1 SV=1
1	2	NaN	132 932 pyruvate kinase [Crassostrea gigas]
1	2	NaN	170 1375 PREDICTED: sialate O-acetylesterase-like [Oreochromis niloticus]
1	1	NaN	84 425 minus strand Transcription elongation factor B polypeptide 2 [Crassostrea gigas]; 84 425 minus strand Trans
1	2	NaN	143 247 minus strand dynein light chain [Aplysia californica] gi
1	8	NaN	" 223 423 ADPATP carrier protein; K05863 solute carrier family 25 (mitochondrial adenine nucleotide translocator), n
1	1	NaN	79 2202 minus strand PREDICTED: dolichyl-diphosphooligosaccharide–protein glycosyltransferase subunit STT3A-
1	10	NaN	230 334 protein disulfide isomerase [Conus virgo]
1	1	NaN	1180 1521 minus strand coronin [Schistosoma mansoni]
1	2	NaN	" 166 705 minus strand ADP-ribosylation factor, partial [Antheraea yamamai]; 166 705 minus strand ADP-ribosylatio
1	1	NaN	212 1336 6-phosphofructokinase [Crassostrea gigas]; 208 1332 6-phosphofructokinase [Crassostrea gigas]
1	3	NaN	82 1449 hypothetical protein BRAFLDRAFT_127120 [Branchiostoma floridae] gi
1	2	NaN	235 1038 Translocon-associated protein subunit alpha [Crassostrea gigas]
1	1	NaN	142 789 60S ribosomal protein L6 [Crassostrea gigas]
1	1	NaN	1050 1307 calreticulin precursor [Aplysia californica] gi
1	1	NaN	2 349 minus strand hypothetical protein BRAFLDRAFT_206907 [Branchiostoma floridae] gi
1	1	NaN	1 111 glycogen synthase [Crassostrea gigas]
1	1	NaN	219 554 minus strand ribosomal protein P1 [Biomphalaria glabrata]
1	1	NaN	172 678 Myosin regulatory light chain sqh [Crassostrea gigas]
1	6	NaN	174 1556 minus strand aspartyl-tRNA synthetase [Xenopus laevis] gi; 174 1721 minus strand aspartyl-tRNA syntheta
1	3	NaN	80 589 minus strand PREDICTED: adenine phosphoribosyltransferase-like [Strongylocentrotus purpuratus]; 328 468
1	2	NaN	244 837 PREDICTED: ras-like GTP-binding protein Rho1-like [Megachile rotundata]; 650 883 hypothetical protein SIN
1	2	NaN	114 731 minus strand Proteasome subunit beta type-6 [Crassostrea gigas]
1	4	NaN	16 939 hypothetical protein BRAFLDRAFT_87838 [Branchiostoma floridae] gi
1	3	NaN	5 301 PREDICTED: hypothetical protein [Saccoglossus kowalevskii]
1	4	NaN	1237 1374 minus strand putative 60S ribosomal protein L23 [Maconellicoccus hirsutus]
1	1	NaN	" 1362 1544 minus strand Phosphoenolpyruvate carboxykinase, cytosolic [GTP] [Crassostrea gigas]"
1	2	NaN	971 1963 Spectrin alpha chain [Crassostrea gigas]; 972 1979 Spectrin alpha chain [Crassostrea gigas]
1	2	NaN	254 1093 uncharacterized protein LOC432187 precursor [Xenopus laevis] gi; 343 561 minus strand cathepsin Z prec
1	2	NaN	1215 1694 Pikachurin [Crassostrea gigas]
1	2	NaN	313 1908 minus strand Heterogeneous nuclear ribonucleoprotein L [Crassostrea gigas]; 313 1833 minus strand Hete
1	1	NaN	93 368 60S ribosomal protein L37A [Branchiostoma belcheri] gi; 93 395 60S ribosomal protein L37A [Branchiostoma
1	1	NaN	85 1632 minus strand Alpha-aminoadipic semialdehyde dehydrogenase [Crassostrea gigas]
1	1	NaN	54 1076 PREDICTED: UPF0553 protein C9orf64-like [Anolis carolinensis]; 71 1093 PREDICTED: UPF0553 protein C9o
1	1	NaN	144 1307 minus strand PREDICTED: similar to AGAP005845-PA [Tribolium castaneum]; 146 1309 minus strand PRED
1	1	NaN	165 635 hypothetical protein CGI_10007639 [Crassostrea gigas]
1	1	NaN	390 908 Cysteine proteinase 5 OS=Dictyostelium discoideum GN=cprE PE=2 SV=2
1	1	NaN	78 2003 Chromatin assembly factor 1 subunit A [Crassostrea gigas]
1	1	NaN	" 11 427 PREDICTED: similar to syntaxin, partial [Hydra magnipapillata]"
1	1	NaN	" 174 569 NADH dehydrogenase [ubiquinone] iron-sulfur protein 4, mitochondrial [Crassostrea gigas]"
1	2	NaN	189 353 40S ribosomal protein S12 [Crassostrea gigas]
1	1	NaN	143 865 minus strand predicted protein [Nematostella vectensis] gi
1	1	NaN	13 585 UDP-glucose:glycoprotein glucosyltransferase 1 [Crassostrea gigas]
1	3	NaN	447 977 Expansin-yoaJ [Crassostrea gigas]
1	6	NaN	174 1721 minus strand aspartyl-tRNA synthetase [Xenopus laevis] gi
1	1	NaN	249 845 sigma class glutathione-s-transferase 2 [Haliotis discus discus]; 252 848 sigma class glutathione-s-transfer
1	1	NaN	52 1653 minus strand PREDICTED: eukaryotic translation initiation factor 3 subunit 6 interacting protein-like [Sacco
1	1	NaN	181 684 hypothetical protein BRAFLDRAFT_131834 [Branchiostoma floridae] gi
1	1	NaN	382 972 minus strand Muscle-specific protein 20 [Crassostrea gigas]; 807 1397 minus strand Muscle-specific protein
1	2	NaN	4 234 ribosomal protein rpl14 [Arenicola marina]
1	2	NaN	7825 8754 minus strand H2 [Haliotis tuberculata]
1	1	NaN	33 422 minus strand PREDICTED: heat shock 70 kDa protein cognate 4-like [Megachile rotundata]
1	8	NaN	1027 1440 minus strand hemocyanin isoform 1 [Haliotis diversicolor]
1	1	NaN	245 1243 Prostaglandin reductase 1 [Crassostrea gigas]
1	1	NaN	1 456 minus strand Putative sodium/potassium-transporting ATPase subunit beta-2 [Crassostrea gigas]; 688 1251 m
1	1	NaN	14 502 minus strand Ufm1-conjugating enzyme 1 [Crassostrea gigas]
1	2	NaN	2764 3420 PREDICTED: filamin-C-like [Nasonia vitripennis]
1	1	NaN	" 61 573 steroid membrane receptor Hpr6.6/25-Dx, putative [Ixodes scapularis] gi"
1	1	NaN	23 409 minus strand CDGSH iron sulfur domain-containing protein 2 [Crassostrea gigas]; 1 207 minus strand CDGS
1	1	NaN	121 1452 minus strand Hexokinase type 2 [Crassostrea gigas]; 245 1576 minus strand Hexokinase type 2 [Crassostr
1	1	NaN	" 451 1026 PREDICTED: GTP-binding protein SAR1B, putative-like [Saccoglossus kowalevskii]"
1	1	NaN	231 902 Sodium- and chloride-dependent GABA transporter ine [Crassostrea gigas]
1	2	NaN	155 3214 sarco/endoplasmic reticulum calcium ATPase isoform B [Pinctada fucata]; 132 3110 sarco/endoplasmic ret
1	1	NaN	729 1541 troponin T [Mizuhopecten yessoensis]

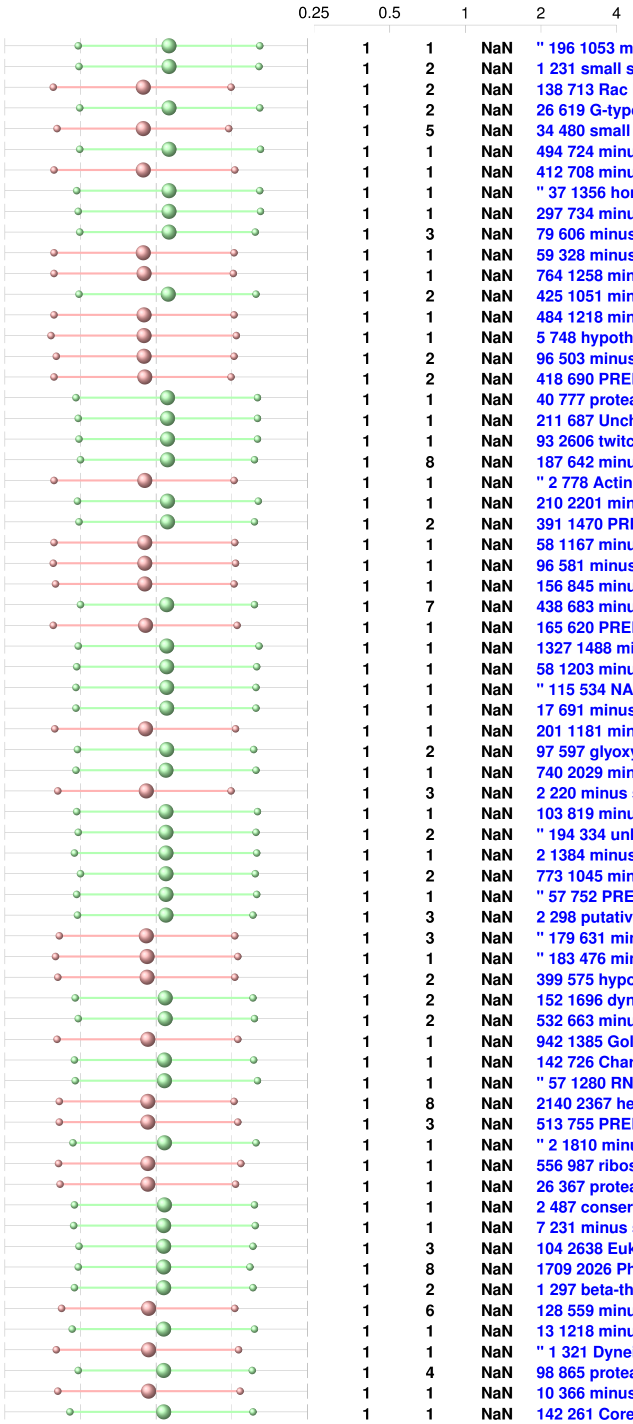

Peptides	Spectra	Coverage	Protein
1	1	NaN	" 196 1053 minus strand Cytochrome b-c1 complex subunit 2, mitochondrial OS=Homo sapiens GN=UQCRC2 PE=1
1	2	NaN	1 231 small subunit ribosomal protein S11e [Clonorchis sinensis]; 1 171 40S ribosomal protein S11 OS=Canis famili
1	2	NaN	138 713 Rac [Aplysia californica] gi; 125 691 Rac [Aplysia californica] gi; 149 871 minus strand Rac [Aplysia californ
1	2	NaN	26 619 G-type lysozyme [Physella acuta]
1	5	NaN	34 480 small subunit ribosomal protein S18e [Clonorchis sinensis]; 431 799 SJCHGC01887 protein [Schistosoma jap
1	1	NaN	494 724 minus strand PREDICTED: SH3 domain-binding glutamic acid-rich-like protein 3-like [Amphimedon queensl
1	1	NaN	412 708 minus strand heat shock protein beta-1 [Trichinella spiralis] gi; 459 755 minus strand heat shock protein bet
1	1	NaN	" 37 1356 homogentisate 1,2-dioxygenase [Danio rerio] gi; 54 1361 homogentisate 1,2-dioxygenase [Danio rerio] gi"
1	1	NaN	297 734 minus strand PREDICTED: calmodulin [Canis lupus familiaris]; 297 734 PREDICTED: calmodulin [Canis lupu
1	3	NaN	79 606 minus strand PREDICTED: similar to Ras-like protein 3 precursor (Protein roughened) [Tribolium castaneum]
1	1	NaN	59 328 minus strand PREDICTED: similar to predicted protein [Hydra magnipapillata]; 59 328 PREDICTED: similar to
1	1	NaN	764 1258 minus strand Caprin-2 [Crassostrea gigas]
1	2	NaN	425 1051 minus strand unknown [Schistosoma japonicum]
1	1	NaN	484 1218 minus strand alginate lyase [Littorina brevicula]; 484 1218 minus strand alginate lyase [Littorina brevicula]
1	1	NaN	5 748 hypothetical protein SINV_09458 [Solenopsis invicta]
1	2	NaN	96 503 minus strand mitochondrial ATP synthase delta chain [Haliotis discus discus]; 111 551 minus strand mitocho
1	2	NaN	418 690 PREDICTED: protein disulfide isomerase A4-like [Saccoglossus kowalevskii]
1	1	NaN	40 777 proteasome subunit N3 [Haliotis discus discus]; 40 777 proteasome subunit N3 [Haliotis discus discus]
1	1	NaN	211 687 Uncharacterized protein CG16817 [Harpegnathos saltator]; 200 676 Uncharacterized protein CG16817 [Harp
1	1	NaN	93 2606 twitchin [Mytilus galloprovincialis]
1	8	NaN	187 642 minus strand PREDICTED: similar to pol-like protein [Tribolium castaneum]
1	1	NaN	" 2 778 Actin, gamma-enteric smooth muscle OS=Rattus norvegicus GN=Actg2 PE=2 SV=1"
1	1	NaN	210 2201 minus strand Glycyl-tRNA synthetase [Crassostrea gigas]; 210 2201 minus strand Glycyl-tRNA synthetase
1	2	NaN	391 1470 PREDICTED: heparanase-like [Amphimedon queenslandica]; 391 1470 PREDICTED: heparanase-like [Amp
1	1	NaN	58 1167 minus strand PREDICTED: arylacetamide deacetylase-like 4-like [Gallus gallus]; 76 1239 minus strand PRED
1	1	NaN	96 581 minus strand thioredoxin 2 [Haliotis discus discus]; 1682 2167 minus strand thioredoxin 2 [Haliotis discus di
1	1	NaN	156 845 minus strand histone H1 [Mytilus californianus]; 156 845 minus strand histone H1 [Mytilus californianus]; 15
1	7	NaN	438 683 minus strand Protein lethal(2)essential for life OS=Drosophila melanogaster GN=l(2)efl PE=1 SV=1
1	1	NaN	165 620 PREDICTED: uncharacterized protein LOC575072 isoform 2 [Strongylocentrotus purpuratus] gi
1	1	NaN	1327 1488 minus strand Ubiquinol-cytochrome C reductase complex [Xenopus laevis] gi
1	1	NaN	58 1203 minus strand Obg-like ATPase 1 [Crassostrea gigas]
1	1	NaN	" 115 534 NADH:ubiquinone oxidoreductase, NDUFB6/B17 subunit, putative [Ixodes scapularis] gi"
1	1	NaN	17 691 minus strand Cleavage and polyadenylation specificity factor subunit 5 [Crassostrea gigas]
1	1	NaN	201 1181 minus strand Serine/threonine-protein phosphatase PP1-alpha catalytic subunit [Crassostrea gigas]
1	2	NaN	97 597 glyoxylate reductase/hydroxypyruvate reductase [Ictalurus punctatus] gi
1	1	NaN	740 2029 minus strand PREDICTED: protein alan shepard-like isoform 2 [Bombus terrestris]
1	3	NaN	2 220 minus strand haemocyanin [Aplysia californica]
1	1	NaN	103 819 minus strand PREDICTED: l-xylulose reductase-like [Monodelphis domestica]
1	2	NaN	" 194 334 unknown, partial [Lymnaea stagnalis] gi; 207 347 unknown, partial [Lymnaea stagnalis] gi; 384 524 unknow
1	1	NaN	2 1384 minus strand twitchin [Mytilus galloprovincialis]; 2 1384 minus strand twitchin [Mytilus galloprovincialis]; 2 1
1	2	NaN	773 1045 minus strand serine protease inhibitor 2 [Latrodectus hesperus]
1	1	NaN	" 57 752 PREDICTED: adenylate kinase 2, mitochondrial-like isoform 2 [Callithrix jacchus]"
1	3	NaN	2 298 putative 60S ribosomal protein RPL24 [Novocrania anomala]
1	3	NaN	" 179 631 minus strand 60S ribosomal protein L7/L12, partial [Ostrea edulis]"
1	1	NaN	" 183 476 minus strand guanine nucleotide-binding protein G(q), alpha subunit [Aplysia californica] gi"
1	2	NaN	399 575 hypothetical protein BRAFLDRAFT_69739 [Branchiostoma floridae] gi
1	2	NaN	152 1696 dynamin isoform A [Lymnaea stagnalis]
1	2	NaN	532 663 minus strand 40S ribosomal protein S23 [Ascaris suum]; 526 654 minus strand RecName: Full=40S ribosom
1	1	NaN	942 1385 Golgi-associated plant pathogenesis-related protein 1 [Crassostrea gigas]; 536 979 Golgi-associated plant
1	1	NaN	142 726 Charged multivesicular body protein 4b [Crassostrea gigas]
1	1	NaN	" 57 1280 RNA binding motif protein, X-linked [Mustela putorius furo]"
1	8	NaN	2140 2367 heat shock cognate 71 [Mytilus galloprovincialis]
1	3	NaN	513 755 PREDICTED: 40S ribosomal protein S16-like [Papio anubis]; 513 755 PREDICTED: 40S ribosomal protein S1
1	1	NaN	" 2 1810 minus strand Glutaminyl-tRNA synthetase, partial [Crassostrea gigas]"
1	1	NaN	556 987 ribosomal protein rps15 [Lineus viridis]
1	1	NaN	26 367 proteasome alpha type 2 [Haliotis discus discus]
1	1	NaN	2 487 conserved hypothetical protein [Ixodes scapularis] gi
1	1	NaN	7 231 minus strand AGAP006879-PA [Anopheles gambiae str. PEST] gi
1	3	NaN	104 2638 Eukaryotic translation initiation factor 3 subunit C [Crassostrea gigas]
1	8	NaN	1709 2026 Phosphoenolpyruvate carboxykinase [GTP] [Crassostrea gigas]
1	2	NaN	1 297 beta-thymosin domain repeat protein CSP24KDa_v1 [Hermissenda crassicornis] gi
1	6	NaN	128 559 minus strand predicted protein [Nematostella vectensis] gi
1	1	NaN	13 1218 minus strand PREDICTED: eukaryotic initiation factor 4A-III-like [Bombus terrestris] gi
1	1	NaN	" 1 321 Dynein heavy chain, cytoplasmic [Crassostrea gigas]"
1	4	NaN	98 865 proteasome (prosome macropain) subunit alpha type 1 [Xenopus (Silurana) tropicalis]; 98 865 proteasome (p
1	1	NaN	10 366 minus strand hypothetical protein TRIADDRAFT_25239 [Trichoplax adhaerens] gi
1	1	NaN	142 261 Core histone macro-H2A.2 OS=Mus musculus GN=H2afy2 PE=1 SV=3

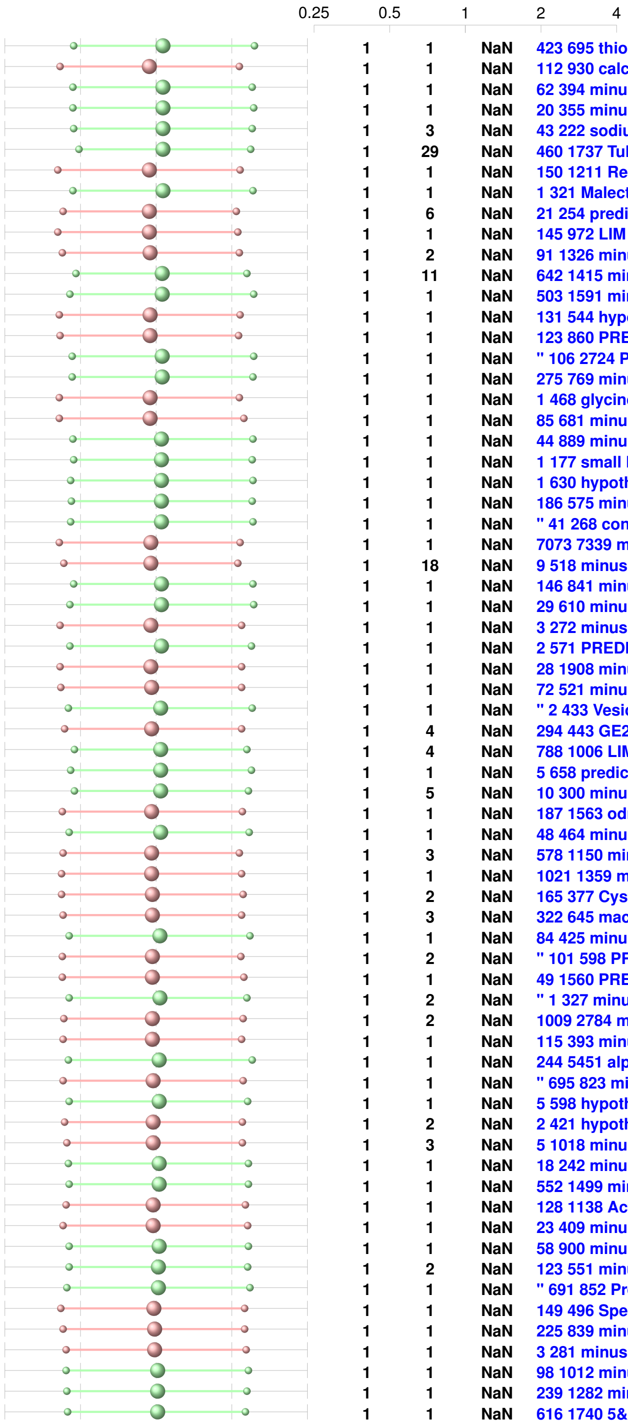

Peptides	Spectra	Coverage	Protein
1	1	NaN	423 695 thioredoxin peroxidase 2 [Haliotis discus discus]
1	1	NaN	112 930 calcineurin A [Haliotis discus discus]
1	1	NaN	62 394 minus strand PREDICTED: NADH dehydrogenase [ubiquinone] 1 alpha subcomplex subunit 5 [Saimiri bolivie
1	1	NaN	20 355 minus strand PREDICTED: uroporphyrinogen decarboxylase-like [Monodelphis domestica]
1	3	NaN	43 222 sodium-calcium exchanger [Doryteuthis pealeii]
1	29	NaN	460 1737 Tubulin beta-4B chain OS=Mus musculus GN=Tubb4b PE=1 SV=1
1	1	NaN	150 1211 RecName: Full=Guanine nucleotide-binding protein G(i) subunit alpha; AltName: Full=Adenylate cyclase-in
1	1	NaN	1 321 Malectin [Crassostrea gigas]
1	6	NaN	21 254 predicted protein [Nematostella vectensis] gi
1	1	NaN	145 972 LIM and SH3 domain protein F42H10.3 OS=Caenorhabditis elegans GN=F42H10.3 PE=2 SV=3
1	2	NaN	91 1326 minus strand Adipocyte plasma membrane-associated protein [Crassostrea gigas]; 107 1273 minus strand
1	11	NaN	642 1415 minus strand cytochrome c oxidase subunit II [Oncomelania hupensis hupensis]
1	1	NaN	503 1591 minus strand Septin-2 [Crassostrea gigas]; 648 1736 minus strand Septin-2 [Crassostrea gigas]
1	1	NaN	131 544 hypothetical protein CGI_10001749 [Crassostrea gigas]
1	1	NaN	123 860 PREDICTED: 3-hydroxybutyrate dehydrogenase type 2-like isoform 1 [Sus scrofa] gi; 32 901 3-hydroxybutyr
1	1	NaN	" 106 2724 PREDICTED: 2-oxoglutarate dehydrogenase, mitochondrial isoform 1 [Sarcophilus harrisii]; 492 3473 PR
1	1	NaN	275 769 minus strand hypothetical protein CGI_10025135 [Crassostrea gigas]; 216 683 hypothetical protein CGI_100
1	1	NaN	1 468 glycine cleavage system H protein [Rhipicephalus sanguineus]
1	1	NaN	85 681 minus strand 40S ribosomal protein S8 OS=Xenopus laevis GN=rps8 PE=2 SV=3
1	1	NaN	44 889 minus strand hypothetical protein CGI_10015025 [Crassostrea gigas]
1	1	NaN	1 177 small heat shock protein [Trichinella pseudospiralis]
1	1	NaN	1 630 hypothetical protein [Trichomonas vaginalis G3] gi
1	1	NaN	186 575 minus strand predicted protein [Nematostella vectensis] gi
1	1	NaN	" 41 268 conserved hypothetical protein [Perkinsus marinus ATCC 50983] gi; 29 238 PREDICTED: succinyl-CoA ligas
1	1	NaN	7073 7339 minus strand Collagen alpha-3(VI) chain [Crassostrea gigas]
1	18	NaN	9 518 minus strand hemocyanin isoform 1 [Nucula nucleus]
1	1	NaN	146 841 minus strand hypothetical protein CGI_10006609 [Crassostrea gigas]; 146 841 minus strand hypothetical pr
1	1	NaN	29 610 minus strand 40S ribosomal protein S7 [Crassostrea gigas]
1	1	NaN	3 272 minus strand ADP/ATP carrier [Clonorchis sinensis]
1	1	NaN	2 571 PREDICTED: copper homeostasis protein cutC homolog [Anolis carolinensis]
1	1	NaN	28 1908 minus strand Mitochondrial inner membrane protein [Crassostrea gigas]; 10 1902 Mitochondrial inner mem
1	1	NaN	72 521 minus strand GF14593 [Drosophila ananassae] gi; 60 392 GD23124 [Drosophila simulans] gi
1	1	NaN	" 2 433 Vesicle-fusing ATPase, partial [Ascaris suum]"
1	4	NaN	294 443 GE25988 gene product from transcript GE25988-RA; K02980 small subunit ribosomal protein S29e; 292 441
1	4	NaN	788 1006 LIM protein [Haliotis discus discus]
1	1	NaN	5 658 predicted protein [Nematostella vectensis] gi
1	5	NaN	10 300 minus strand unnamed protein product [Tetraodon nigroviridis]
1	1	NaN	187 1563 odr-4-like protein [Crassostrea gigas]
1	1	NaN	48 464 minus strand Acyl-coenzyme A thioesterase 13 [Crassostrea gigas]
1	3	NaN	578 1150 minus strand expressed hypothetical protein [Trichoplax adhaerens] gi; 376 948 minus strand expressed h
1	1	NaN	1021 1359 minus strand RecName: Full=40S ribosomal protein S26 gi|6015599|emb|CAB57819.1| ribosomal protein
1	2	NaN	165 377 Cysteine and glycine-rich protein 3 [Crassostrea gigas]; 135 344 Cysteine and glycine-rich protein 3 [Crasso
1	3	NaN	322 645 macrophage migration inhibitory-like factor protein [Chlamys farreri]; 437 682 macrophage migration inhibit
1	1	NaN	84 425 minus strand thioester-containing protein [Euphaedusa tau]
1	2	NaN	" 101 598 PREDICTED: aminomethyltransferase, mitochondrial-like [Oreochromis niloticus]"
1	1	NaN	49 1560 PREDICTED: tRNA-splicing ligase RtcB homolog [Megachile rotundata]
1	2	NaN	" 1 327 minus strand endo-beta-1,4-glucanase [Ampullaria crossean]"
1	2	NaN	1009 2784 minus strand Collagen alpha-3(VI) chain [Crassostrea gigas]
1	1	NaN	115 393 minus strand Yolk ferritin OS=Lymnaea stagnalis PE=1 SV=1; 132 413 RecName: Full=Yolk ferritin; Flags: Pr
1	1	NaN	244 5451 alpha macroglobulin [Chlamys farreri]
1	1	NaN	" 695 823 minus strand Aldehyde dehydrogenase family 16 member A1, partial [Crassostrea gigas]"
1	1	NaN	5 598 hypothetical protein DAPPUDRAFT_344656 [Daphnia pulex]
1	2	NaN	2 421 hypothetical protein BRAFLDRAFT_119044 [Branchiostoma floridae] gi
1	3	NaN	5 1018 minus strand Glutaredoxin-3 [Crassostrea gigas]; 5 1018 Glutaredoxin-3 [Crassostrea gigas]
1	1	NaN	18 242 minus strand translation elongation factor 1B gamma subunit [Strongylocentrotus purpuratus] gi
1	1	NaN	552 1499 minus strand uncharacterized protein LOC100158466 [Xenopus (Silurana) tropicalis] gi; 552 1499 minus st
1	1	NaN	128 1138 Actin-related protein 2/3 complex subunit 1A OS=Homo sapiens GN=ARPC1A PE=1 SV=2
1	1	NaN	23 409 minus strand CDGSH iron sulfur domain-containing protein 2 [Crassostrea gigas]
1	1	NaN	58 900 minus strand Toll-interacting protein [Crassostrea gigas]
1	2	NaN	123 551 minus strand Transcription factor BTF3-like protein 4 [Crassostrea gigas]
1	1	NaN	" 691 852 Protein MLT-11, isoform e [Caenorhabditis elegans] gi"
1	1	NaN	149 496 Spectrin alpha chain [Crassostrea gigas]
1	1	NaN	225 839 minus strand Metallo-beta-lactamase domain-containing protein 1 [Crassostrea gigas]; 201 851 minus stran
1	1	NaN	3 281 minus strand hypothetical protein CGI_10015501 [Crassostrea gigas]
1	1	NaN	98 1012 minus strand RecName: Full=Substrate-specific endoprotease Tex31; Flags: Precursor gi|32187775|emb|C
1	1	NaN	239 1282 minus strand PREDICTED: hypothetical protein LOC100122475 [Nasonia vitripennis]
1	1	NaN	616 1740 5'-AMP-activated protein kinase subunit beta-2 [Crassostrea gigas]; 641 1762 minus strand 5'

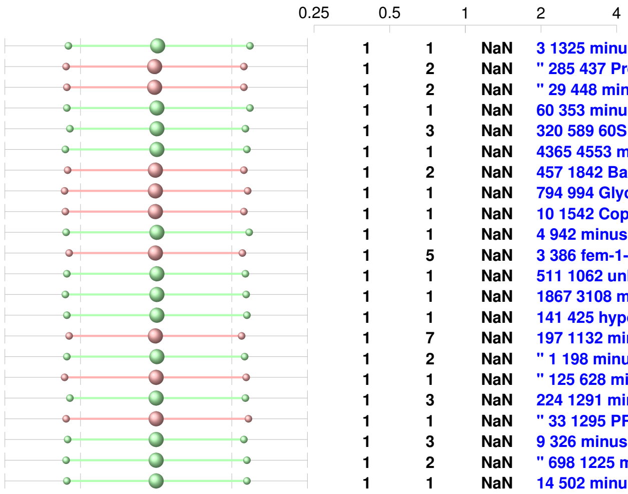

Peptides	Spectra	Coverage	Protein
1	1	NaN	3 1325 minus strand Eukaryotic translation initiation factor 3 subunit B [Crassostrea gigas]
1	2	NaN	" 285 437 Propionyl-CoA carboxylase alpha chain, mitochondrial [Crassostrea gigas]"
1	2	NaN	" 29 448 minus strand PREDICTED: similar to NADH:ubiquinone dehydrogenase, putative [Tribolium castaneum] gi;
1	1	NaN	60 353 minus strand Histone H3.3 [Harpegnathos saltator]
1	3	NaN	320 589 60S ribosomal protein L34 OS=Aedes triseriatus GN=RpL34 PE=2 SV=3
1	1	NaN	4365 4553 minus strand Basement membrane-specific heparan sulfate proteoglycan core protein [Crassostrea gigas
1	2	NaN	457 1842 Basement membrane-specific heparan sulfate proteoglycan core protein [Crassostrea gigas]; 457 1842 Ba
1	1	NaN	794 994 Glycogenin-1 [Crassostrea gigas]; 1425 1739 minus strand Glycogenin-1 [Crassostrea gigas]
1	1	NaN	10 1542 Copine-8 [Crassostrea gigas]
1	1	NaN	4 942 minus strand KTEL motif-containing protein 1 [Crassostrea gigas]
1	5	NaN	3 386 fem-1-like protein C [Crassostrea gigas]
1	1	NaN	511 1062 unknown [Dendroctonus ponderosae]; 511 1062 unknown [Dendroctonus ponderosae]
1	1	NaN	1867 3108 minus strand roundabout homolog 1 precursor [Rattus norvegicus] gi; 1867 3108 minus strand roundabo
1	1	NaN	141 425 hypothetical protein BRAFLDRAFT_216227 [Branchiostoma floridae] gi; 141 425 minus strand hypothetical
1	7	NaN	197 1132 minus strand Pancreatic lipase-related protein 2 [Crassostrea gigas]
1	2	NaN	" 1 198 minus strand Chain A, Rep1-Nxsq Fatty Acid Transporter; 1 231 minus strand Chain A, Rep1-Nxsq Fatty Acid
1	1	NaN	" 125 628 minus strand collectin sub-family member 12, partial [Oncorhynchus mykiss]"
1	3	NaN	224 1291 minus strand coproporphirynogen oxidase [Aplysia californica] gi
1	1	NaN	" 33 1295 PREDICTED: LOW QUALITY PROTEIN: selenide, water dikinase-like [Bombus impatiens]"
1	3	NaN	9 326 minus strand catalase [Paracyclopina nana]; 9 209 Catalase OS=Cavia porcellus GN=CAT PE=2 SV=4
1	2	NaN	" 698 1225 minus strand actin related protein 2/3 complex, 21 kDa subunit [Crassostrea gigas]"
1	1	NaN	14 502 minus strand ependymin related protein-1 precursor [Haliotis discus discus]; 14 547 ependymin related prote

5 Protein Details

A detailed summary of each protein is given below. These sections include peptide relative expression estimates in addition to protein-level estimates.

5.1 2881 3411 myosin heavy chain [Todarodes pacificus]; 2915 3445 myosin heavy chain [Todarodes pacificus]

Protein Accession	Unigene19024$_{H1}$ Unigene19021$_{H1}$
Mean Expression Ratio	2.81
Median Expression Ratio	2.81
Credible Interval	(1.7, 4.73)
Associated Peptides	11
Associated Spectra	47
Coverage	NaN

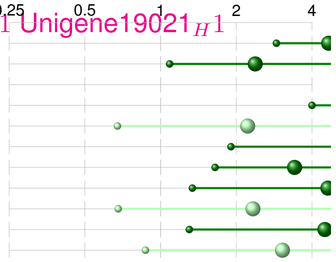

A	B	2.5	50	97.5	Sequence
3	11	2.9	4.7	7.5	ALDSMQASLEAEAK
2	2	1.1	2.4	5.2	IQEKEEEFENTR
2	7	5	9	16	IQEKEEEFENTRR
1	7	4	7.3	13	KLQQQVIETQQHVEEEAR
1	0	0.67	2.2	7.6	GKAELEK
0	2	1.9	5.2	15	CTALTGEVEELR
0	5	1.7	3.4	7.1	KLEQDINELEIALDQANR
0	1	1.3	4.6	16	LQQQVIETQQHVEEEAR
0	1	0.68	2.3	8.3	RCTALTGEVEELR
0	1	1.3	4.5	16	RIQEKEEEFENTR
0	1	0.87	3.1	11	ALDSMQASLEAEAKGKAEAMR

5.2 764 1015 minus strand heat shock protein beta-1 [Trichinella spiralis] gi

Protein Accession	CL46983.Contig3$_{H}1$
Mean Expression Ratio	2.51
Median Expression Ratio	2.5
Credible Interval	(1.43, 4.41)
Associated Peptides	7
Associated Spectra	77
Coverage	NaN

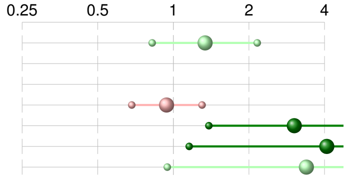

A	B	2.5	50	97.5	Sequence
7	6	0.83	1.3	2.2	SYVLPK
5	5	5.5	9.4	16	SYVLPKDVDPK
4	12	26	41	64	TVTSSLSQNGVLTLKAPK
10	22	0.68	0.94	1.3	TVTSSLSQNGVLTLK
0	4	1.4	3	6.7	VVINAKHEERPDEHGYIMR
0	1	1.2	4.1	15	SSLSQNGVLTLKAPK
0	1	0.94	3.4	12	MALEAPQEQV

5.3 1592 1768 minus strand beta-actin [Diabolocatantops pinguis]

Protein Accession	CL22741.Contig2$_{H}1$
Mean Expression Ratio	2.29
Median Expression Ratio	2.29
Credible Interval	(1.26, 4.15)
Associated Peptides	6
Associated Spectra	46
Coverage	NaN

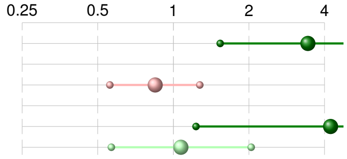

A	B	2.5	50	97.5	Sequence
3	1	1.5	3.4	7.7	VIAPPERK
1	7	12	22	40	IKVIAPPERK
8	11	0.56	0.84	1.3	VIAPPER
0	7	6.2	12	22	IKVIAPPER
0	1	1.2	4.2	15	GDQKEISALAPPTMK
0	7	0.57	1.1	2	EISALAPPTMK

5.4 2 370 hemocyanin alphaD-subunit [Helix lucorum]

Protein Accession	CL46908.Contig1$_{H}1$
Mean Expression Ratio	0.44
Median Expression Ratio	0.441
Credible Interval	(0.253, 0.767)
Associated Peptides	8
Associated Spectra	41
Coverage	NaN

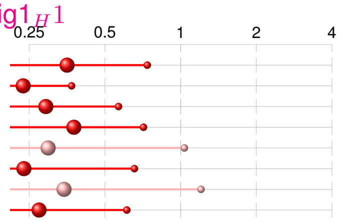

A	B	2.5	50	97.5	Sequence
2	3	0.17	0.35	0.74	AFAVFK
4	11	0.15	0.24	0.37	LLGIGTSAEVK
0	6	0.15	0.29	0.57	NEMPWQFER
0	7	0.2	0.38	0.71	SFYLDVSEAVVR
0	1	0.085	0.3	1	SPHCEGAK
0	2	0.087	0.24	0.66	LASNVLPK
0	1	0.099	0.34	1.2	LYNMLETR
0	4	0.12	0.27	0.61	SFFILGGK

5.5 2 256 Polyubiquitin (Fragment) OS=Xenopus laevis PE=1 SV=2; 20 388 large subunit ribosomal protein L40e [Clonorchis sinensis]

Protein Accession	CL47507.Contig2$_{H}1$ CL39010.Contig1$_{H}1$
Mean Expression Ratio	0.455
Median Expression Ratio	0.457
Credible Interval	(0.238, 0.853)
Associated Peptides	5
Associated Spectra	14
Coverage	NaN

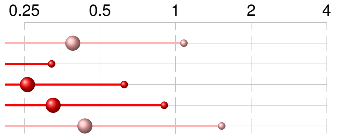

A	B	2.5	50	97.5	Sequence
2	0	0.14	0.39	1.1	IQDKEGIPPDQQR
2	3	0.058	0.14	0.32	TITLEVEPSDTIENVK
2	2	0.11	0.26	0.62	TLSDYNIQK
2	0	0.12	0.32	0.9	LIFAGK
1	0	0.13	0.43	1.5	MQIFVK

5.6 405 1373 paramyosin [Haliotis discus discus]

Protein Accession	CL43455.Contig4$_H$1
Mean Expression Ratio	2.17
Median Expression Ratio	2.16
Credible Interval	(1.58, 2.97)
Associated Peptides	37
Associated Spectra	290
Coverage	NaN

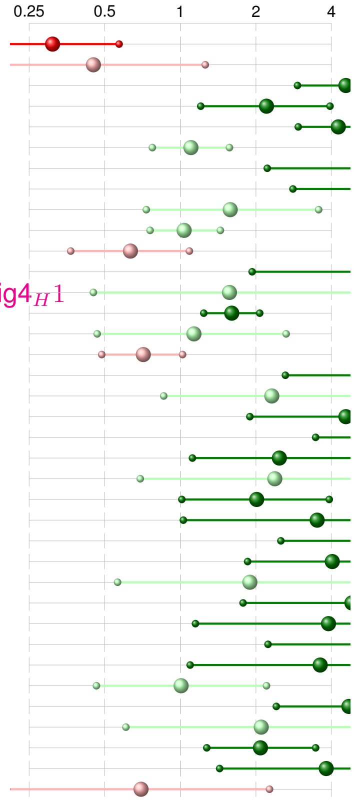

A	B	2.5	50	97.5	Sequence
1	6	0.16	0.31	0.57	ELEDALDAER
2	0	0.16	0.45	1.3	FDKELAAK
3	12	2.9	4.6	7.2	HTEAVNDLSDQVDYLTK
1	7	1.2	2.2	4	IRELEDALDAER
9	15	3	4.3	6.1	LKGQVDDLTR
20	6	0.78	1.1	1.6	LSLESR
1	2	2.2	5.4	13	QKLSLESR
1	2	2.8	6.7	16	QLNDLTALKAR
1	3	0.73	1.6	3.5	TRFDKELAAK
20	12	0.76	1	1.4	TVAELSFR
4	6	0.37	0.63	1.1	LDEEAETSSNLR
1	1	1.9	5.1	14	FEAAEASLR
1	0	0.45	1.6	5.3	ESEIYK
24	29	1.2	1.6	2.1	QLNDLTALK
2	1	0.47	1.1	2.6	SQLQNQNDDLKR
15	8	0.49	0.71	1	VSADYAALK
0	3	2.6	6.3	15	AKSQLQNQNDDLKR
0	2	0.86	2.3	6.3	DLELATAQ
0	3	1.9	4.6	11	ELEDALDAERDGR
0	16	3.4	5.3	8.3	IRELEDALDAERDGR
0	4	1.1	2.5	5.5	IRELEDALDAERDGRVR
0	1	0.69	2.4	8	ITELEDSCEQLR
0	6	1	2	3.9	KDLELATAQFEAAEASLR
0	1	1	3.5	12	LDQVQDSLDEQGSSTVAQIELNKKR
0	2	2.5	6.8	19	LSLESRVDAAEQSAAR
0	4	1.8	4	8.9	LTQENFDLQHQVQELDSSNAALAK
0	1	0.56	1.9	6.3	RELEDALDAERDGR
0	2	1.8	4.8	14	ESEIYKLR
0	1	1.1	3.9	13	ESEIYKLRK
0	1	2.2	7.7	26	KRESEIYK
0	1	1.1	3.6	12	QFEAAEASLR
0	4	0.46	1	2.2	DEQGSSTVAQIELNK
0	6	2.4	4.7	9.3	DLELATAQFEAAEASLR
0	1	0.61	2.1	7	LDEEAETSSNLRNSLSK
0	12	1.3	2.1	3.5	LDQVQDSLDEQGSSTVAQIELNK
0	2	1.4	3.8	11	QTLVIELDGLSGQLDSITK
0	1	0.2	0.7	2.3	TEELEETR

5.7 167 679 tropomyosin [Haliotis discus discus]

Protein Accession	CL2925.Contig3$_H$1
Mean Expression Ratio	2.14
Median Expression Ratio	2.14
Credible Interval	(1.33, 3.5)
Associated Peptides	14
Associated Spectra	54
Coverage	NaN

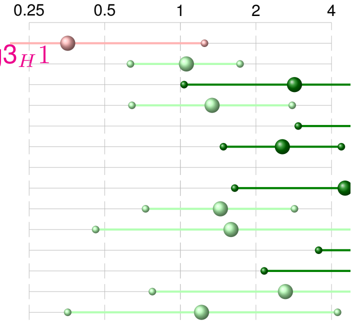

A	B	2.5	50	97.5	Sequence
1	0	0.1	0.36	1.2	ANEGLIEANTK
7	5	0.63	1.1	1.7	LRDTEVQK
1	1	1	2.8	7.8	MDAIK
1	4	0.64	1.3	2.8	RIKLLEEDLER
1	3	2.9	6.5	14	KMLAMK
7	3	1.5	2.5	4.4	MLAMK
2	2	7.9	17	38	MDAIKKK
1	1	1.6	4.5	12	MDAIKK
0	6	0.72	1.4	2.9	IKLLEEDLER
0	1	0.46	1.6	5.6	MDKENAMDK
0	4	3.6	7.8	17	MDKENAMDKAEQMEQK
0	1	2.2	7.5	26	YANLENEYDKANEGLIEANTKLEASEK
0	1	0.77	2.6	9.2	AMDKAEQMEQK
0	1	0.36	1.2	4.2	SKLEEDLNPLQK

5.8 160 588 hypothetical protein CGI_10001640 [Crassostrea gigas]

Protein Accession	CL2347.Contig8$_{H}1$
Mean Expression Ratio	0.484
Median Expression Ratio	0.483
Credible Interval	(0.245, 0.955)
Associated Peptides	2
Associated Spectra	13
Coverage	NaN

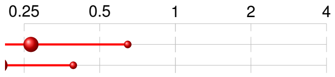

A	B	2.5	50	97.5	Sequence
2	2	0.11	0.27	0.65	AFLEQLGSK
5	4	0.1	0.2	0.39	ANLIVIGSR

5.9 254 1648 Tektin-3 [Crassostrea gigas]; 254 1648 Tektin-3 [Crassostrea gigas]

Protein Accession	CL6717.Contig3$_{H}1$ CL6717.Contig2$_{H}1$
Mean Expression Ratio	0.484
Median Expression Ratio	0.483
Credible Interval	(0.261, 0.891)
Associated Peptides	8
Associated Spectra	9
Coverage	NaN

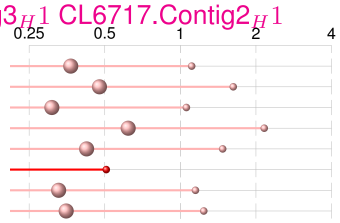

A	B	2.5	50	97.5	Sequence
1	1	0.12	0.37	1.1	AAQHALEIDAK
1	0	0.13	0.48	1.6	ATLEHDISVK
1	0	0.085	0.31	1.1	ILPYVTDK
1	0	0.18	0.62	2.2	KGFPMDPK
1	0	0.12	0.42	1.5	NVELLR
1	0	0.041	0.14	0.51	EVDIIR
1	0	0.093	0.33	1.1	EVAEIR
1	0	0.097	0.35	1.2	EAFMK

5.10 507 1277 alpha tubulin [Notothenia coriiceps]

Protein Accession	Unigene56691$_{H}1$
Mean Expression Ratio	0.49
Median Expression Ratio	0.489
Credible Interval	(0.237, 1.01)
Associated Peptides	2
Associated Spectra	13
Coverage	NaN

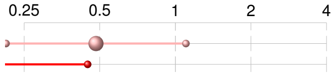

A	B	2.5	50	97.5	Sequence
3	2	0.21	0.48	1.1	LSVDYGKK
1	7	0.072	0.18	0.45	LSVDYGK

5.11 16 288 SJCHGC01950 protein [Schistosoma japonicum] gi

Protein Accession	Unigene16338$_{H}1$
Mean Expression Ratio	0.492
Median Expression Ratio	0.492
Credible Interval	(0.227, 1.06)
Associated Peptides	2
Associated Spectra	4
Coverage	NaN

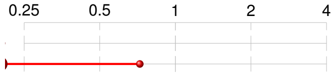

A	B	2.5	50	97.5	Sequence
1	2	0.034	0.082	0.2	DIAAFIK
1	0	0.056	0.2	0.72	YNIEK

5.12 3591 4526 minus strand myosin heavy chain cardiac muscle specific isoform 1 [Argopecten irradians]

Protein Accession	CL49594.Contig2$_{H}$1
Mean Expression Ratio	2.01
Median Expression Ratio	2.01
Credible Interval	(1.35, 3.04)
Associated Peptides	21
Associated Spectra	99
Coverage	NaN

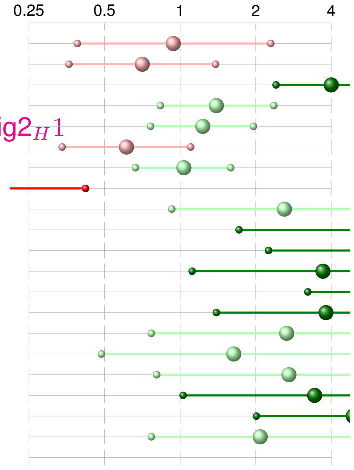

A	B	2.5	50	97.5	Sequence
1	2	0.39	0.94	2.3	ATQANVEDLER
5	1	0.36	0.71	1.4	KLEQDLK
4	7	2.4	4	6.8	LQQETANLTR
4	7	0.84	1.4	2.4	QLEEAEHK
6	8	0.76	1.2	2	QVEAQLAELSAK
4	4	0.34	0.61	1.1	SEVDDLQMQLQNVGK
9	7	0.66	1	1.6	TINDLQTQK
3	0	0.072	0.17	0.42	VGALTK
0	2	0.93	2.6	7.1	AKLEQTLDELEDNLER
0	1	1.7	5.8	20	AQLKSEVDDLQMQLQNVGK
0	3	2.2	5.4	13	KLEQDLKATQANVEDLER
0	1	1.1	3.7	13	NRLQQETANLTR
0	3	3.2	7.8	18	QLEEAEHKVGALTKEK
0	2	1.4	3.8	11	QVEAQLAELSAKVEEGNR
0	1	0.77	2.7	9.2	TMEDLQAEEDKVNHLNK
0	1	0.49	1.6	5.6	AATSRSRTLQHEAQVSALR
0	1	0.8	2.7	9.4	VKRELEENVR
0	1	1	3.4	12	RKLEQDLK
0	3	2	4.9	12	ATSRSRTLQHEAQVSALR
0	2	0.77	2.1	5.7	LEQTLDELEDNLEREK
0	6	8.7	17	34	QLEEAEHKVGALTK

5.13 40 1098 minus strand major egg antigen [Clonorchis sinensis]

Protein Accession	CL2613.Contig4$_{H}$1
Mean Expression Ratio	0.509
Median Expression Ratio	0.51
Credible Interval	(0.217, 1.17)
Associated Peptides	1
Associated Spectra	1
Coverage	NaN

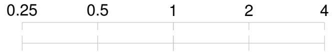

A	B	2.5	50	97.5	Sequence
1	0	0.011	0.039	0.14	LESHLTK

5.14 209 781 minus strand high mobility group protein 1 [Biomphalaria glabrata]

Protein Accession	CL25076.Contig4$_{H}$1
Mean Expression Ratio	0.51
Median Expression Ratio	0.511
Credible Interval	(0.254, 1)
Associated Peptides	3
Associated Spectra	10
Coverage	NaN

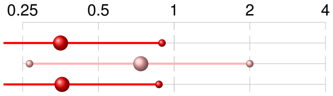

A	B	2.5	50	97.5	Sequence
2	1	0.14	0.35	0.9	KPFEDMAAK
1	2	0.27	0.74	2	KYEAEMAEYR
2	2	0.15	0.36	0.87	LAGEEWR

5.15 2 328 cytoskeletal actin 2 [Molgula oculata]

Protein Accession	Unigene2743$_{H}1$
Mean Expression Ratio	1.93
Median Expression Ratio	1.93
Credible Interval	(0.896, 4.19)
Associated Peptides	2
Associated Spectra	6
Coverage	NaN

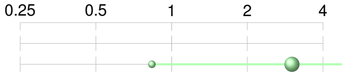

A	B	2.5	50	97.5	Sequence
1	4	7.4	15	32	MQKDITALAPPTMK
0	1	0.84	3	11	MQKDITALAPP

5.16 " 223 423 ADPATP carrier protein; K05863 solute carrier family 25 (mitochondrial adenine nucleotide translocator), member 4/5/6/31; 237 455 minus strand ADP,ATP carrier protein [Crassostrea gigas]"

Protein Accession	CL27196.Contig3$_{H}1$ CL27196.Contig2$_{H}1$
Mean Expression Ratio	1.93
Median Expression Ratio	1.92
Credible Interval	(0.966, 3.93)
Associated Peptides	3
Associated Spectra	10
Coverage	NaN

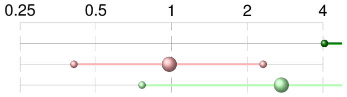

A	B	2.5	50	97.5	Sequence
0	1	4.1	14	51	VKLLVQNQNEMIK
6	2	0.41	0.98	2.3	LLVQNQNEMIK
0	1	0.76	2.7	9.7	AENFALSGLVLPLSSPK

5.17 195 455 60S ribosomal protein L23a OS=Rattus norvegicus GN=Rpl23a PE=2 SV=1; 195 512 60S ribosomal protein L23a OS=Rattus norvegicus GN=Rpl23a PE=2 SV=1; 16 465 60S ribosomal protein L23a OS=Rattus norvegicus GN=Rpl23a PE=2 SV=1

Protein Accession	CL27563.Contig3$_{H}1$ CL27563.Contig2$_{H}1$ CL27563.Contig1$_{H}1$
Mean Expression Ratio	1.92
Median Expression Ratio	1.92
Credible Interval	(0.988, 3.78)
Associated Peptides	3
Associated Spectra	11
Coverage	NaN

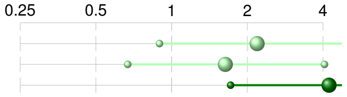

A	B	2.5	50	97.5	Sequence
2	2	0.9	2.2	5.3	ANKPLIK
2	2	0.67	1.6	4.1	SLYLYDVDIAK
0	3	1.7	4.2	10	VNTLIRPDGEKK

5.18 266 607 minus strand H2 [Haliotis tuberculata]

Protein Accession	Unigene62350$_H$1
Mean Expression Ratio	0.523
Median Expression Ratio	0.525
Credible Interval	(0.253, 1.08)
Associated Peptides	3
Associated Spectra	16
Coverage	NaN

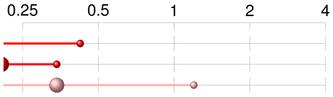

A	B	2.5	50	97.5	Sequence
2	1	0.071	0.17	0.42	KDINTLTDEEIK
6	6	0.13	0.21	0.34	YAVAGIGVR
0	1	0.097	0.34	1.2	DINTLTDEEIK

5.19 " 75 446 Actin, cytoplasmic type 8 OS=Xenopus laevis PE=3 SV=1"

Protein Accession	CL31420.Contig6$_H$1
Mean Expression Ratio	1.92
Median Expression Ratio	1.9
Credible Interval	(0.987, 3.73)
Associated Peptides	4
Associated Spectra	79
Coverage	NaN

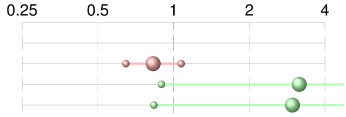

A	B	2.5	50	97.5	Sequence
7	18	14	20	29	MQKEITALAPSTMK
27	25	0.65	0.83	1.1	EITALAPSTMK
0	1	0.89	3.2	11	MQKEITALAPSTMKIK
0	1	0.83	3	11	EITALAPSTMKIK

5.20 118 501 minus strand histone 2A.Z [Aplysia californica]; 15 428 PREDICTED: histone H2A.V-like [Apis florea]

Protein Accession	CL14228.Contig6$_H$1 CL14228.Contig5$_H$1
Mean Expression Ratio	1.85
Median Expression Ratio	1.86
Credible Interval	(1.03, 3.35)
Associated Peptides	5
Associated Spectra	18
Coverage	NaN

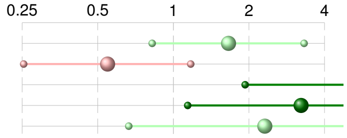

A	B	2.5	50	97.5	Sequence
3	4	0.82	1.7	3.3	ATIAGGGVIPHIHK
3	3	0.25	0.55	1.2	GDEELDSLIK
0	2	1.9	5.4	15	HLQLAIRGDEELDSLIK
1	1	1.1	3.2	9.4	SLIGKK
0	1	0.66	2.3	7.8	GGGVIPHIHK

5.21 222 809 minus strand PREDICTED: 15-hydroxyprostaglandin dehydrogenase [NAD+] [Gallus gallus]

Protein Accession	CL44148.Contig2$_H$1
Mean Expression Ratio	0.542
Median Expression Ratio	0.544
Credible Interval	(0.297, 0.984)
Associated Peptides	6
Associated Spectra	54
Coverage	NaN

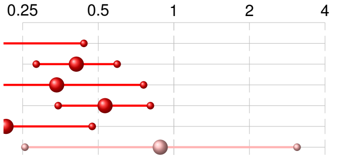

A	B	2.5	50	97.5	Sequence
3	0	0.072	0.18	0.44	AAFEK
9	15	0.28	0.41	0.59	ATVEAFLTK
4	0	0.16	0.34	0.76	AVTSLGR
4	14	0.35	0.53	0.81	VFLTGGAHGIGK
1	3	0.095	0.21	0.47	VMFCDINDK
0	1	0.26	0.88	3.1	RVFLTGGAHGIGK

5.22 205 2490 minus strand glucose-regulated protein 94 [Crassostrea gigas]

Protein Accession	CL5089.Contig1$_H$1
Mean Expression Ratio	0.546
Median Expression Ratio	0.546
Credible Interval	(0.348, 0.856)
Associated Peptides	18
Associated Spectra	39
Coverage	NaN

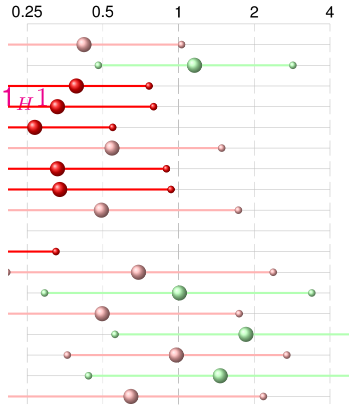

A	B	2.5	50	97.5	Sequence
1	2	0.17	0.42	1	GVVDSDDLPLNVSR
2	1	0.48	1.2	2.8	KTLEINPR
4	2	0.2	0.39	0.76	SGTSDFLAK
3	0	0.14	0.33	0.8	TLEINPR
3	2	0.13	0.27	0.55	NLGTIAR
1	1	0.19	0.54	1.5	KFQNVAK
1	1	0.12	0.33	0.9	DVALADKIEK
1	1	0.12	0.34	0.93	EGLKLDQSEK
1	0	0.14	0.49	1.7	KTLDMIK
1	2	0.027	0.065	0.15	ELISNASDALDKIR
0	1	0.028	0.094	0.32	ELISNASDALDK
0	1	0.21	0.69	2.4	LGVIEDTSNR
0	1	0.29	1	3.4	RVFITDNFEDMLPK
0	1	0.14	0.5	1.7	VFITDNFEDMLPK
0	1	0.56	1.9	6.6	HPLMKELK
0	2	0.36	0.98	2.7	SILFVPK
0	1	0.44	1.5	5	KPGDIDEKEYVDFYK
0	1	0.19	0.65	2.2	GTTVSLHLK

5.23 " 276 659 Fatty acid-binding protein, adipocyte [Crassostrea gigas]; 426 809 Fatty acid-binding protein, adipocyte [Crassostrea gigas]; 277 660 Fatty acid-binding protein, adipocyte [Crassostrea gigas]; 283 666 Fatty acid-binding protein, adipocyte [Crassostrea gigas]; 270 653 Fatty acid-binding protein, adipocyte [Crassostrea gigas]; 283 666 Fatty acid-binding protein, adipocyte [Crassostrea gigas]; 258 641 Fatty acid-binding protein, adipocyte [Crassostrea gigas]; 427 810 Fatty acid-binding protein, adipocyte [Crassostrea gigas]; 277 660 Fatty acid-binding protein, adipocyte [Crassostrea gigas]"

Protein Accession	CL7666.Contig9$_H$1 CL7666.Contig8$_H$1 CL7666.Contig7$_H$1 CL7666.Contig6$_H$1 CL7666.Con
Mean Expression Ratio	1.83
Median Expression Ratio	1.83
Credible Interval	(1.02, 3.31)
Associated Peptides	8
Associated Spectra	18
Coverage	NaN

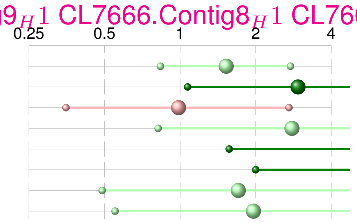

A	B	2.5	50	97.5	Sequence
2	6	0.83	1.5	2.7	AFVDGALK
2	0	1.1	2.9	8.2	AFVDGALKTSMTAK
2	0	0.35	0.98	2.7	QTGEGGEITIR
0	1	0.82	2.8	9.7	AVLTEESGELVER
0	1	1.6	5.5	20	GVTATISFQKA
0	2	2	5.5	15	LTTVAGDKQR
0	1	0.49	1.7	6	TVLAMER
0	1	0.55	2	6.8	GVTATISFQK

5.24 1337 1537 minus strand hemocyanin [Haliotis tuberculata]

Protein Accession	Unigene68371$_{H}1$
Mean Expression Ratio	0.562
Median Expression Ratio	0.563
Credible Interval	(0.259, 1.23)
Associated Peptides	2
Associated Spectra	4
Coverage	NaN

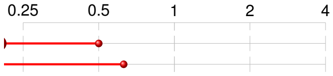

A	B	2.5	50	97.5	Sequence
0	3	0.083	0.2	0.5	DHSTPFDVFNYK
0	1	0.05	0.18	0.63	YDDLQFNGLSISQIQR

5.25 900 1616 minus strand hemocyanin isoform 1 [Haliotis diversicolor]

Protein Accession	Unigene30321$_{H}1$
Mean Expression Ratio	0.565
Median Expression Ratio	0.566
Credible Interval	(0.256, 1.21)
Associated Peptides	2
Associated Spectra	5
Coverage	NaN

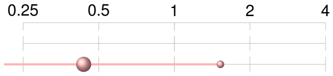

A	B	2.5	50	97.5	Sequence
2	2	0.038	0.085	0.19	VEITDDLK
0	1	0.12	0.44	1.5	LFKVEITDDLK

5.26 27 620 putative mitochondrial ATP synthase [Haliotis discus discus]

Protein Accession	CL13014.Contig1$_{H}1$
Mean Expression Ratio	0.569
Median Expression Ratio	0.567
Credible Interval	(0.3, 1.09)
Associated Peptides	3
Associated Spectra	16
Coverage	NaN

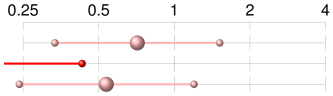

A	B	2.5	50	97.5	Sequence
3	4	0.33	0.71	1.5	LANPALVDQFQK
1	1	0.049	0.15	0.43	VPYPEDK
2	5	0.24	0.54	1.2	SAVDWVAFK

5.27 1971 3368 minus strand Kyphoscoliosis peptidase [Crassostrea gigas]; 1767 3158 minus strand hillarin [Hirudo medicinalis]; 1977 3368 minus strand hillarin [Hirudo medicinalis]

Protein Accession	CL3923.Contig9$_{H}1$ CL3923.Contig8$_{H}1$ CL3923.Contig10$_{H}1$
Mean Expression Ratio	0.568
Median Expression Ratio	0.568
Credible Interval	(0.281, 1.13)
Associated Peptides	4
Associated Spectra	7
Coverage	NaN

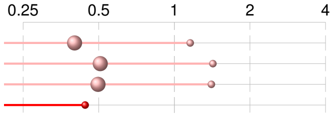

A	B	2.5	50	97.5	Sequence
1	1	0.14	0.4	1.2	GSPEEVLMGLK
1	1	0.18	0.51	1.4	NVLQSVDDHAIQVSK
1	1	0.18	0.5	1.4	EMNFDKVEK
1	0	0.035	0.12	0.44	NITNEIEK

5.28 180 560 calreticulin precursor [Aplysia californica] gi; 180 545 calreticulin precursor [Aplysia californica] gi

Protein Accession CL4813.Contig4$_{H}1$ CL4813.Contig2$_{H}1$
Mean Expression Ratio 0.569
Median Expression Ratio 0.568
Credible Interval (0.329, 0.997)
Associated Peptides 9
Associated Spectra 30
Coverage NaN

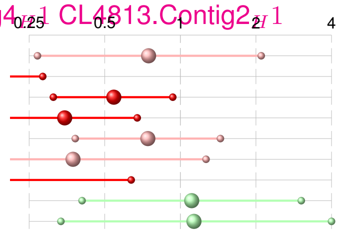

A	B	2.5	50	97.5	Sequence
1	1	0.27	0.75	2.1	DPTVYFK
1	0	0.024	0.082	0.28	FYGDEDKDK
3	7	0.31	0.55	0.93	FYGISAK
1	5	0.18	0.35	0.67	WIPSVHK
2	4	0.38	0.74	1.4	FVWSAGK
0	1	0.11	0.37	1.3	EEFDANWEDR
0	1	0.051	0.18	0.64	TLVIQFTVK
0	2	0.41	1.1	3	FDKFSNEGK
0	1	0.33	1.1	4	HEQNIDCGGGYAK

5.29 1 1350 UPF0663 transmembrane protein C17orf28 [Crassostrea gigas]; 163 1542 UPF0663 transmembrane protein C17orf28 [Crassostrea gigas]; 135 1514 UPF0663 transmembrane protein C17orf28 [Crassostrea gigas]

Protein Accession CL21276.Contig5$_{H}1$ CL21276.Contig4$_{H}1$ CL21276.Contig1$_{H}1$
Mean Expression Ratio 0.567
Median Expression Ratio 0.569
Credible Interval (0.267, 1.18)
Associated Peptides 3
Associated Spectra 4
Coverage NaN

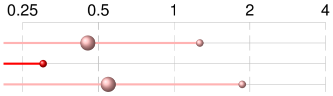

A	B	2.5	50	97.5	Sequence
2	0	0.16	0.45	1.3	EGFQSLLNTK
1	0	0.023	0.084	0.3	GVFLIEPKPK
1	0	0.16	0.55	1.9	KIGFK

5.30 " 2041 3315 minus strand Myosin-10 OS=Mus musculus GN=Myh10 PE=1 SV=2; 2041 3345 minus strand Myosin heavy chain, non-muscle OS=Drosophila melanogaster GN=zip PE=1 SV=2; 2041 3351 minus strand Myosin-10 OS=Mus musculus GN=Myh10 PE=1 SV=2"

Protein Accession Unigene62170$_{H}1$ Unigene62168$_{H}1$ Unigene62165$_{H}1$
Mean Expression Ratio 0.568
Median Expression Ratio 0.57
Credible Interval (0.284, 1.13)
Associated Peptides 4
Associated Spectra 8
Coverage NaN

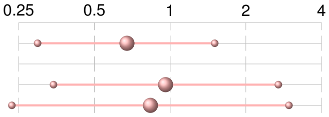

A	B	2.5	50	97.5	Sequence
1	3	0.3	0.67	1.5	AGVLAHLEEER
1	0	0.0066	0.023	0.082	TVGQLYKEQLNK
1	1	0.34	0.96	2.7	IDSPLVLDQLR
0	1	0.23	0.84	3	MIASLELDSNLYR

5.31 27 1682 minus strand PREDICTED: nucleolar protein 56 [Oreochromis niloticus]

Protein Accession	Unigene26699$_H1$
Mean Expression Ratio	1.75
Median Expression Ratio	1.75
Credible Interval	(0.99, 3.17)
Associated Peptides	8
Associated Spectra	21
Coverage	NaN

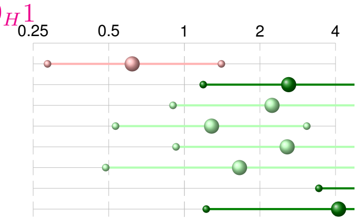

A	B	2.5	50	97.5	Sequence
2	2	0.28	0.62	1.4	LDGLGQAELQK
1	3	1.2	2.6	5.7	VTPLKLDGLGQAELQK
1	2	0.9	2.2	5.5	KLIMQK
1	2	0.53	1.3	3.1	LIAHAGSLTNLAK
0	2	0.93	2.6	7.1	VATLEEEVYDWEIK
0	1	0.49	1.7	5.9	VQAIFDSSK
0	3	3.4	8.4	20	LVKDLHSK
0	1	1.2	4.1	15	KGLGGLSTEKK

5.32 2473 2898 RecName: Full=Paramyosin gi|397603|emb|CAA79849.1| paramyosin [Echinococcus granulosus]

Protein Accession	CL43455.Contig1$_H1$
Mean Expression Ratio	1.73
Median Expression Ratio	1.73
Credible Interval	(1.08, 2.81)
Associated Peptides	12
Associated Spectra	66
Coverage	NaN

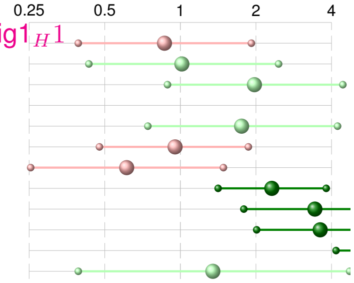

A	B	2.5	50	97.5	Sequence
4	0	0.39	0.87	1.9	AQTLIEEAENR
2	1	0.43	1	2.5	EILAQTEEERR
2	2	0.89	2	4.4	INKEILAQTEEER
1	8	5.9	10	18	INKEILAQTEEERR
1	2	0.74	1.8	4.2	KQLEIEIR
3	3	0.48	0.95	1.9	QLEIEIR
0	3	0.25	0.61	1.5	EILAQTEEER
0	12	1.4	2.3	3.8	KAQTLIEEAENR
0	7	1.8	3.4	6.6	QLAEFASLNEQLNLR
0	8	2	3.6	6.6	QLEEAEDVANPTMNK
0	6	4.2	8.1	16	QLEEAEDVANPTMNKYR
0	1	0.39	1.3	4.7	RQLAEFASLNEQLNLR

5.33 503 1090 minus strand myosin regulatory light chain [Fasciola hepatica]; 278 865 minus strand myosin regulatory light chain [Fasciola hepatica]

Protein Accession	CL43006.Contig3$_H1$ CL43006.Contig4$_H1$
Mean Expression Ratio	0.576
Median Expression Ratio	0.579
Credible Interval	(0.335, 0.986)
Associated Peptides	10
Associated Spectra	31
Coverage	NaN

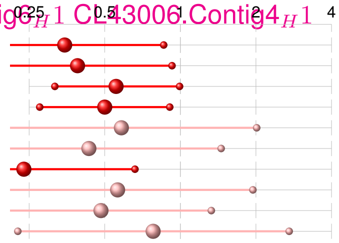

A	B	2.5	50	97.5	Sequence
3	0	0.14	0.35	0.86	DGVIDIEDLK
2	1	0.16	0.39	0.93	EAFTMIDQNR
4	5	0.32	0.56	0.99	IPPDDELKEMLK
5	3	0.27	0.5	0.91	QAQVQEFK
1	0	0.17	0.58	2	EAPLEK
1	0	0.12	0.43	1.5	IPPDDELK
1	1	0.085	0.24	0.66	NAFAMFDDEGK
0	1	0.17	0.56	1.9	GYLPEEYIK
0	2	0.18	0.48	1.3	LSGTDPEDTIR
0	1	0.23	0.78	2.7	DGVIDIEDLKDIYSNLGR

5.34 390 2267 neurofilament protein NF70 [Helix aspersa]; 318 2195 neurofilament protein NF70 [Helix aspersa]

Protein Accession	CL1353.Contig5$_H1$ CL1353.Contig4$_H1$
Mean Expression Ratio	1.72
Median Expression Ratio	1.72
Credible Interval	(0.812, 3.69)
Associated Peptides	2
Associated Spectra	4
Coverage	NaN

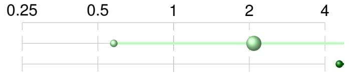

A	B	2.5	50	97.5	Sequence
0	1	0.58	2.1	7.5	KDVNLMNWTLQR
0	3	4.6	11	28	VGQLEELVDDLQR

5.35 44 1465 minus strand Adenylyl cyclase-associated protein 1 [Crassostrea gigas]; 52 1473 minus strand Adenylyl cyclase-associated protein 1 [Crassostrea gigas]; 48 1469 Adenylyl cyclase-associated protein 1 [Crassostrea gigas]

Protein Accession	CL19470.Contig9$_H1$ CL19470.Contig8$_H1$ CL19470.Contig1$_H1$
Mean Expression Ratio	0.583
Median Expression Ratio	0.583
Credible Interval	(0.318, 1.06)
Associated Peptides	7
Associated Spectra	13
Coverage	NaN

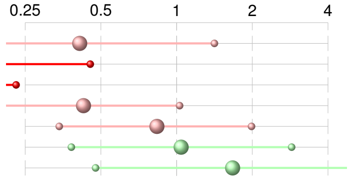

A	B	2.5	50	97.5	Sequence
1	0	0.12	0.41	1.4	AQLDIVNAAFK
1	1	0.06	0.16	0.45	DSLDTEIVTAK
1	0	0.019	0.066	0.23	EFALPEQFK
1	2	0.17	0.43	1	NIVLDNVELK
1	2	0.34	0.84	2	QFLEVASK
1	1	0.38	1	2.9	SKQPPQADLPK
0	1	0.48	1.7	5.8	VEAAKPPLTELQNKK

5.36 48 356 DEC-3 [Lymnaea stagnalis]

Protein Accession	CL26259.Contig1$_H1$
Mean Expression Ratio	0.585
Median Expression Ratio	0.584
Credible Interval	(0.268, 1.25)
Associated Peptides	2
Associated Spectra	5
Coverage	NaN

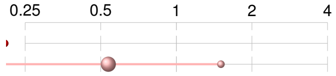

A	B	2.5	50	97.5	Sequence
2	1	0.034	0.085	0.21	SEFDTAFR
0	2	0.19	0.54	1.5	EFSFNWR

5.37 118 501 minus strand histone 2A.Z [Aplysia californica]

Protein Accession	CL14228.Contig6$_H1$
Mean Expression Ratio	0.589
Median Expression Ratio	0.587
Credible Interval	(0.333, 1.04)
Associated Peptides	6
Associated Spectra	36
Coverage	NaN

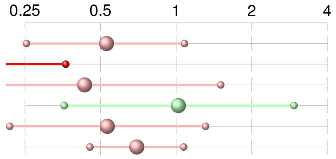

A	B	2.5	50	97.5	Sequence
3	4	0.25	0.53	1.1	ATIAGGGVIPHIHK
3	3	0.082	0.17	0.36	GDEELDSLIK
1	0	0.12	0.43	1.5	HLQLAIR
1	1	0.36	1	2.9	SLIGKK
3	0	0.22	0.53	1.3	SLIGK
17	0	0.46	0.7	1.1	AGLQFPVGR

5.38 372 860 minus strand small heat shock protein [Tegillarca granosa]

Protein Accession	CL18190.Contig4$_H$1
Mean Expression Ratio	0.587
Median Expression Ratio	0.59
Credible Interval	(0.326, 1.03)
Associated Peptides	8
Associated Spectra	24
Coverage	NaN

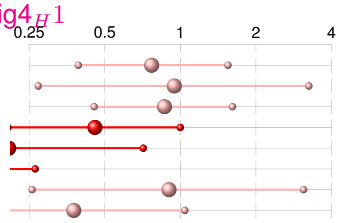

A	B	2.5	50	97.5	Sequence
2	4	0.39	0.77	1.5	IDKDGLSIK
1	0	0.27	0.95	3.3	KSDEYSFVSR
2	5	0.45	0.86	1.6	LTEDGHLIISAR
1	3	0.21	0.46	1	MNVSHYEPDELSVK
1	0	0.059	0.21	0.71	TFGPASIEQQMK
1	1	0.036	0.097	0.26	TIAIESDK
0	1	0.26	0.9	3.1	AFDWPAEGHGENAEVVQAK
0	2	0.14	0.38	1	EFLIPEEIDAETLGSR

5.39 " 1240 1911 PREDICTED: ATP synthase subunit alpha, mitochondrial-like [Anolis carolinensis]"

Protein Accession	Unigene30533$_H$1
Mean Expression Ratio	0.593
Median Expression Ratio	0.594
Credible Interval	(0.287, 1.23)
Associated Peptides	3
Associated Spectra	8
Coverage	NaN

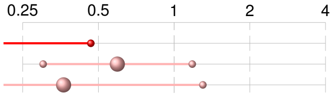

A	B	2.5	50	97.5	Sequence
1	0	0.038	0.13	0.47	LELAQYR
3	3	0.3	0.6	1.2	SSQQELLK
1	0	0.1	0.36	1.3	LTELLK

5.40 813 1244 Probable protein disulfide-isomerase A6 OS=Caenorhabditis elegans GN=tag-320 PE=2 SV=1

Protein Accession	CL39540.Contig4$_H$1
Mean Expression Ratio	0.601
Median Expression Ratio	0.597
Credible Interval	(0.269, 1.37)
Associated Peptides	1
Associated Spectra	3
Coverage	NaN

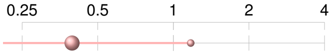

A	B	2.5	50	97.5	Sequence
1	2	0.13	0.39	1.2	TASDIINWATEK

5.41 210 779 GH20056 [Drosophila grimshawi] gi; 216 785 minus strand GH20056 [Drosophila grimshawi] gi; 210 779 GH20056 [Drosophila grimshawi] gi; 216 785 GH20056 [Drosophila grimshawi] gi

Protein Accession	CL34497.Contig4$_H$1 CL34497.Contig3$_H$1 CL34497.Contig2$_H$1 CL34497.Contig1$_H$1
Mean Expression Ratio	0.597
Median Expression Ratio	0.599
Credible Interval	(0.283, 1.26)
Associated Peptides	3
Associated Spectra	3
Coverage	NaN

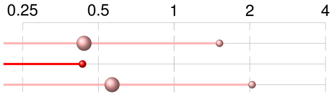

A	B	2.5	50	97.5	Sequence
1	0	0.12	0.44	1.5	QLSQEEVSNFLK
1	0	0.033	0.12	0.43	EVSNFLK
0	1	0.16	0.57	2	KQPFEFQIGVGQVIK

5.42 " 28 1104 minus strand Pyruvate dehydrogenase E1 component subunit beta, mitochondrial [Crassostrea gigas]"

Protein Accession	CL18015.Contig14$_H$1
Mean Expression Ratio	1.66
Median Expression Ratio	1.66
Credible Interval	(0.881, 3.09)
Associated Peptides	7
Associated Spectra	10
Coverage	NaN

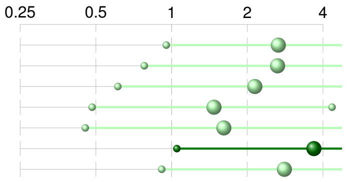

A	B	2.5	50	97.5	Sequence
0	2	0.95	2.7	7.6	DALNSAMDEEIARDER
0	1	0.78	2.6	9.4	ALDAAKELEGK
0	1	0.61	2.1	7.3	SLRPVDDETIIK
1	1	0.48	1.5	4.3	SLEEAALPQPVNVIR
0	1	0.45	1.6	5.5	IEREGK
0	1	1	3.7	13	VSKGLWK
1	1	0.92	2.8	8.5	VTGADVPMPYAK

5.43 116 352 minus strand troponin I 4 [Clonorchis sinensis]

Protein Accession	Unigene63816$_H$1
Mean Expression Ratio	1.66
Median Expression Ratio	1.66
Credible Interval	(0.787, 3.47)
Associated Peptides	3
Associated Spectra	7
Coverage	NaN

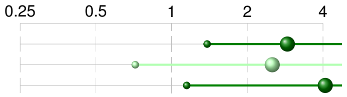

A	B	2.5	50	97.5	Sequence
0	5	1.4	2.9	6.1	FIKPVLR
0	1	0.72	2.5	9.1	QSPTRGNLKSTGR
0	1	1.1	4.1	15	GKFIKPVLR

5.44 373 579 myosin heavy chain [Argopecten irradians] gi

Protein Accession	Unigene64289$_H$1
Mean Expression Ratio	1.66
Median Expression Ratio	1.66
Credible Interval	(0.915, 2.97)
Associated Peptides	8
Associated Spectra	19
Coverage	NaN

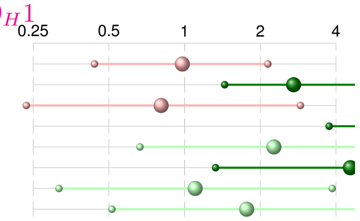

A	B	2.5	50	97.5	Sequence
1	3	0.44	0.98	2.1	KVQNELEDAEER
1	6	1.4	2.7	5.1	QVEEAEEIAAVNLAK
1	0	0.23	0.81	2.9	QELVDK
0	3	3.7	9.2	22	KVQNELEDAEERADSAEGTLSK
0	1	0.66	2.3	7.9	RQVEEAEEIAAVNLAK
0	1	1.3	4.6	16	VQNELEDAEERADSAEGTLSK
0	1	0.32	1.1	3.9	ELEDAEER
0	1	0.51	1.8	6.2	VQNELEDAEER

5.45 161 301 Heat shock protein beta-6 OS=Mus musculus GN=Hspb6 PE=1 SV=1

Protein Accession	CL46983.Contig2$_H$1
Mean Expression Ratio	1.66
Median Expression Ratio	1.66
Credible Interval	(0.86, 3.23)
Associated Peptides	4
Associated Spectra	31
Coverage	NaN

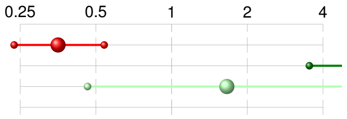

A	B	2.5	50	97.5	Sequence
5	14	0.24	0.35	0.54	VNMDVQHFKPEEIK
0	5	3.5	7.3	15	VNMDVQHFKPEEIKITTKDNR
0	1	0.46	1.7	5.6	VVISAK
0	6	5.9	12	23	VNMDVQHFKPEEIKITTK

5.46 35 370 Ganglioside GM2 activator [Crassostrea gigas]; 2 487 minus strand PREDICTED: hypothetical protein LOC100699750 [Oreochromis niloticus]; 41 535 minus strand PREDICTED: hypothetical protein LOC100699750 [Oreochromis niloticus]

Protein Accession	CL7266.Contig6$_H$1 CL7266.Contig5$_H$1 CL7266.Contig3$_H$1
Mean Expression Ratio	0.602
Median Expression Ratio	0.604
Credible Interval	(0.279, 1.28)
Associated Peptides	1
Associated Spectra	5
Coverage	NaN

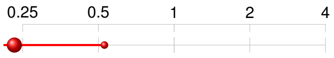

A	B	2.5	50	97.5	Sequence
**3**	**2**	**0.1**	**0.23**	**0.53**	**NVALVVSVK**

5.47 316 1512 Alpha-N-acetylgalactosaminidase [Crassostrea gigas]; 282 1478 Alpha-N-acetylgalactosaminidase [Crassostrea gigas]

Protein Accession	CL2428.Contig4$_H$1 CL2428.Contig1$_H$1
Mean Expression Ratio	0.607
Median Expression Ratio	0.607
Credible Interval	(0.321, 1.15)
Associated Peptides	6
Associated Spectra	12
Coverage	NaN

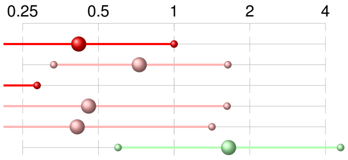

A	B	2.5	50	97.5	Sequence
**1**	**2**	**0.17**	**0.42**	**1**	**LGIYEDFGSK**
**2**	**2**	**0.33**	**0.73**	**1.6**	**LVELGLR**
**1**	**0**	**0.023**	**0.082**	**0.29**	**YGYPVMQR**
**1**	**0**	**0.13**	**0.46**	**1.6**	**LYMAQANK**
**1**	**0**	**0.12**	**0.41**	**1.4**	**VDMVK**
**0**	**2**	**0.6**	**1.7**	**4.6**	**AGYRARELFAGK**

5.48 91 2658 minus strand puromycin-sensitive aminopeptidase [Saccoglossus kowalevskii]

Protein Accession	CL6708.Contig2$_H$1
Mean Expression Ratio	0.607
Median Expression Ratio	0.608
Credible Interval	(0.335, 1.1)
Associated Peptides	8
Associated Spectra	12
Coverage	NaN

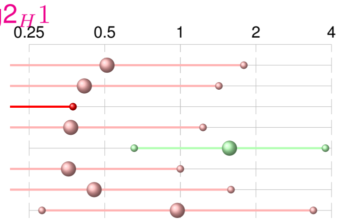

A	B	2.5	50	97.5	Sequence
**1**	**0**	**0.15**	**0.51**	**1.8**	**GLTVQQEK**
**1**	**0**	**0.12**	**0.41**	**1.4**	**VILSNMPVK**
**1**	**1**	**0.05**	**0.14**	**0.37**	**TLDFALSDK**
**1**	**0**	**0.11**	**0.37**	**1.2**	**VYTPVGK**
**1**	**2**	**0.66**	**1.6**	**3.8**	**SLGVNDDPELIKR**
**1**	**1**	**0.13**	**0.36**	**1**	**MIELYEK**
**1**	**0**	**0.13**	**0.45**	**1.6**	**LVTYR**
**0**	**1**	**0.28**	**0.97**	**3.4**	**TIQQSLENIMLNKR**

5.49 111 1226 gelsolin [Aplysia californica] gi; 42 1139 minus strand gelsolin [Aplysia californica] gi

Protein Accession	Unigene39723$_H$1 Unigene32782$_H$1
Mean Expression Ratio	1.64
Median Expression Ratio	1.64
Credible Interval	(0.909, 2.93)
Associated Peptides	9
Associated Spectra	12
Coverage	NaN

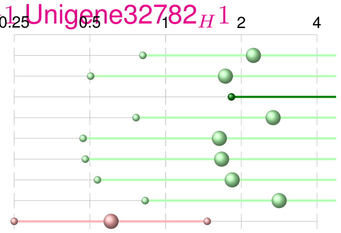

A	B	2.5	50	97.5	Sequence
0	2	0.81	2.2	6.2	AMQFIGEIK
0	1	0.5	1.7	6	DLVTSDDVYILDTGLK
0	1	1.8	6.5	23	SKAMQFIGEIK
0	1	0.76	2.7	9.2	TVELDTKLNDKPIQHR
0	1	0.47	1.6	5.6	QFDTALAA
0	1	0.48	1.7	6	KNGLPLAHNYLR
0	1	0.53	1.8	6.5	EVQGHESDLFK
0	1	0.83	2.8	10	ESGNPWRPVTVIK
0	3	0.25	0.61	1.5	SDDVFIFDSGK

5.50 346 702 minus strand PREDICTED: galectin-4-like [Anolis carolinensis]; 367 669 minus strand PREDICTED: galectin-7-like [Cavia porcellus]; 599 964 C. briggsae CBR-LEC-4 protein [Caenorhabditis briggsae]

Protein Accession	Unigene42203$_H$1 Unigene41782$_H$1 CL22459.Contig3$_H$1
Mean Expression Ratio	1.64
Median Expression Ratio	1.64
Credible Interval	(0.858, 3.11)
Associated Peptides	4
Associated Spectra	11
Coverage	NaN

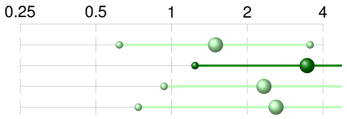

A	B	2.5	50	97.5	Sequence
3	2	0.62	1.5	3.6	AEATLEVR
0	2	1.2	3.5	9.7	KSVPHGLVK
1	2	0.93	2.3	5.9	SVPHGLVK
0	1	0.74	2.6	9.1	VKDSGFK

5.51 442 2595 minus strand Amiloride-sensitive amine oxidase [copper-containing] [Crassostrea gigas]

Protein Accession	Unigene105$_H$1
Mean Expression Ratio	1.64
Median Expression Ratio	1.64
Credible Interval	(0.791, 3.46)
Associated Peptides	3
Associated Spectra	4
Coverage	NaN

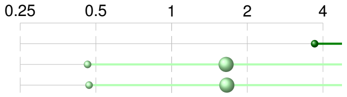

A	B	2.5	50	97.5	Sequence
1	1	3.7	11	30	QQVAVTVQKDEER
0	1	0.46	1.6	6	ILPTGMSPQLLK
0	1	0.47	1.7	5.8	VTDFVDSGALIGTHSK

5.52 22 870 minus strand PREDICTED: alcohol dehydrogenase class-3 [Sarcophilus harrisii]

Protein Accession	Unigene27690$_{H}1$
Mean Expression Ratio	0.606
Median Expression Ratio	0.611
Credible Interval	(0.293, 1.24)
Associated Peptides	3
Associated Spectra	9
Coverage	NaN

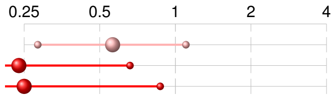

A	B	2.5	50	97.5	Sequence
4	2	0.28	0.56	1.1	AAVAWEAK
1	1	0.084	0.24	0.66	IIGIDVNQDK
1	0	0.069	0.25	0.87	FAMAK

5.53 3 500 Tumor protein D54 [Crassostrea gigas]; 3 464 Tumor protein D54 [Crassostrea gigas]; 3 464 Tumor protein D54 [Crassostrea gigas]; 3 500 Tumor protein D54 [Crassostrea gigas]; 6 440 Tumor protein D54 [Crassostrea gigas]; 3 464 Tumor protein D54 [Crassostrea gigas]; 3 500 Tumor protein D54 [Crassostrea gigas]

Protein Accession	CL25616.Contig8$_{H}1$ CL25616.Contig6$_{H}1$ CL25616.Contig5$_{H}1$ CL25616.Contig4$_{H}1$ CL2561
Mean Expression Ratio	0.612
Median Expression Ratio	0.612
Credible Interval	(0.275, 1.35)
Associated Peptides	2
Associated Spectra	2
Coverage	NaN

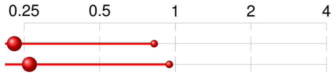

A	B	2.5	50	97.5	Sequence
1	0	0.065	0.23	0.82	STDDIDTAFK
1	0	0.072	0.26	0.94	NLGITPFK

5.54 195 455 60S ribosomal protein L23a OS=Rattus norvegicus GN=Rpl23a PE=2 SV=1; 16 465 60S ribosomal protein L23a OS=Rattus norvegicus GN=Rpl23a PE=2 SV=1

Protein Accession	CL27563.Contig3$_{H}1$ CL27563.Contig1$_{H}1$
Mean Expression Ratio	0.617
Median Expression Ratio	0.617
Credible Interval	(0.31, 1.21)
Associated Peptides	3
Associated Spectra	9
Coverage	NaN

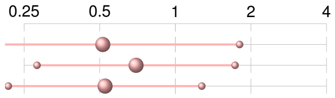

A	B	2.5	50	97.5	Sequence
1	0	0.15	0.51	1.8	VNTLIRPDGEK
2	2	0.28	0.7	1.7	ANKPLIK
2	2	0.22	0.52	1.3	SLYLYDVDIAK

5.55 718 1155 Annexin A7 [Crassostrea gigas]

Protein Accession	CL15539.Contig2$_{H}$1
Mean Expression Ratio	0.62
Median Expression Ratio	0.618
Credible Interval	(0.351, 1.11)
Associated Peptides	8
Associated Spectra	18
Coverage	NaN

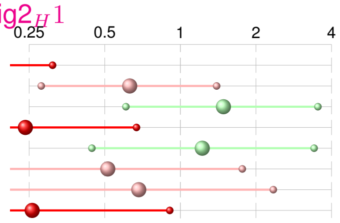

A	B	2.5	50	97.5	Sequence
3	1	0.061	0.14	0.31	ATFDAYQQIAGK
2	2	0.28	0.63	1.4	NVPGYFAQR
1	2	0.61	1.5	3.5	KLGTDEETFNR
2	0	0.088	0.24	0.67	SEVDLGATK
1	1	0.44	1.2	3.4	SFPQLR
1	0	0.15	0.51	1.8	AQEAQELLQAGVK
0	1	0.2	0.68	2.3	SEMSGNLEEGFLAIVR
0	1	0.075	0.26	0.9	DIESAIK

5.56 349 885 juvenile hormone diol kinase [Danaus plexippus]; 349 885 juvenile hormone diol kinase [Danaus plexippus]; 349 885 juvenile hormone diol kinase [Danaus plexippus]; 349 885 minus strand juvenile hormone diol kinase [Danaus plexippus]; 349 885 juvenile hormone diol kinase [Danaus plexippus]; 349 885 juvenile hormone diol kinase [Danaus plexippus]; 349 885 juvenile hormone diol kinase [Danaus plexippus]; 349 885 juvenile hormone diol kinase [Danaus plexippus]; 349 885 juvenile hormone diol kinase [Danaus plexippus]; 349 885 juvenile hormone diol kinase [Danaus plexippus]

Protein Accession	Unigene38920$_{H}$1 Unigene38919$_{H}$1 CL7590.Contig1$_{H}$1 CL7590.Contig16$_{H}$1 CL7590.Contig
Mean Expression Ratio	0.617
Median Expression Ratio	0.619
Credible Interval	(0.28, 1.36)
Associated Peptides	2
Associated Spectra	2
Coverage	NaN

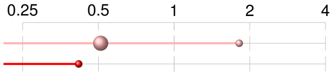

A	B	2.5	50	97.5	Sequence
1	0	0.14	0.51	1.8	IFGNSEADSNK
1	0	0.033	0.12	0.42	DGIIDKNDFK

5.57 5 193 minus strand PREDICTED: 40S ribosomal protein S28-like [Strongylocentrotus purpuratus]; 3 122 PREDICTED: 40S ribosomal protein S28-like [Strongylocentrotus purpuratus]

Protein Accession	CL49462.Contig2$_{H}$1 CL49462.Contig1$_{H}$1
Mean Expression Ratio	1.61
Median Expression Ratio	1.61
Credible Interval	(0.762, 3.38)
Associated Peptides	2
Associated Spectra	5
Coverage	NaN

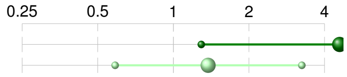

A	B	2.5	50	97.5	Sequence
0	1	1.3	4.6	16	NVKGPVREGDILTLLESER
1	3	0.59	1.4	3.2	VEFLDDSNR

5.58 1 852 beta actin-like 1 protein [Macrostomum lignano]

Protein Accession	Unigene31438$_{H}1$
Mean Expression Ratio	1.61
Median Expression Ratio	1.61
Credible Interval	(1.14, 2.29)
Associated Peptides	26
Associated Spectra	323
Coverage	NaN

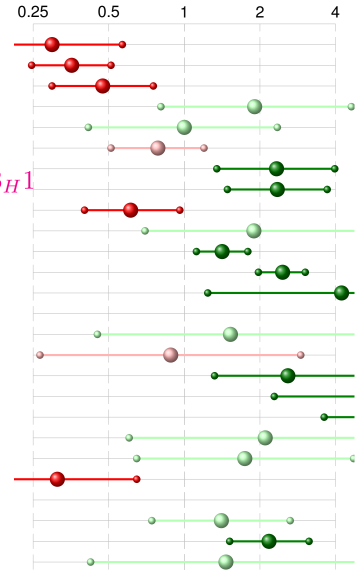

A	B	2.5	50	97.5	Sequence
4	3	0.16	0.3	0.57	AVFPSIVGR
10	14	0.25	0.36	0.51	GILTLK
3	11	0.3	0.47	0.76	YPIEHGIVTNWDDMEK
1	2	0.81	1.9	4.6	VAPEEHPVLL
2	1	0.42	1	2.4	TEAPLNPK
5	12	0.51	0.78	1.2	RGILTLK
4	6	1.4	2.3	4	VAPEEHPVLLTEA
9	6	1.5	2.3	3.7	AGFAGDDAPR
6	10	0.4	0.61	0.96	DSYVGDEAQSK
1	1	0.7	1.9	5.2	HQGVMVGM
31	33	1.1	1.4	1.8	HQGVMVGMGQK
18	59	2	2.5	3	VAPEEHPVLLTEAPLNPK
0	1	1.2	4.2	14	APEEHPVLLTEAPLNPK
0	5	5.5	11	24	HQGVMVGMGQKDSYVGDEAQSK
0	1	0.45	1.5	5.2	SYELPDGQVITIGN
0	1	0.27	0.88	2.9	TTGIVLDSGD
0	6	1.3	2.6	5.1	VAPEEHPVLLTEAPLN
0	3	2.3	5.5	14	VAPEEHPVLLTEAPLNPKANR
0	6	3.6	7.1	14	VAPEEHPVLLTEAPLNPKANREK
0	1	0.6	2.1	7.1	VAPEEHPVL
0	2	0.65	1.7	4.7	AVFPSIVGRPR
0	5	0.15	0.31	0.65	GYSFTTTAER
0	8	13	24	44	HQGVMVGMGQKDSYVGDEAQSKR
0	7	0.74	1.4	2.6	IWHHTFYNELR
0	24	1.5	2.2	3.1	SYELPDGQVITIGNER
0	1	0.42	1.5	5.1	TTGIVLDSGDGVTHTVPIYEGYALPHAILRLDLAG

5.59 2 532 heat shock protein 70 [Echinostoma caproni]

Protein Accession	Unigene17002$_{H}1$
Mean Expression Ratio	1.6
Median Expression Ratio	1.6
Credible Interval	(0.747, 3.48)
Associated Peptides	2
Associated Spectra	3
Coverage	NaN

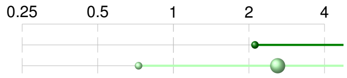

A	B	2.5	50	97.5	Sequence
0	2	2.1	5.8	16	ARFEELNADLFR
0	1	0.73	2.6	9.6	ARFEELNADLFRGTLEPVEK

5.60 1070 1888 minus strand galectin [Pinctada fucata]; 1371 2189 minus strand galectin [Pinctada fucata]

Protein Accession	CL7245.Contig4$_{H}1$ CL7245.Contig3$_{H}1$
Mean Expression Ratio	0.628
Median Expression Ratio	0.627
Credible Interval	(0.343, 1.13)
Associated Peptides	4
Associated Spectra	28
Coverage	NaN

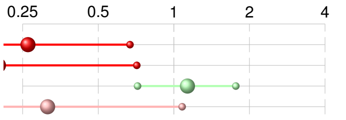

A	B	2.5	50	97.5	Sequence
1	2	0.1	0.26	0.67	EIPQGLEK
1	0	0.058	0.2	0.71	GEAFIYEVK
13	10	0.72	1.1	1.8	FNEGVTVR
1	0	0.091	0.31	1.1	NDLVVIK

5.61 501 1838 Hsp90A [Haliotis asinina]

Protein Accession	CL5184.Contig2$_{H}$1
Mean Expression Ratio	0.629
Median Expression Ratio	0.631
Credible Interval	(0.373, 1.06)
Associated Peptides	11
Associated Spectra	24
Coverage	NaN

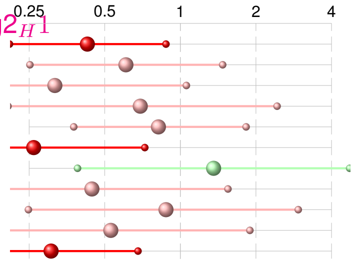

A	B	2.5	50	97.5	Sequence
2	3	0.21	0.43	0.88	EMLQQSK
1	2	0.25	0.61	1.5	SLTNDWEDHLAVK
1	0	0.093	0.32	1.1	GVVDSEDLPLNISR
0	1	0.21	0.69	2.4	AIYYITGESLDAVK
0	4	0.38	0.82	1.8	HFSVEGQLEFR
0	2	0.093	0.26	0.72	KHFEINPDHPIIK
0	1	0.39	1.4	4.7	RAPFDLFENKK
0	1	0.13	0.44	1.5	YHSSQAGDELVTLK
0	1	0.25	0.87	3	KLAEFLR
0	1	0.15	0.53	1.9	TKPLWTR
0	4	0.14	0.31	0.68	LAEFLR

5.62 " 166 1248 PREDICTED: probable pyruvate dehydrogenase E1 component subunit alpha, mitochondrial-like [Metaseiulus occidentalis]; 166 1248 PREDICTED: probable pyruvate dehydrogenase E1 component subunit alpha, mitochondrial-like [Metaseiulus occidentalis]; 166 1248 PREDICTED: probable pyruvate dehydrogenase E1 component subunit alpha, mitochondrial-like [Metaseiulus occidentalis]; 166 1248 PREDICTED: probable pyruvate dehydrogenase E1 component subunit alpha, mitochondrial-like [Metaseiulus occidentalis]; 166 1248 PREDICTED: probable pyruvate dehydrogenase E1 component subunit alpha, mitochondrial-like [Metaseiulus occidentalis]; 166 1248 minus strand PREDICTED: probable pyruvate dehydrogenase E1 component subunit alpha, mitochondrial-like [Metaseiulus occidentalis]; 166 1248 minus strand PREDICTED: probable pyruvate dehydrogenase E1 component subunit alpha, mitochondrial-like [Metaseiulus occidentalis]; 166 1248 PREDICTED: probable pyruvate dehydrogenase E1 component subunit alpha, mitochondrial-like [Metaseiulus occidentalis]; 166 1248 PREDICTED: probable pyruvate dehydrogenase E1 component subunit alpha, mitochondrial-like [Metaseiulus occidentalis]"

Protein Accession	Unigene34082$_{H}$1 Unigene34081$_{H}$1 Unigene34079$_{H}$1 Unigene34077$_{H}$1 Unigene34075$_{H}$1 U
Mean Expression Ratio	0.628
Median Expression Ratio	0.631
Credible Interval	(0.299, 1.32)
Associated Peptides	3
Associated Spectra	4
Coverage	NaN

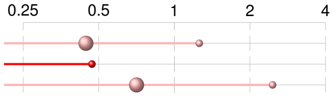

A	B	2.5	50	97.5	Sequence
2	0	0.16	0.45	1.3	TGLATEEELKK
1	0	0.04	0.14	0.47	DMQVIR
1	0	0.2	0.71	2.5	ATEATFK

5.63 366 1049 hypothetical protein CGI_10004855 [Crassostrea gigas]

Protein Accession	CL43590.Contig5$_H1$
Mean Expression Ratio	1.57
Median Expression Ratio	1.58
Credible Interval	(0.881, 2.78)
Associated Peptides	8
Associated Spectra	18
Coverage	NaN

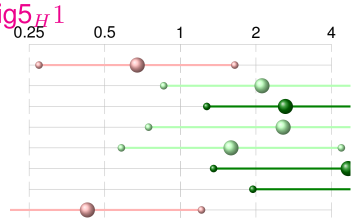

A	B	2.5	50	97.5	Sequence
2	1	0.27	0.67	1.6	TTVQLHK
2	1	0.86	2.1	5.2	VKVPGAK
0	5	1.3	2.6	5.4	GGDLWQGEVSLEQHR
0	1	0.75	2.6	9.1	GKNVDVKLNAR
0	2	0.58	1.6	4.4	SAEFSTNDNIFDIEMK
0	1	1.4	4.7	17	TPQPLKTTVQLHK
0	1	1.9	6.6	23	GKNVDVK
0	2	0.15	0.43	1.2	DEWTHLTAK

5.64 6 650 minus strand glutathione S-transferase mu class protein [Crassostrea ariakensis]; 117 761 glutathione S-transferase mu class protein [Crassostrea ariakensis]; 122 766 glutathione S-transferase mu class protein [Crassostrea ariakensis]; 122 766 glutathione S-transferase mu class protein [Crassostrea ariakensis]

Protein Accession	Unigene56987$_H1$ CL6887.Contig3$_H1$ CL6887.Contig2$_H1$ CL6887.Contig1$_H1$
Mean Expression Ratio	0.633
Median Expression Ratio	0.635
Credible Interval	(0.358, 1.11)
Associated Peptides	8
Associated Spectra	24
Coverage	NaN

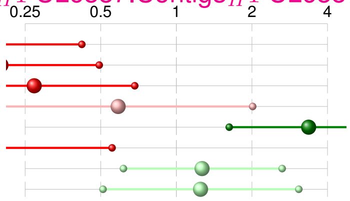

A	B	2.5	50	97.5	Sequence
3	0	0.07	0.17	0.42	GLAQPIR
3	0	0.082	0.2	0.49	ITQSNAILR
2	1	0.11	0.27	0.68	LSQFVK
1	0	0.17	0.59	2	AYMSSDK
0	5	1.6	3.4	7	KHDLLGKTDKEK
0	1	0.043	0.15	0.55	IMIPGILDDYPK
0	5	0.62	1.3	2.6	DAWFNVK
0	3	0.51	1.3	3.1	PALGYWK

5.65 " 3 227 minus strand alpha tubulin, putative [Ixodes scapularis] gi"

Protein Accession	Unigene31690$_H1$
Mean Expression Ratio	1.57
Median Expression Ratio	1.57
Credible Interval	(0.783, 3.12)
Associated Peptides	2
Associated Spectra	13
Coverage	NaN

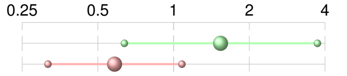

A	B	2.5	50	97.5	Sequence
3	2	0.64	1.5	3.7	LSVDYGKK
1	7	0.32	0.58	1.1	LSVDYGK

5.66 " 9 125 minus strand Hemocyanin, beta-C chain unit D (Fragment) OS=Helix pomatia PE=1 SV=2"

Protein Accession	CL7906.Contig6$_{H}1$
Mean Expression Ratio	0.637
Median Expression Ratio	0.639
Credible Interval	(0.28, 1.41)
Associated Peptides	1
Associated Spectra	3
Coverage	NaN

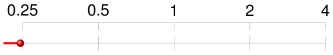

A	B	2.5	50	97.5	Sequence
**2**	**1**	**0.027**	**0.083**	**0.25**	**YDISDAVK**

5.67 14 277 minus strand Histidine triad nucleotide-binding protein 1 OS=Oryctolagus cuniculus GN=HINT1 PE=1 SV=2

Protein Accession	CL25563.Contig1$_{H}1$
Mean Expression Ratio	0.638
Median Expression Ratio	0.641
Credible Interval	(0.292, 1.36)
Associated Peptides	2
Associated Spectra	4
Coverage	NaN

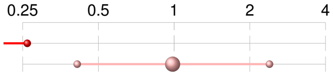

A	B	2.5	50	97.5	Sequence
**1**	**0**	**0.02**	**0.074**	**0.26**	**LAEAVEADEK**
**1**	**2**	**0.41**	**0.99**	**2.4**	**AHLAKPGGDTIFGK**

5.68 185 742 Transmembrane protein Tmp21 [Crassostrea gigas]

Protein Accession	CL3732.Contig3$_{H}1$
Mean Expression Ratio	1.56
Median Expression Ratio	1.56
Credible Interval	(0.81, 3.08)
Associated Peptides	5
Associated Spectra	7
Coverage	NaN

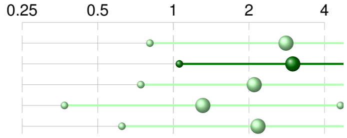

A	B	2.5	50	97.5	Sequence
**0**	**1**	**0.8**	**2.8**	**9.9**	**SIVDDFAYMR**
**0**	**2**	**1.1**	**3**	**8.5**	**SYDELAKAEK**
**0**	**2**	**0.74**	**2.1**	**5.8**	**RLEDLSK**
**0**	**1**	**0.37**	**1.3**	**4.6**	**VLYLR**
**0**	**1**	**0.63**	**2.2**	**7.8**	**GHILYSK**

5.69 " 116 1168 minus strand hypothetical protein CGI_10014553, partial [Crassostrea gigas]"

Protein Accession	CL19215.Contig5$_{H}1$
Mean Expression Ratio	0.64
Median Expression Ratio	0.642
Credible Interval	(0.318, 1.29)
Associated Peptides	4
Associated Spectra	6
Coverage	NaN

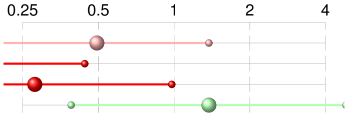

A	B	2.5	50	97.5	Sequence
**1**	**1**	**0.18**	**0.49**	**1.4**	**FGPTTSEFEK**
**1**	**1**	**0.058**	**0.17**	**0.44**	**SVIALGEGAGANIVAR**
**1**	**0**	**0.08**	**0.28**	**0.98**	**VLEER**
**0**	**1**	**0.39**	**1.4**	**4.8**	**AQNKDQLQK**

5.70 " 4146 6107 minus strand nonmuscle myosin II, partial [Aplysia californica]"

Protein Accession	Unigene30363$_{H}1$
Mean Expression Ratio	1.55
Median Expression Ratio	1.55
Credible Interval	(1.08, 2.21)
Associated Peptides	31
Associated Spectra	64
Coverage	NaN

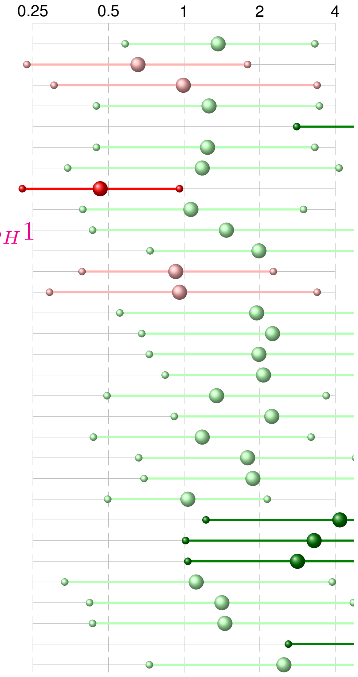

A	B	2.5	50	97.5	Sequence
1	2	0.58	1.4	3.3	ALEATVQEQR
2	0	0.24	0.65	1.8	ATIAALEGK
1	0	0.3	0.99	3.4	DKLAELEGQSR
1	1	0.45	1.3	3.5	LAELEGQSR
3	3	2.8	5.5	11	TKATIAALEGK
1	1	0.45	1.2	3.3	ALTLAQR
1	0	0.34	1.2	4.1	LKEDAIK
3	2	0.23	0.46	0.96	NLEAELVK
1	1	0.4	1.1	3	IEQLER
1	0	0.43	1.5	5.2	AALARTNR
0	2	0.73	2	5.5	DLEQVSTQLEQVETK
0	3	0.39	0.93	2.3	ELDDQLEQNDVLVR
0	1	0.29	0.96	3.4	ELQIQAEDER
0	1	0.56	1.9	6.6	EMEAELEEER
0	1	0.68	2.2	7.8	EMEQLHAQLEEAR
0	2	0.72	2	5.5	IANLEEQLDQEAKER
0	3	0.84	2.1	5	ILNLSHAMEEMQGR
0	2	0.49	1.3	3.7	KAQIAMEQMATDLAAER
0	3	0.91	2.2	5.3	LKELQIQAEDERR
0	2	0.44	1.2	3.2	LQEDLAAAER
0	2	0.66	1.8	4.8	NAEAERDELADEIGSSATSK
0	2	0.69	1.9	5.2	QVDEAEEEVAR
0	5	0.5	1	2.1	VQIEELEDELQAAEDAK
0	1	1.2	4.2	14	IAAQMKDFQR
0	1	1	3.3	11	LALEKDNIER
0	2	1	2.8	7.6	SADQYKEQLEK
0	1	0.34	1.1	3.9	QSLLDEK
0	1	0.42	1.4	4.7	RKLQAEVEDLTITLDNQR
0	1	0.43	1.4	5	LDEKRRLEAR
0	2	2.6	6.9	19	SVSSKLENQR
0	1	0.73	2.5	8.6	QKLAVQSK

5.71 " 90 1085 squid, variant A [Blattella germanica]"

Protein Accession	CL14696.Contig5$_{H}1$
Mean Expression Ratio	0.641
Median Expression Ratio	0.645
Credible Interval	(0.324, 1.26)
Associated Peptides	3
Associated Spectra	9
Coverage	NaN

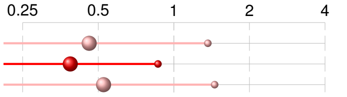

A	B	2.5	50	97.5	Sequence
1	1	0.15	0.46	1.4	IMEVTTHK
3	2	0.17	0.39	0.87	LGGQEVDVK
2	0	0.19	0.52	1.5	IFVGR

5.72 594 1253 peroxiredoxin 6 [Eriocheir sinensis]

Protein Accession	CL21894.Contig2$_{H}1$
Mean Expression Ratio	0.653
Median Expression Ratio	0.656
Credible Interval	(0.393, 1.07)
Associated Peptides	11
Associated Spectra	44
Coverage	NaN

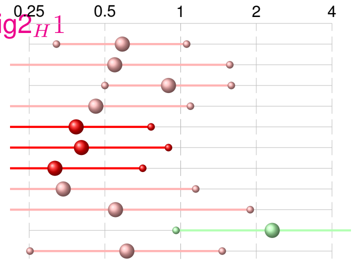

A	B	2.5	50	97.5	Sequence
4	4	0.32	0.59	1.1	VATPVDWK
1	1	0.2	0.55	1.6	AVFIIGPDKK
2	7	0.5	0.89	1.6	AVFIIGPDK
2	1	0.19	0.46	1.1	LFPNYK
5	1	0.19	0.38	0.76	QLTATQK
1	3	0.18	0.4	0.9	LLPEFEK
1	3	0.14	0.32	0.71	VLDSLQLTATQK
0	1	0.1	0.34	1.1	ELAVQLGMVDPDEK
0	1	0.16	0.55	1.9	NFDEILR
0	3	0.96	2.3	5.6	KVATPVDWK
0	3	0.25	0.61	1.5	VNLGDVFPDFK

5.73 871 1371 ribosomal protein L11 [Triatoma infestans]; 343 843 minus strand ribosomal protein L11 [Triatoma infestans]

Protein Accession	CL20979.Contig2$_{H}1$ CL20979.Contig1$_{H}1$
Mean Expression Ratio	0.655
Median Expression Ratio	0.657
Credible Interval	(0.298, 1.43)
Associated Peptides	2
Associated Spectra	3
Coverage	NaN

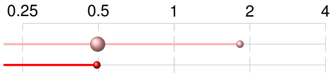

A	B	2.5	50	97.5	Sequence
1	0	0.14	0.5	1.8	ITKEEAIK
2	0	0.062	0.18	0.49	AEEILER

5.74 3 272 minus strand ribosomal protein L28 [Haliotis asinina]

Protein Accession	CL403.Contig16$_{H}1$
Mean Expression Ratio	0.661
Median Expression Ratio	0.658
Credible Interval	(0.307, 1.4)
Associated Peptides	2
Associated Spectra	7
Coverage	NaN

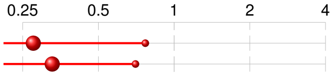

A	B	2.5	50	97.5	Sequence
2	0	0.1	0.28	0.77	GVVLVTK
5	0	0.16	0.33	0.7	TVGVEPSKDGK

5.75 " 220 627 Fatty acid-binding protein, brain [Crassostrea gigas]"

Protein Accession	CL9041.Contig2$_{H}1$
Mean Expression Ratio	0.658
Median Expression Ratio	0.66
Credible Interval	(0.349, 1.23)
Associated Peptides	5
Associated Spectra	26
Coverage	NaN

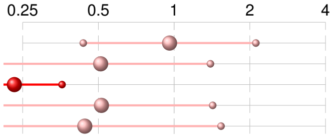

A	B	2.5	50	97.5	Sequence
1	3	0.43	0.96	2.1	EVDGGVMTSTTTFDGK
1	1	0.18	0.51	1.4	IVENYKLEDK
10	7	0.15	0.23	0.36	LTITVEGGK
0	2	0.19	0.52	1.4	LGETFDYNSIDGTKPK
0	1	0.13	0.44	1.5	VDLASTTGLEEFGTAIGFSEEK

5.76 55 795 Elongation factor 1-delta OS=Bos taurus GN=EEF1D PE=2 SV=2; 49 747 PREDICTED: elongation factor 1-delta-like [Strongylocentrotus purpuratus]; 49 819 PREDICTED: elongation factor 1-delta-like isoform 2 [Strongylocentrotus purpuratus]; 49 795 PREDICTED: elongation factor 1-delta-like isoform 2 [Strongylocentrotus purpuratus]

Protein Accession	CL10790.Contig9$_H$1 CL10790.Contig13$_H$1 CL10790.Contig12$_H$1 CL10790.Contig11$_H$1
Mean Expression Ratio	1.51
Median Expression Ratio	1.51
Credible Interval	(0.831, 2.78)
Associated Peptides	7
Associated Spectra	12
Coverage	NaN

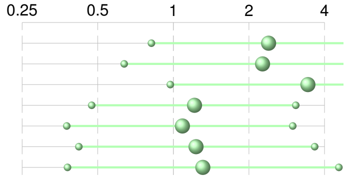

A	B	2.5	50	97.5	Sequence
1	1	0.82	2.4	7	KPVVIAK
0	1	0.64	2.3	7.8	QKFEEAEAHFYAR
0	1	0.98	3.4	12	TIQKDGLTWGAAK
1	2	0.47	1.2	3.1	VAALEVVGTGGASPK
0	2	0.38	1.1	3	KVTSDLR
1	1	0.42	1.2	3.6	LLEEYAAK
0	1	0.38	1.3	4.6	RLEEIEKENK

5.77 194 904 Aldo-keto reductase family 1 member B10 [Crassostrea gigas]

Protein Accession	CL3558.Contig4$_H$1
Mean Expression Ratio	0.662
Median Expression Ratio	0.665
Credible Interval	(0.294, 1.44)
Associated Peptides	1
Associated Spectra	3
Coverage	NaN

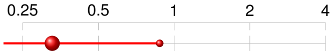

A	B	2.5	50	97.5	Sequence
2	1	0.13	0.33	0.88	ALEVLVSEGK

5.78 156 1163 cathepsin B [Crassostrea ariakensis]

Protein Accession	CL26836.Contig9$_H$1
Mean Expression Ratio	1.5
Median Expression Ratio	1.5
Credible Interval	(0.761, 2.97)
Associated Peptides	5
Associated Spectra	7
Coverage	NaN

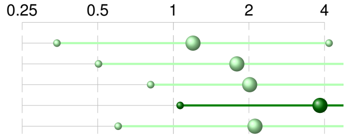

A	B	2.5	50	97.5	Sequence
0	1	0.34	1.2	4.2	GEQNIMEELYTR
0	1	0.5	1.8	6.4	TKWPNCPTLKEVR
0	3	0.81	2	4.9	DLPDSFDPR
0	1	1.1	3.8	13	LTDEEIFFINK
0	1	0.6	2.1	7.5	LPDSFDPR

5.79 196 657 superoxide dismutase [Aplysia californica] gi; 690 1151 superoxide dismutase [Aplysia californica] gi

Protein Accession	Unigene18046$_{H}1$ Unigene150$_{H}1$
Mean Expression Ratio	1.5
Median Expression Ratio	1.5
Credible Interval	(0.794, 2.88)
Associated Peptides	4
Associated Spectra	12
Coverage	NaN

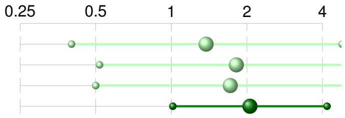

A	B	2.5	50	97.5	Sequence
0	1	0.4	1.4	4.8	THGGPDDVERHAGDLGNIR
0	1	0.52	1.8	6.4	AVCVLK
0	1	0.5	1.7	6	AGDLGNIR
5	4	1	2.1	4.2	SLVVHEK

5.80 3 116 Paramyosin [Crassostrea gigas]; 3 107 minus strand Paramyosin [Crassostrea gigas]

Protein Accession	CL43455.Contig7$_{H}1$ CL43455.Contig13$_{H}1$
Mean Expression Ratio	1.49
Median Expression Ratio	1.5
Credible Interval	(0.684, 3.32)
Associated Peptides	2
Associated Spectra	2
Coverage	NaN

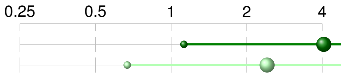

A	B	2.5	50	97.5	Sequence
0	1	1.1	4.1	15	AQTLIEEAENRADSAEK
0	1	0.67	2.4	8.4	NLQVVR

5.81 14 1642 minus strand chaperonin containing tcp1 [Haliotis discus discus]; 15 1619 chaperonin containing tcp1 [Haliotis discus discus]; 15 1619 chaperonin containing tcp1 [Haliotis discus discus]; 14 1462 minus strand chaperonin containing tcp1 [Haliotis discus discus]; 14 1582 minus strand chaperonin containing tcp1 [Haliotis discus discus]

Protein Accession	CL16298.Contig4$_{H}1$ CL16298.Contig2$_{H}1$ CL16298.Contig1$_{H}1$ CL16298.Contig15$_{H}1$ CL162
Mean Expression Ratio	0.674
Median Expression Ratio	0.677
Credible Interval	(0.308, 1.45)
Associated Peptides	2
Associated Spectra	3
Coverage	NaN

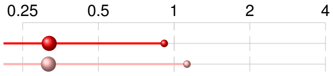

A	B	2.5	50	97.5	Sequence
2	0	0.11	0.32	0.91	QVEGDITK
1	0	0.09	0.32	1.1	NAEELMSFSK

5.82 194 1996 minus strand Neutral and basic amino acid transport protein rBAT [Crassostrea gigas]; 265 2067 minus strand Neutral and basic amino acid transport protein rBAT [Crassostrea gigas]; 194 1996 minus strand Neutral and basic amino acid transport protein rBAT [Crassostrea gigas]; 194 1996 minus strand Neutral and basic amino acid transport protein rBAT [Crassostrea gigas]; 265 2067 minus strand Neutral and basic amino acid transport protein rBAT [Crassostrea gigas]

Protein Accession	CL9047.Contig9$_H$1 CL9047.Contig8$_H$1 CL9047.Contig6$_H$1 CL9047.Contig15$_H$1 CL9047.Co
Mean Expression Ratio	1.48
Median Expression Ratio	1.48
Credible Interval	(0.719, 3)
Associated Peptides	3
Associated Spectra	8
Coverage	NaN

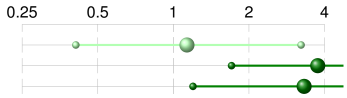

A	B	2.5	50	97.5	Sequence
1	1	0.41	1.1	3.2	NFAGNLDVVDALR
0	4	1.7	3.8	8.4	TAVDDLDNKPGREK
0	2	1.2	3.3	9.2	TITPMQWSK

5.83 3 434 minus strand hypothetical protein CGI_10015501 [Crassostrea gigas]

Protein Accession	Unigene6481$_H$1
Mean Expression Ratio	0.675
Median Expression Ratio	0.679
Credible Interval	(0.286, 1.57)
Associated Peptides	1
Associated Spectra	1
Coverage	NaN

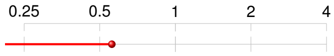

A	B	2.5	50	97.5	Sequence
1	0	0.044	0.16	0.56	NDFIGGLDVMK

5.84 1271 1663 minus strand PREDICTED: similar to AGAP007393-PB [Tribolium castaneum] gi

Protein Accession	CL28563.Contig3$_H$1
Mean Expression Ratio	1.48
Median Expression Ratio	1.47
Credible Interval	(0.683, 3.25)
Associated Peptides	2
Associated Spectra	2
Coverage	NaN

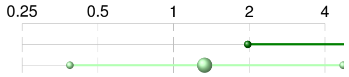

A	B	2.5	50	97.5	Sequence
0	1	2	6.8	24	KYEGGREVDEFITYLAR
0	1	0.39	1.3	4.7	EVDEFITYLAR

5.85 1 864 minus strand Pyruvate kinase isozymes M1/M2 [Crassostrea gigas]; 3 959 minus strand Pyruvate kinase isozymes M1/M2 [Crassostrea gigas]; 24 899 minus strand Pyruvate kinase isozymes M1/M2 [Crassostrea gigas]

Protein Accession	CL24469.Contig6$_{H}1$ CL24469.Contig3$_{H}1$ CL24469.Contig2$_{H}1$
Mean Expression Ratio	0.678
Median Expression Ratio	0.68
Credible Interval	(0.289, 1.57)
Associated Peptides	1
Associated Spectra	1
Coverage	NaN

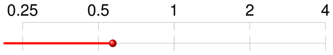

A	B	2.5	50	97.5	Sequence
1	0	0.045	0.16	0.57	NFDEILK

5.86 109 510 minus strand hypothetical protein DAPPUDRAFT_231281 [Daphnia pulex]

Protein Accession	Unigene4945$_{H}1$
Mean Expression Ratio	1.46
Median Expression Ratio	1.47
Credible Interval	(0.82, 2.59)
Associated Peptides	7
Associated Spectra	32
Coverage	NaN

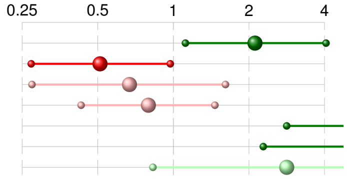

A	B	2.5	50	97.5	Sequence
1	6	1.1	2.1	4.1	LAGIEDPFEMNVK
2	5	0.27	0.51	0.97	VSMPDTLGHAVGIER
1	2	0.27	0.67	1.6	GTVDEPTLIPSLYEK
3	5	0.43	0.79	1.5	YELLAK
0	3	2.8	6.9	17	AKGTVDEPTLIPSLYEK
0	3	2.3	5.5	13	IASDKVSMPDTLGHAVGIER
0	1	0.83	2.8	9.8	LAGIEDPFEMNVKK

5.87 137 1369 hypothetical protein TcasGA2_TC002962 [Tribolium castaneum]

Protein Accession	CL20015.Contig2$_{H}1$
Mean Expression Ratio	1.47
Median Expression Ratio	1.46
Credible Interval	(0.685, 3.23)
Associated Peptides	2
Associated Spectra	4
Coverage	NaN

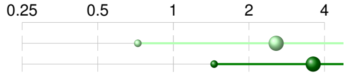

A	B	2.5	50	97.5	Sequence
0	1	0.72	2.6	9.5	AAAAEPAVVENK
0	3	1.5	3.6	8.9	AEAAEAK

5.88 362 874 minus strand Transgelin-2 [Crassostrea gigas]

Protein Accession	CL445.Contig3$_{H}1$
Mean Expression Ratio	1.46
Median Expression Ratio	1.46
Credible Interval	(0.833, 2.6)
Associated Peptides	8
Associated Spectra	31
Coverage	NaN

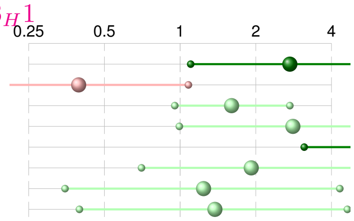

A	B	2.5	50	97.5	Sequence
2	1	1.1	2.7	6.5	KAGLGAEIEK
2	0	0.14	0.39	1.1	LLESEGKPK
6	5	0.95	1.6	2.7	AGLGAEIEK
0	2	0.99	2.8	8	AKKAGLGAEIEK
0	9	3.1	5.5	9.8	LINFD
0	2	0.7	1.9	5.3	LINKLLESEGKPK
0	1	0.35	1.2	4.3	SLGFLANSK
0	1	0.4	1.4	4.6	GFQPSYTGSQSR

5.89 135 2630 Filamin-A [Crassostrea gigas]; 118 2613 Filamin-A [Crassostrea gigas]; 157 2652 Filamin-A [Crassostrea gigas]

Protein Accession	CL393.Contig15$_H$1 CL393.Contig13$_H$1 CL393.Contig12$_H$1
Mean Expression Ratio	1.46
Median Expression Ratio	1.46
Credible Interval	(0.858, 2.48)
Associated Peptides	12
Associated Spectra	19
Coverage	NaN

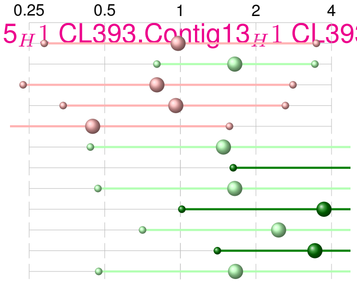

A	B	2.5	50	97.5	Sequence
1	0	0.29	0.98	3.5	ENGVHYVDVK
2	3	0.81	1.6	3.4	MNGIHTLSVR
1	0	0.24	0.81	2.8	VQVGGPGLEK
1	1	0.34	0.96	2.6	VINFQIETK
1	0	0.13	0.45	1.6	VYGVRY
0	1	0.44	1.5	5.2	AQIFLEERPNGFLGVQYK
0	1	1.6	5.6	19	SKGEYKVNVTR
0	1	0.47	1.6	5.8	YGNIPIAGSPYK
0	1	1	3.7	13	YIPKENGVHYVDVK
0	1	0.71	2.5	8.3	EAGPGTLTVAVEGPGK
0	3	1.4	3.4	8.1	IMFTPK
0	1	0.47	1.7	5.7	YKGNPIK

5.90 " 19 1167 minus strand ureidopropionase, beta [Xenopus (Silurana) tropicalis] gi"

Protein Accession	CL15113.Contig4$_H$1
Mean Expression Ratio	0.683
Median Expression Ratio	0.685
Credible Interval	(0.349, 1.36)
Associated Peptides	5
Associated Spectra	7
Coverage	NaN

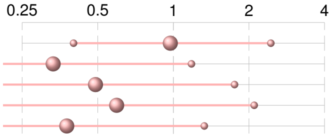

A	B	2.5	50	97.5	Sequence
2	1	0.4	0.97	2.4	KLAVPER
1	0	0.092	0.33	1.2	AESLALER
0	1	0.14	0.49	1.8	YVPLEELGSLK
0	1	0.17	0.6	2.1	MAGQIENIEQIIEK
0	1	0.11	0.38	1.3	AGGQIENIEQIIEK

5.91 350 910 Peptidyl-prolyl cis-trans isomerase B [Crassostrea gigas]; 488 1039 Peptidyl-prolyl cis-trans isomerase B [Crassostrea gigas]; 488 1039 Peptidyl-prolyl cis-trans isomerase B [Crassostrea gigas]

Protein Accession	CL49248.Contig5$_H$1 CL49248.Contig2$_H$1 CL49248.Contig1$_H$1
Mean Expression Ratio	0.688
Median Expression Ratio	0.686
Credible Interval	(0.33, 1.46)
Associated Peptides	3
Associated Spectra	3
Coverage	NaN

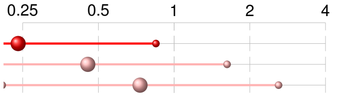

A	B	2.5	50	97.5	Sequence
0	1	0.068	0.24	0.85	HVAFGQIIDK
0	1	0.13	0.45	1.6	ASLEFLDK
0	1	0.21	0.73	2.6	AVLDFLIKN

5.92 252 848 sigma class glutathione-s-transferase 2 [Haliotis discus discus]

Protein Accession	CL19285.Contig21$_{H}1$
Mean Expression Ratio	0.687
Median Expression Ratio	0.687
Credible Interval	(0.326, 1.43)
Associated Peptides	2
Associated Spectra	7
Coverage	NaN

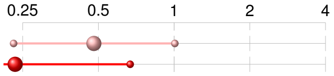

A	B	2.5	50	97.5	Sequence
1	4	0.23	0.48	1	LQDVDKDYPEVQK
2	0	0.083	0.23	0.67	LGNELTMA

5.93 95 2506 Transitional endoplasmic reticulum ATPase [Crassostrea gigas]

Protein Accession	CL15973.Contig2$_{H}1$
Mean Expression Ratio	0.689
Median Expression Ratio	0.689
Credible Interval	(0.422, 1.14)
Associated Peptides	16
Associated Spectra	22
Coverage	NaN

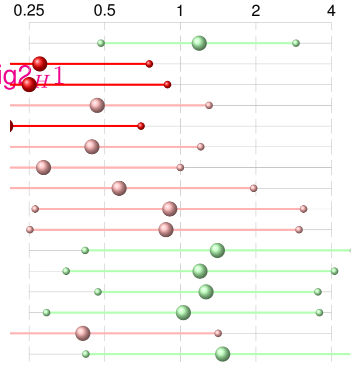

A	B	2.5	50	97.5	Sequence
1	2	0.48	1.2	2.9	AHFEEAMK
1	1	0.1	0.28	0.75	DIDLDYLAK
1	0	0.072	0.25	0.89	EMVELPLR
1	1	0.17	0.47	1.3	MAIEAEIR
1	0	0.059	0.2	0.7	AIGVKPPR
1	1	0.16	0.44	1.2	HPQLFK
1	0	0.084	0.29	1	GFGGNFR
1	0	0.17	0.57	2	LQQIR
0	1	0.26	0.91	3.1	AEVQNEDDLATAILK
0	1	0.25	0.88	3	GILLYGPPGTGK
0	1	0.42	1.4	4.9	KYEMFAQTLQQSR
0	1	0.35	1.2	4.1	MDELQLFR
0	2	0.47	1.3	3.5	KGDIFLVR
0	1	0.29	1	3.6	KAFEEAEK
0	1	0.12	0.41	1.4	EVDIGIPDATGR
0	1	0.42	1.5	5.1	GGNVGDGGGAADR

5.94 186 1778 predicted protein [Nematostella vectensis] gi

Protein Accession	CL20488.Contig9$_{H}1$
Mean Expression Ratio	0.69
Median Expression Ratio	0.689
Credible Interval	(0.388, 1.24)
Associated Peptides	8
Associated Spectra	17
Coverage	NaN

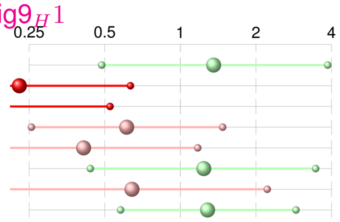

A	B	2.5	50	97.5	Sequence
1	1	0.49	1.4	3.9	ATNLENKDGLLGK
2	0	0.082	0.23	0.63	FAQQAQQQEK
1	0	0.042	0.15	0.52	TKDGEVSWPVIDPK
1	2	0.25	0.61	1.5	SDPYLQISSK
1	1	0.15	0.41	1.2	EGTENVEMHFR
0	2	0.44	1.2	3.4	AKDGTTHVIWK
0	1	0.19	0.64	2.2	DGTTHVIWK
0	4	0.58	1.3	2.9	AEELKEGTENVEMHFR

5.95 11 244 minus strand Tubulin alpha chain (Fragment) OS=Lytechinus pictus PE=3 SV=1

Protein Accession	CL3047.Contig6$_{H}1$
Mean Expression Ratio	0.686
Median Expression Ratio	0.69
Credible Interval	(0.312, 1.48)
Associated Peptides	2
Associated Spectra	5
Coverage	NaN

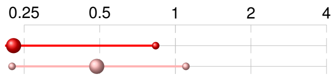

A	B	2.5	50	97.5	Sequence
0	1	0.063	0.23	0.83	EDLAALEK
0	4	0.22	0.49	1.1	FDLMYAK

5.96 27 515 heat shock protein 70 [Capsaspora owczarzaki ATCC 30864]; 30 464 minus strand hypothetical protein [Trichinella spiralis]; 27 491 minus strand heat shock protein 70 [Capsaspora owczarzaki ATCC 30864]

Protein Accession	CL14449.Contig4$_{H}1$ CL14449.Contig2$_{H}1$ CL14449.Contig1$_{H}1$
Mean Expression Ratio	0.692
Median Expression Ratio	0.692
Credible Interval	(0.336, 1.44)
Associated Peptides	3
Associated Spectra	7
Coverage	NaN

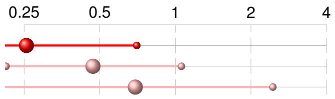

A	B	2.5	50	97.5	Sequence
2	0	0.091	0.26	0.7	FDDPSVQSDMK
3	1	0.21	0.47	1.1	NQVAMNPENTVFDAK
0	1	0.19	0.69	2.5	RFDDPSVQSDMK

5.97 160 1218 Galectin-9 [Crassostrea gigas]

Protein Accession	Unigene165$_{H}1$
Mean Expression Ratio	0.69
Median Expression Ratio	0.692
Credible Interval	(0.339, 1.38)
Associated Peptides	3
Associated Spectra	10
Coverage	NaN

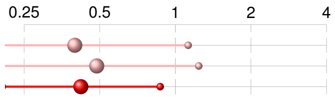

A	B	2.5	50	97.5	Sequence
1	1	0.14	0.4	1.1	EATALVVTGDVR
1	2	0.2	0.49	1.2	IAVNNQHLVEFR
3	2	0.2	0.42	0.87	SGIPGGLAPGR

5.98 " 242 646 minus strand Fatty acid-binding protein, heart [Crassostrea gigas]; 227 631 minus strand Fatty acid-binding protein, heart [Crassostrea gigas]; 265 669 Fatty acid-binding protein, heart [Crassostrea gigas]"

Protein Accession	CL7643.Contig9$_{H}$1 CL7643.Contig11$_{H}$1 CL7643.Contig10$_{H}$1
Mean Expression Ratio	1.45
Median Expression Ratio	1.44
Credible Interval	(0.833, 2.56)
Associated Peptides	8
Associated Spectra	43
Coverage	NaN

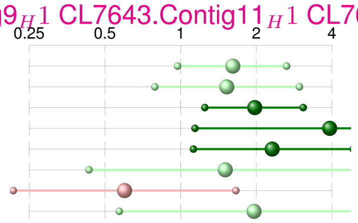

A	B	2.5	50	97.5	Sequence
3	9	0.98	1.6	2.6	AINNGQMIMTMK
3	3	0.79	1.5	3	ATSGVEFESR
6	9	1.2	2	3.1	CGDVTSIR
1	0	1.1	3.9	13	YLDGREIK
2	3	1.1	2.3	4.8	KYDKV
0	1	0.43	1.5	5.2	TVFRVEGDTLIEDVSGPFSTSIVR
0	2	0.22	0.6	1.7	VEGDTLIEDVSGPFSTSIVR
0	1	0.57	2	6.9	AALNGKWK

5.99 103 684 EF-hand domain-containing protein D2 [Crassostrea gigas]

Protein Accession	CL20544.Contig1$_{H}$1
Mean Expression Ratio	0.687
Median Expression Ratio	0.694
Credible Interval	(0.313, 1.47)
Associated Peptides	2
Associated Spectra	3
Coverage	NaN

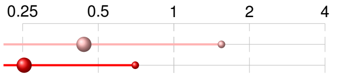

A	B	2.5	50	97.5	Sequence
1	0	0.12	0.44	1.5	VPQTHLALK
2	0	0.092	0.25	0.7	KQIQDFQK

5.100 124 390 PREDICTED: 60S ribosomal protein L23a-like [Callithrix jacchus]

Protein Accession	CL27563.Contig5$_{H}$1
Mean Expression Ratio	1.44
Median Expression Ratio	1.44
Credible Interval	(0.674, 3.08)
Associated Peptides	2
Associated Spectra	4
Coverage	NaN

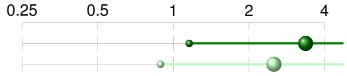

A	B	2.5	50	97.5	Sequence
0	2	1.2	3.4	9.5	AAPKPAVK
0	2	0.89	2.5	7	VNRLDAFAIVK

5.101 248 1018 Aldo-keto reductase family 1 member B10 [Crassostrea gigas]; 44 730 minus strand Aldo-keto reductase family 1 member B10 [Crassostrea gigas]; 194 904 Aldo-keto reductase family 1 member B10 [Crassostrea gigas]; 580 1287 Aldo-keto reductase family 1 member B10 [Crassostrea gigas]; 675 1382 minus strand Aldo-keto reductase family 1 member B10 [Crassostrea gigas]

Protein Accession	Unigene68551$_{H}$1 Unigene37546$_{H}$1 CL3558.Contig4$_{H}$1 CL3558.Contig3$_{H}$1 CL3558.Contig1
Mean Expression Ratio	1.44
Median Expression Ratio	1.44
Credible Interval	(0.67, 3.1)
Associated Peptides	2
Associated Spectra	4
Coverage	NaN

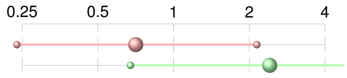

A	B	2.5	50	97.5	Sequence
2	1	0.24	0.71	2.1	ALEVLVSEGK
0	1	0.68	2.4	8.8	DEGYR

5.102 209 781 minus strand high mobility group protein 1 [Biomphalaria glabrata]; 277 771 high mobility group protein 1 [Biomphalaria glabrata]; 277 867 minus strand high mobility group protein 1 [Biomphalaria glabrata]; 277 870 minus strand high mobility group protein 1 [Biomphalaria glabrata]

Protein Accession	CL25076.Contig4$_{H}$1 CL25076.Contig3$_{H}$1 CL25076.Contig2$_{H}$1 CL25076.Contig1$_{H}$1
Mean Expression Ratio	1.44
Median Expression Ratio	1.44
Credible Interval	(0.754, 2.68)
Associated Peptides	5
Associated Spectra	12
Coverage	NaN

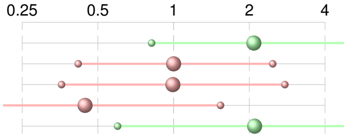

A	B	2.5	50	97.5	Sequence
1	2	0.82	2.1	5.2	KYEAEMAEYR
2	2	0.42	1	2.5	LAGEEWR
2	1	0.36	1	2.8	KPFEDMAAK
0	1	0.13	0.44	1.5	LGDFR
0	1	0.6	2.1	7.3	ELPAGQKKPFEDMAAKDKAR

5.103 80 589 minus strand PREDICTED: adenine phosphoribosyltransferase-like [Strongylocentrotus purpuratus]

Protein Accession	CL8682.Contig6$_{H}$1
Mean Expression Ratio	1.44
Median Expression Ratio	1.43
Credible Interval	(0.676, 3.06)
Associated Peptides	2
Associated Spectra	4
Coverage	NaN

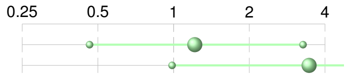

A	B	2.5	50	97.5	Sequence
1	2	0.46	1.2	3.3	AAVELVEK
0	1	0.98	3.5	12	GKLTVPVESLIKY

5.104 " 53 1291 Acetyl-CoA acetyltransferase B, mitochondrial [Crassostrea gigas]"

Protein Accession	CL1322.Contig1$_{H}1$
Mean Expression Ratio	1.43
Median Expression Ratio	1.42
Credible Interval	(0.664, 3.14)
Associated Peptides	2
Associated Spectra	3
Coverage	NaN

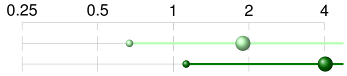

A	B	2.5	50	97.5	Sequence
**0**	**2**	**0.67**	**1.9**	**5.3**	**TLKPVFVK**
**0**	**1**	**1.1**	**4**	**14**	**LGSVAIKEAVSR**

5.105 119 481 Glutamate receptor U1 [Crassostrea gigas]; 113 328 glutamate receptor subunit protein GluR2 precursor [Aplysia californica] gi; 122 328 PREDICTED: glutamate receptor 3 [Ornithorhynchus anatinus]

Protein Accession	CL570.Contig5$_{H}1$ CL570.Contig4$_{H}1$ CL570.Contig2$_{H}1$
Mean Expression Ratio	0.704
Median Expression Ratio	0.703
Credible Interval	(0.312, 1.59)
Associated Peptides	1
Associated Spectra	2
Coverage	NaN

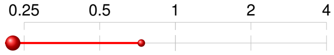

A	B	2.5	50	97.5	Sequence
**1**	**1**	**0.07**	**0.22**	**0.73**	**GTANIGAGALTVTSER**

5.106 816 1211 H2 [Haliotis tuberculata]

Protein Accession	CL11519.Contig5$_{H}1$
Mean Expression Ratio	0.698
Median Expression Ratio	0.703
Credible Interval	(0.342, 1.4)
Associated Peptides	3
Associated Spectra	10
Coverage	NaN

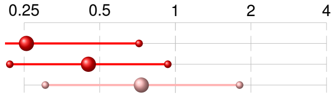

A	B	2.5	50	97.5	Sequence
**1**	**1**	**0.09**	**0.26**	**0.72**	**LDSSADFDLQVK**
**1**	**4**	**0.22**	**0.45**	**0.93**	**SLILAMR**
**1**	**2**	**0.3**	**0.73**	**1.8**	**KNVLSLTPAER**

5.107 " 42 905 PREDICTED: enoyl-CoA hydratase, mitochondrial-like isoform 1 [Oreochromis niloticus]"

Protein Accession	CL6477.Contig7$_{H}1$
Mean Expression Ratio	0.704
Median Expression Ratio	0.703
Credible Interval	(0.356, 1.37)
Associated Peptides	4
Associated Spectra	13
Coverage	NaN

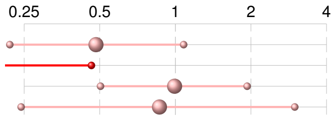

A	B	2.5	50	97.5	Sequence
**2**	**2**	**0.22**	**0.48**	**1.1**	**AFAAGADIK**
**1**	**1**	**0.057**	**0.16**	**0.46**	**LLDEAVK**
**4**	**2**	**0.5**	**0.99**	**1.9**	**SVVLQASR**
**0**	**1**	**0.24**	**0.86**	**3**	**AMEMVLTGER**

5.108 " 45 506 minus strand PREDICTED: dihydrolipoyl dehydrogenase, mitochondrial [Monodelphis domestica]"

Protein Accession	CL24292.Contig3$_{H}1$
Mean Expression Ratio	0.703
Median Expression Ratio	0.705
Credible Interval	(0.325, 1.5)
Associated Peptides	2
Associated Spectra	3
Coverage	NaN

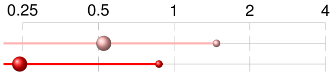

A	B	2.5	50	97.5	Sequence
1	1	0.19	0.52	1.5	GIEIDGLR
1	0	0.071	0.24	0.87	AAQLGMK

5.109 1009 2784 minus strand Collagen alpha-3(VI) chain [Crassostrea gigas]; 1009 2787 minus strand Collagen alpha-3(VI) chain [Crassostrea gigas]

Protein Accession	Unigene26554$_{H}1$ Unigene26550$_{H}1$
Mean Expression Ratio	0.705
Median Expression Ratio	0.705
Credible Interval	(0.375, 1.35)
Associated Peptides	6
Associated Spectra	10
Coverage	NaN

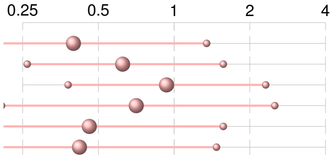

A	B	2.5	50	97.5	Sequence
1	0	0.11	0.4	1.3	ELEGNVHSIR
3	0	0.26	0.62	1.6	IITVGIGEANR
3	0	0.38	0.93	2.3	SDAAAALIR
1	0	0.21	0.7	2.5	VAMSNFR
1	0	0.13	0.46	1.6	MFIPVR
1	0	0.12	0.42	1.5	LTTVR

5.110 111 530 minus strand Nucleoside diphosphate kinase B OS=Homo sapiens GN=NME2 PE=1 SV=1

Protein Accession	CL22280.Contig3$_{H}1$
Mean Expression Ratio	1.41
Median Expression Ratio	1.41
Credible Interval	(0.709, 2.83)
Associated Peptides	4
Associated Spectra	7
Coverage	NaN

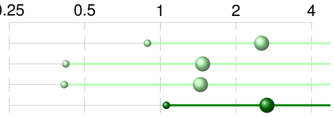

A	B	2.5	50	97.5	Sequence
0	2	0.89	2.5	7.4	GDFCIEMGR
0	1	0.42	1.5	5.2	NICHGSDEVK
0	1	0.41	1.4	5	NICHGSDEVKNAEKEIK
0	3	1.1	2.7	6.6	VMLGETNPLNSKPGTIR

5.111 112 819 pyruvate kinase [Crassostrea gigas]; 132 932 pyruvate kinase [Crassostrea gigas]

Protein Accession	CL31920.Contig3$_{H}1$ CL31920.Contig1$_{H}1$
Mean Expression Ratio	0.705
Median Expression Ratio	0.708
Credible Interval	(0.323, 1.52)
Associated Peptides	2
Associated Spectra	3
Coverage	NaN

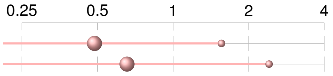

A	B	2.5	50	97.5	Sequence
1	1	0.16	0.49	1.6	ITIDDSYKEK
1	0	0.18	0.65	2.4	MIMEGMNIAR

5.112 316 1368 minus strand hypothetical protein CGI_10006559 [Crassostrea gigas]

Protein Accession	Unigene26912$_H$1
Mean Expression Ratio	0.706
Median Expression Ratio	0.709
Credible Interval	(0.319, 1.54)
Associated Peptides	2
Associated Spectra	2
Coverage	NaN

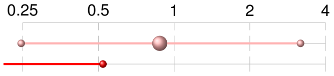

A	B	2.5	50	97.5	Sequence
0	1	0.25	0.87	3.2	TLDDLVR
0	1	0.041	0.15	0.52	IVGSWLAGQER

5.113 150 473 40S ribosomal protein S16 OS=Heteropneustes fossilis GN=rps16 PE=2 SV=1

Protein Accession	CL14629.Contig4$_H$1
Mean Expression Ratio	0.705
Median Expression Ratio	0.709
Credible Interval	(0.37, 1.32)
Associated Peptides	5
Associated Spectra	13
Coverage	NaN

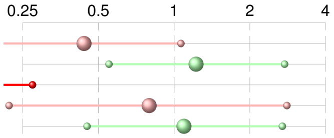

A	B	2.5	50	97.5	Sequence
2	1	0.18	0.44	1.1	SLVAYYQK
2	2	0.55	1.2	2.7	AVQVFGR
2	0	0.035	0.096	0.27	ILRAK
1	0	0.22	0.79	2.8	AKLEEPVR
0	3	0.45	1.1	2.7	VNGRPLDQVEPQILR

5.114 736 1287 60S ribosomal protein L3 OS=Rattus norvegicus GN=Rpl3 PE=1 SV=3

Protein Accession	Unigene22848$_H$1
Mean Expression Ratio	0.707
Median Expression Ratio	0.71
Credible Interval	(0.334, 1.46)
Associated Peptides	3
Associated Spectra	4
Coverage	NaN

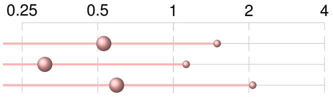

A	B	2.5	50	97.5	Sequence
1	1	0.19	0.53	1.5	VQFTVAR
1	0	0.084	0.31	1.1	EIETDLNK
1	0	0.17	0.59	2.1	IGAGIHTK

5.115 9 722 minus strand actin related protein [Lumbricus rubellus]

Protein Accession	CL45325.Contig2$_H$1
Mean Expression Ratio	1.4
Median Expression Ratio	1.41
Credible Interval	(0.633, 3.09)
Associated Peptides	2
Associated Spectra	2
Coverage	NaN

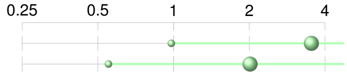

A	B	2.5	50	97.5	Sequence
0	1	0.98	3.5	13	ISAEEFLKNF
0	1	0.55	2	7	LAVNSRAR

5.116 587 811 hemocyanin 2 [Megathura crenulata]

Protein Accession	Unigene4334$_{H}1$
Mean Expression Ratio	0.705
Median Expression Ratio	0.71
Credible Interval	(0.326, 1.51)
Associated Peptides	2
Associated Spectra	5
Coverage	NaN

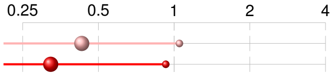

A	B	2.5	50	97.5	Sequence
2	1	0.18	0.43	1.1	AIDDGTFR
0	2	0.11	0.32	0.93	SVEDLTAGEAESLK

5.117 401 1174 minus strand Mechanosensory protein 2 [Crassostrea gigas]

Protein Accession	Unigene7703$_{H}1$
Mean Expression Ratio	1.4
Median Expression Ratio	1.4
Credible Interval	(0.735, 2.7)
Associated Peptides	5
Associated Spectra	10
Coverage	NaN

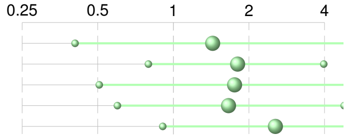

A	B	2.5	50	97.5	Sequence
1	0	0.4	1.4	4.9	AMAAEAEASR
2	2	0.79	1.8	4	VIAAEGEQK
1	0	0.51	1.8	6.3	AMAAEAEASREAR
1	1	0.6	1.7	4.8	LPVQLQR
1	1	0.91	2.6	7	AKVIAAEGEQK

5.118 2 436 minus strand PREDICTED: stress-induced-phosphoprotein 1 [Sarcophilus harrisii]

Protein Accession	CL38065.Contig3$_{H}1$
Mean Expression Ratio	1.39
Median Expression Ratio	1.4
Credible Interval	(0.678, 2.84)
Associated Peptides	3
Associated Spectra	7
Coverage	NaN

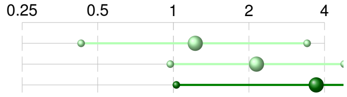

A	B	2.5	50	97.5	Sequence
1	1	0.43	1.2	3.4	IGNSYMR
0	4	0.97	2.1	4.8	ADFTAIAK
0	1	1	3.7	13	LAYVNP

5.119 2019 2723 minus strand Kyphoscoliosis peptidase [Crassostrea gigas]; 2060 2764 minus strand Kyphoscoliosis peptidase [Crassostrea gigas]; 1074 1778 minus strand Kyphoscoliosis peptidase [Crassostrea gigas]; 2063 2767 minus strand Kyphoscoliosis peptidase [Crassostrea gigas]; 2044 2748 minus strand Kyphoscoliosis peptidase [Crassostrea gigas]; 2044 2748 minus strand Kyphoscoliosis peptidase [Crassostrea gigas]

Protein Accession	CL3923.Contig7$_H$1 CL3923.Contig6$_H$1 CL3923.Contig5$_H$1 CL3923.Contig4$_H$1 CL3923.Con
Mean Expression Ratio	1.4
Median Expression Ratio	1.4
Credible Interval	(0.648, 3.03)
Associated Peptides	2
Associated Spectra	5
Coverage	NaN

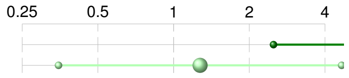

A	B	2.5	50	97.5	Sequence
**2**	**2**	**2.5**	**5.6**	**13**	**TMGQAPPTVPKDFQLK**
**0**	**1**	**0.35**	**1.3**	**4.7**	**TMGQAPPTVPK**

5.120 551 871 minus strand Protein disulfide-isomerase A4 [Crassostrea gigas]

Protein Accession	CL20258.Contig1$_H$1
Mean Expression Ratio	0.713
Median Expression Ratio	0.716
Credible Interval	(0.305, 1.61)
Associated Peptides	1
Associated Spectra	1
Coverage	NaN

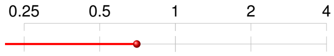

A	B	2.5	50	97.5	Sequence
**1**	**0**	**0.054**	**0.19**	**0.7**	**EGPEPILLAK**

5.121 362 553 putative RNA-binding protein [Haliotis diversicolor]

Protein Accession	CL41007.Contig2$_H$1
Mean Expression Ratio	1.39
Median Expression Ratio	1.39
Credible Interval	(0.73, 2.62)
Associated Peptides	5
Associated Spectra	16
Coverage	NaN

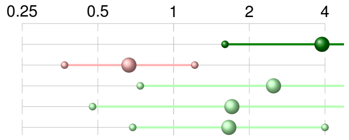

A	B	2.5	50	97.5	Sequence
**1**	**2**	**1.6**	**3.9**	**9.4**	**VRELQDETGCR**
**2**	**6**	**0.37**	**0.67**	**1.2**	**EVMHVDSSFVGR**
**0**	**1**	**0.74**	**2.5**	**8.7**	**DGGAKEVMHVDSSFVGR**
**0**	**1**	**0.48**	**1.7**	**5.9**	**DGGRDGGAKEVMHVDSSFVGR**
**0**	**3**	**0.69**	**1.7**	**4**	**ELQDETGCR**

5.122 187 696 minus strand putative ribosomal protein S10 [Sipunculus nudus]

Protein Accession	Unigene1233$_{H1}$
Mean Expression Ratio	1.39
Median Expression Ratio	1.39
Credible Interval	(0.72, 2.66)
Associated Peptides	5
Associated Spectra	12
Coverage	NaN

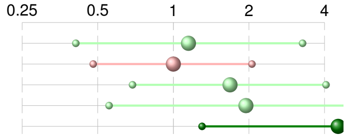

A	B	2.5	50	97.5	Sequence
0	2	0.41	1.1	3.3	ADVGAGATESFQFR
0	5	0.48	1	2.1	HQEIDVPNLHVIK
0	3	0.69	1.7	4	KDFFSPK
0	1	0.55	1.9	6.9	ALTSIK
0	1	1.3	4.5	16	KAGGPPGGDKKADVGAGATESFQFR

5.123 2295 2510 beta-thymosin domain repeat protein CSP24KDa_v1 [Hermissenda crassicornis] gi

Protein Accession	CL9548.Contig1$_{H1}$
Mean Expression Ratio	1.39
Median Expression Ratio	1.39
Credible Interval	(0.656, 2.97)
Associated Peptides	3
Associated Spectra	3
Coverage	NaN

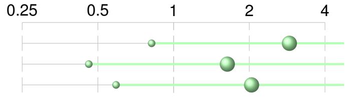

A	B	2.5	50	97.5	Sequence
0	1	0.82	2.9	10	ESVAAFDKSHLK
0	1	0.46	1.6	5.8	ESVAAFDK
0	1	0.59	2	7.2	AFDKSHLK

5.124 195 2201 minus strand hypothetical protein DAPPUDRAFT_315656 [Daphnia pulex]

Protein Accession	Unigene38538$_{H1}$
Mean Expression Ratio	0.721
Median Expression Ratio	0.721
Credible Interval	(0.384, 1.34)
Associated Peptides	6
Associated Spectra	12
Coverage	NaN

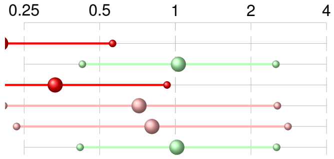

A	B	2.5	50	97.5	Sequence
2	0	0.071	0.2	0.56	GPQFEEIGK
1	2	0.43	1	2.5	VALSPAATQALVK
1	1	0.12	0.33	0.93	VQIEAGAGEEAK
1	0	0.21	0.72	2.5	EIFPNER
1	0	0.23	0.81	2.8	NMGAIVR
1	2	0.42	1	2.5	GSIVLHDGK

5.125 4 219 hypothetical protein BRAFLDRAFT_66351 [Branchiostoma floridae] gi; 3 239 hypothetical protein BRAFLDRAFT_66351 [Branchiostoma floridae] gi

Protein Accession	CL31114.Contig2$_{H1}$ CL31114.Contig1$_{H1}$
Mean Expression Ratio	1.39
Median Expression Ratio	1.38
Credible Interval	(0.67, 2.99)
Associated Peptides	3
Associated Spectra	3
Coverage	NaN

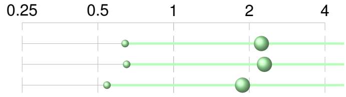

A	B	2.5	50	97.5	Sequence
0	1	0.64	2.2	8.1	KGSENVLAK
0	1	0.65	2.3	8.3	KLEEVSGELR
0	1	0.54	1.9	6.5	NNQLNGDLK

5.126 " 213 1499 minus strand Citrate synthase, mitochondrial [Crassostrea gigas]"

Protein Accession	CL992.Contig2$_H1$
Mean Expression Ratio	0.721
Median Expression Ratio	0.723
Credible Interval	(0.436, 1.18)
Associated Peptides	12
Associated Spectra	33
Coverage	NaN

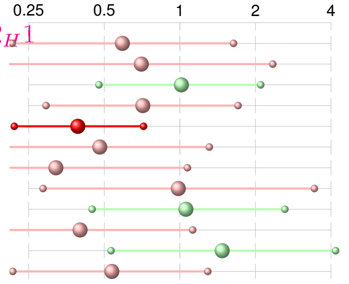

A	B	2.5	50	97.5	Sequence
1	1	0.21	0.59	1.6	ALGLPIERPK
1	0	0.2	0.7	2.3	AYSEGVNK
3	2	0.48	1	2.1	SFDTPSLR
1	2	0.29	0.71	1.7	SGQVVPGYGHAVLR
4	4	0.22	0.39	0.72	VVPDILIEQGK
1	1	0.17	0.48	1.3	DVLSQIIPEK
1	0	0.094	0.32	1.1	EVSEFR
1	0	0.28	0.98	3.4	AIIYR
0	3	0.45	1.1	2.6	ALVTETSVLDPEEGIR
0	2	0.14	0.4	1.1	HLPNDPMFK
0	2	0.53	1.5	4.2	VGEVTVDMMYGGMR
0	3	0.22	0.54	1.3	LISQLYK

5.127 151 1599 PREDICTED: galactosamine (N-acetyl)-6-sulfate sulfatase-like [Saccoglossus kowalevskii]

Protein Accession	CL13278.Contig2$_H1$
Mean Expression Ratio	1.39
Median Expression Ratio	1.38
Credible Interval	(0.653, 2.96)
Associated Peptides	2
Associated Spectra	3
Coverage	NaN

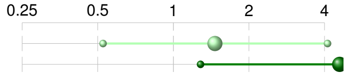

A	B	2.5	50	97.5	Sequence
0	2	0.52	1.5	4.1	GLVPSLISK
0	1	1.3	4.6	16	TTLWEGGTR

5.128 18 647 minus strand ATP synthase-like protein [Biomphalaria glabrata]

Protein Accession	CL42054.Contig4$_H1$
Mean Expression Ratio	1.38
Median Expression Ratio	1.38
Credible Interval	(0.782, 2.43)
Associated Peptides	9
Associated Spectra	17
Coverage	NaN

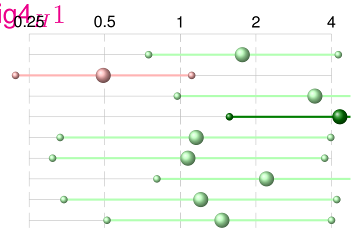

A	B	2.5	50	97.5	Sequence
1	2	0.75	1.8	4.3	YATALYSAASK
2	2	0.22	0.49	1.1	AVLEQALK
0	1	0.98	3.4	11	AVLEQALKQQK
0	2	1.6	4.3	12	GKLVTTPIQVFGIDGR
0	1	0.33	1.2	4	LAEFVANPSLQR
0	1	0.31	1.1	3.8	NFQNLLK
0	2	0.81	2.2	6.1	GEVVCTVK
0	1	0.34	1.2	4.2	KLAEFVANPSLQR
0	2	0.51	1.5	4	GALQGFLK

5.129 148 462 hypothetical protein DAPPUDRAFT_306954 [Daphnia pulex]; 148 462 hypothetical protein DAPPUDRAFT_306954 [Daphnia pulex]; 148 462 hypothetical protein DAPPUDRAFT_306954 [Daphnia pulex]; 148 462 hypothetical protein DAPPUDRAFT_306954 [Daphnia pulex]; 173 487 hypothetical protein DAPPUDRAFT_306954 [Daphnia pulex]

Protein Accession CL48682.Contig7$_{H}$1 CL48682.Contig6$_{H}$1 CL48682.Contig5$_{H}$1 CL48682.Contig4$_{H}$1 CL4868
Mean Expression Ratio 0.724
Median Expression Ratio 0.724
Credible Interval (0.346, 1.5)
Associated Peptides 3
Associated Spectra 4
Coverage NaN

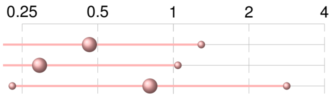

A	B	2.5	50	97.5	Sequence
1	1	0.17	0.46	1.3	ATNFDHFK
1	0	0.085	0.29	1	GAHDGGVIFDPAVQR
1	0	0.23	0.81	2.8	AGEVPYKDR

5.130 280 726 minus strand RecName: Full=Calmodulin; Short=CaM gi|14588595|dbj|BAB61794.1| calmodulin [Metridium senile] gi

Protein Accession Unigene4486$_{H}$1
Mean Expression Ratio 0.727
Median Expression Ratio 0.726
Credible Interval (0.339, 1.55)
Associated Peptides 1
Associated Spectra 6
Coverage NaN

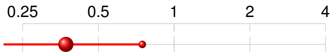

A	B	2.5	50	97.5	Sequence
5	1	0.18	0.37	0.75	ELGTVMR

5.131 448 1626 matrilin [Biomphalaria glabrata]

Protein Accession CL7510.Contig3$_{H}$1
Mean Expression Ratio 1.37
Median Expression Ratio 1.38
Credible Interval (0.65, 2.96)
Associated Peptides 2
Associated Spectra 5
Coverage NaN

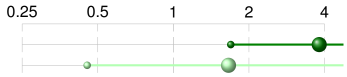

A	B	2.5	50	97.5	Sequence
3	1	1.7	3.8	8.5	VSAIKYGR
0	1	0.45	1.7	5.8	VSNTELLNIAGDQER

5.132 " 189 2099 Hsp70, putative [Ixodes scapularis] gi"

Protein Accession	CL16113.Contig2$_H1$
Mean Expression Ratio	1.38
Median Expression Ratio	1.38
Credible Interval	(0.695, 2.73)
Associated Peptides	4
Associated Spectra	7
Coverage	NaN

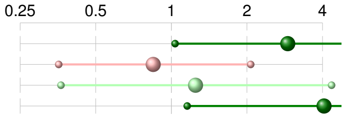

A	B	2.5	50	97.5	Sequence
1	1	1	2.9	8	LVGMPAKR
1	2	0.36	0.85	2.1	ALQDAEVK
0	1	0.36	1.3	4.3	ASNGDAWVEAQGK
0	1	1.2	4	15	RFEDPEVQKDMK

5.133 25 405 minus strand Elongation factor 1-delta [Anoplopoma fimbria]

Protein Accession	CL10502.Contig1$_H1$
Mean Expression Ratio	1.37
Median Expression Ratio	1.38
Credible Interval	(0.708, 2.72)
Associated Peptides	5
Associated Spectra	7
Coverage	NaN

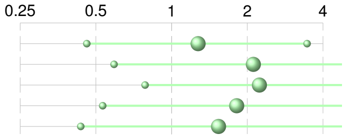

A	B	2.5	50	97.5	Sequence
1	1	0.46	1.3	3.5	KPVLIAK
0	1	0.59	2.1	7.3	SKKPVLIAK
0	2	0.78	2.2	6.2	VQTDGLVWGASK
0	1	0.53	1.8	6.4	RVQTDGLVWGASK
0	1	0.43	1.5	5.4	LVPVGYGINK

5.134 118 1866 Lamin Dm0 [Crassostrea gigas]

Protein Accession	CL4079.Contig7$_H1$
Mean Expression Ratio	1.37
Median Expression Ratio	1.37
Credible Interval	(0.883, 2.14)
Associated Peptides	19
Associated Spectra	36
Coverage	NaN

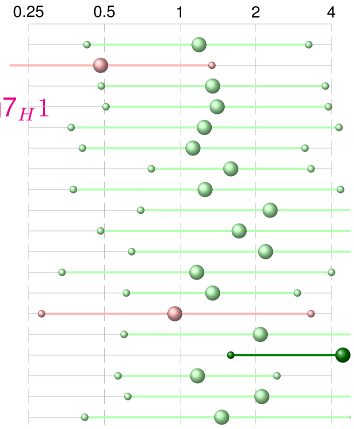

A	B	2.5	50	97.5	Sequence
1	1	0.43	1.2	3.3	KQLEEETLLR
1	1	0.17	0.48	1.3	LLEGEESR
1	1	0.49	1.3	3.8	LSEVEAPR
1	1	0.51	1.4	3.9	SLVIKPGK
1	0	0.37	1.2	4.3	LLEALR
0	2	0.41	1.1	3.1	EKHEFDLQTVR
0	5	0.77	1.6	3.3	GGIDIEEVDPEGK
0	1	0.38	1.3	4.3	LRAESSGFESR
0	1	0.7	2.3	7.8	RVETLEAQVAELTAK
0	1	0.48	1.7	5.7	SLYETELADAR
0	1	0.64	2.2	7.5	SLYETELADARR
0	1	0.34	1.2	4	VQSLKEDLHFK
0	4	0.61	1.3	2.9	WFVADEMK
0	1	0.28	0.95	3.3	YKTEYEDLLAK
0	1	0.6	2.1	7.2	IQIEANKYK
0	2	1.6	4.4	12	LLDETAKEK
0	5	0.57	1.2	2.4	IQIEANK
0	1	0.62	2.1	7.2	RLALKDDEIADLR
0	1	0.42	1.5	4.9	ADDLSAELAK

5.135 " 99 497 RecName: Full=Histone H2B, gonadal"

Protein Accession	CL29794.Contig1$_{H}$1
Mean Expression Ratio	1.38
Median Expression Ratio	1.37
Credible Interval	(0.795, 2.4)
Associated Peptides	5
Associated Spectra	44
Coverage	NaN

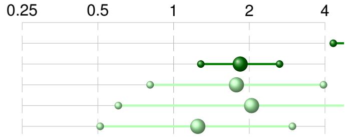

A	B	2.5	50	97.5	Sequence
3	3	4.3	9	18	AVTKYTSSK
8	21	1.3	1.8	2.6	LLLPGELAK
3	2	0.8	1.8	3.9	STITSREIQTAVR
0	1	0.6	2	7.1	LLPGELAK
0	3	0.51	1.3	3	KESYAIYIYK

5.136 110 2365 N-acetylated-alpha-linked acidic dipeptidase 2 [Crassostrea gigas]

Protein Accession	CL8322.Contig4$_{H}$1
Mean Expression Ratio	0.729
Median Expression Ratio	0.732
Credible Interval	(0.338, 1.6)
Associated Peptides	2
Associated Spectra	2
Coverage	NaN

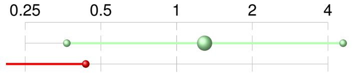

A	B	2.5	50	97.5	Sequence
0	1	0.37	1.3	4.6	ALGTPLMYR
0	1	0.035	0.12	0.44	AQRGTILNADGDPLTPGYPAVR

5.137 51 890 prohibitin [Octopus tankahkeei]

Protein Accession	CL26635.Contig2$_{H}$1
Mean Expression Ratio	1.37
Median Expression Ratio	1.36
Credible Interval	(0.771, 2.45)
Associated Peptides	9
Associated Spectra	12
Coverage	NaN

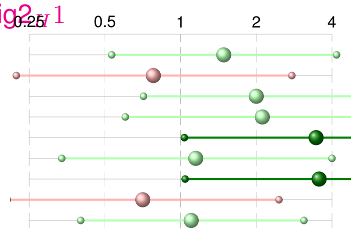

A	B	2.5	50	97.5	Sequence
1	1	0.53	1.5	4.2	AFTEAGEGLVELR
1	0	0.22	0.78	2.8	EFTEAVEMK
0	2	0.71	2	5.5	FLVEKEEQK
0	1	0.6	2.1	7.3	KIEAAEDIAYQLSR
0	1	1	3.5	12	NVPVITGSKDLQNVNITLR
0	1	0.34	1.2	4	VSEELTDR
0	1	1	3.6	12	FLVEKEEQKKR
0	1	0.2	0.71	2.5	AAVISAEGDSLAAELLAK
0	2	0.4	1.1	3.1	NVPVITGSK

5.138 " 10 609 lethal(2)essential for life protein, l2efl [Aedes aegypti] gi"

Protein Accession	Unigene67975$_{H}$1
Mean Expression Ratio	0.734
Median Expression Ratio	0.734
Credible Interval	(0.394, 1.38)
Associated Peptides	5
Associated Spectra	21
Coverage	NaN

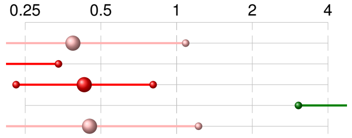

A	B	2.5	50	97.5	Sequence
1	1	0.14	0.39	1.1	ALEAPGETVIPITFEGK
4	0	0.067	0.15	0.34	MQMGETGK
7	0	0.23	0.43	0.81	INMDVK
0	6	3.1	6	12	KALEAPGETVIPITFEGKGDGK
0	2	0.16	0.45	1.2	KALEAPGETVIPITFEGK

5.139 140 283 minus strand elongation factor 1 alpha [Angiostrongylus cantonensis]

Protein Accession	CL16758.Contig7$_H$1
Mean Expression Ratio	0.735
Median Expression Ratio	0.735
Credible Interval	(0.336, 1.61)
Associated Peptides	2
Associated Spectra	3
Coverage	NaN

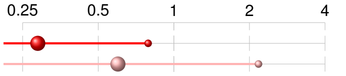

A	B	2.5	50	97.5	Sequence
2	0	0.1	0.29	0.79	VIEKFEK
1	0	0.17	0.6	2.2	STTTGHLIYK

5.140 227 3073 minus strand Lysosomal alpha-mannosidase [Crassostrea gigas]

Protein Accession	CL2930.Contig2$_H$1
Mean Expression Ratio	1.36
Median Expression Ratio	1.36
Credible Interval	(0.856, 2.17)
Associated Peptides	14
Associated Spectra	33
Coverage	NaN

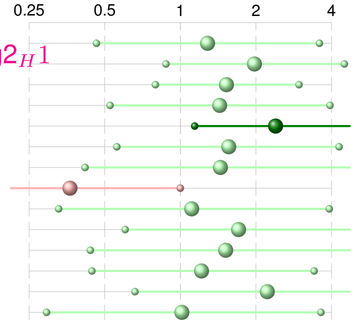

A	B	2.5	50	97.5	Sequence
0	2	0.46	1.3	3.6	ALPDNVHLLTLEQFR
1	3	0.88	2	4.5	ASLTWSGVR
2	5	0.8	1.5	3	EQASLFAR
0	2	0.52	1.4	3.9	GSHYVMLEPVSSAAK
1	4	1.1	2.4	5	IFIRDEAK
0	2	0.56	1.6	4.3	LANGVMEGQK
0	1	0.42	1.4	4.9	SQGGSSLLDGQIELMVHRR
0	2	0.13	0.36	1	VASELIPITPDTFR
0	1	0.33	1.1	3.9	VGWQIDPFGHSR
1	1	0.6	1.7	5	VVNDALKK
0	1	0.44	1.5	5.1	QAVAYDYAER
0	2	0.44	1.2	3.4	QVNNFLQVVK
0	1	0.66	2.2	7.7	LQWNYPH
0	1	0.29	1	3.6	VVNDALK

5.141 40 1752 minus strand heat shock protein 60 [Biomphalaria glabrata]; 40 1752 minus strand heat shock protein 60 [Biomphalaria glabrata]

Protein Accession	CL5583.Contig6$_H$1 CL5583.Contig5$_H$1
Mean Expression Ratio	1.36
Median Expression Ratio	1.36
Credible Interval	(0.822, 2.23)
Associated Peptides	14
Associated Spectra	33
Coverage	NaN

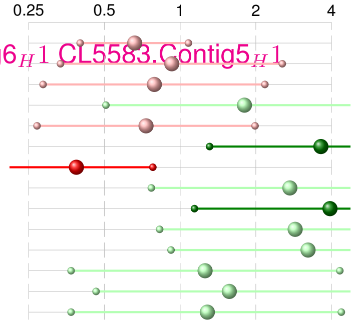

A	B	2.5	50	97.5	Sequence
3	9	0.4	0.66	1.1	AVGDLISSAMK
1	1	0.34	0.93	2.5	DKYQNIGAK
2	0	0.29	0.79	2.2	GANPIEIR
1	0	0.51	1.8	6.2	LSNGVAVLK
1	1	0.27	0.73	2	VGGTSEVEVNEK
1	1	1.3	3.6	10	ITKDGVTVAK
3	2	0.19	0.39	0.78	VGLQVVAVK
0	1	0.77	2.7	9.6	AVGDLISSAMKK
0	1	1.1	4	14	VGGTSEVEVNEKKDR
0	1	0.83	2.9	9.7	AIELKDKYQNIGAK
0	1	0.92	3.2	11	AIAKEGFDKISK
0	1	0.37	1.3	4.3	CEFQDALVLLSEK
0	1	0.46	1.6	5.3	GYISPYFINTSK
0	1	0.37	1.3	4.4	TLRDELEVIEGMK

5.142 95 1579 minus strand hypothetical protein BRAFLDRAFT_113974 [Branchiostoma floridae] gi

Protein Accession	CL19753.Contig5$_{H}$1
Mean Expression Ratio	1.36
Median Expression Ratio	1.36
Credible Interval	(0.757, 2.44)
Associated Peptides	9
Associated Spectra	15
Coverage	NaN

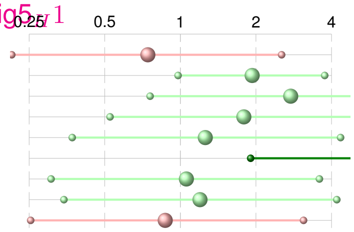

A	B	2.5	50	97.5	Sequence
1	0	0.21	0.74	2.5	TFPVVNPATEK
1	5	0.98	1.9	3.8	DINEVIERANR
0	1	0.76	2.8	9.5	FKDINEVIER
0	1	0.53	1.8	6.2	TSYGLAAGVITK
0	1	0.37	1.3	4.3	TVGDPFDAVTMQGPQVDQEQFEK
0	2	1.9	5.3	15	TVTIKLPSK
0	1	0.31	1.1	3.6	ADLRPDPNIK
0	1	0.34	1.2	4.2	ELGEYALK
0	1	0.25	0.87	3.1	DEIFGPVQSIIK

5.143 1 825 Spectrin alpha chain [Crassostrea gigas]

Protein Accession	CL10282.Contig3$_{H}$1
Mean Expression Ratio	1.36
Median Expression Ratio	1.36
Credible Interval	(0.69, 2.71)
Associated Peptides	5
Associated Spectra	6
Coverage	NaN

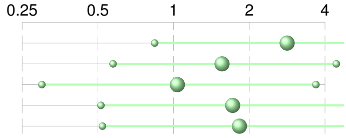

A	B	2.5	50	97.5	Sequence
0	1	0.84	2.8	10	KIEDLGAAMEER
0	2	0.57	1.6	4.4	MQHNLEQQIEAR
0	1	0.3	1	3.7	AIPDSYDYVDFTR
0	1	0.52	1.7	6.3	RKATEVR
0	1	0.52	1.8	6.3	MKPYVDK

5.144 366 935 minus strand 40S ribosomal protein S2 OS=Urechis caupo GN=RPS2 PE=2 SV=1

Protein Accession	CL2084.Contig7$_{H}$1
Mean Expression Ratio	0.737
Median Expression Ratio	0.74
Credible Interval	(0.375, 1.43)
Associated Peptides	5
Associated Spectra	8
Coverage	NaN

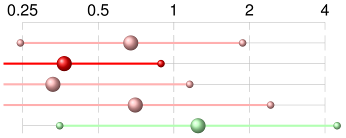

A	B	2.5	50	97.5	Sequence
2	0	0.24	0.67	1.9	GTGIVSAPVPK
2	1	0.15	0.37	0.89	LSVIPVR
1	0	0.094	0.33	1.2	EFTDYLMK
1	0	0.2	0.7	2.4	LIPAPR
0	1	0.35	1.2	4.5	GATATLGNFAK

5.145 157 459 minus strand Synaptojanin-2-binding protein [Crassostrea gigas]

Protein Accession	CL12494.Contig1$_{H}$1
Mean Expression Ratio	1.35
Median Expression Ratio	1.35
Credible Interval	(0.727, 2.51)
Associated Peptides	6
Associated Spectra	20
Coverage	NaN

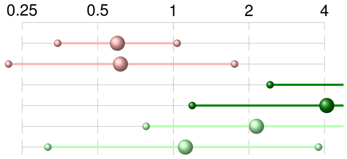

A	B	2.5	50	97.5	Sequence
3	7	0.35	0.6	1	GDSGLGFNIR
2	0	0.22	0.61	1.8	LKEDGAAFK
0	4	2.4	5.4	12	LKEGDKILEVNGK
0	1	1.2	4.1	15	LKEDGAAFKDGR
0	2	0.78	2.2	6	GGTDIPHVPGDTGIFVTR
0	1	0.32	1.1	3.8	ILEVNGK

5.146 51 371 minus strand putative mitochondrial ATP synthase F chain [Haliotis discus discus]; 51 371 minus strand putative mitochondrial ATP synthase F chain [Haliotis discus discus]

Protein Accession	CL18283.Contig4$_H$1 CL18283.Contig2$_H$1
Mean Expression Ratio	0.743
Median Expression Ratio	0.741
Credible Interval	(0.329, 1.64)
Associated Peptides	1
Associated Spectra	3
Coverage	NaN

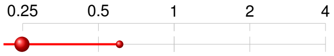

A	B	2.5	50	97.5	Sequence
**3**	**0**	**0.1**	**0.25**	**0.61**	**ADLPLGQVK**

5.147 1 219 minus strand PREDICTED: nucleoside diphosphate kinase 3-like [Pan troglodytes]

Protein Accession	Unigene5384$_H$1
Mean Expression Ratio	0.739
Median Expression Ratio	0.742
Credible Interval	(0.339, 1.57)
Associated Peptides	2
Associated Spectra	4
Coverage	NaN

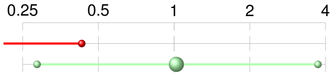

A	B	2.5	50	97.5	Sequence
**1**	**2**	**0.07**	**0.17**	**0.43**	**GLVGEIIK**
**0**	**1**	**0.29**	**1**	**3.7**	**GLVGEIIKR**

5.148 102 887 hypothetical protein DAPPUDRAFT_304712 [Daphnia pulex]

Protein Accession	CL6869.Contig1$_H$1
Mean Expression Ratio	0.743
Median Expression Ratio	0.742
Credible Interval	(0.399, 1.39)
Associated Peptides	6
Associated Spectra	15
Coverage	NaN

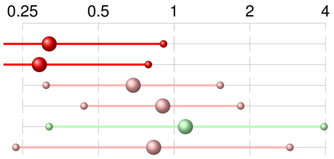

A	B	2.5	50	97.5	Sequence
**1**	**1**	**0.11**	**0.32**	**0.91**	**TAEEIAAEEK**
**2**	**0**	**0.1**	**0.29**	**0.79**	**VDLSQLR**
**2**	**2**	**0.31**	**0.69**	**1.5**	**LADIPEGK**
**3**	**2**	**0.44**	**0.9**	**1.8**	**AFAIQAEK**
**0**	**1**	**0.32**	**1.1**	**4**	**LALTSYAMSK**
**0**	**1**	**0.24**	**0.83**	**2.9**	**HPEADAER**

5.149 " 343 1179 ATP synthase subunit alpha, mitochondrial OS=Pongo abelii GN=ATP5A1 PE=2 SV=1"

Protein Accession	Unigene30534$_H$1
Mean Expression Ratio	1.35
Median Expression Ratio	1.35
Credible Interval	(0.824, 2.19)
Associated Peptides	11
Associated Spectra	63
Coverage	NaN

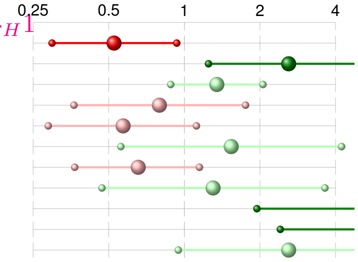

A	B	2.5	50	97.5	Sequence
7	2	0.3	0.53	0.93	STVAQLVK
2	3	1.2	2.6	5.4	TSVKEPMQTGIK
6	12	0.88	1.3	2.1	VLSIGDGIAR
1	3	0.36	0.8	1.8	VVDALGTPIDGK
3	3	0.29	0.57	1.1	APGIIPR
1	1	0.56	1.5	4.2	VYGLK
3	6	0.37	0.66	1.1	TAVAIDTIINQK
0	2	0.47	1.3	3.6	NIQAEEMVEFSSGLK
0	2	1.9	5.5	15	VGLKAPGIIPR
0	4	2.4	5.3	12	VVDALGTPIDGKGPITSK
0	2	0.95	2.6	7.1	TAVAIDTIINQKR

5.150 260 892 minus strand calponin [Haliotis diversicolor]

Protein Accession	CL48702.Contig1$_H$1
Mean Expression Ratio	1.35
Median Expression Ratio	1.35
Credible Interval	(0.701, 2.58)
Associated Peptides	5
Associated Spectra	10
Coverage	NaN

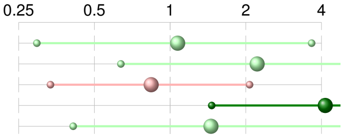

A	B	2.5	50	97.5	Sequence
0	1	0.3	1.1	3.7	AATGQQFPIEESNER
0	1	0.64	2.2	7.6	AFGIEVESHPSYTGQR
2	2	0.33	0.84	2.1	VESSFDPEDAKK
2	1	1.5	4.1	11	NFSEDKLR
0	1	0.41	1.5	5.1	SGLRAPK

5.151 94 489 Sortilin-related receptor [Crassostrea gigas]

Protein Accession	CL15197.Contig3$_H$1
Mean Expression Ratio	0.739
Median Expression Ratio	0.743
Credible Interval	(0.321, 1.66)
Associated Peptides	1
Associated Spectra	2
Coverage	NaN

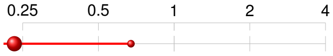

A	B	2.5	50	97.5	Sequence
2	0	0.082	0.23	0.67	CVPIEFR

5.152 45 407 minus strand 60S ribosomal protein L31 OS=Xenopus laevis GN=rpl31 PE=2 SV=1

Protein Accession	CL4728.Contig1$_H$1
Mean Expression Ratio	1.35
Median Expression Ratio	1.34
Credible Interval	(0.623, 2.9)
Associated Peptides	2
Associated Spectra	4
Coverage	NaN

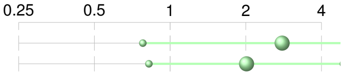

A	B	2.5	50	97.5	Sequence
0	1	0.78	2.8	10	IHGVSFKK
0	3	0.83	2	4.9	IHGVSFK

5.153 221 1450 minus strand PREDICTED: dihydrolipoamide branched chain transacylase E2 [Taeniopygia guttata]; 221 1609 minus strand PREDICTED: dihydrolipoamide branched chain transacylase E2 [Taeniopygia guttata]

Protein Accession	CL25782.Contig5$_H$1 CL25782.Contig3$_H$1
Mean Expression Ratio	1.35
Median Expression Ratio	1.34
Credible Interval	(0.597, 3.12)
Associated Peptides	1
Associated Spectra	2
Coverage	NaN

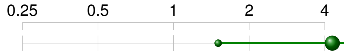

A	B	2.5	50	97.5	Sequence
0	2	1.5	4.3	12	VLATPAVR

5.154 97 477 minus strand PREDICTED: complement C1q tumor necrosis factor-related protein 3-like [Oreochromis niloticus]; 97 447 minus strand PREDICTED: complement C1q tumor necrosis factor-related protein 3-like [Oreochromis niloticus]

Protein Accession	CL370.Contig3$_H$1 CL370.Contig2$_H$1
Mean Expression Ratio	1.34
Median Expression Ratio	1.34
Credible Interval	(0.869, 2.09)
Associated Peptides	16
Associated Spectra	115
Coverage	NaN

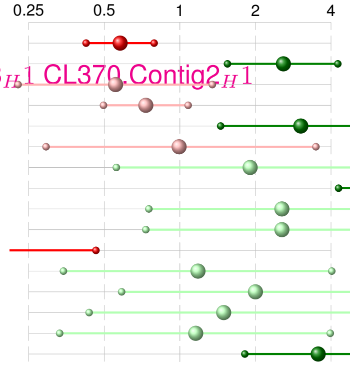

A	B	2.5	50	97.5	Sequence
8	25	0.42	0.58	0.79	QMVDALKDEQQR
3	9	1.5	2.6	4.2	QTSDISRLENR
1	2	0.23	0.56	1.3	PIAFSK
0	21	0.5	0.73	1.1	LESGVRPPVYRPDLESK
0	5	1.5	3	6.2	QMVDALKDEQQRQQEVINR
0	1	0.29	1	3.5	QSQQIEKQTSDISR
0	1	0.56	1.9	6.6	QSQQIEKQTSDISRLENR
0	25	4.3	6.1	8.7	VTKLESGVRPPVYRPDLESK
0	1	0.75	2.6	8.7	VTKLESGVRPP
0	1	0.73	2.6	8.8	LENRVTK
0	2	0.061	0.17	0.46	VAFTAH
0	1	0.34	1.2	4	MVDALKDEQQR
0	1	0.59	2	6.8	RPIAFSK
0	1	0.43	1.5	5.1	DALKDEQQR
0	1	0.33	1.2	4	QMVDALK
0	6	1.8	3.6	7	VAFTAHFSQGGSMDIGTSRPIAFSK

5.155 3 491 hemocyanin [Haliotis tuberculata]

Protein Accession	CL40281.Contig3$_H$1
Mean Expression Ratio	0.746
Median Expression Ratio	0.745
Credible Interval	(0.385, 1.45)
Associated Peptides	4
Associated Spectra	15
Coverage	NaN

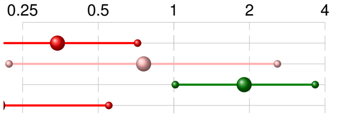

A	B	2.5	50	97.5	Sequence
2	3	0.17	0.34	0.72	DINPFALTK
0	1	0.22	0.76	2.6	GKPFTSAQCSVELFSK
0	7	1	1.9	3.7	TLEPFDRDINPFALTK
0	2	0.072	0.2	0.55	TLEPFDR

5.156 273 1304 Guanine nucleotide-binding protein subunit beta [Crassostrea gigas]

Protein Accession	CL15682.Contig1$_{H}$1
Mean Expression Ratio	1.34
Median Expression Ratio	1.34
Credible Interval	(0.653, 2.75)
Associated Peptides	4
Associated Spectra	4
Coverage	NaN

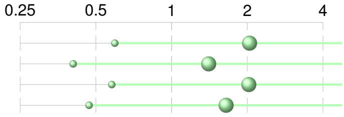

A	B	2.5	50	97.5	Sequence
0	1	0.6	2	7.3	TFVSGACDASAK
0	1	0.41	1.4	5	KAAGDTTLAQATAQCEPVGR
0	1	0.58	2	7.1	TSELDALRQETEQLKNQIR
0	1	0.47	1.6	5.9	IYAMHWASDSR

5.157 178 582 hypothetical protein CGI_10001640 [Crassostrea gigas]

Protein Accession	CL2347.Contig2$_{H}$1
Mean Expression Ratio	1.34
Median Expression Ratio	1.34
Credible Interval	(0.714, 2.51)
Associated Peptides	4
Associated Spectra	15
Coverage	NaN

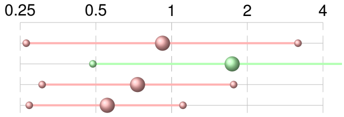

A	B	2.5	50	97.5	Sequence
0	1	0.26	0.92	3.2	LKSHGIGGK
0	1	0.49	1.7	5.9	SVGGNAGEVICK
2	2	0.31	0.73	1.8	AFLEQLGSK
5	4	0.27	0.56	1.1	ANLIVIGSR

5.158 77 502 hypothetical protein CGI_10008263 [Crassostrea gigas]

Protein Accession	CL38960.Contig1$_{H}$1
Mean Expression Ratio	1.34
Median Expression Ratio	1.34
Credible Interval	(0.666, 2.68)
Associated Peptides	3
Associated Spectra	12
Coverage	NaN

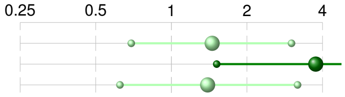

A	B	2.5	50	97.5	Sequence
0	5	0.69	1.4	3	IFVAIDGSK
0	3	1.5	3.8	9	KIFVAIDGSK
0	4	0.63	1.4	3.2	ANIEAITK

5.159 280 690 Histone H3.3 [Harpegnathos saltator]

Protein Accession	Unigene26607$_{H}$1
Mean Expression Ratio	0.75
Median Expression Ratio	0.747
Credible Interval	(0.439, 1.27)
Associated Peptides	9
Associated Spectra	81
Coverage	NaN

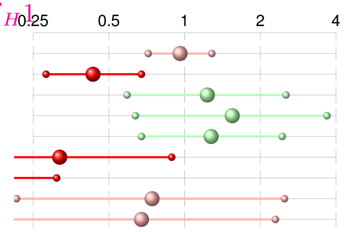

A	B	2.5	50	97.5	Sequence
30	10	0.72	0.96	1.3	DIQLAR
16	0	0.28	0.43	0.67	STELLIR
2	3	0.59	1.2	2.5	VTIMPK
3	0	0.64	1.5	3.7	KQLATK
3	4	0.67	1.3	2.4	RVTIMPK
2	0	0.12	0.32	0.89	KLPFQR
6	0	0.079	0.16	0.31	EIAQDFK
0	1	0.22	0.74	2.5	RVTIMPKDIQLAR
0	1	0.19	0.67	2.3	VTIMPKDIQLAR

5.160 77 2278 Far upstream element-binding protein 3 [Crassostrea gigas]

Protein Accession	CL5030.Contig3$_H$1
Mean Expression Ratio	0.75
Median Expression Ratio	0.749
Credible Interval	(0.341, 1.62)
Associated Peptides	2
Associated Spectra	2
Coverage	NaN

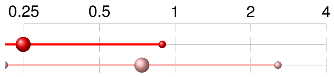

A	B	2.5	50	97.5	Sequence
1	0	0.071	0.25	0.89	LFNIR
1	0	0.21	0.74	2.6	QAVGLVIGK

5.161 2000 2641 Polyadenylate-binding protein 4 [Crassostrea gigas]

Protein Accession	Unigene7641$_H$1
Mean Expression Ratio	1.34
Median Expression Ratio	1.34
Credible Interval	(0.626, 2.95)
Associated Peptides	2
Associated Spectra	3
Coverage	NaN

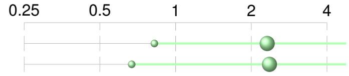

A	B	2.5	50	97.5	Sequence
1	1	0.82	2.3	6.5	KEFSQFGTITSAK
0	1	0.67	2.4	8.2	NIDDSIDDERLR

5.162 129 299 glycogen phosphorylase [Crassostrea gigas]

Protein Accession	Unigene20640$_H$1
Mean Expression Ratio	1.33
Median Expression Ratio	1.33
Credible Interval	(0.583, 3.05)
Associated Peptides	1
Associated Spectra	1
Coverage	NaN

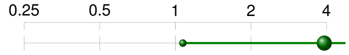

A	B	2.5	50	97.5	Sequence
0	1	1.1	3.9	14	GIAPVENVGTIKK

5.163 42 434 cytochrome b5 [Haliotis discus discus]; 42 434 cytochrome b5 [Haliotis discus discus]

Protein Accession	CL6985.Contig9$_H$1 CL6985.Contig5$_H$1
Mean Expression Ratio	1.33
Median Expression Ratio	1.33
Credible Interval	(0.64, 2.87)
Associated Peptides	3
Associated Spectra	4
Coverage	NaN

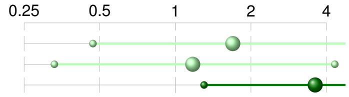

A	B	2.5	50	97.5	Sequence
0	1	0.47	1.7	6.1	KTFETNTGPVGR
0	1	0.33	1.2	4.3	ELMKDYLIGK
0	2	1.3	3.6	10	VFRSEEVKSHKDAK

5.164 " 75 803 hypothetical protein DAPPUDRAFT_231350 [Daphnia pulex]; 82 612 ATP synthase B chain, putative [Ixodes scapularis] gi"

Protein Accession CL10537.Contig4$_{H}1$ CL10537.Contig13$_{H}1$
Mean Expression Ratio 0.75
Median Expression Ratio 0.751
Credible Interval (0.33, 1.68)
Associated Peptides 1
Associated Spectra 3
Coverage NaN

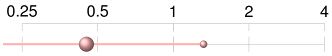

A	B	2.5	50	97.5	Sequence
1	2	0.15	0.45	1.3	SITPQQEAESIK

5.165 REVERSED 216 2639 minus strand polycystin 2 [Gallus gallus]

Protein Accession RRRRRCL818.Contig5$_{H}1$
Mean Expression Ratio 0.747
Median Expression Ratio 0.752
Credible Interval (0.331, 1.66)
Associated Peptides 1
Associated Spectra 2
Coverage NaN

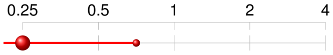

A	B	2.5	50	97.5	Sequence
2	0	0.089	0.25	0.71	DGLIIR

5.166 71 535 ribosomal protein rpl14 [Arenicola marina]

Protein Accession Unigene142$_{H}1$
Mean Expression Ratio 0.752
Median Expression Ratio 0.753
Credible Interval (0.389, 1.46)
Associated Peptides 4
Associated Spectra 14
Coverage NaN

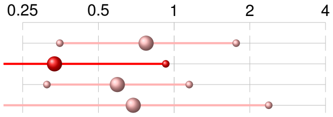

A	B	2.5	50	97.5	Sequence
2	2	0.35	0.78	1.8	IINVEYGK
2	0	0.12	0.34	0.92	AQLTDFER
4	3	0.31	0.59	1.1	FVEIGR
1	0	0.2	0.69	2.4	KAWEK

5.167 " 144 1145 PREDICTED: RNA-binding protein squid-like isoform 1 [Megachile rotundata]; 90 1085 squid, variant A [Blattella germanica]; 144 1145 PREDICTED: RNA-binding protein squid-like isoform 1 [Megachile rotundata]"

Protein Accession CL14696.Contig7$_{H}1$ CL14696.Contig5$_{H}1$ CL14696.Contig4$_{H}1$
Mean Expression Ratio 1.32
Median Expression Ratio 1.33
Credible Interval (0.664, 2.65)
Associated Peptides 3
Associated Spectra 8
Coverage NaN

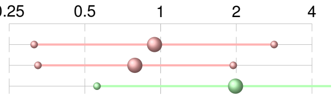

A	B	2.5	50	97.5	Sequence
1	1	0.31	0.94	2.8	IMEVTTHK
3	2	0.32	0.79	1.9	LGGQEVDVK
0	1	0.56	2	6.9	KIMEVTTHK

5.168 " 3 587 elongation factor 1 alpha, partial [Phorbas sp. nov. SB-2012]"

Protein Accession	Unigene20048$_{H}1$
Mean Expression Ratio	0.754
Median Expression Ratio	0.755
Credible Interval	(0.368, 1.54)
Associated Peptides	3
Associated Spectra	17
Coverage	NaN

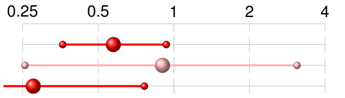

A	B	2.5	50	97.5	Sequence
6	8	0.36	0.57	0.93	IGGIGTVPVGR
1	0	0.25	0.9	3.1	GISTEVK
0	2	0.1	0.28	0.76	LPLQDVYK

5.169 " 27 974 PREDICTED: succinyl-CoA ligase [GDP-forming] subunit alpha, mitochondrial-like [Oreochromis niloticus]; 27 974 minus strand PREDICTED: succinyl-CoA ligase [GDP-forming] subunit alpha, mitochondrial-like [Oreochromis niloticus]"

Protein Accession	CL10674.Contig2$_{H}1$ CL10674.Contig1$_{H}1$
Mean Expression Ratio	1.33
Median Expression Ratio	1.32
Credible Interval	(0.629, 2.72)
Associated Peptides	3
Associated Spectra	5
Coverage	NaN

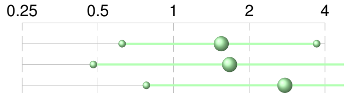

A	B	2.5	50	97.5	Sequence
0	3	0.63	1.6	3.7	MGHAGAIIAGGK
0	1	0.48	1.7	6.1	IYMATR
0	1	0.78	2.8	9.8	GGAKEKIEILK

5.170 521 6844 hypothetical protein CGI_10014469 [Crassostrea gigas]

Protein Accession	CL12911.Contig1$_{H}1$
Mean Expression Ratio	0.754
Median Expression Ratio	0.757
Credible Interval	(0.424, 1.35)
Associated Peptides	7
Associated Spectra	33
Coverage	NaN

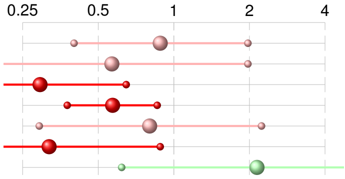

A	B	2.5	50	97.5	Sequence
2	2	0.4	0.89	2	VAAVQAFR
1	0	0.16	0.57	2	LQTIK
3	1	0.13	0.29	0.64	LTRIQV
4	15	0.38	0.57	0.86	IDAALVLK
0	2	0.29	0.8	2.2	KLFDLNPLK
0	2	0.11	0.32	0.88	LFDLNPLK
0	1	0.62	2.1	7.5	RSPWQLPKDLSLTLK

5.171 235 777 RNA-binding protein 4.1 [Clonorchis sinensis]

Protein Accession	CL49317.Contig6$_{H}1$
Mean Expression Ratio	1.32
Median Expression Ratio	1.32
Credible Interval	(0.606, 2.83)
Associated Peptides	2
Associated Spectra	4
Coverage	NaN

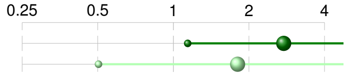

A	B	2.5	50	97.5	Sequence
1	2	1.1	2.7	6.6	VELSTGK
0	1	0.5	1.8	6.4	DRLPPFAEDR

5.172 73 837 minus strand Electron transfer flavoprotein subunit beta [Crassostrea gigas]; 73 837 minus strand Electron transfer flavoprotein subunit beta [Crassostrea gigas]

Protein Accession	CL33148.Contig2$_H$1 CL33148.Contig1$_H$1
Mean Expression Ratio	0.76
Median Expression Ratio	0.759
Credible Interval	(0.391, 1.49)
Associated Peptides	5
Associated Spectra	9
Coverage	NaN

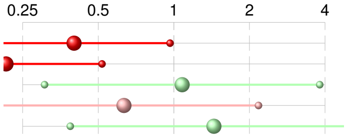

A	B	2.5	50	97.5	Sequence
2	1	0.16	0.4	0.97	AIHVDVDSK
2	1	0.089	0.21	0.52	EIDGGLENVLVK
0	1	0.3	1.1	3.8	HSMNPFDEIAVEEAVR
0	1	0.18	0.63	2.2	VLSVEEPPVR
0	1	0.39	1.4	5.1	YATLPNIMK

5.173 232 1401 RecName: Full=Guanine nucleotide-binding protein G(s) subunit alpha; AltName: Full=Adenylate cyclase-stimulating G alpha protein gi|9635|emb|CAA78808.1| GTP-binding protein alpha-s subunit [Lymnaea stagnalis]; 232 1356 RecName: Full=Guanine nucleotide-binding protein G(s) subunit alpha; AltName: Full=Adenylate cyclase-stimulating G alpha protein gi|9635|emb|CAA78808.1| GTP-binding protein alpha-s subunit [Lymnaea stagnalis]

Protein Accession	CL8653.Contig3$_H$1 CL8653.Contig1$_H$1
Mean Expression Ratio	1.32
Median Expression Ratio	1.32
Credible Interval	(0.631, 2.71)
Associated Peptides	3
Associated Spectra	5
Coverage	NaN

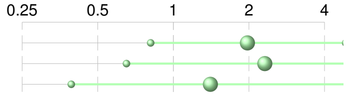

A	B	2.5	50	97.5	Sequence
0	3	0.81	2	4.8	LLLLGAGESGK
0	1	0.65	2.3	8.1	QKIEDIK
0	1	0.39	1.4	5	EQDILR

5.174 166 2364 Dipeptidyl-peptidase 3 [Crassostrea gigas]

Protein Accession	CL9071.Contig2$_H$1
Mean Expression Ratio	0.753
Median Expression Ratio	0.759
Credible Interval	(0.351, 1.6)
Associated Peptides	3
Associated Spectra	3
Coverage	NaN

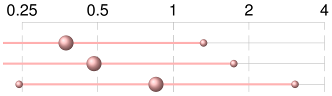

A	B	2.5	50	97.5	Sequence
0	1	0.1	0.37	1.3	DAEIAVEFMTSK
0	1	0.13	0.48	1.7	FLSLR
0	1	0.24	0.85	3.1	VETIILK

5.175 153 953 minus strand ribosomal protein rpl7 [Arenicola marina]

Protein Accession	CL20943.Contig3$_{H}1$
Mean Expression Ratio	0.761
Median Expression Ratio	0.759
Credible Interval	(0.353, 1.66)
Associated Peptides	2
Associated Spectra	2
Coverage	NaN

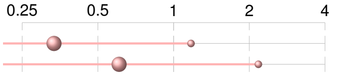

A	B	2.5	50	97.5	Sequence
1	0	0.093	0.33	1.2	VPETILK
1	0	0.17	0.61	2.2	ATLNMLK

5.176 62 1093 Heterogeneous nuclear ribonucleoprotein A2-like protein 1 [Crassostrea gigas]

Protein Accession	CL47083.Contig4$_{H}1$
Mean Expression Ratio	1.33
Median Expression Ratio	1.31
Credible Interval	(0.715, 2.47)
Associated Peptides	7
Associated Spectra	12
Coverage	NaN

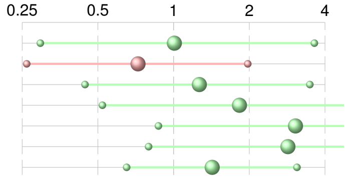

A	B	2.5	50	97.5	Sequence
1	0	0.3	1	3.6	GGGRDYGGGR
0	2	0.26	0.72	2	ETFSPFGTISQIDLITDK
0	2	0.44	1.3	3.5	MFVGGIKEDTTEDMIR
0	1	0.52	1.8	6.4	KLFIGGLSYETDENSLR
0	1	0.87	3	11	KAMSK
0	1	0.8	2.8	9.9	MPGYR
0	4	0.65	1.4	3.1	GFGFITYK

5.177 188 3946 Vigilin [Crassostrea gigas]; 188 4135 Vigilin [Crassostrea gigas]

Protein Accession	CL846.Contig4$_{H}1$ CL846.Contig3$_{H}1$
Mean Expression Ratio	0.76
Median Expression Ratio	0.762
Credible Interval	(0.407, 1.41)
Associated Peptides	7
Associated Spectra	8
Coverage	NaN

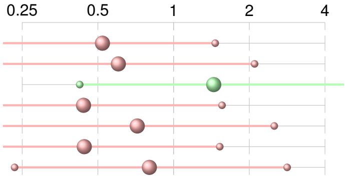

A	B	2.5	50	97.5	Sequence
0	2	0.19	0.52	1.5	EVSIDIPHR
0	1	0.17	0.6	2.1	NIAEEFGGVTVSFPR
0	1	0.42	1.4	5.1	IKNETGVSIR
0	1	0.13	0.44	1.6	AVIDDLK
0	1	0.2	0.72	2.5	LQELELATSTK
0	1	0.12	0.44	1.5	GFQLQVEVDPR
0	1	0.23	0.8	2.8	FLIGR

5.178 311 928 minus strand peroxiredoxin 3 [Clonorchis sinensis] gi; 288 905 minus strand peroxiredoxin 3 [Clonorchis sinensis] gi

Protein Accession	CL2043.Contig4$_{H}1$ CL2043.Contig1$_{H}1$
Mean Expression Ratio	0.763
Median Expression Ratio	0.762
Credible Interval	(0.373, 1.56)
Associated Peptides	3
Associated Spectra	9
Coverage	NaN

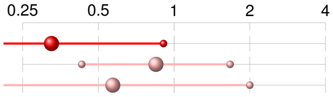

A	B	2.5	50	97.5	Sequence
1	1	0.12	0.32	0.91	SVDEILR
3	3	0.43	0.85	1.7	QITINDLPVGR
0	1	0.17	0.57	2	VQHPAPDFK

5.179 78 560 Sorting nexin-12 [Camponotus floridanus]; 78 578 Sorting nexin-12 [Camponotus floridanus]; 132 605 Sorting nexin-12 [Camponotus floridanus]; 276 623 Sorting nexin-12 [Camponotus floridanus]; 276 629 Sorting nexin-12 [Camponotus floridanus]

Protein Accession	CL2450.Contig5$_{H}1$ CL2450.Contig4$_{H}1$ CL2450.Contig3$_{H}1$ CL2450.Contig2$_{H}1$ CL2450.Con
Mean Expression Ratio	1.31
Median Expression Ratio	1.31
Credible Interval	(0.602, 2.87)
Associated Peptides	2
Associated Spectra	2
Coverage	NaN

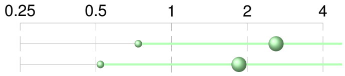

A	B	2.5	50	97.5	Sequence
**0**	**1**	**0.74**	**2.6**	**9.3**	**MKTNLPVFK**
**0**	**1**	**0.52**	**1.9**	**6.5**	**IVVPPLPGK**

5.180 87 764 Enolase-phosphatase E1 [Crassostrea gigas]; 87 1058 Enolase-phosphatase E1 [Crassostrea gigas]; 87 764 Enolase-phosphatase E1 [Crassostrea gigas]

Protein Accession	CL25175.Contig7$_{H}1$ CL25175.Contig6$_{H}1$ CL25175.Contig4$_{H}1$
Mean Expression Ratio	0.767
Median Expression Ratio	0.764
Credible Interval	(0.355, 1.64)
Associated Peptides	1
Associated Spectra	4
Coverage	NaN

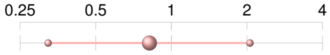

A	B	2.5	50	97.5	Sequence
**2**	**2**	**0.32**	**0.82**	**2.1**	**SAAEAESLLVGVK**

5.181 788 1006 LIM protein [Haliotis discus discus]; 768 980 muscle LIM protein isoform 1 [Danaus plexippus]

Protein Accession	CL29824.Contig2$_{H}1$ CL29824.Contig1$_{H}1$
Mean Expression Ratio	1.3
Median Expression Ratio	1.31
Credible Interval	(0.607, 2.79)
Associated Peptides	1
Associated Spectra	4
Coverage	NaN

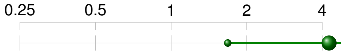

A	B	2.5	50	97.5	Sequence
**2**	**2**	**1.7**	**4.3**	**11**	**AIGVNTPWHK**

5.182 104 445 minus strand macrophage migration inhibitory factor [Ascaris suum]

Protein Accession	CL34536.Contig3$_H$1
Mean Expression Ratio	1.31
Median Expression Ratio	1.31
Credible Interval	(0.647, 2.7)
Associated Peptides	3
Associated Spectra	6
Coverage	NaN

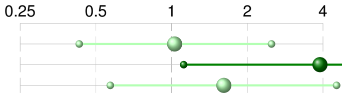

A	B	2.5	50	97.5	Sequence
1	2	0.43	1	2.5	HIEEALGIPQDR
0	1	1.1	3.9	14	AFLKDTSK
0	2	0.57	1.6	4.5	LVVPFMK

5.183 227 2920 minus strand Lysosomal alpha-mannosidase [Crassostrea gigas]; 227 3073 minus strand Lysosomal alpha-mannosidase [Crassostrea gigas]; 193 2886 minus strand Lysosomal alpha-mannosidase [Crassostrea gigas]

Protein Accession	CL2930.Contig3$_H$1 CL2930.Contig2$_H$1 CL2930.Contig1$_H$1
Mean Expression Ratio	0.763
Median Expression Ratio	0.764
Credible Interval	(0.401, 1.45)
Associated Peptides	4
Associated Spectra	18
Coverage	NaN

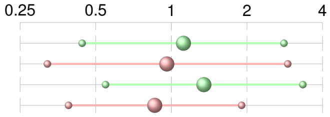

A	B	2.5	50	97.5	Sequence
1	3	0.44	1.1	2.8	ASLTWSGVR
1	1	0.32	0.96	2.9	VVNDALKK
1	4	0.55	1.3	3.3	IFIRDEAK
2	5	0.39	0.86	1.9	EQASLFAR

5.184 94 933 four and A half lim domains [Schistosoma mansoni]

Protein Accession	CL7046.Contig6$_H$1
Mean Expression Ratio	1.31
Median Expression Ratio	1.31
Credible Interval	(0.559, 3)
Associated Peptides	1
Associated Spectra	1
Coverage	NaN

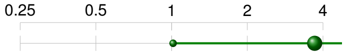

A	B	2.5	50	97.5	Sequence
1	0	1	3.7	13	CTKPITGIGGTK

5.185 61 723 minus strand 40S ribosomal protein S3-A OS=Xenopus laevis GN=rps3-a PE=2 SV=2

Protein Accession	Unigene19057$_H$1
Mean Expression Ratio	0.765
Median Expression Ratio	0.766
Credible Interval	(0.382, 1.53)
Associated Peptides	4
Associated Spectra	6
Coverage	NaN

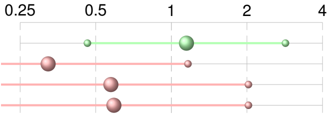

A	B	2.5	50	97.5	Sequence
1	2	0.46	1.2	2.8	ELAEDGYSGVEVR
1	0	0.09	0.32	1.2	TQNVLGEK
1	0	0.16	0.58	2	DEPAPLHPYSEQK
0	1	0.16	0.59	2	QGVLGIK

5.186 635 1294 minus strand Kyphoscoliosis peptidase [Crassostrea gigas]; 643 1302 minus strand Kyphoscoliosis peptidase [Crassostrea gigas]; 635 1294 minus strand Kyphoscoliosis peptidase [Crassostrea gigas]; 634 1293 minus strand Kyphoscoliosis peptidase [Crassostrea gigas]; 634 1293 minus strand Kyphoscoliosis peptidase [Crassostrea gigas]; 634 1293 minus strand Kyphoscoliosis peptidase [Crassostrea gigas]; 639 1298 minus strand Kyphoscoliosis peptidase [Crassostrea gigas]

Protein Accession	CL7772.Contig20$_H$1 CL7772.Contig1$_H$1 CL7772.Contig19$_H$1 CL7772.Contig16$_H$1 CL7772.C
Mean Expression Ratio	1.31
Median Expression Ratio	1.3
Credible Interval	(0.639, 2.68)
Associated Peptides	3
Associated Spectra	6
Coverage	NaN

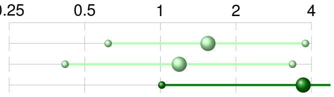

A	B	2.5	50	97.5	Sequence
2	1	0.62	1.6	3.8	LSNVTFK
0	2	0.42	1.2	3.4	GAGEVWEGTAALDK
0	1	1	3.7	13	GAGEVWEGTAALDKHR

5.187 1425 2018 minus strand Ankyrin-2 [Crassostrea gigas]; 1425 2018 minus strand Ankyrin-2 [Crassostrea gigas]

Protein Accession	CL45186.Contig15$_H$1 CL45186.Contig14$_H$1
Mean Expression Ratio	1.3
Median Expression Ratio	1.3
Credible Interval	(0.601, 2.85)
Associated Peptides	2
Associated Spectra	3
Coverage	NaN

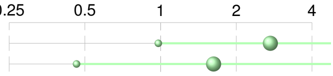

A	B	2.5	50	97.5	Sequence
1	1	0.98	2.7	7.8	VAFMKEPK
0	1	0.46	1.6	5.8	SGDQLHINFK

5.188 2 838 Alanine aminotransferase 2 [Crassostrea gigas]

Protein Accession	CL37048.Contig1$_{H}1$
Mean Expression Ratio	0.765
Median Expression Ratio	0.769
Credible Interval	(0.331, 1.75)
Associated Peptides	1
Associated Spectra	1
Coverage	NaN

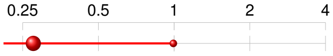

A	B	2.5	50	97.5	Sequence
1	0	0.077	0.28	1	QVLTK

5.189 " 65 1576 minus strand Aldehyde dehydrogenase, mitochondrial [Crassostrea gigas]"

Protein Accession	CL5835.Contig1$_{H}1$
Mean Expression Ratio	0.77
Median Expression Ratio	0.769
Credible Interval	(0.477, 1.25)
Associated Peptides	14
Associated Spectra	37
Coverage	NaN

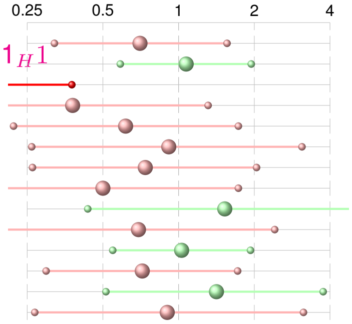

A	B	2.5	50	97.5	Sequence
2	2	0.32	0.7	1.6	SAAALPKPLTSPEIK
4	4	0.59	1.1	1.9	TFPTVDPR
1	2	0.065	0.15	0.38	LADLLER
1	0	0.11	0.38	1.3	TVTFR
1	1	0.22	0.62	1.7	FKDVK
1	0	0.26	0.92	3.1	LGSPWR
1	1	0.26	0.74	2	FVDSVSGK
0	1	0.15	0.5	1.7	ALYLSNSLR
0	1	0.44	1.5	5.4	IYNEFVER
0	1	0.2	0.69	2.4	LAEQTPLSGLYVAK
0	7	0.55	1	1.9	LIPVMAAK
0	3	0.3	0.72	1.7	TLHIGGDHFAYTR
0	2	0.51	1.4	3.8	IFVEEK
0	1	0.27	0.9	3.1	LADLLERDR

5.190 179 2140 minus strand PREDICTED: hypothetical protein [Saccoglossus kowalevskii]

Protein Accession	CL13749.Contig6$_{H}1$
Mean Expression Ratio	1.3
Median Expression Ratio	1.3
Credible Interval	(0.603, 2.86)
Associated Peptides	2
Associated Spectra	3
Coverage	NaN

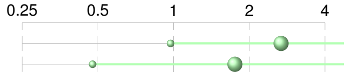

A	B	2.5	50	97.5	Sequence
0	2	0.97	2.7	7.4	KFDGIGGLSGGGATSK
0	1	0.48	1.8	6.2	VGGTYNFDDSNGLGR

5.191 593 862 minus strand mitochondrial ATP synthase coupling factor 6 precursor [Ochlerotatus taeniorhynchus]

Protein Accession	Unigene4216$_{H}1$
Mean Expression Ratio	0.764
Median Expression Ratio	0.77
Credible Interval	(0.347, 1.63)
Associated Peptides	2
Associated Spectra	2
Coverage	NaN

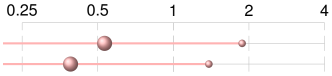

A	B	2.5	50	97.5	Sequence
**0**	**1**	**0.15**	**0.53**	**1.9**	**GSTVTDPIQQLFLDK**
**0**	**1**	**0.11**	**0.39**	**1.4**	**LVDASPTVQAALKEELEK**

5.192 801 971 RecName: Full=Tropomyosin-2; AltName: Full=Tropomyosin II; Short=BgTMII; Short=TMII gi|155940|gb|AAA27816.1| tropomyosin [Biomphalaria glabrata]

Protein Accession	CL2925.Contig4$_{H}1$
Mean Expression Ratio	1.3
Median Expression Ratio	1.3
Credible Interval	(0.566, 2.94)
Associated Peptides	1
Associated Spectra	1
Coverage	NaN

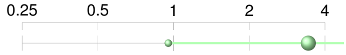

A	B	2.5	50	97.5	Sequence
**0**	**1**	**0.95**	**3.4**	**13**	**ASQREDSYEETIRDLTQR**

5.193 128 532 minus strand mitochondrial ATPase inhibitor [Ornithodoros parkeri]

Protein Accession	Unigene1376$_{H}1$
Mean Expression Ratio	1.3
Median Expression Ratio	1.3
Credible Interval	(0.667, 2.57)
Associated Peptides	4
Associated Spectra	10
Coverage	NaN

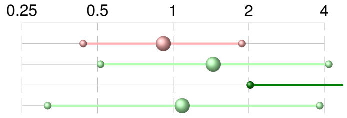

A	B	2.5	50	97.5	Sequence
**4**	**1**	**0.44**	**0.92**	**1.9**	**EAGGAFGK**
**1**	**1**	**0.51**	**1.4**	**4.2**	**MEAAHEELYFK**
**0**	**2**	**2**	**5.6**	**16**	**KLEREQIAK**
**0**	**1**	**0.32**	**1.1**	**3.8**	**SGIGEFGSGAGK**

5.194 108 401 60S ribosomal L7a-like protein [Phragmatopoma lapidosa]; 108 401 60S ribosomal L7a-like protein [Phragmatopoma lapidosa]; 108 431 60S ribosomal L7a-like protein [Phragmatopoma lapidosa]

Protein Accession	CL1313.Contig6$_{H}1$ CL1313.Contig5$_{H}1$ CL1313.Contig1$_{H}1$
Mean Expression Ratio	0.768
Median Expression Ratio	0.771
Credible Interval	(0.351, 1.65)
Associated Peptides	2
Associated Spectra	3
Coverage	NaN

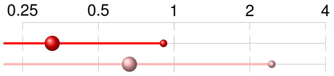

A	B	2.5	50	97.5	Sequence
2	0	0.11	0.33	0.91	QTATQIFR
1	0	0.18	0.66	2.4	SGVNTVTALVESK

5.195 1 381 hypothetical protein [Eimeria tenella]

Protein Accession	CL17786.Contig1$_{H}1$
Mean Expression Ratio	1.3
Median Expression Ratio	1.3
Credible Interval	(0.583, 2.89)
Associated Peptides	1
Associated Spectra	4
Coverage	NaN

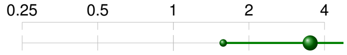

A	B	2.5	50	97.5	Sequence
0	4	1.6	3.5	8	IINEPTAAAIAYGLDKK

5.196 1714 2121 Phosphoenolpyruvate carboxykinase [GTP] [Crassostrea gigas]

Protein Accession	CL25948.Contig1$_{H}1$
Mean Expression Ratio	0.771
Median Expression Ratio	0.772
Credible Interval	(0.351, 1.7)
Associated Peptides	1
Associated Spectra	5
Coverage	NaN

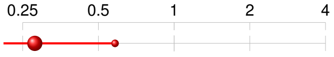

A	B	2.5	50	97.5	Sequence
3	2	0.13	0.28	0.58	SEATAAAEFK

5.197 114 1001 Vinculin [Crassostrea gigas]

Protein Accession	CL18499.Contig1$_{H}1$
Mean Expression Ratio	1.29
Median Expression Ratio	1.29
Credible Interval	(0.633, 2.76)
Associated Peptides	3
Associated Spectra	3
Coverage	NaN

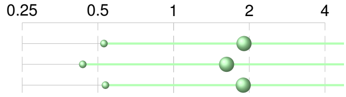

A	B	2.5	50	97.5	Sequence
1	0	0.53	1.9	6.9	PVFHTK
0	1	0.43	1.6	5.7	TIESILEPVAQQVSR
0	1	0.53	1.9	6.6	KMSDEIHEIIR

5.198 531 665 minus strand Proteasome subunit beta type-6 [Crassostrea gigas]

Protein Accession	CL12961.Contig2$_H1$
Mean Expression Ratio	0.77
Median Expression Ratio	0.774
Credible Interval	(0.342, 1.71)
Associated Peptides	1
Associated Spectra	2
Coverage	NaN

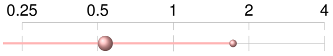

A	B	2.5	50	97.5	Sequence
1	1	0.17	0.54	1.7	FTLTGDDLPK

5.199 " 59 2365 minus strand Aconitate hydratase, mitochondrial [Crassostrea gigas]"

Protein Accession	CL13509.Contig1$_H1$
Mean Expression Ratio	1.29
Median Expression Ratio	1.29
Credible Interval	(0.725, 2.29)
Associated Peptides	10
Associated Spectra	11
Coverage	NaN

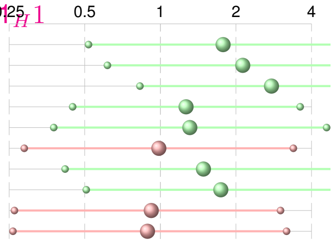

A	B	2.5	50	97.5	Sequence
0	1	0.52	1.8	6.2	HTMNEGQIEWFR
0	1	0.62	2.1	7.6	KGETNTIVTSYNR
0	1	0.83	2.8	9.7	SKFTITPGSEQIR
0	2	0.45	1.3	3.6	WDGKDLVDMPVLIK
0	1	0.38	1.3	4.6	KNGWPLNIR
0	1	0.29	0.99	3.4	VAGILTVK
0	1	0.42	1.5	5.1	LGRPLTLSEK
0	1	0.51	1.7	6	MQDYLK
0	1	0.26	0.92	3	IHETNLKK
0	1	0.26	0.89	3.2	LDDPKNEK

5.200 182 1846 minus strand hypothetical protein DAPPUDRAFT_302108 [Daphnia pulex]; 110 1774 minus strand hypothetical protein DAPPUDRAFT_302108 [Daphnia pulex]

Protein Accession	CL11561.Contig6$_H1$ CL11561.Contig1$_H1$
Mean Expression Ratio	1.3
Median Expression Ratio	1.29
Credible Interval	(0.652, 2.61)
Associated Peptides	4
Associated Spectra	5
Coverage	NaN

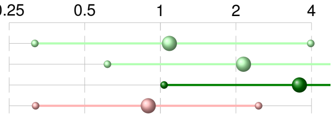

A	B	2.5	50	97.5	Sequence
0	1	0.32	1.1	4	TDGGIILTASHNPGGK
0	1	0.62	2.1	7.6	VATSPFEGQKPGTSGLR
0	1	1	3.6	12	IVYRLSGTGSSGATIR
0	2	0.32	0.89	2.5	LFLPGGLK

5.201 31 777 Proteasome subunit alpha type-7-like protein [Crassostrea gigas]

Protein Accession	CL35094.Contig4$_H1$
Mean Expression Ratio	0.777
Median Expression Ratio	0.775
Credible Interval	(0.343, 1.76)
Associated Peptides	1
Associated Spectra	1
Coverage	NaN

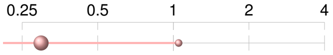

A	B	2.5	50	97.5	Sequence
1	0	0.082	0.3	1	GDNLVVLGVEK

5.202 " 158 355 Protein NAS-37, isoform b [Caenorhabditis elegans] gi; 15 227 Hemicentin-1 [Crassostrea gigas]"

Protein Accession	Unigene57180$_{H1}$ CL8016.Contig2$_{H1}$
Mean Expression Ratio	1.3
Median Expression Ratio	1.29
Credible Interval	(0.569, 2.97)
Associated Peptides	1
Associated Spectra	1
Coverage	NaN

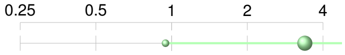

A	B	2.5	50	97.5	Sequence
**0**	**1**	**0.95**	**3.4**	**12**	**FKGSYGGVSR**

5.203 155 3214 sarco/endoplasmic reticulum calcium ATPase isoform B [Pinctada fucata]; 155 3214 minus strand sarco/endoplasmic reticulum calcium ATPase isoform B [Pinctada fucata]

Protein Accession	CL1323.Contig9$_{H1}$ CL1323.Contig1$_{H1}$
Mean Expression Ratio	1.28
Median Expression Ratio	1.29
Credible Interval	(0.651, 2.51)
Associated Peptides	4
Associated Spectra	7
Coverage	NaN

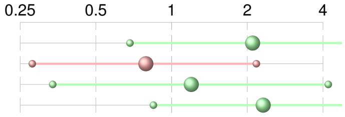

A	B	2.5	50	97.5	Sequence
**1**	**1**	**0.68**	**2.1**	**6.5**	**KAEIGIAMGSGTAVAK**
**0**	**2**	**0.28**	**0.79**	**2.2**	**VGEATETALTVLVEK**
**0**	**1**	**0.34**	**1.2**	**4.2**	**GAPEGLLDR**
**0**	**2**	**0.85**	**2.3**	**6.4**	**MDQAFTK**

5.204 1 309 PREDICTED: intersectin-1-like [Sus scrofa]

Protein Accession	Unigene37688$_{H1}$
Mean Expression Ratio	1.29
Median Expression Ratio	1.29
Credible Interval	(0.566, 3.05)
Associated Peptides	1
Associated Spectra	1
Coverage	NaN

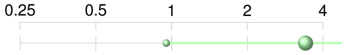

A	B	2.5	50	97.5	Sequence
**0**	**1**	**0.95**	**3.4**	**12**	**VDIQPGPKSSK**

5.205 130 1731 T-complex protein 1 subunit epsilon [Crassostrea gigas]; 130 1731 T-complex protein 1 subunit epsilon [Crassostrea gigas]

Protein Accession	CL1223.Contig8$_H$1 CL1223.Contig2$_H$1
Mean Expression Ratio	1.28
Median Expression Ratio	1.29
Credible Interval	(0.715, 2.26)
Associated Peptides	9
Associated Spectra	14
Coverage	NaN

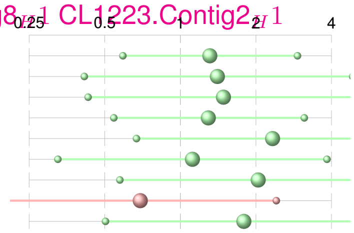

A	B	2.5	50	97.5	Sequence
1	3	0.59	1.3	2.9	LTGIDAHK
0	1	0.42	1.4	4.9	GVVVDKDMSHPQMPK
0	1	0.43	1.5	5.2	HKLDVTSVEDYR
0	3	0.54	1.3	3.1	LMVQLSK
0	1	0.67	2.3	8.3	HKLDVTSVEDYRK
0	1	0.32	1.1	3.8	MLVIEECK
0	1	0.58	2	7.2	IVPRFEELTPEK
0	1	0.2	0.69	2.4	IAVETLDK
0	1	0.5	1.8	6.1	SHILAAK

5.206 433 669 minus strand Superoxide dismutase [Cu-Zn] OS=Prionace glauca GN=sod1 PE=1 SV=1

Protein Accession	Unigene63976$_H$1
Mean Expression Ratio	0.777
Median Expression Ratio	0.778
Credible Interval	(0.381, 1.57)
Associated Peptides	1
Associated Spectra	9
Coverage	NaN

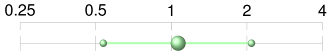

A	B	2.5	50	97.5	Sequence
5	4	0.54	1.1	2.1	SLVVHEK

5.207 95 2278 hypothetical protein CGI_10017740 [Crassostrea gigas]

Protein Accession	CL48637.Contig1$_H$1
Mean Expression Ratio	0.775
Median Expression Ratio	0.778
Credible Interval	(0.363, 1.63)
Associated Peptides	3
Associated Spectra	3
Coverage	NaN

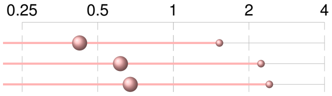

A	B	2.5	50	97.5	Sequence
1	0	0.12	0.42	1.5	VAMYR
0	1	0.17	0.61	2.2	SISYALR
0	1	0.19	0.68	2.4	AGLELVPK

5.208 11 187 PREDICTED: 40S ribosomal protein S28-like [Strongylocentrotus purpuratus]

Protein Accession	Unigene696$_H$1
Mean Expression Ratio	0.778
Median Expression Ratio	0.778
Credible Interval	(0.347, 1.72)
Associated Peptides	1
Associated Spectra	4
Coverage	NaN

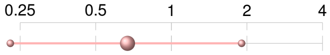

A	B	2.5	50	97.5	Sequence
1	3	0.23	0.67	1.9	VEFLDDSNR

5.209 1292 1471 minus strand hemocyanin isoform 1 [Haliotis diversicolor]

Protein Accession	Unigene65233$_{H}1$
Mean Expression Ratio	0.779
Median Expression Ratio	0.779
Credible Interval	(0.367, 1.66)
Associated Peptides	2
Associated Spectra	5
Coverage	NaN

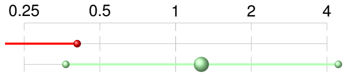

A	B	2.5	50	97.5	Sequence
1	3	0.081	0.18	0.41	LYTVQFEDALR
0	1	0.37	1.3	4.4	LYTVQFEDALRR

5.210 91 1392 minus strand hypothetical protein AND_03103 [Anopheles darlingi]

Protein Accession	CL10598.Contig5$_{H}1$
Mean Expression Ratio	0.777
Median Expression Ratio	0.779
Credible Interval	(0.363, 1.65)
Associated Peptides	3
Associated Spectra	3
Coverage	NaN

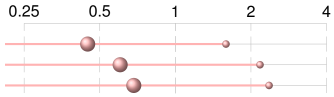

A	B	2.5	50	97.5	Sequence
0	1	0.13	0.45	1.6	APGFAHLAGLDK
0	1	0.18	0.6	2.2	LPPFNEHALVQEK
0	1	0.19	0.68	2.4	LPVMYPDEESSK

5.211 244 837 PREDICTED: ras-like GTP-binding protein Rho1-like [Megachile rotundata]

Protein Accession	Unigene11792$_{H}1$
Mean Expression Ratio	1.28
Median Expression Ratio	1.28
Credible Interval	(0.587, 2.8)
Associated Peptides	2
Associated Spectra	3
Coverage	NaN

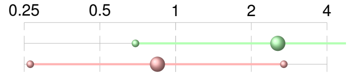

A	B	2.5	50	97.5	Sequence
0	1	0.69	2.5	9.1	EGVREVFETATR
1	1	0.26	0.84	2.7	EVFETATR

5.212 72 1400 minus strand hypothetical protein RCJMB04_22i13 [Gallus gallus]

Protein Accession	CL44755.Contig5$_{H}1$
Mean Expression Ratio	0.782
Median Expression Ratio	0.782
Credible Interval	(0.443, 1.39)
Associated Peptides	9
Associated Spectra	18
Coverage	NaN

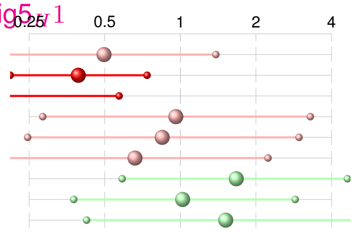

A	B	2.5	50	97.5	Sequence
1	1	0.18	0.5	1.4	EMGFSEGSIDK
5	2	0.21	0.39	0.74	LVFSADEAK
1	0	0.047	0.16	0.57	AQVLAGGR
1	0	0.28	0.96	3.3	AVFQMR
1	0	0.25	0.85	3	EQAVYVAR
1	0	0.19	0.66	2.2	GKGTWDSGLK
0	2	0.59	1.7	4.6	FLVASTPDEALQHAAELGK
0	2	0.38	1	2.9	LSGIPDVFVK
0	1	0.42	1.5	5.2	ILACDNLDEAASMVVK

5.213 " 1 1887 Pyruvate carboxylase, mitochondrial [Crassostrea gigas]"

Protein Accession	CL26073.Contig5$_H$1
Mean Expression Ratio	1.28
Median Expression Ratio	1.28
Credible Interval	(0.613, 2.68)
Associated Peptides	3
Associated Spectra	4
Coverage	NaN

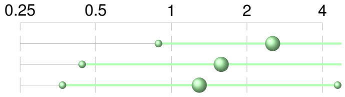

A	B	2.5	50	97.5	Sequence
0	2	0.89	2.5	7.2	HLEDVEENFKR
0	1	0.44	1.6	5.6	AGTHILGIK
0	1	0.37	1.3	4.6	IEVFR

5.214 633 806 Peroxiredoxin-1 [Crassostrea gigas]; 648 821 Peroxiredoxin-1 [Crassostrea gigas]; 666 839 Peroxiredoxin-1 [Crassostrea gigas]; 625 798 Peroxiredoxin-1 [Crassostrea gigas]; 633 824 Peroxiredoxin-1 [Crassostrea gigas]

Protein Accession	CL3160.Contig18$_H$1 CL3160.Contig17$_H$1 CL3160.Contig16$_H$1 CL3160.Contig15$_H$1 CL3160
Mean Expression Ratio	0.783
Median Expression Ratio	0.784
Credible Interval	(0.351, 1.75)
Associated Peptides	1
Associated Spectra	5
Coverage	NaN

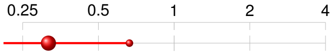

A	B	2.5	50	97.5	Sequence
0	5	0.15	0.32	0.66	LVQAFQFTDK

5.215 160 387 small nuclear ribonucleoprotein polypeptide G protein [Crassostrea ariakensis]; 180 407 small nuclear ribonucleoprotein polypeptide G protein [Crassostrea ariakensis]

Protein Accession	CL1272.Contig8$_H$1 CL1272.Contig7$_H$1
Mean Expression Ratio	1.27
Median Expression Ratio	1.27
Credible Interval	(0.665, 2.46)
Associated Peptides	4
Associated Spectra	18
Coverage	NaN

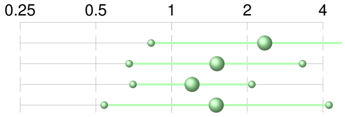

A	B	2.5	50	97.5	Sequence
1	1	0.83	2.4	6.7	AHPPELK
3	1	0.68	1.5	3.3	SIGMVVVR
5	5	0.7	1.2	2.1	TITGILR
1	1	0.54	1.5	4.2	AHPPELKK

5.216 201 779 minus strand sigma class glutathione-s-transferase 2 [Haliotis discus discus]

Protein Accession	Unigene22998$_{H}1$
Mean Expression Ratio	0.786
Median Expression Ratio	0.785
Credible Interval	(0.408, 1.52)
Associated Peptides	6
Associated Spectra	8
Coverage	NaN

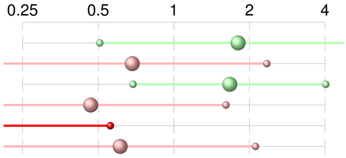

A	B	2.5	50	97.5	Sequence
0	1	0.51	1.8	6.4	IKNYVANR
0	1	0.19	0.68	2.3	LVLALADQK
0	3	0.69	1.7	4	ALYENVANNPR
0	1	0.14	0.47	1.6	YFGMFQK
0	1	0.047	0.16	0.56	KLEEEEFPK
0	1	0.18	0.61	2.1	VINIYK

5.217 3 263 calponin-like protein [Taenia asiatica]

Protein Accession	CL26318.Contig4$_{H}1$
Mean Expression Ratio	1.28
Median Expression Ratio	1.27
Credible Interval	(0.561, 2.97)
Associated Peptides	1
Associated Spectra	2
Coverage	NaN

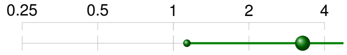

A	B	2.5	50	97.5	Sequence
0	2	1.1	3.3	9.2	HIADIKVDDMSAASK

5.218 811 1407 minus strand mitochondrial malate dehydrogenase 2 [Eospalax baileyi]

Protein Accession	CL10426.Contig13$_{H}1$
Mean Expression Ratio	0.787
Median Expression Ratio	0.785
Credible Interval	(0.394, 1.59)
Associated Peptides	3
Associated Spectra	16
Coverage	NaN

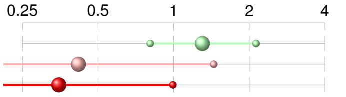

A	B	2.5	50	97.5	Sequence
6	7	0.81	1.3	2.1	IQNAGTEVVEAK
1	0	0.12	0.42	1.4	NLGMGK
0	2	0.12	0.35	0.99	AAMPELIANISK

5.219 " 64 1332 Flotillin-1, putative [Pediculus humanus corporis] gi"

Protein Accession	CL33952.Contig1$_{H}1$
Mean Expression Ratio	1.27
Median Expression Ratio	1.27
Credible Interval	(0.656, 2.45)
Associated Peptides	6
Associated Spectra	7
Coverage	NaN

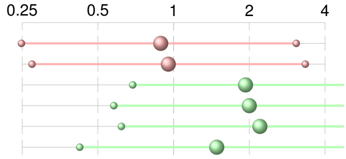

A	B	2.5	50	97.5	Sequence
1	0	0.25	0.89	3.1	AVAQETLEGHQR
1	0	0.27	0.96	3.3	VAAEIAQPLSNAK
1	1	0.69	1.9	5.5	FLNDIEIAK
0	1	0.58	2	6.9	AQADLAYDLQAAK
0	1	0.62	2.2	7.6	GQAIQVQEQEIIRR
0	1	0.42	1.5	5.1	VKGEAEAFAIEAK

5.220 " 347 847 minus strand ADP,ATP carrier protein 1 OS=Anopheles gambiae GN=AGAP006782 PE=2 SV=2"

Protein Accession	CL27196.Contig1$_{H}1$
Mean Expression Ratio	0.785
Median Expression Ratio	0.788
Credible Interval	(0.345, 1.76)
Associated Peptides	1
Associated Spectra	1
Coverage	NaN

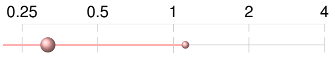

A	B	2.5	50	97.5	Sequence
1	0	0.088	0.32	1.1	SNPGESYAVK

5.221 613 2421 PREDICTED: lysosomal alpha-glucosidase [Danio rerio]

Protein Accession	CL5792.Contig1$_{H}1$
Mean Expression Ratio	1.27
Median Expression Ratio	1.27
Credible Interval	(0.674, 2.39)
Associated Peptides	5
Associated Spectra	18
Coverage	NaN

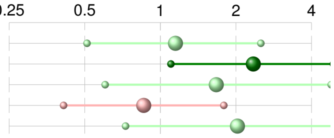

A	B	2.5	50	97.5	Sequence
2	2	0.51	1.1	2.5	ATMGGLQK
1	4	1.1	2.3	4.9	LPPATTTNIMR
2	0	0.6	1.7	4.8	GGSIIPR
0	5	0.41	0.86	1.8	GLPDIVDDLHSHNQR
0	2	0.73	2	5.7	SFVLSR

5.222 74 2734 26S proteasome non-ATPase regulatory subunit 2 [Crassostrea gigas]

Protein Accession	CL32689.Contig3$_{H}1$
Mean Expression Ratio	1.26
Median Expression Ratio	1.27
Credible Interval	(0.55, 2.89)
Associated Peptides	1
Associated Spectra	1
Coverage	NaN

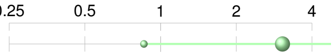

A	B	2.5	50	97.5	Sequence
0	1	0.86	3.1	11	VGQAVDVVGQAGKPK

5.223 680 991 minus strand PDZ and LIM domain protein 1 [Crassostrea gigas]; 553 864 minus strand PDZ and LIM domain protein 1 [Crassostrea gigas]

Protein Accession	Unigene7664$_{H}1$ Unigene7663$_{H}1$
Mean Expression Ratio	1.27
Median Expression Ratio	1.27
Credible Interval	(0.567, 2.87)
Associated Peptides	1
Associated Spectra	2
Coverage	NaN

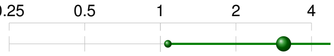

A	B	2.5	50	97.5	Sequence
0	2	1.1	3.1	8.9	QLTHMQAHQLIK

5.224 4 783 minus strand hemocyanin isoform 2 [Nucula nucleus]

Protein Accession	CL3697.Contig2$_{H}1$
Mean Expression Ratio	0.79
Median Expression Ratio	0.79
Credible Interval	(0.382, 1.66)
Associated Peptides	3
Associated Spectra	4
Coverage	NaN

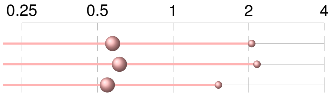

A	B	2.5	50	97.5	Sequence
**0**	**1**	**0.17**	**0.58**	**2.1**	**ELSEEQIK**
**0**	**1**	**0.18**	**0.61**	**2.2**	**DVMDYK**
**0**	**2**	**0.19**	**0.55**	**1.5**	**SGTWLK**

5.225 28 516 minus strand Signal peptidase complex subunit 3 [Crassostrea gigas]; 30 554 minus strand Signal peptidase complex subunit 3 [Crassostrea gigas]

Protein Accession	Unigene59022$_{H}1$ Unigene26504$_{H}1$
Mean Expression Ratio	0.787
Median Expression Ratio	0.79
Credible Interval	(0.341, 1.76)
Associated Peptides	1
Associated Spectra	2
Coverage	NaN

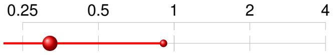

A	B	2.5	50	97.5	Sequence
**1**	**1**	**0.11**	**0.32**	**0.91**	**SDLGLVTFDLK**

5.226 56 937 Chloride intracellular channel exc-4 [Crassostrea gigas]

Protein Accession	CL34620.Contig3$_{H}1$
Mean Expression Ratio	1.27
Median Expression Ratio	1.27
Credible Interval	(0.598, 2.72)
Associated Peptides	2
Associated Spectra	4
Coverage	NaN

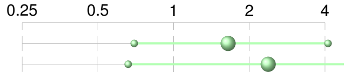

A	B	2.5	50	97.5	Sequence
**1**	**2**	**0.7**	**1.6**	**4.1**	**KVQGPLVK**
**0**	**1**	**0.66**	**2.4**	**8.3**	**QKFELYIK**

5.227 163 1059 minus strand Heterogeneous nuclear ribonucleoprotein A2-like protein 1 [Crassostrea gigas]; 99 995 minus strand Heterogeneous nuclear ribonucleoprotein A2-like protein 1 [Crassostrea gigas]; 71 967 minus strand Heterogeneous nuclear ribonucleoprotein A2-like protein 1 [Crassostrea gigas]; 71 967 minus strand Heterogeneous nuclear ribonucleoprotein A2-like protein 1 [Crassostrea gigas]

Protein Accession	Unigene65$_{H}1$ Unigene63$_{H}1$ Unigene38787$_{H}1$ Unigene38786$_{H}1$
Mean Expression Ratio	0.788
Median Expression Ratio	0.79
Credible Interval	(0.364, 1.66)
Associated Peptides	2
Associated Spectra	3
Coverage	NaN

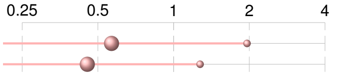

A	B	2.5	50	97.5	Sequence
1	0	0.16	0.57	2	IENVEMIK
2	0	0.16	0.45	1.3	IENVEMIKDK

5.228 689 1984 hypothetical protein BRAFLDRAFT_78257 [Branchiostoma floridae] gi

Protein Accession	Unigene34076$_{H}1$
Mean Expression Ratio	1.27
Median Expression Ratio	1.27
Credible Interval	(0.706, 2.32)
Associated Peptides	7
Associated Spectra	13
Coverage	NaN

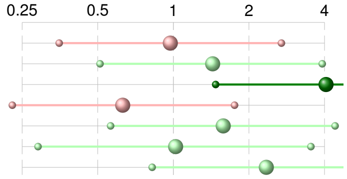

A	B	2.5	50	97.5	Sequence
1	1	0.35	0.97	2.7	VQQKPEPSPAEGEVLIR
1	1	0.51	1.4	3.9	AWAELVAV
0	2	1.5	4	11	ILLDPSKAPTPK
0	2	0.23	0.63	1.8	ILLDPSK
0	2	0.56	1.6	4.4	SFFSFAK
0	1	0.29	1	3.5	GISILKPMGK
0	2	0.82	2.3	6.5	SLVLTGFGSMK

5.229 2939 3208 minus strand beta-thymosin domain repeat protein CSP24KDa_v1 [Hermissenda crassicornis] gi

Protein Accession	CL9548.Contig3$_{H}1$
Mean Expression Ratio	0.791
Median Expression Ratio	0.791
Credible Interval	(0.421, 1.52)
Associated Peptides	6
Associated Spectra	9
Coverage	NaN

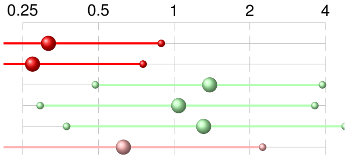

A	B	2.5	50	97.5	Sequence
1	1	0.12	0.32	0.89	NPLPSTDAIAAEK
1	1	0.096	0.27	0.75	SIGEFDK
1	1	0.49	1.4	3.9	SIGEFDKGA
0	1	0.29	1	3.6	KAETEEKNPLPTKEVIQQEK
0	1	0.37	1.3	4.8	SIGEFDKGALK
0	1	0.18	0.63	2.2	AETEEKNPLPTK

5.230 71 409 minus strand heat shock protein 108 [Alligator mississippiensis]

Protein Accession	CL5089.Contig3$_{H}1$
Mean Expression Ratio	1.27
Median Expression Ratio	1.26
Credible Interval	(0.548, 2.94)
Associated Peptides	1
Associated Spectra	1
Coverage	NaN

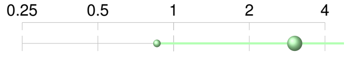

A	B	2.5	50	97.5	Sequence
0	1	0.86	3	11	QETVDDDLGKTMEGSR

5.231 122 1195 minus strand Severin [Crassostrea gigas]

Protein Accession	CL7486.Contig12$_{H}1$
Mean Expression Ratio	1.26
Median Expression Ratio	1.26
Credible Interval	(0.662, 2.47)
Associated Peptides	6
Associated Spectra	8
Coverage	NaN

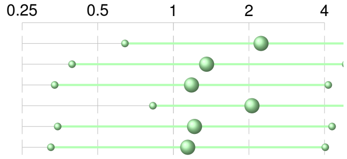

A	B	2.5	50	97.5	Sequence
0	1	0.64	2.2	7.7	TDHPLVPITCLTEGTK
0	1	0.39	1.4	4.8	TVELDTFLNDVPVQHR
0	1	0.34	1.2	4.1	SVKPEEYRPR
0	3	0.83	2.1	5.1	EVQGHESDLFR
0	1	0.35	1.2	4.3	LLHFHGDR
0	1	0.33	1.1	4	SDFISEDVFILDTK

5.232 1 753 hypothetical protein BRAFLDRAFT_131251 [Branchiostoma floridae] gi

Protein Accession	CL18526.Contig3$_{H}1$
Mean Expression Ratio	0.795
Median Expression Ratio	0.794
Credible Interval	(0.408, 1.53)
Associated Peptides	5
Associated Spectra	7
Coverage	NaN

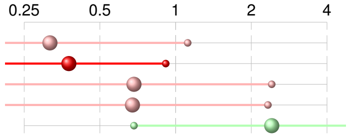

A	B	2.5	50	97.5	Sequence
1	0	0.091	0.32	1.1	GVFEIDSEK
3	0	0.15	0.38	0.91	GLGPFPR
0	1	0.19	0.69	2.4	GAGEPIDADYLLR
0	1	0.19	0.67	2.3	NVVDMIITEK
0	1	0.68	2.4	8.4	VIKGEKYEK

5.233 112 1050 Enolase-phosphatase E1 [Crassostrea gigas]; 112 1050 Enolase-phosphatase E1 [Crassostrea gigas]

Protein Accession	CL25175.Contig3$_{H}1$ CL25175.Contig2$_{H}1$
Mean Expression Ratio	1.26
Median Expression Ratio	1.26
Credible Interval	(0.595, 2.64)
Associated Peptides	2
Associated Spectra	5
Coverage	NaN

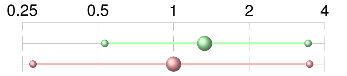

A	B	2.5	50	97.5	Sequence
2	2	0.53	1.3	3.4	SAAEAESLLVGVK
0	1	0.27	1	3.5	QLQGHIWR

5.234 3 434 minus strand myosin essential light chain [Haliotis discus discus]

Protein Accession	CL17177.Contig8$_{H}$1
Mean Expression Ratio	1.26
Median Expression Ratio	1.26
Credible Interval	(0.712, 2.24)
Associated Peptides	7
Associated Spectra	25
Coverage	NaN

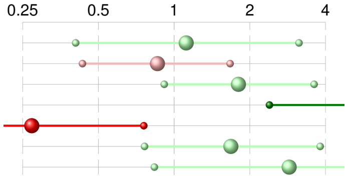

A	B	2.5	50	97.5	Sequence
1	1	0.41	1.1	3.1	EGQGFMSAAELR
3	3	0.43	0.86	1.7	LTDQQVTDVIK
3	3	0.91	1.8	3.6	FTDTREDLEGNVK
2	2	2.4	5.3	12	YADFIEK
1	1	0.099	0.27	0.76	DGDVDAFK
1	3	0.76	1.7	3.8	HVLTGLGEK
0	1	0.84	2.9	10	LSDREGQGFMSAAELR

5.235 3 416 minus strand myosin essential light chain [Crassostrea gigas]

Protein Accession	Unigene47974$_{H}$1
Mean Expression Ratio	1.26
Median Expression Ratio	1.26
Credible Interval	(0.564, 2.88)
Associated Peptides	1
Associated Spectra	2
Coverage	NaN

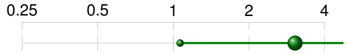

A	B	2.5	50	97.5	Sequence
0	2	1.1	3	8.6	CVGLNPTLATIK

5.236 " 431 880 Myosin essential light chain, striated adductor muscle OS=Aequipecten irradians PE=1 SV=2"

Protein Accession	CL36.Contig2$_{H}$1
Mean Expression Ratio	0.794
Median Expression Ratio	0.795
Credible Interval	(0.409, 1.54)
Associated Peptides	4
Associated Spectra	24
Coverage	NaN

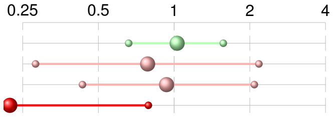

A	B	2.5	50	97.5	Sequence
8	9	0.66	1	1.6	VGDLLR
0	2	0.28	0.79	2.2	LSDEEVDQIMK
0	4	0.43	0.93	2.1	LTDITIDLEGNLK
0	1	0.062	0.22	0.79	VDQIMK

5.237 397 510 PREDICTED: actin-5C-like [Megachile rotundata]

Protein Accession	CL48442.Contig2$_{H}$1
Mean Expression Ratio	0.794
Median Expression Ratio	0.795
Credible Interval	(0.36, 1.77)
Associated Peptides	1
Associated Spectra	9
Coverage	NaN

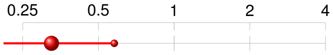

A	B	2.5	50	97.5	Sequence
0	9	0.18	0.33	0.58	DLTDYLMK

5.238 " 193 333 minus strand RecName: Full=Globin; AltName: Full=Myoglobin gi|299274|gb|AAB25743.1| myoglobin [Nassa mutabilis=marine gastropods, radular muscles, Peptide, 147 aa] gi; 193 333 minus strand RecName: Full=Globin; AltName: Full=Myoglobin gi|299274|gb|AAB25743.1| myoglobin [Nassa mutabilis=marine gastropods, radular muscles, Peptide, 147 aa] gi"

Protein Accession	CL8458.Contig5$_H1$ CL8458.Contig1$_H1$
Mean Expression Ratio	0.793
Median Expression Ratio	0.795
Credible Interval	(0.37, 1.7)
Associated Peptides	2
Associated Spectra	6
Coverage	NaN

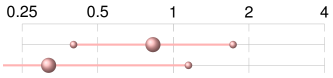

A	B	2.5	50	97.5	Sequence
4	1	0.4	0.83	1.7	AAINSTWPK
1	0	0.088	0.32	1.1	LSADKETMENNGAR

5.239 1 264 Spectrin beta chain [Crassostrea gigas]

Protein Accession	Unigene36334$_H1$
Mean Expression Ratio	1.25
Median Expression Ratio	1.26
Credible Interval	(0.585, 2.71)
Associated Peptides	2
Associated Spectra	2
Coverage	NaN

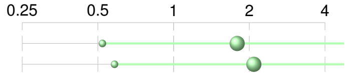

A	B	2.5	50	97.5	Sequence
0	1	0.52	1.8	6.3	QLLQVEHPNAAEVVQR
0	1	0.58	2.1	7.5	VAVVNQLAR

5.240 69 731 hypoxanthine phosphoribosyltransferase 1 [Haliotis discus discus]

Protein Accession	Unigene30480$_H1$
Mean Expression Ratio	0.795
Median Expression Ratio	0.796
Credible Interval	(0.369, 1.71)
Associated Peptides	2
Associated Spectra	3
Coverage	NaN

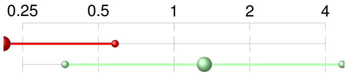

A	B	2.5	50	97.5	Sequence
1	1	0.074	0.21	0.58	TLEAVHPK
0	1	0.37	1.3	4.7	LDKIK

5.241 51 1295 PREDICTED: phosphoglycerate kinase-like [Nasonia vitripennis]; 97 1341 PREDICTED: phosphoglycerate kinase-like [Nasonia vitripennis]

Protein Accession	CL14417.Contig13$_{H}1$ CL14417.Contig12$_{H}1$
Mean Expression Ratio	1.26
Median Expression Ratio	1.26
Credible Interval	(0.842, 1.88)
Associated Peptides	22
Associated Spectra	66
Coverage	NaN

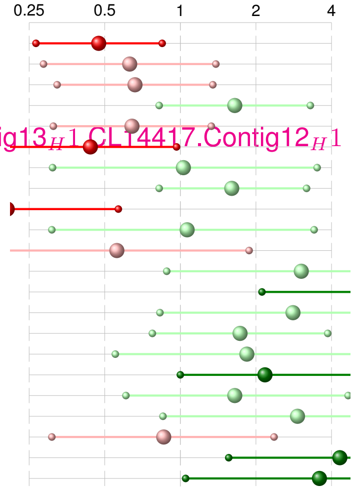

A	B	2.5	50	97.5	Sequence
4	5	0.27	0.47	0.85	ALMDAVVEATK
2	2	0.29	0.63	1.4	ATQEDIDKFR
3	2	0.32	0.66	1.3	FHVEEEGK
2	4	0.83	1.6	3.3	LSIDKVDVK
2	3	0.31	0.64	1.3	VDFNVPLKDGK
3	1	0.2	0.44	0.97	FTDAVK
1	0	0.31	1	3.5	VLNNMEIGK
1	5	0.82	1.6	3.2	VLPGVAALTDA
1	1	0.074	0.21	0.57	YALENGAK
0	1	0.31	1.1	3.4	AHSSMVGCQLPIR
0	1	0.16	0.56	1.9	ALNNPDRPFLAILGGAK
0	1	0.88	3	10	ASGFLLKK
0	3	2.1	5.2	12	FHVEEEGKGVAPDGTK
0	1	0.83	2.8	9.6	FHVEEEGKGVAPDGTKLK
0	4	0.77	1.7	3.9	IVAALPTIK
0	1	0.55	1.8	6.3	LGDIYVNDAFGTAHR
0	4	1	2.2	4.9	SGIPADWMGLDIGPK
0	2	0.61	1.7	4.6	SVVLMSHLGRPDGTK
0	1	0.85	2.9	10	VDFNVPLKDGKITNNQR
0	2	0.31	0.86	2.4	ASGFLLK
0	2	1.6	4.3	12	FTDAVKR
0	1	1.1	3.6	12	LNKLSIDK

5.242 238 687 60S ribosomal protein L6 [Crassostrea gigas]; 142 789 60S ribosomal protein L6 [Crassostrea gigas]

Protein Accession	Unigene30349$_{H}1$ CL6501.Contig1$_{H}1$
Mean Expression Ratio	0.795
Median Expression Ratio	0.797
Credible Interval	(0.348, 1.83)
Associated Peptides	1
Associated Spectra	1
Coverage	NaN

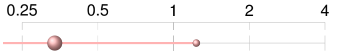

A	B	2.5	50	97.5	Sequence
0	1	0.097	0.34	1.2	AVDTQLLEVIR

5.243 313 2763 NCAM-related cell adhesion molecule precursor [Aplysia californica] gi

Protein Accession	Unigene11449$_{H}1$
Mean Expression Ratio	1.26
Median Expression Ratio	1.26
Credible Interval	(0.661, 2.43)
Associated Peptides	5
Associated Spectra	9
Coverage	NaN

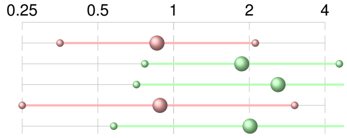

A	B	2.5	50	97.5	Sequence
2	1	0.35	0.86	2.1	SAGEPLLDAVTK
1	2	0.77	1.9	4.6	QILYFR
0	1	0.71	2.6	9.1	KTSVAEDYKELTVDR
0	1	0.25	0.88	3	EALEVDTSK
0	1	0.58	2	7	IGSSDPTVFVFK

5.244 31 375 minus strand 40S ribosomal protein S19 OS=Mya arenaria GN=RPS19 PE=2 SV=1

Protein Accession	CL8932.Contig2$_{H}1$
Mean Expression Ratio	1.26
Median Expression Ratio	1.26
Credible Interval	(0.55, 2.94)
Associated Peptides	1
Associated Spectra	1
Coverage	NaN

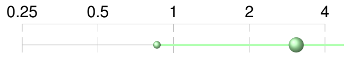

A	B	2.5	50	97.5	Sequence
**0**	**1**	**0.86**	**3.1**	**11**	**GISVKDVNGHEFTK**

5.245 360 764 minus strand Peptidyl-prolyl cis-trans isomerase A OS=Dictyostelium discoideum GN=ppiA PE=1 SV=1

Protein Accession	Unigene64429$_{H}1$
Mean Expression Ratio	0.794
Median Expression Ratio	0.797
Credible Interval	(0.354, 1.84)
Associated Peptides	1
Associated Spectra	2
Coverage	NaN

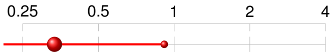

A	B	2.5	50	97.5	Sequence
**0**	**2**	**0.12**	**0.33**	**0.91**	**HVVFGQVVEGLDVVR**

5.246 132 932 pyruvate kinase [Crassostrea gigas]

Protein Accession	CL31920.Contig1$_{H}1$
Mean Expression Ratio	1.26
Median Expression Ratio	1.25
Credible Interval	(0.558, 2.87)
Associated Peptides	1
Associated Spectra	2
Coverage	NaN

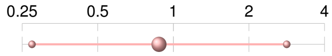

A	B	2.5	50	97.5	Sequence
**1**	**1**	**0.27**	**0.87**	**2.8**	**ITIDDSYKEK**

5.247 170 1375 PREDICTED: sialate O-acetylesterase-like [Oreochromis niloticus]

Protein Accession	CL26143.Contig1$_{H}1$
Mean Expression Ratio	0.795
Median Expression Ratio	0.798
Credible Interval	(0.348, 1.78)
Associated Peptides	1
Associated Spectra	2
Coverage	NaN

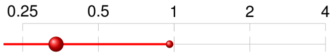

A	B	2.5	50	97.5	Sequence
**2**	**0**	**0.12**	**0.34**	**0.96**	**DKLDVAQR**

5.248 18 1613 minus strand chaperonin containing T-complex polypeptide subunit zeta [Haliotis discus hannai]; 527 2089 minus strand chaperonin containing T-complex polypeptide subunit zeta [Haliotis discus hannai]; 18 1586 minus strand chaperonin containing T-complex polypeptide subunit zeta [Haliotis discus hannai]

Protein Accession	CL23722.Contig7$_{H}1$ CL23722.Contig5$_{H}1$ CL23722.Contig4$_{H}1$
Mean Expression Ratio	1.25
Median Expression Ratio	1.25
Credible Interval	(0.625, 2.52)
Associated Peptides	4
Associated Spectra	5
Coverage	NaN

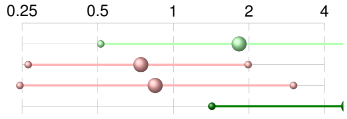

A	B	2.5	50	97.5	Sequence
0	1	0.52	1.8	6.5	AAQALAVNISAAK
0	2	0.26	0.74	2	GIDPFSLDMFAK
0	1	0.24	0.85	3	TDMDSTLVR
0	1	1.4	5	18	AQALAVNISAAK

5.249 6 317 myosin heavy chain cardiac muscle specific isoform 2 [Argopecten irradians]

Protein Accession	Unigene19023$_{H}1$
Mean Expression Ratio	1.25
Median Expression Ratio	1.25
Credible Interval	(0.8, 1.92)
Associated Peptides	16
Associated Spectra	58
Coverage	NaN

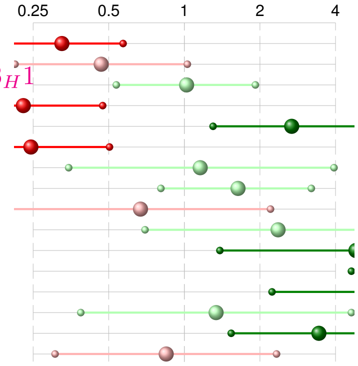

A	B	2.5	50	97.5	Sequence
7	2	0.18	0.33	0.57	ALETQLK
2	2	0.21	0.47	1	ELEMELDSEQR
4	3	0.54	1	1.9	KALETQLK
2	3	0.11	0.23	0.47	LDEAEAQALK
1	4	1.3	2.7	5.5	LDEAEAQALKGGK
5	0	0.12	0.24	0.5	LADELR
1	0	0.35	1.2	3.9	ELAFQSDEDRK
1	5	0.81	1.6	3.2	LADELRQEQEHSLQVEK
0	1	0.19	0.67	2.2	ELAFQSDEDR
0	1	0.7	2.4	8.2	ELEMELDSEQRR
0	1	1.4	4.8	17	KALETQLKDMQAR
0	4	4.6	10	23	KIIQKLEQR
0	2	2.2	6.3	17	LADELRQEQEHSLQVEKLR
0	1	0.39	1.3	4.6	VRELEMELDSEQR
0	4	1.5	3.4	7.7	VRELEMELDSEQRR
0	2	0.3	0.85	2.3	LKELAFQSDEDRK

5.250 " 4 423 minus strand PREDICTED: similar to NADH dehydrogenase, putative [Tribolium castaneum] gi"

Protein Accession	CL4111.Contig2$_{H}1$
Mean Expression Ratio	0.798
Median Expression Ratio	0.802
Credible Interval	(0.362, 1.73)
Associated Peptides	2
Associated Spectra	2
Coverage	NaN

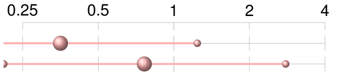

A	B	2.5	50	97.5	Sequence
1	0	0.1	0.35	1.2	QVKPILSTNPAEAK
1	0	0.21	0.77	2.8	IKQVK

5.251 145 1020 minus strand PREDICTED: inorganic pyrophosphatase-like [Saccoglossus kowalevskii]

Protein Accession	CL12968.Contig14$_{H}1$
Mean Expression Ratio	0.803
Median Expression Ratio	0.802
Credible Interval	(0.43, 1.5)
Associated Peptides	7
Associated Spectra	10
Coverage	NaN

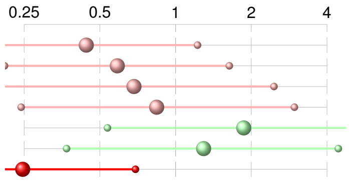

A	B	2.5	50	97.5	Sequence
1	1	0.16	0.44	1.2	IIAIDVTDPLASK
2	0	0.21	0.58	1.6	SLMADPSVGK
0	1	0.19	0.68	2.5	LNDIDDVHR
0	1	0.24	0.84	3	MEINKEEK
0	1	0.53	1.9	6.6	GSVIQVK
0	1	0.37	1.3	4.4	LNPLKQDVK
0	2	0.088	0.25	0.69	AVIDQSVNK

5.252 89 283 hypothetical protein CGI_10017178 [Crassostrea gigas]

Protein Accession	Unigene58795$_{H}1$
Mean Expression Ratio	1.25
Median Expression Ratio	1.24
Credible Interval	(0.64, 2.46)
Associated Peptides	3
Associated Spectra	21
Coverage	NaN

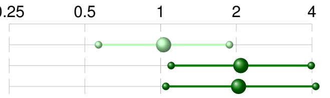

A	B	2.5	50	97.5	Sequence
3	5	0.56	1	1.9	AGIIASAK
0	7	1.1	2.1	4	NMSVIAHVDHGK
0	6	1	2	4.2	VNFTIDQIR

5.253 14 1642 minus strand chaperonin containing tcp1 [Haliotis discus discus]; 14 1462 minus strand chaperonin containing tcp1 [Haliotis discus discus]; 14 1582 minus strand chaperonin containing tcp1 [Haliotis discus discus]

Protein Accession	CL16298.Contig4$_{H}1$ CL16298.Contig15$_{H}1$ CL16298.Contig14$_{H}1$
Mean Expression Ratio	1.24
Median Expression Ratio	1.24
Credible Interval	(0.605, 2.53)
Associated Peptides	4
Associated Spectra	4
Coverage	NaN

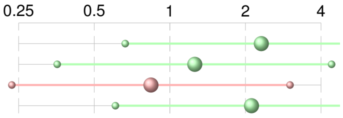

A	B	2.5	50	97.5	Sequence
0	1	0.66	2.3	7.8	FAESLEVIPR
0	1	0.35	1.3	4.4	MVINHLEK
0	1	0.24	0.84	3	ACVSILPAK
0	1	0.61	2.1	7.5	HFSGLEEAVYR

5.254 59 379 hypothetical protein DAPPUDRAFT_230756 [Daphnia pulex]; 117 443 minus strand hypothetical protein DAPPUDRAFT_230756 [Daphnia pulex]; 120 473 minus strand hypothetical protein DAPPUDRAFT_230756 [Daphnia pulex]

Protein Accession	CL2137.Contig6$_{H}1$ CL2137.Contig5$_{H}1$ CL2137.Contig4$_{H}1$
Mean Expression Ratio	0.804
Median Expression Ratio	0.805
Credible Interval	(0.379, 1.69)
Associated Peptides	2
Associated Spectra	7
Coverage	NaN

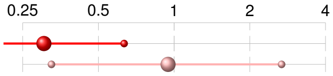

A	B	2.5	50	97.5	Sequence
3	2	0.15	0.3	0.63	AASTLAVK
0	2	0.33	0.95	2.7	LVNSLTASGTGALK

5.255 84 425 minus strand Transcription elongation factor B polypeptide 2 [Crassostrea gigas]; 84 425 minus strand Transcription elongation factor B polypeptide 2 [Crassostrea gigas]; 84 425 minus strand Transcription elongation factor B polypeptide 2 [Crassostrea gigas]; 112 366 Transcription elongation factor B polypeptide 2 OS=Rattus norvegicus GN=Tceb2 PE=1 SV=1; 101 442 minus strand Transcription elongation factor B polypeptide 2 [Crassostrea gigas]; 101 442 minus strand Transcription elongation factor B polypeptide 2 [Crassostrea gigas]; 84 425 minus strand Transcription elongation factor B polypeptide 2 [Crassostrea gigas]

Protein Accession	CL19448.Contig9$_{H}1$ CL19448.Contig8$_{H}1$ CL19448.Contig7$_{H}1$ CL19448.Contig4$_{H}1$ CL1944
Mean Expression Ratio	0.801
Median Expression Ratio	0.805
Credible Interval	(0.345, 1.82)
Associated Peptides	1
Associated Spectra	1
Coverage	NaN

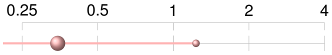

A	B	2.5	50	97.5	Sequence
1	0	0.095	0.35	1.2	AQAPATLGLVYK

5.256 313 1908 minus strand Heterogeneous nuclear ribonucleoprotein L [Crassostrea gigas]

Protein Accession	CL19412.Contig8$_{H}1$
Mean Expression Ratio	1.24
Median Expression Ratio	1.24
Credible Interval	(0.602, 2.65)
Associated Peptides	3
Associated Spectra	4
Coverage	NaN

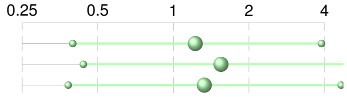

A	B	2.5	50	97.5	Sequence
1	1	0.4	1.2	3.9	SSTGLLEFESK
0	1	0.44	1.5	5.4	GQPLLQEPR
0	1	0.38	1.3	4.7	TPFIFK

5.257 253 765 cytochrome c oxidase subunit IV [Urechis caupo]

Protein Accession	CL38636.Contig3$_H1$
Mean Expression Ratio	0.809
Median Expression Ratio	0.806
Credible Interval	(0.419, 1.57)
Associated Peptides	4
Associated Spectra	17
Coverage	NaN

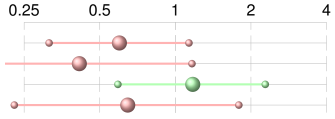

A	B	2.5	50	97.5	Sequence
3	4	0.32	0.6	1.1	IEGLSSK
2	0	0.15	0.41	1.2	SLTLEDKK
1	5	0.59	1.2	2.3	FKENTPDVMALR
0	2	0.23	0.65	1.8	TEAALDQTARDRIHPK

5.258 152 1930 hypothetical protein BRAFLDRAFT_223365 [Branchiostoma floridae] gi

Protein Accession	CL49005.Contig4$_H1$
Mean Expression Ratio	0.808
Median Expression Ratio	0.807
Credible Interval	(0.423, 1.53)
Associated Peptides	6
Associated Spectra	9
Coverage	NaN

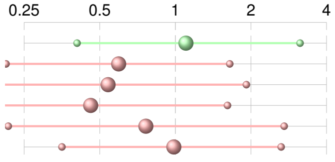

A	B	2.5	50	97.5	Sequence
1	1	0.41	1.1	3.1	QYSAGVSQLSLR
1	1	0.21	0.59	1.6	VTLLR
0	1	0.15	0.54	1.9	ALGLPAATSFK
0	1	0.13	0.46	1.6	LSALGLR
0	1	0.22	0.76	2.7	EVSDGVIAPGYEAAALEILK
0	2	0.35	0.99	2.6	AVIDNSLFGNIVTK

5.259 116 1321 PREDICTED: similar to aspartate aminotransferase [Tribolium castaneum] gi

Protein Accession	CL11692.Contig6$_H1$
Mean Expression Ratio	0.806
Median Expression Ratio	0.807
Credible Interval	(0.482, 1.34)
Associated Peptides	13
Associated Spectra	27
Coverage	NaN

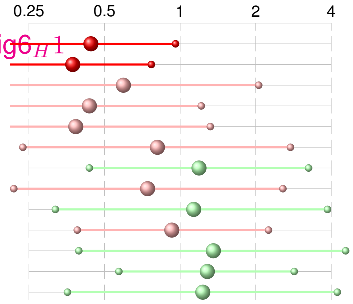

A	B	2.5	50	97.5	Sequence
2	2	0.2	0.44	0.96	ILSTPALR
3	2	0.18	0.37	0.77	ISVAGLSSK
1	0	0.17	0.59	2	NMGLYGER
1	1	0.16	0.44	1.2	DAFALR
1	0	0.11	0.39	1.3	TQWLGEVK
1	0	0.24	0.81	2.7	DDNGKPYVLPSVLK
0	2	0.43	1.2	3.3	AEKEIIDAK
0	1	0.22	0.74	2.6	DGHQVALAQSFAK
0	1	0.32	1.1	3.9	MDKEYAGITGVPEFTK
0	3	0.39	0.93	2.3	VGAFTIICDNKDEAAR
0	1	0.39	1.4	4.6	NVGYLAK
0	4	0.57	1.3	2.9	IGAAFLAK
0	1	0.36	1.2	4.2	VMSQVK

5.260 143 247 minus strand dynein light chain [Aplysia californica] gi

Protein Accession	CL13886.Contig1$_{H}1$
Mean Expression Ratio	0.807
Median Expression Ratio	0.807
Credible Interval	(0.36, 1.83)
Associated Peptides	1
Associated Spectra	2
Coverage	NaN

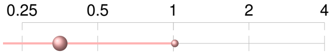

A	B	2.5	50	97.5	Sequence
0	2	0.13	0.35	1	NADMAEDMQQDAVDCATQALEK

5.261 1200 1547 protein disulfide isomerase [Conus eburneus]

Protein Accession	CL3770.Contig1$_{H}1$
Mean Expression Ratio	0.811
Median Expression Ratio	0.807
Credible Interval	(0.462, 1.44)
Associated Peptides	7
Associated Spectra	49
Coverage	NaN

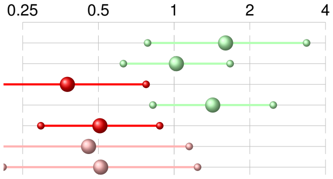

A	B	2.5	50	97.5	Sequence
2	3	0.78	1.6	3.4	TLSAFVK
4	9	0.63	1	1.7	VQSFPTLK
4	1	0.18	0.38	0.77	YKDSNDIVIAK
1	9	0.82	1.4	2.5	IDSTANDVENIR
2	8	0.3	0.51	0.88	NFNDVAFDK
3	0	0.19	0.46	1.1	VLVGK
0	3	0.21	0.51	1.2	QLAPIWDELAEK

5.262 " 223 423 ADPATP carrier protein; K05863 solute carrier family 25 (mitochondrial adenine nucleotide translocator), member 4/5/6/31"

Protein Accession	CL27196.Contig3$_{H}1$
Mean Expression Ratio	0.808
Median Expression Ratio	0.808
Credible Interval	(0.396, 1.67)
Associated Peptides	1
Associated Spectra	8
Coverage	NaN

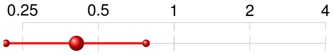

A	B	2.5	50	97.5	Sequence
6	2	0.21	0.41	0.77	LLVQNQNEMIK

5.263 3 1460 Myosin-VI [Crassostrea gigas]

Protein Accession	CL6618.Contig5$_{H}1$
Mean Expression Ratio	0.806
Median Expression Ratio	0.808
Credible Interval	(0.369, 1.78)
Associated Peptides	2
Associated Spectra	2
Coverage	NaN

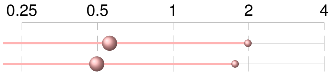

A	B	2.5	50	97.5	Sequence
1	0	0.16	0.56	2	AIEEAAEK
1	0	0.14	0.5	1.8	LGIAK

5.264 503 1219 heat shock cognate 71 [Mytilus galloprovincialis]

Protein Accession	CL23109.Contig5$_{H}1$
Mean Expression Ratio	0.809
Median Expression Ratio	0.809
Credible Interval	(0.38, 1.7)
Associated Peptides	2
Associated Spectra	9
Coverage	NaN

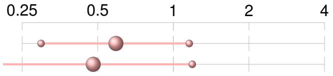

A	B	2.5	50	97.5	Sequence
6	0	0.3	0.59	1.2	GTLEPVEK
0	3	0.19	0.48	1.2	SAIHEIVLVGGSTR

5.265 " 87 287 Chain A, Structure Of Deoxygenated Hemocyanin From Rapana Thomasiana gi|31615505|pdb|1LNL|B Chain B, Structure Of Deoxygenated Hemocyanin From Rapana Thomasiana gi|31615506|pdb|1LNL|C Chain C, Structure Of Deoxygenated Hemocyanin From Rapana Thomasiana"

Protein Accession	Unigene36631$_{H}1$
Mean Expression Ratio	0.808
Median Expression Ratio	0.809
Credible Interval	(0.428, 1.52)
Associated Peptides	4
Associated Spectra	29
Coverage	NaN

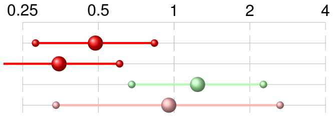

A	B	2.5	50	97.5	Sequence
3	7	0.28	0.49	0.84	QLQEDGSAGGFQK
6	3	0.2	0.35	0.61	SLVEAMR
0	8	0.68	1.2	2.3	KNILELTESEQR
0	2	0.34	0.95	2.6	NILELTESEQR

5.266 85 633 hypothetical protein [Amblyomma maculatum]

Protein Accession	CL2221.Contig1$_{H}1$
Mean Expression Ratio	0.809
Median Expression Ratio	0.81
Credible Interval	(0.388, 1.7)
Associated Peptides	3
Associated Spectra	5
Coverage	NaN

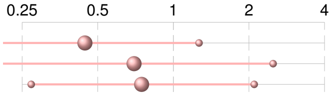

A	B	2.5	50	97.5	Sequence
0	2	0.17	0.45	1.3	IFYTEHLHADEEIR
0	1	0.19	0.7	2.5	DANDEWIR
0	2	0.27	0.75	2.1	EKLPDYDNK

5.267 " 134 2047 PREDICTED: succinate dehydrogenase [ubiquinone] flavoprotein subunit, mitochondrial-like isoform 3, partial [Strongylocentrotus purpuratus]; 134 2047 PREDICTED: succinate dehydrogenase [ubiquinone] flavoprotein subunit, mitochondrial-like isoform 3, partial [Strongylocentrotus purpuratus]; 134 2047 PREDICTED: succinate dehydrogenase [ubiquinone] flavoprotein subunit, mitochondrial-like isoform 3, partial [Strongylocentrotus purpuratus]"

Protein Accession	CL47322.Contig4$_{H}1$ CL47322.Contig3$_{H}1$ CL47322.Contig2$_{H}1$
Mean Expression Ratio	0.809
Median Expression Ratio	0.81
Credible Interval	(0.493, 1.33)
Associated Peptides	13
Associated Spectra	32
Coverage	NaN

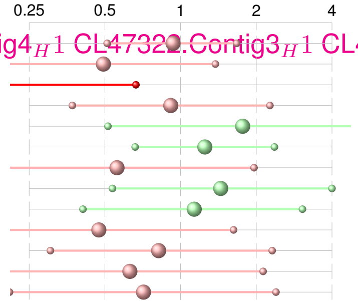

A	B	2.5	50	97.5	Sequence
2	6	0.51	0.93	1.7	AAFGLAEEGFK
2	0	0.18	0.49	1.4	ACANTIIEENKPGDR
1	0	0.056	0.19	0.67	DGPTLQAGVR
2	1	0.37	0.92	2.3	AFGGQSLK
1	0	0.51	1.8	5.9	AFGGQSLKYGK
4	3	0.66	1.2	2.4	AITIEIR
0	1	0.17	0.56	2	IYQQMEDLK
0	2	0.54	1.4	4	KHTLAYTNVDTGK
0	2	0.41	1.1	3.1	NTILATGGYGR
0	1	0.14	0.47	1.6	QDAGEASVGNLDK
0	2	0.3	0.82	2.3	TTLDYRPVIDTTLDEHDCPQVKPAIR
0	1	0.18	0.63	2.1	WHFYDTVK
0	1	0.21	0.71	2.4	HTLAYTNVDTGK

5.268 102 872 RecName: Full=Gelsolin-like protein 1; AltName: Full=Actin-modulator; Short=EWAM; Short=EWAM-P1 gi|157362355|dbj|BAA06219.2| actin-modulator [Lumbricus terrestris]; 102 872 RecName: Full=Gelsolin-like protein 1; AltName: Full=Actin-modulator; Short=EWAM; Short=EWAM-P1 gi|157362355|dbj|BAA06219.2| actin-modulator [Lumbricus terrestris]

Protein Accession	Unigene39721$_{H}1$ Unigene39720$_{H}1$
Mean Expression Ratio	1.24
Median Expression Ratio	1.23
Credible Interval	(0.567, 2.76)
Associated Peptides	2
Associated Spectra	2
Coverage	NaN

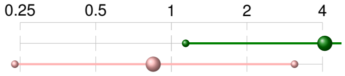

A	B	2.5	50	97.5	Sequence
1	0	1.1	4.1	15	PKGLVK
1	0	0.24	0.85	3.1	SVPAEEYK

5.269 79 2202 minus strand PREDICTED: dolichyl-diphosphooligosaccharide–protein glycosyltransferase subunit STT3A-like isoform 2 [Strongylocentrotus purpuratus]; 79 2202 PREDICTED: dolichyl-diphosphooligosaccharide–protein glycosyltransferase subunit STT3A-like isoform 2 [Strongylocentrotus purpuratus]; 79 2202 PREDICTED: dolichyl-diphosphooligosaccharide–protein glycosyltransferase subunit STT3A-like isoform 2 [Strongylocentrotus purpuratus]

Protein Accession	CL5867.Contig2$_{H}1$ CL5867.Contig11$_{H}1$ CL5867.Contig10$_{H}1$
Mean Expression Ratio	0.806
Median Expression Ratio	0.811
Credible Interval	(0.345, 1.86)
Associated Peptides	1
Associated Spectra	1
Coverage	NaN

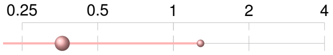

A	B	2.5	50	97.5	Sequence
1	0	0.1	0.36	1.3	VGQAMASTEEK

5.270 137 853 14-3-3 protein [Heterodera glycines]

Protein Accession	CL9040.Contig4$_{H}1$
Mean Expression Ratio	1.23
Median Expression Ratio	1.23
Credible Interval	(0.581, 2.61)
Associated Peptides	2
Associated Spectra	5
Coverage	NaN

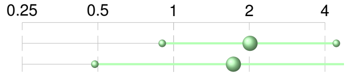

A	B	2.5	50	97.5	Sequence
2	2	0.9	2	4.4	VSEQAERYDDMAK
0	1	0.49	1.7	6.2	AIECANQAYQK

5.271 1166 1939 minus strand Calcium-binding mitochondrial carrier protein Aralar2 OS=Homo sapiens GN=SLC25A13 PE=1 SV=2

Protein Accession	Unigene34297$_{H}1$
Mean Expression Ratio	0.814
Median Expression Ratio	0.812
Credible Interval	(0.366, 1.78)
Associated Peptides	2
Associated Spectra	2
Coverage	NaN

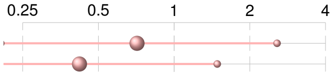

A	B	2.5	50	97.5	Sequence
1	0	0.2	0.71	2.6	LATATFDGVESK
1	0	0.12	0.42	1.5	SAARVLR

5.272 152 1207 haemocyanin precursor [Nautilus pompilius]

Protein Accession	Unigene27031$_{H}1$
Mean Expression Ratio	1.23
Median Expression Ratio	1.23
Credible Interval	(0.577, 2.64)
Associated Peptides	2
Associated Spectra	6
Coverage	NaN

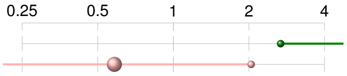

A	B	2.5	50	97.5	Sequence
1	4	2.7	5.5	11	AVDDRLFQK
0	1	0.15	0.58	2	DPNGGMGLK

5.273 " 172 867 minus strand complement C1q-like protein 4 precursor, partial [Oryzias melastigma]"

Protein Accession	Unigene7884$_{H}1$
Mean Expression Ratio	1.23
Median Expression Ratio	1.23
Credible Interval	(0.658, 2.32)
Associated Peptides	5
Associated Spectra	25
Coverage	NaN

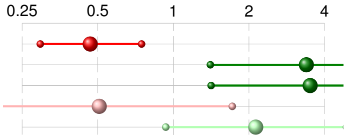

A	B	2.5	50	97.5	Sequence
2	12	0.29	0.47	0.75	DLQQAVESMRPSLK
1	2	1.4	3.4	8.3	EDQIKDLQDK
1	2	1.4	3.5	8.6	EDQIKDLQDKQK
0	1	0.14	0.51	1.7	DLQQAVESMR
0	4	0.94	2.1	4.9	DVKDLQQAVESMRPSLK

5.274 " 703 1173 60S acidic ribosomal protein P0, putative [Ixodes scapularis] gi"

Protein Accession	Unigene34489$_{H}1$
Mean Expression Ratio	0.811
Median Expression Ratio	0.816
Credible Interval	(0.393, 1.64)
Associated Peptides	3
Associated Spectra	6
Coverage	NaN

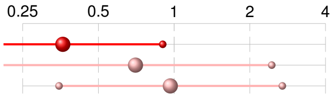

A	B	2.5	50	97.5	Sequence
1	2	0.15	0.36	0.9	EYLKDPSK
0	1	0.2	0.7	2.5	FAAVAAVAAPAAAK
0	2	0.35	0.96	2.7	KSFLEGVR

5.275 3 614 PREDICTED: 60S ribosomal protein L8-like [Papio anubis]

Protein Accession	CL18365.Contig1$_{H}1$
Mean Expression Ratio	1.23
Median Expression Ratio	1.23
Credible Interval	(0.563, 2.72)
Associated Peptides	2
Associated Spectra	2
Coverage	NaN

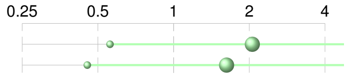

A	B	2.5	50	97.5	Sequence
0	1	0.56	2.1	7.6	KVGLIAAR
0	1	0.45	1.6	5.6	VGLIAARR

5.276 65 523 40S ribosomal protein S9 [Crassostrea gigas]; 65 523 40S ribosomal protein S9 [Crassostrea gigas]

Protein Accession	Unigene72778$_H1$ Unigene72208$_H1$
Mean Expression Ratio	1.22
Median Expression Ratio	1.22
Credible Interval	(0.553, 2.72)
Associated Peptides	2
Associated Spectra	2
Coverage	NaN

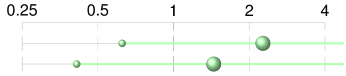

A	B	2.5	50	97.5	Sequence
**0**	**1**	**0.62**	**2.3**	**7.8**	**VKYTLGK**
**0**	**1**	**0.41**	**1.4**	**5.2**	**LIGEYGLR**

5.277 49 819 PREDICTED: elongation factor 1-delta-like isoform 2 [Strongylocentrotus purpuratus]

Protein Accession	CL10790.Contig12$_H1$
Mean Expression Ratio	0.815
Median Expression Ratio	0.816
Credible Interval	(0.403, 1.67)
Associated Peptides	3
Associated Spectra	7
Coverage	NaN

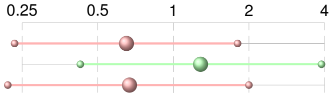

A	B	2.5	50	97.5	Sequence
**1**	**2**	**0.23**	**0.65**	**1.8**	**VAALEVVGTGGASPK**
**1**	**1**	**0.43**	**1.3**	**3.9**	**KPVVIAK**
**1**	**1**	**0.22**	**0.67**	**2**	**LLEEYAAK**

5.278 230 334 protein disulfide isomerase [Conus virgo]

Protein Accession	Unigene67026$_H1$
Mean Expression Ratio	0.82
Median Expression Ratio	0.817
Credible Interval	(0.37, 1.84)
Associated Peptides	1
Associated Spectra	10
Coverage	NaN

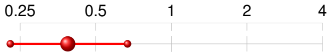

A	B	2.5	50	97.5	Sequence
**3**	**7**	**0.23**	**0.39**	**0.67**	**VDATVENDLASK**

5.279 1180 1521 minus strand coronin [Schistosoma mansoni]

Protein Accession	CL11779.Contig9$_H1$
Mean Expression Ratio	0.815
Median Expression Ratio	0.818
Credible Interval	(0.357, 1.86)
Associated Peptides	1
Associated Spectra	1
Coverage	NaN

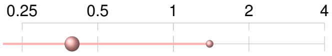

A	B	2.5	50	97.5	Sequence
**1**	**0**	**0.11**	**0.4**	**1.4**	**DANPILVSLK**

5.280 55 1569 minus strand Putative aminopeptidase W07G4.4 [Crassostrea gigas]; 228 1742 Putative aminopeptidase W07G4.4 [Crassostrea gigas]

Protein Accession	CL1078.Contig5$_{H}1$ CL1078.Contig1$_{H}1$
Mean Expression Ratio	1.22
Median Expression Ratio	1.22
Credible Interval	(0.609, 2.38)
Associated Peptides	5
Associated Spectra	5
Coverage	NaN

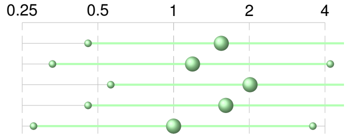

A	B	2.5	50	97.5	Sequence
0	1	0.46	1.5	5.6	AVGGLSFVR
0	1	0.33	1.2	4.2	KLTGSLQPLAEVLNK
0	1	0.56	2	7	LIFSPTGPLDRDQDDVRR
0	1	0.46	1.6	5.8	WSETDKGFEK
0	1	0.28	1	3.6	AVGEQYSIIMDNGPAR

5.281 254 1648 Tektin-3 [Crassostrea gigas]

Protein Accession	CL6717.Contig3$_{H}1$
Mean Expression Ratio	1.22
Median Expression Ratio	1.22
Credible Interval	(0.585, 2.59)
Associated Peptides	3
Associated Spectra	4
Coverage	NaN

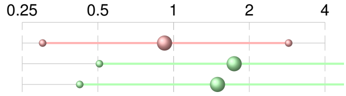

A	B	2.5	50	97.5	Sequence
1	1	0.3	0.92	2.9	AAQHALEIDAK
0	1	0.51	1.7	6.1	ILPYVTDKTISR
0	1	0.42	1.5	5.1	NYFNPATR

5.282 771 1505 O-methyltransferase family 3 [Dicentrarchus labrax]

Protein Accession	Unigene19132$_{H}1$
Mean Expression Ratio	0.816
Median Expression Ratio	0.819
Credible Interval	(0.41, 1.62)
Associated Peptides	5
Associated Spectra	6
Coverage	NaN

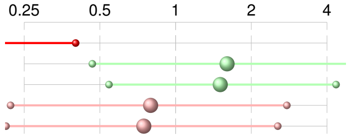

A	B	2.5	50	97.5	Sequence
1	0	0.032	0.11	0.4	VLIPVR
0	1	0.47	1.6	5.9	AEATQADPEVIEGLKR
0	2	0.54	1.5	4.3	FGASYLPR
0	1	0.22	0.79	2.8	IAETTLTHPWR
0	1	0.21	0.75	2.6	VFGEHVINDQR

5.283 " 166 705 minus strand ADP-ribosylation factor, partial [Antheraea yamamai]; 166 705 minus strand ADP-ribosylation factor, partial [Antheraea yamamai]; 166 705 minus strand ADP-ribosylation factor, partial [Antheraea yamamai]; 166 705 minus strand ADP-ribosylation factor, partial [Antheraea yamamai]; 166 705 minus strand ADP-ribosylation factor, partial [Antheraea yamamai]"

Protein Accession	Unigene39229$_{H}$1 Unigene39228$_{H}$1 Unigene39227$_{H}$1 Unigene39226$_{H}$1 Unigene39225$_{H}$1
Mean Expression Ratio	1.23
Median Expression Ratio	1.22
Credible Interval	(0.546, 2.76)
Associated Peptides	1
Associated Spectra	2
Coverage	NaN

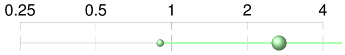

A	B	2.5	50	97.5	Sequence
0	2	0.9	2.7	7.6	IGEARDELQR

5.284 133 354 minus strand 60S ribosomal protein L7a OS=Rattus norvegicus GN=Rpl7a PE=1 SV=2

Protein Accession	CL20520.Contig1$_{H}$1
Mean Expression Ratio	1.22
Median Expression Ratio	1.22
Credible Interval	(0.573, 2.65)
Associated Peptides	2
Associated Spectra	3
Coverage	NaN

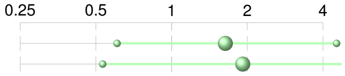

A	B	2.5	50	97.5	Sequence
0	2	0.61	1.6	4.5	KVVNPLFEK
0	1	0.53	1.9	6.8	NFGIGQDIQPK

5.285 " 95 1279 2-amino-3-ketobutyrate coenzyme A ligase, mitochondrial, partial [Crassostrea gigas]"

Protein Accession	CL33712.Contig1$_{H}$1
Mean Expression Ratio	1.22
Median Expression Ratio	1.22
Credible Interval	(0.578, 2.57)
Associated Peptides	3
Associated Spectra	3
Coverage	NaN

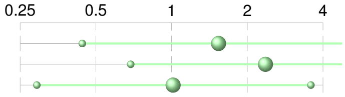

A	B	2.5	50	97.5	Sequence
0	1	0.44	1.5	5.6	GTEEHFNIMGR
0	1	0.69	2.4	8.6	VIQSPQGPSIR
0	1	0.29	1	3.6	LAATLR

5.286 64 393 Tubulin beta chain OS=Paracentrotus lividus PE=2 SV=1; 109 438 Tubulin beta chain OS=Paracentrotus lividus PE=2 SV=1; 160 489 Tubulin beta chain OS=Paracentrotus lividus PE=2 SV=1

Protein Accession	Unigene62663$_{H}1$ Unigene62661$_{H}1$ Unigene62660$_{H}1$
Mean Expression Ratio	1.22
Median Expression Ratio	1.22
Credible Interval	(0.579, 2.56)
Associated Peptides	2
Associated Spectra	14
Coverage	NaN

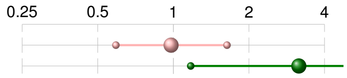

A	B	2.5	50	97.5	Sequence
**5**	**7**	**0.59**	**0.98**	**1.6**	**INVYFNEATGGK**
**0**	**2**	**1.2**	**3.2**	**8.8**	**INVYFNEATGGKYVPR**

5.287 212 1336 6-phosphofructokinase [Crassostrea gigas]; 208 1332 6-phosphofructokinase [Crassostrea gigas]

Protein Accession	CL48438.Contig4$_{H}1$ CL48438.Contig3$_{H}1$
Mean Expression Ratio	0.823
Median Expression Ratio	0.823
Credible Interval	(0.361, 1.86)
Associated Peptides	1
Associated Spectra	1
Coverage	NaN

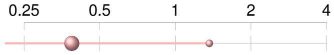

A	B	2.5	50	97.5	Sequence
**1**	**0**	**0.11**	**0.39**	**1.4**	**LGLTQGYR**

5.288 1519 2583 Proliferation-associated protein 2G4 [Crassostrea gigas]; 565 1629 Proliferation-associated protein 2G4 [Crassostrea gigas]

Protein Accession	CL14279.Contig3$_{H}1$ CL14279.Contig2$_{H}1$
Mean Expression Ratio	0.819
Median Expression Ratio	0.824
Credible Interval	(0.369, 1.79)
Associated Peptides	2
Associated Spectra	2
Coverage	NaN

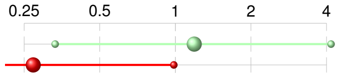

A	B	2.5	50	97.5	Sequence
**0**	**1**	**0.33**	**1.2**	**4.2**	**ALLSASASSK**
**0**	**1**	**0.075**	**0.27**	**0.98**	**ILEETGK**

5.289 44 910 PREDICTED: probable ATP-dependent RNA helicase DDX10 [Cricetulus griseus] gi

Protein Accession	CL33109.Contig1$_H$1
Mean Expression Ratio	1.22
Median Expression Ratio	1.21
Credible Interval	(0.552, 2.65)
Associated Peptides	2
Associated Spectra	2
Coverage	NaN

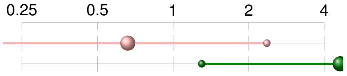

A	B	2.5	50	97.5	Sequence
1	0	0.19	0.66	2.4	GIDVTK
1	0	1.3	4.6	16	AKYLK

5.290 2 811 voltage-dependent anion channel 2-like protein [Haliotis diversicolor]; 2 811 voltage-dependent anion channel 2-like protein [Haliotis diversicolor]; 2 811 voltage-dependent anion channel 2-like protein [Haliotis diversicolor]

Protein Accession	CL2477.Contig3$_H$1 CL2477.Contig2$_H$1 CL2477.Contig1$_H$1
Mean Expression Ratio	1.21
Median Expression Ratio	1.21
Credible Interval	(0.742, 1.98)
Associated Peptides	13
Associated Spectra	48
Coverage	NaN

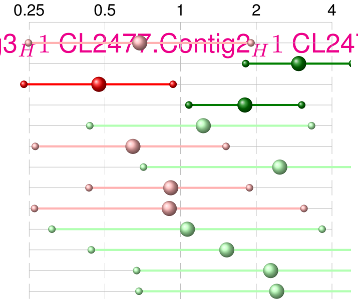

A	B	2.5	50	97.5	Sequence
1	1	0.25	0.69	1.9	GYNYGFFK
4	9	1.8	3	4.8	LAFDTSFAPQTGKK
3	3	0.24	0.47	0.94	VSGSLETK
4	7	1.1	1.8	3	YTLDSSASVSAK
1	1	0.44	1.2	3.3	FALGTK
1	2	0.26	0.64	1.5	LAFDTSFAPQTGK
0	1	0.71	2.5	8.7	GYNYGFFKLEAK
0	5	0.43	0.91	1.9	VGLGLDVEA
0	1	0.26	0.9	3.1	WSEYGLTFTEK
0	1	0.31	1.1	3.7	WNTDNVLNTEIAIEDQIAK
0	1	0.44	1.5	5.3	DIFSK
0	1	0.67	2.3	7.9	KLEAK
0	1	0.68	2.4	8.2	SINQGGHKVGLGLDVEA

5.291 1 1284 minus strand Thioredoxin reductase 3 [Crassostrea gigas]

Protein Accession	CL3500.Contig1$_H$1
Mean Expression Ratio	1.22
Median Expression Ratio	1.21
Credible Interval	(0.561, 2.64)
Associated Peptides	2
Associated Spectra	4
Coverage	NaN

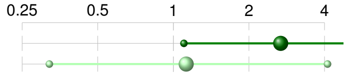

A	B	2.5	50	97.5	Sequence
0	3	1.1	2.7	6.7	GAVPSQIEQLEEGKPGR
0	1	0.32	1.1	4.1	LLVQRL

5.292 82 1449 hypothetical protein BRAFLDRAFT_127120 [Branchiostoma floridae] gi

Protein Accession	CL41883.Contig1$_{H}$1
Mean Expression Ratio	1.21
Median Expression Ratio	1.21
Credible Interval	(0.544, 2.73)
Associated Peptides	1
Associated Spectra	3
Coverage	NaN

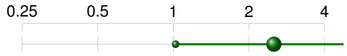

A	B	2.5	50	97.5	Sequence
**0**	**3**	**1**	**2.5**	**6.2**	**ILQEQEDKFLK**

5.293 408 1010 glutathione S-transferase sigma 2 [Mytilus galloprovincialis]; 166 768 glutathione S-transferase sigma 2 [Mytilus galloprovincialis]

Protein Accession	CL22038.Contig5$_{H}$1 CL22038.Contig4$_{H}$1
Mean Expression Ratio	0.825
Median Expression Ratio	0.825
Credible Interval	(0.455, 1.49)
Associated Peptides	7
Associated Spectra	17
Coverage	NaN

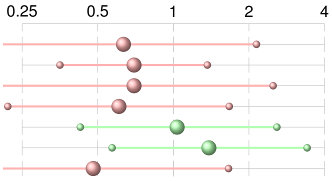

A	B	2.5	50	97.5	Sequence
**1**	**0**	**0.18**	**0.63**	**2.1**	**LVLEAAGR**
**2**	**4**	**0.35**	**0.7**	**1.4**	**VFSEATPK**
**1**	**0**	**0.2**	**0.7**	**2.5**	**KAELYEK**
**2**	**0**	**0.22**	**0.61**	**1.7**	**ELGLYGK**
**0**	**3**	**0.43**	**1**	**2.6**	**LVYFDLR**
**0**	**3**	**0.57**	**1.4**	**3.4**	**STVESHPFLK**
**0**	**1**	**0.14**	**0.48**	**1.7**	**YLGFFQK**

5.294 235 1038 Translocon-associated protein subunit alpha [Crassostrea gigas]

Protein Accession	CL36450.Contig6$_{H}$1
Mean Expression Ratio	0.825
Median Expression Ratio	0.826
Credible Interval	(0.374, 1.85)
Associated Peptides	1
Associated Spectra	2
Coverage	NaN

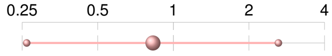

A	B	2.5	50	97.5	Sequence
**1**	**1**	**0.26**	**0.83**	**2.6**	**ELPAGKPVR**

5.295 " 10 1191 3-ketoacyl-CoA thiolase, mitochondrial [Crassostrea gigas]"

Protein Accession	CL29653.Contig1$_{H}$1
Mean Expression Ratio	0.826
Median Expression Ratio	0.826
Credible Interval	(0.437, 1.56)
Associated Peptides	6
Associated Spectra	10
Coverage	NaN

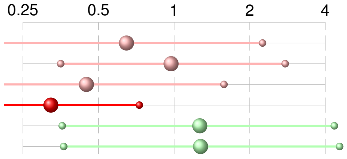

A	B	2.5	50	97.5	Sequence
**1**	**0**	**0.19**	**0.65**	**2.2**	**FNDEIVPVQVK**
**1**	**1**	**0.35**	**0.98**	**2.8**	**KGPEDFNVDEHPR**
**1**	**0**	**0.13**	**0.45**	**1.6**	**IVAAK**
**0**	**4**	**0.15**	**0.32**	**0.72**	**ITTNLVHEIR**
**0**	**1**	**0.36**	**1.3**	**4.3**	**VMGIGPVPASK**
**0**	**1**	**0.36**	**1.3**	**4.5**	**TQAPYSVR**

5.296 43 1623 minus strand MGC84105 protein precursor [Xenopus laevis] gi; 43 1506 minus strand MGC84105 protein precursor [Xenopus laevis] gi

Protein Accession	CL16647.Contig5$_{H}1$ CL16647.Contig4$_{H}1$
Mean Expression Ratio	1.21
Median Expression Ratio	1.21
Credible Interval	(0.593, 2.51)
Associated Peptides	3
Associated Spectra	4
Coverage	NaN

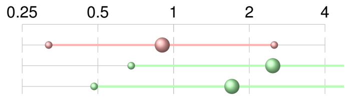

A	B	2.5	50	97.5	Sequence
1	1	0.32	0.9	2.5	LIDAADSAR
0	1	0.68	2.5	8.5	QLPELIAVRDEMQK
0	1	0.48	1.7	6	LSELEK

5.297 142 789 60S ribosomal protein L6 [Crassostrea gigas]

Protein Accession	CL6501.Contig1$_{H}1$
Mean Expression Ratio	0.827
Median Expression Ratio	0.828
Credible Interval	(0.356, 1.88)
Associated Peptides	1
Associated Spectra	1
Coverage	NaN

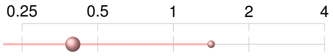

A	B	2.5	50	97.5	Sequence
1	0	0.11	0.4	1.4	HAEGEIFDTK

5.298 162 785 minus strand Ropporin-1-like protein OS=Xenopus laevis GN=ropn1l PE=2 SV=1

Protein Accession	CL618.Contig4$_{H}1$
Mean Expression Ratio	0.831
Median Expression Ratio	0.828
Credible Interval	(0.41, 1.7)
Associated Peptides	3
Associated Spectra	6
Coverage	NaN

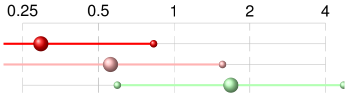

A	B	2.5	50	97.5	Sequence
1	1	0.11	0.3	0.83	EQFDDLVR
0	2	0.2	0.56	1.6	TVALTVIEEK
0	2	0.59	1.7	4.7	TDTGLTPGLLK

5.299 157 1422 Tektin-2 [Crassostrea gigas]

Protein Accession	Unigene17501$_{H}1$
Mean Expression Ratio	1.21
Median Expression Ratio	1.21
Credible Interval	(0.562, 2.6)
Associated Peptides	2
Associated Spectra	3
Coverage	NaN

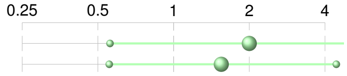

A	B	2.5	50	97.5	Sequence
0	1	0.56	2	7.2	ADAEMLAANR
0	2	0.55	1.5	4.4	NLTITGIERPR

5.300 113 1402 minus strand S-adenosylhomocysteine hydrolase [Crassostrea ariakensis]; 113 1402 minus strand S-adenosylhomocysteine hydrolase [Crassostrea ariakensis]

Protein Accession	CL7405.Contig2$_H1$ CL7405.Contig1$_H1$
Mean Expression Ratio	1.21
Median Expression Ratio	1.21
Credible Interval	(0.655, 2.28)
Associated Peptides	8
Associated Spectra	8
Coverage	NaN

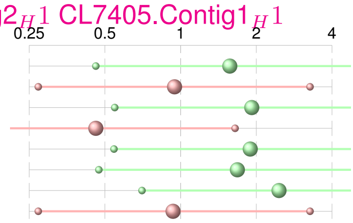

A	B	2.5	50	97.5	Sequence
1	0	0.46	1.6	5.5	VAVVAGYGDVGK
1	0	0.27	0.94	3.3	TGVPVYAWK
0	1	0.55	1.9	6.6	GLSEETTTGVHNLYR
0	1	0.13	0.46	1.6	HIIVLAEGR
0	1	0.54	1.9	6.6	LKVPAINVNDSVTK
0	1	0.47	1.7	5.8	VADISLAEMGR
0	1	0.7	2.5	8.6	LTKLDEDQAK
0	1	0.27	0.93	3.3	YLGIPQEGPFKPEHYR

5.301 459 755 minus strand heat shock protein beta-1 [Trichinella spiralis] gi; 464 763 minus strand heat shock protein beta-1 [Trichinella spiralis] gi; 437 736 minus strand heat shock protein beta-1 [Trichinella spiralis] gi; 437 736 minus strand heat shock protein beta-1 [Trichinella spiralis] gi; 437 736 minus strand heat shock protein beta-1 [Trichinella spiralis] gi

Protein Accession	CL17234.Contig5$_H1$ CL17234.Contig4$_H1$ CL17234.Contig3$_H1$ CL17234.Contig2$_H1$ CL1723
Mean Expression Ratio	1.21
Median Expression Ratio	1.21
Credible Interval	(0.584, 2.52)
Associated Peptides	2
Associated Spectra	13
Coverage	NaN

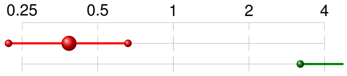

A	B	2.5	50	97.5	Sequence
0	10	0.22	0.38	0.66	DVEPTTVTSSLNADGVLTLK
0	3	3.2	8	20	TVTSSLNADGVLTLKAPK

5.302 " 1 1815 Glycogen phosphorylase, muscle form [Crassostrea gigas]"

Protein Accession	CL20630.Contig5$_H1$
Mean Expression Ratio	1.21
Median Expression Ratio	1.21
Credible Interval	(0.579, 2.54)
Associated Peptides	2
Associated Spectra	4
Coverage	NaN

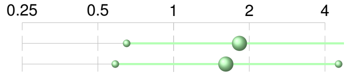

A	B	2.5	50	97.5	Sequence
0	2	0.65	1.8	5.1	IEELEEIEEDAGL
0	2	0.59	1.6	4.5	FGNPWEK

5.303 9 161 PREDICTED: tubulin alpha-1C chain-like [Acyrthosiphon pisum]; 9 161 Tubulin alpha-3 chain (Fragments) OS=Mesocricetus auratus PE=1 SV=1

Protein Accession	Unigene74551$_{H}$1 Unigene70816$_{H}$1
Mean Expression Ratio	1.21
Median Expression Ratio	1.2
Credible Interval	(0.573, 2.59)
Associated Peptides	3
Associated Spectra	3
Coverage	NaN

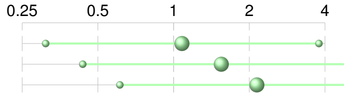

A	B	2.5	50	97.5	Sequence
0	1	0.31	1.1	3.8	IHFPLAT
0	1	0.44	1.6	5.6	IHFPLA
0	1	0.61	2.1	7.5	IHFPLATYAPVISAEK

5.304 92 1813 minus strand Dihydropyrimidinase [Crassostrea gigas]

Protein Accession	CL7386.Contig6$_{H}$1
Mean Expression Ratio	0.826
Median Expression Ratio	0.83
Credible Interval	(0.417, 1.61)
Associated Peptides	5
Associated Spectra	5
Coverage	NaN

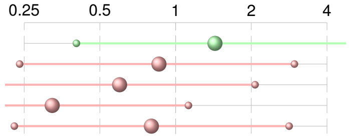

A	B	2.5	50	97.5	Sequence
1	0	0.4	1.4	4.9	SSADVISAAR
1	0	0.24	0.86	3	AENGQIIALK
1	0	0.17	0.6	2.1	LPVVR
1	0	0.093	0.32	1.1	IENGIIK
0	1	0.23	0.8	2.8	IAEGSDADVVVWDPEQAR

5.305 613 846 cytochrome c oxidase subunit Va [Antheraea yamamai] gi

Protein Accession	Unigene3832$_{H}$1
Mean Expression Ratio	1.2
Median Expression Ratio	1.2
Credible Interval	(0.6, 2.41)
Associated Peptides	4
Associated Spectra	5
Coverage	NaN

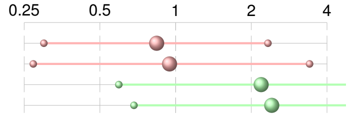

A	B	2.5	50	97.5	Sequence
0	2	0.3	0.84	2.3	AMNDLQGEDLVPEPK
0	1	0.27	0.95	3.4	YETFFSRPDIDGWEIR
0	1	0.59	2.2	8	KAMNDLQGEDLVPEPK
0	1	0.68	2.4	8.5	YETFFSRPDIDGWEIRK

5.306 63 1865 Dolichyl-diphosphooligosaccharide–protein glycosyltransferase subunit 1 [Crassostrea gigas]; 63 1865 Dolichyl-diphosphooligosaccharide–protein glycosyltransferase subunit 1 [Crassostrea gigas]

Protein Accession	CL41703.Contig2$_{H}$1 CL41703.Contig1$_{H}$1
Mean Expression Ratio	1.21
Median Expression Ratio	1.2
Credible Interval	(0.771, 1.9)
Associated Peptides	19
Associated Spectra	33
Coverage	NaN

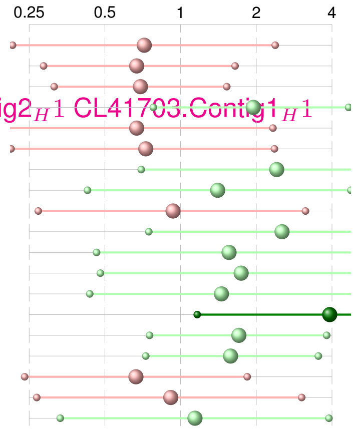

A	B	2.5	50	97.5	Sequence
1	0	0.21	0.72	2.4	APFSEAQLR
1	2	0.28	0.67	1.6	QQYLDAEAGVTSK
2	2	0.31	0.69	1.5	VILPEGVQNIEVR
1	2	0.78	1.9	4.7	VVPTTVPGHSDKELYR
1	0	0.2	0.67	2.3	GAVLK
1	0	0.21	0.73	2.4	LVAGK
1	0	0.7	2.4	8.2	TPFEVKR
0	1	0.42	1.4	4.7	DLNAFQVAR
0	1	0.27	0.93	3.1	ELDDSVDIELRPR
0	1	0.75	2.5	8.6	FKTSKDLNAFQVAR
0	1	0.46	1.6	5.5	HSGAVLKGPFSR
0	1	0.48	1.7	5.9	KVDIATHIVK
0	1	0.43	1.5	4.9	SALYQSYDDAINR
0	1	1.2	3.9	13	TSKDLNAFQVAR
0	4	0.75	1.7	3.8	VASLVEEVQSAQDKR
0	4	0.72	1.6	3.5	VQLPFNLDAGK
0	2	0.24	0.67	1.8	LSFISAVTK
0	1	0.27	0.91	3	LPAAAR
0	1	0.33	1.1	3.9	FVDHIFDDMVIDEAVVK

5.307 1050 1307 calreticulin precursor [Aplysia californica] gi

Protein Accession	CL4813.Contig1$_{H}$1
Mean Expression Ratio	0.829
Median Expression Ratio	0.831
Credible Interval	(0.362, 1.87)
Associated Peptides	1
Associated Spectra	1
Coverage	NaN

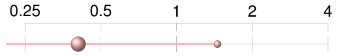

A	B	2.5	50	97.5	Sequence
0	1	0.11	0.41	1.5	EFGDSTWGK

5.308 365 2530 minus strand calpain B [Gecarcinus lateralis]

Protein Accession	CL34074.Contig5$_{H}$1
Mean Expression Ratio	1.2
Median Expression Ratio	1.2
Credible Interval	(0.576, 2.46)
Associated Peptides	3
Associated Spectra	5
Coverage	NaN

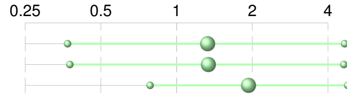

A	B	2.5	50	97.5	Sequence
0	1	0.37	1.3	4.6	SLQSLVMR
0	1	0.38	1.3	4.6	SLFFSQSMR
0	3	0.79	1.9	4.8	MMDIK

5.309 496 2151 NADP-dependent malic enzyme [Crassostrea gigas]

Protein Accession	CL26274.Contig1$_{H}$1
Mean Expression Ratio	1.21
Median Expression Ratio	1.2
Credible Interval	(0.73, 2.03)
Associated Peptides	12
Associated Spectra	33
Coverage	NaN

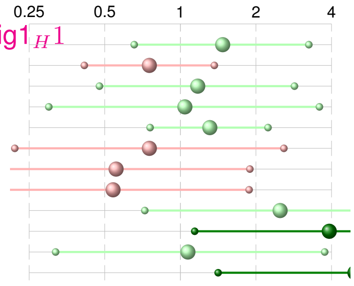

A	B	2.5	50	97.5	Sequence
1	3	0.66	1.5	3.3	AIVVTDGER
4	4	0.41	0.75	1.4	GIDILR
1	2	0.48	1.2	2.8	IWMVDSK
1	0	0.3	1	3.6	EGMASAYPEPQDKR
2	8	0.76	1.3	2.2	VLVNFR
1	0	0.22	0.75	2.6	GLLVK
1	0	0.16	0.55	1.9	APFAK
0	1	0.15	0.54	1.9	AECTAEEAYTLTEGR
0	1	0.72	2.5	8.7	LNKGTAFSLK
0	1	1.1	3.9	14	GIDILRNPK
0	1	0.32	1.1	3.8	GTAFSLK
0	1	1.4	5	17	LNKGTAFSLKER

5.310 2 349 minus strand hypothetical protein BRAFLDRAFT_206907 [Branchiostoma floridae] gi

Protein Accession	Unigene875$_{H}$1
Mean Expression Ratio	0.832
Median Expression Ratio	0.832
Credible Interval	(0.353, 1.92)
Associated Peptides	1
Associated Spectra	1
Coverage	NaN

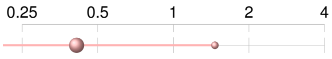

A	B	2.5	50	97.5	Sequence
1	0	0.12	0.41	1.5	YTPTFR

5.311 " 1252 1812 minus strand Alanine–glyoxylate aminotransferase 2, mitochondrial [Crassostrea gigas]"

Protein Accession	CL10973.Contig3$_{H}$1
Mean Expression Ratio	0.832
Median Expression Ratio	0.833
Credible Interval	(0.419, 1.63)
Associated Peptides	5
Associated Spectra	6
Coverage	NaN

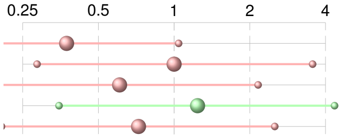

A	B	2.5	50	97.5	Sequence
1	1	0.14	0.37	1	IKPPMCISK
0	1	0.28	1	3.6	ADADFAINVLR
0	1	0.17	0.61	2.2	KPLPADQVAAIWEETK
0	1	0.35	1.2	4.4	LRDEYEVVGDVR
0	1	0.21	0.72	2.5	SADVGTHMLNELLK

5.312 90 677 minus strand PREDICTED: 15-hydroxyprostaglandin dehydrogenase [NAD+] [Gallus gallus]; 90 683 minus strand PREDICTED: 15-hydroxyprostaglandin dehydrogenase [NAD+] [Gallus gallus]; 69 776 PREDICTED: 15-hydroxyprostaglandin dehydrogenase [NAD+] [Gallus gallus]; 69 779 PREDICTED: 15-hydroxyprostaglandin dehydrogenase [NAD+] [Gallus gallus]

Protein Accession	Unigene58786$_{H}1$ Unigene57240$_{H}1$ Unigene48682$_{H}1$ CL44148.Contig4$_{H}1$
Mean Expression Ratio	1.21
Median Expression Ratio	1.2
Credible Interval	(0.559, 2.63)
Associated Peptides	2
Associated Spectra	3
Coverage	NaN

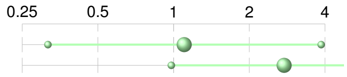

A	B	2.5	50	97.5	Sequence
**0**	**1**	**0.32**	**1.1**	**3.9**	**AETFSETDMEK**
**0**	**2**	**0.98**	**2.8**	**7.8**	**AITEAFLAK**

5.313 957 2147 haemocyanin [Aplysia californica]

Protein Accession	Unigene30320$_{H}1$
Mean Expression Ratio	0.834
Median Expression Ratio	0.834
Credible Interval	(0.446, 1.58)
Associated Peptides	5
Associated Spectra	17
Coverage	NaN

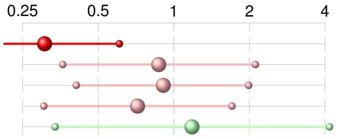

A	B	2.5	50	97.5	Sequence
**2**	**4**	**0.16**	**0.31**	**0.61**	**LLTVQFER**
**0**	**3**	**0.36**	**0.87**	**2.1**	**GAQVGVPYWDWTRPGK**
**0**	**4**	**0.41**	**0.91**	**2**	**TFHISGVNQDITR**
**0**	**3**	**0.3**	**0.71**	**1.7**	**YEVSELSLEQVSNLR**
**0**	**1**	**0.34**	**1.2**	**4.2**	**EKGAQVGVPYWDWTRPGK**

5.314 1 111 glycogen synthase [Crassostrea gigas]

Protein Accession	Unigene78448$_{H}1$
Mean Expression Ratio	0.831
Median Expression Ratio	0.835
Credible Interval	(0.358, 1.88)
Associated Peptides	1
Associated Spectra	1
Coverage	NaN

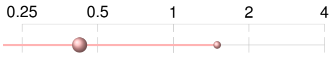

A	B	2.5	50	97.5	Sequence
**0**	**1**	**0.11**	**0.42**	**1.5**	**NVEPDLDDDYLDR**

5.315 3 551 minus strand Spectrin beta chain [Crassostrea gigas]

Protein Accession	Unigene35960$_{H}1$
Mean Expression Ratio	0.838
Median Expression Ratio	0.835
Credible Interval	(0.392, 1.81)
Associated Peptides	2
Associated Spectra	4
Coverage	NaN

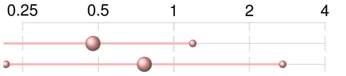

A	B	2.5	50	97.5	Sequence
**1**	**2**	**0.2**	**0.48**	**1.2**	**LISDINK**
**0**	**1**	**0.21**	**0.77**	**2.7**	**LVSQDNFGADLAAVEAATK**

5.316 269 1504 Ribosome-binding protein 1 [Crassostrea gigas]; 265 1500 Ribosome-binding protein 1 [Crassostrea gigas]

Protein Accession	CL8282.Contig5$_{H}1$ CL8282.Contig2$_{H}1$
Mean Expression Ratio	1.2
Median Expression Ratio	1.2
Credible Interval	(0.623, 2.31)
Associated Peptides	6
Associated Spectra	6
Coverage	NaN

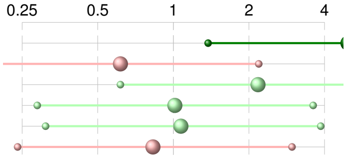

A	B	2.5	50	97.5	Sequence
**0**	**1**	**1.4**	**5**	**18**	**AMEALEKAEK**
**0**	**1**	**0.17**	**0.62**	**2.2**	**DAIIEVEK**
**0**	**1**	**0.62**	**2.2**	**7.6**	**LKEDKETLEAR**
**0**	**1**	**0.29**	**1**	**3.6**	**QFTQLHAENER**
**0**	**1**	**0.31**	**1.1**	**3.9**	**LVQQLQEQLSEK**
**0**	**1**	**0.24**	**0.83**	**3**	**AMEALEK**

5.317 1 945 hemocyanin isoform 1 [Haliotis diversicolor]

Protein Accession	Unigene30318$_{H}1$
Mean Expression Ratio	0.833
Median Expression Ratio	0.835
Credible Interval	(0.383, 1.8)
Associated Peptides	2
Associated Spectra	3
Coverage	NaN

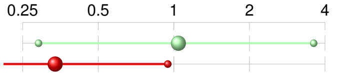

A	B	2.5	50	97.5	Sequence
**0**	**1**	**0.29**	**1**	**3.6**	**LFVVEVEDELRAR**
**0**	**2**	**0.12**	**0.34**	**0.94**	**VEITDVLK**

5.318 197 913 PREDICTED: l-xylulose reductase-like [Monodelphis domestica]; 197 913 PREDICTED: l-xylulose reductase-like [Monodelphis domestica]; 197 913 PREDICTED: l-xylulose reductase-like [Monodelphis domestica]; 197 913 PREDICTED: l-xylulose reductase-like [Monodelphis domestica]; 197 913 PREDICTED: l-xylulose reductase-like [Monodelphis domestica]; 197 913 PREDICTED: l-xylulose reductase-like [Monodelphis domestica]; 203 919 minus strand PREDICTED: l-xylulose reductase-like [Monodelphis domestica]; 200 916 PREDICTED: l-xylulose reductase-like [Monodelphis domestica]; 200 916 PREDICTED: l-xylulose reductase-like [Monodelphis domestica]; 203 919 minus strand PREDICTED: l-xylulose reductase-like [Monodelphis domestica]

Protein Accession CL13128.Contig8$_{H}1$ CL13128.Contig7$_{H}1$ CL13128.Contig6$_{H}1$ CL13128.Contig5$_{H}1$ CL1312
Mean Expression Ratio 1.2
Median Expression Ratio 1.2
Credible Interval (0.617, 2.32)
Associated Peptides 5
Associated Spectra 8
Coverage NaN

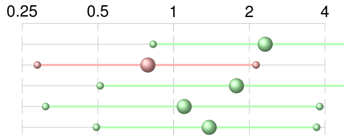

A	B	2.5	50	97.5	Sequence
1	1	0.83	2.3	6.6	MGTFAAEHPEGK
0	2	0.29	0.79	2.1	AIAEALYQHGAR
0	1	0.51	1.8	6.4	GGSIVNVSSISGK
0	1	0.31	1.1	3.8	VNSVNPTCVMTK
0	2	0.49	1.4	3.7	VVALSR

5.319 43 591 minus strand cyclophilin type peptidyl-prolyl cis-trans isomerase [Glossina morsitans morsitans]

Protein Accession CL39270.Contig1$_{H}1$
Mean Expression Ratio 0.834
Median Expression Ratio 0.836
Credible Interval (0.452, 1.54)
Associated Peptides 5
Associated Spectra 30
Coverage NaN

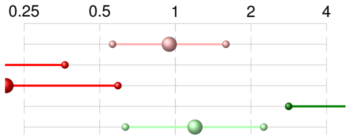

A	B	2.5	50	97.5	Sequence
5	6	0.56	0.94	1.6	ITMELR
1	2	0.062	0.15	0.36	FADENFTLK
2	0	0.077	0.21	0.59	GFGYK
0	7	2.8	5.3	10	KIESYGSKNGATSQK
0	7	0.63	1.2	2.3	VFFDMTADGVPLGR

5.320 835 1083 minus strand Calmodulin [Crassostrea gigas]

Protein Accession CL49568.Contig2$_{H}1$
Mean Expression Ratio 0.834
Median Expression Ratio 0.836
Credible Interval (0.385, 1.8)
Associated Peptides 2
Associated Spectra 4
Coverage NaN

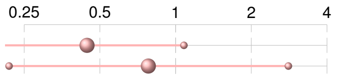

A	B	2.5	50	97.5	Sequence
0	3	0.18	0.44	1.1	VDFTEFYK
0	1	0.22	0.78	2.8	GFIANEEMK

5.321 99 3011 Eukaryotic translation initiation factor 3 subunit A [Crassostrea gigas]

Protein Accession	CL36771.Contig2$_{H}1$
Mean Expression Ratio	1.2
Median Expression Ratio	1.19
Credible Interval	(0.605, 2.36)
Associated Peptides	5
Associated Spectra	5
Coverage	NaN

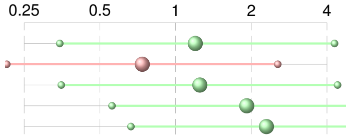

A	B	2.5	50	97.5	Sequence
**0**	**1**	**0.35**	**1.2**	**4.3**	**IHEPIMER**
**0**	**1**	**0.21**	**0.74**	**2.5**	**AVEDIHGLISLSK**
**0**	**1**	**0.35**	**1.2**	**4.4**	**ELALETK**
**0**	**1**	**0.56**	**1.9**	**6.6**	**KVDYLAR**
**0**	**1**	**0.67**	**2.3**	**8**	**KEQLENLNDAR**

5.322 219 554 minus strand ribosomal protein P1 [Biomphalaria glabrata]

Protein Accession	CL45629.Contig2$_{H}1$
Mean Expression Ratio	0.834
Median Expression Ratio	0.838
Credible Interval	(0.361, 1.92)
Associated Peptides	1
Associated Spectra	1
Coverage	NaN

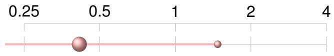

A	B	2.5	50	97.5	Sequence
**1**	**0**	**0.12**	**0.41**	**1.5**	**ALEGVNIK**

5.323 172 678 Myosin regulatory light chain sqh [Crassostrea gigas]

Protein Accession	CL23773.Contig1$_{H}1$
Mean Expression Ratio	1.19
Median Expression Ratio	1.19
Credible Interval	(0.522, 2.72)
Associated Peptides	1
Associated Spectra	1
Coverage	NaN

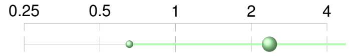

A	B	2.5	50	97.5	Sequence
**0**	**1**	**0.66**	**2.4**	**8.4**	**GNFDYNEFTR**

5.324 174 1556 minus strand aspartyl-tRNA synthetase [Xenopus laevis] gi; 174 1721 minus strand aspartyl-tRNA synthetase [Xenopus laevis] gi

Protein Accession	CL9865.Contig2$_{H}1$ CL9865.Contig1$_{H}1$
Mean Expression Ratio	1.19
Median Expression Ratio	1.19
Credible Interval	(0.566, 2.5)
Associated Peptides	1
Associated Spectra	6
Coverage	NaN

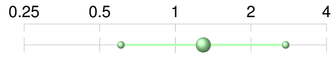

A	B	2.5	50	97.5	Sequence
**2**	**4**	**0.61**	**1.3**	**2.8**	**IHDPDFLSQR**

5.325 546 995 Hypothetical protein CBG12002 [Caenorhabditis briggsae]; 543 995 minus strand PREDICTED: tubulin polymerization-promoting protein family member 2-like [Anolis carolinensis]; 546 995 Hypothetical protein CBG12002 [Caenorhabditis briggsae]; 546 995 minus strand Hypothetical protein CBG12002 [Caenorhabditis briggsae]

Protein Accession	CL6849.Contig7$_H$1 CL6849.Contig6$_H$1 CL6849.Contig2$_H$1 CL6849.Contig1$_H$1
Mean Expression Ratio	1.2
Median Expression Ratio	1.19
Credible Interval	(0.604, 2.36)
Associated Peptides	4
Associated Spectra	7
Coverage	NaN

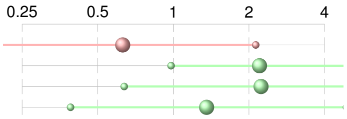

A	B	2.5	50	97.5	Sequence
1	0	0.18	0.63	2.1	LLPQLAADK
0	4	0.98	2.2	5	LLPQLAADKAK
0	1	0.64	2.2	7.8	LKDNVAK
0	1	0.39	1.4	4.9	ADFLNK

5.326 192 1820 PREDICTED: copine IV-like [Saccoglossus kowalevskii]; 125 1753 PREDICTED: copine IV-like [Saccoglossus kowalevskii]

Protein Accession	CL15074.Contig3$_H$1 CL15074.Contig1$_H$1
Mean Expression Ratio	0.842
Median Expression Ratio	0.84
Credible Interval	(0.395, 1.8)
Associated Peptides	2
Associated Spectra	5
Coverage	NaN

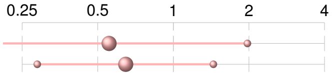

A	B	2.5	50	97.5	Sequence
1	0	0.16	0.56	2	LLNAK
1	3	0.29	0.64	1.4	HVLAEVPK

5.327 177 791 Ras-related protein Rab-1A [Haliotis discus discus]

Protein Accession	CL21389.Contig6$_H$1
Mean Expression Ratio	1.19
Median Expression Ratio	1.19
Credible Interval	(0.587, 2.4)
Associated Peptides	3
Associated Spectra	8
Coverage	NaN

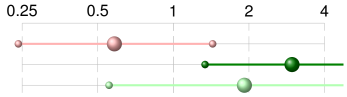

A	B	2.5	50	97.5	Sequence
2	1	0.24	0.58	1.4	KVVDYTTAK
1	3	1.3	3	6.6	MGPVTSASENKPSVK
1	0	0.56	1.9	6.6	INSSTPVK

5.328 80 589 minus strand PREDICTED: adenine phosphoribosyltransferase-like [Strongylocentrotus purpuratus]; 328 468 minus strand PREDICTED: adenine phosphoribosyltransferase [Apis mellifera]

Protein Accession	CL8682.Contig6$_{H}1$ CL8682.Contig4$_{H}1$
Mean Expression Ratio	0.838
Median Expression Ratio	0.841
Credible Interval	(0.375, 1.86)
Associated Peptides	1
Associated Spectra	3
Coverage	NaN

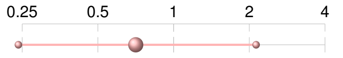

A	B	2.5	50	97.5	Sequence
1	2	0.24	0.71	2.1	AAVELVEK

5.329 " 241 1110 minus strand N(G),N(G)-dimethylarginine dimethylaminohydrolase 1 [Crassostrea gigas]; 241 1110 minus strand N(G),N(G)-dimethylarginine dimethylaminohydrolase 1 [Crassostrea gigas]; 241 1110 minus strand N(G),N(G)-dimethylarginine dimethylaminohydrolase 1 [Crassostrea gigas]"

Protein Accession	Unigene30451$_{H}1$ CL7107.Contig3$_{H}1$ CL7107.Contig1$_{H}1$
Mean Expression Ratio	1.19
Median Expression Ratio	1.19
Credible Interval	(0.544, 2.66)
Associated Peptides	2
Associated Spectra	2
Coverage	NaN

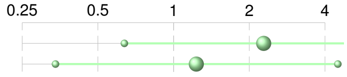

A	B	2.5	50	97.5	Sequence
0	1	0.64	2.3	8.3	LGEVGEIRR
0	1	0.34	1.2	4.5	LGEVGEIR

5.330 244 837 PREDICTED: ras-like GTP-binding protein Rho1-like [Megachile rotundata]; 650 883 hypothetical protein SINV_15432 [Solenopsis invicta]; 534 767 hypothetical protein SINV_15432 [Solenopsis invicta]

Protein Accession	Unigene11792$_{H}1$ Unigene11791$_{H}1$ Unigene11790$_{H}1$
Mean Expression Ratio	0.84
Median Expression Ratio	0.841
Credible Interval	(0.378, 1.86)
Associated Peptides	1
Associated Spectra	2
Coverage	NaN

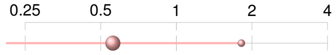

A	B	2.5	50	97.5	Sequence
1	1	0.17	0.56	1.8	EVFETATR

5.331 " 2 1174 endo-1,3-beta-D-glucanase [Littorina sitkana]"

Protein Accession	CL31231.Contig3$_H$1
Mean Expression Ratio	1.19
Median Expression Ratio	1.19
Credible Interval	(0.62, 2.28)
Associated Peptides	4
Associated Spectra	22
Coverage	NaN

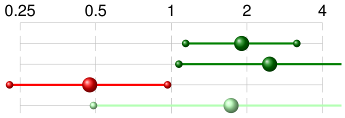

A	B	2.5	50	97.5	Sequence
5	7	1.1	1.9	3.2	FGTLEVR
1	3	1.1	2.5	5.5	SGEIDIMESR
5	0	0.23	0.47	0.97	AVIFR
0	1	0.49	1.7	6.2	NGMLPPVMSGK

5.332 " 371 820 minus strand Peroxiredoxin-5, mitochondrial [Crassostrea gigas]; 91 528 minus strand Peroxiredoxin-5, mitochondrial [Crassostrea gigas]; 113 550 Peroxiredoxin-5, mitochondrial [Crassostrea gigas]; 453 902 Peroxiredoxin-5, mitochondrial [Crassostrea gigas]; 87 530 Peroxiredoxin-5, mitochondrial [Crassostrea gigas]"

Protein Accession	CL5795.Contig8$_H$1 CL5795.Contig7$_H$1 CL5795.Contig6$_H$1 CL5795.Contig5$_H$1 CL5795.Con
Mean Expression Ratio	1.19
Median Expression Ratio	1.19
Credible Interval	(0.545, 2.62)
Associated Peptides	2
Associated Spectra	2
Coverage	NaN

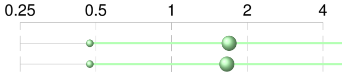

A	B	2.5	50	97.5	Sequence
0	1	0.47	1.7	6	ADLGIDLSSVLGNVR
0	1	0.47	1.7	6	FVLVAQDGVVK

5.333 114 731 minus strand Proteasome subunit beta type-6 [Crassostrea gigas]

Protein Accession	CL12961.Contig1$_H$1
Mean Expression Ratio	1.18
Median Expression Ratio	1.19
Credible Interval	(0.517, 2.72)
Associated Peptides	1
Associated Spectra	2
Coverage	NaN

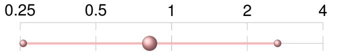

A	B	2.5	50	97.5	Sequence
1	1	0.26	0.82	2.6	FTLTGDDLPK

5.334 184 438 PREDICTED: similar to CG14235 CG14235-PA [Tribolium castaneum] gi

Protein Accession	Unigene638$_{H}1$
Mean Expression Ratio	0.842
Median Expression Ratio	0.843
Credible Interval	(0.428, 1.64)
Associated Peptides	5
Associated Spectra	7
Coverage	NaN

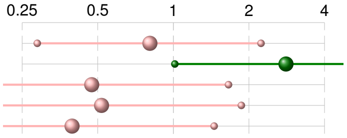

A	B	2.5	50	97.5	Sequence
2	0	0.29	0.8	2.2	TAPFDAR
1	1	1	2.8	8.1	GDEQKYYQLK
1	0	0.13	0.47	1.7	YYQLK
0	1	0.15	0.52	1.9	WDDQVANGTFAGKI
0	1	0.12	0.39	1.5	WDDQVANGTFAGK

5.335 16 939 hypothetical protein BRAFLDRAFT_87838 [Branchiostoma floridae] gi

Protein Accession	CL38761.Contig1$_{H}1$
Mean Expression Ratio	0.84
Median Expression Ratio	0.843
Credible Interval	(0.38, 1.84)
Associated Peptides	1
Associated Spectra	4
Coverage	NaN

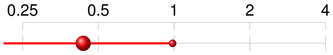

A	B	2.5	50	97.5	Sequence
4	0	0.19	0.44	0.99	ALLLR

5.336 299 7507 minus strand filamin [Clonorchis sinensis]

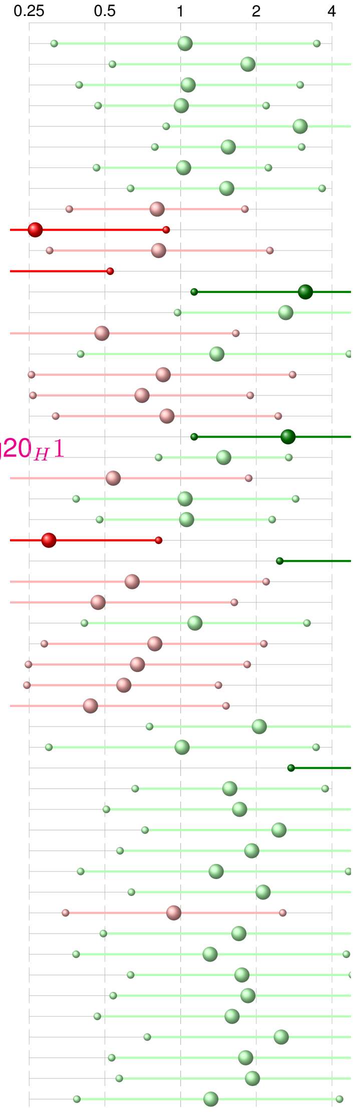

A	B	2.5	50	97.5	Sequence
1	0	0.31	1	3.5	AQLSVGSSSEVSLK
1	0	0.53	1.8	6.2	AYGPGLEPK
1	1	0.4	1.1	3	DVPGAPFPVK
1	3	0.47	1	2.2	ETGEHLVNVFR
1	0	0.88	3	10	GAGKGGLGLGIEGPVEAK
2	4	0.79	1.5	3	GGLGLGIEGPVEAK
2	2	0.46	1	2.2	GTLEVTLLNPK
1	2	0.63	1.5	3.7	IMVGESELGNASK
2	2	0.36	0.81	1.8	KISISALQDK
1	0	0.076	0.26	0.88	LANGHLGITFTPR
1	1	0.3	0.82	2.3	NIMGSPFTAK
1	0	0.045	0.16	0.53	VDAVPDTGIPK
1	1	1.1	3.1	8.9	VLPGVDASKVK
1	1	0.97	2.6	7.1	VQNPVDASKVK
1	0	0.14	0.49	1.7	VYVSEPTNPAK
1	0	0.4	1.4	4.7	TTNVPVQDLVDKK
1	0	0.25	0.85	2.8	VSEELITR
1	1	0.26	0.7	1.9	SPVDELTVK
1	1	0.32	0.88	2.4	FNDEHIPDSPYR
1	2	1.1	2.7	6.4	VKPAALDVSGVK
4	4	0.82	1.5	2.7	VLPGVDASK
1	0	0.16	0.54	1.9	QVEVQVK
1	1	0.38	1	2.9	HIANSPFK
1	3	0.48	1.1	2.3	VTETDITDLTASIR
1	1	0.11	0.3	0.82	EEPCLLK
1	2	2.5	6.1	15	VIAKGPGLEK
1	0	0.18	0.64	2.2	IMVGK
1	0	0.14	0.47	1.6	NNAKEAMDAAEK
2	0	0.42	1.1	3.2	VQNPVDASK
1	1	0.29	0.79	2.1	EETKPGSLGVTVEGPK
1	1	0.25	0.67	1.8	ELADDAQWK
1	2	0.24	0.59	1.4	VEVGPFWK
0	1	0.13	0.44	1.5	AEIDFQDR
0	2	0.75	2.1	5.6	AYGPGLEGGITNQPAVFTVDMK
0	1	0.3	1	3.5	EMGIHTVSVK
0	4	2.8	6.1	13	FADKNIMGSPFTAK
0	3	0.66	1.6	3.8	GLQVGKPAAFVVNFNGAK
0	1	0.51	1.7	5.8	INVTHADKPIPGSPFK
0	1	0.72	2.5	8.2	TQKLENVTMVLK
0	1	0.57	1.9	6.4	VKDSADFYVHTK
0	1	0.4	1.4	4.7	VQVSPSIGDAR
0	1	0.64	2.1	7.4	VTYRPTEPGTYIINIK
0	2	0.35	0.94	2.6	WANEHLK
0	1	0.49	1.7	5.7	WLDVPQLIKPDEMTNPK
0	1	0.39	1.3	4.5	FLEEDEGIR
0	2	0.63	1.8	4.8	HKDMHIPGSPFQFTVGPIAGGGAHK
0	1	0.54	1.9	6.3	VTGPGVQGTTPASLPAVFK
0	1	0.47	1.6	5.6	FSGIQK
0	1	0.74	2.5	8.6	SPSGREEPCLLKR
0	1	0.53	1.8	6.6	NGKHIANSPFK
0	1	0.57	1.9	6.5	FIVNTVNAGA
0	1	0.39	1.3	4.3	YAGVNIAGSPFK

Protein Accession	CL4145.Contig20$_{H}1$
Mean Expression Ratio	1.19
Median Expression Ratio	1.19
Credible Interval	(0.884, 1.6)
Associated Peptides	52
Associated Spectra	103
Coverage	NaN

5.337 557 2329 minus strand Collagen alpha-3(VI) chain [Crassostrea gigas]

Protein Accession	CL47586.Contig7$_{H}1$
Mean Expression Ratio	1.18
Median Expression Ratio	1.19
Credible Interval	(0.56, 2.42)
Associated Peptides	3
Associated Spectra	4
Coverage	NaN

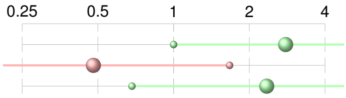

A	B	2.5	50	97.5	Sequence
0	2	1	2.8	7.6	KLDVVVAFHASPR
0	1	0.14	0.48	1.7	VALGFFNK
0	1	0.68	2.4	8.5	KADYSAALGKLPK

5.338 " 130 1161 minus strand Isocitrate dehydrogenase [NAD] subunit alpha, mitochondrial [Crassostrea gigas]; 198 1229 minus strand Isocitrate dehydrogenase [NAD] subunit alpha, mitochondrial [Crassostrea gigas]"

Protein Accession	CL5384.Contig8$_{H}1$ CL5384.Contig7$_{H}1$
Mean Expression Ratio	0.841
Median Expression Ratio	0.844
Credible Interval	(0.384, 1.83)
Associated Peptides	2
Associated Spectra	2
Coverage	NaN

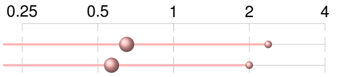

A	B	2.5	50	97.5	Sequence
1	0	0.18	0.65	2.4	SLNLALR
1	0	0.16	0.57	2	VAEYAFK

5.339 552 1886 minus strand intermediate filament protein [Biomphalaria glabrata]

Protein Accession	Unigene26795$_{H}1$
Mean Expression Ratio	0.846
Median Expression Ratio	0.845
Credible Interval	(0.525, 1.36)
Associated Peptides	14
Associated Spectra	48
Coverage	NaN

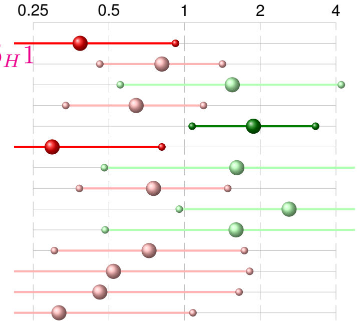

A	B	2.5	50	97.5	Sequence
2	1	0.16	0.38	0.92	KLLEGEENR
3	6	0.46	0.81	1.4	KVITEFSTQR
1	1	0.55	1.6	4.2	SATGISEFR
4	3	0.34	0.64	1.2	WLEAQNK
3	6	1.1	1.9	3.3	YAAQMDQLR
0	2	0.11	0.3	0.81	ADLDAETAAHIENEVLAQTK
0	1	0.48	1.6	5.7	CGSLEDELAK
0	6	0.38	0.75	1.5	KLEAELEALR
0	2	0.95	2.6	7.2	KQEDWKPIRDMYEGELAQAR
0	1	0.48	1.6	5.7	MSLGFNPGVYSK
0	3	0.3	0.72	1.7	KQEDWKPIR
0	1	0.15	0.52	1.8	LLEGEENR
0	1	0.14	0.46	1.6	DMYEGELAQAR
0	1	0.091	0.32	1.1	SLELEIACYR

5.340 77 511 cytochrome c oxidase subunit VIIa polypeptide 2 like [Xenopus (Silurana) tropicalis]; 77 511 cytochrome c oxidase subunit VIIa polypeptide 2 like [Xenopus (Silurana) tropicalis]; 77 511 cytochrome c oxidase subunit VIIa polypeptide 2 like [Xenopus (Silurana) tropicalis]; 545 979 cytochrome c oxidase subunit VIIa polypeptide 2 like [Xenopus (Silurana) tropicalis]

Protein Accession	CL17601.Contig8$_H1$ CL17601.Contig7$_H1$ CL17601.Contig6$_H1$ CL17601.Contig2$_H1$
Mean Expression Ratio	0.845
Median Expression Ratio	0.846
Credible Interval	(0.408, 1.71)
Associated Peptides	3
Associated Spectra	6
Coverage	NaN

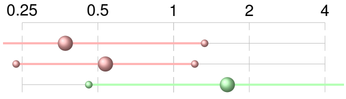

A	B	2.5	50	97.5	Sequence
1	0	0.11	0.37	1.3	EPSVIIFDSK
1	3	0.24	0.53	1.2	QDGLLVWQK
0	1	0.46	1.6	5.8	TTKPAVAPPAPMK

5.341 36 770 minus strand Endoplasmic reticulum protein ERp29 [Crassostrea gigas]

Protein Accession	CL37188.Contig1$_H1$
Mean Expression Ratio	0.845
Median Expression Ratio	0.846
Credible Interval	(0.443, 1.6)
Associated Peptides	5
Associated Spectra	13
Coverage	NaN

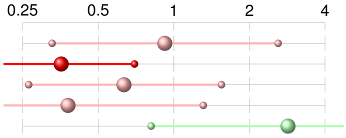

A	B	2.5	50	97.5	Sequence
2	0	0.33	0.92	2.6	AQTAAAALTK
3	3	0.18	0.36	0.7	KPDFPVYK
1	2	0.26	0.63	1.5	EADKESADIYIK
1	0	0.1	0.38	1.3	QDPIPYTGDVK
0	1	0.81	2.9	10	GSINLNSGVFDKVIAK

5.342 125 550 minus strand ribosomal protein S20 [Chlamys farreri]; 125 550 minus strand ribosomal protein S20 [Chlamys farreri]; 125 550 minus strand ribosomal protein S20 [Chlamys farreri]

Protein Accession	CL6833.Contig3$_H1$ CL6833.Contig2$_H1$ CL6833.Contig1$_H1$
Mean Expression Ratio	1.18
Median Expression Ratio	1.18
Credible Interval	(0.588, 2.37)
Associated Peptides	3
Associated Spectra	10
Coverage	NaN

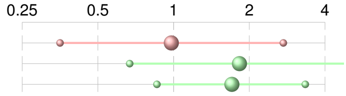

A	B	2.5	50	97.5	Sequence
1	1	0.35	0.98	2.7	KTPCGEGSK
1	1	0.67	1.8	5.2	ITLTSR
0	6	0.86	1.7	3.3	IIDLHSPSEIVK

5.343 5 301 PREDICTED: hypothetical protein [Saccoglossus kowalevskii]

Protein Accession Unigene21633$_{H}$1
Mean Expression Ratio 0.847
Median Expression Ratio 0.847
Credible Interval (0.379, 1.94)
Associated Peptides 1
Associated Spectra 3
Coverage NaN

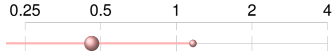

A	B	2.5	50	97.5	Sequence
**2**	**1**	**0.19**	**0.46**	**1.2**	**AYIPIQMR**

5.344 4 2400 IQ motif containing GTPase activating protein 2 [Xenopus laevis] gi; 4 2169 IQ motif containing GTPase activating protein 2 [Xenopus laevis] gi

Protein Accession CL875.Contig2$_{H}$1 CL875.Contig1$_{H}$1
Mean Expression Ratio 1.19
Median Expression Ratio 1.18
Credible Interval (0.541, 2.61)
Associated Peptides 2
Associated Spectra 2
Coverage NaN

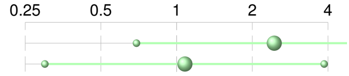

A	B	2.5	50	97.5	Sequence
**0**	**1**	**0.69**	**2.4**	**8.8**	**ITLQDVVTHNR**
**0**	**1**	**0.3**	**1.1**	**3.9**	**TEVLEK**

5.345 94 519 minus strand hypothetical protein BRAFLDRAFT_115029 [Branchiostoma floridae] gi

Protein Accession Unigene52953$_{H}$1
Mean Expression Ratio 0.847
Median Expression Ratio 0.848
Credible Interval (0.399, 1.73)
Associated Peptides 3
Associated Spectra 5
Coverage NaN

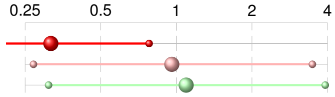

A	B	2.5	50	97.5	Sequence
**0**	**3**	**0.13**	**0.32**	**0.78**	**SLDTDGDGTVSAAELR**
**0**	**1**	**0.27**	**0.96**	**3.5**	**YGVVFK**
**0**	**1**	**0.31**	**1.1**	**3.9**	**FINKK**

5.346 2 352 Ribosome-binding protein 1 [Crassostrea gigas]

Protein Accession Unigene24847$_{H}$1
Mean Expression Ratio 0.846
Median Expression Ratio 0.848
Credible Interval (0.387, 1.85)
Associated Peptides 2
Associated Spectra 2
Coverage NaN

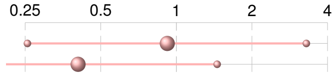

A	B	2.5	50	97.5	Sequence
**0**	**1**	**0.26**	**0.92**	**3.3**	**LVAHQLELEALQAR**
**0**	**1**	**0.11**	**0.41**	**1.5**	**LQPLEQSTDAALVQK**

5.347 1237 1374 minus strand putative 60S ribosomal protein L23 [Maconellicoccus hirsutus]

Protein Accession	CL834.Contig9$_{H}1$
Mean Expression Ratio	1.18
Median Expression Ratio	1.18
Credible Interval	(0.531, 2.67)
Associated Peptides	1
Associated Spectra	4
Coverage	NaN

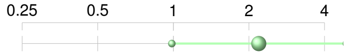

A	B	2.5	50	97.5	Sequence
**0**	**4**	**0.98**	**2.2**	**4.9**	**IASNASSIA**

5.348 " 1362 1544 minus strand Phosphoenolpyruvate carboxykinase, cytosolic [GTP] [Crassostrea gigas]"

Protein Accession	CL1008.Contig7$_{H}1$
Mean Expression Ratio	0.85
Median Expression Ratio	0.849
Credible Interval	(0.366, 1.97)
Associated Peptides	1
Associated Spectra	1
Coverage	NaN

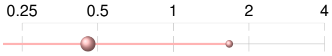

A	B	2.5	50	97.5	Sequence
**0**	**1**	**0.12**	**0.46**	**1.7**	**KVQHYVEEYAR**

5.349 " 131 544 Histone H2B, gonadal OS=Patella granatina PE=1 SV=2"

Protein Accession	CL29794.Contig2$_{H}1$
Mean Expression Ratio	1.18
Median Expression Ratio	1.18
Credible Interval	(0.687, 2.03)
Associated Peptides	5
Associated Spectra	47
Coverage	NaN

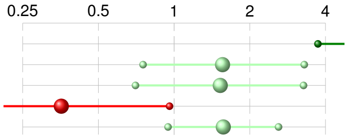

A	B	2.5	50	97.5	Sequence
**3**	**3**	**3.7**	**7.7**	**16**	**AVTKYTSSK**
**5**	**0**	**0.75**	**1.6**	**3.3**	**EIQTAVR**
**3**	**2**	**0.7**	**1.5**	**3.3**	**STITSREIQTAVR**
**2**	**0**	**0.13**	**0.36**	**0.96**	**LIFER**
**8**	**21**	**0.95**	**1.6**	**2.6**	**LLLPGELAK**

5.350 971 1963 Spectrin alpha chain [Crassostrea gigas]; 972 1979 Spectrin alpha chain [Crassostrea gigas]

Protein Accession	CL2195.Contig6$_{H}1$ CL2195.Contig4$_{H}1$
Mean Expression Ratio	1.18
Median Expression Ratio	1.18
Credible Interval	(0.518, 2.66)
Associated Peptides	1
Associated Spectra	2
Coverage	NaN

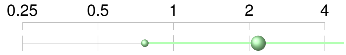

A	B	2.5	50	97.5	Sequence
**0**	**2**	**0.77**	**2.2**	**6.1**	**EAFLAGDTVDGDNVETLIK**

5.351 254 1093 uncharacterized protein LOC432187 precursor [Xenopus laevis] gi; 343 561 minus strand cathepsin Z precursor [Danio rerio] gi

Protein Accession: CL7800.Contig7$_H$1 CL7800.Contig1$_H$1
Mean Expression Ratio: 1.17
Median Expression Ratio: 1.18
Credible Interval: (0.523, 2.63)
Associated Peptides: 1
Associated Spectra: 2
Coverage: NaN

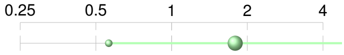

A	B	2.5	50	97.5	Sequence
1	1	0.56	1.8	5.9	GINFASTTR

5.352 1215 1694 Pikachurin [Crassostrea gigas]

Protein Accession: CL4889.Contig4$_H$1
Mean Expression Ratio: 0.85
Median Expression Ratio: 0.85
Credible Interval: (0.373, 1.9)
Associated Peptides: 1
Associated Spectra: 2
Coverage: NaN

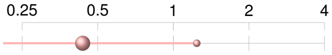

A	B	2.5	50	97.5	Sequence
0	2	0.15	0.44	1.2	HGLGVNELPIASPK

5.353 259 1296 minus strand GD20916 [Drosophila simulans] gi

Protein Accession: CL25607.Contig2$_H$1
Mean Expression Ratio: 1.17
Median Expression Ratio: 1.18
Credible Interval: (0.548, 2.51)
Associated Peptides: 2
Associated Spectra: 4
Coverage: NaN

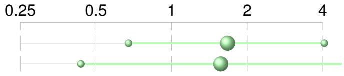

A	B	2.5	50	97.5	Sequence
0	3	0.68	1.7	4.1	QIVWEHPEVK
0	1	0.43	1.6	5.5	YAPWPYKK

5.354 304 1830 minus strand Methenyltetrahydrofolate synthetase domain-containing protein [Crassostrea gigas]

Protein Accession: CL45489.Contig2$_H$1
Mean Expression Ratio: 1.18
Median Expression Ratio: 1.17
Credible Interval: (0.595, 2.38)
Associated Peptides: 4
Associated Spectra: 6
Coverage: NaN

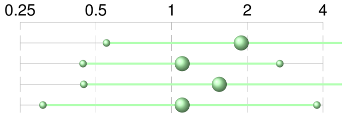

A	B	2.5	50	97.5	Sequence
0	1	0.55	1.9	6.7	KTLLVPTPR
0	3	0.45	1.1	2.7	FLTLEAK
0	1	0.45	1.6	5.6	TGLFNR
0	1	0.31	1.1	3.8	IPVLR

5.355 527 1321 minus strand troponin T [Mizuhopecten yessoensis]; 305 1099 minus strand troponin T [Mizuhopecten yessoensis]; 305 1099 minus strand troponin T [Mizuhopecten yessoensis]

Protein Accession	Unigene65787$_{H}1$ CL2214.Contig4$_{H}1$ CL2214.Contig3$_{H}1$
Mean Expression Ratio	1.18
Median Expression Ratio	1.17
Credible Interval	(0.633, 2.21)
Associated Peptides	5
Associated Spectra	24
Coverage	NaN

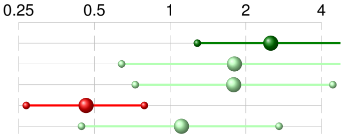

A	B	2.5	50	97.5	Sequence
2	4	1.3	2.5	4.9	KGPNFVITK
1	1	0.64	1.8	5	LDSVMSKEELQK
3	0	0.73	1.8	4.4	AILSQR
10	0	0.27	0.46	0.79	LTELR
0	3	0.45	1.1	2.7	GAEILALK

5.356 80 250 minus strand 40S ribosomal protein S29 [Ornithodoros parkeri]

Protein Accession	CL11390.Contig2$_{H}1$
Mean Expression Ratio	1.17
Median Expression Ratio	1.17
Credible Interval	(0.595, 2.32)
Associated Peptides	3
Associated Spectra	9
Coverage	NaN

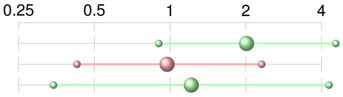

A	B	2.5	50	97.5	Sequence
0	4	0.9	2	4.6	GFQTTWYSHPR
1	3	0.43	0.98	2.3	QFAPDIGFK
0	1	0.34	1.2	4.3	KYGLNICR

5.357 313 1908 minus strand Heterogeneous nuclear ribonucleoprotein L [Crassostrea gigas]; 313 1833 minus strand Heterogeneous nuclear ribonucleoprotein L [Crassostrea gigas]

Protein Accession	CL19412.Contig8$_{H}1$ CL19412.Contig7$_{H}1$
Mean Expression Ratio	0.855
Median Expression Ratio	0.854
Credible Interval	(0.369, 1.94)
Associated Peptides	1
Associated Spectra	2
Coverage	NaN

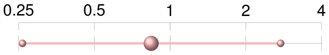

A	B	2.5	50	97.5	Sequence
1	1	0.26	0.84	2.7	SSTGLLEFESK

5.358 93 368 60S ribosomal protein L37A [Branchiostoma belcheri] gi; 93 395 60S ribosomal protein L37A [Branchiostoma belcheri] gi; 90 272 ribosomal protein L37ae [Wuchereria bancrofti]; 44 319 minus strand 60S ribosomal protein L37A [Branchiostoma belcheri] gi; 44 319 minus strand 60S ribosomal protein L37A [Branchiostoma belcheri] gi

Protein Accession	Unigene66574$_{H}1$ CL6728.Contig4$_{H}1$ CL6728.Contig3$_{H}1$ CL6728.Contig2$_{H}1$ CL6728.Contig
Mean Expression Ratio	1.18
Median Expression Ratio	1.17
Credible Interval	(0.52, 2.71)
Associated Peptides	1
Associated Spectra	1
Coverage	NaN

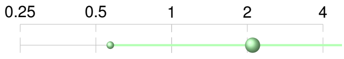

A	B	2.5	50	97.5	Sequence
**0**	**1**	**0.57**	**2.1**	**7.7**	**VGIVGK**

5.359 85 1632 minus strand Alpha-aminoadipic semialdehyde dehydrogenase [Crassostrea gigas]

Protein Accession	Unigene19467$_{H}1$
Mean Expression Ratio	1.18
Median Expression Ratio	1.17
Credible Interval	(0.507, 2.75)
Associated Peptides	1
Associated Spectra	1
Coverage	NaN

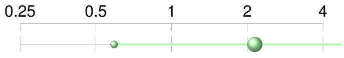

A	B	2.5	50	97.5	Sequence
**0**	**1**	**0.59**	**2.1**	**7.6**	**ELPLAQGIKFE**

5.360 89 361 40S ribosomal protein S18 OS=Rattus norvegicus GN=Rps18 PE=1 SV=3

Protein Accession	CL28311.Contig1$_{H}1$
Mean Expression Ratio	1.17
Median Expression Ratio	1.17
Credible Interval	(0.535, 2.57)
Associated Peptides	2
Associated Spectra	3
Coverage	NaN

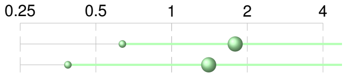

A	B	2.5	50	97.5	Sequence
**0**	**2**	**0.64**	**1.8**	**5**	**AGELTEDEVER**
**0**	**1**	**0.39**	**1.4**	**5**	**LNRQKDVK**

5.361 54 1076 PREDICTED: UPF0553 protein C9orf64-like [Anolis carolinensis]; 71 1093 PREDICTED: UPF0553 protein C9orf64-like [Anolis carolinensis]; 33 1055 PREDICTED: UPF0553 protein C9orf64-like [Anolis carolinensis]; 54 1076 PREDICTED: UPF0553 protein C9orf64-like [Anolis carolinensis]

Protein Accession	CL26820.Contig7$_{H}1$ CL26820.Contig6$_{H}1$ CL26820.Contig5$_{H}1$ CL26820.Contig4$_{H}1$
Mean Expression Ratio	0.853
Median Expression Ratio	0.855
Credible Interval	(0.365, 1.97)
Associated Peptides	1
Associated Spectra	1
Coverage	NaN

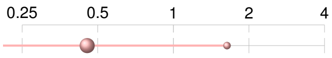

A	B	2.5	50	97.5	Sequence
0	1	0.13	0.45	1.6	SKELLDILDK

5.362 144 1307 minus strand PREDICTED: similar to AGAP005845-PA [Tribolium castaneum]; 146 1309 minus strand PREDICTED: similar to AGAP005845-PA [Tribolium castaneum]; 144 1307 PREDICTED: similar to AGAP005845-PA [Tribolium castaneum]

Protein Accession	CL8394.Contig21$_{H}1$ CL8394.Contig20$_{H}1$ CL8394.Contig14$_{H}1$
Mean Expression Ratio	1.17
Median Expression Ratio	1.17
Credible Interval	(0.513, 2.66)
Associated Peptides	1
Associated Spectra	1
Coverage	NaN

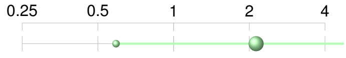

A	B	2.5	50	97.5	Sequence
0	1	0.59	2.1	7.4	KATGSLLTR

5.363 " 303 500 minus strand protein lethal, putative [Pediculus humanus corporis] gi"

Protein Accession	CL15038.Contig1$_{H}1$
Mean Expression Ratio	1.17
Median Expression Ratio	1.17
Credible Interval	(0.6, 2.3)
Associated Peptides	3
Associated Spectra	26
Coverage	NaN

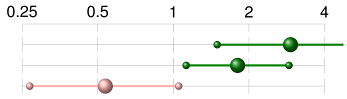

A	B	2.5	50	97.5	Sequence
3	3	1.5	2.9	5.7	VPSRQDMLATGR
4	10	1.1	1.8	2.9	QMPMLGR
3	3	0.27	0.53	1.1	QDMLATGR

5.364 95 271 minus strand hypothetical protein EAI_01530 [Harpegnathos saltator]

Protein Accession	CL9369.Contig9$_H$1
Mean Expression Ratio	1.16
Median Expression Ratio	1.17
Credible Interval	(0.537, 2.56)
Associated Peptides	2
Associated Spectra	2
Coverage	NaN

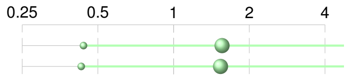

A	B	2.5	50	97.5	Sequence
0	1	0.44	1.6	5.7	YPYTISSK
0	1	0.43	1.5	5.4	VAQFPFR

5.365 430 759 hypothetical protein [Amblyomma maculatum]; 336 665 hypothetical protein [Amblyomma maculatum]

Protein Accession	CL4062.Contig3$_H$1 CL4062.Contig2$_H$1
Mean Expression Ratio	1.17
Median Expression Ratio	1.17
Credible Interval	(0.612, 2.25)
Associated Peptides	5
Associated Spectra	10
Coverage	NaN

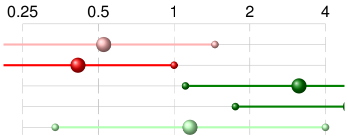

A	B	2.5	50	97.5	Sequence
1	1	0.19	0.52	1.5	KYIPGTK
0	3	0.17	0.42	1	ETLFEYLADPK
0	2	1.1	3.1	8.9	ETLFEYLADPKK
0	2	1.8	5	14	MVFAGIKKPQER
0	1	0.34	1.2	4	ADIPDGDFEK

5.366 40 609 minus strand ependymin related protein-1 precursor [Haliotis discus discus]

Protein Accession	CL31954.Contig1$_H$1
Mean Expression Ratio	0.858
Median Expression Ratio	0.858
Credible Interval	(0.393, 1.88)
Associated Peptides	2
Associated Spectra	2
Coverage	NaN

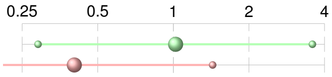

A	B	2.5	50	97.5	Sequence
0	1	0.29	1	3.6	EIEEIQIGSDQEFYDVLR
0	1	0.11	0.4	1.4	QQPKPCTSPPQFVAR

5.367 165 635 hypothetical protein CGI_10007639 [Crassostrea gigas]

Protein Accession	CL36715.Contig1$_H$1
Mean Expression Ratio	1.16
Median Expression Ratio	1.17
Credible Interval	(0.506, 2.69)
Associated Peptides	1
Associated Spectra	1
Coverage	NaN

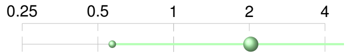

A	B	2.5	50	97.5	Sequence
0	1	0.57	2	7.2	YVDDQTFTFSSK

5.368 390 908 Cysteine proteinase 5 OS=Dictyostelium discoideum GN=cprE PE=2 SV=2

Protein Accession	CL6665.Contig2$_{H}1$
Mean Expression Ratio	0.852
Median Expression Ratio	0.858
Credible Interval	(0.358, 1.93)
Associated Peptides	1
Associated Spectra	1
Coverage	NaN

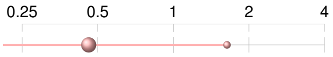

A	B	2.5	50	97.5	Sequence
**0**	**1**	**0.13**	**0.46**	**1.6**	**GPVSVGIDASHK**

5.369 118 1629 PREDICTED: similar to tektin 3 [Ciona intestinalis]

Protein Accession	Unigene11521$_{H}1$
Mean Expression Ratio	0.861
Median Expression Ratio	0.858
Credible Interval	(0.437, 1.7)
Associated Peptides	4
Associated Spectra	8
Coverage	NaN

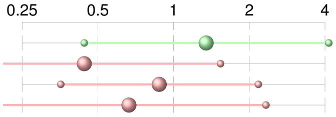

A	B	2.5	50	97.5	Sequence
**1**	**1**	**0.44**	**1.3**	**4.1**	**GLTFHNGVQR**
**1**	**0**	**0.13**	**0.44**	**1.5**	**SALEIDLAVK**
**2**	**2**	**0.36**	**0.88**	**2.2**	**QTIDLVHDAPEK**
**1**	**0**	**0.19**	**0.67**	**2.3**	**EVDTIKR**

5.370 11 1462 coronin [Schistosoma mansoni]

Protein Accession	CL11779.Contig2$_{H}1$
Mean Expression Ratio	0.861
Median Expression Ratio	0.859
Credible Interval	(0.392, 1.91)
Associated Peptides	2
Associated Spectra	2
Coverage	NaN

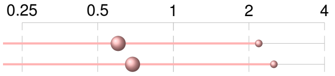

A	B	2.5	50	97.5	Sequence
**0**	**1**	**0.17**	**0.6**	**2.2**	**VPMDAPVVAGHK**
**0**	**1**	**0.2**	**0.69**	**2.5**	**HVFGKPEK**

5.371 78 2003 Chromatin assembly factor 1 subunit A [Crassostrea gigas]

Protein Accession	CL14511.Contig5$_{H}1$
Mean Expression Ratio	1.16
Median Expression Ratio	1.16
Credible Interval	(0.517, 2.61)
Associated Peptides	1
Associated Spectra	1
Coverage	NaN

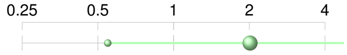

A	B	2.5	50	97.5	Sequence
**1**	**0**	**0.55**	**2**	**7.2**	**IAKELK**

5.372 " 11 427 PREDICTED: similar to syntaxin, partial [Hydra magnipapillata]"

Protein Accession	CL41814.Contig1$_H1$
Mean Expression Ratio	0.859
Median Expression Ratio	0.859
Credible Interval	(0.371, 1.97)
Associated Peptides	1
Associated Spectra	1
Coverage	NaN

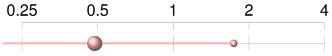

A	B	2.5	50	97.5	Sequence
0	1	0.13	0.48	1.7	HKEMLALEK

5.373 " 519 3182 minus strand Alpha-actinin, sarcomeric [Crassostrea gigas]"

Protein Accession	CL28960.Contig2$_H1$
Mean Expression Ratio	0.86
Median Expression Ratio	0.859
Credible Interval	(0.537, 1.38)
Associated Peptides	17
Associated Spectra	30
Coverage	NaN

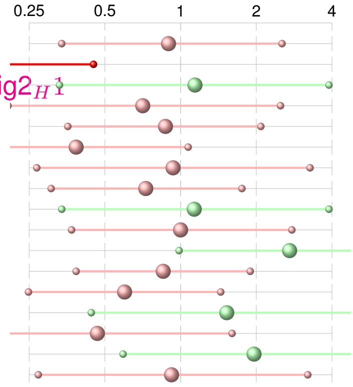

A	B	2.5	50	97.5	Sequence
1	1	0.34	0.9	2.5	NINEVENQILTR
1	0	0.038	0.13	0.45	LGQLTVK
1	0	0.33	1.1	3.9	HLDIPR
1	0	0.21	0.71	2.5	MSFNHFDK
2	1	0.36	0.87	2.1	RDQTLQQEMMR
1	1	0.14	0.39	1.1	LDEFR
0	1	0.27	0.94	3.3	ACLISLGYK
0	3	0.31	0.73	1.8	AIIQYKPHIDELER
0	1	0.34	1.1	3.9	DNPIENLNTAFDIAEK
0	2	0.37	1	2.8	ETADTDTAEQVMQSFK
0	2	0.98	2.7	7.4	GISEDQMNEFR
0	4	0.38	0.85	1.9	IDQLHLEFAK
0	3	0.25	0.6	1.4	ILAGDKPYITAQILR
0	1	0.44	1.5	5.2	LSNRPAYLPTEGK
0	1	0.13	0.47	1.6	ALDFIASK
0	1	0.59	2	6.7	MSFNHFDKNR
0	1	0.27	0.92	3.2	EALEEAER

5.374 126 704 minus strand ribosomal protein L12 [Ixodes scapularis]

Protein Accession	CL24053.Contig3$_H1$
Mean Expression Ratio	1.17
Median Expression Ratio	1.16
Credible Interval	(0.652, 2.11)
Associated Peptides	6
Associated Spectra	30
Coverage	NaN

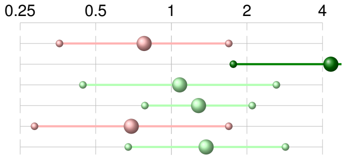

A	B	2.5	50	97.5	Sequence
1	3	0.36	0.78	1.7	AVGGEVPATSALAPK
1	2	1.8	4.3	10	INAKEIEIPDE
1	2	0.45	1.1	2.6	KVGDDIAK
5	7	0.78	1.3	2.1	IGPLGLSPK
1	2	0.29	0.69	1.7	EIEIPDE
1	4	0.67	1.4	2.8	SNPHVIIEK

5.375 143 634 minus strand ribosomal protein S14 [Solea senegalensis]

Protein Accession	CL17182.Contig1$_H$1
Mean Expression Ratio	1.16
Median Expression Ratio	1.16
Credible Interval	(0.54, 2.52)
Associated Peptides	2
Associated Spectra	3
Coverage	NaN

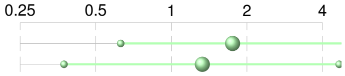

A	B	2.5	50	97.5	Sequence
**0**	**2**	**0.63**	**1.7**	**4.9**	**TVQQEQVVLGPQAK**
**0**	**1**	**0.37**	**1.3**	**4.6**	**QLGITALHIK**

5.376 75 455 minus strand PREDICTED: calnexin [Strongylocentrotus purpuratus]

Protein Accession	CL46825.Contig1$_H$1
Mean Expression Ratio	0.856
Median Expression Ratio	0.86
Credible Interval	(0.396, 1.82)
Associated Peptides	2
Associated Spectra	3
Coverage	NaN

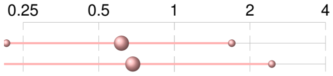

A	B	2.5	50	97.5	Sequence
**1**	**1**	**0.21**	**0.62**	**1.7**	**SNALDGDLGLILK**
**0**	**1**	**0.19**	**0.68**	**2.4**	**APVFFHEPFDSK**

5.377 " 174 569 NADH dehydrogenase [ubiquinone] iron-sulfur protein 4, mitochondrial [Crassostrea gigas]"

Protein Accession	CL4564.Contig1$_H$1
Mean Expression Ratio	0.857
Median Expression Ratio	0.861
Credible Interval	(0.368, 1.98)
Associated Peptides	1
Associated Spectra	1
Coverage	NaN

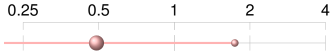

A	B	2.5	50	97.5	Sequence
**0**	**1**	**0.13**	**0.49**	**1.7**	**VDISCLTGVPEEHIK**

5.378 696 4010 Lethal(2) giant larvae-like protein 1 [Crassostrea gigas]; 657 3971 minus strand Lethal(2) giant larvae-like protein 1 [Crassostrea gigas]; 696 3749 minus strand Lethal(2) giant larvae-like protein 1 [Crassostrea gigas]; 156 3338 Lethal(2) giant larvae-like protein 1 [Crassostrea gigas]; 156 3209 Lethal(2) giant larvae-like protein 1 [Crassostrea gigas]; 156 3470 Lethal(2) giant larvae-like protein 1 [Crassostrea gigas]

Protein Accession	CL3717.Contig4$_H1$ CL3717.Contig3$_H1$ CL3717.Contig2$_H1$ CL3717.Contig19$_H1$ CL3717.Co
Mean Expression Ratio	1.16
Median Expression Ratio	1.16
Credible Interval	(0.523, 2.55)
Associated Peptides	2
Associated Spectra	2
Coverage	NaN

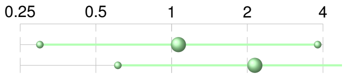

A	B	2.5	50	97.5	Sequence
0	1	0.3	1.1	3.8	VLPESLEVIHER
0	1	0.61	2.1	7.7	NLAKEPTTK

5.379 98 673 transforming protein Rho-like [Acyrthosiphon pisum]

Protein Accession	Unigene30464$_H1$
Mean Expression Ratio	1.16
Median Expression Ratio	1.16
Credible Interval	(0.527, 2.6)
Associated Peptides	2
Associated Spectra	2
Coverage	NaN

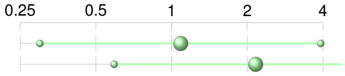

A	B	2.5	50	97.5	Sequence
0	1	0.3	1.1	3.9	EVFEQATR
0	1	0.59	2.2	7.8	EGVREVFEQATR

5.380 15 1361 hypothetical protein BRAFLDRAFT_231908 [Branchiostoma floridae] gi

Protein Accession	CL24447.Contig2$_H1$
Mean Expression Ratio	1.16
Median Expression Ratio	1.16
Credible Interval	(0.542, 2.52)
Associated Peptides	2
Associated Spectra	3
Coverage	NaN

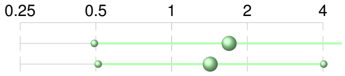

A	B	2.5	50	97.5	Sequence
0	1	0.49	1.7	6.1	GLGLDDALEAR
0	2	0.51	1.4	4	GITRIR

5.381 189 353 40S ribosomal protein S12 [Crassostrea gigas]

Protein Accession	Unigene385$_{H}1$
Mean Expression Ratio	1.16
Median Expression Ratio	1.16
Credible Interval	(0.51, 2.62)
Associated Peptides	1
Associated Spectra	2
Coverage	NaN

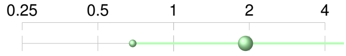

A	B	2.5	50	97.5	Sequence
0	2	0.69	1.9	5.6	TALIHDGLAR

5.382 34 1122 Fructose-bisphosphate aldolase [Crassostrea gigas]

Protein Accession	CL24228.Contig1$_{H}1$
Mean Expression Ratio	1.16
Median Expression Ratio	1.16
Credible Interval	(0.769, 1.75)
Associated Peptides	21
Associated Spectra	65
Coverage	NaN

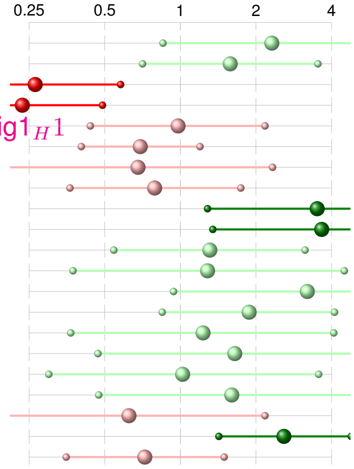

A	B	2.5	50	97.5	Sequence
1	1	0.85	2.3	6.4	AKANSEAALGK
2	2	0.71	1.6	3.5	GAQDELLKR
2	2	0.12	0.26	0.58	GIIPGIK
5	0	0.11	0.24	0.49	GILAADESVGSMGK
1	3	0.44	0.98	2.2	RLEGIGLENTEENR
7	3	0.4	0.69	1.2	ANSEAALGK
1	0	0.2	0.68	2.3	DSQGTPFVK
0	4	0.36	0.79	1.7	ALQASVLAAWK
0	2	1.3	3.5	9.9	ALQASVLAAWKGDDAK
0	2	1.3	3.7	9.9	ALQASVLAAWKGDDAKVK
0	3	0.54	1.3	3.1	RLEGIGLENTEENRR
0	1	0.37	1.3	4.5	RLEGIGLENTEENRRR
0	1	0.94	3.2	11	VKGAQDELLKR
0	4	0.85	1.9	4.1	QIANSIVAPGK
0	1	0.37	1.2	4.1	KDGAQFAK
0	1	0.47	1.6	5.6	GIIPGIKVDK
0	1	0.3	1	3.6	GKNPWAVTF
0	1	0.47	1.6	5.3	PVIAQYLTKQQEDELR
0	1	0.18	0.62	2.2	VKGAQDELLK
0	8	1.4	2.6	4.8	PVIAQYLTK
0	5	0.35	0.72	1.5	VTEQVLAFTYK

5.383 151 471 minus strand ribosomal protein rps25 [Arenicola marina]

Protein Accession	CL17623.Contig4$_{H}1$
Mean Expression Ratio	1.16
Median Expression Ratio	1.16
Credible Interval	(0.546, 2.46)
Associated Peptides	2
Associated Spectra	4
Coverage	NaN

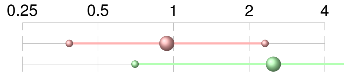

A	B	2.5	50	97.5	Sequence
1	2	0.39	0.94	2.3	LITPSVVSER
0	1	0.7	2.5	9.1	MLKEVPSYK

5.384 " 110 1717 PREDICTED: glutamate dehydrogenase, mitochondrial-like [Oreochromis niloticus]; 110 1717 PREDICTED: glutamate dehydrogenase, mitochondrial-like [Oreochromis niloticus]; 110 1717 PREDICTED: glutamate dehydrogenase, mitochondrial-like [Oreochromis niloticus]"

Protein Accession	CL16377.Contig9$_{H}$1 CL16377.Contig8$_{H}$1 CL16377.Contig10$_{H}$1
Mean Expression Ratio	1.16
Median Expression Ratio	1.16
Credible Interval	(0.688, 2)
Associated Peptides	13
Associated Spectra	15
Coverage	NaN

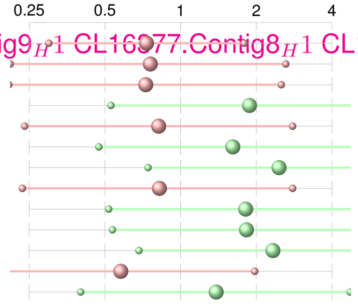

A	B	2.5	50	97.5	Sequence
1	2	0.3	0.73	1.8	DGSIYNPVDGIHPR
1	0	0.21	0.76	2.6	FTVELAK
1	0	0.21	0.73	2.5	PFGGAK
0	1	0.53	1.9	6.5	ACVTGKPIPQGGIHGR
0	1	0.24	0.82	2.8	CAIVDVPFGGAK
0	1	0.47	1.6	5.6	CDILVPAAGER
0	1	0.74	2.5	8.7	IAGASEKDIVHSGLEYTMER
0	1	0.23	0.82	2.8	IIAEGANGPTTAGADEIFLK
0	1	0.52	1.8	6.4	KGFIGPGIDVPAPDMGTGER
0	1	0.54	1.8	6.3	INPANYSENELEK
0	1	0.68	2.3	7.9	LTFKYEK
0	1	0.17	0.58	2	EAGLTFT
0	1	0.4	1.4	4.7	DSNFHLLQSVQR

5.385 76 522 minus strand Troponin C [Crassostrea gigas]; 173 619 minus strand Troponin C [Crassostrea gigas]; 173 619 minus strand Troponin C [Crassostrea gigas]

Protein Accession	Unigene53263$_{H}$1 Unigene11674$_{H}$1 CL13001.Contig4$_{H}$1
Mean Expression Ratio	0.864
Median Expression Ratio	0.864
Credible Interval	(0.427, 1.74)
Associated Peptides	4
Associated Spectra	7
Coverage	NaN

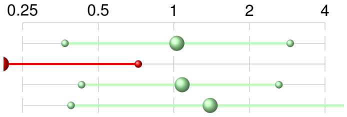

A	B	2.5	50	97.5	Sequence
1	1	0.37	1	2.9	GVIPVEDLR
1	0	0.057	0.21	0.72	SLGLQVEDEK
2	1	0.43	1.1	2.6	ELANVFK
1	0	0.39	1.4	4.9	EAFRVLDK

5.386 35 1861 hypothetical protein DAPPUDRAFT_309082 [Daphnia pulex]

Protein Accession	CL49314.Contig5$_{H}$1
Mean Expression Ratio	0.864
Median Expression Ratio	0.864
Credible Interval	(0.464, 1.6)
Associated Peptides	6
Associated Spectra	17
Coverage	NaN

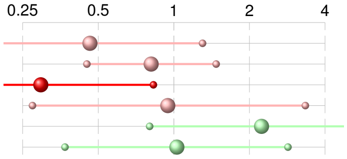

A	B	2.5	50	97.5	Sequence
1	1	0.17	0.46	1.3	LINHSVPDTIDER
5	3	0.45	0.81	1.5	YAIGLAR
1	1	0.11	0.29	0.83	KVELVK
0	1	0.27	0.95	3.3	ISNFGDDIKDSEAYVHLLHR
0	2	0.8	2.2	6.3	DIGHTFKK
0	2	0.37	1	2.8	LNDGIIIAK

5.387 3 1352 nervous system adducin [Aplysia californica] gi; 3 1418 Protein hu-li tai shao [Crassostrea gigas]; 3 1214 nervous system adducin [Aplysia californica] gi; 3 1319 minus strand nervous system adducin [Aplysia californica] gi; 3 1523 minus strand Protein hu-li tai shao [Crassostrea gigas]; 3 1439 minus strand Protein hu-li tai shao [Crassostrea gigas]; 3 1562 minus strand Protein hu-li tai shao [Crassostrea gigas]

Protein Accession	CL3889.Contig6$_{H}$1 CL3889.Contig5$_{H}$1 CL3889.Contig4$_{H}$1 CL3889.Contig14$_{H}$1 CL3889.Co
Mean Expression Ratio	1.16
Median Expression Ratio	1.16
Credible Interval	(0.613, 2.23)
Associated Peptides	5
Associated Spectra	8
Coverage	NaN

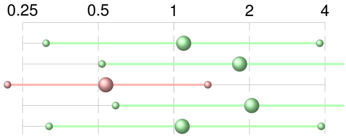

A	B	2.5	50	97.5	Sequence
1	0	0.31	1.1	3.8	WVPESETQGK
1	0	0.52	1.8	6.2	LISTEER
2	2	0.22	0.54	1.4	VMLLR
1	0	0.59	2	7.1	GDQTV
1	0	0.32	1.1	3.9	KTEIEEIGTTTPK

5.388 201 4058 PREDICTED: xanthine dehydrogenase/oxidase-like [Amphimedon queenslandica]

Protein Accession	CL17390.Contig11$_{H}$1
Mean Expression Ratio	0.864
Median Expression Ratio	0.865
Credible Interval	(0.395, 1.87)
Associated Peptides	2
Associated Spectra	2
Coverage	NaN

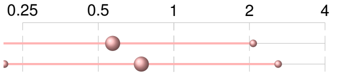

A	B	2.5	50	97.5	Sequence
1	0	0.16	0.57	2.1	AVTDLQEAISNK
1	0	0.21	0.74	2.6	MSLIR

5.389 26 1909 Dolichyl-diphosphooligosaccharide–protein glycosyltransferase subunit 2 [Crassostrea gigas]

Protein Accession	CL6515.Contig1$_{H}$1
Mean Expression Ratio	0.863
Median Expression Ratio	0.865
Credible Interval	(0.533, 1.4)
Associated Peptides	14
Associated Spectra	30
Coverage	NaN

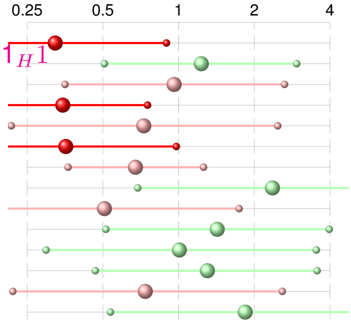

A	B	2.5	50	97.5	Sequence
2	0	0.12	0.32	0.89	APAIAEDK
1	2	0.51	1.2	2.9	HLVDEAIVLSK
1	1	0.35	0.96	2.6	LIGTSGAEVEVK
2	2	0.15	0.35	0.75	NLGLAVDEK
1	0	0.22	0.73	2.5	KPEIK
1	1	0.13	0.36	0.98	KQFTASASDK
2	5	0.36	0.67	1.3	LLTDAISAK
0	1	0.69	2.4	8	HLVDEAIVLSKK
0	1	0.14	0.51	1.7	TAYQFVSVIK
0	2	0.51	1.4	4	VSVENVEIGVADKDQTAAAR
0	1	0.3	1	3.5	VSVTNLMGGDLGQLTVTADSAR
0	2	0.47	1.3	3.6	LTISVAPK
0	1	0.22	0.74	2.6	TLYELNFMEAK
0	1	0.54	1.8	6.4	LTPSTFLTTLDQQR

5.390 143 865 minus strand predicted protein [Nematostella vectensis] gi

Protein Accession	CL5421.Contig1$_H$1
Mean Expression Ratio	1.16
Median Expression Ratio	1.16
Credible Interval	(0.51, 2.68)
Associated Peptides	1
Associated Spectra	1
Coverage	NaN

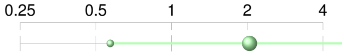

A	B	2.5	50	97.5	Sequence
0	1	0.57	2	7.6	AIGSGSEGAQQALQEQYHK

5.391 13 585 UDP-glucose:glycoprotein glucosyltransferase 1 [Crassostrea gigas]

Protein Accession	CL48318.Contig1$_H$1
Mean Expression Ratio	0.869
Median Expression Ratio	0.866
Credible Interval	(0.378, 2.02)
Associated Peptides	1
Associated Spectra	1
Coverage	NaN

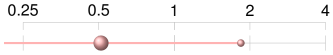

A	B	2.5	50	97.5	Sequence
0	1	0.14	0.51	1.8	FLDLPHK

5.392 101 1693 CALNUC [Spodoptera frugiperda]; 141 1325 Nucleobindin-2 [Crassostrea gigas]; 141 1289 Nucleobindin-2 [Crassostrea gigas]

Protein Accession	CL1888.Contig9$_H$1 CL1888.Contig6$_H$1 CL1888.Contig5$_H$1
Mean Expression Ratio	1.15
Median Expression Ratio	1.15
Credible Interval	(0.528, 2.49)
Associated Peptides	2
Associated Spectra	2
Coverage	NaN

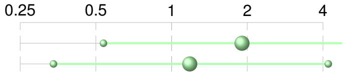

A	B	2.5	50	97.5	Sequence
0	1	0.54	1.9	6.9	LKELPEVER
0	1	0.34	1.2	4.2	LETANISDIK

5.393 102 1475 minus strand Cytosolic non-specific dipeptidase [Crassostrea gigas]

Protein Accession	CL4706.Contig1$_H$1
Mean Expression Ratio	1.16
Median Expression Ratio	1.15
Credible Interval	(0.594, 2.24)
Associated Peptides	5
Associated Spectra	8
Coverage	NaN

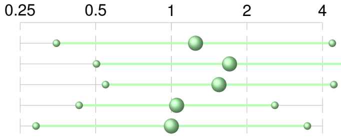

A	B	2.5	50	97.5	Sequence
0	1	0.35	1.2	4.4	EGGSIPVTLTFQEATGK
0	1	0.5	1.7	6.1	EHVEKVFAER
0	2	0.55	1.5	4.4	LVPDQEPHVIESLVR
0	3	0.43	1	2.6	LSEAVAIK
0	1	0.29	1	3.5	AELQELGEQTLHDGSK

5.394 447 977 Expansin-yoaJ [Crassostrea gigas]

Protein Accession	CL6172.Contig2$_{H}$1
Mean Expression Ratio	0.867
Median Expression Ratio	0.866
Credible Interval	(0.385, 1.97)
Associated Peptides	1
Associated Spectra	3
Coverage	NaN

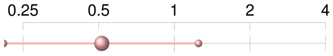

A	B	2.5	50	97.5	Sequence
**0**	**3**	**0.21**	**0.51**	**1.2**	**AGDVDLGVNGDGR**

5.395 174 1721 minus strand aspartyl-tRNA synthetase [Xenopus laevis] gi

Protein Accession	CL9865.Contig1$_{H}$1
Mean Expression Ratio	0.868
Median Expression Ratio	0.867
Credible Interval	(0.401, 1.86)
Associated Peptides	1
Associated Spectra	6
Coverage	NaN

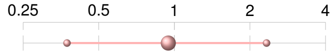

A	B	2.5	50	97.5	Sequence
**2**	**4**	**0.37**	**0.94**	**2.3**	**IHDPDFLSQR**

5.396 249 845 sigma class glutathione-s-transferase 2 [Haliotis discus discus]; 252 848 sigma class glutathione-s-transferase 2 [Haliotis discus discus]

Protein Accession	CL19285.Contig32$_{H}$1 CL19285.Contig20$_{H}$1
Mean Expression Ratio	0.865
Median Expression Ratio	0.867
Credible Interval	(0.377, 1.96)
Associated Peptides	1
Associated Spectra	1
Coverage	NaN

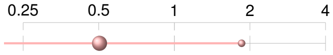

A	B	2.5	50	97.5	Sequence
**1**	**0**	**0.14**	**0.5**	**1.9**	**VKTENLPR**

5.397 48 479 minus strand 40S ribosomal protein S24 OS=Macaca fascicularis GN=RPS24 PE=2 SV=1

Protein Accession	CL40647.Contig4$_{H}$1
Mean Expression Ratio	1.16
Median Expression Ratio	1.15
Credible Interval	(0.575, 2.38)
Associated Peptides	3
Associated Spectra	9
Coverage	NaN

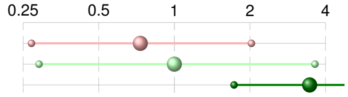

A	B	2.5	50	97.5	Sequence
**0**	**2**	**0.27**	**0.73**	**2**	**HGLLELK**
**0**	**1**	**0.29**	**1**	**3.6**	**TTGFGLVYDTLESAK**
**0**	**6**	**1.7**	**3.5**	**6.8**	**HGLLELKK**

5.398 108 1040 minus strand PREDICTED: Na(+)/H(+) exchange regulatory cofactor NHE-RF3-like [Metaseiulus occidentalis]; 108 1259 minus strand PREDICTED: Na(+)/H(+) exchange regulatory cofactor NHE-RF3-like [Metaseiulus occidentalis]; 108 1190 minus strand PREDICTED: Na(+)/H(+) exchange regulatory cofactor NHE-RF3-like [Metaseiulus occidentalis]; 108 971 minus strand PREDICTED: Na(+)/H(+) exchange regulatory cofactor NHE-RF3-like [Metaseiulus occidentalis]

Protein Accession	CL26023.Contig8$_{H}1$ CL26023.Contig7$_{H}1$ CL26023.Contig6$_{H}1$ CL26023.Contig5$_{H}1$
Mean Expression Ratio	1.15
Median Expression Ratio	1.15
Credible Interval	(0.525, 2.47)
Associated Peptides	2
Associated Spectra	3
Coverage	NaN

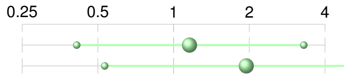

A	B	2.5	50	97.5	Sequence
0	2	0.41	1.2	3.3	GDLPEVLHLTAVR
0	1	0.53	1.9	7.1	IVKGDL

5.399 23 214 minus strand hemocyanin isoform 1 [Nucula nucleus]

Protein Accession	Unigene58881$_{H}1$
Mean Expression Ratio	1.16
Median Expression Ratio	1.15
Credible Interval	(0.586, 2.3)
Associated Peptides	3
Associated Spectra	22
Coverage	NaN

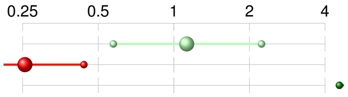

A	B	2.5	50	97.5	Sequence
2	4	0.58	1.1	2.2	GKIPNLNIYTSR
4	7	0.15	0.26	0.44	QEINPFFR
2	3	4.6	9.4	19	TAKQEINPFFR

5.400 68 694 minus strand hypothetical protein BRAFLDRAFT_272416 [Branchiostoma floridae] gi; 68 667 minus strand hypothetical protein BRAFLDRAFT_272416 [Branchiostoma floridae] gi; 68 697 minus strand hypothetical protein BRAFLDRAFT_272416 [Branchiostoma floridae] gi; 68 697 minus strand hypothetical protein BRAFLDRAFT_272416 [Branchiostoma floridae] gi; 68 667 minus strand hypothetical protein BRAFLDRAFT_272416 [Branchiostoma floridae] gi

Protein Accession	CL37759.Contig7$_{H}1$ CL37759.Contig4$_{H}1$ CL37759.Contig3$_{H}1$ CL37759.Contig2$_{H}1$ CL3775
Mean Expression Ratio	1.15
Median Expression Ratio	1.15
Credible Interval	(0.532, 2.49)
Associated Peptides	2
Associated Spectra	3
Coverage	NaN

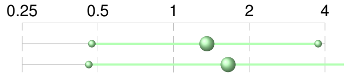

A	B	2.5	50	97.5	Sequence
0	2	0.47	1.4	3.8	TLEEDEDVLLQLR
0	1	0.46	1.7	5.9	LLAIR

5.401 52 1653 minus strand PREDICTED: eukaryotic translation initiation factor 3 subunit 6 interacting protein-like [Saccoglossus kowalevskii]

Protein Accession	CL23174.Contig2$_{H}1$
Mean Expression Ratio	0.868
Median Expression Ratio	0.869
Credible Interval	(0.372, 1.99)
Associated Peptides	1
Associated Spectra	1
Coverage	NaN

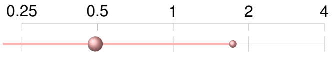

A	B	2.5	50	97.5	Sequence
1	0	0.14	0.49	1.7	NMIHVADTK

5.402 104 283 minus strand RecName: Full=Hemocyanin type 2 unit a; AltName: Full=Hemocyanin RHSS2 subunit; AltName: Full=Hemocyanin Rta; AltName: Full=Hemocyanin heavy structural subunit; AltName: Full=RtH2-a gi|7515001|pir||JC5632 hemocyanin dioxygen-binding functional unit - grosse

Protein Accession	Unigene21935$_{H}1$
Mean Expression Ratio	0.873
Median Expression Ratio	0.87
Credible Interval	(0.405, 1.87)
Associated Peptides	2
Associated Spectra	4
Coverage	NaN

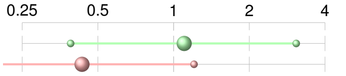

A	B	2.5	50	97.5	Sequence
0	2	0.39	1.1	3.1	KNVASLSDDEVLTLQSVLR
0	2	0.15	0.43	1.2	NVASLSDDEVLTLQSVLR

5.403 119 481 Glutamate receptor U1 [Crassostrea gigas]

Protein Accession	CL570.Contig5$_{H}1$
Mean Expression Ratio	1.15
Median Expression Ratio	1.15
Credible Interval	(0.575, 2.36)
Associated Peptides	3
Associated Spectra	8
Coverage	NaN

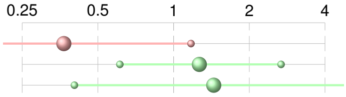

A	B	2.5	50	97.5	Sequence
1	1	0.12	0.37	1.2	GTANIGAGALTVTSER
0	5	0.61	1.3	2.7	RTPDGWSGIIGDVVK
0	1	0.4	1.4	5.1	TPDGWSGIIGDVVK

5.404 21 341 minus strand hemocyanin isoform 1 [Nucula nucleus]

Protein Accession	CL40281.Contig2$_{H}$1
Mean Expression Ratio	0.871
Median Expression Ratio	0.871
Credible Interval	(0.433, 1.77)
Associated Peptides	3
Associated Spectra	15
Coverage	NaN

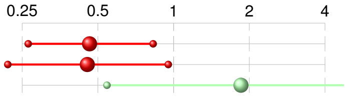

A	B	2.5	50	97.5	Sequence
7	2	0.26	0.46	0.83	DSNPIALTR
1	4	0.22	0.46	0.95	GDELFSYLK
0	1	0.54	1.9	6.6	KPLEPFNRDSNPIALTR

5.405 242 1834 Regulator of differentiation 1 [Crassostrea gigas]; 458 2050 minus strand Regulator of differentiation 1 [Crassostrea gigas]; 503 2095 Regulator of differentiation 1 [Crassostrea gigas]; 434 1942 Regulator of differentiation 1 [Crassostrea gigas]

Protein Accession	CL11169.Contig7$_{H}$1 CL11169.Contig5$_{H}$1 CL11169.Contig4$_{H}$1 CL11169.Contig1$_{H}$1
Mean Expression Ratio	1.15
Median Expression Ratio	1.15
Credible Interval	(0.569, 2.33)
Associated Peptides	4
Associated Spectra	5
Coverage	NaN

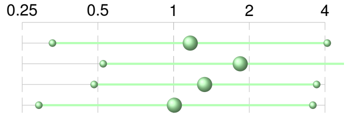

A	B	2.5	50	97.5	Sequence
0	1	0.33	1.2	4.1	DFLNSPLHR
0	1	0.52	1.8	6.4	SLPHEVAETDIVQLGMPFGK
0	2	0.48	1.3	3.7	MTNVLVLK
0	1	0.29	1	3.6	LTNLNVK

5.406 181 684 hypothetical protein BRAFLDRAFT_131834 [Branchiostoma floridae] gi

Protein Accession	CL10218.Contig4$_{H}$1
Mean Expression Ratio	0.873
Median Expression Ratio	0.871
Credible Interval	(0.375, 2.01)
Associated Peptides	1
Associated Spectra	1
Coverage	NaN

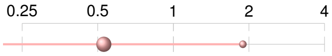

A	B	2.5	50	97.5	Sequence
1	0	0.15	0.53	1.9	LSGEGLETSK

5.407 416 1717 minus strand T-complex protein 1 subunit delta OS=Aedes triseriatus PE=2 SV=1

Protein Accession	CL8566.Contig1$_{H}1$
Mean Expression Ratio	0.872
Median Expression Ratio	0.872
Credible Interval	(0.407, 1.85)
Associated Peptides	2
Associated Spectra	6
Coverage	NaN

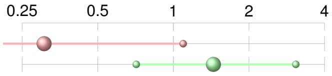

A	B	2.5	50	97.5	Sequence
**1**	**0**	**0.088**	**0.31**	**1.1**	**LVLEEADR**
**1**	**4**	**0.71**	**1.4**	**3.1**	**HAAGELTAGINVR**

5.408 382 972 minus strand Muscle-specific protein 20 [Crassostrea gigas]; 807 1397 minus strand Muscle-specific protein 20 [Crassostrea gigas]

Protein Accession	Unigene366$_{H}1$ CL6243.Contig1$_{H}1$
Mean Expression Ratio	0.878
Median Expression Ratio	0.873
Credible Interval	(0.392, 2.03)
Associated Peptides	1
Associated Spectra	1
Coverage	NaN

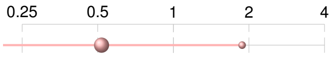

A	B	2.5	50	97.5	Sequence
**1**	**0**	**0.14**	**0.52**	**1.9**	**GFQGPTLGPK**

5.409 4 234 ribosomal protein rpl14 [Arenicola marina]

Protein Accession	CL2715.Contig2$_{H}1$
Mean Expression Ratio	1.14
Median Expression Ratio	1.14
Credible Interval	(0.5, 2.57)
Associated Peptides	1
Associated Spectra	2
Coverage	NaN

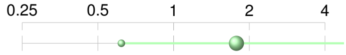

A	B	2.5	50	97.5	Sequence
**0**	**2**	**0.62**	**1.8**	**5.1**	**ALHLTQFVIK**

5.410 182 832 minus strand qm-like protein [Crassostrea ariakensis]

Protein Accession	CL13773.Contig2$_{H}1$
Mean Expression Ratio	1.14
Median Expression Ratio	1.14
Credible Interval	(0.62, 2.11)
Associated Peptides	6
Associated Spectra	19
Coverage	NaN

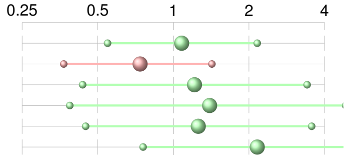

A	B	2.5	50	97.5	Sequence
**2**	**4**	**0.55**	**1.1**	**2.2**	**VHIGQPIMSVR**
**3**	**3**	**0.37**	**0.74**	**1.4**	**IFDLGR**
**0**	**2**	**0.44**	**1.2**	**3.4**	**EQHEAAIIEALR**
**0**	**1**	**0.39**	**1.4**	**4.9**	**EQHEAAIIEALRR**
**0**	**2**	**0.45**	**1.3**	**3.5**	**AREQHEAAIIEALR**
**0**	**2**	**0.76**	**2.2**	**5.9**	**GAFGKPQGTVAR**

5.411 7825 8754 minus strand H2 [Haliotis tuberculata]

Protein Accession	CL7906.Contig3$_H$1
Mean Expression Ratio	0.874
Median Expression Ratio	0.875
Credible Interval	(0.396, 1.99)
Associated Peptides	1
Associated Spectra	2
Coverage	NaN

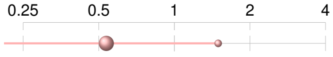

A	B	2.5	50	97.5	Sequence
0	2	0.19	0.54	1.5	ALPSLFTDPYDSNPFK

5.412 835 1116 tropomyosin [Ilyanassa obsoleta]

Protein Accession	CL2925.Contig8$_H$1
Mean Expression Ratio	1.15
Median Expression Ratio	1.14
Credible Interval	(0.671, 1.98)
Associated Peptides	10
Associated Spectra	23
Coverage	NaN

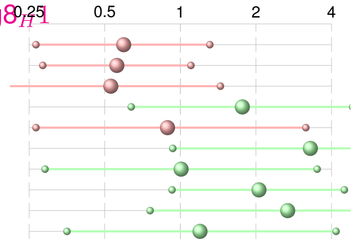

A	B	2.5	50	97.5	Sequence
1	3	0.27	0.6	1.3	LEDVETELR
2	4	0.28	0.56	1.1	QLELYEEK
1	1	0.19	0.53	1.4	ELEEQSHALTNSLK
0	2	0.64	1.8	4.9	KLAVMEAELEKAEDR
0	1	0.27	0.89	3.2	LAVMEAELEK
0	1	0.93	3.3	12	LYAGDERLEDVETELR
0	1	0.29	1	3.5	VRELEEQSHALTNSLK
0	4	0.93	2.1	4.5	KLAVMEAELEKAEDRADSSERR
0	1	0.76	2.7	9.4	QAAQEWEARYEEAAR
0	1	0.35	1.2	4.2	KLAVMEAELEKAEDRADSSER

5.413 333 770 USP-like protein isoform 2 [Crassostrea ariakensis]

Protein Accession	Unigene7710$_H$1
Mean Expression Ratio	1.14
Median Expression Ratio	1.14
Credible Interval	(0.584, 2.21)
Associated Peptides	4
Associated Spectra	12
Coverage	NaN

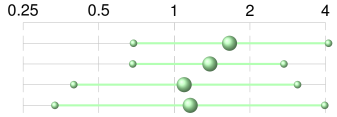

A	B	2.5	50	97.5	Sequence
1	2	0.69	1.7	4.1	KIVIAMDGSK
2	4	0.68	1.4	2.7	MSANTPGEAVIK
0	2	0.4	1.1	3.1	DIGADLIITGTR
0	1	0.34	1.2	4	LQNLLTENGMNGNVVR

5.414 33 422 minus strand PREDICTED: heat shock 70 kDa protein cognate 4-like [Megachile rotundata]

Protein Accession	CL15750.Contig1$_H$1
Mean Expression Ratio	0.877
Median Expression Ratio	0.877
Credible Interval	(0.385, 2)
Associated Peptides	1
Associated Spectra	1
Coverage	NaN

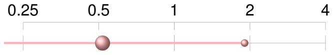

A	B	2.5	50	97.5	Sequence
1	0	0.15	0.52	1.9	VVNEGDKPK

5.415 1027 1440 minus strand hemocyanin isoform 1 [Haliotis diversicolor]

Protein Accession	CL40281.Contig1$_{H}$1
Mean Expression Ratio	0.88
Median Expression Ratio	0.877
Credible Interval	(0.395, 1.97)
Associated Peptides	1
Associated Spectra	8
Coverage	NaN

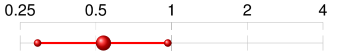

A	B	2.5	50	97.5	Sequence
**0**	**8**	**0.29**	**0.53**	**0.97**	**LFVVQVEDELK**

5.416 96 503 minus strand hypothetical protein DAPPUDRAFT_230378 [Daphnia pulex]

Protein Accession	CL14922.Contig5$_{H}$1
Mean Expression Ratio	1.14
Median Expression Ratio	1.14
Credible Interval	(0.547, 2.39)
Associated Peptides	3
Associated Spectra	3
Coverage	NaN

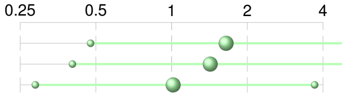

A	B	2.5	50	97.5	Sequence
**0**	**1**	**0.48**	**1.7**	**5.8**	**HLQLAIRNDEELNK**
**0**	**1**	**0.4**	**1.4**	**5.1**	**KTHTKPTTGAGSAAAGKA**
**0**	**1**	**0.29**	**1**	**3.7**	**QLAIRNDEELNK**

5.417 " 112 1320 Aspartate aminotransferase, cytoplasmic [Crassostrea gigas]"

Protein Accession	CL44136.Contig1$_{H}$1
Mean Expression Ratio	0.877
Median Expression Ratio	0.878
Credible Interval	(0.502, 1.56)
Associated Peptides	10
Associated Spectra	14
Coverage	NaN

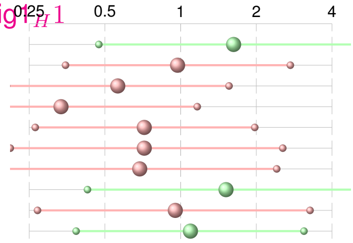

A	B	2.5	50	97.5	Sequence
**1**	**0**	**0.47**	**1.6**	**5.6**	**SQMEIIVR**
**1**	**1**	**0.35**	**0.97**	**2.7**	**ASIFADVK**
**1**	**1**	**0.2**	**0.56**	**1.6**	**IADVMEAK**
**1**	**0**	**0.096**	**0.33**	**1.2**	**LAAEFLK**
**0**	**2**	**0.26**	**0.72**	**2**	**IGNLCIVTK**
**0**	**1**	**0.21**	**0.72**	**2.5**	**TLANALILGK**
**0**	**1**	**0.2**	**0.69**	**2.4**	**VNLGVGAYR**
**0**	**1**	**0.43**	**1.5**	**5.2**	**LAAEFLKR**
**0**	**1**	**0.27**	**0.95**	**3.3**	**ISMAGVTNK**
**0**	**2**	**0.38**	**1.1**	**3.1**	**VEGIQVQGGTGGIR**

5.418 245 1243 Prostaglandin reductase 1 [Crassostrea gigas]

Protein Accession	CL37890.Contig1$_{H}$1
Mean Expression Ratio	0.883
Median Expression Ratio	0.878
Credible Interval	(0.388, 2.03)
Associated Peptides	1
Associated Spectra	1
Coverage	NaN

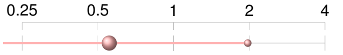

A	B	2.5	50	97.5	Sequence
**0**	**1**	**0.15**	**0.56**	**2**	**TLAVLHPDMPR**

5.419 1 456 minus strand Putative sodium/potassium-transporting ATPase subunit beta-2 [Crassostrea gigas]; 688 1251 minus strand Putative sodium/potassium-transporting ATPase subunit beta-2 [Crassostrea gigas]

Protein Accession	CL42979.Contig3$_{H}1$ CL42979.Contig2$_{H}1$
Mean Expression Ratio	1.14
Median Expression Ratio	1.14
Credible Interval	(0.5, 2.58)
Associated Peptides	1
Associated Spectra	1
Coverage	NaN

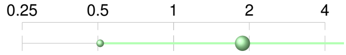

A	B	2.5	50	97.5	Sequence
0	1	0.51	1.9	6.7	NYMSPLVFVK

5.420 72 839 minus strand 60S ribosomal protein L8 OS=Spodoptera frugiperda GN=RpL8 PE=2 SV=1

Protein Accession	Unigene15547$_{H}1$
Mean Expression Ratio	1.14
Median Expression Ratio	1.14
Credible Interval	(0.597, 2.22)
Associated Peptides	5
Associated Spectra	8
Coverage	NaN

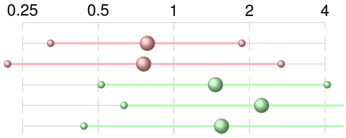

A	B	2.5	50	97.5	Sequence
1	2	0.32	0.78	1.9	AMVGIVAGGGR
1	0	0.22	0.76	2.7	IDKPILK
0	2	0.51	1.5	4.1	GVIKDIVHDPGR
0	1	0.63	2.2	7.9	KGAGSVFK
0	1	0.44	1.5	5.3	VAFRDPYR

5.421 82 816 PREDICTED: triosephosphate isomerase B [Oreochromis niloticus]

Protein Accession	CL26247.Contig4$_{H}1$
Mean Expression Ratio	1.14
Median Expression Ratio	1.14
Credible Interval	(0.594, 2.19)
Associated Peptides	6
Associated Spectra	6
Coverage	NaN

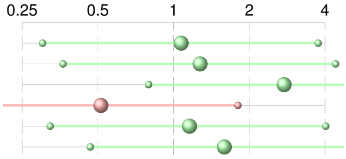

A	B	2.5	50	97.5	Sequence
0	1	0.3	1.1	3.8	TEFITIINSK
0	1	0.36	1.3	4.4	QFFVGGNWK
0	1	0.8	2.8	9.8	NVSKDVANTVR
0	1	0.15	0.51	1.8	SVDEIIK
0	1	0.32	1.2	4	AIADNVNDWK
0	1	0.47	1.6	5.5	AIADNVNDWKR

5.422 212 1783 PREDICTED: d-3-phosphoglycerate dehydrogenase-like [Strongylocentrotus purpuratus]

Protein Accession	CL34337.Contig3$_{H}1$
Mean Expression Ratio	1.14
Median Expression Ratio	1.14
Credible Interval	(0.54, 2.4)
Associated Peptides	3
Associated Spectra	3
Coverage	NaN

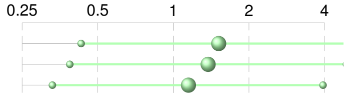

A	B	2.5	50	97.5	Sequence
0	1	0.43	1.5	5.3	GGIIDEDALLR
0	1	0.39	1.4	4.9	VTSTPHLGANTEEAQSR
0	1	0.33	1.2	3.9	LSASLFAGYLR

5.423 " 109 1632 minus strand Seryl-tRNA synthetase, cytoplasmic [Crassostrea gigas]; 109 1614 minus strand Seryl-tRNA synthetase, cytoplasmic [Crassostrea gigas]"

Protein Accession	CL11056.Contig9$_{H}1$ CL11056.Contig1$_{H}1$
Mean Expression Ratio	1.14
Median Expression Ratio	1.14
Credible Interval	(0.566, 2.31)
Associated Peptides	4
Associated Spectra	6
Coverage	NaN

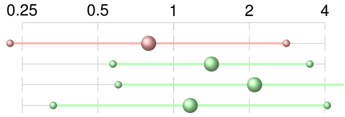

A	B	2.5	50	97.5	Sequence
1	0	0.22	0.8	2.8	ELTVTQIK
0	3	0.58	1.4	3.5	LVDTVVDQDAAWR
0	1	0.6	2.1	7.5	VLDIELFR
0	1	0.33	1.2	4.1	GGSPDKVR

5.424 14 502 minus strand Ufm1-conjugating enzyme 1 [Crassostrea gigas]

Protein Accession	CL15276.Contig2$_{H}1$
Mean Expression Ratio	1.14
Median Expression Ratio	1.14
Credible Interval	(0.497, 2.62)
Associated Peptides	1
Associated Spectra	1
Coverage	NaN

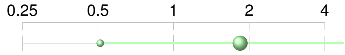

A	B	2.5	50	97.5	Sequence
0	1	0.51	1.8	6.7	TLAQIPLLK

5.425 2764 3420 PREDICTED: filamin-C-like [Nasonia vitripennis]

Protein Accession	CL4145.Contig1$_{H}1$
Mean Expression Ratio	0.884
Median Expression Ratio	0.88
Credible Interval	(0.394, 2)
Associated Peptides	1
Associated Spectra	2
Coverage	NaN

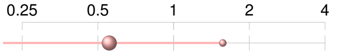

A	B	2.5	50	97.5	Sequence
1	1	0.2	0.56	1.6	GVDVPVNTIDNK

5.426 18 1406 AGAP006996-PA [Anopheles gambiae str. PEST] gi

Protein Accession	CL48173.Contig5$_{H}1$
Mean Expression Ratio	1.13
Median Expression Ratio	1.14
Credible Interval	(0.52, 2.49)
Associated Peptides	2
Associated Spectra	2
Coverage	NaN

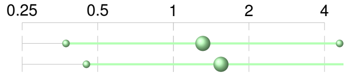

A	B	2.5	50	97.5	Sequence
0	1	0.37	1.3	4.6	VVLNLR
0	1	0.45	1.5	5.6	TTLTAAITK

5.427 16 1560 V-type proton ATPase subunit B [Crassostrea gigas]

Protein Accession	CL1031.Contig1$_H$1
Mean Expression Ratio	0.883
Median Expression Ratio	0.881
Credible Interval	(0.466, 1.64)
Associated Peptides	7
Associated Spectra	8
Coverage	NaN

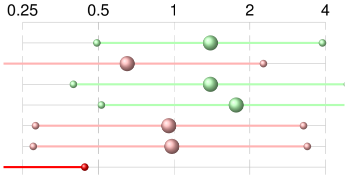

A	B	2.5	50	97.5	Sequence
0	2	0.49	1.4	3.9	AVVQVFEGTSGIDAK
0	1	0.19	0.65	2.3	FAEIVNLTLADGSK
0	1	0.4	1.4	4.9	HTTCEFTGDILR
0	1	0.51	1.8	6.1	MPTSEDMLGR
0	1	0.28	0.95	3.3	NFISQGHYENR
0	1	0.27	0.98	3.4	SLNGPLNDAAAR
0	1	0.037	0.13	0.44	ATIYERAGR

5.428 174 1649 minus strand Collagen alpha-1(XII) chain [Crassostrea gigas]; 123 1490 minus strand Collagen alpha-1(XII) chain [Crassostrea gigas]; 135 1610 Collagen alpha-1(XII) chain [Crassostrea gigas]

Protein Accession	CL5340.Contig13$_H$1 CL5340.Contig12$_H$1 CL5340.Contig11$_H$1
Mean Expression Ratio	1.13
Median Expression Ratio	1.13
Credible Interval	(0.513, 2.47)
Associated Peptides	2
Associated Spectra	2
Coverage	NaN

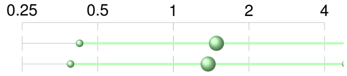

A	B	2.5	50	97.5	Sequence
0	1	0.42	1.5	5.6	ADVVFIVDSSR
0	1	0.39	1.4	4.8	SIQHMQGGLTETYEALR

5.429 " 61 573 steroid membrane receptor Hpr6.6/25-Dx, putative [Ixodes scapularis] gi"

Protein Accession	CL29406.Contig1$_H$1
Mean Expression Ratio	0.883
Median Expression Ratio	0.883
Credible Interval	(0.381, 2.01)
Associated Peptides	1
Associated Spectra	1
Coverage	NaN

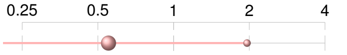

A	B	2.5	50	97.5	Sequence
1	0	0.15	0.55	2	ILIAVNGK

5.430 176 526 minus strand Translocon-associated protein subunit delta [Crassostrea gigas]

Protein Accession	Unigene63010$_H$1
Mean Expression Ratio	1.13
Median Expression Ratio	1.13
Credible Interval	(0.519, 2.47)
Associated Peptides	2
Associated Spectra	3
Coverage	NaN

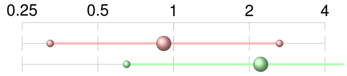

A	B	2.5	50	97.5	Sequence
1	1	0.32	0.91	2.6	KLPSGNYQVR
0	1	0.65	2.2	8	VFDEEGYALLR

5.431 23 409 minus strand CDGSH iron sulfur domain-containing protein 2 [Crassostrea gigas]; 1 207 minus strand CDGSH iron sulfur domain-containing protein 2 [Crassostrea gigas]; 22 135 minus strand CDGSH iron-sulfur domain-containing protein 2 OS=Lithobates catesbeiana GN=cisd2 PE=2 SV=1; 22 207 minus strand CDGSH iron-sulfur domain-containing protein 2 homolog B OS=Branchiostoma floridae GN=BRAFLDRAFT_274541 PE=3 SV=1

Protein Accession	CL36832.Contig6$_H1$ CL36832.Contig3$_H1$ CL36832.Contig2$_H1$ CL36832.Contig1$_H1$
Mean Expression Ratio	0.883
Median Expression Ratio	0.885
Credible Interval	(0.378, 2.04)
Associated Peptides	1
Associated Spectra	1
Coverage	NaN

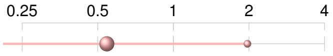

A	B	2.5	50	97.5	Sequence
0	1	0.15	0.54	2	VVNMVDIEDLGDK

5.432 26 550 Translocon-associated protein subunit gamma [Crassostrea gigas]

Protein Accession	CL32873.Contig1$_H1$
Mean Expression Ratio	1.13
Median Expression Ratio	1.13
Credible Interval	(0.514, 2.46)
Associated Peptides	2
Associated Spectra	2
Coverage	NaN

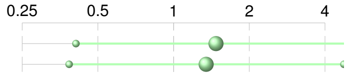

A	B	2.5	50	97.5	Sequence
0	1	0.41	1.5	5.1	QLSREEELLLQDFSR
0	1	0.38	1.3	4.7	KLADDKK

5.433 241 561 minus strand thioredoxin [Crassostrea ariakensis]

Protein Accession	CL33159.Contig3$_H1$
Mean Expression Ratio	0.879
Median Expression Ratio	0.886
Credible Interval	(0.431, 1.78)
Associated Peptides	3
Associated Spectra	6
Coverage	NaN

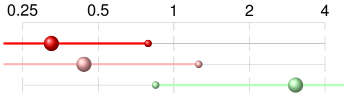

A	B	2.5	50	97.5	Sequence
1	2	0.13	0.33	0.79	IETQEDFEK
1	1	0.15	0.44	1.3	EVGEVVGASEEEIKK
0	1	0.85	3.1	11	VKIETQEDFEK

5.434 121 1452 minus strand Hexokinase type 2 [Crassostrea gigas]; 245 1576 minus strand Hexokinase type 2 [Crassostrea gigas]; 151 1455 minus strand Hexokinase type 2 [Crassostrea gigas]; 121 1452 minus strand Hexokinase type 2 [Crassostrea gigas]; 121 1452 minus strand Hexokinase type 2 [Crassostrea gigas]; 161 1513 minus strand Hexokinase type 2 [Crassostrea gigas]; 113 1471 minus strand Hexokinase type 2 [Crassostrea gigas]; 225 1577 minus strand Hexokinase type 2 [Crassostrea gigas]; 121 1452 minus strand Hexokinase type 2 [Crassostrea gigas]

Protein Accession	CL350.Contig9$_{H}1$ CL350.Contig8$_{H}1$ CL350.Contig7$_{H}1$ CL350.Contig6$_{H}1$ CL350.Contig5$_{H}1$
Mean Expression Ratio	1.13
Median Expression Ratio	1.13
Credible Interval	(0.5, 2.58)
Associated Peptides	1
Associated Spectra	1
Coverage	NaN

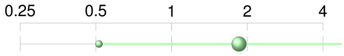

A	B	2.5	50	97.5	Sequence
**1**	**0**	**0.51**	**1.9**	**6.6**	**MYPTYVR**

5.435 " 451 1026 PREDICTED: GTP-binding protein SAR1B, putative-like [Saccoglossus kowalevskii]"

Protein Accession	Unigene4124$_{H}1$
Mean Expression Ratio	1.13
Median Expression Ratio	1.13
Credible Interval	(0.502, 2.62)
Associated Peptides	1
Associated Spectra	1
Coverage	NaN

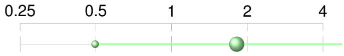

A	B	2.5	50	97.5	Sequence
**0**	**1**	**0.5**	**1.8**	**6.4**	**FTTFDLGGHR**

5.436 203 1942 ezrin/radixin/moesin [Aplysia californica] gi; 243 1982 minus strand ezrin/radixin/moesin [Aplysia californica] gi

Protein Accession	CL13506.Contig5$_H$1 CL13506.Contig4$_H$1
Mean Expression Ratio	1.13
Median Expression Ratio	1.13
Credible Interval	(0.756, 1.68)
Associated Peptides	25
Associated Spectra	46
Coverage	NaN

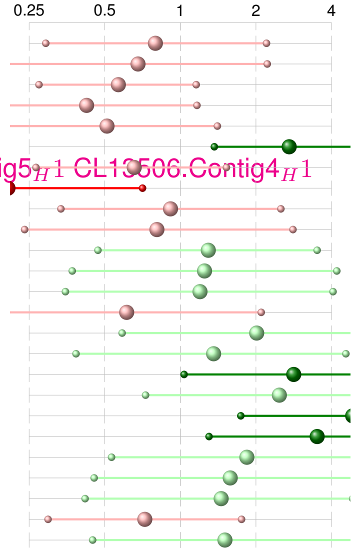

A	B	2.5	50	97.5	Sequence
1	1	0.29	0.8	2.2	KESPLQFK
1	0	0.2	0.68	2.2	KVLSQDVR
3	2	0.27	0.57	1.2	NISFNDKK
2	0	0.15	0.42	1.2	QLFDQVVK
1	1	0.19	0.51	1.4	FVIKPIDK
2	4	1.4	2.7	5.3	RKPDTIEVQQMK
1	2	0.27	0.65	1.5	ESPLQFK
1	0	0.06	0.21	0.71	APDFVFYAPR
2	0	0.34	0.91	2.5	LNEIVYK
1	0	0.24	0.81	2.8	LLPQR
1	1	0.47	1.3	3.5	DMADLQAR
1	0	0.37	1.2	4.2	LLALR
1	0	0.35	1.2	4.1	VLSQDVR
0	1	0.17	0.61	2.1	DDAMMEYLK
0	1	0.59	2	6.9	KAPDFVFYAPR
0	1	0.39	1.4	4.6	LADELETAQR
0	2	1	2.8	7.8	LGENGLTKNDELHAENVR
0	1	0.73	2.5	8.7	MAELLALREELDAER
0	2	1.7	4.9	13	QKLADELETAQR
0	2	1.3	3.5	9.7	VEEERLTEAEKNQR
0	1	0.53	1.8	6.5	FVIKPIDKK
0	1	0.45	1.6	5.4	QLEMLK
0	1	0.42	1.5	4.9	AEELRLEMER
0	3	0.3	0.72	1.8	SWWGEHK
0	1	0.45	1.5	5.2	MAELLALREELDAERR

5.437 " 132 1544 minus strand Serine hydroxymethyltransferase, cytosolic [Crassostrea gigas]"

Protein Accession	CL22573.Contig2$_H$1
Mean Expression Ratio	1.12
Median Expression Ratio	1.13
Credible Interval	(0.514, 2.46)
Associated Peptides	2
Associated Spectra	2
Coverage	NaN

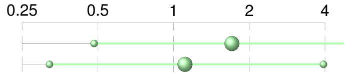

A	B	2.5	50	97.5	Sequence
0	1	0.48	1.7	6	IGTPALTSR
0	1	0.32	1.1	3.9	SGIIFYR

5.438 231 902 Sodium- and chloride-dependent GABA transporter ine [Crassostrea gigas]

Protein Accession	CL11289.Contig3$_H$1
Mean Expression Ratio	0.886
Median Expression Ratio	0.888
Credible Interval	(0.386, 2.07)
Associated Peptides	1
Associated Spectra	1
Coverage	NaN

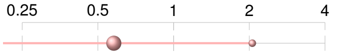

A	B	2.5	50	97.5	Sequence
0	1	0.16	0.58	2.1	EAFELSFK

5.439 155 3214 sarco/endoplasmic reticulum calcium ATPase isoform B [Pinctada fucata]; 132 3110 sarco/endoplasmic reticulum calcium ATPase isoform C [Pinctada fucata]; 132 3110 minus strand sarco/endoplasmic reticulum calcium ATPase isoform C [Pinctada fucata]; 155 3133 sarco/endoplasmic reticulum calcium ATPase isoform C [Pinctada fucata]; 155 3133 sarco/endoplasmic reticulum calcium ATPase isoform C [Pinctada fucata]; 155 3214 minus strand sarco/endoplasmic reticulum calcium ATPase isoform B [Pinctada fucata]

Protein Accession	CL1323.Contig9$_{H}1$ CL1323.Contig8$_{H}1$ CL1323.Contig6$_{H}1$ CL1323.Contig21$_{H}1$ CL1323.Co
Mean Expression Ratio	0.892
Median Expression Ratio	0.889
Credible Interval	(0.399, 2.02)
Associated Peptides	1
Associated Spectra	2
Coverage	NaN

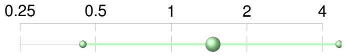

A	B	2.5	50	97.5	Sequence
1	1	0.45	1.5	4.6	KAEIGIAMGSGTAVAK

5.440 729 1541 troponin T [Mizuhopecten yessoensis]

Protein Accession	Unigene34289$_{H}1$
Mean Expression Ratio	1.12
Median Expression Ratio	1.13
Credible Interval	(0.488, 2.56)
Associated Peptides	1
Associated Spectra	1
Coverage	NaN

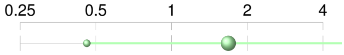

A	B	2.5	50	97.5	Sequence
0	1	0.46	1.7	6.1	QQYDMIELAER

5.441 3 1670 Spectrin alpha chain [Crassostrea gigas]

Protein Accession	CL2195.Contig7$_{H}1$
Mean Expression Ratio	1.12
Median Expression Ratio	1.13
Credible Interval	(0.605, 2.11)
Associated Peptides	7
Associated Spectra	11
Coverage	NaN

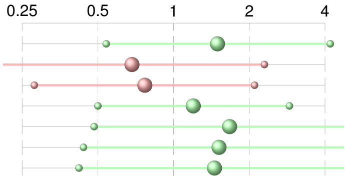

A	B	2.5	50	97.5	Sequence
1	1	0.54	1.5	4.2	KLDPSLSASR
1	0	0.19	0.68	2.3	DLAALGEK
0	2	0.28	0.77	2.1	EAAELAAWITEK
0	3	0.5	1.2	2.9	HQALQAEIAGHEPR
0	1	0.48	1.7	5.8	KHQALQAEIAGHEPR
0	1	0.44	1.5	5.3	QGFVPAAYVK
0	1	0.42	1.5	5	VAALYDYTEK

5.442 " 926 1189 minus strand Myosin heavy chain, striated muscle [Crassostrea gigas]"

Protein Accession	Unigene11948$_{H}1$
Mean Expression Ratio	1.12
Median Expression Ratio	1.12
Credible Interval	(0.556, 2.22)
Associated Peptides	4
Associated Spectra	9
Coverage	NaN

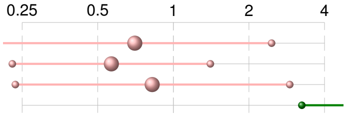

A	B	2.5	50	97.5	Sequence
1	0	0.2	0.7	2.5	TFLDK
2	1	0.23	0.57	1.4	LNGNHLGK
0	1	0.23	0.82	2.9	TFLDKLNGNHLGK
0	4	3.3	7.3	16	ASDKTFLDKLNGNHLGK

5.443 " 196 1053 minus strand Cytochrome b-c1 complex subunit 2, mitochondrial OS=Homo sapiens GN=UQCRC2 PE=1 SV=3"

Protein Accession	CL7847.Contig5$_{H}1$
Mean Expression Ratio	1.12
Median Expression Ratio	1.12
Credible Interval	(0.49, 2.58)
Associated Peptides	1
Associated Spectra	1
Coverage	NaN

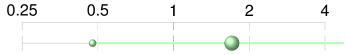

A	B	2.5	50	97.5	Sequence
0	1	0.48	1.7	6.1	VGKYNAEQLLHYMK

5.444 1 231 small subunit ribosomal protein S11e [Clonorchis sinensis]; 1 171 40S ribosomal protein S11 OS=Canis familiaris GN=RPS11 PE=1 SV=2

Protein Accession	CL39695.Contig2$_{H}1$ CL39695.Contig1$_{H}1$
Mean Expression Ratio	1.12
Median Expression Ratio	1.12
Credible Interval	(0.495, 2.57)
Associated Peptides	1
Associated Spectra	2
Coverage	NaN

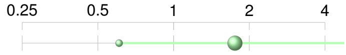

A	B	2.5	50	97.5	Sequence
0	2	0.61	1.8	5.1	TIVVRRDYLK

5.445 424 1347 minus strand Tubulin alpha chain [Ascaris suum]

Protein Accession	Unigene7901$_{H}1$
Mean Expression Ratio	1.13
Median Expression Ratio	1.12
Credible Interval	(0.556, 2.27)
Associated Peptides	3
Associated Spectra	10
Coverage	NaN

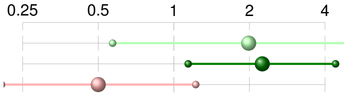

A	B	2.5	50	97.5	Sequence
0	1	0.57	2	6.9	VDLEPTVVDEVR
0	6	1.1	2.2	4.4	NTFFNETGSGKHVPRAIFVDLEPTVVDEVR
0	3	0.21	0.5	1.2	QLFHPEQLITGK

5.446 138 713 Rac [Aplysia californica] gi; 125 691 Rac [Aplysia californica] gi; 149 871 minus strand Rac [Aplysia californica] gi; 101 676 Rac [Aplysia californica] gi

Protein Accession	CL11009.Contig9$_{H}1$ CL11009.Contig8$_{H}1$ CL11009.Contig4$_{H}1$ CL11009.Contig1$_{H}1$
Mean Expression Ratio	0.884
Median Expression Ratio	0.89
Credible Interval	(0.391, 1.99)
Associated Peptides	1
Associated Spectra	2
Coverage	NaN

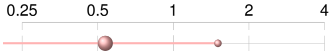

A	B	2.5	50	97.5	Sequence
1	1	0.19	0.53	1.5	NVFDEAIR

5.447 366 1220 Ectoine hydroxylase [Crassostrea gigas]

Protein Accession	CL2342.Contig2$_{H}1$
Mean Expression Ratio	1.13
Median Expression Ratio	1.12
Credible Interval	(0.559, 2.27)
Associated Peptides	4
Associated Spectra	5
Coverage	NaN

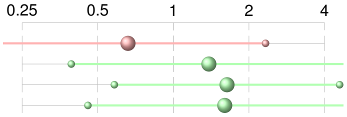

A	B	2.5	50	97.5	Sequence
1	0	0.18	0.66	2.3	IDHDLVAGQQR
0	1	0.39	1.4	5	AFDDDGYILIK
0	2	0.58	1.6	4.6	GFLDQEELDNLRK
0	1	0.46	1.6	5.8	TVEDSDLFR

5.448 26 619 G-type lysozyme [Physella acuta]

Protein Accession	CL43984.Contig1$_{H}1$
Mean Expression Ratio	1.13
Median Expression Ratio	1.12
Credible Interval	(0.496, 2.58)
Associated Peptides	1
Associated Spectra	2
Coverage	NaN

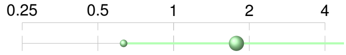

A	B	2.5	50	97.5	Sequence
0	2	0.63	1.8	5	GHFAGWPIER

5.449 34 480 small subunit ribosomal protein S18e [Clonorchis sinensis]; 431 799 SJCHGC01887 protein [Schistosoma japonicum] gi

Protein Accession	CL10131.Contig2$_{H}1$ CL10131.Contig1$_{H}1$
Mean Expression Ratio	0.885
Median Expression Ratio	0.89
Credible Interval	(0.402, 1.95)
Associated Peptides	1
Associated Spectra	5
Coverage	NaN

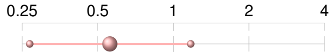

A	B	2.5	50	97.5	Sequence
2	3	0.27	0.56	1.2	LVTVMSNPR

5.450 494 724 minus strand PREDICTED: SH3 domain-binding glutamic acid-rich-like protein 3-like [Amphimedon queenslandica]; 424 819 minus strand PREDICTED: SH3 domain-binding glutamic acid-rich-like protein 3-like [Macaca mulatta]; 236 631 minus strand PREDICTED: SH3 domain-binding glutamic acid-rich-like protein 3-like [Macaca mulatta]

Protein Accession	CL49318.Contig4$_{H1}$ CL49318.Contig3$_{H1}$ CL49318.Contig2$_{H1}$
Mean Expression Ratio	1.13
Median Expression Ratio	1.12
Credible Interval	(0.495, 2.6)
Associated Peptides	1
Associated Spectra	1
Coverage	NaN

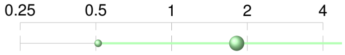

A	B	2.5	50	97.5	Sequence
**0**	**1**	**0.51**	**1.8**	**6.5**	**GDEYLGDYER**

5.451 412 708 minus strand heat shock protein beta-1 [Trichinella spiralis] gi; 459 755 minus strand heat shock protein beta-1 [Trichinella spiralis] gi; 464 763 minus strand heat shock protein beta-1 [Trichinella spiralis] gi; 437 736 minus strand heat shock protein beta-1 [Trichinella spiralis] gi; 437 736 minus strand heat shock protein beta-1 [Trichinella spiralis] gi; 437 736 minus strand heat shock protein beta-1 [Trichinella spiralis] gi

Protein Accession	CL17234.Contig6$_{H1}$ CL17234.Contig5$_{H1}$ CL17234.Contig4$_{H1}$ CL17234.Contig3$_{H1}$ CL1723
Mean Expression Ratio	0.892
Median Expression Ratio	0.89
Credible Interval	(0.392, 2.05)
Associated Peptides	1
Associated Spectra	1
Coverage	NaN

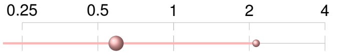

A	B	2.5	50	97.5	Sequence
**1**	**0**	**0.16**	**0.59**	**2.1**	**TVTSSLNADGVLTLK**

5.452 " 37 1356 homogentisate 1,2-dioxygenase [Danio rerio] gi; 54 1361 homogentisate 1,2-dioxygenase [Danio rerio] gi"

Protein Accession	CL16355.Contig5$_{H1}$ CL16355.Contig1$_{H1}$
Mean Expression Ratio	1.12
Median Expression Ratio	1.12
Credible Interval	(0.485, 2.59)
Associated Peptides	1
Associated Spectra	1
Coverage	NaN

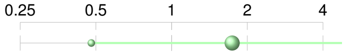

A	B	2.5	50	97.5	Sequence
**0**	**1**	**0.48**	**1.7**	**6.1**	**SWLYR**

5.453 " 28 1104 minus strand Pyruvate dehydrogenase E1 component subunit beta, mitochondrial [Crassostrea gigas]; 1087 1581 Pyruvate dehydrogenase E1 component subunit beta, mitochondrial [Crassostrea gigas]"

Protein Accession	CL18015.Contig14$_H$1 CL18015.Contig13$_H$1
Mean Expression Ratio	1.12
Median Expression Ratio	1.12
Credible Interval	(0.527, 2.44)
Associated Peptides	2
Associated Spectra	4
Coverage	NaN

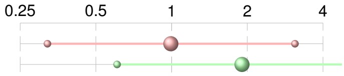

A	B	2.5	50	97.5	Sequence
**1**	**1**	**0.32**	**1**	**3.1**	**SLEEAALPQPVNVIR**
**1**	**1**	**0.61**	**1.9**	**6**	**VTGADVPMPYAK**

5.454 297 734 minus strand PREDICTED: calmodulin [Canis lupus familiaris]; 297 734 PREDICTED: calmodulin [Canis lupus familiaris]; 143 508 minus strand Calmodulin [Crassostrea gigas]

Protein Accession	Unigene26719$_H$1 Unigene17836$_H$1 Unigene10331$_H$1
Mean Expression Ratio	1.13
Median Expression Ratio	1.12
Credible Interval	(0.489, 2.61)
Associated Peptides	1
Associated Spectra	1
Coverage	NaN

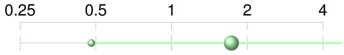

A	B	2.5	50	97.5	Sequence
**0**	**1**	**0.48**	**1.7**	**6.3**	**TLITEFENR**

5.455 79 606 minus strand PREDICTED: similar to Ras-like protein 3 precursor (Protein roughened) [Tribolium castaneum] gi

Protein Accession	Unigene4246$_H$1
Mean Expression Ratio	1.12
Median Expression Ratio	1.12
Credible Interval	(0.495, 2.49)
Associated Peptides	1
Associated Spectra	3
Coverage	NaN

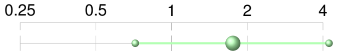

A	B	2.5	50	97.5	Sequence
**0**	**3**	**0.72**	**1.8**	**4.2**	**LVVLGSGGVGK**

5.456 188 1021 hypothetical protein BRAFLDRAFT_214346 [Branchiostoma floridae] gi

Protein Accession	CL38498.Contig2$_{H}1$
Mean Expression Ratio	1.12
Median Expression Ratio	1.12
Credible Interval	(0.516, 2.45)
Associated Peptides	2
Associated Spectra	2
Coverage	NaN

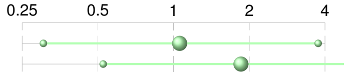

A	B	2.5	50	97.5	Sequence
**0**	**1**	**0.3**	**1.1**	**3.8**	**AANGGAYPPDLSYIVQAR**
**0**	**1**	**0.53**	**1.8**	**6.6**	**KIAFK**

5.457 59 328 minus strand PREDICTED: similar to predicted protein [Hydra magnipapillata]; 59 328 PREDICTED: similar to predicted protein [Hydra magnipapillata]; 89 364 minus strand PREDICTED: similar to predicted protein [Hydra magnipapillata]; 89 364 minus strand PREDICTED: similar to predicted protein [Hydra magnipapillata]

Protein Accession	Unigene53643$_{H}1$ Unigene39171$_{H}1$ Unigene11519$_{H}1$ Unigene11518$_{H}1$
Mean Expression Ratio	0.893
Median Expression Ratio	0.892
Credible Interval	(0.394, 2.05)
Associated Peptides	1
Associated Spectra	1
Coverage	NaN

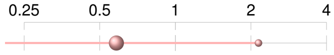

A	B	2.5	50	97.5	Sequence
**0**	**1**	**0.16**	**0.58**	**2.1**	**DLDPQNDLTFLR**

5.458 254 1093 uncharacterized protein LOC432187 precursor [Xenopus laevis] gi

Protein Accession	CL7800.Contig7$_{H}1$
Mean Expression Ratio	0.89
Median Expression Ratio	0.892
Credible Interval	(0.41, 1.95)
Associated Peptides	2
Associated Spectra	3
Coverage	NaN

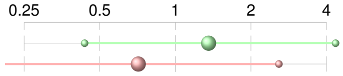

A	B	2.5	50	97.5	Sequence
**1**	**1**	**0.44**	**1.4**	**4.4**	**GINFASTTR**
**1**	**0**	**0.2**	**0.71**	**2.6**	**LVTSK**

5.459 274 666 Calmodulin-4 [Heterocephalus glaber]

Protein Accession	Unigene19050$_{H}1$
Mean Expression Ratio	0.89
Median Expression Ratio	0.892
Credible Interval	(0.435, 1.83)
Associated Peptides	3
Associated Spectra	9
Coverage	NaN

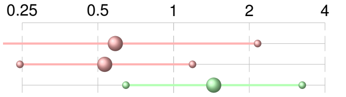

A	B	2.5	50	97.5	Sequence
1	0	0.17	0.59	2.2	SGFITLDELK
2	2	0.25	0.53	1.2	VLLSVGR
2	2	0.65	1.4	3.2	TLDELK

5.460 764 1258 minus strand Caprin-2 [Crassostrea gigas]

Protein Accession	CL23459.Contig2$_{H}1$
Mean Expression Ratio	0.892
Median Expression Ratio	0.893
Credible Interval	(0.392, 2.03)
Associated Peptides	1
Associated Spectra	1
Coverage	NaN

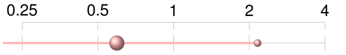

A	B	2.5	50	97.5	Sequence
0	1	0.16	0.6	2.2	FAVLDSEVR

5.461 " 448 1365 Nitrile-specifier protein 5, partial [Crassostrea gigas]"

Protein Accession	CL8618.Contig4$_{H}1$
Mean Expression Ratio	1.12
Median Expression Ratio	1.12
Credible Interval	(0.533, 2.36)
Associated Peptides	3
Associated Spectra	5
Coverage	NaN

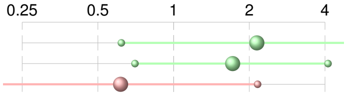

A	B	2.5	50	97.5	Sequence
0	1	0.62	2.1	7.6	KLPTFDAILPR
0	3	0.7	1.7	4.1	LLDTHGATVPPR
0	1	0.17	0.62	2.2	LYVFGGFTGK

5.462 99 437 minus strand V-type proton ATPase subunit G [Crassostrea gigas]

Protein Accession	CL20812.Contig1$_{H}1$
Mean Expression Ratio	1.12
Median Expression Ratio	1.12
Credible Interval	(0.515, 2.49)
Associated Peptides	2
Associated Spectra	3
Coverage	NaN

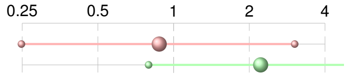

A	B	2.5	50	97.5	Sequence
0	1	0.25	0.88	3	ASQTQGIQQLLAAEK
0	2	0.79	2.2	6.1	GDLEAKIEASTR

5.463 284 1318 hsc70-interacting protein [Gallus gallus] gi; 88 1137 hsc70-interacting protein [Gallus gallus] gi

Protein Accession	CL11847.Contig4$_{H}1$ CL11847.Contig2$_{H}1$
Mean Expression Ratio	0.892
Median Expression Ratio	0.893
Credible Interval	(0.413, 1.89)
Associated Peptides	2
Associated Spectra	5
Coverage	NaN

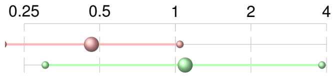

A	B	2.5	50	97.5	Sequence
**4**	**0**	**0.21**	**0.46**	**1**	**AQLDLLK**
**0**	**1**	**0.3**	**1.1**	**3.8**	**LNPTSALLYAK**

5.464 425 1051 minus strand unknown [Schistosoma japonicum]

Protein Accession	CL11292.Contig9$_{H}1$
Mean Expression Ratio	1.12
Median Expression Ratio	1.12
Credible Interval	(0.494, 2.5)
Associated Peptides	1
Associated Spectra	2
Coverage	NaN

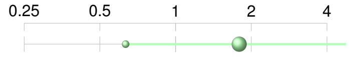

A	B	2.5	50	97.5	Sequence
**0**	**2**	**0.63**	**1.8**	**5**	**AGNDVDLVVQR**

5.465 484 1218 minus strand alginate lyase [Littorina brevicula]; 484 1218 minus strand alginate lyase [Littorina brevicula]

Protein Accession	CL8738.Contig15$_{H}1$ CL8738.Contig13$_{H}1$
Mean Expression Ratio	0.897
Median Expression Ratio	0.895
Credible Interval	(0.392, 2.05)
Associated Peptides	1
Associated Spectra	1
Coverage	NaN

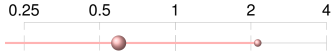

A	B	2.5	50	97.5	Sequence
**0**	**1**	**0.16**	**0.59**	**2.1**	**VFLDGHK**

5.466 " 460 1944 PREDICTED: succinyl-CoA:3-ketoacid-coenzyme A transferase 1, mitochondrial [Canis lupus familiaris]"

Protein Accession	Unigene4332$_{H}1$
Mean Expression Ratio	0.894
Median Expression Ratio	0.896
Credible Interval	(0.535, 1.49)
Associated Peptides	12
Associated Spectra	24
Coverage	NaN

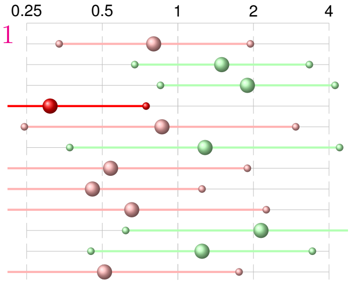

A	B	2.5	50	97.5	Sequence
1	2	0.34	0.8	1.9	EVAEGETAESIAK
1	3	0.67	1.5	3.3	ITGVEFR
2	2	0.85	1.9	4.2	NFNPPMAR
1	2	0.13	0.31	0.75	EVVNMIVTEK
1	0	0.24	0.87	3	VFLGK
0	1	0.37	1.3	4.4	ADKLGNLTFR
0	1	0.15	0.54	1.9	AVFDVDNK
0	2	0.17	0.46	1.3	LGNLTFR
0	1	0.19	0.66	2.2	VMGMGGAMDLVSADK
0	1	0.62	2.1	7.4	ILKEVTLPK
0	2	0.45	1.2	3.4	FNPDGTILHGSAGR
0	1	0.15	0.51	1.8	TGDVALVK

5.467 " 3 2693 PREDICTED: similar to coatomer protein complex, subunit beta 2 (beta prime) [Taeniopygia guttata]"

Protein Accession	CL19504.Contig2$_{H}1$
Mean Expression Ratio	1.11
Median Expression Ratio	1.12
Credible Interval	(0.532, 2.37)
Associated Peptides	3
Associated Spectra	3
Coverage	NaN

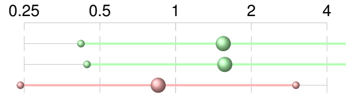

A	B	2.5	50	97.5	Sequence
0	1	0.42	1.6	5.4	RLPEAAFFAR
0	1	0.44	1.6	5.5	TYLPSQVSR
0	1	0.24	0.85	3	VVSLWK

5.468 57 2378 minus strand Heterogeneous nuclear ribonucleoprotein U-like protein 1 [Crassostrea gigas]

Protein Accession	CL15560.Contig5$_{H}1$
Mean Expression Ratio	1.12
Median Expression Ratio	1.12
Credible Interval	(0.523, 2.37)
Associated Peptides	3
Associated Spectra	3
Coverage	NaN

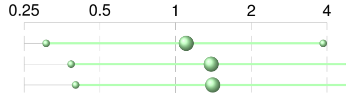

A	B	2.5	50	97.5	Sequence
0	1	0.31	1.1	3.9	VTELRDELK
0	1	0.38	1.4	4.9	LRDELK
0	1	0.4	1.4	5.2	RSRSPPPRKVE

5.469 5 748 hypothetical protein SINV_09458 [Solenopsis invicta]

Protein Accession	CL25819.Contig2$_{H}1$
Mean Expression Ratio	0.898
Median Expression Ratio	0.896
Credible Interval	(0.382, 2.09)
Associated Peptides	1
Associated Spectra	1
Coverage	NaN

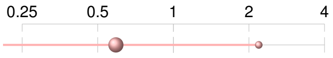

A	B	2.5	50	97.5	Sequence
**0**	**1**	**0.16**	**0.59**	**2.2**	**LVSPAASAR**

5.470 96 503 minus strand mitochondrial ATP synthase delta chain [Haliotis discus discus]; 111 551 minus strand mitochondrial ATP synthase delta chain [Haliotis discus discus]; 96 542 minus strand mitochondrial ATP synthase delta chain [Haliotis discus discus]; 202 642 minus strand mitochondrial ATP synthase delta chain [Haliotis discus discus]; 44 529 mitochondrial ATP synthase delta chain [Haliotis discus discus]

Protein Accession	Unigene42244$_{H}1$ CL2187.Contig3$_{H}1$ CL2187.Contig2$_{H}1$ CL2187.Contig1$_{H}1$ CL2187.Contig
Mean Expression Ratio	0.898
Median Expression Ratio	0.896
Credible Interval	(0.4, 2.04)
Associated Peptides	1
Associated Spectra	2
Coverage	NaN

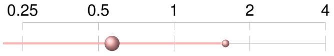

A	B	2.5	50	97.5	Sequence
**2**	**0**	**0.2**	**0.57**	**1.6**	**FDAQAIR**

5.471 346 702 minus strand PREDICTED: galectin-4-like [Anolis carolinensis]

Protein Accession	Unigene42203$_{H}1$
Mean Expression Ratio	0.897
Median Expression Ratio	0.897
Credible Interval	(0.441, 1.82)
Associated Peptides	2
Associated Spectra	8
Coverage	NaN

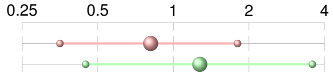

A	B	2.5	50	97.5	Sequence
**3**	**2**	**0.35**	**0.81**	**1.8**	**AEATLEVR**
**1**	**2**	**0.45**	**1.3**	**3.6**	**SVPHGLVK**

5.472 102 1685 minus strand predicted protein [Nematostella vectensis] gi

Protein Accession	CL44087.Contig1$_{H}$1
Mean Expression Ratio	0.896
Median Expression Ratio	0.897
Credible Interval	(0.438, 1.8)
Associated Peptides	4
Associated Spectra	4
Coverage	NaN

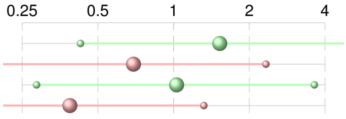

A	B	2.5	50	97.5	Sequence
0	1	0.43	1.5	5.3	IGGNMGLAMK
0	1	0.2	0.69	2.3	LNPQTAFFQGK
0	1	0.28	1	3.6	ADTTISMSDADLLSLMSGK
0	1	0.11	0.39	1.3	GVFLFK

5.473 " 283 1602 minus strand 4-aminobutyrate aminotransferase, mitochondrial [Crassostrea gigas]"

Protein Accession	CL30906.Contig1$_{H}$1
Mean Expression Ratio	1.11
Median Expression Ratio	1.11
Credible Interval	(0.54, 2.32)
Associated Peptides	3
Associated Spectra	4
Coverage	NaN

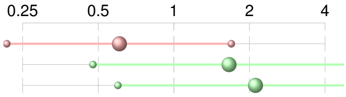

A	B	2.5	50	97.5	Sequence
1	1	0.22	0.61	1.7	AMFMTYQR
0	1	0.48	1.7	5.9	TMGALALTHTK
0	1	0.6	2.1	7.4	IIEAFKNPK

5.474 136 675 minus strand predicted protein [Nematostella vectensis] gi

Protein Accession	CL17411.Contig1$_{H}$1
Mean Expression Ratio	1.11
Median Expression Ratio	1.11
Credible Interval	(0.528, 2.34)
Associated Peptides	3
Associated Spectra	4
Coverage	NaN

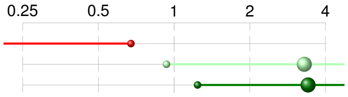

A	B	2.5	50	97.5	Sequence
1	0	0.05	0.18	0.67	MLNEDELR
0	1	0.93	3.3	11	MLNEDELRDAK
0	2	1.2	3.4	9.8	VSEARDELQR

5.475 418 690 PREDICTED: protein disulfide isomerase A4-like [Saccoglossus kowalevskii]

Protein Accession	CL20258.Contig2$_{H}$1
Mean Expression Ratio	0.892
Median Expression Ratio	0.898
Credible Interval	(0.391, 1.99)
Associated Peptides	1
Associated Spectra	2
Coverage	NaN

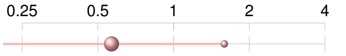

A	B	2.5	50	97.5	Sequence
0	2	0.2	0.57	1.6	TFEQIVLDK

5.476 58 732 glutathione-s-transferase [Haliotis discus discus]

Protein Accession	CL4861.Contig1$_{H}1$
Mean Expression Ratio	0.898
Median Expression Ratio	0.899
Credible Interval	(0.463, 1.74)
Associated Peptides	4
Associated Spectra	11
Coverage	NaN

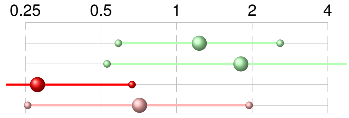

A	B	2.5	50	97.5	Sequence
3	2	0.59	1.2	2.6	GQVPTFR
1	0	0.53	1.8	6.4	LLPKEAGGR
0	3	0.11	0.28	0.67	IDKEYLEVK
0	2	0.25	0.71	1.9	LISFSANEHK

5.477 40 777 proteasome subunit N3 [Haliotis discus discus]; 40 777 proteasome subunit N3 [Haliotis discus discus]

Protein Accession	CL39106.Contig2$_{H}1$ CL39106.Contig1$_{H}1$
Mean Expression Ratio	1.11
Median Expression Ratio	1.11
Credible Interval	(0.479, 2.53)
Associated Peptides	1
Associated Spectra	1
Coverage	NaN

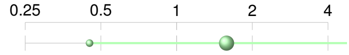

A	B	2.5	50	97.5	Sequence
0	1	0.45	1.6	5.8	YQIAIVTSK

5.478 211 687 Uncharacterized protein CG16817 [Harpegnathos saltator]; 200 676 Uncharacterized protein CG16817 [Harpegnathos saltator]; 294 770 Uncharacterized protein CG16817 [Harpegnathos saltator]; 211 687 Uncharacterized protein CG16817 [Harpegnathos saltator]

Protein Accession	Unigene6191$_{H}1$ Unigene53279$_{H}1$ Unigene41893$_{H}1$ Unigene11183$_{H}1$
Mean Expression Ratio	1.12
Median Expression Ratio	1.11
Credible Interval	(0.491, 2.53)
Associated Peptides	1
Associated Spectra	1
Coverage	NaN

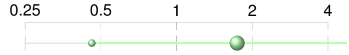

A	B	2.5	50	97.5	Sequence
0	1	0.46	1.7	6.2	KFHFLK

5.479 93 2606 twitchin [Mytilus galloprovincialis]

Protein Accession	Unigene71883$_{H}1$
Mean Expression Ratio	1.11
Median Expression Ratio	1.11
Credible Interval	(0.493, 2.53)
Associated Peptides	1
Associated Spectra	1
Coverage	NaN

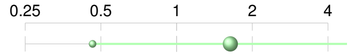

A	B	2.5	50	97.5	Sequence
0	1	0.46	1.6	6	LNPDEIVK

5.480 " 3475 4188 minus strand Heat shock 70 kDa protein cognate, putative [Pediculus humanus corporis] gi"

Protein Accession	CL5924.Contig14$_H$1
Mean Expression Ratio	0.895
Median Expression Ratio	0.899
Credible Interval	(0.42, 1.93)
Associated Peptides	2
Associated Spectra	5
Coverage	NaN

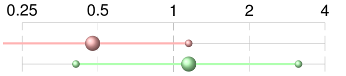

A	B	2.5	50	97.5	Sequence
**1**	**2**	**0.2**	**0.47**	**1.2**	**STIEDEKLK**
**0**	**2**	**0.41**	**1.1**	**3.1**	**NSLESYAFHMK**

5.481 444 719 minus strand 60S ribosomal protein L9 [Laodelphax striatella]

Protein Accession	CL1530.Contig3$_H$1
Mean Expression Ratio	1.12
Median Expression Ratio	1.11
Credible Interval	(0.557, 2.27)
Associated Peptides	3
Associated Spectra	9
Coverage	NaN

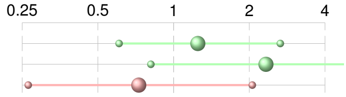

A	B	2.5	50	97.5	Sequence
**2**	**3**	**0.61**	**1.2**	**2.7**	**SAALIQQSTTVK**
**1**	**1**	**0.81**	**2.3**	**6.5**	**SAALIQQSTTVKK**
**1**	**1**	**0.26**	**0.73**	**2**	**FLDGIYVSEK**

5.482 187 642 minus strand PREDICTED: similar to pol-like protein [Tribolium castaneum]

Protein Accession	CL5644.Contig3$_H$1
Mean Expression Ratio	1.11
Median Expression Ratio	1.11
Credible Interval	(0.499, 2.47)
Associated Peptides	1
Associated Spectra	8
Coverage	NaN

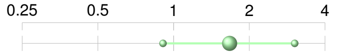

A	B	2.5	50	97.5	Sequence
**0**	**8**	**0.91**	**1.7**	**3**	**IILEQEELK**

5.483 " 1 2508 Alanyl-tRNA synthetase, cytoplasmic [Crassostrea gigas]; 1 2508 Alanyl-tRNA synthetase, cytoplasmic [Crassostrea gigas]; 1 2508 minus strand Alanyl-tRNA synthetase, cytoplasmic [Crassostrea gigas]"

Protein Accession	CL11594.Contig2$_H$1 CL11594.Contig1$_H$1 CL11594.Contig11$_H$1
Mean Expression Ratio	0.899
Median Expression Ratio	0.901
Credible Interval	(0.423, 1.88)
Associated Peptides	3
Associated Spectra	3
Coverage	NaN

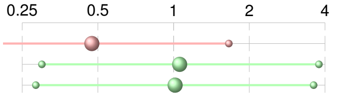

A	B	2.5	50	97.5	Sequence
**1**	**0**	**0.13**	**0.47**	**1.7**	**GLTVDNEGFEK**
**1**	**0**	**0.3**	**1.1**	**3.8**	**SIQGLR**
**1**	**0**	**0.28**	**1**	**3.6**	**VIALR**

5.484 " 2 778 Actin, gamma-enteric smooth muscle OS=Rattus norvegicus GN=Actg2 PE=2 SV=1"

Protein Accession: Unigene64184$_{H}$1
Mean Expression Ratio: 0.901
Median Expression Ratio: 0.902
Credible Interval: (0.391, 2.04)
Associated Peptides: 1
Associated Spectra: 1
Coverage: NaN

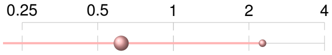

A	B	2.5	50	97.5	Sequence
**1**	**0**	**0.17**	**0.62**	**2.3**	**EITSLAPSTMK**

5.485 210 2201 minus strand Glycyl-tRNA synthetase [Crassostrea gigas]; 210 2201 minus strand Glycyl-tRNA synthetase [Crassostrea gigas]; 210 2201 minus strand Glycyl-tRNA synthetase [Crassostrea gigas]; 210 2201 minus strand Glycyl-tRNA synthetase [Crassostrea gigas]; 210 2201 minus strand Glycyl-tRNA synthetase [Crassostrea gigas]; 210 2201 minus strand Glycyl-tRNA synthetase [Crassostrea gigas]

Protein Accession: CL4073.Contig8$_{H}$1 CL4073.Contig7$_{H}$1 CL4073.Contig6$_{H}$1 CL4073.Contig5$_{H}$1 CL4073.Con
Mean Expression Ratio: 1.1
Median Expression Ratio: 1.11
Credible Interval: (0.487, 2.55)
Associated Peptides: 1
Associated Spectra: 1
Coverage: NaN

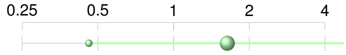

A	B	2.5	50	97.5	Sequence
**1**	**0**	**0.46**	**1.6**	**5.9**	**ATGVKLVAER**

5.486 43 525 Reticulon-4 [Crassostrea gigas]; 143 757 Reticulon-4 [Crassostrea gigas]; 43 564 Reticulon-4 [Crassostrea gigas]; 302 583 Reticulon-4 OS=Rattus norvegicus GN=Rtn4 PE=1 SV=1

Protein Accession: CL29775.Contig4$_{H}$1 CL29775.Contig3$_{H}$1 CL29775.Contig2$_{H}$1 CL29775.Contig1$_{H}$1
Mean Expression Ratio: 0.904
Median Expression Ratio: 0.902
Credible Interval: (0.412, 1.95)
Associated Peptides: 2
Associated Spectra: 3
Coverage: NaN

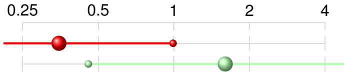

A	B	2.5	50	97.5	Sequence
**1**	**1**	**0.12**	**0.35**	**1**	**VTQTILQHVNR**
**0**	**1**	**0.46**	**1.6**	**5.6**	**LAAVQK**

5.487 132 590 minus strand Non-neuronal cytoplasmic intermediate filament protein OS=Helix aspersa PE=3 SV=2

Protein Accession Unigene7770$_{H}1$
Mean Expression Ratio 1.1
Median Expression Ratio 1.11
Credible Interval (0.515, 2.35)
Associated Peptides 2
Associated Spectra 4
Coverage NaN

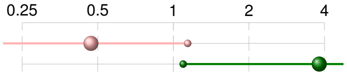

A	B	2.5	50	97.5	Sequence
**2**	**1**	**0.2**	**0.47**	**1.1**	**TMAEVPLGEK**
**0**	**1**	**1.1**	**3.8**	**14**	**VTKEVSGK**

5.488 48 1523 minus strand Spermatogenesis-associated protein 18-like protein [Crassostrea gigas]

Protein Accession CL6432.Contig3$_{H}1$
Mean Expression Ratio 1.11
Median Expression Ratio 1.11
Credible Interval (0.513, 2.41)
Associated Peptides 2
Associated Spectra 2
Coverage NaN

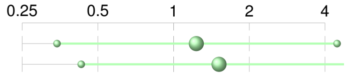

A	B	2.5	50	97.5	Sequence
**0**	**1**	**0.34**	**1.2**	**4.5**	**IDLDRRLR**
**0**	**1**	**0.43**	**1.5**	**5.4**	**SLSPALR**

5.489 391 1470 PREDICTED: heparanase-like [Amphimedon queenslandica]; 391 1470 PREDICTED: heparanase-like [Amphimedon queenslandica]; 391 1470 PREDICTED: heparanase-like [Amphimedon queenslandica]; 391 1470 PREDICTED: heparanase-like [Amphimedon queenslandica]; 391 1755 PREDICTED: heparanase-like [Amphimedon queenslandica]; 418 1365 PREDICTED: similar to heparanase-like protein [Tribolium castaneum]; 391 1755 PREDICTED: heparanase-like [Amphimedon queenslandica]; 391 1470 PREDICTED: heparanase-like [Amphimedon queenslandica]

Protein Accession CL2227.Contig4$_{H}1$ CL2227.Contig3$_{H}1$ CL2227.Contig2$_{H}1$ CL2227.Contig1$_{H}1$ CL2227.Con
Mean Expression Ratio 1.11
Median Expression Ratio 1.11
Credible Interval (0.492, 2.47)
Associated Peptides 1
Associated Spectra 2
Coverage NaN

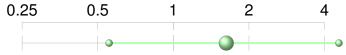

A	B	2.5	50	97.5	Sequence
**1**	**1**	**0.55**	**1.6**	**4.6**	**ALSPAYFR**

5.490 58 1167 minus strand PREDICTED: arylacetamide deacetylase-like 4-like [Gallus gallus]; 76 1239 minus strand PREDICTED: arylacetamide deacetylase-like 4-like [Gallus gallus]

Protein Accession	CL44820.Contig3$_{H}1$ CL44820.Contig1$_{H}1$
Mean Expression Ratio	0.905
Median Expression Ratio	0.903
Credible Interval	(0.391, 2.05)
Associated Peptides	1
Associated Spectra	1
Coverage	NaN

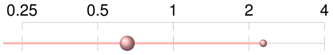

A	B	2.5	50	97.5	Sequence
**0**	**1**	**0.18**	**0.66**	**2.3**	**NSHDVLLPR**

5.491 " 235 1038 Translocon-associated protein subunit alpha [Crassostrea gigas]; 309 725 hypothetical protein [Amblyomma maculatum]; 309 590 translocon-associated complex TRAP, alpha subunit, putative [Ixodes scapularis] gi; 309 725 hypothetical protein [Amblyomma maculatum]"

Protein Accession	CL36450.Contig6$_{H}1$ CL36450.Contig5$_{H}1$ CL36450.Contig2$_{H}1$ CL36450.Contig1$_{H}1$
Mean Expression Ratio	1.11
Median Expression Ratio	1.11
Credible Interval	(0.526, 2.33)
Associated Peptides	2
Associated Spectra	4
Coverage	NaN

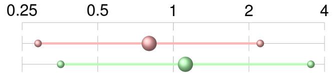

A	B	2.5	50	97.5	Sequence
**0**	**2**	**0.29**	**0.8**	**2.2**	**LLVGFTNK**
**1**	**1**	**0.36**	**1.1**	**3.5**	**ELPAGKPVR**

5.492 96 581 minus strand thioredoxin 2 [Haliotis discus discus]; 1682 2167 minus strand thioredoxin 2 [Haliotis discus discus]; 191 676 minus strand thioredoxin 2 [Haliotis discus discus]

Protein Accession	CL10014.Contig7$_{H}1$ CL10014.Contig3$_{H}1$ CL10014.Contig1$_{H}1$
Mean Expression Ratio	0.904
Median Expression Ratio	0.904
Credible Interval	(0.39, 2.07)
Associated Peptides	1
Associated Spectra	1
Coverage	NaN

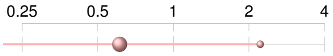

A	B	2.5	50	97.5	Sequence
**0**	**1**	**0.18**	**0.61**	**2.2**	**LETIVSGEK**

5.493 125 754 minus strand peptidyl prolyl cis-trans isomerase B [Conus novaehollandiae]

Protein Accession	Unigene19558$_{H}1$
Mean Expression Ratio	1.1
Median Expression Ratio	1.11
Credible Interval	(0.561, 2.15)
Associated Peptides	4
Associated Spectra	12
Coverage	NaN

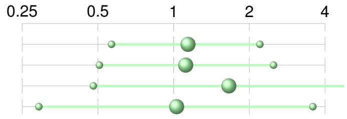

A	B	2.5	50	97.5	Sequence
1	5	0.57	1.1	2.2	VLSGMDVVR
0	4	0.51	1.1	2.5	HLALGDKEDGGVK
0	1	0.48	1.7	6	SIYGEKFPDENFK
0	1	0.29	1	3.6	TDSRDRPEK

5.494 1067 3010 minus strand PREDICTED: nucleolin-like isoform 2 [Oreochromis niloticus]

Protein Accession	CL7031.Contig7$_{H}1$
Mean Expression Ratio	0.899
Median Expression Ratio	0.904
Credible Interval	(0.422, 1.89)
Associated Peptides	3
Associated Spectra	3
Coverage	NaN

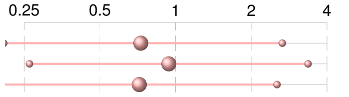

A	B	2.5	50	97.5	Sequence
1	0	0.21	0.73	2.7	KGIAVSDVR
1	0	0.26	0.94	3.4	KISIEAAAVVASAK
1	0	0.2	0.72	2.5	RFAYADLENPDDLDK

5.495 2 226 minus strand hypothetical protein CGI_10015501 [Crassostrea gigas]

Protein Accession	Unigene73065$_{H}1$
Mean Expression Ratio	1.11
Median Expression Ratio	1.11
Credible Interval	(0.526, 2.32)
Associated Peptides	2
Associated Spectra	11
Coverage	NaN

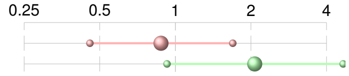

A	B	2.5	50	97.5	Sequence
0	7	0.46	0.88	1.7	SSVAADALR
0	4	0.93	2.1	4.7	SSVAADALRK

5.496 156 845 minus strand histone H1 [Mytilus californianus]; 156 845 minus strand histone H1 [Mytilus californianus]; 156 845 minus strand histone H1 [Mytilus californianus]; 156 845 minus strand histone H1 [Mytilus californianus]

Protein Accession	CL26709.Contig6$_{H}1$ CL26709.Contig5$_{H}1$ CL26709.Contig4$_{H}1$ CL26709.Contig3$_{H}1$
Mean Expression Ratio	0.904
Median Expression Ratio	0.904
Credible Interval	(0.397, 2.05)
Associated Peptides	1
Associated Spectra	1
Coverage	NaN

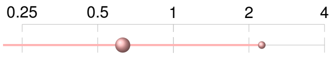

A	B	2.5	50	97.5	Sequence
1	0	0.18	0.63	2.2	DAITALKER

5.497 304 2952 minus strand hypothetical protein CGI_10026535 [Crassostrea gigas]

Protein Accession	CL21819.Contig2$_{H}1$
Mean Expression Ratio	1.1
Median Expression Ratio	1.11
Credible Interval	(0.566, 2.19)
Associated Peptides	5
Associated Spectra	5
Coverage	NaN

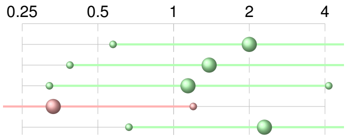

A	B	2.5	50	97.5	Sequence
0	1	0.57	2	7.1	LVQLEETAK
0	1	0.39	1.4	5	VADIAGDIPK
0	1	0.32	1.1	4.1	LVGMFSK
0	1	0.095	0.33	1.2	ESPLHK
0	1	0.67	2.3	8.4	AEYSLAAVALPPDNDAK

5.498 " 3195 3968 Spectrin alpha chain, putative [Pediculus humanus corporis] gi"

Protein Accession	CL2195.Contig1$_{H}1$
Mean Expression Ratio	0.904
Median Expression Ratio	0.904
Credible Interval	(0.439, 1.9)
Associated Peptides	3
Associated Spectra	3
Coverage	NaN

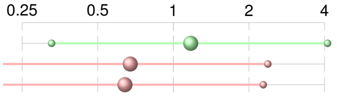

A	B	2.5	50	97.5	Sequence
0	1	0.33	1.2	4.1	LIDEGNHNADGIVER
0	1	0.19	0.67	2.4	LSELEAAASQR
0	1	0.19	0.64	2.3	TDVVDSWIADKETQVR

5.499 " 1 681 40S ribosomal protein S4, X isoform [Crassostrea gigas]"

Protein Accession CL2560.Contig4$_H$1
Mean Expression Ratio 0.905
Median Expression Ratio 0.904
Credible Interval (0.51, 1.62)
Associated Peptides 8
Associated Spectra 23
Coverage NaN

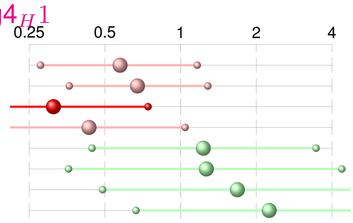

A	B	2.5	50	97.5	Sequence
2	3	0.28	0.57	1.2	QWVSLPK
1	6	0.36	0.68	1.3	VNDTVVLDIASGK
2	1	0.13	0.31	0.74	LTIAEER
1	2	0.18	0.43	1	GVPYIVTHDGR
1	1	0.45	1.2	3.5	IKVDGK
0	1	0.36	1.3	4.4	FDSGNMVMVTGGHNLGR
0	1	0.49	1.7	5.9	MKDYIK
0	1	0.66	2.2	7.9	LIKVDGK

5.500 36 1310 minus strand elongation factor-1 gamma [Diguetia canities]

Protein Accession CL14269.Contig5$_H$1
Mean Expression Ratio 0.907
Median Expression Ratio 0.904
Credible Interval (0.509, 1.63)
Associated Peptides 9
Associated Spectra 14
Coverage NaN

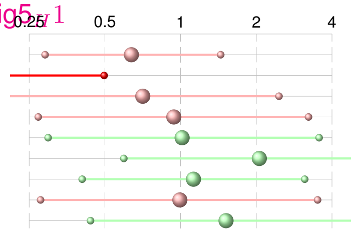

A	B	2.5	50	97.5	Sequence
1	3	0.29	0.64	1.4	LAETMAQFDAK
2	0	0.064	0.18	0.5	TYLVGEK
1	0	0.2	0.71	2.5	FVGNK
0	1	0.27	0.94	3.2	YSGADVQVDPNFK
0	1	0.3	1	3.6	GNFDMDEWK
0	1	0.59	2	7	GNFDMDEWKR
0	2	0.41	1.1	3.1	SADFLAK
0	1	0.28	0.99	3.5	ESKDPFLAFPK
0	1	0.44	1.5	5.2	LDPDDPETK

5.501 438 683 minus strand Protein lethal(2)essential for life OS=Drosophila melanogaster GN=l(2)efl PE=1 SV=1

Protein Accession CL17234.Contig7$_H$1
Mean Expression Ratio 1.11
Median Expression Ratio 1.11
Credible Interval (0.501, 2.47)
Associated Peptides 1
Associated Spectra 7
Coverage NaN

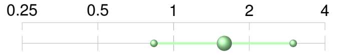

A	B	2.5	50	97.5	Sequence
1	6	0.83	1.6	3	ESGTSEVINNDKEFQIK

5.502 165 620 PREDICTED: uncharacterized protein LOC575072 isoform 2 [Strongylocentrotus purpuratus] gi

Protein Accession CL20552.Contig4$_H$1
Mean Expression Ratio 0.909
Median Expression Ratio 0.905
Credible Interval (0.391, 2.1)
Associated Peptides 1
Associated Spectra 1
Coverage NaN

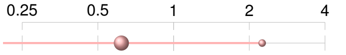

A	B	2.5	50	97.5	Sequence
1	0	0.17	0.62	2.2	IKPTPLNDR

5.503 " 1 1125 PREDICTED: trifunctional enzyme subunit beta, mitochondrial-like [Oreochromis niloticus]"

Protein Accession	CL19853.Contig2$_H$1
Mean Expression Ratio	1.1
Median Expression Ratio	1.1
Credible Interval	(0.505, 2.4)
Associated Peptides	2
Associated Spectra	2
Coverage	NaN

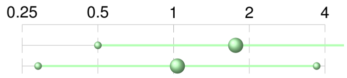

A	B	2.5	50	97.5	Sequence
0	1	0.5	1.8	6.3	KEQDEFALR
0	1	0.29	1	3.7	LMLSLNK

5.504 124 747 ribosomal protein S10 [Crassostrea gigas] gi

Protein Accession	CL18986.Contig1$_H$1
Mean Expression Ratio	0.905
Median Expression Ratio	0.905
Credible Interval	(0.417, 1.98)
Associated Peptides	2
Associated Spectra	2
Coverage	NaN

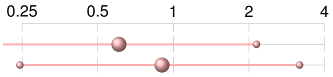

A	B	2.5	50	97.5	Sequence
1	0	0.17	0.61	2.1	ALTSLK
1	0	0.24	0.9	3.2	MLIPK

5.505 1327 1488 minus strand Ubiquinol-cytochrome C reductase complex [Xenopus laevis] gi

Protein Accession	CL7847.Contig1$_H$1
Mean Expression Ratio	1.11
Median Expression Ratio	1.1
Credible Interval	(0.491, 2.56)
Associated Peptides	1
Associated Spectra	1
Coverage	NaN

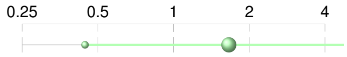

A	B	2.5	50	97.5	Sequence
0	1	0.45	1.7	6	LVDSITSSDINNIAK

5.506 154 621 minus strand Stress response protein nhaX [Crassostrea gigas]

Protein Accession	CL4265.Contig1$_H$1
Mean Expression Ratio	0.906
Median Expression Ratio	0.906
Credible Interval	(0.474, 1.71)
Associated Peptides	4
Associated Spectra	16
Coverage	NaN

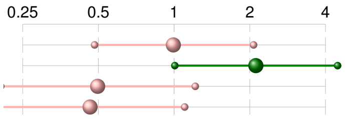

A	B	2.5	50	97.5	Sequence
3	2	0.48	0.99	2.1	SVAAAKPGEGILK
2	3	1	2.1	4.5	GLGVMR
0	3	0.21	0.5	1.2	IQHDLEK
0	3	0.19	0.46	1.1	EVLEAMLKDEQK

5.507 125 601 minus strand omega class glutathione-s-transferase [Chlamys farreri]; 113 808 minus strand omega class glutathione-s-transferase [Chlamys farreri]; 335 1045 minus strand omega class glutathione-s-transferase [Chlamys farreri]

Protein Accession	Unigene32974$_{H}1$ CL8096.Contig3$_{H}1$ CL8096.Contig2$_{H}1$
Mean Expression Ratio	0.904
Median Expression Ratio	0.906
Credible Interval	(0.459, 1.79)
Associated Peptides	5
Associated Spectra	6
Coverage	NaN

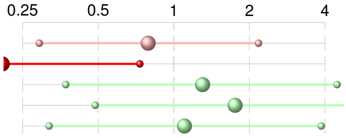

A	B	2.5	50	97.5	Sequence
1	1	0.29	0.79	2.2	HPLGLVPTLEQDSK
0	1	0.058	0.21	0.73	DAMLIDFFGNK
0	1	0.37	1.3	4.5	FIPNYYK
0	1	0.49	1.8	6.3	LTPVDPYRK
0	1	0.32	1.1	3.9	LVLEHK

5.508 " 81 1349 minus strand PREDICTED: LOW QUALITY PROTEIN: dolichyl-diphosphooligosaccharide–protein glycosyltransferase 48 kDa subunit-like, partial [Cavia porcellus]; 81 1349 minus strand PREDICTED: LOW QUALITY PROTEIN: dolichyl-diphosphooligosaccharide–protein glycosyltransferase 48 kDa subunit-like, partial [Cavia porcellus]"

Protein Accession	CL49524.Contig2$_{H}1$ CL49524.Contig1$_{H}1$
Mean Expression Ratio	0.909
Median Expression Ratio	0.906
Credible Interval	(0.432, 1.92)
Associated Peptides	3
Associated Spectra	3
Coverage	NaN

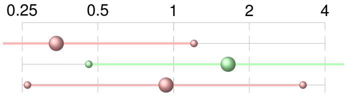

A	B	2.5	50	97.5	Sequence
0	1	0.097	0.34	1.2	DLGFDLTFK
0	1	0.46	1.7	6	KGNSPFLFR
0	1	0.26	0.93	3.3	NLAQWVFHQK

5.509 187 1323 minus strand gelsolin [Aplysia californica] gi

Protein Accession	CL10220.Contig1$_{H}1$
Mean Expression Ratio	1.11
Median Expression Ratio	1.1
Credible Interval	(0.535, 2.29)
Associated Peptides	3
Associated Spectra	5
Coverage	NaN

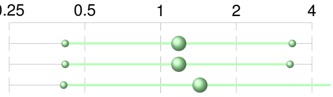

A	B	2.5	50	97.5	Sequence
0	2	0.42	1.2	3.3	AAQVLQDLK
0	2	0.42	1.2	3.3	LSDASGELDFK
0	1	0.41	1.4	5.1	SDVETIDEEEGGAALEK

5.510 200 2128 minus strand heat shock protein 70 [Cristaria plicata]

Protein Accession	CL8787.Contig2$_{H}$1
Mean Expression Ratio	0.906
Median Expression Ratio	0.907
Credible Interval	(0.41, 1.98)
Associated Peptides	2
Associated Spectra	2
Coverage	NaN

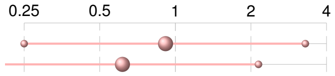

A	B	2.5	50	97.5	Sequence
0	1	0.25	0.91	3.3	LTEVQK
0	1	0.17	0.61	2.1	LSKEEIDR

5.511 462 671 enhancer protein [Homo sapiens]

Protein Accession	CL3160.Contig12$_{H}$1
Mean Expression Ratio	0.906
Median Expression Ratio	0.907
Credible Interval	(0.43, 1.88)
Associated Peptides	2
Associated Spectra	11
Coverage	NaN

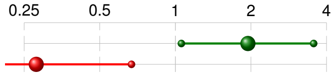

A	B	2.5	50	97.5	Sequence
0	8	1.1	1.9	3.6	GLFIIDDKGNLR
0	3	0.11	0.28	0.67	GLFIIDDK

5.512 2 487 minus strand PREDICTED: hypothetical protein LOC100699750 [Oreochromis niloticus]; 41 535 minus strand PREDICTED: hypothetical protein LOC100699750 [Oreochromis niloticus]

Protein Accession	CL7266.Contig5$_{H}$1 CL7266.Contig3$_{H}$1
Mean Expression Ratio	1.1
Median Expression Ratio	1.1
Credible Interval	(0.549, 2.21)
Associated Peptides	3
Associated Spectra	7
Coverage	NaN

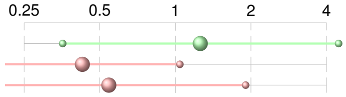

A	B	2.5	50	97.5	Sequence
0	1	0.36	1.3	4.5	VVNINNEAVTFASPR
3	2	0.17	0.43	1	NVALVVSVK
0	1	0.15	0.54	1.9	IPGDGTLTLHGSNGDTAFDR

5.513 187 942 Testis-specific gene 10 protein OS=Mus musculus GN=Tsga10 PE=1 SV=1; 212 967 Testis-specific gene 10 protein OS=Mus musculus GN=Tsga10 PE=1 SV=1; 214 870 Testis-specific gene 10 protein OS=Mus musculus GN=Tsga10 PE=1 SV=1; 134 793 Testis-specific gene 10 protein OS=Mus musculus GN=Tsga10 PE=1 SV=1

Protein Accession	CL11641.Contig6$_H1$ CL11641.Contig5$_H1$ CL11641.Contig4$_H1$ CL11641.Contig3$_H1$
Mean Expression Ratio	1.1
Median Expression Ratio	1.1
Credible Interval	(0.529, 2.28)
Associated Peptides	3
Associated Spectra	4
Coverage	NaN

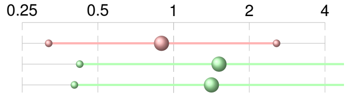

A	B	2.5	50	97.5	Sequence
0	2	0.32	0.9	2.6	RVENERDDAIADLHR
0	1	0.42	1.5	5.6	LLVEEAEK
0	1	0.4	1.4	5.2	ALEEQTK

5.514 " 39 968 l-3-hydroxyacyl-coenzyme a dehydrogenase, short chain [Haliotis discus discus]; 39 968 l-3-hydroxyacyl-coenzyme a dehydrogenase, short chain [Haliotis discus discus]"

Protein Accession	CL29042.Contig8$_H1$ CL29042.Contig1$_H1$
Mean Expression Ratio	0.904
Median Expression Ratio	0.908
Credible Interval	(0.432, 1.86)
Associated Peptides	3
Associated Spectra	4
Coverage	NaN

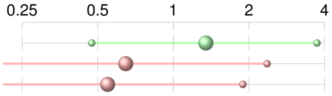

A	B	2.5	50	97.5	Sequence
1	1	0.47	1.3	3.7	ISTSTDAATAAK
1	0	0.18	0.65	2.4	STASIQQSLQR
0	1	0.16	0.55	1.9	QTTFVK

5.515 246 887 rab11-2 [Limulus polyphemus]

Protein Accession	CL3557.Contig1$_H1$
Mean Expression Ratio	0.91
Median Expression Ratio	0.908
Credible Interval	(0.433, 1.93)
Associated Peptides	3
Associated Spectra	3
Coverage	NaN

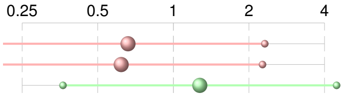

A	B	2.5	50	97.5	Sequence
1	0	0.19	0.66	2.3	NEFNLESK
1	0	0.17	0.62	2.3	GAVGALLVYDIAK
0	1	0.36	1.3	4.5	VVLIGDSGVGK

5.516 213 875 es1 protein [Haliotis discus discus]

Protein Accession	CL35629.Contig1$_{H}1$
Mean Expression Ratio	0.907
Median Expression Ratio	0.908
Credible Interval	(0.413, 1.97)
Associated Peptides	2
Associated Spectra	2
Coverage	NaN

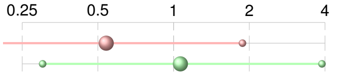

A	B	2.5	50	97.5	Sequence
1	0	0.15	0.54	1.9	NLSNFAFDAK
0	1	0.3	1.1	3.9	IARGPVNL

5.517 " 103 981 PREDICTED: ATP synthase, H+ transporting, mitochondrial F1 complex, gamma subunit isoform H (heart)-like [Saccoglossus kowalevskii]"

Protein Accession	CL25149.Contig1$_{H}1$
Mean Expression Ratio	1.1
Median Expression Ratio	1.1
Credible Interval	(0.601, 2.01)
Associated Peptides	7
Associated Spectra	14
Coverage	NaN

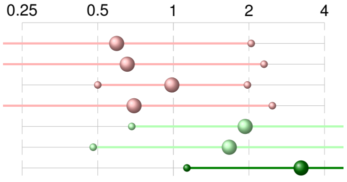

A	B	2.5	50	97.5	Sequence
1	0	0.17	0.6	2	NAGEMISK
1	0	0.19	0.65	2.3	AILGEK
2	4	0.5	0.99	2	LTLTFNR
1	0	0.2	0.7	2.5	AFFEK
0	2	0.68	1.9	5.3	ELKPARPYGAGAK
0	1	0.48	1.7	5.6	LIVLGDKAR
0	2	1.1	3.2	9.2	ATLKDIR

5.518 238 1521 26S protease regulatory subunit 6A [Crassostrea gigas]

Protein Accession	CL11449.Contig8$_{H}1$
Mean Expression Ratio	1.1
Median Expression Ratio	1.1
Credible Interval	(0.53, 2.28)
Associated Peptides	3
Associated Spectra	4
Coverage	NaN

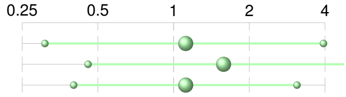

A	B	2.5	50	97.5	Sequence
0	1	0.31	1.1	3.9	AIWEDGEEALGEEVLR
0	1	0.46	1.6	5.6	LLDNEIK
0	2	0.4	1.1	3.1	KIEFPHPNEEAR

5.519 58 1203 minus strand Obg-like ATPase 1 [Crassostrea gigas]

Protein Accession	CL33105.Contig1$_{H}1$
Mean Expression Ratio	1.1
Median Expression Ratio	1.1
Credible Interval	(0.483, 2.52)
Associated Peptides	1
Associated Spectra	1
Coverage	NaN

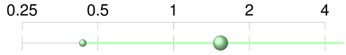

A	B	2.5	50	97.5	Sequence
0	1	0.43	1.5	5.5	IHTDFEK

5.520 " 115 534 NADH:ubiquinone oxidoreductase, NDUFB6/B17 subunit, putative [Ixodes scapularis] gi"

Protein Accession	CL16455.Contig5$_H1$
Mean Expression Ratio	1.1
Median Expression Ratio	1.1
Credible Interval	(0.48, 2.49)
Associated Peptides	1
Associated Spectra	1
Coverage	NaN

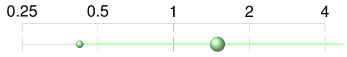

A	B	2.5	50	97.5	Sequence
**0**	**1**	**0.42**	**1.5**	**5.2**	**LAGTGMTDEDR**

5.521 17 691 minus strand Cleavage and polyadenylation specificity factor subunit 5 [Crassostrea gigas]

Protein Accession	CL17041.Contig3$_H1$
Mean Expression Ratio	1.1
Median Expression Ratio	1.1
Credible Interval	(0.479, 2.5)
Associated Peptides	1
Associated Spectra	1
Coverage	NaN

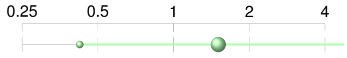

A	B	2.5	50	97.5	Sequence
**0**	**1**	**0.42**	**1.5**	**5.4**	**ALFAVPR**

5.522 201 1181 minus strand Serine/threonine-protein phosphatase PP1-alpha catalytic subunit [Crassostrea gigas]

Protein Accession	CL28655.Contig4$_H1$
Mean Expression Ratio	0.906
Median Expression Ratio	0.91
Credible Interval	(0.396, 2.07)
Associated Peptides	1
Associated Spectra	1
Coverage	NaN

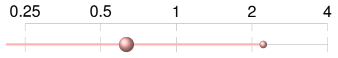

A	B	2.5	50	97.5	Sequence
**0**	**1**	**0.17**	**0.63**	**2.2**	**AHQVVEDGYEFFAK**

5.523 97 597 glyoxylate reductase/hydroxypyruvate reductase [Ictalurus punctatus] gi

Protein Accession	CL18211.Contig2$_H1$
Mean Expression Ratio	1.09
Median Expression Ratio	1.1
Credible Interval	(0.485, 2.45)
Associated Peptides	1
Associated Spectra	2
Coverage	NaN

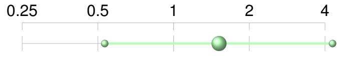

A	B	2.5	50	97.5	Sequence
**0**	**2**	**0.53**	**1.5**	**4.3**	**KIPAIGIDLLTR**

5.524 3 4517 minus strand Clathrin heavy chain 1 [Crassostrea gigas]

Protein Accession	CL16446.Contig1$_{H}1$
Mean Expression Ratio	0.908
Median Expression Ratio	0.91
Credible Interval	(0.506, 1.62)
Associated Peptides	9
Associated Spectra	12
Coverage	NaN

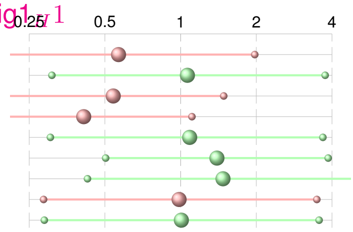

A	B	2.5	50	97.5	Sequence
1	0	0.17	0.57	2	KFEVNTSAIK
1	0	0.31	1.1	3.8	VHEGVTEPATHNALAK
1	1	0.2	0.54	1.5	EAIDSYIK
0	2	0.15	0.41	1.1	LHVIEVGSPPQGNQPFAK
0	1	0.3	1.1	3.7	TNQVPLVKPYLR
0	2	0.51	1.4	3.9	AVNFFIK
0	1	0.43	1.5	5.1	AQLNQGMVK
0	1	0.29	0.99	3.5	IAAYLYK
0	1	0.29	1	3.6	ELVEEVEK

5.525 3512 6661 hypothetical protein BRAFLDRAFT_255626 [Branchiostoma floridae] gi

Protein Accession	CL1959.Contig8$_{H}1$
Mean Expression Ratio	1.1
Median Expression Ratio	1.1
Credible Interval	(0.606, 1.98)
Associated Peptides	9
Associated Spectra	11
Coverage	NaN

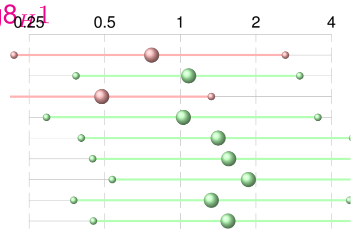

A	B	2.5	50	97.5	Sequence
1	0	0.22	0.77	2.6	SGVKIDVSDAEAQAR
1	1	0.38	1.1	3	LDEAFLSK
0	2	0.17	0.48	1.3	FDRPAQLHMAFR
0	1	0.29	1	3.5	IDVSDAEAQAR
0	1	0.4	1.4	4.9	NVVLGGVK
0	1	0.45	1.6	5.3	VKAALPSPEGLK
0	1	0.53	1.9	6.6	SVTIHDLGK
0	1	0.38	1.3	4.7	GGNVVQVKMPK
0	1	0.45	1.5	5.4	FIVAETNGLFGK

5.526 635 1561 Uncharacterized protein yfeX [Crassostrea gigas]

Protein Accession	CL12046.Contig4$_{H}1$
Mean Expression Ratio	1.1
Median Expression Ratio	1.1
Credible Interval	(0.557, 2.18)
Associated Peptides	5
Associated Spectra	7
Coverage	NaN

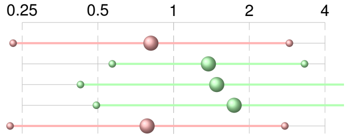

A	B	2.5	50	97.5	Sequence
1	0	0.23	0.81	2.9	ATGGSYVITQK
1	2	0.57	1.4	3.3	TLESWVGR
0	1	0.43	1.5	5.2	MVGAGGDGHADDIMR
0	1	0.49	1.7	6	KAFQIVR
0	1	0.22	0.79	2.8	WIHDLHVINTEKEK

5.527 740 2029 minus strand PREDICTED: protein alan shepard-like isoform 2 [Bombus terrestris]

Protein Accession	CL693.Contig2$_{H}1$
Mean Expression Ratio	1.1
Median Expression Ratio	1.1
Credible Interval	(0.479, 2.49)
Associated Peptides	1
Associated Spectra	1
Coverage	NaN

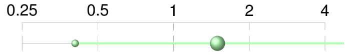

A	B	2.5	50	97.5	Sequence
0	1	0.41	1.5	5.4	GAQGIQAQMAK

5.528 2 220 minus strand haemocyanin [Aplysia californica]

Protein Accession	CL7906.Contig5$_{H}1$
Mean Expression Ratio	0.908
Median Expression Ratio	0.911
Credible Interval	(0.405, 1.99)
Associated Peptides	1
Associated Spectra	3
Coverage	NaN

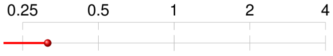

A	B	2.5	50	97.5	Sequence
2	1	0.044	0.12	0.31	YDISDAVK

5.529 290 2458 Putative beta-D-xylosidase 5 [Crassostrea gigas]

Protein Accession	CL49577.Contig2$_{H}1$
Mean Expression Ratio	0.909
Median Expression Ratio	0.911
Credible Interval	(0.525, 1.55)
Associated Peptides	11
Associated Spectra	19
Coverage	NaN

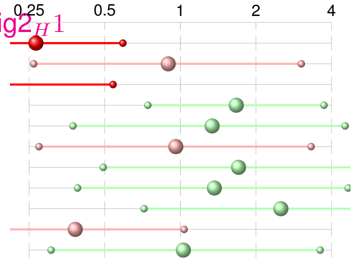

A	B	2.5	50	97.5	Sequence
2	2	0.12	0.27	0.59	VAEAAGVEVR
1	0	0.26	0.89	3	VQDLVSR
1	1	0.072	0.2	0.54	EIQEQLAR
1	3	0.74	1.7	3.7	VTPLFYTR
0	1	0.37	1.3	4.5	HFDAYAGPENVPTSR
0	1	0.27	0.96	3.3	LTDYDWR
0	1	0.49	1.7	5.8	SAPSQVLVQPFTIK
0	1	0.39	1.4	4.7	NNGILPLDPR
0	1	0.72	2.5	8.8	LEALEQGK
0	2	0.14	0.38	1	QLLTDILR
0	1	0.3	1	3.6	SFVLLK

5.530 62 292 PREDICTED: heterogeneous nuclear ribonucleoprotein F-like [Otolemur garnettii]

Protein Accession	CL30592.Contig1$_{H}1$
Mean Expression Ratio	1.1
Median Expression Ratio	1.1
Credible Interval	(0.504, 2.39)
Associated Peptides	2
Associated Spectra	2
Coverage	NaN

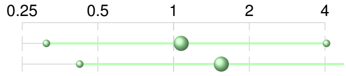

A	B	2.5	50	97.5	Sequence
0	1	0.31	1.1	4	GLPWSTTPEDIVK
0	1	0.42	1.6	5.5	FFDESEIAGGEK

5.531 388 1653 Tektin-3 [Crassostrea gigas]

Protein Accession	CL28380.Contig1$_{H}1$
Mean Expression Ratio	1.09
Median Expression Ratio	1.1
Credible Interval	(0.549, 2.2)
Associated Peptides	3
Associated Spectra	7
Coverage	NaN

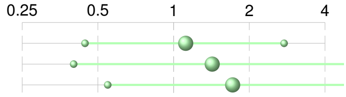

A	B	2.5	50	97.5	Sequence
2	2	0.45	1.1	2.8	QTIDLVHDAPEK
0	1	0.4	1.4	5.1	RQTIDLVHDAPEK
1	1	0.55	1.7	5.1	GLTFHNGVQR

5.532 103 819 minus strand PREDICTED: l-xylulose reductase-like [Monodelphis domestica]

Protein Accession	Unigene30376$_{H}$1
Mean Expression Ratio	1.1
Median Expression Ratio	1.1
Credible Interval	(0.482, 2.54)
Associated Peptides	1
Associated Spectra	1
Coverage	NaN

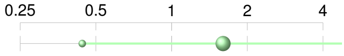

A	B	2.5	50	97.5	Sequence
**0**	**1**	**0.44**	**1.6**	**5.8**	**GSGGAIVNISSQASK**

5.533 6043 6174 myosin heavy chain [Mytilus galloprovincialis]; 5869 6000 myosin heavy chain [Mytilus galloprovincialis]

Protein Accession	Unigene19025$_{H}$1 CL49594.Contig1$_{H}$1
Mean Expression Ratio	1.1
Median Expression Ratio	1.1
Credible Interval	(0.516, 2.4)
Associated Peptides	2
Associated Spectra	5
Coverage	NaN

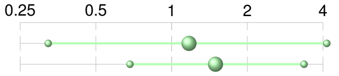

A	B	2.5	50	97.5	Sequence
**0**	**1**	**0.32**	**1.2**	**4.1**	**AMQTDLDEMNGELR**
**0**	**4**	**0.68**	**1.5**	**3.4**	**KLEADIQAMQTDLDEMNGELR**

5.534 62 505 Myosin-2 essential light chain [Crassostrea gigas]

Protein Accession	CL47077.Contig1$_{H}$1
Mean Expression Ratio	0.913
Median Expression Ratio	0.913
Credible Interval	(0.45, 1.84)
Associated Peptides	3
Associated Spectra	12
Coverage	NaN

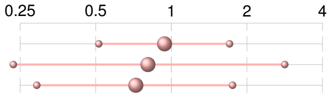

A	B	2.5	50	97.5	Sequence
**4**	**4**	**0.51**	**0.94**	**1.7**	**IAASQLGDVLR**
**1**	**0**	**0.23**	**0.81**	**2.8**	**SLNQNPTEQEIR**
**0**	**3**	**0.29**	**0.72**	**1.8**	**HILTSLGEK**

5.535 " 194 334 unknown, partial [Lymnaea stagnalis] gi; 207 347 unknown, partial [Lymnaea stagnalis] gi; 384 524 unknown, partial [Lymnaea stagnalis] gi"

Protein Accession	Unigene62752$_{H}$1 Unigene62751$_{H}$1 Unigene62750$_{H}$1
Mean Expression Ratio	1.1
Median Expression Ratio	1.09
Credible Interval	(0.491, 2.5)
Associated Peptides	1
Associated Spectra	2
Coverage	NaN

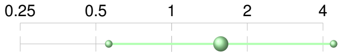

A	B	2.5	50	97.5	Sequence
**0**	**2**	**0.56**	**1.6**	**4.4**	**AFGFGMR**

5.536 2 1384 minus strand twitchin [Mytilus galloprovincialis]; 2 1384 minus strand twitchin [Mytilus galloprovincialis]; 2 1384 minus strand twitchin [Mytilus galloprovincialis]; 2 1384 minus strand twitchin [Mytilus galloprovincialis]; 2 1384 minus strand twitchin [Mytilus galloprovincialis]; 2 1384 minus strand twitchin [Mytilus galloprovincialis]

Protein Accession	Unigene62659$_{H}1$ Unigene62658$_{H}1$ Unigene62657$_{H}1$ Unigene62656$_{H}1$ Unigene62655$_{H}1$ U
Mean Expression Ratio	1.1
Median Expression Ratio	1.09
Credible Interval	(0.473, 2.51)
Associated Peptides	1
Associated Spectra	1
Coverage	NaN

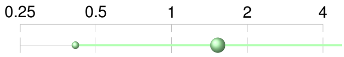

A	B	2.5	50	97.5	Sequence
**0**	**1**	**0.42**	**1.5**	**5.5**	**LEGDTIIIQQDITSK**

5.537 773 1045 minus strand serine protease inhibitor 2 [Latrodectus hesperus]

Protein Accession	Unigene4107$_{H}1$
Mean Expression Ratio	1.1
Median Expression Ratio	1.09
Credible Interval	(0.499, 2.47)
Associated Peptides	1
Associated Spectra	2
Coverage	NaN

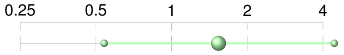

A	B	2.5	50	97.5	Sequence
**0**	**2**	**0.54**	**1.5**	**4.4**	**KFYFNK**

5.538 " 57 752 PREDICTED: adenylate kinase 2, mitochondrial-like isoform 2 [Callithrix jacchus]"

Protein Accession	CL4149.Contig1$_{H}1$
Mean Expression Ratio	1.09
Median Expression Ratio	1.09
Credible Interval	(0.482, 2.52)
Associated Peptides	1
Associated Spectra	1
Coverage	NaN

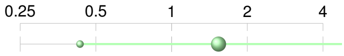

A	B	2.5	50	97.5	Sequence
**0**	**1**	**0.43**	**1.5**	**5.5**	**AVISSGSALGKK**

5.539 40 315 minus strand 60S ribosomal protein L18a OS=Homo sapiens GN=RPL18A PE=1 SV=2

Protein Accession	Unigene26823$_{H}1$
Mean Expression Ratio	1.09
Median Expression Ratio	1.09
Credible Interval	(0.524, 2.28)
Associated Peptides	2
Associated Spectra	7
Coverage	NaN

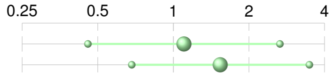

A	B	2.5	50	97.5	Sequence
2	1	0.46	1.1	2.7	IFAPDQATAK
2	2	0.68	1.5	3.5	TPTLYQMR

5.540 333 1799 Alba-like protein C9orf23-like protein [Crassostrea gigas]

Protein Accession	Unigene30413$_{H}1$
Mean Expression Ratio	0.914
Median Expression Ratio	0.916
Credible Interval	(0.468, 1.78)
Associated Peptides	5
Associated Spectra	7
Coverage	NaN

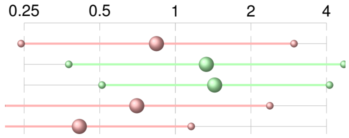

A	B	2.5	50	97.5	Sequence
0	1	0.24	0.84	3	AASWLGMR
0	1	0.38	1.3	4.7	GKGALELAQR
0	2	0.51	1.4	4.1	TINPATEAETQGK
0	1	0.2	0.7	2.4	EIMTELSR
0	2	0.15	0.42	1.2	ELNDFMTK

5.541 2 298 putative 60S ribosomal protein RPL24 [Novocrania anomala]

Protein Accession	Unigene278$_{H}1$
Mean Expression Ratio	1.09
Median Expression Ratio	1.09
Credible Interval	(0.485, 2.43)
Associated Peptides	1
Associated Spectra	3
Coverage	NaN

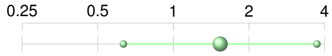

A	B	2.5	50	97.5	Sequence
0	3	0.64	1.5	3.7	AITGVSLSEIIAK

5.542 5 451 minus strand Spectrin beta chain [Crassostrea gigas]

Protein Accession	CL19757.Contig1$_{H}1$
Mean Expression Ratio	0.911
Median Expression Ratio	0.916
Credible Interval	(0.411, 1.99)
Associated Peptides	2
Associated Spectra	2
Coverage	NaN

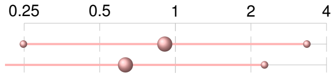

A	B	2.5	50	97.5	Sequence
0	1	0.25	0.91	3.3	HSLLEADIHVVGER
0	1	0.17	0.63	2.3	HLMGVEDLLQK

5.543 100 834 vesicle-associated membrane protein/synaptobrevin-binding protein [Aplysia californica] gi

Protein Accession	CL36367.Contig1$_{H}1$
Mean Expression Ratio	1.1
Median Expression Ratio	1.09
Credible Interval	(0.492, 2.43)
Associated Peptides	2
Associated Spectra	2
Coverage	NaN

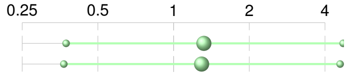

A	B	2.5	50	97.5	Sequence
0	1	0.37	1.3	4.7	FKGPFTDVVTAELK
0	1	0.37	1.3	4.6	TEQALQLEPANELR

5.544 " 179 631 minus strand 60S ribosomal protein L7/L12, partial [Ostrea edulis]"

Protein Accession	CL34109.Contig5$_{H}1$
Mean Expression Ratio	0.917
Median Expression Ratio	0.917
Credible Interval	(0.412, 2.06)
Associated Peptides	1
Associated Spectra	3
Coverage	NaN

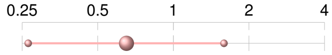

A	B	2.5	50	97.5	Sequence
2	1	0.26	0.65	1.6	KFVESVPQVVK

5.545 91 2298 hypothetical protein BRAFLDRAFT_267717 [Branchiostoma floridae] gi; 84 2291 hypothetical protein BRAFLDRAFT_267717 [Branchiostoma floridae] gi; 71 2278 hypothetical protein BRAFLDRAFT_267717 [Branchiostoma floridae] gi; 81 2288 hypothetical protein BRAFLDRAFT_267717 [Branchiostoma floridae] gi; 58 2268 hypothetical protein BRAFLDRAFT_267717 [Branchiostoma floridae] gi; 62 2245 hypothetical protein BRAFLDRAFT_267717 [Branchiostoma floridae] gi

Protein Accession	CL26176.Contig5$_{H}1$ CL26176.Contig4$_{H}1$ CL26176.Contig3$_{H}1$ CL26176.Contig2$_{H}1$ CL2617
Mean Expression Ratio	1.1
Median Expression Ratio	1.09
Credible Interval	(0.582, 2.04)
Associated Peptides	7
Associated Spectra	9
Coverage	NaN

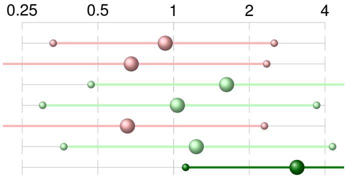

A	B	2.5	50	97.5	Sequence
1	1	0.33	0.93	2.5	VGAVEEASFLK
1	0	0.19	0.68	2.3	NIGPLIK
1	0	0.47	1.6	5.7	VESIR
1	0	0.3	1	3.7	LTTPFVK
0	1	0.18	0.66	2.3	FANEVAGVEDLGTTGR
0	1	0.37	1.2	4.3	VFNVLQR
0	2	1.1	3.1	8.7	YTDIAYTGKR

5.546 326 724 hypothetical protein [Amblyomma maculatum]

Protein Accession	Unigene1454$_{H1}$
Mean Expression Ratio	1.09
Median Expression Ratio	1.09
Credible Interval	(0.529, 2.27)
Associated Peptides	3
Associated Spectra	5
Coverage	NaN

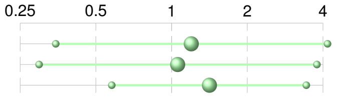

A	B	2.5	50	97.5	Sequence
0	1	0.35	1.2	4.2	EAYLELER
0	1	0.3	1.1	3.8	KLEEHFADTK
0	3	0.58	1.4	3.4	EAYLELERR

5.547 " 142 1284 minus strand Chain O, Crsytal Structure Of Human Liver Gapdh gi|67464044|pdb|1ZNQ|P Chain P, Crsytal Structure Of Human Liver Gapdh gi|67464045|pdb|1ZNQ|R Chain R, Crsytal Structure Of Human Liver Gapdh gi|67464046|pdb|1ZNQ|Q Chain Q, Crsytal Structure Of Human Liver Gapdh"

Protein Accession	CL34318.Contig1$_{H1}$
Mean Expression Ratio	0.916
Median Expression Ratio	0.917
Credible Interval	(0.467, 1.78)
Associated Peptides	4
Associated Spectra	10
Coverage	NaN

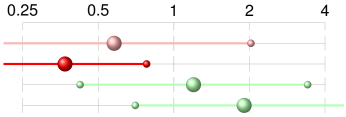

A	B	2.5	50	97.5	Sequence
1	0	0.17	0.58	2	DPTAIPWR
3	2	0.18	0.37	0.78	VIPELNGK
0	2	0.42	1.2	3.4	INVYQEKDPTAIPWR
0	2	0.7	1.9	5.4	VIPELNGKLTGMAFR

5.548 " 183 476 minus strand guanine nucleotide-binding protein G(q), alpha subunit [Aplysia californica] gi"

Protein Accession	CL36147.Contig1$_{H1}$
Mean Expression Ratio	0.916
Median Expression Ratio	0.918
Credible Interval	(0.399, 2.11)
Associated Peptides	1
Associated Spectra	1
Coverage	NaN

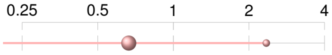

A	B	2.5	50	97.5	Sequence
0	1	0.18	0.66	2.3	LLLLGTGESGK

5.549 977 1852 PREDICTED: similar to prohibitin [Tribolium castaneum] gi

Protein Accession	CL8219.Contig4$_H$1
Mean Expression Ratio	1.09
Median Expression Ratio	1.09
Credible Interval	(0.575, 2.11)
Associated Peptides	5
Associated Spectra	10
Coverage	NaN

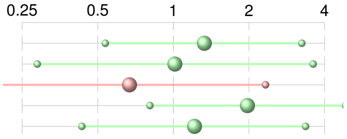

A	B	2.5	50	97.5	Sequence
1	2	0.54	1.3	3.3	VVQAEGEAAAAK
1	0	0.29	1	3.6	AQFVVEK
1	0	0.19	0.67	2.3	SLLIR
1	2	0.81	2	4.8	KLGDLAGR
0	2	0.43	1.2	3.4	LIGQAVSANPGFLK

5.550 " 1 1563 Putative methylmalonate-semialdehyde dehydrogenase [acylating], mitochondrial [Crassostrea gigas]"

Protein Accession	Unigene56839$_H$1
Mean Expression Ratio	1.09
Median Expression Ratio	1.09
Credible Interval	(0.659, 1.8)
Associated Peptides	14
Associated Spectra	26
Coverage	NaN

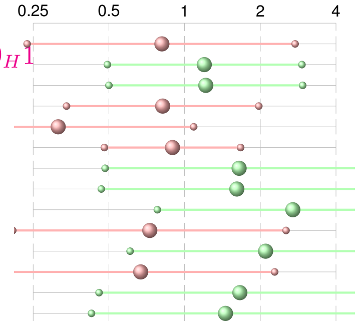

A	B	2.5	50	97.5	Sequence
1	0	0.24	0.81	2.8	GADLGPLISPESK
1	2	0.49	1.2	2.9	IAANITEQQGK
1	2	0.5	1.2	3	TLVDAEGDVLR
1	2	0.34	0.82	2	TVTQMWR
1	0	0.094	0.31	1.1	YQQLIK
2	5	0.48	0.9	1.7	GNFVGPTILHK
0	1	0.48	1.6	5.8	AISFVGSDQAGQYIYER
0	1	0.47	1.6	5.6	LFINNEFVESK
0	1	0.78	2.7	9.2	NIKVPGYEK
0	1	0.21	0.73	2.5	NWIPELVER
0	1	0.61	2.1	7.2	QGVQFYTQVK
0	1	0.19	0.67	2.3	WIDVHNPATNEVVTR
0	1	0.46	1.7	5.5	QQIMFR
0	1	0.43	1.5	5	FINNEFVESK

5.551 399 575 hypothetical protein BRAFLDRAFT_69739 [Branchiostoma floridae] gi

Protein Accession	CL7219.Contig3$_H$1
Mean Expression Ratio	0.92
Median Expression Ratio	0.918
Credible Interval	(0.407, 2.05)
Associated Peptides	1
Associated Spectra	2
Coverage	NaN

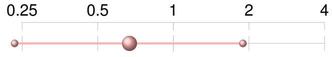

A	B	2.5	50	97.5	Sequence
0	2	0.23	0.67	1.9	HFLQPSGDLVSYPGK

5.552 17 1681 minus strand Glucose-6-phosphate isomerase [Crassostrea gigas]

Protein Accession: CL34850.Contig1$_{H}1$
Mean Expression Ratio: 0.917
Median Expression Ratio: 0.918
Credible Interval: (0.464, 1.8)
Associated Peptides: 5
Associated Spectra: 7
Coverage: NaN

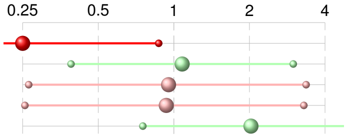

A	B	2.5	50	97.5	Sequence
1	0	0.07	0.25	0.87	INFTEDR
0	2	0.39	1.1	3	INMAQMFK
0	1	0.26	0.95	3.4	SGMNQEQISHILPHK
0	1	0.26	0.93	3.3	EAAEDELRK
0	2	0.75	2	5.6	VHFISNVDGTHLAK

5.553 8 1636 minus strand V-type proton ATPase catalytic subunit A [Crassostrea gigas]; 8 1636 minus strand V-type proton ATPase catalytic subunit A [Crassostrea gigas]; 8 1636 minus strand V-type proton ATPase catalytic subunit A [Crassostrea gigas]

Protein Accession: CL33671.Contig3$_{H}1$ CL33671.Contig2$_{H}1$ CL33671.Contig1$_{H}1$
Mean Expression Ratio: 0.922
Median Expression Ratio: 0.919
Credible Interval: (0.482, 1.76)
Associated Peptides: 5
Associated Spectra: 9
Coverage: NaN

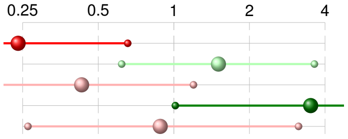

A	B	2.5	50	97.5	Sequence
2	0	0.086	0.24	0.65	GINTPALDANK
2	1	0.62	1.5	3.6	IMLNPNAR
2	0	0.15	0.43	1.2	TVGMLR
1	0	1	3.5	12	FRNLED
0	1	0.26	0.88	3.1	LAANHPLLTGQR

5.554 71 1321 minus strand Vinculin [Crassostrea gigas]

Protein Accession: Unigene23324$_{H}1$
Mean Expression Ratio: 1.09
Median Expression Ratio: 1.09
Credible Interval: (0.518, 2.3)
Associated Peptides: 3
Associated Spectra: 3
Coverage: NaN

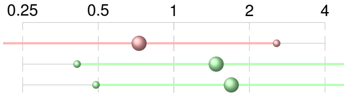

A	B	2.5	50	97.5	Sequence
0	1	0.2	0.73	2.6	GLVDEAVDTAALIR
0	1	0.41	1.5	5.3	KPAPTVDGK
0	1	0.49	1.7	6	LINAVGGVR

5.555 219 1559 Programmed cell death protein 4 [Crassostrea gigas]

Protein Accession: CL35470.Contig4$_{H}1$
Mean Expression Ratio: 1.08
Median Expression Ratio: 1.09
Credible Interval: (0.501, 2.34)
Associated Peptides: 2
Associated Spectra: 3
Coverage: NaN

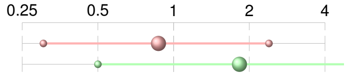

A	B	2.5	50	97.5	Sequence
0	2	0.3	0.87	2.4	MSDLVIDAPDATSVVGK
0	1	0.5	1.8	6.4	GRKRFVSEGDGGAVKQ

5.556 167 2713 minus strand Major vault protein [Crassostrea gigas]

Protein Accession	CL18286.Contig3$_H$1
Mean Expression Ratio	1.09
Median Expression Ratio	1.09
Credible Interval	(0.645, 1.85)
Associated Peptides	13
Associated Spectra	16
Coverage	NaN

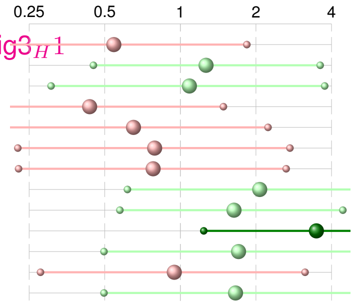

A	B	2.5	50	97.5	Sequence
1	0	0.16	0.54	1.8	GAVAQVQFDDFHK
1	1	0.45	1.3	3.6	IEGEAAVEQAK
1	0	0.31	1.1	3.7	IVDEAEAEK
1	0	0.13	0.43	1.5	LKAEAMK
1	0	0.19	0.65	2.2	VTGEEWLAK
1	0	0.23	0.79	2.7	TYTDDFNK
1	0	0.23	0.78	2.6	DPLADRSER
0	1	0.62	2.1	7	KEVVVEETIR
0	2	0.57	1.6	4.4	NADNEVVMDAAGQAK
0	2	1.2	3.5	9.5	QKIVDEAEAEK
0	1	0.5	1.7	6	VVPANSALR
0	1	0.28	0.95	3.1	IEAESELER
0	1	0.5	1.7	5.7	VVTFR

5.557 152 1696 dynamin isoform A [Lymnaea stagnalis]

Protein Accession	CL22122.Contig2$_H$1
Mean Expression Ratio	1.09
Median Expression Ratio	1.09
Credible Interval	(0.476, 2.42)
Associated Peptides	1
Associated Spectra	2
Coverage	NaN

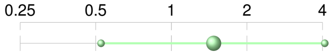

A	B	2.5	50	97.5	Sequence
0	2	0.53	1.5	4.1	KFFLSHPAYR

5.558 532 663 minus strand 40S ribosomal protein S23 [Ascaris suum]; 526 654 minus strand RecName: Full=40S ribosomal protein S23 gi|1754684|gb|AAC47632.1| ribosomal protein S23 [Brugia malayi]

Protein Accession	CL10408.Contig3$_H$1 CL10408.Contig2$_H$1
Mean Expression Ratio	1.09
Median Expression Ratio	1.09
Credible Interval	(0.488, 2.47)
Associated Peptides	1
Associated Spectra	2
Coverage	NaN

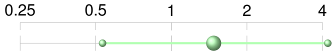

A	B	2.5	50	97.5	Sequence
0	2	0.53	1.5	4.2	KGHAVGDIPGVR

5.559 98 850 minus strand 14-3-3 protein epsilon [Schistocerca gregaria]

Protein Accession	CL27652.Contig3$_H$1
Mean Expression Ratio	1.08
Median Expression Ratio	1.09
Credible Interval	(0.596, 2)
Associated Peptides	7
Associated Spectra	16
Coverage	NaN

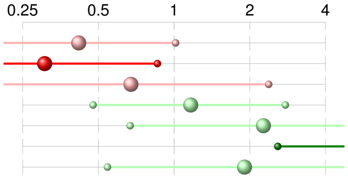

A	B	2.5	50	97.5	Sequence
2	1	0.17	0.42	1	EAAENSLVAYK
1	1	0.11	0.31	0.86	YDEMVEAMK
1	0	0.19	0.67	2.4	IISSMEQK
0	3	0.48	1.2	2.8	AASDISMTELPPTHPIR
0	1	0.67	2.3	7.8	LAEQAERYDEMVEAMK
0	5	2.6	5.3	11	LAEQAERYDEMVEAMKK
0	1	0.54	1.9	6.5	VFYYK

5.560 282 683 PREDICTED: ribonuclease UK114-like [Oreochromis niloticus]

Protein Accession	CL39557.Contig5$_H$1
Mean Expression Ratio	1.09
Median Expression Ratio	1.09
Credible Interval	(0.502, 2.45)
Associated Peptides	2
Associated Spectra	2
Coverage	NaN

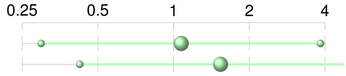

A	B	2.5	50	97.5	Sequence
0	1	0.3	1.1	3.8	LVPGGIQAETDQTLK
0	1	0.42	1.5	5.6	YFSDNKPAR

5.561 156 1106 ELAV 2-like protein [Aplysia californica] gi

Protein Accession	CL1500.Contig1$_H$1
Mean Expression Ratio	1.09
Median Expression Ratio	1.08
Credible Interval	(0.563, 2.09)
Associated Peptides	5
Associated Spectra	9
Coverage	NaN

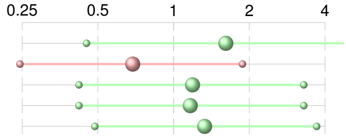

A	B	2.5	50	97.5	Sequence
1	0	0.45	1.6	5.7	GANLYVR
1	1	0.24	0.69	1.9	VLVDLQTGVSK
0	2	0.42	1.2	3.3	NLPIPWTENELNK
0	2	0.42	1.2	3.3	SMFLSIGPIK
0	2	0.49	1.3	3.7	LFDPYGK

5.562 " 169 792 minus strand rho GDP dissociation inhibitor, putative [Ixodes scapularis] gi"

Protein Accession	CL39798.Contig1$_H$1
Mean Expression Ratio	0.921
Median Expression Ratio	0.923
Credible Interval	(0.428, 2)
Associated Peptides	2
Associated Spectra	3
Coverage	NaN

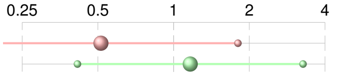

A	B	2.5	50	97.5	Sequence
1	0	0.15	0.51	1.8	YKEQLLAGAGSGEK
0	2	0.42	1.2	3.3	IIIDDSNPLNVIVK

5.563 636 1232 sigma class glutathione-s-transferase 3 [Haliotis discus discus]; 963 1559 sigma class glutathione-s-transferase 3 [Haliotis discus discus]; 907 1503 sigma class glutathione-s-transferase 3 [Haliotis discus discus]; 673 1269 sigma class glutathione-s-transferase 3 [Haliotis discus discus]; 177 773 sigma class glutathione-s-transferase 3 [Haliotis discus discus]; 208 804 sigma class glutathione-s-transferase 3 [Haliotis discus discus]; 327 923 sigma class glutathione-s-transferase 3 [Haliotis discus discus]

Protein Accession	Unigene22829$_{H}$1 CL19285.Contig31$_{H}$1 CL19285.Contig30$_{H}$1 CL19285.Contig27$_{H}$1 CL192[illegible]
Mean Expression Ratio	1.09
Median Expression Ratio	1.08
Credible Interval	(0.532, 2.27)
Associated Peptides	3
Associated Spectra	5
Coverage	NaN

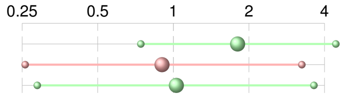

A	B	2.5	50	97.5	Sequence
0	3	0.74	1.8	4.4	LLLAASGKDWEDVR
0	1	0.26	0.9	3.2	LQGFDKDYPEVQK
0	1	0.29	1	3.6	FSDEDWPK

5.564 942 1385 Golgi-associated plant pathogenesis-related protein 1 [Crassostrea gigas]; 536 979 Golgi-associated plant pathogenesis-related protein 1 [Crassostrea gigas]

Protein Accession	Unigene391$_{H}$1 CL15263.Contig11$_{H}$1
Mean Expression Ratio	0.922
Median Expression Ratio	0.924
Credible Interval	(0.402, 2.11)
Associated Peptides	1
Associated Spectra	1
Coverage	NaN

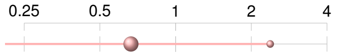

A	B	2.5	50	97.5	Sequence
0	1	0.18	0.66	2.4	IITEETIIK

5.565 142 726 Charged multivesicular body protein 4b [Crassostrea gigas]

Protein Accession	CL35875.Contig1$_{H}$1
Mean Expression Ratio	1.08
Median Expression Ratio	1.08
Credible Interval	(0.473, 2.45)
Associated Peptides	1
Associated Spectra	1
Coverage	NaN

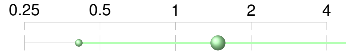

A	B	2.5	50	97.5	Sequence
0	1	0.41	1.5	5.2	KIEHELATAK

5.566 177 2369 ATPase family AAA domain-containing protein 2B [Crassostrea gigas]; 177 2369 ATPase family AAA domain-containing protein 2B [Crassostrea gigas]

Protein Accession	CL3021.Contig3$_{H}$1 CL3021.Contig2$_{H}$1
Mean Expression Ratio	1.08
Median Expression Ratio	1.08
Credible Interval	(0.595, 1.94)
Associated Peptides	8
Associated Spectra	11
Coverage	NaN

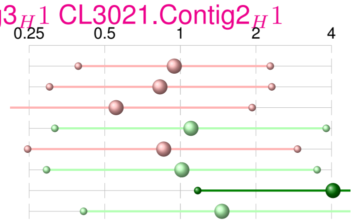

A	B	2.5	50	97.5	Sequence
2	1	0.39	0.95	2.3	FGGGDVNLLK
2	0	0.3	0.83	2.3	ILEVVR
1	0	0.16	0.56	1.9	IAVADRK
0	1	0.32	1.1	3.8	MTSGLYPAPLK
0	1	0.25	0.86	2.9	TKEEVIELSQK
0	1	0.29	1	3.5	YLDLHGAAPLVK
0	1	1.2	4.1	14	IAEASKRPEK
0	1	0.41	1.5	5.2	KITSFER

5.567 1278 1643 Fatty acid-binding-like protein 5 [Crassostrea gigas]

Protein Accession	CL11559.Contig3$_{H}$1
Mean Expression Ratio	0.926
Median Expression Ratio	0.925
Credible Interval	(0.441, 1.94)
Associated Peptides	2
Associated Spectra	5
Coverage	NaN

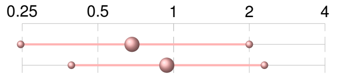

A	B	2.5	50	97.5	Sequence
1	1	0.25	0.68	2	AAGVGLVTR
1	2	0.39	0.94	2.3	EILPDGR

5.568 781 1212 minus strand cathepsin L2 cysteine protease [Pinctada fucata]

Protein Accession	CL7881.Contig5$_{H}$1
Mean Expression Ratio	1.08
Median Expression Ratio	1.08
Credible Interval	(0.51, 2.27)
Associated Peptides	2
Associated Spectra	5
Coverage	NaN

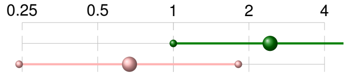

A	B	2.5	50	97.5	Sequence
2	1	1	2.4	5.9	GIYNEPACSAK
1	1	0.24	0.67	1.8	NTVGATLSSYK

5.569 " 57 1280 RNA binding motif protein, X-linked [Mustela putorius furo]"

Protein Accession	CL2752.Contig1$_{H}$1
Mean Expression Ratio	1.09
Median Expression Ratio	1.08
Credible Interval	(0.476, 2.53)
Associated Peptides	1
Associated Spectra	1
Coverage	NaN

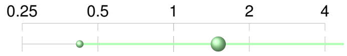

A	B	2.5	50	97.5	Sequence
0	1	0.42	1.5	5.3	LMDGKEVHGK

5.570 2140 2367 heat shock cognate 71 [Mytilus galloprovincialis]

Protein Accession	CL5924.Contig13$_{H}1$
Mean Expression Ratio	0.925
Median Expression Ratio	0.926
Credible Interval	(0.41, 2.05)
Associated Peptides	1
Associated Spectra	8
Coverage	NaN

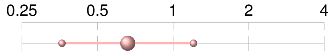

A	B	2.5	50	97.5	Sequence
**6**	**2**	**0.36**	**0.66**	**1.2**	**LIGDAAK**

5.571 513 755 PREDICTED: 40S ribosomal protein S16-like [Papio anubis]; 513 755 PREDICTED: 40S ribosomal protein S16-like [Papio anubis]

Protein Accession	CL14629.Contig2$_{H}1$ CL14629.Contig1$_{H}1$
Mean Expression Ratio	0.925
Median Expression Ratio	0.926
Credible Interval	(0.411, 2.11)
Associated Peptides	1
Associated Spectra	3
Coverage	NaN

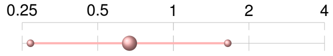

A	B	2.5	50	97.5	Sequence
**3**	**0**	**0.27**	**0.67**	**1.6**	**SLLVADPR**

5.572 2 2572 Glycogen debranching enzyme [Crassostrea gigas]

Protein Accession	CL45503.Contig1$_{H}1$
Mean Expression Ratio	0.927
Median Expression Ratio	0.927
Credible Interval	(0.424, 2.02)
Associated Peptides	2
Associated Spectra	2
Coverage	NaN

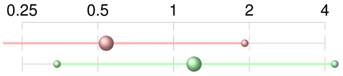

A	B	2.5	50	97.5	Sequence
**0**	**1**	**0.15**	**0.54**	**1.9**	**QMLNNLHYEMAR**
**0**	**1**	**0.34**	**1.2**	**4.4**	**SVEVQGVVDEVMFEGR**

5.573 3 263 minus strand beta-actin [Urechis unicinctus]

Protein Accession	CL31420.Contig5$_{H}1$
Mean Expression Ratio	1.08
Median Expression Ratio	1.08
Credible Interval	(0.54, 2.16)
Associated Peptides	4
Associated Spectra	15
Coverage	NaN

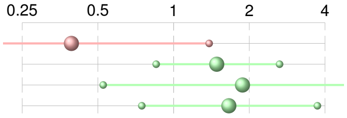

A	B	2.5	50	97.5	Sequence
**1**	**0**	**0.11**	**0.39**	**1.4**	**EITALAPPTMK**
**0**	**9**	**0.85**	**1.5**	**2.6**	**DLYANTVLSGGSTMYPGIADR**
**0**	**1**	**0.52**	**1.9**	**6.5**	**MQKEITALAPPTMK**
**0**	**4**	**0.75**	**1.7**	**3.7**	**KDLYANTVLSGGSTMYPGIADR**

5.574 " 2 1810 minus strand Glutaminyl-tRNA synthetase, partial [Crassostrea gigas]"

Protein Accession CL16949.Contig1$_{H}$1
Mean Expression Ratio 1.08
Median Expression Ratio 1.08
Credible Interval (0.467, 2.49)
Associated Peptides 1
Associated Spectra 1
Coverage NaN

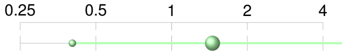

A	B	2.5	50	97.5	Sequence
**0**	**1**	**0.4**	**1.5**	**5.2**	**IAEGEATLR**

5.575 556 987 ribosomal protein rps15 [Lineus viridis]

Protein Accession CL7683.Contig9$_{H}$1
Mean Expression Ratio 0.933
Median Expression Ratio 0.927
Credible Interval (0.41, 2.17)
Associated Peptides 1
Associated Spectra 1
Coverage NaN

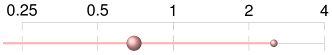

A	B	2.5	50	97.5	Sequence
**0**	**1**	**0.2**	**0.7**	**2.5**	**EAGPLEKPEVVK**

5.576 26 367 proteasome alpha type 2 [Haliotis discus discus]

Protein Accession CL43771.Contig1$_{H}$1
Mean Expression Ratio 0.925
Median Expression Ratio 0.928
Credible Interval (0.415, 2.07)
Associated Peptides 1
Associated Spectra 1
Coverage NaN

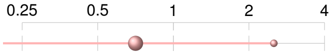

A	B	2.5	50	97.5	Sequence
**1**	**0**	**0.19**	**0.71**	**2.5**	**ATNGVVLATEK**

5.577 1155 1649 78 kDa glucose-regulated protein OS=Aplysia californica PE=1 SV=1

Protein Accession CL41594.Contig1$_{H}$1
Mean Expression Ratio 1.08
Median Expression Ratio 1.08
Credible Interval (0.531, 2.14)
Associated Peptides 3
Associated Spectra 7
Coverage NaN

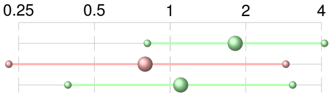

A	B	2.5	50	97.5	Sequence
**0**	**4**	**0.81**	**1.8**	**4.1**	**AKFEELNMDLFR**
**0**	**1**	**0.23**	**0.8**	**2.9**	**STVKPVK**
**0**	**2**	**0.39**	**1.1**	**3.1**	**VEVESLFDGEDFSEQLTR**

5.578 247 1440 minus strand Septin-2 [Crassostrea gigas]

Protein Accession	CL14404.Contig1$_H$1
Mean Expression Ratio	0.928
Median Expression Ratio	0.928
Credible Interval	(0.438, 1.97)
Associated Peptides	2
Associated Spectra	5
Coverage	NaN

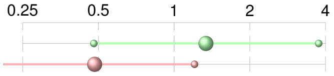

A	B	2.5	50	97.5	Sequence
**0**	**2**	**0.48**	**1.3**	**3.7**	**HKLEEMGFSDSETR**
**0**	**3**	**0.2**	**0.48**	**1.2**	**ALDLVTMK**

5.579 76 627 minus strand Heme-binding protein 2 [Crassostrea gigas]; 296 847 minus strand Heme-binding protein 2 [Crassostrea gigas]; 271 822 minus strand Heme-binding protein 2 [Crassostrea gigas]; 367 918 minus strand Heme-binding protein 2 [Crassostrea gigas]; 282 554 minus strand PREDICTED: heme-binding protein 2-like [Strongylocentrotus purpuratus]; 279 878 minus strand Heme-binding protein 2 [Crassostrea gigas]

Protein Accession	CL8470.Contig9$_H$1 CL8470.Contig8$_H$1 CL8470.Contig6$_H$1 CL8470.Contig5$_H$1 CL8470.Con
Mean Expression Ratio	1.08
Median Expression Ratio	1.08
Credible Interval	(0.488, 2.37)
Associated Peptides	2
Associated Spectra	2
Coverage	NaN

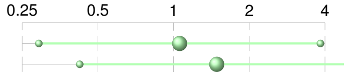

A	B	2.5	50	97.5	Sequence
**0**	**1**	**0.29**	**1.1**	**3.8**	**GVEFDEAENK**
**0**	**1**	**0.42**	**1.5**	**5.3**	**IAMTVPVLTK**

5.580 2 487 conserved hypothetical protein [Ixodes scapularis] gi

Protein Accession	CL12002.Contig4$_H$1
Mean Expression Ratio	1.08
Median Expression Ratio	1.08
Credible Interval	(0.473, 2.47)
Associated Peptides	1
Associated Spectra	1
Coverage	NaN

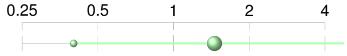

A	B	2.5	50	97.5	Sequence
**0**	**1**	**0.4**	**1.5**	**5.2**	**DNIAPETLVTIK**

5.581 120 833 B-cell receptor-associated protein 31 [Crassostrea gigas]; 238 951 B-cell receptor-associated protein 31 [Crassostrea gigas]; 88 801 B-cell receptor-associated protein 31 [Crassostrea gigas]; 88 801 B-cell receptor-associated protein 31 [Crassostrea gigas]

Protein Accession	CL9442.Contig7$_H$1 CL9442.Contig5$_H$1 CL9442.Contig4$_H$1 CL9442.Contig3$_H$1
Mean Expression Ratio	1.08
Median Expression Ratio	1.08
Credible Interval	(0.524, 2.2)
Associated Peptides	3
Associated Spectra	7
Coverage	NaN

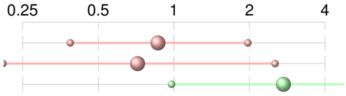

A	B	2.5	50	97.5	Sequence
1	3	0.39	0.87	2	LLEEHAQLQK
1	0	0.21	0.72	2.5	LGGLK
0	2	0.98	2.7	7.8	IEFSSMKK

5.582 18 1613 minus strand chaperonin containing T-complex polypeptide subunit zeta [Haliotis discus hannai]; 527 2089 minus strand chaperonin containing T-complex polypeptide subunit zeta [Haliotis discus hannai]

Protein Accession	CL23722.Contig7$_H$1 CL23722.Contig5$_H$1
Mean Expression Ratio	0.926
Median Expression Ratio	0.928
Credible Interval	(0.423, 2.01)
Associated Peptides	2
Associated Spectra	3
Coverage	NaN

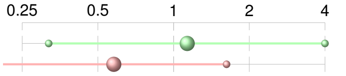

A	B	2.5	50	97.5	Sequence
1	0	0.32	1.1	4	AGMSSLKGD
2	0	0.2	0.58	1.6	EGIIALR

5.583 130 2145 minus strand Glucosamine–fructose-6-phosphate aminotransferase [isomerizing] 1 [Crassostrea gigas]; 130 2121 minus strand Glucosamine–fructose-6-phosphate aminotransferase [isomerizing] 1 [Crassostrea gigas]

Protein Accession	CL2589.Contig3$_H$1 CL2589.Contig2$_H$1
Mean Expression Ratio	1.08
Median Expression Ratio	1.08
Credible Interval	(0.514, 2.26)
Associated Peptides	3
Associated Spectra	3
Coverage	NaN

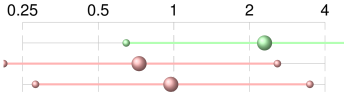

A	B	2.5	50	97.5	Sequence
1	0	0.65	2.3	8.1	GSEPQIIKQTGK
1	0	0.21	0.73	2.6	SLLVMGR
0	1	0.28	0.97	3.5	VANLEAQIK

5.584 30 536 putative mitochondrial ATP synthase [Haliotis discus discus]; 27 620 putative mitochondrial ATP synthase [Haliotis discus discus]

Protein Accession	CL13014.Contig4$_{H}$1 CL13014.Contig1$_{H}$1
Mean Expression Ratio	1.08
Median Expression Ratio	1.08
Credible Interval	(0.628, 1.85)
Associated Peptides	8
Associated Spectra	24
Coverage	NaN

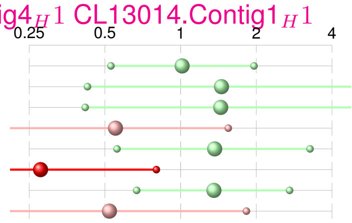

A	B	2.5	50	97.5	Sequence
2	5	0.53	1	2	SAVDWVAFK
0	1	0.43	1.5	5	SDSFVNKVHQYPAELPK
0	1	0.42	1.4	5	SNALKDIAAEEK
0	2	0.2	0.55	1.6	VHQYPAELPK
0	3	0.56	1.4	3.3	IDFSAYK
1	1	0.097	0.28	0.8	VPYPEDK
3	4	0.67	1.4	2.7	LANPALVDQFQK
0	1	0.15	0.52	1.8	VHQYPAEL

5.585 8 937 minus strand galectin [Crassostrea virginica]

Protein Accession	CL7245.Contig6$_{H}$1
Mean Expression Ratio	1.07
Median Expression Ratio	1.08
Credible Interval	(0.538, 2.18)
Associated Peptides	3
Associated Spectra	13
Coverage	NaN

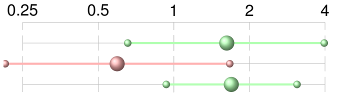

A	B	2.5	50	97.5	Sequence
1	2	0.66	1.6	4	LGGEWGPEQR
1	1	0.21	0.59	1.7	QGDNIGLHFNPR
3	5	0.93	1.7	3.1	QDQGDVVLNAK

5.586 87 485 Sex-regulated protein janus-A [Crassostrea gigas]

Protein Accession	CL13131.Contig3$_{H}$1
Mean Expression Ratio	1.08
Median Expression Ratio	1.08
Credible Interval	(0.49, 2.36)
Associated Peptides	2
Associated Spectra	2
Coverage	NaN

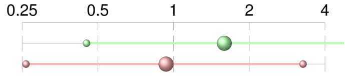

A	B	2.5	50	97.5	Sequence
0	1	0.45	1.6	5.8	ADHSITQTLLLR
0	1	0.26	0.93	3.3	VVGEIESK

5.587 7 231 minus strand AGAP006879-PA [Anopheles gambiae str. PEST] gi

Protein Accession	Unigene16066$_{H}$1
Mean Expression Ratio	1.08
Median Expression Ratio	1.08
Credible Interval	(0.471, 2.49)
Associated Peptides	1
Associated Spectra	1
Coverage	NaN

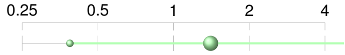

A	B	2.5	50	97.5	Sequence
0	1	0.39	1.4	5.2	NVSPLIR

5.588 104 2638 Eukaryotic translation initiation factor 3 subunit C [Crassostrea gigas]

Protein Accession	CL904.Contig3$_{H}1$
Mean Expression Ratio	1.08
Median Expression Ratio	1.07
Credible Interval	(0.492, 2.42)
Associated Peptides	1
Associated Spectra	3
Coverage	NaN

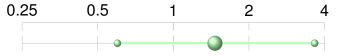

A	B	2.5	50	97.5	Sequence
**2**	**1**	**0.6**	**1.5**	**3.7**	**GAPLIVEKPK**

5.589 5 841 minus strand Spectrin alpha chain [Crassostrea gigas]

Protein Accession	CL44897.Contig1$_{H}1$
Mean Expression Ratio	1.07
Median Expression Ratio	1.07
Credible Interval	(0.492, 2.3)
Associated Peptides	2
Associated Spectra	3
Coverage	NaN

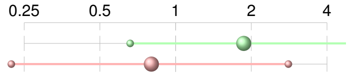

A	B	2.5	50	97.5	Sequence
**1**	**1**	**0.66**	**1.9**	**5.3**	**HQNFEAEIEANR**
**0**	**1**	**0.22**	**0.8**	**2.8**	**HALLEADVAAHQDR**

5.590 2 292 minus strand H2 [Haliotis tuberculata]

Protein Accession	Unigene10170$_{H}1$
Mean Expression Ratio	0.932
Median Expression Ratio	0.931
Credible Interval	(0.437, 1.96)
Associated Peptides	2
Associated Spectra	5
Coverage	NaN

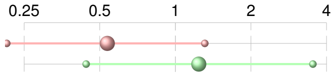

A	B	2.5	50	97.5	Sequence
**1**	**2**	**0.21**	**0.53**	**1.3**	**NLESLSEGEVESLK**
**0**	**2**	**0.44**	**1.2**	**3.5**	**RNLESLSEGEVESLK**

5.591 585 944 minus strand Protein disulfide-isomerase A6 [Crassostrea gigas]

Protein Accession	CL39540.Contig7$_{H}1$
Mean Expression Ratio	1.07
Median Expression Ratio	1.07
Credible Interval	(0.551, 2.08)
Associated Peptides	4
Associated Spectra	10
Coverage	NaN

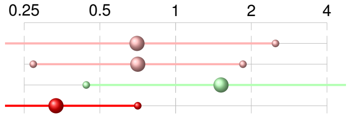

A	B	2.5	50	97.5	Sequence
**0**	**1**	**0.2**	**0.7**	**2.5**	**AGNPEDVVEVTDSNFQK**
**1**	**2**	**0.27**	**0.71**	**1.8**	**TASDIINWATEK**
**0**	**1**	**0.44**	**1.5**	**5.3**	**NLAPHWAQAATELKGK**
**0**	**5**	**0.16**	**0.34**	**0.71**	**NLAPHWAQAATELK**

5.592 89 838 Putative phosphoglycerate mutase [Crassostrea gigas]; 89 838 Putative phosphoglycerate mutase [Crassostrea gigas]; 89 835 Putative phosphoglycerate mutase [Crassostrea gigas]; 89 838 Putative phosphoglycerate mutase [Crassostrea gigas]

Protein Accession	CL6793.Contig6$_{H}1$ CL6793.Contig5$_{H}1$ CL6793.Contig4$_{H}1$ CL6793.Contig3$_{H}1$
Mean Expression Ratio	1.07
Median Expression Ratio	1.07
Credible Interval	(0.536, 2.14)
Associated Peptides	3
Associated Spectra	9
Coverage	NaN

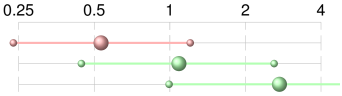

A	B	2.5	50	97.5	Sequence
2	2	0.24	0.53	1.2	EAQYAGELIK
2	1	0.45	1.1	2.6	VLIAAHGNSLR
0	2	0.99	2.7	7.8	HYGGLQGLNKAETAAK

5.593 1709 2026 Phosphoenolpyruvate carboxykinase [GTP] [Crassostrea gigas]

Protein Accession	CL25948.Contig2$_{H}1$
Mean Expression Ratio	1.07
Median Expression Ratio	1.07
Credible Interval	(0.491, 2.36)
Associated Peptides	1
Associated Spectra	8
Coverage	NaN

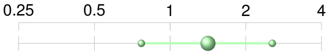

A	B	2.5	50	97.5	Sequence
5	3	0.77	1.4	2.6	GVPLVYEAK

5.594 1 297 beta-thymosin domain repeat protein CSP24KDa_v1 [Hermissenda crassicornis] gi

Protein Accession	Unigene8923$_{H}1$
Mean Expression Ratio	1.07
Median Expression Ratio	1.07
Credible Interval	(0.474, 2.43)
Associated Peptides	1
Associated Spectra	2
Coverage	NaN

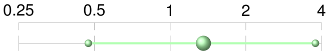

A	B	2.5	50	97.5	Sequence
0	2	0.47	1.4	3.8	ELLHHVVPEEK

5.595 65 490 PREDICTED: cofilin-2 [Otolemur garnettii]

Protein Accession	CL15471.Contig3$_{H}1$
Mean Expression Ratio	1.08
Median Expression Ratio	1.07
Credible Interval	(0.542, 2.12)
Associated Peptides	4
Associated Spectra	12
Coverage	NaN

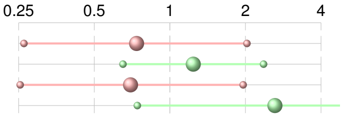

A	B	2.5	50	97.5	Sequence
1	1	0.26	0.74	2	MLYSSSSSAVK
2	5	0.65	1.2	2.4	IVMENEFAK
0	2	0.25	0.7	2	FVIWDLDVDTK
0	1	0.74	2.6	9.2	ALVFK

5.596 " 153 536 GST omega, partial [Reishia clavigera]"

Protein Accession	CL2384.Contig3$_H$1
Mean Expression Ratio	1.07
Median Expression Ratio	1.07
Credible Interval	(0.494, 2.35)
Associated Peptides	2
Associated Spectra	3
Coverage	NaN

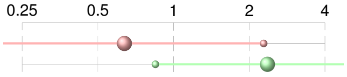

A	B	2.5	50	97.5	Sequence
0	1	0.18	0.64	2.3	EKPEWFLEK
0	2	0.85	2.4	6.6	ALLPSDPYRR

5.597 128 559 minus strand predicted protein [Nematostella vectensis] gi

Protein Accession	CL19343.Contig1$_H$1
Mean Expression Ratio	0.932
Median Expression Ratio	0.933
Credible Interval	(0.42, 2.06)
Associated Peptides	1
Associated Spectra	6
Coverage	NaN

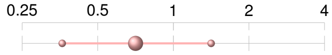

A	B	2.5	50	97.5	Sequence
1	5	0.36	0.71	1.4	TVILGDLTGSK

5.598 13 1218 minus strand PREDICTED: eukaryotic initiation factor 4A-III-like [Bombus terrestris] gi

Protein Accession	CL12065.Contig7$_H$1
Mean Expression Ratio	1.07
Median Expression Ratio	1.07
Credible Interval	(0.464, 2.46)
Associated Peptides	1
Associated Spectra	1
Coverage	NaN

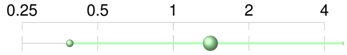

A	B	2.5	50	97.5	Sequence
0	1	0.39	1.4	4.9	GIYAYGFEKPSAIQQR

5.599 60 1190 hypothetical protein BRAFLDRAFT_109688 [Branchiostoma floridae] gi; 51 1184 hypothetical protein BRAFLDRAFT_109688 [Branchiostoma floridae] gi

Protein Accession	CL25572.Contig3$_H$1 CL25572.Contig1$_H$1
Mean Expression Ratio	1.07
Median Expression Ratio	1.07
Credible Interval	(0.495, 2.33)
Associated Peptides	2
Associated Spectra	2
Coverage	NaN

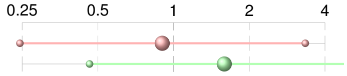

A	B	2.5	50	97.5	Sequence
0	1	0.24	0.9	3.3	AGHFQDEIIPVVIQSR
0	1	0.46	1.6	5.6	SVVLGAMAVQ

5.600 214 1878 PREDICTED: lysyl-tRNA synthetase-like [Oreochromis niloticus]

Protein Accession	CL25588.Contig2$_H1$
Mean Expression Ratio	0.929
Median Expression Ratio	0.933
Credible Interval	(0.435, 1.96)
Associated Peptides	3
Associated Spectra	3
Coverage	NaN

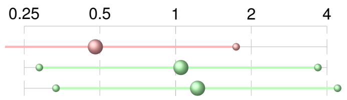

A	B	2.5	50	97.5	Sequence
0	1	0.13	0.48	1.7	ISLISDLEK
0	1	0.29	1.1	3.7	IAPELYLK
0	1	0.34	1.2	4.4	IQIMANAK

5.601 " 1 321 Dynein heavy chain, cytoplasmic [Crassostrea gigas]"

Protein Accession	Unigene35195$_H1$
Mean Expression Ratio	0.933
Median Expression Ratio	0.934
Credible Interval	(0.402, 2.13)
Associated Peptides	1
Associated Spectra	1
Coverage	NaN

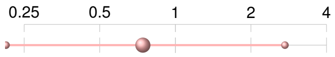

A	B	2.5	50	97.5	Sequence
1	0	0.21	0.74	2.7	EILAVIQK

5.602 98 865 proteasome (prosome macropain) subunit alpha type 1 [Xenopus (Silurana) tropicalis]; 98 865 proteasome (prosome macropain) subunit alpha type 1 [Xenopus (Silurana) tropicalis]

Protein Accession	CL5647.Contig8$_H1$ CL5647.Contig7$_H1$
Mean Expression Ratio	1.08
Median Expression Ratio	1.07
Credible Interval	(0.489, 2.41)
Associated Peptides	1
Associated Spectra	4
Coverage	NaN

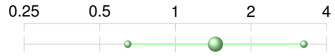

A	B	2.5	50	97.5	Sequence
0	4	0.65	1.4	3.2	LVSVVGNK

5.603 406 1392 minus strand hypothetical protein CGI_10007007 [Crassostrea gigas]

Protein Accession	CL18045.Contig1$_H1$
Mean Expression Ratio	1.07
Median Expression Ratio	1.07
Credible Interval	(0.488, 2.35)
Associated Peptides	2
Associated Spectra	2
Coverage	NaN

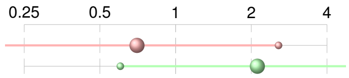

A	B	2.5	50	97.5	Sequence
1	0	0.19	0.7	2.6	TLGVDLKK
0	1	0.6	2.1	7.6	WQRPDKPVGFYDLK

5.604 10 366 minus strand hypothetical protein TRIADDRAFT_25239 [Trichoplax adhaerens] gi

Protein Accession	CL32844.Contig2$_{H}1$
Mean Expression Ratio	0.937
Median Expression Ratio	0.934
Credible Interval	(0.406, 2.16)
Associated Peptides	1
Associated Spectra	1
Coverage	NaN

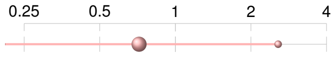

A	B	2.5	50	97.5	Sequence
1	0	0.2	0.72	2.6	TAVEDVDLK

5.605 90 344 PREDICTED: diazepam binding inhibitor-like [Saccoglossus kowalevskii]

Protein Accession	CL28509.Contig1$_{H}1$
Mean Expression Ratio	0.936
Median Expression Ratio	0.935
Credible Interval	(0.498, 1.76)
Associated Peptides	6
Associated Spectra	10
Coverage	NaN

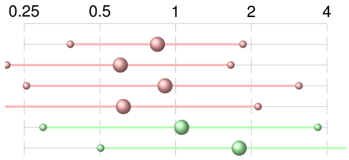

A	B	2.5	50	97.5	Sequence
1	3	0.38	0.84	1.9	AEYVLLVK
0	2	0.21	0.6	1.7	ADEAFNQAAEDVK
0	1	0.25	0.91	3.1	WNAWNTLK
0	1	0.18	0.62	2.1	VYGLFK
0	1	0.3	1.1	3.7	KPTDDEMLK
0	1	0.5	1.8	6.3	ADEAFNQAAEDVKK

5.606 73 525 minus strand Actin-related protein 2/3 complex subunit 5-like protein [Salmo salar] gi; 73 525 Actin-related protein 2/3 complex subunit 5-like protein [Salmo salar] gi; 73 525 minus strand Actin-related protein 2/3 complex subunit 5-like protein [Salmo salar] gi

Protein Accession	CL14637.Contig6$_{H}1$ CL14637.Contig5$_{H}1$ CL14637.Contig4$_{H}1$
Mean Expression Ratio	0.937
Median Expression Ratio	0.935
Credible Interval	(0.444, 1.99)
Associated Peptides	3
Associated Spectra	3
Coverage	NaN

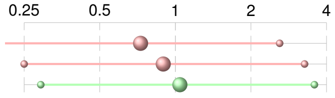

A	B	2.5	50	97.5	Sequence
0	1	0.2	0.73	2.6	LVINSTPFGSK
0	1	0.25	0.89	3.3	EVDILMK
0	1	0.29	1	3.6	VLLSFK

5.607 266 1123 minus strand mitochondrial malate dehydrogenase precursor [Nucella lapillus]

Protein Accession	CL10426.Contig11$_{H}1$
Mean Expression Ratio	1.07
Median Expression Ratio	1.07
Credible Interval	(0.498, 2.32)
Associated Peptides	2
Associated Spectra	4
Coverage	NaN

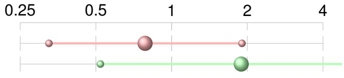

A	B	2.5	50	97.5	Sequence
1	2	0.33	0.79	1.9	SLIEALNGMEGR
0	1	0.52	1.9	6.7	VRSATLSMAYAAAEFSK

5.608 142 261 Core histone macro-H2A.2 OS=Mus musculus GN=H2afy2 PE=1 SV=3

Protein Accession	Unigene14198$_{H}1$
Mean Expression Ratio	1.07
Median Expression Ratio	1.07
Credible Interval	(0.455, 2.43)
Associated Peptides	1
Associated Spectra	1
Coverage	NaN

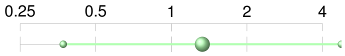

A	B	2.5	50	97.5	Sequence
0	1	0.37	1.3	4.8	AGVLFPVAR

5.609 169 1200 PREDICTED: nidogen-2-like isoform 1 [Strongylocentrotus purpuratus]

Protein Accession	CL478.Contig2$_{H}1$
Mean Expression Ratio	1.06
Median Expression Ratio	1.07
Credible Interval	(0.505, 2.22)
Associated Peptides	3
Associated Spectra	3
Coverage	NaN

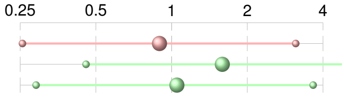

A	B	2.5	50	97.5	Sequence
0	1	0.26	0.89	3.1	AIVLDINGGK
0	1	0.46	1.6	5.6	LVYAR
0	1	0.29	1	3.6	TLFNTDMVNPR

5.610 123 860 PREDICTED: proteasome subunit alpha type-6-like [Cavia porcellus]; 1164 1901 PREDICTED: proteasome subunit alpha type-6-like [Cavia porcellus]

Protein Accession	Unigene21398$_H$1 Unigene13394$_H$1
Mean Expression Ratio	1.07
Median Expression Ratio	1.07
Credible Interval	(0.504, 2.29)
Associated Peptides	2
Associated Spectra	4
Coverage	NaN

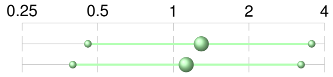

A	B	2.5	50	97.5	Sequence
**1**	**1**	**0.46**	**1.3**	**3.6**	**QLEASGFLEK**
**0**	**2**	**0.4**	**1.1**	**3.2**	**VLTEQEIDVHLTSIAEKD**

5.611 " 133 1077 PREDICTED: electron transfer flavoprotein, alpha polypeptide-like isoform 2 [Saccoglossus kowalevskii]"

Protein Accession	CL6589.Contig3$_H$1
Mean Expression Ratio	0.936
Median Expression Ratio	0.937
Credible Interval	(0.446, 1.97)
Associated Peptides	3
Associated Spectra	4
Coverage	NaN

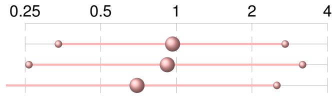

A	B	2.5	50	97.5	Sequence
**0**	**2**	**0.34**	**0.97**	**2.7**	**AAVDAGFVSNDLQIGQTGK**
**0**	**1**	**0.26**	**0.92**	**3.2**	**SDRPELASANIVIAGGR**
**0**	**1**	**0.2**	**0.7**	**2.5**	**GTAFEAAK**

5.612 423 695 thioredoxin peroxidase 2 [Haliotis discus discus]

Protein Accession	CL3160.Contig13$_H$1
Mean Expression Ratio	1.07
Median Expression Ratio	1.07
Credible Interval	(0.469, 2.45)
Associated Peptides	1
Associated Spectra	1
Coverage	NaN

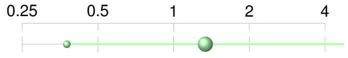

A	B	2.5	50	97.5	Sequence
**0**	**1**	**0.38**	**1.3**	**4.9**	**KIGGLGNMNIPLLEDKTHEIAR**

5.613 2 256 Polyubiquitin (Fragment) OS=Xenopus laevis PE=1 SV=2

Protein Accession	CL47507.Contig2$_H$1
Mean Expression Ratio	1.06
Median Expression Ratio	1.07
Credible Interval	(0.547, 2.02)
Associated Peptides	3
Associated Spectra	12
Coverage	NaN

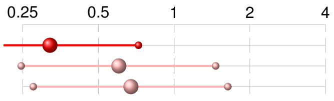

A	B	2.5	50	97.5	Sequence
**2**	**3**	**0.14**	**0.32**	**0.72**	**TITLEVEPSDTIENVK**
**2**	**2**	**0.25**	**0.6**	**1.5**	**TLSDYNIQK**
**0**	**3**	**0.28**	**0.68**	**1.6**	**ESTLHLVLR**

5.614 112 930 calcineurin A [Haliotis discus discus]

Protein Accession	CL3137.Contig1$_{H}$1
Mean Expression Ratio	0.938
Median Expression Ratio	0.939
Credible Interval	(0.414, 2.14)
Associated Peptides	1
Associated Spectra	1
Coverage	NaN

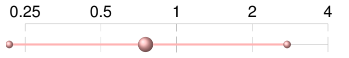

A	B	2.5	50	97.5	Sequence
1	0	0.22	0.75	2.8	QHFILEGR

5.615 62 394 minus strand PREDICTED: NADH dehydrogenase [ubiquinone] 1 alpha subcomplex subunit 5 [Saimiri boliviensis boliviensis]; 62 394 minus strand PREDICTED: NADH dehydrogenase [ubiquinone] 1 alpha subcomplex subunit 5 [Saimiri boliviensis boliviensis]

Protein Accession	CL6313.Contig4$_{H}$1 CL6313.Contig3$_{H}$1
Mean Expression Ratio	1.07
Median Expression Ratio	1.07
Credible Interval	(0.466, 2.42)
Associated Peptides	1
Associated Spectra	1
Coverage	NaN

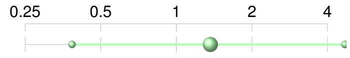

A	B	2.5	50	97.5	Sequence
0	1	0.38	1.4	4.7	LAIVNSEPDPGRLEMK

5.616 3 224 PREDICTED: 40S ribosomal protein S14-like [Sarcophilus harrisii]

Protein Accession	Unigene48834$_{H}$1
Mean Expression Ratio	0.937
Median Expression Ratio	0.939
Credible Interval	(0.445, 1.95)
Associated Peptides	3
Associated Spectra	3
Coverage	NaN

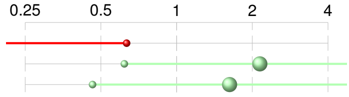

A	B	2.5	50	97.5	Sequence
0	1	0.049	0.18	0.63	IEDVTPIPSDSTR
0	1	0.62	2.1	7.5	IGRIEDVTPIPSDSTR
0	1	0.46	1.6	5.9	IGRIEDVTPIPSDSTRR

5.617 212 1405 minus strand unnamed protein product [Tetraodon nigroviridis]

Protein Accession	CL37546.Contig3$_{H}$1
Mean Expression Ratio	1.07
Median Expression Ratio	1.06
Credible Interval	(0.516, 2.23)
Associated Peptides	2
Associated Spectra	7
Coverage	NaN

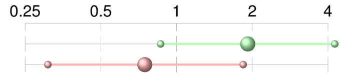

A	B	2.5	50	97.5	Sequence
2	2	0.87	1.9	4.3	GLVVPVIR
1	2	0.31	0.75	1.8	TIAELGEK

5.618 238 681 minus strand Globin OS=Cerithidea rhizophorarum PE=1 SV=1

Protein Accession	CL8458.Contig2$_{H}1$
Mean Expression Ratio	1.06
Median Expression Ratio	1.06
Credible Interval	(0.543, 2.08)
Associated Peptides	4
Associated Spectra	66
Coverage	NaN

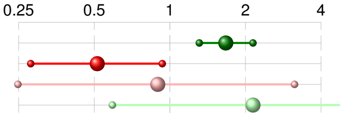

A	B	2.5	50	97.5	Sequence
13	43	1.3	1.7	2.1	AAITSTWSK
0	8	0.28	0.51	0.93	AYFPFLPGASEGDIK
0	1	0.25	0.89	3.1	LASDKATMENNGAK
0	1	0.59	2.1	7.6	AITSTWSK

5.619 20 355 minus strand PREDICTED: uroporphyrinogen decarboxylase-like [Monodelphis domestica]

Protein Accession	CL11915.Contig5$_{H}1$
Mean Expression Ratio	1.06
Median Expression Ratio	1.06
Credible Interval	(0.467, 2.44)
Associated Peptides	1
Associated Spectra	1
Coverage	NaN

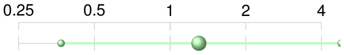

A	B	2.5	50	97.5	Sequence
0	1	0.37	1.3	4.8	SKPFPPLQNDLILR

5.620 43 222 sodium-calcium exchanger [Doryteuthis pealeii]

Protein Accession	Unigene7579$_{H}1$
Mean Expression Ratio	1.07
Median Expression Ratio	1.06
Credible Interval	(0.469, 2.41)
Associated Peptides	1
Associated Spectra	3
Coverage	NaN

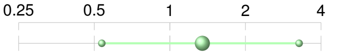

A	B	2.5	50	97.5	Sequence
1	2	0.54	1.3	3.3	TVGVGMVMR

5.621 21 1007 minus strand cathepsin L2 cysteine protease [Pinctada fucata]; 21 1007 minus strand cathepsin L2 cysteine protease [Pinctada fucata]

Protein Accession	CL7881.Contig4$_{H}1$ CL7881.Contig3$_{H}1$
Mean Expression Ratio	1.06
Median Expression Ratio	1.06
Credible Interval	(0.482, 2.31)
Associated Peptides	2
Associated Spectra	2
Coverage	NaN

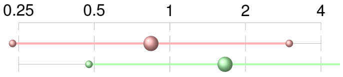

A	B	2.5	50	97.5	Sequence
1	0	0.24	0.84	3	NLDLIIR
1	0	0.48	1.7	5.9	DLPDSVDWR

5.622 460 1737 Tubulin beta-4B chain OS=Mus musculus GN=Tubb4b PE=1 SV=1

Protein Accession	Unigene34147$_{H1}$
Mean Expression Ratio	1.07
Median Expression Ratio	1.06
Credible Interval	(0.492, 2.39)
Associated Peptides	1
Associated Spectra	29
Coverage	NaN

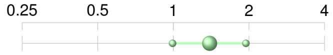

A	B	2.5	50	97.5	Sequence
**5**	**24**	**0.99**	**1.4**	**1.9**	**AVLVDLEPGTMDSVR**

5.623 116 337 minus strand Golgi-associated plant pathogenesis-related protein 1 [Crassostrea gigas]; 538 759 minus strand Golgi-associated plant pathogenesis-related protein 1 [Crassostrea gigas]; 116 337 minus strand Golgi-associated plant pathogenesis-related protein 1 [Crassostrea gigas]; 261 482 minus strand Golgi-associated plant pathogenesis-related protein 1 [Crassostrea gigas]; 116 337 minus strand Golgi-associated plant pathogenesis-related protein 1 [Crassostrea gigas]; 116 337 minus strand Golgi-associated plant pathogenesis-related protein 1 [Crassostrea gigas]

Protein Accession	CL15263.Contig9$_{H1}$ CL15263.Contig8$_{H1}$ CL15263.Contig7$_{H1}$ CL15263.Contig5$_{H1}$ CL1526
Mean Expression Ratio	0.941
Median Expression Ratio	0.94
Credible Interval	(0.431, 2.05)
Associated Peptides	2
Associated Spectra	2
Coverage	NaN

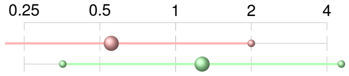

A	B	2.5	50	97.5	Sequence
**0**	**1**	**0.16**	**0.56**	**2**	**FIDEMLEAHNK**
**0**	**1**	**0.36**	**1.3**	**4.6**	**KEHQVQPLVINK**

5.624 219 428 Peptidyl-prolyl cis-trans isomerase OS=Hemicentrotus pulcherrimus PE=2 SV=1

Protein Accession	CL39270.Contig2$_{H1}$
Mean Expression Ratio	1.06
Median Expression Ratio	1.06
Credible Interval	(0.498, 2.25)
Associated Peptides	2
Associated Spectra	9
Coverage	NaN

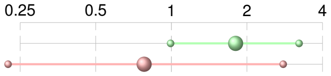

A	B	2.5	50	97.5	Sequence
**2**	**6**	**0.99**	**1.8**	**3.2**	**IIIANSGQV**
**1**	**0**	**0.22**	**0.78**	**2.8**	**IIIANSGQ**

5.625 1371 2189 minus strand galectin [Pinctada fucata]

Protein Accession	CL7245.Contig3$_H$1
Mean Expression Ratio	0.944
Median Expression Ratio	0.941
Credible Interval	(0.518, 1.71)
Associated Peptides	4
Associated Spectra	28
Coverage	NaN

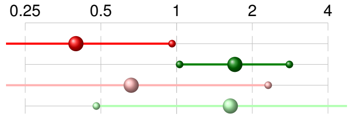

A	B	2.5	50	97.5	Sequence
1	2	0.16	0.4	0.96	EIPQGLEK
13	10	1	1.7	2.8	FNEGVTVR
0	1	0.19	0.66	2.3	LGGAWGGEER
0	1	0.48	1.6	5.7	RNEGQVVFNSK

5.626 150 1211 RecName: Full=Guanine nucleotide-binding protein G(i) subunit alpha; AltName: Full=Adenylate cyclase-inhibiting G alpha protein gi|9631|emb|CAA78807.1| GTP-binding protein alpha-i subunit [Lymnaea stagnalis]

Protein Accession	CL13885.Contig6$_H$1
Mean Expression Ratio	0.941
Median Expression Ratio	0.941
Credible Interval	(0.405, 2.16)
Associated Peptides	1
Associated Spectra	1
Coverage	NaN

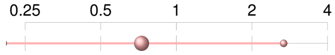

A	B	2.5	50	97.5	Sequence
1	0	0.21	0.73	2.7	IAIIR

5.627 1 321 Malectin [Crassostrea gigas]

Protein Accession	Unigene56360$_H$1
Mean Expression Ratio	1.06
Median Expression Ratio	1.06
Credible Interval	(0.468, 2.42)
Associated Peptides	1
Associated Spectra	1
Coverage	NaN

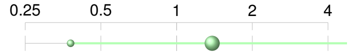

A	B	2.5	50	97.5	Sequence
0	1	0.38	1.4	5	GVAHDEIVPFSIK

5.628 17 703 Dihydropteridine reductase [Crassostrea gigas]

Protein Accession	CL125.Contig5$_H$1
Mean Expression Ratio	0.939
Median Expression Ratio	0.941
Credible Interval	(0.431, 2.03)
Associated Peptides	2
Associated Spectra	3
Coverage	NaN

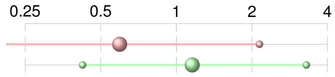

A	B	2.5	50	97.5	Sequence
0	1	0.17	0.59	2.1	VAEALGEEK
0	2	0.42	1.2	3.3	AAVHHLVQSLGAPK

5.629 113 367 hypothetical protein DAPPUDRAFT_329128 [Daphnia pulex]; 113 367 hypothetical protein DAPPUDRAFT_329128 [Daphnia pulex]; 113 367 hypothetical protein DAPPUDRAFT_329128 [Daphnia pulex]

Protein Accession	CL9011.Contig3$_H$1 CL9011.Contig2$_H$1 CL9011.Contig1$_H$1
Mean Expression Ratio	1.06
Median Expression Ratio	1.06
Credible Interval	(0.515, 2.18)
Associated Peptides	3
Associated Spectra	5
Coverage	NaN

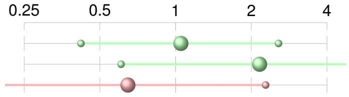

A	B	2.5	50	97.5	Sequence
2	1	0.42	1	2.6	KPMVSGAIIK
0	1	0.61	2.2	7.7	ILAIEDYIR
0	1	0.19	0.64	2.3	LHPEDFPEEEPK

5.630 21 254 predicted protein [Nematostella vectensis] gi

Protein Accession	CL43392.Contig2$_H$1
Mean Expression Ratio	0.943
Median Expression Ratio	0.942
Credible Interval	(0.427, 2.09)
Associated Peptides	1
Associated Spectra	6
Coverage	NaN

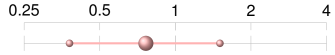

A	B	2.5	50	97.5	Sequence
4	2	0.38	0.76	1.5	AADAVVAEIK

5.631 178 480 minus strand PREDICTED: lipoxygenase homology domain-containing protein 1-like [Danio rerio]

Protein Accession	CL41319.Contig2$_H$1
Mean Expression Ratio	0.942
Median Expression Ratio	0.943
Credible Interval	(0.432, 2.06)
Associated Peptides	2
Associated Spectra	2
Coverage	NaN

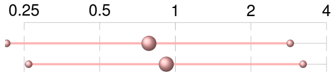

A	B	2.5	50	97.5	Sequence
0	1	0.21	0.78	2.9	ILLDVVGENNFR
0	1	0.26	0.92	3.2	VSLKPEVFPLVK

5.632 145 972 LIM and SH3 domain protein F42H10.3 OS=Caenorhabditis elegans GN=F42H10.3 PE=2 SV=3

Protein Accession	CL49642.Contig8$_H$1
Mean Expression Ratio	0.937
Median Expression Ratio	0.943
Credible Interval	(0.406, 2.12)
Associated Peptides	1
Associated Spectra	1
Coverage	NaN

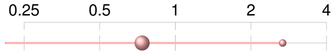

A	B	2.5	50	97.5	Sequence
0	1	0.2	0.74	2.7	TVYPTEELK

5.633 85 546 minus strand RecName: Full=Eukaryotic translation initiation factor 5A; Short=eIF-5A gi|51702279|sp|P62925.1|IF5A_SPOFR RecName: Full=Eukaryotic translation initiation factor 5A; Short=eIF-5A gi|6469515|gb|AAF13315.1|AF109730_1 translation initiation factor 5A [Spodoptera exigua] gi

Protein Accession	CL40725.Contig2$_{H}1$
Mean Expression Ratio	1.06
Median Expression Ratio	1.06
Credible Interval	(0.583, 1.89)
Associated Peptides	7
Associated Spectra	21
Coverage	NaN

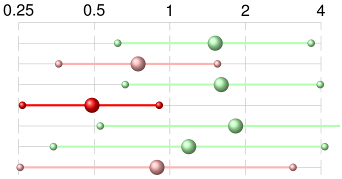

A	B	2.5	50	97.5	Sequence
1	2	0.62	1.5	3.7	AMGEECVVAVK
3	2	0.36	0.74	1.6	IVEMSTSK
1	2	0.67	1.6	4	KNEFVVIK
3	4	0.26	0.49	0.91	EDSLAAEIQK
0	1	0.53	1.8	6.3	EDSLAAEIQKK
0	1	0.34	1.2	4.1	KYEDICPSTHNMEVPVVTR
0	1	0.25	0.89	3.1	NEFVVIK

5.634 194 838 glutathione S-transferase class mu [Cyphoma gibbosum]

Protein Accession	CL8626.Contig6$_{H}1$
Mean Expression Ratio	1.06
Median Expression Ratio	1.06
Credible Interval	(0.473, 2.33)
Associated Peptides	2
Associated Spectra	2
Coverage	NaN

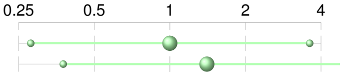

A	B	2.5	50	97.5	Sequence
0	1	0.28	1	3.6	KHDLLGQTEK
0	1	0.38	1.4	5	PTLGYWK

5.635 91 1326 minus strand Adipocyte plasma membrane-associated protein [Crassostrea gigas]; 107 1273 minus strand Adipocyte plasma membrane-associated protein [Crassostrea gigas]; 224 1267 Adipocyte plasma membrane-associated protein [Crassostrea gigas]; 209 1267 minus strand Adipocyte plasma membrane-associated protein [Crassostrea gigas]; 195 1253 minus strand Adipocyte plasma membrane-associated protein [Crassostrea gigas]

Protein Accession	CL11239.Contig2$_{H}1$ CL11239.Contig1$_{H}1$ CL11239.Contig12$_{H}1$ CL11239.Contig11$_{H}1$ CL11
Mean Expression Ratio	0.944
Median Expression Ratio	0.944
Credible Interval	(0.422, 2.15)
Associated Peptides	1
Associated Spectra	2
Coverage	NaN

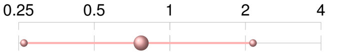

A	B	2.5	50	97.5	Sequence
0	2	0.26	0.77	2.1	YGLIAELDKEGK

5.636 260 892 minus strand calponin [Haliotis diversicolor]

Protein Accession	CL48702.Contig2$_{H}1$
Mean Expression Ratio	0.95
Median Expression Ratio	0.944
Credible Interval	(0.468, 1.93)
Associated Peptides	2
Associated Spectra	7
Coverage	NaN

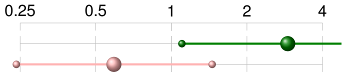

A	B	2.5	50	97.5	Sequence
**2**	**1**	**1.1**	**2.9**	**7.6**	**NFSEDKLR**
**2**	**2**	**0.24**	**0.59**	**1.5**	**VESSFDPEDAKK**

5.637 642 1415 minus strand cytochrome c oxidase subunit II [Oncomelania hupensis hupensis]

Protein Accession	CL6764.Contig3$_{H}1$
Mean Expression Ratio	1.06
Median Expression Ratio	1.06
Credible Interval	(0.48, 2.3)
Associated Peptides	1
Associated Spectra	11
Coverage	NaN

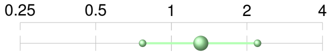

A	B	2.5	50	97.5	Sequence
**3**	**8**	**0.77**	**1.3**	**2.2**	**IVLPTHTDIR**

5.638 503 1591 minus strand Septin-2 [Crassostrea gigas]; 648 1736 minus strand Septin-2 [Crassostrea gigas]

Protein Accession	CL9569.Contig7$_{H}1$ CL9569.Contig6$_{H}1$
Mean Expression Ratio	1.06
Median Expression Ratio	1.06
Credible Interval	(0.453, 2.45)
Associated Peptides	1
Associated Spectra	1
Coverage	NaN

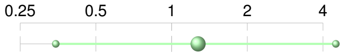

A	B	2.5	50	97.5	Sequence
**0**	**1**	**0.35**	**1.3**	**4.5**	**VNIVPVLAK**

5.639 27 377 minus strand ribosomal protein LP2 [Papilio xuthus] gi; 27 377 minus strand ribosomal protein LP2 [Papilio xuthus] gi; 27 377 minus strand ribosomal protein LP2 [Papilio xuthus] gi; 1538 1891 minus strand ribosomal protein LP2 [Papilio xuthus] gi

Protein Accession	Unigene53017$_{H}1$ Unigene53016$_{H}1$ Unigene53015$_{H}1$ CL49012.Contig9$_{H}1$
Mean Expression Ratio	1.06
Median Expression Ratio	1.06
Credible Interval	(0.598, 1.86)
Associated Peptides	7
Associated Spectra	29
Coverage	NaN

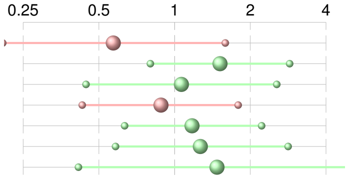

A	B	2.5	50	97.5	Sequence
1	1	0.21	0.57	1.6	ILSSVGIEAEADK
1	6	0.8	1.5	2.9	SGKEPTAADLEK
1	2	0.44	1.1	2.6	SIDQLIAEGSR
1	4	0.43	0.88	1.8	VVSQLSGK
2	5	0.63	1.2	2.2	KVVSQLSGK
0	4	0.58	1.3	2.8	ILSSVGIEAEADKVK
0	1	0.42	1.5	5.2	SVGIEAEADKVK

5.640 131 544 hypothetical protein CGI_10001749 [Crassostrea gigas]

Protein Accession	CL36022.Contig1$_{H}1$
Mean Expression Ratio	0.946
Median Expression Ratio	0.944
Credible Interval	(0.413, 2.16)
Associated Peptides	1
Associated Spectra	1
Coverage	NaN

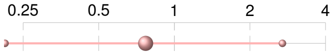

A	B	2.5	50	97.5	Sequence
0	1	0.21	0.77	2.7	LIVADGTNHR

5.641 37 885 glyceraldehyde 3-phosphate dehydrogenase [Dugesia japonica]

Protein Accession	CL47424.Contig2$_{H}1$
Mean Expression Ratio	1.06
Median Expression Ratio	1.06
Credible Interval	(0.501, 2.25)
Associated Peptides	2
Associated Spectra	18
Coverage	NaN

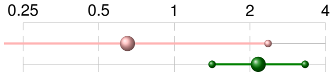

A	B	2.5	50	97.5	Sequence
1	0	0.18	0.65	2.4	VGINGFGR
5	12	1.4	2.2	3.3	GAAQNIIPASTGAAK

5.642 54 1406 minus strand Protein henna [Crassostrea gigas]; 54 1406 minus strand Protein henna [Crassostrea gigas]

Protein Accession	CL24330.Contig4$_{H}1$ CL24330.Contig1$_{H}1$
Mean Expression Ratio	1.05
Median Expression Ratio	1.06
Credible Interval	(0.498, 2.17)
Associated Peptides	3
Associated Spectra	4
Coverage	NaN

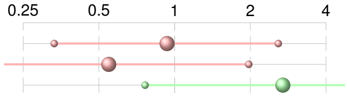

A	B	2.5	50	97.5	Sequence
0	2	0.33	0.93	2.6	GDLSLLEDALK
0	1	0.15	0.55	2	TLHLQILSR
0	1	0.76	2.7	9.8	VSAYAATIPRPFSV

5.643 185 481 minus strand Zgc:56530 [Danio rerio]

Protein Accession	CL27088.Contig2$_H$1
Mean Expression Ratio	0.945
Median Expression Ratio	0.947
Credible Interval	(0.465, 1.88)
Associated Peptides	4
Associated Spectra	6
Coverage	NaN

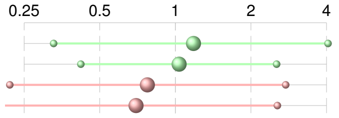

A	B	2.5	50	97.5	Sequence
0	1	0.33	1.2	4.1	TQVVAGTNYFIK
0	3	0.42	1	2.5	TFTDFK
0	1	0.22	0.77	2.8	TADDELEYF
0	1	0.2	0.7	2.5	ALSYK

5.644 123 860 PREDICTED: 3-hydroxybutyrate dehydrogenase type 2-like isoform 1 [Sus scrofa] gi; 32 901 3-hydroxybutyrate dehydrogenase type 2 [Rattus norvegicus]

Protein Accession	CL33059.Contig4$_H$1 CL33059.Contig2$_H$1
Mean Expression Ratio	0.94
Median Expression Ratio	0.947
Credible Interval	(0.414, 2.13)
Associated Peptides	1
Associated Spectra	1
Coverage	NaN

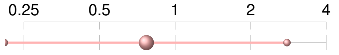

A	B	2.5	50	97.5	Sequence
1	0	0.21	0.77	2.8	ILDVLDTEK

5.645 75 803 hypothetical protein DAPPUDRAFT_231350 [Daphnia pulex]

Protein Accession	CL10537.Contig4$_H$1
Mean Expression Ratio	0.947
Median Expression Ratio	0.948
Credible Interval	(0.486, 1.83)
Associated Peptides	5
Associated Spectra	7
Coverage	NaN

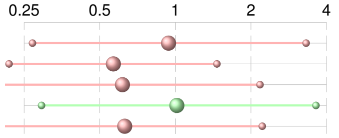

A	B	2.5	50	97.5	Sequence
0	1	0.27	0.94	3.3	NFPHPVMPETSGK
1	2	0.22	0.57	1.5	SITPQQEAESIK
0	1	0.17	0.62	2.2	ENVALQLEASYR
0	1	0.29	1	3.6	ATTLLSK
0	1	0.18	0.63	2.2	VCIAQLK

5.646 484 1104 Calponin-2 [Crassostrea gigas]; 309 938 Calponin-2 [Crassostrea gigas]

Protein Accession	CL30203.Contig3$_H$1 CL21394.Contig2$_H$1
Mean Expression Ratio	1.06
Median Expression Ratio	1.05
Credible Interval	(0.589, 1.92)
Associated Peptides	6
Associated Spectra	21
Coverage	NaN

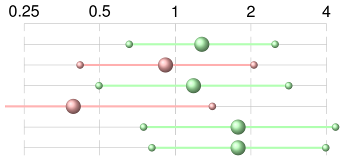

A	B	2.5	50	97.5	Sequence
2	4	0.66	1.3	2.5	MNTLAAPFK
1	3	0.42	0.91	2.1	QMENIEVFLK
1	2	0.5	1.2	2.8	QLTGEDIGSGAVQVEK
1	0	0.11	0.39	1.4	NGQILCR
0	3	0.75	1.8	4.4	YDTGAEYEVR
0	4	0.8	1.8	4	AGQGIIGLQAGTNK

5.647 211 1413 Alpha-N-acetylgalactosaminidase [Crassostrea gigas]

Protein Accession	CL5989.Contig1$_H$1
Mean Expression Ratio	1.05
Median Expression Ratio	1.05
Credible Interval	(0.484, 2.26)
Associated Peptides	2
Associated Spectra	3
Coverage	NaN

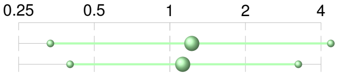

A	B	2.5	50	97.5	Sequence
**0**	**1**	**0.33**	**1.2**	**4.4**	**ALADYMHDR**
**0**	**2**	**0.4**	**1.1**	**3.2**	**ALLLNR**

5.648 " 106 2724 PREDICTED: 2-oxoglutarate dehydrogenase, mitochondrial isoform 1 [Sarcophilus harrisii]; 492 3473 PREDICTED: 2-oxoglutarate dehydrogenase, mitochondrial isoform 1 [Sarcophilus harrisii]; 492 3458 PREDICTED: 2-oxoglutarate dehydrogenase, mitochondrial isoform 1 [Sarcophilus harrisii]"

Protein Accession	CL2200.Contig3$_H$1 CL2200.Contig1$_H$1 CL2200.Contig16$_H$1
Mean Expression Ratio	1.06
Median Expression Ratio	1.05
Credible Interval	(0.462, 2.44)
Associated Peptides	1
Associated Spectra	1
Coverage	NaN

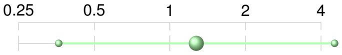

A	B	2.5	50	97.5	Sequence
**0**	**1**	**0.36**	**1.3**	**4.5**	**LAVVANPSHLEAVDPVVQGK**

5.649 275 769 minus strand hypothetical protein CGI_10025135 [Crassostrea gigas]; 216 683 hypothetical protein CGI_10025135 [Crassostrea gigas]

Protein Accession	CL14194.Contig2$_H$1 CL14194.Contig1$_H$1
Mean Expression Ratio	1.05
Median Expression Ratio	1.05
Credible Interval	(0.463, 2.42)
Associated Peptides	1
Associated Spectra	1
Coverage	NaN

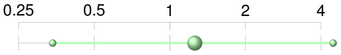

A	B	2.5	50	97.5	Sequence
**0**	**1**	**0.34**	**1.3**	**4.5**	**ILQPYVDQLKK**

5.650 996 1463 minus strand catalase [Cristaria plicata] gi

Protein Accession	CL6841.Contig3$_{H}1$
Mean Expression Ratio	1.06
Median Expression Ratio	1.05
Credible Interval	(0.482, 2.35)
Associated Peptides	2
Associated Spectra	2
Coverage	NaN

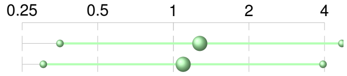

A	B	2.5	50	97.5	Sequence
**0**	**1**	**0.35**	**1.3**	**4.7**	**YLECPQHFSGDVQR**
**0**	**1**	**0.3**	**1.1**	**3.9**	**LFAYSDTHR**

5.651 1 468 glycine cleavage system H protein [Rhipicephalus sanguineus]

Protein Accession	CL42651.Contig1$_{H}1$
Mean Expression Ratio	0.947
Median Expression Ratio	0.949
Credible Interval	(0.411, 2.14)
Associated Peptides	1
Associated Spectra	1
Coverage	NaN

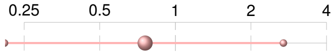

A	B	2.5	50	97.5	Sequence
**0**	**1**	**0.21**	**0.76**	**2.7**	**SPLDDGWVFK**

5.652 85 681 minus strand 40S ribosomal protein S8 OS=Xenopus laevis GN=rps8 PE=2 SV=3

Protein Accession	Unigene34347$_{H}1$
Mean Expression Ratio	0.949
Median Expression Ratio	0.949
Credible Interval	(0.413, 2.23)
Associated Peptides	1
Associated Spectra	1
Coverage	NaN

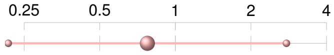

A	B	2.5	50	97.5	Sequence
**0**	**1**	**0.22**	**0.77**	**2.8**	**VEPALDEQFSSGR**

5.653 44 889 minus strand hypothetical protein CGI_10015025 [Crassostrea gigas]

Protein Accession	CL40973.Contig3$_{H}1$
Mean Expression Ratio	1.05
Median Expression Ratio	1.05
Credible Interval	(0.466, 2.43)
Associated Peptides	1
Associated Spectra	1
Coverage	NaN

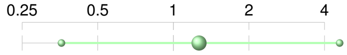

A	B	2.5	50	97.5	Sequence
**0**	**1**	**0.36**	**1.3**	**4.6**	**NVELEGDIER**

5.654 26 667 ribosomal protein L15 [Acipenser sinensis] gi; 26 667 ribosomal protein L15 [Acipenser sinensis] gi; 26 667 ribosomal protein L15 [Acipenser sinensis] gi

Protein Accession	Unigene63676$_{H1}$ CL22.Contig2$_{H1}$ CL22.Contig1$_{H1}$
Mean Expression Ratio	0.951
Median Expression Ratio	0.95
Credible Interval	(0.45, 1.99)
Associated Peptides	2
Associated Spectra	7
Coverage	NaN

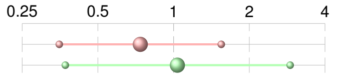

A	B	2.5	50	97.5	Sequence
3	2	0.35	0.74	1.5	NTLSLLR
0	2	0.37	1	2.9	TIGGSRR

5.655 204 791 minus strand PREDICTED: 15-hydroxyprostaglandin dehydrogenase [NAD+] [Gallus gallus]

Protein Accession	Unigene3818$_{H1}$
Mean Expression Ratio	0.948
Median Expression Ratio	0.95
Credible Interval	(0.583, 1.54)
Associated Peptides	12
Associated Spectra	104
Coverage	NaN

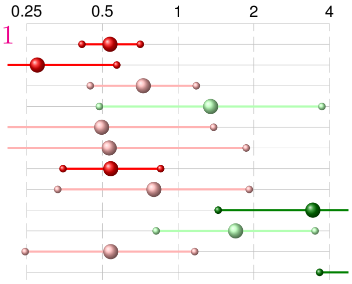

A	B	2.5	50	97.5	Sequence
20	26	0.41	0.54	0.7	AVTEALLAK
1	4	0.13	0.28	0.57	HFISAFEK
8	5	0.45	0.73	1.2	IAFVSGGAMGTGR
1	1	0.49	1.3	3.7	MGTTTDKELK
1	1	0.18	0.5	1.4	VMFCDIDEK
1	0	0.15	0.53	1.9	DITDK
4	12	0.35	0.54	0.85	AITMWGK
1	2	0.33	0.8	1.9	MDITDK
0	3	1.4	3.4	8.3	DITDKIAFVSGGAMGTGR
0	5	0.82	1.7	3.5	MDITDKIAFVSGGAMGTGR
0	4	0.25	0.54	1.2	VDIMVNNAEIFSETDMEK
0	4	3.7	8	18	AVTEALLAKGAK

5.656 1 177 small heat shock protein [Trichinella pseudospiralis]

Protein Accession	Unigene29352$_{H1}$
Mean Expression Ratio	1.06
Median Expression Ratio	1.05
Credible Interval	(0.469, 2.41)
Associated Peptides	1
Associated Spectra	1
Coverage	NaN

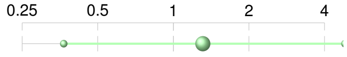

A	B	2.5	50	97.5	Sequence
0	1	0.37	1.3	4.8	ISMDVQHFKPEEIK

5.657 1 630 hypothetical protein [Trichomonas vaginalis G3] gi

Protein Accession	CL4495.Contig3$_{H}1$
Mean Expression Ratio	1.05
Median Expression Ratio	1.05
Credible Interval	(0.457, 2.44)
Associated Peptides	1
Associated Spectra	1
Coverage	NaN

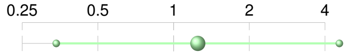

A	B	2.5	50	97.5	Sequence
**0**	**1**	**0.34**	**1.3**	**4.6**	**TLTEEKENLNK**

5.658 186 575 minus strand predicted protein [Nematostella vectensis] gi

Protein Accession	CL1734.Contig5$_{H}1$
Mean Expression Ratio	1.05
Median Expression Ratio	1.05
Credible Interval	(0.461, 2.42)
Associated Peptides	1
Associated Spectra	1
Coverage	NaN

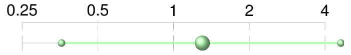

A	B	2.5	50	97.5	Sequence
**0**	**1**	**0.36**	**1.3**	**4.6**	**EATESDVETAAQAAR**

5.659 " 41 268 conserved hypothetical protein [Perkinsus marinus ATCC 50983] gi; 29 238 PREDICTED: succinyl-CoA ligase [GDP-forming] subunit beta, mitochondrial-like, partial [Meleagris gallopavo]"

Protein Accession	CL48047.Contig2$_{H}1$ CL48047.Contig1$_{H}1$
Mean Expression Ratio	1.05
Median Expression Ratio	1.05
Credible Interval	(0.458, 2.42)
Associated Peptides	1
Associated Spectra	1
Coverage	NaN

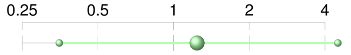

A	B	2.5	50	97.5	Sequence
**1**	**0**	**0.35**	**1.2**	**4.5**	**FVVASTPEDASK**

5.660 140 1564 selenium-binding protein [Haliotis discus hannai]

Protein Accession	CL3868.Contig7$_{H}1$
Mean Expression Ratio	0.953
Median Expression Ratio	0.951
Credible Interval	(0.439, 2.12)
Associated Peptides	2
Associated Spectra	2
Coverage	NaN

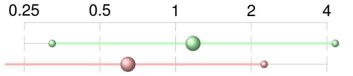

A	B	2.5	50	97.5	Sequence
**0**	**1**	**0.32**	**1.2**	**4.3**	**VVEAVDIHR**
**0**	**1**	**0.19**	**0.64**	**2.3**	**SLNVWNWK**

5.661 116 1882 Actin-interacting protein 1 [Crassostrea gigas]

Protein Accession	CL12416.Contig1$_H$1
Mean Expression Ratio	0.947
Median Expression Ratio	0.951
Credible Interval	(0.501, 1.75)
Associated Peptides	7
Associated Spectra	9
Coverage	NaN

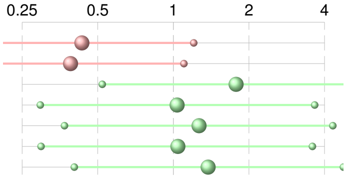

A	B	2.5	50	97.5	Sequence
1	1	0.16	0.43	1.2	QFLDGEVK
1	1	0.14	0.39	1.1	TLDELDNKEIGAPGAHK
0	1	0.52	1.8	6.2	FSPDGEVAVSGGADGK
0	1	0.3	1	3.7	GHMKPITAMALSDDR
0	1	0.37	1.3	4.3	LVSIGVDDVVR
0	1	0.3	1	3.6	QILTASGDASAK
0	1	0.4	1.4	4.8	VFDVQSGSLVQEFK

5.662 7073 7339 minus strand Collagen alpha-3(VI) chain [Crassostrea gigas]

Protein Accession	Unigene26547$_H$1
Mean Expression Ratio	0.947
Median Expression Ratio	0.951
Credible Interval	(0.411, 2.15)
Associated Peptides	1
Associated Spectra	1
Coverage	NaN

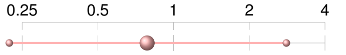

A	B	2.5	50	97.5	Sequence
0	1	0.22	0.78	2.8	YDTPQQVLEAIATTR

5.663 9 518 minus strand hemocyanin isoform 1 [Nucula nucleus]

Protein Accession	Unigene71735$_H$1
Mean Expression Ratio	0.95
Median Expression Ratio	0.952
Credible Interval	(0.429, 2.12)
Associated Peptides	1
Associated Spectra	18
Coverage	NaN

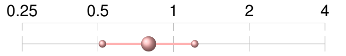

A	B	2.5	50	97.5	Sequence
9	9	0.52	0.79	1.2	IDFEHADVAR

5.664 146 841 minus strand hypothetical protein CGI_10006609 [Crassostrea gigas]; 146 841 minus strand hypothetical protein CGI_10006609 [Crassostrea gigas]; 146 841 minus strand hypothetical protein CGI_10006609 [Crassostrea gigas]

Protein Accession	CL6681.Contig3$_H$1 CL6681.Contig2$_H$1 CL6681.Contig1$_H$1
Mean Expression Ratio	1.06
Median Expression Ratio	1.05
Credible Interval	(0.471, 2.43)
Associated Peptides	1
Associated Spectra	1
Coverage	NaN

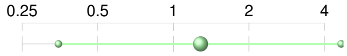

A	B	2.5	50	97.5	Sequence
0	1	0.35	1.3	4.7	GHVEGLEVLPAR

5.665 29 610 minus strand 40S ribosomal protein S7 [Crassostrea gigas]

Protein Accession	CL8074.Contig11$_H$1
Mean Expression Ratio	1.05
Median Expression Ratio	1.05
Credible Interval	(0.455, 2.44)
Associated Peptides	1
Associated Spectra	1
Coverage	NaN

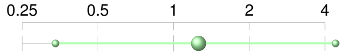

A	B	2.5	50	97.5	Sequence
0	1	0.34	1.3	4.4	TSLHELYITGAK

5.666 604 1236 minus strand selenium-dependent glutathione peroxidase [Crassostrea gigas]; 138 770 minus strand selenium-dependent glutathione peroxidase [Crassostrea gigas]; 138 770 minus strand selenium-dependent glutathione peroxidase [Crassostrea gigas]; 147 779 minus strand selenium-dependent glutathione peroxidase [Crassostrea gigas]; 149 781 minus strand selenium-dependent glutathione peroxidase [Crassostrea gigas]; 144 776 minus strand selenium-dependent glutathione peroxidase [Crassostrea gigas]; 141 773 minus strand selenium-dependent glutathione peroxidase [Crassostrea gigas]

Protein Accession	CL12854.Contig7$_H$1 CL12854.Contig20$_H$1 CL12854.Contig19$_H$1 CL12854.Contig18$_H$1 CL1
Mean Expression Ratio	1.05
Median Expression Ratio	1.05
Credible Interval	(0.533, 2.06)
Associated Peptides	4
Associated Spectra	10
Coverage	NaN

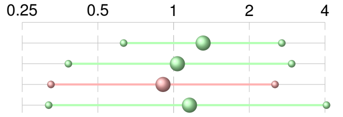

A	B	2.5	50	97.5	Sequence
3	2	0.63	1.3	2.7	SGVQGLSVPR
0	2	0.38	1	2.9	ALGLQDELGPLLEGR
0	2	0.33	0.91	2.5	FLVGQDGLPVAR
0	1	0.32	1.2	4.1	SDVNGQNELPLYR

5.667 214 1284 glutamine synthetase [Tegillarca granosa]

Protein Accession	CL6905.Contig5$_H$1
Mean Expression Ratio	0.953
Median Expression Ratio	0.953
Credible Interval	(0.446, 2.05)
Associated Peptides	2
Associated Spectra	3
Coverage	NaN

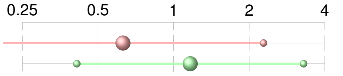

A	B	2.5	50	97.5	Sequence
0	1	0.18	0.63	2.3	GGLAVIEEAIAK
0	2	0.41	1.2	3.3	QPGGLAVIEEAIAK

5.668 2 244 minus strand hypothetical protein KGM_08771 [Danaus plexippus]

Protein Accession	CL49466.Contig2$_{H}1$
Mean Expression Ratio	1.05
Median Expression Ratio	1.05
Credible Interval	(0.489, 2.34)
Associated Peptides	2
Associated Spectra	2
Coverage	NaN

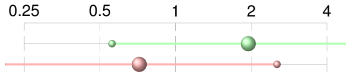

A	B	2.5	50	97.5	Sequence
0	1	0.56	1.9	7.1	AVFIDLEPTVVDEVR
0	1	0.2	0.72	2.5	TVGGGDDSFNTFFSETGAGK

5.669 3 272 minus strand ADP/ATP carrier [Clonorchis sinensis]

Protein Accession	Unigene4627$_{H}1$
Mean Expression Ratio	0.952
Median Expression Ratio	0.954
Credible Interval	(0.416, 2.19)
Associated Peptides	1
Associated Spectra	1
Coverage	NaN

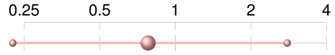

A	B	2.5	50	97.5	Sequence
0	1	0.23	0.78	2.8	MMMTSGQAVK

5.670 " 31 2061 minus strand 1,4-alpha-glucan-branching enzyme [Crassostrea gigas]"

Protein Accession	CL12555.Contig7$_{H}1$
Mean Expression Ratio	0.957
Median Expression Ratio	0.954
Credible Interval	(0.456, 1.98)
Associated Peptides	3
Associated Spectra	4
Coverage	NaN

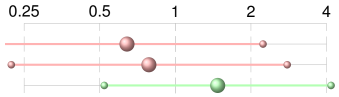

A	B	2.5	50	97.5	Sequence
0	1	0.18	0.64	2.2	GTHDLWDSR
0	1	0.22	0.79	2.8	LFKLDGYLR
0	2	0.52	1.5	4.2	LDFPR

5.671 2 571 PREDICTED: copper homeostasis protein cutC homolog [Anolis carolinensis]

Protein Accession	CL43087.Contig1$_{H}1$
Mean Expression Ratio	1.05
Median Expression Ratio	1.05
Credible Interval	(0.454, 2.39)
Associated Peptides	1
Associated Spectra	1
Coverage	NaN

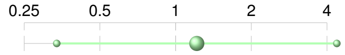

A	B	2.5	50	97.5	Sequence
0	1	0.34	1.2	4.4	IHQDSEMLFQK

5.672 3 218 beta-tubulin [Fasciola hepatica] gi

Protein Accession	Unigene7955$_H$1
Mean Expression Ratio	1.05
Median Expression Ratio	1.05
Credible Interval	(0.555, 1.95)
Associated Peptides	5
Associated Spectra	17
Coverage	NaN

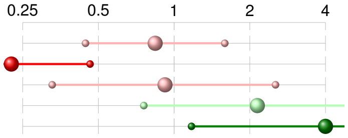

A	B	2.5	50	97.5	Sequence
2	5	0.45	0.84	1.6	TAVCDIPPR
2	3	0.11	0.23	0.46	EVDEQMLNVQNK
0	2	0.33	0.92	2.5	NSSYFVEWIPNNVK
0	2	0.76	2.1	6	TAVCDIPPRGLK
0	1	1.2	4	14	MSMKEVDEQMLNVQNK

5.673 28 1908 minus strand Mitochondrial inner membrane protein [Crassostrea gigas]; 10 1902 Mitochondrial inner membrane protein [Crassostrea gigas]; 10 1902 Mitochondrial inner membrane protein [Crassostrea gigas]; 10 1902 Mitochondrial inner membrane protein [Crassostrea gigas]; 10 1902 Mitochondrial inner membrane protein [Crassostrea gigas]; 10 1902 Mitochondrial inner membrane protein [Crassostrea gigas]; 10 1902 Mitochondrial inner membrane protein [Crassostrea gigas]

Protein Accession	CL6414.Contig9$_H$1 CL6414.Contig8$_H$1 CL6414.Contig7$_H$1 CL6414.Contig6$_H$1 CL6414.Con
Mean Expression Ratio	0.958
Median Expression Ratio	0.956
Credible Interval	(0.416, 2.19)
Associated Peptides	1
Associated Spectra	1
Coverage	NaN

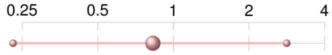

A	B	2.5	50	97.5	Sequence
0	1	0.23	0.83	2.8	EIHTQLLQER

5.674 72 521 minus strand GF14593 [Drosophila ananassae] gi; 60 392 GD23124 [Drosophila simulans] gi

Protein Accession	CL38325.Contig2$_H$1 CL38325.Contig1$_H$1
Mean Expression Ratio	0.957
Median Expression Ratio	0.957
Credible Interval	(0.416, 2.19)
Associated Peptides	1
Associated Spectra	1
Coverage	NaN

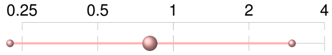

A	B	2.5	50	97.5	Sequence
0	1	0.22	0.81	3	LTADVHGK

5.675 30 1088 arginine kinase [Conus novaehollandiae]

Protein Accession	CL5308.Contig5$_{H}1$
Mean Expression Ratio	0.959
Median Expression Ratio	0.957
Credible Interval	(0.691, 1.33)
Associated Peptides	34
Associated Spectra	122
Coverage	NaN

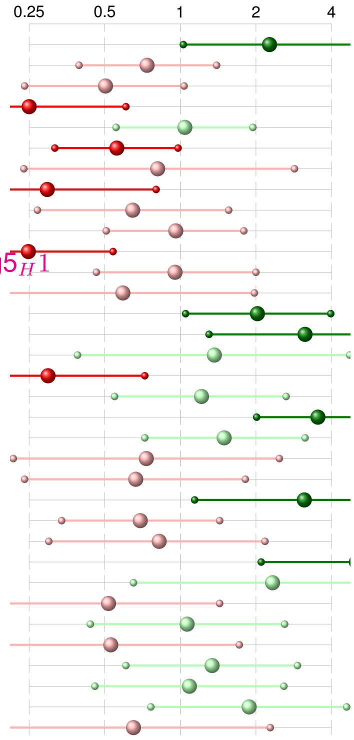

A	B	2.5	50	97.5	Sequence
1	3	1	2.3	5	AAGGYDDWPVGR
4	3	0.39	0.74	1.4	FNFPDLDPSGK
2	3	0.24	0.5	1	GTYYPLLGMDK
2	1	0.1	0.25	0.61	LAALPEFK
4	3	0.55	1	1.9	YIVSTR
4	5	0.32	0.56	0.98	LTFAR
1	0	0.24	0.81	2.9	VNMEK
1	1	0.11	0.3	0.8	GIFHNPDK
1	2	0.27	0.64	1.6	GGNLTQVYQR
4	3	0.51	0.96	1.8	HPAPDFGEK
4	0	0.11	0.25	0.54	IISMQK
1	4	0.46	0.95	2	LVSGIHALEK
1	0	0.17	0.59	2	RFNFPDLDPSGK
0	6	1.1	2	4	ATVEALNTFEGELK
0	3	1.3	3.1	7.6	FNFPDLDPSGKYIVSTR
0	1	0.39	1.4	4.7	GTYYPLLGMDKATEK
0	3	0.12	0.3	0.72	KLIDDHFLFK
0	4	0.55	1.2	2.6	KLIDDHFLFKDDDR
0	9	2	3.5	6.2	KLIDDHFLFKDDDRFLK
0	5	0.72	1.5	3.1	LGLSEFDAIMEMK
0	1	0.22	0.73	2.5	LIDDHFLFKDDDR
0	2	0.24	0.67	1.8	NGVEEILKK
0	2	1.1	3.1	8.6	RFNFPDLDPSGKYIVSTR
0	5	0.34	0.69	1.4	SHASYGFPPVLSK
0	2	0.3	0.83	2.2	TFLVWVNEEDHIR
0	3	2.1	5	12	VAEVKHPAPDFGEK
0	1	0.65	2.3	7.8	VAEVKHPAPDFGEKR
0	2	0.19	0.52	1.4	NGVEEILK
0	3	0.44	1.1	2.6	KLTFAR
0	1	0.16	0.53	1.7	DYHKVAEVK
0	4	0.61	1.3	2.9	GIHGEHTESVGGVYDISNK
0	3	0.46	1.1	2.6	GIHGEHTESVGGVYDISNKR
0	3	0.76	1.9	4.6	GIHGEHTESVGGVYDISNKRR
0	1	0.19	0.65	2.3	LIDDHFLFK

5.676 " 2 433 Vesicle-fusing ATPase, partial [Ascaris suum]"

Protein Accession	CL20981.Contig2$_{H}1$
Mean Expression Ratio	1.04
Median Expression Ratio	1.04
Credible Interval	(0.447, 2.41)
Associated Peptides	1
Associated Spectra	1
Coverage	NaN

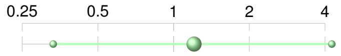

A	B	2.5	50	97.5	Sequence
1	0	0.33	1.2	4.3	LENSNLNLIGK

5.677 294 443 GE25988 gene product from transcript GE25988-RA; K02980 small subunit ribosomal protein S29e; 292 441 GE25988 gene product from transcript GE25988-RA; K02980 small subunit ribosomal protein S29e; 308 457 GE25988 gene product from transcript GE25988-RA; K02980 small subunit ribosomal protein S29e; 80 250 minus strand 40S ribosomal protein S29 [Ornithodoros parkeri]

Protein Accession	CL11390.Contig8$_H$1 CL11390.Contig4$_H$1 CL11390.Contig3$_H$1 CL11390.Contig2$_H$1
Mean Expression Ratio	0.96
Median Expression Ratio	0.958
Credible Interval	(0.431, 2.18)
Associated Peptides	1
Associated Spectra	4
Coverage	NaN

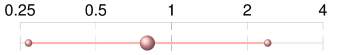

A	B	2.5	50	97.5	Sequence
**1**	**3**	**0.27**	**0.8**	**2.4**	**QFAPDIGFK**

5.678 49 219 haemocyanin precursor [Nautilus pompilius]

Protein Accession	Unigene32601$_H$1
Mean Expression Ratio	1.04
Median Expression Ratio	1.04
Credible Interval	(0.524, 2.05)
Associated Peptides	3
Associated Spectra	15
Coverage	NaN

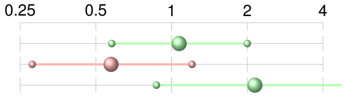

A	B	2.5	50	97.5	Sequence
**2**	**5**	**0.58**	**1.1**	**2**	**GYIDFENGYTVR**
**3**	**2**	**0.28**	**0.58**	**1.2**	**NTQPELFK**
**2**	**1**	**0.87**	**2.1**	**5.1**	**NTQPELFKK**

5.679 788 1006 LIM protein [Haliotis discus discus]

Protein Accession	CL29824.Contig2$_H$1
Mean Expression Ratio	1.04
Median Expression Ratio	1.04
Credible Interval	(0.472, 2.3)
Associated Peptides	1
Associated Spectra	4
Coverage	NaN

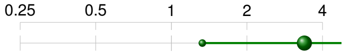

A	B	2.5	50	97.5	Sequence
**2**	**2**	**1.3**	**3.4**	**8.8**	**AIGVNTPWHK**

5.680 5 658 predicted protein [Nematostella vectensis] gi

Protein Accession	CL36575.Contig6$_{H}$1
Mean Expression Ratio	1.05
Median Expression Ratio	1.04
Credible Interval	(0.456, 2.4)
Associated Peptides	1
Associated Spectra	1
Coverage	NaN

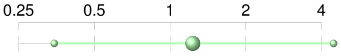

A	B	2.5	50	97.5	Sequence
**0**	**1**	**0.35**	**1.2**	**4.5**	**KVQIGNIAAGK**

5.681 10 300 minus strand unnamed protein product [Tetraodon nigroviridis]

Protein Accession	CL14559.Contig2$_{H}$1
Mean Expression Ratio	1.04
Median Expression Ratio	1.04
Credible Interval	(0.473, 2.33)
Associated Peptides	1
Associated Spectra	5
Coverage	NaN

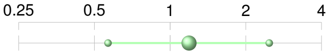

A	B	2.5	50	97.5	Sequence
**0**	**5**	**0.57**	**1.2**	**2.5**	**ILVQEGIHDR**

5.682 187 1563 odr-4-like protein [Crassostrea gigas]

Protein Accession	CL15156.Contig3$_{H}$1
Mean Expression Ratio	0.959
Median Expression Ratio	0.959
Credible Interval	(0.423, 2.2)
Associated Peptides	1
Associated Spectra	1
Coverage	NaN

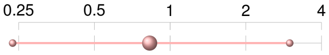

A	B	2.5	50	97.5	Sequence
**0**	**1**	**0.24**	**0.83**	**3**	**TLDAADELWVATHAK**

5.683 48 464 minus strand Acyl-coenzyme A thioesterase 13 [Crassostrea gigas]

Protein Accession	CL38770.Contig1$_{H}$1
Mean Expression Ratio	1.04
Median Expression Ratio	1.04
Credible Interval	(0.45, 2.35)
Associated Peptides	1
Associated Spectra	1
Coverage	NaN

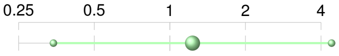

A	B	2.5	50	97.5	Sequence
**1**	**0**	**0.34**	**1.2**	**4.4**	**TALPGETVVAEAK**

5.684 158 1318 hypothetical protein BRAFLDRAFT_98992 [Branchiostoma floridae] gi

Protein Accession	CL14371.Contig5$_{H}1$
Mean Expression Ratio	1.04
Median Expression Ratio	1.04
Credible Interval	(0.482, 2.23)
Associated Peptides	2
Associated Spectra	2
Coverage	NaN

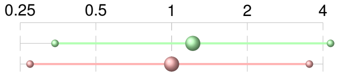

A	B	2.5	50	97.5	Sequence
0	1	0.34	1.2	4.3	AFTGFIVDGDSK
0	1	0.27	1	3.5	LAVMR

5.685 " 2057 2497 Phosphate carrier protein, mitochondrial [Crassostrea gigas]"

Protein Accession	CL2712.Contig11$_{H}1$
Mean Expression Ratio	0.962
Median Expression Ratio	0.96
Credible Interval	(0.439, 2.08)
Associated Peptides	2
Associated Spectra	2
Coverage	NaN

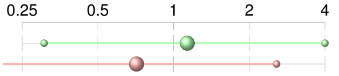

A	B	2.5	50	97.5	Sequence
0	1	0.3	1.1	4	LPRPPPPEMPESLKR
0	1	0.2	0.71	2.6	YVVPKPR

5.686 578 1150 minus strand expressed hypothetical protein [Trichoplax adhaerens] gi; 376 948 minus strand expressed hypothetical protein [Trichoplax adhaerens] gi; 176 748 minus strand expressed hypothetical protein [Trichoplax adhaerens] gi; 176 748 minus strand expressed hypothetical protein [Trichoplax adhaerens] gi; 307 879 minus strand expressed hypothetical protein [Trichoplax adhaerens] gi; 307 879 minus strand expressed hypothetical protein [Trichoplax adhaerens] gi; 307 879 minus strand expressed hypothetical protein [Trichoplax adhaerens] gi

Protein Accession	CL11851.Contig7$_{H}1$ CL11851.Contig6$_{H}1$ CL11851.Contig5$_{H}1$ CL11851.Contig4$_{H}1$ CL1185
Mean Expression Ratio	0.958
Median Expression Ratio	0.96
Credible Interval	(0.427, 2.15)
Associated Peptides	1
Associated Spectra	3
Coverage	NaN

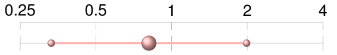

A	B	2.5	50	97.5	Sequence
0	3	0.33	0.81	2	GLLDIEAAVK

5.687 158 862 X-box binding protein [Haliotis discus discus]

Protein Accession	CL6396.Contig3$_{H}1$
Mean Expression Ratio	0.953
Median Expression Ratio	0.96
Credible Interval	(0.494, 1.83)
Associated Peptides	5
Associated Spectra	12
Coverage	NaN

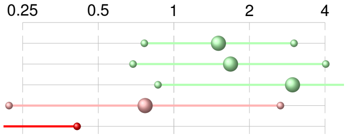

A	B	2.5	50	97.5	Sequence
4	2	0.76	1.5	3	SYYIGAR
0	3	0.69	1.7	4	VFPDFYFGAGDDKLDAR
0	1	0.86	3	11	ACIPDKAK
0	1	0.22	0.77	2.7	KYSGTNSTGRLYIVDLAK
0	1	0.033	0.12	0.41	VFPDFYFGAGDDK

5.688 325 642 minus strand Protein phosphatase 1B [Crassostrea gigas]

Protein Accession	Unigene268$_{H}1$
Mean Expression Ratio	1.04
Median Expression Ratio	1.04
Credible Interval	(0.478, 2.27)
Associated Peptides	2
Associated Spectra	3
Coverage	NaN

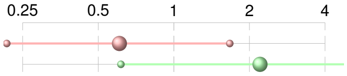

A	B	2.5	50	97.5	Sequence
1	1	0.22	0.61	1.7	VEMEDAHTAVVSIPHAR
1	0	0.62	2.2	7.5	GAFLDKPK

5.689 " 492 3458 PREDICTED: 2-oxoglutarate dehydrogenase, mitochondrial isoform 1 [Sarcophilus harrisii]"

Protein Accession	CL2200.Contig16$_{H}1$
Mean Expression Ratio	0.959
Median Expression Ratio	0.96
Credible Interval	(0.442, 2.11)
Associated Peptides	2
Associated Spectra	2
Coverage	NaN

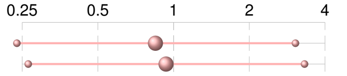

A	B	2.5	50	97.5	Sequence
1	0	0.24	0.85	3.1	IAKEPSVLTK
1	0	0.26	0.93	3.3	FRAKK

5.690 131 541 minus strand nervous system adducin [Aplysia californica] gi

Protein Accession	CL27337.Contig1$_{H}1$
Mean Expression Ratio	1.04
Median Expression Ratio	1.04
Credible Interval	(0.481, 2.23)
Associated Peptides	2
Associated Spectra	3
Coverage	NaN

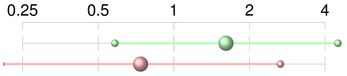

A	B	2.5	50	97.5	Sequence
1	1	0.58	1.6	4.5	GIGASFGR
0	1	0.2	0.74	2.7	FIDTIDPDDPEYIR

5.691 113 445 ribosomal protein rps19 [Lineus viridis]

Protein Accession	CL8932.Contig6$_H$1
Mean Expression Ratio	0.956
Median Expression Ratio	0.96
Credible Interval	(0.468, 1.94)
Associated Peptides	3
Associated Spectra	6
Coverage	NaN

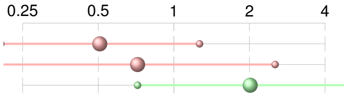

A	B	2.5	50	97.5	Sequence
1	2	0.2	0.51	1.3	QPEWADIVK
1	0	0.2	0.72	2.5	LKQPEWADIVK
0	2	0.72	2	5.7	APAGVGAFTK

5.692 1021 1359 minus strand RecName: Full=40S ribosomal protein S26 gi|6015599|emb|CAB57819.1| ribosomal protein S26 [Octopus vulgaris]

Protein Accession	Unigene11569$_H$1
Mean Expression Ratio	0.956
Median Expression Ratio	0.96
Credible Interval	(0.42, 2.19)
Associated Peptides	1
Associated Spectra	1
Coverage	NaN

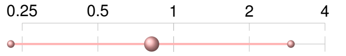

A	B	2.5	50	97.5	Sequence
1	0	0.23	0.82	2.9	NIVEAAAVR

5.693 230 1189 hypothetical protein [Amblyomma maculatum]

Protein Accession	CL11202.Contig4$_H$1
Mean Expression Ratio	0.958
Median Expression Ratio	0.962
Credible Interval	(0.436, 2.12)
Associated Peptides	2
Associated Spectra	2
Coverage	NaN

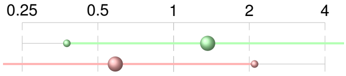

A	B	2.5	50	97.5	Sequence
0	1	0.38	1.4	4.9	KFAVDAVK
0	1	0.16	0.59	2.1	LEDMLR

5.694 915 1505 minus strand Collagen alpha-5(VI) chain [Crassostrea gigas]

Protein Accession	CL40183.Contig3$_H$1
Mean Expression Ratio	0.964
Median Expression Ratio	0.962
Credible Interval	(0.516, 1.82)
Associated Peptides	6
Associated Spectra	10
Coverage	NaN

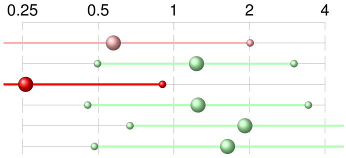

A	B	2.5	50	97.5	Sequence
1	0	0.16	0.57	2	EVQMAK
1	2	0.49	1.2	3	HGLATNTGAGIK
1	0	0.073	0.26	0.9	LEAIPWR
0	2	0.45	1.2	3.4	VGEQLDAEELLNIAGDPK
0	2	0.67	1.9	5.2	VGEQLDAEELLNIAGDPKR
0	1	0.48	1.6	5.6	HGLATNTGAG

5.695 151 1449 rab GDP-dissociation inhibitor [Branchiostoma belcheri]

Protein Accession CL30719.Contig3$_H$1
Mean Expression Ratio 1.04
Median Expression Ratio 1.04
Credible Interval (0.529, 2.07)
Associated Peptides 4
Associated Spectra 7
Coverage NaN

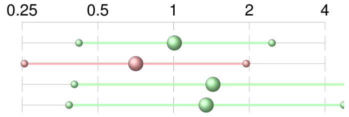

A	B	2.5	50	97.5	Sequence
1	2	0.42	1	2.5	ITGEAFDFSK
1	1	0.25	0.71	1.9	LLVATGVTR
0	1	0.4	1.4	5	TLYEPVDMGETSK
0	1	0.38	1.4	4.8	AICILNHPIKDTK

5.696 76 822 14-3-3 protein [Heterodera glycines]; 76 822 minus strand 14-3-3 protein [Heterodera glycines]; 76 822 minus strand 14-3-3 protein [Heterodera glycines]

Protein Accession Unigene34590$_H$1 Unigene29843$_H$1 Unigene28010$_H$1
Mean Expression Ratio 1.04
Median Expression Ratio 1.04
Credible Interval (0.557, 1.95)
Associated Peptides 6
Associated Spectra 20
Coverage NaN

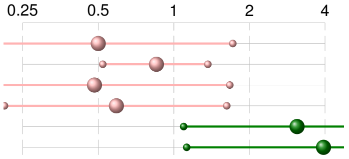

A	B	2.5	50	97.5	Sequence
1	0	0.14	0.5	1.7	YLSEVSQGEQR
7	6	0.52	0.85	1.4	VVSSIEQK
1	0	0.14	0.48	1.7	KLEMAK
0	2	0.21	0.59	1.6	IAQEQMPTTHPIR
0	2	1.1	3.1	8.7	LAEQAERYDDMAK
0	1	1.1	4	14	SMDAYKK

5.697 " 143 451 minus strand PREDICTED: 10 kDa heat shock protein, mitochondrial isoform 1 [Canis lupus familiaris]"

Protein Accession CL48287.Contig1$_H$1
Mean Expression Ratio 1.04
Median Expression Ratio 1.04
Credible Interval (0.555, 1.97)
Associated Peptides 6
Associated Spectra 10
Coverage NaN

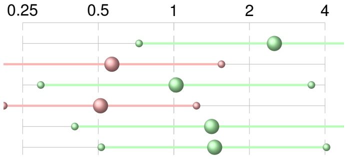

A	B	2.5	50	97.5	Sequence
1	0	0.73	2.5	8.7	GGIMIPEKSQGK
2	0	0.2	0.57	1.6	TKGGIMIPEK
1	0	0.3	1	3.5	SIPETK
3	0	0.21	0.51	1.2	GGIMIPEK
0	1	0.4	1.4	4.9	AQEAVVVAVGSGSR
0	2	0.51	1.5	4.1	VLLPEYGGTK

5.698 100 2307 minus strand Xanthine dehydrogenase/oxidase [Crassostrea gigas]

Protein Accession Unigene10743$_H$1
Mean Expression Ratio 1.04
Median Expression Ratio 1.04
Credible Interval (0.476, 2.26)
Associated Peptides 2
Associated Spectra 3
Coverage NaN

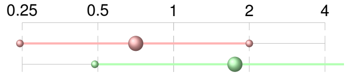

A	B	2.5	50	97.5	Sequence
1	1	0.25	0.71	2	VLSTDIVMDVGK
0	1	0.49	1.8	6.3	TLNIPQNK

5.699 35 2953 hypothetical protein CGI_10020867 [Crassostrea gigas]; 97 3015 minus strand hypothetical protein CGI_10020867 [Crassostrea gigas]

Protein Accession	CL2537.Contig6$_H$1 CL2537.Contig4$_H$1
Mean Expression Ratio	0.968
Median Expression Ratio	0.964
Credible Interval	(0.448, 2.09)
Associated Peptides	2
Associated Spectra	4
Coverage	NaN

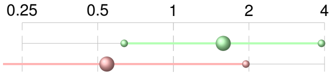

A	B	2.5	50	97.5	Sequence
**0**	**3**	**0.64**	**1.6**	**3.9**	**GGGQIIPTAR**
**0**	**1**	**0.15**	**0.54**	**1.9**	**LLDATIAAEPIHR**

5.700 165 377 Cysteine and glycine-rich protein 3 [Crassostrea gigas]; 135 344 Cysteine and glycine-rich protein 3 [Crassostrea gigas]

Protein Accession	CL38347.Contig2$_H$1 CL38347.Contig1$_H$1
Mean Expression Ratio	0.963
Median Expression Ratio	0.964
Credible Interval	(0.42, 2.23)
Associated Peptides	1
Associated Spectra	2
Coverage	NaN

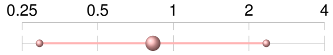

A	B	2.5	50	97.5	Sequence
**1**	**1**	**0.29**	**0.83**	**2.3**	**VEAGNIPFHK**

5.701 " 599 907 minus strand Tryptophanyl-tRNA synthetase, cytoplasmic [Heterocephalus glaber]"

Protein Accession	Unigene26755$_H$1
Mean Expression Ratio	0.962
Median Expression Ratio	0.964
Credible Interval	(0.463, 2)
Associated Peptides	3
Associated Spectra	5
Coverage	NaN

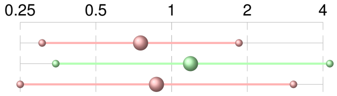

A	B	2.5	50	97.5	Sequence
**0**	**3**	**0.3**	**0.75**	**1.9**	**DMDPLLDLYEK**
**0**	**1**	**0.35**	**1.2**	**4.2**	**KTPFYLYTGR**
**0**	**1**	**0.25**	**0.87**	**3.1**	**ITDELK**

5.702 52 1599 minus strand ATP synthase beta subunit [Pinctada fucata]

Protein Accession	CL34251.Contig5$_H$1
Mean Expression Ratio	1.04
Median Expression Ratio	1.04
Credible Interval	(0.665, 1.62)
Associated Peptides	15
Associated Spectra	47
Coverage	NaN

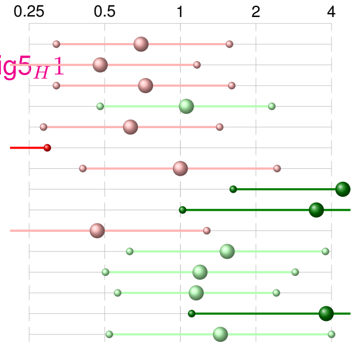

A	B	2.5	50	97.5	Sequence
1	3	0.32	0.7	1.6	EAAKPAAAGNLAVGR
1	2	0.2	0.48	1.2	IINVIGEPIDER
2	2	0.32	0.72	1.6	VISLTDDTSK
1	3	0.48	1.1	2.3	VSLVYGQMNEPPGAR
2	2	0.29	0.63	1.4	IPVGPK
1	5	0.075	0.15	0.29	ILQDFK
0	3	0.41	1	2.4	AHGGYSVFAGVGER
0	2	1.6	4.4	12	AKEAAKPAAAGNLAVGR
0	1	1	3.5	12	GQACKDTGTSIRIPVGPK
0	2	0.17	0.47	1.3	IGLFGGAGVGK
0	3	0.63	1.5	3.8	ILDPNVVGMEHYK
0	3	0.5	1.2	2.9	TIAMDGTEGLVR
0	5	0.56	1.2	2.4	VVDMLAPYAR
0	1	1.1	3.8	13	DTGTSIRIPVGPK
0	2	0.52	1.4	4	TREGNDLYHEMITTK

5.703 322 645 macrophage migration inhibitory-like factor protein [Chlamys farreri]; 437 682 macrophage migration inhibitory-like factor protein [Chlamys farreri]; 437 682 macrophage migration inhibitory-like factor protein [Chlamys farreri]; 468 713 macrophage migration inhibitory-like factor protein [Chlamys farreri]

Protein Accession	CL8506.Contig6$_H$1 CL8506.Contig5$_H$1 CL8506.Contig3$_H$1 CL8506.Contig2$_H$1
Mean Expression Ratio	0.965
Median Expression Ratio	0.965
Credible Interval	(0.427, 2.18)
Associated Peptides	1
Associated Spectra	3
Coverage	NaN

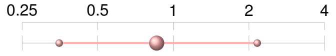

A	B	2.5	50	97.5	Sequence
1	2	0.35	0.86	2.2	ITLTIHSGLR

5.704 84 425 minus strand thioester-containing protein [Euphaedusa tau]

Protein Accession	Unigene24513$_H$1
Mean Expression Ratio	1.04
Median Expression Ratio	1.03
Credible Interval	(0.451, 2.37)
Associated Peptides	1
Associated Spectra	1
Coverage	NaN

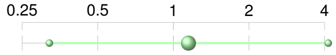

A	B	2.5	50	97.5	Sequence
0	1	0.32	1.1	4.1	TYVILAPAK

5.705 " 101 598 PREDICTED: aminomethyltransferase, mitochondrial-like [Oreochromis niloticus]"

Protein Accession	CL10455.Contig5$_{H}1$
Mean Expression Ratio	0.967
Median Expression Ratio	0.968
Credible Interval	(0.424, 2.18)
Associated Peptides	1
Associated Spectra	2
Coverage	NaN

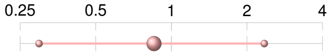

A	B	2.5	50	97.5	Sequence
**1**	**1**	**0.3**	**0.85**	**2.4**	**TTITEEHLQVR**

5.706 49 1560 PREDICTED: tRNA-splicing ligase RtcB homolog [Megachile rotundata]

Protein Accession	CL21686.Contig3$_{H}1$
Mean Expression Ratio	0.967
Median Expression Ratio	0.968
Credible Interval	(0.424, 2.23)
Associated Peptides	1
Associated Spectra	1
Coverage	NaN

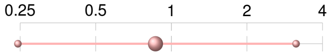

A	B	2.5	50	97.5	Sequence
**0**	**1**	**0.24**	**0.87**	**3.1**	**GLGHQVATDALVVMEK**

5.707 4 399 Spectrin beta chain [Crassostrea gigas]

Protein Accession	Unigene17190$_{H}1$
Mean Expression Ratio	0.965
Median Expression Ratio	0.968
Credible Interval	(0.454, 2.03)
Associated Peptides	3
Associated Spectra	3
Coverage	NaN

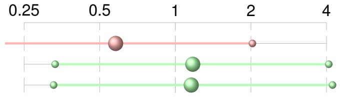

A	B	2.5	50	97.5	Sequence
**0**	**1**	**0.16**	**0.58**	**2**	**DGLAFNAIIHK**
**0**	**1**	**0.33**	**1.2**	**4.1**	**VHLENMGAHDIVDGSAR**
**0**	**1**	**0.33**	**1.2**	**4.2**	**HRPDLIQYDK**

5.708 " 1 327 minus strand endo-beta-1,4-glucanase [Ampullaria crossean]"

Protein Accession	CL17318.Contig3$_{H}1$
Mean Expression Ratio	1.03
Median Expression Ratio	1.03
Credible Interval	(0.452, 2.29)
Associated Peptides	1
Associated Spectra	2
Coverage	NaN

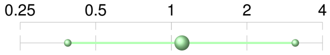

A	B	2.5	50	97.5	Sequence
**0**	**2**	**0.39**	**1.1**	**3.1**	**CHMDGGVLK**

5.709 1009 2784 minus strand Collagen alpha-3(VI) chain [Crassostrea gigas]

Protein Accession	Unigene46961$_H1$
Mean Expression Ratio	0.968
Median Expression Ratio	0.968
Credible Interval	(0.43, 2.21)
Associated Peptides	1
Associated Spectra	2
Coverage	NaN

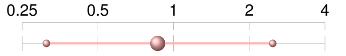

A	B	2.5	50	97.5	Sequence
0	2	0.31	0.87	2.5	ELEGIASPPAR

5.710 4 675 Laminin subunit gamma-1 [Crassostrea gigas]

Protein Accession	CL35712.Contig1$_H1$
Mean Expression Ratio	1.03
Median Expression Ratio	1.03
Credible Interval	(0.498, 2.14)
Associated Peptides	3
Associated Spectra	4
Coverage	NaN

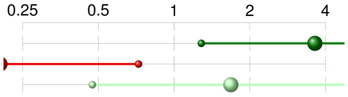

A	B	2.5	50	97.5	Sequence
0	2	1.3	3.6	10	LAQLAEDTAIQASAEAR
0	1	0.058	0.2	0.72	DALARVPEIEK
0	1	0.47	1.7	5.8	SKVNEALAK

5.711 2008 2715 Protein disulfide-isomerase A5 [Crassostrea gigas]

Protein Accession	CL7820.Contig3$_H1$
Mean Expression Ratio	0.968
Median Expression Ratio	0.968
Credible Interval	(0.495, 1.91)
Associated Peptides	5
Associated Spectra	7
Coverage	NaN

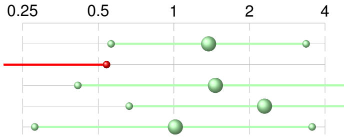

A	B	2.5	50	97.5	Sequence
1	2	0.56	1.4	3.4	LKQESPTAK
1	0	0.046	0.16	0.54	FGGLASK
0	1	0.42	1.5	5.2	MKPAYMSAAAR
0	1	0.66	2.3	8.2	FGGLASKYEVK
0	1	0.28	1	3.5	SKEDLVAFMKDPKPK

5.712 260 1597 isocitrate dehydrogenase [Mytilus galloprovincialis]; 260 1597 isocitrate dehydrogenase [Mytilus galloprovincialis]; 260 1597 isocitrate dehydrogenase [Mytilus galloprovincialis]

Protein Accession	CL10046.Contig8$_H1$ CL10046.Contig7$_H1$ CL10046.Contig6$_H1$
Mean Expression Ratio	1.03
Median Expression Ratio	1.03
Credible Interval	(0.551, 1.91)
Associated Peptides	6
Associated Spectra	11
Coverage	NaN

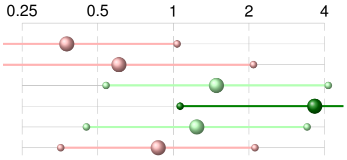

A	B	2.5	50	97.5	Sequence
2	0	0.14	0.38	1	VKEFNLK
1	0	0.17	0.61	2.1	KYNVGIK
0	2	0.54	1.5	4.1	TIEAEAAHGTVTR
0	1	1.1	3.7	13	GKLDGNKDLQR
0	2	0.45	1.2	3.4	SWPLYMSTK
0	3	0.36	0.87	2.1	LIDDMVAQALK

5.713 28 1455 minus strand PREDICTED: probable serine carboxypeptidase CPVL-like [Anolis carolinensis]

Protein Accession	CL34087.Contig4$_H$1
Mean Expression Ratio	0.968
Median Expression Ratio	0.968
Credible Interval	(0.457, 2.04)
Associated Peptides	3
Associated Spectra	5
Coverage	NaN

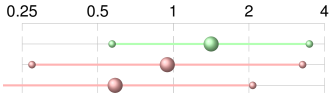

A	B	2.5	50	97.5	Sequence
1	2	0.57	1.4	3.5	YVPAISYK
0	1	0.27	0.95	3.3	GMAIGDGLCDPVTMMPK
0	1	0.17	0.59	2.1	VNPDDVEVAGWVR

5.714 243 2165 minus strand Elongation factor 2 OS=Drosophila melanogaster GN=Ef2b PE=2 SV=4

Protein Accession	Unigene11527$_H$1
Mean Expression Ratio	0.965
Median Expression Ratio	0.969
Credible Interval	(0.453, 2.05)
Associated Peptides	2
Associated Spectra	6
Coverage	NaN

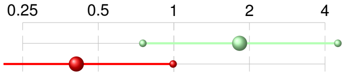

A	B	2.5	50	97.5	Sequence
0	3	0.75	1.8	4.5	ALPLQEGLPEAIDKGDIAPR
0	3	0.16	0.41	1	TQEDTLALADK

5.715 93 2876 Hypoxia up-regulated protein 1 [Crassostrea gigas]

Protein Accession	CL16620.Contig1$_H$1
Mean Expression Ratio	0.967
Median Expression Ratio	0.969
Credible Interval	(0.471, 1.98)
Associated Peptides	4
Associated Spectra	4
Coverage	NaN

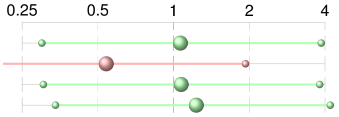

A	B	2.5	50	97.5	Sequence
0	1	0.3	1.1	3.9	MDESGILHLER
0	1	0.15	0.54	1.9	VESVFEK
0	1	0.3	1.1	3.8	IHEILLEK
0	1	0.34	1.2	4.2	IVAFR

5.716 274 1242 minus strand cathepsin D [Pinctada maxima]; 290 1387 cathepsin D [Pinctada maxima]; 290 1402 minus strand cathepsin D [Pinctada maxima]; 290 1480 minus strand cathepsin D [Pinctada maxima]

Protein Accession	CL1722.Contig9$_H$1 CL1722.Contig2$_H$1 CL1722.Contig1$_H$1 CL1722.Contig10$_H$1
Mean Expression Ratio	1.04
Median Expression Ratio	1.03
Credible Interval	(0.481, 2.23)
Associated Peptides	2
Associated Spectra	5
Coverage	NaN

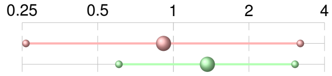

A	B	2.5	50	97.5	Sequence
0	1	0.26	0.92	3.2	VVFDTGSSNLWVPSKK
0	4	0.61	1.4	3.1	EGYWQIK

5.717 115 393 minus strand Yolk ferritin OS=Lymnaea stagnalis PE=1 SV=1; 132 413 RecName: Full=Yolk ferritin; Flags: Precursor gi|9655|emb|CAA40097.1| snail yolk ferritin [Lymnaea stagnalis] gi; 2 430 RecName: Full=Yolk ferritin; Flags: Precursor gi|9655|emb|CAA40097.1| snail yolk ferritin [Lymnaea stagnalis] gi; 3 389 RecName: Full=Yolk ferritin; Flags: Precursor gi|9655|emb|CAA40097.1| snail yolk ferritin [Lymnaea stagnalis] gi

Protein Accession	CL33796.Contig4$_H$1 CL33796.Contig3$_H$1 CL33796.Contig2$_H$1 CL33796.Contig1$_H$1
Mean Expression Ratio	0.97
Median Expression Ratio	0.969
Credible Interval	(0.428, 2.19)
Associated Peptides	1
Associated Spectra	1
Coverage	NaN

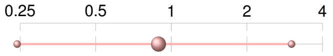

A	B	2.5	50	97.5	Sequence
**0**	**1**	**0.24**	**0.89**	**3**	**QYGLQAMEDALALER**

5.718 244 5451 alpha macroglobulin [Chlamys farreri]

Protein Accession	CL20671.Contig5$_H$1
Mean Expression Ratio	1.04
Median Expression Ratio	1.03
Credible Interval	(0.448, 2.41)
Associated Peptides	1
Associated Spectra	1
Coverage	NaN

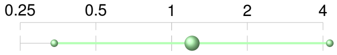

A	B	2.5	50	97.5	Sequence
**0**	**1**	**0.34**	**1.2**	**4.3**	**HLALDVEQLNR**

5.719 " 695 823 minus strand Aldehyde dehydrogenase family 16 member A1, partial [Crassostrea gigas]"

Protein Accession	CL17427.Contig5$_H$1
Mean Expression Ratio	0.972
Median Expression Ratio	0.971
Credible Interval	(0.426, 2.22)
Associated Peptides	1
Associated Spectra	1
Coverage	NaN

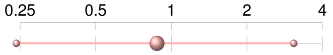

A	B	2.5	50	97.5	Sequence
**0**	**1**	**0.24**	**0.87**	**3.1**	**TVWIPAGEIFAN**

5.720 5 598 hypothetical protein DAPPUDRAFT_344656 [Daphnia pulex]

Protein Accession	Unigene24559$_H1$
Mean Expression Ratio	1.03
Median Expression Ratio	1.03
Credible Interval	(0.452, 2.32)
Associated Peptides	1
Associated Spectra	1
Coverage	NaN

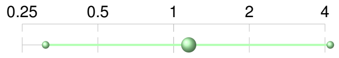

A	B	2.5	50	97.5	Sequence
0	1	0.31	1.2	4.2	WHLAEFVR

5.721 479 1732 minus strand Multifunctional protein ADE2 [Crassostrea gigas]; 239 1501 minus strand Multifunctional protein ADE2 [Crassostrea gigas]

Protein Accession	CL4210.Contig3$_H1$ CL4210.Contig1$_H1$
Mean Expression Ratio	0.968
Median Expression Ratio	0.972
Credible Interval	(0.447, 2.07)
Associated Peptides	2
Associated Spectra	3
Coverage	NaN

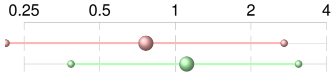

A	B	2.5	50	97.5	Sequence
1	0	0.21	0.76	2.7	VPLLSPKPK
2	0	0.38	1.1	3.1	ELQEVTK

5.722 150 716 calponin/transgelin [Papilio xuthus]

Protein Accession	CL11747.Contig2$_H1$
Mean Expression Ratio	1.03
Median Expression Ratio	1.03
Credible Interval	(0.473, 2.27)
Associated Peptides	2
Associated Spectra	2
Coverage	NaN

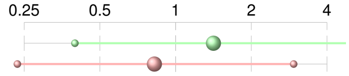

A	B	2.5	50	97.5	Sequence
0	1	0.4	1.4	5.2	MAFQQMEAIEK
0	1	0.23	0.83	3	DFTEEQLAQGK

5.723 275 1372 Cystathionine gamma-lyase OS=Bos taurus GN=CTH PE=2 SV=1

Protein Accession	CL16415.Contig5$_H1$
Mean Expression Ratio	0.975
Median Expression Ratio	0.972
Credible Interval	(0.503, 1.92)
Associated Peptides	5
Associated Spectra	7
Coverage	NaN

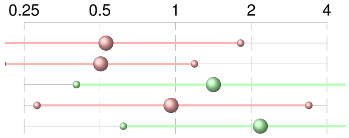

A	B	2.5	50	97.5	Sequence
1	0	0.15	0.53	1.8	GGMTEASNFLK
1	2	0.2	0.5	1.2	LVDIEAVSK
0	1	0.4	1.4	5	SMAVVPPISTSTTFK
0	1	0.28	0.96	3.4	VIHPGLESHPQYELAK
0	1	0.62	2.2	7.5	GLVVARFLEK

5.724 105 503 Transgelin-2 [Crassostrea gigas]

Protein Accession	CL445.Contig2$_{H}1$
Mean Expression Ratio	1.03
Median Expression Ratio	1.03
Credible Interval	(0.475, 2.22)
Associated Peptides	2
Associated Spectra	4
Coverage	NaN

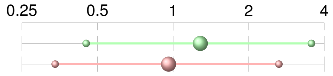

A	B	2.5	50	97.5	Sequence
1	1	0.45	1.3	3.6	SGLGLEIDK
2	0	0.34	0.96	2.6	SGLGLEIDKK

5.725 2 421 hypothetical protein BRAFLDRAFT_119044 [Branchiostoma floridae] gi

Protein Accession	CL14721.Contig1$_{H}1$
Mean Expression Ratio	0.976
Median Expression Ratio	0.973
Credible Interval	(0.431, 2.2)
Associated Peptides	1
Associated Spectra	2
Coverage	NaN

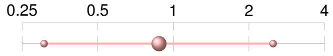

A	B	2.5	50	97.5	Sequence
0	2	0.31	0.88	2.5	DLEMDLAK

5.726 6 581 PREDICTED: mammalian ependymin-related protein 1 [Gallus gallus]

Protein Accession	CL15402.Contig1$_{H}1$
Mean Expression Ratio	0.969
Median Expression Ratio	0.973
Credible Interval	(0.449, 2.1)
Associated Peptides	2
Associated Spectra	4
Coverage	NaN

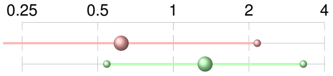

A	B	2.5	50	97.5	Sequence
0	1	0.17	0.62	2.2	ALFEEQNVITPGR
0	3	0.54	1.3	3.3	WYISYDALNK

5.727 5 1018 minus strand Glutaredoxin-3 [Crassostrea gigas]; 5 1018 Glutaredoxin-3 [Crassostrea gigas]

Protein Accession	CL7734.Contig9$_{H}1$ CL7734.Contig10$_{H}1$
Mean Expression Ratio	0.975
Median Expression Ratio	0.973
Credible Interval	(0.441, 2.2)
Associated Peptides	1
Associated Spectra	3
Coverage	NaN

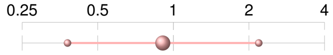

A	B	2.5	50	97.5	Sequence
1	2	0.38	0.91	2.2	VAAEDLAEVSHR

5.728 712 831 haemocyanin [Aplysia californica]

Protein Accession	Unigene65929$_{H}1$
Mean Expression Ratio	1.03
Median Expression Ratio	1.03
Credible Interval	(0.509, 2.11)
Associated Peptides	3
Associated Spectra	11
Coverage	NaN

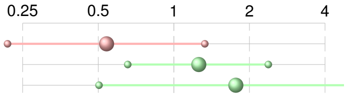

A	B	2.5	50	97.5	Sequence
0	3	0.22	0.54	1.3	YDVTDVFNQMR
0	7	0.66	1.3	2.4	YHFNVK
0	1	0.5	1.8	6.3	LRPDSKYHFNVK

5.729 18 242 minus strand translation elongation factor 1B gamma subunit [Strongylocentrotus purpuratus] gi

Protein Accession	CL14269.Contig2$_{H}1$
Mean Expression Ratio	1.02
Median Expression Ratio	1.03
Credible Interval	(0.449, 2.32)
Associated Peptides	1
Associated Spectra	1
Coverage	NaN

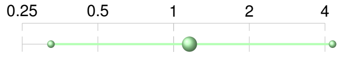

A	B	2.5	50	97.5	Sequence
1	0	0.32	1.2	4.3	ALIAAK

5.730 552 1499 minus strand uncharacterized protein LOC100158466 [Xenopus (Silurana) tropicalis] gi; 552 1499 minus strand uncharacterized protein LOC100158466 [Xenopus (Silurana) tropicalis] gi; 552 1499 minus strand uncharacterized protein LOC100158466 [Xenopus (Silurana) tropicalis] gi

Protein Accession	Unigene57645$_{H}1$ Unigene52825$_{H}1$ Unigene48504$_{H}1$
Mean Expression Ratio	1.03
Median Expression Ratio	1.03
Credible Interval	(0.451, 2.34)
Associated Peptides	1
Associated Spectra	1
Coverage	NaN

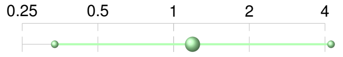

A	B	2.5	50	97.5	Sequence
0	1	0.34	1.2	4.2	TGEPVEVDGELATLLAR

5.731 26 250 minus strand fructose-bisphosphate aldolase [Haliotis discus discus]

Protein Accession	Unigene16711$_{H}1$
Mean Expression Ratio	1.02
Median Expression Ratio	1.03
Credible Interval	(0.486, 2.17)
Associated Peptides	3
Associated Spectra	3
Coverage	NaN

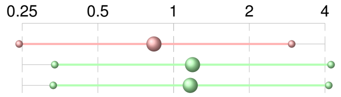

A	B	2.5	50	97.5	Sequence
0	1	0.24	0.84	2.9	RLEGIGVENNEENRR
0	1	0.34	1.2	4.2	IANAIVAPGK
0	1	0.33	1.2	4.2	ELRRIANAIVAPGK

5.732 145 459 fk506-binding protein 2 [Papilio xuthus]; 237 551 fk506-binding protein 2 [Papilio xuthus]

Protein Accession	Unigene7883$_{H}1$ CL4621.Contig4$_{H}1$
Mean Expression Ratio	1.03
Median Expression Ratio	1.03
Credible Interval	(0.555, 1.9)
Associated Peptides	5
Associated Spectra	24
Coverage	NaN

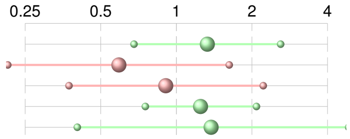

A	B	2.5	50	97.5	Sequence
3	3	0.68	1.3	2.6	GVDVETISEGDKK
1	1	0.21	0.59	1.6	GWDEGVAQMSK
1	2	0.37	0.91	2.2	IGKGEVIK
6	6	0.75	1.2	2.1	NKPFQFK
0	1	0.4	1.4	4.9	GVDVETISEGDKKNYPK

5.733 128 1138 Actin-related protein 2/3 complex subunit 1A OS=Homo sapiens GN=ARPC1A PE=1 SV=2

Protein Accession	CL49279.Contig6$_{H}1$
Mean Expression Ratio	0.98
Median Expression Ratio	0.975
Credible Interval	(0.438, 2.27)
Associated Peptides	1
Associated Spectra	1
Coverage	NaN

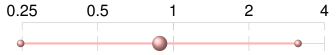

A	B	2.5	50	97.5	Sequence
0	1	0.25	0.89	3.1	TQHQNTITQVSVYEGTK

5.734 " 134 1567 minus strand 6-phosphogluconate dehydrogenase, decarboxylating [Crassostrea gigas]; 134 1573 minus strand 6-phosphogluconate dehydrogenase, decarboxylating [Crassostrea gigas]"

Protein Accession	CL5140.Contig5$_{H}1$ CL5140.Contig4$_{H}1$
Mean Expression Ratio	1.02
Median Expression Ratio	1.03
Credible Interval	(0.516, 1.99)
Associated Peptides	5
Associated Spectra	5
Coverage	NaN

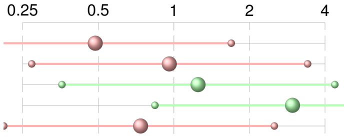

A	B	2.5	50	97.5	Sequence
1	0	0.14	0.49	1.7	FKDTDGSPLVEK
1	0	0.27	0.96	3.4	AFVEDIKR
0	1	0.36	1.3	4.4	SLEDMVDKLK
0	1	0.84	3	11	TVSKVDEFLSNEAK
0	1	0.21	0.74	2.5	NIFQSISAK

5.735 23 409 minus strand CDGSH iron sulfur domain-containing protein 2 [Crassostrea gigas]

Protein Accession	CL36832.Contig6$_{H}1$
Mean Expression Ratio	0.982
Median Expression Ratio	0.976
Credible Interval	(0.426, 2.31)
Associated Peptides	1
Associated Spectra	1
Coverage	NaN

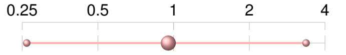

A	B	2.5	50	97.5	Sequence
1	0	0.26	0.95	3.4	VSLPTYLK

5.736 58 900 minus strand Toll-interacting protein [Crassostrea gigas]

Protein Accession	CL6342.Contig9$_{H}1$
Mean Expression Ratio	1.03
Median Expression Ratio	1.02
Credible Interval	(0.451, 2.33)
Associated Peptides	1
Associated Spectra	1
Coverage	NaN

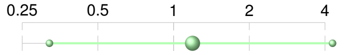

A	B	2.5	50	97.5	Sequence
0	1	0.32	1.2	4.3	VMLGDLPSDFLR

5.737 369 746 stanniocalcin [Haliotis diversicolor supertexta] gi

Protein Accession	CL10391.Contig1$_{H}1$
Mean Expression Ratio	0.977
Median Expression Ratio	0.977
Credible Interval	(0.443, 2.14)
Associated Peptides	2
Associated Spectra	2
Coverage	NaN

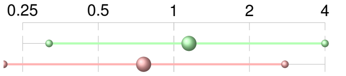

A	B	2.5	50	97.5	Sequence
0	1	0.32	1.1	4	VLQLDDLFR
0	1	0.21	0.76	2.8	FARALNNFTSQGQEFLR

5.738 87 1997 Transketolase-like protein 2 [Crassostrea gigas]; 87 1997 Transketolase-like protein 2 [Crassostrea gigas]; 87 1997 Transketolase-like protein 2 [Crassostrea gigas]; 87 1997 Transketolase-like protein 2 [Crassostrea gigas]; 87 1997 Transketolase-like protein 2 [Crassostrea gigas]; 87 1997 Transketolase-like protein 2 [Crassostrea gigas]; 87 1997 Transketolase-like protein 2 [Crassostrea gigas]

Protein Accession	CL1127.Contig6$_H$1 CL1127.Contig5$_H$1 CL1127.Contig4$_H$1 CL1127.Contig3$_H$1 CL1127.Con
Mean Expression Ratio	1.02
Median Expression Ratio	1.02
Credible Interval	(0.543, 1.95)
Associated Peptides	6
Associated Spectra	8
Coverage	NaN

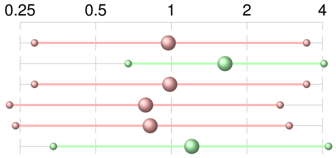

A	B	2.5	50	97.5	Sequence
**0**	**1**	**0.29**	**0.97**	**3.5**	**FTSDLEGHPTPR**
**0**	**3**	**0.68**	**1.6**	**4.1**	**LAYGDALVK**
**0**	**1**	**0.29**	**0.99**	**3.5**	**SAVQVLR**
**0**	**1**	**0.23**	**0.79**	**2.7**	**GIEGIEDLDNWHGK**
**0**	**1**	**0.24**	**0.82**	**3**	**VISLDGDVK**
**0**	**1**	**0.34**	**1.2**	**4.2**	**IIDPFTIKPIDADTIIK**

5.739 123 551 minus strand Transcription factor BTF3-like protein 4 [Crassostrea gigas]

Protein Accession	Unigene19288$_H$1
Mean Expression Ratio	1.03
Median Expression Ratio	1.02
Credible Interval	(0.452, 2.32)
Associated Peptides	1
Associated Spectra	2
Coverage	NaN

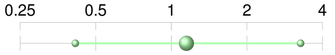

A	B	2.5	50	97.5	Sequence
**0**	**2**	**0.42**	**1.1**	**3.3**	**VQASLAANTFAITGHAETK**

5.740 " 691 852 Protein MLT-11, isoform e [Caenorhabditis elegans] gi"

Protein Accession	CL7753.Contig1$_H$1
Mean Expression Ratio	1.02
Median Expression Ratio	1.02
Credible Interval	(0.441, 2.36)
Associated Peptides	1
Associated Spectra	1
Coverage	NaN

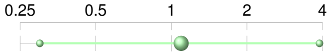

A	B	2.5	50	97.5	Sequence
**0**	**1**	**0.3**	**1.1**	**3.9**	**YYFNYHSR**

5.741 " 353 781 alpha-B-crystallin, putative [Ixodes scapularis] gi"

Protein Accession	CL37349.Contig1$_{H}1$
Mean Expression Ratio	1.02
Median Expression Ratio	1.02
Credible Interval	(0.586, 1.76)
Associated Peptides	7
Associated Spectra	55
Coverage	NaN

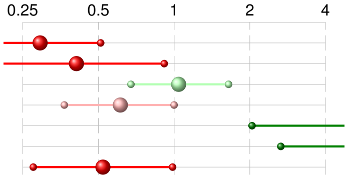

A	B	2.5	50	97.5	Sequence
7	3	0.17	0.29	0.51	IIINAK
2	2	0.18	0.41	0.91	LALEAPK
5	10	0.67	1	1.6	LQESGTSEVVNNDKEFR
3	9	0.37	0.61	1	VAMDVQHFKPEELK
0	2	2	5.5	15	VAMDVQHFKPEELKITTK
0	5	2.7	5.6	12	VAMDVQHFKPEELKITTKDNR
0	7	0.28	0.52	0.99	TVTSSLSPSGVLTLK

5.742 172 612 minus strand putative polyadenylate-binding protein 1 [Haliotis diversicolor]; 172 612 putative polyadenylate-binding protein 1 [Haliotis diversicolor]

Protein Accession	CL48895.Contig3$_{H}1$ CL48895.Contig2$_{H}1$
Mean Expression Ratio	1.02
Median Expression Ratio	1.02
Credible Interval	(0.463, 2.21)
Associated Peptides	2
Associated Spectra	2
Coverage	NaN

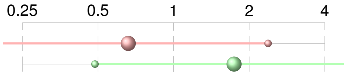

A	B	2.5	50	97.5	Sequence
1	0	0.19	0.66	2.4	ALDTMNFDTVK
0	1	0.49	1.7	6.2	KSGVGNVFIK

5.743 149 496 Spectrin alpha chain [Crassostrea gigas]

Protein Accession	CL2014.Contig1$_{H}1$
Mean Expression Ratio	0.981
Median Expression Ratio	0.98
Credible Interval	(0.419, 2.25)
Associated Peptides	1
Associated Spectra	1
Coverage	NaN

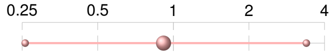

A	B	2.5	50	97.5	Sequence
0	1	0.26	0.92	3.4	DADELESWILEK

5.744 176 670 hypothetical protein BRAFLDRAFT_122621 [Branchiostoma floridae] gi

Protein Accession	Unigene180$_{H}1$
Mean Expression Ratio	1.02
Median Expression Ratio	1.02
Credible Interval	(0.558, 1.88)
Associated Peptides	5
Associated Spectra	34
Coverage	NaN

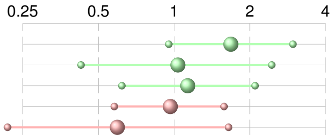

A	B	2.5	50	97.5	Sequence
2	7	0.95	1.7	3	VFFDITADGQPLGR
1	2	0.43	1	2.4	ADVVPK
2	6	0.62	1.1	2.1	FADENFLLK
6	6	0.58	0.96	1.6	IVMELR
0	2	0.22	0.6	1.6	HVVFGQVVEGMDVVR

5.745 " 34 1656 Chaperonin containing TCP1, subunit 7 (eta) [Danio rerio] gi; 34 1656 Chaperonin containing TCP1, subunit 7 (eta) [Danio rerio] gi"

Protein Accession	CL24061.Contig2$_{H}1$ CL24061.Contig1$_{H}1$
Mean Expression Ratio	0.981
Median Expression Ratio	0.98
Credible Interval	(0.511, 1.89)
Associated Peptides	5
Associated Spectra	10
Coverage	NaN

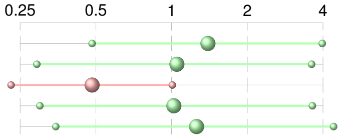

A	B	2.5	50	97.5	Sequence
1	1	0.48	1.4	4	TLDIVHPAAK
1	0	0.29	1	3.6	TLVDIAR
2	3	0.23	0.48	1	SIAVSIKK
0	1	0.3	1	3.6	SLHDAIMIVR
0	1	0.35	1.3	4.4	MQAPIILLK

5.746 247 726 Annexin A7 [Crassostrea gigas]; 286 675 Annexin A7 [Crassostrea gigas]

Protein Accession	CL15539.Contig3$_{H}1$ CL15539.Contig1$_{H}1$
Mean Expression Ratio	0.982
Median Expression Ratio	0.981
Credible Interval	(0.484, 1.96)
Associated Peptides	3
Associated Spectra	9
Coverage	NaN

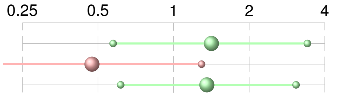

A	B	2.5	50	97.5	Sequence
1	2	0.57	1.4	3.4	GLGTDEAAVIR
1	1	0.17	0.47	1.3	DLVSDLK
0	4	0.61	1.4	3.1	ILTSHNNMQR

5.747 284 700 Ganglioside GM2 activator [Crassostrea gigas]

Protein Accession	Unigene16455$_{H}1$
Mean Expression Ratio	1.02
Median Expression Ratio	1.02
Credible Interval	(0.541, 1.87)
Associated Peptides	6
Associated Spectra	18
Coverage	NaN

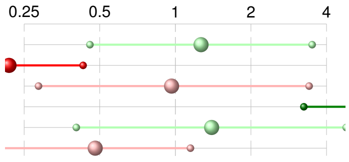

A	B	2.5	50	97.5	Sequence
1	1	0.46	1.3	3.5	ATVDSPLKAEVYLGK
4	2	0.11	0.22	0.43	AEVYLGK
1	0	0.28	0.96	3.4	VGSIWVK
0	5	3.2	6.8	14	ATVDSPLKAEVYLGKK
0	1	0.4	1.4	4.8	TFQFTNCLPK
0	3	0.19	0.48	1.2	ATVDSPLK

5.748 40 1182 4-hydroxyphenylpyruvate dioxygenase [Crassostrea gigas]

Protein Accession	CL7585.Contig2$_{H}1$
Mean Expression Ratio	1.02
Median Expression Ratio	1.02
Credible Interval	(0.579, 1.8)
Associated Peptides	10
Associated Spectra	15
Coverage	NaN

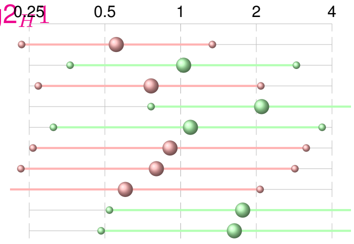

A	B	2.5	50	97.5	Sequence
1	2	0.23	0.55	1.3	SIVVTNYEETIK
1	1	0.36	1	2.9	TYGDTTHTLIDR
1	1	0.27	0.76	2.1	QVAAHVVK
0	2	0.76	2.1	5.8	DVAFEVEDLDAIFKR
0	1	0.31	1.1	3.6	QIHTDYSALR
0	1	0.26	0.91	3.2	SLFEAIELDQAER
0	1	0.23	0.8	2.8	VQIKEDLDELQK
0	1	0.17	0.6	2.1	FWSVDDK
0	1	0.52	1.8	6.1	MPINEPAPGKR
0	1	0.48	1.6	5.7	TSNIIQSINR

5.749 1 987 minus strand RecName: Full=Tubulin beta chain; AltName: Full=Beta-tubulin

Protein Accession Unigene7954$_H1$
Mean Expression Ratio 0.981
Median Expression Ratio 0.983
Credible Interval (0.54, 1.79)
Associated Peptides 7
Associated Spectra 16
Coverage NaN

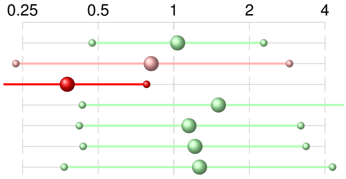

A	B	2.5	50	97.5	Sequence
2	2	0.47	1	2.3	ALTVPELTQQMFDAK
0	1	0.24	0.81	2.9	AILVDLEPGTMDSVR
0	5	0.18	0.38	0.78	IMNTFSVVPSPK
0	1	0.43	1.5	5.4	FWEVISDEHGIDPTGTYHGDSDLQLER
0	2	0.42	1.1	3.2	INVYYNEATGGK
0	2	0.43	1.2	3.4	PELTQQMFDAK
0	1	0.37	1.3	4.3	VAAIFR

5.750 103 1356 minus strand PREDICTED: actin-related protein 3-like [Oreochromis niloticus]

Protein Accession CL20855.Contig4$_H1$
Mean Expression Ratio 0.983
Median Expression Ratio 0.983
Credible Interval (0.456, 2.17)
Associated Peptides 2
Associated Spectra 3
Coverage NaN

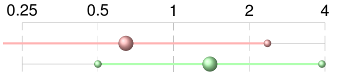

A	B	2.5	50	97.5	Sequence
1	0	0.18	0.64	2.4	LPAVVVDTGTGYTK
1	1	0.5	1.4	3.9	HNPVFGAMA

5.751 225 839 minus strand Metallo-beta-lactamase domain-containing protein 1 [Crassostrea gigas]; 201 851 minus strand Metallo-beta-lactamase domain-containing protein 1 [Crassostrea gigas]

Protein Accession CL35672.Contig2$_H1$ CL35672.Contig1$_H1$
Mean Expression Ratio 0.981
Median Expression Ratio 0.983
Credible Interval (0.426, 2.26)
Associated Peptides 1
Associated Spectra 1
Coverage NaN

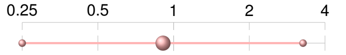

A	B	2.5	50	97.5	Sequence
0	1	0.25	0.91	3.3	EADFIVPGHGPMFR

5.752 3 281 minus strand hypothetical protein CGI_10015501 [Crassostrea gigas]

Protein Accession	Unigene26367$_{H}1$
Mean Expression Ratio	0.992
Median Expression Ratio	0.983
Credible Interval	(0.438, 2.28)
Associated Peptides	1
Associated Spectra	1
Coverage	NaN

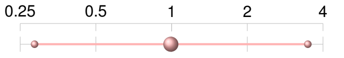

A	B	2.5	50	97.5	Sequence
0	1	0.28	0.99	3.5	AGNDITADQVIDDVK

5.753 91 3270 Valyl-tRNA synthetase [Crassostrea gigas]

Protein Accession	CL23212.Contig7$_{H}1$
Mean Expression Ratio	1.01
Median Expression Ratio	1.02
Credible Interval	(0.463, 2.24)
Associated Peptides	2
Associated Spectra	2
Coverage	NaN

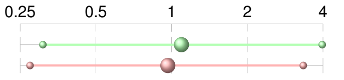

A	B	2.5	50	97.5	Sequence
0	1	0.31	1.1	4	FVQHPFFDR
0	1	0.27	0.96	3.3	SFGTGAVK

5.754 206 316 minus strand ribosomal protein S17 [Branchiostoma belcheri]

Protein Accession	Unigene31494$_{H}1$
Mean Expression Ratio	1.02
Median Expression Ratio	1.02
Credible Interval	(0.482, 2.13)
Associated Peptides	3
Associated Spectra	5
Coverage	NaN

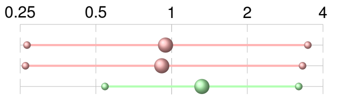

A	B	2.5	50	97.5	Sequence
0	1	0.27	0.95	3.5	LTLDFHTNK
0	1	0.26	0.92	3.3	VCEEIAIIPSK
0	3	0.54	1.3	3.2	LTLDFHTNKR

5.755 266 934 minus strand Kyphoscoliosis peptidase [Crassostrea gigas]

Protein Accession	CL7490.Contig16$_{H}1$
Mean Expression Ratio	1.01
Median Expression Ratio	1.02
Credible Interval	(0.463, 2.21)
Associated Peptides	2
Associated Spectra	2
Coverage	NaN

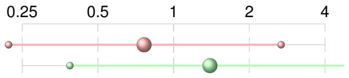

A	B	2.5	50	97.5	Sequence
0	1	0.22	0.76	2.7	DLDNVHFK
0	1	0.39	1.4	5	SRPELPHGYLGAQSK

5.756 98 1012 minus strand RecName: Full=Substrate-specific endoprotease Tex31; Flags: Precursor gi|32187775|emb|CAD36507.1| substrate-specific endoprotease [Conus textile]

Protein Accession	CL13681.Contig1$_H$1
Mean Expression Ratio	1.02
Median Expression Ratio	1.02
Credible Interval	(0.438, 2.34)
Associated Peptides	1
Associated Spectra	1
Coverage	NaN

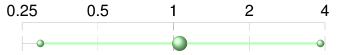

A	B	2.5	50	97.5	Sequence
**0**	**1**	**0.3**	**1.1**	**3.8**	**NIPLSASDISAIVER**

5.757 293 1795 Amidase [Crassostrea gigas]

Protein Accession	CL5248.Contig6$_H$1
Mean Expression Ratio	1.01
Median Expression Ratio	1.02
Credible Interval	(0.508, 2)
Associated Peptides	4
Associated Spectra	7
Coverage	NaN

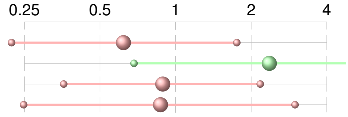

A	B	2.5	50	97.5	Sequence
**1**	**1**	**0.22**	**0.62**	**1.8**	**EVSVPMHK**
**0**	**1**	**0.68**	**2.4**	**8.3**	**ILDAGGHIMGK**
**0**	**3**	**0.36**	**0.89**	**2.2**	**LSTIGVTHFDR**
**0**	**1**	**0.25**	**0.87**	**3**	**YAHPQDLHPMIK**

5.758 239 1282 minus strand PREDICTED: hypothetical protein LOC100122475 [Nasonia vitripennis]

Protein Accession	CL45186.Contig4$_H$1
Mean Expression Ratio	1.01
Median Expression Ratio	1.02
Credible Interval	(0.44, 2.3)
Associated Peptides	1
Associated Spectra	1
Coverage	NaN

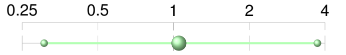

A	B	2.5	50	97.5	Sequence
**1**	**0**	**0.31**	**1.1**	**3.7**	**EQQTPLHIASR**

5.759 227 1201 PREDICTED: quinone oxidoreductase-like isoform 1 [Ornithorhynchus anatinus]; 109 1083 PREDICTED: quinone oxidoreductase-like isoform 1 [Ornithorhynchus anatinus]; 164 1138 PREDICTED: quinone oxidoreductase-like isoform 1 [Ornithorhynchus anatinus]

Protein Accession	CL11899.Contig2$_H$1 CL11899.Contig1$_H$1 CL11899.Contig13$_H$1
Mean Expression Ratio	0.982
Median Expression Ratio	0.985
Credible Interval	(0.505, 1.91)
Associated Peptides	5
Associated Spectra	8
Coverage	NaN

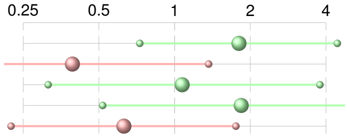

A	B	2.5	50	97.5	Sequence
1	2	0.72	1.8	4.4	VHASGVNPVDTYIR
0	1	0.11	0.39	1.4	EGLELVK
0	1	0.31	1.1	3.8	ILGTAGTKEGLELVK
0	1	0.52	1.8	6.4	LVLKL
0	2	0.22	0.63	1.7	VHQWGGPEVLK

5.760 54 638 minus strand ubiquitin carboxyl-terminal hydrolase [Aplysia californica] gi

Protein Accession	CL20666.Contig3$_H$1
Mean Expression Ratio	0.985
Median Expression Ratio	0.986
Credible Interval	(0.464, 2.13)
Associated Peptides	3
Associated Spectra	3
Coverage	NaN

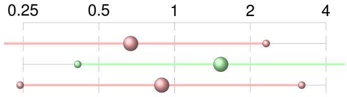

A	B	2.5	50	97.5	Sequence
0	1	0.19	0.67	2.3	THADTLLEDTVK
0	1	0.41	1.5	5.4	KDAPVCHGK
0	1	0.24	0.89	3.2	FLDETK

5.761 154 261 ribosomal protein S18 [Stenella coeruleoalba]

Protein Accession	CL15120.Contig1$_H$1
Mean Expression Ratio	1.02
Median Expression Ratio	1.01
Credible Interval	(0.472, 2.21)
Associated Peptides	2
Associated Spectra	4
Coverage	NaN

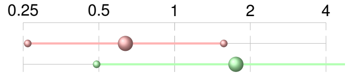

A	B	2.5	50	97.5	Sequence
1	2	0.26	0.64	1.6	VLNTNIDGR
0	1	0.49	1.8	6.2	VLNTNIDGRR

5.762 440 1645 Tektin-1 [Crassostrea gigas]

Protein Accession	CL18799.Contig2$_{H}1$
Mean Expression Ratio	1.02
Median Expression Ratio	1.01
Credible Interval	(0.491, 2.13)
Associated Peptides	3
Associated Spectra	3
Coverage	NaN

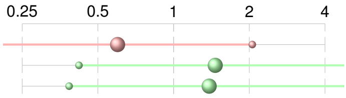

A	B	2.5	50	97.5	Sequence
**0**	**1**	**0.17**	**0.6**	**2.1**	**EVETLEGVQALLQR**
**0**	**1**	**0.42**	**1.5**	**5.1**	**SLQATEEPLHIAR**
**0**	**1**	**0.38**	**1.4**	**4.9**	**FTHEDWTR**

5.763 96 497 AAEL009772-PB [Aedes aegypti]

Protein Accession	CL27854.Contig2$_{H}1$
Mean Expression Ratio	1.01
Median Expression Ratio	1.01
Credible Interval	(0.463, 2.21)
Associated Peptides	2
Associated Spectra	2
Coverage	NaN

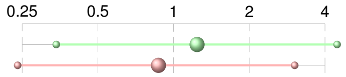

A	B	2.5	50	97.5	Sequence
**0**	**1**	**0.34**	**1.2**	**4.5**	**LVEEFPMGNIK**
**0**	**1**	**0.24**	**0.87**	**3**	**MNPGFDTIGK**

5.764 128 1519 minus strand V-type proton ATPase subunit H [Crassostrea gigas]; 128 1519 minus strand V-type proton ATPase subunit H [Crassostrea gigas]; 128 1519 minus strand V-type proton ATPase subunit H [Crassostrea gigas]; 128 1519 minus strand V-type proton ATPase subunit H [Crassostrea gigas]

Protein Accession	Unigene39559$_{H}1$ Unigene2219$_{H}1$ CL1883.Contig8$_{H}1$ CL1883.Contig6$_{H}1$
Mean Expression Ratio	0.986
Median Expression Ratio	0.986
Credible Interval	(0.451, 2.14)
Associated Peptides	2
Associated Spectra	3
Coverage	NaN

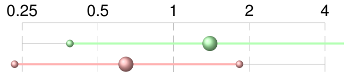

A	B	2.5	50	97.5	Sequence
**0**	**1**	**0.39**	**1.4**	**5**	**ANVVNWK**
**0**	**2**	**0.23**	**0.65**	**1.8**	**SGNLEWSPVHR**

5.765 616 1740 5'-AMP-activated protein kinase subunit beta-2 [Crassostrea gigas]; 641 1762 minus strand 5'-AMP-activated protein kinase subunit beta-2 [Crassostrea gigas]; 626 1786 5'-AMP-activated protein kinase subunit beta-2 [Crassostrea gigas]; 431 1591 5'-AMP-activated protein kinase subunit beta-2 [Crassostrea gigas]

Protein Accession	Unigene52845$_H$1 Unigene41754$_H$1 Unigene36145$_H$1 CL5821.Contig3$_H$1
Mean Expression Ratio	1.01
Median Expression Ratio	1.01
Credible Interval	(0.445, 2.26)
Associated Peptides	1
Associated Spectra	1
Coverage	NaN

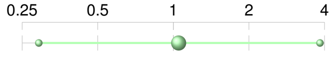

A	B	2.5	50	97.5	Sequence
0	1	0.29	1	3.8	FIVDEQWVVSK

5.766 424 3507 minus strand putative Na+/K+-ATPase alpha subunit [Paroctopus digueti]; 424 3507 minus strand putative Na+/K+-ATPase alpha subunit [Paroctopus digueti]

Protein Accession	Unigene11494$_H$1 Unigene11493$_H$1
Mean Expression Ratio	0.982
Median Expression Ratio	0.987
Credible Interval	(0.585, 1.63)
Associated Peptides	14
Associated Spectra	21
Coverage	NaN

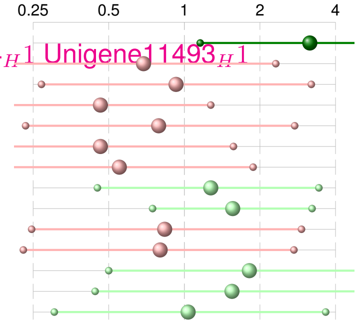

A	B	2.5	50	97.5	Sequence
2	0	1.2	3.2	8.8	GIPVEEVDPREAK
1	0	0.19	0.69	2.3	KVVEIPFNSTNK
1	0	0.27	0.93	3.2	SAEFTHENPLETR
1	1	0.17	0.46	1.3	VIVAHGFK
1	0	0.23	0.79	2.8	GVGIISEGSK
1	0	0.13	0.46	1.6	AENDIMK
0	1	0.16	0.55	1.9	AEELVLGDVVEVK
0	2	0.45	1.3	3.4	DMTPAQIDEILR
0	5	0.75	1.6	3.2	FGDRVPADLR
0	1	0.25	0.84	2.9	GIPVEEVDPR
0	1	0.23	0.8	2.7	VIMVTGDHPITAK
0	1	0.5	1.8	6.1	AKEILLR
0	1	0.44	1.6	5.5	NLDELK
0	1	0.3	1	3.7	YLLVMK

5.767 " 3341 4240 minus strand nonmuscle myosin II, partial [Aplysia californica]"

Protein Accession	CL19183.Contig2$_H$1
Mean Expression Ratio	0.988
Median Expression Ratio	0.987
Credible Interval	(0.563, 1.75)
Associated Peptides	9
Associated Spectra	16
Coverage	NaN

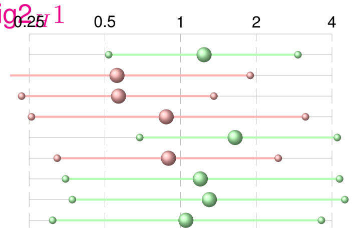

A	B	2.5	50	97.5	Sequence
1	2	0.52	1.2	2.9	QKQDVEQLEAR
1	0	0.17	0.56	1.9	RQQVEELQAQLAK
2	1	0.23	0.57	1.4	TATMLEEK
1	0	0.26	0.88	3.1	YAQMIEEK
0	3	0.69	1.6	4.2	ELQNQMTELNEDLETER
0	2	0.32	0.89	2.4	LHTKEEELK
0	1	0.35	1.2	4.3	RKEELEDILHEMELR
0	1	0.37	1.3	4.5	KLEEDLALNEDTNQK
0	1	0.31	1	3.6	LSEAMAQIVEEEEK

5.768 32 193 minus strand Ubiquinol-cytochrome C reductase complex [Xenopus laevis] gi; 32 193 minus strand Ubiquinol-cytochrome C reductase complex [Xenopus laevis] gi

Protein Accession	CL7847.Contig7$_H$1 CL7847.Contig4$_H$1
Mean Expression Ratio	1.02
Median Expression Ratio	1.01
Credible Interval	(0.485, 2.16)
Associated Peptides	2
Associated Spectra	8
Coverage	NaN

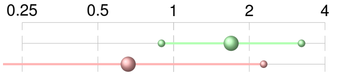

A	B	2.5	50	97.5	Sequence
3	4	0.89	1.7	3.2	AFLKPQAPK
1	0	0.19	0.66	2.3	AGPSIFR

5.769 242 1510 minus strand hypothetical protein BRAFLDRAFT_276673 [Branchiostoma floridae] gi

Protein Accession	Unigene23008$_H$1
Mean Expression Ratio	1.02
Median Expression Ratio	1.01
Credible Interval	(0.48, 2.21)
Associated Peptides	2
Associated Spectra	3
Coverage	NaN

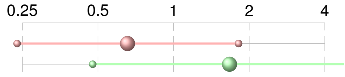

A	B	2.5	50	97.5	Sequence
2	0	0.24	0.66	1.8	TVFITGASR
0	1	0.47	1.7	6	DGANIVIAAK

5.770 288 1487 minus strand putative procollagen type VI alpha 4 [Haliotis diversicolor]

Protein Accession	CL6899.Contig2$_H$1
Mean Expression Ratio	0.985
Median Expression Ratio	0.987
Credible Interval	(0.569, 1.7)
Associated Peptides	9
Associated Spectra	26
Coverage	NaN

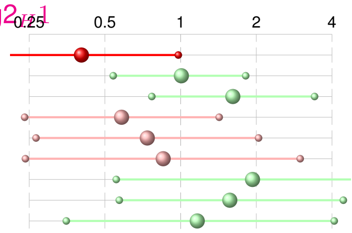

A	B	2.5	50	97.5	Sequence
1	2	0.16	0.4	0.98	IGALTFSDDVK
4	4	0.54	1	1.8	TGVTNTHLAIR
2	3	0.77	1.6	3.4	FRDDITK
2	1	0.24	0.58	1.4	QVLQFVSQVITR
0	2	0.27	0.74	2	AEVLAAIDQVR
0	1	0.24	0.85	3	MGFDVDQFR
0	1	0.55	1.9	6.8	VMVVITDGSSR
0	2	0.57	1.6	4.4	LRENLK
0	1	0.35	1.2	4.1	QLAAAARSLR

5.771 70 705 RecName: Full=Ras-related protein Rab-2 gi|288938|emb|CAA51234.1| RAB2 [Lymnaea stagnalis]; 70 705 RecName: Full=Ras-related protein Rab-2 gi|288938|emb|CAA51234.1| RAB2 [Lymnaea stagnalis]

Protein Accession	Unigene59013$_{H}1$ Unigene19254$_{H}1$
Mean Expression Ratio	0.983
Median Expression Ratio	0.987
Credible Interval	(0.466, 2.08)
Associated Peptides	3
Associated Spectra	3
Coverage	NaN

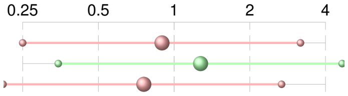

A	B	2.5	50	97.5	Sequence
0	1	0.25	0.9	3.2	TAANVEEAFINTAK
0	1	0.35	1.3	4.6	KEEGEAFAR
0	1	0.21	0.76	2.7	EHGLIFMETSAK

5.772 49 1038 Glyceraldehyde-3-phosphate dehydrogenase [Crotalus adamanteus]

Protein Accession	Unigene7744$_{H}1$
Mean Expression Ratio	1.02
Median Expression Ratio	1.01
Credible Interval	(0.561, 1.84)
Associated Peptides	6
Associated Spectra	25
Coverage	NaN

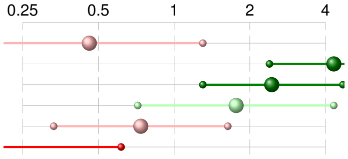

A	B	2.5	50	97.5	Sequence
2	0	0.17	0.46	1.3	AGIQLSDKFVK
2	6	2.4	4.3	7.8	TVDGPSNKDWR
1	6	1.3	2.5	4.7	SSIFDAK
0	3	0.72	1.8	4.3	GAYQNIIPSSTGAAK
0	4	0.33	0.74	1.6	VIPALNGK
0	1	0.051	0.18	0.62	FGIEEALMTTVHSATATQK

5.773 3 1325 minus strand Eukaryotic translation initiation factor 3 subunit B [Crassostrea gigas]

Protein Accession	CL19479.Contig9$_{H}1$
Mean Expression Ratio	1.02
Median Expression Ratio	1.01
Credible Interval	(0.448, 2.36)
Associated Peptides	1
Associated Spectra	1
Coverage	NaN

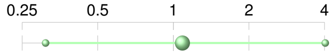

A	B	2.5	50	97.5	Sequence
0	1	0.31	1.1	4	FAFIHGESPR

5.774 " 285 437 Propionyl-CoA carboxylase alpha chain, mitochondrial [Crassostrea gigas]"

Protein Accession	CL45581.Contig2$_H$1
Mean Expression Ratio	0.992
Median Expression Ratio	0.989
Credible Interval	(0.44, 2.23)
Associated Peptides	1
Associated Spectra	2
Coverage	NaN

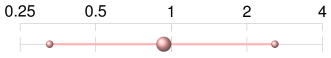

A	B	2.5	50	97.5	Sequence
**0**	**2**	**0.33**	**0.93**	**2.6**	**GVTHNIPLLR**

5.775 " 29 448 minus strand PREDICTED: similar to NADH:ubiquinone dehydrogenase, putative [Tribolium castaneum] gi; 19 408 minus strand hypothetical protein KGM_18901 [Danaus plexippus]; 29 448 minus strand PREDICTED: similar to NADH:ubiquinone dehydrogenase, putative [Tribolium castaneum] gi"

Protein Accession	CL2684.Contig8$_H$1 CL2684.Contig7$_H$1 CL2684.Contig6$_H$1
Mean Expression Ratio	0.991
Median Expression Ratio	0.989
Credible Interval	(0.441, 2.23)
Associated Peptides	1
Associated Spectra	2
Coverage	NaN

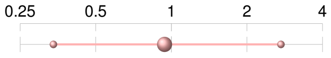

A	B	2.5	50	97.5	Sequence
**0**	**2**	**0.34**	**0.94**	**2.7**	**LLQDGEKELFLK**

5.776 2 478 Glyoxylate reductase/hydroxypyruvate reductase [Crassostrea gigas]

Protein Accession	CL29479.Contig5$_H$1
Mean Expression Ratio	1.01
Median Expression Ratio	1.01
Credible Interval	(0.48, 2.14)
Associated Peptides	3
Associated Spectra	3
Coverage	NaN

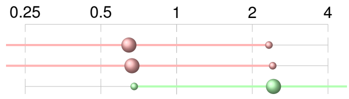

A	B	2.5	50	97.5	Sequence
**1**	**0**	**0.18**	**0.65**	**2.3**	**VGFGVAR**
**0**	**1**	**0.19**	**0.67**	**2.4**	**GSIVDQDALDEALK**
**0**	**1**	**0.68**	**2.4**	**8.7**	**MKEGSILINTAR**

5.777 77 1561 retinal dehydrogenase [Lymnaea stagnalis]; 69 1553 retinal dehydrogenase [Lymnaea stagnalis]

Protein Accession	CL13284.Contig7$_{H}$1 CL13284.Contig2$_{H}$1
Mean Expression Ratio	1.01
Median Expression Ratio	1.01
Credible Interval	(0.655, 1.55)
Associated Peptides	19
Associated Spectra	48
Coverage	NaN

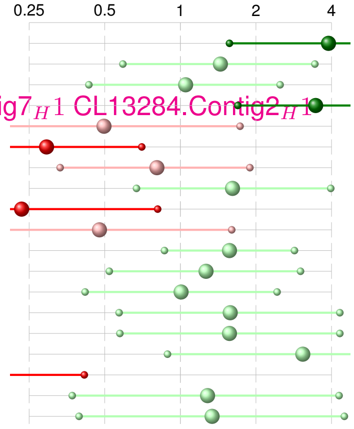

A	B	2.5	50	97.5	Sequence
2	1	1.6	3.9	9.3	NVVIKIPQKNS
1	2	0.59	1.4	3.4	SFPEPIRNPEVK
1	2	0.43	1	2.5	TFPTINPATGK
3	2	1.7	3.4	7.1	TFPTINPATGKK
1	0	0.15	0.5	1.7	TAELANKR
1	2	0.12	0.29	0.7	AVEVAQDAFK
1	2	0.33	0.8	1.9	IYDEFVR
1	2	0.67	1.6	4	LGSAWR
1	0	0.07	0.23	0.81	NVVIK
1	0	0.14	0.48	1.6	GALLYK
1	7	0.87	1.6	2.9	ADVDKAVEVAQDAFK
1	2	0.52	1.3	3	KTAELANK
0	3	0.42	1	2.4	ELGEYGLQQYVEVK
0	2	0.57	1.6	4.3	FSTVEEAIER
0	2	0.58	1.6	4.3	KTAELANKR
0	1	0.89	3.1	11	NVVIKIPQK
0	1	0.035	0.12	0.41	LADLMEK
0	1	0.37	1.3	4.3	RGALLYK
0	1	0.4	1.3	4.5	TTLELGGK

5.778 " 1482 3320 minus strand Phosphoenolpyruvate carboxykinase, cytosolic [GTP] [Crassostrea gigas]; 1326 3164 minus strand Phosphoenolpyruvate carboxykinase, cytosolic [GTP] [Crassostrea gigas]; 303 2102 minus strand Phosphoenolpyruvate carboxykinase, cytosolic [GTP] [Crassostrea gigas]"

Protein Accession	CL1008.Contig5$_{H}$1 CL1008.Contig3$_{H}$1 CL1008.Contig2$_{H}$1
Mean Expression Ratio	1.01
Median Expression Ratio	1.01
Credible Interval	(0.49, 2.07)
Associated Peptides	4
Associated Spectra	4
Coverage	NaN

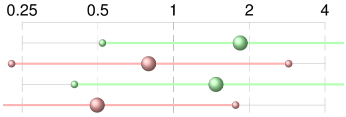

A	B	2.5	50	97.5	Sequence
0	1	0.52	1.8	6.5	AINPENGFFGVAPGTSVK
0	1	0.23	0.79	2.9	YFDEQVNEDLPR
0	1	0.4	1.5	5.1	NLHLPK
0	1	0.14	0.5	1.8	GGNALLGK

5.779 356 820 minus strand calmodulin-1 [Trichinella spiralis] gi

Protein Accession	CL672.Contig3$_{H}$1
Mean Expression Ratio	1.01
Median Expression Ratio	1.01
Credible Interval	(0.492, 2.09)
Associated Peptides	3
Associated Spectra	8
Coverage	NaN

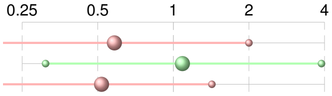

A	B	2.5	50	97.5	Sequence
0	1	0.16	0.58	2	ADQLTEEQIAEFK
0	1	0.31	1.1	3.9	VFDKDGNGFISAAELR
5	1	0.19	0.52	1.4	ELGTVMR

5.780 60 353 minus strand Histone H3.3 [Harpegnathos saltator]

Protein Accession	Unigene31518$_H$1
Mean Expression Ratio	1.01
Median Expression Ratio	1.01
Credible Interval	(0.441, 2.36)
Associated Peptides	1
Associated Spectra	1
Coverage	NaN

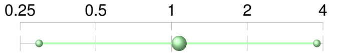

A	B	2.5	50	97.5	Sequence
**0**	**1**	**0.3**	**1.1**	**3.8**	**KSAPSTGGVK**

5.781 1573 2385 minus strand Spectrin beta chain [Crassostrea gigas]

Protein Accession	CL5904.Contig4$_H$1
Mean Expression Ratio	1.01
Median Expression Ratio	1.01
Credible Interval	(0.464, 2.26)
Associated Peptides	2
Associated Spectra	2
Coverage	NaN

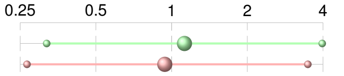

A	B	2.5	50	97.5	Sequence
**0**	**1**	**0.32**	**1.1**	**4**	**LIQEEHPESDQIAVR**
**0**	**1**	**0.27**	**0.94**	**3.5**	**RLIQEEHPESDQIAVR**

5.782 628 1140 paramyosin [Haliotis discus discus]

Protein Accession	CL43455.Contig3$_H$1
Mean Expression Ratio	1.01
Median Expression Ratio	1.01
Credible Interval	(0.476, 2.14)
Associated Peptides	2
Associated Spectra	21
Coverage	NaN

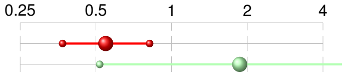

A	B	2.5	50	97.5	Sequence
**8**	**12**	**0.37**	**0.55**	**0.82**	**LEEAEAFAVK**
**1**	**0**	**0.52**	**1.9**	**6.4**	**EITRVVKV**

5.783 320 589 60S ribosomal protein L34 OS=Aedes triseriatus GN=RpL34 PE=2 SV=3

Protein Accession	CL29774.Contig2$_H$1
Mean Expression Ratio	1.01
Median Expression Ratio	1.01
Credible Interval	(0.454, 2.26)
Associated Peptides	1
Associated Spectra	3
Coverage	NaN

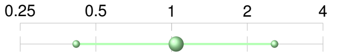

A	B	2.5	50	97.5	Sequence
**1**	**2**	**0.42**	**1**	**2.6**	**AFLIEEQK**

5.784 4365 4553 minus strand Basement membrane-specific heparan sulfate proteoglycan core protein [Crassostrea gigas]; 2502 2696 minus strand hypothetical protein DAPPUDRAFT_186952 [Daphnia pulex]

Protein Accession	CL24300.Contig6$_{H}1$ CL24300.Contig2$_{H}1$
Mean Expression Ratio	1
Median Expression Ratio	1.01
Credible Interval	(0.436, 2.3)
Associated Peptides	1
Associated Spectra	1
Coverage	NaN

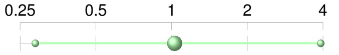

A	B	2.5	50	97.5	Sequence
0	1	0.29	1	3.9	DGSIIVDGQEVK

5.785 323 1192 cytosolic malate dehydrogenase [Lottia scabra]; 323 1192 cytosolic malate dehydrogenase [Lottia scabra]; 323 1192 cytosolic malate dehydrogenase [Lottia scabra]; 323 1192 cytosolic malate dehydrogenase [Lottia scabra]; 2474 3343 cytosolic malate dehydrogenase [Lottia scabra]; 116 1111 minus strand cytosolic malate dehydrogenase [Lottia scabra]

Protein Accession	CL280.Contig21$_{H}1$ CL280.Contig20$_{H}1$ CL280.Contig19$_{H}1$ CL280.Contig18$_{H}1$ CL280.Conti
Mean Expression Ratio	0.992
Median Expression Ratio	0.991
Credible Interval	(0.66, 1.49)
Associated Peptides	21
Associated Spectra	110
Coverage	NaN

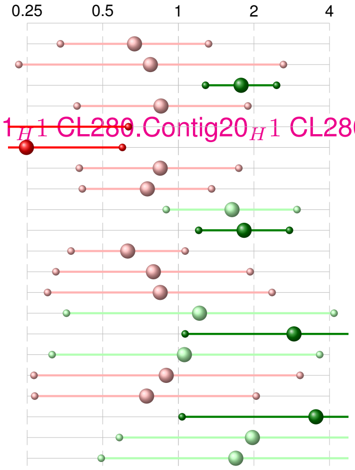

A	B	2.5	50	97.5	Sequence
4	2	0.34	0.67	1.3	DLLAANIK
1	0	0.23	0.77	2.6	GAEVIK
11	19	1.3	1.8	2.5	KLSSAMSAAK
2	2	0.4	0.86	1.9	LSSAMSAAK
1	0	0.055	0.19	0.63	EVIPTSDEK
2	1	0.1	0.25	0.6	ELLEER
1	4	0.4	0.85	1.7	QVTIVEGLSISDFAR
2	6	0.42	0.75	1.4	EMAEQILAA
1	7	0.9	1.6	3	KDLLAANIK
5	13	1.2	1.8	2.8	LGIPNNAVSK
5	6	0.37	0.63	1.1	SQGANLDAVAK
0	3	0.33	0.79	1.9	DCDAAFLVGAMPR
0	2	0.3	0.85	2.4	EAVKDDNWINNEFISTVQK
0	1	0.36	1.2	4.2	ELLEEREMAEQILAA
0	2	1.1	2.9	8.1	MDDTAKELLEER
0	1	0.31	1.1	3.7	NKQVTIVEGLSISDFAR
0	1	0.27	0.9	3	VVIVGNPANTNALICAK
0	2	0.27	0.75	2	YGIPEGVMYSYPVQIK
0	1	1	3.5	12	SQGANLDAVAKK
0	1	0.58	2	6.4	GAEVIKMR
0	1	0.49	1.7	5.9	VTIWGNHSSTQFPDVDHAVAVVNGKPHPVR

5.786 457 1842 Basement membrane-specific heparan sulfate proteoglycan core protein [Crassostrea gigas]; 457 1842 Basement membrane-specific heparan sulfate proteoglycan core protein [Crassostrea gigas]

Protein Accession	CL24300.Contig9$_{H}1$ CL24300.Contig10$_{H}1$
Mean Expression Ratio	0.989
Median Expression Ratio	0.991
Credible Interval	(0.443, 2.24)
Associated Peptides	1
Associated Spectra	2
Coverage	NaN

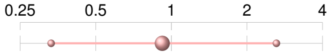

A	B	2.5	50	97.5	Sequence
1	1	0.33	0.92	2.6	KDGELPVEHK

5.787 794 994 Glycogenin-1 [Crassostrea gigas]; 1425 1739 minus strand Glycogenin-1 [Crassostrea gigas]

Protein Accession	CL18608.Contig3$_{H}1$ CL18608.Contig2$_{H}1$
Mean Expression Ratio	0.991
Median Expression Ratio	0.991
Credible Interval	(0.432, 2.32)
Associated Peptides	1
Associated Spectra	1
Coverage	NaN

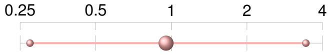

A	B	2.5	50	97.5	Sequence
0	1	0.27	0.95	3.4	IAAPVLSFK

5.788 87 1454 hypothetical protein BRAFLDRAFT_287788 [Branchiostoma floridae] gi; 117 1469 hypothetical protein BRAFLDRAFT_287788 [Branchiostoma floridae] gi; 117 1469 hypothetical protein BRAFLDRAFT_287788 [Branchiostoma floridae] gi

Protein Accession	Unigene48935$_{H}1$ Unigene21344$_{H}1$ CL945.Contig6$_{H}1$
Mean Expression Ratio	0.995
Median Expression Ratio	0.992
Credible Interval	(0.582, 1.71)
Associated Peptides	9
Associated Spectra	29
Coverage	NaN

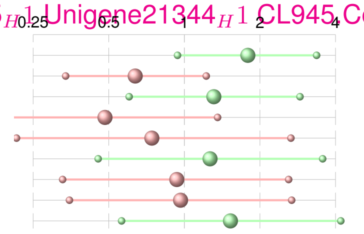

A	B	2.5	50	97.5	Sequence
4	3	0.94	1.8	3.4	ISSLDNGLR
3	4	0.34	0.64	1.2	RIPLPELEAR
2	2	0.6	1.3	2.9	TILGPTQNIR
1	1	0.17	0.48	1.4	FTGSEIR
1	0	0.21	0.74	2.7	IDAVNAK
0	2	0.45	1.3	3.5	AATLEQTILNVPETR
0	2	0.33	0.93	2.6	MVLAAAGGVNHGELVDLAK
0	2	0.35	0.97	2.7	SAMYWLR
0	2	0.56	1.5	4.2	EQTVYYAK

5.789 181 744 unknown [Dendroctonus ponderosae]

Protein Accession	CL25948.Contig3$_H$1
Mean Expression Ratio	1
Median Expression Ratio	1.01
Credible Interval	(0.515, 1.94)
Associated Peptides	4
Associated Spectra	18
Coverage	NaN

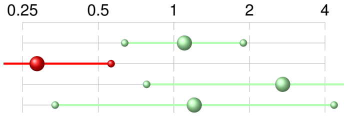

A	B	2.5	50	97.5	Sequence
**6**	**4**	**0.64**	**1.1**	**1.9**	**VGHVPVAK**
**5**	**1**	**0.14**	**0.29**	**0.56**	**GTLTQLTK**
**0**	**1**	**0.78**	**2.7**	**9.7**	**GDFSKLPR**
**0**	**1**	**0.34**	**1.2**	**4.3**	**SMGPIGSPMSK**

5.790 10 1542 Copine-8 [Crassostrea gigas]

Protein Accession	CL9423.Contig1$_H$1
Mean Expression Ratio	0.986
Median Expression Ratio	0.993
Credible Interval	(0.435, 2.24)
Associated Peptides	1
Associated Spectra	1
Coverage	NaN

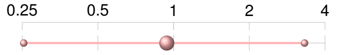

A	B	2.5	50	97.5	Sequence
**0**	**1**	**0.25**	**0.94**	**3.3**	**SYRGQYAER**

5.791 4 942 minus strand KTEL motif-containing protein 1 [Crassostrea gigas]

Protein Accession	CL11353.Contig3$_H$1
Mean Expression Ratio	1.01
Median Expression Ratio	1.01
Credible Interval	(0.438, 2.34)
Associated Peptides	1
Associated Spectra	1
Coverage	NaN

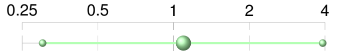

A	B	2.5	50	97.5	Sequence
**0**	**1**	**0.3**	**1.1**	**3.9**	**GIHYQIVDHK**

5.792 3 386 fem-1-like protein C [Crassostrea gigas]

Protein Accession	Unigene19937$_H$1
Mean Expression Ratio	0.992
Median Expression Ratio	0.993
Credible Interval	(0.45, 2.2)
Associated Peptides	1
Associated Spectra	5
Coverage	NaN

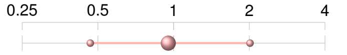

A	B	2.5	50	97.5	Sequence
**0**	**5**	**0.47**	**0.95**	**2**	**MQSLLIR**

5.793 8 271 minus strand Laminin subunit gamma-1 [Crassostrea gigas]

Protein Accession	Unigene36706$_{H}$1
Mean Expression Ratio	0.987
Median Expression Ratio	0.993
Credible Interval	(0.443, 2.16)
Associated Peptides	2
Associated Spectra	2
Coverage	NaN

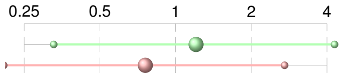

A	B	2.5	50	97.5	Sequence
0	1	0.33	1.2	4.3	ALDLYR
0	1	0.21	0.76	2.7	HMQEAAQIDNIAR

5.794 511 1062 unknown [Dendroctonus ponderosae]; 511 1062 unknown [Dendroctonus ponderosae]

Protein Accession	CL18443.Contig3$_{H}$1 CL18443.Contig1$_{H}$1
Mean Expression Ratio	1
Median Expression Ratio	1.01
Credible Interval	(0.441, 2.26)
Associated Peptides	1
Associated Spectra	1
Coverage	NaN

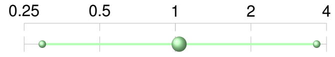

A	B	2.5	50	97.5	Sequence
0	1	0.29	1	3.6	FKDEIEMHLK

5.795 1867 3108 minus strand roundabout homolog 1 precursor [Rattus norvegicus] gi; 1867 3108 minus strand roundabout homolog 1 precursor [Rattus norvegicus] gi; 1835 2956 minus strand roundabout homolog 1 precursor [Rattus norvegicus] gi; 1867 3108 minus strand roundabout homolog 1 precursor [Rattus norvegicus] gi

Protein Accession	CL2792.Contig8$_{H}$1 CL2792.Contig5$_{H}$1 CL2792.Contig11$_{H}$1 CL2792.Contig10$_{H}$1
Mean Expression Ratio	1.01
Median Expression Ratio	1.01
Credible Interval	(0.437, 2.28)
Associated Peptides	1
Associated Spectra	1
Coverage	NaN

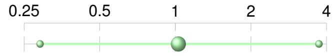

A	B	2.5	50	97.5	Sequence
0	1	0.29	1	3.7	VAHLDRVFQQEPVR

5.796 3 1418 Protein hu-li tai shao [Crassostrea gigas]; 3 1523 minus strand Protein hu-li tai shao [Crassostrea gigas]; 3 1562 minus strand Protein hu-li tai shao [Crassostrea gigas]

Protein Accession	CL3889.Contig5$_H$1 CL3889.Contig13$_H$1 CL3889.Contig11$_H$1
Mean Expression Ratio	1.01
Median Expression Ratio	1.01
Credible Interval	(0.487, 2.11)
Associated Peptides	2
Associated Spectra	5
Coverage	NaN

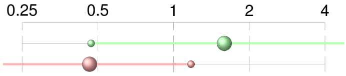

A	B	2.5	50	97.5	Sequence
**0**	**1**	**0.47**	**1.6**	**5.7**	**ILEGLSWEEAAR**
**2**	**2**	**0.18**	**0.46**	**1.2**	**VMLLR**

5.797 129 1628 beta-hexosaminidase subunit beta precursor [Danio rerio] gi

Protein Accession	CL21114.Contig2$_H$1
Mean Expression Ratio	0.991
Median Expression Ratio	0.994
Credible Interval	(0.46, 2.15)
Associated Peptides	2
Associated Spectra	2
Coverage	NaN

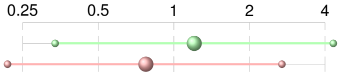

A	B	2.5	50	97.5	Sequence
**0**	**1**	**0.34**	**1.2**	**4.3**	**EYQTNGNVNTIDAASFR**
**0**	**1**	**0.22**	**0.78**	**2.7**	**LWSPQDVNFPQIAEPR**

5.798 141 425 hypothetical protein BRAFLDRAFT_216227 [Branchiostoma floridae] gi; 141 425 minus strand hypothetical protein BRAFLDRAFT_216227 [Branchiostoma floridae] gi; 141 425 minus strand hypothetical protein BRAFLDRAFT_216227 [Branchiostoma floridae] gi

Protein Accession	CL8062.Contig5$_H$1 CL8062.Contig4$_H$1 CL8062.Contig2$_H$1
Mean Expression Ratio	1.01
Median Expression Ratio	1.01
Credible Interval	(0.442, 2.3)
Associated Peptides	1
Associated Spectra	1
Coverage	NaN

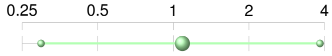

A	B	2.5	50	97.5	Sequence
**0**	**1**	**0.3**	**1.1**	**3.8**	**KVEVDNPDPETNLLMFLR**

5.799 197 1132 minus strand Pancreatic lipase-related protein 2 [Crassostrea gigas]

Protein Accession	CL8259.Contig1$_H$1
Mean Expression Ratio	0.996
Median Expression Ratio	0.994
Credible Interval	(0.451, 2.19)
Associated Peptides	1
Associated Spectra	7
Coverage	NaN

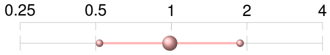

A	B	2.5	50	97.5	Sequence
**0**	**7**	**0.52**	**0.98**	**1.9**	**ITGLDPAGPK**

5.800 1363 1527 Protein disulfide-isomerase A3 [Crassostrea gigas]

Protein Accession	CL28563.Contig1$_H$1
Mean Expression Ratio	1.01
Median Expression Ratio	1.01
Credible Interval	(0.512, 2.03)
Associated Peptides	3
Associated Spectra	15
Coverage	NaN

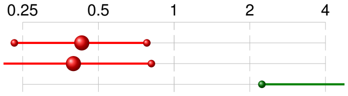

A	B	2.5	50	97.5	Sequence
**3**	**5**	**0.23**	**0.43**	**0.78**	**LKDEPEITIAK**
**2**	**3**	**0.19**	**0.4**	**0.81**	**FEELAEK**
**0**	**2**	**2.2**	**6.3**	**18**	**ALEPKFEELAEK**

5.801 175 1416 Ubiquinone biosynthesis monooxygenase COQ6 [Crassostrea gigas]

Protein Accession	Unigene197$_H$1
Mean Expression Ratio	1.01
Median Expression Ratio	1
Credible Interval	(0.466, 2.19)
Associated Peptides	2
Associated Spectra	2
Coverage	NaN

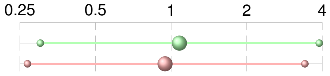

A	B	2.5	50	97.5	Sequence
**0**	**1**	**0.3**	**1.1**	**3.9**	**VALIGDAAHR**
**0**	**1**	**0.27**	**0.95**	**3.4**	**PTACGVVEK**

5.802 " 1 198 minus strand Chain A, Rep1-Nxsq Fatty Acid Transporter; 1 231 minus strand Chain A, Rep1-Nxsq Fatty Acid Transporter; 1 198 minus strand Chain A, Rep1-Nxsq Fatty Acid Transporter"

Protein Accession	CL14182.Contig4$_H$1 CL14182.Contig3$_H$1 CL14182.Contig2$_H$1
Mean Expression Ratio	1
Median Expression Ratio	1
Credible Interval	(0.441, 2.26)
Associated Peptides	1
Associated Spectra	2
Coverage	NaN

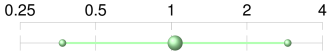

A	B	2.5	50	97.5	Sequence
**1**	**1**	**0.37**	**1**	**2.9**	**GSPDSLIVR**

5.803 95 1870 PREDICTED: acetylcholinesterase-like [Equus caballus]; 99 1874 PREDICTED: acetylcholinesterase-like [Equus caballus]

Protein Accession	CL10104.Contig9$_{H1}$ CL10104.Contig10$_{H1}$
Mean Expression Ratio	0.999
Median Expression Ratio	0.997
Credible Interval	(0.461, 2.18)
Associated Peptides	2
Associated Spectra	2
Coverage	NaN

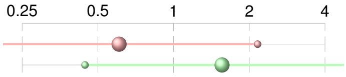

A	B	2.5	50	97.5	Sequence
**1**	**0**	**0.17**	**0.61**	**2.2**	**SFGNEPTEIK**
**0**	**1**	**0.45**	**1.6**	**5.7**	**VDGDFLPDDPAR**

5.804 727 2022 RecName: Full=Enolase; AltName: Full=2-phospho-D-glycerate hydro-lyase; AltName: Full=2-phosphoglycerate dehydratase gi|1911573|gb|AAB50731.1| enolase [Doryteuthis pealeii]; 690 1985 RecName: Full=Enolase; AltName: Full=2-phospho-D-glycerate hydro-lyase; AltName: Full=2-phosphoglycerate dehydratase gi|1911573|gb|AAB50731.1| enolase [Doryteuthis pealeii]; 213 1508 RecName: Full=Enolase; AltName: Full=2-phospho-D-glycerate hydro-lyase; AltName: Full=2-phosphoglycerate dehydratase gi|1911573|gb|AAB50731.1| enolase [Doryteuthis pealeii]; 246 1541 RecName: Full=Enolase; AltName: Full=2-phospho-D-glycerate hydro-lyase; AltName: Full=2-phosphoglycerate dehydratase gi|1911573|gb|AAB50731.1| enolase [Doryteuthis pealeii]

Protein Accession	CL15687.Contig6$_H$1 CL15687.Contig5$_H$1 CL15687.Contig3$_H$1 CL15687.Contig2$_H$1
Mean Expression Ratio	1
Median Expression Ratio	1
Credible Interval	(0.692, 1.44)
Associated Peptides	27
Associated Spectra	101
Coverage	NaN

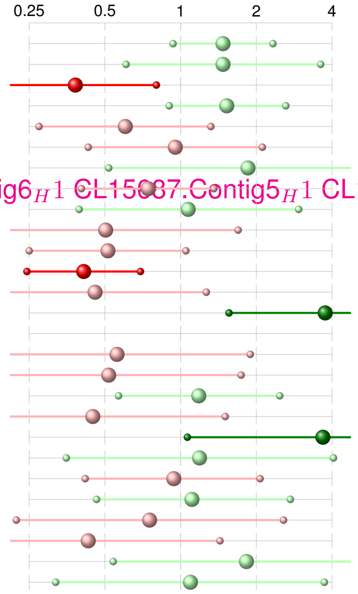

A	B	2.5	50	97.5	Sequence
4	11	0.93	1.5	2.3	AAGWGVMVSHR
1	2	0.61	1.5	3.6	FQIVGDDLLVTNPK
1	4	0.18	0.38	0.8	IMLDLDGTPNKEK
8	3	0.9	1.5	2.6	QIFDSR
1	3	0.27	0.6	1.3	TLDLADQTAMDK
2	2	0.43	0.95	2.1	VQMGIDR
1	0	0.52	1.9	6.3	VIKSIK
4	4	0.4	0.74	1.4	YNQILR
1	1	0.39	1.1	2.9	VAIEK
1	0	0.14	0.5	1.7	AYLGK
2	3	0.25	0.51	1	GVPLYR
5	6	0.24	0.41	0.69	IAPAFVGK
0	2	0.16	0.46	1.3	AAVPSGASTGIYEALELR
0	3	1.6	3.8	9.1	AAVPSGASTGIYEALELRDNDPK
0	5	5.2	11	22	AVSNINDKIAPAFVGK
0	1	0.16	0.56	1.9	FQIVGDDLLVTN
0	1	0.15	0.52	1.7	HIADLCGNK
0	5	0.57	1.2	2.5	IEEELGVDAFYAGK
0	1	0.14	0.45	1.5	IEIGMDVAASEFCK
0	1	1.1	3.7	12	LAKYNQILR
0	1	0.35	1.2	4.1	SYPIVTIEDPFDQDDWDAYTK
0	4	0.42	0.94	2.1	TSQQMIDMYHDFVK
0	3	0.46	1.1	2.7	VNQIGSVSESIQAFK
0	1	0.22	0.75	2.6	DGKYDLDFK
0	1	0.13	0.43	1.4	YDLDFK
0	1	0.54	1.8	6.3	NINDKIAPAFVGK
0	1	0.32	1.1	3.7	MGSETYHHLK

5.805 " 125 628 minus strand collectin sub-family member 12, partial [Oncorhynchus mykiss]"

Protein Accession	CL46744.Contig5$_{H1}$
Mean Expression Ratio	0.999
Median Expression Ratio	0.997
Credible Interval	(0.433, 2.28)
Associated Peptides	1
Associated Spectra	1
Coverage	NaN

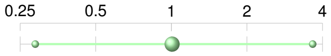

A	B	2.5	50	97.5	Sequence
0	1	0.29	1	3.7	WVQGDNRPLAYTR

5.806 224 1291 minus strand coproporphirynogen oxidase [Aplysia californica] gi

Protein Accession	CL8705.Contig1$_{H1}$
Mean Expression Ratio	1.01
Median Expression Ratio	1
Credible Interval	(0.455, 2.27)
Associated Peptides	1
Associated Spectra	3
Coverage	NaN

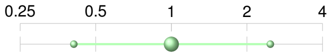

A	B	2.5	50	97.5	Sequence
0	3	0.41	1	2.5	NPHVPTMHFNYR

5.807 198 821 minus strand sigma class glutathione-s-transferase 2 [Haliotis discus discus]

Protein Accession	CL1352.Contig4$_{H1}$
Mean Expression Ratio	0.997
Median Expression Ratio	0.998
Credible Interval	(0.48, 2.04)
Associated Peptides	3
Associated Spectra	5
Coverage	NaN

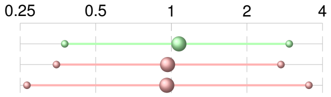

A	B	2.5	50	97.5	Sequence
1	1	0.38	1.1	3	AAGQPFDDNVVDR
1	1	0.35	0.97	2.7	ASWGPDCEAK
0	1	0.27	0.96	3.5	AAPYGQLPYVIYK

5.808 " 115 1104 minus strand PREDICTED: KH domain-containing, RNA-binding, signal transduction-associated protein 3 [Monodelphis domestica]"

Protein Accession	Unigene26700$_{H1}$
Mean Expression Ratio	1
Median Expression Ratio	1
Credible Interval	(0.475, 2.1)
Associated Peptides	3
Associated Spectra	4
Coverage	NaN

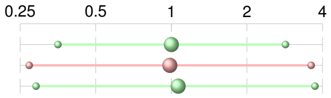

A	B	2.5	50	97.5	Sequence
1	1	0.35	1	2.9	LLAQEIER
1	0	0.27	0.99	3.6	GRGGGVP
0	1	0.29	1.1	3.7	LQEETGCK

5.809 " 33 1295 PREDICTED: LOW QUALITY PROTEIN: selenide, water dikinase-like [Bombus impatiens]"

Protein Accession	CL14673.Contig3$_{H}1$
Mean Expression Ratio	1.01
Median Expression Ratio	0.999
Credible Interval	(0.439, 2.32)
Associated Peptides	1
Associated Spectra	1
Coverage	NaN

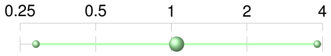

A	B	2.5	50	97.5	Sequence
**0**	**1**	**0.29**	**1**	**3.8**	**RPFDPVAHELDPNFR**

5.810 9 326 minus strand catalase [Paracyclopina nana]; 9 209 Catalase OS=Cavia porcellus GN=CAT PE=2 SV=4

Protein Accession	CL6745.Contig2$_{H}1$ CL6745.Contig1$_{H}1$
Mean Expression Ratio	1
Median Expression Ratio	1
Credible Interval	(0.444, 2.23)
Associated Peptides	1
Associated Spectra	3
Coverage	NaN

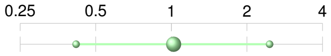

A	B	2.5	50	97.5	Sequence
**1**	**2**	**0.42**	**1**	**2.5**	**LATMTVGPR**

5.811 106 1701 T-complex protein 1 subunit beta [Crassostrea gigas]

Protein Accession	CL8176.Contig1$_{H}1$
Mean Expression Ratio	1
Median Expression Ratio	1
Credible Interval	(0.472, 2.13)
Associated Peptides	2
Associated Spectra	3
Coverage	NaN

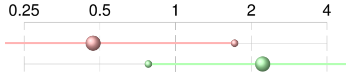

A	B	2.5	50	97.5	Sequence
**0**	**1**	**0.13**	**0.47**	**1.7**	**EAESLIGQK**
**0**	**2**	**0.78**	**2.2**	**6.3**	**VAELELAEKEK**

5.812 2 2248 PREDICTED: heat shock 70 kDa protein 4-like [Oreochromis niloticus]; 2 2227 PREDICTED: heat shock 70 kDa protein 4-like [Oreochromis niloticus]

Protein Accession	CL12749.Contig5$_{H}1$ CL12749.Contig4$_{H}1$
Mean Expression Ratio	1
Median Expression Ratio	1
Credible Interval	(0.458, 2.21)
Associated Peptides	2
Associated Spectra	2
Coverage	NaN

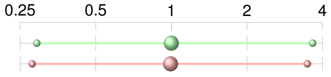

A	B	2.5	50	97.5	Sequence
**0**	**1**	**0.29**	**1**	**3.7**	**LLQECEK**
**0**	**1**	**0.28**	**0.99**	**3.5**	**TAWWTPK**

5.813 " 698 1225 minus strand actin related protein 2/3 complex, 21 kDa subunit [Crassostrea gigas]"

Protein Accession	CL17389.Contig2$_{H}1$
Mean Expression Ratio	1
Median Expression Ratio	1
Credible Interval	(0.437, 2.3)
Associated Peptides	1
Associated Spectra	2
Coverage	NaN

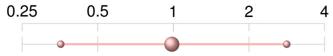

A	B	2.5	50	97.5	Sequence
**0**	**2**	**0.36**	**0.98**	**2.8**	**ANIFFR**

5.814 " 132 2075 PREDICTED: arginyl-tRNA synthetase, cytoplasmic [Ornithorhynchus anatinus]; 132 2090 minus strand PREDICTED: arginyl-tRNA synthetase, cytoplasmic [Ornithorhynchus anatinus]"

Protein Accession	CL15019.Contig4$_{H}1$ CL15019.Contig3$_{H}1$
Mean Expression Ratio	1
Median Expression Ratio	1
Credible Interval	(0.474, 2.1)
Associated Peptides	3
Associated Spectra	3
Coverage	NaN

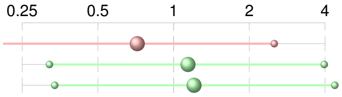

A	B	2.5	50	97.5	Sequence
**0**	**1**	**0.2**	**0.72**	**2.5**	**LVDLLDEGVK**
**0**	**1**	**0.32**	**1.1**	**4**	**LSYQLNHLKR**
**0**	**1**	**0.34**	**1.2**	**4.4**	**QRTGAVSLQRMLEDVFR**

5.815 14 502 minus strand ependymin related protein-1 precursor [Haliotis discus discus]; 14 547 ependymin related protein-1 precursor [Haliotis discus discus]

Protein Accession	CL3749.Contig3$_{H}1$ CL3749.Contig1$_{H}1$
Mean Expression Ratio	1.01
Median Expression Ratio	1
Credible Interval	(0.44, 2.3)
Associated Peptides	1
Associated Spectra	1
Coverage	NaN

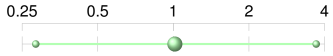

A	B	2.5	50	97.5	Sequence
**0**	**1**	**0.28**	**1**	**3.7**	**IWYDAENQR**

iTRAQ Data Analysis Report

Javier, Body 7d versus control

April 7, 2014

1 Introduction

This document summarizes an analysis of relative protein expression using iTRAQ. The reporter ion peak area measurements supplied by the ABI software are used to estimate treatment-dependent peptide and protein relative expression. Estimation is accomplished using a Bayesian approach with the model given below. The document includes a protein relative expression summary and a per-protein detailed analysis. The document is internally hyperlinked and linked externally to NCBI.

2 Experiment and Model Description

2.1 Experiment Design

The report summarizes data from one or more iTRAQ experiments addressing a common comparison. The experiment design, used in this analysis, is given in the table below.

	Experiment	Treatment	Channel	Sample
1	A	control	113	s1
2	A	test	115	s1
3	B	control	113	s2
4	B	test	115	s2

2.2 Input Files

Data for this analysis was extracted from the following tandem mass spectra (MSMS) summary files.

Experiment	MSMS Summary File
A	body1.txt
B	body2.txt

2.3 Statistical Model

The following statistical model was used to estimate the treatment-dependent effects.

LogIntensity ˜ Channel + Spectrum + Protein + Peptide + Protein:Treatment + Peptide:Treatment

3 Data Summary

The data supplied in the MSMS summary is filtered to remove unidentified proteins, contaminants, and peptides containing selected modifications. The following table sumarizes the data provided and used in the analysis.

	A	B	Combined
Supplied Spectra	11560	18985	30545
Unidentified Spectra	6008	8178	14186
Disallowed Modifications	36	201	237
Spectra from Contaminants	0	0	0
Missing Data	1836	3375	5211
Low Confidence Spectra	6008	8178	14186
Degenerate Peptides	1480	2288	3768
Remaining Spectra			7143
Unique Proteins			800
Unique Peptides			2737
Model R^2			0.787

4 Protein Summary

Each protein identified in one or more of the MSMS summaries is listed below in decreasing order of expression change magnitude. The median and estimated credible interval for each protein is given to the left in the table. Proteins identified by a single peptide are listed in a separate table.

4.1 Identified Proteins

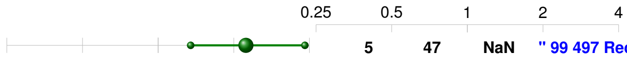

Peptides	Spectra	Coverage	Protein
5	47	NaN	" 99 497 RecName: Full=Histone H2B, gonadal"

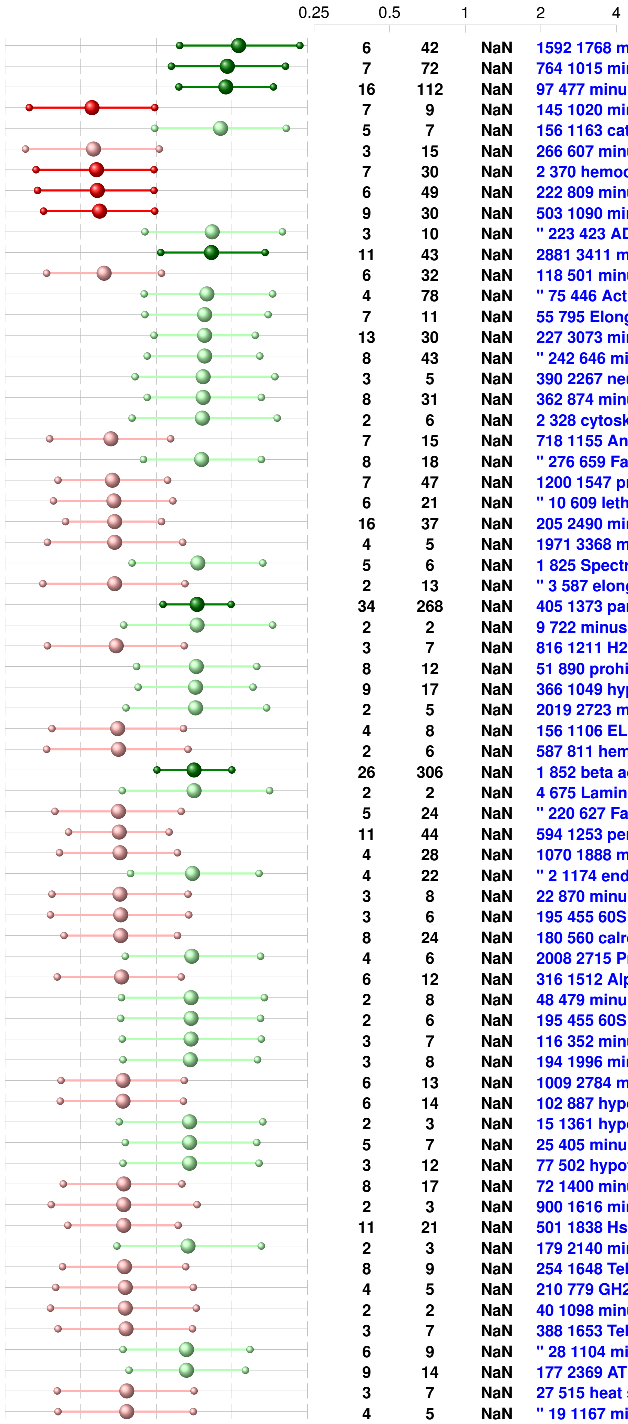

Peptides	Spectra	Coverage	Protein
6	42	NaN	1592 1768 minus strand beta-actin [Diabolocatantops pinguis]
7	72	NaN	764 1015 minus strand heat shock protein beta-1 [Trichinella spiralis] gi
16	112	NaN	97 477 minus strand PREDICTED: complement C1q tumor necrosis factor-related protein 3-like [Oreochromis niloti
7	9	NaN	145 1020 minus strand PREDICTED: inorganic pyrophosphatase-like [Saccoglossus kowalevskii]
5	7	NaN	156 1163 cathepsin B [Crassostrea ariakensis]
3	15	NaN	266 607 minus strand H2 [Haliotis tuberculata]
7	30	NaN	2 370 hemocyanin alphaD-subunit [Helix lucorum]
6	49	NaN	222 809 minus strand PREDICTED: 15-hydroxyprostaglandin dehydrogenase [NAD+] [Gallus gallus]
9	30	NaN	503 1090 minus strand myosin regulatory light chain [Fasciola hepatica]; 278 865 minus strand myosin regulatory li
3	10	NaN	" 223 423 ADPATP carrier protein; K05863 solute carrier family 25 (mitochondrial adenine nucleotide translocator), m
11	43	NaN	2881 3411 myosin heavy chain [Todarodes pacificus]; 2915 3445 myosin heavy chain [Todarodes pacificus]
6	32	NaN	118 501 minus strand histone 2A.Z [Aplysia californica]
4	78	NaN	" 75 446 Actin, cytoplasmic type 8 OS=Xenopus laevis PE=3 SV=1"
7	11	NaN	55 795 Elongation factor 1-delta OS=Bos taurus GN=EEF1D PE=2 SV=2; 49 747 PREDICTED: elongation factor 1-del
13	30	NaN	227 3073 minus strand Lysosomal alpha-mannosidase [Crassostrea gigas]
8	43	NaN	" 242 646 minus strand Fatty acid-binding protein, heart [Crassostrea gigas]; 227 631 minus strand Fatty acid-bindir
3	5	NaN	390 2267 neurofilament protein NF70 [Helix aspersa]; 318 2195 neurofilament protein NF70 [Helix aspersa]
8	31	NaN	362 874 minus strand Transgelin-2 [Crassostrea gigas]
2	6	NaN	2 328 cytoskeletal actin 2 [Molgula oculata]
7	15	NaN	718 1155 Annexin A7 [Crassostrea gigas]
8	18	NaN	" 276 659 Fatty acid-binding protein, adipocyte [Crassostrea gigas]; 426 809 Fatty acid-binding protein, adipocyte [C
7	47	NaN	1200 1547 protein disulfide isomerase [Conus eburneus]
6	21	NaN	" 10 609 lethal(2)essential for life protein, l2efl [Aedes aegypti] gi"
16	37	NaN	205 2490 minus strand glucose-regulated protein 94 [Crassostrea gigas]
4	5	NaN	1971 3368 minus strand Kyphoscoliosis peptidase [Crassostrea gigas]; 1767 3158 minus strand hillarin [Hirudo med
5	6	NaN	1 825 Spectrin alpha chain [Crassostrea gigas]
2	13	NaN	" 3 587 elongation factor 1 alpha, partial [Phorbas sp. nov. SB-2012]"
34	268	NaN	405 1373 paramyosin [Haliotis discus discus]
2	2	NaN	9 722 minus strand actin related protein [Lumbricus rubellus]
3	7	NaN	816 1211 H2 [Haliotis tuberculata]
8	12	NaN	51 890 prohibitin [Octopus tankahkeei]
9	17	NaN	366 1049 hypothetical protein CGI_10004855 [Crassostrea gigas]
2	5	NaN	2019 2723 minus strand Kyphoscoliosis peptidase [Crassostrea gigas]; 2060 2764 minus strand Kyphoscoliosis pep
4	8	NaN	156 1106 ELAV 2-like protein [Aplysia californica] gi
2	6	NaN	587 811 hemocyanin 2 [Megathura crenulata]
26	306	NaN	1 852 beta actin-like 1 protein [Macrostomum lignano]
2	2	NaN	4 675 Laminin subunit gamma-1 [Crassostrea gigas]
5	24	NaN	" 220 627 Fatty acid-binding protein, brain [Crassostrea gigas]"
11	44	NaN	594 1253 peroxiredoxin 6 [Eriocheir sinensis]
4	28	NaN	1070 1888 minus strand galectin [Pinctada fucata]; 1371 2189 minus strand galectin [Pinctada fucata]
4	22	NaN	" 2 1174 endo-1,3-beta-D-glucanase [Littorina sitkana]"
3	8	NaN	22 870 minus strand PREDICTED: alcohol dehydrogenase class-3 [Sarcophilus harrisii]
3	6	NaN	195 455 60S ribosomal protein L23a OS=Rattus norvegicus GN=Rpl23a PE=2 SV=1; 16 465 60S ribosomal protein L2
8	24	NaN	180 560 calreticulin precursor [Aplysia californica] gi; 180 545 calreticulin precursor [Aplysia californica] gi
4	6	NaN	2008 2715 Protein disulfide-isomerase A5 [Crassostrea gigas]
6	12	NaN	316 1512 Alpha-N-acetylgalactosaminidase [Crassostrea gigas]; 282 1478 Alpha-N-acetylgalactosaminidase [Crasso
2	8	NaN	48 479 minus strand 40S ribosomal protein S24 OS=Macaca fascicularis GN=RPS24 PE=2 SV=1
2	6	NaN	195 455 60S ribosomal protein L23a OS=Rattus norvegicus GN=Rpl23a PE=2 SV=1; 195 512 60S ribosomal protein L
3	7	NaN	116 352 minus strand troponin I 4 [Clonorchis sinensis]
3	8	NaN	194 1996 minus strand Neutral and basic amino acid transport protein rBAT [Crassostrea gigas]; 265 2067 minus str
6	13	NaN	1009 2784 minus strand Collagen alpha-3(VI) chain [Crassostrea gigas]; 1009 2787 minus strand Collagen alpha-3(V
6	14	NaN	102 887 hypothetical protein DAPPUDRAFT_304712 [Daphnia pulex]
2	3	NaN	15 1361 hypothetical protein BRAFLDRAFT_231908 [Branchiostoma floridae] gi
5	7	NaN	25 405 minus strand Elongation factor 1-delta [Anoplopoma fimbria]
3	12	NaN	77 502 hypothetical protein CGI_10008263 [Crassostrea gigas]
8	17	NaN	72 1400 minus strand hypothetical protein RCJMB04_22i13 [Gallus gallus]
2	3	NaN	900 1616 minus strand hemocyanin isoform 1 [Haliotis diversicolor]
11	21	NaN	501 1838 Hsp90A [Haliotis asinina]
2	3	NaN	179 2140 minus strand PREDICTED: hypothetical protein [Saccoglossus kowalevskii]
8	9	NaN	254 1648 Tektin-3 [Crassostrea gigas]; 254 1648 Tektin-3 [Crassostrea gigas]
4	5	NaN	210 779 GH20056 [Drosophila grimshawi] gi; 216 785 minus strand GH20056 [Drosophila grimshawi] gi; 210 779 GH2
2	2	NaN	40 1098 minus strand major egg antigen [Clonorchis sinensis]
3	7	NaN	388 1653 Tektin-3 [Crassostrea gigas]
6	9	NaN	" 28 1104 minus strand Pyruvate dehydrogenase E1 component subunit beta, mitochondrial [Crassostrea gigas]"
9	14	NaN	177 2369 ATPase family AAA domain-containing protein 2B [Crassostrea gigas]; 177 2369 ATPase family AAA doma
3	7	NaN	27 515 heat shock protein 70 [Capsaspora owczarzaki ATCC 30864]; 30 464 minus strand hypothetical protein [Trich
4	5	NaN	" 19 1167 minus strand ureidopropionase, beta [Xenopus (Silurana) tropicalis] gi"

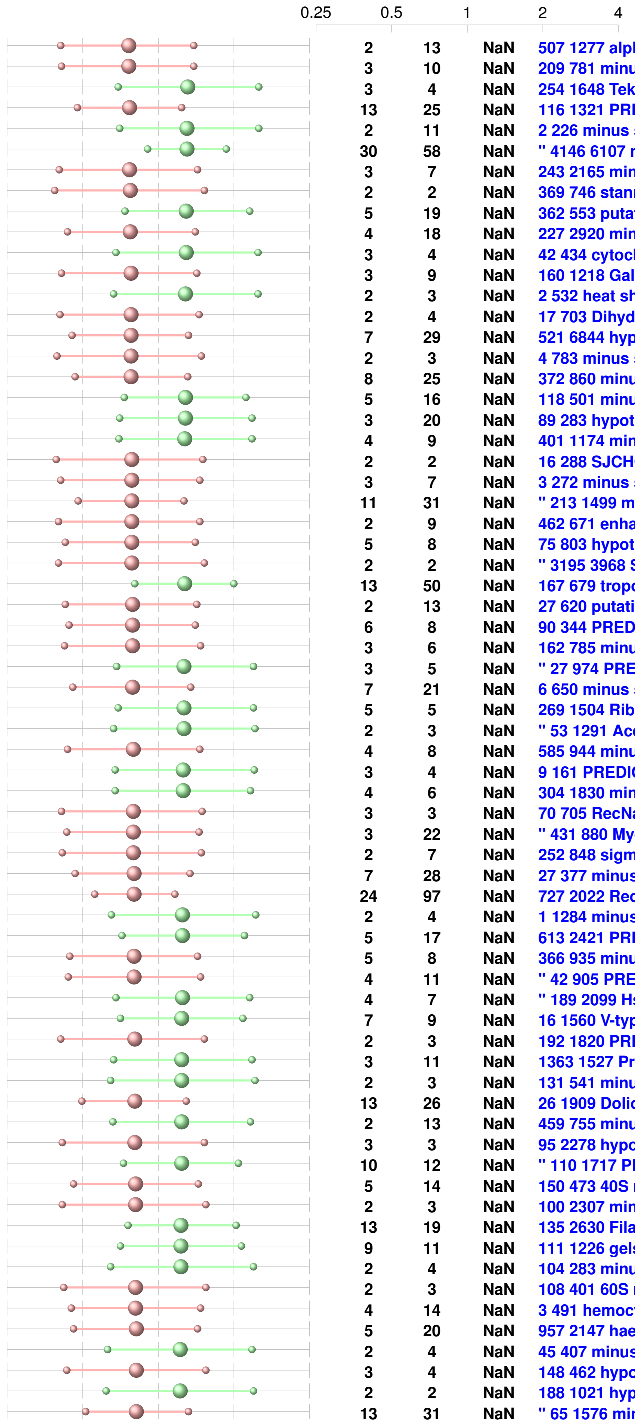

Peptides	Spectra	Coverage	Protein
2	13	NaN	507 1277 alpha tubulin [Notothenia coriiceps]
3	10	NaN	209 781 minus strand high mobility group protein 1 [Biomphalaria glabrata]
3	4	NaN	254 1648 Tektin-3 [Crassostrea gigas]
13	25	NaN	116 1321 PREDICTED: similar to aspartate aminotransferase [Tribolium castaneum] gi
2	11	NaN	2 226 minus strand hypothetical protein CGI_10015501 [Crassostrea gigas]
30	58	NaN	" 4146 6107 minus strand nonmuscle myosin II, partial [Aplysia californica]"
3	7	NaN	243 2165 minus strand Elongation factor 2 OS=Drosophila melanogaster GN=Ef2b PE=2 SV=4
2	2	NaN	369 746 stanniocalcin [Haliotis diversicolor supertexta] gi
5	19	NaN	362 553 putative RNA-binding protein [Haliotis diversicolor]
4	18	NaN	227 2920 minus strand Lysosomal alpha-mannosidase [Crassostrea gigas]; 227 3073 minus strand Lysosomal alpha
3	4	NaN	42 434 cytochrome b5 [Haliotis discus discus]; 42 434 cytochrome b5 [Haliotis discus discus]
3	9	NaN	160 1218 Galectin-9 [Crassostrea gigas]
2	3	NaN	2 532 heat shock protein 70 [Echinostoma caproni]
2	4	NaN	17 703 Dihydropteridine reductase [Crassostrea gigas]
7	29	NaN	521 6844 hypothetical protein CGI_10014469 [Crassostrea gigas]
2	3	NaN	4 783 minus strand hemocyanin isoform 2 [Nucula nucleus]
8	25	NaN	372 860 minus strand small heat shock protein [Tegillarca granosa]
5	16	NaN	118 501 minus strand histone 2A.Z [Aplysia californica]; 15 428 PREDICTED: histone H2A.V-like [Apis florea]
3	20	NaN	89 283 hypothetical protein CGI_10017178 [Crassostrea gigas]
4	9	NaN	401 1174 minus strand Mechanosensory protein 2 [Crassostrea gigas]
2	2	NaN	16 288 SJCHGC01950 protein [Schistosoma japonicum] gi
3	7	NaN	3 272 minus strand ribosomal protein L28 [Haliotis asinina]
11	31	NaN	" 213 1499 minus strand Citrate synthase, mitochondrial [Crassostrea gigas]"
2	9	NaN	462 671 enhancer protein [Homo sapiens]
5	8	NaN	75 803 hypothetical protein DAPPUDRAFT_231350 [Daphnia pulex]
2	2	NaN	" 3195 3968 Spectrin alpha chain, putative [Pediculus humanus corporis] gi"
13	50	NaN	167 679 tropomyosin [Haliotis discus discus]
2	13	NaN	27 620 putative mitochondrial ATP synthase [Haliotis discus discus]
6	8	NaN	90 344 PREDICTED: diazepam binding inhibitor-like [Saccoglossus kowalevskii]
3	6	NaN	162 785 minus strand Ropporin-1-like protein OS=Xenopus laevis GN=ropn1l PE=2 SV=1
3	5	NaN	" 27 974 PREDICTED: succinyl-CoA ligase [GDP-forming] subunit alpha, mitochondrial-like [Oreochromis niloticus];
7	21	NaN	6 650 minus strand glutathione S-transferase mu class protein [Crassostrea ariakensis]; 117 761 glutathione S-trans
5	5	NaN	269 1504 Ribosome-binding protein 1 [Crassostrea gigas]; 265 1500 Ribosome-binding protein 1 [Crassostrea gigas
2	3	NaN	" 53 1291 Acetyl-CoA acetyltransferase B, mitochondrial [Crassostrea gigas]"
4	8	NaN	585 944 minus strand Protein disulfide-isomerase A6 [Crassostrea gigas]
3	4	NaN	9 161 PREDICTED: tubulin alpha-1C chain-like [Acyrthosiphon pisum]; 9 161 Tubulin alpha-3 chain (Fragments) OS=
4	6	NaN	304 1830 minus strand Methenyltetrahydrofolate synthetase domain-containing protein [Crassostrea gigas]
3	3	NaN	70 705 RecName: Full=Ras-related protein Rab-2 gi|288938|emb|CAA51234.1| RAB2 [Lymnaea stagnalis]; 70 705 Re
3	22	NaN	" 431 880 Myosin essential light chain, striated adductor muscle OS=Aequipecten irradians PE=1 SV=2"
2	7	NaN	252 848 sigma class glutathione-s-transferase 2 [Haliotis discus discus]
7	28	NaN	27 377 minus strand ribosomal protein LP2 [Papilio xuthus] gi; 27 377 minus strand ribosomal protein LP2 [Papilio x
24	97	NaN	727 2022 RecName: Full=Enolase; AltName: Full=2-phospho-D-glycerate hydro-lyase; AltName: Full=2-phosphoglyc
2	4	NaN	1 1284 minus strand Thioredoxin reductase 3 [Crassostrea gigas]
5	17	NaN	613 2421 PREDICTED: lysosomal alpha-glucosidase [Danio rerio]
5	8	NaN	366 935 minus strand 40S ribosomal protein S2 OS=Urechis caupo GN=RPS2 PE=2 SV=1
4	11	NaN	" 42 905 PREDICTED: enoyl-CoA hydratase, mitochondrial-like isoform 1 [Oreochromis niloticus]"
4	7	NaN	" 189 2099 Hsp70, putative [Ixodes scapularis] gi"
7	9	NaN	16 1560 V-type proton ATPase subunit B [Crassostrea gigas]
2	3	NaN	192 1820 PREDICTED: copine IV-like [Saccoglossus kowalevskii]; 125 1753 PREDICTED: copine IV-like [Saccoglossu
3	11	NaN	1363 1527 Protein disulfide-isomerase A3 [Crassostrea gigas]
2	3	NaN	131 541 minus strand nervous system adducin [Aplysia californica] gi
13	26	NaN	26 1909 Dolichyl-diphosphooligosaccharide–protein glycosyltransferase subunit 2 [Crassostrea gigas]
2	13	NaN	459 755 minus strand heat shock protein beta-1 [Trichinella spiralis] gi; 464 763 minus strand heat shock protein bet
3	3	NaN	95 2278 hypothetical protein CGI_10017740 [Crassostrea gigas]
10	12	NaN	" 110 1717 PREDICTED: glutamate dehydrogenase, mitochondrial-like [Oreochromis niloticus]; 110 1717 PREDICTE
5	14	NaN	150 473 40S ribosomal protein S16 OS=Heteropneustes fossilis GN=rps16 PE=2 SV=1
2	3	NaN	100 2307 minus strand Xanthine dehydrogenase/oxidase [Crassostrea gigas]
13	19	NaN	135 2630 Filamin-A [Crassostrea gigas]; 118 2613 Filamin-A [Crassostrea gigas]; 157 2652 Filamin-A [Crassostrea g
9	11	NaN	111 1226 gelsolin [Aplysia californica] gi; 42 1139 minus strand gelsolin [Aplysia californica] gi
2	4	NaN	104 283 minus strand RecName: Full=Hemocyanin type 2 unit a; AltName: Full=Hemocyanin RHSS2 subunit; AltNam
2	3	NaN	108 401 60S ribosomal L7a-like protein [Phragmatopoma lapidosa]; 108 401 60S ribosomal L7a-like protein [Phragm
4	14	NaN	3 491 hemocyanin [Haliotis tuberculata]
5	20	NaN	957 2147 haemocyanin [Aplysia californica]
2	4	NaN	45 407 minus strand 60S ribosomal protein L31 OS=Xenopus laevis GN=rpl31 PE=2 SV=1
3	4	NaN	148 462 hypothetical protein DAPPUDRAFT_306954 [Daphnia pulex]; 148 462 hypothetical protein DAPPUDRAFT_30
2	2	NaN	188 1021 hypothetical protein BRAFLDRAFT_214346 [Branchiostoma floridae] gi
13	31	NaN	" 65 1576 minus strand Aldehyde dehydrogenase, mitochondrial [Crassostrea gigas]"

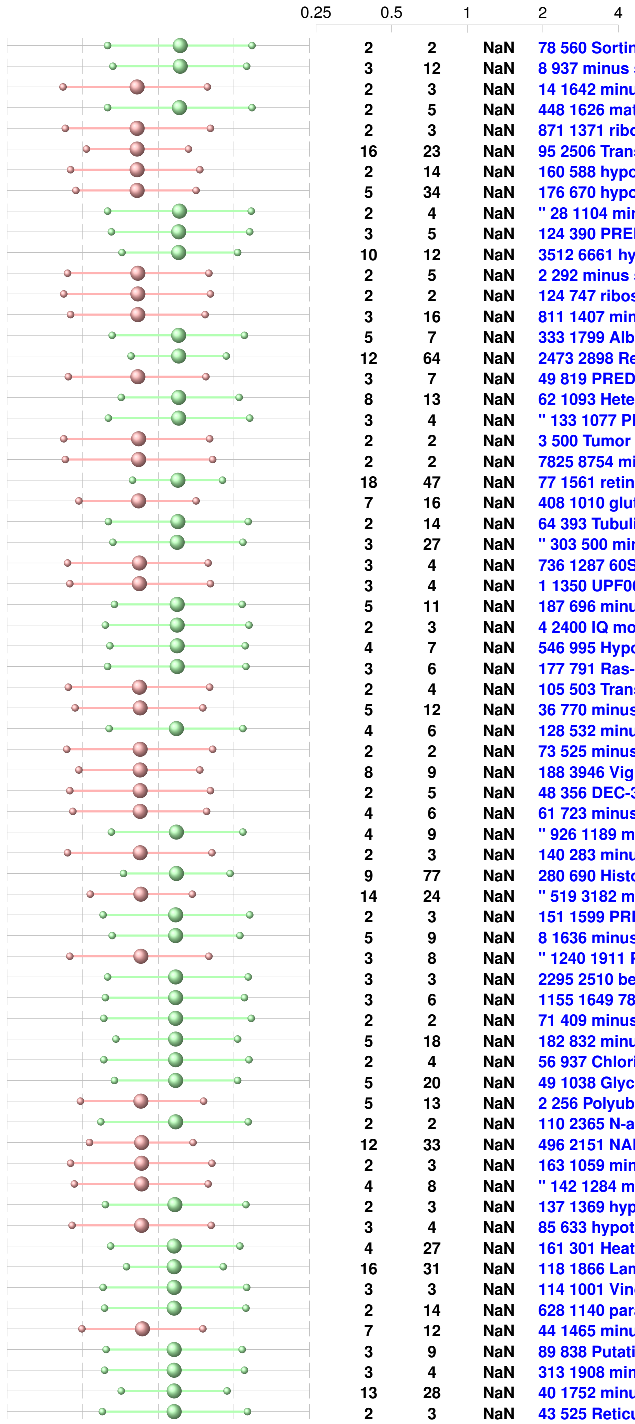

Peptides	Spectra	Coverage	Protein
2	2	NaN	78 560 Sorting nexin-12 [Camponotus floridanus]; 78 578 Sorting nexin-12 [Camponotus floridanus]; 132 605 Sortin
3	12	NaN	8 937 minus strand galectin [Crassostrea virginica]
2	3	NaN	14 1642 minus strand chaperonin containing tcp1 [Haliotis discus discus]; 15 1619 chaperonin containing tcp1 [Hali
2	5	NaN	448 1626 matrilin [Biomphalaria glabrata]
2	3	NaN	871 1371 ribosomal protein L11 [Triatoma infestans]; 343 843 minus strand ribosomal protein L11 [Triatoma infestan
16	23	NaN	95 2506 Transitional endoplasmic reticulum ATPase [Crassostrea gigas]
2	14	NaN	160 588 hypothetical protein CGI_10001640 [Crassostrea gigas]
5	34	NaN	176 670 hypothetical protein BRAFLDRAFT_122621 [Branchiostoma floridae] gi
2	4	NaN	" 28 1104 minus strand Pyruvate dehydrogenase E1 component subunit beta, mitochondrial [Crassostrea gigas]; 10
3	5	NaN	124 390 PREDICTED: 60S ribosomal protein L23a-like [Callithrix jacchus]
10	12	NaN	3512 6661 hypothetical protein BRAFLDRAFT_255626 [Branchiostoma floridae] gi
2	5	NaN	2 292 minus strand H2 [Haliotis tuberculata]
2	2	NaN	124 747 ribosomal protein S10 [Crassostrea gigas] gi
3	16	NaN	811 1407 minus strand mitochondrial malate dehydrogenase 2 [Eospalax baileyi]
5	7	NaN	333 1799 Alba-like protein C9orf23-like protein [Crassostrea gigas]
12	64	NaN	2473 2898 RecName: Full=Paramyosin gi|397603|emb|CAA79849.1| paramyosin [Echinococcus granulosus]
3	7	NaN	49 819 PREDICTED: elongation factor 1-delta-like isoform 2 [Strongylocentrotus purpuratus]
8	13	NaN	62 1093 Heterogeneous nuclear ribonucleoprotein A2-like protein 1 [Crassostrea gigas]
3	4	NaN	" 133 1077 PREDICTED: electron transfer flavoprotein, alpha polypeptide-like isoform 2 [Saccoglossus kowalevskii]"
2	2	NaN	3 500 Tumor protein D54 [Crassostrea gigas]; 3 464 Tumor protein D54 [Crassostrea gigas]; 3 464 Tumor protein D54
2	2	NaN	7825 8754 minus strand H2 [Haliotis tuberculata]
18	47	NaN	77 1561 retinal dehydrogenase [Lymnaea stagnalis]; 69 1553 retinal dehydrogenase [Lymnaea stagnalis]
7	16	NaN	408 1010 glutathione S-transferase sigma 2 [Mytilus galloprovincialis]; 166 768 glutathione S-transferase sigma 2 [M
2	14	NaN	64 393 Tubulin beta chain OS=Paracentrotus lividus PE=2 SV=1; 109 438 Tubulin beta chain OS=Paracentrotus lividu
3	27	NaN	" 303 500 minus strand protein lethal, putative [Pediculus humanus corporis] gi"
3	4	NaN	736 1287 60S ribosomal protein L3 OS=Rattus norvegicus GN=Rpl3 PE=1 SV=3
3	4	NaN	1 1350 UPF0663 transmembrane protein C17orf28 [Crassostrea gigas]; 163 1542 UPF0663 transmembrane protein C
5	11	NaN	187 696 minus strand putative ribosomal protein S10 [Sipunculus nudus]
2	3	NaN	4 2400 IQ motif containing GTPase activating protein 2 [Xenopus laevis] gi; 4 2169 IQ motif containing GTPase activ
4	7	NaN	546 995 Hypothetical protein CBG12002 [Caenorhabditis briggsae]; 543 995 minus strand PREDICTED: tubulin polyr
3	6	NaN	177 791 Ras-related protein Rab-1A [Haliotis discus discus]
2	4	NaN	105 503 Transgelin-2 [Crassostrea gigas]
5	12	NaN	36 770 minus strand Endoplasmic reticulum protein ERp29 [Crassostrea gigas]
4	6	NaN	128 532 minus strand mitochondrial ATPase inhibitor [Ornithodoros parkeri]
2	2	NaN	73 525 minus strand Actin-related protein 2/3 complex subunit 5-like protein [Salmo salar] gi; 73 525 Actin-related pr
8	9	NaN	188 3946 Vigilin [Crassostrea gigas]; 188 4135 Vigilin [Crassostrea gigas]
2	5	NaN	48 356 DEC-3 [Lymnaea stagnalis]
4	6	NaN	61 723 minus strand 40S ribosomal protein S3-A OS=Xenopus laevis GN=rps3-a PE=2 SV=2
4	9	NaN	" 926 1189 minus strand Myosin heavy chain, striated muscle [Crassostrea gigas]"
2	3	NaN	140 283 minus strand elongation factor 1 alpha [Angiostrongylus cantonensis]
9	77	NaN	280 690 Histone H3.3 [Harpegnathos saltator]
14	24	NaN	" 519 3182 minus strand Alpha-actinin, sarcomeric [Crassostrea gigas]"
2	3	NaN	151 1599 PREDICTED: galactosamine (N-acetyl)-6-sulfate sulfatase-like [Saccoglossus kowalevskii]
5	9	NaN	8 1636 minus strand V-type proton ATPase catalytic subunit A [Crassostrea gigas]; 8 1636 minus strand V-type proto
3	8	NaN	" 1240 1911 PREDICTED: ATP synthase subunit alpha, mitochondrial-like [Anolis carolinensis]"
3	3	NaN	2295 2510 beta-thymosin domain repeat protein CSP24KDa_v1 [Hermissenda crassicornis] gi
3	6	NaN	1155 1649 78 kDa glucose-regulated protein OS=Aplysia californica PE=1 SV=1
2	2	NaN	71 409 minus strand heat shock protein 108 [Alligator mississippiensis]
5	18	NaN	182 832 minus strand qm-like protein [Crassostrea ariakensis]
2	4	NaN	56 937 Chloride intracellular channel exc-4 [Crassostrea gigas]
5	20	NaN	49 1038 Glyceraldehyde-3-phosphate dehydrogenase [Crotalus adamanteus]
5	13	NaN	2 256 Polyubiquitin (Fragment) OS=Xenopus laevis PE=1 SV=2; 20 388 large subunit ribosomal protein L40e [Clonor
2	2	NaN	110 2365 N-acetylated-alpha-linked acidic dipeptidase 2 [Crassostrea gigas]
12	33	NaN	496 2151 NADP-dependent malic enzyme [Crassostrea gigas]
2	3	NaN	163 1059 minus strand Heterogeneous nuclear ribonucleoprotein A2-like protein 1 [Crassostrea gigas]; 99 995 minu
4	8	NaN	" 142 1284 minus strand Chain O, Crsytal Structure Of Human Liver Gapdh gi|67464044|pdb|1ZNQ|P Chain P, Crsyta
2	3	NaN	137 1369 hypothetical protein TcasGA2_TC002962 [Tribolium castaneum]
3	4	NaN	85 633 hypothetical protein [Amblyomma maculatum]
4	27	NaN	161 301 Heat shock protein beta-6 OS=Mus musculus GN=Hspb6 PE=1 SV=1
16	31	NaN	118 1866 Lamin Dm0 [Crassostrea gigas]
3	3	NaN	114 1001 Vinculin [Crassostrea gigas]
2	14	NaN	628 1140 paramyosin [Haliotis discus discus]
7	12	NaN	44 1465 minus strand Adenylyl cyclase-associated protein 1 [Crassostrea gigas]; 52 1473 minus strand Adenylyl cyc
3	9	NaN	89 838 Putative phosphoglycerate mutase [Crassostrea gigas]; 89 838 Putative phosphoglycerate mutase [Crassost
3	4	NaN	313 1908 minus strand Heterogeneous nuclear ribonucleoprotein L [Crassostrea gigas]
13	28	NaN	40 1752 minus strand heat shock protein 60 [Biomphalaria glabrata]; 40 1752 minus strand heat shock protein 60 [B
2	3	NaN	43 525 Reticulon-4 [Crassostrea gigas]; 143 757 Reticulon-4 [Crassostrea gigas]; 43 564 Reticulon-4 [Crassostrea gi

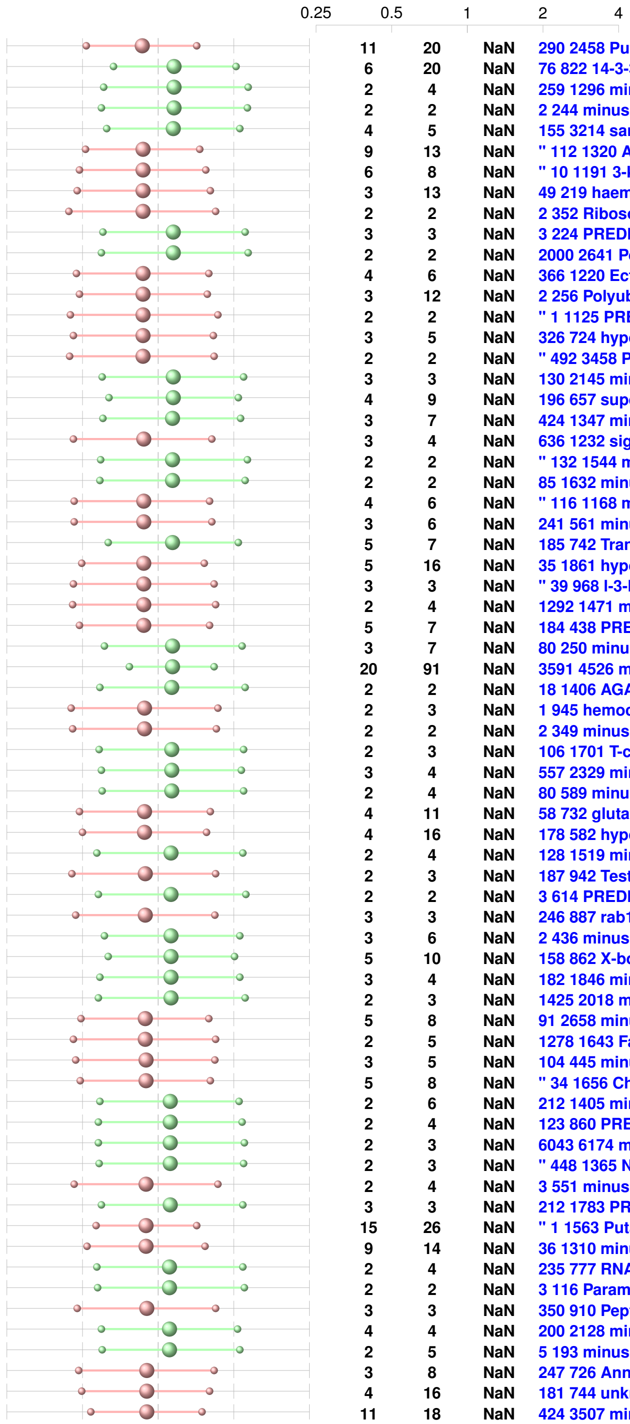

Peptides	Spectra	Coverage	Protein
11	20	NaN	290 2458 Putative beta-D-xylosidase 5 [Crassostrea gigas]
6	20	NaN	76 822 14-3-3 protein [Heterodera glycines]; 76 822 minus strand 14-3-3 protein [Heterodera glycines]; 76 822 minus
2	4	NaN	259 1296 minus strand GD20916 [Drosophila simulans] gi
2	2	NaN	2 244 minus strand hypothetical protein KGM_08771 [Danaus plexippus]
4	5	NaN	155 3214 sarco/endoplasmic reticulum calcium ATPase isoform B [Pinctada fucata]; 155 3214 minus strand sarco/er
9	13	NaN	" 112 1320 Aspartate aminotransferase, cytoplasmic [Crassostrea gigas]"
6	8	NaN	" 10 1191 3-ketoacyl-CoA thiolase, mitochondrial [Crassostrea gigas]"
3	13	NaN	49 219 haemocyanin precursor [Nautilus pompilius]
2	2	NaN	2 352 Ribosome-binding protein 1 [Crassostrea gigas]
3	3	NaN	3 224 PREDICTED: 40S ribosomal protein S14-like [Sarcophilus harrisii]
2	2	NaN	2000 2641 Polyadenylate-binding protein 4 [Crassostrea gigas]
4	6	NaN	366 1220 Ectoine hydroxylase [Crassostrea gigas]
3	12	NaN	2 256 Polyubiquitin (Fragment) OS=Xenopus laevis PE=1 SV=2
2	2	NaN	" 1 1125 PREDICTED: trifunctional enzyme subunit beta, mitochondrial-like [Oreochromis niloticus]"
3	5	NaN	326 724 hypothetical protein [Amblyomma maculatum]
2	2	NaN	" 492 3458 PREDICTED: 2-oxoglutarate dehydrogenase, mitochondrial isoform 1 [Sarcophilus harrisii]"
3	3	NaN	130 2145 minus strand Glucosamine–fructose-6-phosphate aminotransferase [isomerizing] 1 [Crassostrea gigas]; 1
4	9	NaN	196 657 superoxide dismutase [Aplysia californica] gi; 690 1151 superoxide dismutase [Aplysia californica] gi
3	7	NaN	424 1347 minus strand Tubulin alpha chain [Ascaris suum]
3	4	NaN	636 1232 sigma class glutathione-s-transferase 3 [Haliotis discus discus]; 963 1559 sigma class glutathione-s-transf
2	2	NaN	" 132 1544 minus strand Serine hydroxymethyltransferase, cytosolic [Crassostrea gigas]"
2	2	NaN	85 1632 minus strand Alpha-aminoadipic semialdehyde dehydrogenase [Crassostrea gigas]
4	6	NaN	" 116 1168 minus strand hypothetical protein CGI_10014553, partial [Crassostrea gigas]"
3	6	NaN	241 561 minus strand thioredoxin [Crassostrea ariakensis]
5	7	NaN	185 742 Transmembrane protein Tmp21 [Crassostrea gigas]
5	16	NaN	35 1861 hypothetical protein DAPPUDRAFT_309082 [Daphnia pulex]
3	3	NaN	" 39 968 l-3-hydroxyacyl-coenzyme a dehydrogenase, short chain [Haliotis discus discus]; 39 968 l-3-hydroxyacyl-co
2	4	NaN	1292 1471 minus strand hemocyanin isoform 1 [Haliotis diversicolor]
5	7	NaN	184 438 PREDICTED: similar to CG14235 CG14235-PA [Tribolium castaneum] gi
3	7	NaN	80 250 minus strand 40S ribosomal protein S29 [Ornithodoros parkeri]
20	91	NaN	3591 4526 minus strand myosin heavy chain cardiac muscle specific isoform 1 [Argopecten irradians]
2	2	NaN	18 1406 AGAP006996-PA [Anopheles gambiae str. PEST] gi
2	3	NaN	1 945 hemocyanin isoform 1 [Haliotis diversicolor]
2	2	NaN	2 349 minus strand hypothetical protein BRAFLDRAFT_206907 [Branchiostoma floridae] gi
2	3	NaN	106 1701 T-complex protein 1 subunit beta [Crassostrea gigas]
3	4	NaN	557 2329 minus strand Collagen alpha-3(VI) chain [Crassostrea gigas]
2	4	NaN	80 589 minus strand PREDICTED: adenine phosphoribosyltransferase-like [Strongylocentrotus purpuratus]
4	11	NaN	58 732 glutathione-s-transferase [Haliotis discus discus]
4	16	NaN	178 582 hypothetical protein CGI_10001640 [Crassostrea gigas]
2	4	NaN	128 1519 minus strand V-type proton ATPase subunit H [Crassostrea gigas]; 128 1519 minus strand V-type proton A
2	3	NaN	187 942 Testis-specific gene 10 protein OS=Mus musculus GN=Tsga10 PE=1 SV=1; 212 967 Testis-specific gene 10 p
2	2	NaN	3 614 PREDICTED: 60S ribosomal protein L8-like [Papio anubis]
3	3	NaN	246 887 rab11-2 [Limulus polyphemus]
3	6	NaN	2 436 minus strand PREDICTED: stress-induced-phosphoprotein 1 [Sarcophilus harrisii]
5	10	NaN	158 862 X-box binding protein [Haliotis discus discus]
3	4	NaN	182 1846 minus strand hypothetical protein DAPPUDRAFT_302108 [Daphnia pulex]; 110 1774 minus strand hypothe
2	3	NaN	1425 2018 minus strand Ankyrin-2 [Crassostrea gigas]; 1425 2018 minus strand Ankyrin-2 [Crassostrea gigas]
5	8	NaN	91 2658 minus strand puromycin-sensitive aminopeptidase [Saccoglossus kowalevskii]
2	5	NaN	1278 1643 Fatty acid-binding-like protein 5 [Crassostrea gigas]
3	5	NaN	104 445 minus strand macrophage migration inhibitory factor [Ascaris suum]
5	8	NaN	" 34 1656 Chaperonin containing TCP1, subunit 7 (eta) [Danio rerio] gi; 34 1656 Chaperonin containing TCP1, subun
2	6	NaN	212 1405 minus strand unnamed protein product [Tetraodon nigroviridis]
2	4	NaN	123 860 PREDICTED: proteasome subunit alpha type-6-like [Cavia porcellus]; 1164 1901 PREDICTED: proteasome su
2	3	NaN	6043 6174 myosin heavy chain [Mytilus galloprovincialis]; 5869 6000 myosin heavy chain [Mytilus galloprovincialis]
2	3	NaN	" 448 1365 Nitrile-specifier protein 5, partial [Crassostrea gigas]"
2	4	NaN	3 551 minus strand Spectrin beta chain [Crassostrea gigas]
3	3	NaN	212 1783 PREDICTED: d-3-phosphoglycerate dehydrogenase-like [Strongylocentrotus purpuratus]
15	26	NaN	" 1 1563 Putative methylmalonate-semialdehyde dehydrogenase [acylating], mitochondrial [Crassostrea gigas]"
9	14	NaN	36 1310 minus strand elongation factor-1 gamma [Diguetia canities]
2	4	NaN	235 777 RNA-binding protein 4.1 [Clonorchis sinensis]
2	2	NaN	3 116 Paramyosin [Crassostrea gigas]; 3 107 minus strand Paramyosin [Crassostrea gigas]
3	3	NaN	350 910 Peptidyl-prolyl cis-trans isomerase B [Crassostrea gigas]; 488 1039 Peptidyl-prolyl cis-trans isomerase B [C
4	4	NaN	200 2128 minus strand heat shock protein 70 [Cristaria plicata]
2	5	NaN	5 193 minus strand PREDICTED: 40S ribosomal protein S28-like [Strongylocentrotus purpuratus]; 3 122 PREDICTED
3	8	NaN	247 726 Annexin A7 [Crassostrea gigas]; 286 675 Annexin A7 [Crassostrea gigas]
4	16	NaN	181 744 unknown [Dendroctonus ponderosae]
11	18	NaN	424 3507 minus strand putative Na+/K+-ATPase alpha subunit [Paroctopus digueti]; 424 3507 minus strand putative

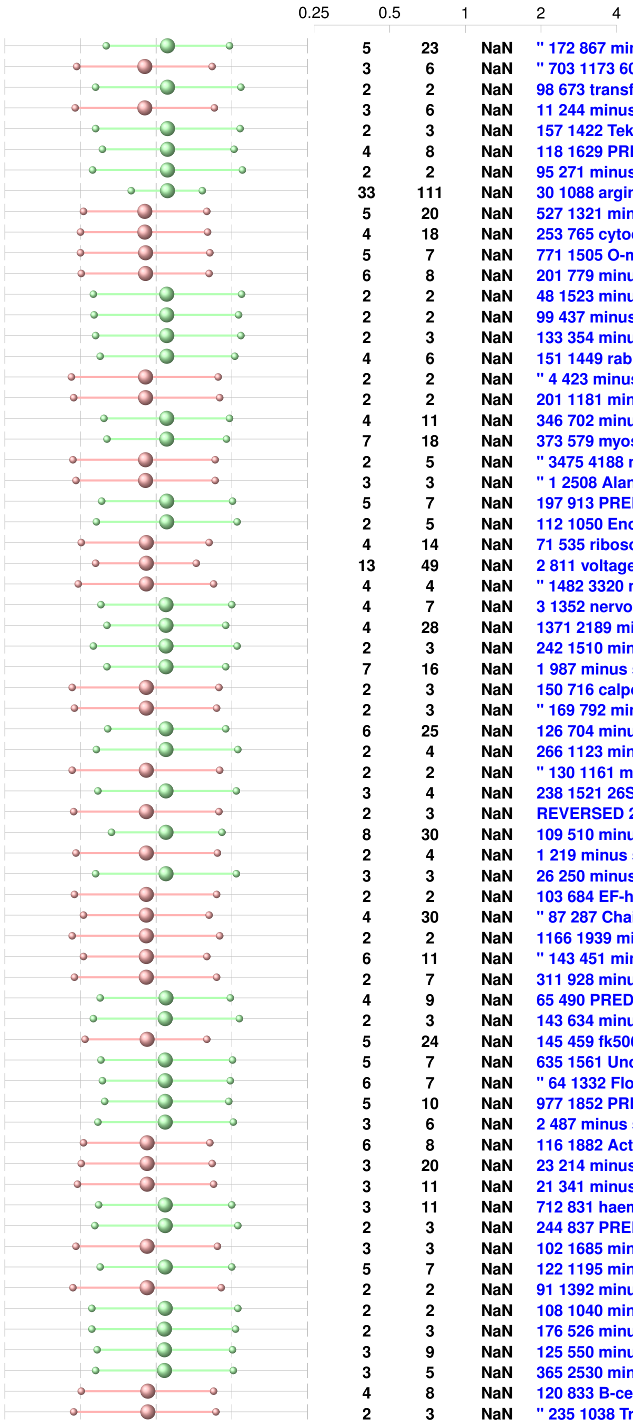

Peptides	Spectra	Coverage	Protein
5	23	NaN	" 172 867 minus strand complement C1q-like protein 4 precursor, partial [Oryzias melastigma]"
3	6	NaN	" 703 1173 60S acidic ribosomal protein P0, putative [Ixodes scapularis] gi"
2	2	NaN	98 673 transforming protein Rho-like [Acyrthosiphon pisum]
3	6	NaN	11 244 minus strand Tubulin alpha chain (Fragment) OS=Lytechinus pictus PE=3 SV=1
2	3	NaN	157 1422 Tektin-2 [Crassostrea gigas]
4	8	NaN	118 1629 PREDICTED: similar to tektin 3 [Ciona intestinalis]
2	2	NaN	95 271 minus strand hypothetical protein EAI_01530 [Harpegnathos saltator]
33	111	NaN	30 1088 arginine kinase [Conus novaehollandiae]
5	20	NaN	527 1321 minus strand troponin T [Mizuhopecten yessoensis]; 305 1099 minus strand troponin T [Mizuhopecten yes
4	18	NaN	253 765 cytochrome c oxidase subunit IV [Urechis caupo]
5	7	NaN	771 1505 O-methyltransferase family 3 [Dicentrarchus labrax]
6	8	NaN	201 779 minus strand sigma class glutathione-s-transferase 2 [Haliotis discus discus]
2	2	NaN	48 1523 minus strand Spermatogenesis-associated protein 18-like protein [Crassostrea gigas]
2	2	NaN	99 437 minus strand V-type proton ATPase subunit G [Crassostrea gigas]
2	3	NaN	133 354 minus strand 60S ribosomal protein L7a OS=Rattus norvegicus GN=Rpl7a PE=1 SV=2
4	6	NaN	151 1449 rab GDP-dissociation inhibitor [Branchiostoma belcheri]
2	2	NaN	" 4 423 minus strand PREDICTED: similar to NADH dehydrogenase, putative [Tribolium castaneum] gi"
2	2	NaN	201 1181 minus strand Serine/threonine-protein phosphatase PP1-alpha catalytic subunit [Crassostrea gigas]
4	11	NaN	346 702 minus strand PREDICTED: galectin-4-like [Anolis carolinensis]; 367 669 minus strand PREDICTED: galectin
7	18	NaN	373 579 myosin heavy chain [Argopecten irradians] gi
2	5	NaN	" 3475 4188 minus strand Heat shock 70 kDa protein cognate, putative [Pediculus humanus corporis] gi"
3	3	NaN	" 1 2508 Alanyl-tRNA synthetase, cytoplasmic [Crassostrea gigas]; 1 2508 Alanyl-tRNA synthetase, cytoplasmic [Cra
5	7	NaN	197 913 PREDICTED: l-xylulose reductase-like [Monodelphis domestica]; 197 913 PREDICTED: l-xylulose reductase-
2	5	NaN	112 1050 Enolase-phosphatase E1 [Crassostrea gigas]; 112 1050 Enolase-phosphatase E1 [Crassostrea gigas]
4	14	NaN	71 535 ribosomal protein rpl14 [Arenicola marina]
13	49	NaN	2 811 voltage-dependent anion channel 2-like protein [Haliotis diversicolor]; 2 811 voltage-dependent anion channel
4	4	NaN	" 1482 3320 minus strand Phosphoenolpyruvate carboxykinase, cytosolic [GTP] [Crassostrea gigas]; 1326 3164 min
4	7	NaN	3 1352 nervous system adducin [Aplysia californica] gi; 3 1418 Protein hu-li tai shao [Crassostrea gigas]; 3 1214 ner
4	28	NaN	1371 2189 minus strand galectin [Pinctada fucata]
2	3	NaN	242 1510 minus strand hypothetical protein BRAFLDRAFT_276673 [Branchiostoma floridae] gi
7	16	NaN	1 987 minus strand RecName: Full=Tubulin beta chain; AltName: Full=Beta-tubulin
2	3	NaN	150 716 calponin/transgelin [Papilio xuthus]
2	3	NaN	" 169 792 minus strand rho GDP dissociation inhibitor, putative [Ixodes scapularis] gi"
6	25	NaN	126 704 minus strand ribosomal protein L12 [Ixodes scapularis]
2	4	NaN	266 1123 minus strand mitochondrial malate dehydrogenase precursor [Nucella lapillus]
2	2	NaN	" 130 1161 minus strand Isocitrate dehydrogenase [NAD] subunit alpha, mitochondrial [Crassostrea gigas]; 198 1229
3	4	NaN	238 1521 26S protease regulatory subunit 6A [Crassostrea gigas]
2	3	NaN	REVERSED 216 2639 minus strand polycystin 2 [Gallus gallus]
8	30	NaN	109 510 minus strand hypothetical protein DAPPUDRAFT_231281 [Daphnia pulex]
2	4	NaN	1 219 minus strand PREDICTED: nucleoside diphosphate kinase 3-like [Pan troglodytes]
3	3	NaN	26 250 minus strand fructose-bisphosphate aldolase [Haliotis discus discus]
2	2	NaN	103 684 EF-hand domain-containing protein D2 [Crassostrea gigas]
4	30	NaN	" 87 287 Chain A, Structure Of Deoxygenated Hemocyanin From Rapana Thomasiana gi|31615505|pdb|1LNL|B Chai
2	2	NaN	1166 1939 minus strand Calcium-binding mitochondrial carrier protein Aralar2 OS=Homo sapiens GN=SLC25A13 PE
6	11	NaN	" 143 451 minus strand PREDICTED: 10 kDa heat shock protein, mitochondrial isoform 1 [Canis lupus familiaris]"
2	7	NaN	311 928 minus strand peroxiredoxin 3 [Clonorchis sinensis] gi; 288 905 minus strand peroxiredoxin 3 [Clonorchis si
4	9	NaN	65 490 PREDICTED: cofilin-2 [Otolemur garnettii]
2	3	NaN	143 634 minus strand ribosomal protein S14 [Solea senegalensis]
5	24	NaN	145 459 fk506-binding protein 2 [Papilio xuthus]; 237 551 fk506-binding protein 2 [Papilio xuthus]
5	7	NaN	635 1561 Uncharacterized protein yfeX [Crassostrea gigas]
6	7	NaN	" 64 1332 Flotillin-1, putative [Pediculus humanus corporis] gi"
5	10	NaN	977 1852 PREDICTED: similar to prohibitin [Tribolium castaneum] gi
3	6	NaN	2 487 minus strand PREDICTED: hypothetical protein LOC100699750 [Oreochromis niloticus]; 41 535 minus strand I
6	8	NaN	116 1882 Actin-interacting protein 1 [Crassostrea gigas]
3	20	NaN	23 214 minus strand hemocyanin isoform 1 [Nucula nucleus]
3	11	NaN	21 341 minus strand hemocyanin isoform 1 [Nucula nucleus]
3	11	NaN	712 831 haemocyanin [Aplysia californica]
2	3	NaN	244 837 PREDICTED: ras-like GTP-binding protein Rho1-like [Megachile rotundata]
3	3	NaN	102 1685 minus strand predicted protein [Nematostella vectensis] gi
5	7	NaN	122 1195 minus strand Severin [Crassostrea gigas]
2	2	NaN	91 1392 minus strand hypothetical protein AND_03103 [Anopheles darlingi]
2	2	NaN	108 1040 minus strand PREDICTED: Na(+)/H(+) exchange regulatory cofactor NHE-RF3-like [Metaseiulus occidentali
2	3	NaN	176 526 minus strand Translocon-associated protein subunit delta [Crassostrea gigas]
3	9	NaN	125 550 minus strand ribosomal protein S20 [Chlamys farreri]; 125 550 minus strand ribosomal protein S20 [Chlamy
3	5	NaN	365 2530 minus strand calpain B [Gecarcinus lateralis]
4	8	NaN	120 833 B-cell receptor-associated protein 31 [Crassostrea gigas]; 238 951 B-cell receptor-associated protein 31 [Cr
2	3	NaN	" 235 1038 Translocon-associated protein subunit alpha [Crassostrea gigas]; 309 725 hypothetical protein [Amblyom

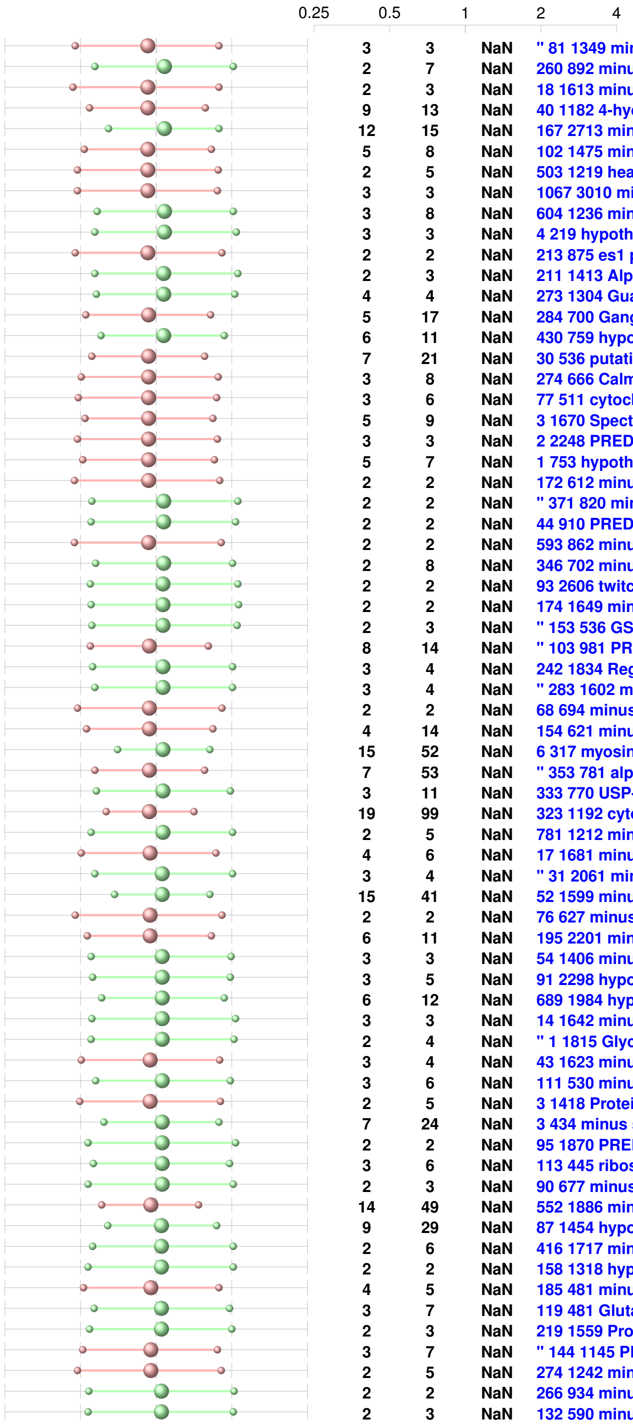

Peptides	Spectra	Coverage	Protein
3	3	NaN	" 81 1349 minus strand PREDICTED: LOW QUALITY PROTEIN: dolichyl-diphosphooligosaccharide–protein glycosyl
2	7	NaN	260 892 minus strand calponin [Haliotis diversicolor]
2	3	NaN	18 1613 minus strand chaperonin containing T-complex polypeptide subunit zeta [Haliotis discus hannai]; 527 2089
9	13	NaN	40 1182 4-hydroxyphenylpyruvate dioxygenase [Crassostrea gigas]
12	15	NaN	167 2713 minus strand Major vault protein [Crassostrea gigas]
5	8	NaN	102 1475 minus strand Cytosolic non-specific dipeptidase [Crassostrea gigas]
2	5	NaN	503 1219 heat shock cognate 71 [Mytilus galloprovincialis]
3	3	NaN	1067 3010 minus strand PREDICTED: nucleolin-like isoform 2 [Oreochromis niloticus]
3	8	NaN	604 1236 minus strand selenium-dependent glutathione peroxidase [Crassostrea gigas]; 138 770 minus strand seler
3	3	NaN	4 219 hypothetical protein BRAFLDRAFT_66351 [Branchiostoma floridae] gi; 3 239 hypothetical protein BRAFLDRAI
2	2	NaN	213 875 es1 protein [Haliotis discus discus]
2	3	NaN	211 1413 Alpha-N-acetylgalactosaminidase [Crassostrea gigas]
4	4	NaN	273 1304 Guanine nucleotide-binding protein subunit beta [Crassostrea gigas]
5	17	NaN	284 700 Ganglioside GM2 activator [Crassostrea gigas]
6	11	NaN	430 759 hypothetical protein [Amblyomma maculatum]; 336 665 hypothetical protein [Amblyomma maculatum]
7	21	NaN	30 536 putative mitochondrial ATP synthase [Haliotis discus discus]; 27 620 putative mitochondrial ATP synthase [H
3	8	NaN	274 666 Calmodulin-4 [Heterocephalus glaber]
3	6	NaN	77 511 cytochrome c oxidase subunit VIIa polypeptide 2 like [Xenopus (Silurana) tropicalis]; 77 511 cytochrome c ox
5	9	NaN	3 1670 Spectrin alpha chain [Crassostrea gigas]
3	3	NaN	2 2248 PREDICTED: heat shock 70 kDa protein 4-like [Oreochromis niloticus]; 2 2227 PREDICTED: heat shock 70 kD
5	7	NaN	1 753 hypothetical protein BRAFLDRAFT_131251 [Branchiostoma floridae] gi
2	2	NaN	172 612 minus strand putative polyadenylate-binding protein 1 [Haliotis diversicolor]; 172 612 putative polyadenylat
2	2	NaN	" 371 820 minus strand Peroxiredoxin-5, mitochondrial [Crassostrea gigas]; 91 528 minus strand Peroxiredoxin-5, m
2	2	NaN	44 910 PREDICTED: probable ATP-dependent RNA helicase DDX10 [Cricetulus griseus] gi
2	2	NaN	593 862 minus strand mitochondrial ATP synthase coupling factor 6 precursor [Ochlerotatus taeniorhynchus]
2	8	NaN	346 702 minus strand PREDICTED: galectin-4-like [Anolis carolinensis]
2	2	NaN	93 2606 twitchin [Mytilus galloprovincialis]
2	2	NaN	174 1649 minus strand Collagen alpha-1(XII) chain [Crassostrea gigas]; 123 1490 minus strand Collagen alpha-1(XII)
2	3	NaN	" 153 536 GST omega, partial [Reishia clavigera]"
8	14	NaN	" 103 981 PREDICTED: ATP synthase, H+ transporting, mitochondrial F1 complex, gamma subunit isoform H (heart)
3	4	NaN	242 1834 Regulator of differentiation 1 [Crassostrea gigas]; 458 2050 minus strand Regulator of differentiation 1 [Cr
3	4	NaN	" 283 1602 minus strand 4-aminobutyrate aminotransferase, mitochondrial [Crassostrea gigas]"
2	2	NaN	68 694 minus strand hypothetical protein BRAFLDRAFT_272416 [Branchiostoma floridae] gi; 68 667 minus strand h
4	14	NaN	154 621 minus strand Stress response protein nhaX [Crassostrea gigas]
15	52	NaN	6 317 myosin heavy chain cardiac muscle specific isoform 2 [Argopecten irradians]
7	53	NaN	" 353 781 alpha-B-crystallin, putative [Ixodes scapularis] gi"
3	11	NaN	333 770 USP-like protein isoform 2 [Crassostrea ariakensis]
19	99	NaN	323 1192 cytosolic malate dehydrogenase [Lottia scabra]; 323 1192 cytosolic malate dehydrogenase [Lottia scabra];
2	5	NaN	781 1212 minus strand cathepsin L2 cysteine protease [Pinctada fucata]
4	6	NaN	17 1681 minus strand Glucose-6-phosphate isomerase [Crassostrea gigas]
3	4	NaN	" 31 2061 minus strand 1,4-alpha-glucan-branching enzyme [Crassostrea gigas]"
15	41	NaN	52 1599 minus strand ATP synthase beta subunit [Pinctada fucata]
2	2	NaN	76 627 minus strand Heme-binding protein 2 [Crassostrea gigas]; 296 847 minus strand Heme-binding protein 2 [Cra
6	11	NaN	195 2201 minus strand hypothetical protein DAPPUDRAFT_315656 [Daphnia pulex]
3	3	NaN	54 1406 minus strand Protein henna [Crassostrea gigas]; 54 1406 minus strand Protein henna [Crassostrea gigas]
3	5	NaN	91 2298 hypothetical protein BRAFLDRAFT_267717 [Branchiostoma floridae] gi; 84 2291 hypothetical protein BRAFI
6	12	NaN	689 1984 hypothetical protein BRAFLDRAFT_78257 [Branchiostoma floridae] gi
3	3	NaN	14 1642 minus strand chaperonin containing tcp1 [Haliotis discus discus]; 14 1462 minus strand chaperonin contair
2	4	NaN	" 1 1815 Glycogen phosphorylase, muscle form [Crassostrea gigas]"
3	4	NaN	43 1623 minus strand MGC84105 protein precursor [Xenopus laevis] gi; 43 1506 minus strand MGC84105 protein pre
3	6	NaN	111 530 minus strand Nucleoside diphosphate kinase B OS=Homo sapiens GN=NME2 PE=1 SV=1
2	5	NaN	3 1418 Protein hu-li tai shao [Crassostrea gigas]; 3 1523 minus strand Protein hu-li tai shao [Crassostrea gigas]; 3 1
7	24	NaN	3 434 minus strand myosin essential light chain [Haliotis discus discus]
2	2	NaN	95 1870 PREDICTED: acetylcholinesterase-like [Equus caballus]; 99 1874 PREDICTED: acetylcholinesterase-like [Eq
3	6	NaN	113 445 ribosomal protein rps19 [Lineus viridis]
2	3	NaN	90 677 minus strand PREDICTED: 15-hydroxyprostaglandin dehydrogenase [NAD+] [Gallus gallus]; 90 683 minus str
14	49	NaN	552 1886 minus strand intermediate filament protein [Biomphalaria glabrata]
9	29	NaN	87 1454 hypothetical protein BRAFLDRAFT_287788 [Branchiostoma floridae] gi; 117 1469 hypothetical protein BRAI
2	6	NaN	416 1717 minus strand T-complex protein 1 subunit delta OS=Aedes triseriatus PE=2 SV=1
2	2	NaN	158 1318 hypothetical protein BRAFLDRAFT_98992 [Branchiostoma floridae] gi
4	5	NaN	185 481 minus strand Zgc:56530 [Danio rerio]
3	7	NaN	119 481 Glutamate receptor U1 [Crassostrea gigas]
2	3	NaN	219 1559 Programmed cell death protein 4 [Crassostrea gigas]
3	7	NaN	" 144 1145 PREDICTED: RNA-binding protein squid-like isoform 1 [Megachile rotundata]; 90 1085 squid, variant A [B
2	5	NaN	274 1242 minus strand cathepsin D [Pinctada maxima]; 290 1387 cathepsin D [Pinctada maxima]; 290 1402 minus st
2	2	NaN	266 934 minus strand Kyphoscoliosis peptidase [Crassostrea gigas]
2	3	NaN	132 590 minus strand Non-neuronal cytoplasmic intermediate filament protein OS=Helix aspersa PE=3 SV=2

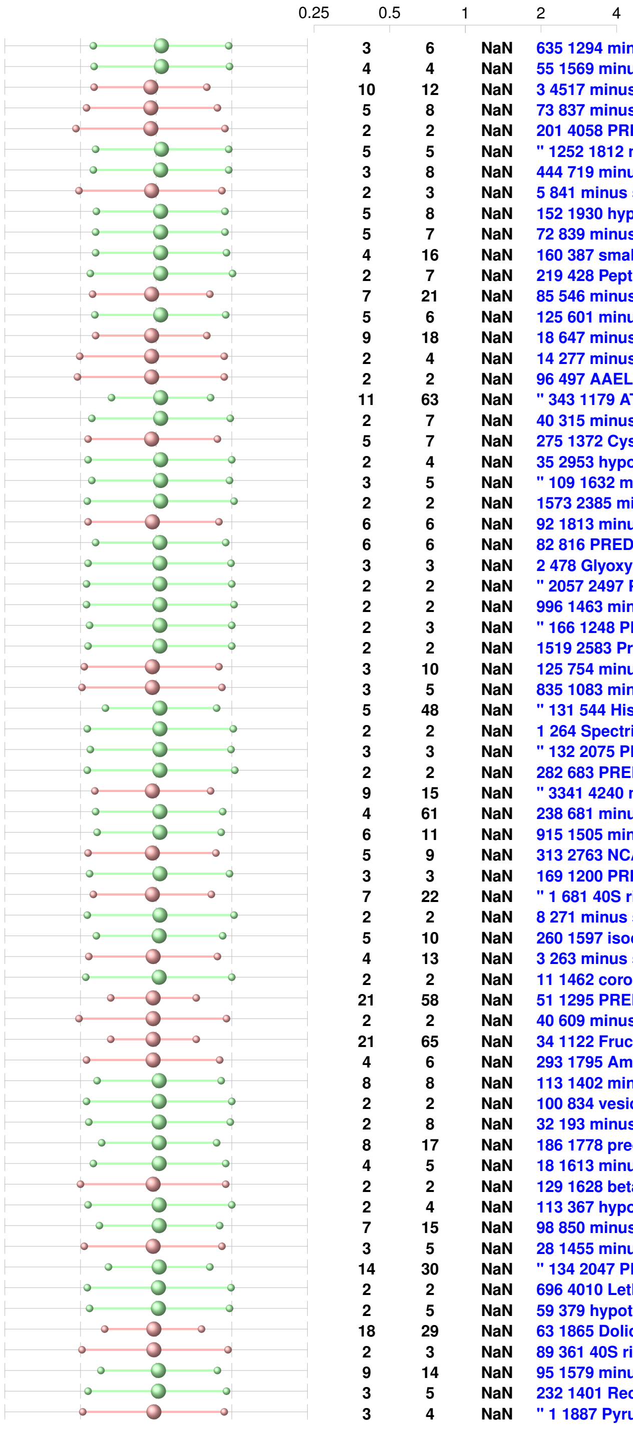

Peptides	Spectra	Coverage	Protein
3	6	NaN	635 1294 minus strand Kyphoscoliosis peptidase [Crassostrea gigas]; 643 1302 minus strand Kyphoscoliosis peptic
4	4	NaN	55 1569 minus strand Putative aminopeptidase W07G4.4 [Crassostrea gigas]; 228 1742 Putative aminopeptidase W0
10	12	NaN	3 4517 minus strand Clathrin heavy chain 1 [Crassostrea gigas]
5	8	NaN	73 837 minus strand Electron transfer flavoprotein subunit beta [Crassostrea gigas]; 73 837 minus strand Electron tr
2	2	NaN	201 4058 PREDICTED: xanthine dehydrogenase/oxidase-like [Amphimedon queenslandica]
5	5	NaN	" 1252 1812 minus strand Alanine–glyoxylate aminotransferase 2, mitochondrial [Crassostrea gigas]"
3	8	NaN	444 719 minus strand 60S ribosomal protein L9 [Laodelphax striatella]
2	3	NaN	5 841 minus strand Spectrin alpha chain [Crassostrea gigas]
5	8	NaN	152 1930 hypothetical protein BRAFLDRAFT_223365 [Branchiostoma floridae] gi
5	7	NaN	72 839 minus strand 60S ribosomal protein L8 OS=Spodoptera frugiperda GN=RpL8 PE=2 SV=1
4	16	NaN	160 387 small nuclear ribonucleoprotein polypeptide G protein [Crassostrea ariakensis]; 180 407 small nuclear ribor
2	7	NaN	219 428 Peptidyl-prolyl cis-trans isomerase OS=Hemicentrotus pulcherrimus PE=2 SV=1
7	21	NaN	85 546 minus strand RecName: Full=Eukaryotic translation initiation factor 5A; Short=eIF-5A gi|51702279|sp|P6292
5	6	NaN	125 601 minus strand omega class glutathione-s-transferase [Chlamys farreri]; 113 808 minus strand omega class g
9	18	NaN	18 647 minus strand ATP synthase-like protein [Biomphalaria glabrata]
2	4	NaN	14 277 minus strand Histidine triad nucleotide-binding protein 1 OS=Oryctolagus cuniculus GN=HINT1 PE=1 SV=2
2	2	NaN	96 497 AAEL009772-PB [Aedes aegypti]
11	63	NaN	" 343 1179 ATP synthase subunit alpha, mitochondrial OS=Pongo abelii GN=ATP5A1 PE=2 SV=1"
2	7	NaN	40 315 minus strand 60S ribosomal protein L18a OS=Homo sapiens GN=RPL18A PE=1 SV=2
5	7	NaN	275 1372 Cystathionine gamma-lyase OS=Bos taurus GN=CTH PE=2 SV=1
2	4	NaN	35 2953 hypothetical protein CGI_10020867 [Crassostrea gigas]; 97 3015 minus strand hypothetical protein CGI_100
3	5	NaN	" 109 1632 minus strand Seryl-tRNA synthetase, cytoplasmic [Crassostrea gigas]; 109 1614 minus strand Seryl-tRNA
2	2	NaN	1573 2385 minus strand Spectrin beta chain [Crassostrea gigas]
6	6	NaN	92 1813 minus strand Dihydropyrimidinase [Crassostrea gigas]
6	6	NaN	82 816 PREDICTED: triosephosphate isomerase B [Oreochromis niloticus]
3	3	NaN	2 478 Glyoxylate reductase/hydroxypyruvate reductase [Crassostrea gigas]
2	2	NaN	" 2057 2497 Phosphate carrier protein, mitochondrial [Crassostrea gigas]"
2	2	NaN	996 1463 minus strand catalase [Cristaria plicata] gi
2	3	NaN	" 166 1248 PREDICTED: probable pyruvate dehydrogenase E1 component subunit alpha, mitochondrial-like [Metase
2	2	NaN	1519 2583 Proliferation-associated protein 2G4 [Crassostrea gigas]; 565 1629 Proliferation-associated protein 2G4 [
3	10	NaN	125 754 minus strand peptidyl prolyl cis-trans isomerase B [Conus novaehollandiae]
3	5	NaN	835 1083 minus strand Calmodulin [Crassostrea gigas]
5	48	NaN	" 131 544 Histone H2B, gonadal OS=Patella granatina PE=1 SV=2"
2	2	NaN	1 264 Spectrin beta chain [Crassostrea gigas]
3	3	NaN	" 132 2075 PREDICTED: arginyl-tRNA synthetase, cytoplasmic [Ornithorhynchus anatinus]; 132 2090 minus strand F
2	2	NaN	282 683 PREDICTED: ribonuclease UK114-like [Oreochromis niloticus]
9	15	NaN	" 3341 4240 minus strand nonmuscle myosin II, partial [Aplysia californica]"
4	61	NaN	238 681 minus strand Globin OS=Cerithidea rhizophorarum PE=1 SV=1
6	11	NaN	915 1505 minus strand Collagen alpha-5(VI) chain [Crassostrea gigas]
5	9	NaN	313 2763 NCAM-related cell adhesion molecule precursor [Aplysia californica] gi
3	3	NaN	169 1200 PREDICTED: nidogen-2-like isoform 1 [Strongylocentrotus purpuratus]
7	22	NaN	" 1 681 40S ribosomal protein S4, X isoform [Crassostrea gigas]"
2	2	NaN	8 271 minus strand Laminin subunit gamma-1 [Crassostrea gigas]
5	10	NaN	260 1597 isocitrate dehydrogenase [Mytilus galloprovincialis]; 260 1597 isocitrate dehydrogenase [Mytilus galloprov
4	13	NaN	3 263 minus strand beta-actin [Urechis unicinctus]
2	2	NaN	11 1462 coronin [Schistosoma mansoni]
21	58	NaN	51 1295 PREDICTED: phosphoglycerate kinase-like [Nasonia vitripennis]; 97 1341 PREDICTED: phosphoglycerate k
2	2	NaN	40 609 minus strand ependymin related protein-1 precursor [Haliotis discus discus]
21	65	NaN	34 1122 Fructose-bisphosphate aldolase [Crassostrea gigas]
4	6	NaN	293 1795 Amidase [Crassostrea gigas]
8	8	NaN	113 1402 minus strand S-adenosylhomocysteine hydrolase [Crassostrea ariakensis]; 113 1402 minus strand S-aden
2	2	NaN	100 834 vesicle-associated membrane protein/synaptobrevin-binding protein [Aplysia californica] gi
2	8	NaN	32 193 minus strand Ubiquinol-cytochrome C reductase complex [Xenopus laevis] gi; 32 193 minus strand Ubiquino
8	17	NaN	186 1778 predicted protein [Nematostella vectensis] gi
4	5	NaN	18 1613 minus strand chaperonin containing T-complex polypeptide subunit zeta [Haliotis discus hannai]; 527 2089
2	2	NaN	129 1628 beta-hexosaminidase subunit beta precursor [Danio rerio] gi
2	4	NaN	113 367 hypothetical protein DAPPUDRAFT_329128 [Daphnia pulex]; 113 367 hypothetical protein DAPPUDRAFT_32
7	15	NaN	98 850 minus strand 14-3-3 protein epsilon [Schistocerca gregaria]
3	5	NaN	28 1455 minus strand PREDICTED: probable serine carboxypeptidase CPVL-like [Anolis carolinensis]
14	30	NaN	" 134 2047 PREDICTED: succinate dehydrogenase [ubiquinone] flavoprotein subunit, mitochondrial-like isoform 3, p
2	2	NaN	696 4010 Lethal(2) giant larvae-like protein 1 [Crassostrea gigas]; 657 3971 minus strand Lethal(2) giant larvae-like p
2	5	NaN	59 379 hypothetical protein DAPPUDRAFT_230756 [Daphnia pulex]; 117 443 minus strand hypothetical protein DAPP
18	29	NaN	63 1865 Dolichyl-diphosphooligosaccharide–protein glycosyltransferase subunit 1 [Crassostrea gigas]; 63 1865 Dol
2	3	NaN	89 361 40S ribosomal protein S18 OS=Rattus norvegicus GN=Rps18 PE=1 SV=3
9	14	NaN	95 1579 minus strand hypothetical protein BRAFLDRAFT_113974 [Branchiostoma floridae] gi
3	5	NaN	232 1401 RecName: Full=Guanine nucleotide-binding protein G(s) subunit alpha; AltName: Full=Adenylate cyclase-s
3	4	NaN	" 1 1887 Pyruvate carboxylase, mitochondrial [Crassostrea gigas]"

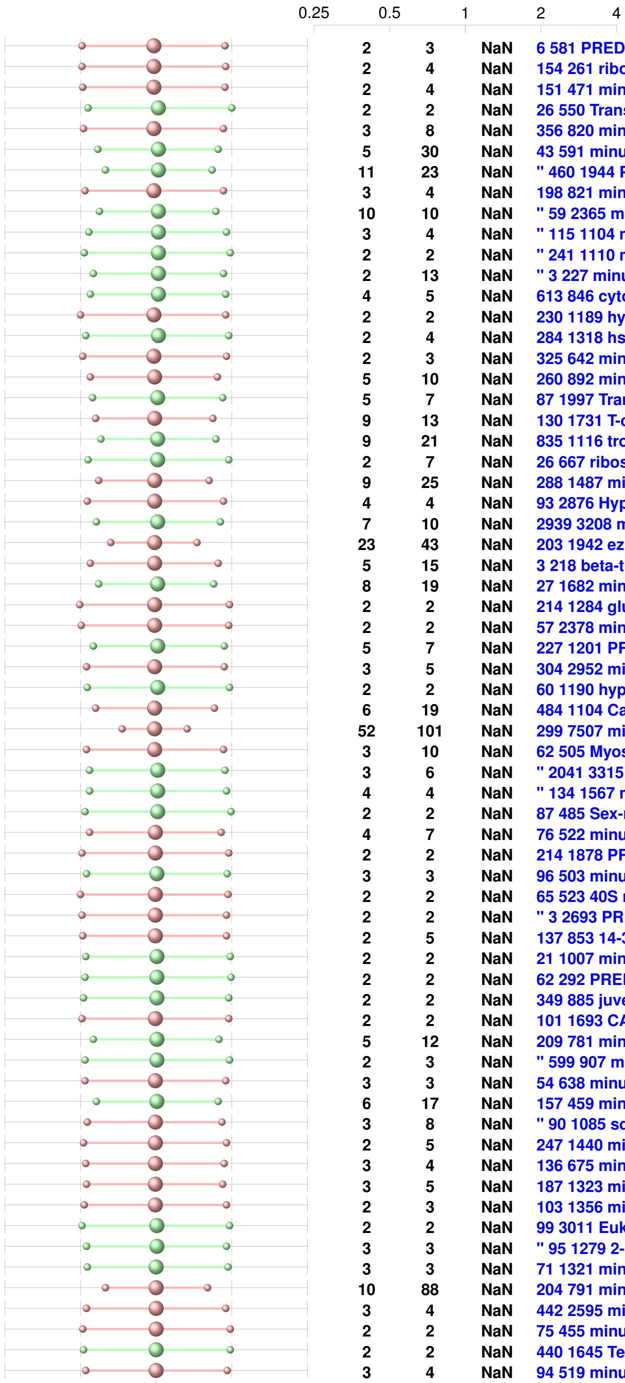

Peptides	Spectra	Coverage	Protein
2	3	NaN	6 581 PREDICTED: mammalian ependymin-related protein 1 [Gallus gallus]
2	4	NaN	154 261 ribosomal protein S18 [Stenella coeruleoalba]
2	4	NaN	151 471 minus strand ribosomal protein rps25 [Arenicola marina]
2	2	NaN	26 550 Translocon-associated protein subunit gamma [Crassostrea gigas]
3	8	NaN	356 820 minus strand calmodulin-1 [Trichinella spiralis] gi
5	30	NaN	43 591 minus strand cyclophilin type peptidyl-prolyl cis-trans isomerase [Glossina morsitans morsitans]
11	23	NaN	" 460 1944 PREDICTED: succinyl-CoA:3-ketoacid-coenzyme A transferase 1, mitochondrial [Canis lupus familiaris]"
3	4	NaN	198 821 minus strand sigma class glutathione-s-transferase 2 [Haliotis discus discus]
10	10	NaN	" 59 2365 minus strand Aconitate hydratase, mitochondrial [Crassostrea gigas]"
3	4	NaN	" 115 1104 minus strand PREDICTED: KH domain-containing, RNA-binding, signal transduction-associated protein 3
2	2	NaN	" 241 1110 minus strand N(G),N(G)-dimethylarginine dimethylaminohydrolase 1 [Crassostrea gigas]; 241 1110 minus
2	13	NaN	" 3 227 minus strand alpha tubulin, putative [Ixodes scapularis] gi"
4	5	NaN	613 846 cytochrome c oxidase subunit Va [Antheraea yamamai] gi
2	2	NaN	230 1189 hypothetical protein [Amblyomma maculatum]
2	4	NaN	284 1318 hsc70-interacting protein [Gallus gallus] gi; 88 1137 hsc70-interacting protein [Gallus gallus] gi
2	3	NaN	325 642 minus strand Protein phosphatase 1B [Crassostrea gigas]
5	10	NaN	260 892 minus strand calponin [Haliotis diversicolor]
5	7	NaN	87 1997 Transketolase-like protein 2 [Crassostrea gigas]; 87 1997 Transketolase-like protein 2 [Crassostrea gigas]; 8
9	13	NaN	130 1731 T-complex protein 1 subunit epsilon [Crassostrea gigas]; 130 1731 T-complex protein 1 subunit epsilon [Cr
9	21	NaN	835 1116 tropomyosin [Ilyanassa obsoleta]
2	7	NaN	26 667 ribosomal protein L15 [Acipenser sinensis] gi; 26 667 ribosomal protein L15 [Acipenser sinensis] gi; 26 667 r
9	25	NaN	288 1487 minus strand putative procollagen type VI alpha 4 [Haliotis diversicolor]
4	4	NaN	93 2876 Hypoxia up-regulated protein 1 [Crassostrea gigas]
7	10	NaN	2939 3208 minus strand beta-thymosin domain repeat protein CSP24KDa_v1 [Hermissenda crassicornis] gi
23	43	NaN	203 1942 ezrin/radixin/moesin [Aplysia californica] gi; 243 1982 minus strand ezrin/radixin/moesin [Aplysia californic
5	15	NaN	3 218 beta-tubulin [Fasciola hepatica] gi
8	19	NaN	27 1682 minus strand PREDICTED: nucleolar protein 56 [Oreochromis niloticus]
2	2	NaN	214 1284 glutamine synthetase [Tegillarca granosa]
2	2	NaN	57 2378 minus strand Heterogeneous nuclear ribonucleoprotein U-like protein 1 [Crassostrea gigas]
5	7	NaN	227 1201 PREDICTED: quinone oxidoreductase-like isoform 1 [Ornithorhynchus anatinus]; 109 1083 PREDICTED: qu
3	5	NaN	304 2952 minus strand hypothetical protein CGI_10026535 [Crassostrea gigas]
2	2	NaN	60 1190 hypothetical protein BRAFLDRAFT_109688 [Branchiostoma floridae] gi; 51 1184 hypothetical protein BRAFL
6	19	NaN	484 1104 Calponin-2 [Crassostrea gigas]; 309 938 Calponin-2 [Crassostrea gigas]
52	101	NaN	299 7507 minus strand filamin [Clonorchis sinensis]
3	10	NaN	62 505 Myosin-2 essential light chain [Crassostrea gigas]
3	6	NaN	" 2041 3315 minus strand Myosin-10 OS=Mus musculus GN=Myh10 PE=1 SV=2; 2041 3345 minus strand Myosin hea
4	4	NaN	" 134 1567 minus strand 6-phosphogluconate dehydrogenase, decarboxylating [Crassostrea gigas]; 134 1573 minus
2	2	NaN	87 485 Sex-regulated protein janus-A [Crassostrea gigas]
4	7	NaN	76 522 minus strand Troponin C [Crassostrea gigas]; 173 619 minus strand Troponin C [Crassostrea gigas]; 173 619
2	2	NaN	214 1878 PREDICTED: lysyl-tRNA synthetase-like [Oreochromis niloticus]
3	3	NaN	96 503 minus strand hypothetical protein DAPPUDRAFT_230378 [Daphnia pulex]
2	2	NaN	65 523 40S ribosomal protein S9 [Crassostrea gigas]; 65 523 40S ribosomal protein S9 [Crassostrea gigas]
2	2	NaN	" 3 2693 PREDICTED: similar to coatomer protein complex, subunit beta 2 (beta prime) [Taeniopygia guttata]"
2	5	NaN	137 853 14-3-3 protein [Heterodera glycines]
2	2	NaN	21 1007 minus strand cathepsin L2 cysteine protease [Pinctada fucata]; 21 1007 minus strand cathepsin L2 cysteine
2	2	NaN	62 292 PREDICTED: heterogeneous nuclear ribonucleoprotein F-like [Otolemur garnettii]
2	2	NaN	349 885 juvenile hormone diol kinase [Danaus plexippus]; 349 885 juvenile hormone diol kinase [Danaus plexippus]
2	2	NaN	101 1693 CALNUC [Spodoptera frugiperda]; 141 1325 Nucleobindin-2 [Crassostrea gigas]; 141 1289 Nucleobindin-2
5	12	NaN	209 781 minus strand high mobility group protein 1 [Biomphalaria glabrata]; 277 771 high mobility group protein 1 [B
2	3	NaN	" 599 907 minus strand Tryptophanyl-tRNA synthetase, cytoplasmic [Heterocephalus glaber]"
3	3	NaN	54 638 minus strand ubiquitin carboxyl-terminal hydrolase [Aplysia californica] gi
6	17	NaN	157 459 minus strand Synaptojanin-2-binding protein [Crassostrea gigas]
3	8	NaN	" 90 1085 squid, variant A [Blattella germanica]"
2	5	NaN	247 1440 minus strand Septin-2 [Crassostrea gigas]
3	4	NaN	136 675 minus strand predicted protein [Nematostella vectensis] gi
3	5	NaN	187 1323 minus strand gelsolin [Aplysia californica] gi
2	3	NaN	103 1356 minus strand PREDICTED: actin-related protein 3-like [Oreochromis niloticus]
2	2	NaN	99 3011 Eukaryotic translation initiation factor 3 subunit A [Crassostrea gigas]
3	3	NaN	" 95 1279 2-amino-3-ketobutyrate coenzyme A ligase, mitochondrial, partial [Crassostrea gigas]"
3	3	NaN	71 1321 minus strand Vinculin [Crassostrea gigas]
10	88	NaN	204 791 minus strand PREDICTED: 15-hydroxyprostaglandin dehydrogenase [NAD+] [Gallus gallus]
3	4	NaN	442 2595 minus strand Amiloride-sensitive amine oxidase [copper-containing] [Crassostrea gigas]
2	2	NaN	75 455 minus strand PREDICTED: calnexin [Strongylocentrotus purpuratus]
2	2	NaN	440 1645 Tektin-1 [Crassostrea gigas]
3	4	NaN	94 519 minus strand hypothetical protein BRAFLDRAFT_115029 [Branchiostoma floridae] gi

4.2 Proteins Identified by a Single Peptide

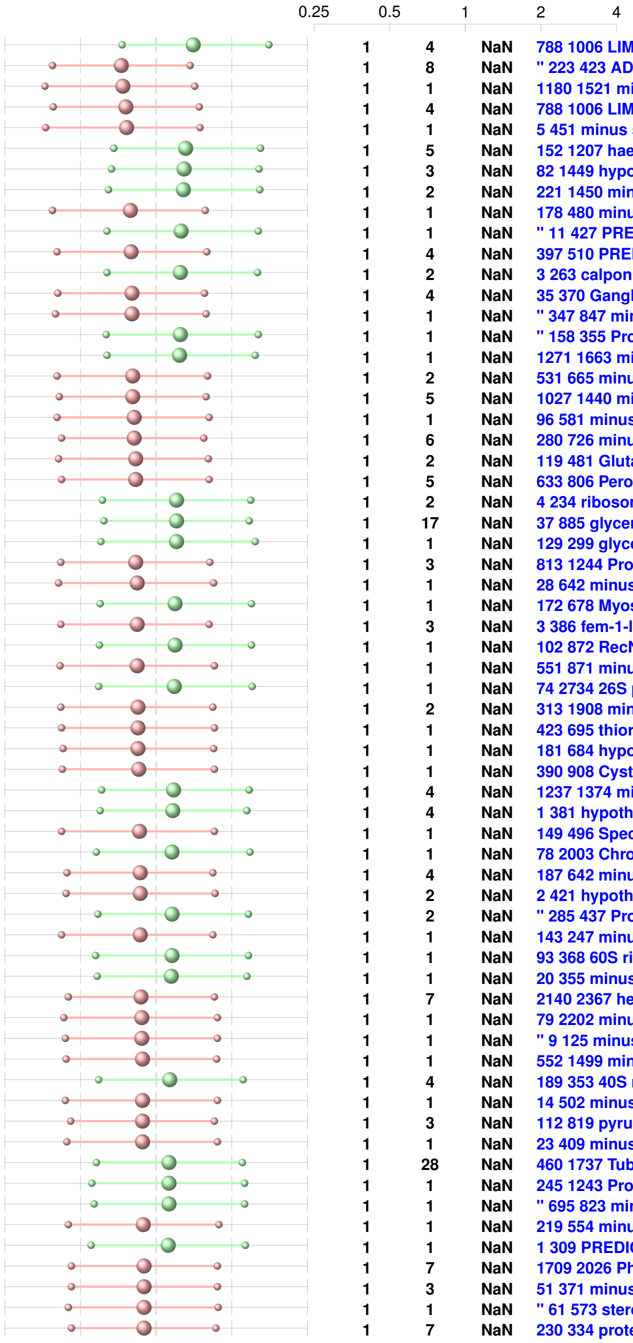

Peptides	Spectra	Coverage	Protein
1	4	NaN	788 1006 LIM protein [Haliotis discus discus]; 768 980 muscle LIM protein isoform 1 [Danaus plexippus]
1	8	NaN	" 223 423 ADPATP carrier protein; K05863 solute carrier family 25 (mitochondrial adenine nucleotide translocator), n
1	1	NaN	1180 1521 minus strand coronin [Schistosoma mansoni]
1	4	NaN	788 1006 LIM protein [Haliotis discus discus]
1	1	NaN	5 451 minus strand Spectrin beta chain [Crassostrea gigas]
1	5	NaN	152 1207 haemocyanin precursor [Nautilus pompilius]
1	3	NaN	82 1449 hypothetical protein BRAFLDRAFT_127120 [Branchiostoma floridae] gi
1	2	NaN	221 1450 minus strand PREDICTED: dihydrolipoamide branched chain transacylase E2 [Taeniopygia guttata]; 221 16
1	1	NaN	178 480 minus strand PREDICTED: lipoxygenase homology domain-containing protein 1-like [Danio rerio]
1	1	NaN	" 11 427 PREDICTED: similar to syntaxin, partial [Hydra magnipapillata]"
1	4	NaN	397 510 PREDICTED: actin-5C-like [Megachile rotundata]
1	2	NaN	3 263 calponin-like protein [Taenia asiatica]
1	4	NaN	35 370 Ganglioside GM2 activator [Crassostrea gigas]; 2 487 minus strand PREDICTED: hypothetical protein LOC10
1	1	NaN	" 347 847 minus strand ADP,ATP carrier protein 1 OS=Anopheles gambiae GN=AGAP006782 PE=2 SV=2"
1	1	NaN	" 158 355 Protein NAS-37, isoform b [Caenorhabditis elegans] gi; 15 227 Hemicentin-1 [Crassostrea gigas]"
1	1	NaN	1271 1663 minus strand PREDICTED: similar to AGAP007393-PB [Tribolium castaneum] gi
1	2	NaN	531 665 minus strand Proteasome subunit beta type-6 [Crassostrea gigas]
1	5	NaN	1027 1440 minus strand hemocyanin isoform 1 [Haliotis diversicolor]
1	1	NaN	96 581 minus strand thioredoxin 2 [Haliotis discus discus]; 1682 2167 minus strand thioredoxin 2 [Haliotis discus di
1	6	NaN	280 726 minus strand RecName: Full=Calmodulin; Short=CaM gi|14588595|dbj|BAB61794.1| calmodulin [Metridium
1	2	NaN	119 481 Glutamate receptor U1 [Crassostrea gigas]; 113 328 glutamate receptor subunit protein GluR2 precursor [A
1	5	NaN	633 806 Peroxiredoxin-1 [Crassostrea gigas]; 648 821 Peroxiredoxin-1 [Crassostrea gigas]; 666 839 Peroxiredoxin-1
1	2	NaN	4 234 ribosomal protein rpl14 [Arenicola marina]
1	17	NaN	37 885 glyceraldehyde 3-phosphate dehydrogenase [Dugesia japonica]
1	1	NaN	129 299 glycogen phosphorylase [Crassostrea gigas]
1	3	NaN	813 1244 Probable protein disulfide-isomerase A6 OS=Caenorhabditis elegans GN=tag-320 PE=2 SV=1
1	1	NaN	28 642 minus strand Transmembrane emp24 domain-containing protein 9 [Crassostrea gigas]; 236 709 minus strand
1	1	NaN	172 678 Myosin regulatory light chain sqh [Crassostrea gigas]
1	3	NaN	3 386 fem-1-like protein C [Crassostrea gigas]
1	1	NaN	102 872 RecName: Full=Gelsolin-like protein 1; AltName: Full=Actin-modulator; Short=EWAM; Short=EWAM-P1 gi|1
1	1	NaN	551 871 minus strand Protein disulfide-isomerase A4 [Crassostrea gigas]
1	1	NaN	74 2734 26S proteasome non-ATPase regulatory subunit 2 [Crassostrea gigas]
1	2	NaN	313 1908 minus strand Heterogeneous nuclear ribonucleoprotein L [Crassostrea gigas]; 313 1833 minus strand Hete
1	1	NaN	423 695 thioredoxin peroxidase 2 [Haliotis discus discus]
1	1	NaN	181 684 hypothetical protein BRAFLDRAFT_131834 [Branchiostoma floridae] gi
1	1	NaN	390 908 Cysteine proteinase 5 OS=Dictyostelium discoideum GN=cprE PE=2 SV=2
1	4	NaN	1237 1374 minus strand putative 60S ribosomal protein L23 [Maconellicoccus hirsutus]
1	4	NaN	1 381 hypothetical protein [Eimeria tenella]
1	1	NaN	149 496 Spectrin alpha chain [Crassostrea gigas]
1	1	NaN	78 2003 Chromatin assembly factor 1 subunit A [Crassostrea gigas]
1	4	NaN	187 642 minus strand PREDICTED: similar to pol-like protein [Tribolium castaneum]
1	2	NaN	2 421 hypothetical protein BRAFLDRAFT_119044 [Branchiostoma floridae] gi
1	2	NaN	" 285 437 Propionyl-CoA carboxylase alpha chain, mitochondrial [Crassostrea gigas]"
1	1	NaN	143 247 minus strand dynein light chain [Aplysia californica] gi
1	1	NaN	93 368 60S ribosomal protein L37A [Branchiostoma belcheri] gi; 93 395 60S ribosomal protein L37A [Branchiostoma
1	1	NaN	20 355 minus strand PREDICTED: uroporphyrinogen decarboxylase-like [Monodelphis domestica]
1	7	NaN	2140 2367 heat shock cognate 71 [Mytilus galloprovincialis]
1	1	NaN	79 2202 minus strand PREDICTED: dolichyl-diphosphooligosaccharide–protein glycosyltransferase subunit STT3A-
1	1	NaN	" 9 125 minus strand Hemocyanin, beta-C chain unit D (Fragment) OS=Helix pomatia PE=1 SV=2"
1	1	NaN	552 1499 minus strand uncharacterized protein LOC100158466 [Xenopus (Silurana) tropicalis] gi; 552 1499 minus st
1	4	NaN	189 353 40S ribosomal protein S12 [Crassostrea gigas]
1	1	NaN	14 502 minus strand ependymin related protein-1 precursor [Haliotis discus discus]; 14 547 ependymin related prote
1	3	NaN	112 819 pyruvate kinase [Crassostrea gigas]; 132 932 pyruvate kinase [Crassostrea gigas]
1	1	NaN	23 409 minus strand CDGSH iron sulfur domain-containing protein 2 [Crassostrea gigas]; 1 207 minus strand CDGS
1	28	NaN	460 1737 Tubulin beta-4B chain OS=Mus musculus GN=Tubb4b PE=1 SV=1
1	1	NaN	245 1243 Prostaglandin reductase 1 [Crassostrea gigas]
1	1	NaN	" 695 823 minus strand Aldehyde dehydrogenase family 16 member A1, partial [Crassostrea gigas]"
1	1	NaN	219 554 minus strand ribosomal protein P1 [Biomphalaria glabrata]
1	1	NaN	1 309 PREDICTED: intersectin-1-like [Sus scrofa]
1	7	NaN	1709 2026 Phosphoenolpyruvate carboxykinase [GTP] [Crassostrea gigas]
1	3	NaN	51 371 minus strand putative mitochondrial ATP synthase F chain [Haliotis discus discus]; 51 371 minus strand puta
1	1	NaN	" 61 573 steroid membrane receptor Hpr6.6/25-Dx, putative [Ixodes scapularis] gi"
1	7	NaN	230 334 protein disulfide isomerase [Conus virgo]

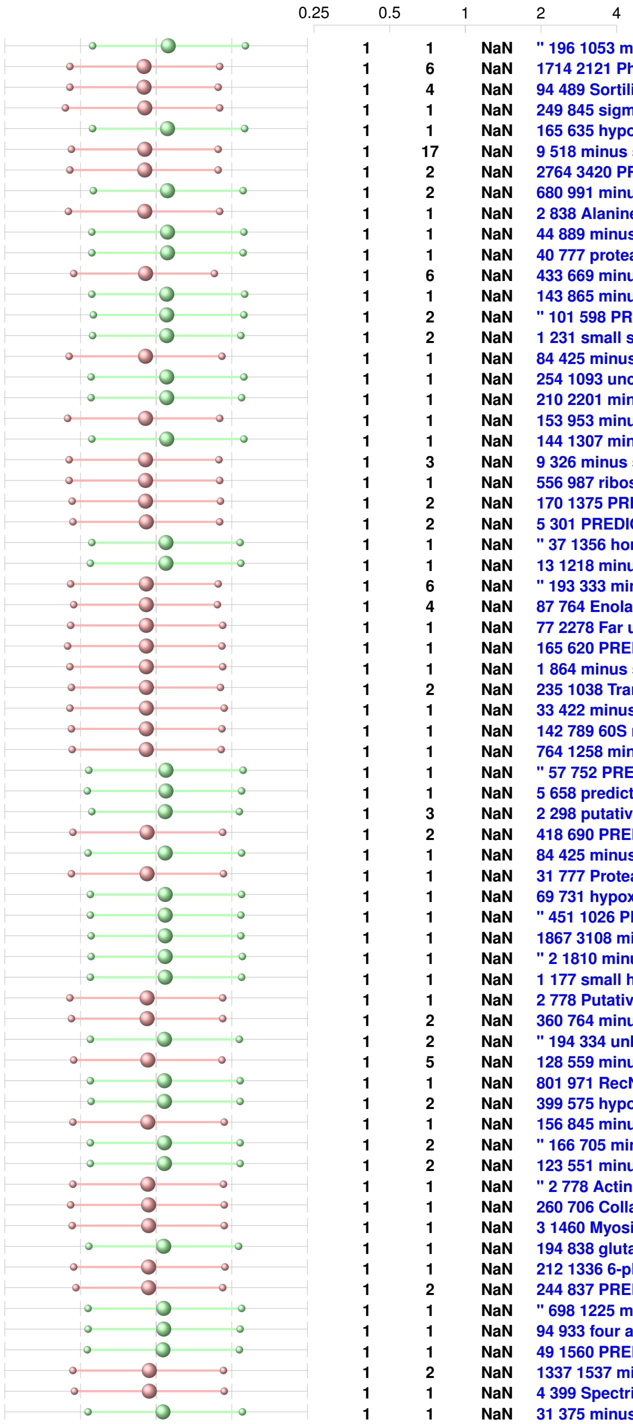

Peptides	Spectra	Coverage	Protein
1	1	NaN	" 196 1053 minus strand Cytochrome b-c1 complex subunit 2, mitochondrial OS=Homo sapiens GN=UQCRC2 PE=1
1	6	NaN	1714 2121 Phosphoenolpyruvate carboxykinase [GTP] [Crassostrea gigas]
1	4	NaN	94 489 Sortilin-related receptor [Crassostrea gigas]
1	1	NaN	249 845 sigma class glutathione-s-transferase 2 [Haliotis discus discus]; 252 848 sigma class glutathione-s-transfer
1	1	NaN	165 635 hypothetical protein CGI_10007639 [Crassostrea gigas]
1	17	NaN	9 518 minus strand hemocyanin isoform 1 [Nucula nucleus]
1	2	NaN	2764 3420 PREDICTED: filamin-C-like [Nasonia vitripennis]
1	2	NaN	680 991 minus strand PDZ and LIM domain protein 1 [Crassostrea gigas]; 553 864 minus strand PDZ and LIM domai
1	1	NaN	2 838 Alanine aminotransferase 2 [Crassostrea gigas]
1	1	NaN	44 889 minus strand hypothetical protein CGI_10015025 [Crassostrea gigas]
1	1	NaN	40 777 proteasome subunit N3 [Haliotis discus discus]; 40 777 proteasome subunit N3 [Haliotis discus discus]
1	6	NaN	433 669 minus strand Superoxide dismutase [Cu-Zn] OS=Prionace glauca GN=sod1 PE=1 SV=1
1	1	NaN	143 865 minus strand predicted protein [Nematostella vectensis] gi
1	2	NaN	" 101 598 PREDICTED: aminomethyltransferase, mitochondrial-like [Oreochromis niloticus]"
1	2	NaN	1 231 small subunit ribosomal protein S11e [Clonorchis sinensis]; 1 171 40S ribosomal protein S11 OS=Canis famili
1	1	NaN	84 425 minus strand Transcription elongation factor B polypeptide 2 [Crassostrea gigas]; 84 425 minus strand Trans
1	1	NaN	254 1093 uncharacterized protein LOC432187 precursor [Xenopus laevis] gi; 343 561 minus strand cathepsin Z prec
1	1	NaN	210 2201 minus strand Glycyl-tRNA synthetase [Crassostrea gigas]; 210 2201 minus strand Glycyl-tRNA synthetase
1	1	NaN	153 953 minus strand ribosomal protein rpl7 [Arenicola marina]
1	1	NaN	144 1307 minus strand PREDICTED: similar to AGAP005845-PA [Tribolium castaneum]; 146 1309 minus strand PRED
1	3	NaN	9 326 minus strand catalase [Paracyclopina nana]; 9 209 Catalase OS=Cavia porcellus GN=CAT PE=2 SV=4
1	1	NaN	556 987 ribosomal protein rps15 [Lineus viridis]
1	2	NaN	170 1375 PREDICTED: sialate O-acetylesterase-like [Oreochromis niloticus]
1	2	NaN	5 301 PREDICTED: hypothetical protein [Saccoglossus kowalevskii]
1	1	NaN	" 37 1356 homogentisate 1,2-dioxygenase [Danio rerio] gi; 54 1361 homogentisate 1,2-dioxygenase [Danio rerio] gi"
1	1	NaN	13 1218 minus strand PREDICTED: eukaryotic initiation factor 4A-III-like [Bombus terrestris] gi
1	6	NaN	" 193 333 minus strand RecName: Full=Globin; AltName: Full=Myoglobin gi|299274|gb|AAB25743.1| myoglobin [Na
1	4	NaN	87 764 Enolase-phosphatase E1 [Crassostrea gigas]; 87 1058 Enolase-phosphatase E1 [Crassostrea gigas]; 87 764
1	1	NaN	77 2278 Far upstream element-binding protein 3 [Crassostrea gigas]
1	1	NaN	165 620 PREDICTED: uncharacterized protein LOC575072 isoform 2 [Strongylocentrotus purpuratus] gi
1	1	NaN	1 864 minus strand Pyruvate kinase isozymes M1/M2 [Crassostrea gigas]; 3 959 minus strand Pyruvate kinase isozy
1	2	NaN	235 1038 Translocon-associated protein subunit alpha [Crassostrea gigas]
1	1	NaN	33 422 minus strand PREDICTED: heat shock 70 kDa protein cognate 4-like [Megachile rotundata]
1	1	NaN	142 789 60S ribosomal protein L6 [Crassostrea gigas]
1	1	NaN	764 1258 minus strand Caprin-2 [Crassostrea gigas]
1	1	NaN	" 57 752 PREDICTED: adenylate kinase 2, mitochondrial-like isoform 2 [Callithrix jacchus]"
1	1	NaN	5 658 predicted protein [Nematostella vectensis] gi
1	3	NaN	2 298 putative 60S ribosomal protein RPL24 [Novocrania anomala]
1	2	NaN	418 690 PREDICTED: protein disulfide isomerase A4-like [Saccoglossus kowalevskii]
1	1	NaN	84 425 minus strand thioester-containing protein [Euphaedusa tau]
1	1	NaN	31 777 Proteasome subunit alpha type-7-like protein [Crassostrea gigas]
1	1	NaN	69 731 hypoxanthine phosphoribosyltransferase 1 [Haliotis discus discus]
1	1	NaN	" 451 1026 PREDICTED: GTP-binding protein SAR1B, putative-like [Saccoglossus kowalevskii]"
1	1	NaN	1867 3108 minus strand roundabout homolog 1 precursor [Rattus norvegicus] gi; 1867 3108 minus strand roundabo
1	1	NaN	" 2 1810 minus strand Glutaminyl-tRNA synthetase, partial [Crassostrea gigas]"
1	1	NaN	1 177 small heat shock protein [Trichinella pseudospiralis]
1	1	NaN	2 778 Putative adenosylhomocysteinase 3 [Crassostrea gigas]
1	2	NaN	360 764 minus strand Peptidyl-prolyl cis-trans isomerase A OS=Dictyostelium discoideum GN=ppiA PE=1 SV=1
1	2	NaN	" 194 334 unknown, partial [Lymnaea stagnalis] gi; 207 347 unknown, partial [Lymnaea stagnalis] gi; 384 524 unknow
1	5	NaN	128 559 minus strand predicted protein [Nematostella vectensis] gi
1	1	NaN	801 971 RecName: Full=Tropomyosin-2; AltName: Full=Tropomyosin II; Short=BgTMII; Short=TMII gi|155940|gb|AA
1	2	NaN	399 575 hypothetical protein BRAFLDRAFT_69739 [Branchiostoma floridae] gi
1	1	NaN	156 845 minus strand histone H1 [Mytilus californianus]; 156 845 minus strand histone H1 [Mytilus californianus]; 1
1	2	NaN	" 166 705 minus strand ADP-ribosylation factor, partial [Antheraea yamamai]; 166 705 minus strand ADP-ribosylatio
1	2	NaN	123 551 minus strand Transcription factor BTF3-like protein 4 [Crassostrea gigas]
1	1	NaN	" 2 778 Actin, gamma-enteric smooth muscle OS=Rattus norvegicus GN=Actg2 PE=2 SV=1"
1	1	NaN	260 706 Collagen alpha-1(XII) chain (Fragment) OS=Notophthalmus viridescens PE=2 SV=1
1	1	NaN	3 1460 Myosin-VI [Crassostrea gigas]
1	1	NaN	194 838 glutathione S-transferase class mu [Cyphoma gibbosum]
1	1	NaN	212 1336 6-phosphofructokinase [Crassostrea gigas]; 208 1332 6-phosphofructokinase [Crassostrea gigas]
1	2	NaN	244 837 PREDICTED: ras-like GTP-binding protein Rho1-like [Megachile rotundata]; 650 883 hypothetical protein SIN
1	1	NaN	" 698 1225 minus strand actin related protein 2/3 complex, 21 kDa subunit [Crassostrea gigas]"
1	1	NaN	94 933 four and A half lim domains [Schistosoma mansoni]
1	1	NaN	49 1560 PREDICTED: tRNA-splicing ligase RtcB homolog [Megachile rotundata]
1	2	NaN	1337 1537 minus strand hemocyanin [Haliotis tuberculata]
1	1	NaN	4 399 Spectrin beta chain [Crassostrea gigas]
1	1	NaN	31 375 minus strand 40S ribosomal protein S19 OS=Mya arenaria GN=RPS19 PE=2 SV=1

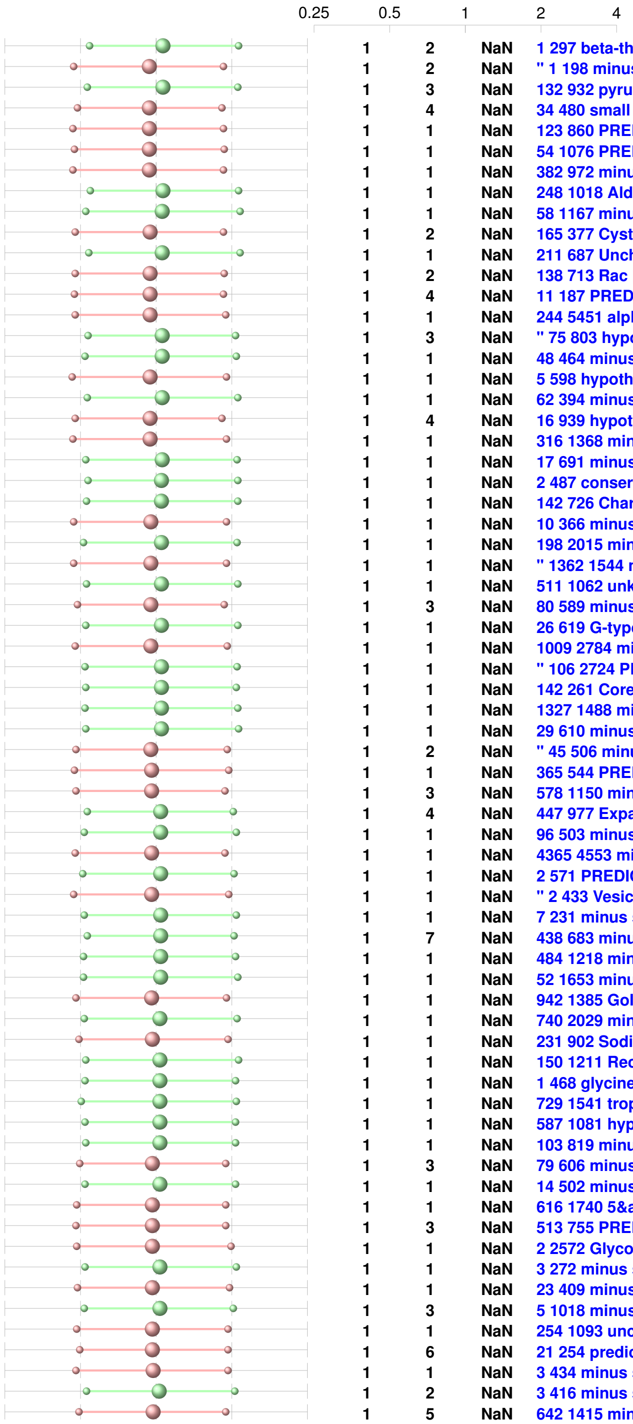

Peptides	Spectra	Coverage	Protein
1	2	NaN	1 297 beta-thymosin domain repeat protein CSP24KDa_v1 [Hermissenda crassicornis] gi
1	2	NaN	" 1 198 minus strand Chain A, Rep1-Nxsq Fatty Acid Transporter; 1 231 minus strand Chain A, Rep1-Nxsq Fatty Acid
1	3	NaN	132 932 pyruvate kinase [Crassostrea gigas]
1	4	NaN	34 480 small subunit ribosomal protein S18e [Clonorchis sinensis]; 431 799 SJCHGC01887 protein [Schistosoma jap
1	1	NaN	123 860 PREDICTED: 3-hydroxybutyrate dehydrogenase type 2-like isoform 1 [Sus scrofa] gi; 32 901 3-hydroxybutyr
1	1	NaN	54 1076 PREDICTED: UPF0553 protein C9orf64-like [Anolis carolinensis]; 71 1093 PREDICTED: UPF0553 protein C9o
1	1	NaN	382 972 minus strand Muscle-specific protein 20 [Crassostrea gigas]; 807 1397 minus strand Muscle-specific protein
1	1	NaN	248 1018 Aldo-keto reductase family 1 member B10 [Crassostrea gigas]; 44 730 minus strand Aldo-keto reductase fa
1	1	NaN	58 1167 minus strand PREDICTED: arylacetamide deacetylase-like 4-like [Gallus gallus]; 76 1239 minus strand PRED
1	2	NaN	165 377 Cysteine and glycine-rich protein 3 [Crassostrea gigas]; 135 344 Cysteine and glycine-rich protein 3 [Crasso
1	1	NaN	211 687 Uncharacterized protein CG16817 [Harpegnathos saltator]; 200 676 Uncharacterized protein CG16817 [Harp
1	2	NaN	138 713 Rac [Aplysia californica] gi; 125 691 Rac [Aplysia californica] gi; 149 871 minus strand Rac [Aplysia californ
1	4	NaN	11 187 PREDICTED: 40S ribosomal protein S28-like [Strongylocentrotus purpuratus]
1	1	NaN	244 5451 alpha macroglobulin [Chlamys farreri]
1	3	NaN	" 75 803 hypothetical protein DAPPUDRAFT_231350 [Daphnia pulex]; 82 612 ATP synthase B chain, putative [Ixodes
1	1	NaN	48 464 minus strand Acyl-coenzyme A thioesterase 13 [Crassostrea gigas]
1	1	NaN	5 598 hypothetical protein DAPPUDRAFT_344656 [Daphnia pulex]
1	1	NaN	62 394 minus strand PREDICTED: NADH dehydrogenase [ubiquinone] 1 alpha subcomplex subunit 5 [Saimiri bolivie
1	4	NaN	16 939 hypothetical protein BRAFLDRAFT_87838 [Branchiostoma floridae] gi
1	1	NaN	316 1368 minus strand hypothetical protein CGI_10006559 [Crassostrea gigas]
1	1	NaN	17 691 minus strand Cleavage and polyadenylation specificity factor subunit 5 [Crassostrea gigas]
1	1	NaN	2 487 conserved hypothetical protein [Ixodes scapularis] gi
1	1	NaN	142 726 Charged multivesicular body protein 4b [Crassostrea gigas]
1	1	NaN	10 366 minus strand hypothetical protein TRIADDRAFT_25239 [Trichoplax adhaerens] gi
1	1	NaN	198 2015 minus strand Xaa-Pro aminopeptidase 1 [Crassostrea gigas]
1	1	NaN	" 1362 1544 minus strand Phosphoenolpyruvate carboxykinase, cytosolic [GTP] [Crassostrea gigas]"
1	1	NaN	511 1062 unknown [Dendroctonus ponderosae]; 511 1062 unknown [Dendroctonus ponderosae]
1	3	NaN	80 589 minus strand PREDICTED: adenine phosphoribosyltransferase-like [Strongylocentrotus purpuratus]; 328 468
1	1	NaN	26 619 G-type lysozyme [Physella acuta]
1	1	NaN	1009 2784 minus strand Collagen alpha-3(VI) chain [Crassostrea gigas]
1	1	NaN	" 106 2724 PREDICTED: 2-oxoglutarate dehydrogenase, mitochondrial isoform 1 [Sarcophilus harrisii]; 492 3473 PR
1	1	NaN	142 261 Core histone macro-H2A.2 OS=Mus musculus GN=H2afy2 PE=1 SV=3
1	1	NaN	1327 1488 minus strand Ubiquinol-cytochrome C reductase complex [Xenopus laevis] gi
1	1	NaN	29 610 minus strand 40S ribosomal protein S7 [Crassostrea gigas]
1	2	NaN	" 45 506 minus strand PREDICTED: dihydrolipoyl dehydrogenase, mitochondrial [Monodelphis domestica]"
1	1	NaN	365 544 PREDICTED: cofilin-2 [Otolemur garnettii]
1	3	NaN	578 1150 minus strand expressed hypothetical protein [Trichoplax adhaerens] gi; 376 948 minus strand expressed h
1	4	NaN	447 977 Expansin-yoaJ [Crassostrea gigas]
1	1	NaN	96 503 minus strand mitochondrial ATP synthase delta chain [Haliotis discus discus]; 111 551 minus strand mitocho
1	1	NaN	4365 4553 minus strand Basement membrane-specific heparan sulfate proteoglycan core protein [Crassostrea gigas
1	1	NaN	2 571 PREDICTED: copper homeostasis protein cutC homolog [Anolis carolinensis]
1	1	NaN	" 2 433 Vesicle-fusing ATPase, partial [Ascaris suum]"
1	1	NaN	7 231 minus strand AGAP006879-PA [Anopheles gambiae str. PEST] gi
1	7	NaN	438 683 minus strand Protein lethal(2)essential for life OS=Drosophila melanogaster GN=l(2)efl PE=1 SV=1
1	1	NaN	484 1218 minus strand alginate lyase [Littorina brevicula]; 484 1218 minus strand alginate lyase [Littorina brevicula]
1	1	NaN	52 1653 minus strand PREDICTED: eukaryotic translation initiation factor 3 subunit 6 interacting protein-like [Sacco
1	1	NaN	942 1385 Golgi-associated plant pathogenesis-related protein 1 [Crassostrea gigas]; 536 979 Golgi-associated plant
1	1	NaN	740 2029 minus strand PREDICTED: protein alan shepard-like isoform 2 [Bombus terrestris]
1	1	NaN	231 902 Sodium- and chloride-dependent GABA transporter ine [Crassostrea gigas]
1	1	NaN	150 1211 RecName: Full=Guanine nucleotide-binding protein G(i) subunit alpha; AltName: Full=Adenylate cyclase-in
1	1	NaN	1 468 glycine cleavage system H protein [Rhipicephalus sanguineus]
1	1	NaN	729 1541 troponin T [Mizuhopecten yessoensis]
1	1	NaN	587 1081 hypothetical protein CGI_10026502 [Crassostrea gigas]
1	1	NaN	103 819 minus strand PREDICTED: l-xylulose reductase-like [Monodelphis domestica]
1	3	NaN	79 606 minus strand PREDICTED: similar to Ras-like protein 3 precursor (Protein roughened) [Tribolium castaneum]
1	1	NaN	14 502 minus strand Ufm1-conjugating enzyme 1 [Crassostrea gigas]
1	1	NaN	616 1740 5'-AMP-activated protein kinase subunit beta-2 [Crassostrea gigas]; 641 1762 minus strand 5'
1	3	NaN	513 755 PREDICTED: 40S ribosomal protein S16-like [Papio anubis]; 513 755 PREDICTED: 40S ribosomal protein S1
1	1	NaN	2 2572 Glycogen debranching enzyme [Crassostrea gigas]
1	1	NaN	3 272 minus strand ADP/ATP carrier [Clonorchis sinensis]
1	1	NaN	23 409 minus strand CDGSH iron sulfur domain-containing protein 2 [Crassostrea gigas]
1	3	NaN	5 1018 minus strand Glutaredoxin-3 [Crassostrea gigas]; 5 1018 Glutaredoxin-3 [Crassostrea gigas]
1	1	NaN	254 1093 uncharacterized protein LOC432187 precursor [Xenopus laevis] gi
1	6	NaN	21 254 predicted protein [Nematostella vectensis] gi
1	1	NaN	3 434 minus strand hypothetical protein CGI_10015501 [Crassostrea gigas]
1	2	NaN	3 416 minus strand myosin essential light chain [Crassostrea gigas]
1	5	NaN	642 1415 minus strand cytochrome c oxidase subunit II [Oncomelania hupensis hupensis]

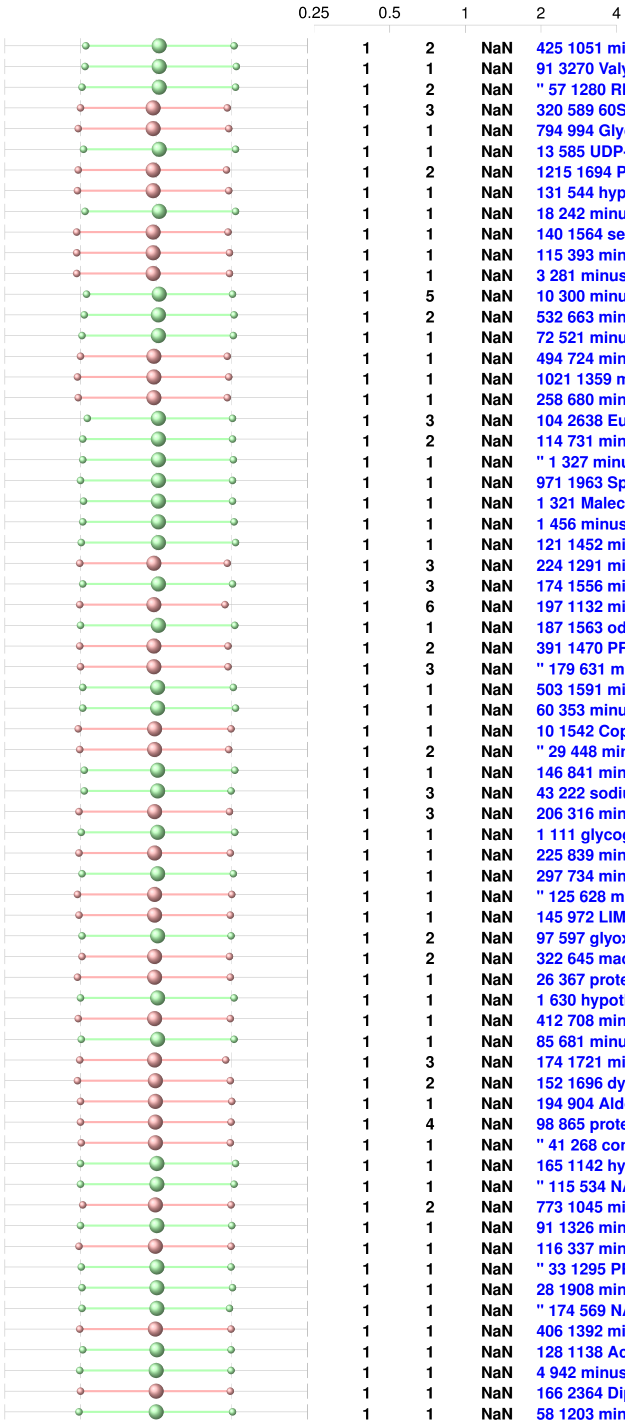

Peptides	Spectra	Coverage	Protein
1	2	NaN	425 1051 minus strand unknown [Schistosoma japonicum]
1	1	NaN	91 3270 Valyl-tRNA synthetase [Crassostrea gigas]
1	2	NaN	" 57 1280 RNA binding motif protein, X-linked [Mustela putorius furo]"
1	3	NaN	320 589 60S ribosomal protein L34 OS=Aedes triseriatus GN=RpL34 PE=2 SV=3
1	1	NaN	794 994 Glycogenin-1 [Crassostrea gigas]; 1425 1739 minus strand Glycogenin-1 [Crassostrea gigas]
1	1	NaN	13 585 UDP-glucose:glycoprotein glucosyltransferase 1 [Crassostrea gigas]
1	2	NaN	1215 1694 Pikachurin [Crassostrea gigas]
1	1	NaN	131 544 hypothetical protein CGI_10001749 [Crassostrea gigas]
1	1	NaN	18 242 minus strand translation elongation factor 1B gamma subunit [Strongylocentrotus purpuratus] gi
1	1	NaN	140 1564 selenium-binding protein [Haliotis discus hannai]
1	1	NaN	115 393 minus strand Yolk ferritin OS=Lymnaea stagnalis PE=1 SV=1; 132 413 RecName: Full=Yolk ferritin; Flags: P
1	1	NaN	3 281 minus strand hypothetical protein CGI_10015501 [Crassostrea gigas]
1	5	NaN	10 300 minus strand unnamed protein product [Tetraodon nigroviridis]
1	2	NaN	532 663 minus strand 40S ribosomal protein S23 [Ascaris suum]; 526 654 minus strand RecName: Full=40S ribosom
1	1	NaN	72 521 minus strand GF14593 [Drosophila ananassae] gi; 60 392 GD23124 [Drosophila simulans] gi
1	1	NaN	494 724 minus strand PREDICTED: SH3 domain-binding glutamic acid-rich-like protein 3-like [Amphimedon queensl
1	1	NaN	1021 1359 minus strand RecName: Full=40S ribosomal protein S26 gi|6015599|emb|CAB57819.1| ribosomal protein
1	1	NaN	258 680 minus strand synapse-associated protein [Aplysia californica] gi
1	3	NaN	104 2638 Eukaryotic translation initiation factor 3 subunit C [Crassostrea gigas]
1	2	NaN	114 731 minus strand Proteasome subunit beta type-6 [Crassostrea gigas]
1	1	NaN	" 1 327 minus strand endo-beta-1,4-glucanase [Ampullaria crossean]"
1	1	NaN	971 1963 Spectrin alpha chain [Crassostrea gigas]; 972 1979 Spectrin alpha chain [Crassostrea gigas]
1	1	NaN	1 321 Malectin [Crassostrea gigas]
1	1	NaN	1 456 minus strand Putative sodium/potassium-transporting ATPase subunit beta-2 [Crassostrea gigas]; 688 1251 m
1	1	NaN	121 1452 minus strand Hexokinase type 2 [Crassostrea gigas]; 245 1576 minus strand Hexokinase type 2 [Crassostr
1	3	NaN	224 1291 minus strand coproporphirynogen oxidase [Aplysia californica] gi
1	3	NaN	174 1556 minus strand aspartyl-tRNA synthetase [Xenopus laevis] gi; 174 1721 minus strand aspartyl-tRNA synthet
1	6	NaN	197 1132 minus strand Pancreatic lipase-related protein 2 [Crassostrea gigas]
1	1	NaN	187 1563 odr-4-like protein [Crassostrea gigas]
1	2	NaN	391 1470 PREDICTED: heparanase-like [Amphimedon queenslandica]; 391 1470 PREDICTED: heparanase-like [Amp
1	3	NaN	" 179 631 minus strand 60S ribosomal protein L7/L12, partial [Ostrea edulis]"
1	1	NaN	503 1591 minus strand Septin-2 [Crassostrea gigas]; 648 1736 minus strand Septin-2 [Crassostrea gigas]
1	1	NaN	60 353 minus strand Histone H3.3 [Harpegnathos saltator]
1	1	NaN	10 1542 Copine-8 [Crassostrea gigas]
1	2	NaN	" 29 448 minus strand PREDICTED: similar to NADH:ubiquinone dehydrogenase, putative [Tribolium castaneum] gi;
1	1	NaN	146 841 minus strand hypothetical protein CGI_10006609 [Crassostrea gigas]; 146 841 minus strand hypothetical pr
1	3	NaN	43 222 sodium-calcium exchanger [Doryteuthis pealeii]
1	3	NaN	206 316 minus strand ribosomal protein S17 [Branchiostoma belcheri]
1	1	NaN	1 111 glycogen synthase [Crassostrea gigas]
1	1	NaN	225 839 minus strand Metallo-beta-lactamase domain-containing protein 1 [Crassostrea gigas]; 201 851 minus stran
1	1	NaN	297 734 minus strand PREDICTED: calmodulin [Canis lupus familiaris]; 297 734 PREDICTED: calmodulin [Canis lupu
1	1	NaN	" 125 628 minus strand collectin sub-family member 12, partial [Oncorhynchus mykiss]"
1	1	NaN	145 972 LIM and SH3 domain protein F42H10.3 OS=Caenorhabditis elegans GN=F42H10.3 PE=2 SV=3
1	2	NaN	97 597 glyoxylate reductase/hydroxypyruvate reductase [Ictalurus punctatus] gi
1	2	NaN	322 645 macrophage migration inhibitory-like factor protein [Chlamys farreri]; 437 682 macrophage migration inhibit
1	1	NaN	26 367 proteasome alpha type 2 [Haliotis discus discus]
1	1	NaN	1 630 hypothetical protein [Trichomonas vaginalis G3] gi
1	1	NaN	412 708 minus strand heat shock protein beta-1 [Trichinella spiralis] gi; 459 755 minus strand heat shock protein bet
1	1	NaN	85 681 minus strand 40S ribosomal protein S8 OS=Xenopus laevis GN=rps8 PE=2 SV=3
1	3	NaN	174 1721 minus strand aspartyl-tRNA synthetase [Xenopus laevis] gi
1	2	NaN	152 1696 dynamin isoform A [Lymnaea stagnalis]
1	1	NaN	194 904 Aldo-keto reductase family 1 member B10 [Crassostrea gigas]
1	4	NaN	98 865 proteasome (prosome macropain) subunit alpha type 1 [Xenopus (Silurana) tropicalis]; 98 865 proteasome (p
1	1	NaN	" 41 268 conserved hypothetical protein [Perkinsus marinus ATCC 50983] gi; 29 238 PREDICTED: succinyl-CoA ligas
1	1	NaN	165 1142 hypothetical protein CGI_10023942 [Crassostrea gigas]
1	1	NaN	" 115 534 NADH:ubiquinone oxidoreductase, NDUFB6/B17 subunit, putative [Ixodes scapularis] gi"
1	2	NaN	773 1045 minus strand serine protease inhibitor 2 [Latrodectus hesperus]
1	1	NaN	91 1326 minus strand Adipocyte plasma membrane-associated protein [Crassostrea gigas]; 107 1273 minus strand
1	1	NaN	116 337 minus strand Golgi-associated plant pathogenesis-related protein 1 [Crassostrea gigas]; 538 759 minus stra
1	1	NaN	" 33 1295 PREDICTED: LOW QUALITY PROTEIN: selenide, water dikinase-like [Bombus impatiens]"
1	1	NaN	28 1908 minus strand Mitochondrial inner membrane protein [Crassostrea gigas]; 10 1902 Mitochondrial inner meml
1	1	NaN	" 174 569 NADH dehydrogenase [ubiquinone] iron-sulfur protein 4, mitochondrial [Crassostrea gigas]"
1	1	NaN	406 1392 minus strand hypothetical protein CGI_10007007 [Crassostrea gigas]
1	1	NaN	128 1138 Actin-related protein 2/3 complex subunit 1A OS=Homo sapiens GN=ARPC1A PE=1 SV=2
1	1	NaN	4 942 minus strand KTEL motif-containing protein 1 [Crassostrea gigas]
1	1	NaN	166 2364 Dipeptidyl-peptidase 3 [Crassostrea gigas]
1	1	NaN	58 1203 minus strand Obg-like ATPase 1 [Crassostrea gigas]

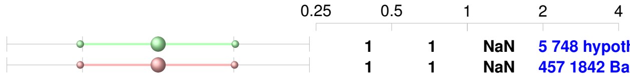

Peptides	Spectra	Coverage	Protein
1	1	NaN	5 748 hypothetical protein SINV_09458 [Solenopsis invicta]
1	1	NaN	457 1842 Basement membrane-specific heparan sulfate proteoglycan core protein [Crassostrea gigas]; 457 1842 Ba

5 Protein Details

A detailed summary of each protein is given below. These sections include peptide relative expression estimates in addition to protein-level estimates.

5.1 " 99 497 RecName: Full=Histone H2B, gonadal"

Protein Accession	CL29794.Contig1$_H$1
Mean Expression Ratio	2.24
Median Expression Ratio	2.24
Credible Interval	(1.35, 3.85)
Associated Peptides	5
Associated Spectra	47
Coverage	NaN

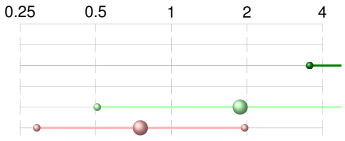

A	B	2.5	50	97.5	Sequence
3	3	12	25	55	AVTKYTSSK
9	23	3.6	5.4	7.9	LLLPGELAK
3	2	13	30	71	STITSREIQTAVR
0	1	0.51	1.9	6.8	LLPGELAK
0	3	0.29	0.75	2	KESYAIYIYK

5.2 1592 1768 minus strand beta-actin [Diabolocatantops pinguis]

Protein Accession	CL22741.Contig2$_H$1
Mean Expression Ratio	2.13
Median Expression Ratio	2.12
Credible Interval	(1.24, 3.73)
Associated Peptides	6
Associated Spectra	42
Coverage	NaN

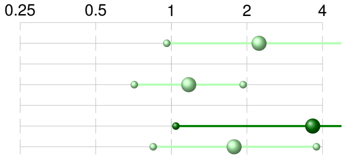

A	B	2.5	50	97.5	Sequence
3	1	0.96	2.2	5.4	VIAPPERK
1	7	12	23	44	IKVIAPPERK
7	9	0.71	1.2	1.9	VIAPPER
0	7	6.6	13	27	IKVIAPPER
0	1	1	3.6	13	GDQKEISALAPPTMK
0	6	0.85	1.8	3.8	EISALAPPTMK

5.3 764 1015 minus strand heat shock protein beta-1 [Trichinella spiralis] gi

Protein Accession	CL46983.Contig3$_H$1
Mean Expression Ratio	1.93
Median Expression Ratio	1.92
Credible Interval	(1.15, 3.27)
Associated Peptides	7
Associated Spectra	72
Coverage	NaN

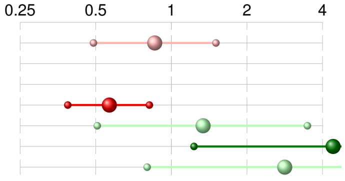

A	B	2.5	50	97.5	Sequence
6	6	0.49	0.86	1.5	SYVLPK
5	5	5.6	10	18	SYVLPKDVDPK
4	12	24	40	66	TVTSSLSQNGVLTLKAPK
8	21	0.39	0.56	0.82	TVTSSLSQNGVLTLK
0	3	0.51	1.3	3.5	VVINAKHEERPDEHGYIMR
0	1	1.2	4.4	16	SSLSQNGVLTLKAPK
0	1	0.8	2.8	9.9	MALEAPQEQV

5.4 97 477 minus strand PREDICTED: complement C1q tumor necrosis factor-related protein 3-like [Oreochromis niloticus]; 97 447 minus strand PREDICTED: complement C1q tumor necrosis factor-related protein 3-like [Oreochromis niloticus]

Protein Accession	CL370.Contig3$_H$1 CL370.Contig2$_H$1
Mean Expression Ratio	1.9
Median Expression Ratio	1.9
Credible Interval	(1.23, 2.93)
Associated Peptides	16
Associated Spectra	112
Coverage	NaN

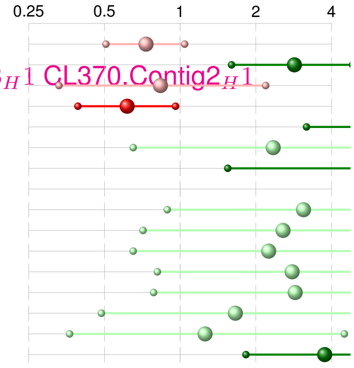

A	B	2.5	50	97.5	Sequence
8	24	0.51	0.73	1	QMVDALKDEQQR
3	9	1.6	2.8	4.9	QTSDISRLENR
1	2	0.33	0.84	2.2	PIAFSK
0	20	0.39	0.62	0.96	LESGVRPPVYRPDLESK
0	5	3.2	7.2	16	QMVDALKDEQQRQQEVINR
0	1	0.65	2.3	8.6	QSQQIEKQTSDISR
0	1	1.5	5.7	20	QSQQIEKQTSDISRLENR
0	25	5.3	7.9	12	VTKLESGVRPPVYRPDLESK
0	1	0.89	3.1	11	VTKLESGVRPP
0	1	0.71	2.6	8.9	LENRVTK
0	1	0.65	2.3	8.4	MVDALKDEQQR
0	1	0.81	2.8	10	RPIAFSK
0	1	0.79	2.9	10	DALKDEQQR
0	1	0.48	1.7	5.9	QMVDALK
0	1	0.36	1.3	4.5	VAFTAH
0	6	1.8	3.8	7.9	VAFTAHFSQGGSMDIGTSRPIAFSK

5.5 145 1020 minus strand PREDICTED: inorganic pyrophosphatase-like [Saccoglossus kowalevskii]

Protein Accession	CL12968.Contig14$_H$1
Mean Expression Ratio	0.554
Median Expression Ratio	0.555
Credible Interval	(0.312, 0.987)
Associated Peptides	7
Associated Spectra	9
Coverage	NaN

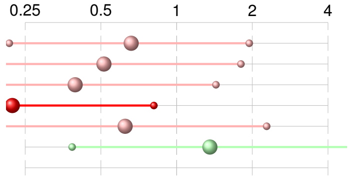

A	B	2.5	50	97.5	Sequence
1	1	0.22	0.66	2	LNPLKQDVK
1	0	0.14	0.51	1.8	SLMADPSVGK
0	1	0.11	0.4	1.4	IIAIDVTDPLASK
0	1	0.06	0.22	0.81	LNDIDDVHR
0	1	0.17	0.62	2.3	MEINKEEK
0	1	0.38	1.4	4.9	GSVIQVK
0	2	0.0094	0.028	0.083	AVIDQSVNK

5.6 156 1163 cathepsin B [Crassostrea ariakensis]

Protein Accession	CL26836.Contig9$_H$1
Mean Expression Ratio	1.8
Median Expression Ratio	1.8
Credible Interval	(0.989, 3.3)
Associated Peptides	5
Associated Spectra	7
Coverage	NaN

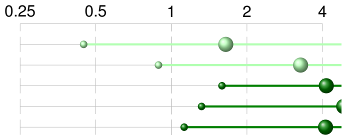

A	B	2.5	50	97.5	Sequence
0	1	0.45	1.6	5.8	GEQNIMEELYTR
0	1	0.89	3.3	12	TKWPNCPTLKEVR
0	3	1.6	4.2	11	DLPDSFDPR
0	1	1.3	4.8	18	LTDEEIFFINK
0	1	1.1	4.1	15	LPDSFDPR

5.7 266 607 minus strand H2 [Haliotis tuberculata]

Protein Accession	Unigene62350$_{H}$1
Mean Expression Ratio	0.562
Median Expression Ratio	0.564
Credible Interval	(0.301, 1.03)
Associated Peptides	3
Associated Spectra	15
Coverage	NaN

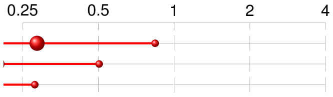

A	B	2.5	50	97.5	Sequence
1	1	0.095	0.28	0.84	DINTLTDEEIK
2	1	0.076	0.2	0.5	KDINTLTDEEIK
4	6	0.082	0.15	0.28	YAVAGIGVR

5.8 2 370 hemocyanin alphaD-subunit [Helix lucorum]

Protein Accession	CL46908.Contig1$_{H}$1
Mean Expression Ratio	0.577
Median Expression Ratio	0.578
Credible Interval	(0.332, 0.982)
Associated Peptides	7
Associated Spectra	30
Coverage	NaN

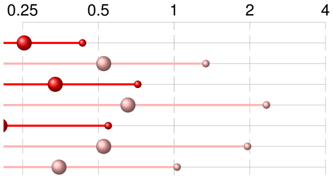

A	B	2.5	50	97.5	Sequence
4	10	0.15	0.25	0.43	LLGIGTSAEVK
1	2	0.2	0.52	1.3	SFYLDVSEAVVR
0	6	0.16	0.34	0.72	NEMPWQFER
0	1	0.18	0.65	2.3	LASNVLPK
0	3	0.079	0.2	0.55	AFAVFK
0	1	0.15	0.53	2	LYNMLETR
0	2	0.12	0.35	1	SFFILGGK

5.9 222 809 minus strand PREDICTED: 15-hydroxyprostaglandin dehydrogenase [NAD+] [Gallus gallus]

Protein Accession	CL44148.Contig2$_{H}$1
Mean Expression Ratio	0.58
Median Expression Ratio	0.582
Credible Interval	(0.337, 0.98)
Associated Peptides	6
Associated Spectra	49
Coverage	NaN

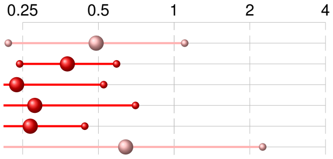

A	B	2.5	50	97.5	Sequence
5	0	0.22	0.49	1.1	AVTSLGR
6	14	0.24	0.38	0.59	VFLTGGAHGIGK
2	3	0.11	0.24	0.53	VMFCDINDK
3	0	0.11	0.28	0.7	AAFEK
8	7	0.16	0.27	0.44	ATVEAFLTK
0	1	0.18	0.64	2.3	RVFLTGGAHGIGK

5.10 503 1090 minus strand myosin regulatory light chain [Fasciola hepatica]; 278 865 minus strand myosin regulatory light chain [Fasciola hepatica]

Protein Accession	CL43006.Contig6$_{H}$1 CL43006.Contig4$_{H}$1
Mean Expression Ratio	0.595
Median Expression Ratio	0.596
Credible Interval	(0.357, 0.988)
Associated Peptides	9
Associated Spectra	30
Coverage	NaN

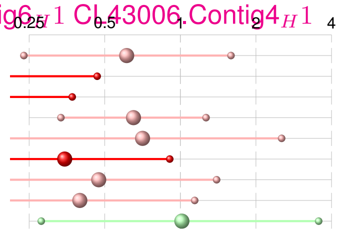

A	B	2.5	50	97.5	Sequence
3	0	0.24	0.61	1.6	DGVIDIEDLK
1	1	0.055	0.16	0.47	GYLPEEYIK
4	4	0.097	0.19	0.37	IPPDDELKEMLK
5	3	0.34	0.65	1.3	QAQVQEFK
1	0	0.2	0.71	2.5	IPPDDELK
2	1	0.13	0.35	0.91	EAFTMIDQNR
1	1	0.16	0.47	1.4	NAFAMFDDEGK
0	2	0.14	0.4	1.1	LSGTDPEDTIR
0	1	0.28	1	3.6	DGVIDIEDLKDIYSNLGR

5.11 " 223 423 ADPATP carrier protein; K05863 solute carrier family 25 (mitochondrial adenine nucleotide translocator), member 4/5/6/31; 237 455 minus strand ADP,ATP carrier protein [Crassostrea gigas]"

Protein Accession	CL27196.Contig3$_{H}1$ CL27196.Contig2$_{H}1$
Mean Expression Ratio	1.67
Median Expression Ratio	1.67
Credible Interval	(0.904, 3.19)
Associated Peptides	3
Associated Spectra	10
Coverage	NaN

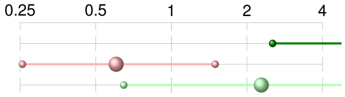

A	B	2.5	50	97.5	Sequence
0	1	2.5	9.2	33	VKLLVQNQNEMIK
6	2	0.25	0.6	1.5	LLVQNQNEMIK
0	1	0.65	2.3	8.5	AENFALSGLVLPLSSPK

5.12 2881 3411 myosin heavy chain [Todarodes pacificus]; 2915 3445 myosin heavy chain [Todarodes pacificus]

Protein Accession	Unigene19024$_{H}1$ Unigene19021$_{H}1$
Mean Expression Ratio	1.67
Median Expression Ratio	1.66
Credible Interval	(1.04, 2.71)
Associated Peptides	11
Associated Spectra	43
Coverage	NaN

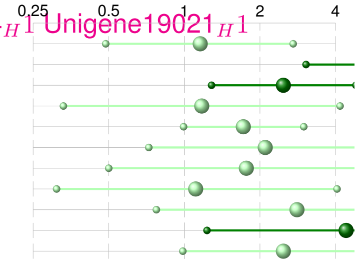

A	B	2.5	50	97.5	Sequence
2	2	0.49	1.2	2.7	IQEKEEEFENTR
2	7	3.1	5.7	11	IQEKEEEFENTRR
1	7	1.3	2.5	4.8	KLQQQVIETQQHVEEEAR
1	0	0.33	1.2	4.2	GKAELEK
1	11	0.99	1.7	3	ALDSMQASLEAEAK
0	2	0.72	2.1	6.2	CTALTGEVEELR
0	1	0.5	1.8	6.3	LQQQVIETQQHVEEEAR
0	1	0.31	1.1	4	RCTALTGEVEELR
0	1	0.77	2.8	9.8	RIQEKEEEFENTR
0	1	1.2	4.4	16	ALDSMQASLEAEAKGKAEAMR
0	3	0.99	2.5	6.4	KLEQDINELEIALDQANR

5.13 118 501 minus strand histone 2A.Z [Aplysia californica]

Protein Accession	CL14228.Contig6$_{H}1$
Mean Expression Ratio	0.619
Median Expression Ratio	0.619
Credible Interval	(0.365, 1.05)
Associated Peptides	6
Associated Spectra	32
Coverage	NaN

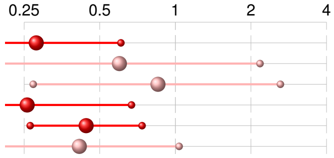

A	B	2.5	50	97.5	Sequence
3	4	0.12	0.28	0.61	ATIAGGGVIPHIHK
1	0	0.17	0.6	2.2	HLQLAIR
1	1	0.27	0.85	2.6	SLIGKK
3	0	0.098	0.26	0.67	SLIGK
15	0	0.26	0.44	0.74	AGLQFPVGR
3	1	0.17	0.41	1	GDEELDSLIK

5.14 " 75 446 Actin, cytoplasmic type 8 OS=Xenopus laevis PE=3 SV=1"

Protein Accession	CL31420.Contig6$_{H}$1
Mean Expression Ratio	1.59
Median Expression Ratio	1.59
Credible Interval	(0.896, 2.92)
Associated Peptides	4
Associated Spectra	78
Coverage	NaN

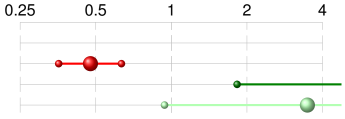

A	B	2.5	50	97.5	Sequence
6	17	5	7.5	11	MQKEITALAPSTMK
26	27	0.35	0.47	0.63	EITALAPSTMK
0	1	1.8	6.4	23	MQKEITALAPSTMKIK
0	1	0.94	3.5	13	EITALAPSTMKIK

5.15 55 795 Elongation factor 1-delta OS=Bos taurus GN=EEF1D PE=2 SV=2; 49 747 PREDICTED: elongation factor 1-delta-like [Strongylocentrotus purpuratus]; 49 819 PREDICTED: elongation factor 1-delta-like isoform 2 [Strongylocentrotus purpuratus]; 49 795 PREDICTED: elongation factor 1-delta-like isoform 2 [Strongylocentrotus purpuratus]

Protein Accession	CL10790.Contig9$_{H}$1 CL10790.Contig13$_{H}$1 CL10790.Contig12$_{H}$1 CL10790.Contig11$_{H}$1
Mean Expression Ratio	1.57
Median Expression Ratio	1.56
Credible Interval	(0.9, 2.79)
Associated Peptides	7
Associated Spectra	11
Coverage	NaN

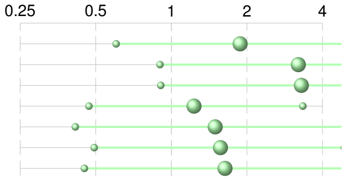

A	B	2.5	50	97.5	Sequence
1	1	0.6	1.9	5.8	KPVVIAK
0	1	0.9	3.2	12	QKFEEAEAHFYAR
0	1	0.91	3.3	12	TIQKDGLTWGAAK
1	2	0.47	1.2	3.3	VAALEVVGTGGASPK
0	1	0.42	1.5	5.5	KVTSDLR
1	1	0.49	1.6	4.9	LLEEYAAK
0	1	0.45	1.6	5.9	RLEEIEKENK

5.16 227 3073 minus strand Lysosomal alpha-mannosidase [Crassostrea gigas]

Protein Accession	CL2930.Contig2$_{H}$1
Mean Expression Ratio	1.56
Median Expression Ratio	1.56
Credible Interval	(0.977, 2.48)
Associated Peptides	13
Associated Spectra	30
Coverage	NaN

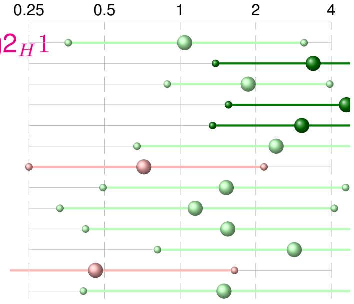

A	B	2.5	50	97.5	Sequence
0	2	0.36	1	3.1	ALPDNVHLLTLEQFR
1	3	1.4	3.4	8.2	ASLTWSGVR
2	5	0.89	1.9	3.9	EQASLFAR
0	2	1.6	4.6	13	GSHYVMLEPVSSAAK
1	4	1.3	3.1	6.9	IFIRDEAK
0	1	0.67	2.4	8.4	SQGGSSLLDGQIELMVHRR
0	2	0.25	0.72	2.2	VASELIPITPDTFR
1	1	0.49	1.5	4.6	VVNDALKK
0	1	0.33	1.1	4.1	QAVAYDYAER
0	1	0.42	1.6	5.4	QVNNFLQVVK
0	1	0.81	2.8	10	LQWNYPH
0	1	0.13	0.46	1.7	LANGVMEGQK
0	1	0.41	1.5	5.3	VVNDALK

5.17 " 242 646 minus strand Fatty acid-binding protein, heart [Crassostrea gigas]; 227 631 minus strand Fatty acid-binding protein, heart [Crassostrea gigas]; 265 669 Fatty acid-binding protein, heart [Crassostrea gigas]"

Protein Accession	CL7643.Contig9$_H$1 CL7643.Contig11$_H$1 CL7643.Contig10$_H$1
Mean Expression Ratio	1.56
Median Expression Ratio	1.56
Credible Interval	(0.922, 2.58)
Associated Peptides	8
Associated Spectra	43
Coverage	NaN

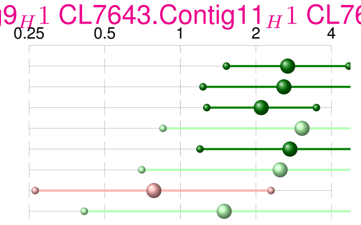

A	B	2.5	50	97.5	Sequence
3	9	1.5	2.7	4.7	AINNGQMIMTMK
3	3	1.2	2.6	5.4	ATSGVEFESR
6	9	1.3	2.1	3.5	CGDVTSIR
1	0	0.86	3.1	11	YLDGREIK
2	3	1.2	2.7	6.1	KYDKV
0	1	0.7	2.5	9.1	TVFRVEGDTLIEDVSGPFSTSIVR
0	2	0.26	0.79	2.3	VEGDTLIEDVSGPFSTSIVR
0	1	0.41	1.5	5.4	AALNGKWK

5.18 390 2267 neurofilament protein NF70 [Helix aspersa]; 318 2195 neurofilament protein NF70 [Helix aspersa]

Protein Accession	CL1353.Contig5$_H$1 CL1353.Contig4$_H$1
Mean Expression Ratio	1.54
Median Expression Ratio	1.53
Credible Interval	(0.823, 2.96)
Associated Peptides	3
Associated Spectra	5
Coverage	NaN

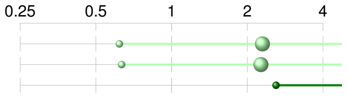

A	B	2.5	50	97.5	Sequence
0	1	0.62	2.3	8.1	KDVNLMNWTLQR
0	1	0.63	2.3	8.3	LEEARK
0	3	2.6	6.8	18	VGQLEELVDDLQR

5.19 362 874 minus strand Transgelin-2 [Crassostrea gigas]

Protein Accession	CL445.Contig3$_H$1
Mean Expression Ratio	1.53
Median Expression Ratio	1.53
Credible Interval	(0.918, 2.61)
Associated Peptides	8
Associated Spectra	31
Coverage	NaN

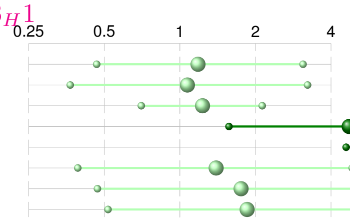

A	B	2.5	50	97.5	Sequence
2	1	0.47	1.2	3.1	KAGLGAEIEK
2	0	0.37	1.1	3.2	LLESEGKPK
7	5	0.7	1.2	2.1	AGLGAEIEK
0	2	1.6	4.7	14	AKKAGLGAEIEK
0	9	4.6	8.6	16	LINFD
0	1	0.39	1.4	4.9	SLGFLANSK
0	1	0.47	1.8	6.3	GFQPSYTGSQSR
0	1	0.52	1.9	6.8	LINKLLESEGKPK

5.20 2 328 cytoskeletal actin 2 [Molgula oculata]

Protein Accession	Unigene2743$_H$1
Mean Expression Ratio	1.53
Median Expression Ratio	1.53
Credible Interval	(0.802, 3.03)
Associated Peptides	2
Associated Spectra	6
Coverage	NaN

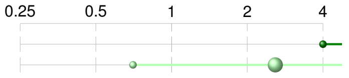

A	B	2.5	50	97.5	Sequence
1	4	4	8.9	20	MQKDITALAPPTMK
0	1	0.7	2.6	9.3	MQKDITALAPP

5.21 718 1155 Annexin A7 [Crassostrea gigas]

Protein Accession	CL15539.Contig2$_{H}$1
Mean Expression Ratio	0.656
Median Expression Ratio	0.658
Credible Interval	(0.377, 1.14)
Associated Peptides	7
Associated Spectra	15
Coverage	NaN

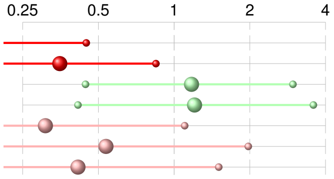

A	B	2.5	50	97.5	Sequence
2	1	0.066	0.17	0.45	ATFDAYQQIAGK
2	2	0.15	0.35	0.85	NVPGYFAQR
1	2	0.44	1.2	3	KLGTDEETFNR
1	1	0.41	1.2	3.6	SFPQLR
1	0	0.082	0.31	1.1	AQEAQELLQAGVK
1	0	0.15	0.54	2	SEVDLGATK
0	1	0.11	0.41	1.5	SEMSGNLEEGFLAIVR

5.22 " 276 659 Fatty acid-binding protein, adipocyte [Crassostrea gigas]; 426 809 Fatty acid-binding protein, adipocyte [Crassostrea gigas]; 277 660 Fatty acid-binding protein, adipocyte [Crassostrea gigas]; 283 666 Fatty acid-binding protein, adipocyte [Crassostrea gigas]; 270 653 Fatty acid-binding protein, adipocyte [Crassostrea gigas]; 283 666 Fatty acid-binding protein, adipocyte [Crassostrea gigas]; 258 641 Fatty acid-binding protein, adipocyte [Crassostrea gigas]; 427 810 Fatty acid-binding protein, adipocyte [Crassostrea gigas]; 277 660 Fatty acid-binding protein, adipocyte [Crassostrea gigas]"

Protein Accession	CL7666.Contig9$_{H}$1 CL7666.Contig8$_{H}$1 CL7666.Contig7$_{H}$1 CL7666.Contig6$_{H}$1 CL7666.Con
Mean Expression Ratio	1.52
Median Expression Ratio	1.51
Credible Interval	(0.888, 2.63)
Associated Peptides	8
Associated Spectra	18
Coverage	NaN

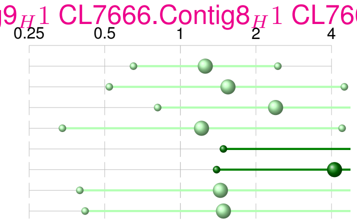

A	B	2.5	50	97.5	Sequence
2	6	0.65	1.3	2.4	AFVDGALK
2	0	0.52	1.5	4.5	AFVDGALKTSMTAK
2	0	0.81	2.4	7	QTGEGGEITIR
0	1	0.34	1.2	4.4	AVLTEESGELVER
0	1	1.5	5.3	19	GVTATISFQKA
0	2	1.4	4.1	12	LTTVAGDKQR
0	1	0.4	1.4	5.2	TVLAMER
0	1	0.42	1.5	5.5	GVTATISFQK

5.23 1200 1547 protein disulfide isomerase [Conus eburneus]

Protein Accession	CL3770.Contig1$_{H}$1
Mean Expression Ratio	0.669
Median Expression Ratio	0.669
Credible Interval	(0.405, 1.11)
Associated Peptides	7
Associated Spectra	47
Coverage	NaN

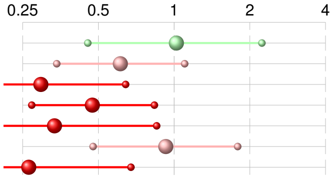

A	B	2.5	50	97.5	Sequence
2	3	0.45	1	2.2	TLSAFVK
3	8	0.34	0.61	1.1	VQSFPTLK
4	1	0.13	0.29	0.64	YKDSNDIVIAK
5	7	0.27	0.47	0.83	NFNDVAFDK
3	0	0.13	0.33	0.85	VLVGK
0	8	0.48	0.93	1.8	IDSTANDVENIR
0	3	0.1	0.26	0.67	QLAPIWDELAEK

5.24 " 10 609 lethal(2)essential for life protein, l2efl [Aedes aegypti] gi"

Protein Accession	Unigene67975$_{H}1$
Mean Expression Ratio	0.679
Median Expression Ratio	0.678
Credible Interval	(0.39, 1.16)
Associated Peptides	6
Associated Spectra	21
Coverage	NaN

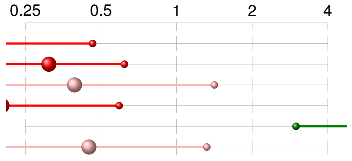

A	B	2.5	50	97.5	Sequence
3	0	0.069	0.18	0.46	MQMGETGK
7	0	0.15	0.31	0.62	INMDVK
1	0	0.11	0.39	1.4	SEVVNDDKEFR
0	2	0.07	0.2	0.59	ALEAPGETVIPITFEGK
0	6	3	6.5	13	KALEAPGETVIPITFEGKGDGK
0	2	0.15	0.45	1.3	KALEAPGETVIPITFEGK

5.25 205 2490 minus strand glucose-regulated protein 94 [Crassostrea gigas]

Protein Accession	CL5089.Contig1$_{H}1$
Mean Expression Ratio	0.679
Median Expression Ratio	0.681
Credible Interval	(0.436, 1.05)
Associated Peptides	16
Associated Spectra	37
Coverage	NaN

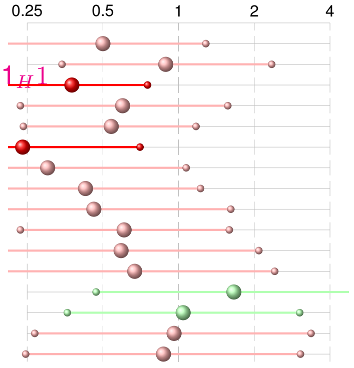

A	B	2.5	50	97.5	Sequence
1	2	0.2	0.5	1.3	GVVDSDDLPLNVSR
2	1	0.34	0.89	2.3	KTLEINPR
4	3	0.19	0.38	0.75	SGTSDFLAK
3	0	0.24	0.6	1.6	TLEINPR
3	2	0.24	0.54	1.2	NLGTIAR
1	1	0.082	0.24	0.7	KFQNVAK
1	0	0.084	0.3	1.1	DVALADKIEK
1	1	0.15	0.43	1.2	EGLKLDQSEK
1	0	0.13	0.46	1.6	KTLDMIK
1	2	0.24	0.61	1.6	ELISNASDALDKIR
0	1	0.17	0.59	2.1	ELISNASDALDK
0	1	0.18	0.67	2.4	LGVIEDTSNR
0	1	0.47	1.7	6	HPLMKELK
0	2	0.36	1	3	SILFVPK
0	1	0.27	0.96	3.4	KPGDIDEKEYVDFYK
0	1	0.25	0.87	3.1	GTTVSLHLK

5.26 1971 3368 minus strand Kyphoscoliosis peptidase [Crassostrea gigas]; 1767 3158 minus strand hillarin [Hirudo medicinalis]; 1977 3368 minus strand hillarin [Hirudo medicinalis]

Protein Accession	CL3923.Contig9$_{H}1$ CL3923.Contig8$_{H}1$ CL3923.Contig10$_{H}1$
Mean Expression Ratio	0.684
Median Expression Ratio	0.683
Credible Interval	(0.369, 1.27)
Associated Peptides	4
Associated Spectra	5
Coverage	NaN

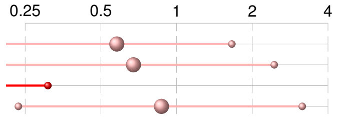

A	B	2.5	50	97.5	Sequence
1	1	0.19	0.58	1.7	EMNFDKVEK
1	0	0.18	0.67	2.4	NITNEIEK
0	1	0.024	0.083	0.31	GSPEEVLMGLK
0	1	0.24	0.87	3.2	NVLQSVDDHAIQVSK

5.27 1 825 Spectrin alpha chain [Crassostrea gigas]

Protein Accession	CL10282.Contig3$_{H}$1
Mean Expression Ratio	1.46
Median Expression Ratio	1.46
Credible Interval	(0.8, 2.66)
Associated Peptides	5
Associated Spectra	6
Coverage	NaN

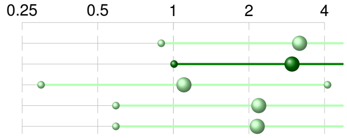

A	B	2.5	50	97.5	Sequence
0	1	0.9	3.2	11	KIEDLGAAMEER
0	2	1	3	8.8	MQHNLEQQIEAR
0	1	0.3	1.1	4.1	AIPDSYDYVDFTR
0	1	0.59	2.2	8	RKATEVR
0	1	0.59	2.2	8	MKPYVDK

5.28 " 3 587 elongation factor 1 alpha, partial [Phorbas sp. nov. SB-2012]"

Protein Accession	Unigene20048$_{H}$1
Mean Expression Ratio	0.684
Median Expression Ratio	0.684
Credible Interval	(0.354, 1.3)
Associated Peptides	2
Associated Spectra	13
Coverage	NaN

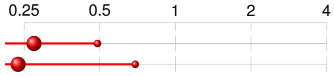

A	B	2.5	50	97.5	Sequence
6	5	0.15	0.27	0.49	IGGIGTVPVGR
1	1	0.079	0.24	0.69	LPLQDVYK

5.29 405 1373 paramyosin [Haliotis discus discus]

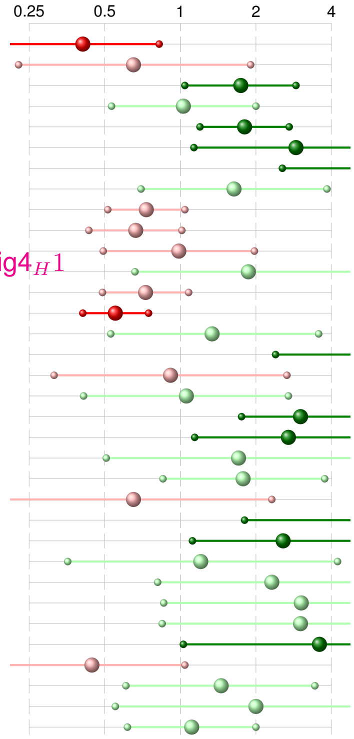

A	B	2.5	50	97.5	Sequence
1	6	0.2	0.41	0.83	ELEDALDAER
2	0	0.23	0.65	1.9	FDKELAAK
3	12	1	1.7	2.9	HTEAVNDLSDQVDYLTK
1	7	0.53	1	2	IRELEDALDAER
9	15	1.2	1.8	2.7	LKGQVDDLTR
1	2	1.1	2.9	7.3	QKLSLESR
1	2	2.5	6.5	17	QLNDLTALKAR
1	3	0.7	1.6	3.8	TRFDKELAAK
21	12	0.52	0.73	1	TVAELSFR
13	9	0.43	0.66	1	VSADYAALK
3	4	0.49	0.98	2	LDEEAETSSNLR
1	1	0.66	1.9	5.2	FEAAEASLR
19	6	0.49	0.73	1.1	LSLESR
19	27	0.41	0.55	0.75	QLNDLTALK
2	1	0.53	1.3	3.6	SQLQNQNDDLKR
0	2	2.4	7.1	21	AKSQLQNQNDDLKR
0	2	0.31	0.91	2.7	DLELATAQ
0	3	0.41	1.1	2.7	ELEDALDAERDGR
0	13	1.8	3	5.1	IRELEDALDAERDGR
0	4	1.1	2.7	6.5	IRELEDALDAERDGRVR
0	1	0.51	1.7	6	ITELEDSCEQLR
0	6	0.85	1.8	3.8	KDLELATAQFEAAEASLR
0	1	0.18	0.65	2.3	LDQVQDSLDEQGSSTVAQIELNKKR
0	2	1.8	5.3	15	LSLESRVDAAEQSAAR
0	4	1.1	2.6	6	LTQENFDLQHQVQELDSSNAALAK
0	1	0.36	1.2	4.2	RELEDALDAERDGR
0	2	0.81	2.3	6.9	ESEIYKLR
0	1	0.86	3	11	ESEIYKLRK
0	1	0.85	3	11	KRESEIYK
0	1	1	3.6	12	QFEAAEASLR
0	4	0.19	0.44	1	DEQGSSTVAQIELNK
0	4	0.61	1.5	3.4	DLELATAQFEAAEASLR
0	1	0.55	2	7	LDEEAETSSNLRNSLSK
0	11	0.61	1.1	2	LDQVQDSLDEQGSSTVAQIELNK

Protein Accession	CL43455.Contig4$_H$1
Mean Expression Ratio	1.45
Median Expression Ratio	1.45
Credible Interval	(1.06, 1.99)
Associated Peptides	34
Associated Spectra	268
Coverage	NaN

5.30 9 722 minus strand actin related protein [Lumbricus rubellus]

Protein Accession	CL45325.Contig2$_H$1
Mean Expression Ratio	1.45
Median Expression Ratio	1.45
Credible Interval	(0.743, 2.91)
Associated Peptides	2
Associated Spectra	2
Coverage	NaN

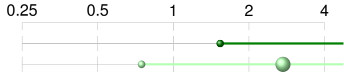

A	B	2.5	50	97.5	Sequence
0	1	1.5	5.6	20	ISAEEFLKNF
0	1	0.75	2.7	10	LAVNSRAR

5.31 816 1211 H2 [Haliotis tuberculata]

Protein Accession	CL11519.Contig5$_{H}1$
Mean Expression Ratio	0.69
Median Expression Ratio	0.691
Credible Interval	(0.368, 1.29)
Associated Peptides	3
Associated Spectra	7
Coverage	NaN

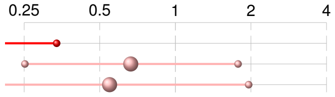

A	B	2.5	50	97.5	Sequence
1	2	0.05	0.13	0.34	SLILAMR
1	2	0.25	0.66	1.8	KNVLSLTPAER
0	1	0.15	0.55	2	LDSSADFDLQVK

5.32 51 890 prohibitin [Octopus tankahkeei]

Protein Accession	CL26635.Contig2$_{H}1$
Mean Expression Ratio	1.44
Median Expression Ratio	1.44
Credible Interval	(0.836, 2.51)
Associated Peptides	8
Associated Spectra	12
Coverage	NaN

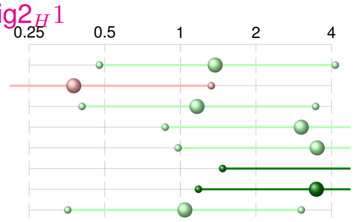

A	B	2.5	50	97.5	Sequence
1	1	0.48	1.4	4.1	AFTEAGEGLVELR
1	0	0.11	0.38	1.3	EFTEAVEMK
0	2	0.41	1.2	3.5	FLVEKEEQK
0	1	0.87	3	11	KIEAAEDIAYQLSR
0	1	0.98	3.5	12	NVPVITGSKDLQNVNITLR
0	1	1.5	5.4	20	FLVEKEEQKKR
0	2	1.2	3.5	10	AAVISAEGDSLAAELLAK
0	2	0.36	1	3	NVPVITGSK

5.33 366 1049 hypothetical protein CGI_10004855 [Crassostrea gigas]

Protein Accession	CL43590.Contig5$_{H}1$
Mean Expression Ratio	1.44
Median Expression Ratio	1.44
Credible Interval	(0.847, 2.43)
Associated Peptides	9
Associated Spectra	17
Coverage	NaN

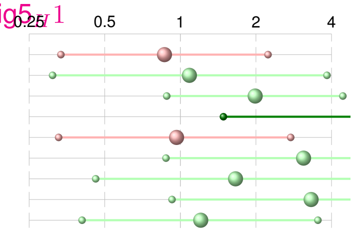

A	B	2.5	50	97.5	Sequence
2	1	0.34	0.87	2.2	TTVQLHK
1	0	0.31	1.1	3.8	KVFPYPK
0	5	0.88	2	4.4	GGDLWQGEVSLEQHR
0	1	1.5	5.2	19	GKNVDVKLNAR
0	2	0.33	0.97	2.8	SAEFSTNDNIFDIEMK
0	1	0.88	3.1	11	TPQPLKTTVQLHK
0	1	0.46	1.7	6.2	VKVPGAK
0	1	0.93	3.3	12	GKNVDVK
0	2	0.41	1.2	3.5	DEWTHLTAK

5.34 2019 2723 minus strand Kyphoscoliosis peptidase [Crassostrea gigas]; 2060 2764 minus strand Kyphoscoliosis peptidase [Crassostrea gigas]; 1074 1778 minus strand Kyphoscoliosis peptidase [Crassostrea gigas]; 2063 2767 minus strand Kyphoscoliosis peptidase [Crassostrea gigas]; 2044 2748 minus strand Kyphoscoliosis peptidase [Crassostrea gigas]; 2044 2748 minus strand Kyphoscoliosis peptidase [Crassostrea gigas]

Protein Accession	CL3923.Contig7$_H1$ CL3923.Contig6$_H1$ CL3923.Contig5$_H1$ CL3923.Contig4$_H1$ CL3923.Con
Mean Expression Ratio	1.43
Median Expression Ratio	1.43
Credible Interval	(0.757, 2.75)
Associated Peptides	2
Associated Spectra	5
Coverage	NaN

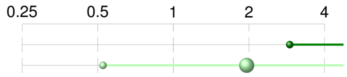

A	B	2.5	50	97.5	Sequence
2	2	2.9	7	17	TMGQAPPTVPKDFQLK
0	1	0.53	2	7.1	TMGQAPPTVPK

5.35 156 1106 ELAV 2-like protein [Aplysia californica] gi

Protein Accession	CL1500.Contig1$_H1$
Mean Expression Ratio	0.703
Median Expression Ratio	0.702
Credible Interval	(0.386, 1.28)
Associated Peptides	4
Associated Spectra	8
Coverage	NaN

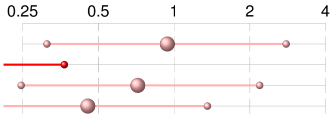

A	B	2.5	50	97.5	Sequence
1	1	0.31	0.94	2.8	VLVDLQTGVSK
0	2	0.042	0.12	0.37	NLPIPWTENELNK
0	2	0.25	0.72	2.2	SMFLSIGPIK
0	2	0.15	0.45	1.4	LFDPYGK

5.36 587 811 hemocyanin 2 [Megathura crenulata]

Protein Accession	Unigene4334$_H1$
Mean Expression Ratio	0.703
Median Expression Ratio	0.707
Credible Interval	(0.366, 1.34)
Associated Peptides	2
Associated Spectra	6
Coverage	NaN

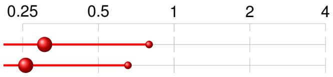

A	B	2.5	50	97.5	Sequence
2	1	0.12	0.31	0.8	AIDDGTFR
1	2	0.096	0.26	0.66	SVEDLTAGEAESLK

5.37 1 852 beta actin-like 1 protein [Macrostomum lignano]

Protein Accession	Unigene31438$_H$1
Mean Expression Ratio	1.42
Median Expression Ratio	1.41
Credible Interval	(1.01, 2)
Associated Peptides	26
Associated Spectra	306
Coverage	NaN

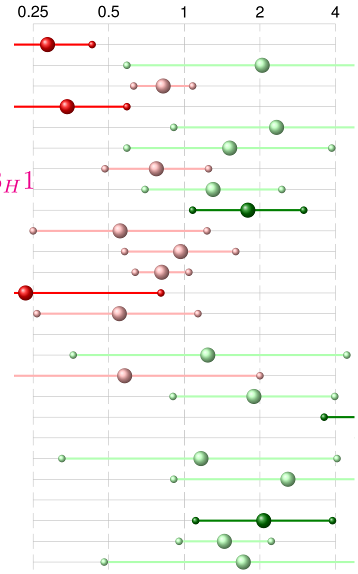

A	B	2.5	50	97.5	Sequence
10	14	0.19	0.28	0.43	GILTLK
1	0	0.59	2	7.3	HQGVMVGM
25	34	0.63	0.83	1.1	HQGVMVGMGQK
3	9	0.2	0.34	0.59	YPIEHGIVTNWDDMEK
1	2	0.9	2.3	6	VAPEEHPVLL
2	1	0.59	1.5	3.9	TEAPLNPK
5	12	0.48	0.77	1.2	RGILTLK
3	6	0.7	1.3	2.5	VAPEEHPVLLTEA
9	6	1.1	1.8	3	AGFAGDDAPR
4	1	0.25	0.56	1.2	AVFPSIVGR
5	10	0.58	0.97	1.6	DSYVGDEAQSK
16	54	0.64	0.81	1	VAPEEHPVLLTEAPLNPK
0	1	0.067	0.23	0.8	APEEHPVLLTEAPLNPK
0	6	0.26	0.55	1.1	GYSFTTTAER
0	5	5.5	12	27	HQGVMVGMGQKDSYVGDEAQSK
0	1	0.36	1.2	4.4	SYELPDGQVITIGN
0	1	0.16	0.58	2	TTGIVLDSGD
0	6	0.9	1.9	4	VAPEEHPVLLTEAPLN
0	3	3.6	9.4	25	VAPEEHPVLLTEAPLNPKANR
0	6	4.9	10	21	VAPEEHPVLLTEAPLNPKANREK
0	1	0.32	1.2	4.1	VAPEEHPVL
0	2	0.91	2.6	7.4	AVFPSIVGRPR
0	9	19	36	69	HQGVMVGMGQKDSYVGDEAQSKR
0	9	1.1	2.1	3.9	IWHHTFYNELR
0	22	0.95	1.4	2.2	SYELPDGQVITIGNER
0	1	0.48	1.7	5.9	TTGIVLDSGDGVTHTVPIYEGYALPHAILRLDLA[illegible]

5.38 4 675 Laminin subunit gamma-1 [Crassostrea gigas]

Protein Accession	CL35712.Contig1$_H$1
Mean Expression Ratio	1.42
Median Expression Ratio	1.41
Credible Interval	(0.734, 2.83)
Associated Peptides	2
Associated Spectra	2
Coverage	NaN

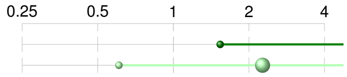

A	B	2.5	50	97.5	Sequence
0	1	1.5	5.6	20	SKVNEALAK
0	1	0.61	2.3	8.4	LAQLAEDTAIQASAEAR

5.39 " 220 627 Fatty acid-binding protein, brain [Crassostrea gigas]"

Protein Accession	CL9041.Contig2$_H$1
Mean Expression Ratio	0.705
Median Expression Ratio	0.708
Credible Interval	(0.394, 1.25)
Associated Peptides	5
Associated Spectra	24
Coverage	NaN

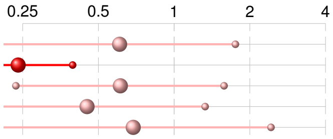

A	B	2.5	50	97.5	Sequence
1	1	0.2	0.61	1.7	IVENYKLEDK
10	6	0.15	0.24	0.39	LTITVEGGK
0	3	0.23	0.61	1.6	EVDGGVMTSTTTFDGK
0	2	0.15	0.45	1.3	LGETFDYNSIDGTKPK
0	1	0.19	0.69	2.4	VDLASTTGLEEFGTAIGFSEEK

5.40 594 1253 peroxiredoxin 6 [Eriocheir sinensis]

Protein Accession	CL21894.Contig2$_{H}1$
Mean Expression Ratio	0.711
Median Expression Ratio	0.711
Credible Interval	(0.448, 1.12)
Associated Peptides	11
Associated Spectra	44
Coverage	NaN

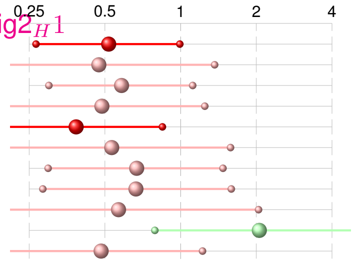

A	B	2.5	50	97.5	Sequence
4	4	0.27	0.52	0.99	VATPVDWK
1	1	0.16	0.47	1.4	NFDEILR
2	6	0.3	0.58	1.1	AVFIIGPDK
2	1	0.19	0.49	1.2	LFPNYK
4	1	0.18	0.38	0.84	QLTATQK
1	1	0.18	0.53	1.6	AVFIIGPDKK
1	4	0.3	0.67	1.5	LLPEFEK
1	3	0.28	0.67	1.6	VLDSLQLTATQK
0	1	0.16	0.57	2	ELAVQLGMVDPDEK
0	3	0.79	2.1	5.3	KVATPVDWK
0	3	0.19	0.48	1.2	VNLGDVFPDFK

5.41 788 1006 LIM protein [Haliotis discus discus]; 768 980 muscle LIM protein isoform 1 [Danaus plexippus]

Protein Accession	CL29824.Contig2$_{H}1$ CL29824.Contig1$_{H}1$
Mean Expression Ratio	1.41
Median Expression Ratio	1.4
Credible Interval	(0.733, 2.82)
Associated Peptides	1
Associated Spectra	4
Coverage	NaN

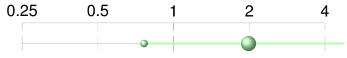

A	B	2.5	50	97.5	Sequence
2	2	0.76	2	5.3	AIGVNTPWHK

5.42 1070 1888 minus strand galectin [Pinctada fucata]; 1371 2189 minus strand galectin [Pinctada fucata]

Protein Accession	CL7245.Contig4$_{H}1$ CL7245.Contig3$_{H}1$
Mean Expression Ratio	0.712
Median Expression Ratio	0.715
Credible Interval	(0.413, 1.21)
Associated Peptides	4
Associated Spectra	28
Coverage	NaN

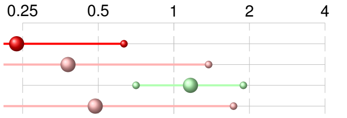

A	B	2.5	50	97.5	Sequence
2	1	0.091	0.24	0.63	EIPQGLEK
1	0	0.1	0.38	1.4	GEAFIYEVK
13	10	0.71	1.2	1.9	FNEGVTVR
1	0	0.14	0.49	1.7	NDLVVIK

5.43 " 2 1174 endo-1,3-beta-D-glucanase [Littorina sitkana]"

Protein Accession	CL31231.Contig3$_{H}1$
Mean Expression Ratio	1.4
Median Expression Ratio	1.4
Credible Interval	(0.79, 2.56)
Associated Peptides	4
Associated Spectra	22
Coverage	NaN

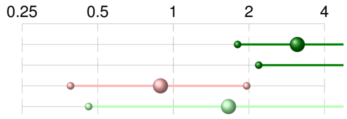

A	B	2.5	50	97.5	Sequence
5	7	1.8	3.1	5.5	FGTLEVR
1	3	2.2	5.2	12	SGEIDIMESR
5	0	0.39	0.89	2	AVIFR
0	1	0.46	1.7	6.1	NGMLPPVMSGK

5.44 22 870 minus strand PREDICTED: alcohol dehydrogenase class-3 [Sarcophilus harrisii]

Protein Accession	Unigene27690$_H$1
Mean Expression Ratio	0.717
Median Expression Ratio	0.718
Credible Interval	(0.383, 1.33)
Associated Peptides	3
Associated Spectra	8
Coverage	NaN

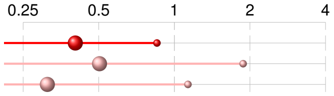

A	B	2.5	50	97.5	Sequence
4	2	0.19	0.4	0.85	AAVAWEAK
1	0	0.14	0.5	1.9	FAMAK
0	1	0.083	0.31	1.1	IIGIDVNQDK

5.45 195 455 60S ribosomal protein L23a OS=Rattus norvegicus GN=Rpl23a PE=2 SV=1; 16 465 60S ribosomal protein L23a OS=Rattus norvegicus GN=Rpl23a PE=2 SV=1

Protein Accession	CL27563.Contig3$_H$1 CL27563.Contig1$_H$1
Mean Expression Ratio	0.72
Median Expression Ratio	0.72
Credible Interval	(0.379, 1.35)
Associated Peptides	3
Associated Spectra	6
Coverage	NaN

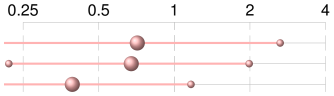

A	B	2.5	50	97.5	Sequence
1	0	0.2	0.71	2.6	VNTLIRPDGEK
1	2	0.22	0.68	2	ANKPLIK
2	0	0.13	0.39	1.2	SLYLYDVDIAK

5.46 180 560 calreticulin precursor [Aplysia californica] gi; 180 545 calreticulin precursor [Aplysia californica] gi

Protein Accession	CL4813.Contig4$_H$1 CL4813.Contig2$_H$1
Mean Expression Ratio	0.721
Median Expression Ratio	0.721
Credible Interval	(0.431, 1.21)
Associated Peptides	8
Associated Spectra	24
Coverage	NaN

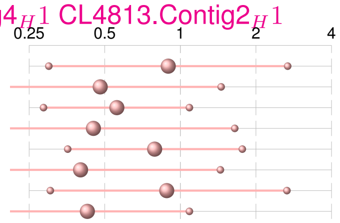

A	B	2.5	50	97.5	Sequence
1	1	0.3	0.89	2.7	DPTVYFK
1	1	0.17	0.48	1.4	FYGDEDKDK
1	7	0.28	0.56	1.1	FYGISAK
0	1	0.13	0.45	1.6	EEFDANWEDR
0	5	0.36	0.79	1.8	WIPSVHK
0	1	0.11	0.4	1.4	TLVIQFTVK
0	2	0.3	0.88	2.7	FDKFSNEGK
0	3	0.16	0.43	1.1	FVWSAGK

5.47 2008 2715 Protein disulfide-isomerase A5 [Crassostrea gigas]

Protein Accession	CL7820.Contig3$_H$1
Mean Expression Ratio	1.39
Median Expression Ratio	1.38
Credible Interval	(0.751, 2.6)
Associated Peptides	4
Associated Spectra	6
Coverage	NaN

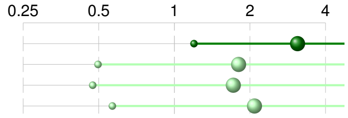

A	B	2.5	50	97.5	Sequence
1	2	1.2	3.1	8.1	LKQESPTAK
0	1	0.5	1.8	6.6	MKPAYMSAAAR
0	1	0.47	1.7	6.2	FGGLASKYEVK
0	1	0.57	2.1	7.6	SKEDLVAFMKDPKPK

5.48 316 1512 Alpha-N-acetylgalactosaminidase [Crassostrea gigas]; 282 1478 Alpha-N-acetylgalactosaminidase [Crassostrea gigas]

Protein Accession	CL2428.Contig4$_{H}1$ CL2428.Contig1$_{H}1$
Mean Expression Ratio	0.723
Median Expression Ratio	0.726
Credible Interval	(0.405, 1.26)
Associated Peptides	6
Associated Spectra	12
Coverage	NaN

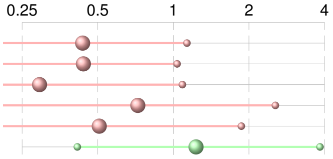

A	B	2.5	50	97.5	Sequence
1	2	0.17	0.43	1.1	LGIYEDFGSK
2	2	0.19	0.44	1	LVELGLR
1	0	0.079	0.29	1.1	YGYPVMQR
1	0	0.19	0.72	2.5	LYMAQANK
1	0	0.14	0.51	1.9	VDMVK
0	2	0.42	1.2	3.8	AGYRARELFAGK

5.49 48 479 minus strand 40S ribosomal protein S24 OS=Macaca fascicularis GN=RPS24 PE=2 SV=1

Protein Accession	CL40647.Contig4$_{H}1$
Mean Expression Ratio	1.38
Median Expression Ratio	1.38
Credible Interval	(0.729, 2.69)
Associated Peptides	2
Associated Spectra	8
Coverage	NaN

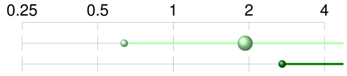

A	B	2.5	50	97.5	Sequence
0	2	0.64	1.9	5.7	HGLLELK
0	6	2.7	5.7	12	HGLLELKK

5.50 " 223 423 ADPATP carrier protein; K05863 solute carrier family 25 (mitochondrial adenine nucleotide translocator), member 4/5/6/31"

Protein Accession	CL27196.Contig3$_{H}1$
Mean Expression Ratio	0.726
Median Expression Ratio	0.728
Credible Interval	(0.387, 1.36)
Associated Peptides	1
Associated Spectra	8
Coverage	NaN

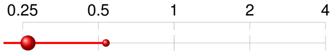

A	B	2.5	50	97.5	Sequence
6	2	0.13	0.26	0.54	LLVQNQNEMIK

5.51 195 455 60S ribosomal protein L23a OS=Rattus norvegicus GN=Rpl23a PE=2 SV=1; 195 512 60S ribosomal protein L23a OS=Rattus norvegicus GN=Rpl23a PE=2 SV=1; 16 465 60S ribosomal protein L23a OS=Rattus norvegicus GN=Rpl23a PE=2 SV=1

Protein Accession	CL27563.Contig3$_{H}1$ CL27563.Contig2$_{H}1$ CL27563.Contig1$_{H}1$
Mean Expression Ratio	1.37
Median Expression Ratio	1.37
Credible Interval	(0.721, 2.61)
Associated Peptides	2
Associated Spectra	6
Coverage	NaN

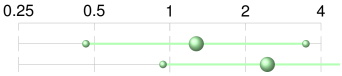

A	B	2.5	50	97.5	Sequence
1	2	0.46	1.3	3.5	ANKPLIK
0	3	0.94	2.4	6.5	VNTLIRPDGEKK

5.52 116 352 minus strand troponin I 4 [Clonorchis sinensis]

Protein Accession	Unigene63816$_{H}1$
Mean Expression Ratio	1.38
Median Expression Ratio	1.37
Credible Interval	(0.731, 2.62)
Associated Peptides	3
Associated Spectra	7
Coverage	NaN

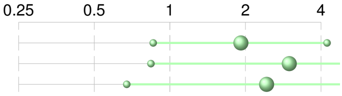

A	B	2.5	50	97.5	Sequence
0	5	0.86	1.9	4.2	FIKPVLR
0	1	0.84	3	11	QSPTRGNLKSTGR
0	1	0.67	2.4	8.9	GKFIKPVLR

5.53 194 1996 minus strand Neutral and basic amino acid transport protein rBAT [Crassostrea gigas]; 265 2067 minus strand Neutral and basic amino acid transport protein rBAT [Crassostrea gigas]; 194 1996 minus strand Neutral and basic amino acid transport protein rBAT [Crassostrea gigas]; 194 1996 minus strand Neutral and basic amino acid transport protein rBAT [Crassostrea gigas]; 265 2067 minus strand Neutral and basic amino acid transport protein rBAT [Crassostrea gigas]

Protein Accession	CL9047.Contig9$_{H}1$ CL9047.Contig8$_{H}1$ CL9047.Contig6$_{H}1$ CL9047.Contig15$_{H}1$ CL9047.Co
Mean Expression Ratio	1.37
Median Expression Ratio	1.37
Credible Interval	(0.736, 2.53)
Associated Peptides	3
Associated Spectra	8
Coverage	NaN

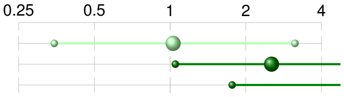

A	B	2.5	50	97.5	Sequence
1	1	0.35	1	3.1	NFAGNLDVVDALR
0	4	1	2.5	6	TAVDDLDNKPGREK
0	2	1.8	5.2	16	TITPMQWSK

5.54 1009 2784 minus strand Collagen alpha-3(VI) chain [Crassostrea gigas]; 1009 2787 minus strand Collagen alpha-3(VI) chain [Crassostrea gigas]

Protein Accession	Unigene26554$_{H}1$ Unigene26550$_{H}1$
Mean Expression Ratio	0.735
Median Expression Ratio	0.735
Credible Interval	(0.419, 1.29)
Associated Peptides	6
Associated Spectra	13
Coverage	NaN

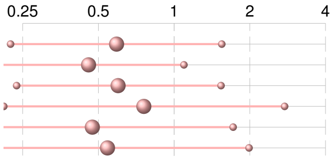

A	B	2.5	50	97.5	Sequence
3	0	0.22	0.59	1.5	ELEGNVHSIR
4	0	0.19	0.46	1.1	IITVGIGEANR
3	0	0.24	0.6	1.5	SDAAAALIR
1	0	0.21	0.76	2.8	VAMSNFR
1	0	0.13	0.47	1.7	MFIPVR
1	0	0.15	0.54	2	LTTVR

5.55 102 887 hypothetical protein DAPPUDRAFT_304712 [Daphnia pulex]

Protein Accession	CL6869.Contig1$_{H}1$
Mean Expression Ratio	0.735
Median Expression Ratio	0.736
Credible Interval	(0.416, 1.28)
Associated Peptides	6
Associated Spectra	14
Coverage	NaN

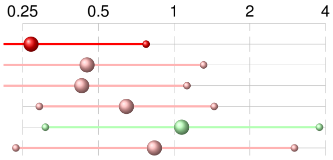

A	B	2.5	50	97.5	Sequence
1	1	0.092	0.27	0.78	TAEEIAAEEK
2	0	0.15	0.45	1.3	VDLSQLR
1	2	0.17	0.43	1.1	LADIPEGK
3	2	0.29	0.65	1.4	AFAIQAEK
0	1	0.31	1.1	3.8	AHTDVQVPDFSYYR
0	1	0.23	0.83	3	LALTSYAMSK

5.56 1180 1521 minus strand coronin [Schistosoma mansoni]

Protein Accession	CL11779.Contig9$_{H}1$
Mean Expression Ratio	0.732
Median Expression Ratio	0.736
Credible Interval	(0.361, 1.43)
Associated Peptides	1
Associated Spectra	1
Coverage	NaN

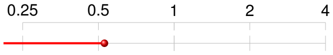

A	B	2.5	50	97.5	Sequence
1	0	0.04	0.14	0.53	DANPILVSLK

5.57 15 1361 hypothetical protein BRAFLDRAFT_231908 [Branchiostoma floridae] gi

Protein Accession	CL24447.Contig2$_{H}1$
Mean Expression Ratio	1.36
Median Expression Ratio	1.36
Credible Interval	(0.711, 2.66)
Associated Peptides	2
Associated Spectra	3
Coverage	NaN

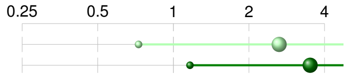

A	B	2.5	50	97.5	Sequence
0	1	0.73	2.6	9.8	GLGLDDALEAR
0	2	1.2	3.5	11	GITRIR

5.58 25 405 minus strand Elongation factor 1-delta [Anoplopoma fimbria]

Protein Accession	CL10502.Contig1$_H1$
Mean Expression Ratio	1.36
Median Expression Ratio	1.36
Credible Interval	(0.752, 2.51)
Associated Peptides	5
Associated Spectra	7
Coverage	NaN

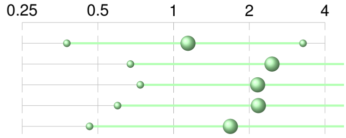

A	B	2.5	50	97.5	Sequence
1	1	0.38	1.1	3.3	KPVLIAK
0	1	0.67	2.5	9.1	SKKPVLIAK
0	2	0.74	2.2	6.5	VQTDGLVWGASK
0	1	0.6	2.2	8.1	RVQTDGLVWGASK
0	1	0.46	1.7	6.3	LVPVGYGINK

5.59 77 502 hypothetical protein CGI_10008263 [Crassostrea gigas]

Protein Accession	CL38960.Contig1$_H1$
Mean Expression Ratio	1.36
Median Expression Ratio	1.35
Credible Interval	(0.738, 2.56)
Associated Peptides	3
Associated Spectra	12
Coverage	NaN

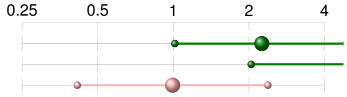

A	B	2.5	50	97.5	Sequence
0	5	1	2.3	4.9	IFVAIDGSK
0	3	2	5.3	14	KIFVAIDGSK
0	4	0.41	1	2.4	ANIEAITK

5.60 72 1400 minus strand hypothetical protein RCJMB04_22i13 [Gallus gallus]

Protein Accession	CL44755.Contig5$_H1$
Mean Expression Ratio	0.737
Median Expression Ratio	0.74
Credible Interval	(0.425, 1.26)
Associated Peptides	8
Associated Spectra	17
Coverage	NaN

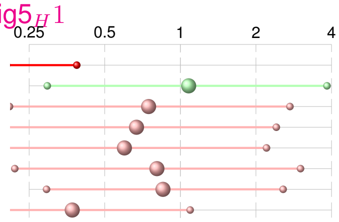

A	B	2.5	50	97.5	Sequence
5	3	0.1	0.2	0.39	LVFSADEAK
1	0	0.3	1.1	3.8	AVFQMR
1	0	0.21	0.75	2.7	EQAVYVAR
1	0	0.18	0.67	2.4	GKGTWDSGLK
1	0	0.17	0.6	2.2	AQVLAGGR
0	1	0.22	0.81	3	EMGFSEGSIDK
0	2	0.29	0.85	2.6	FLVASTPDEALQHAAELGK
0	2	0.13	0.37	1.1	LSGIPDVFVK

5.61 900 1616 minus strand hemocyanin isoform 1 [Haliotis diversicolor]

Protein Accession	Unigene30321$_H1$
Mean Expression Ratio	0.745
Median Expression Ratio	0.744
Credible Interval	(0.382, 1.45)
Associated Peptides	2
Associated Spectra	3
Coverage	NaN

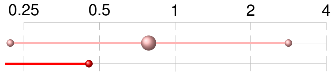

A	B	2.5	50	97.5	Sequence
0	1	0.22	0.79	2.8	LFKVEITDDLK
0	2	0.049	0.15	0.45	VEITDDLK

5.62 501 1838 Hsp90A [Haliotis asinina]

Protein Accession	CL5184.Contig2$_H$1
Mean Expression Ratio	0.742
Median Expression Ratio	0.745
Credible Interval	(0.446, 1.22)
Associated Peptides	11
Associated Spectra	21
Coverage	NaN

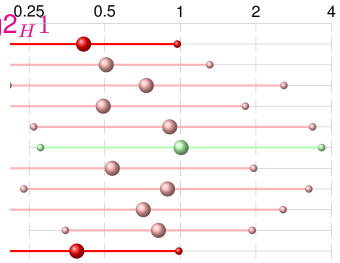

A	B	2.5	50	97.5	Sequence
1	3	0.17	0.41	0.97	EMLQQSK
1	2	0.2	0.51	1.3	SLTNDWEDHLAVK
1	0	0.21	0.73	2.6	GVVDSEDLPLNISR
0	1	0.14	0.49	1.8	AIYYITGESLDAVK
0	1	0.26	0.91	3.4	KHFEINPDHPIIK
0	1	0.28	1	3.7	RAPFDLFENKK
0	1	0.15	0.54	2	YHSSQAGDELVTLK
0	1	0.24	0.89	3.3	KLAEFLR
0	1	0.2	0.71	2.6	TKPLWTR
0	4	0.35	0.82	1.9	HFSVEGQLEFR
0	3	0.15	0.39	0.99	LAEFLR

5.63 179 2140 minus strand PREDICTED: hypothetical protein [Saccoglossus kowalevskii]

Protein Accession	CL13749.Contig6$_H$1
Mean Expression Ratio	1.34
Median Expression Ratio	1.34
Credible Interval	(0.7, 2.63)
Associated Peptides	2
Associated Spectra	3
Coverage	NaN

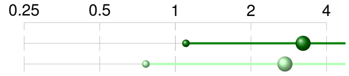

A	B	2.5	50	97.5	Sequence
0	2	1.1	3.2	9.6	KFDGIGGLSGGGATSK
0	1	0.76	2.7	10	VGGTYNFDDSNGLGR

5.64 254 1648 Tektin-3 [Crassostrea gigas]; 254 1648 Tektin-3 [Crassostrea gigas]

Protein Accession	CL6717.Contig3$_H$1 CL6717.Contig2$_H$1
Mean Expression Ratio	0.749
Median Expression Ratio	0.748
Credible Interval	(0.424, 1.31)
Associated Peptides	8
Associated Spectra	9
Coverage	NaN

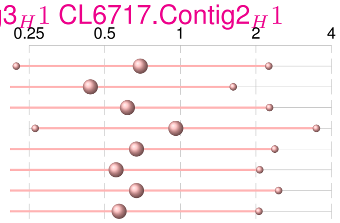

A	B	2.5	50	97.5	Sequence
1	1	0.22	0.69	2.2	AAQHALEIDAK
1	0	0.12	0.44	1.6	ATLEHDISVK
1	0	0.17	0.61	2.3	ILPYVTDK
1	0	0.26	0.96	3.5	KGFPMDPK
1	0	0.19	0.67	2.4	NVELLR
1	0	0.16	0.55	2.1	EVDIIR
1	0	0.19	0.67	2.5	EVAEIR
1	0	0.16	0.57	2.1	EAFMK

5.65 210 779 GH20056 [Drosophila grimshawi] gi; 216 785 minus strand GH20056 [Drosophila grimshawi] gi; 210 779 GH20056 [Drosophila grimshawi] gi; 216 785 GH20056 [Drosophila grimshawi] gi

Protein Accession	CL34497.Contig4$_{H}$1 CL34497.Contig3$_{H}$1 CL34497.Contig2$_{H}$1 CL34497.Contig1$_{H}$1
Mean Expression Ratio	0.754
Median Expression Ratio	0.754
Credible Interval	(0.399, 1.4)
Associated Peptides	4
Associated Spectra	5
Coverage	NaN

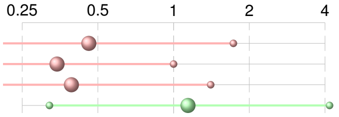

A	B	2.5	50	97.5	Sequence
1	0	0.12	0.46	1.7	QLSQEEVSNFLK
2	0	0.12	0.34	1	EVSNFLK
0	1	0.1	0.39	1.4	GLLDMCAGEK
0	1	0.32	1.1	4.2	KQPFEFQIGVGQVIK

5.66 40 1098 minus strand major egg antigen [Clonorchis sinensis]

Protein Accession	CL2613.Contig4$_{H}$1
Mean Expression Ratio	0.753
Median Expression Ratio	0.755
Credible Interval	(0.38, 1.45)
Associated Peptides	2
Associated Spectra	2
Coverage	NaN

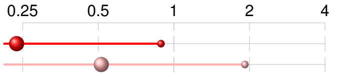

A	B	2.5	50	97.5	Sequence
1	0	0.065	0.24	0.89	AIVLKPTGK
1	0	0.14	0.52	1.9	LESHLTK

5.67 388 1653 Tektin-3 [Crassostrea gigas]

Protein Accession	CL28380.Contig1$_{H}$1
Mean Expression Ratio	0.757
Median Expression Ratio	0.757
Credible Interval	(0.405, 1.39)
Associated Peptides	3
Associated Spectra	7
Coverage	NaN

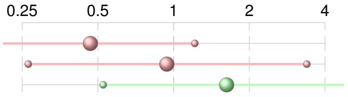

A	B	2.5	50	97.5	Sequence
2	2	0.18	0.47	1.2	QTIDLVHDAPEK
0	1	0.26	0.94	3.4	RQTIDLVHDAPEK
1	1	0.53	1.6	5.1	GLTFHNGVQR

5.68 " 28 1104 minus strand Pyruvate dehydrogenase E1 component subunit beta, mitochondrial [Crassostrea gigas]"

Protein Accession	CL18015.Contig14$_{H}$1
Mean Expression Ratio	1.32
Median Expression Ratio	1.32
Credible Interval	(0.737, 2.36)
Associated Peptides	6
Associated Spectra	9
Coverage	NaN

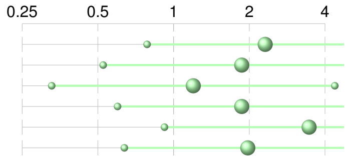

A	B	2.5	50	97.5	Sequence
0	2	0.79	2.3	6.8	DALNSAMDEEIARDER
0	1	0.53	1.9	6.8	ALDAAKELEGK
0	1	0.33	1.2	4.4	SLRPVDDETIIK
1	1	0.6	1.9	5.9	SLEEAALPQPVNVIR
0	1	0.92	3.5	13	VSKGLWK
1	1	0.64	2	6.2	VTGADVPMPYAK

5.69 788 1006 LIM protein [Haliotis discus discus]

Protein Accession	CL29824.Contig2$_{H}1$
Mean Expression Ratio	0.757
Median Expression Ratio	0.758
Credible Interval	(0.391, 1.49)
Associated Peptides	1
Associated Spectra	4
Coverage	NaN

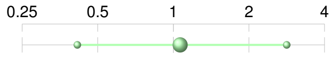

A	B	2.5	50	97.5	Sequence
**2**	**2**	**0.41**	**1.1**	**2.8**	**AIGVNTPWHK**

5.70 177 2369 ATPase family AAA domain-containing protein 2B [Crassostrea gigas]; 177 2369 ATPase family AAA domain-containing protein 2B [Crassostrea gigas]

Protein Accession	CL3021.Contig3$_{H}1$ CL3021.Contig2$_{H}1$
Mean Expression Ratio	1.32
Median Expression Ratio	1.32
Credible Interval	(0.777, 2.27)
Associated Peptides	9
Associated Spectra	14
Coverage	NaN

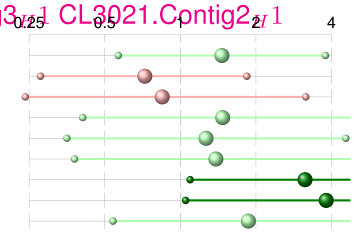

A	B	2.5	50	97.5	Sequence
**2**	**1**	**0.56**	**1.5**	**3.8**	**FGGGDVNLLK**
**2**	**1**	**0.28**	**0.72**	**1.8**	**ILEVVR**
**1**	**0**	**0.24**	**0.84**	**3.2**	**IAVADRK**
**0**	**1**	**0.41**	**1.5**	**5.3**	**MTSGLYPAPLK**
**0**	**1**	**0.35**	**1.3**	**4.6**	**TKEEVIELSQK**
**0**	**1**	**0.38**	**1.4**	**5.1**	**VIGMHYFSPVDK**
**0**	**2**	**1.1**	**3.1**	**9.1**	**YLDLHGAAPLVK**
**0**	**1**	**1.1**	**3.8**	**13**	**IAEASKRPEK**
**0**	**1**	**0.54**	**1.9**	**6.9**	**KITSFER**

5.71 5 451 minus strand Spectrin beta chain [Crassostrea gigas]

Protein Accession	CL19757.Contig1$_{H}1$
Mean Expression Ratio	0.755
Median Expression Ratio	0.761
Credible Interval	(0.365, 1.5)
Associated Peptides	1
Associated Spectra	1
Coverage	NaN

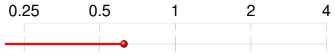

A	B	2.5	50	97.5	Sequence
**0**	**1**	**0.046**	**0.17**	**0.62**	**HSLLEADIHVVGER**

5.72 27 515 heat shock protein 70 [Capsaspora owczarzaki ATCC 30864]; 30 464 minus strand hypothetical protein [Trichinella spiralis]; 27 491 minus strand heat shock protein 70 [Capsaspora owczarzaki ATCC 30864]

Protein Accession	CL14449.Contig4$_H$1 CL14449.Contig2$_H$1 CL14449.Contig1$_H$1
Mean Expression Ratio	0.759
Median Expression Ratio	0.762
Credible Interval	(0.402, 1.42)
Associated Peptides	3
Associated Spectra	7
Coverage	NaN

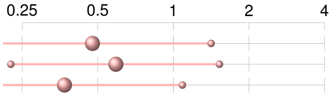

A	B	2.5	50	97.5	Sequence
2	0	0.16	0.48	1.4	FDDPSVQSDMK
2	1	0.23	0.59	1.5	NQVAMNPENTVFDAK
1	1	0.12	0.37	1.1	RFDDPSVQSDMK

5.73 " 19 1167 minus strand ureidopropionase, beta [Xenopus (Silurana) tropicalis] gi"

Protein Accession	CL15113.Contig4$_H$1
Mean Expression Ratio	0.761
Median Expression Ratio	0.762
Credible Interval	(0.407, 1.41)
Associated Peptides	4
Associated Spectra	5
Coverage	NaN

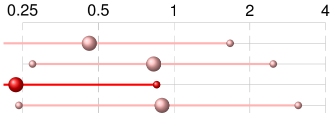

A	B	2.5	50	97.5	Sequence
1	0	0.13	0.46	1.7	AESLALER
1	1	0.27	0.83	2.5	KLAVPER
0	1	0.065	0.24	0.85	YVPLEELGSLK
0	1	0.24	0.89	3.1	MAGQIENIEQIIEK

5.74 507 1277 alpha tubulin [Notothenia coriiceps]

Protein Accession	Unigene56691$_H$1
Mean Expression Ratio	0.761
Median Expression Ratio	0.763
Credible Interval	(0.408, 1.39)
Associated Peptides	2
Associated Spectra	13
Coverage	NaN

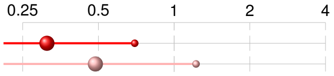

A	B	2.5	50	97.5	Sequence
3	6	0.15	0.31	0.7	LSVDYGK
2	2	0.19	0.49	1.2	LSVDYGKK

5.75 152 1207 haemocyanin precursor [Nautilus pompilius]

Protein Accession	Unigene27031$_H$1
Mean Expression Ratio	1.31
Median Expression Ratio	1.31
Credible Interval	(0.677, 2.6)
Associated Peptides	1
Associated Spectra	5
Coverage	NaN

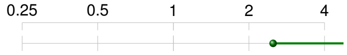

A	B	2.5	50	97.5	Sequence
1	4	2.5	5.6	13	AVDDRLFQK

5.76 209 781 minus strand high mobility group protein 1 [Biomphalaria glabrata]

Protein Accession	CL25076.Contig4$_{H}1$
Mean Expression Ratio	0.763
Median Expression Ratio	0.764
Credible Interval	(0.412, 1.39)
Associated Peptides	3
Associated Spectra	10
Coverage	NaN

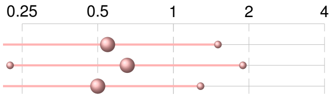

A	B	2.5	50	97.5	Sequence
2	1	0.2	0.55	1.5	KPFEDMAAK
1	2	0.22	0.65	1.9	KYEAEMAEYR
2	2	0.19	0.5	1.3	LAGEEWR

5.77 254 1648 Tektin-3 [Crassostrea gigas]

Protein Accession	CL6717.Contig3$_{H}1$
Mean Expression Ratio	1.31
Median Expression Ratio	1.31
Credible Interval	(0.69, 2.51)
Associated Peptides	3
Associated Spectra	4
Coverage	NaN

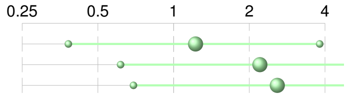

A	B	2.5	50	97.5	Sequence
1	1	0.38	1.2	3.8	AAQHALEIDAK
0	1	0.62	2.2	8.2	ILPYVTDKTISR
0	1	0.69	2.6	9.3	NYFNPATR

5.78 116 1321 PREDICTED: similar to aspartate aminotransferase [Tribolium castaneum] gi

Protein Accession	CL11692.Contig6$_{H}1$
Mean Expression Ratio	0.767
Median Expression Ratio	0.767
Credible Interval	(0.475, 1.24)
Associated Peptides	13
Associated Spectra	25
Coverage	NaN

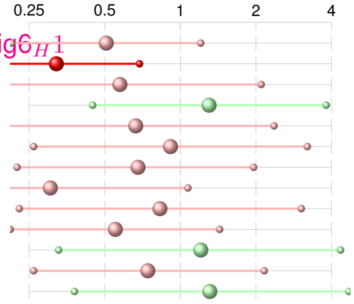

A	B	2.5	50	97.5	Sequence
2	2	0.21	0.51	1.2	ILSTPALR
3	2	0.14	0.32	0.69	ISVAGLSSK
1	0	0.16	0.58	2.1	NMGLYGER
1	1	0.45	1.3	3.8	DAFALR
1	0	0.19	0.67	2.4	TQWLGEVK
1	0	0.26	0.92	3.2	DDNGKPYVLPSVLK
1	1	0.22	0.68	2	DGHQVALAQSFAK
0	1	0.085	0.3	1.1	AEKEIIDAK
0	1	0.23	0.83	3	MDKEYAGITGVPEFTK
0	3	0.21	0.55	1.4	VGAFTIICDNKDEAAR
0	1	0.33	1.2	4.3	NVGYLAK
0	2	0.26	0.74	2.2	IGAAFLAK
0	1	0.38	1.3	4.7	VMSQVK

5.79 2 226 minus strand hypothetical protein CGI_10015501 [Crassostrea gigas]

Protein Accession	Unigene73065$_{H}1$
Mean Expression Ratio	1.31
Median Expression Ratio	1.3
Credible Interval	(0.704, 2.51)
Associated Peptides	2
Associated Spectra	11
Coverage	NaN

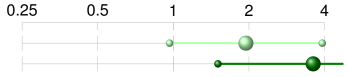

A	B	2.5	50	97.5	Sequence
0	7	0.97	2	3.9	SSVAADALR
0	4	1.5	3.6	8.7	SSVAADALRK

5.80 " 4146 6107 minus strand nonmuscle myosin II, partial [Aplysia californica]"

Protein Accession	Unigene30363$_H1$
Mean Expression Ratio	1.3
Median Expression Ratio	1.3
Credible Interval	(0.908, 1.87)
Associated Peptides	30
Associated Spectra	58
Coverage	NaN

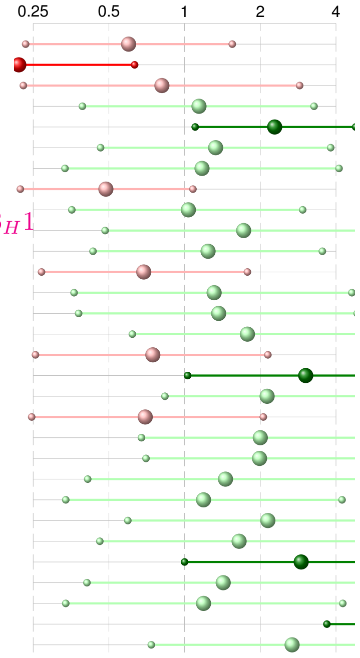

A	B	2.5	50	97.5	Sequence
1	2	0.23	0.6	1.5	ALEATVQEQR
2	0	0.076	0.22	0.63	ATIAALEGK
1	0	0.23	0.81	2.9	DKLAELEGQSR
1	1	0.39	1.1	3.3	LAELEGQSR
3	3	1.1	2.3	4.8	TKATIAALEGK
1	1	0.46	1.3	3.8	ALTLAQR
1	0	0.34	1.2	4.1	LKEDAIK
3	2	0.22	0.49	1.1	NLEAELVK
1	1	0.36	1	2.9	IEQLER
1	0	0.48	1.7	6.1	AALARTNR
0	2	0.43	1.2	3.5	DLEQVSTQLEQVETK
0	3	0.27	0.69	1.8	ELDDQLEQNDVLVR
0	1	0.36	1.3	4.6	EMEAELEEER
0	1	0.38	1.4	4.9	EMEQLHAQLEEAR
0	2	0.62	1.8	5.2	IANLEEQLDQEAKER
0	2	0.26	0.75	2.1	ILNLSHAMEEMQGR
0	2	1	3	8.6	KAQIAMEQMATDLAAER
0	3	0.84	2.1	5.5	LKELQIQAEDERR
0	2	0.25	0.7	2.1	LQEDLAAAER
0	2	0.68	2	5.8	NAEAERDELADEIGSSATSK
0	2	0.7	2	5.9	QVDEAEEEVAR
0	1	0.41	1.5	5	VQIEELEDELQAAEDAK
0	1	0.34	1.2	4.2	VSSLEAQLADSQELMEEETR
0	1	0.59	2.1	7.4	IAAQMKDFQR
0	1	0.46	1.7	5.9	LALEKDNIER
0	2	1	2.9	8.4	SADQYKEQLEK
0	1	0.41	1.4	5.2	QSLLDEK
0	1	0.34	1.2	4.3	RKLQAEVEDLTITLDNQR
0	2	3.7	11	31	SVSSKLENQR
0	1	0.74	2.7	9.3	QKLAVQSK

5.81 243 2165 minus strand Elongation factor 2 OS=Drosophila melanogaster GN=Ef2b PE=2 SV=4

Protein Accession	Unigene11527$_H1$
Mean Expression Ratio	0.769
Median Expression Ratio	0.771
Credible Interval	(0.404, 1.44)
Associated Peptides	3
Associated Spectra	7
Coverage	NaN

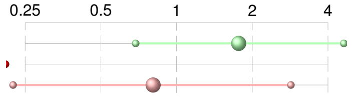

A	B	2.5	50	97.5	Sequence
0	3	0.69	1.8	4.6	ALPLQEGLPEAIDKGDIAPR
0	3	0.031	0.08	0.21	TQEDTLALADK
0	1	0.22	0.8	2.8	FNMEEVK

5.82 369 746 stanniocalcin [Haliotis diversicolor supertexta] gi

Protein Accession	CL10391.Contig1$_H1$
Mean Expression Ratio	0.772
Median Expression Ratio	0.772
Credible Interval	(0.386, 1.52)
Associated Peptides	2
Associated Spectra	2
Coverage	NaN

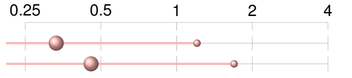

A	B	2.5	50	97.5	Sequence
0	1	0.092	0.33	1.2	VLQLDDLFR
0	1	0.13	0.46	1.7	FARALNNFTSQGQEFLR

5.83 362 553 putative RNA-binding protein [Haliotis diversicolor]

Protein Accession CL41007.Contig2$_{H}$1
Mean Expression Ratio 1.29
Median Expression Ratio 1.3
Credible Interval (0.738, 2.31)
Associated Peptides 5
Associated Spectra 19
Coverage NaN

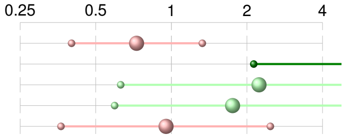

A	B	2.5	50	97.5	Sequence
2	8	0.4	0.73	1.3	EVMHVDSSFVGR
1	2	2.1	5.4	14	VRELQDETGCR
0	1	0.63	2.2	8.1	DGGAKEVMHVDSSFVGR
0	2	0.59	1.7	5.1	DGGRDGGAKEVMHVDSSFVGR
0	3	0.36	0.95	2.5	ELQDETGCR

5.84 227 2920 minus strand Lysosomal alpha-mannosidase [Crassostrea gigas]; 227 3073 minus strand Lysosomal alpha-mannosidase [Crassostrea gigas]; 193 2886 minus strand Lysosomal alpha-mannosidase [Crassostrea gigas]

Protein Accession CL2930.Contig3$_{H}$1 CL2930.Contig2$_{H}$1 CL2930.Contig1$_{H}$1
Mean Expression Ratio 0.777
Median Expression Ratio 0.773
Credible Interval (0.435, 1.4)
Associated Peptides 4
Associated Spectra 18
Coverage NaN

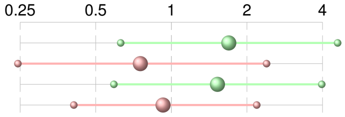

A	B	2.5	50	97.5	Sequence
1	3	0.63	1.7	4.6	ASLTWSGVR
1	1	0.25	0.75	2.4	VVNDALKK
1	4	0.59	1.5	4	IFIRDEAK
2	5	0.41	0.93	2.2	EQASLFAR

5.85 42 434 cytochrome b5 [Haliotis discus discus]; 42 434 cytochrome b5 [Haliotis discus discus]

Protein Accession CL6985.Contig9$_{H}$1 CL6985.Contig5$_{H}$1
Mean Expression Ratio 1.29
Median Expression Ratio 1.29
Credible Interval (0.68, 2.49)
Associated Peptides 3
Associated Spectra 4
Coverage NaN

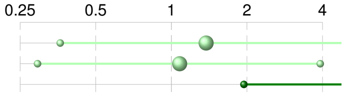

A	B	2.5	50	97.5	Sequence
0	1	0.36	1.4	5.1	KTFETNTGPVGR
0	1	0.29	1.1	3.9	ELMKDYLIGK
0	2	2	5.7	17	VFRSEEVKSHKDAK

5.86 82 1449 hypothetical protein BRAFLDRAFT_127120 [Branchiostoma floridae] gi

Protein Accession CL41883.Contig1$_{H}$1
Mean Expression Ratio 1.29
Median Expression Ratio 1.29
Credible Interval (0.663, 2.56)
Associated Peptides 1
Associated Spectra 3
Coverage NaN

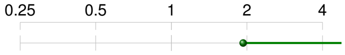

A	B	2.5	50	97.5	Sequence
0	3	1.9	5.1	13	ILQEQEDKFLK

5.87 160 1218 Galectin-9 [Crassostrea gigas]

Protein Accession	Unigene165$_{H}1$
Mean Expression Ratio	0.774
Median Expression Ratio	0.777
Credible Interval	(0.412, 1.43)
Associated Peptides	3
Associated Spectra	9
Coverage	NaN

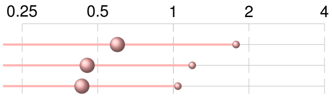

A	B	2.5	50	97.5	Sequence
1	1	0.2	0.6	1.8	EATALVVTGDVR
1	2	0.17	0.45	1.2	IAVNNQHLVEFR
2	2	0.18	0.43	1	SGIPGGLAPGR

5.88 2 532 heat shock protein 70 [Echinostoma caproni]

Protein Accession	Unigene17002$_{H}1$
Mean Expression Ratio	1.28
Median Expression Ratio	1.29
Credible Interval	(0.665, 2.49)
Associated Peptides	2
Associated Spectra	3
Coverage	NaN

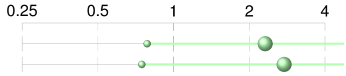

A	B	2.5	50	97.5	Sequence
0	2	0.78	2.3	7	ARFEELNADLFR
0	1	0.75	2.8	10	ARFEELNADLFRGTLEPVEK

5.89 17 703 Dihydropteridine reductase [Crassostrea gigas]

Protein Accession	CL125.Contig5$_{H}1$
Mean Expression Ratio	0.776
Median Expression Ratio	0.778
Credible Interval	(0.405, 1.45)
Associated Peptides	2
Associated Spectra	4
Coverage	NaN

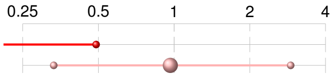

A	B	2.5	50	97.5	Sequence
1	1	0.054	0.16	0.49	VAEALGEEK
0	2	0.33	0.96	2.9	AAVHHLVQSLGAPK

5.90 521 6844 hypothetical protein CGI_10014469 [Crassostrea gigas]

Protein Accession	CL12911.Contig1$_{H}1$
Mean Expression Ratio	0.778
Median Expression Ratio	0.778
Credible Interval	(0.455, 1.32)
Associated Peptides	7
Associated Spectra	29
Coverage	NaN

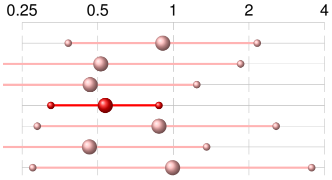

A	B	2.5	50	97.5	Sequence
2	2	0.38	0.9	2.2	VAAVQAFR
1	0	0.14	0.51	1.9	LQTIK
2	1	0.18	0.47	1.2	LTRIQV
4	12	0.33	0.54	0.88	IDAALVLK
0	2	0.29	0.88	2.6	KLFDLNPLK
0	2	0.16	0.46	1.4	LFDLNPLK
0	1	0.28	0.99	3.6	RSPWQLPKDLSLTLK

5.91 4 783 minus strand hemocyanin isoform 2 [Nucula nucleus]

Protein Accession	CL3697.Contig2$_{H}1$
Mean Expression Ratio	0.774
Median Expression Ratio	0.778
Credible Interval	(0.396, 1.48)
Associated Peptides	2
Associated Spectra	3
Coverage	NaN

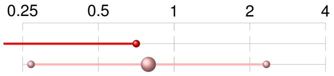

A	B	2.5	50	97.5	Sequence
0	1	0.052	0.19	0.71	DVMDYK
0	2	0.27	0.79	2.3	SGTWLK

5.92 221 1450 minus strand PREDICTED: dihydrolipoamide branched chain transacylase E2 [Taeniopygia guttata]; 221 1609 minus strand PREDICTED: dihydrolipoamide branched chain transacylase E2 [Taeniopygia guttata]

Protein Accession	CL25782.Contig5$_{H}1$ CL25782.Contig3$_{H}1$
Mean Expression Ratio	1.28
Median Expression Ratio	1.28
Credible Interval	(0.644, 2.59)
Associated Peptides	1
Associated Spectra	2
Coverage	NaN

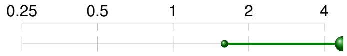

A	B	2.5	50	97.5	Sequence
0	2	1.6	4.7	14	VLATPAVR

5.93 372 860 minus strand small heat shock protein [Tegillarca granosa]

Protein Accession	CL18190.Contig4$_{H}1$
Mean Expression Ratio	0.781
Median Expression Ratio	0.781
Credible Interval	(0.467, 1.31)
Associated Peptides	8
Associated Spectra	25
Coverage	NaN

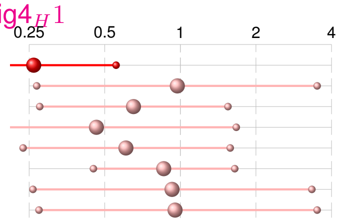

A	B	2.5	50	97.5	Sequence
2	4	0.12	0.26	0.55	IDKDGLSIK
1	0	0.27	0.97	3.5	KSDEYSFVSR
1	3	0.28	0.65	1.5	MNVSHYEPDELSVK
1	0	0.13	0.46	1.7	TFGPASIEQQMK
2	1	0.24	0.61	1.6	TIAIESDK
2	6	0.45	0.86	1.6	LTEDGHLIISAR
0	1	0.26	0.92	3.4	AFDWPAEGHGENAEVVQAK
0	1	0.27	0.96	3.5	EFLIPEEIDAETLGSR

5.94 118 501 minus strand histone 2A.Z [Aplysia californica]; 15 428 PREDICTED: histone H2A.V-like [Apis florea]

Protein Accession	CL14228.Contig6$_{H}1$ CL14228.Contig5$_{H}1$
Mean Expression Ratio	1.28
Median Expression Ratio	1.28
Credible Interval	(0.733, 2.23)
Associated Peptides	5
Associated Spectra	16
Coverage	NaN

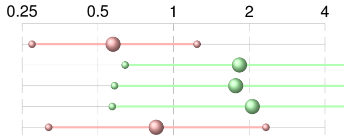

A	B	2.5	50	97.5	Sequence
3	4	0.27	0.57	1.2	ATIAGGGVIPHIHK
0	2	0.64	1.8	5.3	HLQLAIRGDEELDSLIK
1	1	0.58	1.8	5.5	SLIGKK
0	1	0.57	2.1	7.4	GGGVIPHIHK
3	1	0.32	0.85	2.3	GDEELDSLIK

5.95 89 283 hypothetical protein CGI_10017178 [Crassostrea gigas]

Protein Accession Unigene58795$_{H}1$
Mean Expression Ratio 1.28
Median Expression Ratio 1.28
Credible Interval (0.705, 2.36)
Associated Peptides 3
Associated Spectra 20
Coverage NaN

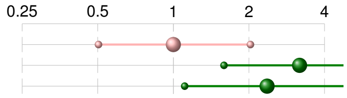

A	B	2.5	50	97.5	Sequence
2	5	0.5	1	2	AGIIASAK
0	7	1.6	3.2	6.4	NMSVIAHVDHGK
0	6	1.1	2.4	4.9	VNFTIDQIR

5.96 401 1174 minus strand Mechanosensory protein 2 [Crassostrea gigas]

Protein Accession Unigene7703$_{H}1$
Mean Expression Ratio 1.28
Median Expression Ratio 1.28
Credible Interval (0.7, 2.36)
Associated Peptides 4
Associated Spectra 9
Coverage NaN

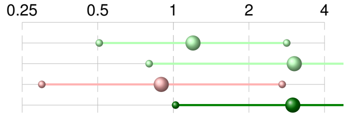

A	B	2.5	50	97.5	Sequence
2	2	0.51	1.2	2.8	VIAAEGEQK
1	0	0.8	3	11	AMAAEAEASREAR
1	1	0.3	0.9	2.7	LPVQLQR
1	1	1	3	9	AKVIAAEGEQK

5.97 16 288 SJCHGC01950 protein [Schistosoma japonicum] gi

Protein Accession Unigene16338$_{H}1$
Mean Expression Ratio 0.78
Median Expression Ratio 0.782
Credible Interval (0.393, 1.51)
Associated Peptides 2
Associated Spectra 2
Coverage NaN

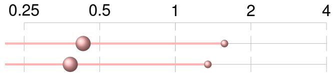

A	B	2.5	50	97.5	Sequence
1	0	0.12	0.43	1.6	YNIEK
0	1	0.11	0.38	1.3	DIAAFIK

5.98 3 272 minus strand ribosomal protein L28 [Haliotis asinina]

Protein Accession CL403.Contig16$_{H}1$
Mean Expression Ratio 0.781
Median Expression Ratio 0.783
Credible Interval (0.408, 1.47)
Associated Peptides 3
Associated Spectra 7
Coverage NaN

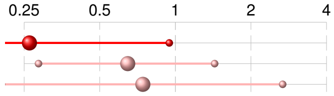

A	B	2.5	50	97.5	Sequence
1	0	0.07	0.26	0.95	GVVLVTK
5	0	0.29	0.65	1.4	TVGVEPSKDGK
1	0	0.2	0.74	2.7	DLGMAAIR

5.99 " 213 1499 minus strand Citrate synthase, mitochondrial [Crassostrea gigas]"

Protein Accession	CL992.Contig2$_{H}1$
Mean Expression Ratio	0.783
Median Expression Ratio	0.783
Credible Interval	(0.48, 1.26)
Associated Peptides	11
Associated Spectra	31
Coverage	NaN

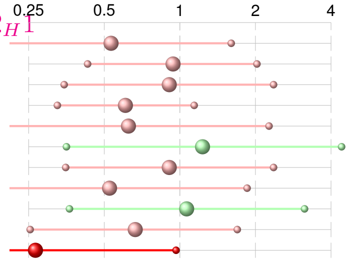

A	B	2.5	50	97.5	Sequence
1	1	0.18	0.53	1.6	ALGLPIERPK
3	2	0.43	0.94	2	SFDTPSLR
1	2	0.35	0.91	2.4	SGQVVPGYGHAVLR
6	3	0.33	0.61	1.1	VVPDILIEQGK
1	0	0.17	0.62	2.3	EVSEFR
1	0	0.35	1.2	4.4	AIIYR
0	3	0.35	0.91	2.4	ALVTETSVLDPEEGIR
0	1	0.15	0.52	1.9	DVLSQIIPEK
0	2	0.36	1.1	3.1	VGEVTVDMMYGGMR
0	3	0.25	0.66	1.7	LISQLYK
0	1	0.075	0.27	0.97	HLPNDPMFK

5.100 462 671 enhancer protein [Homo sapiens]

Protein Accession	CL3160.Contig12$_{H}1$
Mean Expression Ratio	0.782
Median Expression Ratio	0.784
Credible Interval	(0.401, 1.47)
Associated Peptides	2
Associated Spectra	9
Coverage	NaN

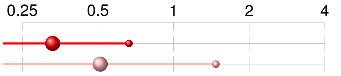

A	B	2.5	50	97.5	Sequence
0	7	0.16	0.33	0.66	GLFIIDDKGNLR
0	2	0.17	0.51	1.5	GLFIIDDK

5.101 75 803 hypothetical protein DAPPUDRAFT_231350 [Daphnia pulex]

Protein Accession	CL10537.Contig4$_{H}1$
Mean Expression Ratio	0.782
Median Expression Ratio	0.784
Credible Interval	(0.426, 1.41)
Associated Peptides	5
Associated Spectra	8
Coverage	NaN

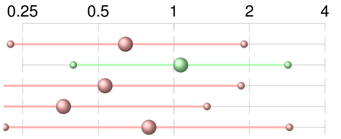

A	B	2.5	50	97.5	Sequence
0	2	0.22	0.64	1.9	NFPHPVMPETSGK
1	2	0.4	1.1	2.9	SITPQQEAESIK
0	1	0.15	0.53	1.9	ENVALQLEASYR
0	1	0.099	0.36	1.4	ATTLLSK
0	1	0.21	0.79	2.9	VCIAQLK

5.102 " 3195 3968 Spectrin alpha chain, putative [Pediculus humanus corporis] gi"

Protein Accession	CL2195.Contig1$_{H}1$
Mean Expression Ratio	0.789
Median Expression Ratio	0.786
Credible Interval	(0.401, 1.53)
Associated Peptides	2
Associated Spectra	2
Coverage	NaN

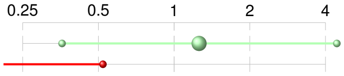

A	B	2.5	50	97.5	Sequence
0	1	0.36	1.3	4.4	LIDEGNHNADGIVER
0	1	0.038	0.14	0.52	LSELEAAASQR

5.103 167 679 tropomyosin [Haliotis discus discus]

Protein Accession CL2925.Contig3$_{H}1$
Mean Expression Ratio 1.27
Median Expression Ratio 1.27
Credible Interval (0.807, 2)
Associated Peptides 13
Associated Spectra 50
Coverage NaN

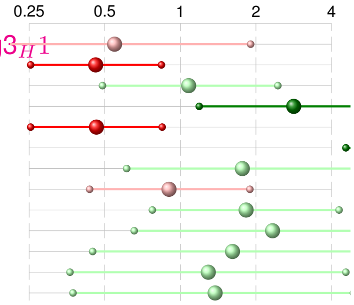

A	B	2.5	50	97.5	Sequence
1	0	0.15	0.55	1.9	ANEGLIEANTK
7	3	0.25	0.46	0.84	LRDTEVQK
1	4	0.49	1.1	2.5	RIKLLEEDLER
1	3	1.2	2.8	6.6	KMLAMK
7	3	0.25	0.46	0.85	MLAMK
2	2	4.6	11	26	MDAIKKK
1	1	0.61	1.8	5.1	MDAIKK
0	6	0.44	0.9	1.9	IKLLEEDLER
0	4	0.78	1.8	4.3	MDKENAMDKAEQMEQK
0	1	0.65	2.3	8.1	YANLENEYDKANEGLIEANTKLEASEK
0	1	0.45	1.6	5.9	AMDKAEQMEQK
0	1	0.36	1.3	4.6	SKLEEDLNPLQK
0	1	0.37	1.4	4.9	MDAIK

5.104 27 620 putative mitochondrial ATP synthase [Haliotis discus discus]

Protein Accession CL13014.Contig1$_{H}1$
Mean Expression Ratio 0.785
Median Expression Ratio 0.789
Credible Interval (0.428, 1.42)
Associated Peptides 2
Associated Spectra 13
Coverage NaN

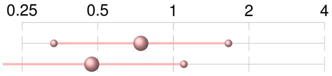

A	B	2.5	50	97.5	Sequence
3	4	0.34	0.74	1.7	LANPALVDQFQK
2	4	0.2	0.47	1.1	SAVDWVAFK

5.105 178 480 minus strand PREDICTED: lipoxygenase homology domain-containing protein 1-like [Danio rerio]

Protein Accession CL41319.Contig2$_{H}1$
Mean Expression Ratio 0.788
Median Expression Ratio 0.789
Credible Interval (0.388, 1.56)
Associated Peptides 1
Associated Spectra 1
Coverage NaN

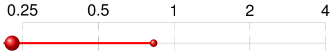

A	B	2.5	50	97.5	Sequence
0	1	0.062	0.23	0.83	VSLKPEVFPLVK

5.106 90 344 PREDICTED: diazepam binding inhibitor-like [Saccoglossus kowalevskii]

Protein Accession	CL28509.Contig1$_H$1
Mean Expression Ratio	0.788
Median Expression Ratio	0.79
Credible Interval	(0.443, 1.41)
Associated Peptides	6
Associated Spectra	8
Coverage	NaN

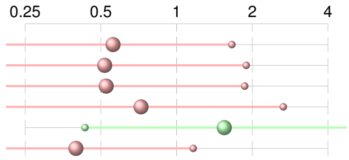

A	B	2.5	50	97.5	Sequence
0	2	0.19	0.56	1.7	ADEAFNQAAEDVK
0	1	0.15	0.52	1.9	WNAWNTLK
0	1	0.15	0.53	1.9	VYGLFK
0	1	0.2	0.72	2.7	KPTDDEMLK
0	1	0.43	1.5	5.9	ADEAFNQAAEDVKK
0	2	0.13	0.4	1.2	AEYVLLVK

5.107 162 785 minus strand Ropporin-1-like protein OS=Xenopus laevis GN=ropn1l PE=2 SV=1

Protein Accession	CL618.Contig4$_H$1
Mean Expression Ratio	0.788
Median Expression Ratio	0.791
Credible Interval	(0.424, 1.48)
Associated Peptides	3
Associated Spectra	6
Coverage	NaN

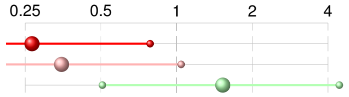

A	B	2.5	50	97.5	Sequence
1	1	0.089	0.27	0.78	EQFDDLVR
0	2	0.12	0.35	1	TVALTVIEEK
0	2	0.51	1.5	4.4	TDTGLTPGLLK

5.108 " 27 974 PREDICTED: succinyl-CoA ligase [GDP-forming] subunit alpha, mitochondrial-like [Oreochromis niloticus]; 27 974 minus strand PREDICTED: succinyl-CoA ligase [GDP-forming] subunit alpha, mitochondrial-like [Oreochromis niloticus]"

Protein Accession	CL10674.Contig2$_H$1 CL10674.Contig1$_H$1
Mean Expression Ratio	1.27
Median Expression Ratio	1.26
Credible Interval	(0.683, 2.4)
Associated Peptides	3
Associated Spectra	5
Coverage	NaN

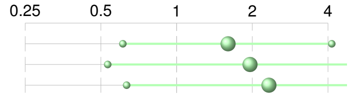

A	B	2.5	50	97.5	Sequence
0	3	0.61	1.6	4.1	MGHAGAIIAGGK
0	1	0.53	2	7.3	IYMATR
0	1	0.63	2.3	8.2	GGAKEKIEILK

5.109 6 650 minus strand glutathione S-transferase mu class protein [Crassostrea ariakensis]; 117 761 glutathione S-transferase mu class protein [Crassostrea ariakensis]; 122 766 glutathione S-transferase mu class protein [Crassostrea ariakensis]; 122 766 glutathione S-transferase mu class protein [Crassostrea ariakensis]

Protein Accession	Unigene56987$_{H}1$ CL6887.Contig3$_{H}1$ CL6887.Contig2$_{H}1$ CL6887.Contig1$_{H}1$
Mean Expression Ratio	0.793
Median Expression Ratio	0.791
Credible Interval	(0.458, 1.35)
Associated Peptides	7
Associated Spectra	21
Coverage	NaN

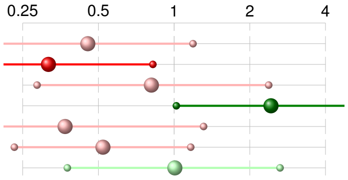

A	B	2.5	50	97.5	Sequence
3	0	0.18	0.45	1.2	GLAQPIR
3	0	0.12	0.32	0.82	ITQSNAILR
1	1	0.28	0.81	2.4	LSQFVK
0	4	1	2.4	5.7	KHDLLGKTDKEK
0	1	0.1	0.37	1.3	IMIPGILDDYPK
0	5	0.23	0.52	1.2	DAWFNVK
0	3	0.38	1	2.6	PALGYWK

5.110 269 1504 Ribosome-binding protein 1 [Crassostrea gigas]; 265 1500 Ribosome-binding protein 1 [Crassostrea gigas]

Protein Accession	CL8282.Contig5$_{H}1$ CL8282.Contig2$_{H}1$
Mean Expression Ratio	1.27
Median Expression Ratio	1.26
Credible Interval	(0.691, 2.39)
Associated Peptides	5
Associated Spectra	5
Coverage	NaN

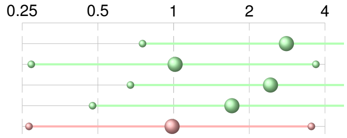

A	B	2.5	50	97.5	Sequence
0	1	0.75	2.8	10	AMEALEKAEK
0	1	0.27	1	3.7	DAIIEVEK
0	1	0.67	2.4	8.6	LKEDKETLEAR
0	1	0.48	1.7	6.3	QFTQLHAENER
0	1	0.27	0.99	3.5	LVQQLQEQLSEK

5.111 " 53 1291 Acetyl-CoA acetyltransferase B, mitochondrial [Crassostrea gigas]"

Protein Accession	CL1322.Contig1$_{H}1$
Mean Expression Ratio	1.26
Median Expression Ratio	1.26
Credible Interval	(0.662, 2.44)
Associated Peptides	2
Associated Spectra	3
Coverage	NaN

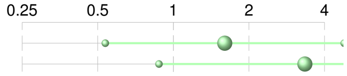

A	B	2.5	50	97.5	Sequence
0	2	0.54	1.6	4.8	TLKPVFVK
0	1	0.88	3.3	12	LGSVAIKEAVSR

5.112 585 944 minus strand Protein disulfide-isomerase A6 [Crassostrea gigas]

Protein Accession	CL39540.Contig7$_H$1
Mean Expression Ratio	0.793
Median Expression Ratio	0.793
Credible Interval	(0.434, 1.46)
Associated Peptides	4
Associated Spectra	8
Coverage	NaN

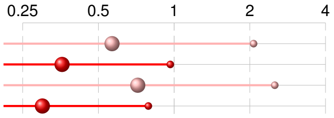

A	B	2.5	50	97.5	Sequence
0	1	0.16	0.57	2.1	AGNPEDVVEVTDSNFQK
1	2	0.13	0.36	0.96	TASDIINWATEK
0	1	0.19	0.72	2.5	NLAPHWAQAATELKGK
0	3	0.12	0.3	0.79	NLAPHWAQAATELK

5.113 9 161 PREDICTED: tubulin alpha-1C chain-like [Acyrthosiphon pisum]; 9 161 Tubulin alpha-3 chain (Fragments) OS=Mesocricetus auratus PE=1 SV=1

Protein Accession	Unigene74551$_H$1 Unigene70816$_H$1
Mean Expression Ratio	1.27
Median Expression Ratio	1.26
Credible Interval	(0.675, 2.41)
Associated Peptides	3
Associated Spectra	4
Coverage	NaN

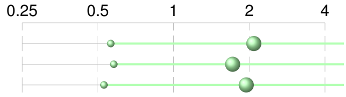

A	B	2.5	50	97.5	Sequence
0	1	0.56	2.1	7.4	IHFPLAT
0	2	0.58	1.7	5.1	IHFPLA
0	1	0.53	1.9	7.3	IHFPLATYAPVISAEK

5.114 304 1830 minus strand Methenyltetrahydrofolate synthetase domain-containing protein [Crassostrea gigas]

Protein Accession	CL45489.Contig2$_H$1
Mean Expression Ratio	1.25
Median Expression Ratio	1.26
Credible Interval	(0.671, 2.33)
Associated Peptides	4
Associated Spectra	6
Coverage	NaN

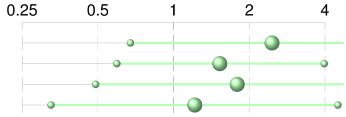

A	B	2.5	50	97.5	Sequence
0	1	0.67	2.5	8.9	KTLLVPTPR
0	3	0.6	1.5	4	FLTLEAK
0	1	0.49	1.8	6.6	TGLFNR
0	1	0.33	1.2	4.5	IPVLR

5.115 70 705 RecName: Full=Ras-related protein Rab-2 gi|288938|emb|CAA51234.1| RAB2 [Lymnaea stagnalis]; 70 705 RecName: Full=Ras-related protein Rab-2 gi|288938|emb|CAA51234.1| RAB2 [Lymnaea stagnalis]

Protein Accession	Unigene59013$_{H}$1 Unigene19254$_{H}$1
Mean Expression Ratio	0.794
Median Expression Ratio	0.796
Credible Interval	(0.411, 1.5)
Associated Peptides	3
Associated Spectra	3
Coverage	NaN

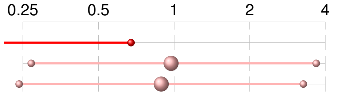

A	B	2.5	50	97.5	Sequence
0	1	0.048	0.18	0.67	TAANVEEAFINTAK
0	1	0.27	0.98	3.7	KEEGEAFAR
0	1	0.24	0.89	3.3	EHGLIFMETSAK

5.116 " 431 880 Myosin essential light chain, striated adductor muscle OS=Aequipecten irradians PE=1 SV=2"

Protein Accession	CL36.Contig2$_{H}$1
Mean Expression Ratio	0.795
Median Expression Ratio	0.797
Credible Interval	(0.433, 1.45)
Associated Peptides	3
Associated Spectra	22
Coverage	NaN

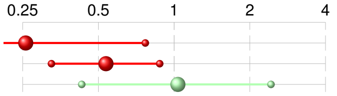

A	B	2.5	50	97.5	Sequence
1	1	0.088	0.26	0.77	LSDEEVDQIMK
8	8	0.33	0.54	0.88	VGDLLR
0	4	0.43	1	2.4	LTDITIDLEGNLK

5.117 252 848 sigma class glutathione-s-transferase 2 [Haliotis discus discus]

Protein Accession	CL19285.Contig21$_{H}$1
Mean Expression Ratio	0.793
Median Expression Ratio	0.798
Credible Interval	(0.415, 1.48)
Associated Peptides	2
Associated Spectra	7
Coverage	NaN

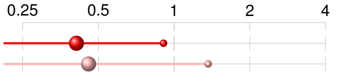

A	B	2.5	50	97.5	Sequence
1	4	0.19	0.41	0.91	LQDVDKDYPEVQK
2	0	0.16	0.46	1.4	LGNELTMA

5.118 " 11 427 PREDICTED: similar to syntaxin, partial [Hydra magnipapillata]"

Protein Accession	CL41814.Contig1$_{H}$1
Mean Expression Ratio	1.26
Median Expression Ratio	1.25
Credible Interval	(0.637, 2.55)
Associated Peptides	1
Associated Spectra	1
Coverage	NaN

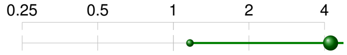

A	B	2.5	50	97.5	Sequence
0	1	1.2	4.2	16	HKEMLALEK

5.119 397 510 PREDICTED: actin-5C-like [Megachile rotundata]

Protein Accession	CL48442.Contig2$_{H}1$
Mean Expression Ratio	0.798
Median Expression Ratio	0.798
Credible Interval	(0.402, 1.59)
Associated Peptides	1
Associated Spectra	4
Coverage	NaN

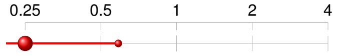

A	B	2.5	50	97.5	Sequence
**0**	**4**	**0.1**	**0.25**	**0.59**	**DLTDYLMK**

5.120 3 263 calponin-like protein [Taenia asiatica]

Protein Accession	CL26318.Contig4$_{H}1$
Mean Expression Ratio	1.26
Median Expression Ratio	1.25
Credible Interval	(0.639, 2.54)
Associated Peptides	1
Associated Spectra	2
Coverage	NaN

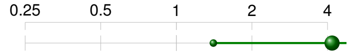

A	B	2.5	50	97.5	Sequence
**0**	**2**	**1.4**	**4.2**	**12**	**HIADIKVDDMSAASK**

5.121 35 370 Ganglioside GM2 activator [Crassostrea gigas]; 2 487 minus strand PREDICTED: hypothetical protein LOC100699750 [Oreochromis niloticus]; 41 535 minus strand PREDICTED: hypothetical protein LOC100699750 [Oreochromis niloticus]

Protein Accession	CL7266.Contig6$_{H}1$ CL7266.Contig5$_{H}1$ CL7266.Contig3$_{H}1$
Mean Expression Ratio	0.799
Median Expression Ratio	0.8
Credible Interval	(0.406, 1.55)
Associated Peptides	1
Associated Spectra	4
Coverage	NaN

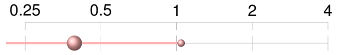

A	B	2.5	50	97.5	Sequence
**2**	**2**	**0.15**	**0.39**	**1**	**NVALVVSVK**

5.122 27 377 minus strand ribosomal protein LP2 [Papilio xuthus] gi; 27 377 minus strand ribosomal protein LP2 [Papilio xuthus] gi; 27 377 minus strand ribosomal protein LP2 [Papilio xuthus] gi; 1538 1891 minus strand ribosomal protein LP2 [Papilio xuthus] gi

Protein Accession	Unigene53017$_{H}1$ Unigene53016$_{H}1$ Unigene53015$_{H}1$ CL49012.Contig9$_{H}1$
Mean Expression Ratio	0.797
Median Expression Ratio	0.8
Credible Interval	(0.468, 1.33)
Associated Peptides	7
Associated Spectra	28
Coverage	NaN

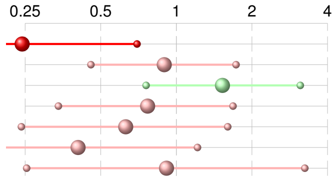

A	B	2.5	50	97.5	Sequence
1	1	0.082	0.24	0.7	ILSSVGIEAEADK
3	5	0.46	0.89	1.7	KVVSQLSGK
2	5	0.76	1.5	3.1	SGKEPTAADLEK
1	4	0.34	0.77	1.7	VVSQLSGK
0	3	0.24	0.63	1.6	ILSSVGIEAEADKVK
0	2	0.14	0.41	1.2	SIDQLIAEGSR
0	1	0.25	0.91	3.2	SVGIEAEADKVK

5.123 727 2022 RecName: Full=Enolase; AltName: Full=2-phospho-D-glycerate hydro-lyase; AltName: Full=2-phosphoglycerate dehydratase gi|1911573|gb|AAB50731.1| enolase [Doryteuthis pealeii]; 690 1985 RecName: Full=Enolase; AltName: Full=2-phospho-D-glycerate hydro-lyase; AltName: Full=2-phosphoglycerate dehydratase gi|1911573|gb|AAB50731.1| enolase [Doryteuthis pealeii]; 213 1508 RecName: Full=Enolase; AltName: Full=2-phospho-D-glycerate hydro-lyase; AltName: Full=2-phosphoglycerate dehydratase gi|1911573|gb|AAB50731.1| enolase [Doryteuthis pealeii]; 246 1541 RecName: Full=Enolase; AltName: Full=2-phospho-D-glycerate hydro-lyase; AltName: Full=2-phosphoglycerate dehydratase gi|1911573|gb|AAB50731.1| enolase [Doryteuthis pealeii]

Protein Accession	CL15687.Contig6$_H$1 CL15687.Contig5$_H$1 CL15687.Contig3$_H$1 CL15687.Contig2$_H$1
Mean Expression Ratio	0.802
Median Expression Ratio	0.8
Credible Interval	(0.558, 1.17)
Associated Peptides	24
Associated Spectra	97
Coverage	NaN

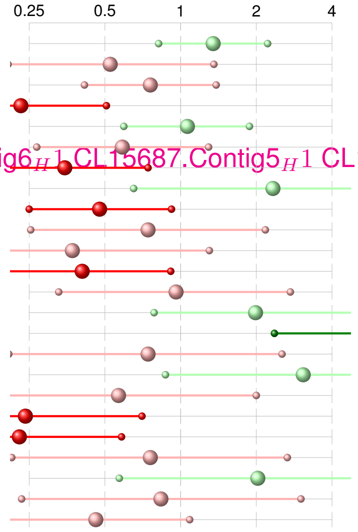

A	B	2.5	50	97.5	Sequence
4	11	0.82	1.3	2.2	AAGWGVMVSHR
1	2	0.21	0.53	1.4	FQIVGDDLLVTNPK
5	5	0.41	0.76	1.4	IAPAFVGK
1	4	0.1	0.23	0.51	IMLDLDGTPNKEK
8	3	0.59	1.1	1.9	QIFDSR
3	2	0.27	0.59	1.3	VQMGIDR
3	3	0.17	0.35	0.74	GVPLYR
1	0	0.65	2.3	8.1	VIKSIK
4	4	0.25	0.48	0.92	YNQILR
1	1	0.25	0.74	2.2	VAIEK
1	0	0.1	0.37	1.3	AYLGK
2	3	0.18	0.41	0.91	TLDLADQTAMDK
0	2	0.33	0.96	2.7	AAVPSGASTGIYEALELR
0	3	0.78	2	5.1	AAVPSGASTGIYEALELRDNDPK
0	5	2.4	5.2	12	AVSNINDKIAPAFVGK
0	1	0.21	0.74	2.5	HIADLCGNK
0	1	0.87	3.1	11	LAKYNQILR
0	1	0.16	0.57	2	SYPIVTIEDPFDQDDWDAYTK
0	2	0.083	0.24	0.7	TSQQMIDMYHDFVK
0	3	0.089	0.23	0.58	VNQIGSVSESIQAFK
0	1	0.21	0.76	2.7	DGKYDLDFK
0	1	0.57	2	7.2	NINDKIAPAFVGK
0	1	0.23	0.84	3	MGSETYHHLK
0	4	0.2	0.46	1.1	IEEELGVDAFYAGK

5.124 1 1284 minus strand Thioredoxin reductase 3 [Crassostrea gigas]

Protein Accession	CL3500.Contig1$_H$1
Mean Expression Ratio	1.26
Median Expression Ratio	1.25
Credible Interval	(0.652, 2.45)
Associated Peptides	2
Associated Spectra	4
Coverage	NaN

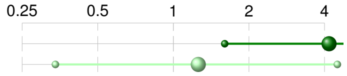

A	B	2.5	50	97.5	Sequence
0	3	1.6	4.2	11	GAVPSQIEQLEEGKPGR
0	1	0.34	1.3	4.5	LLVQRL

5.125 " 347 847 minus strand ADP,ATP carrier protein 1 OS=Anopheles gambiae GN=AGAP006782 PE=2 SV=2"

Protein Accession	CL27196.Contig1$_{H}$1
Mean Expression Ratio	0.799
Median Expression Ratio	0.801
Credible Interval	(0.399, 1.58)
Associated Peptides	1
Associated Spectra	1
Coverage	NaN

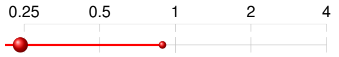

A	B	2.5	50	97.5	Sequence
1	0	0.065	0.24	0.89	SNPGESYAVK

5.126 " 158 355 Protein NAS-37, isoform b [Caenorhabditis elegans] gi; 15 227 Hemicentin-1 [Crassostrea gigas]"

Protein Accession	Unigene57180$_{H}$1 CL8016.Contig2$_{H}$1
Mean Expression Ratio	1.26
Median Expression Ratio	1.25
Credible Interval	(0.633, 2.54)
Associated Peptides	1
Associated Spectra	1
Coverage	NaN

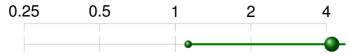

A	B	2.5	50	97.5	Sequence
0	1	1.1	4.2	15	FKGSYGGVSR

5.127 613 2421 PREDICTED: lysosomal alpha-glucosidase [Danio rerio]

Protein Accession	CL5792.Contig1$_{H}$1
Mean Expression Ratio	1.25
Median Expression Ratio	1.25
Credible Interval	(0.715, 2.2)
Associated Peptides	5
Associated Spectra	17
Coverage	NaN

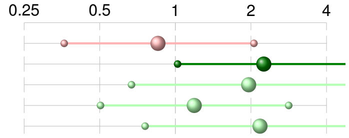

A	B	2.5	50	97.5	Sequence
2	2	0.36	0.85	2.1	ATMGGLQK
1	4	1	2.3	5	LPPATTTNIMR
2	0	0.67	2	5.8	GGSIIPR
0	4	0.5	1.2	2.8	GLPDIVDDLHSHNQR
0	2	0.76	2.2	6.6	SFVLSR

5.128 366 935 minus strand 40S ribosomal protein S2 OS=Urechis caupo GN=RPS2 PE=2 SV=1

Protein Accession	CL2084.Contig7$_{H}$1
Mean Expression Ratio	0.802
Median Expression Ratio	0.803
Credible Interval	(0.444, 1.43)
Associated Peptides	5
Associated Spectra	8
Coverage	NaN

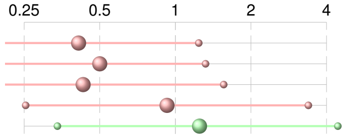

A	B	2.5	50	97.5	Sequence
2	0	0.14	0.41	1.2	GTGIVSAPVPK
2	1	0.19	0.5	1.3	LSVIPVR
1	0	0.12	0.43	1.6	EFTDYLMK
1	0	0.25	0.93	3.4	LIPAPR
0	1	0.34	1.2	4.4	GATATLGNFAK

5.129 " 42 905 PREDICTED: enoyl-CoA hydratase, mitochondrial-like isoform 1 [Oreochromis niloticus]"

Protein Accession CL6477.Contig7$_{H}$1
Mean Expression Ratio 0.803
Median Expression Ratio 0.803
Credible Interval (0.438, 1.48)
Associated Peptides 4
Associated Spectra 11
Coverage NaN

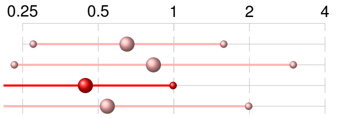

A	B	2.5	50	97.5	Sequence
2	2	0.27	0.65	1.6	AFAAGADIK
1	0	0.23	0.83	3	LLDEAVK
4	1	0.2	0.44	0.99	SVVLQASR
0	1	0.16	0.54	2	AMEMVLTGER

5.130 " 189 2099 Hsp70, putative [Ixodes scapularis] gi"

Protein Accession CL16113.Contig2$_{H}$1
Mean Expression Ratio 1.25
Median Expression Ratio 1.24
Credible Interval (0.68, 2.31)
Associated Peptides 4
Associated Spectra 7
Coverage NaN

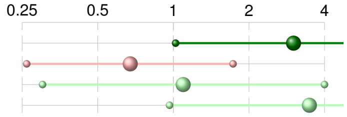

A	B	2.5	50	97.5	Sequence
1	1	1	3	9	LVGMPAKR
1	2	0.26	0.67	1.7	ALQDAEVK
0	1	0.3	1.1	4	ASNGDAWVEAQGK
0	1	0.96	3.5	13	RFEDPEVQKDMK

5.131 1271 1663 minus strand PREDICTED: similar to AGAP007393-PB [Tribolium castaneum] gi

Protein Accession CL28563.Contig3$_{H}$1
Mean Expression Ratio 1.25
Median Expression Ratio 1.24
Credible Interval (0.639, 2.48)
Associated Peptides 1
Associated Spectra 1
Coverage NaN

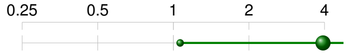

A	B	2.5	50	97.5	Sequence
0	1	1.1	3.9	15	KYEGGREVDEFITYLAR

5.132 16 1560 V-type proton ATPase subunit B [Crassostrea gigas]

Protein Accession CL1031.Contig1$_{H}$1
Mean Expression Ratio 1.24
Median Expression Ratio 1.24
Credible Interval (0.707, 2.17)
Associated Peptides 7
Associated Spectra 9
Coverage NaN

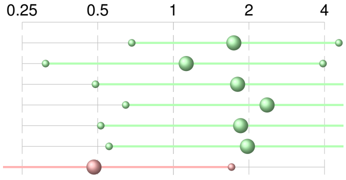

A	B	2.5	50	97.5	Sequence
1	2	0.68	1.7	4.6	AVVQVFEGTSGIDAK
0	1	0.31	1.1	3.9	FAEIVNLTLADGSK
0	1	0.49	1.8	6.5	HTTCEFTGDILR
0	1	0.65	2.4	8.5	MPTSEDMLGR
0	1	0.51	1.9	6.9	NFISQGHYENR
0	1	0.55	2	7.2	SLNGPLNDAAAR
0	1	0.13	0.48	1.7	ATIYERAGR

5.133 192 1820 PREDICTED: copine IV-like [Saccoglossus kowalevskii]; 125 1753 PREDICTED: copine IV-like [Saccoglossus kowalevskii]

Protein Accession	CL15074.Contig3$_H1$ CL15074.Contig1$_H1$
Mean Expression Ratio	0.801
Median Expression Ratio	0.805
Credible Interval	(0.41, 1.53)
Associated Peptides	2
Associated Spectra	3
Coverage	NaN

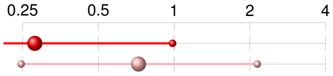

A	B	2.5	50	97.5	Sequence
1	0	0.075	0.28	0.99	LLNAK
0	2	0.25	0.72	2.1	HVLAEVPK

5.134 1363 1527 Protein disulfide-isomerase A3 [Crassostrea gigas]

Protein Accession	CL28563.Contig1$_H1$
Mean Expression Ratio	1.25
Median Expression Ratio	1.24
Credible Interval	(0.666, 2.36)
Associated Peptides	3
Associated Spectra	11
Coverage	NaN

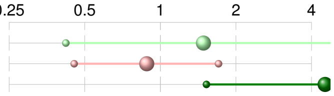

A	B	2.5	50	97.5	Sequence
1	0	0.42	1.5	5.5	FEELAEK
3	5	0.45	0.88	1.7	LKDEPEITIAK
0	2	1.5	4.5	14	ALEPKFEELAEK

5.135 131 541 minus strand nervous system adducin [Aplysia californica] gi

Protein Accession	CL27337.Contig1$_H1$
Mean Expression Ratio	1.25
Median Expression Ratio	1.24
Credible Interval	(0.645, 2.43)
Associated Peptides	2
Associated Spectra	3
Coverage	NaN

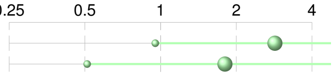

A	B	2.5	50	97.5	Sequence
1	1	0.95	2.9	8.4	GIGASFGR
0	1	0.51	1.8	6.6	FIDTIDPDDPEYIR

5.136 26 1909 Dolichyl-diphosphooligosaccharide–protein glycosyltransferase subunit 2 [Crassostrea gigas]

Protein Accession	CL6515.Contig1$_H$1
Mean Expression Ratio	0.804
Median Expression Ratio	0.805
Credible Interval	(0.496, 1.29)
Associated Peptides	13
Associated Spectra	26
Coverage	NaN

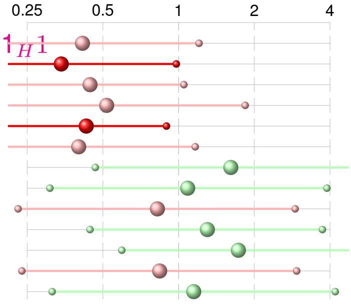

A	B	2.5	50	97.5	Sequence
2	0	0.14	0.41	1.2	APAIAEDK
1	1	0.12	0.34	0.98	LIGTSGAEVEVK
2	2	0.19	0.44	1	NLGLAVDEK
1	0	0.15	0.52	1.8	KPEIK
1	5	0.2	0.43	0.89	LLTDAISAK
0	2	0.14	0.4	1.2	HLVDEAIVLSK
0	1	0.47	1.6	6	HLVDEAIVLSKK
0	1	0.31	1.1	3.9	KQFTASASDK
0	1	0.23	0.82	2.9	TAYQFVSVIK
0	2	0.44	1.3	3.7	VSVENVEIGVADKDQTAAAR
0	2	0.6	1.7	5.1	LTISVAPK
0	1	0.24	0.84	3	TLYELNFMEAK
0	1	0.31	1.1	4.2	LTPSTFLTTLDQQR

5.137 459 755 minus strand heat shock protein beta-1 [Trichinella spiralis] gi; 464 763 minus strand heat shock protein beta-1 [Trichinella spiralis] gi; 437 736 minus strand heat shock protein beta-1 [Trichinella spiralis] gi; 437 736 minus strand heat shock protein beta-1 [Trichinella spiralis] gi; 437 736 minus strand heat shock protein beta-1 [Trichinella spiralis] gi

Protein Accession	CL17234.Contig5$_H$1 CL17234.Contig4$_H$1 CL17234.Contig3$_H$1 CL17234.Contig2$_H$1 CL1723
Mean Expression Ratio	1.24
Median Expression Ratio	1.24
Credible Interval	(0.66, 2.32)
Associated Peptides	2
Associated Spectra	13
Coverage	NaN

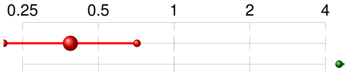

A	B	2.5	50	97.5	Sequence
0	10	0.21	0.39	0.71	DVEPTTVTSSLNADGVLTLK
0	3	4.5	12	31	TVTSSLNADGVLTLKAPK

5.138 95 2278 hypothetical protein CGI_10017740 [Crassostrea gigas]

Protein Accession	CL48637.Contig1$_H$1
Mean Expression Ratio	0.801
Median Expression Ratio	0.806
Credible Interval	(0.416, 1.52)
Associated Peptides	3
Associated Spectra	3
Coverage	NaN

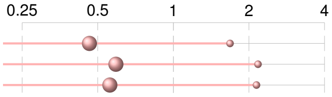

A	B	2.5	50	97.5	Sequence
1	0	0.13	0.46	1.7	VAMYR
0	1	0.16	0.59	2.2	SISYALR
0	1	0.16	0.56	2.1	AGLELVPK

5.139 531 665 minus strand Proteasome subunit beta type-6 [Crassostrea gigas]

Protein Accession	CL12961.Contig2$_H1$
Mean Expression Ratio	0.806
Median Expression Ratio	0.807
Credible Interval	(0.403, 1.6)
Associated Peptides	1
Associated Spectra	2
Coverage	NaN

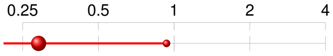

A	B	2.5	50	97.5	Sequence
**1**	**1**	**0.087**	**0.29**	**0.93**	**FTLTGDDLPK**

5.140 1027 1440 minus strand hemocyanin isoform 1 [Haliotis diversicolor]

Protein Accession	CL40281.Contig1$_H1$
Mean Expression Ratio	0.804
Median Expression Ratio	0.808
Credible Interval	(0.411, 1.58)
Associated Peptides	1
Associated Spectra	5
Coverage	NaN

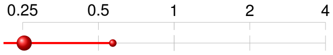

A	B	2.5	50	97.5	Sequence
**0**	**5**	**0.11**	**0.25**	**0.57**	**LFVVQVEDELK**

5.141 " 110 1717 PREDICTED: glutamate dehydrogenase, mitochondrial-like [Oreochromis niloticus]; 110 1717 PREDICTED: glutamate dehydrogenase, mitochondrial-like [Oreochromis niloticus]; 110 1717 PREDICTED: glutamate dehydrogenase, mitochondrial-like [Oreochromis niloticus]"

Protein Accession	CL16377.Contig9$_H1$ CL16377.Contig8$_H1$ CL16377.Contig10$_H1$
Mean Expression Ratio	1.24
Median Expression Ratio	1.24
Credible Interval	(0.726, 2.09)
Associated Peptides	10
Associated Spectra	12
Coverage	NaN

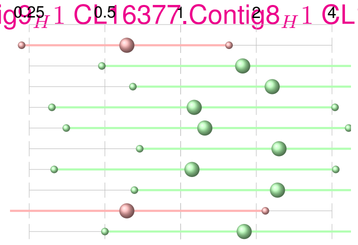

A	B	2.5	50	97.5	Sequence
**1**	**2**	**0.23**	**0.61**	**1.6**	**DGSIYNPVDGIHPR**
**1**	**0**	**0.49**	**1.8**	**6.5**	**AANIIEK**
**0**	**1**	**0.64**	**2.3**	**8.1**	**ACVTGKPIPQGGIHGR**
**0**	**1**	**0.31**	**1.1**	**4.1**	**CAIVDVPFGGAK**
**0**	**1**	**0.35**	**1.3**	**4.7**	**CDILVPAAGER**
**0**	**1**	**0.69**	**2.5**	**8.7**	**IAGASEKDIVHSGLEYTMER**
**0**	**1**	**0.31**	**1.1**	**4.1**	**IIAEGANGPTTAGADEIFLK**
**0**	**1**	**0.66**	**2.4**	**8.5**	**LTFKYEK**
**0**	**1**	**0.17**	**0.61**	**2.2**	**EAGLTFT**
**0**	**1**	**0.5**	**1.8**	**6.5**	**DSNFHLLQSVQR**

5.142 150 473 40S ribosomal protein S16 OS=Heteropneustes fossilis GN=rps16 PE=2 SV=1

Protein Accession	CL14629.Contig4$_H$1
Mean Expression Ratio	0.81
Median Expression Ratio	0.81
Credible Interval	(0.46, 1.44)
Associated Peptides	5
Associated Spectra	14
Coverage	NaN

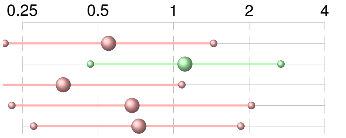

A	B	2.5	50	97.5	Sequence
2	1	0.21	0.55	1.4	SLVAYYQK
2	2	0.47	1.1	2.7	AVQVFGR
2	0	0.12	0.36	1.1	ILRAK
1	1	0.23	0.68	2	AKLEEPVR
0	3	0.28	0.73	1.8	VNGRPLDQVEPQILR

5.143 100 2307 minus strand Xanthine dehydrogenase/oxidase [Crassostrea gigas]

Protein Accession	Unigene10743$_H$1
Mean Expression Ratio	0.806
Median Expression Ratio	0.812
Credible Interval	(0.416, 1.54)
Associated Peptides	2
Associated Spectra	3
Coverage	NaN

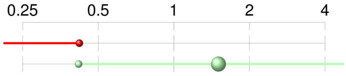

A	B	2.5	50	97.5	Sequence
1	1	0.046	0.14	0.42	VLSTDIVMDVGK
0	1	0.42	1.5	5.5	TLNIPQNK

5.144 135 2630 Filamin-A [Crassostrea gigas]; 118 2613 Filamin-A [Crassostrea gigas]; 157 2652 Filamin-A [Crassostrea gigas]

Protein Accession	CL393.Contig15$_H$1 CL393.Contig13$_H$1 CL393.Contig12$_H$1
Mean Expression Ratio	1.23
Median Expression Ratio	1.23
Credible Interval	(0.757, 2.04)
Associated Peptides	13
Associated Spectra	19
Coverage	NaN

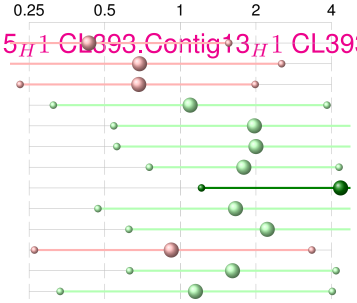

A	B	2.5	50	97.5	Sequence
1	0	0.12	0.43	1.6	ENGVHYVDVK
1	0	0.19	0.69	2.5	VQVGGPGLEK
1	1	0.23	0.68	2	VINFQIETK
1	0	0.31	1.1	3.8	VYGVRY
0	1	0.54	2	7.1	AQIFLEERPNGFLGVQYK
0	1	0.56	2	7.4	IGDSELGHAAGCK
0	4	0.75	1.8	4.3	MNGIHTLSVR
0	1	1.2	4.4	16	SKGEYKVNVTR
0	1	0.47	1.7	5.9	YGNIPIAGSPYK
0	1	0.63	2.2	7.9	YIPKENGVHYVDVK
0	1	0.26	0.92	3.3	EAGPGTLTVAVEGPGK
0	3	0.63	1.6	4.2	IMFTPK
0	1	0.33	1.1	4	YKGNPIK

5.145 111 1226 gelsolin [Aplysia californica] gi; 42 1139 minus strand gelsolin [Aplysia californica] gi

Protein Accession	Unigene39723$_{H}$1 Unigene32782$_{H}$1
Mean Expression Ratio	1.23
Median Expression Ratio	1.23
Credible Interval	(0.706, 2.14)
Associated Peptides	9
Associated Spectra	11
Coverage	NaN

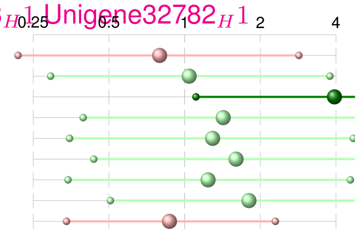

A	B	2.5	50	97.5	Sequence
0	1	0.22	0.79	2.8	AMQFIGEIK
0	1	0.29	1	3.8	DLVTSDDVYILDTGLK
0	1	1.1	4	14	SKAMQFIGEIK
0	1	0.4	1.4	5.1	TVELDTKLNDKPIQHR
0	1	0.35	1.3	4.7	QFDTALAA
0	1	0.43	1.6	5.8	KNGLPLAHNYLR
0	1	0.34	1.2	4.5	EVQGHESDLFK
0	1	0.51	1.8	6.5	ESGNPWRPVTVIK
0	3	0.34	0.87	2.3	SDDVFIFDSGK

5.146 104 283 minus strand RecName: Full=Hemocyanin type 2 unit a; AltName: Full=Hemocyanin RHSS2 subunit; AltName: Full=Hemocyanin Rta; AltName: Full=Hemocyanin heavy structural subunit; AltName: Full=RtH2-a gi|7515001|pir||JC5632 hemocyanin dioxygen-binding functional unit - grosse

Protein Accession	Unigene21935$_{H}$1
Mean Expression Ratio	1.24
Median Expression Ratio	1.23
Credible Interval	(0.646, 2.4)
Associated Peptides	2
Associated Spectra	4
Coverage	NaN

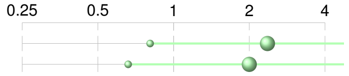

A	B	2.5	50	97.5	Sequence
0	2	0.81	2.4	6.8	KNVASLSDDEVLTLQSVLR
0	2	0.66	2	6.1	NVASLSDDEVLTLQSVLR

5.147 108 401 60S ribosomal L7a-like protein [Phragmatopoma lapidosa]; 108 401 60S ribosomal L7a-like protein [Phragmatopoma lapidosa]; 108 431 60S ribosomal L7a-like protein [Phragmatopoma lapidosa]

Protein Accession	CL1313.Contig6$_{H}$1 CL1313.Contig5$_{H}$1 CL1313.Contig1$_{H}$1
Mean Expression Ratio	0.811
Median Expression Ratio	0.814
Credible Interval	(0.42, 1.54)
Associated Peptides	2
Associated Spectra	3
Coverage	NaN

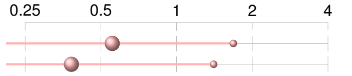

A	B	2.5	50	97.5	Sequence
2	0	0.18	0.55	1.7	QTATQIFR
1	0	0.1	0.38	1.4	SGVNTVTALVESK

5.148 3 491 hemocyanin [Haliotis tuberculata]

Protein Accession	CL40281.Contig3$_{H}1$
Mean Expression Ratio	0.814
Median Expression Ratio	0.815
Credible Interval	(0.451, 1.47)
Associated Peptides	4
Associated Spectra	14
Coverage	NaN

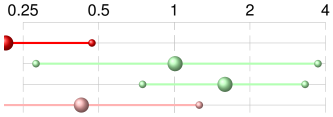

A	B	2.5	50	97.5	Sequence
2	3	0.096	0.21	0.47	DINPFALTK
0	1	0.28	1	3.7	GKPFTSAQCSVELFSK
0	6	0.75	1.6	3.3	TLEPFDRDINPFALTK
0	2	0.14	0.43	1.3	TLEPFDR

5.149 957 2147 haemocyanin [Aplysia californica]

Protein Accession	Unigene30320$_{H}1$
Mean Expression Ratio	0.815
Median Expression Ratio	0.816
Credible Interval	(0.459, 1.43)
Associated Peptides	5
Associated Spectra	20
Coverage	NaN

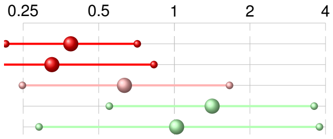

A	B	2.5	50	97.5	Sequence
5	5	0.21	0.39	0.71	LLTVQFER
1	2	0.12	0.33	0.83	YEVSELSLEQVSNLR
0	3	0.25	0.63	1.7	GAQVGVPYWDWTRPGK
0	3	0.55	1.4	3.6	TFHISGVNQDITR
0	1	0.29	1	3.8	EKGAQVGVPYWDWTRPGK

5.150 45 407 minus strand 60S ribosomal protein L31 OS=Xenopus laevis GN=rpl31 PE=2 SV=1

Protein Accession	CL4728.Contig1$_{H}1$
Mean Expression Ratio	1.22
Median Expression Ratio	1.22
Credible Interval	(0.628, 2.36)
Associated Peptides	2
Associated Spectra	4
Coverage	NaN

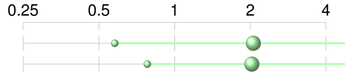

A	B	2.5	50	97.5	Sequence
0	1	0.58	2.1	7.6	IHGVSFKK
0	3	0.78	2	5.4	IHGVSFK

5.151 148 462 hypothetical protein DAPPUDRAFT_306954 [Daphnia pulex]; 148 462 hypothetical protein DAPPUDRAFT_306954 [Daphnia pulex]; 148 462 hypothetical protein DAPPUDRAFT_306954 [Daphnia pulex]; 148 462 hypothetical protein DAPPUDRAFT_306954 [Daphnia pulex]; 173 487 hypothetical protein DAPPUDRAFT_306954 [Daphnia pulex]

Protein Accession	CL48682.Contig7$_{H}1$ CL48682.Contig6$_{H}1$ CL48682.Contig5$_{H}1$ CL48682.Contig4$_{H}1$ CL4868
Mean Expression Ratio	0.818
Median Expression Ratio	0.818
Credible Interval	(0.431, 1.54)
Associated Peptides	3
Associated Spectra	4
Coverage	NaN

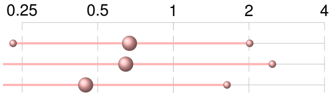

A	B	2.5	50	97.5	Sequence
1	1	0.23	0.67	2	ATNFDHFK
1	0	0.18	0.64	2.5	GAHDGGVIFDPAVQR
1	0	0.12	0.45	1.6	AGEVPYKDR

5.152 188 1021 hypothetical protein BRAFLDRAFT_214346 [Branchiostoma floridae] gi

Protein Accession	CL38498.Contig2$_{H}1$
Mean Expression Ratio	1.22
Median Expression Ratio	1.22
Credible Interval	(0.62, 2.39)
Associated Peptides	2
Associated Spectra	2
Coverage	NaN

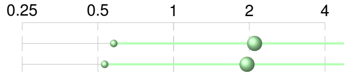

A	B	2.5	50	97.5	Sequence
0	1	0.58	2.1	7.8	AANGGAYPPDLSYIVQAR
0	1	0.53	2	7	KIAFK

5.153 96 581 minus strand thioredoxin 2 [Haliotis discus discus]; 1682 2167 minus strand thioredoxin 2 [Haliotis discus discus]; 191 676 minus strand thioredoxin 2 [Haliotis discus discus]

Protein Accession	CL10014.Contig7$_{H}1$ CL10014.Contig3$_{H}1$ CL10014.Contig1$_{H}1$
Mean Expression Ratio	0.814
Median Expression Ratio	0.819
Credible Interval	(0.403, 1.62)
Associated Peptides	1
Associated Spectra	1
Coverage	NaN

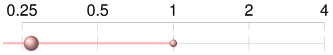

A	B	2.5	50	97.5	Sequence
0	1	0.076	0.27	1	LETIVSGEK

5.154 280 726 minus strand RecName: Full=Calmodulin; Short=CaM gi|14588595|dbj|BAB61794.1| calmodulin [Metridium senile] gi

Protein Accession	Unigene4486$_H$1
Mean Expression Ratio	0.816
Median Expression Ratio	0.82
Credible Interval	(0.421, 1.55)
Associated Peptides	1
Associated Spectra	6
Coverage	NaN

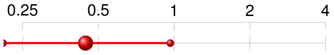

A	B	2.5	50	97.5	Sequence
5	1	0.21	0.44	0.97	ELGTVMR

5.155 " 65 1576 minus strand Aldehyde dehydrogenase, mitochondrial [Crassostrea gigas]"

Protein Accession	CL5835.Contig1$_H$1
Mean Expression Ratio	0.821
Median Expression Ratio	0.82
Credible Interval	(0.514, 1.32)
Associated Peptides	13
Associated Spectra	31
Coverage	NaN

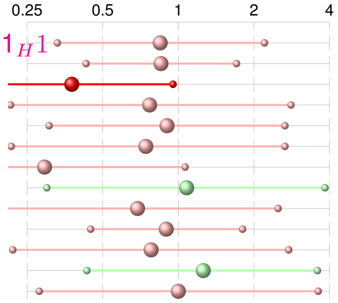

A	B	2.5	50	97.5	Sequence
1	2	0.33	0.85	2.2	SAAALPKPLTSPEIK
4	3	0.43	0.85	1.7	TFPTVDPR
1	2	0.15	0.38	0.95	LADLLER
1	0	0.21	0.77	2.8	TVTFR
1	1	0.31	0.9	2.7	FKDVK
1	0	0.22	0.74	2.7	LGSPWR
1	0	0.083	0.29	1.1	FVDSVSGK
0	1	0.3	1.1	3.8	IYNEFVER
0	1	0.18	0.69	2.5	LAEQTPLSGLYVAK
0	7	0.45	0.89	1.8	LIPVMAAK
0	1	0.22	0.78	2.7	TLHIGGDHFAYTR
0	2	0.43	1.3	3.6	IFVEEK
0	1	0.28	1	3.6	LADLLERDR

5.156 78 560 Sorting nexin-12 [Camponotus floridanus]; 78 578 Sorting nexin-12 [Camponotus floridanus]; 132 605 Sorting nexin-12 [Camponotus floridanus]; 276 623 Sorting nexin-12 [Camponotus floridanus]; 276 629 Sorting nexin-12 [Camponotus floridanus]

Protein Accession	CL2450.Contig5$_H$1 CL2450.Contig4$_H$1 CL2450.Contig3$_H$1 CL2450.Contig2$_H$1 CL2450.Con
Mean Expression Ratio	1.22
Median Expression Ratio	1.22
Credible Interval	(0.63, 2.37)
Associated Peptides	2
Associated Spectra	2
Coverage	NaN

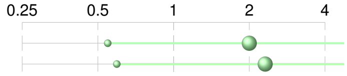

A	B	2.5	50	97.5	Sequence
0	1	0.55	2	7.3	MKTNLPVFK
0	1	0.6	2.3	8.2	IVVPPLPGK

5.157 8 937 minus strand galectin [Crassostrea virginica]

Protein Accession	CL7245.Contig6$_{H}1$
Mean Expression Ratio	1.22
Median Expression Ratio	1.22
Credible Interval	(0.661, 2.25)
Associated Peptides	3
Associated Spectra	12
Coverage	NaN

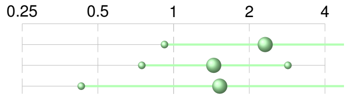

A	B	2.5	50	97.5	Sequence
1	2	0.92	2.3	6.2	LGGEWGPEQR
2	6	0.75	1.4	2.8	QDQGDVVLNAK
0	1	0.43	1.5	5.7	QGDNIGLHFNPR

5.158 14 1642 minus strand chaperonin containing tcp1 [Haliotis discus discus]; 15 1619 chaperonin containing tcp1 [Haliotis discus discus]; 15 1619 chaperonin containing tcp1 [Haliotis discus discus]; 14 1462 minus strand chaperonin containing tcp1 [Haliotis discus discus]; 14 1582 minus strand chaperonin containing tcp1 [Haliotis discus discus]

Protein Accession	CL16298.Contig4$_{H}1$ CL16298.Contig2$_{H}1$ CL16298.Contig1$_{H}1$ CL16298.Contig15$_{H}1$ CL162
Mean Expression Ratio	0.819
Median Expression Ratio	0.821
Credible Interval	(0.417, 1.57)
Associated Peptides	2
Associated Spectra	3
Coverage	NaN

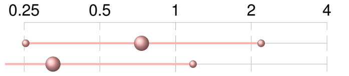

A	B	2.5	50	97.5	Sequence
2	0	0.25	0.73	2.2	QVEGDITK
1	0	0.088	0.32	1.2	NAEELMSFSK

5.159 448 1626 matrilin [Biomphalaria glabrata]

Protein Accession	CL7510.Contig3$_{H}1$
Mean Expression Ratio	1.22
Median Expression Ratio	1.22
Credible Interval	(0.63, 2.36)
Associated Peptides	2
Associated Spectra	5
Coverage	NaN

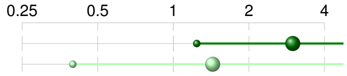

A	B	2.5	50	97.5	Sequence
3	1	1.2	3	7	VSAIKYGR
0	1	0.4	1.4	5.2	VSNTELLNIAGDQER

5.160 871 1371 ribosomal protein L11 [Triatoma infestans]; 343 843 minus strand ribosomal protein L11 [Triatoma infestans]

Protein Accession	CL20979.Contig2$_{H}1$ CL20979.Contig1$_{H}1$
Mean Expression Ratio	0.824
Median Expression Ratio	0.822
Credible Interval	(0.426, 1.61)
Associated Peptides	2
Associated Spectra	3
Coverage	NaN

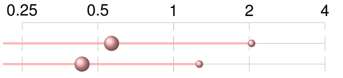

A	B	2.5	50	97.5	Sequence
1	0	0.15	0.57	2	ITKEEAIK
2	0	0.14	0.43	1.3	AEEILER

5.161 95 2506 Transitional endoplasmic reticulum ATPase [Crassostrea gigas]

Protein Accession	CL15973.Contig2$_{H}1$
Mean Expression Ratio	0.824
Median Expression Ratio	0.824
Credible Interval	(0.518, 1.32)
Associated Peptides	16
Associated Spectra	23
Coverage	NaN

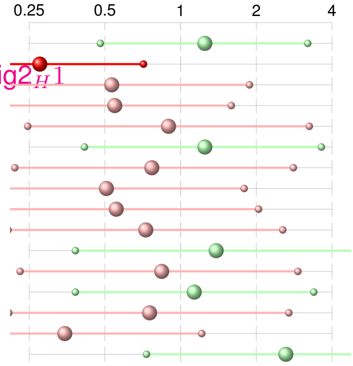

A	B	2.5	50	97.5	Sequence
1	2	0.48	1.2	3.2	AHFEEAMK
2	1	0.11	0.27	0.71	DIDLDYLAK
1	0	0.15	0.53	1.9	EMVELPLR
1	1	0.19	0.55	1.6	MAIEAEIR
1	0	0.25	0.9	3.2	AIGVKPPR
1	1	0.41	1.2	3.6	HPQLFK
1	0	0.22	0.77	2.8	GFGGNFR
1	0	0.14	0.51	1.8	LQQIR
0	1	0.15	0.56	2	AEVQNEDDLATAILK
0	1	0.21	0.73	2.6	GILLYGPPGTGK
0	1	0.38	1.4	4.9	KYEMFAQTLQQSR
0	1	0.23	0.84	2.9	MDELQLFR
0	2	0.38	1.1	3.4	KGDIFLVR
0	1	0.21	0.75	2.7	KAFEEAEK
0	1	0.097	0.35	1.2	EVDIGIPDATGR
0	1	0.73	2.6	9.2	GGNVGDGGGAADR

5.162 160 588 hypothetical protein CGI_10001640 [Crassostrea gigas]

Protein Accession	CL2347.Contig8$_{H}1$
Mean Expression Ratio	0.821
Median Expression Ratio	0.825
Credible Interval	(0.449, 1.46)
Associated Peptides	2
Associated Spectra	14
Coverage	NaN

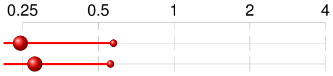

A	B	2.5	50	97.5	Sequence
3	2	0.1	0.24	0.57	AFLEQLGSK
5	4	0.14	0.28	0.56	ANLIVIGSR

5.163 176 670 hypothetical protein BRAFLDRAFT_122621 [Branchiostoma floridae] gi

Protein Accession	Unigene180$_{H}1$
Mean Expression Ratio	0.823
Median Expression Ratio	0.825
Credible Interval	(0.468, 1.41)
Associated Peptides	5
Associated Spectra	34
Coverage	NaN

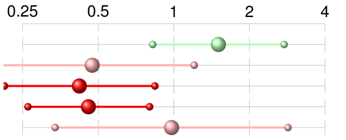

A	B	2.5	50	97.5	Sequence
3	7	0.82	1.5	2.8	VFFDITADGQPLGR
1	2	0.18	0.47	1.2	ADVVPK
2	5	0.21	0.42	0.84	FADENFLLK
6	6	0.26	0.46	0.8	IVMELR
0	2	0.34	0.98	2.9	HVVFGQVVEGMDVVR

5.164 " 28 1104 minus strand Pyruvate dehydrogenase E1 component subunit beta, mitochondrial [Crassostrea gigas]; 1087 1581 Pyruvate dehydrogenase E1 component subunit beta, mitochondrial [Crassostrea gigas]"

Protein Accession	CL18015.Contig14$_{H}1$ CL18015.Contig13$_{H}1$
Mean Expression Ratio	1.21
Median Expression Ratio	1.21
Credible Interval	(0.627, 2.35)
Associated Peptides	2
Associated Spectra	4
Coverage	NaN

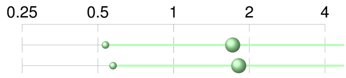

A	B	2.5	50	97.5	Sequence
1	1	0.54	1.7	5.4	SLEEAALPQPVNVIR
1	1	0.57	1.8	5.8	VTGADVPMPYAK

5.165 124 390 PREDICTED: 60S ribosomal protein L23a-like [Callithrix jacchus]

Protein Accession	CL27563.Contig5$_{H}1$
Mean Expression Ratio	1.21
Median Expression Ratio	1.21
Credible Interval	(0.653, 2.31)
Associated Peptides	3
Associated Spectra	5
Coverage	NaN

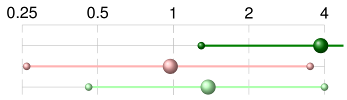

A	B	2.5	50	97.5	Sequence
0	2	1.3	3.9	11	AAPKPAVK
0	1	0.26	0.97	3.5	LDAFAIVK
0	2	0.46	1.4	4	VNRLDAFAIVK

5.166 3512 6661 hypothetical protein BRAFLDRAFT_255626 [Branchiostoma floridae] gi

Protein Accession	CL1959.Contig8$_{H}1$
Mean Expression Ratio	1.21
Median Expression Ratio	1.21
Credible Interval	(0.717, 2.07)
Associated Peptides	10
Associated Spectra	12
Coverage	NaN

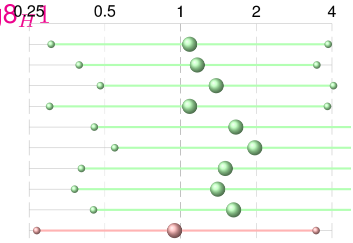

A	B	2.5	50	97.5	Sequence
1	0	0.31	1.1	3.9	SGVKIDVSDAEAQAR
1	1	0.39	1.2	3.5	LDEAFLSK
0	2	0.48	1.4	4.1	FDRPAQLHMAFR
0	1	0.3	1.1	3.8	IDVSDAEAQAR
0	1	0.45	1.7	5.9	NVVLGGVK
0	1	0.55	2	7.2	VKAALPSPEGLK
0	1	0.4	1.5	5.3	QLYVLGHEAMR
0	1	0.38	1.4	5	SVTIHDLGK
0	1	0.45	1.6	5.7	GGNVVQVKMPK
0	1	0.27	0.95	3.5	FIVAETNGLFGK

5.167 2 292 minus strand H2 [Haliotis tuberculata]

Protein Accession	Unigene10170$_{H}1$
Mean Expression Ratio	0.828
Median Expression Ratio	0.828
Credible Interval	(0.435, 1.59)
Associated Peptides	2
Associated Spectra	5
Coverage	NaN

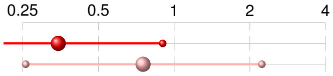

A	B	2.5	50	97.5	Sequence
1	2	0.13	0.35	0.9	NLESLSEGEVESLK
0	2	0.26	0.76	2.2	RNLESLSEGEVESLK

5.168 119 481 Glutamate receptor U1 [Crassostrea gigas]; 113 328 glutamate receptor subunit protein GluR2 precursor [Aplysia californica] gi; 122 328 PREDICTED: glutamate receptor 3 [Ornithorhynchus anatinus]

Protein Accession	CL570.Contig5$_{H}1$ CL570.Contig4$_{H}1$ CL570.Contig2$_{H}1$
Mean Expression Ratio	0.825
Median Expression Ratio	0.828
Credible Interval	(0.41, 1.61)
Associated Peptides	1
Associated Spectra	2
Coverage	NaN

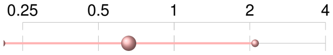

A	B	2.5	50	97.5	Sequence
1	1	0.21	0.66	2.1	GTANIGAGALTVTSER

5.169 633 806 Peroxiredoxin-1 [Crassostrea gigas]; 648 821 Peroxiredoxin-1 [Crassostrea gigas]; 666 839 Peroxiredoxin-1 [Crassostrea gigas]; 625 798 Peroxiredoxin-1 [Crassostrea gigas]; 633 824 Peroxiredoxin-1 [Crassostrea gigas]

Protein Accession	CL3160.Contig18$_{H}1$ CL3160.Contig17$_{H}1$ CL3160.Contig16$_{H}1$ CL3160.Contig15$_{H}1$ CL3160
Mean Expression Ratio	0.829
Median Expression Ratio	0.828
Credible Interval	(0.42, 1.62)
Associated Peptides	1
Associated Spectra	5
Coverage	NaN

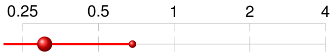

A	B	2.5	50	97.5	Sequence
0	5	0.14	0.31	0.68	LVQAFQFTDK

5.170 4 234 ribosomal protein rpl14 [Arenicola marina]

Protein Accession	CL2715.Contig2$_{H}1$
Mean Expression Ratio	1.21
Median Expression Ratio	1.21
Credible Interval	(0.611, 2.37)
Associated Peptides	1
Associated Spectra	2
Coverage	NaN

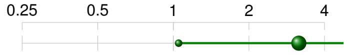

A	B	2.5	50	97.5	Sequence
0	2	1	3.2	9.5	ALHLTQFVIK

5.171 124 747 ribosomal protein S10 [Crassostrea gigas] gi

Protein Accession	CL18986.Contig1$_{H}1$
Mean Expression Ratio	0.827
Median Expression Ratio	0.829
Credible Interval	(0.42, 1.61)
Associated Peptides	2
Associated Spectra	2
Coverage	NaN

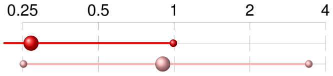

A	B	2.5	50	97.5	Sequence
1	0	0.073	0.27	1	ALTSLK
1	0	0.25	0.9	3.4	MLIPK

5.172 811 1407 minus strand mitochondrial malate dehydrogenase 2 [Eospalax baileyi]

Protein Accession	CL10426.Contig13$_{H}1$
Mean Expression Ratio	0.832
Median Expression Ratio	0.829
Credible Interval	(0.448, 1.53)
Associated Peptides	3
Associated Spectra	16
Coverage	NaN

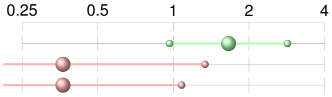

A	B	2.5	50	97.5	Sequence
6	7	0.97	1.7	2.9	IQNAGTEVVEAK
1	0	0.099	0.36	1.3	NLGMGK
0	2	0.13	0.36	1.1	AAMPELIANISK

5.173 37 885 glyceraldehyde 3-phosphate dehydrogenase [Dugesia japonica]

Protein Accession	CL47424.Contig2$_{H}$1
Mean Expression Ratio	1.2
Median Expression Ratio	1.21
Credible Interval	(0.621, 2.34)
Associated Peptides	1
Associated Spectra	17
Coverage	NaN

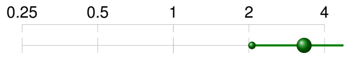

A	B	2.5	50	97.5	Sequence
5	12	2.1	3.3	5.3	GAAQNIIPASTGAAK

5.174 333 1799 Alba-like protein C9orf23-like protein [Crassostrea gigas]

Protein Accession	Unigene30413$_{H}$1
Mean Expression Ratio	1.2
Median Expression Ratio	1.2
Credible Interval	(0.657, 2.21)
Associated Peptides	5
Associated Spectra	7
Coverage	NaN

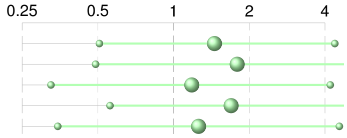

A	B	2.5	50	97.5	Sequence
0	2	0.51	1.5	4.4	AASWLGMR
0	1	0.49	1.8	6.4	GKGALELAQR
0	1	0.33	1.2	4.2	SPLYVDEGNILMDVR
0	2	0.56	1.7	5	TINPATEAETQGK
0	1	0.35	1.3	4.6	EIMTELSR

5.175 2473 2898 RecName: Full=Paramyosin gi|397603|emb|CAA79849.1| paramyosin [Echinococcus granulosus]

Protein Accession	CL43455.Contig1$_{H}$1
Mean Expression Ratio	1.21
Median Expression Ratio	1.2
Credible Interval	(0.779, 1.87)
Associated Peptides	12
Associated Spectra	64
Coverage	NaN

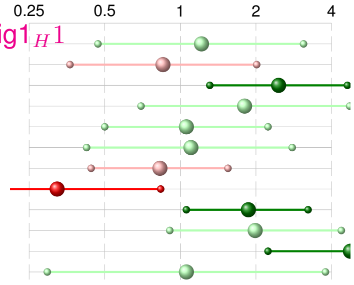

A	B	2.5	50	97.5	Sequence
2	1	0.47	1.2	3.1	EILAQTEEERR
2	2	0.36	0.85	2	INKEILAQTEEER
1	8	1.3	2.5	4.6	INKEILAQTEEERR
1	2	0.7	1.8	4.7	KQLEIEIR
3	3	0.5	1.1	2.2	QLEIEIR
3	0	0.42	1.1	2.8	AQTLIEEAENR
1	8	0.44	0.83	1.5	QLEEAEDVANPTMNK
0	3	0.13	0.32	0.83	EILAQTEEER
0	12	1.1	1.9	3.2	KAQTLIEEAENR
0	5	0.91	2	4.4	QLAEFASLNEQLNLR
0	6	2.2	4.7	10	QLEEAEDVANPTMNKYR
0	1	0.3	1.1	3.8	RQLAEFASLNEQLNLR

5.176 49 819 PREDICTED: elongation factor 1-delta-like isoform 2 [Strongylocentrotus purpuratus]

Protein Accession	CL10790.Contig12$_{H}$1
Mean Expression Ratio	0.827
Median Expression Ratio	0.831
Credible Interval	(0.44, 1.55)
Associated Peptides	3
Associated Spectra	7
Coverage	NaN

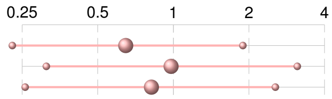

A	B	2.5	50	97.5	Sequence
1	2	0.23	0.65	1.9	VAALEVVGTGGASPK
1	1	0.31	0.98	3.1	KPVVIAK
1	1	0.26	0.82	2.5	LLEEYAAK

5.177 129 299 glycogen phosphorylase [Crassostrea gigas]

Protein Accession	Unigene20640$_{H}1$
Mean Expression Ratio	1.21
Median Expression Ratio	1.2
Credible Interval	(0.601, 2.47)
Associated Peptides	1
Associated Spectra	1
Coverage	NaN

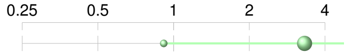

A	B	2.5	50	97.5	Sequence
0	1	0.92	3.3	12	GIAPVENVGTIKK

5.178 813 1244 Probable protein disulfide-isomerase A6 OS=Caenorhabditis elegans GN=tag-320 PE=2 SV=1

Protein Accession	CL39540.Contig4$_{H}1$
Mean Expression Ratio	0.833
Median Expression Ratio	0.832
Credible Interval	(0.417, 1.63)
Associated Peptides	1
Associated Spectra	3
Coverage	NaN

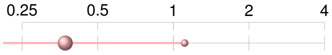

A	B	2.5	50	97.5	Sequence
1	2	0.13	0.37	1.1	TASDIINWATEK

5.179 62 1093 Heterogeneous nuclear ribonucleoprotein A2-like protein 1 [Crassostrea gigas]

Protein Accession	CL47083.Contig4$_{H}1$
Mean Expression Ratio	1.21
Median Expression Ratio	1.2
Credible Interval	(0.71, 2.11)
Associated Peptides	8
Associated Spectra	13
Coverage	NaN

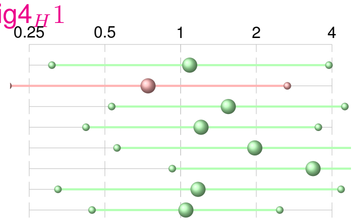

A	B	2.5	50	97.5	Sequence
1	0	0.31	1.1	3.9	GGGRDYGGGR
0	1	0.21	0.74	2.7	ETFSPFGTISQIDLITDK
0	2	0.53	1.6	4.5	HELNGRQVEVK
0	2	0.42	1.2	3.5	MFVGGIKEDTTEDMIR
0	1	0.56	2	7.2	KLFIGGLSYETDENSLR
0	1	0.92	3.4	12	KAMSK
0	1	0.33	1.2	4.3	MPGYR
0	4	0.44	1.1	2.5	GFGFITYK

5.180 " 133 1077 PREDICTED: electron transfer flavoprotein, alpha polypeptide-like isoform 2 [Saccoglossus kowalevskii]"

Protein Accession	CL6589.Contig3$_{H}1$
Mean Expression Ratio	1.2
Median Expression Ratio	1.2
Credible Interval	(0.635, 2.31)
Associated Peptides	3
Associated Spectra	4
Coverage	NaN

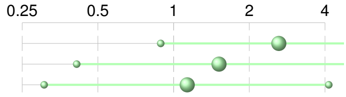

A	B	2.5	50	97.5	Sequence
0	2	0.89	2.6	7.9	AAVDAGFVSNDLQIGQTGK
0	1	0.41	1.5	5.6	SDRPELASANIVIAGGR
0	1	0.31	1.1	4.1	GTAFEAAK

5.181 3 500 Tumor protein D54 [Crassostrea gigas]; 3 464 Tumor protein D54 [Crassostrea gigas]; 3 464 Tumor protein D54 [Crassostrea gigas]; 3 500 Tumor protein D54 [Crassostrea gigas]; 6 440 Tumor protein D54 [Crassostrea gigas]; 3 464 Tumor protein D54 [Crassostrea gigas]; 3 500 Tumor protein D54 [Crassostrea gigas]

Protein Accession	CL25616.Contig8$_H$1 CL25616.Contig6$_H$1 CL25616.Contig5$_H$1 CL25616.Contig4$_H$1 CL2561
Mean Expression Ratio	0.833
Median Expression Ratio	0.835
Credible Interval	(0.419, 1.61)
Associated Peptides	2
Associated Spectra	2
Coverage	NaN

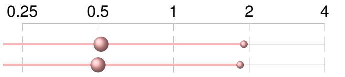

A	B	2.5	50	97.5	Sequence
1	0	0.14	0.52	1.9	STDDIDTAFK
1	0	0.13	0.5	1.8	NLGITPFK

5.182 7825 8754 minus strand H2 [Haliotis tuberculata]

Protein Accession	CL7906.Contig3$_H$1
Mean Expression Ratio	0.836
Median Expression Ratio	0.835
Credible Interval	(0.426, 1.64)
Associated Peptides	2
Associated Spectra	2
Coverage	NaN

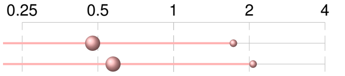

A	B	2.5	50	97.5	Sequence
1	0	0.13	0.48	1.7	ISSTGQYITR
0	1	0.16	0.57	2.1	ALPSLFTDPYDSNPFK

5.183 77 1561 retinal dehydrogenase [Lymnaea stagnalis]; 69 1553 retinal dehydrogenase [Lymnaea stagnalis]

Protein Accession	CL13284.Contig7$_H$1 CL13284.Contig2$_H$1
Mean Expression Ratio	1.2
Median Expression Ratio	1.2
Credible Interval	(0.79, 1.8)
Associated Peptides	18
Associated Spectra	47
Coverage	NaN

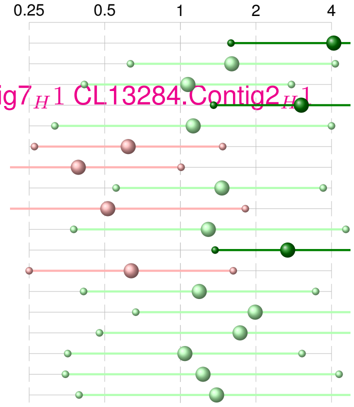

A	B	2.5	50	97.5	Sequence
2	1	1.6	4.1	11	NVVIKIPQKNS
1	2	0.63	1.6	4.1	SFPEPIRNPEVK
1	2	0.42	1.1	2.8	TFPTINPATGK
3	2	1.4	3	6.7	TFPTINPATGKK
1	0	0.32	1.1	4	TAELANKR
2	2	0.26	0.62	1.5	AVEVAQDAFK
1	2	0.15	0.39	1	IYDEFVR
1	2	0.56	1.5	3.7	LGSAWR
1	0	0.14	0.51	1.8	NVVIK
1	0	0.38	1.3	4.6	GALLYK
1	7	1.4	2.7	5.2	ADVDKAVEVAQDAFK
0	3	0.25	0.64	1.6	ELGEYGLQQYVEVK
0	2	0.41	1.2	3.5	FSTVEEAIER
0	2	0.67	2	5.8	KTAELANKR
0	1	0.48	1.7	6.1	NVVIKIPQK
0	2	0.36	1	3.1	KTAELANK
0	1	0.35	1.2	4.3	RGALLYK
0	1	0.39	1.4	5	TTLELGGK

5.184 408 1010 glutathione S-transferase sigma 2 [Mytilus galloprovincialis]; 166 768 glutathione S-transferase sigma 2 [Mytilus galloprovincialis]

Protein Accession	CL22038.Contig5$_{H}$1 CL22038.Contig4$_{H}$1
Mean Expression Ratio	0.836
Median Expression Ratio	0.836
Credible Interval	(0.481, 1.42)
Associated Peptides	7
Associated Spectra	16
Coverage	NaN

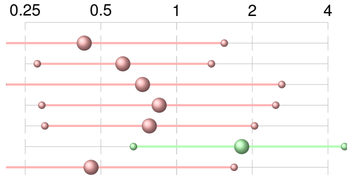

A	B	2.5	50	97.5	Sequence
1	0	0.12	0.43	1.5	LVLEAAGR
2	3	0.28	0.61	1.4	VFSEATPK
1	0	0.2	0.73	2.6	KAELYEK
2	0	0.29	0.85	2.5	ELGLYGK
0	3	0.3	0.78	2	LVYFDLR
0	3	0.67	1.8	4.7	STVESHPFLK
0	1	0.12	0.46	1.7	YLGFFQK

5.185 64 393 Tubulin beta chain OS=Paracentrotus lividus PE=2 SV=1; 109 438 Tubulin beta chain OS=Paracentrotus lividus PE=2 SV=1; 160 489 Tubulin beta chain OS=Paracentrotus lividus PE=2 SV=1

Protein Accession	Unigene62663$_{H}$1 Unigene62661$_{H}$1 Unigene62660$_{H}$1
Mean Expression Ratio	1.2
Median Expression Ratio	1.19
Credible Interval	(0.633, 2.28)
Associated Peptides	2
Associated Spectra	14
Coverage	NaN

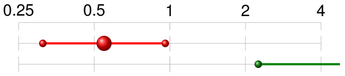

A	B	2.5	50	97.5	Sequence
5	7	0.31	0.55	0.96	INVYFNEATGGK
0	2	2.3	6.6	20	INVYFNEATGGKYVPR

5.186 " 303 500 minus strand protein lethal, putative [Pediculus humanus corporis] gi"

Protein Accession	CL15038.Contig1$_{H}$1
Mean Expression Ratio	1.2
Median Expression Ratio	1.19
Credible Interval	(0.662, 2.17)
Associated Peptides	3
Associated Spectra	27
Coverage	NaN

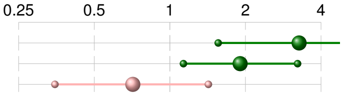

A	B	2.5	50	97.5	Sequence
3	3	1.6	3.3	7	VPSRQDMLATGR
4	10	1.1	1.9	3.2	QMPMLGR
3	4	0.35	0.71	1.4	QDMLATGR

5.187 736 1287 60S ribosomal protein L3 OS=Rattus norvegicus GN=Rpl3 PE=1 SV=3

Protein Accession	Unigene22848$_{H}1$
Mean Expression Ratio	0.836
Median Expression Ratio	0.839
Credible Interval	(0.435, 1.58)
Associated Peptides	3
Associated Spectra	4
Coverage	NaN

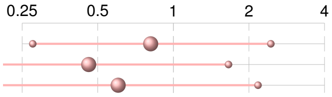

A	B	2.5	50	97.5	Sequence
1	1	0.28	0.81	2.4	VQFTVAR
1	0	0.13	0.46	1.7	EIETDLNK
1	0	0.16	0.6	2.2	IGAGIHTK

5.188 1 1350 UPF0663 transmembrane protein C17orf28 [Crassostrea gigas]; 163 1542 UPF0663 transmembrane protein C17orf28 [Crassostrea gigas]; 135 1514 UPF0663 transmembrane protein C17orf28 [Crassostrea gigas]

Protein Accession	CL21276.Contig5$_{H}1$ CL21276.Contig4$_{H}1$ CL21276.Contig1$_{H}1$
Mean Expression Ratio	0.839
Median Expression Ratio	0.839
Credible Interval	(0.444, 1.62)
Associated Peptides	3
Associated Spectra	4
Coverage	NaN

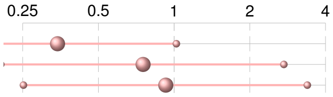

A	B	2.5	50	97.5	Sequence
2	0	0.12	0.34	1	EGFQSLLNTK
1	0	0.21	0.75	2.7	GVFLIEPKPK
1	0	0.25	0.92	3.4	KIGFK

5.189 187 696 minus strand putative ribosomal protein S10 [Sipunculus nudus]

Protein Accession	Unigene1233$_{H}1$
Mean Expression Ratio	1.2
Median Expression Ratio	1.19
Credible Interval	(0.671, 2.16)
Associated Peptides	5
Associated Spectra	11
Coverage	NaN

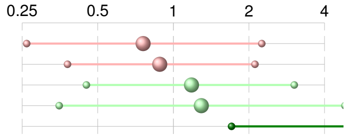

A	B	2.5	50	97.5	Sequence
0	2	0.26	0.76	2.2	ADVGAGATESFQFR
0	4	0.38	0.89	2.1	HQEIDVPNLHVIK
0	3	0.45	1.2	3	KDFFSPK
0	1	0.35	1.3	4.8	ALTSIK
0	1	1.7	6	22	KAGGPPGGDKKADVGAGATESFQFR

5.190 28 642 minus strand Transmembrane emp24 domain-containing protein 9 [Crassostrea gigas]; 236 709 minus strand Transmembrane emp24 domain-containing protein 9 OS=Rattus norvegicus GN=Tmed9 PE=1 SV=1; 239 742 minus strand Transmembrane emp24 domain-containing protein eca OS=Drosophila erecta GN=eca PE=3 SV=1; 236 709 minus strand Transmembrane emp24 domain-containing protein 9 OS=Rattus norvegicus GN=Tmed9 PE=1 SV=1; 236 709 minus strand Transmembrane emp24 domain-containing protein 9 OS=Rattus norvegicus GN=Tmed9 PE=1 SV=1; 236 709 minus strand Transmembrane emp24 domain-containing protein 9 OS=Rattus norvegicus GN=Tmed9 PE=1 SV=1; 44 658 minus strand Transmembrane emp24 domain-containing protein 9 [Crassostrea gigas]

Protein Accession	CL2503.Contig8$_{H}1$ CL2503.Contig7$_{H}1$ CL2503.Contig5$_{H}1$ CL2503.Contig4$_{H}1$ CL2503.Con
Mean Expression Ratio	0.839
Median Expression Ratio	0.839
Credible Interval	(0.409, 1.69)
Associated Peptides	1
Associated Spectra	1
Coverage	NaN

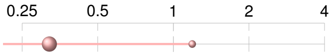

A	B	2.5	50	97.5	Sequence
1	0	0.086	0.32	1.2	QLLDQVEQITK

5.191 172 678 Myosin regulatory light chain sqh [Crassostrea gigas]

Protein Accession	CL23773.Contig1$_{H}1$
Mean Expression Ratio	1.19
Median Expression Ratio	1.19
Credible Interval	(0.597, 2.39)
Associated Peptides	1
Associated Spectra	1
Coverage	NaN

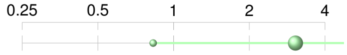

A	B	2.5	50	97.5	Sequence
0	1	0.83	3	11	GNFDYNEFTR

5.192 4 2400 IQ motif containing GTPase activating protein 2 [Xenopus laevis] gi; 4 2169 IQ motif containing GTPase activating protein 2 [Xenopus laevis] gi

Protein Accession	CL875.Contig2$_{H}1$ CL875.Contig1$_{H}1$
Mean Expression Ratio	1.19
Median Expression Ratio	1.19
Credible Interval	(0.616, 2.3)
Associated Peptides	2
Associated Spectra	3
Coverage	NaN

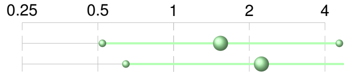

A	B	2.5	50	97.5	Sequence
0	2	0.52	1.5	4.6	ITLQDVVTHNR
0	1	0.65	2.2	8.4	TEVLEK

5.193 546 995 Hypothetical protein CBG12002 [Caenorhabditis briggsae]; 543 995 minus strand PREDICTED: tubulin polymerization-promoting protein family member 2-like [Anolis carolinensis]; 546 995 Hypothetical protein CBG12002 [Caenorhabditis briggsae]; 546 995 minus strand Hypothetical protein CBG12002 [Caenorhabditis briggsae]

Protein Accession	CL6849.Contig7$_{H}$1 CL6849.Contig6$_{H}$1 CL6849.Contig2$_{H}$1 CL6849.Contig1$_{H}$1
Mean Expression Ratio	1.19
Median Expression Ratio	1.19
Credible Interval	(0.641, 2.22)
Associated Peptides	4
Associated Spectra	7
Coverage	NaN

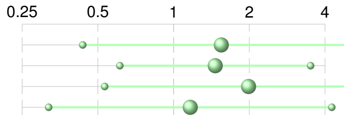

A	B	2.5	50	97.5	Sequence
1	0	0.43	1.5	5.5	LLPQLAADK
0	4	0.61	1.5	3.5	LLPQLAADKAK
0	1	0.53	2	7.3	LKDNVAK
0	1	0.32	1.2	4.3	ADFLNK

5.194 177 791 Ras-related protein Rab-1A [Haliotis discus discus]

Protein Accession	CL21389.Contig6$_{H}$1
Mean Expression Ratio	1.19
Median Expression Ratio	1.19
Credible Interval	(0.631, 2.24)
Associated Peptides	3
Associated Spectra	6
Coverage	NaN

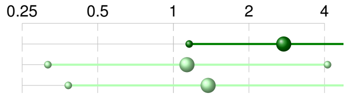

A	B	2.5	50	97.5	Sequence
1	3	1.2	2.7	6.5	MGPVTSASENKPSVK
1	0	0.32	1.1	4.1	INSSTPVK
0	1	0.38	1.4	4.9	KVVDYTTAK

5.195 3 386 fem-1-like protein C [Crassostrea gigas]

Protein Accession	Unigene19937$_{H}$1
Mean Expression Ratio	0.837
Median Expression Ratio	0.841
Credible Interval	(0.418, 1.63)
Associated Peptides	1
Associated Spectra	3
Coverage	NaN

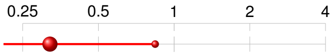

A	B	2.5	50	97.5	Sequence
0	3	0.12	0.32	0.84	MQSLLIR

5.196 105 503 Transgelin-2 [Crassostrea gigas]

Protein Accession	CL445.Contig2$_{H}$1
Mean Expression Ratio	0.845
Median Expression Ratio	0.842
Credible Interval	(0.44, 1.6)
Associated Peptides	2
Associated Spectra	4
Coverage	NaN

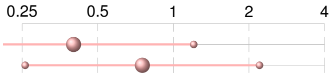

A	B	2.5	50	97.5	Sequence
1	1	0.13	0.4	1.2	SGLGLEIDK
2	0	0.26	0.75	2.2	SGLGLEIDKK

5.197 102 872 RecName: Full=Gelsolin-like protein 1; AltName: Full=Actin-modulator; Short=EWAM; Short=EWAM-P1 gi|157362355|dbj|BAA06219.2| actin-modulator [Lumbricus terrestris]; 102 872 RecName: Full=Gelsolin-like protein 1; AltName: Full=Actin-modulator; Short=EWAM; Short=EWAM-P1 gi|157362355|dbj|BAA06219.2| actin-modulator [Lumbricus terrestris]

Protein Accession	Unigene39721$_H1$ Unigene39720$_H1$
Mean Expression Ratio	1.19
Median Expression Ratio	1.19
Credible Interval	(0.596, 2.39)
Associated Peptides	1
Associated Spectra	1
Coverage	NaN

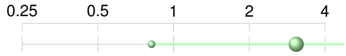

A	B	2.5	50	97.5	Sequence
1	0	0.82	3.1	11	PKGLVK

5.198 551 871 minus strand Protein disulfide-isomerase A4 [Crassostrea gigas]

Protein Accession	CL20258.Contig1$_H1$
Mean Expression Ratio	0.845
Median Expression Ratio	0.844
Credible Interval	(0.415, 1.7)
Associated Peptides	1
Associated Spectra	1
Coverage	NaN

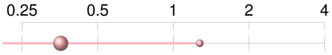

A	B	2.5	50	97.5	Sequence
1	0	0.096	0.36	1.3	EGPEPILLAK

5.199 36 770 minus strand Endoplasmic reticulum protein ERp29 [Crassostrea gigas]

Protein Accession	CL37188.Contig1$_H1$
Mean Expression Ratio	0.841
Median Expression Ratio	0.844
Credible Interval	(0.467, 1.51)
Associated Peptides	5
Associated Spectra	12
Coverage	NaN

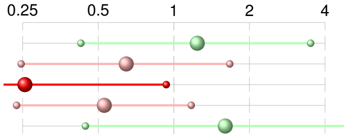

A	B	2.5	50	97.5	Sequence
2	0	0.43	1.2	3.5	AQTAAAALTK
1	2	0.25	0.65	1.7	EADKESADIYIK
1	0	0.07	0.25	0.94	QDPIPYTGDVK
2	3	0.24	0.53	1.2	KPDFPVYK
0	1	0.45	1.6	5.7	GSINLNSGVFDKVIAK

5.200 128 532 minus strand mitochondrial ATPase inhibitor [Ornithodoros parkeri]

Protein Accession	Unigene1376$_H$1
Mean Expression Ratio	1.19
Median Expression Ratio	1.18
Credible Interval	(0.638, 2.17)
Associated Peptides	4
Associated Spectra	6
Coverage	NaN

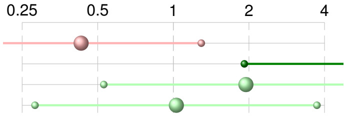

A	B	2.5	50	97.5	Sequence
2	0	0.15	0.43	1.3	EAGGAFGK
0	2	1.9	5.7	17	KLEREQIAK
0	1	0.53	1.9	7.1	SGIGEFGSGAGK
0	1	0.28	1	3.7	MEAAHEELYFK

5.201 74 2734 26S proteasome non-ATPase regulatory subunit 2 [Crassostrea gigas]

Protein Accession	CL32689.Contig3$_H$1
Mean Expression Ratio	1.18
Median Expression Ratio	1.18
Credible Interval	(0.592, 2.4)
Associated Peptides	1
Associated Spectra	1
Coverage	NaN

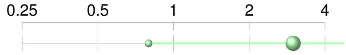

A	B	2.5	50	97.5	Sequence
0	1	0.79	3	11	VGQAVDVVGQAGKPK

5.202 73 525 minus strand Actin-related protein 2/3 complex subunit 5-like protein [Salmo salar] gi; 73 525 Actin-related protein 2/3 complex subunit 5-like protein [Salmo salar] gi; 73 525 minus strand Actin-related protein 2/3 complex subunit 5-like protein [Salmo salar] gi

Protein Accession	CL14637.Contig6$_H$1 CL14637.Contig5$_H$1 CL14637.Contig4$_H$1
Mean Expression Ratio	0.842
Median Expression Ratio	0.845
Credible Interval	(0.433, 1.64)
Associated Peptides	2
Associated Spectra	2
Coverage	NaN

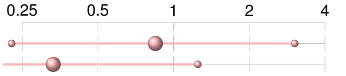

A	B	2.5	50	97.5	Sequence
0	1	0.23	0.85	3	LVINSTPFGSK
0	1	0.089	0.33	1.2	EVDILMK

5.203 188 3946 Vigilin [Crassostrea gigas]; 188 4135 Vigilin [Crassostrea gigas]

Protein Accession	CL846.Contig4$_H$1 CL846.Contig3$_H$1
Mean Expression Ratio	0.845
Median Expression Ratio	0.845
Credible Interval	(0.482, 1.47)
Associated Peptides	8
Associated Spectra	9
Coverage	NaN

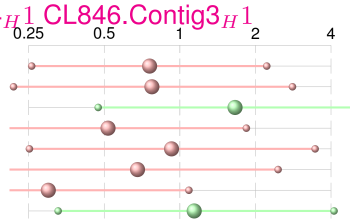

A	B	2.5	50	97.5	Sequence
0	2	0.26	0.76	2.2	EVSIDIPHR
0	1	0.22	0.77	2.8	NIAEEFGGVTVSFPR
0	1	0.47	1.7	6.1	IKNETGVSIR
0	1	0.14	0.52	1.8	AVIDDLK
0	1	0.25	0.93	3.5	VHEVGIK
0	1	0.19	0.68	2.5	LQELELATSTK
0	1	0.083	0.3	1.1	GFQLQVEVDPR
0	1	0.33	1.1	4.1	FLIGR

5.204 48 356 DEC-3 [Lymnaea stagnalis]

Protein Accession	CL26259.Contig1$_H$1
Mean Expression Ratio	0.845
Median Expression Ratio	0.846
Credible Interval	(0.445, 1.61)
Associated Peptides	2
Associated Spectra	5
Coverage	NaN

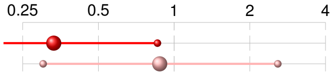

A	B	2.5	50	97.5	Sequence
2	1	0.13	0.33	0.86	SEFDTAFR
0	2	0.3	0.87	2.6	EFSFNWR

5.205 313 1908 minus strand Heterogeneous nuclear ribonucleoprotein L [Crassostrea gigas]; 313 1833 minus strand Heterogeneous nuclear ribonucleoprotein L [Crassostrea gigas]

Protein Accession	CL19412.Contig8$_H$1 CL19412.Contig7$_H$1
Mean Expression Ratio	0.845
Median Expression Ratio	0.846
Credible Interval	(0.418, 1.68)
Associated Peptides	1
Associated Spectra	2
Coverage	NaN

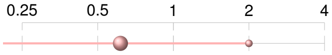

A	B	2.5	50	97.5	Sequence
1	1	0.19	0.62	2	SSTGLLEFESK

5.206 61 723 minus strand 40S ribosomal protein S3-A OS=Xenopus laevis GN=rps3-a PE=2 SV=2

Protein Accession	Unigene19057$_H$1
Mean Expression Ratio	0.848
Median Expression Ratio	0.847
Credible Interval	(0.456, 1.56)
Associated Peptides	4
Associated Spectra	6
Coverage	NaN

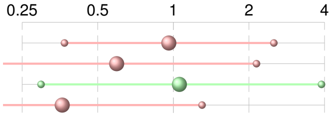

A	B	2.5	50	97.5	Sequence
1	2	0.37	0.96	2.5	ELAEDGYSGVEVR
1	0	0.16	0.59	2.1	TQNVLGEK
1	0	0.3	1.1	3.9	DEPAPLHPYSEQK
0	1	0.098	0.36	1.3	QGVLGIK

5.207 " 926 1189 minus strand Myosin heavy chain, striated muscle [Crassostrea gigas]"

Protein Accession	Unigene11948$_H1$
Mean Expression Ratio	1.18
Median Expression Ratio	1.18
Credible Interval	(0.649, 2.17)
Associated Peptides	4
Associated Spectra	9
Coverage	NaN

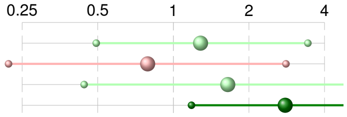

A	B	2.5	50	97.5	Sequence
3	0	0.49	1.3	3.4	LNGNHLGK
1	0	0.22	0.79	2.8	TFLDK
0	1	0.44	1.6	5.9	TFLDKLNGNHLGK
0	4	1.2	2.8	6.8	ASDKTFLDKLNGNHLGK

5.208 140 283 minus strand elongation factor 1 alpha [Angiostrongylus cantonensis]

Protein Accession	CL16758.Contig7$_H1$
Mean Expression Ratio	0.845
Median Expression Ratio	0.848
Credible Interval	(0.435, 1.64)
Associated Peptides	2
Associated Spectra	3
Coverage	NaN

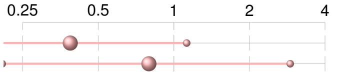

A	B	2.5	50	97.5	Sequence
2	0	0.13	0.39	1.1	VIEKFEK
1	0	0.21	0.79	2.9	STTTGHLIYK

5.209 280 690 Histone H3.3 [Harpegnathos saltator]

Protein Accession	Unigene26607$_H1$
Mean Expression Ratio	1.18
Median Expression Ratio	1.18
Credible Interval	(0.726, 1.94)
Associated Peptides	9
Associated Spectra	77
Coverage	NaN

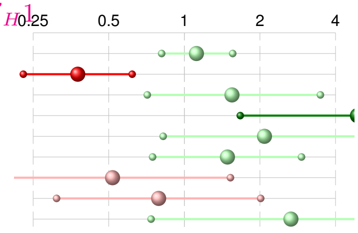

A	B	2.5	50	97.5	Sequence
28	10	0.81	1.1	1.6	DIQLAR
16	0	0.23	0.38	0.62	STELLIR
2	3	0.71	1.5	3.5	VTIMPK
1	1	1.7	4.9	15	VTIMPKDIQLAR
3	0	0.82	2.1	5.3	KQLATK
3	4	0.75	1.5	2.9	RVTIMPK
2	0	0.17	0.52	1.5	KLPFQR
3	0	0.31	0.79	2	EIAQDFK
0	1	0.74	2.7	9.7	RVTIMPKDIQLAR

5.210 423 695 thioredoxin peroxidase 2 [Haliotis discus discus]

Protein Accession	CL3160.Contig13$_H1$
Mean Expression Ratio	0.85
Median Expression Ratio	0.849
Credible Interval	(0.422, 1.71)
Associated Peptides	1
Associated Spectra	1
Coverage	NaN

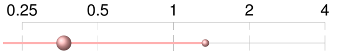

A	B	2.5	50	97.5	Sequence
0	1	0.1	0.37	1.3	KIGGLGNMNIPLLEDKTHEIAR

5.211 181 684 hypothetical protein BRAFLDRAFT_131834 [Branchiostoma floridae] gi

Protein Accession	CL10218.Contig4$_H$1
Mean Expression Ratio	0.848
Median Expression Ratio	0.849
Credible Interval	(0.425, 1.69)
Associated Peptides	1
Associated Spectra	1
Coverage	NaN

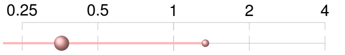

A	B	2.5	50	97.5	Sequence
**1**	**0**	**0.096**	**0.36**	**1.3**	**LSGEGLETSK**

5.212 " 519 3182 minus strand Alpha-actinin, sarcomeric [Crassostrea gigas]"

Protein Accession	CL28960.Contig2$_H$1
Mean Expression Ratio	0.85
Median Expression Ratio	0.85
Credible Interval	(0.537, 1.37)
Associated Peptides	14
Associated Spectra	24
Coverage	NaN

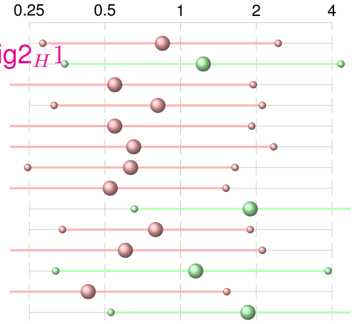

A	B	2.5	50	97.5	Sequence
**1**	**1**	**0.28**	**0.84**	**2.4**	**NINEVENQILTR**
**1**	**0**	**0.35**	**1.2**	**4.3**	**HLDIPR**
**1**	**0**	**0.15**	**0.55**	**1.9**	**MSFNHFDK**
**2**	**1**	**0.31**	**0.81**	**2.1**	**RDQTLQQEMMR**
**1**	**0**	**0.15**	**0.55**	**1.9**	**LERLDHLAQK**
**0**	**1**	**0.18**	**0.65**	**2.3**	**ACLISLGYK**
**0**	**3**	**0.25**	**0.63**	**1.6**	**AIIQYKPHIDELER**
**0**	**2**	**0.17**	**0.52**	**1.5**	**ETADTDTAEQVMQSFK**
**0**	**2**	**0.66**	**1.9**	**5.5**	**GISEDQMNEFR**
**0**	**4**	**0.34**	**0.79**	**1.9**	**IDQLHLEFAK**
**0**	**1**	**0.17**	**0.6**	**2.1**	**ILAGDKPYITAQILR**
**0**	**1**	**0.32**	**1.1**	**3.9**	**LSNRPAYLPTEGK**
**0**	**1**	**0.12**	**0.43**	**1.5**	**LDEFR**
**0**	**1**	**0.53**	**1.9**	**6.8**	**MSFNHFDKNR**

5.213 151 1599 PREDICTED: galactosamine (N-acetyl)-6-sulfate sulfatase-like [Saccoglossus kowalevskii]

Protein Accession	CL13278.Contig2$_H$1
Mean Expression Ratio	1.18
Median Expression Ratio	1.18
Credible Interval	(0.604, 2.31)
Associated Peptides	2
Associated Spectra	3
Coverage	NaN

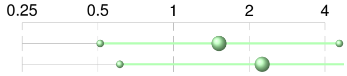

A	B	2.5	50	97.5	Sequence
**0**	**2**	**0.51**	**1.5**	**4.6**	**GLVPSLISK**
**0**	**1**	**0.61**	**2.2**	**8.1**	**TTLWEGGTR**

5.214 8 1636 minus strand V-type proton ATPase catalytic subunit A [Crassostrea gigas]; 8 1636 minus strand V-type proton ATPase catalytic subunit A [Crassostrea gigas]; 8 1636 minus strand V-type proton ATPase catalytic subunit A [Crassostrea gigas]

Protein Accession	CL33671.Contig3$_H$1 CL33671.Contig2$_H$1 CL33671.Contig1$_H$1
Mean Expression Ratio	1.17
Median Expression Ratio	1.17
Credible Interval	(0.657, 2.12)
Associated Peptides	5
Associated Spectra	9
Coverage	NaN

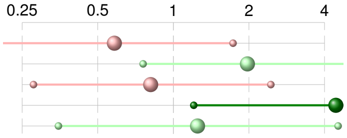

A	B	2.5	50	97.5	Sequence
2	0	0.2	0.58	1.7	GINTPALDANK
2	1	0.76	2	5	IMLNPNAR
2	0	0.28	0.81	2.4	TVGMLR
1	0	1.2	4.5	16	FRNLED
0	1	0.35	1.2	4.5	LAANHPLLTGQR

5.215 " 1240 1911 PREDICTED: ATP synthase subunit alpha, mitochondrial-like [Anolis carolinensis]"

Protein Accession	Unigene30533$_H$1
Mean Expression Ratio	0.849
Median Expression Ratio	0.851
Credible Interval	(0.445, 1.59)
Associated Peptides	3
Associated Spectra	8
Coverage	NaN

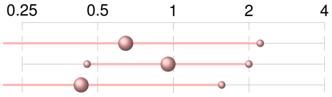

A	B	2.5	50	97.5	Sequence
1	0	0.18	0.64	2.2	LELAQYR
3	3	0.45	0.95	2	SSQQELLK
1	0	0.12	0.43	1.6	LTELLK

5.216 2295 2510 beta-thymosin domain repeat protein CSP24KDa_v1 [Hermissenda crassicornis] gi

Protein Accession	CL9548.Contig1$_H$1
Mean Expression Ratio	1.18
Median Expression Ratio	1.17
Credible Interval	(0.628, 2.28)
Associated Peptides	3
Associated Spectra	3
Coverage	NaN

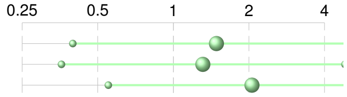

A	B	2.5	50	97.5	Sequence
0	1	0.4	1.5	5.4	ESVAAFDKSHLK
0	1	0.36	1.3	4.8	ESVAAFDK
0	1	0.55	2	7.4	AFDKSHLK

5.217 1155 1649 78 kDa glucose-regulated protein OS=Aplysia californica PE=1 SV=1

Protein Accession	CL41594.Contig1$_{H}$1
Mean Expression Ratio	1.17
Median Expression Ratio	1.17
Credible Interval	(0.616, 2.2)
Associated Peptides	3
Associated Spectra	6
Coverage	NaN

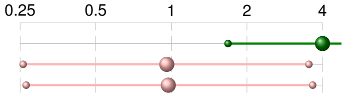

A	B	2.5	50	97.5	Sequence
0	4	1.7	4	9.6	AKFEELNMDLFR
0	1	0.26	0.96	3.5	STVKPVK
0	1	0.26	0.97	3.6	VEVESLFDGEDFSEQLTR

5.218 71 409 minus strand heat shock protein 108 [Alligator mississippiensis]

Protein Accession	CL5089.Contig3$_{H}$1
Mean Expression Ratio	1.18
Median Expression Ratio	1.17
Credible Interval	(0.609, 2.34)
Associated Peptides	2
Associated Spectra	2
Coverage	NaN

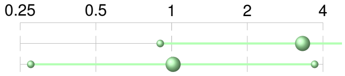

A	B	2.5	50	97.5	Sequence
0	1	0.9	3.3	12	QETVDDDLGKTMEGSR
0	1	0.28	1	3.7	QETVDDDLGK

5.219 182 832 minus strand qm-like protein [Crassostrea ariakensis]

Protein Accession	CL13773.Contig2$_{H}$1
Mean Expression Ratio	1.18
Median Expression Ratio	1.17
Credible Interval	(0.676, 2.07)
Associated Peptides	5
Associated Spectra	18
Coverage	NaN

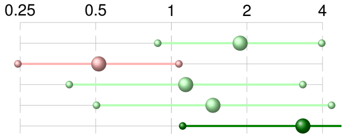

A	B	2.5	50	97.5	Sequence
2	4	0.89	1.9	4	VHIGQPIMSVR
3	3	0.24	0.52	1.1	IFDLGR
0	2	0.39	1.1	3.3	EQHEAAIIEALR
0	2	0.5	1.5	4.3	AREQHEAAIIEALR
0	2	1.1	3.3	9.6	GAFGKPQGTVAR

5.220 56 937 Chloride intracellular channel exc-4 [Crassostrea gigas]

Protein Accession	CL34620.Contig3$_{H}$1
Mean Expression Ratio	1.17
Median Expression Ratio	1.17
Credible Interval	(0.607, 2.3)
Associated Peptides	2
Associated Spectra	4
Coverage	NaN

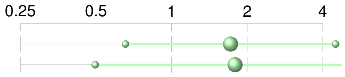

A	B	2.5	50	97.5	Sequence
1	2	0.65	1.7	4.5	KVQGPLVK
0	1	0.5	1.8	6.6	QKFELYIK

5.221 390 908 Cysteine proteinase 5 OS=Dictyostelium discoideum GN=cprE PE=2 SV=2

Protein Accession	CL6665.Contig2$_{H}$1
Mean Expression Ratio	0.854
Median Expression Ratio	0.854
Credible Interval	(0.423, 1.71)
Associated Peptides	1
Associated Spectra	1
Coverage	NaN

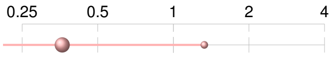

A	B	2.5	50	97.5	Sequence
0	1	0.1	0.36	1.3	GPVSVGIDASHK

5.222 1237 1374 minus strand putative 60S ribosomal protein L23 [Maconellicoccus hirsutus]

Protein Accession	CL834.Contig9$_{H}$1
Mean Expression Ratio	1.17
Median Expression Ratio	1.17
Credible Interval	(0.608, 2.35)
Associated Peptides	1
Associated Spectra	4
Coverage	NaN

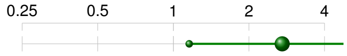

A	B	2.5	50	97.5	Sequence
0	4	1.2	2.7	6.5	IASNASSIA

5.223 49 1038 Glyceraldehyde-3-phosphate dehydrogenase [Crotalus adamanteus]

Protein Accession	Unigene7744$_{H}$1
Mean Expression Ratio	1.17
Median Expression Ratio	1.17
Credible Interval	(0.669, 2.07)
Associated Peptides	5
Associated Spectra	20
Coverage	NaN

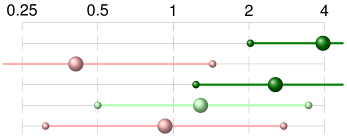

A	B	2.5	50	97.5	Sequence
2	6	2	3.9	7.6	TVDGPSNKDWR
1	0	0.11	0.41	1.4	AGIQLSDKFVK
1	5	1.2	2.6	5.4	SSIFDAK
0	3	0.5	1.3	3.5	GAYQNIIPSSTGAAK
0	2	0.31	0.92	2.7	VIPALNGK

5.224 2 256 Polyubiquitin (Fragment) OS=Xenopus laevis PE=1 SV=2; 20 388 large subunit ribosomal protein L40e [Clonorchis sinensis]

Protein Accession	CL47507.Contig2$_{H}$1 CL39010.Contig1$_{H}$1
Mean Expression Ratio	0.856
Median Expression Ratio	0.854
Credible Interval	(0.488, 1.51)
Associated Peptides	5
Associated Spectra	13
Coverage	NaN

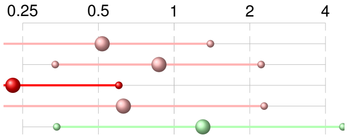

A	B	2.5	50	97.5	Sequence
1	3	0.19	0.52	1.4	TITLEVEPSDTIENVK
2	2	0.34	0.87	2.2	TLSDYNIQK
3	0	0.088	0.23	0.6	LIFAGK
1	0	0.17	0.63	2.3	IQDKEGIPPDQQR
1	0	0.34	1.3	4.7	MQIFVK

5.225 110 2365 N-acetylated-alpha-linked acidic dipeptidase 2 [Crassostrea gigas]

Protein Accession	CL8322.Contig4$_H$1
Mean Expression Ratio	1.17
Median Expression Ratio	1.17
Credible Interval	(0.589, 2.27)
Associated Peptides	2
Associated Spectra	2
Coverage	NaN

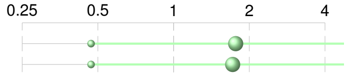

A	B	2.5	50	97.5	Sequence
0	1	0.47	1.8	6.6	ALGTPLMYR
0	1	0.47	1.7	6.4	AQRGTILNADGDPLTPGYPAVR

5.226 496 2151 NADP-dependent malic enzyme [Crassostrea gigas]

Protein Accession	CL26274.Contig1$_H$1
Mean Expression Ratio	0.858
Median Expression Ratio	0.857
Credible Interval	(0.533, 1.37)
Associated Peptides	12
Associated Spectra	33
Coverage	NaN

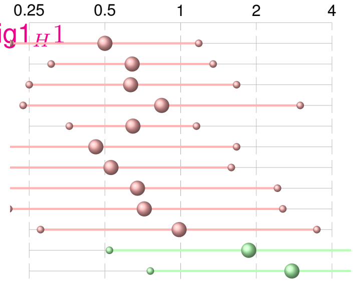

A	B	2.5	50	97.5	Sequence
1	3	0.21	0.5	1.2	AIVVTDGER
3	3	0.31	0.64	1.3	GIDILR
1	2	0.25	0.63	1.7	IWMVDSK
1	0	0.24	0.84	3	EGMASAYPEPQDKR
2	9	0.36	0.65	1.2	VLVNFR
1	0	0.13	0.46	1.7	GLLVK
1	1	0.18	0.53	1.6	GTAFSLK
1	0	0.19	0.67	2.4	APFAK
0	1	0.21	0.72	2.6	AECTAEEAYTLTEGR
0	1	0.28	0.98	3.5	LNKGTAFSLK
0	1	0.52	1.9	6.4	GIDILRNPK
0	1	0.76	2.8	10	LNKGTAFSLKER

5.227 1 381 hypothetical protein [Eimeria tenella]

Protein Accession	CL17786.Contig1$_H$1
Mean Expression Ratio	1.17
Median Expression Ratio	1.17
Credible Interval	(0.599, 2.29)
Associated Peptides	1
Associated Spectra	4
Coverage	NaN

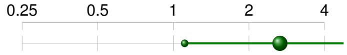

A	B	2.5	50	97.5	Sequence
0	4	1.1	2.7	6.4	IINEPTAAAIAYGLDKK

5.228 163 1059 minus strand Heterogeneous nuclear ribonucleoprotein A2-like protein 1 [Crassostrea gigas]; 99 995 minus strand Heterogeneous nuclear ribonucleoprotein A2-like protein 1 [Crassostrea gigas]; 71 967 minus strand Heterogeneous nuclear ribonucleoprotein A2-like protein 1 [Crassostrea gigas]; 71 967 minus strand Heterogeneous nuclear ribonucleoprotein A2-like protein 1 [Crassostrea gigas]

Protein Accession	Unigene65$_{H}1$ Unigene63$_{H}1$ Unigene38787$_{H}1$ Unigene38786$_{H}1$
Mean Expression Ratio	0.856
Median Expression Ratio	0.857
Credible Interval	(0.448, 1.63)
Associated Peptides	2
Associated Spectra	3
Coverage	NaN

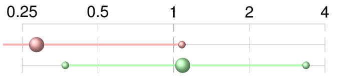

A	B	2.5	50	97.5	Sequence
1	0	0.076	0.29	1.1	IENVEMIK
2	0	0.37	1.1	3.4	IENVEMIKDK

5.229 " 142 1284 minus strand Chain O, Crsytal Structure Of Human Liver Gapdh gi|67464044|pdb|1ZNQ|P Chain P, Crsytal Structure Of Human Liver Gapdh gi|67464045|pdb|1ZNQ|R Chain R, Crsytal Structure Of Human Liver Gapdh gi|67464046|pdb|1ZNQ|Q Chain Q, Crsytal Structure Of Human Liver Gapdh"

Protein Accession	CL34318.Contig1$_{H}1$
Mean Expression Ratio	0.858
Median Expression Ratio	0.858
Credible Interval	(0.465, 1.58)
Associated Peptides	4
Associated Spectra	8
Coverage	NaN

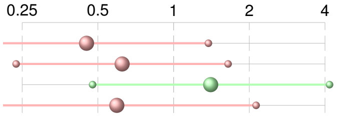

A	B	2.5	50	97.5	Sequence
1	1	0.15	0.45	1.4	DPTAIPWR
1	2	0.24	0.62	1.6	VIPELNGK
0	2	0.48	1.4	4.2	INVYQEKDPTAIPWR
0	1	0.16	0.6	2.1	VIPELNGKLTGMAFR

5.230 137 1369 hypothetical protein TcasGA2_TC002962 [Tribolium castaneum]

Protein Accession	CL20015.Contig2$_{H}1$
Mean Expression Ratio	1.17
Median Expression Ratio	1.17
Credible Interval	(0.615, 2.23)
Associated Peptides	2
Associated Spectra	3
Coverage	NaN

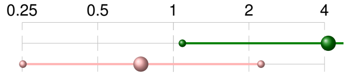

A	B	2.5	50	97.5	Sequence
0	1	1.1	4.2	15	AAAAEPAVVENK
0	2	0.25	0.74	2.2	AEAAEAK

5.231 149 496 Spectrin alpha chain [Crassostrea gigas]

Protein Accession	CL2014.Contig1$_{H}1$
Mean Expression Ratio	0.855
Median Expression Ratio	0.86
Credible Interval	(0.422, 1.7)
Associated Peptides	1
Associated Spectra	1
Coverage	NaN

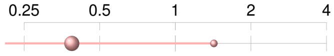

A	B	2.5	50	97.5	Sequence
0	1	0.1	0.39	1.4	DADELESWILEK

5.232 85 633 hypothetical protein [Amblyomma maculatum]

Protein Accession	CL2221.Contig1$_{H}1$
Mean Expression Ratio	0.86
Median Expression Ratio	0.861
Credible Interval	(0.454, 1.62)
Associated Peptides	3
Associated Spectra	4
Coverage	NaN

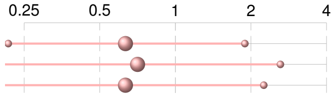

A	B	2.5	50	97.5	Sequence
0	2	0.22	0.63	1.9	IFYTEHLHADEEIR
0	1	0.2	0.71	2.6	DANDEWIR
0	1	0.17	0.63	2.2	EKLPDYDNK

5.233 161 301 Heat shock protein beta-6 OS=Mus musculus GN=Hspb6 PE=1 SV=1

Protein Accession	CL46983.Contig2$_{H}1$
Mean Expression Ratio	1.16
Median Expression Ratio	1.16
Credible Interval	(0.646, 2.11)
Associated Peptides	4
Associated Spectra	27
Coverage	NaN

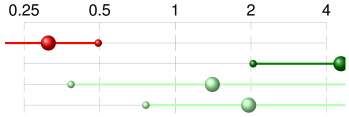

A	B	2.5	50	97.5	Sequence
5	13	0.19	0.31	0.49	VNMDVQHFKPEEIK
0	5	2	4.6	10	VNMDVQHFKPEEIKITTKDNR
0	1	0.38	1.4	5.1	VVISAK
0	3	0.76	2	5.1	VNMDVQHFKPEEIKITTK

5.234 118 1866 Lamin Dm0 [Crassostrea gigas]

Protein Accession	CL4079.Contig7$_{H}$1
Mean Expression Ratio	1.16
Median Expression Ratio	1.16
Credible Interval	(0.747, 1.81)
Associated Peptides	16
Associated Spectra	31
Coverage	NaN

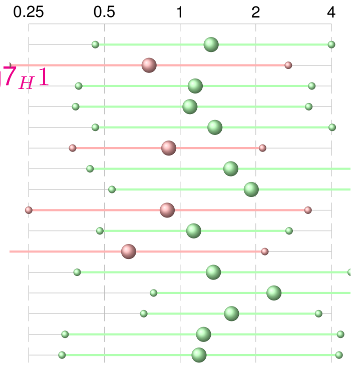

A	B	2.5	50	97.5	Sequence
1	1	0.46	1.3	4	KQLEEETLLR
1	0	0.21	0.75	2.7	LLEGEESR
1	1	0.4	1.1	3.3	LSEVEAPR
1	1	0.38	1.1	3.2	SLVIKPGK
0	2	0.46	1.4	4	EKHEFDLQTVR
0	4	0.37	0.9	2.1	GGIDIEEVDPEGK
0	1	0.44	1.6	5.6	SLYETELADAR
0	1	0.53	1.9	6.9	SLYETELADARR
0	1	0.25	0.89	3.2	VQSLKEDLHFK
0	4	0.48	1.1	2.7	WFVADEMK
0	1	0.17	0.62	2.2	YKTEYEDLLAK
0	1	0.39	1.4	4.8	IQIEANKYK
0	2	0.78	2.4	6.9	LLDETAKEK
0	5	0.72	1.6	3.6	IQIEANK
0	1	0.35	1.2	4.3	RLALKDDEIADLR
0	1	0.34	1.2	4.3	ADDLSAELAK

5.235 114 1001 Vinculin [Crassostrea gigas]

Protein Accession	CL18499.Contig1$_{H}$1
Mean Expression Ratio	1.16
Median Expression Ratio	1.16
Credible Interval	(0.605, 2.24)
Associated Peptides	3
Associated Spectra	3
Coverage	NaN

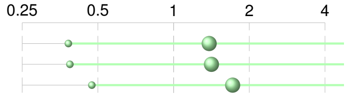

A	B	2.5	50	97.5	Sequence
1	0	0.38	1.4	5	PVFHTK
0	1	0.39	1.4	5.2	TIESILEPVAQQVSR
0	1	0.47	1.7	6.3	KMSDEIHEIIR

5.236 628 1140 paramyosin [Haliotis discus discus]

Protein Accession	CL43455.Contig3$_{H}$1
Mean Expression Ratio	1.16
Median Expression Ratio	1.16
Credible Interval	(0.613, 2.23)
Associated Peptides	2
Associated Spectra	14
Coverage	NaN

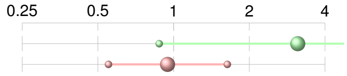

A	B	2.5	50	97.5	Sequence
1	0	0.88	3.1	12	EITRVVKV
2	11	0.55	0.95	1.6	LEEAEAFAVK

5.237 44 1465 minus strand Adenylyl cyclase-associated protein 1 [Crassostrea gigas]; 52 1473 minus strand Adenylyl cyclase-associated protein 1 [Crassostrea gigas]; 48 1469 Adenylyl cyclase-associated protein 1 [Crassostrea gigas]

Protein Accession	CL19470.Contig9$_H$1 CL19470.Contig8$_H$1 CL19470.Contig1$_H$1
Mean Expression Ratio	0.862
Median Expression Ratio	0.863
Credible Interval	(0.498, 1.51)
Associated Peptides	7
Associated Spectra	12
Coverage	NaN

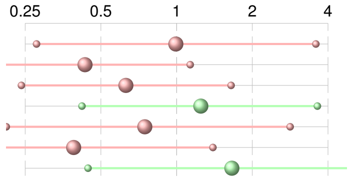

A	B	2.5	50	97.5	Sequence
1	0	0.28	0.99	3.6	AQLDIVNAAFK
1	2	0.17	0.43	1.1	NIVLDNVELK
1	2	0.24	0.63	1.7	QFLEVASK
1	1	0.42	1.2	3.6	SKQPPQADLPK
1	0	0.21	0.75	2.8	AQIGGDLK
0	1	0.1	0.39	1.4	DSLDTEIVTAK
0	1	0.44	1.7	6	VEAAKPPLTELQNKK

5.238 89 838 Putative phosphoglycerate mutase [Crassostrea gigas]; 89 838 Putative phosphoglycerate mutase [Crassostrea gigas]; 89 835 Putative phosphoglycerate mutase [Crassostrea gigas]; 89 838 Putative phosphoglycerate mutase [Crassostrea gigas]

Protein Accession	CL6793.Contig6$_H$1 CL6793.Contig5$_H$1 CL6793.Contig4$_H$1 CL6793.Contig3$_H$1
Mean Expression Ratio	1.16
Median Expression Ratio	1.16
Credible Interval	(0.62, 2.15)
Associated Peptides	3
Associated Spectra	9
Coverage	NaN

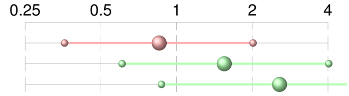

A	B	2.5	50	97.5	Sequence
2	2	0.36	0.85	2	EAQYAGELIK
2	1	0.61	1.5	4	VLIAAHGNSLR
0	2	0.87	2.6	7.7	HYGGLQGLNKAETAAK

5.239 313 1908 minus strand Heterogeneous nuclear ribonucleoprotein L [Crassostrea gigas]

Protein Accession	CL19412.Contig8$_H$1
Mean Expression Ratio	1.16
Median Expression Ratio	1.16
Credible Interval	(0.611, 2.2)
Associated Peptides	3
Associated Spectra	4
Coverage	NaN

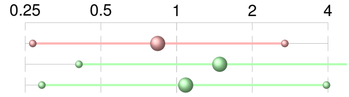

A	B	2.5	50	97.5	Sequence
1	1	0.27	0.84	2.7	SSTGLLEFESK
0	1	0.41	1.5	5.4	GQPLLQEPR
0	1	0.29	1.1	3.9	TPFIFK

5.240 40 1752 minus strand heat shock protein 60 [Biomphalaria glabrata]; 40 1752 minus strand heat shock protein 60 [Biomphalaria glabrata]

Protein Accession	CL5583.Contig6$_{H}1$ CL5583.Contig5$_{H}1$
Mean Expression Ratio	1.16
Median Expression Ratio	1.16
Credible Interval	(0.714, 1.88)
Associated Peptides	13
Associated Spectra	28
Coverage	NaN

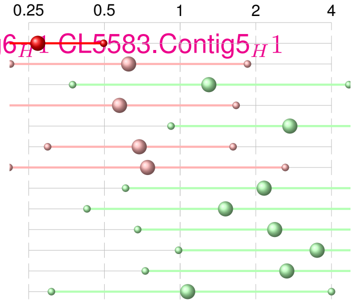

A	B	2.5	50	97.5	Sequence
3	7	0.15	0.27	0.5	AVGDLISSAMK
2	0	0.21	0.62	1.9	GANPIEIR
1	0	0.37	1.3	4.7	LSNGVAVLK
1	1	0.2	0.57	1.7	VGGTSEVEVNEK
1	1	0.92	2.7	8.1	ITKDGVTVAK
2	2	0.3	0.69	1.6	VGLQVVAVK
0	1	0.21	0.74	2.6	AAVEQGIVAGGGTALLR
0	1	0.61	2.2	7.9	AVGDLISSAMKK
0	1	0.43	1.5	5.3	DKYQNIGAK
0	1	0.68	2.4	8.7	VGGTSEVEVNEKKDR
0	1	0.99	3.5	12	AIELKDKYQNIGAK
0	1	0.73	2.7	9.4	AIAKEGFDKISK
0	1	0.31	1.1	4	GYISPYFINTSK

5.241 43 525 Reticulon-4 [Crassostrea gigas]; 143 757 Reticulon-4 [Crassostrea gigas]; 43 564 Reticulon-4 [Crassostrea gigas]; 302 583 Reticulon-4 OS=Rattus norvegicus GN=Rtn4 PE=1 SV=1

Protein Accession	CL29775.Contig4$_{H}1$ CL29775.Contig3$_{H}1$ CL29775.Contig2$_{H}1$ CL29775.Contig1$_{H}1$
Mean Expression Ratio	1.16
Median Expression Ratio	1.16
Credible Interval	(0.597, 2.27)
Associated Peptides	2
Associated Spectra	3
Coverage	NaN

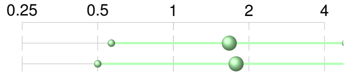

A	B	2.5	50	97.5	Sequence
1	1	0.57	1.7	4.9	VTQTILQHVNR
0	1	0.5	1.8	6.8	LAAVQK

5.242 290 2458 Putative beta-D-xylosidase 5 [Crassostrea gigas]

Protein Accession	CL49577.Contig2$_{H}1$
Mean Expression Ratio	0.863
Median Expression Ratio	0.864
Credible Interval	(0.519, 1.42)
Associated Peptides	11
Associated Spectra	20
Coverage	NaN

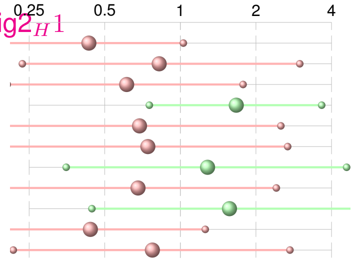

A	B	2.5	50	97.5	Sequence
2	2	0.18	0.43	1	VAEAAGVEVR
1	0	0.23	0.82	3	VQDLVSR
1	1	0.21	0.61	1.8	EIQEQLAR
2	3	0.76	1.7	3.7	VTPLFYTR
0	1	0.2	0.69	2.5	HFDAYAGPENVPTSR
0	1	0.2	0.74	2.7	LTDYDWR
0	1	0.35	1.3	4.6	SAPSQVLVQPFTIK
0	1	0.18	0.68	2.4	NNGILPLDPR
0	1	0.44	1.6	5.5	LEALEQGK
0	2	0.14	0.44	1.3	QLLTDILR
0	1	0.22	0.77	2.7	SFVLLK

5.243 78 2003 Chromatin assembly factor 1 subunit A [Crassostrea gigas]

Protein Accession	CL14511.Contig5$_{H}1$
Mean Expression Ratio	1.16
Median Expression Ratio	1.16
Credible Interval	(0.578, 2.36)
Associated Peptides	1
Associated Spectra	1
Coverage	NaN

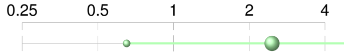

A	B	2.5	50	97.5	Sequence
1	0	0.65	2.5	8.9	IAKELK

5.244 76 822 14-3-3 protein [Heterodera glycines]; 76 822 minus strand 14-3-3 protein [Heterodera glycines]; 76 822 minus strand 14-3-3 protein [Heterodera glycines]

Protein Accession	Unigene34590$_{H}1$ Unigene29843$_{H}1$ Unigene28010$_{H}1$
Mean Expression Ratio	1.16
Median Expression Ratio	1.16
Credible Interval	(0.665, 2.05)
Associated Peptides	6
Associated Spectra	20
Coverage	NaN

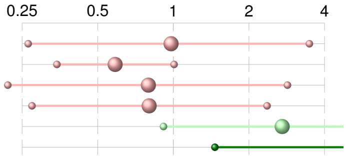

A	B	2.5	50	97.5	Sequence
1	0	0.26	0.98	3.5	YLSEVSQGEQR
7	6	0.34	0.59	1	VVSSIEQK
1	0	0.22	0.8	2.8	KLEMAK
0	2	0.27	0.8	2.4	IAQEQMPTTHPIR
0	2	0.91	2.7	8.2	LAEQAERYDDMAK
0	1	1.5	5.2	19	SMDAYKK

5.245 187 642 minus strand PREDICTED: similar to pol-like protein [Tribolium castaneum]

Protein Accession	CL5644.Contig3$_{H}1$
Mean Expression Ratio	0.865
Median Expression Ratio	0.865
Credible Interval	(0.442, 1.69)
Associated Peptides	1
Associated Spectra	4
Coverage	NaN

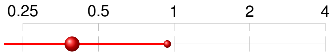

A	B	2.5	50	97.5	Sequence
0	4	0.16	0.39	0.94	IILEQEELK

5.246 2 421 hypothetical protein BRAFLDRAFT_119044 [Branchiostoma floridae] gi

Protein Accession	CL14721.Contig1$_{H}1$
Mean Expression Ratio	0.87
Median Expression Ratio	0.866
Credible Interval	(0.439, 1.72)
Associated Peptides	1
Associated Spectra	2
Coverage	NaN

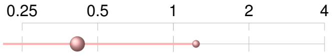

A	B	2.5	50	97.5	Sequence
0	2	0.14	0.41	1.2	DLEMDLAK

5.247 " 285 437 Propionyl-CoA carboxylase alpha chain, mitochondrial [Crassostrea gigas]"

Protein Accession	CL45581.Contig2$_{H}$1
Mean Expression Ratio	1.16
Median Expression Ratio	1.16
Credible Interval	(0.585, 2.33)
Associated Peptides	1
Associated Spectra	2
Coverage	NaN

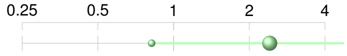

A	B	2.5	50	97.5	Sequence
**0**	**2**	**0.82**	**2.4**	**7.2**	**GVTHNIPLLR**

5.248 143 247 minus strand dynein light chain [Aplysia californica] gi

Protein Accession	CL13886.Contig1$_{H}$1
Mean Expression Ratio	0.858
Median Expression Ratio	0.866
Credible Interval	(0.42, 1.69)
Associated Peptides	1
Associated Spectra	1
Coverage	NaN

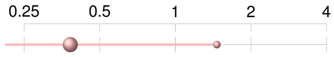

A	B	2.5	50	97.5	Sequence
**0**	**1**	**0.1**	**0.38**	**1.5**	**NADMAEDMQQDAVDCATQALEK**

5.249 259 1296 minus strand GD20916 [Drosophila simulans] gi

Protein Accession	CL25607.Contig2$_{H}$1
Mean Expression Ratio	1.16
Median Expression Ratio	1.15
Credible Interval	(0.606, 2.29)
Associated Peptides	2
Associated Spectra	4
Coverage	NaN

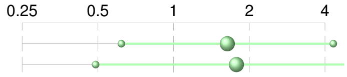

A	B	2.5	50	97.5	Sequence
**0**	**3**	**0.62**	**1.6**	**4.3**	**QIVWEHPEVK**
**0**	**1**	**0.49**	**1.8**	**6.4**	**YAPWPYKK**

5.250 93 368 60S ribosomal protein L37A [Branchiostoma belcheri] gi; 93 395 60S ribosomal protein L37A [Branchiostoma belcheri] gi; 90 272 ribosomal protein L37ae [Wuchereria bancrofti]; 44 319 minus strand 60S ribosomal protein L37A [Branchiostoma belcheri] gi; 44 319 minus strand 60S ribosomal protein L37A [Branchiostoma belcheri] gi

Protein Accession	Unigene66574$_{H}1$ CL6728.Contig4$_{H}1$ CL6728.Contig3$_{H}1$ CL6728.Contig2$_{H}1$ CL6728.Contig
Mean Expression Ratio	1.15
Median Expression Ratio	1.15
Credible Interval	(0.575, 2.33)
Associated Peptides	1
Associated Spectra	1
Coverage	NaN

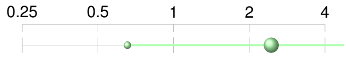

A	B	2.5	50	97.5	Sequence
0	1	0.66	2.4	9.4	VGIVGK

5.251 2 244 minus strand hypothetical protein KGM_08771 [Danaus plexippus]

Protein Accession	CL49466.Contig2$_{H}1$
Mean Expression Ratio	1.15
Median Expression Ratio	1.15
Credible Interval	(0.596, 2.26)
Associated Peptides	2
Associated Spectra	2
Coverage	NaN

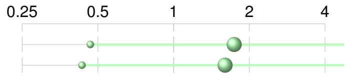

A	B	2.5	50	97.5	Sequence
0	1	0.47	1.7	6.5	AVFIDLEPTVVDEVR
0	1	0.43	1.6	6	QLFHPEQLVTGK

5.252 155 3214 sarco/endoplasmic reticulum calcium ATPase isoform B [Pinctada fucata]; 155 3214 minus strand sarco/endoplasmic reticulum calcium ATPase isoform B [Pinctada fucata]

Protein Accession	CL1323.Contig9$_{H}1$ CL1323.Contig1$_{H}1$
Mean Expression Ratio	1.15
Median Expression Ratio	1.15
Credible Interval	(0.624, 2.11)
Associated Peptides	4
Associated Spectra	5
Coverage	NaN

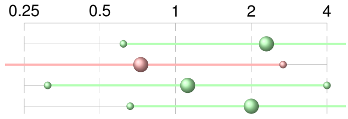

A	B	2.5	50	97.5	Sequence
0	1	0.62	2.3	8.4	KAEIGIAMGSGTAVAK
0	1	0.2	0.73	2.7	VGEATETALTVLVEK
0	1	0.31	1.1	4	GAPEGLLDR
0	2	0.66	2	5.9	MDQAFTK

5.253 " 112 1320 Aspartate aminotransferase, cytoplasmic [Crassostrea gigas]"

Protein Accession	CL44136.Contig1$_H$1
Mean Expression Ratio	0.867
Median Expression Ratio	0.869
Credible Interval	(0.512, 1.47)
Associated Peptides	9
Associated Spectra	13
Coverage	NaN

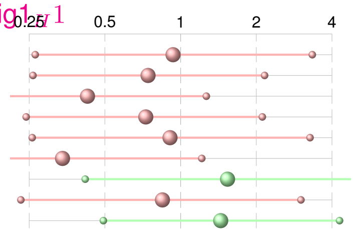

A	B	2.5	50	97.5	Sequence
1	0	0.26	0.94	3.3	SQMEIIVR
1	1	0.26	0.74	2.2	ASIFADVK
1	1	0.14	0.43	1.3	IADVMEAK
0	2	0.24	0.73	2.1	IGNLCIVTK
0	1	0.26	0.91	3.3	TLANALILGK
0	1	0.092	0.34	1.2	VNLGVGAYR
0	1	0.42	1.5	5.4	LAAEFLKR
0	1	0.23	0.84	3	ISMAGVTNK
0	2	0.49	1.4	4.3	VEGIQVQGGTGGIR

5.254 20 355 minus strand PREDICTED: uroporphyrinogen decarboxylase-like [Monodelphis domestica]

Protein Accession	CL11915.Contig5$_H$1
Mean Expression Ratio	1.15
Median Expression Ratio	1.15
Credible Interval	(0.581, 2.3)
Associated Peptides	1
Associated Spectra	1
Coverage	NaN

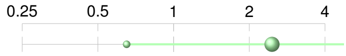

A	B	2.5	50	97.5	Sequence
0	1	0.65	2.5	9	SKPFPPLQNDLILR

5.255 " 10 1191 3-ketoacyl-CoA thiolase, mitochondrial [Crassostrea gigas]"

Protein Accession	CL29653.Contig1$_H$1
Mean Expression Ratio	0.869
Median Expression Ratio	0.869
Credible Interval	(0.488, 1.55)
Associated Peptides	6
Associated Spectra	8
Coverage	NaN

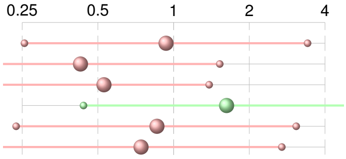

A	B	2.5	50	97.5	Sequence
1	0	0.25	0.93	3.4	ELGLDPAK
1	0	0.12	0.43	1.5	IVAAK
0	3	0.2	0.53	1.4	ITTNLVHEIR
0	1	0.44	1.6	6	KGPEDFNVDEHPR
0	1	0.24	0.86	3.1	VMGIGPVPASK
0	1	0.2	0.74	2.7	TQAPYSVR

5.256 49 219 haemocyanin precursor [Nautilus pompilius]

Protein Accession	Unigene32601$_H$1
Mean Expression Ratio	0.872
Median Expression Ratio	0.87
Credible Interval	(0.478, 1.61)
Associated Peptides	3
Associated Spectra	13
Coverage	NaN

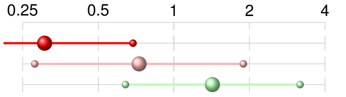

A	B	2.5	50	97.5	Sequence
3	2	0.14	0.31	0.69	NTQPELFK
2	1	0.28	0.73	1.9	NTQPELFKK
0	5	0.64	1.4	3.2	GYIDFENGYTVR

5.257 2 352 Ribosome-binding protein 1 [Crassostrea gigas]

Protein Accession	Unigene24847$_{H}1$
Mean Expression Ratio	0.873
Median Expression Ratio	0.87
Credible Interval	(0.441, 1.7)
Associated Peptides	2
Associated Spectra	2
Coverage	NaN

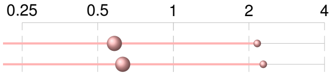

A	B	2.5	50	97.5	Sequence
0	1	0.16	0.58	2.2	LVAHQLELEALQAR
0	1	0.17	0.63	2.3	LQPLEQSTDAALVQK

5.258 3 224 PREDICTED: 40S ribosomal protein S14-like [Sarcophilus harrisii]

Protein Accession	Unigene48834$_{H}1$
Mean Expression Ratio	1.15
Median Expression Ratio	1.15
Credible Interval	(0.602, 2.21)
Associated Peptides	3
Associated Spectra	3
Coverage	NaN

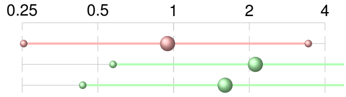

A	B	2.5	50	97.5	Sequence
0	1	0.25	0.95	3.4	IEDVTPIPSDSTR
0	1	0.57	2.1	7.7	IGRIEDVTPIPSDSTR
0	1	0.44	1.6	5.9	IGRIEDVTPIPSDSTRR

5.259 2000 2641 Polyadenylate-binding protein 4 [Crassostrea gigas]

Protein Accession	Unigene7641$_{H}1$
Mean Expression Ratio	1.15
Median Expression Ratio	1.15
Credible Interval	(0.593, 2.27)
Associated Peptides	2
Associated Spectra	2
Coverage	NaN

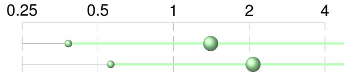

A	B	2.5	50	97.5	Sequence
0	1	0.38	1.4	5.1	NIDDSIDDERLR
0	1	0.56	2.1	7.8	KEFSQFGTITSAK

5.260 366 1220 Ectoine hydroxylase [Crassostrea gigas]

Protein Accession	CL2342.Contig2$_{H}1$
Mean Expression Ratio	0.87
Median Expression Ratio	0.871
Credible Interval	(0.473, 1.59)
Associated Peptides	4
Associated Spectra	6
Coverage	NaN

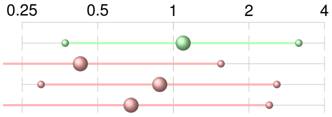

A	B	2.5	50	97.5	Sequence
2	0	0.37	1.1	3.2	IDHDLVAGQQR
0	1	0.12	0.42	1.5	AFDDDGYILIK
0	2	0.3	0.88	2.6	GFLDQEELDNLRK
0	1	0.19	0.68	2.4	TVEDSDLFR

5.261 2 256 Polyubiquitin (Fragment) OS=Xenopus laevis PE=1 SV=2

Protein Accession	CL47507.Contig2$_H$1
Mean Expression Ratio	0.873
Median Expression Ratio	0.872
Credible Interval	(0.486, 1.57)
Associated Peptides	3
Associated Spectra	12
Coverage	NaN

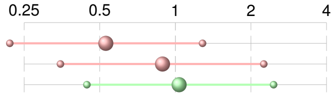

A	B	2.5	50	97.5	Sequence
**1**	**3**	**0.22**	**0.53**	**1.3**	**TITLEVEPSDTIENVK**
**2**	**2**	**0.35**	**0.89**	**2.2**	**TLSDYNIQK**
**0**	**4**	**0.45**	**1**	**2.5**	**ESTLHLVLR**

5.262 " 1 1125 PREDICTED: trifunctional enzyme subunit beta, mitochondrial-like [Oreochromis niloticus]"

Protein Accession	CL19853.Contig2$_H$1
Mean Expression Ratio	0.875
Median Expression Ratio	0.872
Credible Interval	(0.447, 1.73)
Associated Peptides	2
Associated Spectra	2
Coverage	NaN

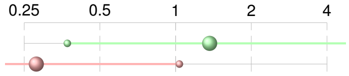

A	B	2.5	50	97.5	Sequence
**0**	**1**	**0.37**	**1.4**	**5.1**	**KEQDEFALR**
**0**	**1**	**0.077**	**0.28**	**1**	**LMLSLNK**

5.263 326 724 hypothetical protein [Amblyomma maculatum]

Protein Accession	Unigene1454$_H$1
Mean Expression Ratio	0.87
Median Expression Ratio	0.872
Credible Interval	(0.461, 1.66)
Associated Peptides	3
Associated Spectra	5
Coverage	NaN

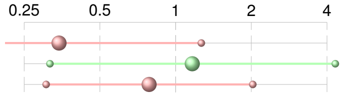

A	B	2.5	50	97.5	Sequence
**0**	**1**	**0.094**	**0.34**	**1.3**	**EAYLELER**
**0**	**1**	**0.32**	**1.2**	**4.3**	**KLEEHFADTK**
**0**	**3**	**0.31**	**0.79**	**2**	**EAYLELERR**

5.264 " 492 3458 PREDICTED: 2-oxoglutarate dehydrogenase, mitochondrial isoform 1 [Sarcophilus harrisii]"

Protein Accession	CL2200.Contig16$_H$1
Mean Expression Ratio	0.869
Median Expression Ratio	0.873
Credible Interval	(0.443, 1.68)
Associated Peptides	2
Associated Spectra	2
Coverage	NaN

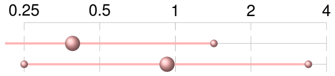

A	B	2.5	50	97.5	Sequence
**1**	**0**	**0.1**	**0.39**	**1.4**	**IAKEPSVLTK**
**1**	**0**	**0.25**	**0.93**	**3.4**	**FRAKK**

5.265 130 2145 minus strand Glucosamine–fructose-6-phosphate aminotransferase [isomerizing] 1 [Crassostrea gigas]; 130 2121 minus strand Glucosamine–fructose-6-phosphate aminotransferase [isomerizing] 1 [Crassostrea gigas]

Protein Accession	CL2589.Contig3$_{H}1$ CL2589.Contig2$_{H}1$
Mean Expression Ratio	1.15
Median Expression Ratio	1.15
Credible Interval	(0.597, 2.19)
Associated Peptides	3
Associated Spectra	3
Coverage	NaN

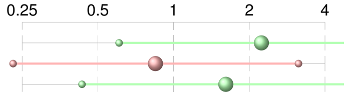

A	B	2.5	50	97.5	Sequence
1	0	0.61	2.2	8.3	GSEPQIIKQTGK
1	0	0.23	0.85	3.1	SLLVMGR
0	1	0.43	1.6	6	VANLEAQIK

5.266 196 657 superoxide dismutase [Aplysia californica] gi; 690 1151 superoxide dismutase [Aplysia californica] gi

Protein Accession	Unigene18046$_{H}1$ Unigene150$_{H}1$
Mean Expression Ratio	1.15
Median Expression Ratio	1.15
Credible Interval	(0.635, 2.08)
Associated Peptides	4
Associated Spectra	9
Coverage	NaN

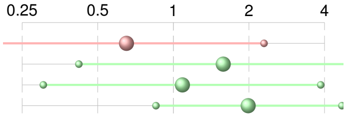

A	B	2.5	50	97.5	Sequence
0	1	0.18	0.65	2.3	THGGPDDVERHAGDLGNIR
0	1	0.42	1.6	5.6	AVCVLK
0	1	0.3	1.1	3.9	AGDLGNIR
3	3	0.85	2	4.7	SLVVHEK

5.267 2140 2367 heat shock cognate 71 [Mytilus galloprovincialis]

Protein Accession	CL5924.Contig13$_{H}1$
Mean Expression Ratio	0.872
Median Expression Ratio	0.873
Credible Interval	(0.448, 1.7)
Associated Peptides	1
Associated Spectra	7
Coverage	NaN

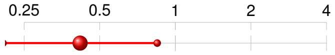

A	B	2.5	50	97.5	Sequence
6	1	0.21	0.42	0.85	LIGDAAK

5.268 424 1347 minus strand Tubulin alpha chain [Ascaris suum]

Protein Accession	Unigene7901$_{H}1$
Mean Expression Ratio	1.14
Median Expression Ratio	1.14
Credible Interval	(0.603, 2.13)
Associated Peptides	3
Associated Spectra	7
Coverage	NaN

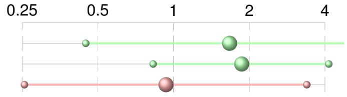

A	B	2.5	50	97.5	Sequence
0	1	0.45	1.7	6	VDLEPTVVDEVR
0	5	0.83	1.9	4.1	NTFFNETGSGKHVPRAIFVDLEPTVVDEVR
0	1	0.26	0.93	3.4	QLFHPEQLITGK

5.269 636 1232 sigma class glutathione-s-transferase 3 [Haliotis discus discus]; 963 1559 sigma class glutathione-s-transferase 3 [Haliotis discus discus]; 907 1503 sigma class glutathione-s-transferase 3 [Haliotis discus discus]; 673 1269 sigma class glutathione-s-transferase 3 [Haliotis discus discus]; 177 773 sigma class glutathione-s-transferase 3 [Haliotis discus discus]; 208 804 sigma class glutathione-s-transferase 3 [Haliotis discus discus]; 327 923 sigma class glutathione-s-transferase 3 [Haliotis discus discus]

Protein Accession	Unigene22829$_{H}1$ CL19285.Contig31$_{H}1$ CL19285.Contig30$_{H}1$ CL19285.Contig27$_{H}1$ CL192[illegible]
Mean Expression Ratio	0.871
Median Expression Ratio	0.874
Credible Interval	(0.461, 1.63)
Associated Peptides	3
Associated Spectra	4
Coverage	NaN

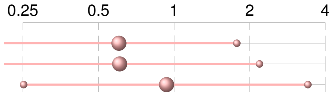

A	B	2.5	50	97.5	Sequence
**0**	**2**	**0.2**	**0.6**	**1.8**	**LLLAASGKDWEDVR**
**0**	**1**	**0.17**	**0.61**	**2.2**	**LQGFDKDYPEVQK**
**0**	**1**	**0.25**	**0.94**	**3.4**	**FSDEDWPK**

5.270 " 132 1544 minus strand Serine hydroxymethyltransferase, cytosolic [Crassostrea gigas]"

Protein Accession	CL22573.Contig2$_{H}1$
Mean Expression Ratio	1.14
Median Expression Ratio	1.14
Credible Interval	(0.589, 2.26)
Associated Peptides	2
Associated Spectra	2
Coverage	NaN

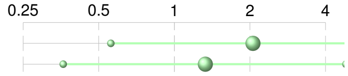

A	B	2.5	50	97.5	Sequence
**0**	**1**	**0.56**	**2.1**	**7.3**	**IGTPALTSR**
**0**	**1**	**0.36**	**1.3**	**4.8**	**SGIIFYR**

5.271 85 1632 minus strand Alpha-aminoadipic semialdehyde dehydrogenase [Crassostrea gigas]

Protein Accession	Unigene19467$_{H}1$
Mean Expression Ratio	1.14
Median Expression Ratio	1.14
Credible Interval	(0.587, 2.22)
Associated Peptides	2
Associated Spectra	2
Coverage	NaN

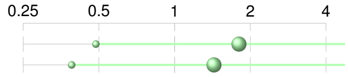

A	B	2.5	50	97.5	Sequence
**0**	**1**	**0.49**	**1.8**	**6.7**	**ELPLAQGIKFE**
**0**	**1**	**0.39**	**1.4**	**5.3**	**MFAGHVFPSER**

5.272 " 116 1168 minus strand hypothetical protein CGI_10014553, partial [Crassostrea gigas]"

Protein Accession	CL19215.Contig5$_{H}1$
Mean Expression Ratio	0.871
Median Expression Ratio	0.875
Credible Interval	(0.463, 1.6)
Associated Peptides	4
Associated Spectra	6
Coverage	NaN

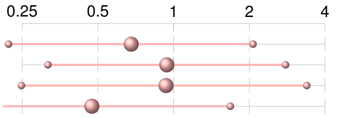

A	B	2.5	50	97.5	Sequence
1	1	0.22	0.68	2.1	FGPTTSEFEK
1	1	0.32	0.94	2.8	SVIALGEGAGANIVAR
1	0	0.25	0.93	3.4	VLEER
0	1	0.13	0.47	1.7	AQNKDQLQK

5.273 241 561 minus strand thioredoxin [Crassostrea ariakensis]

Protein Accession	CL33159.Contig3$_{H}1$
Mean Expression Ratio	0.874
Median Expression Ratio	0.875
Credible Interval	(0.463, 1.64)
Associated Peptides	3
Associated Spectra	6
Coverage	NaN

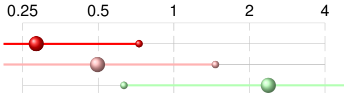

A	B	2.5	50	97.5	Sequence
2	1	0.11	0.28	0.73	IETQEDFEK
1	1	0.17	0.5	1.5	EVGEVVGASEEEIKK
0	1	0.63	2.4	8.8	VKIETQEDFEK

5.274 185 742 Transmembrane protein Tmp21 [Crassostrea gigas]

Protein Accession	CL3732.Contig3$_{H}1$
Mean Expression Ratio	1.14
Median Expression Ratio	1.14
Credible Interval	(0.635, 2.08)
Associated Peptides	5
Associated Spectra	7
Coverage	NaN

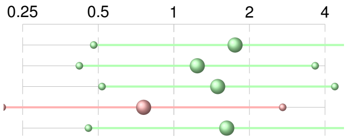

A	B	2.5	50	97.5	Sequence
0	1	0.48	1.8	6.3	SIVDDFAYMR
0	2	0.42	1.2	3.7	SYDELAKAEK
0	2	0.52	1.5	4.4	RLEDLSK
0	1	0.21	0.76	2.7	VLYLR
0	1	0.46	1.6	6	GHILYSK

5.275 35 1861 hypothetical protein DAPPUDRAFT_309082 [Daphnia pulex]

Protein Accession	CL49314.Contig5$_{H}1$
Mean Expression Ratio	0.875
Median Expression Ratio	0.876
Credible Interval	(0.496, 1.53)
Associated Peptides	5
Associated Spectra	16
Coverage	NaN

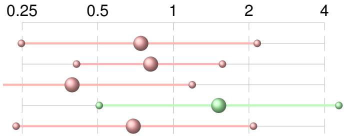

A	B	2.5	50	97.5	Sequence
1	1	0.25	0.74	2.2	LINHSVPDTIDER
5	3	0.41	0.81	1.6	YAIGLAR
1	1	0.13	0.4	1.2	KVELVK
0	2	0.51	1.5	4.6	DIGHTFKK
0	2	0.24	0.69	2.1	LNDGIIIAK

5.276 " 39 968 l-3-hydroxyacyl-coenzyme a dehydrogenase, short chain [Haliotis discus discus]; 39 968 l-3-hydroxyacyl-coenzyme a dehydrogenase, short chain [Haliotis discus discus]"

Protein Accession	CL29042.Contig8$_{H}$1 CL29042.Contig1$_{H}$1
Mean Expression Ratio	0.876
Median Expression Ratio	0.877
Credible Interval	(0.462, 1.67)
Associated Peptides	3
Associated Spectra	3
Coverage	NaN

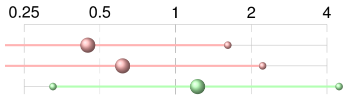

A	B	2.5	50	97.5	Sequence
1	0	0.12	0.45	1.6	ISTSTDAATAAK
1	0	0.17	0.62	2.2	STASIQQSLQR
0	1	0.33	1.2	4.5	LFTALDAAAPK

5.277 1292 1471 minus strand hemocyanin isoform 1 [Haliotis diversicolor]

Protein Accession	Unigene65233$_{H}$1
Mean Expression Ratio	0.875
Median Expression Ratio	0.877
Credible Interval	(0.456, 1.69)
Associated Peptides	2
Associated Spectra	4
Coverage	NaN

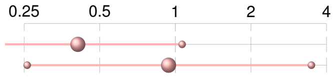

A	B	2.5	50	97.5	Sequence
1	2	0.16	0.41	1.1	LYTVQFEDALR
0	1	0.26	0.94	3.5	LYTVQFEDALRR

5.278 184 438 PREDICTED: similar to CG14235 CG14235-PA [Tribolium castaneum] gi

Protein Accession	Unigene638$_{H}$1
Mean Expression Ratio	0.877
Median Expression Ratio	0.877
Credible Interval	(0.486, 1.6)
Associated Peptides	5
Associated Spectra	7
Coverage	NaN

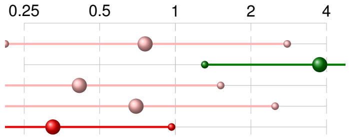

A	B	2.5	50	97.5	Sequence
1	0	0.21	0.76	2.8	TAPFDAR
1	1	1.3	3.8	11	GDEQKYYQLK
1	0	0.11	0.41	1.5	YYQLK
0	1	0.19	0.7	2.5	WDDQVANGTFAGKI
0	2	0.11	0.33	0.96	WDDQVANGTFAGK

5.279 79 2202 minus strand PREDICTED: dolichyl-diphosphooligosaccharide–protein glycosyltransferase subunit STT3A-like isoform 2 [Strongylocentrotus purpuratus]; 79 2202 PREDICTED: dolichyl-diphosphooligosaccharide–protein glycosyltransferase subunit STT3A-like isoform 2 [Strongylocentrotus purpuratus]; 79 2202 PREDICTED: dolichyl-diphosphooligosaccharide–protein glycosyltransferase subunit STT3A-like isoform 2 [Strongylocentrotus purpuratus]

Protein Accession	CL5867.Contig2$_{H}1$ CL5867.Contig11$_{H}1$ CL5867.Contig10$_{H}1$
Mean Expression Ratio	0.875
Median Expression Ratio	0.877
Credible Interval	(0.428, 1.75)
Associated Peptides	1
Associated Spectra	1
Coverage	NaN

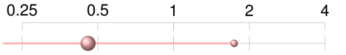

A	B	2.5	50	97.5	Sequence
1	0	0.12	0.46	1.7	VGQAMASTEEK

5.280 80 250 minus strand 40S ribosomal protein S29 [Ornithodoros parkeri]

Protein Accession	CL11390.Contig2$_{H}1$
Mean Expression Ratio	1.14
Median Expression Ratio	1.14
Credible Interval	(0.611, 2.15)
Associated Peptides	3
Associated Spectra	7
Coverage	NaN

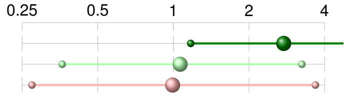

A	B	2.5	50	97.5	Sequence
0	4	1.2	2.8	6.6	GFQTTWYSHPR
0	2	0.36	1.1	3.3	QFAPDIGFK
0	1	0.27	0.99	3.7	KYGLNICR

5.281 " 9 125 minus strand Hemocyanin, beta-C chain unit D (Fragment) OS=Helix pomatia PE=1 SV=2"

Protein Accession	CL7906.Contig6$_{H}1$
Mean Expression Ratio	0.878
Median Expression Ratio	0.879
Credible Interval	(0.436, 1.75)
Associated Peptides	1
Associated Spectra	1
Coverage	NaN

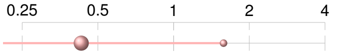

A	B	2.5	50	97.5	Sequence
0	1	0.12	0.43	1.6	YDISDAVK

5.282 3591 4526 minus strand myosin heavy chain cardiac muscle specific isoform 1 [Argopecten irradians]

Protein Accession	CL49594.Contig2$_{H}$1
Mean Expression Ratio	1.14
Median Expression Ratio	1.14
Credible Interval	(0.77, 1.67)
Associated Peptides	20
Associated Spectra	91
Coverage	NaN

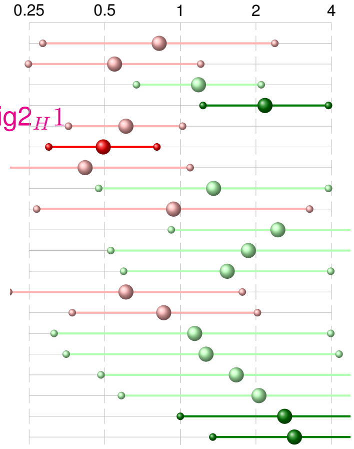

A	B	2.5	50	97.5	Sequence
1	1	0.28	0.82	2.4	ATQANVEDLER
4	1	0.25	0.55	1.2	KLEQDLK
4	7	0.67	1.2	2.1	LQQETANLTR
5	6	1.2	2.2	3.9	QLEEAEHK
6	8	0.36	0.61	1	QVEAQLAELSAK
9	7	0.3	0.49	0.81	TINDLQTQK
3	0	0.16	0.42	1.1	VGALTK
0	2	0.47	1.4	3.9	AKLEQTLDELEDNLER
0	1	0.27	0.94	3.3	AQLKSEVDDLQMQLQNVGK
0	3	0.92	2.4	6.3	KLEQDLKATQANVEDLER
0	1	0.53	1.9	6.4	NRLQQETANLTR
0	3	0.6	1.5	4	QLEEAEHKVGALTKEK
0	2	0.21	0.61	1.8	QVEAQLAELSAKVEEGNR
0	4	0.37	0.86	2	SEVDDLQMQLQNVGK
0	1	0.32	1.1	4	TMEDLQAEEDKVNHLNK
0	1	0.35	1.3	4.3	AATSRSRTLQHEAQVSALR
0	1	0.48	1.7	6	VKRELEENVR
0	1	0.58	2.1	7.3	RKLEQDLK
0	3	1	2.6	6.7	ATSRSRTLQHEAQVSALR
0	6	1.3	2.8	5.9	QLEEAEHKVGALTK

5.283 18 1406 AGAP006996-PA [Anopheles gambiae str. PEST] gi

Protein Accession	CL48173.Contig5$_{H}$1
Mean Expression Ratio	1.14
Median Expression Ratio	1.14
Credible Interval	(0.586, 2.22)
Associated Peptides	2
Associated Spectra	2
Coverage	NaN

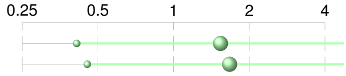

A	B	2.5	50	97.5	Sequence
0	1	0.41	1.5	5.5	VVLNLR
0	1	0.46	1.7	6.3	TTLTAAITK

5.284 1 945 hemocyanin isoform 1 [Haliotis diversicolor]

Protein Accession	Unigene30318$_{H}$1
Mean Expression Ratio	0.881
Median Expression Ratio	0.882
Credible Interval	(0.45, 1.73)
Associated Peptides	2
Associated Spectra	3
Coverage	NaN

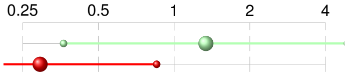

A	B	2.5	50	97.5	Sequence
0	1	0.36	1.3	4.9	LFVVEVEDELRAR
0	2	0.097	0.29	0.85	VEITDVLK

5.285 552 1499 minus strand uncharacterized protein LOC100158466 [Xenopus (Silurana) tropicalis] gi; 552 1499 minus strand uncharacterized protein LOC100158466 [Xenopus (Silurana) tropicalis] gi; 552 1499 minus strand uncharacterized protein LOC100158466 [Xenopus (Silurana) tropicalis] gi

Protein Accession	Unigene57645$_H1$ Unigene52825$_H1$ Unigene48504$_H1$
Mean Expression Ratio	0.881
Median Expression Ratio	0.882
Credible Interval	(0.437, 1.74)
Associated Peptides	1
Associated Spectra	1
Coverage	NaN

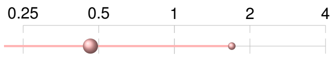

A	B	2.5	50	97.5	Sequence
**0**	**1**	**0.12**	**0.46**	**1.7**	**TGEPVEVDGELATLLAR**

5.286 2 349 minus strand hypothetical protein BRAFLDRAFT_206907 [Branchiostoma floridae] gi

Protein Accession	Unigene875$_H1$
Mean Expression Ratio	0.883
Median Expression Ratio	0.882
Credible Interval	(0.457, 1.71)
Associated Peptides	2
Associated Spectra	2
Coverage	NaN

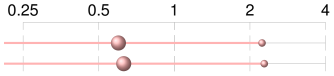

A	B	2.5	50	97.5	Sequence
**1**	**0**	**0.16**	**0.6**	**2.2**	**AQAIIGQHDK**
**1**	**0**	**0.17**	**0.63**	**2.3**	**YTPTFR**

5.287 189 353 40S ribosomal protein S12 [Crassostrea gigas]

Protein Accession	Unigene385$_H1$
Mean Expression Ratio	1.14
Median Expression Ratio	1.13
Credible Interval	(0.59, 2.22)
Associated Peptides	1
Associated Spectra	4
Coverage	NaN

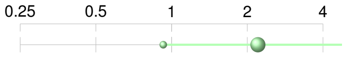

A	B	2.5	50	97.5	Sequence
**0**	**4**	**0.92**	**2.2**	**5.2**	**TALIHDGLAR**

5.288 14 502 minus strand ependymin related protein-1 precursor [Haliotis discus discus]; 14 547 ependymin related protein-1 precursor [Haliotis discus discus]

Protein Accession	CL3749.Contig3$_H1$ CL3749.Contig1$_H1$
Mean Expression Ratio	0.882
Median Expression Ratio	0.883
Credible Interval	(0.434, 1.75)
Associated Peptides	1
Associated Spectra	1
Coverage	NaN

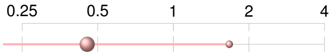

A	B	2.5	50	97.5	Sequence
**0**	**1**	**0.12**	**0.45**	**1.7**	**IWYDAENQR**

5.289 106 1701 T-complex protein 1 subunit beta [Crassostrea gigas]

Protein Accession	CL8176.Contig1$_H1$
Mean Expression Ratio	1.13
Median Expression Ratio	1.13
Credible Interval	(0.584, 2.18)
Associated Peptides	2
Associated Spectra	3
Coverage	NaN

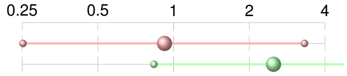

A	B	2.5	50	97.5	Sequence
**0**	**1**	**0.25**	**0.92**	**3.3**	**EAESLIGQK**
**0**	**2**	**0.83**	**2.5**	**7.5**	**VAELELAEKEK**

5.290 112 819 pyruvate kinase [Crassostrea gigas]; 132 932 pyruvate kinase [Crassostrea gigas]

Protein Accession	CL31920.Contig3$_H1$ CL31920.Contig1$_H1$
Mean Expression Ratio	0.883
Median Expression Ratio	0.884
Credible Interval	(0.456, 1.71)
Associated Peptides	1
Associated Spectra	3
Coverage	NaN

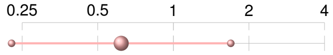

A	B	2.5	50	97.5	Sequence
**2**	**1**	**0.23**	**0.62**	**1.7**	**ITIDDSYKEK**

5.291 557 2329 minus strand Collagen alpha-3(VI) chain [Crassostrea gigas]

Protein Accession	CL47586.Contig7$_H1$
Mean Expression Ratio	1.13
Median Expression Ratio	1.13
Credible Interval	(0.594, 2.14)
Associated Peptides	3
Associated Spectra	4
Coverage	NaN

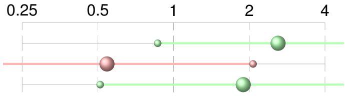

A	B	2.5	50	97.5	Sequence
**0**	**2**	**0.87**	**2.6**	**7.8**	**KLDVVVAFHASPR**
**0**	**1**	**0.15**	**0.54**	**2.1**	**VALGFFNK**
**0**	**1**	**0.51**	**1.9**	**7.1**	**KADYSAALGKLPK**

5.292 80 589 minus strand PREDICTED: adenine phosphoribosyltransferase-like [Strongylocentrotus purpuratus]

Protein Accession	CL8682.Contig6$_H$1
Mean Expression Ratio	1.13
Median Expression Ratio	1.13
Credible Interval	(0.599, 2.19)
Associated Peptides	2
Associated Spectra	4
Coverage	NaN

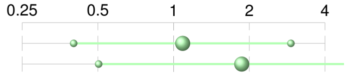

A	B	2.5	50	97.5	Sequence
1	2	0.4	1.1	2.9	AAVELVEK
0	1	0.5	1.9	7	GKLTVPVESLIKY

5.293 58 732 glutathione-s-transferase [Haliotis discus discus]

Protein Accession	CL4861.Contig1$_H$1
Mean Expression Ratio	0.888
Median Expression Ratio	0.885
Credible Interval	(0.487, 1.61)
Associated Peptides	4
Associated Spectra	11
Coverage	NaN

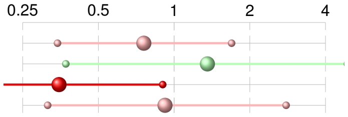

A	B	2.5	50	97.5	Sequence
3	2	0.35	0.76	1.7	GQVPTFR
1	0	0.37	1.4	4.9	LLPKEAGGR
0	3	0.13	0.35	0.9	IDKEYLEVK
0	2	0.31	0.92	2.8	LISFSANEHK

5.294 23 409 minus strand CDGSH iron sulfur domain-containing protein 2 [Crassostrea gigas]; 1 207 minus strand CDGSH iron sulfur domain-containing protein 2 [Crassostrea gigas]; 22 135 minus strand CDGSH iron-sulfur domain-containing protein 2 OS=Lithobates catesbeiana GN=cisd2 PE=2 SV=1; 22 207 minus strand CDGSH iron-sulfur domain-containing protein 2 homolog B OS=Branchiostoma floridae GN=BRAFLDRAFT_274541 PE=3 SV=1

Protein Accession	CL36832.Contig6$_H$1 CL36832.Contig3$_H$1 CL36832.Contig2$_H$1 CL36832.Contig1$_H$1
Mean Expression Ratio	0.885
Median Expression Ratio	0.885
Credible Interval	(0.441, 1.76)
Associated Peptides	1
Associated Spectra	1
Coverage	NaN

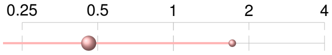

A	B	2.5	50	97.5	Sequence
0	1	0.13	0.46	1.7	VVNMVDIEDLGDK

5.295 178 582 hypothetical protein CGI_10001640 [Crassostrea gigas]

Protein Accession	CL2347.Contig2$_{H}1$
Mean Expression Ratio	0.883
Median Expression Ratio	0.886
Credible Interval	(0.5, 1.55)
Associated Peptides	4
Associated Spectra	16
Coverage	NaN

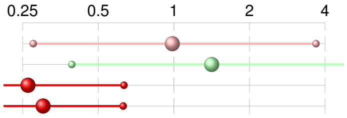

A	B	2.5	50	97.5	Sequence
**0**	**1**	**0.28**	**0.99**	**3.7**	**LKSHGIGGK**
**0**	**1**	**0.39**	**1.4**	**5.1**	**SVGGNAGEVICK**
**3**	**2**	**0.11**	**0.26**	**0.63**	**AFLEQLGSK**
**5**	**4**	**0.14**	**0.3**	**0.63**	**ANLIVIGSR**

5.296 128 1519 minus strand V-type proton ATPase subunit H [Crassostrea gigas]; 128 1519 minus strand V-type proton ATPase subunit H [Crassostrea gigas]; 128 1519 minus strand V-type proton ATPase subunit H [Crassostrea gigas]; 128 1519 minus strand V-type proton ATPase subunit H [Crassostrea gigas]

Protein Accession	Unigene39559$_{H}1$ Unigene2219$_{H}1$ CL1883.Contig8$_{H}1$ CL1883.Contig6$_{H}1$
Mean Expression Ratio	1.13
Median Expression Ratio	1.13
Credible Interval	(0.571, 2.18)
Associated Peptides	2
Associated Spectra	4
Coverage	NaN

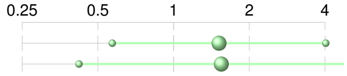

A	B	2.5	50	97.5	Sequence
**0**	**3**	**0.57**	**1.5**	**4**	**SGNLEWSPVHR**
**0**	**1**	**0.42**	**1.6**	**5.6**	**ANVVNWK**

5.297 460 1737 Tubulin beta-4B chain OS=Mus musculus GN=Tubb4b PE=1 SV=1

Protein Accession	Unigene34147$_{H}1$
Mean Expression Ratio	1.13
Median Expression Ratio	1.13
Credible Interval	(0.577, 2.21)
Associated Peptides	1
Associated Spectra	28
Coverage	NaN

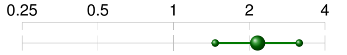

A	B	2.5	50	97.5	Sequence
**5**	**23**	**1.5**	**2.2**	**3.2**	**AVLVDLEPGTMDSVR**

5.298 245 1243 Prostaglandin reductase 1 [Crassostrea gigas]

Protein Accession	CL37890.Contig1$_{H}1$
Mean Expression Ratio	1.12
Median Expression Ratio	1.13
Credible Interval	(0.556, 2.25)
Associated Peptides	1
Associated Spectra	1
Coverage	NaN

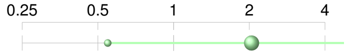

A	B	2.5	50	97.5	Sequence
**0**	**1**	**0.55**	**2**	**7.5**	**TLAVLHPDMPR**

5.299 187 942 Testis-specific gene 10 protein OS=Mus musculus GN=Tsga10 PE=1 SV=1; 212 967 Testis-specific gene 10 protein OS=Mus musculus GN=Tsga10 PE=1 SV=1; 214 870 Testis-specific gene 10 protein OS=Mus musculus GN=Tsga10 PE=1 SV=1; 134 793 Testis-specific gene 10 protein OS=Mus musculus GN=Tsga10 PE=1 SV=1

Protein Accession	CL11641.Contig6$_{H}1$ CL11641.Contig5$_{H}1$ CL11641.Contig4$_{H}1$ CL11641.Contig3$_{H}1$
Mean Expression Ratio	0.884
Median Expression Ratio	0.887
Credible Interval	(0.454, 1.69)
Associated Peptides	2
Associated Spectra	3
Coverage	NaN

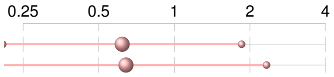

A	B	2.5	50	97.5	Sequence
**0**	**2**	**0.21**	**0.62**	**1.8**	**RVENERDDAIADLHR**
**0**	**1**	**0.18**	**0.64**	**2.3**	**ALEEQTK**

5.300 3 614 PREDICTED: 60S ribosomal protein L8-like [Papio anubis]

Protein Accession	CL18365.Contig1$_{H}1$
Mean Expression Ratio	1.14
Median Expression Ratio	1.13
Credible Interval	(0.58, 2.24)
Associated Peptides	2
Associated Spectra	2
Coverage	NaN

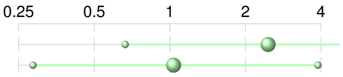

A	B	2.5	50	97.5	Sequence
**0**	**1**	**0.66**	**2.5**	**9**	**KVGLIAAR**
**0**	**1**	**0.28**	**1**	**3.9**	**VGLIAARR**

5.301 " 695 823 minus strand Aldehyde dehydrogenase family 16 member A1, partial [Crassostrea gigas]"

Protein Accession	CL17427.Contig5$_{H}1$
Mean Expression Ratio	1.12
Median Expression Ratio	1.13
Credible Interval	(0.567, 2.26)
Associated Peptides	1
Associated Spectra	1
Coverage	NaN

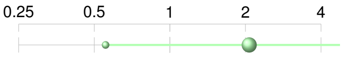

A	B	2.5	50	97.5	Sequence
**0**	**1**	**0.55**	**2.1**	**7.6**	**TVWIPAGEIFAN**

5.302 246 887 rab11-2 [Limulus polyphemus]

Protein Accession	CL3557.Contig1$_{H}$1
Mean Expression Ratio	0.889
Median Expression Ratio	0.888
Credible Interval	(0.469, 1.69)
Associated Peptides	3
Associated Spectra	3
Coverage	NaN

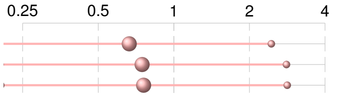

A	B	2.5	50	97.5	Sequence
1	0	0.19	0.66	2.5	NEFNLESK
1	0	0.2	0.75	2.8	GAVGALLVYDIAK
0	1	0.2	0.76	2.8	VVLIGDSGVGK

5.303 2 436 minus strand PREDICTED: stress-induced-phosphoprotein 1 [Sarcophilus harrisii]

Protein Accession	CL38065.Contig3$_{H}$1
Mean Expression Ratio	1.13
Median Expression Ratio	1.13
Credible Interval	(0.613, 2.11)
Associated Peptides	3
Associated Spectra	6
Coverage	NaN

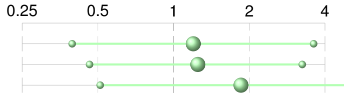

A	B	2.5	50	97.5	Sequence
1	1	0.39	1.2	3.6	IGNSYMR
0	3	0.46	1.2	3.3	ADFTAIAK
0	1	0.51	1.9	6.9	LAYVNP

5.304 158 862 X-box binding protein [Haliotis discus discus]

Protein Accession	CL6396.Contig3$_{H}$1
Mean Expression Ratio	1.12
Median Expression Ratio	1.13
Credible Interval	(0.634, 2.02)
Associated Peptides	5
Associated Spectra	10
Coverage	NaN

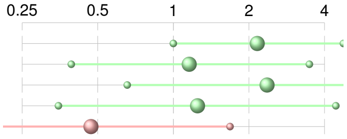

A	B	2.5	50	97.5	Sequence
3	2	1	2.2	4.8	SYYIGAR
0	2	0.39	1.2	3.5	VFPDFYFGAGDDKLDAR
0	1	0.66	2.4	8.6	ACIPDKAK
0	1	0.35	1.3	4.5	KYSGTNSTGRLYIVDLAK
0	1	0.13	0.47	1.7	VFPDFYFGAGDDK

5.305 182 1846 minus strand hypothetical protein DAPPUDRAFT_302108 [Daphnia pulex]; 110 1774 minus strand hypothetical protein DAPPUDRAFT_302108 [Daphnia pulex]

Protein Accession	CL11561.Contig6$_{H}$1 CL11561.Contig1$_{H}$1
Mean Expression Ratio	1.13
Median Expression Ratio	1.13
Credible Interval	(0.586, 2.12)
Associated Peptides	3
Associated Spectra	4
Coverage	NaN

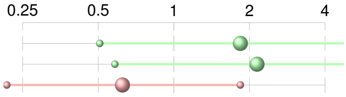

A	B	2.5	50	97.5	Sequence
0	1	0.51	1.8	6.8	VATSPFEGQKPGTSGLR
0	1	0.58	2.1	8	IVYRLSGTGSSGATIR
0	2	0.22	0.62	1.8	LFLPGGLK

5.306 1425 2018 minus strand Ankyrin-2 [Crassostrea gigas]; 1425 2018 minus strand Ankyrin-2 [Crassostrea gigas]

Protein Accession	CL45186.Contig15$_{H}1$ CL45186.Contig14$_{H}1$
Mean Expression Ratio	1.13
Median Expression Ratio	1.12
Credible Interval	(0.578, 2.22)
Associated Peptides	2
Associated Spectra	3
Coverage	NaN

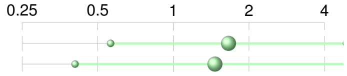

A	B	2.5	50	97.5	Sequence
1	1	0.56	1.7	4.9	VAFMKEPK
0	1	0.41	1.5	5.2	SGDQLHINFK

5.307 219 554 minus strand ribosomal protein P1 [Biomphalaria glabrata]

Protein Accession	CL45629.Contig2$_{H}1$
Mean Expression Ratio	0.89
Median Expression Ratio	0.89
Credible Interval	(0.446, 1.77)
Associated Peptides	1
Associated Spectra	1
Coverage	NaN

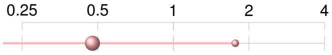

A	B	2.5	50	97.5	Sequence
1	0	0.13	0.48	1.8	ALEGVNIK

5.308 91 2658 minus strand puromycin-sensitive aminopeptidase [Saccoglossus kowalevskii]

Protein Accession	CL6708.Contig2$_{H}1$
Mean Expression Ratio	0.889
Median Expression Ratio	0.89
Credible Interval	(0.493, 1.59)
Associated Peptides	5
Associated Spectra	8
Coverage	NaN

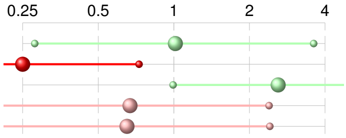

A	B	2.5	50	97.5	Sequence
1	0	0.28	1	3.6	GLTVQQEK
1	1	0.085	0.25	0.73	TLDFALSDK
1	2	1	2.6	6.8	SLGVNDDPELIKR
1	0	0.19	0.67	2.4	LVTYR
0	1	0.18	0.65	2.4	TIQQSLENIMLNKR

5.309 1278 1643 Fatty acid-binding-like protein 5 [Crassostrea gigas]

Protein Accession	CL11559.Contig3$_{H}1$
Mean Expression Ratio	0.887
Median Expression Ratio	0.89
Credible Interval	(0.461, 1.7)
Associated Peptides	2
Associated Spectra	5
Coverage	NaN

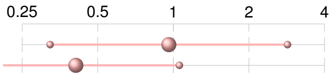

A	B	2.5	50	97.5	Sequence
1	1	0.32	0.96	2.8	AAGVGLVTR
1	2	0.16	0.41	1.1	EILPDGR

5.310 1 309 PREDICTED: intersectin-1-like [Sus scrofa]

Protein Accession	Unigene37688$_{H1}$
Mean Expression Ratio	1.12
Median Expression Ratio	1.12
Credible Interval	(0.552, 2.27)
Associated Peptides	1
Associated Spectra	1
Coverage	NaN

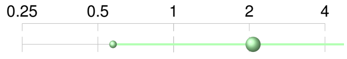

A	B	2.5	50	97.5	Sequence
**0**	**1**	**0.57**	**2.1**	**7.8**	**VDIQPGPKSSK**

5.311 104 445 minus strand macrophage migration inhibitory factor [Ascaris suum]

Protein Accession	CL34536.Contig3$_{H1}$
Mean Expression Ratio	0.89
Median Expression Ratio	0.892
Credible Interval	(0.47, 1.68)
Associated Peptides	3
Associated Spectra	5
Coverage	NaN

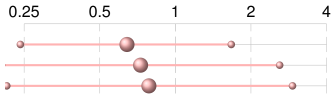

A	B	2.5	50	97.5	Sequence
**1**	**2**	**0.24**	**0.64**	**1.7**	**HIEEALGIPQDR**
**0**	**1**	**0.2**	**0.73**	**2.6**	**AFLKDTSK**
**0**	**1**	**0.21**	**0.78**	**2.9**	**LVVPFMK**

5.312 1709 2026 Phosphoenolpyruvate carboxykinase [GTP] [Crassostrea gigas]

Protein Accession	CL25948.Contig2$_{H1}$
Mean Expression Ratio	0.896
Median Expression Ratio	0.893
Credible Interval	(0.461, 1.75)
Associated Peptides	1
Associated Spectra	7
Coverage	NaN

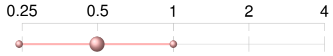

A	B	2.5	50	97.5	Sequence
**4**	**3**	**0.24**	**0.49**	**1**	**GVPLVYEAK**

5.313 " 34 1656 Chaperonin containing TCP1, subunit 7 (eta) [Danio rerio] gi; 34 1656 Chaperonin containing TCP1, subunit 7 (eta) [Danio rerio] gi"

Protein Accession	CL24061.Contig2$_{H1}$ CL24061.Contig1$_{H1}$
Mean Expression Ratio	0.892
Median Expression Ratio	0.893
Credible Interval	(0.491, 1.62)
Associated Peptides	5
Associated Spectra	8
Coverage	NaN

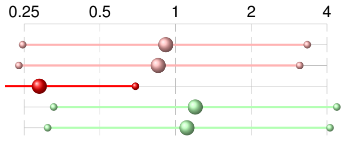

A	B	2.5	50	97.5	Sequence
**1**	**0**	**0.25**	**0.91**	**3.3**	**TLDIVHPAAK**
**1**	**0**	**0.24**	**0.86**	**3.1**	**TLVDIAR**
**1**	**3**	**0.12**	**0.29**	**0.69**	**SIAVSIKK**
**0**	**1**	**0.33**	**1.2**	**4.4**	**SLHDAIMIVR**
**0**	**1**	**0.31**	**1.1**	**4.1**	**MQAPIILLK**

5.314 51 371 minus strand putative mitochondrial ATP synthase F chain [Haliotis discus discus]; 51 371 minus strand putative mitochondrial ATP synthase F chain [Haliotis discus discus]

Protein Accession	CL18283.Contig4$_{H}1$ CL18283.Contig2$_{H}1$
Mean Expression Ratio	0.89
Median Expression Ratio	0.893
Credible Interval	(0.46, 1.75)
Associated Peptides	1
Associated Spectra	3
Coverage	NaN

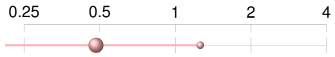

A	B	2.5	50	97.5	Sequence
**3**	**0**	**0.18**	**0.48**	**1.3**	**ADLPLGQVK**

5.315 212 1405 minus strand unnamed protein product [Tetraodon nigroviridis]

Protein Accession	CL37546.Contig3$_{H}1$
Mean Expression Ratio	1.12
Median Expression Ratio	1.12
Credible Interval	(0.588, 2.1)
Associated Peptides	2
Associated Spectra	6
Coverage	NaN

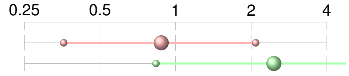

A	B	2.5	50	97.5	Sequence
**2**	**2**	**0.36**	**0.88**	**2.1**	**TIAELGEK**
**0**	**2**	**0.83**	**2.5**	**7.3**	**GLVVPVIR**

5.316 " 61 573 steroid membrane receptor Hpr6.6/25-Dx, putative [Ixodes scapularis] gi"

Protein Accession	CL29406.Contig1$_{H}1$
Mean Expression Ratio	0.894
Median Expression Ratio	0.895
Credible Interval	(0.446, 1.76)
Associated Peptides	1
Associated Spectra	1
Coverage	NaN

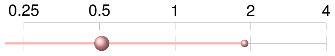

A	B	2.5	50	97.5	Sequence
**1**	**0**	**0.14**	**0.51**	**1.9**	**ILIAVNGK**

5.317 123 860 PREDICTED: proteasome subunit alpha type-6-like [Cavia porcellus]; 1164 1901 PREDICTED: proteasome subunit alpha type-6-like [Cavia porcellus]

Protein Accession	Unigene21398$_{H}1$ Unigene13394$_{H}1$
Mean Expression Ratio	1.12
Median Expression Ratio	1.12
Credible Interval	(0.58, 2.17)
Associated Peptides	2
Associated Spectra	4
Coverage	NaN

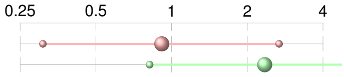

A	B	2.5	50	97.5	Sequence
1	1	0.31	0.91	2.7	QLEASGFLEK
0	2	0.82	2.3	6.9	VLTEQEIDVHLTSIAEKD

5.318 6043 6174 myosin heavy chain [Mytilus galloprovincialis]; 5869 6000 myosin heavy chain [Mytilus galloprovincialis]

Protein Accession	Unigene19025$_{H}1$ CL49594.Contig1$_{H}1$
Mean Expression Ratio	1.12
Median Expression Ratio	1.12
Credible Interval	(0.58, 2.2)
Associated Peptides	2
Associated Spectra	3
Coverage	NaN

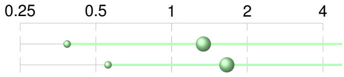

A	B	2.5	50	97.5	Sequence
0	1	0.39	1.3	4.9	AMQTDLDEMNGELR
0	2	0.56	1.7	4.9	KLEADIQAMQTDLDEMNGELR

5.319 230 334 protein disulfide isomerase [Conus virgo]

Protein Accession	Unigene67026$_{H}1$
Mean Expression Ratio	0.897
Median Expression Ratio	0.896
Credible Interval	(0.461, 1.73)
Associated Peptides	1
Associated Spectra	7
Coverage	NaN

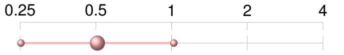

A	B	2.5	50	97.5	Sequence
1	6	0.25	0.51	1	VDATVENDLASK

5.320 " 448 1365 Nitrile-specifier protein 5, partial [Crassostrea gigas]"

Protein Accession	CL8618.Contig4$_{H}1$
Mean Expression Ratio	1.12
Median Expression Ratio	1.12
Credible Interval	(0.581, 2.18)
Associated Peptides	2
Associated Spectra	3
Coverage	NaN

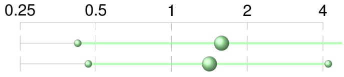

A	B	2.5	50	97.5	Sequence
0	1	0.42	1.6	5.9	KLPTFDAILPR
0	2	0.47	1.4	4.2	LLDTHGATVPPR

5.321 3 551 minus strand Spectrin beta chain [Crassostrea gigas]

Protein Accession	Unigene35960$_{H}1$
Mean Expression Ratio	0.896
Median Expression Ratio	0.896
Credible Interval	(0.464, 1.73)
Associated Peptides	2
Associated Spectra	4
Coverage	NaN

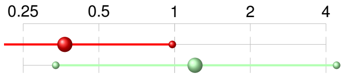

A	B	2.5	50	97.5	Sequence
1	2	0.14	0.37	0.98	LISDINK
0	1	0.34	1.2	4.4	LVSQDNFGADLAAVEAATK

5.322 " 196 1053 minus strand Cytochrome b-c1 complex subunit 2, mitochondrial OS=Homo sapiens GN=UQCRC2 PE=1 SV=3"

Protein Accession	CL7847.Contig5$_{H}1$
Mean Expression Ratio	1.12
Median Expression Ratio	1.12
Credible Interval	(0.559, 2.27)
Associated Peptides	1
Associated Spectra	1
Coverage	NaN

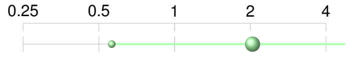

A	B	2.5	50	97.5	Sequence
0	1	0.56	2	7.6	VGKYNAEQLLHYMK

5.323 212 1783 PREDICTED: d-3-phosphoglycerate dehydrogenase-like [Strongylocentrotus purpuratus]

Protein Accession	CL34337.Contig3$_{H}1$
Mean Expression Ratio	1.12
Median Expression Ratio	1.12
Credible Interval	(0.595, 2.17)
Associated Peptides	3
Associated Spectra	3
Coverage	NaN

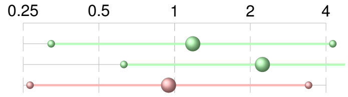

A	B	2.5	50	97.5	Sequence
0	1	0.32	1.2	4.3	GGIIDEDALLR
0	1	0.63	2.2	8.3	VTSTPHLGANTEEAQSR
0	1	0.27	0.95	3.4	LSASLFAGYLR

5.324 " 1 1563 Putative methylmalonate-semialdehyde dehydrogenase [acylating], mitochondrial [Crassostrea gigas]"

Protein Accession	Unigene56839$_{H}1$
Mean Expression Ratio	0.897
Median Expression Ratio	0.897
Credible Interval	(0.568, 1.43)
Associated Peptides	15
Associated Spectra	26
Coverage	NaN

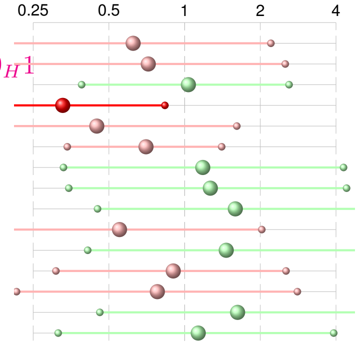

A	B	2.5	50	97.5	Sequence
1	0	0.17	0.62	2.2	AEMDAAVAAAK
1	0	0.19	0.72	2.5	GADLGPLISPESK
1	2	0.39	1	2.6	IAANITEQQGK
1	2	0.13	0.33	0.84	TVTQMWR
1	0	0.13	0.45	1.6	YQQLIK
2	5	0.34	0.7	1.4	GNFVGPTILHK
0	1	0.33	1.2	4.3	AISFVGSDQAGQYIYER
0	1	0.35	1.3	4.4	LFINNEFVESK
0	1	0.45	1.6	5.7	NIKVPGYEK
0	1	0.15	0.55	2	NWIPELVER
0	1	0.41	1.5	5.2	QGVQFYTQVK
0	2	0.31	0.9	2.5	TLVDAEGDVLR
0	1	0.22	0.78	2.8	WIDVHNPATNEVVTR
0	1	0.46	1.6	5.9	QQIMFR
0	1	0.31	1.1	3.9	FINNEFVESK

5.325 36 1310 minus strand elongation factor-1 gamma [Diguetia canities]

Protein Accession	CL14269.Contig5$_{H}1$
Mean Expression Ratio	0.896
Median Expression Ratio	0.897
Credible Interval	(0.523, 1.54)
Associated Peptides	9
Associated Spectra	14
Coverage	NaN

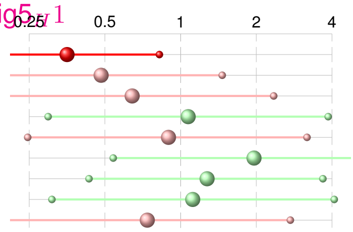

A	B	2.5	50	97.5	Sequence
1	3	0.14	0.35	0.82	LAETMAQFDAK
2	0	0.17	0.48	1.5	TYLVGEK
1	0	0.18	0.64	2.4	FVGNK
0	1	0.3	1.1	3.9	YSGADVQVDPNFK
0	1	0.25	0.9	3.2	GNFDMDEWK
0	1	0.54	2	7.1	GNFDMDEWKR
0	2	0.43	1.3	3.7	SADFLAK
0	1	0.31	1.1	4.1	ESKDPFLAFPK
0	1	0.2	0.74	2.7	LDPDDPETK

5.326 1714 2121 Phosphoenolpyruvate carboxykinase [GTP] [Crassostrea gigas]

Protein Accession	CL25948.Contig1$_{H}1$
Mean Expression Ratio	0.896
Median Expression Ratio	0.898
Credible Interval	(0.453, 1.79)
Associated Peptides	1
Associated Spectra	6
Coverage	NaN

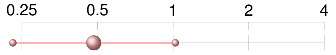

A	B	2.5	50	97.5	Sequence
4	2	0.23	0.48	1	SEATAAAEFK

5.327 94 489 Sortilin-related receptor [Crassostrea gigas]

Protein Accession	CL15197.Contig3$_{H}1$
Mean Expression Ratio	0.898
Median Expression Ratio	0.898
Credible Interval	(0.455, 1.76)
Associated Peptides	1
Associated Spectra	4
Coverage	NaN

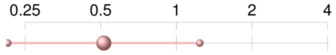

A	B	2.5	50	97.5	Sequence
**4**	**0**	**0.21**	**0.51**	**1.2**	**CVPIEFR**

5.328 235 777 RNA-binding protein 4.1 [Clonorchis sinensis]

Protein Accession	CL49317.Contig6$_{H}1$
Mean Expression Ratio	1.11
Median Expression Ratio	1.11
Credible Interval	(0.57, 2.18)
Associated Peptides	2
Associated Spectra	4
Coverage	NaN

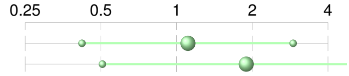

A	B	2.5	50	97.5	Sequence
**1**	**2**	**0.42**	**1.1**	**2.9**	**VELSTGK**
**0**	**1**	**0.51**	**1.9**	**6.9**	**DRLPPFAEDR**

5.329 3 116 Paramyosin [Crassostrea gigas]; 3 107 minus strand Paramyosin [Crassostrea gigas]

Protein Accession	CL43455.Contig7$_{H}1$ CL43455.Contig13$_{H}1$
Mean Expression Ratio	1.12
Median Expression Ratio	1.11
Credible Interval	(0.573, 2.21)
Associated Peptides	2
Associated Spectra	2
Coverage	NaN

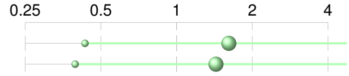

A	B	2.5	50	97.5	Sequence
**0**	**1**	**0.43**	**1.6**	**6**	**AQTLIEEAENRADSAEK**
**0**	**1**	**0.4**	**1.4**	**5.3**	**NLQVVR**

5.330 350 910 Peptidyl-prolyl cis-trans isomerase B [Crassostrea gigas]; 488 1039 Peptidyl-prolyl cis-trans isomerase B [Crassostrea gigas]; 488 1039 Peptidyl-prolyl cis-trans isomerase B [Crassostrea gigas]

Protein Accession	CL49248.Contig5$_{H}1$ CL49248.Contig2$_{H}1$ CL49248.Contig1$_{H}1$
Mean Expression Ratio	0.899
Median Expression Ratio	0.899
Credible Interval	(0.476, 1.7)
Associated Peptides	3
Associated Spectra	3
Coverage	NaN

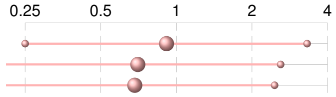

A	B	2.5	50	97.5	Sequence
**0**	**1**	**0.25**	**0.92**	**3.3**	**HVAFGQIIDK**
**0**	**1**	**0.19**	**0.7**	**2.6**	**ASLEFLDK**
**0**	**1**	**0.19**	**0.68**	**2.5**	**AVLDFLIKN**

5.331 200 2128 minus strand heat shock protein 70 [Cristaria plicata]

Protein Accession	CL8787.Contig2$_{H}$1
Mean Expression Ratio	1.11
Median Expression Ratio	1.11
Credible Interval	(0.593, 2.07)
Associated Peptides	4
Associated Spectra	4
Coverage	NaN

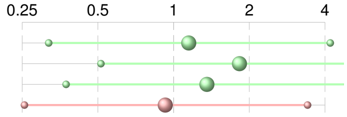

A	B	2.5	50	97.5	Sequence
**0**	**1**	**0.32**	**1.1**	**4.2**	**MVQHFAEEFKR**
**0**	**1**	**0.52**	**1.8**	**6.8**	**TLAPEEISSMVLVK**
**0**	**1**	**0.37**	**1.4**	**4.9**	**LTEVQK**
**0**	**1**	**0.26**	**0.93**	**3.4**	**LSKEEIDR**

5.332 249 845 sigma class glutathione-s-transferase 2 [Haliotis discus discus]; 252 848 sigma class glutathione-s-transferase 2 [Haliotis discus discus]

Protein Accession	CL19285.Contig32$_{H}$1 CL19285.Contig20$_{H}$1
Mean Expression Ratio	0.895
Median Expression Ratio	0.899
Credible Interval	(0.436, 1.79)
Associated Peptides	1
Associated Spectra	1
Coverage	NaN

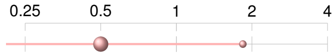

A	B	2.5	50	97.5	Sequence
**1**	**0**	**0.13**	**0.5**	**1.8**	**VKTENLPR**

5.333 5 193 minus strand PREDICTED: 40S ribosomal protein S28-like [Strongylocentrotus purpuratus]; 3 122 PREDICTED: 40S ribosomal protein S28-like [Strongylocentrotus purpuratus]

Protein Accession	CL49462.Contig2$_{H}$1 CL49462.Contig1$_{H}$1
Mean Expression Ratio	1.12
Median Expression Ratio	1.11
Credible Interval	(0.597, 2.11)
Associated Peptides	2
Associated Spectra	5
Coverage	NaN

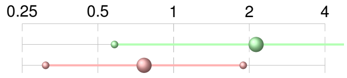

A	B	2.5	50	97.5	Sequence
**0**	**1**	**0.58**	**2.1**	**7.6**	**NVKGPVREGDILTLLESER**
**1**	**3**	**0.31**	**0.76**	**1.9**	**VEFLDDSNR**

5.334 165 635 hypothetical protein CGI_10007639 [Crassostrea gigas]

Protein Accession	CL36715.Contig1$_{H}$1
Mean Expression Ratio	1.11
Median Expression Ratio	1.11
Credible Interval	(0.557, 2.24)
Associated Peptides	1
Associated Spectra	1
Coverage	NaN

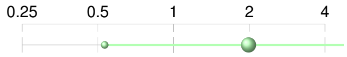

A	B	2.5	50	97.5	Sequence
**0**	**1**	**0.53**	**2**	**7.4**	**YVDDQTFTFSSK**

5.335 9 518 minus strand hemocyanin isoform 1 [Nucula nucleus]

Protein Accession	Unigene71735$_H1$
Mean Expression Ratio	0.897
Median Expression Ratio	0.899
Credible Interval	(0.459, 1.76)
Associated Peptides	1
Associated Spectra	17
Coverage	NaN

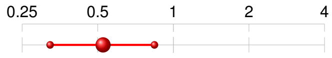

A	B	2.5	50	97.5	Sequence
**8**	**9**	**0.32**	**0.52**	**0.84**	**IDFEHADVAR**

5.336 247 726 Annexin A7 [Crassostrea gigas]; 286 675 Annexin A7 [Crassostrea gigas]

Protein Accession	CL15539.Contig3$_H1$ CL15539.Contig1$_H1$
Mean Expression Ratio	0.898
Median Expression Ratio	0.899
Credible Interval	(0.482, 1.67)
Associated Peptides	3
Associated Spectra	8
Coverage	NaN

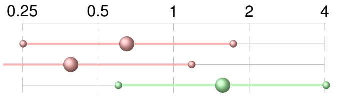

A	B	2.5	50	97.5	Sequence
**1**	**2**	**0.25**	**0.65**	**1.7**	**GLGTDEAAVIR**
**1**	**1**	**0.13**	**0.39**	**1.2**	**DLVSDLK**
**0**	**3**	**0.6**	**1.6**	**4.1**	**ILTSHNNMQR**

5.337 181 744 unknown [Dendroctonus ponderosae]

Protein Accession	CL25948.Contig3$_H1$
Mean Expression Ratio	0.9
Median Expression Ratio	0.899
Credible Interval	(0.496, 1.6)
Associated Peptides	4
Associated Spectra	16
Coverage	NaN

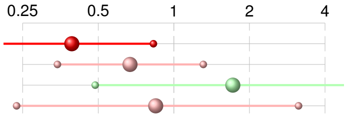

A	B	2.5	50	97.5	Sequence
**5**	**1**	**0.19**	**0.39**	**0.83**	**GTLTQLTK**
**4**	**4**	**0.34**	**0.67**	**1.3**	**VGHVPVAK**
**0**	**1**	**0.49**	**1.7**	**6.3**	**GDFSKLPR**
**0**	**1**	**0.24**	**0.84**	**3.1**	**SMGPIGSPMSK**

5.338 424 3507 minus strand putative Na+/K+-ATPase alpha subunit [Paroctopus digueti]; 424 3507 minus strand putative Na+/K+-ATPase alpha subunit [Paroctopus digueti]

Protein Accession	Unigene11494$_{H}1$ Unigene11493$_{H}1$
Mean Expression Ratio	0.9
Median Expression Ratio	0.899
Credible Interval	(0.541, 1.49)
Associated Peptides	11
Associated Spectra	18
Coverage	NaN

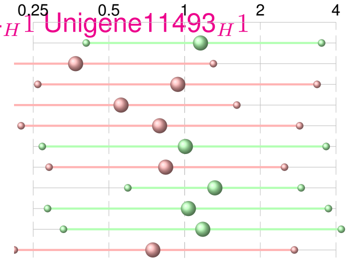

A	B	2.5	50	97.5	Sequence
2	0	0.41	1.2	3.5	GIPVEEVDPREAK
1	0	0.1	0.37	1.3	KVVEIPFNSTNK
1	0	0.26	0.94	3.4	SAEFTHENPLETR
1	1	0.19	0.56	1.6	VIVAHGFK
1	0	0.22	0.79	2.9	GVGIISEGSK
0	1	0.27	1	3.7	AEELVLGDVVEVK
0	2	0.29	0.84	2.5	DMTPAQIDEILR
0	5	0.59	1.3	2.9	FGDRVPADLR
0	1	0.28	1	3.7	GIPVEEVDPR
0	1	0.33	1.2	4.2	AKEILLR
0	1	0.21	0.74	2.7	YLLVMK

5.339 " 172 867 minus strand complement C1q-like protein 4 precursor, partial [Oryzias melastigma]"

Protein Accession	Unigene7884$_{H}1$
Mean Expression Ratio	1.11
Median Expression Ratio	1.11
Credible Interval	(0.633, 1.96)
Associated Peptides	5
Associated Spectra	23
Coverage	NaN

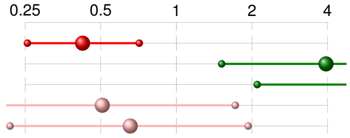

A	B	2.5	50	97.5	Sequence
2	12	0.25	0.42	0.71	DLQQAVESMRPSLK
1	2	1.5	4	10	EDQIKDLQDK
1	2	2.1	5.5	14	EDQIKDLQDKQK
0	1	0.14	0.51	1.7	DLQQAVESMR
0	2	0.22	0.66	1.9	DVKDLQQAVESMRPSLK

5.340 2764 3420 PREDICTED: filamin-C-like [Nasonia vitripennis]

Protein Accession	CL4145.Contig1$_{H}1$
Mean Expression Ratio	0.898
Median Expression Ratio	0.9
Credible Interval	(0.452, 1.77)
Associated Peptides	1
Associated Spectra	2
Coverage	NaN

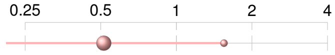

A	B	2.5	50	97.5	Sequence
1	1	0.17	0.51	1.5	GVDVPVNTIDNK

5.341 " 703 1173 60S acidic ribosomal protein P0, putative [Ixodes scapularis] gi"

Protein Accession	Unigene34489$_{H}1$
Mean Expression Ratio	0.899
Median Expression Ratio	0.901
Credible Interval	(0.483, 1.67)
Associated Peptides	3
Associated Spectra	6
Coverage	NaN

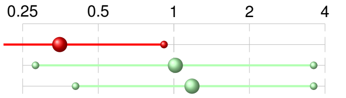

A	B	2.5	50	97.5	Sequence
1	2	0.14	0.35	0.92	EYLKDPSK
0	1	0.28	1	3.6	FAAVAAVAAPAAAK
0	2	0.41	1.2	3.6	KSFLEGVR

5.342 98 673 transforming protein Rho-like [Acyrthosiphon pisum]

Protein Accession	Unigene30464$_{H}1$
Mean Expression Ratio	1.11
Median Expression Ratio	1.11
Credible Interval	(0.575, 2.17)
Associated Peptides	2
Associated Spectra	2
Coverage	NaN

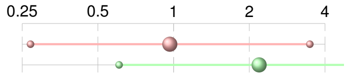

A	B	2.5	50	97.5	Sequence
0	1	0.27	0.97	3.5	EVFEQATR
0	1	0.61	2.2	8	EGVREVFEQATR

5.343 11 244 minus strand Tubulin alpha chain (Fragment) OS=Lytechinus pictus PE=3 SV=1

Protein Accession	CL3047.Contig6$_{H}1$
Mean Expression Ratio	0.902
Median Expression Ratio	0.901
Credible Interval	(0.477, 1.7)
Associated Peptides	3
Associated Spectra	6
Coverage	NaN

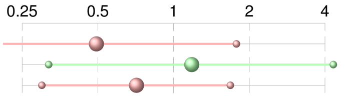

A	B	2.5	50	97.5	Sequence
0	1	0.13	0.49	1.8	EDLAALEK
0	1	0.32	1.2	4.3	LDHKFDLMYAK
0	4	0.3	0.71	1.7	FDLMYAK

5.344 680 991 minus strand PDZ and LIM domain protein 1 [Crassostrea gigas]; 553 864 minus strand PDZ and LIM domain protein 1 [Crassostrea gigas]

Protein Accession	Unigene7664$_{H}1$ Unigene7663$_{H}1$
Mean Expression Ratio	1.11
Median Expression Ratio	1.11
Credible Interval	(0.564, 2.21)
Associated Peptides	1
Associated Spectra	2
Coverage	NaN

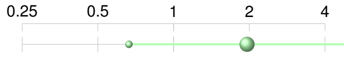

A	B	2.5	50	97.5	Sequence
0	2	0.66	2	5.8	QLTHMQAHQLIK

5.345 157 1422 Tektin-2 [Crassostrea gigas]

Protein Accession	Unigene17501$_H1$
Mean Expression Ratio	1.11
Median Expression Ratio	1.11
Credible Interval	(0.573, 2.16)
Associated Peptides	2
Associated Spectra	3
Coverage	NaN

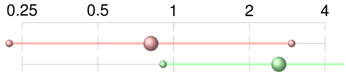

A	B	2.5	50	97.5	Sequence
**0**	**1**	**0.22**	**0.81**	**3**	**ADAEMLAANR**
**0**	**2**	**0.9**	**2.6**	**7.6**	**NLTITGIERPR**

5.346 2 838 Alanine aminotransferase 2 [Crassostrea gigas]

Protein Accession	CL37048.Contig1$_H1$
Mean Expression Ratio	0.899
Median Expression Ratio	0.902
Credible Interval	(0.448, 1.79)
Associated Peptides	1
Associated Spectra	1
Coverage	NaN

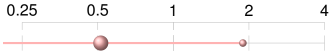

A	B	2.5	50	97.5	Sequence
**1**	**0**	**0.14**	**0.51**	**1.9**	**QVLTK**

5.347 118 1629 PREDICTED: similar to tektin 3 [Ciona intestinalis]

Protein Accession	Unigene11521$_H1$
Mean Expression Ratio	1.11
Median Expression Ratio	1.11
Credible Interval	(0.61, 2.04)
Associated Peptides	4
Associated Spectra	8
Coverage	NaN

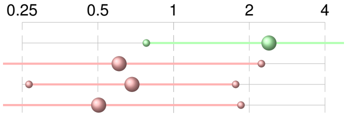

A	B	2.5	50	97.5	Sequence
**1**	**1**	**0.78**	**2.4**	**7.7**	**GLTFHNGVQR**
**1**	**0**	**0.16**	**0.61**	**2.2**	**SALEIDLAVK**
**2**	**2**	**0.27**	**0.68**	**1.8**	**QTIDLVHDAPEK**
**1**	**0**	**0.14**	**0.51**	**1.9**	**EVDTIKR**

5.348 44 889 minus strand hypothetical protein CGI_10015025 [Crassostrea gigas]

Protein Accession	CL40973.Contig3$_H1$
Mean Expression Ratio	1.11
Median Expression Ratio	1.11
Credible Interval	(0.554, 2.24)
Associated Peptides	1
Associated Spectra	1
Coverage	NaN

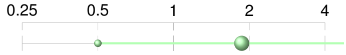

A	B	2.5	50	97.5	Sequence
**0**	**1**	**0.5**	**1.9**	**6.8**	**NVELEGDIER**

5.349 40 777 proteasome subunit N3 [Haliotis discus discus]; 40 777 proteasome subunit N3 [Haliotis discus discus]

Protein Accession	CL39106.Contig2$_H1$ CL39106.Contig1$_H1$
Mean Expression Ratio	1.11
Median Expression Ratio	1.11
Credible Interval	(0.554, 2.22)
Associated Peptides	1
Associated Spectra	1
Coverage	NaN

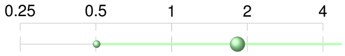

A	B	2.5	50	97.5	Sequence
**0**	**1**	**0.5**	**1.8**	**6.7**	**YQIAIVTSK**

5.350 95 271 minus strand hypothetical protein EAI_01530 [Harpegnathos saltator]

Protein Accession	CL9369.Contig9$_H1$
Mean Expression Ratio	1.11
Median Expression Ratio	1.11
Credible Interval	(0.56, 2.2)
Associated Peptides	2
Associated Spectra	2
Coverage	NaN

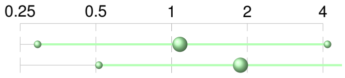

A	B	2.5	50	97.5	Sequence
**0**	**1**	**0.29**	**1.1**	**4.2**	**YPYTISSK**
**0**	**1**	**0.51**	**1.9**	**6.9**	**VAQFPFR**

5.351 30 1088 arginine kinase [Conus novaehollandiae]

Protein Accession	CL5308.Contig5$_H$1
Mean Expression Ratio	1.11
Median Expression Ratio	1.11
Credible Interval	(0.797, 1.53)
Associated Peptides	33
Associated Spectra	111
Coverage	NaN

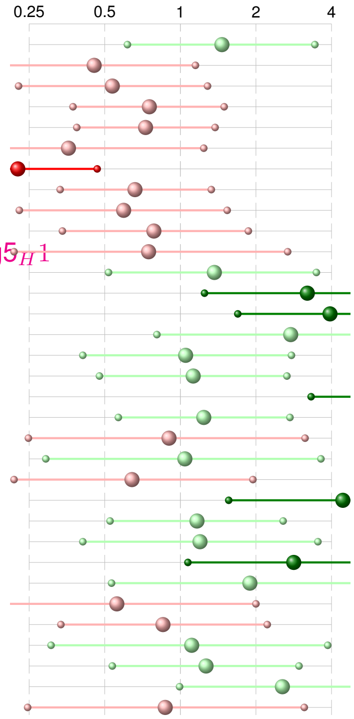

A	B	2.5	50	97.5	Sequence
1	3	0.62	1.5	3.4	AAGGYDDWPVGR
2	1	0.18	0.45	1.1	GGNLTQVYQR
2	2	0.23	0.53	1.3	GTYYPLLGMDK
5	2	0.37	0.75	1.5	YIVSTR
4	5	0.39	0.73	1.4	LTFAR
1	0	0.1	0.36	1.2	VNMEK
3	3	0.11	0.23	0.47	FNFPDLDPSGK
4	3	0.33	0.66	1.3	HPAPDFGEK
3	0	0.23	0.59	1.5	IISMQK
1	3	0.34	0.79	1.9	LVSGIHALEK
1	0	0.22	0.75	2.7	RFNFPDLDPSGK
0	3	0.52	1.4	3.5	ATVEALNTFEGELK
0	3	1.2	3.2	8.3	FNFPDLDPSGKYIVSTR
0	4	1.7	4	9.4	GIHGEHTESVGGVYDISNKRR
0	1	0.81	2.8	9.5	GTYYPLLGMDKATEK
0	3	0.41	1	2.8	KLIDDHFLFK
0	4	0.48	1.1	2.7	KLIDDHFLFKDDDR
0	9	3.3	6.2	12	KLIDDHFLFKDDDRFLK
0	5	0.57	1.2	2.7	LGLSEFDAIMEMK
0	1	0.25	0.9	3.1	LIDDHFLFK
0	1	0.29	1	3.6	LIDDHFLFKDDDR
0	2	0.22	0.64	1.9	NGVEEILKK
0	2	1.6	4.4	13	RFNFPDLDPSGKYIVSTR
0	5	0.52	1.2	2.6	SHASYGFPPVLSK
0	2	0.41	1.2	3.5	TFLVWVNEEDHIR
0	3	1.1	2.8	7.1	VAEVKHPAPDFGEK
0	1	0.53	1.9	6.7	VAEVKHPAPDFGEKR
0	1	0.16	0.56	2	LAALPEFK
0	3	0.33	0.85	2.2	KLTFAR
0	1	0.31	1.1	3.9	DYHKVAEVK
0	4	0.54	1.3	3	GIHGEHTESVGGVYDISNK
0	3	0.99	2.6	6.6	GIHGEHTESVGGVYDISNKR
0	1	0.25	0.87	3.1	NGVEEILK

5.352 527 1321 minus strand troponin T [Mizuhopecten yessoensis]; 305 1099 minus strand troponin T [Mizuhopecten yessoensis]; 305 1099 minus strand troponin T [Mizuhopecten yessoensis]

Protein Accession	Unigene65787$_H$1 CL2214.Contig4$_H$1 CL2214.Contig3$_H$1
Mean Expression Ratio	0.904
Median Expression Ratio	0.904
Credible Interval	(0.514, 1.59)
Associated Peptides	5
Associated Spectra	20
Coverage	NaN

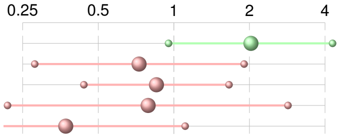

A	B	2.5	50	97.5	Sequence
2	4	0.95	2	4.3	KGPNFVITK
3	0	0.28	0.73	1.9	AILSQR
8	0	0.44	0.85	1.7	LTELR
0	1	0.22	0.79	2.9	LDSVMSKEELQK
0	2	0.12	0.37	1.1	GAEILALK

5.353 253 765 cytochrome c oxidase subunit IV [Urechis caupo]

Protein Accession	CL38636.Contig3$_H$1
Mean Expression Ratio	0.902
Median Expression Ratio	0.904
Credible Interval	(0.5, 1.6)
Associated Peptides	4
Associated Spectra	18
Coverage	NaN

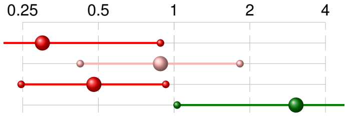

A	B	2.5	50	97.5	Sequence
2	0	0.1	0.3	0.88	SLTLEDKK
1	5	0.42	0.88	1.8	FKENTPDVMALR
4	4	0.25	0.48	0.93	IEGLSSK
0	2	1	3	8.9	TEAALDQTARDRIHPK

5.354 771 1505 O-methyltransferase family 3 [Dicentrarchus labrax]

Protein Accession	Unigene19132$_H$1
Mean Expression Ratio	0.907
Median Expression Ratio	0.905
Credible Interval	(0.502, 1.63)
Associated Peptides	5
Associated Spectra	7
Coverage	NaN

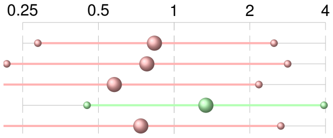

A	B	2.5	50	97.5	Sequence
1	1	0.29	0.83	2.5	VFGEHVINDQR
1	0	0.22	0.78	2.8	VLIPVR
0	1	0.16	0.58	2.2	AEATQADPEVIEGLKR
0	2	0.45	1.3	3.9	FGASYLPR
0	1	0.2	0.74	2.7	IAETTLTHPWR

5.355 201 779 minus strand sigma class glutathione-s-transferase 2 [Haliotis discus discus]

Protein Accession	Unigene22998$_H$1
Mean Expression Ratio	0.904
Median Expression Ratio	0.905
Credible Interval	(0.503, 1.62)
Associated Peptides	6
Associated Spectra	8
Coverage	NaN

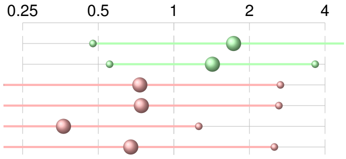

A	B	2.5	50	97.5	Sequence
0	1	0.48	1.7	6.2	IKNYVANR
0	3	0.55	1.4	3.7	ALYENVANNPR
0	1	0.2	0.73	2.7	KKLEEEEFPK
0	1	0.2	0.74	2.6	YFGMFQK
0	1	0.1	0.36	1.3	KLEEEEFPK
0	1	0.18	0.67	2.5	VINIYK

5.356 433 669 minus strand Superoxide dismutase [Cu-Zn] OS=Prionace glauca GN=sod1 PE=1 SV=1

Protein Accession	Unigene63976$_H$1
Mean Expression Ratio	0.907
Median Expression Ratio	0.905
Credible Interval	(0.468, 1.71)
Associated Peptides	1
Associated Spectra	6
Coverage	NaN

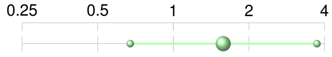

A	B	2.5	50	97.5	Sequence
3	3	0.67	1.6	3.7	SLVVHEK

5.357 48 1523 minus strand Spermatogenesis-associated protein 18-like protein [Crassostrea gigas]

Protein Accession	CL6432.Contig3$_H1$
Mean Expression Ratio	1.11
Median Expression Ratio	1.1
Credible Interval	(0.561, 2.19)
Associated Peptides	2
Associated Spectra	2
Coverage	NaN

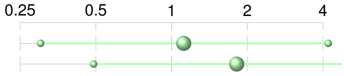

A	B	2.5	50	97.5	Sequence
**0**	**1**	**0.3**	**1.1**	**4.2**	**NTDLVDVPGLVSDVIR**
**0**	**1**	**0.49**	**1.8**	**6.7**	**SLSPALR**

5.358 143 865 minus strand predicted protein [Nematostella vectensis] gi

Protein Accession	CL5421.Contig1$_H1$
Mean Expression Ratio	1.11
Median Expression Ratio	1.1
Credible Interval	(0.556, 2.25)
Associated Peptides	1
Associated Spectra	1
Coverage	NaN

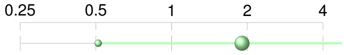

A	B	2.5	50	97.5	Sequence
**0**	**1**	**0.51**	**1.9**	**7.1**	**AIGSGSEGAQQALQEQYHK**

5.359 " 101 598 PREDICTED: aminomethyltransferase, mitochondrial-like [Oreochromis niloticus]"

Protein Accession	CL10455.Contig5$_H1$
Mean Expression Ratio	1.11
Median Expression Ratio	1.1
Credible Interval	(0.562, 2.23)
Associated Peptides	1
Associated Spectra	2
Coverage	NaN

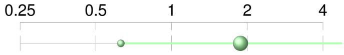

A	B	2.5	50	97.5	Sequence
**1**	**1**	**0.63**	**1.9**	**5.5**	**TTITEEHLQVR**

5.360 99 437 minus strand V-type proton ATPase subunit G [Crassostrea gigas]

Protein Accession	CL20812.Contig1$_H1$
Mean Expression Ratio	1.1
Median Expression Ratio	1.1
Credible Interval	(0.567, 2.13)
Associated Peptides	2
Associated Spectra	2
Coverage	NaN

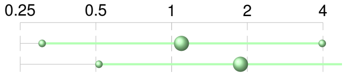

A	B	2.5	50	97.5	Sequence
**0**	**1**	**0.3**	**1.1**	**4**	**ASQTQGIQQLLAAEK**
**0**	**1**	**0.51**	**1.9**	**6.8**	**GDLEAKIEASTR**

5.361 1 231 small subunit ribosomal protein S11e [Clonorchis sinensis]; 1 171 40S ribosomal protein S11 OS=Canis familiaris GN=RPS11 PE=1 SV=2

Protein Accession	CL39695.Contig2$_H1$ CL39695.Contig1$_H1$
Mean Expression Ratio	1.1
Median Expression Ratio	1.1
Credible Interval	(0.559, 2.18)
Associated Peptides	1
Associated Spectra	2
Coverage	NaN

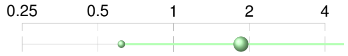

A	B	2.5	50	97.5	Sequence
0	2	0.62	1.9	5.5	TIVVRRDYLK

5.362 133 354 minus strand 60S ribosomal protein L7a OS=Rattus norvegicus GN=Rpl7a PE=1 SV=2

Protein Accession	CL20520.Contig1$_H1$
Mean Expression Ratio	1.11
Median Expression Ratio	1.1
Credible Interval	(0.573, 2.17)
Associated Peptides	2
Associated Spectra	3
Coverage	NaN

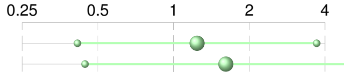

A	B	2.5	50	97.5	Sequence
0	2	0.41	1.2	3.7	KVVNPLFEK
0	1	0.45	1.6	6	NFGIGQDIQPK

5.363 151 1449 rab GDP-dissociation inhibitor [Branchiostoma belcheri]

Protein Accession	CL30719.Contig3$_H1$
Mean Expression Ratio	1.1
Median Expression Ratio	1.1
Credible Interval	(0.597, 2.05)
Associated Peptides	4
Associated Spectra	6
Coverage	NaN

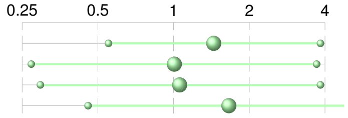

A	B	2.5	50	97.5	Sequence
1	2	0.55	1.4	3.8	ITGEAFDFSK
0	1	0.27	1	3.7	LLVATGVTR
0	1	0.3	1.1	3.8	TLYEPVDMGETSK
0	1	0.46	1.7	6	AICILNHPIKDTK

5.364 " 4 423 minus strand PREDICTED: similar to NADH dehydrogenase, putative [Tribolium castaneum] gi"

Protein Accession	CL4111.Contig2$_H1$
Mean Expression Ratio	0.905
Median Expression Ratio	0.907
Credible Interval	(0.46, 1.77)
Associated Peptides	2
Associated Spectra	2
Coverage	NaN

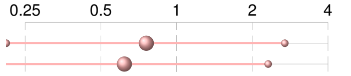

A	B	2.5	50	97.5	Sequence
1	0	0.21	0.76	2.7	QVKPILSTNPAEAK
1	0	0.18	0.62	2.3	IKQVK

5.365 201 1181 minus strand Serine/threonine-protein phosphatase PP1-alpha catalytic subunit [Crassostrea gigas]

Protein Accession	CL28655.Contig4$_H$1
Mean Expression Ratio	0.911
Median Expression Ratio	0.907
Credible Interval	(0.468, 1.79)
Associated Peptides	2
Associated Spectra	2
Coverage	NaN

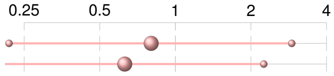

A	B	2.5	50	97.5	Sequence
0	1	0.22	0.8	2.9	ADTDKLNIDSIIAR
0	1	0.17	0.63	2.3	AHQVVEDGYEFFAK

5.366 84 425 minus strand Transcription elongation factor B polypeptide 2 [Crassostrea gigas]; 84 425 minus strand Transcription elongation factor B polypeptide 2 [Crassostrea gigas]; 84 425 minus strand Transcription elongation factor B polypeptide 2 [Crassostrea gigas]; 112 366 Transcription elongation factor B polypeptide 2 OS=Rattus norvegicus GN=Tceb2 PE=1 SV=1; 101 442 minus strand Transcription elongation factor B polypeptide 2 [Crassostrea gigas]; 101 442 minus strand Transcription elongation factor B polypeptide 2 [Crassostrea gigas]; 84 425 minus strand Transcription elongation factor B polypeptide 2 [Crassostrea gigas]

Protein Accession	CL19448.Contig9$_H$1 CL19448.Contig8$_H$1 CL19448.Contig7$_H$1 CL19448.Contig4$_H$1 CL1944
Mean Expression Ratio	0.906
Median Expression Ratio	0.908
Credible Interval	(0.45, 1.83)
Associated Peptides	1
Associated Spectra	1
Coverage	NaN

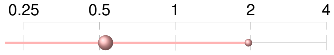

A	B	2.5	50	97.5	Sequence
1	0	0.14	0.53	2	AQAPATLGLVYK

5.367 254 1093 uncharacterized protein LOC432187 precursor [Xenopus laevis] gi; 343 561 minus strand cathepsin Z precursor [Danio rerio] gi

Protein Accession	CL7800.Contig7$_H$1 CL7800.Contig1$_H$1
Mean Expression Ratio	1.1
Median Expression Ratio	1.1
Credible Interval	(0.551, 2.24)
Associated Peptides	1
Associated Spectra	1
Coverage	NaN

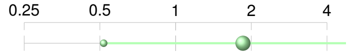

A	B	2.5	50	97.5	Sequence
0	1	0.52	1.9	7	GINFASTTR

5.368 346 702 minus strand PREDICTED: galectin-4-like [Anolis carolinensis]; 367 669 minus strand PREDICTED: galectin-7-like [Cavia porcellus]; 599 964 C. briggsae CBR-LEC-4 protein [Caenorhabditis briggsae]

Protein Accession	Unigene42203$_H$1 Unigene41782$_H$1 CL22459.Contig3$_H$1
Mean Expression Ratio	1.1
Median Expression Ratio	1.1
Credible Interval	(0.62, 1.96)
Associated Peptides	4
Associated Spectra	11
Coverage	NaN

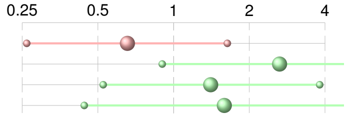

A	B	2.5	50	97.5	Sequence
3	2	0.26	0.65	1.6	AEATLEVR
0	2	0.9	2.6	7.7	KSVPHGLVK
1	2	0.52	1.4	3.8	SVPHGLVK
0	1	0.44	1.6	5.7	VKDSGFK

5.369 210 2201 minus strand Glycyl-tRNA synthetase [Crassostrea gigas]; 210 2201 minus strand Glycyl-tRNA synthetase [Crassostrea gigas]; 210 2201 minus strand Glycyl-tRNA synthetase [Crassostrea gigas]; 210 2201 minus strand Glycyl-tRNA synthetase [Crassostrea gigas]; 210 2201 minus strand Glycyl-tRNA synthetase [Crassostrea gigas]; 210 2201 minus strand Glycyl-tRNA synthetase [Crassostrea gigas]

Protein Accession	CL4073.Contig8$_H$1 CL4073.Contig7$_H$1 CL4073.Contig6$_H$1 CL4073.Contig5$_H$1 CL4073.Con
Mean Expression Ratio	1.1
Median Expression Ratio	1.1
Credible Interval	(0.552, 2.19)
Associated Peptides	1
Associated Spectra	1
Coverage	NaN

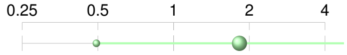

A	B	2.5	50	97.5	Sequence
1	0	0.49	1.8	6.7	ATGVKLVAER

5.370 153 953 minus strand ribosomal protein rpl7 [Arenicola marina]

Protein Accession	CL20943.Contig3$_H$1
Mean Expression Ratio	0.904
Median Expression Ratio	0.908
Credible Interval	(0.444, 1.79)
Associated Peptides	1
Associated Spectra	1
Coverage	NaN

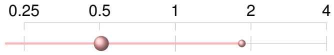

A	B	2.5	50	97.5	Sequence
1	0	0.15	0.51	1.8	VPETILK

5.371 144 1307 minus strand PREDICTED: similar to AGAP005845-PA [Tribolium castaneum]; 146 1309 minus strand PREDICTED: similar to AGAP005845-PA [Tribolium castaneum]; 144 1307 PREDICTED: similar to AGAP005845-PA [Tribolium castaneum]

Protein Accession	CL8394.Contig21$_H$1 CL8394.Contig20$_H$1 CL8394.Contig14$_H$1
Mean Expression Ratio	1.1
Median Expression Ratio	1.1
Credible Interval	(0.556, 2.23)
Associated Peptides	1
Associated Spectra	1
Coverage	NaN

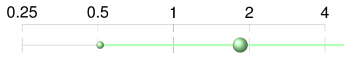

A	B	2.5	50	97.5	Sequence
**0**	**1**	**0.51**	**1.8**	**6.6**	**KATGSLLTR**

5.372 373 579 myosin heavy chain [Argopecten irradians] gi

Protein Accession	Unigene64289$_H$1
Mean Expression Ratio	1.1
Median Expression Ratio	1.1
Credible Interval	(0.636, 1.91)
Associated Peptides	7
Associated Spectra	18
Coverage	NaN

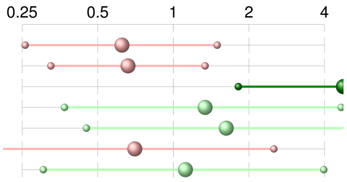

A	B	2.5	50	97.5	Sequence
**1**	**3**	**0.26**	**0.62**	**1.5**	**KVQNELEDAEER**
**1**	**6**	**0.33**	**0.66**	**1.3**	**QVEEAEEIAAVNLAK**
**0**	**3**	**1.8**	**4.7**	**12**	**KVQNELEDAEERADSAEGTLSK**
**0**	**1**	**0.37**	**1.3**	**4.7**	**RQVEEAEEIAAVNLAK**
**0**	**1**	**0.45**	**1.6**	**5.9**	**VQNELEDAEERADSAEGTLSK**
**0**	**1**	**0.19**	**0.7**	**2.5**	**ELEDAEER**
**0**	**1**	**0.3**	**1.1**	**4**	**VQNELEDAEER**

5.373 9 326 minus strand catalase [Paracyclopina nana]; 9 209 Catalase OS=Cavia porcellus GN=CAT PE=2 SV=4

Protein Accession	CL6745.Contig2$_H$1 CL6745.Contig1$_H$1
Mean Expression Ratio	0.902
Median Expression Ratio	0.908
Credible Interval	(0.449, 1.78)
Associated Peptides	1
Associated Spectra	3
Coverage	NaN

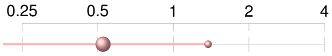

A	B	2.5	50	97.5	Sequence
**1**	**2**	**0.2**	**0.53**	**1.4**	**LATMTVGPR**

5.374 " 3475 4188 minus strand Heat shock 70 kDa protein cognate, putative [Pediculus humanus corporis] gi"

Protein Accession	CL5924.Contig14$_{H1}$
Mean Expression Ratio	0.905
Median Expression Ratio	0.909
Credible Interval	(0.467, 1.71)
Associated Peptides	2
Associated Spectra	5
Coverage	NaN

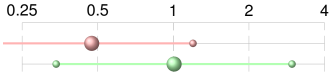

A	B	2.5	50	97.5	Sequence
2	1	0.18	0.47	1.2	STIEDEKLK
0	2	0.34	1	3	NSLESYAFHMK

5.375 " 1 2508 Alanyl-tRNA synthetase, cytoplasmic [Crassostrea gigas]; 1 2508 Alanyl-tRNA synthetase, cytoplasmic [Crassostrea gigas]; 1 2508 minus strand Alanyl-tRNA synthetase, cytoplasmic [Crassostrea gigas]"

Protein Accession	CL11594.Contig2$_{H1}$ CL11594.Contig1$_{H1}$ CL11594.Contig11$_{H1}$
Mean Expression Ratio	0.91
Median Expression Ratio	0.909
Credible Interval	(0.478, 1.72)
Associated Peptides	3
Associated Spectra	3
Coverage	NaN

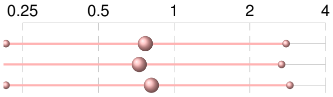

A	B	2.5	50	97.5	Sequence
1	0	0.21	0.77	2.8	GLTVDNEGFEK
1	0	0.2	0.73	2.7	SIQGLR
1	0	0.21	0.81	2.9	VIALR

5.376 556 987 ribosomal protein rps15 [Lineus viridis]

Protein Accession	CL7683.Contig9$_{H1}$
Mean Expression Ratio	0.906
Median Expression Ratio	0.91
Credible Interval	(0.451, 1.79)
Associated Peptides	1
Associated Spectra	1
Coverage	NaN

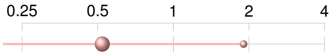

A	B	2.5	50	97.5	Sequence
0	1	0.14	0.52	1.9	EAGPLEKPEVVK

5.377 197 913 PREDICTED: l-xylulose reductase-like [Monodelphis domestica]; 197 913 PREDICTED: l-xylulose reductase-like [Monodelphis domestica]; 197 913 PREDICTED: l-xylulose reductase-like [Monodelphis domestica]; 197 913 PREDICTED: l-xylulose reductase-like [Monodelphis domestica]; 197 913 PREDICTED: l-xylulose reductase-like [Monodelphis domestica]; 197 913 PREDICTED: l-xylulose reductase-like [Monodelphis domestica]; 203 919 minus strand PREDICTED: l-xylulose reductase-like [Monodelphis domestica]; 200 916 PREDICTED: l-xylulose reductase-like [Monodelphis domestica]; 200 916 PREDICTED: l-xylulose reductase-like [Monodelphis domestica]; 203 919 minus strand PREDICTED: l-xylulose reductase-like [Monodelphis domestica]

Protein Accession	CL13128.Contig8$_H$1 CL13128.Contig7$_H$1 CL13128.Contig6$_H$1 CL13128.Contig5$_H$1 CL1312
Mean Expression Ratio	1.1
Median Expression Ratio	1.1
Credible Interval	(0.608, 2.02)
Associated Peptides	5
Associated Spectra	7
Coverage	NaN

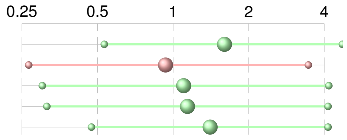

A	B	2.5	50	97.5	Sequence
1	1	0.53	1.6	4.7	MGTFAAEHPEGK
0	1	0.27	0.93	3.5	AIAEALYQHGAR
0	1	0.3	1.1	4.2	GGSIVNVSSISGK
0	1	0.31	1.1	4.1	VNSVNPTCVMTK
0	2	0.47	1.4	4.1	VVALSR

5.378 170 1375 PREDICTED: sialate O-acetylesterase-like [Oreochromis niloticus]

Protein Accession	CL26143.Contig1$_H$1
Mean Expression Ratio	0.913
Median Expression Ratio	0.91
Credible Interval	(0.462, 1.81)
Associated Peptides	1
Associated Spectra	2
Coverage	NaN

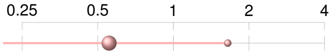

A	B	2.5	50	97.5	Sequence
2	0	0.19	0.56	1.6	DKLDVAQR

5.379 112 1050 Enolase-phosphatase E1 [Crassostrea gigas]; 112 1050 Enolase-phosphatase E1 [Crassostrea gigas]

Protein Accession	CL25175.Contig3$_H$1 CL25175.Contig2$_H$1
Mean Expression Ratio	1.1
Median Expression Ratio	1.1
Credible Interval	(0.58, 2.1)
Associated Peptides	2
Associated Spectra	5
Coverage	NaN

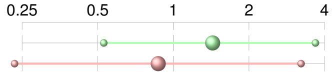

A	B	2.5	50	97.5	Sequence
2	2	0.53	1.4	3.7	SAAEAESLLVGVK
0	1	0.23	0.87	3.2	QLQGHIWR

5.380 71 535 ribosomal protein rpl14 [Arenicola marina]

Protein Accession	Unigene142$_H1$
Mean Expression Ratio	0.911
Median Expression Ratio	0.911
Credible Interval	(0.504, 1.62)
Associated Peptides	4
Associated Spectra	14
Coverage	NaN

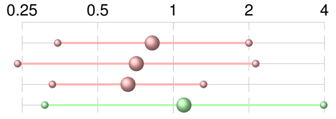

A	B	2.5	50	97.5	Sequence
2	2	0.35	0.82	2	IINVEYGK
2	0	0.24	0.71	2.1	AQLTDFER
4	3	0.33	0.66	1.3	FVEIGR
1	0	0.31	1.1	4	KAWEK

5.381 2 811 voltage-dependent anion channel 2-like protein [Haliotis diversicolor]; 2 811 voltage-dependent anion channel 2-like protein [Haliotis diversicolor]; 2 811 voltage-dependent anion channel 2-like protein [Haliotis diversicolor]

Protein Accession	CL2477.Contig3$_H1$ CL2477.Contig2$_H1$ CL2477.Contig1$_H1$
Mean Expression Ratio	0.91
Median Expression Ratio	0.911
Credible Interval	(0.576, 1.45)
Associated Peptides	13
Associated Spectra	49
Coverage	NaN

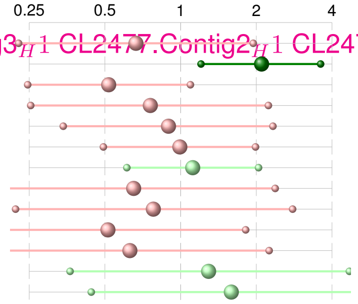

A	B	2.5	50	97.5	Sequence
1	1	0.23	0.66	1.9	GYNYGFFK
4	9	1.2	2.1	3.6	LAFDTSFAPQTGKK
3	3	0.25	0.52	1.1	VSGSLETK
1	1	0.25	0.76	2.2	FALGTK
1	2	0.34	0.89	2.3	LAFDTSFAPQTGK
1	6	0.49	0.99	2	VGLGLDVEA
3	7	0.61	1.1	2	YTLDSSASVSAK
0	1	0.18	0.65	2.4	GYNYGFFKLEAK
0	1	0.22	0.78	2.8	WSEYGLTFTEK
0	1	0.14	0.51	1.8	WNTDNVLNTEIAIEDQIAK
0	1	0.18	0.63	2.3	DIFSK
0	1	0.36	1.3	4.7	KLEAK
0	1	0.44	1.6	5.7	SINQGGHKVGLGLDVEA

5.382 5 301 PREDICTED: hypothetical protein [Saccoglossus kowalevskii]

Protein Accession	Unigene21633$_H1$
Mean Expression Ratio	0.913
Median Expression Ratio	0.911
Credible Interval	(0.466, 1.79)
Associated Peptides	1
Associated Spectra	2
Coverage	NaN

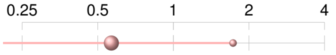

A	B	2.5	50	97.5	Sequence
1	1	0.19	0.57	1.7	AYIPIQMR

5.383 " 1482 3320 minus strand Phosphoenolpyruvate carboxykinase, cytosolic [GTP] [Crassostrea gigas]; 1326 3164 minus strand Phosphoenolpyruvate carboxykinase, cytosolic [GTP] [Crassostrea gigas]; 303 2102 minus strand Phosphoenolpyruvate carboxykinase, cytosolic [GTP] [Crassostrea gigas]"

Protein Accession	CL1008.Contig5$_{H}1$ CL1008.Contig3$_{H}1$ CL1008.Contig2$_{H}1$
Mean Expression Ratio	0.912
Median Expression Ratio	0.912
Credible Interval	(0.49, 1.69)
Associated Peptides	4
Associated Spectra	4
Coverage	NaN

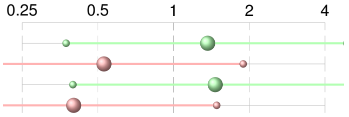

A	B	2.5	50	97.5	Sequence
0	1	0.37	1.4	4.9	AINPENGFFGVAPGTSVK
0	1	0.14	0.53	1.9	YFDEQVNEDLPR
0	1	0.4	1.5	5.2	NLHLPK
0	1	0.11	0.4	1.5	GGNALLGK

5.384 3 1352 nervous system adducin [Aplysia californica] gi; 3 1418 Protein hu-li tai shao [Crassostrea gigas]; 3 1214 nervous system adducin [Aplysia californica] gi; 3 1319 minus strand nervous system adducin [Aplysia californica] gi; 3 1523 minus strand Protein hu-li tai shao [Crassostrea gigas]; 3 1439 minus strand Protein hu-li tai shao [Crassostrea gigas]; 3 1562 minus strand Protein hu-li tai shao [Crassostrea gigas]

Protein Accession	CL3889.Contig6$_{H}1$ CL3889.Contig5$_{H}1$ CL3889.Contig4$_{H}1$ CL3889.Contig14$_{H}1$ CL3889.Co
Mean Expression Ratio	1.1
Median Expression Ratio	1.1
Credible Interval	(0.602, 2)
Associated Peptides	4
Associated Spectra	7
Coverage	NaN

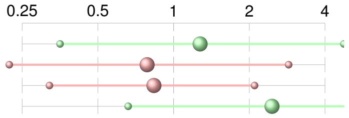

A	B	2.5	50	97.5	Sequence
1	0	0.35	1.3	4.7	WVPESETQGK
1	0	0.22	0.78	2.9	LISTEER
2	2	0.32	0.84	2.1	VMLLR
1	0	0.66	2.5	9.2	KTEIEEIGTTTPK

5.385 " 37 1356 homogentisate 1,2-dioxygenase [Danio rerio] gi; 54 1361 homogentisate 1,2-dioxygenase [Danio rerio] gi"

Protein Accession	CL16355.Contig5$_{H}1$ CL16355.Contig1$_{H}1$
Mean Expression Ratio	1.1
Median Expression Ratio	1.1
Credible Interval	(0.554, 2.17)
Associated Peptides	1
Associated Spectra	1
Coverage	NaN

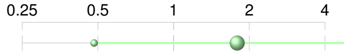

A	B	2.5	50	97.5	Sequence
0	1	0.48	1.8	6.5	SWLYR

5.386 1371 2189 minus strand galectin [Pinctada fucata]

Protein Accession	CL7245.Contig3$_{H}1$
Mean Expression Ratio	1.1
Median Expression Ratio	1.1
Credible Interval	(0.638, 1.9)
Associated Peptides	4
Associated Spectra	28
Coverage	NaN

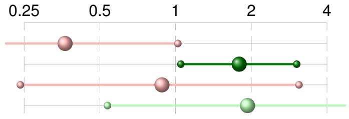

A	B	2.5	50	97.5	Sequence
2	1	0.13	0.36	1	EIPQGLEK
13	10	1	1.8	3	FNEGVTVR
0	1	0.24	0.88	3.1	LGGAWGGEER
0	1	0.54	1.9	7.2	RNEGQVVFNSK

5.387 242 1510 minus strand hypothetical protein BRAFLDRAFT_276673 [Branchiostoma floridae] gi

Protein Accession	Unigene23008$_{H}1$
Mean Expression Ratio	1.09
Median Expression Ratio	1.1
Credible Interval	(0.562, 2.1)
Associated Peptides	2
Associated Spectra	3
Coverage	NaN

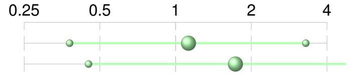

A	B	2.5	50	97.5	Sequence
2	0	0.38	1.1	3.3	TVFITGASR
0	1	0.45	1.7	6.4	DGANIVIAAK

5.388 1 987 minus strand RecName: Full=Tubulin beta chain; AltName: Full=Beta-tubulin

Protein Accession	Unigene7954$_{H}1$
Mean Expression Ratio	1.1
Median Expression Ratio	1.1
Credible Interval	(0.638, 1.89)
Associated Peptides	7
Associated Spectra	16
Coverage	NaN

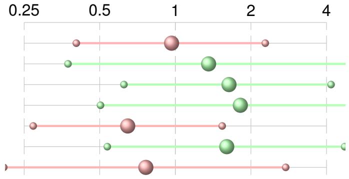

A	B	2.5	50	97.5	Sequence
2	2	0.4	0.97	2.3	ALTVPELTQQMFDAK
0	1	0.37	1.4	4.9	AILVDLEPGTMDSVR
0	3	0.63	1.6	4.2	PELTQQMFDAK
0	1	0.5	1.8	6.4	FWEVISDEHGIDPTGTYHGDSDLQLER
0	4	0.27	0.65	1.5	IMNTFSVVPSPK
0	2	0.54	1.6	4.7	INVYYNEATGGK
0	1	0.21	0.76	2.8	VAAIFR

5.389 150 716 calponin/transgelin [Papilio xuthus]

Protein Accession	CL11747.Contig2$_{H}1$
Mean Expression Ratio	0.91
Median Expression Ratio	0.912
Credible Interval	(0.463, 1.78)
Associated Peptides	2
Associated Spectra	3
Coverage	NaN

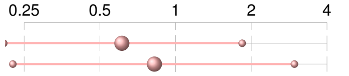

A	B	2.5	50	97.5	Sequence
1	1	0.21	0.61	1.8	MAFQQMEAIEK
0	1	0.23	0.83	3	DFTEEQLAQGK

5.390 " 169 792 minus strand rho GDP dissociation inhibitor, putative [Ixodes scapularis] gi"

Protein Accession	CL39798.Contig1$_{H}1$
Mean Expression Ratio	0.91
Median Expression Ratio	0.912
Credible Interval	(0.472, 1.74)
Associated Peptides	2
Associated Spectra	3
Coverage	NaN

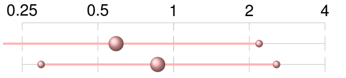

A	B	2.5	50	97.5	Sequence
1	0	0.16	0.59	2.2	YKEQLLAGAGSGEK
0	2	0.3	0.87	2.6	IIIDDSNPLNVIVK

5.391 126 704 minus strand ribosomal protein L12 [Ixodes scapularis]

Protein Accession	CL24053.Contig3$_{H}1$
Mean Expression Ratio	1.1
Median Expression Ratio	1.1
Credible Interval	(0.64, 1.89)
Associated Peptides	6
Associated Spectra	25
Coverage	NaN

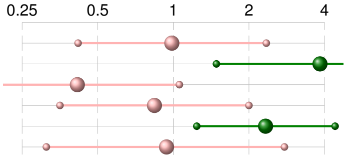

A	B	2.5	50	97.5	Sequence
1	3	0.42	0.98	2.3	AVGGEVPATSALAPK
1	2	1.5	3.8	10	INAKEIEIPDE
1	2	0.16	0.42	1.1	KVGDDIAK
2	2	0.35	0.84	2	SNPHVIIEK
2	7	1.2	2.3	4.4	IGPLGLSPK
0	2	0.31	0.94	2.8	EIEIPDE

5.392 13 1218 minus strand PREDICTED: eukaryotic initiation factor 4A-III-like [Bombus terrestris] gi

Protein Accession	CL12065.Contig7$_{H}1$
Mean Expression Ratio	1.1
Median Expression Ratio	1.1
Credible Interval	(0.548, 2.17)
Associated Peptides	1
Associated Spectra	1
Coverage	NaN

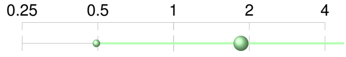

A	B	2.5	50	97.5	Sequence
0	1	0.49	1.9	6.8	GIYAYGFEKPSAIQQR

5.393 " 193 333 minus strand RecName: Full=Globin; AltName: Full=Myoglobin gi|299274|gb|AAB25743.1| myoglobin [Nassa mutabilis=marine gastropods, radular muscles, Peptide, 147 aa] gi; 193 333 minus strand RecName: Full=Globin; AltName: Full=Myoglobin gi|299274|gb|AAB25743.1| myoglobin [Nassa mutabilis=marine gastropods, radular muscles, Peptide, 147 aa] gi"

Protein Accession	CL8458.Contig5$_{H}1$ CL8458.Contig1$_{H}1$
Mean Expression Ratio	0.906
Median Expression Ratio	0.913
Credible Interval	(0.456, 1.77)
Associated Peptides	1
Associated Spectra	6
Coverage	NaN

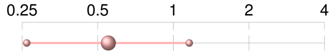

A	B	2.5	50	97.5	Sequence
**4**	**2**	**0.26**	**0.55**	**1.2**	**AAINSTWPK**

5.394 87 764 Enolase-phosphatase E1 [Crassostrea gigas]; 87 1058 Enolase-phosphatase E1 [Crassostrea gigas]; 87 764 Enolase-phosphatase E1 [Crassostrea gigas]

Protein Accession	CL25175.Contig7$_{H}1$ CL25175.Contig6$_{H}1$ CL25175.Contig4$_{H}1$
Mean Expression Ratio	0.907
Median Expression Ratio	0.913
Credible Interval	(0.468, 1.75)
Associated Peptides	1
Associated Spectra	4
Coverage	NaN

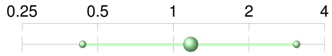

A	B	2.5	50	97.5	Sequence
**2**	**2**	**0.44**	**1.2**	**3.1**	**SAAEAESLLVGVK**

5.395 266 1123 minus strand mitochondrial malate dehydrogenase precursor [Nucella lapillus]

Protein Accession	CL10426.Contig11$_{H}1$
Mean Expression Ratio	1.1
Median Expression Ratio	1.1
Credible Interval	(0.577, 2.11)
Associated Peptides	2
Associated Spectra	4
Coverage	NaN

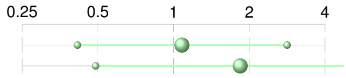

A	B	2.5	50	97.5	Sequence
**1**	**2**	**0.42**	**1.1**	**2.8**	**SLIEALNGMEGR**
**0**	**1**	**0.49**	**1.8**	**6.7**	**VRSATLSMAYAAAEFSK**

5.396 " 130 1161 minus strand Isocitrate dehydrogenase [NAD] subunit alpha, mitochondrial [Crassostrea gigas]; 198 1229 minus strand Isocitrate dehydrogenase [NAD] subunit alpha, mitochondrial [Crassostrea gigas]"

Protein Accession	CL5384.Contig8$_{H}$1 CL5384.Contig7$_{H}$1
Mean Expression Ratio	0.912
Median Expression Ratio	0.913
Credible Interval	(0.465, 1.79)
Associated Peptides	2
Associated Spectra	2
Coverage	NaN

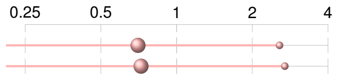

A	B	2.5	50	97.5	Sequence
**1**	**0**	**0.2**	**0.7**	**2.6**	**SLNLALR**
**1**	**0**	**0.2**	**0.72**	**2.7**	**VAEYAFK**

5.397 77 2278 Far upstream element-binding protein 3 [Crassostrea gigas]

Protein Accession	CL5030.Contig3$_{H}$1
Mean Expression Ratio	0.913
Median Expression Ratio	0.913
Credible Interval	(0.457, 1.84)
Associated Peptides	1
Associated Spectra	1
Coverage	NaN

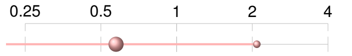

A	B	2.5	50	97.5	Sequence
**1**	**0**	**0.16**	**0.57**	**2.1**	**QAVGLVIGK**

5.398 238 1521 26S protease regulatory subunit 6A [Crassostrea gigas]

Protein Accession	CL11449.Contig8$_{H}$1
Mean Expression Ratio	1.1
Median Expression Ratio	1.09
Credible Interval	(0.585, 2.09)
Associated Peptides	3
Associated Spectra	4
Coverage	NaN

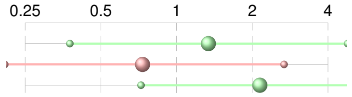

A	B	2.5	50	97.5	Sequence
**0**	**1**	**0.38**	**1.3**	**4.8**	**AIWEDGEEALGEEVLR**
**0**	**1**	**0.21**	**0.73**	**2.7**	**LLDNEIK**
**0**	**2**	**0.72**	**2.1**	**6.5**	**KIEFPHPNEEAR**

5.399 165 620 PREDICTED: uncharacterized protein LOC575072 isoform 2 [Strongylocentrotus purpuratus] gi

Protein Accession	CL20552.Contig4$_{H}$1
Mean Expression Ratio	0.911
Median Expression Ratio	0.914
Credible Interval	(0.445, 1.83)
Associated Peptides	1
Associated Spectra	1
Coverage	NaN

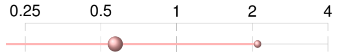

A	B	2.5	50	97.5	Sequence
**1**	**0**	**0.15**	**0.57**	**2.1**	**IKPTPLNDR**

5.400 REVERSED 216 2639 minus strand polycystin 2 [Gallus gallus]

Protein Accession	RRRRRCL818.Contig5$_H1$
Mean Expression Ratio	0.916
Median Expression Ratio	0.914
Credible Interval	(0.47, 1.77)
Associated Peptides	2
Associated Spectra	3
Coverage	NaN

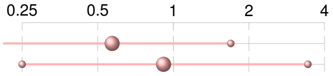

A	B	2.5	50	97.5	Sequence
2	0	0.19	0.57	1.7	DGLIIR
1	0	0.25	0.91	3.4	QIDVIK

5.401 109 510 minus strand hypothetical protein DAPPUDRAFT_231281 [Daphnia pulex]

Protein Accession	Unigene4945$_H1$
Mean Expression Ratio	1.1
Median Expression Ratio	1.09
Credible Interval	(0.663, 1.83)
Associated Peptides	8
Associated Spectra	30
Coverage	NaN

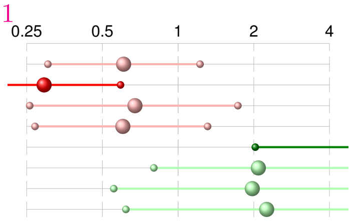

A	B	2.5	50	97.5	Sequence
1	6	0.3	0.61	1.2	LAGIEDPFEMNVK
2	5	0.15	0.29	0.59	VSMPDTLGHAVGIER
1	2	0.26	0.67	1.7	GTVDEPTLIPSLYEK
1	4	0.27	0.6	1.3	YELLAK
0	3	2	5.3	14	AKGTVDEPTLIPSLYEK
0	3	0.8	2.1	5.3	IASDKVSMPDTLGHAVGIER
0	1	0.55	2	6.9	LAGIEDPFEMNVKK
0	1	0.62	2.2	7.8	TLGHAVGIER

5.402 1 219 minus strand PREDICTED: nucleoside diphosphate kinase 3-like [Pan troglodytes]

Protein Accession	Unigene5384$_H1$
Mean Expression Ratio	0.913
Median Expression Ratio	0.915
Credible Interval	(0.479, 1.76)
Associated Peptides	2
Associated Spectra	4
Coverage	NaN

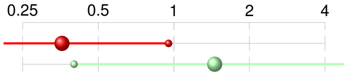

A	B	2.5	50	97.5	Sequence
2	1	0.14	0.36	0.95	GLVGEIIK
0	1	0.4	1.5	5.3	GLVGEIIKR

5.403 26 250 minus strand fructose-bisphosphate aldolase [Haliotis discus discus]

Protein Accession	Unigene16711$_H1$
Mean Expression Ratio	1.09
Median Expression Ratio	1.09
Credible Interval	(0.575, 2.09)
Associated Peptides	3
Associated Spectra	3
Coverage	NaN

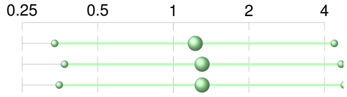

A	B	2.5	50	97.5	Sequence
0	1	0.34	1.2	4.4	RLEGIGVENNEENRR
0	1	0.37	1.3	4.6	IANAIVAPGK
0	1	0.35	1.3	4.8	ELRRIANAIVAPGK

5.404 103 684 EF-hand domain-containing protein D2 [Crassostrea gigas]

Protein Accession	CL20544.Contig1$_{H}1$
Mean Expression Ratio	0.912
Median Expression Ratio	0.915
Credible Interval	(0.471, 1.74)
Associated Peptides	2
Associated Spectra	2
Coverage	NaN

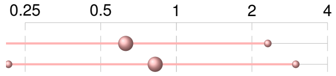

A	B	2.5	50	97.5	Sequence
**1**	**0**	**0.17**	**0.63**	**2.3**	**VPQTHLALK**
**1**	**0**	**0.22**	**0.82**	**3**	**KQIQDFQK**

5.405 " 87 287 Chain A, Structure Of Deoxygenated Hemocyanin From Rapana Thomasiana gi|31615505|pdb|1LNL|B Chain B, Structure Of Deoxygenated Hemocyanin From Rapana Thomasiana gi|31615506|pdb|1LNL|C Chain C, Structure Of Deoxygenated Hemocyanin From Rapana Thomasiana"

Protein Accession	Unigene36631$_{H}1$
Mean Expression Ratio	0.913
Median Expression Ratio	0.915
Credible Interval	(0.514, 1.62)
Associated Peptides	4
Associated Spectra	30
Coverage	NaN

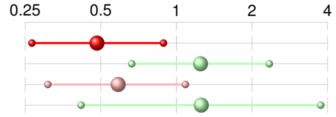

A	B	2.5	50	97.5	Sequence
**7**	**3**	**0.27**	**0.48**	**0.89**	**SLVEAMR**
**1**	**8**	**0.67**	**1.3**	**2.3**	**KNILELTESEQR**
**2**	**7**	**0.31**	**0.59**	**1.1**	**QLQEDGSAGGFQK**
**0**	**2**	**0.42**	**1.3**	**3.7**	**NILELTESEQR**

5.406 1 864 minus strand Pyruvate kinase isozymes M1/M2 [Crassostrea gigas]; 3 959 minus strand Pyruvate kinase isozymes M1/M2 [Crassostrea gigas]; 24 899 minus strand Pyruvate kinase isozymes M1/M2 [Crassostrea gigas]

Protein Accession	CL24469.Contig6$_{H}1$ CL24469.Contig3$_{H}1$ CL24469.Contig2$_{H}1$
Mean Expression Ratio	0.916
Median Expression Ratio	0.915
Credible Interval	(0.453, 1.84)
Associated Peptides	1
Associated Spectra	1
Coverage	NaN

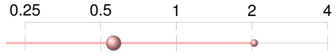

A	B	2.5	50	97.5	Sequence
**1**	**0**	**0.15**	**0.56**	**2**	**NFDEILK**

5.407 1166 1939 minus strand Calcium-binding mitochondrial carrier protein Aralar2 OS=Homo sapiens GN=SLC25A13 PE=1 SV=2

Protein Accession	Unigene34297$_{H}1$
Mean Expression Ratio	0.914
Median Expression Ratio	0.915
Credible Interval	(0.464, 1.79)
Associated Peptides	2
Associated Spectra	2
Coverage	NaN

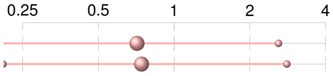

A	B	2.5	50	97.5	Sequence
1	0	0.19	0.71	2.6	LATATFDGVESK
1	0	0.21	0.74	2.8	SAARVLR

5.408 235 1038 Translocon-associated protein subunit alpha [Crassostrea gigas]

Protein Accession	CL36450.Contig6$_{H}1$
Mean Expression Ratio	0.912
Median Expression Ratio	0.916
Credible Interval	(0.459, 1.8)
Associated Peptides	1
Associated Spectra	2
Coverage	NaN

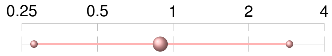

A	B	2.5	50	97.5	Sequence
1	1	0.28	0.89	2.9	ELPAGKPVR

5.409 33 422 minus strand PREDICTED: heat shock 70 kDa protein cognate 4-like [Megachile rotundata]

Protein Accession	CL15750.Contig1$_{H}1$
Mean Expression Ratio	0.917
Median Expression Ratio	0.916
Credible Interval	(0.455, 1.86)
Associated Peptides	1
Associated Spectra	1
Coverage	NaN

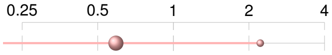

A	B	2.5	50	97.5	Sequence
1	0	0.16	0.59	2.2	VVNEGDKPK

5.410 " 143 451 minus strand PREDICTED: 10 kDa heat shock protein, mitochondrial isoform 1 [Canis lupus familiaris]"

Protein Accession	CL48287.Contig1$_{H}1$
Mean Expression Ratio	0.913
Median Expression Ratio	0.916
Credible Interval	(0.515, 1.6)
Associated Peptides	6
Associated Spectra	11
Coverage	NaN

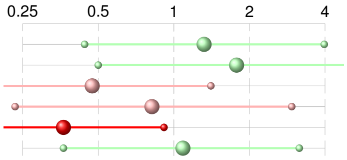

A	B	2.5	50	97.5	Sequence
1	1	0.44	1.3	4	AQEAVVVAVGSGSR
1	0	0.5	1.8	6.3	GGIMIPEKSQGK
2	0	0.16	0.47	1.4	TKGGIMIPEK
1	0	0.23	0.82	3	SIPETK
3	0	0.14	0.36	0.91	GGIMIPEK
0	2	0.36	1.1	3.2	VLLPEYGGTK

5.411 311 928 minus strand peroxiredoxin 3 [Clonorchis sinensis] gi; 288 905 minus strand peroxiredoxin 3 [Clonorchis sinensis] gi

Protein Accession	CL2043.Contig4$_{H}$1 CL2043.Contig1$_{H}$1
Mean Expression Ratio	0.913
Median Expression Ratio	0.916
Credible Interval	(0.472, 1.74)
Associated Peptides	2
Associated Spectra	7
Coverage	NaN

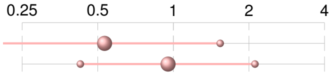

A	B	2.5	50	97.5	Sequence
**1**	**1**	**0.18**	**0.53**	**1.5**	**SVDEILR**
**2**	**3**	**0.43**	**0.95**	**2.1**	**QITINDLPVGR**

5.412 142 789 60S ribosomal protein L6 [Crassostrea gigas]

Protein Accession	CL6501.Contig1$_{H}$1
Mean Expression Ratio	0.915
Median Expression Ratio	0.916
Credible Interval	(0.462, 1.83)
Associated Peptides	1
Associated Spectra	1
Coverage	NaN

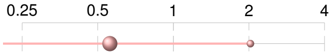

A	B	2.5	50	97.5	Sequence
**1**	**0**	**0.16**	**0.56**	**2**	**HAEGEIFDTK**

5.413 764 1258 minus strand Caprin-2 [Crassostrea gigas]

Protein Accession	CL23459.Contig2$_{H}$1
Mean Expression Ratio	0.919
Median Expression Ratio	0.917
Credible Interval	(0.465, 1.81)
Associated Peptides	1
Associated Spectra	1
Coverage	NaN

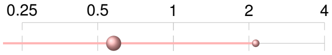

A	B	2.5	50	97.5	Sequence
**0**	**1**	**0.16**	**0.58**	**2.1**	**FAVLDSEVR**

5.414 " 57 752 PREDICTED: adenylate kinase 2, mitochondrial-like isoform 2 [Callithrix jacchus]"

Protein Accession	CL4149.Contig1$_{H}$1
Mean Expression Ratio	1.09
Median Expression Ratio	1.09
Credible Interval	(0.541, 2.22)
Associated Peptides	1
Associated Spectra	1
Coverage	NaN

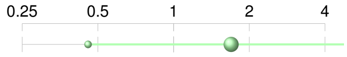

A	B	2.5	50	97.5	Sequence
**0**	**1**	**0.46**	**1.7**	**6.3**	**AVISSGSALGKK**

5.415 65 490 PREDICTED: cofilin-2 [Otolemur garnettii]

Protein Accession	CL15471.Contig3$_H$1
Mean Expression Ratio	1.09
Median Expression Ratio	1.09
Credible Interval	(0.598, 1.98)
Associated Peptides	4
Associated Spectra	9
Coverage	NaN

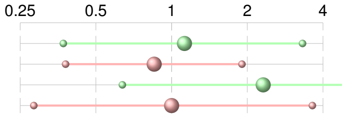

A	B	2.5	50	97.5	Sequence
1	1	0.37	1.1	3.3	MLYSSSSSAVK
1	4	0.38	0.85	1.9	IVMENEFAK
0	1	0.64	2.3	8.4	ALVFK
0	1	0.28	1	3.6	FVIWDLDVDTK

5.416 5 658 predicted protein [Nematostella vectensis] gi

Protein Accession	CL36575.Contig6$_H$1
Mean Expression Ratio	1.09
Median Expression Ratio	1.09
Credible Interval	(0.533, 2.2)
Associated Peptides	1
Associated Spectra	1
Coverage	NaN

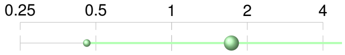

A	B	2.5	50	97.5	Sequence
0	1	0.46	1.7	6.5	KVQIGNIAAGK

5.417 143 634 minus strand ribosomal protein S14 [Solea senegalensis]

Protein Accession	CL17182.Contig1$_H$1
Mean Expression Ratio	1.09
Median Expression Ratio	1.09
Credible Interval	(0.563, 2.14)
Associated Peptides	2
Associated Spectra	3
Coverage	NaN

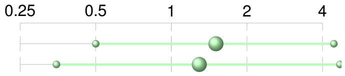

A	B	2.5	50	97.5	Sequence
0	2	0.5	1.5	4.5	TVQQEQVVLGPQAK
0	1	0.35	1.3	4.7	QLGITALHIK

5.418 2 298 putative 60S ribosomal protein RPL24 [Novocrania anomala]

Protein Accession	Unigene278$_H$1
Mean Expression Ratio	1.09
Median Expression Ratio	1.09
Credible Interval	(0.554, 2.15)
Associated Peptides	1
Associated Spectra	3
Coverage	NaN

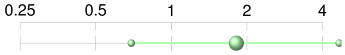

A	B	2.5	50	97.5	Sequence
0	3	0.69	1.8	4.7	AITGVSLSEIIAK

5.419 145 459 fk506-binding protein 2 [Papilio xuthus]; 237 551 fk506-binding protein 2 [Papilio xuthus]

Protein Accession	Unigene7883$_H$1 CL4621.Contig4$_H$1
Mean Expression Ratio	0.916
Median Expression Ratio	0.918
Credible Interval	(0.521, 1.59)
Associated Peptides	5
Associated Spectra	24
Coverage	NaN

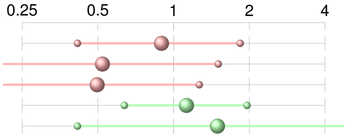

A	B	2.5	50	97.5	Sequence
3	3	0.42	0.9	1.8	GVDVETISEGDKK
1	1	0.18	0.52	1.5	GWDEGVAQMSK
1	2	0.19	0.5	1.3	IGKGEVIK
6	6	0.64	1.1	2	NKPFQFK
0	1	0.41	1.5	5.5	GVDVETISEGDKKNYPK

5.420 418 690 PREDICTED: protein disulfide isomerase A4-like [Saccoglossus kowalevskii]

Protein Accession	CL20258.Contig2$_H$1
Mean Expression Ratio	0.921
Median Expression Ratio	0.918
Credible Interval	(0.468, 1.84)
Associated Peptides	1
Associated Spectra	2
Coverage	NaN

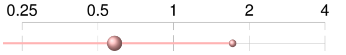

A	B	2.5	50	97.5	Sequence
0	2	0.2	0.58	1.7	TFEQIVLDK

5.421 635 1561 Uncharacterized protein yfeX [Crassostrea gigas]

Protein Accession	CL12046.Contig4$_H$1
Mean Expression Ratio	1.09
Median Expression Ratio	1.09
Credible Interval	(0.605, 2.02)
Associated Peptides	5
Associated Spectra	7
Coverage	NaN

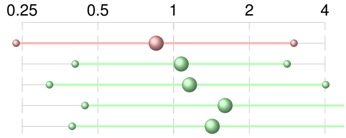

A	B	2.5	50	97.5	Sequence
1	0	0.24	0.86	3	ATGGSYVITQK
1	2	0.41	1.1	2.8	TLESWVGR
0	1	0.32	1.2	4	MVGAGGDGHADDIMR
0	1	0.44	1.6	5.8	KAFQIVR
0	1	0.39	1.4	5.3	WIHDLHVINTEKEK

5.422 " 64 1332 Flotillin-1, putative [Pediculus humanus corporis] gi"

Protein Accession	CL33952.Contig1$_H$1
Mean Expression Ratio	1.09
Median Expression Ratio	1.09
Credible Interval	(0.613, 1.97)
Associated Peptides	6
Associated Spectra	7
Coverage	NaN

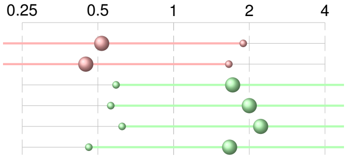

A	B	2.5	50	97.5	Sequence
1	0	0.14	0.52	1.9	AVAQETLEGHQR
1	0	0.12	0.45	1.7	VAAEIAQPLSNAK
1	1	0.59	1.7	5.2	FLNDIEIAK
0	1	0.56	2	7.2	AQADLAYDLQAAK
0	1	0.63	2.2	8.1	GQAIQVQEQEIIRR
0	1	0.46	1.7	6	VKGEAEAFAIEAK

5.423 977 1852 PREDICTED: similar to prohibitin [Tribolium castaneum] gi

Protein Accession CL8219.Contig4$_{H}1$
Mean Expression Ratio 1.09
Median Expression Ratio 1.09
Credible Interval (0.623, 1.95)
Associated Peptides 5
Associated Spectra 10
Coverage NaN

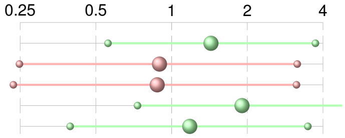

A	B	2.5	50	97.5	Sequence
1	2	0.56	1.4	3.7	VVQAEGEAAAAK
1	0	0.25	0.89	3.2	AQFVVEK
1	0	0.24	0.88	3.1	SLLIR
1	2	0.73	1.9	5	KLGDLAGR
0	2	0.39	1.2	3.5	LIGQAVSANPGFLK

5.424 2 487 minus strand PREDICTED: hypothetical protein LOC100699750 [Oreochromis niloticus]; 41 535 minus strand PREDICTED: hypothetical protein LOC100699750 [Oreochromis niloticus]

Protein Accession CL7266.Contig5$_{H}1$ CL7266.Contig3$_{H}1$
Mean Expression Ratio 1.09
Median Expression Ratio 1.09
Credible Interval (0.585, 2.03)
Associated Peptides 3
Associated Spectra 6
Coverage NaN

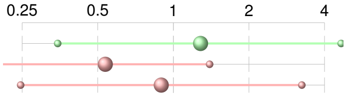

A	B	2.5	50	97.5	Sequence
0	1	0.35	1.3	4.6	VVNINNEAVTFASPR
2	2	0.2	0.53	1.4	NVALVVSVK
0	1	0.25	0.9	3.2	IPGDGTLTLHGSNGDTAFDR

5.425 84 425 minus strand thioester-containing protein [Euphaedusa tau]

Protein Accession Unigene24513$_{H}1$
Mean Expression Ratio 1.09
Median Expression Ratio 1.09
Credible Interval (0.534, 2.2)
Associated Peptides 1
Associated Spectra 1
Coverage NaN

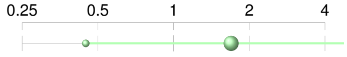

A	B	2.5	50	97.5	Sequence
0	1	0.45	1.7	6.3	TYVILAPAK

5.426 116 1882 Actin-interacting protein 1 [Crassostrea gigas]

Protein Accession CL12416.Contig1$_{H}1$
Mean Expression Ratio 0.92
Median Expression Ratio 0.919
Credible Interval (0.515, 1.63)
Associated Peptides 6
Associated Spectra 8
Coverage NaN

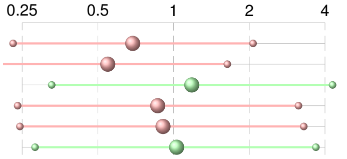

A	B	2.5	50	97.5	Sequence
1	1	0.23	0.69	2.1	QFLDGEVK
1	1	0.19	0.55	1.6	TLDELDNKEIGAPGAHK
0	1	0.33	1.2	4.3	FSPDGEVAVSGGADGK
0	1	0.24	0.86	3.1	GHMKPITAMALSDDR
0	1	0.24	0.91	3.3	QILTASGDASAK
0	1	0.28	1	3.7	VFDVQSGSLVQEFK

5.427 23 214 minus strand hemocyanin isoform 1 [Nucula nucleus]

Protein Accession	Unigene58881$_{H}$1
Mean Expression Ratio	0.921
Median Expression Ratio	0.919
Credible Interval	(0.503, 1.67)
Associated Peptides	3
Associated Spectra	20
Coverage	NaN

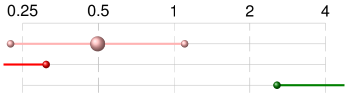

A	B	2.5	50	97.5	Sequence
2	3	0.22	0.5	1.1	GKIPNLNIYTSR
4	7	0.097	0.17	0.31	QEINPFFR
1	3	2.6	6.1	15	TAKQEINPFFR

5.428 31 777 Proteasome subunit alpha type-7-like protein [Crassostrea gigas]

Protein Accession	CL35094.Contig4$_{H}$1
Mean Expression Ratio	0.921
Median Expression Ratio	0.92
Credible Interval	(0.46, 1.85)
Associated Peptides	1
Associated Spectra	1
Coverage	NaN

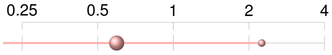

A	B	2.5	50	97.5	Sequence
1	0	0.16	0.6	2.2	GDNLVVLGVEK

5.429 69 731 hypoxanthine phosphoribosyltransferase 1 [Haliotis discus discus]

Protein Accession	Unigene30480$_{H}$1
Mean Expression Ratio	1.09
Median Expression Ratio	1.09
Credible Interval	(0.547, 2.19)
Associated Peptides	1
Associated Spectra	1
Coverage	NaN

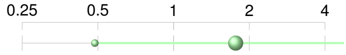

A	B	2.5	50	97.5	Sequence
0	1	0.49	1.8	6.4	LDKIK

5.430 21 341 minus strand hemocyanin isoform 1 [Nucula nucleus]

Protein Accession	CL40281.Contig2$_{H}$1
Mean Expression Ratio	0.919
Median Expression Ratio	0.92
Credible Interval	(0.488, 1.69)
Associated Peptides	3
Associated Spectra	11
Coverage	NaN

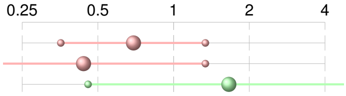

A	B	2.5	50	97.5	Sequence
6	2	0.36	0.69	1.3	DSNPIALTR
0	2	0.15	0.44	1.3	GDELFSYLK
0	1	0.46	1.7	6	KPLEPFNRDSNPIALTR

5.431 712 831 haemocyanin [Aplysia californica]

Protein Accession	Unigene65929$_{H}1$
Mean Expression Ratio	1.09
Median Expression Ratio	1.09
Credible Interval	(0.592, 2.01)
Associated Peptides	3
Associated Spectra	11
Coverage	NaN

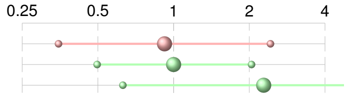

A	B	2.5	50	97.5	Sequence
0	3	0.35	0.92	2.4	YDVTDVFNQMR
0	7	0.5	1	2	YHFNVK
0	1	0.63	2.3	8.3	LRPDSKYHFNVK

5.432 " 451 1026 PREDICTED: GTP-binding protein SAR1B, putative-like [Saccoglossus kowalevskii]"

Protein Accession	Unigene4124$_{H}1$
Mean Expression Ratio	1.09
Median Expression Ratio	1.09
Credible Interval	(0.546, 2.17)
Associated Peptides	1
Associated Spectra	1
Coverage	NaN

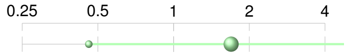

A	B	2.5	50	97.5	Sequence
0	1	0.46	1.7	6.3	FTTFDLGGHR

5.433 244 837 PREDICTED: ras-like GTP-binding protein Rho1-like [Megachile rotundata]

Protein Accession	Unigene11792$_{H}1$
Mean Expression Ratio	1.09
Median Expression Ratio	1.09
Credible Interval	(0.572, 2.11)
Associated Peptides	2
Associated Spectra	3
Coverage	NaN

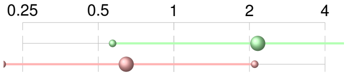

A	B	2.5	50	97.5	Sequence
0	1	0.57	2.2	8	EGVREVFETATR
1	1	0.21	0.65	2.1	EVFETATR

5.434 1867 3108 minus strand roundabout homolog 1 precursor [Rattus norvegicus] gi; 1867 3108 minus strand roundabout homolog 1 precursor [Rattus norvegicus] gi; 1835 2956 minus strand roundabout homolog 1 precursor [Rattus norvegicus] gi; 1867 3108 minus strand roundabout homolog 1 precursor [Rattus norvegicus] gi

Protein Accession	CL2792.Contig8$_H$1 CL2792.Contig5$_H$1 CL2792.Contig11$_H$1 CL2792.Contig10$_H$1
Mean Expression Ratio	1.09
Median Expression Ratio	1.09
Credible Interval	(0.55, 2.18)
Associated Peptides	1
Associated Spectra	1
Coverage	NaN

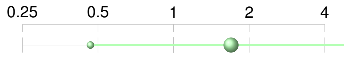

A	B	2.5	50	97.5	Sequence
**0**	**1**	**0.47**	**1.7**	**6.3**	**VAHLDRVFQQEPVR**

5.435 " 2 1810 minus strand Glutaminyl-tRNA synthetase, partial [Crassostrea gigas]"

Protein Accession	CL16949.Contig1$_H$1
Mean Expression Ratio	1.09
Median Expression Ratio	1.09
Credible Interval	(0.549, 2.21)
Associated Peptides	1
Associated Spectra	1
Coverage	NaN

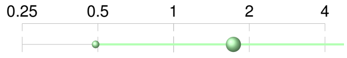

A	B	2.5	50	97.5	Sequence
**0**	**1**	**0.49**	**1.7**	**6.4**	**IAEGEATLR**

5.436 102 1685 minus strand predicted protein [Nematostella vectensis] gi

Protein Accession	CL44087.Contig1$_H$1
Mean Expression Ratio	0.918
Median Expression Ratio	0.922
Credible Interval	(0.479, 1.75)
Associated Peptides	3
Associated Spectra	3
Coverage	NaN

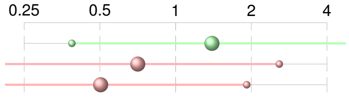

A	B	2.5	50	97.5	Sequence
**0**	**1**	**0.39**	**1.4**	**5**	**IGGNMGLAMK**
**0**	**1**	**0.18**	**0.7**	**2.6**	**LNPQTAFFQGK**
**0**	**1**	**0.14**	**0.5**	**1.9**	**GVFLFK**

5.437 122 1195 minus strand Severin [Crassostrea gigas]

Protein Accession	CL7486.Contig12$_{H}1$
Mean Expression Ratio	1.09
Median Expression Ratio	1.08
Credible Interval	(0.598, 2)
Associated Peptides	5
Associated Spectra	7
Coverage	NaN

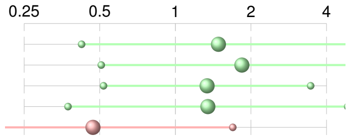

A	B	2.5	50	97.5	Sequence
**0**	**1**	**0.42**	**1.5**	**5.4**	**TVELDTFLNDVPVQHR**
**0**	**1**	**0.51**	**1.8**	**6.8**	**SVKPEEYRPR**
**0**	**3**	**0.52**	**1.3**	**3.4**	**EVQGHESDLFR**
**0**	**1**	**0.37**	**1.4**	**4.8**	**LLHFHGDR**
**0**	**1**	**0.13**	**0.47**	**1.7**	**SDFISEDVFILDTK**

5.438 1 177 small heat shock protein [Trichinella pseudospiralis]

Protein Accession	Unigene29352$_{H}1$
Mean Expression Ratio	1.09
Median Expression Ratio	1.08
Credible Interval	(0.547, 2.19)
Associated Peptides	1
Associated Spectra	1
Coverage	NaN

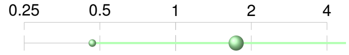

A	B	2.5	50	97.5	Sequence
**0**	**1**	**0.47**	**1.7**	**6.6**	**ISMDVQHFKPEEIK**

5.439 91 1392 minus strand hypothetical protein AND_03103 [Anopheles darlingi]

Protein Accession	CL10598.Contig5$_{H}1$
Mean Expression Ratio	0.923
Median Expression Ratio	0.922
Credible Interval	(0.466, 1.82)
Associated Peptides	2
Associated Spectra	2
Coverage	NaN

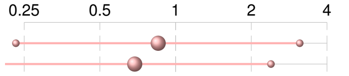

A	B	2.5	50	97.5	Sequence
**0**	**1**	**0.23**	**0.85**	**3.1**	**LPPFNEHALVQEK**
**0**	**1**	**0.18**	**0.69**	**2.4**	**LPVMYPDEESSK**

5.440 108 1040 minus strand PREDICTED: Na(+)/H(+) exchange regulatory cofactor NHE-RF3-like [Metaseiulus occidentalis]; 108 1259 minus strand PREDICTED: Na(+)/H(+) exchange regulatory cofactor NHE-RF3-like [Metaseiulus occidentalis]; 108 1190 minus strand PREDICTED: Na(+)/H(+) exchange regulatory cofactor NHE-RF3-like [Metaseiulus occidentalis]; 108 971 minus strand PREDICTED: Na(+)/H(+) exchange regulatory cofactor NHE-RF3-like [Metaseiulus occidentalis]

Protein Accession	CL26023.Contig8$_H$1 CL26023.Contig7$_H$1 CL26023.Contig6$_H$1 CL26023.Contig5$_H$1
Mean Expression Ratio	1.08
Median Expression Ratio	1.08
Credible Interval	(0.556, 2.12)
Associated Peptides	2
Associated Spectra	2
Coverage	NaN

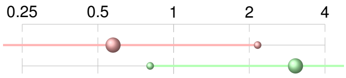

A	B	2.5	50	97.5	Sequence
0	1	0.15	0.57	2.2	GDLPEVLHLTAVR
0	1	0.81	3	11	IVKGDL

5.441 2 778 Putative adenosylhomocysteinase 3 [Crassostrea gigas]

Protein Accession	CL23756.Contig1$_H$1
Mean Expression Ratio	0.923
Median Expression Ratio	0.923
Credible Interval	(0.454, 1.84)
Associated Peptides	1
Associated Spectra	1
Coverage	NaN

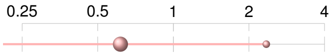

A	B	2.5	50	97.5	Sequence
1	0	0.17	0.62	2.3	TPELTWEK

5.442 360 764 minus strand Peptidyl-prolyl cis-trans isomerase A OS=Dictyostelium discoideum GN=ppiA PE=1 SV=1

Protein Accession	Unigene64429$_H$1
Mean Expression Ratio	0.921
Median Expression Ratio	0.923
Credible Interval	(0.461, 1.84)
Associated Peptides	1
Associated Spectra	2
Coverage	NaN

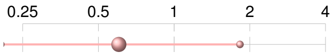

A	B	2.5	50	97.5	Sequence
0	2	0.2	0.6	1.8	HVVFGQVVEGLDVVR

5.443 176 526 minus strand Translocon-associated protein subunit delta [Crassostrea gigas]

Protein Accession	Unigene63010$_{H}1$
Mean Expression Ratio	1.08
Median Expression Ratio	1.08
Credible Interval	(0.555, 2.07)
Associated Peptides	2
Associated Spectra	3
Coverage	NaN

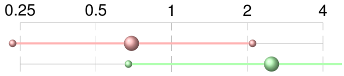

A	B	2.5	50	97.5	Sequence
**1**	**1**	**0.23**	**0.69**	**2.1**	**KLPSGNYQVR**
**0**	**1**	**0.67**	**2.5**	**9.2**	**VFDEEGYALLR**

5.444 125 550 minus strand ribosomal protein S20 [Chlamys farreri]; 125 550 minus strand ribosomal protein S20 [Chlamys farreri]; 125 550 minus strand ribosomal protein S20 [Chlamys farreri]

Protein Accession	CL6833.Contig3$_{H}1$ CL6833.Contig2$_{H}1$ CL6833.Contig1$_{H}1$
Mean Expression Ratio	1.08
Median Expression Ratio	1.08
Credible Interval	(0.583, 2.01)
Associated Peptides	3
Associated Spectra	9
Coverage	NaN

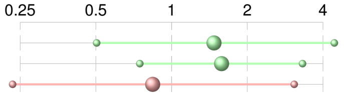

A	B	2.5	50	97.5	Sequence
**1**	**1**	**0.5**	**1.5**	**4.4**	**ITLTSR**
**0**	**6**	**0.75**	**1.6**	**3.3**	**IIDLHSPSEIVK**
**0**	**1**	**0.23**	**0.84**	**3.1**	**KTPCGEGSK**

5.445 " 194 334 unknown, partial [Lymnaea stagnalis] gi; 207 347 unknown, partial [Lymnaea stagnalis] gi; 384 524 unknown, partial [Lymnaea stagnalis] gi"

Protein Accession	Unigene62752$_{H}1$ Unigene62751$_{H}1$ Unigene62750$_{H}1$
Mean Expression Ratio	1.09
Median Expression Ratio	1.08
Credible Interval	(0.547, 2.15)
Associated Peptides	1
Associated Spectra	2
Coverage	NaN

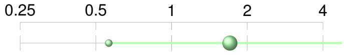

A	B	2.5	50	97.5	Sequence
**0**	**2**	**0.56**	**1.7**	**5.1**	**AFGFGMR**

5.446 365 2530 minus strand calpain B [Gecarcinus lateralis]

Protein Accession	CL34074.Contig5$_H$1
Mean Expression Ratio	1.08
Median Expression Ratio	1.08
Credible Interval	(0.573, 2.03)
Associated Peptides	3
Associated Spectra	5
Coverage	NaN

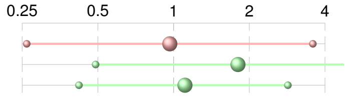

A	B	2.5	50	97.5	Sequence
0	1	0.26	0.96	3.6	SLQSLVMR
0	1	0.49	1.8	6.7	SLFFSQSMR
0	3	0.42	1.1	2.9	MMDIK

5.447 120 833 B-cell receptor-associated protein 31 [Crassostrea gigas]; 238 951 B-cell receptor-associated protein 31 [Crassostrea gigas]; 88 801 B-cell receptor-associated protein 31 [Crassostrea gigas]; 88 801 B-cell receptor-associated protein 31 [Crassostrea gigas]

Protein Accession	CL9442.Contig7$_H$1 CL9442.Contig5$_H$1 CL9442.Contig4$_H$1 CL9442.Contig3$_H$1
Mean Expression Ratio	0.922
Median Expression Ratio	0.925
Credible Interval	(0.502, 1.69)
Associated Peptides	4
Associated Spectra	8
Coverage	NaN

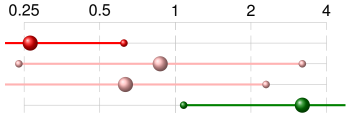

A	B	2.5	50	97.5	Sequence
1	3	0.11	0.26	0.62	LLEEHAQLQK
1	0	0.24	0.87	3.2	TELSDEMK
1	0	0.18	0.63	2.3	LGGLK
0	2	1.1	3.2	9.2	IEFSSMKK

5.448 128 559 minus strand predicted protein [Nematostella vectensis] gi

Protein Accession	CL19343.Contig1$_H$1
Mean Expression Ratio	0.924
Median Expression Ratio	0.925
Credible Interval	(0.47, 1.83)
Associated Peptides	1
Associated Spectra	5
Coverage	NaN

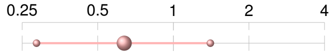

A	B	2.5	50	97.5	Sequence
0	5	0.28	0.64	1.4	TVILGDLTGSK

5.449 " 235 1038 Translocon-associated protein subunit alpha [Crassostrea gigas]; 309 725 hypothetical protein [Amblyomma maculatum]; 309 590 translocon-associated complex TRAP, alpha subunit, putative [Ixodes scapularis] gi; 309 725 hypothetical protein [Amblyomma maculatum]"

Protein Accession	CL36450.Contig6$_{H1}$ CL36450.Contig5$_{H1}$ CL36450.Contig2$_{H1}$ CL36450.Contig1$_{H1}$
Mean Expression Ratio	0.916
Median Expression Ratio	0.925
Credible Interval	(0.469, 1.73)
Associated Peptides	2
Associated Spectra	3
Coverage	NaN

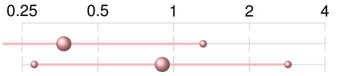

A	B	2.5	50	97.5	Sequence
0	1	0.099	0.37	1.3	LLVGFTNK
1	1	0.28	0.9	2.9	ELPAGKPVR

5.450 " 81 1349 minus strand PREDICTED: LOW QUALITY PROTEIN: dolichyl-diphosphooligosaccharide–protein glycosyltransferase 48 kDa subunit-like, partial [Cavia porcellus]; 81 1349 minus strand PREDICTED: LOW QUALITY PROTEIN: dolichyl-diphosphooligosaccharide–protein glycosyltransferase 48 kDa subunit-like, partial [Cavia porcellus]"

Protein Accession	CL49524.Contig2$_{H1}$ CL49524.Contig1$_{H1}$
Mean Expression Ratio	0.923
Median Expression Ratio	0.926
Credible Interval	(0.475, 1.78)
Associated Peptides	3
Associated Spectra	3
Coverage	NaN

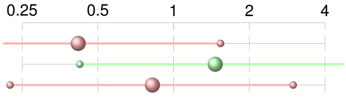

A	B	2.5	50	97.5	Sequence
0	1	0.12	0.42	1.5	DLGFDLTFK
0	1	0.42	1.5	5.4	KGNSPFLFR
0	1	0.22	0.83	3	IDPFVR

5.451 260 892 minus strand calponin [Haliotis diversicolor]

Protein Accession	CL48702.Contig2$_{H1}$
Mean Expression Ratio	1.08
Median Expression Ratio	1.08
Credible Interval	(0.57, 2.03)
Associated Peptides	2
Associated Spectra	7
Coverage	NaN

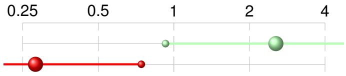

A	B	2.5	50	97.5	Sequence
2	1	0.93	2.5	6.9	NFSEDKLR
2	2	0.11	0.28	0.74	VESSFDPEDAKK

5.452 18 1613 minus strand chaperonin containing T-complex polypeptide subunit zeta [Haliotis discus hannai]; 527 2089 minus strand chaperonin containing T-complex polypeptide subunit zeta [Haliotis discus hannai]

Protein Accession	CL23722.Contig7$_{H}1$ CL23722.Contig5$_{H}1$
Mean Expression Ratio	0.92
Median Expression Ratio	0.926
Credible Interval	(0.468, 1.78)
Associated Peptides	2
Associated Spectra	3
Coverage	NaN

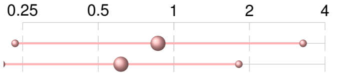

A	B	2.5	50	97.5	Sequence
1	0	0.23	0.87	3.3	AGMSSLKGD
2	0	0.21	0.61	1.8	EGIIALR

5.453 801 971 RecName: Full=Tropomyosin-2; AltName: Full=Tropomyosin II; Short=BgTMII; Short=TMII gi|155940|gb|AAA27816.1| tropomyosin [Biomphalaria glabrata]

Protein Accession	CL2925.Contig4$_{H}1$
Mean Expression Ratio	1.08
Median Expression Ratio	1.08
Credible Interval	(0.546, 2.15)
Associated Peptides	1
Associated Spectra	1
Coverage	NaN

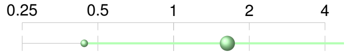

A	B	2.5	50	97.5	Sequence
0	1	0.44	1.6	6	ASQREDSYEETIRDLTQR

5.454 40 1182 4-hydroxyphenylpyruvate dioxygenase [Crassostrea gigas]

Protein Accession	CL7585.Contig2$_{H}1$
Mean Expression Ratio	0.926
Median Expression Ratio	0.926
Credible Interval	(0.544, 1.56)
Associated Peptides	9
Associated Spectra	13
Coverage	NaN

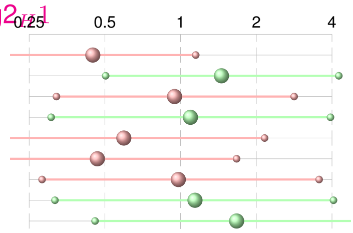

A	B	2.5	50	97.5	Sequence
1	2	0.17	0.45	1.1	SIVVTNYEETIK
1	1	0.5	1.4	4.3	TYGDTTHTLIDR
1	1	0.32	0.95	2.8	QVAAHVVK
0	1	0.31	1.1	4	QIHTDYSALR
0	1	0.16	0.59	2.2	SLFEAIELDQAER
0	1	0.13	0.47	1.7	VQIKEDLDELQK
0	1	0.28	0.98	3.6	FWSVDDK
0	1	0.32	1.1	4.1	MPINEPAPGKR
0	1	0.46	1.7	6	TSNIIQSINR

5.455 167 2713 minus strand Major vault protein [Crassostrea gigas]

Protein Accession	CL18286.Contig3$_{H}1$
Mean Expression Ratio	1.08
Median Expression Ratio	1.08
Credible Interval	(0.647, 1.77)
Associated Peptides	12
Associated Spectra	15
Coverage	NaN

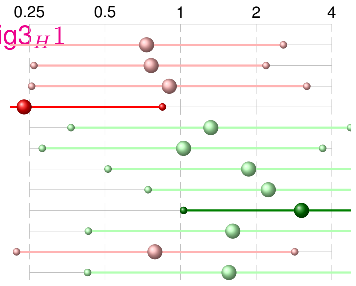

A	B	2.5	50	97.5	Sequence
1	0	0.2	0.73	2.6	GAVAQVQFDDFHK
1	1	0.26	0.76	2.2	IEGEAAVEQAK
1	0	0.25	0.9	3.2	LKAEAMK
1	0	0.067	0.24	0.85	VTGEEWLAK
1	0	0.37	1.3	4.8	TYTDDFNK
1	0	0.28	1	3.7	DPLADRSER
0	1	0.51	1.9	6.6	KEVVVEETIR
0	2	0.74	2.2	6.8	NADNEVVMDAAGQAK
0	2	1	3	9	QKIVDEAEAEK
0	1	0.43	1.6	5.7	VVPANSALR
0	1	0.22	0.79	2.9	IEAESELER
0	1	0.43	1.6	5.7	VVTFR

5.456 102 1475 minus strand Cytosolic non-specific dipeptidase [Crassostrea gigas]

Protein Accession	CL4706.Contig1$_{H}1$
Mean Expression Ratio	0.925
Median Expression Ratio	0.927
Credible Interval	(0.518, 1.66)
Associated Peptides	5
Associated Spectra	8
Coverage	NaN

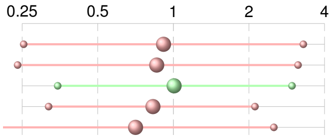

A	B	2.5	50	97.5	Sequence
0	1	0.25	0.92	3.3	EGGSIPVTLTFQEATGK
0	1	0.24	0.86	3.1	EHVEKVFAER
0	2	0.35	1	3	LVPDQEPHVIESLVR
0	3	0.32	0.83	2.1	LSEAVAIK
0	1	0.2	0.71	2.5	AELQELGEQTLHDGSK

5.457 399 575 hypothetical protein BRAFLDRAFT_69739 [Branchiostoma floridae] gi

Protein Accession	CL7219.Contig3$_{H}1$
Mean Expression Ratio	1.08
Median Expression Ratio	1.08
Credible Interval	(0.55, 2.15)
Associated Peptides	1
Associated Spectra	2
Coverage	NaN

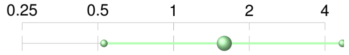

A	B	2.5	50	97.5	Sequence
0	2	0.53	1.6	4.7	HFLQPSGDLVSYPGK

5.458 503 1219 heat shock cognate 71 [Mytilus galloprovincialis]

Protein Accession	CL23109.Contig5$_{H}1$
Mean Expression Ratio	0.926
Median Expression Ratio	0.928
Credible Interval	(0.486, 1.77)
Associated Peptides	2
Associated Spectra	5
Coverage	NaN

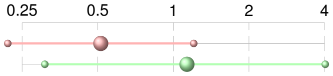

A	B	2.5	50	97.5	Sequence
4	0	0.22	0.51	1.2	GTLEPVEK
0	1	0.31	1.1	4	SAIHEIVLVGGSTR

5.459 1067 3010 minus strand PREDICTED: nucleolin-like isoform 2 [Oreochromis niloticus]

Protein Accession	CL7031.Contig7$_H1$
Mean Expression Ratio	0.931
Median Expression Ratio	0.928
Credible Interval	(0.485, 1.76)
Associated Peptides	3
Associated Spectra	3
Coverage	NaN

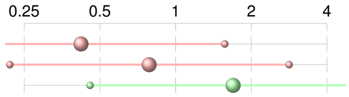

A	B	2.5	50	97.5	Sequence
**1**	**0**	**0.12**	**0.42**	**1.6**	**KGIAVSDVR**
**1**	**0**	**0.22**	**0.78**	**2.8**	**KISIEAAAVVASAK**
**1**	**0**	**0.46**	**1.7**	**6.2**	**RFAYADLENPDDLDK**

5.460 156 845 minus strand histone H1 [Mytilus californianus]; 156 845 minus strand histone H1 [Mytilus californianus]; 156 845 minus strand histone H1 [Mytilus californianus]; 156 845 minus strand histone H1 [Mytilus californianus]

Protein Accession	CL26709.Contig6$_H1$ CL26709.Contig5$_H1$ CL26709.Contig4$_H1$ CL26709.Contig3$_H1$
Mean Expression Ratio	0.927
Median Expression Ratio	0.928
Credible Interval	(0.465, 1.86)
Associated Peptides	1
Associated Spectra	1
Coverage	NaN

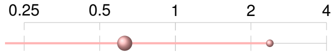

A	B	2.5	50	97.5	Sequence
**1**	**0**	**0.17**	**0.63**	**2.4**	**DAITALKER**

5.461 " 166 705 minus strand ADP-ribosylation factor, partial [Antheraea yamamai]; 166 705 minus strand ADP-ribosylation factor, partial [Antheraea yamamai]; 166 705 minus strand ADP-ribosylation factor, partial [Antheraea yamamai]; 166 705 minus strand ADP-ribosylation factor, partial [Antheraea yamamai]; 166 705 minus strand ADP-ribosylation factor, partial [Antheraea yamamai]"

Protein Accession	Unigene39229$_H1$ Unigene39228$_H1$ Unigene39227$_H1$ Unigene39226$_H1$ Unigene39225$_H1$
Mean Expression Ratio	1.08
Median Expression Ratio	1.08
Credible Interval	(0.548, 2.15)
Associated Peptides	1
Associated Spectra	2
Coverage	NaN

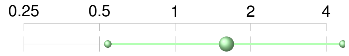

A	B	2.5	50	97.5	Sequence
**0**	**2**	**0.54**	**1.6**	**4.7**	**IGEARDELQR**

5.462 123 551 minus strand Transcription factor BTF3-like protein 4 [Crassostrea gigas]

Protein Accession	Unigene19288$_{H}1$
Mean Expression Ratio	1.08
Median Expression Ratio	1.08
Credible Interval	(0.549, 2.15)
Associated Peptides	1
Associated Spectra	2
Coverage	NaN

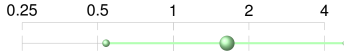

A	B	2.5	50	97.5	Sequence
**0**	**2**	**0.54**	**1.6**	**4.9**	**VQASLAANTFAITGHAETK**

5.463 604 1236 minus strand selenium-dependent glutathione peroxidase [Crassostrea gigas]; 138 770 minus strand selenium-dependent glutathione peroxidase [Crassostrea gigas]; 138 770 minus strand selenium-dependent glutathione peroxidase [Crassostrea gigas]; 147 779 minus strand selenium-dependent glutathione peroxidase [Crassostrea gigas]; 149 781 minus strand selenium-dependent glutathione peroxidase [Crassostrea gigas]; 144 776 minus strand selenium-dependent glutathione peroxidase [Crassostrea gigas]; 141 773 minus strand selenium-dependent glutathione peroxidase [Crassostrea gigas]

Protein Accession	CL12854.Contig7$_{H}1$ CL12854.Contig20$_{H}1$ CL12854.Contig19$_{H}1$ CL12854.Contig18$_{H}1$ CL1
Mean Expression Ratio	1.08
Median Expression Ratio	1.08
Credible Interval	(0.582, 2.03)
Associated Peptides	3
Associated Spectra	8
Coverage	NaN

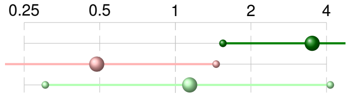

A	B	2.5	50	97.5	Sequence
**3**	**2**	**1.5**	**3.5**	**7.8**	**SGVQGLSVPR**
**0**	**2**	**0.17**	**0.49**	**1.5**	**FLVGQDGLPVAR**
**0**	**1**	**0.3**	**1.1**	**4.1**	**SDVNGQNELPLYR**

5.464 " 2 778 Actin, gamma-enteric smooth muscle OS=Rattus norvegicus GN=Actg2 PE=2 SV=1"

Protein Accession	Unigene64184$_{H}1$
Mean Expression Ratio	0.928
Median Expression Ratio	0.929
Credible Interval	(0.466, 1.85)
Associated Peptides	1
Associated Spectra	1
Coverage	NaN

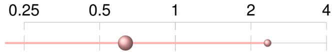

A	B	2.5	50	97.5	Sequence
**1**	**0**	**0.17**	**0.63**	**2.3**	**EITSLAPSTMK**

5.465 4 219 hypothetical protein BRAFLDRAFT_66351 [Branchiostoma floridae] gi; 3 239 hypothetical protein BRAFLDRAFT_66351 [Branchiostoma floridae] gi

Protein Accession	CL31114.Contig2$_{H}$1 CL31114.Contig1$_{H}$1
Mean Expression Ratio	1.08
Median Expression Ratio	1.08
Credible Interval	(0.572, 2.09)
Associated Peptides	3
Associated Spectra	3
Coverage	NaN

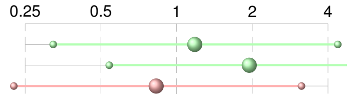

A	B	2.5	50	97.5	Sequence
0	1	0.32	1.2	4.4	KGSENVLAK
0	1	0.54	1.9	7.3	KLEEVSGELR
0	1	0.23	0.83	3.1	NNQLNGDLK

5.466 213 875 es1 protein [Haliotis discus discus]

Protein Accession	CL35629.Contig1$_{H}$1
Mean Expression Ratio	0.931
Median Expression Ratio	0.93
Credible Interval	(0.478, 1.83)
Associated Peptides	2
Associated Spectra	2
Coverage	NaN

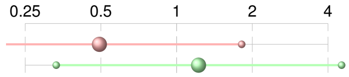

A	B	2.5	50	97.5	Sequence
1	0	0.13	0.49	1.8	NLSNFAFDAK
0	1	0.33	1.2	4.5	IARGPVNL

5.467 211 1413 Alpha-N-acetylgalactosaminidase [Crassostrea gigas]

Protein Accession	CL5989.Contig1$_{H}$1
Mean Expression Ratio	1.08
Median Expression Ratio	1.08
Credible Interval	(0.569, 2.11)
Associated Peptides	2
Associated Spectra	3
Coverage	NaN

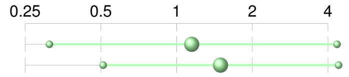

A	B	2.5	50	97.5	Sequence
0	1	0.31	1.1	4.3	ALADYMHDR
0	2	0.51	1.5	4.4	ALLLNR

5.468 273 1304 Guanine nucleotide-binding protein subunit beta [Crassostrea gigas]

Protein Accession	CL15682.Contig1$_{H}$1
Mean Expression Ratio	1.08
Median Expression Ratio	1.08
Credible Interval	(0.58, 2.06)
Associated Peptides	4
Associated Spectra	4
Coverage	NaN

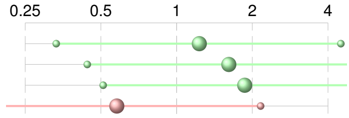

A	B	2.5	50	97.5	Sequence
0	1	0.33	1.2	4.5	TFVSGACDASAK
0	1	0.44	1.6	5.9	KAAGDTTLAQATAQCEPVGR
0	1	0.51	1.9	6.9	TSELDALRQETEQLKNQIR
0	1	0.16	0.58	2.2	IYAMHWASDSR

5.469 260 706 Collagen alpha-1(XII) chain (Fragment) OS=Notophthalmus viridescens PE=2 SV=1

Protein Accession	CL284.Contig5$_{H}1$
Mean Expression Ratio	0.929
Median Expression Ratio	0.931
Credible Interval	(0.456, 1.86)
Associated Peptides	1
Associated Spectra	1
Coverage	NaN

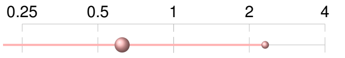

A	B	2.5	50	97.5	Sequence
1	0	0.17	0.62	2.3	VSLVTFSNEANAK

5.470 3 1460 Myosin-VI [Crassostrea gigas]

Protein Accession	CL6618.Contig5$_{H}1$
Mean Expression Ratio	0.934
Median Expression Ratio	0.931
Credible Interval	(0.464, 1.86)
Associated Peptides	1
Associated Spectra	1
Coverage	NaN

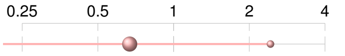

A	B	2.5	50	97.5	Sequence
1	0	0.18	0.67	2.4	LGIAK

5.471 284 700 Ganglioside GM2 activator [Crassostrea gigas]

Protein Accession	Unigene16455$_{H}1$
Mean Expression Ratio	0.928
Median Expression Ratio	0.931
Credible Interval	(0.524, 1.65)
Associated Peptides	5
Associated Spectra	17
Coverage	NaN

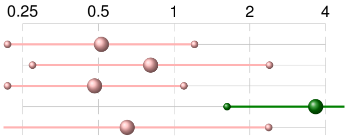

A	B	2.5	50	97.5	Sequence
1	3	0.22	0.52	1.2	ATVDSPLK
1	1	0.27	0.81	2.4	ATVDSPLKAEVYLGK
3	2	0.22	0.48	1.1	AEVYLGK
0	5	1.6	3.6	8.1	ATVDSPLKAEVYLGKK
0	1	0.18	0.65	2.4	TFQFTNCLPK

5.472 430 759 hypothetical protein [Amblyomma maculatum]; 336 665 hypothetical protein [Amblyomma maculatum]

Protein Accession	CL4062.Contig3$_{H}1$ CL4062.Contig2$_{H}1$
Mean Expression Ratio	1.07
Median Expression Ratio	1.07
Credible Interval	(0.604, 1.86)
Associated Peptides	6
Associated Spectra	11
Coverage	NaN

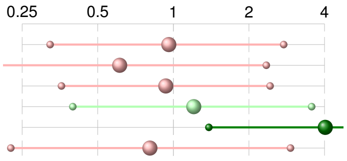

A	B	2.5	50	97.5	Sequence
1	1	0.32	0.96	2.7	KYIPGTK
1	0	0.17	0.61	2.3	MVFAGIK
0	3	0.36	0.93	2.4	ETLFEYLADPK
0	2	0.4	1.2	3.6	ETLFEYLADPKK
0	2	1.4	4	12	MVFAGIKKPQER
0	1	0.23	0.81	2.9	ADIPDGDFEK

5.473 30 536 putative mitochondrial ATP synthase [Haliotis discus discus]; 27 620 putative mitochondrial ATP synthase [Haliotis discus discus]

Protein Accession	CL13014.Contig4$_{H}1$ CL13014.Contig1$_{H}1$
Mean Expression Ratio	0.934
Median Expression Ratio	0.933
Credible Interval	(0.553, 1.56)
Associated Peptides	7
Associated Spectra	21
Coverage	NaN

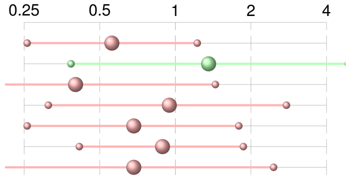

A	B	2.5	50	97.5	Sequence
2	4	0.26	0.56	1.2	SAVDWVAFK
0	1	0.38	1.4	4.9	SDSFVNKVHQYPAELPK
0	1	0.11	0.4	1.4	SNALKDIAAEEK
0	2	0.31	0.94	2.8	VHQYPAELPK
0	3	0.26	0.68	1.8	IDFSAYK
3	4	0.41	0.89	1.9	LANPALVDQFQK
0	1	0.19	0.68	2.5	VHQYPAEL

5.474 274 666 Calmodulin-4 [Heterocephalus glaber]

Protein Accession	Unigene19050$_{H}1$
Mean Expression Ratio	0.937
Median Expression Ratio	0.933
Credible Interval	(0.502, 1.77)
Associated Peptides	3
Associated Spectra	8
Coverage	NaN

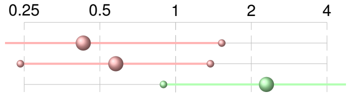

A	B	2.5	50	97.5	Sequence
1	0	0.12	0.43	1.5	SGFITLDELK
2	2	0.24	0.58	1.4	VLLSVGR
1	2	0.89	2.3	6	TLDELK

5.475 77 511 cytochrome c oxidase subunit VIIa polypeptide 2 like [Xenopus (Silurana) tropicalis]; 77 511 cytochrome c oxidase subunit VIIa polypeptide 2 like [Xenopus (Silurana) tropicalis]; 77 511 cytochrome c oxidase subunit VIIa polypeptide 2 like [Xenopus (Silurana) tropicalis]; 545 979 cytochrome c oxidase subunit VIIa polypeptide 2 like [Xenopus (Silurana) tropicalis]

Protein Accession	CL17601.Contig8$_{H}1$ CL17601.Contig7$_{H}1$ CL17601.Contig6$_{H}1$ CL17601.Contig2$_{H}1$
Mean Expression Ratio	0.934
Median Expression Ratio	0.934
Credible Interval	(0.491, 1.75)
Associated Peptides	3
Associated Spectra	6
Coverage	NaN

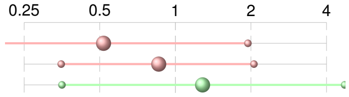

A	B	2.5	50	97.5	Sequence
1	0	0.14	0.52	1.9	EPSVIIFDSK
1	3	0.35	0.86	2.1	QDGLLVWQK
0	1	0.35	1.3	4.7	TTKPAVAPPAPMK

5.476 3 1670 Spectrin alpha chain [Crassostrea gigas]

Protein Accession	CL2195.Contig7$_H$1
Mean Expression Ratio	0.933
Median Expression Ratio	0.934
Credible Interval	(0.522, 1.68)
Associated Peptides	5
Associated Spectra	9
Coverage	NaN

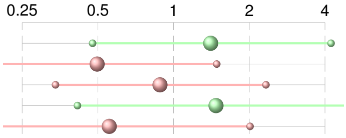

A	B	2.5	50	97.5	Sequence
1	1	0.48	1.4	4.2	KLDPSLSASR
0	2	0.17	0.5	1.5	EAAELAAWITEK
0	3	0.34	0.88	2.3	HQALQAEIAGHEPR
0	1	0.41	1.5	5.5	QGFVPAAYVK
0	1	0.15	0.56	2	VAALYDYTEK

5.477 194 838 glutathione S-transferase class mu [Cyphoma gibbosum]

Protein Accession	CL8626.Contig6$_H$1
Mean Expression Ratio	1.07
Median Expression Ratio	1.07
Credible Interval	(0.538, 2.12)
Associated Peptides	1
Associated Spectra	1
Coverage	NaN

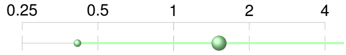

A	B	2.5	50	97.5	Sequence
0	1	0.41	1.5	5.5	PTLGYWK

5.478 212 1336 6-phosphofructokinase [Crassostrea gigas]; 208 1332 6-phosphofructokinase [Crassostrea gigas]

Protein Accession	CL48438.Contig4$_H$1 CL48438.Contig3$_H$1
Mean Expression Ratio	0.933
Median Expression Ratio	0.934
Credible Interval	(0.471, 1.88)
Associated Peptides	1
Associated Spectra	1
Coverage	NaN

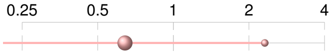

A	B	2.5	50	97.5	Sequence
1	0	0.17	0.64	2.3	LGLTQGYR

5.479 2 2248 PREDICTED: heat shock 70 kDa protein 4-like [Oreochromis niloticus]; 2 2227 PREDICTED: heat shock 70 kDa protein 4-like [Oreochromis niloticus]

Protein Accession	CL12749.Contig5$_H$1 CL12749.Contig4$_H$1
Mean Expression Ratio	0.93
Median Expression Ratio	0.934
Credible Interval	(0.485, 1.75)
Associated Peptides	3
Associated Spectra	3
Coverage	NaN

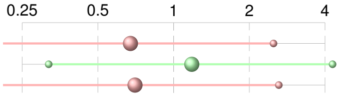

A	B	2.5	50	97.5	Sequence
0	1	0.19	0.67	2.5	LLQECEK
0	1	0.32	1.2	4.3	TAWWTPK
0	1	0.19	0.7	2.6	EMLNLLIEK

5.480 1 753 hypothetical protein BRAFLDRAFT_131251 [Branchiostoma floridae] gi

Protein Accession	CL18526.Contig3$_{H}1$
Mean Expression Ratio	0.934
Median Expression Ratio	0.934
Credible Interval	(0.51, 1.71)
Associated Peptides	5
Associated Spectra	7
Coverage	NaN

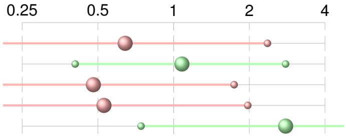

A	B	2.5	50	97.5	Sequence
1	0	0.17	0.64	2.4	GVFEIDSEK
3	0	0.41	1.1	2.8	GLGPFPR
0	1	0.14	0.48	1.7	GAGEPIDADYLLR
0	1	0.15	0.53	2	NVVDMIITEK
0	1	0.74	2.8	10	VIKGEKYEK

5.481 172 612 minus strand putative polyadenylate-binding protein 1 [Haliotis diversicolor]; 172 612 putative polyadenylate-binding protein 1 [Haliotis diversicolor]

Protein Accession	CL48895.Contig3$_{H}1$ CL48895.Contig2$_{H}1$
Mean Expression Ratio	0.93
Median Expression Ratio	0.935
Credible Interval	(0.472, 1.79)
Associated Peptides	2
Associated Spectra	2
Coverage	NaN

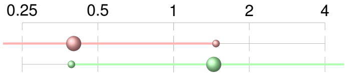

A	B	2.5	50	97.5	Sequence
1	0	0.11	0.4	1.5	ALDTMNFDTVK
0	1	0.39	1.4	5.3	KSGVGNVFIK

5.482 " 371 820 minus strand Peroxiredoxin-5, mitochondrial [Crassostrea gigas]; 91 528 minus strand Peroxiredoxin-5, mitochondrial [Crassostrea gigas]; 113 550 Peroxiredoxin-5, mitochondrial [Crassostrea gigas]; 453 902 Peroxiredoxin-5, mitochondrial [Crassostrea gigas]; 87 530 Peroxiredoxin-5, mitochondrial [Crassostrea gigas]"

Protein Accession	CL5795.Contig8$_{H}1$ CL5795.Contig7$_{H}1$ CL5795.Contig6$_{H}1$ CL5795.Contig5$_{H}1$ CL5795.Con
Mean Expression Ratio	1.07
Median Expression Ratio	1.07
Credible Interval	(0.553, 2.11)
Associated Peptides	2
Associated Spectra	2
Coverage	NaN

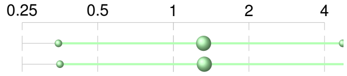

A	B	2.5	50	97.5	Sequence
0	1	0.35	1.3	4.7	ADLGIDLSSVLGNVR
0	1	0.35	1.3	4.9	FVLVAQDGVVK

5.483 244 837 PREDICTED: ras-like GTP-binding protein Rho1-like [Megachile rotundata]; 650 883 hypothetical protein SINV_15432 [Solenopsis invicta]; 534 767 hypothetical protein SINV_15432 [Solenopsis invicta]

Protein Accession	Unigene11792$_H$1 Unigene11791$_H$1 Unigene11790$_H$1
Mean Expression Ratio	0.938
Median Expression Ratio	0.935
Credible Interval	(0.48, 1.84)
Associated Peptides	1
Associated Spectra	2
Coverage	NaN

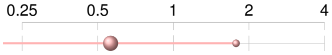

A	B	2.5	50	97.5	Sequence
**1**	**1**	**0.18**	**0.56**	**1.8**	**EVFETATR**

5.484 " 698 1225 minus strand actin related protein 2/3 complex, 21 kDa subunit [Crassostrea gigas]"

Protein Accession	CL17389.Contig2$_H$1
Mean Expression Ratio	1.07
Median Expression Ratio	1.07
Credible Interval	(0.535, 2.19)
Associated Peptides	1
Associated Spectra	1
Coverage	NaN

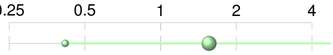

A	B	2.5	50	97.5	Sequence
**0**	**1**	**0.42**	**1.6**	**5.7**	**ANIFFR**

5.485 94 933 four and A half lim domains [Schistosoma mansoni]

Protein Accession	CL7046.Contig6$_H$1
Mean Expression Ratio	1.07
Median Expression Ratio	1.07
Credible Interval	(0.534, 2.16)
Associated Peptides	1
Associated Spectra	1
Coverage	NaN

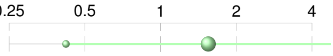

A	B	2.5	50	97.5	Sequence
**1**	**0**	**0.42**	**1.6**	**5.6**	**CTKPITGIGGTK**

5.486 44 910 PREDICTED: probable ATP-dependent RNA helicase DDX10 [Cricetulus griseus] gi

Protein Accession	CL33109.Contig1$_H$1
Mean Expression Ratio	1.07
Median Expression Ratio	1.07
Credible Interval	(0.552, 2.06)
Associated Peptides	2
Associated Spectra	2
Coverage	NaN

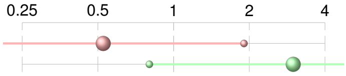

A	B	2.5	50	97.5	Sequence
**1**	**0**	**0.14**	**0.52**	**1.9**	**GIDVTK**
**1**	**0**	**0.8**	**3**	**11**	**AKYLK**

5.487 593 862 minus strand mitochondrial ATP synthase coupling factor 6 precursor [Ochlerotatus taeniorhynchus]

Protein Accession	Unigene4216$_H$1
Mean Expression Ratio	0.932
Median Expression Ratio	0.936
Credible Interval	(0.474, 1.81)
Associated Peptides	2
Associated Spectra	2
Coverage	NaN

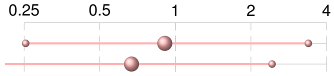

A	B	2.5	50	97.5	Sequence
0	1	0.25	0.91	3.4	GSTVTDPIQQLFLDK
0	1	0.18	0.67	2.4	LVDASPTVQAALKEELEK

5.488 346 702 minus strand PREDICTED: galectin-4-like [Anolis carolinensis]

Protein Accession	Unigene42203$_H$1
Mean Expression Ratio	1.07
Median Expression Ratio	1.07
Credible Interval	(0.574, 2.02)
Associated Peptides	2
Associated Spectra	8
Coverage	NaN

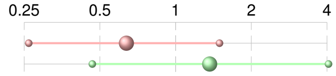

A	B	2.5	50	97.5	Sequence
3	2	0.26	0.64	1.5	AEATLEVR
1	2	0.47	1.4	4	SVPHGLVK

5.489 49 1560 PREDICTED: tRNA-splicing ligase RtcB homolog [Megachile rotundata]

Protein Accession	CL21686.Contig3$_H$1
Mean Expression Ratio	1.07
Median Expression Ratio	1.07
Credible Interval	(0.534, 2.15)
Associated Peptides	1
Associated Spectra	1
Coverage	NaN

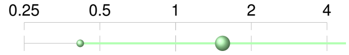

A	B	2.5	50	97.5	Sequence
0	1	0.42	1.5	5.6	GLGHQVATDALVVMEK

5.490 1337 1537 minus strand hemocyanin [Haliotis tuberculata]

Protein Accession	Unigene68371$_H$1
Mean Expression Ratio	0.93
Median Expression Ratio	0.937
Credible Interval	(0.465, 1.86)
Associated Peptides	1
Associated Spectra	2
Coverage	NaN

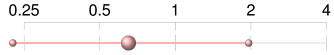

A	B	2.5	50	97.5	Sequence
0	2	0.23	0.65	2	DHSTPFDVFNYK

5.491 93 2606 twitchin [Mytilus galloprovincialis]

Protein Accession	Unigene71883$_{H}1$
Mean Expression Ratio	1.07
Median Expression Ratio	1.07
Credible Interval	(0.548, 2.12)
Associated Peptides	2
Associated Spectra	2
Coverage	NaN

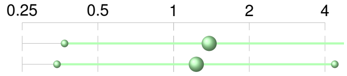

A	B	2.5	50	97.5	Sequence
0	1	0.37	1.4	5	IEINIMNQLHHPK
0	1	0.34	1.2	4.4	LNPDEIVK

5.492 174 1649 minus strand Collagen alpha-1(XII) chain [Crassostrea gigas]; 123 1490 minus strand Collagen alpha-1(XII) chain [Crassostrea gigas]; 135 1610 Collagen alpha-1(XII) chain [Crassostrea gigas]

Protein Accession	CL5340.Contig13$_{H}1$ CL5340.Contig12$_{H}1$ CL5340.Contig11$_{H}1$
Mean Expression Ratio	1.07
Median Expression Ratio	1.07
Credible Interval	(0.55, 2.13)
Associated Peptides	2
Associated Spectra	2
Coverage	NaN

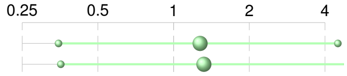

A	B	2.5	50	97.5	Sequence
0	1	0.35	1.3	4.5	ADVVFIVDSSR
0	1	0.36	1.3	4.9	SIQHMQGGLTETYEALR

5.493 " 153 536 GST omega, partial [Reishia clavigera]"

Protein Accession	CL2384.Contig3$_{H}1$
Mean Expression Ratio	1.07
Median Expression Ratio	1.07
Credible Interval	(0.555, 2.1)
Associated Peptides	2
Associated Spectra	3
Coverage	NaN

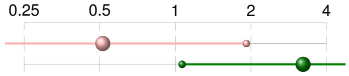

A	B	2.5	50	97.5	Sequence
0	1	0.14	0.51	1.9	EKPEWFLEK
0	2	1.1	3.2	9.4	ALLPSDPYRR

5.494 " 103 981 PREDICTED: ATP synthase, H+ transporting, mitochondrial F1 complex, gamma subunit isoform H (heart)-like [Saccoglossus kowalevskii]"

Protein Accession	CL25149.Contig1$_{H}1$
Mean Expression Ratio	0.941
Median Expression Ratio	0.938
Credible Interval	(0.546, 1.62)
Associated Peptides	8
Associated Spectra	14
Coverage	NaN

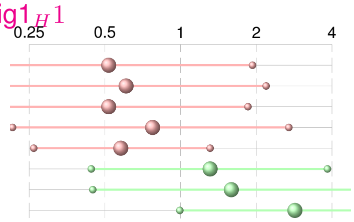

A	B	2.5	50	97.5	Sequence
1	0	0.14	0.52	1.9	LIVLGDK
1	0	0.17	0.61	2.2	NAGEMISK
1	0	0.14	0.52	1.9	AILGEK
1	0	0.21	0.78	2.7	AFFEK
1	4	0.26	0.58	1.3	LTLTFNR
0	2	0.44	1.3	3.8	ELKPARPYGAGAK
0	1	0.45	1.6	5.8	LIVLGDKAR
0	2	0.99	2.9	8.3	ATLKDIR

5.495 4 399 Spectrin beta chain [Crassostrea gigas]

Protein Accession Unigene17190$_{H}1$
Mean Expression Ratio 0.936
Median Expression Ratio 0.938
Credible Interval (0.473, 1.87)
Associated Peptides 1
Associated Spectra 1
Coverage NaN

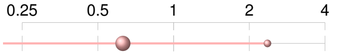

A	B	2.5	50	97.5	Sequence
0	1	0.17	0.63	2.4	DGLAFNAIIHK

5.496 242 1834 Regulator of differentiation 1 [Crassostrea gigas]; 458 2050 minus strand Regulator of differentiation 1 [Crassostrea gigas]; 503 2095 Regulator of differentiation 1 [Crassostrea gigas]; 434 1942 Regulator of differentiation 1 [Crassostrea gigas]

Protein Accession CL11169.Contig7$_{H}1$ CL11169.Contig5$_{H}1$ CL11169.Contig4$_{H}1$ CL11169.Contig1$_{H}1$
Mean Expression Ratio 1.06
Median Expression Ratio 1.07
Credible Interval (0.56, 2.01)
Associated Peptides 3
Associated Spectra 4
Coverage NaN

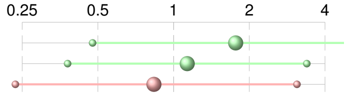

A	B	2.5	50	97.5	Sequence
0	1	0.48	1.8	6.5	SLPHEVAETDIVQLGMPFGK
0	2	0.38	1.1	3.4	MTNVLVLK
0	1	0.24	0.84	3.1	LTNLNVK

5.497 31 375 minus strand 40S ribosomal protein S19 OS=Mya arenaria GN=RPS19 PE=2 SV=1

Protein Accession CL8932.Contig2$_{H}1$
Mean Expression Ratio 1.07
Median Expression Ratio 1.07
Credible Interval (0.535, 2.21)
Associated Peptides 1
Associated Spectra 1
Coverage NaN

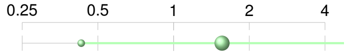

A	B	2.5	50	97.5	Sequence
0	1	0.43	1.6	5.9	GISVKDVNGHEFTK

5.498 " 283 1602 minus strand 4-aminobutyrate aminotransferase, mitochondrial [Crassostrea gigas]"

Protein Accession	CL30906.Contig1$_{H}$1
Mean Expression Ratio	1.07
Median Expression Ratio	1.07
Credible Interval	(0.57, 2.01)
Associated Peptides	3
Associated Spectra	4
Coverage	NaN

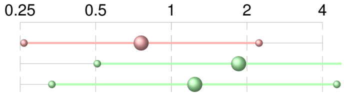

A	B	2.5	50	97.5	Sequence
**1**	**1**	**0.26**	**0.76**	**2.2**	**AMFMTYQR**
**0**	**1**	**0.51**	**1.9**	**6.8**	**TMGALALTHTK**
**0**	**1**	**0.34**	**1.2**	**4.6**	**IIEAFKNPK**

5.499 1 297 beta-thymosin domain repeat protein CSP24KDa_v1 [Hermissenda crassicornis] gi

Protein Accession	Unigene8923$_{H}$1
Mean Expression Ratio	1.07
Median Expression Ratio	1.06
Credible Interval	(0.543, 2.13)
Associated Peptides	1
Associated Spectra	2
Coverage	NaN

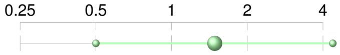

A	B	2.5	50	97.5	Sequence
**0**	**2**	**0.5**	**1.5**	**4.4**	**ELLHHVVPEEK**

5.500 " 1 198 minus strand Chain A, Rep1-Nxsq Fatty Acid Transporter; 1 231 minus strand Chain A, Rep1-Nxsq Fatty Acid Transporter; 1 198 minus strand Chain A, Rep1-Nxsq Fatty Acid Transporter"

Protein Accession	CL14182.Contig4$_{H}$1 CL14182.Contig3$_{H}$1 CL14182.Contig2$_{H}$1
Mean Expression Ratio	0.938
Median Expression Ratio	0.939
Credible Interval	(0.47, 1.85)
Associated Peptides	1
Associated Spectra	2
Coverage	NaN

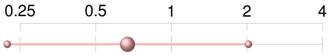

A	B	2.5	50	97.5	Sequence
**1**	**1**	**0.22**	**0.67**	**2**	**GSPDSLIVR**

5.501 68 694 minus strand hypothetical protein BRAFLDRAFT_272416 [Branchiostoma floridae] gi; 68 667 minus strand hypothetical protein BRAFLDRAFT_272416 [Branchiostoma floridae] gi; 68 697 minus strand hypothetical protein BRAFLDRAFT_272416 [Branchiostoma floridae] gi; 68 697 minus strand hypothetical protein BRAFLDRAFT_272416 [Branchiostoma floridae] gi; 68 667 minus strand hypothetical protein BRAFLDRAFT_272416 [Branchiostoma floridae] gi

Protein Accession	CL37759.Contig7$_H$1 CL37759.Contig4$_H$1 CL37759.Contig3$_H$1 CL37759.Contig2$_H$1 CL3775
Mean Expression Ratio	0.942
Median Expression Ratio	0.94
Credible Interval	(0.488, 1.83)
Associated Peptides	2
Associated Spectra	2
Coverage	NaN

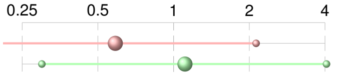

A	B	2.5	50	97.5	Sequence
0	1	0.16	0.59	2.1	LLAIR
0	1	0.3	1.1	4.1	TLEEDEDVLLQLR

5.502 154 621 minus strand Stress response protein nhaX [Crassostrea gigas]

Protein Accession	CL4265.Contig1$_H$1
Mean Expression Ratio	0.941
Median Expression Ratio	0.94
Credible Interval	(0.528, 1.69)
Associated Peptides	4
Associated Spectra	14
Coverage	NaN

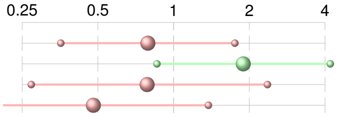

A	B	2.5	50	97.5	Sequence
3	2	0.36	0.79	1.8	SVAAAKPGEGILK
2	3	0.86	1.9	4.2	GLGVMR
0	2	0.27	0.79	2.4	IQHDLEK
0	2	0.16	0.48	1.4	EVLEAMLKDEQK

5.503 6 317 myosin heavy chain cardiac muscle specific isoform 2 [Argopecten irradians]

Protein Accession	Unigene19023$_H$1
Mean Expression Ratio	1.07
Median Expression Ratio	1.06
Credible Interval	(0.701, 1.64)
Associated Peptides	15
Associated Spectra	52
Coverage	NaN

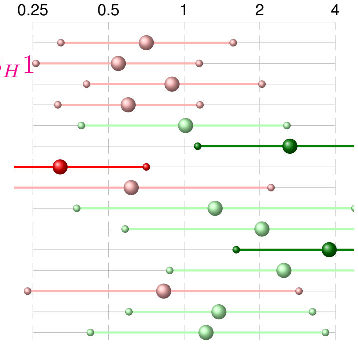

A	B	2.5	50	97.5	Sequence
1	4	0.32	0.71	1.6	LDEAEAQALKGGK
6	0	0.26	0.55	1.1	LADELR
1	4	0.41	0.9	2	LADELRQEQEHSLQVEK
6	2	0.31	0.6	1.2	ALETQLK
1	2	0.39	1	2.6	ELEMELDSEQR
1	3	1.1	2.6	6.1	KALETQLK
2	3	0.14	0.32	0.71	LDEAEAQALK
0	1	0.17	0.62	2.2	ELAFQSDEDR
0	1	0.37	1.3	4.8	ELEMELDSEQRR
0	1	0.58	2	7.3	KALETQLKDMQAR
0	4	1.6	3.8	8.9	KIIQKLEQR
0	2	0.88	2.5	7.2	LADELRQEQEHSLQVEKLR
0	1	0.24	0.83	2.9	VRELEMELDSEQR
0	4	0.6	1.4	3.3	VRELEMELDSEQRR
0	2	0.42	1.2	3.6	LKELAFQSDEDRK

5.504 132 932 pyruvate kinase [Crassostrea gigas]

Protein Accession	CL31920.Contig1$_{H}1$
Mean Expression Ratio	1.06
Median Expression Ratio	1.06
Credible Interval	(0.532, 2.12)
Associated Peptides	1
Associated Spectra	3
Coverage	NaN

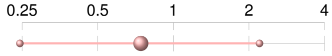

A	B	2.5	50	97.5	Sequence
2	1	0.25	0.74	2.2	ITIDDSYKEK

5.505 " 353 781 alpha-B-crystallin, putative [Ixodes scapularis] gi"

Protein Accession	CL37349.Contig1$_{H}1$
Mean Expression Ratio	0.943
Median Expression Ratio	0.94
Credible Interval	(0.57, 1.55)
Associated Peptides	7
Associated Spectra	53
Coverage	NaN

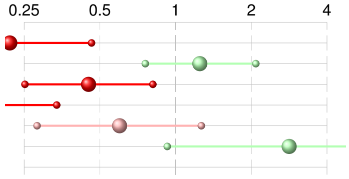

A	B	2.5	50	97.5	Sequence
4	2	0.11	0.22	0.46	LALEAPK
5	10	0.76	1.2	2.1	LQESGTSEVVNNDKEFR
2	9	0.25	0.45	0.81	VAMDVQHFKPEELK
5	3	0.089	0.17	0.34	IIINAK
0	6	0.28	0.6	1.3	TVTSSLSPSGVLTLK
0	2	0.93	2.8	8.2	VAMDVQHFKPEELKITTK
0	5	6.3	14	32	VAMDVQHFKPEELKITTKDNR

5.506 333 770 USP-like protein isoform 2 [Crassostrea ariakensis]

Protein Accession	Unigene7710$_{H}1$
Mean Expression Ratio	1.06
Median Expression Ratio	1.06
Credible Interval	(0.578, 1.97)
Associated Peptides	3
Associated Spectra	11
Coverage	NaN

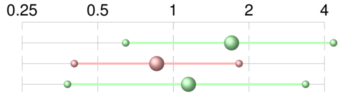

A	B	2.5	50	97.5	Sequence
1	2	0.65	1.7	4.3	KIVIAMDGSK
2	4	0.4	0.86	1.8	MSANTPGEAVIK
0	2	0.38	1.1	3.4	DIGADLIITGTR

5.507 34 480 small subunit ribosomal protein S18e [Clonorchis sinensis]; 431 799 SJCHGC01887 protein [Schistosoma japonicum] gi

Protein Accession	CL10131.Contig2$_{H}1$ CL10131.Contig1$_{H}1$
Mean Expression Ratio	0.942
Median Expression Ratio	0.941
Credible Interval	(0.486, 1.82)
Associated Peptides	1
Associated Spectra	4
Coverage	NaN

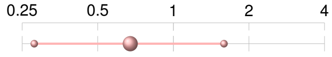

A	B	2.5	50	97.5	Sequence
2	2	0.28	0.67	1.6	LVTVMSNPR

5.508 123 860 PREDICTED: 3-hydroxybutyrate dehydrogenase type 2-like isoform 1 [Sus scrofa] gi; 32 901 3-hydroxybutyrate dehydrogenase type 2 [Rattus norvegicus]

Protein Accession	CL33059.Contig4$_H$1 CL33059.Contig2$_H$1
Mean Expression Ratio	0.942
Median Expression Ratio	0.941
Credible Interval	(0.468, 1.86)
Associated Peptides	1
Associated Spectra	1
Coverage	NaN

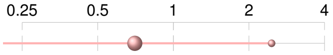

A	B	2.5	50	97.5	Sequence
**1**	**0**	**0.19**	**0.7**	**2.5**	**ILDVLDTEK**

5.509 54 1076 PREDICTED: UPF0553 protein C9orf64-like [Anolis carolinensis]; 71 1093 PREDICTED: UPF0553 protein C9orf64-like [Anolis carolinensis]; 33 1055 PREDICTED: UPF0553 protein C9orf64-like [Anolis carolinensis]; 54 1076 PREDICTED: UPF0553 protein C9orf64-like [Anolis carolinensis]

Protein Accession	CL26820.Contig7$_H$1 CL26820.Contig6$_H$1 CL26820.Contig5$_H$1 CL26820.Contig4$_H$1
Mean Expression Ratio	0.941
Median Expression Ratio	0.941
Credible Interval	(0.472, 1.87)
Associated Peptides	1
Associated Spectra	1
Coverage	NaN

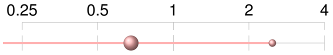

A	B	2.5	50	97.5	Sequence
**0**	**1**	**0.18**	**0.68**	**2.5**	**SKELLDILDK**

5.510 382 972 minus strand Muscle-specific protein 20 [Crassostrea gigas]; 807 1397 minus strand Muscle-specific protein 20 [Crassostrea gigas]

Protein Accession	Unigene366$_H$1 CL6243.Contig1$_H$1
Mean Expression Ratio	0.939
Median Expression Ratio	0.941
Credible Interval	(0.465, 1.85)
Associated Peptides	1
Associated Spectra	1
Coverage	NaN

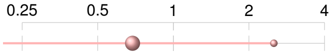

A	B	2.5	50	97.5	Sequence
**1**	**0**	**0.18**	**0.69**	**2.5**	**GFQGPTLGPK**

5.511 248 1018 Aldo-keto reductase family 1 member B10 [Crassostrea gigas]; 44 730 minus strand Aldo-keto reductase family 1 member B10 [Crassostrea gigas]; 194 904 Aldo-keto reductase family 1 member B10 [Crassostrea gigas]; 580 1287 Aldo-keto reductase family 1 member B10 [Crassostrea gigas]; 675 1382 minus strand Aldo-keto reductase family 1 member B10 [Crassostrea gigas]

Protein Accession	Unigene68551 $_{H}$1 Unigene37546 $_{H}$1 CL3558.Contig4 $_{H}$1 CL3558.Contig3 $_{H}$1 CL3558.Contig1
Mean Expression Ratio	1.07
Median Expression Ratio	1.06
Credible Interval	(0.546, 2.14)
Associated Peptides	1
Associated Spectra	1
Coverage	NaN

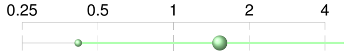

A	B	2.5	50	97.5	Sequence
0	1	0.42	1.5	5.7	DEGYR

5.512 323 1192 cytosolic malate dehydrogenase [Lottia scabra]; 323 1192 cytosolic malate dehydrogenase [Lottia scabra]; 323 1192 cytosolic malate dehydrogenase [Lottia scabra]; 323 1192 cytosolic malate dehydrogenase [Lottia scabra]; 2474 3343 cytosolic malate dehydrogenase [Lottia scabra]; 116 1111 minus strand cytosolic malate dehydrogenase [Lottia scabra]

Protein Accession	CL280.Contig21 $_{H}$1 CL280.Contig20 $_{H}$1 CL280.Contig19 $_{H}$1 CL280.Contig18 $_{H}$1 CL280.Conti
Mean Expression Ratio	0.944
Median Expression Ratio	0.942
Credible Interval	(0.635, 1.41)
Associated Peptides	19
Associated Spectra	99
Coverage	NaN

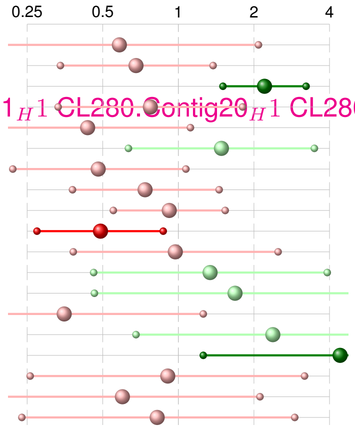

A	B	2.5	50	97.5	Sequence
1	0	0.17	0.58	2.1	GAEVIK
2	5	0.34	0.68	1.4	KDLLAANIK
11	19	1.5	2.2	3.2	KLSSAMSAAK
2	2	0.33	0.77	1.8	LSSAMSAAK
3	0	0.17	0.43	1.1	ELLEER
1	3	0.63	1.5	3.5	QVTIVEGLSISDFAR
3	2	0.22	0.48	1.1	DLLAANIK
2	5	0.38	0.74	1.5	EMAEQILAA
4	11	0.55	0.92	1.5	LGIPNNAVSK
5	6	0.27	0.49	0.87	SQGANLDAVAK
0	3	0.38	0.98	2.5	DCDAAFLVGAMPR
0	2	0.46	1.3	3.9	MDDTAKELLEER
0	1	0.46	1.7	6	NKQVTIVEGLSISDFAR
0	1	0.1	0.35	1.3	VVIVGNPANTNALICAK
0	1	0.68	2.4	8.5	SQGANLDAVAKK
0	1	1.3	4.4	16	GAEVIKMR
0	1	0.26	0.9	3.2	EAVKDDNWINNEFISTVQK
0	1	0.17	0.6	2.1	VTIWGNHSSTQFPDVDHAVAVVNGKPHPVR
0	1	0.24	0.82	2.9	YGIPEGVMYSYPVQIK

5.513 781 1212 minus strand cathepsin L2 cysteine protease [Pinctada fucata]

Protein Accession	CL7881.Contig5$_H1$
Mean Expression Ratio	1.06
Median Expression Ratio	1.06
Credible Interval	(0.55, 2.02)
Associated Peptides	2
Associated Spectra	5
Coverage	NaN

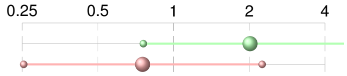

A	B	2.5	50	97.5	Sequence
**2**	**1**	**0.76**	**2**	**5.2**	**GIYNEPACSAK**
**1**	**1**	**0.25**	**0.75**	**2.3**	**NTVGATLSSYK**

5.514 58 1167 minus strand PREDICTED: arylacetamide deacetylase-like 4-like [Gallus gallus]; 76 1239 minus strand PREDICTED: arylacetamide deacetylase-like 4-like [Gallus gallus]

Protein Accession	CL44820.Contig3$_H1$ CL44820.Contig1$_H1$
Mean Expression Ratio	1.06
Median Expression Ratio	1.06
Credible Interval	(0.523, 2.16)
Associated Peptides	1
Associated Spectra	1
Coverage	NaN

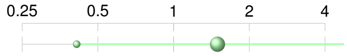

A	B	2.5	50	97.5	Sequence
**0**	**1**	**0.41**	**1.5**	**5.5**	**NSHDVLLPR**

5.515 165 377 Cysteine and glycine-rich protein 3 [Crassostrea gigas]; 135 344 Cysteine and glycine-rich protein 3 [Crassostrea gigas]

Protein Accession	CL38347.Contig2$_H1$ CL38347.Contig1$_H1$
Mean Expression Ratio	0.945
Median Expression Ratio	0.943
Credible Interval	(0.477, 1.86)
Associated Peptides	1
Associated Spectra	2
Coverage	NaN

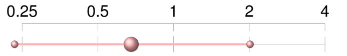

A	B	2.5	50	97.5	Sequence
**1**	**1**	**0.23**	**0.68**	**2**	**VEAGNIPFHK**

5.516 17 1681 minus strand Glucose-6-phosphate isomerase [Crassostrea gigas]

Protein Accession	CL34850.Contig1$_H1$
Mean Expression Ratio	0.945
Median Expression Ratio	0.944
Credible Interval	(0.503, 1.73)
Associated Peptides	4
Associated Spectra	6
Coverage	NaN

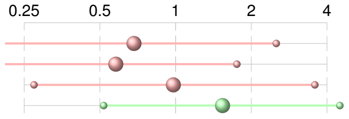

A	B	2.5	50	97.5	Sequence
**1**	**0**	**0.18**	**0.68**	**2.5**	**INFTEDR**
**0**	**2**	**0.2**	**0.58**	**1.8**	**INMAQMFK**
**0**	**1**	**0.27**	**0.98**	**3.6**	**EAAEDELRK**
**0**	**2**	**0.52**	**1.5**	**4.5**	**VHFISNVDGTHLAK**

5.517 " 31 2061 minus strand 1,4-alpha-glucan-branching enzyme [Crassostrea gigas]"

Protein Accession	CL12555.Contig7$_{H}$1
Mean Expression Ratio	1.06
Median Expression Ratio	1.06
Credible Interval	(0.568, 2.02)
Associated Peptides	3
Associated Spectra	4
Coverage	NaN

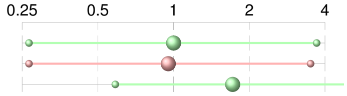

A	B	2.5	50	97.5	Sequence
0	1	0.27	1	3.7	GTHDLWDSR
0	1	0.27	0.95	3.5	LFKLDGYLR
0	2	0.59	1.7	5.1	LDFPR

5.518 52 1599 minus strand ATP synthase beta subunit [Pinctada fucata]

Protein Accession	CL34251.Contig5$_{H}$1
Mean Expression Ratio	1.06
Median Expression Ratio	1.06
Credible Interval	(0.683, 1.64)
Associated Peptides	15
Associated Spectra	41
Coverage	NaN

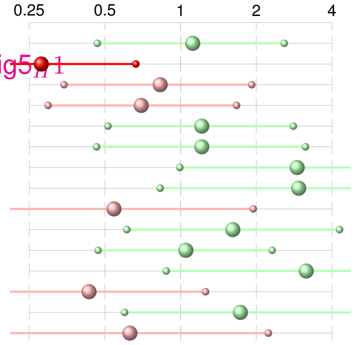

A	B	2.5	50	97.5	Sequence
1	3	0.47	1.1	2.6	EAAKPAAAGNLAVGR
2	2	0.12	0.28	0.67	VISLTDDTSK
1	3	0.34	0.83	1.9	VSLVYGQMNEPPGAR
2	2	0.3	0.7	1.7	IPVGPK
1	3	0.52	1.2	2.8	ILDPNVVGMEHYK
0	3	0.46	1.2	3.1	AHGGYSVFAGVGER
0	2	0.99	2.9	8.5	AKEAAKPAAAGNLAVGR
0	1	0.83	2.9	11	GQACKDTGTSIRIPVGPK
0	1	0.15	0.54	1.9	IGLFGGAGVGK
0	3	0.61	1.6	4.3	TIAMDGTEGLVR
0	5	0.47	1.1	2.3	VVDMLAPYAR
0	1	0.88	3.2	11	DTGTSIRIPVGPK
0	2	0.15	0.43	1.3	ILQDFK
0	2	0.6	1.7	5	TREGNDLYHEMITTK
0	1	0.18	0.63	2.2	IINVIGEPIDER

5.519 76 627 minus strand Heme-binding protein 2 [Crassostrea gigas]; 296 847 minus strand Heme-binding protein 2 [Crassostrea gigas]; 271 822 minus strand Heme-binding protein 2 [Crassostrea gigas]; 367 918 minus strand Heme-binding protein 2 [Crassostrea gigas]; 282 554 minus strand PREDICTED: heme-binding protein 2-like [Strongylocentrotus purpuratus]; 279 878 minus strand Heme-binding protein 2 [Crassostrea gigas]

Protein Accession	CL8470.Contig9$_{H}$1 CL8470.Contig8$_{H}$1 CL8470.Contig6$_{H}$1 CL8470.Contig5$_{H}$1 CL8470.Con
Mean Expression Ratio	0.941
Median Expression Ratio	0.944
Credible Interval	(0.475, 1.83)
Associated Peptides	2
Associated Spectra	2
Coverage	NaN

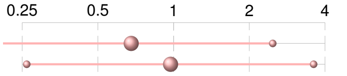

A	B	2.5	50	97.5	Sequence
0	1	0.18	0.68	2.5	GVEFDEAENK
0	1	0.26	0.97	3.6	IAMTVPVLTK

5.520 195 2201 minus strand hypothetical protein DAPPUDRAFT_315656 [Daphnia pulex]

Protein Accession	Unigene38538$_{H}1$
Mean Expression Ratio	0.943
Median Expression Ratio	0.945
Credible Interval	(0.531, 1.66)
Associated Peptides	6
Associated Spectra	11
Coverage	NaN

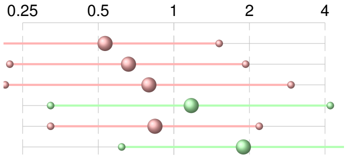

A	B	2.5	50	97.5	Sequence
2	0	0.18	0.53	1.5	GPQFEEIGK
1	1	0.22	0.66	1.9	VQIEAGAGEEAK
1	0	0.21	0.79	2.9	EIFPNER
1	0	0.32	1.2	4.2	NMGAIVR
1	2	0.32	0.84	2.2	GSIVLHDGK
0	2	0.62	1.9	5.7	VALSPAATQALVK

5.521 211 687 Uncharacterized protein CG16817 [Harpegnathos saltator]; 200 676 Uncharacterized protein CG16817 [Harpegnathos saltator]; 294 770 Uncharacterized protein CG16817 [Harpegnathos saltator]; 211 687 Uncharacterized protein CG16817 [Harpegnathos saltator]

Protein Accession	Unigene6191$_{H}1$ Unigene53279$_{H}1$ Unigene41893$_{H}1$ Unigene11183$_{H}1$
Mean Expression Ratio	1.06
Median Expression Ratio	1.06
Credible Interval	(0.538, 2.16)
Associated Peptides	1
Associated Spectra	1
Coverage	NaN

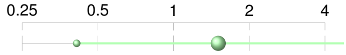

A	B	2.5	50	97.5	Sequence
0	1	0.41	1.5	5.6	KFHFLK

5.522 54 1406 minus strand Protein henna [Crassostrea gigas]; 54 1406 minus strand Protein henna [Crassostrea gigas]

Protein Accession	CL24330.Contig4$_{H}1$ CL24330.Contig1$_{H}1$
Mean Expression Ratio	1.05
Median Expression Ratio	1.06
Credible Interval	(0.552, 1.99)
Associated Peptides	3
Associated Spectra	3
Coverage	NaN

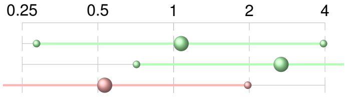

A	B	2.5	50	97.5	Sequence
0	1	0.29	1.1	3.9	TLHLQILSR
0	1	0.71	2.7	9.9	VSAYAATIPRPFSV
0	1	0.15	0.53	2	GDLSLLEDALK

5.523 138 713 Rac [Aplysia californica] gi; 125 691 Rac [Aplysia californica] gi; 149 871 minus strand Rac [Aplysia californica] gi; 101 676 Rac [Aplysia californica] gi

Protein Accession	CL11009.Contig9$_{H}$1 CL11009.Contig8$_{H}$1 CL11009.Contig4$_{H}$1 CL11009.Contig1$_{H}$1
Mean Expression Ratio	0.945
Median Expression Ratio	0.945
Credible Interval	(0.478, 1.87)
Associated Peptides	1
Associated Spectra	2
Coverage	NaN

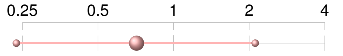

A	B	2.5	50	97.5	Sequence
1	1	0.24	0.71	2.1	NVFDEAIR

5.524 91 2298 hypothetical protein BRAFLDRAFT_267717 [Branchiostoma floridae] gi; 84 2291 hypothetical protein BRAFLDRAFT_267717 [Branchiostoma floridae] gi; 71 2278 hypothetical protein BRAFLDRAFT_267717 [Branchiostoma floridae] gi; 81 2288 hypothetical protein BRAFLDRAFT_267717 [Branchiostoma floridae] gi; 58 2268 hypothetical protein BRAFLDRAFT_267717 [Branchiostoma floridae] gi; 62 2245 hypothetical protein BRAFLDRAFT_267717 [Branchiostoma floridae] gi

Protein Accession	CL26176.Contig5$_{H}$1 CL26176.Contig4$_{H}$1 CL26176.Contig3$_{H}$1 CL26176.Contig2$_{H}$1 CL2617
Mean Expression Ratio	1.06
Median Expression Ratio	1.06
Credible Interval	(0.561, 1.97)
Associated Peptides	3
Associated Spectra	5
Coverage	NaN

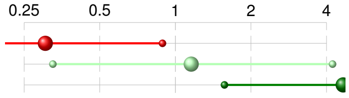

A	B	2.5	50	97.5	Sequence
1	1	0.1	0.3	0.89	VGAVEEASFLK
0	1	0.33	1.2	4.2	FANEVAGVEDLGTTGR
0	2	1.6	4.7	14	YTDIAYTGKR

5.525 689 1984 hypothetical protein BRAFLDRAFT_78257 [Branchiostoma floridae] gi

Protein Accession	Unigene34076$_{H}$1
Mean Expression Ratio	1.06
Median Expression Ratio	1.06
Credible Interval	(0.607, 1.87)
Associated Peptides	6
Associated Spectra	12
Coverage	NaN

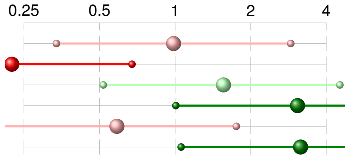

A	B	2.5	50	97.5	Sequence
1	1	0.34	0.98	2.9	VQQKPEPSPAEGEVLIR
1	1	0.075	0.22	0.67	ILLDPSK
1	1	0.52	1.6	4.5	AWAELVAV
0	2	1	3.1	9.2	ILLDPSKAPTPK
0	2	0.2	0.59	1.8	SFFSFAK
0	2	1.1	3.2	9.6	SLVLTGFGSMK

5.526 11 187 PREDICTED: 40S ribosomal protein S28-like [Strongylocentrotus purpuratus]

Protein Accession	Unigene696$_{H1}$
Mean Expression Ratio	0.942
Median Expression Ratio	0.945
Credible Interval	(0.472, 1.86)
Associated Peptides	1
Associated Spectra	4
Coverage	NaN

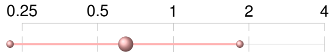

A	B	2.5	50	97.5	Sequence
1	3	0.22	0.65	1.8	VEFLDDSNR

5.527 14 1642 minus strand chaperonin containing tcp1 [Haliotis discus discus]; 14 1462 minus strand chaperonin containing tcp1 [Haliotis discus discus]; 14 1582 minus strand chaperonin containing tcp1 [Haliotis discus discus]

Protein Accession	CL16298.Contig4$_{H1}$ CL16298.Contig15$_{H1}$ CL16298.Contig14$_{H1}$
Mean Expression Ratio	1.06
Median Expression Ratio	1.06
Credible Interval	(0.556, 2.07)
Associated Peptides	3
Associated Spectra	3
Coverage	NaN

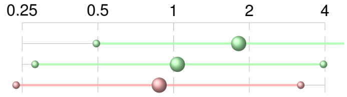

A	B	2.5	50	97.5	Sequence
0	1	0.49	1.8	6.5	FAESLEVIPR
0	1	0.28	1	3.9	MVINHLEK
0	1	0.24	0.88	3.2	ACVSILPAK

5.528 " 1 1815 Glycogen phosphorylase, muscle form [Crassostrea gigas]"

Protein Accession	CL20630.Contig5$_{H1}$
Mean Expression Ratio	1.06
Median Expression Ratio	1.06
Credible Interval	(0.55, 2.04)
Associated Peptides	2
Associated Spectra	4
Coverage	NaN

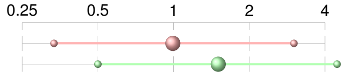

A	B	2.5	50	97.5	Sequence
0	2	0.33	0.99	3	IEELEEIEEDAGL
0	2	0.5	1.5	4.5	FGNPWEK

5.529 43 1623 minus strand MGC84105 protein precursor [Xenopus laevis] gi; 43 1506 minus strand MGC84105 protein precursor [Xenopus laevis] gi

Protein Accession	CL16647.Contig5$_{H1}$ CL16647.Contig4$_{H1}$
Mean Expression Ratio	0.946
Median Expression Ratio	0.946
Credible Interval	(0.504, 1.79)
Associated Peptides	3
Associated Spectra	4
Coverage	NaN

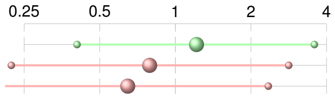

A	B	2.5	50	97.5	Sequence
1	1	0.41	1.2	3.6	LIDAADSAR
0	1	0.22	0.79	2.8	QLPELIAVRDEMQK
0	1	0.18	0.65	2.4	LSELEK

5.530 244 5451 alpha macroglobulin [Chlamys farreri]

Protein Accession	CL20671.Contig5$_{H}$1
Mean Expression Ratio	0.946
Median Expression Ratio	0.946
Credible Interval	(0.477, 1.89)
Associated Peptides	1
Associated Spectra	1
Coverage	NaN

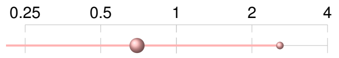

A	B	2.5	50	97.5	Sequence
**0**	**1**	**0.19**	**0.7**	**2.6**	**HLALDVEQLNR**

5.531 111 530 minus strand Nucleoside diphosphate kinase B OS=Homo sapiens GN=NME2 PE=1 SV=1

Protein Accession	CL22280.Contig3$_{H}$1
Mean Expression Ratio	1.06
Median Expression Ratio	1.06
Credible Interval	(0.575, 1.97)
Associated Peptides	3
Associated Spectra	6
Coverage	NaN

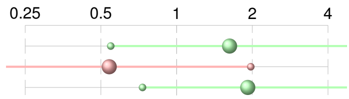

A	B	2.5	50	97.5	Sequence
**0**	**2**	**0.55**	**1.6**	**4.9**	**GDFCIEMGR**
**0**	**1**	**0.14**	**0.54**	**2**	**NICHGSDEVK**
**0**	**3**	**0.73**	**1.9**	**5**	**VMLGETNPLNSKPGTIR**

5.532 " 75 803 hypothetical protein DAPPUDRAFT_231350 [Daphnia pulex]; 82 612 ATP synthase B chain, putative [Ixodes scapularis] gi"

Protein Accession	CL10537.Contig4$_{H}$1 CL10537.Contig13$_{H}$1
Mean Expression Ratio	1.06
Median Expression Ratio	1.06
Credible Interval	(0.536, 2.07)
Associated Peptides	1
Associated Spectra	3
Coverage	NaN

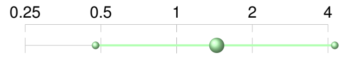

A	B	2.5	50	97.5	Sequence
**1**	**2**	**0.48**	**1.4**	**4.3**	**SITPQQEAESIK**

5.533 48 464 minus strand Acyl-coenzyme A thioesterase 13 [Crassostrea gigas]

Protein Accession	CL38770.Contig1$_{H}$1
Mean Expression Ratio	1.05
Median Expression Ratio	1.06
Credible Interval	(0.521, 2.09)
Associated Peptides	1
Associated Spectra	1
Coverage	NaN

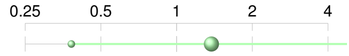

A	B	2.5	50	97.5	Sequence
**1**	**0**	**0.38**	**1.4**	**5**	**TALPGETVVAEAK**

5.534 5 598 hypothetical protein DAPPUDRAFT_344656 [Daphnia pulex]

Protein Accession	Unigene24559$_H1$
Mean Expression Ratio	0.944
Median Expression Ratio	0.947
Credible Interval	(0.465, 1.91)
Associated Peptides	1
Associated Spectra	1
Coverage	NaN

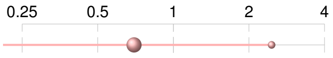

A	B	2.5	50	97.5	Sequence
**0**	**1**	**0.19**	**0.7**	**2.5**	**WHLAEFVR**

5.535 62 394 minus strand PREDICTED: NADH dehydrogenase [ubiquinone] 1 alpha subcomplex subunit 5 [Saimiri boliviensis boliviensis]; 62 394 minus strand PREDICTED: NADH dehydrogenase [ubiquinone] 1 alpha subcomplex subunit 5 [Saimiri boliviensis boliviensis]

Protein Accession	CL6313.Contig4$_H1$ CL6313.Contig3$_H1$
Mean Expression Ratio	1.06
Median Expression Ratio	1.06
Credible Interval	(0.531, 2.11)
Associated Peptides	1
Associated Spectra	1
Coverage	NaN

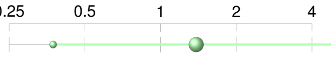

A	B	2.5	50	97.5	Sequence
**0**	**1**	**0.37**	**1.4**	**5.1**	**LAIVNSEPDPGRLEMK**

5.536 3 1418 Protein hu-li tai shao [Crassostrea gigas]; 3 1523 minus strand Protein hu-li tai shao [Crassostrea gigas]; 3 1562 minus strand Protein hu-li tai shao [Crassostrea gigas]

Protein Accession	CL3889.Contig5$_H1$ CL3889.Contig13$_H1$ CL3889.Contig11$_H1$
Mean Expression Ratio	0.943
Median Expression Ratio	0.947
Credible Interval	(0.496, 1.8)
Associated Peptides	2
Associated Spectra	5
Coverage	NaN

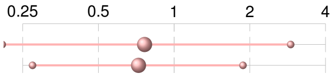

A	B	2.5	50	97.5	Sequence
**0**	**1**	**0.21**	**0.76**	**2.9**	**ILEGLSWEEAAR**
**2**	**2**	**0.27**	**0.72**	**1.9**	**VMLLR**

5.537 3 434 minus strand myosin essential light chain [Haliotis discus discus]

Protein Accession	CL17177.Contig8$_{H}$1
Mean Expression Ratio	1.06
Median Expression Ratio	1.05
Credible Interval	(0.619, 1.78)
Associated Peptides	7
Associated Spectra	24
Coverage	NaN

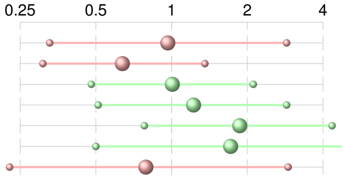

A	B	2.5	50	97.5	Sequence
1	1	0.33	0.97	2.9	EGQGFMSAAELR
3	3	0.31	0.64	1.4	LTDQQVTDVIK
3	3	0.48	1	2.1	FTDTREDLEGNVK
2	2	0.51	1.2	2.9	YADFIEK
1	3	0.78	1.9	4.3	HVLTGLGEK
0	1	0.5	1.7	6.1	LSDREGQGFMSAAELR
0	1	0.23	0.79	2.9	DGDVDAFK

5.538 95 1870 PREDICTED: acetylcholinesterase-like [Equus caballus]; 99 1874 PREDICTED: acetylcholinesterase-like [Equus caballus]

Protein Accession	CL10104.Contig9$_{H}$1 CL10104.Contig10$_{H}$1
Mean Expression Ratio	1.05
Median Expression Ratio	1.05
Credible Interval	(0.535, 2.07)
Associated Peptides	2
Associated Spectra	2
Coverage	NaN

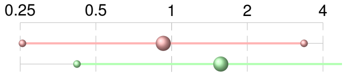

A	B	2.5	50	97.5	Sequence
1	0	0.26	0.93	3.4	SFGNEPTEIK
0	1	0.42	1.6	5.6	VDGDFLPDDPAR

5.539 16 939 hypothetical protein BRAFLDRAFT_87838 [Branchiostoma floridae] gi

Protein Accession	CL38761.Contig1$_{H}$1
Mean Expression Ratio	0.946
Median Expression Ratio	0.949
Credible Interval	(0.475, 1.83)
Associated Peptides	1
Associated Spectra	4
Coverage	NaN

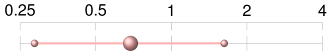

A	B	2.5	50	97.5	Sequence
4	0	0.29	0.69	1.6	ALLLR

5.540 316 1368 minus strand hypothetical protein CGI_10006559 [Crassostrea gigas]

Protein Accession	Unigene26912$_{H}$1
Mean Expression Ratio	0.949
Median Expression Ratio	0.949
Credible Interval	(0.467, 1.91)
Associated Peptides	1
Associated Spectra	1
Coverage	NaN

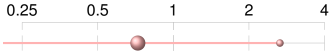

A	B	2.5	50	97.5	Sequence
0	1	0.2	0.72	2.7	TLDDLVR

5.541 113 445 ribosomal protein rps19 [Lineus viridis]

Protein Accession	CL8932.Contig6$_{H}$1
Mean Expression Ratio	1.05
Median Expression Ratio	1.05
Credible Interval	(0.562, 1.95)
Associated Peptides	3
Associated Spectra	6
Coverage	NaN

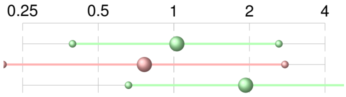

A	B	2.5	50	97.5	Sequence
1	2	0.4	1	2.6	QPEWADIVK
1	0	0.21	0.76	2.8	LKQPEWADIVK
0	2	0.66	1.9	5.7	APAGVGAFTK

5.542 17 691 minus strand Cleavage and polyadenylation specificity factor subunit 5 [Crassostrea gigas]

Protein Accession	CL17041.Contig3$_{H}$1
Mean Expression Ratio	1.05
Median Expression Ratio	1.05
Credible Interval	(0.524, 2.1)
Associated Peptides	1
Associated Spectra	1
Coverage	NaN

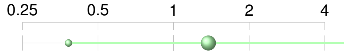

A	B	2.5	50	97.5	Sequence
0	1	0.38	1.4	5.1	ALFAVPR

5.543 90 677 minus strand PREDICTED: 15-hydroxyprostaglandin dehydrogenase [NAD+] [Gallus gallus]; 90 683 minus strand PREDICTED: 15-hydroxyprostaglandin dehydrogenase [NAD+] [Gallus gallus]; 69 776 PREDICTED: 15-hydroxyprostaglandin dehydrogenase [NAD+] [Gallus gallus]; 69 779 PREDICTED: 15-hydroxyprostaglandin dehydrogenase [NAD+] [Gallus gallus]

Protein Accession	Unigene58786$_{H}$1 Unigene57240$_{H}$1 Unigene48682$_{H}$1 CL44148.Contig4$_{H}$1
Mean Expression Ratio	1.05
Median Expression Ratio	1.05
Credible Interval	(0.536, 2.02)
Associated Peptides	2
Associated Spectra	3
Coverage	NaN

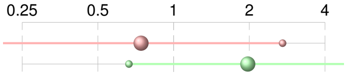

A	B	2.5	50	97.5	Sequence
0	1	0.2	0.74	2.7	AETFSETDMEK
0	2	0.67	2	5.8	AITEAFLAK

5.544 2 487 conserved hypothetical protein [Ixodes scapularis] gi

Protein Accession	CL12002.Contig4$_H$1
Mean Expression Ratio	1.06
Median Expression Ratio	1.05
Credible Interval	(0.536, 2.12)
Associated Peptides	1
Associated Spectra	1
Coverage	NaN

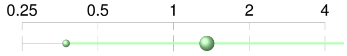

A	B	2.5	50	97.5	Sequence
0	1	0.37	1.4	5	DNIAPETLVTIK

5.545 552 1886 minus strand intermediate filament protein [Biomphalaria glabrata]

Protein Accession	Unigene26795$_H$1
Mean Expression Ratio	0.95
Median Expression Ratio	0.95
Credible Interval	(0.607, 1.47)
Associated Peptides	14
Associated Spectra	49
Coverage	NaN

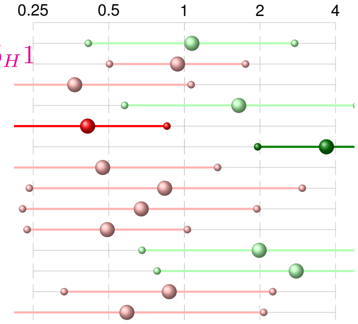

A	B	2.5	50	97.5	Sequence
2	1	0.42	1.1	2.8	KLLEGEENR
3	6	0.5	0.94	1.8	KVITEFSTQR
1	1	0.12	0.37	1.1	LLEGEENR
1	1	0.58	1.6	4.9	SATGISEFR
3	3	0.2	0.41	0.86	WLEAQNK
3	6	2	3.7	6.9	YAAQMDQLR
0	2	0.17	0.47	1.4	ADLDAETAAHIENEVLAQTK
0	1	0.24	0.84	3	CGSLEDELAK
0	2	0.23	0.67	2	DMYEGELAQAR
0	6	0.24	0.49	1	KLEAELEALR
0	2	0.68	2	6	KQEDWKPIRDMYEGELAQAR
0	1	0.78	2.8	9.6	MSLGFNPGVYSK
0	3	0.33	0.87	2.3	KQEDWKPIR
0	1	0.17	0.59	2.1	SLELEIACYR

5.546 87 1454 hypothetical protein BRAFLDRAFT_287788 [Branchiostoma floridae] gi; 117 1469 hypothetical protein BRAFLDRAFT_287788 [Branchiostoma floridae] gi; 117 1469 hypothetical protein BRAFLDRAFT_287788 [Branchiostoma floridae] gi

Protein Accession	Unigene48935$_H$1 Unigene21344$_H$1 CL945.Contig6$_H$1
Mean Expression Ratio	1.05
Median Expression Ratio	1.05
Credible Interval	(0.64, 1.74)
Associated Peptides	9
Associated Spectra	29
Coverage	NaN

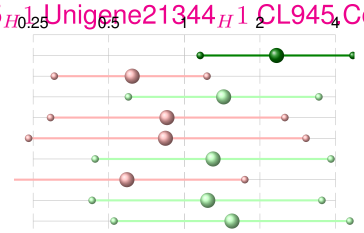

A	B	2.5	50	97.5	Sequence
4	3	1.2	2.3	4.7	ISSLDNGLR
3	4	0.3	0.62	1.2	RIPLPELEAR
2	2	0.6	1.4	3.4	TILGPTQNIR
1	1	0.29	0.85	2.5	FTGSEIR
1	0	0.24	0.84	3.1	IDAVNAK
0	2	0.44	1.3	3.8	AATLEQTILNVPETR
0	2	0.2	0.59	1.7	MVLAAAGGVNHGELVDLAK
0	2	0.43	1.2	3.5	SAMYWLR
0	2	0.53	1.6	4.6	EQTVYYAK

5.547 142 726 Charged multivesicular body protein 4b [Crassostrea gigas]

Protein Accession	CL35875.Contig1$_H$1
Mean Expression Ratio	1.05
Median Expression Ratio	1.05
Credible Interval	(0.529, 2.11)
Associated Peptides	1
Associated Spectra	1
Coverage	NaN

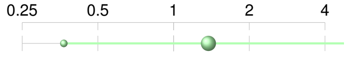

A	B	2.5	50	97.5	Sequence
**0**	**1**	**0.37**	**1.4**	**5**	**KIEHELATAK**

5.548 10 366 minus strand hypothetical protein TRIADDRAFT_25239 [Trichoplax adhaerens] gi

Protein Accession	CL32844.Contig2$_H$1
Mean Expression Ratio	0.952
Median Expression Ratio	0.95
Credible Interval	(0.471, 1.91)
Associated Peptides	1
Associated Spectra	1
Coverage	NaN

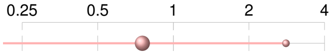

A	B	2.5	50	97.5	Sequence
**1**	**0**	**0.2**	**0.75**	**2.8**	**TAVEDVDLK**

5.549 416 1717 minus strand T-complex protein 1 subunit delta OS=Aedes triseriatus PE=2 SV=1

Protein Accession	CL8566.Contig1$_H$1
Mean Expression Ratio	1.06
Median Expression Ratio	1.05
Credible Interval	(0.557, 2.02)
Associated Peptides	2
Associated Spectra	6
Coverage	NaN

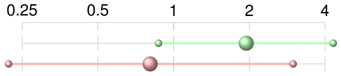

A	B	2.5	50	97.5	Sequence
**2**	**3**	**0.87**	**1.9**	**4.3**	**HAAGELTAGINVR**
**1**	**0**	**0.22**	**0.81**	**3**	**LVLEEADR**

5.550 158 1318 hypothetical protein BRAFLDRAFT_98992 [Branchiostoma floridae] gi

Protein Accession	CL14371.Contig5$_H$1
Mean Expression Ratio	1.05
Median Expression Ratio	1.05
Credible Interval	(0.536, 2.03)
Associated Peptides	2
Associated Spectra	2
Coverage	NaN

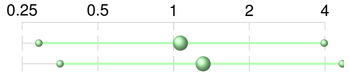

A	B	2.5	50	97.5	Sequence
**0**	**1**	**0.29**	**1.1**	**4**	**AFTGFIVDGDSK**
**0**	**1**	**0.35**	**1.3**	**4.7**	**LAVMR**

5.551 198 2015 minus strand Xaa-Pro aminopeptidase 1 [Crassostrea gigas]

Protein Accession	Unigene11641$_H1$
Mean Expression Ratio	1.05
Median Expression Ratio	1.05
Credible Interval	(0.515, 2.1)
Associated Peptides	1
Associated Spectra	1
Coverage	NaN

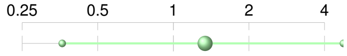

A	B	2.5	50	97.5	Sequence
0	1	0.36	1.3	4.8	APLWEVGLEYR

5.552 185 481 minus strand Zgc:56530 [Danio rerio]

Protein Accession	CL27088.Contig2$_H1$
Mean Expression Ratio	0.953
Median Expression Ratio	0.952
Credible Interval	(0.515, 1.78)
Associated Peptides	4
Associated Spectra	5
Coverage	NaN

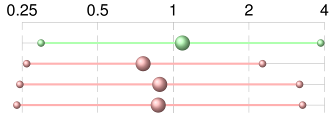

A	B	2.5	50	97.5	Sequence
0	1	0.3	1.1	3.9	TQVVAGTNYFIK
0	2	0.26	0.76	2.3	TFTDFK
0	1	0.25	0.88	3.2	TADDELEYF
0	1	0.24	0.87	3.3	ALSYK

5.553 " 1362 1544 minus strand Phosphoenolpyruvate carboxykinase, cytosolic [GTP] [Crassostrea gigas]"

Protein Accession	CL1008.Contig7$_H1$
Mean Expression Ratio	0.951
Median Expression Ratio	0.952
Credible Interval	(0.47, 1.9)
Associated Peptides	1
Associated Spectra	1
Coverage	NaN

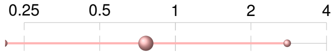

A	B	2.5	50	97.5	Sequence
0	1	0.21	0.76	2.8	KVQHYVEEYAR

5.554 119 481 Glutamate receptor U1 [Crassostrea gigas]

Protein Accession	CL570.Contig5$_H1$
Mean Expression Ratio	1.05
Median Expression Ratio	1.05
Credible Interval	(0.565, 1.95)
Associated Peptides	3
Associated Spectra	7
Coverage	NaN

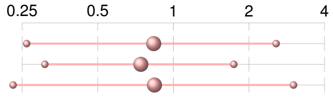

A	B	2.5	50	97.5	Sequence
1	1	0.26	0.84	2.6	GTANIGAGALTVTSER
0	4	0.31	0.74	1.7	RTPDGWSGIIGDVVK
0	1	0.23	0.84	3	TPDGWSGIIGDVVK

5.555 219 1559 Programmed cell death protein 4 [Crassostrea gigas]

Protein Accession	CL35470.Contig4$_H1$
Mean Expression Ratio	1.05
Median Expression Ratio	1.05
Credible Interval	(0.543, 2.01)
Associated Peptides	2
Associated Spectra	3
Coverage	NaN

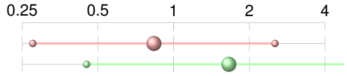

A	B	2.5	50	97.5	Sequence
0	2	0.28	0.84	2.5	MSDLVIDAPDATSVVGK
0	1	0.45	1.7	5.9	GRKRFVSEGDGGAVKQ

5.556 " 144 1145 PREDICTED: RNA-binding protein squid-like isoform 1 [Megachile rotundata]; 90 1085 squid, variant A [Blattella germanica]; 144 1145 PREDICTED: RNA-binding protein squid-like isoform 1 [Megachile rotundata]"

Protein Accession	CL14696.Contig7$_H1$ CL14696.Contig5$_H1$ CL14696.Contig4$_H1$
Mean Expression Ratio	0.949
Median Expression Ratio	0.953
Credible Interval	(0.511, 1.75)
Associated Peptides	3
Associated Spectra	7
Coverage	NaN

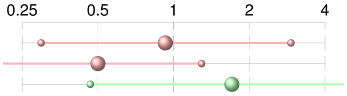

A	B	2.5	50	97.5	Sequence
1	1	0.3	0.93	2.9	IMEVTTHK
2	2	0.19	0.5	1.3	LGGQEVDVK
0	1	0.47	1.7	6.2	KIMEVTTHK

5.557 274 1242 minus strand cathepsin D [Pinctada maxima]; 290 1387 cathepsin D [Pinctada maxima]; 290 1402 minus strand cathepsin D [Pinctada maxima]; 290 1480 minus strand cathepsin D [Pinctada maxima]

Protein Accession	CL1722.Contig9$_H1$ CL1722.Contig2$_H1$ CL1722.Contig1$_H1$ CL1722.Contig10$_H1$
Mean Expression Ratio	0.951
Median Expression Ratio	0.953
Credible Interval	(0.485, 1.82)
Associated Peptides	2
Associated Spectra	5
Coverage	NaN

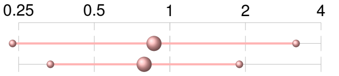

A	B	2.5	50	97.5	Sequence
0	1	0.24	0.86	3.2	VVFDTGSSNLWVPSKK
0	4	0.33	0.79	1.9	EGYWQIK

5.558 511 1062 unknown [Dendroctonus ponderosae]; 511 1062 unknown [Dendroctonus ponderosae]

Protein Accession	CL18443.Contig3$_{H}1$ CL18443.Contig1$_{H}1$
Mean Expression Ratio	1.05
Median Expression Ratio	1.05
Credible Interval	(0.528, 2.12)
Associated Peptides	1
Associated Spectra	1
Coverage	NaN

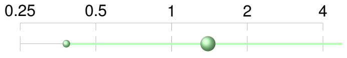

A	B	2.5	50	97.5	Sequence
**0**	**1**	**0.38**	**1.4**	**5.1**	**FKDEIEMHLK**

5.559 80 589 minus strand PREDICTED: adenine phosphoribosyltransferase-like [Strongylocentrotus purpuratus]; 328 468 minus strand PREDICTED: adenine phosphoribosyltransferase [Apis mellifera]

Protein Accession	CL8682.Contig6$_{H}1$ CL8682.Contig4$_{H}1$
Mean Expression Ratio	0.954
Median Expression Ratio	0.953
Credible Interval	(0.486, 1.87)
Associated Peptides	1
Associated Spectra	3
Coverage	NaN

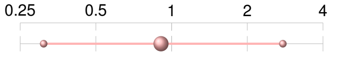

A	B	2.5	50	97.5	Sequence
**1**	**2**	**0.31**	**0.91**	**2.8**	**AAVELVEK**

5.560 266 934 minus strand Kyphoscoliosis peptidase [Crassostrea gigas]

Protein Accession	CL7490.Contig16$_{H}1$
Mean Expression Ratio	1.05
Median Expression Ratio	1.05
Credible Interval	(0.54, 2.04)
Associated Peptides	2
Associated Spectra	2
Coverage	NaN

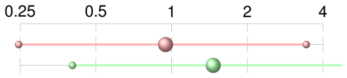

A	B	2.5	50	97.5	Sequence
**0**	**1**	**0.25**	**0.95**	**3.4**	**DLDNVHFK**
**0**	**1**	**0.4**	**1.5**	**5.4**	**SRPELPHGYLGAQSK**

5.561 26 619 G-type lysozyme [Physella acuta]

Protein Accession	CL43984.Contig1$_{H}1$
Mean Expression Ratio	1.05
Median Expression Ratio	1.05
Credible Interval	(0.524, 2.11)
Associated Peptides	1
Associated Spectra	1
Coverage	NaN

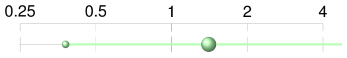

A	B	2.5	50	97.5	Sequence
**0**	**1**	**0.38**	**1.4**	**5.2**	**GHFAGWPIER**

5.562 132 590 minus strand Non-neuronal cytoplasmic intermediate filament protein OS=Helix aspersa PE=3 SV=2

Protein Accession	Unigene7770$_{H}1$
Mean Expression Ratio	1.05
Median Expression Ratio	1.05
Credible Interval	(0.535, 2.03)
Associated Peptides	2
Associated Spectra	3
Coverage	NaN

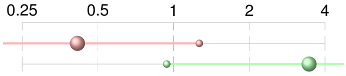

A	B	2.5	50	97.5	Sequence
1	1	0.14	0.41	1.3	TMAEVPLGEK
0	1	0.94	3.5	13	VTKEVSGK

5.563 635 1294 minus strand Kyphoscoliosis peptidase [Crassostrea gigas]; 643 1302 minus strand Kyphoscoliosis peptidase [Crassostrea gigas]; 635 1294 minus strand Kyphoscoliosis peptidase [Crassostrea gigas]; 634 1293 minus strand Kyphoscoliosis peptidase [Crassostrea gigas]; 634 1293 minus strand Kyphoscoliosis peptidase [Crassostrea gigas]; 634 1293 minus strand Kyphoscoliosis peptidase [Crassostrea gigas]; 639 1298 minus strand Kyphoscoliosis peptidase [Crassostrea gigas]

Protein Accession	CL7772.Contig20$_{H}1$ CL7772.Contig1$_{H}1$ CL7772.Contig19$_{H}1$ CL7772.Contig16$_{H}1$ CL7772.C
Mean Expression Ratio	1.05
Median Expression Ratio	1.05
Credible Interval	(0.563, 1.95)
Associated Peptides	3
Associated Spectra	6
Coverage	NaN

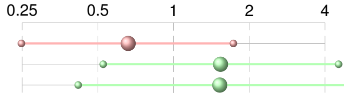

A	B	2.5	50	97.5	Sequence
2	1	0.25	0.66	1.7	LSNVTFK
0	2	0.52	1.5	4.5	GAGEVWEGTAALDK
0	1	0.42	1.5	5.5	GAGEVWEGTAALDKHR

5.564 55 1569 minus strand Putative aminopeptidase W07G4.4 [Crassostrea gigas]; 228 1742 Putative aminopeptidase W07G4.4 [Crassostrea gigas]

Protein Accession	CL1078.Contig5$_{H}1$ CL1078.Contig1$_{H}1$
Mean Expression Ratio	1.05
Median Expression Ratio	1.05
Credible Interval	(0.565, 1.96)
Associated Peptides	4
Associated Spectra	4
Coverage	NaN

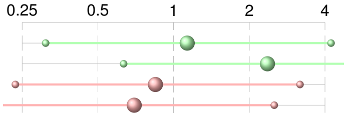

A	B	2.5	50	97.5	Sequence
0	1	0.31	1.1	4.2	AVGGLSFVR
0	1	0.63	2.4	8.7	LIFSPTGPLDRDQDDVRR
0	1	0.23	0.85	3.2	WSETDKGFEK
0	1	0.19	0.7	2.5	AVGEQYSIIMDNGPAR

5.565 1009 2784 minus strand Collagen alpha-3(VI) chain [Crassostrea gigas]

Protein Accession	Unigene46961$_{H}1$
Mean Expression Ratio	0.954
Median Expression Ratio	0.954
Credible Interval	(0.476, 1.92)
Associated Peptides	1
Associated Spectra	1
Coverage	NaN

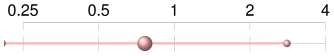

A	B	2.5	50	97.5	Sequence
0	1	0.21	0.76	2.8	ELEGIASPPAR

5.566 3 4517 minus strand Clathrin heavy chain 1 [Crassostrea gigas]

Protein Accession	CL16446.Contig1$_{H}1$
Mean Expression Ratio	0.953
Median Expression Ratio	0.954
Credible Interval	(0.566, 1.59)
Associated Peptides	10
Associated Spectra	12
Coverage	NaN

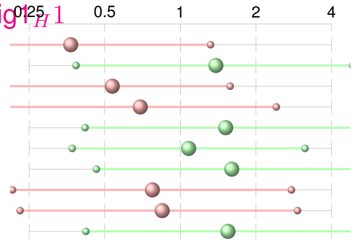

A	B	2.5	50	97.5	Sequence
1	0	0.099	0.37	1.3	KFEVNTSAIK
1	0	0.38	1.4	4.8	VHEGVTEPATHNALAK
1	1	0.18	0.54	1.6	EAIDSYIK
0	1	0.19	0.69	2.4	IYIDSNNNPER
0	1	0.42	1.5	5.2	TNQVPLVKPYLR
0	2	0.37	1.1	3.1	AVNFFIK
0	1	0.46	1.6	5.9	AQLNQGMVK
0	1	0.21	0.77	2.8	IAAYLYK
0	1	0.23	0.85	2.9	ELVEEVEK
0	1	0.42	1.5	5.6	LHVIEVGSPPQGNQPFAK

5.567 " 106 2724 PREDICTED: 2-oxoglutarate dehydrogenase, mitochondrial isoform 1 [Sarcophilus harrisii]; 492 3473 PREDICTED: 2-oxoglutarate dehydrogenase, mitochondrial isoform 1 [Sarcophilus harrisii]; 492 3458 PREDICTED: 2-oxoglutarate dehydrogenase, mitochondrial isoform 1 [Sarcophilus harrisii]"

Protein Accession	CL2200.Contig3$_{H}1$ CL2200.Contig1$_{H}1$ CL2200.Contig16$_{H}1$
Mean Expression Ratio	1.05
Median Expression Ratio	1.05
Credible Interval	(0.521, 2.1)
Associated Peptides	1
Associated Spectra	1
Coverage	NaN

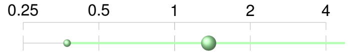

A	B	2.5	50	97.5	Sequence
0	1	0.37	1.4	5	LAVVANPSHLEAVDPVVQGK

5.568 142 261 Core histone macro-H2A.2 OS=Mus musculus GN=H2afy2 PE=1 SV=3

Protein Accession	Unigene14198$_H$1
Mean Expression Ratio	1.05
Median Expression Ratio	1.05
Credible Interval	(0.526, 2.08)
Associated Peptides	1
Associated Spectra	1
Coverage	NaN

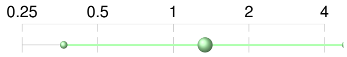

A	B	2.5	50	97.5	Sequence
**0**	**1**	**0.37**	**1.3**	**4.9**	**AGVLFPVAR**

5.569 1327 1488 minus strand Ubiquinol-cytochrome C reductase complex [Xenopus laevis] gi

Protein Accession	CL7847.Contig1$_H$1
Mean Expression Ratio	1.05
Median Expression Ratio	1.05
Credible Interval	(0.523, 2.11)
Associated Peptides	1
Associated Spectra	1
Coverage	NaN

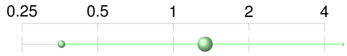

A	B	2.5	50	97.5	Sequence
**0**	**1**	**0.36**	**1.3**	**4.9**	**LVDSITSSDINNIAK**

5.570 29 610 minus strand 40S ribosomal protein S7 [Crassostrea gigas]

Protein Accession	CL8074.Contig11$_H$1
Mean Expression Ratio	1.05
Median Expression Ratio	1.05
Credible Interval	(0.525, 2.12)
Associated Peptides	1
Associated Spectra	1
Coverage	NaN

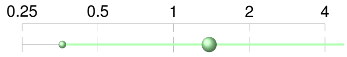

A	B	2.5	50	97.5	Sequence
**0**	**1**	**0.36**	**1.4**	**5.1**	**TSLHELYITGAK**

5.571 73 837 minus strand Electron transfer flavoprotein subunit beta [Crassostrea gigas]; 73 837 minus strand Electron transfer flavoprotein subunit beta [Crassostrea gigas]

Protein Accession	CL33148.Contig2$_H$1 CL33148.Contig1$_H$1
Mean Expression Ratio	0.956
Median Expression Ratio	0.955
Credible Interval	(0.529, 1.75)
Associated Peptides	5
Associated Spectra	8
Coverage	NaN

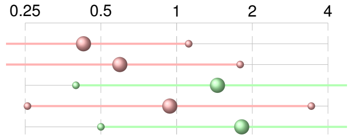

A	B	2.5	50	97.5	Sequence
**2**	**1**	**0.16**	**0.43**	**1.1**	**AIHVDVDSK**
**1**	**1**	**0.2**	**0.6**	**1.8**	**EIDGGLENVLVK**
**0**	**1**	**0.4**	**1.5**	**5.3**	**HSMNPFDEIAVEEAVR**
**0**	**1**	**0.25**	**0.94**	**3.4**	**VLSVEEPPVR**
**0**	**1**	**0.5**	**1.8**	**6.5**	**YATLPNIMK**

5.572 201 4058 PREDICTED: xanthine dehydrogenase/oxidase-like [Amphimedon queenslandica]

Protein Accession	CL17390.Contig11$_H$1
Mean Expression Ratio	0.956
Median Expression Ratio	0.955
Credible Interval	(0.481, 1.88)
Associated Peptides	2
Associated Spectra	2
Coverage	NaN

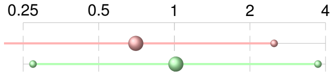

A	B	2.5	50	97.5	Sequence
**1**	**0**	**0.19**	**0.7**	**2.5**	**AVTDLQEAISNK**
**1**	**0**	**0.27**	**1**	**3.7**	**MSLIR**

5.573 " 45 506 minus strand PREDICTED: dihydrolipoyl dehydrogenase, mitochondrial [Monodelphis domestica]"

Protein Accession	CL24292.Contig3$_H$1
Mean Expression Ratio	0.956
Median Expression Ratio	0.956
Credible Interval	(0.479, 1.91)
Associated Peptides	1
Associated Spectra	2
Coverage	NaN

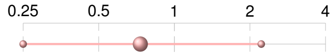

A	B	2.5	50	97.5	Sequence
**1**	**1**	**0.25**	**0.73**	**2.2**	**GIEIDGLR**

5.574 " 1252 1812 minus strand Alanine–glyoxylate aminotransferase 2, mitochondrial [Crassostrea gigas]"

Protein Accession	CL10973.Contig3$_H$1
Mean Expression Ratio	1.05
Median Expression Ratio	1.05
Credible Interval	(0.575, 1.94)
Associated Peptides	5
Associated Spectra	5
Coverage	NaN

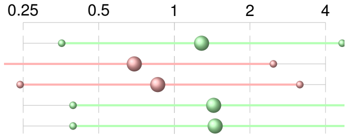

A	B	2.5	50	97.5	Sequence
**0**	**1**	**0.36**	**1.3**	**4.7**	**ADADFAINVLR**
**0**	**1**	**0.19**	**0.69**	**2.5**	**IKPPMCISK**
**0**	**1**	**0.24**	**0.86**	**3.2**	**KPLPADQVAAIWEETK**
**0**	**1**	**0.4**	**1.4**	**5.2**	**LRDEYEVVGDVR**
**0**	**1**	**0.4**	**1.5**	**5.4**	**SADVGTHMLNELLK**

5.575 444 719 minus strand 60S ribosomal protein L9 [Laodelphax striatella]

Protein Accession	CL1530.Contig3$_H$1
Mean Expression Ratio	1.04
Median Expression Ratio	1.05
Credible Interval	(0.563, 1.95)
Associated Peptides	3
Associated Spectra	8
Coverage	NaN

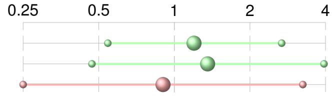

A	B	2.5	50	97.5	Sequence
**2**	**3**	**0.54**	**1.2**	**2.7**	**SAALIQQSTTVK**
**1**	**1**	**0.47**	**1.4**	**3.9**	**SAALIQQSTTVKK**
**0**	**1**	**0.25**	**0.9**	**3.2**	**FLDGIYVSEK**

5.576 365 544 PREDICTED: cofilin-2 [Otolemur garnettii]

Protein Accession	CL10440.Contig9$_{H}1$
Mean Expression Ratio	0.959
Median Expression Ratio	0.956
Credible Interval	(0.474, 1.94)
Associated Peptides	1
Associated Spectra	1
Coverage	NaN

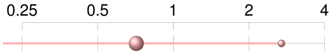

A	B	2.5	50	97.5	Sequence
1	0	0.2	0.71	2.7	MLYASSK

5.577 5 841 minus strand Spectrin alpha chain [Crassostrea gigas]

Protein Accession	CL44897.Contig1$_{H}1$
Mean Expression Ratio	0.954
Median Expression Ratio	0.956
Credible Interval	(0.492, 1.82)
Associated Peptides	2
Associated Spectra	3
Coverage	NaN

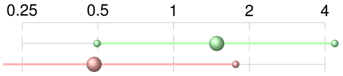

A	B	2.5	50	97.5	Sequence
1	1	0.5	1.5	4.4	HQNFEAEIEANR
0	1	0.13	0.48	1.8	HALLEADVAAHQDR

5.578 152 1930 hypothetical protein BRAFLDRAFT_223365 [Branchiostoma floridae] gi

Protein Accession	CL49005.Contig4$_{H}1$
Mean Expression Ratio	1.04
Median Expression Ratio	1.05
Credible Interval	(0.578, 1.88)
Associated Peptides	5
Associated Spectra	8
Coverage	NaN

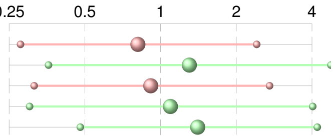

A	B	2.5	50	97.5	Sequence
1	1	0.28	0.81	2.4	QYSAGVSQLSLR
1	0	0.36	1.3	4.7	LSALGLR
1	1	0.31	0.91	2.7	VTLLR
0	1	0.3	1.1	4	ALGLPAATSFK
0	2	0.48	1.4	4.2	AVIDNSLFGNIVTK

5.579 72 839 minus strand 60S ribosomal protein L8 OS=Spodoptera frugiperda GN=RpL8 PE=2 SV=1

Protein Accession	Unigene15547$_{H}1$
Mean Expression Ratio	1.04
Median Expression Ratio	1.05
Credible Interval	(0.576, 1.87)
Associated Peptides	5
Associated Spectra	7
Coverage	NaN

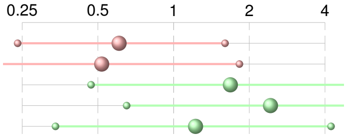

A	B	2.5	50	97.5	Sequence
1	2	0.24	0.61	1.6	AMVGIVAGGGR
1	0	0.14	0.52	1.8	IDKPILK
0	1	0.47	1.7	6.2	GVIKDIVHDPGR
0	1	0.65	2.4	8.7	KGAGSVFK
0	1	0.34	1.2	4.2	VAFRDPYR

5.580 578 1150 minus strand expressed hypothetical protein [Trichoplax adhaerens] gi; 376 948 minus strand expressed hypothetical protein [Trichoplax adhaerens] gi; 176 748 minus strand expressed hypothetical protein [Trichoplax adhaerens] gi; 176 748 minus strand expressed hypothetical protein [Trichoplax adhaerens] gi; 307 879 minus strand expressed hypothetical protein [Trichoplax adhaerens] gi; 307 879 minus strand expressed hypothetical protein [Trichoplax adhaerens] gi; 307 879 minus strand expressed hypothetical protein [Trichoplax adhaerens] gi

Protein Accession	CL11851.Contig7$_{H1}$ CL11851.Contig6$_{H1}$ CL11851.Contig5$_{H1}$ CL11851.Contig4$_{H1}$ CL1185
Mean Expression Ratio	0.957
Median Expression Ratio	0.957
Credible Interval	(0.479, 1.88)
Associated Peptides	1
Associated Spectra	3
Coverage	NaN

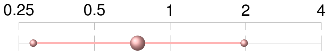

A	B	2.5	50	97.5	Sequence
0	3	0.28	0.74	2	GLLDIEAAVK

5.581 160 387 small nuclear ribonucleoprotein polypeptide G protein [Crassostrea ariakensis]; 180 407 small nuclear ribonucleoprotein polypeptide G protein [Crassostrea ariakensis]

Protein Accession	CL1272.Contig8$_{H1}$ CL1272.Contig7$_{H1}$
Mean Expression Ratio	1.04
Median Expression Ratio	1.04
Credible Interval	(0.574, 1.9)
Associated Peptides	4
Associated Spectra	16
Coverage	NaN

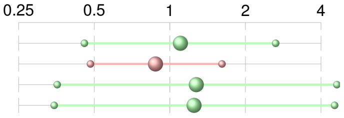

A	B	2.5	50	97.5	Sequence
3	1	0.46	1.1	2.6	SIGMVVVR
5	5	0.48	0.88	1.6	TITGILR
0	1	0.35	1.3	4.6	AHPPELKK
0	1	0.35	1.2	4.5	AHPPELK

5.582 447 977 Expansin-yoaJ [Crassostrea gigas]

Protein Accession	CL6172.Contig2$_{H1}$
Mean Expression Ratio	1.05
Median Expression Ratio	1.04
Credible Interval	(0.534, 2.03)
Associated Peptides	1
Associated Spectra	4
Coverage	NaN

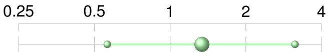

A	B	2.5	50	97.5	Sequence
0	4	0.56	1.3	3.1	AGDVDLGVNGDGR

5.583 96 503 minus strand mitochondrial ATP synthase delta chain [Haliotis discus discus]; 111 551 minus strand mitochondrial ATP synthase delta chain [Haliotis discus discus]; 96 542 minus strand mitochondrial ATP synthase delta chain [Haliotis discus discus]; 202 642 minus strand mitochondrial ATP synthase delta chain [Haliotis discus discus]; 44 529 mitochondrial ATP synthase delta chain [Haliotis discus discus]

Protein Accession	Unigene42244$_{H}1$ CL2187.Contig3$_{H}1$ CL2187.Contig2$_{H}1$ CL2187.Contig1$_{H}1$ CL2187.Contig
Mean Expression Ratio	1.04
Median Expression Ratio	1.04
Credible Interval	(0.519, 2.08)
Associated Peptides	1
Associated Spectra	1
Coverage	NaN

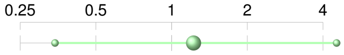

A	B	2.5	50	97.5	Sequence
1	0	0.34	1.2	4.5	FDAQAIR

5.584 219 428 Peptidyl-prolyl cis-trans isomerase OS=Hemicentrotus pulcherrimus PE=2 SV=1

Protein Accession	CL39270.Contig2$_{H}1$
Mean Expression Ratio	1.04
Median Expression Ratio	1.04
Credible Interval	(0.549, 2.01)
Associated Peptides	2
Associated Spectra	7
Coverage	NaN

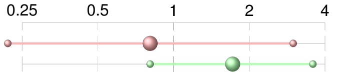

A	B	2.5	50	97.5	Sequence
1	0	0.22	0.81	3	IIIANSGQ
0	6	0.81	1.7	3.6	IIIANSGQV

5.585 85 546 minus strand RecName: Full=Eukaryotic translation initiation factor 5A; Short=eIF-5A gi|51702279|sp|P62925.1|IF5A_SPOFR RecName: Full=Eukaryotic translation initiation factor 5A; Short=eIF-5A gi|6469515|gb|AAF13315.1|AF109730_1 translation initiation factor 5A [Spodoptera exigua] gi

Protein Accession	CL40725.Contig2$_{H}1$
Mean Expression Ratio	0.959
Median Expression Ratio	0.959
Credible Interval	(0.558, 1.64)
Associated Peptides	7
Associated Spectra	21
Coverage	NaN

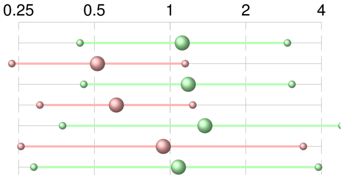

A	B	2.5	50	97.5	Sequence
1	2	0.44	1.1	2.9	AMGEECVVAVK
3	2	0.24	0.51	1.2	IVEMSTSK
1	2	0.45	1.2	3	KNEFVVIK
4	3	0.3	0.61	1.2	EDSLAAEIQK
0	1	0.37	1.4	4.8	EDSLAAEIQKK
0	1	0.26	0.94	3.4	KYEDICPSTHNMEVPVVTR
0	1	0.29	1.1	3.9	NEFVVIK

5.586 125 601 minus strand omega class glutathione-s-transferase [Chlamys farreri]; 113 808 minus strand omega class glutathione-s-transferase [Chlamys farreri]; 335 1045 minus strand omega class glutathione-s-transferase [Chlamys farreri]

Protein Accession	Unigene32974$_{H}1$ CL8096.Contig3$_{H}1$ CL8096.Contig2$_{H}1$
Mean Expression Ratio	1.04
Median Expression Ratio	1.04
Credible Interval	(0.572, 1.89)
Associated Peptides	5
Associated Spectra	6
Coverage	NaN

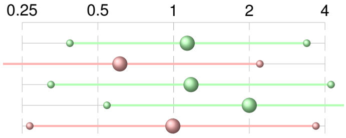

A	B	2.5	50	97.5	Sequence
1	1	0.39	1.1	3.4	HPLGLVPTLEQDSK
0	1	0.17	0.61	2.2	DAMLIDFFGNK
0	1	0.33	1.2	4.2	FIPNYYK
0	1	0.54	2	7.1	LTPVDPYRK
0	1	0.27	0.99	3.7	LVLEHK

5.587 18 647 minus strand ATP synthase-like protein [Biomphalaria glabrata]

Protein Accession	CL42054.Contig4$_{H}1$
Mean Expression Ratio	0.958
Median Expression Ratio	0.959
Credible Interval	(0.573, 1.59)
Associated Peptides	9
Associated Spectra	18
Coverage	NaN

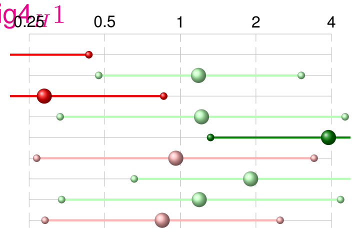

A	B	2.5	50	97.5	Sequence
2	2	0.077	0.18	0.43	AVLEQALK
1	2	0.47	1.2	3	YATALYSAASK
1	1	0.097	0.29	0.86	NFQNLLK
0	1	0.33	1.2	4.5	AVLEQALKQQK
0	2	1.3	3.9	12	GKLVTTPIQVFGIDGR
0	1	0.27	0.96	3.4	LAEFVANPSLQR
0	2	0.66	1.9	5.6	GEVVCTVK
0	1	0.34	1.2	4.3	KLAEFVANPSLQR
0	2	0.29	0.84	2.5	GALQGFLK

5.588 4365 4553 minus strand Basement membrane-specific heparan sulfate proteoglycan core protein [Crassostrea gigas]; 2502 2696 minus strand hypothetical protein DAPPUDRAFT_186952 [Daphnia pulex]

Protein Accession	CL24300.Contig6$_{H}1$ CL24300.Contig2$_{H}1$
Mean Expression Ratio	0.956
Median Expression Ratio	0.959
Credible Interval	(0.478, 1.88)
Associated Peptides	1
Associated Spectra	1
Coverage	NaN

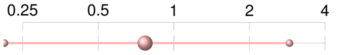

A	B	2.5	50	97.5	Sequence
0	1	0.21	0.77	2.9	DGSIIVDGQEVK

5.589 14 277 minus strand Histidine triad nucleotide-binding protein 1 OS=Oryctolagus cuniculus GN=HINT1 PE=1 SV=2

Protein Accession	CL25563.Contig1$_{H}1$
Mean Expression Ratio	0.962
Median Expression Ratio	0.959
Credible Interval	(0.496, 1.87)
Associated Peptides	2
Associated Spectra	4
Coverage	NaN

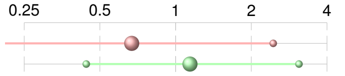

A	B	2.5	50	97.5	Sequence
**1**	**0**	**0.18**	**0.67**	**2.4**	**LAEAVEADEK**
**1**	**2**	**0.44**	**1.1**	**3.1**	**AHLAKPGGDTIFGK**

5.590 96 497 AAEL009772-PB [Aedes aegypti]

Protein Accession	CL27854.Contig2$_{H}1$
Mean Expression Ratio	0.955
Median Expression Ratio	0.959
Credible Interval	(0.487, 1.86)
Associated Peptides	2
Associated Spectra	2
Coverage	NaN

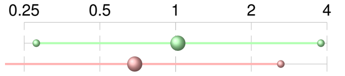

A	B	2.5	50	97.5	Sequence
**0**	**1**	**0.28**	**1**	**3.8**	**LVEEFPMGNIK**
**0**	**1**	**0.19**	**0.69**	**2.6**	**MNPGFDTIGK**

5.591 2 571 PREDICTED: copper homeostasis protein cutC homolog [Anolis carolinensis]

Protein Accession	CL43087.Contig1$_{H}1$
Mean Expression Ratio	1.04
Median Expression Ratio	1.04
Credible Interval	(0.511, 2.05)
Associated Peptides	1
Associated Spectra	1
Coverage	NaN

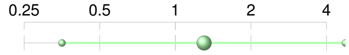

A	B	2.5	50	97.5	Sequence
**0**	**1**	**0.35**	**1.3**	**4.7**	**IHQDSEMLFQK**

5.592 " 2 433 Vesicle-fusing ATPase, partial [Ascaris suum]"

Protein Accession	CL20981.Contig2$_{H}1$
Mean Expression Ratio	0.96
Median Expression Ratio	0.96
Credible Interval	(0.471, 1.94)
Associated Peptides	1
Associated Spectra	1
Coverage	NaN

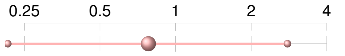

A	B	2.5	50	97.5	Sequence
**1**	**0**	**0.21**	**0.78**	**2.8**	**LENSNLNLIGK**

5.593 " 343 1179 ATP synthase subunit alpha, mitochondrial OS=Pongo abelii GN=ATP5A1 PE=2 SV=1"

Protein Accession	Unigene30534$_{H1}$
Mean Expression Ratio	1.04
Median Expression Ratio	1.04
Credible Interval	(0.662, 1.64)
Associated Peptides	11
Associated Spectra	63
Coverage	NaN

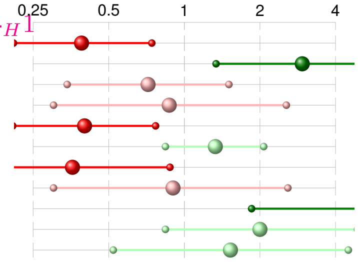

A	B	2.5	50	97.5	Sequence
7	2	0.21	0.39	0.74	STVAQLVK
2	3	1.3	3	6.5	TSVKEPMQTGIK
3	3	0.34	0.72	1.5	APGIIPR
1	1	0.3	0.87	2.5	VYGLK
2	6	0.21	0.4	0.77	TAVAIDTIINQK
6	13	0.84	1.3	2.1	VLSIGDGIAR
1	3	0.15	0.36	0.87	VVDALGTPIDGK
0	2	0.3	0.9	2.6	NIQAEEMVEFSSGLK
0	2	1.8	5.4	16	VGLKAPGIIPR
0	4	0.84	2	4.9	VVDALGTPIDGKGPITSK
0	2	0.52	1.5	4.5	TAVAIDTIINQKR

5.594 7 231 minus strand AGAP006879-PA [Anopheles gambiae str. PEST] gi

Protein Accession	Unigene16066$_{H1}$
Mean Expression Ratio	1.04
Median Expression Ratio	1.04
Credible Interval	(0.517, 2.08)
Associated Peptides	1
Associated Spectra	1
Coverage	NaN

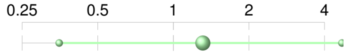

A	B	2.5	50	97.5	Sequence
0	1	0.35	1.3	4.7	NVSPLIR

5.595 40 315 minus strand 60S ribosomal protein L18a OS=Homo sapiens GN=RPL18A PE=1 SV=2

Protein Accession	Unigene26823$_{H1}$
Mean Expression Ratio	1.05
Median Expression Ratio	1.04
Credible Interval	(0.554, 1.97)
Associated Peptides	2
Associated Spectra	7
Coverage	NaN

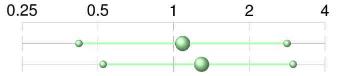

A	B	2.5	50	97.5	Sequence
2	1	0.42	1.1	2.8	IFAPDQATAK
2	2	0.52	1.3	3	TPTLYQMR

5.596 438 683 minus strand Protein lethal(2)essential for life OS=Drosophila melanogaster GN=l(2)efl PE=1 SV=1

Protein Accession	CL17234.Contig7$_H$1
Mean Expression Ratio	1.04
Median Expression Ratio	1.04
Credible Interval	(0.533, 2.05)
Associated Peptides	1
Associated Spectra	7
Coverage	NaN

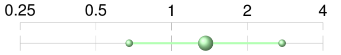

A	B	2.5	50	97.5	Sequence
**1**	**6**	**0.68**	**1.4**	**2.8**	**ESGTSEVINNDKEFQIK**

5.597 484 1218 minus strand alginate lyase [Littorina brevicula]; 484 1218 minus strand alginate lyase [Littorina brevicula]

Protein Accession	CL8738.Contig15$_H$1 CL8738.Contig13$_H$1
Mean Expression Ratio	1.04
Median Expression Ratio	1.04
Credible Interval	(0.515, 2.07)
Associated Peptides	1
Associated Spectra	1
Coverage	NaN

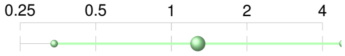

A	B	2.5	50	97.5	Sequence
**0**	**1**	**0.34**	**1.3**	**4.8**	**VFLDGHK**

5.598 275 1372 Cystathionine gamma-lyase OS=Bos taurus GN=CTH PE=2 SV=1

Protein Accession	CL16415.Contig5$_H$1
Mean Expression Ratio	0.961
Median Expression Ratio	0.961
Credible Interval	(0.534, 1.75)
Associated Peptides	5
Associated Spectra	7
Coverage	NaN

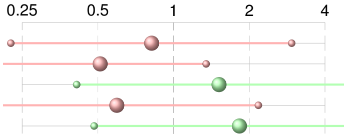

A	B	2.5	50	97.5	Sequence
**1**	**0**	**0.23**	**0.82**	**3**	**GGMTEASNFLK**
**1**	**2**	**0.2**	**0.51**	**1.3**	**LVDIEAVSK**
**0**	**1**	**0.41**	**1.5**	**5.8**	**SMAVVPPISTSTTFK**
**0**	**1**	**0.16**	**0.59**	**2.2**	**VIHPGLESHPQYELAK**
**0**	**1**	**0.48**	**1.8**	**6.7**	**GLVVARFLEK**

5.599 52 1653 minus strand PREDICTED: eukaryotic translation initiation factor 3 subunit 6 interacting protein-like [Saccoglossus kowalevskii]

Protein Accession	CL23174.Contig2$_H$1
Mean Expression Ratio	1.04
Median Expression Ratio	1.04
Credible Interval	(0.515, 2.12)
Associated Peptides	1
Associated Spectra	1
Coverage	NaN

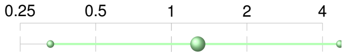

A	B	2.5	50	97.5	Sequence
**1**	**0**	**0.33**	**1.3**	**4.7**	**NMIHVADTK**

5.600 35 2953 hypothetical protein CGI_10020867 [Crassostrea gigas]; 97 3015 minus strand hypothetical protein CGI_10020867 [Crassostrea gigas]

Protein Accession	CL2537.Contig6$_{H}1$ CL2537.Contig4$_{H}1$
Mean Expression Ratio	1.03
Median Expression Ratio	1.04
Credible Interval	(0.534, 2)
Associated Peptides	2
Associated Spectra	4
Coverage	NaN

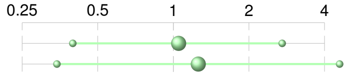

A	B	2.5	50	97.5	Sequence
0	3	0.4	1	2.7	GGGQIIPTAR
0	1	0.34	1.3	4.6	LLDATIAAEPIHR

5.601 " 109 1632 minus strand Seryl-tRNA synthetase, cytoplasmic [Crassostrea gigas]; 109 1614 minus strand Seryl-tRNA synthetase, cytoplasmic [Crassostrea gigas]"

Protein Accession	CL11056.Contig9$_{H}1$ CL11056.Contig1$_{H}1$
Mean Expression Ratio	1.04
Median Expression Ratio	1.04
Credible Interval	(0.553, 1.95)
Associated Peptides	3
Associated Spectra	5
Coverage	NaN

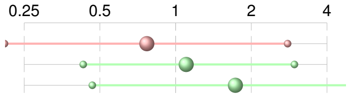

A	B	2.5	50	97.5	Sequence
1	0	0.21	0.77	2.8	ELTVTQIK
0	3	0.43	1.1	3	LVDTVVDQDAAWR
0	1	0.46	1.7	6.5	VLDIELFR

5.602 1573 2385 minus strand Spectrin beta chain [Crassostrea gigas]

Protein Accession	CL5904.Contig4$_{H}1$
Mean Expression Ratio	1.04
Median Expression Ratio	1.04
Credible Interval	(0.533, 2.04)
Associated Peptides	2
Associated Spectra	2
Coverage	NaN

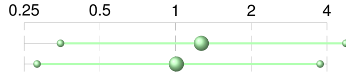

A	B	2.5	50	97.5	Sequence
0	1	0.35	1.3	4.8	LIQEEHPESDQIAVR
0	1	0.28	1	3.8	RLIQEEHPESDQIAVR

5.603 942 1385 Golgi-associated plant pathogenesis-related protein 1 [Crassostrea gigas]; 536 979 Golgi-associated plant pathogenesis-related protein 1 [Crassostrea gigas]

Protein Accession	Unigene391$_{H}1$ CL15263.Contig11$_{H}1$
Mean Expression Ratio	0.96
Median Expression Ratio	0.962
Credible Interval	(0.479, 1.9)
Associated Peptides	1
Associated Spectra	1
Coverage	NaN

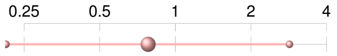

A	B	2.5	50	97.5	Sequence
**0**	**1**	**0.21**	**0.78**	**2.9**	**IITEETIIK**

5.604 740 2029 minus strand PREDICTED: protein alan shepard-like isoform 2 [Bombus terrestris]

Protein Accession	CL693.Contig2$_{H}1$
Mean Expression Ratio	1.04
Median Expression Ratio	1.04
Credible Interval	(0.517, 2.09)
Associated Peptides	1
Associated Spectra	1
Coverage	NaN

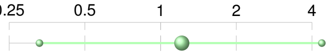

A	B	2.5	50	97.5	Sequence
**0**	**1**	**0.33**	**1.2**	**4.4**	**GAQGIQAQMAK**

5.605 231 902 Sodium- and chloride-dependent GABA transporter ine [Crassostrea gigas]

Protein Accession	CL11289.Contig3$_{H}1$
Mean Expression Ratio	0.964
Median Expression Ratio	0.963
Credible Interval	(0.494, 1.93)
Associated Peptides	1
Associated Spectra	1
Coverage	NaN

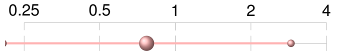

A	B	2.5	50	97.5	Sequence
**0**	**1**	**0.21**	**0.77**	**2.9**	**EAFELSFK**

5.606 150 1211 RecName: Full=Guanine nucleotide-binding protein G(i) subunit alpha; AltName: Full=Adenylate cyclase-inhibiting G alpha protein gi|9631|emb|CAA78807.1| GTP-binding protein alpha-i subunit [Lymnaea stagnalis]

Protein Accession	CL13885.Contig6$_{H}$1
Mean Expression Ratio	1.04
Median Expression Ratio	1.04
Credible Interval	(0.525, 2.13)
Associated Peptides	1
Associated Spectra	1
Coverage	NaN

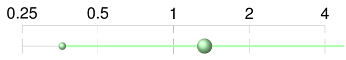

A	B	2.5	50	97.5	Sequence
1	0	0.36	1.3	4.9	IAIIR

5.607 1 468 glycine cleavage system H protein [Rhipicephalus sanguineus]

Protein Accession	CL42651.Contig1$_{H}$1
Mean Expression Ratio	1.04
Median Expression Ratio	1.04
Credible Interval	(0.52, 2.06)
Associated Peptides	1
Associated Spectra	1
Coverage	NaN

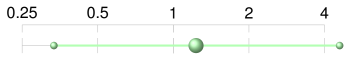

A	B	2.5	50	97.5	Sequence
0	1	0.33	1.2	4.6	SPLDDGWVFK

5.608 92 1813 minus strand Dihydropyrimidinase [Crassostrea gigas]

Protein Accession	CL7386.Contig6$_{H}$1
Mean Expression Ratio	0.969
Median Expression Ratio	0.964
Credible Interval	(0.537, 1.77)
Associated Peptides	6
Associated Spectra	6
Coverage	NaN

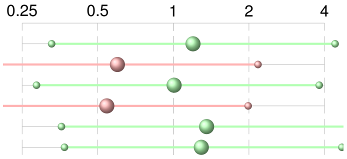

A	B	2.5	50	97.5	Sequence
1	0	0.33	1.2	4.4	SSADVISAAR
1	0	0.17	0.6	2.2	AENGQIIALK
1	0	0.28	1	3.8	LPVVR
1	0	0.15	0.54	2	IENGIIK
0	1	0.36	1.4	5	IAEGSDADVVVWDPEQAR
0	1	0.37	1.3	4.7	NIPNGVNGVEDR

5.609 82 816 PREDICTED: triosephosphate isomerase B [Oreochromis niloticus]

Protein Accession	CL26247.Contig4$_{H}$1
Mean Expression Ratio	1.04
Median Expression Ratio	1.04
Credible Interval	(0.575, 1.9)
Associated Peptides	6
Associated Spectra	6
Coverage	NaN

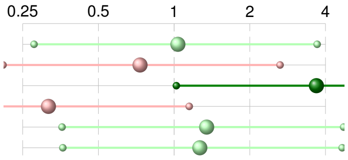

A	B	2.5	50	97.5	Sequence
0	1	0.28	1	3.7	TEFITIINSK
0	1	0.21	0.73	2.6	QFFVGGNWK
0	1	1	3.7	14	NVSKDVANTVR
0	1	0.089	0.32	1.1	SVDEIIK
0	1	0.36	1.3	4.7	AIADNVNDWK
0	1	0.36	1.3	4.7	AIADNVNDWKR

5.610 2 478 Glyoxylate reductase/hydroxypyruvate reductase [Crassostrea gigas]

Protein Accession	CL29479.Contig5$_{H1}$
Mean Expression Ratio	1.04
Median Expression Ratio	1.04
Credible Interval	(0.538, 1.99)
Associated Peptides	3
Associated Spectra	3
Coverage	NaN

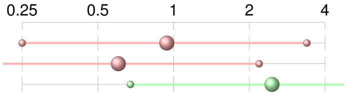

A	B	2.5	50	97.5	Sequence
1	0	0.25	0.94	3.4	VGFGVAR
0	1	0.16	0.6	2.2	GSIVDQDALDEALK
0	1	0.67	2.5	9.1	MKEGSILINTAR

5.611 " 2057 2497 Phosphate carrier protein, mitochondrial [Crassostrea gigas]"

Protein Accession	CL2712.Contig11$_{H1}$
Mean Expression Ratio	1.03
Median Expression Ratio	1.04
Credible Interval	(0.529, 2)
Associated Peptides	2
Associated Spectra	2
Coverage	NaN

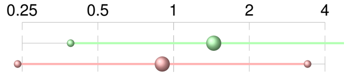

A	B	2.5	50	97.5	Sequence
0	1	0.39	1.4	5.2	LPRPPPPEMPESLKR
0	1	0.24	0.9	3.4	YVVPKPR

5.612 996 1463 minus strand catalase [Cristaria plicata] gi

Protein Accession	CL6841.Contig3$_{H1}$
Mean Expression Ratio	1.04
Median Expression Ratio	1.04
Credible Interval	(0.527, 2.04)
Associated Peptides	2
Associated Spectra	2
Coverage	NaN

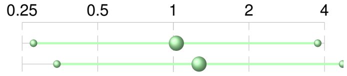

A	B	2.5	50	97.5	Sequence
0	1	0.28	1	3.8	YLECPQHFSGDVQR
0	1	0.34	1.3	4.7	LFAYSDTHR

5.613 729 1541 troponin T [Mizuhopecten yessoensis]

Protein Accession	Unigene34289$_{H1}$
Mean Expression Ratio	1.03
Median Expression Ratio	1.04
Credible Interval	(0.502, 2.08)
Associated Peptides	1
Associated Spectra	1
Coverage	NaN

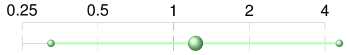

A	B	2.5	50	97.5	Sequence
0	1	0.33	1.2	4.6	QQYDMIELAER

5.614 " 166 1248 PREDICTED: probable pyruvate dehydrogenase E1 component subunit alpha, mitochondrial-like [Metaseiulus occidentalis]; 166 1248 PREDICTED: probable pyruvate dehydrogenase E1 component subunit alpha, mitochondrial-like [Metaseiulus occidentalis]; 166 1248 PREDICTED: probable pyruvate dehydrogenase E1 component subunit alpha, mitochondrial-like [Metaseiulus occidentalis]; 166 1248 PREDICTED: probable pyruvate dehydrogenase E1 component subunit alpha, mitochondrial-like [Metaseiulus occidentalis]; 166 1248 PREDICTED: probable pyruvate dehydrogenase E1 component subunit alpha, mitochondrial-like [Metaseiulus occidentalis]; 166 1248 minus strand PREDICTED: probable pyruvate dehydrogenase E1 component subunit alpha, mitochondrial-like [Metaseiulus occidentalis]; 166 1248 minus strand PREDICTED: probable pyruvate dehydrogenase E1 component subunit alpha, mitochondrial-like [Metaseiulus occidentalis]; 166 1248 PREDICTED: probable pyruvate dehydrogenase E1 component subunit alpha, mitochondrial-like [Metaseiulus occidentalis]; 166 1248 PREDICTED: probable pyruvate dehydrogenase E1 component subunit alpha, mitochondrial-like [Metaseiulus occidentalis]"

Protein Accession	Unigene34082$_H$1 Unigene34081$_H$1 Unigene34079$_H$1 Unigene34077$_H$1 Unigene34075$_H$1 U
Mean Expression Ratio	1.04
Median Expression Ratio	1.04
Credible Interval	(0.543, 2)
Associated Peptides	2
Associated Spectra	3
Coverage	NaN

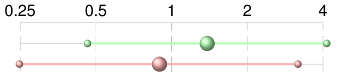

A	B	2.5	50	97.5	Sequence
**2**	**0**	**0.46**	**1.4**	**4.1**	**TGLATEEELKK**
**1**	**0**	**0.25**	**0.9**	**3.2**	**DMQVIR**

5.615 1519 2583 Proliferation-associated protein 2G4 [Crassostrea gigas]; 565 1629 Proliferation-associated protein 2G4 [Crassostrea gigas]

Protein Accession	CL14279.Contig3$_H$1 CL14279.Contig2$_H$1
Mean Expression Ratio	1.03
Median Expression Ratio	1.04
Credible Interval	(0.534, 2)
Associated Peptides	2
Associated Spectra	2
Coverage	NaN

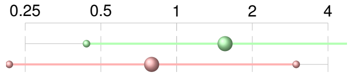

A	B	2.5	50	97.5	Sequence
**0**	**1**	**0.44**	**1.6**	**6**	**ALLSASASSK**
**0**	**1**	**0.22**	**0.8**	**3**	**ILEETGK**

5.616 587 1081 hypothetical protein CGI_10026502 [Crassostrea gigas]

Protein Accession	Unigene22889$_H$1
Mean Expression Ratio	1.04
Median Expression Ratio	1.04
Credible Interval	(0.522, 2.07)
Associated Peptides	1
Associated Spectra	1
Coverage	NaN

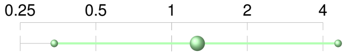

A	B	2.5	50	97.5	Sequence
1	0	0.34	1.3	4.6	LRRTLLGS

5.617 103 819 minus strand PREDICTED: l-xylulose reductase-like [Monodelphis domestica]

Protein Accession	Unigene30376$_H$1
Mean Expression Ratio	1.04
Median Expression Ratio	1.04
Credible Interval	(0.524, 2.07)
Associated Peptides	1
Associated Spectra	1
Coverage	NaN

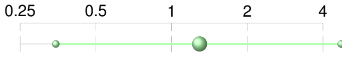

A	B	2.5	50	97.5	Sequence
0	1	0.35	1.3	4.7	GSGGAIVNISSQASK

5.618 79 606 minus strand PREDICTED: similar to Ras-like protein 3 precursor (Protein roughened) [Tribolium castaneum] gi

Protein Accession	Unigene4246$_H$1
Mean Expression Ratio	0.964
Median Expression Ratio	0.966
Credible Interval	(0.496, 1.9)
Associated Peptides	1
Associated Spectra	3
Coverage	NaN

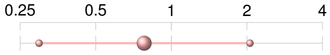

A	B	2.5	50	97.5	Sequence
0	3	0.3	0.78	2.1	LVVLGSGGVGK

5.619 125 754 minus strand peptidyl prolyl cis-trans isomerase B [Conus novaehollandiae]

Protein Accession	Unigene19558$_H$1
Mean Expression Ratio	0.964
Median Expression Ratio	0.966
Credible Interval	(0.518, 1.77)
Associated Peptides	3
Associated Spectra	10
Coverage	NaN

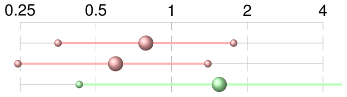

A	B	2.5	50	97.5	Sequence
1	4	0.35	0.79	1.8	VLSGMDVVR
0	4	0.24	0.6	1.4	HLALGDKEDGGVK
0	1	0.43	1.5	5.6	SIYGEKFPDENFK

5.620 14 502 minus strand Ufm1-conjugating enzyme 1 [Crassostrea gigas]

Protein Accession	CL15276.Contig2$_H$1
Mean Expression Ratio	1.04
Median Expression Ratio	1.03
Credible Interval	(0.52, 2.08)
Associated Peptides	1
Associated Spectra	1
Coverage	NaN

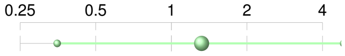

A	B	2.5	50	97.5	Sequence
**0**	**1**	**0.35**	**1.3**	**4.9**	**TLAQIPLLK**

5.621 835 1083 minus strand Calmodulin [Crassostrea gigas]

Protein Accession	CL49568.Contig2$_H$1
Mean Expression Ratio	0.963
Median Expression Ratio	0.966
Credible Interval	(0.507, 1.82)
Associated Peptides	3
Associated Spectra	5
Coverage	NaN

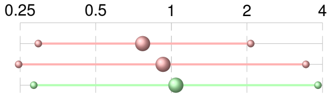

A	B	2.5	50	97.5	Sequence
**0**	**3**	**0.3**	**0.77**	**2.1**	**VDFTEFYK**
**0**	**1**	**0.25**	**0.93**	**3.4**	**HLLTDKDEETCYK**
**0**	**1**	**0.28**	**1**	**3.8**	**GFIANEEMK**

5.622 616 1740 5'-AMP-activated protein kinase subunit beta-2 [Crassostrea gigas]; 641 1762 minus strand 5'-AMP-activated protein kinase subunit beta-2 [Crassostrea gigas]; 626 1786 5'-AMP-activated protein kinase subunit beta-2 [Crassostrea gigas]; 431 1591 5'-AMP-activated protein kinase subunit beta-2 [Crassostrea gigas]

Protein Accession	Unigene52845$_H$1 Unigene41754$_H$1 Unigene36145$_H$1 CL5821.Contig3$_H$1
Mean Expression Ratio	0.963
Median Expression Ratio	0.966
Credible Interval	(0.482, 1.9)
Associated Peptides	1
Associated Spectra	1
Coverage	NaN

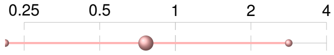

A	B	2.5	50	97.5	Sequence
**0**	**1**	**0.21**	**0.76**	**2.8**	**FIVDEQWVVSK**

5.623 " 131 544 Histone H2B, gonadal OS=Patella granatina PE=1 SV=2"

Protein Accession	CL29794.Contig2$_{H}$1
Mean Expression Ratio	1.04
Median Expression Ratio	1.03
Credible Interval	(0.63, 1.74)
Associated Peptides	5
Associated Spectra	48
Coverage	NaN

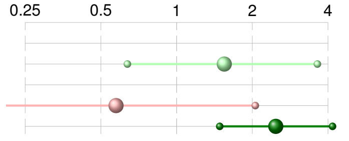

A	B	2.5	50	97.5	Sequence
3	3	5.2	12	25	AVTKYTSSK
4	0	0.64	1.5	3.6	EIQTAVR
3	2	6.2	14	32	STITSREIQTAVR
1	0	0.16	0.58	2.1	LIFER
9	23	1.5	2.5	4.2	LLLPGELAK

5.624 1 264 Spectrin beta chain [Crassostrea gigas]

Protein Accession	Unigene36334$_{H}$1
Mean Expression Ratio	1.03
Median Expression Ratio	1.03
Credible Interval	(0.531, 2.03)
Associated Peptides	2
Associated Spectra	2
Coverage	NaN

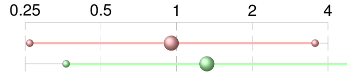

A	B	2.5	50	97.5	Sequence
0	1	0.26	0.95	3.5	QLLQVEHPNAAEVVQR
0	1	0.36	1.3	4.9	VAVVNQLAR

5.625 513 755 PREDICTED: 40S ribosomal protein S16-like [Papio anubis]; 513 755 PREDICTED: 40S ribosomal protein S16-like [Papio anubis]

Protein Accession	CL14629.Contig2$_{H}$1 CL14629.Contig1$_{H}$1
Mean Expression Ratio	0.962
Median Expression Ratio	0.967
Credible Interval	(0.48, 1.89)
Associated Peptides	1
Associated Spectra	3
Coverage	NaN

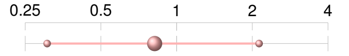

A	B	2.5	50	97.5	Sequence
3	0	0.31	0.82	2.1	SLLVADPR

5.626 " 132 2075 PREDICTED: arginyl-tRNA synthetase, cytoplasmic [Ornithorhynchus anatinus]; 132 2090 minus strand PREDICTED: arginyl-tRNA synthetase, cytoplasmic [Ornithorhynchus anatinus]"

Protein Accession	CL15019.Contig4$_{H}$1 CL15019.Contig3$_{H}$1
Mean Expression Ratio	1.04
Median Expression Ratio	1.03
Credible Interval	(0.547, 1.99)
Associated Peptides	3
Associated Spectra	3
Coverage	NaN

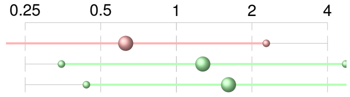

A	B	2.5	50	97.5	Sequence
0	1	0.17	0.63	2.3	LVDLLDEGVK
0	1	0.35	1.3	4.7	LSYQLNHLKR
0	1	0.44	1.6	5.9	QRTGAVSLQRMLEDVFR

5.627 2 2572 Glycogen debranching enzyme [Crassostrea gigas]

Protein Accession CL45503.Contig1$_H$1
Mean Expression Ratio 0.971
Median Expression Ratio 0.967
Credible Interval (0.484, 1.98)
Associated Peptides 1
Associated Spectra 1
Coverage NaN

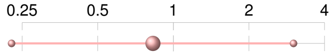

A	B	2.5	50	97.5	Sequence
0	1	0.23	0.83	3	QMLNNLHYEMAR

5.628 282 683 PREDICTED: ribonuclease UK114-like [Oreochromis niloticus]

Protein Accession CL39557.Contig5$_H$1
Mean Expression Ratio 1.04
Median Expression Ratio 1.03
Credible Interval (0.531, 2.06)
Associated Peptides 2
Associated Spectra 2
Coverage NaN

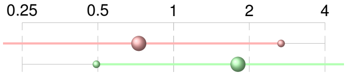

A	B	2.5	50	97.5	Sequence
0	1	0.2	0.73	2.7	LVPGGIQAETDQTLK
0	1	0.49	1.8	6.8	YFSDNKPAR

5.629 3 272 minus strand ADP/ATP carrier [Clonorchis sinensis]

Protein Accession Unigene4627$_H$1
Mean Expression Ratio 1.03
Median Expression Ratio 1.03
Credible Interval (0.523, 2.09)
Associated Peptides 1
Associated Spectra 1
Coverage NaN

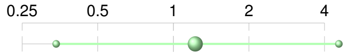

A	B	2.5	50	97.5	Sequence
0	1	0.34	1.2	4.6	MMMTSGQAVK

5.630 " 3341 4240 minus strand nonmuscle myosin II, partial [Aplysia californica]"

Protein Accession CL19183.Contig2$_H$1
Mean Expression Ratio 0.967
Median Expression Ratio 0.968
Credible Interval (0.572, 1.65)
Associated Peptides 9
Associated Spectra 15
Coverage NaN

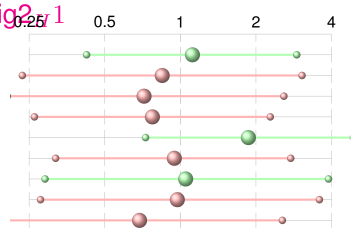

A	B	2.5	50	97.5	Sequence
1	2	0.42	1.1	2.9	QKQDVEQLEAR
1	0	0.23	0.85	3	RQQVEELQAQLAK
1	0	0.2	0.71	2.6	YAQMIEEK
1	1	0.26	0.77	2.3	TATMLEEK
0	3	0.73	1.9	4.9	ELQNQMTELNEDLETER
0	2	0.32	0.95	2.7	LHTKEEELK
0	1	0.29	1	3.9	RKEELEDILHEMELR
0	1	0.28	0.97	3.6	KLEEDLALNEDTNQK
0	1	0.2	0.69	2.5	LSEAMAQIVEEEEK

5.631 238 681 minus strand Globin OS=Cerithidea rhizophorarum PE=1 SV=1

Protein Accession	CL8458.Contig2$_H$1
Mean Expression Ratio	1.03
Median Expression Ratio	1.03
Credible Interval	(0.573, 1.85)
Associated Peptides	4
Associated Spectra	61
Coverage	NaN

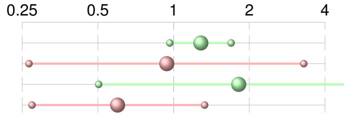

A	B	2.5	50	97.5	Sequence
11	43	0.97	1.3	1.7	AAITSTWSK
0	1	0.27	0.94	3.3	LASDKATMENNGAK
0	1	0.5	1.8	6.6	AITSTWSK
0	5	0.27	0.6	1.3	AYFPFLPGASEGDIK

5.632 915 1505 minus strand Collagen alpha-5(VI) chain [Crassostrea gigas]

Protein Accession	CL40183.Contig3$_H$1
Mean Expression Ratio	1.04
Median Expression Ratio	1.03
Credible Interval	(0.583, 1.82)
Associated Peptides	6
Associated Spectra	11
Coverage	NaN

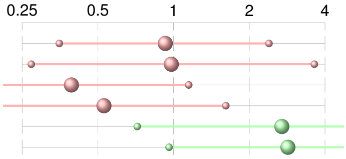

A	B	2.5	50	97.5	Sequence
1	2	0.35	0.92	2.4	HGLATNTGAGIK
1	0	0.27	0.98	3.6	LEAIPWR
0	2	0.13	0.39	1.2	VGEQLDAEELLNIAGDPK
0	2	0.18	0.53	1.6	VGEQLDAEELLNIAGDPKR
0	1	0.72	2.7	9.7	HGLATNTGAG
0	2	0.96	2.9	8.4	VVTAENYAHLNLVK

5.633 23 409 minus strand CDGSH iron sulfur domain-containing protein 2 [Crassostrea gigas]

Protein Accession	CL36832.Contig6$_H$1
Mean Expression Ratio	0.972
Median Expression Ratio	0.968
Credible Interval	(0.487, 1.96)
Associated Peptides	1
Associated Spectra	1
Coverage	NaN

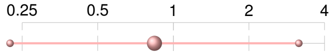

A	B	2.5	50	97.5	Sequence
1	0	0.22	0.84	3.1	VSLPTYLK

5.634 5 1018 minus strand Glutaredoxin-3 [Crassostrea gigas]; 5 1018 Glutaredoxin-3 [Crassostrea gigas]

Protein Accession	CL7734.Contig9$_H$1 CL7734.Contig10$_H$1
Mean Expression Ratio	1.03
Median Expression Ratio	1.03
Credible Interval	(0.518, 2.02)
Associated Peptides	1
Associated Spectra	3
Coverage	NaN

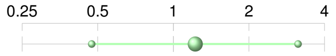

A	B	2.5	50	97.5	Sequence
1	2	0.47	1.2	3.1	VAAEDLAEVSHR

5.635 254 1093 uncharacterized protein LOC432187 precursor [Xenopus laevis] gi

Protein Accession	CL7800.Contig7$_H$1
Mean Expression Ratio	0.965
Median Expression Ratio	0.969
Credible Interval	(0.484, 1.93)
Associated Peptides	1
Associated Spectra	1
Coverage	NaN

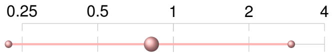

A	B	2.5	50	97.5	Sequence
**1**	**0**	**0.22**	**0.82**	**2.9**	**LVTSK**

5.636 313 2763 NCAM-related cell adhesion molecule precursor [Aplysia californica] gi

Protein Accession	Unigene11449$_H$1
Mean Expression Ratio	0.968
Median Expression Ratio	0.969
Credible Interval	(0.536, 1.73)
Associated Peptides	5
Associated Spectra	9
Coverage	NaN

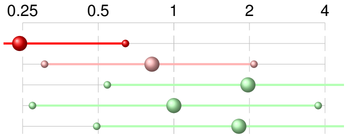

A	B	2.5	50	97.5	Sequence
**2**	**1**	**0.091**	**0.24**	**0.64**	**SAGEPLLDAVTK**
**1**	**2**	**0.31**	**0.82**	**2.1**	**QILYFR**
**0**	**1**	**0.54**	**2**	**7.2**	**KTSVAEDYKELTVDR**
**0**	**1**	**0.27**	**1**	**3.8**	**EALEVDTSK**
**0**	**1**	**0.49**	**1.8**	**6.6**	**IGSSDPTVFVFK**

5.637 169 1200 PREDICTED: nidogen-2-like isoform 1 [Strongylocentrotus purpuratus]

Protein Accession	CL478.Contig2$_H$1
Mean Expression Ratio	1.04
Median Expression Ratio	1.03
Credible Interval	(0.542, 1.96)
Associated Peptides	3
Associated Spectra	3
Coverage	NaN

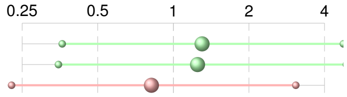

A	B	2.5	50	97.5	Sequence
**0**	**1**	**0.36**	**1.3**	**4.8**	**AIVLDINGGK**
**0**	**1**	**0.35**	**1.2**	**4.9**	**LVYAR**
**0**	**1**	**0.23**	**0.82**	**3.1**	**TLFNTDMVNPR**

5.638 21 254 predicted protein [Nematostella vectensis] gi

Protein Accession	CL43392.Contig2$_H$1
Mean Expression Ratio	0.975
Median Expression Ratio	0.969
Credible Interval	(0.498, 1.93)
Associated Peptides	1
Associated Spectra	6
Coverage	NaN

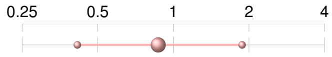

A	B	2.5	50	97.5	Sequence
**4**	**2**	**0.42**	**0.87**	**1.9**	**AADAVVAEIK**

5.639 " 1 681 40S ribosomal protein S4, X isoform [Crassostrea gigas]"

Protein Accession	CL2560.Contig4$_H1$
Mean Expression Ratio	0.972
Median Expression Ratio	0.969
Credible Interval	(0.564, 1.66)
Associated Peptides	7
Associated Spectra	22
Coverage	NaN

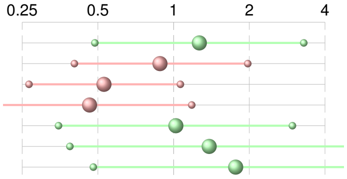

A	B	2.5	50	97.5	Sequence
1	2	0.49	1.3	3.3	GVPYIVTHDGR
2	3	0.4	0.88	2	QWVSLPK
1	6	0.27	0.53	1.1	VNDTVVLDIASGK
2	1	0.17	0.46	1.2	LTIAEER
1	1	0.35	1	3	IKVDGK
0	1	0.39	1.4	5	MKDYIK
0	1	0.48	1.8	6.3	LIKVDGK

5.640 8 271 minus strand Laminin subunit gamma-1 [Crassostrea gigas]

Protein Accession	Unigene36706$_H1$
Mean Expression Ratio	1.03
Median Expression Ratio	1.03
Credible Interval	(0.533, 2.04)
Associated Peptides	2
Associated Spectra	2
Coverage	NaN

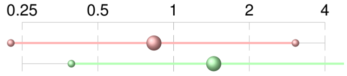

A	B	2.5	50	97.5	Sequence
0	1	0.23	0.83	3.1	ALDLYR
0	1	0.39	1.4	5.2	HMQEAAQIDNIAR

5.641 260 1597 isocitrate dehydrogenase [Mytilus galloprovincialis]; 260 1597 isocitrate dehydrogenase [Mytilus galloprovincialis]; 260 1597 isocitrate dehydrogenase [Mytilus galloprovincialis]

Protein Accession	CL10046.Contig8$_H1$ CL10046.Contig7$_H1$ CL10046.Contig6$_H1$
Mean Expression Ratio	1.03
Median Expression Ratio	1.03
Credible Interval	(0.578, 1.84)
Associated Peptides	5
Associated Spectra	10
Coverage	NaN

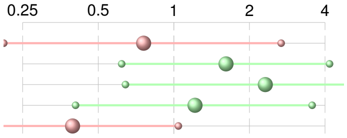

A	B	2.5	50	97.5	Sequence
1	0	0.21	0.76	2.7	KYNVGIK
1	2	0.62	1.6	4.2	TIEAEAAHGTVTR
0	1	0.64	2.3	8.4	GKLDGNKDLQR
0	2	0.41	1.2	3.6	SWPLYMSTK
0	3	0.15	0.39	1	LIDDMVAQALK

5.642 3 434 minus strand hypothetical protein CGI_10015501 [Crassostrea gigas]

Protein Accession	Unigene6481$_H1$
Mean Expression Ratio	0.971
Median Expression Ratio	0.97
Credible Interval	(0.48, 1.93)
Associated Peptides	1
Associated Spectra	1
Coverage	NaN

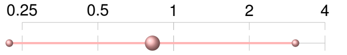

A	B	2.5	50	97.5	Sequence
1	0	0.22	0.82	3	NDFIGGLDVMK

5.643 3 263 minus strand beta-actin [Urechis unicinctus]

Protein Accession	CL31420.Contig5$_H$1
Mean Expression Ratio	0.972
Median Expression Ratio	0.97
Credible Interval	(0.539, 1.75)
Associated Peptides	4
Associated Spectra	13
Coverage	NaN

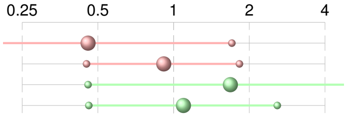

A	B	2.5	50	97.5	Sequence
1	0	0.13	0.46	1.7	EITALAPPTMK
0	7	0.45	0.91	1.8	DLYANTVLSGGSTMYPGIADR
0	1	0.46	1.7	6.2	MQKEITALAPPTMK
0	4	0.46	1.1	2.6	KDLYANTVLSGGSTMYPGIADR

5.644 3 416 minus strand myosin essential light chain [Crassostrea gigas]

Protein Accession	Unigene47974$_H$1
Mean Expression Ratio	1.03
Median Expression Ratio	1.03
Credible Interval	(0.528, 2.05)
Associated Peptides	1
Associated Spectra	2
Coverage	NaN

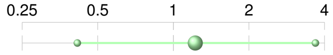

A	B	2.5	50	97.5	Sequence
0	2	0.41	1.2	3.7	CVGLNPTLATIK

5.645 11 1462 coronin [Schistosoma mansoni]

Protein Accession	CL11779.Contig2$_H$1
Mean Expression Ratio	1.03
Median Expression Ratio	1.03
Credible Interval	(0.524, 1.99)
Associated Peptides	2
Associated Spectra	2
Coverage	NaN

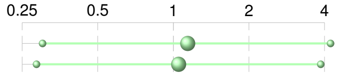

A	B	2.5	50	97.5	Sequence
0	1	0.3	1.1	4.2	VPMDAPVVAGHK
0	1	0.29	1.1	3.9	HVFGKPEK

5.646 51 1295 PREDICTED: phosphoglycerate kinase-like [Nasonia vitripennis]; 97 1341 PREDICTED: phosphoglycerate kinase-like [Nasonia vitripennis]

Protein Accession	CL14417.Contig13$_{H}1$ CL14417.Contig12$_{H}1$
Mean Expression Ratio	0.972
Median Expression Ratio	0.971
Credible Interval	(0.659, 1.45)
Associated Peptides	21
Associated Spectra	58
Coverage	NaN

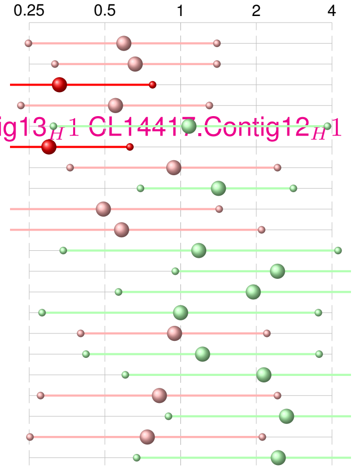

A	B	2.5	50	97.5	Sequence
2	2	0.25	0.59	1.4	ATQEDIDKFR
2	4	0.32	0.66	1.4	LSIDKVDVK
2	2	0.14	0.33	0.77	VDFNVPLKDGK
3	1	0.23	0.55	1.3	FTDAVK
1	0	0.31	1.1	3.8	VLNNMEIGK
1	5	0.14	0.3	0.63	ALMDAVVEATK
1	2	0.36	0.94	2.4	FHVEEEGK
2	5	0.69	1.4	2.8	VLPGVAALTDA
1	1	0.17	0.49	1.4	YALENGAK
0	1	0.16	0.58	2.1	AHSSMVGCQLPIR
0	1	0.34	1.2	4.2	ASGFLLKK
0	3	0.95	2.4	6.3	FHVEEEGKGVAPDGTK
0	1	0.56	1.9	6.9	FHVEEEGKGVAPDGTKLK
0	1	0.28	1	3.5	LGDIYVNDAFGTAHR
0	4	0.4	0.95	2.2	SGIPADWMGLDIGPK
0	2	0.42	1.2	3.5	SVVLMSHLGRPDGTK
0	1	0.6	2.1	7.7	VDFNVPLKDGKITNNQR
0	2	0.28	0.83	2.4	ASGFLLK
0	2	0.89	2.6	7.7	FTDAVKR
0	2	0.25	0.74	2.1	IVAALPTIK
0	1	0.67	2.4	9	LNKLSIDK

5.647 40 609 minus strand ependymin related protein-1 precursor [Haliotis discus discus]

Protein Accession	CL31954.Contig1$_{H}1$
Mean Expression Ratio	0.972
Median Expression Ratio	0.971
Credible Interval	(0.492, 1.91)
Associated Peptides	2
Associated Spectra	2
Coverage	NaN

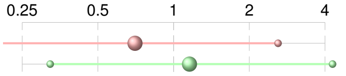

A	B	2.5	50	97.5	Sequence
0	1	0.19	0.7	2.6	EIEEIQIGSDQEFYDVLR
0	1	0.32	1.2	4.3	QQPKPCTSPPQFVAR

5.648 642 1415 minus strand cytochrome c oxidase subunit II [Oncomelania hupensis hupensis]

Protein Accession	CL6764.Contig3$_{H}1$
Mean Expression Ratio	0.968
Median Expression Ratio	0.971
Credible Interval	(0.493, 1.9)
Associated Peptides	1
Associated Spectra	5
Coverage	NaN

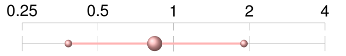

A	B	2.5	50	97.5	Sequence
0	5	0.38	0.84	1.9	IVLPTHTDIR

5.649 425 1051 minus strand unknown [Schistosoma japonicum]

Protein Accession	CL11292.Contig9$_{H}1$
Mean Expression Ratio	1.03
Median Expression Ratio	1.03
Credible Interval	(0.526, 2.04)
Associated Peptides	1
Associated Spectra	2
Coverage	NaN

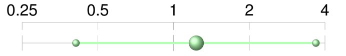

A	B	2.5	50	97.5	Sequence
0	2	0.41	1.2	3.7	AGNDVDLVVQR

5.650 91 3270 Valyl-tRNA synthetase [Crassostrea gigas]

Protein Accession	CL23212.Contig7$_{H}1$
Mean Expression Ratio	1.04
Median Expression Ratio	1.03
Credible Interval	(0.522, 2.08)
Associated Peptides	1
Associated Spectra	1
Coverage	NaN

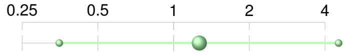

A	B	2.5	50	97.5	Sequence
0	1	0.35	1.3	4.5	SFGTGAVK

5.651 34 1122 Fructose-bisphosphate aldolase [Crassostrea gigas]

Protein Accession	CL24228.Contig1$_{H}1$
Mean Expression Ratio	0.975
Median Expression Ratio	0.972
Credible Interval	(0.659, 1.45)
Associated Peptides	21
Associated Spectra	65
Coverage	NaN

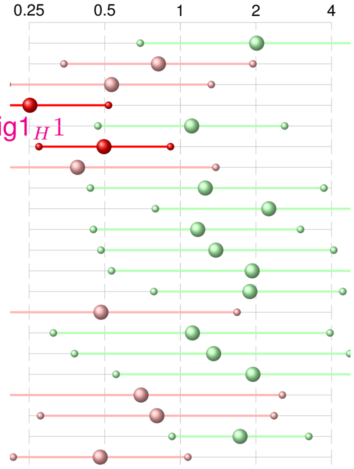

A	B	2.5	50	97.5	Sequence
1	1	0.69	2	5.9	AKANSEAALGK
2	2	0.34	0.82	1.9	GAQDELLKR
1	2	0.2	0.53	1.3	GIIPGIK
6	0	0.12	0.25	0.52	GILAADESVGSMGK
1	3	0.47	1.1	2.6	RLEGIGLENTEENR
7	3	0.27	0.5	0.91	ANSEAALGK
1	0	0.11	0.39	1.4	DSQGTPFVK
0	2	0.44	1.3	3.7	ALQASVLAAWKGDDAK
0	2	0.8	2.3	6.6	ALQASVLAAWKGDDAKVK
0	3	0.45	1.2	3	RLEGIGLENTEENRR
0	2	0.48	1.4	4.1	RLEGIGLENTEENRRR
0	1	0.53	1.9	6.9	VKGAQDELLKR
0	4	0.78	1.9	4.5	QIANSIVAPGK
0	1	0.14	0.48	1.7	KDGAQFAK
0	1	0.31	1.1	3.9	GIIPGIKVDK
0	1	0.38	1.4	4.7	GKNPWAVTF
0	1	0.55	1.9	7	PVIAQYLTKQQEDELR
0	1	0.2	0.7	2.5	VKGAQDELLK
0	2	0.28	0.81	2.4	ALQASVLAAWK
0	9	0.93	1.7	3.2	PVIAQYLTK
0	5	0.22	0.48	1.1	VTEQVLAFTYK

5.652 " 57 1280 RNA binding motif protein, X-linked [Mustela putorius furo]"

Protein Accession	CL2752.Contig1$_{H}$1
Mean Expression Ratio	1.02
Median Expression Ratio	1.03
Credible Interval	(0.507, 2.06)
Associated Peptides	1
Associated Spectra	2
Coverage	NaN

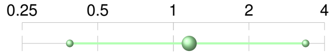

A	B	2.5	50	97.5	Sequence
0	2	0.39	1.2	3.4	LMDGKEVHGK

5.653 320 589 60S ribosomal protein L34 OS=Aedes triseriatus GN=RpL34 PE=2 SV=3

Protein Accession	CL29774.Contig2$_{H}$1
Mean Expression Ratio	0.973
Median Expression Ratio	0.973
Credible Interval	(0.501, 1.91)
Associated Peptides	1
Associated Spectra	3
Coverage	NaN

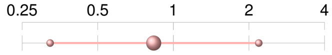

A	B	2.5	50	97.5	Sequence
2	1	0.32	0.84	2.2	AFLIEEQK

5.654 293 1795 Amidase [Crassostrea gigas]

Protein Accession	CL5248.Contig6$_{H}$1
Mean Expression Ratio	0.973
Median Expression Ratio	0.973
Credible Interval	(0.528, 1.79)
Associated Peptides	4
Associated Spectra	6
Coverage	NaN

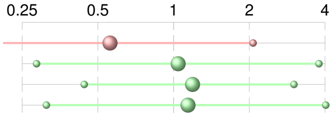

A	B	2.5	50	97.5	Sequence
1	0	0.15	0.56	2.1	EVSVPMHK
0	1	0.28	1	3.8	ILDAGGHIMGK
0	3	0.44	1.2	3	LSTIGVTHFDR
0	1	0.31	1.1	4	YAHPQDLHPMIK

5.655 794 994 Glycogenin-1 [Crassostrea gigas]; 1425 1739 minus strand Glycogenin-1 [Crassostrea gigas]

Protein Accession	CL18608.Contig3$_{H}$1 CL18608.Contig2$_{H}$1
Mean Expression Ratio	0.973
Median Expression Ratio	0.973
Credible Interval	(0.488, 1.95)
Associated Peptides	1
Associated Spectra	1
Coverage	NaN

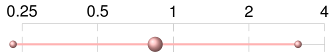

A	B	2.5	50	97.5	Sequence
0	1	0.23	0.85	3.1	IAAPVLSFK

5.656 13 585 UDP-glucose:glycoprotein glucosyltransferase 1 [Crassostrea gigas]

Protein Accession	CL48318.Contig1$_H$1
Mean Expression Ratio	1.03
Median Expression Ratio	1.03
Credible Interval	(0.514, 2.07)
Associated Peptides	1
Associated Spectra	1
Coverage	NaN

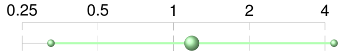

A	B	2.5	50	97.5	Sequence
0	1	0.32	1.2	4.3	FLDLPHK

5.657 1215 1694 Pikachurin [Crassostrea gigas]

Protein Accession	CL4889.Contig4$_H$1
Mean Expression Ratio	0.972
Median Expression Ratio	0.973
Credible Interval	(0.49, 1.91)
Associated Peptides	1
Associated Spectra	2
Coverage	NaN

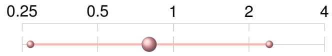

A	B	2.5	50	97.5	Sequence
0	2	0.27	0.8	2.4	HGLGVNELPIASPK

5.658 113 1402 minus strand S-adenosylhomocysteine hydrolase [Crassostrea ariakensis]; 113 1402 minus strand S-adenosylhomocysteine hydrolase [Crassostrea ariakensis]

Protein Accession	CL7405.Contig2$_H$1 CL7405.Contig1$_H$1
Mean Expression Ratio	1.03
Median Expression Ratio	1.03
Credible Interval	(0.584, 1.81)
Associated Peptides	8
Associated Spectra	8
Coverage	NaN

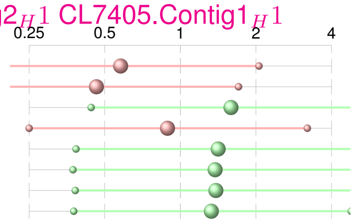

A	B	2.5	50	97.5	Sequence
1	0	0.16	0.58	2.1	VAVVAGYGDVGK
1	0	0.13	0.46	1.7	TGVPVYAWK
0	1	0.44	1.6	5.7	GLSEETTTGVHNLYR
0	1	0.25	0.89	3.2	HIIVLAEGR
0	1	0.39	1.4	5	LKVPAINVNDSVTK
0	1	0.37	1.4	5	VADISLAEMGR
0	1	0.38	1.4	5.1	LTKLDEDQAK
0	1	0.38	1.3	4.8	YLGIPQEGPFKPEHYR

5.659 131 544 hypothetical protein CGI_10001749 [Crassostrea gigas]

Protein Accession	CL36022.Contig1$_H$1
Mean Expression Ratio	0.975
Median Expression Ratio	0.974
Credible Interval	(0.486, 1.94)
Associated Peptides	1
Associated Spectra	1
Coverage	NaN

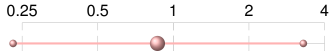

A	B	2.5	50	97.5	Sequence
0	1	0.23	0.86	3.3	LIVADGTNHR

5.660 18 242 minus strand translation elongation factor 1B gamma subunit [Strongylocentrotus purpuratus] gi

Protein Accession	CL14269.Contig2$_{H}1$
Mean Expression Ratio	1.03
Median Expression Ratio	1.03
Credible Interval	(0.521, 2.08)
Associated Peptides	1
Associated Spectra	1
Coverage	NaN

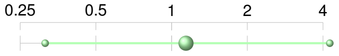

A	B	2.5	50	97.5	Sequence
**1**	**0**	**0.32**	**1.1**	**4.2**	**ALIAAK**

5.661 140 1564 selenium-binding protein [Haliotis discus hannai]

Protein Accession	CL3868.Contig7$_{H}1$
Mean Expression Ratio	0.975
Median Expression Ratio	0.974
Credible Interval	(0.483, 1.93)
Associated Peptides	1
Associated Spectra	1
Coverage	NaN

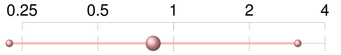

A	B	2.5	50	97.5	Sequence
**0**	**1**	**0.22**	**0.83**	**3.1**	**VVEAVDIHR**

5.662 100 834 vesicle-associated membrane protein/synaptobrevin-binding protein [Aplysia californica] gi

Protein Accession	CL36367.Contig1$_{H}1$
Mean Expression Ratio	1.03
Median Expression Ratio	1.03
Credible Interval	(0.527, 2.01)
Associated Peptides	2
Associated Spectra	2
Coverage	NaN

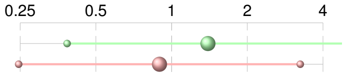

A	B	2.5	50	97.5	Sequence
**0**	**1**	**0.38**	**1.4**	**5.3**	**FKGPFTDVVTAELK**
**0**	**1**	**0.25**	**0.89**	**3.2**	**TEQALQLEPANELR**

5.663 32 193 minus strand Ubiquinol-cytochrome C reductase complex [Xenopus laevis] gi; 32 193 minus strand Ubiquinol-cytochrome C reductase complex [Xenopus laevis] gi

Protein Accession	CL7847.Contig7$_{H}1$ CL7847.Contig4$_{H}1$
Mean Expression Ratio	1.03
Median Expression Ratio	1.03
Credible Interval	(0.541, 1.98)
Associated Peptides	2
Associated Spectra	8
Coverage	NaN

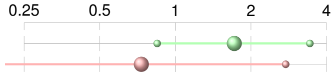

A	B	2.5	50	97.5	Sequence
**3**	**4**	**0.85**	**1.7**	**3.4**	**AFLKPQAPK**
**1**	**0**	**0.2**	**0.73**	**2.8**	**AGPSIFR**

5.664 186 1778 predicted protein [Nematostella vectensis] gi

Protein Accession	CL20488.Contig9$_{H}1$
Mean Expression Ratio	1.03
Median Expression Ratio	1.03
Credible Interval	(0.609, 1.74)
Associated Peptides	8
Associated Spectra	17
Coverage	NaN

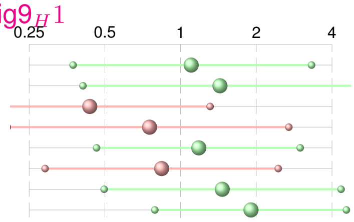

A	B	2.5	50	97.5	Sequence
1	1	0.37	1.1	3.3	ATNLENKDGLLGK
1	0	0.41	1.4	5.1	DGTTHVIWK
2	0	0.15	0.44	1.3	FAQQAQQQEK
1	0	0.21	0.75	2.7	TKDGEVSWPVIDPK
1	2	0.46	1.2	3	SDPYLQISSK
1	1	0.29	0.84	2.5	EGTENVEMHFR
0	2	0.5	1.5	4.4	AKDGTTHVIWK
0	4	0.79	1.9	4.5	AEELKEGTENVEMHFR

5.665 115 393 minus strand Yolk ferritin OS=Lymnaea stagnalis PE=1 SV=1; 132 413 RecName: Full=Yolk ferritin; Flags: Precursor gi|9655|emb|CAA40097.1| snail yolk ferritin [Lymnaea stagnalis] gi; 2 430 RecName: Full=Yolk ferritin; Flags: Precursor gi|9655|emb|CAA40097.1| snail yolk ferritin [Lymnaea stagnalis] gi; 3 389 RecName: Full=Yolk ferritin; Flags: Precursor gi|9655|emb|CAA40097.1| snail yolk ferritin [Lymnaea stagnalis] gi

Protein Accession	CL33796.Contig4$_{H}1$ CL33796.Contig3$_{H}1$ CL33796.Contig2$_{H}1$ CL33796.Contig1$_{H}1$
Mean Expression Ratio	0.979
Median Expression Ratio	0.974
Credible Interval	(0.483, 1.96)
Associated Peptides	1
Associated Spectra	1
Coverage	NaN

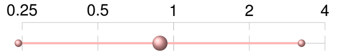

A	B	2.5	50	97.5	Sequence
0	1	0.24	0.88	3.2	QYGLQAMEDALALER

5.666 18 1613 minus strand chaperonin containing T-complex polypeptide subunit zeta [Haliotis discus hannai]; 527 2089 minus strand chaperonin containing T-complex polypeptide subunit zeta [Haliotis discus hannai]; 18 1586 minus strand chaperonin containing T-complex polypeptide subunit zeta [Haliotis discus hannai]

Protein Accession	CL23722.Contig7$_{H}1$ CL23722.Contig5$_{H}1$ CL23722.Contig4$_{H}1$
Mean Expression Ratio	1.03
Median Expression Ratio	1.03
Credible Interval	(0.561, 1.89)
Associated Peptides	4
Associated Spectra	5
Coverage	NaN

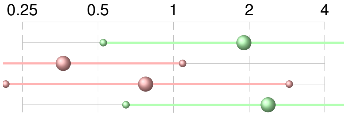

A	B	2.5	50	97.5	Sequence
0	1	0.52	1.9	6.8	AAQALAVNISAAK
0	2	0.12	0.36	1.1	GIDPFSLDMFAK
0	1	0.22	0.77	2.9	TDMDSTLVR
0	1	0.65	2.4	8.5	AQALAVNISAAK

5.667 129 1628 beta-hexosaminidase subunit beta precursor [Danio rerio] gi

Protein Accession	CL21114.Contig2$_{H}1$
Mean Expression Ratio	0.974
Median Expression Ratio	0.975
Credible Interval	(0.498, 1.89)
Associated Peptides	2
Associated Spectra	2
Coverage	NaN

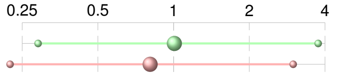

A	B	2.5	50	97.5	Sequence
0	1	0.29	1	3.8	EYQTNGNVNTIDAASFR
0	1	0.22	0.81	3	LWSPQDVNFPQIAEPR

5.668 113 367 hypothetical protein DAPPUDRAFT_329128 [Daphnia pulex]; 113 367 hypothetical protein DAPPUDRAFT_329128 [Daphnia pulex]; 113 367 hypothetical protein DAPPUDRAFT_329128 [Daphnia pulex]

Protein Accession	CL9011.Contig3$_{H}1$ CL9011.Contig2$_{H}1$ CL9011.Contig1$_{H}1$
Mean Expression Ratio	1.03
Median Expression Ratio	1.03
Credible Interval	(0.536, 2)
Associated Peptides	2
Associated Spectra	4
Coverage	NaN

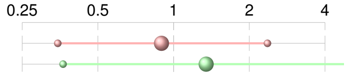

A	B	2.5	50	97.5	Sequence
2	1	0.35	0.89	2.4	KPMVSGAIIK
0	1	0.36	1.3	5.1	ILAIEDYIR

5.669 98 850 minus strand 14-3-3 protein epsilon [Schistocerca gregaria]

Protein Accession	CL27652.Contig3$_{H}1$
Mean Expression Ratio	1.03
Median Expression Ratio	1.02
Credible Interval	(0.593, 1.79)
Associated Peptides	7
Associated Spectra	15
Coverage	NaN

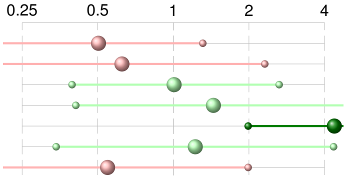

A	B	2.5	50	97.5	Sequence
2	1	0.19	0.5	1.3	EAAENSLVAYK
1	0	0.17	0.62	2.3	IISSMEQK
0	3	0.4	1	2.6	AASDISMTELPPTHPIR
0	1	0.41	1.4	5.3	LAEQAERYDEMVEAMK
0	5	2	4.4	9.8	LAEQAERYDEMVEAMKK
0	1	0.34	1.2	4.4	VFYYK
0	1	0.15	0.55	2	YDEMVEAMK

5.670 3 281 minus strand hypothetical protein CGI_10015501 [Crassostrea gigas]

Protein Accession	Unigene26367$_{H}1$
Mean Expression Ratio	0.974
Median Expression Ratio	0.976
Credible Interval	(0.482, 1.95)
Associated Peptides	1
Associated Spectra	1
Coverage	NaN

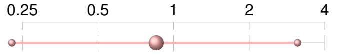

A	B	2.5	50	97.5	Sequence
0	1	0.23	0.85	3.1	AGNDITADQVIDDVK

5.671 28 1455 minus strand PREDICTED: probable serine carboxypeptidase CPVL-like [Anolis carolinensis]

Protein Accession	CL34087.Contig4$_H$1
Mean Expression Ratio	0.974
Median Expression Ratio	0.976
Credible Interval	(0.518, 1.83)
Associated Peptides	3
Associated Spectra	5
Coverage	NaN

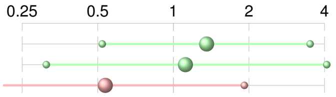

A	B	2.5	50	97.5	Sequence
1	2	0.52	1.4	3.5	YVPAISYK
0	1	0.31	1.1	4.1	GMAIGDGLCDPVTMMPK
0	1	0.15	0.53	1.9	VNPDDVEVAGWVR

5.672 10 300 minus strand unnamed protein product [Tetraodon nigroviridis]

Protein Accession	CL14559.Contig2$_H$1
Mean Expression Ratio	1.02
Median Expression Ratio	1.02
Credible Interval	(0.528, 2.01)
Associated Peptides	1
Associated Spectra	5
Coverage	NaN

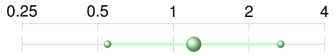

A	B	2.5	50	97.5	Sequence
0	5	0.55	1.2	2.7	ILVQEGIHDR

5.673 " 134 2047 PREDICTED: succinate dehydrogenase [ubiquinone] flavoprotein subunit, mitochondrial-like isoform 3, partial [Strongylocentrotus purpuratus]; 134 2047 PREDICTED: succinate dehydrogenase [ubiquinone] flavoprotein subunit, mitochondrial-like isoform 3, partial [Strongylocentrotus purpuratus]; 134 2047 PREDICTED: succinate dehydrogenase [ubiquinone] flavoprotein subunit, mitochondrial-like isoform 3, partial [Strongylocentrotus purpuratus]"

Protein Accession	CL47322.Contig4$_H$1 CL47322.Contig3$_H$1 CL47322.Contig2$_H$1
Mean Expression Ratio	1.03
Median Expression Ratio	1.02
Credible Interval	(0.645, 1.64)
Associated Peptides	14
Associated Spectra	30
Coverage	NaN

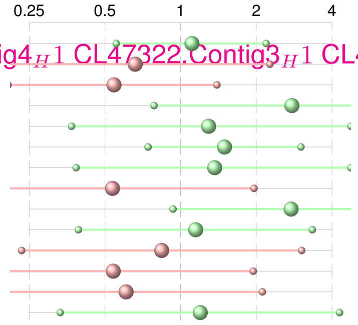

A	B	2.5	50	97.5	Sequence
1	6	0.56	1.1	2.2	AAFGLAEEGFK
1	0	0.18	0.66	2.3	DGPTLQAGVR
2	1	0.21	0.54	1.4	AFGGQSLK
1	0	0.79	2.8	10	AFGGQSLKYGK
1	0	0.37	1.3	4.8	ACANTIIEENKPGDR
4	3	0.74	1.5	3	AITIEIR
0	1	0.38	1.4	4.8	AVIELENYGMPFSR
0	1	0.15	0.54	2	IYQQMEDLK
0	2	0.94	2.8	7.9	KHTLAYTNVDTGK
0	2	0.39	1.1	3.3	NTILATGGYGR
0	1	0.23	0.84	3	QDAGEASVGNLDK
0	1	0.15	0.54	1.9	WHFYDTVK
0	1	0.17	0.61	2.1	HTLAYTNVDTGK
0	1	0.33	1.2	4.3	TTLDYRPVIDTTLDEHDCPQVKPAIR

5.674 696 4010 Lethal(2) giant larvae-like protein 1 [Crassostrea gigas]; 657 3971 minus strand Lethal(2) giant larvae-like protein 1 [Crassostrea gigas]; 696 3749 minus strand Lethal(2) giant larvae-like protein 1 [Crassostrea gigas]; 156 3338 Lethal(2) giant larvae-like protein 1 [Crassostrea gigas]; 156 3209 Lethal(2) giant larvae-like protein 1 [Crassostrea gigas]; 156 3470 Lethal(2) giant larvae-like protein 1 [Crassostrea gigas]

Protein Accession	CL3717.Contig4$_H$1 CL3717.Contig3$_H$1 CL3717.Contig2$_H$1 CL3717.Contig19$_H$1 CL3717.Co
Mean Expression Ratio	1.03
Median Expression Ratio	1.02
Credible Interval	(0.533, 1.99)
Associated Peptides	2
Associated Spectra	2
Coverage	NaN

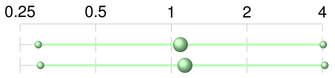

A	B	2.5	50	97.5	Sequence
0	1	0.29	1.1	4	VLPESLEVIHER
0	1	0.3	1.1	4.1	NLAKEPTTK

5.675 59 379 hypothetical protein DAPPUDRAFT_230756 [Daphnia pulex]; 117 443 minus strand hypothetical protein DAPPUDRAFT_230756 [Daphnia pulex]; 120 473 minus strand hypothetical protein DAPPUDRAFT_230756 [Daphnia pulex]

Protein Accession	CL2137.Contig6$_H$1 CL2137.Contig5$_H$1 CL2137.Contig4$_H$1
Mean Expression Ratio	1.03
Median Expression Ratio	1.02
Credible Interval	(0.542, 1.95)
Associated Peptides	2
Associated Spectra	5
Coverage	NaN

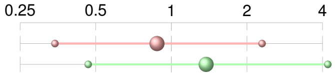

A	B	2.5	50	97.5	Sequence
1	2	0.34	0.87	2.3	AASTLAVK
0	2	0.47	1.4	4.2	LVNSLTASGTGALK

5.676 63 1865 Dolichyl-diphosphooligosaccharide–protein glycosyltransferase subunit 1 [Crassostrea gigas]; 63 1865 Dolichyl-diphosphooligosaccharide–protein glycosyltransferase subunit 1 [Crassostrea gigas]

Protein Accession	CL41703.Contig2$_H$1 CL41703.Contig1$_H$1
Mean Expression Ratio	0.975
Median Expression Ratio	0.976
Credible Interval	(0.626, 1.51)
Associated Peptides	18
Associated Spectra	29
Coverage	NaN

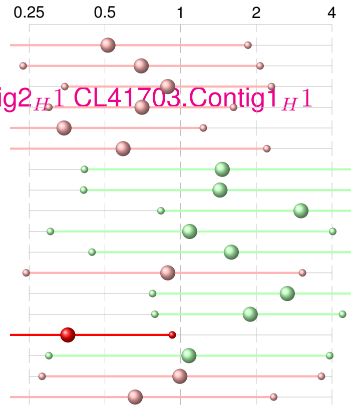

A	B	2.5	50	97.5	Sequence
1	0	0.14	0.52	1.9	APFSEAQLR
1	1	0.24	0.7	2.1	ELDDSVDIELRPR
1	2	0.35	0.89	2.3	QQYLDAEAGVTSK
2	2	0.3	0.7	1.6	VILPEGVQNIEVR
1	0	0.092	0.34	1.2	GAVLK
1	0	0.17	0.59	2.2	LVAGK
1	0	0.41	1.5	5.3	TPFEVKR
0	1	0.41	1.4	5.1	DLNAFQVAR
0	1	0.84	3	11	FKTSKDLNAFQVAR
0	1	0.3	1.1	4	HSGAVLKGPFSR
0	1	0.45	1.6	5.7	KVDIATHIVK
0	1	0.24	0.89	3.1	SALYQSYDDAINR
0	1	0.78	2.7	9.7	TSKDLNAFQVAR
0	4	0.79	1.9	4.4	VASLVEEVQSAQDKR
0	3	0.14	0.36	0.93	VQLPFNLDAGK
0	1	0.3	1.1	3.9	VVPTTVPGHSDKELYR
0	1	0.28	0.99	3.6	LPAAAR
0	1	0.19	0.66	2.3	LSFISAVTK

5.677 532 663 minus strand 40S ribosomal protein S23 [Ascaris suum]; 526 654 minus strand RecName: Full=40S ribosomal protein S23 gi|1754684|gb|AAC47632.1| ribosomal protein S23 [Brugia malayi]

Protein Accession	CL10408.Contig3$_H$1 CL10408.Contig2$_H$1
Mean Expression Ratio	1.02
Median Expression Ratio	1.02
Credible Interval	(0.516, 2.05)
Associated Peptides	1
Associated Spectra	2
Coverage	NaN

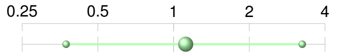

A	B	2.5	50	97.5	Sequence
0	2	0.37	1.1	3.3	KGHAVGDIPGVR

5.678 72 521 minus strand GF14593 [Drosophila ananassae] gi; 60 392 GD23124 [Drosophila simulans] gi

Protein Accession	CL38325.Contig2$_H$1 CL38325.Contig1$_H$1
Mean Expression Ratio	1.02
Median Expression Ratio	1.02
Credible Interval	(0.509, 2.03)
Associated Peptides	1
Associated Spectra	1
Coverage	NaN

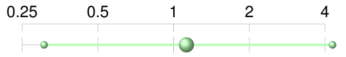

A	B	2.5	50	97.5	Sequence
0	1	0.3	1.1	4.3	LTADVHGK

5.679 494 724 minus strand PREDICTED: SH3 domain-binding glutamic acid-rich-like protein 3-like [Amphimedon queenslandica]; 424 819 minus strand PREDICTED: SH3 domain-binding glutamic acid-rich-like protein 3-like [Macaca mulatta]; 236 631 minus strand PREDICTED: SH3 domain-binding glutamic acid-rich-like protein 3-like [Macaca mulatta]

Protein Accession	CL49318.Contig4$_{H}1$ CL49318.Contig3$_{H}1$ CL49318.Contig2$_{H}1$
Mean Expression Ratio	0.981
Median Expression Ratio	0.978
Credible Interval	(0.499, 1.92)
Associated Peptides	1
Associated Spectra	1
Coverage	NaN

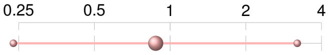

A	B	2.5	50	97.5	Sequence
**0**	**1**	**0.24**	**0.88**	**3.2**	**GDEYLGDYER**

5.680 1021 1359 minus strand RecName: Full=40S ribosomal protein S26 gi|6015599|emb|CAB57819.1| ribosomal protein S26 [Octopus vulgaris]

Protein Accession	Unigene11569$_{H}1$
Mean Expression Ratio	0.98
Median Expression Ratio	0.978
Credible Interval	(0.488, 1.95)
Associated Peptides	1
Associated Spectra	1
Coverage	NaN

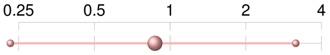

A	B	2.5	50	97.5	Sequence
**1**	**0**	**0.23**	**0.87**	**3.2**	**NIVEAAAVR**

5.681 258 680 minus strand synapse-associated protein [Aplysia californica] gi

Protein Accession	CL37287.Contig1$_{H}1$
Mean Expression Ratio	0.974
Median Expression Ratio	0.978
Credible Interval	(0.491, 1.92)
Associated Peptides	1
Associated Spectra	1
Coverage	NaN

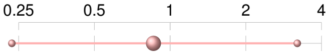

A	B	2.5	50	97.5	Sequence
**0**	**1**	**0.23**	**0.86**	**3.2**	**ALELLEDYHTR**

5.682 89 361 40S ribosomal protein S18 OS=Rattus norvegicus GN=Rps18 PE=1 SV=3

Protein Accession	CL28311.Contig1$_{H}$1
Mean Expression Ratio	0.979
Median Expression Ratio	0.978
Credible Interval	(0.507, 1.93)
Associated Peptides	2
Associated Spectra	3
Coverage	NaN

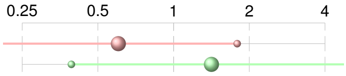

A	B	2.5	50	97.5	Sequence
0	2	0.2	0.6	1.8	AGELTEDEVER
0	1	0.39	1.4	5.1	LNRQKDVK

5.683 95 1579 minus strand hypothetical protein BRAFLDRAFT_113974 [Branchiostoma floridae] gi

Protein Accession	CL19753.Contig5$_{H}$1
Mean Expression Ratio	1.02
Median Expression Ratio	1.02
Credible Interval	(0.603, 1.75)
Associated Peptides	9
Associated Spectra	14
Coverage	NaN

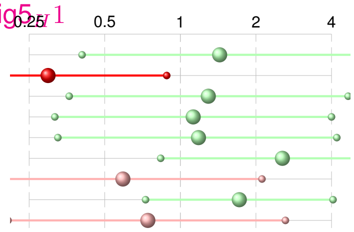

A	B	2.5	50	97.5	Sequence
1	0	0.41	1.4	5.3	TFPVVNPATEK
1	1	0.1	0.3	0.88	ELGEYALK
0	1	0.36	1.3	4.7	FKDINEVIER
0	1	0.32	1.1	4	TSYGLAAGVITK
0	1	0.32	1.2	4.2	TVGDPFDAVTMQGPQVDQEQFEK
0	2	0.84	2.5	7.5	TVTIKLPSK
0	1	0.17	0.59	2.1	ADLRPDPNIK
0	4	0.73	1.7	4.1	DINEVIERANR
0	1	0.21	0.74	2.6	DEIFGPVQSIIK

5.684 232 1401 RecName: Full=Guanine nucleotide-binding protein G(s) subunit alpha; AltName: Full=Adenylate cyclase-stimulating G alpha protein gi|9635|emb|CAA78808.1| GTP-binding protein alpha-s subunit [Lymnaea stagnalis]; 232 1356 RecName: Full=Guanine nucleotide-binding protein G(s) subunit alpha; AltName: Full=Adenylate cyclase-stimulating G alpha protein gi|9635|emb|CAA78808.1| GTP-binding protein alpha-s subunit [Lymnaea stagnalis]

Protein Accession	CL8653.Contig3$_{H}$1 CL8653.Contig1$_{H}$1
Mean Expression Ratio	1.02
Median Expression Ratio	1.02
Credible Interval	(0.536, 1.9)
Associated Peptides	3
Associated Spectra	5
Coverage	NaN

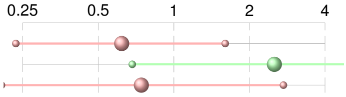

A	B	2.5	50	97.5	Sequence
0	3	0.24	0.62	1.6	LLLLGAGESGK
0	1	0.68	2.5	9.1	QKIEDIK
0	1	0.21	0.74	2.7	EQDILR

5.685 " 1 1887 Pyruvate carboxylase, mitochondrial [Crassostrea gigas]"

Protein Accession	CL26073.Contig5$_{H}1$
Mean Expression Ratio	0.98
Median Expression Ratio	0.979
Credible Interval	(0.512, 1.87)
Associated Peptides	3
Associated Spectra	4
Coverage	NaN

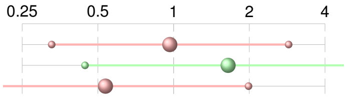

A	B	2.5	50	97.5	Sequence
**0**	**2**	**0.33**	**0.97**	**2.9**	**HLEDVEENFKR**
**0**	**1**	**0.45**	**1.6**	**6.1**	**AGTHILGIK**
**0**	**1**	**0.14**	**0.53**	**2**	**IEVFR**

5.686 104 2638 Eukaryotic translation initiation factor 3 subunit C [Crassostrea gigas]

Protein Accession	CL904.Contig3$_{H}1$
Mean Expression Ratio	1.02
Median Expression Ratio	1.02
Credible Interval	(0.532, 2.01)
Associated Peptides	1
Associated Spectra	3
Coverage	NaN

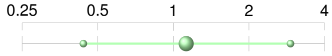

A	B	2.5	50	97.5	Sequence
**2**	**1**	**0.44**	**1.1**	**2.9**	**GAPLIVEKPK**

5.687 6 581 PREDICTED: mammalian ependymin-related protein 1 [Gallus gallus]

Protein Accession	CL15402.Contig1$_{H}1$
Mean Expression Ratio	0.978
Median Expression Ratio	0.979
Credible Interval	(0.506, 1.88)
Associated Peptides	2
Associated Spectra	3
Coverage	NaN

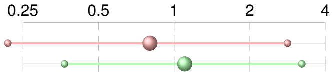

A	B	2.5	50	97.5	Sequence
**0**	**1**	**0.22**	**0.8**	**2.8**	**ALFEEQNVITPGR**
**0**	**2**	**0.37**	**1.1**	**3.2**	**WYISYDALNK**

5.688 114 731 minus strand Proteasome subunit beta type-6 [Crassostrea gigas]

Protein Accession	CL12961.Contig1$_{H}1$
Mean Expression Ratio	1.02
Median Expression Ratio	1.02
Credible Interval	(0.51, 2.01)
Associated Peptides	1
Associated Spectra	2
Coverage	NaN

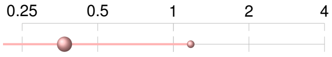

A	B	2.5	50	97.5	Sequence
**1**	**1**	**0.11**	**0.37**	**1.2**	**FTLTGDDLPK**

5.689 154 261 ribosomal protein S18 [Stenella coeruleoalba]

Protein Accession	CL15120.Contig1$_H$1
Mean Expression Ratio	0.979
Median Expression Ratio	0.979
Credible Interval	(0.506, 1.89)
Associated Peptides	2
Associated Spectra	4
Coverage	NaN

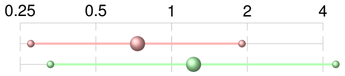

A	B	2.5	50	97.5	Sequence
**1**	**2**	**0.28**	**0.73**	**1.9**	**VLNTNIDGR**
**0**	**1**	**0.33**	**1.2**	**4.5**	**VLNTNIDGRR**

5.690 " 1 327 minus strand endo-beta-1,4-glucanase [Ampullaria crossean]"

Protein Accession	CL17318.Contig3$_H$1
Mean Expression Ratio	1.02
Median Expression Ratio	1.02
Credible Interval	(0.51, 2.03)
Associated Peptides	1
Associated Spectra	1
Coverage	NaN

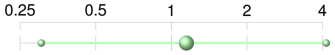

A	B	2.5	50	97.5	Sequence
**0**	**1**	**0.3**	**1.1**	**4.1**	**CHMDGGVLK**

5.691 971 1963 Spectrin alpha chain [Crassostrea gigas]; 972 1979 Spectrin alpha chain [Crassostrea gigas]

Protein Accession	CL2195.Contig6$_H$1 CL2195.Contig4$_H$1
Mean Expression Ratio	1.02
Median Expression Ratio	1.02
Credible Interval	(0.501, 2.01)
Associated Peptides	1
Associated Spectra	1
Coverage	NaN

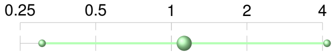

A	B	2.5	50	97.5	Sequence
**0**	**1**	**0.31**	**1.1**	**4.2**	**EAFLAGDTVDGDNVETLIK**

5.692 151 471 minus strand ribosomal protein rps25 [Arenicola marina]

Protein Accession	CL17623.Contig4$_H$1
Mean Expression Ratio	0.978
Median Expression Ratio	0.979
Credible Interval	(0.512, 1.88)
Associated Peptides	2
Associated Spectra	4
Coverage	NaN

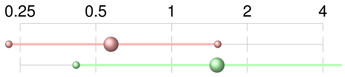

A	B	2.5	50	97.5	Sequence
**1**	**2**	**0.23**	**0.57**	**1.5**	**LITPSVVSER**
**0**	**1**	**0.42**	**1.5**	**5.5**	**MLKEVPSYK**

5.693 26 550 Translocon-associated protein subunit gamma [Crassostrea gigas]

Protein Accession	CL32873.Contig1$_{H}1$
Mean Expression Ratio	1.02
Median Expression Ratio	1.02
Credible Interval	(0.535, 2)
Associated Peptides	2
Associated Spectra	2
Coverage	NaN

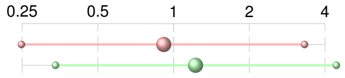

A	B	2.5	50	97.5	Sequence
**0**	**1**	**0.25**	**0.91**	**3.3**	**QLSREEELLLQDFSR**
**0**	**1**	**0.34**	**1.2**	**4.4**	**KLADDKK**

5.694 1 321 Malectin [Crassostrea gigas]

Protein Accession	Unigene56360$_{H}1$
Mean Expression Ratio	1.02
Median Expression Ratio	1.02
Credible Interval	(0.514, 2.01)
Associated Peptides	1
Associated Spectra	1
Coverage	NaN

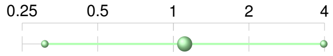

A	B	2.5	50	97.5	Sequence
**0**	**1**	**0.31**	**1.1**	**4**	**GVAHDEIVPFSIK**

5.695 356 820 minus strand calmodulin-1 [Trichinella spiralis] gi

Protein Accession	CL672.Contig3$_{H}1$
Mean Expression Ratio	0.98
Median Expression Ratio	0.98
Credible Interval	(0.513, 1.85)
Associated Peptides	3
Associated Spectra	8
Coverage	NaN

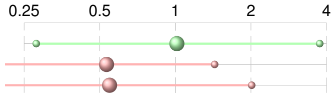

A	B	2.5	50	97.5	Sequence
**0**	**1**	**0.28**	**1**	**3.8**	**VFDKDGNGFISAAELR**
**5**	**1**	**0.2**	**0.53**	**1.4**	**ELGTVMR**
**0**	**1**	**0.15**	**0.55**	**2**	**ADQLTEEQIAEFK**

5.696 43 591 minus strand cyclophilin type peptidyl-prolyl cis-trans isomerase [Glossina morsitans morsitans]

Protein Accession	CL39270.Contig1$_{H}1$
Mean Expression Ratio	1.02
Median Expression Ratio	1.02
Credible Interval	(0.586, 1.77)
Associated Peptides	5
Associated Spectra	30
Coverage	NaN

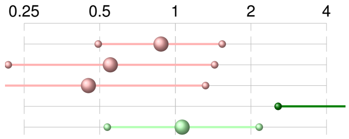

A	B	2.5	50	97.5	Sequence
**5**	**6**	**0.49**	**0.87**	**1.5**	**ITMELR**
**1**	**2**	**0.22**	**0.55**	**1.4**	**FADENFTLK**
**2**	**0**	**0.15**	**0.45**	**1.3**	**GFGYK**
**0**	**7**	**2.6**	**5.1**	**10**	**KIESYGSKNGATSQK**
**0**	**7**	**0.54**	**1.1**	**2.2**	**VFFDMTADGVPLGR**

5.697 " 460 1944 PREDICTED: succinyl-CoA:3-ketoacid-coenzyme A transferase 1, mitochondrial [Canis lupus familiaris]"

Protein Accession	Unigene4332$_{H}1$
Mean Expression Ratio	1.02
Median Expression Ratio	1.02
Credible Interval	(0.627, 1.67)
Associated Peptides	11
Associated Spectra	23
Coverage	NaN

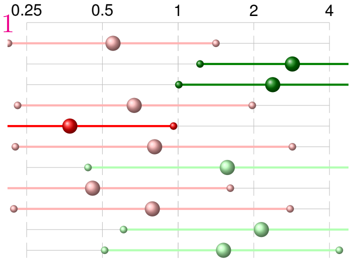

A	B	2.5	50	97.5	Sequence
1	2	0.21	0.55	1.4	EVAEGETAESIAK
1	3	1.2	2.9	6.8	ITGVEFR
2	2	1	2.4	5.7	NFNPPMAR
1	1	0.23	0.67	2	LGNLTFR
1	2	0.14	0.37	0.96	EVVNMIVTEK
1	0	0.23	0.81	2.8	VFLGK
0	1	0.44	1.6	5.7	ADKLGNLTFR
0	1	0.13	0.46	1.6	AVFDVDNK
0	1	0.22	0.79	2.8	VMGMGGAMDLVSADK
0	1	0.61	2.1	7.5	ILKEVTLPK
0	2	0.51	1.5	4.4	FNPDGTILHGSAGR

5.698 198 821 minus strand sigma class glutathione-s-transferase 2 [Haliotis discus discus]

Protein Accession	CL1352.Contig4$_{H}1$
Mean Expression Ratio	0.982
Median Expression Ratio	0.981
Credible Interval	(0.522, 1.85)
Associated Peptides	3
Associated Spectra	4
Coverage	NaN

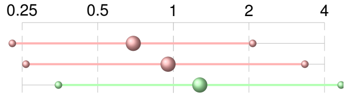

A	B	2.5	50	97.5	Sequence
1	1	0.23	0.69	2.1	ASWGPDCEAK
0	1	0.26	0.95	3.3	AAGQPFDDNVVDR
0	1	0.35	1.3	4.7	AAPYGQLPYVIYK

5.699 1 456 minus strand Putative sodium/potassium-transporting ATPase subunit beta-2 [Crassostrea gigas]; 688 1251 minus strand Putative sodium/potassium-transporting ATPase subunit beta-2 [Crassostrea gigas]

Protein Accession	CL42979.Contig3$_{H}1$ CL42979.Contig2$_{H}1$
Mean Expression Ratio	1.02
Median Expression Ratio	1.02
Credible Interval	(0.509, 2.05)
Associated Peptides	1
Associated Spectra	1
Coverage	NaN

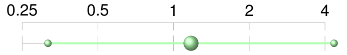

A	B	2.5	50	97.5	Sequence
0	1	0.32	1.2	4.3	NYMSPLVFVK

5.700 121 1452 minus strand Hexokinase type 2 [Crassostrea gigas]; 245 1576 minus strand Hexokinase type 2 [Crassostrea gigas]; 151 1455 minus strand Hexokinase type 2 [Crassostrea gigas]; 121 1452 minus strand Hexokinase type 2 [Crassostrea gigas]; 121 1452 minus strand Hexokinase type 2 [Crassostrea gigas]; 161 1513 minus strand Hexokinase type 2 [Crassostrea gigas]; 113 1471 minus strand Hexokinase type 2 [Crassostrea gigas]; 225 1577 minus strand Hexokinase type 2 [Crassostrea gigas]; 121 1452 minus strand Hexokinase type 2 [Crassostrea gigas]

Protein Accession	CL350.Contig9$_{H}$1 CL350.Contig8$_{H}$1 CL350.Contig7$_{H}$1 CL350.Contig6$_{H}$1 CL350.Contig5$_{H}$1
Mean Expression Ratio	1.02
Median Expression Ratio	1.02
Credible Interval	(0.505, 2.07)
Associated Peptides	1
Associated Spectra	1
Coverage	NaN

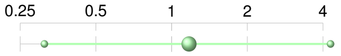

A	B	2.5	50	97.5	Sequence
1	0	0.31	1.2	4.3	MYPTYVR

5.701 " 59 2365 minus strand Aconitate hydratase, mitochondrial [Crassostrea gigas]"

Protein Accession	CL13509.Contig1$_{H}$1
Mean Expression Ratio	1.02
Median Expression Ratio	1.02
Credible Interval	(0.593, 1.73)
Associated Peptides	10
Associated Spectra	10
Coverage	NaN

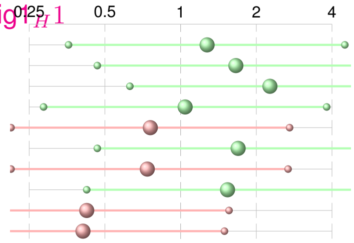

A	B	2.5	50	97.5	Sequence
0	1	0.36	1.3	4.5	HTMNEGQIEWFR
0	1	0.47	1.7	6	KGETNTIVTSYNR
0	1	0.63	2.3	8.1	SKFTITPGSEQIR
0	1	0.29	1	3.8	KNGWPLNIR
0	1	0.21	0.76	2.7	VAGILTVK
0	1	0.47	1.7	6.1	LGRPLTLSEK
0	1	0.21	0.74	2.7	MQDYLK
0	1	0.42	1.5	5.6	IHETNLKK
0	1	0.12	0.42	1.6	LDDPKNEK
0	1	0.11	0.41	1.5	WDGKDLVDMPVLIK

5.702 224 1291 minus strand coproporphirynogen oxidase [Aplysia californica] gi

Protein Accession	CL8705.Contig1$_{H}$1
Mean Expression Ratio	0.98
Median Expression Ratio	0.981
Credible Interval	(0.496, 1.92)
Associated Peptides	1
Associated Spectra	3
Coverage	NaN

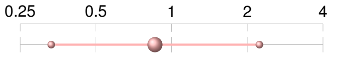

A	B	2.5	50	97.5	Sequence
0	3	0.33	0.86	2.2	NPHVPTMHFNYR

5.703 174 1556 minus strand aspartyl-tRNA synthetase [Xenopus laevis] gi; 174 1721 minus strand aspartyl-tRNA synthetase [Xenopus laevis] gi

Protein Accession	CL9865.Contig2$_{H}1$ CL9865.Contig1$_{H}1$
Mean Expression Ratio	1.02
Median Expression Ratio	1.02
Credible Interval	(0.51, 2.01)
Associated Peptides	1
Associated Spectra	3
Coverage	NaN

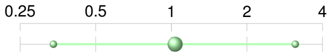

A	B	2.5	50	97.5	Sequence
**2**	**1**	**0.34**	**1**	**3.1**	**IHDPDFLSQR**

5.704 197 1132 minus strand Pancreatic lipase-related protein 2 [Crassostrea gigas]

Protein Accession	CL8259.Contig1$_{H}1$
Mean Expression Ratio	0.98
Median Expression Ratio	0.982
Credible Interval	(0.497, 1.88)
Associated Peptides	1
Associated Spectra	6
Coverage	NaN

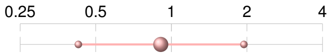

A	B	2.5	50	97.5	Sequence
**0**	**6**	**0.43**	**0.91**	**1.9**	**ITGLDPAGPK**

5.705 " 115 1104 minus strand PREDICTED: KH domain-containing, RNA-binding, signal transduction-associated protein 3 [Monodelphis domestica]"

Protein Accession	Unigene26700$_{H}1$
Mean Expression Ratio	1.02
Median Expression Ratio	1.02
Credible Interval	(0.538, 1.9)
Associated Peptides	3
Associated Spectra	4
Coverage	NaN

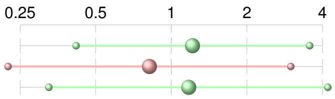

A	B	2.5	50	97.5	Sequence
**1**	**1**	**0.42**	**1.2**	**3.6**	**LLAQEIER**
**1**	**0**	**0.22**	**0.82**	**3**	**GRGGGVP**
**0**	**1**	**0.33**	**1.2**	**4.2**	**LQEETGCK**

5.706 " 241 1110 minus strand N(G),N(G)-dimethylarginine dimethylaminohydrolase 1 [Crassostrea gigas]; 241 1110 minus strand N(G),N(G)-dimethylarginine dimethylaminohydrolase 1 [Crassostrea gigas]; 241 1110 minus strand N(G),N(G)-dimethylarginine dimethylaminohydrolase 1 [Crassostrea gigas]"

Protein Accession	Unigene30451$_{H}1$ CL7107.Contig3$_{H}1$ CL7107.Contig1$_{H}1$
Mean Expression Ratio	1.02
Median Expression Ratio	1.02
Credible Interval	(0.519, 1.97)
Associated Peptides	2
Associated Spectra	2
Coverage	NaN

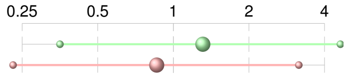

A	B	2.5	50	97.5	Sequence
0	1	0.35	1.3	4.6	LGEVGEIRR
0	1	0.23	0.86	3.2	LGEVGEIR

5.707 " 3 227 minus strand alpha tubulin, putative [Ixodes scapularis] gi"

Protein Accession	Unigene31690$_{H}1$
Mean Expression Ratio	1.02
Median Expression Ratio	1.02
Credible Interval	(0.561, 1.86)
Associated Peptides	2
Associated Spectra	13
Coverage	NaN

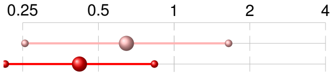

A	B	2.5	50	97.5	Sequence
2	2	0.26	0.65	1.7	LSVDYGKK
3	6	0.21	0.42	0.83	LSVDYGK

5.708 613 846 cytochrome c oxidase subunit Va [Antheraea yamamai] gi

Protein Accession	Unigene3832$_{H}1$
Mean Expression Ratio	1.02
Median Expression Ratio	1.02
Credible Interval	(0.548, 1.9)
Associated Peptides	4
Associated Spectra	5
Coverage	NaN

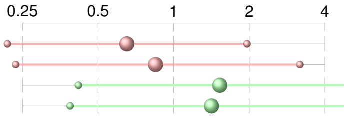

A	B	2.5	50	97.5	Sequence
0	2	0.22	0.65	2	AMNDLQGEDLVPEPK
0	1	0.24	0.85	3.2	YETFFSRPDIDGWEIR
0	1	0.42	1.5	5.6	KAMNDLQGEDLVPEPK
0	1	0.39	1.4	5.2	YETFFSRPDIDGWEIRK

5.709 230 1189 hypothetical protein [Amblyomma maculatum]

Protein Accession	CL11202.Contig4$_{H}1$
Mean Expression Ratio	0.982
Median Expression Ratio	0.982
Credible Interval	(0.501, 1.89)
Associated Peptides	2
Associated Spectra	2
Coverage	NaN

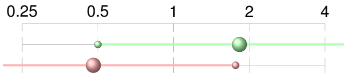

A	B	2.5	50	97.5	Sequence
0	1	0.5	1.8	6.4	KFAVDAVK
0	1	0.13	0.48	1.8	LEDMLR

5.710 187 1563 odr-4-like protein [Crassostrea gigas]

Protein Accession	CL15156.Contig3$_{H}1$
Mean Expression Ratio	1.02
Median Expression Ratio	1.02
Credible Interval	(0.499, 2.05)
Associated Peptides	1
Associated Spectra	1
Coverage	NaN

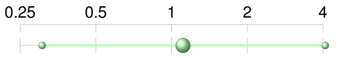

A	B	2.5	50	97.5	Sequence
**0**	**1**	**0.31**	**1.1**	**4.1**	**TLDAADELWVATHAK**

5.711 284 1318 hsc70-interacting protein [Gallus gallus] gi; 88 1137 hsc70-interacting protein [Gallus gallus] gi

Protein Accession	CL11847.Contig4$_{H}1$ CL11847.Contig2$_{H}1$
Mean Expression Ratio	1.01
Median Expression Ratio	1.02
Credible Interval	(0.525, 1.95)
Associated Peptides	2
Associated Spectra	4
Coverage	NaN

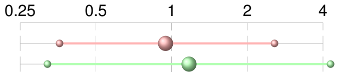

A	B	2.5	50	97.5	Sequence
**2**	**1**	**0.36**	**0.95**	**2.6**	**AQLDLLK**
**0**	**1**	**0.32**	**1.2**	**4.3**	**LNPTSALLYAK**

5.712 325 642 minus strand Protein phosphatase 1B [Crassostrea gigas]

Protein Accession	Unigene268$_{H}1$
Mean Expression Ratio	0.982
Median Expression Ratio	0.983
Credible Interval	(0.51, 1.91)
Associated Peptides	2
Associated Spectra	3
Coverage	NaN

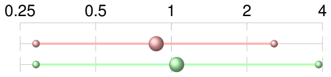

A	B	2.5	50	97.5	Sequence
**1**	**1**	**0.29**	**0.87**	**2.6**	**VEMEDAHTAVVSIPHAR**
**1**	**0**	**0.29**	**1**	**3.9**	**GAFLDKPK**

5.713 391 1470 PREDICTED: heparanase-like [Amphimedon queenslandica]; 391 1470 PREDICTED: heparanase-like [Amphimedon queenslandica]; 391 1470 PREDICTED: heparanase-like [Amphimedon queenslandica]; 391 1470 PREDICTED: heparanase-like [Amphimedon queenslandica]; 391 1755 PREDICTED: heparanase-like [Amphimedon queenslandica]; 418 1365 PREDICTED: similar to heparanase-like protein [Tribolium castaneum]; 391 1755 PREDICTED: heparanase-like [Amphimedon queenslandica]; 391 1470 PREDICTED: heparanase-like [Amphimedon queenslandica]

Protein Accession CL2227.Contig4$_{H}$1 CL2227.Contig3$_{H}$1 CL2227.Contig2$_{H}$1 CL2227.Contig1$_{H}$1 CL2227.Con
Mean Expression Ratio 0.984
Median Expression Ratio 0.983
Credible Interval (0.498, 1.93)
Associated Peptides 1
Associated Spectra 2
Coverage NaN

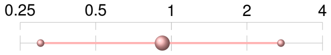

A	B	2.5	50	97.5	Sequence
**1**	**1**	**0.3**	**0.92**	**2.7**	**ALSPAYFR**

5.714 " 179 631 minus strand 60S ribosomal protein L7/L12, partial [Ostrea edulis]"

Protein Accession CL34109.Contig5$_{H}$1
Mean Expression Ratio 0.982
Median Expression Ratio 0.983
Credible Interval (0.498, 1.93)
Associated Peptides 1
Associated Spectra 3
Coverage NaN

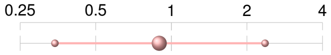

A	B	2.5	50	97.5	Sequence
**2**	**1**	**0.34**	**0.89**	**2.4**	**KFVESVPQVVK**

5.715 503 1591 minus strand Septin-2 [Crassostrea gigas]; 648 1736 minus strand Septin-2 [Crassostrea gigas]

Protein Accession CL9569.Contig7$_{H}$1 CL9569.Contig6$_{H}$1
Mean Expression Ratio 1.02
Median Expression Ratio 1.02
Credible Interval (0.509, 2.03)
Associated Peptides 1
Associated Spectra 1
Coverage NaN

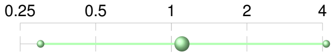

A	B	2.5	50	97.5	Sequence
**0**	**1**	**0.3**	**1.1**	**4.1**	**VNIVPVLAK**

5.716 60 353 minus strand Histone H3.3 [Harpegnathos saltator]

Protein Accession	Unigene31518$_{H}1$
Mean Expression Ratio	1.02
Median Expression Ratio	1.02
Credible Interval	(0.511, 2.07)
Associated Peptides	1
Associated Spectra	1
Coverage	NaN

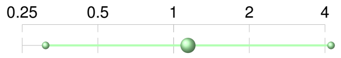

A	B	2.5	50	97.5	Sequence
0	1	0.31	1.1	4.2	KSAPSTGGVK

5.717 260 892 minus strand calponin [Haliotis diversicolor]

Protein Accession	CL48702.Contig1$_{H}1$
Mean Expression Ratio	0.979
Median Expression Ratio	0.983
Credible Interval	(0.545, 1.75)
Associated Peptides	5
Associated Spectra	10
Coverage	NaN

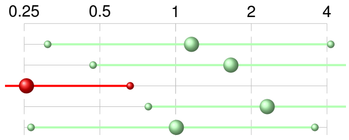

A	B	2.5	50	97.5	Sequence
0	1	0.31	1.2	4.1	AATGQQFPIEESNER
0	1	0.47	1.7	6.1	AFGIEVESHPSYTGQR
2	2	0.099	0.26	0.66	VESSFDPEDAKK
2	1	0.78	2.3	6.5	NFSEDKLR
0	1	0.27	1	3.6	SGLRAPK

5.718 10 1542 Copine-8 [Crassostrea gigas]

Protein Accession	CL9423.Contig1$_{H}1$
Mean Expression Ratio	0.985
Median Expression Ratio	0.984
Credible Interval	(0.491, 1.98)
Associated Peptides	1
Associated Spectra	1
Coverage	NaN

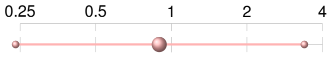

A	B	2.5	50	97.5	Sequence
0	1	0.24	0.89	3.4	SYRGQYAER

5.719 87 1997 Transketolase-like protein 2 [Crassostrea gigas]; 87 1997 Transketolase-like protein 2 [Crassostrea gigas]; 87 1997 Transketolase-like protein 2 [Crassostrea gigas]; 87 1997 Transketolase-like protein 2 [Crassostrea gigas]; 87 1997 Transketolase-like protein 2 [Crassostrea gigas]; 87 1997 Transketolase-like protein 2 [Crassostrea gigas]; 87 1997 Transketolase-like protein 2 [Crassostrea gigas]

Protein Accession	CL1127.Contig6$_H$1 CL1127.Contig5$_H$1 CL1127.Contig4$_H$1 CL1127.Contig3$_H$1 CL1127.Con
Mean Expression Ratio	1.01
Median Expression Ratio	1.02
Credible Interval	(0.557, 1.85)
Associated Peptides	5
Associated Spectra	7
Coverage	NaN

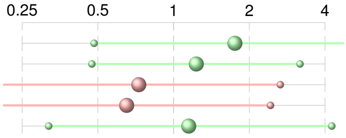

A	B	2.5	50	97.5	Sequence
0	1	0.48	1.8	6.3	FTSDLEGHPTPR
0	3	0.47	1.2	3.2	LAYGDALVK
0	1	0.2	0.73	2.7	SAVQVLR
0	1	0.19	0.65	2.4	GIEGIEDLDNWHGK
0	1	0.32	1.1	4.3	VISLDGDVK

5.720 " 29 448 minus strand PREDICTED: similar to NADH:ubiquinone dehydrogenase, putative [Tribolium castaneum] gi; 19 408 minus strand hypothetical protein KGM_18901 [Danaus plexippus]; 29 448 minus strand PREDICTED: similar to NADH:ubiquinone dehydrogenase, putative [Tribolium castaneum] gi"

Protein Accession	CL2684.Contig8$_H$1 CL2684.Contig7$_H$1 CL2684.Contig6$_H$1
Mean Expression Ratio	0.987
Median Expression Ratio	0.984
Credible Interval	(0.496, 1.95)
Associated Peptides	1
Associated Spectra	2
Coverage	NaN

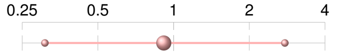

A	B	2.5	50	97.5	Sequence
0	2	0.31	0.92	2.8	LLQDGEKELFLK

5.721 130 1731 T-complex protein 1 subunit epsilon [Crassostrea gigas]; 130 1731 T-complex protein 1 subunit epsilon [Crassostrea gigas]

Protein Accession	CL1223.Contig8$_H$1 CL1223.Contig2$_H$1
Mean Expression Ratio	0.983
Median Expression Ratio	0.984
Credible Interval	(0.576, 1.68)
Associated Peptides	9
Associated Spectra	13
Coverage	NaN

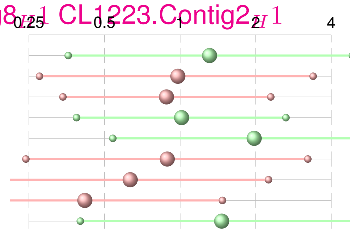

A	B	2.5	50	97.5	Sequence
0	1	0.36	1.3	4.9	GVVVDKDMSHPQMPK
0	1	0.28	0.98	3.4	HKLDVTSVEDYR
0	3	0.34	0.89	2.3	LTGIDAHK
0	3	0.39	1	2.6	LMVQLSK
0	1	0.54	2	6.9	HKLDVTSVEDYRK
0	1	0.24	0.89	3.2	MLVIEECK
0	1	0.18	0.63	2.3	IVPRFEELTPEK
0	1	0.11	0.42	1.5	IAVETLDK
0	1	0.4	1.5	5.2	SHILAAK

5.722 835 1116 tropomyosin [Ilyanassa obsoleta]

Protein Accession	CL2925.Contig8$_{H}$1
Mean Expression Ratio	1.02
Median Expression Ratio	1.02
Credible Interval	(0.602, 1.72)
Associated Peptides	9
Associated Spectra	21
Coverage	NaN

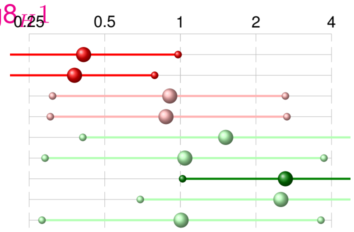

A	B	2.5	50	97.5	Sequence
1	3	0.17	0.41	0.98	LEDVETELR
2	4	0.18	0.38	0.79	QLELYEEK
1	1	0.31	0.91	2.6	ELEEQSHALTNSLK
0	2	0.3	0.88	2.7	KLAVMEAELEKAEDR
0	1	0.41	1.5	5.6	LYAGDERLEDVETELR
0	1	0.29	1	3.7	VRELEEQSHALTNSLK
0	3	1	2.6	6.6	KLAVMEAELEKAEDRADSSERR
0	1	0.69	2.5	8.9	QAAQEWEARYEEAAR
0	1	0.28	1	3.6	KLAVMEAELEKAEDRADSSER

5.723 146 841 minus strand hypothetical protein CGI_10006609 [Crassostrea gigas]; 146 841 minus strand hypothetical protein CGI_10006609 [Crassostrea gigas]; 146 841 minus strand hypothetical protein CGI_10006609 [Crassostrea gigas]

Protein Accession	CL6681.Contig3$_{H}$1 CL6681.Contig2$_{H}$1 CL6681.Contig1$_{H}$1
Mean Expression Ratio	1.02
Median Expression Ratio	1.02
Credible Interval	(0.518, 2.05)
Associated Peptides	1
Associated Spectra	1
Coverage	NaN

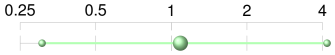

A	B	2.5	50	97.5	Sequence
0	1	0.31	1.1	4.2	GHVEGLEVLPAR

5.724 26 667 ribosomal protein L15 [Acipenser sinensis] gi; 26 667 ribosomal protein L15 [Acipenser sinensis] gi; 26 667 ribosomal protein L15 [Acipenser sinensis] gi

Protein Accession	Unigene63676$_{H}$1 CL22.Contig2$_{H}$1 CL22.Contig1$_{H}$1
Mean Expression Ratio	1.02
Median Expression Ratio	1.02
Credible Interval	(0.538, 1.95)
Associated Peptides	2
Associated Spectra	7
Coverage	NaN

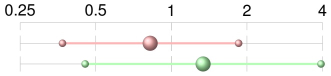

A	B	2.5	50	97.5	Sequence
3	2	0.37	0.82	1.9	NTLSLLR
0	2	0.45	1.3	4	TIGGSRR

5.725 43 222 sodium-calcium exchanger [Doryteuthis pealeii]

Protein Accession	Unigene7579$_{H}$1
Mean Expression Ratio	1.02
Median Expression Ratio	1.02
Credible Interval	(0.518, 1.99)
Associated Peptides	1
Associated Spectra	3
Coverage	NaN

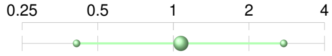

A	B	2.5	50	97.5	Sequence
1	2	0.41	1.1	2.8	TVGVGMVMR

5.726 206 316 minus strand ribosomal protein S17 [Branchiostoma belcheri]

Protein Accession	Unigene31494$_{H}$1
Mean Expression Ratio	0.986
Median Expression Ratio	0.985
Credible Interval	(0.495, 1.95)
Associated Peptides	1
Associated Spectra	3
Coverage	NaN

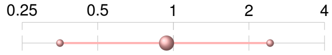

A	B	2.5	50	97.5	Sequence
0	3	0.35	0.94	2.4	LTLDFHTNKR

5.727 288 1487 minus strand putative procollagen type VI alpha 4 [Haliotis diversicolor]

Protein Accession	CL6899.Contig2$_{H}$1
Mean Expression Ratio	0.984
Median Expression Ratio	0.985
Credible Interval	(0.59, 1.63)
Associated Peptides	9
Associated Spectra	25
Coverage	NaN

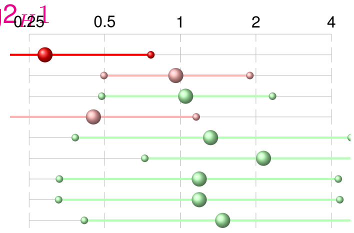

A	B	2.5	50	97.5	Sequence
1	2	0.11	0.29	0.76	IGALTFSDDVK
4	4	0.5	0.96	1.9	TGVTNTHLAIR
2	3	0.49	1.1	2.3	FRDDITK
2	1	0.18	0.45	1.2	QVLQFVSQVITR
0	1	0.38	1.3	4.8	VMVVITDGSSR
0	2	0.72	2.1	6.3	LRENLK
0	1	0.33	1.2	4.2	QLAAAARSLR
0	1	0.33	1.2	4.3	AIASDPDNDYLFNITAFR
0	1	0.41	1.5	5.3	AEVLAAIDQVR

5.728 93 2876 Hypoxia up-regulated protein 1 [Crassostrea gigas]

Protein Accession	CL16620.Contig1$_{H}$1
Mean Expression Ratio	0.988
Median Expression Ratio	0.985
Credible Interval	(0.534, 1.85)
Associated Peptides	4
Associated Spectra	4
Coverage	NaN

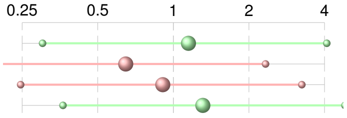

A	B	2.5	50	97.5	Sequence
0	1	0.3	1.1	4.1	MDESGILHLER
0	1	0.18	0.64	2.3	VESVFEK
0	1	0.25	0.91	3.2	IHEILLEK
0	1	0.36	1.3	4.8	IVAFR

5.729 2939 3208 minus strand beta-thymosin domain repeat protein CSP24KDa_v1 [Hermissenda crassicornis] gi

Protein Accession	CL9548.Contig3$_H$1
Mean Expression Ratio	1.01
Median Expression Ratio	1.01
Credible Interval	(0.578, 1.8)
Associated Peptides	7
Associated Spectra	10
Coverage	NaN

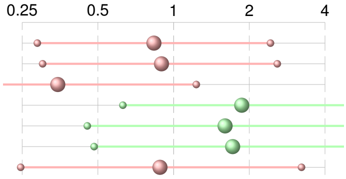

A	B	2.5	50	97.5	Sequence
1	1	0.29	0.83	2.4	NPLPSTDAIAAEK
1	1	0.3	0.89	2.6	SIGEFDK
1	0	0.094	0.35	1.2	NPLPTK
1	1	0.63	1.9	5.5	SIGEFDKGA
0	1	0.46	1.6	5.9	KAETEEKNPLPTKEVIQQEK
0	1	0.48	1.7	6.2	SIGEFDKGALK
0	1	0.25	0.88	3.2	AETEEKNPLPTK

5.730 203 1942 ezrin/radixin/moesin [Aplysia californica] gi; 243 1982 minus strand ezrin/radixin/moesin [Aplysia californica] gi

Protein Accession	CL13506.Contig5$_H$1 CL13506.Contig4$_H$1
Mean Expression Ratio	0.987
Median Expression Ratio	0.986
Credible Interval	(0.662, 1.46)
Associated Peptides	23
Associated Spectra	43
Coverage	NaN

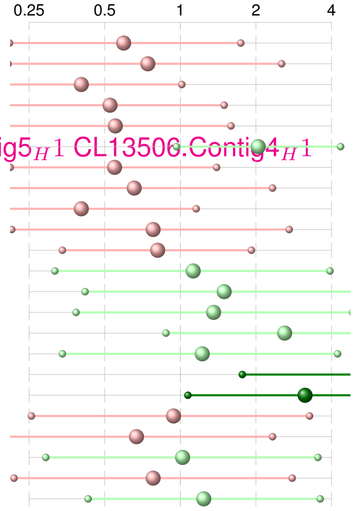

A	B	2.5	50	97.5	Sequence
1	1	0.21	0.59	1.7	KESPLQFK
1	0	0.21	0.74	2.5	KVLSQDVR
3	0	0.16	0.4	1	QLFDQVVK
1	1	0.18	0.52	1.5	DDAMMEYLK
1	1	0.19	0.55	1.6	FVIKPIDK
2	4	0.97	2	4.3	RKPDTIEVQQMK
1	2	0.21	0.55	1.4	ESPLQFK
1	0	0.18	0.66	2.3	APDFVFYAPR
2	0	0.14	0.4	1.2	LNEIVYK
1	0	0.21	0.78	2.7	LLPQR
2	2	0.34	0.81	1.9	NISFNDKK
1	0	0.32	1.1	3.9	VLSQDVR
0	1	0.42	1.5	5.4	KAPDFVFYAPR
0	1	0.38	1.4	4.8	LADELETAQR
0	2	0.88	2.6	7.5	LGENGLTKNDELHAENVR
0	1	0.34	1.2	4.2	MAELLALREELDAER
0	2	1.8	5.1	15	QKLADELETAQR
0	2	1.1	3.1	9.1	VEEERLTEAEKNQR
0	1	0.25	0.94	3.3	FVIKPIDKK
0	1	0.18	0.67	2.3	QLEMLK
0	1	0.29	1	3.5	AEELRLEMER
0	1	0.22	0.78	2.8	DMADLQAR
0	2	0.43	1.2	3.6	SWWGEHK

5.731 1 111 glycogen synthase [Crassostrea gigas]

Protein Accession	Unigene78448$_H$1
Mean Expression Ratio	1.01
Median Expression Ratio	1.01
Credible Interval	(0.502, 2.05)
Associated Peptides	1
Associated Spectra	1
Coverage	NaN

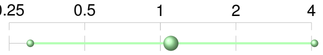

A	B	2.5	50	97.5	Sequence
0	1	0.3	1.1	4.1	NVEPDLDDDYLDR

5.732 225 839 minus strand Metallo-beta-lactamase domain-containing protein 1 [Crassostrea gigas]; 201 851 minus strand Metallo-beta-lactamase domain-containing protein 1 [Crassostrea gigas]

Protein Accession	CL35672.Contig2$_H$1 CL35672.Contig1$_H$1
Mean Expression Ratio	0.984
Median Expression Ratio	0.986
Credible Interval	(0.493, 1.97)
Associated Peptides	1
Associated Spectra	1
Coverage	NaN

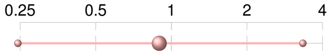

A	B	2.5	50	97.5	Sequence
**0**	**1**	**0.24**	**0.9**	**3.3**	**EADFIVPGHGPMFR**

5.733 297 734 minus strand PREDICTED: calmodulin [Canis lupus familiaris]; 297 734 PREDICTED: calmodulin [Canis lupus familiaris]; 143 508 minus strand Calmodulin [Crassostrea gigas]

Protein Accession	Unigene26719$_H$1 Unigene17836$_H$1 Unigene10331$_H$1
Mean Expression Ratio	1.01
Median Expression Ratio	1.01
Credible Interval	(0.507, 2.03)
Associated Peptides	1
Associated Spectra	1
Coverage	NaN

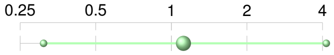

A	B	2.5	50	97.5	Sequence
**0**	**1**	**0.31**	**1.1**	**4.2**	**TLITEFENR**

5.734 " 125 628 minus strand collectin sub-family member 12, partial [Oncorhynchus mykiss]"

Protein Accession	CL46744.Contig5$_H$1
Mean Expression Ratio	0.986
Median Expression Ratio	0.986
Credible Interval	(0.488, 2)
Associated Peptides	1
Associated Spectra	1
Coverage	NaN

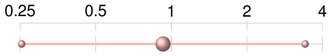

A	B	2.5	50	97.5	Sequence
**0**	**1**	**0.25**	**0.92**	**3.4**	**WVQGDNRPLAYTR**

5.735 145 972 LIM and SH3 domain protein F42H10.3 OS=Caenorhabditis elegans GN=F42H10.3 PE=2 SV=3

Protein Accession	CL49642.Contig8$_{H1}$
Mean Expression Ratio	0.985
Median Expression Ratio	0.986
Credible Interval	(0.492, 1.98)
Associated Peptides	1
Associated Spectra	1
Coverage	NaN

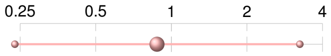

A	B	2.5	50	97.5	Sequence
**0**	**1**	**0.24**	**0.88**	**3.2**	**TVYPTEELK**

5.736 97 597 glyoxylate reductase/hydroxypyruvate reductase [Ictalurus punctatus] gi

Protein Accession	CL18211.Contig2$_{H1}$
Mean Expression Ratio	1.01
Median Expression Ratio	1.01
Credible Interval	(0.507, 1.99)
Associated Peptides	1
Associated Spectra	2
Coverage	NaN

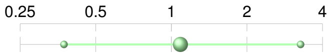

A	B	2.5	50	97.5	Sequence
**0**	**2**	**0.37**	**1.1**	**3.3**	**KIPAIGIDLLTR**

5.737 3 218 beta-tubulin [Fasciola hepatica] gi

Protein Accession	Unigene7955$_{H1}$
Mean Expression Ratio	0.985
Median Expression Ratio	0.987
Credible Interval	(0.547, 1.76)
Associated Peptides	5
Associated Spectra	15
Coverage	NaN

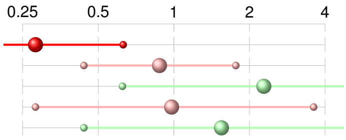

A	B	2.5	50	97.5	Sequence
**2**	**3**	**0.13**	**0.28**	**0.63**	**EVDEQMLNVQNK**
**2**	**5**	**0.44**	**0.88**	**1.8**	**TAVCDIPPR**
**0**	**1**	**0.62**	**2.3**	**8**	**MSMKEVDEQMLNVQNK**
**0**	**1**	**0.28**	**0.98**	**3.6**	**NSSYFVEWIPNNVK**
**0**	**1**	**0.44**	**1.6**	**5.4**	**TAVCDIPPRGLK**

5.738 322 645 macrophage migration inhibitory-like factor protein [Chlamys farreri]; 437 682 macrophage migration inhibitory-like factor protein [Chlamys farreri]; 437 682 macrophage migration inhibitory-like factor protein [Chlamys farreri]; 468 713 macrophage migration inhibitory-like factor protein [Chlamys farreri]

Protein Accession	CL8506.Contig6$_{H}$1 CL8506.Contig5$_{H}$1 CL8506.Contig3$_{H}$1 CL8506.Contig2$_{H}$1
Mean Expression Ratio	0.991
Median Expression Ratio	0.987
Credible Interval	(0.506, 1.96)
Associated Peptides	1
Associated Spectra	2
Coverage	NaN

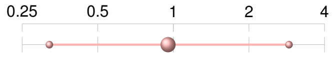

A	B	2.5	50	97.5	Sequence
0	2	0.32	0.95	2.9	ITLTIHSGLR

5.739 26 367 proteasome alpha type 2 [Haliotis discus discus]

Protein Accession	CL43771.Contig1$_{H}$1
Mean Expression Ratio	0.988
Median Expression Ratio	0.987
Credible Interval	(0.487, 1.97)
Associated Peptides	1
Associated Spectra	1
Coverage	NaN

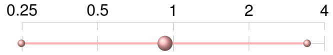

A	B	2.5	50	97.5	Sequence
1	0	0.25	0.92	3.4	ATNGVVLATEK

5.740 27 1682 minus strand PREDICTED: nucleolar protein 56 [Oreochromis niloticus]

Protein Accession	Unigene26699$_{H}$1
Mean Expression Ratio	1.01
Median Expression Ratio	1.01
Credible Interval	(0.591, 1.7)
Associated Peptides	8
Associated Spectra	19
Coverage	NaN

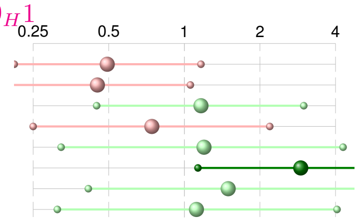

A	B	2.5	50	97.5	Sequence
2	2	0.21	0.49	1.2	LDGLGQAELQK
1	3	0.19	0.45	1.1	VTPLKLDGLGQAELQK
1	2	0.45	1.2	3	KLIMQK
0	2	0.25	0.74	2.2	VATLEEEVYDWEIK
0	1	0.32	1.2	4.3	VQAIFDSSK
0	3	1.1	2.9	7.6	LVKDLHSK
0	1	0.41	1.5	5.3	KGLGGLSTEKK
0	1	0.31	1.1	4.1	LIAHAGSLTNLAK

5.741 214 1284 glutamine synthetase [Tegillarca granosa]

Protein Accession	CL6905.Contig5$_H$1
Mean Expression Ratio	0.984
Median Expression Ratio	0.988
Credible Interval	(0.496, 1.95)
Associated Peptides	2
Associated Spectra	2
Coverage	NaN

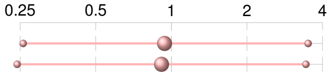

A	B	2.5	50	97.5	Sequence
**1**	**0**	**0.26**	**0.94**	**3.5**	**QPGGLAVIEEAIAK**
**0**	**1**	**0.24**	**0.91**	**3.4**	**GGLAVIEEAIAK**

5.742 1 630 hypothetical protein [Trichomonas vaginalis G3] gi

Protein Accession	CL4495.Contig3$_H$1
Mean Expression Ratio	1.01
Median Expression Ratio	1.01
Credible Interval	(0.5, 2.04)
Associated Peptides	1
Associated Spectra	1
Coverage	NaN

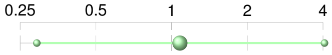

A	B	2.5	50	97.5	Sequence
**0**	**1**	**0.29**	**1.1**	**4**	**TLTEEKENLNK**

5.743 57 2378 minus strand Heterogeneous nuclear ribonucleoprotein U-like protein 1 [Crassostrea gigas]

Protein Accession	CL15560.Contig5$_H$1
Mean Expression Ratio	0.987
Median Expression Ratio	0.988
Credible Interval	(0.505, 1.94)
Associated Peptides	2
Associated Spectra	2
Coverage	NaN

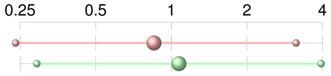

A	B	2.5	50	97.5	Sequence
**0**	**1**	**0.24**	**0.85**	**3.1**	**LRDELK**
**0**	**1**	**0.29**	**1.1**	**4**	**RSRSPPPRKVE**

5.744 412 708 minus strand heat shock protein beta-1 [Trichinella spiralis] gi; 459 755 minus strand heat shock protein beta-1 [Trichinella spiralis] gi; 464 763 minus strand heat shock protein beta-1 [Trichinella spiralis] gi; 437 736 minus strand heat shock protein beta-1 [Trichinella spiralis] gi; 437 736 minus strand heat shock protein beta-1 [Trichinella spiralis] gi; 437 736 minus strand heat shock protein beta-1 [Trichinella spiralis] gi

Protein Accession	CL17234.Contig6$_{H}1$ CL17234.Contig5$_{H}1$ CL17234.Contig4$_{H}1$ CL17234.Contig3$_{H}1$ CL1723
Mean Expression Ratio	0.988
Median Expression Ratio	0.988
Credible Interval	(0.489, 1.98)
Associated Peptides	1
Associated Spectra	1
Coverage	NaN

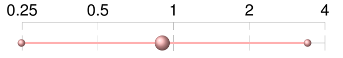

A	B	2.5	50	97.5	Sequence
1	0	0.25	0.9	3.4	TVTSSLNADGVLTLK

5.745 227 1201 PREDICTED: quinone oxidoreductase-like isoform 1 [Ornithorhynchus anatinus]; 109 1083 PREDICTED: quinone oxidoreductase-like isoform 1 [Ornithorhynchus anatinus]; 164 1138 PREDICTED: quinone oxidoreductase-like isoform 1 [Ornithorhynchus anatinus]

Protein Accession	CL11899.Contig2$_{H}1$ CL11899.Contig1$_{H}1$ CL11899.Contig13$_{H}1$
Mean Expression Ratio	1.02
Median Expression Ratio	1.01
Credible Interval	(0.563, 1.86)
Associated Peptides	5
Associated Spectra	7
Coverage	NaN

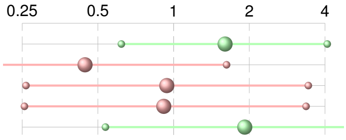

A	B	2.5	50	97.5	Sequence
1	2	0.62	1.6	4.1	VHASGVNPVDTYIR
1	0	0.12	0.44	1.6	EGLELVK
1	0	0.26	0.94	3.4	VMGLR
0	1	0.26	0.92	3.4	ILGTAGTKEGLELVK
0	1	0.53	1.9	7	LVLKL

5.746 304 2952 minus strand hypothetical protein CGI_10026535 [Crassostrea gigas]

Protein Accession	CL21819.Contig2$_{H}1$
Mean Expression Ratio	0.989
Median Expression Ratio	0.988
Credible Interval	(0.527, 1.86)
Associated Peptides	3
Associated Spectra	5
Coverage	NaN

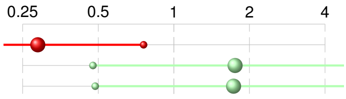

A	B	2.5	50	97.5	Sequence
2	1	0.11	0.29	0.76	VADIAGDIPK
0	1	0.48	1.8	6.5	LVQLEETAK
0	1	0.49	1.7	6.3	AEYSLAAVALPPDNDAK

5.747 85 681 minus strand 40S ribosomal protein S8 OS=Xenopus laevis GN=rps8 PE=2 SV=3

Protein Accession	Unigene34347$_{H}1$
Mean Expression Ratio	1.01
Median Expression Ratio	1.01
Credible Interval	(0.505, 2.04)
Associated Peptides	1
Associated Spectra	1
Coverage	NaN

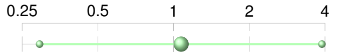

A	B	2.5	50	97.5	Sequence
**0**	**1**	**0.29**	**1.1**	**3.9**	**VEPALDEQFSSGR**

5.748 60 1190 hypothetical protein BRAFLDRAFT_109688 [Branchiostoma floridae] gi; 51 1184 hypothetical protein BRAFLDRAFT_109688 [Branchiostoma floridae] gi

Protein Accession	CL25572.Contig3$_{H}1$ CL25572.Contig1$_{H}1$
Mean Expression Ratio	1.01
Median Expression Ratio	1.01
Credible Interval	(0.53, 1.96)
Associated Peptides	2
Associated Spectra	2
Coverage	NaN

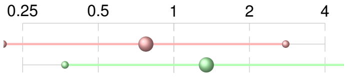

A	B	2.5	50	97.5	Sequence
**0**	**1**	**0.21**	**0.77**	**2.8**	**AGHFQDEIIPVVIQSR**
**0**	**1**	**0.37**	**1.3**	**4.9**	**SVVLGAMAVQ**

5.749 484 1104 Calponin-2 [Crassostrea gigas]; 309 938 Calponin-2 [Crassostrea gigas]

Protein Accession	CL30203.Contig3$_{H}1$ CL21394.Contig2$_{H}1$
Mean Expression Ratio	0.989
Median Expression Ratio	0.989
Credible Interval	(0.576, 1.7)
Associated Peptides	6
Associated Spectra	19
Coverage	NaN

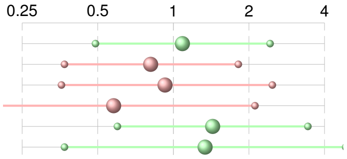

A	B	2.5	50	97.5	Sequence
**1**	**4**	**0.49**	**1.1**	**2.4**	**MNTLAAPFK**
**1**	**4**	**0.37**	**0.81**	**1.8**	**QMENIEVFLK**
**1**	**2**	**0.36**	**0.93**	**2.5**	**QLTGEDIGSGAVQVEK**
**1**	**0**	**0.16**	**0.58**	**2.1**	**NGQILCR**
**0**	**4**	**0.6**	**1.4**	**3.4**	**AGQGIIGLQAGTNK**
**0**	**1**	**0.37**	**1.3**	**4.8**	**YDTGAEYEVR**

5.750 299 7507 minus strand filamin [Clonorchis sinensis]

Protein Accession	CL4145.Contig20$_H$1
Mean Expression Ratio	0.988
Median Expression Ratio	0.989
Credible Interval	(0.733, 1.33)
Associated Peptides	52
Associated Spectra	101
Coverage	NaN

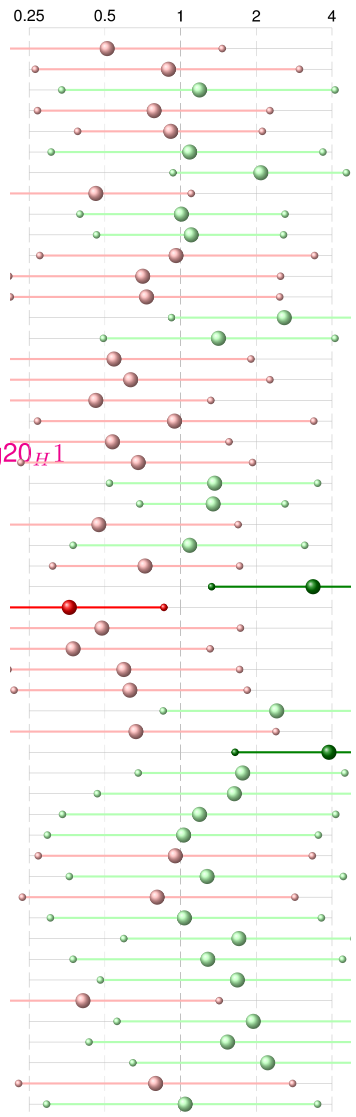

A	B	2.5	50	97.5	Sequence
1	1	0.17	0.51	1.5	AEIDFQDR
1	0	0.26	0.9	3	AQLSVGSSSEVSLK
1	0	0.34	1.2	4.1	AYGPGLEPK
1	1	0.27	0.78	2.3	DVPGAPFPVK
1	3	0.39	0.92	2.1	ETGEHLVNVFR
1	0	0.3	1.1	3.7	GAGKGGLGLGIEGPVEAK
1	4	0.93	2.1	4.6	GGLGLGIEGPVEAK
2	2	0.2	0.46	1.1	GTLEVTLLNPK
1	2	0.4	1	2.6	IMVGESELGNASK
2	2	0.46	1.1	2.6	KISISALQDK
1	0	0.28	0.96	3.4	LANGHLGITFTPR
1	0	0.21	0.71	2.5	LENVTMVLK
1	0	0.21	0.73	2.5	VDAVPDTGIPK
1	1	0.92	2.6	7.5	VLPGVDASKVK
1	1	0.49	1.4	4.1	VQNPVDASKVK
1	0	0.15	0.54	1.9	VYVSEPTNPAK
1	0	0.17	0.63	2.3	TTNVPVQDLVDKK
1	1	0.16	0.46	1.3	ELADDAQWK
1	0	0.27	0.95	3.4	VSEELITR
1	1	0.19	0.54	1.6	SPVDELTVK
1	1	0.23	0.68	1.9	FNDEHIPDSPYR
1	2	0.52	1.4	3.5	VKPAALDVSGVK
4	4	0.69	1.3	2.6	VLPGVDASK
1	0	0.14	0.47	1.7	QVEVQVK
1	1	0.37	1.1	3.1	HIANSPFK
1	3	0.31	0.72	1.7	VTETDITDLTASIR
1	2	1.3	3.4	8.8	VIAKGPGLEK
2	2	0.15	0.36	0.86	VEVGPFWK
1	0	0.14	0.49	1.7	IMVGK
1	0	0.11	0.37	1.3	NNAKEAMDAAEK
2	0	0.21	0.59	1.7	VQNPVDASK
1	1	0.22	0.63	1.8	EETKPGSLGVTVEGPK
0	2	0.86	2.4	7	AYGPGLEGGITNQPAVFTVDMK
0	1	0.19	0.66	2.4	EMGIHTVSVK
0	4	1.6	3.9	9.3	FADKNIMGSPFTAK
0	3	0.68	1.8	4.5	GLQVGKPAAFVVNFNGAK
0	1	0.47	1.6	5.6	INVTHADKPIPGSPFK
0	1	0.34	1.2	4.1	TQKLENVTMVLK
0	1	0.29	1	3.5	VKDSADFYVHTK
0	1	0.27	0.95	3.4	VQVSPSIGDAR
0	1	0.36	1.3	4.4	VTYRPTEPGTYIINIK
0	1	0.23	0.8	2.9	FLEEDEGIR
0	1	0.3	1	3.6	NIMGSPFTAK
0	2	0.59	1.7	4.9	HKDMHIPGSPFQFTVGPIAGGGAHK
0	1	0.37	1.3	4.4	VTGPGVQGTTPASLPAVFK
0	1	0.48	1.7	5.9	FSGIQK
0	1	0.12	0.41	1.4	EEPCLLK
0	1	0.56	1.9	6.8	SPSGREEPCLLKR
0	1	0.43	1.5	5.6	NGKHIANSPFK
0	1	0.65	2.2	7.8	FIVNTVNAGA
0	1	0.23	0.8	2.8	VEGAGQPSGVQEHASVVVETVTK
0	1	0.29	1	3.5	YAGVNIAGSPFK

5.751 62 505 Myosin-2 essential light chain [Crassostrea gigas]

Protein Accession	CL47077.Contig1$_{H}1$
Mean Expression Ratio	0.987
Median Expression Ratio	0.989
Credible Interval	(0.53, 1.85)
Associated Peptides	3
Associated Spectra	10
Coverage	NaN

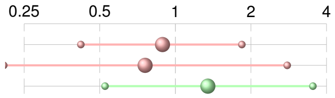

A	B	2.5	50	97.5	Sequence
3	3	0.42	0.89	1.8	IAASQLGDVLR
1	0	0.21	0.76	2.8	SLNQNPTEQEIR
0	3	0.52	1.3	3.5	HILTSLGEK

5.752 " 2041 3315 minus strand Myosin-10 OS=Mus musculus GN=Myh10 PE=1 SV=2; 2041 3345 minus strand Myosin heavy chain, non-muscle OS=Drosophila melanogaster GN=zip PE=1 SV=2; 2041 3351 minus strand Myosin-10 OS=Mus musculus GN=Myh10 PE=1 SV=2"

Protein Accession	Unigene62170$_{H}1$ Unigene62168$_{H}1$ Unigene62165$_{H}1$
Mean Expression Ratio	1.01
Median Expression Ratio	1.01
Credible Interval	(0.543, 1.87)
Associated Peptides	3
Associated Spectra	6
Coverage	NaN

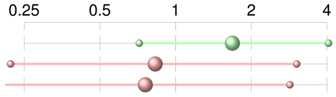

A	B	2.5	50	97.5	Sequence
1	3	0.71	1.7	4.1	AGVLAHLEEER
1	0	0.22	0.83	3	TVGQLYKEQLNK
0	1	0.2	0.76	2.8	IDSPLVLDQLR

5.753 174 1721 minus strand aspartyl-tRNA synthetase [Xenopus laevis] gi

Protein Accession	CL9865.Contig1$_{H}1$
Mean Expression Ratio	0.983
Median Expression Ratio	0.99
Credible Interval	(0.496, 1.9)
Associated Peptides	1
Associated Spectra	3
Coverage	NaN

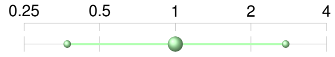

A	B	2.5	50	97.5	Sequence
2	1	0.37	1	2.8	IHDPDFLSQR

5.754 " 134 1567 minus strand 6-phosphogluconate dehydrogenase, decarboxylating [Crassostrea gigas]; 134 1573 minus strand 6-phosphogluconate dehydrogenase, decarboxylating [Crassostrea gigas]"

Protein Accession	CL5140.Contig5$_H$1 CL5140.Contig4$_H$1
Mean Expression Ratio	1.01
Median Expression Ratio	1.01
Credible Interval	(0.544, 1.91)
Associated Peptides	4
Associated Spectra	4
Coverage	NaN

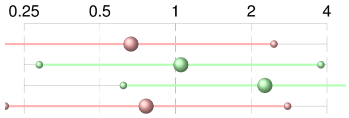

A	B	2.5	50	97.5	Sequence
1	0	0.18	0.67	2.5	FKDTDGSPLVEK
1	0	0.29	1	3.8	AFVEDIKR
0	1	0.62	2.3	8.2	TVSKVDEFLSNEAK
0	1	0.21	0.76	2.8	NIFQSISAK

5.755 87 485 Sex-regulated protein janus-A [Crassostrea gigas]

Protein Accession	CL13131.Contig3$_H$1
Mean Expression Ratio	1.01
Median Expression Ratio	1.01
Credible Interval	(0.52, 1.98)
Associated Peptides	2
Associated Spectra	2
Coverage	NaN

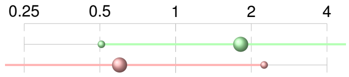

A	B	2.5	50	97.5	Sequence
0	1	0.51	1.8	6.7	ADHSITQTLLLR
0	1	0.17	0.6	2.2	VVGEIESK

5.756 76 522 minus strand Troponin C [Crassostrea gigas]; 173 619 minus strand Troponin C [Crassostrea gigas]; 173 619 minus strand Troponin C [Crassostrea gigas]

Protein Accession	Unigene53263$_H$1 Unigene11674$_H$1 CL13001.Contig4$_H$1
Mean Expression Ratio	0.993
Median Expression Ratio	0.99
Credible Interval	(0.544, 1.82)
Associated Peptides	4
Associated Spectra	7
Coverage	NaN

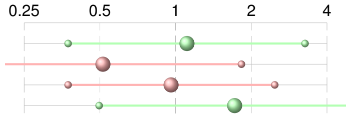

A	B	2.5	50	97.5	Sequence
1	1	0.37	1.1	3.3	GVIPVEDLR
1	0	0.14	0.51	1.8	SLGLQVEDEK
2	1	0.37	0.96	2.5	ELANVFK
1	0	0.5	1.7	6.2	EAFRVLDK

5.757 152 1696 dynamin isoform A [Lymnaea stagnalis]

Protein Accession	CL22122.Contig2$_H$1
Mean Expression Ratio	0.992
Median Expression Ratio	0.99
Credible Interval	(0.487, 1.98)
Associated Peptides	1
Associated Spectra	2
Coverage	NaN

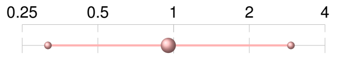

A	B	2.5	50	97.5	Sequence
**0**	**2**	**0.32**	**0.95**	**2.9**	**KFFLSHPAYR**

5.758 214 1878 PREDICTED: lysyl-tRNA synthetase-like [Oreochromis niloticus]

Protein Accession	CL25588.Contig2$_H$1
Mean Expression Ratio	0.996
Median Expression Ratio	0.99
Credible Interval	(0.506, 1.95)
Associated Peptides	2
Associated Spectra	2
Coverage	NaN

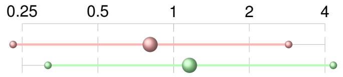

A	B	2.5	50	97.5	Sequence
**0**	**1**	**0.23**	**0.81**	**2.9**	**ISLISDLEK**
**0**	**1**	**0.32**	**1.2**	**4.3**	**IQIMANAK**

5.759 96 503 minus strand hypothetical protein DAPPUDRAFT_230378 [Daphnia pulex]

Protein Accession	CL14922.Contig5$_H$1
Mean Expression Ratio	1.01
Median Expression Ratio	1.01
Credible Interval	(0.527, 1.91)
Associated Peptides	3
Associated Spectra	3
Coverage	NaN

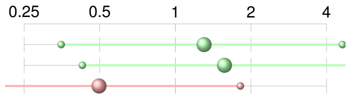

A	B	2.5	50	97.5	Sequence
**0**	**1**	**0.35**	**1.3**	**4.6**	**HLQLAIRNDEELNK**
**0**	**1**	**0.43**	**1.6**	**5.7**	**KTHTKPTTGAGSAAAGKA**
**0**	**1**	**0.14**	**0.5**	**1.8**	**QLAIRNDEELNK**

5.760 65 523 40S ribosomal protein S9 [Crassostrea gigas]; 65 523 40S ribosomal protein S9 [Crassostrea gigas]

Protein Accession	Unigene72778$_H$1 Unigene72208$_H$1
Mean Expression Ratio	0.989
Median Expression Ratio	0.991
Credible Interval	(0.501, 1.93)
Associated Peptides	2
Associated Spectra	2
Coverage	NaN

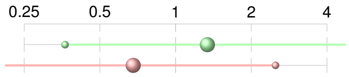

A	B	2.5	50	97.5	Sequence
**0**	**1**	**0.36**	**1.3**	**5**	**VKYTLGK**
**0**	**1**	**0.18**	**0.68**	**2.5**	**LIGEYGLR**

5.761 " 3 2693 PREDICTED: similar to coatomer protein complex, subunit beta 2 (beta prime) [Taeniopygia guttata]"

Protein Accession	CL19504.Contig2$_H$1
Mean Expression Ratio	0.988
Median Expression Ratio	0.991
Credible Interval	(0.508, 1.91)
Associated Peptides	2
Associated Spectra	2
Coverage	NaN

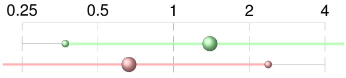

A	B	2.5	50	97.5	Sequence
0	1	0.37	1.4	5	TYLPSQVSR
0	1	0.17	0.67	2.4	VVSLWK

5.762 137 853 14-3-3 protein [Heterodera glycines]

Protein Accession	CL9040.Contig4$_H$1
Mean Expression Ratio	0.991
Median Expression Ratio	0.991
Credible Interval	(0.512, 1.91)
Associated Peptides	2
Associated Spectra	5
Coverage	NaN

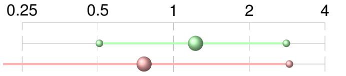

A	B	2.5	50	97.5	Sequence
2	2	0.51	1.2	2.8	VSEQAERYDDMAK
0	1	0.2	0.76	2.9	AIECANQAYQK

5.763 194 904 Aldo-keto reductase family 1 member B10 [Crassostrea gigas]

Protein Accession	CL3558.Contig4$_H$1
Mean Expression Ratio	0.989
Median Expression Ratio	0.991
Credible Interval	(0.499, 2)
Associated Peptides	1
Associated Spectra	1
Coverage	NaN

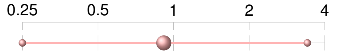

A	B	2.5	50	97.5	Sequence
1	0	0.25	0.91	3.4	ALEVLVSEGK

5.764 98 865 proteasome (prosome macropain) subunit alpha type 1 [Xenopus (Silurana) tropicalis]; 98 865 proteasome (prosome macropain) subunit alpha type 1 [Xenopus (Silurana) tropicalis]

Protein Accession	CL5647.Contig8$_H$1 CL5647.Contig7$_H$1
Mean Expression Ratio	0.991
Median Expression Ratio	0.991
Credible Interval	(0.5, 1.99)
Associated Peptides	1
Associated Spectra	4
Coverage	NaN

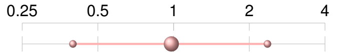

A	B	2.5	50	97.5	Sequence
0	4	0.4	0.98	2.4	LVSVVGNK

5.765 " 41 268 conserved hypothetical protein [Perkinsus marinus ATCC 50983] gi; 29 238 PREDICTED: succinyl-CoA ligase [GDP-forming] subunit beta, mitochondrial-like, partial [Meleagris gallopavo]"

Protein Accession	CL48047.Contig2$_{H}1$ CL48047.Contig1$_{H}1$
Mean Expression Ratio	0.992
Median Expression Ratio	0.991
Credible Interval	(0.502, 1.98)
Associated Peptides	1
Associated Spectra	1
Coverage	NaN

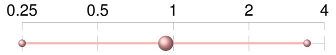

A	B	2.5	50	97.5	Sequence
**1**	**0**	**0.25**	**0.93**	**3.4**	**FVVASTPEDASK**

5.766 21 1007 minus strand cathepsin L2 cysteine protease [Pinctada fucata]; 21 1007 minus strand cathepsin L2 cysteine protease [Pinctada fucata]

Protein Accession	CL7881.Contig4$_{H}1$ CL7881.Contig3$_{H}1$
Mean Expression Ratio	1.01
Median Expression Ratio	1.01
Credible Interval	(0.523, 1.97)
Associated Peptides	2
Associated Spectra	2
Coverage	NaN

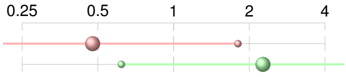

A	B	2.5	50	97.5	Sequence
**1**	**0**	**0.12**	**0.48**	**1.8**	**NLDLIIR**
**1**	**0**	**0.62**	**2.3**	**8.5**	**DLPDSVDWR**

5.767 165 1142 hypothetical protein CGI_10023942 [Crassostrea gigas]

Protein Accession	CL38025.Contig3$_{H}1$
Mean Expression Ratio	1.01
Median Expression Ratio	1.01
Credible Interval	(0.501, 2.07)
Associated Peptides	1
Associated Spectra	1
Coverage	NaN

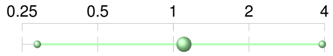

A	B	2.5	50	97.5	Sequence
**1**	**0**	**0.29**	**1.1**	**3.9**	**MVPGYTGYVPR**

5.768 " 115 534 NADH:ubiquinone oxidoreductase, NDUFB6/B17 subunit, putative [Ixodes scapularis] gi"

Protein Accession	CL16455.Contig5$_{H}1$
Mean Expression Ratio	1.01
Median Expression Ratio	1.01
Credible Interval	(0.501, 2.04)
Associated Peptides	1
Associated Spectra	1
Coverage	NaN

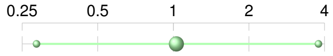

A	B	2.5	50	97.5	Sequence
**0**	**1**	**0.28**	**1**	**3.8**	**LAGTGMTDEDR**

5.769 62 292 PREDICTED: heterogeneous nuclear ribonucleoprotein F-like [Otolemur garnettii]

Protein Accession	CL30592.Contig1$_{H}$1
Mean Expression Ratio	1.01
Median Expression Ratio	1.01
Credible Interval	(0.52, 1.98)
Associated Peptides	2
Associated Spectra	2
Coverage	NaN

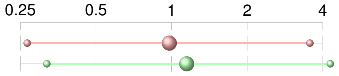

A	B	2.5	50	97.5	Sequence
**0**	**1**	**0.27**	**0.98**	**3.5**	**GLPWSTTPEDIVK**
**0**	**1**	**0.32**	**1.1**	**4.3**	**FFDESEIAGGEK**

5.770 349 885 juvenile hormone diol kinase [Danaus plexippus]; 349 885 juvenile hormone diol kinase [Danaus plexippus]; 349 885 juvenile hormone diol kinase [Danaus plexippus]; 349 885 minus strand juvenile hormone diol kinase [Danaus plexippus]; 349 885 juvenile hormone diol kinase [Danaus plexippus]; 349 885 juvenile hormone diol kinase [Danaus plexippus]; 349 885 juvenile hormone diol kinase [Danaus plexippus]; 349 885 juvenile hormone diol kinase [Danaus plexippus]; 349 885 juvenile hormone diol kinase [Danaus plexippus]; 349 885 juvenile hormone diol kinase [Danaus plexippus]

Protein Accession	Unigene38920$_{H}$1 Unigene38919$_{H}$1 CL7590.Contig1$_{H}$1 CL7590.Contig16$_{H}$1 CL7590.Contig
Mean Expression Ratio	1.01
Median Expression Ratio	1.01
Credible Interval	(0.514, 1.95)
Associated Peptides	2
Associated Spectra	2
Coverage	NaN

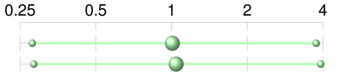

A	B	2.5	50	97.5	Sequence
**1**	**0**	**0.28**	**1**	**3.8**	**IFGNSEADSNK**
**1**	**0**	**0.28**	**1**	**3.9**	**DGIIDKNDFK**

5.771 773 1045 minus strand serine protease inhibitor 2 [Latrodectus hesperus]

Protein Accession	Unigene4107$_{H}$1
Mean Expression Ratio	0.996
Median Expression Ratio	0.992
Credible Interval	(0.509, 1.99)
Associated Peptides	1
Associated Spectra	2
Coverage	NaN

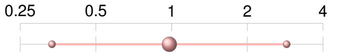

A	B	2.5	50	97.5	Sequence
**0**	**2**	**0.33**	**0.98**	**2.9**	**KFYFNK**

5.772 91 1326 minus strand Adipocyte plasma membrane-associated protein [Crassostrea gigas]; 107 1273 minus strand Adipocyte plasma membrane-associated protein [Crassostrea gigas]; 224 1267 Adipocyte plasma membrane-associated protein [Crassostrea gigas]; 209 1267 minus strand Adipocyte plasma membrane-associated protein [Crassostrea gigas]; 195 1253 minus strand Adipocyte plasma membrane-associated protein [Crassostrea gigas]

Protein Accession	CL11239.Contig2$_H$1 CL11239.Contig1$_H$1 CL11239.Contig12$_H$1 CL11239.Contig11$_H$1 CL11
Mean Expression Ratio	1.01
Median Expression Ratio	1.01
Credible Interval	(0.5, 2)
Associated Peptides	1
Associated Spectra	1
Coverage	NaN

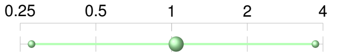

A	B	2.5	50	97.5	Sequence
**0**	**1**	**0.28**	**1**	**3.7**	**YGLIAELDKEGK**

5.773 101 1693 CALNUC [Spodoptera frugiperda]; 141 1325 Nucleobindin-2 [Crassostrea gigas]; 141 1289 Nucleobindin-2 [Crassostrea gigas]

Protein Accession	CL1888.Contig9$_H$1 CL1888.Contig6$_H$1 CL1888.Contig5$_H$1
Mean Expression Ratio	0.993
Median Expression Ratio	0.992
Credible Interval	(0.506, 1.94)
Associated Peptides	2
Associated Spectra	2
Coverage	NaN

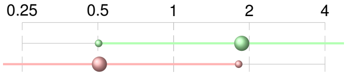

A	B	2.5	50	97.5	Sequence
**0**	**1**	**0.5**	**1.9**	**6.9**	**LKELPEVER**
**0**	**1**	**0.14**	**0.51**	**1.8**	**LETANISDIK**

5.774 209 781 minus strand high mobility group protein 1 [Biomphalaria glabrata]; 277 771 high mobility group protein 1 [Biomphalaria glabrata]; 277 867 minus strand high mobility group protein 1 [Biomphalaria glabrata]; 277 870 minus strand high mobility group protein 1 [Biomphalaria glabrata]

Protein Accession	CL25076.Contig4$_H$1 CL25076.Contig3$_H$1 CL25076.Contig2$_H$1 CL25076.Contig1$_H$1
Mean Expression Ratio	1.01
Median Expression Ratio	1.01
Credible Interval	(0.561, 1.77)
Associated Peptides	5
Associated Spectra	12
Coverage	NaN

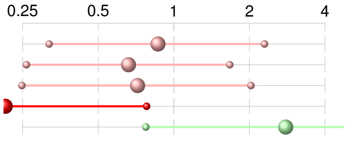

A	B	2.5	50	97.5	Sequence
**1**	**2**	**0.32**	**0.87**	**2.3**	**KYEAEMAEYR**
**2**	**2**	**0.26**	**0.66**	**1.7**	**LAGEEWR**
**2**	**1**	**0.25**	**0.72**	**2**	**KPFEDMAAK**
**0**	**1**	**0.059**	**0.21**	**0.78**	**LGDFR**
**0**	**1**	**0.77**	**2.8**	**10**	**ELPAGQKKPFEDMAAKDKAR**

5.775 " 599 907 minus strand Tryptophanyl-tRNA synthetase, cytoplasmic [Heterocephalus glaber]"

Protein Accession	Unigene26755$_{H}1$
Mean Expression Ratio	1.01
Median Expression Ratio	1.01
Credible Interval	(0.522, 1.96)
Associated Peptides	2
Associated Spectra	3
Coverage	NaN

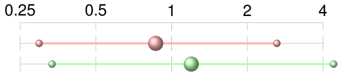

A	B	2.5	50	97.5	Sequence
**0**	**2**	**0.3**	**0.86**	**2.6**	**DMDPLLDLYEK**
**0**	**1**	**0.33**	**1.2**	**4.4**	**KTPFYLYTGR**

5.776 116 337 minus strand Golgi-associated plant pathogenesis-related protein 1 [Crassostrea gigas]; 538 759 minus strand Golgi-associated plant pathogenesis-related protein 1 [Crassostrea gigas]; 116 337 minus strand Golgi-associated plant pathogenesis-related protein 1 [Crassostrea gigas]; 261 482 minus strand Golgi-associated plant pathogenesis-related protein 1 [Crassostrea gigas]; 116 337 minus strand Golgi-associated plant pathogenesis-related protein 1 [Crassostrea gigas]; 116 337 minus strand Golgi-associated plant pathogenesis-related protein 1 [Crassostrea gigas]

Protein Accession	CL15263.Contig9$_{H}1$ CL15263.Contig8$_{H}1$ CL15263.Contig7$_{H}1$ CL15263.Contig5$_{H}1$ CL1526
Mean Expression Ratio	0.993
Median Expression Ratio	0.993
Credible Interval	(0.494, 1.98)
Associated Peptides	1
Associated Spectra	1
Coverage	NaN

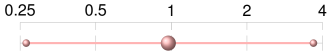

A	B	2.5	50	97.5	Sequence
**0**	**1**	**0.26**	**0.97**	**3.7**	**KEHQVQPLVINK**

5.777 " 33 1295 PREDICTED: LOW QUALITY PROTEIN: selenide, water dikinase-like [Bombus impatiens]"

Protein Accession	CL14673.Contig3$_{H}1$
Mean Expression Ratio	1.01
Median Expression Ratio	1.01
Credible Interval	(0.505, 1.99)
Associated Peptides	1
Associated Spectra	1
Coverage	NaN

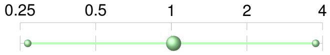

A	B	2.5	50	97.5	Sequence
**0**	**1**	**0.27**	**1**	**3.7**	**RPFDPVAHELDPNFR**

5.778 28 1908 minus strand Mitochondrial inner membrane protein [Crassostrea gigas]; 10 1902 Mitochondrial inner membrane protein [Crassostrea gigas]; 10 1902 Mitochondrial inner membrane protein [Crassostrea gigas]; 10 1902 Mitochondrial inner membrane protein [Crassostrea gigas]; 10 1902 Mitochondrial inner membrane protein [Crassostrea gigas]; 10 1902 Mitochondrial inner membrane protein [Crassostrea gigas]; 10 1902 Mitochondrial inner membrane protein [Crassostrea gigas]

Protein Accession	CL6414.Contig9$_{H}1$ CL6414.Contig8$_{H}1$ CL6414.Contig7$_{H}1$ CL6414.Contig6$_{H}1$ CL6414.Con
Mean Expression Ratio	1.01
Median Expression Ratio	1.01
Credible Interval	(0.506, 2.02)
Associated Peptides	1
Associated Spectra	1
Coverage	NaN

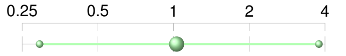

A	B	2.5	50	97.5	Sequence
0	1	0.29	1	3.8	EIHTQLLQER

5.779 54 638 minus strand ubiquitin carboxyl-terminal hydrolase [Aplysia californica] gi

Protein Accession	CL20666.Contig3$_{H}1$
Mean Expression Ratio	0.998
Median Expression Ratio	0.994
Credible Interval	(0.522, 1.89)
Associated Peptides	3
Associated Spectra	3
Coverage	NaN

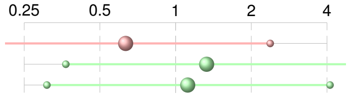

A	B	2.5	50	97.5	Sequence
0	1	0.17	0.63	2.4	THADTLLEDTVK
0	1	0.37	1.3	4.9	KDAPVCHGK
0	1	0.31	1.1	4.1	FLDETK

5.780 157 459 minus strand Synaptojanin-2-binding protein [Crassostrea gigas]

Protein Accession	CL12494.Contig1$_{H}1$
Mean Expression Ratio	1.01
Median Expression Ratio	1.01
Credible Interval	(0.578, 1.76)
Associated Peptides	6
Associated Spectra	17
Coverage	NaN

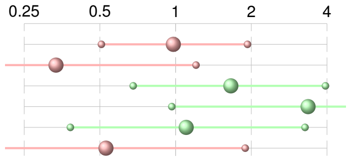

A	B	2.5	50	97.5	Sequence
3	5	0.51	0.98	1.9	GDSGLGFNIR
1	0	0.093	0.33	1.2	LKEDGAAFK
0	4	0.68	1.7	3.9	LKEGDKILEVNGK
0	1	0.96	3.4	12	LKEDGAAFKDGR
0	2	0.38	1.1	3.3	GGTDIPHVPGDTGIFVTR
0	1	0.14	0.53	1.9	ILEVNGK

5.781 " 90 1085 squid, variant A [Blattella germanica]"

Protein Accession CL14696.Contig5$_H1$
Mean Expression Ratio 0.989
Median Expression Ratio 0.994
Credible Interval (0.533, 1.83)
Associated Peptides 3
Associated Spectra 8
Coverage NaN

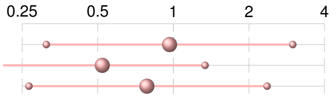

A	B	2.5	50	97.5	Sequence
**1**	**1**	**0.31**	**0.97**	**3**	**IMEVTTHK**
**2**	**2**	**0.2**	**0.52**	**1.3**	**LGGQEVDVK**
**2**	**0**	**0.27**	**0.79**	**2.4**	**IFVGR**

5.782 247 1440 minus strand Septin-2 [Crassostrea gigas]

Protein Accession CL14404.Contig1$_H1$
Mean Expression Ratio 0.995
Median Expression Ratio 0.994
Credible Interval (0.515, 1.91)
Associated Peptides 2
Associated Spectra 5
Coverage NaN

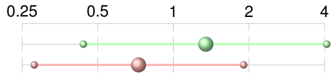

A	B	2.5	50	97.5	Sequence
**0**	**2**	**0.44**	**1.4**	**4.1**	**HKLEEMGFSDSETR**
**0**	**3**	**0.28**	**0.73**	**1.9**	**ALDLVTMK**

5.783 136 675 minus strand predicted protein [Nematostella vectensis] gi

Protein Accession CL17411.Contig1$_H1$
Mean Expression Ratio 0.997
Median Expression Ratio 0.994
Credible Interval (0.524, 1.87)
Associated Peptides 3
Associated Spectra 4
Coverage NaN

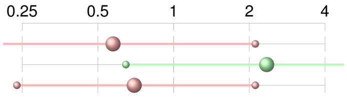

A	B	2.5	50	97.5	Sequence
**1**	**0**	**0.16**	**0.58**	**2.1**	**MLNEDELR**
**0**	**1**	**0.64**	**2.3**	**8.7**	**MLNEDELRDAK**
**0**	**2**	**0.24**	**0.7**	**2.1**	**VSEARDELQR**

5.784 187 1323 minus strand gelsolin [Aplysia californica] gi

Protein Accession CL10220.Contig1$_H1$
Mean Expression Ratio 0.994
Median Expression Ratio 0.995
Credible Interval (0.528, 1.84)
Associated Peptides 3
Associated Spectra 5
Coverage NaN

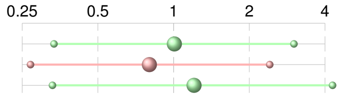

A	B	2.5	50	97.5	Sequence
**0**	**2**	**0.33**	**1**	**3**	**AAQVLQDLK**
**0**	**2**	**0.27**	**0.8**	**2.4**	**LSDASGELDFK**
**0**	**1**	**0.33**	**1.2**	**4.3**	**SDVETIDEEEGGAALEK**

5.785 103 1356 minus strand PREDICTED: actin-related protein 3-like [Oreochromis niloticus]

Protein Accession	CL20855.Contig4$_{H}$1
Mean Expression Ratio	0.996
Median Expression Ratio	0.995
Credible Interval	(0.516, 1.91)
Associated Peptides	2
Associated Spectra	3
Coverage	NaN

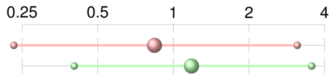

A	B	2.5	50	97.5	Sequence
**1**	**0**	**0.23**	**0.84**	**3.1**	**LPAVVVDTGTGYTK**
**0**	**2**	**0.4**	**1.2**	**3.6**	**HNPVFGAMA**

5.786 " 174 569 NADH dehydrogenase [ubiquinone] iron-sulfur protein 4, mitochondrial [Crassostrea gigas]"

Protein Accession	CL4564.Contig1$_{H}$1
Mean Expression Ratio	1
Median Expression Ratio	1.01
Credible Interval	(0.506, 1.96)
Associated Peptides	1
Associated Spectra	1
Coverage	NaN

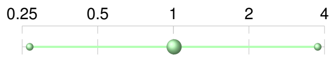

A	B	2.5	50	97.5	Sequence
**0**	**1**	**0.27**	**1**	**3.8**	**VDISCLTGVPEEHIK**

5.787 406 1392 minus strand hypothetical protein CGI_10007007 [Crassostrea gigas]

Protein Accession	CL18045.Contig1$_{H}$1
Mean Expression Ratio	0.995
Median Expression Ratio	0.996
Credible Interval	(0.498, 1.98)
Associated Peptides	1
Associated Spectra	1
Coverage	NaN

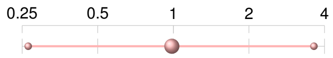

A	B	2.5	50	97.5	Sequence
**0**	**1**	**0.26**	**0.98**	**3.6**	**WQRPDKPVGFYDLK**

5.788 99 3011 Eukaryotic translation initiation factor 3 subunit A [Crassostrea gigas]

Protein Accession	CL36771.Contig2$_{H}$1
Mean Expression Ratio	1
Median Expression Ratio	1
Credible Interval	(0.508, 1.96)
Associated Peptides	2
Associated Spectra	2
Coverage	NaN

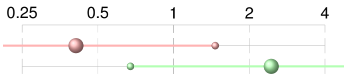

A	B	2.5	50	97.5	Sequence
**0**	**1**	**0.11**	**0.41**	**1.5**	**ELALETK**
**0**	**1**	**0.67**	**2.4**	**8.9**	**KVDYLAR**

5.789 128 1138 Actin-related protein 2/3 complex subunit 1A OS=Homo sapiens GN=ARPC1A PE=1 SV=2

Protein Accession	CL49279.Contig6$_{H}1$
Mean Expression Ratio	1
Median Expression Ratio	1
Credible Interval	(0.509, 1.99)
Associated Peptides	1
Associated Spectra	1
Coverage	NaN

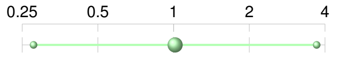

A	B	2.5	50	97.5	Sequence
**0**	**1**	**0.28**	**1**	**3.7**	**TQHQNTITQVSVYEGTK**

5.790 " 95 1279 2-amino-3-ketobutyrate coenzyme A ligase, mitochondrial, partial [Crassostrea gigas]"

Protein Accession	CL33712.Contig1$_{H}1$
Mean Expression Ratio	1
Median Expression Ratio	1
Credible Interval	(0.528, 1.9)
Associated Peptides	3
Associated Spectra	3
Coverage	NaN

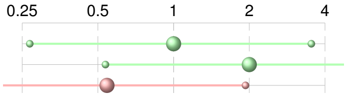

A	B	2.5	50	97.5	Sequence
**0**	**1**	**0.27**	**1**	**3.5**	**GTEEHFNIMGR**
**0**	**1**	**0.54**	**2**	**7.4**	**VIQSPQGPSIR**
**0**	**1**	**0.15**	**0.54**	**1.9**	**LAATLR**

5.791 4 942 minus strand KTEL motif-containing protein 1 [Crassostrea gigas]

Protein Accession	CL11353.Contig3$_{H}1$
Mean Expression Ratio	1
Median Expression Ratio	1
Credible Interval	(0.497, 1.98)
Associated Peptides	1
Associated Spectra	1
Coverage	NaN

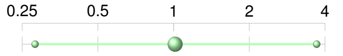

A	B	2.5	50	97.5	Sequence
**0**	**1**	**0.28**	**1**	**3.7**	**GIHYQIVDHK**

5.792 71 1321 minus strand Vinculin [Crassostrea gigas]

Protein Accession	Unigene23324$_{H}1$
Mean Expression Ratio	1.01
Median Expression Ratio	1
Credible Interval	(0.532, 1.93)
Associated Peptides	3
Associated Spectra	3
Coverage	NaN

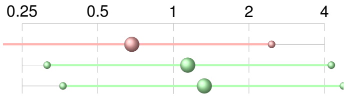

A	B	2.5	50	97.5	Sequence
**0**	**1**	**0.19**	**0.68**	**2.5**	**GLVDEAVDTAALIR**
**0**	**1**	**0.32**	**1.1**	**4.3**	**KPAPTVDGK**
**0**	**1**	**0.36**	**1.3**	**4.7**	**LINAVGGVR**

5.793 204 791 minus strand PREDICTED: 15-hydroxyprostaglandin dehydrogenase [NAD+] [Gallus gallus]

Protein Accession	Unigene3818$_{H}$1
Mean Expression Ratio	0.996
Median Expression Ratio	0.997
Credible Interval	(0.628, 1.6)
Associated Peptides	10
Associated Spectra	88
Coverage	NaN

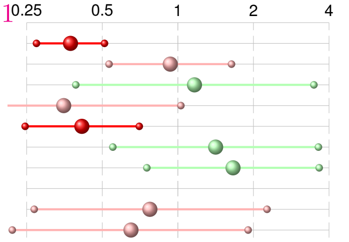

A	B	2.5	50	97.5	Sequence
17	25	0.27	0.37	0.51	AVTEALLAK
7	5	0.53	0.93	1.6	IAFVSGGAMGTGR
1	1	0.39	1.2	3.5	MGTTTDKELK
1	1	0.12	0.35	1	VMFCDIDEK
3	11	0.25	0.41	0.7	AITMWGK
0	3	0.55	1.4	3.6	DITDKIAFVSGGAMGTGR
0	5	0.75	1.7	3.6	MDITDKIAFVSGGAMGTGR
0	4	5.9	14	33	AVTEALLAKGAK
0	2	0.27	0.78	2.3	MDITDK
0	2	0.22	0.65	1.9	HFISAFEK

5.794 442 2595 minus strand Amiloride-sensitive amine oxidase [copper-containing] [Crassostrea gigas]

Protein Accession	Unigene105$_{H}$1
Mean Expression Ratio	1
Median Expression Ratio	0.997
Credible Interval	(0.529, 1.9)
Associated Peptides	3
Associated Spectra	4
Coverage	NaN

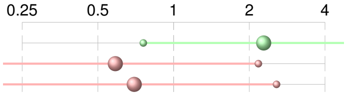

A	B	2.5	50	97.5	Sequence
1	1	0.76	2.3	6.9	QQVAVTVQKDEER
0	1	0.16	0.59	2.2	ILPTGMSPQLLK
0	1	0.2	0.7	2.6	VTDFVDSGALIGTHSK

5.795 166 2364 Dipeptidyl-peptidase 3 [Crassostrea gigas]

Protein Accession	CL9071.Contig2$_{H}$1
Mean Expression Ratio	0.995
Median Expression Ratio	0.998
Credible Interval	(0.5, 1.97)
Associated Peptides	1
Associated Spectra	1
Coverage	NaN

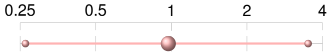

A	B	2.5	50	97.5	Sequence
0	1	0.26	0.97	3.5	VETIILK

5.796 58 1203 minus strand Obg-like ATPase 1 [Crassostrea gigas]

Protein Accession	CL33105.Contig1$_{H}$1
Mean Expression Ratio	1
Median Expression Ratio	1
Credible Interval	(0.493, 2.01)
Associated Peptides	1
Associated Spectra	1
Coverage	NaN

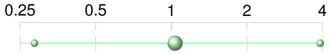

A	B	2.5	50	97.5	Sequence
0	1	0.29	1	3.9	IHTDFEK

5.797 5 748 hypothetical protein SINV_09458 [Solenopsis invicta]

Protein Accession	CL25819.Contig2$_{H}1$
Mean Expression Ratio	1
Median Expression Ratio	1
Credible Interval	(0.49, 2.03)
Associated Peptides	1
Associated Spectra	1
Coverage	NaN

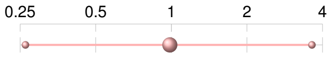

A	B	2.5	50	97.5	Sequence
**0**	**1**	**0.26**	**0.98**	**3.6**	**LVSPAASAR**

5.798 457 1842 Basement membrane-specific heparan sulfate proteoglycan core protein [Crassostrea gigas]; 457 1842 Basement membrane-specific heparan sulfate proteoglycan core protein [Crassostrea gigas]

Protein Accession	CL24300.Contig9$_{H}1$ CL24300.Contig10$_{H}1$
Mean Expression Ratio	0.996
Median Expression Ratio	0.999
Credible Interval	(0.489, 2.01)
Associated Peptides	1
Associated Spectra	1
Coverage	NaN

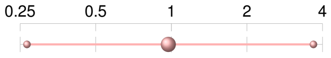

A	B	2.5	50	97.5	Sequence
**0**	**1**	**0.27**	**0.97**	**3.7**	**KDGELPVEHK**

5.799 75 455 minus strand PREDICTED: calnexin [Strongylocentrotus purpuratus]

Protein Accession	CL46825.Contig1$_{H}1$
Mean Expression Ratio	1
Median Expression Ratio	0.999
Credible Interval	(0.512, 1.97)
Associated Peptides	2
Associated Spectra	2
Coverage	NaN

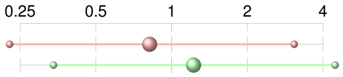

A	B	2.5	50	97.5	Sequence
**0**	**1**	**0.23**	**0.82**	**3.1**	**APVFFHEPFDSK**
**0**	**1**	**0.34**	**1.2**	**4.5**	**SNALDGDLGLILK**

5.800 440 1645 Tektin-1 [Crassostrea gigas]

Protein Accession	CL18799.Contig2$_{H}1$
Mean Expression Ratio	0.998
Median Expression Ratio	1
Credible Interval	(0.514, 1.97)
Associated Peptides	2
Associated Spectra	2
Coverage	NaN

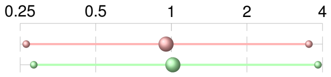

A	B	2.5	50	97.5	Sequence
**0**	**1**	**0.26**	**0.95**	**3.5**	**SLQATEEPLHIAR**
**0**	**1**	**0.28**	**1**	**3.8**	**FTHEDWTR**

5.801 94 519 minus strand hypothetical protein BRAFLDRAFT_115029 [Branchiostoma floridae] gi

Protein Accession	Unigene52953$_{H}1$
Mean Expression Ratio	1
Median Expression Ratio	1
Credible Interval	(0.525, 1.92)
Associated Peptides	3
Associated Spectra	4
Coverage	NaN

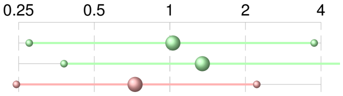

A	B	2.5	50	97.5	Sequence
0	1	0.27	1	3.8	YGVVFK
0	1	0.38	1.4	5.1	FINKK
0	2	0.24	0.72	2.2	SLDTDGDGTVSAAELR

iTRAQ Data Analysis Report

Javier, Body 14d versus control

April 7, 2014

1 Introduction

This document summarizes an analysis of relative protein expression using iTRAQ. The reporter ion peak area measurements supplied by the ABI software are used to estimate treatment-dependent peptide and protein relative expression. Estimation is accomplished using a Bayesian approach with the model given below. The document includes a protein relative expression summary and a per-protein detailed analysis. The document is internally hyperlinked and linked externally to NCBI.

2 Experiment and Model Description

2.1 Experiment Design

The report summarizes data from one or more iTRAQ experiments addressing a common comparison. The experiment design, used in this analysis, is given in the table below.

	Experiment	Treatment	Channel	Sample
1	A	control	113	s1
2	A	test	116	s1
3	B	control	113	s2
4	B	test	116	s2

2.2 Input Files

Data for this analysis was extracted from the following tandem mass spectra (MSMS) summary files.

Experiment	MSMS Summary File
A	body1.txt
B	body2.txt

2.3 Statistical Model

The following statistical model was used to estimate the treatment-dependent effects.

LogIntensity ~ Channel + Spectrum + Protein + Peptide + Protein:Treatment + Peptide:Treatment

3 Data Summary

The data supplied in the MSMS summary is filtered to remove unidentified proteins, contaminants, and peptides containing selected modifications. The following table sumarizes the data provided and used in the analysis.

	A	B	Combined
Supplied Spectra	11560	18985	30545
Unidentified Spectra	6008	8178	14186
Disallowed Modifications	41	210	251
Spectra from Contaminants	0	0	0
Missing Data	1521	2675	4196
Low Confidence Spectra	6008	8178	14186
Degenerate Peptides	1596	2504	4100
Remaining Spectra			7812
Unique Proteins			820
Unique Peptides			2898
Model R^2			0.764

4 Protein Summary

Each protein identified in one or more of the MSMS summaries is listed below in decreasing order of expression change magnitude. The median and estimated credible interval for each protein is given to the left in the table. Proteins identified by a single peptide are listed in a separate table.

4.1 Identified Proteins

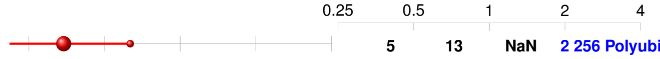

	Peptides	Spectra	Coverage	Protein
	5	13	NaN	2 256 Polyubiquitin (Fragment) OS=Xenopus laevis PE=1 SV=2; 20 388 large subunit ribosomal protein L40e [Clonor

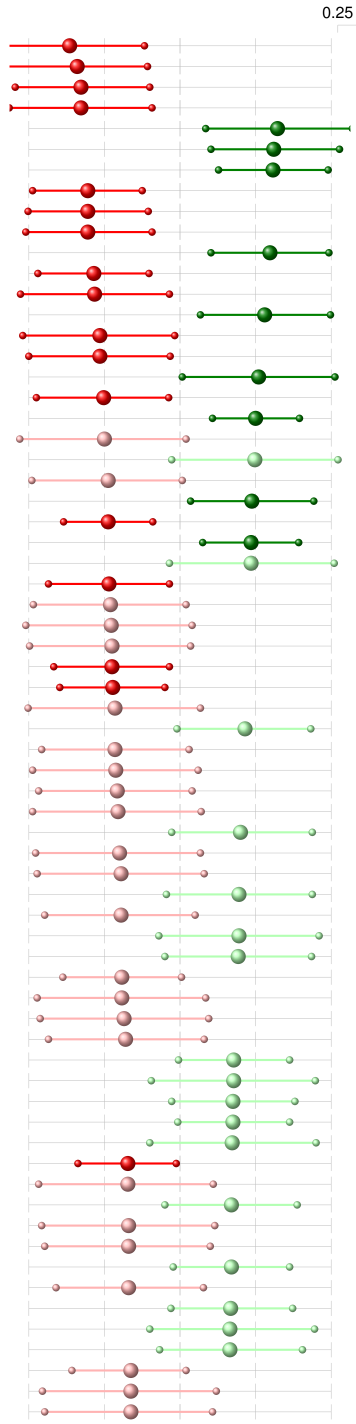

Peptides	Spectra	Coverage	Protein
2	15	NaN	507 1277 alpha tubulin [Notothenia coriiceps]
2	14	NaN	160 588 hypothetical protein CGI_10001640 [Crassostrea gigas]
8	9	NaN	254 1648 Tektin-3 [Crassostrea gigas]; 254 1648 Tektin-3 [Crassostrea gigas]
4	18	NaN	266 607 minus strand H2 [Haliotis tuberculata]
3	11	NaN	195 455 60S ribosomal protein L23a OS=Rattus norvegicus GN=Rpl23a PE=2 SV=1; 195 512 60S ribosomal protein L
5	17	NaN	118 501 minus strand histone 2A.Z [Aplysia californica]; 15 428 PREDICTED: histone H2A.V-like [Apis florea]
7	76	NaN	764 1015 minus strand heat shock protein beta-1 [Trichinella spiralis] gi
8	45	NaN	2 370 hemocyanin alphaD-subunit [Helix lucorum]
6	34	NaN	118 501 minus strand histone 2A.Z [Aplysia californica]
4	29	NaN	1070 1888 minus strand galectin [Pinctada fucata]; 1371 2189 minus strand galectin [Pinctada fucata]
6	46	NaN	1592 1768 minus strand beta-actin [Diabolocatantops pinguis]
9	32	NaN	503 1090 minus strand myosin regulatory light chain [Fasciola hepatica]; 278 865 minus strand myosin regulatory li
3	9	NaN	195 455 60S ribosomal protein L23a OS=Rattus norvegicus GN=Rpl23a PE=2 SV=1; 16 465 60S ribosomal protein L2
5	25	NaN	" 172 867 minus strand complement C1q-like protein 4 precursor, partial [Oryzias melastigma]"
3	10	NaN	209 781 minus strand high mobility group protein 1 [Biomphalaria glabrata]
4	17	NaN	27 620 putative mitochondrial ATP synthase [Haliotis discus discus]
3	10	NaN	" 223 423 ADPATP carrier protein; K05863 solute carrier family 25 (mitochondrial adenine nucleotide translocator), m
6	12	NaN	1009 2784 minus strand Collagen alpha-3(VI) chain [Crassostrea gigas]; 1009 2787 minus strand Collagen alpha-3(V
16	114	NaN	97 477 minus strand PREDICTED: complement C1q tumor necrosis factor-related protein 3-like [Oreochromis niloti
2	3	NaN	140 283 minus strand elongation factor 1 alpha [Angiostrongylus cantonensis]
2	6	NaN	2 328 cytoskeletal actin 2 [Molgula oculata]
3	8	NaN	3 272 minus strand ribosomal protein L28 [Haliotis asinina]
8	17	NaN	" 276 659 Fatty acid-binding protein, adipocyte [Crassostrea gigas]; 426 809 Fatty acid-binding protein, adipocyte [C
18	39	NaN	205 2490 minus strand glucose-regulated protein 94 [Crassostrea gigas]
14	33	NaN	227 3073 minus strand Lysosomal alpha-mannosidase [Crassostrea gigas]
2	4	NaN	80 589 minus strand PREDICTED: adenine phosphoribosyltransferase-like [Strongylocentrotus purpuratus]
6	24	NaN	" 10 609 lethal(2)essential for life protein, l2efl [Aedes aegypti] gi"
3	8	NaN	" 1240 1911 PREDICTED: ATP synthase subunit alpha, mitochondrial-like [Anolis carolinensis]"
2	4	NaN	16 288 SJCHGC01950 protein [Schistosoma japonicum] gi
3	4	NaN	1 1350 UPF0663 transmembrane protein C17orf28 [Crassostrea gigas]; 163 1542 UPF0663 transmembrane protein C
6	56	NaN	222 809 minus strand PREDICTED: 15-hydroxyprostaglandin dehydrogenase [NAD+] [Gallus gallus]
7	56	NaN	" 353 781 alpha-B-crystallin, putative [Ixodes scapularis] gi"
2	2	NaN	181 684 hypothetical protein BRAFLDRAFT_131834 [Branchiostoma floridae] gi
4	31	NaN	161 301 Heat shock protein beta-6 OS=Mus musculus GN=Hspb6 PE=1 SV=1
3	9	NaN	" 90 1085 squid, variant A [Blattella germanica]"
2	4	NaN	1 945 hemocyanin isoform 1 [Haliotis diversicolor]
4	4	NaN	210 779 GH20056 [Drosophila grimshawi] gi; 216 785 minus strand GH20056 [Drosophila grimshawi] gi; 210 779 GH2
2	3	NaN	108 401 60S ribosomal L7a-like protein [Phragmatopoma lapidosa]; 108 401 60S ribosomal L7a-like protein [Phragm
4	11	NaN	346 702 minus strand PREDICTED: galectin-4-like [Anolis carolinensis]; 367 669 minus strand PREDICTED: galectin
2	4	NaN	112 819 pyruvate kinase [Crassostrea gigas]; 132 932 pyruvate kinase [Crassostrea gigas]
2	3	NaN	14 1642 minus strand chaperonin containing tcp1 [Haliotis discus discus]; 15 1619 chaperonin containing tcp1 [Hali
2	15	NaN	" 3 227 minus strand alpha tubulin, putative [Ixodes scapularis] gi"
3	9	NaN	22 870 minus strand PREDICTED: alcohol dehydrogenase class-3 [Sarcophilus harrisii]
2	5	NaN	5 193 minus strand PREDICTED: 40S ribosomal protein S28-like [Strongylocentrotus purpuratus]; 3 122 PREDICTED
5	7	NaN	156 1163 cathepsin B [Crassostrea ariakensis]
9	18	NaN	72 1400 minus strand hypothetical protein RCJMB04_22i13 [Gallus gallus]
2	3	NaN	REVERSED 216 2639 minus strand polycystin 2 [Gallus gallus]
2	3	NaN	871 1371 ribosomal protein L11 [Triatoma infestans]; 343 843 minus strand ribosomal protein L11 [Triatoma infestan
3	4	NaN	736 1287 60S ribosomal protein L3 OS=Rattus norvegicus GN=Rpl3 PE=1 SV=3
6	46	NaN	" 99 497 RecName: Full=Histone H2B, gonadal"
2	5	NaN	2019 2723 minus strand Kyphoscoliosis peptidase [Crassostrea gigas]; 2060 2764 minus strand Kyphoscoliosis pep
10	11	NaN	" 59 2365 minus strand Aconitate hydratase, mitochondrial [Crassostrea gigas]"
8	43	NaN	" 242 646 minus strand Fatty acid-binding protein, heart [Crassostrea gigas]; 227 631 minus strand Fatty acid-bindin
2	3	NaN	" 53 1291 Acetyl-CoA acetyltransferase B, mitochondrial [Crassostrea gigas]"
11	44	NaN	594 1253 peroxiredoxin 6 [Eriocheir sinensis]
2	2	NaN	163 1059 minus strand Heterogeneous nuclear ribonucleoprotein A2-like protein 1 [Crassostrea gigas]; 99 995 minu
7	12	NaN	55 795 Elongation factor 1-delta OS=Bos taurus GN=EEF1D PE=2 SV=2; 49 747 PREDICTED: elongation factor 1-del
2	2	NaN	3 500 Tumor protein D54 [Crassostrea gigas]; 3 464 Tumor protein D54 [Crassostrea gigas]; 3 464 Tumor protein D54
2	4	NaN	1337 1537 minus strand hemocyanin [Haliotis tuberculata]
8	24	NaN	30 536 putative mitochondrial ATP synthase [Haliotis discus discus]; 27 620 putative mitochondrial ATP synthase [H
3	8	NaN	27 515 heat shock protein 70 [Capsaspora owczarzaki ATCC 30864]; 30 464 minus strand hypothetical protein [Trich
10	13	NaN	111 1226 gelsolin [Aplysia californica] gi; 42 1139 minus strand gelsolin [Aplysia californica] gi
2	4	NaN	390 2267 neurofilament protein NF70 [Helix aspersa]; 318 2195 neurofilament protein NF70 [Helix aspersa]
5	7	NaN	185 742 Transmembrane protein Tmp21 [Crassostrea gigas]
8	25	NaN	372 860 minus strand small heat shock protein [Tegillarca granosa]
2	2	NaN	2 349 minus strand hypothetical protein BRAFLDRAFT_206907 [Branchiostoma floridae] gi
2	3	NaN	103 684 EF-hand domain-containing protein D2 [Crassostrea gigas]

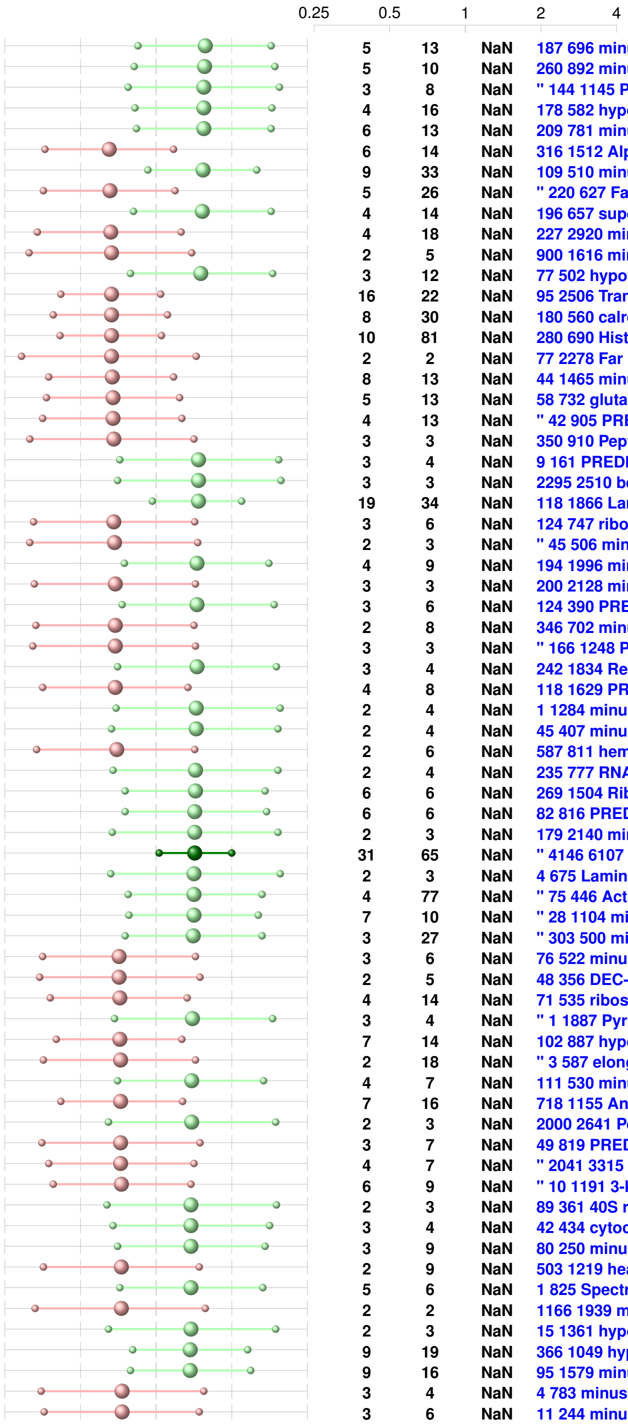

Peptides	Spectra	Coverage	Protein
5	13	NaN	187 696 minus strand putative ribosomal protein S10 [Sipunculus nudus]
5	10	NaN	260 892 minus strand calponin [Haliotis diversicolor]
3	8	NaN	" 144 1145 PREDICTED: RNA-binding protein squid-like isoform 1 [Megachile rotundata]; 90 1085 squid, variant A [B
4	16	NaN	178 582 hypothetical protein CGI_10001640 [Crassostrea gigas]
6	13	NaN	209 781 minus strand high mobility group protein 1 [Biomphalaria glabrata]; 277 771 high mobility group protein 1 [
6	14	NaN	316 1512 Alpha-N-acetylgalactosaminidase [Crassostrea gigas]; 282 1478 Alpha-N-acetylgalactosaminidase [Crasso
9	33	NaN	109 510 minus strand hypothetical protein DAPPUDRAFT_231281 [Daphnia pulex]
5	26	NaN	" 220 627 Fatty acid-binding protein, brain [Crassostrea gigas]"
4	14	NaN	196 657 superoxide dismutase [Aplysia californica] gi; 690 1151 superoxide dismutase [Aplysia californica] gi
4	18	NaN	227 2920 minus strand Lysosomal alpha-mannosidase [Crassostrea gigas]; 227 3073 minus strand Lysosomal alpha
2	5	NaN	900 1616 minus strand hemocyanin isoform 1 [Haliotis diversicolor]
3	12	NaN	77 502 hypothetical protein CGI_10008263 [Crassostrea gigas]
16	22	NaN	95 2506 Transitional endoplasmic reticulum ATPase [Crassostrea gigas]
8	30	NaN	180 560 calreticulin precursor [Aplysia californica] gi; 180 545 calreticulin precursor [Aplysia californica] gi
10	81	NaN	280 690 Histone H3.3 [Harpegnathos saltator]
2	2	NaN	77 2278 Far upstream element-binding protein 3 [Crassostrea gigas]
8	13	NaN	44 1465 minus strand Adenylyl cyclase-associated protein 1 [Crassostrea gigas]; 52 1473 minus strand Adenylyl cyc
5	13	NaN	58 732 glutathione-s-transferase [Haliotis discus discus]
4	13	NaN	" 42 905 PREDICTED: enoyl-CoA hydratase, mitochondrial-like isoform 1 [Oreochromis niloticus]"
3	3	NaN	350 910 Peptidyl-prolyl cis-trans isomerase B [Crassostrea gigas]; 488 1039 Peptidyl-prolyl cis-trans isomerase B [C
3	4	NaN	9 161 PREDICTED: tubulin alpha-1C chain-like [Acyrthosiphon pisum]; 9 161 Tubulin alpha-3 chain (Fragments) OS=
3	3	NaN	2295 2510 beta-thymosin domain repeat protein CSP24KDa_v1 [Hermissenda crassicornis] gi
19	34	NaN	118 1866 Lamin Dm0 [Crassostrea gigas]
3	6	NaN	124 747 ribosomal protein S10 [Crassostrea gigas] gi
2	3	NaN	" 45 506 minus strand PREDICTED: dihydrolipoyl dehydrogenase, mitochondrial [Monodelphis domestica]"
4	9	NaN	194 1996 minus strand Neutral and basic amino acid transport protein rBAT [Crassostrea gigas]; 265 2067 minus str
3	3	NaN	200 2128 minus strand heat shock protein 70 [Cristaria plicata]
3	6	NaN	124 390 PREDICTED: 60S ribosomal protein L23a-like [Callithrix jacchus]
2	8	NaN	346 702 minus strand PREDICTED: galectin-4-like [Anolis carolinensis]
3	3	NaN	" 166 1248 PREDICTED: probable pyruvate dehydrogenase E1 component subunit alpha, mitochondrial-like [Metase
3	4	NaN	242 1834 Regulator of differentiation 1 [Crassostrea gigas]; 458 2050 minus strand Regulator of differentiation 1 [Cr
4	8	NaN	118 1629 PREDICTED: similar to tektin 3 [Ciona intestinalis]
2	4	NaN	1 1284 minus strand Thioredoxin reductase 3 [Crassostrea gigas]
2	4	NaN	45 407 minus strand 60S ribosomal protein L31 OS=Xenopus laevis GN=rpl31 PE=2 SV=1
2	6	NaN	587 811 hemocyanin 2 [Megathura crenulata]
2	4	NaN	235 777 RNA-binding protein 4.1 [Clonorchis sinensis]
6	6	NaN	269 1504 Ribosome-binding protein 1 [Crassostrea gigas]; 265 1500 Ribosome-binding protein 1 [Crassostrea gigas
6	6	NaN	82 816 PREDICTED: triosephosphate isomerase B [Oreochromis niloticus]
2	3	NaN	179 2140 minus strand PREDICTED: hypothetical protein [Saccoglossus kowalevskii]
31	65	NaN	" 4146 6107 minus strand nonmuscle myosin II, partial [Aplysia californica]"
2	3	NaN	4 675 Laminin subunit gamma-1 [Crassostrea gigas]
4	77	NaN	" 75 446 Actin, cytoplasmic type 8 OS=Xenopus laevis PE=3 SV=1"
7	10	NaN	" 28 1104 minus strand Pyruvate dehydrogenase E1 component subunit beta, mitochondrial [Crassostrea gigas]"
3	27	NaN	" 303 500 minus strand protein lethal, putative [Pediculus humanus corporis] gi"
3	6	NaN	76 522 minus strand Troponin C [Crassostrea gigas]; 173 619 minus strand Troponin C [Crassostrea gigas]; 173 619
2	5	NaN	48 356 DEC-3 [Lymnaea stagnalis]
4	14	NaN	71 535 ribosomal protein rpl14 [Arenicola marina]
3	4	NaN	" 1 1887 Pyruvate carboxylase, mitochondrial [Crassostrea gigas]"
7	14	NaN	102 887 hypothetical protein DAPPUDRAFT_304712 [Daphnia pulex]
2	18	NaN	" 3 587 elongation factor 1 alpha, partial [Phorbas sp. nov. SB-2012]"
4	7	NaN	111 530 minus strand Nucleoside diphosphate kinase B OS=Homo sapiens GN=NME2 PE=1 SV=1
7	16	NaN	718 1155 Annexin A7 [Crassostrea gigas]
2	3	NaN	2000 2641 Polyadenylate-binding protein 4 [Crassostrea gigas]
3	7	NaN	49 819 PREDICTED: elongation factor 1-delta-like isoform 2 [Strongylocentrotus purpuratus]
4	7	NaN	" 2041 3315 minus strand Myosin-10 OS=Mus musculus GN=Myh10 PE=1 SV=2; 2041 3345 minus strand Myosin hea
6	9	NaN	" 10 1191 3-ketoacyl-CoA thiolase, mitochondrial [Crassostrea gigas]"
2	3	NaN	89 361 40S ribosomal protein S18 OS=Rattus norvegicus GN=Rps18 PE=1 SV=3
3	4	NaN	42 434 cytochrome b5 [Haliotis discus discus]; 42 434 cytochrome b5 [Haliotis discus discus]
3	9	NaN	80 250 minus strand 40S ribosomal protein S29 [Ornithodoros parkeri]
2	9	NaN	503 1219 heat shock cognate 71 [Mytilus galloprovincialis]
5	6	NaN	1 825 Spectrin alpha chain [Crassostrea gigas]
2	2	NaN	1166 1939 minus strand Calcium-binding mitochondrial carrier protein Aralar2 OS=Homo sapiens GN=SLC25A13 PE
2	3	NaN	15 1361 hypothetical protein BRAFLDRAFT_231908 [Branchiostoma floridae] gi
9	19	NaN	366 1049 hypothetical protein CGI_10004855 [Crassostrea gigas]
9	16	NaN	95 1579 minus strand hypothetical protein BRAFLDRAFT_113974 [Branchiostoma floridae] gi
3	4	NaN	4 783 minus strand hemocyanin isoform 2 [Nucula nucleus]
3	6	NaN	11 244 minus strand Tubulin alpha chain (Fragment) OS=Lytechinus pictus PE=3 SV=1

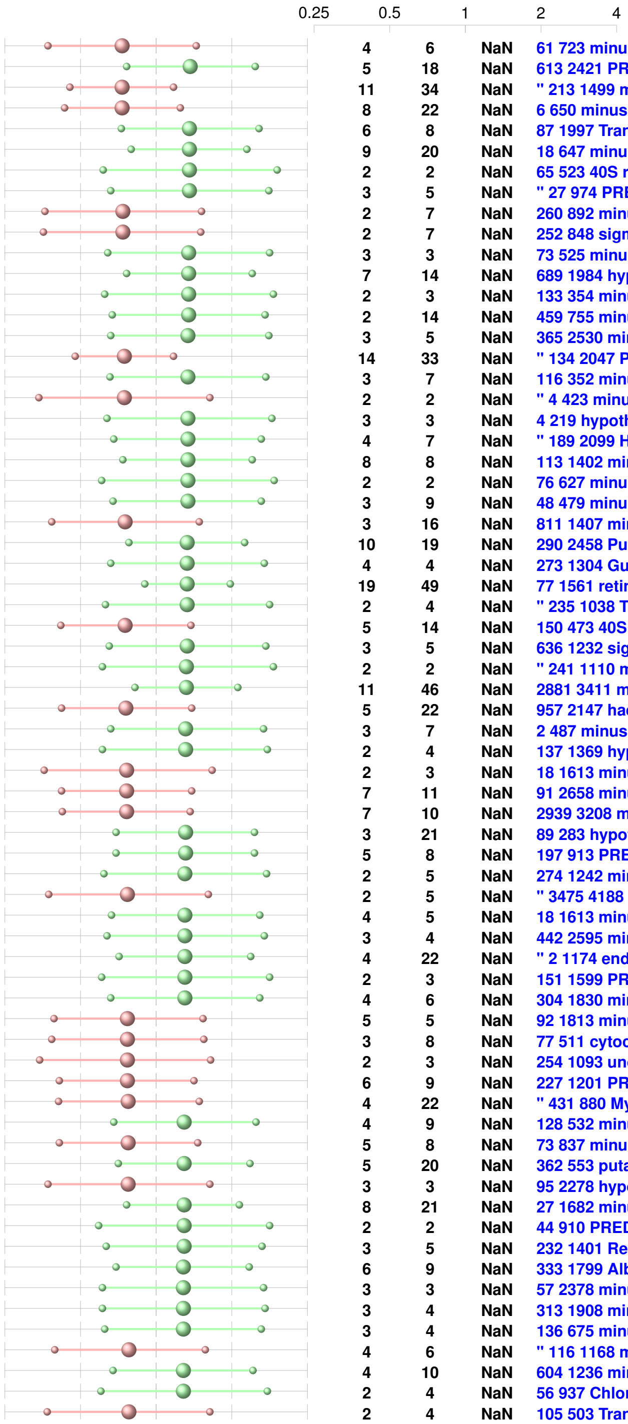

Peptides	Spectra	Coverage	Protein
4	6	NaN	61 723 minus strand 40S ribosomal protein S3-A OS=Xenopus laevis GN=rps3-a PE=2 SV=2
5	18	NaN	613 2421 PREDICTED: lysosomal alpha-glucosidase [Danio rerio]
11	34	NaN	" 213 1499 minus strand Citrate synthase, mitochondrial [Crassostrea gigas]"
8	22	NaN	6 650 minus strand glutathione S-transferase mu class protein [Crassostrea ariakensis]; 117 761 glutathione S-trans
6	8	NaN	87 1997 Transketolase-like protein 2 [Crassostrea gigas]; 87 1997 Transketolase-like protein 2 [Crassostrea gigas]; 8
9	20	NaN	18 647 minus strand ATP synthase-like protein [Biomphalaria glabrata]
2	2	NaN	65 523 40S ribosomal protein S9 [Crassostrea gigas]; 65 523 40S ribosomal protein S9 [Crassostrea gigas]
3	5	NaN	" 27 974 PREDICTED: succinyl-CoA ligase [GDP-forming] subunit alpha, mitochondrial-like [Oreochromis niloticus];
2	7	NaN	260 892 minus strand calponin [Haliotis diversicolor]
2	7	NaN	252 848 sigma class glutathione-s-transferase 2 [Haliotis discus discus]
3	3	NaN	73 525 minus strand Actin-related protein 2/3 complex subunit 5-like protein [Salmo salar] gi; 73 525 Actin-related pr
7	14	NaN	689 1984 hypothetical protein BRAFLDRAFT_78257 [Branchiostoma floridae] gi
2	3	NaN	133 354 minus strand 60S ribosomal protein L7a OS=Rattus norvegicus GN=Rpl7a PE=1 SV=2
2	14	NaN	459 755 minus strand heat shock protein beta-1 [Trichinella spiralis] gi; 464 763 minus strand heat shock protein bet
3	5	NaN	365 2530 minus strand calpain B [Gecarcinus lateralis]
14	33	NaN	" 134 2047 PREDICTED: succinate dehydrogenase [ubiquinone] flavoprotein subunit, mitochondrial-like isoform 3, p
3	7	NaN	116 352 minus strand troponin I 4 [Clonorchis sinensis]
2	2	NaN	" 4 423 minus strand PREDICTED: similar to NADH dehydrogenase, putative [Tribolium castaneum] gi"
3	3	NaN	4 219 hypothetical protein BRAFLDRAFT_66351 [Branchiostoma floridae] gi; 3 239 hypothetical protein BRAFLDRAI
4	7	NaN	" 189 2099 Hsp70, putative [Ixodes scapularis] gi"
8	8	NaN	113 1402 minus strand S-adenosylhomocysteine hydrolase [Crassostrea ariakensis]; 113 1402 minus strand S-aden
2	2	NaN	76 627 minus strand Heme-binding protein 2 [Crassostrea gigas]; 296 847 minus strand Heme-binding protein 2 [Cra
3	9	NaN	48 479 minus strand 40S ribosomal protein S24 OS=Macaca fascicularis GN=RPS24 PE=2 SV=1
3	16	NaN	811 1407 minus strand mitochondrial malate dehydrogenase 2 [Eospalax baileyi]
10	19	NaN	290 2458 Putative beta-D-xylosidase 5 [Crassostrea gigas]
4	4	NaN	273 1304 Guanine nucleotide-binding protein subunit beta [Crassostrea gigas]
19	49	NaN	77 1561 retinal dehydrogenase [Lymnaea stagnalis]; 69 1553 retinal dehydrogenase [Lymnaea stagnalis]
2	4	NaN	" 235 1038 Translocon-associated protein subunit alpha [Crassostrea gigas]; 309 725 hypothetical protein [Amblyon
5	14	NaN	150 473 40S ribosomal protein S16 OS=Heteropneustes fossilis GN=rps16 PE=2 SV=1
3	5	NaN	636 1232 sigma class glutathione-s-transferase 3 [Haliotis discus discus]; 963 1559 sigma class glutathione-s-transf
2	2	NaN	" 241 1110 minus strand N(G),N(G)-dimethylarginine dimethylaminohydrolase 1 [Crassostrea gigas]; 241 1110 minus
11	46	NaN	2881 3411 myosin heavy chain [Todarodes pacificus]; 2915 3445 myosin heavy chain [Todarodes pacificus]
5	22	NaN	957 2147 haemocyanin [Aplysia californica]
3	7	NaN	2 487 minus strand PREDICTED: hypothetical protein LOC100699750 [Oreochromis niloticus]; 41 535 minus strand I
2	4	NaN	137 1369 hypothetical protein TcasGA2_TC002962 [Tribolium castaneum]
2	3	NaN	18 1613 minus strand chaperonin containing T-complex polypeptide subunit zeta [Haliotis discus hannai]; 527 2089
7	11	NaN	91 2658 minus strand puromycin-sensitive aminopeptidase [Saccoglossus kowalevskii]
7	10	NaN	2939 3208 minus strand beta-thymosin domain repeat protein CSP24KDa_v1 [Hermissenda crassicornis] gi
3	21	NaN	89 283 hypothetical protein CGI_10017178 [Crassostrea gigas]
5	8	NaN	197 913 PREDICTED: l-xylulose reductase-like [Monodelphis domestica]; 197 913 PREDICTED: l-xylulose reductase
2	5	NaN	274 1242 minus strand cathepsin D [Pinctada maxima]; 290 1387 cathepsin D [Pinctada maxima]; 290 1402 minus st
2	5	NaN	" 3475 4188 minus strand Heat shock 70 kDa protein cognate, putative [Pediculus humanus corporis] gi"
4	5	NaN	18 1613 minus strand chaperonin containing T-complex polypeptide subunit zeta [Haliotis discus hannai]; 527 2089
3	4	NaN	442 2595 minus strand Amiloride-sensitive amine oxidase [copper-containing] [Crassostrea gigas]
4	22	NaN	" 2 1174 endo-1,3-beta-D-glucanase [Littorina sitkana]"
2	3	NaN	151 1599 PREDICTED: galactosamine (N-acetyl)-6-sulfate sulfatase-like [Saccoglossus kowalevskii]
4	6	NaN	304 1830 minus strand Methenyltetrahydrofolate synthetase domain-containing protein [Crassostrea gigas]
5	5	NaN	92 1813 minus strand Dihydropyrimidinase [Crassostrea gigas]
3	8	NaN	77 511 cytochrome c oxidase subunit VIIa polypeptide 2 like [Xenopus (Silurana) tropicalis]; 77 511 cytochrome c ox
2	3	NaN	254 1093 uncharacterized protein LOC432187 precursor [Xenopus laevis] gi
6	9	NaN	227 1201 PREDICTED: quinone oxidoreductase-like isoform 1 [Ornithorhynchus anatinus]; 109 1083 PREDICTED: qu
4	22	NaN	" 431 880 Myosin essential light chain, striated adductor muscle OS=Aequipecten irradians PE=1 SV=2"
4	9	NaN	128 532 minus strand mitochondrial ATPase inhibitor [Ornithodoros parkeri]
5	8	NaN	73 837 minus strand Electron transfer flavoprotein subunit beta [Crassostrea gigas]; 73 837 minus strand Electron tr
5	20	NaN	362 553 putative RNA-binding protein [Haliotis diversicolor]
3	3	NaN	95 2278 hypothetical protein CGI_10017740 [Crassostrea gigas]
8	21	NaN	27 1682 minus strand PREDICTED: nucleolar protein 56 [Oreochromis niloticus]
2	2	NaN	44 910 PREDICTED: probable ATP-dependent RNA helicase DDX10 [Cricetulus griseus] gi
3	5	NaN	232 1401 RecName: Full=Guanine nucleotide-binding protein G(s) subunit alpha; AltName: Full=Adenylate cyclase-s
6	9	NaN	333 1799 Alba-like protein C9orf23-like protein [Crassostrea gigas]
3	3	NaN	57 2378 minus strand Heterogeneous nuclear ribonucleoprotein U-like protein 1 [Crassostrea gigas]
3	4	NaN	313 1908 minus strand Heterogeneous nuclear ribonucleoprotein L [Crassostrea gigas]
3	4	NaN	136 675 minus strand predicted protein [Nematostella vectensis] gi
4	6	NaN	" 116 1168 minus strand hypothetical protein CGI_10014553, partial [Crassostrea gigas]"
4	10	NaN	604 1236 minus strand selenium-dependent glutathione peroxidase [Crassostrea gigas]; 138 770 minus strand seler
2	4	NaN	56 937 Chloride intracellular channel exc-4 [Crassostrea gigas]
2	4	NaN	105 503 Transgelin-2 [Crassostrea gigas]

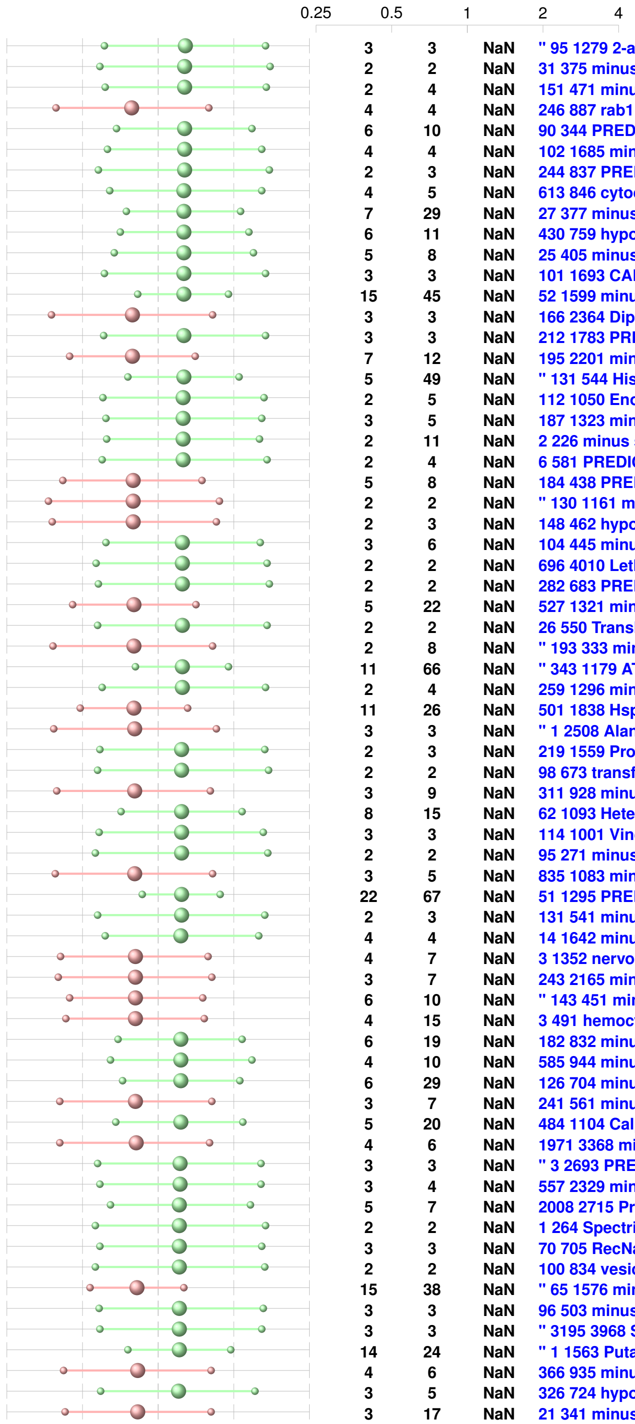

Peptides	Spectra	Coverage	Protein
3	3	NaN	" 95 1279 2-amino-3-ketobutyrate coenzyme A ligase, mitochondrial, partial [Crassostrea gigas]"
2	2	NaN	31 375 minus strand 40S ribosomal protein S19 OS=Mya arenaria GN=RPS19 PE=2 SV=1
2	4	NaN	151 471 minus strand ribosomal protein rps25 [Arenicola marina]
4	4	NaN	246 887 rab11-2 [Limulus polyphemus]
6	10	NaN	90 344 PREDICTED: diazepam binding inhibitor-like [Saccoglossus kowalevskii]
4	4	NaN	102 1685 minus strand predicted protein [Nematostella vectensis] gi
2	3	NaN	244 837 PREDICTED: ras-like GTP-binding protein Rho1-like [Megachile rotundata]
4	5	NaN	613 846 cytochrome c oxidase subunit Va [Antheraea yamamai] gi
7	29	NaN	27 377 minus strand ribosomal protein LP2 [Papilio xuthus] gi; 27 377 minus strand ribosomal protein LP2 [Papilio x
6	11	NaN	430 759 hypothetical protein [Amblyomma maculatum]; 336 665 hypothetical protein [Amblyomma maculatum]
5	8	NaN	25 405 minus strand Elongation factor 1-delta [Anoplopoma fimbria]
3	3	NaN	101 1693 CALNUC [Spodoptera frugiperda]; 141 1325 Nucleobindin-2 [Crassostrea gigas]; 141 1289 Nucleobindin-2
15	45	NaN	52 1599 minus strand ATP synthase beta subunit [Pinctada fucata]
3	3	NaN	166 2364 Dipeptidyl-peptidase 3 [Crassostrea gigas]
3	3	NaN	212 1783 PREDICTED: d-3-phosphoglycerate dehydrogenase-like [Strongylocentrotus purpuratus]
7	12	NaN	195 2201 minus strand hypothetical protein DAPPUDRAFT_315656 [Daphnia pulex]
5	49	NaN	" 131 544 Histone H2B, gonadal OS=Patella granatina PE=1 SV=2"
2	5	NaN	112 1050 Enolase-phosphatase E1 [Crassostrea gigas]; 112 1050 Enolase-phosphatase E1 [Crassostrea gigas]
3	5	NaN	187 1323 minus strand gelsolin [Aplysia californica] gi
2	11	NaN	2 226 minus strand hypothetical protein CGI_10015501 [Crassostrea gigas]
2	4	NaN	6 581 PREDICTED: mammalian ependymin-related protein 1 [Gallus gallus]
5	8	NaN	184 438 PREDICTED: similar to CG14235 CG14235-PA [Tribolium castaneum] gi
2	2	NaN	" 130 1161 minus strand Isocitrate dehydrogenase [NAD] subunit alpha, mitochondrial [Crassostrea gigas]; 198 122
2	3	NaN	148 462 hypothetical protein DAPPUDRAFT_306954 [Daphnia pulex]; 148 462 hypothetical protein DAPPUDRAFT_30
3	6	NaN	104 445 minus strand macrophage migration inhibitory factor [Ascaris suum]
2	2	NaN	696 4010 Lethal(2) giant larvae-like protein 1 [Crassostrea gigas]; 657 3971 minus strand Lethal(2) giant larvae-like p
2	2	NaN	282 683 PREDICTED: ribonuclease UK114-like [Oreochromis niloticus]
5	22	NaN	527 1321 minus strand troponin T [Mizuhopecten yessoensis]; 305 1099 minus strand troponin T [Mizuhopecten yes
2	2	NaN	26 550 Translocon-associated protein subunit gamma [Crassostrea gigas]
2	8	NaN	" 193 333 minus strand RecName: Full=Globin; AltName: Full=Myoglobin gi|299274|gb|AAB25743.1| myoglobin [Na
11	66	NaN	" 343 1179 ATP synthase subunit alpha, mitochondrial OS=Pongo abelii GN=ATP5A1 PE=2 SV=1"
2	4	NaN	259 1296 minus strand GD20916 [Drosophila simulans] gi
11	26	NaN	501 1838 Hsp90A [Haliotis asinina]
3	3	NaN	" 1 2508 Alanyl-tRNA synthetase, cytoplasmic [Crassostrea gigas]; 1 2508 Alanyl-tRNA synthetase, cytoplasmic [Cra
2	3	NaN	219 1559 Programmed cell death protein 4 [Crassostrea gigas]
2	2	NaN	98 673 transforming protein Rho-like [Acyrthosiphon pisum]
3	9	NaN	311 928 minus strand peroxiredoxin 3 [Clonorchis sinensis] gi; 288 905 minus strand peroxiredoxin 3 [Clonorchis si
8	15	NaN	62 1093 Heterogeneous nuclear ribonucleoprotein A2-like protein 1 [Crassostrea gigas]
3	3	NaN	114 1001 Vinculin [Crassostrea gigas]
2	2	NaN	95 271 minus strand hypothetical protein EAI_01530 [Harpegnathos saltator]
3	5	NaN	835 1083 minus strand Calmodulin [Crassostrea gigas]
22	67	NaN	51 1295 PREDICTED: phosphoglycerate kinase-like [Nasonia vitripennis]; 97 1341 PREDICTED: phosphoglycerate k
2	3	NaN	131 541 minus strand nervous system adducin [Aplysia californica] gi
4	4	NaN	14 1642 minus strand chaperonin containing tcp1 [Haliotis discus discus]; 14 1462 minus strand chaperonin contain
4	7	NaN	3 1352 nervous system adducin [Aplysia californica] gi; 3 1418 Protein hu-li tai shao [Crassostrea gigas]; 3 1214 ner
3	7	NaN	243 2165 minus strand Elongation factor 2 OS=Drosophila melanogaster GN=Ef2b PE=2 SV=4
6	10	NaN	" 143 451 minus strand PREDICTED: 10 kDa heat shock protein, mitochondrial isoform 1 [Canis lupus familiaris]"
4	15	NaN	3 491 hemocyanin [Haliotis tuberculata]
6	19	NaN	182 832 minus strand qm-like protein [Crassostrea ariakensis]
4	10	NaN	585 944 minus strand Protein disulfide-isomerase A6 [Crassostrea gigas]
6	29	NaN	126 704 minus strand ribosomal protein L12 [Ixodes scapularis]
3	7	NaN	241 561 minus strand thioredoxin [Crassostrea ariakensis]
5	20	NaN	484 1104 Calponin-2 [Crassostrea gigas]; 309 938 Calponin-2 [Crassostrea gigas]
4	6	NaN	1971 3368 minus strand Kyphoscoliosis peptidase [Crassostrea gigas]; 1767 3158 minus strand hillarin [Hirudo med
3	3	NaN	" 3 2693 PREDICTED: similar to coatomer protein complex, subunit beta 2 (beta prime) [Taeniopygia guttata]"
3	4	NaN	557 2329 minus strand Collagen alpha-3(VI) chain [Crassostrea gigas]
5	7	NaN	2008 2715 Protein disulfide-isomerase A5 [Crassostrea gigas]
2	2	NaN	1 264 Spectrin beta chain [Crassostrea gigas]
3	3	NaN	70 705 RecName: Full=Ras-related protein Rab-2 gi|288938|emb|CAA51234.1| RAB2 [Lymnaea stagnalis]; 70 705 Re
2	2	NaN	100 834 vesicle-associated membrane protein/synaptobrevin-binding protein [Aplysia californica] gi
15	38	NaN	" 65 1576 minus strand Aldehyde dehydrogenase, mitochondrial [Crassostrea gigas]"
3	3	NaN	96 503 minus strand hypothetical protein DAPPUDRAFT_230378 [Daphnia pulex]
3	3	NaN	" 3195 3968 Spectrin alpha chain, putative [Pediculus humanus corporis] gi"
14	24	NaN	" 1 1563 Putative methylmalonate-semialdehyde dehydrogenase [acylating], mitochondrial [Crassostrea gigas]"
4	6	NaN	366 935 minus strand 40S ribosomal protein S2 OS=Urechis caupo GN=RPS2 PE=2 SV=1
3	5	NaN	326 724 hypothetical protein [Amblyomma maculatum]
3	17	NaN	21 341 minus strand hemocyanin isoform 1 [Nucula nucleus]

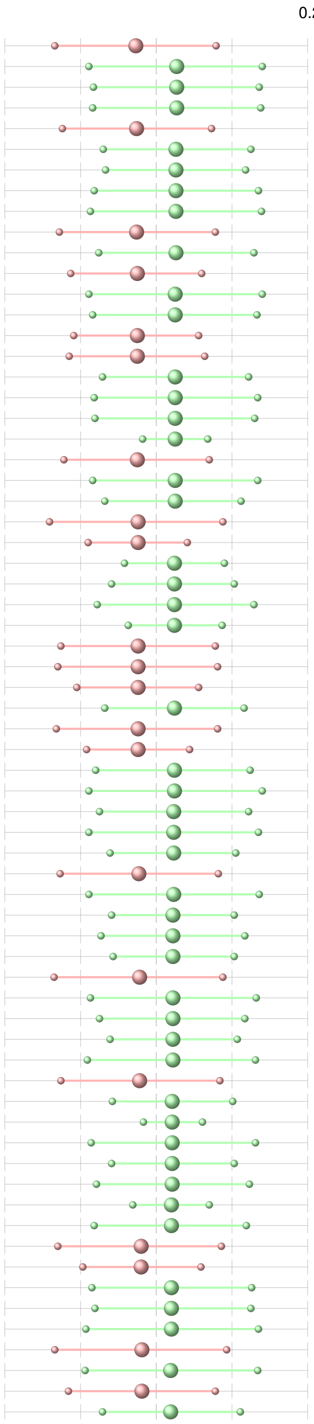

Peptides	Spectra	Coverage	Protein
2	7	NaN	284 1318 hsc70-interacting protein [Gallus gallus] gi; 88 1137 hsc70-interacting protein [Gallus gallus] gi
2	2	NaN	26 250 minus strand fructose-bisphosphate aldolase [Haliotis discus discus]
2	3	NaN	" 31 2061 minus strand 1,4-alpha-glucan-branching enzyme [Crassostrea gigas]"
2	3	NaN	157 1422 Tektin-2 [Crassostrea gigas]
3	9	NaN	160 1218 Galectin-9 [Crassostrea gigas]
4	7	NaN	155 3214 sarco/endoplasmic reticulum calcium ATPase isoform B [Pinctada fucata]; 155 3214 minus strand sarco/er
6	7	NaN	" 64 1332 Flotillin-1, putative [Pediculus humanus corporis] gi"
2	4	NaN	" 1 1815 Glycogen phosphorylase, muscle form [Crassostrea gigas]"
2	3	NaN	143 634 minus strand ribosomal protein S14 [Solea senegalensis]
2	21	NaN	628 1140 paramyosin [Haliotis discus discus]
3	4	NaN	182 1846 minus strand hypothetical protein DAPPUDRAFT_302108 [Daphnia pulex]; 110 1774 minus strand hypothe
6	20	NaN	76 822 14-3-3 protein [Heterodera glycines]; 76 822 minus strand 14-3-3 protein [Heterodera glycines]; 76 822 minus
2	2	NaN	91 3270 Valyl-tRNA synthetase [Crassostrea gigas]
2	3	NaN	2 532 heat shock protein 70 [Echinostoma caproni]
7	17	NaN	408 1010 glutathione S-transferase sigma 2 [Mytilus galloprovincialis]; 166 768 glutathione S-transferase sigma 2 [M
4	16	NaN	253 765 cytochrome c oxidase subunit IV [Urechis caupo]
4	7	NaN	151 1449 rab GDP-dissociation inhibitor [Branchiostoma belcheri]
2	3	NaN	176 526 minus strand Translocon-associated protein subunit delta [Crassostrea gigas]
3	4	NaN	" 283 1602 minus strand 4-aminobutyrate aminotransferase, mitochondrial [Crassostrea gigas]"
26	328	NaN	1 852 beta actin-like 1 protein [Macrostomum lignano]
3	11	NaN	816 1211 H2 [Haliotis tuberculata]
3	3	NaN	71 1321 minus strand Vinculin [Crassostrea gigas]
5	9	NaN	3 1670 Spectrin alpha chain [Crassostrea gigas]
2	2	NaN	213 875 es1 protein [Haliotis discus discus]
15	30	NaN	26 1909 Dolichyl-diphosphooligosaccharide–protein glycosyltransferase subunit 2 [Crassostrea gigas]
15	33	NaN	40 1752 minus strand heat shock protein 60 [Biomphalaria glabrata]; 40 1752 minus strand heat shock protein 60 [B
9	11	NaN	3512 6661 hypothetical protein BRAFLDRAFT_255626 [Branchiostoma floridae] gi
3	5	NaN	" 448 1365 Nitrile-specifier protein 5, partial [Crassostrea gigas]"
13	52	NaN	167 679 tropomyosin [Haliotis discus discus]
3	6	NaN	" 703 1173 60S acidic ribosomal protein P0, putative [Ixodes scapularis] gi"
2	7	NaN	416 1717 minus strand T-complex protein 1 subunit delta OS=Aedes triseriatus PE=2 SV=1
7	20	NaN	3 434 minus strand myosin essential light chain [Haliotis discus discus]
5	9	NaN	313 2763 NCAM-related cell adhesion molecule precursor [Aplysia californica] gi
3	3	NaN	2 2248 PREDICTED: heat shock 70 kDa protein 4-like [Oreochromis niloticus]; 2 2227 PREDICTED: heat shock 70 kD
8	53	NaN	1200 1547 protein disulfide isomerase [Conus eburneus]
3	6	NaN	635 1294 minus strand Kyphoscoliosis peptidase [Crassostrea gigas]; 643 1302 minus strand Kyphoscoliosis peptic
2	2	NaN	18 1406 AGAP006996-PA [Anopheles gambiae str. PEST] gi
4	7	NaN	546 995 Hypothetical protein CBG12002 [Caenorhabditis briggsae]; 543 995 minus strand PREDICTED: tubulin polyr
2	2	NaN	" 132 1544 minus strand Serine hydroxymethyltransferase, cytosolic [Crassostrea gigas]"
4	29	NaN	1371 2189 minus strand galectin [Pinctada fucata]
3	4	NaN	" 39 968 l-3-hydroxyacyl-coenzyme a dehydrogenase, short chain [Haliotis discus discus]; 39 968 l-3-hydroxyacyl-co
2	2	NaN	3 614 PREDICTED: 60S ribosomal protein L8-like [Papio anubis]
7	16	NaN	98 850 minus strand 14-3-3 protein epsilon [Schistocerca gregaria]
3	12	NaN	1363 1527 Protein disulfide-isomerase A3 [Crassostrea gigas]
8	19	NaN	373 579 myosin heavy chain [Argopecten irradians] gi
2	2	NaN	593 862 minus strand mitochondrial ATP synthase coupling factor 6 precursor [Ochlerotatus taeniorhynchus]
2	3	NaN	108 1040 minus strand PREDICTED: Na(+)/H(+) exchange regulatory cofactor NHE-RF3-like [Metaseiulus occidentali
4	7	NaN	156 1106 ELAV 2-like protein [Aplysia californica] gi
7	12	NaN	915 1505 minus strand Collagen alpha-5(VI) chain [Crassostrea gigas]
2	3	NaN	211 1413 Alpha-N-acetylgalactosaminidase [Crassostrea gigas]
4	4	NaN	" 81 1349 minus strand PREDICTED: LOW QUALITY PROTEIN: dolichyl-diphosphooligosaccharide–protein glycosyl
9	12	NaN	51 890 prohibitin [Octopus tankahkeei]
36	291	NaN	405 1373 paramyosin [Haliotis discus discus]
2	3	NaN	8 271 minus strand Laminin subunit gamma-1 [Crassostrea gigas]
9	13	NaN	130 1731 T-complex protein 1 subunit epsilon [Crassostrea gigas]; 130 1731 T-complex protein 1 subunit epsilon [Cr
3	7	NaN	177 791 Ras-related protein Rab-1A [Haliotis discus discus]
21	99	NaN	3591 4526 minus strand myosin heavy chain cardiac muscle specific isoform 1 [Argopecten irradians]
2	11	NaN	462 671 enhancer protein [Homo sapiens]
2	5	NaN	1 219 minus strand PREDICTED: nucleoside diphosphate kinase 3-like [Pan troglodytes]
10	13	NaN	3 4517 minus strand Clathrin heavy chain 1 [Crassostrea gigas]
2	5	NaN	137 853 14-3-3 protein [Heterodera glycines]
4	4	NaN	130 2145 minus strand Glucosamine–fructose-6-phosphate aminotransferase [isomerizing] 1 [Crassostrea gigas]; 1
2	2	NaN	62 292 PREDICTED: heterogeneous nuclear ribonucleoprotein F-like [Otolemur garnettii]
2	2	NaN	5 451 minus strand Spectrin beta chain [Crassostrea gigas]
2	2	NaN	194 838 glutathione S-transferase class mu [Cyphoma gibbosum]
3	9	NaN	274 666 Calmodulin-4 [Heterocephalus glaber]
3	21	NaN	23 214 minus strand hemocyanin isoform 1 [Nucula nucleus]

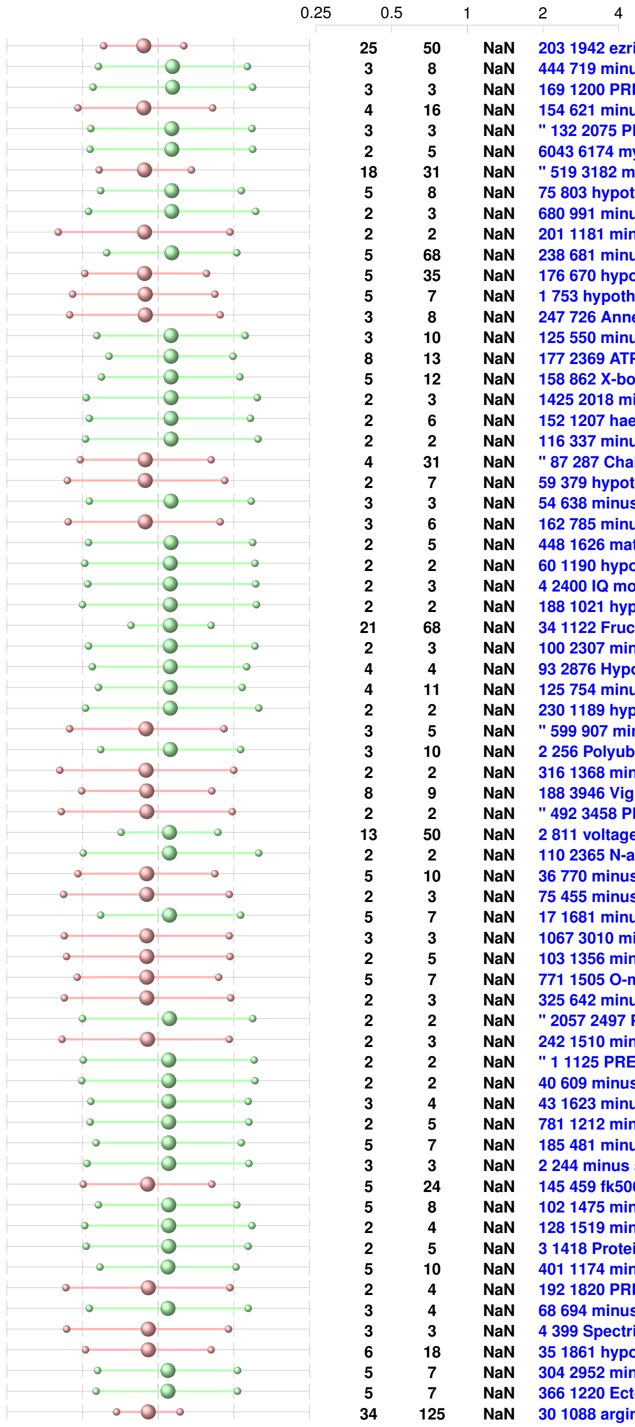

Peptides	Spectra	Coverage	Protein
25	50	NaN	203 1942 ezrin/radixin/moesin [Aplysia californica] gi; 243 1982 minus strand ezrin/radixin/moesin [Aplysia californic
3	8	NaN	444 719 minus strand 60S ribosomal protein L9 [Laodelphax striatella]
3	3	NaN	169 1200 PREDICTED: nidogen-2-like isoform 1 [Strongylocentrotus purpuratus]
4	16	NaN	154 621 minus strand Stress response protein nhaX [Crassostrea gigas]
3	3	NaN	" 132 2075 PREDICTED: arginyl-tRNA synthetase, cytoplasmic [Ornithorhynchus anatinus]; 132 2090 minus strand P
2	5	NaN	6043 6174 myosin heavy chain [Mytilus galloprovincialis]; 5869 6000 myosin heavy chain [Mytilus galloprovincialis]
18	31	NaN	" 519 3182 minus strand Alpha-actinin, sarcomeric [Crassostrea gigas]"
5	8	NaN	75 803 hypothetical protein DAPPUDRAFT_231350 [Daphnia pulex]
2	3	NaN	680 991 minus strand PDZ and LIM domain protein 1 [Crassostrea gigas]; 553 864 minus strand PDZ and LIM domai
2	2	NaN	201 1181 minus strand Serine/threonine-protein phosphatase PP1-alpha catalytic subunit [Crassostrea gigas]
5	68	NaN	238 681 minus strand Globin OS=Cerithidea rhizophorarum PE=1 SV=1
5	35	NaN	176 670 hypothetical protein BRAFLDRAFT_122621 [Branchiostoma floridae] gi
5	7	NaN	1 753 hypothetical protein BRAFLDRAFT_131251 [Branchiostoma floridae] gi
3	8	NaN	247 726 Annexin A7 [Crassostrea gigas]; 286 675 Annexin A7 [Crassostrea gigas]
3	10	NaN	125 550 minus strand ribosomal protein S20 [Chlamys farreri]; 125 550 minus strand ribosomal protein S20 [Chlamy
8	13	NaN	177 2369 ATPase family AAA domain-containing protein 2B [Crassostrea gigas]; 177 2369 ATPase family AAA doma
5	12	NaN	158 862 X-box binding protein [Haliotis discus discus]
2	3	NaN	1425 2018 minus strand Ankyrin-2 [Crassostrea gigas]; 1425 2018 minus strand Ankyrin-2 [Crassostrea gigas]
2	6	NaN	152 1207 haemocyanin precursor [Nautilus pompilius]
2	2	NaN	116 337 minus strand Golgi-associated plant pathogenesis-related protein 1 [Crassostrea gigas]; 538 759 minus stra
4	31	NaN	" 87 287 Chain A, Structure Of Deoxygenated Hemocyanin From Rapana Thomasiana gi|31615505|pdb|1LNL|B Chai
2	7	NaN	59 379 hypothetical protein DAPPUDRAFT_230756 [Daphnia pulex]; 117 443 minus strand hypothetical protein DAPP
3	3	NaN	54 638 minus strand ubiquitin carboxyl-terminal hydrolase [Aplysia californica] gi
3	6	NaN	162 785 minus strand Ropporin-1-like protein OS=Xenopus laevis GN=ropn1l PE=2 SV=1
2	5	NaN	448 1626 matrilin [Biomphalaria glabrata]
2	2	NaN	60 1190 hypothetical protein BRAFLDRAFT_109688 [Branchiostoma floridae] gi; 51 1184 hypothetical protein BRAFL
2	3	NaN	4 2400 IQ motif containing GTPase activating protein 2 [Xenopus laevis] gi; 4 2169 IQ motif containing GTPase activa
2	2	NaN	188 1021 hypothetical protein BRAFLDRAFT_214346 [Branchiostoma floridae] gi
21	68	NaN	34 1122 Fructose-bisphosphate aldolase [Crassostrea gigas]
2	3	NaN	100 2307 minus strand Xanthine dehydrogenase/oxidase [Crassostrea gigas]
4	4	NaN	93 2876 Hypoxia up-regulated protein 1 [Crassostrea gigas]
4	11	NaN	125 754 minus strand peptidyl prolyl cis-trans isomerase B [Conus novaehollandiae]
2	2	NaN	230 1189 hypothetical protein [Amblyomma maculatum]
3	5	NaN	" 599 907 minus strand Tryptophanyl-tRNA synthetase, cytoplasmic [Heterocephalus glaber]"
3	10	NaN	2 256 Polyubiquitin (Fragment) OS=Xenopus laevis PE=1 SV=2
2	2	NaN	316 1368 minus strand hypothetical protein CGI_10006559 [Crassostrea gigas]
8	9	NaN	188 3946 Vigilin [Crassostrea gigas]; 188 4135 Vigilin [Crassostrea gigas]
2	2	NaN	" 492 3458 PREDICTED: 2-oxoglutarate dehydrogenase, mitochondrial isoform 1 [Sarcophilus harrisii]"
13	50	NaN	2 811 voltage-dependent anion channel 2-like protein [Haliotis diversicolor]; 2 811 voltage-dependent anion channel
2	2	NaN	110 2365 N-acetylated-alpha-linked acidic dipeptidase 2 [Crassostrea gigas]
5	10	NaN	36 770 minus strand Endoplasmic reticulum protein ERp29 [Crassostrea gigas]
2	3	NaN	75 455 minus strand PREDICTED: calnexin [Strongylocentrotus purpuratus]
5	7	NaN	17 1681 minus strand Glucose-6-phosphate isomerase [Crassostrea gigas]
3	3	NaN	1067 3010 minus strand PREDICTED: nucleolin-like isoform 2 [Oreochromis niloticus]
2	5	NaN	103 1356 minus strand PREDICTED: actin-related protein 3-like [Oreochromis niloticus]
5	7	NaN	771 1505 O-methyltransferase family 3 [Dicentrarchus labrax]
2	3	NaN	325 642 minus strand Protein phosphatase 1B [Crassostrea gigas]
2	2	NaN	" 2057 2497 Phosphate carrier protein, mitochondrial [Crassostrea gigas]"
2	3	NaN	242 1510 minus strand hypothetical protein BRAFLDRAFT_276673 [Branchiostoma floridae] gi
2	2	NaN	" 1 1125 PREDICTED: trifunctional enzyme subunit beta, mitochondrial-like [Oreochromis niloticus]"
2	2	NaN	40 609 minus strand ependymin related protein-1 precursor [Haliotis discus discus]
3	4	NaN	43 1623 minus strand MGC84105 protein precursor [Xenopus laevis] gi; 43 1506 minus strand MGC84105 protein pre
2	5	NaN	781 1212 minus strand cathepsin L2 cysteine protease [Pinctada fucata]
5	7	NaN	185 481 minus strand Zgc:56530 [Danio rerio]
3	3	NaN	2 244 minus strand hypothetical protein KGM_08771 [Danaus plexippus]
5	24	NaN	145 459 fk506-binding protein 2 [Papilio xuthus]; 237 551 fk506-binding protein 2 [Papilio xuthus]
5	8	NaN	102 1475 minus strand Cytosolic non-specific dipeptidase [Crassostrea gigas]
2	4	NaN	128 1519 minus strand V-type proton ATPase subunit H [Crassostrea gigas]; 128 1519 minus strand V-type proton AT
2	5	NaN	3 1418 Protein hu-li tai shao [Crassostrea gigas]; 3 1523 minus strand Protein hu-li tai shao [Crassostrea gigas]; 3 1
5	10	NaN	401 1174 minus strand Mechanosensory protein 2 [Crassostrea gigas]
2	4	NaN	192 1820 PREDICTED: copine IV-like [Saccoglossus kowalevskii]; 125 1753 PREDICTED: copine IV-like [Saccoglossu
3	4	NaN	68 694 minus strand hypothetical protein BRAFLDRAFT_272416 [Branchiostoma floridae] gi; 68 667 minus strand hy
3	3	NaN	4 399 Spectrin beta chain [Crassostrea gigas]
6	18	NaN	35 1861 hypothetical protein DAPPUDRAFT_309082 [Daphnia pulex]
5	7	NaN	304 2952 minus strand hypothetical protein CGI_10026535 [Crassostrea gigas]
5	7	NaN	366 1220 Ectoine hydroxylase [Crassostrea gigas]
34	125	NaN	30 1088 arginine kinase [Conus novaehollandiae]

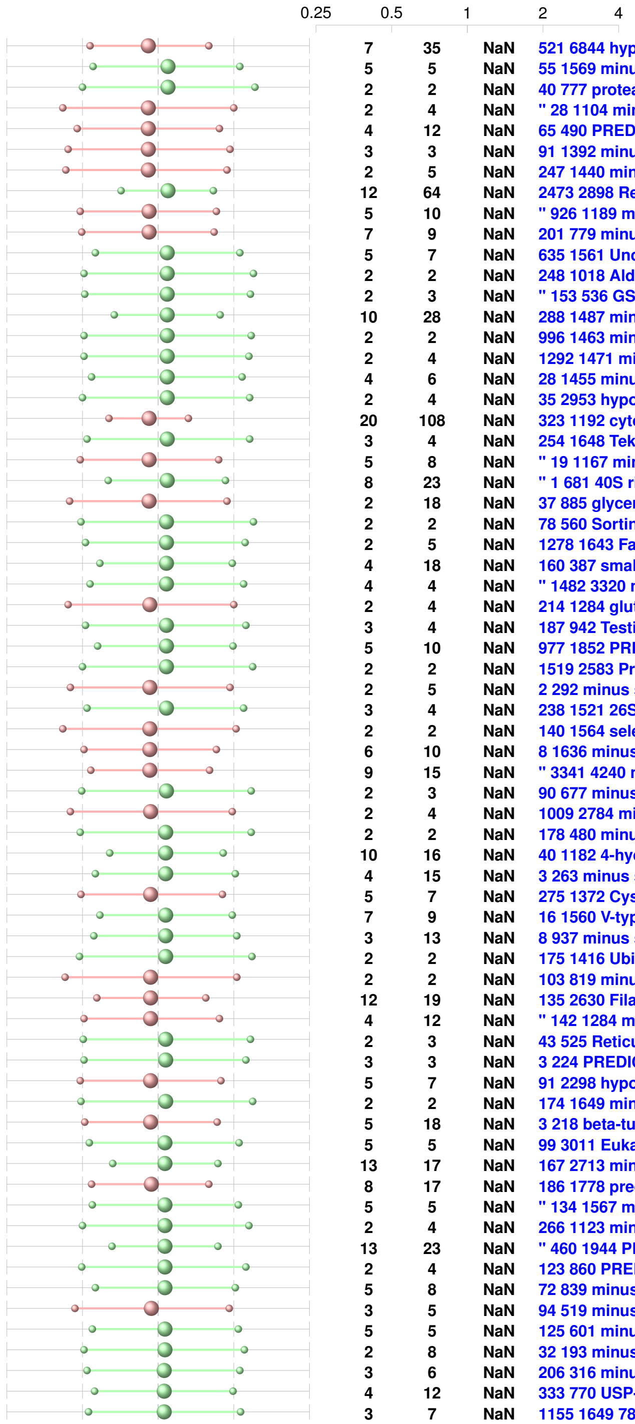

Peptides	Spectra	Coverage	Protein
7	35	NaN	521 6844 hypothetical protein CGI_10014469 [Crassostrea gigas]
5	5	NaN	55 1569 minus strand Putative aminopeptidase W07G4.4 [Crassostrea gigas]; 228 1742 Putative aminopeptidase W0
2	2	NaN	40 777 proteasome subunit N3 [Haliotis discus discus]; 40 777 proteasome subunit N3 [Haliotis discus discus]
2	4	NaN	" 28 1104 minus strand Pyruvate dehydrogenase E1 component subunit beta, mitochondrial [Crassostrea gigas]; 10
4	12	NaN	65 490 PREDICTED: cofilin-2 [Otolemur garnettii]
3	3	NaN	91 1392 minus strand hypothetical protein AND_03103 [Anopheles darlingi]
2	5	NaN	247 1440 minus strand Septin-2 [Crassostrea gigas]
12	64	NaN	2473 2898 RecName: Full=Paramyosin gi|397603|emb|CAA79849.1| paramyosin [Echinococcus granulosus]
5	10	NaN	" 926 1189 minus strand Myosin heavy chain, striated muscle [Crassostrea gigas]"
7	9	NaN	201 779 minus strand sigma class glutathione-s-transferase 2 [Haliotis discus discus]
5	7	NaN	635 1561 Uncharacterized protein yfeX [Crassostrea gigas]
2	2	NaN	248 1018 Aldo-keto reductase family 1 member B10 [Crassostrea gigas]; 44 730 minus strand Aldo-keto reductase fa
2	3	NaN	" 153 536 GST omega, partial [Reishia clavigera]"
10	28	NaN	288 1487 minus strand putative procollagen type VI alpha 4 [Haliotis diversicolor]
2	2	NaN	996 1463 minus strand catalase [Cristaria plicata] gi
2	4	NaN	1292 1471 minus strand hemocyanin isoform 1 [Haliotis diversicolor]
4	6	NaN	28 1455 minus strand PREDICTED: probable serine carboxypeptidase CPVL-like [Anolis carolinensis]
2	4	NaN	35 2953 hypothetical protein CGI_10020867 [Crassostrea gigas]; 97 3015 minus strand hypothetical protein CGI_100
20	108	NaN	323 1192 cytosolic malate dehydrogenase [Lottia scabra]; 323 1192 cytosolic malate dehydrogenase [Lottia scabra];
3	4	NaN	254 1648 Tektin-3 [Crassostrea gigas]
5	8	NaN	" 19 1167 minus strand ureidopropionase, beta [Xenopus (Silurana) tropicalis] gi"
8	23	NaN	" 1 681 40S ribosomal protein S4, X isoform [Crassostrea gigas]"
2	18	NaN	37 885 glyceraldehyde 3-phosphate dehydrogenase [Dugesia japonica]
2	2	NaN	78 560 Sorting nexin-12 [Camponotus floridanus]; 78 578 Sorting nexin-12 [Camponotus floridanus]; 132 605 Sorting
2	5	NaN	1278 1643 Fatty acid-binding-like protein 5 [Crassostrea gigas]
4	18	NaN	160 387 small nuclear ribonucleoprotein polypeptide G protein [Crassostrea ariakensis]; 180 407 small nuclear ribor
4	4	NaN	" 1482 3320 minus strand Phosphoenolpyruvate carboxykinase, cytosolic [GTP] [Crassostrea gigas]; 1326 3164 min
2	4	NaN	214 1284 glutamine synthetase [Tegillarca granosa]
3	4	NaN	187 942 Testis-specific gene 10 protein OS=Mus musculus GN=Tsga10 PE=1 SV=1; 212 967 Testis-specific gene 10 p
5	10	NaN	977 1852 PREDICTED: similar to prohibitin [Tribolium castaneum] gi
2	2	NaN	1519 2583 Proliferation-associated protein 2G4 [Crassostrea gigas]; 565 1629 Proliferation-associated protein 2G4 [C
2	5	NaN	2 292 minus strand H2 [Haliotis tuberculata]
3	4	NaN	238 1521 26S protease regulatory subunit 6A [Crassostrea gigas]
2	2	NaN	140 1564 selenium-binding protein [Haliotis discus hannai]
6	10	NaN	8 1636 minus strand V-type proton ATPase catalytic subunit A [Crassostrea gigas]; 8 1636 minus strand V-type proto
9	15	NaN	" 3341 4240 minus strand nonmuscle myosin II, partial [Aplysia californica]"
2	3	NaN	90 677 minus strand PREDICTED: 15-hydroxyprostaglandin dehydrogenase [NAD+] [Gallus gallus]; 90 683 minus str
2	4	NaN	1009 2784 minus strand Collagen alpha-3(VI) chain [Crassostrea gigas]
2	2	NaN	178 480 minus strand PREDICTED: lipoxygenase homology domain-containing protein 1-like [Danio rerio]
10	16	NaN	40 1182 4-hydroxyphenylpyruvate dioxygenase [Crassostrea gigas]
4	15	NaN	3 263 minus strand beta-actin [Urechis unicinctus]
5	7	NaN	275 1372 Cystathionine gamma-lyase OS=Bos taurus GN=CTH PE=2 SV=1
7	9	NaN	16 1560 V-type proton ATPase subunit B [Crassostrea gigas]
3	13	NaN	8 937 minus strand galectin [Crassostrea virginica]
2	2	NaN	175 1416 Ubiquinone biosynthesis monooxygenase COQ6 [Crassostrea gigas]
2	2	NaN	103 819 minus strand PREDICTED: l-xylulose reductase-like [Monodelphis domestica]
12	19	NaN	135 2630 Filamin-A [Crassostrea gigas]; 118 2613 Filamin-A [Crassostrea gigas]; 157 2652 Filamin-A [Crassostrea gi
4	12	NaN	" 142 1284 minus strand Chain O, Crsytal Structure Of Human Liver Gapdh gi|67464044|pdb|1ZNQ|P Chain P, Crsyta
2	3	NaN	43 525 Reticulon-4 [Crassostrea gigas]; 143 757 Reticulon-4 [Crassostrea gigas]; 43 564 Reticulon-4 [Crassostrea gi
3	3	NaN	3 224 PREDICTED: 40S ribosomal protein S14-like [Sarcophilus harrisii]
5	7	NaN	91 2298 hypothetical protein BRAFLDRAFT_267717 [Branchiostoma floridae] gi; 84 2291 hypothetical protein BRAFL
2	2	NaN	174 1649 minus strand Collagen alpha-1(XII) chain [Crassostrea gigas]; 123 1490 minus strand Collagen alpha-1(XII)
5	18	NaN	3 218 beta-tubulin [Fasciola hepatica] gi
5	5	NaN	99 3011 Eukaryotic translation initiation factor 3 subunit A [Crassostrea gigas]
13	17	NaN	167 2713 minus strand Major vault protein [Crassostrea gigas]
8	17	NaN	186 1778 predicted protein [Nematostella vectensis] gi
5	5	NaN	" 134 1567 minus strand 6-phosphogluconate dehydrogenase, decarboxylating [Crassostrea gigas]; 134 1573 minus
2	4	NaN	266 1123 minus strand mitochondrial malate dehydrogenase precursor [Nucella lapillus]
13	23	NaN	" 460 1944 PREDICTED: succinyl-CoA:3-ketoacid-coenzyme A transferase 1, mitochondrial [Canis lupus familiaris]"
2	4	NaN	123 860 PREDICTED: proteasome subunit alpha type-6-like [Cavia porcellus]; 1164 1901 PREDICTED: proteasome su
5	8	NaN	72 839 minus strand 60S ribosomal protein L8 OS=Spodoptera frugiperda GN=RpL8 PE=2 SV=1
3	5	NaN	94 519 minus strand hypothetical protein BRAFLDRAFT_115029 [Branchiostoma floridae] gi
5	5	NaN	125 601 minus strand omega class glutathione-s-transferase [Chlamys farreri]; 113 808 minus strand omega class g
2	8	NaN	32 193 minus strand Ubiquinol-cytochrome C reductase complex [Xenopus laevis] gi; 32 193 minus strand Ubiquino
3	6	NaN	206 316 minus strand ribosomal protein S17 [Branchiostoma belcheri]
4	12	NaN	333 770 USP-like protein isoform 2 [Crassostrea ariakensis]
3	7	NaN	1155 1649 78 kDa glucose-regulated protein OS=Aplysia californica PE=1 SV=1

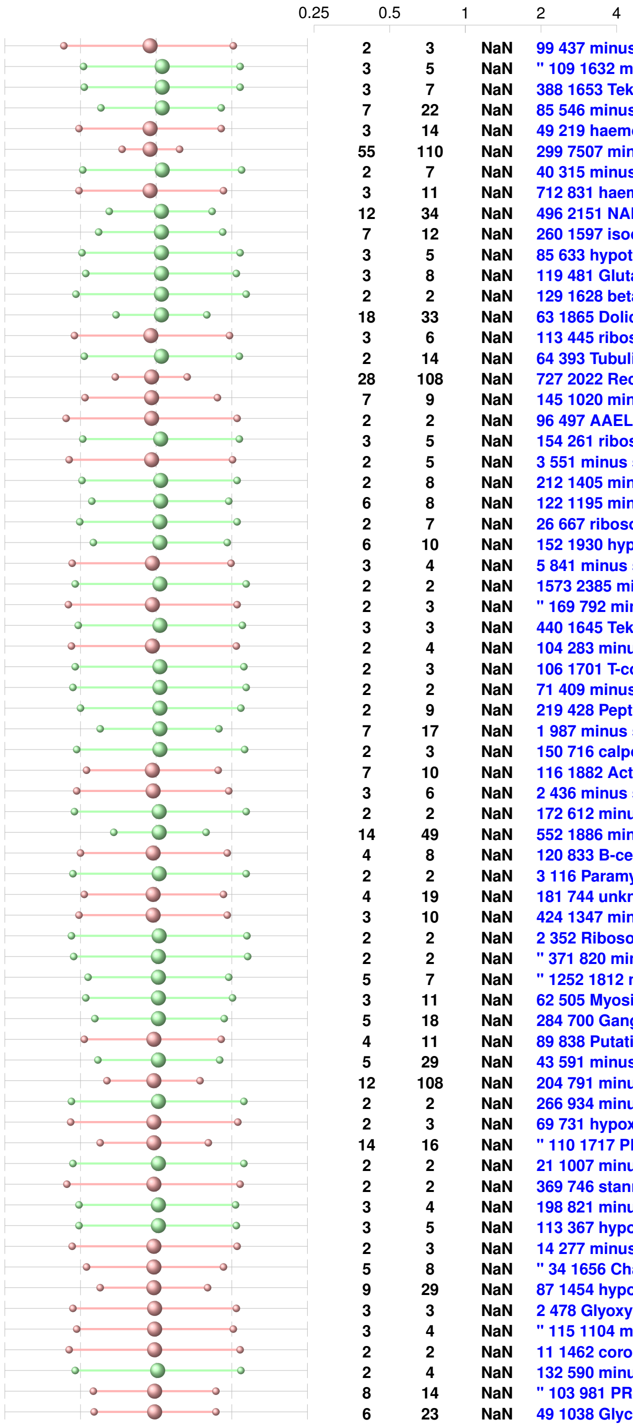

Peptides	Spectra	Coverage	Protein
2	3	NaN	99 437 minus strand V-type proton ATPase subunit G [Crassostrea gigas]
3	5	NaN	" 109 1632 minus strand Seryl-tRNA synthetase, cytoplasmic [Crassostrea gigas]; 109 1614 minus strand Seryl-tRN/
3	7	NaN	388 1653 Tektin-3 [Crassostrea gigas]
7	22	NaN	85 546 minus strand RecName: Full=Eukaryotic translation initiation factor 5A; Short=eIF-5A gi|51702279|sp|P6292
3	14	NaN	49 219 haemocyanin precursor [Nautilus pompilius]
55	110	NaN	299 7507 minus strand filamin [Clonorchis sinensis]
2	7	NaN	40 315 minus strand 60S ribosomal protein L18a OS=Homo sapiens GN=RPL18A PE=1 SV=2
3	11	NaN	712 831 haemocyanin [Aplysia californica]
12	34	NaN	496 2151 NADP-dependent malic enzyme [Crassostrea gigas]
7	12	NaN	260 1597 isocitrate dehydrogenase [Mytilus galloprovincialis]; 260 1597 isocitrate dehydrogenase [Mytilus galloprov
3	5	NaN	85 633 hypothetical protein [Amblyomma maculatum]
3	8	NaN	119 481 Glutamate receptor U1 [Crassostrea gigas]
2	2	NaN	129 1628 beta-hexosaminidase subunit beta precursor [Danio rerio] gi
18	33	NaN	63 1865 Dolichyl-diphosphooligosaccharide–protein glycosyltransferase subunit 1 [Crassostrea gigas]; 63 1865 Dol
3	6	NaN	113 445 ribosomal protein rps19 [Lineus viridis]
2	14	NaN	64 393 Tubulin beta chain OS=Paracentrotus lividus PE=2 SV=1; 109 438 Tubulin beta chain OS=Paracentrotus lividu
28	108	NaN	727 2022 RecName: Full=Enolase; AltName: Full=2-phospho-D-glycerate hydro-lyase; AltName: Full=2-phosphoglyc
7	9	NaN	145 1020 minus strand PREDICTED: inorganic pyrophosphatase-like [Saccoglossus kowalevskii]
2	2	NaN	96 497 AAEL009772-PB [Aedes aegypti]
3	5	NaN	154 261 ribosomal protein S18 [Stenella coeruleoalba]
2	5	NaN	3 551 minus strand Spectrin beta chain [Crassostrea gigas]
2	8	NaN	212 1405 minus strand unnamed protein product [Tetraodon nigroviridis]
6	8	NaN	122 1195 minus strand Severin [Crassostrea gigas]
2	7	NaN	26 667 ribosomal protein L15 [Acipenser sinensis] gi; 26 667 ribosomal protein L15 [Acipenser sinensis] gi; 26 667 r
6	10	NaN	152 1930 hypothetical protein BRAFLDRAFT_223365 [Branchiostoma floridae] gi
3	4	NaN	5 841 minus strand Spectrin alpha chain [Crassostrea gigas]
2	2	NaN	1573 2385 minus strand Spectrin beta chain [Crassostrea gigas]
2	3	NaN	" 169 792 minus strand rho GDP dissociation inhibitor, putative [Ixodes scapularis] gi"
3	3	NaN	440 1645 Tektin-1 [Crassostrea gigas]
2	4	NaN	104 283 minus strand RecName: Full=Hemocyanin type 2 unit a; AltName: Full=Hemocyanin RHSS2 subunit; AltNan
2	3	NaN	106 1701 T-complex protein 1 subunit beta [Crassostrea gigas]
2	2	NaN	71 409 minus strand heat shock protein 108 [Alligator mississippiensis]
2	9	NaN	219 428 Peptidyl-prolyl cis-trans isomerase OS=Hemicentrotus pulcherrimus PE=2 SV=1
7	17	NaN	1 987 minus strand RecName: Full=Tubulin beta chain; AltName: Full=Beta-tubulin
2	3	NaN	150 716 calponin/transgelin [Papilio xuthus]
7	10	NaN	116 1882 Actin-interacting protein 1 [Crassostrea gigas]
3	6	NaN	2 436 minus strand PREDICTED: stress-induced-phosphoprotein 1 [Sarcophilus harrisii]
2	2	NaN	172 612 minus strand putative polyadenylate-binding protein 1 [Haliotis diversicolor]; 172 612 putative polyadenylat
14	49	NaN	552 1886 minus strand intermediate filament protein [Biomphalaria glabrata]
4	8	NaN	120 833 B-cell receptor-associated protein 31 [Crassostrea gigas]; 238 951 B-cell receptor-associated protein 31 [Cr
2	2	NaN	3 116 Paramyosin [Crassostrea gigas]; 3 107 minus strand Paramyosin [Crassostrea gigas]
4	19	NaN	181 744 unknown [Dendroctonus ponderosae]
3	10	NaN	424 1347 minus strand Tubulin alpha chain [Ascaris suum]
2	2	NaN	2 352 Ribosome-binding protein 1 [Crassostrea gigas]
2	2	NaN	" 371 820 minus strand Peroxiredoxin-5, mitochondrial [Crassostrea gigas]; 91 528 minus strand Peroxiredoxin-5, m
5	7	NaN	" 1252 1812 minus strand Alanine–glyoxylate aminotransferase 2, mitochondrial [Crassostrea gigas]"
3	11	NaN	62 505 Myosin-2 essential light chain [Crassostrea gigas]
5	18	NaN	284 700 Ganglioside GM2 activator [Crassostrea gigas]
4	11	NaN	89 838 Putative phosphoglycerate mutase [Crassostrea gigas]; 89 838 Putative phosphoglycerate mutase [Crassost
5	29	NaN	43 591 minus strand cyclophilin type peptidyl-prolyl cis-trans isomerase [Glossina morsitans morsitans]
12	108	NaN	204 791 minus strand PREDICTED: 15-hydroxyprostaglandin dehydrogenase [NAD+] [Gallus gallus]
2	2	NaN	266 934 minus strand Kyphoscoliosis peptidase [Crassostrea gigas]
2	3	NaN	69 731 hypoxanthine phosphoribosyltransferase 1 [Haliotis discus discus]
14	16	NaN	" 110 1717 PREDICTED: glutamate dehydrogenase, mitochondrial-like [Oreochromis niloticus]; 110 1717 PREDICTE
2	2	NaN	21 1007 minus strand cathepsin L2 cysteine protease [Pinctada fucata]; 21 1007 minus strand cathepsin L2 cysteine
2	2	NaN	369 746 stanniocalcin [Haliotis diversicolor supertexta] gi
3	4	NaN	198 821 minus strand sigma class glutathione-s-transferase 2 [Haliotis discus discus]
3	5	NaN	113 367 hypothetical protein DAPPUDRAFT_329128 [Daphnia pulex]; 113 367 hypothetical protein DAPPUDRAFT_32
2	3	NaN	14 277 minus strand Histidine triad nucleotide-binding protein 1 OS=Oryctolagus cuniculus GN=HINT1 PE=1 SV=2
5	8	NaN	" 34 1656 Chaperonin containing TCP1, subunit 7 (eta) [Danio rerio] gi; 34 1656 Chaperonin containing TCP1, subun
9	29	NaN	87 1454 hypothetical protein BRAFLDRAFT_287788 [Branchiostoma floridae] gi; 117 1469 hypothetical protein BRAI
3	3	NaN	2 478 Glyoxylate reductase/hydroxypyruvate reductase [Crassostrea gigas]
3	4	NaN	" 115 1104 minus strand PREDICTED: KH domain-containing, RNA-binding, signal transduction-associated protein :
2	2	NaN	11 1462 coronin [Schistosoma mansoni]
2	4	NaN	132 590 minus strand Non-neuronal cytoplasmic intermediate filament protein OS=Helix aspersa PE=3 SV=2
8	14	NaN	" 103 981 PREDICTED: ATP synthase, H+ transporting, mitochondrial F1 complex, gamma subunit isoform H (heart)
6	23	NaN	49 1038 Glyceraldehyde-3-phosphate dehydrogenase [Crotalus adamanteus]

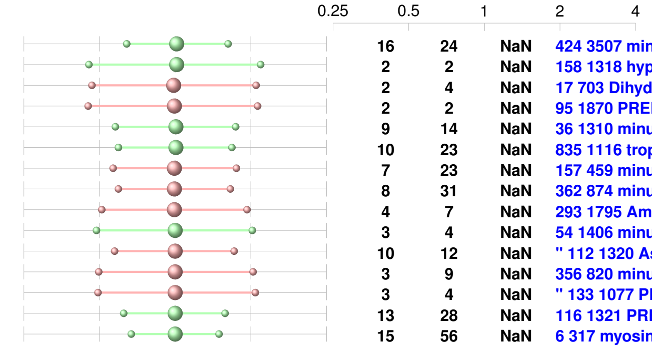

Peptides	Spectra	Coverage	Protein
16	24	NaN	424 3507 minus strand putative Na+/K+-ATPase alpha subunit [Paroctopus digueti]; 424 3507 minus strand putative
2	2	NaN	158 1318 hypothetical protein BRAFLDRAFT_98992 [Branchiostoma floridae] gi
2	4	NaN	17 703 Dihydropteridine reductase [Crassostrea gigas]
2	2	NaN	95 1870 PREDICTED: acetylcholinesterase-like [Equus caballus]; 99 1874 PREDICTED: acetylcholinesterase-like [Eq
9	14	NaN	36 1310 minus strand elongation factor-1 gamma [Diguetia canities]
10	23	NaN	835 1116 tropomyosin [Ilyanassa obsoleta]
7	23	NaN	157 459 minus strand Synaptojanin-2-binding protein [Crassostrea gigas]
8	31	NaN	362 874 minus strand Transgelin-2 [Crassostrea gigas]
4	7	NaN	293 1795 Amidase [Crassostrea gigas]
3	4	NaN	54 1406 minus strand Protein henna [Crassostrea gigas]; 54 1406 minus strand Protein henna [Crassostrea gigas]
10	12	NaN	" 112 1320 Aspartate aminotransferase, cytoplasmic [Crassostrea gigas]"
3	9	NaN	356 820 minus strand calmodulin-1 [Trichinella spiralis] gi
3	4	NaN	" 133 1077 PREDICTED: electron transfer flavoprotein, alpha polypeptide-like isoform 2 [Saccoglossus kowalevskii]"
13	28	NaN	116 1321 PREDICTED: similar to aspartate aminotransferase [Tribolium castaneum] gi
15	56	NaN	6 317 myosin heavy chain cardiac muscle specific isoform 2 [Argopecten irradians]

4.2 Proteins Identified by a Single Peptide

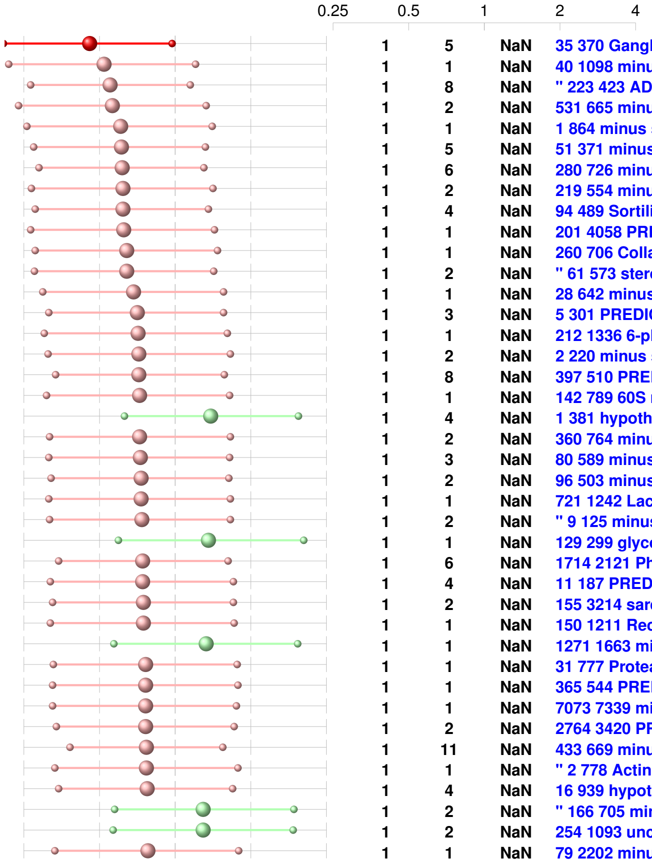

Peptides	Spectra	Coverage	Protein
1	5	NaN	35 370 Ganglioside GM2 activator [Crassostrea gigas]; 2 487 minus strand PREDICTED: hypothetical protein LOC10
1	1	NaN	40 1098 minus strand major egg antigen [Clonorchis sinensis]
1	8	NaN	" 223 423 ADPATP carrier protein; K05863 solute carrier family 25 (mitochondrial adenine nucleotide translocator), n
1	2	NaN	531 665 minus strand Proteasome subunit beta type-6 [Crassostrea gigas]
1	1	NaN	1 864 minus strand Pyruvate kinase isozymes M1/M2 [Crassostrea gigas]; 3 959 minus strand Pyruvate kinase isozy
1	5	NaN	51 371 minus strand putative mitochondrial ATP synthase F chain [Haliotis discus discus]; 51 371 minus strand puta
1	6	NaN	280 726 minus strand RecName: Full=Calmodulin; Short=CaM gi|14588595|dbj|BAB61794.1| calmodulin [Metridium
1	2	NaN	219 554 minus strand ribosomal protein P1 [Biomphalaria glabrata]
1	4	NaN	94 489 Sortilin-related receptor [Crassostrea gigas]
1	1	NaN	201 4058 PREDICTED: xanthine dehydrogenase/oxidase-like [Amphimedon queenslandica]
1	1	NaN	260 706 Collagen alpha-1(XII) chain (Fragment) OS=Notophthalmus viridescens PE=2 SV=1
1	2	NaN	" 61 573 steroid membrane receptor Hpr6.6/25-Dx, putative [Ixodes scapularis] gi"
1	1	NaN	28 642 minus strand Transmembrane emp24 domain-containing protein 9 [Crassostrea gigas]; 236 709 minus strand
1	3	NaN	5 301 PREDICTED: hypothetical protein [Saccoglossus kowalevskii]
1	1	NaN	212 1336 6-phosphofructokinase [Crassostrea gigas]; 208 1332 6-phosphofructokinase [Crassostrea gigas]
1	2	NaN	2 220 minus strand haemocyanin [Aplysia californica]
1	8	NaN	397 510 PREDICTED: actin-5C-like [Megachile rotundata]
1	1	NaN	142 789 60S ribosomal protein L6 [Crassostrea gigas]
1	4	NaN	1 381 hypothetical protein [Eimeria tenella]
1	2	NaN	360 764 minus strand Peptidyl-prolyl cis-trans isomerase A OS=Dictyostelium discoideum GN=ppiA PE=1 SV=1
1	3	NaN	80 589 minus strand PREDICTED: adenine phosphoribosyltransferase-like [Strongylocentrotus purpuratus]; 328 468
1	2	NaN	96 503 minus strand mitochondrial ATP synthase delta chain [Haliotis discus discus]; 111 551 minus strand mitocho
1	1	NaN	721 1242 Lactoylglutathione lyase [Crassostrea gigas]; 113 634 Lactoylglutathione lyase [Crassostrea gigas]
1	2	NaN	" 9 125 minus strand Hemocyanin, beta-C chain unit D (Fragment) OS=Helix pomatia PE=1 SV=2"
1	1	NaN	129 299 glycogen phosphorylase [Crassostrea gigas]
1	6	NaN	1714 2121 Phosphoenolpyruvate carboxykinase [GTP] [Crassostrea gigas]
1	4	NaN	11 187 PREDICTED: 40S ribosomal protein S28-like [Strongylocentrotus purpuratus]
1	2	NaN	155 3214 sarco/endoplasmic reticulum calcium ATPase isoform B [Pinctada fucata]; 132 3110 sarco/endoplasmic ret
1	1	NaN	150 1211 RecName: Full=Guanine nucleotide-binding protein G(i) subunit alpha; AltName: Full=Adenylate cyclase-in
1	1	NaN	1271 1663 minus strand PREDICTED: similar to AGAP007393-PB [Tribolium castaneum] gi
1	1	NaN	31 777 Proteasome subunit alpha type-7-like protein [Crassostrea gigas]
1	1	NaN	365 544 PREDICTED: cofilin-2 [Otolemur garnettii]
1	1	NaN	7073 7339 minus strand Collagen alpha-3(VI) chain [Crassostrea gigas]
1	2	NaN	2764 3420 PREDICTED: filamin-C-like [Nasonia vitripennis]
1	11	NaN	433 669 minus strand Superoxide dismutase [Cu-Zn] OS=Prionace glauca GN=sod1 PE=1 SV=1
1	1	NaN	" 2 778 Actin, gamma-enteric smooth muscle OS=Rattus norvegicus GN=Actg2 PE=2 SV=1"
1	4	NaN	16 939 hypothetical protein BRAFLDRAFT_87838 [Branchiostoma floridae] gi
1	2	NaN	" 166 705 minus strand ADP-ribosylation factor, partial [Antheraea yamamai]; 166 705 minus strand ADP-ribosylatio
1	2	NaN	254 1093 uncharacterized protein LOC432187 precursor [Xenopus laevis] gi; 343 561 minus strand cathepsin Z prec
1	1	NaN	79 2202 minus strand PREDICTED: dolichyl-diphosphooligosaccharide–protein glycosyltransferase subunit STT3A-

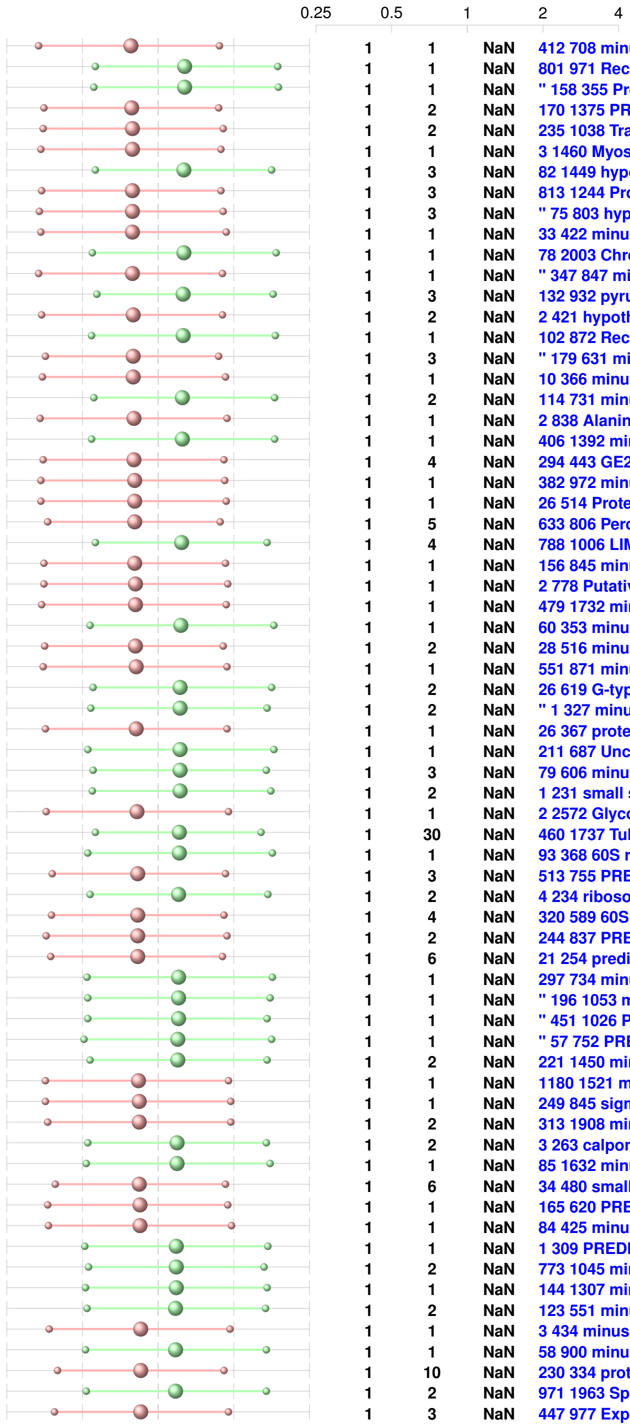

Peptides	Spectra	Coverage	Protein
1	1	NaN	412 708 minus strand heat shock protein beta-1 [Trichinella spiralis] gi; 459 755 minus strand heat shock protein bet
1	1	NaN	801 971 RecName: Full=Tropomyosin-2; AltName: Full=Tropomyosin II; Short=BgTMII; Short=TMII gi|155940|gb|AA
1	1	NaN	" 158 355 Protein NAS-37, isoform b [Caenorhabditis elegans] gi; 15 227 Hemicentin-1 [Crassostrea gigas]"
1	2	NaN	170 1375 PREDICTED: sialate O-acetylesterase-like [Oreochromis niloticus]
1	2	NaN	235 1038 Translocon-associated protein subunit alpha [Crassostrea gigas]
1	1	NaN	3 1460 Myosin-VI [Crassostrea gigas]
1	3	NaN	82 1449 hypothetical protein BRAFLDRAFT_127120 [Branchiostoma floridae] gi
1	3	NaN	813 1244 Probable protein disulfide-isomerase A6 OS=Caenorhabditis elegans GN=tag-320 PE=2 SV=1
1	3	NaN	" 75 803 hypothetical protein DAPPUDRAFT_231350 [Daphnia pulex]; 82 612 ATP synthase B chain, putative [Ixodes
1	1	NaN	33 422 minus strand PREDICTED: heat shock 70 kDa protein cognate 4-like [Megachile rotundata]
1	1	NaN	78 2003 Chromatin assembly factor 1 subunit A [Crassostrea gigas]
1	1	NaN	" 347 847 minus strand ADP,ATP carrier protein 1 OS=Anopheles gambiae GN=AGAP006782 PE=2 SV=2"
1	3	NaN	132 932 pyruvate kinase [Crassostrea gigas]
1	2	NaN	2 421 hypothetical protein BRAFLDRAFT_119044 [Branchiostoma floridae] gi
1	1	NaN	102 872 RecName: Full=Gelsolin-like protein 1; AltName: Full=Actin-modulator; Short=EWAM; Short=EWAM-P1 gi|1
1	3	NaN	" 179 631 minus strand 60S ribosomal protein L7/L12, partial [Ostrea edulis]"
1	1	NaN	10 366 minus strand hypothetical protein TRIADDRAFT_25239 [Trichoplax adhaerens] gi
1	2	NaN	114 731 minus strand Proteasome subunit beta type-6 [Crassostrea gigas]
1	1	NaN	2 838 Alanine aminotransferase 2 [Crassostrea gigas]
1	1	NaN	406 1392 minus strand hypothetical protein CGI_10007007 [Crassostrea gigas]
1	4	NaN	294 443 GE25988 gene product from transcript GE25988-RA; K02980 small subunit ribosomal protein S29e; 292 441
1	1	NaN	382 972 minus strand Muscle-specific protein 20 [Crassostrea gigas]; 807 1397 minus strand Muscle-specific protein
1	1	NaN	26 514 Protein phosphatase inhibitor 2 [Crassostrea gigas]
1	5	NaN	633 806 Peroxiredoxin-1 [Crassostrea gigas]; 648 821 Peroxiredoxin-1 [Crassostrea gigas]; 666 839 Peroxiredoxin-1
1	4	NaN	788 1006 LIM protein [Haliotis discus discus]; 768 980 muscle LIM protein isoform 1 [Danaus plexippus]
1	1	NaN	156 845 minus strand histone H1 [Mytilus californianus]; 156 845 minus strand histone H1 [Mytilus californianus]; 15
1	1	NaN	2 778 Putative adenosylhomocysteinase 3 [Crassostrea gigas]
1	1	NaN	479 1732 minus strand Multifunctional protein ADE2 [Crassostrea gigas]; 239 1501 minus strand Multifunctional pro
1	1	NaN	60 353 minus strand Histone H3.3 [Harpegnathos saltator]
1	2	NaN	28 516 minus strand Signal peptidase complex subunit 3 [Crassostrea gigas]; 30 554 minus strand Signal peptidase
1	1	NaN	551 871 minus strand Protein disulfide-isomerase A4 [Crassostrea gigas]
1	2	NaN	26 619 G-type lysozyme [Physella acuta]
1	2	NaN	" 1 327 minus strand endo-beta-1,4-glucanase [Ampullaria crossean]"
1	1	NaN	26 367 proteasome alpha type 2 [Haliotis discus discus]
1	1	NaN	211 687 Uncharacterized protein CG16817 [Harpegnathos saltator]; 200 676 Uncharacterized protein CG16817 [Harp
1	3	NaN	79 606 minus strand PREDICTED: similar to Ras-like protein 3 precursor (Protein roughened) [Tribolium castaneum]
1	2	NaN	1 231 small subunit ribosomal protein S11e [Clonorchis sinensis]; 1 171 40S ribosomal protein S11 OS=Canis famili
1	1	NaN	2 2572 Glycogen debranching enzyme [Crassostrea gigas]
1	30	NaN	460 1737 Tubulin beta-4B chain OS=Mus musculus GN=Tubb4b PE=1 SV=1
1	1	NaN	93 368 60S ribosomal protein L37A [Branchiostoma belcheri] gi; 93 395 60S ribosomal protein L37A [Branchiostoma
1	3	NaN	513 755 PREDICTED: 40S ribosomal protein S16-like [Papio anubis]; 513 755 PREDICTED: 40S ribosomal protein S1
1	2	NaN	4 234 ribosomal protein rpl14 [Arenicola marina]
1	4	NaN	320 589 60S ribosomal protein L34 OS=Aedes triseriatus GN=RpL34 PE=2 SV=3
1	2	NaN	244 837 PREDICTED: ras-like GTP-binding protein Rho1-like [Megachile rotundata]; 650 883 hypothetical protein SIN
1	6	NaN	21 254 predicted protein [Nematostella vectensis] gi
1	1	NaN	297 734 minus strand PREDICTED: calmodulin [Canis lupus familiaris]; 297 734 PREDICTED: calmodulin [Canis lupu
1	1	NaN	" 196 1053 minus strand Cytochrome b-c1 complex subunit 2, mitochondrial OS=Homo sapiens GN=UQCRC2 PE=1
1	1	NaN	" 451 1026 PREDICTED: GTP-binding protein SAR1B, putative-like [Saccoglossus kowalevskii]"
1	1	NaN	" 57 752 PREDICTED: adenylate kinase 2, mitochondrial-like isoform 2 [Callithrix jacchus]"
1	2	NaN	221 1450 minus strand PREDICTED: dihydrolipoamide branched chain transacylase E2 [Taeniopygia guttata]; 221 16
1	1	NaN	1180 1521 minus strand coronin [Schistosoma mansoni]
1	1	NaN	249 845 sigma class glutathione-s-transferase 2 [Haliotis discus discus]; 252 848 sigma class glutathione-s-transfer
1	2	NaN	313 1908 minus strand Heterogeneous nuclear ribonucleoprotein L [Crassostrea gigas]; 313 1833 minus strand Hete
1	2	NaN	3 263 calponin-like protein [Taenia asiatica]
1	1	NaN	85 1632 minus strand Alpha-aminoadipic semialdehyde dehydrogenase [Crassostrea gigas]
1	6	NaN	34 480 small subunit ribosomal protein S18e [Clonorchis sinensis]; 431 799 SJCHGC01887 protein [Schistosoma jap
1	1	NaN	165 620 PREDICTED: uncharacterized protein LOC575072 isoform 2 [Strongylocentrotus purpuratus] gi
1	1	NaN	84 425 minus strand Transcription elongation factor B polypeptide 2 [Crassostrea gigas]; 84 425 minus strand Trans
1	1	NaN	1 309 PREDICTED: intersectin-1-like [Sus scrofa]
1	2	NaN	773 1045 minus strand serine protease inhibitor 2 [Latrodectus hesperus]
1	1	NaN	144 1307 minus strand PREDICTED: similar to AGAP005845-PA [Tribolium castaneum]; 146 1309 minus strand PRED
1	2	NaN	123 551 minus strand Transcription factor BTF3-like protein 4 [Crassostrea gigas]
1	1	NaN	3 434 minus strand hypothetical protein CGI_10015501 [Crassostrea gigas]
1	1	NaN	58 900 minus strand Toll-interacting protein [Crassostrea gigas]
1	10	NaN	230 334 protein disulfide isomerase [Conus virgo]
1	2	NaN	971 1963 Spectrin alpha chain [Crassostrea gigas]; 972 1979 Spectrin alpha chain [Crassostrea gigas]
1	3	NaN	447 977 Expansin-yoaJ [Crassostrea gigas]

0.25 0.5 1 2 4

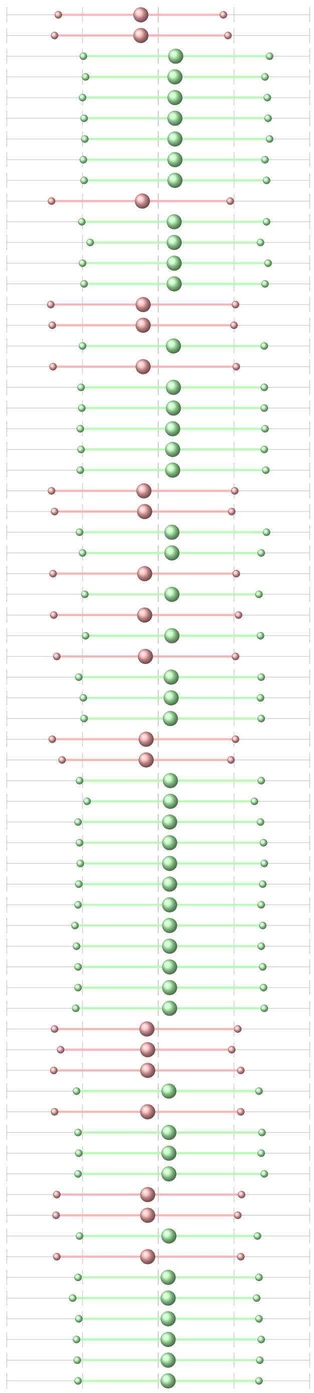

Peptides	Spectra	Coverage	Protein
1	8	NaN	2140 2367 heat shock cognate 71 [Mytilus galloprovincialis]
1	4	NaN	788 1006 LIM protein [Haliotis discus discus]
1	1	NaN	58 1203 minus strand Obg-like ATPase 1 [Crassostrea gigas]
1	2	NaN	" 57 1280 RNA binding motif protein, X-linked [Mustela putorius furo]"
1	1	NaN	" 37 1356 homogentisate 1,2-dioxygenase [Danio rerio] gi; 54 1361 homogentisate 1,2-dioxygenase [Danio rerio] gi"
1	1	NaN	740 2029 minus strand PREDICTED: protein alan shepard-like isoform 2 [Bombus terrestris]
1	1	NaN	165 635 hypothetical protein CGI_10007639 [Crassostrea gigas]
1	1	NaN	74 2734 26S proteasome non-ATPase regulatory subunit 2 [Crassostrea gigas]
1	1	NaN	494 724 minus strand PREDICTED: SH3 domain-binding glutamic acid-rich-like protein 3-like [Amphimedon queensl
1	2	NaN	399 575 hypothetical protein BRAFLDRAFT_69739 [Branchiostoma floridae] gi
1	1	NaN	214 1878 PREDICTED: lysyl-tRNA synthetase-like [Oreochromis niloticus]
1	4	NaN	1237 1374 minus strand putative 60S ribosomal protein L23 [Maconellicoccus hirsutus]
1	1	NaN	172 678 Myosin regulatory light chain sqh [Crassostrea gigas]
1	1	NaN	9 722 minus strand actin related protein [Lumbricus rubellus]
1	1	NaN	128 1138 Actin-related protein 2/3 complex subunit 1A OS=Homo sapiens GN=ARPC1A PE=1 SV=2
1	1	NaN	5 598 hypothetical protein DAPPUDRAFT_344656 [Daphnia pulex]
1	1	NaN	13 1218 minus strand PREDICTED: eukaryotic initiation factor 4A-III-like [Bombus terrestris] gi
1	1	NaN	52 1653 minus strand PREDICTED: eukaryotic translation initiation factor 3 subunit 6 interacting protein-like [Sacco
1	1	NaN	275 769 minus strand hypothetical protein CGI_10025135 [Crassostrea gigas]; 216 683 hypothetical protein CGI_100
1	1	NaN	84 425 minus strand thioester-containing protein [Euphaedusa tau]
1	1	NaN	143 865 minus strand predicted protein [Nematostella vectensis] gi
1	1	NaN	17 691 minus strand Cleavage and polyadenylation specificity factor subunit 5 [Crassostrea gigas]
1	1	NaN	616 1740 5'-AMP-activated protein kinase subunit beta-2 [Crassostrea gigas]; 641 1762 minus strand 5'
1	1	NaN	153 953 minus strand ribosomal protein rpl7 [Arenicola marina]
1	2	NaN	418 690 PREDICTED: protein disulfide isomerase A4-like [Saccoglossus kowalevskii]
1	1	NaN	1 177 small heat shock protein [Trichinella pseudospiralis]
1	2	NaN	" 194 334 unknown, partial [Lymnaea stagnalis] gi; 207 347 unknown, partial [Lymnaea stagnalis] gi; 384 524 unknow
1	1	NaN	" 1362 1544 minus strand Phosphoenolpyruvate carboxykinase, cytosolic [GTP] [Crassostrea gigas]"
1	4	NaN	98 865 proteasome (prosome macropain) subunit alpha type 1 [Xenopus (Silurana) tropicalis]; 98 865 proteasome (p
1	1	NaN	231 902 Sodium- and chloride-dependent GABA transporter ine [Crassostrea gigas]
1	3	NaN	2 298 putative 60S ribosomal protein RPL24 [Novocrania anomala]
1	2	NaN	138 713 Rac [Aplysia californica] gi; 125 691 Rac [Aplysia californica] gi; 149 871 minus strand Rac [Aplysia californ
1	1	NaN	1 456 minus strand Putative sodium/potassium-transporting ATPase subunit beta-2 [Crassostrea gigas]; 688 1251 m
1	3	NaN	91 1326 minus strand Adipocyte plasma membrane-associated protein [Crassostrea gigas]; 107 1273 minus strand /
1	2	NaN	" 29 448 minus strand PREDICTED: similar to NADH:ubiquinone dehydrogenase, putative [Tribolium castaneum] gi;
1	1	NaN	764 1258 minus strand Caprin-2 [Crassostrea gigas]
1	8	NaN	187 642 minus strand PREDICTED: similar to pol-like protein [Tribolium castaneum]
1	1	NaN	44 889 minus strand hypothetical protein CGI_10015025 [Crassostrea gigas]
1	7	NaN	438 683 minus strand Protein lethal(2)essential for life OS=Drosophila melanogaster GN=l(2)efl PE=1 SV=1
1	1	NaN	5 658 predicted protein [Nematostella vectensis] gi
1	1	NaN	49 1560 PREDICTED: tRNA-splicing ligase RtcB homolog [Megachile rotundata]
1	1	NaN	503 1591 minus strand Septin-2 [Crassostrea gigas]; 648 1736 minus strand Septin-2 [Crassostrea gigas]
1	1	NaN	2 487 conserved hypothetical protein [Ixodes scapularis] gi
1	1	NaN	4365 4553 minus strand Basement membrane-specific heparan sulfate proteoglycan core protein [Crassostrea gigas
1	1	NaN	29 610 minus strand 40S ribosomal protein S7 [Crassostrea gigas]
1	1	NaN	794 994 Glycogenin-1 [Crassostrea gigas]; 1425 1739 minus strand Glycogenin-1 [Crassostrea gigas]
1	1	NaN	14 502 minus strand ependymin related protein-1 precursor [Haliotis discus discus]; 14 547 ependymin related prote
1	1	NaN	62 394 minus strand PREDICTED: NADH dehydrogenase [ubiquinone] 1 alpha subcomplex subunit 5 [Saimiri bolivie
1	1	NaN	423 695 thioredoxin peroxidase 2 [Haliotis discus discus]
1	1	NaN	1 468 glycine cleavage system H protein [Rhipicephalus sanguineus]
1	4	NaN	87 764 Enolase-phosphatase E1 [Crassostrea gigas]; 87 1058 Enolase-phosphatase E1 [Crassostrea gigas]; 87 764 I
1	1	NaN	1021 1359 minus strand RecName: Full=40S ribosomal protein S26 gi|6015599|emb|CAB57819.1| ribosomal protein
1	1	NaN	115 393 minus strand Yolk ferritin OS=Lymnaea stagnalis PE=1 SV=1; 132 413 RecName: Full=Yolk ferritin; Flags: Pr
1	1	NaN	123 860 PREDICTED: 3-hydroxybutyrate dehydrogenase type 2-like isoform 1 [Sus scrofa] gi; 32 901 3-hydroxybutyr
1	1	NaN	" 2 1810 minus strand Glutaminyl-tRNA synthetase, partial [Crassostrea gigas]"
1	1	NaN	942 1385 Golgi-associated plant pathogenesis-related protein 1 [Crassostrea gigas]; 536 979 Golgi-associated plant
1	1	NaN	2 1384 minus strand twitchin [Mytilus galloprovincialis]; 2 1384 minus strand twitchin [Mytilus galloprovincialis]; 2 1
1	1	NaN	236 733 Spectrin alpha chain [Crassostrea gigas]
1	1	NaN	" 695 823 minus strand Aldehyde dehydrogenase family 16 member A1, partial [Crassostrea gigas]"
1	2	NaN	532 663 minus strand 40S ribosomal protein S23 [Ascaris suum]; 526 654 minus strand RecName: Full=40S ribosom
1	1	NaN	13 585 UDP-glucose:glycoprotein glucosyltransferase 1 [Crassostrea gigas]
1	2	NaN	1 297 beta-thymosin domain repeat protein CSP24KDa_v1 [Hermissenda crassicornis] gi
1	1	NaN	1 630 hypothetical protein [Trichomonas vaginalis G3] gi
1	1	NaN	" 125 628 minus strand collectin sub-family member 12, partial [Oncorhynchus mykiss]"
1	1	NaN	511 1062 unknown [Dendroctonus ponderosae]; 511 1062 unknown [Dendroctonus ponderosae]
1	1	NaN	48 1523 minus strand Spermatogenesis-associated protein 18-like protein [Crassostrea gigas]
1	1	NaN	93 2606 twitchin [Mytilus galloprovincialis]

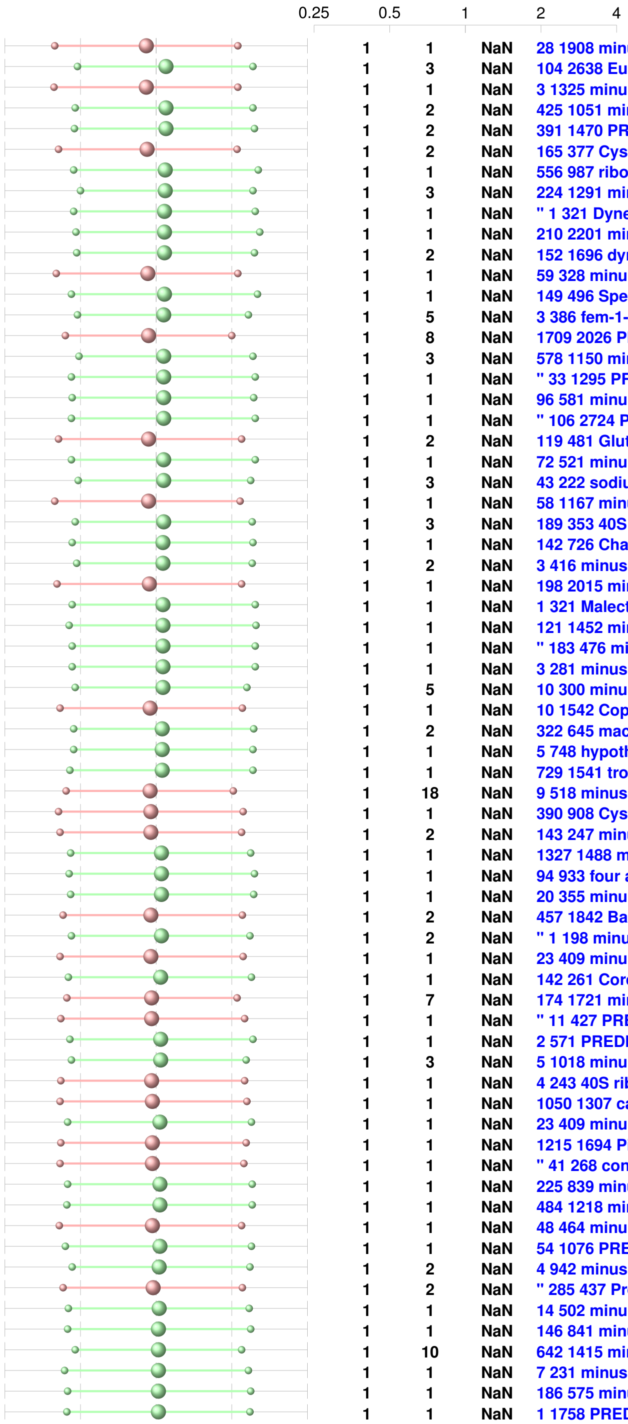

Peptides	Spectra	Coverage	Protein
1	1	NaN	28 1908 minus strand Mitochondrial inner membrane protein [Crassostrea gigas]; 10 1902 Mitochondrial inner meml
1	3	NaN	104 2638 Eukaryotic translation initiation factor 3 subunit C [Crassostrea gigas]
1	1	NaN	3 1325 minus strand Eukaryotic translation initiation factor 3 subunit B [Crassostrea gigas]
1	2	NaN	425 1051 minus strand unknown [Schistosoma japonicum]
1	2	NaN	391 1470 PREDICTED: heparanase-like [Amphimedon queenslandica]; 391 1470 PREDICTED: heparanase-like [Amp
1	2	NaN	165 377 Cysteine and glycine-rich protein 3 [Crassostrea gigas]; 135 344 Cysteine and glycine-rich protein 3 [Crasso
1	1	NaN	556 987 ribosomal protein rps15 [Lineus viridis]
1	3	NaN	224 1291 minus strand coproporphyrinogen oxidase [Aplysia californica] gi
1	1	NaN	" 1 321 Dynein heavy chain, cytoplasmic [Crassostrea gigas]"
1	1	NaN	210 2201 minus strand Glycyl-tRNA synthetase [Crassostrea gigas]; 210 2201 minus strand Glycyl-tRNA synthetase
1	2	NaN	152 1696 dynamin isoform A [Lymnaea stagnalis]
1	1	NaN	59 328 minus strand PREDICTED: similar to predicted protein [Hydra magnipapillata]; 59 328 PREDICTED: similar to
1	1	NaN	149 496 Spectrin alpha chain [Crassostrea gigas]
1	5	NaN	3 386 fem-1-like protein C [Crassostrea gigas]
1	8	NaN	1709 2026 Phosphoenolpyruvate carboxykinase [GTP] [Crassostrea gigas]
1	3	NaN	578 1150 minus strand expressed hypothetical protein [Trichoplax adhaerens] gi; 376 948 minus strand expressed h
1	1	NaN	" 33 1295 PREDICTED: LOW QUALITY PROTEIN: selenide, water dikinase-like [Bombus impatiens]"
1	1	NaN	96 581 minus strand thioredoxin 2 [Haliotis discus discus]; 1682 2167 minus strand thioredoxin 2 [Haliotis discus di
1	1	NaN	" 106 2724 PREDICTED: 2-oxoglutarate dehydrogenase, mitochondrial isoform 1 [Sarcophilus harrisii]; 492 3473 PR
1	2	NaN	119 481 Glutamate receptor U1 [Crassostrea gigas]; 113 328 glutamate receptor subunit protein GluR2 precursor [A
1	1	NaN	72 521 minus strand GF14593 [Drosophila ananassae] gi; 60 392 GD23124 [Drosophila simulans] gi
1	3	NaN	43 222 sodium-calcium exchanger [Doryteuthis pealeii]
1	1	NaN	58 1167 minus strand PREDICTED: arylacetamide deacetylase-like 4-like [Gallus gallus]; 76 1239 minus strand PRED
1	3	NaN	189 353 40S ribosomal protein S12 [Crassostrea gigas]
1	1	NaN	142 726 Charged multivesicular body protein 4b [Crassostrea gigas]
1	2	NaN	3 416 minus strand myosin essential light chain [Crassostrea gigas]
1	1	NaN	198 2015 minus strand Xaa-Pro aminopeptidase 1 [Crassostrea gigas]
1	1	NaN	1 321 Malectin [Crassostrea gigas]
1	1	NaN	121 1452 minus strand Hexokinase type 2 [Crassostrea gigas]; 245 1576 minus strand Hexokinase type 2 [Crassostr
1	1	NaN	" 183 476 minus strand guanine nucleotide-binding protein G(q), alpha subunit [Aplysia californica] gi"
1	1	NaN	3 281 minus strand hypothetical protein CGI_10015501 [Crassostrea gigas]
1	5	NaN	10 300 minus strand unnamed protein product [Tetraodon nigroviridis]
1	1	NaN	10 1542 Copine-8 [Crassostrea gigas]
1	2	NaN	322 645 macrophage migration inhibitory-like factor protein [Chlamys farreri]; 437 682 macrophage migration inhibit
1	1	NaN	5 748 hypothetical protein SINV_09458 [Solenopsis invicta]
1	1	NaN	729 1541 troponin T [Mizuhopecten yessoensis]
1	18	NaN	9 518 minus strand hemocyanin isoform 1 [Nucula nucleus]
1	1	NaN	390 908 Cysteine proteinase 5 OS=Dictyostelium discoideum GN=cprE PE=2 SV=2
1	2	NaN	143 247 minus strand dynein light chain [Aplysia californica] gi
1	1	NaN	1327 1488 minus strand Ubiquinol-cytochrome C reductase complex [Xenopus laevis] gi
1	1	NaN	94 933 four and A half lim domains [Schistosoma mansoni]
1	1	NaN	20 355 minus strand PREDICTED: uroporphyrinogen decarboxylase-like [Monodelphis domestica]
1	2	NaN	457 1842 Basement membrane-specific heparan sulfate proteoglycan core protein [Crassostrea gigas]; 457 1842 Ba
1	2	NaN	" 1 198 minus strand Chain A, Rep1-Nxsq Fatty Acid Transporter; 1 231 minus strand Chain A, Rep1-Nxsq Fatty Acid
1	1	NaN	23 409 minus strand CDGSH iron sulfur domain-containing protein 2 [Crassostrea gigas]; 1 207 minus strand CDGS
1	1	NaN	142 261 Core histone macro-H2A.2 OS=Mus musculus GN=H2afy2 PE=1 SV=3
1	7	NaN	174 1721 minus strand aspartyl-tRNA synthetase [Xenopus laevis] gi
1	1	NaN	" 11 427 PREDICTED: similar to syntaxin, partial [Hydra magnipapillata]"
1	1	NaN	2 571 PREDICTED: copper homeostasis protein cutC homolog [Anolis carolinensis]
1	3	NaN	5 1018 minus strand Glutaredoxin-3 [Crassostrea gigas]; 5 1018 Glutaredoxin-3 [Crassostrea gigas]
1	1	NaN	4 243 40S ribosomal protein S13 OS=Caenorhabditis elegans GN=rps-13 PE=2 SV=2; 4 243 40S ribosomal protein S1
1	1	NaN	1050 1307 calreticulin precursor [Aplysia californica] gi
1	1	NaN	23 409 minus strand CDGSH iron sulfur domain-containing protein 2 [Crassostrea gigas]
1	1	NaN	1215 1694 Pikachurin [Crassostrea gigas]
1	1	NaN	" 41 268 conserved hypothetical protein [Perkinsus marinus ATCC 50983] gi; 29 238 PREDICTED: succinyl-CoA ligas
1	1	NaN	225 839 minus strand Metallo-beta-lactamase domain-containing protein 1 [Crassostrea gigas]; 201 851 minus stran
1	1	NaN	484 1218 minus strand alginate lyase [Littorina brevicula]; 484 1218 minus strand alginate lyase [Littorina brevicula]
1	1	NaN	48 464 minus strand Acyl-coenzyme A thioesterase 13 [Crassostrea gigas]
1	1	NaN	54 1076 PREDICTED: UPF0553 protein C9orf64-like [Anolis carolinensis]; 71 1093 PREDICTED: UPF0553 protein C9o
1	2	NaN	4 942 minus strand KTEL motif-containing protein 1 [Crassostrea gigas]
1	2	NaN	" 285 437 Propionyl-CoA carboxylase alpha chain, mitochondrial [Crassostrea gigas]"
1	1	NaN	14 502 minus strand Ufm1-conjugating enzyme 1 [Crassostrea gigas]
1	1	NaN	146 841 minus strand hypothetical protein CGI_10006609 [Crassostrea gigas]; 146 841 minus strand hypothetical pr
1	10	NaN	642 1415 minus strand cytochrome c oxidase subunit II [Oncomelania hupensis hupensis]
1	1	NaN	7 231 minus strand AGAP006879-PA [Anopheles gambiae str. PEST] gi
1	1	NaN	186 575 minus strand predicted protein [Nematostella vectensis] gi
1	1	NaN	1 1758 PREDICTED: polyprotein-like [Saccoglossus kowalevskii]

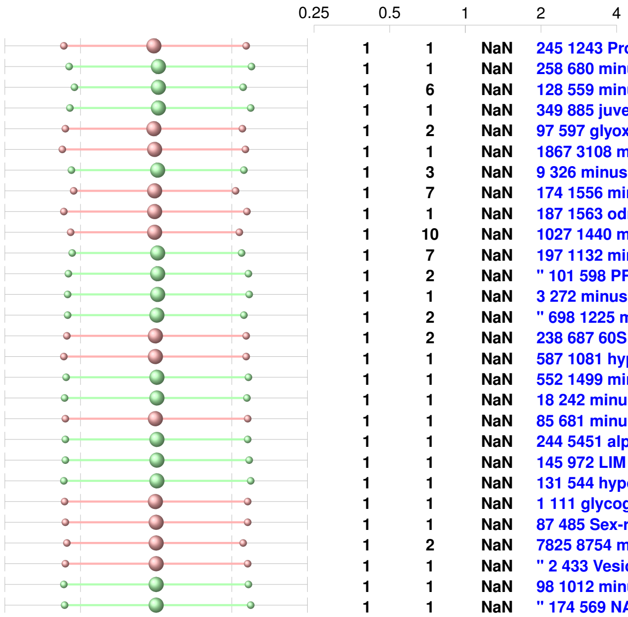

Peptides	Spectra	Coverage	Protein
1	1	NaN	245 1243 Prostaglandin reductase 1 [Crassostrea gigas]
1	1	NaN	258 680 minus strand synapse-associated protein [Aplysia californica] gi
1	6	NaN	128 559 minus strand predicted protein [Nematostella vectensis] gi
1	1	NaN	349 885 juvenile hormone diol kinase [Danaus plexippus]; 349 885 juvenile hormone diol kinase [Danaus plexippus]
1	2	NaN	97 597 glyoxylate reductase/hydroxypyruvate reductase [Ictalurus punctatus] gi
1	1	NaN	1867 3108 minus strand roundabout homolog 1 precursor [Rattus norvegicus] gi; 1867 3108 minus strand roundabo
1	3	NaN	9 326 minus strand catalase [Paracyclopina nana]; 9 209 Catalase OS=Cavia porcellus GN=CAT PE=2 SV=4
1	7	NaN	174 1556 minus strand aspartyl-tRNA synthetase [Xenopus laevis] gi; 174 1721 minus strand aspartyl-tRNA synthet
1	1	NaN	187 1563 odr-4-like protein [Crassostrea gigas]
1	10	NaN	1027 1440 minus strand hemocyanin isoform 1 [Haliotis diversicolor]
1	7	NaN	197 1132 minus strand Pancreatic lipase-related protein 2 [Crassostrea gigas]
1	2	NaN	" 101 598 PREDICTED: aminomethyltransferase, mitochondrial-like [Oreochromis niloticus]"
1	1	NaN	3 272 minus strand ADP/ATP carrier [Clonorchis sinensis]
1	2	NaN	" 698 1225 minus strand actin related protein 2/3 complex, 21 kDa subunit [Crassostrea gigas]"
1	2	NaN	238 687 60S ribosomal protein L6 [Crassostrea gigas]; 142 789 60S ribosomal protein L6 [Crassostrea gigas]
1	1	NaN	587 1081 hypothetical protein CGI_10026502 [Crassostrea gigas]
1	1	NaN	552 1499 minus strand uncharacterized protein LOC100158466 [Xenopus (Silurana) tropicalis] gi; 552 1499 minus st
1	1	NaN	18 242 minus strand translation elongation factor 1B gamma subunit [Strongylocentrotus purpuratus] gi
1	1	NaN	85 681 minus strand 40S ribosomal protein S8 OS=Xenopus laevis GN=rps8 PE=2 SV=3
1	1	NaN	244 5451 alpha macroglobulin [Chlamys farreri]
1	1	NaN	145 972 LIM and SH3 domain protein F42H10.3 OS=Caenorhabditis elegans GN=F42H10.3 PE=2 SV=3
1	1	NaN	131 544 hypothetical protein CGI_10001749 [Crassostrea gigas]
1	1	NaN	1 111 glycogen synthase [Crassostrea gigas]
1	1	NaN	87 485 Sex-regulated protein janus-A [Crassostrea gigas]
1	2	NaN	7825 8754 minus strand H2 [Haliotis tuberculata]
1	1	NaN	" 2 433 Vesicle-fusing ATPase, partial [Ascaris suum]"
1	1	NaN	98 1012 minus strand RecName: Full=Substrate-specific endoprotease Tex31; Flags: Precursor gi|32187775|emb|C
1	1	NaN	" 174 569 NADH dehydrogenase [ubiquinone] iron-sulfur protein 4, mitochondrial [Crassostrea gigas]"

5 Protein Details

A detailed summary of each protein is given below. These sections include peptide relative expression estimates in addition to protein-level estimates.

5.1 2 256 Polyubiquitin (Fragment) OS=Xenopus laevis PE=1 SV=2; 20 388 large subunit ribosomal protein L40e [Clonorchis sinensis]

Protein Accession	CL47507.Contig2$_{H}1$ CL39010.Contig1$_{H}1$
Mean Expression Ratio	0.342
Median Expression Ratio	0.345
Credible Interval	(0.18, 0.634)
Associated Peptides	5
Associated Spectra	13
Coverage	NaN

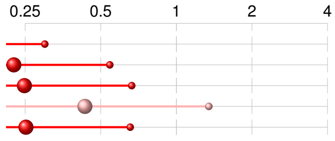

A	B	2.5	50	97.5	Sequence
2	0	0.042	0.11	0.3	IQDKEGIPPDQQR
2	2	0.094	0.22	0.54	TLSDYNIQK
2	0	0.091	0.25	0.67	LIFAGK
1	0	0.13	0.43	1.4	MQIFVK
1	3	0.095	0.25	0.66	TITLEVEPSDTIENVK

5.2 507 1277 alpha tubulin [Notothenia coriiceps]

Protein Accession	Unigene56691$_H$1
Mean Expression Ratio	0.364
Median Expression Ratio	0.364
Credible Interval	(0.181, 0.723)
Associated Peptides	2
Associated Spectra	15
Coverage	NaN

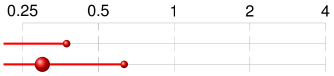

A	B	2.5	50	97.5	Sequence
2	7	0.071	0.17	0.37	LSVDYGK
4	2	0.14	0.3	0.63	LSVDYGKK

5.3 160 588 hypothetical protein CGI_10001640 [Crassostrea gigas]

Protein Accession	CL2347.Contig8$_H$1
Mean Expression Ratio	0.388
Median Expression Ratio	0.389
Credible Interval	(0.201, 0.744)
Associated Peptides	2
Associated Spectra	14
Coverage	NaN

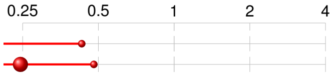

A	B	2.5	50	97.5	Sequence
3	2	0.086	0.19	0.43	AFLEQLGSK
5	4	0.12	0.25	0.48	ANLIVIGSR

5.4 254 1648 Tektin-3 [Crassostrea gigas]; 254 1648 Tektin-3 [Crassostrea gigas]

Protein Accession	CL6717.Contig3$_H$1 CL6717.Contig2$_H$1
Mean Expression Ratio	0.406
Median Expression Ratio	0.404
Credible Interval	(0.221, 0.757)
Associated Peptides	8
Associated Spectra	9
Coverage	NaN

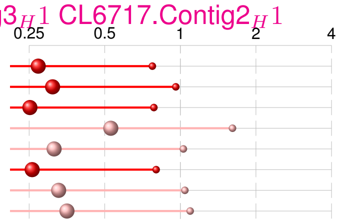

A	B	2.5	50	97.5	Sequence
1	1	0.095	0.27	0.77	AAQHALEIDAK
1	0	0.099	0.31	0.96	ATLEHDISVK
1	0	0.079	0.25	0.79	ILPYVTDK
1	0	0.17	0.53	1.6	KGFPMDPK
1	0	0.1	0.31	1	NVELLR
1	0	0.082	0.26	0.8	EVDIIR
1	0	0.11	0.33	1	EVAEIR
1	0	0.11	0.35	1.1	EAFMK

5.5 266 607 minus strand H2 [Haliotis tuberculata]

Protein Accession	Unigene62350$_H$1
Mean Expression Ratio	0.404
Median Expression Ratio	0.404
Credible Interval	(0.209, 0.773)
Associated Peptides	4
Associated Spectra	18
Coverage	NaN

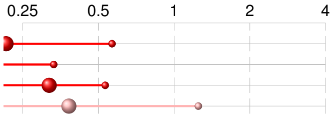

A	B	2.5	50	97.5	Sequence
1	1	0.078	0.22	0.57	DINTLTDEEIK
2	1	0.056	0.14	0.33	KDINTLTDEEIK
6	6	0.19	0.32	0.53	YAVAGIGVR
0	1	0.12	0.38	1.2	DIDGASPLSFEQIAEFHGYPGK

5.6 195 455 60S ribosomal protein L23a OS=Rattus norvegicus GN=Rpl23a PE=2 SV=1; 195 512 60S ribosomal protein L23a OS=Rattus norvegicus GN=Rpl23a PE=2 SV=1; 16 465 60S ribosomal protein L23a OS=Rattus norvegicus GN=Rpl23a PE=2 SV=1

Protein Accession	CL27563.Contig3$_{H}$1 CL27563.Contig2$_{H}$1 CL27563.Contig1$_{H}$1
Mean Expression Ratio	2.45
Median Expression Ratio	2.44
Credible Interval	(1.27, 4.84)
Associated Peptides	3
Associated Spectra	11
Coverage	NaN

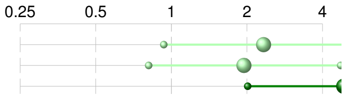

A	B	2.5	50	97.5	Sequence
2	2	0.94	2.3	5.6	ANKPLIK
2	2	0.81	1.9	4.7	SLYLYDVDIAK
0	3	2	4.8	12	VNTLIRPDGEKK

5.7 118 501 minus strand histone 2A.Z [Aplysia californica]; 15 428 PREDICTED: histone H2A.V-like [Apis florea]

Protein Accession	CL14228.Contig6$_{H}$1 CL14228.Contig5$_{H}$1
Mean Expression Ratio	2.36
Median Expression Ratio	2.37
Credible Interval	(1.33, 4.31)
Associated Peptides	5
Associated Spectra	17
Coverage	NaN

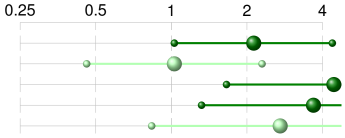

A	B	2.5	50	97.5	Sequence
3	4	1	2.1	4.4	ATIAGGGVIPHIHK
2	3	0.46	1	2.3	GDEELDSLIK
0	2	1.7	4.4	12	HLQLAIRGDEELDSLIK
1	1	1.3	3.7	10	SLIGKK
0	1	0.83	2.7	8.5	GGGVIPHIHK

5.8 764 1015 minus strand heat shock protein beta-1 [Trichinella spiralis] gi

Protein Accession	CL46983.Contig3$_{H}$1
Mean Expression Ratio	2.34
Median Expression Ratio	2.35
Credible Interval	(1.42, 3.9)
Associated Peptides	7
Associated Spectra	76
Coverage	NaN

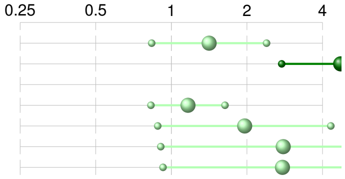

A	B	2.5	50	97.5	Sequence
6	6	0.83	1.4	2.4	SYVLPK
5	5	2.7	4.7	8.3	SYVLPKDVDPK
4	12	13	21	33	TVTSSLSQNGVLTLKAPK
10	22	0.83	1.2	1.6	TVTSSLSQNGVLTLK
0	4	0.88	2	4.3	VVINAKHEERPDEHGYIMR
0	1	0.91	2.8	8.6	SSLSQNGVLTLKAPK
0	1	0.92	2.8	8.5	MALEAPQEQV

5.9 2 370 hemocyanin alphaD-subunit [Helix lucorum]

Protein Accession	CL46908.Contig1$_{H}$1
Mean Expression Ratio	0.429
Median Expression Ratio	0.429
Credible Interval	(0.259, 0.707)
Associated Peptides	8
Associated Spectra	45
Coverage	NaN

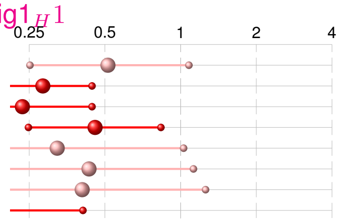

A	B	2.5	50	97.5	Sequence
2	3	0.25	0.51	1.1	AFAVFK
5	12	0.18	0.28	0.44	LLGIGTSAEVK
1	6	0.12	0.23	0.44	NEMPWQFER
1	7	0.25	0.46	0.83	SFYLDVSEAVVR
0	1	0.1	0.32	1	SPHCEGAK
0	2	0.16	0.43	1.1	LASNVLPK
0	1	0.14	0.41	1.3	LYNMLETR
0	4	0.085	0.19	0.41	SFFILGGK

5.10 118 501 minus strand histone 2A.Z [Aplysia californica]

Protein Accession	CL14228.Contig6$_{H}$1
Mean Expression Ratio	0.43
Median Expression Ratio	0.429
Credible Interval	(0.248, 0.747)
Associated Peptides	6
Associated Spectra	34
Coverage	NaN

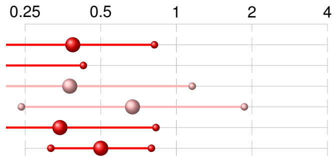

A	B	2.5	50	97.5	Sequence
3	4	0.18	0.39	0.82	ATIAGGGVIPHIHK
2	3	0.081	0.19	0.43	GDEELDSLIK
1	0	0.12	0.38	1.2	HLQLAIR
1	1	0.24	0.67	1.9	SLIGKK
3	0	0.14	0.34	0.83	SLIGK
16	0	0.32	0.5	0.79	AGLQFPVGR

5.11 1070 1888 minus strand galectin [Pinctada fucata]; 1371 2189 minus strand galectin [Pinctada fucata]

Protein Accession	CL7245.Contig4$_{H}$1 CL7245.Contig3$_{H}$1
Mean Expression Ratio	0.429
Median Expression Ratio	0.43
Credible Interval	(0.243, 0.773)
Associated Peptides	4
Associated Spectra	29
Coverage	NaN

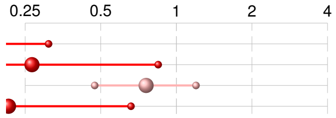

A	B	2.5	50	97.5	Sequence
2	2	0.057	0.13	0.31	EIPQGLEK
1	0	0.085	0.27	0.85	GEAFIYEVK
13	10	0.47	0.76	1.2	FNEGVTVR
1	0	0.067	0.21	0.66	NDLVVIK

5.12 1592 1768 minus strand beta-actin [Diabolocatantops pinguis]

Protein Accession	CL22741.Contig2$_{H}$1
Mean Expression Ratio	2.28
Median Expression Ratio	2.28
Credible Interval	(1.33, 3.91)
Associated Peptides	6
Associated Spectra	46
Coverage	NaN

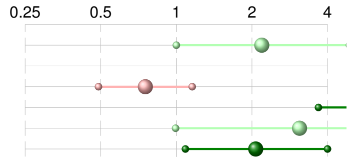

A	B	2.5	50	97.5	Sequence
3	1	1	2.2	4.9	VIAPPERK
1	7	6.4	12	22	IKVIAPPERK
8	11	0.49	0.75	1.2	VIAPPER
0	7	3.7	7	13	IKVIAPPER
0	1	0.99	3.1	9.3	GDQKEISALAPPTMK
0	7	1.1	2.1	4	EISALAPPTMK

5.13 503 1090 minus strand myosin regulatory light chain [Fasciola hepatica]; 278 865 minus strand myosin regulatory light chain [Fasciola hepatica]

Protein Accession	CL43006.Contig6$_{H}$1 CL43006.Contig4$_{H}$1
Mean Expression Ratio	0.455
Median Expression Ratio	0.454
Credible Interval	(0.272, 0.751)
Associated Peptides	9
Associated Spectra	32
Coverage	NaN

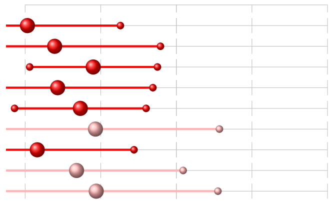

A	B	2.5	50	97.5	Sequence
3	0	0.11	0.25	0.6	DGVIDIEDLK
1	1	0.13	0.33	0.87	GYLPEEYIK
4	5	0.26	0.47	0.84	IPPDDELKEMLK
2	1	0.14	0.34	0.81	NAFAMFDDEGK
5	3	0.23	0.41	0.76	QAQVQEFK
1	0	0.15	0.48	1.5	IPPDDELK
2	1	0.12	0.28	0.68	EAFTMIDQNR
0	2	0.15	0.4	1.1	LSGTDPEDTIR
0	1	0.15	0.48	1.5	DGVIDIEDLKDIYSNLGR

5.14 35 370 Ganglioside GM2 activator [Crassostrea gigas]; 2 487 minus strand PREDICTED: hypothetical protein LOC100699750 [Oreochromis niloticus]; 41 535 minus strand PREDICTED: hypothetical protein LOC100699750 [Oreochromis niloticus]

Protein Accession	CL7266.Contig6$_{H}1$ CL7266.Contig5$_{H}1$ CL7266.Contig3$_{H}1$
Mean Expression Ratio	0.455
Median Expression Ratio	0.456
Credible Interval	(0.207, 0.975)
Associated Peptides	1
Associated Spectra	5
Coverage	NaN

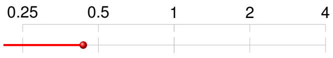

A	B	2.5	50	97.5	Sequence
3	2	0.081	0.19	0.44	NVALVVSVK

5.15 195 455 60S ribosomal protein L23a OS=Rattus norvegicus GN=Rpl23a PE=2 SV=1; 16 465 60S ribosomal protein L23a OS=Rattus norvegicus GN=Rpl23a PE=2 SV=1

Protein Accession	CL27563.Contig3$_{H}1$ CL27563.Contig1$_{H}1$
Mean Expression Ratio	0.457
Median Expression Ratio	0.457
Credible Interval	(0.231, 0.905)
Associated Peptides	3
Associated Spectra	9
Coverage	NaN

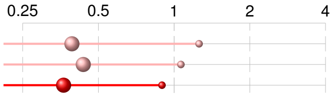

A	B	2.5	50	97.5	Sequence
1	0	0.12	0.39	1.3	VNTLIRPDGEK
2	2	0.18	0.43	1.1	ANKPLIK
2	2	0.15	0.36	0.9	SLYLYDVDIAK

5.16 " 172 867 minus strand complement C1q-like protein 4 precursor, partial [Oryzias melastigma]"

Protein Accession	Unigene7884$_{H}1$
Mean Expression Ratio	2.18
Median Expression Ratio	2.18
Credible Interval	(1.21, 3.97)
Associated Peptides	5
Associated Spectra	25
Coverage	NaN

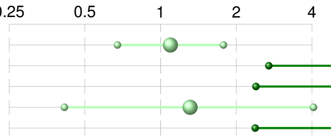

A	B	2.5	50	97.5	Sequence
2	12	0.67	1.1	1.8	DLQQAVESMRPSLK
1	2	2.7	6.4	15	EDQIKDLQDK
1	2	2.4	5.8	14	EDQIKDLQDKQK
0	1	0.41	1.3	4.1	DLQQAVESMR
0	4	2.4	5.3	12	DVKDLQQAVESMRPSLK

5.17 209 781 minus strand high mobility group protein 1 [Biomphalaria glabrata]

Protein Accession	CL25076.Contig4$_H$1
Mean Expression Ratio	0.478
Median Expression Ratio	0.479
Credible Interval	(0.236, 0.955)
Associated Peptides	3
Associated Spectra	10
Coverage	NaN

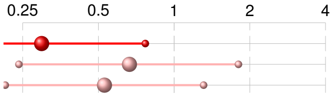

A	B	2.5	50	97.5	Sequence
2	1	0.12	0.3	0.77	KPFEDMAAK
1	2	0.24	0.67	1.8	KYEAEMAEYR
2	2	0.21	0.53	1.3	LAGEEWR

5.18 27 620 putative mitochondrial ATP synthase [Haliotis discus discus]

Protein Accession	CL13014.Contig1$_H$1
Mean Expression Ratio	0.481
Median Expression Ratio	0.48
Credible Interval	(0.25, 0.911)
Associated Peptides	4
Associated Spectra	17
Coverage	NaN

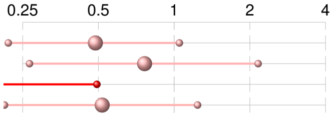

A	B	2.5	50	97.5	Sequence
3	4	0.22	0.49	1	LANPALVDQFQK
1	1	0.27	0.76	2.2	SNALKDIAAEEK
1	1	0.062	0.18	0.49	VPYPEDK
1	5	0.21	0.52	1.2	SAVDWVAFK

5.19 " 223 423 ADPATP carrier protein; K05863 solute carrier family 25 (mitochondrial adenine nucleotide translocator), member 4/5/6/31; 237 455 minus strand ADP,ATP carrier protein [Crassostrea gigas]"

Protein Accession	CL27196.Contig3$_H$1 CL27196.Contig2$_H$1
Mean Expression Ratio	2.06
Median Expression Ratio	2.05
Credible Interval	(1.02, 4.14)
Associated Peptides	3
Associated Spectra	10
Coverage	NaN

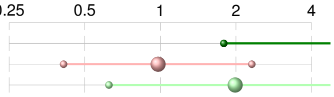

A	B	2.5	50	97.5	Sequence
0	1	1.8	5.8	20	VKLLVQNQNEMIK
6	2	0.41	0.98	2.3	LLVQNQNEMIK
0	1	0.62	2	6.5	AENFALSGLVLPLSSPK

5.20 1009 2784 minus strand Collagen alpha-3(VI) chain [Crassostrea gigas]; 1009 2787 minus strand Collagen alpha-3(VI) chain [Crassostrea gigas]

Protein Accession	Unigene26554$_H$1 Unigene26550$_H$1
Mean Expression Ratio	0.495
Median Expression Ratio	0.497
Credible Interval	(0.269, 0.903)
Associated Peptides	6
Associated Spectra	12
Coverage	NaN

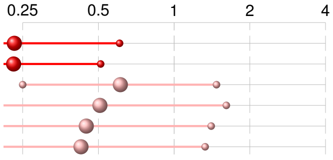

A	B	2.5	50	97.5	Sequence
2	0	0.086	0.23	0.61	ELEGNVHSIR
4	0	0.11	0.23	0.51	IITVGIGEANR
3	0	0.25	0.61	1.5	SDAAAALIR
1	0	0.16	0.51	1.6	VAMSNFR
1	0	0.14	0.45	1.4	MFIPVR
1	0	0.13	0.43	1.3	LTTVR

5.21 97 477 minus strand PREDICTED: complement C1q tumor necrosis factor-related protein 3-like [Oreochromis niloticus]; 97 447 minus strand PREDICTED: complement C1q tumor necrosis factor-related protein 3-like [Oreochromis niloticus]

Protein Accession	CL370.Contig3$_H$1 CL370.Contig2$_H$1
Mean Expression Ratio	2
Median Expression Ratio	2
Credible Interval	(1.34, 2.99)
Associated Peptides	16
Associated Spectra	114
Coverage	NaN

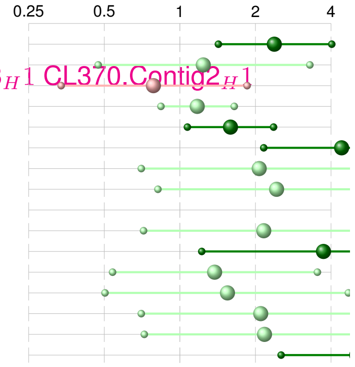

A	B	2.5	50	97.5	Sequence
3	9	1.4	2.4	4	QTSDISRLENR
1	1	0.47	1.2	3.3	QMVDALK
1	2	0.34	0.78	1.9	PIAFSK
5	26	0.84	1.2	1.6	QMVDALKDEQQR
0	21	1.1	1.6	2.4	LESGVRPPVYRPDLESK
0	5	2.2	4.4	9.1	QMVDALKDEQQRQQEVINR
0	1	0.7	2.1	6.4	QSQQIEKQTSDISR
0	1	0.82	2.4	7.3	QSQQIEKQTSDISRLENR
0	25	5.3	7.8	11	VTKLESGVRPPVYRPDLESK
0	1	0.72	2.2	6.5	VTKLESGVRPP
0	1	1.2	3.7	11	LENRVTK
0	2	0.54	1.4	3.5	VAFTAH
0	1	0.5	1.6	4.7	MVDALKDEQQR
0	1	0.7	2.1	6.4	RPIAFSK
0	1	0.72	2.2	6.5	DALKDEQQR
0	6	2.5	5.1	10	VAFTAHFSQGGSMDIGTSRPIAFSK

5.22 140 283 minus strand elongation factor 1 alpha [Angiostrongylus cantonensis]

Protein Accession	CL16758.Contig7$_H$1
Mean Expression Ratio	0.5
Median Expression Ratio	0.5
Credible Interval	(0.229, 1.06)
Associated Peptides	2
Associated Spectra	3
Coverage	NaN

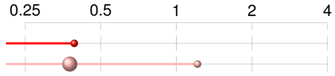

A	B	2.5	50	97.5	Sequence
2	0	0.052	0.14	0.39	VIEKFEK
1	0	0.11	0.38	1.2	STTTGHLIYK

5.23 2 328 cytoskeletal actin 2 [Molgula oculata]

Protein Accession	Unigene2743$_H$1
Mean Expression Ratio	1.98
Median Expression Ratio	1.98
Credible Interval	(0.93, 4.25)
Associated Peptides	2
Associated Spectra	6
Coverage	NaN

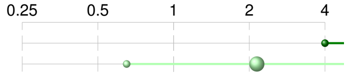

A	B	2.5	50	97.5	Sequence
1	4	4	8.4	17	MQKDITALAPPTMK
0	1	0.65	2.1	7.1	MQKDITALAPP

5.24 3 272 minus strand ribosomal protein L28 [Haliotis asinina]

Protein Accession	CL403.Contig16$_{H}1$
Mean Expression Ratio	0.516
Median Expression Ratio	0.517
Credible Interval	(0.258, 1.02)
Associated Peptides	3
Associated Spectra	8
Coverage	NaN

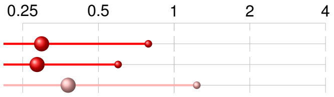

A	B	2.5	50	97.5	Sequence
2	0	0.11	0.3	0.79	GVVLVTK
5	0	0.14	0.28	0.6	TVGVEPSKDGK
1	0	0.12	0.38	1.2	DLGMAAIR

5.25 " 276 659 Fatty acid-binding protein, adipocyte [Crassostrea gigas]; 426 809 Fatty acid-binding protein, adipocyte [Crassostrea gigas]; 277 660 Fatty acid-binding protein, adipocyte [Crassostrea gigas]; 283 666 Fatty acid-binding protein, adipocyte [Crassostrea gigas]; 270 653 Fatty acid-binding protein, adipocyte [Crassostrea gigas]; 283 666 Fatty acid-binding protein, adipocyte [Crassostrea gigas]; 258 641 Fatty acid-binding protein, adipocyte [Crassostrea gigas]; 427 810 Fatty acid-binding protein, adipocyte [Crassostrea gigas]; 277 660 Fatty acid-binding protein, adipocyte [Crassostrea gigas]"

Protein Accession	CL7666.Contig9$_{H}1$ CL7666.Contig8$_{H}1$ CL7666.Contig7$_{H}1$ CL7666.Contig6$_{H}1$ CL7666.Con
Mean Expression Ratio	1.94
Median Expression Ratio	1.93
Credible Interval	(1.1, 3.4)
Associated Peptides	8
Associated Spectra	17
Coverage	NaN

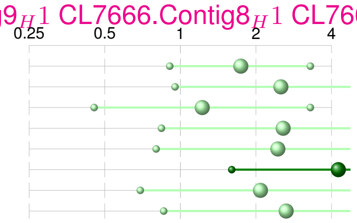

A	B	2.5	50	97.5	Sequence
2	5	0.91	1.7	3.3	AFVDGALK
2	0	0.95	2.5	6.6	AFVDGALKTSMTAK
2	0	0.45	1.2	3.3	QTGEGGEITIR
0	1	0.84	2.6	8.2	AVLTEESGELVER
0	1	0.8	2.5	8.1	GVTATISFQKA
0	2	1.6	4.2	11	LTTVAGDKQR
0	1	0.69	2.1	6.6	TVLAMER
0	1	0.86	2.6	8.1	GVTATISFQK

5.26 205 2490 minus strand glucose-regulated protein 94 [Crassostrea gigas]

Protein Accession	CL5089.Contig1$_H$1
Mean Expression Ratio	0.518
Median Expression Ratio	0.518
Credible Interval	(0.343, 0.779)
Associated Peptides	18
Associated Spectra	39
Coverage	NaN

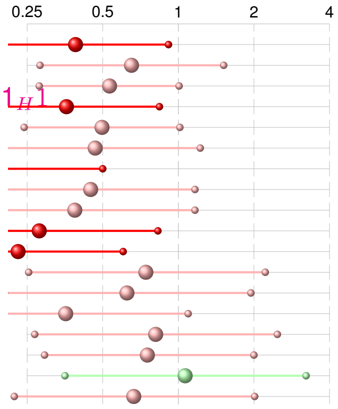

A	B	2.5	50	97.5	Sequence
1	2	0.16	0.39	0.91	GVVDSDDLPLNVSR
2	1	0.28	0.65	1.5	KTLEINPR
4	3	0.28	0.53	1	SGTSDFLAK
3	0	0.15	0.36	0.84	TLEINPR
3	2	0.24	0.5	1	NLGTIAR
1	1	0.18	0.47	1.2	KFQNVAK
1	1	0.073	0.19	0.5	DVALADKIEK
1	1	0.17	0.45	1.2	EGLKLDQSEK
1	0	0.13	0.39	1.2	KTLDMIK
0	1	0.09	0.28	0.83	ELISNASDALDK
0	2	0.088	0.23	0.6	ELISNASDALDKIR
0	1	0.25	0.74	2.2	LGVIEDTSNR
0	1	0.2	0.62	1.9	RVFITDNFEDMLPK
0	1	0.12	0.36	1.1	VFITDNFEDMLPK
0	1	0.27	0.81	2.5	HPLMKELK
0	2	0.29	0.75	2	SILFVPK
0	1	0.35	1.1	3.2	KPGDIDEKEYVDFYK
0	1	0.22	0.67	2	GTTVSLHLK

5.27 40 1098 minus strand major egg antigen [Clonorchis sinensis]

Protein Accession	CL2613.Contig4$_H$1
Mean Expression Ratio	0.516
Median Expression Ratio	0.52
Credible Interval	(0.217, 1.21)
Associated Peptides	1
Associated Spectra	1
Coverage	NaN

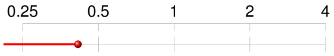

A	B	2.5	50	97.5	Sequence
1	0	0.036	0.12	0.42	AIVLKPTGK

5.28 227 3073 minus strand Lysosomal alpha-mannosidase [Crassostrea gigas]

Protein Accession	CL2930.Contig2$_H$1
Mean Expression Ratio	1.92
Median Expression Ratio	1.92
Credible Interval	(1.23, 2.98)
Associated Peptides	14
Associated Spectra	33
Coverage	NaN

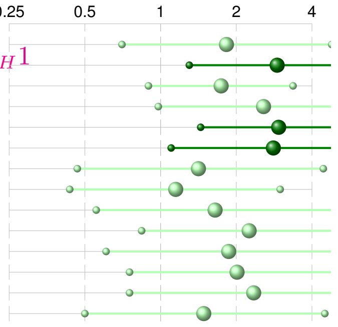

A	B	2.5	50	97.5	Sequence
0	2	0.7	1.8	4.8	ALPDNVHLLTLEQFR
1	3	1.3	2.9	6.5	ASLTWSGVR
2	5	0.89	1.7	3.4	EQASLFAR
0	2	0.98	2.6	6.7	GSHYVMLEPVSSAAK
1	4	1.4	3	6.2	IFIRDEAK
0	2	1.1	2.8	7.2	LANGVMEGQK
0	1	0.47	1.4	4.4	SQGGSSLLDGQIELMVHRR
0	2	0.44	1.1	3	VASELIPITPDTFR
0	1	0.55	1.6	5.1	VGWQIDPFGHSR
1	1	0.84	2.2	6	VVNDALKK
0	1	0.61	1.9	5.8	QAVAYDYAER
0	2	0.75	2	5.2	QVNNFLQVVK
0	1	0.75	2.3	7.2	LQWNYPH
0	1	0.5	1.5	4.5	VVNDALK

5.29 80 589 minus strand PREDICTED: adenine phosphoribosyltransferase-like [Strongylocentrotus purpuratus]

Protein Accession	CL8682.Contig6$_{H}1$
Mean Expression Ratio	1.93
Median Expression Ratio	1.92
Credible Interval	(0.91, 4.1)
Associated Peptides	2
Associated Spectra	4
Coverage	NaN

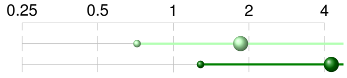

A	B	2.5	50	97.5	Sequence
1	2	0.72	1.8	5	AAVELVEK
0	1	1.3	4.3	14	GKLTVPVESLIKY

5.30 " 10 609 lethal(2)essential for life protein, l2efl [Aedes aegypti] gi"

Protein Accession	Unigene67975$_{H}1$
Mean Expression Ratio	0.522
Median Expression Ratio	0.522
Credible Interval	(0.3, 0.907)
Associated Peptides	6
Associated Spectra	24
Coverage	NaN

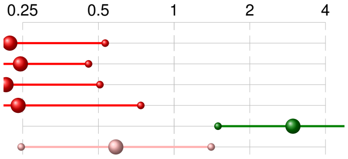

A	B	2.5	50	97.5	Sequence
1	2	0.093	0.22	0.53	ALEAPGETVIPITFEGK
8	0	0.13	0.25	0.46	INMDVK
3	0	0.091	0.21	0.51	MQMGETGK
1	0	0.078	0.24	0.74	SEVVNDDKEFR
0	6	1.5	3	5.9	KALEAPGETVIPITFEGKGDGK
0	3	0.25	0.58	1.4	KALEAPGETVIPITFEGK

5.31 " 1240 1911 PREDICTED: ATP synthase subunit alpha, mitochondrial-like [Anolis carolinensis]"

Protein Accession	Unigene30533$_{H}1$
Mean Expression Ratio	0.526
Median Expression Ratio	0.527
Credible Interval	(0.26, 1.06)
Associated Peptides	3
Associated Spectra	8
Coverage	NaN

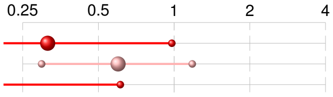

A	B	2.5	50	97.5	Sequence
1	0	0.095	0.31	0.98	LELAQYR
3	3	0.3	0.6	1.2	SSQQELLK
1	0	0.06	0.19	0.61	LTELLK

5.32 16 288 SJCHGC01950 protein [Schistosoma japonicum] gi

Protein Accession	Unigene16338$_{H}1$
Mean Expression Ratio	0.528
Median Expression Ratio	0.53
Credible Interval	(0.243, 1.11)
Associated Peptides	2
Associated Spectra	4
Coverage	NaN

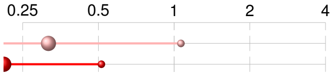

A	B	2.5	50	97.5	Sequence
1	0	0.096	0.32	1.1	YNIEK
0	3	0.087	0.21	0.52	DIAAFIK

5.33 1 1350 UPF0663 transmembrane protein C17orf28 [Crassostrea gigas]; 163 1542 UPF0663 transmembrane protein C17orf28 [Crassostrea gigas]; 135 1514 UPF0663 transmembrane protein C17orf28 [Crassostrea gigas]

Protein Accession	CL21276.Contig5$_H$1 CL21276.Contig4$_H$1 CL21276.Contig1$_H$1
Mean Expression Ratio	0.537
Median Expression Ratio	0.536
Credible Interval	(0.251, 1.1)
Associated Peptides	3
Associated Spectra	4
Coverage	NaN

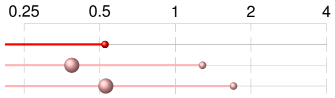

A	B	2.5	50	97.5	Sequence
2	0	0.07	0.19	0.52	EGFQSLLNTK
1	0	0.12	0.39	1.3	GVFLIEPKPK
1	0	0.16	0.53	1.7	KIGFK

5.34 222 809 minus strand PREDICTED: 15-hydroxyprostaglandin dehydrogenase [NAD+] [Gallus gallus]

Protein Accession	CL44148.Contig2$_H$1
Mean Expression Ratio	0.536
Median Expression Ratio	0.537
Credible Interval	(0.315, 0.905)
Associated Peptides	6
Associated Spectra	56
Coverage	NaN

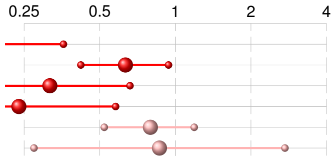

A	B	2.5	50	97.5	Sequence
6	0	0.092	0.18	0.36	AVTSLGR
7	14	0.42	0.63	0.94	VFLTGGAHGIGK
2	3	0.15	0.32	0.66	VMFCDINDK
3	0	0.097	0.24	0.58	AAFEK
5	15	0.52	0.79	1.2	ATVEAFLTK
0	1	0.27	0.86	2.7	RVFLTGGAHGIGK

5.35 " 353 781 alpha-B-crystallin, putative [Ixodes scapularis] gi"

Protein Accession	CL37349.Contig1$_H$1
Mean Expression Ratio	0.541
Median Expression Ratio	0.541
Credible Interval	(0.333, 0.873)
Associated Peptides	7
Associated Spectra	56
Coverage	NaN

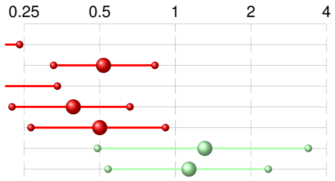

A	B	2.5	50	97.5	Sequence
4	2	0.061	0.12	0.24	LALEAPK
5	10	0.33	0.52	0.83	LQESGTSEVVNNDKEFR
6	3	0.11	0.19	0.34	IIINAK
2	9	0.22	0.39	0.66	VAMDVQHFKPEELK
0	8	0.27	0.5	0.91	TVTSSLSPSGVLTLK
0	2	0.49	1.3	3.4	VAMDVQHFKPEELKITTK
0	5	0.54	1.1	2.3	VAMDVQHFKPEELKITTKDNR

5.36 181 684 hypothetical protein BRAFLDRAFT_131834 [Branchiostoma floridae] gi

Protein Accession	CL10218.Contig4$_H$1
Mean Expression Ratio	0.549
Median Expression Ratio	0.55
Credible Interval	(0.248, 1.2)
Associated Peptides	2
Associated Spectra	2
Coverage	NaN

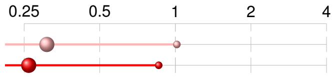

A	B	2.5	50	97.5	Sequence
1	0	0.093	0.31	1	LSGEGLETSK
1	0	0.08	0.26	0.86	AIVYQFR

5.37 " 223 423 ADPATP carrier protein; K05863 solute carrier family 25 (mitochondrial adenine nucleotide translocator), member 4/5/6/31"

Protein Accession	CL27196.Contig3$_{H}1$
Mean Expression Ratio	0.55
Median Expression Ratio	0.551
Credible Interval	(0.267, 1.15)
Associated Peptides	1
Associated Spectra	8
Coverage	NaN

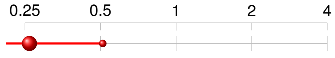

A	B	2.5	50	97.5	Sequence
6	2	0.13	0.26	0.51	LLVQNQNEMIK

5.38 161 301 Heat shock protein beta-6 OS=Mus musculus GN=Hspb6 PE=1 SV=1

Protein Accession	CL46983.Contig2$_{H}1$
Mean Expression Ratio	1.82
Median Expression Ratio	1.81
Credible Interval	(0.973, 3.33)
Associated Peptides	4
Associated Spectra	31
Coverage	NaN

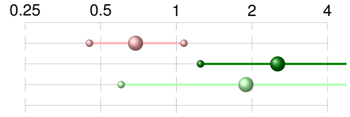

A	B	2.5	50	97.5	Sequence
5	14	0.45	0.69	1.1	VNMDVQHFKPEEIK
0	5	1.2	2.5	5.3	VNMDVQHFKPEEIKITTKDNR
0	1	0.6	1.9	6.1	VVISAK
0	6	6.1	12	24	VNMDVQHFKPEEIKITTK

5.39 " 90 1085 squid, variant A [Blattella germanica]"

Protein Accession	CL14696.Contig5$_{H}1$
Mean Expression Ratio	0.553
Median Expression Ratio	0.552
Credible Interval	(0.282, 1.09)
Associated Peptides	3
Associated Spectra	9
Coverage	NaN

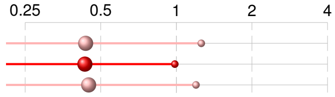

A	B	2.5	50	97.5	Sequence
1	1	0.15	0.44	1.3	IMEVTTHK
3	2	0.19	0.43	0.99	LGGQEVDVK
2	0	0.16	0.45	1.2	IFVGR

5.40 1 945 hemocyanin isoform 1 [Haliotis diversicolor]

Protein Accession	Unigene30318$_{H}1$
Mean Expression Ratio	0.552
Median Expression Ratio	0.554
Credible Interval	(0.258, 1.18)
Associated Peptides	2
Associated Spectra	4
Coverage	NaN

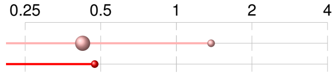

A	B	2.5	50	97.5	Sequence
0	1	0.13	0.42	1.4	LFVVEVEDELRAR
0	3	0.08	0.19	0.47	VEITDVLK

5.41 210 779 GH20056 [Drosophila grimshawi] gi; 216 785 minus strand GH20056 [Drosophila grimshawi] gi; 210 779 GH20056 [Drosophila grimshawi] gi; 216 785 GH20056 [Drosophila grimshawi] gi

Protein Accession	CL34497.Contig4$_{H}1$ CL34497.Contig3$_{H}1$ CL34497.Contig2$_{H}1$ CL34497.Contig1$_{H}1$
Mean Expression Ratio	0.559
Median Expression Ratio	0.561
Credible Interval	(0.274, 1.12)
Associated Peptides	4
Associated Spectra	4
Coverage	NaN

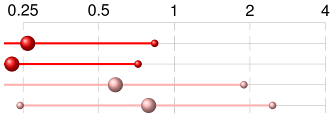

A	B	2.5	50	97.5	Sequence
**1**	**0**	**0.081**	**0.26**	**0.83**	**QLSQEEVSNFLK**
**1**	**0**	**0.068**	**0.22**	**0.72**	**EVSNFLK**
**0**	**1**	**0.18**	**0.58**	**1.9**	**GLLDMCAGEK**
**0**	**1**	**0.24**	**0.79**	**2.5**	**KQPFEFQIGVGQVIK**

5.42 531 665 minus strand Proteasome subunit beta type-6 [Crassostrea gigas]

Protein Accession	CL12961.Contig2$_{H}1$
Mean Expression Ratio	0.565
Median Expression Ratio	0.564
Credible Interval	(0.239, 1.33)
Associated Peptides	1
Associated Spectra	2
Coverage	NaN

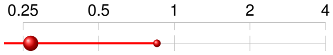

A	B	2.5	50	97.5	Sequence
**1**	**1**	**0.086**	**0.27**	**0.85**	**FTLTGDDLPK**

5.43 108 401 60S ribosomal L7a-like protein [Phragmatopoma lapidosa]; 108 401 60S ribosomal L7a-like protein [Phragmatopoma lapidosa]; 108 431 60S ribosomal L7a-like protein [Phragmatopoma lapidosa]

Protein Accession	CL1313.Contig6$_{H}1$ CL1313.Contig5$_{H}1$ CL1313.Contig1$_{H}1$
Mean Expression Ratio	0.564
Median Expression Ratio	0.566
Credible Interval	(0.258, 1.21)
Associated Peptides	2
Associated Spectra	3
Coverage	NaN

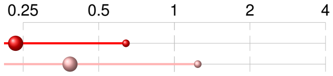

A	B	2.5	50	97.5	Sequence
**2**	**0**	**0.086**	**0.23**	**0.64**	**QTATQIFR**
**1**	**0**	**0.11**	**0.38**	**1.2**	**SGVNTVTALVESK**

5.44 346 702 minus strand PREDICTED: galectin-4-like [Anolis carolinensis]; 367 669 minus strand PREDICTED: galectin-7-like [Cavia porcellus]; 599 964 C. briggsae CBR-LEC-4 protein [Caenorhabditis briggsae]

Protein Accession	Unigene42203$_{H}1$ Unigene41782$_{H}1$ CL22459.Contig3$_{H}1$
Mean Expression Ratio	1.75
Median Expression Ratio	1.75
Credible Interval	(0.927, 3.37)
Associated Peptides	4
Associated Spectra	11
Coverage	NaN

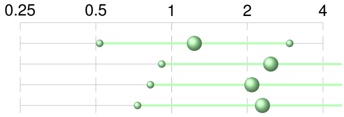

A	B	2.5	50	97.5	Sequence
3	2	0.52	1.2	3	AEATLEVR
0	2	0.91	2.5	6.7	KSVPHGLVK
1	2	0.82	2.1	5.2	SVPHGLVK
0	1	0.73	2.3	7.1	VKDSGFK

5.45 112 819 pyruvate kinase [Crassostrea gigas]; 132 932 pyruvate kinase [Crassostrea gigas]

Protein Accession	CL31920.Contig3$_{H}1$ CL31920.Contig1$_{H}1$
Mean Expression Ratio	0.573
Median Expression Ratio	0.575
Credible Interval	(0.266, 1.21)
Associated Peptides	2
Associated Spectra	4
Coverage	NaN

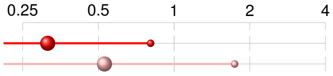

A	B	2.5	50	97.5	Sequence
2	1	0.12	0.31	0.8	ITIDDSYKEK
1	0	0.17	0.53	1.7	MIMEGMNIAR

5.46 14 1642 minus strand chaperonin containing tcp1 [Haliotis discus discus]; 15 1619 chaperonin containing tcp1 [Haliotis discus discus]; 15 1619 chaperonin containing tcp1 [Haliotis discus discus]; 14 1462 minus strand chaperonin containing tcp1 [Haliotis discus discus]; 14 1582 minus strand chaperonin containing tcp1 [Haliotis discus discus]

Protein Accession	CL16298.Contig4$_{H}1$ CL16298.Contig2$_{H}1$ CL16298.Contig1$_{H}1$ CL16298.Contig15$_{H}1$ CL162
Mean Expression Ratio	0.583
Median Expression Ratio	0.581
Credible Interval	(0.269, 1.24)
Associated Peptides	2
Associated Spectra	3
Coverage	NaN

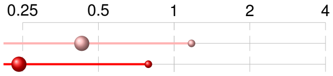

A	B	2.5	50	97.5	Sequence
2	0	0.16	0.43	1.2	QVEGDITK
1	0	0.073	0.24	0.79	NAEELMSFSK

5.47 " 3 227 minus strand alpha tubulin, putative [Ixodes scapularis] gi"

Protein Accession	Unigene31690$_{H}1$
Mean Expression Ratio	1.72
Median Expression Ratio	1.72
Credible Interval	(0.88, 3.36)
Associated Peptides	2
Associated Spectra	15
Coverage	NaN

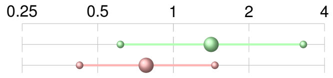

A	B	2.5	50	97.5	Sequence
**4**	**2**	**0.61**	**1.4**	**3.3**	**LSVDYGKK**
**2**	**7**	**0.42**	**0.78**	**1.5**	**LSVDYGK**

5.48 22 870 minus strand PREDICTED: alcohol dehydrogenase class-3 [Sarcophilus harrisii]

Protein Accession	Unigene27690$_{H}1$
Mean Expression Ratio	0.583
Median Expression Ratio	0.583
Credible Interval	(0.289, 1.15)
Associated Peptides	3
Associated Spectra	9
Coverage	NaN

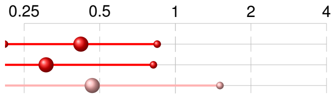

A	B	2.5	50	97.5	Sequence
**4**	**2**	**0.21**	**0.42**	**0.85**	**AAVAWEAK**
**1**	**1**	**0.11**	**0.31**	**0.82**	**IIGIDVNQDK**
**1**	**0**	**0.15**	**0.46**	**1.5**	**FAMAK**

5.49 5 193 minus strand PREDICTED: 40S ribosomal protein S28-like [Strongylocentrotus purpuratus]; 3 122 PREDICTED: 40S ribosomal protein S28-like [Strongylocentrotus purpuratus]

Protein Accession	CL49462.Contig2$_{H}1$ CL49462.Contig1$_{H}1$
Mean Expression Ratio	1.72
Median Expression Ratio	1.71
Credible Interval	(0.825, 3.59)
Associated Peptides	2
Associated Spectra	5
Coverage	NaN

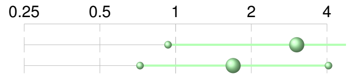

A	B	2.5	50	97.5	Sequence
**0**	**1**	**0.93**	**3**	**9.6**	**NVKGPVREGDILTLLESER**
**1**	**3**	**0.72**	**1.7**	**4.1**	**VEFLDDSNR**

5.50 156 1163 cathepsin B [Crassostrea ariakensis]

Protein Accession	CL26836.Contig9$_{H}1$
Mean Expression Ratio	1.7
Median Expression Ratio	1.7
Credible Interval	(0.871, 3.35)
Associated Peptides	5
Associated Spectra	7
Coverage	NaN

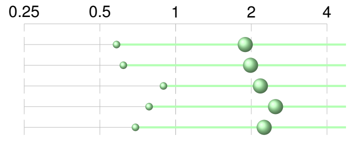

A	B	2.5	50	97.5	Sequence
**0**	**1**	**0.58**	**1.9**	**6**	**GEQNIMEELYTR**
**0**	**1**	**0.62**	**2**	**6.5**	**TKWPNCPTLKEVR**
**0**	**3**	**0.89**	**2.2**	**5.2**	**DLPDSFDPR**
**0**	**1**	**0.78**	**2.5**	**8**	**LTDEEIFFINK**
**0**	**1**	**0.69**	**2.3**	**7.1**	**LPDSFDPR**

5.51 72 1400 minus strand hypothetical protein RCJMB04_22i13 [Gallus gallus]

Protein Accession	CL44755.Contig5$_{H}1$
Mean Expression Ratio	0.588
Median Expression Ratio	0.588
Credible Interval	(0.341, 1.02)
Associated Peptides	9
Associated Spectra	18
Coverage	NaN

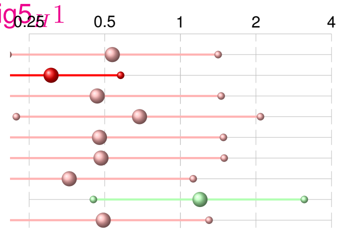

A	B	2.5	50	97.5	Sequence
1	1	0.21	0.54	1.4	EMGFSEGSIDK
4	3	0.16	0.3	0.58	LVFSADEAK
1	0	0.15	0.47	1.4	EAAVDVK
1	0	0.22	0.69	2.1	AVFQMR
1	0	0.15	0.48	1.5	EQAVYVAR
1	0	0.15	0.48	1.5	GKGTWDSGLK
1	0	0.12	0.36	1.1	AQVLAGGR
0	2	0.45	1.2	3.1	FLVASTPDEALQHAAELGK
0	2	0.18	0.49	1.3	LSGIPDVFVK

5.52 REVERSED 216 2639 minus strand polycystin 2 [Gallus gallus]

Protein Accession	RRRRRCL818.Contig5$_{H}1$
Mean Expression Ratio	0.587
Median Expression Ratio	0.588
Credible Interval	(0.271, 1.26)
Associated Peptides	2
Associated Spectra	3
Coverage	NaN

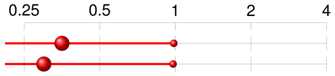

A	B	2.5	50	97.5	Sequence
2	0	0.13	0.35	0.99	DGLIIR
1	0	0.09	0.3	0.98	QIDVIK

5.53 871 1371 ribosomal protein L11 [Triatoma infestans]; 343 843 minus strand ribosomal protein L11 [Triatoma infestans]

Protein Accession	CL20979.Contig2$_{H}1$ CL20979.Contig1$_{H}1$
Mean Expression Ratio	0.601
Median Expression Ratio	0.6
Credible Interval	(0.276, 1.3)
Associated Peptides	2
Associated Spectra	3
Coverage	NaN

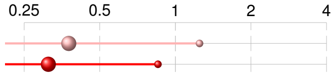

A	B	2.5	50	97.5	Sequence
1	0	0.11	0.38	1.2	ITKEEAIK
2	0	0.11	0.31	0.85	AEEILER

5.54 1 864 minus strand Pyruvate kinase isozymes M1/M2 [Crassostrea gigas]; 3 959 minus strand Pyruvate kinase isozymes M1/M2 [Crassostrea gigas]; 24 899 minus strand Pyruvate kinase isozymes M1/M2 [Crassostrea gigas]

Protein Accession	CL24469.Contig6$_{H}1$ CL24469.Contig3$_{H}1$ CL24469.Contig2$_{H}1$
Mean Expression Ratio	0.605
Median Expression Ratio	0.606
Credible Interval	(0.256, 1.41)
Associated Peptides	1
Associated Spectra	1
Coverage	NaN

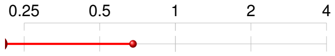

A	B	2.5	50	97.5	Sequence
1	0	0.06	0.2	0.68	NFDEILK

5.55 736 1287 60S ribosomal protein L3 OS=Rattus norvegicus GN=Rpl3 PE=1 SV=3

Protein Accession	Unigene22848$_{H}1$
Mean Expression Ratio	0.611
Median Expression Ratio	0.609
Credible Interval	(0.298, 1.25)
Associated Peptides	3
Associated Spectra	4
Coverage	NaN

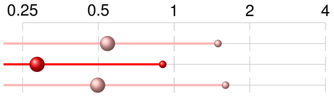

A	B	2.5	50	97.5	Sequence
1	1	0.2	0.54	1.5	VQFTVAR
1	0	0.09	0.29	0.9	EIETDLNK
1	0	0.15	0.5	1.6	IGAGIHTK

5.56 51 371 minus strand putative mitochondrial ATP synthase F chain [Haliotis discus discus]; 51 371 minus strand putative mitochondrial ATP synthase F chain [Haliotis discus discus]

Protein Accession	CL18283.Contig4$_{H}1$ CL18283.Contig2$_{H}1$
Mean Expression Ratio	0.61
Median Expression Ratio	0.612
Credible Interval	(0.273, 1.32)
Associated Peptides	1
Associated Spectra	5
Coverage	NaN

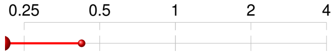

A	B	2.5	50	97.5	Sequence
5	0	0.097	0.21	0.42	ADLPLGQVK

5.57 " 99 497 RecName: Full=Histone H2B, gonadal"

Protein Accession	CL29794.Contig1$_{H}1$
Mean Expression Ratio	1.63
Median Expression Ratio	1.63
Credible Interval	(0.984, 2.73)
Associated Peptides	6
Associated Spectra	46
Coverage	NaN

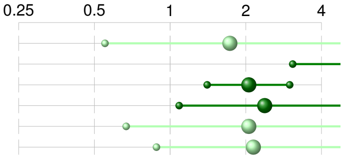

A	B	2.5	50	97.5	Sequence
0	1	0.55	1.7	5.4	AMSIMNSFVNDIFER
3	3	3.1	6.4	13	AVTKYTSSK
9	21	1.4	2.1	3	LLLPGELAK
3	2	1.1	2.4	5.2	STITSREIQTAVR
0	1	0.67	2.1	6.3	LLPGELAK
0	3	0.88	2.1	5.1	KESYAIYIYK

5.58 2019 2723 minus strand Kyphoscoliosis peptidase [Crassostrea gigas]; 2060 2764 minus strand Kyphoscoliosis peptidase [Crassostrea gigas]; 1074 1778 minus strand Kyphoscoliosis peptidase [Crassostrea gigas]; 2063 2767 minus strand Kyphoscoliosis peptidase [Crassostrea gigas]; 2044 2748 minus strand Kyphoscoliosis peptidase [Crassostrea gigas]; 2044 2748 minus strand Kyphoscoliosis peptidase [Crassostrea gigas]

Protein Accession	CL3923.Contig7$_{H}$1 CL3923.Contig6$_{H}$1 CL3923.Contig5$_{H}$1 CL3923.Contig4$_{H}$1 CL3923.Con
Mean Expression Ratio	1.63
Median Expression Ratio	1.63
Credible Interval	(0.77, 3.47)
Associated Peptides	2
Associated Spectra	5
Coverage	NaN

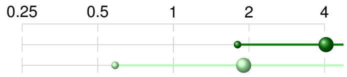

A	B	2.5	50	97.5	Sequence
2	2	1.8	4.1	9.1	TMGQAPPTVPKDFQLK
0	1	0.59	1.9	6.2	TMGQAPPTVPK

5.59 280 726 minus strand RecName: Full=Calmodulin; Short=CaM gi|14588595|dbj|BAB61794.1| calmodulin [Metridium senile] gi

Protein Accession	Unigene4486$_{H}$1
Mean Expression Ratio	0.614
Median Expression Ratio	0.616
Credible Interval	(0.288, 1.3)
Associated Peptides	1
Associated Spectra	6
Coverage	NaN

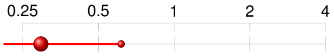

A	B	2.5	50	97.5	Sequence
5	1	0.14	0.3	0.61	ELGTVMR

5.60 " 59 2365 minus strand Aconitate hydratase, mitochondrial [Crassostrea gigas]"

Protein Accession	CL13509.Contig1$_{H}$1
Mean Expression Ratio	1.63
Median Expression Ratio	1.62
Credible Interval	(0.929, 2.88)
Associated Peptides	10
Associated Spectra	11
Coverage	NaN

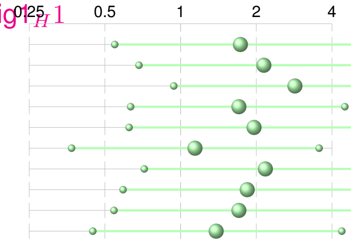

A	B	2.5	50	97.5	Sequence
0	1	0.55	1.7	5.4	HTMNEGQIEWFR
0	1	0.68	2.1	6.5	KGETNTIVTSYNR
0	1	0.94	2.8	9.1	SKFTITPGSEQIR
0	2	0.63	1.7	4.5	WDGKDLVDMPVLIK
0	1	0.62	2	6.1	KNGWPLNIR
0	1	0.37	1.1	3.5	VAGILTVK
0	1	0.72	2.2	6.8	LGRPLTLSEK
0	1	0.59	1.8	5.7	MQDYLK
0	1	0.54	1.7	5.3	IHETNLKK
0	1	0.45	1.4	4.4	LDDPKNEK

5.61 " 242 646 minus strand Fatty acid-binding protein, heart [Crassostrea gigas]; 227 631 minus strand Fatty acid-binding protein, heart [Crassostrea gigas]; 265 669 Fatty acid-binding protein, heart [Crassostrea gigas]"

Protein Accession	CL7643.Contig9$_H1$ CL7643.Contig11$_H1$ CL7643.Contig10$_H1$
Mean Expression Ratio	1.63
Median Expression Ratio	1.62
Credible Interval	(0.977, 2.73)
Associated Peptides	8
Associated Spectra	43
Coverage	NaN

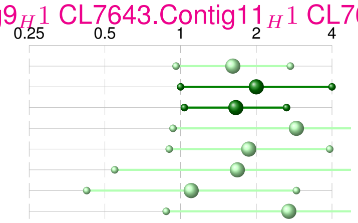

A	B	2.5	50	97.5	Sequence
3	9	0.96	1.6	2.7	AINNGQMIMTMK
3	3	1	2	4	ATSGVEFESR
6	9	1	1.7	2.6	CGDVTSIR
1	0	0.93	2.9	8.9	YLDGREIK
2	3	0.9	1.9	3.9	KYDKV
0	1	0.55	1.7	5.3	TVFRVEGDTLIEDVSGPFSTSIVR
0	2	0.42	1.1	2.9	VEGDTLIEDVSGPFSTSIVR
0	1	0.87	2.7	8.2	AALNGKWK

5.62 " 53 1291 Acetyl-CoA acetyltransferase B, mitochondrial [Crassostrea gigas]"

Protein Accession	CL1322.Contig1$_H1$
Mean Expression Ratio	1.61
Median Expression Ratio	1.62
Credible Interval	(0.757, 3.48)
Associated Peptides	2
Associated Spectra	3
Coverage	NaN

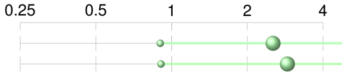

A	B	2.5	50	97.5	Sequence
0	2	0.9	2.5	7.1	TLKPVFVK
0	1	0.91	2.9	9.5	LGSVAIKEAVSR

5.63 219 554 minus strand ribosomal protein P1 [Biomphalaria glabrata]

Protein Accession	CL45629.Contig2$_H1$
Mean Expression Ratio	0.618
Median Expression Ratio	0.618
Credible Interval	(0.267, 1.41)
Associated Peptides	1
Associated Spectra	2
Coverage	NaN

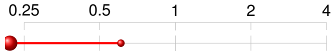

A	B	2.5	50	97.5	Sequence
2	0	0.08	0.22	0.61	ALEGVNIK

5.64 594 1253 peroxiredoxin 6 [Eriocheir sinensis]

Protein Accession	CL21894.Contig2$_{H}$1
Mean Expression Ratio	0.619
Median Expression Ratio	0.621
Credible Interval	(0.394, 0.966)
Associated Peptides	11
Associated Spectra	44
Coverage	NaN

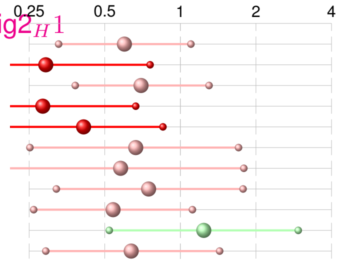

A	B	2.5	50	97.5	Sequence
4	4	0.33	0.6	1.1	VATPVDWK
1	1	0.11	0.29	0.76	NFDEILR
2	6	0.38	0.7	1.3	AVFIIGPDK
2	1	0.12	0.28	0.67	LFPNYK
4	1	0.2	0.41	0.85	QLTATQK
1	1	0.25	0.66	1.7	AVFIIGPDKK
0	1	0.19	0.58	1.8	ELAVQLGMVDPDEK
0	3	0.32	0.75	1.8	VLDSLQLTATQK
0	5	0.26	0.54	1.1	LLPEFEK
0	3	0.52	1.2	2.9	KVATPVDWK
0	4	0.29	0.64	1.4	VNLGDVFPDFK

5.65 163 1059 minus strand Heterogeneous nuclear ribonucleoprotein A2-like protein 1 [Crassostrea gigas]; 99 995 minus strand Heterogeneous nuclear ribonucleoprotein A2-like protein 1 [Crassostrea gigas]; 71 967 minus strand Heterogeneous nuclear ribonucleoprotein A2-like protein 1 [Crassostrea gigas]; 71 967 minus strand Heterogeneous nuclear ribonucleoprotein A2-like protein 1 [Crassostrea gigas]

Protein Accession	Unigene65$_{H}$1 Unigene63$_{H}$1 Unigene38787$_{H}$1 Unigene38786$_{H}$1
Mean Expression Ratio	0.617
Median Expression Ratio	0.622
Credible Interval	(0.274, 1.35)
Associated Peptides	2
Associated Spectra	2
Coverage	NaN

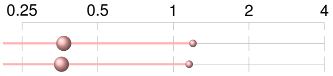

A	B	2.5	50	97.5	Sequence
1	0	0.11	0.37	1.2	IENVEMIK
1	0	0.11	0.36	1.2	IENVEMIKDK

5.66 94 489 Sortilin-related receptor [Crassostrea gigas]

Protein Accession	CL15197.Contig3$_{H}$1
Mean Expression Ratio	0.621
Median Expression Ratio	0.622
Credible Interval	(0.278, 1.36)
Associated Peptides	1
Associated Spectra	4
Coverage	NaN

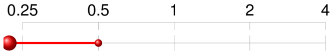

A	B	2.5	50	97.5	Sequence
4	0	0.099	0.22	0.5	CVPIEFR

5.67 201 4058 PREDICTED: xanthine dehydrogenase/oxidase-like [Amphimedon queenslandica]

Protein Accession	CL17390.Contig11$_{H}$1
Mean Expression Ratio	0.622
Median Expression Ratio	0.623
Credible Interval	(0.265, 1.43)
Associated Peptides	1
Associated Spectra	1
Coverage	NaN

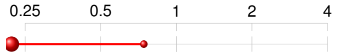

A	B	2.5	50	97.5	Sequence
1	0	0.064	0.22	0.74	AVTDLQEAISNK

5.68 55 795 Elongation factor 1-delta OS=Bos taurus GN=EEF1D PE=2 SV=2; 49 747 PREDICTED: elongation factor 1-delta-like [Strongylocentrotus purpuratus]; 49 819 PREDICTED: elongation factor 1-delta-like isoform 2 [Strongylocentrotus purpuratus]; 49 795 PREDICTED: elongation factor 1-delta-like isoform 2 [Strongylocentrotus purpuratus]

Protein Accession	CL10790.Contig9$_{H}$1 CL10790.Contig13$_{H}$1 CL10790.Contig12$_{H}$1 CL10790.Contig11$_{H}$1
Mean Expression Ratio	1.6
Median Expression Ratio	1.6
Credible Interval	(0.869, 2.92)
Associated Peptides	7
Associated Spectra	12
Coverage	NaN

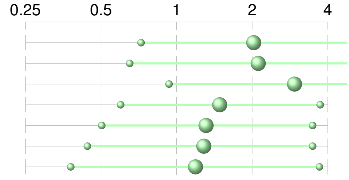

A	B	2.5	50	97.5	Sequence
1	1	0.72	2	5.7	KPVVIAK
0	1	0.65	2.1	6.5	QKFEEAEAHFYAR
0	1	0.93	3	9.3	TIQKDGLTWGAAK
1	2	0.6	1.5	3.7	VAALEVVGTGGASPK
0	2	0.5	1.3	3.5	KVTSDLR
1	1	0.44	1.3	3.5	LLEEYAAK
0	1	0.38	1.2	3.7	RLEEIEKENK

5.69 3 500 Tumor protein D54 [Crassostrea gigas]; 3 464 Tumor protein D54 [Crassostrea gigas]; 3 464 Tumor protein D54 [Crassostrea gigas]; 3 500 Tumor protein D54 [Crassostrea gigas]; 6 440 Tumor protein D54 [Crassostrea gigas]; 3 464 Tumor protein D54 [Crassostrea gigas]; 3 500 Tumor protein D54 [Crassostrea gigas]

Protein Accession	CL25616.Contig8$_{H}$1 CL25616.Contig6$_{H}$1 CL25616.Contig5$_{H}$1 CL25616.Contig4$_{H}$1 CL2561
Mean Expression Ratio	0.624
Median Expression Ratio	0.625
Credible Interval	(0.282, 1.38)
Associated Peptides	2
Associated Spectra	2
Coverage	NaN

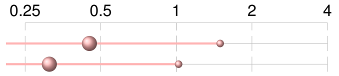

A	B	2.5	50	97.5	Sequence
1	0	0.14	0.45	1.5	STDDIDTAFK
1	0	0.093	0.31	1	NLGITPFK

5.70 1337 1537 minus strand hemocyanin [Haliotis tuberculata]

Protein Accession	Unigene68371$_H1$
Mean Expression Ratio	0.625
Median Expression Ratio	0.625
Credible Interval	(0.289, 1.32)
Associated Peptides	2
Associated Spectra	4
Coverage	NaN

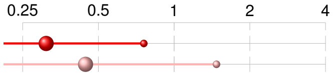

A	B	2.5	50	97.5	Sequence
0	3	0.13	0.31	0.76	DHSTPFDVFNYK
0	1	0.13	0.44	1.5	YDDLQFNGLSISQIQR

5.71 30 536 putative mitochondrial ATP synthase [Haliotis discus discus]; 27 620 putative mitochondrial ATP synthase [Haliotis discus discus]

Protein Accession	CL13014.Contig4$_H1$ CL13014.Contig1$_H1$
Mean Expression Ratio	1.6
Median Expression Ratio	1.6
Credible Interval	(0.939, 2.72)
Associated Peptides	8
Associated Spectra	24
Coverage	NaN

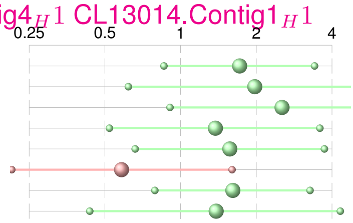

A	B	2.5	50	97.5	Sequence
1	5	0.86	1.7	3.4	SAVDWVAFK
0	1	0.62	2	6.1	SDSFVNKVHQYPAELPK
1	1	0.91	2.5	7	SNALKDIAAEEK
0	2	0.52	1.4	3.6	VHQYPAELPK
0	3	0.66	1.6	3.7	IDFSAYK
1	1	0.21	0.58	1.6	VPYPEDK
3	4	0.79	1.6	3.3	LANPALVDQFQK
0	1	0.43	1.4	4.3	VHQYPAEL

5.72 27 515 heat shock protein 70 [Capsaspora owczarzaki ATCC 30864]; 30 464 minus strand hypothetical protein [Trichinella spiralis]; 27 491 minus strand heat shock protein 70 [Capsaspora owczarzaki ATCC 30864]

Protein Accession	CL14449.Contig4$_H1$ CL14449.Contig2$_H1$ CL14449.Contig1$_H1$
Mean Expression Ratio	0.625
Median Expression Ratio	0.626
Credible Interval	(0.32, 1.24)
Associated Peptides	3
Associated Spectra	8
Coverage	NaN

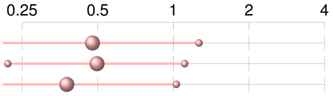

A	B	2.5	50	97.5	Sequence
2	0	0.17	0.48	1.3	FDDPSVQSDMK
3	1	0.22	0.5	1.1	NQVAMNPENTVFDAK
1	1	0.14	0.38	1	RFDDPSVQSDMK

5.73 111 1226 gelsolin [Aplysia californica] gi; 42 1139 minus strand gelsolin [Aplysia californica] gi

Protein Accession	Unigene39723$_{H}1$ Unigene32782$_{H}1$
Mean Expression Ratio	1.59
Median Expression Ratio	1.59
Credible Interval	(0.917, 2.81)
Associated Peptides	10
Associated Spectra	13
Coverage	NaN

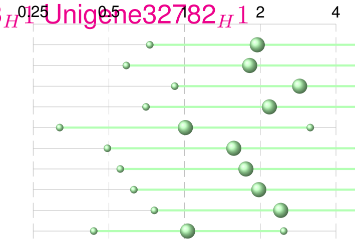

A	B	2.5	50	97.5	Sequence
0	2	0.73	2	5.2	AMQFIGEIK
0	1	0.58	1.8	5.5	DLVTSDDVYILDTGLK
0	1	0.91	2.9	9	SKAMQFIGEIK
0	1	0.7	2.2	6.9	TVELDTKLNDKPIQHR
0	1	0.32	1	3.2	SVPAEEYK
0	1	0.49	1.6	4.9	QFDTALAA
0	1	0.56	1.8	5.5	KNGLPLAHNYLR
0	1	0.63	2	6.1	EVQGHESDLFK
0	1	0.76	2.4	7.4	ESGNPWRPVTVIK
0	3	0.44	1	2.5	SDDVFIFDSGK

5.74 390 2267 neurofilament protein NF70 [Helix aspersa]; 318 2195 neurofilament protein NF70 [Helix aspersa]

Protein Accession	CL1353.Contig5$_{H}1$ CL1353.Contig4$_{H}1$
Mean Expression Ratio	1.59
Median Expression Ratio	1.58
Credible Interval	(0.758, 3.44)
Associated Peptides	2
Associated Spectra	4
Coverage	NaN

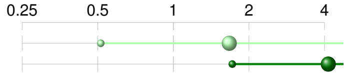

A	B	2.5	50	97.5	Sequence
0	1	0.51	1.7	5.6	KDVNLMNWTLQR
0	3	1.7	4.1	10	VGQLEELVDDLQR

5.75 185 742 Transmembrane protein Tmp21 [Crassostrea gigas]

Protein Accession	CL3732.Contig3$_{H}1$
Mean Expression Ratio	1.59
Median Expression Ratio	1.58
Credible Interval	(0.828, 3.08)
Associated Peptides	5
Associated Spectra	7
Coverage	NaN

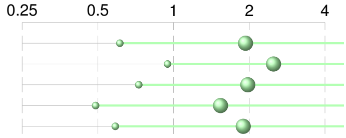

A	B	2.5	50	97.5	Sequence
0	1	0.61	1.9	6.2	SIVDDFAYMR
0	2	0.95	2.5	6.6	SYDELAKAEK
0	2	0.73	2	5.3	RLEDLSK
0	1	0.49	1.5	5	VLYLR
0	1	0.59	1.9	6.1	GHILYSK

5.76 372 860 minus strand small heat shock protein [Tegillarca granosa]

Protein Accession	CL18190.Contig4$_{H}1$
Mean Expression Ratio	0.634
Median Expression Ratio	0.637
Credible Interval	(0.37, 1.06)
Associated Peptides	8
Associated Spectra	25
Coverage	NaN

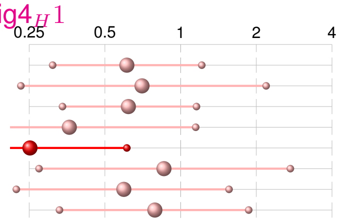

A	B	2.5	50	97.5	Sequence
2	4	0.31	0.61	1.2	IDKDGLSIK
1	0	0.23	0.7	2.2	KSDEYSFVSR
3	5	0.34	0.62	1.2	LTEDGHLIISAR
1	0	0.12	0.36	1.1	TFGPASIEQQMK
2	1	0.1	0.25	0.61	TIAIESDK
0	1	0.27	0.86	2.7	AFDWPAEGHGENAEVVQAK
0	2	0.22	0.59	1.6	EFLIPEEIDAETLGSR
0	3	0.33	0.79	1.9	MNVSHYEPDELSVK

5.77 2 349 minus strand hypothetical protein BRAFLDRAFT_206907 [Branchiostoma floridae] gi

Protein Accession	Unigene875$_H$1
Mean Expression Ratio	0.635
Median Expression Ratio	0.637
Credible Interval	(0.283, 1.39)
Associated Peptides	2
Associated Spectra	2
Coverage	NaN

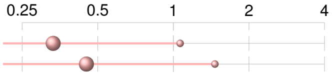

A	B	2.5	50	97.5	Sequence
1	0	0.1	0.33	1.1	AQAIIGQHDK
1	0	0.14	0.45	1.5	YTPTFR

5.78 103 684 EF-hand domain-containing protein D2 [Crassostrea gigas]

Protein Accession	CL20544.Contig1$_H$1
Mean Expression Ratio	0.634
Median Expression Ratio	0.638
Credible Interval	(0.29, 1.34)
Associated Peptides	2
Associated Spectra	3
Coverage	NaN

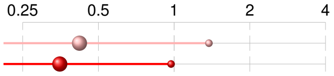

A	B	2.5	50	97.5	Sequence
1	0	0.13	0.42	1.4	VPQTHLALK
2	0	0.13	0.35	0.97	KQIQDFQK

5.79 187 696 minus strand putative ribosomal protein S10 [Sipunculus nudus]

Protein Accession	Unigene1233$_H$1
Mean Expression Ratio	1.56
Median Expression Ratio	1.57
Credible Interval	(0.846, 2.87)
Associated Peptides	5
Associated Spectra	13
Coverage	NaN

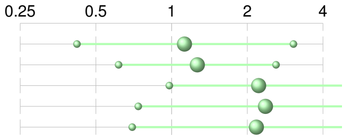

A	B	2.5	50	97.5	Sequence
0	2	0.42	1.1	3	ADVGAGATESFQFR
0	5	0.61	1.3	2.6	HQEIDVPNLHVIK
1	3	0.98	2.2	5.1	KDFFSPK
0	1	0.74	2.4	7.7	ALTSIK
0	1	0.7	2.2	7.2	KAGGPPGGDKKADVGAGATESFQFR

5.80 260 892 minus strand calponin [Haliotis diversicolor]

Protein Accession	CL48702.Contig1$_H$1
Mean Expression Ratio	1.56
Median Expression Ratio	1.56
Credible Interval	(0.815, 2.96)
Associated Peptides	5
Associated Spectra	10
Coverage	NaN

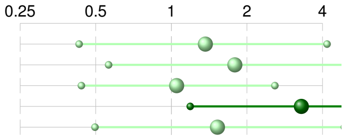

A	B	2.5	50	97.5	Sequence
0	1	0.43	1.4	4.2	AATGQQFPIEESNER
0	1	0.56	1.8	5.5	AFGIEVESHPSYTGQR
2	2	0.44	1.1	2.6	VESSFDPEDAKK
2	1	1.2	3.3	8.9	NFSEDKLR
0	1	0.5	1.5	4.9	SGLRAPK

5.81 260 706 Collagen alpha-1(XII) chain (Fragment) OS=Notophthalmus viridescens PE=2 SV=1

Protein Accession	CL284.Contig5$_H1$
Mean Expression Ratio	0.642
Median Expression Ratio	0.642
Credible Interval	(0.278, 1.48)
Associated Peptides	1
Associated Spectra	1
Coverage	NaN

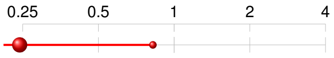

A	B	2.5	50	97.5	Sequence
**1**	**0**	**0.071**	**0.24**	**0.82**	**VSLVTFSNEANAK**

5.82 " 61 573 steroid membrane receptor Hpr6.6/25-Dx, putative [Ixodes scapularis] gi"

Protein Accession	CL29406.Contig1$_H1$
Mean Expression Ratio	0.636
Median Expression Ratio	0.643
Credible Interval	(0.276, 1.43)
Associated Peptides	1
Associated Spectra	2
Coverage	NaN

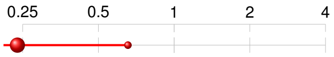

A	B	2.5	50	97.5	Sequence
**2**	**0**	**0.085**	**0.24**	**0.65**	**ILIAVNGK**

5.83 " 144 1145 PREDICTED: RNA-binding protein squid-like isoform 1 [Megachile rotundata]; 90 1085 squid, variant A [Blattella germanica]; 144 1145 PREDICTED: RNA-binding protein squid-like isoform 1 [Megachile rotundata]"

Protein Accession	CL14696.Contig7$_H1$ CL14696.Contig5$_H1$ CL14696.Contig4$_H1$
Mean Expression Ratio	1.55
Median Expression Ratio	1.55
Credible Interval	(0.774, 3.1)
Associated Peptides	3
Associated Spectra	8
Coverage	NaN

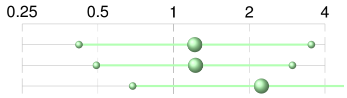

A	B	2.5	50	97.5	Sequence
**1**	**1**	**0.42**	**1.2**	**3.5**	**IMEVTTHK**
**3**	**2**	**0.49**	**1.2**	**3**	**LGGQEVDVK**
**0**	**1**	**0.69**	**2.2**	**7.2**	**KIMEVTTHK**

5.84 178 582 hypothetical protein CGI_10001640 [Crassostrea gigas]

Protein Accession	CL2347.Contig2$_{H}1$
Mean Expression Ratio	1.55
Median Expression Ratio	1.55
Credible Interval	(0.822, 2.89)
Associated Peptides	4
Associated Spectra	16
Coverage	NaN

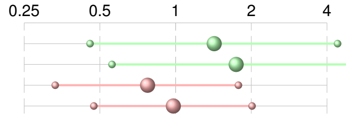

A	B	2.5	50	97.5	Sequence
0	1	0.46	1.4	4.4	LKSHGIGGK
0	1	0.56	1.7	5.5	SVGGNAGEVICK
3	2	0.33	0.77	1.8	AFLEQLGSK
5	4	0.47	0.98	2	ANLIVIGSR

5.85 209 781 minus strand high mobility group protein 1 [Biomphalaria glabrata]; 277 771 high mobility group protein 1 [Biomphalaria glabrata]; 277 867 minus strand high mobility group protein 1 [Biomphalaria glabrata]; 277 870 minus strand high mobility group protein 1 [Biomphalaria glabrata]

Protein Accession	CL25076.Contig4$_{H}1$ CL25076.Contig3$_{H}1$ CL25076.Contig2$_{H}1$ CL25076.Contig1$_{H}1$
Mean Expression Ratio	1.54
Median Expression Ratio	1.54
Credible Interval	(0.835, 2.86)
Associated Peptides	6
Associated Spectra	13
Coverage	NaN

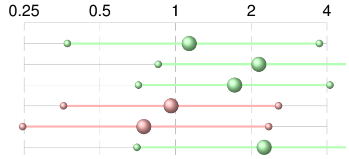

A	B	2.5	50	97.5	Sequence
0	1	0.37	1.1	3.7	GIDHKEILR
1	2	0.85	2.1	5.4	KYEAEMAEYR
2	2	0.71	1.7	4.1	LAGEEWR
2	1	0.36	0.96	2.6	KPFEDMAAK
0	1	0.25	0.75	2.4	LGDFR
0	1	0.7	2.3	7	ELPAGQKKPFEDMAAKDKAR

5.86 316 1512 Alpha-N-acetylgalactosaminidase [Crassostrea gigas]; 282 1478 Alpha-N-acetylgalactosaminidase [Crassostrea gigas]

Protein Accession	CL2428.Contig4$_{H}1$ CL2428.Contig1$_{H}1$
Mean Expression Ratio	0.649
Median Expression Ratio	0.649
Credible Interval	(0.36, 1.17)
Associated Peptides	6
Associated Spectra	14
Coverage	NaN

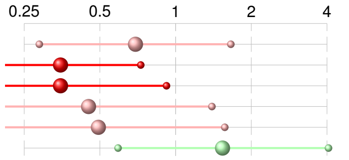

A	B	2.5	50	97.5	Sequence
1	2	0.29	0.69	1.7	LGIYEDFGSK
2	3	0.17	0.35	0.73	LVELGLR
2	0	0.13	0.35	0.92	YGYPVMQR
1	0	0.14	0.45	1.4	LYMAQANK
1	0	0.15	0.49	1.6	VDMVK
0	2	0.59	1.5	4.1	AGYRARELFAGK

5.87 109 510 minus strand hypothetical protein DAPPUDRAFT_231281 [Daphnia pulex]

Protein Accession	Unigene4945$_H$1
Mean Expression Ratio	1.54
Median Expression Ratio	1.54
Credible Interval	(0.923, 2.51)
Associated Peptides	9
Associated Spectra	33
Coverage	NaN

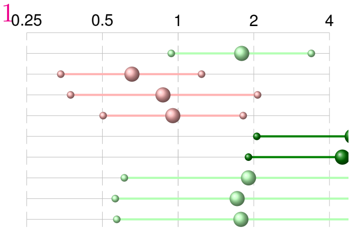

A	B	2.5	50	97.5	Sequence
1	6	0.94	1.8	3.4	LAGIEDPFEMNVK
2	5	0.34	0.66	1.2	VSMPDTLGHAVGIER
1	2	0.37	0.87	2.1	GTVDEPTLIPSLYEK
2	5	0.5	0.95	1.8	YELLAK
0	3	2.1	4.9	12	AKGTVDEPTLIPSLYEK
0	3	1.9	4.5	11	IASDKVSMPDTLGHAVGIER
0	1	0.61	1.9	6	LAGIEDPFEMNVKK
0	1	0.56	1.7	5.3	TLGHAVGIER
0	1	0.57	1.8	5.6	GKIASDKVSMPDTLGHAVGIER

5.88 " 220 627 Fatty acid-binding protein, brain [Crassostrea gigas]"

Protein Accession	CL9041.Contig2$_H$1
Mean Expression Ratio	0.654
Median Expression Ratio	0.654
Credible Interval	(0.355, 1.19)
Associated Peptides	5
Associated Spectra	26
Coverage	NaN

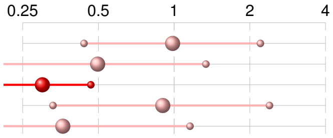

A	B	2.5	50	97.5	Sequence
1	3	0.44	0.99	2.2	EVDGGVMTSTTTFDGK
1	1	0.18	0.5	1.3	IVENYKLEDK
10	7	0.19	0.3	0.47	LTITVEGGK
0	2	0.33	0.9	2.4	LGETFDYNSIDGTKPK
0	1	0.11	0.36	1.2	VDLASTTGLEEFGTAIGFSEEK

5.89 196 657 superoxide dismutase [Aplysia californica] gi; 690 1151 superoxide dismutase [Aplysia californica] gi

Protein Accession	Unigene18046$_H$1 Unigene150$_H$1
Mean Expression Ratio	1.53
Median Expression Ratio	1.52
Credible Interval	(0.812, 2.87)
Associated Peptides	4
Associated Spectra	14
Coverage	NaN

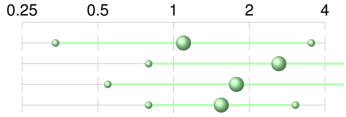

A	B	2.5	50	97.5	Sequence
0	1	0.34	1.1	3.5	THGGPDDVERHAGDLGNIR
0	1	0.8	2.6	8	AVCVLK
0	1	0.55	1.8	5.6	AGDLGNIR
6	5	0.79	1.5	3.1	SLVVHEK

5.90 227 2920 minus strand Lysosomal alpha-mannosidase [Crassostrea gigas]; 227 3073 minus strand Lysosomal alpha-mannosidase [Crassostrea gigas]; 193 2886 minus strand Lysosomal alpha-mannosidase [Crassostrea gigas]

Protein Accession	CL2930.Contig3$_H1$ CL2930.Contig2$_H1$ CL2930.Contig1$_H1$
Mean Expression Ratio	0.657
Median Expression Ratio	0.661
Credible Interval	(0.338, 1.26)
Associated Peptides	4
Associated Spectra	18
Coverage	NaN

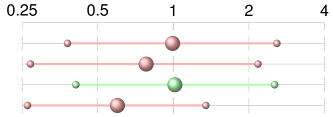

A	B	2.5	50	97.5	Sequence
1	3	0.38	1	2.6	ASLTWSGVR
1	1	0.27	0.78	2.2	VVNDALKK
1	4	0.41	1	2.5	IFIRDEAK
2	5	0.26	0.6	1.3	EQASLFAR

5.91 900 1616 minus strand hemocyanin isoform 1 [Haliotis diversicolor]

Protein Accession	Unigene30321$_H1$
Mean Expression Ratio	0.662
Median Expression Ratio	0.663
Credible Interval	(0.312, 1.38)
Associated Peptides	2
Associated Spectra	5
Coverage	NaN

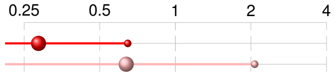

A	B	2.5	50	97.5	Sequence
2	2	0.13	0.29	0.64	VEITDDLK
0	1	0.19	0.64	2.1	LFKVEITDDLK

5.92 77 502 hypothetical protein CGI_10008263 [Crassostrea gigas]

Protein Accession	CL38960.Contig1$_H1$
Mean Expression Ratio	1.52
Median Expression Ratio	1.51
Credible Interval	(0.789, 2.92)
Associated Peptides	3
Associated Spectra	12
Coverage	NaN

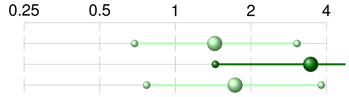

A	B	2.5	50	97.5	Sequence
0	5	0.69	1.4	3	IFVAIDGSK
0	3	1.4	3.5	8.5	KIFVAIDGSK
0	4	0.77	1.7	3.8	ANIEAITK

5.93 95 2506 Transitional endoplasmic reticulum ATPase [Crassostrea gigas]

Protein Accession	CL15973.Contig2$_H$1
Mean Expression Ratio	0.662
Median Expression Ratio	0.663
Credible Interval	(0.417, 1.04)
Associated Peptides	16
Associated Spectra	22
Coverage	NaN

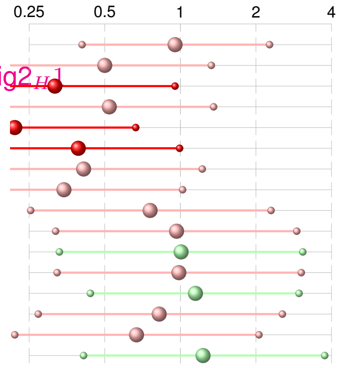

A	B	2.5	50	97.5	Sequence
1	2	0.41	0.95	2.3	AHFEEAMK
1	1	0.19	0.5	1.3	DIDLDYLAK
1	0	0.1	0.32	0.95	EMVELPLR
1	1	0.2	0.52	1.4	MAIEAEIR
1	0	0.072	0.22	0.66	AIGVKPPR
1	1	0.15	0.39	0.99	HPQLFK
1	0	0.13	0.41	1.2	GFGGNFR
1	0	0.11	0.34	1	LQQIR
0	1	0.25	0.76	2.3	AEVQNEDDLATAILK
0	1	0.32	0.96	2.9	GILLYGPPGTGK
0	1	0.33	1	3.1	KYEMFAQTLQQSR
0	1	0.32	0.99	3	MDELQLFR
0	2	0.44	1.1	3	KGDIFLVR
0	1	0.27	0.82	2.5	KAFEEAEK
0	1	0.22	0.67	2.1	EVDIGIPDATGR
0	1	0.41	1.2	3.8	GGNVGDGGGAADR

5.94 180 560 calreticulin precursor [Aplysia californica] gi; 180 545 calreticulin precursor [Aplysia californica] gi

Protein Accession	CL4813.Contig4$_H$1 CL4813.Contig2$_H$1
Mean Expression Ratio	0.66
Median Expression Ratio	0.663
Credible Interval	(0.388, 1.11)
Associated Peptides	8
Associated Spectra	30
Coverage	NaN

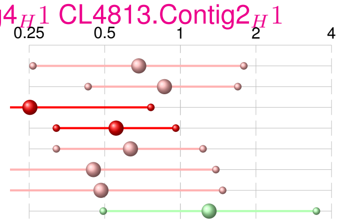

A	B	2.5	50	97.5	Sequence
1	1	0.26	0.68	1.8	DPTVYFK
2	4	0.43	0.87	1.7	FVWSAGK
1	0	0.08	0.25	0.77	FYGDEDKDK
4	7	0.32	0.55	0.96	FYGISAK
1	5	0.32	0.63	1.2	WIPSVHK
0	1	0.15	0.45	1.4	EEFDANWEDR
0	1	0.15	0.48	1.5	TLVIQFTVK
0	2	0.49	1.3	3.5	FDKFSNEGK

5.95 280 690 Histone H3.3 [Harpegnathos saltator]

Protein Accession	Unigene26607$_H$1
Mean Expression Ratio	0.662
Median Expression Ratio	0.663
Credible Interval	(0.415, 1.05)
Associated Peptides	10
Associated Spectra	81
Coverage	NaN

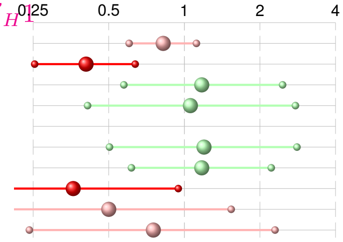

A	B	2.5	50	97.5	Sequence
28	10	0.6	0.83	1.1	DIQLAR
16	0	0.25	0.4	0.64	STELLIR
2	3	0.57	1.2	2.5	VTIMPK
1	1	0.41	1.1	2.8	VTIMPKDIQLAR
6	0	0.043	0.087	0.17	EIAQDFK
3	0	0.5	1.2	2.8	KQLATK
3	4	0.62	1.2	2.2	RVTIMPK
2	0	0.14	0.36	0.95	KLPFQR
1	0	0.16	0.5	1.5	EIAQDFKTDLR
0	1	0.24	0.75	2.3	RVTIMPKDIQLAR

5.96 77 2278 Far upstream element-binding protein 3 [Crassostrea gigas]

Protein Accession	CL5030.Contig3$_H$1
Mean Expression Ratio	0.662
Median Expression Ratio	0.663
Credible Interval	(0.291, 1.44)
Associated Peptides	2
Associated Spectra	2
Coverage	NaN

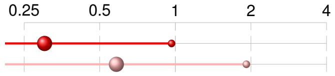

A	B	2.5	50	97.5	Sequence
1	0	0.093	0.3	0.97	LFNIR
1	0	0.18	0.58	1.9	QAVGLVIGK

5.97 44 1465 minus strand Adenylyl cyclase-associated protein 1 [Crassostrea gigas]; 52 1473 minus strand Adenylyl cyclase-associated protein 1 [Crassostrea gigas]; 48 1469 Adenylyl cyclase-associated protein 1 [Crassostrea gigas]

Protein Accession	CL19470.Contig9$_H$1 CL19470.Contig8$_H$1 CL19470.Contig1$_H$1
Mean Expression Ratio	0.667
Median Expression Ratio	0.669
Credible Interval	(0.375, 1.17)
Associated Peptides	8
Associated Spectra	13
Coverage	NaN

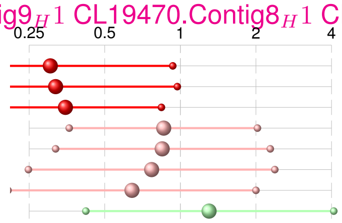

A	B	2.5	50	97.5	Sequence
1	0	0.097	0.3	0.93	AQLDIVNAAFK
1	0	0.1	0.32	0.97	EFALPEQFK
1	2	0.15	0.35	0.84	NIVLDNVELK
1	2	0.36	0.86	2	QFLEVASK
1	1	0.32	0.85	2.3	SKQPPQADLPK
1	0	0.25	0.77	2.4	AQIGGDLK
0	1	0.21	0.64	2	DSLDTEIVTAK
0	1	0.42	1.3	4.1	VEAAKPPLTELQNKK

5.98 58 732 glutathione-s-transferase [Haliotis discus discus]

Protein Accession	CL4861.Contig1$_H$1
Mean Expression Ratio	0.672
Median Expression Ratio	0.673
Credible Interval	(0.366, 1.24)
Associated Peptides	5
Associated Spectra	13
Coverage	NaN

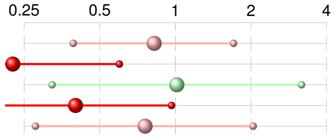

A	B	2.5	50	97.5	Sequence
3	2	0.39	0.82	1.7	GQVPTFR
1	1	0.084	0.22	0.6	SEEILK
1	0	0.32	1	3.2	LLPKEAGGR
0	3	0.17	0.4	0.97	IDKEYLEVK
0	2	0.28	0.76	2	LISFSANEHK

5.99 " 42 905 PREDICTED: enoyl-CoA hydratase, mitochondrial-like isoform 1 [Oreochromis niloticus]"

Protein Accession	CL6477.Contig7$_H$1
Mean Expression Ratio	0.673
Median Expression Ratio	0.673
Credible Interval	(0.354, 1.27)
Associated Peptides	4
Associated Spectra	13
Coverage	NaN

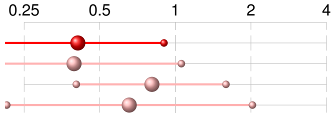

A	B	2.5	50	97.5	Sequence
2	2	0.18	0.41	0.9	AFAAGADIK
1	1	0.15	0.39	1.1	LLDEAVK
4	2	0.4	0.81	1.6	SVVLQASR
0	1	0.21	0.66	2	AMEMVLTGER

5.100 350 910 Peptidyl-prolyl cis-trans isomerase B [Crassostrea gigas]; 488 1039 Peptidyl-prolyl cis-trans isomerase B [Crassostrea gigas]; 488 1039 Peptidyl-prolyl cis-trans isomerase B [Crassostrea gigas]

Protein Accession	CL49248.Contig5$_{H}1$ CL49248.Contig2$_{H}1$ CL49248.Contig1$_{H}1$
Mean Expression Ratio	0.676
Median Expression Ratio	0.677
Credible Interval	(0.315, 1.42)
Associated Peptides	3
Associated Spectra	3
Coverage	NaN

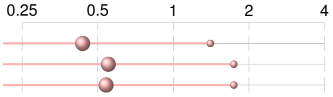

A	B	2.5	50	97.5	Sequence
0	1	0.14	0.44	1.4	HVAFGQIIDK
0	1	0.16	0.55	1.7	ASLEFLDK
0	1	0.17	0.54	1.7	AVLDFLIKN

5.101 9 161 PREDICTED: tubulin alpha-1C chain-like [Acyrthosiphon pisum]; 9 161 Tubulin alpha-3 chain (Fragments) OS=Mesocricetus auratus PE=1 SV=1

Protein Accession	Unigene74551$_{H}1$ Unigene70816$_{H}1$
Mean Expression Ratio	1.47
Median Expression Ratio	1.47
Credible Interval	(0.718, 3.08)
Associated Peptides	3
Associated Spectra	4
Coverage	NaN

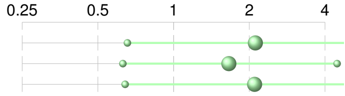

A	B	2.5	50	97.5	Sequence
0	1	0.65	2.1	7.1	IHFPLAT
0	2	0.63	1.7	4.5	IHFPLA
0	1	0.64	2.1	7	IHFPLATYAPVISAEK

5.102 2295 2510 beta-thymosin domain repeat protein CSP24KDa_v1 [Hermissenda crassicornis] gi

Protein Accession	CL9548.Contig1$_{H}1$
Mean Expression Ratio	1.48
Median Expression Ratio	1.47
Credible Interval	(0.704, 3.14)
Associated Peptides	3
Associated Spectra	3
Coverage	NaN

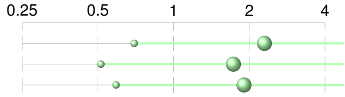

A	B	2.5	50	97.5	Sequence
0	1	0.7	2.3	7.6	ESVAAFDKSHLK
0	1	0.51	1.7	5.5	ESVAAFDK
0	1	0.59	1.9	6.1	AFDKSHLK

5.103 118 1866 Lamin Dm0 [Crassostrea gigas]

Protein Accession	CL4079.Contig7$_H$1
Mean Expression Ratio	1.47
Median Expression Ratio	1.47
Credible Interval	(0.965, 2.2)
Associated Peptides	19
Associated Spectra	34
Coverage	NaN

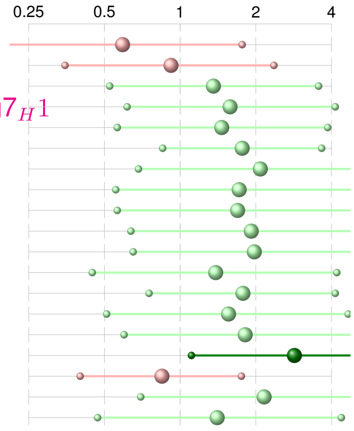

A	B	2.5	50	97.5	Sequence
1	0	0.2	0.59	1.8	AEIAHLEK
1	1	0.35	0.92	2.4	LLEGEESR
1	1	0.52	1.4	3.6	LSEVEAPR
1	1	0.62	1.6	4.1	SLVIKPGK
0	2	0.56	1.5	3.9	EKHEFDLQTVR
0	5	0.85	1.8	3.6	GGIDIEEVDPEGK
0	1	0.68	2.1	6.5	KQLEEETLLR
0	1	0.55	1.7	5.3	LRAESSGFESR
0	1	0.56	1.7	5.1	RVETLEAQVAELTAK
0	1	0.64	1.9	5.7	SLYETELADAR
0	1	0.65	2	6	SLYETELADARR
0	1	0.45	1.4	4.2	VQSLKEDLHFK
0	3	0.75	1.8	4.1	WFVADEMK
0	1	0.51	1.6	4.7	YKTEYEDLLAK
0	1	0.6	1.8	5.6	IQIEANKYK
0	2	1.1	2.9	7.4	LLDETAKEK
0	5	0.4	0.85	1.7	IQIEANK
0	1	0.7	2.2	6.5	RLALKDDEIADLR
0	1	0.47	1.4	4.4	ADDLSAELAK

5.104 124 747 ribosomal protein S10 [Crassostrea gigas] gi

Protein Accession	CL18986.Contig1$_H$1
Mean Expression Ratio	0.681
Median Expression Ratio	0.681
Credible Interval	(0.325, 1.42)
Associated Peptides	3
Associated Spectra	6
Coverage	NaN

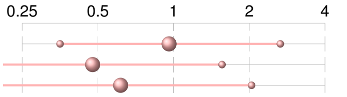

A	B	2.5	50	97.5	Sequence
1	3	0.35	0.96	2.7	KDFFSPK
1	0	0.15	0.48	1.6	ALTSLK
1	0	0.19	0.61	2	MLIPK

5.105 28 642 minus strand Transmembrane emp24 domain-containing protein 9 [Crassostrea gigas]; 236 709 minus strand Transmembrane emp24 domain-containing protein 9 OS=Rattus norvegicus GN=Tmed9 PE=1 SV=1; 239 742 minus strand Transmembrane emp24 domain-containing protein eca OS=Drosophila erecta GN=eca PE=3 SV=1; 236 709 minus strand Transmembrane emp24 domain-containing protein 9 OS=Rattus norvegicus GN=Tmed9 PE=1 SV=1; 236 709 minus strand Transmembrane emp24 domain-containing protein 9 OS=Rattus norvegicus GN=Tmed9 PE=1 SV=1; 236 709 minus strand Transmembrane emp24 domain-containing protein 9 OS=Rattus norvegicus GN=Tmed9 PE=1 SV=1; 44 658 minus strand Transmembrane emp24 domain-containing protein 9 [Crassostrea gigas]

Protein Accession	CL2503.Contig8$_H$1 CL2503.Contig7$_H$1 CL2503.Contig5$_H$1 CL2503.Contig4$_H$1 CL2503.Con
Mean Expression Ratio	0.678
Median Expression Ratio	0.682
Credible Interval	(0.297, 1.56)
Associated Peptides	1
Associated Spectra	1
Coverage	NaN

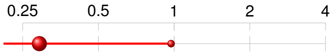

A	B	2.5	50	97.5	Sequence
**1**	**0**	**0.086**	**0.29**	**0.97**	**QLLDQVEQITK**

5.106 " 45 506 minus strand PREDICTED: dihydrolipoyl dehydrogenase, mitochondrial [Monodelphis domestica]"

Protein Accession	CL24292.Contig3$_H$1
Mean Expression Ratio	0.683
Median Expression Ratio	0.682
Credible Interval	(0.314, 1.46)
Associated Peptides	2
Associated Spectra	3
Coverage	NaN

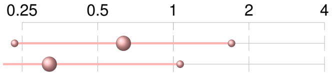

A	B	2.5	50	97.5	Sequence
**1**	**1**	**0.23**	**0.63**	**1.7**	**GIEIDGLR**
**1**	**0**	**0.098**	**0.32**	**1.1**	**AAQLGMK**

5.107 194 1996 minus strand Neutral and basic amino acid transport protein rBAT [Crassostrea gigas]; 265 2067 minus strand Neutral and basic amino acid transport protein rBAT [Crassostrea gigas]; 194 1996 minus strand Neutral and basic amino acid transport protein rBAT [Crassostrea gigas]; 194 1996 minus strand Neutral and basic amino acid transport protein rBAT [Crassostrea gigas]; 265 2067 minus strand Neutral and basic amino acid transport protein rBAT [Crassostrea gigas]

Protein Accession	CL9047.Contig9$_{H}$1 CL9047.Contig8$_{H}$1 CL9047.Contig6$_{H}$1 CL9047.Contig15$_{H}$1 CL9047.Co
Mean Expression Ratio	1.45
Median Expression Ratio	1.45
Credible Interval	(0.746, 2.81)
Associated Peptides	4
Associated Spectra	9
Coverage	NaN

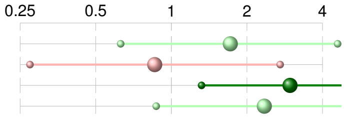

A	B	2.5	50	97.5	Sequence
1	1	0.63	1.7	4.6	NFAGNLDVVDALR
1	0	0.27	0.86	2.7	TAVDDLDNKPGR
0	4	1.3	3	6.7	TAVDDLDNKPGREK
0	2	0.87	2.3	6.2	TITPMQWSK

5.108 200 2128 minus strand heat shock protein 70 [Cristaria plicata]

Protein Accession	CL8787.Contig2$_{H}$1
Mean Expression Ratio	0.685
Median Expression Ratio	0.688
Credible Interval	(0.327, 1.44)
Associated Peptides	3
Associated Spectra	3
Coverage	NaN

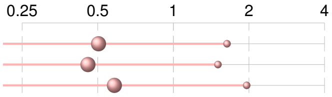

A	B	2.5	50	97.5	Sequence
0	1	0.16	0.5	1.6	TLAPEEISSMVLVK
0	1	0.14	0.46	1.5	LTEVQK
0	1	0.18	0.58	2	LSKEEIDR

5.109 124 390 PREDICTED: 60S ribosomal protein L23a-like [Callithrix jacchus]

Protein Accession	CL27563.Contig5$_{H}$1
Mean Expression Ratio	1.46
Median Expression Ratio	1.45
Credible Interval	(0.731, 2.95)
Associated Peptides	3
Associated Spectra	6
Coverage	NaN

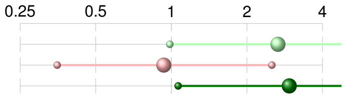

A	B	2.5	50	97.5	Sequence
0	2	0.98	2.7	7.1	AAPKPAVK
0	2	0.35	0.93	2.5	LDAFAIVK
0	2	1.1	3	8.1	VNRLDAFAIVK

5.110 346 702 minus strand PREDICTED: galectin-4-like [Anolis carolinensis]

Protein Accession	Unigene42203$_H$1
Mean Expression Ratio	0.692
Median Expression Ratio	0.689
Credible Interval	(0.332, 1.42)
Associated Peptides	2
Associated Spectra	8
Coverage	NaN

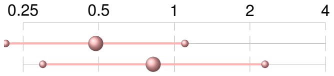

A	B	2.5	50	97.5	Sequence
**3**	**2**	**0.21**	**0.49**	**1.1**	**AEATLEVR**
**1**	**2**	**0.3**	**0.83**	**2.3**	**SVPHGLVK**

5.111 " 166 1248 PREDICTED: probable pyruvate dehydrogenase E1 component subunit alpha, mitochondrial-like [Metaseiulus occidentalis]; 166 1248 PREDICTED: probable pyruvate dehydrogenase E1 component subunit alpha, mitochondrial-like [Metaseiulus occidentalis]; 166 1248 PREDICTED: probable pyruvate dehydrogenase E1 component subunit alpha, mitochondrial-like [Metaseiulus occidentalis]; 166 1248 PREDICTED: probable pyruvate dehydrogenase E1 component subunit alpha, mitochondrial-like [Metaseiulus occidentalis]; 166 1248 minus strand PREDICTED: probable pyruvate dehydrogenase E1 component subunit alpha, mitochondrial-like [Metaseiulus occidentalis]; 166 1248 minus strand PREDICTED: probable pyruvate dehydrogenase E1 component subunit alpha, mitochondrial-like [Metaseiulus occidentalis]; 166 1248 PREDICTED: probable pyruvate dehydrogenase E1 component subunit alpha, mitochondrial-like [Metaseiulus occidentalis]; 166 1248 PREDICTED: probable pyruvate dehydrogenase E1 component subunit alpha, mitochondrial-like [Metaseiulus occidentalis]"

Protein Accession	Unigene34082$_H$1 Unigene34081$_H$1 Unigene34079$_H$1 Unigene34077$_H$1 Unigene34075$_H$1 U
Mean Expression Ratio	0.686
Median Expression Ratio	0.689
Credible Interval	(0.323, 1.44)
Associated Peptides	3
Associated Spectra	3
Coverage	NaN

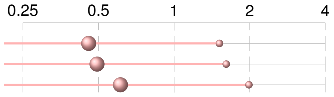

A	B	2.5	50	97.5	Sequence
**1**	**0**	**0.14**	**0.46**	**1.5**	**DMQVIR**
**1**	**0**	**0.15**	**0.49**	**1.6**	**ATEATFK**
**1**	**0**	**0.19**	**0.61**	**2**	**TGLATEEELKK**

5.112 242 1834 Regulator of differentiation 1 [Crassostrea gigas]; 458 2050 minus strand Regulator of differentiation 1 [Crassostrea gigas]; 503 2095 Regulator of differentiation 1 [Crassostrea gigas]; 434 1942 Regulator of differentiation 1 [Crassostrea gigas]

Protein Accession	CL11169.Contig7$_H$1 CL11169.Contig5$_H$1 CL11169.Contig4$_H$1 CL11169.Contig1$_H$1
Mean Expression Ratio	1.45
Median Expression Ratio	1.45
Credible Interval	(0.7, 3.01)
Associated Peptides	3
Associated Spectra	4
Coverage	NaN

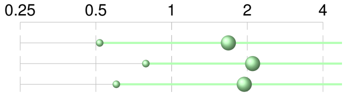

A	B	2.5	50	97.5	Sequence
0	1	0.52	1.7	5.5	SLPHEVAETDIVQLGMPFGK
0	2	0.79	2.1	5.7	MTNVLVLK
0	1	0.6	1.9	6.4	LTNLNVK

5.113 118 1629 PREDICTED: similar to tektin 3 [Ciona intestinalis]

Protein Accession	Unigene11521$_H$1
Mean Expression Ratio	0.688
Median Expression Ratio	0.69
Credible Interval	(0.353, 1.34)
Associated Peptides	4
Associated Spectra	8
Coverage	NaN

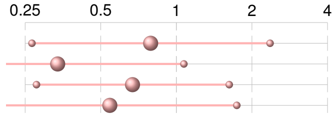

A	B	2.5	50	97.5	Sequence
1	1	0.27	0.79	2.4	GLTFHNGVQR
1	0	0.11	0.34	1.1	SALEIDLAVK
2	2	0.28	0.67	1.6	QTIDLVHDAPEK
1	0	0.17	0.54	1.7	EVDTIKR

5.114 1 1284 minus strand Thioredoxin reductase 3 [Crassostrea gigas]

Protein Accession	CL3500.Contig1$_H$1
Mean Expression Ratio	1.45
Median Expression Ratio	1.45
Credible Interval	(0.692, 3.12)
Associated Peptides	2
Associated Spectra	4
Coverage	NaN

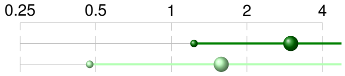

A	B	2.5	50	97.5	Sequence
0	3	1.2	3	7.3	GAVPSQIEQLEEGKPGR
0	1	0.47	1.6	5.2	LLVQRL

5.115 45 407 minus strand 60S ribosomal protein L31 OS=Xenopus laevis GN=rpl31 PE=2 SV=1

Protein Accession	CL4728.Contig1$_H$1
Mean Expression Ratio	1.44
Median Expression Ratio	1.44
Credible Interval	(0.665, 3.06)
Associated Peptides	2
Associated Spectra	4
Coverage	NaN

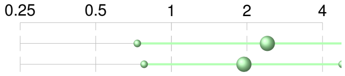

A	B	2.5	50	97.5	Sequence
0	1	0.73	2.4	7.7	IHGVSFKK
0	3	0.78	1.9	4.8	IHGVSFK

5.116 587 811 hemocyanin 2 [Megathura crenulata]

Protein Accession	Unigene4334$_{H}1$
Mean Expression Ratio	0.692
Median Expression Ratio	0.696
Credible Interval	(0.334, 1.42)
Associated Peptides	2
Associated Spectra	6
Coverage	NaN

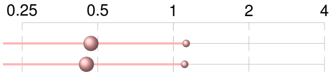

A	B	2.5	50	97.5	Sequence
2	1	0.19	0.47	1.1	AIDDGTFR
1	2	0.18	0.45	1.1	SVEDLTAGEAESLK

5.117 235 777 RNA-binding protein 4.1 [Clonorchis sinensis]

Protein Accession	CL49317.Contig6$_{H}1$
Mean Expression Ratio	1.43
Median Expression Ratio	1.43
Credible Interval	(0.674, 3.05)
Associated Peptides	2
Associated Spectra	4
Coverage	NaN

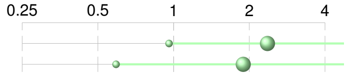

A	B	2.5	50	97.5	Sequence
1	2	0.96	2.4	5.8	VELSTGK
0	1	0.59	1.9	6.2	DRLPPFAEDR

5.118 269 1504 Ribosome-binding protein 1 [Crassostrea gigas]; 265 1500 Ribosome-binding protein 1 [Crassostrea gigas]

Protein Accession	CL8282.Contig5$_{H}1$ CL8282.Contig2$_{H}1$
Mean Expression Ratio	1.43
Median Expression Ratio	1.43
Credible Interval	(0.752, 2.71)
Associated Peptides	6
Associated Spectra	6
Coverage	NaN

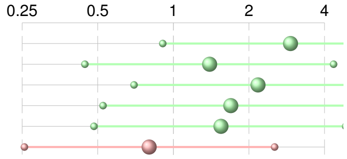

A	B	2.5	50	97.5	Sequence
0	1	0.91	2.9	9.1	AMEALEKAEK
0	1	0.44	1.4	4.4	DAIIEVEK
0	1	0.7	2.2	6.9	LKEDKETLEAR
0	1	0.52	1.7	5.3	QFTQLHAENER
0	1	0.48	1.5	4.8	LVQQLQEQLSEK
0	1	0.26	0.8	2.5	AMEALEK

5.119 82 816 PREDICTED: triosephosphate isomerase B [Oreochromis niloticus]

Protein Accession	CL26247.Contig4$_{H}1$
Mean Expression Ratio	1.43
Median Expression Ratio	1.43
Credible Interval	(0.751, 2.75)
Associated Peptides	6
Associated Spectra	6
Coverage	NaN

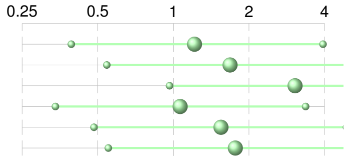

A	B	2.5	50	97.5	Sequence
0	1	0.39	1.2	4	TEFITIINSK
0	1	0.54	1.7	5.4	QFFVGGNWK
0	1	0.97	3.1	9.6	NVSKDVANTVR
0	1	0.34	1.1	3.4	SVDEIIK
0	1	0.48	1.6	4.9	AIADNVNDWK
0	1	0.55	1.8	5.6	AIADNVNDWKR

5.120 179 2140 minus strand PREDICTED: hypothetical protein [Saccoglossus kowalevskii]

Protein Accession	CL13749.Contig6$_{H}$1
Mean Expression Ratio	1.42
Median Expression Ratio	1.42
Credible Interval	(0.671, 3.05)
Associated Peptides	2
Associated Spectra	3
Coverage	NaN

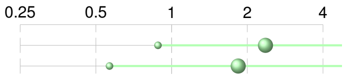

A	B	2.5	50	97.5	Sequence
0	2	0.88	2.4	6.4	KFDGIGGLSGGGATSK
0	1	0.57	1.8	6.1	VGGTYNFDDSNGLGR

5.121 " 4146 6107 minus strand nonmuscle myosin II, partial [Aplysia californica]"

Protein Accession	Unigene30363$_{H}$1
Mean Expression Ratio	1.43
Median Expression Ratio	1.42
Credible Interval	(1.03, 1.99)
Associated Peptides	31
Associated Spectra	65
Coverage	NaN

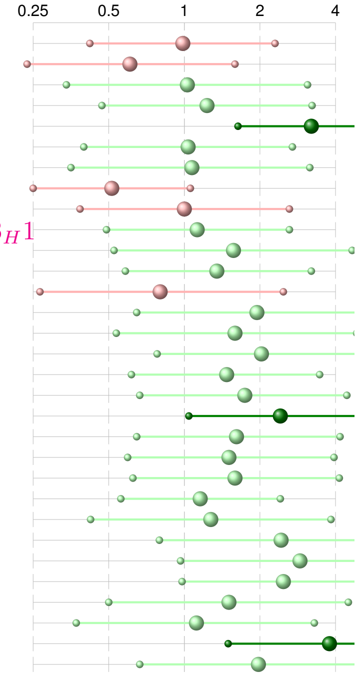

A	B	2.5	50	97.5	Sequence
1	2	0.42	0.99	2.3	ALEATVQEQR
2	0	0.24	0.61	1.6	ATIAALEGK
1	0	0.34	1	3.1	DKLAELEGQSR
1	1	0.47	1.2	3.2	LAELEGQSR
3	3	1.6	3.2	6.3	TKATIAALEGK
1	1	0.4	1	2.7	ALTLAQR
1	0	0.35	1.1	3.2	LKEDAIK
3	2	0.25	0.51	1.1	NLEAELVK
1	1	0.38	1	2.6	IEQLER
1	2	0.49	1.1	2.6	DLEQVSTQLEQVETK
1	0	0.53	1.6	4.7	AALARTNR
0	3	0.58	1.3	3.2	ELDDQLEQNDVLVR
0	1	0.27	0.8	2.5	ELQIQAEDER
0	1	0.65	1.9	5.9	EMEAELEEER
0	1	0.54	1.6	4.8	EMEQLHAQLEEAR
0	2	0.78	2	5.1	IANLEEQLDQEAKER
0	3	0.62	1.5	3.5	ILNLSHAMEEMQGR
0	2	0.66	1.7	4.4	KAQIAMEQMATDLAAER
0	3	1	2.4	5.7	LKELQIQAEDERR
0	2	0.64	1.6	4.2	LQEDLAAAER
0	2	0.59	1.5	3.9	NAEAERDELADEIGSSATSK
0	2	0.63	1.6	4.1	QVDEAEEEVAR
0	5	0.56	1.2	2.4	VQIEELEDELQAAEDAK
0	1	0.42	1.3	3.8	VSSLEAQLADSQELMEEETR
0	1	0.79	2.4	7.4	IAAQMKDFQR
0	1	0.96	2.9	8.8	LALEKDNIER
0	2	0.98	2.5	6.4	SADQYKEQLEK
0	1	0.5	1.5	4.5	QSLLDEK
0	1	0.37	1.1	3.3	RKLQAEVEDLTITLDNQR
0	2	1.5	3.8	9.9	SVSSKLENQR
0	1	0.67	2	5.9	QKLAVQSK

5.122 4 675 Laminin subunit gamma-1 [Crassostrea gigas]

Protein Accession	CL35712.Contig1$_H$1
Mean Expression Ratio	1.42
Median Expression Ratio	1.42
Credible Interval	(0.66, 3.11)
Associated Peptides	2
Associated Spectra	3
Coverage	NaN

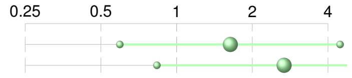

A	B	2.5	50	97.5	Sequence
0	2	0.59	1.6	4.5	LAQLAEDTAIQASAEAR
0	1	0.84	2.7	8.6	SKVNEALAK

5.123 5 301 PREDICTED: hypothetical protein [Saccoglossus kowalevskii]

Protein Accession	Unigene21633$_H$1
Mean Expression Ratio	0.705
Median Expression Ratio	0.706
Credible Interval	(0.314, 1.56)
Associated Peptides	1
Associated Spectra	3
Coverage	NaN

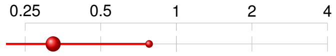

A	B	2.5	50	97.5	Sequence
2	1	0.13	0.32	0.78	AYIPIQMR

5.124 " 75 446 Actin, cytoplasmic type 8 OS=Xenopus laevis PE=3 SV=1"

Protein Accession	CL31420.Contig6$_H$1
Mean Expression Ratio	1.41
Median Expression Ratio	1.41
Credible Interval	(0.774, 2.64)
Associated Peptides	4
Associated Spectra	77
Coverage	NaN

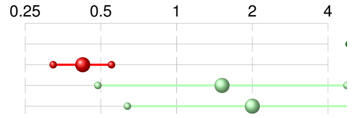

A	B	2.5	50	97.5	Sequence
6	17	4.9	7.1	11	MQKEITALAPSTMK
28	24	0.32	0.42	0.55	EITALAPSTMK
0	1	0.49	1.5	4.7	MQKEITALAPSTMKIK
0	1	0.64	2	6.3	EITALAPSTMKIK

5.125 " 28 1104 minus strand Pyruvate dehydrogenase E1 component subunit beta, mitochondrial [Crassostrea gigas]"

Protein Accession	CL18015.Contig14$_H$1
Mean Expression Ratio	1.41
Median Expression Ratio	1.41
Credible Interval	(0.778, 2.56)
Associated Peptides	7
Associated Spectra	10
Coverage	NaN

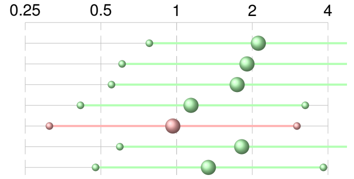

A	B	2.5	50	97.5	Sequence
0	2	0.78	2.1	5.7	DALNSAMDEEIARDER
0	1	0.61	1.9	6.1	ALDAAKELEGK
0	1	0.55	1.7	5.6	SLRPVDDETIIK
1	1	0.41	1.1	3.2	SLEEAALPQPVNVIR
0	1	0.31	0.97	3	IEREGK
0	1	0.6	1.8	5.8	VSKGLWK
1	1	0.48	1.3	3.8	VTGADVPMPYAK

5.126 " 303 500 minus strand protein lethal, putative [Pediculus humanus corporis] gi"

Protein Accession	CL15038.Contig1$_{H}1$
Mean Expression Ratio	1.41
Median Expression Ratio	1.41
Credible Interval	(0.751, 2.64)
Associated Peptides	3
Associated Spectra	27
Coverage	NaN

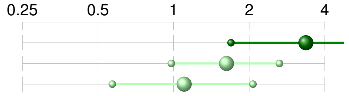

A	B	2.5	50	97.5	Sequence
**3**	**3**	**1.7**	**3.4**	**6.8**	**VPSRQDMLATGR**
**4**	**10**	**0.98**	**1.6**	**2.6**	**QMPMLGR**
**3**	**4**	**0.57**	**1.1**	**2.1**	**QDMLATGR**

5.127 76 522 minus strand Troponin C [Crassostrea gigas]; 173 619 minus strand Troponin C [Crassostrea gigas]; 173 619 minus strand Troponin C [Crassostrea gigas]

Protein Accession	Unigene53263$_{H}1$ Unigene11674$_{H}1$ CL13001.Contig4$_{H}1$
Mean Expression Ratio	0.713
Median Expression Ratio	0.71
Credible Interval	(0.354, 1.43)
Associated Peptides	3
Associated Spectra	6
Coverage	NaN

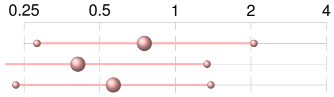

A	B	2.5	50	97.5	Sequence
**1**	**1**	**0.28**	**0.75**	**2.1**	**GVIPVEDLR**
**1**	**0**	**0.13**	**0.41**	**1.3**	**SLGLQVEDEK**
**2**	**1**	**0.23**	**0.57**	**1.4**	**ELANVFK**

5.128 212 1336 6-phosphofructokinase [Crassostrea gigas]; 208 1332 6-phosphofructokinase [Crassostrea gigas]

Protein Accession	CL48438.Contig4$_{H}1$ CL48438.Contig3$_{H}1$
Mean Expression Ratio	0.707
Median Expression Ratio	0.711
Credible Interval	(0.302, 1.61)
Associated Peptides	1
Associated Spectra	1
Coverage	NaN

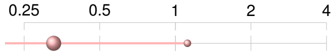

A	B	2.5	50	97.5	Sequence
**1**	**0**	**0.099**	**0.33**	**1.1**	**LGLTQGYR**

5.129 48 356 DEC-3 [Lymnaea stagnalis]

Protein Accession	CL26259.Contig1$_{H}1$
Mean Expression Ratio	0.714
Median Expression Ratio	0.713
Credible Interval	(0.343, 1.49)
Associated Peptides	2
Associated Spectra	5
Coverage	NaN

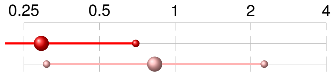

A	B	2.5	50	97.5	Sequence
**2**	**1**	**0.12**	**0.29**	**0.7**	**SEFDTAFR**
**0**	**2**	**0.31**	**0.83**	**2.3**	**EFSFNWR**

5.130 2 220 minus strand haemocyanin [Aplysia californica]

Protein Accession	CL7906.Contig5$_{H}1$
Mean Expression Ratio	0.718
Median Expression Ratio	0.716
Credible Interval	(0.312, 1.66)
Associated Peptides	1
Associated Spectra	2
Coverage	NaN

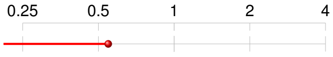

A	B	2.5	50	97.5	Sequence
1	1	0.056	0.18	0.55	YDISDAVK

5.131 71 535 ribosomal protein rpl14 [Arenicola marina]

Protein Accession	Unigene142$_{H}1$
Mean Expression Ratio	0.713
Median Expression Ratio	0.717
Credible Interval	(0.379, 1.33)
Associated Peptides	4
Associated Spectra	14
Coverage	NaN

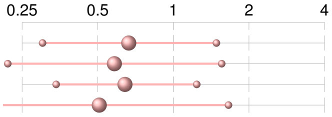

A	B	2.5	50	97.5	Sequence
2	2	0.3	0.66	1.5	IINVEYGK
2	0	0.22	0.58	1.6	AQLTDFER
4	3	0.34	0.64	1.2	FVEIGR
1	0	0.16	0.51	1.7	KAWEK

5.132 " 1 1887 Pyruvate carboxylase, mitochondrial [Crassostrea gigas]"

Protein Accession	CL26073.Contig5$_{H}1$
Mean Expression Ratio	1.39
Median Expression Ratio	1.39
Credible Interval	(0.682, 2.9)
Associated Peptides	3
Associated Spectra	4
Coverage	NaN

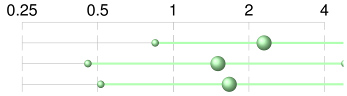

A	B	2.5	50	97.5	Sequence
0	2	0.84	2.3	6.2	HLEDVEENFKR
0	1	0.46	1.5	4.8	AGTHILGIK
0	1	0.51	1.7	5.6	IEVFR

5.133 397 510 PREDICTED: actin-5C-like [Megachile rotundata]

Protein Accession	CL48442.Contig2$_{H}1$
Mean Expression Ratio	0.718
Median Expression Ratio	0.718
Credible Interval	(0.334, 1.57)
Associated Peptides	1
Associated Spectra	8
Coverage	NaN

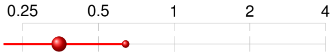

A	B	2.5	50	97.5	Sequence
0	8	0.19	0.35	0.64	DLTDYLMK

5.134 102 887 hypothetical protein DAPPUDRAFT_304712 [Daphnia pulex]

Protein Accession	CL6869.Contig1$_H$1
Mean Expression Ratio	0.718
Median Expression Ratio	0.719
Credible Interval	(0.402, 1.27)
Associated Peptides	7
Associated Spectra	14
Coverage	NaN

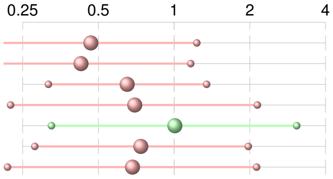

A	B	2.5	50	97.5	Sequence
1	1	0.17	0.47	1.2	TAEEIAAEEK
2	0	0.16	0.42	1.2	VDLSQLR
3	2	0.32	0.65	1.3	AFAIQAEK
0	1	0.22	0.7	2.1	AHTDVQVPDFSYYR
0	1	0.33	1	3.1	LALTSYAMSK
0	2	0.28	0.74	2	LADIPEGK
0	1	0.22	0.68	2.1	HPEADAER

5.135 " 3 587 elongation factor 1 alpha, partial [Phorbas sp. nov. SB-2012]"

Protein Accession	Unigene20048$_H$1
Mean Expression Ratio	0.721
Median Expression Ratio	0.72
Credible Interval	(0.356, 1.43)
Associated Peptides	2
Associated Spectra	18
Coverage	NaN

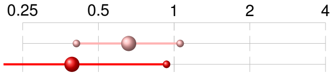

A	B	2.5	50	97.5	Sequence
7	8	0.41	0.66	1.1	IGGIGTVPVGR
1	2	0.16	0.39	0.93	LPLQDVYK

5.136 111 530 minus strand Nucleoside diphosphate kinase B OS=Homo sapiens GN=NME2 PE=1 SV=1

Protein Accession	CL22280.Contig3$_H$1
Mean Expression Ratio	1.39
Median Expression Ratio	1.39
Credible Interval	(0.7, 2.68)
Associated Peptides	4
Associated Spectra	7
Coverage	NaN

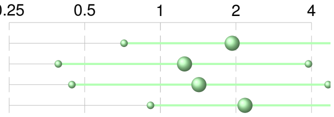

A	B	2.5	50	97.5	Sequence
0	2	0.72	1.9	5.1	GDFCIEMGR
0	1	0.39	1.3	3.9	NICHGSDEVK
0	1	0.44	1.4	4.6	NICHGSDEVKNAEKEIK
0	3	0.91	2.2	5.3	VMLGETNPLNSKPGTIR

5.137 718 1155 Annexin A7 [Crassostrea gigas]

Protein Accession	CL15539.Contig2$_H$1
Mean Expression Ratio	0.723
Median Expression Ratio	0.723
Credible Interval	(0.417, 1.28)
Associated Peptides	7
Associated Spectra	16
Coverage	NaN

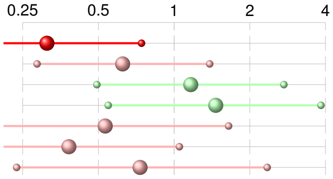

A	B	2.5	50	97.5	Sequence
2	1	0.13	0.31	0.74	ATFDAYQQIAGK
2	2	0.29	0.62	1.4	NVPGYFAQR
1	2	0.49	1.2	2.7	KLGTDEETFNR
1	1	0.55	1.5	3.8	SFPQLR
1	0	0.17	0.53	1.6	AQEAQELLQAGVK
1	1	0.14	0.38	1	DIESAIK
0	1	0.24	0.73	2.4	SEMSGNLEEGFLAIVR

5.138 2000 2641 Polyadenylate-binding protein 4 [Crassostrea gigas]

Protein Accession	Unigene7641$_{H}$1
Mean Expression Ratio	1.39
Median Expression Ratio	1.38
Credible Interval	(0.644, 2.98)
Associated Peptides	2
Associated Spectra	3
Coverage	NaN

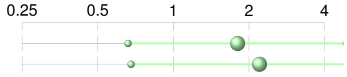

A	B	2.5	50	97.5	Sequence
1	1	0.66	1.8	4.9	KEFSQFGTITSAK
0	1	0.68	2.2	7.1	NIDDSIDDERLR

5.139 49 819 PREDICTED: elongation factor 1-delta-like isoform 2 [Strongylocentrotus purpuratus]

Protein Accession	CL10790.Contig12$_{H}$1
Mean Expression Ratio	0.725
Median Expression Ratio	0.723
Credible Interval	(0.351, 1.49)
Associated Peptides	3
Associated Spectra	7
Coverage	NaN

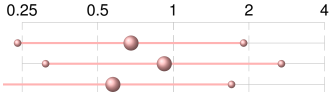

A	B	2.5	50	97.5	Sequence
1	2	0.24	0.68	1.9	VAALEVVGTGGASPK
1	1	0.31	0.92	2.7	KPVVIAK
1	1	0.2	0.57	1.7	LLEEYAAK

5.140 142 789 60S ribosomal protein L6 [Crassostrea gigas]

Protein Accession	CL6501.Contig1$_{H}$1
Mean Expression Ratio	0.724
Median Expression Ratio	0.723
Credible Interval	(0.308, 1.65)
Associated Peptides	1
Associated Spectra	1
Coverage	NaN

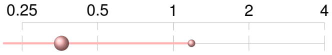

A	B	2.5	50	97.5	Sequence
1	0	0.11	0.36	1.2	HAEGEIFDTK

5.141 1 381 hypothetical protein [Eimeria tenella]

Protein Accession	CL17786.Contig1$_{H}$1
Mean Expression Ratio	1.38
Median Expression Ratio	1.38
Credible Interval	(0.627, 3.09)
Associated Peptides	1
Associated Spectra	4
Coverage	NaN

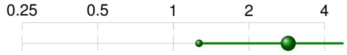

A	B	2.5	50	97.5	Sequence
0	4	1.3	2.9	6.4	IINEPTAAAIAYGLDKK

5.142 " 2041 3315 minus strand Myosin-10 OS=Mus musculus GN=Myh10 PE=1 SV=2; 2041 3345 minus strand Myosin heavy chain, non-muscle OS=Drosophila melanogaster GN=zip PE=1 SV=2; 2041 3351 minus strand Myosin-10 OS=Mus musculus GN=Myh10 PE=1 SV=2"

Protein Accession	Unigene62170$_{H}1$ Unigene62168$_{H}1$ Unigene62165$_{H}1$
Mean Expression Ratio	0.723
Median Expression Ratio	0.724
Credible Interval	(0.373, 1.41)
Associated Peptides	4
Associated Spectra	7
Coverage	NaN

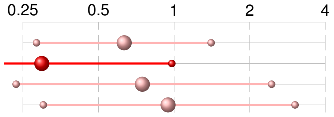

A	B	2.5	50	97.5	Sequence
**1**	**3**	**0.28**	**0.63**	**1.4**	**AGVLAHLEEER**
**1**	**0**	**0.093**	**0.3**	**0.98**	**TVGQLYKEQLNK**
**0**	**1**	**0.24**	**0.75**	**2.4**	**IDSPLVLDQLR**
**0**	**1**	**0.3**	**0.95**	**3**	**MIASLELDSNLYR**

5.143 360 764 minus strand Peptidyl-prolyl cis-trans isomerase A OS=Dictyostelium discoideum GN=ppiA PE=1 SV=1

Protein Accession	Unigene64429$_{H}1$
Mean Expression Ratio	0.724
Median Expression Ratio	0.724
Credible Interval	(0.316, 1.65)
Associated Peptides	1
Associated Spectra	2
Coverage	NaN

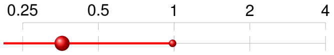

A	B	2.5	50	97.5	Sequence
**0**	**2**	**0.13**	**0.36**	**0.98**	**HVVFGQVVEGLDVVR**

5.144 " 10 1191 3-ketoacyl-CoA thiolase, mitochondrial [Crassostrea gigas]"

Protein Accession	CL29653.Contig1$_{H}1$
Mean Expression Ratio	0.727
Median Expression Ratio	0.724
Credible Interval	(0.389, 1.37)
Associated Peptides	6
Associated Spectra	9
Coverage	NaN

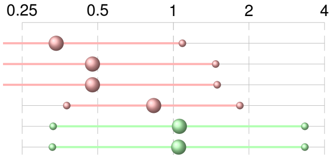

A	B	2.5	50	97.5	Sequence
**1**	**0**	**0.11**	**0.34**	**1.1**	**ELGLDPAK**
**1**	**0**	**0.15**	**0.48**	**1.5**	**FNDEIVPVQVK**
**1**	**0**	**0.15**	**0.48**	**1.5**	**IVAAK**
**0**	**4**	**0.38**	**0.84**	**1.8**	**ITTNLVHEIR**
**0**	**1**	**0.33**	**1.1**	**3.3**	**KGPEDFNVDEHPR**
**0**	**1**	**0.33**	**1.1**	**3.3**	**VMGIGPVPASK**

5.145 89 361 40S ribosomal protein S18 OS=Rattus norvegicus GN=Rps18 PE=1 SV=3

Protein Accession	CL28311.Contig1$_H$1
Mean Expression Ratio	1.38
Median Expression Ratio	1.38
Credible Interval	(0.639, 3.01)
Associated Peptides	2
Associated Spectra	3
Coverage	NaN

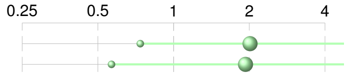

A	B	2.5	50	97.5	Sequence
0	2	0.74	2	5.5	AGELTEDEVER
0	1	0.57	1.9	6.4	LNRQKDVK

5.146 42 434 cytochrome b5 [Haliotis discus discus]; 42 434 cytochrome b5 [Haliotis discus discus]

Protein Accession	CL6985.Contig9$_H$1 CL6985.Contig5$_H$1
Mean Expression Ratio	1.38
Median Expression Ratio	1.38
Credible Interval	(0.673, 2.83)
Associated Peptides	3
Associated Spectra	4
Coverage	NaN

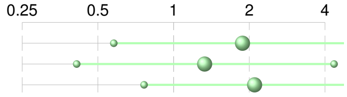

A	B	2.5	50	97.5	Sequence
0	1	0.58	1.9	6.1	KTFETNTGPVGR
0	1	0.41	1.3	4.3	ELMKDYLIGK
0	2	0.76	2.1	5.7	VFRSEEVKSHKDAK

5.147 80 250 minus strand 40S ribosomal protein S29 [Ornithodoros parkeri]

Protein Accession	CL11390.Contig2$_H$1
Mean Expression Ratio	1.38
Median Expression Ratio	1.38
Credible Interval	(0.702, 2.72)
Associated Peptides	3
Associated Spectra	9
Coverage	NaN

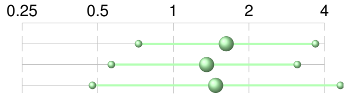

A	B	2.5	50	97.5	Sequence
0	4	0.73	1.6	3.7	GFQTTWYSHPR
1	3	0.57	1.4	3.1	QFAPDIGFK
0	1	0.48	1.5	4.6	KYGLNICR

5.148 503 1219 heat shock cognate 71 [Mytilus galloprovincialis]

Protein Accession	CL23109.Contig5$_H$1
Mean Expression Ratio	0.727
Median Expression Ratio	0.726
Credible Interval	(0.356, 1.48)
Associated Peptides	2
Associated Spectra	9
Coverage	NaN

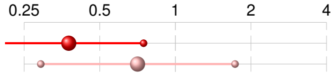

A	B	2.5	50	97.5	Sequence
6	0	0.19	0.38	0.75	GTLEPVEK
0	3	0.29	0.7	1.7	SAIHEIVLVGGSTR

5.149 1 825 Spectrin alpha chain [Crassostrea gigas]

Protein Accession	CL10282.Contig3$_H$1
Mean Expression Ratio	1.37
Median Expression Ratio	1.38
Credible Interval	(0.718, 2.65)
Associated Peptides	5
Associated Spectra	6
Coverage	NaN

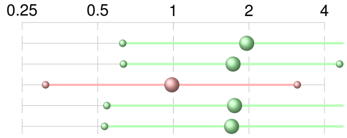

A	B	2.5	50	97.5	Sequence
0	1	0.63	2	6.1	KIEDLGAAMEER
0	2	0.63	1.7	4.6	MQHNLEQQIEAR
0	1	0.31	0.99	3.1	AIPDSYDYVDFTR
0	1	0.54	1.8	5.6	RKATEVR
0	1	0.53	1.7	5.5	MKPYVDK

5.150 1166 1939 minus strand Calcium-binding mitochondrial carrier protein Aralar2 OS=Homo sapiens GN=SLC25A13 PE=1 SV=2

Protein Accession	Unigene34297$_H$1
Mean Expression Ratio	0.723
Median Expression Ratio	0.727
Credible Interval	(0.33, 1.57)
Associated Peptides	2
Associated Spectra	2
Coverage	NaN

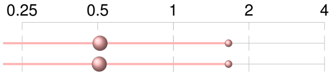

A	B	2.5	50	97.5	Sequence
1	0	0.15	0.51	1.7	LATATFDGVESK
1	0	0.16	0.51	1.7	SAARVLR

5.151 80 589 minus strand PREDICTED: adenine phosphoribosyltransferase-like [Strongylocentrotus purpuratus]; 328 468 minus strand PREDICTED: adenine phosphoribosyltransferase [Apis mellifera]

Protein Accession	CL8682.Contig6$_H$1 CL8682.Contig4$_H$1
Mean Expression Ratio	0.727
Median Expression Ratio	0.728
Credible Interval	(0.315, 1.65)
Associated Peptides	1
Associated Spectra	3
Coverage	NaN

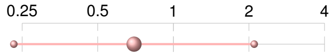

A	B	2.5	50	97.5	Sequence
1	2	0.23	0.7	2.1	AAVELVEK

5.152 15 1361 hypothetical protein BRAFLDRAFT_231908 [Branchiostoma floridae] gi

Protein Accession	CL24447.Contig2$_H$1
Mean Expression Ratio	1.37
Median Expression Ratio	1.37
Credible Interval	(0.645, 3)
Associated Peptides	2
Associated Spectra	3
Coverage	NaN

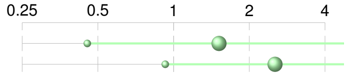

A	B	2.5	50	97.5	Sequence
0	1	0.45	1.5	4.9	GLGLDDALEAR
0	2	0.93	2.5	7.1	GITRIR

5.153 366 1049 hypothetical protein CGI_10004855 [Crassostrea gigas]

Protein Accession	CL43590.Contig5$_H$1
Mean Expression Ratio	1.37
Median Expression Ratio	1.37
Credible Interval	(0.809, 2.31)
Associated Peptides	9
Associated Spectra	19
Coverage	NaN

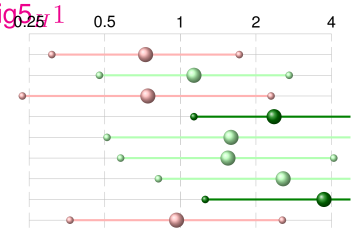

A	B	2.5	50	97.5	Sequence
2	1	0.31	0.73	1.7	TTVQLHK
2	1	0.48	1.1	2.7	VKVPGAK
1	0	0.24	0.74	2.3	KVFPYPK
0	5	1.1	2.4	4.9	GGDLWQGEVSLEQHR
0	1	0.51	1.6	5	GKNVDVKLNAR
0	2	0.58	1.6	4.1	SAEFSTNDNIFDIEMK
0	1	0.82	2.6	8.1	TPQPLKTTVQLHK
0	1	1.3	3.7	12	GKNVDVK
0	2	0.36	0.97	2.5	DEWTHLTAK

5.154 95 1579 minus strand hypothetical protein BRAFLDRAFT_113974 [Branchiostoma floridae] gi

Protein Accession	CL19753.Contig5$_H$1
Mean Expression Ratio	1.37
Median Expression Ratio	1.37
Credible Interval	(0.789, 2.37)
Associated Peptides	9
Associated Spectra	16
Coverage	NaN

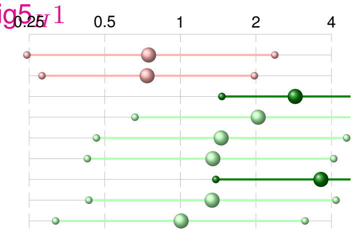

A	B	2.5	50	97.5	Sequence
1	0	0.24	0.75	2.4	TFPVVNPATEK
1	1	0.28	0.74	2	ELGEYALK
1	5	1.5	2.9	5.8	DINEVIERANR
0	1	0.66	2	6.7	FKDINEVIER
0	1	0.46	1.5	4.6	TSYGLAAGVITK
0	1	0.43	1.3	4.1	TVGDPFDAVTMQGPQVDQEQFEK
0	2	1.4	3.6	9.6	TVTIKLPSK
0	1	0.43	1.3	4.2	ADLRPDPNIK
0	1	0.32	1	3.1	DEIFGPVQSIIK

5.155 96 503 minus strand mitochondrial ATP synthase delta chain [Haliotis discus discus]; 111 551 minus strand mitochondrial ATP synthase delta chain [Haliotis discus discus]; 96 542 minus strand mitochondrial ATP synthase delta chain [Haliotis discus discus]; 202 642 minus strand mitochondrial ATP synthase delta chain [Haliotis discus discus]; 44 529 mitochondrial ATP synthase delta chain [Haliotis discus discus]

Protein Accession	Unigene42244$_H$1 CL2187.Contig3$_H$1 CL2187.Contig2$_H$1 CL2187.Contig1$_H$1 CL2187.Contig
Mean Expression Ratio	0.732
Median Expression Ratio	0.73
Credible Interval	(0.321, 1.63)
Associated Peptides	1
Associated Spectra	2
Coverage	NaN

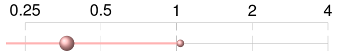

A	B	2.5	50	97.5	Sequence
2	0	0.13	0.37	1	FDAQAIR

5.156 4 783 minus strand hemocyanin isoform 2 [Nucula nucleus]

Protein Accession	CL3697.Contig2$_H$1
Mean Expression Ratio	0.732
Median Expression Ratio	0.73
Credible Interval	(0.348, 1.54)
Associated Peptides	3
Associated Spectra	4
Coverage	NaN

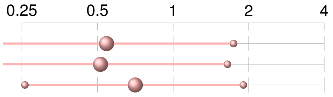

A	B	2.5	50	97.5	Sequence
0	1	0.16	0.54	1.7	ELSEEQIK
0	1	0.16	0.51	1.6	DVMDYK
0	2	0.26	0.71	1.9	SGTWLK

5.157 11 244 minus strand Tubulin alpha chain (Fragment) OS=Lytechinus pictus PE=3 SV=1

Protein Accession	CL3047.Contig6$_H$1
Mean Expression Ratio	0.729
Median Expression Ratio	0.73
Credible Interval	(0.358, 1.48)
Associated Peptides	3
Associated Spectra	6
Coverage	NaN

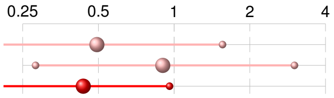

A	B	2.5	50	97.5	Sequence
0	1	0.15	0.49	1.6	EDLAALEK
0	1	0.28	0.9	3	LDHKFDLMYAK
0	4	0.2	0.44	0.96	FDLMYAK

5.158 61 723 minus strand 40S ribosomal protein S3-A OS=Xenopus laevis GN=rps3-a PE=2 SV=2

Protein Accession	Unigene19057$_H$1
Mean Expression Ratio	0.734
Median Expression Ratio	0.731
Credible Interval	(0.371, 1.44)
Associated Peptides	4
Associated Spectra	6
Coverage	NaN

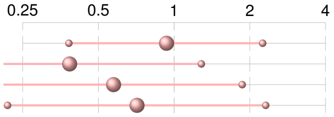

A	B	2.5	50	97.5	Sequence
1	2	0.38	0.93	2.3	ELAEDGYSGVEVR
1	0	0.12	0.39	1.3	TQNVLGEK
1	0	0.18	0.57	1.9	DEPAPLHPYSEQK
0	1	0.22	0.71	2.3	QGVLGIK

5.159 613 2421 PREDICTED: lysosomal alpha-glucosidase [Danio rerio]

Protein Accession	CL5792.Contig1$_H$1
Mean Expression Ratio	1.37
Median Expression Ratio	1.37
Credible Interval	(0.764, 2.49)
Associated Peptides	5
Associated Spectra	18
Coverage	NaN

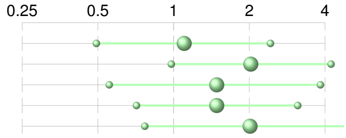

A	B	2.5	50	97.5	Sequence
2	2	0.49	1.1	2.4	ATMGGLQK
1	4	0.98	2	4.2	LPPATTTNIMR
2	0	0.56	1.5	3.8	GGSIIPR
0	5	0.71	1.5	3.1	GLPDIVDDLHSHNQR
0	2	0.77	2	5.4	SFVLSR

5.160 721 1242 Lactoylglutathione lyase [Crassostrea gigas]; 113 634 Lactoylglutathione lyase [Crassostrea gigas]

Protein Accession	Unigene26921$_H$1 CL10946.Contig1$_H$1
Mean Expression Ratio	0.729
Median Expression Ratio	0.733
Credible Interval	(0.314, 1.66)
Associated Peptides	1
Associated Spectra	1
Coverage	NaN

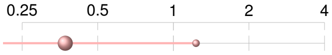

A	B	2.5	50	97.5	Sequence
0	1	0.11	0.37	1.2	FESLGVEFIK

5.161 " 213 1499 minus strand Citrate synthase, mitochondrial [Crassostrea gigas]"

Protein Accession	CL992.Contig2$_H$1
Mean Expression Ratio	0.733
Median Expression Ratio	0.733
Credible Interval	(0.453, 1.17)
Associated Peptides	11
Associated Spectra	34
Coverage	NaN

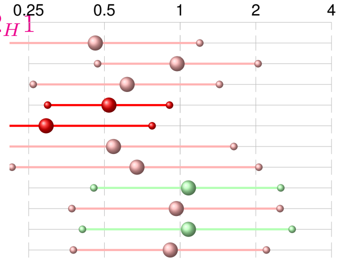

A	B	2.5	50	97.5	Sequence
1	1	0.18	0.46	1.2	ALGLPIERPK
3	2	0.47	0.97	2	SFDTPSLR
1	2	0.26	0.62	1.4	SGQVVPGYGHAVLR
6	4	0.3	0.52	0.91	VVPDILIEQGK
1	1	0.11	0.29	0.77	DVLSQIIPEK
1	0	0.18	0.54	1.6	EVSEFR
1	0	0.22	0.67	2.1	AIIYR
0	3	0.45	1.1	2.5	ALVTETSVLDPEEGIR
0	2	0.37	0.97	2.5	HLPNDPMFK
0	2	0.41	1.1	2.8	VGEVTVDMMYGGMR
0	3	0.38	0.92	2.2	LISQLYK

5.162 6 650 minus strand glutathione S-transferase mu class protein [Crassostrea ariakensis]; 117 761 glutathione S-transferase mu class protein [Crassostrea ariakensis]; 122 766 glutathione S-transferase mu class protein [Crassostrea ariakensis]; 122 766 glutathione S-transferase mu class protein [Crassostrea ariakensis]

Protein Accession	Unigene56987$_H$1 CL6887.Contig3$_H$1 CL6887.Contig2$_H$1 CL6887.Contig1$_H$1
Mean Expression Ratio	0.734
Median Expression Ratio	0.734
Credible Interval	(0.433, 1.25)
Associated Peptides	8
Associated Spectra	22
Coverage	NaN

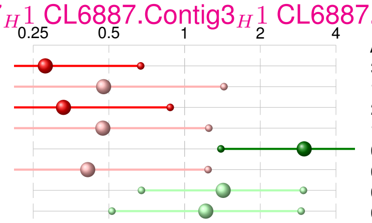

A	B	2.5	50	97.5	Sequence
3	0	0.12	0.28	0.67	ITQSNAILR
1	0	0.15	0.48	1.4	AYMSSDK
2	0	0.13	0.33	0.88	GLAQPIR
1	1	0.18	0.47	1.3	LSQFVK
0	5	1.4	3	6.2	KHDLLGKTDKEK
0	1	0.13	0.41	1.2	IMIPGILDDYPK
0	5	0.67	1.4	3	DAWFNVK
0	3	0.51	1.2	2.9	PALGYWK

5.163 87 1997 Transketolase-like protein 2 [Crassostrea gigas]; 87 1997 Transketolase-like protein 2 [Crassostrea gigas]; 87 1997 Transketolase-like protein 2 [Crassostrea gigas]; 87 1997 Transketolase-like protein 2 [Crassostrea gigas]; 87 1997 Transketolase-like protein 2 [Crassostrea gigas]; 87 1997 Transketolase-like protein 2 [Crassostrea gigas]; 87 1997 Transketolase-like protein 2 [Crassostrea gigas]

Protein Accession	CL1127.Contig6$_H$1 CL1127.Contig5$_H$1 CL1127.Contig4$_H$1 CL1127.Contig3$_H$1 CL1127.Con
Mean Expression Ratio	1.36
Median Expression Ratio	1.36
Credible Interval	(0.729, 2.57)
Associated Peptides	6
Associated Spectra	8
Coverage	NaN

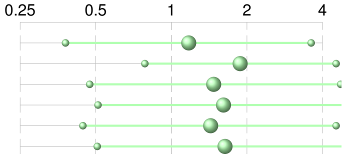

A	B	2.5	50	97.5	Sequence
0	1	0.38	1.2	3.6	FTSDLEGHPTPR
0	3	0.79	1.9	4.5	LAYGDALVK
0	1	0.47	1.5	4.7	SAVQVLR
0	1	0.51	1.6	5.1	GIEGIEDLDNWHGK
0	1	0.44	1.4	4.5	VISLDGDVK
0	1	0.51	1.6	5.2	IIDPFTIKPIDADTIIK

5.164 18 647 minus strand ATP synthase-like protein [Biomphalaria glabrata]

Protein Accession	CL42054.Contig4$_H$1
Mean Expression Ratio	1.35
Median Expression Ratio	1.36
Credible Interval	(0.796, 2.3)
Associated Peptides	9
Associated Spectra	20
Coverage	NaN

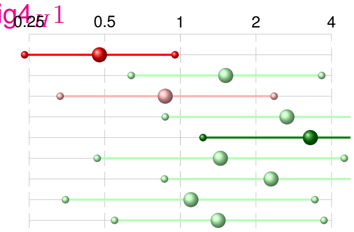

A	B	2.5	50	97.5	Sequence
3	3	0.24	0.48	0.95	AVLEQALK
1	2	0.64	1.5	3.7	YATALYSAASK
1	1	0.33	0.87	2.4	NFQNLLK
0	1	0.87	2.6	8.3	AVLEQALKQQK
0	2	1.2	3.3	8.6	GKLVTTPIQVFGIDGR
0	1	0.47	1.4	4.5	LAEFVANPSLQR
0	2	0.87	2.3	6	GEVVCTVK
0	1	0.35	1.1	3.4	KLAEFVANPSLQR
0	2	0.55	1.4	3.7	GALQGFLK

5.165 " 9 125 minus strand Hemocyanin, beta-C chain unit D (Fragment) OS=Helix pomatia PE=1 SV=2"

Protein Accession	CL7906.Contig6$_H$1
Mean Expression Ratio	0.731
Median Expression Ratio	0.735
Credible Interval	(0.318, 1.66)
Associated Peptides	1
Associated Spectra	2
Coverage	NaN

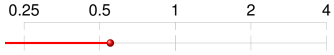

A	B	2.5	50	97.5	Sequence
1	1	0.058	0.18	0.55	YDISDAVK

5.166 65 523 40S ribosomal protein S9 [Crassostrea gigas]; 65 523 40S ribosomal protein S9 [Crassostrea gigas]

Protein Accession	Unigene72778$_{H}1$ Unigene72208$_{H}1$
Mean Expression Ratio	1.36
Median Expression Ratio	1.36
Credible Interval	(0.614, 3.03)
Associated Peptides	2
Associated Spectra	2
Coverage	NaN

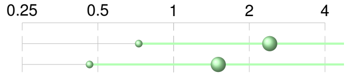

A	B	2.5	50	97.5	Sequence
0	1	0.73	2.4	7.9	VKYTLGK
0	1	0.46	1.5	5.1	LIGEYGLR

5.167 " 27 974 PREDICTED: succinyl-CoA ligase [GDP-forming] subunit alpha, mitochondrial-like [Oreochromis niloticus]; 27 974 minus strand PREDICTED: succinyl-CoA ligase [GDP-forming] subunit alpha, mitochondrial-like [Oreochromis niloticus]"

Protein Accession	CL10674.Contig2$_{H}1$ CL10674.Contig1$_{H}1$
Mean Expression Ratio	1.36
Median Expression Ratio	1.35
Credible Interval	(0.662, 2.81)
Associated Peptides	3
Associated Spectra	5
Coverage	NaN

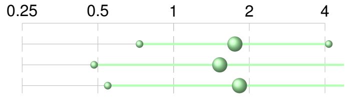

A	B	2.5	50	97.5	Sequence
0	3	0.73	1.8	4.1	MGHAGAIIAGGK
0	1	0.48	1.5	5	IYMATR
0	1	0.55	1.8	5.8	GGAKEKIEILK

5.168 260 892 minus strand calponin [Haliotis diversicolor]

Protein Accession	CL48702.Contig2$_{H}1$
Mean Expression Ratio	0.739
Median Expression Ratio	0.738
Credible Interval	(0.362, 1.52)
Associated Peptides	2
Associated Spectra	7
Coverage	NaN

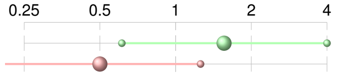

A	B	2.5	50	97.5	Sequence
2	1	0.61	1.6	4	NFSEDKLR
2	2	0.2	0.5	1.3	VESSFDPEDAKK

5.169 129 299 glycogen phosphorylase [Crassostrea gigas]

Protein Accession	Unigene20640$_{H}1$
Mean Expression Ratio	1.36
Median Expression Ratio	1.35
Credible Interval	(0.596, 3.25)
Associated Peptides	1
Associated Spectra	1
Coverage	NaN

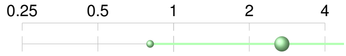

A	B	2.5	50	97.5	Sequence
0	1	0.81	2.7	9.1	GIAPVENVGTIKK

5.170 252 848 sigma class glutathione-s-transferase 2 [Haliotis discus discus]

Protein Accession	CL19285.Contig21$_{H}1$
Mean Expression Ratio	0.736
Median Expression Ratio	0.739
Credible Interval	(0.356, 1.5)
Associated Peptides	2
Associated Spectra	7
Coverage	NaN

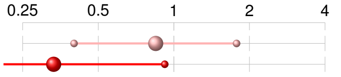

A	B	2.5	50	97.5	Sequence
1	4	0.4	0.85	1.8	LQDVDKDYPEVQK
2	0	0.12	0.33	0.92	LGNELTMA

5.171 73 525 minus strand Actin-related protein 2/3 complex subunit 5-like protein [Salmo salar] gi; 73 525 Actin-related protein 2/3 complex subunit 5-like protein [Salmo salar] gi; 73 525 minus strand Actin-related protein 2/3 complex subunit 5-like protein [Salmo salar] gi

Protein Accession	CL14637.Contig6$_{H}1$ CL14637.Contig5$_{H}1$ CL14637.Contig4$_{H}1$
Mean Expression Ratio	1.35
Median Expression Ratio	1.35
Credible Interval	(0.642, 2.83)
Associated Peptides	3
Associated Spectra	3
Coverage	NaN

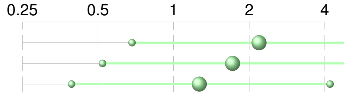

A	B	2.5	50	97.5	Sequence
0	1	0.68	2.2	7.1	LVINSTPFGSK
0	1	0.52	1.7	5.5	EVDILMK
0	1	0.39	1.3	4.2	VLLSFK

5.172 689 1984 hypothetical protein BRAFLDRAFT_78257 [Branchiostoma floridae] gi

Protein Accession	Unigene34076$_{H}1$
Mean Expression Ratio	1.35
Median Expression Ratio	1.35
Credible Interval	(0.764, 2.41)
Associated Peptides	7
Associated Spectra	14
Coverage	NaN

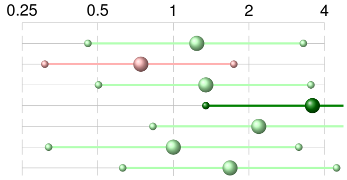

A	B	2.5	50	97.5	Sequence
1	1	0.46	1.2	3.3	VQQKPEPSPAEGEVLIR
1	2	0.31	0.74	1.7	ILLDPSK
1	1	0.5	1.3	3.5	AWAELVAV
0	2	1.3	3.6	9.5	ILLDPSKAPTPK
0	2	0.83	2.2	5.8	SFFSFAK
0	1	0.32	1	3.1	GISILKPMGK
0	2	0.63	1.7	4.5	SLVLTGFGSMK

5.173 133 354 minus strand 60S ribosomal protein L7a OS=Rattus norvegicus GN=Rpl7a PE=1 SV=2

Protein Accession	CL20520.Contig1$_H$1
Mean Expression Ratio	1.35
Median Expression Ratio	1.35
Credible Interval	(0.622, 2.92)
Associated Peptides	2
Associated Spectra	3
Coverage	NaN

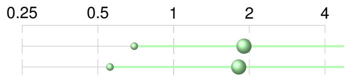

A	B	2.5	50	97.5	Sequence
0	2	0.7	1.9	5.1	KVVNPLFEK
0	1	0.56	1.8	5.9	NFGIGQDIQPK

5.174 459 755 minus strand heat shock protein beta-1 [Trichinella spiralis] gi; 464 763 minus strand heat shock protein beta-1 [Trichinella spiralis] gi; 437 736 minus strand heat shock protein beta-1 [Trichinella spiralis] gi; 437 736 minus strand heat shock protein beta-1 [Trichinella spiralis] gi; 437 736 minus strand heat shock protein beta-1 [Trichinella spiralis] gi

Protein Accession	CL17234.Contig5$_H$1 CL17234.Contig4$_H$1 CL17234.Contig3$_H$1 CL17234.Contig2$_H$1 CL1723[illegible]
Mean Expression Ratio	1.35
Median Expression Ratio	1.35
Credible Interval	(0.67, 2.72)
Associated Peptides	2
Associated Spectra	14
Coverage	NaN

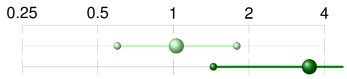

A	B	2.5	50	97.5	Sequence
0	11	0.6	1	1.8	DVEPTTVTSSLNADGVLTLK
0	3	1.4	3.5	8.3	TVTSSLNADGVLTLKAPK

5.175 1714 2121 Phosphoenolpyruvate carboxykinase [GTP] [Crassostrea gigas]

Protein Accession	CL25948.Contig1$_H$1
Mean Expression Ratio	0.744
Median Expression Ratio	0.743
Credible Interval	(0.343, 1.63)
Associated Peptides	1
Associated Spectra	6
Coverage	NaN

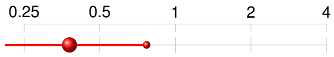

A	B	2.5	50	97.5	Sequence
4	2	0.19	0.38	0.77	SEATAAAEFK

5.176 11 187 PREDICTED: 40S ribosomal protein S28-like [Strongylocentrotus purpuratus]

Protein Accession	Unigene696$_H$1
Mean Expression Ratio	0.743
Median Expression Ratio	0.744
Credible Interval	(0.32, 1.71)
Associated Peptides	1
Associated Spectra	4
Coverage	NaN

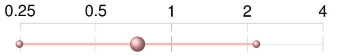

A	B	2.5	50	97.5	Sequence
1	3	0.25	0.73	2.2	VEFLDDSNR

5.177 365 2530 minus strand calpain B [Gecarcinus lateralis]

Protein Accession	CL34074.Contig5$_H$1
Mean Expression Ratio	1.35
Median Expression Ratio	1.34
Credible Interval	(0.659, 2.8)
Associated Peptides	3
Associated Spectra	5
Coverage	NaN

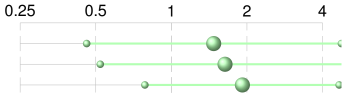

A	B	2.5	50	97.5	Sequence
0	1	0.46	1.5	4.8	SLQSLVMR
0	1	0.52	1.6	5.2	SLFFSQSMR
0	3	0.78	1.9	4.7	MMDIK

5.178 " 134 2047 PREDICTED: succinate dehydrogenase [ubiquinone] flavoprotein subunit, mitochondrial-like isoform 3, partial [Strongylocentrotus purpuratus]; 134 2047 PREDICTED: succinate dehydrogenase [ubiquinone] flavoprotein subunit, mitochondrial-like isoform 3, partial [Strongylocentrotus purpuratus]; 134 2047 PREDICTED: succinate dehydrogenase [ubiquinone] flavoprotein subunit, mitochondrial-like isoform 3, partial [Strongylocentrotus purpuratus]"

Protein Accession	CL47322.Contig4$_H$1 CL47322.Contig3$_H$1 CL47322.Contig2$_H$1
Mean Expression Ratio	0.747
Median Expression Ratio	0.746
Credible Interval	(0.475, 1.17)
Associated Peptides	14
Associated Spectra	33
Coverage	NaN

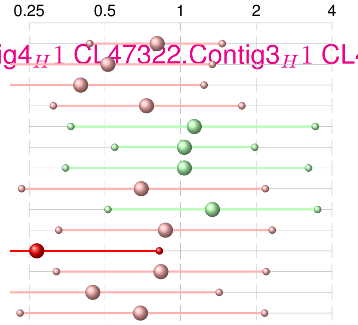

A	B	2.5	50	97.5	Sequence
2	6	0.43	0.8	1.5	AAFGLAEEGFK
2	0	0.2	0.51	1.3	ACANTIIEENKPGDR
1	0	0.13	0.4	1.2	DGPTLQAGVR
2	1	0.31	0.73	1.7	AFGGQSLK
1	0	0.37	1.1	3.4	AFGGQSLKYGK
4	3	0.55	1	2	AITIEIR
0	1	0.35	1	3.2	AVIELENYGMPFSR
0	1	0.23	0.7	2.2	IYQQMEDLK
0	2	0.51	1.3	3.5	KHTLAYTNVDTGK
0	2	0.33	0.87	2.3	NTILATGGYGR
0	1	0.086	0.27	0.82	QDAGEASVGNLDK
0	2	0.32	0.84	2.2	TTLDYRPVIDTTLDEHDCPQVKPAIR
0	1	0.15	0.45	1.4	WHFYDTVK
0	1	0.23	0.69	2.2	HTLAYTNVDTGK

5.179 155 3214 sarco/endoplasmic reticulum calcium ATPase isoform B [Pinctada fucata]; 132 3110 sarco/endoplasmic reticulum calcium ATPase isoform C [Pinctada fucata]; 132 3110 minus strand sarco/endoplasmic reticulum calcium ATPase isoform C [Pinctada fucata]; 155 3133 sarco/endoplasmic reticulum calcium ATPase isoform C [Pinctada fucata]; 155 3133 sarco/endoplasmic reticulum calcium ATPase isoform C [Pinctada fucata]; 155 3214 minus strand sarco/endoplasmic reticulum calcium ATPase isoform B [Pinctada fucata]

Protein Accession	CL1323.Contig9$_{H}1$ CL1323.Contig8$_{H}1$ CL1323.Contig6$_{H}1$ CL1323.Contig21$_{H}1$ CL1323.Co
Mean Expression Ratio	0.746
Median Expression Ratio	0.747
Credible Interval	(0.326, 1.7)
Associated Peptides	1
Associated Spectra	2
Coverage	NaN

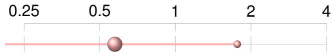

A	B	2.5	50	97.5	Sequence
**1**	**1**	**0.18**	**0.57**	**1.8**	**KAEIGIAMGSGTAVAK**

5.180 116 352 minus strand troponin I 4 [Clonorchis sinensis]

Protein Accession	Unigene63816$_{H}1$
Mean Expression Ratio	1.34
Median Expression Ratio	1.34
Credible Interval	(0.657, 2.74)
Associated Peptides	3
Associated Spectra	7
Coverage	NaN

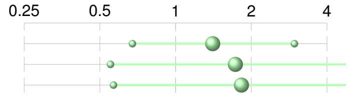

A	B	2.5	50	97.5	Sequence
**0**	**5**	**0.67**	**1.4**	**3**	**FIKPVLR**
**0**	**1**	**0.55**	**1.7**	**5.8**	**QSPTRGNLKSTGR**
**0**	**1**	**0.57**	**1.8**	**6**	**GKFIKPVLR**

5.181 150 1211 RecName: Full=Guanine nucleotide-binding protein G(i) subunit alpha; AltName: Full=Adenylate cyclase-inhibiting G alpha protein gi|9631|emb|CAA78807.1| GTP-binding protein alpha-i subunit [Lymnaea stagnalis]

Protein Accession	CL13885.Contig6$_{H}1$
Mean Expression Ratio	0.745
Median Expression Ratio	0.747
Credible Interval	(0.319, 1.71)
Associated Peptides	1
Associated Spectra	1
Coverage	NaN

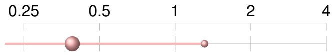

A	B	2.5	50	97.5	Sequence
**1**	**0**	**0.12**	**0.39**	**1.3**	**IAIIR**

5.182 " 4 423 minus strand PREDICTED: similar to NADH dehydrogenase, putative [Tribolium castaneum] gi"

Protein Accession: CL4111.Contig2$_{H}$1
Mean Expression Ratio: 0.75
Median Expression Ratio: 0.748
Credible Interval: (0.341, 1.63)
Associated Peptides: 2
Associated Spectra: 2
Coverage: NaN

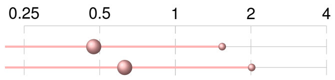

A	B	2.5	50	97.5	Sequence
1	0	0.14	0.47	1.5	QVKPILSTNPAEAK
1	0	0.19	0.63	2	IKQVK

5.183 4 219 hypothetical protein BRAFLDRAFT_66351 [Branchiostoma floridae] gi; 3 239 hypothetical protein BRAFLDRAFT_66351 [Branchiostoma floridae] gi

Protein Accession: CL31114.Contig2$_{H}$1 CL31114.Contig1$_{H}$1
Mean Expression Ratio: 1.34
Median Expression Ratio: 1.34
Credible Interval: (0.638, 2.89)
Associated Peptides: 3
Associated Spectra: 3
Coverage: NaN

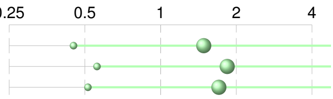

A	B	2.5	50	97.5	Sequence
0	1	0.45	1.5	5	KGSENVLAK
0	1	0.56	1.8	5.8	KLEEVSGELR
0	1	0.51	1.7	5.6	NNQLNGDLK

5.184 " 189 2099 Hsp70, putative [Ixodes scapularis] gi"

Protein Accession: CL16113.Contig2$_{H}$1
Mean Expression Ratio: 1.33
Median Expression Ratio: 1.33
Credible Interval: (0.677, 2.63)
Associated Peptides: 4
Associated Spectra: 7
Coverage: NaN

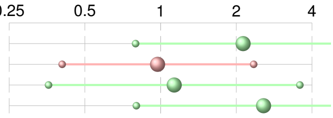

A	B	2.5	50	97.5	Sequence
1	1	0.8	2.1	5.8	LVGMPAKR
1	2	0.41	0.97	2.3	ALQDAEVK
0	1	0.36	1.1	3.6	ASNGDAWVEAQGK
0	1	0.8	2.6	8.4	RFEDPEVQKDMK

5.185 113 1402 minus strand S-adenosylhomocysteine hydrolase [Crassostrea ariakensis]; 113 1402 minus strand S-adenosylhomocysteine hydrolase [Crassostrea ariakensis]

Protein Accession	CL7405.Contig2$_{H}$1 CL7405.Contig1$_{H}$1
Mean Expression Ratio	1.33
Median Expression Ratio	1.33
Credible Interval	(0.739, 2.41)
Associated Peptides	8
Associated Spectra	8
Coverage	NaN

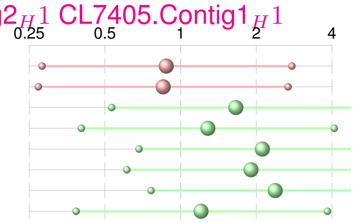

A	B	2.5	50	97.5	Sequence
1	0	0.28	0.88	2.8	VAVVAGYGDVGK
1	0	0.27	0.85	2.7	TGVPVYAWK
0	1	0.53	1.7	5.2	GLSEETTTGVHNLYR
0	1	0.4	1.3	4.1	HIIVLAEGR
0	1	0.69	2.1	6.7	LKVPAINVNDSVTK
0	1	0.61	1.9	6.1	VADISLAEMGR
0	1	0.76	2.4	7.7	LTKLDEDQAK
0	1	0.39	1.2	3.8	YLGIPQEGPFKPEHYR

5.186 76 627 minus strand Heme-binding protein 2 [Crassostrea gigas]; 296 847 minus strand Heme-binding protein 2 [Crassostrea gigas]; 271 822 minus strand Heme-binding protein 2 [Crassostrea gigas]; 367 918 minus strand Heme-binding protein 2 [Crassostrea gigas]; 282 554 minus strand PREDICTED: heme-binding protein 2-like [Strongylocentrotus purpuratus]; 279 878 minus strand Heme-binding protein 2 [Crassostrea gigas]

Protein Accession	CL8470.Contig9$_{H}$1 CL8470.Contig8$_{H}$1 CL8470.Contig6$_{H}$1 CL8470.Contig5$_{H}$1 CL8470.Con
Mean Expression Ratio	1.34
Median Expression Ratio	1.33
Credible Interval	(0.607, 2.96)
Associated Peptides	2
Associated Spectra	2
Coverage	NaN

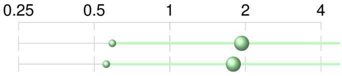

A	B	2.5	50	97.5	Sequence
0	1	0.59	1.9	6.3	GVEFDEAENK
0	1	0.56	1.8	6	IAMTVPVLTK

5.187 48 479 minus strand 40S ribosomal protein S24 OS=Macaca fascicularis GN=RPS24 PE=2 SV=1

Protein Accession	CL40647.Contig4$_{H}$1
Mean Expression Ratio	1.33
Median Expression Ratio	1.33
Credible Interval	(0.674, 2.63)
Associated Peptides	3
Associated Spectra	9
Coverage	NaN

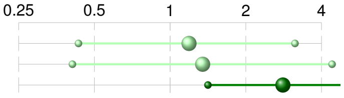

A	B	2.5	50	97.5	Sequence
0	2	0.43	1.2	3.1	HGLLELK
0	1	0.41	1.3	4.4	TTGFGLVYDTLESAK
0	6	1.4	2.8	5.6	HGLLELKK

5.188 811 1407 minus strand mitochondrial malate dehydrogenase 2 [Eospalax baileyi]

Protein Accession	CL10426.Contig13$_H$1
Mean Expression Ratio	0.752
Median Expression Ratio	0.75
Credible Interval	(0.383, 1.48)
Associated Peptides	3
Associated Spectra	16
Coverage	NaN

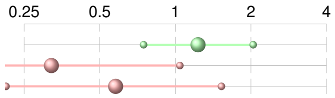

A	B	2.5	50	97.5	Sequence
6	7	0.75	1.2	2	IQNAGTEVVEAK
1	0	0.098	0.32	1	NLGMGK
0	2	0.21	0.58	1.5	AAMPELIANISK

5.189 290 2458 Putative beta-D-xylosidase 5 [Crassostrea gigas]

Protein Accession	CL49577.Contig2$_H$1
Mean Expression Ratio	1.33
Median Expression Ratio	1.33
Credible Interval	(0.782, 2.25)
Associated Peptides	10
Associated Spectra	19
Coverage	NaN

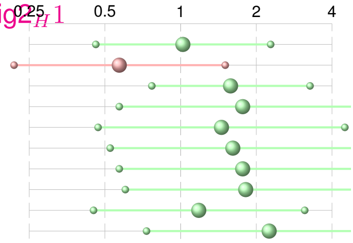

A	B	2.5	50	97.5	Sequence
2	2	0.46	1	2.3	VAEAAGVEVR
1	1	0.22	0.57	1.5	EIQEQLAR
2	3	0.77	1.6	3.3	VTPLFYTR
0	1	0.57	1.8	5.4	HFDAYAGPENVPTSR
0	1	0.47	1.5	4.5	LTDYDWR
0	1	0.52	1.6	5.1	SAPSQVLVQPFTIK
0	1	0.57	1.8	5.3	NNGILPLDPR
0	1	0.6	1.8	5.6	LEALEQGK
0	2	0.45	1.2	3.1	QLLTDILR
0	1	0.73	2.3	7	SFVLLK

5.190 273 1304 Guanine nucleotide-binding protein subunit beta [Crassostrea gigas]

Protein Accession	CL15682.Contig1$_H$1
Mean Expression Ratio	1.33
Median Expression Ratio	1.33
Credible Interval	(0.66, 2.7)
Associated Peptides	4
Associated Spectra	4
Coverage	NaN

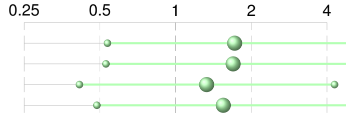

A	B	2.5	50	97.5	Sequence
0	1	0.54	1.7	5.5	TFVSGACDASAK
0	1	0.53	1.7	5.4	KAAGDTTLAQATAQCEPVGR
0	1	0.41	1.3	4.3	TSELDALRQETEQLKNQIR
0	1	0.49	1.5	5	IYAMHWASDSR

5.191 77 1561 retinal dehydrogenase [Lymnaea stagnalis]; 69 1553 retinal dehydrogenase [Lymnaea stagnalis]

Protein Accession	CL13284.Contig7$_{H}$1 CL13284.Contig2$_{H}$1
Mean Expression Ratio	1.33
Median Expression Ratio	1.33
Credible Interval	(0.903, 1.97)
Associated Peptides	19
Associated Spectra	49
Coverage	NaN

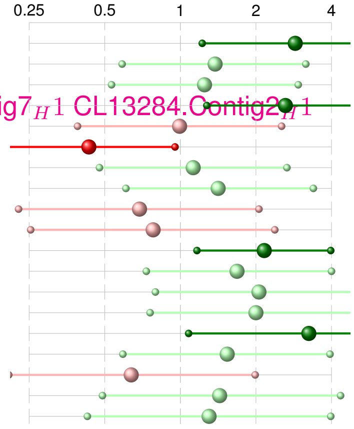

A	B	2.5	50	97.5	Sequence
2	1	1.2	2.9	6.8	NVVIKIPQKNS
1	2	0.59	1.4	3.2	SFPEPIRNPEVK
1	2	0.53	1.3	2.9	TFPTINPATGK
3	2	1.3	2.6	5.3	TFPTINPATGKK
1	1	0.39	0.99	2.5	TAELANKR
2	2	0.2	0.43	0.95	AVEVAQDAFK
1	2	0.47	1.1	2.7	IYDEFVR
1	2	0.61	1.4	3.4	LGSAWR
1	0	0.23	0.69	2.1	NVVIK
1	0	0.25	0.78	2.4	GALLYK
1	7	1.2	2.2	4	ADVDKAVEVAQDAFK
0	3	0.73	1.7	4	ELGEYGLQQYVEVK
0	2	0.8	2.1	5.4	FSTVEEAIER
0	2	0.76	2	5.3	KTAELANKR
0	1	1.1	3.2	9.9	NVVIKIPQK
0	2	0.59	1.5	3.9	KTAELANK
0	1	0.21	0.64	2	LADLMEK
0	1	0.49	1.4	4.3	RGALLYK
0	1	0.43	1.3	4	TTLELGGK

5.192 " 235 1038 Translocon-associated protein subunit alpha [Crassostrea gigas]; 309 725 hypothetical protein [Amblyomma maculatum]; 309 590 translocon-associated complex TRAP, alpha subunit, putative [Ixodes scapularis] gi; 309 725 hypothetical protein [Amblyomma maculatum]"

Protein Accession	CL36450.Contig6$_{H}$1 CL36450.Contig5$_{H}$1 CL36450.Contig2$_{H}$1 CL36450.Contig1$_{H}$1
Mean Expression Ratio	1.33
Median Expression Ratio	1.33
Credible Interval	(0.63, 2.84)
Associated Peptides	2
Associated Spectra	4
Coverage	NaN

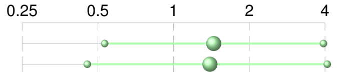

A	B	2.5	50	97.5	Sequence
0	2	0.53	1.4	3.9	LLVGFTNK
1	1	0.45	1.4	4.1	ELPAGKPVR

5.193 150 473 40S ribosomal protein S16 OS=Heteropneustes fossilis GN=rps16 PE=2 SV=1

Protein Accession	CL14629.Contig4$_{H}$1
Mean Expression Ratio	0.754
Median Expression Ratio	0.754
Credible Interval	(0.419, 1.37)
Associated Peptides	5
Associated Spectra	14
Coverage	NaN

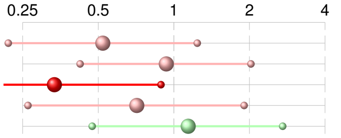

A	B	2.5	50	97.5	Sequence
2	1	0.22	0.52	1.2	SLVAYYQK
2	2	0.42	0.93	2	AVQVFGR
2	0	0.12	0.33	0.89	ILRAK
1	1	0.26	0.71	1.9	AKLEEPVR
0	3	0.47	1.1	2.7	VNGRPLDQVEPQILR

5.194 1271 1663 minus strand PREDICTED: similar to AGAP007393-PB [Tribolium castaneum] gi

Protein Accession	CL28563.Contig3$_H$1
Mean Expression Ratio	1.33
Median Expression Ratio	1.32
Credible Interval	(0.57, 3.07)
Associated Peptides	1
Associated Spectra	1
Coverage	NaN

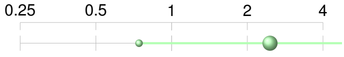

A	B	2.5	50	97.5	Sequence
**0**	**1**	**0.74**	**2.5**	**8.4**	**KYEGGREVDEFITYLAR**

5.195 636 1232 sigma class glutathione-s-transferase 3 [Haliotis discus discus]; 963 1559 sigma class glutathione-s-transferase 3 [Haliotis discus discus]; 907 1503 sigma class glutathione-s-transferase 3 [Haliotis discus discus]; 673 1269 sigma class glutathione-s-transferase 3 [Haliotis discus discus]; 177 773 sigma class glutathione-s-transferase 3 [Haliotis discus discus]; 208 804 sigma class glutathione-s-transferase 3 [Haliotis discus discus]; 327 923 sigma class glutathione-s-transferase 3 [Haliotis discus discus]

Protein Accession	Unigene22829$_H$1 CL19285.Contig31$_H$1 CL19285.Contig30$_H$1 CL19285.Contig27$_H$1 CL192[illegible]
Mean Expression Ratio	1.33
Median Expression Ratio	1.32
Credible Interval	(0.649, 2.74)
Associated Peptides	3
Associated Spectra	5
Coverage	NaN

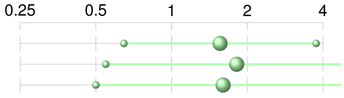

A	B	2.5	50	97.5	Sequence
**0**	**3**	**0.65**	**1.6**	**3.8**	**LLLAASGKDWEDVR**
**0**	**1**	**0.55**	**1.8**	**5.9**	**LQGFDKDYPEVQK**
**0**	**1**	**0.5**	**1.6**	**5.2**	**FSDEDWPK**

5.196 " 241 1110 minus strand N(G),N(G)-dimethylarginine dimethylaminohydrolase 1 [Crassostrea gigas]; 241 1110 minus strand N(G),N(G)-dimethylarginine dimethylaminohydrolase 1 [Crassostrea gigas]; 241 1110 minus strand N(G),N(G)-dimethylarginine dimethylaminohydrolase 1 [Crassostrea gigas]"

Protein Accession	Unigene30451$_H$1 CL7107.Contig3$_H$1 CL7107.Contig1$_H$1
Mean Expression Ratio	1.32
Median Expression Ratio	1.32
Credible Interval	(0.611, 2.93)
Associated Peptides	2
Associated Spectra	2
Coverage	NaN

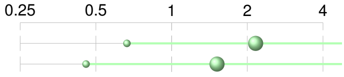

A	B	2.5	50	97.5	Sequence
**0**	**1**	**0.66**	**2.2**	**7.2**	**LGEVGEIRR**
**0**	**1**	**0.46**	**1.5**	**5.1**	**LGEVGEIR**

5.197 2881 3411 myosin heavy chain [Todarodes pacificus]; 2915 3445 myosin heavy chain [Todarodes pacificus]

Protein Accession	Unigene19024$_H$1 Unigene19021$_H$1
Mean Expression Ratio	1.31
Median Expression Ratio	1.32
Credible Interval	(0.823, 2.12)
Associated Peptides	11
Associated Spectra	46
Coverage	NaN

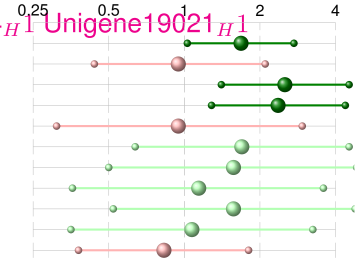

A	B	2.5	50	97.5	Sequence
3	11	1	1.7	2.7	ALDSMQASLEAEAK
2	2	0.44	0.94	2.1	IQEKEEEFENTR
2	7	1.4	2.5	4.5	IQEKEEEFENTRR
1	7	1.3	2.4	4.4	KLQQQVIETQQHVEEEAR
1	0	0.31	0.95	3	GKAELEK
0	2	0.64	1.7	4.5	CTALTGEVEELR
0	1	0.5	1.6	4.9	LQQQVIETQQHVEEEAR
0	1	0.36	1.1	3.6	RCTALTGEVEELR
0	1	0.52	1.6	4.8	RIQEKEEEFENTR
0	1	0.35	1.1	3.2	ALDSMQASLEAEAKGKAEAMR
0	4	0.38	0.83	1.8	KLEQDINELEIALDQANR

5.198 957 2147 haemocyanin [Aplysia californica]

Protein Accession	Unigene30320$_H$1
Mean Expression Ratio	0.759
Median Expression Ratio	0.76
Credible Interval	(0.421, 1.38)
Associated Peptides	5
Associated Spectra	22
Coverage	NaN

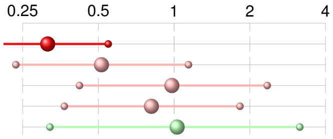

A	B	2.5	50	97.5	Sequence
5	5	0.18	0.32	0.55	LLTVQFER
1	3	0.23	0.52	1.1	YEVSELSLEQVSNLR
0	3	0.42	0.98	2.3	GAQVGVPYWDWTRPGK
0	4	0.37	0.81	1.8	TFHISGVNQDITR
0	1	0.32	1	3.2	EKGAQVGVPYWDWTRPGK

5.199 2 487 minus strand PREDICTED: hypothetical protein LOC100699750 [Oreochromis niloticus]; 41 535 minus strand PREDICTED: hypothetical protein LOC100699750 [Oreochromis niloticus]

Protein Accession	CL7266.Contig5$_H$1 CL7266.Contig3$_H$1
Mean Expression Ratio	1.32
Median Expression Ratio	1.31
Credible Interval	(0.659, 2.68)
Associated Peptides	3
Associated Spectra	7
Coverage	NaN

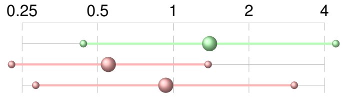

A	B	2.5	50	97.5	Sequence
0	1	0.44	1.4	4.4	VVNINNEAVTFASPR
3	2	0.23	0.55	1.4	NVALVVSVK
0	1	0.28	0.93	3	IPGDGTLTLHGSNGDTAFDR

5.200 137 1369 hypothetical protein TcasGA2_TC002962 [Tribolium castaneum]

Protein Accession	CL20015.Contig2$_H$1
Mean Expression Ratio	1.31
Median Expression Ratio	1.31
Credible Interval	(0.613, 2.77)
Associated Peptides	2
Associated Spectra	4
Coverage	NaN

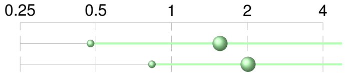

A	B	2.5	50	97.5	Sequence
0	1	0.48	1.6	5.2	AAAAEPAVVENK
0	3	0.83	2	5	AEAAAEAK

5.201 18 1613 minus strand chaperonin containing T-complex polypeptide subunit zeta [Haliotis discus hannai]; 527 2089 minus strand chaperonin containing T-complex polypeptide subunit zeta [Haliotis discus hannai]

Protein Accession	CL23722.Contig7$_H$1 CL23722.Contig5$_H$1
Mean Expression Ratio	0.766
Median Expression Ratio	0.762
Credible Interval	(0.359, 1.66)
Associated Peptides	2
Associated Spectra	3
Coverage	NaN

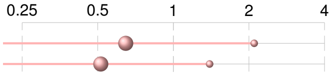

A	B	2.5	50	97.5	Sequence
1	0	0.19	0.65	2.1	AGMSSLKGD
2	0	0.19	0.52	1.4	EGIIALR

5.202 91 2658 minus strand puromycin-sensitive aminopeptidase [Saccoglossus kowalevskii]

Protein Accession	CL6708.Contig2$_H$1
Mean Expression Ratio	0.763
Median Expression Ratio	0.762
Credible Interval	(0.42, 1.38)
Associated Peptides	7
Associated Spectra	11
Coverage	NaN

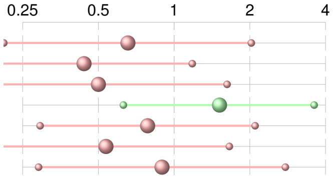

A	B	2.5	50	97.5	Sequence
1	0	0.21	0.65	2	GLTVQQEK
1	1	0.16	0.44	1.2	TLDFALSDK
1	0	0.16	0.5	1.6	VYTPVGK
1	2	0.63	1.5	3.6	SLGVNDDPELIKR
1	1	0.29	0.79	2.1	MIELYEK
1	0	0.17	0.53	1.7	LVTYR
0	1	0.29	0.9	2.8	TIQQSLENIMLNKR

5.203 2939 3208 minus strand beta-thymosin domain repeat protein CSP24KDa_v1 [Hermissenda crassicornis] gi

Protein Accession	CL9548.Contig3$_H$1
Mean Expression Ratio	0.764
Median Expression Ratio	0.764
Credible Interval	(0.424, 1.37)
Associated Peptides	7
Associated Spectra	10
Coverage	NaN

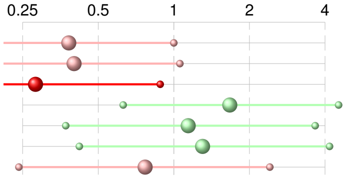

A	B	2.5	50	97.5	Sequence
1	1	0.14	0.38	1	NPLPSTDAIAAEK
1	1	0.15	0.4	1.1	SIGEFDK
1	0	0.089	0.28	0.88	NPLPTK
1	1	0.63	1.7	4.5	SIGEFDKGA
0	1	0.37	1.1	3.7	KAETEEKNPLPTKEVIQQEK
0	1	0.42	1.3	4.2	SIGEFDKGALK
0	1	0.24	0.77	2.4	AETEEKNPLPTK

5.204 89 283 hypothetical protein CGI_10017178 [Crassostrea gigas]

Protein Accession	Unigene58795$_H1$
Mean Expression Ratio	1.31
Median Expression Ratio	1.31
Credible Interval	(0.694, 2.46)
Associated Peptides	3
Associated Spectra	21
Coverage	NaN

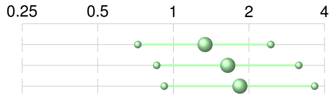

A	B	2.5	50	97.5	Sequence
**3**	**5**	**0.72**	**1.3**	**2.4**	**AGIIASAK**
**0**	**7**	**0.86**	**1.7**	**3.2**	**NMSVIAHVDHGK**
**0**	**6**	**0.92**	**1.8**	**3.7**	**VNFTIDQIR**

5.205 31 777 Proteasome subunit alpha type-7-like protein [Crassostrea gigas]

Protein Accession	CL35094.Contig4$_H1$
Mean Expression Ratio	0.763
Median Expression Ratio	0.764
Credible Interval	(0.327, 1.77)
Associated Peptides	1
Associated Spectra	1
Coverage	NaN

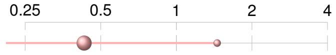

A	B	2.5	50	97.5	Sequence
**1**	**0**	**0.13**	**0.43**	**1.5**	**GDNLVVLGVEK**

5.206 365 544 PREDICTED: cofilin-2 [Otolemur garnettii]

Protein Accession	CL10440.Contig9$_H1$
Mean Expression Ratio	0.764
Median Expression Ratio	0.764
Credible Interval	(0.326, 1.78)
Associated Peptides	1
Associated Spectra	1
Coverage	NaN

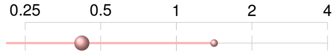

A	B	2.5	50	97.5	Sequence
**1**	**0**	**0.12**	**0.42**	**1.4**	**MLYASSK**

5.207 7073 7339 minus strand Collagen alpha-3(VI) chain [Crassostrea gigas]

Protein Accession	Unigene26547$_H1$
Mean Expression Ratio	0.763
Median Expression Ratio	0.764
Credible Interval	(0.325, 1.76)
Associated Peptides	1
Associated Spectra	1
Coverage	NaN

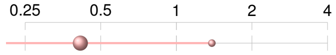

A	B	2.5	50	97.5	Sequence
**0**	**1**	**0.12**	**0.41**	**1.4**	**YDTPQQVLEAIATTR**

5.208 197 913 PREDICTED: l-xylulose reductase-like [Monodelphis domestica]; 197 913 PREDICTED: l-xylulose reductase-like [Monodelphis domestica]; 197 913 PREDICTED: l-xylulose reductase-like [Monodelphis domestica]; 197 913 PREDICTED: l-xylulose reductase-like [Monodelphis domestica]; 197 913 PREDICTED: l-xylulose reductase-like [Monodelphis domestica]; 197 913 PREDICTED: l-xylulose reductase-like [Monodelphis domestica]; 203 919 minus strand PREDICTED: l-xylulose reductase-like [Monodelphis domestica]; 200 916 PREDICTED: l-xylulose reductase-like [Monodelphis domestica]; 200 916 PREDICTED: l-xylulose reductase-like [Monodelphis domestica]; 203 919 minus strand PREDICTED: l-xylulose reductase-like [Monodelphis domestica]

Protein Accession	CL13128.Contig8$_{H}1$ CL13128.Contig7$_{H}1$ CL13128.Contig6$_{H}1$ CL13128.Contig5$_{H}1$ CL1312
Mean Expression Ratio	1.31
Median Expression Ratio	1.31
Credible Interval	(0.69, 2.46)
Associated Peptides	5
Associated Spectra	8
Coverage	NaN

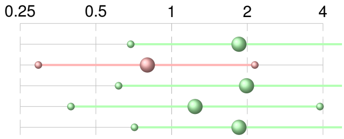

A	B	2.5	50	97.5	Sequence
1	1	0.69	1.9	5	MGTFAAEHPEGK
0	2	0.29	0.8	2.1	AIAEALYQHGAR
0	1	0.62	2	6.1	GGSIVNVSSISGK
0	1	0.4	1.2	3.9	VNSVNPTCVMTK
0	2	0.71	1.9	5	VVALSR

5.209 2764 3420 PREDICTED: filamin-C-like [Nasonia vitripennis]

Protein Accession	CL4145.Contig1$_{H}1$
Mean Expression Ratio	0.767
Median Expression Ratio	0.765
Credible Interval	(0.337, 1.72)
Associated Peptides	1
Associated Spectra	2
Coverage	NaN

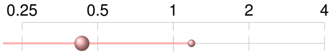

A	B	2.5	50	97.5	Sequence
1	1	0.16	0.43	1.2	GVDVPVNTIDNK

5.210 274 1242 minus strand cathepsin D [Pinctada maxima]; 290 1387 cathepsin D [Pinctada maxima]; 290 1402 minus strand cathepsin D [Pinctada maxima]; 290 1480 minus strand cathepsin D [Pinctada maxima]

Protein Accession	CL1722.Contig9$_{H}1$ CL1722.Contig2$_{H}1$ CL1722.Contig1$_{H}1$ CL1722.Contig10$_{H}1$
Mean Expression Ratio	1.31
Median Expression Ratio	1.31
Credible Interval	(0.62, 2.75)
Associated Peptides	2
Associated Spectra	5
Coverage	NaN

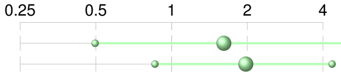

A	B	2.5	50	97.5	Sequence
0	1	0.5	1.6	5.4	VVFDTGSSNLWVPSKK
0	4	0.86	2	4.3	EGYWQIK

5.211 " 3475 4188 minus strand Heat shock 70 kDa protein cognate, putative [Pediculus humanus corporis] gi"

Protein Accession	CL5924.Contig14$_{H}1$
Mean Expression Ratio	0.769
Median Expression Ratio	0.767
Credible Interval	(0.375, 1.61)
Associated Peptides	2
Associated Spectra	5
Coverage	NaN

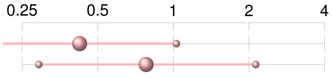

A	B	2.5	50	97.5	Sequence
1	2	0.18	0.42	1	STIEDEKLK
0	2	0.29	0.78	2.1	NSLESYAFHMK

5.212 18 1613 minus strand chaperonin containing T-complex polypeptide subunit zeta [Haliotis discus hannai]; 527 2089 minus strand chaperonin containing T-complex polypeptide subunit zeta [Haliotis discus hannai]; 18 1586 minus strand chaperonin containing T-complex polypeptide subunit zeta [Haliotis discus hannai]

Protein Accession	CL23722.Contig7$_{H}1$ CL23722.Contig5$_{H}1$ CL23722.Contig4$_{H}1$
Mean Expression Ratio	1.31
Median Expression Ratio	1.3
Credible Interval	(0.666, 2.58)
Associated Peptides	4
Associated Spectra	5
Coverage	NaN

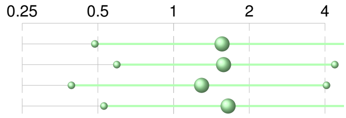

A	B	2.5	50	97.5	Sequence
0	1	0.49	1.6	5.1	AAQALAVNISAAK
0	2	0.59	1.6	4.4	GIDPFSLDMFAK
0	1	0.39	1.3	4	TDMDSTLVR
0	1	0.53	1.6	5.3	AQALAVNISAAK

5.213 442 2595 minus strand Amiloride-sensitive amine oxidase [copper-containing] [Crassostrea gigas]

Protein Accession	Unigene105$_{H}1$
Mean Expression Ratio	1.31
Median Expression Ratio	1.3
Credible Interval	(0.639, 2.7)
Associated Peptides	3
Associated Spectra	4
Coverage	NaN

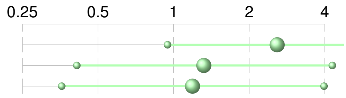

A	B	2.5	50	97.5	Sequence
1	1	0.94	2.6	7	QQVAVTVQKDEER
0	1	0.41	1.3	4.3	ILPTGMSPQLLK
0	1	0.36	1.2	4	VTDFVDSGALIGTHSK

5.214 " 2 1174 endo-1,3-beta-D-glucanase [Littorina sitkana]"

Protein Accession	CL31231.Contig3$_H$1
Mean Expression Ratio	1.3
Median Expression Ratio	1.3
Credible Interval	(0.713, 2.38)
Associated Peptides	4
Associated Spectra	22
Coverage	NaN

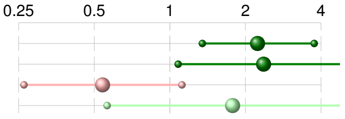

A	B	2.5	50	97.5	Sequence
5	7	1.3	2.2	3.8	FGTLEVR
1	3	1.1	2.4	5.3	SGEIDIMESR
5	0	0.26	0.54	1.1	AVIFR
0	1	0.56	1.8	5.5	NGMLPPVMSGK

5.215 151 1599 PREDICTED: galactosamine (N-acetyl)-6-sulfate sulfatase-like [Saccoglossus kowalevskii]

Protein Accession	CL13278.Contig2$_H$1
Mean Expression Ratio	1.3
Median Expression Ratio	1.3
Credible Interval	(0.605, 2.82)
Associated Peptides	2
Associated Spectra	3
Coverage	NaN

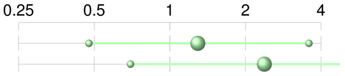

A	B	2.5	50	97.5	Sequence
0	2	0.48	1.3	3.6	GLVPSLISK
0	1	0.7	2.4	7.9	TTLWEGGTR

5.216 304 1830 minus strand Methenyltetrahydrofolate synthetase domain-containing protein [Crassostrea gigas]

Protein Accession	CL45489.Contig2$_H$1
Mean Expression Ratio	1.3
Median Expression Ratio	1.3
Credible Interval	(0.661, 2.58)
Associated Peptides	4
Associated Spectra	6
Coverage	NaN

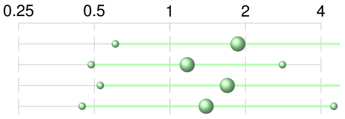

A	B	2.5	50	97.5	Sequence
0	1	0.61	1.9	6	KTLLVPTPR
0	3	0.48	1.2	2.8	FLTLEAK
0	1	0.53	1.7	5.3	TGLFNR
0	1	0.45	1.4	4.5	IPVLR

5.217 433 669 minus strand Superoxide dismutase [Cu-Zn] OS=Prionace glauca GN=sod1 PE=1 SV=1

Protein Accession	Unigene63976$_H$1
Mean Expression Ratio	0.767
Median Expression Ratio	0.77
Credible Interval	(0.382, 1.55)
Associated Peptides	1
Associated Spectra	11
Coverage	NaN

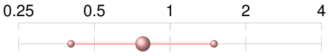

A	B	2.5	50	97.5	Sequence
6	5	0.4	0.78	1.5	SLVVHEK

5.218 92 1813 minus strand Dihydropyrimidinase [Crassostrea gigas]

Protein Accession	CL7386.Contig6$_{H}1$
Mean Expression Ratio	0.772
Median Expression Ratio	0.77
Credible Interval	(0.394, 1.53)
Associated Peptides	5
Associated Spectra	5
Coverage	NaN

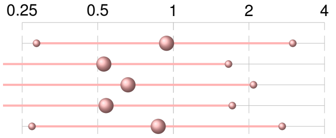

A	B	2.5	50	97.5	Sequence
1	0	0.28	0.94	3	SSADVISAAR
1	0	0.16	0.53	1.7	AENGQIIALK
1	0	0.2	0.66	2.1	LPVVR
1	0	0.17	0.54	1.7	IENGIIK
0	1	0.27	0.87	2.7	IAEGSDADVVVWDPEQAR

5.219 77 511 cytochrome c oxidase subunit VIIa polypeptide 2 like [Xenopus (Silurana) tropicalis]; 77 511 cytochrome c oxidase subunit VIIa polypeptide 2 like [Xenopus (Silurana) tropicalis]; 77 511 cytochrome c oxidase subunit VIIa polypeptide 2 like [Xenopus (Silurana) tropicalis]; 545 979 cytochrome c oxidase subunit VIIa polypeptide 2 like [Xenopus (Silurana) tropicalis]

Protein Accession	CL17601.Contig8$_{H}1$ CL17601.Contig7$_{H}1$ CL17601.Contig6$_{H}1$ CL17601.Contig2$_{H}1$
Mean Expression Ratio	0.771
Median Expression Ratio	0.77
Credible Interval	(0.385, 1.54)
Associated Peptides	3
Associated Spectra	8
Coverage	NaN

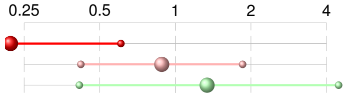

A	B	2.5	50	97.5	Sequence
2	0	0.083	0.22	0.61	EPSVIIFDSK
1	4	0.42	0.88	1.9	QDGLLVWQK
0	1	0.41	1.3	4.5	TTKPAVAPPAPMK

5.220 254 1093 uncharacterized protein LOC432187 precursor [Xenopus laevis] gi

Protein Accession	CL7800.Contig7$_{H}1$
Mean Expression Ratio	0.766
Median Expression Ratio	0.771
Credible Interval	(0.344, 1.65)
Associated Peptides	2
Associated Spectra	3
Coverage	NaN

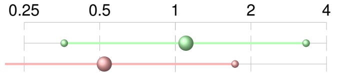

A	B	2.5	50	97.5	Sequence
1	1	0.36	1.1	3.3	GINFASTTR
1	0	0.16	0.52	1.7	LVTSK

5.221 227 1201 PREDICTED: quinone oxidoreductase-like isoform 1 [Ornithorhynchus anatinus]; 109 1083 PREDICTED: quinone oxidoreductase-like isoform 1 [Ornithorhynchus anatinus]; 164 1138 PREDICTED: quinone oxidoreductase-like isoform 1 [Ornithorhynchus anatinus]

Protein Accession	CL11899.Contig2$_{H}1$ CL11899.Contig1$_{H}1$ CL11899.Contig13$_{H}1$
Mean Expression Ratio	0.769
Median Expression Ratio	0.771
Credible Interval	(0.412, 1.42)
Associated Peptides	6
Associated Spectra	9
Coverage	NaN

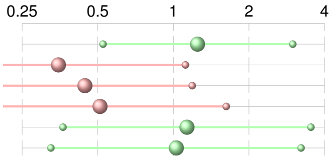

A	B	2.5	50	97.5	Sequence
1	2	0.52	1.2	3	VHASGVNPVDTYIR
1	0	0.11	0.35	1.1	VMGLR
0	2	0.16	0.44	1.2	VHQWGGPEVLK
0	1	0.16	0.51	1.6	EGLELVK
0	1	0.36	1.1	3.5	ILGTAGTKEGLELVK
0	1	0.32	1	3.2	LVLKL

5.222 " 2 778 Actin, gamma-enteric smooth muscle OS=Rattus norvegicus GN=Actg2 PE=2 SV=1"

Protein Accession	Unigene64184$_{H}1$
Mean Expression Ratio	0.769
Median Expression Ratio	0.771
Credible Interval	(0.332, 1.78)
Associated Peptides	1
Associated Spectra	1
Coverage	NaN

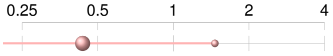

A	B	2.5	50	97.5	Sequence
1	0	0.13	0.44	1.5	EITSLAPSTMK

5.223 " 431 880 Myosin essential light chain, striated adductor muscle OS=Aequipecten irradians PE=1 SV=2"

Protein Accession	CL36.Contig2$_{H}1$
Mean Expression Ratio	0.776
Median Expression Ratio	0.772
Credible Interval	(0.408, 1.48)
Associated Peptides	4
Associated Spectra	22
Coverage	NaN

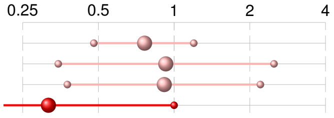

A	B	2.5	50	97.5	Sequence
7	9	0.48	0.76	1.2	VGDLLR
0	2	0.35	0.93	2.5	LSDEEVDQIMK
0	3	0.38	0.91	2.2	LTDITIDLEGNLK
0	1	0.099	0.32	1	VDQIMK

5.224 128 532 minus strand mitochondrial ATPase inhibitor [Ornithodoros parkeri]

Protein Accession	Unigene1376$_H1$
Mean Expression Ratio	1.3
Median Expression Ratio	1.29
Credible Interval	(0.678, 2.49)
Associated Peptides	4
Associated Spectra	9
Coverage	NaN

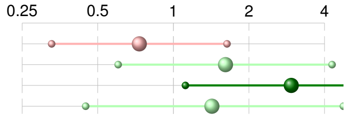

A	B	2.5	50	97.5	Sequence
4	0	0.33	0.73	1.6	EAGGAFGK
1	1	0.6	1.6	4.3	MEAAHEELYFK
0	2	1.1	3	8	KLEREQIAK
0	1	0.45	1.4	4.7	SGIGEFGSGAGK

5.225 73 837 minus strand Electron transfer flavoprotein subunit beta [Crassostrea gigas]; 73 837 minus strand Electron transfer flavoprotein subunit beta [Crassostrea gigas]

Protein Accession	CL33148.Contig2$_H1$ CL33148.Contig1$_H1$
Mean Expression Ratio	0.774
Median Expression Ratio	0.773
Credible Interval	(0.412, 1.46)
Associated Peptides	5
Associated Spectra	8
Coverage	NaN

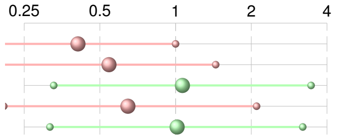

A	B	2.5	50	97.5	Sequence
2	1	0.17	0.41	1	AIHVDVDSK
1	1	0.2	0.54	1.4	EIDGGLENVLVK
0	1	0.33	1.1	3.5	HSMNPFDEIAVEEAVR
0	1	0.21	0.65	2.1	VLSVEEPPVR
0	1	0.32	1	3.2	YATLPNIMK

5.226 16 939 hypothetical protein BRAFLDRAFT_87838 [Branchiostoma floridae] gi

Protein Accession	CL38761.Contig1$_H1$
Mean Expression Ratio	0.769
Median Expression Ratio	0.773
Credible Interval	(0.344, 1.69)
Associated Peptides	1
Associated Spectra	4
Coverage	NaN

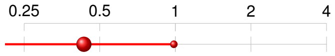

A	B	2.5	50	97.5	Sequence
4	0	0.19	0.43	0.98	ALLLR

5.227 " 166 705 minus strand ADP-ribosylation factor, partial [Antheraea yamamai]; 166 705 minus strand ADP-ribosylation factor, partial [Antheraea yamamai]; 166 705 minus strand ADP-ribosylation factor, partial [Antheraea yamamai]; 166 705 minus strand ADP-ribosylation factor, partial [Antheraea yamamai]; 166 705 minus strand ADP-ribosylation factor, partial [Antheraea yamamai]"

Protein Accession	Unigene39229$_{H}1$ Unigene39228$_{H}1$ Unigene39227$_{H}1$ Unigene39226$_{H}1$ Unigene39225$_{H}1$
Mean Expression Ratio	1.29
Median Expression Ratio	1.29
Credible Interval	(0.573, 2.97)
Associated Peptides	1
Associated Spectra	2
Coverage	NaN

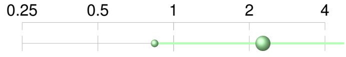

A	B	2.5	50	97.5	Sequence
0	2	0.84	2.3	6.2	IGEARDELQR

5.228 362 553 putative RNA-binding protein [Haliotis diversicolor]

Protein Accession	CL41007.Contig2$_{H}1$
Mean Expression Ratio	1.29
Median Expression Ratio	1.29
Credible Interval	(0.708, 2.36)
Associated Peptides	5
Associated Spectra	20
Coverage	NaN

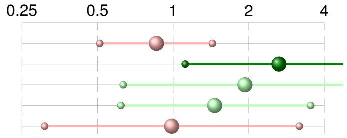

A	B	2.5	50	97.5	Sequence
4	8	0.51	0.86	1.4	EVMHVDSSFVGR
1	2	1.1	2.6	6.2	VRELQDETGCR
0	1	0.63	1.9	6.2	DGGAKEVMHVDSSFVGR
0	3	0.62	1.5	3.5	ELQDETGCR
0	1	0.31	0.99	3.2	DGGRDGGAKEVMHVDSSFVGR

5.229 95 2278 hypothetical protein CGI_10017740 [Crassostrea gigas]

Protein Accession	CL48637.Contig1$_{H}1$
Mean Expression Ratio	0.776
Median Expression Ratio	0.776
Credible Interval	(0.37, 1.63)
Associated Peptides	3
Associated Spectra	3
Coverage	NaN

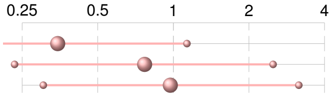

A	B	2.5	50	97.5	Sequence
1	0	0.11	0.35	1.1	VAMYR
0	1	0.23	0.77	2.5	SISYALR
0	1	0.3	0.97	3.2	AGLELVPK

5.230 27 1682 minus strand PREDICTED: nucleolar protein 56 [Oreochromis niloticus]

Protein Accession	Unigene26699$_{H}1$
Mean Expression Ratio	1.29
Median Expression Ratio	1.29
Credible Interval	(0.761, 2.15)
Associated Peptides	8
Associated Spectra	21
Coverage	NaN

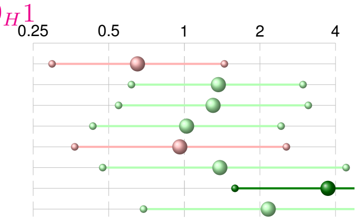

A	B	2.5	50	97.5	Sequence
2	2	0.3	0.65	1.4	LDGLGQAELQK
1	3	0.62	1.4	3	VTPLKLDGLGQAELQK
1	2	0.55	1.3	3.1	KLIMQK
1	2	0.43	1	2.4	LIAHAGSLTNLAK
0	2	0.37	0.96	2.5	VATLEEEVYDWEIK
0	1	0.47	1.4	4.4	VQAIFDSSK
0	3	1.6	3.7	8.7	LVKDLHSK
0	1	0.69	2.2	6.8	KGLGGLSTEKK

5.231 44 910 PREDICTED: probable ATP-dependent RNA helicase DDX10 [Cricetulus griseus] gi

Protein Accession	CL33109.Contig1$_{H}1$
Mean Expression Ratio	1.28
Median Expression Ratio	1.29
Credible Interval	(0.589, 2.82)
Associated Peptides	2
Associated Spectra	2
Coverage	NaN

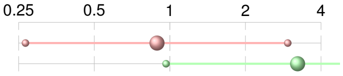

A	B	2.5	50	97.5	Sequence
1	0	0.27	0.89	2.9	GIDVTK
1	0	0.97	3.2	11	AKYLK

5.232 254 1093 uncharacterized protein LOC432187 precursor [Xenopus laevis] gi; 343 561 minus strand cathepsin Z precursor [Danio rerio] gi

Protein Accession	CL7800.Contig7$_{H}1$ CL7800.Contig1$_{H}1$
Mean Expression Ratio	1.29
Median Expression Ratio	1.29
Credible Interval	(0.567, 2.95)
Associated Peptides	1
Associated Spectra	2
Coverage	NaN

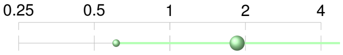

A	B	2.5	50	97.5	Sequence
1	1	0.61	1.8	5.7	GINFASTTR

5.233 232 1401 RecName: Full=Guanine nucleotide-binding protein G(s) subunit alpha; AltName: Full=Adenylate cyclase-stimulating G alpha protein gi|9635|emb|CAA78808.1| GTP-binding protein alpha-s subunit [Lymnaea stagnalis]; 232 1356 RecName: Full=Guanine nucleotide-binding protein G(s) subunit alpha; AltName: Full=Adenylate cyclase-stimulating G alpha protein gi|9635|emb|CAA78808.1| GTP-binding protein alpha-s subunit [Lymnaea stagnalis]

Protein Accession	CL8653.Contig3$_{H}1$ CL8653.Contig1$_{H}1$
Mean Expression Ratio	1.29
Median Expression Ratio	1.29
Credible Interval	(0.631, 2.63)
Associated Peptides	3
Associated Spectra	5
Coverage	NaN

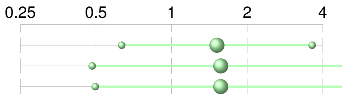

A	B	2.5	50	97.5	Sequence
0	3	0.63	1.5	3.6	LLLLGAGESGK
0	1	0.48	1.6	5	QKIEDIK
0	1	0.5	1.6	5.2	EQDILR

5.234 333 1799 Alba-like protein C9orf23-like protein [Crassostrea gigas]

Protein Accession	Unigene30413$_{H}1$
Mean Expression Ratio	1.28
Median Expression Ratio	1.29
Credible Interval	(0.691, 2.35)
Associated Peptides	6
Associated Spectra	9
Coverage	NaN

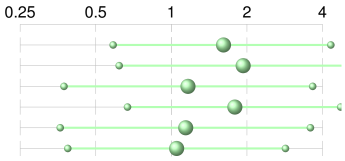

A	B	2.5	50	97.5	Sequence
0	2	0.59	1.6	4.3	AASWLGMR
0	1	0.62	1.9	6.2	GKGALELAQR
0	1	0.37	1.2	3.6	SPLYVDEGNILMDVR
0	2	0.67	1.8	4.7	TINPATEAETQGK
0	1	0.36	1.1	3.6	EIMTELSR
0	2	0.39	1	2.8	ELNDFMTK

5.235 57 2378 minus strand Heterogeneous nuclear ribonucleoprotein U-like protein 1 [Crassostrea gigas]

Protein Accession	CL15560.Contig5$_{H}1$
Mean Expression Ratio	1.28
Median Expression Ratio	1.29
Credible Interval	(0.612, 2.68)
Associated Peptides	3
Associated Spectra	3
Coverage	NaN

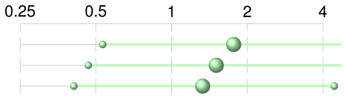

A	B	2.5	50	97.5	Sequence
0	1	0.53	1.8	5.8	VTELRDELK
0	1	0.47	1.5	5	LRDELK
0	1	0.41	1.3	4.4	RSRSPPPRKVE

5.236 313 1908 minus strand Heterogeneous nuclear ribonucleoprotein L [Crassostrea gigas]

Protein Accession	CL19412.Contig8$_H$1
Mean Expression Ratio	1.28
Median Expression Ratio	1.29
Credible Interval	(0.613, 2.71)
Associated Peptides	3
Associated Spectra	4
Coverage	NaN

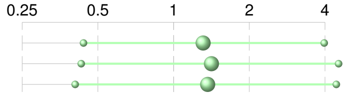

A	B	2.5	50	97.5	Sequence
1	1	0.44	1.3	4	SSTGLLEFESK
0	1	0.43	1.4	4.5	GQPLLQEPR
0	1	0.41	1.4	4.4	TPFIFK

5.237 136 675 minus strand predicted protein [Nematostella vectensis] gi

Protein Accession	CL17411.Contig1$_H$1
Mean Expression Ratio	1.29
Median Expression Ratio	1.28
Credible Interval	(0.625, 2.62)
Associated Peptides	3
Associated Spectra	4
Coverage	NaN

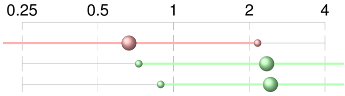

A	B	2.5	50	97.5	Sequence
1	0	0.2	0.67	2.2	MLNEDELR
0	1	0.72	2.4	7.5	MLNEDELRDAK
0	2	0.89	2.4	6.6	VSEARDELQR

5.238 " 116 1168 minus strand hypothetical protein CGI_10014553, partial [Crassostrea gigas]"

Protein Accession	CL19215.Contig5$_H$1
Mean Expression Ratio	0.783
Median Expression Ratio	0.78
Credible Interval	(0.394, 1.57)
Associated Peptides	4
Associated Spectra	6
Coverage	NaN

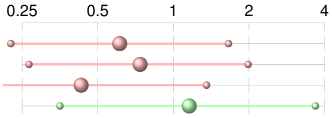

A	B	2.5	50	97.5	Sequence
1	1	0.22	0.61	1.7	FGPTTSEFEK
1	1	0.27	0.74	2	SVIALGEGAGANIVAR
1	0	0.14	0.43	1.4	VLEER
0	1	0.35	1.2	3.7	AQNKDQLQK

5.239 79 2202 minus strand PREDICTED: dolichyl-diphosphooligosaccharide–protein glycosyltransferase subunit STT3A-like isoform 2 [Strongylocentrotus purpuratus]; 79 2202 PREDICTED: dolichyl-diphosphooligosaccharide–protein glycosyltransferase subunit STT3A-like isoform 2 [Strongylocentrotus purpuratus]; 79 2202 PREDICTED: dolichyl-diphosphooligosaccharide–protein glycosyltransferase subunit STT3A-like isoform 2 [Strongylocentrotus purpuratus]

Protein Accession	CL5867.Contig2$_H$1 CL5867.Contig11$_H$1 CL5867.Contig10$_H$1
Mean Expression Ratio	0.775
Median Expression Ratio	0.781
Credible Interval	(0.331, 1.79)
Associated Peptides	1
Associated Spectra	1
Coverage	NaN

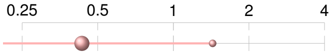

A	B	2.5	50	97.5	Sequence
1	0	0.13	0.43	1.4	VGQAMASTEEK

5.240 604 1236 minus strand selenium-dependent glutathione peroxidase [Crassostrea gigas]; 138 770 minus strand selenium-dependent glutathione peroxidase [Crassostrea gigas]; 138 770 minus strand selenium-dependent glutathione peroxidase [Crassostrea gigas]; 147 779 minus strand selenium-dependent glutathione peroxidase [Crassostrea gigas]; 149 781 minus strand selenium-dependent glutathione peroxidase [Crassostrea gigas]; 144 776 minus strand selenium-dependent glutathione peroxidase [Crassostrea gigas]; 141 773 minus strand selenium-dependent glutathione peroxidase [Crassostrea gigas]

Protein Accession	CL12854.Contig7$_H$1 CL12854.Contig20$_H$1 CL12854.Contig19$_H$1 CL12854.Contig18$_H$1 CL1
Mean Expression Ratio	1.28
Median Expression Ratio	1.28
Credible Interval	(0.674, 2.43)
Associated Peptides	4
Associated Spectra	10
Coverage	NaN

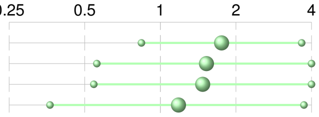

A	B	2.5	50	97.5	Sequence
3	2	0.84	1.7	3.6	SGVQGLSVPR
0	2	0.56	1.5	4	ALGLQDELGPLLEGR
0	2	0.54	1.5	4	FLVGQDGLPVAR
0	1	0.36	1.2	3.7	SDVNGQNELPLYR

5.241 412 708 minus strand heat shock protein beta-1 [Trichinella spiralis] gi; 459 755 minus strand heat shock protein beta-1 [Trichinella spiralis] gi; 464 763 minus strand heat shock protein beta-1 [Trichinella spiralis] gi; 437 736 minus strand heat shock protein beta-1 [Trichinella spiralis] gi; 437 736 minus strand heat shock protein beta-1 [Trichinella spiralis] gi; 437 736 minus strand heat shock protein beta-1 [Trichinella spiralis] gi

Protein Accession	CL17234.Contig6$_{H}1$ CL17234.Contig5$_{H}1$ CL17234.Contig4$_{H}1$ CL17234.Contig3$_{H}1$ CL1723
Mean Expression Ratio	0.779
Median Expression Ratio	0.781
Credible Interval	(0.335, 1.75)
Associated Peptides	1
Associated Spectra	1
Coverage	NaN

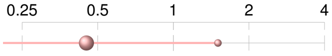

A	B	2.5	50	97.5	Sequence
**1**	**0**	**0.14**	**0.45**	**1.5**	**TVTSSLNADGVLTLK**

5.242 56 937 Chloride intracellular channel exc-4 [Crassostrea gigas]

Protein Accession	CL34620.Contig3$_{H}1$
Mean Expression Ratio	1.29
Median Expression Ratio	1.28
Credible Interval	(0.603, 2.77)
Associated Peptides	2
Associated Spectra	4
Coverage	NaN

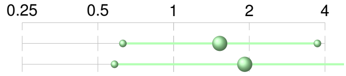

A	B	2.5	50	97.5	Sequence
**1**	**2**	**0.63**	**1.5**	**3.7**	**KVQGPLVK**
**0**	**1**	**0.58**	**1.9**	**6.1**	**QKFELYIK**

5.243 105 503 Transgelin-2 [Crassostrea gigas]

Protein Accession	CL445.Contig2$_{H}1$
Mean Expression Ratio	0.779
Median Expression Ratio	0.782
Credible Interval	(0.368, 1.64)
Associated Peptides	2
Associated Spectra	4
Coverage	NaN

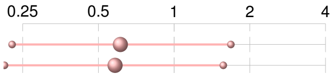

A	B	2.5	50	97.5	Sequence
**1**	**1**	**0.23**	**0.61**	**1.7**	**SGLGLEIDK**
**2**	**0**	**0.21**	**0.58**	**1.6**	**SGLGLEIDKK**

5.244 " 95 1279 2-amino-3-ketobutyrate coenzyme A ligase, mitochondrial, partial [Crassostrea gigas]"

Protein Accession	CL33712.Contig1$_H1$
Mean Expression Ratio	1.28
Median Expression Ratio	1.28
Credible Interval	(0.609, 2.68)
Associated Peptides	3
Associated Spectra	3
Coverage	NaN

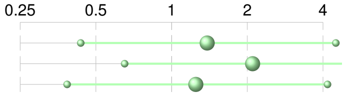

A	B	2.5	50	97.5	Sequence
0	1	0.43	1.4	4.5	GTEEHFNIMGR
0	1	0.65	2.1	6.8	VIQSPQGPSIR
0	1	0.39	1.2	4.2	LAATLR

5.245 31 375 minus strand 40S ribosomal protein S19 OS=Mya arenaria GN=RPS19 PE=2 SV=1

Protein Accession	CL8932.Contig2$_H1$
Mean Expression Ratio	1.28
Median Expression Ratio	1.28
Credible Interval	(0.588, 2.78)
Associated Peptides	2
Associated Spectra	2
Coverage	NaN

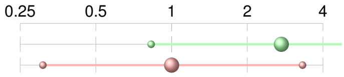

A	B	2.5	50	97.5	Sequence
0	1	0.83	2.7	9	GISVKDVNGHEFTK
0	1	0.31	1	3.3	ALAAFLK

5.246 801 971 RecName: Full=Tropomyosin-2; AltName: Full=Tropomyosin II; Short=BgTMII; Short=TMII gi|155940|gb|AAA27816.1| tropomyosin [Biomphalaria glabrata]

Protein Accession	CL2925.Contig4$_H1$
Mean Expression Ratio	1.28
Median Expression Ratio	1.28
Credible Interval	(0.561, 2.99)
Associated Peptides	1
Associated Spectra	1
Coverage	NaN

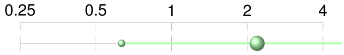

A	B	2.5	50	97.5	Sequence
0	1	0.63	2.2	7.5	ASQREDSYEETIRDLTQR

5.247 151 471 minus strand ribosomal protein rps25 [Arenicola marina]

Protein Accession	CL17623.Contig4$_H1$
Mean Expression Ratio	1.28
Median Expression Ratio	1.28
Credible Interval	(0.614, 2.7)
Associated Peptides	2
Associated Spectra	4
Coverage	NaN

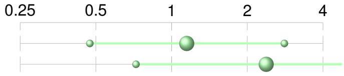

A	B	2.5	50	97.5	Sequence
1	2	0.47	1.1	2.8	LITPSVVSER
0	1	0.72	2.4	7.7	MLKEVPSYK

5.248 246 887 rab11-2 [Limulus polyphemus]

Protein Accession	CL3557.Contig1$_{H}$1
Mean Expression Ratio	0.786
Median Expression Ratio	0.784
Credible Interval	(0.391, 1.59)
Associated Peptides	4
Associated Spectra	4
Coverage	NaN

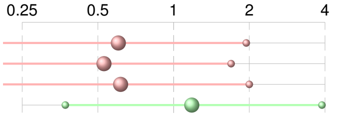

A	B	2.5	50	97.5	Sequence
1	0	0.19	0.6	1.9	NEFNLESK
1	0	0.16	0.53	1.7	GAVGALLVYDIAK
0	1	0.19	0.62	2	AITSAYYR
0	1	0.37	1.2	3.9	VVLIGDSGVGK

5.249 90 344 PREDICTED: diazepam binding inhibitor-like [Saccoglossus kowalevskii]

Protein Accession	CL28509.Contig1$_{H}$1
Mean Expression Ratio	1.27
Median Expression Ratio	1.27
Credible Interval	(0.685, 2.37)
Associated Peptides	6
Associated Spectra	10
Coverage	NaN

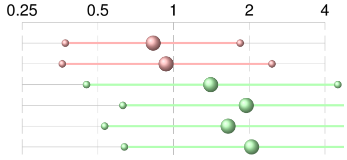

A	B	2.5	50	97.5	Sequence
1	3	0.37	0.83	1.8	AEYVLLVK
0	2	0.36	0.93	2.5	ADEAFNQAAEDVK
0	1	0.45	1.4	4.5	WNAWNTLK
0	1	0.63	1.9	6.2	VYGLFK
0	1	0.53	1.7	5.3	KPTDDEMLK
0	1	0.64	2	6.4	ADEAFNQAAEDVKK

5.250 102 1685 minus strand predicted protein [Nematostella vectensis] gi

Protein Accession	CL44087.Contig1$_{H}$1
Mean Expression Ratio	1.27
Median Expression Ratio	1.27
Credible Interval	(0.629, 2.59)
Associated Peptides	4
Associated Spectra	4
Coverage	NaN

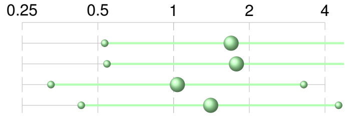

A	B	2.5	50	97.5	Sequence
0	1	0.53	1.7	5.6	IGGNMGLAMK
0	1	0.54	1.8	5.7	LNPQTAFFQGK
0	1	0.33	1	3.3	ADTTISMSDADLLSLMSGK
0	1	0.43	1.4	4.5	GVFLFK

5.251 " 158 355 Protein NAS-37, isoform b [Caenorhabditis elegans] gi; 15 227 Hemicentin-1 [Crassostrea gigas]"

Protein Accession	Unigene57180$_{H}$1 CL8016.Contig2$_{H}$1
Mean Expression Ratio	1.28
Median Expression Ratio	1.27
Credible Interval	(0.554, 3.01)
Associated Peptides	1
Associated Spectra	1
Coverage	NaN

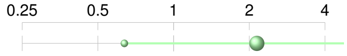

A	B	2.5	50	97.5	Sequence
0	1	0.64	2.1	7.2	FKGSYGGVSR

5.252 244 837 PREDICTED: ras-like GTP-binding protein Rho1-like [Megachile rotundata]

Protein Accession	Unigene11792$_H$1
Mean Expression Ratio	1.27
Median Expression Ratio	1.27
Credible Interval	(0.577, 2.78)
Associated Peptides	2
Associated Spectra	3
Coverage	NaN

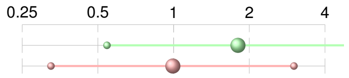

A	B	2.5	50	97.5	Sequence
**0**	**1**	**0.54**	**1.8**	**5.9**	**EGVREVFETATR**
**1**	**1**	**0.32**	**1**	**3**	**EVFETATR**

5.253 170 1375 PREDICTED: sialate O-acetylesterase-like [Oreochromis niloticus]

Protein Accession	CL26143.Contig1$_H$1
Mean Expression Ratio	0.783
Median Expression Ratio	0.787
Credible Interval	(0.35, 1.74)
Associated Peptides	1
Associated Spectra	2
Coverage	NaN

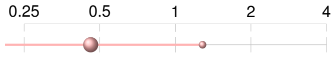

A	B	2.5	50	97.5	Sequence
**2**	**0**	**0.17**	**0.46**	**1.3**	**DKLDVAQR**

5.254 613 846 cytochrome c oxidase subunit Va [Antheraea yamamai] gi

Protein Accession	Unigene3832$_H$1
Mean Expression Ratio	1.27
Median Expression Ratio	1.27
Credible Interval	(0.643, 2.59)
Associated Peptides	4
Associated Spectra	5
Coverage	NaN

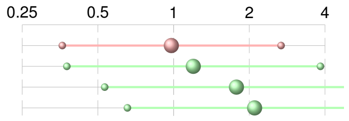

A	B	2.5	50	97.5	Sequence
**0**	**2**	**0.36**	**0.98**	**2.7**	**AMNDLQGEDLVPEPK**
**0**	**1**	**0.38**	**1.2**	**3.8**	**YETFFSRPDIDGWEIR**
**0**	**1**	**0.53**	**1.8**	**5.9**	**KAMNDLQGEDLVPEPK**
**0**	**1**	**0.65**	**2.1**	**6.6**	**YETFFSRPDIDGWEIRK**

5.255 235 1038 Translocon-associated protein subunit alpha [Crassostrea gigas]

Protein Accession	CL36450.Contig6$_H$1
Mean Expression Ratio	0.786
Median Expression Ratio	0.788
Credible Interval	(0.35, 1.82)
Associated Peptides	1
Associated Spectra	2
Coverage	NaN

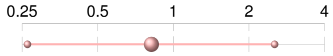

A	B	2.5	50	97.5	Sequence
**1**	**1**	**0.26**	**0.82**	**2.5**	**ELPAGKPVR**

5.256 27 377 minus strand ribosomal protein LP2 [Papilio xuthus] gi; 27 377 minus strand ribosomal protein LP2 [Papilio xuthus] gi; 27 377 minus strand ribosomal protein LP2 [Papilio xuthus] gi; 1538 1891 minus strand ribosomal protein LP2 [Papilio xuthus] gi

Protein Accession	Unigene53017$_{H}1$ Unigene53016$_{H}1$ Unigene53015$_{H}1$ CL49012.Contig9$_{H}1$
Mean Expression Ratio	1.27
Median Expression Ratio	1.27
Credible Interval	(0.746, 2.14)
Associated Peptides	7
Associated Spectra	29
Coverage	NaN

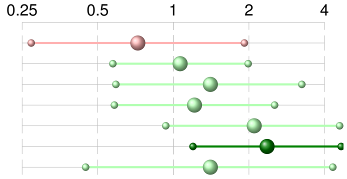

A	B	2.5	50	97.5	Sequence
1	1	0.27	0.72	1.9	ILSSVGIEAEADK
3	5	0.58	1.1	2	KVVSQLSGK
1	2	0.59	1.4	3.3	SIDQLIAEGSR
1	4	0.58	1.2	2.5	VVSQLSGK
0	4	0.93	2.1	4.6	ILSSVGIEAEADKVK
0	6	1.2	2.4	4.7	SGKEPTAADLEK
0	1	0.45	1.4	4.3	SVGIEAEADKVK

5.257 3 1460 Myosin-VI [Crassostrea gigas]

Protein Accession	CL6618.Contig5$_{H}1$
Mean Expression Ratio	0.787
Median Expression Ratio	0.789
Credible Interval	(0.342, 1.78)
Associated Peptides	1
Associated Spectra	1
Coverage	NaN

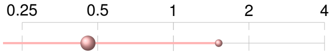

A	B	2.5	50	97.5	Sequence
1	0	0.14	0.46	1.5	LGIAK

5.258 430 759 hypothetical protein [Amblyomma maculatum]; 336 665 hypothetical protein [Amblyomma maculatum]

Protein Accession	CL4062.Contig3$_{H}1$ CL4062.Contig2$_{H}1$
Mean Expression Ratio	1.27
Median Expression Ratio	1.27
Credible Interval	(0.709, 2.3)
Associated Peptides	6
Associated Spectra	11
Coverage	NaN

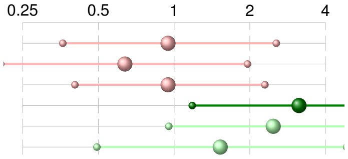

A	B	2.5	50	97.5	Sequence
1	1	0.36	0.95	2.6	KYIPGTK
1	0	0.2	0.64	2	MVFAGIK
0	3	0.4	0.95	2.3	ETLFEYLADPK
0	2	1.2	3.1	8.3	ETLFEYLADPKK
0	2	0.95	2.5	6.7	MVFAGIKKPQER
0	1	0.49	1.5	4.9	ADIPDGDFEK

5.259 82 1449 hypothetical protein BRAFLDRAFT_127120 [Branchiostoma floridae] gi

Protein Accession	CL41883.Contig1$_H$1
Mean Expression Ratio	1.27
Median Expression Ratio	1.27
Credible Interval	(0.563, 2.84)
Associated Peptides	1
Associated Spectra	3
Coverage	NaN

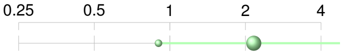

A	B	2.5	50	97.5	Sequence
0	3	0.9	2.2	5.3	ILQEQEDKFLK

5.260 25 405 minus strand Elongation factor 1-delta [Anoplopoma fimbria]

Protein Accession	CL10502.Contig1$_H$1
Mean Expression Ratio	1.27
Median Expression Ratio	1.27
Credible Interval	(0.669, 2.39)
Associated Peptides	5
Associated Spectra	8
Coverage	NaN

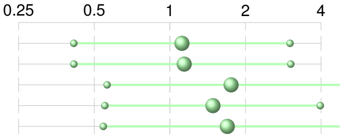

A	B	2.5	50	97.5	Sequence
1	1	0.42	1.1	3	LVPVGYGINK
1	1	0.42	1.1	3	KPVLIAK
0	1	0.56	1.8	5.4	SKKPVLIAK
0	2	0.55	1.5	4	VQTDGLVWGASK
0	1	0.54	1.7	5.3	RVQTDGLVWGASK

5.261 101 1693 CALNUC [Spodoptera frugiperda]; 141 1325 Nucleobindin-2 [Crassostrea gigas]; 141 1289 Nucleobindin-2 [Crassostrea gigas]

Protein Accession	CL1888.Contig9$_H$1 CL1888.Contig6$_H$1 CL1888.Contig5$_H$1
Mean Expression Ratio	1.27
Median Expression Ratio	1.27
Credible Interval	(0.612, 2.68)
Associated Peptides	3
Associated Spectra	3
Coverage	NaN

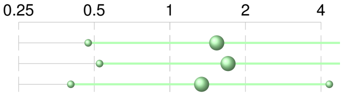

A	B	2.5	50	97.5	Sequence
0	1	0.47	1.5	5	MDIPGHLDLR
0	1	0.53	1.7	5.7	LKELPEVER
0	1	0.4	1.3	4.3	LETANISDIK

5.262 813 1244 Probable protein disulfide-isomerase A6 OS=Caenorhabditis elegans GN=tag-320 PE=2 SV=1

Protein Accession	CL39540.Contig4$_H$1
Mean Expression Ratio	0.791
Median Expression Ratio	0.79
Credible Interval	(0.345, 1.77)
Associated Peptides	1
Associated Spectra	3
Coverage	NaN

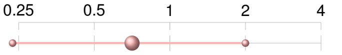

A	B	2.5	50	97.5	Sequence
1	2	0.24	0.7	2	TASDIINWATEK

5.263 " 75 803 hypothetical protein DAPPUDRAFT_231350 [Daphnia pulex]; 82 612 ATP synthase B chain, putative [Ixodes scapularis] gi"

Protein Accession	CL10537.Contig4$_H1$ CL10537.Contig13$_H1$
Mean Expression Ratio	0.789
Median Expression Ratio	0.79
Credible Interval	(0.336, 1.81)
Associated Peptides	1
Associated Spectra	3
Coverage	NaN

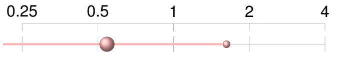

A	B	2.5	50	97.5	Sequence
1	2	0.18	0.54	1.6	SITPQQEAESIK

5.264 52 1599 minus strand ATP synthase beta subunit [Pinctada fucata]

Protein Accession	CL34251.Contig5$_H1$
Mean Expression Ratio	1.27
Median Expression Ratio	1.27
Credible Interval	(0.832, 1.91)
Associated Peptides	15
Associated Spectra	45
Coverage	NaN

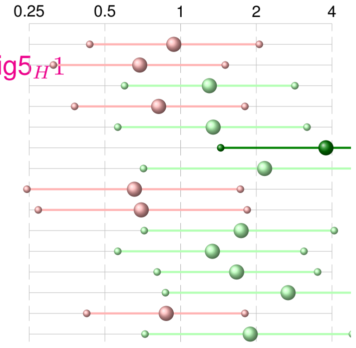

A	B	2.5	50	97.5	Sequence
1	3	0.43	0.94	2.1	EAAKPAAAGNLAVGR
2	2	0.31	0.69	1.5	VISLTDDTSK
1	3	0.6	1.3	2.8	VSLVYGQMNEPPGAR
2	2	0.38	0.82	1.8	IPVGPK
0	3	0.56	1.4	3.2	AHGGYSVFAGVGER
0	2	1.4	3.8	9.8	AKEAAKPAAAGNLAVGR
0	1	0.71	2.2	6.5	GQACKDTGTSIRIPVGPK
0	2	0.24	0.65	1.7	IGLFGGAGVGK
0	2	0.27	0.7	1.8	IINVIGEPIDER
0	3	0.72	1.7	4.1	ILDPNVVGMEHYK
0	3	0.56	1.3	3.1	TIAMDGTEGLVR
0	5	0.81	1.7	3.5	VVDMLAPYAR
0	1	0.87	2.7	8.2	DTGTSIRIPVGPK
0	5	0.42	0.88	1.8	ILQDFK
0	2	0.72	1.9	4.8	TREGNDLYHEMITTK

5.265 33 422 minus strand PREDICTED: heat shock 70 kDa protein cognate 4-like [Megachile rotundata]

Protein Accession	CL15750.Contig1$_H1$
Mean Expression Ratio	0.794
Median Expression Ratio	0.791
Credible Interval	(0.34, 1.87)
Associated Peptides	1
Associated Spectra	1
Coverage	NaN

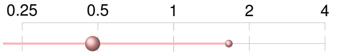

A	B	2.5	50	97.5	Sequence
1	0	0.14	0.48	1.7	VVNEGDKPK

5.266 78 2003 Chromatin assembly factor 1 subunit A [Crassostrea gigas]

Protein Accession	CL14511.Contig5$_{H}1$
Mean Expression Ratio	1.27
Median Expression Ratio	1.26
Credible Interval	(0.549, 2.95)
Associated Peptides	1
Associated Spectra	1
Coverage	NaN

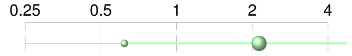

A	B	2.5	50	97.5	Sequence
**1**	**0**	**0.62**	**2.1**	**6.9**	**IAKELK**

5.267 166 2364 Dipeptidyl-peptidase 3 [Crassostrea gigas]

Protein Accession	CL9071.Contig2$_{H}1$
Mean Expression Ratio	0.793
Median Expression Ratio	0.791
Credible Interval	(0.377, 1.65)
Associated Peptides	3
Associated Spectra	3
Coverage	NaN

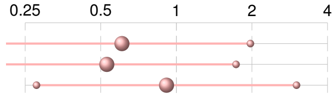

A	B	2.5	50	97.5	Sequence
**0**	**1**	**0.19**	**0.61**	**2**	**DAEIAVEFMTSK**
**0**	**1**	**0.16**	**0.53**	**1.7**	**FLSLR**
**0**	**1**	**0.28**	**0.91**	**3**	**VETIILK**

5.268 212 1783 PREDICTED: d-3-phosphoglycerate dehydrogenase-like [Strongylocentrotus purpuratus]

Protein Accession	CL34337.Contig3$_{H}1$
Mean Expression Ratio	1.27
Median Expression Ratio	1.26
Credible Interval	(0.608, 2.67)
Associated Peptides	3
Associated Spectra	3
Coverage	NaN

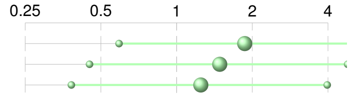

A	B	2.5	50	97.5	Sequence
**0**	**1**	**0.59**	**1.9**	**6.2**	**GGIIDEDALLR**
**0**	**1**	**0.45**	**1.5**	**4.8**	**VTSTPHLGANTEEAQSR**
**0**	**1**	**0.38**	**1.3**	**4**	**LSASLFAGYLR**

5.269 " 347 847 minus strand ADP,ATP carrier protein 1 OS=Anopheles gambiae GN=AGAP006782 PE=2 SV=2"

Protein Accession	CL27196.Contig1$_{H}1$
Mean Expression Ratio	0.79
Median Expression Ratio	0.792
Credible Interval	(0.334, 1.8)
Associated Peptides	1
Associated Spectra	1
Coverage	NaN

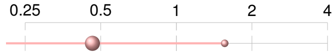

A	B	2.5	50	97.5	Sequence
**1**	**0**	**0.14**	**0.46**	**1.6**	**SNPGESYAVK**

5.270 195 2201 minus strand hypothetical protein DAPPUDRAFT_315656 [Daphnia pulex]

Protein Accession	Unigene38538$_H$1
Mean Expression Ratio	0.79
Median Expression Ratio	0.792
Credible Interval	(0.444, 1.4)
Associated Peptides	7
Associated Spectra	12
Coverage	NaN

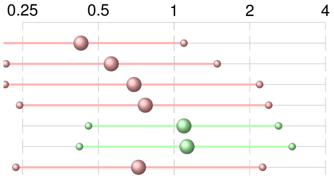

A	B	2.5	50	97.5	Sequence
2	0	0.16	0.43	1.1	GPQFEEIGK
1	1	0.21	0.56	1.5	VQIEAGAGEEAK
1	0	0.21	0.69	2.2	EIFPNER
1	0	0.24	0.77	2.4	NMGAIVR
1	2	0.46	1.1	2.6	GSIVLHDGK
0	2	0.42	1.1	2.9	VALSPAATQALVK
0	1	0.24	0.72	2.2	ISGITAVGGMLLMG

5.271 " 131 544 Histone H2B, gonadal OS=Patella granatina PE=1 SV=2"

Protein Accession	CL29794.Contig2$_H$1
Mean Expression Ratio	1.26
Median Expression Ratio	1.26
Credible Interval	(0.759, 2.11)
Associated Peptides	5
Associated Spectra	49
Coverage	NaN

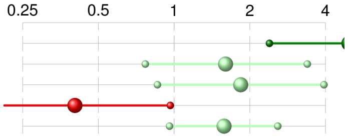

A	B	2.5	50	97.5	Sequence
3	3	2.4	5	10	AVTKYTSSK
5	0	0.77	1.6	3.4	EIQTAVR
3	2	0.86	1.8	4	STITSREIQTAVR
3	0	0.17	0.4	0.97	LIFER
9	21	0.96	1.6	2.6	LLLPGELAK

5.272 112 1050 Enolase-phosphatase E1 [Crassostrea gigas]; 112 1050 Enolase-phosphatase E1 [Crassostrea gigas]

Protein Accession	CL25175.Contig3$_H$1 CL25175.Contig2$_H$1
Mean Expression Ratio	1.26
Median Expression Ratio	1.26
Credible Interval	(0.602, 2.64)
Associated Peptides	2
Associated Spectra	5
Coverage	NaN

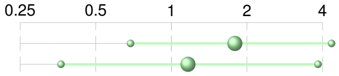

A	B	2.5	50	97.5	Sequence
2	2	0.69	1.8	4.4	SAAEAESLLVGVK
0	1	0.36	1.2	3.8	QLQGHIWR

5.273 132 932 pyruvate kinase [Crassostrea gigas]

Protein Accession	CL31920.Contig1$_H$1
Mean Expression Ratio	1.27
Median Expression Ratio	1.26
Credible Interval	(0.569, 2.87)
Associated Peptides	1
Associated Spectra	3
Coverage	NaN

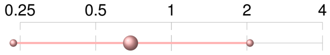

A	B	2.5	50	97.5	Sequence
2	1	0.23	0.69	2.1	ITIDDSYKEK

5.274 187 1323 minus strand gelsolin [Aplysia californica] gi

Protein Accession	CL10220.Contig1$_H$1
Mean Expression Ratio	1.26
Median Expression Ratio	1.26
Credible Interval	(0.62, 2.58)
Associated Peptides	3
Associated Spectra	5
Coverage	NaN

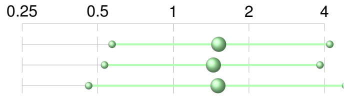

A	B	2.5	50	97.5	Sequence
**0**	**2**	**0.57**	**1.5**	**4.2**	**AAQVLQDLK**
**0**	**2**	**0.53**	**1.4**	**3.8**	**LSDASGELDFK**
**0**	**1**	**0.46**	**1.5**	**4.8**	**SDVETIDEEEGGAALEK**

5.275 2 226 minus strand hypothetical protein CGI_10015501 [Crassostrea gigas]

Protein Accession	Unigene73065$_H$1
Mean Expression Ratio	1.26
Median Expression Ratio	1.26
Credible Interval	(0.625, 2.53)
Associated Peptides	2
Associated Spectra	11
Coverage	NaN

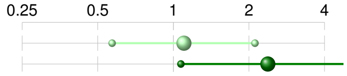

A	B	2.5	50	97.5	Sequence
**0**	**7**	**0.57**	**1.1**	**2.1**	**SSVAADALR**
**0**	**4**	**1.1**	**2.4**	**5.4**	**SSVAADALRK**

5.276 6 581 PREDICTED: mammalian ependymin-related protein 1 [Gallus gallus]

Protein Accession	CL15402.Contig1$_H$1
Mean Expression Ratio	1.26
Median Expression Ratio	1.26
Credible Interval	(0.597, 2.71)
Associated Peptides	2
Associated Spectra	4
Coverage	NaN

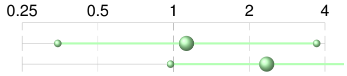

A	B	2.5	50	97.5	Sequence
**0**	**1**	**0.35**	**1.1**	**3.7**	**ALFEEQNVITPGR**
**0**	**3**	**0.97**	**2.3**	**5.6**	**WYISYDALNK**

5.277 2 421 hypothetical protein BRAFLDRAFT_119044 [Branchiostoma floridae] gi

Protein Accession	CL14721.Contig1$_H$1
Mean Expression Ratio	0.79
Median Expression Ratio	0.794
Credible Interval	(0.344, 1.8)
Associated Peptides	1
Associated Spectra	2
Coverage	NaN

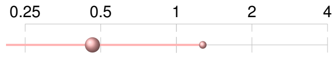

A	B	2.5	50	97.5	Sequence
**0**	**2**	**0.17**	**0.46**	**1.3**	**DLEMDLAK**

5.278 102 872 RecName: Full=Gelsolin-like protein 1; AltName: Full=Actin-modulator; Short=EWAM; Short=EWAM-P1 gi|157362355|dbj|BAA06219.2| actin-modulator [Lumbricus terrestris]; 102 872 RecName: Full=Gelsolin-like protein 1; AltName: Full=Actin-modulator; Short=EWAM; Short=EWAM-P1 gi|157362355|dbj|BAA06219.2| actin-modulator [Lumbricus terrestris]

Protein Accession	Unigene39721$_H$1 Unigene39720$_H$1
Mean Expression Ratio	1.26
Median Expression Ratio	1.26
Credible Interval	(0.542, 2.94)
Associated Peptides	1
Associated Spectra	1
Coverage	NaN

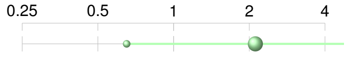

A	B	2.5	50	97.5	Sequence
**1**	**0**	**0.65**	**2.1**	**7.3**	**PKGLVK**

5.279 " 179 631 minus strand 60S ribosomal protein L7/L12, partial [Ostrea edulis]"

Protein Accession	CL34109.Contig5$_H$1
Mean Expression Ratio	0.791
Median Expression Ratio	0.795
Credible Interval	(0.356, 1.74)
Associated Peptides	1
Associated Spectra	3
Coverage	NaN

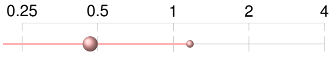

A	B	2.5	50	97.5	Sequence
**2**	**1**	**0.19**	**0.47**	**1.2**	**KFVESVPQVVK**

5.280 184 438 PREDICTED: similar to CG14235 CG14235-PA [Tribolium castaneum] gi

Protein Accession	Unigene638$_H$1
Mean Expression Ratio	0.796
Median Expression Ratio	0.796
Credible Interval	(0.418, 1.49)
Associated Peptides	5
Associated Spectra	8
Coverage	NaN

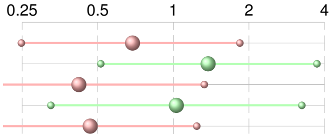

A	B	2.5	50	97.5	Sequence
**2**	**0**	**0.25**	**0.69**	**1.8**	**TAPFDAR**
**1**	**1**	**0.51**	**1.4**	**3.7**	**GDEQKYYQLK**
**1**	**0**	**0.13**	**0.42**	**1.3**	**YYQLK**
**0**	**1**	**0.32**	**1**	**3.3**	**WDDQVANGTFAGKI**
**0**	**2**	**0.17**	**0.47**	**1.2**	**WDDQVANGTFAGK**

5.281 " 130 1161 minus strand Isocitrate dehydrogenase [NAD] subunit alpha, mitochondrial [Crassostrea gigas]; 198 1229 minus strand Isocitrate dehydrogenase [NAD] subunit alpha, mitochondrial [Crassostrea gigas]"

Protein Accession	CL5384.Contig8$_H$1 CL5384.Contig7$_H$1
Mean Expression Ratio	0.797
Median Expression Ratio	0.796
Credible Interval	(0.367, 1.75)
Associated Peptides	2
Associated Spectra	2
Coverage	NaN

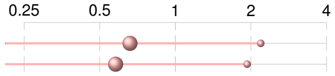

A	B	2.5	50	97.5	Sequence
**1**	**0**	**0.2**	**0.66**	**2.2**	**SLNLALR**
**1**	**0**	**0.17**	**0.58**	**1.9**	**VAEYAFK**

5.282 10 366 minus strand hypothetical protein TRIADDRAFT_25239 [Trichoplax adhaerens] gi

Protein Accession	CL32844.Contig2$_H$1
Mean Expression Ratio	0.797
Median Expression Ratio	0.797
Credible Interval	(0.347, 1.86)
Associated Peptides	1
Associated Spectra	1
Coverage	NaN

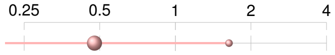

A	B	2.5	50	97.5	Sequence
**1**	**0**	**0.14**	**0.48**	**1.6**	**TAVEDVDLK**

5.283 148 462 hypothetical protein DAPPUDRAFT_306954 [Daphnia pulex]; 148 462 hypothetical protein DAPPUDRAFT_306954 [Daphnia pulex]; 148 462 hypothetical protein DAPPUDRAFT_306954 [Daphnia pulex]; 148 462 hypothetical protein DAPPUDRAFT_306954 [Daphnia pulex]; 173 487 hypothetical protein DAPPUDRAFT_306954 [Daphnia pulex]

Protein Accession	CL48682.Contig7$_H$1 CL48682.Contig6$_H$1 CL48682.Contig5$_H$1 CL48682.Contig4$_H$1 CL4868
Mean Expression Ratio	0.8
Median Expression Ratio	0.798
Credible Interval	(0.378, 1.7)
Associated Peptides	2
Associated Spectra	3
Coverage	NaN

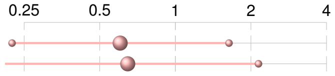

A	B	2.5	50	97.5	Sequence
**1**	**1**	**0.22**	**0.6**	**1.6**	**ATNFDHFK**
**1**	**0**	**0.2**	**0.65**	**2.1**	**AGEVPYKDR**

5.284 114 731 minus strand Proteasome subunit beta type-6 [Crassostrea gigas]

Protein Accession	CL12961.Contig1$_{H}1$
Mean Expression Ratio	1.26
Median Expression Ratio	1.25
Credible Interval	(0.553, 2.91)
Associated Peptides	1
Associated Spectra	2
Coverage	NaN

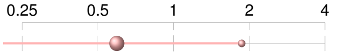

A	B	2.5	50	97.5	Sequence
1	1	0.19	0.59	1.9	FTLTGDDLPK

5.285 104 445 minus strand macrophage migration inhibitory factor [Ascaris suum]

Protein Accession	CL34536.Contig3$_{H}1$
Mean Expression Ratio	1.25
Median Expression Ratio	1.25
Credible Interval	(0.621, 2.54)
Associated Peptides	3
Associated Spectra	6
Coverage	NaN

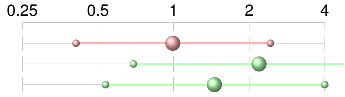

A	B	2.5	50	97.5	Sequence
1	2	0.41	0.99	2.4	HIEEALGIPQDR
0	1	0.69	2.2	7.1	AFLKDTSK
0	2	0.54	1.5	4	LVVPFMK

5.286 696 4010 Lethal(2) giant larvae-like protein 1 [Crassostrea gigas]; 657 3971 minus strand Lethal(2) giant larvae-like protein 1 [Crassostrea gigas]; 696 3749 minus strand Lethal(2) giant larvae-like protein 1 [Crassostrea gigas]; 156 3338 Lethal(2) giant larvae-like protein 1 [Crassostrea gigas]; 156 3209 Lethal(2) giant larvae-like protein 1 [Crassostrea gigas]; 156 3470 Lethal(2) giant larvae-like protein 1 [Crassostrea gigas]

Protein Accession	CL3717.Contig4$_{H}1$ CL3717.Contig3$_{H}1$ CL3717.Contig2$_{H}1$ CL3717.Contig19$_{H}1$ CL3717.Co
Mean Expression Ratio	1.25
Median Expression Ratio	1.25
Credible Interval	(0.567, 2.72)
Associated Peptides	2
Associated Spectra	2
Coverage	NaN

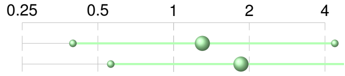

A	B	2.5	50	97.5	Sequence
0	1	0.4	1.3	4.4	VLPESLEVIHER
0	1	0.56	1.8	6.2	NLAKEPTTK

5.287 282 683 PREDICTED: ribonuclease UK114-like [Oreochromis niloticus]

Protein Accession	CL39557.Contig5$_{H}1$
Mean Expression Ratio	1.25
Median Expression Ratio	1.25
Credible Interval	(0.58, 2.78)
Associated Peptides	2
Associated Spectra	2
Coverage	NaN

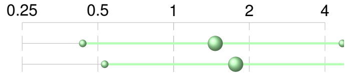

A	B	2.5	50	97.5	Sequence
0	1	0.44	1.5	4.7	LVPGGIQAETDQTLK
0	1	0.53	1.8	5.9	YFSDNKPAR

5.288 2 838 Alanine aminotransferase 2 [Crassostrea gigas]

Protein Accession	CL37048.Contig1$_{H}1$
Mean Expression Ratio	0.798
Median Expression Ratio	0.801
Credible Interval	(0.34, 1.88)
Associated Peptides	1
Associated Spectra	1
Coverage	NaN

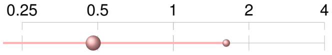

A	B	2.5	50	97.5	Sequence
1	0	0.14	0.48	1.6	QVLTK

5.289 527 1321 minus strand troponin T [Mizuhopecten yessoensis]; 305 1099 minus strand troponin T [Mizuhopecten yessoensis]; 305 1099 minus strand troponin T [Mizuhopecten yessoensis]

Protein Accession	Unigene65787$_{H}1$ CL2214.Contig4$_{H}1$ CL2214.Contig3$_{H}1$
Mean Expression Ratio	0.802
Median Expression Ratio	0.802
Credible Interval	(0.457, 1.41)
Associated Peptides	5
Associated Spectra	22
Coverage	NaN

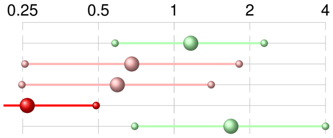

A	B	2.5	50	97.5	Sequence
2	4	0.58	1.2	2.3	KGPNFVITK
1	1	0.25	0.68	1.8	LDSVMSKEELQK
3	0	0.25	0.59	1.4	AILSQR
8	0	0.14	0.26	0.49	LTELR
0	3	0.7	1.7	4	GAEILALK

5.290 26 550 Translocon-associated protein subunit gamma [Crassostrea gigas]

Protein Accession	CL32873.Contig1$_{H}1$
Mean Expression Ratio	1.25
Median Expression Ratio	1.25
Credible Interval	(0.576, 2.71)
Associated Peptides	2
Associated Spectra	2
Coverage	NaN

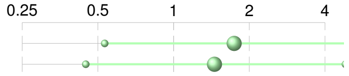

A	B	2.5	50	97.5	Sequence
0	1	0.53	1.7	5.6	QLSREEELLLQDFSR
0	1	0.45	1.5	4.8	KLADDKK

5.291 " 193 333 minus strand RecName: Full=Globin; AltName: Full=Myoglobin gi|299274|gb|AAB25743.1| myoglobin [Nassa mutabilis=marine gastropods, radular muscles, Peptide, 147 aa] gi; 193 333 minus strand RecName: Full=Globin; AltName: Full=Myoglobin gi|299274|gb|AAB25743.1| myoglobin [Nassa mutabilis=marine gastropods, radular muscles, Peptide, 147 aa] gi"

Protein Accession	CL8458.Contig5$_{H}1$ CL8458.Contig1$_{H}1$
Mean Expression Ratio	0.802
Median Expression Ratio	0.803
Credible Interval	(0.383, 1.65)
Associated Peptides	2
Associated Spectra	8
Coverage	NaN

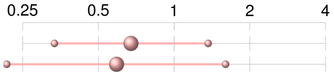

A	B	2.5	50	97.5	Sequence
4	2	0.33	0.67	1.4	AAINSTWPK
1	1	0.22	0.59	1.6	LSADKETMENNGAR

5.292 " 343 1179 ATP synthase subunit alpha, mitochondrial OS=Pongo abelii GN=ATP5A1 PE=2 SV=1"

Protein Accession	Unigene30534$_{H}1$
Mean Expression Ratio	1.25
Median Expression Ratio	1.25
Credible Interval	(0.813, 1.91)
Associated Peptides	11
Associated Spectra	66
Coverage	NaN

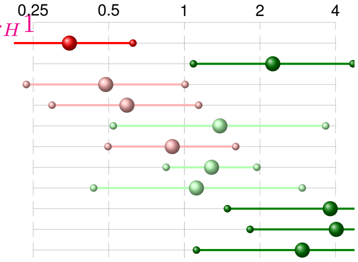

A	B	2.5	50	97.5	Sequence
7	2	0.19	0.35	0.62	STVAQLVK
2	3	1.1	2.2	4.7	TSVKEPMQTGIK
2	3	0.24	0.49	1	VVDALGTPIDGK
3	3	0.3	0.59	1.1	APGIIPR
1	1	0.52	1.4	3.7	VYGLK
3	6	0.5	0.9	1.6	TAVAIDTIINQK
6	14	0.85	1.3	1.9	VLSIGDGIAR
0	2	0.44	1.1	2.9	NIQAEEMVEFSSGLK
0	2	1.5	3.8	10	VGLKAPGIIPR
0	4	1.8	4	8.9	VVDALGTPIDGKGPITSK
0	2	1.1	3	7.7	TAVAIDTIINQKR

5.293 406 1392 minus strand hypothetical protein CGI_10007007 [Crassostrea gigas]

Protein Accession	CL18045.Contig1$_{H}1$
Mean Expression Ratio	1.25
Median Expression Ratio	1.25
Credible Interval	(0.543, 2.91)
Associated Peptides	1
Associated Spectra	1
Coverage	NaN

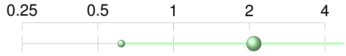

A	B	2.5	50	97.5	Sequence
0	1	0.62	2.1	6.9	WQRPDKPVGFYDLK

5.294 259 1296 minus strand GD20916 [Drosophila simulans] gi

Protein Accession	CL25607.Contig2$_{H}1$
Mean Expression Ratio	1.25
Median Expression Ratio	1.24
Credible Interval	(0.597, 2.67)
Associated Peptides	2
Associated Spectra	4
Coverage	NaN

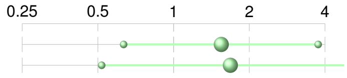

A	B	2.5	50	97.5	Sequence
0	3	0.63	1.5	3.8	QIVWEHPEVK
0	1	0.52	1.7	5.5	YAPWPYKK

5.295 501 1838 Hsp90A [Haliotis asinina]

Protein Accession	CL5184.Contig2$_{H}1$
Mean Expression Ratio	0.803
Median Expression Ratio	0.803
Credible Interval	(0.49, 1.31)
Associated Peptides	11
Associated Spectra	26
Coverage	NaN

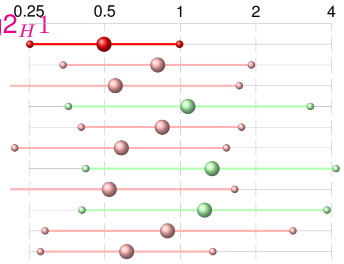

A	B	2.5	50	97.5	Sequence
3	3	0.25	0.5	0.99	EMLQQSK
1	2	0.34	0.81	1.9	SLTNDWEDHLAVK
1	0	0.18	0.55	1.7	GVVDSEDLPLNISR
0	1	0.36	1.1	3.3	AIYYITGESLDAVK
0	5	0.4	0.85	1.7	HFSVEGQLEFR
0	2	0.22	0.58	1.5	KHFEINPDHPIIK
0	1	0.42	1.3	4.2	RAPFDLFENKK
0	1	0.16	0.52	1.6	YHSSQAGDELVTLK
0	1	0.41	1.2	3.8	KLAEFLR
0	1	0.29	0.89	2.8	TKPLWTR
0	4	0.28	0.61	1.3	LAEFLR

5.296 294 443 GE25988 gene product from transcript GE25988-RA; K02980 small subunit ribosomal protein S29e; 292 441 GE25988 gene product from transcript GE25988-RA; K02980 small subunit ribosomal protein S29e; 308 457 GE25988 gene product from transcript GE25988-RA; K02980 small subunit ribosomal protein S29e; 80 250 minus strand 40S ribosomal protein S29 [Ornithodoros parkeri]

Protein Accession	CL11390.Contig8$_{H}1$ CL11390.Contig4$_{H}1$ CL11390.Contig3$_{H}1$ CL11390.Contig2$_{H}1$
Mean Expression Ratio	0.802
Median Expression Ratio	0.804
Credible Interval	(0.348, 1.83)
Associated Peptides	1
Associated Spectra	4
Coverage	NaN

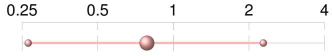

A	B	2.5	50	97.5	Sequence
1	3	0.26	0.78	2.3	QFAPDIGFK

5.297 " 1 2508 Alanyl-tRNA synthetase, cytoplasmic [Crassostrea gigas]; 1 2508 Alanyl-tRNA synthetase, cytoplasmic [Crassostrea gigas]; 1 2508 minus strand Alanyl-tRNA synthetase, cytoplasmic [Crassostrea gigas]"

Protein Accession	CL11594.Contig2$_{H}$1 CL11594.Contig1$_{H}$1 CL11594.Contig11$_{H}$1
Mean Expression Ratio	0.806
Median Expression Ratio	0.804
Credible Interval	(0.384, 1.7)
Associated Peptides	3
Associated Spectra	3
Coverage	NaN

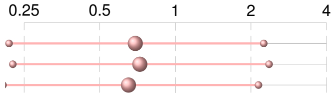

A	B	2.5	50	97.5	Sequence
**1**	**0**	**0.22**	**0.69**	**2.2**	**GLTVDNEGFEK**
**1**	**0**	**0.22**	**0.72**	**2.4**	**SIQGLR**
**1**	**0**	**0.21**	**0.65**	**2.1**	**VIALR**

5.298 382 972 minus strand Muscle-specific protein 20 [Crassostrea gigas]; 807 1397 minus strand Muscle-specific protein 20 [Crassostrea gigas]

Protein Accession	Unigene366$_{H}$1 CL6243.Contig1$_{H}$1
Mean Expression Ratio	0.8
Median Expression Ratio	0.804
Credible Interval	(0.341, 1.84)
Associated Peptides	1
Associated Spectra	1
Coverage	NaN

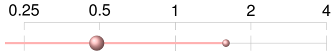

A	B	2.5	50	97.5	Sequence
**1**	**0**	**0.14**	**0.49**	**1.6**	**GFQGPTLGPK**

5.299 26 514 Protein phosphatase inhibitor 2 [Crassostrea gigas]

Protein Accession	CL43517.Contig2$_{H}$1
Mean Expression Ratio	0.802
Median Expression Ratio	0.804
Credible Interval	(0.342, 1.87)
Associated Peptides	1
Associated Spectra	1
Coverage	NaN

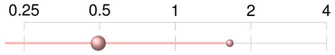

A	B	2.5	50	97.5	Sequence
**0**	**1**	**0.15**	**0.49**	**1.6**	**HVGLDPTQLASK**

5.300 633 806 Peroxiredoxin-1 [Crassostrea gigas]; 648 821 Peroxiredoxin-1 [Crassostrea gigas]; 666 839 Peroxiredoxin-1 [Crassostrea gigas]; 625 798 Peroxiredoxin-1 [Crassostrea gigas]; 633 824 Peroxiredoxin-1 [Crassostrea gigas]

Protein Accession: CL3160.Contig18$_H$1 CL3160.Contig17$_H$1 CL3160.Contig16$_H$1 CL3160.Contig15$_H$1 CL3160
Mean Expression Ratio: 0.803
Median Expression Ratio: 0.805
Credible Interval: (0.364, 1.77)
Associated Peptides: 1
Associated Spectra: 5
Coverage: NaN

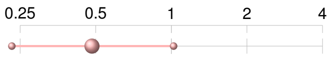

A	B	2.5	50	97.5	Sequence
0	5	0.23	0.48	1	LVQAFQFTDK

5.301 219 1559 Programmed cell death protein 4 [Crassostrea gigas]

Protein Accession: CL35470.Contig4$_H$1
Mean Expression Ratio: 1.24
Median Expression Ratio: 1.24
Credible Interval: (0.586, 2.66)
Associated Peptides: 2
Associated Spectra: 3
Coverage: NaN

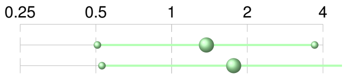

A	B	2.5	50	97.5	Sequence
0	2	0.51	1.4	3.7	MSDLVIDAPDATSVVGK
0	1	0.53	1.8	5.8	GRKRFVSEGDGGAVKQ

5.302 98 673 transforming protein Rho-like [Acyrthosiphon pisum]

Protein Accession: Unigene30464$_H$1
Mean Expression Ratio: 1.25
Median Expression Ratio: 1.24
Credible Interval: (0.576, 2.75)
Associated Peptides: 2
Associated Spectra: 2
Coverage: NaN

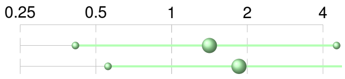

A	B	2.5	50	97.5	Sequence
0	1	0.42	1.4	4.5	EVFEQATR
0	1	0.56	1.8	6.2	EGVREVFEQATR

5.303 311 928 minus strand peroxiredoxin 3 [Clonorchis sinensis] gi; 288 905 minus strand peroxiredoxin 3 [Clonorchis sinensis] gi

Protein Accession: CL2043.Contig4$_H$1 CL2043.Contig1$_H$1
Mean Expression Ratio: 0.802
Median Expression Ratio: 0.806
Credible Interval: (0.396, 1.62)
Associated Peptides: 3
Associated Spectra: 9
Coverage: NaN

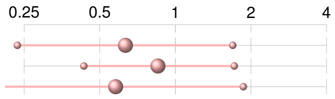

A	B	2.5	50	97.5	Sequence
1	1	0.23	0.63	1.7	SVDEILR
3	3	0.43	0.85	1.7	QITINDLPVGR
0	1	0.18	0.58	1.9	VQHPAPDFK

5.304 788 1006 LIM protein [Haliotis discus discus]; 768 980 muscle LIM protein isoform 1 [Danaus plexippus]

Protein Accession	CL29824.Contig2$_{H}1$ CL29824.Contig1$_{H}1$
Mean Expression Ratio	1.24
Median Expression Ratio	1.24
Credible Interval	(0.564, 2.7)
Associated Peptides	1
Associated Spectra	4
Coverage	NaN

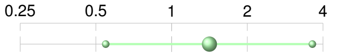

A	B	2.5	50	97.5	Sequence
2	2	0.55	1.4	3.6	AIGVNTPWHK

5.305 62 1093 Heterogeneous nuclear ribonucleoprotein A2-like protein 1 [Crassostrea gigas]

Protein Accession	CL47083.Contig4$_{H}1$
Mean Expression Ratio	1.24
Median Expression Ratio	1.24
Credible Interval	(0.714, 2.16)
Associated Peptides	8
Associated Spectra	15
Coverage	NaN

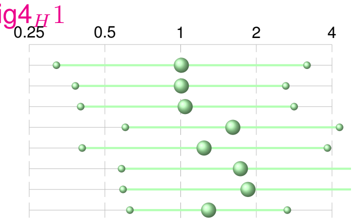

A	B	2.5	50	97.5	Sequence
1	0	0.32	1	3.2	GGGRDYGGGR
0	2	0.38	1	2.6	ETFSPFGTISQIDLITDK
0	2	0.4	1	2.8	HELNGRQVEVK
0	2	0.6	1.6	4.3	MFVGGIKEDTTEDMIR
0	1	0.41	1.2	3.8	KLFIGGLSYETDENSLR
0	1	0.58	1.7	5.5	KAMSK
0	1	0.59	1.8	5.9	MPGYR
0	5	0.63	1.3	2.7	GFGFITYK

5.306 114 1001 Vinculin [Crassostrea gigas]

Protein Accession	CL18499.Contig1$_{H}1$
Mean Expression Ratio	1.24
Median Expression Ratio	1.24
Credible Interval	(0.582, 2.63)
Associated Peptides	3
Associated Spectra	3
Coverage	NaN

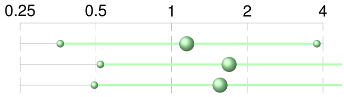

A	B	2.5	50	97.5	Sequence
1	0	0.36	1.1	3.8	PVFHTK
0	1	0.52	1.7	5.6	TIESILEPVAQQVSR
0	1	0.49	1.6	5	KMSDEIHEIIR

5.307 95 271 minus strand hypothetical protein EAI_01530 [Harpegnathos saltator]

Protein Accession	CL9369.Contig9$_{H}1$
Mean Expression Ratio	1.24
Median Expression Ratio	1.24
Credible Interval	(0.563, 2.74)
Associated Peptides	2
Associated Spectra	2
Coverage	NaN

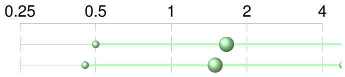

A	B	2.5	50	97.5	Sequence
0	1	0.5	1.7	5.6	YPYTISSK
0	1	0.45	1.5	4.8	VAQFPFR

5.308 835 1083 minus strand Calmodulin [Crassostrea gigas]

Protein Accession	CL49568.Contig2$_{H}$1
Mean Expression Ratio	0.804
Median Expression Ratio	0.808
Credible Interval	(0.39, 1.64)
Associated Peptides	3
Associated Spectra	5
Coverage	NaN

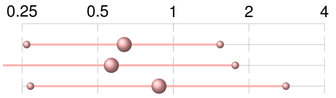

A	B	2.5	50	97.5	Sequence
0	3	0.26	0.64	1.5	VDFTEFYK
0	1	0.18	0.57	1.8	HLLTDKDEETCYK
0	1	0.27	0.88	2.8	GFIANEEMK

5.309 156 845 minus strand histone H1 [Mytilus californianus]; 156 845 minus strand histone H1 [Mytilus californianus]; 156 845 minus strand histone H1 [Mytilus californianus]; 156 845 minus strand histone H1 [Mytilus californianus]

Protein Accession	CL26709.Contig6$_{H}$1 CL26709.Contig5$_{H}$1 CL26709.Contig4$_{H}$1 CL26709.Contig3$_{H}$1
Mean Expression Ratio	0.812
Median Expression Ratio	0.809
Credible Interval	(0.351, 1.85)
Associated Peptides	1
Associated Spectra	1
Coverage	NaN

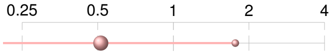

A	B	2.5	50	97.5	Sequence
1	0	0.15	0.51	1.8	DAITALKER

5.310 51 1295 PREDICTED: phosphoglycerate kinase-like [Nasonia vitripennis]; 97 1341 PREDICTED: phosphoglycerate kinase-like [Nasonia vitripennis]

Protein Accession	CL14417.Contig13$_{H}$1 CL14417.Contig12$_{H}$1
Mean Expression Ratio	1.24
Median Expression Ratio	1.24
Credible Interval	(0.863, 1.76)
Associated Peptides	22
Associated Spectra	67
Coverage	NaN

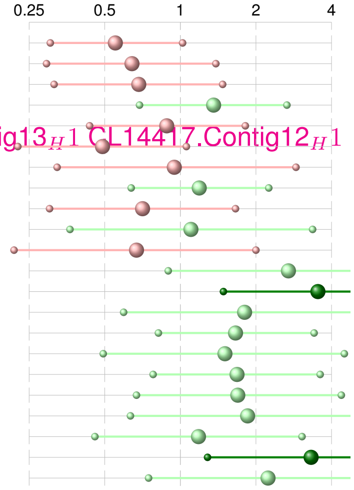

A	B	2.5	50	97.5	Sequence
3	5	0.3	0.55	1	ALMDAVVEATK
2	2	0.29	0.64	1.4	ATQEDIDKFR
2	2	0.31	0.68	1.5	FHVEEEGK
2	4	0.69	1.4	2.7	LSIDKVDVK
2	3	0.44	0.88	1.8	VDFNVPLKDGK
3	1	0.22	0.49	1.1	FTDAVK
1	0	0.32	0.95	2.9	VLNNMEIGK
2	5	0.64	1.2	2.3	VLPGVAALTDA
1	2	0.3	0.71	1.7	YALENGAK
0	1	0.36	1.1	3.4	AHSSMVGCQLPIR
0	1	0.22	0.67	2	ALNNPDRPFLAILGGAK
0	1	0.89	2.7	8.1	ASGFLLKK
0	3	1.5	3.5	8.2	FHVEEEGKGVAPDGTK
0	1	0.6	1.8	5.4	FHVEEEGKGVAPDGTKLK
0	5	0.82	1.7	3.4	IVAALPTIK
0	1	0.49	1.5	4.5	LGDIYVNDAFGTAHR
0	4	0.78	1.7	3.6	SGIPADWMGLDIGPK
0	2	0.67	1.7	4.4	SVVLMSHLGRPDGTK
0	1	0.63	1.9	5.6	VDFNVPLKDGKITNNQR
0	2	0.46	1.2	3	ASGFLLK
0	2	1.3	3.3	8.9	FTDAVKR
0	1	0.75	2.2	6.8	LNKLSIDK

5.311 131 541 minus strand nervous system adducin [Aplysia californica] gi

Protein Accession	CL27337.Contig1$_{H}$1
Mean Expression Ratio	1.24
Median Expression Ratio	1.24
Credible Interval	(0.575, 2.66)
Associated Peptides	2
Associated Spectra	3
Coverage	NaN

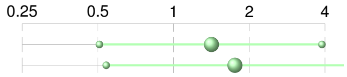

A	B	2.5	50	97.5	Sequence
1	1	0.51	1.4	3.9	GIGASFGR
0	1	0.54	1.8	5.8	FIDTIDPDDPEYIR

5.312 14 1642 minus strand chaperonin containing tcp1 [Haliotis discus discus]; 14 1462 minus strand chaperonin containing tcp1 [Haliotis discus discus]; 14 1582 minus strand chaperonin containing tcp1 [Haliotis discus discus]

Protein Accession	CL16298.Contig4$_{H}$1 CL16298.Contig15$_{H}$1 CL16298.Contig14$_{H}$1
Mean Expression Ratio	1.24
Median Expression Ratio	1.23
Credible Interval	(0.616, 2.51)
Associated Peptides	4
Associated Spectra	4
Coverage	NaN

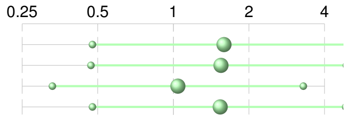

A	B	2.5	50	97.5	Sequence
0	1	0.48	1.6	5.2	FAESLEVIPR
0	1	0.47	1.5	4.9	MVINHLEK
0	1	0.33	1	3.3	ACVSILPAK
0	1	0.48	1.5	4.9	HFSGLEEAVYR

5.313 3 1352 nervous system adducin [Aplysia californica] gi; 3 1418 Protein hu-li tai shao [Crassostrea gigas]; 3 1214 nervous system adducin [Aplysia californica] gi; 3 1319 minus strand nervous system adducin [Aplysia californica] gi; 3 1523 minus strand Protein hu-li tai shao [Crassostrea gigas]; 3 1439 minus strand Protein hu-li tai shao [Crassostrea gigas]; 3 1562 minus strand Protein hu-li tai shao [Crassostrea gigas]

Protein Accession	CL3889.Contig6$_{H}$1 CL3889.Contig5$_{H}$1 CL3889.Contig4$_{H}$1 CL3889.Contig14$_{H}$1 CL3889.Co
Mean Expression Ratio	0.808
Median Expression Ratio	0.811
Credible Interval	(0.409, 1.58)
Associated Peptides	4
Associated Spectra	7
Coverage	NaN

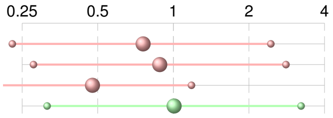

A	B	2.5	50	97.5	Sequence
1	0	0.23	0.76	2.5	WVPESETQGK
1	0	0.28	0.88	2.8	LISTEER
2	2	0.19	0.48	1.2	VMLLR
1	0	0.31	1	3.2	GDQTV

5.314 243 2165 minus strand Elongation factor 2 OS=Drosophila melanogaster GN=Ef2b PE=2 SV=4

Protein Accession	Unigene11527$_H$1
Mean Expression Ratio	0.813
Median Expression Ratio	0.812
Credible Interval	(0.401, 1.64)
Associated Peptides	3
Associated Spectra	7
Coverage	NaN

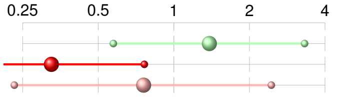

A	B	2.5	50	97.5	Sequence
0	3	0.57	1.4	3.3	ALPLQEGLPEAIDKGDIAPR
0	3	0.14	0.33	0.76	TQEDTLALADK
0	1	0.23	0.76	2.4	FNMEEVK

5.315 " 143 451 minus strand PREDICTED: 10 kDa heat shock protein, mitochondrial isoform 1 [Canis lupus familiaris]"

Protein Accession	CL48287.Contig1$_H$1
Mean Expression Ratio	0.813
Median Expression Ratio	0.812
Credible Interval	(0.445, 1.5)
Associated Peptides	6
Associated Spectra	10
Coverage	NaN

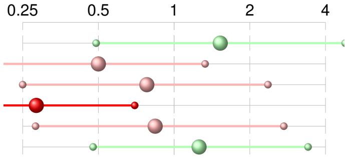

A	B	2.5	50	97.5	Sequence
1	0	0.49	1.5	4.8	GGIMIPEKSQGK
2	0	0.19	0.5	1.3	TKGGIMIPEK
1	0	0.25	0.78	2.4	SIPETK
3	0	0.12	0.28	0.7	GGIMIPEK
0	1	0.28	0.84	2.7	AQEAVVVAVGSGSR
0	2	0.48	1.3	3.4	VLLPEYGGTK

5.316 3 491 hemocyanin [Haliotis tuberculata]

Protein Accession	CL40281.Contig3$_H$1
Mean Expression Ratio	0.811
Median Expression Ratio	0.813
Credible Interval	(0.43, 1.53)
Associated Peptides	4
Associated Spectra	15
Coverage	NaN

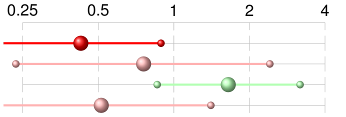

A	B	2.5	50	97.5	Sequence
2	3	0.2	0.43	0.89	DINPFALTK
0	1	0.23	0.76	2.4	GKPFTSAQCSVELFSK
0	7	0.86	1.6	3.2	TLEPFDRDINPFALTK
0	2	0.19	0.52	1.4	TLEPFDR

5.317 182 832 minus strand qm-like protein [Crassostrea ariakensis]

Protein Accession	CL13773.Contig2$_H$1
Mean Expression Ratio	1.23
Median Expression Ratio	1.23
Credible Interval	(0.693, 2.16)
Associated Peptides	6
Associated Spectra	19
Coverage	NaN

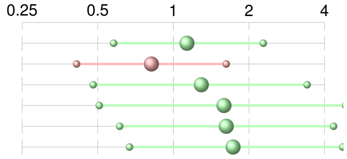

A	B	2.5	50	97.5	Sequence
2	4	0.58	1.1	2.3	VHIGQPIMSVR
3	3	0.41	0.82	1.6	IFDLGR
0	2	0.48	1.3	3.4	EQHEAAIIEALR
0	1	0.51	1.6	4.8	EQHEAAIIEALRR
0	2	0.61	1.6	4.3	AREQHEAAIIEALR
0	2	0.67	1.7	4.7	GAFGKPQGTVAR

5.318 585 944 minus strand Protein disulfide-isomerase A6 [Crassostrea gigas]

Protein Accession	CL39540.Contig7$_{H}1$
Mean Expression Ratio	1.23
Median Expression Ratio	1.23
Credible Interval	(0.648, 2.36)
Associated Peptides	4
Associated Spectra	10
Coverage	NaN

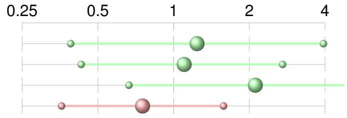

A	B	2.5	50	97.5	Sequence
0	1	0.39	1.2	3.9	AGNPEDVVEVTDSNFQK
1	2	0.43	1.1	2.7	TASDIINWATEK
0	1	0.66	2.1	6.7	NLAPHWAQAATELKGK
0	5	0.36	0.75	1.6	NLAPHWAQAATELK

5.319 2 778 Putative adenosylhomocysteinase 3 [Crassostrea gigas]

Protein Accession	CL23756.Contig1$_{H}1$
Mean Expression Ratio	0.812
Median Expression Ratio	0.813
Credible Interval	(0.352, 1.89)
Associated Peptides	1
Associated Spectra	1
Coverage	NaN

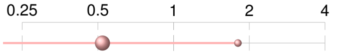

A	B	2.5	50	97.5	Sequence
1	0	0.15	0.52	1.8	TPELTWEK

5.320 479 1732 minus strand Multifunctional protein ADE2 [Crassostrea gigas]; 239 1501 minus strand Multifunctional protein ADE2 [Crassostrea gigas]

Protein Accession	CL4210.Contig3$_{H}1$ CL4210.Contig1$_{H}1$
Mean Expression Ratio	0.811
Median Expression Ratio	0.813
Credible Interval	(0.343, 1.86)
Associated Peptides	1
Associated Spectra	1
Coverage	NaN

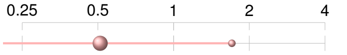

A	B	2.5	50	97.5	Sequence
1	0	0.15	0.51	1.7	VPLLSPKPK

5.321 126 704 minus strand ribosomal protein L12 [Ixodes scapularis]

Protein Accession	CL24053.Contig3$_{H}1$
Mean Expression Ratio	1.23
Median Expression Ratio	1.23
Credible Interval	(0.722, 2.12)
Associated Peptides	6
Associated Spectra	29
Coverage	NaN

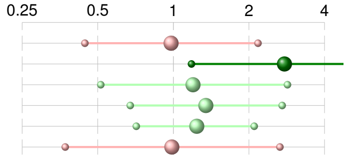

A	B	2.5	50	97.5	Sequence
1	3	0.44	0.98	2.2	AVGGEVPATSALAPK
1	2	1.2	2.8	6.7	INAKEIEIPDE
1	2	0.51	1.2	2.8	KVGDDIAK
2	4	0.67	1.3	2.7	SNPHVIIEK
4	7	0.71	1.2	2.1	IGPLGLSPK
0	2	0.37	0.98	2.7	EIEIPDE

5.322 241 561 minus strand thioredoxin [Crassostrea ariakensis]

Protein Accession	CL33159.Contig3$_{H}$1
Mean Expression Ratio	0.814
Median Expression Ratio	0.814
Credible Interval	(0.407, 1.63)
Associated Peptides	3
Associated Spectra	7
Coverage	NaN

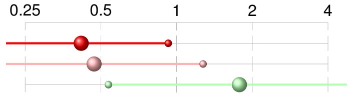

A	B	2.5	50	97.5	Sequence
2	2	0.19	0.42	0.93	IETQEDFEK
1	1	0.17	0.47	1.3	EVGEVVGASEEEIKK
0	1	0.54	1.8	5.6	VKIETQEDFEK

5.323 60 353 minus strand Histone H3.3 [Harpegnathos saltator]

Protein Accession	Unigene31518$_{H}$1
Mean Expression Ratio	1.23
Median Expression Ratio	1.23
Credible Interval	(0.535, 2.88)
Associated Peptides	1
Associated Spectra	1
Coverage	NaN

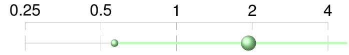

A	B	2.5	50	97.5	Sequence
0	1	0.57	1.9	6.5	KSAPSTGGVK

5.324 28 516 minus strand Signal peptidase complex subunit 3 [Crassostrea gigas]; 30 554 minus strand Signal peptidase complex subunit 3 [Crassostrea gigas]

Protein Accession	Unigene59022$_{H}$1 Unigene26504$_{H}$1
Mean Expression Ratio	0.812
Median Expression Ratio	0.814
Credible Interval	(0.354, 1.81)
Associated Peptides	1
Associated Spectra	2
Coverage	NaN

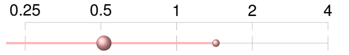

A	B	2.5	50	97.5	Sequence
1	1	0.18	0.51	1.4	SDLGLVTFDLK

5.325 484 1104 Calponin-2 [Crassostrea gigas]; 309 938 Calponin-2 [Crassostrea gigas]

Protein Accession	CL30203.Contig3$_{H}$1 CL21394.Contig2$_{H}$1
Mean Expression Ratio	1.23
Median Expression Ratio	1.23
Credible Interval	(0.681, 2.17)
Associated Peptides	5
Associated Spectra	20
Coverage	NaN

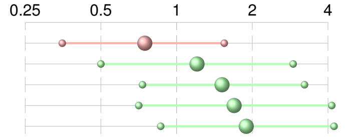

A	B	2.5	50	97.5	Sequence
1	4	0.35	0.74	1.6	QMENIEVFLK
1	2	0.5	1.2	2.9	QLTGEDIGSGAVQVEK
1	4	0.73	1.5	3.2	MNTLAAPFK
0	3	0.71	1.7	4.1	YDTGAEYEVR
0	4	0.86	1.9	4.2	AGQGIIGLQAGTNK

5.326 551 871 minus strand Protein disulfide-isomerase A4 [Crassostrea gigas]

Protein Accession	CL20258.Contig1$_H$1
Mean Expression Ratio	0.817
Median Expression Ratio	0.816
Credible Interval	(0.348, 1.89)
Associated Peptides	1
Associated Spectra	1
Coverage	NaN

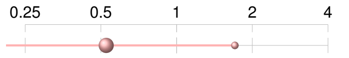

A	B	2.5	50	97.5	Sequence
**1**	**0**	**0.16**	**0.52**	**1.7**	**EGPEPILLAK**

5.327 26 619 G-type lysozyme [Physella acuta]

Protein Accession	CL43984.Contig1$_H$1
Mean Expression Ratio	1.22
Median Expression Ratio	1.23
Credible Interval	(0.55, 2.83)
Associated Peptides	1
Associated Spectra	2
Coverage	NaN

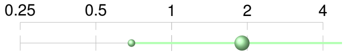

A	B	2.5	50	97.5	Sequence
**0**	**2**	**0.69**	**1.9**	**5.3**	**GHFAGWPIER**

5.328 " 1 327 minus strand endo-beta-1,4-glucanase [Ampullaria crossean]"

Protein Accession	CL17318.Contig3$_H$1
Mean Expression Ratio	1.22
Median Expression Ratio	1.22
Credible Interval	(0.539, 2.71)
Associated Peptides	1
Associated Spectra	2
Coverage	NaN

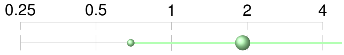

A	B	2.5	50	97.5	Sequence
**0**	**2**	**0.69**	**1.9**	**5.3**	**CHMDGGVLK**

5.329 26 367 proteasome alpha type 2 [Haliotis discus discus]

Protein Accession	CL43771.Contig1$_H$1
Mean Expression Ratio	0.819
Median Expression Ratio	0.818
Credible Interval	(0.355, 1.88)
Associated Peptides	1
Associated Spectra	1
Coverage	NaN

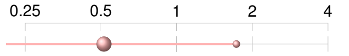

A	B	2.5	50	97.5	Sequence
**1**	**0**	**0.16**	**0.51**	**1.7**	**ATNGVVLATEK**

5.330 211 687 Uncharacterized protein CG16817 [Harpegnathos saltator]; 200 676 Uncharacterized protein CG16817 [Harpegnathos saltator]; 294 770 Uncharacterized protein CG16817 [Harpegnathos saltator]; 211 687 Uncharacterized protein CG16817 [Harpegnathos saltator]

Protein Accession	Unigene6191$_H$1 Unigene53279$_H$1 Unigene41893$_H$1 Unigene11183$_H$1
Mean Expression Ratio	1.23
Median Expression Ratio	1.22
Credible Interval	(0.524, 2.88)
Associated Peptides	1
Associated Spectra	1
Coverage	NaN

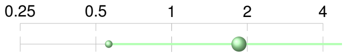

A	B	2.5	50	97.5	Sequence
**0**	**1**	**0.56**	**1.9**	**6.3**	**KFHFLK**

5.331 79 606 minus strand PREDICTED: similar to Ras-like protein 3 precursor (Protein roughened) [Tribolium castaneum] gi

Protein Accession	Unigene4246$_H$1
Mean Expression Ratio	1.22
Median Expression Ratio	1.22
Credible Interval	(0.551, 2.7)
Associated Peptides	1
Associated Spectra	3
Coverage	NaN

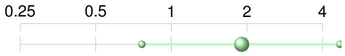

A	B	2.5	50	97.5	Sequence
**0**	**3**	**0.77**	**1.9**	**4.7**	**LVVLGSGGVGK**

5.332 1 231 small subunit ribosomal protein S11e [Clonorchis sinensis]; 1 171 40S ribosomal protein S11 OS=Canis familiaris GN=RPS11 PE=1 SV=2

Protein Accession	CL39695.Contig2$_H$1 CL39695.Contig1$_H$1
Mean Expression Ratio	1.23
Median Expression Ratio	1.22
Credible Interval	(0.546, 2.81)
Associated Peptides	1
Associated Spectra	2
Coverage	NaN

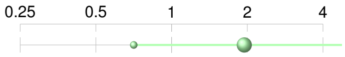

A	B	2.5	50	97.5	Sequence
**0**	**2**	**0.71**	**1.9**	**5.3**	**TIVVRRDYLK**

5.333 1971 3368 minus strand Kyphoscoliosis peptidase [Crassostrea gigas]; 1767 3158 minus strand hillarin [Hirudo medicinalis]; 1977 3368 minus strand hillarin [Hirudo medicinalis]

Protein Accession	CL3923.Contig9$_{H}1$ CL3923.Contig8$_{H}1$ CL3923.Contig10$_{H}1$
Mean Expression Ratio	0.816
Median Expression Ratio	0.818
Credible Interval	(0.407, 1.6)
Associated Peptides	4
Associated Spectra	6
Coverage	NaN

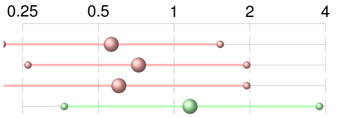

A	B	2.5	50	97.5	Sequence
1	1	0.21	0.56	1.5	GSPEEVLMGLK
1	1	0.26	0.72	2	EMNFDKVEK
1	0	0.18	0.6	1.9	NITNEIEK
0	1	0.37	1.2	3.8	NVLQSVDDHAIQVSK

5.334 " 3 2693 PREDICTED: similar to coatomer protein complex, subunit beta 2 (beta prime) [Taeniopygia guttata]"

Protein Accession	CL19504.Contig2$_{H}1$
Mean Expression Ratio	1.22
Median Expression Ratio	1.22
Credible Interval	(0.576, 2.57)
Associated Peptides	3
Associated Spectra	3
Coverage	NaN

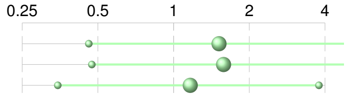

A	B	2.5	50	97.5	Sequence
0	1	0.46	1.5	5.1	RLPEAAFFAR
0	1	0.47	1.6	5.1	TYLPSQVSR
0	1	0.35	1.2	3.8	VVSLWK

5.335 557 2329 minus strand Collagen alpha-3(VI) chain [Crassostrea gigas]

Protein Accession	CL47586.Contig7$_{H}1$
Mean Expression Ratio	1.22
Median Expression Ratio	1.22
Credible Interval	(0.587, 2.57)
Associated Peptides	3
Associated Spectra	4
Coverage	NaN

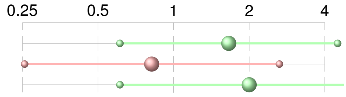

A	B	2.5	50	97.5	Sequence
0	2	0.61	1.7	4.5	KLDVVVAFHASPR
0	1	0.25	0.82	2.6	VALGFFNK
0	1	0.61	2	6.7	KADYSAALGKLPK

5.336 2008 2715 Protein disulfide-isomerase A5 [Crassostrea gigas]

Protein Accession	CL7820.Contig3$_{H}1$
Mean Expression Ratio	1.22
Median Expression Ratio	1.22
Credible Interval	(0.645, 2.34)
Associated Peptides	5
Associated Spectra	7
Coverage	NaN

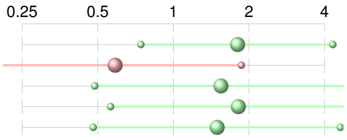

A	B	2.5	50	97.5	Sequence
1	2	0.74	1.8	4.3	LKQESPTAK
1	0	0.19	0.59	1.9	FGGLASK
0	1	0.49	1.5	5	MKPAYMSAAAR
0	1	0.56	1.8	5.8	FGGLASKYEVK
0	1	0.48	1.5	4.6	SKEDLVAFMKDPKPK

5.337 1 264 Spectrin beta chain [Crassostrea gigas]

Protein Accession	Unigene36334$_{H}1$
Mean Expression Ratio	1.22
Median Expression Ratio	1.22
Credible Interval	(0.563, 2.67)
Associated Peptides	2
Associated Spectra	2
Coverage	NaN

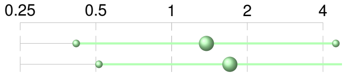

A	B	2.5	50	97.5	Sequence
0	1	0.42	1.4	4.5	QLLQVEHPNAAEVVQR
0	1	0.51	1.7	5.5	VAVVNQLAR

5.338 70 705 RecName: Full=Ras-related protein Rab-2 gi|288938|emb|CAA51234.1| RAB2 [Lymnaea stagnalis]; 70 705 RecName: Full=Ras-related protein Rab-2 gi|288938|emb|CAA51234.1| RAB2 [Lymnaea stagnalis]

Protein Accession	Unigene59013$_{H}1$ Unigene19254$_{H}1$
Mean Expression Ratio	1.22
Median Expression Ratio	1.22
Credible Interval	(0.585, 2.59)
Associated Peptides	3
Associated Spectra	3
Coverage	NaN

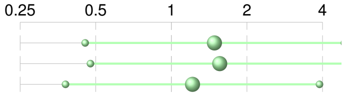

A	B	2.5	50	97.5	Sequence
0	1	0.45	1.5	4.9	TAANVEEAFINTAK
0	1	0.48	1.6	5.1	KEEGEAFAR
0	1	0.38	1.2	3.9	EHGLIFMETSAK

5.339 100 834 vesicle-associated membrane protein/synaptobrevin-binding protein [Aplysia californica] gi

Protein Accession	CL36367.Contig1$_{H}1$
Mean Expression Ratio	1.22
Median Expression Ratio	1.22
Credible Interval	(0.562, 2.65)
Associated Peptides	2
Associated Spectra	2
Coverage	NaN

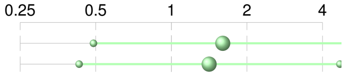

A	B	2.5	50	97.5	Sequence
0	1	0.49	1.6	5.3	FKGPFTDVVTAELK
0	1	0.43	1.4	4.7	TEQALQLEPANELR

5.340 " 65 1576 minus strand Aldehyde dehydrogenase, mitochondrial [Crassostrea gigas]"

Protein Accession	CL5835.Contig1$_H$1
Mean Expression Ratio	0.82
Median Expression Ratio	0.822
Credible Interval	(0.535, 1.27)
Associated Peptides	15
Associated Spectra	38
Coverage	NaN

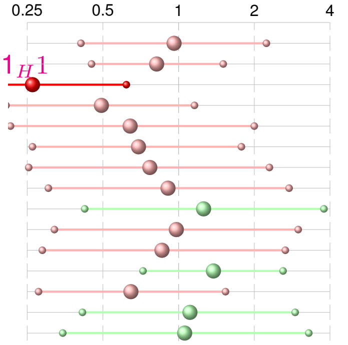

A	B	2.5	50	97.5	Sequence
1	2	0.41	0.96	2.2	SAAALPKPLTSPEIK
4	4	0.45	0.82	1.5	TFPTVDPR
1	2	0.11	0.26	0.62	LADLLER
2	1	0.21	0.49	1.2	FVDSVSGK
1	0	0.21	0.64	2	TVTFR
1	1	0.26	0.69	1.8	FKDVK
1	0	0.25	0.77	2.3	LGSPWR
0	1	0.3	0.91	2.8	ALYLSNSLR
0	1	0.42	1.3	3.8	IYNEFVER
0	1	0.32	0.98	3	LAEQTPLSGLYVAK
0	1	0.29	0.86	2.7	LGPALAMGNTVVMK
0	7	0.72	1.4	2.6	LIPVMAAK
0	3	0.28	0.64	1.5	TLHIGGDHFAYTR
0	2	0.42	1.1	2.9	IFVEEK
0	1	0.35	1.1	3.3	LADLLERDR

5.341 96 503 minus strand hypothetical protein DAPPUDRAFT_230378 [Daphnia pulex]

Protein Accession	CL14922.Contig5$_H$1
Mean Expression Ratio	1.22
Median Expression Ratio	1.22
Credible Interval	(0.582, 2.62)
Associated Peptides	3
Associated Spectra	3
Coverage	NaN

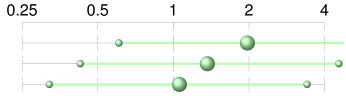

A	B	2.5	50	97.5	Sequence
0	1	0.61	2	6.4	HLQLAIRNDEELNK
0	1	0.43	1.4	4.6	KTHTKPTTGAGSAAAGKA
0	1	0.32	1.1	3.4	QLAIRNDEELNK

5.342 " 3195 3968 Spectrin alpha chain, putative [Pediculus humanus corporis] gi"

Protein Accession	CL2195.Contig1$_H$1
Mean Expression Ratio	1.22
Median Expression Ratio	1.21
Credible Interval	(0.588, 2.58)
Associated Peptides	3
Associated Spectra	3
Coverage	NaN

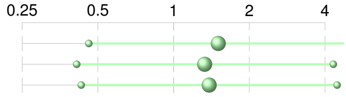

A	B	2.5	50	97.5	Sequence
0	1	0.46	1.5	5	LIDEGNHNADGIVER
0	1	0.41	1.3	4.3	LSELEAAASQR
0	1	0.43	1.4	4.5	TDVVDSWIADKETQVR

5.343 2 2572 Glycogen debranching enzyme [Crassostrea gigas]

Protein Accession	CL45503.Contig1$_H$1
Mean Expression Ratio	0.827
Median Expression Ratio	0.825
Credible Interval	(0.358, 1.91)
Associated Peptides	1
Associated Spectra	1
Coverage	NaN

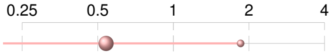

A	B	2.5	50	97.5	Sequence
**0**	**1**	**0.16**	**0.54**	**1.9**	**QMLNNLHYEMAR**

5.344 460 1737 Tubulin beta-4B chain OS=Mus musculus GN=Tubb4b PE=1 SV=1

Protein Accession	Unigene34147$_H$1
Mean Expression Ratio	1.21
Median Expression Ratio	1.21
Credible Interval	(0.564, 2.56)
Associated Peptides	1
Associated Spectra	30
Coverage	NaN

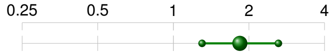

A	B	2.5	50	97.5	Sequence
**5**	**25**	**1.3**	**1.8**	**2.6**	**AVLVDLEPGTMDSVR**

5.345 93 368 60S ribosomal protein L37A [Branchiostoma belcheri] gi; 93 395 60S ribosomal protein L37A [Branchiostoma belcheri] gi; 90 272 ribosomal protein L37ae [Wuchereria bancrofti]; 44 319 minus strand 60S ribosomal protein L37A [Branchiostoma belcheri] gi; 44 319 minus strand 60S ribosomal protein L37A [Branchiostoma belcheri] gi

Protein Accession	Unigene66574$_H$1 CL6728.Contig4$_H$1 CL6728.Contig3$_H$1 CL6728.Contig2$_H$1 CL6728.Contig
Mean Expression Ratio	1.21
Median Expression Ratio	1.21
Credible Interval	(0.524, 2.85)
Associated Peptides	1
Associated Spectra	1
Coverage	NaN

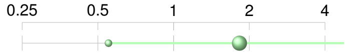

A	B	2.5	50	97.5	Sequence
**0**	**1**	**0.55**	**1.8**	**6.2**	**VGIVGK**

5.346 " 1 1563 Putative methylmalonate-semialdehyde dehydrogenase [acylating], mitochondrial [Crassostrea gigas]"

Protein Accession	Unigene56839$_H$1
Mean Expression Ratio	1.21
Median Expression Ratio	1.21
Credible Interval	(0.758, 1.95)
Associated Peptides	14
Associated Spectra	24
Coverage	NaN

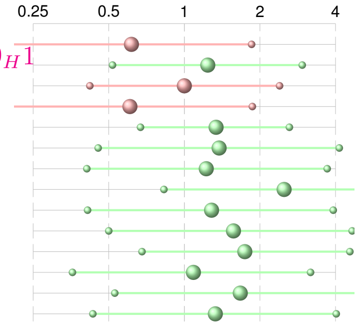

A	B	2.5	50	97.5	Sequence
1	0	0.2	0.62	1.9	GADLGPLISPESK
1	2	0.52	1.2	2.9	IAANITEQQGK
1	2	0.42	1	2.4	TVTQMWR
1	0	0.19	0.61	1.9	YQQLIK
1	5	0.67	1.3	2.6	GNFVGPTILHK
0	1	0.45	1.4	4.1	AISFVGSDQAGQYIYER
0	1	0.41	1.2	3.7	LFINNEFVESK
0	1	0.83	2.5	7.8	NIKVPGYEK
0	1	0.41	1.3	3.9	NWIPELVER
0	1	0.5	1.6	4.7	QGVQFYTQVK
0	2	0.68	1.7	4.6	TLVDAEGDVLR
0	1	0.36	1.1	3.2	WIDVHNPATNEVVTR
0	1	0.53	1.7	5.2	QQIMFR
0	1	0.43	1.3	4	FINNEFVESK

5.347 366 935 minus strand 40S ribosomal protein S2 OS=Urechis caupo GN=RPS2 PE=2 SV=1

Protein Accession	CL2084.Contig7$_H$1
Mean Expression Ratio	0.828
Median Expression Ratio	0.827
Credible Interval	(0.42, 1.63)
Associated Peptides	4
Associated Spectra	6
Coverage	NaN

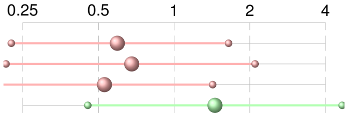

A	B	2.5	50	97.5	Sequence
2	0	0.22	0.59	1.7	GTGIVSAPVPK
1	0	0.21	0.68	2.1	LIPAPR
1	1	0.2	0.53	1.4	LSVIPVR
0	1	0.45	1.5	4.7	GATATLGNFAK

5.348 513 755 PREDICTED: 40S ribosomal protein S16-like [Papio anubis]; 513 755 PREDICTED: 40S ribosomal protein S16-like [Papio anubis]

Protein Accession	CL14629.Contig2$_H$1 CL14629.Contig1$_H$1
Mean Expression Ratio	0.829
Median Expression Ratio	0.827
Credible Interval	(0.378, 1.85)
Associated Peptides	1
Associated Spectra	3
Coverage	NaN

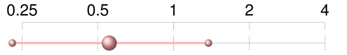

A	B	2.5	50	97.5	Sequence
3	0	0.23	0.55	1.4	SLLVADPR

5.349 4 234 ribosomal protein rpl14 [Arenicola marina]

Protein Accession	CL2715.Contig2$_{H}1$
Mean Expression Ratio	1.21
Median Expression Ratio	1.21
Credible Interval	(0.535, 2.74)
Associated Peptides	1
Associated Spectra	2
Coverage	NaN

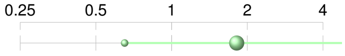

A	B	2.5	50	97.5	Sequence
**0**	**2**	**0.65**	**1.8**	**5.1**	**ALHLTQFVIK**

5.350 326 724 hypothetical protein [Amblyomma maculatum]

Protein Accession	Unigene1454$_{H}1$
Mean Expression Ratio	1.21
Median Expression Ratio	1.21
Credible Interval	(0.592, 2.43)
Associated Peptides	3
Associated Spectra	5
Coverage	NaN

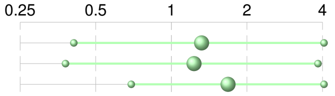

A	B	2.5	50	97.5	Sequence
**0**	**1**	**0.41**	**1.3**	**4.1**	**EAYLELER**
**0**	**1**	**0.38**	**1.2**	**3.8**	**KLEEHFADTK**
**0**	**3**	**0.69**	**1.7**	**4.1**	**EAYLELERR**

5.351 320 589 60S ribosomal protein L34 OS=Aedes triseriatus GN=RpL34 PE=2 SV=3

Protein Accession	CL29774.Contig2$_{H}1$
Mean Expression Ratio	0.83
Median Expression Ratio	0.828
Credible Interval	(0.378, 1.83)
Associated Peptides	1
Associated Spectra	4
Coverage	NaN

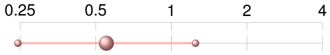

A	B	2.5	50	97.5	Sequence
**2**	**2**	**0.24**	**0.55**	**1.3**	**AFLIEEQK**

5.352 244 837 PREDICTED: ras-like GTP-binding protein Rho1-like [Megachile rotundata]; 650 883 hypothetical protein SINV_15432 [Solenopsis invicta]; 534 767 hypothetical protein SINV_15432 [Solenopsis invicta]

Protein Accession	Unigene11792$_{H}1$ Unigene11791$_{H}1$ Unigene11790$_{H}1$
Mean Expression Ratio	0.824
Median Expression Ratio	0.829
Credible Interval	(0.358, 1.88)
Associated Peptides	1
Associated Spectra	2
Coverage	NaN

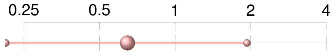

A	B	2.5	50	97.5	Sequence
**1**	**1**	**0.21**	**0.65**	**1.9**	**EVFETATR**

5.353 21 341 minus strand hemocyanin isoform 1 [Nucula nucleus]

Protein Accession	CL40281.Contig2$_{H}1$
Mean Expression Ratio	0.83
Median Expression Ratio	0.829
Credible Interval	(0.426, 1.62)
Associated Peptides	3
Associated Spectra	17
Coverage	NaN

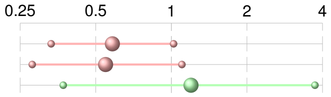

A	B	2.5	50	97.5	Sequence
8	2	0.33	0.58	1	DSNPIALTR
2	4	0.28	0.55	1.1	GDELFSYLK
0	1	0.37	1.2	3.7	KPLEPFNRDSNPIALTR

5.354 21 254 predicted protein [Nematostella vectensis] gi

Protein Accession	CL43392.Contig2$_{H}1$
Mean Expression Ratio	0.83
Median Expression Ratio	0.83
Credible Interval	(0.374, 1.8)
Associated Peptides	1
Associated Spectra	6
Coverage	NaN

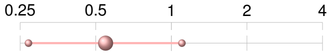

A	B	2.5	50	97.5	Sequence
4	2	0.27	0.55	1.1	AADAVVAEIK

5.355 297 734 minus strand PREDICTED: calmodulin [Canis lupus familiaris]; 297 734 PREDICTED: calmodulin [Canis lupus familiaris]; 143 508 minus strand Calmodulin [Crassostrea gigas]

Protein Accession	Unigene26719$_{H}1$ Unigene17836$_{H}1$ Unigene10331$_{H}1$
Mean Expression Ratio	1.2
Median Expression Ratio	1.21
Credible Interval	(0.52, 2.84)
Associated Peptides	1
Associated Spectra	1
Coverage	NaN

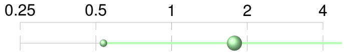

A	B	2.5	50	97.5	Sequence
0	1	0.53	1.8	5.9	TLITEFENR

5.356 " 196 1053 minus strand Cytochrome b-c1 complex subunit 2, mitochondrial OS=Homo sapiens GN=UQCRC2 PE=1 SV=3"

Protein Accession	CL7847.Contig5$_{H}1$
Mean Expression Ratio	1.21
Median Expression Ratio	1.21
Credible Interval	(0.526, 2.78)
Associated Peptides	1
Associated Spectra	1
Coverage	NaN

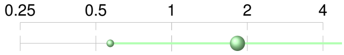

A	B	2.5	50	97.5	Sequence
0	1	0.57	1.8	6	VGKYNAEQLLHYMK

5.357 284 1318 hsc70-interacting protein [Gallus gallus] gi; 88 1137 hsc70-interacting protein [Gallus gallus] gi

Protein Accession	CL11847.Contig4$_{H}1$ CL11847.Contig2$_{H}1$
Mean Expression Ratio	0.829
Median Expression Ratio	0.83
Credible Interval	(0.396, 1.73)
Associated Peptides	2
Associated Spectra	7
Coverage	NaN

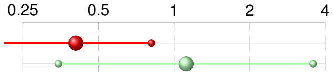

A	B	2.5	50	97.5	Sequence
**5**	**1**	**0.2**	**0.41**	**0.81**	**AQLDLLK**
**0**	**1**	**0.35**	**1.1**	**3.6**	**LNPTSALLYAK**

5.358 " 451 1026 PREDICTED: GTP-binding protein SAR1B, putative-like [Saccoglossus kowalevskii]"

Protein Accession	Unigene4124$_{H}1$
Mean Expression Ratio	1.2
Median Expression Ratio	1.2
Credible Interval	(0.523, 2.72)
Associated Peptides	1
Associated Spectra	1
Coverage	NaN

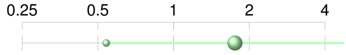

A	B	2.5	50	97.5	Sequence
**0**	**1**	**0.54**	**1.7**	**6.1**	**FTTFDLGGHR**

5.359 26 250 minus strand fructose-bisphosphate aldolase [Haliotis discus discus]

Protein Accession	Unigene16711$_{H}1$
Mean Expression Ratio	1.2
Median Expression Ratio	1.2
Credible Interval	(0.541, 2.63)
Associated Peptides	2
Associated Spectra	2
Coverage	NaN

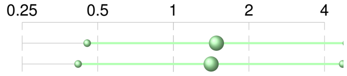

A	B	2.5	50	97.5	Sequence
**0**	**1**	**0.45**	**1.5**	**4.9**	**IANAIVAPGK**
**0**	**1**	**0.42**	**1.4**	**4.7**	**ELRRIANAIVAPGK**

5.360 " 31 2061 minus strand 1,4-alpha-glucan-branching enzyme [Crassostrea gigas]"

Protein Accession	CL12555.Contig7$_{H}1$
Mean Expression Ratio	1.21
Median Expression Ratio	1.2
Credible Interval	(0.563, 2.58)
Associated Peptides	2
Associated Spectra	3
Coverage	NaN

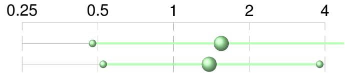

A	B	2.5	50	97.5	Sequence
**0**	**1**	**0.48**	**1.5**	**5.1**	**LFKLDGYLR**
**0**	**2**	**0.52**	**1.4**	**3.8**	**LDFPR**

5.361 157 1422 Tektin-2 [Crassostrea gigas]

Protein Accession	Unigene17501$_H$1
Mean Expression Ratio	1.2
Median Expression Ratio	1.2
Credible Interval	(0.557, 2.6)
Associated Peptides	2
Associated Spectra	3
Coverage	NaN

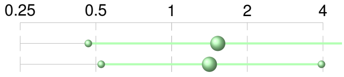

A	B	2.5	50	97.5	Sequence
**0**	**1**	**0.47**	**1.5**	**5**	**ADAEMLAANR**
**0**	**2**	**0.53**	**1.4**	**3.9**	**NLTITGIERPR**

5.362 160 1218 Galectin-9 [Crassostrea gigas]

Protein Accession	Unigene165$_H$1
Mean Expression Ratio	0.833
Median Expression Ratio	0.832
Credible Interval	(0.422, 1.66)
Associated Peptides	3
Associated Spectra	9
Coverage	NaN

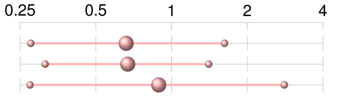

A	B	2.5	50	97.5	Sequence
**1**	**2**	**0.28**	**0.66**	**1.6**	**IAVNNQHLVEFR**
**3**	**2**	**0.31**	**0.67**	**1.4**	**SGIPGGLAPGR**
**0**	**1**	**0.27**	**0.89**	**2.8**	**EATALVVTGDVR**

5.363 " 57 752 PREDICTED: adenylate kinase 2, mitochondrial-like isoform 2 [Callithrix jacchus]"

Protein Accession	CL4149.Contig1$_H$1
Mean Expression Ratio	1.21
Median Expression Ratio	1.2
Credible Interval	(0.506, 2.82)
Associated Peptides	1
Associated Spectra	1
Coverage	NaN

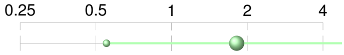

A	B	2.5	50	97.5	Sequence
**0**	**1**	**0.55**	**1.8**	**5.9**	**AVISSGSALGKK**

5.364 155 3214 sarco/endoplasmic reticulum calcium ATPase isoform B [Pinctada fucata]; 155 3214 minus strand sarco/endoplasmic reticulum calcium ATPase isoform B [Pinctada fucata]

Protein Accession	CL1323.Contig9$_H$1 CL1323.Contig1$_H$1
Mean Expression Ratio	1.2
Median Expression Ratio	1.2
Credible Interval	(0.616, 2.37)
Associated Peptides	4
Associated Spectra	7
Coverage	NaN

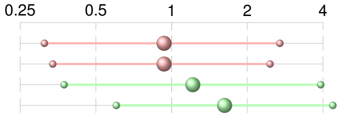

A	B	2.5	50	97.5	Sequence
**1**	**1**	**0.31**	**0.93**	**2.7**	**KAEIGIAMGSGTAVAK**
**0**	**2**	**0.34**	**0.93**	**2.5**	**VGEATETALTVLVEK**
**0**	**1**	**0.37**	**1.2**	**3.9**	**GAPEGLLDR**
**0**	**2**	**0.6**	**1.6**	**4.4**	**MDQAFTK**

5.365 221 1450 minus strand PREDICTED: dihydrolipoamide branched chain transacylase E2 [Taeniopygia guttata]; 221 1609 minus strand PREDICTED: dihydrolipoamide branched chain transacylase E2 [Taeniopygia guttata]

Protein Accession	CL25782.Contig5$_{H}1$ CL25782.Contig3$_{H}1$
Mean Expression Ratio	1.2
Median Expression Ratio	1.2
Credible Interval	(0.537, 2.72)
Associated Peptides	1
Associated Spectra	2
Coverage	NaN

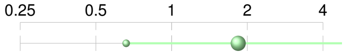

A	B	2.5	50	97.5	Sequence
0	2	0.66	1.8	5.1	VLATPAVR

5.366 1180 1521 minus strand coronin [Schistosoma mansoni]

Protein Accession	CL11779.Contig9$_{H}1$
Mean Expression Ratio	0.834
Median Expression Ratio	0.835
Credible Interval	(0.357, 1.9)
Associated Peptides	1
Associated Spectra	1
Coverage	NaN

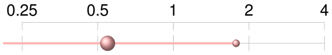

A	B	2.5	50	97.5	Sequence
1	0	0.16	0.55	1.8	DANPILVSLK

5.367 " 64 1332 Flotillin-1, putative [Pediculus humanus corporis] gi"

Protein Accession	CL33952.Contig1$_{H}1$
Mean Expression Ratio	1.2
Median Expression Ratio	1.2
Credible Interval	(0.63, 2.27)
Associated Peptides	6
Associated Spectra	7
Coverage	NaN

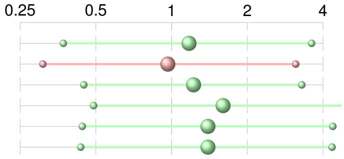

A	B	2.5	50	97.5	Sequence
1	0	0.37	1.2	3.6	AVAQETLEGHQR
1	0	0.31	0.97	3.1	VAAEIAQPLSNAK
1	1	0.45	1.2	3.3	FLNDIEIAK
0	1	0.49	1.6	5.1	AQADLAYDLQAAK
0	1	0.44	1.4	4.4	GQAIQVQEQEIIRR
0	1	0.44	1.4	4.3	VKGEAEAFAIEAK

5.368 " 1 1815 Glycogen phosphorylase, muscle form [Crassostrea gigas]"

Protein Accession	CL20630.Contig5$_{H}1$
Mean Expression Ratio	1.19
Median Expression Ratio	1.2
Credible Interval	(0.565, 2.54)
Associated Peptides	2
Associated Spectra	4
Coverage	NaN

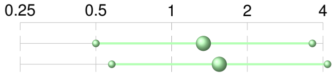

A	B	2.5	50	97.5	Sequence
0	2	0.5	1.3	3.6	IEELEEIEEDAGL
0	2	0.58	1.5	4.2	FGNPWEK

5.369 143 634 minus strand ribosomal protein S14 [Solea senegalensis]

Protein Accession	CL17182.Contig1$_H$1
Mean Expression Ratio	1.19
Median Expression Ratio	1.19
Credible Interval	(0.549, 2.62)
Associated Peptides	2
Associated Spectra	3
Coverage	NaN

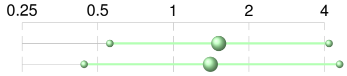

A	B	2.5	50	97.5	Sequence
0	2	0.56	1.5	4.2	TVQQEQVVLGPQAK
0	1	0.44	1.4	4.6	QLGITALHIK

5.370 628 1140 paramyosin [Haliotis discus discus]

Protein Accession	CL43455.Contig3$_H$1
Mean Expression Ratio	0.84
Median Expression Ratio	0.838
Credible Interval	(0.412, 1.71)
Associated Peptides	2
Associated Spectra	21
Coverage	NaN

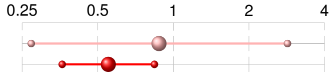

A	B	2.5	50	97.5	Sequence
1	0	0.27	0.88	2.9	EITRVVKV
7	13	0.36	0.55	0.84	LEEAEAFAVK

5.371 182 1846 minus strand hypothetical protein DAPPUDRAFT_302108 [Daphnia pulex]; 110 1774 minus strand hypothetical protein DAPPUDRAFT_302108 [Daphnia pulex]

Protein Accession	CL11561.Contig6$_H$1 CL11561.Contig1$_H$1
Mean Expression Ratio	1.2
Median Expression Ratio	1.19
Credible Interval	(0.592, 2.45)
Associated Peptides	3
Associated Spectra	4
Coverage	NaN

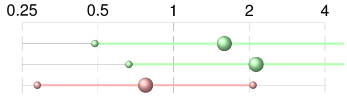

A	B	2.5	50	97.5	Sequence
0	1	0.49	1.6	5	VATSPFEGQKPGTSGLR
0	1	0.66	2.1	6.8	IVYRLSGTGSSGATIR
0	2	0.29	0.77	2.1	LFLPGGLK

5.372 76 822 14-3-3 protein [Heterodera glycines]; 76 822 minus strand 14-3-3 protein [Heterodera glycines]; 76 822 minus strand 14-3-3 protein [Heterodera glycines]

Protein Accession	Unigene34590$_H$1 Unigene29843$_H$1 Unigene28010$_H$1
Mean Expression Ratio	0.835
Median Expression Ratio	0.838
Credible Interval	(0.456, 1.51)
Associated Peptides	6
Associated Spectra	20
Coverage	NaN

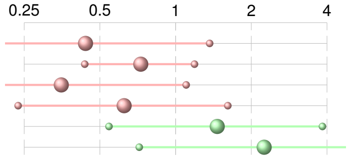

A	B	2.5	50	97.5	Sequence
1	0	0.14	0.44	1.4	YLSEVSQGEQR
7	6	0.44	0.73	1.2	VVSSIEQK
1	0	0.11	0.35	1.1	KLEMAK
0	2	0.24	0.62	1.6	IAQEQMPTTHPIR
0	2	0.54	1.5	3.8	LAEQAERYDDMAK
0	1	0.72	2.2	7	SMDAYKK

5.373 91 3270 Valyl-tRNA synthetase [Crassostrea gigas]

Protein Accession	CL23212.Contig7$_{H}1$
Mean Expression Ratio	1.2
Median Expression Ratio	1.19
Credible Interval	(0.54, 2.65)
Associated Peptides	2
Associated Spectra	2
Coverage	NaN

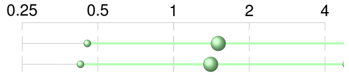

A	B	2.5	50	97.5	Sequence
**0**	**1**	**0.45**	**1.5**	**5.2**	**FVQHPFFDR**
**0**	**1**	**0.43**	**1.4**	**4.8**	**SFGTGAVK**

5.374 249 845 sigma class glutathione-s-transferase 2 [Haliotis discus discus]; 252 848 sigma class glutathione-s-transferase 2 [Haliotis discus discus]

Protein Accession	CL19285.Contig32$_{H}1$ CL19285.Contig20$_{H}1$
Mean Expression Ratio	0.838
Median Expression Ratio	0.839
Credible Interval	(0.357, 1.95)
Associated Peptides	1
Associated Spectra	1
Coverage	NaN

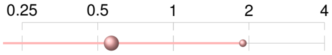

A	B	2.5	50	97.5	Sequence
**1**	**0**	**0.17**	**0.56**	**1.9**	**VKTENLPR**

5.375 2 532 heat shock protein 70 [Echinostoma caproni]

Protein Accession	Unigene17002$_{H}1$
Mean Expression Ratio	1.19
Median Expression Ratio	1.19
Credible Interval	(0.56, 2.52)
Associated Peptides	2
Associated Spectra	3
Coverage	NaN

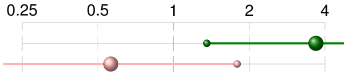

A	B	2.5	50	97.5	Sequence
**0**	**2**	**1.4**	**3.7**	**9.9**	**ARFEELNADLFR**
**0**	**1**	**0.17**	**0.56**	**1.8**	**ARFEELNADLFRGTLEPVEK**

5.376 313 1908 minus strand Heterogeneous nuclear ribonucleoprotein L [Crassostrea gigas]; 313 1833 minus strand Heterogeneous nuclear ribonucleoprotein L [Crassostrea gigas]

Protein Accession	CL19412.Contig8$_{H}1$ CL19412.Contig7$_{H}1$
Mean Expression Ratio	0.837
Median Expression Ratio	0.839
Credible Interval	(0.363, 1.94)
Associated Peptides	1
Associated Spectra	2
Coverage	NaN

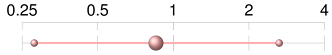

A	B	2.5	50	97.5	Sequence
**1**	**1**	**0.28**	**0.85**	**2.6**	**SSTGLLEFESK**

5.377 3 263 calponin-like protein [Taenia asiatica]

Protein Accession	CL26318.Contig4$_{H}1$
Mean Expression Ratio	1.19
Median Expression Ratio	1.19
Credible Interval	(0.526, 2.7)
Associated Peptides	1
Associated Spectra	2
Coverage	NaN

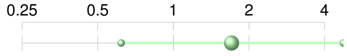

A	B	2.5	50	97.5	Sequence
**0**	**2**	**0.62**	**1.7**	**4.8**	**HIADIKVDDMSAASK**

5.378 408 1010 glutathione S-transferase sigma 2 [Mytilus galloprovincialis]; 166 768 glutathione S-transferase sigma 2 [Mytilus galloprovincialis]

Protein Accession	CL22038.Contig5$_{H}1$ CL22038.Contig4$_{H}1$
Mean Expression Ratio	0.842
Median Expression Ratio	0.841
Credible Interval	(0.471, 1.48)
Associated Peptides	7
Associated Spectra	17
Coverage	NaN

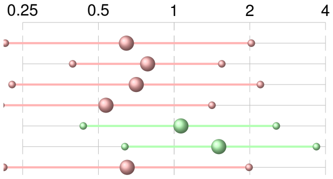

A	B	2.5	50	97.5	Sequence
**1**	**0**	**0.21**	**0.65**	**2**	**LVLEAAGR**
**2**	**4**	**0.4**	**0.78**	**1.5**	**VFSEATPK**
**1**	**0**	**0.23**	**0.71**	**2.2**	**KAELYEK**
**2**	**0**	**0.2**	**0.53**	**1.4**	**ELGLYGK**
**0**	**3**	**0.44**	**1.1**	**2.5**	**LVYFDLR**
**0**	**3**	**0.64**	**1.5**	**3.7**	**STVESHPFLK**
**0**	**1**	**0.21**	**0.65**	**2**	**YLGFFQK**

5.379 85 1632 minus strand Alpha-aminoadipic semialdehyde dehydrogenase [Crassostrea gigas]

Protein Accession	Unigene19467$_{H}1$
Mean Expression Ratio	1.19
Median Expression Ratio	1.19
Credible Interval	(0.518, 2.79)
Associated Peptides	1
Associated Spectra	1
Coverage	NaN

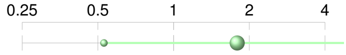

A	B	2.5	50	97.5	Sequence
**0**	**1**	**0.53**	**1.8**	**5.9**	**ELPLAQGIKFE**

5.380 253 765 cytochrome c oxidase subunit IV [Urechis caupo]

Protein Accession	CL38636.Contig3$_{H}1$
Mean Expression Ratio	0.841
Median Expression Ratio	0.841
Credible Interval	(0.451, 1.56)
Associated Peptides	4
Associated Spectra	16
Coverage	NaN

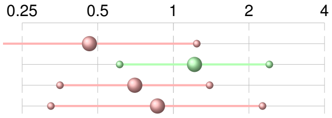

A	B	2.5	50	97.5	Sequence
**2**	**0**	**0.17**	**0.46**	**1.2**	**SLTLEDKK**
**1**	**5**	**0.61**	**1.2**	**2.4**	**FKENTPDVMALR**
**2**	**4**	**0.35**	**0.7**	**1.4**	**IEGLSSK**
**0**	**2**	**0.33**	**0.86**	**2.3**	**TEAALDQTARDRIHPK**

5.381 151 1449 rab GDP-dissociation inhibitor [Branchiostoma belcheri]

Protein Accession	CL30719.Contig3$_H$1
Mean Expression Ratio	1.19
Median Expression Ratio	1.19
Credible Interval	(0.611, 2.33)
Associated Peptides	4
Associated Spectra	7
Coverage	NaN

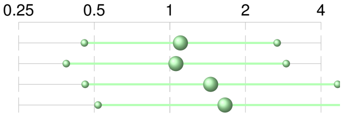

A	B	2.5	50	97.5	Sequence
1	2	0.46	1.1	2.7	ITGEAFDFSK
1	1	0.39	1.1	2.9	LLVATGVTR
0	1	0.46	1.5	4.7	TLYEPVDMGETSK
0	1	0.52	1.7	5.3	AICILNHPIKDTK

5.382 176 526 minus strand Translocon-associated protein subunit delta [Crassostrea gigas]

Protein Accession	Unigene63010$_H$1
Mean Expression Ratio	1.19
Median Expression Ratio	1.19
Credible Interval	(0.567, 2.53)
Associated Peptides	2
Associated Spectra	3
Coverage	NaN

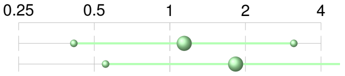

A	B	2.5	50	97.5	Sequence
1	1	0.41	1.1	3.1	KLPSGNYQVR
0	1	0.56	1.8	6.1	VFDEEGYALLR

5.383 " 283 1602 minus strand 4-aminobutyrate aminotransferase, mitochondrial [Crassostrea gigas]"

Protein Accession	CL30906.Contig1$_H$1
Mean Expression Ratio	1.18
Median Expression Ratio	1.19
Credible Interval	(0.569, 2.46)
Associated Peptides	3
Associated Spectra	4
Coverage	NaN

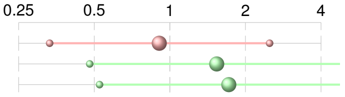

A	B	2.5	50	97.5	Sequence
1	1	0.33	0.91	2.5	AMFMTYQR
0	1	0.48	1.5	5	TMGALALTHTK
0	1	0.53	1.7	5.7	IIEAFKNPK

5.384 1 852 beta actin-like 1 protein [Macrostomum lignano]

Protein Accession Unigene31438$_{H}1$
Mean Expression Ratio 1.19
Median Expression Ratio 1.19
Credible Interval (0.88, 1.6)
Associated Peptides 26
Associated Spectra 328
Coverage NaN

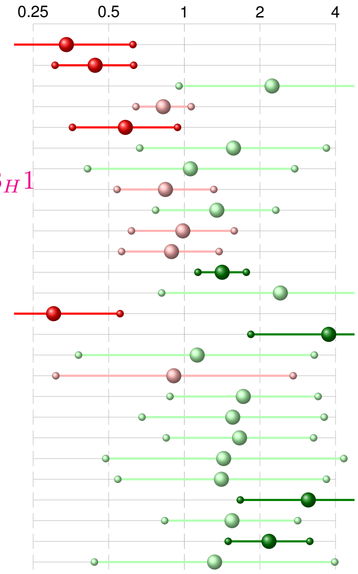

A	B	2.5	50	97.5	Sequence
4	4	0.19	0.34	0.62	AVFPSIVGR
13	14	0.31	0.44	0.63	GILTLK
2	1	0.95	2.2	5.2	HQGVMVGM
27	33	0.64	0.82	1.1	HQGVMVGMGQK
3	11	0.36	0.58	0.94	YPIEHGIVTNWDDMEK
1	2	0.67	1.6	3.7	VAPEEHPVLL
1	1	0.41	1.1	2.7	TEAPLNPK
5	12	0.54	0.84	1.3	RGILTLK
4	6	0.77	1.3	2.3	VAPEEHPVLLTEA
9	6	0.61	0.98	1.6	AGFAGDDAPR
5	12	0.56	0.89	1.4	DSYVGDEAQSK
18	58	1.1	1.4	1.8	VAPEEHPVLLTEAPLNPK
0	1	0.81	2.4	7.2	APEEHPVLLTEAPLNPK
0	8	0.16	0.3	0.55	GYSFTTTAER
0	5	1.8	3.8	7.8	HQGVMVGMGQKDSYVGDEAQSK
0	1	0.38	1.1	3.3	SYELPDGQVITIGN
0	1	0.31	0.91	2.7	TTGIVLDSGD
0	6	0.88	1.7	3.4	VAPEEHPVLLTEAPLN
0	3	0.68	1.6	3.6	VAPEEHPVLLTEAPLNPKANR
0	6	0.85	1.7	3.3	VAPEEHPVLLTEAPLNPKANREK
0	1	0.49	1.4	4.3	VAPEEHPVL
0	2	0.54	1.4	3.7	AVFPSIVGRPR
0	8	1.7	3.1	5.7	HQGVMVGMGQKDSYVGDEAQSKR
0	8	0.84	1.5	2.8	IWHHTFYNELR
0	25	1.5	2.2	3.2	SYELPDGQVITIGNER
0	1	0.44	1.3	4	TTGIVLDSGDGVTHTVPIYEGYALPHAILRLDLAG

5.385 816 1211 H2 [Haliotis tuberculata]

Protein Accession CL11519.Contig5$_{H}1$
Mean Expression Ratio 0.839
Median Expression Ratio 0.842
Credible Interval (0.43, 1.63)
Associated Peptides 3
Associated Spectra 11
Coverage NaN

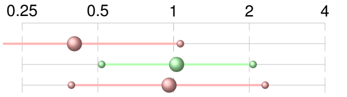

A	B	2.5	50	97.5	Sequence
1	1	0.15	0.4	1.1	LDSSADFDLQVK
1	5	0.52	1	2.1	SLILAMR
1	2	0.39	0.96	2.3	KNVLSLTPAER

5.386 71 1321 minus strand Vinculin [Crassostrea gigas]

Protein Accession Unigene23324$_{H}1$
Mean Expression Ratio 1.18
Median Expression Ratio 1.19
Credible Interval (0.559, 2.53)
Associated Peptides 3
Associated Spectra 3
Coverage NaN

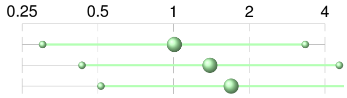

A	B	2.5	50	97.5	Sequence
0	1	0.3	1	3.4	GLVDEAVDTAALIR
0	1	0.43	1.4	4.6	KPAPTVDGK
0	1	0.51	1.7	5.5	LINAVGGVR

5.387 3 1670 Spectrin alpha chain [Crassostrea gigas]

Protein Accession	CL2195.Contig7$_{H}1$
Mean Expression Ratio	1.18
Median Expression Ratio	1.19
Credible Interval	(0.623, 2.17)
Associated Peptides	5
Associated Spectra	9
Coverage	NaN

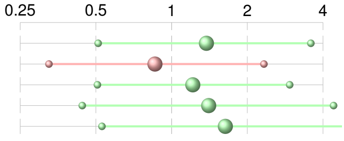

A	B	2.5	50	97.5	Sequence
1	1	0.51	1.4	3.6	KLDPSLSASR
0	2	0.32	0.86	2.3	EAAELAAWITEK
0	3	0.51	1.2	3	HQALQAEIAGHEPR
0	1	0.44	1.4	4.4	QGFVPAAYVK
0	1	0.53	1.6	5.2	VAALYDYTEK

5.388 34 480 small subunit ribosomal protein S18e [Clonorchis sinensis]; 431 799 SJCHGC01887 protein [Schistosoma japonicum] gi

Protein Accession	CL10131.Contig2$_{H}1$ CL10131.Contig1$_{H}1$
Mean Expression Ratio	0.846
Median Expression Ratio	0.843
Credible Interval	(0.39, 1.85)
Associated Peptides	1
Associated Spectra	6
Coverage	NaN

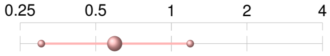

A	B	2.5	50	97.5	Sequence
3	3	0.3	0.6	1.2	LVTVMSNPR

5.389 165 620 PREDICTED: uncharacterized protein LOC575072 isoform 2 [Strongylocentrotus purpuratus] gi

Protein Accession	CL20552.Contig4$_{H}1$
Mean Expression Ratio	0.84
Median Expression Ratio	0.845
Credible Interval	(0.362, 1.89)
Associated Peptides	1
Associated Spectra	1
Coverage	NaN

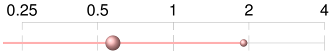

A	B	2.5	50	97.5	Sequence
1	0	0.18	0.58	1.9	IKPTPLNDR

5.390 213 875 es1 protein [Haliotis discus discus]

Protein Accession	CL35629.Contig1$_{H}1$
Mean Expression Ratio	0.841
Median Expression Ratio	0.845
Credible Interval	(0.377, 1.85)
Associated Peptides	2
Associated Spectra	2
Coverage	NaN

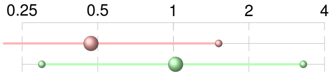

A	B	2.5	50	97.5	Sequence
1	0	0.15	0.47	1.5	NLSNFAFDAK
0	1	0.3	1	3.3	IARGPVNL

5.391 26 1909 Dolichyl-diphosphooligosaccharide–protein glycosyltransferase subunit 2 [Crassostrea gigas]

Protein Accession	CL6515.Contig1$_{H}$1
Mean Expression Ratio	0.848
Median Expression Ratio	0.846
Credible Interval	(0.536, 1.33)
Associated Peptides	15
Associated Spectra	30
Coverage	NaN

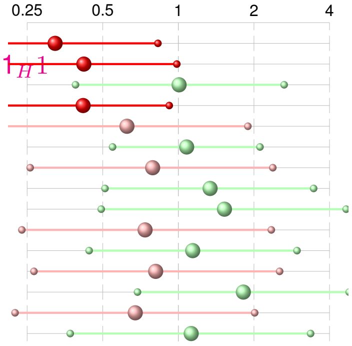

A	B	2.5	50	97.5	Sequence
2	0	0.12	0.32	0.83	APAIAEDK
2	1	0.18	0.42	0.99	KQFTASASDK
1	1	0.39	1	2.6	LIGTSGAEVEVK
2	2	0.19	0.42	0.92	NLGLAVDEK
1	0	0.2	0.62	1.9	KPEIK
1	5	0.55	1.1	2.1	LLTDAISAK
0	1	0.26	0.79	2.4	AVSSALLDALK
0	2	0.51	1.3	3.5	HLVDEAIVLSK
0	1	0.49	1.5	4.7	HLVDEAIVLSKK
0	1	0.24	0.74	2.3	TAYQFVSVIK
0	2	0.44	1.1	3	VSVENVEIGVADKDQTAAAR
0	1	0.27	0.81	2.5	VSVTNLMGGDLGQLTVTADSAR
0	2	0.69	1.8	4.8	LTISVAPK
0	1	0.22	0.67	2	TLYELNFMEAK
0	1	0.37	1.1	3.4	LTPSTFLTTLDQQR

5.392 40 1752 minus strand heat shock protein 60 [Biomphalaria glabrata]; 40 1752 minus strand heat shock protein 60 [Biomphalaria glabrata]

Protein Accession	CL5583.Contig6$_{H}$1 CL5583.Contig5$_{H}$1
Mean Expression Ratio	1.18
Median Expression Ratio	1.18
Credible Interval	(0.745, 1.86)
Associated Peptides	15
Associated Spectra	33
Coverage	NaN

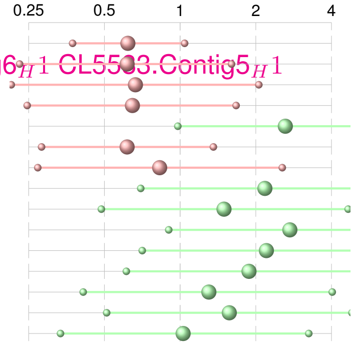

A	B	2.5	50	97.5	Sequence
4	9	0.37	0.62	1	AVGDLISSAMK
2	0	0.23	0.62	1.6	GANPIEIR
1	0	0.21	0.66	2.1	LSNGVAVLK
1	1	0.25	0.64	1.7	VGGTSEVEVNEK
1	1	0.98	2.6	6.8	ITKDGVTVAK
2	2	0.28	0.61	1.4	VGLQVVAVK
0	1	0.27	0.83	2.6	AAVEQGIVAGGGTALLR
0	1	0.7	2.2	6.7	AVGDLISSAMKK
0	1	0.49	1.5	4.7	DKYQNIGAK
0	1	0.9	2.7	8.7	VGGTSEVEVNEKKDR
0	1	0.71	2.2	6.8	AIELKDKYQNIGAK
0	1	0.61	1.9	5.6	AIAKEGFDKISK
0	1	0.41	1.3	4	CEFQDALVLLSEK
0	1	0.51	1.6	4.8	GYISPYFINTSK
0	1	0.33	1	3.3	TLRDELEVIEGMK

5.393 3512 6661 hypothetical protein BRAFLDRAFT_255626 [Branchiostoma floridae] gi

Protein Accession	CL1959.Contig8$_{H}$1
Mean Expression Ratio	1.18
Median Expression Ratio	1.18
Credible Interval	(0.665, 2.04)
Associated Peptides	9
Associated Spectra	11
Coverage	NaN

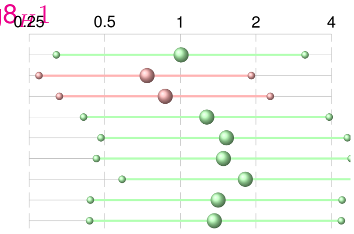

A	B	2.5	50	97.5	Sequence
1	0	0.32	1	3.1	SGVKIDVSDAEAQAR
1	1	0.27	0.73	1.9	LDEAFLSK
0	2	0.33	0.87	2.3	FDRPAQLHMAFR
0	1	0.41	1.3	3.9	IDVSDAEAQAR
0	1	0.48	1.5	4.6	NVVLGGVK
0	1	0.46	1.5	4.8	VKAALPSPEGLK
0	1	0.59	1.8	5.8	SVTIHDLGK
0	1	0.44	1.4	4.4	GGNVVQVKMPK
0	1	0.44	1.4	4.4	FIVAETNGLFGK

5.394 " 448 1365 Nitrile-specifier protein 5, partial [Crassostrea gigas]"

Protein Accession CL8618.Contig4$_{H}$1
Mean Expression Ratio 1.19
Median Expression Ratio 1.18
Credible Interval (0.584, 2.45)
Associated Peptides 3
Associated Spectra 5
Coverage NaN

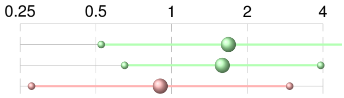

A	B	2.5	50	97.5	Sequence
0	1	0.52	1.7	5.6	KLPTFDAILPR
0	3	0.65	1.6	3.9	LLDTHGATVPPR
0	1	0.28	0.9	2.9	LYVFGGFTGK

5.395 167 679 tropomyosin [Haliotis discus discus]

Protein Accession CL2925.Contig3$_{H}$1
Mean Expression Ratio 1.18
Median Expression Ratio 1.18
Credible Interval (0.771, 1.82)
Associated Peptides 13
Associated Spectra 52
Coverage NaN

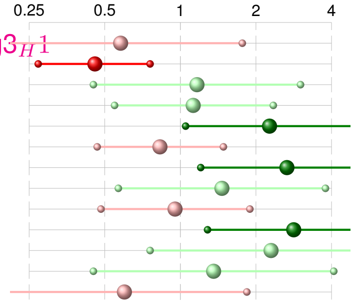

A	B	2.5	50	97.5	Sequence
1	0	0.19	0.58	1.8	ANEGLIEANTK
7	5	0.27	0.46	0.76	LRDTEVQK
1	1	0.45	1.2	3	MDAIK
1	4	0.55	1.1	2.3	RIKLLEEDLER
1	3	1.1	2.3	4.9	KMLAMK
6	3	0.47	0.83	1.5	MLAMK
2	2	1.2	2.7	5.9	MDAIKKK
1	1	0.57	1.5	3.8	MDAIKK
0	6	0.48	0.95	1.9	IKLLEEDLER
0	4	1.3	2.8	6.2	MDKENAMDKAEQMEQK
0	1	0.76	2.3	6.9	YANLENEYDKANEGLIEANTKLEASEK
0	1	0.45	1.4	4.1	AMDKAEQMEQK
0	1	0.2	0.6	1.8	SKLEEDLNPLQK

5.396 " 703 1173 60S acidic ribosomal protein P0, putative [Ixodes scapularis] gi"

Protein Accession Unigene34489$_{H}$1
Mean Expression Ratio 0.847
Median Expression Ratio 0.847
Credible Interval (0.419, 1.72)
Associated Peptides 3
Associated Spectra 6
Coverage NaN

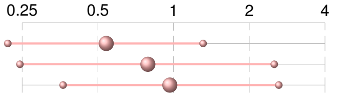

A	B	2.5	50	97.5	Sequence
1	2	0.22	0.54	1.3	EYLKDPSK
0	1	0.25	0.79	2.5	FAAVAAVAAPAAAK
0	2	0.36	0.97	2.6	KSFLEGVR

5.397 416 1717 minus strand T-complex protein 1 subunit delta OS=Aedes triseriatus PE=2 SV=1

Protein Accession CL8566.Contig1$_{H}$1
Mean Expression Ratio 0.842
Median Expression Ratio 0.847
Credible Interval (0.406, 1.75)
Associated Peptides 2
Associated Spectra 7
Coverage NaN

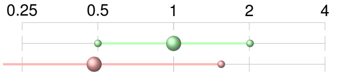

A	B	2.5	50	97.5	Sequence
2	4	0.5	1	2	HAAGELTAGINVR
1	0	0.15	0.48	1.6	LVLEEADR

5.398 84 425 minus strand Transcription elongation factor B polypeptide 2 [Crassostrea gigas]; 84 425 minus strand Transcription elongation factor B polypeptide 2 [Crassostrea gigas]; 84 425 minus strand Transcription elongation factor B polypeptide 2 [Crassostrea gigas]; 112 366 Transcription elongation factor B polypeptide 2 OS=Rattus norvegicus GN=Tceb2 PE=1 SV=1; 101 442 minus strand Transcription elongation factor B polypeptide 2 [Crassostrea gigas]; 101 442 minus strand Transcription elongation factor B polypeptide 2 [Crassostrea gigas]; 84 425 minus strand Transcription elongation factor B polypeptide 2 [Crassostrea gigas]

Protein Accession	CL19448.Contig9$_{H}1$ CL19448.Contig8$_{H}1$ CL19448.Contig7$_{H}1$ CL19448.Contig4$_{H}1$ CL1944
Mean Expression Ratio	0.847
Median Expression Ratio	0.847
Credible Interval	(0.367, 1.95)
Associated Peptides	1
Associated Spectra	1
Coverage	NaN

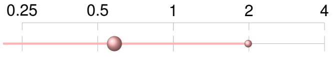

A	B	2.5	50	97.5	Sequence
1	0	0.17	0.58	2	AQAPATLGLVYK

5.399 3 434 minus strand myosin essential light chain [Haliotis discus discus]

Protein Accession	CL17177.Contig8$_{H}1$
Mean Expression Ratio	0.848
Median Expression Ratio	0.848
Credible Interval	(0.483, 1.47)
Associated Peptides	7
Associated Spectra	20
Coverage	NaN

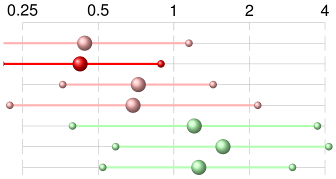

A	B	2.5	50	97.5	Sequence
1	1	0.16	0.44	1.2	EGQGFMSAAELR
2	3	0.2	0.42	0.89	LTDQQVTDVIK
3	3	0.36	0.72	1.4	FTDTREDLEGNVK
1	0	0.22	0.69	2.2	DGDVDAFK
0	1	0.39	1.2	3.7	LSDREGQGFMSAAELR
0	2	0.59	1.6	4.1	YADFIEK
0	3	0.52	1.3	3	HVLTGLGEK

5.400 313 2763 NCAM-related cell adhesion molecule precursor [Aplysia californica] gi

Protein Accession	Unigene11449$_{H}1$
Mean Expression Ratio	1.17
Median Expression Ratio	1.18
Credible Interval	(0.622, 2.23)
Associated Peptides	5
Associated Spectra	9
Coverage	NaN

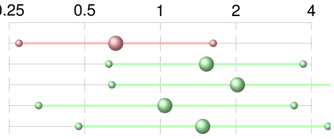

A	B	2.5	50	97.5	Sequence
2	1	0.27	0.66	1.6	SAGEPLLDAVTK
1	2	0.62	1.5	3.7	QILYFR
0	1	0.64	2	6.2	KTSVAEDYKELTVDR
0	1	0.33	1	3.4	EALEVDTSK
0	1	0.47	1.5	4.7	IGSSDPTVFVFK

5.401 1 309 PREDICTED: intersectin-1-like [Sus scrofa]

Protein Accession	Unigene37688$_H1$
Mean Expression Ratio	1.18
Median Expression Ratio	1.18
Credible Interval	(0.512, 2.73)
Associated Peptides	1
Associated Spectra	1
Coverage	NaN

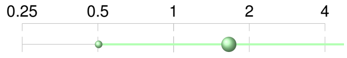

A	B	2.5	50	97.5	Sequence
**0**	**1**	**0.5**	**1.7**	**5.6**	**VDIQPGPKSSK**

5.402 2 2248 PREDICTED: heat shock 70 kDa protein 4-like [Oreochromis niloticus]; 2 2227 PREDICTED: heat shock 70 kDa protein 4-like [Oreochromis niloticus]

Protein Accession	CL12749.Contig5$_H1$ CL12749.Contig4$_H1$
Mean Expression Ratio	0.845
Median Expression Ratio	0.848
Credible Interval	(0.401, 1.76)
Associated Peptides	3
Associated Spectra	3
Coverage	NaN

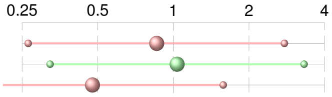

A	B	2.5	50	97.5	Sequence
**0**	**1**	**0.26**	**0.86**	**2.8**	**LLQECEK**
**0**	**1**	**0.32**	**1**	**3.3**	**TAWWTPK**
**0**	**1**	**0.15**	**0.48**	**1.6**	**EMLNLLIEK**

5.403 773 1045 minus strand serine protease inhibitor 2 [Latrodectus hesperus]

Protein Accession	Unigene4107$_H1$
Mean Expression Ratio	1.18
Median Expression Ratio	1.18
Credible Interval	(0.527, 2.64)
Associated Peptides	1
Associated Spectra	2
Coverage	NaN

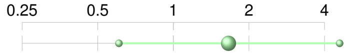

A	B	2.5	50	97.5	Sequence
**0**	**2**	**0.61**	**1.7**	**4.6**	**KFYFNK**

5.404 1200 1547 protein disulfide isomerase [Conus eburneus]

Protein Accession	CL3770.Contig1$_H1$
Mean Expression Ratio	0.848
Median Expression Ratio	0.849
Credible Interval	(0.527, 1.35)
Associated Peptides	8
Associated Spectra	53
Coverage	NaN

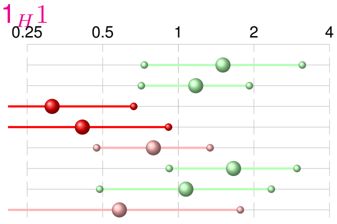

A	B	2.5	50	97.5	Sequence
**2**	**3**	**0.73**	**1.5**	**3.1**	**TLSAFVK**
**4**	**9**	**0.71**	**1.2**	**1.9**	**VQSFPTLK**
**4**	**1**	**0.15**	**0.31**	**0.67**	**YKDSNDIVIAK**
**4**	**0**	**0.19**	**0.41**	**0.91**	**VLVGK**
**4**	**8**	**0.47**	**0.8**	**1.3**	**NFNDVAFDK**
**0**	**9**	**0.92**	**1.7**	**3**	**IDSTANDVENIR**
**0**	**4**	**0.48**	**1.1**	**2.3**	**QLAPIWDELAEK**
**0**	**1**	**0.19**	**0.58**	**1.8**	**VVDYNGER**

5.405 635 1294 minus strand Kyphoscoliosis peptidase [Crassostrea gigas]; 643 1302 minus strand Kyphoscoliosis peptidase [Crassostrea gigas]; 635 1294 minus strand Kyphoscoliosis peptidase [Crassostrea gigas]; 634 1293 minus strand Kyphoscoliosis peptidase [Crassostrea gigas]; 634 1293 minus strand Kyphoscoliosis peptidase [Crassostrea gigas]; 634 1293 minus strand Kyphoscoliosis peptidase [Crassostrea gigas]; 639 1298 minus strand Kyphoscoliosis peptidase [Crassostrea gigas]

Protein Accession	CL7772.Contig20$_H$1 CL7772.Contig1$_H$1 CL7772.Contig19$_H$1 CL7772.Contig16$_H$1 CL7772.C
Mean Expression Ratio	1.17
Median Expression Ratio	1.18
Credible Interval	(0.575, 2.36)
Associated Peptides	3
Associated Spectra	6
Coverage	NaN

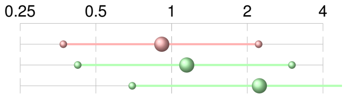

A	B	2.5	50	97.5	Sequence
2	1	0.37	0.91	2.2	LSNVTFK
0	2	0.42	1.1	3	GAGEVWEGTAALDK
0	1	0.7	2.2	7.2	GAGEVWEGTAALDKHR

5.406 18 1406 AGAP006996-PA [Anopheles gambiae str. PEST] gi

Protein Accession	CL48173.Contig5$_H$1
Mean Expression Ratio	1.18
Median Expression Ratio	1.18
Credible Interval	(0.54, 2.64)
Associated Peptides	2
Associated Spectra	2
Coverage	NaN

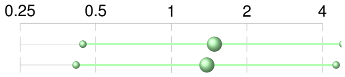

A	B	2.5	50	97.5	Sequence
0	1	0.45	1.5	4.8	VVLNLR
0	1	0.42	1.4	4.5	TTLTAAITK

5.407 144 1307 minus strand PREDICTED: similar to AGAP005845-PA [Tribolium castaneum]; 146 1309 minus strand PREDICTED: similar to AGAP005845-PA [Tribolium castaneum]; 144 1307 PREDICTED: similar to AGAP005845-PA [Tribolium castaneum]

Protein Accession	CL8394.Contig21$_H$1 CL8394.Contig20$_H$1 CL8394.Contig14$_H$1
Mean Expression Ratio	1.18
Median Expression Ratio	1.18
Credible Interval	(0.515, 2.71)
Associated Peptides	1
Associated Spectra	1
Coverage	NaN

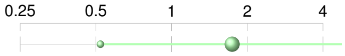

A	B	2.5	50	97.5	Sequence
0	1	0.52	1.7	5.6	KATGSLLTR

5.408 123 551 minus strand Transcription factor BTF3-like protein 4 [Crassostrea gigas]

Protein Accession	Unigene19288$_H$1
Mean Expression Ratio	1.18
Median Expression Ratio	1.18
Credible Interval	(0.519, 2.68)
Associated Peptides	1
Associated Spectra	2
Coverage	NaN

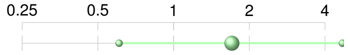

A	B	2.5	50	97.5	Sequence
**0**	**2**	**0.61**	**1.7**	**4.7**	**VQASLAANTFAITGHAETK**

5.409 546 995 Hypothetical protein CBG12002 [Caenorhabditis briggsae]; 543 995 minus strand PREDICTED: tubulin polymerization-promoting protein family member 2-like [Anolis carolinensis]; 546 995 Hypothetical protein CBG12002 [Caenorhabditis briggsae]; 546 995 minus strand Hypothetical protein CBG12002 [Caenorhabditis briggsae]

Protein Accession	CL6849.Contig7$_H$1 CL6849.Contig6$_H$1 CL6849.Contig2$_H$1 CL6849.Contig1$_H$1
Mean Expression Ratio	1.17
Median Expression Ratio	1.18
Credible Interval	(0.595, 2.32)
Associated Peptides	4
Associated Spectra	7
Coverage	NaN

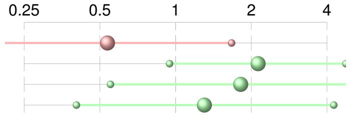

A	B	2.5	50	97.5	Sequence
**1**	**0**	**0.16**	**0.53**	**1.7**	**LLPQLAADK**
**0**	**4**	**0.95**	**2.1**	**4.7**	**LLPQLAADKAK**
**0**	**1**	**0.55**	**1.8**	**5.7**	**LKDNVAK**
**0**	**1**	**0.4**	**1.3**	**4.3**	**ADFLNK**

5.410 3 434 minus strand hypothetical protein CGI_10015501 [Crassostrea gigas]

Protein Accession	Unigene6481$_H$1
Mean Expression Ratio	0.85
Median Expression Ratio	0.851
Credible Interval	(0.369, 1.93)
Associated Peptides	1
Associated Spectra	1
Coverage	NaN

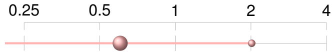

A	B	2.5	50	97.5	Sequence
**1**	**0**	**0.18**	**0.6**	**2**	**NDFIGGLDVMK**

5.411 58 900 minus strand Toll-interacting protein [Crassostrea gigas]

Protein Accession	CL6342.Contig9$_H$1
Mean Expression Ratio	1.17
Median Expression Ratio	1.18
Credible Interval	(0.514, 2.69)
Associated Peptides	1
Associated Spectra	1
Coverage	NaN

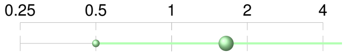

A	B	2.5	50	97.5	Sequence
**0**	**1**	**0.5**	**1.7**	**5.6**	**VMLGDLPSDFLR**

5.412 230 334 protein disulfide isomerase [Conus virgo]

Protein Accession	Unigene67026$_H$1
Mean Expression Ratio	0.854
Median Expression Ratio	0.851
Credible Interval	(0.398, 1.83)
Associated Peptides	1
Associated Spectra	10
Coverage	NaN

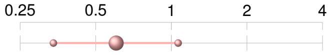

A	B	2.5	50	97.5	Sequence
**3**	**7**	**0.34**	**0.6**	**1.1**	**VDATVENDLASK**

5.413 " 132 1544 minus strand Serine hydroxymethyltransferase, cytosolic [Crassostrea gigas]"

Protein Accession	CL22573.Contig2$_H$1
Mean Expression Ratio	1.18
Median Expression Ratio	1.17
Credible Interval	(0.538, 2.55)
Associated Peptides	2
Associated Spectra	2
Coverage	NaN

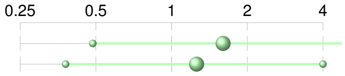

A	B	2.5	50	97.5	Sequence
**0**	**1**	**0.49**	**1.6**	**5.3**	**IGTPALTSR**
**0**	**1**	**0.38**	**1.3**	**4**	**SGIIFYR**

5.414 971 1963 Spectrin alpha chain [Crassostrea gigas]; 972 1979 Spectrin alpha chain [Crassostrea gigas]

Protein Accession	CL2195.Contig6$_H$1 CL2195.Contig4$_H$1
Mean Expression Ratio	1.17
Median Expression Ratio	1.17
Credible Interval	(0.516, 2.69)
Associated Peptides	1
Associated Spectra	2
Coverage	NaN

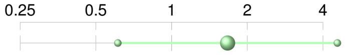

A	B	2.5	50	97.5	Sequence
**0**	**2**	**0.61**	**1.7**	**4.6**	**EAFLAGDTVDGDNVETLIK**

5.415 447 977 Expansin-yoaJ [Crassostrea gigas]

Protein Accession	CL6172.Contig2$_{H}1$
Mean Expression Ratio	0.853
Median Expression Ratio	0.852
Credible Interval	(0.387, 1.9)
Associated Peptides	1
Associated Spectra	3
Coverage	NaN

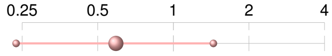

A	B	2.5	50	97.5	Sequence
**0**	**3**	**0.24**	**0.59**	**1.4**	**AGDVDLGVNGDGR**

5.416 2140 2367 heat shock cognate 71 [Mytilus galloprovincialis]

Protein Accession	CL5924.Contig13$_{H}1$
Mean Expression Ratio	0.852
Median Expression Ratio	0.852
Credible Interval	(0.4, 1.81)
Associated Peptides	1
Associated Spectra	8
Coverage	NaN

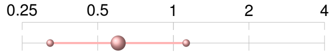

A	B	2.5	50	97.5	Sequence
**6**	**2**	**0.32**	**0.6**	**1.1**	**LIGDAAK**

5.417 1371 2189 minus strand galectin [Pinctada fucata]

Protein Accession	CL7245.Contig3$_{H}1$
Mean Expression Ratio	1.17
Median Expression Ratio	1.17
Credible Interval	(0.656, 2.07)
Associated Peptides	4
Associated Spectra	29
Coverage	NaN

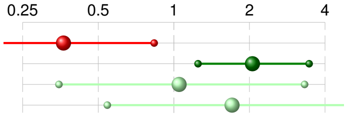

A	B	2.5	50	97.5	Sequence
**2**	**2**	**0.16**	**0.36**	**0.83**	**EIPQGLEK**
**13**	**10**	**1.2**	**2.1**	**3.5**	**FNEGVTVR**
**0**	**1**	**0.35**	**1**	**3.3**	**LGGAWGGEER**
**0**	**1**	**0.54**	**1.7**	**5.3**	**RNEGQVVFNSK**

5.418 " 39 968 l-3-hydroxyacyl-coenzyme a dehydrogenase, short chain [Haliotis discus discus]; 39 968 l-3-hydroxyacyl-coenzyme a dehydrogenase, short chain [Haliotis discus discus]"

Protein Accession	CL29042.Contig8$_{H}1$ CL29042.Contig1$_{H}1$
Mean Expression Ratio	0.85
Median Expression Ratio	0.853
Credible Interval	(0.414, 1.77)
Associated Peptides	3
Associated Spectra	4
Coverage	NaN

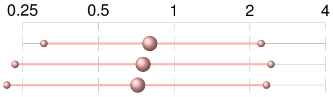

A	B	2.5	50	97.5	Sequence
**1**	**1**	**0.3**	**0.8**	**2.2**	**ISTSTDAATAAK**
**1**	**0**	**0.23**	**0.75**	**2.4**	**STASIQQSLQR**
**0**	**1**	**0.22**	**0.72**	**2.3**	**QTTFVK**

5.419 788 1006 LIM protein [Haliotis discus discus]

Protein Accession	CL29824.Contig2$_{H}$1
Mean Expression Ratio	0.857
Median Expression Ratio	0.853
Credible Interval	(0.386, 1.89)
Associated Peptides	1
Associated Spectra	4
Coverage	NaN

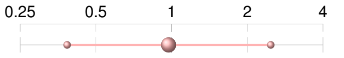

A	B	2.5	50	97.5	Sequence
**2**	**2**	**0.39**	**0.97**	**2.5**	**AIGVNTPWHK**

5.420 58 1203 minus strand Obg-like ATPase 1 [Crassostrea gigas]

Protein Accession	CL33105.Contig1$_{H}$1
Mean Expression Ratio	1.17
Median Expression Ratio	1.17
Credible Interval	(0.504, 2.76)
Associated Peptides	1
Associated Spectra	1
Coverage	NaN

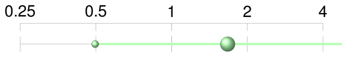

A	B	2.5	50	97.5	Sequence
**0**	**1**	**0.5**	**1.7**	**5.6**	**IHTDFEK**

5.421 3 614 PREDICTED: 60S ribosomal protein L8-like [Papio anubis]

Protein Accession	CL18365.Contig1$_{H}$1
Mean Expression Ratio	1.17
Median Expression Ratio	1.17
Credible Interval	(0.54, 2.56)
Associated Peptides	2
Associated Spectra	2
Coverage	NaN

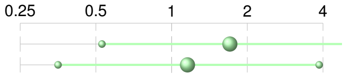

A	B	2.5	50	97.5	Sequence
**0**	**1**	**0.53**	**1.7**	**5.6**	**KVGLIAAR**
**0**	**1**	**0.35**	**1.2**	**3.9**	**VGLIAARR**

5.422 98 850 minus strand 14-3-3 protein epsilon [Schistocerca gregaria]

Protein Accession	CL27652.Contig3$_{H}$1
Mean Expression Ratio	1.17
Median Expression Ratio	1.17
Credible Interval	(0.666, 2.05)
Associated Peptides	7
Associated Spectra	16
Coverage	NaN

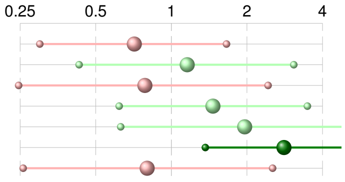

A	B	2.5	50	97.5	Sequence
**2**	**1**	**0.3**	**0.71**	**1.7**	**EAAENSLVAYK**
**1**	**1**	**0.43**	**1.2**	**3.1**	**VFYYK**
**1**	**0**	**0.25**	**0.79**	**2.4**	**IISSMEQK**
**0**	**3**	**0.62**	**1.5**	**3.5**	**AASDISMTELPPTHPIR**
**0**	**1**	**0.63**	**2**	**6.2**	**LAEQAERYDEMVEAMK**
**0**	**5**	**1.4**	**2.8**	**5.9**	**LAEQAERYDEMVEAMKK**
**0**	**1**	**0.26**	**0.8**	**2.5**	**YDEMVEAMK**

5.423 1363 1527 Protein disulfide-isomerase A3 [Crassostrea gigas]

Protein Accession	CL28563.Contig1$_{H}$1
Mean Expression Ratio	1.17
Median Expression Ratio	1.17
Credible Interval	(0.604, 2.25)
Associated Peptides	3
Associated Spectra	12
Coverage	NaN

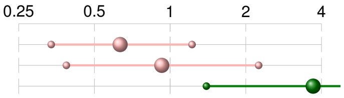

A	B	2.5	50	97.5	Sequence
2	5	0.34	0.63	1.2	LKDEPEITIAK
0	3	0.39	0.93	2.3	FEELAEK
0	2	1.4	3.7	10	ALEPKFEELAEK

5.424 " 57 1280 RNA binding motif protein, X-linked [Mustela putorius furo]"

Protein Accession	CL2752.Contig1$_{H}$1
Mean Expression Ratio	1.17
Median Expression Ratio	1.17
Credible Interval	(0.515, 2.67)
Associated Peptides	1
Associated Spectra	2
Coverage	NaN

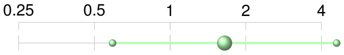

A	B	2.5	50	97.5	Sequence
0	2	0.59	1.6	4.6	LMDGKEVHGK

5.425 " 37 1356 homogentisate 1,2-dioxygenase [Danio rerio] gi; 54 1361 homogentisate 1,2-dioxygenase [Danio rerio] gi"

Protein Accession	CL16355.Contig5$_{H}$1 CL16355.Contig1$_{H}$1
Mean Expression Ratio	1.17
Median Expression Ratio	1.17
Credible Interval	(0.5, 2.72)
Associated Peptides	1
Associated Spectra	1
Coverage	NaN

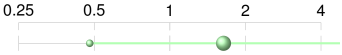

A	B	2.5	50	97.5	Sequence
0	1	0.48	1.6	5.5	SWLYR

5.426 373 579 myosin heavy chain [Argopecten irradians] gi

Protein Accession	Unigene64289$_{H}$1
Mean Expression Ratio	1.17
Median Expression Ratio	1.17
Credible Interval	(0.672, 2.05)
Associated Peptides	8
Associated Spectra	19
Coverage	NaN

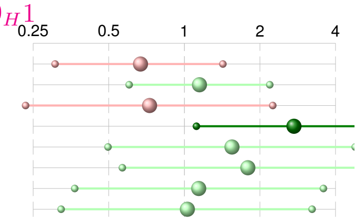

A	B	2.5	50	97.5	Sequence
1	3	0.31	0.67	1.4	KVQNELEDAEER
1	6	0.6	1.1	2.2	QVEEAEEIAAVNLAK
1	0	0.23	0.73	2.2	QELVDK
0	3	1.1	2.7	6.5	KVQNELEDAEERADSAEGTLSK
0	1	0.5	1.6	4.8	RQVEEAEEIAAVNLAK
0	1	0.57	1.8	5.4	VQNELEDAEERADSAEGTLSK
0	1	0.37	1.1	3.6	ELEDAEER
0	1	0.32	1	3.2	VQNELEDAEER

5.427 740 2029 minus strand PREDICTED: protein alan shepard-like isoform 2 [Bombus terrestris]

Protein Accession	CL693.Contig2$_{H}1$
Mean Expression Ratio	1.17
Median Expression Ratio	1.17
Credible Interval	(0.507, 2.73)
Associated Peptides	1
Associated Spectra	1
Coverage	NaN

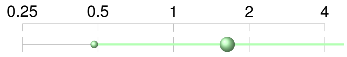

A	B	2.5	50	97.5	Sequence
**0**	**1**	**0.48**	**1.6**	**5.5**	**GAQGIQAQMAK**

5.428 593 862 minus strand mitochondrial ATP synthase coupling factor 6 precursor [Ochlerotatus taeniorhynchus]

Protein Accession	Unigene4216$_{H}1$
Mean Expression Ratio	0.856
Median Expression Ratio	0.858
Credible Interval	(0.393, 1.84)
Associated Peptides	2
Associated Spectra	2
Coverage	NaN

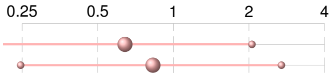

A	B	2.5	50	97.5	Sequence
**0**	**1**	**0.2**	**0.64**	**2.1**	**GSTVTDPIQQLFLDK**
**0**	**1**	**0.25**	**0.83**	**2.7**	**LVDASPTVQAALKEELEK**

5.429 165 635 hypothetical protein CGI_10007639 [Crassostrea gigas]

Protein Accession	CL36715.Contig1$_{H}1$
Mean Expression Ratio	1.17
Median Expression Ratio	1.16
Credible Interval	(0.51, 2.77)
Associated Peptides	1
Associated Spectra	1
Coverage	NaN

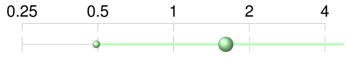

A	B	2.5	50	97.5	Sequence
**0**	**1**	**0.49**	**1.6**	**5.5**	**YVDDQTFTFSSK**

5.430 108 1040 minus strand PREDICTED: Na(+)/H(+) exchange regulatory cofactor NHE-RF3-like [Metaseiulus occidentalis]; 108 1259 minus strand PREDICTED: Na(+)/H(+) exchange regulatory cofactor NHE-RF3-like [Metaseiulus occidentalis]; 108 1190 minus strand PREDICTED: Na(+)/H(+) exchange regulatory cofactor NHE-RF3-like [Metaseiulus occidentalis]; 108 971 minus strand PREDICTED: Na(+)/H(+) exchange regulatory cofactor NHE-RF3-like [Metaseiulus occidentalis]

Protein Accession	CL26023.Contig8$_{H}1$ CL26023.Contig7$_{H}1$ CL26023.Contig6$_{H}1$ CL26023.Contig5$_{H}1$
Mean Expression Ratio	1.17
Median Expression Ratio	1.16
Credible Interval	(0.546, 2.49)
Associated Peptides	2
Associated Spectra	3
Coverage	NaN

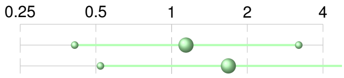

A	B	2.5	50	97.5	Sequence
0	2	0.41	1.1	3.2	GDLPEVLHLTAVR
0	1	0.52	1.7	5.6	IVKGDL

5.431 156 1106 ELAV 2-like protein [Aplysia californica] gi

Protein Accession	CL1500.Contig1$_{H}1$
Mean Expression Ratio	1.16
Median Expression Ratio	1.16
Credible Interval	(0.596, 2.26)
Associated Peptides	4
Associated Spectra	7
Coverage	NaN

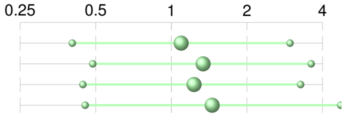

A	B	2.5	50	97.5	Sequence
0	2	0.4	1.1	3	NLPIPWTENELNK
0	2	0.49	1.3	3.6	SMFLSIGPIK
0	2	0.44	1.2	3.3	LFDPYGK
0	1	0.45	1.5	4.7	VLVDLQTGVSK

5.432 915 1505 minus strand Collagen alpha-5(VI) chain [Crassostrea gigas]

Protein Accession	CL40183.Contig3$_{H}1$
Mean Expression Ratio	1.17
Median Expression Ratio	1.16
Credible Interval	(0.653, 2.11)
Associated Peptides	7
Associated Spectra	12
Coverage	NaN

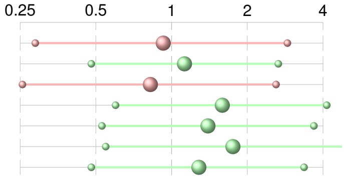

A	B	2.5	50	97.5	Sequence
1	0	0.29	0.93	2.9	EVQMAK
1	2	0.48	1.1	2.7	HGLATNTGAGIK
1	0	0.25	0.82	2.6	LEAIPWR
0	2	0.6	1.6	4.2	VGEQLDAEELLNIAGDPK
0	2	0.53	1.4	3.7	VGEQLDAEELLNIAGDPKR
0	1	0.55	1.8	5.4	HGLATNTGAG
0	2	0.48	1.3	3.4	VVTAENYAHLNLVK

5.433 74 2734 26S proteasome non-ATPase regulatory subunit 2 [Crassostrea gigas]

Protein Accession CL32689.Contig3$_{H}1$
Mean Expression Ratio 1.16
Median Expression Ratio 1.16
Credible Interval (0.504, 2.66)
Associated Peptides 1
Associated Spectra 1
Coverage NaN

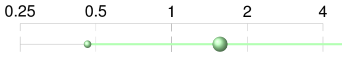

A	B	2.5	50	97.5	Sequence
0	1	0.46	1.6	5.3	VGQAVDVVGQAGKPK

5.434 211 1413 Alpha-N-acetylgalactosaminidase [Crassostrea gigas]

Protein Accession CL5989.Contig1$_{H}1$
Mean Expression Ratio 1.16
Median Expression Ratio 1.16
Credible Interval (0.534, 2.48)
Associated Peptides 2
Associated Spectra 3
Coverage NaN

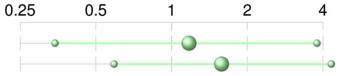

A	B	2.5	50	97.5	Sequence
0	1	0.34	1.2	3.8	ALADYMHDR
0	2	0.59	1.6	4.3	ALLLNR

5.435 " 81 1349 minus strand PREDICTED: LOW QUALITY PROTEIN: dolichyl-diphosphooligosaccharide–protein glycosyltransferase 48 kDa subunit-like, partial [Cavia porcellus]; 81 1349 minus strand PREDICTED: LOW QUALITY PROTEIN: dolichyl-diphosphooligosaccharide–protein glycosyltransferase 48 kDa subunit-like, partial [Cavia porcellus]"

Protein Accession CL49524.Contig2$_{H}1$ CL49524.Contig1$_{H}1$
Mean Expression Ratio 0.86
Median Expression Ratio 0.861
Credible Interval (0.417, 1.79)
Associated Peptides 4
Associated Spectra 4
Coverage NaN

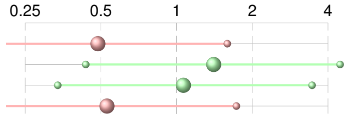

A	B	2.5	50	97.5	Sequence
0	1	0.15	0.49	1.6	DLGFDLTFK
0	1	0.43	1.4	4.5	KGNSPFLFR
0	1	0.34	1.1	3.5	NLAQWVFHQK
0	1	0.17	0.53	1.7	IDPFVR

5.436 494 724 minus strand PREDICTED: SH3 domain-binding glutamic acid-rich-like protein 3-like [Amphimedon queenslandica]; 424 819 minus strand PREDICTED: SH3 domain-binding glutamic acid-rich-like protein 3-like [Macaca mulatta]; 236 631 minus strand PREDICTED: SH3 domain-binding glutamic acid-rich-like protein 3-like [Macaca mulatta]

Protein Accession	CL49318.Contig4$_{H}1$ CL49318.Contig3$_{H}1$ CL49318.Contig2$_{H}1$
Mean Expression Ratio	1.16
Median Expression Ratio	1.16
Credible Interval	(0.505, 2.7)
Associated Peptides	1
Associated Spectra	1
Coverage	NaN

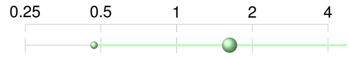

A	B	2.5	50	97.5	Sequence
**0**	**1**	**0.47**	**1.6**	**5.3**	**GDEYLGDYER**

5.437 51 890 prohibitin [Octopus tankahkeei]

Protein Accession	CL26635.Contig2$_{H}1$
Mean Expression Ratio	1.16
Median Expression Ratio	1.16
Credible Interval	(0.671, 2.01)
Associated Peptides	9
Associated Spectra	12
Coverage	NaN

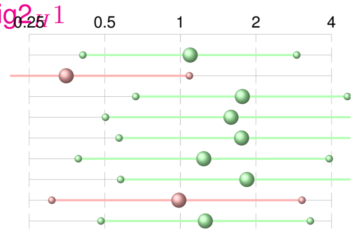

A	B	2.5	50	97.5	Sequence
**1**	**1**	**0.41**	**1.1**	**2.9**	**AFTEAGEGLVELR**
**1**	**0**	**0.11**	**0.35**	**1.1**	**EFTEAVEMK**
**0**	**2**	**0.67**	**1.8**	**4.6**	**FLVEKEEQK**
**0**	**1**	**0.5**	**1.6**	**5.1**	**KIEAAEDIAYQLSR**
**0**	**1**	**0.57**	**1.8**	**5.4**	**NVPVITGSKDLQNVNITLR**
**0**	**1**	**0.39**	**1.2**	**3.9**	**VSEELTDR**
**0**	**1**	**0.58**	**1.8**	**5.7**	**FLVEKEEQKKR**
**0**	**1**	**0.31**	**0.98**	**3**	**AAVISAEGDSLAAELLAK**
**0**	**2**	**0.48**	**1.3**	**3.3**	**NVPVITGSK**

5.438 399 575 hypothetical protein BRAFLDRAFT_69739 [Branchiostoma floridae] gi

Protein Accession	CL7219.Contig3$_{H}1$
Mean Expression Ratio	0.862
Median Expression Ratio	0.862
Credible Interval	(0.377, 1.93)
Associated Peptides	1
Associated Spectra	2
Coverage	NaN

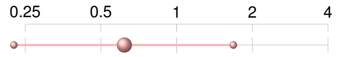

A	B	2.5	50	97.5	Sequence
**0**	**2**	**0.23**	**0.62**	**1.7**	**HFLQPSGDLVSYPGK**

5.439 214 1878 PREDICTED: lysyl-tRNA synthetase-like [Oreochromis niloticus]

Protein Accession	CL25588.Contig2$_{H}1$
Mean Expression Ratio	1.16
Median Expression Ratio	1.16
Credible Interval	(0.498, 2.69)
Associated Peptides	1
Associated Spectra	1
Coverage	NaN

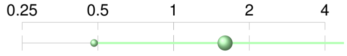

A	B	2.5	50	97.5	Sequence
0	1	0.48	1.6	5.3	IQIMANAK

5.440 405 1373 paramyosin [Haliotis discus discus]

Protein Accession	CL43455.Contig4$_{H}1$
Mean Expression Ratio	1.16
Median Expression Ratio	1.16
Credible Interval	(0.89, 1.53)
Associated Peptides	36
Associated Spectra	291
Coverage	NaN

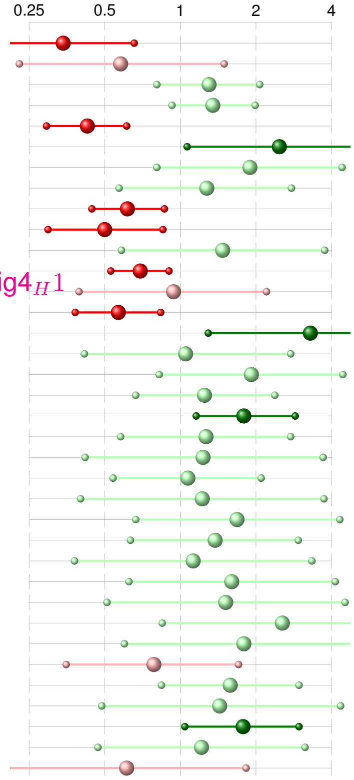

A	B	2.5	50	97.5	Sequence
1	6	0.18	0.34	0.65	ELEDALDAER
2	0	0.23	0.58	1.5	FDKELAAK
3	12	0.81	1.3	2.1	HTEAVNDLSDQVDYLTK
9	15	0.93	1.4	2	LKGQVDDLTR
20	6	0.29	0.43	0.61	LSLESR
1	2	1.1	2.5	5.7	QKLSLESR
1	2	0.81	1.9	4.4	QLNDLTALKAR
1	3	0.57	1.3	2.8	TRFDKELAAK
21	12	0.44	0.62	0.86	TVAELSFR
5	6	0.3	0.5	0.85	LDEEAETSSNLR
1	1	0.58	1.5	3.8	FEAAEASLR
23	31	0.53	0.69	0.9	QLNDLTALK
2	1	0.4	0.94	2.2	SQLQNQNDDLKR
14	9	0.38	0.57	0.83	VSADYAALK
0	2	1.3	3.3	8.7	AKSQLQNQNDDLKR
0	2	0.42	1	2.7	DLELATAQ
0	3	0.82	1.9	4.4	ELEDALDAERDGR
0	7	0.67	1.3	2.4	IRELEDALDAER
0	16	1.2	1.8	2.9	IRELEDALDAERDGR
0	4	0.58	1.3	2.7	IRELEDALDAERDGRVR
0	1	0.42	1.2	3.7	ITELEDSCEQLR
0	6	0.54	1.1	2.1	KDLELATAQFEAAEASLR
0	1	0.4	1.2	3.7	LDQVQDSLDEQGSSTVAQIELNKKR
0	2	0.67	1.7	4.3	LSLESRVDAAEQSAAR
0	4	0.63	1.4	2.9	LTQENFDLQHQVQELDSSNAALAK
0	1	0.38	1.1	3.4	RELEDALDAERDGR
0	2	0.63	1.6	4.2	ESEIYKLR
0	1	0.51	1.5	4.5	ESEIYKLRK
0	1	0.85	2.5	7.5	KRESEIYK
0	1	0.6	1.8	5.4	QFEAAEASLR
0	4	0.35	0.78	1.7	DEQGSSTVAQIELNK
0	7	0.84	1.6	3	DLELATAQFEAAEASLR
0	1	0.49	1.4	4.4	LDEEAETSSNLRNSLSK
0	12	1	1.8	3	LDQVQDSLDEQGSSTVAQIELNK
0	2	0.47	1.2	3.1	QTLVIELDGLSGQLDSITK
0	1	0.2	0.61	1.8	TEELEETR

5.441 8 271 minus strand Laminin subunit gamma-1 [Crassostrea gigas]

Protein Accession	Unigene36706$_{H}1$
Mean Expression Ratio	1.16
Median Expression Ratio	1.16
Credible Interval	(0.551, 2.47)
Associated Peptides	2
Associated Spectra	3
Coverage	NaN

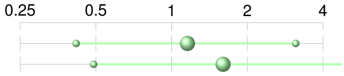

A	B	2.5	50	97.5	Sequence
0	2	0.42	1.2	3.1	HMQEAAQIDNIAR
0	1	0.49	1.6	5.3	ALDLYR

5.442 1237 1374 minus strand putative 60S ribosomal protein L23 [Maconellicoccus hirsutus]

Protein Accession	CL834.Contig9$_{H}1$
Mean Expression Ratio	1.16
Median Expression Ratio	1.16
Credible Interval	(0.536, 2.54)
Associated Peptides	1
Associated Spectra	4
Coverage	NaN

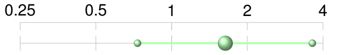

A	B	2.5	50	97.5	Sequence
0	4	0.73	1.6	3.6	IASNASSIA

5.443 172 678 Myosin regulatory light chain sqh [Crassostrea gigas]

Protein Accession	CL23773.Contig1$_{H}1$
Mean Expression Ratio	1.16
Median Expression Ratio	1.16
Credible Interval	(0.499, 2.73)
Associated Peptides	1
Associated Spectra	1
Coverage	NaN

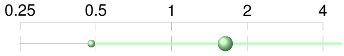

A	B	2.5	50	97.5	Sequence
0	1	0.48	1.6	5.4	GNFDYNEFTR

5.444 130 1731 T-complex protein 1 subunit epsilon [Crassostrea gigas]; 130 1731 T-complex protein 1 subunit epsilon [Crassostrea gigas]

Protein Accession	CL1223.Contig8$_{H}1$ CL1223.Contig2$_{H}1$
Mean Expression Ratio	1.16
Median Expression Ratio	1.16
Credible Interval	(0.663, 2.04)
Associated Peptides	9
Associated Spectra	13
Coverage	NaN

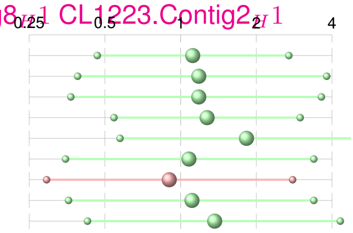

A	B	2.5	50	97.5	Sequence
1	2	0.47	1.1	2.7	LTGIDAHK
0	1	0.39	1.2	3.8	GVVVDKDMSHPQMPK
0	1	0.36	1.2	3.6	HKLDVTSVEDYR
0	3	0.54	1.3	3	LMVQLSK
0	1	0.57	1.8	5.5	HKLDVTSVEDYRK
0	1	0.35	1.1	3.4	MLVIEECK
0	1	0.29	0.9	2.8	IVPRFEELTPEK
0	1	0.36	1.1	3.4	IAVETLDK
0	1	0.43	1.4	4.3	SHILAAK

5.445 177 791 Ras-related protein Rab-1A [Haliotis discus discus]

Protein Accession	CL21389.Contig6$_{H}1$
Mean Expression Ratio	1.15
Median Expression Ratio	1.15
Credible Interval	(0.579, 2.35)
Associated Peptides	3
Associated Spectra	7
Coverage	NaN

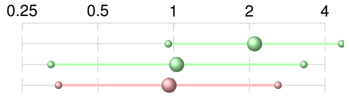

A	B	2.5	50	97.5	Sequence
1	3	0.95	2.1	4.7	MGPVTSASENKPSVK
1	0	0.33	1	3.3	INSSTPVK
1	1	0.35	0.96	2.6	KVVDYTTAK

5.446 9 722 minus strand actin related protein [Lumbricus rubellus]

Protein Accession	CL45325.Contig2$_{H}1$
Mean Expression Ratio	1.16
Median Expression Ratio	1.15
Credible Interval	(0.508, 2.66)
Associated Peptides	1
Associated Spectra	1
Coverage	NaN

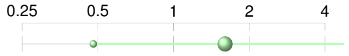

A	B	2.5	50	97.5	Sequence
0	1	0.48	1.6	5.3	LAVNSRAR

5.447 128 1138 Actin-related protein 2/3 complex subunit 1A OS=Homo sapiens GN=ARPC1A PE=1 SV=2

Protein Accession	CL49279.Contig6$_{H}1$
Mean Expression Ratio	0.869
Median Expression Ratio	0.868
Credible Interval	(0.375, 2.03)
Associated Peptides	1
Associated Spectra	1
Coverage	NaN

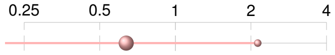

A	B	2.5	50	97.5	Sequence
0	1	0.19	0.64	2.1	TQHQNTITQVSVYEGTK

5.448 5 598 hypothetical protein DAPPUDRAFT_344656 [Daphnia pulex]

Protein Accession	Unigene24559$_{H}1$
Mean Expression Ratio	0.869
Median Expression Ratio	0.868
Credible Interval	(0.38, 2.01)
Associated Peptides	1
Associated Spectra	1
Coverage	NaN

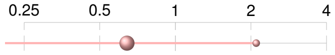

A	B	2.5	50	97.5	Sequence
0	1	0.19	0.64	2.1	WHLAEFVR

5.449 3591 4526 minus strand myosin heavy chain cardiac muscle specific isoform 1 [Argopecten irradians]

Protein Accession	CL49594.Contig2$_{H}1$
Mean Expression Ratio	1.15
Median Expression Ratio	1.15
Credible Interval	(0.805, 1.63)
Associated Peptides	21
Associated Spectra	99
Coverage	NaN

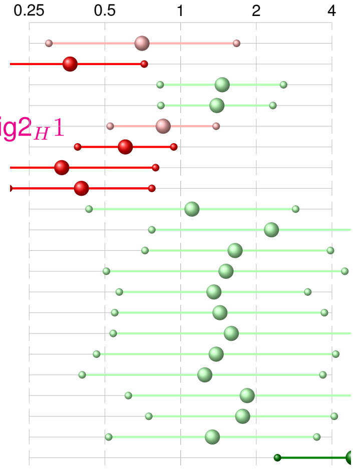

A	B	2.5	50	97.5	Sequence
1	2	0.3	0.7	1.7	ATQANVEDLER
5	1	0.18	0.36	0.72	KLEQDLK
3	7	0.83	1.5	2.6	LQQETANLTR
5	7	0.84	1.4	2.3	QLEEAEHK
6	8	0.52	0.85	1.4	QVEAQLAELSAK
10	7	0.39	0.6	0.94	TINDLQTQK
3	0	0.15	0.34	0.8	VGALTK
3	4	0.21	0.4	0.77	SEVDDLQMQLQNVGK
0	2	0.43	1.1	2.9	AKLEQTLDELEDNLER
0	1	0.77	2.3	7	AQLKSEVDDLQMQLQNVGK
0	3	0.72	1.6	4	KLEQDLKATQANVEDLER
0	1	0.51	1.5	4.5	NRLQQETANLTR
0	3	0.57	1.4	3.2	QLEEAEHKVGALTKEK
0	2	0.55	1.4	3.7	QVEAQLAELSAKVEEGNR
0	1	0.54	1.6	4.9	TMEDLQAEEDKVNHLNK
0	1	0.46	1.4	4.1	AATSRSRTLQHEAQVSALR
0	1	0.41	1.3	3.7	VKRELEENVR
0	1	0.62	1.8	5.6	RKLEQDLK
0	3	0.75	1.8	4.1	ATSRSRTLQHEAQVSALR
0	2	0.52	1.3	3.5	LEQTLDELEDNLEREK
0	6	2.4	4.8	9.5	QLEEAEHKVGALTK

5.450 462 671 enhancer protein [Homo sapiens]

Protein Accession	CL3160.Contig12$_{H}1$
Mean Expression Ratio	1.15
Median Expression Ratio	1.15
Credible Interval	(0.565, 2.29)
Associated Peptides	2
Associated Spectra	11
Coverage	NaN

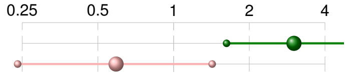

A	B	2.5	50	97.5	Sequence
0	8	1.6	3	5.7	GLFIIDDKGNLR
0	3	0.24	0.59	1.4	GLFIIDDK

5.451 1 219 minus strand PREDICTED: nucleoside diphosphate kinase 3-like [Pan troglodytes]

Protein Accession	Unigene5384$_{H}1$
Mean Expression Ratio	0.867
Median Expression Ratio	0.868
Credible Interval	(0.407, 1.81)
Associated Peptides	2
Associated Spectra	5
Coverage	NaN

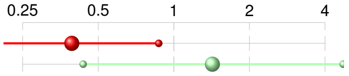

A	B	2.5	50	97.5	Sequence
2	2	0.17	0.39	0.87	GLVGEIIK
0	1	0.44	1.4	4.7	GLVGEIIKR

5.452 3 4517 minus strand Clathrin heavy chain 1 [Crassostrea gigas]

Protein Accession	CL16446.Contig1$_{H}1$
Mean Expression Ratio	0.872
Median Expression Ratio	0.869
Credible Interval	(0.512, 1.51)
Associated Peptides	10
Associated Spectra	13
Coverage	NaN

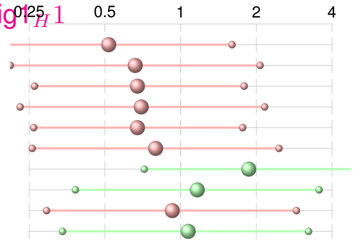

A	B	2.5	50	97.5	Sequence
1	0	0.17	0.52	1.6	KFEVNTSAIK
1	0	0.21	0.66	2.1	VHEGVTEPATHNALAK
1	1	0.26	0.67	1.8	EAIDSYIK
0	1	0.23	0.7	2.2	IYIDSNNNPER
0	2	0.26	0.67	1.8	LHVIEVGSPPQGNQPFAK
0	1	0.26	0.79	2.5	TNQVPLVKPYLR
0	2	0.72	1.9	5	AVNFFIK
0	1	0.38	1.2	3.6	AQLNQGMVK
0	1	0.29	0.93	2.9	IAAYLYK
0	1	0.34	1.1	3.2	ELVEEVEK

5.453 13 1218 minus strand PREDICTED: eukaryotic initiation factor 4A-III-like [Bombus terrestris] gi

Protein Accession	CL12065.Contig7$_{H}1$
Mean Expression Ratio	1.15
Median Expression Ratio	1.15
Credible Interval	(0.501, 2.64)
Associated Peptides	1
Associated Spectra	1
Coverage	NaN

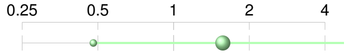

A	B	2.5	50	97.5	Sequence
0	1	0.48	1.6	5.3	GIYAYGFEKPSAIQQR

5.454 52 1653 minus strand PREDICTED: eukaryotic translation initiation factor 3 subunit 6 interacting protein-like [Saccoglossus kowalevskii]

Protein Accession	CL23174.Contig2$_{H}1$
Mean Expression Ratio	0.875
Median Expression Ratio	0.87
Credible Interval	(0.382, 2.05)
Associated Peptides	1
Associated Spectra	1
Coverage	NaN

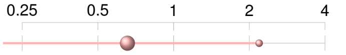

A	B	2.5	50	97.5	Sequence
1	0	0.2	0.66	2.2	NMIHVADTK

5.455 137 853 14-3-3 protein [Heterodera glycines]

Protein Accession	CL9040.Contig4$_{H}1$
Mean Expression Ratio	1.15
Median Expression Ratio	1.15
Credible Interval	(0.555, 2.39)
Associated Peptides	2
Associated Spectra	5
Coverage	NaN

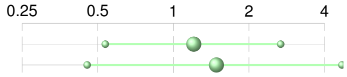

A	B	2.5	50	97.5	Sequence
2	2	0.54	1.2	2.7	VSEQAERYDDMAK
0	1	0.46	1.5	4.7	AIECANQAYQK

5.456 275 769 minus strand hypothetical protein CGI_10025135 [Crassostrea gigas]; 216 683 hypothetical protein CGI_10025135 [Crassostrea gigas]

Protein Accession	CL14194.Contig2$_{H}1$ CL14194.Contig1$_{H}1$
Mean Expression Ratio	1.15
Median Expression Ratio	1.15
Credible Interval	(0.493, 2.64)
Associated Peptides	1
Associated Spectra	1
Coverage	NaN

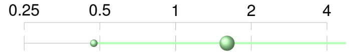

A	B	2.5	50	97.5	Sequence
**0**	**1**	**0.47**	**1.6**	**5.3**	**ILQPYVDQLKK**

5.457 130 2145 minus strand Glucosamine–fructose-6-phosphate aminotransferase [isomerizing] 1 [Crassostrea gigas]; 130 2121 minus strand Glucosamine–fructose-6-phosphate aminotransferase [isomerizing] 1 [Crassostrea gigas]

Protein Accession	CL2589.Contig3$_{H}1$ CL2589.Contig2$_{H}1$
Mean Expression Ratio	1.15
Median Expression Ratio	1.15
Credible Interval	(0.57, 2.37)
Associated Peptides	4
Associated Spectra	4
Coverage	NaN

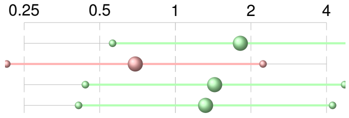

A	B	2.5	50	97.5	Sequence
**1**	**0**	**0.56**	**1.8**	**5.7**	**GSEPQIIKQTGK**
**1**	**0**	**0.21**	**0.69**	**2.2**	**SLLVMGR**
**0**	**1**	**0.44**	**1.4**	**4.7**	**LTTDHIPVLYSK**
**0**	**1**	**0.41**	**1.3**	**4.2**	**VANLEAQIK**

5.458 62 292 PREDICTED: heterogeneous nuclear ribonucleoprotein F-like [Otolemur garnettii]

Protein Accession	CL30592.Contig1$_{H}1$
Mean Expression Ratio	1.15
Median Expression Ratio	1.15
Credible Interval	(0.524, 2.54)
Associated Peptides	2
Associated Spectra	2
Coverage	NaN

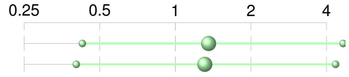

A	B	2.5	50	97.5	Sequence
**0**	**1**	**0.43**	**1.4**	**4.7**	**GLPWSTTPEDIVK**
**0**	**1**	**0.4**	**1.3**	**4.3**	**FFDESEIAGGEK**

5.459 84 425 minus strand thioester-containing protein [Euphaedusa tau]

Protein Accession	Unigene24513$_H$1
Mean Expression Ratio	1.15
Median Expression Ratio	1.14
Credible Interval	(0.498, 2.65)
Associated Peptides	1
Associated Spectra	1
Coverage	NaN

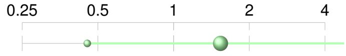

A	B	2.5	50	97.5	Sequence
**0**	**1**	**0.45**	**1.5**	**5.1**	**TYVILAPAK**

5.460 143 865 minus strand predicted protein [Nematostella vectensis] gi

Protein Accession	CL5421.Contig1$_H$1
Mean Expression Ratio	1.15
Median Expression Ratio	1.14
Credible Interval	(0.491, 2.66)
Associated Peptides	1
Associated Spectra	1
Coverage	NaN

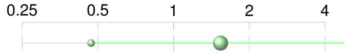

A	B	2.5	50	97.5	Sequence
**0**	**1**	**0.47**	**1.5**	**5.2**	**AIGSGSEGAQQALQEQYHK**

5.461 5 451 minus strand Spectrin beta chain [Crassostrea gigas]

Protein Accession	CL19757.Contig1$_H$1
Mean Expression Ratio	0.873
Median Expression Ratio	0.874
Credible Interval	(0.395, 1.91)
Associated Peptides	2
Associated Spectra	2
Coverage	NaN

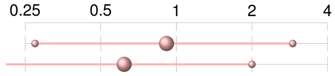

A	B	2.5	50	97.5	Sequence
**0**	**1**	**0.27**	**0.91**	**2.9**	**HSLLEADIHVVGER**
**0**	**1**	**0.19**	**0.62**	**2**	**HLMGVEDLLQK**

5.462 194 838 glutathione S-transferase class mu [Cyphoma gibbosum]

Protein Accession	CL8626.Contig6$_H$1
Mean Expression Ratio	1.15
Median Expression Ratio	1.14
Credible Interval	(0.521, 2.53)
Associated Peptides	2
Associated Spectra	2
Coverage	NaN

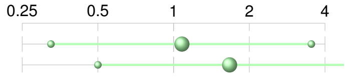

A	B	2.5	50	97.5	Sequence
**0**	**1**	**0.32**	**1.1**	**3.5**	**KHDLLGQTEK**
**0**	**1**	**0.5**	**1.7**	**5.4**	**PTLGYWK**

5.463 274 666 Calmodulin-4 [Heterocephalus glaber]

Protein Accession Unigene19050$_{H}1$
Mean Expression Ratio 0.877
Median Expression Ratio 0.875
Credible Interval (0.447, 1.72)
Associated Peptides 3
Associated Spectra 9
Coverage NaN

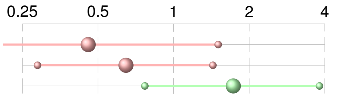

A	B	2.5	50	97.5	Sequence
1	0	0.14	0.46	1.5	SGFITLDELK
2	2	0.29	0.65	1.4	VLLSVGR
2	2	0.77	1.7	3.8	TLDELK

5.464 23 214 minus strand hemocyanin isoform 1 [Nucula nucleus]

Protein Accession Unigene58881$_{H}1$
Mean Expression Ratio 1.14
Median Expression Ratio 1.14
Credible Interval (0.61, 2.16)
Associated Peptides 3
Associated Spectra 21
Coverage NaN

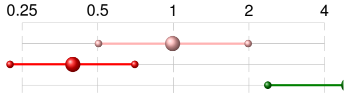

A	B	2.5	50	97.5	Sequence
2	4	0.51	0.99	2	GKIPNLNIYTSR
4	6	0.22	0.4	0.7	QEINPFFR
2	3	2.4	5	11	TAKQEINPFFR

5.465 203 1942 ezrin/radixin/moesin [Aplysia californica] gi; 243 1982 minus strand ezrin/radixin/moesin [Aplysia californica] gi

Protein Accession CL13506.Contig5$_{H}1$ CL13506.Contig4$_{H}1$
Mean Expression Ratio 0.875
Median Expression Ratio 0.876
Credible Interval (0.606, 1.27)
Associated Peptides 25
Associated Spectra 50
Coverage NaN

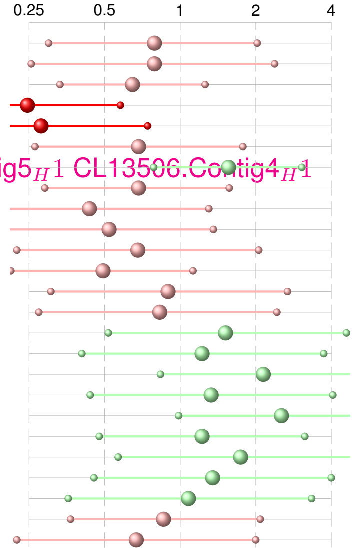

A	B	2.5	50	97.5	Sequence
1	1	0.3	0.79	2	KESPLQFK
1	0	0.26	0.79	2.4	KVLSQDVR
4	2	0.33	0.65	1.3	NISFNDKK
3	0	0.1	0.25	0.58	QLFDQVVK
1	1	0.11	0.28	0.74	DDAMMEYLK
1	1	0.26	0.68	1.8	FVIKPIDK
2	4	0.78	1.6	3.1	RKPDTIEVQQMK
1	2	0.29	0.68	1.6	ESPLQFK
1	0	0.14	0.43	1.3	APDFVFYAPR
2	0	0.2	0.52	1.4	LNEIVYK
1	0	0.22	0.68	2.1	LLPQR
1	2	0.21	0.49	1.1	DMADLQAR
1	0	0.3	0.89	2.7	LLALR
1	0	0.27	0.83	2.4	VLSQDVR
0	1	0.52	1.5	4.6	KAPDFVFYAPR
0	1	0.41	1.2	3.7	LADELETAQR
0	2	0.83	2.1	5.4	LGENGLTKNDELHAENVR
0	1	0.44	1.3	4	MAELLALREELDAER
0	2	0.99	2.5	6.6	QKLADELETAQR
0	2	0.48	1.2	3.1	VEEERLTEAEKNQR
0	1	0.57	1.7	5.4	FVIKPIDKK
0	1	0.45	1.4	4	QLEMLK
0	1	0.36	1.1	3.3	AEELRLEMER
0	3	0.37	0.86	2.1	SWWGEHK
0	1	0.22	0.67	2	MAELLALREELDAERR

5.466 17 691 minus strand Cleavage and polyadenylation specificity factor subunit 5 [Crassostrea gigas]

Protein Accession	CL17041.Contig3$_{H}1$
Mean Expression Ratio	1.14
Median Expression Ratio	1.14
Credible Interval	(0.494, 2.65)
Associated Peptides	1
Associated Spectra	1
Coverage	NaN

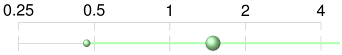

A	B	2.5	50	97.5	Sequence
**0**	**1**	**0.47**	**1.5**	**5**	**ALFAVPR**

5.467 444 719 minus strand 60S ribosomal protein L9 [Laodelphax striatella]

Protein Accession	CL1530.Contig3$_{H}1$
Mean Expression Ratio	1.14
Median Expression Ratio	1.14
Credible Interval	(0.579, 2.27)
Associated Peptides	3
Associated Spectra	8
Coverage	NaN

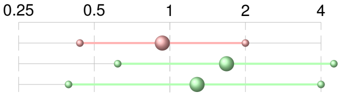

A	B	2.5	50	97.5	Sequence
**2**	**3**	**0.44**	**0.93**	**2**	**SAALIQQSTTVK**
**1**	**1**	**0.62**	**1.7**	**4.5**	**SAALIQQSTTVKK**
**0**	**1**	**0.4**	**1.3**	**4**	**FLDGIYVSEK**

5.468 169 1200 PREDICTED: nidogen-2-like isoform 1 [Strongylocentrotus purpuratus]

Protein Accession	CL478.Contig2$_{H}1$
Mean Expression Ratio	1.14
Median Expression Ratio	1.14
Credible Interval	(0.552, 2.38)
Associated Peptides	3
Associated Spectra	3
Coverage	NaN

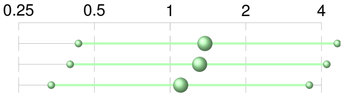

A	B	2.5	50	97.5	Sequence
**0**	**1**	**0.43**	**1.4**	**4.6**	**AIVLDINGGK**
**0**	**1**	**0.4**	**1.3**	**4.2**	**LVYAR**
**0**	**1**	**0.34**	**1.1**	**3.6**	**TLFNTDMVNPR**

5.469 616 1740 5'-AMP-activated protein kinase subunit beta-2 [Crassostrea gigas]; 641 1762 minus strand 5'-AMP-activated protein kinase subunit beta-2 [Crassostrea gigas]; 626 1786 5'-AMP-activated protein kinase subunit beta-2 [Crassostrea gigas]; 431 1591 5'-AMP-activated protein kinase subunit beta-2 [Crassostrea gigas]

Protein Accession	Unigene52845$_{H}1$ Unigene41754$_{H}1$ Unigene36145$_{H}1$ CL5821.Contig3$_{H}1$
Mean Expression Ratio	1.14
Median Expression Ratio	1.14
Credible Interval	(0.488, 2.68)
Associated Peptides	1
Associated Spectra	1
Coverage	NaN

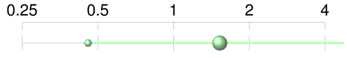

A	B	2.5	50	97.5	Sequence
**0**	**1**	**0.46**	**1.5**	**5**	**FIVDEQWVVSK**

5.470 154 621 minus strand Stress response protein nhaX [Crassostrea gigas]

Protein Accession	CL4265.Contig1$_{H}1$
Mean Expression Ratio	0.88
Median Expression Ratio	0.879
Credible Interval	(0.48, 1.64)
Associated Peptides	4
Associated Spectra	16
Coverage	NaN

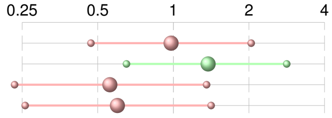

A	B	2.5	50	97.5	Sequence
**3**	**2**	**0.47**	**0.98**	**2**	**SVAAAKPGEGILK**
**2**	**3**	**0.65**	**1.4**	**2.8**	**GLGVMR**
**0**	**3**	**0.23**	**0.56**	**1.4**	**IQHDLEK**
**0**	**3**	**0.26**	**0.6**	**1.4**	**EVLEAMLKDEQK**

5.471 153 953 minus strand ribosomal protein rpl7 [Arenicola marina]

Protein Accession	CL20943.Contig3$_{H}1$
Mean Expression Ratio	0.874
Median Expression Ratio	0.879
Credible Interval	(0.377, 2.01)
Associated Peptides	1
Associated Spectra	1
Coverage	NaN

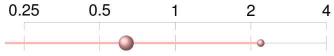

A	B	2.5	50	97.5	Sequence
**1**	**0**	**0.19**	**0.64**	**2.2**	**VPETILK**

5.472 418 690 PREDICTED: protein disulfide isomerase A4-like [Saccoglossus kowalevskii]

Protein Accession	CL20258.Contig2$_H$1
Mean Expression Ratio	0.879
Median Expression Ratio	0.88
Credible Interval	(0.386, 1.96)
Associated Peptides	1
Associated Spectra	2
Coverage	NaN

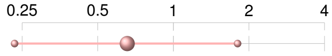

A	B	2.5	50	97.5	Sequence
**0**	**2**	**0.23**	**0.66**	**1.8**	**TFEQIVLDK**

5.473 1 177 small heat shock protein [Trichinella pseudospiralis]

Protein Accession	Unigene29352$_H$1
Mean Expression Ratio	1.13
Median Expression Ratio	1.14
Credible Interval	(0.488, 2.69)
Associated Peptides	1
Associated Spectra	1
Coverage	NaN

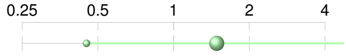

A	B	2.5	50	97.5	Sequence
**0**	**1**	**0.45**	**1.5**	**4.9**	**ISMDVQHFKPEEIK**

5.474 " 194 334 unknown, partial [Lymnaea stagnalis] gi; 207 347 unknown, partial [Lymnaea stagnalis] gi; 384 524 unknown, partial [Lymnaea stagnalis] gi"

Protein Accession	Unigene62752$_H$1 Unigene62751$_H$1 Unigene62750$_H$1
Mean Expression Ratio	1.14
Median Expression Ratio	1.14
Credible Interval	(0.501, 2.57)
Associated Peptides	1
Associated Spectra	2
Coverage	NaN

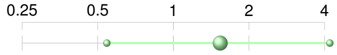

A	B	2.5	50	97.5	Sequence
**0**	**2**	**0.54**	**1.5**	**4.2**	**AFGFGMR**

5.475 " 1362 1544 minus strand Phosphoenolpyruvate carboxykinase, cytosolic [GTP] [Crassostrea gigas]"

Protein Accession	CL1008.Contig7$_H$1
Mean Expression Ratio	0.883
Median Expression Ratio	0.883
Credible Interval	(0.381, 2.04)
Associated Peptides	1
Associated Spectra	1
Coverage	NaN

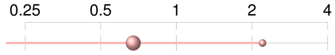

A	B	2.5	50	97.5	Sequence
**0**	**1**	**0.2**	**0.68**	**2.2**	**KVQHYVEEYAR**

5.476 98 865 proteasome (prosome macropain) subunit alpha type 1 [Xenopus (Silurana) tropicalis]; 98 865 proteasome (prosome macropain) subunit alpha type 1 [Xenopus (Silurana) tropicalis]

Protein Accession	CL5647.Contig8$_H$1 CL5647.Contig7$_H$1
Mean Expression Ratio	1.13
Median Expression Ratio	1.13
Credible Interval	(0.51, 2.51)
Associated Peptides	1
Associated Spectra	4
Coverage	NaN

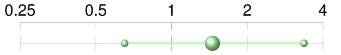

A	B	2.5	50	97.5	Sequence
**0**	**4**	**0.65**	**1.5**	**3.4**	**LVSVVGNK**

5.477 " 132 2075 PREDICTED: arginyl-tRNA synthetase, cytoplasmic [Ornithorhynchus anatinus]; 132 2090 minus strand PREDICTED: arginyl-tRNA synthetase, cytoplasmic [Ornithorhynchus anatinus]"

Protein Accession	CL15019.Contig4$_H$1 CL15019.Contig3$_H$1
Mean Expression Ratio	1.13
Median Expression Ratio	1.13
Credible Interval	(0.541, 2.37)
Associated Peptides	3
Associated Spectra	3
Coverage	NaN

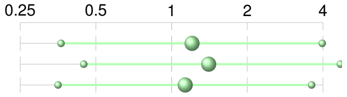

A	B	2.5	50	97.5	Sequence
**0**	**1**	**0.36**	**1.2**	**4**	**LVDLLDEGVK**
**0**	**1**	**0.45**	**1.4**	**4.7**	**LSYQLNHLKR**
**0**	**1**	**0.35**	**1.1**	**3.6**	**QRTGAVSLQRMLEDVFR**

5.478 6043 6174 myosin heavy chain [Mytilus galloprovincialis]; 5869 6000 myosin heavy chain [Mytilus galloprovincialis]

Protein Accession	Unigene19025$_H$1 CL49594.Contig1$_H$1
Mean Expression Ratio	1.13
Median Expression Ratio	1.13
Credible Interval	(0.537, 2.37)
Associated Peptides	2
Associated Spectra	5
Coverage	NaN

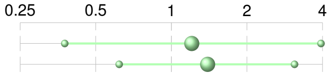

A	B	2.5	50	97.5	Sequence
**0**	**1**	**0.38**	**1.2**	**4**	**AMQTDLDEMNGELR**
**0**	**4**	**0.62**	**1.4**	**3.1**	**KLEADIQAMQTDLDEMNGELR**

5.479 231 902 Sodium- and chloride-dependent GABA transporter ine [Crassostrea gigas]

Protein Accession	CL11289.Contig3$_H$1
Mean Expression Ratio	0.888
Median Expression Ratio	0.884
Credible Interval	(0.384, 2.09)
Associated Peptides	1
Associated Spectra	1
Coverage	NaN

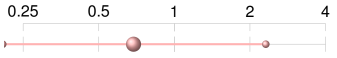

A	B	2.5	50	97.5	Sequence
0	1	0.21	0.69	2.3	EAFELSFK

5.480 " 519 3182 minus strand Alpha-actinin, sarcomeric [Crassostrea gigas]"

Protein Accession	CL28960.Contig2$_H$1
Mean Expression Ratio	0.886
Median Expression Ratio	0.884
Credible Interval	(0.581, 1.35)
Associated Peptides	18
Associated Spectra	31
Coverage	NaN

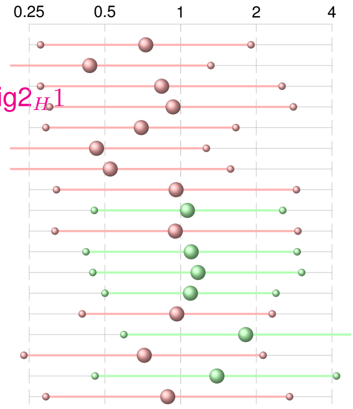

A	B	2.5	50	97.5	Sequence
1	1	0.28	0.73	1.9	NINEVENQILTR
1	0	0.14	0.43	1.3	LGQLTVK
1	0	0.28	0.84	2.5	HLDIPR
1	0	0.3	0.93	2.8	MSFNHFDK
2	1	0.29	0.7	1.7	RDQTLQQEMMR
1	1	0.18	0.46	1.3	LDEFR
1	0	0.18	0.52	1.6	LERLDHLAQK
0	1	0.32	0.96	2.9	ACLISLGYK
0	3	0.45	1.1	2.5	AIIQYKPHIDELER
0	1	0.32	0.95	2.9	DNPIENLNTAFDIAEK
0	2	0.42	1.1	2.9	ETADTDTAEQVMQSFK
0	2	0.45	1.2	3	GISEDQMNEFR
0	4	0.5	1.1	2.4	IDQLHLEFAK
0	3	0.4	0.97	2.3	ILAGDKPYITAQILR
0	1	0.59	1.8	5.5	LSNRPAYLPTEGK
0	1	0.24	0.72	2.1	ALDFIASK
0	1	0.46	1.4	4.2	MSFNHFDKNR
0	1	0.29	0.89	2.7	EALEEAER

5.481 2 298 putative 60S ribosomal protein RPL24 [Novocrania anomala]

Protein Accession	Unigene278$_H$1
Mean Expression Ratio	1.13
Median Expression Ratio	1.13
Credible Interval	(0.515, 2.55)
Associated Peptides	1
Associated Spectra	3
Coverage	NaN

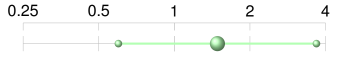

A	B	2.5	50	97.5	Sequence
0	3	0.6	1.5	3.7	AITGVSLSEIIAK

5.482 75 803 hypothetical protein DAPPUDRAFT_231350 [Daphnia pulex]

Protein Accession	CL10537.Contig4$_H$1
Mean Expression Ratio	1.13
Median Expression Ratio	1.13
Credible Interval	(0.592, 2.15)
Associated Peptides	5
Associated Spectra	8
Coverage	NaN

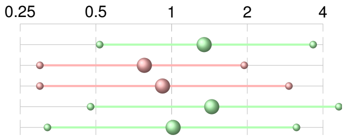

A	B	2.5	50	97.5	Sequence
0	2	0.52	1.3	3.7	NFPHPVMPETSGK
1	2	0.3	0.78	2	SITPQQEAESIK
0	1	0.3	0.92	2.9	ENVALQLEASYR
0	1	0.47	1.4	4.6	ATTLLSK
0	1	0.32	1	3.1	VCIAQLK

5.483 680 991 minus strand PDZ and LIM domain protein 1 [Crassostrea gigas]; 553 864 minus strand PDZ and LIM domain protein 1 [Crassostrea gigas]

Protein Accession	Unigene7664$_H$1 Unigene7663$_H$1
Mean Expression Ratio	1.13
Median Expression Ratio	1.13
Credible Interval	(0.528, 2.44)
Associated Peptides	2
Associated Spectra	3
Coverage	NaN

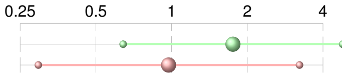

A	B	2.5	50	97.5	Sequence
0	2	0.64	1.8	4.8	QLTHMQAHQLIK
0	1	0.29	0.97	3.2	SAGTQLQLTVLR

5.484 201 1181 minus strand Serine/threonine-protein phosphatase PP1-alpha catalytic subunit [Crassostrea gigas]

Protein Accession	CL28655.Contig4$_H$1
Mean Expression Ratio	0.885
Median Expression Ratio	0.885
Credible Interval	(0.402, 1.94)
Associated Peptides	2
Associated Spectra	2
Coverage	NaN

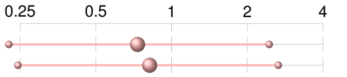

A	B	2.5	50	97.5	Sequence
0	1	0.23	0.73	2.4	ADTDKLNIDSIIAR
0	1	0.24	0.82	2.7	AHQVVEDGYEFFAK

5.485 238 681 minus strand Globin OS=Cerithidea rhizophorarum PE=1 SV=1

Protein Accession	CL8458.Contig2$_H$1
Mean Expression Ratio	1.13
Median Expression Ratio	1.13
Credible Interval	(0.623, 2.06)
Associated Peptides	5
Associated Spectra	68
Coverage	NaN

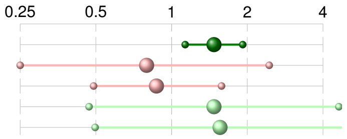

A	B	2.5	50	97.5	Sequence
13	43	1.1	1.5	1.9	AAITSTWSK
1	0	0.25	0.8	2.4	EVFGSFLEATLGGAATPDVK
0	9	0.49	0.87	1.6	AYFPFLPGASEGDIK
0	1	0.47	1.5	4.6	LASDKATMENNGAK
0	1	0.5	1.6	5	AITSTWSK

5.486 176 670 hypothetical protein BRAFLDRAFT_122621 [Branchiostoma floridae] gi

Protein Accession	Unigene180$_{H}1$
Mean Expression Ratio	0.887
Median Expression Ratio	0.886
Credible Interval	(0.51, 1.56)
Associated Peptides	5
Associated Spectra	35
Coverage	NaN

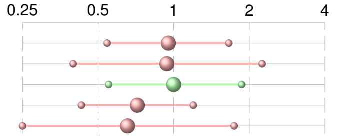

A	B	2.5	50	97.5	Sequence
2	8	0.54	0.95	1.7	VFFDITADGQPLGR
1	2	0.4	0.94	2.2	ADVVPK
2	6	0.55	1	1.9	FADENFLLK
6	6	0.43	0.71	1.2	IVMELR
0	2	0.25	0.66	1.7	HVVFGQVVEGMDVVR

5.487 1 753 hypothetical protein BRAFLDRAFT_131251 [Branchiostoma floridae] gi

Protein Accession	CL18526.Contig3$_{H}1$
Mean Expression Ratio	0.883
Median Expression Ratio	0.887
Credible Interval	(0.456, 1.68)
Associated Peptides	5
Associated Spectra	7
Coverage	NaN

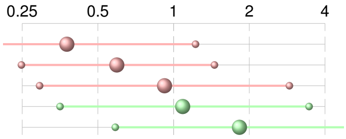

A	B	2.5	50	97.5	Sequence
1	0	0.12	0.38	1.2	GVFEIDSEK
3	0	0.25	0.6	1.5	GLGPFPR
0	1	0.29	0.92	2.9	GAGEPIDADYLLR
0	1	0.35	1.1	3.5	NVVDMIITEK
0	1	0.59	1.8	5.9	VIKGEKYEK

5.488 247 726 Annexin A7 [Crassostrea gigas]; 286 675 Annexin A7 [Crassostrea gigas]

Protein Accession	CL15539.Contig3$_{H}1$ CL15539.Contig1$_{H}1$
Mean Expression Ratio	0.888
Median Expression Ratio	0.887
Credible Interval	(0.446, 1.76)
Associated Peptides	3
Associated Spectra	8
Coverage	NaN

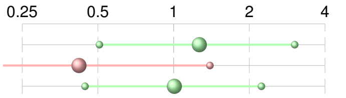

A	B	2.5	50	97.5	Sequence
1	2	0.51	1.3	3	GLGTDEAAVIR
0	1	0.13	0.42	1.4	DLVSDLK
0	4	0.44	1	2.2	ILTSHNNMQR

5.489 125 550 minus strand ribosomal protein S20 [Chlamys farreri]; 125 550 minus strand ribosomal protein S20 [Chlamys farreri]; 125 550 minus strand ribosomal protein S20 [Chlamys farreri]

Protein Accession	CL6833.Contig3$_{H}1$ CL6833.Contig2$_{H}1$ CL6833.Contig1$_{H}1$
Mean Expression Ratio	1.13
Median Expression Ratio	1.13
Credible Interval	(0.57, 2.22)
Associated Peptides	3
Associated Spectra	10
Coverage	NaN

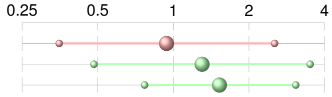

A	B	2.5	50	97.5	Sequence
1	1	0.35	0.94	2.5	KTPCGEGSK
1	1	0.48	1.3	3.5	ITLTSR
0	6	0.77	1.5	3.1	IIDLHSPSEIVK

5.490 177 2369 ATPase family AAA domain-containing protein 2B [Crassostrea gigas]; 177 2369 ATPase family AAA domain-containing protein 2B [Crassostrea gigas]

Protein Accession	CL3021.Contig3$_{H}1$ CL3021.Contig2$_{H}1$
Mean Expression Ratio	1.12
Median Expression Ratio	1.12
Credible Interval	(0.638, 1.98)
Associated Peptides	8
Associated Spectra	13
Coverage	NaN

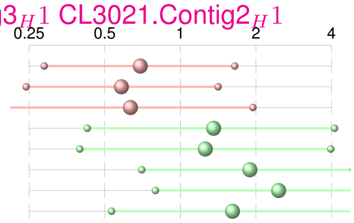

A	B	2.5	50	97.5	Sequence
2	1	0.29	0.69	1.7	FGGGDVNLLK
2	1	0.24	0.58	1.4	ILEVVR
1	0	0.2	0.63	1.9	IAVADRK
0	1	0.43	1.4	4.1	MTSGLYPAPLK
0	1	0.4	1.3	4	TKEEVIELSQK
0	2	0.7	1.9	4.9	YLDLHGAAPLVK
0	1	0.8	2.5	7.8	IAEASKRPEK
0	1	0.53	1.6	5.1	KITSFER

5.491 158 862 X-box binding protein [Haliotis discus discus]

Protein Accession	CL6396.Contig3$_{H}1$
Mean Expression Ratio	1.12
Median Expression Ratio	1.12
Credible Interval	(0.594, 2.11)
Associated Peptides	5
Associated Spectra	12
Coverage	NaN

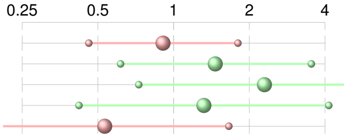

A	B	2.5	50	97.5	Sequence
4	2	0.46	0.91	1.8	SYYIGAR
0	3	0.61	1.5	3.5	VFPDFYFGAGDDKLDAR
0	1	0.73	2.3	7.2	ACIPDKAK
0	1	0.42	1.3	4.1	KYSGTNSTGRLYIVDLAK
0	1	0.17	0.53	1.7	VFPDFYFGAGDDK

5.492 138 713 Rac [Aplysia californica] gi; 125 691 Rac [Aplysia californica] gi; 149 871 minus strand Rac [Aplysia californica] gi; 101 676 Rac [Aplysia californica] gi

Protein Accession	CL11009.Contig9$_H$1 CL11009.Contig8$_H$1 CL11009.Contig4$_H$1 CL11009.Contig1$_H$1
Mean Expression Ratio	0.888
Median Expression Ratio	0.89
Credible Interval	(0.396, 2.02)
Associated Peptides	1
Associated Spectra	2
Coverage	NaN

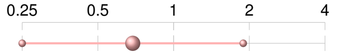

A	B	2.5	50	97.5	Sequence
**1**	**1**	**0.25**	**0.69**	**1.9**	**NVFDEAIR**

5.493 1425 2018 minus strand Ankyrin-2 [Crassostrea gigas]; 1425 2018 minus strand Ankyrin-2 [Crassostrea gigas]

Protein Accession	CL45186.Contig15$_H$1 CL45186.Contig14$_H$1
Mean Expression Ratio	1.12
Median Expression Ratio	1.12
Credible Interval	(0.519, 2.47)
Associated Peptides	2
Associated Spectra	3
Coverage	NaN

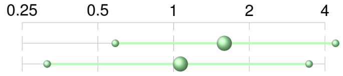

A	B	2.5	50	97.5	Sequence
**1**	**1**	**0.59**	**1.6**	**4.4**	**VAFMKEPK**
**0**	**1**	**0.31**	**1.1**	**3.5**	**SGDQLHINFK**

5.494 152 1207 haemocyanin precursor [Nautilus pompilius]

Protein Accession	Unigene27031$_H$1
Mean Expression Ratio	1.12
Median Expression Ratio	1.12
Credible Interval	(0.533, 2.34)
Associated Peptides	2
Associated Spectra	6
Coverage	NaN

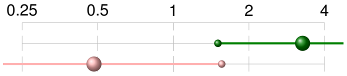

A	B	2.5	50	97.5	Sequence
**1**	**4**	**1.5**	**3.3**	**6.8**	**AVDDRLFQK**
**0**	**1**	**0.14**	**0.48**	**1.6**	**DPNGGMGLK**

5.495 1 456 minus strand Putative sodium/potassium-transporting ATPase subunit beta-2 [Crassostrea gigas]; 688 1251 minus strand Putative sodium/potassium-transporting ATPase subunit beta-2 [Crassostrea gigas]

Protein Accession	CL42979.Contig3$_H1$ CL42979.Contig2$_H1$
Mean Expression Ratio	1.12
Median Expression Ratio	1.12
Credible Interval	(0.482, 2.57)
Associated Peptides	1
Associated Spectra	1
Coverage	NaN

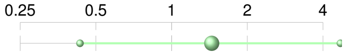

A	B	2.5	50	97.5	Sequence
**0**	**1**	**0.43**	**1.4**	**4.7**	**NYMSPLVFVK**

5.496 116 337 minus strand Golgi-associated plant pathogenesis-related protein 1 [Crassostrea gigas]; 538 759 minus strand Golgi-associated plant pathogenesis-related protein 1 [Crassostrea gigas]; 116 337 minus strand Golgi-associated plant pathogenesis-related protein 1 [Crassostrea gigas]; 261 482 minus strand Golgi-associated plant pathogenesis-related protein 1 [Crassostrea gigas]; 116 337 minus strand Golgi-associated plant pathogenesis-related protein 1 [Crassostrea gigas]; 116 337 minus strand Golgi-associated plant pathogenesis-related protein 1 [Crassostrea gigas]

Protein Accession	CL15263.Contig9$_H1$ CL15263.Contig8$_H1$ CL15263.Contig7$_H1$ CL15263.Contig5$_H1$ CL1526
Mean Expression Ratio	1.12
Median Expression Ratio	1.12
Credible Interval	(0.514, 2.49)
Associated Peptides	2
Associated Spectra	2
Coverage	NaN

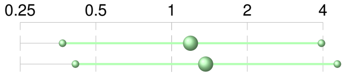

A	B	2.5	50	97.5	Sequence
**0**	**1**	**0.37**	**1.2**	**3.9**	**FIDEMLEAHNK**
**0**	**1**	**0.42**	**1.4**	**4.6**	**KEHQVQPLVINK**

5.497 91 1326 minus strand Adipocyte plasma membrane-associated protein [Crassostrea gigas]; 107 1273 minus strand Adipocyte plasma membrane-associated protein [Crassostrea gigas]; 224 1267 Adipocyte plasma membrane-associated protein [Crassostrea gigas]; 209 1267 minus strand Adipocyte plasma membrane-associated protein [Crassostrea gigas]; 195 1253 minus strand Adipocyte plasma membrane-associated protein [Crassostrea gigas]

Protein Accession	CL11239.Contig2$_{H}$1 CL11239.Contig1$_{H}$1 CL11239.Contig12$_{H}$1 CL11239.Contig11$_{H}$1 CL11
Mean Expression Ratio	1.12
Median Expression Ratio	1.12
Credible Interval	(0.504, 2.55)
Associated Peptides	1
Associated Spectra	3
Coverage	NaN

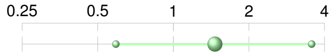

A	B	2.5	50	97.5	Sequence
0	3	0.59	1.5	3.6	YGLIAELDKEGK

5.498 " 87 287 Chain A, Structure Of Deoxygenated Hemocyanin From Rapana Thomasiana gi|31615505|pdb|1LNL|B Chain B, Structure Of Deoxygenated Hemocyanin From Rapana Thomasiana gi|31615506|pdb|1LNL|C Chain C, Structure Of Deoxygenated Hemocyanin From Rapana Thomasiana"

Protein Accession	Unigene36631$_{H}$1
Mean Expression Ratio	0.892
Median Expression Ratio	0.891
Credible Interval	(0.49, 1.62)
Associated Peptides	4
Associated Spectra	31
Coverage	NaN

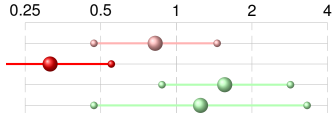

A	B	2.5	50	97.5	Sequence
3	7	0.47	0.83	1.5	QLQEDGSAGGFQK
7	3	0.18	0.31	0.55	SLVEAMR
1	8	0.88	1.6	2.8	KNILELTESEQR
0	2	0.47	1.2	3.3	NILELTESEQR

5.499 59 379 hypothetical protein DAPPUDRAFT_230756 [Daphnia pulex]; 117 443 minus strand hypothetical protein DAPPUDRAFT_230756 [Daphnia pulex]; 120 473 minus strand hypothetical protein DAPPUDRAFT_230756 [Daphnia pulex]

Protein Accession	CL2137.Contig6$_{H}$1 CL2137.Contig5$_{H}$1 CL2137.Contig4$_{H}$1
Mean Expression Ratio	0.893
Median Expression Ratio	0.891
Credible Interval	(0.436, 1.84)
Associated Peptides	2
Associated Spectra	7
Coverage	NaN

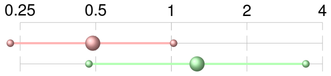

A	B	2.5	50	97.5	Sequence
3	2	0.23	0.49	1	AASTLAVK
0	2	0.47	1.3	3.4	LVNSLTASGTGALK

5.500 54 638 minus strand ubiquitin carboxyl-terminal hydrolase [Aplysia californica] gi

Protein Accession CL20666.Contig3$_{H}1$
Mean Expression Ratio 1.13
Median Expression Ratio 1.12
Credible Interval (0.533, 2.34)
Associated Peptides 3
Associated Spectra 3
Coverage NaN

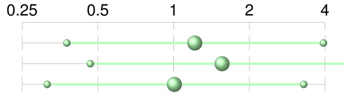

A	B	2.5	50	97.5	Sequence
0	1	0.38	1.2	3.9	THADTLLEDTVK
0	1	0.47	1.6	5	KDAPVCHGK
0	1	0.31	1	3.3	FLDETK

5.501 162 785 minus strand Ropporin-1-like protein OS=Xenopus laevis GN=ropn1l PE=2 SV=1

Protein Accession CL618.Contig4$_{H}1$
Mean Expression Ratio 0.888
Median Expression Ratio 0.891
Credible Interval (0.44, 1.77)
Associated Peptides 3
Associated Spectra 6
Coverage NaN

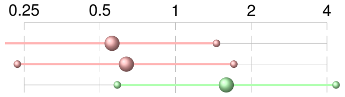

A	B	2.5	50	97.5	Sequence
1	1	0.2	0.56	1.4	EQFDDLVR
0	2	0.23	0.64	1.7	TVALTVIEEK
0	2	0.59	1.6	4.4	TDTGLTPGLLK

5.502 448 1626 matrilin [Biomphalaria glabrata]

Protein Accession CL7510.Contig3$_{H}1$
Mean Expression Ratio 1.12
Median Expression Ratio 1.12
Credible Interval (0.528, 2.38)
Associated Peptides 2
Associated Spectra 5
Coverage NaN

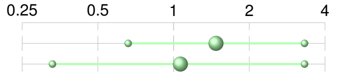

A	B	2.5	50	97.5	Sequence
3	1	0.66	1.5	3.3	VSAIKYGR
0	1	0.33	1.1	3.3	VSNTELLNIAGDQER

5.503 60 1190 hypothetical protein BRAFLDRAFT_109688 [Branchiostoma floridae] gi; 51 1184 hypothetical protein BRAFLDRAFT_109688 [Branchiostoma floridae] gi

Protein Accession CL25572.Contig3$_{H}1$ CL25572.Contig1$_{H}1$
Mean Expression Ratio 1.11
Median Expression Ratio 1.12
Credible Interval (0.51, 2.43)
Associated Peptides 2
Associated Spectra 2
Coverage NaN

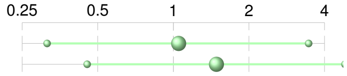

A	B	2.5	50	97.5	Sequence
0	1	0.31	1	3.5	AGHFQDEIIPVVIQSR
0	1	0.45	1.5	4.8	SVVLGAMAVQ

5.504 " 29 448 minus strand PREDICTED: similar to NADH:ubiquinone dehydrogenase, putative [Tribolium castaneum] gi; 19 408 minus strand hypothetical protein KGM_18901 [Danaus plexippus]; 29 448 minus strand PREDICTED: similar to NADH:ubiquinone dehydrogenase, putative [Tribolium castaneum] gi"

Protein Accession	CL2684.Contig8$_H$1 CL2684.Contig7$_H$1 CL2684.Contig6$_H$1
Mean Expression Ratio	1.13
Median Expression Ratio	1.12
Credible Interval	(0.507, 2.56)
Associated Peptides	1
Associated Spectra	2
Coverage	NaN

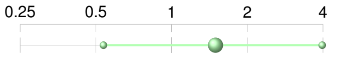

A	B	2.5	50	97.5	Sequence
**0**	**2**	**0.54**	**1.5**	**4**	**LLQDGEKELFLK**

5.505 4 2400 IQ motif containing GTPase activating protein 2 [Xenopus laevis] gi; 4 2169 IQ motif containing GTPase activating protein 2 [Xenopus laevis] gi

Protein Accession	CL875.Contig2$_H$1 CL875.Contig1$_H$1
Mean Expression Ratio	1.12
Median Expression Ratio	1.12
Credible Interval	(0.524, 2.44)
Associated Peptides	2
Associated Spectra	3
Coverage	NaN

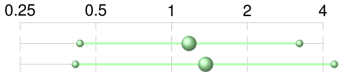

A	B	2.5	50	97.5	Sequence
**0**	**2**	**0.43**	**1.2**	**3.2**	**ITLQDVVTHNR**
**0**	**1**	**0.41**	**1.4**	**4.4**	**TEVLEK**

5.506 188 1021 hypothetical protein BRAFLDRAFT_214346 [Branchiostoma floridae] gi

Protein Accession	CL38498.Contig2$_H$1
Mean Expression Ratio	1.12
Median Expression Ratio	1.12
Credible Interval	(0.499, 2.47)
Associated Peptides	2
Associated Spectra	2
Coverage	NaN

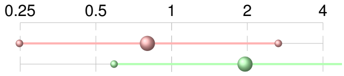

A	B	2.5	50	97.5	Sequence
**0**	**1**	**0.25**	**0.8**	**2.7**	**AANGGAYPPDLSYIVQAR**
**0**	**1**	**0.59**	**2**	**6.6**	**KIAFK**

5.507 34 1122 Fructose-bisphosphate aldolase [Crassostrea gigas]

Protein Accession	CL24228.Contig1$_H1$
Mean Expression Ratio	1.12
Median Expression Ratio	1.12
Credible Interval	(0.781, 1.63)
Associated Peptides	21
Associated Spectra	68
Coverage	NaN

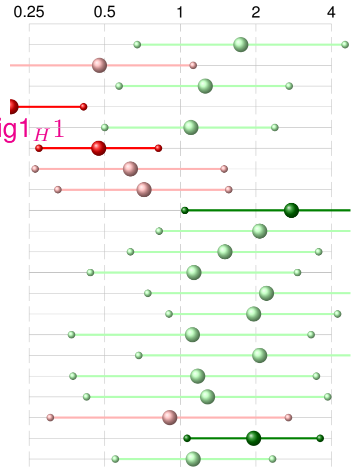

A	B	2.5	50	97.5	Sequence
1	1	0.67	1.7	4.5	AKANSEAALGK
3	0	0.2	0.48	1.1	DSQGTPFVK
2	2	0.57	1.3	2.7	GAQDELLKR
6	0	0.11	0.21	0.41	GILAADESVGSMGK
1	3	0.5	1.1	2.4	RLEGIGLENTEENR
7	3	0.27	0.47	0.82	ANSEAALGK
1	2	0.26	0.63	1.5	GIIPGIK
0	4	0.32	0.72	1.6	ALQASVLAAWK
0	2	1	2.8	7.2	ALQASVLAAWKGDDAK
0	2	0.82	2.1	5.3	ALQASVLAAWKGDDAKVK
0	3	0.63	1.5	3.5	RLEGIGLENTEENRR
0	2	0.44	1.1	2.9	RLEGIGLENTEENRRR
0	1	0.74	2.2	6.7	VKGAQDELLKR
0	4	0.9	2	4.2	QIANSIVAPGK
0	1	0.37	1.1	3.3	KDGAQFAK
0	1	0.69	2.1	6.3	GIIPGIKVDK
0	1	0.37	1.2	3.5	GKNPWAVTF
0	1	0.42	1.3	3.9	PVIAQYLTKQQEDELR
0	1	0.3	0.91	2.7	VKGAQDELLK
0	8	1.1	2	3.6	PVIAQYLTK
0	5	0.55	1.1	2.3	VTEQVLAFTYK

5.508 764 1258 minus strand Caprin-2 [Crassostrea gigas]

Protein Accession	CL23459.Contig2$_H1$
Mean Expression Ratio	0.891
Median Expression Ratio	0.893
Credible Interval	(0.378, 2.03)
Associated Peptides	1
Associated Spectra	1
Coverage	NaN

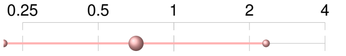

A	B	2.5	50	97.5	Sequence
0	1	0.21	0.71	2.3	FAVLDSEVR

5.509 100 2307 minus strand Xanthine dehydrogenase/oxidase [Crassostrea gigas]

Protein Accession	Unigene10743$_H1$
Mean Expression Ratio	1.12
Median Expression Ratio	1.12
Credible Interval	(0.528, 2.42)
Associated Peptides	2
Associated Spectra	3
Coverage	NaN

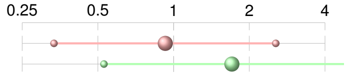

A	B	2.5	50	97.5	Sequence
1	1	0.33	0.93	2.6	VLSTDIVMDVGK
0	1	0.53	1.7	5.6	TLNIPQNK

5.510 93 2876 Hypoxia up-regulated protein 1 [Crassostrea gigas]

Protein Accession	CL16620.Contig1$_H1$
Mean Expression Ratio	1.12
Median Expression Ratio	1.12
Credible Interval	(0.547, 2.25)
Associated Peptides	4
Associated Spectra	4
Coverage	NaN

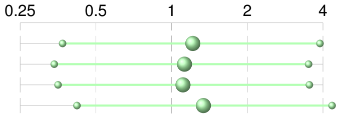

A	B	2.5	50	97.5	Sequence
0	1	0.37	1.2	3.9	MDESGILHLER
0	1	0.34	1.1	3.5	VESVFEK
0	1	0.35	1.1	3.5	IHEILLEK
0	1	0.42	1.3	4.3	IVAFR

5.511 187 642 minus strand PREDICTED: similar to pol-like protein [Tribolium castaneum]

Protein Accession	CL5644.Contig3$_H1$
Mean Expression Ratio	0.896
Median Expression Ratio	0.894
Credible Interval	(0.414, 1.94)
Associated Peptides	1
Associated Spectra	8
Coverage	NaN

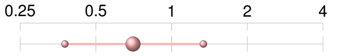

A	B	2.5	50	97.5	Sequence
0	8	0.38	0.7	1.3	IILEQEELK

5.512 44 889 minus strand hypothetical protein CGI_10015025 [Crassostrea gigas]

Protein Accession	CL40973.Contig3$_H1$
Mean Expression Ratio	1.11
Median Expression Ratio	1.12
Credible Interval	(0.487, 2.56)
Associated Peptides	1
Associated Spectra	1
Coverage	NaN

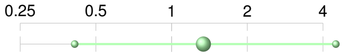

A	B	2.5	50	97.5	Sequence
0	1	0.41	1.3	4.5	NVELEGDIER

5.513 125 754 minus strand peptidyl prolyl cis-trans isomerase B [Conus novaehollandiae]

Protein Accession	Unigene19558$_H1$
Mean Expression Ratio	1.12
Median Expression Ratio	1.12
Credible Interval	(0.58, 2.16)
Associated Peptides	4
Associated Spectra	11
Coverage	NaN

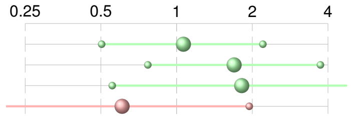

A	B	2.5	50	97.5	Sequence
1	4	0.5	1.1	2.2	VLSGMDVVR
0	4	0.77	1.7	3.7	HLALGDKEDGGVK
0	1	0.56	1.8	6	SIYGEKFPDENFK
0	1	0.19	0.61	1.9	TDSRDRPEK

5.514 438 683 minus strand Protein lethal(2)essential for life OS=Drosophila melanogaster GN=l(2)efl PE=1 SV=1

Protein Accession	CL17234.Contig7$_H$1
Mean Expression Ratio	1.12
Median Expression Ratio	1.12
Credible Interval	(0.52, 2.42)
Associated Peptides	1
Associated Spectra	7
Coverage	NaN

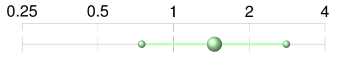

A	B	2.5	50	97.5	Sequence
1	6	0.75	1.5	2.8	ESGTSEVINNDKEFQIK

5.515 230 1189 hypothetical protein [Amblyomma maculatum]

Protein Accession	CL11202.Contig4$_H$1
Mean Expression Ratio	1.12
Median Expression Ratio	1.12
Credible Interval	(0.515, 2.52)
Associated Peptides	2
Associated Spectra	2
Coverage	NaN

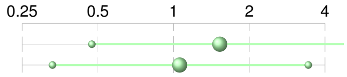

A	B	2.5	50	97.5	Sequence
0	1	0.47	1.5	5	KFAVDAVK
0	1	0.33	1.1	3.4	LEDMLR

5.516 " 599 907 minus strand Tryptophanyl-tRNA synthetase, cytoplasmic [Heterocephalus glaber]"

Protein Accession	Unigene26755$_H$1
Mean Expression Ratio	0.899
Median Expression Ratio	0.896
Credible Interval	(0.444, 1.83)
Associated Peptides	3
Associated Spectra	5
Coverage	NaN

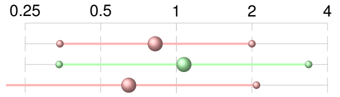

A	B	2.5	50	97.5	Sequence
0	3	0.34	0.82	2	DMDPLLDLYEK
0	1	0.34	1.1	3.4	KTPFYLYTGR
0	1	0.2	0.65	2.1	ITDELK

5.517 2 256 Polyubiquitin (Fragment) OS=Xenopus laevis PE=1 SV=2

Protein Accession	CL47507.Contig2$_H$1
Mean Expression Ratio	1.12
Median Expression Ratio	1.12
Credible Interval	(0.589, 2.12)
Associated Peptides	3
Associated Spectra	10
Coverage	NaN

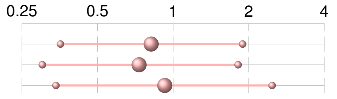

A	B	2.5	50	97.5	Sequence
1	3	0.36	0.82	1.9	TITLEVEPSDTIENVK
2	2	0.3	0.73	1.8	TLSDYNIQK
0	2	0.34	0.92	2.5	ESTLHLVLR

5.518 316 1368 minus strand hypothetical protein CGI_10006559 [Crassostrea gigas]

Protein Accession	Unigene26912$_{H1}$
Mean Expression Ratio	0.897
Median Expression Ratio	0.898
Credible Interval	(0.407, 2)
Associated Peptides	2
Associated Spectra	2
Coverage	NaN

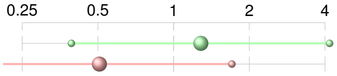

A	B	2.5	50	97.5	Sequence
0	1	0.39	1.3	4.2	TLDDLVR
0	1	0.15	0.51	1.7	IVGSWLAGQER

5.519 188 3946 Vigilin [Crassostrea gigas]; 188 4135 Vigilin [Crassostrea gigas]

Protein Accession	CL846.Contig4$_{H1}$ CL846.Contig3$_{H1}$
Mean Expression Ratio	0.901
Median Expression Ratio	0.899
Credible Interval	(0.498, 1.64)
Associated Peptides	8
Associated Spectra	9
Coverage	NaN

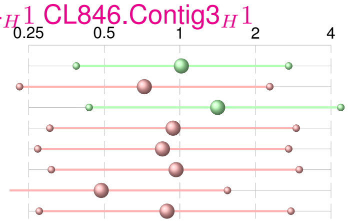

A	B	2.5	50	97.5	Sequence
0	2	0.39	1	2.7	EVSIDIPHR
0	1	0.23	0.72	2.3	NIAEEFGGVTVSFPR
0	1	0.43	1.4	4.4	IKNETGVSIR
0	1	0.3	0.94	2.9	AVIDDLK
0	1	0.27	0.85	2.7	VHEVGIK
0	1	0.31	0.97	3	LQELELATSTK
0	1	0.15	0.49	1.5	GFQLQVEVDPR
0	1	0.27	0.89	2.8	FLIGR

5.520 5 658 predicted protein [Nematostella vectensis] gi

Protein Accession	CL36575.Contig6$_{H1}$
Mean Expression Ratio	1.12
Median Expression Ratio	1.11
Credible Interval	(0.481, 2.55)
Associated Peptides	1
Associated Spectra	1
Coverage	NaN

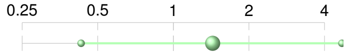

A	B	2.5	50	97.5	Sequence
0	1	0.43	1.4	4.7	KVQIGNIAAGK

5.521 49 1560 PREDICTED: tRNA-splicing ligase RtcB homolog [Megachile rotundata]

Protein Accession	CL21686.Contig3$_{H1}$
Mean Expression Ratio	1.12
Median Expression Ratio	1.11
Credible Interval	(0.485, 2.63)
Associated Peptides	1
Associated Spectra	1
Coverage	NaN

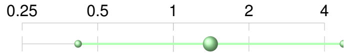

A	B	2.5	50	97.5	Sequence
0	1	0.42	1.4	4.8	GLGHQVATDALVVMEK

5.522 " 492 3458 PREDICTED: 2-oxoglutarate dehydrogenase, mitochondrial isoform 1 [Sarcophilus harrisii]"

Protein Accession	CL2200.Contig16$_H1$
Mean Expression Ratio	0.898
Median Expression Ratio	0.9
Credible Interval	(0.41, 1.97)
Associated Peptides	2
Associated Spectra	2
Coverage	NaN

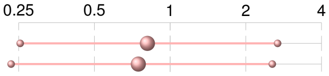

A	B	2.5	50	97.5	Sequence
1	0	0.25	0.81	2.7	IAKEPSVLTK
1	0	0.23	0.75	2.5	FRAKK

5.523 2 811 voltage-dependent anion channel 2-like protein [Haliotis diversicolor]; 2 811 voltage-dependent anion channel 2-like protein [Haliotis diversicolor]; 2 811 voltage-dependent anion channel 2-like protein [Haliotis diversicolor]

Protein Accession	CL2477.Contig3$_H1$ CL2477.Contig2$_H1$ CL2477.Contig1$_H1$
Mean Expression Ratio	1.11
Median Expression Ratio	1.11
Credible Interval	(0.711, 1.73)
Associated Peptides	13
Associated Spectra	50
Coverage	NaN

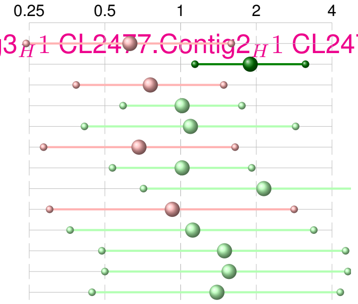

A	B	2.5	50	97.5	Sequence
1	1	0.24	0.63	1.6	GYNYGFFK
4	9	1.1	1.9	3.1	LAFDTSFAPQTGKK
2	4	0.38	0.76	1.5	VSGSLETK
4	7	0.59	1	1.8	YTLDSSASVSAK
1	1	0.42	1.1	2.9	FALGTK
1	2	0.29	0.68	1.6	LAFDTSFAPQTGK
1	6	0.53	1	1.9	VGLGLDVEA
0	1	0.71	2.1	6.6	GYNYGFFKLEAK
0	1	0.3	0.92	2.8	WSEYGLTFTEK
0	1	0.36	1.1	3.4	WNTDNVLNTEIAIEDQIAK
0	1	0.49	1.5	4.5	DIFSK
0	1	0.5	1.6	4.6	KLEAK
0	1	0.44	1.4	4.3	SINQGGHKVGLGLDVEA

5.524 110 2365 N-acetylated-alpha-linked acidic dipeptidase 2 [Crassostrea gigas]

Protein Accession	CL8322.Contig4$_H1$
Mean Expression Ratio	1.11
Median Expression Ratio	1.11
Credible Interval	(0.502, 2.51)
Associated Peptides	2
Associated Spectra	2
Coverage	NaN

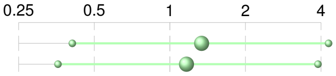

A	B	2.5	50	97.5	Sequence
0	1	0.41	1.3	4.3	ALGTPLMYR
0	1	0.36	1.2	3.9	AQRGTILNADGDPLTPGYPAVR

5.525 36 770 minus strand Endoplasmic reticulum protein ERp29 [Crassostrea gigas]

Protein Accession CL37188.Contig1$_H$1
Mean Expression Ratio 0.901
Median Expression Ratio 0.9
Credible Interval (0.479, 1.68)
Associated Peptides 5
Associated Spectra 10
Coverage NaN

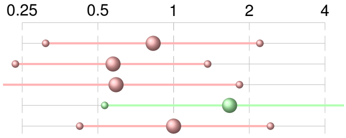

A	B	2.5	50	97.5	Sequence
2	0	0.31	0.83	2.2	AQTAAAALTK
1	2	0.24	0.57	1.4	EADKESADIYIK
1	0	0.19	0.59	1.8	QDPIPYTGDVK
0	1	0.53	1.7	5.4	GSINLNSGVFDKVIAK
0	3	0.42	1	2.4	KPDFPVYK

5.526 75 455 minus strand PREDICTED: calnexin [Strongylocentrotus purpuratus]

Protein Accession CL46825.Contig1$_H$1
Mean Expression Ratio 0.898
Median Expression Ratio 0.9
Credible Interval (0.421, 1.92)
Associated Peptides 2
Associated Spectra 3
Coverage NaN

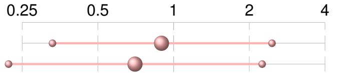

A	B	2.5	50	97.5	Sequence
1	1	0.33	0.9	2.5	SNALDGDLGLILK
0	1	0.22	0.7	2.3	APVFFHEPFDSK

5.527 503 1591 minus strand Septin-2 [Crassostrea gigas]; 648 1736 minus strand Septin-2 [Crassostrea gigas]

Protein Accession CL9569.Contig7$_H$1 CL9569.Contig6$_H$1
Mean Expression Ratio 1.12
Median Expression Ratio 1.11
Credible Interval (0.49, 2.64)
Associated Peptides 1
Associated Spectra 1
Coverage NaN

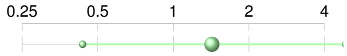

A	B	2.5	50	97.5	Sequence
0	1	0.44	1.4	4.8	VNIVPVLAK

5.528 17 1681 minus strand Glucose-6-phosphate isomerase [Crassostrea gigas]

Protein Accession CL34850.Contig1$_H$1
Mean Expression Ratio 1.11
Median Expression Ratio 1.11
Credible Interval (0.59, 2.13)
Associated Peptides 5
Associated Spectra 7
Coverage NaN

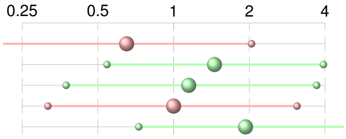

A	B	2.5	50	97.5	Sequence
1	0	0.2	0.65	2	INFTEDR
0	2	0.55	1.5	3.9	INMAQMFK
0	1	0.37	1.2	3.7	SGMNQEQISHILPHK
0	1	0.32	1	3.1	EAAEDELRK
0	2	0.73	1.9	5.2	VHFISNVDGTHLAK

5.529 1067 3010 minus strand PREDICTED: nucleolin-like isoform 2 [Oreochromis niloticus]

Protein Accession	CL7031.Contig7$_{H}1$
Mean Expression Ratio	0.901
Median Expression Ratio	0.9
Credible Interval	(0.425, 1.91)
Associated Peptides	3
Associated Spectra	3
Coverage	NaN

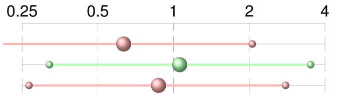

A	B	2.5	50	97.5	Sequence
1	0	0.19	0.63	2.1	KGIAVSDVR
1	0	0.32	1.1	3.5	KISIEAAAVVASAK
1	0	0.27	0.87	2.8	RFAYADLENPDDLDK

5.530 2 487 conserved hypothetical protein [Ixodes scapularis] gi

Protein Accession	CL12002.Contig4$_{H}1$
Mean Expression Ratio	1.11
Median Expression Ratio	1.11
Credible Interval	(0.483, 2.6)
Associated Peptides	1
Associated Spectra	1
Coverage	NaN

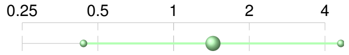

A	B	2.5	50	97.5	Sequence
0	1	0.44	1.4	4.6	DNIAPETLVTIK

5.531 4365 4553 minus strand Basement membrane-specific heparan sulfate proteoglycan core protein [Crassostrea gigas]; 2502 2696 minus strand hypothetical protein DAPPUDRAFT_186952 [Daphnia pulex]

Protein Accession	CL24300.Contig6$_{H}1$ CL24300.Contig2$_{H}1$
Mean Expression Ratio	1.11
Median Expression Ratio	1.11
Credible Interval	(0.481, 2.58)
Associated Peptides	1
Associated Spectra	1
Coverage	NaN

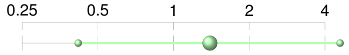

A	B	2.5	50	97.5	Sequence
0	1	0.42	1.4	4.6	DGSIIVDGQEVK

5.532 29 610 minus strand 40S ribosomal protein S7 [Crassostrea gigas]

Protein Accession	CL8074.Contig11$_{H}1$
Mean Expression Ratio	1.11
Median Expression Ratio	1.11
Credible Interval	(0.468, 2.61)
Associated Peptides	1
Associated Spectra	1
Coverage	NaN

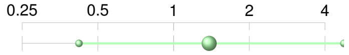

A	B	2.5	50	97.5	Sequence
0	1	0.42	1.4	4.7	TSLHELYITGAK

5.533 794 994 Glycogenin-1 [Crassostrea gigas]; 1425 1739 minus strand Glycogenin-1 [Crassostrea gigas]

Protein Accession	CL18608.Contig3$_H1$ CL18608.Contig2$_H1$
Mean Expression Ratio	1.11
Median Expression Ratio	1.11
Credible Interval	(0.471, 2.56)
Associated Peptides	1
Associated Spectra	1
Coverage	NaN

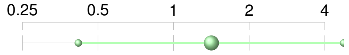

A	B	2.5	50	97.5	Sequence
**0**	**1**	**0.42**	**1.4**	**4.7**	**IAAPVLSFK**

5.534 103 1356 minus strand PREDICTED: actin-related protein 3-like [Oreochromis niloticus]

Protein Accession	CL20855.Contig4$_H1$
Mean Expression Ratio	0.908
Median Expression Ratio	0.903
Credible Interval	(0.433, 1.93)
Associated Peptides	2
Associated Spectra	5
Coverage	NaN

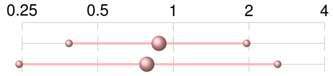

A	B	2.5	50	97.5	Sequence
**2**	**2**	**0.38**	**0.88**	**2**	**HNPVFGAMA**
**1**	**0**	**0.24**	**0.78**	**2.6**	**LPAVVVDTGTGYTK**

5.535 771 1505 O-methyltransferase family 3 [Dicentrarchus labrax]

Protein Accession	Unigene19132$_H1$
Mean Expression Ratio	0.907
Median Expression Ratio	0.903
Credible Interval	(0.476, 1.74)
Associated Peptides	5
Associated Spectra	7
Coverage	NaN

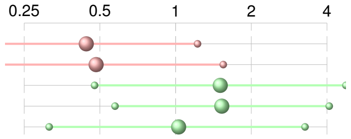

A	B	2.5	50	97.5	Sequence
**1**	**1**	**0.16**	**0.44**	**1.2**	**VFGEHVINDQR**
**1**	**0**	**0.15**	**0.48**	**1.5**	**VLIPVR**
**0**	**1**	**0.48**	**1.5**	**4.8**	**AEATQADPEVIEGLKR**
**0**	**2**	**0.57**	**1.5**	**4.1**	**FGASYLPR**
**0**	**1**	**0.31**	**1**	**3.3**	**IAETTLTHPWR**

5.536 14 502 minus strand ependymin related protein-1 precursor [Haliotis discus discus]; 14 547 ependymin related protein-1 precursor [Haliotis discus discus]

Protein Accession	CL3749.Contig3$_H1$ CL3749.Contig1$_H1$
Mean Expression Ratio	1.11
Median Expression Ratio	1.11
Credible Interval	(0.479, 2.6)
Associated Peptides	1
Associated Spectra	1
Coverage	NaN

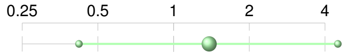

A	B	2.5	50	97.5	Sequence
**0**	**1**	**0.42**	**1.4**	**4.5**	**IWYDAENQR**

5.537 325 642 minus strand Protein phosphatase 1B [Crassostrea gigas]

Protein Accession	Unigene268$_H$1
Mean Expression Ratio	0.904
Median Expression Ratio	0.904
Credible Interval	(0.424, 1.95)
Associated Peptides	2
Associated Spectra	3
Coverage	NaN

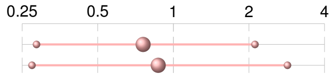

A	B	2.5	50	97.5	Sequence
**1**	**1**	**0.29**	**0.76**	**2.1**	**VEMEDAHTAVVSIPHAR**
**1**	**0**	**0.27**	**0.87**	**2.9**	**GAFLDKPK**

5.538 62 394 minus strand PREDICTED: NADH dehydrogenase [ubiquinone] 1 alpha subcomplex subunit 5 [Saimiri boliviensis boliviensis]; 62 394 minus strand PREDICTED: NADH dehydrogenase [ubiquinone] 1 alpha subcomplex subunit 5 [Saimiri boliviensis boliviensis]

Protein Accession	CL6313.Contig4$_H$1 CL6313.Contig3$_H$1
Mean Expression Ratio	1.11
Median Expression Ratio	1.11
Credible Interval	(0.48, 2.62)
Associated Peptides	1
Associated Spectra	1
Coverage	NaN

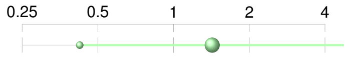

A	B	2.5	50	97.5	Sequence
**0**	**1**	**0.42**	**1.4**	**5**	**LAIVNSEPDPGRLEMK**

5.539 423 695 thioredoxin peroxidase 2 [Haliotis discus discus]

Protein Accession	CL3160.Contig13$_H$1
Mean Expression Ratio	1.11
Median Expression Ratio	1.11
Credible Interval	(0.469, 2.64)
Associated Peptides	1
Associated Spectra	1
Coverage	NaN

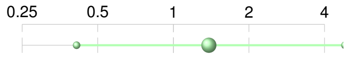

A	B	2.5	50	97.5	Sequence
**0**	**1**	**0.41**	**1.4**	**4.8**	**KIGGLGNMNIPLLEDKTHEIAR**

5.540 1 468 glycine cleavage system H protein [Rhipicephalus sanguineus]

Protein Accession	CL42651.Contig1$_H$1
Mean Expression Ratio	0.901
Median Expression Ratio	0.904
Credible Interval	(0.387, 2.07)
Associated Peptides	1
Associated Spectra	1
Coverage	NaN

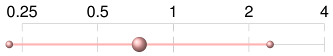

A	B	2.5	50	97.5	Sequence
**0**	**1**	**0.22**	**0.73**	**2.4**	**SPLDDGWVFK**

5.541 " 2057 2497 Phosphate carrier protein, mitochondrial [Crassostrea gigas]"

Protein Accession CL2712.Contig11$_{H}1$
Mean Expression Ratio 1.1
Median Expression Ratio 1.11
Credible Interval (0.5, 2.39)
Associated Peptides 2
Associated Spectra 2
Coverage NaN

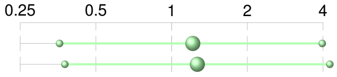

A	B	2.5	50	97.5	Sequence
**0**	**1**	**0.36**	**1.2**	**4**	**LPRPPPPEMPESLKR**
**0**	**1**	**0.38**	**1.3**	**4.2**	**YVVPKPR**

5.542 87 764 Enolase-phosphatase E1 [Crassostrea gigas]; 87 1058 Enolase-phosphatase E1 [Crassostrea gigas]; 87 764 Enolase-phosphatase E1 [Crassostrea gigas]

Protein Accession CL25175.Contig7$_{H}1$ CL25175.Contig6$_{H}1$ CL25175.Contig4$_{H}1$
Mean Expression Ratio 0.903
Median Expression Ratio 0.905
Credible Interval (0.41, 1.96)
Associated Peptides 1
Associated Spectra 4
Coverage NaN

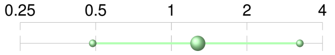

A	B	2.5	50	97.5	Sequence
**2**	**2**	**0.49**	**1.3**	**3.3**	**SAAEAESLLVGVK**

5.543 1021 1359 minus strand RecName: Full=40S ribosomal protein S26 gi|6015599|emb|CAB57819.1| ribosomal protein S26 [Octopus vulgaris]

Protein Accession Unigene11569$_{H}1$
Mean Expression Ratio 0.904
Median Expression Ratio 0.905
Credible Interval (0.385, 2.13)
Associated Peptides 1
Associated Spectra 1
Coverage NaN

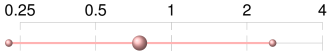

A	B	2.5	50	97.5	Sequence
**1**	**0**	**0.23**	**0.75**	**2.5**	**NIVEAAAVR**

5.544 115 393 minus strand Yolk ferritin OS=Lymnaea stagnalis PE=1 SV=1; 132 413 RecName: Full=Yolk ferritin; Flags: Precursor gi|9655|emb|CAA40097.1| snail yolk ferritin [Lymnaea stagnalis] gi; 2 430 RecName: Full=Yolk ferritin; Flags: Precursor gi|9655|emb|CAA40097.1| snail yolk ferritin [Lymnaea stagnalis] gi; 3 389 RecName: Full=Yolk ferritin; Flags: Precursor gi|9655|emb|CAA40097.1| snail yolk ferritin [Lymnaea stagnalis] gi

Protein Accession	CL33796.Contig4$_H$1 CL33796.Contig3$_H$1 CL33796.Contig2$_H$1 CL33796.Contig1$_H$1
Mean Expression Ratio	1.1
Median Expression Ratio	1.1
Credible Interval	(0.472, 2.51)
Associated Peptides	1
Associated Spectra	1
Coverage	NaN

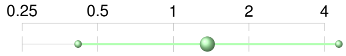

A	B	2.5	50	97.5	Sequence
**0**	**1**	**0.42**	**1.4**	**4.6**	**QYGLQAMEDALALER**

5.545 123 860 PREDICTED: 3-hydroxybutyrate dehydrogenase type 2-like isoform 1 [Sus scrofa] gi; 32 901 3-hydroxybutyrate dehydrogenase type 2 [Rattus norvegicus]

Protein Accession	CL33059.Contig4$_H$1 CL33059.Contig2$_H$1
Mean Expression Ratio	0.902
Median Expression Ratio	0.906
Credible Interval	(0.386, 2.13)
Associated Peptides	1
Associated Spectra	1
Coverage	NaN

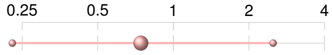

A	B	2.5	50	97.5	Sequence
**1**	**0**	**0.23**	**0.74**	**2.5**	**ILDVLDTEK**

5.546 242 1510 minus strand hypothetical protein BRAFLDRAFT_276673 [Branchiostoma floridae] gi

Protein Accession	Unigene23008$_H$1
Mean Expression Ratio	0.9
Median Expression Ratio	0.906
Credible Interval	(0.414, 1.92)
Associated Peptides	2
Associated Spectra	3
Coverage	NaN

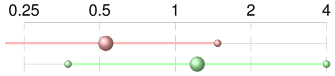

A	B	2.5	50	97.5	Sequence
**2**	**0**	**0.2**	**0.53**	**1.5**	**TVFITGASR**
**0**	**1**	**0.37**	**1.2**	**4**	**DGANIVIAAK**

5.547 " 1 1125 PREDICTED: trifunctional enzyme subunit beta, mitochondrial-like [Oreochromis niloticus]"

Protein Accession	CL19853.Contig2$_{H}1$
Mean Expression Ratio	1.1
Median Expression Ratio	1.1
Credible Interval	(0.503, 2.42)
Associated Peptides	2
Associated Spectra	2
Coverage	NaN

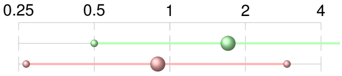

A	B	2.5	50	97.5	Sequence
**0**	**1**	**0.5**	**1.7**	**5.6**	**KEQDEFALR**
**0**	**1**	**0.27**	**0.9**	**2.9**	**LMLSLNK**

5.548 " 2 1810 minus strand Glutaminyl-tRNA synthetase, partial [Crassostrea gigas]"

Protein Accession	CL16949.Contig1$_{H}1$
Mean Expression Ratio	1.11
Median Expression Ratio	1.1
Credible Interval	(0.48, 2.58)
Associated Peptides	1
Associated Spectra	1
Coverage	NaN

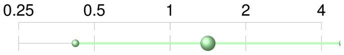

A	B	2.5	50	97.5	Sequence
**0**	**1**	**0.42**	**1.4**	**4.9**	**IAEGEATLR**

5.549 942 1385 Golgi-associated plant pathogenesis-related protein 1 [Crassostrea gigas]; 536 979 Golgi-associated plant pathogenesis-related protein 1 [Crassostrea gigas]

Protein Accession	Unigene391$_{H}1$ CL15263.Contig11$_{H}1$
Mean Expression Ratio	1.11
Median Expression Ratio	1.1
Credible Interval	(0.483, 2.57)
Associated Peptides	1
Associated Spectra	1
Coverage	NaN

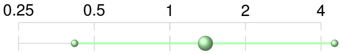

A	B	2.5	50	97.5	Sequence
**0**	**1**	**0.42**	**1.4**	**4.5**	**IITEETIIK**

5.550 40 609 minus strand ependymin related protein-1 precursor [Haliotis discus discus]

Protein Accession CL31954.Contig1$_{H}1$
Mean Expression Ratio 1.1
Median Expression Ratio 1.1
Credible Interval (0.496, 2.43)
Associated Peptides 2
Associated Spectra 2
Coverage NaN

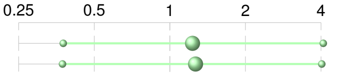

A	B	2.5	50	97.5	Sequence
**0**	**1**	**0.38**	**1.2**	**4.1**	**EIEEIQIGSDQEFYDVLR**
**0**	**1**	**0.37**	**1.3**	**4**	**QQPKPCTSPPQFVAR**

5.551 2 1384 minus strand twitchin [Mytilus galloprovincialis]; 2 1384 minus strand twitchin [Mytilus galloprovincialis]; 2 1384 minus strand twitchin [Mytilus galloprovincialis]; 2 1384 minus strand twitchin [Mytilus galloprovincialis]; 2 1384 minus strand twitchin [Mytilus galloprovincialis]; 2 1384 minus strand twitchin [Mytilus galloprovincialis]

Protein Accession Unigene62659$_{H}1$ Unigene62658$_{H}1$ Unigene62657$_{H}1$ Unigene62656$_{H}1$ Unigene62655$_{H}1$ U
Mean Expression Ratio 1.1
Median Expression Ratio 1.1
Credible Interval (0.479, 2.63)
Associated Peptides 1
Associated Spectra 1
Coverage NaN

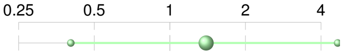

A	B	2.5	50	97.5	Sequence
**0**	**1**	**0.4**	**1.4**	**4.6**	**LEGDTIIIQQDITSK**

5.552 43 1623 minus strand MGC84105 protein precursor [Xenopus laevis] gi; 43 1506 minus strand MGC84105 protein precursor [Xenopus laevis] gi

Protein Accession CL16647.Contig5$_{H}1$ CL16647.Contig4$_{H}1$
Mean Expression Ratio 1.1
Median Expression Ratio 1.1
Credible Interval (0.538, 2.28)
Associated Peptides 3
Associated Spectra 4
Coverage NaN

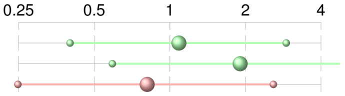

A	B	2.5	50	97.5	Sequence
**1**	**1**	**0.4**	**1.1**	**2.9**	**LIDAADSAR**
**0**	**1**	**0.59**	**1.9**	**6.1**	**QLPELIAVRDEMQK**
**0**	**1**	**0.25**	**0.81**	**2.6**	**LSELEK**

5.553 236 733 Spectrin alpha chain [Crassostrea gigas]

Protein Accession	Unigene61767$_{H}1$
Mean Expression Ratio	0.912
Median Expression Ratio	0.908
Credible Interval	(0.394, 2.14)
Associated Peptides	1
Associated Spectra	1
Coverage	NaN

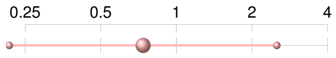

A	B	2.5	50	97.5	Sequence
0	1	0.22	0.74	2.5	LNEANTMHQFLR

5.554 781 1212 minus strand cathepsin L2 cysteine protease [Pinctada fucata]

Protein Accession	CL7881.Contig5$_{H}1$
Mean Expression Ratio	1.1
Median Expression Ratio	1.1
Credible Interval	(0.538, 2.29)
Associated Peptides	2
Associated Spectra	5
Coverage	NaN

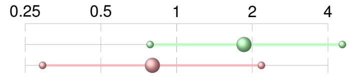

A	B	2.5	50	97.5	Sequence
2	1	0.78	1.9	4.5	GIYNEPACSAK
1	1	0.29	0.8	2.2	NTVGATLSSYK

5.555 185 481 minus strand Zgc:56530 [Danio rerio]

Protein Accession	CL27088.Contig2$_{H}1$
Mean Expression Ratio	1.1
Median Expression Ratio	1.1
Credible Interval	(0.566, 2.14)
Associated Peptides	5
Associated Spectra	7
Coverage	NaN

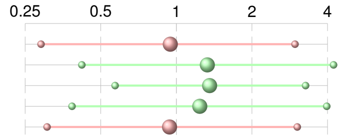

A	B	2.5	50	97.5	Sequence
0	1	0.29	0.94	3	EATQEIQEICDQIR
0	1	0.42	1.3	4.2	TQVVAGTNYFIK
0	3	0.57	1.4	3.3	TFTDFK
0	1	0.38	1.2	4	TADDELEYF
0	1	0.31	0.94	3	ALSYK

5.556 2 244 minus strand hypothetical protein KGM_08771 [Danaus plexippus]

Protein Accession	CL49466.Contig2$_{H}1$
Mean Expression Ratio	1.1
Median Expression Ratio	1.1
Credible Interval	(0.519, 2.3)
Associated Peptides	3
Associated Spectra	3
Coverage	NaN

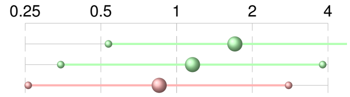

A	B	2.5	50	97.5	Sequence
0	1	0.54	1.7	5.5	AVFIDLEPTVVDEVR
0	1	0.35	1.2	3.8	TVGGGDDSFNTFFSETGAGK
0	1	0.26	0.85	2.8	QLFHPEQLVTGK

5.557 145 459 fk506-binding protein 2 [Papilio xuthus]; 237 551 fk506-binding protein 2 [Papilio xuthus]

Protein Accession	Unigene7883$_{H}$1 CL4621.Contig4$_{H}$1
Mean Expression Ratio	0.909
Median Expression Ratio	0.909
Credible Interval	(0.505, 1.64)
Associated Peptides	5
Associated Spectra	24
Coverage	NaN

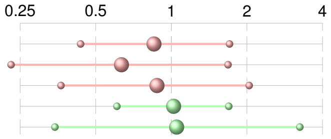

A	B	2.5	50	97.5	Sequence
3	3	0.44	0.85	1.7	GVDVETISEGDKK
1	1	0.23	0.63	1.7	GWDEGVAQMSK
1	2	0.36	0.87	2	IGKGEVIK
6	6	0.61	1	1.7	NKPFQFK
0	1	0.34	1.1	3.3	GVDVETISEGDKKNYPK

5.558 " 695 823 minus strand Aldehyde dehydrogenase family 16 member A1, partial [Crassostrea gigas]"

Protein Accession	CL17427.Contig5$_{H}$1
Mean Expression Ratio	0.908
Median Expression Ratio	0.91
Credible Interval	(0.392, 2.07)
Associated Peptides	1
Associated Spectra	1
Coverage	NaN

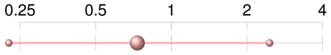

A	B	2.5	50	97.5	Sequence
0	1	0.23	0.73	2.5	TVWIPAGEIFAN

5.559 532 663 minus strand 40S ribosomal protein S23 [Ascaris suum]; 526 654 minus strand RecName: Full=40S ribosomal protein S23 gi|1754684|gb|AAC47632.1| ribosomal protein S23 [Brugia malayi]

Protein Accession	CL10408.Contig3$_{H}$1 CL10408.Contig2$_{H}$1
Mean Expression Ratio	1.11
Median Expression Ratio	1.1
Credible Interval	(0.488, 2.47)
Associated Peptides	1
Associated Spectra	2
Coverage	NaN

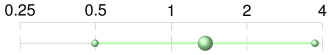

A	B	2.5	50	97.5	Sequence
0	2	0.5	1.4	3.7	KGHAVGDIPGVR

5.560 102 1475 minus strand Cytosolic non-specific dipeptidase [Crassostrea gigas]

Protein Accession	CL4706.Contig1$_{H}1$
Mean Expression Ratio	1.1
Median Expression Ratio	1.1
Credible Interval	(0.577, 2.06)
Associated Peptides	5
Associated Spectra	8
Coverage	NaN

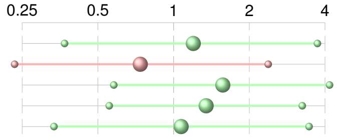

A	B	2.5	50	97.5	Sequence
0	1	0.37	1.2	3.7	EGGSIPVTLTFQEATGK
0	1	0.23	0.74	2.4	EHVEKVFAER
0	2	0.58	1.6	4.2	LVPDQEPHVIESLVR
0	3	0.56	1.3	3.2	LSEAVAIK
0	1	0.33	1.1	3.5	AELQELGEQTLHDGSK

5.561 128 1519 minus strand V-type proton ATPase subunit H [Crassostrea gigas]; 128 1519 minus strand V-type proton ATPase subunit H [Crassostrea gigas]; 128 1519 minus strand V-type proton ATPase subunit H [Crassostrea gigas]; 128 1519 minus strand V-type proton ATPase subunit H [Crassostrea gigas]

Protein Accession	Unigene39559$_{H}1$ Unigene2219$_{H}1$ CL1883.Contig8$_{H}1$ CL1883.Contig6$_{H}1$
Mean Expression Ratio	1.1
Median Expression Ratio	1.1
Credible Interval	(0.509, 2.36)
Associated Peptides	2
Associated Spectra	4
Coverage	NaN

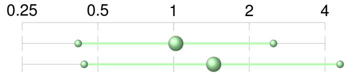

A	B	2.5	50	97.5	Sequence
0	3	0.42	1	2.5	SGNLEWSPVHR
0	1	0.44	1.4	4.6	ANVVNWK

5.562 13 585 UDP-glucose:glycoprotein glucosyltransferase 1 [Crassostrea gigas]

Protein Accession	CL48318.Contig1$_{H}1$
Mean Expression Ratio	0.911
Median Expression Ratio	0.91
Credible Interval	(0.394, 2.13)
Associated Peptides	1
Associated Spectra	1
Coverage	NaN

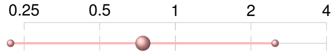

A	B	2.5	50	97.5	Sequence
0	1	0.22	0.74	2.5	FLDLPHK

5.563 3 1418 Protein hu-li tai shao [Crassostrea gigas]; 3 1523 minus strand Protein hu-li tai shao [Crassostrea gigas]; 3 1562 minus strand Protein hu-li tai shao [Crassostrea gigas]

Protein Accession	CL3889.Contig5$_H1$ CL3889.Contig13$_H1$ CL3889.Contig11$_H1$
Mean Expression Ratio	1.1
Median Expression Ratio	1.1
Credible Interval	(0.516, 2.28)
Associated Peptides	2
Associated Spectra	5
Coverage	NaN

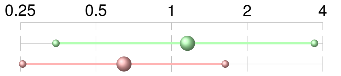

A	B	2.5	50	97.5	Sequence
**0**	**1**	**0.35**	**1.2**	**3.7**	**ILEGLSWEEAAR**
**2**	**2**	**0.25**	**0.64**	**1.6**	**VMLLR**

5.564 401 1174 minus strand Mechanosensory protein 2 [Crassostrea gigas]

Protein Accession	Unigene7703$_H1$
Mean Expression Ratio	1.09
Median Expression Ratio	1.1
Credible Interval	(0.587, 2.04)
Associated Peptides	5
Associated Spectra	10
Coverage	NaN

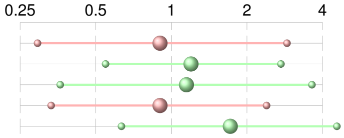

A	B	2.5	50	97.5	Sequence
**1**	**0**	**0.29**	**0.9**	**2.9**	**AMAAEAEASR**
**2**	**2**	**0.55**	**1.2**	**2.7**	**VIAAEGEQK**
**1**	**0**	**0.36**	**1.1**	**3.6**	**AMAAEAEASREAR**
**1**	**1**	**0.33**	**0.9**	**2.4**	**LPVQLQR**
**1**	**1**	**0.63**	**1.7**	**4.6**	**AKVIAAEGEQK**

5.565 192 1820 PREDICTED: copine IV-like [Saccoglossus kowalevskii]; 125 1753 PREDICTED: copine IV-like [Saccoglossus kowalevskii]

Protein Accession	CL15074.Contig3$_H1$ CL15074.Contig1$_H1$
Mean Expression Ratio	0.91
Median Expression Ratio	0.911
Credible Interval	(0.429, 1.93)
Associated Peptides	2
Associated Spectra	4
Coverage	NaN

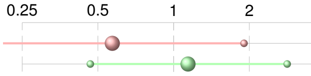

A	B	2.5	50	97.5	Sequence
**1**	**0**	**0.18**	**0.57**	**1.9**	**LLNAK**
**0**	**3**	**0.47**	**1.1**	**2.8**	**HVLAEVPK**

5.566 68 694 minus strand hypothetical protein BRAFLDRAFT_272416 [Branchiostoma floridae] gi; 68 667 minus strand hypothetical protein BRAFLDRAFT_272416 [Branchiostoma floridae] gi; 68 697 minus strand hypothetical protein BRAFLDRAFT_272416 [Branchiostoma floridae] gi; 68 697 minus strand hypothetical protein BRAFLDRAFT_272416 [Branchiostoma floridae] gi; 68 667 minus strand hypothetical protein BRAFLDRAFT_272416 [Branchiostoma floridae] gi

Protein Accession	CL37759.Contig7$_{H}1$ CL37759.Contig4$_{H}1$ CL37759.Contig3$_{H}1$ CL37759.Contig2$_{H}1$ CL3775
Mean Expression Ratio	1.1
Median Expression Ratio	1.1
Credible Interval	(0.534, 2.27)
Associated Peptides	3
Associated Spectra	4
Coverage	NaN

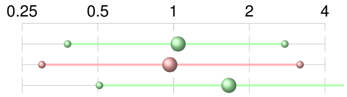

A	B	2.5	50	97.5	Sequence
0	2	0.38	1	2.8	TLEEDEDVLLQLR
0	1	0.3	0.97	3.2	VLLQLR
0	1	0.51	1.7	5.5	LLAIR

5.567 4 399 Spectrin beta chain [Crassostrea gigas]

Protein Accession	Unigene17190$_{H}1$
Mean Expression Ratio	0.91
Median Expression Ratio	0.912
Credible Interval	(0.433, 1.9)
Associated Peptides	3
Associated Spectra	3
Coverage	NaN

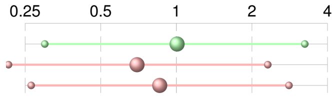

A	B	2.5	50	97.5	Sequence
0	1	0.3	1	3.2	DGLAFNAIIHK
0	1	0.21	0.7	2.3	VHLENMGAHDIVDGSAR
0	1	0.26	0.86	2.8	HRPDLIQYDK

5.568 1 297 beta-thymosin domain repeat protein CSP24KDa_v1 [Hermissenda crassicornis] gi

Protein Accession	Unigene8923$_{H}1$
Mean Expression Ratio	1.09
Median Expression Ratio	1.1
Credible Interval	(0.479, 2.51)
Associated Peptides	1
Associated Spectra	2
Coverage	NaN

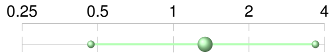

A	B	2.5	50	97.5	Sequence
0	2	0.47	1.3	3.7	ELLHHVVPEEK

5.569 1 630 hypothetical protein [Trichomonas vaginalis G3] gi

Protein Accession	CL4495.Contig3$_H$1
Mean Expression Ratio	1.09
Median Expression Ratio	1.1
Credible Interval	(0.457, 2.47)
Associated Peptides	1
Associated Spectra	1
Coverage	NaN

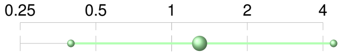

A	B	2.5	50	97.5	Sequence
0	1	0.4	1.3	4.4	TLTEEKENLNK

5.570 " 125 628 minus strand collectin sub-family member 12, partial [Oncorhynchus mykiss]"

Protein Accession	CL46744.Contig5$_H$1
Mean Expression Ratio	1.1
Median Expression Ratio	1.1
Credible Interval	(0.482, 2.51)
Associated Peptides	1
Associated Spectra	1
Coverage	NaN

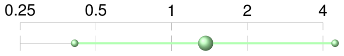

A	B	2.5	50	97.5	Sequence
0	1	0.41	1.4	4.5	WVQGDNRPLAYTR

5.571 511 1062 unknown [Dendroctonus ponderosae]; 511 1062 unknown [Dendroctonus ponderosae]

Protein Accession	CL18443.Contig3$_H$1 CL18443.Contig1$_H$1
Mean Expression Ratio	1.09
Median Expression Ratio	1.1
Credible Interval	(0.473, 2.57)
Associated Peptides	1
Associated Spectra	1
Coverage	NaN

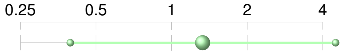

A	B	2.5	50	97.5	Sequence
0	1	0.4	1.3	4.5	FKDEIEMHLK

5.572 35 1861 hypothetical protein DAPPUDRAFT_309082 [Daphnia pulex]

Protein Accession	CL49314.Contig5$_H$1
Mean Expression Ratio	0.909
Median Expression Ratio	0.913
Credible Interval	(0.513, 1.62)
Associated Peptides	6
Associated Spectra	18
Coverage	NaN

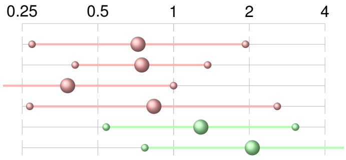

A	B	2.5	50	97.5	Sequence
1	1	0.27	0.72	1.9	LINHSVPDTIDER
5	3	0.4	0.75	1.4	YAIGLAR
1	1	0.14	0.38	1	KVELVK
0	1	0.27	0.84	2.6	ISNFGDDIKDSEAYVHLLHR
0	3	0.54	1.3	3.1	LNDGIIIAK
0	2	0.77	2.1	5.5	DIGHTFKK

5.573 304 2952 minus strand hypothetical protein CGI_10026535 [Crassostrea gigas]

Protein Accession	CL21819.Contig2$_{H}1$
Mean Expression Ratio	1.1
Median Expression Ratio	1.1
Credible Interval	(0.575, 2.07)
Associated Peptides	5
Associated Spectra	7
Coverage	NaN

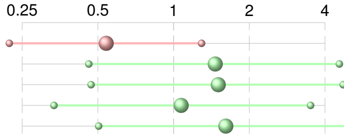

A	B	2.5	50	97.5	Sequence
2	1	0.22	0.54	1.3	VADIAGDIPK
0	1	0.46	1.5	4.6	LVQLEETAK
0	1	0.47	1.5	4.7	LVGMFSK
0	1	0.34	1.1	3.5	ESPLHK
0	1	0.51	1.6	5.2	AEYSLAAVALPPDNDAK

5.574 48 1523 minus strand Spermatogenesis-associated protein 18-like protein [Crassostrea gigas]

Protein Accession	CL6432.Contig3$_{H}1$
Mean Expression Ratio	1.1
Median Expression Ratio	1.09
Credible Interval	(0.476, 2.53)
Associated Peptides	1
Associated Spectra	1
Coverage	NaN

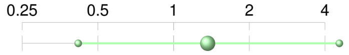

A	B	2.5	50	97.5	Sequence
0	1	0.42	1.4	4.6	SLSPALR

5.575 93 2606 twitchin [Mytilus galloprovincialis]

Protein Accession	Unigene71883$_{H}1$
Mean Expression Ratio	1.09
Median Expression Ratio	1.09
Credible Interval	(0.48, 2.51)
Associated Peptides	1
Associated Spectra	1
Coverage	NaN

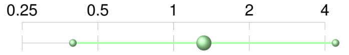

A	B	2.5	50	97.5	Sequence
0	1	0.4	1.3	4.4	LNPDEIVK

5.576 366 1220 Ectoine hydroxylase [Crassostrea gigas]

Protein Accession	CL2342.Contig2$_{H}1$
Mean Expression Ratio	1.09
Median Expression Ratio	1.09
Credible Interval	(0.568, 2.06)
Associated Peptides	5
Associated Spectra	7
Coverage	NaN

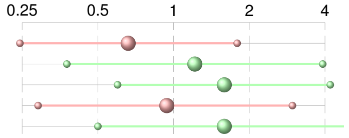

A	B	2.5	50	97.5	Sequence
1	1	0.24	0.66	1.8	IDHDLVAGQQR
0	1	0.38	1.2	3.9	AFDDDGYILIK
0	2	0.6	1.6	4.2	GFLDQEELDNLRK
0	1	0.29	0.94	3	LLGGEVYHYHTK
0	1	0.5	1.6	5	TVEDSDLFR

5.577 28 1908 minus strand Mitochondrial inner membrane protein [Crassostrea gigas]; 10 1902 Mitochondrial inner membrane protein [Crassostrea gigas]; 10 1902 Mitochondrial inner membrane protein [Crassostrea gigas]; 10 1902 Mitochondrial inner membrane protein [Crassostrea gigas]; 10 1902 Mitochondrial inner membrane protein [Crassostrea gigas]; 10 1902 Mitochondrial inner membrane protein [Crassostrea gigas]; 10 1902 Mitochondrial inner membrane protein [Crassostrea gigas]

Protein Accession	CL6414.Contig9$_H1$ CL6414.Contig8$_H1$ CL6414.Contig7$_H1$ CL6414.Contig6$_H1$ CL6414.Con
Mean Expression Ratio	0.919
Median Expression Ratio	0.914
Credible Interval	(0.394, 2.12)
Associated Peptides	1
Associated Spectra	1
Coverage	NaN

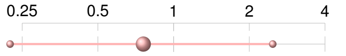

A	B	2.5	50	97.5	Sequence
0	1	0.22	0.76	2.5	EIHTQLLQER

5.578 30 1088 arginine kinase [Conus novaehollandiae]

Protein Accession	CL5308.Contig5$_H1$
Mean Expression Ratio	0.914
Median Expression Ratio	0.914
Credible Interval	(0.685, 1.22)
Associated Peptides	34
Associated Spectra	125
Coverage	NaN

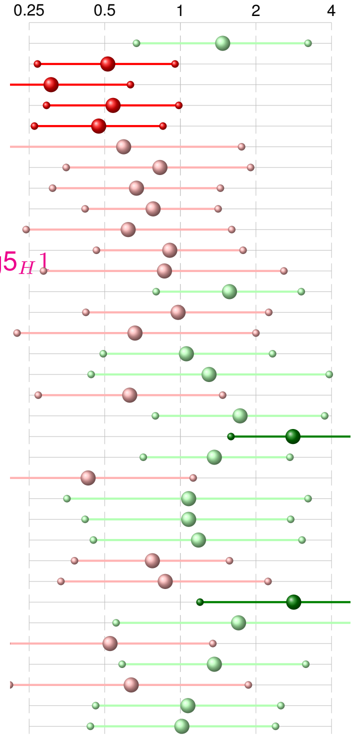

A	B	2.5	50	97.5	Sequence
1	3	0.67	1.5	3.2	AAGGYDDWPVGR
4	3	0.27	0.51	0.96	FNFPDLDPSGK
5	0	0.15	0.31	0.63	IISMQK
5	3	0.29	0.54	0.99	YIVSTR
4	5	0.26	0.47	0.85	LTFAR
1	0	0.2	0.59	1.7	VNMEK
1	2	0.35	0.83	1.9	GGNLTQVYQR
1	3	0.31	0.67	1.4	GTYYPLLGMDK
5	3	0.42	0.78	1.4	HPAPDFGEK
1	1	0.24	0.62	1.6	LAALPEFK
1	5	0.46	0.91	1.8	LVSGIHALEK
1	0	0.29	0.87	2.6	RFNFPDLDPSGK
0	6	0.8	1.6	3	ATVEALNTFEGELK
0	3	0.42	0.98	2.3	FNFPDLDPSGKYIVSTR
0	1	0.22	0.66	2	GIFHNPDK
0	4	0.49	1.1	2.3	GIHGEHTESVGGVYDISNKRR
0	1	0.44	1.3	3.9	GTYYPLLGMDKATEK
0	3	0.27	0.63	1.5	KLIDDHFLFK
0	4	0.8	1.7	3.8	KLIDDHFLFKDDDR
0	9	1.6	2.8	5	KLIDDHFLFKDDDRFLK
0	6	0.71	1.4	2.7	LGLSEFDAIMEMK
0	2	0.17	0.43	1.1	LIDDHFLFK
0	1	0.35	1.1	3.2	LIDDHFLFKDDDR
0	2	0.42	1.1	2.7	NGVEEILKK
0	2	0.45	1.2	3.1	RFNFPDLDPSGKYIVSTR
0	5	0.38	0.77	1.6	SHASYGFPPVLSK
0	2	0.34	0.87	2.2	TFLVWVNEEDHIR
0	3	1.2	2.8	6.6	VAEVKHPAPDFGEK
0	1	0.55	1.7	5.1	VAEVKHPAPDFGEKR
0	2	0.2	0.53	1.3	NGVEEILK
0	3	0.59	1.4	3.2	KLTFAR
0	1	0.21	0.64	1.9	DYHKVAEVK
0	3	0.46	1.1	2.5	GIHGEHTESVGGVYDISNK
0	3	0.44	1	2.4	GIHGEHTESVGGVYDISNKR

5.579 521 6844 hypothetical protein CGI_10014469 [Crassostrea gigas]

Protein Accession	CL12911.Contig1$_{H}1$
Mean Expression Ratio	0.915
Median Expression Ratio	0.914
Credible Interval	(0.534, 1.59)
Associated Peptides	7
Associated Spectra	35
Coverage	NaN

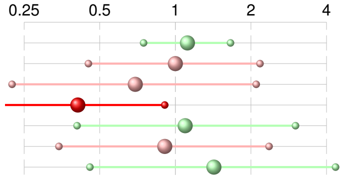

A	B	2.5	50	97.5	Sequence
5	16	0.75	1.1	1.7	IDAALVLK
2	2	0.45	1	2.2	VAAVQAFR
1	0	0.22	0.69	2.1	LQTIK
3	1	0.18	0.41	0.91	LTRIQV
0	2	0.41	1.1	3	KLFDLNPLK
0	2	0.34	0.91	2.4	LFDLNPLK
0	1	0.46	1.4	4.3	RSPWQLPKDLSLTLK

5.580 104 2638 Eukaryotic translation initiation factor 3 subunit C [Crassostrea gigas]

Protein Accession	CL904.Contig3$_{H}1$
Mean Expression Ratio	1.09
Median Expression Ratio	1.09
Credible Interval	(0.485, 2.44)
Associated Peptides	1
Associated Spectra	3
Coverage	NaN

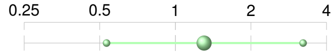

A	B	2.5	50	97.5	Sequence
2	1	0.53	1.3	3.2	GAPLIVEKPK

5.581 3 1325 minus strand Eukaryotic translation initiation factor 3 subunit B [Crassostrea gigas]

Protein Accession	CL19479.Contig9$_{H}1$
Mean Expression Ratio	0.912
Median Expression Ratio	0.915
Credible Interval	(0.392, 2.11)
Associated Peptides	1
Associated Spectra	1
Coverage	NaN

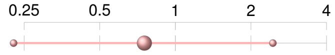

A	B	2.5	50	97.5	Sequence
0	1	0.23	0.75	2.4	FAFIHGESPR

5.582 55 1569 minus strand Putative aminopeptidase W07G4.4 [Crassostrea gigas]; 228 1742 Putative aminopeptidase W07G4.4 [Crassostrea gigas]

Protein Accession	CL1078.Contig5$_{H}1$ CL1078.Contig1$_{H}1$
Mean Expression Ratio	1.09
Median Expression Ratio	1.09
Credible Interval	(0.553, 2.12)
Associated Peptides	5
Associated Spectra	5
Coverage	NaN

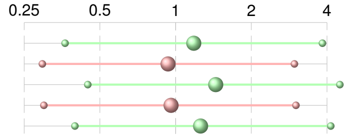

A	B	2.5	50	97.5	Sequence
0	1	0.36	1.2	3.8	AVGGLSFVR
0	1	0.3	0.94	3	KLTGSLQPLAEVLNK
0	1	0.45	1.4	4.5	LIFSPTGPLDRDQDDVRR
0	1	0.3	0.96	3	WSETDKGFEK
0	1	0.4	1.3	4.1	AVGEQYSIIMDNGPAR

5.583 40 777 proteasome subunit N3 [Haliotis discus discus]; 40 777 proteasome subunit N3 [Haliotis discus discus]

Protein Accession	CL39106.Contig2$_{H}1$ CL39106.Contig1$_{H}1$
Mean Expression Ratio	1.09
Median Expression Ratio	1.09
Credible Interval	(0.501, 2.43)
Associated Peptides	2
Associated Spectra	2
Coverage	NaN

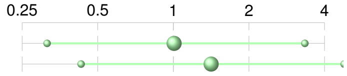

A	B	2.5	50	97.5	Sequence
0	1	0.32	1	3.3	TLMPSVTTTSVLGLK
0	1	0.43	1.4	4.8	YQIAIVTSK

5.584 " 28 1104 minus strand Pyruvate dehydrogenase E1 component subunit beta, mitochondrial [Crassostrea gigas]; 1087 1581 Pyruvate dehydrogenase E1 component subunit beta, mitochondrial [Crassostrea gigas]"

Protein Accession	CL18015.Contig14$_{H}1$ CL18015.Contig13$_{H}1$
Mean Expression Ratio	0.913
Median Expression Ratio	0.915
Credible Interval	(0.417, 2)
Associated Peptides	2
Associated Spectra	4
Coverage	NaN

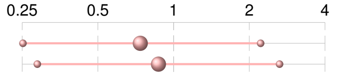

A	B	2.5	50	97.5	Sequence
1	1	0.25	0.74	2.2	SLEEAALPQPVNVIR
1	1	0.29	0.87	2.6	VTGADVPMPYAK

5.585 65 490 PREDICTED: cofilin-2 [Otolemur garnettii]

Protein Accession	CL15471.Contig3$_{H}1$
Mean Expression Ratio	0.913
Median Expression Ratio	0.915
Credible Interval	(0.475, 1.75)
Associated Peptides	4
Associated Spectra	12
Coverage	NaN

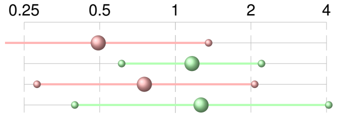

A	B	2.5	50	97.5	Sequence
1	1	0.18	0.49	1.4	MLYSSSSSAVK
2	5	0.61	1.2	2.2	IVMENEFAK
0	2	0.28	0.75	2.1	FVIWDLDVDTK
0	1	0.4	1.3	4.1	ALVFK

5.586 425 1051 minus strand unknown [Schistosoma japonicum]

Protein Accession	CL11292.Contig9$_{H}1$
Mean Expression Ratio	1.09
Median Expression Ratio	1.09
Credible Interval	(0.475, 2.43)
Associated Peptides	1
Associated Spectra	2
Coverage	NaN

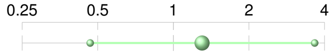

A	B	2.5	50	97.5	Sequence
0	2	0.47	1.3	3.7	AGNDVDLVVQR

5.587 91 1392 minus strand hypothetical protein AND_03103 [Anopheles darlingi]

Protein Accession	CL10598.Contig5$_{H}1$
Mean Expression Ratio	0.915
Median Expression Ratio	0.916
Credible Interval	(0.437, 1.93)
Associated Peptides	3
Associated Spectra	3
Coverage	NaN

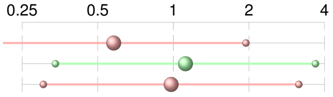

A	B	2.5	50	97.5	Sequence
0	1	0.18	0.58	1.9	APGFAHLAGLDK
0	1	0.34	1.1	3.7	LPPFNEHALVQEK
0	1	0.3	0.98	3.2	LPVMYPDEESSK

5.588 391 1470 PREDICTED: heparanase-like [Amphimedon queenslandica]; 391 1470 PREDICTED: heparanase-like [Amphimedon queenslandica]; 391 1470 PREDICTED: heparanase-like [Amphimedon queenslandica]; 391 1470 PREDICTED: heparanase-like [Amphimedon queenslandica]; 391 1755 PREDICTED: heparanase-like [Amphimedon queenslandica]; 418 1365 PREDICTED: similar to heparanase-like protein [Tribolium castaneum]; 391 1755 PREDICTED: heparanase-like [Amphimedon queenslandica]; 391 1470 PREDICTED: heparanase-like [Amphimedon queenslandica]

Protein Accession	CL2227.Contig4$_{H}1$ CL2227.Contig3$_{H}1$ CL2227.Contig2$_{H}1$ CL2227.Contig1$_{H}1$ CL2227.Con
Mean Expression Ratio	1.09
Median Expression Ratio	1.09
Credible Interval	(0.474, 2.47)
Associated Peptides	1
Associated Spectra	2
Coverage	NaN

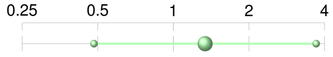

A	B	2.5	50	97.5	Sequence
1	1	0.48	1.3	3.7	ALSPAYFR

5.589 247 1440 minus strand Septin-2 [Crassostrea gigas]

Protein Accession	CL14404.Contig1$_{H}1$
Mean Expression Ratio	0.912
Median Expression Ratio	0.916
Credible Interval	(0.43, 1.88)
Associated Peptides	2
Associated Spectra	5
Coverage	NaN

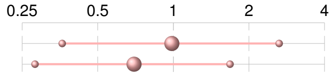

A	B	2.5	50	97.5	Sequence
0	2	0.36	0.99	2.6	HKLEEMGFSDSETR
0	3	0.28	0.7	1.7	ALDLVTMK

5.590 2473 2898 RecName: Full=Paramyosin gi|397603|emb|CAA79849.1| paramyosin [Echinococcus granulosus]

Protein Accession	CL43455.Contig1$_H$1
Mean Expression Ratio	1.09
Median Expression Ratio	1.09
Credible Interval	(0.712, 1.66)
Associated Peptides	12
Associated Spectra	64
Coverage	NaN

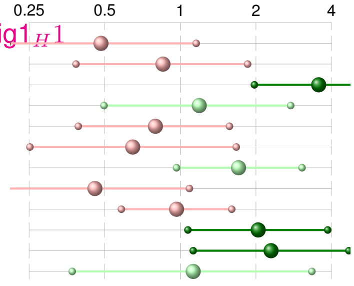

A	B	2.5	50	97.5	Sequence
2	1	0.21	0.48	1.2	EILAQTEEERR
2	2	0.38	0.85	1.9	INKEILAQTEEER
1	8	2	3.5	6.3	INKEILAQTEEERR
1	2	0.5	1.2	2.7	KQLEIEIR
3	3	0.39	0.79	1.6	QLEIEIR
2	0	0.25	0.65	1.7	AQTLIEEAENR
1	8	0.96	1.7	3	QLEEAEDVANPTMNK
0	3	0.2	0.46	1.1	EILAQTEEER
0	12	0.58	0.97	1.6	KAQTLIEEAENR
0	7	1.1	2	3.9	QLAEFASLNEQLNLR
0	5	1.1	2.3	4.7	QLEEAEDVANPTMNKYR
0	1	0.37	1.1	3.3	RQLAEFASLNEQLNLR

5.591 " 926 1189 minus strand Myosin heavy chain, striated muscle [Crassostrea gigas]"

Protein Accession	Unigene11948$_H$1
Mean Expression Ratio	0.917
Median Expression Ratio	0.917
Credible Interval	(0.489, 1.71)
Associated Peptides	5
Associated Spectra	10
Coverage	NaN

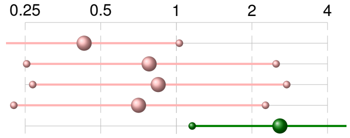

A	B	2.5	50	97.5	Sequence
2	1	0.18	0.43	1	LNGNHLGK
1	0	0.25	0.78	2.5	TFLDK
0	1	0.27	0.85	2.7	KATQAPAHFELH
0	1	0.22	0.71	2.3	TFLDKLNGNHLGK
0	4	1.2	2.6	5.7	ASDKTFLDKLNGNHLGK

5.592 201 779 minus strand sigma class glutathione-s-transferase 2 [Haliotis discus discus]

Protein Accession	Unigene22998$_H$1
Mean Expression Ratio	0.916
Median Expression Ratio	0.917
Credible Interval	(0.495, 1.68)
Associated Peptides	7
Associated Spectra	9
Coverage	NaN

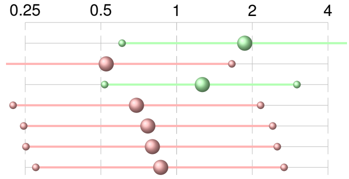

A	B	2.5	50	97.5	Sequence
0	1	0.61	1.9	5.9	IKNYVANR
0	1	0.17	0.53	1.7	LVLALADQK
0	3	0.52	1.3	3	ALYENVANNPR
0	1	0.22	0.69	2.2	KKLEEEEFPK
0	1	0.25	0.77	2.4	YFGMFQK
0	1	0.25	0.8	2.5	KLEEEEFPK
0	1	0.28	0.86	2.7	VINIYK

5.593 635 1561 Uncharacterized protein yfeX [Crassostrea gigas]

Protein Accession	CL12046.Contig4$_H 1$
Mean Expression Ratio	1.09
Median Expression Ratio	1.09
Credible Interval	(0.562, 2.12)
Associated Peptides	5
Associated Spectra	7
Coverage	NaN

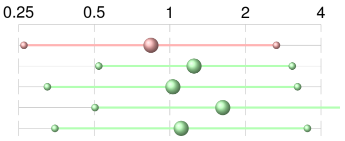

A	B	2.5	50	97.5	Sequence
1	0	0.26	0.84	2.7	ATGGSYVITQK
1	2	0.52	1.3	3.1	TLESWVGR
0	1	0.32	1	3.2	MVGAGGDGHADDIMR
0	1	0.5	1.6	5.2	KAFQIVR
0	1	0.35	1.1	3.5	WIHDLHVINTEKEK

5.594 248 1018 Aldo-keto reductase family 1 member B10 [Crassostrea gigas]; 44 730 minus strand Aldo-keto reductase family 1 member B10 [Crassostrea gigas]; 194 904 Aldo-keto reductase family 1 member B10 [Crassostrea gigas]; 580 1287 Aldo-keto reductase family 1 member B10 [Crassostrea gigas]; 675 1382 minus strand Aldo-keto reductase family 1 member B10 [Crassostrea gigas]

Protein Accession	Unigene68551$_H 1$ Unigene37546$_H 1$ CL3558.Contig4$_H 1$ CL3558.Contig3$_H 1$ CL3558.Contig1
Mean Expression Ratio	1.09
Median Expression Ratio	1.09
Credible Interval	(0.506, 2.39)
Associated Peptides	2
Associated Spectra	2
Coverage	NaN

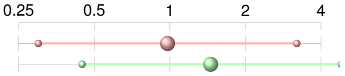

A	B	2.5	50	97.5	Sequence
0	1	0.3	0.98	3.2	ALEVLVSEGK
0	1	0.45	1.5	4.8	DEGYR

5.595 " 153 536 GST omega, partial [Reishia clavigera]"

Protein Accession	CL2384.Contig3$_H 1$
Mean Expression Ratio	1.09
Median Expression Ratio	1.09
Credible Interval	(0.512, 2.34)
Associated Peptides	2
Associated Spectra	3
Coverage	NaN

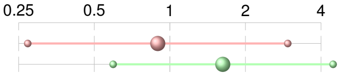

A	B	2.5	50	97.5	Sequence
0	1	0.27	0.89	2.9	EKPEWFLEK
0	2	0.59	1.6	4.5	ALLPSDPYRR

5.596 288 1487 minus strand putative procollagen type VI alpha 4 [Haliotis diversicolor]

Protein Accession	CL6899.Contig2$_H$1
Mean Expression Ratio	1.09
Median Expression Ratio	1.09
Credible Interval	(0.667, 1.77)
Associated Peptides	10
Associated Spectra	28
Coverage	NaN

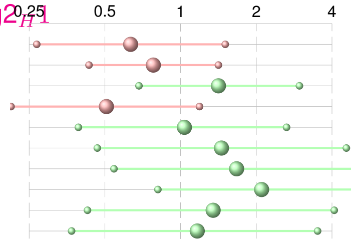

A	B	2.5	50	97.5	Sequence
1	2	0.27	0.63	1.5	IGALTFSDDVK
5	4	0.43	0.78	1.4	TGVTNTHLAIR
2	3	0.68	1.4	3	FRDDITK
2	1	0.21	0.51	1.2	QVLQFVSQVITR
0	2	0.39	1	2.6	AEVLAAIDQVR
0	1	0.47	1.5	4.6	MGFDVDQFR
0	1	0.54	1.7	5.1	VMVVITDGSSR
0	2	0.81	2.1	5.6	LRENLK
0	1	0.43	1.4	4.1	QLAAAARSLR
0	1	0.37	1.2	3.5	AIASDPDNDYLFNITAFR

5.597 996 1463 minus strand catalase [Cristaria plicata] gi

Protein Accession	CL6841.Contig3$_H$1
Mean Expression Ratio	1.09
Median Expression Ratio	1.09
Credible Interval	(0.506, 2.35)
Associated Peptides	2
Associated Spectra	2
Coverage	NaN

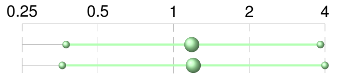

A	B	2.5	50	97.5	Sequence
0	1	0.37	1.2	3.8	YLECPQHFSGDVQR
0	1	0.36	1.2	4	LFAYSDTHR

5.598 1292 1471 minus strand hemocyanin isoform 1 [Haliotis diversicolor]

Protein Accession	Unigene65233$_H$1
Mean Expression Ratio	1.08
Median Expression Ratio	1.09
Credible Interval	(0.508, 2.3)
Associated Peptides	2
Associated Spectra	4
Coverage	NaN

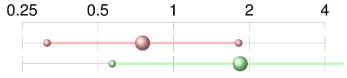

A	B	2.5	50	97.5	Sequence
0	3	0.31	0.75	1.8	LYTVQFEDALR
0	1	0.57	1.8	6	LYTVQFEDALRR

5.599 28 1455 minus strand PREDICTED: probable serine carboxypeptidase CPVL-like [Anolis carolinensis]

Protein Accession	CL34087.Contig4$_H$1
Mean Expression Ratio	1.09
Median Expression Ratio	1.09
Credible Interval	(0.544, 2.16)
Associated Peptides	4
Associated Spectra	6
Coverage	NaN

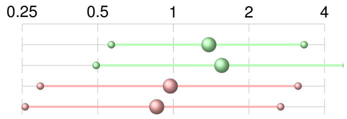

A	B	2.5	50	97.5	Sequence
1	2	0.57	1.4	3.3	YVPAISYK
0	1	0.49	1.6	4.9	GMAIGDGLCDPVTMMPK
0	1	0.3	0.97	3.1	NFYQVIVR
0	1	0.26	0.86	2.7	VNPDDVEVAGWVR

5.600 165 377 Cysteine and glycine-rich protein 3 [Crassostrea gigas]; 135 344 Cysteine and glycine-rich protein 3 [Crassostrea gigas]

Protein Accession	CL38347.Contig2$_{H}1$ CL38347.Contig1$_{H}1$
Mean Expression Ratio	0.919
Median Expression Ratio	0.92
Credible Interval	(0.408, 2.1)
Associated Peptides	1
Associated Spectra	2
Coverage	NaN

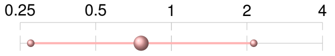

A	B	2.5	50	97.5	Sequence
**1**	**1**	**0.28**	**0.76**	**2.1**	**VEAGNIPFHK**

5.601 556 987 ribosomal protein rps15 [Lineus viridis]

Protein Accession	CL7683.Contig9$_{H}1$
Mean Expression Ratio	1.09
Median Expression Ratio	1.09
Credible Interval	(0.471, 2.55)
Associated Peptides	1
Associated Spectra	1
Coverage	NaN

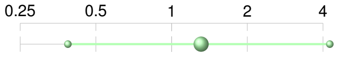

A	B	2.5	50	97.5	Sequence
**0**	**1**	**0.39**	**1.3**	**4.3**	**EAGPLEKPEVVK**

5.602 35 2953 hypothetical protein CGI_10020867 [Crassostrea gigas]; 97 3015 minus strand hypothetical protein CGI_10020867 [Crassostrea gigas]

Protein Accession	CL2537.Contig6$_{H}1$ CL2537.Contig4$_{H}1$
Mean Expression Ratio	1.08
Median Expression Ratio	1.09
Credible Interval	(0.5, 2.32)
Associated Peptides	2
Associated Spectra	4
Coverage	NaN

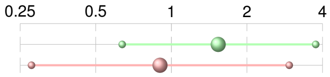

A	B	2.5	50	97.5	Sequence
**0**	**3**	**0.64**	**1.5**	**3.8**	**GGGQIIPTAR**
**0**	**1**	**0.28**	**0.9**	**2.9**	**LLDATIAAEPIHR**

5.603 323 1192 cytosolic malate dehydrogenase [Lottia scabra]; 323 1192 cytosolic malate dehydrogenase [Lottia scabra]; 323 1192 cytosolic malate dehydrogenase [Lottia scabra]; 323 1192 cytosolic malate dehydrogenase [Lottia scabra]; 2474 3343 cytosolic malate dehydrogenase [Lottia scabra]; 116 1111 minus strand cytosolic malate dehydrogenase [Lottia scabra]

Protein Accession	CL280.Contig21$_{H}$1 CL280.Contig20$_{H}$1 CL280.Contig19$_{H}$1 CL280.Contig18$_{H}$1 CL280.Conti
Mean Expression Ratio	0.918
Median Expression Ratio	0.92
Credible Interval	(0.639, 1.32)
Associated Peptides	20
Associated Spectra	108
Coverage	NaN

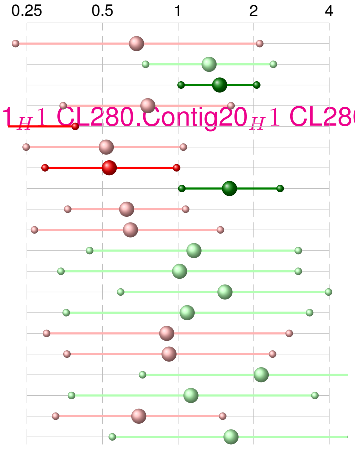

A	B	2.5	50	97.5	Sequence
1	0	0.23	0.68	2.1	GAEVIK
2	7	0.74	1.3	2.4	KDLLAANIK
11	19	1	1.5	2.1	KLSSAMSAAK
2	2	0.35	0.76	1.6	LSSAMSAAK
3	1	0.081	0.18	0.39	ELLEER
3	2	0.25	0.52	1.1	DLLAANIK
2	6	0.29	0.53	0.99	EMAEQILAA
4	13	1	1.6	2.5	LGIPNNAVSK
5	6	0.36	0.63	1.1	SQGANLDAVAK
0	3	0.27	0.65	1.5	DCDAAFLVGAMPR
0	2	0.45	1.2	3	EAVKDDNWINNEFISTVQK
0	1	0.34	1	3	ELLEEREMAEQILAA
0	2	0.59	1.5	4	MDDTAKELLEER
0	1	0.36	1.1	3.3	NKQVTIVEGLSISDFAR
0	1	0.3	0.9	2.8	VVIVGNPANTNALICAK
0	2	0.36	0.92	2.4	YGIPEGVMYSYPVQIK
0	1	0.72	2.2	6.5	SQGANLDAVAKK
0	1	0.38	1.1	3.5	GAEVIKMR
0	4	0.33	0.7	1.5	QVTIVEGLSISDFAR
0	1	0.55	1.6	4.9	VTIWGNHSSTQFPDVDHAVAVVNGKPHPVR

5.604 224 1291 minus strand coproporphirynogen oxidase [Aplysia californica] gi

Protein Accession	CL8705.Contig1$_{H}$1
Mean Expression Ratio	1.09
Median Expression Ratio	1.09
Credible Interval	(0.5, 2.43)
Associated Peptides	1
Associated Spectra	3
Coverage	NaN

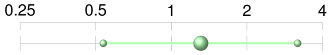

A	B	2.5	50	97.5	Sequence
0	3	0.53	1.3	3.2	NPHVPTMHFNYR

5.605 254 1648 Tektin-3 [Crassostrea gigas]

Protein Accession	CL6717.Contig3$_{H}$1
Mean Expression Ratio	1.08
Median Expression Ratio	1.09
Credible Interval	(0.52, 2.31)
Associated Peptides	3
Associated Spectra	4
Coverage	NaN

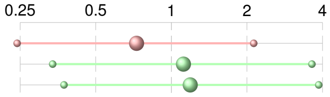

A	B	2.5	50	97.5	Sequence
1	1	0.24	0.72	2.1	AAQHALEIDAK
0	1	0.34	1.1	3.6	ILPYVTDKTISR
0	1	0.37	1.2	3.9	NYFNPATR

5.606 " 19 1167 minus strand ureidopropionase, beta [Xenopus (Silurana) tropicalis] gi"

Protein Accession	CL15113.Contig4$_{H}1$
Mean Expression Ratio	0.922
Median Expression Ratio	0.921
Credible Interval	(0.49, 1.74)
Associated Peptides	5
Associated Spectra	8
Coverage	NaN

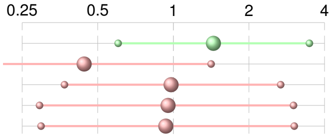

A	B	2.5	50	97.5	Sequence
2	1	0.6	1.4	3.5	KLAVPER
1	0	0.14	0.44	1.4	AESLALER
0	2	0.37	0.98	2.7	AGQIENIEQIIEK
0	1	0.29	0.95	3	YVPLEELGSLK
0	1	0.3	0.94	3	MAGQIENIEQIIEK

5.607 " 1 681 40S ribosomal protein S4, X isoform [Crassostrea gigas]"

Protein Accession	CL2560.Contig4$_{H}1$
Mean Expression Ratio	1.08
Median Expression Ratio	1.09
Credible Interval	(0.633, 1.85)
Associated Peptides	8
Associated Spectra	23
Coverage	NaN

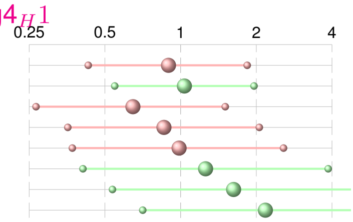

A	B	2.5	50	97.5	Sequence
2	3	0.43	0.89	1.8	QWVSLPK
1	6	0.55	1	2	VNDTVVLDIASGK
2	1	0.27	0.64	1.5	LTIAEER
1	2	0.36	0.86	2.1	GVPYIVTHDGR
1	1	0.37	0.99	2.6	IKVDGK
0	1	0.41	1.3	3.9	FDSGNMVMVTGGHNLGR
0	1	0.53	1.6	5	MKDYIK
0	1	0.71	2.2	6.8	LIKVDGK

5.608 37 885 glyceraldehyde 3-phosphate dehydrogenase [Dugesia japonica]

Protein Accession	CL47424.Contig2$_{H}1$
Mean Expression Ratio	0.919
Median Expression Ratio	0.922
Credible Interval	(0.444, 1.88)
Associated Peptides	2
Associated Spectra	18
Coverage	NaN

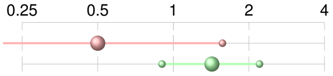

A	B	2.5	50	97.5	Sequence
1	0	0.15	0.5	1.6	VGINGFGR
5	12	0.9	1.4	2.2	GAAQNIIPASTGAAK

5.609 " 1 321 Dynein heavy chain, cytoplasmic [Crassostrea gigas]"

Protein Accession	Unigene35195$_{H}1$
Mean Expression Ratio	1.08
Median Expression Ratio	1.08
Credible Interval	(0.471, 2.47)
Associated Peptides	1
Associated Spectra	1
Coverage	NaN

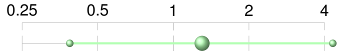

A	B	2.5	50	97.5	Sequence
1	0	0.39	1.3	4.3	EILAVIQK

5.610 78 560 Sorting nexin-12 [Camponotus floridanus]; 78 578 Sorting nexin-12 [Camponotus floridanus]; 132 605 Sorting nexin-12 [Camponotus floridanus]; 276 623 Sorting nexin-12 [Camponotus floridanus]; 276 629 Sorting nexin-12 [Camponotus floridanus]

Protein Accession	CL2450.Contig5$_{H}$1 CL2450.Contig4$_{H}$1 CL2450.Contig3$_{H}$1 CL2450.Contig2$_{H}$1 CL2450.Con
Mean Expression Ratio	1.08
Median Expression Ratio	1.08
Credible Interval	(0.491, 2.39)
Associated Peptides	2
Associated Spectra	2
Coverage	NaN

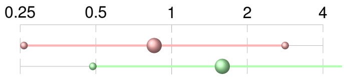

A	B	2.5	50	97.5	Sequence
**0**	**1**	**0.26**	**0.85**	**2.8**	**MKTNLPVFK**
**0**	**1**	**0.49**	**1.6**	**5.3**	**IVVPPLPGK**

5.611 1278 1643 Fatty acid-binding-like protein 5 [Crassostrea gigas]

Protein Accession	CL11559.Contig3$_{H}$1
Mean Expression Ratio	1.08
Median Expression Ratio	1.08
Credible Interval	(0.515, 2.23)
Associated Peptides	2
Associated Spectra	5
Coverage	NaN

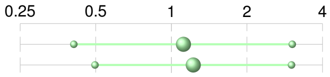

A	B	2.5	50	97.5	Sequence
**1**	**1**	**0.41**	**1.1**	**3**	**AAGVGLVTR**
**1**	**2**	**0.5**	**1.2**	**3**	**EILPDGR**

5.612 160 387 small nuclear ribonucleoprotein polypeptide G protein [Crassostrea ariakensis]; 180 407 small nuclear ribonucleoprotein polypeptide G protein [Crassostrea ariakensis]

Protein Accession	CL1272.Contig8$_{H}$1 CL1272.Contig7$_{H}$1
Mean Expression Ratio	1.08
Median Expression Ratio	1.08
Credible Interval	(0.587, 1.98)
Associated Peptides	4
Associated Spectra	18
Coverage	NaN

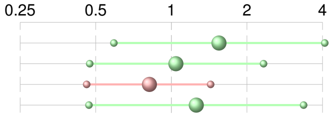

A	B	2.5	50	97.5	Sequence
**1**	**1**	**0.59**	**1.6**	**4.1**	**AHPPELK**
**3**	**1**	**0.47**	**1**	**2.3**	**SIGMVVVR**
**5**	**5**	**0.46**	**0.82**	**1.4**	**TITGILR**
**1**	**1**	**0.47**	**1.3**	**3.4**	**AHPPELKK**

5.613 " 1482 3320 minus strand Phosphoenolpyruvate carboxykinase, cytosolic [GTP] [Crassostrea gigas]; 1326 3164 minus strand Phosphoenolpyruvate carboxykinase, cytosolic [GTP] [Crassostrea gigas]; 303 2102 minus strand Phosphoenolpyruvate carboxykinase, cytosolic [GTP] [Crassostrea gigas]"

Protein Accession	CL1008.Contig5$_H1$ CL1008.Contig3$_H1$ CL1008.Contig2$_H1$
Mean Expression Ratio	1.08
Median Expression Ratio	1.08
Credible Interval	(0.535, 2.19)
Associated Peptides	4
Associated Spectra	4
Coverage	NaN

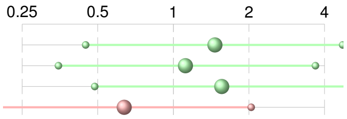

A	B	2.5	50	97.5	Sequence
0	1	0.45	1.5	4.7	AINPENGFFGVAPGTSVK
0	1	0.35	1.1	3.7	YFDEQVNEDLPR
0	1	0.49	1.6	5	NLHLPK
0	1	0.19	0.64	2	GGNALLGK

5.614 210 2201 minus strand Glycyl-tRNA synthetase [Crassostrea gigas]; 210 2201 minus strand Glycyl-tRNA synthetase [Crassostrea gigas]; 210 2201 minus strand Glycyl-tRNA synthetase [Crassostrea gigas]; 210 2201 minus strand Glycyl-tRNA synthetase [Crassostrea gigas]; 210 2201 minus strand Glycyl-tRNA synthetase [Crassostrea gigas]; 210 2201 minus strand Glycyl-tRNA synthetase [Crassostrea gigas]

Protein Accession	CL4073.Contig8$_H1$ CL4073.Contig7$_H1$ CL4073.Contig6$_H1$ CL4073.Contig5$_H1$ CL4073.Con
Mean Expression Ratio	1.09
Median Expression Ratio	1.08
Credible Interval	(0.479, 2.58)
Associated Peptides	1
Associated Spectra	1
Coverage	NaN

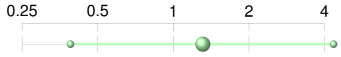

A	B	2.5	50	97.5	Sequence
1	0	0.39	1.3	4.3	ATGVKLVAER

5.615 152 1696 dynamin isoform A [Lymnaea stagnalis]

Protein Accession	CL22122.Contig2$_H1$
Mean Expression Ratio	1.09
Median Expression Ratio	1.08
Credible Interval	(0.484, 2.46)
Associated Peptides	1
Associated Spectra	2
Coverage	NaN

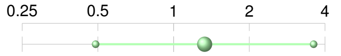

A	B	2.5	50	97.5	Sequence
0	2	0.49	1.3	3.6	KFFLSHPAYR

5.616 214 1284 glutamine synthetase [Tegillarca granosa]

Protein Accession	CL6905.Contig5$_{H}1$
Mean Expression Ratio	0.931
Median Expression Ratio	0.926
Credible Interval	(0.438, 2)
Associated Peptides	2
Associated Spectra	4
Coverage	NaN

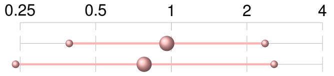

A	B	2.5	50	97.5	Sequence
1	2	0.39	0.96	2.4	QPGGLAVIEEAIAK
0	1	0.24	0.78	2.6	GGLAVIEEAIAK

5.617 187 942 Testis-specific gene 10 protein OS=Mus musculus GN=Tsga10 PE=1 SV=1; 212 967 Testis-specific gene 10 protein OS=Mus musculus GN=Tsga10 PE=1 SV=1; 214 870 Testis-specific gene 10 protein OS=Mus musculus GN=Tsga10 PE=1 SV=1; 134 793 Testis-specific gene 10 protein OS=Mus musculus GN=Tsga10 PE=1 SV=1

Protein Accession	CL11641.Contig6$_{H}1$ CL11641.Contig5$_{H}1$ CL11641.Contig4$_{H}1$ CL11641.Contig3$_{H}1$
Mean Expression Ratio	1.08
Median Expression Ratio	1.08
Credible Interval	(0.515, 2.23)
Associated Peptides	3
Associated Spectra	4
Coverage	NaN

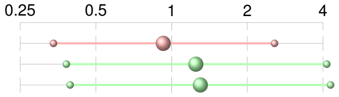

A	B	2.5	50	97.5	Sequence
0	2	0.34	0.93	2.6	RVENERDDAIADLHR
0	1	0.38	1.3	4.1	LLVEEAEK
0	1	0.4	1.3	4.3	ALEEQTK

5.618 59 328 minus strand PREDICTED: similar to predicted protein [Hydra magnipapillata]; 59 328 PREDICTED: similar to predicted protein [Hydra magnipapillata]; 89 364 minus strand PREDICTED: similar to predicted protein [Hydra magnipapillata]; 89 364 minus strand PREDICTED: similar to predicted protein [Hydra magnipapillata]

Protein Accession	Unigene53643$_{H}1$ Unigene39171$_{H}1$ Unigene11519$_{H}1$ Unigene11518$_{H}1$
Mean Expression Ratio	0.929
Median Expression Ratio	0.928
Credible Interval	(0.4, 2.11)
Associated Peptides	1
Associated Spectra	1
Coverage	NaN

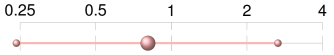

A	B	2.5	50	97.5	Sequence
0	1	0.24	0.8	2.7	DLDPQNDLTFLR

5.619 977 1852 PREDICTED: similar to prohibitin [Tribolium castaneum] gi

Protein Accession	CL8219.Contig4$_H1$
Mean Expression Ratio	1.08
Median Expression Ratio	1.08
Credible Interval	(0.574, 1.99)
Associated Peptides	5
Associated Spectra	10
Coverage	NaN

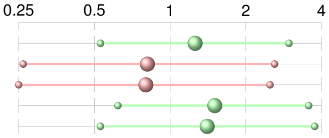

A	B	2.5	50	97.5	Sequence
1	2	0.53	1.3	3	VVQAEGEAAAAK
1	0	0.26	0.81	2.6	AQFVVEK
1	0	0.25	0.8	2.5	SLLIR
1	2	0.62	1.5	3.5	KLGDLAGR
0	2	0.53	1.4	3.7	LIGQAVSANPGFLK

5.620 1519 2583 Proliferation-associated protein 2G4 [Crassostrea gigas]; 565 1629 Proliferation-associated protein 2G4 [Crassostrea gigas]

Protein Accession	CL14279.Contig3$_H1$ CL14279.Contig2$_H1$
Mean Expression Ratio	1.08
Median Expression Ratio	1.08
Credible Interval	(0.501, 2.38)
Associated Peptides	2
Associated Spectra	2
Coverage	NaN

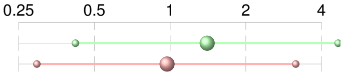

A	B	2.5	50	97.5	Sequence
0	1	0.42	1.4	4.6	ALLSASASSK
0	1	0.29	0.97	3.2	ILEETGK

5.621 2 292 minus strand H2 [Haliotis tuberculata]

Protein Accession	Unigene10170$_H1$
Mean Expression Ratio	0.93
Median Expression Ratio	0.928
Credible Interval	(0.447, 1.93)
Associated Peptides	2
Associated Spectra	5
Coverage	NaN

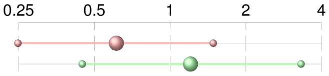

A	B	2.5	50	97.5	Sequence
1	2	0.25	0.61	1.5	NLESLSEGEVESLK
0	2	0.45	1.2	3.3	RNLESLSEGEVESLK

5.622 238 1521 26S protease regulatory subunit 6A [Crassostrea gigas]

Protein Accession	CL11449.Contig8$_H1$
Mean Expression Ratio	1.08
Median Expression Ratio	1.08
Credible Interval	(0.523, 2.2)
Associated Peptides	3
Associated Spectra	4
Coverage	NaN

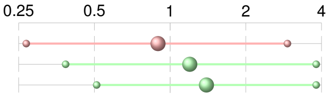

A	B	2.5	50	97.5	Sequence
0	1	0.27	0.89	2.9	AIWEDGEEALGEEVLR
0	1	0.38	1.2	3.8	LLDNEIK
0	2	0.51	1.4	3.8	KIEFPHPNEEAR

5.623 140 1564 selenium-binding protein [Haliotis discus hannai]

Protein Accession	CL3868.Contig7$_{H}1$
Mean Expression Ratio	0.924
Median Expression Ratio	0.928
Credible Interval	(0.418, 2.04)
Associated Peptides	2
Associated Spectra	2
Coverage	NaN

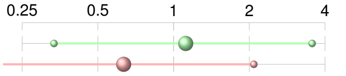

A	B	2.5	50	97.5	Sequence
0	1	0.34	1.1	3.6	VVEAVDIHR
0	1	0.19	0.63	2.1	SLNVWNWK

5.624 8 1636 minus strand V-type proton ATPase catalytic subunit A [Crassostrea gigas]; 8 1636 minus strand V-type proton ATPase catalytic subunit A [Crassostrea gigas]; 8 1636 minus strand V-type proton ATPase catalytic subunit A [Crassostrea gigas]

Protein Accession	CL33671.Contig3$_{H}1$ CL33671.Contig2$_{H}1$ CL33671.Contig1$_{H}1$
Mean Expression Ratio	0.929
Median Expression Ratio	0.928
Credible Interval	(0.507, 1.71)
Associated Peptides	6
Associated Spectra	10
Coverage	NaN

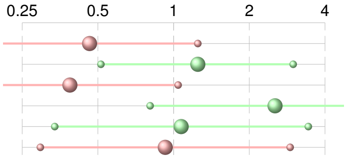

A	B	2.5	50	97.5	Sequence
2	0	0.18	0.46	1.3	GINTPALDANK
2	1	0.52	1.2	3	IMLNPNAR
2	0	0.15	0.39	1	TVGMLR
1	0	0.81	2.5	8	FRNLED
0	1	0.34	1.1	3.4	LAANHPLLTGQR
0	1	0.29	0.93	2.9	ITLEVAK

5.625 " 3341 4240 minus strand nonmuscle myosin II, partial [Aplysia californica]"

Protein Accession	CL19183.Contig2$_{H}1$
Mean Expression Ratio	0.933
Median Expression Ratio	0.929
Credible Interval	(0.541, 1.6)
Associated Peptides	9
Associated Spectra	15
Coverage	NaN

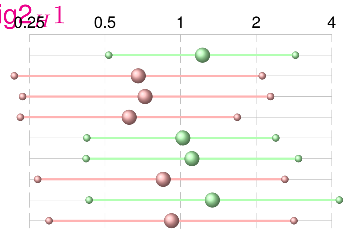

A	B	2.5	50	97.5	Sequence
1	2	0.52	1.2	2.9	QKQDVEQLEAR
1	0	0.22	0.68	2.1	RQQVEELQAQLAK
1	0	0.23	0.72	2.3	YAQMIEEK
1	1	0.23	0.62	1.7	TATMLEEK
0	3	0.42	1	2.4	ELQNQMTELNEDLETER
0	2	0.42	1.1	2.9	LHTKEEELK
0	1	0.27	0.86	2.6	RKEELEDILHEMELR
0	1	0.43	1.3	4.3	KLEEDLALNEDTNQK
0	1	0.3	0.92	2.8	LSEAMAQIVEEEEK

5.626 149 496 Spectrin alpha chain [Crassostrea gigas]

Protein Accession	CL2014.Contig1$_H$1
Mean Expression Ratio	1.08
Median Expression Ratio	1.08
Credible Interval	(0.461, 2.53)
Associated Peptides	1
Associated Spectra	1
Coverage	NaN

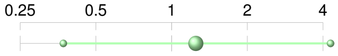

A	B	2.5	50	97.5	Sequence
**0**	**1**	**0.37**	**1.2**	**4.3**	**DADELESWILEK**

5.627 90 677 minus strand PREDICTED: 15-hydroxyprostaglandin dehydrogenase [NAD+] [Gallus gallus]; 90 683 minus strand PREDICTED: 15-hydroxyprostaglandin dehydrogenase [NAD+] [Gallus gallus]; 69 776 PREDICTED: 15-hydroxyprostaglandin dehydrogenase [NAD+] [Gallus gallus]; 69 779 PREDICTED: 15-hydroxyprostaglandin dehydrogenase [NAD+] [Gallus gallus]

Protein Accession	Unigene58786$_H$1 Unigene57240$_H$1 Unigene48682$_H$1 CL44148.Contig4$_H$1
Mean Expression Ratio	1.08
Median Expression Ratio	1.08
Credible Interval	(0.497, 2.34)
Associated Peptides	2
Associated Spectra	3
Coverage	NaN

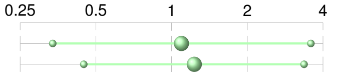

A	B	2.5	50	97.5	Sequence
**0**	**1**	**0.34**	**1.1**	**3.6**	**AETFSETDMEK**
**0**	**2**	**0.45**	**1.2**	**3.4**	**AITEAFLAK**

5.628 1009 2784 minus strand Collagen alpha-3(VI) chain [Crassostrea gigas]

Protein Accession	Unigene46961$_H$1
Mean Expression Ratio	0.932
Median Expression Ratio	0.93
Credible Interval	(0.447, 1.97)
Associated Peptides	2
Associated Spectra	4
Coverage	NaN

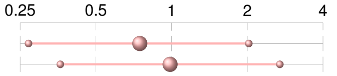

A	B	2.5	50	97.5	Sequence
**0**	**2**	**0.27**	**0.75**	**2**	**ADVPNVAIVITDGVSNIDAER**
**0**	**2**	**0.36**	**0.99**	**2.7**	**ELEGIASPPAR**

5.629 178 480 minus strand PREDICTED: lipoxygenase homology domain-containing protein 1-like [Danio rerio]

Protein Accession	CL41319.Contig2$_{H}1$
Mean Expression Ratio	1.07
Median Expression Ratio	1.07
Credible Interval	(0.49, 2.34)
Associated Peptides	2
Associated Spectra	2
Coverage	NaN

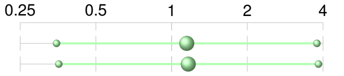

A	B	2.5	50	97.5	Sequence
0	1	0.35	1.1	3.8	ILLDVVGENNFR
0	1	0.36	1.2	3.8	VSLKPEVFPLVK

5.630 3 386 fem-1-like protein C [Crassostrea gigas]

Protein Accession	Unigene19937$_{H}1$
Mean Expression Ratio	1.07
Median Expression Ratio	1.07
Credible Interval	(0.488, 2.32)
Associated Peptides	1
Associated Spectra	5
Coverage	NaN

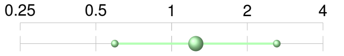

A	B	2.5	50	97.5	Sequence
0	5	0.59	1.2	2.6	MQSLLIR

5.631 40 1182 4-hydroxyphenylpyruvate dioxygenase [Crassostrea gigas]

Protein Accession	CL7585.Contig2$_{H}1$
Mean Expression Ratio	1.08
Median Expression Ratio	1.07
Credible Interval	(0.642, 1.82)
Associated Peptides	10
Associated Spectra	16
Coverage	NaN

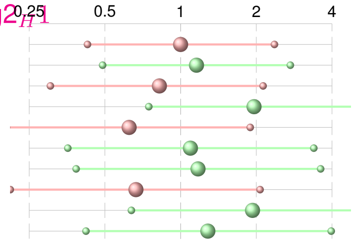

A	B	2.5	50	97.5	Sequence
1	2	0.43	1	2.4	SIVVTNYEETIK
1	2	0.49	1.2	2.7	TYGDTTHTLIDR
1	1	0.3	0.82	2.1	QVAAHVVK
0	2	0.75	2	5.1	DVAFEVEDLDAIFKR
0	1	0.2	0.63	1.9	QIHTDYSALR
0	1	0.36	1.1	3.4	SLFEAIELDQAER
0	1	0.38	1.2	3.6	VQIKEDLDELQK
0	1	0.21	0.66	2.1	FWSVDDK
0	1	0.64	1.9	6.1	MPINEPAPGKR
0	1	0.42	1.3	4	TSNIIQSINR

5.632 1709 2026 Phosphoenolpyruvate carboxykinase [GTP] [Crassostrea gigas]

Protein Accession	CL25948.Contig2$_{H}1$
Mean Expression Ratio	0.932
Median Expression Ratio	0.932
Credible Interval	(0.436, 2)
Associated Peptides	1
Associated Spectra	8
Coverage	NaN

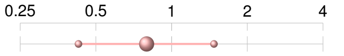

A	B	2.5	50	97.5	Sequence
5	3	0.43	0.8	1.5	GVPLVYEAK

5.633 578 1150 minus strand expressed hypothetical protein [Trichoplax adhaerens] gi; 376 948 minus strand expressed hypothetical protein [Trichoplax adhaerens] gi; 176 748 minus strand expressed hypothetical protein [Trichoplax adhaerens] gi; 176 748 minus strand expressed hypothetical protein [Trichoplax adhaerens] gi; 307 879 minus strand expressed hypothetical protein [Trichoplax adhaerens] gi; 307 879 minus strand expressed hypothetical protein [Trichoplax adhaerens] gi; 307 879 minus strand expressed hypothetical protein [Trichoplax adhaerens] gi

Protein Accession	CL11851.Contig7$_H$1 CL11851.Contig6$_H$1 CL11851.Contig5$_H$1 CL11851.Contig4$_H$1 CL1185
Mean Expression Ratio	1.08
Median Expression Ratio	1.07
Credible Interval	(0.495, 2.43)
Associated Peptides	1
Associated Spectra	3
Coverage	NaN

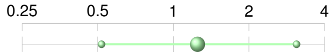

A	B	2.5	50	97.5	Sequence
0	3	0.52	1.3	3.1	GLLDIEAAVK

5.634 3 263 minus strand beta-actin [Urechis unicinctus]

Protein Accession	CL31420.Contig5$_H$1
Mean Expression Ratio	1.07
Median Expression Ratio	1.07
Credible Interval	(0.562, 2.03)
Associated Peptides	4
Associated Spectra	15
Coverage	NaN

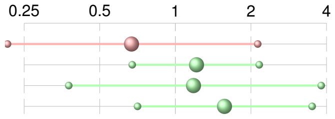

A	B	2.5	50	97.5	Sequence
1	0	0.21	0.67	2.1	EITALAPPTMK
0	9	0.67	1.2	2.2	DLYANTVLSGGSTMYPGIADR
0	1	0.38	1.2	3.8	MQKEITALAPPTMK
0	4	0.71	1.6	3.5	KDLYANTVLSGGSTMYPGIADR

5.635 " 33 1295 PREDICTED: LOW QUALITY PROTEIN: selenide, water dikinase-like [Bombus impatiens]"

Protein Accession	CL14673.Contig3$_H$1
Mean Expression Ratio	1.07
Median Expression Ratio	1.07
Credible Interval	(0.459, 2.48)
Associated Peptides	1
Associated Spectra	1
Coverage	NaN

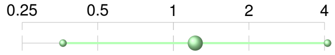

A	B	2.5	50	97.5	Sequence
0	1	0.36	1.2	4.1	RPFDPVAHELDPNFR

5.636 96 581 minus strand thioredoxin 2 [Haliotis discus discus]; 1682 2167 minus strand thioredoxin 2 [Haliotis discus discus]; 191 676 minus strand thioredoxin 2 [Haliotis discus discus]

Protein Accession	CL10014.Contig7$_H$1 CL10014.Contig3$_H$1 CL10014.Contig1$_H$1
Mean Expression Ratio	1.07
Median Expression Ratio	1.07
Credible Interval	(0.462, 2.44)
Associated Peptides	1
Associated Spectra	1
Coverage	NaN

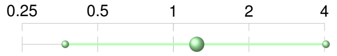

A	B	2.5	50	97.5	Sequence
**0**	**1**	**0.37**	**1.2**	**4.1**	**LETIVSGEK**

5.637 " 106 2724 PREDICTED: 2-oxoglutarate dehydrogenase, mitochondrial isoform 1 [Sarcophilus harrisii]; 492 3473 PREDICTED: 2-oxoglutarate dehydrogenase, mitochondrial isoform 1 [Sarcophilus harrisii]; 492 3458 PREDICTED: 2-oxoglutarate dehydrogenase, mitochondrial isoform 1 [Sarcophilus harrisii]"

Protein Accession	CL2200.Contig3$_H$1 CL2200.Contig1$_H$1 CL2200.Contig16$_H$1
Mean Expression Ratio	1.07
Median Expression Ratio	1.07
Credible Interval	(0.459, 2.47)
Associated Peptides	1
Associated Spectra	1
Coverage	NaN

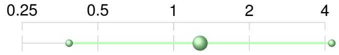

A	B	2.5	50	97.5	Sequence
**0**	**1**	**0.38**	**1.3**	**4.2**	**LAVVANPSHLEAVDPVVQGK**

5.638 275 1372 Cystathionine gamma-lyase OS=Bos taurus GN=CTH PE=2 SV=1

Protein Accession	CL16415.Contig5$_H$1
Mean Expression Ratio	0.935
Median Expression Ratio	0.933
Credible Interval	(0.493, 1.8)
Associated Peptides	5
Associated Spectra	7
Coverage	NaN

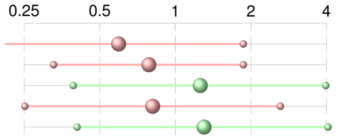

A	B	2.5	50	97.5	Sequence
**1**	**0**	**0.19**	**0.59**	**1.9**	**GGMTEASNFLK**
**1**	**2**	**0.33**	**0.79**	**1.9**	**LVDIEAVSK**
**0**	**1**	**0.39**	**1.3**	**4**	**SMAVVPPISTSTTFK**
**0**	**1**	**0.25**	**0.81**	**2.6**	**VIHPGLESHPQYELAK**
**0**	**1**	**0.41**	**1.3**	**4**	**GLVVARFLEK**

5.639 16 1560 V-type proton ATPase subunit B [Crassostrea gigas]

Protein Accession	CL1031.Contig1$_{H}1$
Mean Expression Ratio	1.07
Median Expression Ratio	1.07
Credible Interval	(0.586, 1.97)
Associated Peptides	7
Associated Spectra	9
Coverage	NaN

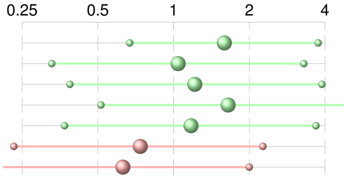

A	B	2.5	50	97.5	Sequence
1	2	0.67	1.6	3.7	AVVQVFEGTSGIDAK
0	1	0.33	1	3.3	FAEIVNLTLADGSK
0	1	0.39	1.2	3.9	HTTCEFTGDILR
0	1	0.51	1.6	5.2	MPTSEDMLGR
0	1	0.37	1.2	3.7	NFISQGHYENR
0	1	0.23	0.74	2.3	SLNGPLNDAAAR
0	1	0.2	0.63	2	ATIYERAGR

5.640 8 937 minus strand galectin [Crassostrea virginica]

Protein Accession	CL7245.Contig6$_{H}1$
Mean Expression Ratio	1.07
Median Expression Ratio	1.07
Credible Interval	(0.554, 2.06)
Associated Peptides	3
Associated Spectra	13
Coverage	NaN

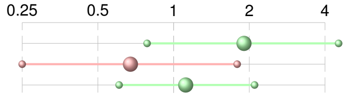

A	B	2.5	50	97.5	Sequence
1	2	0.78	1.9	4.5	LGGEWGPEQR
1	1	0.25	0.67	1.8	QGDNIGLHFNPR
3	5	0.61	1.1	2.1	QDQGDVVLNAK

5.641 175 1416 Ubiquinone biosynthesis monooxygenase COQ6 [Crassostrea gigas]

Protein Accession	Unigene197$_{H}1$
Mean Expression Ratio	1.07
Median Expression Ratio	1.07
Credible Interval	(0.486, 2.37)
Associated Peptides	2
Associated Spectra	2
Coverage	NaN

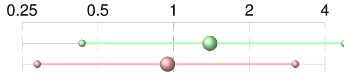

A	B	2.5	50	97.5	Sequence
0	1	0.43	1.4	4.8	VALIGDAAHR
0	1	0.29	0.95	3.1	PTACGVVEK

5.642 119 481 Glutamate receptor U1 [Crassostrea gigas]; 113 328 glutamate receptor subunit protein GluR2 precursor [Aplysia californica] gi; 122 328 PREDICTED: glutamate receptor 3 [Ornithorhynchus anatinus]

Protein Accession	CL570.Contig5$_{H}1$ CL570.Contig4$_{H}1$ CL570.Contig2$_{H}1$
Mean Expression Ratio	0.94
Median Expression Ratio	0.934
Credible Interval	(0.41, 2.19)
Associated Peptides	1
Associated Spectra	2
Coverage	NaN

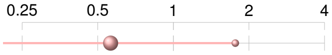

A	B	2.5	50	97.5	Sequence
1	1	0.18	0.56	1.8	GTANIGAGALTVTSER

5.643 103 819 minus strand PREDICTED: l-xylulose reductase-like [Monodelphis domestica]

Protein Accession	Unigene30376$_{H}1$
Mean Expression Ratio	0.935
Median Expression Ratio	0.934
Credible Interval	(0.428, 2.06)
Associated Peptides	2
Associated Spectra	2
Coverage	NaN

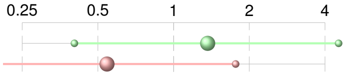

A	B	2.5	50	97.5	Sequence
0	1	0.4	1.4	4.5	GSGGAIVNISSQASK
0	1	0.17	0.54	1.8	AAVDSLTQVMALELGPHK

5.644 135 2630 Filamin-A [Crassostrea gigas]; 118 2613 Filamin-A [Crassostrea gigas]; 157 2652 Filamin-A [Crassostrea gigas]

Protein Accession	CL393.Contig15$_{H}1$ CL393.Contig13$_{H}1$ CL393.Contig12$_{H}1$
Mean Expression Ratio	0.934
Median Expression Ratio	0.935
Credible Interval	(0.571, 1.55)
Associated Peptides	12
Associated Spectra	19
Coverage	NaN

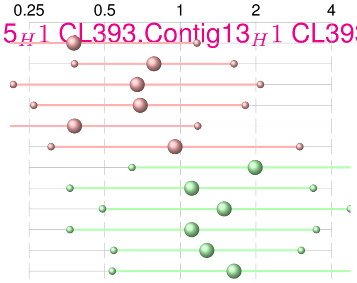

A	B	2.5	50	97.5	Sequence
1	0	0.12	0.38	1.2	ENGVHYVDVK
2	3	0.38	0.78	1.6	MNGIHTLSVR
1	0	0.22	0.68	2.1	VQVGGPGLEK
1	1	0.26	0.69	1.8	VINFQIETK
1	0	0.12	0.38	1.2	VYGVRY
0	1	0.31	0.95	3	AQIFLEERPNGFLGVQYK
0	1	0.64	2	6	SKGEYKVNVTR
0	1	0.36	1.1	3.4	YGNIPIAGSPYK
0	1	0.49	1.5	4.8	YIPKENGVHYVDVK
0	1	0.36	1.1	3.5	EAGPGTLTVAVEGPGK
0	3	0.54	1.3	3	IMFTPK
0	1	0.54	1.6	5	YKGNPIK

5.645 72 521 minus strand GF14593 [Drosophila ananassae] gi; 60 392 GD23124 [Drosophila simulans] gi

Protein Accession	CL38325.Contig2$_{H}1$ CL38325.Contig1$_{H}1$
Mean Expression Ratio	1.07
Median Expression Ratio	1.07
Credible Interval	(0.461, 2.48)
Associated Peptides	1
Associated Spectra	1
Coverage	NaN

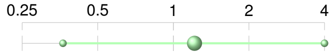

A	B	2.5	50	97.5	Sequence
0	1	0.36	1.2	4	LTADVHGK

5.646 " 142 1284 minus strand Chain O, Crsytal Structure Of Human Liver Gapdh gi|67464044|pdb|1ZNQ|P Chain P, Crsytal Structure Of Human Liver Gapdh gi|67464045|pdb|1ZNQ|R Chain R, Crsytal Structure Of Human Liver Gapdh gi|67464046|pdb|1ZNQ|Q Chain Q, Crsytal Structure Of Human Liver Gapdh"

Protein Accession CL34318.Contig1$_H$1
Mean Expression Ratio 0.939
Median Expression Ratio 0.935
Credible Interval (0.509, 1.76)
Associated Peptides 4
Associated Spectra 12
Coverage NaN

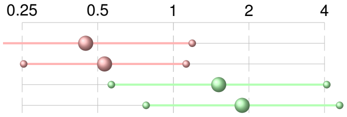

A	B	2.5	50	97.5	Sequence
1	1	0.17	0.45	1.2	DPTAIPWR
3	2	0.25	0.53	1.1	VIPELNGK
0	2	0.57	1.5	4.1	INVYQEKDPTAIPWR
0	3	0.78	1.9	4.6	VIPELNGKLTGMAFR

5.647 43 222 sodium-calcium exchanger [Doryteuthis pealeii]

Protein Accession Unigene7579$_H$1
Mean Expression Ratio 1.07
Median Expression Ratio 1.07
Credible Interval (0.49, 2.38)
Associated Peptides 1
Associated Spectra 3
Coverage NaN

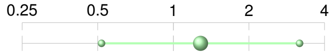

A	B	2.5	50	97.5	Sequence
1	2	0.52	1.3	3.2	TVGVGMVMR

5.648 58 1167 minus strand PREDICTED: arylacetamide deacetylase-like 4-like [Gallus gallus]; 76 1239 minus strand PREDICTED: arylacetamide deacetylase-like 4-like [Gallus gallus]

Protein Accession CL44820.Contig3$_H$1 CL44820.Contig1$_H$1
Mean Expression Ratio 0.931
Median Expression Ratio 0.935
Credible Interval (0.395, 2.17)
Associated Peptides 1
Associated Spectra 1
Coverage NaN

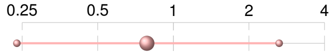

A	B	2.5	50	97.5	Sequence
0	1	0.24	0.79	2.6	NSHDVLLPR

5.649 43 525 Reticulon-4 [Crassostrea gigas]; 143 757 Reticulon-4 [Crassostrea gigas]; 43 564 Reticulon-4 [Crassostrea gigas]; 302 583 Reticulon-4 OS=Rattus norvegicus GN=Rtn4 PE=1 SV=1

Protein Accession	CL29775.Contig4$_H1$ CL29775.Contig3$_H1$ CL29775.Contig2$_H1$ CL29775.Contig1$_H1$
Mean Expression Ratio	1.08
Median Expression Ratio	1.07
Credible Interval	(0.504, 2.33)
Associated Peptides	2
Associated Spectra	3
Coverage	NaN

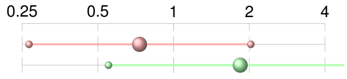

A	B	2.5	50	97.5	Sequence
**1**	**1**	**0.27**	**0.73**	**2**	**VTQTILQHVNR**
**0**	**1**	**0.55**	**1.8**	**6**	**LAAVQK**

5.650 3 224 PREDICTED: 40S ribosomal protein S14-like [Sarcophilus harrisii]

Protein Accession	Unigene48834$_H1$
Mean Expression Ratio	1.07
Median Expression Ratio	1.07
Credible Interval	(0.506, 2.23)
Associated Peptides	3
Associated Spectra	3
Coverage	NaN

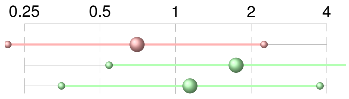

A	B	2.5	50	97.5	Sequence
**0**	**1**	**0.21**	**0.7**	**2.3**	**IEDVTPIPSDSTR**
**0**	**1**	**0.54**	**1.7**	**5.7**	**IGRIEDVTPIPSDSTR**
**0**	**1**	**0.35**	**1.1**	**3.8**	**IGRIEDVTPIPSDSTRR**

5.651 189 353 40S ribosomal protein S12 [Crassostrea gigas]

Protein Accession	Unigene385$_H1$
Mean Expression Ratio	1.07
Median Expression Ratio	1.07
Credible Interval	(0.476, 2.41)
Associated Peptides	1
Associated Spectra	3
Coverage	NaN

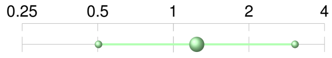

A	B	2.5	50	97.5	Sequence
**0**	**3**	**0.5**	**1.2**	**3.1**	**TALIHDGLAR**

5.652 91 2298 hypothetical protein BRAFLDRAFT_267717 [Branchiostoma floridae] gi; 84 2291 hypothetical protein BRAFLDRAFT_267717 [Branchiostoma floridae] gi; 71 2278 hypothetical protein BRAFLDRAFT_267717 [Branchiostoma floridae] gi; 81 2288 hypothetical protein BRAFLDRAFT_267717 [Branchiostoma floridae] gi; 58 2268 hypothetical protein BRAFLDRAFT_267717 [Branchiostoma floridae] gi; 62 2245 hypothetical protein BRAFLDRAFT_267717 [Branchiostoma floridae] gi

Protein Accession	CL26176.Contig5$_H$1 CL26176.Contig4$_H$1 CL26176.Contig3$_H$1 CL26176.Contig2$_H$1 CL2617
Mean Expression Ratio	0.935
Median Expression Ratio	0.935
Credible Interval	(0.491, 1.77)
Associated Peptides	5
Associated Spectra	7
Coverage	NaN

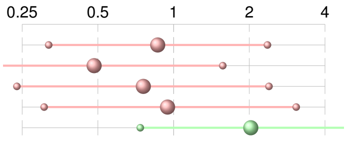

A	B	2.5	50	97.5	Sequence
1	1	0.32	0.86	2.4	VGAVEEASFLK
1	0	0.16	0.48	1.6	NIGPLIK
1	0	0.24	0.76	2.4	LTTPFVK
0	1	0.31	0.95	3.1	FANEVAGVEDLGTTGR
0	2	0.74	2	5.4	YTDIAYTGKR

5.653 174 1649 minus strand Collagen alpha-1(XII) chain [Crassostrea gigas]; 123 1490 minus strand Collagen alpha-1(XII) chain [Crassostrea gigas]; 135 1610 Collagen alpha-1(XII) chain [Crassostrea gigas]

Protein Accession	CL5340.Contig13$_H$1 CL5340.Contig12$_H$1 CL5340.Contig11$_H$1
Mean Expression Ratio	1.07
Median Expression Ratio	1.07
Credible Interval	(0.493, 2.37)
Associated Peptides	2
Associated Spectra	2
Coverage	NaN

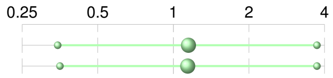

A	B	2.5	50	97.5	Sequence
0	1	0.35	1.1	3.7	ADVVFIVDSSR
0	1	0.35	1.1	3.7	SIQHMQGGLTETYEALR

5.654 3 218 beta-tubulin [Fasciola hepatica] gi

Protein Accession	Unigene7955$_H$1
Mean Expression Ratio	0.935
Median Expression Ratio	0.936
Credible Interval	(0.512, 1.72)
Associated Peptides	5
Associated Spectra	18
Coverage	NaN

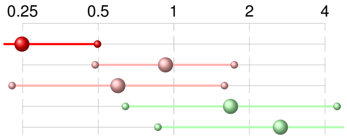

A	B	2.5	50	97.5	Sequence
3	3	0.12	0.25	0.5	EVDEQMLNVQNK
2	5	0.49	0.93	1.7	TAVCDIPPR
0	2	0.23	0.6	1.6	NSSYFVEWIPNNVK
0	2	0.64	1.7	4.5	TAVCDIPPRGLK
0	1	0.86	2.7	8.5	MSMKEVDEQMLNVQNK

5.655 99 3011 Eukaryotic translation initiation factor 3 subunit A [Crassostrea gigas]

Protein Accession	CL36771.Contig2$_H$1
Mean Expression Ratio	1.07
Median Expression Ratio	1.07
Credible Interval	(0.531, 2.1)
Associated Peptides	5
Associated Spectra	5
Coverage	NaN

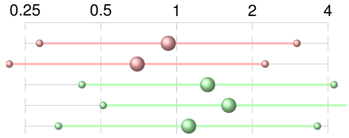

A	B	2.5	50	97.5	Sequence
0	1	0.28	0.93	3	IHEPIMER
0	1	0.22	0.7	2.3	AVEDIHGLISLSK
0	1	0.42	1.3	4.2	ELALETK
0	1	0.51	1.6	5.2	KVDYLAR
0	1	0.34	1.1	3.6	KEQLENLNDAR

5.656 142 726 Charged multivesicular body protein 4b [Crassostrea gigas]

Protein Accession	CL35875.Contig1$_H$1
Mean Expression Ratio	1.06
Median Expression Ratio	1.07
Credible Interval	(0.462, 2.43)
Associated Peptides	1
Associated Spectra	1
Coverage	NaN

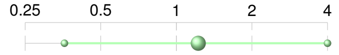

A	B	2.5	50	97.5	Sequence
0	1	0.36	1.2	4	KIEHELATAK

5.657 3 416 minus strand myosin essential light chain [Crassostrea gigas]

Protein Accession	Unigene47974$_H$1
Mean Expression Ratio	1.07
Median Expression Ratio	1.07
Credible Interval	(0.483, 2.41)
Associated Peptides	1
Associated Spectra	2
Coverage	NaN

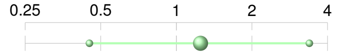

A	B	2.5	50	97.5	Sequence
0	2	0.45	1.3	3.4	CVGLNPTLATIK

5.658 198 2015 minus strand Xaa-Pro aminopeptidase 1 [Crassostrea gigas]

Protein Accession	Unigene11641$_H$1
Mean Expression Ratio	0.937
Median Expression Ratio	0.938
Credible Interval	(0.402, 2.19)
Associated Peptides	1
Associated Spectra	1
Coverage	NaN

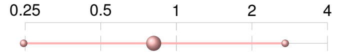

A	B	2.5	50	97.5	Sequence
0	1	0.25	0.81	2.7	APLWEVGLEYR

5.659 167 2713 minus strand Major vault protein [Crassostrea gigas]

Protein Accession	CL18286.Contig8$_{H}$1
Mean Expression Ratio	1.07
Median Expression Ratio	1.07
Credible Interval	(0.658, 1.73)
Associated Peptides	13
Associated Spectra	17
Coverage	NaN

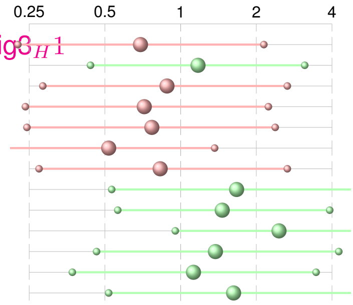

A	B	2.5	50	97.5	Sequence
1	0	0.22	0.69	2.1	GAVAQVQFDDFHK
1	1	0.44	1.2	3.1	IEGEAAVEQAK
1	0	0.28	0.89	2.7	IVDEAEAEK
1	0	0.24	0.72	2.2	LKAEAMK
1	0	0.25	0.77	2.4	VTGEEWLAK
1	1	0.2	0.52	1.4	TYTDDFNK
1	0	0.27	0.83	2.7	DPLADRSER
0	1	0.53	1.7	5.3	KEVVVEETIR
0	2	0.56	1.5	3.9	NADNEVVMDAAGQAK
0	2	0.96	2.5	6.4	QKIVDEAEAEK
0	1	0.46	1.4	4.3	VVPANSALR
0	1	0.37	1.1	3.5	IEAESELER
0	1	0.52	1.6	5	VVTFR

5.660 186 1778 predicted protein [Nematostella vectensis] gi

Protein Accession	CL20488.Contig9$_{H}$1
Mean Expression Ratio	0.934
Median Expression Ratio	0.938
Credible Interval	(0.543, 1.6)
Associated Peptides	8
Associated Spectra	17
Coverage	NaN

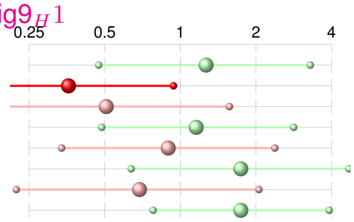

A	B	2.5	50	97.5	Sequence
1	1	0.47	1.3	3.3	ATNLENKDGLLGK
2	0	0.13	0.36	0.94	FAQQAQQQEK
1	0	0.17	0.51	1.6	TKDGEVSWPVIDPK
1	2	0.49	1.2	2.8	SDPYLQISSK
1	1	0.34	0.89	2.4	EGTENVEMHFR
0	2	0.64	1.7	4.7	AKDGTTHVIWK
0	1	0.22	0.69	2.1	DGTTHVIWK
0	4	0.78	1.7	3.9	AEELKEGTENVEMHFR

5.661 " 134 1567 minus strand 6-phosphogluconate dehydrogenase, decarboxylating [Crassostrea gigas]; 134 1573 minus strand 6-phosphogluconate dehydrogenase, decarboxylating [Crassostrea gigas]"

Protein Accession	CL5140.Contig5$_{H}$1 CL5140.Contig4$_{H}$1
Mean Expression Ratio	1.07
Median Expression Ratio	1.07
Credible Interval	(0.546, 2.08)
Associated Peptides	5
Associated Spectra	5
Coverage	NaN

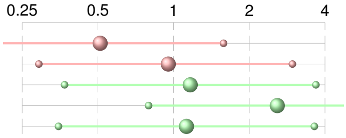

A	B	2.5	50	97.5	Sequence
1	0	0.16	0.51	1.6	FKDTDGSPLVEK
1	0	0.29	0.95	3	AFVEDIKR
0	1	0.37	1.2	3.7	SLEDMVDKLK
0	1	0.79	2.6	8.2	TVSKVDEFLSNEAK
0	1	0.35	1.1	3.6	NIFQSISAK

5.662 266 1123 minus strand mitochondrial malate dehydrogenase precursor [Nucella lapillus]

Protein Accession	CL10426.Contig11$_H$1
Mean Expression Ratio	1.06
Median Expression Ratio	1.07
Credible Interval	(0.501, 2.3)
Associated Peptides	2
Associated Spectra	4
Coverage	NaN

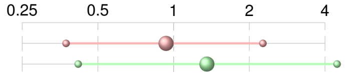

A	B	2.5	50	97.5	Sequence
1	2	0.37	0.94	2.3	SLIEALNGMEGR
0	1	0.42	1.4	4.5	VRSATLSMAYAAAEFSK

5.663 " 460 1944 PREDICTED: succinyl-CoA:3-ketoacid-coenzyme A transferase 1, mitochondrial [Canis lupus familiaris]"

Protein Accession	Unigene4332$_H$1
Mean Expression Ratio	1.06
Median Expression Ratio	1.07
Credible Interval	(0.657, 1.73)
Associated Peptides	13
Associated Spectra	23
Coverage	NaN

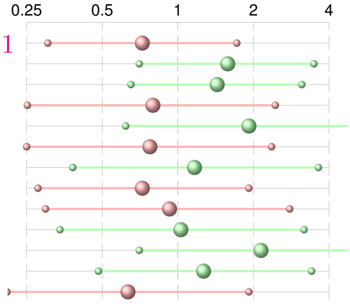

A	B	2.5	50	97.5	Sequence
1	2	0.3	0.72	1.7	EVAEGETAESIAK
1	3	0.7	1.6	3.5	ITGVEFR
2	2	0.65	1.4	3.1	NFNPPMAR
1	0	0.25	0.8	2.4	VFLGK
0	1	0.62	1.9	6.1	ADKLGNLTFR
0	1	0.25	0.77	2.4	AVFDVDNK
0	1	0.38	1.2	3.6	AWKADKLGNLTFR
0	2	0.28	0.72	1.9	EVVNMIVTEK
0	1	0.3	0.93	2.8	LGNLTFR
0	1	0.34	1	3.2	VMGMGGAMDLVSADK
0	1	0.7	2.1	6.9	ILKEVTLPK
0	2	0.48	1.3	3.4	FNPDGTILHGSAGR
0	1	0.21	0.63	1.9	TGDVALVK

5.664 1 321 Malectin [Crassostrea gigas]

Protein Accession	Unigene56360$_H$1
Mean Expression Ratio	1.07
Median Expression Ratio	1.07
Credible Interval	(0.464, 2.48)
Associated Peptides	1
Associated Spectra	1
Coverage	NaN

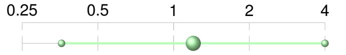

A	B	2.5	50	97.5	Sequence
0	1	0.36	1.2	4	GVAHDEIVPFSIK

5.665 123 860 PREDICTED: proteasome subunit alpha type-6-like [Cavia porcellus]; 1164 1901 PREDICTED: proteasome subunit alpha type-6-like [Cavia porcellus]

Protein Accession	Unigene21398$_{H}1$ Unigene13394$_{H}1$
Mean Expression Ratio	1.07
Median Expression Ratio	1.06
Credible Interval	(0.496, 2.23)
Associated Peptides	2
Associated Spectra	4
Coverage	NaN

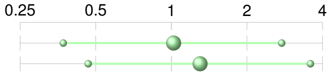

A	B	2.5	50	97.5	Sequence
1	1	0.37	1	2.7	QLEASGFLEK
0	2	0.47	1.3	3.6	VLTEQEIDVHLTSIAEKD

5.666 121 1452 minus strand Hexokinase type 2 [Crassostrea gigas]; 245 1576 minus strand Hexokinase type 2 [Crassostrea gigas]; 151 1455 minus strand Hexokinase type 2 [Crassostrea gigas]; 121 1452 minus strand Hexokinase type 2 [Crassostrea gigas]; 121 1452 minus strand Hexokinase type 2 [Crassostrea gigas]; 161 1513 minus strand Hexokinase type 2 [Crassostrea gigas]; 113 1471 minus strand Hexokinase type 2 [Crassostrea gigas]; 225 1577 minus strand Hexokinase type 2 [Crassostrea gigas]; 121 1452 minus strand Hexokinase type 2 [Crassostrea gigas]

Protein Accession	CL350.Contig9$_{H}1$ CL350.Contig8$_{H}1$ CL350.Contig7$_{H}1$ CL350.Contig6$_{H}1$ CL350.Contig5$_{H}1$
Mean Expression Ratio	1.06
Median Expression Ratio	1.06
Credible Interval	(0.451, 2.49)
Associated Peptides	1
Associated Spectra	1
Coverage	NaN

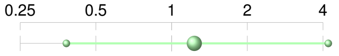

A	B	2.5	50	97.5	Sequence
1	0	0.38	1.2	4.2	MYPTYVR

5.667 72 839 minus strand 60S ribosomal protein L8 OS=Spodoptera frugiperda GN=RpL8 PE=2 SV=1

Protein Accession	Unigene15547$_{H}1$
Mean Expression Ratio	1.06
Median Expression Ratio	1.06
Credible Interval	(0.563, 2.03)
Associated Peptides	5
Associated Spectra	8
Coverage	NaN

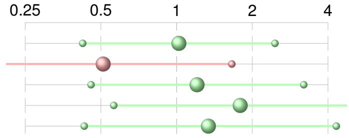

A	B	2.5	50	97.5	Sequence
1	2	0.42	1	2.5	AMVGIVAGGGR
1	0	0.16	0.51	1.7	IDKPILK
0	2	0.46	1.2	3.2	GVIKDIVHDPGR
0	1	0.56	1.8	5.7	KGAGSVFK
0	1	0.43	1.3	4.3	VAFRDPYR

5.668 94 519 minus strand hypothetical protein BRAFLDRAFT_115029 [Branchiostoma floridae] gi

Protein Accession	Unigene52953$_H$1
Mean Expression Ratio	0.943
Median Expression Ratio	0.941
Credible Interval	(0.467, 1.92)
Associated Peptides	3
Associated Spectra	5
Coverage	NaN

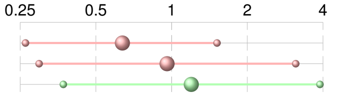

A	B	2.5	50	97.5	Sequence
0	3	0.26	0.64	1.5	SLDTDGDGTVSAAELR
0	1	0.3	0.96	3.1	YGVVFK
0	1	0.37	1.2	3.9	FINKK

5.669 " 183 476 minus strand guanine nucleotide-binding protein G(q), alpha subunit [Aplysia californica] gi"

Protein Accession	CL36147.Contig1$_H$1
Mean Expression Ratio	1.07
Median Expression Ratio	1.06
Credible Interval	(0.464, 2.47)
Associated Peptides	1
Associated Spectra	1
Coverage	NaN

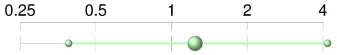

A	B	2.5	50	97.5	Sequence
0	1	0.39	1.2	4.2	LLLLGTGESGK

5.670 125 601 minus strand omega class glutathione-s-transferase [Chlamys farreri]; 113 808 minus strand omega class glutathione-s-transferase [Chlamys farreri]; 335 1045 minus strand omega class glutathione-s-transferase [Chlamys farreri]

Protein Accession	Unigene32974$_H$1 CL8096.Contig3$_H$1 CL8096.Contig2$_H$1
Mean Expression Ratio	1.06
Median Expression Ratio	1.06
Credible Interval	(0.547, 2.09)
Associated Peptides	5
Associated Spectra	5
Coverage	NaN

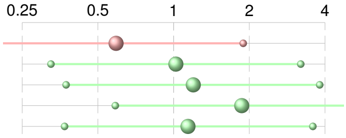

A	B	2.5	50	97.5	Sequence
0	1	0.18	0.59	1.9	DAMLIDFFGNK
0	1	0.33	1	3.2	HPLGLVPTLEQDSK
0	1	0.37	1.2	3.8	FIPNYYK
0	1	0.59	1.9	5.8	LTPVDPYRK
0	1	0.37	1.1	3.6	LVLEHK

5.671 3 281 minus strand hypothetical protein CGI_10015501 [Crassostrea gigas]

Protein Accession	Unigene26367$_{H}1$
Mean Expression Ratio	1.07
Median Expression Ratio	1.06
Credible Interval	(0.462, 2.48)
Associated Peptides	1
Associated Spectra	1
Coverage	NaN

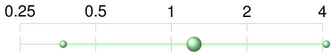

A	B	2.5	50	97.5	Sequence
**0**	**1**	**0.37**	**1.2**	**4.1**	**AGNDITADQVIDDVK**

5.672 10 300 minus strand unnamed protein product [Tetraodon nigroviridis]

Protein Accession	CL14559.Contig2$_{H}1$
Mean Expression Ratio	1.06
Median Expression Ratio	1.06
Credible Interval	(0.477, 2.29)
Associated Peptides	1
Associated Spectra	5
Coverage	NaN

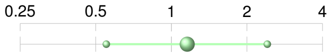

A	B	2.5	50	97.5	Sequence
**0**	**5**	**0.55**	**1.2**	**2.4**	**ILVQEGIHDR**

5.673 32 193 minus strand Ubiquinol-cytochrome C reductase complex [Xenopus laevis] gi; 32 193 minus strand Ubiquinol-cytochrome C reductase complex [Xenopus laevis] gi

Protein Accession	CL7847.Contig7$_{H}1$ CL7847.Contig4$_{H}1$
Mean Expression Ratio	1.06
Median Expression Ratio	1.06
Credible Interval	(0.506, 2.2)
Associated Peptides	2
Associated Spectra	8
Coverage	NaN

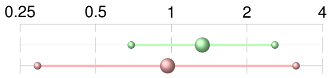

A	B	2.5	50	97.5	Sequence
**3**	**4**	**0.69**	**1.3**	**2.6**	**AFLKPQAPK**
**1**	**0**	**0.29**	**0.97**	**3.1**	**AGPSIFR**

5.674 206 316 minus strand ribosomal protein S17 [Branchiostoma belcheri]

Protein Accession	Unigene31494$_{H}1$
Mean Expression Ratio	1.06
Median Expression Ratio	1.06
Credible Interval	(0.521, 2.11)
Associated Peptides	3
Associated Spectra	6
Coverage	NaN

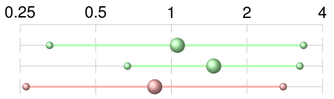

A	B	2.5	50	97.5	Sequence
**0**	**1**	**0.33**	**1.1**	**3.4**	**LTLDFHTNK**
**0**	**4**	**0.67**	**1.5**	**3.2**	**LTLDFHTNKR**
**0**	**1**	**0.26**	**0.86**	**2.8**	**VCEEIAIIPSK**

5.675 333 770 USP-like protein isoform 2 [Crassostrea ariakensis]

Protein Accession	Unigene7710$_H$1
Mean Expression Ratio	1.06
Median Expression Ratio	1.06
Credible Interval	(0.56, 1.99)
Associated Peptides	4
Associated Spectra	12
Coverage	NaN

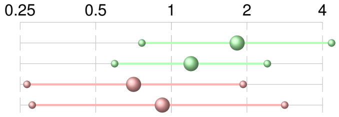

A	B	2.5	50	97.5	Sequence
1	2	0.76	1.8	4.4	KIVIAMDGSK
2	4	0.6	1.2	2.4	MSANTPGEAVIK
0	2	0.27	0.71	1.9	DIGADLIITGTR
0	1	0.28	0.92	2.8	LQNLLTENGMNGNVVR

5.676 1155 1649 78 kDa glucose-regulated protein OS=Aplysia californica PE=1 SV=1

Protein Accession	CL41594.Contig1$_H$1
Mean Expression Ratio	1.06
Median Expression Ratio	1.06
Credible Interval	(0.529, 2.13)
Associated Peptides	3
Associated Spectra	7
Coverage	NaN

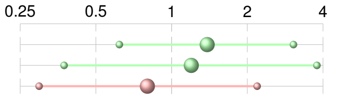

A	B	2.5	50	97.5	Sequence
0	4	0.62	1.4	3	AKFEELNMDLFR
0	1	0.37	1.2	3.8	STVKPVK
0	2	0.3	0.8	2.2	VEVESLFDGEDFSEQLTR

5.677 10 1542 Copine-8 [Crassostrea gigas]

Protein Accession	CL9423.Contig1$_H$1
Mean Expression Ratio	0.949
Median Expression Ratio	0.945
Credible Interval	(0.413, 2.21)
Associated Peptides	1
Associated Spectra	1
Coverage	NaN

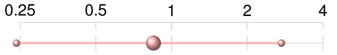

A	B	2.5	50	97.5	Sequence
0	1	0.24	0.85	2.7	SYRGQYAER

5.678 99 437 minus strand V-type proton ATPase subunit G [Crassostrea gigas]

Protein Accession	CL20812.Contig1$_H$1
Mean Expression Ratio	0.947
Median Expression Ratio	0.945
Credible Interval	(0.43, 2.03)
Associated Peptides	2
Associated Spectra	3
Coverage	NaN

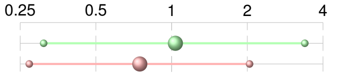

A	B	2.5	50	97.5	Sequence
0	1	0.31	1	3.4	ASQTQGIQQLLAAEK
0	2	0.27	0.75	2	GDLEAKIEASTR

5.679 322 645 macrophage migration inhibitory-like factor protein [Chlamys farreri]; 437 682 macrophage migration inhibitory-like factor protein [Chlamys farreri]; 437 682 macrophage migration inhibitory-like factor protein [Chlamys farreri]; 468 713 macrophage migration inhibitory-like factor protein [Chlamys farreri]

Protein Accession	CL8506.Contig6$_H$1 CL8506.Contig5$_H$1 CL8506.Contig3$_H$1 CL8506.Contig2$_H$1
Mean Expression Ratio	1.06
Median Expression Ratio	1.06
Credible Interval	(0.47, 2.45)
Associated Peptides	1
Associated Spectra	2
Coverage	NaN

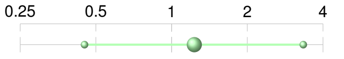

A	B	2.5	50	97.5	Sequence
0	2	0.45	1.2	3.3	ITLTIHSGLR

5.680 " 109 1632 minus strand Seryl-tRNA synthetase, cytoplasmic [Crassostrea gigas]; 109 1614 minus strand Seryl-tRNA synthetase, cytoplasmic [Crassostrea gigas]"

Protein Accession	CL11056.Contig9$_H$1 CL11056.Contig1$_H$1
Mean Expression Ratio	1.06
Median Expression Ratio	1.06
Credible Interval	(0.513, 2.16)
Associated Peptides	3
Associated Spectra	5
Coverage	NaN

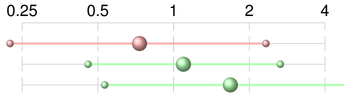

A	B	2.5	50	97.5	Sequence
1	0	0.22	0.73	2.3	ELTVTQIK
0	3	0.46	1.1	2.7	LVDTVVDQDAAWR
0	1	0.53	1.7	5.5	VLDIELFR

5.681 388 1653 Tektin-3 [Crassostrea gigas]

Protein Accession	CL28380.Contig1$_H$1
Mean Expression Ratio	1.06
Median Expression Ratio	1.06
Credible Interval	(0.519, 2.15)
Associated Peptides	3
Associated Spectra	7
Coverage	NaN

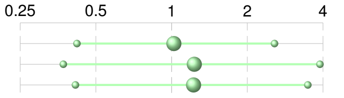

A	B	2.5	50	97.5	Sequence
2	2	0.42	1	2.6	QTIDLVHDAPEK
0	1	0.37	1.2	3.9	RQTIDLVHDAPEK
1	1	0.41	1.2	3.5	GLTFHNGVQR

5.682 5 748 hypothetical protein SINV_09458 [Solenopsis invicta]

Protein Accession	CL25819.Contig2$_{H}$1
Mean Expression Ratio	1.06
Median Expression Ratio	1.06
Credible Interval	(0.47, 2.42)
Associated Peptides	1
Associated Spectra	1
Coverage	NaN

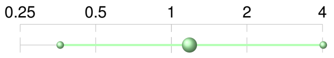

A	B	2.5	50	97.5	Sequence
0	1	0.36	1.2	4	LVSPAASAR

5.683 85 546 minus strand RecName: Full=Eukaryotic translation initiation factor 5A; Short=eIF-5A gi|51702279|sp|P62925.1|IF5A_SPOFR RecName: Full=Eukaryotic translation initiation factor 5A; Short=eIF-5A gi|6469515|gb|AAF13315.1|AF109730_1 translation initiation factor 5A [Spodoptera exigua] gi

Protein Accession	CL40725.Contig2$_{H}$1
Mean Expression Ratio	1.05
Median Expression Ratio	1.06
Credible Interval	(0.602, 1.82)
Associated Peptides	7
Associated Spectra	22
Coverage	NaN

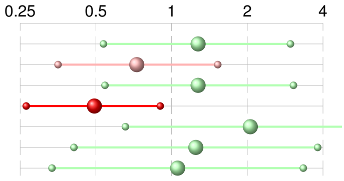

A	B	2.5	50	97.5	Sequence
1	2	0.53	1.3	3	AMGEECVVAVK
3	2	0.35	0.73	1.5	IVEMSTSK
1	2	0.54	1.3	3.1	KNEFVVIK
4	4	0.26	0.49	0.9	EDSLAAEIQK
0	1	0.65	2.1	6.3	EDSLAAEIQKK
0	1	0.41	1.2	3.8	KYEDICPSTHNMEVPVVTR
0	1	0.33	1.1	3.3	NEFVVIK

5.684 729 1541 troponin T [Mizuhopecten yessoensis]

Protein Accession	Unigene34289$_{H}$1
Mean Expression Ratio	1.05
Median Expression Ratio	1.05
Credible Interval	(0.453, 2.43)
Associated Peptides	1
Associated Spectra	1
Coverage	NaN

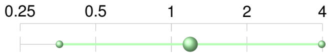

A	B	2.5	50	97.5	Sequence
0	1	0.36	1.2	4	QQYDMIELAER

5.685 49 219 haemocyanin precursor [Nautilus pompilius]

Protein Accession	Unigene32601$_{H}$1
Mean Expression Ratio	0.945
Median Expression Ratio	0.948
Credible Interval	(0.493, 1.81)
Associated Peptides	3
Associated Spectra	14
Coverage	NaN

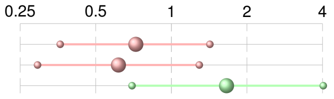

A	B	2.5	50	97.5	Sequence
1	5	0.36	0.72	1.4	GYIDFENGYTVR
3	2	0.29	0.62	1.3	NTQPELFK
2	1	0.7	1.7	4	NTQPELFKK

5.686 299 7507 minus strand filamin [Clonorchis sinensis]

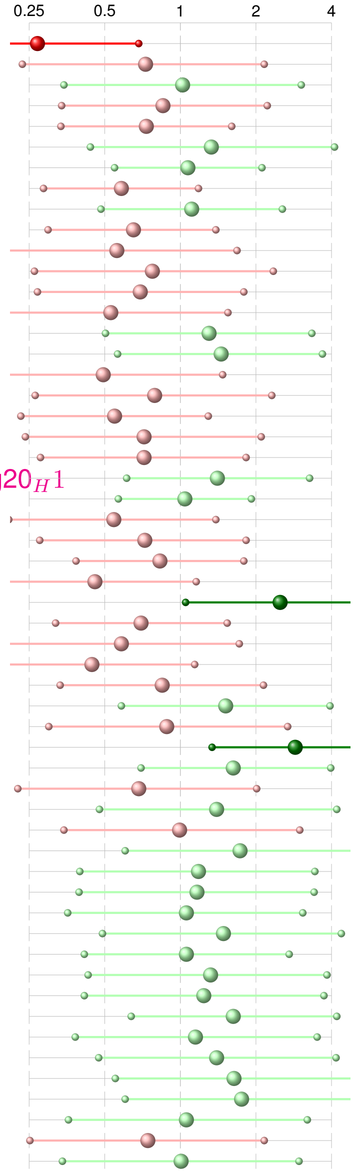

A	B	2.5	50	97.5	Sequence
1	1	0.1	0.27	0.68	AEIDFQDR
1	0	0.23	0.73	2.2	AQLSVGSSSEVSLK
1	0	0.34	1	3	AYGPGLEPK
1	1	0.34	0.85	2.2	DVPGAPFPVK
1	3	0.33	0.73	1.6	ETGEHLVNVFR
1	0	0.44	1.3	4.1	GAGKGGLGLGIEGPVEAK
2	4	0.55	1.1	2.1	GGLGLGIEGPVEAK
3	2	0.28	0.58	1.2	GTLEVTLLNPK
1	2	0.48	1.1	2.6	IMVGESELGNASK
2	2	0.3	0.65	1.4	KISISALQDK
1	0	0.19	0.56	1.7	LANGHLGITFTPR
1	0	0.26	0.77	2.3	LENVTMVLK
1	1	0.27	0.69	1.8	NIMGSPFTAK
1	0	0.17	0.53	1.5	VDAVPDTGIPK
1	1	0.5	1.3	3.3	VLPGVDASKVK
1	1	0.56	1.5	3.7	VQNPVDASKVK
1	0	0.17	0.49	1.5	VYVSEPTNPAK
1	0	0.26	0.79	2.3	TTNVPVQDLVDKK
2	1	0.23	0.55	1.3	ELADDAQWK
1	0	0.24	0.72	2.1	VSEELITR
1	1	0.28	0.72	1.8	FNDEHIPDSPYR
1	2	0.61	1.4	3.3	VKPAALDVSGVK
4	4	0.57	1	1.9	VLPGVDASK
1	1	0.21	0.54	1.4	QVEVQVK
1	1	0.28	0.72	1.8	HIANSPFK
1	3	0.38	0.83	1.8	VTETDITDLTASIR
1	1	0.18	0.46	1.2	EEPCLLK
1	2	1	2.5	5.9	VIAKGPGLEK
2	2	0.32	0.7	1.5	VEVGPFWK
1	0	0.2	0.58	1.7	IMVGK
2	0	0.17	0.44	1.1	VQNPVDASK
1	1	0.33	0.84	2.1	EETKPGSLGVTVEGPK
0	2	0.58	1.5	4	AYGPGLEGGITNQPAVFTVDMK
0	1	0.3	0.89	2.7	EMGIHTVSVK
0	4	1.3	2.9	6.2	FADKNIMGSPFTAK
0	3	0.7	1.6	4	GLQVGKPAAFVVNFNGAK
0	1	0.23	0.68	2	GLTEGMANEINEFIVDTR
0	1	0.48	1.4	4.2	INVTHADKPIPGSPFK
0	1	0.34	0.99	3	SPVDELTVK
0	1	0.6	1.7	5.1	TQKLENVTMVLK
0	1	0.4	1.2	3.4	VKDSADFYVHTK
0	1	0.4	1.2	3.4	VQVSPSIGDAR
0	1	0.36	1.1	3.1	VTAYGEGLHTGTTGQISK
0	1	0.49	1.5	4.4	VTYRPTEPGTYIINIK
0	2	0.42	1.1	2.7	WANEHLK
0	1	0.43	1.3	3.8	WLDVPQLIKPDEMTNPK
0	1	0.41	1.2	3.7	FLEEDEGIR
0	2	0.64	1.6	4.2	HKDMHIPGSPFQFTVGPIAGGGAHK
0	1	0.38	1.1	3.5	VTGPGVQGTTPASLPAVFK
0	1	0.47	1.4	4.2	FSGIQK
0	1	0.55	1.6	5	SPSGREEPCLLKR
0	1	0.6	1.8	5.2	NGKHIANSPFK
0	1	0.36	1.1	3.2	FIVNTVNAGA
0	1	0.25	0.74	2.2	VEGAGQPSGVQEHASVVVETVTK
0	1	0.34	1	3	YAGVNIAGSPFK

Protein Accession	CL4145.Contig20$_H$1
Mean Expression Ratio	0.949
Median Expression Ratio	0.948
Credible Interval	(0.731, 1.24)
Associated Peptides	55
Associated Spectra	110
Coverage	NaN

5.687 40 315 minus strand 60S ribosomal protein L18a OS=Homo sapiens GN=RPL18A PE=1 SV=2

Protein Accession	Unigene26823$_H1$
Mean Expression Ratio	1.05
Median Expression Ratio	1.05
Credible Interval	(0.509, 2.2)
Associated Peptides	2
Associated Spectra	7
Coverage	NaN

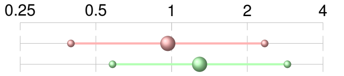

A	B	2.5	50	97.5	Sequence
2	1	0.4	0.97	2.4	IFAPDQATAK
2	2	0.58	1.3	2.9	TPTLYQMR

5.688 9 518 minus strand hemocyanin isoform 1 [Nucula nucleus]

Protein Accession	Unigene71735$_H1$
Mean Expression Ratio	0.95
Median Expression Ratio	0.948
Credible Interval	(0.437, 2.02)
Associated Peptides	1
Associated Spectra	18
Coverage	NaN

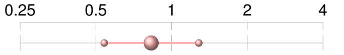

A	B	2.5	50	97.5	Sequence
9	9	0.54	0.83	1.3	IDFEHADVAR

5.689 712 831 haemocyanin [Aplysia californica]

Protein Accession	Unigene65929$_H1$
Mean Expression Ratio	0.95
Median Expression Ratio	0.949
Credible Interval	(0.492, 1.85)
Associated Peptides	3
Associated Spectra	11
Coverage	NaN

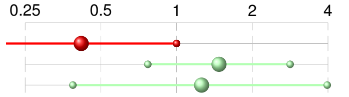

A	B	2.5	50	97.5	Sequence
0	3	0.17	0.42	1	YDVTDVFNQMR
0	7	0.77	1.5	2.8	YHFNVK
0	1	0.39	1.3	4	LRPDSKYHFNVK

5.690 496 2151 NADP-dependent malic enzyme [Crassostrea gigas]

Protein Accession	CL26274.Contig1$_H1$
Mean Expression Ratio	1.05
Median Expression Ratio	1.05
Credible Interval	(0.651, 1.67)
Associated Peptides	12
Associated Spectra	34
Coverage	NaN

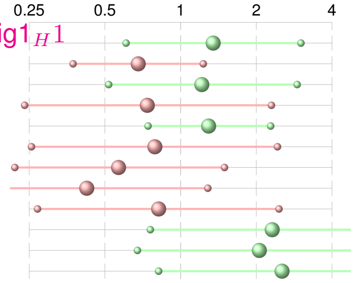

A	B	2.5	50	97.5	Sequence
1	3	0.61	1.4	3	AIVVTDGER
4	4	0.37	0.68	1.2	GIDILR
1	2	0.52	1.2	2.9	IWMVDSK
1	0	0.24	0.74	2.3	EGMASAYPEPQDKR
1	9	0.74	1.3	2.3	VLVNFR
1	0	0.26	0.79	2.4	GLLVK
1	1	0.22	0.57	1.5	GTAFSLK
1	0	0.14	0.42	1.3	APFAK
0	1	0.27	0.82	2.5	AECTAEEAYTLTEGR
0	1	0.76	2.3	7.1	LNKGTAFSLK
0	1	0.67	2.1	6.4	GIDILRNPK
0	1	0.82	2.5	7.9	LNKGTAFSLKER

5.691 390 908 Cysteine proteinase 5 OS=Dictyostelium discoideum GN=cprE PE=2 SV=2

Protein Accession	CL6665.Contig2$_{H}1$
Mean Expression Ratio	0.952
Median Expression Ratio	0.95
Credible Interval	(0.409, 2.22)
Associated Peptides	1
Associated Spectra	1
Coverage	NaN

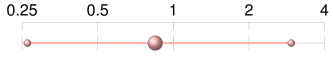

A	B	2.5	50	97.5	Sequence
**0**	**1**	**0.26**	**0.85**	**3**	**GPVSVGIDASHK**

5.692 260 1597 isocitrate dehydrogenase [Mytilus galloprovincialis]; 260 1597 isocitrate dehydrogenase [Mytilus galloprovincialis]; 260 1597 isocitrate dehydrogenase [Mytilus galloprovincialis]

Protein Accession	CL10046.Contig8$_{H}1$ CL10046.Contig7$_{H}1$ CL10046.Contig6$_{H}1$
Mean Expression Ratio	1.05
Median Expression Ratio	1.05
Credible Interval	(0.591, 1.84)
Associated Peptides	7
Associated Spectra	12
Coverage	NaN

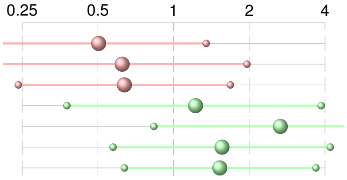

A	B	2.5	50	97.5	Sequence
**2**	**0**	**0.19**	**0.5**	**1.4**	**VKEFNLK**
**1**	**0**	**0.2**	**0.63**	**2**	**KYNVGIK**
**2**	**0**	**0.24**	**0.64**	**1.7**	**TIEAEAAHGTVTR**
**0**	**1**	**0.38**	**1.2**	**3.9**	**NILGGTVFR**
**0**	**1**	**0.84**	**2.7**	**8.3**	**GKLDGNKDLQR**
**0**	**2**	**0.58**	**1.6**	**4.2**	**SWPLYMSTK**
**0**	**3**	**0.64**	**1.5**	**3.7**	**LIDDMVAQALK**

5.693 143 247 minus strand dynein light chain [Aplysia californica] gi

Protein Accession	CL13886.Contig1$_{H}1$
Mean Expression Ratio	0.953
Median Expression Ratio	0.951
Credible Interval	(0.415, 2.18)
Associated Peptides	1
Associated Spectra	2
Coverage	NaN

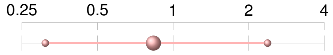

A	B	2.5	50	97.5	Sequence
**0**	**2**	**0.31**	**0.84**	**2.4**	**NADMAEDMQQDAVDCATQALEK**

5.694 1327 1488 minus strand Ubiquinol-cytochrome C reductase complex [Xenopus laevis] gi

Protein Accession	CL7847.Contig1$_{H}1$
Mean Expression Ratio	1.05
Median Expression Ratio	1.05
Credible Interval	(0.456, 2.38)
Associated Peptides	1
Associated Spectra	1
Coverage	NaN

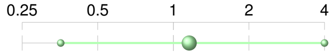

A	B	2.5	50	97.5	Sequence
**0**	**1**	**0.36**	**1.2**	**4**	**LVDSITSSDINNIAK**

5.695 94 933 four and A half lim domains [Schistosoma mansoni]

Protein Accession	CL7046.Contig6$_H$1
Mean Expression Ratio	1.05
Median Expression Ratio	1.05
Credible Interval	(0.45, 2.46)
Associated Peptides	1
Associated Spectra	1
Coverage	NaN

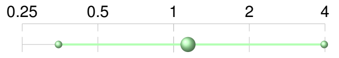

A	B	2.5	50	97.5	Sequence
**1**	**0**	**0.35**	**1.1**	**4**	**CTKPITGIGGTK**

5.696 85 633 hypothetical protein [Amblyomma maculatum]

Protein Accession	CL2221.Contig1$_H$1
Mean Expression Ratio	1.05
Median Expression Ratio	1.05
Credible Interval	(0.508, 2.16)
Associated Peptides	3
Associated Spectra	5
Coverage	NaN

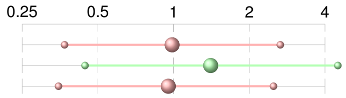

A	B	2.5	50	97.5	Sequence
**0**	**2**	**0.37**	**0.99**	**2.7**	**IFYTEHLHADEEIR**
**0**	**1**	**0.44**	**1.4**	**4.5**	**DANDEWIR**
**0**	**2**	**0.35**	**0.96**	**2.5**	**EKLPDYDNK**

5.697 119 481 Glutamate receptor U1 [Crassostrea gigas]

Protein Accession	CL570.Contig5$_H$1
Mean Expression Ratio	1.05
Median Expression Ratio	1.05
Credible Interval	(0.526, 2.09)
Associated Peptides	3
Associated Spectra	8
Coverage	NaN

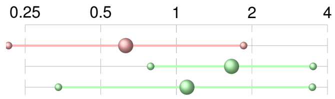

A	B	2.5	50	97.5	Sequence
**1**	**1**	**0.21**	**0.63**	**1.9**	**GTANIGAGALTVTSER**
**0**	**5**	**0.79**	**1.7**	**3.5**	**RTPDGWSGIIGDVVK**
**0**	**1**	**0.34**	**1.1**	**3.5**	**TPDGWSGIIGDVVK**

5.698 20 355 minus strand PREDICTED: uroporphyrinogen decarboxylase-like [Monodelphis domestica]

Protein Accession	CL11915.Contig5$_H$1
Mean Expression Ratio	1.05
Median Expression Ratio	1.05
Credible Interval	(0.457, 2.45)
Associated Peptides	1
Associated Spectra	1
Coverage	NaN

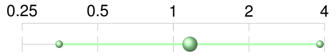

A	B	2.5	50	97.5	Sequence
**0**	**1**	**0.35**	**1.2**	**3.8**	**SKPFPPLQNDLILR**

5.699 457 1842 Basement membrane-specific heparan sulfate proteoglycan core protein [Crassostrea gigas]; 457 1842 Basement membrane-specific heparan sulfate proteoglycan core protein [Crassostrea gigas]

Protein Accession	CL24300.Contig9$_H1$ CL24300.Contig10$_H1$
Mean Expression Ratio	0.959
Median Expression Ratio	0.953
Credible Interval	(0.427, 2.21)
Associated Peptides	1
Associated Spectra	2
Coverage	NaN

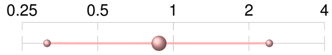

A	B	2.5	50	97.5	Sequence
1	1	0.32	0.87	2.4	KDGELPVEHK

5.700 129 1628 beta-hexosaminidase subunit beta precursor [Danio rerio] gi

Protein Accession	CL21114.Contig2$_H1$
Mean Expression Ratio	1.05
Median Expression Ratio	1.05
Credible Interval	(0.479, 2.28)
Associated Peptides	2
Associated Spectra	2
Coverage	NaN

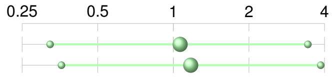

A	B	2.5	50	97.5	Sequence
0	1	0.32	1.1	3.4	EYQTNGNVNTIDAASFR
0	1	0.36	1.2	3.9	LWSPQDVNFPQIAEPR

5.701 63 1865 Dolichyl-diphosphooligosaccharide–protein glycosyltransferase subunit 1 [Crassostrea gigas]; 63 1865 Dolichyl-diphosphooligosaccharide–protein glycosyltransferase subunit 1 [Crassostrea gigas]

Protein Accession	CL41703.Contig2$_H1$ CL41703.Contig1$_H1$
Mean Expression Ratio	1.05
Median Expression Ratio	1.05
Credible Interval	(0.695, 1.59)
Associated Peptides	18
Associated Spectra	33
Coverage	NaN

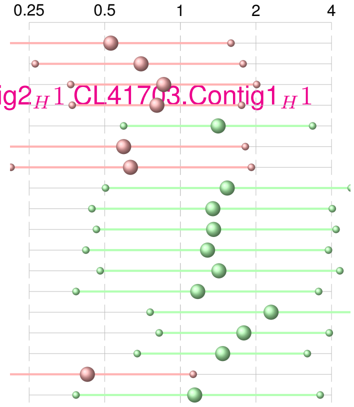

A	B	2.5	50	97.5	Sequence
1	0	0.17	0.53	1.6	APFSEAQLR
1	1	0.26	0.7	1.8	ELDDSVDIELRPR
1	2	0.32	0.86	2	QQYLDAEAGVTSK
2	2	0.37	0.81	1.8	VILPEGVQNIEVR
1	2	0.6	1.4	3.4	VVPTTVPGHSDKELYR
1	0	0.2	0.6	1.8	GAVLK
1	0	0.21	0.63	1.9	LVAGK
1	0	0.5	1.5	4.8	TPFEVKR
0	1	0.45	1.4	4	DLNAFQVAR
0	1	0.46	1.4	4.2	FKTSKDLNAFQVAR
0	1	0.42	1.3	3.9	HSGAVLKGPFSR
0	1	0.48	1.4	4.3	KVDIATHIVK
0	1	0.38	1.2	3.6	SALYQSYDDAINR
0	1	0.76	2.3	7	TSKDLNAFQVAR
0	4	0.82	1.8	3.9	VASLVEEVQSAQDKR
0	4	0.67	1.5	3.2	VQLPFNLDAGK
0	2	0.16	0.43	1.1	LSFISAVTK
0	1	0.38	1.1	3.6	LPAAAR

5.702 " 1 198 minus strand Chain A, Rep1-Nxsq Fatty Acid Transporter; 1 231 minus strand Chain A, Rep1-Nxsq Fatty Acid Transporter; 1 198 minus strand Chain A, Rep1-Nxsq Fatty Acid Transporter"

Protein Accession	CL14182.Contig4$_{H}1$ CL14182.Contig3$_{H}1$ CL14182.Contig2$_{H}1$
Mean Expression Ratio	1.05
Median Expression Ratio	1.05
Credible Interval	(0.462, 2.37)
Associated Peptides	1
Associated Spectra	2
Coverage	NaN

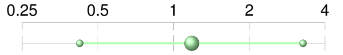

A	B	2.5	50	97.5	Sequence
1	1	0.42	1.2	3.3	GSPDSLIVR

5.703 113 445 ribosomal protein rps19 [Lineus viridis]

Protein Accession	CL8932.Contig6$_{H}1$
Mean Expression Ratio	0.957
Median Expression Ratio	0.955
Credible Interval	(0.474, 1.96)
Associated Peptides	3
Associated Spectra	6
Coverage	NaN

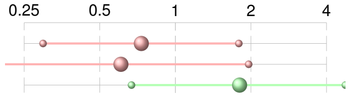

A	B	2.5	50	97.5	Sequence
1	2	0.3	0.73	1.8	QPEWADIVK
1	0	0.19	0.61	2	LKQPEWADIVK
0	2	0.67	1.8	4.8	APAGVGAFTK

5.704 23 409 minus strand CDGSH iron sulfur domain-containing protein 2 [Crassostrea gigas]; 1 207 minus strand CDGSH iron sulfur domain-containing protein 2 [Crassostrea gigas]; 22 135 minus strand CDGSH iron-sulfur domain-containing protein 2 OS=Lithobates catesbeiana GN=cisd2 PE=2 SV=1; 22 207 minus strand CDGSH iron-sulfur domain-containing protein 2 homolog B OS=Branchiostoma floridae GN=BRAFLDRAFT_274541 PE=3 SV=1

Protein Accession	CL36832.Contig6$_{H}1$ CL36832.Contig3$_{H}1$ CL36832.Contig2$_{H}1$ CL36832.Contig1$_{H}1$
Mean Expression Ratio	0.955
Median Expression Ratio	0.956
Credible Interval	(0.416, 2.23)
Associated Peptides	1
Associated Spectra	1
Coverage	NaN

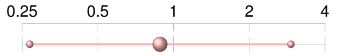

A	B	2.5	50	97.5	Sequence
0	1	0.27	0.88	2.9	VVNMVDIEDLGDK

5.705 64 393 Tubulin beta chain OS=Paracentrotus lividus PE=2 SV=1; 109 438 Tubulin beta chain OS=Paracentrotus lividus PE=2 SV=1; 160 489 Tubulin beta chain OS=Paracentrotus lividus PE=2 SV=1

Protein Accession	Unigene62663$_{H}1$ Unigene62661$_{H}1$ Unigene62660$_{H}1$
Mean Expression Ratio	1.05
Median Expression Ratio	1.05
Credible Interval	(0.518, 2.14)
Associated Peptides	2
Associated Spectra	14
Coverage	NaN

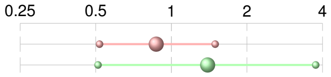

A	B	2.5	50	97.5	Sequence
**5**	**7**	**0.52**	**0.87**	**1.5**	**INVYFNEATGGK**
**0**	**2**	**0.51**	**1.4**	**3.8**	**INVYFNEATGGKYVPR**

5.706 142 261 Core histone macro-H2A.2 OS=Mus musculus GN=H2afy2 PE=1 SV=3

Protein Accession	Unigene14198$_{H}1$
Mean Expression Ratio	1.04
Median Expression Ratio	1.05
Credible Interval	(0.447, 2.39)
Associated Peptides	1
Associated Spectra	1
Coverage	NaN

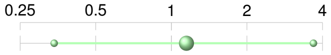

A	B	2.5	50	97.5	Sequence
**0**	**1**	**0.34**	**1.1**	**3.7**	**AGVLFPVAR**

5.707 727 2022 RecName: Full=Enolase; AltName: Full=2-phospho-D-glycerate hydro-lyase; AltName: Full=2-phosphoglycerate dehydratase gi|1911573|gb|AAB50731.1| enolase [Doryteuthis pealeii]; 690 1985 RecName: Full=Enolase; AltName: Full=2-phospho-D-glycerate hydro-lyase; AltName: Full=2-phosphoglycerate dehydratase gi|1911573|gb|AAB50731.1| enolase [Doryteuthis pealeii]; 213 1508 RecName: Full=Enolase; AltName: Full=2-phospho-D-glycerate hydro-lyase; AltName: Full=2-phosphoglycerate dehydratase gi|1911573|gb|AAB50731.1| enolase [Doryteuthis pealeii]; 246 1541 RecName: Full=Enolase; AltName: Full=2-phospho-D-glycerate hydro-lyase; AltName: Full=2-phosphoglycerate dehydratase gi|1911573|gb|AAB50731.1| enolase [Doryteuthis pealeii]

Protein Accession	CL15687.Contig6$_H$1 CL15687.Contig5$_H$1 CL15687.Contig3$_H$1 CL15687.Contig2$_H$1
Mean Expression Ratio	0.957
Median Expression Ratio	0.957
Credible Interval	(0.689, 1.33)
Associated Peptides	28
Associated Spectra	108
Coverage	NaN

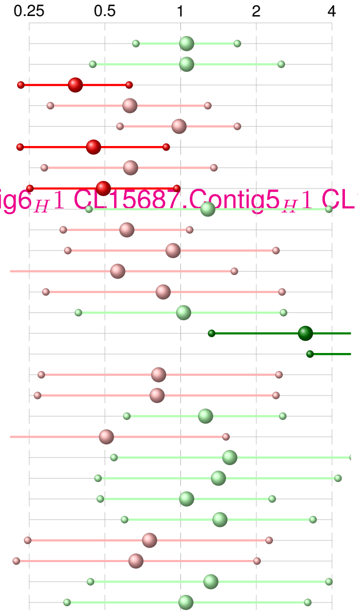

A	B	2.5	50	97.5	Sequence
4	11	0.67	1.1	1.7	AAGWGVMVSHR
1	2	0.45	1.1	2.5	FQIVGDDLLVTNPK
7	6	0.23	0.38	0.62	IAPAFVGK
1	4	0.3	0.63	1.3	IMLDLDGTPNKEK
8	3	0.57	0.98	1.7	QIFDSR
3	3	0.23	0.45	0.88	TLDLADQTAMDK
2	2	0.29	0.63	1.4	VQMGIDR
3	3	0.25	0.49	0.97	GVPLYR
1	0	0.43	1.3	3.9	VIKSIK
5	4	0.34	0.61	1.1	YNQILR
1	1	0.36	0.93	2.4	VAIEK
1	0	0.19	0.56	1.6	AYLGK
1	0	0.29	0.85	2.5	EGLELLK
0	2	0.39	1	2.6	AAVPSGASTGIYEALELR
0	3	1.3	3.1	7.4	AAVPSGASTGIYEALELRDNDPK
0	5	3.3	6.7	14	AVSNINDKIAPAFVGK
0	1	0.28	0.82	2.5	FQIVGDDLLVTN
0	1	0.27	0.81	2.4	HIADLCGNK
0	5	0.61	1.3	2.6	IEEELGVDAFYAGK
0	1	0.16	0.51	1.5	IEIGMDVAASEFCK
0	1	0.54	1.6	4.8	LAKYNQILR
0	1	0.47	1.4	4.2	SYPIVTIEDPFDQDDWDAYTK
0	4	0.48	1.1	2.3	TSQQMIDMYHDFVK
0	3	0.6	1.4	3.4	VNQIGSVSESIQAFK
0	1	0.25	0.76	2.3	DGKYDLDFK
0	1	0.22	0.67	2	YDLDFK
0	1	0.44	1.3	3.9	NINDKIAPAFVGK
0	1	0.35	1.1	3.2	MGSETYHHLK

5.708 174 1721 minus strand aspartyl-tRNA synthetase [Xenopus laevis] gi

Protein Accession	CL9865.Contig1$_H$1
Mean Expression Ratio	0.957
Median Expression Ratio	0.958
Credible Interval	(0.441, 2.1)
Associated Peptides	1
Associated Spectra	7
Coverage	NaN

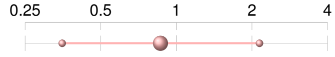

A	B	2.5	50	97.5	Sequence
2	5	0.35	0.86	2.1	IHDPDFLSQR

5.709 145 1020 minus strand PREDICTED: inorganic pyrophosphatase-like [Saccoglossus kowalevskii]

Protein Accession	CL12968.Contig14$_{H}1$
Mean Expression Ratio	0.957
Median Expression Ratio	0.958
Credible Interval	(0.522, 1.75)
Associated Peptides	7
Associated Spectra	9
Coverage	NaN

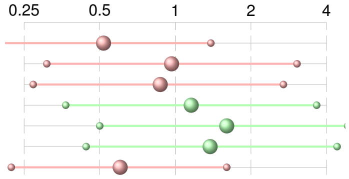

A	B	2.5	50	97.5	Sequence
2	0	0.2	0.52	1.4	SLMADPSVGK
0	1	0.31	0.97	3	IIAIDVTDPLASK
0	1	0.27	0.87	2.7	LNDIDDVHR
0	1	0.37	1.2	3.7	MEINKEEK
0	1	0.5	1.6	4.9	GSVIQVK
0	1	0.44	1.4	4.4	LNPLKQDVK
0	2	0.22	0.6	1.6	AVIDQSVNK

5.710 " 11 427 PREDICTED: similar to syntaxin, partial [Hydra magnipapillata]"

Protein Accession	CL41814.Contig1$_{H}1$
Mean Expression Ratio	0.964
Median Expression Ratio	0.96
Credible Interval	(0.418, 2.26)
Associated Peptides	1
Associated Spectra	1
Coverage	NaN

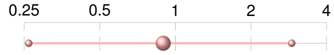

A	B	2.5	50	97.5	Sequence
0	1	0.26	0.89	2.9	HKEMLALEK

5.711 96 497 AAEL009772-PB [Aedes aegypti]

Protein Accession	CL27854.Contig2$_{H}1$
Mean Expression Ratio	0.959
Median Expression Ratio	0.96
Credible Interval	(0.439, 2.1)
Associated Peptides	2
Associated Spectra	2
Coverage	NaN

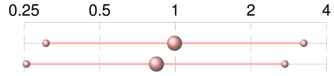

A	B	2.5	50	97.5	Sequence
0	1	0.31	0.99	3.3	LVEEFPMGNIK
0	1	0.25	0.84	2.7	MNPGFDTIGK

5.712 154 261 ribosomal protein S18 [Stenella coeruleoalba]

Protein Accession	CL15120.Contig1$_{H}1$
Mean Expression Ratio	1.04
Median Expression Ratio	1.04
Credible Interval	(0.512, 2.15)
Associated Peptides	3
Associated Spectra	5
Coverage	NaN

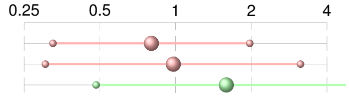

A	B	2.5	50	97.5	Sequence
1	2	0.32	0.8	2	VLNTNIDGR
0	1	0.3	0.98	3.1	INFAITAIK
0	1	0.48	1.6	5.2	VLNTNIDGRR

5.713 2 571 PREDICTED: copper homeostasis protein cutC homolog [Anolis carolinensis]

Protein Accession CL43087.Contig1$_{H}1$
Mean Expression Ratio 1.05
Median Expression Ratio 1.04
Credible Interval (0.454, 2.43)
Associated Peptides 1
Associated Spectra 1
Coverage NaN

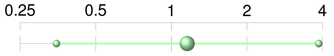

A	B	2.5	50	97.5	Sequence
0	1	0.35	1.2	3.9	IHQDSEMLFQK

5.714 3 551 minus strand Spectrin beta chain [Crassostrea gigas]

Protein Accession Unigene35960$_{H}1$
Mean Expression Ratio 0.958
Median Expression Ratio 0.961
Credible Interval (0.45, 2.01)
Associated Peptides 2
Associated Spectra 5
Coverage NaN

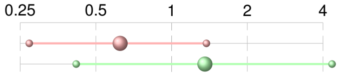

A	B	2.5	50	97.5	Sequence
1	3	0.27	0.62	1.4	LISDINK
0	1	0.42	1.4	4.3	LVSQDNFGADLAAVEAATK

5.715 5 1018 minus strand Glutaredoxin-3 [Crassostrea gigas]; 5 1018 Glutaredoxin-3 [Crassostrea gigas]

Protein Accession CL7734.Contig9$_{H}1$ CL7734.Contig10$_{H}1$
Mean Expression Ratio 1.04
Median Expression Ratio 1.04
Credible Interval (0.46, 2.28)
Associated Peptides 1
Associated Spectra 3
Coverage NaN

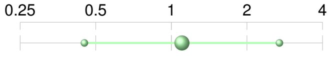

A	B	2.5	50	97.5	Sequence
1	2	0.45	1.1	2.7	VAAEDLAEVSHR

5.716 212 1405 minus strand unnamed protein product [Tetraodon nigroviridis]

Protein Accession CL37546.Contig3$_{H}1$
Mean Expression Ratio 1.04
Median Expression Ratio 1.04
Credible Interval (0.508, 2.09)
Associated Peptides 2
Associated Spectra 8
Coverage NaN

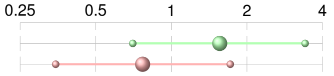

A	B	2.5	50	97.5	Sequence
2	2	0.7	1.6	3.4	GLVVPVIR
2	2	0.35	0.77	1.7	TIAELGEK

5.717 122 1195 minus strand Severin [Crassostrea gigas]

Protein Accession	CL7486.Contig12$_H$1
Mean Expression Ratio	1.04
Median Expression Ratio	1.04
Credible Interval	(0.553, 1.94)
Associated Peptides	6
Associated Spectra	8
Coverage	NaN

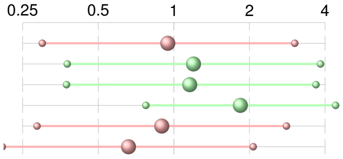

A	B	2.5	50	97.5	Sequence
0	1	0.3	0.95	3	TDHPLVPITCLTEGTK
0	1	0.38	1.2	3.8	TVELDTFLNDVPVQHR
0	1	0.37	1.2	3.7	SVKPEEYRPR
0	3	0.77	1.8	4.4	EVQGHESDLFR
0	1	0.28	0.9	2.8	LLHFHGDR
0	1	0.21	0.66	2.1	SDFISEDVFILDTK

5.718 4 243 40S ribosomal protein S13 OS=Caenorhabditis elegans GN=rps-13 PE=2 SV=2; 4 243 40S ribosomal protein S13 OS=Caenorhabditis elegans GN=rps-13 PE=2 SV=2; 1 252 40S ribosomal protein S13 OS=Xenopus laevis GN=rps13 PE=3 SV=2; 1 213 40S ribosomal protein S13 OS=Xenopus laevis GN=rps13 PE=3 SV=2

Protein Accession	CL18049.Contig7$_H$1 CL18049.Contig4$_H$1 CL18049.Contig3$_H$1 CL18049.Contig2$_H$1
Mean Expression Ratio	0.963
Median Expression Ratio	0.962
Credible Interval	(0.418, 2.25)
Associated Peptides	1
Associated Spectra	1
Coverage	NaN

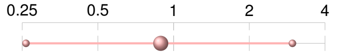

A	B	2.5	50	97.5	Sequence
0	1	0.26	0.89	3	GLAPDIPEDLYSLIK

5.719 1050 1307 calreticulin precursor [Aplysia californica] gi

Protein Accession	CL4813.Contig1$_H$1
Mean Expression Ratio	0.969
Median Expression Ratio	0.963
Credible Interval	(0.415, 2.29)
Associated Peptides	1
Associated Spectra	1
Coverage	NaN

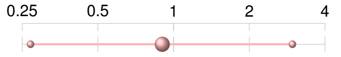

A	B	2.5	50	97.5	Sequence
0	1	0.27	0.9	3	EFGDSTWGK

5.720 26 667 ribosomal protein L15 [Acipenser sinensis] gi; 26 667 ribosomal protein L15 [Acipenser sinensis] gi; 26 667 ribosomal protein L15 [Acipenser sinensis] gi

Protein Accession	Unigene63676$_{H}1$ CL22.Contig2$_{H}1$ CL22.Contig1$_{H}1$
Mean Expression Ratio	1.04
Median Expression Ratio	1.04
Credible Interval	(0.496, 2.09)
Associated Peptides	2
Associated Spectra	7
Coverage	NaN

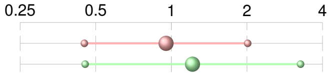

A	B	2.5	50	97.5	Sequence
3	2	0.45	0.95	2	NTLSLLR
0	2	0.45	1.2	3.3	TIGGSRR

5.721 152 1930 hypothetical protein BRAFLDRAFT_223365 [Branchiostoma floridae] gi

Protein Accession	CL49005.Contig4$_{H}1$
Mean Expression Ratio	1.04
Median Expression Ratio	1.04
Credible Interval	(0.561, 1.92)
Associated Peptides	6
Associated Spectra	10
Coverage	NaN

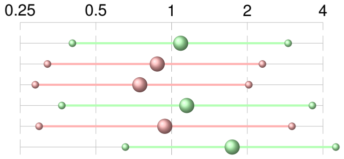

A	B	2.5	50	97.5	Sequence
1	1	0.4	1.1	2.9	QYSAGVSQLSLR
1	1	0.32	0.87	2.3	LSALGLR
1	1	0.29	0.75	2	VTLLR
0	1	0.36	1.1	3.6	ALGLPAATSFK
0	1	0.3	0.94	3	EVSDGVIAPGYEAAALEILK
0	2	0.66	1.7	4.5	AVIDNSLFGNIVTK

5.722 5 841 minus strand Spectrin alpha chain [Crassostrea gigas]

Protein Accession	CL44897.Contig1$_{H}1$
Mean Expression Ratio	0.959
Median Expression Ratio	0.964
Credible Interval	(0.463, 1.99)
Associated Peptides	3
Associated Spectra	4
Coverage	NaN

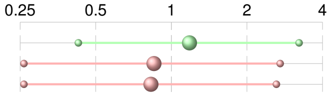

A	B	2.5	50	97.5	Sequence
1	1	0.43	1.2	3.2	HQNFEAEIEANR
0	1	0.26	0.86	2.7	HALLEADVAAHQDR
0	1	0.26	0.83	2.6	KHALLEADVAAHQDR

5.723 23 409 minus strand CDGSH iron sulfur domain-containing protein 2 [Crassostrea gigas]

Protein Accession	CL36832.Contig6$_{H}1$
Mean Expression Ratio	1.04
Median Expression Ratio	1.04
Credible Interval	(0.446, 2.39)
Associated Peptides	1
Associated Spectra	1
Coverage	NaN

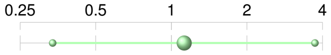

A	B	2.5	50	97.5	Sequence
1	0	0.34	1.1	3.7	VSLPTYLK

5.724 1215 1694 Pikachurin [Crassostrea gigas]

Protein Accession	CL4889.Contig4$_{H}1$
Mean Expression Ratio	0.971
Median Expression Ratio	0.964
Credible Interval	(0.418, 2.28)
Associated Peptides	1
Associated Spectra	1
Coverage	NaN

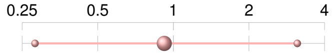

A	B	2.5	50	97.5	Sequence
0	1	0.28	0.92	3.1	HGLGVNELPIASPK

5.725 1573 2385 minus strand Spectrin beta chain [Crassostrea gigas]

Protein Accession	CL5904.Contig4$_{H}1$
Mean Expression Ratio	1.04
Median Expression Ratio	1.04
Credible Interval	(0.475, 2.28)
Associated Peptides	2
Associated Spectra	2
Coverage	NaN

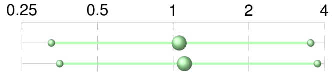

A	B	2.5	50	97.5	Sequence
0	1	0.33	1.1	3.5	LIQEEHPESDQIAVR
0	1	0.35	1.1	3.7	RLIQEEHPESDQIAVR

5.726 " 169 792 minus strand rho GDP dissociation inhibitor, putative [Ixodes scapularis] gi"

Protein Accession	CL39798.Contig1$_{H}1$
Mean Expression Ratio	0.962
Median Expression Ratio	0.964
Credible Interval	(0.448, 2.11)
Associated Peptides	2
Associated Spectra	3
Coverage	NaN

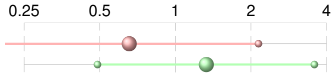

A	B	2.5	50	97.5	Sequence
1	0	0.2	0.65	2.1	YKEQLLAGAGSGEK
0	2	0.49	1.3	3.6	IIIDDSNPLNVIVK

5.727 440 1645 Tektin-1 [Crassostrea gigas]

Protein Accession	CL18799.Contig2$_{H}1$
Mean Expression Ratio	1.04
Median Expression Ratio	1.04
Credible Interval	(0.489, 2.2)
Associated Peptides	3
Associated Spectra	3
Coverage	NaN

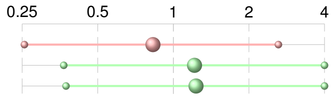

A	B	2.5	50	97.5	Sequence
0	1	0.26	0.83	2.6	EVETLEGVQALLQR
0	1	0.37	1.2	4	SLQATEEPLHIAR
0	1	0.37	1.2	4	FTHEDWTR

5.728 " 41 268 conserved hypothetical protein [Perkinsus marinus ATCC 50983] gi; 29 238 PREDICTED: succinyl-CoA ligase [GDP-forming] subunit beta, mitochondrial-like, partial [Meleagris gallopavo]"

Protein Accession	CL48047.Contig2$_H 1$ CL48047.Contig1$_H 1$
Mean Expression Ratio	0.964
Median Expression Ratio	0.964
Credible Interval	(0.414, 2.24)
Associated Peptides	1
Associated Spectra	1
Coverage	NaN

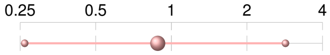

A	B	2.5	50	97.5	Sequence
**1**	**0**	**0.26**	**0.88**	**2.9**	**FVVASTPEDASK**

5.729 225 839 minus strand Metallo-beta-lactamase domain-containing protein 1 [Crassostrea gigas]; 201 851 minus strand Metallo-beta-lactamase domain-containing protein 1 [Crassostrea gigas]

Protein Accession	CL35672.Contig2$_H 1$ CL35672.Contig1$_H 1$
Mean Expression Ratio	1.03
Median Expression Ratio	1.04
Credible Interval	(0.445, 2.41)
Associated Peptides	1
Associated Spectra	1
Coverage	NaN

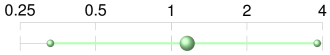

A	B	2.5	50	97.5	Sequence
**0**	**1**	**0.33**	**1.2**	**3.8**	**EADFIVPGHGPMFR**

5.730 484 1218 minus strand alginate lyase [Littorina brevicula]; 484 1218 minus strand alginate lyase [Littorina brevicula]

Protein Accession	CL8738.Contig15$_H 1$ CL8738.Contig13$_H 1$
Mean Expression Ratio	1.03
Median Expression Ratio	1.04
Credible Interval	(0.443, 2.4)
Associated Peptides	1
Associated Spectra	1
Coverage	NaN

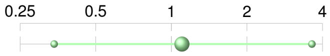

A	B	2.5	50	97.5	Sequence
**0**	**1**	**0.34**	**1.1**	**3.6**	**VFLDGHK**

5.731 104 283 minus strand RecName: Full=Hemocyanin type 2 unit a; AltName: Full=Hemocyanin RHSS2 subunit; AltName: Full=Hemocyanin Rta; AltName: Full=Hemocyanin heavy structural subunit; AltName: Full=RtH2-a gi|7515001|pir||JC5632 hemocyanin dioxygen-binding functional unit - grosse

Protein Accession	Unigene21935$_{H}1$
Mean Expression Ratio	0.968
Median Expression Ratio	0.966
Credible Interval	(0.459, 2.09)
Associated Peptides	2
Associated Spectra	4
Coverage	NaN

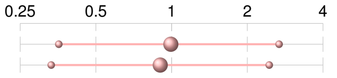

A	B	2.5	50	97.5	Sequence
0	2	0.36	0.99	2.7	KNVASLSDDEVLTLQSVLR
0	2	0.33	0.9	2.4	NVASLSDDEVLTLQSVLR

5.732 48 464 minus strand Acyl-coenzyme A thioesterase 13 [Crassostrea gigas]

Protein Accession	CL38770.Contig1$_{H}1$
Mean Expression Ratio	0.96
Median Expression Ratio	0.966
Credible Interval	(0.413, 2.19)
Associated Peptides	1
Associated Spectra	1
Coverage	NaN

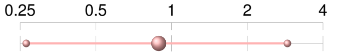

A	B	2.5	50	97.5	Sequence
1	0	0.26	0.89	2.9	TALPGETVVAEAK

5.733 106 1701 T-complex protein 1 subunit beta [Crassostrea gigas]

Protein Accession	CL8176.Contig1$_{H}1$
Mean Expression Ratio	1.03
Median Expression Ratio	1.04
Credible Interval	(0.477, 2.23)
Associated Peptides	2
Associated Spectra	3
Coverage	NaN

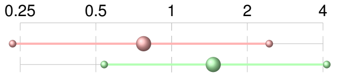

A	B	2.5	50	97.5	Sequence
0	1	0.23	0.77	2.4	EAESLIGQK
0	2	0.54	1.5	4.1	VAELELAEKEK

5.734 71 409 minus strand heat shock protein 108 [Alligator mississippiensis]

Protein Accession	CL5089.Contig3$_{H}1$
Mean Expression Ratio	1.03
Median Expression Ratio	1.04
Credible Interval	(0.466, 2.28)
Associated Peptides	2
Associated Spectra	2
Coverage	NaN

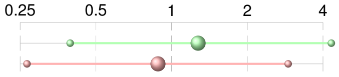

A	B	2.5	50	97.5	Sequence
0	1	0.39	1.3	4.3	QETVDDDLGKTMEGSR
0	1	0.27	0.88	2.9	QETVDDDLGK

5.735 219 428 Peptidyl-prolyl cis-trans isomerase OS=Hemicentrotus pulcherrimus PE=2 SV=1

Protein Accession	CL39270.Contig2$_H1$
Mean Expression Ratio	1.03
Median Expression Ratio	1.03
Credible Interval	(0.5, 2.18)
Associated Peptides	2
Associated Spectra	9
Coverage	NaN

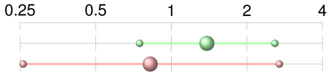

A	B	2.5	50	97.5	Sequence
2	6	0.75	1.4	2.6	IIIANSGQV
1	0	0.26	0.82	2.7	IIIANSGQ

5.736 1 987 minus strand RecName: Full=Tubulin beta chain; AltName: Full=Beta-tubulin

Protein Accession	Unigene7954$_H1$
Mean Expression Ratio	1.03
Median Expression Ratio	1.03
Credible Interval	(0.598, 1.78)
Associated Peptides	7
Associated Spectra	17
Coverage	NaN

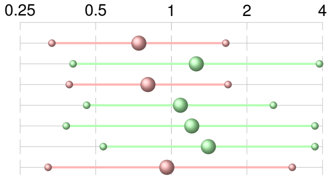

A	B	2.5	50	97.5	Sequence
2	2	0.34	0.74	1.7	ALTVPELTQQMFDAK
0	1	0.41	1.3	3.9	AILVDLEPGTMDSVR
0	5	0.39	0.81	1.7	IMNTFSVVPSPK
0	3	0.46	1.1	2.5	PELTQQMFDAK
0	1	0.38	1.2	3.7	FWEVISDEHGIDPTGTYHGDSDLQLER
0	2	0.53	1.4	3.7	INVYYNEATGGK
0	1	0.32	0.96	3	VAAIFR

5.737 54 1076 PREDICTED: UPF0553 protein C9orf64-like [Anolis carolinensis]; 71 1093 PREDICTED: UPF0553 protein C9orf64-like [Anolis carolinensis]; 33 1055 PREDICTED: UPF0553 protein C9orf64-like [Anolis carolinensis]; 54 1076 PREDICTED: UPF0553 protein C9orf64-like [Anolis carolinensis]

Protein Accession	CL26820.Contig7$_H1$ CL26820.Contig6$_H1$ CL26820.Contig5$_H1$ CL26820.Contig4$_H1$
Mean Expression Ratio	1.03
Median Expression Ratio	1.03
Credible Interval	(0.435, 2.39)
Associated Peptides	1
Associated Spectra	1
Coverage	NaN

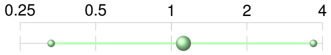

A	B	2.5	50	97.5	Sequence
0	1	0.33	1.1	3.7	SKELLDILDK

5.738 150 716 calponin/transgelin [Papilio xuthus]

Protein Accession	CL11747.Contig2$_{H}1$
Mean Expression Ratio	1.04
Median Expression Ratio	1.03
Credible Interval	(0.483, 2.25)
Associated Peptides	2
Associated Spectra	3
Coverage	NaN

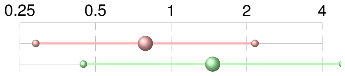

A	B	2.5	50	97.5	Sequence
1	1	0.29	0.79	2.2	MAFQQMEAIEK
0	1	0.45	1.5	4.8	DFTEEQLAQGK

5.739 116 1882 Actin-interacting protein 1 [Crassostrea gigas]

Protein Accession	CL12416.Contig1$_{H}1$
Mean Expression Ratio	0.968
Median Expression Ratio	0.969
Credible Interval	(0.53, 1.76)
Associated Peptides	7
Associated Spectra	10
Coverage	NaN

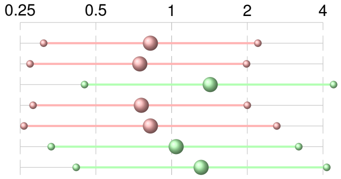

A	B	2.5	50	97.5	Sequence
1	1	0.31	0.83	2.2	QFLDGEVK
1	1	0.27	0.75	2	TLDELDNKEIGAPGAHK
0	1	0.45	1.4	4.4	FSPDGEVAVSGGADGK
0	2	0.28	0.76	2	GHMKPITAMALSDDR
0	1	0.26	0.82	2.6	LVSIGVDDVVR
0	1	0.33	1	3.2	QILTASGDASAK
0	1	0.42	1.3	4.2	VFDVQSGSLVQEFK

5.740 2 436 minus strand PREDICTED: stress-induced-phosphoprotein 1 [Sarcophilus harrisii]

Protein Accession	CL38065.Contig3$_{H}1$
Mean Expression Ratio	0.968
Median Expression Ratio	0.97
Credible Interval	(0.482, 1.95)
Associated Peptides	3
Associated Spectra	6
Coverage	NaN

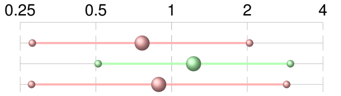

A	B	2.5	50	97.5	Sequence
1	1	0.28	0.76	2	IGNSYMR
0	3	0.51	1.2	3	ADFTAIAK
0	1	0.28	0.89	2.9	LAYVNP

5.741 172 612 minus strand putative polyadenylate-binding protein 1 [Haliotis diversicolor]; 172 612 putative polyadenylate-binding protein 1 [Haliotis diversicolor]

Protein Accession	CL48895.Contig3$_{H}1$ CL48895.Contig2$_{H}1$
Mean Expression Ratio	1.04
Median Expression Ratio	1.03
Credible Interval	(0.473, 2.28)
Associated Peptides	2
Associated Spectra	2
Coverage	NaN

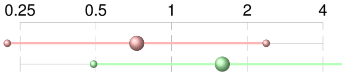

A	B	2.5	50	97.5	Sequence
1	0	0.22	0.73	2.4	ALDTMNFDTVK
0	1	0.49	1.6	5.2	KSGVGNVFIK

5.742 4 942 minus strand KTEL motif-containing protein 1 [Crassostrea gigas]

Protein Accession	CL11353.Contig3$_H$1
Mean Expression Ratio	1.03
Median Expression Ratio	1.03
Credible Interval	(0.463, 2.35)
Associated Peptides	1
Associated Spectra	2
Coverage	NaN

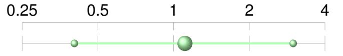

A	B	2.5	50	97.5	Sequence
0	2	0.4	1.1	3	GIHYQIVDHK

5.743 552 1886 minus strand intermediate filament protein [Biomphalaria glabrata]

Protein Accession	Unigene26795$_H$1
Mean Expression Ratio	1.03
Median Expression Ratio	1.03
Credible Interval	(0.676, 1.58)
Associated Peptides	14
Associated Spectra	49
Coverage	NaN

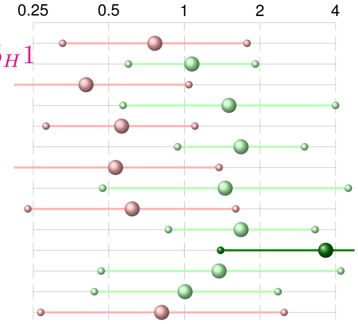

A	B	2.5	50	97.5	Sequence
2	1	0.33	0.76	1.8	KLLEGEENR
3	6	0.6	1.1	1.9	KVITEFSTQR
1	1	0.15	0.4	1	LLEGEENR
1	1	0.57	1.5	4	SATGISEFR
3	3	0.28	0.56	1.1	WLEAQNK
3	6	0.94	1.7	3	YAAQMDQLR
0	2	0.2	0.53	1.4	ADLDAETAAHIENEVLAQTK
0	1	0.47	1.5	4.5	CGSLEDELAK
0	2	0.24	0.62	1.6	DMYEGELAQAR
0	6	0.86	1.7	3.3	KLEAELEALR
0	2	1.4	3.6	9.6	KQEDWKPIRDMYEGELAQAR
0	1	0.47	1.4	4.2	MSLGFNPGVYSK
0	3	0.44	1	2.4	KQEDWKPIR
0	1	0.27	0.81	2.5	SLELEIACYR

5.744 120 833 B-cell receptor-associated protein 31 [Crassostrea gigas]; 238 951 B-cell receptor-associated protein 31 [Crassostrea gigas]; 88 801 B-cell receptor-associated protein 31 [Crassostrea gigas]; 88 801 B-cell receptor-associated protein 31 [Crassostrea gigas]

Protein Accession	CL9442.Contig7$_H$1 CL9442.Contig5$_H$1 CL9442.Contig4$_H$1 CL9442.Contig3$_H$1
Mean Expression Ratio	0.976
Median Expression Ratio	0.973
Credible Interval	(0.5, 1.92)
Associated Peptides	4
Associated Spectra	8
Coverage	NaN

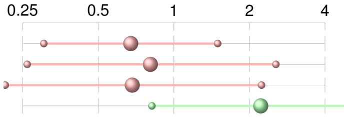

A	B	2.5	50	97.5	Sequence
1	3	0.3	0.67	1.5	LLEEHAQLQK
1	0	0.26	0.8	2.6	TELSDEMK
1	0	0.21	0.68	2.2	LGGLK
0	2	0.82	2.2	6.1	IEFSSMKK

5.745 3 116 Paramyosin [Crassostrea gigas]; 3 107 minus strand Paramyosin [Crassostrea gigas]

Protein Accession	CL43455.Contig7$_{H}1$ CL43455.Contig13$_{H}1$
Mean Expression Ratio	1.03
Median Expression Ratio	1.03
Credible Interval	(0.468, 2.29)
Associated Peptides	2
Associated Spectra	2
Coverage	NaN

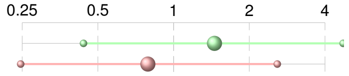

A	B	2.5	50	97.5	Sequence
**0**	**1**	**0.44**	**1.5**	**4.7**	**AQTLIEEAENRADSAEK**
**0**	**1**	**0.25**	**0.79**	**2.6**	**NLQVVR**

5.746 181 744 unknown [Dendroctonus ponderosae]

Protein Accession	CL25948.Contig3$_{H}1$
Mean Expression Ratio	0.974
Median Expression Ratio	0.973
Credible Interval	(0.516, 1.85)
Associated Peptides	4
Associated Spectra	19
Coverage	NaN

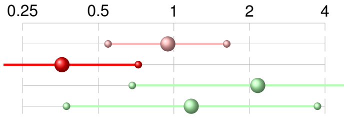

A	B	2.5	50	97.5	Sequence
**7**	**4**	**0.55**	**0.94**	**1.6**	**VGHVPVAK**
**5**	**1**	**0.18**	**0.36**	**0.72**	**GTLTQLTK**
**0**	**1**	**0.68**	**2.2**	**6.9**	**GDFSKLPR**
**0**	**1**	**0.37**	**1.2**	**3.7**	**SMGPIGSPMSK**

5.747 " 285 437 Propionyl-CoA carboxylase alpha chain, mitochondrial [Crassostrea gigas]"

Protein Accession	CL45581.Contig2$_{H}1$
Mean Expression Ratio	0.976
Median Expression Ratio	0.974
Credible Interval	(0.428, 2.2)
Associated Peptides	1
Associated Spectra	2
Coverage	NaN

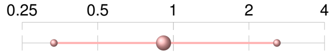

A	B	2.5	50	97.5	Sequence
**0**	**2**	**0.34**	**0.91**	**2.6**	**GVTHNIPLLR**

5.748 14 502 minus strand Ufm1-conjugating enzyme 1 [Crassostrea gigas]

Protein Accession	CL15276.Contig2$_{H}1$
Mean Expression Ratio	1.03
Median Expression Ratio	1.03
Credible Interval	(0.446, 2.34)
Associated Peptides	1
Associated Spectra	1
Coverage	NaN

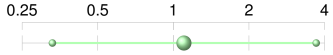

A	B	2.5	50	97.5	Sequence
**0**	**1**	**0.33**	**1.1**	**3.7**	**TLAQIPLLK**

5.749 424 1347 minus strand Tubulin alpha chain [Ascaris suum]

Protein Accession	Unigene7901$_{H}1$
Mean Expression Ratio	0.977
Median Expression Ratio	0.975
Credible Interval	(0.493, 1.92)
Associated Peptides	3
Associated Spectra	10
Coverage	NaN

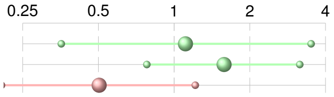

A	B	2.5	50	97.5	Sequence
**0**	**1**	**0.36**	**1.1**	**3.5**	**VDLEPTVVDEVR**
**0**	**6**	**0.78**	**1.6**	**3.2**	**NTFFNETGSGKHVPRAIFVDLEPTVVDEVR**
**0**	**3**	**0.21**	**0.5**	**1.2**	**QLFHPEQLITGK**

5.750 2 352 Ribosome-binding protein 1 [Crassostrea gigas]

Protein Accession	Unigene24847$_{H}1$
Mean Expression Ratio	1.03
Median Expression Ratio	1.02
Credible Interval	(0.46, 2.29)
Associated Peptides	2
Associated Spectra	2
Coverage	NaN

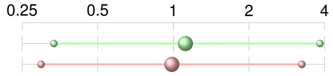

A	B	2.5	50	97.5	Sequence
**0**	**1**	**0.33**	**1.1**	**3.8**	**LVAHQLELEALQAR**
**0**	**1**	**0.3**	**0.99**	**3.3**	**LQPLEQSTDAALVQK**

5.751 146 841 minus strand hypothetical protein CGI_10006609 [Crassostrea gigas]; 146 841 minus strand hypothetical protein CGI_10006609 [Crassostrea gigas]; 146 841 minus strand hypothetical protein CGI_10006609 [Crassostrea gigas]

Protein Accession	CL6681.Contig3$_{H}1$ CL6681.Contig2$_{H}1$ CL6681.Contig1$_{H}1$
Mean Expression Ratio	1.02
Median Expression Ratio	1.02
Credible Interval	(0.443, 2.38)
Associated Peptides	1
Associated Spectra	1
Coverage	NaN

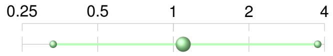

A	B	2.5	50	97.5	Sequence
**0**	**1**	**0.33**	**1.1**	**3.7**	**GHVEGLEVLPAR**

5.752 642 1415 minus strand cytochrome c oxidase subunit II [Oncomelania hupensis hupensis]

Protein Accession	CL6764.Contig3$_{H}1$
Mean Expression Ratio	1.03
Median Expression Ratio	1.02
Credible Interval	(0.477, 2.19)
Associated Peptides	1
Associated Spectra	10
Coverage	NaN

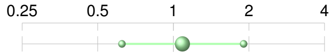

A	B	2.5	50	97.5	Sequence
**3**	**7**	**0.62**	**1.1**	**1.9**	**IVLPTHTDIR**

5.753 " 371 820 minus strand Peroxiredoxin-5, mitochondrial [Crassostrea gigas]; 91 528 minus strand Peroxiredoxin-5, mitochondrial [Crassostrea gigas]; 113 550 Peroxiredoxin-5, mitochondrial [Crassostrea gigas]; 453 902 Peroxiredoxin-5, mitochondrial [Crassostrea gigas]; 87 530 Peroxiredoxin-5, mitochondrial [Crassostrea gigas]"

Protein Accession	CL5795.Contig8$_{H}1$ CL5795.Contig7$_{H}1$ CL5795.Contig6$_{H}1$ CL5795.Contig5$_{H}1$ CL5795.Con
Mean Expression Ratio	1.03
Median Expression Ratio	1.02
Credible Interval	(0.47, 2.31)
Associated Peptides	2
Associated Spectra	2
Coverage	NaN

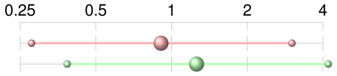

A	B	2.5	50	97.5	Sequence
**0**	**1**	**0.28**	**0.91**	**3**	**ADLGIDLSSVLGNVR**
**0**	**1**	**0.38**	**1.3**	**4.2**	**FVLVAQDGVVK**

5.754 " 1252 1812 minus strand Alanine–glyoxylate aminotransferase 2, mitochondrial [Crassostrea gigas]"

Protein Accession	CL10973.Contig3$_{H}1$
Mean Expression Ratio	1.02
Median Expression Ratio	1.02
Credible Interval	(0.534, 1.95)
Associated Peptides	5
Associated Spectra	7
Coverage	NaN

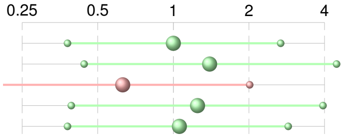

A	B	2.5	50	97.5	Sequence
**1**	**1**	**0.38**	**1**	**2.7**	**IKPPMCISK**
**0**	**1**	**0.44**	**1.4**	**4.5**	**ADADFAINVLR**
**0**	**1**	**0.2**	**0.63**	**2**	**KPLPADQVAAIWEETK**
**0**	**1**	**0.39**	**1.2**	**4**	**LRDEYEVVGDVR**
**0**	**2**	**0.38**	**1.1**	**2.9**	**SADVGTHMLNELLK**

5.755 7 231 minus strand AGAP006879-PA [Anopheles gambiae str. PEST] gi

Protein Accession	Unigene16066$_{H}1$
Mean Expression Ratio	1.02
Median Expression Ratio	1.02
Credible Interval	(0.432, 2.32)
Associated Peptides	1
Associated Spectra	1
Coverage	NaN

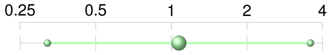

A	B	2.5	50	97.5	Sequence
**0**	**1**	**0.32**	**1.1**	**3.6**	**NVSPLIR**

5.756 62 505 Myosin-2 essential light chain [Crassostrea gigas]

Protein Accession	CL47077.Contig1$_{H}$1
Mean Expression Ratio	1.02
Median Expression Ratio	1.02
Credible Interval	(0.525, 2.02)
Associated Peptides	3
Associated Spectra	11
Coverage	NaN

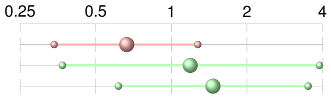

A	B	2.5	50	97.5	Sequence
4	3	0.34	0.66	1.3	IAASQLGDVLR
1	0	0.37	1.2	3.9	SLNQNPTEQEIR
0	3	0.61	1.5	3.5	HILTSLGEK

5.757 284 700 Ganglioside GM2 activator [Crassostrea gigas]

Protein Accession	Unigene16455$_{H}$1
Mean Expression Ratio	1.02
Median Expression Ratio	1.02
Credible Interval	(0.568, 1.87)
Associated Peptides	5
Associated Spectra	18
Coverage	NaN

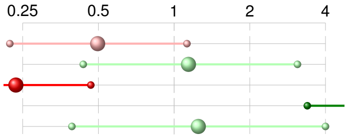

A	B	2.5	50	97.5	Sequence
1	3	0.22	0.5	1.1	ATVDSPLK
1	1	0.44	1.1	3.1	ATVDSPLKAEVYLGK
4	2	0.12	0.23	0.47	AEVYLGK
0	5	3.4	7.1	15	ATVDSPLKAEVYLGKK
0	1	0.39	1.2	4	TFQFTNCLPK

5.758 186 575 minus strand predicted protein [Nematostella vectensis] gi

Protein Accession	CL1734.Contig5$_{H}$1
Mean Expression Ratio	1.02
Median Expression Ratio	1.02
Credible Interval	(0.444, 2.37)
Associated Peptides	1
Associated Spectra	1
Coverage	NaN

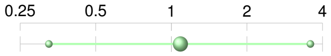

A	B	2.5	50	97.5	Sequence
0	1	0.33	1.1	3.6	EATESDVETAAQAAR

5.759 1 1758 PREDICTED: polyprotein-like [Saccoglossus kowalevskii]

Protein Accession	CL3945.Contig1$_{H}$1
Mean Expression Ratio	1.02
Median Expression Ratio	1.02
Credible Interval	(0.442, 2.36)
Associated Peptides	1
Associated Spectra	1
Coverage	NaN

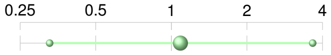

A	B	2.5	50	97.5	Sequence
0	1	0.33	1.1	3.7	ATPHISTGVSPAK

5.760 245 1243 Prostaglandin reductase 1 [Crassostrea gigas]

Protein Accession	CL37890.Contig1$_H$1
Mean Expression Ratio	0.978
Median Expression Ratio	0.979
Credible Interval	(0.428, 2.29)
Associated Peptides	1
Associated Spectra	1
Coverage	NaN

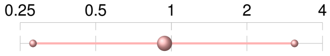

A	B	2.5	50	97.5	Sequence
0	1	0.28	0.94	3.1	TLAVLHPDMPR

5.761 258 680 minus strand synapse-associated protein [Aplysia californica] gi

Protein Accession	CL37287.Contig1$_H$1
Mean Expression Ratio	1.03
Median Expression Ratio	1.02
Credible Interval	(0.452, 2.39)
Associated Peptides	1
Associated Spectra	1
Coverage	NaN

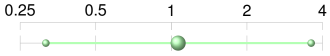

A	B	2.5	50	97.5	Sequence
0	1	0.32	1.1	3.6	ALELLEDYHTR

5.762 89 838 Putative phosphoglycerate mutase [Crassostrea gigas]; 89 838 Putative phosphoglycerate mutase [Crassostrea gigas]; 89 835 Putative phosphoglycerate mutase [Crassostrea gigas]; 89 838 Putative phosphoglycerate mutase [Crassostrea gigas]

Protein Accession	CL6793.Contig6$_H$1 CL6793.Contig5$_H$1 CL6793.Contig4$_H$1 CL6793.Contig3$_H$1
Mean Expression Ratio	0.977
Median Expression Ratio	0.979
Credible Interval	(0.519, 1.81)
Associated Peptides	4
Associated Spectra	11
Coverage	NaN

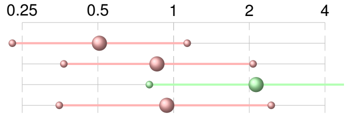

A	B	2.5	50	97.5	Sequence
2	2	0.23	0.51	1.1	EAQYAGELIK
2	1	0.37	0.86	2.1	VLIAAHGNSLR
0	2	0.8	2.1	5.7	HYGGLQGLNKAETAAK
0	2	0.35	0.94	2.4	HGEEQVKIWR

5.763 43 591 minus strand cyclophilin type peptidyl-prolyl cis-trans isomerase [Glossina morsitans morsitans]

Protein Accession	CL39270.Contig1$_H$1
Mean Expression Ratio	1.02
Median Expression Ratio	1.02
Credible Interval	(0.588, 1.79)
Associated Peptides	5
Associated Spectra	29
Coverage	NaN

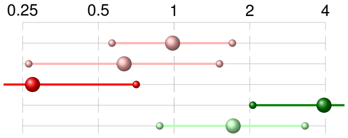

A	B	2.5	50	97.5	Sequence
5	6	0.57	0.98	1.7	ITMELR
1	2	0.26	0.63	1.5	FADENFTLK
2	0	0.1	0.27	0.71	GFGYK
0	7	2.1	4	7.6	KIESYGSKNGATSQK
0	6	0.87	1.7	3.3	VFFDMTADGVPLGR

5.764 204 791 minus strand PREDICTED: 15-hydroxyprostaglandin dehydrogenase [NAD+] [Gallus gallus]

Protein Accession	Unigene3818$_{H}1$
Mean Expression Ratio	0.977
Median Expression Ratio	0.979
Credible Interval	(0.637, 1.49)
Associated Peptides	12
Associated Spectra	108
Coverage	NaN

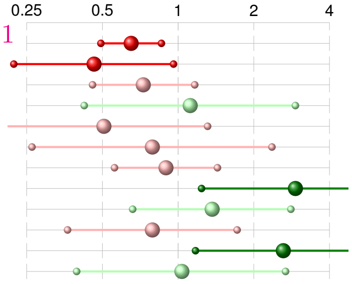

A	B	2.5	50	97.5	Sequence
23	27	0.49	0.65	0.86	AVTEALLAK
1	4	0.22	0.46	0.96	HFISAFEK
9	6	0.46	0.73	1.2	IAFVSGGAMGTGR
1	1	0.42	1.1	2.9	MGTTTDKELK
1	1	0.19	0.51	1.3	VMFCDIDEK
1	0	0.26	0.79	2.4	DITDK
3	12	0.56	0.9	1.4	AITMWGK
0	3	1.2	2.9	7.1	DITDKIAFVSGGAMGTGR
0	5	0.66	1.4	2.8	MDITDKIAFVSGGAMGTGR
0	4	0.36	0.79	1.7	VDIMVNNAEIFSETDMEK
0	4	1.2	2.6	5.7	AVTEALLAKGAK
0	2	0.39	1	2.7	MDITDK

5.765 266 934 minus strand Kyphoscoliosis peptidase [Crassostrea gigas]

Protein Accession	CL7490.Contig16$_{H}1$
Mean Expression Ratio	1.02
Median Expression Ratio	1.02
Credible Interval	(0.459, 2.24)
Associated Peptides	2
Associated Spectra	2
Coverage	NaN

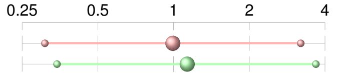

A	B	2.5	50	97.5	Sequence
0	1	0.31	0.99	3.2	DLDNVHFK
0	1	0.34	1.1	3.7	SRPELPHGYLGAQSK

5.766 69 731 hypoxanthine phosphoribosyltransferase 1 [Haliotis discus discus]

Protein Accession	Unigene30480$_{H}1$
Mean Expression Ratio	0.978
Median Expression Ratio	0.98
Credible Interval	(0.458, 2.11)
Associated Peptides	2
Associated Spectra	3
Coverage	NaN

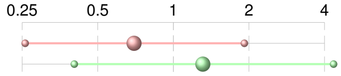

A	B	2.5	50	97.5	Sequence
1	1	0.26	0.7	1.9	TLEAVHPK
0	1	0.4	1.3	4.3	LDKIK

5.767 128 559 minus strand predicted protein [Nematostella vectensis] gi

Protein Accession	CL19343.Contig1$_{H}1$
Mean Expression Ratio	1.02
Median Expression Ratio	1.02
Credible Interval	(0.472, 2.22)
Associated Peptides	1
Associated Spectra	6
Coverage	NaN

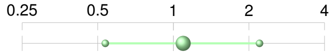

A	B	2.5	50	97.5	Sequence
1	5	0.54	1.1	2.2	TVILGDLTGSK

5.768 " 110 1717 PREDICTED: glutamate dehydrogenase, mitochondrial-like [Oreochromis niloticus]; 110 1717 PREDICTED: glutamate dehydrogenase, mitochondrial-like [Oreochromis niloticus]; 110 1717 PREDICTED: glutamate dehydrogenase, mitochondrial-like [Oreochromis niloticus]"

Protein Accession	CL16377.Contig9$_H$1 CL16377.Contig8$_H$1 CL16377.Contig10$_H$1
Mean Expression Ratio	0.981
Median Expression Ratio	0.98
Credible Interval	(0.6, 1.61)
Associated Peptides	14
Associated Spectra	16
Coverage	NaN

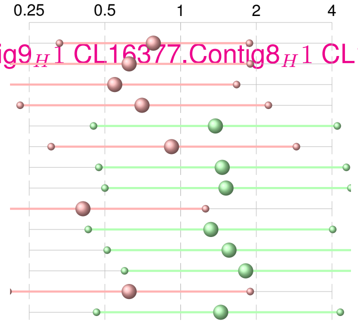

A	B	2.5	50	97.5	Sequence
1	2	0.33	0.78	1.9	DGSIYNPVDGIHPR
1	0	0.2	0.62	1.9	AANIIEK
1	0	0.18	0.55	1.7	FTVELAK
1	0	0.23	0.7	2.2	PFGGAK
0	1	0.45	1.4	4.2	ACVTGKPIPQGGIHGR
0	1	0.31	0.92	2.9	CAIVDVPFGGAK
0	1	0.47	1.5	4.5	CDILVPAAGER
0	1	0.5	1.5	4.8	IAGASEKDIVHSGLEYTMER
0	1	0.13	0.41	1.3	IIAEGANGPTTAGADEIFLK
0	1	0.43	1.3	4	KGFIGPGIDVPAPDMGTGER
0	1	0.51	1.6	4.9	INPANYSENELEK
0	1	0.6	1.8	5.8	LTFKYEK
0	1	0.21	0.62	1.9	EAGLTFT
0	1	0.46	1.4	4.3	DSNFHLLQSVQR

5.769 349 885 juvenile hormone diol kinase [Danaus plexippus]; 349 885 juvenile hormone diol kinase [Danaus plexippus]; 349 885 juvenile hormone diol kinase [Danaus plexippus]; 349 885 minus strand juvenile hormone diol kinase [Danaus plexippus]; 349 885 juvenile hormone diol kinase [Danaus plexippus]; 349 885 juvenile hormone diol kinase [Danaus plexippus]; 349 885 juvenile hormone diol kinase [Danaus plexippus]; 349 885 juvenile hormone diol kinase [Danaus plexippus]; 349 885 juvenile hormone diol kinase [Danaus plexippus]; 349 885 juvenile hormone diol kinase [Danaus plexippus]

Protein Accession	Unigene38920$_H$1 Unigene38919$_H$1 CL7590.Contig1$_H$1 CL7590.Contig16$_H$1 CL7590.Contig
Mean Expression Ratio	1.03
Median Expression Ratio	1.02
Credible Interval	(0.454, 2.38)
Associated Peptides	1
Associated Spectra	1
Coverage	NaN

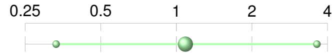

A	B	2.5	50	97.5	Sequence
1	0	0.33	1.1	3.6	IFGNSEADSNK

5.770 21 1007 minus strand cathepsin L2 cysteine protease [Pinctada fucata]; 21 1007 minus strand cathepsin L2 cysteine protease [Pinctada fucata]

Protein Accession	CL7881.Contig4$_{H}$1 CL7881.Contig3$_{H}$1
Mean Expression Ratio	1.02
Median Expression Ratio	1.02
Credible Interval	(0.465, 2.24)
Associated Peptides	2
Associated Spectra	2
Coverage	NaN

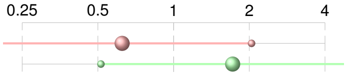

A	B	2.5	50	97.5	Sequence
1	0	0.19	0.63	2	NLDLIIR
1	0	0.52	1.7	5.6	DLPDSVDWR

5.771 369 746 stanniocalcin [Haliotis diversicolor supertexta] gi

Protein Accession	CL10391.Contig1$_{H}$1
Mean Expression Ratio	0.985
Median Expression Ratio	0.981
Credible Interval	(0.441, 2.16)
Associated Peptides	2
Associated Spectra	2
Coverage	NaN

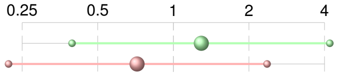

A	B	2.5	50	97.5	Sequence
0	1	0.4	1.3	4.2	VLQLDDLFR
0	1	0.22	0.72	2.4	FARALNNFTSQGQEFLR

5.772 198 821 minus strand sigma class glutathione-s-transferase 2 [Haliotis discus discus]

Protein Accession	CL1352.Contig4$_{H}$1
Mean Expression Ratio	1.02
Median Expression Ratio	1.02
Credible Interval	(0.494, 2.07)
Associated Peptides	3
Associated Spectra	4
Coverage	NaN

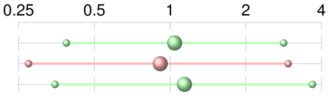

A	B	2.5	50	97.5	Sequence
1	1	0.39	1	2.8	ASWGPDCEAK
0	1	0.27	0.91	2.9	AAGQPFDDNVVDR
0	1	0.35	1.1	3.7	AAPYGGQLPYVIYK

5.773 113 367 hypothetical protein DAPPUDRAFT_329128 [Daphnia pulex]; 113 367 hypothetical protein DAPPUDRAFT_329128 [Daphnia pulex]; 113 367 hypothetical protein DAPPUDRAFT_329128 [Daphnia pulex]

Protein Accession	CL9011.Contig3$_{H}$1 CL9011.Contig2$_{H}$1 CL9011.Contig1$_{H}$1
Mean Expression Ratio	1.02
Median Expression Ratio	1.02
Credible Interval	(0.492, 2.08)
Associated Peptides	3
Associated Spectra	5
Coverage	NaN

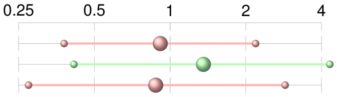

A	B	2.5	50	97.5	Sequence
2	1	0.38	0.91	2.2	KPMVSGAIIK
0	1	0.41	1.4	4.3	ILAIEDYIR
0	1	0.27	0.88	2.9	LHPEDFPEEEPK

5.774 14 277 minus strand Histidine triad nucleotide-binding protein 1 OS=Oryctolagus cuniculus GN=HINT1 PE=1 SV=2

Protein Accession	CL25563.Contig1$_{H}1$
Mean Expression Ratio	0.982
Median Expression Ratio	0.981
Credible Interval	(0.463, 2.1)
Associated Peptides	2
Associated Spectra	3
Coverage	NaN

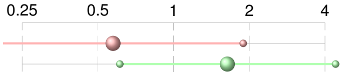

A	B	2.5	50	97.5	Sequence
1	0	0.17	0.57	1.9	LAEAVEADEK
0	2	0.61	1.6	4.4	AHLAKPGGDTIFGK

5.775 " 34 1656 Chaperonin containing TCP1, subunit 7 (eta) [Danio rerio] gi; 34 1656 Chaperonin containing TCP1, subunit 7 (eta) [Danio rerio] gi"

Protein Accession	CL24061.Contig2$_{H}1$ CL24061.Contig1$_{H}1$
Mean Expression Ratio	0.983
Median Expression Ratio	0.982
Credible Interval	(0.527, 1.86)
Associated Peptides	5
Associated Spectra	8
Coverage	NaN

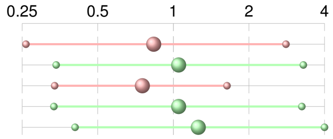

A	B	2.5	50	97.5	Sequence
1	0	0.26	0.84	2.8	TLDIVHPAAK
1	0	0.34	1.1	3.3	TLVDIAR
1	3	0.34	0.75	1.6	SIAVSIKK
0	1	0.33	1	3.2	SLHDAIMIVR
0	1	0.41	1.3	4	MQAPIILLK

5.776 87 1454 hypothetical protein BRAFLDRAFT_287788 [Branchiostoma floridae] gi; 117 1469 hypothetical protein BRAFLDRAFT_287788 [Branchiostoma floridae] gi; 117 1469 hypothetical protein BRAFLDRAFT_287788 [Branchiostoma floridae] gi

Protein Accession	Unigene48935$_{H}1$ Unigene21344$_{H}1$ CL945.Contig6$_{H}1$
Mean Expression Ratio	0.981
Median Expression Ratio	0.982
Credible Interval	(0.597, 1.61)
Associated Peptides	9
Associated Spectra	29
Coverage	NaN

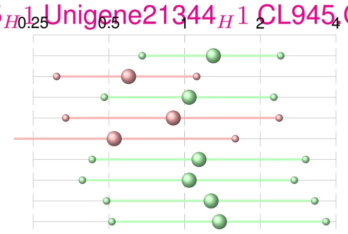

A	B	2.5	50	97.5	Sequence
4	3	0.68	1.3	2.4	ISSLDNGLR
3	4	0.31	0.6	1.1	RIPLPELEAR
2	2	0.48	1	2.3	TILGPTQNIR
1	1	0.34	0.9	2.4	FTGSEIR
1	0	0.17	0.52	1.6	IDAVNAK
0	2	0.43	1.1	3	AATLEQTILNVPETR
0	2	0.39	1	2.7	MVLAAAGGVNHGELVDLAK
0	2	0.49	1.3	3.3	SAMYWLR
0	2	0.51	1.4	3.7	EQTVYYAK

5.777 97 597 glyoxylate reductase/hydroxypyruvate reductase [Ictalurus punctatus] gi

Protein Accession	CL18211.Contig2$_{H}1$
Mean Expression Ratio	0.98
Median Expression Ratio	0.982
Credible Interval	(0.434, 2.21)
Associated Peptides	1
Associated Spectra	2
Coverage	NaN

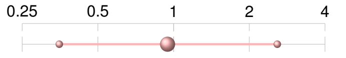

A	B	2.5	50	97.5	Sequence
**0**	**2**	**0.35**	**0.95**	**2.6**	**KIPAIGIDLLTR**

5.778 2 478 Glyoxylate reductase/hydroxypyruvate reductase [Crassostrea gigas]

Protein Accession	CL29479.Contig5$_{H}1$
Mean Expression Ratio	0.985
Median Expression Ratio	0.983
Credible Interval	(0.465, 2.08)
Associated Peptides	3
Associated Spectra	3
Coverage	NaN

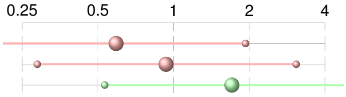

A	B	2.5	50	97.5	Sequence
**1**	**0**	**0.18**	**0.59**	**1.9**	**VGFGVAR**
**0**	**1**	**0.29**	**0.93**	**3.1**	**GSIVDQDALDEALK**
**0**	**1**	**0.53**	**1.7**	**5.4**	**MKEGSILINTAR**

5.779 1867 3108 minus strand roundabout homolog 1 precursor [Rattus norvegicus] gi; 1867 3108 minus strand roundabout homolog 1 precursor [Rattus norvegicus] gi; 1835 2956 minus strand roundabout homolog 1 precursor [Rattus norvegicus] gi; 1867 3108 minus strand roundabout homolog 1 precursor [Rattus norvegicus] gi

Protein Accession	CL2792.Contig8$_{H}1$ CL2792.Contig5$_{H}1$ CL2792.Contig11$_{H}1$ CL2792.Contig10$_{H}1$
Mean Expression Ratio	0.981
Median Expression Ratio	0.984
Credible Interval	(0.425, 2.26)
Associated Peptides	1
Associated Spectra	1
Coverage	NaN

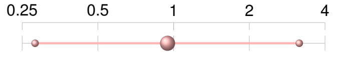

A	B	2.5	50	97.5	Sequence
**0**	**1**	**0.28**	**0.95**	**3.2**	**VAHLDRVFQQEPVR**

5.780 9 326 minus strand catalase [Paracyclopina nana]; 9 209 Catalase OS=Cavia porcellus GN=CAT PE=2 SV=4

Protein Accession	CL6745.Contig2$_H$1 CL6745.Contig1$_H$1
Mean Expression Ratio	1.02
Median Expression Ratio	1.02
Credible Interval	(0.46, 2.24)
Associated Peptides	1
Associated Spectra	3
Coverage	NaN

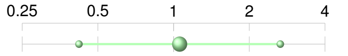

A	B	2.5	50	97.5	Sequence
**1**	**2**	**0.42**	**1.1**	**2.7**	**LATMTVGPR**

5.781 " 115 1104 minus strand PREDICTED: KH domain-containing, RNA-binding, signal transduction-associated protein 3 [Monodelphis domestica]"

Protein Accession	Unigene26700$_H$1
Mean Expression Ratio	0.983
Median Expression Ratio	0.984
Credible Interval	(0.482, 2.03)
Associated Peptides	3
Associated Spectra	4
Coverage	NaN

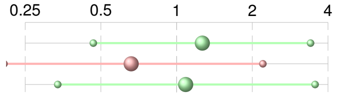

A	B	2.5	50	97.5	Sequence
**1**	**1**	**0.47**	**1.3**	**3.4**	**LLAQEIER**
**1**	**0**	**0.21**	**0.66**	**2.2**	**GRGGGVP**
**0**	**1**	**0.34**	**1.1**	**3.6**	**LQEETGCK**

5.782 11 1462 coronin [Schistosoma mansoni]

Protein Accession	CL11779.Contig2$_H$1
Mean Expression Ratio	0.985
Median Expression Ratio	0.985
Credible Interval	(0.452, 2.16)
Associated Peptides	2
Associated Spectra	2
Coverage	NaN

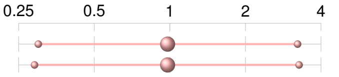

A	B	2.5	50	97.5	Sequence
**0**	**1**	**0.3**	**0.98**	**3.2**	**VPMDAPVVAGHK**
**0**	**1**	**0.29**	**0.98**	**3.3**	**HVFGKPEK**

5.783 132 590 minus strand Non-neuronal cytoplasmic intermediate filament protein OS=Helix aspersa PE=3 SV=2

Protein Accession	Unigene7770$_H$1
Mean Expression Ratio	1.02
Median Expression Ratio	1.02
Credible Interval	(0.476, 2.17)
Associated Peptides	2
Associated Spectra	4
Coverage	NaN

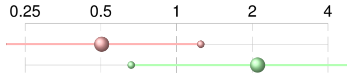

A	B	2.5	50	97.5	Sequence
**2**	**1**	**0.2**	**0.5**	**1.3**	**TMAEVPLGEK**
**0**	**1**	**0.66**	**2.1**	**6.8**	**VTKEVSGK**

5.784 174 1556 minus strand aspartyl-tRNA synthetase [Xenopus laevis] gi; 174 1721 minus strand aspartyl-tRNA synthetase [Xenopus laevis] gi

Protein Accession	CL9865.Contig2$_{H}$1 CL9865.Contig1$_{H}$1
Mean Expression Ratio	0.985
Median Expression Ratio	0.985
Credible Interval	(0.47, 2.06)
Associated Peptides	1
Associated Spectra	7
Coverage	NaN

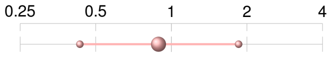

A	B	2.5	50	97.5	Sequence
2	5	0.43	0.89	1.8	IHDPDFLSQR

5.785 " 103 981 PREDICTED: ATP synthase, H+ transporting, mitochondrial F1 complex, gamma subunit isoform H (heart)-like [Saccoglossus kowalevskii]"

Protein Accession	CL25149.Contig1$_{H}$1
Mean Expression Ratio	0.984
Median Expression Ratio	0.985
Credible Interval	(0.564, 1.72)
Associated Peptides	8
Associated Spectra	14
Coverage	NaN

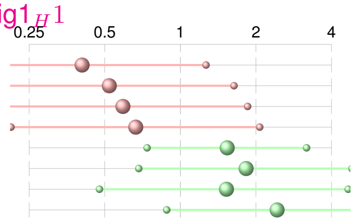

A	B	2.5	50	97.5	Sequence
1	0	0.13	0.41	1.3	LIVLGDK
1	0	0.17	0.52	1.6	NAGEMISK
1	0	0.19	0.59	1.9	AILGEK
1	0	0.21	0.67	2.1	AFFEK
1	4	0.73	1.5	3.2	LTLTFNR
0	2	0.68	1.8	4.8	ELKPARPYGAGAK
0	1	0.48	1.5	4.7	LIVLGDKAR
0	2	0.88	2.4	6.4	ATLKDIR

5.786 187 1563 odr-4-like protein [Crassostrea gigas]

Protein Accession	CL15156.Contig3$_{H}$1
Mean Expression Ratio	0.989
Median Expression Ratio	0.987
Credible Interval	(0.429, 2.3)
Associated Peptides	1
Associated Spectra	1
Coverage	NaN

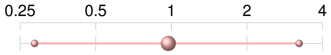

A	B	2.5	50	97.5	Sequence
0	1	0.29	0.97	3.2	TLDAADELWVATHAK

5.787 49 1038 Glyceraldehyde-3-phosphate dehydrogenase [Crotalus adamanteus]

Protein Accession	Unigene7744$_{H}$1
Mean Expression Ratio	0.989
Median Expression Ratio	0.988
Credible Interval	(0.568, 1.73)
Associated Peptides	6
Associated Spectra	23
Coverage	NaN

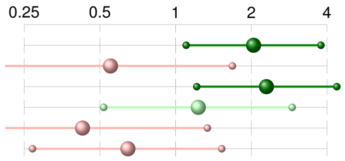

A	B	2.5	50	97.5	Sequence
2	6	1.1	2	3.8	TVDGPSNKDWR
1	0	0.17	0.55	1.7	AGIQLSDKFVK
1	6	1.2	2.3	4.4	SSIFDAK
0	3	0.52	1.2	2.9	GAYQNIIPSSTGAAK
0	1	0.14	0.43	1.3	FGIEEALMTTVHSATATQK
0	3	0.27	0.65	1.5	VIPALNGK

5.788 1027 1440 minus strand hemocyanin isoform 1 [Haliotis diversicolor]

Protein Accession	CL40281.Contig1$_{H}1$
Mean Expression Ratio	0.989
Median Expression Ratio	0.988
Credible Interval	(0.456, 2.15)
Associated Peptides	1
Associated Spectra	10
Coverage	NaN

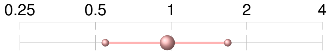

A	B	2.5	50	97.5	Sequence
0	10	0.55	0.97	1.7	LFVVQVEDELK

5.789 197 1132 minus strand Pancreatic lipase-related protein 2 [Crassostrea gigas]

Protein Accession	CL8259.Contig1$_{H}1$
Mean Expression Ratio	1.01
Median Expression Ratio	1.01
Credible Interval	(0.462, 2.19)
Associated Peptides	1
Associated Spectra	7
Coverage	NaN

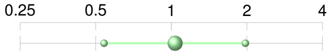

A	B	2.5	50	97.5	Sequence
0	7	0.54	1	2	ITGLDPAGPK

5.790 424 3507 minus strand putative Na+/K+-ATPase alpha subunit [Paroctopus digueti]; 424 3507 minus strand putative Na+/K+-ATPase alpha subunit [Paroctopus digueti]

Protein Accession	Unigene11494$_{H}1$ Unigene11493$_{H}1$
Mean Expression Ratio	1.01
Median Expression Ratio	1.01
Credible Interval	(0.641, 1.62)
Associated Peptides	16
Associated Spectra	24
Coverage	NaN

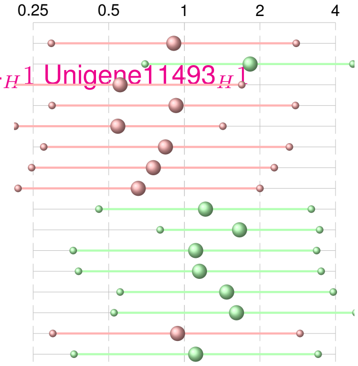

A	B	2.5	50	97.5	Sequence
1	0	0.3	0.91	2.8	AAVIHGGDLR
2	0	0.7	1.8	4.7	GIPVEEVDPREAK
1	0	0.18	0.56	1.7	KVVEIPFNSTNK
1	0	0.3	0.93	2.8	SAEFTHENPLETR
1	1	0.21	0.54	1.4	VIVAHGFK
1	0	0.28	0.84	2.6	GVGIISEGSK
1	0	0.25	0.76	2.3	AENDIMK
0	1	0.22	0.66	2	AEELVLGDVVEVK
0	2	0.46	1.2	3.2	DMTPAQIDEILR
0	5	0.8	1.7	3.5	FGDRVPADLR
0	1	0.36	1.1	3.4	GIPVEEVDPR
0	1	0.38	1.2	3.5	NHSEIVFAR
0	2	0.55	1.5	3.9	VIMVTGDHPITAK
0	1	0.53	1.6	4.8	AKEILLR
0	1	0.3	0.94	2.9	NLDELK
0	1	0.36	1.1	3.4	YLLVMK

5.791 158 1318 hypothetical protein BRAFLDRAFT_98992 [Branchiostoma floridae] gi

Protein Accession	CL14371.Contig5$_{H}$1
Mean Expression Ratio	1.01
Median Expression Ratio	1.01
Credible Interval	(0.455, 2.2)
Associated Peptides	2
Associated Spectra	2
Coverage	NaN

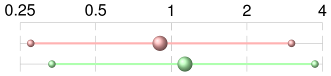

A	B	2.5	50	97.5	Sequence
**0**	**1**	**0.28**	**0.9**	**3**	**AFTGFIVDGDSK**
**0**	**1**	**0.34**	**1.1**	**3.7**	**LAVMR**

5.792 17 703 Dihydropteridine reductase [Crassostrea gigas]

Protein Accession	CL125.Contig5$_{H}$1
Mean Expression Ratio	0.987
Median Expression Ratio	0.989
Credible Interval	(0.467, 2.1)
Associated Peptides	2
Associated Spectra	4
Coverage	NaN

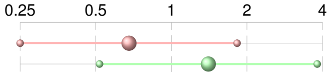

A	B	2.5	50	97.5	Sequence
**1**	**1**	**0.25**	**0.68**	**1.8**	**VAEALGEEK**
**0**	**2**	**0.52**	**1.4**	**3.8**	**AAVHHLVQSLGAPK**

5.793 " 101 598 PREDICTED: aminomethyltransferase, mitochondrial-like [Oreochromis niloticus]"

Protein Accession	CL10455.Contig5$_{H}$1
Mean Expression Ratio	1.01
Median Expression Ratio	1.01
Credible Interval	(0.446, 2.33)
Associated Peptides	1
Associated Spectra	2
Coverage	NaN

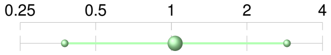

A	B	2.5	50	97.5	Sequence
**1**	**1**	**0.38**	**1**	**2.9**	**TTITEEHLQVR**

5.794 3 272 minus strand ADP/ATP carrier [Clonorchis sinensis]

Protein Accession	Unigene4627$_{H}$1
Mean Expression Ratio	1.01
Median Expression Ratio	1.01
Credible Interval	(0.446, 2.35)
Associated Peptides	1
Associated Spectra	1
Coverage	NaN

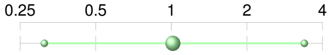

A	B	2.5	50	97.5	Sequence
**0**	**1**	**0.31**	**1**	**3.4**	**MMMTSGQAVK**

5.795 95 1870 PREDICTED: acetylcholinesterase-like [Equus caballus]; 99 1874 PREDICTED: acetylcholinesterase-like [Equus caballus]

Protein Accession	CL10104.Contig9$_H$1 CL10104.Contig10$_H$1
Mean Expression Ratio	0.987
Median Expression Ratio	0.99
Credible Interval	(0.451, 2.13)
Associated Peptides	2
Associated Spectra	2
Coverage	NaN

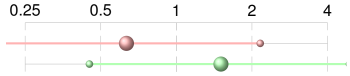

A	B	2.5	50	97.5	Sequence
**1**	**0**	**0.19**	**0.63**	**2.2**	**SFGNEPTEIK**
**0**	**1**	**0.45**	**1.5**	**4.9**	**VDGDFLPDDPAR**

5.796 " 698 1225 minus strand actin related protein 2/3 complex, 21 kDa subunit [Crassostrea gigas]"

Protein Accession	CL17389.Contig2$_H$1
Mean Expression Ratio	1
Median Expression Ratio	1.01
Credible Interval	(0.443, 2.23)
Associated Peptides	1
Associated Spectra	2
Coverage	NaN

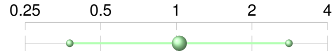

A	B	2.5	50	97.5	Sequence
**0**	**2**	**0.38**	**1**	**2.8**	**ANIFFR**

5.797 238 687 60S ribosomal protein L6 [Crassostrea gigas]; 142 789 60S ribosomal protein L6 [Crassostrea gigas]

Protein Accession	Unigene30349$_H$1 CL6501.Contig1$_H$1
Mean Expression Ratio	0.993
Median Expression Ratio	0.99
Credible Interval	(0.442, 2.28)
Associated Peptides	1
Associated Spectra	2
Coverage	NaN

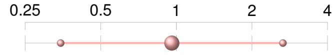

A	B	2.5	50	97.5	Sequence
**0**	**2**	**0.35**	**0.96**	**2.7**	**AVDTQLLEVIR**

5.798 587 1081 hypothetical protein CGI_10026502 [Crassostrea gigas]

Protein Accession	Unigene22889$_H$1
Mean Expression Ratio	0.993
Median Expression Ratio	0.99
Credible Interval	(0.429, 2.28)
Associated Peptides	1
Associated Spectra	1
Coverage	NaN

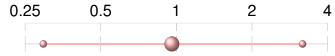

A	B	2.5	50	97.5	Sequence
**1**	**0**	**0.29**	**0.96**	**3.2**	**LRRTLLGS**

5.799 552 1499 minus strand uncharacterized protein LOC100158466 [Xenopus (Silurana) tropicalis] gi; 552 1499 minus strand uncharacterized protein LOC100158466 [Xenopus (Silurana) tropicalis] gi; 552 1499 minus strand uncharacterized protein LOC100158466 [Xenopus (Silurana) tropicalis] gi

Protein Accession	Unigene57645$_{H}1$ Unigene52825$_{H}1$ Unigene48504$_{H}1$
Mean Expression Ratio	1.01
Median Expression Ratio	1.01
Credible Interval	(0.437, 2.33)
Associated Peptides	1
Associated Spectra	1
Coverage	NaN

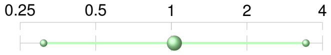

A	B	2.5	50	97.5	Sequence
**0**	**1**	**0.31**	**1**	**3.4**	**TGEPVEVDGELATLLAR**

5.800 18 242 minus strand translation elongation factor 1B gamma subunit [Strongylocentrotus purpuratus] gi

Protein Accession	CL14269.Contig2$_{H}1$
Mean Expression Ratio	1.01
Median Expression Ratio	1.01
Credible Interval	(0.432, 2.29)
Associated Peptides	1
Associated Spectra	1
Coverage	NaN

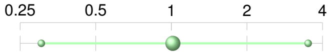

A	B	2.5	50	97.5	Sequence
**1**	**0**	**0.3**	**1**	**3.5**	**ALIAAK**

5.801 85 681 minus strand 40S ribosomal protein S8 OS=Xenopus laevis GN=rps8 PE=2 SV=3

Protein Accession	Unigene34347$_{H}1$
Mean Expression Ratio	0.991
Median Expression Ratio	0.991
Credible Interval	(0.435, 2.32)
Associated Peptides	1
Associated Spectra	1
Coverage	NaN

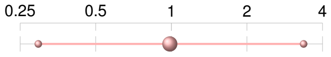

A	B	2.5	50	97.5	Sequence
**0**	**1**	**0.3**	**0.98**	**3.4**	**VEPALDEQFSSGR**

5.802 36 1310 minus strand elongation factor-1 gamma [Diguetia canities]

Protein Accession	CL14269.Contig5$_{H}1$
Mean Expression Ratio	1.01
Median Expression Ratio	1.01
Credible Interval	(0.579, 1.74)
Associated Peptides	9
Associated Spectra	14
Coverage	NaN

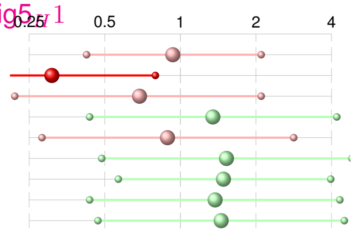

A	B	2.5	50	97.5	Sequence
1	3	0.42	0.93	2.1	LAETMAQFDAK
2	0	0.12	0.31	0.8	TYLVGEK
1	0	0.22	0.69	2.1	FVGNK
0	1	0.43	1.4	4.2	YSGADVQVDPNFK
0	1	0.28	0.89	2.8	GNFDMDEWK
0	1	0.49	1.5	4.8	GNFDMDEWKR
0	2	0.57	1.5	4	SADFLAK
0	1	0.43	1.4	4.3	ESKDPFLAFPK
0	1	0.47	1.5	4.5	LDPDDPETK

5.803 835 1116 tropomyosin [Ilyanassa obsoleta]

Protein Accession	CL2925.Contig8$_{H}1$
Mean Expression Ratio	1.01
Median Expression Ratio	1.01
Credible Interval	(0.596, 1.68)
Associated Peptides	10
Associated Spectra	23
Coverage	NaN

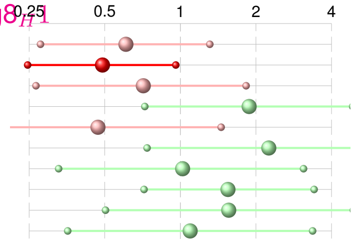

A	B	2.5	50	97.5	Sequence
1	3	0.28	0.61	1.3	LEDVETELR
2	4	0.25	0.49	0.96	QLELYEEK
1	1	0.27	0.71	1.8	ELEEQSHALTNSLK
0	2	0.72	1.9	4.9	KLAVMEAELEKAEDR
0	1	0.16	0.47	1.5	LAVMEAELEK
0	1	0.74	2.3	7.1	LYAGDERLEDVETELR
0	1	0.33	1	3.1	VRELEEQSHALTNSLK
0	4	0.72	1.6	3.4	KLAVMEAELEKAEDRADSSERR
0	1	0.5	1.6	4.9	QAAQEWEARYEEAAR
0	1	0.36	1.1	3.4	KLAVMEAELEKAEDRADSSER

5.804 244 5451 alpha macroglobulin [Chlamys farreri]

Protein Accession	CL20671.Contig5$_{H}1$
Mean Expression Ratio	1.01
Median Expression Ratio	1.01
Credible Interval	(0.436, 2.31)
Associated Peptides	1
Associated Spectra	1
Coverage	NaN

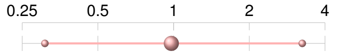

A	B	2.5	50	97.5	Sequence
0	1	0.31	0.98	3.3	HLALDVEQLNR

5.805 145 972 LIM and SH3 domain protein F42H10.3 OS=Caenorhabditis elegans GN=F42H10.3 PE=2 SV=3

Protein Accession	CL49642.Contig8$_{H}1$
Mean Expression Ratio	1
Median Expression Ratio	1.01
Credible Interval	(0.434, 2.35)
Associated Peptides	1
Associated Spectra	1
Coverage	NaN

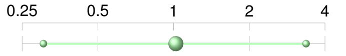

A	B	2.5	50	97.5	Sequence
0	1	0.3	1	3.4	TVYPTEELK

5.806 131 544 hypothetical protein CGI_10001749 [Crassostrea gigas]

Protein Accession	CL36022.Contig1$_{H}1$
Mean Expression Ratio	1.01
Median Expression Ratio	1.01
Credible Interval	(0.429, 2.37)
Associated Peptides	1
Associated Spectra	1
Coverage	NaN

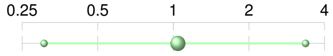

A	B	2.5	50	97.5	Sequence
0	1	0.31	1	3.4	LIVADGTNHR

5.807 1 111 glycogen synthase [Crassostrea gigas]

Protein Accession	Unigene78448$_{H}1$
Mean Expression Ratio	0.998
Median Expression Ratio	0.995
Credible Interval	(0.432, 2.31)
Associated Peptides	1
Associated Spectra	1
Coverage	NaN

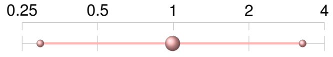

A	B	2.5	50	97.5	Sequence
0	1	0.3	0.99	3.3	NVEPDLDDDYLDR

5.808 157 459 minus strand Synaptojanin-2-binding protein [Crassostrea gigas]

Protein Accession	CL12494.Contig1$_{H}1$
Mean Expression Ratio	0.995
Median Expression Ratio	0.995
Credible Interval	(0.567, 1.75)
Associated Peptides	7
Associated Spectra	23
Coverage	NaN

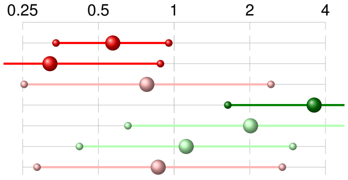

A	B	2.5	50	97.5	Sequence
5	7	0.34	0.57	0.96	GDSGLGFNIR
2	0	0.12	0.32	0.88	LKEDGAAFK
0	1	0.25	0.78	2.4	VQHGAYAELMK
0	4	1.6	3.6	7.8	LKEGDKILEVNGK
0	1	0.66	2	6.4	LKEDGAAFKDGR
0	2	0.42	1.1	3	GGTDIPHVPGDTGIFVTR
0	1	0.28	0.87	2.7	ILEVNGK

5.809 362 874 minus strand Transgelin-2 [Crassostrea gigas]

Protein Accession	CL445.Contig3$_{H}1$
Mean Expression Ratio	0.997
Median Expression Ratio	0.996
Credible Interval	(0.594, 1.66)
Associated Peptides	8
Associated Spectra	31
Coverage	NaN

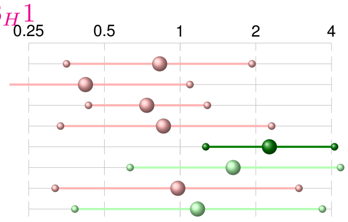

A	B	2.5	50	97.5	Sequence
2	1	0.35	0.83	1.9	KAGLGAEIEK
2	0	0.16	0.42	1.1	LLESEGKPK
6	5	0.43	0.74	1.3	AGLGAEIEK
0	2	0.33	0.86	2.3	AKKAGLGAEIEK
0	9	1.3	2.3	4.1	LINFD
0	2	0.63	1.6	4.3	LINKLLESEGKPK
0	1	0.32	0.98	3	SLGFLANSK
0	1	0.38	1.2	3.7	GFQPSYTGSQSR

5.810 293 1795 Amidase [Crassostrea gigas]

Protein Accession	CL5248.Contig6$_{H}1$
Mean Expression Ratio	0.993
Median Expression Ratio	0.996
Credible Interval	(0.509, 1.93)
Associated Peptides	4
Associated Spectra	7
Coverage	NaN

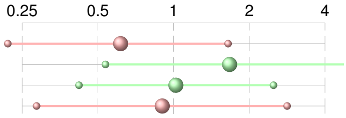

A	B	2.5	50	97.5	Sequence
1	1	0.22	0.61	1.7	EVSVPMHK
0	1	0.53	1.7	5.6	ILDAGGHIMGK
0	3	0.42	1	2.5	LSTIGVTHFDR
0	1	0.29	0.9	2.8	YAHPQDLHPMIK

5.811 54 1406 minus strand Protein henna [Crassostrea gigas]; 54 1406 minus strand Protein henna [Crassostrea gigas]

Protein Accession	CL24330.Contig4$_{H}1$ CL24330.Contig1$_{H}1$
Mean Expression Ratio	1
Median Expression Ratio	1
Credible Interval	(0.488, 2.03)
Associated Peptides	3
Associated Spectra	4
Coverage	NaN

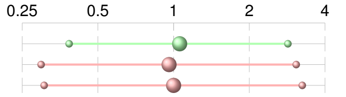

A	B	2.5	50	97.5	Sequence
0	2	0.38	1.1	2.9	GDLSLLEDALK
0	1	0.3	0.96	3.1	TLHLQILSR
0	1	0.31	1	3.3	VSAYAATIPRPFSV

5.812 87 485 Sex-regulated protein janus-A [Crassostrea gigas]

Protein Accession	CL13131.Contig3$_{H}1$
Mean Expression Ratio	1
Median Expression Ratio	0.998
Credible Interval	(0.436, 2.32)
Associated Peptides	1
Associated Spectra	1
Coverage	NaN

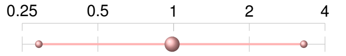

A	B	2.5	50	97.5	Sequence
0	1	0.29	0.99	3.3	VVGEIESK

5.813 " 112 1320 Aspartate aminotransferase, cytoplasmic [Crassostrea gigas]"

Protein Accession	CL44136.Contig1$_{H}1$
Mean Expression Ratio	1
Median Expression Ratio	0.998
Credible Interval	(0.576, 1.72)
Associated Peptides	10
Associated Spectra	12
Coverage	NaN

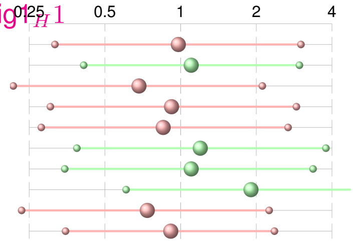

A	B	2.5	50	97.5	Sequence
1	0	0.32	0.98	3	SQMEIIVR
1	1	0.41	1.1	3	ASIFADVK
1	0	0.22	0.68	2.1	LAAEFLK
0	1	0.3	0.92	2.9	IGNLCIVTK
0	1	0.28	0.85	2.7	TLANALILGK
0	1	0.39	1.2	3.8	VNLGVGAYR
0	1	0.35	1.1	3.4	IADVMEAK
0	1	0.61	1.9	5.9	LAAEFLKR
0	1	0.23	0.74	2.3	ISMAGVTNK
0	2	0.35	0.91	2.4	VEGIQVQGGTGGIR

5.814 7825 8754 minus strand H2 [Haliotis tuberculata]

Protein Accession	CL7906.Contig3$_H$1
Mean Expression Ratio	0.991
Median Expression Ratio	0.998
Credible Interval	(0.441, 2.22)
Associated Peptides	1
Associated Spectra	2
Coverage	NaN

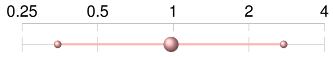

A	B	2.5	50	97.5	Sequence
**0**	**2**	**0.35**	**0.98**	**2.7**	**ALPSLFTDPYDSNPFK**

5.815 356 820 minus strand calmodulin-1 [Trichinella spiralis] gi

Protein Accession	CL672.Contig3$_H$1
Mean Expression Ratio	1
Median Expression Ratio	0.998
Credible Interval	(0.495, 2.05)
Associated Peptides	3
Associated Spectra	9
Coverage	NaN

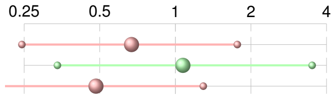

A	B	2.5	50	97.5	Sequence
**0**	**2**	**0.24**	**0.67**	**1.8**	**ADQLTEEQIAEFK**
**0**	**1**	**0.34**	**1.1**	**3.5**	**VFDKDGNGFISAAELR**
**5**	**1**	**0.18**	**0.48**	**1.3**	**ELGTVMR**

5.816 " 133 1077 PREDICTED: electron transfer flavoprotein, alpha polypeptide-like isoform 2 [Saccoglossus kowalevskii]"

Protein Accession	CL6589.Contig3$_H$1
Mean Expression Ratio	1
Median Expression Ratio	0.999
Credible Interval	(0.494, 2.08)
Associated Peptides	3
Associated Spectra	4
Coverage	NaN

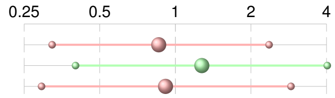

A	B	2.5	50	97.5	Sequence
**0**	**2**	**0.32**	**0.86**	**2.4**	**AAVDAGFVSNDLQIGQTGK**
**0**	**1**	**0.4**	**1.3**	**4**	**SDRPELASANIVIAGGR**
**0**	**1**	**0.29**	**0.91**	**2.9**	**GTAFEAAK**

5.817 116 1321 PREDICTED: similar to aspartate aminotransferase [Tribolium castaneum] gi

Protein Accession	CL11692.Contig6$_H$1
Mean Expression Ratio	1
Median Expression Ratio	1
Credible Interval	(0.625, 1.58)
Associated Peptides	13
Associated Spectra	28
Coverage	NaN

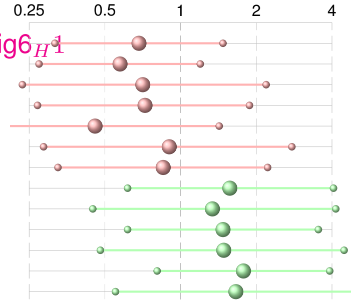

A	B	2.5	50	97.5	Sequence
2	2	0.32	0.68	1.5	ILSTPALR
3	2	0.27	0.57	1.2	ISVAGLSSK
1	0	0.24	0.71	2.2	NMGLYGER
1	1	0.27	0.72	1.9	DAFALR
1	0	0.15	0.46	1.4	TQWLGEVK
1	0	0.29	0.9	2.8	DDNGKPYVLPSVLK
1	1	0.32	0.85	2.2	DGHQVALAQSFAK
0	2	0.62	1.6	4.1	AEKEIIDAK
0	1	0.45	1.3	4.1	MDKEYAGITGVPEFTK
0	3	0.62	1.5	3.5	VGAFTIICDNKDEAAR
0	1	0.48	1.5	4.5	NVGYLAK
0	4	0.8	1.8	3.9	IGAAFLAK
0	1	0.55	1.7	5.1	VMSQVK

5.818 " 2 433 Vesicle-fusing ATPase, partial [Ascaris suum]"

Protein Accession	CL20981.Contig2$_H$1
Mean Expression Ratio	1
Median Expression Ratio	0.999
Credible Interval	(0.436, 2.32)
Associated Peptides	1
Associated Spectra	1
Coverage	NaN

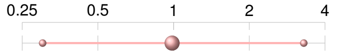

A	B	2.5	50	97.5	Sequence
1	0	0.3	0.99	3.3	LENSNLNLIGK

5.819 6 317 myosin heavy chain cardiac muscle specific isoform 2 [Argopecten irradians]

Protein Accession	Unigene19023$_H$1
Mean Expression Ratio	1
Median Expression Ratio	1
Credible Interval	(0.667, 1.5)
Associated Peptides	15
Associated Spectra	56
Coverage	NaN

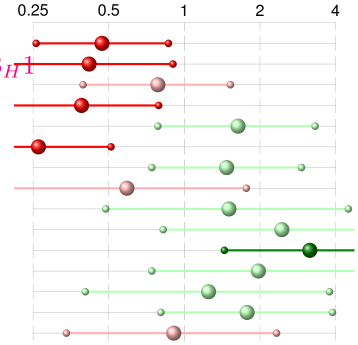

A	B	2.5	50	97.5	Sequence
6	2	0.26	0.47	0.86	ALETQLK
2	2	0.19	0.42	0.9	ELEMELDSEQR
3	3	0.4	0.78	1.5	KALETQLK
2	3	0.19	0.39	0.79	LDEAEAQALK
1	4	0.79	1.6	3.3	LDEAEAQALKGGK
6	0	0.13	0.26	0.51	LADELR
1	5	0.74	1.5	2.9	LADELRQEQEHSLQVEK
0	1	0.2	0.59	1.8	ELAFQSDEDR
0	1	0.49	1.5	4.5	ELEMELDSEQRR
0	1	0.82	2.5	7.4	KALETQLKDMQAR
0	4	1.4	3.2	6.9	KIIQKLEQR
0	2	0.74	2	5	LADELRQEQEHSLQVEKLR
0	1	0.4	1.2	3.8	VRELEMELDSEQR
0	4	0.8	1.8	3.9	VRELEMELDSEQRR
0	2	0.34	0.91	2.3	LKELAFQSDEDRK

5.820 98 1012 minus strand RecName: Full=Substrate-specific endoprotease Tex31; Flags: Precursor gi|32187775|emb|CAD36507.1| substrate-specific endoprotease [Conus textile]

Protein Accession	CL13681.Contig1$_H$1
Mean Expression Ratio	0.997
Median Expression Ratio	1
Credible Interval	(0.428, 2.33)
Associated Peptides	1
Associated Spectra	1
Coverage	NaN

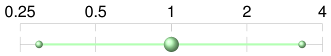

A	B	2.5	50	97.5	Sequence
**0**	**1**	**0.3**	**1**	**3.3**	**NIPLSASDISAIVER**

5.821 " 174 569 NADH dehydrogenase [ubiquinone] iron-sulfur protein 4, mitochondrial [Crassostrea gigas]"

Protein Accession	CL4564.Contig1$_H$1
Mean Expression Ratio	1
Median Expression Ratio	1
Credible Interval	(0.433, 2.37)
Associated Peptides	1
Associated Spectra	1
Coverage	NaN

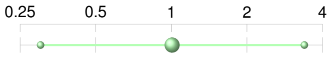

A	B	2.5	50	97.5	Sequence
**0**	**1**	**0.3**	**1**	**3.4**	**VDISCLTGVPEEHIK**

iTRAQ Data Analysis Report

Javier, Body 28d versus control

April 7, 2014

1 Introduction

This document summarizes an analysis of relative protein expression using iTRAQ. The reporter ion peak area measurements supplied by the ABI software are used to estimate treatment-dependent peptide and protein relative expression. Estimation is accomplished using a Bayesian approach with the model given below. The document includes a protein relative expression summary and a per-protein detailed analysis. The document is internally hyperlinked and linked externally to NCBI.

2 Experiment and Model Description

2.1 Experiment Design

The report summarizes data from one or more iTRAQ experiments addressing a common comparison. The experiment design, used in this analysis, is given in the table below.

	Experiment	Treatment	Channel	Sample
1	A	control	113	s1
2	A	test	117	s1
3	B	control	113	s2
4	B	test	117	s2

2.2 Input Files

Data for this analysis was extracted from the following tandem mass spectra (MSMS) summary files.

Experiment	MSMS Summary File
A	body1.txt
B	body2.txt

2.3 Statistical Model

The following statistical model was used to estimate the treatment-dependent effects.

LogIntensity ˜ Channel + Spectrum + Protein + Peptide + Protein:Treatment + Peptide:Treatment

3 Data Summary

The data supplied in the MSMS summary is filtered to remove unidentified proteins, contaminants, and peptides containing selected modifications. The following table sumarizes the data provided and used in the analysis.

	A	B	Combined
Supplied Spectra	11560	18985	30545
Unidentified Spectra	6008	8178	14186
Disallowed Modifications	32	205	237
Spectra from Contaminants	0	0	0
Missing Data	2459	2784	5243
Low Confidence Spectra	6008	8178	14186
Degenerate Peptides	1121	2460	3581
Remaining Spectra			7298
Unique Proteins			809
Unique Peptides			2813
Model R^2			0.67

4 Protein Summary

Each protein identified in one or more of the MSMS summaries is listed below in decreasing order of expression change magnitude. The median and estimated credible interval for each protein is given to the left in the table. Proteins identified by a single peptide are listed in a separate table.

4.1 Identified Proteins

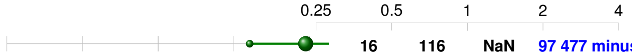

	Peptides	Spectra	Coverage	Protein
	**16**	**116**	**NaN**	**97 477 minus strand PREDICTED: complement C1q tumor necrosis factor-related protein 3-like [Oreochromis nilotic**

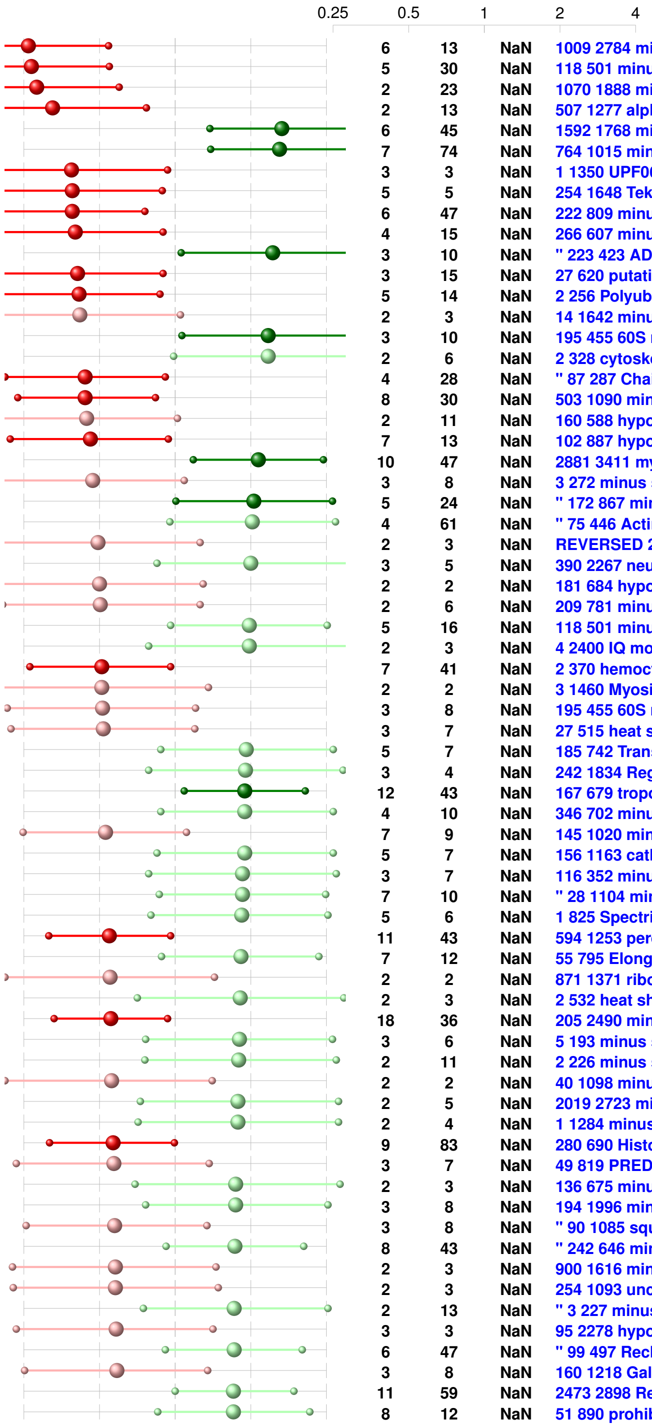

Peptides	Spectra	Coverage	Protein
6	13	NaN	1009 2784 minus strand Collagen alpha-3(VI) chain [Crassostrea gigas]; 1009 2787 minus strand Collagen alpha-3(V
5	30	NaN	118 501 minus strand histone 2A.Z [Aplysia californica]
2	23	NaN	1070 1888 minus strand galectin [Pinctada fucata]; 1371 2189 minus strand galectin [Pinctada fucata]
2	13	NaN	507 1277 alpha tubulin [Notothenia coriiceps]
6	45	NaN	1592 1768 minus strand beta-actin [Diabolocatantops pinguis]
7	74	NaN	764 1015 minus strand heat shock protein beta-1 [Trichinella spiralis] gi
3	3	NaN	1 1350 UPF0663 transmembrane protein C17orf28 [Crassostrea gigas]; 163 1542 UPF0663 transmembrane protein C
5	5	NaN	254 1648 Tektin-3 [Crassostrea gigas]; 254 1648 Tektin-3 [Crassostrea gigas]
6	47	NaN	222 809 minus strand PREDICTED: 15-hydroxyprostaglandin dehydrogenase [NAD+] [Gallus gallus]
4	15	NaN	266 607 minus strand H2 [Haliotis tuberculata]
3	10	NaN	" 223 423 ADPATP carrier protein; K05863 solute carrier family 25 (mitochondrial adenine nucleotide translocator), m
3	15	NaN	27 620 putative mitochondrial ATP synthase [Haliotis discus discus]
5	14	NaN	2 256 Polyubiquitin (Fragment) OS=Xenopus laevis PE=1 SV=2; 20 388 large subunit ribosomal protein L40e [Clonor
2	3	NaN	14 1642 minus strand chaperonin containing tcp1 [Haliotis discus discus]; 15 1619 chaperonin containing tcp1 [Hali
3	10	NaN	195 455 60S ribosomal protein L23a OS=Rattus norvegicus GN=Rpl23a PE=2 SV=1; 195 512 60S ribosomal protein L
2	6	NaN	2 328 cytoskeletal actin 2 [Molgula oculata]
4	28	NaN	" 87 287 Chain A, Structure Of Deoxygenated Hemocyanin From Rapana Thomasiana gi|31615505|pdb|1LNL|B Chai
8	30	NaN	503 1090 minus strand myosin regulatory light chain [Fasciola hepatica]; 278 865 minus strand myosin regulatory li
2	11	NaN	160 588 hypothetical protein CGI_10001640 [Crassostrea gigas]
7	13	NaN	102 887 hypothetical protein DAPPUDRAFT_304712 [Daphnia pulex]
10	47	NaN	2881 3411 myosin heavy chain [Todarodes pacificus]; 2915 3445 myosin heavy chain [Todarodes pacificus]
3	8	NaN	3 272 minus strand ribosomal protein L28 [Haliotis asinina]
5	24	NaN	" 172 867 minus strand complement C1q-like protein 4 precursor, partial [Oryzias melastigma]"
4	61	NaN	" 75 446 Actin, cytoplasmic type 8 OS=Xenopus laevis PE=3 SV=1"
2	3	NaN	REVERSED 216 2639 minus strand polycystin 2 [Gallus gallus]
3	5	NaN	390 2267 neurofilament protein NF70 [Helix aspersa]; 318 2195 neurofilament protein NF70 [Helix aspersa]
2	2	NaN	181 684 hypothetical protein BRAFLDRAFT_131834 [Branchiostoma floridae] gi
2	6	NaN	209 781 minus strand high mobility group protein 1 [Biomphalaria glabrata]
5	16	NaN	118 501 minus strand histone 2A.Z [Aplysia californica]; 15 428 PREDICTED: histone H2A.V-like [Apis florea]
2	3	NaN	4 2400 IQ motif containing GTPase activating protein 2 [Xenopus laevis] gi; 4 2169 IQ motif containing GTPase activ
7	41	NaN	2 370 hemocyanin alphaD-subunit [Helix lucorum]
2	2	NaN	3 1460 Myosin-VI [Crassostrea gigas]
3	8	NaN	195 455 60S ribosomal protein L23a OS=Rattus norvegicus GN=Rpl23a PE=2 SV=1; 16 465 60S ribosomal protein L2
3	7	NaN	27 515 heat shock protein 70 [Capsaspora owczarzaki ATCC 30864]; 30 464 minus strand hypothetical protein [Trich
5	7	NaN	185 742 Transmembrane protein Tmp21 [Crassostrea gigas]
3	4	NaN	242 1834 Regulator of differentiation 1 [Crassostrea gigas]; 458 2050 minus strand Regulator of differentiation 1 [Cr
12	43	NaN	167 679 tropomyosin [Haliotis discus discus]
4	10	NaN	346 702 minus strand PREDICTED: galectin-4-like [Anolis carolinensis]; 367 669 minus strand PREDICTED: galectin
7	9	NaN	145 1020 minus strand PREDICTED: inorganic pyrophosphatase-like [Saccoglossus kowalevskii]
5	7	NaN	156 1163 cathepsin B [Crassostrea ariakensis]
3	7	NaN	116 352 minus strand troponin I 4 [Clonorchis sinensis]
7	10	NaN	" 28 1104 minus strand Pyruvate dehydrogenase E1 component subunit beta, mitochondrial [Crassostrea gigas]"
5	6	NaN	1 825 Spectrin alpha chain [Crassostrea gigas]
11	43	NaN	594 1253 peroxiredoxin 6 [Eriocheir sinensis]
7	12	NaN	55 795 Elongation factor 1-delta OS=Bos taurus GN=EEF1D PE=2 SV=2; 49 747 PREDICTED: elongation factor 1-delt
2	2	NaN	871 1371 ribosomal protein L11 [Triatoma infestans]; 343 843 minus strand ribosomal protein L11 [Triatoma infestan
2	3	NaN	2 532 heat shock protein 70 [Echinostoma caproni]
18	36	NaN	205 2490 minus strand glucose-regulated protein 94 [Crassostrea gigas]
3	6	NaN	5 193 minus strand PREDICTED: 40S ribosomal protein S28-like [Strongylocentrotus purpuratus]; 3 122 PREDICTED
2	11	NaN	2 226 minus strand hypothetical protein CGI_10015501 [Crassostrea gigas]
2	2	NaN	40 1098 minus strand major egg antigen [Clonorchis sinensis]
2	5	NaN	2019 2723 minus strand Kyphoscoliosis peptidase [Crassostrea gigas]; 2060 2764 minus strand Kyphoscoliosis pep
2	4	NaN	1 1284 minus strand Thioredoxin reductase 3 [Crassostrea gigas]
9	83	NaN	280 690 Histone H3.3 [Harpegnathos saltator]
3	7	NaN	49 819 PREDICTED: elongation factor 1-delta-like isoform 2 [Strongylocentrotus purpuratus]
2	3	NaN	136 675 minus strand predicted protein [Nematostella vectensis] gi
3	8	NaN	194 1996 minus strand Neutral and basic amino acid transport protein rBAT [Crassostrea gigas]; 265 2067 minus str
3	8	NaN	" 90 1085 squid, variant A [Blattella germanica]"
8	43	NaN	" 242 646 minus strand Fatty acid-binding protein, heart [Crassostrea gigas]; 227 631 minus strand Fatty acid-bindin
2	3	NaN	900 1616 minus strand hemocyanin isoform 1 [Haliotis diversicolor]
2	3	NaN	254 1093 uncharacterized protein LOC432187 precursor [Xenopus laevis] gi
2	13	NaN	" 3 227 minus strand alpha tubulin, putative [Ixodes scapularis] gi"
3	3	NaN	95 2278 hypothetical protein CGI_10017740 [Crassostrea gigas]
6	47	NaN	" 99 497 RecName: Full=Histone H2B, gonadal"
3	8	NaN	160 1218 Galectin-9 [Crassostrea gigas]
11	59	NaN	2473 2898 RecName: Full=Paramyosin gi|397603|emb|CAA79849.1| paramyosin [Echinococcus granulosus]
8	12	NaN	51 890 prohibitin [Octopus tankahkeei]

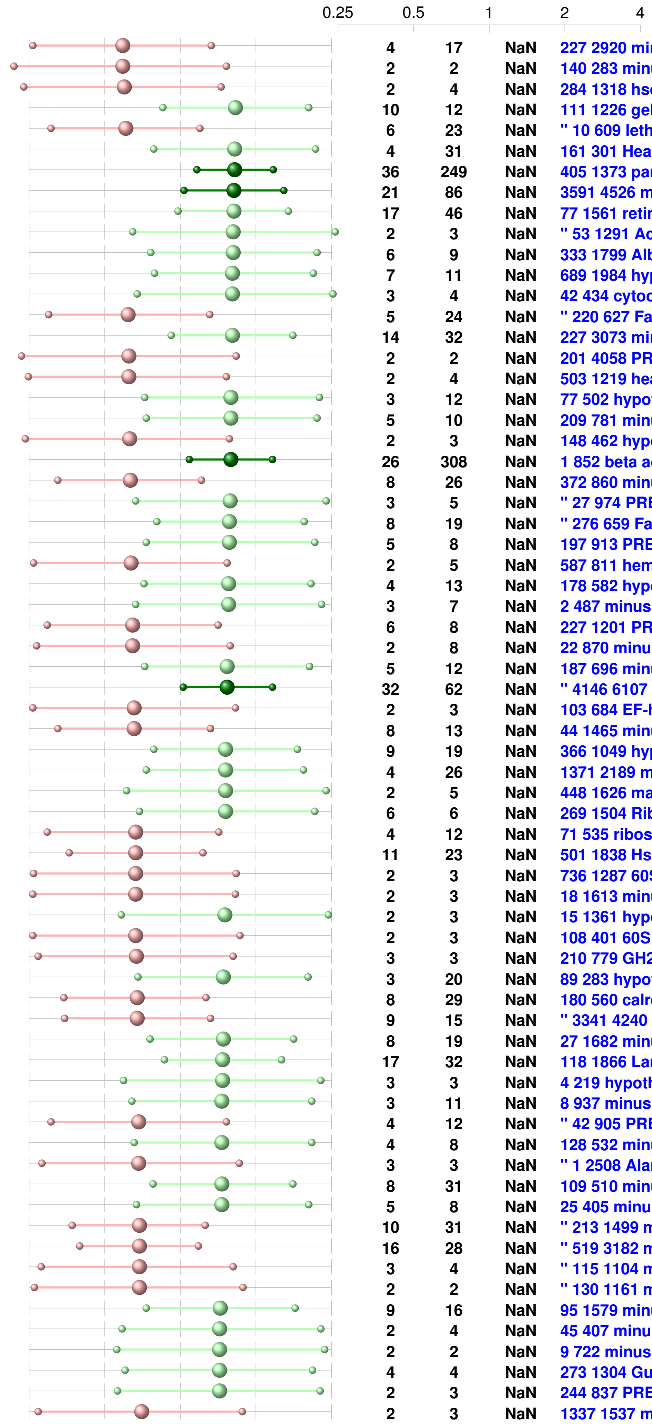

Peptides	Spectra	Coverage	Protein
4	17	NaN	227 2920 minus strand Lysosomal alpha-mannosidase [Crassostrea gigas]; 227 3073 minus strand Lysosomal alpha
2	2	NaN	140 283 minus strand elongation factor 1 alpha [Angiostrongylus cantonensis]
2	4	NaN	284 1318 hsc70-interacting protein [Gallus gallus] gi; 88 1137 hsc70-interacting protein [Gallus gallus] gi
10	12	NaN	111 1226 gelsolin [Aplysia californica] gi; 42 1139 minus strand gelsolin [Aplysia californica] gi
6	23	NaN	" 10 609 lethal(2)essential for life protein, l2efl [Aedes aegypti] gi"
4	31	NaN	161 301 Heat shock protein beta-6 OS=Mus musculus GN=Hspb6 PE=1 SV=1
36	249	NaN	405 1373 paramyosin [Haliotis discus discus]
21	86	NaN	3591 4526 minus strand myosin heavy chain cardiac muscle specific isoform 1 [Argopecten irradians]
17	46	NaN	77 1561 retinal dehydrogenase [Lymnaea stagnalis]; 69 1553 retinal dehydrogenase [Lymnaea stagnalis]
2	3	NaN	" 53 1291 Acetyl-CoA acetyltransferase B, mitochondrial [Crassostrea gigas]"
6	9	NaN	333 1799 Alba-like protein C9orf23-like protein [Crassostrea gigas]
7	11	NaN	689 1984 hypothetical protein BRAFLDRAFT_78257 [Branchiostoma floridae] gi
3	4	NaN	42 434 cytochrome b5 [Haliotis discus discus]; 42 434 cytochrome b5 [Haliotis discus discus]
5	24	NaN	" 220 627 Fatty acid-binding protein, brain [Crassostrea gigas]"
14	32	NaN	227 3073 minus strand Lysosomal alpha-mannosidase [Crassostrea gigas]
2	2	NaN	201 4058 PREDICTED: xanthine dehydrogenase/oxidase-like [Amphimedon queenslandica]
2	4	NaN	503 1219 heat shock cognate 71 [Mytilus galloprovincialis]
3	12	NaN	77 502 hypothetical protein CGI_10008263 [Crassostrea gigas]
5	10	NaN	209 781 minus strand high mobility group protein 1 [Biomphalaria glabrata]; 277 771 high mobility group protein 1 [I
2	3	NaN	148 462 hypothetical protein DAPPUDRAFT_306954 [Daphnia pulex]; 148 462 hypothetical protein DAPPUDRAFT_30
26	308	NaN	1 852 beta actin-like 1 protein [Macrostomum lignano]
8	26	NaN	372 860 minus strand small heat shock protein [Tegillarca granosa]
3	5	NaN	" 27 974 PREDICTED: succinyl-CoA ligase [GDP-forming] subunit alpha, mitochondrial-like [Oreochromis niloticus];
8	19	NaN	" 276 659 Fatty acid-binding protein, adipocyte [Crassostrea gigas]; 426 809 Fatty acid-binding protein, adipocyte [C
5	8	NaN	197 913 PREDICTED: l-xylulose reductase-like [Monodelphis domestica]; 197 913 PREDICTED: l-xylulose reductase-
2	5	NaN	587 811 hemocyanin 2 [Megathura crenulata]
4	13	NaN	178 582 hypothetical protein CGI_10001640 [Crassostrea gigas]
3	7	NaN	2 487 minus strand PREDICTED: hypothetical protein LOC100699750 [Oreochromis niloticus]; 41 535 minus strand I
6	8	NaN	227 1201 PREDICTED: quinone oxidoreductase-like isoform 1 [Ornithorhynchus anatinus]; 109 1083 PREDICTED: qu
2	8	NaN	22 870 minus strand PREDICTED: alcohol dehydrogenase class-3 [Sarcophilus harrisii]
5	12	NaN	187 696 minus strand putative ribosomal protein S10 [Sipunculus nudus]
32	62	NaN	" 4146 6107 minus strand nonmuscle myosin II, partial [Aplysia californica]"
2	3	NaN	103 684 EF-hand domain-containing protein D2 [Crassostrea gigas]
8	13	NaN	44 1465 minus strand Adenylyl cyclase-associated protein 1 [Crassostrea gigas]; 52 1473 minus strand Adenylyl cyc
9	19	NaN	366 1049 hypothetical protein CGI_10004855 [Crassostrea gigas]
4	26	NaN	1371 2189 minus strand galectin [Pinctada fucata]
2	5	NaN	448 1626 matrilin [Biomphalaria glabrata]
6	6	NaN	269 1504 Ribosome-binding protein 1 [Crassostrea gigas]; 265 1500 Ribosome-binding protein 1 [Crassostrea gigas
4	12	NaN	71 535 ribosomal protein rpl14 [Arenicola marina]
11	23	NaN	501 1838 Hsp90A [Haliotis asinina]
2	3	NaN	736 1287 60S ribosomal protein L3 OS=Rattus norvegicus GN=Rpl3 PE=1 SV=3
2	3	NaN	18 1613 minus strand chaperonin containing T-complex polypeptide subunit zeta [Haliotis discus hannai]; 527 2089
2	3	NaN	15 1361 hypothetical protein BRAFLDRAFT_231908 [Branchiostoma floridae] gi
2	3	NaN	108 401 60S ribosomal L7a-like protein [Phragmatopoma lapidosa]; 108 401 60S ribosomal L7a-like protein [Phragm
3	3	NaN	210 779 GH20056 [Drosophila grimshawi] gi; 216 785 minus strand GH20056 [Drosophila grimshawi] gi; 210 779 GH2
3	20	NaN	89 283 hypothetical protein CGI_10017178 [Crassostrea gigas]
8	29	NaN	180 560 calreticulin precursor [Aplysia californica] gi; 180 545 calreticulin precursor [Aplysia californica] gi
9	15	NaN	" 3341 4240 minus strand nonmuscle myosin II, partial [Aplysia californica]"
8	19	NaN	27 1682 minus strand PREDICTED: nucleolar protein 56 [Oreochromis niloticus]
17	32	NaN	118 1866 Lamin Dm0 [Crassostrea gigas]
3	3	NaN	4 219 hypothetical protein BRAFLDRAFT_66351 [Branchiostoma floridae] gi; 3 239 hypothetical protein BRAFLDRAF
3	11	NaN	8 937 minus strand galectin [Crassostrea virginica]
4	12	NaN	" 42 905 PREDICTED: enoyl-CoA hydratase, mitochondrial-like isoform 1 [Oreochromis niloticus]"
4	8	NaN	128 532 minus strand mitochondrial ATPase inhibitor [Ornithodoros parkeri]
3	3	NaN	" 1 2508 Alanyl-tRNA synthetase, cytoplasmic [Crassostrea gigas]; 1 2508 Alanyl-tRNA synthetase, cytoplasmic [Cra
8	31	NaN	109 510 minus strand hypothetical protein DAPPUDRAFT_231281 [Daphnia pulex]
5	8	NaN	25 405 minus strand Elongation factor 1-delta [Anoplopoma fimbria]
10	31	NaN	" 213 1499 minus strand Citrate synthase, mitochondrial [Crassostrea gigas]"
16	28	NaN	" 519 3182 minus strand Alpha-actinin, sarcomeric [Crassostrea gigas]"
3	4	NaN	" 115 1104 minus strand PREDICTED: KH domain-containing, RNA-binding, signal transduction-associated protein 3
2	2	NaN	" 130 1161 minus strand Isocitrate dehydrogenase [NAD] subunit alpha, mitochondrial [Crassostrea gigas]; 198 1229
9	16	NaN	95 1579 minus strand hypothetical protein BRAFLDRAFT_113974 [Branchiostoma floridae] gi
2	4	NaN	45 407 minus strand 60S ribosomal protein L31 OS=Xenopus laevis GN=rpl31 PE=2 SV=1
2	2	NaN	9 722 minus strand actin related protein [Lumbricus rubellus]
4	4	NaN	273 1304 Guanine nucleotide-binding protein subunit beta [Crassostrea gigas]
2	3	NaN	244 837 PREDICTED: ras-like GTP-binding protein Rho1-like [Megachile rotundata]
2	3	NaN	1337 1537 minus strand hemocyanin [Haliotis tuberculata]

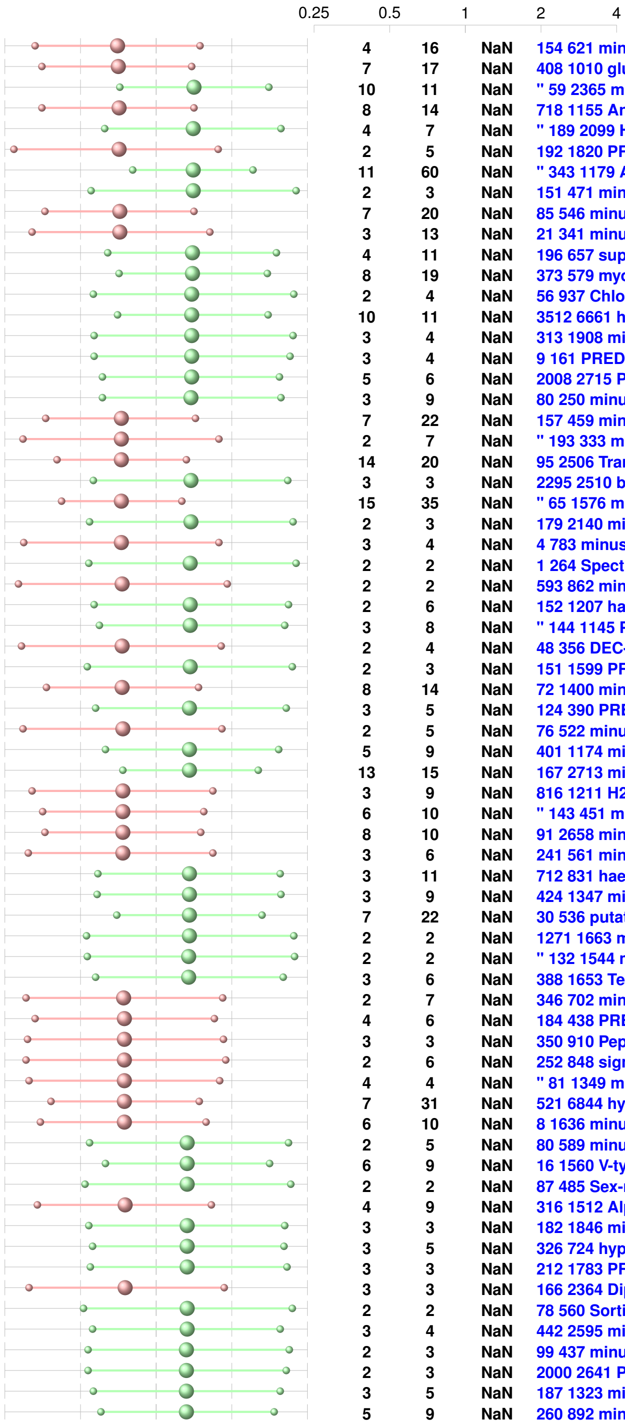

Peptides	Spectra	Coverage	Protein
4	16	NaN	154 621 minus strand Stress response protein nhaX [Crassostrea gigas]
7	17	NaN	408 1010 glutathione S-transferase sigma 2 [Mytilus galloprovincialis]; 166 768 glutathione S-transferase sigma 2 [M
10	11	NaN	" 59 2365 minus strand Aconitate hydratase, mitochondrial [Crassostrea gigas]"
8	14	NaN	718 1155 Annexin A7 [Crassostrea gigas]
4	7	NaN	" 189 2099 Hsp70, putative [Ixodes scapularis] gi"
2	5	NaN	192 1820 PREDICTED: copine IV-like [Saccoglossus kowalevskii]; 125 1753 PREDICTED: copine IV-like [Saccoglossu
11	60	NaN	" 343 1179 ATP synthase subunit alpha, mitochondrial OS=Pongo abelii GN=ATP5A1 PE=2 SV=1"
2	3	NaN	151 471 minus strand ribosomal protein rps25 [Arenicola marina]
7	20	NaN	85 546 minus strand RecName: Full=Eukaryotic translation initiation factor 5A; Short=eIF-5A gi|51702279|sp|P6292
3	13	NaN	21 341 minus strand hemocyanin isoform 1 [Nucula nucleus]
4	11	NaN	196 657 superoxide dismutase [Aplysia californica] gi; 690 1151 superoxide dismutase [Aplysia californica] gi
8	19	NaN	373 579 myosin heavy chain [Argopecten irradians] gi
2	4	NaN	56 937 Chloride intracellular channel exc-4 [Crassostrea gigas]
10	11	NaN	3512 6661 hypothetical protein BRAFLDRAFT_255626 [Branchiostoma floridae] gi
3	4	NaN	313 1908 minus strand Heterogeneous nuclear ribonucleoprotein L [Crassostrea gigas]
3	4	NaN	9 161 PREDICTED: tubulin alpha-1C chain-like [Acyrthosiphon pisum]; 9 161 Tubulin alpha-3 chain (Fragments) OS=
5	6	NaN	2008 2715 Protein disulfide-isomerase A5 [Crassostrea gigas]
3	9	NaN	80 250 minus strand 40S ribosomal protein S29 [Ornithodoros parkeri]
7	22	NaN	157 459 minus strand Synaptojanin-2-binding protein [Crassostrea gigas]
2	7	NaN	" 193 333 minus strand RecName: Full=Globin; AltName: Full=Myoglobin gi|299274|gb|AAB25743.1| myoglobin [Na
14	20	NaN	95 2506 Transitional endoplasmic reticulum ATPase [Crassostrea gigas]
3	3	NaN	2295 2510 beta-thymosin domain repeat protein CSP24KDa_v1 [Hermissenda crassicornis] gi
15	35	NaN	" 65 1576 minus strand Aldehyde dehydrogenase, mitochondrial [Crassostrea gigas]"
2	3	NaN	179 2140 minus strand PREDICTED: hypothetical protein [Saccoglossus kowalevskii]
3	4	NaN	4 783 minus strand hemocyanin isoform 2 [Nucula nucleus]
2	2	NaN	1 264 Spectrin beta chain [Crassostrea gigas]
2	2	NaN	593 862 minus strand mitochondrial ATP synthase coupling factor 6 precursor [Ochlerotatus taeniorhynchus]
2	6	NaN	152 1207 haemocyanin precursor [Nautilus pompilius]
3	8	NaN	" 144 1145 PREDICTED: RNA-binding protein squid-like isoform 1 [Megachile rotundata]; 90 1085 squid, variant A [B
2	4	NaN	48 356 DEC-3 [Lymnaea stagnalis]
2	3	NaN	151 1599 PREDICTED: galactosamine (N-acetyl)-6-sulfate sulfatase-like [Saccoglossus kowalevskii]
8	14	NaN	72 1400 minus strand hypothetical protein RCJMB04_22i13 [Gallus gallus]
3	5	NaN	124 390 PREDICTED: 60S ribosomal protein L23a-like [Callithrix jacchus]
2	5	NaN	76 522 minus strand Troponin C [Crassostrea gigas]; 173 619 minus strand Troponin C [Crassostrea gigas]; 173 619
5	9	NaN	401 1174 minus strand Mechanosensory protein 2 [Crassostrea gigas]
13	15	NaN	167 2713 minus strand Major vault protein [Crassostrea gigas]
3	9	NaN	816 1211 H2 [Haliotis tuberculata]
6	10	NaN	" 143 451 minus strand PREDICTED: 10 kDa heat shock protein, mitochondrial isoform 1 [Canis lupus familiaris]"
8	10	NaN	91 2658 minus strand puromycin-sensitive aminopeptidase [Saccoglossus kowalevskii]
3	6	NaN	241 561 minus strand thioredoxin [Crassostrea ariakensis]
3	11	NaN	712 831 haemocyanin [Aplysia californica]
3	9	NaN	424 1347 minus strand Tubulin alpha chain [Ascaris suum]
7	22	NaN	30 536 putative mitochondrial ATP synthase [Haliotis discus discus]; 27 620 putative mitochondrial ATP synthase [H
2	2	NaN	1271 1663 minus strand PREDICTED: similar to AGAP007393-PB [Tribolium castaneum] gi
2	2	NaN	" 132 1544 minus strand Serine hydroxymethyltransferase, cytosolic [Crassostrea gigas]"
3	6	NaN	388 1653 Tektin-3 [Crassostrea gigas]
2	7	NaN	346 702 minus strand PREDICTED: galectin-4-like [Anolis carolinensis]
4	6	NaN	184 438 PREDICTED: similar to CG14235 CG14235-PA [Tribolium castaneum] gi
3	3	NaN	350 910 Peptidyl-prolyl cis-trans isomerase B [Crassostrea gigas]; 488 1039 Peptidyl-prolyl cis-trans isomerase B [C
2	6	NaN	252 848 sigma class glutathione-s-transferase 2 [Haliotis discus discus]
4	4	NaN	" 81 1349 minus strand PREDICTED: LOW QUALITY PROTEIN: dolichyl-diphosphooligosaccharide–protein glycosyl
7	31	NaN	521 6844 hypothetical protein CGI_10014469 [Crassostrea gigas]
6	10	NaN	8 1636 minus strand V-type proton ATPase catalytic subunit A [Crassostrea gigas]; 8 1636 minus strand V-type proto
2	5	NaN	80 589 minus strand PREDICTED: adenine phosphoribosyltransferase-like [Strongylocentrotus purpuratus]
6	9	NaN	16 1560 V-type proton ATPase subunit B [Crassostrea gigas]
2	2	NaN	87 485 Sex-regulated protein janus-A [Crassostrea gigas]
4	9	NaN	316 1512 Alpha-N-acetylgalactosaminidase [Crassostrea gigas]; 282 1478 Alpha-N-acetylgalactosaminidase [Crasso
3	3	NaN	182 1846 minus strand hypothetical protein DAPPUDRAFT_302108 [Daphnia pulex]; 110 1774 minus strand hypothe
3	5	NaN	326 724 hypothetical protein [Amblyomma maculatum]
3	3	NaN	212 1783 PREDICTED: d-3-phosphoglycerate dehydrogenase-like [Strongylocentrotus purpuratus]
3	3	NaN	166 2364 Dipeptidyl-peptidase 3 [Crassostrea gigas]
2	2	NaN	78 560 Sorting nexin-12 [Camponotus floridanus]; 78 578 Sorting nexin-12 [Camponotus floridanus]; 132 605 Sorting
3	4	NaN	442 2595 minus strand Amiloride-sensitive amine oxidase [copper-containing] [Crassostrea gigas]
2	3	NaN	99 437 minus strand V-type proton ATPase subunit G [Crassostrea gigas]
2	3	NaN	2000 2641 Polyadenylate-binding protein 4 [Crassostrea gigas]
3	5	NaN	187 1323 minus strand gelsolin [Aplysia californica] gi
5	9	NaN	260 892 minus strand calponin [Haliotis diversicolor]

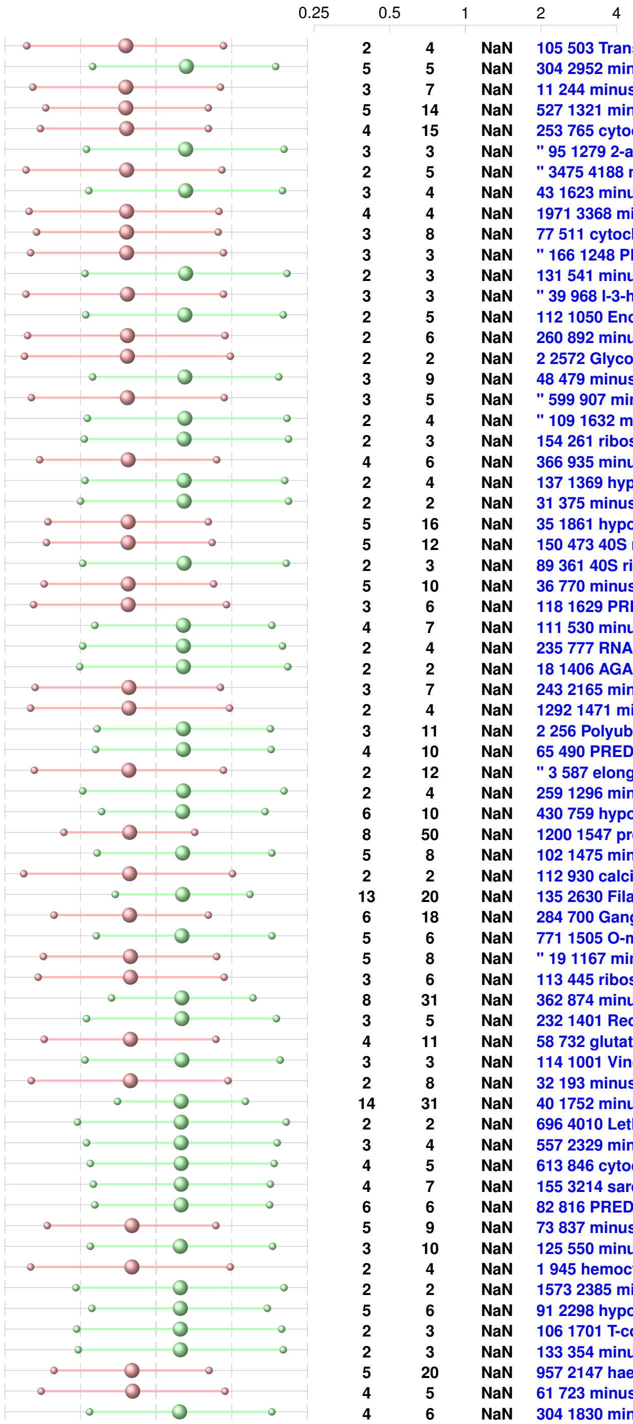

Peptides	Spectra	Coverage	Protein
2	4	NaN	105 503 Transgelin-2 [Crassostrea gigas]
5	5	NaN	304 2952 minus strand hypothetical protein CGI_10026535 [Crassostrea gigas]
3	7	NaN	11 244 minus strand Tubulin alpha chain (Fragment) OS=Lytechinus pictus PE=3 SV=1
5	14	NaN	527 1321 minus strand troponin T [Mizuhopecten yessoensis]; 305 1099 minus strand troponin T [Mizuhopecten yes
4	15	NaN	253 765 cytochrome c oxidase subunit IV [Urechis caupo]
3	3	NaN	" 95 1279 2-amino-3-ketobutyrate coenzyme A ligase, mitochondrial, partial [Crassostrea gigas]"
2	5	NaN	" 3475 4188 minus strand Heat shock 70 kDa protein cognate, putative [Pediculus humanus corporis] gi"
3	4	NaN	43 1623 minus strand MGC84105 protein precursor [Xenopus laevis] gi; 43 1506 minus strand MGC84105 protein pre
4	4	NaN	1971 3368 minus strand Kyphoscoliosis peptidase [Crassostrea gigas]; 1767 3158 minus strand hillarin [Hirudo med
3	8	NaN	77 511 cytochrome c oxidase subunit VIIa polypeptide 2 like [Xenopus (Silurana) tropicalis]; 77 511 cytochrome c ox
3	3	NaN	" 166 1248 PREDICTED: probable pyruvate dehydrogenase E1 component subunit alpha, mitochondrial-like [Metase
2	3	NaN	131 541 minus strand nervous system adducin [Aplysia californica] gi
3	3	NaN	" 39 968 l-3-hydroxyacyl-coenzyme a dehydrogenase, short chain [Haliotis discus discus]; 39 968 l-3-hydroxyacyl-co
2	5	NaN	112 1050 Enolase-phosphatase E1 [Crassostrea gigas]; 112 1050 Enolase-phosphatase E1 [Crassostrea gigas]
2	6	NaN	260 892 minus strand calponin [Haliotis diversicolor]
2	2	NaN	2 2572 Glycogen debranching enzyme [Crassostrea gigas]
3	9	NaN	48 479 minus strand 40S ribosomal protein S24 OS=Macaca fascicularis GN=RPS24 PE=2 SV=1
3	5	NaN	" 599 907 minus strand Tryptophanyl-tRNA synthetase, cytoplasmic [Heterocephalus glaber]"
2	4	NaN	" 109 1632 minus strand Seryl-tRNA synthetase, cytoplasmic [Crassostrea gigas]; 109 1614 minus strand Seryl-tRNA
2	3	NaN	154 261 ribosomal protein S18 [Stenella coeruleoalba]
4	6	NaN	366 935 minus strand 40S ribosomal protein S2 OS=Urechis caupo GN=RPS2 PE=2 SV=1
2	4	NaN	137 1369 hypothetical protein TcasGA2_TC002962 [Tribolium castaneum]
2	2	NaN	31 375 minus strand 40S ribosomal protein S19 OS=Mya arenaria GN=RPS19 PE=2 SV=1
5	16	NaN	35 1861 hypothetical protein DAPPUDRAFT_309082 [Daphnia pulex]
5	12	NaN	150 473 40S ribosomal protein S16 OS=Heteropneustes fossilis GN=rps16 PE=2 SV=1
2	3	NaN	89 361 40S ribosomal protein S18 OS=Rattus norvegicus GN=Rps18 PE=1 SV=3
5	10	NaN	36 770 minus strand Endoplasmic reticulum protein ERp29 [Crassostrea gigas]
3	6	NaN	118 1629 PREDICTED: similar to tektin 3 [Ciona intestinalis]
4	7	NaN	111 530 minus strand Nucleoside diphosphate kinase B OS=Homo sapiens GN=NME2 PE=1 SV=1
2	4	NaN	235 777 RNA-binding protein 4.1 [Clonorchis sinensis]
2	2	NaN	18 1406 AGAP006996-PA [Anopheles gambiae str. PEST] gi
3	7	NaN	243 2165 minus strand Elongation factor 2 OS=Drosophila melanogaster GN=Ef2b PE=2 SV=4
2	4	NaN	1292 1471 minus strand hemocyanin isoform 1 [Haliotis diversicolor]
3	11	NaN	2 256 Polyubiquitin (Fragment) OS=Xenopus laevis PE=1 SV=2
4	10	NaN	65 490 PREDICTED: cofilin-2 [Otolemur garnettii]
2	12	NaN	" 3 587 elongation factor 1 alpha, partial [Phorbas sp. nov. SB-2012]"
2	4	NaN	259 1296 minus strand GD20916 [Drosophila simulans] gi
6	10	NaN	430 759 hypothetical protein [Amblyomma maculatum]; 336 665 hypothetical protein [Amblyomma maculatum]
8	50	NaN	1200 1547 protein disulfide isomerase [Conus eburneus]
5	8	NaN	102 1475 minus strand Cytosolic non-specific dipeptidase [Crassostrea gigas]
2	2	NaN	112 930 calcineurin A [Haliotis discus discus]
13	20	NaN	135 2630 Filamin-A [Crassostrea gigas]; 118 2613 Filamin-A [Crassostrea gigas]; 157 2652 Filamin-A [Crassostrea g
6	18	NaN	284 700 Ganglioside GM2 activator [Crassostrea gigas]
5	6	NaN	771 1505 O-methyltransferase family 3 [Dicentrarchus labrax]
5	8	NaN	" 19 1167 minus strand ureidopropionase, beta [Xenopus (Silurana) tropicalis] gi"
3	6	NaN	113 445 ribosomal protein rps19 [Lineus viridis]
8	31	NaN	362 874 minus strand Transgelin-2 [Crassostrea gigas]
3	5	NaN	232 1401 RecName: Full=Guanine nucleotide-binding protein G(s) subunit alpha; AltName: Full=Adenylate cyclase-s
4	11	NaN	58 732 glutathione-s-transferase [Haliotis discus discus]
3	3	NaN	114 1001 Vinculin [Crassostrea gigas]
2	8	NaN	32 193 minus strand Ubiquinol-cytochrome C reductase complex [Xenopus laevis] gi; 32 193 minus strand Ubiquino
14	31	NaN	40 1752 minus strand heat shock protein 60 [Biomphalaria glabrata]; 40 1752 minus strand heat shock protein 60 [B
2	2	NaN	696 4010 Lethal(2) giant larvae-like protein 1 [Crassostrea gigas]; 657 3971 minus strand Lethal(2) giant larvae-like p
3	4	NaN	557 2329 minus strand Collagen alpha-3(VI) chain [Crassostrea gigas]
4	5	NaN	613 846 cytochrome c oxidase subunit Va [Antheraea yamamai] gi
4	7	NaN	155 3214 sarco/endoplasmic reticulum calcium ATPase isoform B [Pinctada fucata]; 155 3214 minus strand sarco/er
6	6	NaN	82 816 PREDICTED: triosephosphate isomerase B [Oreochromis niloticus]
5	9	NaN	73 837 minus strand Electron transfer flavoprotein subunit beta [Crassostrea gigas]; 73 837 minus strand Electron t
3	10	NaN	125 550 minus strand ribosomal protein S20 [Chlamys farreri]; 125 550 minus strand ribosomal protein S20 [Chlamy
2	4	NaN	1 945 hemocyanin isoform 1 [Haliotis diversicolor]
2	2	NaN	1573 2385 minus strand Spectrin beta chain [Crassostrea gigas]
5	6	NaN	91 2298 hypothetical protein BRAFLDRAFT_267717 [Branchiostoma floridae] gi; 84 2291 hypothetical protein BRAF
2	3	NaN	106 1701 T-complex protein 1 subunit beta [Crassostrea gigas]
2	3	NaN	133 354 minus strand 60S ribosomal protein L7a OS=Rattus norvegicus GN=Rpl7a PE=1 SV=2
5	20	NaN	957 2147 haemocyanin [Aplysia californica]
4	5	NaN	61 723 minus strand 40S ribosomal protein S3-A OS=Xenopus laevis GN=rps3-a PE=2 SV=2
4	6	NaN	304 1830 minus strand Methenyltetrahydrofolate synthetase domain-containing protein [Crassostrea gigas]

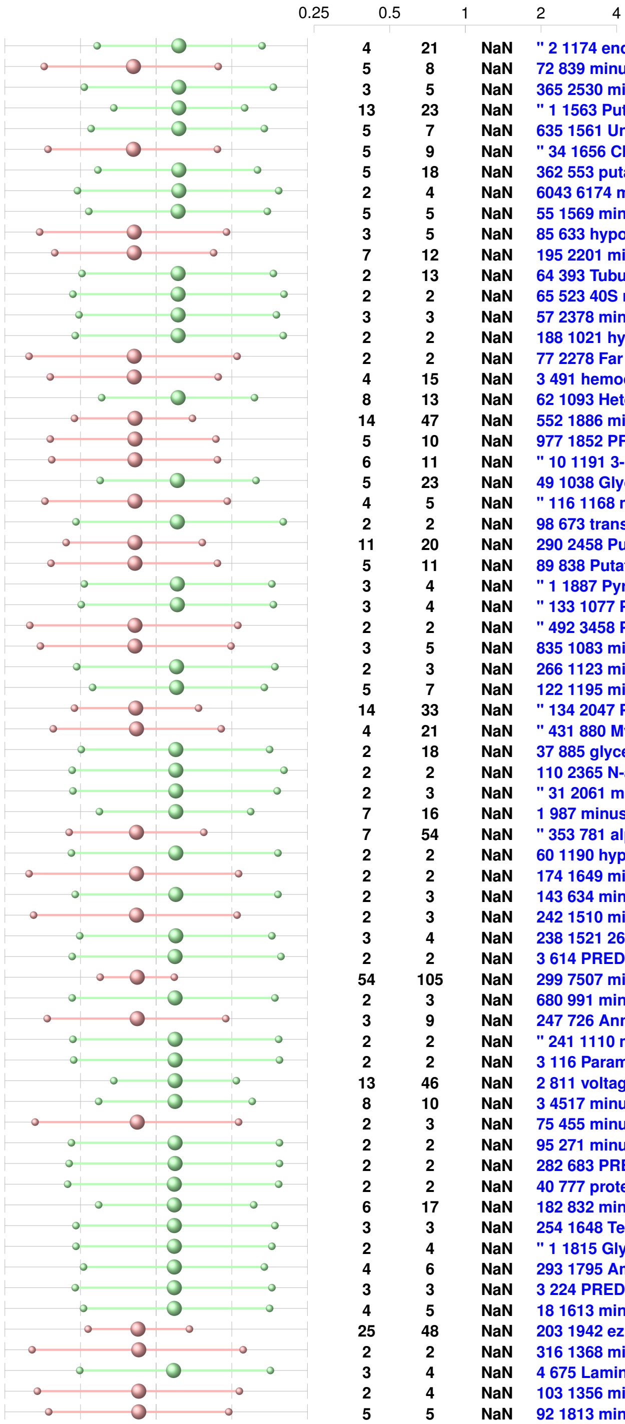

Peptides	Spectra	Coverage	Protein
4	21	NaN	" 2 1174 endo-1,3-beta-D-glucanase [Littorina sitkana]"
5	8	NaN	72 839 minus strand 60S ribosomal protein L8 OS=Spodoptera frugiperda GN=RpL8 PE=2 SV=1
3	5	NaN	365 2530 minus strand calpain B [Gecarcinus lateralis]
13	23	NaN	" 1 1563 Putative methylmalonate-semialdehyde dehydrogenase [acylating], mitochondrial [Crassostrea gigas]"
5	7	NaN	635 1561 Uncharacterized protein yfeX [Crassostrea gigas]
5	9	NaN	" 34 1656 Chaperonin containing TCP1, subunit 7 (eta) [Danio rerio] gi; 34 1656 Chaperonin containing TCP1, subun
5	18	NaN	362 553 putative RNA-binding protein [Haliotis diversicolor]
2	4	NaN	6043 6174 myosin heavy chain [Mytilus galloprovincialis]; 5869 6000 myosin heavy chain [Mytilus galloprovincialis]
5	5	NaN	55 1569 minus strand Putative aminopeptidase W07G4.4 [Crassostrea gigas]; 228 1742 Putative aminopeptidase W0
3	5	NaN	85 633 hypothetical protein [Amblyomma maculatum]
7	12	NaN	195 2201 minus strand hypothetical protein DAPPUDRAFT_315656 [Daphnia pulex]
2	13	NaN	64 393 Tubulin beta chain OS=Paracentrotus lividus PE=2 SV=1; 109 438 Tubulin beta chain OS=Paracentrotus lividu
2	2	NaN	65 523 40S ribosomal protein S9 [Crassostrea gigas]; 65 523 40S ribosomal protein S9 [Crassostrea gigas]
3	3	NaN	57 2378 minus strand Heterogeneous nuclear ribonucleoprotein U-like protein 1 [Crassostrea gigas]
2	2	NaN	188 1021 hypothetical protein BRAFLDRAFT_214346 [Branchiostoma floridae] gi
2	2	NaN	77 2278 Far upstream element-binding protein 3 [Crassostrea gigas]
4	15	NaN	3 491 hemocyanin [Haliotis tuberculata]
8	13	NaN	62 1093 Heterogeneous nuclear ribonucleoprotein A2-like protein 1 [Crassostrea gigas]
14	47	NaN	552 1886 minus strand intermediate filament protein [Biomphalaria glabrata]
5	10	NaN	977 1852 PREDICTED: similar to prohibitin [Tribolium castaneum] gi
6	11	NaN	" 10 1191 3-ketoacyl-CoA thiolase, mitochondrial [Crassostrea gigas]"
5	23	NaN	49 1038 Glyceraldehyde-3-phosphate dehydrogenase [Crotalus adamanteus]
4	5	NaN	" 116 1168 minus strand hypothetical protein CGI_10014553, partial [Crassostrea gigas]"
2	2	NaN	98 673 transforming protein Rho-like [Acyrthosiphon pisum]
11	20	NaN	290 2458 Putative beta-D-xylosidase 5 [Crassostrea gigas]
5	11	NaN	89 838 Putative phosphoglycerate mutase [Crassostrea gigas]; 89 838 Putative phosphoglycerate mutase [Crassost
3	4	NaN	" 1 1887 Pyruvate carboxylase, mitochondrial [Crassostrea gigas]"
3	4	NaN	" 133 1077 PREDICTED: electron transfer flavoprotein, alpha polypeptide-like isoform 2 [Saccoglossus kowalevskii]"
2	2	NaN	" 492 3458 PREDICTED: 2-oxoglutarate dehydrogenase, mitochondrial isoform 1 [Sarcophilus harrisii]"
3	5	NaN	835 1083 minus strand Calmodulin [Crassostrea gigas]
2	3	NaN	266 1123 minus strand mitochondrial malate dehydrogenase precursor [Nucella lapillus]
5	7	NaN	122 1195 minus strand Severin [Crassostrea gigas]
14	33	NaN	" 134 2047 PREDICTED: succinate dehydrogenase [ubiquinone] flavoprotein subunit, mitochondrial-like isoform 3, p
4	21	NaN	" 431 880 Myosin essential light chain, striated adductor muscle OS=Aequipecten irradians PE=1 SV=2"
2	18	NaN	37 885 glyceraldehyde 3-phosphate dehydrogenase [Dugesia japonica]
2	2	NaN	110 2365 N-acetylated-alpha-linked acidic dipeptidase 2 [Crassostrea gigas]
2	3	NaN	" 31 2061 minus strand 1,4-alpha-glucan-branching enzyme [Crassostrea gigas]"
7	16	NaN	1 987 minus strand RecName: Full=Tubulin beta chain; AltName: Full=Beta-tubulin
7	54	NaN	" 353 781 alpha-B-crystallin, putative [Ixodes scapularis] gi"
2	2	NaN	60 1190 hypothetical protein BRAFLDRAFT_109688 [Branchiostoma floridae] gi; 51 1184 hypothetical protein BRAFI
2	2	NaN	174 1649 minus strand Collagen alpha-1(XII) chain [Crassostrea gigas]; 123 1490 minus strand Collagen alpha-1(XII)
2	3	NaN	143 634 minus strand ribosomal protein S14 [Solea senegalensis]
2	3	NaN	242 1510 minus strand hypothetical protein BRAFLDRAFT_276673 [Branchiostoma floridae] gi
3	4	NaN	238 1521 26S protease regulatory subunit 6A [Crassostrea gigas]
2	2	NaN	3 614 PREDICTED: 60S ribosomal protein L8-like [Papio anubis]
54	105	NaN	299 7507 minus strand filamin [Clonorchis sinensis]
2	3	NaN	680 991 minus strand PDZ and LIM domain protein 1 [Crassostrea gigas]; 553 864 minus strand PDZ and LIM domai
3	9	NaN	247 726 Annexin A7 [Crassostrea gigas]; 286 675 Annexin A7 [Crassostrea gigas]
2	2	NaN	" 241 1110 minus strand N(G),N(G)-dimethylarginine dimethylaminohydrolase 1 [Crassostrea gigas]; 241 1110 minus
2	2	NaN	3 116 Paramyosin [Crassostrea gigas]; 3 107 minus strand Paramyosin [Crassostrea gigas]
13	46	NaN	2 811 voltage-dependent anion channel 2-like protein [Haliotis diversicolor]; 2 811 voltage-dependent anion channel
8	10	NaN	3 4517 minus strand Clathrin heavy chain 1 [Crassostrea gigas]
2	3	NaN	75 455 minus strand PREDICTED: calnexin [Strongylocentrotus purpuratus]
2	2	NaN	95 271 minus strand hypothetical protein EAI_01530 [Harpegnathos saltator]
2	2	NaN	282 683 PREDICTED: ribonuclease UK114-like [Oreochromis niloticus]
2	2	NaN	40 777 proteasome subunit N3 [Haliotis discus discus]; 40 777 proteasome subunit N3 [Haliotis discus discus]
6	17	NaN	182 832 minus strand qm-like protein [Crassostrea ariakensis]
3	3	NaN	254 1648 Tektin-3 [Crassostrea gigas]
2	4	NaN	" 1 1815 Glycogen phosphorylase, muscle form [Crassostrea gigas]"
4	6	NaN	293 1795 Amidase [Crassostrea gigas]
3	3	NaN	3 224 PREDICTED: 40S ribosomal protein S14-like [Sarcophilus harrisii]
4	5	NaN	18 1613 minus strand chaperonin containing T-complex polypeptide subunit zeta [Haliotis discus hannai]; 527 2089
25	48	NaN	203 1942 ezrin/radixin/moesin [Aplysia californica] gi; 243 1982 minus strand ezrin/radixin/moesin [Aplysia californic
2	2	NaN	316 1368 minus strand hypothetical protein CGI_10006559 [Crassostrea gigas]
3	4	NaN	4 675 Laminin subunit gamma-1 [Crassostrea gigas]
2	4	NaN	103 1356 minus strand PREDICTED: actin-related protein 3-like [Oreochromis niloticus]
5	5	NaN	92 1813 minus strand Dihydropyrimidinase [Crassostrea gigas]

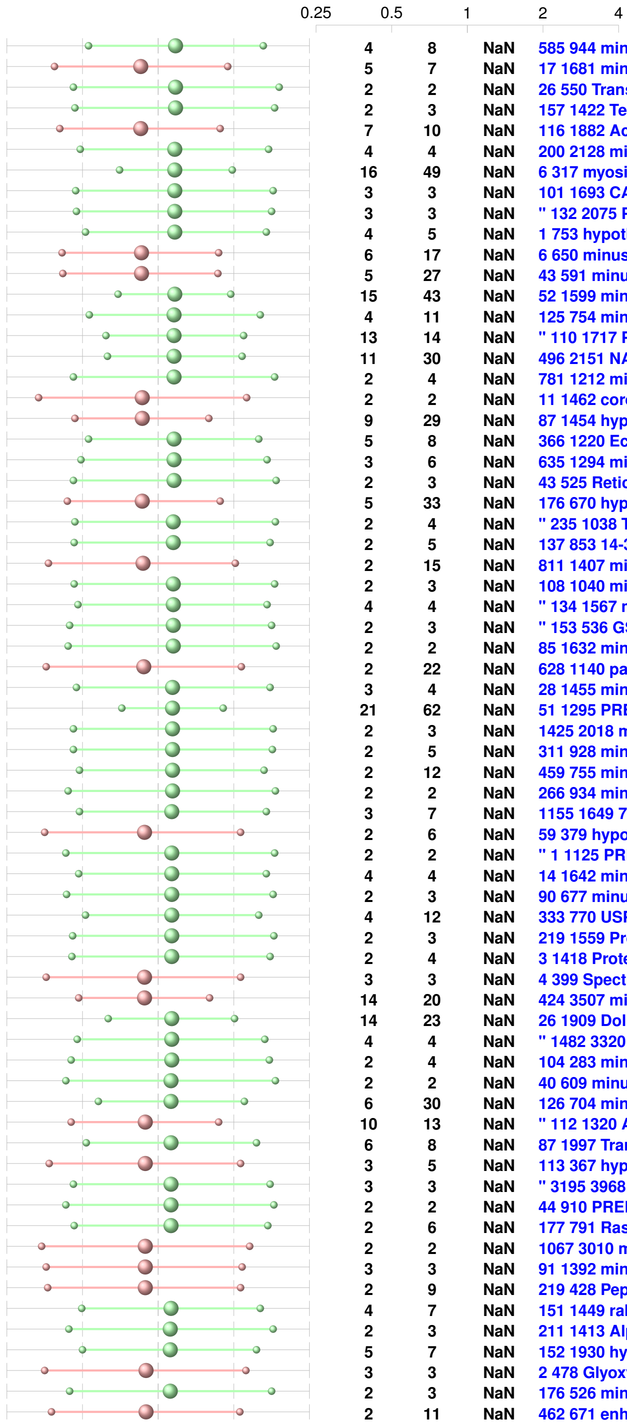

Peptides	Spectra	Coverage	Protein
4	8	NaN	585 944 minus strand Protein disulfide-isomerase A6 [Crassostrea gigas]
5	7	NaN	17 1681 minus strand Glucose-6-phosphate isomerase [Crassostrea gigas]
2	2	NaN	26 550 Translocon-associated protein subunit gamma [Crassostrea gigas]
2	3	NaN	157 1422 Tektin-2 [Crassostrea gigas]
7	10	NaN	116 1882 Actin-interacting protein 1 [Crassostrea gigas]
4	4	NaN	200 2128 minus strand heat shock protein 70 [Cristaria plicata]
16	49	NaN	6 317 myosin heavy chain cardiac muscle specific isoform 2 [Argopecten irradians]
3	3	NaN	101 1693 CALNUC [Spodoptera frugiperda]; 141 1325 Nucleobindin-2 [Crassostrea gigas]; 141 1289 Nucleobindin-2
3	3	NaN	" 132 2075 PREDICTED: arginyl-tRNA synthetase, cytoplasmic [Ornithorhynchus anatinus]; 132 2090 minus strand F
4	5	NaN	1 753 hypothetical protein BRAFLDRAFT_131251 [Branchiostoma floridae] gi
6	17	NaN	6 650 minus strand glutathione S-transferase mu class protein [Crassostrea ariakensis]; 117 761 glutathione S-trans
5	27	NaN	43 591 minus strand cyclophilin type peptidyl-prolyl cis-trans isomerase [Glossina morsitans morsitans]
15	43	NaN	52 1599 minus strand ATP synthase beta subunit [Pinctada fucata]
4	11	NaN	125 754 minus strand peptidyl prolyl cis-trans isomerase B [Conus novaehollandiae]
13	14	NaN	" 110 1717 PREDICTED: glutamate dehydrogenase, mitochondrial-like [Oreochromis niloticus]; 110 1717 PREDICTE
11	30	NaN	496 2151 NADP-dependent malic enzyme [Crassostrea gigas]
2	4	NaN	781 1212 minus strand cathepsin L2 cysteine protease [Pinctada fucata]
2	2	NaN	11 1462 coronin [Schistosoma mansoni]
9	29	NaN	87 1454 hypothetical protein BRAFLDRAFT_287788 [Branchiostoma floridae] gi; 117 1469 hypothetical protein BRAI
5	8	NaN	366 1220 Ectoine hydroxylase [Crassostrea gigas]
3	6	NaN	635 1294 minus strand Kyphoscoliosis peptidase [Crassostrea gigas]; 643 1302 minus strand Kyphoscoliosis peptic
2	3	NaN	43 525 Reticulon-4 [Crassostrea gigas]; 143 757 Reticulon-4 [Crassostrea gigas]; 43 564 Reticulon-4 [Crassostrea gi
5	33	NaN	176 670 hypothetical protein BRAFLDRAFT_122621 [Branchiostoma floridae] gi
2	4	NaN	" 235 1038 Translocon-associated protein subunit alpha [Crassostrea gigas]; 309 725 hypothetical protein [Amblyom
2	5	NaN	137 853 14-3-3 protein [Heterodera glycines]
2	15	NaN	811 1407 minus strand mitochondrial malate dehydrogenase 2 [Eospalax baileyi]
2	3	NaN	108 1040 minus strand PREDICTED: Na(+)/H(+) exchange regulatory cofactor NHE-RF3-like [Metaseiulus occidentali
4	4	NaN	" 134 1567 minus strand 6-phosphogluconate dehydrogenase, decarboxylating [Crassostrea gigas]; 134 1573 minus
2	3	NaN	" 153 536 GST omega, partial [Reishia clavigera]"
2	2	NaN	85 1632 minus strand Alpha-aminoadipic semialdehyde dehydrogenase [Crassostrea gigas]
2	22	NaN	628 1140 paramyosin [Haliotis discus discus]
3	4	NaN	28 1455 minus strand PREDICTED: probable serine carboxypeptidase CPVL-like [Anolis carolinensis]
21	62	NaN	51 1295 PREDICTED: phosphoglycerate kinase-like [Nasonia vitripennis]; 97 1341 PREDICTED: phosphoglycerate k
2	3	NaN	1425 2018 minus strand Ankyrin-2 [Crassostrea gigas]; 1425 2018 minus strand Ankyrin-2 [Crassostrea gigas]
2	5	NaN	311 928 minus strand peroxiredoxin 3 [Clonorchis sinensis] gi; 288 905 minus strand peroxiredoxin 3 [Clonorchis si
2	12	NaN	459 755 minus strand heat shock protein beta-1 [Trichinella spiralis] gi; 464 763 minus strand heat shock protein bet
2	2	NaN	266 934 minus strand Kyphoscoliosis peptidase [Crassostrea gigas]
3	7	NaN	1155 1649 78 kDa glucose-regulated protein OS=Aplysia californica PE=1 SV=1
2	6	NaN	59 379 hypothetical protein DAPPUDRAFT_230756 [Daphnia pulex]; 117 443 minus strand hypothetical protein DAPI
2	2	NaN	" 1 1125 PREDICTED: trifunctional enzyme subunit beta, mitochondrial-like [Oreochromis niloticus]"
4	4	NaN	14 1642 minus strand chaperonin containing tcp1 [Haliotis discus discus]; 14 1462 minus strand chaperonin contair
2	3	NaN	90 677 minus strand PREDICTED: 15-hydroxyprostaglandin dehydrogenase [NAD+] [Gallus gallus]; 90 683 minus str
4	12	NaN	333 770 USP-like protein isoform 2 [Crassostrea ariakensis]
2	3	NaN	219 1559 Programmed cell death protein 4 [Crassostrea gigas]
2	4	NaN	3 1418 Protein hu-li tai shao [Crassostrea gigas]; 3 1523 minus strand Protein hu-li tai shao [Crassostrea gigas]; 3 1
3	3	NaN	4 399 Spectrin beta chain [Crassostrea gigas]
14	20	NaN	424 3507 minus strand putative Na+/K+-ATPase alpha subunit [Paroctopus digueti]; 424 3507 minus strand putative
14	23	NaN	26 1909 Dolichyl-diphosphooligosaccharide–protein glycosyltransferase subunit 2 [Crassostrea gigas]
4	4	NaN	" 1482 3320 minus strand Phosphoenolpyruvate carboxykinase, cytosolic [GTP] [Crassostrea gigas]; 1326 3164 min
2	4	NaN	104 283 minus strand RecName: Full=Hemocyanin type 2 unit a; AltName: Full=Hemocyanin RHSS2 subunit; AltNan
2	2	NaN	40 609 minus strand ependymin related protein-1 precursor [Haliotis discus discus]
6	30	NaN	126 704 minus strand ribosomal protein L12 [Ixodes scapularis]
10	13	NaN	" 112 1320 Aspartate aminotransferase, cytoplasmic [Crassostrea gigas]"
6	8	NaN	87 1997 Transketolase-like protein 2 [Crassostrea gigas]; 87 1997 Transketolase-like protein 2 [Crassostrea gigas]; 8
3	5	NaN	113 367 hypothetical protein DAPPUDRAFT_329128 [Daphnia pulex]; 113 367 hypothetical protein DAPPUDRAFT_32
3	3	NaN	" 3195 3968 Spectrin alpha chain, putative [Pediculus humanus corporis] gi"
2	2	NaN	44 910 PREDICTED: probable ATP-dependent RNA helicase DDX10 [Cricetulus griseus] gi
2	6	NaN	177 791 Ras-related protein Rab-1A [Haliotis discus discus]
2	2	NaN	1067 3010 minus strand PREDICTED: nucleolin-like isoform 2 [Oreochromis niloticus]
3	3	NaN	91 1392 minus strand hypothetical protein AND_03103 [Anopheles darlingi]
2	9	NaN	219 428 Peptidyl-prolyl cis-trans isomerase OS=Hemicentrotus pulcherrimus PE=2 SV=1
4	7	NaN	151 1449 rab GDP-dissociation inhibitor [Branchiostoma belcheri]
2	3	NaN	211 1413 Alpha-N-acetylgalactosaminidase [Crassostrea gigas]
5	7	NaN	152 1930 hypothetical protein BRAFLDRAFT_223365 [Branchiostoma floridae] gi
3	3	NaN	2 478 Glyoxylate reductase/hydroxypyruvate reductase [Crassostrea gigas]
2	3	NaN	176 526 minus strand Translocon-associated protein subunit delta [Crassostrea gigas]
2	11	NaN	462 671 enhancer protein [Homo sapiens]

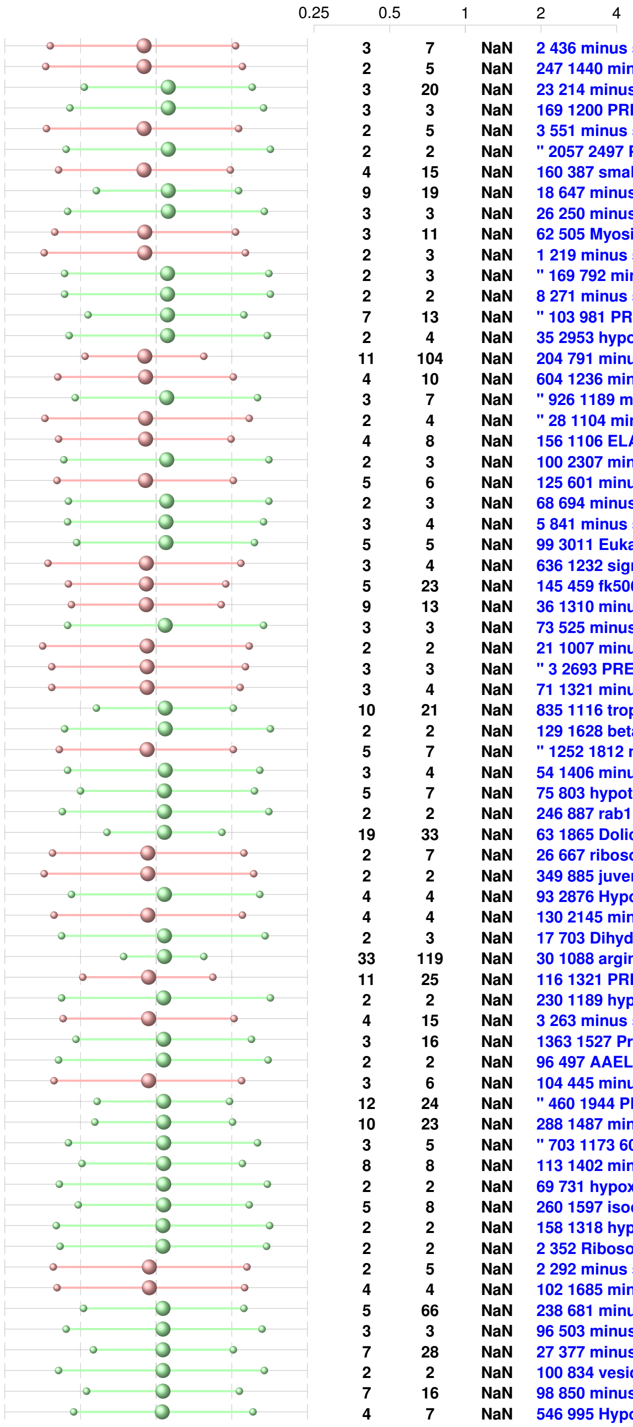

Peptides	Spectra	Coverage	Protein
3	7	NaN	2 436 minus strand PREDICTED: stress-induced-phosphoprotein 1 [Sarcophilus harrisii]
2	5	NaN	247 1440 minus strand Septin-2 [Crassostrea gigas]
3	20	NaN	23 214 minus strand hemocyanin isoform 1 [Nucula nucleus]
3	3	NaN	169 1200 PREDICTED: nidogen-2-like isoform 1 [Strongylocentrotus purpuratus]
2	5	NaN	3 551 minus strand Spectrin beta chain [Crassostrea gigas]
2	2	NaN	" 2057 2497 Phosphate carrier protein, mitochondrial [Crassostrea gigas]"
4	15	NaN	160 387 small nuclear ribonucleoprotein polypeptide G protein [Crassostrea ariakensis]; 180 407 small nuclear ribor
9	19	NaN	18 647 minus strand ATP synthase-like protein [Biomphalaria glabrata]
3	3	NaN	26 250 minus strand fructose-bisphosphate aldolase [Haliotis discus discus]
3	11	NaN	62 505 Myosin-2 essential light chain [Crassostrea gigas]
2	3	NaN	1 219 minus strand PREDICTED: nucleoside diphosphate kinase 3-like [Pan troglodytes]
2	3	NaN	" 169 792 minus strand rho GDP dissociation inhibitor, putative [Ixodes scapularis] gi"
2	2	NaN	8 271 minus strand Laminin subunit gamma-1 [Crassostrea gigas]
7	13	NaN	" 103 981 PREDICTED: ATP synthase, H+ transporting, mitochondrial F1 complex, gamma subunit isoform H (heart)
2	4	NaN	35 2953 hypothetical protein CGI_10020867 [Crassostrea gigas]; 97 3015 minus strand hypothetical protein CGI_100
11	104	NaN	204 791 minus strand PREDICTED: 15-hydroxyprostaglandin dehydrogenase [NAD+] [Gallus gallus]
4	10	NaN	604 1236 minus strand selenium-dependent glutathione peroxidase [Crassostrea gigas]; 138 770 minus strand seler
3	7	NaN	" 926 1189 minus strand Myosin heavy chain, striated muscle [Crassostrea gigas]"
2	4	NaN	" 28 1104 minus strand Pyruvate dehydrogenase E1 component subunit beta, mitochondrial [Crassostrea gigas]; 10
4	8	NaN	156 1106 ELAV 2-like protein [Aplysia californica] gi
2	3	NaN	100 2307 minus strand Xanthine dehydrogenase/oxidase [Crassostrea gigas]
5	6	NaN	125 601 minus strand omega class glutathione-s-transferase [Chlamys farreri]; 113 808 minus strand omega class g
2	3	NaN	68 694 minus strand hypothetical protein BRAFLDRAFT_272416 [Branchiostoma floridae] gi; 68 667 minus strand hy
3	4	NaN	5 841 minus strand Spectrin alpha chain [Crassostrea gigas]
5	5	NaN	99 3011 Eukaryotic translation initiation factor 3 subunit A [Crassostrea gigas]
3	4	NaN	636 1232 sigma class glutathione-s-transferase 3 [Haliotis discus discus]; 963 1559 sigma class glutathione-s-transf
5	23	NaN	145 459 fk506-binding protein 2 [Papilio xuthus]; 237 551 fk506-binding protein 2 [Papilio xuthus]
9	13	NaN	36 1310 minus strand elongation factor-1 gamma [Diguetia canities]
3	3	NaN	73 525 minus strand Actin-related protein 2/3 complex subunit 5-like protein [Salmo salar] gi; 73 525 Actin-related pr
2	2	NaN	21 1007 minus strand cathepsin L2 cysteine protease [Pinctada fucata]; 21 1007 minus strand cathepsin L2 cysteine
3	3	NaN	" 3 2693 PREDICTED: similar to coatomer protein complex, subunit beta 2 (beta prime) [Taeniopygia guttata]"
3	4	NaN	71 1321 minus strand Vinculin [Crassostrea gigas]
10	21	NaN	835 1116 tropomyosin [Ilyanassa obsoleta]
2	2	NaN	129 1628 beta-hexosaminidase subunit beta precursor [Danio rerio] gi
5	7	NaN	" 1252 1812 minus strand Alanine–glyoxylate aminotransferase 2, mitochondrial [Crassostrea gigas]"
3	4	NaN	54 1406 minus strand Protein henna [Crassostrea gigas]; 54 1406 minus strand Protein henna [Crassostrea gigas]
5	7	NaN	75 803 hypothetical protein DAPPUDRAFT_231350 [Daphnia pulex]
2	2	NaN	246 887 rab11-2 [Limulus polyphemus]
19	33	NaN	63 1865 Dolichyl-diphosphooligosaccharide–protein glycosyltransferase subunit 1 [Crassostrea gigas]; 63 1865 Dol
2	7	NaN	26 667 ribosomal protein L15 [Acipenser sinensis] gi; 26 667 ribosomal protein L15 [Acipenser sinensis] gi; 26 667 r
2	2	NaN	349 885 juvenile hormone diol kinase [Danaus plexippus]; 349 885 juvenile hormone diol kinase [Danaus plexippus]
4	4	NaN	93 2876 Hypoxia up-regulated protein 1 [Crassostrea gigas]
4	4	NaN	130 2145 minus strand Glucosamine–fructose-6-phosphate aminotransferase [isomerizing] 1 [Crassostrea gigas]; 1
2	3	NaN	17 703 Dihydropteridine reductase [Crassostrea gigas]
33	119	NaN	30 1088 arginine kinase [Conus novaehollandiae]
11	25	NaN	116 1321 PREDICTED: similar to aspartate aminotransferase [Tribolium castaneum] gi
2	2	NaN	230 1189 hypothetical protein [Amblyomma maculatum]
4	15	NaN	3 263 minus strand beta-actin [Urechis unicinctus]
3	16	NaN	1363 1527 Protein disulfide-isomerase A3 [Crassostrea gigas]
2	2	NaN	96 497 AAEL009772-PB [Aedes aegypti]
3	6	NaN	104 445 minus strand macrophage migration inhibitory factor [Ascaris suum]
12	24	NaN	" 460 1944 PREDICTED: succinyl-CoA:3-ketoacid-coenzyme A transferase 1, mitochondrial [Canis lupus familiaris]"
10	23	NaN	288 1487 minus strand putative procollagen type VI alpha 4 [Haliotis diversicolor]
3	5	NaN	" 703 1173 60S acidic ribosomal protein P0, putative [Ixodes scapularis] gi"
8	8	NaN	113 1402 minus strand S-adenosylhomocysteine hydrolase [Crassostrea ariakensis]; 113 1402 minus strand S-aden
2	2	NaN	69 731 hypoxanthine phosphoribosyltransferase 1 [Haliotis discus discus]
5	8	NaN	260 1597 isocitrate dehydrogenase [Mytilus galloprovincialis]; 260 1597 isocitrate dehydrogenase [Mytilus galloprov
2	2	NaN	158 1318 hypothetical protein BRAFLDRAFT_98992 [Branchiostoma floridae] gi
2	2	NaN	2 352 Ribosome-binding protein 1 [Crassostrea gigas]
2	5	NaN	2 292 minus strand H2 [Haliotis tuberculata]
4	4	NaN	102 1685 minus strand predicted protein [Nematostella vectensis] gi
5	66	NaN	238 681 minus strand Globin OS=Cerithidea rhizophorarum PE=1 SV=1
3	3	NaN	96 503 minus strand hypothetical protein DAPPUDRAFT_230378 [Daphnia pulex]
7	28	NaN	27 377 minus strand ribosomal protein LP2 [Papilio xuthus] gi; 27 377 minus strand ribosomal protein LP2 [Papilio x
2	2	NaN	100 834 vesicle-associated membrane protein/synaptobrevin-binding protein [Aplysia californica] gi
7	16	NaN	98 850 minus strand 14-3-3 protein epsilon [Schistocerca gregaria]
4	7	NaN	546 995 Hypothetical protein CBG12002 [Caenorhabditis briggsae]; 543 995 minus strand PREDICTED: tubulin polyr

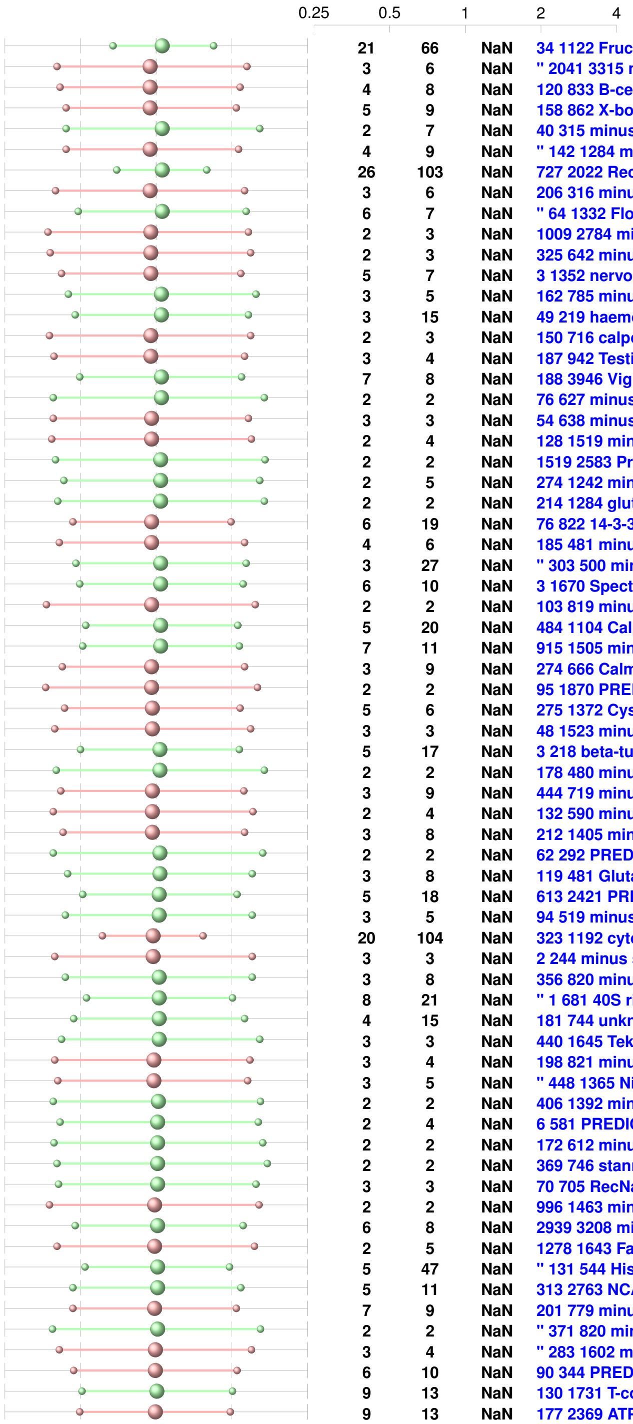

Peptides	Spectra	Coverage	Protein
21	66	NaN	34 1122 Fructose-bisphosphate aldolase [Crassostrea gigas]
3	6	NaN	" 2041 3315 minus strand Myosin-10 OS=Mus musculus GN=Myh10 PE=1 SV=2; 2041 3345 minus strand Myosin hea
4	8	NaN	120 833 B-cell receptor-associated protein 31 [Crassostrea gigas]; 238 951 B-cell receptor-associated protein 31 [Cr
5	9	NaN	158 862 X-box binding protein [Haliotis discus discus]
2	7	NaN	40 315 minus strand 60S ribosomal protein L18a OS=Homo sapiens GN=RPL18A PE=1 SV=2
4	9	NaN	" 142 1284 minus strand Chain O, Crsytal Structure Of Human Liver Gapdh gi|67464044|pdb|1ZNQ|P Chain P, Crsyta
26	103	NaN	727 2022 RecName: Full=Enolase; AltName: Full=2-phospho-D-glycerate hydro-lyase; AltName: Full=2-phosphoglyc
3	6	NaN	206 316 minus strand ribosomal protein S17 [Branchiostoma belcheri]
6	7	NaN	" 64 1332 Flotillin-1, putative [Pediculus humanus corporis] gi"
2	3	NaN	1009 2784 minus strand Collagen alpha-3(VI) chain [Crassostrea gigas]
2	3	NaN	325 642 minus strand Protein phosphatase 1B [Crassostrea gigas]
5	7	NaN	3 1352 nervous system adducin [Aplysia californica] gi; 3 1418 Protein hu-li tai shao [Crassostrea gigas]; 3 1214 ner
3	5	NaN	162 785 minus strand Ropporin-1-like protein OS=Xenopus laevis GN=ropn1l PE=2 SV=1
3	15	NaN	49 219 haemocyanin precursor [Nautilus pompilius]
2	3	NaN	150 716 calponin/transgelin [Papilio xuthus]
3	4	NaN	187 942 Testis-specific gene 10 protein OS=Mus musculus GN=Tsga10 PE=1 SV=1; 212 967 Testis-specific gene 10 p
7	8	NaN	188 3946 Vigilin [Crassostrea gigas]; 188 4135 Vigilin [Crassostrea gigas]
2	2	NaN	76 627 minus strand Heme-binding protein 2 [Crassostrea gigas]; 296 847 minus strand Heme-binding protein 2 [Cra
3	3	NaN	54 638 minus strand ubiquitin carboxyl-terminal hydrolase [Aplysia californica] gi
2	4	NaN	128 1519 minus strand V-type proton ATPase subunit H [Crassostrea gigas]; 128 1519 minus strand V-type proton AT
2	2	NaN	1519 2583 Proliferation-associated protein 2G4 [Crassostrea gigas]; 565 1629 Proliferation-associated protein 2G4 [C
2	5	NaN	274 1242 minus strand cathepsin D [Pinctada maxima]; 290 1387 cathepsin D [Pinctada maxima]; 290 1402 minus st
2	2	NaN	214 1284 glutamine synthetase [Tegillarca granosa]
6	19	NaN	76 822 14-3-3 protein [Heterodera glycines]; 76 822 minus strand 14-3-3 protein [Heterodera glycines]; 76 822 minus
4	6	NaN	185 481 minus strand Zgc:56530 [Danio rerio]
3	27	NaN	" 303 500 minus strand protein lethal, putative [Pediculus humanus corporis] gi"
6	10	NaN	3 1670 Spectrin alpha chain [Crassostrea gigas]
2	2	NaN	103 819 minus strand PREDICTED: l-xylulose reductase-like [Monodelphis domestica]
5	20	NaN	484 1104 Calponin-2 [Crassostrea gigas]; 309 938 Calponin-2 [Crassostrea gigas]
7	11	NaN	915 1505 minus strand Collagen alpha-5(VI) chain [Crassostrea gigas]
3	9	NaN	274 666 Calmodulin-4 [Heterocephalus glaber]
2	2	NaN	95 1870 PREDICTED: acetylcholinesterase-like [Equus caballus]; 99 1874 PREDICTED: acetylcholinesterase-like [Eq
5	6	NaN	275 1372 Cystathionine gamma-lyase OS=Bos taurus GN=CTH PE=2 SV=1
3	3	NaN	48 1523 minus strand Spermatogenesis-associated protein 18-like protein [Crassostrea gigas]
5	17	NaN	3 218 beta-tubulin [Fasciola hepatica] gi
2	2	NaN	178 480 minus strand PREDICTED: lipoxygenase homology domain-containing protein 1-like [Danio rerio]
3	9	NaN	444 719 minus strand 60S ribosomal protein L9 [Laodelphax striatella]
2	4	NaN	132 590 minus strand Non-neuronal cytoplasmic intermediate filament protein OS=Helix aspersa PE=3 SV=2
3	8	NaN	212 1405 minus strand unnamed protein product [Tetraodon nigroviridis]
2	2	NaN	62 292 PREDICTED: heterogeneous nuclear ribonucleoprotein F-like [Otolemur garnettii]
3	8	NaN	119 481 Glutamate receptor U1 [Crassostrea gigas]
5	18	NaN	613 2421 PREDICTED: lysosomal alpha-glucosidase [Danio rerio]
3	5	NaN	94 519 minus strand hypothetical protein BRAFLDRAFT_115029 [Branchiostoma floridae] gi
20	104	NaN	323 1192 cytosolic malate dehydrogenase [Lottia scabra]; 323 1192 cytosolic malate dehydrogenase [Lottia scabra];
3	3	NaN	2 244 minus strand hypothetical protein KGM_08771 [Danaus plexippus]
3	8	NaN	356 820 minus strand calmodulin-1 [Trichinella spiralis] gi
8	21	NaN	" 1 681 40S ribosomal protein S4, X isoform [Crassostrea gigas]"
4	15	NaN	181 744 unknown [Dendroctonus ponderosae]
3	3	NaN	440 1645 Tektin-1 [Crassostrea gigas]
3	4	NaN	198 821 minus strand sigma class glutathione-s-transferase 2 [Haliotis discus discus]
3	5	NaN	" 448 1365 Nitrile-specifier protein 5, partial [Crassostrea gigas]"
2	2	NaN	406 1392 minus strand hypothetical protein CGI_10007007 [Crassostrea gigas]
2	4	NaN	6 581 PREDICTED: mammalian ependymin-related protein 1 [Gallus gallus]
2	2	NaN	172 612 minus strand putative polyadenylate-binding protein 1 [Haliotis diversicolor]; 172 612 putative polyadenylate
2	2	NaN	369 746 stanniocalcin [Haliotis diversicolor supertexta] gi
3	3	NaN	70 705 RecName: Full=Ras-related protein Rab-2 gi|288938|emb|CAA51234.1| RAB2 [Lymnaea stagnalis]; 70 705 Re
2	2	NaN	996 1463 minus strand catalase [Cristaria plicata] gi
6	8	NaN	2939 3208 minus strand beta-thymosin domain repeat protein CSP24KDa_v1 [Hermissenda crassicornis] gi
2	5	NaN	1278 1643 Fatty acid-binding-like protein 5 [Crassostrea gigas]
5	47	NaN	" 131 544 Histone H2B, gonadal OS=Patella granatina PE=1 SV=2"
5	11	NaN	313 2763 NCAM-related cell adhesion molecule precursor [Aplysia californica] gi
7	9	NaN	201 779 minus strand sigma class glutathione-s-transferase 2 [Haliotis discus discus]
2	2	NaN	" 371 820 minus strand Peroxiredoxin-5, mitochondrial [Crassostrea gigas]; 91 528 minus strand Peroxiredoxin-5, m
3	4	NaN	" 283 1602 minus strand 4-aminobutyrate aminotransferase, mitochondrial [Crassostrea gigas]"
6	10	NaN	90 344 PREDICTED: diazepam binding inhibitor-like [Saccoglossus kowalevskii]
9	13	NaN	130 1731 T-complex protein 1 subunit epsilon [Crassostrea gigas]; 130 1731 T-complex protein 1 subunit epsilon [Cr
9	13	NaN	177 2369 ATPase family AAA domain-containing protein 2B [Crassostrea gigas]; 177 2369 ATPase family AAA doma

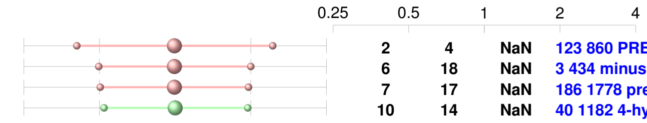

Peptides	Spectra	Coverage	Protein
2	4	NaN	123 860 PREDICTED: proteasome subunit alpha type-6-like [Cavia porcellus]; 1164 1901 PREDICTED: proteasome s
6	18	NaN	3 434 minus strand myosin essential light chain [Haliotis discus discus]
7	17	NaN	186 1778 predicted protein [Nematostella vectensis] gi
10	14	NaN	40 1182 4-hydroxyphenylpyruvate dioxygenase [Crassostrea gigas]

4.2 Proteins Identified by a Single Peptide

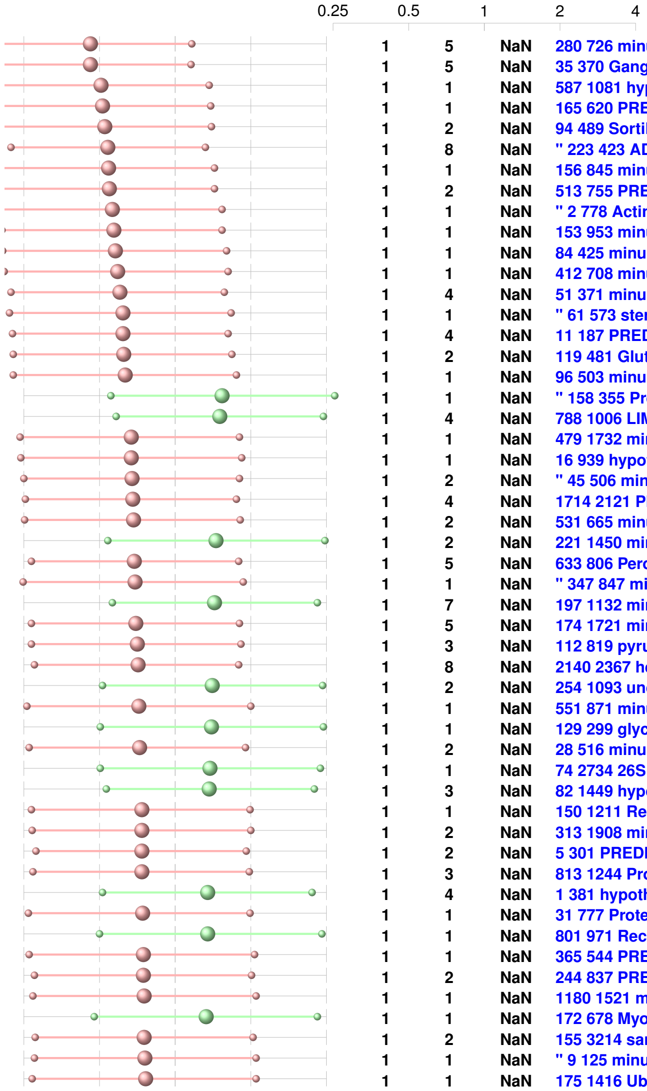

Peptides	Spectra	Coverage	Protein
1	5	NaN	280 726 minus strand RecName: Full=Calmodulin; Short=CaM gi|14588595|dbj|BAB61794.1| calmodulin [Metridium
1	5	NaN	35 370 Ganglioside GM2 activator [Crassostrea gigas]; 2 487 minus strand PREDICTED: hypothetical protein LOC10
1	1	NaN	587 1081 hypothetical protein CGI_10026502 [Crassostrea gigas]
1	1	NaN	165 620 PREDICTED: uncharacterized protein LOC575072 isoform 2 [Strongylocentrotus purpuratus] gi
1	2	NaN	94 489 Sortilin-related receptor [Crassostrea gigas]
1	8	NaN	" 223 423 ADPATP carrier protein; K05863 solute carrier family 25 (mitochondrial adenine nucleotide translocator), n
1	1	NaN	156 845 minus strand histone H1 [Mytilus californianus]; 156 845 minus strand histone H1 [Mytilus californianus]; 15
1	2	NaN	513 755 PREDICTED: 40S ribosomal protein S16-like [Papio anubis]; 513 755 PREDICTED: 40S ribosomal protein S1
1	1	NaN	" 2 778 Actin, gamma-enteric smooth muscle OS=Rattus norvegicus GN=Actg2 PE=2 SV=1"
1	1	NaN	153 953 minus strand ribosomal protein rpl7 [Arenicola marina]
1	1	NaN	84 425 minus strand Transcription elongation factor B polypeptide 2 [Crassostrea gigas]; 84 425 minus strand Trans
1	1	NaN	412 708 minus strand heat shock protein beta-1 [Trichinella spiralis] gi; 459 755 minus strand heat shock protein bet
1	4	NaN	51 371 minus strand putative mitochondrial ATP synthase F chain [Haliotis discus discus]; 51 371 minus strand puta
1	1	NaN	" 61 573 steroid membrane receptor Hpr6.6/25-Dx, putative [Ixodes scapularis] gi"
1	4	NaN	11 187 PREDICTED: 40S ribosomal protein S28-like [Strongylocentrotus purpuratus]
1	2	NaN	119 481 Glutamate receptor U1 [Crassostrea gigas]; 113 328 glutamate receptor subunit protein GluR2 precursor [A
1	1	NaN	96 503 minus strand mitochondrial ATP synthase delta chain [Haliotis discus discus]; 111 551 minus strand mitocho
1	1	NaN	" 158 355 Protein NAS-37, isoform b [Caenorhabditis elegans] gi; 15 227 Hemicentin-1 [Crassostrea gigas]"
1	4	NaN	788 1006 LIM protein [Haliotis discus discus]; 768 980 muscle LIM protein isoform 1 [Danaus plexippus]
1	1	NaN	479 1732 minus strand Multifunctional protein ADE2 [Crassostrea gigas]; 239 1501 minus strand Multifunctional pro
1	1	NaN	16 939 hypothetical protein BRAFLDRAFT_87838 [Branchiostoma floridae] gi
1	2	NaN	" 45 506 minus strand PREDICTED: dihydrolipoyl dehydrogenase, mitochondrial [Monodelphis domestica]"
1	4	NaN	1714 2121 Phosphoenolpyruvate carboxykinase [GTP] [Crassostrea gigas]
1	2	NaN	531 665 minus strand Proteasome subunit beta type-6 [Crassostrea gigas]
1	2	NaN	221 1450 minus strand PREDICTED: dihydrolipoamide branched chain transacylase E2 [Taeniopygia guttata]; 221 16
1	5	NaN	633 806 Peroxiredoxin-1 [Crassostrea gigas]; 648 821 Peroxiredoxin-1 [Crassostrea gigas]; 666 839 Peroxiredoxin-1
1	1	NaN	" 347 847 minus strand ADP,ATP carrier protein 1 OS=Anopheles gambiae GN=AGAP006782 PE=2 SV=2"
1	7	NaN	197 1132 minus strand Pancreatic lipase-related protein 2 [Crassostrea gigas]
1	5	NaN	174 1721 minus strand aspartyl-tRNA synthetase [Xenopus laevis] gi
1	3	NaN	112 819 pyruvate kinase [Crassostrea gigas]; 132 932 pyruvate kinase [Crassostrea gigas]
1	8	NaN	2140 2367 heat shock cognate 71 [Mytilus galloprovincialis]
1	2	NaN	254 1093 uncharacterized protein LOC432187 precursor [Xenopus laevis] gi; 343 561 minus strand cathepsin Z prec
1	1	NaN	551 871 minus strand Protein disulfide-isomerase A4 [Crassostrea gigas]
1	1	NaN	129 299 glycogen phosphorylase [Crassostrea gigas]
1	2	NaN	28 516 minus strand Signal peptidase complex subunit 3 [Crassostrea gigas]; 30 554 minus strand Signal peptidase
1	1	NaN	74 2734 26S proteasome non-ATPase regulatory subunit 2 [Crassostrea gigas]
1	3	NaN	82 1449 hypothetical protein BRAFLDRAFT_127120 [Branchiostoma floridae] gi
1	1	NaN	150 1211 RecName: Full=Guanine nucleotide-binding protein G(i) subunit alpha; AltName: Full=Adenylate cyclase-in
1	2	NaN	313 1908 minus strand Heterogeneous nuclear ribonucleoprotein L [Crassostrea gigas]; 313 1833 minus strand Hete
1	2	NaN	5 301 PREDICTED: hypothetical protein [Saccoglossus kowalevskii]
1	3	NaN	813 1244 Probable protein disulfide-isomerase A6 OS=Caenorhabditis elegans GN=tag-320 PE=2 SV=1
1	4	NaN	1 381 hypothetical protein [Eimeria tenella]
1	1	NaN	31 777 Proteasome subunit alpha type-7-like protein [Crassostrea gigas]
1	1	NaN	801 971 RecName: Full=Tropomyosin-2; AltName: Full=Tropomyosin II; Short=BgTMII; Short=TMII gi|155940|gb|AA
1	1	NaN	365 544 PREDICTED: cofilin-2 [Otolemur garnettii]
1	2	NaN	244 837 PREDICTED: ras-like GTP-binding protein Rho1-like [Megachile rotundata]; 650 883 hypothetical protein SIN
1	1	NaN	1180 1521 minus strand coronin [Schistosoma mansoni]
1	1	NaN	172 678 Myosin regulatory light chain sqh [Crassostrea gigas]
1	2	NaN	155 3214 sarco/endoplasmic reticulum calcium ATPase isoform B [Pinctada fucata]; 132 3110 sarco/endoplasmic ret
1	1	NaN	" 9 125 minus strand Hemocyanin, beta-C chain unit D (Fragment) OS=Helix pomatia PE=1 SV=2"
1	1	NaN	175 1416 Ubiquinone biosynthesis monooxygenase COQ6 [Crassostrea gigas]

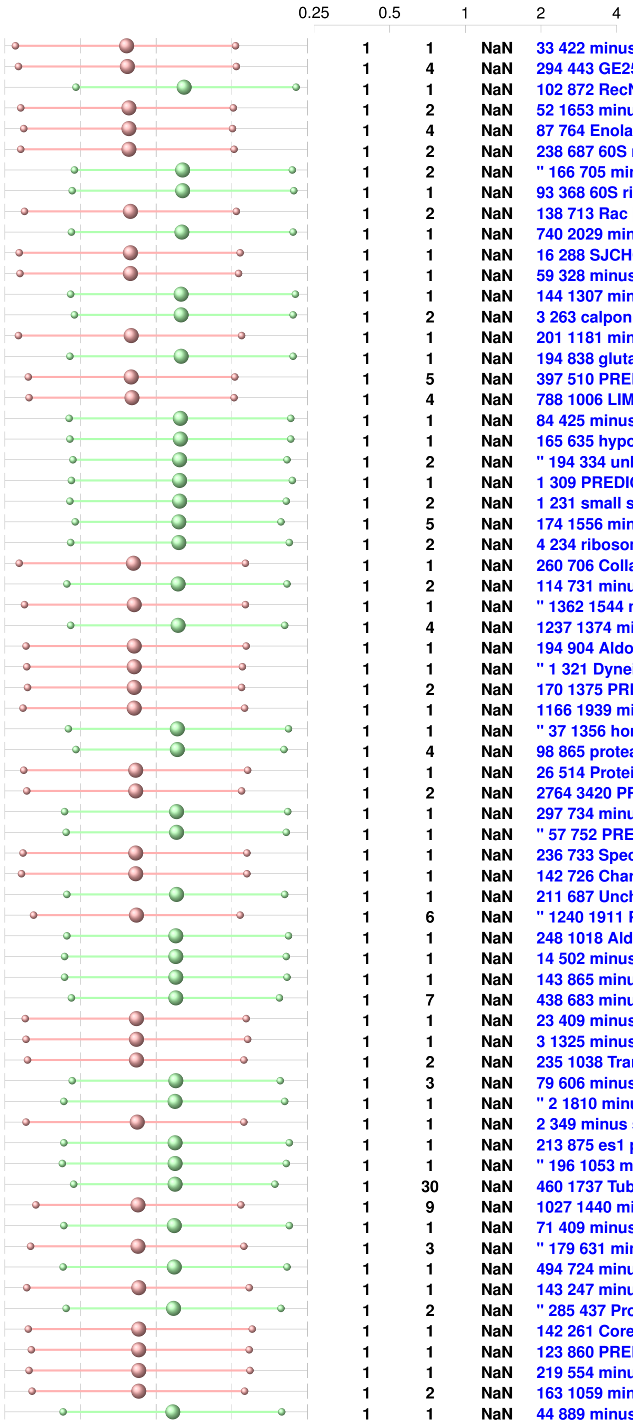

Peptides	Spectra	Coverage	Protein
1	1	NaN	33 422 minus strand PREDICTED: heat shock 70 kDa protein cognate 4-like [Megachile rotundata]
1	4	NaN	294 443 GE25988 gene product from transcript GE25988-RA; K02980 small subunit ribosomal protein S29e; 292 441
1	1	NaN	102 872 RecName: Full=Gelsolin-like protein 1; AltName: Full=Actin-modulator; Short=EWAM; Short=EWAM-P1 gi|1
1	2	NaN	52 1653 minus strand PREDICTED: eukaryotic translation initiation factor 3 subunit 6 interacting protein-like [Sacco
1	4	NaN	87 764 Enolase-phosphatase E1 [Crassostrea gigas]; 87 1058 Enolase-phosphatase E1 [Crassostrea gigas]; 87 764
1	2	NaN	238 687 60S ribosomal protein L6 [Crassostrea gigas]; 142 789 60S ribosomal protein L6 [Crassostrea gigas]
1	2	NaN	" 166 705 minus strand ADP-ribosylation factor, partial [Antheraea yamamai]; 166 705 minus strand ADP-ribosylatio
1	1	NaN	93 368 60S ribosomal protein L37A [Branchiostoma belcheri] gi; 93 395 60S ribosomal protein L37A [Branchiostoma
1	2	NaN	138 713 Rac [Aplysia californica] gi; 125 691 Rac [Aplysia californica] gi; 149 871 minus strand Rac [Aplysia californ
1	1	NaN	740 2029 minus strand PREDICTED: protein alan shepard-like isoform 2 [Bombus terrestris]
1	1	NaN	16 288 SJCHGC01950 protein [Schistosoma japonicum] gi
1	1	NaN	59 328 minus strand PREDICTED: similar to predicted protein [Hydra magnipapillata]; 59 328 PREDICTED: similar to
1	1	NaN	144 1307 minus strand PREDICTED: similar to AGAP005845-PA [Tribolium castaneum]; 146 1309 minus strand PRED
1	2	NaN	3 263 calponin-like protein [Taenia asiatica]
1	1	NaN	201 1181 minus strand Serine/threonine-protein phosphatase PP1-alpha catalytic subunit [Crassostrea gigas]
1	1	NaN	194 838 glutathione S-transferase class mu [Cyphoma gibbosum]
1	5	NaN	397 510 PREDICTED: actin-5C-like [Megachile rotundata]
1	4	NaN	788 1006 LIM protein [Haliotis discus discus]
1	1	NaN	84 425 minus strand thioester-containing protein [Euphaedusa tau]
1	1	NaN	165 635 hypothetical protein CGI_10007639 [Crassostrea gigas]
1	2	NaN	" 194 334 unknown, partial [Lymnaea stagnalis] gi; 207 347 unknown, partial [Lymnaea stagnalis] gi; 384 524 unknow
1	1	NaN	1 309 PREDICTED: intersectin-1-like [Sus scrofa]
1	2	NaN	1 231 small subunit ribosomal protein S11e [Clonorchis sinensis]; 1 171 40S ribosomal protein S11 OS=Canis famili
1	5	NaN	174 1556 minus strand aspartyl-tRNA synthetase [Xenopus laevis] gi; 174 1721 minus strand aspartyl-tRNA synthet
1	2	NaN	4 234 ribosomal protein rpl14 [Arenicola marina]
1	1	NaN	260 706 Collagen alpha-1(XII) chain (Fragment) OS=Notophthalmus viridescens PE=2 SV=1
1	2	NaN	114 731 minus strand Proteasome subunit beta type-6 [Crassostrea gigas]
1	1	NaN	" 1362 1544 minus strand Phosphoenolpyruvate carboxykinase, cytosolic [GTP] [Crassostrea gigas]"
1	4	NaN	1237 1374 minus strand putative 60S ribosomal protein L23 [Maconellicoccus hirsutus]
1	1	NaN	194 904 Aldo-keto reductase family 1 member B10 [Crassostrea gigas]
1	1	NaN	" 1 321 Dynein heavy chain, cytoplasmic [Crassostrea gigas]"
1	2	NaN	170 1375 PREDICTED: sialate O-acetylesterase-like [Oreochromis niloticus]
1	1	NaN	1166 1939 minus strand Calcium-binding mitochondrial carrier protein Aralar2 OS=Homo sapiens GN=SLC25A13 PE
1	1	NaN	" 37 1356 homogentisate 1,2-dioxygenase [Danio rerio] gi; 54 1361 homogentisate 1,2-dioxygenase [Danio rerio] gi"
1	4	NaN	98 865 proteasome (prosome macropain) subunit alpha type 1 [Xenopus (Silurana) tropicalis]; 98 865 proteasome (p
1	1	NaN	26 514 Protein phosphatase inhibitor 2 [Crassostrea gigas]
1	2	NaN	2764 3420 PREDICTED: filamin-C-like [Nasonia vitripennis]
1	1	NaN	297 734 minus strand PREDICTED: calmodulin [Canis lupus familiaris]; 297 734 PREDICTED: calmodulin [Canis lupu
1	1	NaN	" 57 752 PREDICTED: adenylate kinase 2, mitochondrial-like isoform 2 [Callithrix jacchus]"
1	1	NaN	236 733 Spectrin alpha chain [Crassostrea gigas]
1	1	NaN	142 726 Charged multivesicular body protein 4b [Crassostrea gigas]
1	1	NaN	211 687 Uncharacterized protein CG16817 [Harpegnathos saltator]; 200 676 Uncharacterized protein CG16817 [Harp
1	6	NaN	" 1240 1911 PREDICTED: ATP synthase subunit alpha, mitochondrial-like [Anolis carolinensis]"
1	1	NaN	248 1018 Aldo-keto reductase family 1 member B10 [Crassostrea gigas]; 44 730 minus strand Aldo-keto reductase fa
1	1	NaN	14 502 minus strand Ufm1-conjugating enzyme 1 [Crassostrea gigas]
1	1	NaN	143 865 minus strand predicted protein [Nematostella vectensis] gi
1	7	NaN	438 683 minus strand Protein lethal(2)essential for life OS=Drosophila melanogaster GN=l(2)efl PE=1 SV=1
1	1	NaN	23 409 minus strand CDGSH iron sulfur domain-containing protein 2 [Crassostrea gigas]; 1 207 minus strand CDGS
1	1	NaN	3 1325 minus strand Eukaryotic translation initiation factor 3 subunit B [Crassostrea gigas]
1	2	NaN	235 1038 Translocon-associated protein subunit alpha [Crassostrea gigas]
1	3	NaN	79 606 minus strand PREDICTED: similar to Ras-like protein 3 precursor (Protein roughened) [Tribolium castaneum]
1	1	NaN	" 2 1810 minus strand Glutaminyl-tRNA synthetase, partial [Crassostrea gigas]"
1	1	NaN	2 349 minus strand hypothetical protein BRAFLDRAFT_206907 [Branchiostoma floridae] gi
1	1	NaN	213 875 es1 protein [Haliotis discus discus]
1	1	NaN	" 196 1053 minus strand Cytochrome b-c1 complex subunit 2, mitochondrial OS=Homo sapiens GN=UQCRC2 PE=1
1	30	NaN	460 1737 Tubulin beta-4B chain OS=Mus musculus GN=Tubb4b PE=1 SV=1
1	9	NaN	1027 1440 minus strand hemocyanin isoform 1 [Haliotis diversicolor]
1	1	NaN	71 409 minus strand heat shock protein 108 [Alligator mississippiensis]
1	3	NaN	" 179 631 minus strand 60S ribosomal protein L7/L12, partial [Ostrea edulis]"
1	1	NaN	494 724 minus strand PREDICTED: SH3 domain-binding glutamic acid-rich-like protein 3-like [Amphimedon queensl
1	1	NaN	143 247 minus strand dynein light chain [Aplysia californica] gi
1	2	NaN	" 285 437 Propionyl-CoA carboxylase alpha chain, mitochondrial [Crassostrea gigas]"
1	1	NaN	142 261 Core histone macro-H2A.2 OS=Mus musculus GN=H2afy2 PE=1 SV=3
1	1	NaN	123 860 PREDICTED: 3-hydroxybutyrate dehydrogenase type 2-like isoform 1 [Sus scrofa] gi; 32 901 3-hydroxybutyr
1	1	NaN	219 554 minus strand ribosomal protein P1 [Biomphalaria glabrata]
1	2	NaN	163 1059 minus strand Heterogeneous nuclear ribonucleoprotein A2-like protein 1 [Crassostrea gigas]; 99 995 minu
1	1	NaN	44 889 minus strand hypothetical protein CGI_10015025 [Crassostrea gigas]

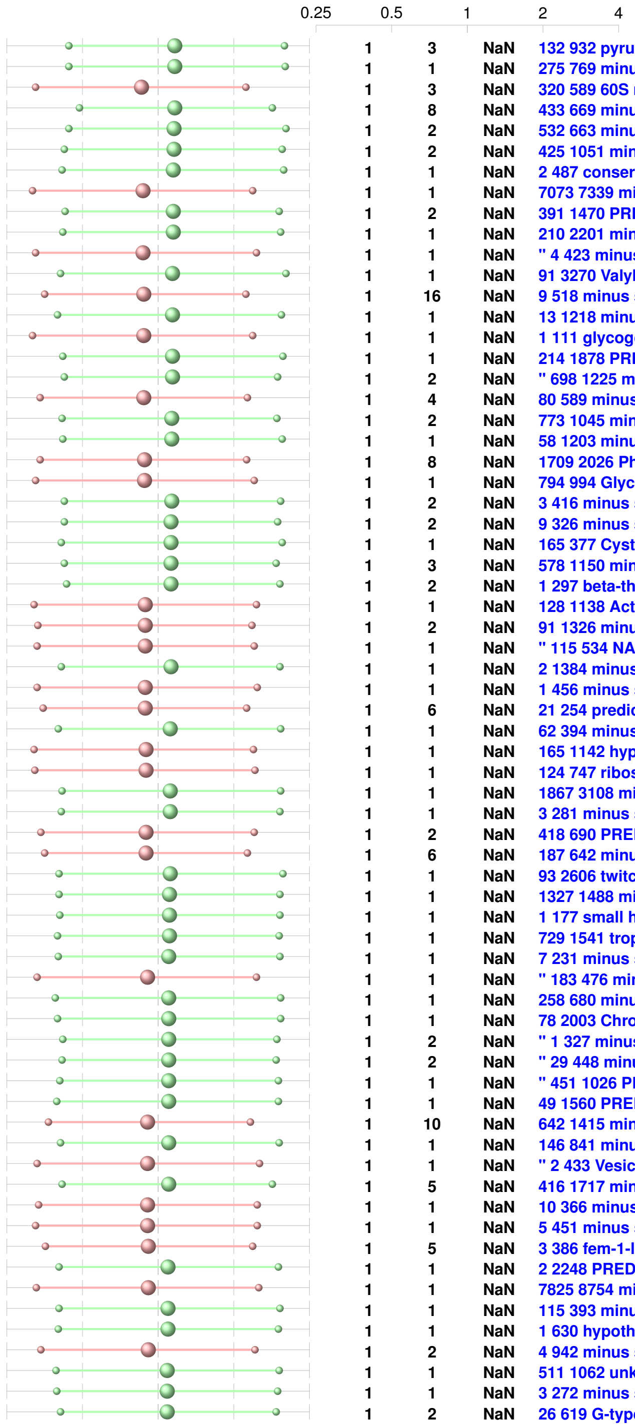

Peptides	Spectra	Coverage	Protein
1	3	NaN	132 932 pyruvate kinase [Crassostrea gigas]
1	1	NaN	275 769 minus strand hypothetical protein CGI_10025135 [Crassostrea gigas]; 216 683 hypothetical protein CGI_100
1	3	NaN	320 589 60S ribosomal protein L34 OS=Aedes triseriatus GN=RpL34 PE=2 SV=3
1	8	NaN	433 669 minus strand Superoxide dismutase [Cu-Zn] OS=Prionace glauca GN=sod1 PE=1 SV=1
1	2	NaN	532 663 minus strand 40S ribosomal protein S23 [Ascaris suum]; 526 654 minus strand RecName: Full=40S ribosom
1	2	NaN	425 1051 minus strand unknown [Schistosoma japonicum]
1	1	NaN	2 487 conserved hypothetical protein [Ixodes scapularis] gi
1	1	NaN	7073 7339 minus strand Collagen alpha-3(VI) chain [Crassostrea gigas]
1	2	NaN	391 1470 PREDICTED: heparanase-like [Amphimedon queenslandica]; 391 1470 PREDICTED: heparanase-like [Amp
1	1	NaN	210 2201 minus strand Glycyl-tRNA synthetase [Crassostrea gigas]; 210 2201 minus strand Glycyl-tRNA synthetase
1	1	NaN	" 4 423 minus strand PREDICTED: similar to NADH dehydrogenase, putative [Tribolium castaneum] gi"
1	1	NaN	91 3270 Valyl-tRNA synthetase [Crassostrea gigas]
1	16	NaN	9 518 minus strand hemocyanin isoform 1 [Nucula nucleus]
1	1	NaN	13 1218 minus strand PREDICTED: eukaryotic initiation factor 4A-III-like [Bombus terrestris] gi
1	1	NaN	1 111 glycogen synthase [Crassostrea gigas]
1	1	NaN	214 1878 PREDICTED: lysyl-tRNA synthetase-like [Oreochromis niloticus]
1	2	NaN	" 698 1225 minus strand actin related protein 2/3 complex, 21 kDa subunit [Crassostrea gigas]"
1	4	NaN	80 589 minus strand PREDICTED: adenine phosphoribosyltransferase-like [Strongylocentrotus purpuratus]; 328 468
1	2	NaN	773 1045 minus strand serine protease inhibitor 2 [Latrodectus hesperus]
1	1	NaN	58 1203 minus strand Obg-like ATPase 1 [Crassostrea gigas]
1	8	NaN	1709 2026 Phosphoenolpyruvate carboxykinase [GTP] [Crassostrea gigas]
1	1	NaN	794 994 Glycogenin-1 [Crassostrea gigas]; 1425 1739 minus strand Glycogenin-1 [Crassostrea gigas]
1	2	NaN	3 416 minus strand myosin essential light chain [Crassostrea gigas]
1	2	NaN	9 326 minus strand catalase [Paracyclopina nana]; 9 209 Catalase OS=Cavia porcellus GN=CAT PE=2 SV=4
1	1	NaN	165 377 Cysteine and glycine-rich protein 3 [Crassostrea gigas]; 135 344 Cysteine and glycine-rich protein 3 [Crasso
1	3	NaN	578 1150 minus strand expressed hypothetical protein [Trichoplax adhaerens] gi; 376 948 minus strand expressed h
1	2	NaN	1 297 beta-thymosin domain repeat protein CSP24KDa_v1 [Hermissenda crassicornis] gi
1	1	NaN	128 1138 Actin-related protein 2/3 complex subunit 1A OS=Homo sapiens GN=ARPC1A PE=1 SV=2
1	2	NaN	91 1326 minus strand Adipocyte plasma membrane-associated protein [Crassostrea gigas]; 107 1273 minus strand A
1	1	NaN	" 115 534 NADH:ubiquinone oxidoreductase, NDUFB6/B17 subunit, putative [Ixodes scapularis] gi"
1	1	NaN	2 1384 minus strand twitchin [Mytilus galloprovincialis]; 2 1384 minus strand twitchin [Mytilus galloprovincialis]; 2 1
1	1	NaN	1 456 minus strand Putative sodium/potassium-transporting ATPase subunit beta-2 [Crassostrea gigas]; 688 1251 m
1	6	NaN	21 254 predicted protein [Nematostella vectensis] gi
1	1	NaN	62 394 minus strand PREDICTED: NADH dehydrogenase [ubiquinone] 1 alpha subcomplex subunit 5 [Saimiri bolivie
1	1	NaN	165 1142 hypothetical protein CGI_10023942 [Crassostrea gigas]
1	1	NaN	124 747 ribosomal protein S10 [Crassostrea gigas] gi
1	1	NaN	1867 3108 minus strand roundabout homolog 1 precursor [Rattus norvegicus] gi; 1867 3108 minus strand roundabo
1	1	NaN	3 281 minus strand hypothetical protein CGI_10015501 [Crassostrea gigas]
1	2	NaN	418 690 PREDICTED: protein disulfide isomerase A4-like [Saccoglossus kowalevskii]
1	6	NaN	187 642 minus strand PREDICTED: similar to pol-like protein [Tribolium castaneum]
1	1	NaN	93 2606 twitchin [Mytilus galloprovincialis]
1	1	NaN	1327 1488 minus strand Ubiquinol-cytochrome C reductase complex [Xenopus laevis] gi
1	1	NaN	1 177 small heat shock protein [Trichinella pseudospiralis]
1	1	NaN	729 1541 troponin T [Mizuhopecten yessoensis]
1	1	NaN	7 231 minus strand AGAP006879-PA [Anopheles gambiae str. PEST] gi
1	1	NaN	" 183 476 minus strand guanine nucleotide-binding protein G(q), alpha subunit [Aplysia californica] gi"
1	1	NaN	258 680 minus strand synapse-associated protein [Aplysia californica] gi
1	1	NaN	78 2003 Chromatin assembly factor 1 subunit A [Crassostrea gigas]
1	2	NaN	" 1 327 minus strand endo-beta-1,4-glucanase [Ampullaria crossean]"
1	2	NaN	" 29 448 minus strand PREDICTED: similar to NADH:ubiquinone dehydrogenase, putative [Tribolium castaneum] gi;
1	1	NaN	" 451 1026 PREDICTED: GTP-binding protein SAR1B, putative-like [Saccoglossus kowalevskii]"
1	1	NaN	49 1560 PREDICTED: tRNA-splicing ligase RtcB homolog [Megachile rotundata]
1	10	NaN	642 1415 minus strand cytochrome c oxidase subunit II [Oncomelania hupensis hupensis]
1	1	NaN	146 841 minus strand hypothetical protein CGI_10006609 [Crassostrea gigas]; 146 841 minus strand hypothetical pr
1	1	NaN	" 2 433 Vesicle-fusing ATPase, partial [Ascaris suum]"
1	5	NaN	416 1717 minus strand T-complex protein 1 subunit delta OS=Aedes triseriatus PE=2 SV=1
1	1	NaN	10 366 minus strand hypothetical protein TRIADDRAFT_25239 [Trichoplax adhaerens] gi
1	1	NaN	5 451 minus strand Spectrin beta chain [Crassostrea gigas]
1	5	NaN	3 386 fem-1-like protein C [Crassostrea gigas]
1	1	NaN	2 2248 PREDICTED: heat shock 70 kDa protein 4-like [Oreochromis niloticus]; 2 2227 PREDICTED: heat shock 70 kD
1	1	NaN	7825 8754 minus strand H2 [Haliotis tuberculata]
1	1	NaN	115 393 minus strand Yolk ferritin OS=Lymnaea stagnalis PE=1 SV=1; 132 413 RecName: Full=Yolk ferritin; Flags: Pr
1	1	NaN	1 630 hypothetical protein [Trichomonas vaginalis G3] gi
1	2	NaN	4 942 minus strand KTEL motif-containing protein 1 [Crassostrea gigas]
1	1	NaN	511 1062 unknown [Dendroctonus ponderosae]; 511 1062 unknown [Dendroctonus ponderosae]
1	1	NaN	3 272 minus strand ADP/ATP carrier [Clonorchis sinensis]
1	2	NaN	26 619 G-type lysozyme [Physella acuta]

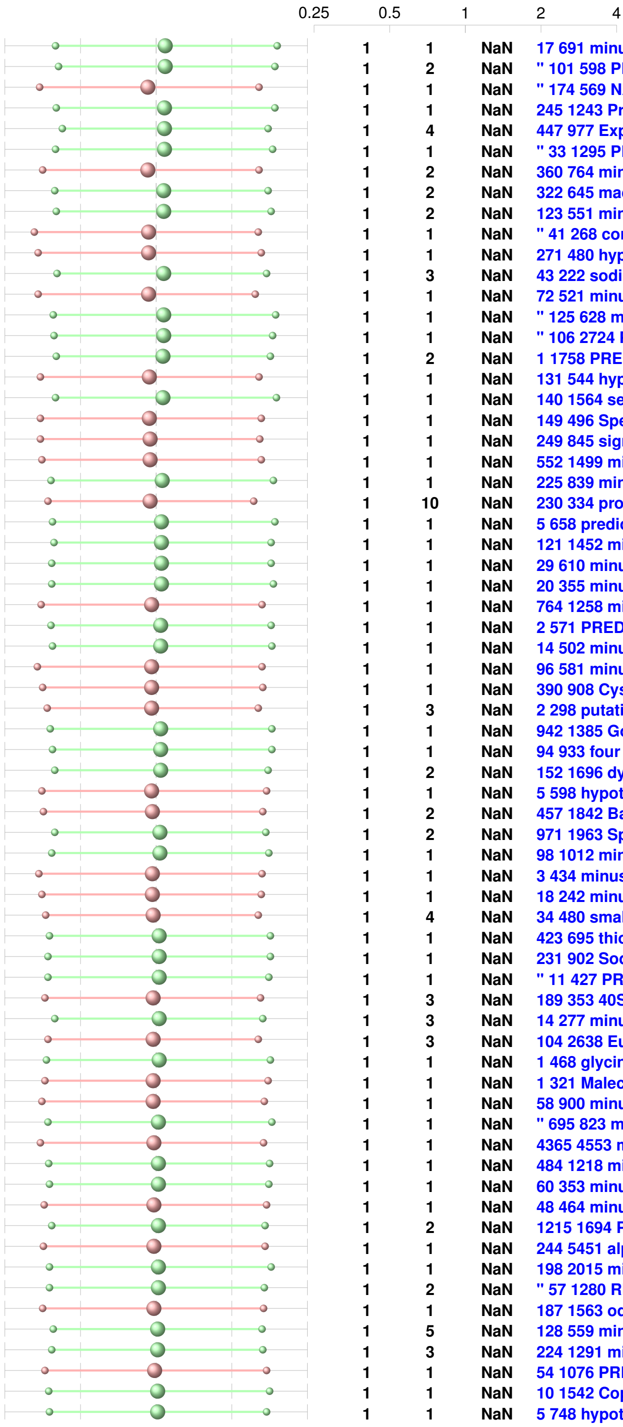

Peptides	Spectra	Coverage	Protein
1	1	NaN	17 691 minus strand Cleavage and polyadenylation specificity factor subunit 5 [Crassostrea gigas]
1	2	NaN	" 101 598 PREDICTED: aminomethyltransferase, mitochondrial-like [Oreochromis niloticus]"
1	1	NaN	" 174 569 NADH dehydrogenase [ubiquinone] iron-sulfur protein 4, mitochondrial [Crassostrea gigas]"
1	1	NaN	245 1243 Prostaglandin reductase 1 [Crassostrea gigas]
1	4	NaN	447 977 Expansin-yoaJ [Crassostrea gigas]
1	1	NaN	" 33 1295 PREDICTED: LOW QUALITY PROTEIN: selenide, water dikinase-like [Bombus impatiens]"
1	2	NaN	360 764 minus strand Peptidyl-prolyl cis-trans isomerase A OS=Dictyostelium discoideum GN=ppiA PE=1 SV=1
1	2	NaN	322 645 macrophage migration inhibitory-like factor protein [Chlamys farreri]; 437 682 macrophage migration inhibit
1	2	NaN	123 551 minus strand Transcription factor BTF3-like protein 4 [Crassostrea gigas]
1	1	NaN	" 41 268 conserved hypothetical protein [Perkinsus marinus ATCC 50983] gi; 29 238 PREDICTED: succinyl-CoA ligas
1	1	NaN	271 480 hypothetical protein BRAFLDRAFT_75518 [Branchiostoma floridae] gi; 219 749 Kynurenine formamidase [C
1	3	NaN	43 222 sodium-calcium exchanger [Doryteuthis pealeii]
1	1	NaN	72 521 minus strand GF14593 [Drosophila ananassae] gi; 60 392 GD23124 [Drosophila simulans] gi
1	1	NaN	" 125 628 minus strand collectin sub-family member 12, partial [Oncorhynchus mykiss]"
1	1	NaN	" 106 2724 PREDICTED: 2-oxoglutarate dehydrogenase, mitochondrial isoform 1 [Sarcophilus harrisii]; 492 3473 PR
1	2	NaN	1 1758 PREDICTED: polyprotein-like [Saccoglossus kowalevskii]
1	1	NaN	131 544 hypothetical protein CGI_10001749 [Crassostrea gigas]
1	1	NaN	140 1564 selenium-binding protein [Haliotis discus hannai]
1	1	NaN	149 496 Spectrin alpha chain [Crassostrea gigas]
1	1	NaN	249 845 sigma class glutathione-s-transferase 2 [Haliotis discus discus]; 252 848 sigma class glutathione-s-transfer
1	1	NaN	552 1499 minus strand uncharacterized protein LOC100158466 [Xenopus (Silurana) tropicalis] gi; 552 1499 minus st
1	1	NaN	225 839 minus strand Metallo-beta-lactamase domain-containing protein 1 [Crassostrea gigas]; 201 851 minus stran
1	10	NaN	230 334 protein disulfide isomerase [Conus virgo]
1	1	NaN	5 658 predicted protein [Nematostella vectensis] gi
1	1	NaN	121 1452 minus strand Hexokinase type 2 [Crassostrea gigas]; 245 1576 minus strand Hexokinase type 2 [Crassostr
1	1	NaN	29 610 minus strand 40S ribosomal protein S7 [Crassostrea gigas]
1	1	NaN	20 355 minus strand PREDICTED: uroporphyrinogen decarboxylase-like [Monodelphis domestica]
1	1	NaN	764 1258 minus strand Caprin-2 [Crassostrea gigas]
1	1	NaN	2 571 PREDICTED: copper homeostasis protein cutC homolog [Anolis carolinensis]
1	1	NaN	14 502 minus strand ependymin related protein-1 precursor [Haliotis discus discus]; 14 547 ependymin related prote
1	1	NaN	96 581 minus strand thioredoxin 2 [Haliotis discus discus]; 1682 2167 minus strand thioredoxin 2 [Haliotis discus di
1	1	NaN	390 908 Cysteine proteinase 5 OS=Dictyostelium discoideum GN=cprE PE=2 SV=2
1	3	NaN	2 298 putative 60S ribosomal protein RPL24 [Novocrania anomala]
1	1	NaN	942 1385 Golgi-associated plant pathogenesis-related protein 1 [Crassostrea gigas]; 536 979 Golgi-associated plant
1	1	NaN	94 933 four and A half lim domains [Schistosoma mansoni]
1	2	NaN	152 1696 dynamin isoform A [Lymnaea stagnalis]
1	1	NaN	5 598 hypothetical protein DAPPUDRAFT_344656 [Daphnia pulex]
1	2	NaN	457 1842 Basement membrane-specific heparan sulfate proteoglycan core protein [Crassostrea gigas]; 457 1842 Ba
1	2	NaN	971 1963 Spectrin alpha chain [Crassostrea gigas]; 972 1979 Spectrin alpha chain [Crassostrea gigas]
1	1	NaN	98 1012 minus strand RecName: Full=Substrate-specific endoprotease Tex31; Flags: Precursor gi|32187775|emb|C
1	1	NaN	3 434 minus strand hypothetical protein CGI_10015501 [Crassostrea gigas]
1	1	NaN	18 242 minus strand translation elongation factor 1B gamma subunit [Strongylocentrotus purpuratus] gi
1	4	NaN	34 480 small subunit ribosomal protein S18e [Clonorchis sinensis]; 431 799 SJCHGC01887 protein [Schistosoma jap
1	1	NaN	423 695 thioredoxin peroxidase 2 [Haliotis discus discus]
1	1	NaN	231 902 Sodium- and chloride-dependent GABA transporter ine [Crassostrea gigas]
1	1	NaN	" 11 427 PREDICTED: similar to syntaxin, partial [Hydra magnipapillata]"
1	3	NaN	189 353 40S ribosomal protein S12 [Crassostrea gigas]
1	3	NaN	14 277 minus strand Histidine triad nucleotide-binding protein 1 OS=Oryctolagus cuniculus GN=HINT1 PE=1 SV=2
1	3	NaN	104 2638 Eukaryotic translation initiation factor 3 subunit C [Crassostrea gigas]
1	1	NaN	1 468 glycine cleavage system H protein [Rhipicephalus sanguineus]
1	1	NaN	1 321 Malectin [Crassostrea gigas]
1	1	NaN	58 900 minus strand Toll-interacting protein [Crassostrea gigas]
1	1	NaN	" 695 823 minus strand Aldehyde dehydrogenase family 16 member A1, partial [Crassostrea gigas]"
1	1	NaN	4365 4553 minus strand Basement membrane-specific heparan sulfate proteoglycan core protein [Crassostrea gigas
1	1	NaN	484 1218 minus strand alginate lyase [Littorina brevicula]; 484 1218 minus strand alginate lyase [Littorina brevicula]
1	1	NaN	60 353 minus strand Histone H3.3 [Harpegnathos saltator]
1	1	NaN	48 464 minus strand Acyl-coenzyme A thioesterase 13 [Crassostrea gigas]
1	2	NaN	1215 1694 Pikachurin [Crassostrea gigas]
1	1	NaN	244 5451 alpha macroglobulin [Chlamys farreri]
1	1	NaN	198 2015 minus strand Xaa-Pro aminopeptidase 1 [Crassostrea gigas]
1	2	NaN	" 57 1280 RNA binding motif protein, X-linked [Mustela putorius furo]"
1	1	NaN	187 1563 odr-4-like protein [Crassostrea gigas]
1	5	NaN	128 559 minus strand predicted protein [Nematostella vectensis] gi
1	3	NaN	224 1291 minus strand coproporphyrynogen oxidase [Aplysia californica] gi
1	1	NaN	54 1076 PREDICTED: UPF0553 protein C9orf64-like [Anolis carolinensis]; 71 1093 PREDICTED: UPF0553 protein C9o
1	1	NaN	10 1542 Copine-8 [Crassostrea gigas]
1	1	NaN	5 748 hypothetical protein SINV_09458 [Solenopsis invicta]

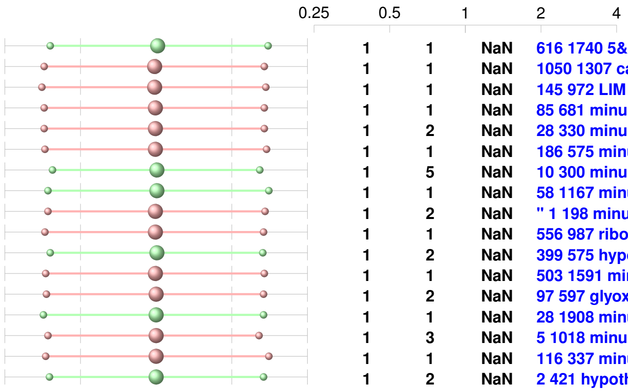

Peptides	Spectra	Coverage	Protein
1	1	NaN	616 1740 5'-AMP-activated protein kinase subunit beta-2 [Crassostrea gigas]; 641 1762 minus strand 5'
1	1	NaN	1050 1307 calreticulin precursor [Aplysia californica] gi
1	1	NaN	145 972 LIM and SH3 domain protein F42H10.3 OS=Caenorhabditis elegans GN=F42H10.3 PE=2 SV=3
1	1	NaN	85 681 minus strand 40S ribosomal protein S8 OS=Xenopus laevis GN=rps8 PE=2 SV=3
1	2	NaN	28 330 minus strand 40S ribosomal protein S17 [Salmo salar] gi; 40 510 minus strand ribosomal protein S17 [Branch
1	1	NaN	186 575 minus strand predicted protein [Nematostella vectensis] gi
1	5	NaN	10 300 minus strand unnamed protein product [Tetraodon nigroviridis]
1	1	NaN	58 1167 minus strand PREDICTED: arylacetamide deacetylase-like 4-like [Gallus gallus]; 76 1239 minus strand PRED
1	2	NaN	" 1 198 minus strand Chain A, Rep1-Nxsq Fatty Acid Transporter; 1 231 minus strand Chain A, Rep1-Nxsq Fatty Acid
1	1	NaN	556 987 ribosomal protein rps15 [Lineus viridis]
1	2	NaN	399 575 hypothetical protein BRAFLDRAFT_69739 [Branchiostoma floridae] gi
1	1	NaN	503 1591 minus strand Septin-2 [Crassostrea gigas]; 648 1736 minus strand Septin-2 [Crassostrea gigas]
1	2	NaN	97 597 glyoxylate reductase/hydroxypyruvate reductase [Ictalurus punctatus] gi
1	1	NaN	28 1908 minus strand Mitochondrial inner membrane protein [Crassostrea gigas]; 10 1902 Mitochondrial inner meml
1	3	NaN	5 1018 minus strand Glutaredoxin-3 [Crassostrea gigas]; 5 1018 Glutaredoxin-3 [Crassostrea gigas]
1	1	NaN	116 337 minus strand Golgi-associated plant pathogenesis-related protein 1 [Crassostrea gigas]; 538 759 minus stra
1	2	NaN	2 421 hypothetical protein BRAFLDRAFT_119044 [Branchiostoma floridae] gi

5 Protein Details

A detailed summary of each protein is given below. These sections include peptide relative expression estimates in addition to protein-level estimates.

5.1 97 477 minus strand PREDICTED: complement C1q tumor necrosis factor-related protein 3-like [Oreochromis niloticus]; 97 447 minus strand PREDICTED: complement C1q tumor necrosis factor-related protein 3-like [Oreochromis niloticus]

Protein Accession	CL370.Contig3$_H$1 CL370.Contig2$_H$1
Mean Expression Ratio	3.87
Median Expression Ratio	3.87
Credible Interval	(2.32, 6.4)
Associated Peptides	16
Associated Spectra	116
Coverage	NaN

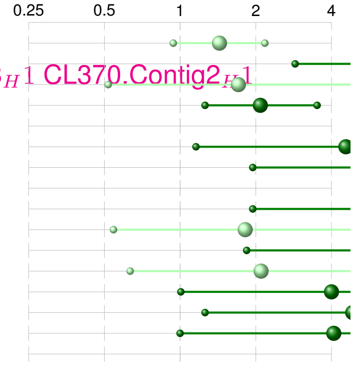

A	B	2.5	50	97.5	Sequence
7	26	0.94	1.4	2.2	QMVDALKDEQQR
3	10	2.9	5.5	10	QTSDISRLENR
1	1	0.52	1.7	5.9	QMVDALK
0	21	1.3	2.1	3.5	LESGVRPPVYRPDLESK
0	5	5.9	15	36	QMVDALKDEQQRQQEVINR
0	1	1.2	4.6	18	QSQQIEKQTSDISR
0	1	1.9	8	33	QSQQIEKQTSDISRLENR
0	25	15	24	39	VTKLESGVRPPVYRPDLESK
0	1	1.9	7.7	31	VTKLESGVRPP
0	2	0.54	1.8	6.1	PIAFSK
0	1	1.8	7.2	29	LENRVTK
0	2	0.63	2.1	7	VAFTAH
0	1	1	4	16	MVDALKDEQQR
0	1	1.3	4.9	20	RPIAFSK
0	1	1	4.1	16	DALKDEQQR
0	6	5.1	12	28	VAFTAHFSQGGSMDIGTSRPIAFSK

5.2 1009 2784 minus strand Collagen alpha-3(VI) chain [Crassostrea gigas]; 1009 2787 minus strand Collagen alpha-3(VI) chain [Crassostrea gigas]

Protein Accession	Unigene26554$_H$1 Unigene26550$_H$1
Mean Expression Ratio	0.259
Median Expression Ratio	0.26
Credible Interval	(0.122, 0.542)
Associated Peptides	6
Associated Spectra	13
Coverage	NaN

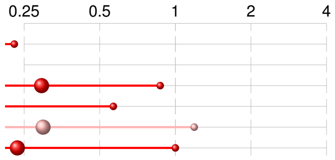

A	B	2.5	50	97.5	Sequence
3	0	0.025	0.075	0.23	ELEGNVHSIR
4	0	0.019	0.053	0.14	IITVGIGEANR
3	0	0.098	0.29	0.87	SDAAAALIR
1	0	0.033	0.14	0.57	VAMSNFR
1	0	0.068	0.3	1.2	MFIPVR
1	0	0.057	0.24	1	LTTVR

5.3 118 501 minus strand histone 2A.Z [Aplysia californica]

Protein Accession	CL14228.Contig6$_H$1
Mean Expression Ratio	0.269
Median Expression Ratio	0.268
Credible Interval	(0.13, 0.547)
Associated Peptides	5
Associated Spectra	30
Coverage	NaN

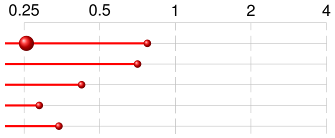

A	B	2.5	50	97.5	Sequence
1	4	0.085	0.25	0.77	ATIAGGGVIPHIHK
1	1	0.055	0.2	0.71	SLIGKK
3	0	0.049	0.15	0.42	SLIGK
14	0	0.083	0.15	0.29	AGLQFPVGR
3	3	0.05	0.13	0.34	GDEELDSLIK

5.4 1070 1888 minus strand galectin [Pinctada fucata]; 1371 2189 minus strand galectin [Pinctada fucata]

Protein Accession	CL7245.Contig4$_H$1 CL7245.Contig3$_H$1
Mean Expression Ratio	0.278
Median Expression Ratio	0.28
Credible Interval	(0.13, 0.597)
Associated Peptides	2
Associated Spectra	23
Coverage	NaN

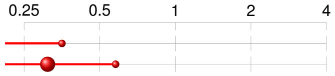

A	B	2.5	50	97.5	Sequence
1	0	0.019	0.082	0.35	GEAFIYEVK
12	10	0.17	0.31	0.58	FNEGVTVR

5.5 507 1277 alpha tubulin [Notothenia coriiceps]

Protein Accession	Unigene56691$_H$1
Mean Expression Ratio	0.321
Median Expression Ratio	0.325
Credible Interval	(0.131, 0.77)
Associated Peptides	2
Associated Spectra	13
Coverage	NaN

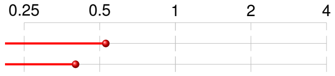

A	B	2.5	50	97.5	Sequence
1	7	0.051	0.17	0.53	LSVDYGK
3	2	0.054	0.15	0.4	LSVDYGKK

5.6 1592 1768 minus strand beta-actin [Diabolocatantops pinguis]

Protein Accession	CL22741.Contig2$_H$1
Mean Expression Ratio	2.66
Median Expression Ratio	2.65
Credible Interval	(1.37, 5.18)
Associated Peptides	6
Associated Spectra	45
Coverage	NaN

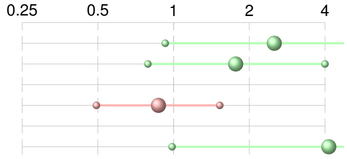

A	B	2.5	50	97.5	Sequence
3	1	0.93	2.5	7	VIAPPERK
1	6	0.79	1.8	4	EISALAPPTMK
1	7	9.2	20	44	IKVIAPPERK
7	11	0.49	0.87	1.5	VIAPPER
0	7	5.3	12	28	IKVIAPPER
0	1	0.99	4.2	18	GDQKEISALAPPTMK

5.7 764 1015 minus strand heat shock protein beta-1 [Trichinella spiralis] gi

Protein Accession	CL46983.Contig3$_H$1
Mean Expression Ratio	2.6
Median Expression Ratio	2.6
Credible Interval	(1.39, 4.91)
Associated Peptides	7
Associated Spectra	74
Coverage	NaN

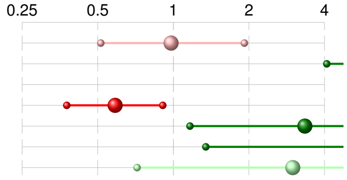

A	B	2.5	50	97.5	Sequence
6	6	0.51	0.98	1.9	SYVLPK
5	5	4.1	8.4	17	SYVLPKDVDPK
4	12	19	33	60	TVTSSLSQNGVLTLKAPK
9	22	0.38	0.58	0.91	TVTSSLSQNGVLTLK
0	3	1.2	3.3	9.8	VVINAKHEERPDEHGYIMR
0	1	1.3	5.6	23	SSLSQNGVLTLKAPK
0	1	0.72	3	12	MALEAPQEQV

5.8 1 1350 UPF0663 transmembrane protein C17orf28 [Crassostrea gigas]; 163 1542 UPF0663 transmembrane protein C17orf28 [Crassostrea gigas]; 135 1514 UPF0663 transmembrane protein C17orf28 [Crassostrea gigas]

Protein Accession	CL21276.Contig5$_H$1 CL21276.Contig4$_H$1 CL21276.Contig1$_H$1
Mean Expression Ratio	0.384
Median Expression Ratio	0.386
Credible Interval	(0.15, 0.932)
Associated Peptides	3
Associated Spectra	3
Coverage	NaN

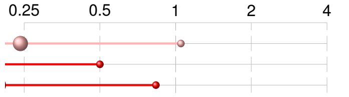

A	B	2.5	50	97.5	Sequence
1	0	0.058	0.24	1.1	EGFQSLLNTK
1	0	0.028	0.12	0.5	GVFLIEPKPK
1	0	0.045	0.2	0.84	KIGFK

5.9 254 1648 Tektin-3 [Crassostrea gigas]; 254 1648 Tektin-3 [Crassostrea gigas]

Protein Accession	CL6717.Contig3$_H$1 CL6717.Contig2$_H$1
Mean Expression Ratio	0.388
Median Expression Ratio	0.389
Credible Interval	(0.171, 0.889)
Associated Peptides	5
Associated Spectra	5
Coverage	NaN

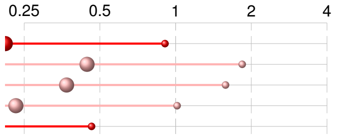

A	B	2.5	50	97.5	Sequence
1	0	0.049	0.21	0.91	ILPYVTDK
1	0	0.1	0.44	1.8	KGFPMDPK
1	0	0.085	0.37	1.6	NVELLR
1	0	0.055	0.23	1	EVAEIR
1	0	0.025	0.11	0.46	EAFMK

5.10 222 809 minus strand PREDICTED: 15-hydroxyprostaglandin dehydrogenase [NAD+] [Gallus gallus]

Protein Accession	CL44148.Contig2$_H$1
Mean Expression Ratio	0.389
Median Expression Ratio	0.39
Credible Interval	(0.202, 0.759)
Associated Peptides	6
Associated Spectra	47
Coverage	NaN

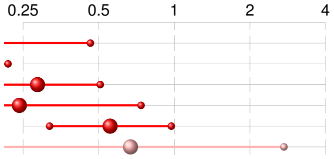

A	B	2.5	50	97.5	Sequence
2	3	0.073	0.18	0.46	VMFCDINDK
3	0	0.025	0.073	0.22	AAFEK
3	14	0.16	0.29	0.51	ATVEAFLTK
3	0	0.084	0.24	0.74	AVTSLGR
4	14	0.32	0.56	0.97	VFLTGGAHGIGK
0	1	0.16	0.67	2.7	RVFLTGGAHGIGK

5.11 266 607 minus strand H2 [Haliotis tuberculata]

Protein Accession	Unigene62350$_H$1
Mean Expression Ratio	0.4
Median Expression Ratio	0.401
Credible Interval	(0.176, 0.894)
Associated Peptides	4
Associated Spectra	15
Coverage	NaN

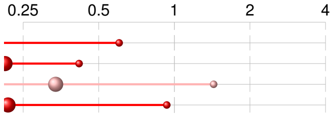

A	B	2.5	50	97.5	Sequence
1	1	0.052	0.18	0.6	DINTLTDEEIK
5	6	0.11	0.21	0.42	YAVAGIGVR
0	1	0.08	0.34	1.4	KDINTLTDEEIK
0	1	0.05	0.22	0.93	DIDGASPLSFEQIAEFHGYPGK

5.12 " 223 423 ADPATP carrier protein; K05863 solute carrier family 25 (mitochondrial adenine nucleotide translocator), member 4/5/6/31; 237 455 minus strand ADP,ATP carrier protein [Crassostrea gigas]"

Protein Accession	CL27196.Contig3$_H$1 CL27196.Contig2$_H$1
Mean Expression Ratio	2.46
Median Expression Ratio	2.45
Credible Interval	(1.06, 5.8)
Associated Peptides	3
Associated Spectra	10
Coverage	NaN

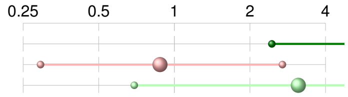

A	B	2.5	50	97.5	Sequence
0	1	2.5	10	46	VKLLVQNQNEMIK
6	2	0.29	0.88	2.7	LLVQNQNEMIK
0	1	0.69	3.1	13	AENFALSGLVLPLSSPK

5.13 27 620 putative mitochondrial ATP synthase [Haliotis discus discus]

Protein Accession	CL13014.Contig1$_H$1
Mean Expression Ratio	0.407
Median Expression Ratio	0.41
Credible Interval	(0.18, 0.894)
Associated Peptides	3
Associated Spectra	15
Coverage	NaN

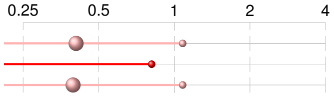

A	B	2.5	50	97.5	Sequence
3	4	0.15	0.41	1.1	LANPALVDQFQK
1	0	0.045	0.19	0.81	VPYPEDK
2	5	0.14	0.4	1.1	SAVDWVAFK

5.14 2 256 Polyubiquitin (Fragment) OS=Xenopus laevis PE=1 SV=2; 20 388 large subunit ribosomal protein L40e [Clonorchis sinensis]

Protein Accession	CL47507.Contig2$_{H}1$ CL39010.Contig1$_{H}1$
Mean Expression Ratio	0.411
Median Expression Ratio	0.414
Credible Interval	(0.195, 0.869)
Associated Peptides	5
Associated Spectra	14
Coverage	NaN

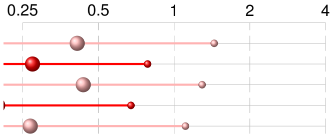

A	B	2.5	50	97.5	Sequence
2	0	0.12	0.41	1.4	IQDKEGIPPDQQR
2	3	0.094	0.27	0.78	TITLEVEPSDTIENVK
2	2	0.15	0.44	1.3	TLSDYNIQK
2	0	0.059	0.2	0.68	LIFAGK
1	0	0.064	0.27	1.1	MQIFVK

5.15 14 1642 minus strand chaperonin containing tcp1 [Haliotis discus discus]; 15 1619 chaperonin containing tcp1 [Haliotis discus discus]; 15 1619 chaperonin containing tcp1 [Haliotis discus discus]; 14 1462 minus strand chaperonin containing tcp1 [Haliotis discus discus]; 14 1582 minus strand chaperonin containing tcp1 [Haliotis discus discus]

Protein Accession	CL16298.Contig4$_{H}1$ CL16298.Contig2$_{H}1$ CL16298.Contig1$_{H}1$ CL16298.Contig15$_{H}1$ CL162
Mean Expression Ratio	0.415
Median Expression Ratio	0.417
Credible Interval	(0.157, 1.05)
Associated Peptides	2
Associated Spectra	3
Coverage	NaN

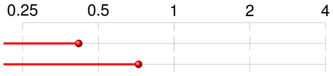

A	B	2.5	50	97.5	Sequence
2	0	0.036	0.12	0.42	QVEGDITK
1	0	0.037	0.16	0.72	NAEELMSFSK

5.16 195 455 60S ribosomal protein L23a OS=Rattus norvegicus GN=Rpl23a PE=2 SV=1; 195 512 60S ribosomal protein L23a OS=Rattus norvegicus GN=Rpl23a PE=2 SV=1; 16 465 60S ribosomal protein L23a OS=Rattus norvegicus GN=Rpl23a PE=2 SV=1

Protein Accession	CL27563.Contig3$_{H}1$ CL27563.Contig2$_{H}1$ CL27563.Contig1$_{H}1$
Mean Expression Ratio	2.36
Median Expression Ratio	2.35
Credible Interval	(1.06, 5.36)
Associated Peptides	3
Associated Spectra	10
Coverage	NaN

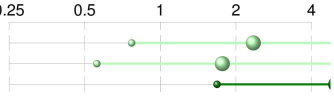

A	B	2.5	50	97.5	Sequence
2	2	0.77	2.4	6.9	ANKPLIK
1	2	0.56	1.8	5.8	SLYLYDVDIAK
0	3	1.7	5	15	VNTLIRPDGEKK

5.17 2 328 cytoskeletal actin 2 [Molgula oculata]

Protein Accession	Unigene2743$_H$1
Mean Expression Ratio	2.37
Median Expression Ratio	2.35
Credible Interval	(0.987, 5.86)
Associated Peptides	2
Associated Spectra	6
Coverage	NaN

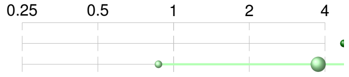

A	B	2.5	50	97.5	Sequence
1	4	4.7	12	30	MQKDITALAPPTMK
0	1	0.87	3.8	16	MQKDITALAPP

5.18 " 87 287 Chain A, Structure Of Deoxygenated Hemocyanin From Rapana Thomasiana gi|31615505|pdb|1LNL|B Chain B, Structure Of Deoxygenated Hemocyanin From Rapana Thomasiana gi|31615506|pdb|1LNL|C Chain C, Structure Of Deoxygenated Hemocyanin From Rapana Thomasiana"

Protein Accession	Unigene36631$_H$1
Mean Expression Ratio	0.438
Median Expression Ratio	0.438
Credible Interval	(0.21, 0.913)
Associated Peptides	4
Associated Spectra	28
Coverage	NaN

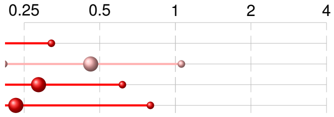

A	B	2.5	50	97.5	Sequence
8	3	0.081	0.16	0.32	SLVEAMR
1	6	0.21	0.46	1.1	KNILELTESEQR
1	7	0.13	0.28	0.62	QLQEDGSAGGFQK
0	2	0.068	0.23	0.8	NILELTESEQR

5.19 503 1090 minus strand myosin regulatory light chain [Fasciola hepatica]; 278 865 minus strand myosin regulatory light chain [Fasciola hepatica]

Protein Accession	CL43006.Contig6$_H$1 CL43006.Contig4$_H$1
Mean Expression Ratio	0.441
Median Expression Ratio	0.439
Credible Interval	(0.236, 0.833)
Associated Peptides	8
Associated Spectra	30
Coverage	NaN

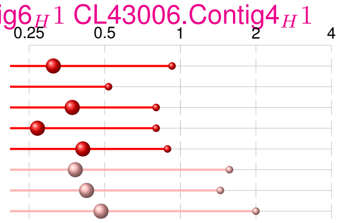

A	B	2.5	50	97.5	Sequence
3	0	0.11	0.31	0.93	DGVIDIEDLK
3	1	0.073	0.19	0.52	EAFTMIDQNR
3	5	0.17	0.37	0.8	IPPDDELKEMLK
2	1	0.09	0.27	0.8	NAFAMFDDEGK
5	3	0.19	0.41	0.89	QAQVQEFK
0	1	0.093	0.38	1.6	GYLPEEYIK
0	2	0.13	0.42	1.4	LSGTDPEDTIR
0	1	0.12	0.48	2	DGVIDIEDLKDIYSNLGR

5.20 160 588 hypothetical protein CGI_10001640 [Crassostrea gigas]

Protein Accession	CL2347.Contig8$_H$1
Mean Expression Ratio	0.441
Median Expression Ratio	0.445
Credible Interval	(0.183, 1.02)
Associated Peptides	2
Associated Spectra	11
Coverage	NaN

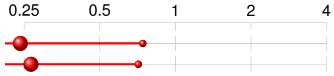

A	B	2.5	50	97.5	Sequence
2	2	0.078	0.24	0.74	AFLEQLGSK
3	4	0.098	0.27	0.71	ANLIVIGSR

5.21 102 887 hypothetical protein DAPPUDRAFT_304712 [Daphnia pulex]

Protein Accession CL6869.Contig1$_H$1
Mean Expression Ratio 0.459
Median Expression Ratio 0.459
Credible Interval (0.22, 0.937)
Associated Peptides 7
Associated Spectra 13
Coverage NaN

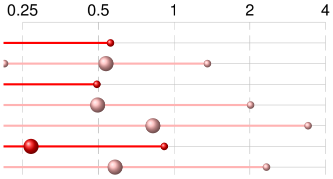

A	B	2.5	50	97.5	Sequence
1	1	0.048	0.16	0.56	TAEEIAAEEK
3	2	0.21	0.54	1.4	AFAIQAEK
1	0	0.028	0.11	0.49	VDLSQLR
0	1	0.12	0.5	2	AHTDVQVPDFSYYR
0	1	0.19	0.82	3.4	LALTSYAMSK
0	2	0.079	0.27	0.91	LADIPEGK
0	1	0.14	0.58	2.3	HPEADAER

5.22 280 726 minus strand RecName: Full=Calmodulin; Short=CaM gi|14588595|dbj|BAB61794.1| calmodulin [Metridium senile] gi

Protein Accession Unigene4486$_H$1
Mean Expression Ratio 0.458
Median Expression Ratio 0.459
Credible Interval (0.176, 1.16)
Associated Peptides 1
Associated Spectra 5
Coverage NaN

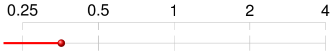

A	B	2.5	50	97.5	Sequence
4	1	0.049	0.14	0.36	ELGTVMR

5.23 35 370 Ganglioside GM2 activator [Crassostrea gigas]; 2 487 minus strand PREDICTED: hypothetical protein LOC100699750 [Oreochromis niloticus]; 41 535 minus strand PREDICTED: hypothetical protein LOC100699750 [Oreochromis niloticus]

Protein Accession CL7266.Contig6$_H$1 CL7266.Contig5$_H$1 CL7266.Contig3$_H$1
Mean Expression Ratio 0.457
Median Expression Ratio 0.459
Credible Interval (0.176, 1.15)
Associated Peptides 1
Associated Spectra 5
Coverage NaN

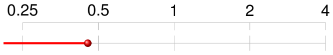

A	B	2.5	50	97.5	Sequence
3	2	0.056	0.16	0.45	NVALVVSVK

5.24 2881 3411 myosin heavy chain [Todarodes pacificus]; 2915 3445 myosin heavy chain [Todarodes pacificus]

Protein Accession	Unigene19024$_H1$ Unigene19021$_H1$
Mean Expression Ratio	2.15
Median Expression Ratio	2.15
Credible Interval	(1.18, 3.88)
Associated Peptides	10
Associated Spectra	47
Coverage	NaN

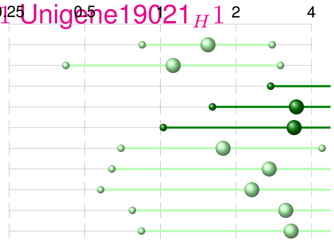

A	B	2.5	50	97.5	Sequence
4	11	0.85	1.6	2.8	ALDSMQASLEAEAK
2	2	0.42	1.1	3	IQEKEEEFENTR
2	7	2.8	5.7	12	IQEKEEEFENTRR
1	7	1.6	3.5	7.6	KLQQQVIETQQHVEEEAR
0	2	1	3.4	11	CTALTGEVEELR
0	5	0.7	1.8	4.4	KLEQDINELEIALDQANR
0	1	0.64	2.7	11	LQQQVIETQQHVEEEAR
0	1	0.58	2.3	9.7	RCTALTGEVEELR
0	1	0.78	3.2	12	RIQEKEEEFENTR
0	1	0.84	3.3	14	ALDSMQASLEAEAKGKAEAMR

5.25 3 272 minus strand ribosomal protein L28 [Haliotis asinina]

Protein Accession	CL403.Contig16$_H1$
Mean Expression Ratio	0.471
Median Expression Ratio	0.47
Credible Interval	(0.201, 1.09)
Associated Peptides	3
Associated Spectra	8
Coverage	NaN

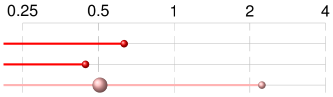

A	B	2.5	50	97.5	Sequence
2	0	0.056	0.19	0.63	GVVLVTK
5	0	0.067	0.17	0.44	TVGVEPSKDGK
1	0	0.12	0.51	2.2	DLGMAAIR

5.26 " 172 867 minus strand complement C1q-like protein 4 precursor, partial [Oryzias melastigma]"

Protein Accession	Unigene7884$_H1$
Mean Expression Ratio	2.06
Median Expression Ratio	2.06
Credible Interval	(1.01, 4.24)
Associated Peptides	5
Associated Spectra	24
Coverage	NaN

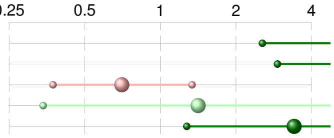

A	B	2.5	50	97.5	Sequence
1	2	2.5	7.5	22	EDQIKDLQDK
1	2	2.9	8.7	26	EDQIKDLQDKQK
1	12	0.37	0.7	1.3	DLQQAVESMRPSLK
0	1	0.34	1.4	5.6	DLQQAVESMR
0	4	1.3	3.4	9.3	DVKDLQQAVESMRPSLK

5.27 " 75 446 Actin, cytoplasmic type 8 OS=Xenopus laevis PE=3 SV=1"

Protein Accession	CL31420.Contig6$_H1$
Mean Expression Ratio	2.04
Median Expression Ratio	2.03
Credible Interval	(0.949, 4.33)
Associated Peptides	4
Associated Spectra	61
Coverage	NaN

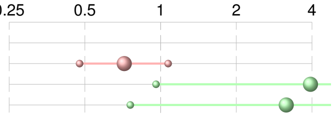

A	B	2.5	50	97.5	Sequence
6	16	6.1	10	17	MQKEITALAPSTMK
12	25	0.47	0.72	1.1	EITALAPSTMK
0	1	0.96	3.9	16	MQKEITALAPSTMKIK
0	1	0.76	3.2	13	EITALAPSTMKIK

5.28 REVERSED 216 2639 minus strand polycystin 2 [Gallus gallus]

Protein Accession	RRRRRCL818.Contig5$_{H}1$
Mean Expression Ratio	0.493
Median Expression Ratio	0.493
Credible Interval	(0.192, 1.25)
Associated Peptides	2
Associated Spectra	3
Coverage	NaN

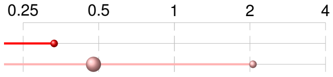

A	B	2.5	50	97.5	Sequence
**2**	**0**	**0.026**	**0.093**	**0.33**	**DGLIIR**
**1**	**0**	**0.11**	**0.47**	**2.1**	**QIDVIK**

5.29 390 2267 neurofilament protein NF70 [Helix aspersa]; 318 2195 neurofilament protein NF70 [Helix aspersa]

Protein Accession	CL1353.Contig5$_{H}1$ CL1353.Contig4$_{H}1$
Mean Expression Ratio	2.02
Median Expression Ratio	2.01
Credible Interval	(0.844, 5)
Associated Peptides	3
Associated Spectra	5
Coverage	NaN

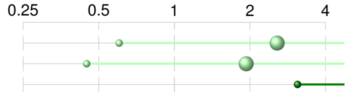

A	B	2.5	50	97.5	Sequence
**0**	**1**	**0.6**	**2.6**	**12**	**KDVNLMNWTLQR**
**0**	**1**	**0.45**	**1.9**	**8.6**	**LEEARK**
**0**	**3**	**3.1**	**9.3**	**29**	**VGQLEELVDDLQR**

5.30 181 684 hypothetical protein BRAFLDRAFT_131834 [Branchiostoma floridae] gi

Protein Accession	CL10218.Contig4$_{H}1$
Mean Expression Ratio	0.498
Median Expression Ratio	0.501
Credible Interval	(0.188, 1.29)
Associated Peptides	2
Associated Spectra	2
Coverage	NaN

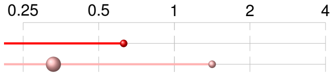

A	B	2.5	50	97.5	Sequence
**1**	**0**	**0.032**	**0.14**	**0.63**	**LSGEGLETSK**
**1**	**0**	**0.074**	**0.33**	**1.4**	**AIVYQFR**

5.31 209 781 minus strand high mobility group protein 1 [Biomphalaria glabrata]

Protein Accession	CL25076.Contig4$_{H}1$
Mean Expression Ratio	0.506
Median Expression Ratio	0.505
Credible Interval	(0.206, 1.25)
Associated Peptides	2
Associated Spectra	6
Coverage	NaN

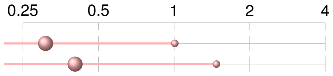

A	B	2.5	50	97.5	Sequence
**2**	**1**	**0.095**	**0.31**	**1**	**KPFEDMAAK**
**1**	**2**	**0.11**	**0.4**	**1.5**	**LAGEEWR**

5.32 587 1081 hypothetical protein CGI_10026502 [Crassostrea gigas]

Protein Accession	Unigene22889$_H$1
Mean Expression Ratio	0.502
Median Expression Ratio	0.506
Credible Interval	(0.176, 1.37)
Associated Peptides	1
Associated Spectra	1
Coverage	NaN

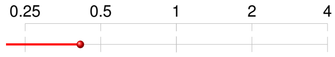

A	B	2.5	50	97.5	Sequence
1	0	0.021	0.094	0.41	LNGDAGHAIVK

5.33 118 501 minus strand histone 2A.Z [Aplysia californica]; 15 428 PREDICTED: histone H2A.V-like [Apis florea]

Protein Accession	CL14228.Contig6$_H$1 CL14228.Contig5$_H$1
Mean Expression Ratio	1.97
Median Expression Ratio	1.97
Credible Interval	(0.961, 4.03)
Associated Peptides	5
Associated Spectra	16
Coverage	NaN

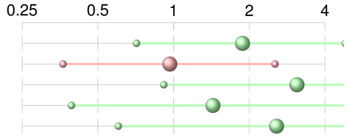

A	B	2.5	50	97.5	Sequence
1	4	0.71	1.9	4.8	ATIAGGGVIPHIHK
3	3	0.36	0.96	2.5	GDEELDSLIK
0	2	0.91	3.1	10	HLQLAIRGDEELDSLIK
1	1	0.39	1.4	5.1	SLIGKK
0	1	0.6	2.6	11	GGGVIPHIHK

5.34 4 2400 IQ motif containing GTPase activating protein 2 [Xenopus laevis] gi; 4 2169 IQ motif containing GTPase activating protein 2 [Xenopus laevis] gi

Protein Accession	CL875.Contig2$_H$1 CL875.Contig1$_H$1
Mean Expression Ratio	1.98
Median Expression Ratio	1.97
Credible Interval	(0.782, 5.2)
Associated Peptides	2
Associated Spectra	3
Coverage	NaN

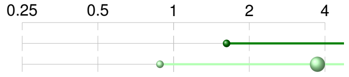

A	B	2.5	50	97.5	Sequence
0	2	1.6	5.7	20	ITLQDVVTHNR
0	1	0.88	3.7	17	TEVLEK

5.35 2 370 hemocyanin alphaD-subunit [Helix lucorum]

Protein Accession	CL46908.Contig1$_H$1
Mean Expression Ratio	0.508
Median Expression Ratio	0.509
Credible Interval	(0.264, 0.957)
Associated Peptides	7
Associated Spectra	41
Coverage	NaN

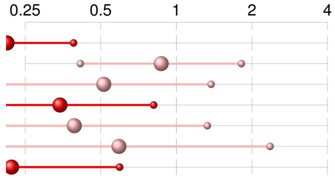

A	B	2.5	50	97.5	Sequence
2	13	0.12	0.21	0.39	LLGIGTSAEVK
1	8	0.42	0.87	1.8	SFYLDVSEAVVR
1	3	0.19	0.51	1.4	AFAVFK
0	6	0.15	0.34	0.81	NEMPWQFER
0	2	0.12	0.39	1.3	LASNVLPK
0	1	0.14	0.59	2.4	LYNMLETR
0	4	0.083	0.22	0.59	SFFILGGK

5.36 3 1460 Myosin-VI [Crassostrea gigas]

Protein Accession	CL6618.Contig5$_H$1
Mean Expression Ratio	0.51
Median Expression Ratio	0.512
Credible Interval	(0.189, 1.36)
Associated Peptides	2
Associated Spectra	2
Coverage	NaN

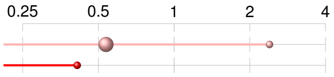

A	B	2.5	50	97.5	Sequence
**1**	**0**	**0.12**	**0.54**	**2.4**	**AIEEAAEK**
**1**	**0**	**0.021**	**0.095**	**0.41**	**LGIAK**

5.37 165 620 PREDICTED: uncharacterized protein LOC575072 isoform 2 [Strongylocentrotus purpuratus] gi

Protein Accession	CL20552.Contig4$_H$1
Mean Expression Ratio	0.508
Median Expression Ratio	0.513
Credible Interval	(0.183, 1.39)
Associated Peptides	1
Associated Spectra	1
Coverage	NaN

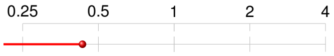

A	B	2.5	50	97.5	Sequence
**1**	**0**	**0.022**	**0.1**	**0.43**	**IKPTPLNDR**

5.38 195 455 60S ribosomal protein L23a OS=Rattus norvegicus GN=Rpl23a PE=2 SV=1; 16 465 60S ribosomal protein L23a OS=Rattus norvegicus GN=Rpl23a PE=2 SV=1

Protein Accession	CL27563.Contig3$_H$1 CL27563.Contig1$_H$1
Mean Expression Ratio	0.514
Median Expression Ratio	0.516
Credible Interval	(0.215, 1.21)
Associated Peptides	3
Associated Spectra	8
Coverage	NaN

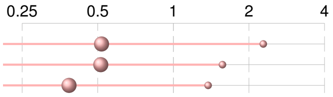

A	B	2.5	50	97.5	Sequence
**1**	**0**	**0.12**	**0.52**	**2.3**	**VNTLIRPDGEK**
**2**	**2**	**0.16**	**0.51**	**1.6**	**ANKPLIK**
**1**	**2**	**0.1**	**0.38**	**1.4**	**SLYLYDVDIAK**

5.39 27 515 heat shock protein 70 [Capsaspora owczarzaki ATCC 30864]; 30 464 minus strand hypothetical protein [Trichinella spiralis]; 27 491 minus strand heat shock protein 70 [Capsaspora owczarzaki ATCC 30864]

Protein Accession	CL14449.Contig4$_H1$ CL14449.Contig2$_H1$ CL14449.Contig1$_H1$
Mean Expression Ratio	0.519
Median Expression Ratio	0.519
Credible Interval	(0.222, 1.2)
Associated Peptides	3
Associated Spectra	7
Coverage	NaN

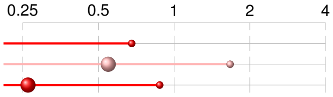

A	B	2.5	50	97.5	Sequence
2	0	0.056	0.19	0.68	FDDPSVQSDMK
2	1	0.18	0.55	1.7	NQVAMNPENTVFDAK
1	1	0.073	0.26	0.88	RFDDPSVQSDMK

5.40 185 742 Transmembrane protein Tmp21 [Crassostrea gigas]

Protein Accession	CL3732.Contig3$_H1$
Mean Expression Ratio	1.93
Median Expression Ratio	1.92
Credible Interval	(0.876, 4.27)
Associated Peptides	5
Associated Spectra	7
Coverage	NaN

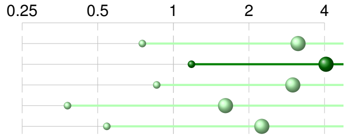

A	B	2.5	50	97.5	Sequence
0	1	0.75	3.1	13	SIVDDFAYMR
0	2	1.2	4.1	14	SYDELAKAEK
0	2	0.86	3	10	RLEDLSK
0	1	0.38	1.6	6.8	VLYLR
0	1	0.54	2.2	9.5	GHILYSK

5.41 242 1834 Regulator of differentiation 1 [Crassostrea gigas]; 458 2050 minus strand Regulator of differentiation 1 [Crassostrea gigas]; 503 2095 Regulator of differentiation 1 [Crassostrea gigas]; 434 1942 Regulator of differentiation 1 [Crassostrea gigas]

Protein Accession	CL11169.Contig7$_H1$ CL11169.Contig5$_H1$ CL11169.Contig4$_H1$ CL11169.Contig1$_H1$
Mean Expression Ratio	1.9
Median Expression Ratio	1.91
Credible Interval	(0.786, 4.65)
Associated Peptides	3
Associated Spectra	4
Coverage	NaN

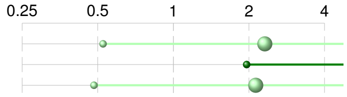

A	B	2.5	50	97.5	Sequence
0	1	0.53	2.3	9.8	SLPHEVAETDIVQLGMPFGK
0	2	2	6.7	24	MTNVLVLK
0	1	0.48	2.1	9	LTNLNVK

5.42 94 489 Sortilin-related receptor [Crassostrea gigas]

Protein Accession	CL15197.Contig3$_H$1
Mean Expression Ratio	0.525
Median Expression Ratio	0.526
Credible Interval	(0.188, 1.4)
Associated Peptides	1
Associated Spectra	2
Coverage	NaN

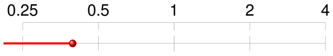

A	B	2.5	50	97.5	Sequence
2	0	0.031	0.11	0.39	CVPIEFR

5.43 167 679 tropomyosin [Haliotis discus discus]

Protein Accession	CL2925.Contig3$_H$1
Mean Expression Ratio	1.9
Median Expression Ratio	1.9
Credible Interval	(1.09, 3.29)
Associated Peptides	12
Associated Spectra	43
Coverage	NaN

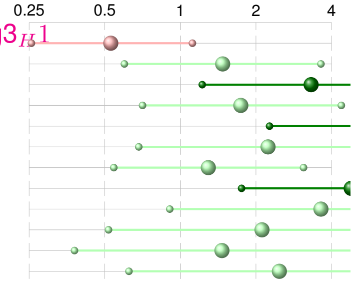

A	B	2.5	50	97.5	Sequence
4	5	0.25	0.53	1.1	LRDTEVQK
1	4	0.6	1.5	3.6	RIKLLEEDLER
1	3	1.2	3.3	8.6	KMLAMK
2	3	0.7	1.7	4.4	MLAMK
2	2	2.3	6.1	16	MDAIKKK
1	1	0.68	2.2	7.5	MDAIKK
0	6	0.54	1.3	3.1	IKLLEEDLER
0	4	1.8	4.8	13	MDKENAMDKAEQMEQK
0	1	0.91	3.6	15	YANLENEYDKANEGLIEANTKLEASEK
0	1	0.52	2.1	8.1	AMDKAEQMEQK
0	1	0.38	1.5	6.1	SKLEEDLNPLQK
0	1	0.62	2.5	10	MDAIK

5.44 346 702 minus strand PREDICTED: galectin-4-like [Anolis carolinensis]; 367 669 minus strand PREDICTED: galectin-7-like [Cavia porcellus]; 599 964 C. briggsae CBR-LEC-4 protein [Caenorhabditis briggsae]

Protein Accession	Unigene42203$_H$1 Unigene41782$_H$1 CL22459.Contig3$_H$1
Mean Expression Ratio	1.9
Median Expression Ratio	1.9
Credible Interval	(0.874, 4.26)
Associated Peptides	4
Associated Spectra	10
Coverage	NaN

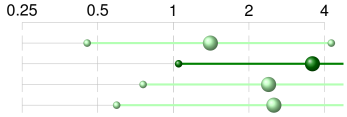

A	B	2.5	50	97.5	Sequence
2	2	0.45	1.4	4.2	AEATLEVR
0	2	1	3.6	12	KSVPHGLVK
1	2	0.76	2.4	7.9	SVPHGLVK
0	1	0.6	2.5	11	VKDSGFK

5.45 145 1020 minus strand PREDICTED: inorganic pyrophosphatase-like [Saccoglossus kowalevskii]

Protein Accession	CL12968.Contig14$_{H}$1
Mean Expression Ratio	0.527
Median Expression Ratio	0.528
Credible Interval	(0.249, 1.11)
Associated Peptides	7
Associated Spectra	9
Coverage	NaN

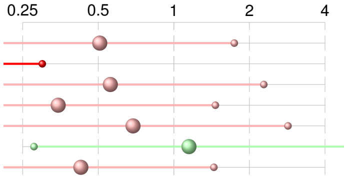

A	B	2.5	50	97.5	Sequence
1	1	0.15	0.51	1.7	LNPLKQDVK
1	0	0.016	0.07	0.3	SLMADPSVGK
0	1	0.13	0.56	2.3	IIAIDVTDPLASK
0	1	0.08	0.35	1.5	LNDIDDVHR
0	1	0.17	0.69	2.8	MEINKEEK
0	1	0.28	1.2	4.9	GSVIQVK
0	2	0.13	0.43	1.4	AVIDQSVNK

5.46 156 1163 cathepsin B [Crassostrea ariakensis]

Protein Accession	CL26836.Contig9$_{H}$1
Mean Expression Ratio	1.9
Median Expression Ratio	1.89
Credible Interval	(0.847, 4.26)
Associated Peptides	5
Associated Spectra	7
Coverage	NaN

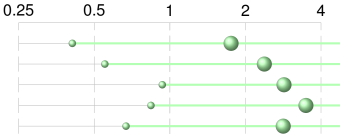

A	B	2.5	50	97.5	Sequence
0	1	0.41	1.8	7.5	GEQNIMEELYTR
0	1	0.55	2.4	9.7	TKWPNCPTLKEVR
0	3	0.94	2.8	8.6	DLPDSFDPR
0	1	0.84	3.5	15	LTDEEIFFINK
0	1	0.67	2.8	12	LPDSFDPR

5.47 " 223 423 ADPATP carrier protein; K05863 solute carrier family 25 (mitochondrial adenine nucleotide translocator), member 4/5/6/31"

Protein Accession	CL27196.Contig3$_{H}$1
Mean Expression Ratio	0.538
Median Expression Ratio	0.538
Credible Interval	(0.222, 1.32)
Associated Peptides	1
Associated Spectra	8
Coverage	NaN

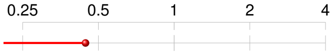

A	B	2.5	50	97.5	Sequence
6	2	0.082	0.19	0.45	LLVQNQNEMIK

5.48 116 352 minus strand troponin I 4 [Clonorchis sinensis]

Protein Accession	Unigene63816$_{H}$1
Mean Expression Ratio	1.85
Median Expression Ratio	1.85
Credible Interval	(0.784, 4.36)
Associated Peptides	3
Associated Spectra	7
Coverage	NaN

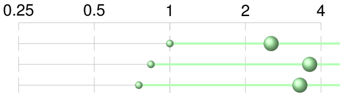

A	B	2.5	50	97.5	Sequence
0	5	1	2.5	6.5	FIKPVLR
0	1	0.84	3.6	15	QSPTRGNLKSTGR
0	1	0.75	3.3	14	GKFIKPVLR

5.49 " 28 1104 minus strand Pyruvate dehydrogenase E1 component subunit beta, mitochondrial [Crassostrea gigas]"

Protein Accession	CL18015.Contig14$_H$1
Mean Expression Ratio	1.85
Median Expression Ratio	1.85
Credible Interval	(0.864, 3.96)
Associated Peptides	7
Associated Spectra	10
Coverage	NaN

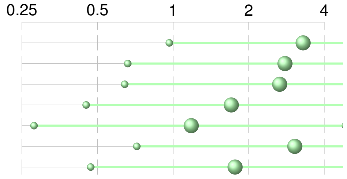

A	B	2.5	50	97.5	Sequence
0	2	0.97	3.3	11	DALNSAMDEEIARDER
0	1	0.66	2.8	12	ALDAAKELEGK
0	1	0.64	2.7	11	SLRPVDDETIIK
1	1	0.45	1.7	6.3	SLEEAALPQPVNVIR
0	1	0.28	1.2	4.8	IEREGK
0	1	0.72	3.1	13	VSKGLWK
1	1	0.47	1.8	6.5	VTGADVPMPYAK

5.50 156 845 minus strand histone H1 [Mytilus californianus]; 156 845 minus strand histone H1 [Mytilus californianus]; 156 845 minus strand histone H1 [Mytilus californianus]; 156 845 minus strand histone H1 [Mytilus californianus]

Protein Accession	CL26709.Contig6$_H$1 CL26709.Contig5$_H$1 CL26709.Contig4$_H$1 CL26709.Contig3$_H$1
Mean Expression Ratio	0.537
Median Expression Ratio	0.544
Credible Interval	(0.191, 1.43)
Associated Peptides	1
Associated Spectra	1
Coverage	NaN

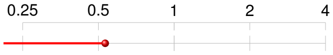

A	B	2.5	50	97.5	Sequence
1	0	0.027	0.12	0.53	DAITALKER

5.51 1 825 Spectrin alpha chain [Crassostrea gigas]

Protein Accession	CL10282.Contig3$_H$1
Mean Expression Ratio	1.83
Median Expression Ratio	1.84
Credible Interval	(0.8, 4.06)
Associated Peptides	5
Associated Spectra	6
Coverage	NaN

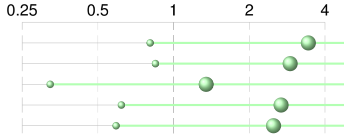

A	B	2.5	50	97.5	Sequence
0	1	0.81	3.4	14	KIEDLGAAMEER
0	2	0.85	2.9	10	MQHNLEQQIEAR
0	1	0.32	1.4	5.4	AIPDSYDYVDFTR
0	1	0.62	2.7	12	RKATEVR
0	1	0.59	2.5	10	MKPYVDK

5.52 513 755 PREDICTED: 40S ribosomal protein S16-like [Papio anubis]; 513 755 PREDICTED: 40S ribosomal protein S16-like [Papio anubis]

Protein Accession	CL14629.Contig2$_H$1 CL14629.Contig1$_H$1
Mean Expression Ratio	0.543
Median Expression Ratio	0.547
Credible Interval	(0.202, 1.44)
Associated Peptides	1
Associated Spectra	2
Coverage	NaN

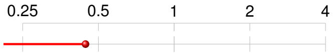

A	B	2.5	50	97.5	Sequence
2	0	0.036	0.13	0.44	SLLVADPR

5.53 594 1253 peroxiredoxin 6 [Eriocheir sinensis]

Protein Accession	CL21894.Contig2$_H$1
Mean Expression Ratio	0.547
Median Expression Ratio	0.548
Credible Interval	(0.315, 0.961)
Associated Peptides	11
Associated Spectra	43
Coverage	NaN

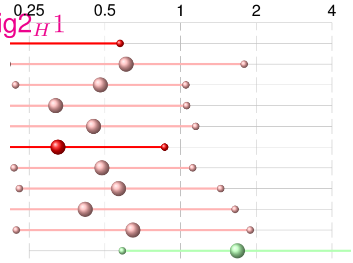

A	B	2.5	50	97.5	Sequence
1	1	0.051	0.17	0.57	NFDEILR
2	1	0.2	0.61	1.8	AVFIIGPDKK
2	6	0.22	0.48	1	AVFIIGPDK
1	1	0.096	0.32	1.1	LFPNYK
1	4	0.18	0.45	1.1	LLPEFEK
3	1	0.12	0.32	0.86	QLTATQK
3	4	0.22	0.49	1.1	VATPVDWK
1	4	0.23	0.57	1.4	VNLGDVFPDFK
0	1	0.1	0.42	1.6	ELAVQLGMVDPDEK
0	3	0.22	0.65	1.9	VLDSLQLTATQK
0	3	0.59	1.7	4.9	KVATPVDWK

5.54 55 795 Elongation factor 1-delta OS=Bos taurus GN=EEF1D PE=2 SV=2; 49 747 PREDICTED: elongation factor 1-delta-like [Strongylocentrotus purpuratus]; 49 819 PREDICTED: elongation factor 1-delta-like isoform 2 [Strongylocentrotus purpuratus]; 49 795 PREDICTED: elongation factor 1-delta-like isoform 2 [Strongylocentrotus purpuratus]

Protein Accession	CL10790.Contig9$_H$1 CL10790.Contig13$_H$1 CL10790.Contig12$_H$1 CL10790.Contig11$_H$1
Mean Expression Ratio	1.82
Median Expression Ratio	1.82
Credible Interval	(0.881, 3.73)
Associated Peptides	7
Associated Spectra	12
Coverage	NaN

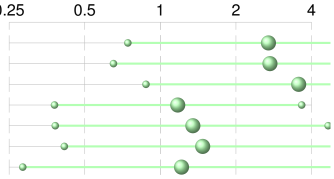

A	B	2.5	50	97.5	Sequence
1	1	0.74	2.7	9.9	KPVVIAK
0	1	0.65	2.7	11	QKFEEAEAHFYAR
0	1	0.88	3.5	15	TIQKDGLTWGAAK
1	2	0.38	1.2	3.7	VAALEVVGTGGASPK
0	2	0.38	1.3	4.7	KVTSDLR
1	1	0.41	1.5	5.6	LLEEYAAK
0	1	0.28	1.2	5.1	RLEEIEKENK

5.55 871 1371 ribosomal protein L11 [Triatoma infestans]; 343 843 minus strand ribosomal protein L11 [Triatoma infestans]

Protein Accession	CL20979.Contig2$_{H}1$ CL20979.Contig1$_{H}1$
Mean Expression Ratio	0.548
Median Expression Ratio	0.55
Credible Interval	(0.21, 1.43)
Associated Peptides	2
Associated Spectra	2
Coverage	NaN

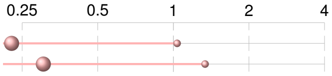

A	B	2.5	50	97.5	Sequence
1	0	0.051	0.23	1	ITKEEAIK
1	0	0.07	0.3	1.3	AEEILER

5.56 2 532 heat shock protein 70 [Echinostoma caproni]

Protein Accession	Unigene17002$_{H}1$
Mean Expression Ratio	1.81
Median Expression Ratio	1.81
Credible Interval	(0.707, 4.69)
Associated Peptides	2
Associated Spectra	3
Coverage	NaN

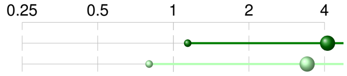

A	B	2.5	50	97.5	Sequence
0	2	1.1	4.1	15	ARFEELNADLFR
0	1	0.8	3.4	15	ARFEELNADLFRGTLEPVEK

5.57 205 2490 minus strand glucose-regulated protein 94 [Crassostrea gigas]

Protein Accession	CL5089.Contig1$_{H}1$
Mean Expression Ratio	0.554
Median Expression Ratio	0.554
Credible Interval	(0.33, 0.935)
Associated Peptides	18
Associated Spectra	36
Coverage	NaN

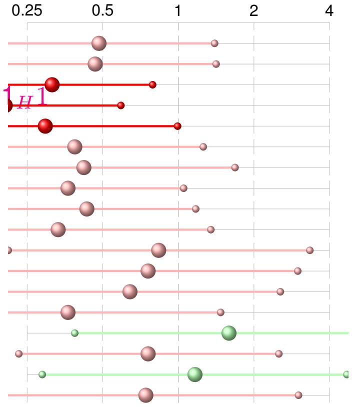

A	B	2.5	50	97.5	Sequence
1	2	0.17	0.48	1.4	GVVDSDDLPLNVSR
2	1	0.16	0.47	1.4	KTLEINPR
3	2	0.13	0.32	0.79	SGTSDFLAK
3	0	0.071	0.2	0.59	TLEINPR
1	1	0.092	0.3	0.99	DVALADKIEK
1	1	0.11	0.39	1.3	EGLKLDQSEK
1	0	0.1	0.42	1.7	KTLDMIK
1	2	0.12	0.36	1.1	ELISNASDALDKIR
2	2	0.16	0.43	1.2	NLGTIAR
0	1	0.083	0.33	1.3	ELISNASDALDK
0	1	0.21	0.83	3.3	KFQNVAK
0	1	0.19	0.76	3	LGVIEDTSNR
0	1	0.16	0.64	2.6	RVFITDNFEDMLPK
0	1	0.089	0.36	1.5	VFITDNFEDMLPK
0	1	0.39	1.6	6.3	HPLMKELK
0	2	0.23	0.76	2.5	SILFVPK
0	1	0.29	1.2	4.7	KPGDIDEKEYVDFYK
0	1	0.18	0.74	3	GTTVSLHLK

5.58 5 193 minus strand PREDICTED: 40S ribosomal protein S28-like [Strongylocentrotus purpuratus]; 3 122 PREDICTED: 40S ribosomal protein S28-like [Strongylocentrotus purpuratus]

Protein Accession	CL49462.Contig2$_{H}1$ CL49462.Contig1$_{H}1$
Mean Expression Ratio	1.8
Median Expression Ratio	1.8
Credible Interval	(0.766, 4.23)
Associated Peptides	3
Associated Spectra	6
Coverage	NaN

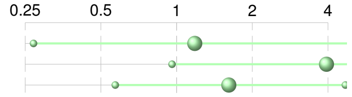

A	B	2.5	50	97.5	Sequence
0	1	0.27	1.2	5.1	EGDILTLLESER
0	1	0.96	3.9	17	NVKGPVREGDILTLLESER
1	3	0.57	1.6	4.7	VEFLDDSNR

5.59 2 226 minus strand hypothetical protein CGI_10015501 [Crassostrea gigas]

Protein Accession	Unigene73065$_{H}1$
Mean Expression Ratio	1.8
Median Expression Ratio	1.79
Credible Interval	(0.757, 4.36)
Associated Peptides	2
Associated Spectra	11
Coverage	NaN

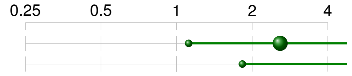

A	B	2.5	50	97.5	Sequence
0	7	1.1	2.6	6	SSVAADALR
0	4	1.8	5.2	15	SSVAADALRK

5.60 40 1098 minus strand major egg antigen [Clonorchis sinensis]

Protein Accession	CL2613.Contig4$_{H}1$
Mean Expression Ratio	0.555
Median Expression Ratio	0.557
Credible Interval	(0.211, 1.41)
Associated Peptides	2
Associated Spectra	2
Coverage	NaN

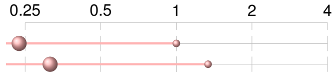

A	B	2.5	50	97.5	Sequence
1	0	0.054	0.24	1	AIVLKPTGK
1	0	0.07	0.31	1.3	LESHLTK

5.61 2019 2723 minus strand Kyphoscoliosis peptidase [Crassostrea gigas]; 2060 2764 minus strand Kyphoscoliosis peptidase [Crassostrea gigas]; 1074 1778 minus strand Kyphoscoliosis peptidase [Crassostrea gigas]; 2063 2767 minus strand Kyphoscoliosis peptidase [Crassostrea gigas]; 2044 2748 minus strand Kyphoscoliosis peptidase [Crassostrea gigas]; 2044 2748 minus strand Kyphoscoliosis peptidase [Crassostrea gigas]

Protein Accession	CL3923.Contig7$_H1$ CL3923.Contig6$_H1$ CL3923.Contig5$_H1$ CL3923.Contig4$_H1$ CL3923.Con
Mean Expression Ratio	1.79
Median Expression Ratio	1.78
Credible Interval	(0.727, 4.47)
Associated Peptides	2
Associated Spectra	5
Coverage	NaN

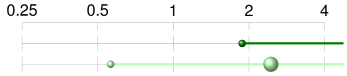

A	B	2.5	50	97.5	Sequence
2	2	1.9	5.2	14	TMGQAPPTVPKDFQLK
0	1	0.56	2.4	11	TMGQAPPTVPK

5.62 " 2 778 Actin, gamma-enteric smooth muscle OS=Rattus norvegicus GN=Actg2 PE=2 SV=1"

Protein Accession	Unigene64184$_H1$
Mean Expression Ratio	0.557
Median Expression Ratio	0.561
Credible Interval	(0.201, 1.53)
Associated Peptides	1
Associated Spectra	1
Coverage	NaN

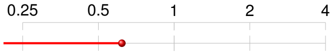

A	B	2.5	50	97.5	Sequence
1	0	0.031	0.14	0.62	EITSLAPSTMK

5.63 1 1284 minus strand Thioredoxin reductase 3 [Crassostrea gigas]

Protein Accession	CL3500.Contig1$_H1$
Mean Expression Ratio	1.78
Median Expression Ratio	1.78
Credible Interval	(0.712, 4.48)
Associated Peptides	2
Associated Spectra	4
Coverage	NaN

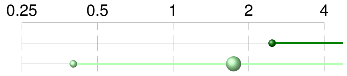

A	B	2.5	50	97.5	Sequence
0	3	2.5	7.5	24	GAVPSQIEQLEEGKPGR
0	1	0.4	1.7	7.5	LLVQRL

5.64 280 690 Histone H3.3 [Harpegnathos saltator]

Protein Accession	Unigene26607$_H$1
Mean Expression Ratio	0.564
Median Expression Ratio	0.565
Credible Interval	(0.315, 0.993)
Associated Peptides	9
Associated Spectra	83
Coverage	NaN

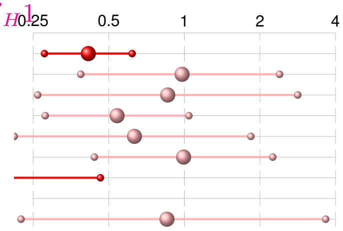

A	B	2.5	50	97.5	Sequence
**28**	**10**	**0.28**	**0.41**	**0.62**	**DIQLAR**
**2**	**3**	**0.39**	**0.98**	**2.4**	**VTIMPK**
**1**	**1**	**0.26**	**0.86**	**2.8**	**VTIMPKDIQLAR**
**12**	**0**	**0.28**	**0.54**	**1**	**EIAQDFK**
**3**	**0**	**0.21**	**0.63**	**1.8**	**KQLATK**
**3**	**4**	**0.44**	**0.99**	**2.2**	**RVTIMPK**
**2**	**0**	**0.042**	**0.14**	**0.46**	**KLPFQR**
**13**	**0**	**0.056**	**0.11**	**0.2**	**STELLIR**
**0**	**1**	**0.22**	**0.85**	**3.7**	**RVTIMPKDIQLAR**

5.65 153 953 minus strand ribosomal protein rpl7 [Arenicola marina]

Protein Accession	CL20943.Contig3$_H$1
Mean Expression Ratio	0.568
Median Expression Ratio	0.569
Credible Interval	(0.205, 1.54)
Associated Peptides	1
Associated Spectra	1
Coverage	NaN

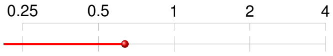

A	B	2.5	50	97.5	Sequence
**1**	**0**	**0.032**	**0.15**	**0.64**	**VPETILK**

5.66 49 819 PREDICTED: elongation factor 1-delta-like isoform 2 [Strongylocentrotus purpuratus]

Protein Accession	CL10790.Contig12$_H$1
Mean Expression Ratio	0.569
Median Expression Ratio	0.572
Credible Interval	(0.233, 1.37)
Associated Peptides	3
Associated Spectra	7
Coverage	NaN

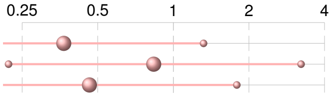

A	B	2.5	50	97.5	Sequence
**1**	**2**	**0.1**	**0.37**	**1.3**	**VAALEVVGTGGASPK**
**1**	**1**	**0.22**	**0.84**	**3.2**	**KPVVIAK**
**1**	**1**	**0.12**	**0.46**	**1.8**	**LLEEYAAK**

5.67 136 675 minus strand predicted protein [Nematostella vectensis] gi

Protein Accession	CL17411.Contig1$_H$1
Mean Expression Ratio	1.75
Median Expression Ratio	1.74
Credible Interval	(0.693, 4.52)
Associated Peptides	2
Associated Spectra	3
Coverage	NaN

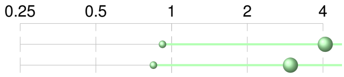

A	B	2.5	50	97.5	Sequence
**0**	**1**	**0.92**	**4.1**	**18**	**MLNEDELRDAK**
**0**	**2**	**0.85**	**3**	**10**	**VSEARDELQR**

5.68 194 1996 minus strand Neutral and basic amino acid transport protein rBAT [Crassostrea gigas]; 265 2067 minus strand Neutral and basic amino acid transport protein rBAT [Crassostrea gigas]; 194 1996 minus strand Neutral and basic amino acid transport protein rBAT [Crassostrea gigas]; 194 1996 minus strand Neutral and basic amino acid transport protein rBAT [Crassostrea gigas]; 265 2067 minus strand Neutral and basic amino acid transport protein rBAT [Crassostrea gigas]

Protein Accession	CL9047.Contig9$_H$1 CL9047.Contig8$_H$1 CL9047.Contig6$_H$1 CL9047.Contig15$_H$1 CL9047.Co
Mean Expression Ratio	1.74
Median Expression Ratio	1.74
Credible Interval	(0.762, 4.05)
Associated Peptides	3
Associated Spectra	8
Coverage	NaN

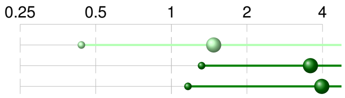

A	B	2.5	50	97.5	Sequence
1	1	0.44	1.5	5	NFAGNLDVVDALR
0	4	1.3	3.6	9.6	TAVDDLDNKPGREK
0	2	1.2	4	13	TITPMQWSK

5.69 " 90 1085 squid, variant A [Blattella germanica]"

Protein Accession	CL14696.Contig5$_H$1
Mean Expression Ratio	0.578
Median Expression Ratio	0.575
Credible Interval	(0.255, 1.34)
Associated Peptides	3
Associated Spectra	8
Coverage	NaN

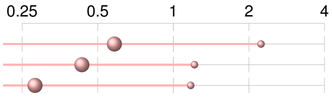

A	B	2.5	50	97.5	Sequence
1	1	0.16	0.58	2.2	IMEVTTHK
3	2	0.15	0.43	1.2	LGGQEVDVK
1	0	0.066	0.28	1.2	IFVGR

5.70 " 242 646 minus strand Fatty acid-binding protein, heart [Crassostrea gigas]; 227 631 minus strand Fatty acid-binding protein, heart [Crassostrea gigas]; 265 669 Fatty acid-binding protein, heart [Crassostrea gigas]"

Protein Accession	CL7643.Contig9$_H$1 CL7643.Contig11$_H$1 CL7643.Contig10$_H$1
Mean Expression Ratio	1.73
Median Expression Ratio	1.73
Credible Interval	(0.919, 3.24)
Associated Peptides	8
Associated Spectra	43
Coverage	NaN

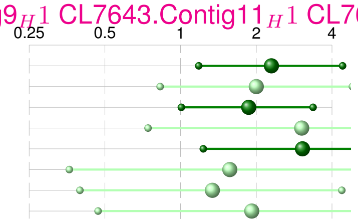

A	B	2.5	50	97.5	Sequence
3	9	1.2	2.3	4.4	AINNGQMIMTMK
3	3	0.83	2	4.8	ATSGVEFESR
6	9	1	1.9	3.4	CGDVTSIR
1	0	0.74	3	13	YLDGREIK
2	3	1.2	3.1	7.8	KYDKV
0	1	0.36	1.6	6.3	TVFRVEGDTLIEDVSGPFSTSIVR
0	2	0.4	1.3	4.4	VEGDTLIEDVSGPFSTSIVR
0	1	0.47	1.9	7.7	AALNGKWK

5.71 900 1616 minus strand hemocyanin isoform 1 [Haliotis diversicolor]

Protein Accession	Unigene30321$_{H}1$
Mean Expression Ratio	0.577
Median Expression Ratio	0.579
Credible Interval	(0.225, 1.46)
Associated Peptides	2
Associated Spectra	3
Coverage	NaN

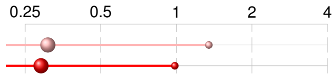

A	B	2.5	50	97.5	Sequence
0	1	0.07	0.31	1.3	LFKVEITDDLK
0	2	0.081	0.29	0.99	VEITDDLK

5.72 254 1093 uncharacterized protein LOC432187 precursor [Xenopus laevis] gi

Protein Accession	CL7800.Contig7$_{H}1$
Mean Expression Ratio	0.577
Median Expression Ratio	0.58
Credible Interval	(0.226, 1.48)
Associated Peptides	2
Associated Spectra	3
Coverage	NaN

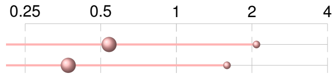

A	B	2.5	50	97.5	Sequence
1	1	0.13	0.54	2.1	GINFASTTR
1	0	0.084	0.37	1.6	LVTSK

5.73 84 425 minus strand Transcription elongation factor B polypeptide 2 [Crassostrea gigas]; 84 425 minus strand Transcription elongation factor B polypeptide 2 [Crassostrea gigas]; 84 425 minus strand Transcription elongation factor B polypeptide 2 [Crassostrea gigas]; 112 366 Transcription elongation factor B polypeptide 2 OS=Rattus norvegicus GN=Tceb2 PE=1 SV=1; 101 442 minus strand Transcription elongation factor B polypeptide 2 [Crassostrea gigas]; 101 442 minus strand Transcription elongation factor B polypeptide 2 [Crassostrea gigas]; 84 425 minus strand Transcription elongation factor B polypeptide 2 [Crassostrea gigas]

Protein Accession	CL19448.Contig9$_{H}1$ CL19448.Contig8$_{H}1$ CL19448.Contig7$_{H}1$ CL19448.Contig4$_{H}1$ CL1944
Mean Expression Ratio	0.577
Median Expression Ratio	0.58
Credible Interval	(0.206, 1.6)
Associated Peptides	1
Associated Spectra	1
Coverage	NaN

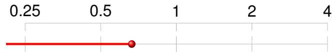

A	B	2.5	50	97.5	Sequence
1	0	0.034	0.15	0.66	AQAPATLGLVYK

5.74 " 3 227 minus strand alpha tubulin, putative [Ixodes scapularis] gi"

Protein Accession	Unigene31690$_H$1
Mean Expression Ratio	1.73
Median Expression Ratio	1.72
Credible Interval	(0.748, 4.05)
Associated Peptides	2
Associated Spectra	13
Coverage	NaN

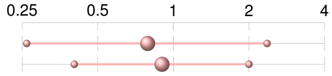

A	B	2.5	50	97.5	Sequence
3	2	0.26	0.79	2.4	LSVDYGKK
1	7	0.4	0.9	2	LSVDYGK

5.75 95 2278 hypothetical protein CGI_10017740 [Crassostrea gigas]

Protein Accession	CL48637.Contig1$_H$1
Mean Expression Ratio	0.578
Median Expression Ratio	0.581
Credible Interval	(0.233, 1.41)
Associated Peptides	3
Associated Spectra	3
Coverage	NaN

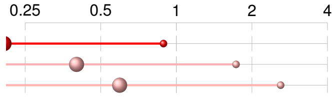

A	B	2.5	50	97.5	Sequence
1	0	0.047	0.21	0.89	VAMYR
0	1	0.096	0.4	1.7	SISYALR
0	1	0.14	0.6	2.6	AGLELVPK

5.76 " 99 497 RecName: Full=Histone H2B, gonadal"

Protein Accession	CL29794.Contig1$_H$1
Mean Expression Ratio	1.71
Median Expression Ratio	1.71
Credible Interval	(0.913, 3.2)
Associated Peptides	6
Associated Spectra	47
Coverage	NaN

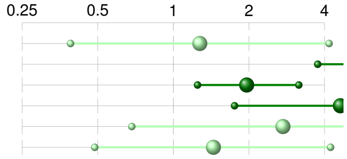

A	B	2.5	50	97.5	Sequence
0	2	0.39	1.3	4.2	AMSIMNSFVNDIFER
3	3	3.8	9.3	23	AVTKYTSSK
7	23	1.2	2	3.2	LLLPGELAK
3	2	1.8	4.6	13	STITSREIQTAVR
0	1	0.68	2.7	11	LLPGELAK
0	3	0.49	1.4	4.2	KESYAIYIYK

5.77 160 1218 Galectin-9 [Crassostrea gigas]

Protein Accession	Unigene165$_H$1
Mean Expression Ratio	0.584
Median Expression Ratio	0.585
Credible Interval	(0.252, 1.34)
Associated Peptides	3
Associated Spectra	8
Coverage	NaN

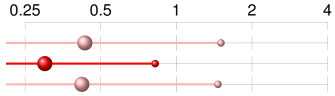

A	B	2.5	50	97.5	Sequence
1	1	0.13	0.43	1.5	EATALVVTGDVR
2	2	0.11	0.3	0.83	SGIPGGLAPGR
0	2	0.12	0.42	1.5	IAVNNQHLVEFR

5.78 2473 2898 RecName: Full=Paramyosin gi|397603|emb|CAA79849.1| paramyosin [Echinococcus granulosus]

Protein Accession	CL43455.Contig1$_{H}$1
Mean Expression Ratio	1.71
Median Expression Ratio	1.71
Credible Interval	(0.997, 2.96)
Associated Peptides	11
Associated Spectra	59
Coverage	NaN

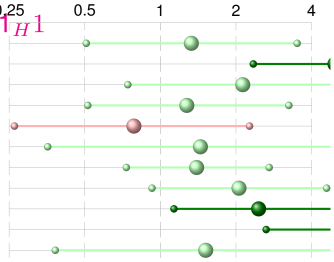

A	B	2.5	50	97.5	Sequence
2	2	0.51	1.3	3.5	INKEILAQTEEER
1	8	2.4	4.9	10	INKEILAQTEEERR
1	2	0.74	2.1	6.2	KQLEIEIR
2	3	0.51	1.3	3.3	QLEIEIR
0	3	0.26	0.79	2.3	EILAQTEEER
0	1	0.36	1.4	6	EILAQTEEERR
0	12	0.73	1.4	2.7	KAQTLIEEAENR
0	7	0.93	2.1	4.6	QLAEFASLNEQLNLR
0	8	1.1	2.5	5.3	QLEEAEDVANPTMNK
0	6	2.6	6.1	15	QLEEAEDVANPTMNKYR
0	1	0.38	1.5	6.3	RQLAEFASLNEQLNLR

5.79 51 890 prohibitin [Octopus tankahkeei]

Protein Accession	CL26635.Contig2$_{H}$1
Mean Expression Ratio	1.7
Median Expression Ratio	1.7
Credible Interval	(0.852, 3.44)
Associated Peptides	8
Associated Spectra	12
Coverage	NaN

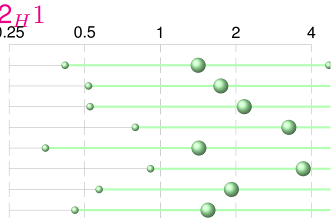

A	B	2.5	50	97.5	Sequence
1	1	0.42	1.4	4.7	AFTEAGEGLVELR
0	2	0.52	1.7	6	FLVEKEEQK
0	1	0.52	2.2	8.8	KIEAAEDIAYQLSR
0	1	0.79	3.2	14	NVPVITGSKDLQNVNITLR
0	1	0.35	1.4	5.9	VSEELTDR
0	1	0.91	3.7	15	FLVEKEEQKKR
0	2	0.57	1.9	6.5	AAVISAEGDSLAAELLAK
0	2	0.46	1.5	5.3	NVPVITGSK

5.80 412 708 minus strand heat shock protein beta-1 [Trichinella spiralis] gi; 459 755 minus strand heat shock protein beta-1 [Trichinella spiralis] gi; 464 763 minus strand heat shock protein beta-1 [Trichinella spiralis] gi; 437 736 minus strand heat shock protein beta-1 [Trichinella spiralis] gi; 437 736 minus strand heat shock protein beta-1 [Trichinella spiralis] gi; 437 736 minus strand heat shock protein beta-1 [Trichinella spiralis] gi

Protein Accession	CL17234.Contig6$_{H}$1 CL17234.Contig5$_{H}$1 CL17234.Contig4$_{H}$1 CL17234.Contig3$_{H}$1 CL1723
Mean Expression Ratio	0.587
Median Expression Ratio	0.589
Credible Interval	(0.209, 1.63)
Associated Peptides	1
Associated Spectra	1
Coverage	NaN

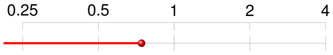

A	B	2.5	50	97.5	Sequence
1	0	0.037	0.16	0.74	TVTSSLNADGVLTLK

5.81 227 2920 minus strand Lysosomal alpha-mannosidase [Crassostrea gigas]; 227 3073 minus strand Lysosomal alpha-mannosidase [Crassostrea gigas]; 193 2886 minus strand Lysosomal alpha-mannosidase [Crassostrea gigas]

Protein Accession	CL2930.Contig3$_{H}$1 CL2930.Contig2$_{H}$1 CL2930.Contig1$_{H}$1
Mean Expression Ratio	0.589
Median Expression Ratio	0.59
Credible Interval	(0.259, 1.33)
Associated Peptides	4
Associated Spectra	17
Coverage	NaN

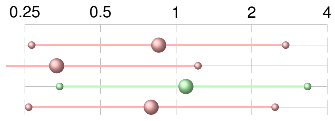

A	B	2.5	50	97.5	Sequence
1	3	0.27	0.85	2.7	ASLTWSGVR
1	1	0.088	0.33	1.2	VVNDALKK
1	4	0.34	1.1	3.3	IFIRDEAK
1	5	0.26	0.8	2.5	EQASLFAR

5.82 140 283 minus strand elongation factor 1 alpha [Angiostrongylus cantonensis]

Protein Accession	CL16758.Contig7$_{H}$1
Mean Expression Ratio	0.587
Median Expression Ratio	0.59
Credible Interval	(0.217, 1.52)
Associated Peptides	2
Associated Spectra	2
Coverage	NaN

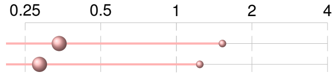

A	B	2.5	50	97.5	Sequence
1	0	0.074	0.34	1.5	VIEKFEK
1	0	0.065	0.28	1.2	STTTGHLIYK

5.83 284 1318 hsc70-interacting protein [Gallus gallus] gi; 88 1137 hsc70-interacting protein [Gallus gallus] gi

Protein Accession	CL11847.Contig4$_{H}$1 CL11847.Contig2$_{H}$1
Mean Expression Ratio	0.597
Median Expression Ratio	0.598
Credible Interval	(0.238, 1.46)
Associated Peptides	2
Associated Spectra	4
Coverage	NaN

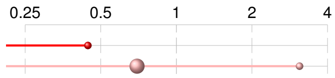

A	B	2.5	50	97.5	Sequence
3	0	0.048	0.15	0.44	AQLDLLK
0	1	0.16	0.7	3.1	LNPTSALLYAK

5.84 51 371 minus strand putative mitochondrial ATP synthase F chain [Haliotis discus discus]; 51 371 minus strand putative mitochondrial ATP synthase F chain [Haliotis discus discus]

Protein Accession	CL18283.Contig4$_{H}$1 CL18283.Contig2$_{H}$1
Mean Expression Ratio	0.602
Median Expression Ratio	0.603
Credible Interval	(0.223, 1.57)
Associated Peptides	1
Associated Spectra	4
Coverage	NaN

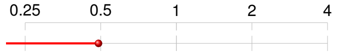

A	B	2.5	50	97.5	Sequence
4	0	0.064	0.18	0.49	ADLPLGQVK

5.85 111 1226 gelsolin [Aplysia californica] gi; 42 1139 minus strand gelsolin [Aplysia californica] gi

Protein Accession	Unigene39723$_{H}$1 Unigene32782$_{H}$1
Mean Expression Ratio	1.67
Median Expression Ratio	1.66
Credible Interval	(0.853, 3.26)
Associated Peptides	10
Associated Spectra	12
Coverage	NaN

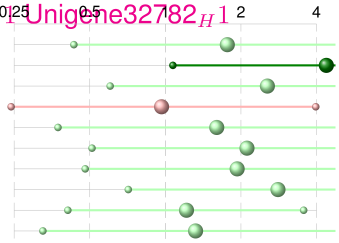

A	B	2.5	50	97.5	Sequence
0	1	0.43	1.8	7.3	DLVTSDDVYILDTGLK
0	1	1.1	4.4	18	SKAMQFIGEIK
0	1	0.6	2.6	10	TVELDTKLNDKPIQHR
0	1	0.24	0.97	4	SVPAEEYK
0	1	0.37	1.6	6.5	QFDTALAA
0	1	0.51	2.1	9	KNGLPLAHNYLR
0	1	0.48	1.9	8	EVQGHESDLFK
0	1	0.71	2.8	12	ESGNPWRPVTVIK
0	3	0.41	1.2	3.5	SDDVFIFDSGK
0	1	0.33	1.3	5.5	AMQFIGEIK

5.86 " 10 609 lethal(2)essential for life protein, l2efl [Aedes aegypti] gi"

Protein Accession	Unigene67975$_{H}$1
Mean Expression Ratio	0.605
Median Expression Ratio	0.606
Credible Interval	(0.305, 1.2)
Associated Peptides	6
Associated Spectra	23
Coverage	NaN

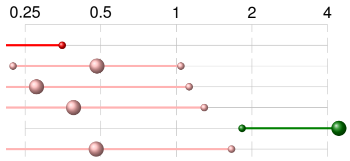

A	B	2.5	50	97.5	Sequence
4	0	0.048	0.13	0.35	MQMGETGK
8	0	0.22	0.48	1	INMDVK
1	0	0.066	0.28	1.1	SEVVNDDKEFR
0	2	0.11	0.39	1.3	ALEAPGETVIPITFEGK
0	6	1.8	4.4	10	KALEAPGETVIPITFEGKGDGK
0	2	0.14	0.48	1.7	KALEAPGETVIPITFEGK

5.87 161 301 Heat shock protein beta-6 OS=Mus musculus GN=Hspb6 PE=1 SV=1

Protein Accession	CL46983.Contig2$_{H}1$
Mean Expression Ratio	1.65
Median Expression Ratio	1.65
Credible Interval	(0.787, 3.46)
Associated Peptides	4
Associated Spectra	31
Coverage	NaN

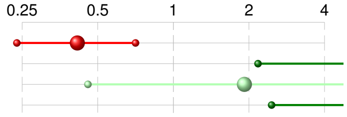

A	B	2.5	50	97.5	Sequence
5	14	0.24	0.41	0.71	VNMDVQHFKPEEIK
0	5	2.2	5.5	14	VNMDVQHFKPEEIKITTKDNR
0	1	0.46	1.9	7.9	VVISAK
0	6	2.5	5.8	14	VNMDVQHFKPEEIKITTK

5.88 405 1373 paramyosin [Haliotis discus discus]

Protein Accession	CL43455.Contig4$_{H}1$
Mean Expression Ratio	1.65
Median Expression Ratio	1.65
Credible Interval	(1.16, 2.34)
Associated Peptides	36
Associated Spectra	249
Coverage	NaN

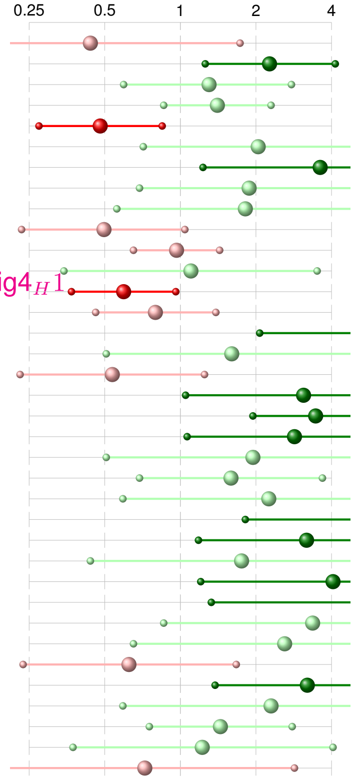

A	B	2.5	50	97.5	Sequence
1	0	0.11	0.44	1.7	FDKELAAK
3	12	1.3	2.3	4.1	HTEAVNDLSDQVDYLTK
1	7	0.6	1.3	2.8	IRELEDALDAER
9	15	0.86	1.4	2.3	LKGQVDDLTR
11	6	0.27	0.48	0.85	LSLESR
1	2	0.71	2	6	QKLSLESR
1	2	1.2	3.6	10	QLNDLTALKAR
1	3	0.69	1.9	5	TRFDKELAAK
1	1	0.56	1.8	5.9	FEAAEASLR
3	6	0.23	0.5	1	LDEEAETSSNLR
9	30	0.65	0.96	1.4	QLNDLTALK
1	1	0.34	1.1	3.5	SQLQNQNDDLKR
13	12	0.37	0.59	0.96	TVAELSFR
9	9	0.46	0.8	1.4	VSADYAALK
0	2	2.1	6.9	23	AKSQLQNQNDDLKR
0	2	0.51	1.6	5.3	DLELATAQ
0	6	0.23	0.54	1.3	ELEDALDAER
0	3	1	3.1	9.2	ELEDALDAERDGR
0	16	1.9	3.5	6.1	IRELEDALDAERDGR
0	4	1.1	2.9	7.5	IRELEDALDAERDGRVR
0	1	0.51	1.9	7.5	ITELEDSCEQLR
0	6	0.69	1.6	3.7	KDLELATAQFEAAEASLR
0	1	0.59	2.3	8.9	LDQVQDSLDEQGGSSTVAQIELNKKR
0	2	1.8	6.1	20	LSLESRVDAAEQSAAR
0	4	1.2	3.2	8.1	LTQENFDLQHQVQELDSSNAALAK
0	1	0.44	1.8	6.9	RELEDALDAERDGR
0	2	1.2	4	13	ESEIYKLR
0	1	1.3	5.2	20	ESEIYKLRK
0	1	0.86	3.4	13	KRESEIYK
0	1	0.65	2.6	10	QFEAAEASLR
0	4	0.24	0.62	1.7	DEQGGSSTVAQIELNK
0	6	1.4	3.2	7.4	DLELATAQFEAAEASLR
0	1	0.59	2.3	8.8	LDEEAETSSNLRNSLSK
0	12	0.75	1.4	2.8	LDQVQDSLDEQGGSSTVAQIELNK
0	2	0.37	1.2	4.1	QTLVIELDGLSGQLDSITK
0	1	0.18	0.72	2.8	TEELEETR

5.89 3591 4526 minus strand myosin heavy chain cardiac muscle specific isoform 1 [Argopecten irradians]

Protein Accession	CL49594.Contig2$_{H}$1
Mean Expression Ratio	1.64
Median Expression Ratio	1.64
Credible Interval	(1.04, 2.58)
Associated Peptides	21
Associated Spectra	86
Coverage	NaN

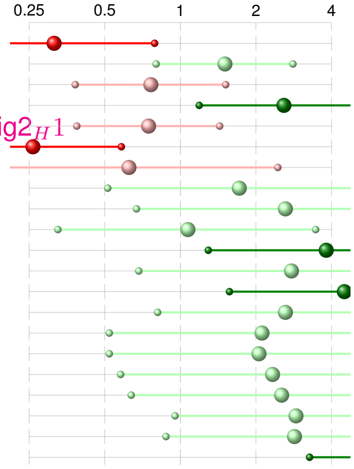

A	B	2.5	50	97.5	Sequence
4	1	0.13	0.32	0.79	KLEQDLK
6	7	0.8	1.5	2.8	QLEEAEHK
4	7	0.38	0.76	1.5	TINDLQTQK
1	7	1.2	2.6	5.6	LQQETANLTR
4	8	0.39	0.75	1.4	QVEAQLAELSAK
3	4	0.11	0.26	0.58	SEVDDLQMQLQNVGK
1	0	0.16	0.62	2.4	VGALTK
0	2	0.51	1.7	5.8	AKLEQTLDELEDNLER
0	1	0.67	2.6	11	AQLKSEVDDLQMQLQNVGK
0	2	0.32	1.1	3.5	ATQANVEDLER
0	3	1.3	3.8	11	KLEQDLKATQANVEDLER
0	1	0.68	2.8	11	NRLQQETANLTR
0	3	1.6	4.5	13	QLEEAEHKVGALTKEK
0	2	0.81	2.6	8.5	QVEAQLAELSAKVEEGNR
0	1	0.52	2.1	8.3	TMEDLQAEEDKVNHLNK
0	1	0.52	2.1	8	AATSRSRTLQHEAQVSALR
0	1	0.58	2.3	9.3	VKRELEENVR
0	1	0.64	2.5	10	RKLEQDLK
0	3	0.95	2.9	8.4	ATSRSRTLQHEAQVSALR
0	2	0.88	2.9	9.2	LEQTLDELEDNLEREK
0	6	3.3	7.8	18	QLEEAEHKVGALTK

5.90 77 1561 retinal dehydrogenase [Lymnaea stagnalis]; 69 1553 retinal dehydrogenase [Lymnaea stagnalis]

Protein Accession	CL13284.Contig7$_{H}$1 CL13284.Contig2$_{H}$1
Mean Expression Ratio	1.63
Median Expression Ratio	1.63
Credible Interval	(0.982, 2.69)
Associated Peptides	17
Associated Spectra	46
Coverage	NaN

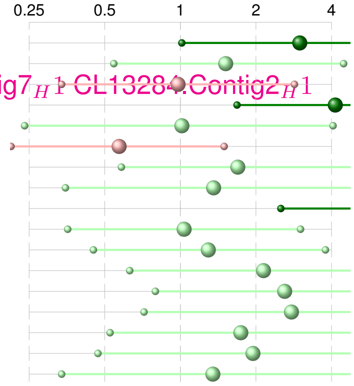

A	B	2.5	50	97.5	Sequence
2	1	1	3	8.6	NVVIKIPQKNS
1	2	0.54	1.5	4.5	SFPEPIRNPEVK
1	2	0.34	0.98	2.8	TFPTINPATGK
3	2	1.7	4.1	10	TFPTINPATGKK
1	0	0.24	1	4.1	TAELANKR
2	2	0.21	0.57	1.5	AVEVAQDAFK
1	2	0.58	1.7	4.9	LGSAWR
1	0	0.35	1.4	5.3	GALLYK
1	7	2.5	5.4	12	ADVDKAVEVAQDAFK
1	2	0.36	1	3	KTAELANK
0	3	0.45	1.3	3.8	ELGEYGLQQYVEVK
0	2	0.63	2.2	7	FSTVEEAIER
0	2	0.79	2.6	8.7	KTAELANKR
0	1	0.72	2.8	11	NVVIKIPQK
0	2	0.52	1.7	5.8	IYDEFVR
0	1	0.47	1.9	8	RGALLYK
0	1	0.34	1.4	5.3	TTLELGGK

5.91 " 53 1291 Acetyl-CoA acetyltransferase B, mitochondrial [Crassostrea gigas]"

Protein Accession	CL1322.Contig1$_{H}$1
Mean Expression Ratio	1.63
Median Expression Ratio	1.62
Credible Interval	(0.646, 4.15)
Associated Peptides	2
Associated Spectra	3
Coverage	NaN

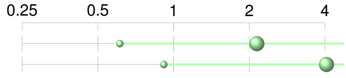

A	B	2.5	50	97.5	Sequence
0	2	0.61	2.1	7.8	TLKPVFVK
0	1	0.92	4.1	18	LGSVAIKEAVSR

5.92 333 1799 Alba-like protein C9orf23-like protein [Crassostrea gigas]

Protein Accession	Unigene30413$_{H}$1
Mean Expression Ratio	1.62
Median Expression Ratio	1.62
Credible Interval	(0.764, 3.5)
Associated Peptides	6
Associated Spectra	9
Coverage	NaN

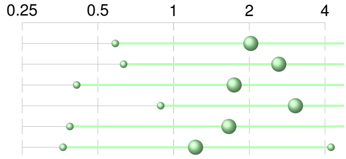

A	B	2.5	50	97.5	Sequence
0	2	0.59	2	7.1	AASWLGMR
0	1	0.63	2.6	11	GKGALELAQR
0	1	0.41	1.7	7.2	SPLYVDEGNILMDVR
0	2	0.89	3.1	10	TINPATEAETQGK
0	1	0.39	1.7	7	EIMTELSR
0	2	0.36	1.2	4.2	ELNDFMTK

5.93 " 61 573 steroid membrane receptor Hpr6.6/25-Dx, putative [Ixodes scapularis] gi"

Protein Accession	CL29406.Contig1$_{H}$1
Mean Expression Ratio	0.616
Median Expression Ratio	0.618
Credible Interval	(0.22, 1.67)
Associated Peptides	1
Associated Spectra	1
Coverage	NaN

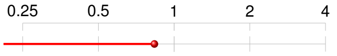

A	B	2.5	50	97.5	Sequence
1	0	0.043	0.19	0.83	ILIAVNGK

5.94 689 1984 hypothetical protein BRAFLDRAFT_78257 [Branchiostoma floridae] gi

Protein Accession	Unigene34076$_{H}$1
Mean Expression Ratio	1.62
Median Expression Ratio	1.61
Credible Interval	(0.789, 3.39)
Associated Peptides	7
Associated Spectra	11
Coverage	NaN

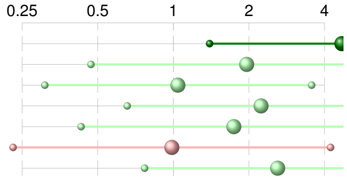

A	B	2.5	50	97.5	Sequence
0	2	1.4	4.7	16	ILLDPSKAPTPK
0	1	0.47	2	8.1	VQQKPEPSPAEGEVLIR
0	2	0.31	1	3.6	ILLDPSK
0	2	0.65	2.2	7.6	SFFSFAK
0	1	0.43	1.7	7.5	AWAELVAV
0	1	0.23	0.98	4.2	GISILKPMGK
0	2	0.77	2.6	9	SLVLTGFGSMK

5.95 42 434 cytochrome b5 [Haliotis discus discus]; 42 434 cytochrome b5 [Haliotis discus discus]

Protein Accession	CL6985.Contig9$_H$1 CL6985.Contig5$_H$1
Mean Expression Ratio	1.62
Median Expression Ratio	1.61
Credible Interval	(0.674, 4.05)
Associated Peptides	3
Associated Spectra	4
Coverage	NaN

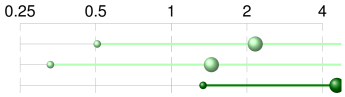

A	B	2.5	50	97.5	Sequence
0	1	0.51	2.2	9.7	KTFETNTGPVGR
0	1	0.33	1.4	6.3	ELMKDYLIGK
0	2	1.3	4.6	17	VFRSEEVKSHKDAK

5.96 " 220 627 Fatty acid-binding protein, brain [Crassostrea gigas]"

Protein Accession	CL9041.Contig2$_H$1
Mean Expression Ratio	0.623
Median Expression Ratio	0.62
Credible Interval	(0.299, 1.31)
Associated Peptides	5
Associated Spectra	24
Coverage	NaN

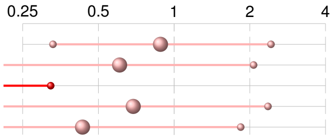

A	B	2.5	50	97.5	Sequence
1	3	0.33	0.88	2.4	EVDGGVMTSTTTFDGK
1	1	0.18	0.61	2.1	IVENYKLEDK
8	7	0.099	0.18	0.32	LTITVEGGK
0	2	0.2	0.69	2.4	LGETFDYNSIDGTKPK
0	1	0.1	0.43	1.8	VDLASTTGLEEFGTAIGFSEEK

5.97 227 3073 minus strand Lysosomal alpha-mannosidase [Crassostrea gigas]

Protein Accession	CL2930.Contig2$_H$1
Mean Expression Ratio	1.61
Median Expression Ratio	1.61
Credible Interval	(0.92, 2.8)
Associated Peptides	14
Associated Spectra	32
Coverage	NaN

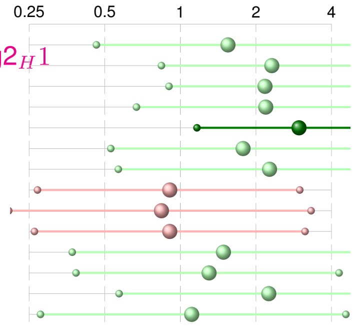

A	B	2.5	50	97.5	Sequence
0	2	0.46	1.6	5	ALPDNVHLLTLEQFR
1	3	0.84	2.3	6.3	ASLTWSGVR
1	5	0.9	2.2	5.2	EQASLFAR
0	2	0.67	2.2	7.3	GSHYVMLEPVSSAAK
1	4	1.2	3	7.6	IFIRDEAK
0	2	0.53	1.8	5.8	LANGVMEGQK
0	1	0.57	2.3	9	SQGGSSLLDGQIELMVHRR
0	2	0.27	0.91	3	VASELIPITPDTFR
0	1	0.21	0.84	3.3	VGWQIDPFGHSR
1	1	0.26	0.91	3.1	VVNDALKK
0	1	0.37	1.5	6.1	QAVAYDYAER
0	2	0.39	1.3	4.3	QVNNFLQVVK
0	1	0.57	2.3	9.6	LQWNYPH
0	1	0.28	1.1	4.6	VVNDALK

5.98 11 187 PREDICTED: 40S ribosomal protein S28-like [Strongylocentrotus purpuratus]

Protein Accession	Unigene696$_H$1
Mean Expression Ratio	0.617
Median Expression Ratio	0.621
Credible Interval	(0.225, 1.63)
Associated Peptides	1
Associated Spectra	4
Coverage	NaN

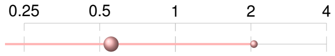

A	B	2.5	50	97.5	Sequence
**1**	**3**	**0.15**	**0.56**	**2.1**	**VEFLDDSNR**

5.99 201 4058 PREDICTED: xanthine dehydrogenase/oxidase-like [Amphimedon queenslandica]

Protein Accession	CL17390.Contig11$_H$1
Mean Expression Ratio	0.622
Median Expression Ratio	0.623
Credible Interval	(0.233, 1.66)
Associated Peptides	2
Associated Spectra	2
Coverage	NaN

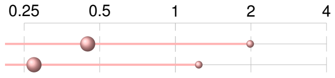

A	B	2.5	50	97.5	Sequence
**1**	**0**	**0.099**	**0.45**	**2**	**AVTDLQEAISNK**
**1**	**0**	**0.06**	**0.27**	**1.2**	**MSLIR**

5.100 503 1219 heat shock cognate 71 [Mytilus galloprovincialis]

Protein Accession	CL23109.Contig5$_H$1
Mean Expression Ratio	0.626
Median Expression Ratio	0.624
Credible Interval	(0.248, 1.53)
Associated Peptides	2
Associated Spectra	4
Coverage	NaN

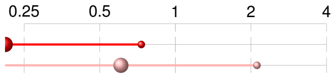

A	B	2.5	50	97.5	Sequence
**2**	**0**	**0.059**	**0.21**	**0.73**	**GTLEPVEK**
**0**	**2**	**0.17**	**0.61**	**2.1**	**SAIHEIVLVGGSTR**

5.101 119 481 Glutamate receptor U1 [Crassostrea gigas]; 113 328 glutamate receptor subunit protein GluR2 precursor [Aplysia californica] gi; 122 328 PREDICTED: glutamate receptor 3 [Ornithorhynchus anatinus]

Protein Accession	CL570.Contig5$_H$1 CL570.Contig4$_H$1 CL570.Contig2$_H$1
Mean Expression Ratio	0.625
Median Expression Ratio	0.624
Credible Interval	(0.228, 1.68)
Associated Peptides	1
Associated Spectra	2
Coverage	NaN

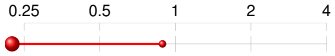

A	B	2.5	50	97.5	Sequence
**1**	**1**	**0.056**	**0.22**	**0.89**	**GTANIGAGALTVTSER**

5.102 77 502 hypothetical protein CGI_10008263 [Crassostrea gigas]

Protein Accession	CL38960.Contig1$_{H}1$
Mean Expression Ratio	1.59
Median Expression Ratio	1.59
Credible Interval	(0.721, 3.58)
Associated Peptides	3
Associated Spectra	12
Coverage	NaN

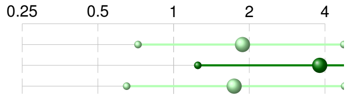

A	B	2.5	50	97.5	Sequence
0	5	0.72	1.9	4.7	IFVAIDGSK
0	3	1.2	3.8	12	KIFVAIDGSK
0	4	0.65	1.7	4.8	ANIEAITK

5.103 209 781 minus strand high mobility group protein 1 [Biomphalaria glabrata]; 277 771 high mobility group protein 1 [Biomphalaria glabrata]; 277 867 minus strand high mobility group protein 1 [Biomphalaria glabrata]; 277 870 minus strand high mobility group protein 1 [Biomphalaria glabrata]

Protein Accession	CL25076.Contig4$_{H}1$ CL25076.Contig3$_{H}1$ CL25076.Contig2$_{H}1$ CL25076.Contig1$_{H}1$
Mean Expression Ratio	1.59
Median Expression Ratio	1.59
Credible Interval	(0.734, 3.5)
Associated Peptides	5
Associated Spectra	10
Coverage	NaN

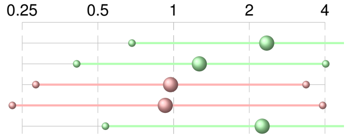

A	B	2.5	50	97.5	Sequence
0	2	0.68	2.3	8.2	KYEAEMAEYR
1	2	0.41	1.3	4	LAGEEWR
2	1	0.28	0.97	3.4	KPFEDMAAK
0	1	0.23	0.93	3.9	LGDFR
0	1	0.54	2.3	9.2	ELPAGQKKPFEDMAAKDKAR

5.104 148 462 hypothetical protein DAPPUDRAFT_306954 [Daphnia pulex]; 148 462 hypothetical protein DAPPUDRAFT_306954 [Daphnia pulex]; 148 462 hypothetical protein DAPPUDRAFT_306954 [Daphnia pulex]; 148 462 hypothetical protein DAPPUDRAFT_306954 [Daphnia pulex]; 173 487 hypothetical protein DAPPUDRAFT_306954 [Daphnia pulex]

Protein Accession	CL48682.Contig7$_{H}1$ CL48682.Contig6$_{H}1$ CL48682.Contig5$_{H}1$ CL48682.Contig4$_{H}1$ CL4868
Mean Expression Ratio	0.626
Median Expression Ratio	0.629
Credible Interval	(0.242, 1.57)
Associated Peptides	2
Associated Spectra	3
Coverage	NaN

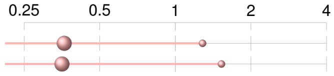

A	B	2.5	50	97.5	Sequence
1	1	0.1	0.36	1.3	ATNFDHFK
1	0	0.079	0.35	1.5	GAHDGGVIFDPAVQR

5.105 1 852 beta actin-like 1 protein [Macrostomum lignano]

Protein Accession	Unigene31438$_{H}1$
Mean Expression Ratio	1.59
Median Expression Ratio	1.59
Credible Interval	(1.08, 2.34)
Associated Peptides	26
Associated Spectra	308
Coverage	NaN

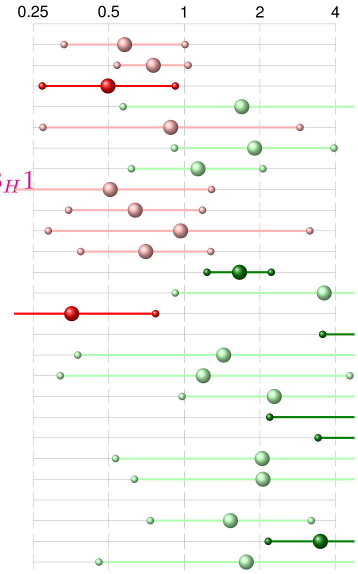

A	B	2.5	50	97.5	Sequence
4	14	0.33	0.58	1	GILTLK
26	32	0.54	0.75	1	HQGVMVGMGQK
3	11	0.27	0.5	0.92	YPIEHGIVTNWDDMEK
1	2	0.57	1.7	4.9	VAPEEHPVLL
1	1	0.27	0.88	2.9	TEAPLNPK
3	6	0.91	1.9	4	VAPEEHPVLLTEA
9	6	0.62	1.1	2.1	AGFAGDDAPR
1	4	0.2	0.51	1.3	AVFPSIVGR
4	10	0.35	0.64	1.2	DSYVGDEAQSK
1	1	0.29	0.97	3.2	HQGVMVGM
3	12	0.39	0.7	1.3	RGILTLK
16	59	1.2	1.7	2.2	VAPEEHPVLLTEAPLNPK
0	1	0.92	3.6	14	APEEHPVLLTEAPLNPK
0	8	0.17	0.36	0.77	GYSFTTTAER
0	5	3.6	8.7	22	HQGVMVGMGQKDSYVGDEAQSK
0	1	0.38	1.4	5.7	SYELPDGQVITIGN
0	1	0.32	1.2	4.6	TTGIVLDSGD
0	6	0.98	2.3	5.5	VAPEEHPVLLTEAPLN
0	3	2.2	6.2	18	VAPEEHPVLLTEAPLNPKANR
0	6	3.4	8.1	19	VAPEEHPVLLTEAPLNPKANREK
0	1	0.53	2	8.3	VAPEEHPVL
0	2	0.63	2	6.8	AVFPSIVGRPR
0	9	11	23	48	HQGVMVGMGQKDSYVGDEAQSKR
0	9	0.73	1.5	3.2	IWHHTFYNELR
0	25	2.2	3.5	5.6	SYELPDGQVITIGNER
0	1	0.46	1.8	7.1	TTGIVLDSGDGVTHTVPIYEGYALPHAILRLDLAG

5.106 372 860 minus strand small heat shock protein [Tegillarca granosa]

Protein Accession	CL18190.Contig4$_{H}1$
Mean Expression Ratio	0.629
Median Expression Ratio	0.631
Credible Interval	(0.324, 1.21)
Associated Peptides	8
Associated Spectra	26
Coverage	NaN

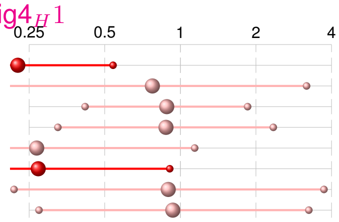

A	B	2.5	50	97.5	Sequence
2	4	0.093	0.23	0.54	IDKDGLSIK
1	0	0.18	0.77	3.2	KSDEYSFVSR
3	6	0.42	0.88	1.9	LTEDGHLIISAR
1	3	0.32	0.87	2.4	MNVSHYEPDELSVK
1	0	0.066	0.27	1.1	TFGPASIEQQMK
1	1	0.079	0.27	0.9	TIAIESDK
0	1	0.22	0.89	3.7	AFDWPAEGHGENAEVVQAK
0	2	0.27	0.93	3.3	EFLIPEEIDAETLGSR

5.107 " 27 974 PREDICTED: succinyl-CoA ligase [GDP-forming] subunit alpha, mitochondrial-like [Oreochromis niloticus]; 27 974 minus strand PREDICTED: succinyl-CoA ligase [GDP-forming] subunit alpha, mitochondrial-like [Oreochromis niloticus]"

Protein Accession	CL10674.Contig2$_{H}1$ CL10674.Contig1$_{H}1$
Mean Expression Ratio	1.58
Median Expression Ratio	1.58
Credible Interval	(0.665, 3.82)
Associated Peptides	3
Associated Spectra	5
Coverage	NaN

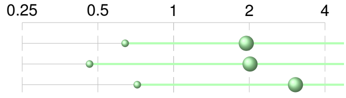

A	B	2.5	50	97.5	Sequence
0	3	0.64	1.9	5.9	MGHAGAIIAGGK
0	1	0.46	2	8.7	IYMATR
0	1	0.72	3.1	13	GGAKEKIEILK

5.108 96 503 minus strand mitochondrial ATP synthase delta chain [Haliotis discus discus]; 111 551 minus strand mitochondrial ATP synthase delta chain [Haliotis discus discus]; 96 542 minus strand mitochondrial ATP synthase delta chain [Haliotis discus discus]; 202 642 minus strand mitochondrial ATP synthase delta chain [Haliotis discus discus]; 44 529 mitochondrial ATP synthase delta chain [Haliotis discus discus]

Protein Accession	Unigene42244$_H$1 CL2187.Contig3$_H$1 CL2187.Contig2$_H$1 CL2187.Contig1$_H$1 CL2187.Contig
Mean Expression Ratio	0.635
Median Expression Ratio	0.635
Credible Interval	(0.227, 1.75)
Associated Peptides	1
Associated Spectra	1
Coverage	NaN

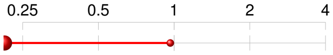

A	B	2.5	50	97.5	Sequence
1	0	0.047	0.21	0.96	FDAQAIR

5.109 " 276 659 Fatty acid-binding protein, adipocyte [Crassostrea gigas]; 426 809 Fatty acid-binding protein, adipocyte [Crassostrea gigas]; 277 660 Fatty acid-binding protein, adipocyte [Crassostrea gigas]; 283 666 Fatty acid-binding protein, adipocyte [Crassostrea gigas]; 270 653 Fatty acid-binding protein, adipocyte [Crassostrea gigas]; 283 666 Fatty acid-binding protein, adipocyte [Crassostrea gigas]; 258 641 Fatty acid-binding protein, adipocyte [Crassostrea gigas]; 427 810 Fatty acid-binding protein, adipocyte [Crassostrea gigas]; 277 660 Fatty acid-binding protein, adipocyte [Crassostrea gigas]"

Protein Accession	CL7666.Contig9$_H$1 CL7666.Contig8$_H$1 CL7666.Contig7$_H$1 CL7666.Contig6$_H$1 CL7666.Con
Mean Expression Ratio	1.57
Median Expression Ratio	1.57
Credible Interval	(0.804, 3.12)
Associated Peptides	8
Associated Spectra	19
Coverage	NaN

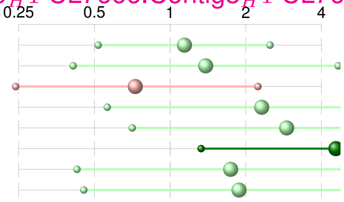

A	B	2.5	50	97.5	Sequence
2	6	0.52	1.1	2.5	AFVDGALK
2	0	0.41	1.4	4.7	AFVDGALKTSMTAK
3	0	0.24	0.73	2.2	QTGEGGEITIR
0	1	0.56	2.3	9.1	AVLTEESGELVER
0	1	0.71	2.9	12	GVTATISFQKA
0	2	1.3	4.6	15	LTTVAGDKQR
0	1	0.43	1.7	7.2	TVLAMER
0	1	0.46	1.9	7.8	GVTATISFQK

5.110 197 913 PREDICTED: l-xylulose reductase-like [Monodelphis domestica]; 197 913 PREDICTED: l-xylulose reductase-like [Monodelphis domestica]; 197 913 PREDICTED: l-xylulose reductase-like [Monodelphis domestica]; 197 913 PREDICTED: l-xylulose reductase-like [Monodelphis domestica]; 197 913 PREDICTED: l-xylulose reductase-like [Monodelphis domestica]; 197 913 PREDICTED: l-xylulose reductase-like [Monodelphis domestica]; 203 919 minus strand PREDICTED: l-xylulose reductase-like [Monodelphis domestica]; 200 916 PREDICTED: l-xylulose reductase-like [Monodelphis domestica]; 200 916 PREDICTED: l-xylulose reductase-like [Monodelphis domestica]; 203 919 minus strand PREDICTED: l-xylulose reductase-like [Monodelphis domestica]

Protein Accession	CL13128.Contig8$_H$1 CL13128.Contig7$_H$1 CL13128.Contig6$_H$1 CL13128.Contig5$_H$1 CL1312
Mean Expression Ratio	1.57
Median Expression Ratio	1.57
Credible Interval	(0.731, 3.43)
Associated Peptides	5
Associated Spectra	8
Coverage	NaN

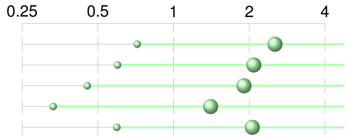

A	B	2.5	50	97.5	Sequence
1	1	0.72	2.5	8.8	MGTFAAEHPEGK
0	2	0.6	2.1	7.3	AIAEALYQHGAR
0	1	0.45	1.9	8	GGSIVNVSSISGK
0	1	0.33	1.4	6	VNSVNPTCVMTK
0	2	0.59	2.1	7.1	VVALSR

5.111 587 811 hemocyanin 2 [Megathura crenulata]

Protein Accession	Unigene4334$_H$1
Mean Expression Ratio	0.637
Median Expression Ratio	0.639
Credible Interval	(0.261, 1.54)
Associated Peptides	2
Associated Spectra	5
Coverage	NaN

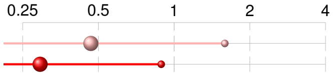

A	B	2.5	50	97.5	Sequence
1	1	0.13	0.47	1.6	AIDDGTFR
1	2	0.096	0.29	0.89	SVEDLTAGEAESLK

5.112 178 582 hypothetical protein CGI_10001640 [Crassostrea gigas]

Protein Accession	CL2347.Contig2$_H$1
Mean Expression Ratio	1.55
Median Expression Ratio	1.56
Credible Interval	(0.716, 3.33)
Associated Peptides	4
Associated Spectra	13
Coverage	NaN

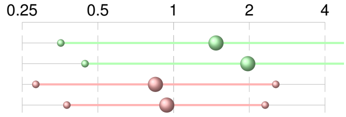

A	B	2.5	50	97.5	Sequence
0	1	0.36	1.5	6.3	LKSHGIGGK
0	1	0.44	2	8.4	SVGGNAGEVICK
2	2	0.28	0.85	2.5	AFLEQLGSK
3	4	0.38	0.94	2.3	ANLIVIGSR

5.113 2 487 minus strand PREDICTED: hypothetical protein LOC100699750 [Oreochromis niloticus]; 41 535 minus strand PREDICTED: hypothetical protein LOC100699750 [Oreochromis niloticus]

Protein Accession	CL7266.Contig5$_{H}1$ CL7266.Contig3$_{H}1$
Mean Expression Ratio	1.56
Median Expression Ratio	1.56
Credible Interval	(0.664, 3.67)
Associated Peptides	3
Associated Spectra	7
Coverage	NaN

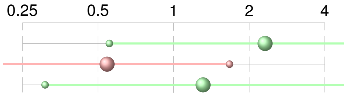

A	B	2.5	50	97.5	Sequence
0	1	0.55	2.3	10	VVNINNEAVTFASPR
3	2	0.18	0.54	1.7	NVALVVSVK
0	1	0.31	1.3	5.5	IPGDGTLTLHGSNGDTAFDR

5.114 227 1201 PREDICTED: quinone oxidoreductase-like isoform 1 [Ornithorhynchus anatinus]; 109 1083 PREDICTED: quinone oxidoreductase-like isoform 1 [Ornithorhynchus anatinus]; 164 1138 PREDICTED: quinone oxidoreductase-like isoform 1 [Ornithorhynchus anatinus]

Protein Accession	CL11899.Contig2$_{H}1$ CL11899.Contig1$_{H}1$ CL11899.Contig13$_{H}1$
Mean Expression Ratio	0.647
Median Expression Ratio	0.645
Credible Interval	(0.296, 1.41)
Associated Peptides	6
Associated Spectra	8
Coverage	NaN

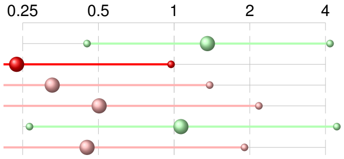

A	B	2.5	50	97.5	Sequence
1	2	0.45	1.4	4.2	VHASGVNPVDTYIR
1	0	0.053	0.24	0.97	VMGLR
0	1	0.079	0.33	1.4	EGLELVK
0	1	0.12	0.5	2.2	ILGTAGTKEGLELVK
0	1	0.27	1.1	4.4	LVLKL
0	1	0.11	0.45	1.9	VHQWGGPEVLK

5.115 22 870 minus strand PREDICTED: alcohol dehydrogenase class-3 [Sarcophilus harrisii]

Protein Accession	Unigene27690$_{H}1$
Mean Expression Ratio	0.648
Median Expression Ratio	0.646
Credible Interval	(0.268, 1.58)
Associated Peptides	2
Associated Spectra	8
Coverage	NaN

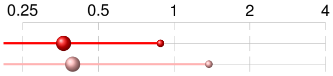

A	B	2.5	50	97.5	Sequence
4	2	0.15	0.36	0.88	AAVAWEAK
1	1	0.11	0.4	1.4	IIGIDVNQDK

5.116 187 696 minus strand putative ribosomal protein S10 [Sipunculus nudus]

Protein Accession	Unigene1233$_H$1
Mean Expression Ratio	1.54
Median Expression Ratio	1.54
Credible Interval	(0.72, 3.28)
Associated Peptides	5
Associated Spectra	12
Coverage	NaN

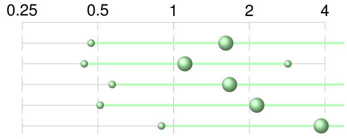

A	B	2.5	50	97.5	Sequence
0	2	0.47	1.6	5.5	ADVGAGATESFQFR
0	5	0.44	1.1	2.8	HQEIDVPNLHVIK
0	3	0.57	1.7	4.9	KDFFSPK
0	1	0.51	2.1	9	ALTSIK
0	1	0.89	3.9	16	KAGGPPGGDKKADVGAGATESFQFR

5.117 " 4146 6107 minus strand nonmuscle myosin II, partial [Aplysia californica]"

Protein Accession	Unigene30363$_H$1
Mean Expression Ratio	1.54
Median Expression Ratio	1.54
Credible Interval	(1.03, 2.33)
Associated Peptides	32
Associated Spectra	62
Coverage	NaN

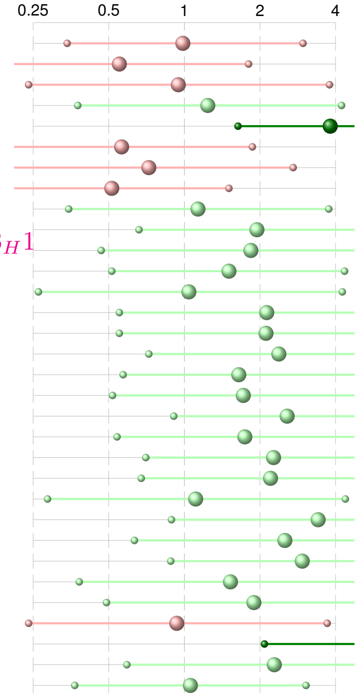

A	B	2.5	50	97.5	Sequence
1	2	0.34	0.99	3	ALEATVQEQR
2	0	0.17	0.55	1.8	ATIAALEGK
1	0	0.24	0.94	3.8	DKLAELEGGQSR
1	1	0.38	1.2	4.2	LAELEGGQSR
3	3	1.6	3.8	9.1	TKATIAALEGK
1	1	0.17	0.56	1.9	ALTLAQR
1	0	0.19	0.72	2.7	LKEDAIK
1	2	0.18	0.51	1.5	NLEAELVK
1	1	0.35	1.1	3.8	IEQLER
1	2	0.66	1.9	5.7	DLEQVSTQLEQVETK
1	0	0.47	1.8	7.2	AALARTNR
0	3	0.51	1.5	4.3	ELDDQLEQNDVLVR
0	1	0.26	1	4.3	ELQIQAEDER
0	1	0.55	2.1	8.8	EMEAELEEER
0	1	0.55	2.1	8.2	EMEQLHAQLEEAR
0	2	0.72	2.4	7.7	IANLEEQLDQEAKER
0	3	0.57	1.7	4.9	ILNLSHAMEEMQGR
0	2	0.52	1.7	5.7	KAQIAMEQMATDLAAER
0	3	0.91	2.6	7.5	LKELQIQAEDERR
0	2	0.54	1.7	5.8	LQEDLAAAER
0	2	0.7	2.3	7.5	NAEAERDELADEIGSSATSK
0	2	0.68	2.2	7.4	QVDEAEEEVAR
0	1	0.29	1.1	4.4	VSSLEAQLADSQELMEEETR
0	1	0.89	3.4	14	IAAQMKDFQR
0	1	0.63	2.5	9.9	LALEKDNIER
0	2	0.88	3	9.7	SADQYKEQLEK
0	1	0.38	1.5	6	QSLLDEK
0	1	0.49	1.9	7.7	RKLQAEVEDLTITLDNQR
0	1	0.24	0.93	3.7	LDEKRRLEAR
0	2	2.1	6.9	23	SVSSKLENQR
0	1	0.59	2.3	8.9	QKLAVQSK
0	3	0.37	1.1	3	VQIEELEDELQAAEDAK

5.118 " 158 355 Protein NAS-37, isoform b [Caenorhabditis elegans] gi; 15 227 Hemicentin-1 [Crassostrea gigas]"

Protein Accession	Unigene57180$_H$1 CL8016.Contig2$_H$1
Mean Expression Ratio	1.55
Median Expression Ratio	1.53
Credible Interval	(0.555, 4.33)
Associated Peptides	1
Associated Spectra	1
Coverage	NaN

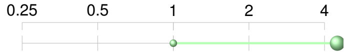

A	B	2.5	50	97.5	Sequence
**0**	**1**	**1**	**4.5**	**19**	**FKGSYGGVSR**

5.119 103 684 EF-hand domain-containing protein D2 [Crassostrea gigas]

Protein Accession	CL20544.Contig1$_H$1
Mean Expression Ratio	0.656
Median Expression Ratio	0.653
Credible Interval	(0.258, 1.66)
Associated Peptides	2
Associated Spectra	3
Coverage	NaN

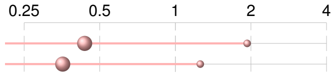

A	B	2.5	50	97.5	Sequence
**1**	**0**	**0.098**	**0.44**	**1.9**	**VPQTHLALK**
**2**	**0**	**0.1**	**0.36**	**1.3**	**KQIQDFQK**

5.120 44 1465 minus strand Adenylyl cyclase-associated protein 1 [Crassostrea gigas]; 52 1473 minus strand Adenylyl cyclase-associated protein 1 [Crassostrea gigas]; 48 1469 Adenylyl cyclase-associated protein 1 [Crassostrea gigas]

Protein Accession	CL19470.Contig9$_H$1 CL19470.Contig8$_H$1 CL19470.Contig1$_H$1
Mean Expression Ratio	0.653
Median Expression Ratio	0.654
Credible Interval	(0.325, 1.32)
Associated Peptides	8
Associated Spectra	13
Coverage	NaN

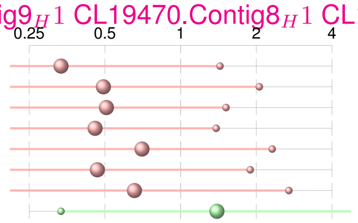

A	B	2.5	50	97.5	Sequence
**1**	**0**	**0.082**	**0.33**	**1.4**	**AQLDIVNAAFK**
**1**	**0**	**0.12**	**0.49**	**2.1**	**EFALPEQFK**
**1**	**2**	**0.17**	**0.51**	**1.5**	**NIVLDNVELK**
**1**	**2**	**0.15**	**0.46**	**1.4**	**QFLEVASK**
**1**	**1**	**0.2**	**0.7**	**2.3**	**SKQPPQADLPK**
**1**	**0**	**0.11**	**0.47**	**1.9**	**AQIGGDLK**
**0**	**1**	**0.16**	**0.65**	**2.7**	**DSLDTEIVTAK**
**0**	**1**	**0.33**	**1.4**	**5.8**	**VEAAKPPLTELQNKK**

5.121 366 1049 hypothetical protein CGI_10004855 [Crassostrea gigas]

Protein Accession	CL43590.Contig5$_{H}$1
Mean Expression Ratio	1.52
Median Expression Ratio	1.52
Credible Interval	(0.787, 2.93)
Associated Peptides	9
Associated Spectra	19
Coverage	NaN

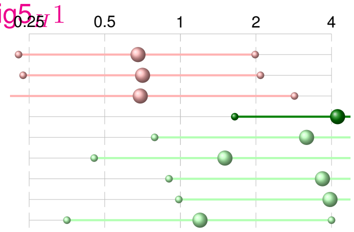

A	B	2.5	50	97.5	Sequence
2	1	0.23	0.68	2	TTVQLHK
2	1	0.24	0.71	2.1	VKVPGAK
1	0	0.16	0.69	2.8	KVFPYPK
0	5	1.7	4.2	11	GGDLWQGEVSLEQHR
0	1	0.79	3.2	13	GKNVDVKLNAR
0	2	0.46	1.5	5	SAEFSTNDNIFDIEMK
0	1	0.9	3.7	15	TPQPLKTTVQLHK
0	1	0.98	3.9	16	GKNVDVK
0	2	0.35	1.2	4	DEWTHLTAK

5.122 1371 2189 minus strand galectin [Pinctada fucata]

Protein Accession	CL7245.Contig3$_{H}$1
Mean Expression Ratio	1.51
Median Expression Ratio	1.52
Credible Interval	(0.73, 3.1)
Associated Peptides	4
Associated Spectra	26
Coverage	NaN

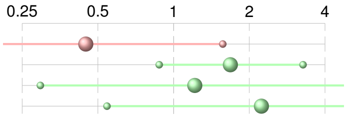

A	B	2.5	50	97.5	Sequence
0	2	0.13	0.45	1.6	EIPQGLEK
12	10	0.87	1.7	3.3	FNEGVTVR
0	1	0.3	1.2	5.2	LGGAWGGEER
0	1	0.54	2.2	9.5	RNEGQVVFNSK

5.123 448 1626 matrilin [Biomphalaria glabrata]

Protein Accession	CL7510.Contig3$_{H}$1
Mean Expression Ratio	1.52
Median Expression Ratio	1.51
Credible Interval	(0.61, 3.82)
Associated Peptides	2
Associated Spectra	5
Coverage	NaN

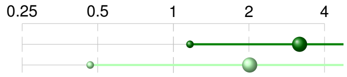

A	B	2.5	50	97.5	Sequence
3	1	1.2	3.2	8.8	VSAIKYGR
0	1	0.47	2	8.6	VSNTELLNIAGDQER

5.124 269 1504 Ribosome-binding protein 1 [Crassostrea gigas]; 265 1500 Ribosome-binding protein 1 [Crassostrea gigas]

Protein Accession	CL8282.Contig5$_{H}$1 CL8282.Contig2$_{H}$1
Mean Expression Ratio	1.52
Median Expression Ratio	1.51
Credible Interval	(0.69, 3.43)
Associated Peptides	6
Associated Spectra	6
Coverage	NaN

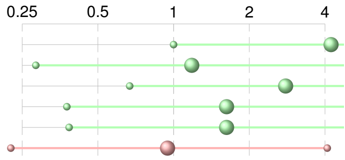

A	B	2.5	50	97.5	Sequence
0	1	1	4.2	18	AMEALEKAEK
0	1	0.28	1.2	5	DAIIEVEK
0	1	0.67	2.8	12	LKEDKETLEAR
0	1	0.38	1.6	6.8	QFTQLHAENER
0	1	0.38	1.6	6.9	LVQQLQEQLSEK
0	1	0.23	0.95	4.1	AMEALEK

5.125 71 535 ribosomal protein rpl14 [Arenicola marina]

Protein Accession	Unigene142$_H$1
Mean Expression Ratio	0.661
Median Expression Ratio	0.663
Credible Interval	(0.296, 1.43)
Associated Peptides	4
Associated Spectra	12
Coverage	NaN

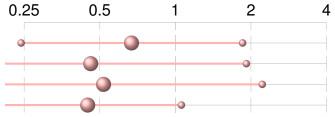

A	B	2.5	50	97.5	Sequence
2	2	0.24	0.67	1.9	IINVEYGK
1	0	0.11	0.46	1.9	KAWEK
1	0	0.12	0.52	2.2	AQLTDFER
3	3	0.19	0.45	1.1	FVEIGR

5.126 788 1006 LIM protein [Haliotis discus discus]; 768 980 muscle LIM protein isoform 1 [Danaus plexippus]

Protein Accession	CL29824.Contig2$_H$1 CL29824.Contig1$_H$1
Mean Expression Ratio	1.52
Median Expression Ratio	1.51
Credible Interval	(0.581, 3.89)
Associated Peptides	1
Associated Spectra	4
Coverage	NaN

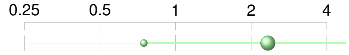

A	B	2.5	50	97.5	Sequence
2	2	0.75	2.3	7.8	AIGVNTPWHK

5.127 501 1838 Hsp90A [Haliotis asinina]

Protein Accession	CL5184.Contig2$_H$1
Mean Expression Ratio	0.664
Median Expression Ratio	0.665
Credible Interval	(0.36, 1.23)
Associated Peptides	11
Associated Spectra	23
Coverage	NaN

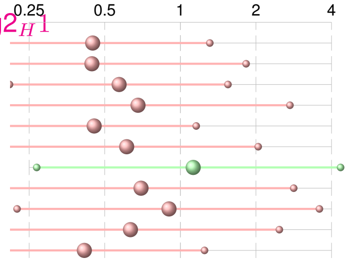

A	B	2.5	50	97.5	Sequence
1	2	0.15	0.45	1.3	SLTNDWEDHLAVK
1	0	0.1	0.44	1.8	GVVDSEDLPLNISR
1	3	0.21	0.57	1.5	EMLQQSK
0	1	0.17	0.68	2.7	AIYYITGESLDAVK
0	5	0.18	0.45	1.2	HFSVEGQLEFR
0	2	0.18	0.61	2	KHFEINPDHPIIK
0	1	0.27	1.1	4.4	RAPFDLFENKK
0	1	0.17	0.7	2.8	YHSSQAGDELVTLK
0	1	0.22	0.9	3.6	KLAEFLR
0	1	0.15	0.63	2.5	TKPLWTR
0	3	0.14	0.41	1.2	LAEFLR

5.128 736 1287 60S ribosomal protein L3 OS=Rattus norvegicus GN=Rpl3 PE=1 SV=3

Protein Accession	Unigene22848$_H$1
Mean Expression Ratio	0.662
Median Expression Ratio	0.665
Credible Interval	(0.26, 1.67)
Associated Peptides	2
Associated Spectra	3
Coverage	NaN

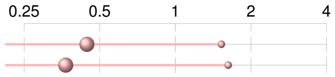

A	B	2.5	50	97.5	Sequence
1	1	0.13	0.45	1.5	VQFTVAR
1	0	0.083	0.37	1.6	IGAGIHTK

5.129 18 1613 minus strand chaperonin containing T-complex polypeptide subunit zeta [Haliotis discus hannai]; 527 2089 minus strand chaperonin containing T-complex polypeptide subunit zeta [Haliotis discus hannai]

Protein Accession	CL23722.Contig7$_{H}$1 CL23722.Contig5$_{H}$1
Mean Expression Ratio	0.661
Median Expression Ratio	0.666
Credible Interval	(0.258, 1.66)
Associated Peptides	2
Associated Spectra	3
Coverage	NaN

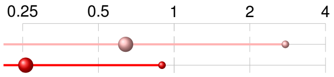

A	B	2.5	50	97.5	Sequence
1	0	0.15	0.64	2.8	AGMSSLKGD
2	0	0.072	0.26	0.9	EGIIALR

5.130 15 1361 hypothetical protein BRAFLDRAFT_231908 [Branchiostoma floridae] gi

Protein Accession	CL24447.Contig2$_{H}$1
Mean Expression Ratio	1.5
Median Expression Ratio	1.5
Credible Interval	(0.581, 3.9)
Associated Peptides	2
Associated Spectra	3
Coverage	NaN

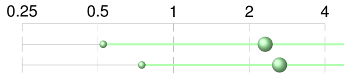

A	B	2.5	50	97.5	Sequence
0	1	0.52	2.3	10	GLGLDDALEAR
0	2	0.74	2.6	9.3	GITRIR

5.131 108 401 60S ribosomal L7a-like protein [Phragmatopoma lapidosa]; 108 401 60S ribosomal L7a-like protein [Phragmatopoma lapidosa]; 108 431 60S ribosomal L7a-like protein [Phragmatopoma lapidosa]

Protein Accession	CL1313.Contig6$_{H}$1 CL1313.Contig5$_{H}$1 CL1313.Contig1$_{H}$1
Mean Expression Ratio	0.667
Median Expression Ratio	0.666
Credible Interval	(0.258, 1.72)
Associated Peptides	2
Associated Spectra	3
Coverage	NaN

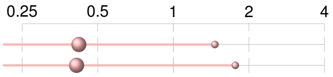

A	B	2.5	50	97.5	Sequence
2	0	0.12	0.42	1.5	QTATQIFR
1	0	0.097	0.41	1.8	SGVNTVTALVESK

5.132 210 779 GH20056 [Drosophila grimshawi] gi; 216 785 minus strand GH20056 [Drosophila grimshawi] gi; 210 779 GH20056 [Drosophila grimshawi] gi; 216 785 GH20056 [Drosophila grimshawi] gi

Protein Accession	CL34497.Contig4$_{H}1$ CL34497.Contig3$_{H}1$ CL34497.Contig2$_{H}1$ CL34497.Contig1$_{H}1$
Mean Expression Ratio	0.67
Median Expression Ratio	0.671
Credible Interval	(0.271, 1.63)
Associated Peptides	3
Associated Spectra	3
Coverage	NaN

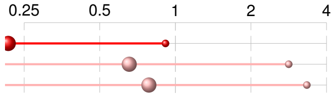

A	B	2.5	50	97.5	Sequence
1	0	0.049	0.22	0.91	EVSNFLK
0	1	0.15	0.66	2.8	GLLDMCAGEK
0	1	0.18	0.79	3.3	KQPFEFQIGVGQVIK

5.133 479 1732 minus strand Multifunctional protein ADE2 [Crassostrea gigas]; 239 1501 minus strand Multifunctional protein ADE2 [Crassostrea gigas]

Protein Accession	CL4210.Contig3$_{H}1$ CL4210.Contig1$_{H}1$
Mean Expression Ratio	0.668
Median Expression Ratio	0.671
Credible Interval	(0.241, 1.8)
Associated Peptides	1
Associated Spectra	1
Coverage	NaN

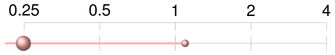

A	B	2.5	50	97.5	Sequence
1	0	0.055	0.25	1.1	VPLLSPKPK

5.134 16 939 hypothetical protein BRAFLDRAFT_87838 [Branchiostoma floridae] gi

Protein Accession	CL38761.Contig1$_{H}1$
Mean Expression Ratio	0.672
Median Expression Ratio	0.671
Credible Interval	(0.242, 1.84)
Associated Peptides	1
Associated Spectra	1
Coverage	NaN

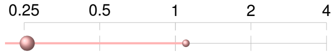

A	B	2.5	50	97.5	Sequence
1	0	0.057	0.26	1.1	ALLLR

5.135 89 283 hypothetical protein CGI_10017178 [Crassostrea gigas]

Protein Accession	Unigene58795$_{H}1$
Mean Expression Ratio	1.48
Median Expression Ratio	1.48
Credible Interval	(0.678, 3.22)
Associated Peptides	3
Associated Spectra	20
Coverage	NaN

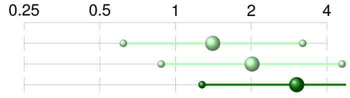

A	B	2.5	50	97.5	Sequence
2	5	0.62	1.4	3.2	AGIIASAK
0	7	0.88	2	4.6	NMSVIAHVDHGK
0	6	1.3	3	7.5	VNFTIDQIR

5.136 " 45 506 minus strand PREDICTED: dihydrolipoyl dehydrogenase, mitochondrial [Monodelphis domestica]"

Protein Accession	CL24292.Contig3$_{H}1$
Mean Expression Ratio	0.671
Median Expression Ratio	0.674
Credible Interval	(0.249, 1.8)
Associated Peptides	1
Associated Spectra	2
Coverage	NaN

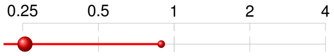

A	B	2.5	50	97.5	Sequence
**1**	**1**	**0.071**	**0.25**	**0.89**	**GIEIDGLR**

5.137 180 560 calreticulin precursor [Aplysia californica] gi; 180 545 calreticulin precursor [Aplysia californica] gi

Protein Accession	CL4813.Contig4$_{H}1$ CL4813.Contig2$_{H}1$
Mean Expression Ratio	0.67
Median Expression Ratio	0.674
Credible Interval	(0.345, 1.27)
Associated Peptides	8
Associated Spectra	29
Coverage	NaN

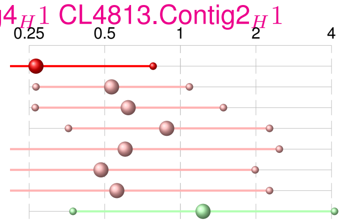

A	B	2.5	50	97.5	Sequence
**2**	**1**	**0.09**	**0.27**	**0.78**	**DPTVYFK**
**3**	**7**	**0.27**	**0.53**	**1.1**	**FYGISAK**
**1**	**5**	**0.26**	**0.62**	**1.5**	**WIPSVHK**
**1**	**4**	**0.36**	**0.88**	**2.3**	**FVWSAGK**
**0**	**1**	**0.15**	**0.6**	**2.5**	**EEFDANWEDR**
**0**	**1**	**0.12**	**0.48**	**2**	**FYGDEDKDK**
**0**	**1**	**0.13**	**0.56**	**2.3**	**TLVIQFTVK**
**0**	**2**	**0.37**	**1.2**	**4.1**	**FDKFSNEGK**

5.138 " 3341 4240 minus strand nonmuscle myosin II, partial [Aplysia californica]"

Protein Accession	CL19183.Contig2$_{H}1$
Mean Expression Ratio	0.675
Median Expression Ratio	0.675
Credible Interval	(0.345, 1.32)
Associated Peptides	9
Associated Spectra	15
Coverage	NaN

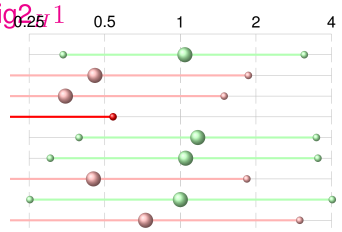

A	B	2.5	50	97.5	Sequence
**1**	**2**	**0.34**	**1**	**3.1**	**QKQDVEQLEAR**
**1**	**0**	**0.11**	**0.46**	**1.9**	**RQQVEELQAQLAK**
**1**	**0**	**0.083**	**0.35**	**1.5**	**YAQMIEEK**
**1**	**1**	**0.047**	**0.16**	**0.54**	**TATMLEEK**
**0**	**3**	**0.39**	**1.2**	**3.5**	**ELQNQMTELNEDLETER**
**0**	**2**	**0.3**	**1**	**3.5**	**LHTKEEELK**
**0**	**1**	**0.11**	**0.45**	**1.8**	**RKEELEDILHEMELR**
**0**	**1**	**0.25**	**1**	**4**	**KLEEDLALNEDTNQK**
**0**	**1**	**0.18**	**0.73**	**3**	**LSEAMAQIVEEEEK**

5.139 27 1682 minus strand PREDICTED: nucleolar protein 56 [Oreochromis niloticus]

Protein Accession	Unigene26699$_{H1}$
Mean Expression Ratio	1.48
Median Expression Ratio	1.48
Credible Interval	(0.757, 2.83)
Associated Peptides	8
Associated Spectra	19
Coverage	NaN

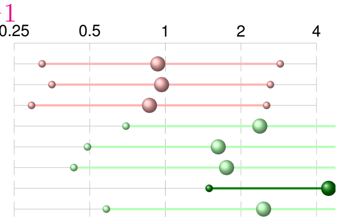

A	B	2.5	50	97.5	Sequence
1	2	0.32	0.94	2.9	LDGLGQAELQK
1	3	0.35	0.96	2.6	VTPLKLDGLGQAELQK
1	2	0.29	0.86	2.5	LIAHAGSLTNLAK
0	2	0.7	2.4	8.2	KLIMQK
0	2	0.49	1.6	5.3	VATLEEEVYDWEIK
0	1	0.43	1.8	7.2	VQAIFDSSK
0	3	1.5	4.5	13	LVKDLHSK
0	1	0.58	2.5	10	KGLGGLSTEKK

5.140 118 1866 Lamin Dm0 [Crassostrea gigas]

Protein Accession	CL4079.Contig7$_{H1}$
Mean Expression Ratio	1.48
Median Expression Ratio	1.48
Credible Interval	(0.863, 2.53)
Associated Peptides	17
Associated Spectra	32
Coverage	NaN

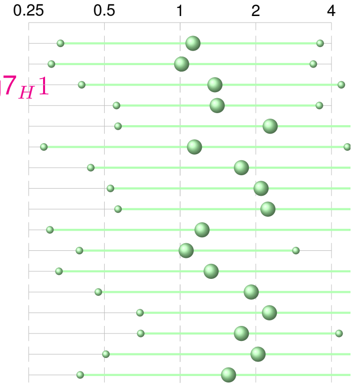

A	B	2.5	50	97.5	Sequence
1	1	0.33	1.1	3.6	LSEVEAPR
1	1	0.31	1	3.4	SLVIKPGK
0	2	0.41	1.4	4.4	EKHEFDLQTVR
0	5	0.56	1.4	3.6	GGIDIEEVDPEGK
0	1	0.57	2.3	9	KQLEEETLLR
0	1	0.29	1.1	4.6	LLEGEESR
0	1	0.44	1.8	7.2	RVETLEAQVAELTAK
0	1	0.53	2.1	8.1	SLYETELADAR
0	1	0.56	2.2	8.9	SLYETELADARR
0	1	0.3	1.2	4.9	VQSLKEDLHFK
0	4	0.4	1.1	2.9	WFVADEMK
0	1	0.33	1.3	5.3	YKTEYEDLLAK
0	1	0.47	1.9	7.9	IQIEANKYK
0	2	0.69	2.3	7.6	LLDETAKEK
0	5	0.7	1.7	4.3	IQIEANK
0	1	0.51	2	8.4	RLALKDDEIADLR
0	1	0.4	1.6	6.2	ADDLSAELAK

5.141 4 219 hypothetical protein BRAFLDRAFT_66351 [Branchiostoma floridae] gi; 3 239 hypothetical protein BRAFLDRAFT_66351 [Branchiostoma floridae] gi

Protein Accession	CL31114.Contig2$_{H1}$ CL31114.Contig1$_{H1}$
Mean Expression Ratio	1.47
Median Expression Ratio	1.47
Credible Interval	(0.593, 3.63)
Associated Peptides	3
Associated Spectra	3
Coverage	NaN

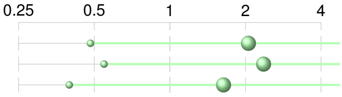

A	B	2.5	50	97.5	Sequence
0	1	0.48	2.1	8.7	KGSENVLAK
0	1	0.55	2.4	10	KLEEVSGELR
0	1	0.4	1.6	7	NNQLNGDLK

5.142 1714 2121 Phosphoenolpyruvate carboxykinase [GTP] [Crassostrea gigas]

Protein Accession	CL25948.Contig1$_{H}$1
Mean Expression Ratio	0.679
Median Expression Ratio	0.68
Credible Interval	(0.254, 1.76)
Associated Peptides	1
Associated Spectra	4
Coverage	NaN

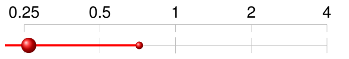

A	B	2.5	50	97.5	Sequence
2	2	0.092	0.26	0.72	SEATAAAEFK

5.143 8 937 minus strand galectin [Crassostrea virginica]

Protein Accession	CL7245.Contig6$_{H}$1
Mean Expression Ratio	1.47
Median Expression Ratio	1.47
Credible Interval	(0.641, 3.33)
Associated Peptides	3
Associated Spectra	11
Coverage	NaN

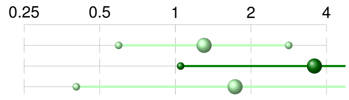

A	B	2.5	50	97.5	Sequence
2	6	0.6	1.3	2.8	QDQGDVVLNAK
0	2	1	3.6	12	LGGEWGPEQR
0	1	0.4	1.7	7.4	QGDNIGLHFNPR

5.144 " 42 905 PREDICTED: enoyl-CoA hydratase, mitochondrial-like isoform 1 [Oreochromis niloticus]"

Protein Accession	CL6477.Contig7$_{H}$1
Mean Expression Ratio	0.682
Median Expression Ratio	0.681
Credible Interval	(0.305, 1.53)
Associated Peptides	4
Associated Spectra	12
Coverage	NaN

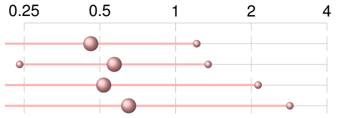

A	B	2.5	50	97.5	Sequence
2	2	0.17	0.46	1.2	AFAAGADIK
4	2	0.24	0.57	1.3	SVVLQASR
0	1	0.12	0.52	2.1	LLDEAVK
0	1	0.16	0.65	2.8	AMEMVLTGER

5.145 128 532 minus strand mitochondrial ATPase inhibitor [Ornithodoros parkeri]

Protein Accession	Unigene1376$_{H}$1
Mean Expression Ratio	1.46
Median Expression Ratio	1.47
Credible Interval	(0.655, 3.35)
Associated Peptides	4
Associated Spectra	8
Coverage	NaN

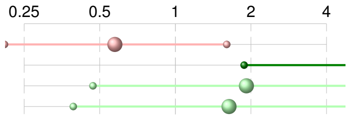

A	B	2.5	50	97.5	Sequence
3	1	0.21	0.58	1.6	EAGGAFGK
0	2	1.9	6.5	22	KLEREQIAK
0	1	0.47	1.9	8	SGIGEFGSGAGK
0	1	0.39	1.6	6.9	MEAAHEELYFK

5.146 " 1 2508 Alanyl-tRNA synthetase, cytoplasmic [Crassostrea gigas]; 1 2508 Alanyl-tRNA synthetase, cytoplasmic [Crassostrea gigas]; 1 2508 minus strand Alanyl-tRNA synthetase, cytoplasmic [Crassostrea gigas]"

Protein Accession	CL11594.Contig2$_{H}$1 CL11594.Contig1$_{H}$1 CL11594.Contig11$_{H}$1
Mean Expression Ratio	0.687
Median Expression Ratio	0.682
Credible Interval	(0.282, 1.72)
Associated Peptides	3
Associated Spectra	3
Coverage	NaN

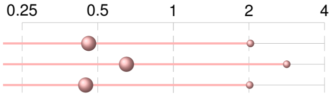

A	B	2.5	50	97.5	Sequence
1	0	0.1	0.46	2	GLTVDNEGFEK
1	0	0.15	0.65	2.8	SIQGLR
1	0	0.1	0.45	2	VIALR

5.147 531 665 minus strand Proteasome subunit beta type-6 [Crassostrea gigas]

Protein Accession	CL12961.Contig2$_{H}$1
Mean Expression Ratio	0.683
Median Expression Ratio	0.682
Credible Interval	(0.251, 1.81)
Associated Peptides	1
Associated Spectra	2
Coverage	NaN

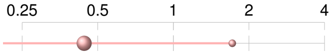

A	B	2.5	50	97.5	Sequence
1	1	0.11	0.44	1.7	FTLTGDDLPK

5.148 109 510 minus strand hypothetical protein DAPPUDRAFT_231281 [Daphnia pulex]

Protein Accession	Unigene4945$_{H}$1
Mean Expression Ratio	1.47
Median Expression Ratio	1.46
Credible Interval	(0.777, 2.8)
Associated Peptides	8
Associated Spectra	31
Coverage	NaN

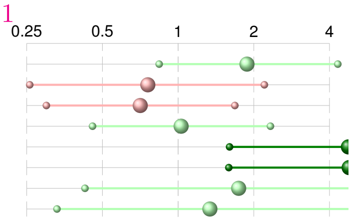

A	B	2.5	50	97.5	Sequence
1	6	0.84	1.9	4.3	LAGIEDPFEMNVK
1	2	0.26	0.76	2.2	GTVDEPTLIPSLYEK
1	5	0.3	0.71	1.7	VSMPDTLGHAVGIER
2	5	0.46	1	2.3	YELLAK
0	3	1.6	4.7	14	AKGTVDEPTLIPSLYEK
0	3	1.6	4.8	15	IASDKVSMPDTLGHAVGIER
0	1	0.43	1.7	7.1	LAGIEDPFEMNVKK
0	1	0.33	1.3	5.5	TLGHAVGIER

5.149 25 405 minus strand Elongation factor 1-delta [Anoplopoma fimbria]

Protein Accession	CL10502.Contig1$_{H}$1
Mean Expression Ratio	1.47
Median Expression Ratio	1.46
Credible Interval	(0.671, 3.24)
Associated Peptides	5
Associated Spectra	8
Coverage	NaN

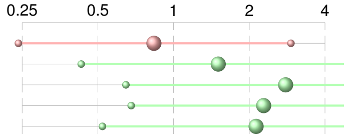

A	B	2.5	50	97.5	Sequence
1	1	0.24	0.84	2.9	LVPVGYGINK
1	1	0.43	1.5	5.1	KPVLIAK
0	1	0.65	2.8	11	SKKPVLIAK
0	2	0.68	2.3	7.6	VQTDGLVWGASK
0	1	0.52	2.1	9.3	RVQTDGLVWGASK

5.150 " 213 1499 minus strand Citrate synthase, mitochondrial [Crassostrea gigas]"

Protein Accession	CL992.Contig2$_H$1
Mean Expression Ratio	0.684
Median Expression Ratio	0.686
Credible Interval	(0.371, 1.26)
Associated Peptides	10
Associated Spectra	31
Coverage	NaN

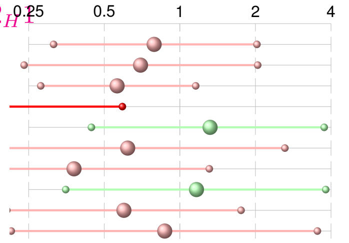

A	B	2.5	50	97.5	Sequence
3	2	0.32	0.79	2	SFDTPSLR
1	2	0.24	0.7	2	SGQVVPGYGHAVLR
6	4	0.28	0.56	1.2	VVPDILIEQGK
1	0	0.035	0.14	0.59	EVSEFR
0	3	0.44	1.3	3.8	ALVTETSVLDPEEGIR
0	1	0.16	0.62	2.6	DVLSQIIPEK
0	2	0.11	0.38	1.3	HLPNDPMFK
0	2	0.35	1.2	3.8	VGEVTVDMMYGGMR
0	3	0.2	0.6	1.8	LISQLYK
0	1	0.21	0.87	3.5	ALGLPIERPK

5.151 " 519 3182 minus strand Alpha-actinin, sarcomeric [Crassostrea gigas]"

Protein Accession	CL28960.Contig2$_H$1
Mean Expression Ratio	0.687
Median Expression Ratio	0.688
Credible Interval	(0.397, 1.18)
Associated Peptides	16
Associated Spectra	28
Coverage	NaN

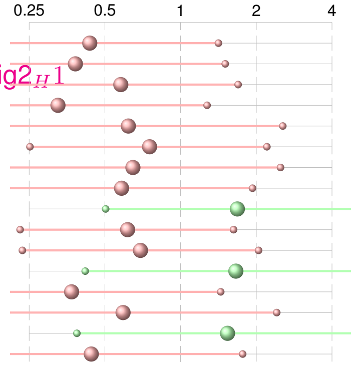

A	B	2.5	50	97.5	Sequence
1	1	0.13	0.43	1.4	NINEVENQILTR
1	0	0.094	0.38	1.5	HLDIPR
2	1	0.2	0.58	1.7	RDQTLQQEMMR
1	0	0.08	0.32	1.3	LERLDHLAQK
0	1	0.16	0.62	2.6	ACLISLGYK
0	3	0.25	0.75	2.2	AIIQYKPHIDELER
0	1	0.16	0.65	2.5	DNPIENLNTAFDIAEK
0	2	0.18	0.58	1.9	ETADTDTAEQVMQSFK
0	2	0.5	1.7	5.7	GISEDQMNEFR
0	4	0.23	0.62	1.6	IDQLHLEFAK
0	3	0.23	0.69	2	ILAGDKPYITAQILR
0	1	0.42	1.7	6.7	LSNRPAYLPTEGK
0	1	0.094	0.37	1.4	ALDFIASK
0	1	0.15	0.59	2.4	LDEFR
0	1	0.39	1.5	6.3	MSFNHFDKNR
0	1	0.11	0.44	1.8	EALEEAER

5.152 " 115 1104 minus strand PREDICTED: KH domain-containing, RNA-binding, signal transduction-associated protein 3 [Monodelphis domestica]"

Protein Accession	Unigene26700$_H$1
Mean Expression Ratio	0.684
Median Expression Ratio	0.688
Credible Interval	(0.28, 1.63)
Associated Peptides	3
Associated Spectra	4
Coverage	NaN

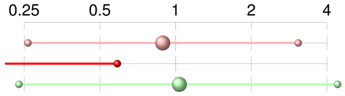

A	B	2.5	50	97.5	Sequence
1	1	0.26	0.89	3.1	LLAQEIER
1	0	0.031	0.13	0.58	GRGGGVP
0	1	0.24	1	4.4	LQEETGCK

5.153 221 1450 minus strand PREDICTED: dihydrolipoamide branched chain transacylase E2 [Taeniopygia guttata]; 221 1609 minus strand PREDICTED: dihydrolipoamide branched chain transacylase E2 [Taeniopygia guttata]

Protein Accession	CL25782.Contig5$_H1$ CL25782.Contig3$_H1$
Mean Expression Ratio	1.44
Median Expression Ratio	1.45
Credible Interval	(0.539, 3.95)
Associated Peptides	1
Associated Spectra	2
Coverage	NaN

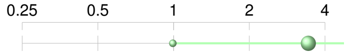

A	B	2.5	50	97.5	Sequence
**0**	**2**	**0.99**	**3.4**	**13**	**VLATPAVR**

5.154 " 130 1161 minus strand Isocitrate dehydrogenase [NAD] subunit alpha, mitochondrial [Crassostrea gigas]; 198 1229 minus strand Isocitrate dehydrogenase [NAD] subunit alpha, mitochondrial [Crassostrea gigas]"

Protein Accession	CL5384.Contig8$_H1$ CL5384.Contig7$_H1$
Mean Expression Ratio	0.684
Median Expression Ratio	0.688
Credible Interval	(0.263, 1.78)
Associated Peptides	2
Associated Spectra	2
Coverage	NaN

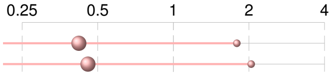

A	B	2.5	50	97.5	Sequence
**1**	**0**	**0.096**	**0.42**	**1.8**	**SLNLALR**
**1**	**0**	**0.11**	**0.46**	**2**	**VAEYAFK**

5.155 633 806 Peroxiredoxin-1 [Crassostrea gigas]; 648 821 Peroxiredoxin-1 [Crassostrea gigas]; 666 839 Peroxiredoxin-1 [Crassostrea gigas]; 625 798 Peroxiredoxin-1 [Crassostrea gigas]; 633 824 Peroxiredoxin-1 [Crassostrea gigas]

Protein Accession	CL3160.Contig18$_H1$ CL3160.Contig17$_H1$ CL3160.Contig16$_H1$ CL3160.Contig15$_H1$ CL3160
Mean Expression Ratio	0.691
Median Expression Ratio	0.689
Credible Interval	(0.268, 1.79)
Associated Peptides	1
Associated Spectra	5
Coverage	NaN

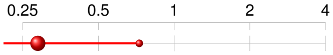

A	B	2.5	50	97.5	Sequence
**0**	**5**	**0.11**	**0.29**	**0.73**	**LVQAFQFTDK**

5.156 95 1579 minus strand hypothetical protein BRAFLDRAFT_113974 [Branchiostoma floridae] gi

Protein Accession	CL19753.Contig5$_{H}$1
Mean Expression Ratio	1.44
Median Expression Ratio	1.45
Credible Interval	(0.734, 2.86)
Associated Peptides	9
Associated Spectra	16
Coverage	NaN

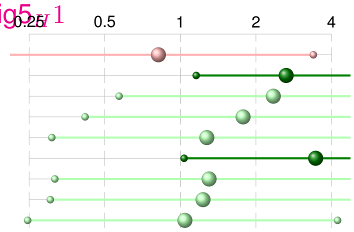

A	B	2.5	50	97.5	Sequence
1	0	0.19	0.82	3.4	TFPVVNPATEK
2	5	1.2	2.6	6	DINEVIERANR
0	1	0.57	2.4	10	FKDINEVIER
0	1	0.42	1.8	7.5	TSYGLAAGVITK
0	1	0.31	1.3	5.2	TVGDPFDAVTMQGPQVDQEQFEK
0	2	1	3.5	12	TVTIKLPSK
0	1	0.32	1.3	5.5	ADLRPDPNIK
0	1	0.3	1.2	5	ELGEYALK
0	1	0.25	1	4.2	DEIFGPVQSIIK

5.157 " 347 847 minus strand ADP,ATP carrier protein 1 OS=Anopheles gambiae GN=AGAP006782 PE=2 SV=2"

Protein Accession	CL27196.Contig1$_{H}$1
Mean Expression Ratio	0.69
Median Expression Ratio	0.692
Credible Interval	(0.248, 1.87)
Associated Peptides	1
Associated Spectra	1
Coverage	NaN

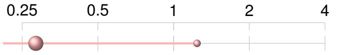

A	B	2.5	50	97.5	Sequence
1	0	0.065	0.28	1.2	SNPGESYAVK

5.158 45 407 minus strand 60S ribosomal protein L31 OS=Xenopus laevis GN=rpl31 PE=2 SV=1

Protein Accession	CL4728.Contig1$_{H}$1
Mean Expression Ratio	1.44
Median Expression Ratio	1.44
Credible Interval	(0.586, 3.63)
Associated Peptides	2
Associated Spectra	4
Coverage	NaN

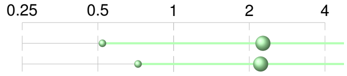

A	B	2.5	50	97.5	Sequence
0	1	0.52	2.3	9.7	IHGVSFKK
0	3	0.72	2.2	6.9	IHGVSFK

5.159 9 722 minus strand actin related protein [Lumbricus rubellus]

Protein Accession	CL45325.Contig2$_{H}$1
Mean Expression Ratio	1.44
Median Expression Ratio	1.44
Credible Interval	(0.557, 3.77)
Associated Peptides	2
Associated Spectra	2
Coverage	NaN

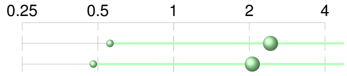

A	B	2.5	50	97.5	Sequence
0	1	0.56	2.4	11	ISAEEFLKNF
0	1	0.48	2.1	9.4	LAVNSRAR

5.160 197 1132 minus strand Pancreatic lipase-related protein 2 [Crassostrea gigas]

Protein Accession	CL8259.Contig1$_H1$
Mean Expression Ratio	1.44
Median Expression Ratio	1.44
Credible Interval	(0.562, 3.69)
Associated Peptides	1
Associated Spectra	7
Coverage	NaN

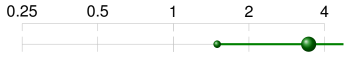

A	B	2.5	50	97.5	Sequence
**0**	**7**	**1.5**	**3.4**	**8**	**ITGLDPAGPK**

5.161 273 1304 Guanine nucleotide-binding protein subunit beta [Crassostrea gigas]

Protein Accession	CL15682.Contig1$_H1$
Mean Expression Ratio	1.43
Median Expression Ratio	1.43
Credible Interval	(0.605, 3.37)
Associated Peptides	4
Associated Spectra	4
Coverage	NaN

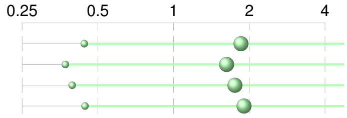

A	B	2.5	50	97.5	Sequence
**0**	**1**	**0.44**	**1.9**	**7.9**	**TFVSGACDASAK**
**0**	**1**	**0.37**	**1.6**	**7.1**	**KAAGDTTLAQATAQCEPVGR**
**0**	**1**	**0.4**	**1.8**	**7.5**	**TSELDALRQETEQLKNQIR**
**0**	**1**	**0.44**	**1.9**	**8.1**	**IYAMHWASDSR**

5.162 244 837 PREDICTED: ras-like GTP-binding protein Rho1-like [Megachile rotundata]

Protein Accession	Unigene11792$_H1$
Mean Expression Ratio	1.43
Median Expression Ratio	1.43
Credible Interval	(0.561, 3.61)
Associated Peptides	2
Associated Spectra	3
Coverage	NaN

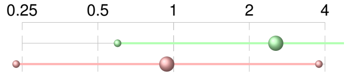

A	B	2.5	50	97.5	Sequence
**0**	**1**	**0.6**	**2.6**	**11**	**EGVREVFETATR**
**1**	**1**	**0.24**	**0.94**	**3.8**	**EVFETATR**

5.163 174 1721 minus strand aspartyl-tRNA synthetase [Xenopus laevis] gi

Protein Accession	CL9865.Contig1$_H1$
Mean Expression Ratio	0.701
Median Expression Ratio	0.699
Credible Interval	(0.267, 1.8)
Associated Peptides	1
Associated Spectra	5
Coverage	NaN

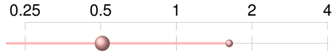

A	B	2.5	50	97.5	Sequence
**2**	**3**	**0.16**	**0.51**	**1.6**	**IHDPDFLSQR**

5.164 1337 1537 minus strand hemocyanin [Haliotis tuberculata]

Protein Accession	Unigene68371$_{H}$1
Mean Expression Ratio	0.696
Median Expression Ratio	0.702
Credible Interval	(0.272, 1.77)
Associated Peptides	2
Associated Spectra	3
Coverage	NaN

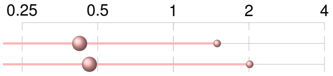

A	B	2.5	50	97.5	Sequence
0	2	0.12	0.42	1.5	DHSTPFDVFNYK
0	1	0.1	0.46	2	YDDLQFNGLSISQIQR

5.165 154 621 minus strand Stress response protein nhaX [Crassostrea gigas]

Protein Accession	CL4265.Contig1$_{H}$1
Mean Expression Ratio	0.702
Median Expression Ratio	0.704
Credible Interval	(0.33, 1.49)
Associated Peptides	4
Associated Spectra	16
Coverage	NaN

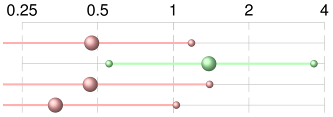

A	B	2.5	50	97.5	Sequence
3	2	0.19	0.47	1.2	SVAAAKPGEGILK
2	3	0.55	1.4	3.6	GLGVMR
0	3	0.16	0.47	1.4	IQHDLEK
0	3	0.11	0.34	1	EVLEAMLKDEQK

5.166 408 1010 glutathione S-transferase sigma 2 [Mytilus galloprovincialis]; 166 768 glutathione S-transferase sigma 2 [Mytilus galloprovincialis]

Protein Accession	CL22038.Contig5$_{H}$1 CL22038.Contig4$_{H}$1
Mean Expression Ratio	0.704
Median Expression Ratio	0.705
Credible Interval	(0.352, 1.38)
Associated Peptides	7
Associated Spectra	17
Coverage	NaN

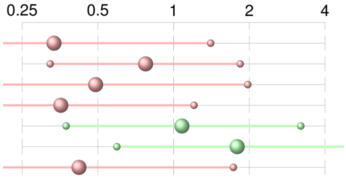

A	B	2.5	50	97.5	Sequence
1	0	0.079	0.34	1.4	LVLEAAGR
2	4	0.32	0.77	1.8	VFSEATPK
1	0	0.12	0.49	2	KAELYEK
2	0	0.11	0.36	1.2	ELGLYGK
0	3	0.37	1.1	3.2	LVYFDLR
0	3	0.59	1.8	5.2	STVESHPFLK
0	1	0.1	0.42	1.7	YLGFFQK

5.167 112 819 pyruvate kinase [Crassostrea gigas]; 132 932 pyruvate kinase [Crassostrea gigas]

Protein Accession	CL31920.Contig3$_{H}$1 CL31920.Contig1$_{H}$1
Mean Expression Ratio	0.705
Median Expression Ratio	0.706
Credible Interval	(0.268, 1.83)
Associated Peptides	1
Associated Spectra	3
Coverage	NaN

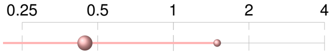

A	B	2.5	50	97.5	Sequence
2	1	0.13	0.44	1.5	ITIDDSYKEK

5.168 " 59 2365 minus strand Aconitate hydratase, mitochondrial [Crassostrea gigas]"

Protein Accession	CL13509.Contig1$_{H}$1
Mean Expression Ratio	1.41
Median Expression Ratio	1.41
Credible Interval	(0.716, 2.8)
Associated Peptides	10
Associated Spectra	11
Coverage	NaN

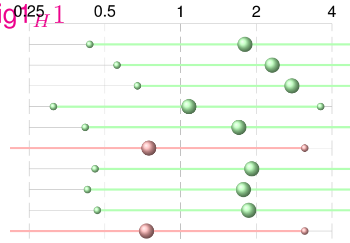

A	B	2.5	50	97.5	Sequence
0	1	0.44	1.8	7.2	HTMNEGQIEWFR
0	1	0.56	2.3	9.5	KGETNTIVTSYNR
0	1	0.67	2.8	12	SKFTITPGSEQIR
0	2	0.31	1.1	3.6	WDGKDLVDMPVLIK
0	1	0.42	1.7	6.9	KNGWPLNIR
0	1	0.18	0.75	3.1	VAGILTVK
0	1	0.46	1.9	8.1	LGRPLTLSEK
0	1	0.43	1.8	7.2	MQDYLK
0	1	0.47	1.9	8	IHETNLKK
0	1	0.17	0.73	3.1	LDDPKNEK

5.169 718 1155 Annexin A7 [Crassostrea gigas]

Protein Accession	CL15539.Contig2$_{H}$1
Mean Expression Ratio	0.708
Median Expression Ratio	0.71
Credible Interval	(0.351, 1.42)
Associated Peptides	8
Associated Spectra	14
Coverage	NaN

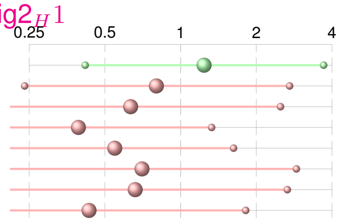

A	B	2.5	50	97.5	Sequence
1	2	0.42	1.2	3.7	KLGTDEETFNR
1	1	0.24	0.8	2.7	SFPQLR
1	0	0.15	0.63	2.5	AQEAQELLQAGVK
1	1	0.12	0.39	1.3	ATFDAYQQIAGK
1	2	0.18	0.55	1.6	NVPGYFAQR
1	0	0.17	0.7	2.9	SEVDLGATK
0	1	0.16	0.66	2.7	SEMSGNLEEGFLAIVR
0	1	0.11	0.43	1.8	DIESAIK

5.170 " 189 2099 Hsp70, putative [Ixodes scapularis] gi"

Protein Accession	CL16113.Contig2$_{H}$1
Mean Expression Ratio	1.4
Median Expression Ratio	1.41
Credible Interval	(0.625, 3.15)
Associated Peptides	4
Associated Spectra	7
Coverage	NaN

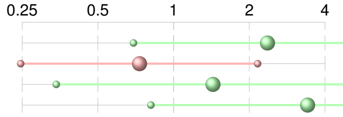

A	B	2.5	50	97.5	Sequence
1	1	0.69	2.4	8.1	LVGMPAKR
1	2	0.25	0.73	2.2	ALQDAEVK
0	1	0.34	1.4	6.1	ASNGDAWVEAQGK
0	1	0.81	3.4	14	RFEDPEVQKDMK

5.171 2140 2367 heat shock cognate 71 [Mytilus galloprovincialis]

Protein Accession	CL5924.Contig13$_{H}$1
Mean Expression Ratio	0.709
Median Expression Ratio	0.71
Credible Interval	(0.276, 1.79)
Associated Peptides	1
Associated Spectra	8
Coverage	NaN

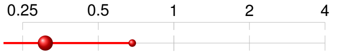

A	B	2.5	50	97.5	Sequence
6	2	0.14	0.31	0.68	LIGDAAK

5.172 192 1820 PREDICTED: copine IV-like [Saccoglossus kowalevskii]; 125 1753 PREDICTED: copine IV-like [Saccoglossus kowalevskii]

Protein Accession	CL15074.Contig3$_{H}1$ CL15074.Contig1$_{H}1$
Mean Expression Ratio	0.704
Median Expression Ratio	0.71
Credible Interval	(0.271, 1.76)
Associated Peptides	2
Associated Spectra	5
Coverage	NaN

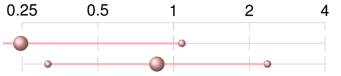

A	B	2.5	50	97.5	Sequence
1	0	0.057	0.25	1.1	LLNAK
1	3	0.32	0.86	2.4	HVLAEVPK

5.173 " 343 1179 ATP synthase subunit alpha, mitochondrial OS=Pongo abelii GN=ATP5A1 PE=2 SV=1"

Protein Accession	Unigene30534$_{H}1$
Mean Expression Ratio	1.41
Median Expression Ratio	1.41
Credible Interval	(0.806, 2.42)
Associated Peptides	11
Associated Spectra	60
Coverage	NaN

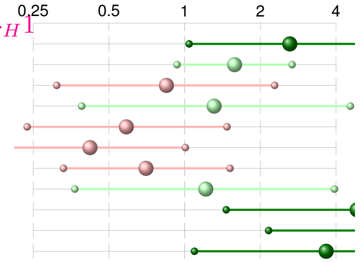

A	B	2.5	50	97.5	Sequence
2	3	1	2.6	6.4	TSVKEPMQTGIK
7	13	0.94	1.6	2.7	VLSIGDGIAR
1	3	0.31	0.84	2.3	VVDALGTPIDGK
1	1	0.39	1.3	4.6	VYGLK
2	3	0.24	0.59	1.5	APGIIPR
4	2	0.17	0.42	1	STVAQLVK
2	6	0.33	0.7	1.5	TAVAIDTIINQK
0	2	0.37	1.2	3.9	NIQAEEMVEFSSGLK
0	2	1.5	4.9	16	VGLKAPGIIPR
0	4	2.2	5.7	15	VVDALGTPIDGKGPITSK
0	2	1.1	3.7	12	TAVAIDTIINQKR

5.174 254 1093 uncharacterized protein LOC432187 precursor [Xenopus laevis] gi; 343 561 minus strand cathepsin Z precursor [Danio rerio] gi

Protein Accession	CL7800.Contig7$_{H}1$ CL7800.Contig1$_{H}1$
Mean Expression Ratio	1.4
Median Expression Ratio	1.4
Credible Interval	(0.516, 3.85)
Associated Peptides	1
Associated Spectra	2
Coverage	NaN

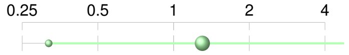

A	B	2.5	50	97.5	Sequence
1	1	0.32	1.3	5.4	GINFASTTR

5.175 151 471 minus strand ribosomal protein rps25 [Arenicola marina]

Protein Accession CL17623.Contig4$_H$1
Mean Expression Ratio 1.4
Median Expression Ratio 1.4
Credible Interval (0.55, 3.6)
Associated Peptides 2
Associated Spectra 3
Coverage NaN

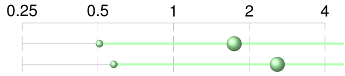

A	B	2.5	50	97.5	Sequence
**0**	**2**	**0.51**	**1.7**	**6.1**	**LITPSVVSER**
**0**	**1**	**0.58**	**2.6**	**11**	**MLKEVPSYK**

5.176 551 871 minus strand Protein disulfide-isomerase A4 [Crassostrea gigas]

Protein Accession CL20258.Contig1$_H$1
Mean Expression Ratio 0.715
Median Expression Ratio 0.715
Credible Interval (0.256, 2)
Associated Peptides 1
Associated Spectra 1
Coverage NaN

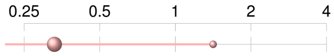

A	B	2.5	50	97.5	Sequence
**1**	**0**	**0.072**	**0.33**	**1.4**	**EGPEPILLAK**

5.177 85 546 minus strand RecName: Full=Eukaryotic translation initiation factor 5A; Short=eIF-5A gi|51702279|sp|P62925.1|IF5A_SPOFR RecName: Full=Eukaryotic translation initiation factor 5A; Short=eIF-5A gi|6469515|gb|AAF13315.1|AF109730_1 translation initiation factor 5A [Spodoptera exigua] gi

Protein Accession CL40725.Contig2$_H$1
Mean Expression Ratio 0.714
Median Expression Ratio 0.715
Credible Interval (0.361, 1.42)
Associated Peptides 7
Associated Spectra 20
Coverage NaN

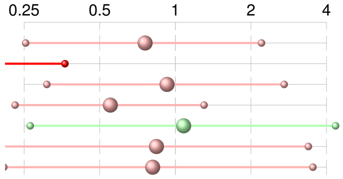

A	B	2.5	50	97.5	Sequence
**1**	**2**	**0.25**	**0.76**	**2.2**	**AMGEECVVAVK**
**3**	**2**	**0.057**	**0.14**	**0.36**	**IVEMSTSK**
**1**	**2**	**0.31**	**0.92**	**2.7**	**KNEFVVIK**
**2**	**4**	**0.23**	**0.55**	**1.3**	**EDSLAAEIQK**
**0**	**1**	**0.26**	**1.1**	**4.3**	**EDSLAAEIQKK**
**0**	**1**	**0.2**	**0.84**	**3.4**	**KYEDICPSTHNMEVPVVTR**
**0**	**1**	**0.21**	**0.81**	**3.5**	**NEFVVIK**

5.178 129 299 glycogen phosphorylase [Crassostrea gigas]

Protein Accession Unigene20640$_H$1
Mean Expression Ratio 1.39
Median Expression Ratio 1.4
Credible Interval (0.505, 3.9)
Associated Peptides 1
Associated Spectra 1
Coverage NaN

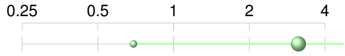

A	B	2.5	50	97.5	Sequence
**0**	**1**	**0.69**	**3.1**	**14**	**GIAPVENVGTIKK**

5.179 21 341 minus strand hemocyanin isoform 1 [Nucula nucleus]

Protein Accession	CL40281.Contig2$_{H}1$
Mean Expression Ratio	0.718
Median Expression Ratio	0.717
Credible Interval	(0.321, 1.64)
Associated Peptides	3
Associated Spectra	13
Coverage	NaN

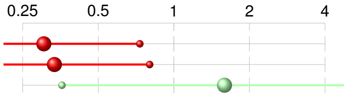

A	B	2.5	50	97.5	Sequence
4	2	0.13	0.3	0.73	DSNPIALTR
2	4	0.14	0.33	0.8	GDELFSYLK
0	1	0.36	1.6	6.7	KPLEPFNRDSNPIALTR

5.180 196 657 superoxide dismutase [Aplysia californica] gi; 690 1151 superoxide dismutase [Aplysia californica] gi

Protein Accession	Unigene18046$_{H}1$ Unigene150$_{H}1$
Mean Expression Ratio	1.39
Median Expression Ratio	1.39
Credible Interval	(0.643, 3.02)
Associated Peptides	4
Associated Spectra	11
Coverage	NaN

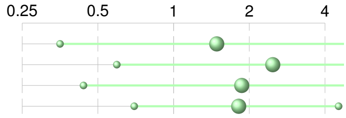

A	B	2.5	50	97.5	Sequence
0	1	0.35	1.5	6.3	THGGPDDVERHAGDLGNIR
0	1	0.59	2.5	10	AVCVLK
0	1	0.44	1.9	7.6	AGDLGNIR
4	4	0.7	1.8	4.5	SLVVHEK

5.181 373 579 myosin heavy chain [Argopecten irradians] gi

Protein Accession	Unigene64289$_{H}1$
Mean Expression Ratio	1.4
Median Expression Ratio	1.39
Credible Interval	(0.714, 2.77)
Associated Peptides	8
Associated Spectra	19
Coverage	NaN

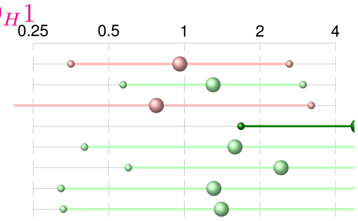

A	B	2.5	50	97.5	Sequence
1	3	0.35	0.96	2.6	KVQNELEDAEER
1	6	0.57	1.3	3	QVEEAEEIAAVNLAK
1	0	0.18	0.78	3.2	QELVDK
0	3	1.7	4.9	15	KVQNELEDAEERADSAEGTLSK
0	1	0.4	1.6	6.5	RQVEEAEEIAAVNLAK
0	1	0.6	2.4	10	VQNELEDAEERADSAEGTLSK
0	1	0.32	1.3	5.4	ELEDAEER
0	1	0.33	1.4	5.8	VQNELEDAEER

5.182 56 937 Chloride intracellular channel exc-4 [Crassostrea gigas]

Protein Accession	CL34620.Contig3$_{H}1$
Mean Expression Ratio	1.39
Median Expression Ratio	1.39
Credible Interval	(0.564, 3.53)
Associated Peptides	2
Associated Spectra	4
Coverage	NaN

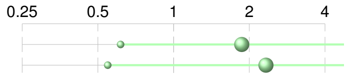

A	B	2.5	50	97.5	Sequence
1	2	0.62	1.9	5.8	KVQGPLVK
0	1	0.55	2.3	10	QKFELYIK

5.183 3512 6661 hypothetical protein BRAFLDRAFT_255626 [Branchiostoma floridae] gi

Protein Accession	CL1959.Contig8$_H$1
Mean Expression Ratio	1.39
Median Expression Ratio	1.39
Credible Interval	(0.704, 2.78)
Associated Peptides	10
Associated Spectra	11
Coverage	NaN

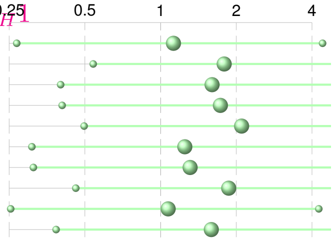

A	B	2.5	50	97.5	Sequence
1	0	0.27	1.1	4.4	SGVKIDVSDAEAQAR
0	2	0.54	1.8	6.1	FDRPAQLHMAFR
0	1	0.4	1.6	6.6	IDVSDAEAQAR
0	1	0.41	1.7	7.3	NVVLGGVK
0	1	0.5	2.1	8.4	VKAALPSPEGLK
0	1	0.31	1.3	5.1	LDEAFLSK
0	1	0.31	1.3	5.4	QLYVLGHEAMR
0	1	0.46	1.9	8.1	SVTIHDLGK
0	1	0.25	1.1	4.3	GGNVVQVKMPK
0	1	0.38	1.6	6.6	FIVAETNGLFGK

5.184 28 516 minus strand Signal peptidase complex subunit 3 [Crassostrea gigas]; 30 554 minus strand Signal peptidase complex subunit 3 [Crassostrea gigas]

Protein Accession	Unigene59022$_H$1 Unigene26504$_H$1
Mean Expression Ratio	0.72
Median Expression Ratio	0.721
Credible Interval	(0.262, 1.91)
Associated Peptides	1
Associated Spectra	2
Coverage	NaN

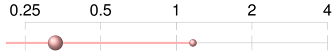

A	B	2.5	50	97.5	Sequence
1	1	0.093	0.33	1.2	SDLGLVTFDLK

5.185 313 1908 minus strand Heterogeneous nuclear ribonucleoprotein L [Crassostrea gigas]

Protein Accession	CL19412.Contig8$_H$1
Mean Expression Ratio	1.39
Median Expression Ratio	1.39
Credible Interval	(0.566, 3.5)
Associated Peptides	3
Associated Spectra	4
Coverage	NaN

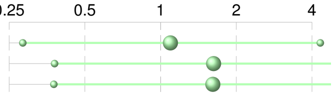

A	B	2.5	50	97.5	Sequence
1	1	0.28	1.1	4.3	SSTGLLEFESK
0	1	0.38	1.6	7	GQPLLQEPR
0	1	0.38	1.6	7	TPFIFK

5.186 9 161 PREDICTED: tubulin alpha-1C chain-like [Acyrthosiphon pisum]; 9 161 Tubulin alpha-3 chain (Fragments) OS=Mesocricetus auratus PE=1 SV=1

Protein Accession	Unigene74551$_{H}1$ Unigene70816$_{H}1$
Mean Expression Ratio	1.39
Median Expression Ratio	1.38
Credible Interval	(0.568, 3.4)
Associated Peptides	3
Associated Spectra	4
Coverage	NaN

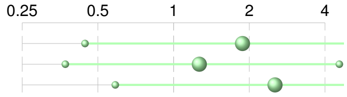

A	B	2.5	50	97.5	Sequence
0	1	0.45	1.9	8.2	IHFPLAT
0	2	0.37	1.3	4.6	IHFPLA
0	1	0.58	2.5	11	IHFPLATYAPVISAEK

5.187 2008 2715 Protein disulfide-isomerase A5 [Crassostrea gigas]

Protein Accession	CL7820.Contig3$_{H}1$
Mean Expression Ratio	1.38
Median Expression Ratio	1.38
Credible Interval	(0.611, 3.09)
Associated Peptides	5
Associated Spectra	6
Coverage	NaN

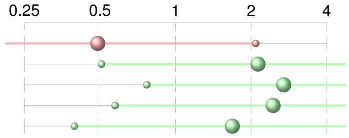

A	B	2.5	50	97.5	Sequence
1	0	0.11	0.49	2.1	FGGLASK
0	1	0.51	2.1	8.9	MKPAYMSAAAR
0	2	0.77	2.7	9.3	LKQESPTAK
0	1	0.57	2.4	10	FGGLASKYEVK
0	1	0.4	1.7	7.1	SKEDLVAFMKDPKPK

5.188 74 2734 26S proteasome non-ATPase regulatory subunit 2 [Crassostrea gigas]

Protein Accession	CL32689.Contig3$_{H}1$
Mean Expression Ratio	1.37
Median Expression Ratio	1.38
Credible Interval	(0.504, 3.78)
Associated Peptides	1
Associated Spectra	1
Coverage	NaN

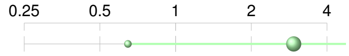

A	B	2.5	50	97.5	Sequence
0	1	0.65	3	14	VGQAVDVVGQAGKPK

5.189 80 250 minus strand 40S ribosomal protein S29 [Ornithodoros parkeri]

Protein Accession	CL11390.Contig2$_{H}1$
Mean Expression Ratio	1.38
Median Expression Ratio	1.38
Credible Interval	(0.61, 3.15)
Associated Peptides	3
Associated Spectra	9
Coverage	NaN

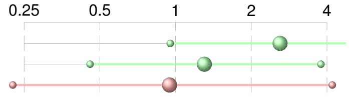

A	B	2.5	50	97.5	Sequence
0	4	0.95	2.6	7.2	GFQTTWYSHPR
1	3	0.46	1.3	3.8	QFAPDIGFK
0	1	0.23	0.94	4.2	KYGLNICR

5.190 157 459 minus strand Synaptojanin-2-binding protein [Crassostrea gigas]

Protein Accession	CL12494.Contig1$_{H}$1
Mean Expression Ratio	0.726
Median Expression Ratio	0.727
Credible Interval	(0.363, 1.43)
Associated Peptides	7
Associated Spectra	22
Coverage	NaN

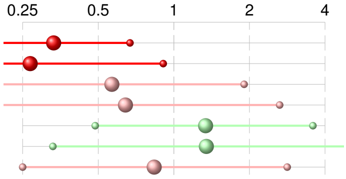

A	B	2.5	50	97.5	Sequence
4	6	0.16	0.33	0.67	GDSGLGFNIR
2	0	0.078	0.27	0.91	LKEDGAAFK
1	1	0.17	0.57	1.9	ILEVNGK
0	1	0.16	0.64	2.6	VQHGAYAELMK
0	4	0.49	1.3	3.6	LKEGDKILEVNGK
0	1	0.33	1.3	5.4	LKEDGAAFKDGR
0	2	0.25	0.84	2.8	GGTDIPHVPGDTGIFVTR

5.191 " 193 333 minus strand RecName: Full=Globin; AltName: Full=Myoglobin gi|299274|gb|AAB25743.1| myoglobin [Nassa mutabilis=marine gastropods, radular muscles, Peptide, 147 aa] gi; 193 333 minus strand RecName: Full=Globin; AltName: Full=Myoglobin gi|299274|gb|AAB25743.1| myoglobin [Nassa mutabilis=marine gastropods, radular muscles, Peptide, 147 aa] gi"

Protein Accession	CL8458.Contig5$_{H}$1 CL8458.Contig1$_{H}$1
Mean Expression Ratio	0.725
Median Expression Ratio	0.729
Credible Interval	(0.295, 1.78)
Associated Peptides	2
Associated Spectra	7
Coverage	NaN

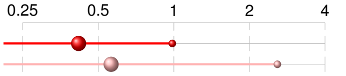

A	B	2.5	50	97.5	Sequence
4	2	0.17	0.42	0.98	AAINSTWPK
0	1	0.13	0.56	2.6	LSADKETMENNGAR

5.192 95 2506 Transitional endoplasmic reticulum ATPase [Crassostrea gigas]

Protein Accession	CL15973.Contig2$_{H}$1
Mean Expression Ratio	0.731
Median Expression Ratio	0.729
Credible Interval	(0.405, 1.32)
Associated Peptides	14
Associated Spectra	20
Coverage	NaN

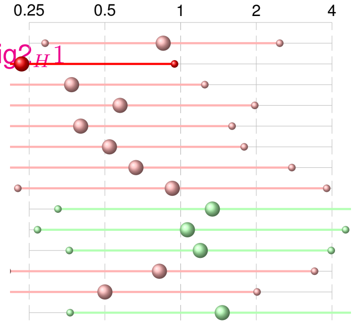

A	B	2.5	50	97.5	Sequence
1	2	0.29	0.85	2.5	AHFEEAMK
1	0	0.056	0.23	0.95	EMVELPLR
1	1	0.11	0.37	1.2	MAIEAEIR
1	1	0.17	0.57	2	HPQLFK
1	0	0.095	0.4	1.6	GFGGNFR
1	1	0.15	0.52	1.8	DIDLDYLAK
0	1	0.17	0.66	2.8	AEVQNEDDLATAILK
0	1	0.23	0.93	3.8	GILLYGPPGTGK
0	1	0.32	1.3	5.5	KYEMFAQTLQQSR
0	1	0.27	1.1	4.5	MDELQLFR
0	2	0.36	1.2	4	KGDIFLVR
0	1	0.2	0.83	3.4	KAFEEAEK
0	1	0.12	0.5	2	EVDIGIPDATGR
0	1	0.36	1.5	6.2	GGNVGDGGGAADR

5.193 2295 2510 beta-thymosin domain repeat protein CSP24KDa_v1 [Hermissenda crassicornis] gi

Protein Accession	CL9548.Contig1$_H$1
Mean Expression Ratio	1.38
Median Expression Ratio	1.37
Credible Interval	(0.561, 3.34)
Associated Peptides	3
Associated Spectra	3
Coverage	NaN

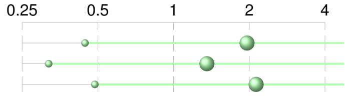

A	B	2.5	50	97.5	Sequence
0	1	0.45	2	8.7	ESVAAFDKSHLK
0	1	0.32	1.4	5.7	ESVAAFDK
0	1	0.48	2.1	9.4	AFDKSHLK

5.194 " 65 1576 minus strand Aldehyde dehydrogenase, mitochondrial [Crassostrea gigas]"

Protein Accession	CL5835.Contig1$_H$1
Mean Expression Ratio	0.73
Median Expression Ratio	0.729
Credible Interval	(0.42, 1.27)
Associated Peptides	15
Associated Spectra	35
Coverage	NaN

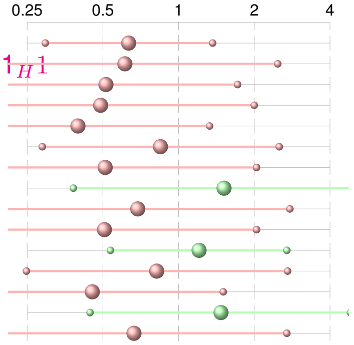

A	B	2.5	50	97.5	Sequence
4	4	0.3	0.63	1.4	TFPTVDPR
1	0	0.15	0.61	2.5	TVTFR
1	1	0.15	0.51	1.7	FKDVK
1	0	0.12	0.49	2	LGSPWR
1	1	0.12	0.4	1.3	FVDSVSGK
1	2	0.29	0.85	2.5	SAAALPKPLTSPEIK
0	1	0.13	0.51	2	ALYLSNSLR
0	1	0.38	1.5	6.2	IYNEFVER
0	1	0.17	0.69	2.8	LAEQTPLSGLYVAK
0	1	0.12	0.51	2	LGPALAMGNTVVMK
0	7	0.54	1.2	2.7	LIPVMAAK
0	2	0.25	0.82	2.7	TLHIGGDHFAYTR
0	2	0.14	0.45	1.5	LADLLER
0	2	0.44	1.5	4.8	IFVEEK
0	1	0.17	0.66	2.7	LADLLERDR

5.195 179 2140 minus strand PREDICTED: hypothetical protein [Saccoglossus kowalevskii]

Protein Accession	CL13749.Contig6$_H$1
Mean Expression Ratio	1.37
Median Expression Ratio	1.37
Credible Interval	(0.542, 3.5)
Associated Peptides	2
Associated Spectra	3
Coverage	NaN

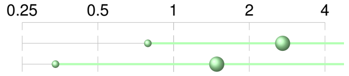

A	B	2.5	50	97.5	Sequence
0	2	0.79	2.7	9.4	KFDGIGGLSGGGATSK
0	1	0.34	1.5	6.4	VGGTYNFDDSNGLGR

5.196 4 783 minus strand hemocyanin isoform 2 [Nucula nucleus]

Protein Accession	CL3697.Contig2$_{H}1$
Mean Expression Ratio	0.73
Median Expression Ratio	0.729
Credible Interval	(0.297, 1.78)
Associated Peptides	3
Associated Spectra	4
Coverage	NaN

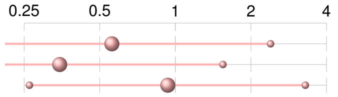

A	B	2.5	50	97.5	Sequence
0	1	0.13	0.56	2.4	ELSEEQIK
0	1	0.081	0.35	1.5	DVMDYK
0	2	0.26	0.93	3.3	SGTWLK

5.197 1 264 Spectrin beta chain [Crassostrea gigas]

Protein Accession	Unigene36334$_{H}1$
Mean Expression Ratio	1.38
Median Expression Ratio	1.37
Credible Interval	(0.54, 3.6)
Associated Peptides	2
Associated Spectra	2
Coverage	NaN

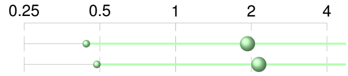

A	B	2.5	50	97.5	Sequence
0	1	0.44	1.9	8.4	QLLQVEHPNAAEVVQR
0	1	0.49	2.1	9.7	VAVVNQLAR

5.198 82 1449 hypothetical protein BRAFLDRAFT_127120 [Branchiostoma floridae] gi

Protein Accession	CL41883.Contig1$_{H}1$
Mean Expression Ratio	1.37
Median Expression Ratio	1.37
Credible Interval	(0.533, 3.59)
Associated Peptides	1
Associated Spectra	3
Coverage	NaN

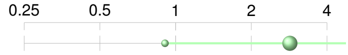

A	B	2.5	50	97.5	Sequence
0	3	0.91	2.9	8.6	ILQEQEDKFLK

5.199 593 862 minus strand mitochondrial ATP synthase coupling factor 6 precursor [Ochlerotatus taeniorhynchus]

Protein Accession	Unigene4216$_{H}1$
Mean Expression Ratio	0.732
Median Expression Ratio	0.73
Credible Interval	(0.283, 1.92)
Associated Peptides	2
Associated Spectra	2
Coverage	NaN

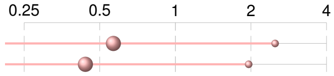

A	B	2.5	50	97.5	Sequence
0	1	0.13	0.57	2.5	GSTVTDPIQQLFLDK
0	1	0.1	0.44	2	LVDASPTVQAALKEELEK

5.200 152 1207 haemocyanin precursor [Nautilus pompilius]

Protein Accession	Unigene27031$_H1$
Mean Expression Ratio	1.37
Median Expression Ratio	1.37
Credible Interval	(0.566, 3.37)
Associated Peptides	2
Associated Spectra	6
Coverage	NaN

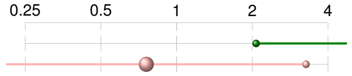

A	B	2.5	50	97.5	Sequence
1	4	2.1	5.3	13	AVDDRLFQK
0	1	0.17	0.76	3.3	DPNGGMGLK

5.201 " 144 1145 PREDICTED: RNA-binding protein squid-like isoform 1 [Megachile rotundata]; 90 1085 squid, variant A [Blattella germanica]; 144 1145 PREDICTED: RNA-binding protein squid-like isoform 1 [Megachile rotundata]"

Protein Accession	CL14696.Contig7$_H1$ CL14696.Contig5$_H1$ CL14696.Contig4$_H1$
Mean Expression Ratio	1.37
Median Expression Ratio	1.37
Credible Interval	(0.593, 3.25)
Associated Peptides	3
Associated Spectra	8
Coverage	NaN

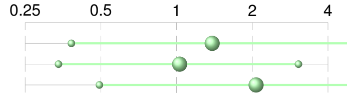

A	B	2.5	50	97.5	Sequence
1	1	0.38	1.4	5.4	IMEVTTHK
3	2	0.34	1	3.1	LGGQEVDVK
0	1	0.49	2.1	9.1	KIMEVTTHK

5.202 48 356 DEC-3 [Lymnaea stagnalis]

Protein Accession	CL26259.Contig1$_H1$
Mean Expression Ratio	0.731
Median Expression Ratio	0.732
Credible Interval	(0.29, 1.81)
Associated Peptides	2
Associated Spectra	4
Coverage	NaN

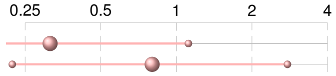

A	B	2.5	50	97.5	Sequence
1	1	0.088	0.31	1.1	SEFDTAFR
0	2	0.22	0.8	2.8	EFSFNWR

5.203 151 1599 PREDICTED: galactosamine (N-acetyl)-6-sulfate sulfatase-like [Saccoglossus kowalevskii]

Protein Accession	CL13278.Contig2$_H1$
Mean Expression Ratio	1.36
Median Expression Ratio	1.36
Credible Interval	(0.532, 3.48)
Associated Peptides	2
Associated Spectra	3
Coverage	NaN

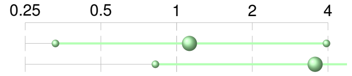

A	B	2.5	50	97.5	Sequence
0	2	0.33	1.1	3.9	GLVPSLISK
0	1	0.83	3.6	15	TTLWEGGTR

5.204 72 1400 minus strand hypothetical protein RCJMB04_22i13 [Gallus gallus]

Protein Accession	CL44755.Contig5$_H1$
Mean Expression Ratio	0.735
Median Expression Ratio	0.733
Credible Interval	(0.365, 1.48)
Associated Peptides	8
Associated Spectra	14
Coverage	NaN

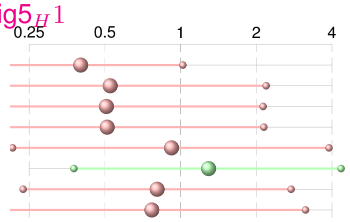

A	B	2.5	50	97.5	Sequence
2	3	0.16	0.4	1	LVFSADEAK
1	0	0.13	0.52	2.2	EAAVDVK
1	0	0.12	0.51	2.1	AVFQMR
1	0	0.13	0.51	2.1	EQAVYVAR
0	1	0.21	0.92	3.9	EMGFSEGSIDK
0	2	0.38	1.3	4.4	FLVASTPDEALQHAAELGK
0	2	0.24	0.8	2.8	LSGIPDVFVK
0	1	0.2	0.77	3.1	ILACDNLDEAASMVVK

5.205 124 390 PREDICTED: 60S ribosomal protein L23a-like [Callithrix jacchus]

Protein Accession	CL27563.Contig5$_H1$
Mean Expression Ratio	1.37
Median Expression Ratio	1.36
Credible Interval	(0.573, 3.29)
Associated Peptides	3
Associated Spectra	5
Coverage	NaN

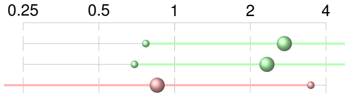

A	B	2.5	50	97.5	Sequence
0	2	0.77	2.7	9.4	AAPKPAVK
0	2	0.69	2.3	8.1	VNRLDAFAIVK
0	1	0.19	0.85	3.5	LDAFAIVK

5.206 150 1211 RecName: Full=Guanine nucleotide-binding protein G(i) subunit alpha; AltName: Full=Adenylate cyclase-inhibiting G alpha protein gi|9631|emb|CAA78807.1| GTP-binding protein alpha-i subunit [Lymnaea stagnalis]

Protein Accession	CL13885.Contig6$_H1$
Mean Expression Ratio	0.735
Median Expression Ratio	0.735
Credible Interval	(0.268, 1.98)
Associated Peptides	1
Associated Spectra	1
Coverage	NaN

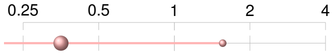

A	B	2.5	50	97.5	Sequence
1	0	0.074	0.35	1.6	IAIIR

5.207 76 522 minus strand Troponin C [Crassostrea gigas]; 173 619 minus strand Troponin C [Crassostrea gigas]; 173 619 minus strand Troponin C [Crassostrea gigas]

Protein Accession	Unigene53263$_H1$ Unigene11674$_H1$ CL13001.Contig4$_H1$
Mean Expression Ratio	0.733
Median Expression Ratio	0.735
Credible Interval	(0.295, 1.83)
Associated Peptides	2
Associated Spectra	5
Coverage	NaN

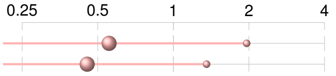

A	B	2.5	50	97.5	Sequence
1	1	0.16	0.56	2	GVIPVEDLR
2	1	0.15	0.45	1.4	ELANVFK

5.208 401 1174 minus strand Mechanosensory protein 2 [Crassostrea gigas]

Protein Accession	Unigene7703$_H1$
Mean Expression Ratio	1.37
Median Expression Ratio	1.36
Credible Interval	(0.627, 3.07)
Associated Peptides	5
Associated Spectra	9
Coverage	NaN

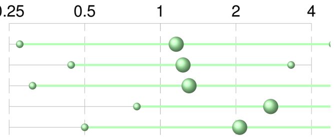

A	B	2.5	50	97.5	Sequence
1	0	0.28	1.2	4.9	AMAAEAEASR
2	2	0.44	1.2	3.3	VIAAEGEQK
1	0	0.31	1.3	5.6	AMAAEAEASREAR
1	1	0.8	2.7	9.3	AKVIAAEGEQK
0	1	0.5	2.1	8.9	LPVQLQR

5.209 167 2713 minus strand Major vault protein [Crassostrea gigas]

Protein Accession	CL18286.Contig3$_H1$
Mean Expression Ratio	1.36
Median Expression Ratio	1.36
Credible Interval	(0.739, 2.55)
Associated Peptides	13
Associated Spectra	15
Coverage	NaN

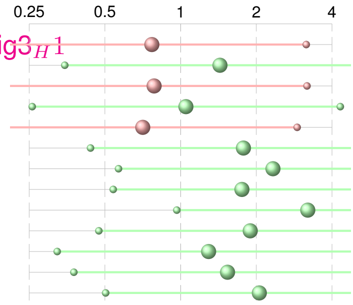

A	B	2.5	50	97.5	Sequence
1	0	0.19	0.77	3.2	GAVAQVQFDDFHK
1	0	0.35	1.4	5.7	IVDEAEAEK
1	0	0.19	0.79	3.2	LKAEAMK
1	0	0.26	1	4.3	VTGEEWLAK
1	0	0.17	0.71	2.9	DPLADRSER
0	1	0.44	1.8	7.1	IEGEAAVEQAK
0	1	0.57	2.3	9.2	KEVVVEETIR
0	2	0.54	1.8	5.9	NADNEVVMDAAGQAK
0	2	0.96	3.2	11	QKIVDEAEAEK
0	1	0.47	1.9	7.9	VVPANSALR
0	1	0.32	1.3	5.1	IEAESELER
0	1	0.38	1.5	6.4	TYTDDFNK
0	1	0.5	2.1	8.5	VVTFR

5.210 313 1908 minus strand Heterogeneous nuclear ribonucleoprotein L [Crassostrea gigas]; 313 1833 minus strand Heterogeneous nuclear ribonucleoprotein L [Crassostrea gigas]

Protein Accession	CL19412.Contig8$_{H}$1 CL19412.Contig7$_{H}$1
Mean Expression Ratio	0.734
Median Expression Ratio	0.737
Credible Interval	(0.27, 1.99)
Associated Peptides	1
Associated Spectra	2
Coverage	NaN

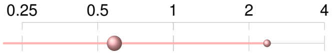

A	B	2.5	50	97.5	Sequence
1	1	0.14	0.58	2.4	SSTGLLEFESK

5.211 816 1211 H2 [Haliotis tuberculata]

Protein Accession	CL11519.Contig5$_{H}$1
Mean Expression Ratio	0.736
Median Expression Ratio	0.737
Credible Interval	(0.322, 1.68)
Associated Peptides	3
Associated Spectra	9
Coverage	NaN

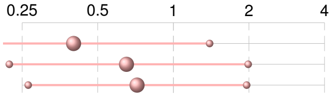

A	B	2.5	50	97.5	Sequence
1	1	0.12	0.4	1.4	LDSSADFDLQVK
1	2	0.22	0.65	2	KNVLSLTPAER
0	4	0.26	0.72	2	SLILAMR

5.212 " 143 451 minus strand PREDICTED: 10 kDa heat shock protein, mitochondrial isoform 1 [Canis lupus familiaris]"

Protein Accession	CL48287.Contig1$_{H}$1
Mean Expression Ratio	0.739
Median Expression Ratio	0.737
Credible Interval	(0.355, 1.55)
Associated Peptides	6
Associated Spectra	10
Coverage	NaN

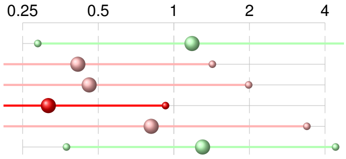

A	B	2.5	50	97.5	Sequence
1	0	0.29	1.2	5.2	GGIMIPEKSQGK
2	0	0.12	0.42	1.4	TKGGIMIPEK
1	0	0.11	0.46	2	SIPETK
3	0	0.11	0.32	0.93	GGIMIPEK
0	1	0.2	0.81	3.4	AQEAVVVAVGSGSR
0	2	0.37	1.3	4.4	VLLPEYGGTK

5.213 91 2658 minus strand puromycin-sensitive aminopeptidase [Saccoglossus kowalevskii]

Protein Accession	CL6708.Contig2$_{H}$1
Mean Expression Ratio	0.738
Median Expression Ratio	0.737
Credible Interval	(0.361, 1.5)
Associated Peptides	8
Associated Spectra	10
Coverage	NaN

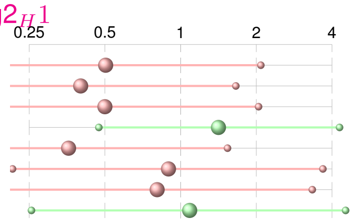

A	B	2.5	50	97.5	Sequence
1	0	0.12	0.5	2.1	GLTVQQEK
1	0	0.092	0.4	1.7	VILSNMPVK
1	0	0.12	0.5	2	VYTPVGK
1	2	0.47	1.4	4.3	SLGVNDDPELIKR
1	0	0.087	0.36	1.5	LVTYR
0	1	0.21	0.9	3.7	TIQQSLENIMLNKR
0	1	0.19	0.81	3.3	TLDFALSDK
0	1	0.26	1.1	4.5	MIELYEK

5.214 241 561 minus strand thioredoxin [Crassostrea ariakensis]

Protein Accession	CL33159.Contig3$_{H1}$
Mean Expression Ratio	0.736
Median Expression Ratio	0.738
Credible Interval	(0.31, 1.68)
Associated Peptides	3
Associated Spectra	6
Coverage	NaN

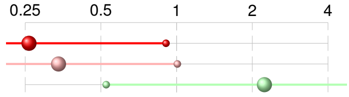

A	B	2.5	50	97.5	Sequence
**1**	**1**	**0.078**	**0.26**	**0.91**	**EVGEVVGASEEEIKK**
**1**	**2**	**0.11**	**0.34**	**1**	**IETQEDFEK**
**0**	**1**	**0.52**	**2.2**	**9.5**	**VKIETQEDFEK**

5.215 5 301 PREDICTED: hypothetical protein [Saccoglossus kowalevskii]

Protein Accession	Unigene21633$_{H1}$
Mean Expression Ratio	0.736
Median Expression Ratio	0.738
Credible Interval	(0.28, 1.91)
Associated Peptides	1
Associated Spectra	2
Coverage	NaN

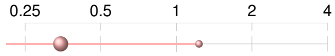

A	B	2.5	50	97.5	Sequence
**1**	**1**	**0.1**	**0.35**	**1.2**	**AYIPIQMR**

5.216 712 831 haemocyanin [Aplysia californica]

Protein Accession	Unigene65929$_{H1}$
Mean Expression Ratio	1.36
Median Expression Ratio	1.35
Credible Interval	(0.586, 3.12)
Associated Peptides	3
Associated Spectra	11
Coverage	NaN

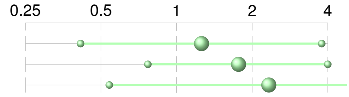

A	B	2.5	50	97.5	Sequence
**0**	**3**	**0.42**	**1.3**	**3.8**	**YDVTDVFNQMR**
**0**	**7**	**0.77**	**1.8**	**4**	**YHFNVK**
**0**	**1**	**0.54**	**2.3**	**9.7**	**LRPDSKYHFNVK**

5.217 813 1244 Probable protein disulfide-isomerase A6 OS=Caenorhabditis elegans GN=tag-320 PE=2 SV=1

Protein Accession	CL39540.Contig4$_{H1}$
Mean Expression Ratio	0.738
Median Expression Ratio	0.738
Credible Interval	(0.272, 1.98)
Associated Peptides	1
Associated Spectra	3
Coverage	NaN

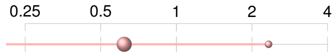

A	B	2.5	50	97.5	Sequence
**1**	**2**	**0.16**	**0.62**	**2.3**	**TASDIINWATEK**

5.218 424 1347 minus strand Tubulin alpha chain [Ascaris suum]

Protein Accession	Unigene7901$_H$1
Mean Expression Ratio	1.35
Median Expression Ratio	1.35
Credible Interval	(0.583, 3.15)
Associated Peptides	3
Associated Spectra	9
Coverage	NaN

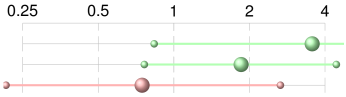

A	B	2.5	50	97.5	Sequence
0	1	0.84	3.6	15	VDLEPTVVDEVR
0	6	0.76	1.9	4.4	NTFFNETGSGKHVPRAIFVDLEPTVVDEVR
0	2	0.21	0.75	2.7	QLFHPEQLITGK

5.219 30 536 putative mitochondrial ATP synthase [Haliotis discus discus]; 27 620 putative mitochondrial ATP synthase [Haliotis discus discus]

Protein Accession	CL13014.Contig4$_H$1 CL13014.Contig1$_H$1
Mean Expression Ratio	1.35
Median Expression Ratio	1.35
Credible Interval	(0.695, 2.64)
Associated Peptides	7
Associated Spectra	22
Coverage	NaN

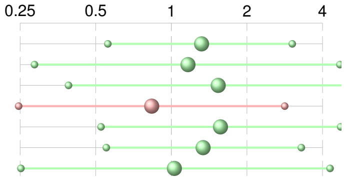

A	B	2.5	50	97.5	Sequence
2	5	0.56	1.3	3	SAVDWVAFK
0	1	0.29	1.2	4.7	SDSFVNKVHQYPAELPK
0	1	0.39	1.5	6.1	SNALKDIAAEEK
0	2	0.25	0.83	2.8	VHQYPAELPK
0	3	0.53	1.6	4.7	IDFSAYK
3	4	0.55	1.3	3.3	LANPALVDQFQK
0	1	0.25	1	4.3	VHQYPAEL

5.220 1271 1663 minus strand PREDICTED: similar to AGAP007393-PB [Tribolium castaneum] gi

Protein Accession	CL28563.Contig3$_H$1
Mean Expression Ratio	1.35
Median Expression Ratio	1.35
Credible Interval	(0.528, 3.53)
Associated Peptides	2
Associated Spectra	2
Coverage	NaN

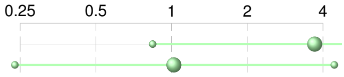

A	B	2.5	50	97.5	Sequence
0	1	0.84	3.7	16	KYEGGREVDEFITYLAR
0	1	0.24	1	4.4	EVDEFITYLAR

5.221 " 132 1544 minus strand Serine hydroxymethyltransferase, cytosolic [Crassostrea gigas]"

Protein Accession	CL22573.Contig2$_H$1
Mean Expression Ratio	1.36
Median Expression Ratio	1.35
Credible Interval	(0.533, 3.56)
Associated Peptides	2
Associated Spectra	2
Coverage	NaN

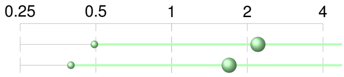

A	B	2.5	50	97.5	Sequence
0	1	0.49	2.2	9.8	IGTPALTSR
0	1	0.4	1.7	7.8	SGIIFYR

5.222 1 381 hypothetical protein [Eimeria tenella]

Protein Accession	CL17786.Contig1$_{H}1$
Mean Expression Ratio	1.36
Median Expression Ratio	1.35
Credible Interval	(0.513, 3.5)
Associated Peptides	1
Associated Spectra	4
Coverage	NaN

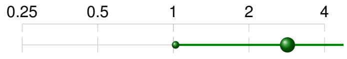

A	B	2.5	50	97.5	Sequence
**0**	**4**	**1**	**2.8**	**7.8**	**IINEPTAAAIAYGLDKK**

5.223 388 1653 Tektin-3 [Crassostrea gigas]

Protein Accession	CL28380.Contig1$_{H}1$
Mean Expression Ratio	1.35
Median Expression Ratio	1.35
Credible Interval	(0.574, 3.2)
Associated Peptides	3
Associated Spectra	6
Coverage	NaN

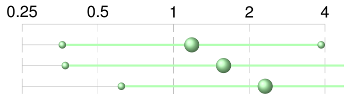

A	B	2.5	50	97.5	Sequence
**1**	**2**	**0.36**	**1.2**	**3.9**	**QTIDLVHDAPEK**
**0**	**1**	**0.37**	**1.6**	**6.7**	**RQTIDLVHDAPEK**
**1**	**1**	**0.62**	**2.3**	**8.8**	**GLTFHNGVQR**

5.224 346 702 minus strand PREDICTED: galectin-4-like [Anolis carolinensis]

Protein Accession	Unigene42203$_{H}1$
Mean Expression Ratio	0.743
Median Expression Ratio	0.744
Credible Interval	(0.303, 1.84)
Associated Peptides	2
Associated Spectra	7
Coverage	NaN

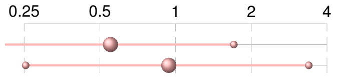

A	B	2.5	50	97.5	Sequence
**2**	**2**	**0.17**	**0.55**	**1.7**	**AEATLEVR**
**1**	**2**	**0.25**	**0.94**	**3.4**	**SVPHGLVK**

5.225 31 777 Proteasome subunit alpha type-7-like protein [Crassostrea gigas]

Protein Accession	CL35094.Contig4$_{H}1$
Mean Expression Ratio	0.739
Median Expression Ratio	0.744
Credible Interval	(0.261, 1.98)
Associated Peptides	1
Associated Spectra	1
Coverage	NaN

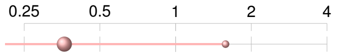

A	B	2.5	50	97.5	Sequence
**1**	**0**	**0.08**	**0.36**	**1.6**	**GDNLVVLGVEK**

5.226 801 971 RecName: Full=Tropomyosin-2; AltName: Full=Tropomyosin II; Short=BgTMII; Short=TMII gi|155940|gb|AAA27816.1| tropomyosin [Biomphalaria glabrata]

Protein Accession	CL2925.Contig4$_H$1
Mean Expression Ratio	1.35
Median Expression Ratio	1.34
Credible Interval	(0.501, 3.85)
Associated Peptides	1
Associated Spectra	1
Coverage	NaN

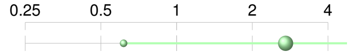

A	B	2.5	50	97.5	Sequence
**0**	**1**	**0.62**	**2.7**	**13**	**ASQREDSYEETIRDLTQR**

5.227 365 544 PREDICTED: cofilin-2 [Otolemur garnettii]

Protein Accession	CL10440.Contig9$_H$1
Mean Expression Ratio	0.743
Median Expression Ratio	0.746
Credible Interval	(0.263, 2.07)
Associated Peptides	1
Associated Spectra	1
Coverage	NaN

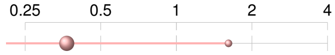

A	B	2.5	50	97.5	Sequence
**1**	**0**	**0.084**	**0.37**	**1.6**	**MLYASSK**

5.228 184 438 PREDICTED: similar to CG14235 CG14235-PA [Tribolium castaneum] gi

Protein Accession	Unigene638$_H$1
Mean Expression Ratio	0.747
Median Expression Ratio	0.746
Credible Interval	(0.329, 1.71)
Associated Peptides	4
Associated Spectra	6
Coverage	NaN

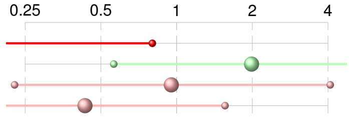

A	B	2.5	50	97.5	Sequence
**1**	**0**	**0.043**	**0.19**	**0.8**	**TAPFDAR**
**1**	**1**	**0.56**	**2**	**6.9**	**GDEQKYYQLK**
**0**	**1**	**0.23**	**0.95**	**4.1**	**WDDQVANGTFAGKI**
**0**	**2**	**0.12**	**0.43**	**1.6**	**WDDQVANGTFAGK**

5.229 350 910 Peptidyl-prolyl cis-trans isomerase B [Crassostrea gigas]; 488 1039 Peptidyl-prolyl cis-trans isomerase B [Crassostrea gigas]; 488 1039 Peptidyl-prolyl cis-trans isomerase B [Crassostrea gigas]

Protein Accession	CL49248.Contig5$_{H}1$ CL49248.Contig2$_{H}1$ CL49248.Contig1$_{H}1$
Mean Expression Ratio	0.749
Median Expression Ratio	0.747
Credible Interval	(0.307, 1.85)
Associated Peptides	3
Associated Spectra	3
Coverage	NaN

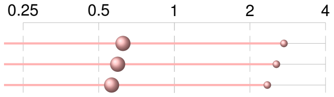

A	B	2.5	50	97.5	Sequence
0	1	0.14	0.62	2.7	HVAFGQIIDK
0	1	0.14	0.6	2.6	ASLEFLDK
0	1	0.13	0.56	2.3	AVLDFLIKN

5.230 244 837 PREDICTED: ras-like GTP-binding protein Rho1-like [Megachile rotundata]; 650 883 hypothetical protein SINV_15432 [Solenopsis invicta]; 534 767 hypothetical protein SINV_15432 [Solenopsis invicta]

Protein Accession	Unigene11792$_{H}1$ Unigene11791$_{H}1$ Unigene11790$_{H}1$
Mean Expression Ratio	0.747
Median Expression Ratio	0.747
Credible Interval	(0.275, 2.02)
Associated Peptides	1
Associated Spectra	2
Coverage	NaN

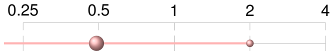

A	B	2.5	50	97.5	Sequence
1	1	0.12	0.49	2	EVFETATR

5.231 252 848 sigma class glutathione-s-transferase 2 [Haliotis discus discus]

Protein Accession	CL19285.Contig21$_{H}1$
Mean Expression Ratio	0.75
Median Expression Ratio	0.748
Credible Interval	(0.304, 1.9)
Associated Peptides	2
Associated Spectra	6
Coverage	NaN

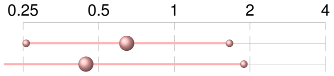

A	B	2.5	50	97.5	Sequence
1	4	0.26	0.64	1.7	LQDVDKDYPEVQK
1	0	0.1	0.45	1.9	LGNELTMA

5.232 " 81 1349 minus strand PREDICTED: LOW QUALITY PROTEIN: dolichyl-diphosphooligosaccharide–protein glycosyltransferase 48 kDa subunit-like, partial [Cavia porcellus]; 81 1349 minus strand PREDICTED: LOW QUALITY PROTEIN: dolichyl-diphosphooligosaccharide–protein glycosyltransferase 48 kDa subunit-like, partial [Cavia porcellus]"

Protein Accession	CL49524.Contig2$_{H}1$ CL49524.Contig1$_{H}1$
Mean Expression Ratio	0.748
Median Expression Ratio	0.748
Credible Interval	(0.312, 1.79)
Associated Peptides	4
Associated Spectra	4
Coverage	NaN

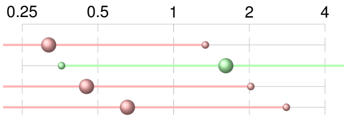

A	B	2.5	50	97.5	Sequence
0	1	0.074	0.32	1.3	DLGFDLTFK
0	1	0.36	1.6	7	KGNSPFLFR
0	1	0.1	0.45	2	NLAQWVFHQK
0	1	0.15	0.65	2.8	IDPFVR

5.233 521 6844 hypothetical protein CGI_10014469 [Crassostrea gigas]

Protein Accession	CL12911.Contig1$_{H}1$
Mean Expression Ratio	0.75
Median Expression Ratio	0.748
Credible Interval	(0.382, 1.48)
Associated Peptides	7
Associated Spectra	31
Coverage	NaN

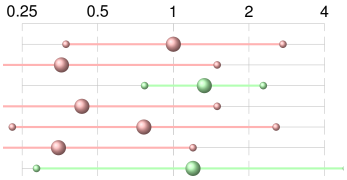

A	B	2.5	50	97.5	Sequence
2	2	0.37	1	2.7	VAAVQAFR
1	0	0.087	0.36	1.5	LQTIK
3	16	0.77	1.3	2.3	IDAALVLK
1	1	0.13	0.43	1.5	LTRIQV
0	2	0.23	0.76	2.6	KLFDLNPLK
0	2	0.1	0.35	1.2	LFDLNPLK
0	1	0.29	1.2	4.9	RSPWQLPKDLSLTLK

5.234 8 1636 minus strand V-type proton ATPase catalytic subunit A [Crassostrea gigas]; 8 1636 minus strand V-type proton ATPase catalytic subunit A [Crassostrea gigas]; 8 1636 minus strand V-type proton ATPase catalytic subunit A [Crassostrea gigas]

Protein Accession	CL33671.Contig3$_{H}1$ CL33671.Contig2$_{H}1$ CL33671.Contig1$_{H}1$
Mean Expression Ratio	0.749
Median Expression Ratio	0.748
Credible Interval	(0.347, 1.58)
Associated Peptides	6
Associated Spectra	10
Coverage	NaN

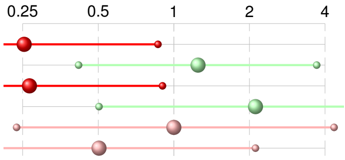

A	B	2.5	50	97.5	Sequence
2	0	0.072	0.25	0.86	GINTPALDANK
2	1	0.42	1.3	3.7	IMLNPNAR
2	0	0.079	0.27	0.9	TVGMLR
1	0	0.5	2.1	9	FRNLED
0	1	0.24	1	4.4	LAANHPLLTGQR
0	1	0.12	0.5	2.1	ITLEVAK

5.235 80 589 minus strand PREDICTED: adenine phosphoribosyltransferase-like [Strongylocentrotus purpuratus]

Protein Accession	CL8682.Contig6$_{H}$1
Mean Expression Ratio	1.34
Median Expression Ratio	1.33
Credible Interval	(0.544, 3.36)
Associated Peptides	2
Associated Spectra	5
Coverage	NaN

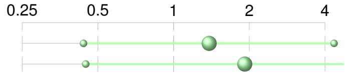

A	B	2.5	50	97.5	Sequence
2	2	0.44	1.4	4.4	AAVELVEK
0	1	0.45	1.9	8.5	GKLTVPVESLIKY

5.236 16 1560 V-type proton ATPase subunit B [Crassostrea gigas]

Protein Accession	CL1031.Contig1$_{H}$1
Mean Expression Ratio	1.34
Median Expression Ratio	1.33
Credible Interval	(0.629, 2.83)
Associated Peptides	6
Associated Spectra	9
Coverage	NaN

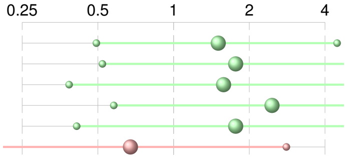

A	B	2.5	50	97.5	Sequence
1	2	0.49	1.5	4.5	AVVQVFEGTSGIDAK
1	1	0.52	1.8	6	SLNGPLNDAAAR
0	1	0.38	1.6	6.5	HTTCEFTGDILR
0	1	0.58	2.5	10	MPTSEDMLGR
0	1	0.41	1.8	7.4	NFISQGHYENR
0	1	0.16	0.68	2.8	ATIYERAGR

5.237 87 485 Sex-regulated protein janus-A [Crassostrea gigas]

Protein Accession	CL13131.Contig3$_{H}$1
Mean Expression Ratio	1.33
Median Expression Ratio	1.33
Credible Interval	(0.52, 3.44)
Associated Peptides	2
Associated Spectra	2
Coverage	NaN

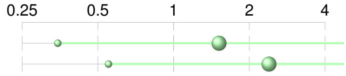

A	B	2.5	50	97.5	Sequence
0	1	0.35	1.5	6.7	ADHSITQTLLLR
0	1	0.55	2.4	11	VVGEIESK

5.238 316 1512 Alpha-N-acetylgalactosaminidase [Crassostrea gigas]; 282 1478 Alpha-N-acetylgalactosaminidase [Crassostrea gigas]

Protein Accession	CL2428.Contig4$_{H}$1 CL2428.Contig1$_{H}$1
Mean Expression Ratio	0.749
Median Expression Ratio	0.751
Credible Interval	(0.337, 1.66)
Associated Peptides	4
Associated Spectra	9
Coverage	NaN

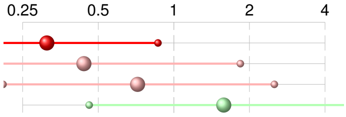

A	B	2.5	50	97.5	Sequence
1	3	0.12	0.31	0.86	LVELGLR
1	0	0.1	0.44	1.8	LYMAQANK
0	2	0.21	0.72	2.5	LGIYEDFGSK
0	2	0.46	1.6	5.6	AGYRARELFAGK
[truncated: 562,873 more chars]
