# Supplementary material for: Data set from the proteomic analysis of Bithynia siamensis goniomphalos snails upon infection with the carcinogenic liver fluke Opisthorchis viverrini
Source: Data Brief. 2014 Nov 6;2:16–20. doi: 10.1016/j.dib.2014.09.005 (PMC4459563; doi:10.1016/j.dib.2014.09.005)
Supplement: Supplementary file 2 — Document generated by iQuantitator summarizing the analysis of relative protein expression using iTRAQ in the headfoot of infected Bithynia siamensis goniomphalos. The reporter ion peak area measurements supplied by the ProteinPilot software are used to estimate treatment-dependent peptide and protein relative expression. The document includes a protein relative expression summary and a per-protein detailed analysis of all time points studied. [file mmc2.pdf]

iTRAQ Data Analysis Report

Javier, Head 1d versus control

April 7, 2014

1 Introduction

This document summarizes an analysis of relative protein expression using iTRAQ. The reporter ion peak area measurements supplied by the ABI software are used to estimate treatment-dependent peptide and protein relative expression. Estimation is accomplished using a Bayesian approach with the model given below. The document includes a protein relative expression summary and a per-protein detailed analysis. The document is internally hyperlinked and linked externally to NCBI.

2 Experiment and Model Description

2.1 Experiment Design

The report summarizes data from one or more iTRAQ experiments addressing a common comparison. The experiment design, used in this analysis, is given in the table below.

	Experiment	Treatment	Channel	Sample
1	A	control	115	s1
2	A	test	116	s1
3	B	control	115	s2
4	B	test	116	s2

2.2 Input Files

Data for this analysis was extracted from the following tandem mass spectra (MSMS) summary files.

Experiment	MSMS Summary File
A	head2.txt
B	head3.txt

2.3 Statistical Model

The following statistical model was used to estimate the treatment-dependent effects.

LogIntensity ˜ Channel + Spectrum + Protein + Peptide + Protein:Treatment + Peptide:Treatment

3 Data Summary

The data supplied in the MSMS summary is filtered to remove unidentified proteins, contaminants, and peptides containing selected modifications. The following table sumarizes the data provided and used in the analysis.

	A	B	Combined
Supplied Spectra	21342	14837	36179
Unidentified Spectra	8154	6352	14506
Disallowed Modifications	360	182	542
Spectra from Contaminants	0	0	0
Missing Data	3175	2197	5372
Low Confidence Spectra	8154	6352	14506
Degenerate Peptides	3591	2236	5827
Remaining Spectra			9932
Unique Proteins			655
Unique Peptides			2359
Model R^2			0.939

4 Protein Summary

Each protein identified in one or more of the MSMS summaries is listed below in decreasing order of expression change magnitude. The median and estimated credible interval for each protein is given to the left in the table. Proteins identified by a single peptide are listed in a separate table.

4.1 Identified Proteins

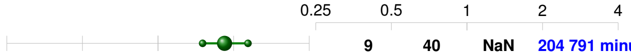

Peptides	Spectra	Coverage	Protein
9	40	NaN	204 791 minus strand PREDICTED: 15-hydroxyprostaglandin dehydrogenase [NAD+] [Gallus gallus]

0.25 0.5 1 2 4

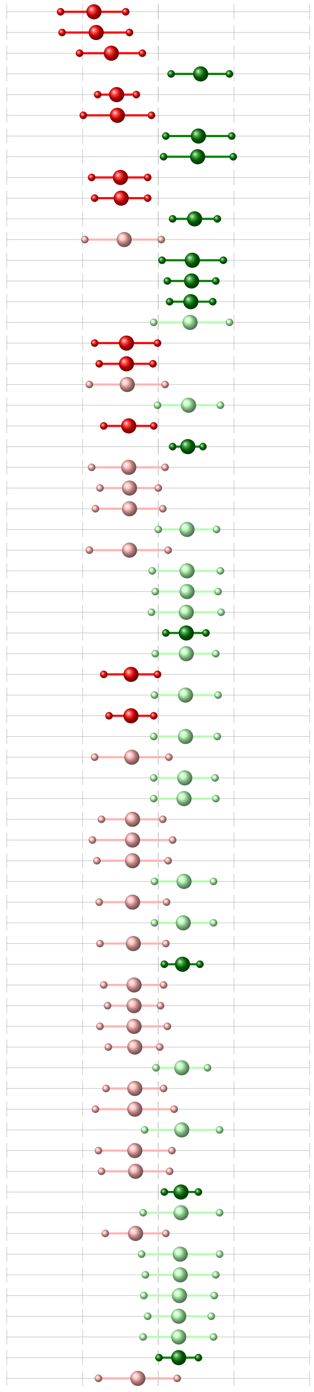

Peptides	Spectra	Coverage	Protein
4	7	NaN	" 353 781 alpha-B-crystallin, putative [Ixodes scapularis] gi"
4	6	NaN	" 10 609 lethal(2)essential for life protein, l2efl [Aedes aegypti] gi"
4	7	NaN	372 860 minus strand small heat shock protein [Tegillarca granosa]; 651 998 minus strand small heat shock protein
2	38	NaN	REVERSED 6 395 PREDICTED: sister chromatid cohesion protein PDS5 homolog B-A isoform 1 [Nasonia vitripennis
13	34	NaN	" 460 1944 PREDICTED: succinyl-CoA:3-ketoacid-coenzyme A transferase 1, mitochondrial [Canis lupus familiaris]"
3	6	NaN	200 2128 minus strand heat shock protein 70 [Cristaria plicata]
4	6	NaN	557 2329 minus strand Collagen alpha-3(VI) chain [Crassostrea gigas]
2	6	NaN	2996 3340 minus strand Collagen alpha-5(VI) chain [Crassostrea gigas]
6	10	NaN	51 890 prohibitin [Octopus tankahkeei]
7	14	NaN	501 1838 Hsp90A [Haliotis asinina]
10	23	NaN	95 1579 minus strand hypothetical protein BRAFLDRAFT_113974 [Branchiostoma floridae] gi
2	3	NaN	1 945 hemocyanin isoform 1 [Haliotis diversicolor]
3	11	NaN	801 971 RecName: Full=Tropomyosin-2; AltName: Full=Tropomyosin II; Short=BgTMII; Short=TMII gi|155940|gb|AA
8	21	NaN	1009 2784 minus strand Collagen alpha-3(VI) chain [Crassostrea gigas]
8	44	NaN	915 1505 minus strand Collagen alpha-5(VI) chain [Crassostrea gigas]
3	3	NaN	103 684 EF-hand domain-containing protein D2 [Crassostrea gigas]
3	8	NaN	3 491 hemocyanin [Haliotis tuberculata]
6	15	NaN	280 690 Histone H3.3 [Harpegnathos saltator]
3	3	NaN	40 1182 4-hydroxyphenylpyruvate dioxygenase [Crassostrea gigas]
4	7	NaN	230 1189 hypothetical protein [Amblyomma maculatum]
8	15	NaN	4726 5571 minus strand twitchin [Mytilus galloprovincialis]
18	76	NaN	111 1226 gelsolin [Aplysia californica] gi; 42 1139 minus strand gelsolin [Aplysia californica] gi
2	4	NaN	712 831 haemocyanin [Aplysia californica]
5	23	NaN	118 501 minus strand histone 2A.Z [Aplysia californica]
2	6	NaN	21 341 minus strand hemocyanin isoform 1 [Nucula nucleus]
5	10	NaN	684 1250 minus strand Collagen alpha-5(VI) chain [Crassostrea gigas]
2	2	NaN	696 4010 Lethal(2) giant larvae-like protein 1 [Crassostrea gigas]; 657 3971 minus strand Lethal(2) giant larvae-like p
3	5	NaN	" 110 1717 PREDICTED: glutamate dehydrogenase, mitochondrial-like [Oreochromis niloticus]; 110 1717 PREDICTE
4	7	NaN	503 1219 heat shock cognate 71 [Mytilus galloprovincialis]
4	5	NaN	244 837 PREDICTED: ras-like GTP-binding protein Rho1-like [Megachile rotundata]
7	84	NaN	222 809 minus strand PREDICTED: 15-hydroxyprostaglandin dehydrogenase [NAD+] [Gallus gallus]
4	10	NaN	31 735 hypothetical protein BRAFLDRAFT_78603 [Branchiostoma floridae] gi; 55 735 hypothetical protein BRAFLDF
7	13	NaN	957 2147 haemocyanin [Aplysia californica]
3	15	NaN	174 1649 minus strand Collagen alpha-1(XII) chain [Crassostrea gigas]; 123 1490 minus strand Collagen alpha-1(XII)
7	27	NaN	27 1682 minus strand PREDICTED: nucleolar protein 56 [Oreochromis niloticus]
3	8	NaN	150 716 calponin/transgelin [Papilio xuthus]
2	3	NaN	160 1218 Galectin-9 [Crassostrea gigas]
4	8	NaN	146 727 Adenylate kinase isoenzyme 1 [Crassostrea gigas]
4	8	NaN	127 657 ADP ribosylation factor 4 [Marsupenaeus japonicus]
4	7	NaN	430 759 hypothetical protein [Amblyomma maculatum]; 336 665 hypothetical protein [Amblyomma maculatum]
2	2	NaN	" 109 1632 minus strand Seryl-tRNA synthetase, cytoplasmic [Crassostrea gigas]; 109 1614 minus strand Seryl-tRN
2	4	NaN	23 214 minus strand hemocyanin isoform 1 [Nucula nucleus]
5	10	NaN	80 589 minus strand PREDICTED: adenine phosphoribosyltransferase-like [Strongylocentrotus purpuratus]
4	5	NaN	169 1200 PREDICTED: nidogen-2-like isoform 1 [Strongylocentrotus purpuratus]
2	15	NaN	105 503 Transgelin-2 [Crassostrea gigas]
2	9	NaN	" 303 500 minus strand protein lethal, putative [Pediculus humanus corporis] gi"
15	41	NaN	7 1722 minus strand Membrane metallo-endopeptidase-like 1 OS=Mus musculus GN=Mmel1 PE=1 SV=1
2	18	NaN	" 131 544 Histone H2B, gonadal OS=Patella granatina PE=1 SV=2"
7	14	NaN	366 1049 hypothetical protein CGI_10004855 [Crassostrea gigas]
2	8	NaN	" 599 907 minus strand Tryptophanyl-tRNA synthetase, cytoplasmic [Heterocephalus glaber]"
5	20	NaN	93 2606 twitchin [Mytilus galloprovincialis]
6	18	NaN	247 1308 RecName: Full=Guanine nucleotide-binding protein G(o) subunit alpha gi|9633|emb|CAA78806.1| GTP-bin
6	9	NaN	" 59 2365 minus strand Aconitate hydratase, mitochondrial [Crassostrea gigas]"
2	2	NaN	57 2378 minus strand Heterogeneous nuclear ribonucleoprotein U-like protein 1 [Crassostrea gigas]
2	3	NaN	2 772 minus strand arylsulfatase [Haliotis discus discus]
3	8	NaN	459 755 minus strand heat shock protein beta-1 [Trichinella spiralis] gi; 464 763 minus strand heat shock protein bet
5	5	NaN	424 3507 minus strand putative Na+/K+-ATPase alpha subunit [Paroctopus digueti]
11	107	NaN	260 892 minus strand calponin [Haliotis diversicolor]
2	3	NaN	" 81 1349 minus strand PREDICTED: LOW QUALITY PROTEIN: dolichyl-diphosphooligosaccharide–protein glycosyl
5	8	NaN	793 1269 PREDICTED: calmodulin-like [Saccoglossus kowalevskii]
2	2	NaN	3 503 minus strand elongation factor-1 alpha [Pomacea bridgesi]
3	4	NaN	186 1778 predicted protein [Nematostella vectensis] gi
2	5	NaN	57 1688 Talin-1 [Camponotus floridanus]; 57 1865 Talin-1 [Camponotus floridanus]; 57 1835 Talin-1 [Camponotus flo
4	7	NaN	353 2161 Bifunctional 3'-phosphoadenosine 5'-phosphosulfate synthetase [Crassostrea gigas]; 141 19
3	4	NaN	124 711 glutathione S-transferase sigma 3 [Mytilus galloprovincialis]; 124 711 glutathione S-transferase sigma 3 [My
7	51	NaN	" 142 1284 minus strand Chain O, Crsytal Structure Of Human Liver Gapdh gi|67464044|pdb|1ZNQ|P Chain P, Crsyta
2	2	NaN	" 17 283 Glycerol-3-phosphate dehydrogenase [NAD+], cytoplasmic [Crassostrea gigas]"

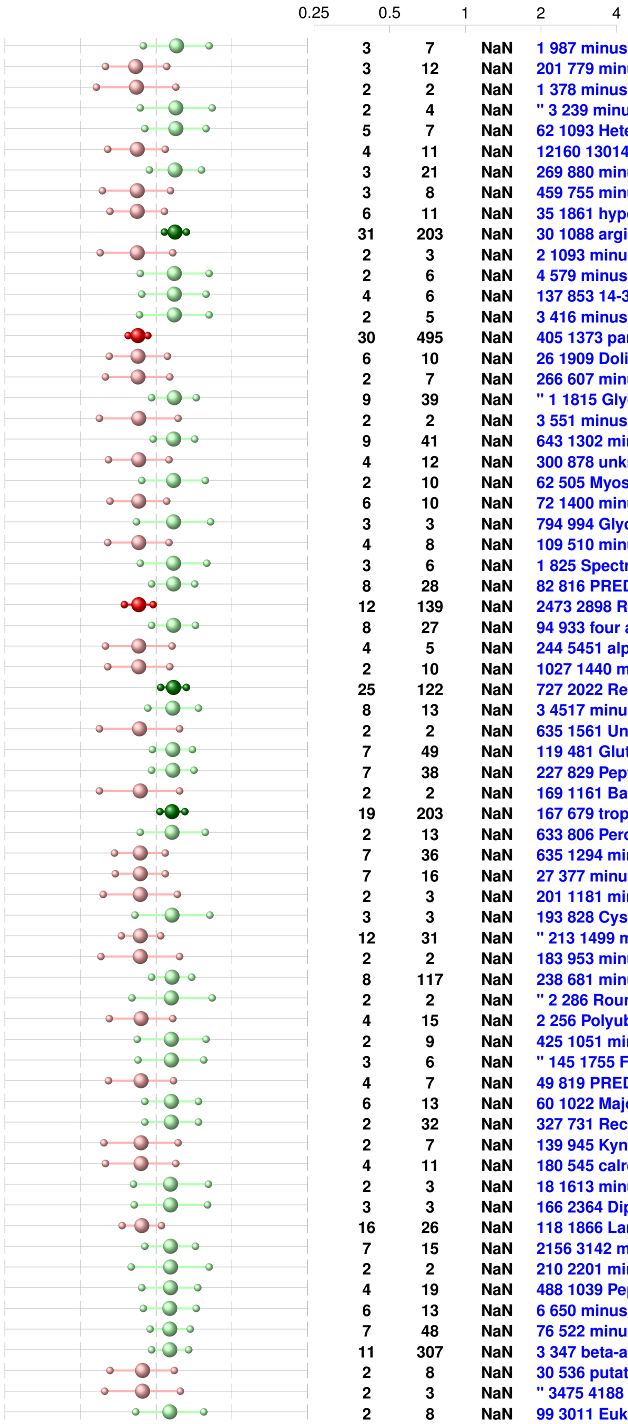

	Peptides	Spectra	Coverage	Protein
	3	7	NaN	1 987 minus strand RecName: Full=Tubulin beta chain; AltName: Full=Beta-tubulin
	3	12	NaN	201 779 minus strand sigma class glutathione-s-transferase 2 [Haliotis discus discus]
	2	2	NaN	1 378 minus strand PREDICTED: CG10098-like [Saccoglossus kowalevskii]
	2	4	NaN	" 3 239 minus strand laminin beta 1 chain, putative [Ixodes scapularis] gi"
	5	7	NaN	62 1093 Heterogeneous nuclear ribonucleoprotein A2-like protein 1 [Crassostrea gigas]
	4	11	NaN	12160 13014 twitchin [Mytilus galloprovincialis]
	3	21	NaN	269 880 minus strand myosin regulatory light chain [Fasciola hepatica]
	3	8	NaN	459 755 minus strand heat shock protein beta-1 [Trichinella spiralis] gi
	6	11	NaN	35 1861 hypothetical protein DAPPUDRAFT_309082 [Daphnia pulex]
	31	203	NaN	30 1088 arginine kinase [Conus novaehollandiae]
	2	3	NaN	2 1093 minus strand Neurexin-1-alpha [Crassostrea gigas]
	2	6	NaN	4 579 minus strand Collagen alpha-3(VI) chain [Crassostrea gigas]
	4	6	NaN	137 853 14-3-3 protein [Heterodera glycines]
	2	5	NaN	3 416 minus strand myosin essential light chain [Crassostrea gigas]
	30	495	NaN	405 1373 paramyosin [Haliotis discus discus]
	6	10	NaN	26 1909 Dolichyl-diphosphooligosaccharide–protein glycosyltransferase subunit 2 [Crassostrea gigas]
	2	7	NaN	266 607 minus strand H2 [Haliotis tuberculata]
	9	39	NaN	" 1 1815 Glycogen phosphorylase, muscle form [Crassostrea gigas]"
	2	2	NaN	3 551 minus strand Spectrin beta chain [Crassostrea gigas]
	9	41	NaN	643 1302 minus strand Kyphoscoliosis peptidase [Crassostrea gigas]; 634 1293 minus strand Kyphoscoliosis peptic
	4	12	NaN	300 878 unknown [Schistosoma japonicum]; 567 1145 unknown [Schistosoma japonicum]; 1110 1688 unknown [Sch
	2	10	NaN	62 505 Myosin-2 essential light chain [Crassostrea gigas]
	6	10	NaN	72 1400 minus strand hypothetical protein RCJMB04_22i13 [Gallus gallus]
	3	3	NaN	794 994 Glycogenin-1 [Crassostrea gigas]; 1425 1739 minus strand Glycogenin-1 [Crassostrea gigas]
	4	8	NaN	109 510 minus strand hypothetical protein DAPPUDRAFT_231281 [Daphnia pulex]
	3	6	NaN	1 825 Spectrin alpha chain [Crassostrea gigas]
	8	28	NaN	82 816 PREDICTED: triosephosphate isomerase B [Oreochromis niloticus]
	12	139	NaN	2473 2898 RecName: Full=Paramyosin gi|397603|emb|CAA79849.1| paramyosin [Echinococcus granulosus]
	8	27	NaN	94 933 four and A half lim domains [Schistosoma mansoni]
	4	5	NaN	244 5451 alpha macroglobulin [Chlamys farreri]; 244 5493 alpha macroglobulin [Chlamys farreri]
	2	10	NaN	1027 1440 minus strand hemocyanin isoform 1 [Haliotis diversicolor]
	25	122	NaN	727 2022 RecName: Full=Enolase; AltName: Full=2-phospho-D-glycerate hydro-lyase; AltName: Full=2-phosphoglyc
	8	13	NaN	3 4517 minus strand Clathrin heavy chain 1 [Crassostrea gigas]
	2	2	NaN	635 1561 Uncharacterized protein yfeX [Crassostrea gigas]
	7	49	NaN	119 481 Glutamate receptor U1 [Crassostrea gigas]
	7	38	NaN	227 829 Peptidyl-prolyl cis-trans isomerase B [Crassostrea gigas]
	2	2	NaN	169 1161 Basement membrane-specific heparan sulfate proteoglycan core protein OS=Homo sapiens GN=HSPG2 P
	19	203	NaN	167 679 tropomyosin [Haliotis discus discus]
	2	13	NaN	633 806 Peroxiredoxin-1 [Crassostrea gigas]; 648 821 Peroxiredoxin-1 [Crassostrea gigas]; 666 839 Peroxiredoxin-1
	7	36	NaN	635 1294 minus strand Kyphoscoliosis peptidase [Crassostrea gigas]; 643 1302 minus strand Kyphoscoliosis peptic
	7	16	NaN	27 377 minus strand ribosomal protein LP2 [Papilio xuthus] gi; 27 377 minus strand ribosomal protein LP2 [Papilio x
	2	3	NaN	201 1181 minus strand Serine/threonine-protein phosphatase PP1-alpha catalytic subunit [Crassostrea gigas]
	3	3	NaN	193 828 Cysteine-rich secretory protein LCCL domain-containing 2 [Crassostrea gigas]
	12	31	NaN	" 213 1499 minus strand Citrate synthase, mitochondrial [Crassostrea gigas]"
	2	2	NaN	183 953 minus strand glutathione-S-transferase [Haliotis diversicolor supertexta]; 275 1045 minus strand glutathion
	8	117	NaN	238 681 minus strand Globin OS=Cerithidea rhizophorarum PE=1 SV=1
	2	2	NaN	" 2 286 Roundabout 2, partial [Ascaris suum]"
	4	15	NaN	2 256 Polyubiquitin (Fragment) OS=Xenopus laevis PE=1 SV=2; 20 472 minus strand large subunit ribosomal protein
	2	9	NaN	425 1051 minus strand unknown [Schistosoma japonicum]; 300 878 unknown [Schistosoma japonicum]; 567 1145 u
	3	6	NaN	" 145 1755 Fumarate hydratase class I, aerobic [Crassostrea gigas]"
	4	7	NaN	49 819 PREDICTED: elongation factor 1-delta-like isoform 2 [Strongylocentrotus purpuratus]
	6	13	NaN	60 1022 Major egg antigen [Crassostrea gigas]; 60 1022 Major egg antigen [Crassostrea gigas]
	2	32	NaN	327 731 RecName: Full=Arginine kinase; Short=AK gi|439719|dbj|BAA05100.1| arginine kinase [Haliotis madaka]
	2	7	NaN	139 945 Kynurenine formamidase [Crassostrea gigas]; 145 951 Kynurenine formamidase [Crassostrea gigas]; 220 1(
	4	11	NaN	180 545 calreticulin precursor [Aplysia californica] gi
	2	3	NaN	18 1613 minus strand chaperonin containing T-complex polypeptide subunit zeta [Haliotis discus hannai]; 527 2089
	3	3	NaN	166 2364 Dipeptidyl-peptidase 3 [Crassostrea gigas]
	16	26	NaN	118 1866 Lamin Dm0 [Crassostrea gigas]
	7	15	NaN	2156 3142 minus strand PREDICTED: hemicentin-1 [Xenopus (Silurana) tropicalis]
	2	2	NaN	210 2201 minus strand Glycyl-tRNA synthetase [Crassostrea gigas]; 210 2201 minus strand Glycyl-tRNA synthetase
	4	19	NaN	488 1039 Peptidyl-prolyl cis-trans isomerase B [Crassostrea gigas]; 488 1039 Peptidyl-prolyl cis-trans isomerase B [
	6	13	NaN	6 650 minus strand glutathione S-transferase mu class protein [Crassostrea ariakensis]; 117 761 glutathione S-trans
	7	48	NaN	76 522 minus strand Troponin C [Crassostrea gigas]; 173 619 minus strand Troponin C [Crassostrea gigas]; 173 619
	11	307	NaN	3 347 beta-actin [Meretrix meretrix]; 3 215 Actin-2 OS=Onchocerca volvulus GN=act-2b PE=3 SV=1
	2	8	NaN	30 536 putative mitochondrial ATP synthase [Haliotis discus discus]; 27 620 putative mitochondrial ATP synthase [H
	2	3	NaN	" 3475 4188 minus strand Heat shock 70 kDa protein cognate, putative [Pediculus humanus corporis] gi"
	2	8	NaN	99 3011 Eukaryotic translation initiation factor 3 subunit A [Crassostrea gigas]

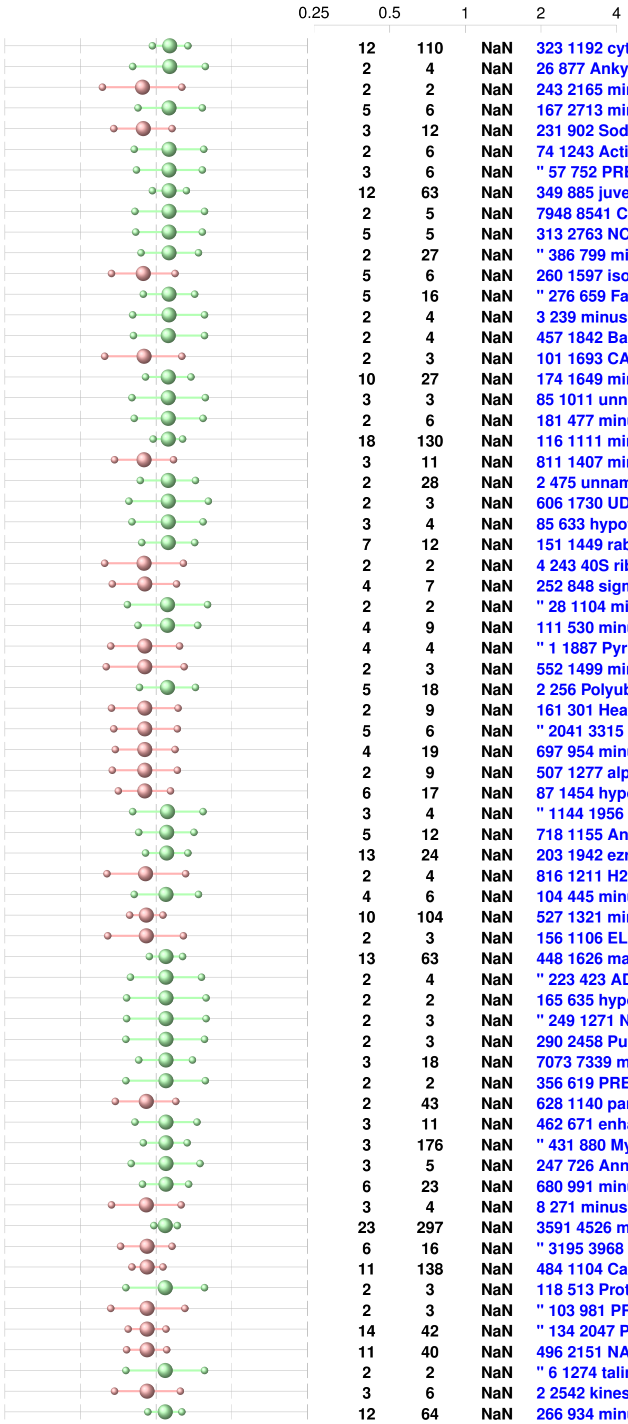

Peptides	Spectra	Coverage	Protein
12	110	NaN	323 1192 cytosolic malate dehydrogenase [Lottia scabra]; 323 1192 cytosolic malate dehydrogenase [Lottia scabra];
2	4	NaN	26 877 Ankyrin-2 [Crassostrea gigas]
2	2	NaN	243 2165 minus strand Elongation factor 2 OS=Drosophila melanogaster GN=Ef2b PE=2 SV=4
5	6	NaN	167 2713 minus strand Major vault protein [Crassostrea gigas]
3	12	NaN	231 902 Sodium- and chloride-dependent GABA transporter ine [Crassostrea gigas]
2	6	NaN	74 1243 Actin-related protein 2 [Crassostrea gigas]
3	6	NaN	" 57 752 PREDICTED: adenylate kinase 2, mitochondrial-like isoform 2 [Callithrix jacchus]"
12	63	NaN	349 885 juvenile hormone diol kinase [Danaus plexippus]; 349 885 juvenile hormone diol kinase [Danaus plexippus]
2	5	NaN	7948 8541 Collagen alpha-3(VI) chain [Crassostrea gigas]
5	5	NaN	313 2763 NCAM-related cell adhesion molecule precursor [Aplysia californica] gi
2	27	NaN	" 386 799 minus strand Myosin regulatory light chain LC-2, mantle muscle OS=Todarodes pacificus PE=1 SV=1; 656
5	6	NaN	260 1597 isocitrate dehydrogenase [Mytilus galloprovincialis]; 260 1597 isocitrate dehydrogenase [Mytilus gallopro
5	16	NaN	" 276 659 Fatty acid-binding protein, adipocyte [Crassostrea gigas]; 426 809 Fatty acid-binding protein, adipocyte [C
2	4	NaN	3 239 minus strand alcohol dehydrogenase class 3 [Branchiostoma lanceolatum]
2	4	NaN	457 1842 Basement membrane-specific heparan sulfate proteoglycan core protein [Crassostrea gigas]; 457 1842 Ba
2	3	NaN	101 1693 CALNUC [Spodoptera frugiperda]; 141 1325 Nucleobindin-2 [Crassostrea gigas]
10	27	NaN	174 1649 minus strand Collagen alpha-1(XII) chain [Crassostrea gigas]; 135 1610 Collagen alpha-1(XII) chain [Crasso
3	3	NaN	85 1011 unnamed protein product [Mus musculus]
2	6	NaN	181 477 minus strand Zgc:56530 [Danio rerio]; 185 481 minus strand Zgc:56530 [Danio rerio]
18	130	NaN	116 1111 minus strand cytosolic malate dehydrogenase [Lottia scabra]
3	11	NaN	811 1407 minus strand mitochondrial malate dehydrogenase 2 [Eospalax baileyi]
2	28	NaN	2 475 unnamed protein product [Tetraodon nigroviridis]
2	3	NaN	606 1730 UDP-glucuronosyltransferase 2C1 precursor [Oryctolagus cuniculus]
3	4	NaN	85 633 hypothetical protein [Amblyomma maculatum]
7	12	NaN	151 1449 rab GDP-dissociation inhibitor [Branchiostoma belcheri]
2	2	NaN	4 243 40S ribosomal protein S13 OS=Caenorhabditis elegans GN=rps-13 PE=2 SV=2; 4 243 40S ribosomal protein S1
4	7	NaN	252 848 sigma class glutathione-s-transferase 2 [Haliotis discus discus]
2	2	NaN	" 28 1104 minus strand Pyruvate dehydrogenase E1 component subunit beta, mitochondrial [Crassostrea gigas]"
4	9	NaN	111 530 minus strand Nucleoside diphosphate kinase B OS=Homo sapiens GN=NME2 PE=1 SV=1
4	4	NaN	" 1 1887 Pyruvate carboxylase, mitochondrial [Crassostrea gigas]"
2	3	NaN	552 1499 minus strand uncharacterized protein LOC100158466 [Xenopus (Silurana) tropicalis] gi
5	18	NaN	2 256 Polyubiquitin (Fragment) OS=Xenopus laevis PE=1 SV=2; 20 388 large subunit ribosomal protein L40e [Clonor
2	9	NaN	161 301 Heat shock protein beta-6 OS=Mus musculus GN=Hspb6 PE=1 SV=1; 167 310 Heat shock protein beta-1 OS=
5	6	NaN	" 2041 3315 minus strand Myosin-10 OS=Mus musculus GN=Myh10 PE=1 SV=2; 2041 3345 minus strand Myosin hea
4	19	NaN	697 954 minus strand Peptidyl-prolyl cis-trans isomerase B [Crassostrea gigas]; 614 877 Peptidyl-prolyl cis-trans iso
2	9	NaN	507 1277 alpha tubulin [Notothenia coriiceps]
6	17	NaN	87 1454 hypothetical protein BRAFLDRAFT_287788 [Branchiostoma floridae] gi; 117 1469 hypothetical protein BRAF
3	4	NaN	" 1144 1956 minus strand Retinol dehydrogenase 12, partial [Crassostrea gigas]"
5	12	NaN	718 1155 Annexin A7 [Crassostrea gigas]
13	24	NaN	203 1942 ezrin/radixin/moesin [Aplysia californica] gi; 243 1982 minus strand ezrin/radixin/moesin [Aplysia californic
2	4	NaN	816 1211 H2 [Haliotis tuberculata]
4	6	NaN	104 445 minus strand macrophage migration inhibitory factor [Ascaris suum]
10	104	NaN	527 1321 minus strand troponin T [Mizuhopecten yessoensis]; 305 1099 minus strand troponin T [Mizuhopecten yes
2	3	NaN	156 1106 ELAV 2-like protein [Aplysia californica] gi
13	63	NaN	448 1626 matrilin [Biomphalaria glabrata]
2	4	NaN	" 223 423 ADPATP carrier protein; K05863 solute carrier family 25 (mitochondrial adenine nucleotide translocator), m
2	2	NaN	165 635 hypothetical protein CGI_10007639 [Crassostrea gigas]
2	3	NaN	" 249 1271 N(G),N(G)-dimethylarginine dimethylaminohydrolase 1 [Crassostrea gigas]"
2	3	NaN	290 2458 Putative beta-D-xylosidase 5 [Crassostrea gigas]; 299 2428 Putative beta-D-xylosidase 2 [Crassostrea giga
3	18	NaN	7073 7339 minus strand Collagen alpha-3(VI) chain [Crassostrea gigas]
2	2	NaN	356 619 PREDICTED: hypothetical protein LOC100698726 [Oreochromis niloticus]
2	43	NaN	628 1140 paramyosin [Haliotis discus discus]
3	11	NaN	462 671 enhancer protein [Homo sapiens]
3	176	NaN	" 431 880 Myosin essential light chain, striated adductor muscle OS=Aequipecten irradians PE=1 SV=2"
3	5	NaN	247 726 Annexin A7 [Crassostrea gigas]; 286 675 Annexin A7 [Crassostrea gigas]
6	23	NaN	680 991 minus strand PDZ and LIM domain protein 1 [Crassostrea gigas]; 553 864 minus strand PDZ and LIM domai
3	4	NaN	8 271 minus strand Laminin subunit gamma-1 [Crassostrea gigas]
23	297	NaN	3591 4526 minus strand myosin heavy chain cardiac muscle specific isoform 1 [Argopecten irradians]
6	16	NaN	" 3195 3968 Spectrin alpha chain, putative [Pediculus humanus corporis] gi"
11	138	NaN	484 1104 Calponin-2 [Crassostrea gigas]; 309 938 Calponin-2 [Crassostrea gigas]
2	3	NaN	118 513 Protein CAL-5 [Caenorhabditis elegans] gi
2	3	NaN	" 103 981 PREDICTED: ATP synthase, H+ transporting, mitochondrial F1 complex, gamma subunit isoform H (heart)
14	42	NaN	" 134 2047 PREDICTED: succinate dehydrogenase [ubiquinone] flavoprotein subunit, mitochondrial-like isoform 3, p
11	40	NaN	496 2151 NADP-dependent malic enzyme [Crassostrea gigas]
2	2	NaN	" 6 1274 talin, putative [Ixodes scapularis] gi"
3	6	NaN	2 2542 kinesin heavy chain 1 [Aplysia californica] gi; 2 2542 kinesin heavy chain 1 [Aplysia californica] gi
12	64	NaN	266 934 minus strand Kyphoscoliosis peptidase [Crassostrea gigas]

0.25 0.5 1 2 4

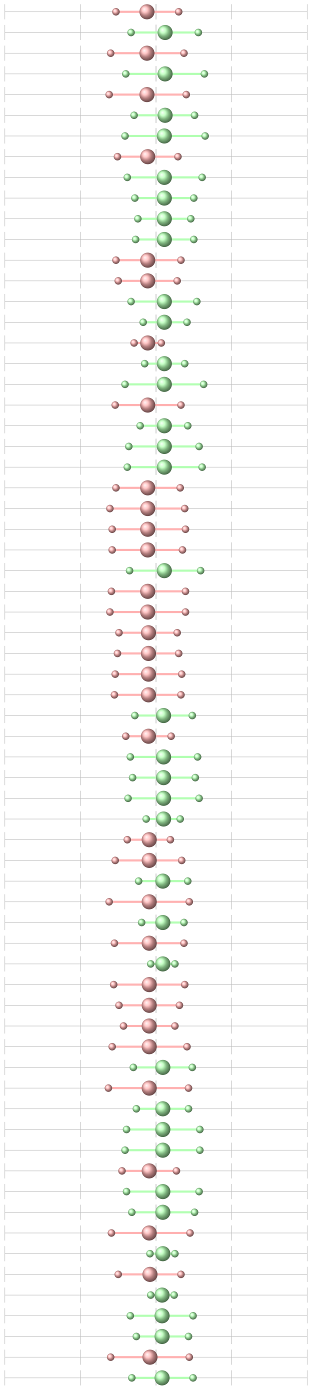

Peptides	Spectra	Coverage	Protein
5	6	NaN	" 65 1576 minus strand Aldehyde dehydrogenase, mitochondrial [Crassostrea gigas]"
3	5	NaN	718 957 minus strand Calponin-1 OS=Gallus gallus GN=CNN1 PE=1 SV=2
2	3	NaN	511 1062 unknown [Dendroctonus ponderosae]; 511 1062 unknown [Dendroctonus ponderosae]
2	2	NaN	1 252 Aquaporin-4 [Crassostrea gigas]
2	5	NaN	316 1512 Alpha-N-acetylgalactosaminidase [Crassostrea gigas]; 282 1478 Alpha-N-acetylgalactosaminidase [Crasso
2	13	NaN	871 1062 Adenylate kinase isoenzyme 5 [Crassostrea gigas]
2	2	NaN	587 811 hemocyanin 2 [Megathura crenulata]
4	8	NaN	2939 3208 minus strand beta-thymosin domain repeat protein CSP24KDa_v1 [Hermissenda crassicornis] gi
2	3	NaN	" 242 1504 Glutamate receptor, ionotropic kainate 3 [Crassostrea gigas]; 239 1501 Glutamate receptor, ionotropic ka
4	9	NaN	79 606 minus strand PREDICTED: similar to Ras-like protein 3 precursor (Protein roughened) [Tribolium castaneum]
6	16	NaN	18 647 minus strand ATP synthase-like protein [Biomphalaria glabrata]
7	9	NaN	185 1258 minus strand Severin [Crassostrea gigas]
2	8	NaN	729 1541 troponin T [Mizuhopecten yessoensis]
2	21	NaN	" 116 352 minus strand troponin I 4 [Clonorchis sinensis]; 491 706 minus strand troponin-I, TnI {C-terminal} [Chlamy
3	6	NaN	247 762 Myophilin [Crassostrea gigas]; 244 759 Myophilin [Crassostrea gigas]; 119 634 Myophilin [Crassostrea giga
8	24	NaN	1200 1547 protein disulfide isomerase [Conus eburneus]
24	81	NaN	51 1295 PREDICTED: phosphoglycerate kinase-like [Nasonia vitripennis]; 97 1341 PREDICTED: phosphoglycerate k
7	64	NaN	176 670 hypothetical protein BRAFLDRAFT_122621 [Branchiostoma floridae] gi
2	2	NaN	228 1553 hypothetical protein CGI_10024545 [Crassostrea gigas]; 228 1553 hypothetical protein CGI_10024545 [Cras
4	5	NaN	27 515 heat shock protein 70 [Capsaspora owczarzaki ATCC 30864]; 30 464 minus strand hypothetical protein [Trich
8	18	NaN	98 850 minus strand 14-3-3 protein epsilon [Schistocerca gregaria]
2	8	NaN	426 896 PREDICTED: calmodulin-like [Metaseiulus occidentalis]
3	3	NaN	182 1846 minus strand hypothetical protein DAPPUDRAFT_302108 [Daphnia pulex]; 110 1774 minus strand hypothe
3	7	NaN	98 1012 minus strand RecName: Full=Substrate-specific endoprotease Tex31; Flags: Precursor gi|32187775|emb|C
2	3	NaN	337 474 minus strand heat shock protein 22 isoform 1 [Ruditapes philippinarum]
3	3	NaN	2 2248 PREDICTED: heat shock 70 kDa protein 4-like [Oreochromis niloticus]; 2 2227 PREDICTED: heat shock 70 kD
3	4	NaN	" 166 1248 PREDICTED: probable pyruvate dehydrogenase E1 component subunit alpha, mitochondrial-like [Metase
2	4	NaN	" 656 787 collagen alpha-1(XII) chain-like; K08132 collagen, type XII, alpha"
2	4	NaN	2302 2547 minus strand hemocyanin isoform 1 [Haliotis diversicolor]; 816 1211 H2 [Haliotis tuberculata]
2	3	NaN	172 678 Myosin regulatory light chain sqh [Crassostrea gigas]
3	13	NaN	311 928 minus strand peroxiredoxin 3 [Clonorchis sinensis] gi; 288 905 minus strand peroxiredoxin 3 [Clonorchis si
2	13	NaN	764 1015 minus strand heat shock protein beta-1 [Trichinella spiralis] gi
4	5	NaN	58 732 glutathione-s-transferase [Haliotis discus discus]
3	6	NaN	387 1064 minus strand PREDICTED: hypothetical protein [Hydra magnipapillata]; 275 952 minus strand PREDICTED
4	11	NaN	145 459 fk506-binding protein 2 [Papilio xuthus]; 237 551 fk506-binding protein 2 [Papilio xuthus]
9	22	NaN	2 811 voltage-dependent anion channel 2-like protein [Haliotis diversicolor]; 2 811 voltage-dependent anion channel
2	6	NaN	410 619 minus strand Guanine nucleotide-binding protein subunit gamma-1 [Crassostrea gigas]
4	7	NaN	75 803 hypothetical protein DAPPUDRAFT_231350 [Daphnia pulex]
3	4	NaN	236 733 Spectrin alpha chain [Crassostrea gigas]
11	69	NaN	3 434 minus strand myosin essential light chain [Haliotis discus discus]
9	35	NaN	" 112 1320 Aspartate aminotransferase, cytoplasmic [Crassostrea gigas]"
5	5	NaN	205 2490 minus strand glucose-regulated protein 94 [Crassostrea gigas]
5	22	NaN	89 838 Putative phosphoglycerate mutase [Crassostrea gigas]; 89 838 Putative phosphoglycerate mutase [Crassost
2	2	NaN	106 1701 T-complex protein 1 subunit beta [Crassostrea gigas]
7	30	NaN	472 1551 minus strand hypothetical protein [Amblyomma maculatum]
2	6	NaN	124 396 minus strand 40S ribosomal protein S10 OS=Lumbricus rubellus GN=RPS10 PE=2 SV=1
22	158	NaN	552 1886 minus strand intermediate filament protein [Biomphalaria glabrata]
3	7	NaN	605 1153 Peptidyl-prolyl cis-trans isomerase B [Crassostrea gigas]; 1046 1630 Peptidyl-prolyl cis-trans isomerase B
5	13	NaN	280 726 minus strand RecName: Full=Calmodulin; Short=CaM gi|14588595|dbj|BAB61794.1| calmodulin [Metridium
6	18	NaN	158 862 X-box binding protein [Haliotis discus discus]
2	3	NaN	212 1336 6-phosphofructokinase [Crassostrea gigas]
5	8	NaN	177 791 Ras-related protein Rab-1A [Haliotis discus discus]
2	2	NaN	11 244 minus strand Tubulin alpha chain (Fragment) OS=Lytechinus pictus PE=3 SV=1
4	17	NaN	274 666 Calmodulin-4 [Heterocephalus glaber]
3	4	NaN	290 2458 Putative beta-D-xylosidase 5 [Crassostrea gigas]
2	3	NaN	77 502 hypothetical protein CGI_10008263 [Crassostrea gigas]
7	12	NaN	" 134 1567 minus strand 6-phosphogluconate dehydrogenase, decarboxylating [Crassostrea gigas]; 134 1573 minus
3	3	NaN	14 1642 minus strand chaperonin containing tcp1 [Haliotis discus discus]
4	7	NaN	144 800 minus strand PREDICTED: protein F37C4.5-like [Amphimedon queenslandica]
2	2	NaN	113 1402 minus strand S-adenosylhomocysteine hydrolase [Crassostrea ariakensis]; 113 1402 minus strand S-aden
20	186	NaN	288 1487 minus strand putative procollagen type VI alpha 4 [Haliotis diversicolor]
2	12	NaN	" 3 587 elongation factor 1 alpha, partial [Phorbas sp. nov. SB-2012]"
35	168	NaN	114 2633 Filamin-A [Crassostrea gigas]; 135 2654 Filamin-A [Crassostrea gigas]
6	6	NaN	4 675 Laminin subunit gamma-1 [Crassostrea gigas]
3	28	NaN	" 926 1189 minus strand Myosin heavy chain, striated muscle [Crassostrea gigas]"
2	2	NaN	68 667 minus strand hypothetical protein BRAFLDRAFT_272416 [Branchiostoma floridae] gi; 68 667 minus strand h
2	13	NaN	160 588 hypothetical protein CGI_10001640 [Crassostrea gigas]

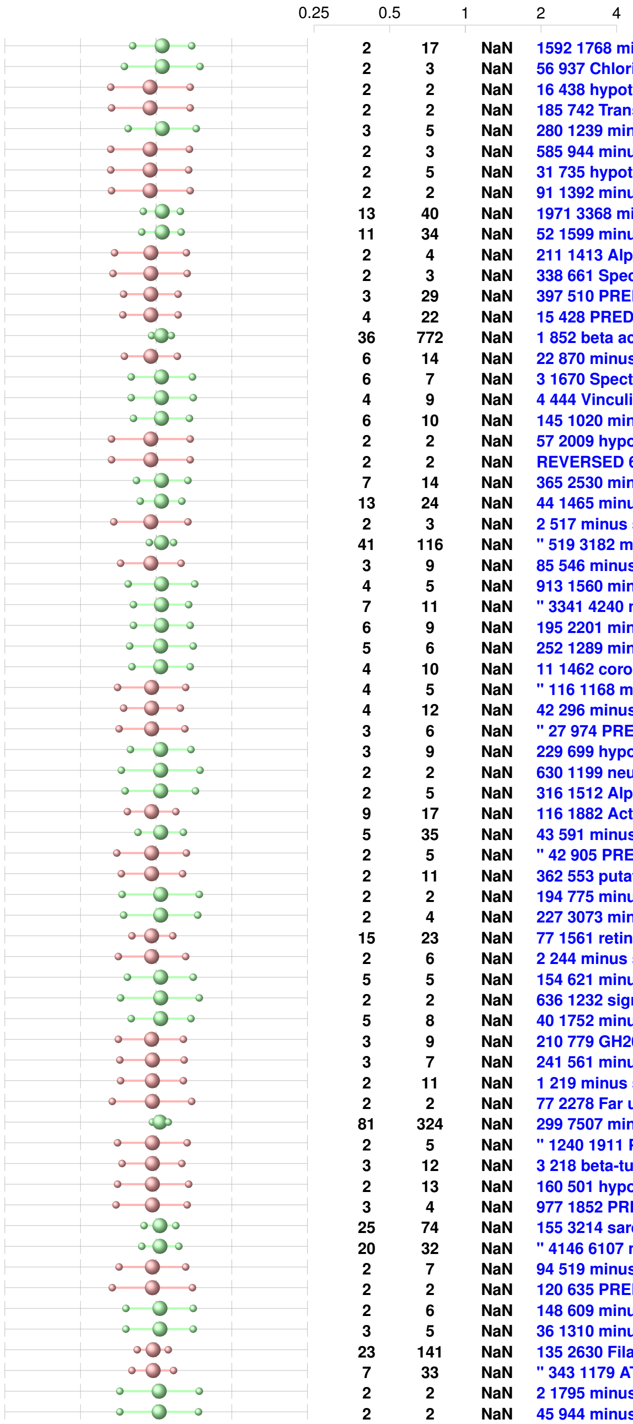

Peptides	Spectra	Coverage	Protein
2	17	NaN	1592 1768 minus strand beta-actin [Diabolocatantops pinguis]
2	3	NaN	56 937 Chloride intracellular channel exc-4 [Crassostrea gigas]
2	2	NaN	16 438 hypothetical protein KGM_06034 [Danaus plexippus]; 16 438 hypothetical protein KGM_06034 [Danaus plexip
2	2	NaN	185 742 Transmembrane protein Tmp21 [Crassostrea gigas]; 1 444 minus strand Transmembrane protein Tmp21 [Cr
3	5	NaN	280 1239 minus strand PREDICTED: smoothelin isoform 2 [Oryctolagus cuniculus]; 280 1239 minus strand PREDICT
2	3	NaN	585 944 minus strand Protein disulfide-isomerase A6 [Crassostrea gigas]
2	5	NaN	31 735 hypothetical protein BRAFLDRAFT_78603 [Branchiostoma floridae] gi; 55 735 hypothetical protein BRAFLDF
2	2	NaN	91 1392 minus strand hypothetical protein AND_03103 [Anopheles darlingi]
13	40	NaN	1971 3368 minus strand Kyphoscoliosis peptidase [Crassostrea gigas]; 1767 3158 minus strand hillarin [Hirudo med
11	34	NaN	52 1599 minus strand ATP synthase beta subunit [Pinctada fucata]
2	4	NaN	211 1413 Alpha-N-acetylgalactosaminidase [Crassostrea gigas]
2	3	NaN	338 661 Spectrin alpha chain [Crassostrea gigas]
3	29	NaN	397 510 PREDICTED: actin-5C-like [Megachile rotundata]
4	22	NaN	15 428 PREDICTED: histone H2A.V-like [Apis florea]
36	772	NaN	1 852 beta actin-like 1 protein [Macrostomum lignano]
6	14	NaN	22 870 minus strand PREDICTED: alcohol dehydrogenase class-3 [Sarcophilus harrisii]
6	7	NaN	3 1670 Spectrin alpha chain [Crassostrea gigas]
4	9	NaN	4 444 Vinculin [Crassostrea gigas]
6	10	NaN	145 1020 minus strand PREDICTED: inorganic pyrophosphatase-like [Saccoglossus kowalevskii]
2	2	NaN	57 2009 hypothetical protein BRAFLDRAFT_285279 [Branchiostoma floridae] gi; 3 1973 hypothetical protein BRAFL
2	2	NaN	REVERSED 68 358 endonuclease-reverse transcriptase [Danaus plexippus]
7	14	NaN	365 2530 minus strand calpain B [Gecarcinus lateralis]
13	24	NaN	44 1465 minus strand Adenylyl cyclase-associated protein 1 [Crassostrea gigas]; 52 1473 minus strand Adenylyl cyc
2	3	NaN	2 517 minus strand Anhydro-N-acetylmuramic acid kinase [Crassostrea gigas]
41	116	NaN	" 519 3182 minus strand Alpha-actinin, sarcomeric [Crassostrea gigas]"
3	9	NaN	85 546 minus strand RecName: Full=Eukaryotic translation initiation factor 5A; Short=eIF-5A gi|51702279|sp|P6292
4	5	NaN	913 1560 minus strand Calcium-dependent protein kinase isoform 2 [Crassostrea gigas]
7	11	NaN	" 3341 4240 minus strand nonmuscle myosin II, partial [Aplysia californica]"
6	9	NaN	195 2201 minus strand hypothetical protein DAPPUDRAFT_315656 [Daphnia pulex]
5	6	NaN	252 1289 minus strand PDZ and LIM domain protein 3 OS=Gallus gallus GN=PDLIM3 PE=1 SV=1
4	10	NaN	11 1462 coronin [Schistosoma mansoni]
4	5	NaN	" 116 1168 minus strand hypothetical protein CGI_10014553, partial [Crassostrea gigas]"
4	12	NaN	42 296 minus strand twitchin [Mytilus galloprovincialis]
3	6	NaN	" 27 974 PREDICTED: succinyl-CoA ligase [GDP-forming] subunit alpha, mitochondrial-like [Oreochromis niloticus];
3	9	NaN	229 699 hypothetical protein CGI_10017744 [Crassostrea gigas]; 199 669 hypothetical protein CGI_10017744 [Crasso
2	2	NaN	630 1199 neuronal calcium sensor-1 [Lymnaea stagnalis]
2	5	NaN	316 1512 Alpha-N-acetylgalactosaminidase [Crassostrea gigas]; 282 1568 Alpha-N-acetylgalactosaminidase [Crasso
9	17	NaN	116 1882 Actin-interacting protein 1 [Crassostrea gigas]
5	35	NaN	43 591 minus strand cyclophilin type peptidyl-prolyl cis-trans isomerase [Glossina morsitans morsitans]
2	5	NaN	" 42 905 PREDICTED: enoyl-CoA hydratase, mitochondrial-like isoform 1 [Oreochromis niloticus]"
2	11	NaN	362 553 putative RNA-binding protein [Haliotis diversicolor]
2	2	NaN	194 775 minus strand Collagen alpha-1(XV) chain [Harpegnathos saltator]
2	4	NaN	227 3073 minus strand Lysosomal alpha-mannosidase [Crassostrea gigas]
15	23	NaN	77 1561 retinal dehydrogenase [Lymnaea stagnalis]; 69 1553 retinal dehydrogenase [Lymnaea stagnalis]; 308 1747 r
2	6	NaN	2 244 minus strand hypothetical protein KGM_08771 [Danaus plexippus]
5	5	NaN	154 621 minus strand Stress response protein nhaX [Crassostrea gigas]
2	2	NaN	636 1232 sigma class glutathione-s-transferase 3 [Haliotis discus discus]; 963 1559 sigma class glutathione-s-transf
5	8	NaN	40 1752 minus strand heat shock protein 60 [Biomphalaria glabrata]; 40 1752 minus strand heat shock protein 60 [B
3	9	NaN	210 779 GH20056 [Drosophila grimshawi] gi; 216 785 minus strand GH20056 [Drosophila grimshawi] gi; 210 779 GH2
3	7	NaN	241 561 minus strand thioredoxin [Crassostrea ariakensis]
2	11	NaN	1 219 minus strand PREDICTED: nucleoside diphosphate kinase 3-like [Pan troglodytes]
2	2	NaN	77 2278 Far upstream element-binding protein 3 [Crassostrea gigas]
81	324	NaN	299 7507 minus strand filamin [Clonorchis sinensis]
2	5	NaN	" 1240 1911 PREDICTED: ATP synthase subunit alpha, mitochondrial-like [Anolis carolinensis]"
3	12	NaN	3 218 beta-tubulin [Fasciola hepatica] gi
2	13	NaN	160 501 hypothetical protein CGI_10001640 [Crassostrea gigas]; 160 588 hypothetical protein CGI_10001640 [Crasso
3	4	NaN	977 1852 PREDICTED: similar to prohibitin [Tribolium castaneum] gi
25	74	NaN	155 3214 sarco/endoplasmic reticulum calcium ATPase isoform B [Pinctada fucata]; 132 3110 sarco/endoplasmic ret
20	32	NaN	" 4146 6107 minus strand nonmuscle myosin II, partial [Aplysia californica]"
2	7	NaN	94 519 minus strand hypothetical protein BRAFLDRAFT_115029 [Branchiostoma floridae] gi
2	2	NaN	120 635 PREDICTED: similar to collagen type VI alpha 6 [Ciona intestinalis]; 120 635 minus strand PREDICTED: simi
2	6	NaN	148 609 minus strand Titin [Crassostrea gigas]
3	5	NaN	36 1310 minus strand elongation factor-1 gamma [Diguetia canities]
23	141	NaN	135 2630 Filamin-A [Crassostrea gigas]; 118 2613 Filamin-A [Crassostrea gigas]; 157 2652 Filamin-A [Crassostrea g
7	33	NaN	" 343 1179 ATP synthase subunit alpha, mitochondrial OS=Pongo abelii GN=ATP5A1 PE=2 SV=1"
2	2	NaN	2 1795 minus strand Plectin-1 [Crassostrea gigas]
2	2	NaN	45 944 minus strand unnamed protein product [Mus musculus] gi; 5 334 minus strand receptor of activated protein l

0.25 0.5 1 2 4

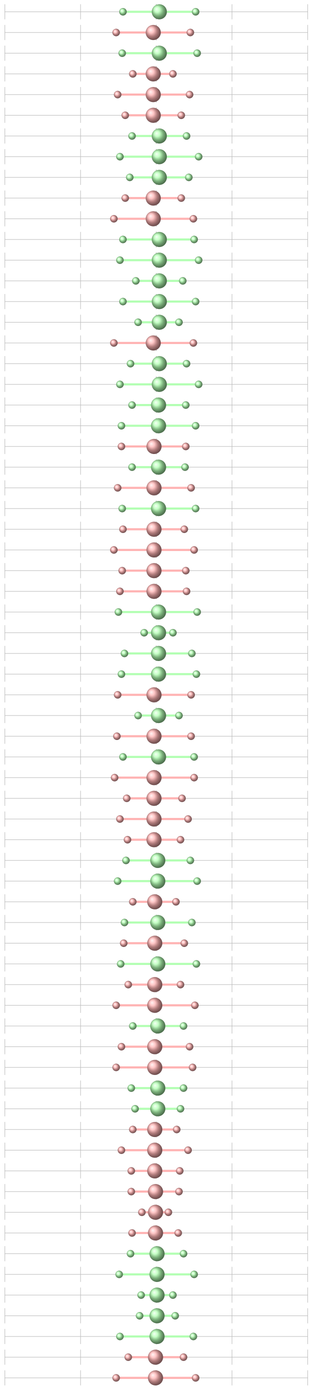

Peptides	Spectra	Coverage	Protein
2	4	NaN	160 393 minus strand PREDICTED: cysteine-rich protein 1-like [Megachile rotundata]; 148 381 PREDICTED: cysteine
2	3	NaN	5 841 minus strand Spectrin alpha chain [Crassostrea gigas]
2	3	NaN	35 271 PREDICTED: SH3 domain-binding glutamic acid-rich-like protein 3-like [Anolis carolinensis]; 35 271 PREDICT
14	59	NaN	155 3214 sarco/endoplasmic reticulum calcium ATPase isoform B [Pinctada fucata]; 155 3214 minus strand sarco/en
2	4	NaN	1139 1378 Titin [Crassostrea gigas]; 1116 1355 Titin [Crassostrea gigas]; 1139 1378 Titin [Crassostrea gigas]; 1139 1
5	13	NaN	" 220 627 Fatty acid-binding protein, brain [Crassostrea gigas]"
6	12	NaN	93 1034 Annexin A7 [Crassostrea gigas]; 73 1014 Annexin A7 [Crassostrea gigas]
2	2	NaN	209 781 minus strand high mobility group protein 1 [Biomphalaria glabrata]; 277 771 high mobility group protein 1 [I
4	11	NaN	65 490 PREDICTED: cofilin-2 [Otolemur garnettii]
5	11	NaN	346 702 minus strand PREDICTED: galectin-4-like [Anolis carolinensis]
2	2	NaN	410 709 calmodulin-like; K02183 calmodulin
2	12	NaN	4 849 minus strand elongation factor 1-alpha [Cryptosporidium muris RN66]
2	2	NaN	138 713 Rac [Aplysia californica] gi; 125 691 Rac [Aplysia californica] gi; 149 871 minus strand Rac [Aplysia californ
6	23	NaN	273 1304 Guanine nucleotide-binding protein subunit beta [Crassostrea gigas]
3	3	NaN	130 2145 minus strand Glucosamine–fructose-6-phosphate aminotransferase [isomerizing] 1 [Crassostrea gigas]; 1
7	48	NaN	835 1083 minus strand Calmodulin [Crassostrea gigas]
2	2	NaN	36 770 minus strand Endoplasmic reticulum protein ERp29 [Crassostrea gigas]
4	16	NaN	" 64 1332 Flotillin-1, putative [Pediculus humanus corporis] gi"
2	2	NaN	170 460 Spectrin beta chain [Crassostrea gigas]
8	11	NaN	114 1001 Vinculin [Crassostrea gigas]
2	3	NaN	324 1982 minus strand Pikachurin [Crassostrea gigas]
3	10	NaN	180 560 calreticulin precursor [Aplysia californica] gi; 180 545 calreticulin precursor [Aplysia californica] gi
7	13	NaN	17 1681 minus strand Glucose-6-phosphate isomerase [Crassostrea gigas]
3	3	NaN	42 434 cytochrome b5 [Haliotis discus discus]; 42 434 cytochrome b5 [Haliotis discus discus]
2	3	NaN	112 930 calcineurin A [Haliotis discus discus]
4	8	NaN	102 887 hypothetical protein DAPPUDRAFT_304712 [Daphnia pulex]
2	2	NaN	22 369 Protein farnesyltransferase/geranylgeranyltransferase type-1 subunit alpha [Crassostrea gigas]
5	8	NaN	227 2920 minus strand Lysosomal alpha-mannosidase [Crassostrea gigas]; 227 3073 minus strand Lysosomal alpha
3	7	NaN	" 698 1225 minus strand actin related protein 2/3 complex, 21 kDa subunit [Crassostrea gigas]"
2	2	NaN	91 2658 minus strand puromycin-sensitive aminopeptidase [Saccoglossus kowalevskii]
14	146	NaN	362 874 minus strand Transgelin-2 [Crassostrea gigas]
3	5	NaN	131 541 minus strand nervous system adducin [Aplysia californica] gi
2	3	NaN	160 387 small nuclear ribonucleoprotein polypeptide G protein [Crassostrea ariakensis]; 180 407 small nuclear ribon
2	6	NaN	43 525 Reticulon-4 [Crassostrea gigas]; 143 757 Reticulon-4 [Crassostrea gigas]; 43 564 Reticulon-4 [Crassostrea gi
7	137	NaN	2 328 cytoskeletal actin 2 [Molgula oculata]
3	3	NaN	188 1021 hypothetical protein BRAFLDRAFT_214346 [Branchiostoma floridae] gi
2	5	NaN	184 438 PREDICTED: similar to CG14235 CG14235-PA [Tribolium castaneum] gi
2	2	NaN	" 40 813 minus strand NADH dehydrogenase [ubiquinone] iron-sulfur protein 3, mitochondrial [Crassostrea gigas]"
5	13	NaN	594 1253 peroxiredoxin 6 [Eriocheir sinensis]
3	5	NaN	" 169 792 minus strand rho GDP dissociation inhibitor, putative [Ixodes scapularis] gi"
7	13	NaN	390 2267 neurofilament protein NF70 [Helix aspersa]; 318 2195 neurofilament protein NF70 [Helix aspersa]
4	6	NaN	582 2135 sulfatase 1A precursor [Haliotis discus discus]
2	2	NaN	" 45 506 minus strand PREDICTED: dihydrolipoyl dehydrogenase, mitochondrial [Monodelphis domestica]"
8	32	NaN	248 2053 PDZ and LIM domain protein 5 [Crassostrea gigas]
3	5	NaN	87 1997 Transketolase-like protein 2 [Crassostrea gigas]; 87 1997 Transketolase-like protein 2 [Crassostrea gigas]; 8
3	11	NaN	900 1142 minus strand heat shock protein 20 [Cyclina sinensis]
2	3	NaN	253 765 cytochrome c oxidase subunit IV [Urechis caupo]
7	13	NaN	783 1463 minus strand twitchin [Mytilus galloprovincialis]; 783 1463 minus strand twitchin [Mytilus galloprovincialis
2	2	NaN	1363 1527 Protein disulfide-isomerase A3 [Crassostrea gigas]
8	14	NaN	689 1984 hypothetical protein BRAFLDRAFT_78257 [Branchiostoma floridae] gi
4	5	NaN	25 405 minus strand Elongation factor 1-delta [Anoplopoma fimbria]
2	3	NaN	1 222 cAMP-dependent protein kinase regulatory subunit [Aplysia californica] gi
4	31	NaN	768 980 muscle LIM protein isoform 1 [Danaus plexippus]
12	20	NaN	71 1321 minus strand Vinculin [Crassostrea gigas]
4	134	NaN	3 263 minus strand beta-actin [Urechis unicinctus]
2	8	NaN	76 822 14-3-3 protein [Heterodera glycines]; 76 822 minus strand 14-3-3 protein [Heterodera glycines]
4	40	NaN	63 443 PREDICTED: RAS protein activator like 1-like [Saccoglossus kowalevskii]
6	20	NaN	429 1013 PDZ domain protein [Ixodes scapularis] gi
16	245	NaN	6 317 myosin heavy chain cardiac muscle specific isoform 2 [Argopecten irradians]
7	23	NaN	181 744 unknown [Dendroctonus ponderosae]
9	11	NaN	95 2506 Transitional endoplasmic reticulum ATPase [Crassostrea gigas]
3	3	NaN	970 1854 fasciclin-like protein precursor [Aplysia californica] gi
18	62	NaN	34 1122 Fructose-bisphosphate aldolase [Crassostrea gigas]
12	107	NaN	260 892 minus strand calponin [Haliotis diversicolor]; 260 892 minus strand calponin [Haliotis diversicolor]
3	3	NaN	771 1505 O-methyltransferase family 3 [Dicentrarchus labrax]
8	10	NaN	116 1321 PREDICTED: similar to aspartate aminotransferase [Tribolium castaneum] gi
2	2	NaN	145 1524 PREDICTED: aspartyl aminopeptidase-like [Oreochromis niloticus]

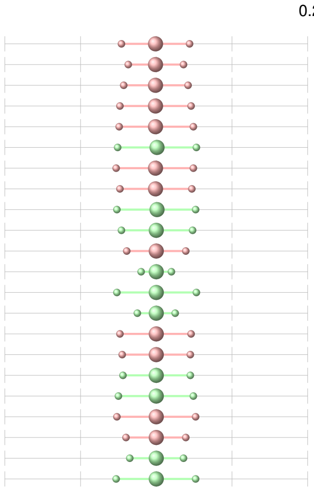

Peptides	Spectra	Coverage	Protein
3	5	NaN	112 1050 Enolase-phosphatase E1 [Crassostrea gigas]; 112 1050 Enolase-phosphatase E1 [Crassostrea gigas]
6	12	NaN	622 2733 PREDICTED: protein-glutamine gamma-glutamyltransferase K isoform 1 [Danio rerio]; 423 2498 PREDICTE
3	7	NaN	981 1379 minus strand Multimerin-1 [Crassostrea gigas]; 998 1396 Multimerin-1 [Crassostrea gigas]; 975 1373 Multir
3	4	NaN	5 514 hypothetical protein KGM_05418 [Danaus plexippus]
2	3	NaN	4365 4553 minus strand Basement membrane-specific heparan sulfate proteoglycan core protein [Crassostrea gigas
2	2	NaN	1 264 Spectrin beta chain [Crassostrea gigas]
2	2	NaN	69 779 PREDICTED: 15-hydroxyprostaglandin dehydrogenase [NAD+] [Gallus gallus]
2	4	NaN	27 722 Spectrin beta chain OS=Drosophila melanogaster GN=beta-Spec PE=1 SV=2; 27 722 Spectrin beta chain OS=
2	2	NaN	" 492 3473 PREDICTED: 2-oxoglutarate dehydrogenase, mitochondrial isoform 1 [Sarcophilus harrisii]; 492 3458 PR
3	4	NaN	150 1511 Tensin OS=Gallus gallus GN=TNS PE=1 SV=2
7	8	NaN	" 1 1563 Putative methylmalonate-semialdehyde dehydrogenase [acylating], mitochondrial [Crassostrea gigas]"
13	135	NaN	2881 3411 myosin heavy chain [Todarodes pacificus]; 2915 3445 myosin heavy chain [Todarodes pacificus]
2	5	NaN	49 747 PREDICTED: elongation factor 1-delta-like [Strongylocentrotus purpuratus]; 49 819 PREDICTED: elongation f
9	62	NaN	373 579 myosin heavy chain [Argopecten irradians] gi
2	6	NaN	605 1153 Peptidyl-prolyl cis-trans isomerase B [Crassostrea gigas]; 1046 1630 Peptidyl-prolyl cis-trans isomerase B
4	5	NaN	125 754 minus strand peptidyl prolyl cis-trans isomerase B [Conus novaehollandiae]
4	5	NaN	63 1865 Dolichyl-diphosphooligosaccharide–protein glycosyltransferase subunit 1 [Crassostrea gigas]; 63 1865 Dol
2	3	NaN	125 550 minus strand ribosomal protein S20 [Chlamys farreri]; 125 550 minus strand ribosomal protein S20 [Chlamy
2	2	NaN	303 1793 PREDICTED: matrix metalloproteinase 19 isoform rasi-1 preproprotein-like [Saccoglossus kowalevskii]
3	10	NaN	549 821 minus strand Calponin-2 [Crassostrea gigas]
4	15	NaN	835 1116 tropomyosin [Ilyanassa obsoleta]
2	2	NaN	242 1834 Regulator of differentiation 1 [Crassostrea gigas]; 458 2050 minus strand Regulator of differentiation 1 [Cra

4.2 Proteins Identified by a Single Peptide

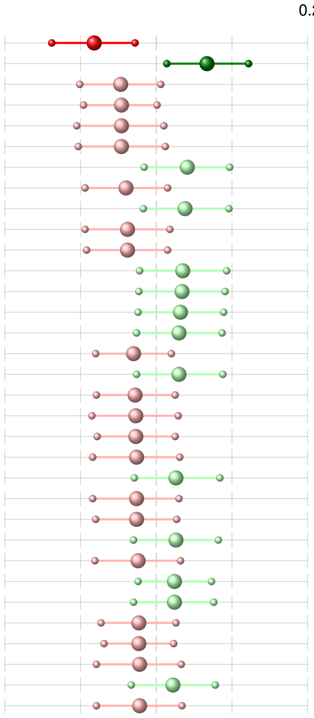

Peptides	Spectra	Coverage	Protein
1	2	NaN	3788 7885 minus strand Filamin-C [Crassostrea gigas]
1	2	NaN	704 817 minus strand expressed hypothetical protein [Trichoplax adhaerens] gi; 688 801 minus strand Lactoylglutat
1	2	NaN	360 764 minus strand Peptidyl-prolyl cis-trans isomerase A OS=Dictyostelium discoideum GN=ppiA PE=1 SV=1
1	5	NaN	361 549 minus strand 32 kDa beta-galactoside-binding lectin [Ascaris suum]
1	2	NaN	118 513 Protein CAL-5 [Caenorhabditis elegans] gi
1	1	NaN	176 667 hypothetical protein CGI_10018452 [Crassostrea gigas]; 176 634 hypothetical protein CGI_10018452 [Crasso
1	1	NaN	66 947 Glyoxalase domain-containing protein 4 [Crassostrea gigas]
1	2	NaN	942 1385 Golgi-associated plant pathogenesis-related protein 1 [Crassostrea gigas]; 338 817 Golgi-associated plant
1	1	NaN	189 353 40S ribosomal protein S12 [Crassostrea gigas]
1	1	NaN	1732 2079 minus strand PREDICTED: annexin A7-like [Ornithorhynchus anatinus]
1	2	NaN	971 1963 Spectrin alpha chain [Crassostrea gigas]; 972 1979 Spectrin alpha chain [Crassostrea gigas]
1	1	NaN	2 2572 Glycogen debranching enzyme [Crassostrea gigas]
1	1	NaN	258 629 Glycogenin-1 OS=Oryctolagus cuniculus GN=GYG1 PE=1 SV=3
1	1	NaN	" 137 1549 minus strand Serine hydroxymethyltransferase, mitochondrial [Crassostrea gigas]; 205 1608 minus stran
1	1	NaN	336 641 minus strand malate dehydrogenase [Schistosoma japonicum]
1	3	NaN	91 1326 minus strand Adipocyte plasma membrane-associated protein [Crassostrea gigas]; 107 1273 minus strand A
1	1	NaN	3 470 minus strand Spectrin beta chain [Crassostrea gigas]; 5 451 minus strand Spectrin beta chain [Crassostrea gi
1	3	NaN	266 1123 minus strand mitochondrial malate dehydrogenase precursor [Nucella lapillus]
1	1	NaN	198 821 minus strand sigma class glutathione-s-transferase 2 [Haliotis discus discus]
1	3	NaN	593 862 minus strand mitochondrial ATP synthase coupling factor 6 precursor [Ochlerotatus taeniorhynchus]
1	1	NaN	194 904 Aldo-keto reductase family 1 member B10 [Crassostrea gigas]
1	1	NaN	232 654 hypothetical protein BRAFLDRAFT_87758 [Branchiostoma floridae] gi
1	1	NaN	38 436 Calcium-dependent protein kinase isoform 2 [Crassostrea gigas]
1	2	NaN	62 1174 Tyrosine-protein kinase CSK [Crassostrea gigas]; 62 1174 minus strand Tyrosine-protein kinase CSK [Crass
1	1	NaN	401 1174 minus strand Mechanosensory protein 2 [Crassostrea gigas]
1	1	NaN	4 2400 IQ motif containing GTPase activating protein 2 [Xenopus laevis] gi; 4 2169 IQ motif containing GTPase activ
1	5	NaN	219 428 Peptidyl-prolyl cis-trans isomerase OS=Hemicentrotus pulcherrimus PE=2 SV=1
1	2	NaN	459 1187 PREDICTED: protein-L-isoaspartate(D-aspartate) O-methyltransferase-like isoform 2 [Anolis carolinensis]
1	6	NaN	233 814 Kynurenine formamidase [Crassostrea gigas]; 233 979 Kynurenine formamidase [Crassostrea gigas]; 139 94
1	8	NaN	460 1737 Tubulin beta-4B chain OS=Mus musculus GN=Tubb4b PE=1 SV=1
1	1	NaN	50 577 hypothetical protein BRAFLDRAFT_119799 [Branchiostoma floridae] gi; 50 577 hypothetical protein BRAFLD
1	1	NaN	176 517 PREDICTED: elongin C-like [Saccoglossus kowalevskii]; 115 456 PREDICTED: elongin C-like [Saccoglossus
1	1	NaN	245 1243 Prostaglandin reductase 1 [Crassostrea gigas]

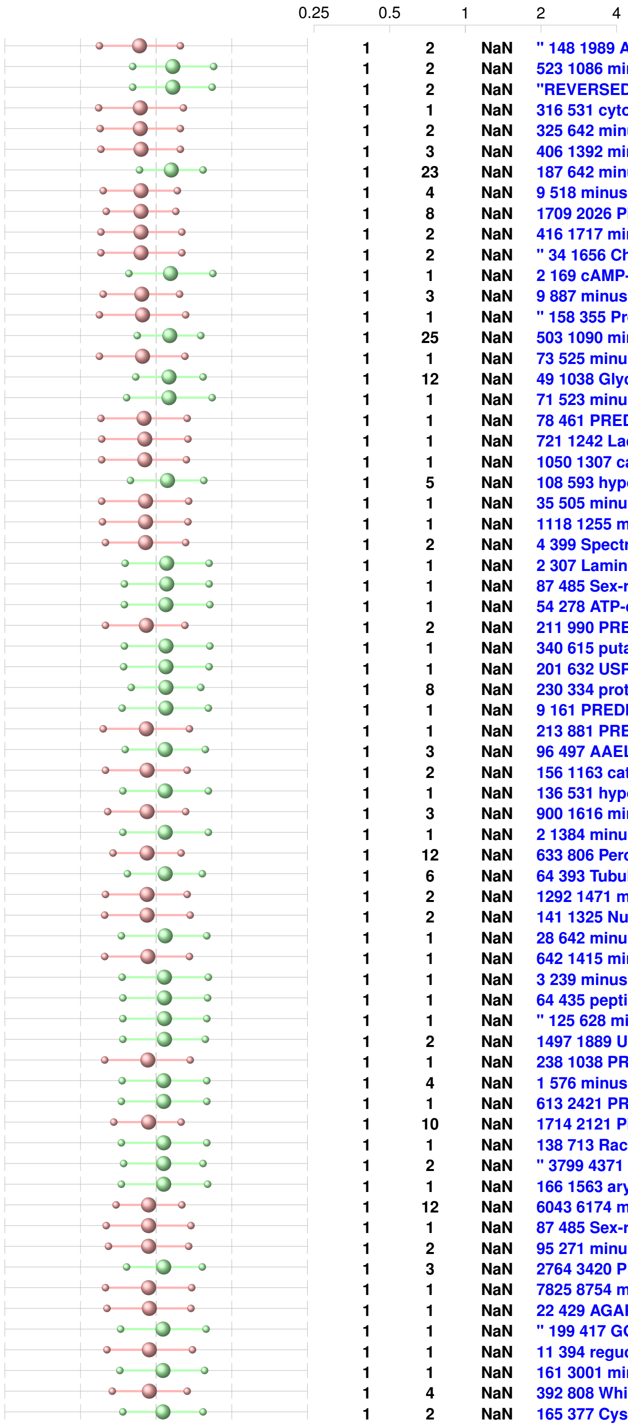

Peptides	Spectra	Coverage	Protein
1	2	NaN	" 148 1989 Acyl-CoA synthetase family member 2, mitochondrial [Crassostrea gigas]; 146 1987 minus strand Acyl-C
1	2	NaN	523 1086 minus strand juvenile hormone diol kinase [Danaus plexippus]
1	2	NaN	"REVERSED 2 1810 minus strand Glutaminyl-tRNA synthetase, partial [Crassostrea gigas]"
1	1	NaN	316 531 cytochrome c oxidase subunit VIIa polypeptide 2 like [Xenopus (Silurana) tropicalis]
1	2	NaN	325 642 minus strand Protein phosphatase 1B [Crassostrea gigas]
1	3	NaN	406 1392 minus strand hypothetical protein CGI_10007007 [Crassostrea gigas]
1	23	NaN	187 642 minus strand PREDICTED: similar to pol-like protein [Tribolium castaneum]
1	4	NaN	9 518 minus strand hemocyanin isoform 1 [Nucula nucleus]
1	8	NaN	1709 2026 Phosphoenolpyruvate carboxykinase [GTP] [Crassostrea gigas]
1	2	NaN	416 1717 minus strand T-complex protein 1 subunit delta OS=Aedes triseriatus PE=2 SV=1
1	2	NaN	" 34 1656 Chaperonin containing TCP1, subunit 7 (eta) [Danio rerio] gi; 34 1656 Chaperonin containing TCP1, subun
1	1	NaN	2 169 cAMP-dependent protein kinase regulatory subunit [Crassostrea gigas]; 125 511 cAMP-dependent protein kina
1	3	NaN	9 887 minus strand hypothetical protein CGI_10006272 [Crassostrea gigas]
1	1	NaN	" 158 355 Protein NAS-37, isoform b [Caenorhabditis elegans] gi; 15 227 Hemicentin-1 [Crassostrea gigas]"
1	25	NaN	503 1090 minus strand myosin regulatory light chain [Fasciola hepatica]; 278 865 minus strand myosin regulatory lig
1	1	NaN	73 525 minus strand Actin-related protein 2/3 complex subunit 5-like protein [Salmo salar] gi; 73 525 Actin-related pr
1	12	NaN	49 1038 Glyceraldehyde-3-phosphate dehydrogenase [Crotalus adamanteus]
1	1	NaN	71 523 minus strand hypothetical protein DAPPUDRAFT_309332 [Daphnia pulex]
1	1	NaN	78 461 PREDICTED: hoi-polloi-like [Saccoglossus kowalevskii]; 103 492 NHP2-like protein 1 OS=Rattus norvegicus (
1	1	NaN	721 1242 Lactoylglutathione lyase [Crassostrea gigas]; 882 1334 Lactoylglutathione lyase OS=Rattus norvegicus GN
1	1	NaN	1050 1307 calreticulin precursor [Aplysia californica] gi
1	5	NaN	108 593 hypothetical protein CGI_10025497 [Crassostrea gigas]
1	1	NaN	35 505 minus strand Basement membrane-specific heparan sulfate proteoglycan core protein [Crassostrea gigas]
1	1	NaN	1118 1255 minus strand equistatin precursor [Actinia equina]
1	2	NaN	4 399 Spectrin beta chain [Crassostrea gigas]
1	1	NaN	2 307 Laminin subunit gamma-1 [Crassostrea gigas]
1	1	NaN	87 485 Sex-regulated protein janus-A [Crassostrea gigas]; 92 310 PREDICTED: hypothetical protein [Saccoglossus k
1	1	NaN	54 278 ATP-citrate synthase [Crassostrea gigas]; 61 465 ATP-citrate synthase [Crassostrea gigas]
1	2	NaN	211 990 PREDICTED: gamma-glutamyl hydrolase-like [Xenopus (Silurana) tropicalis]; 223 1080 PREDICTED: gamma
1	1	NaN	340 615 putative Low-density lipoprotein receptor repeat class B [Trichinella spiralis] gi
1	1	NaN	201 632 USP-like protein isoform 1 [Crassostrea ariakensis] gi; 189 620 minus strand USP-like protein isoform 1 [Cra
1	8	NaN	230 334 protein disulfide isomerase [Conus virgo]
1	1	NaN	9 161 PREDICTED: tubulin alpha-1C chain-like [Acyrthosiphon pisum]; 9 161 Tubulin alpha-3 chain (Fragments) OS=
1	1	NaN	213 881 PREDICTED: hypothetical protein [Saccoglossus kowalevskii]; 260 928 PREDICTED: hypothetical protein [S
1	3	NaN	96 497 AAEL009772-PB [Aedes aegypti]
1	2	NaN	156 1163 cathepsin B [Crassostrea ariakensis]
1	1	NaN	136 531 hypothetical protein CGI_10017533 [Crassostrea gigas]
1	3	NaN	900 1616 minus strand hemocyanin isoform 1 [Haliotis diversicolor]
1	1	NaN	2 1384 minus strand twitchin [Mytilus galloprovincialis]; 2 1384 minus strand twitchin [Mytilus galloprovincialis]; 2 1
1	12	NaN	633 806 Peroxiredoxin-1 [Crassostrea gigas]; 648 821 Peroxiredoxin-1 [Crassostrea gigas]; 666 839 Peroxiredoxin-1 [
1	6	NaN	64 393 Tubulin beta chain OS=Paracentrotus lividus PE=2 SV=1; 109 438 Tubulin beta chain OS=Paracentrotus lividu
1	2	NaN	1292 1471 minus strand hemocyanin isoform 1 [Haliotis diversicolor]
1	2	NaN	141 1325 Nucleobindin-2 [Crassostrea gigas]; 141 1208 minus strand Nucleobindin-2 [Crassostrea gigas]
1	1	NaN	28 642 minus strand Transmembrane emp24 domain-containing protein 9 [Crassostrea gigas]; 236 709 minus strand
1	1	NaN	642 1415 minus strand cytochrome c oxidase subunit II [Oncomelania hupensis hupensis]
1	1	NaN	3 239 minus strand p38 MAPK [Littorina littorea]
1	1	NaN	64 435 peptidoglycan recognition protein S1L [Pomacea canaliculata]
1	1	NaN	" 125 628 minus strand collectin sub-family member 12, partial [Oncorhynchus mykiss]"
1	2	NaN	1497 1889 UDP-glucuronosyltransferase 2C1 (Fragment) OS=Oryctolagus cuniculus GN=UGT2C1 PE=2 SV=1
1	1	NaN	238 1038 PREDICTED: hypothetical protein [Saccoglossus kowalevskii]; 294 992 PREDICTED: hypothetical protein [
1	4	NaN	1 576 minus strand Tubulin beta chain [Crassostrea gigas]
1	1	NaN	613 2421 PREDICTED: lysosomal alpha-glucosidase [Danio rerio]
1	10	NaN	1714 2121 Phosphoenolpyruvate carboxykinase [GTP] [Crassostrea gigas]
1	1	NaN	138 713 Rac [Aplysia californica] gi; 101 676 Rac [Aplysia californica] gi
1	2	NaN	" 3799 4371 papilin, partial [Rhipicephalus microplus]"
1	1	NaN	166 1563 arylsulfatase [Haliotis discus discus]
1	12	NaN	6043 6174 myosin heavy chain [Mytilus galloprovincialis]; 5869 6000 myosin heavy chain [Mytilus galloprovincialis]
1	1	NaN	87 485 Sex-regulated protein janus-A [Crassostrea gigas]
1	2	NaN	95 271 minus strand hypothetical protein EAI_01530 [Harpegnathos saltator]
1	3	NaN	2764 3420 PREDICTED: filamin-C-like [Nasonia vitripennis]
1	1	NaN	7825 8754 minus strand H2 [Haliotis tuberculata]
1	1	NaN	22 429 AGAP006937-PA [Anopheles gambiae str. PEST] gi; 1 474 Ubiquitin-conjugating enzyme E2 variant 2 OS=Rat
1	1	NaN	" 199 417 GG13156 [Drosophila erecta] gi; 253 501 minus strand NADH-ubiquinone oxidoreductase MLRQ subunit-li
1	1	NaN	11 394 regucalcin [Salmo salar] gi
1	1	NaN	161 3001 minus strand E3 ubiquitin-protein ligase MIB2 [Crassostrea gigas]
1	4	NaN	392 808 Whirlin [Crassostrea gigas]
1	2	NaN	165 377 Cysteine and glycine-rich protein 3 [Crassostrea gigas]; 135 344 Cysteine and glycine-rich protein 3 [Crasso

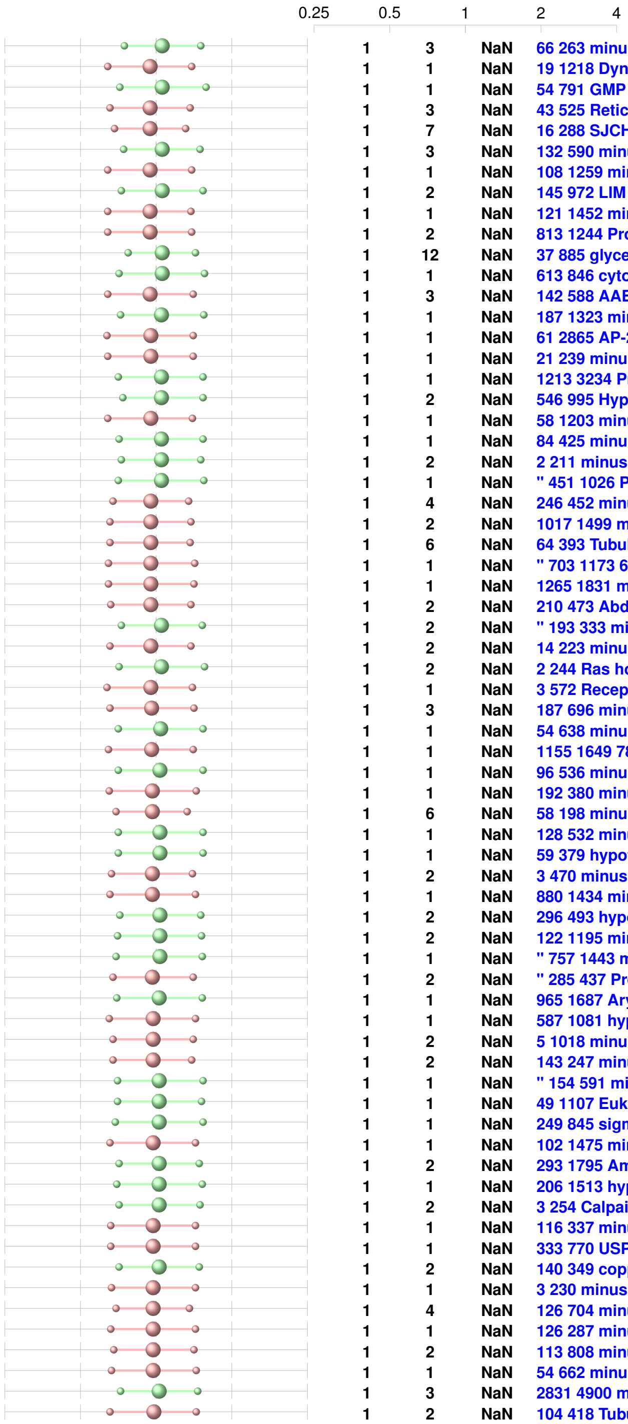

Peptides	Spectra	Coverage	Protein
1	3	NaN	66 263 minus strand twitchin [Mytilus galloprovincialis]; 66 263 minus strand twitchin [Mytilus galloprovincialis]; 66
1	1	NaN	19 1218 Dynactin subunit 2 [Crassostrea gigas]; 48 830 minus strand Dynactin subunit 2 [Crassostrea gigas]
1	1	NaN	54 791 GMP reductase 2 [Crassostrea gigas]
1	3	NaN	43 525 Reticulon-4 [Crassostrea gigas]; 143 757 Reticulon-4 [Crassostrea gigas]; 43 564 Reticulon-4 [Crassostrea gi
1	7	NaN	16 288 SJCHGC01950 protein [Schistosoma japonicum] gi
1	3	NaN	132 590 minus strand Non-neuronal cytoplasmic intermediate filament protein OS=Helix aspersa PE=3 SV=2
1	1	NaN	108 1259 minus strand PREDICTED: Na(+)/H(+) exchange regulatory cofactor NHE-RF3-like [Metaseiulus occidentali
1	2	NaN	145 972 LIM and SH3 domain protein F42H10.3 OS=Caenorhabditis elegans GN=F42H10.3 PE=2 SV=3
1	1	NaN	121 1452 minus strand Hexokinase type 2 [Crassostrea gigas]; 245 1576 minus strand Hexokinase type 2 [Crassostr
1	2	NaN	813 1244 Probable protein disulfide-isomerase A6 OS=Caenorhabditis elegans GN=tag-320 PE=2 SV=1
1	12	NaN	37 885 glyceraldehyde 3-phosphate dehydrogenase [Dugesia japonica]
1	1	NaN	613 846 cytochrome c oxidase subunit Va [Antheraea yamamai] gi
1	3	NaN	142 588 AAEL009772-PB [Aedes aegypti]; 96 497 AAEL009772-PB [Aedes aegypti]
1	1	NaN	187 1323 minus strand gelsolin [Aplysia californica] gi
1	1	NaN	61 2865 AP-2 complex subunit beta [Crassostrea gigas]
1	1	NaN	21 239 minus strand calponin-like protein [Taenia asiatica]
1	1	NaN	1213 3234 Protein-glutamine gamma-glutamyltransferase K [Crassostrea gigas]; 478 2499 Protein-glutamine gamma
1	2	NaN	546 995 Hypothetical protein CBG12002 [Caenorhabditis briggsae]; 543 995 minus strand PREDICTED: tubulin polyr
1	1	NaN	58 1203 minus strand Obg-like ATPase 1 [Crassostrea gigas]
1	1	NaN	84 425 minus strand Transcription elongation factor B polypeptide 2 [Crassostrea gigas]; 84 425 minus strand Trans
1	2	NaN	2 211 minus strand sulfatase 1A precursor [Haliotis discus discus]
1	1	NaN	" 451 1026 PREDICTED: GTP-binding protein SAR1B, putative-like [Saccoglossus kowalevskii]"
1	4	NaN	246 452 minus strand mitochondrial malate dehydrogenase precursor [Nucella lapillus]; 106 312 minus strand mitoc
1	2	NaN	1017 1499 minus strand PREDICTED: leukocyte elastase inhibitor-like [Oreochromis niloticus]
1	6	NaN	64 393 Tubulin beta chain OS=Paracentrotus lividus PE=2 SV=1; 111 398 Tubulin beta chain OS=Paracentrotus lividu
1	1	NaN	" 703 1173 60S acidic ribosomal protein P0, putative [Ixodes scapularis] gi"
1	1	NaN	1265 1831 minus strand Adenosine deaminase CECR1 [Crassostrea gigas]
1	2	NaN	210 473 Abdominal ganglion neuropeptides L5-67 OS=Aplysia californica PE=1 SV=1; 879 1124 abdominal ganglion
1	2	NaN	" 193 333 minus strand RecName: Full=Globin; AltName: Full=Myoglobin gi|299274|gb|AAB25743.1| myoglobin [Na
1	2	NaN	14 223 minus strand Endothelin-converting enzyme 2 OS=Homo sapiens GN=ECE2 PE=1 SV=4
1	2	NaN	2 244 Ras homolog gene family member A [Clonorchis sinensis]
1	1	NaN	3 572 Receptor-type tyrosine-protein phosphatase N2 [Crassostrea gigas]; 3 542 minus strand Receptor-type tyrosir
1	3	NaN	187 696 minus strand putative ribosomal protein S10 [Sipunculus nudus]
1	1	NaN	54 638 minus strand ubiquitin carboxyl-terminal hydrolase [Aplysia californica] gi
1	1	NaN	1155 1649 78 kDa glucose-regulated protein OS=Aplysia californica PE=1 SV=1
1	1	NaN	96 536 minus strand hypothetical protein BRAFLDRAFT_285353 [Branchiostoma floridae] gi
1	1	NaN	192 380 minus strand RAB protein [Haliotis discus discus]
1	6	NaN	58 198 minus strand Protein DJ-1 [Crassostrea gigas]
1	1	NaN	128 532 minus strand mitochondrial ATPase inhibitor [Ornithodoros parkeri]
1	1	NaN	59 379 hypothetical protein DAPPUDRAFT_230756 [Daphnia pulex]; 117 443 minus strand hypothetical protein DAPI
1	2	NaN	3 470 minus strand Spectrin beta chain [Crassostrea gigas]
1	1	NaN	880 1434 minus strand Prohormone-4 [Crassostrea gigas]
1	2	NaN	296 493 hypothetical protein BRAFLDRAFT_66287 [Branchiostoma floridae] gi
1	2	NaN	122 1195 minus strand Severin [Crassostrea gigas]
1	1	NaN	" 757 1443 minus strand nonmuscle myosin II, partial [Aplysia californica]"
1	2	NaN	" 285 437 Propionyl-CoA carboxylase alpha chain, mitochondrial [Crassostrea gigas]"
1	1	NaN	965 1687 Arylsulfatase B [Crassostrea gigas]
1	1	NaN	587 1081 hypothetical protein CGI_10026502 [Crassostrea gigas]
1	2	NaN	5 1018 minus strand Glutaredoxin-3 [Crassostrea gigas]; 5 1018 Glutaredoxin-3 [Crassostrea gigas]
1	2	NaN	143 247 minus strand dynein light chain [Aplysia californica] gi
1	1	NaN	" 154 591 microsomal GST1, partial [Reishia clavigera]; 154 531 microsomal GST1, partial [Reishia clavigera]"
1	1	NaN	49 1107 Eukaryotic translation initiation factor 3 subunit B [Crassostrea gigas]
1	1	NaN	249 845 sigma class glutathione-s-transferase 2 [Haliotis discus discus]; 252 848 sigma class glutathione-s-transfer
1	1	NaN	102 1475 minus strand Cytosolic non-specific dipeptidase [Crassostrea gigas]
1	2	NaN	293 1795 Amidase [Crassostrea gigas]
1	1	NaN	206 1513 hypothetical protein CGI_10019003 [Crassostrea gigas]
1	2	NaN	3 254 Calpain-5 [Crassostrea gigas]; 3 254 minus strand Calpain-5 [Crassostrea gigas]
1	1	NaN	116 337 minus strand Golgi-associated plant pathogenesis-related protein 1 [Crassostrea gigas]; 538 759 minus stra
1	1	NaN	333 770 USP-like protein isoform 2 [Crassostrea ariakensis]
1	2	NaN	140 349 copper chaperone Atox1 [Crassostrea ariakensis]
1	1	NaN	3 230 minus strand RH21460p [Drosophila melanogaster]; 3 230 minus strand RH21460p [Drosophila melanogaster]
1	4	NaN	126 704 minus strand ribosomal protein L12 [Ixodes scapularis]
1	1	NaN	126 287 minus strand Cytochrome b-c1 complex subunit 10 [Cricetulus griseus]; 126 287 minus strand Cytochrome
1	2	NaN	113 808 minus strand omega class glutathione-s-transferase [Chlamys farreri]; 335 1045 minus strand omega class
1	1	NaN	54 662 minus strand Protein DJ-1 [Crassostrea gigas]
1	3	NaN	2831 4900 minus strand Collagen alpha-3(VI) chain [Crassostrea gigas]
1	2	NaN	104 418 Tubulin-specific chaperone A [Crassostrea gigas]; 104 418 minus strand Tubulin-specific chaperone A [Cras

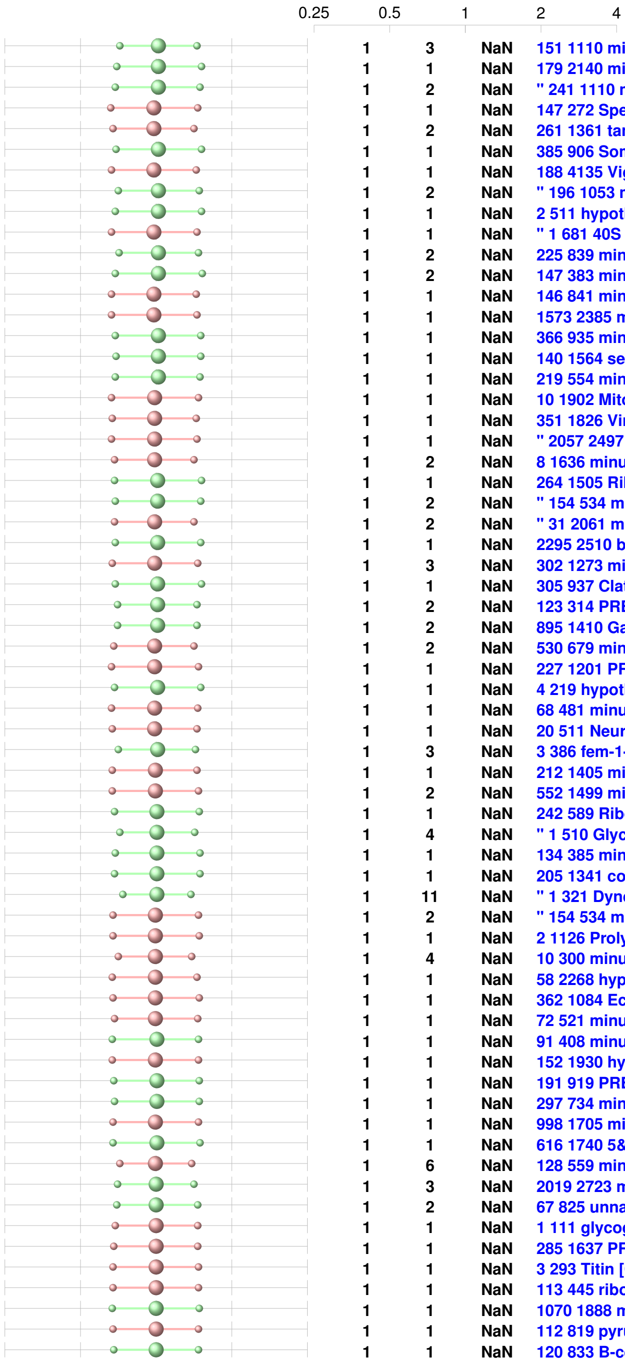

Peptides	Spectra	Coverage	Protein
1	3	NaN	151 1110 minus strand hypothetical protein DAPPUDRAFT_303085 [Daphnia pulex]
1	1	NaN	179 2140 minus strand PREDICTED: hypothetical protein [Saccoglossus kowalevskii]
1	2	NaN	" 241 1110 minus strand N(G),N(G)-dimethylarginine dimethylaminohydrolase 1 [Crassostrea gigas]; 241 1110 minus
1	1	NaN	147 272 Spectrin alpha chain [Crassostrea gigas]
1	2	NaN	261 1361 tandem-repeat galectin [Pinctada fucata]; 261 1361 tandem-repeat galectin [Pinctada fucata]
1	1	NaN	385 906 Soma ferritin OS=Lymnaea stagnalis PE=2 SV=2
1	1	NaN	188 4135 Vigilin [Crassostrea gigas]
1	2	NaN	" 196 1053 minus strand Cytochrome b-c1 complex subunit 2, mitochondrial OS=Homo sapiens GN=UQCRC2 PE=1
1	1	NaN	2 511 hypothetical protein CGI_10006272 [Crassostrea gigas]
1	1	NaN	" 1 681 40S ribosomal protein S4, X isoform [Crassostrea gigas]"
1	2	NaN	225 839 minus strand Metallo-beta-lactamase domain-containing protein 1 [Crassostrea gigas]; 201 851 minus stran
1	2	NaN	147 383 minus strand Tubulin-specific chaperone A OS=Rattus norvegicus GN=Tbca PE=1 SV=1; 104 418 Tubulin-sp
1	1	NaN	146 841 minus strand hypothetical protein CGI_10006609 [Crassostrea gigas]; 146 841 minus strand hypothetical pr
1	1	NaN	1573 2385 minus strand Spectrin beta chain [Crassostrea gigas]
1	1	NaN	366 935 minus strand 40S ribosomal protein S2 OS=Urechis caupo GN=RPS2 PE=2 SV=1
1	1	NaN	140 1564 selenium-binding protein [Haliotis discus hannai]
1	1	NaN	219 554 minus strand ribosomal protein P1 [Biomphalaria glabrata]
1	1	NaN	10 1902 Mitochondrial inner membrane protein [Crassostrea gigas]; 10 1902 Mitochondrial inner membrane protein
1	1	NaN	351 1826 Virulence metalloprotease [Crassostrea gigas]
1	1	NaN	" 2057 2497 Phosphate carrier protein, mitochondrial [Crassostrea gigas]"
1	2	NaN	8 1636 minus strand V-type proton ATPase catalytic subunit A [Crassostrea gigas]; 8 1636 minus strand V-type prot
1	1	NaN	264 1505 Ribosome-binding protein 1 [Crassostrea gigas]; 260 1501 Ribosome-binding protein 1 [Crassostrea gigas
1	2	NaN	" 154 534 minus strand Muscle M-line assembly protein unc-89, partial [Crassostrea gigas]"
1	2	NaN	" 31 2061 minus strand 1,4-alpha-glucan-branching enzyme [Crassostrea gigas]"
1	1	NaN	2295 2510 beta-thymosin domain repeat protein CSP24KDa_v1 [Hermissenda crassicornis] gi
1	3	NaN	302 1273 minus strand hypothetical protein CGI_10007007 [Crassostrea gigas]
1	1	NaN	305 937 Clathrin light chain A [Crassostrea gigas]
1	2	NaN	123 314 PREDICTED: ubiquinol-cytochrome c reductase hinge protein-like [Saccoglossus kowalevskii]; 279 530 PRE
1	2	NaN	895 1410 Galectin-3 OS=Rattus norvegicus GN=Lgals3 PE=1 SV=4; 1067 1405 Galectin-9 [Crassostrea gigas]; 1068 1
1	2	NaN	530 679 minus strand GJ11201 [Drosophila virilis] gi
1	1	NaN	227 1201 PREDICTED: quinone oxidoreductase-like isoform 1 [Ornithorhynchus anatinus]; 109 1083 PREDICTED: qu
1	1	NaN	4 219 hypothetical protein BRAFLDRAFT_66351 [Branchiostoma floridae] gi; 3 239 hypothetical protein BRAFLDRAF
1	1	NaN	68 481 minus strand netrin receptor UNC5A [Danio rerio] gi
1	1	NaN	20 511 Neurexin-2-alpha [Crassostrea gigas]; 20 511 Neurexin-2-alpha [Crassostrea gigas]
1	3	NaN	3 386 fem-1-like protein C [Crassostrea gigas]
1	1	NaN	212 1405 minus strand unnamed protein product [Tetraodon nigroviridis]
1	2	NaN	552 1499 minus strand uncharacterized protein LOC100158466 [Xenopus (Silurana) tropicalis] gi; 552 1499 minus st
1	1	NaN	242 589 Ribonucleoprotein RB97D OS=Drosophila melanogaster GN=Rb97D PE=2 SV=1
1	4	NaN	" 1 510 Glycerol-3-phosphate dehydrogenase [NAD+], cytoplasmic [Crassostrea gigas]"
1	1	NaN	134 385 minus strand CDGSH iron sulfur domain-containing protein 2 [Crassostrea gigas]; 11 511 minus strand CDG
1	1	NaN	205 1341 collagen precursor [Bombyx mori]
1	11	NaN	" 1 321 Dynein heavy chain, cytoplasmic [Crassostrea gigas]"
1	2	NaN	" 154 534 minus strand Muscle M-line assembly protein unc-89, partial [Crassostrea gigas]; 259 690 minus strand M
1	1	NaN	2 1126 Prolyl endopeptidase [Crassostrea gigas]
1	4	NaN	10 300 minus strand unnamed protein product [Tetraodon nigroviridis]
1	1	NaN	58 2268 hypothetical protein BRAFLDRAFT_267717 [Branchiostoma floridae] gi; 62 2245 hypothetical protein BRAF
1	1	NaN	362 1084 Ectoine hydroxylase [Crassostrea gigas]; 362 1084 Ectoine hydroxylase [Crassostrea gigas]; 366 1220 Ect
1	1	NaN	72 521 minus strand GF14593 [Drosophila ananassae] gi
1	1	NaN	91 408 minus strand hypothetical protein [Amblyomma maculatum]
1	1	NaN	152 1930 hypothetical protein BRAFLDRAFT_223365 [Branchiostoma floridae] gi
1	1	NaN	191 919 PREDICTED: 15-hydroxyprostaglandin dehydrogenase [NAD+] [Gallus gallus]
1	1	NaN	297 734 minus strand PREDICTED: calmodulin [Canis lupus familiaris]; 297 734 PREDICTED: calmodulin [Canis lupu
1	1	NaN	998 1705 minus strand Basement membrane-specific heparan sulfate proteoglycan core protein [Crassostrea gigas]
1	1	NaN	616 1740 5'-AMP-activated protein kinase subunit beta-2 [Crassostrea gigas]
1	6	NaN	128 559 minus strand predicted protein [Nematostella vectensis] gi
1	3	NaN	2019 2723 minus strand Kyphoscoliosis peptidase [Crassostrea gigas]; 2060 2764 minus strand Kyphoscoliosis pep
1	2	NaN	67 825 unnamed protein product [Oikopleura dioica]
1	1	NaN	1 111 glycogen synthase [Crassostrea gigas]
1	1	NaN	285 1637 PREDICTED: talin-2-like [Cricetulus griseus]
1	1	NaN	3 293 Titin [Crassostrea gigas]
1	1	NaN	113 445 ribosomal protein rps19 [Lineus viridis]
1	1	NaN	1070 1888 minus strand galectin [Pinctada fucata]; 1104 1922 minus strand galectin [Pinctada fucata]
1	1	NaN	112 819 pyruvate kinase [Crassostrea gigas]; 132 932 pyruvate kinase [Crassostrea gigas]
1	1	NaN	120 833 B-cell receptor-associated protein 31 [Crassostrea gigas]; 238 951 B-cell receptor-associated protein 31 [Cr

5 Protein Details

A detailed summary of each protein is given below. These sections include peptide relative expression estimates in addition to protein-level estimates.

5.1 204 791 minus strand PREDICTED: 15-hydroxyprostaglandin dehydrogenase [NAD+] [Gallus gallus]

Protein Accession	Unigene3818$_{H}$1
Mean Expression Ratio	1.84
Median Expression Ratio	1.84
Credible Interval	(1.5, 2.28)
Associated Peptides	9
Associated Spectra	40
Coverage	NaN

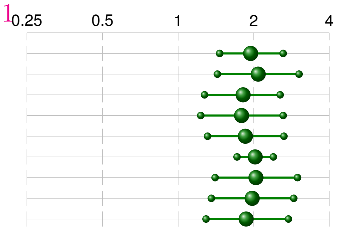

A	B	2.5	50	97.5	Sequence
4	1	1.5	1.9	2.6	AITMWGK
1	0	1.4	2.1	3	MGTTTDKELK
2	0	1.3	1.8	2.6	VDIMVNNAEIFSETDMEK
1	0	1.2	1.8	2.6	NAEIFSETDMEK
1	1	1.3	1.9	2.6	MDITDK
15	11	1.7	2	2.4	AVTEALLAK
1	0	1.4	2	3	IAFVSGGAMGTGR
0	1	1.4	2	2.9	TMTVNVTALMHGTK
0	1	1.3	1.9	2.7	HFISAFEK

5.2 " 353 781 alpha-B-crystallin, putative [Ixodes scapularis] gi"

Protein Accession	CL37349.Contig1$_{H}$1
Mean Expression Ratio	0.554
Median Expression Ratio	0.554
Credible Interval	(0.409, 0.743)
Associated Peptides	4
Associated Spectra	7
Coverage	NaN

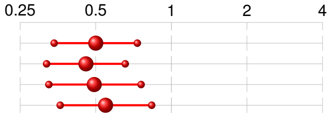

A	B	2.5	50	97.5	Sequence
2	0	0.34	0.5	0.73	LQESGTSEVVNNDKEFR
2	1	0.32	0.46	0.66	VAMDVQHFKPEELK
0	1	0.32	0.49	0.76	IIINAK
0	1	0.36	0.55	0.84	TVTSSLSPSGVLTLK

5.3 3788 7885 minus strand Filamin-C [Crassostrea gigas]

Protein Accession	CL4145.Contig29$_{H}$1
Mean Expression Ratio	0.565
Median Expression Ratio	0.566
Credible Interval	(0.385, 0.826)
Associated Peptides	1
Associated Spectra	2
Coverage	NaN

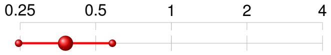

A	B	2.5	50	97.5	Sequence
2	0	0.25	0.38	0.58	WGEEDIPGSPFAVEVI

5.4 " 10 609 lethal(2)essential for life protein, l2efl [Aedes aegypti] gi"

Protein Accession	Unigene67975$_{H}1$
Mean Expression Ratio	0.567
Median Expression Ratio	0.568
Credible Interval	(0.415, 0.766)
Associated Peptides	4
Associated Spectra	6
Coverage	NaN

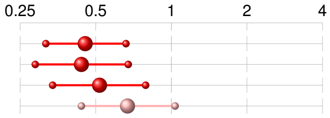

A	B	2.5	50	97.5	Sequence
**1**	**2**	**0.32**	**0.45**	**0.66**	**ALEAPGETVIPITFEGK**
**1**	**0**	**0.29**	**0.44**	**0.67**	**MQMGETGK**
**0**	**1**	**0.34**	**0.52**	**0.79**	**KALEAPGETVIPITFEGKGDGK**
0	1	0.44	0.67	1	KALEAPGETVIPITFEGK

5.5 704 817 minus strand expressed hypothetical protein [Trichoplax adhaerens] gi; 688 801 minus strand Lactoylglutathione lyase [Crassostrea gigas]; 735 848 minus strand expressed hypothetical protein [Trichoplax adhaerens] gi

Protein Accession	Unigene64106$_{H}1$ Unigene64104$_{H}1$ Unigene64103$_{H}1$
Mean Expression Ratio	1.59
Median Expression Ratio	1.59
Credible Interval	(1.1, 2.33)
Associated Peptides	1
Associated Spectra	2
Coverage	NaN

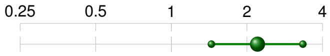

A	B	2.5	50	97.5	Sequence
**1**	**1**	**1.4**	**2.2**	**3.3**	**FESLGVELVK**

5.6 372 860 minus strand small heat shock protein [Tegillarca granosa]; 651 998 minus strand small heat shock protein [Tegillarca granosa]

Protein Accession	CL18190.Contig4$_{H}1$ CL18190.Contig1$_{H}1$
Mean Expression Ratio	0.649
Median Expression Ratio	0.648
Credible Interval	(0.485, 0.865)
Associated Peptides	4
Associated Spectra	7
Coverage	NaN

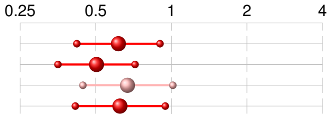

A	B	2.5	50	97.5	Sequence
**1**	**1**	**0.42**	**0.62**	**0.9**	**EFLIPEEIDAETLGSR**
**2**	**1**	**0.35**	**0.5**	**0.72**	**LTEDGHLIISAR**
1	0	0.44	0.67	1	IDKDGLSIK
**0**	**1**	**0.41**	**0.63**	**0.95**	**TIAIESDK**

5.7 REVERSED 6 395 PREDICTED: sister chromatid cohesion protein PDS5 homolog B-A isoform 1 [Nasonia vitripennis]

Protein Accession	RRRRRUnigene51506$_{H}1$
Mean Expression Ratio	1.47
Median Expression Ratio	1.47
Credible Interval	(1.13, 1.92)
Associated Peptides	2
Associated Spectra	38
Coverage	NaN

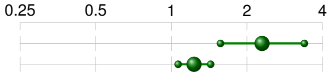

A	B	2.5	50	97.5	Sequence
**2**	**0**	**1.6**	**2.3**	**3.4**	**IGICIDNFR**
**25**	**11**	**1.1**	**1.2**	**1.4**	**LQELVDK**

5.8 " 460 1944 PREDICTED: succinyl-CoA:3-ketoacid-coenzyme A transferase 1, mitochondrial [Canis lupus familiaris]"

Protein Accession	Unigene4332$_{H}$1
Mean Expression Ratio	0.685
Median Expression Ratio	0.685
Credible Interval	(0.573, 0.819)
Associated Peptides	13
Associated Spectra	34
Coverage	NaN

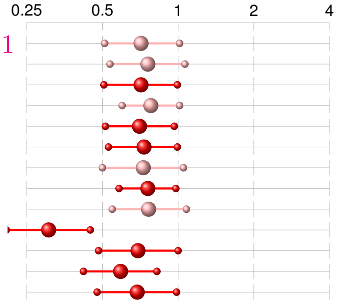

A	B	2.5	50	97.5	Sequence
1	1	0.51	0.71	1	AVFDVDNK
1	1	0.54	0.76	1.1	ECVSSYIGENK
1	1	0.51	0.71	0.99	EVAEGETAESIAK
2	5	0.6	0.78	1	EVVNMIVTEK
2	1	0.51	0.7	0.97	FNPDGTILHGSAGR
2	1	0.53	0.73	0.99	LGNLTFR
1	0	0.5	0.72	1.1	VMGMGGAMDLVSADK
3	4	0.58	0.76	0.98	ITGVEFR
2	0	0.55	0.76	1.1	VTLPK
0	1	0.21	0.31	0.45	AVFDVDNKEGLLLR
0	1	0.48	0.69	1	KGEEDADLINAGK
0	2	0.42	0.59	0.82	IFNGR
0	1	0.48	0.69	0.99	VFLGK

5.9 200 2128 minus strand heat shock protein 70 [Cristaria plicata]

Protein Accession	CL8787.Contig2$_{H}$1
Mean Expression Ratio	0.687
Median Expression Ratio	0.689
Credible Interval	(0.502, 0.94)
Associated Peptides	3
Associated Spectra	6
Coverage	NaN

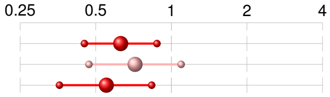

A	B	2.5	50	97.5	Sequence
1	3	0.45	0.63	0.88	IINEPTAAALAYGLDK
1	0	0.47	0.72	1.1	NGQVEIIANDQGQR
1	0	0.36	0.55	0.84	TLAPEEISSMVLVK

5.10 557 2329 minus strand Collagen alpha-3(VI) chain [Crassostrea gigas]

Protein Accession	CL47586.Contig7$_{H}$1
Mean Expression Ratio	1.44
Median Expression Ratio	1.44
Credible Interval	(1.07, 1.96)
Associated Peptides	4
Associated Spectra	6
Coverage	NaN

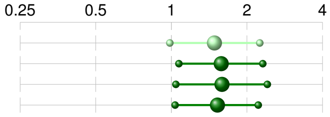

A	B	2.5	50	97.5	Sequence
1	0	0.98	1.5	2.2	KADYSAALGK
1	1	1.1	1.6	2.3	VVAVGVGAVVK
1	0	1	1.6	2.4	KLDVVVAFHASPR
0	2	1	1.5	2.2	VALGFFNK

5.11 2996 3340 minus strand Collagen alpha-5(VI) chain [Crassostrea gigas]

Protein Accession	CL47586.Contig8$_{H}$1
Mean Expression Ratio	1.44
Median Expression Ratio	1.44
Credible Interval	(1.05, 1.99)
Associated Peptides	2
Associated Spectra	6
Coverage	NaN

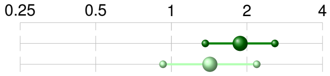

A	B	2.5	50	97.5	Sequence
2	3	1.4	1.9	2.6	ASIFDAIDEIPYR
1	0	0.93	1.4	2.2	YGSTNTADGIK

5.12 51 890 prohibitin [Octopus tankahkeei]

Protein Accession	CL26635.Contig2$_H$1
Mean Expression Ratio	0.706
Median Expression Ratio	0.706
Credible Interval	(0.542, 0.909)
Associated Peptides	6
Associated Spectra	10
Coverage	NaN

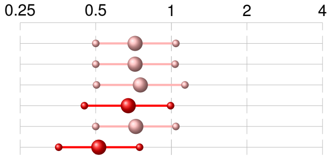

A	B	2.5	50	97.5	Sequence
1	1	0.5	0.72	1	AFTEAGEGLVELR
1	1	0.5	0.72	1	FLVEKEEQK
1	0	0.5	0.75	1.1	NVPVITGSK
1	0	0.45	0.67	0.99	IEAAEDIA
0	2	0.5	0.72	1	AVIFDR
0	2	0.36	0.51	0.75	QVAQQDAERAR

5.13 501 1838 Hsp90A [Haliotis asinina]

Protein Accession	CL5184.Contig2$_H$1
Mean Expression Ratio	0.712
Median Expression Ratio	0.713
Credible Interval	(0.558, 0.909)
Associated Peptides	7
Associated Spectra	14
Coverage	NaN

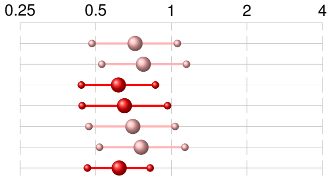

A	B	2.5	50	97.5	Sequence
1	0	0.48	0.72	1.1	AEFEGLCK
1	0	0.53	0.78	1.1	AIYYITGESLDAVK
1	2	0.44	0.61	0.86	GVVDSEDLPLNISR
1	0	0.44	0.65	0.97	NPDDITPEEYAEFYK
1	0	0.47	0.7	1	RAPFDLFENK
1	0	0.52	0.76	1.1	VERVK
2	4	0.46	0.62	0.82	LAEFLR

5.14 95 1579 minus strand hypothetical protein BRAFLDRAFT_113974 [Branchiostoma floridae] gi

Protein Accession	CL19753.Contig5$_H$1
Mean Expression Ratio	1.4
Median Expression Ratio	1.4
Credible Interval	(1.14, 1.71)
Associated Peptides	10
Associated Spectra	23
Coverage	NaN

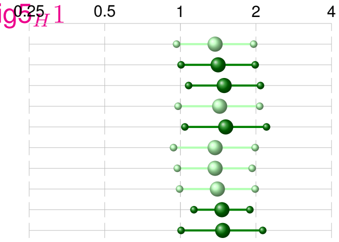

A	B	2.5	50	97.5	Sequence
1	1	0.96	1.4	2	ELGEYALK
1	1	1	1.4	2	FKDINEVIER
2	1	1.1	1.5	2.1	LIMQAAGR
1	0	0.98	1.4	2.1	TFPVVNPATEK
1	0	1	1.5	2.2	TSYGLAAGVITK
1	0	0.94	1.4	2	TVGDPFDAVTMQGPQVDQEQFEK
1	1	0.97	1.4	1.9	ADLRPDPNIK
1	1	0.99	1.4	2	DEIFGPVQSIIK
3	5	1.1	1.5	1.9	FADLVIR
0	1	1	1.5	2.1	VICEVQEADK

5.15 360 764 minus strand Peptidyl-prolyl cis-trans isomerase A OS=Dictyostelium discoideum GN=ppiA PE=1 SV=1

Protein Accession	Unigene64429$_H$1
Mean Expression Ratio	0.723
Median Expression Ratio	0.724
Credible Interval	(0.495, 1.04)
Associated Peptides	1
Associated Spectra	2
Coverage	NaN

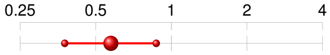

A	B	2.5	50	97.5	Sequence
0	2	0.38	0.57	0.87	HVVFGQVVEGLDVVR

5.16 361 549 minus strand 32 kDa beta-galactoside-binding lectin [Ascaris suum]

Protein Accession	CL5658.Contig3$_{H}1$
Mean Expression Ratio	0.724
Median Expression Ratio	0.725
Credible Interval	(0.516, 1.01)
Associated Peptides	1
Associated Spectra	5
Coverage	NaN

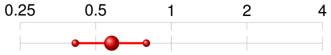

A	B	2.5	50	97.5	Sequence
**3**	**2**	**0.42**	**0.58**	**0.79**	**TIVLNHMQDGR**

5.17 118 513 Protein CAL-5 [Caenorhabditis elegans] gi

Protein Accession	CL18668.Contig1$_{H}1$
Mean Expression Ratio	0.727
Median Expression Ratio	0.727
Credible Interval	(0.483, 1.08)
Associated Peptides	1
Associated Spectra	2
Coverage	NaN

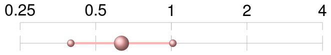

A	B	2.5	50	97.5	Sequence
**1**	**1**	**0.4**	**0.63**	**1**	**EADANGDGYLSIQELK**

5.18 176 667 hypothetical protein CGI_10018452 [Crassostrea gigas]; 176 634 hypothetical protein CGI_10018452 [Crassostrea gigas]

Protein Accession	CL35173.Contig3$_{H}1$ CL35173.Contig1$_{H}1$
Mean Expression Ratio	0.728
Median Expression Ratio	0.728
Credible Interval	(0.489, 1.08)
Associated Peptides	1
Associated Spectra	1
Coverage	NaN

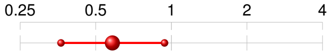

A	B	2.5	50	97.5	Sequence
**0**	**1**	**0.36**	**0.58**	**0.94**	**NFLELTELK**

5.19 1 945 hemocyanin isoform 1 [Haliotis diversicolor]

Protein Accession	Unigene30318$_{H}1$
Mean Expression Ratio	0.728
Median Expression Ratio	0.731
Credible Interval	(0.512, 1.03)
Associated Peptides	2
Associated Spectra	3
Coverage	NaN

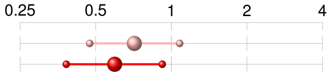

A	B	2.5	50	97.5	Sequence
**1**	**1**	**0.47**	**0.71**	**1.1**	**VEITDVLK**
**0**	**1**	**0.38**	**0.59**	**0.92**	**LFVVEVEDELR**

5.20 801 971 RecName: Full=Tropomyosin-2; AltName: Full=Tropomyosin II; Short=BgTMII; Short=TMII gi|155940|gb|AAA27816.1| tropomyosin [Biomphalaria glabrata]

Protein Accession	CL2925.Contig4$_H$1
Mean Expression Ratio	1.37
Median Expression Ratio	1.37
Credible Interval	(1.04, 1.81)
Associated Peptides	3
Associated Spectra	11
Coverage	NaN

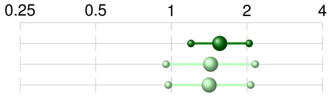

A	B	2.5	50	97.5	Sequence
7	1	1.2	1.6	2	EDSYEETIR
0	1	0.95	1.4	2.2	ISEQEASQR
0	2	0.97	1.4	2.1	QQYEIFGISEQEASQR

5.21 1009 2784 minus strand Collagen alpha-3(VI) chain [Crassostrea gigas]

Protein Accession	Unigene46961$_H$1
Mean Expression Ratio	1.35
Median Expression Ratio	1.35
Credible Interval	(1.09, 1.69)
Associated Peptides	8
Associated Spectra	21
Coverage	NaN

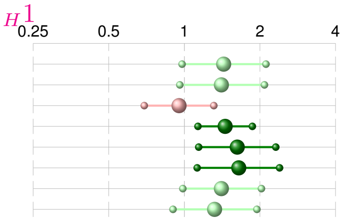

A	B	2.5	50	97.5	Sequence
1	0	0.98	1.4	2.1	FRKSPTLMFNLNEHK
1	0	0.96	1.4	2.1	IITVGIGEANRK
2	2	0.69	0.95	1.3	KSPTLMFNLNEHK
5	4	1.1	1.5	1.9	VAMSNFR
1	1	1.1	1.6	2.3	YADIDGGNVR
1	0	1.1	1.6	2.4	KAFNK
1	1	0.98	1.4	2	MFNLNEHK
0	1	0.9	1.3	1.9	FNLNEHK

5.22 915 1505 minus strand Collagen alpha-5(VI) chain [Crassostrea gigas]

Protein Accession	CL40183.Contig3$_H$1
Mean Expression Ratio	1.35
Median Expression Ratio	1.35
Credible Interval	(1.11, 1.64)
Associated Peptides	8
Associated Spectra	44
Coverage	NaN

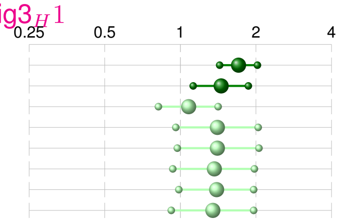

A	B	2.5	50	97.5	Sequence
13	10	1.4	1.7	2	LEAIPWR
4	4	1.1	1.5	1.9	VGEQLDAEELLNIAGDPK
3	4	0.82	1.1	1.4	DFVNAFQINPTAVR
1	0	0.96	1.4	2	HGLATNTGAGIK
1	0	0.97	1.4	2.1	HGLATNTGAGIKYMR
1	0	0.93	1.4	2	VGEQLDAEELL
0	2	0.99	1.4	2	RVVTAENYAHLNLVK
0	1	0.92	1.3	2	VVTAENYAHLNLVK

5.23 103 684 EF-hand domain-containing protein D2 [Crassostrea gigas]

Protein Accession	CL20544.Contig1$_H$1
Mean Expression Ratio	1.35
Median Expression Ratio	1.34
Credible Interval	(0.958, 1.92)
Associated Peptides	3
Associated Spectra	3
Coverage	NaN

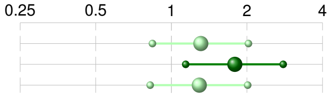

A	B	2.5	50	97.5	Sequence
1	0	0.84	1.3	2	VPQTHLALK
1	0	1.1	1.8	2.8	KFEEEIR
1	0	0.82	1.3	2	FIDFNELK

5.24 3 491 hemocyanin [Haliotis tuberculata]

Protein Accession	CL40281.Contig3$_H1$
Mean Expression Ratio	0.745
Median Expression Ratio	0.746
Credible Interval	(0.559, 0.995)
Associated Peptides	3
Associated Spectra	8
Coverage	NaN

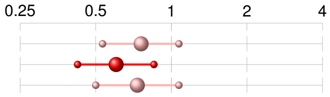

A	B	2.5	50	97.5	Sequence
**2**	**1**	**0.53**	**0.76**	**1.1**	**DINPFALTK**
**1**	**2**	**0.42**	**0.6**	**0.85**	**TLEPFDRDINPFALTK**
**1**	**1**	**0.5**	**0.73**	**1.1**	**TLEPFDR**

5.25 280 690 Histone H3.3 [Harpegnathos saltator]

Protein Accession	Unigene26607$_H1$
Mean Expression Ratio	0.746
Median Expression Ratio	0.746
Credible Interval	(0.583, 0.955)
Associated Peptides	6
Associated Spectra	15
Coverage	NaN

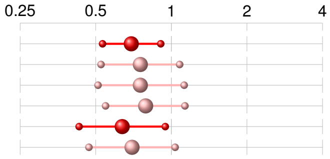

A	B	2.5	50	97.5	Sequence
**4**	**4**	**0.53**	**0.69**	**0.91**	**STELLIR**
**1**	**1**	**0.53**	**0.75**	**1.1**	**RVTIMPK**
**1**	**0**	**0.51**	**0.75**	**1.1**	**RTKQTARK**
**2**	**0**	**0.55**	**0.79**	**1.1**	**MARTKQTARK**
**0**	**1**	**0.43**	**0.64**	**0.95**	**EIAQDFK**
**0**	**1**	**0.47**	**0.7**	**1**	**KLPFQR**

5.26 40 1182 4-hydroxyphenylpyruvate dioxygenase [Crassostrea gigas]

Protein Accession	CL7585.Contig2$_H1$
Mean Expression Ratio	0.755
Median Expression Ratio	0.754
Credible Interval	(0.534, 1.07)
Associated Peptides	3
Associated Spectra	3
Coverage	NaN

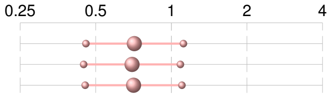

A	B	2.5	50	97.5	Sequence
**1**	**0**	**0.46**	**0.71**	**1.1**	**DVAFEVEDLDAIFK**
**1**	**0**	**0.45**	**0.7**	**1.1**	**TYGDTTHTLIDR**
**0**	**1**	**0.45**	**0.71**	**1.1**	**SLFEAIELDQAER**

5.27 66 947 Glyoxalase domain-containing protein 4 [Crassostrea gigas]

Protein Accession	CL23175.Contig2$_H1$
Mean Expression Ratio	1.32
Median Expression Ratio	1.32
Credible Interval	(0.897, 1.96)
Associated Peptides	1
Associated Spectra	1
Coverage	NaN

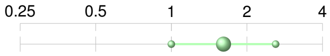

A	B	2.5	50	97.5	Sequence
**0**	**1**	**1**	**1.6**	**2.6**	**LELVEIGQK**

5.28 230 1189 hypothetical protein [Amblyomma maculatum]

Protein Accession	CL11202.Contig4$_{H}1$
Mean Expression Ratio	1.32
Median Expression Ratio	1.32
Credible Interval	(0.992, 1.77)
Associated Peptides	4
Associated Spectra	7
Coverage	NaN

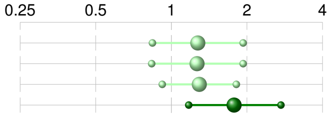

A	B	2.5	50	97.5	Sequence
1	0	0.84	1.3	1.9	KFAVDAVK
1	0	0.83	1.3	1.9	LSFDKEGMVK
0	4	0.92	1.3	1.8	ILEIIPGR
0	1	1.2	1.8	2.7	SFEPLEDPGVR

5.29 942 1385 Golgi-associated plant pathogenesis-related protein 1 [Crassostrea gigas]; 338 817 Golgi-associated plant pathogenesis-related protein 1 [Crassostrea gigas]; 354 833 Golgi-associated plant pathogenesis-related protein 1 [Crassostrea gigas]; 576 1055 Golgi-associated plant pathogenesis-related protein 1 [Crassostrea gigas]; 618 1097 Golgi-associated plant pathogenesis-related protein 1 [Crassostrea gigas]; 536 979 Golgi-associated plant pathogenesis-related protein 1 [Crassostrea gigas]; 740 1219 minus strand Golgi-associated plant pathogenesis-related protein 1 [Crassostrea gigas]

Protein Accession	Unigene391$_{H}1$ CL15263.Contig2$_{H}1$ CL15263.Contig1$_{H}1$ CL15263.Contig13$_{H}1$ CL15263.Co
Mean Expression Ratio	0.76
Median Expression Ratio	0.759
Credible Interval	(0.522, 1.11)
Associated Peptides	1
Associated Spectra	2
Coverage	NaN

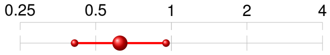

A	B	2.5	50	97.5	Sequence
1	1	0.41	0.63	0.95	IITEETIIK

5.30 4726 5571 minus strand twitchin [Mytilus galloprovincialis]

Protein Accession	CL181.Contig4$_{H}1$
Mean Expression Ratio	0.762
Median Expression Ratio	0.762
Credible Interval	(0.607, 0.957)
Associated Peptides	8
Associated Spectra	15
Coverage	NaN

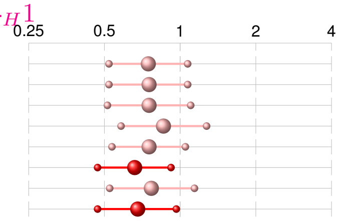

A	B	2.5	50	97.5	Sequence
1	1	0.52	0.75	1.1	EDGGAPITHYIVEK
1	1	0.52	0.75	1.1	GTVEGLQEGKEYEFR
1	0	0.51	0.75	1.1	VCDTDDLEIDVSDLTPGHK
1	0	0.58	0.86	1.3	CALLWDKPK
1	2	0.54	0.75	1	NGTPLNEANLDTETVPK
2	1	0.47	0.66	0.92	GLQEGKEYEFR
0	1	0.53	0.77	1.1	EIFVK
0	2	0.47	0.68	0.97	IPDVQR

5.31 111 1226 gelsolin [Aplysia californica] gi; 42 1139 minus strand gelsolin [Aplysia californica] gi

Protein Accession	Unigene39723$_{H}1$ Unigene32782$_{H}1$
Mean Expression Ratio	1.31
Median Expression Ratio	1.31
Credible Interval	(1.14, 1.51)
Associated Peptides	18
Associated Spectra	76
Coverage	NaN

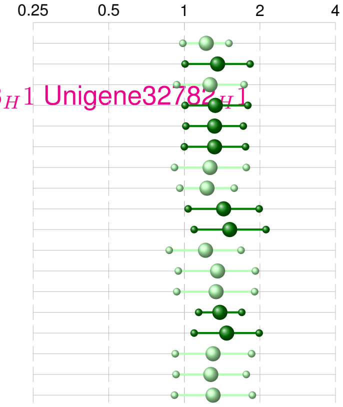

A	B	2.5	50	97.5	Sequence
4	8	0.99	1.2	1.5	AMQFIGEIK
3	1	1	1.4	1.8	DLVTSDDVYILDTGLK
3	0	0.93	1.3	1.7	ESGNPWRPVTVIK
4	0	1	1.3	1.8	EVFVWVGSNASPDEK
5	1	1	1.3	1.7	EVQGHESDLFK
3	2	1	1.3	1.8	KNGLPLAHNYLR
1	1	0.91	1.3	1.8	QGIVTMEGGAASGFR
4	4	0.96	1.2	1.6	SDDVFIFDSGK
1	1	1	1.4	2	TVELDTK
2	0	1.1	1.5	2.1	SVPAEEYK
1	1	0.87	1.2	1.7	QFDTALAA
1	0	0.95	1.4	1.9	KESADSEPAWDGAGQKPGLEIWR
1	0	0.94	1.3	1.9	NSTQDEYGTAAYK
13	3	1.1	1.4	1.7	LFHFAK
3	1	1.1	1.5	2	VVPWPK
0	1	0.92	1.3	1.9	DSNLALFGSDTEK
0	2	0.92	1.3	1.8	LNDFK
0	1	0.92	1.3	1.9	NGLPLAHNYLR

5.32 712 831 haemocyanin [Aplysia californica]

Protein Accession	Unigene65929$_{H}1$
Mean Expression Ratio	0.762
Median Expression Ratio	0.763
Credible Interval	(0.545, 1.06)
Associated Peptides	2
Associated Spectra	4
Coverage	NaN

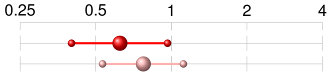

A	B	2.5	50	97.5	Sequence
1	0	0.4	0.62	0.96	YDVTDVFNQMR
3	0	0.53	0.77	1.1	YHFNVK

5.33 118 501 minus strand histone 2A.Z [Aplysia californica]

Protein Accession	CL14228.Contig6$_{H}1$
Mean Expression Ratio	0.766
Median Expression Ratio	0.766
Credible Interval	(0.588, 1)
Associated Peptides	5
Associated Spectra	23
Coverage	NaN

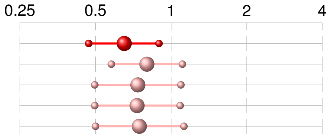

A	B	2.5	50	97.5	Sequence
9	3	0.47	0.65	0.89	AGLQFPVGR
3	3	0.58	0.8	1.1	ATIAGGGVIPHIHK
1	1	0.5	0.74	1.1	GDEELDSLIK
1	1	0.5	0.73	1.1	HLQLAIR
0	1	0.5	0.75	1.1	GGGVIPHIHK

5.34 21 341 minus strand hemocyanin isoform 1 [Nucula nucleus]

Protein Accession	CL40281.Contig2$_{H}1$
Mean Expression Ratio	0.766
Median Expression Ratio	0.766
Credible Interval	(0.561, 1.04)
Associated Peptides	2
Associated Spectra	6
Coverage	NaN

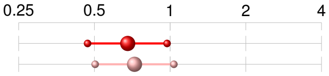

A	B	2.5	50	97.5	Sequence
1	2	0.47	0.68	0.97	GDELFSYLK
2	1	0.5	0.72	1	KPLEPFNR

5.35 189 353 40S ribosomal protein S12 [Crassostrea gigas]

Protein Accession	Unigene385$_{H}1$
Mean Expression Ratio	1.31
Median Expression Ratio	1.3
Credible Interval	(0.89, 1.94)
Associated Peptides	1
Associated Spectra	1
Coverage	NaN

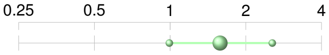

A	B	2.5	50	97.5	Sequence
1	0	0.99	1.6	2.5	TALIHDGLAR

5.36 1732 2079 minus strand PREDICTED: annexin A7-like [Ornithorhynchus anatinus]

Protein Accession	CL9274.Contig2$_{H}1$
Mean Expression Ratio	0.767
Median Expression Ratio	0.768
Credible Interval	(0.52, 1.13)
Associated Peptides	1
Associated Spectra	1
Coverage	NaN

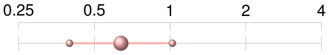

A	B	2.5	50	97.5	Sequence
1	0	0.4	0.64	1	DLLSAISSETSGDFETGLR

5.37 684 1250 minus strand Collagen alpha-5(VI) chain [Crassostrea gigas]

Protein Accession	CL284.Contig4$_{H}1$
Mean Expression Ratio	1.3
Median Expression Ratio	1.3
Credible Interval	(0.998, 1.7)
Associated Peptides	5
Associated Spectra	10
Coverage	NaN

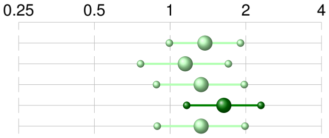

A	B	2.5	50	97.5	Sequence
1	3	0.99	1.4	1.9	HVMDALSAIDHHQGDR
1	0	0.77	1.1	1.7	VCIVITDGQSQEIQQTADEAR
1	0	0.88	1.3	2	EAGITMFAVGVGK
1	2	1.2	1.6	2.3	VAAITFGK
0	1	0.89	1.3	2	EAGITMF

5.38 696 4010 Lethal(2) giant larvae-like protein 1 [Crassostrea gigas]; 657 3971 minus strand Lethal(2) giant larvae-like protein 1 [Crassostrea gigas]; 156 3470 Lethal(2) giant larvae-like protein 1 [Crassostrea gigas]

Protein Accession	CL3717.Contig4$_{H}1$ CL3717.Contig3$_{H}1$ CL3717.Contig16$_{H}1$
Mean Expression Ratio	0.768
Median Expression Ratio	0.77
Credible Interval	(0.534, 1.1)
Associated Peptides	2
Associated Spectra	2
Coverage	NaN

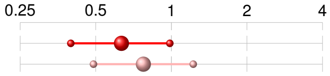

A	B	2.5	50	97.5	Sequence
**0**	**1**	**0.4**	**0.63**	**0.99**	**LDTTADITIDSVR**
**0**	**1**	**0.49**	**0.77**	**1.2**	**VLPESLEVIHER**

5.39 971 1963 Spectrin alpha chain [Crassostrea gigas]; 972 1979 Spectrin alpha chain [Crassostrea gigas]

Protein Accession	CL2195.Contig6$_{H}1$ CL2195.Contig4$_{H}1$
Mean Expression Ratio	0.769
Median Expression Ratio	0.77
Credible Interval	(0.527, 1.11)
Associated Peptides	1
Associated Spectra	2
Coverage	NaN

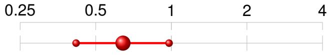

A	B	2.5	50	97.5	Sequence
**1**	**1**	**0.42**	**0.64**	**0.98**	**EAFLAGDTVDGDNVETLIK**

5.40 " 110 1717 PREDICTED: glutamate dehydrogenase, mitochondrial-like [Oreochromis niloticus]; 110 1717 PREDICTED: glutamate dehydrogenase, mitochondrial-like [Oreochromis niloticus]; 110 1717 PREDICTED: glutamate dehydrogenase, mitochondrial-like [Oreochromis niloticus]"

Protein Accession	CL16377.Contig9$_{H}1$ CL16377.Contig8$_{H}1$ CL16377.Contig10$_{H}1$
Mean Expression Ratio	1.3
Median Expression Ratio	1.3
Credible Interval	(0.946, 1.77)
Associated Peptides	3
Associated Spectra	5
Coverage	NaN

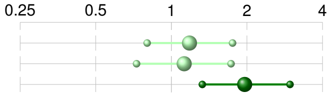

A	B	2.5	50	97.5	Sequence
**1**	**1**	**0.8**	**1.2**	**1.8**	**IIAEGANGPTTAGADEIFLK**
**0**	**1**	**0.73**	**1.1**	**1.7**	**TAAYIAAIEK**
**0**	**2**	**1.3**	**2**	**3**	**EAGLTFT**

5.41 503 1219 heat shock cognate 71 [Mytilus galloprovincialis]

Protein Accession	CL23109.Contig5$_{H}1$
Mean Expression Ratio	1.3
Median Expression Ratio	1.3
Credible Interval	(0.975, 1.73)
Associated Peptides	4
Associated Spectra	7
Coverage	NaN

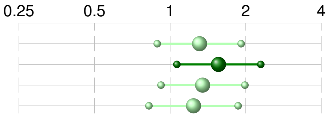

A	B	2.5	50	97.5	Sequence
1	1	0.89	1.3	1.9	FEELNADLFR
2	0	1.1	1.6	2.3	LLQDFFNGK
2	0	0.92	1.3	2	GTLEPVEK
0	1	0.82	1.2	1.9	SAIHEIVLVGGSTR

5.42 244 837 PREDICTED: ras-like GTP-binding protein Rho1-like [Megachile rotundata]

Protein Accession	Unigene11792$_{H}1$
Mean Expression Ratio	1.3
Median Expression Ratio	1.3
Credible Interval	(0.942, 1.78)
Associated Peptides	4
Associated Spectra	5
Coverage	NaN

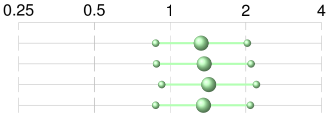

A	B	2.5	50	97.5	Sequence
1	1	0.87	1.3	2	EVFETATR
1	0	0.88	1.4	2.1	KLVIVGDGACGK
1	0	0.92	1.4	2.2	QVELALWDTAGQEDYDR
1	0	0.88	1.4	2.1	LMATVR

5.43 222 809 minus strand PREDICTED: 15-hydroxyprostaglandin dehydrogenase [NAD+] [Gallus gallus]

Protein Accession	CL44148.Contig2$_{H}1$
Mean Expression Ratio	1.29
Median Expression Ratio	1.29
Credible Interval	(1.07, 1.55)
Associated Peptides	7
Associated Spectra	84
Coverage	NaN

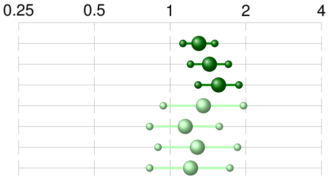

A	B	2.5	50	97.5	Sequence
20	16	1.1	1.3	1.5	ATVEAFLTK
17	6	1.2	1.4	1.7	VFLTGGAHGIGK
13	6	1.3	1.6	1.9	VMFCDINDK
1	0	0.94	1.4	2	RVFLTGGAHGIGK
0	3	0.83	1.2	1.6	TGGAHGIGK
0	1	0.9	1.3	1.9	LTGGAHGIGK
0	1	0.83	1.2	1.7	GGAHGIGK

5.44 31 735 hypothetical protein BRAFLDRAFT_78603 [Branchiostoma floridae] gi; 55 735 hypothetical protein BRAFLDRAFT_78603 [Branchiostoma floridae] gi; 353 1039 hypothetical protein BRAFLDRAFT_78603 [Branchiostoma floridae] gi; 207 893 hypothetical protein BRAFLDRAFT_78603 [Branchiostoma floridae] gi

Protein Accession	CL877.Contig7$_{H1}$ CL877.Contig6$_{H1}$ CL877.Contig4$_{H1}$ CL877.Contig3$_{H1}$
Mean Expression Ratio	1.29
Median Expression Ratio	1.29
Credible Interval	(0.97, 1.7)
Associated Peptides	4
Associated Spectra	10
Coverage	NaN

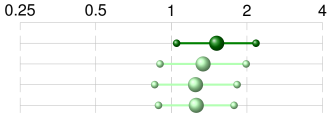

A	B	2.5	50	97.5	Sequence
2	1	1	1.5	2.2	DDQTLVTMTGEVAGR
1	1	0.9	1.3	2	LKLPLDSSNR
2	0	0.86	1.3	1.8	KVIHSIVGPHFAEGR
3	0	0.89	1.3	1.8	VALVPK

5.45 957 2147 haemocyanin [Aplysia californica]

Protein Accession	Unigene30320$_{H1}$
Mean Expression Ratio	0.777
Median Expression Ratio	0.777
Credible Interval	(0.609, 0.991)
Associated Peptides	7
Associated Spectra	13
Coverage	NaN

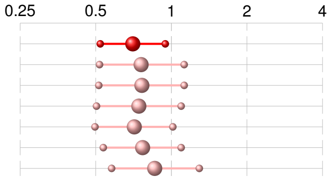

A	B	2.5	50	97.5	Sequence
3	2	0.52	0.7	0.95	LLTVQFER
1	0	0.52	0.76	1.1	YEVSELSLEQVSNLR
1	0	0.52	0.77	1.1	TFHISGVNQDITR
1	0	0.5	0.74	1.1	LLTVQFERAL
0	2	0.5	0.71	1	KAGQNIGDTLK
0	2	0.54	0.77	1.1	IDITDAVK
0	1	0.58	0.86	1.3	ANALPVQVFDTEK

5.46 174 1649 minus strand Collagen alpha-1(XII) chain [Crassostrea gigas]; 123 1490 minus strand Collagen alpha-1(XII) chain [Crassostrea gigas]; 135 1610 Collagen alpha-1(XII) chain [Crassostrea gigas]

Protein Accession	CL5340.Contig13$_{H1}$ CL5340.Contig12$_{H1}$ CL5340.Contig11$_{H1}$
Mean Expression Ratio	1.29
Median Expression Ratio	1.29
Credible Interval	(0.968, 1.72)
Associated Peptides	3
Associated Spectra	15
Coverage	NaN

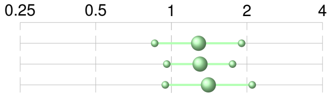

A	B	2.5	50	97.5	Sequence
1	1	0.86	1.3	1.9	DVMSVFHIAR
6	5	0.96	1.3	1.8	DILAFR
1	1	0.94	1.4	2.1	SIWMPDFK

5.47 27 1682 minus strand PREDICTED: nucleolar protein 56 [Oreochromis niloticus]

Protein Accession	Unigene26699$_{H}1$
Mean Expression Ratio	0.782
Median Expression Ratio	0.781
Credible Interval	(0.637, 0.96)
Associated Peptides	7
Associated Spectra	27
Coverage	NaN

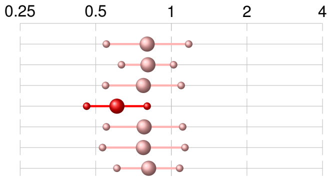

A	B	2.5	50	97.5	Sequence
1	0	0.55	0.8	1.2	KGLGGLSTEKK
7	3	0.63	0.81	1	LDGLGQAELQK
2	0	0.55	0.77	1.1	VATLEEEVYDWEIK
2	4	0.46	0.61	0.8	VTPLKLDGLGQAELQK
1	1	0.55	0.78	1.1	LIMQK
1	0	0.53	0.77	1.1	AAEDLRNEAK
4	1	0.61	0.81	1.1	KLIMQK

5.48 150 716 calponin/transgelin [Papilio xuthus]

Protein Accession	CL11747.Contig2$_{H}1$
Mean Expression Ratio	1.28
Median Expression Ratio	1.28
Credible Interval	(0.963, 1.72)
Associated Peptides	3
Associated Spectra	8
Coverage	NaN

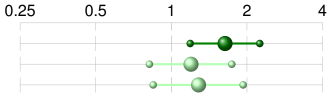

A	B	2.5	50	97.5	Sequence
4	1	1.2	1.6	2.3	GPPLNTPEHSVFATPDLYEK
1	1	0.82	1.2	1.7	MAFQQMEAIEK
1	0	0.85	1.3	1.9	FTTAIK

5.49 2 2572 Glycogen debranching enzyme [Crassostrea gigas]

Protein Accession	CL45503.Contig1$_{H}1$
Mean Expression Ratio	1.28
Median Expression Ratio	1.28
Credible Interval	(0.857, 1.91)
Associated Peptides	1
Associated Spectra	1
Coverage	NaN

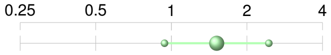

A	B	2.5	50	97.5	Sequence
0	1	0.94	1.5	2.4	IMAVNGWVMGDDPLR

5.50 160 1218 Galectin-9 [Crassostrea gigas]

Protein Accession	Unigene165$_{H}1$
Mean Expression Ratio	0.782
Median Expression Ratio	0.785
Credible Interval	(0.558, 1.1)
Associated Peptides	2
Associated Spectra	3
Coverage	NaN

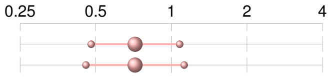

A	B	2.5	50	97.5	Sequence
1	1	0.48	0.72	1.1	IAVNNQHLVEFR
1	0	0.46	0.72	1.1	SGIPGGLAPGR

5.51 146 727 Adenylate kinase isoenzyme 1 [Crassostrea gigas]

Protein Accession	CL42551.Contig1$_{H}$1
Mean Expression Ratio	1.27
Median Expression Ratio	1.27
Credible Interval	(0.962, 1.68)
Associated Peptides	4
Associated Spectra	8
Coverage	NaN

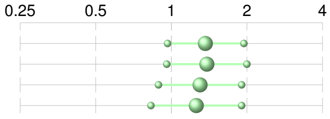

A	B	2.5	50	97.5	Sequence
3	0	0.96	1.4	1.9	DAMIQNITTTK
1	1	0.96	1.4	2	ELEQGLR
0	2	0.89	1.3	1.9	VVFVIGGPGSGK
0	1	0.83	1.3	1.9	LNAIMEK

5.52 127 657 ADP ribosylation factor 4 [Marsupenaeus japonicus]

Protein Accession	Unigene23129$_{H}$1
Mean Expression Ratio	1.27
Median Expression Ratio	1.27
Credible Interval	(0.956, 1.7)
Associated Peptides	4
Associated Spectra	8
Coverage	NaN

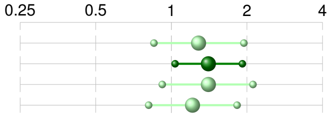

A	B	2.5	50	97.5	Sequence
1	0	0.85	1.3	2	IEEAKDELQK
2	3	1	1.4	1.9	ILMVGLDAAGK
0	1	0.92	1.4	2.1	LLVFANK
0	1	0.81	1.2	1.8	LGLNQLR

5.53 430 759 hypothetical protein [Amblyomma maculatum]; 336 665 hypothetical protein [Amblyomma maculatum]

Protein Accession	CL4062.Contig3$_{H}$1 CL4062.Contig2$_{H}$1
Mean Expression Ratio	0.787
Median Expression Ratio	0.789
Credible Interval	(0.593, 1.04)
Associated Peptides	4
Associated Spectra	7
Coverage	NaN

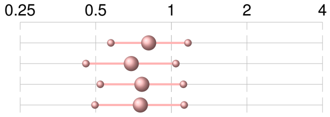

A	B	2.5	50	97.5	Sequence
2	1	0.58	0.81	1.2	ETLFEYLADPK
1	0	0.46	0.69	1	KTGQSAGFSYTDANK
1	1	0.52	0.76	1.1	KYIPGTK
0	1	0.5	0.75	1.1	MVFAGIK

5.54 " 109 1632 minus strand Seryl-tRNA synthetase, cytoplasmic [Crassostrea gigas]; 109 1614 minus strand Seryl-tRNA synthetase, cytoplasmic [Crassostrea gigas]"

Protein Accession	CL11056.Contig9$_{H}$1 CL11056.Contig1$_{H}$1
Mean Expression Ratio	0.789
Median Expression Ratio	0.789
Credible Interval	(0.546, 1.14)
Associated Peptides	2
Associated Spectra	2
Coverage	NaN

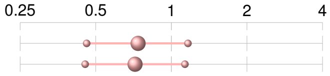

A	B	2.5	50	97.5	Sequence
0	1	0.46	0.73	1.2	LVDTVVDQDAAWR
0	1	0.45	0.72	1.1	SLGIPYR

5.55 23 214 minus strand hemocyanin isoform 1 [Nucula nucleus]

Protein Accession	Unigene58881$_H1$
Mean Expression Ratio	0.79
Median Expression Ratio	0.789
Credible Interval	(0.569, 1.1)
Associated Peptides	2
Associated Spectra	4
Coverage	NaN

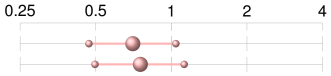

A	B	2.5	50	97.5	Sequence
2	0	0.47	0.7	1	GKIPNLNIYTSR
2	0	0.5	0.75	1.1	QEINPFFR

5.56 80 589 minus strand PREDICTED: adenine phosphoribosyltransferase-like [Strongylocentrotus purpuratus]

Protein Accession	CL8682.Contig6$_H1$
Mean Expression Ratio	1.27
Median Expression Ratio	1.27
Credible Interval	(0.964, 1.66)
Associated Peptides	5
Associated Spectra	10
Coverage	NaN

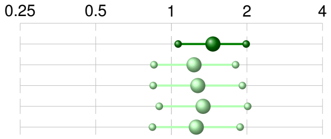

A	B	2.5	50	97.5	Sequence
2	3	1.1	1.5	2	LTVPVESLIK
1	1	0.85	1.2	1.8	AAVELVEK
0	1	0.85	1.3	1.9	GFLFGPMIAEK
0	1	0.89	1.3	2	NAPDADVIIGLESR
0	1	0.84	1.3	1.9	DIFPILR

5.57 258 629 Glycogenin-1 OS=Oryctolagus cuniculus GN=GYG1 PE=1 SV=3

Protein Accession	Unigene77101$_H1$
Mean Expression Ratio	1.27
Median Expression Ratio	1.26
Credible Interval	(0.855, 1.88)
Associated Peptides	1
Associated Spectra	1
Coverage	NaN

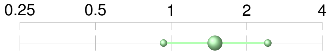

A	B	2.5	50	97.5	Sequence
1	0	0.93	1.5	2.4	IHCWELTQYEK

5.58 169 1200 PREDICTED: nidogen-2-like isoform 1 [Strongylocentrotus purpuratus]

Protein Accession	CL478.Contig2$_H1$
Mean Expression Ratio	0.792
Median Expression Ratio	0.792
Credible Interval	(0.581, 1.08)
Associated Peptides	4
Associated Spectra	5
Coverage	NaN

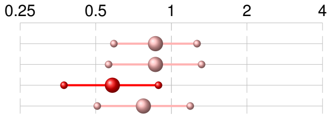

A	B	2.5	50	97.5	Sequence
0	2	0.59	0.86	1.3	LDIVATAR
0	1	0.56	0.87	1.3	LPNGLTFDQVSR
0	1	0.37	0.58	0.89	IECVHR
0	1	0.51	0.77	1.2	VFTVPALSR

5.59 105 503 Transgelin-2 [Crassostrea gigas]

Protein Accession	CL445.Contig2$_{H}1$
Mean Expression Ratio	1.26
Median Expression Ratio	1.26
Credible Interval	(0.963, 1.66)
Associated Peptides	2
Associated Spectra	15
Coverage	NaN

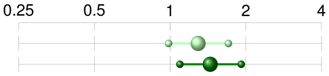

A	B	2.5	50	97.5	Sequence
4	4	0.98	1.3	1.7	SGLGLEIDK
5	2	1.1	1.4	1.9	SGLGLEIDKK

5.60 " 303 500 minus strand protein lethal, putative [Pediculus humanus corporis] gi"

Protein Accession	CL15038.Contig1$_{H}1$
Mean Expression Ratio	0.795
Median Expression Ratio	0.796
Credible Interval	(0.585, 1.07)
Associated Peptides	2
Associated Spectra	9
Coverage	NaN

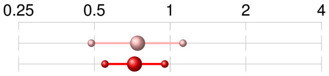

A	B	2.5	50	97.5	Sequence
1	0	0.49	0.74	1.1	QDMLATGR
6	2	0.55	0.72	0.95	QMPMLGR

5.61 7 1722 minus strand Membrane metallo-endopeptidase-like 1 OS=Mus musculus GN=Mmel1 PE=1 SV=1

Protein Accession	CL19557.Contig1$_{H}1$
Mean Expression Ratio	1.25
Median Expression Ratio	1.25
Credible Interval	(1.06, 1.46)
Associated Peptides	15
Associated Spectra	41
Coverage	NaN

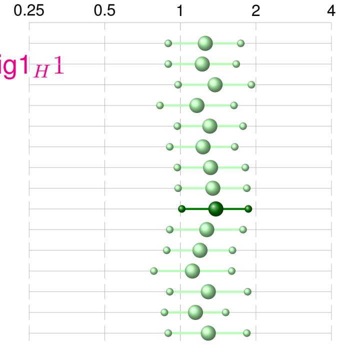

A	B	2.5	50	97.5	Sequence
1	1	0.9	1.3	1.7	AVLIEAIR
3	0	0.89	1.2	1.7	HQVENPNLHEVTSLADLEK
1	1	0.98	1.4	1.9	IGYPDYIMDDLR
1	1	0.83	1.2	1.6	LFVLNSFDQNQR
3	1	0.98	1.3	1.8	QDAQDVADFK
3	1	0.91	1.2	1.6	TLVENIADNAGLK
2	1	0.97	1.3	1.8	WPVSAAAVK
2	1	0.98	1.3	1.8	WLTDASK
4	0	1	1.4	1.9	LFPEVTGR
2	0	0.91	1.3	1.8	GLELFR
2	2	0.88	1.2	1.6	MNIDGYR
1	0	0.78	1.1	1.6	AQALNAK
1	0	0.91	1.3	1.9	QTALDK
2	3	0.86	1.1	1.5	LQLAEISLPK
0	1	0.89	1.3	1.8	LTDASKQTALDK

5.62 " 131 544 Histone H2B, gonadal OS=Patella granatina PE=1 SV=2"

Protein Accession	CL29794.Contig2$_{H}1$
Mean Expression Ratio	0.802
Median Expression Ratio	0.802
Credible Interval	(0.608, 1.05)
Associated Peptides	2
Associated Spectra	18
Coverage	NaN

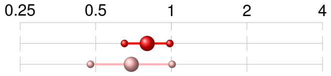

A	B	2.5	50	97.5	Sequence
**9**	**7**	**0.65**	**0.8**	**0.99**	**LLLPGELAK**
**0**	**2**	**0.48**	**0.69**	**1**	**PGELAK**

5.63 " 137 1549 minus strand Serine hydroxymethyltransferase, mitochondrial [Crassostrea gigas]; 205 1608 minus strand Serine hydroxymethyltransferase, mitochondrial [Crassostrea gigas]; 161 1573 minus strand Serine hydroxymethyltransferase, mitochondrial [Crassostrea gigas]; 205 1386 minus strand Serine hydroxymethyltransferase, mitochondrial [Crassostrea gigas]"

Protein Accession	CL22722.Contig6$_{H}1$ CL22722.Contig5$_{H}1$ CL22722.Contig4$_{H}1$ CL22722.Contig1$_{H}1$
Mean Expression Ratio	1.25
Median Expression Ratio	1.25
Credible Interval	(0.847, 1.86)
Associated Peptides	1
Associated Spectra	1
Coverage	NaN

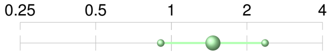

A	B	2.5	50	97.5	Sequence
**1**	**0**	**0.91**	**1.5**	**2.4**	**IIFYR**

5.64 366 1049 hypothetical protein CGI_10004855 [Crassostrea gigas]

Protein Accession	CL43590.Contig5$_{H}1$
Mean Expression Ratio	0.802
Median Expression Ratio	0.802
Credible Interval	(0.63, 1.02)
Associated Peptides	7
Associated Spectra	14
Coverage	NaN

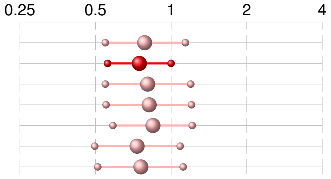

A	B	2.5	50	97.5	Sequence
**1**	**1**	**0.55**	**0.79**	**1.1**	**DEWTHLTAK**
**2**	**4**	**0.56**	**0.75**	**1**	**GGDLWQGEVSLEQHR**
**1**	**0**	**0.55**	**0.81**	**1.2**	**ADSDRPSVSAGIGQKPQFQDLK**
**1**	**0**	**0.55**	**0.82**	**1.2**	**VKVPGAK**
**2**	**0**	**0.59**	**0.85**	**1.2**	**KVFPYPK**
**0**	**1**	**0.5**	**0.73**	**1.1**	**TTVQLHK**
**0**	**1**	**0.51**	**0.76**	**1.1**	**KVEIK**

5.65 " 599 907 minus strand Tryptophanyl-tRNA synthetase, cytoplasmic [Heterocephalus glaber]"

Protein Accession	Unigene26755$_{H1}$
Mean Expression Ratio	0.801
Median Expression Ratio	0.803
Credible Interval	(0.585, 1.09)
Associated Peptides	2
Associated Spectra	8
Coverage	NaN

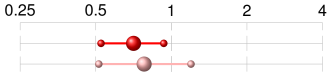

A	B	2.5	50	97.5	Sequence
3	4	0.52	0.71	0.94	DMDPLLDLYEK
1	0	0.51	0.78	1.2	KTPFYLYTGR

5.66 93 2606 twitchin [Mytilus galloprovincialis]

Protein Accession	Unigene71883$_{H1}$
Mean Expression Ratio	0.806
Median Expression Ratio	0.805
Credible Interval	(0.634, 1.02)
Associated Peptides	5
Associated Spectra	20
Coverage	NaN

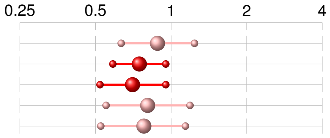

A	B	2.5	50	97.5	Sequence
2	1	0.63	0.88	1.2	FAFHNITGLSPNK
6	4	0.59	0.75	0.95	IIDFGLATR
2	3	0.52	0.7	0.95	FVPQPVEIK
1	0	0.55	0.81	1.2	VIAENFYGR
0	1	0.53	0.78	1.1	LNPDEIVK

5.67 247 1308 RecName: Full=Guanine nucleotide-binding protein G(o) subunit alpha gi|9633|emb|CAA78806.1| GTP-binding protein alpha-o subunit [Lymnaea stagnalis]

Protein Accession	CL26602.Contig4$_{H1}$
Mean Expression Ratio	1.24
Median Expression Ratio	1.24
Credible Interval	(0.977, 1.57)
Associated Peptides	6
Associated Spectra	18
Coverage	NaN

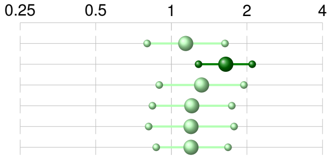

A	B	2.5	50	97.5	Sequence
1	1	0.8	1.1	1.6	IIHEGGFTSEDNK
4	5	1.3	1.6	2.1	LLLLGAGESGK
1	0	0.89	1.3	1.9	QTYEEAAAYIQAQFEAK
1	1	0.84	1.2	1.7	YFLDNLDR
1	0	0.81	1.2	1.8	KDLFEEK
0	3	0.87	1.2	1.7	MVLDVIAR

5.68 " 59 2365 minus strand Aconitate hydratase, mitochondrial [Crassostrea gigas]"

Protein Accession	CL13509.Contig1$_{H1}$
Mean Expression Ratio	0.806
Median Expression Ratio	0.805
Credible Interval	(0.619, 1.05)
Associated Peptides	6
Associated Spectra	9
Coverage	NaN

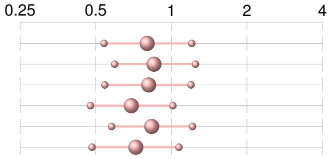

A	B	2.5	50	97.5	Sequence
1	0	0.54	0.8	1.2	HTMNEGQIEWFR
1	1	0.59	0.85	1.3	KNGWPLNIR
1	0	0.55	0.81	1.2	DLKPGAPVVCEIK
1	1	0.48	0.69	1	IHETNLK
0	2	0.58	0.83	1.2	VAGILTVK
0	1	0.48	0.72	1.1	LQLLQPFEK

5.69 57 2378 minus strand Heterogeneous nuclear ribonucleoprotein U-like protein 1 [Crassostrea gigas]

Protein Accession	CL15560.Contig5$_{H}1$
Mean Expression Ratio	0.805
Median Expression Ratio	0.806
Credible Interval	(0.563, 1.16)
Associated Peptides	2
Associated Spectra	2
Coverage	NaN

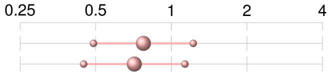

A	B	2.5	50	97.5	Sequence
**0**	**1**	**0.49**	**0.78**	**1.2**	**ALFPHILTK**
**0**	**1**	**0.45**	**0.71**	**1.1**	**VTELRDELK**

5.70 2 772 minus strand arylsulfatase [Haliotis discus discus]

Protein Accession	CL48181.Contig1$_{H}1$
Mean Expression Ratio	1.24
Median Expression Ratio	1.24
Credible Interval	(0.882, 1.76)
Associated Peptides	2
Associated Spectra	3
Coverage	NaN

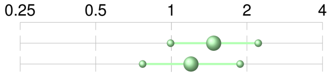

A	B	2.5	50	97.5	Sequence
**1**	**1**	**0.99**	**1.5**	**2.2**	**LLPQLK**
**0**	**1**	**0.77**	**1.2**	**1.9**	**GVGMVVDALK**

5.71 459 755 minus strand heat shock protein beta-1 [Trichinella spiralis] gi; 464 763 minus strand heat shock protein beta-1 [Trichinella spiralis] gi; 437 736 minus strand heat shock protein beta-1 [Trichinella spiralis] gi; 437 736 minus strand heat shock protein beta-1 [Trichinella spiralis] gi; 437 736 minus strand heat shock protein beta-1 [Trichinella spiralis] gi

Protein Accession	CL17234.Contig5$_{H}1$ CL17234.Contig4$_{H}1$ CL17234.Contig3$_{H}1$ CL17234.Contig2$_{H}1$ CL1723
Mean Expression Ratio	0.809
Median Expression Ratio	0.808
Credible Interval	(0.578, 1.13)
Associated Peptides	3
Associated Spectra	8
Coverage	NaN

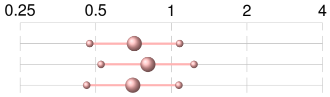

A	B	2.5	50	97.5	Sequence
**2**	**1**	**0.47**	**0.71**	**1.1**	**DVEPTTVTSSLNADGVLTLK**
**2**	**1**	**0.52**	**0.8**	**1.2**	**IVVEGCHEEK**
**1**	**1**	**0.46**	**0.7**	**1.1**	**IVVEGCHEEKQDEHGFITR**

5.72 336 641 minus strand malate dehydrogenase [Schistosoma japonicum]

Protein Accession	CL11441.Contig9$_H$1
Mean Expression Ratio	1.24
Median Expression Ratio	1.23
Credible Interval	(0.837, 1.83)
Associated Peptides	1
Associated Spectra	1
Coverage	NaN

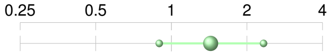

A	B	2.5	50	97.5	Sequence
0	1	0.89	1.4	2.3	ALADLLVAADHR

5.73 424 3507 minus strand putative Na+/K+-ATPase alpha subunit [Paroctopus digueti]

Protein Accession	Unigene11494$_H$1
Mean Expression Ratio	0.81
Median Expression Ratio	0.811
Credible Interval	(0.594, 1.11)
Associated Peptides	5
Associated Spectra	5
Coverage	NaN

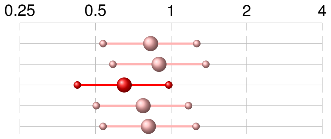

A	B	2.5	50	97.5	Sequence
1	0	0.54	0.83	1.3	SAEFTHENPLETR
0	1	0.59	0.89	1.4	DMTPAQIDEILR
0	1	0.42	0.65	0.98	MTVAHMWFDGR
0	1	0.5	0.77	1.2	VIMVTGDHPITAK
0	1	0.54	0.81	1.3	VIVAHGFK

5.74 91 1326 minus strand Adipocyte plasma membrane-associated protein [Crassostrea gigas]; 107 1273 minus strand Adipocyte plasma membrane-associated protein [Crassostrea gigas]

Protein Accession	CL11239.Contig2$_H$1 CL11239.Contig1$_H$1
Mean Expression Ratio	0.811
Median Expression Ratio	0.811
Credible Interval	(0.573, 1.15)
Associated Peptides	1
Associated Spectra	3
Coverage	NaN

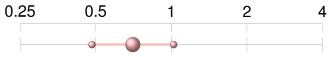

A	B	2.5	50	97.5	Sequence
0	3	0.48	0.7	1	YGLIAELDKEGK

5.75 260 892 minus strand calponin [Haliotis diversicolor]

Protein Accession	CL48702.Contig1$_{H}1$
Mean Expression Ratio	1.23
Median Expression Ratio	1.23
Credible Interval	(1.05, 1.44)
Associated Peptides	11
Associated Spectra	107
Coverage	NaN

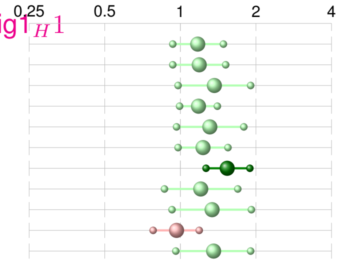

A	B	2.5	50	97.5	Sequence
7	4	0.93	1.2	1.5	AATGQQFPIEESNER
7	3	0.93	1.2	1.5	NFSEDKLR
2	0	0.98	1.4	1.9	TFQPASNLAF
18	8	0.99	1.2	1.4	TFQPASNLAFEAAR
1	3	0.97	1.3	1.8	VESSFDPEDAK
7	5	0.98	1.2	1.5	VESSFDPEDAKK
12	5	1.3	1.5	1.9	VMENFYK
1	1	0.86	1.2	1.7	YKVMENFYK
1	0	0.93	1.3	1.9	AKVESSFDPEDAKK
7	13	0.78	0.96	1.2	IELFIQK
1	1	0.96	1.4	1.9	VLACIR

5.76 " 81 1349 minus strand PREDICTED: LOW QUALITY PROTEIN: dolichyl-diphosphooligosaccharide–protein glycosyltransferase 48 kDa subunit-like, partial [Cavia porcellus]; 81 1349 minus strand PREDICTED: LOW QUALITY PROTEIN: dolichyl-diphosphooligosaccharide–protein glycosyltransferase 48 kDa subunit-like, partial [Cavia porcellus]"

Protein Accession	CL49524.Contig2$_{H}1$ CL49524.Contig1$_{H}1$
Mean Expression Ratio	1.23
Median Expression Ratio	1.23
Credible Interval	(0.873, 1.75)
Associated Peptides	2
Associated Spectra	3
Coverage	NaN

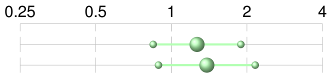

A	B	2.5	50	97.5	Sequence
1	1	0.85	1.3	1.9	DLGFDLTFK
1	0	0.89	1.4	2.2	FPQSGNLELAK

5.77 793 1269 PREDICTED: calmodulin-like [Saccoglossus kowalevskii]

Protein Accession	CL42648.Contig1$_{H}1$
Mean Expression Ratio	0.813
Median Expression Ratio	0.814
Credible Interval	(0.614, 1.07)
Associated Peptides	5
Associated Spectra	8
Coverage	NaN

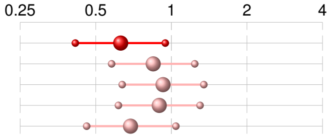

A	B	2.5	50	97.5	Sequence
1	0	0.42	0.63	0.95	EADKDGNGLSIQELASLIR
1	1	0.58	0.85	1.2	KVSLVDITDEEIIK
2	0	0.64	0.93	1.3	KIQESELK
0	2	0.62	0.9	1.3	IFLDLDDNCDR
0	1	0.46	0.69	1	LAEADVDR

5.78 3 470 minus strand Spectrin beta chain [Crassostrea gigas]; 5 451 minus strand Spectrin beta chain [Crassostrea gigas]

Protein Accession	CL19757.Contig2$_H1$ CL19757.Contig1$_H1$
Mean Expression Ratio	1.23
Median Expression Ratio	1.23
Credible Interval	(0.833, 1.84)
Associated Peptides	1
Associated Spectra	1
Coverage	NaN

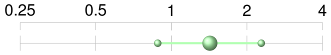

A	B	2.5	50	97.5	Sequence
**0**	**1**	**0.89**	**1.4**	**2.3**	**HLMGVEDLLQK**

5.79 3 503 minus strand elongation factor-1 alpha [Pomacea bridgesi]

Protein Accession	Unigene72143$_H1$
Mean Expression Ratio	1.22
Median Expression Ratio	1.22
Credible Interval	(0.86, 1.76)
Associated Peptides	2
Associated Spectra	2
Coverage	NaN

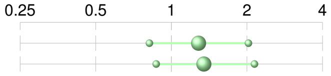

A	B	2.5	50	97.5	Sequence
**0**	**1**	**0.82**	**1.3**	**2**	**QLIICANK**
**0**	**1**	**0.87**	**1.4**	**2.1**	**LVSPR**

5.80 186 1778 predicted protein [Nematostella vectensis] gi

Protein Accession	CL20488.Contig9$_H1$
Mean Expression Ratio	1.22
Median Expression Ratio	1.22
Credible Interval	(0.886, 1.69)
Associated Peptides	3
Associated Spectra	4
Coverage	NaN

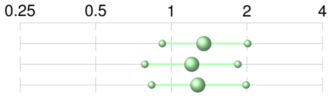

A	B	2.5	50	97.5	Sequence
**1**	**1**	**0.92**	**1.4**	**2**	**EGTENVEMHFR**
**0**	**1**	**0.79**	**1.2**	**1.8**	**DGTTHVIWK**
**0**	**1**	**0.84**	**1.3**	**2**	**TRQLLK**

5.81 57 1688 Talin-1 [Camponotus floridanus]; 57 1865 Talin-1 [Camponotus floridanus]; 57 1835 Talin-1 [Camponotus floridanus]

Protein Accession	CL49468.Contig2$_H1$ CL45858.Contig2$_H1$ CL45858.Contig1$_H1$
Mean Expression Ratio	1.21
Median Expression Ratio	1.21
Credible Interval	(0.877, 1.67)
Associated Peptides	2
Associated Spectra	5
Coverage	NaN

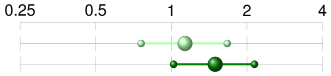

A	B	2.5	50	97.5	Sequence
**2**	**0**	**0.76**	**1.1**	**1.7**	**KAQEPIVYAGK**
**3**	**0**	**1**	**1.5**	**2.1**	**ISIQAR**

5.82 266 1123 minus strand mitochondrial malate dehydrogenase precursor [Nucella lapillus]

Protein Accession	CL10426.Contig11$_{H}1$
Mean Expression Ratio	0.826
Median Expression Ratio	0.826
Credible Interval	(0.579, 1.19)
Associated Peptides	1
Associated Spectra	3
Coverage	NaN

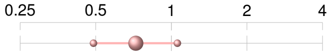

A	B	2.5	50	97.5	Sequence
**1**	**2**	**0.49**	**0.72**	**1.1**	**SLIEALNGMEGR**

5.83 198 821 minus strand sigma class glutathione-s-transferase 2 [Haliotis discus discus]

Protein Accession	CL1352.Contig4$_{H}1$
Mean Expression Ratio	0.824
Median Expression Ratio	0.827
Credible Interval	(0.554, 1.22)
Associated Peptides	1
Associated Spectra	1
Coverage	NaN

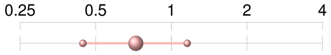

A	B	2.5	50	97.5	Sequence
**0**	**1**	**0.45**	**0.72**	**1.2**	**AAGQPFDDNVVDR**

5.84 353 2161 Bifunctional 3'-phosphoadenosine 5'-phosphosulfate synthetase [Crassostrea gigas]; 141 1943 Bifunctional 3'-phosphoadenosine 5'-phosphosulfate synthetase [Crassostrea gigas]

Protein Accession	CL8992.Contig2$_{H}1$ CL8992.Contig1$_{H}1$
Mean Expression Ratio	1.21
Median Expression Ratio	1.21
Credible Interval	(0.905, 1.62)
Associated Peptides	4
Associated Spectra	7
Coverage	NaN

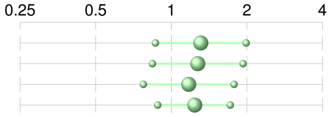

A	B	2.5	50	97.5	Sequence
**1**	**0**	**0.87**	**1.3**	**2**	**QHAAILEEK**
**1**	**0**	**0.84**	**1.3**	**1.9**	**QFGTSNLGHPYVK**
**1**	**0**	**0.78**	**1.2**	**1.8**	**AGPTEVQWHAK**
**2**	**2**	**0.88**	**1.2**	**1.7**	**LDGSAAFALK**

5.85 593 862 minus strand mitochondrial ATP synthase coupling factor 6 precursor [Ochlerotatus taeniorhynchus]

Protein Accession	Unigene4216$_H$1
Mean Expression Ratio	0.828
Median Expression Ratio	0.828
Credible Interval	(0.581, 1.19)
Associated Peptides	1
Associated Spectra	3
Coverage	NaN

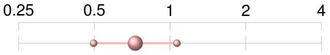

A	B	2.5	50	97.5	Sequence
0	3	0.5	0.72	1.1	LVDASPTVQAALKEELEK

5.86 124 711 glutathione S-transferase sigma 3 [Mytilus galloprovincialis]; 124 711 glutathione S-transferase sigma 3 [Mytilus galloprovincialis]; 110 697 glutathione S-transferase sigma 3 [Mytilus galloprovincialis]

Protein Accession	CL27284.Contig5$_H$1 CL27284.Contig4$_H$1 CL27284.Contig3$_H$1
Mean Expression Ratio	1.21
Median Expression Ratio	1.21
Credible Interval	(0.873, 1.66)
Associated Peptides	3
Associated Spectra	4
Coverage	NaN

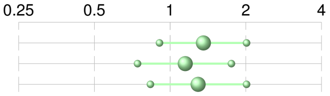

A	B	2.5	50	97.5	Sequence
1	1	0.91	1.4	2	VDEATQGVVDIR
1	0	0.74	1.2	1.8	TALYGYAFEKDEAR
1	0	0.84	1.3	2	LVPEAEK

5.87 " 142 1284 minus strand Chain O, Crsytal Structure Of Human Liver Gapdh gi|67464044|pdb|1ZNQ|P Chain P, Crsytal Structure Of Human Liver Gapdh gi|67464045|pdb|1ZNQ|R Chain R, Crsytal Structure Of Human Liver Gapdh gi|67464046|pdb|1ZNQ|Q Chain Q, Crsytal Structure Of Human Liver Gapdh"

Protein Accession	CL34318.Contig1$_H$1
Mean Expression Ratio	1.21
Median Expression Ratio	1.21
Credible Interval	(1.01, 1.44)
Associated Peptides	7
Associated Spectra	51
Coverage	NaN

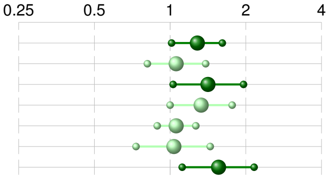

A	B	2.5	50	97.5	Sequence
5	6	1	1.3	1.6	DPTAIPWR
4	2	0.81	1.1	1.4	INVYQEKDPTAIPWR
2	1	1	1.4	2	VIPELNGK
4	1	1	1.3	1.8	INVYQEK
0	21	0.89	1.1	1.3	IGINGFGR
0	2	0.73	1	1.4	VIPELNGKLTGMAFR
0	3	1.1	1.6	2.2	CLAPLAK

5.88 " 17 283 Glycerol-3-phosphate dehydrogenase [NAD+], cytoplasmic [Crassostrea gigas]"

Protein Accession	Unigene9883$_{H}1$
Mean Expression Ratio	0.831
Median Expression Ratio	0.831
Credible Interval	(0.578, 1.19)
Associated Peptides	2
Associated Spectra	2
Coverage	NaN

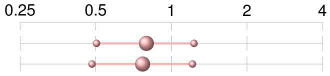

A	B	2.5	50	97.5	Sequence
0	1	0.5	0.79	1.2	KLTEIVNTEHENVK
0	1	0.48	0.77	1.2	LTEIVNTEHENVK

5.89 1 987 minus strand RecName: Full=Tubulin beta chain; AltName: Full=Beta-tubulin

Protein Accession	Unigene7954$_{H}1$
Mean Expression Ratio	1.2
Median Expression Ratio	1.2
Credible Interval	(0.891, 1.62)
Associated Peptides	3
Associated Spectra	7
Coverage	NaN

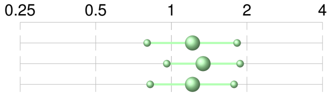

A	B	2.5	50	97.5	Sequence
1	0	0.8	1.2	1.8	AILVDLEPGTMDSVR
4	0	0.96	1.3	1.9	ALTVPELTQQMFDAK
2	0	0.82	1.2	1.8	PELTQQMFDAK

5.90 201 779 minus strand sigma class glutathione-s-transferase 2 [Haliotis discus discus]

Protein Accession	Unigene22998$_{H}1$
Mean Expression Ratio	0.832
Median Expression Ratio	0.832
Credible Interval	(0.63, 1.1)
Associated Peptides	3
Associated Spectra	12
Coverage	NaN

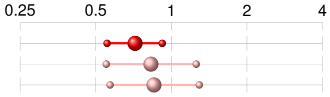

A	B	2.5	50	97.5	Sequence
4	6	0.56	0.72	0.92	LVLALADQK
1	0	0.55	0.83	1.3	KLEEEEFPK
0	1	0.57	0.85	1.3	YFGMFQK

5.91 194 904 Aldo-keto reductase family 1 member B10 [Crassostrea gigas]

Protein Accession	CL3558.Contig4$_{H}1$
Mean Expression Ratio	0.833
Median Expression Ratio	0.833
Credible Interval	(0.56, 1.24)
Associated Peptides	1
Associated Spectra	1
Coverage	NaN

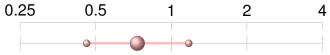

A	B	2.5	50	97.5	Sequence
0	1	0.46	0.73	1.2	ALEVLVSEGK

5.92 232 654 hypothetical protein BRAFLDRAFT_87758 [Branchiostoma floridae] gi

Protein Accession	CL34711.Contig8$_{H}1$
Mean Expression Ratio	1.2
Median Expression Ratio	1.2
Credible Interval	(0.816, 1.79)
Associated Peptides	1
Associated Spectra	1
Coverage	NaN

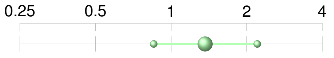

A	B	2.5	50	97.5	Sequence
**1**	**0**	**0.85**	**1.4**	**2.2**	**CVATGQGGYR**

5.93 1 378 minus strand PREDICTED: CG10098-like [Saccoglossus kowalevskii]

Protein Accession	Unigene15933$_{H}1$
Mean Expression Ratio	0.836
Median Expression Ratio	0.836
Credible Interval	(0.577, 1.2)
Associated Peptides	2
Associated Spectra	2
Coverage	NaN

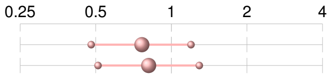

A	B	2.5	50	97.5	Sequence
**1**	**0**	**0.48**	**0.76**	**1.2**	**AFNASFEGLSLSEK**
**1**	**0**	**0.51**	**0.81**	**1.3**	**ALMAEK**

5.94 " 3 239 minus strand laminin beta 1 chain, putative [Ixodes scapularis] gi"

Protein Accession	CL48498.Contig2$_{H}1$
Mean Expression Ratio	1.2
Median Expression Ratio	1.2
Credible Interval	(0.863, 1.67)
Associated Peptides	2
Associated Spectra	4
Coverage	NaN

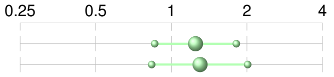

A	B	2.5	50	97.5	Sequence
**2**	**1**	**0.86**	**1.2**	**1.8**	**LAQQAEQLLTTK**
**1**	**0**	**0.84**	**1.3**	**2**	**ARNTSETVR**

5.95 62 1093 Heterogeneous nuclear ribonucleoprotein A2-like protein 1 [Crassostrea gigas]

Protein Accession	CL47083.Contig4$_{H}1$
Mean Expression Ratio	1.2
Median Expression Ratio	1.2
Credible Interval	(0.904, 1.58)
Associated Peptides	5
Associated Spectra	7
Coverage	NaN

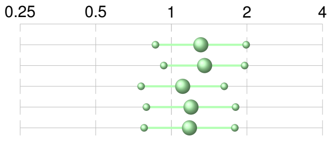

A	B	2.5	50	97.5	Sequence
**1**	**0**	**0.87**	**1.3**	**2**	**ETFSPFGTISQIDLITDK**
**2**	**0**	**0.93**	**1.4**	**2**	**EDTTEDMIR**
**1**	**1**	**0.76**	**1.1**	**1.6**	**MFVGGIKEDTTEDMIR**
**0**	**1**	**0.79**	**1.2**	**1.8**	**GGSRGGR**
**0**	**1**	**0.78**	**1.2**	**1.8**	**GGGRGGSR**

5.96 38 436 Calcium-dependent protein kinase isoform 2 [Crassostrea gigas]

Protein Accession	CL5866.Contig5$_{H}1$
Mean Expression Ratio	0.835
Median Expression Ratio	0.837
Credible Interval	(0.56, 1.23)
Associated Peptides	1
Associated Spectra	1
Coverage	NaN

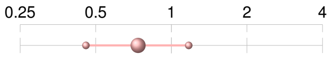

A	B	2.5	50	97.5	Sequence
**1**	**0**	**0.46**	**0.74**	**1.2**	**SHAGLDVEADK**

5.97 62 1174 Tyrosine-protein kinase CSK [Crassostrea gigas]; 62 1174 minus strand Tyrosine-protein kinase CSK [Crassostrea gigas]

Protein Accession	CL22249.Contig5$_{H}1$ CL22249.Contig3$_{H}1$
Mean Expression Ratio	0.837
Median Expression Ratio	0.837
Credible Interval	(0.573, 1.21)
Associated Peptides	1
Associated Spectra	2
Coverage	NaN

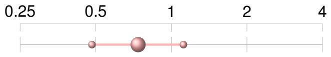

A	B	2.5	50	97.5	Sequence
**0**	**2**	**0.48**	**0.74**	**1.1**	**IPLTDVVMHVER**

5.98 401 1174 minus strand Mechanosensory protein 2 [Crassostrea gigas]

Protein Accession	Unigene7703$_{H}1$
Mean Expression Ratio	1.19
Median Expression Ratio	1.19
Credible Interval	(0.813, 1.77)
Associated Peptides	1
Associated Spectra	1
Coverage	NaN

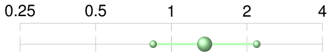

A	B	2.5	50	97.5	Sequence
**1**	**0**	**0.85**	**1.4**	**2.2**	**VIAAEGEQK**

5.99 12160 13014 twitchin [Mytilus galloprovincialis]

Protein Accession	CL181.Contig2$_{H}1$
Mean Expression Ratio	0.837
Median Expression Ratio	0.838
Credible Interval	(0.64, 1.09)
Associated Peptides	4
Associated Spectra	11
Coverage	NaN

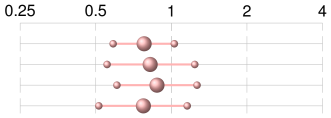

A	B	2.5	50	97.5	Sequence
**1**	**6**	**0.59**	**0.78**	**1**	**ATLTVEPGVEGLR**
**1**	**0**	**0.55**	**0.82**	**1.2**	**AVNSLGEEETSCK**
**1**	**1**	**0.61**	**0.87**	**1.3**	**AFYKPK**
**1**	**0**	**0.52**	**0.77**	**1.2**	**NQLSAEGHTAK**

5.100 269 880 minus strand myosin regulatory light chain [Fasciola hepatica]

Protein Accession	CL43006.Contig5$_{H}1$
Mean Expression Ratio	1.19
Median Expression Ratio	1.19
Credible Interval	(0.938, 1.52)
Associated Peptides	3
Associated Spectra	21
Coverage	NaN

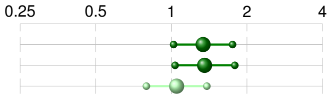

A	B	2.5	50	97.5	Sequence
**5**	**2**	**1**	**1.3**	**1.8**	**ATSNVFAK**
**7**	**0**	**1**	**1.4**	**1.8**	**KLMQEMK**
**4**	**3**	**0.8**	**1.1**	**1.4**	**LMQEMK**

5.101 459 755 minus strand heat shock protein beta-1 [Trichinella spiralis] gi

Protein Accession	CL17234.Contig5$_{H}1$
Mean Expression Ratio	0.838
Median Expression Ratio	0.839
Credible Interval	(0.61, 1.14)
Associated Peptides	3
Associated Spectra	8
Coverage	NaN

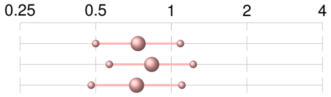

A	B	2.5	50	97.5	Sequence
**2**	**1**	**0.5**	**0.74**	**1.1**	**DVEPTTVTSSLNADGVLTLK**
**2**	**1**	**0.57**	**0.83**	**1.2**	**IVVEGCHEEK**
**1**	**1**	**0.48**	**0.73**	**1.1**	**IVVEGCHEEKQDEHGFITR**

5.102 35 1861 hypothetical protein DAPPUDRAFT_309082 [Daphnia pulex]

Protein Accession	CL49314.Contig5$_{H}1$
Mean Expression Ratio	0.84
Median Expression Ratio	0.84
Credible Interval	(0.653, 1.08)
Associated Peptides	6
Associated Spectra	11
Coverage	NaN

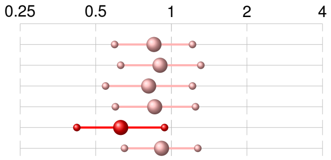

A	B	2.5	50	97.5	Sequence
**2**	**0**	**0.59**	**0.85**	**1.2**	**ELDVALTAVGLK**
**1**	**1**	**0.63**	**0.9**	**1.3**	**LINHSVPDTIDER**
**1**	**0**	**0.55**	**0.81**	**1.2**	**WVNYHLAR**
**1**	**1**	**0.6**	**0.86**	**1.2**	**YAIGLAR**
**1**	**0**	**0.42**	**0.63**	**0.94**	**DIGHTFK**
**0**	**3**	**0.65**	**0.91**	**1.3**	**MADGILLCK**

5.103 30 1088 arginine kinase [Conus novaehollandiae]

Protein Accession	CL5308.Contig5$_{H}1$
Mean Expression Ratio	1.19
Median Expression Ratio	1.19
Credible Interval	(1.08, 1.32)
Associated Peptides	31
Associated Spectra	203
Coverage	NaN

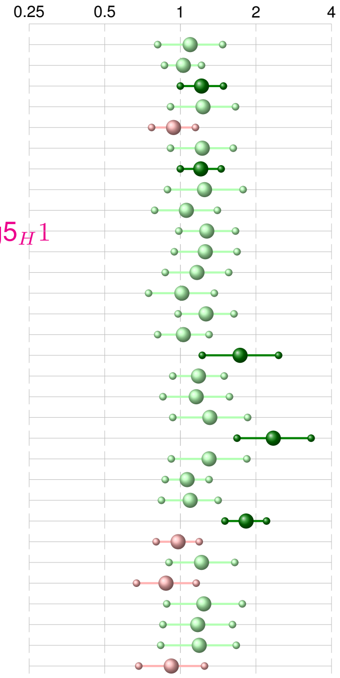

A	B	2.5	50	97.5	Sequence
2	1	0.81	1.1	1.5	AAGGYDDWPVGR
12	10	0.87	1	1.2	ATVEALNTFEGELK
8	8	1	1.2	1.5	FNFPDLDPSGK
2	1	0.91	1.2	1.7	GIHGEHTESVGGVYDISNK
9	5	0.77	0.94	1.2	GTYYPLLGMDK
2	2	0.91	1.2	1.6	KLIDDHFLFKDDDR
11	7	1	1.2	1.5	LAALPEFK
1	0	0.89	1.3	1.8	LGLSEFDAIMEMK
2	2	0.79	1.1	1.4	LIDDHFLFKDDDR
5	1	0.99	1.3	1.7	RFNFPDLDPSGK
3	1	0.95	1.3	1.7	SHASYGFPPVLSK
3	1	0.87	1.2	1.6	TFLVWVNEEDHIR
2	1	0.75	1	1.4	NGVEEILKK
7	0	0.98	1.3	1.6	KLTFAR
5	4	0.81	1	1.3	NGVEEILK
1	0	1.2	1.7	2.5	KLIDDHFLFK
6	3	0.94	1.2	1.5	LTFAR
2	1	0.85	1.2	1.6	EFCDK
1	0	0.93	1.3	1.8	TFEGELK
2	0	1.7	2.3	3.3	GTYYPLLGMDKATEK
1	0	0.92	1.3	1.8	APDFGEK
14	0	0.87	1.1	1.3	GGNLTQVYQR
5	1	0.84	1.1	1.4	HPAPDFGEK
10	7	1.5	1.8	2.2	LVSGIHALEK
7	9	0.8	0.98	1.2	YIVSTR
0	3	0.9	1.2	1.6	LIDDHFLFK
0	5	0.67	0.88	1.2	IISMQK
0	1	0.88	1.2	1.8	VAEVK
0	2	0.85	1.2	1.6	IDDHFLFK
0	1	0.84	1.2	1.7	ATVEALNTFEGE
0	3	0.68	0.92	1.2	GIFHNPDK

5.104 2 1093 minus strand Neurexin-1-alpha [Crassostrea gigas]

Protein Accession	Unigene11591$_{H}1$
Mean Expression Ratio	0.84
Median Expression Ratio	0.841
Credible Interval	(0.597, 1.17)
Associated Peptides	2
Associated Spectra	3
Coverage	NaN

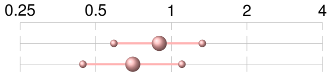

A	B	2.5	50	97.5	Sequence
1	1	0.59	0.89	1.3	IYVGGILDKEEK
0	1	0.44	0.7	1.1	IGDVVEAERPFSLIK

5.105 4 2400 IQ motif containing GTPase activating protein 2 [Xenopus laevis] gi; 4 2169 IQ motif containing GTPase activating protein 2 [Xenopus laevis] gi

Protein Accession: CL875.Contig2$_H$1 CL875.Contig1$_H$1
Mean Expression Ratio: 0.844
Median Expression Ratio: 0.844
Credible Interval: (0.572, 1.25)
Associated Peptides: 1
Associated Spectra: 1
Coverage: NaN

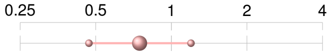

A	B	2.5	50	97.5	Sequence
0	1	0.47	0.75	1.2	ITLQDVVTHNR

5.106 4 579 minus strand Collagen alpha-3(VI) chain [Crassostrea gigas]

Protein Accession: CL46502.Contig3$_H$1
Mean Expression Ratio: 1.19
Median Expression Ratio: 1.18
Credible Interval: (0.864, 1.63)
Associated Peptides: 2
Associated Spectra: 6
Coverage: NaN

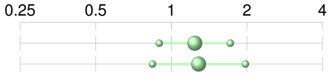

A	B	2.5	50	97.5	Sequence
5	0	0.9	1.2	1.7	FAVQEFSELR
1	0	0.84	1.3	2	LIKDFVK

5.107 137 853 14-3-3 protein [Heterodera glycines]

Protein Accession: CL9040.Contig4$_H$1
Mean Expression Ratio: 1.18
Median Expression Ratio: 1.18
Credible Interval: (0.875, 1.58)
Associated Peptides: 4
Associated Spectra: 6
Coverage: NaN

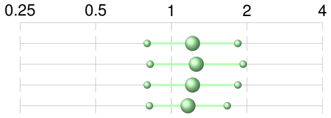

A	B	2.5	50	97.5	Sequence
1	0	0.8	1.2	1.8	AIECANQAYQK
1	0	0.83	1.3	1.9	EAFDEAISELDSLQGEDCK
1	0	0.8	1.2	1.8	NASNEEENVFYLK
3	0	0.82	1.2	1.7	VICMIEQK

5.108 3 416 minus strand myosin essential light chain [Crassostrea gigas]

Protein Accession: Unigene47974$_H$1
Mean Expression Ratio: 1.18
Median Expression Ratio: 1.18
Credible Interval: (0.858, 1.63)
Associated Peptides: 2
Associated Spectra: 5
Coverage: NaN

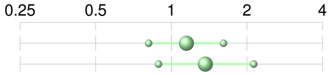

A	B	2.5	50	97.5	Sequence
0	4	0.81	1.1	1.6	CVGLNPTLATIK
0	1	0.89	1.4	2.1	NPTLATIK

5.109 405 1373 paramyosin [Haliotis discus discus]

Protein Accession	CL43455.Contig4$_H$1
Mean Expression Ratio	0.846
Median Expression Ratio	0.846
Credible Interval	(0.771, 0.93)
Associated Peptides	30
Associated Spectra	495
Coverage	NaN

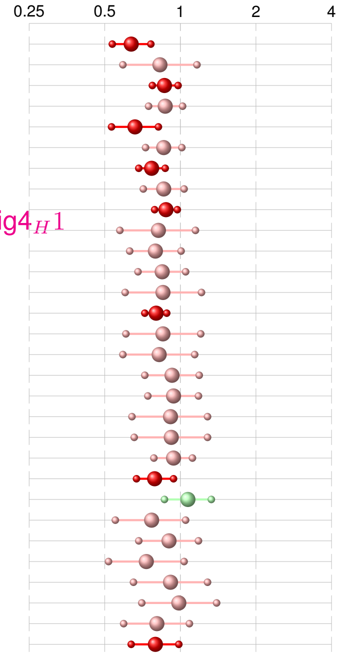

A	B	2.5	50	97.5	Sequence
14	7	0.53	0.64	0.76	DLELATAQFEAAEASLR
1	0	0.59	0.83	1.2	EKQTLVIELDGLSGQLDSITK
44	12	0.77	0.87	0.98	ELEDALDAER
23	5	0.75	0.87	1	HTEAVNDLSDQVDYLTK
5	7	0.53	0.66	0.82	IRELEDALDAER
14	10	0.73	0.86	1	LDEEAETSSNLR
33	19	0.68	0.77	0.87	LKGQVDDLTR
14	4	0.71	0.86	1	LTQENFDLQHQVQELDSSNAALAK
39	37	0.79	0.88	0.97	QLNDLTALK
1	0	0.57	0.82	1.1	QLNDLTALKAR
8	1	0.63	0.8	1	QNLQVNLSALQSDYDSLNAR
9	2	0.68	0.84	1.1	QTLVIELDGLSGQLDSITK
1	0	0.6	0.86	1.2	RHTEAVNDLSDQVDYLTK
45	38	0.72	0.8	0.88	TVAELSFR
1	0	0.61	0.85	1.2	LDQVQDSLDEQGSSTVAQIELNKK
1	1	0.59	0.82	1.1	ELEDALDAERDGR
4	3	0.72	0.93	1.2	KDLELATAQFEAAEASLR
9	1	0.74	0.94	1.2	DEQGSSTVAQIELNK
1	0	0.64	0.91	1.3	DEQGSSTVAQIELNKK
1	0	0.65	0.92	1.3	ESEIYK
20	0	0.79	0.94	1.1	LDQVQDSLDEQGSSTVAQIELNK
11	12	0.67	0.79	0.94	LSLESR
0	12	0.86	1.1	1.3	ITELEDSCEQLR
0	2	0.55	0.77	1	TRFDKELAAK
0	5	0.69	0.9	1.2	FDKELAAK
0	1	0.52	0.73	1	ITELEDSCEQLRV
0	1	0.65	0.91	1.3	ARLDEEAETSSNLR
0	1	0.7	0.99	1.4	LEDALDAER
0	3	0.6	0.8	1.1	TEELEETR
0	12	0.64	0.8	0.99	VSADYAALK

5.110 219 428 Peptidyl-prolyl cis-trans isomerase OS=Hemicentrotus pulcherrimus PE=2 SV=1

Protein Accession	CL39270.Contig2$_H$1
Mean Expression Ratio	1.18
Median Expression Ratio	1.18
Credible Interval	(0.849, 1.65)
Associated Peptides	1
Associated Spectra	5
Coverage	NaN

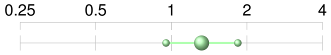

A	B	2.5	50	97.5	Sequence
2	3	0.95	1.3	1.8	IIIANSGQV

5.111 26 1909 Dolichyl-diphosphooligosaccharide–protein glycosyltransferase subunit 2 [Crassostrea gigas]

Protein Accession	CL6515.Contig1$_H$1
Mean Expression Ratio	0.848
Median Expression Ratio	0.848
Credible Interval	(0.649, 1.11)
Associated Peptides	6
Associated Spectra	10
Coverage	NaN

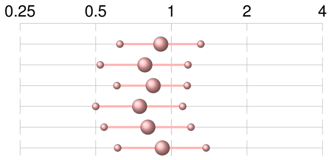

A	B	2.5	50	97.5	Sequence
1	1	0.62	0.9	1.3	AVSSALLDALK
1	0	0.52	0.78	1.2	KQFTASASDK
2	2	0.61	0.85	1.2	NLGLAVDEK
1	0	0.5	0.75	1.1	TLYELNFMEAK
1	0	0.54	0.8	1.2	LLTDAISAK
1	0	0.61	0.92	1.4	VSVTNLMGGDLGQLTVTADSAR

5.112 266 607 minus strand H2 [Haliotis tuberculata]

Protein Accession	Unigene62350$_H$1
Mean Expression Ratio	0.847
Median Expression Ratio	0.848
Credible Interval	(0.628, 1.13)
Associated Peptides	2
Associated Spectra	7
Coverage	NaN

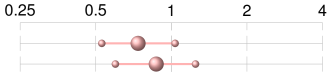

A	B	2.5	50	97.5	Sequence
3	1	0.53	0.74	1	YAVAGIGVR
2	1	0.6	0.87	1.2	KDINTLTDEEIK

5.113 459 1187 PREDICTED: protein-L-isoaspartate(D-aspartate) O-methyltransferase-like isoform 2 [Anolis carolinensis]

Protein Accession	CL3223.Contig3$_H$1
Mean Expression Ratio	1.18
Median Expression Ratio	1.18
Credible Interval	(0.813, 1.69)
Associated Peptides	1
Associated Spectra	2
Coverage	NaN

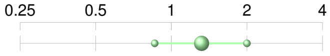

A	B	2.5	50	97.5	Sequence
2	0	0.86	1.3	2	AVGIEHIEELVK

5.114 " 1 1815 Glycogen phosphorylase, muscle form [Crassostrea gigas]"

Protein Accession	CL20630.Contig5$_H$1
Mean Expression Ratio	1.18
Median Expression Ratio	1.18
Credible Interval	(0.961, 1.45)
Associated Peptides	9
Associated Spectra	39
Coverage	NaN

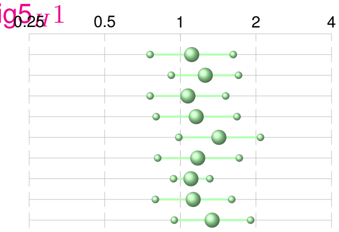

A	B	2.5	50	97.5	Sequence
1	0	0.76	1.1	1.6	VVFLENYR
2	2	0.92	1.3	1.7	VVNNDPIIGDR
1	1	0.76	1.1	1.5	TSFDMFPEK
1	0	0.8	1.2	1.7	WPVSMLER
1	0	0.99	1.4	2.1	DFAQMFPER
1	0	0.81	1.2	1.7	FGNPWEK
16	9	0.94	1.1	1.3	IEELEEIEEDAGL
0	2	0.8	1.1	1.6	AWDLVVR
0	2	0.95	1.3	1.9	LLLCNSGLADVIAEK

5.115 3 551 minus strand Spectrin beta chain [Crassostrea gigas]

Protein Accession	Unigene35960$_{H}$1
Mean Expression Ratio	0.848
Median Expression Ratio	0.85
Credible Interval	(0.594, 1.22)
Associated Peptides	2
Associated Spectra	2
Coverage	NaN

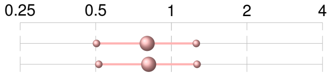

A	B	2.5	50	97.5	Sequence
**0**	**1**	**0.5**	**0.8**	**1.3**	**ELALREELIR**
**0**	**1**	**0.51**	**0.81**	**1.3**	**IVEKPPKFDEK**

5.116 643 1302 minus strand Kyphoscoliosis peptidase [Crassostrea gigas]; 634 1293 minus strand Kyphoscoliosis peptidase [Crassostrea gigas]; 634 1293 minus strand Kyphoscoliosis peptidase [Crassostrea gigas]; 634 1293 minus strand Kyphoscoliosis peptidase [Crassostrea gigas]; 639 1298 minus strand Kyphoscoliosis peptidase [Crassostrea gigas]

Protein Accession	CL7772.Contig1$_{H}$1 CL7772.Contig16$_{H}$1 CL7772.Contig15$_{H}$1 CL7772.Contig14$_{H}$1 CL7772.C
Mean Expression Ratio	1.17
Median Expression Ratio	1.17
Credible Interval	(0.974, 1.42)
Associated Peptides	9
Associated Spectra	41
Coverage	NaN

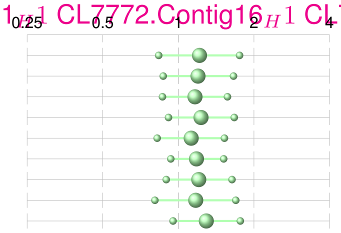

A	B	2.5	50	97.5	Sequence
**1**	**0**	**0.83**	**1.2**	**1.8**	**DGCYLYSPLVLNSSSR**
**2**	**2**	**0.87**	**1.2**	**1.7**	**FQIFALPTSDDSK**
**4**	**1**	**0.86**	**1.2**	**1.6**	**GAGEVWEGTAALDK**
**4**	**0**	**0.91**	**1.2**	**1.7**	**STTNLIQCR**
**3**	**1**	**0.83**	**1.1**	**1.5**	**VTLNACFGEDESK**
**7**	**6**	**0.93**	**1.2**	**1.5**	**LSNVTFK**
**3**	**2**	**0.9**	**1.2**	**1.6**	**QYAPWR**
**1**	**0**	**0.81**	**1.2**	**1.7**	**LELVSHPQAEFTLAQDNEVEIK**
**3**	**1**	**0.95**	**1.3**	**1.8**	**AVYPFPK**

5.117 300 878 unknown [Schistosoma japonicum]; 567 1145 unknown [Schistosoma japonicum]; 1110 1688 unknown [Schistosoma japonicum]; 94 672 unknown [Schistosoma japonicum]; 599 1177 unknown [Schistosoma japonicum]; 336 914 unknown [Schistosoma japonicum]

Protein Accession	CL11292.Contig5$_{H}$1 CL11292.Contig3$_{H}$1 CL11292.Contig2$_{H}$1 CL11292.Contig1$_{H}$1 CL1129
Mean Expression Ratio	0.851
Median Expression Ratio	0.851
Credible Interval	(0.645, 1.12)
Associated Peptides	4
Associated Spectra	12
Coverage	NaN

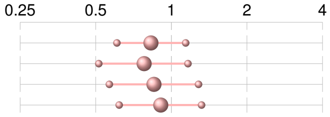

A	B	2.5	50	97.5	Sequence
**4**	**4**	**0.61**	**0.83**	**1.1**	**AGNDVDLVVQR**
**1**	**0**	**0.51**	**0.78**	**1.2**	**GVVNTQSLPQEPEGEKR**
**1**	**0**	**0.57**	**0.85**	**1.3**	**NGDAILQICGESTAAMTHAQAK**
**2**	**0**	**0.62**	**0.91**	**1.3**	**VQLRRYDTSQPWGFR**

5.118 62 505 Myosin-2 essential light chain [Crassostrea gigas]

Protein Accession	CL47077.Contig1$_{H}$1
Mean Expression Ratio	1.17
Median Expression Ratio	1.17
Credible Interval	(0.875, 1.57)
Associated Peptides	2
Associated Spectra	10
Coverage	NaN

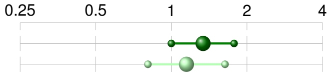

A	B	2.5	50	97.5	Sequence
**5**	**2**	**1**	**1.3**	**1.8**	**IAASQLGDVLR**
**2**	**1**	**0.8**	**1.2**	**1.6**	**HILTSLGEK**

5.119 72 1400 minus strand hypothetical protein RCJMB04_22i13 [Gallus gallus]

Protein Accession	CL44755.Contig5$_{H}$1
Mean Expression Ratio	0.852
Median Expression Ratio	0.852
Credible Interval	(0.655, 1.1)
Associated Peptides	6
Associated Spectra	10
Coverage	NaN

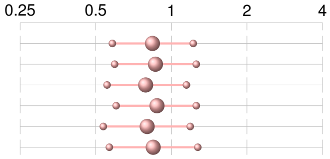

A	B	2.5	50	97.5	Sequence
**2**	**0**	**0.58**	**0.84**	**1.2**	**FLVASTPDEALQHAAELGK**
**1**	**1**	**0.6**	**0.86**	**1.3**	**LVFSADEAK**
**1**	**1**	**0.56**	**0.79**	**1.1**	**AVFQMR**
**1**	**1**	**0.6**	**0.88**	**1.3**	**LSGIPDVFVK**
**0**	**1**	**0.54**	**0.8**	**1.2**	**AIIAASSMK**
**0**	**1**	**0.57**	**0.85**	**1.3**	**LSTIIDLAR**

5.120 794 994 Glycogenin-1 [Crassostrea gigas]; 1425 1739 minus strand Glycogenin-1 [Crassostrea gigas]

Protein Accession	CL18608.Contig3$_{H}$1 CL18608.Contig2$_{H}$1
Mean Expression Ratio	1.18
Median Expression Ratio	1.17
Credible Interval	(0.838, 1.65)
Associated Peptides	3
Associated Spectra	3
Coverage	NaN

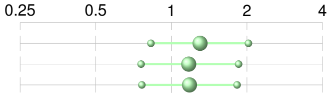

A	B	2.5	50	97.5	Sequence
**1**	**0**	**0.83**	**1.3**	**2**	**VGGGESADYLILDDR**
**1**	**0**	**0.76**	**1.2**	**1.9**	**VGGGESADYLILDDRER**
**0**	**1**	**0.76**	**1.2**	**1.8**	**IAAPVLSFK**

5.121 233 814 Kynurenine formamidase [Crassostrea gigas]; 233 979 Kynurenine formamidase [Crassostrea gigas]; 139 945 Kynurenine formamidase [Crassostrea gigas]; 145 951 Kynurenine formamidase [Crassostrea gigas]; 220 1026 Kynurenine formamidase [Crassostrea gigas]; 39 725 Kynurenine formamidase [Crassostrea gigas]; 198 986 Kynurenine formamidase [Crassostrea gigas]; 39 743 Kynurenine formamidase [Crassostrea gigas]; 123 929 Kynurenine formamidase [Crassostrea gigas]; 39 743 Kynurenine formamidase [Crassostrea gigas]

Protein Accession	Unigene7804$_H$1 Unigene42149$_H$1 CL8132.Contig9$_H$1 CL8132.Contig8$_H$1 CL8132.Contig7$_H$
Mean Expression Ratio	0.851
Median Expression Ratio	0.852
Credible Interval	(0.603, 1.2)
Associated Peptides	1
Associated Spectra	6
Coverage	NaN

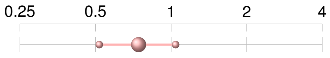

A	B	2.5	50	97.5	Sequence
**4**	**2**	**0.52**	**0.74**	**1**	**LVGPGVVIDVR**

5.122 109 510 minus strand hypothetical protein DAPPUDRAFT_231281 [Daphnia pulex]

Protein Accession	Unigene4945$_H$1
Mean Expression Ratio	0.851
Median Expression Ratio	0.852
Credible Interval	(0.641, 1.13)
Associated Peptides	4
Associated Spectra	8
Coverage	NaN

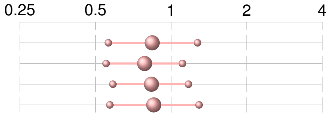

A	B	2.5	50	97.5	Sequence
**1**	**0**	**0.56**	**0.84**	**1.3**	**LAGIEDPFEMNVK**
**2**	**1**	**0.55**	**0.78**	**1.1**	**VSMPDTLGHAVGIER**
**2**	**1**	**0.59**	**0.83**	**1.2**	**YELLAK**
**0**	**1**	**0.57**	**0.85**	**1.3**	**PDTLGHAVGIER**

5.123 1 825 Spectrin alpha chain [Crassostrea gigas]

Protein Accession	CL10282.Contig3$_H$1
Mean Expression Ratio	1.17
Median Expression Ratio	1.17
Credible Interval	(0.864, 1.59)
Associated Peptides	3
Associated Spectra	6
Coverage	NaN

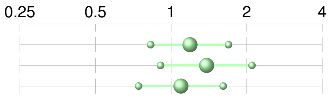

A	B	2.5	50	97.5	Sequence
**2**	**1**	**0.83**	**1.2**	**1.7**	**MQHNLEQQIEAR**
**1**	**0**	**0.9**	**1.4**	**2.1**	**IEDLGAAMEER**
**0**	**2**	**0.74**	**1.1**	**1.6**	**LILDNR**

5.124 82 816 PREDICTED: triosephosphate isomerase B [Oreochromis niloticus]

Protein Accession	CL26247.Contig4$_{H}1$
Mean Expression Ratio	1.17
Median Expression Ratio	1.17
Credible Interval	(0.962, 1.42)
Associated Peptides	8
Associated Spectra	28
Coverage	NaN

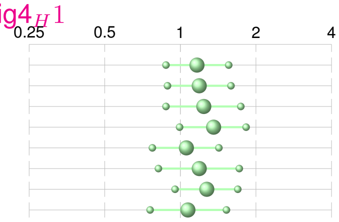

A	B	2.5	50	97.5	Sequence
3	2	0.88	1.2	1.6	AIADNVNDWK
3	2	0.89	1.2	1.6	GAFTGEISPAMIK
2	0	0.87	1.2	1.7	HVFGESDELIGQK
2	2	1	1.4	1.8	QFFVGGNWK
4	0	0.77	1.1	1.4	ELATKPDVDGFLVGGASLK
1	0	0.82	1.2	1.7	IGVAAQNCYKEPK
3	2	0.95	1.3	1.7	TEFITIINSK
0	2	0.76	1.1	1.5	SVDEIIK

5.125 2473 2898 RecName: Full=Paramyosin gi|397603|emb|CAA79849.1| paramyosin [Echinococcus granulosus]

Protein Accession	CL43455.Contig1$_{H}1$
Mean Expression Ratio	0.855
Median Expression Ratio	0.854
Credible Interval	(0.748, 0.975)
Associated Peptides	12
Associated Spectra	139
Coverage	NaN

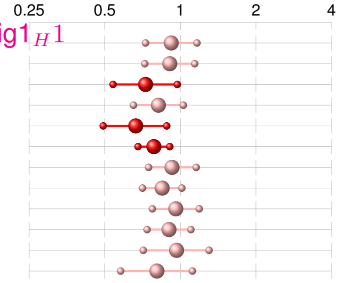

A	B	2.5	50	97.5	Sequence
5	4	0.73	0.92	1.2	AQTLIEEAENR
7	4	0.72	0.91	1.1	EILAQTEEER
2	2	0.54	0.73	0.98	EILAQTEEERR
6	4	0.65	0.82	1	INKEILAQTEEER
3	1	0.49	0.66	0.88	INKEILAQTEEERR
25	11	0.68	0.78	0.91	KAQTLIEEAENR
6	6	0.75	0.93	1.2	KQLEIEIR
14	6	0.71	0.85	1	QLAEFASLNEQLNLR
8	5	0.78	0.96	1.2	QLEEAEDVANPTMNK
9	6	0.74	0.9	1.1	QLEIEIR
1	2	0.71	0.96	1.3	RQLAEFASLNEQLNLR
1	1	0.58	0.81	1.1	RQLEEAEDVANPTMNK

5.126 94 933 four and A half lim domains [Schistosoma mansoni]

Protein Accession	CL7046.Contig6$_{H}1$
Mean Expression Ratio	1.17
Median Expression Ratio	1.17
Credible Interval	(0.96, 1.43)
Associated Peptides	8
Associated Spectra	27
Coverage	NaN

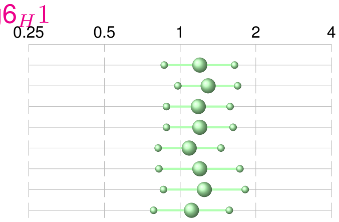

A	B	2.5	50	97.5	Sequence
2	1	0.86	1.2	1.7	CDASMVGR
4	2	0.98	1.3	1.7	CTKPITGIGGTK
3	2	0.88	1.2	1.6	FISFEDR
2	2	0.88	1.2	1.6	SLVDQPFAPK
3	2	0.82	1.1	1.5	SFIPR
1	0	0.82	1.2	1.7	CSGVINK
1	0	0.86	1.2	1.8	KFEYK
1	1	0.78	1.1	1.6	DDQPYCGDCFGELFAK

5.127 244 5451 alpha macroglobulin [Chlamys farreri]; 244 5493 alpha macroglobulin [Chlamys farreri]

Protein Accession	CL20671.Contig5$_{H}1$ CL20671.Contig4$_{H}1$
Mean Expression Ratio	0.854
Median Expression Ratio	0.855
Credible Interval	(0.63, 1.16)
Associated Peptides	4
Associated Spectra	5
Coverage	NaN

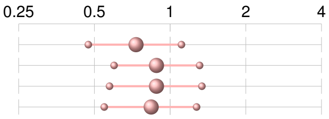

A	B	2.5	50	97.5	Sequence
1	0	0.47	0.73	1.1	DSFSLPLPLDMVPDSGR
1	1	0.6	0.88	1.3	HLALDVEQLNR
0	1	0.57	0.88	1.3	SLKIK
0	1	0.55	0.84	1.3	VAVNGIECINR

5.128 1027 1440 minus strand hemocyanin isoform 1 [Haliotis diversicolor]

Protein Accession	CL40281.Contig1$_H$1
Mean Expression Ratio	0.854
Median Expression Ratio	0.855
Credible Interval	(0.644, 1.13)
Associated Peptides	2
Associated Spectra	10
Coverage	NaN

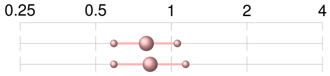

A	B	2.5	50	97.5	Sequence
3	3	0.59	0.79	1.1	LFVVQVEDELK
0	4	0.59	0.82	1.1	HNPFYDADIAFLQK

5.129 727 2022 RecName: Full=Enolase; AltName: Full=2-phospho-D-glycerate hydro-lyase; AltName: Full=2-phosphoglycerate dehydratase gi|1911573|gb|AAB50731.1| enolase [Doryteuthis pealeii]; 690 1985 RecName: Full=Enolase; AltName: Full=2-phospho-D-glycerate hydro-lyase; AltName: Full=2-phosphoglycerate dehydratase gi|1911573|gb|AAB50731.1| enolase [Doryteuthis pealeii]; 213 1508 RecName: Full=Enolase; AltName: Full=2-phospho-D-glycerate hydro-lyase; AltName: Full=2-phosphoglycerate dehydratase gi|1911573|gb|AAB50731.1| enolase [Doryteuthis pealeii]; 246 1541 RecName: Full=Enolase; AltName: Full=2-phospho-D-glycerate hydro-lyase; AltName: Full=2-phosphoglycerate dehydratase gi|1911573|gb|AAB50731.1| enolase [Doryteuthis pealeii]

Protein Accession	CL15687.Contig6$_H$1 CL15687.Contig5$_H$1 CL15687.Contig3$_H$1 CL15687.Contig2$_H$1
Mean Expression Ratio	1.17
Median Expression Ratio	1.17
Credible Interval	(1.04, 1.32)
Associated Peptides	25
Associated Spectra	122
Coverage	NaN

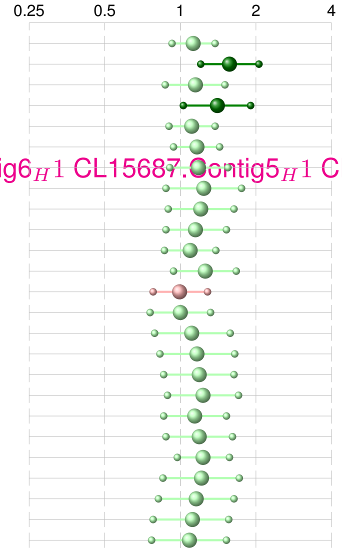

A	B	2.5	50	97.5	Sequence
11	4	0.92	1.1	1.4	AAGWGVMVSHR
3	3	1.2	1.6	2	AAVPSGASTGIYEALELR
3	2	0.87	1.1	1.5	DGKYDLDFK
2	1	1	1.4	1.9	FQIVGDDLLVTNPK
12	1	0.9	1.1	1.4	GVPLYR
9	4	0.94	1.2	1.4	IAPAFVGK
4	2	0.91	1.2	1.5	IEEELGVDAFYAGK
1	0	0.87	1.2	1.8	IEIGMDVAASEFCK
2	1	0.9	1.2	1.6	IMLDLDGTPNK
4	1	0.88	1.2	1.5	IMLDLDGTPNKEK
3	6	0.86	1.1	1.4	TLDLADQTAMDK
3	1	0.94	1.3	1.7	TSQQMIDMYHDFVK
4	4	0.78	1	1.3	VQMGIDR
1	4	0.76	1	1.3	QIFDSR
1	0	0.79	1.1	1.6	SYPIVTIEDPFDQDDWDAYTK
1	0	0.83	1.2	1.6	HIADLCGNKR
1	1	0.86	1.2	1.6	YDLDFK
1	1	0.89	1.2	1.7	LAMQEF
2	2	0.86	1.1	1.5	ACNALLLK
2	1	0.88	1.2	1.6	VNQIGSVSESIQAFK
5	4	0.97	1.2	1.6	YNQILR
0	1	0.85	1.2	1.7	HIADLCGNK
0	1	0.82	1.2	1.6	EGLELLK
0	1	0.78	1.1	1.6	MGSETYHHLK
0	1	0.77	1.1	1.5	ADLVVGLSTGQIK

5.130 460 1737 Tubulin beta-4B chain OS=Mus musculus GN=Tubb4b PE=1 SV=1

Protein Accession	Unigene34147$_H1$
Mean Expression Ratio	0.854
Median Expression Ratio	0.855
Credible Interval	(0.619, 1.17)
Associated Peptides	1
Associated Spectra	8
Coverage	NaN

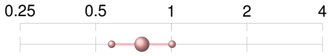

A	B	2.5	50	97.5	Sequence
**3**	**5**	**0.58**	**0.76**	**1**	**AVLVDLEPGTMDSVR**

5.131 50 577 hypothetical protein BRAFLDRAFT_119799 [Branchiostoma floridae] gi; 50 577 hypothetical protein BRAFLDRAFT_119799 [Branchiostoma floridae] gi

Protein Accession	CL5795.Contig4$_H1$ CL5795.Contig3$_H1$
Mean Expression Ratio	0.856
Median Expression Ratio	0.856
Credible Interval	(0.579, 1.26)
Associated Peptides	1
Associated Spectra	1
Coverage	NaN

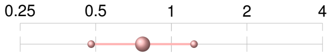

A	B	2.5	50	97.5	Sequence
**0**	**1**	**0.48**	**0.77**	**1.2**	**ADLGIDLSSVLGNVR**

5.132 176 517 PREDICTED: elongin C-like [Saccoglossus kowalevskii]; 115 456 PREDICTED: elongin C-like [Saccoglossus kowalevskii]; 994 1350 PREDICTED: elongin C-like [Saccoglossus kowalevskii]; 126 482 PREDICTED: elongin C-like [Saccoglossus kowalevskii]; 30 371 PREDICTED: elongin C-like [Saccoglossus kowalevskii]

Protein Accession	Unigene38248$_H1$ CL27549.Contig5$_H1$ CL27549.Contig4$_H1$ CL27549.Contig3$_H1$ CL27549.C
Mean Expression Ratio	1.17
Median Expression Ratio	1.17
Credible Interval	(0.797, 1.72)
Associated Peptides	1
Associated Spectra	1
Coverage	NaN

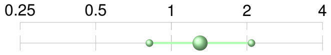

A	B	2.5	50	97.5	Sequence
**1**	**0**	**0.82**	**1.3**	**2.1**	**LISSDGHEFIVK**

5.133 245 1243 Prostaglandin reductase 1 [Crassostrea gigas]

Protein Accession	CL37890.Contig1$_{H}$1
Mean Expression Ratio	0.856
Median Expression Ratio	0.857
Credible Interval	(0.578, 1.27)
Associated Peptides	1
Associated Spectra	1
Coverage	NaN

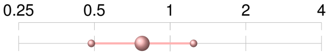

A	B	2.5	50	97.5	Sequence
1	0	0.49	0.78	1.2	TLAVLHPDMPR

5.134 3 4517 minus strand Clathrin heavy chain 1 [Crassostrea gigas]

Protein Accession	CL16446.Contig1$_{H}$1
Mean Expression Ratio	1.17
Median Expression Ratio	1.17
Credible Interval	(0.924, 1.48)
Associated Peptides	8
Associated Spectra	13
Coverage	NaN

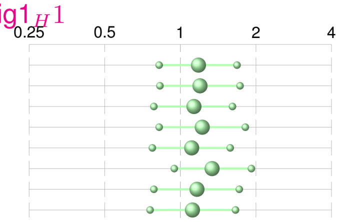

A	B	2.5	50	97.5	Sequence
2	0	0.82	1.2	1.7	KFEVNTSAIK
2	0	0.83	1.2	1.7	AQLNQGMVK
1	1	0.79	1.1	1.6	AGLLQR
1	0	0.83	1.2	1.8	KFNTLFQSGQYQEAAK
1	1	0.77	1.1	1.6	FLQMAR
2	0	0.95	1.3	1.9	VDGAR
1	0	0.78	1.2	1.7	NFLKEAK
0	1	0.76	1.1	1.7	AVNFFIK

5.135 635 1561 Uncharacterized protein yfeX [Crassostrea gigas]

Protein Accession	CL12046.Contig4$_{H}$1
Mean Expression Ratio	0.858
Median Expression Ratio	0.858
Credible Interval	(0.593, 1.24)
Associated Peptides	2
Associated Spectra	2
Coverage	NaN

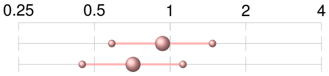

A	B	2.5	50	97.5	Sequence
1	0	0.59	0.93	1.5	TLESWVGR
1	0	0.45	0.71	1.1	LFDLAQLVLK

5.136 " 148 1989 Acyl-CoA synthetase family member 2, mitochondrial [Crassostrea gigas]; 146 1987 minus strand Acyl-CoA synthetase family member 2, mitochondrial [Crassostrea gigas]"

Protein Accession	CL19799.Contig6$_{H}$1 CL19799.Contig5$_{H}$1
Mean Expression Ratio	0.859
Median Expression Ratio	0.859
Credible Interval	(0.593, 1.25)
Associated Peptides	1
Associated Spectra	2
Coverage	NaN

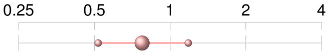

A	B	2.5	50	97.5	Sequence
0	2	0.52	0.77	1.2	LAGPGELGEILVR

5.137 523 1086 minus strand juvenile hormone diol kinase [Danaus plexippus]

Protein Accession	CL7590.Contig5$_H1$
Mean Expression Ratio	1.17
Median Expression Ratio	1.16
Credible Interval	(0.809, 1.69)
Associated Peptides	1
Associated Spectra	2
Coverage	NaN

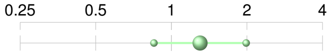

A	B	2.5	50	97.5	Sequence
2	0	0.85	1.3	2	GEFVTVQTIFGNSVEDSHK

5.138 "REVERSED 2 1810 minus strand Glutaminyl-tRNA synthetase, partial [Crassostrea gigas]"

Protein Accession	RRRRRCL16949.Contig1$_H1$
Mean Expression Ratio	1.16
Median Expression Ratio	1.16
Credible Interval	(0.804, 1.67)
Associated Peptides	1
Associated Spectra	2
Coverage	NaN

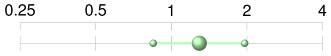

A	B	2.5	50	97.5	Sequence
2	0	0.85	1.3	2	GEELTVK

5.139 119 481 Glutamate receptor U1 [Crassostrea gigas]

Protein Accession	CL570.Contig5$_H1$
Mean Expression Ratio	1.16
Median Expression Ratio	1.16
Credible Interval	(0.963, 1.4)
Associated Peptides	7
Associated Spectra	49
Coverage	NaN

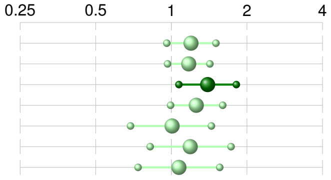

A	B	2.5	50	97.5	Sequence
9	3	0.96	1.2	1.5	GTANIGAGALTVTSER
10	7	0.97	1.2	1.4	RTPDGWSGIIGDVVK
6	1	1.1	1.4	1.8	TPDGWSGIIGDVVK
7	3	0.99	1.3	1.6	FQYTFR
0	1	0.69	1	1.4	AEVVK
0	1	0.82	1.2	1.7	GAGALTVTSER
0	1	0.74	1.1	1.6	GTANIGAGALTVT

5.140 227 829 Peptidyl-prolyl cis-trans isomerase B [Crassostrea gigas]

Protein Accession	CL16665.Contig2$_H1$
Mean Expression Ratio	1.16
Median Expression Ratio	1.16
Credible Interval	(0.958, 1.41)
Associated Peptides	7
Associated Spectra	38
Coverage	NaN

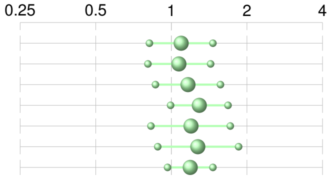

A	B	2.5	50	97.5	Sequence
4	1	0.82	1.1	1.5	DTNGSQFFFTLGPSR
3	2	0.81	1.1	1.4	MGPGADFQSPK
3	1	0.86	1.2	1.6	NYNGQGDDISGK
3	4	0.99	1.3	1.7	TLCDGWVR
1	0	0.83	1.2	1.7	FLDEK
1	0	0.88	1.3	1.9	NFTVTHEATLEIEVK
10	5	0.97	1.2	1.5	GFNFLR

5.141 169 1161 Basement membrane-specific heparan sulfate proteoglycan core protein OS=Homo sapiens GN=HSPG2 PE=1 SV=4

Protein Accession CL5208.Contig1$_{H}$1
Mean Expression Ratio 0.862
Median Expression Ratio 0.862
Credible Interval (0.595, 1.24)
Associated Peptides 2
Associated Spectra 2
Coverage NaN

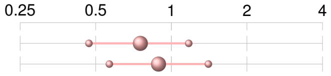

A	B	2.5	50	97.5	Sequence
1	0	0.47	0.75	1.2	GGDLTANHVVQGDVLR
1	0	0.57	0.89	1.4	PTITWSR

5.142 167 679 tropomyosin [Haliotis discus discus]

Protein Accession CL2925.Contig3$_{H}$1
Mean Expression Ratio 1.16
Median Expression Ratio 1.16
Credible Interval (1.03, 1.3)
Associated Peptides 19
Associated Spectra 203
Coverage NaN

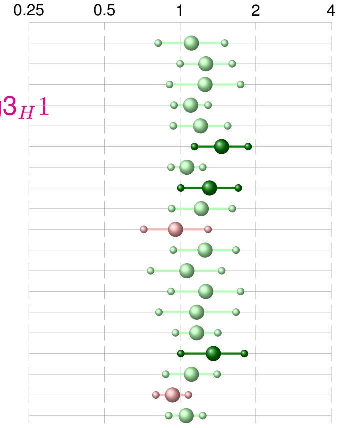

A	B	2.5	50	97.5	Sequence
2	1	0.82	1.1	1.5	AMDKAEQMEQK
6	3	1	1.3	1.6	ANEGLIEANTK
1	1	0.91	1.3	1.7	ENAMDKAEQMEQK
23	6	0.95	1.1	1.3	IKLLEEDLER
6	1	0.94	1.2	1.5	KYANLENEYDKANEGLIEANTK
8	0	1.1	1.5	1.9	MDKENAMDK
24	8	0.92	1.1	1.2	MDKENAMDKAEQMEQK
3	3	1	1.3	1.7	YANLENEYDK
5	0	0.93	1.2	1.6	YANLENEYDKANEGLIEANTK
2	2	0.72	0.96	1.3	KMLAMK
4	0	0.94	1.3	1.7	KYANLENEYDK
1	1	0.76	1.1	1.5	ANEGLIEAN
1	1	0.92	1.3	1.7	SKLEEDLN
1	0	0.82	1.2	1.7	LLEEDLER
3	13	0.96	1.2	1.4	RIKLLEEDLER
4	0	1	1.4	1.8	SKLEEDLNPLQK
8	1	0.88	1.1	1.4	LRDTEVQK
14	17	0.8	0.93	1.1	MDAIK
14	15	0.9	1.1	1.2	MLAMK

5.143 633 806 Peroxiredoxin-1 [Crassostrea gigas]; 648 821 Peroxiredoxin-1 [Crassostrea gigas]; 666 839 Peroxiredoxin-1 [Crassostrea gigas]; 625 798 Peroxiredoxin-1 [Crassostrea gigas]

Protein Accession CL3160.Contig18$_{H}$1 CL3160.Contig17$_{H}$1 CL3160.Contig16$_{H}$1 CL3160.Contig15$_{H}$1
Mean Expression Ratio 1.16
Median Expression Ratio 1.16
Credible Interval (0.862, 1.57)
Associated Peptides 2
Associated Spectra 13
Coverage NaN

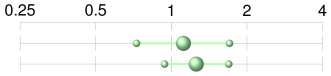

A	B	2.5	50	97.5	Sequence
1	0	0.73	1.1	1.7	EYFSK
6	6	0.94	1.3	1.7	LVQAFQFTDK

5.144 635 1294 minus strand Kyphoscoliosis peptidase [Crassostrea gigas]; 643 1302 minus strand Kyphoscoliosis peptidase [Crassostrea gigas]; 635 1294 minus strand Kyphoscoliosis peptidase [Crassostrea gigas]; 634 1293 minus strand Kyphoscoliosis peptidase [Crassostrea gigas]; 634 1293 minus strand Kyphoscoliosis peptidase [Crassostrea gigas]; 634 1293 minus strand Kyphoscoliosis peptidase [Crassostrea gigas]; 639 1298 minus strand Kyphoscoliosis peptidase [Crassostrea gigas]

Protein Accession	CL7772.Contig20$_{H}1$ CL7772.Contig1$_{H}1$ CL7772.Contig19$_{H}1$ CL7772.Contig16$_{H}1$ CL7772.[illegible]
Mean Expression Ratio	0.864
Median Expression Ratio	0.863
Credible Interval	(0.685, 1.09)
Associated Peptides	7
Associated Spectra	36
Coverage	NaN

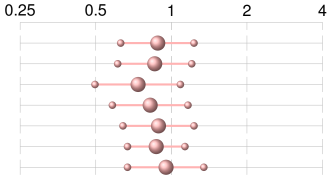

A	B	2.5	50	97.5	Sequence
2	2	0.63	0.88	1.2	FQIFALPTSDDSK
4	1	0.61	0.86	1.2	GAGEVWEGTAALDK
0	1	0.5	0.73	1.1	GAGEVWEGTAALDKHR
3	1	0.58	0.82	1.2	VTLNACFGEDESK
3	2	0.64	0.89	1.2	QYAPWR
7	6	0.67	0.87	1.1	LSNVTFK
3	1	0.67	0.96	1.4	AVYPFPK

5.145 27 377 minus strand ribosomal protein LP2 [Papilio xuthus] gi; 27 377 minus strand ribosomal protein LP2 [Papilio xuthus] gi; 27 377 minus strand ribosomal protein LP2 [Papilio xuthus] gi; 1538 1891 minus strand ribosomal protein LP2 [Papilio xuthus] gi

Protein Accession	Unigene53017$_{H}1$ Unigene53016$_{H}1$ Unigene53015$_{H}1$ CL49012.Contig9$_{H}1$
Mean Expression Ratio	0.865
Median Expression Ratio	0.864
Credible Interval	(0.688, 1.09)
Associated Peptides	7
Associated Spectra	16
Coverage	NaN

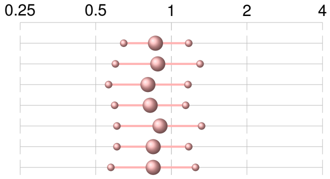

A	B	2.5	50	97.5	Sequence
4	1	0.64	0.87	1.2	KVVSQLSGK
1	0	0.6	0.88	1.3	SIDQLIAEGSR
2	0	0.56	0.81	1.2	VVSQLSGK
3	0	0.59	0.82	1.1	ILSSVGIEAEADK
1	0	0.61	0.9	1.3	AAAPAAEAK
1	2	0.61	0.85	1.2	ILSSVGIEAEADKVK
1	0	0.57	0.85	1.2	SGKEPTAADLEK

5.146 201 1181 minus strand Serine/threonine-protein phosphatase PP1-alpha catalytic subunit [Crassostrea gigas]

Protein Accession	CL28655.Contig4$_{H}1$
Mean Expression Ratio	0.865
Median Expression Ratio	0.864
Credible Interval	(0.614, 1.22)
Associated Peptides	2
Associated Spectra	3
Coverage	NaN

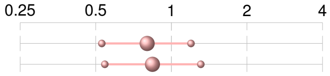

A	B	2.5	50	97.5	Sequence
1	1	0.53	0.8	1.2	ADTDKLNIDSIIAR
0	1	0.54	0.84	1.3	AHQVVEDGYEFFAK

5.147 316 531 cytochrome c oxidase subunit VIIa polypeptide 2 like [Xenopus (Silurana) tropicalis]

Protein Accession	CL17601.Contig5$_{H}$1
Mean Expression Ratio	0.866
Median Expression Ratio	0.864
Credible Interval	(0.589, 1.29)
Associated Peptides	1
Associated Spectra	1
Coverage	NaN

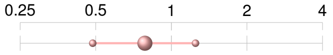

A	B	2.5	50	97.5	Sequence
0	1	0.48	0.78	1.2	QDGLLVWQK

5.148 193 828 Cysteine-rich secretory protein LCCL domain-containing 2 [Crassostrea gigas]

Protein Accession	CL40761.Contig2$_{H}$1
Mean Expression Ratio	1.16
Median Expression Ratio	1.16
Credible Interval	(0.821, 1.64)
Associated Peptides	3
Associated Spectra	3
Coverage	NaN

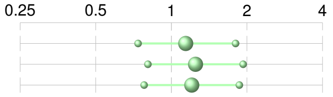

A	B	2.5	50	97.5	Sequence
1	0	0.73	1.1	1.8	LEWDAQMESEATSWAAR
1	0	0.81	1.2	1.9	YSPEGNWVNEKPYEK
1	0	0.78	1.2	1.9	ICQPLR

5.149 " 213 1499 minus strand Citrate synthase, mitochondrial [Crassostrea gigas]"

Protein Accession	CL992.Contig2$_{H}$1
Mean Expression Ratio	0.867
Median Expression Ratio	0.866
Credible Interval	(0.725, 1.04)
Associated Peptides	12
Associated Spectra	31
Coverage	NaN

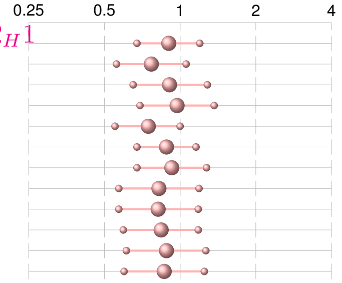

A	B	2.5	50	97.5	Sequence
4	1	0.68	0.9	1.2	ALGLPIERPK
3	0	0.56	0.77	1.1	ALVTETSVLDPEEGIR
1	1	0.65	0.91	1.3	DVLSQIIPEK
2	0	0.69	0.97	1.4	LMESLGGEVSDEQLR
3	1	0.55	0.75	1	SGQVVPGYGHAVLR
4	2	0.68	0.89	1.2	VVPDILIEQGK
3	0	0.67	0.93	1.3	SFDTPSLR
1	0	0.57	0.82	1.2	VGEVTVDMMYGGMR
1	0	0.57	0.82	1.2	EVSEFR
1	1	0.6	0.84	1.2	HLPNDPMFK
0	1	0.61	0.88	1.3	NFIWNTLK
0	1	0.6	0.86	1.2	LISQLYK

5.150 325 642 minus strand Protein phosphatase 1B [Crassostrea gigas]

Protein Accession	Unigene268$_H$1
Mean Expression Ratio	0.867
Median Expression Ratio	0.866
Credible Interval	(0.599, 1.25)
Associated Peptides	1
Associated Spectra	2
Coverage	NaN

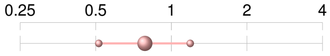

A	B	2.5	50	97.5	Sequence
1	1	0.51	0.78	1.2	VEMEDAHTAVVSIPHAR

5.151 183 953 minus strand glutathione-S-transferase [Haliotis diversicolor supertexta]; 275 1045 minus strand glutathione-S-transferase [Haliotis diversicolor supertexta]; 343 1113 minus strand glutathione-S-transferase [Haliotis diversicolor supertexta]

Protein Accession	Unigene40627$_H$1 CL6878.Contig3$_H$1 CL6878.Contig2$_H$1
Mean Expression Ratio	0.867
Median Expression Ratio	0.866
Credible Interval	(0.603, 1.24)
Associated Peptides	2
Associated Spectra	2
Coverage	NaN

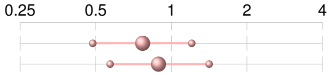

A	B	2.5	50	97.5	Sequence
0	1	0.49	0.77	1.2	LPHDPLPK
0	1	0.57	0.89	1.4	KIDPK

5.152 238 681 minus strand Globin OS=Cerithidea rhizophorarum PE=1 SV=1

Protein Accession	CL8458.Contig2$_H$1
Mean Expression Ratio	1.15
Median Expression Ratio	1.15
Credible Interval	(0.962, 1.38)
Associated Peptides	8
Associated Spectra	117
Coverage	NaN

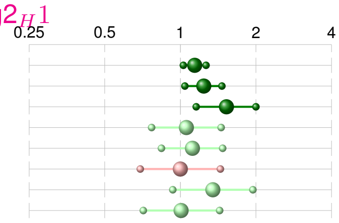

A	B	2.5	50	97.5	Sequence
42	33	1	1.1	1.3	AAITSTWSK
15	9	1	1.2	1.5	AYFPFLPGASEGDIK
4	2	1.2	1.5	2	ILAMDDADCLDGLGTK
0	3	0.77	1.1	1.5	PFLPGASEGDIK
0	5	0.84	1.1	1.5	PGASEGDIK
0	1	0.69	1	1.4	ATMENNGAK
0	1	0.93	1.3	1.9	EVFGSFLEATL
0	2	0.71	1	1.4	EVFGSFLEATLGGAATPDVK

5.153 " 2 286 Roundabout 2, partial [Ascaris suum]"

Protein Accession	CL8589.Contig2$_H$1
Mean Expression Ratio	1.15
Median Expression Ratio	1.15
Credible Interval	(0.803, 1.67)
Associated Peptides	2
Associated Spectra	2
Coverage	NaN

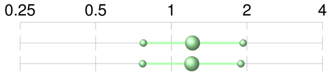

A	B	2.5	50	97.5	Sequence
1	0	0.77	1.2	1.9	GEPTPEITWLK
1	0	0.77	1.2	1.9	KSQTAVLDVIR

5.154 2 256 Polyubiquitin (Fragment) OS=Xenopus laevis PE=1 SV=2; 20 472 minus strand large subunit ribosomal protein L40e [Clonorchis sinensis]; 20 388 large subunit ribosomal protein L40e [Clonorchis sinensis]

Protein Accession	CL47507.Contig2$_{H}1$ CL39010.Contig2$_{H}1$ CL39010.Contig1$_{H}1$
Mean Expression Ratio	0.869
Median Expression Ratio	0.868
Credible Interval	(0.651, 1.17)
Associated Peptides	4
Associated Spectra	15
Coverage	NaN

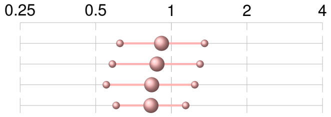

A	B	2.5	50	97.5	Sequence
2	1	0.62	0.92	1.4	TITLEVEPSDTIENVK
1	1	0.58	0.87	1.3	ESTLHLVLR
1	1	0.55	0.84	1.2	MQIFVK
4	4	0.6	0.83	1.1	TLSDYNIQK

5.155 425 1051 minus strand unknown [Schistosoma japonicum]; 300 878 unknown [Schistosoma japonicum]; 567 1145 unknown [Schistosoma japonicum]; 1110 1688 unknown [Schistosoma japonicum]; 94 672 unknown [Schistosoma japonicum]; 599 1177 unknown [Schistosoma japonicum]; 336 914 unknown [Schistosoma japonicum]

Protein Accession	CL11292.Contig9$_{H}1$ CL11292.Contig5$_{H}1$ CL11292.Contig3$_{H}1$ CL11292.Contig2$_{H}1$ CL1129
Mean Expression Ratio	1.15
Median Expression Ratio	1.15
Credible Interval	(0.843, 1.58)
Associated Peptides	2
Associated Spectra	9
Coverage	NaN

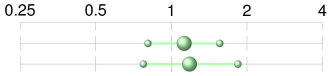

A	B	2.5	50	97.5	Sequence
4	4	0.81	1.1	1.6	AGNDVDLVVQR
0	1	0.77	1.2	1.8	RYDTSQPWGFR

5.156 406 1392 minus strand hypothetical protein CGI_10007007 [Crassostrea gigas]

Protein Accession	CL18045.Contig1$_{H}1$
Mean Expression Ratio	0.869
Median Expression Ratio	0.87
Credible Interval	(0.604, 1.25)
Associated Peptides	1
Associated Spectra	3
Coverage	NaN

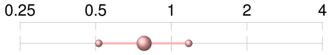

A	B	2.5	50	97.5	Sequence
2	1	0.51	0.78	1.2	IEAEFPGANIILHK

5.157 " 145 1755 Fumarate hydratase class I, aerobic [Crassostrea gigas]"

Protein Accession	CL32374.Contig1$_{H}1$
Mean Expression Ratio	1.15
Median Expression Ratio	1.15
Credible Interval	(0.845, 1.55)
Associated Peptides	3
Associated Spectra	6
Coverage	NaN

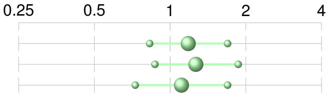

A	B	2.5	50	97.5	Sequence
**2**	**1**	**0.83**	**1.2**	**1.7**	**IGIGAQFGGK**
**1**	**1**	**0.87**	**1.3**	**1.9**	**KVDTPWK**
**0**	**1**	**0.73**	**1.1**	**1.7**	**LEQLE**

5.158 49 819 PREDICTED: elongation factor 1-delta-like isoform 2 [Strongylocentrotus purpuratus]

Protein Accession	CL10790.Contig12$_{H}1$
Mean Expression Ratio	0.87
Median Expression Ratio	0.87
Credible Interval	(0.644, 1.18)
Associated Peptides	4
Associated Spectra	7
Coverage	NaN

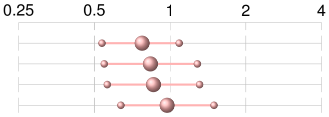

A	B	2.5	50	97.5	Sequence
**1**	**3**	**0.54**	**0.77**	**1.1**	**LVDLAYGIK**
**0**	**1**	**0.55**	**0.84**	**1.3**	**LLEEYAAK**
**0**	**1**	**0.56**	**0.86**	**1.3**	**RLEEIEK**
**0**	**1**	**0.64**	**0.97**	**1.5**	**EISLAR**

5.159 187 642 minus strand PREDICTED: similar to pol-like protein [Tribolium castaneum]

Protein Accession	CL5644.Contig3$_{H}1$
Mean Expression Ratio	1.15
Median Expression Ratio	1.15
Credible Interval	(0.856, 1.54)
Associated Peptides	1
Associated Spectra	23
Coverage	NaN

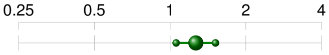

A	B	2.5	50	97.5	Sequence
**23**	**0**	**1.1**	**1.3**	**1.5**	**IILEQEELK**

5.160 9 518 minus strand hemocyanin isoform 1 [Nucula nucleus]

Protein Accession	Unigene71735$_{H}1$
Mean Expression Ratio	0.869
Median Expression Ratio	0.871
Credible Interval	(0.618, 1.21)
Associated Peptides	1
Associated Spectra	4
Coverage	NaN

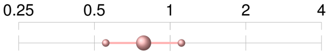

A	B	2.5	50	97.5	Sequence
**2**	**2**	**0.55**	**0.79**	**1.1**	**IDFEHADVAR**

5.161 1709 2026 Phosphoenolpyruvate carboxykinase [GTP] [Crassostrea gigas]

Protein Accession	CL25948.Contig2$_{H}$1
Mean Expression Ratio	0.87
Median Expression Ratio	0.871
Credible Interval	(0.631, 1.2)
Associated Peptides	1
Associated Spectra	8
Coverage	NaN

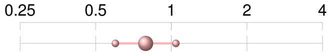

A	B	2.5	50	97.5	Sequence
5	3	0.6	0.79	1	GVPLVYEAK

5.162 60 1022 Major egg antigen [Crassostrea gigas]; 60 1022 Major egg antigen [Crassostrea gigas]

Protein Accession	CL18744.Contig3$_{H}$1 CL18744.Contig2$_{H}$1
Mean Expression Ratio	1.15
Median Expression Ratio	1.15
Credible Interval	(0.903, 1.47)
Associated Peptides	6
Associated Spectra	13
Coverage	NaN

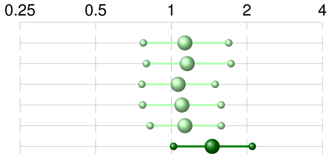

A	B	2.5	50	97.5	Sequence
1	0	0.77	1.1	1.7	QLTIEAPLKPATVQQR
1	0	0.79	1.2	1.7	TAIEVDSEGR
1	2	0.76	1.1	1.5	SEVHIPVQK
2	0	0.77	1.1	1.6	FDVSEFKPEEVSVK
1	3	0.82	1.1	1.6	ETFLPVR
0	2	1	1.5	2.1	LTVDIGEFSPDEIVVK

5.163 416 1717 minus strand T-complex protein 1 subunit delta OS=Aedes triseriatus PE=2 SV=1

Protein Accession	CL8566.Contig1$_{H}$1
Mean Expression Ratio	0.873
Median Expression Ratio	0.872
Credible Interval	(0.602, 1.26)
Associated Peptides	1
Associated Spectra	2
Coverage	NaN

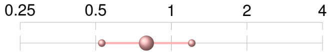

A	B	2.5	50	97.5	Sequence
1	1	0.53	0.8	1.2	HAAGELTAGINVR

5.164 327 731 RecName: Full=Arginine kinase; Short=AK gi|439719|dbj|BAA05100.1| arginine kinase [Haliotis madaka]

Protein Accession	Unigene63526$_{H}$1
Mean Expression Ratio	1.15
Median Expression Ratio	1.15
Credible Interval	(0.901, 1.47)
Associated Peptides	2
Associated Spectra	32
Coverage	NaN

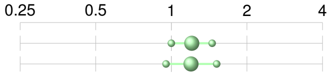

A	B	2.5	50	97.5	Sequence
10	10	1	1.2	1.4	DLYEQLK
7	5	0.95	1.2	1.5	FGGTLAECIR

5.165 " 34 1656 Chaperonin containing TCP1, subunit 7 (eta) [Danio rerio] gi; 34 1656 Chaperonin containing TCP1, subunit 7 (eta) [Danio rerio] gi"

Protein Accession	CL24061.Contig2$_{H}1$ CL24061.Contig1$_{H}1$
Mean Expression Ratio	0.872
Median Expression Ratio	0.873
Credible Interval	(0.601, 1.26)
Associated Peptides	1
Associated Spectra	2
Coverage	NaN

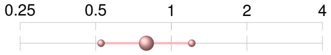

A	B	2.5	50	97.5	Sequence
2	0	0.53	0.8	1.2	SIAVSIKK

5.166 139 945 Kynurenine formamidase [Crassostrea gigas]; 145 951 Kynurenine formamidase [Crassostrea gigas]; 220 1026 Kynurenine formamidase [Crassostrea gigas]; 39 743 Kynurenine formamidase [Crassostrea gigas]; 123 929 Kynurenine formamidase [Crassostrea gigas]; 39 743 Kynurenine formamidase [Crassostrea gigas]

Protein Accession	CL8132.Contig9$_{H}1$ CL8132.Contig8$_{H}1$ CL8132.Contig7$_{H}1$ CL8132.Contig3$_{H}1$ CL8132.Con
Mean Expression Ratio	0.87
Median Expression Ratio	0.873
Credible Interval	(0.619, 1.22)
Associated Peptides	2
Associated Spectra	7
Coverage	NaN

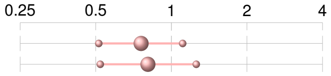

A	B	2.5	50	97.5	Sequence
4	2	0.52	0.76	1.1	LVGPGVVIDVR
0	1	0.52	0.81	1.3	WLTTNR

5.167 180 545 calreticulin precursor [Aplysia californica] gi

Protein Accession	CL4813.Contig2$_{H}1$
Mean Expression Ratio	0.872
Median Expression Ratio	0.873
Credible Interval	(0.63, 1.2)
Associated Peptides	4
Associated Spectra	11
Coverage	NaN

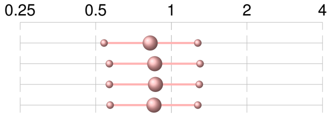

A	B	2.5	50	97.5	Sequence
0	1	0.54	0.82	1.3	EEFDANWEDR
1	1	0.56	0.86	1.3	FYGISAK
2	1	0.56	0.86	1.3	FVWSAGK
4	1	0.57	0.85	1.3	WIPSVHK

5.168 18 1613 minus strand chaperonin containing T-complex polypeptide subunit zeta [Haliotis discus hannai]; 527 2089 minus strand chaperonin containing T-complex polypeptide subunit zeta [Haliotis discus hannai]

Protein Accession	CL23722.Contig7$_H$1 CL23722.Contig5$_H$1
Mean Expression Ratio	1.14
Median Expression Ratio	1.14
Credible Interval	(0.814, 1.61)
Associated Peptides	2
Associated Spectra	3
Coverage	NaN

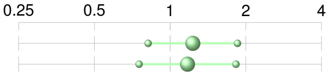

A	B	2.5	50	97.5	Sequence
**2**	**0**	**0.82**	**1.2**	**1.9**	**LITEGFELAR**
**1**	**0**	**0.75**	**1.2**	**1.8**	**AAQALAVNISAA**

5.169 166 2364 Dipeptidyl-peptidase 3 [Crassostrea gigas]

Protein Accession	CL9071.Contig2$_H$1
Mean Expression Ratio	1.14
Median Expression Ratio	1.14
Credible Interval	(0.82, 1.6)
Associated Peptides	3
Associated Spectra	3
Coverage	NaN

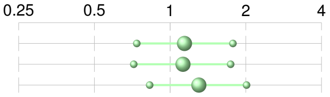

A	B	2.5	50	97.5	Sequence
**1**	**0**	**0.73**	**1.1**	**1.8**	**SFETGSIPAHK**
**1**	**0**	**0.72**	**1.1**	**1.7**	**YVILR**
**1**	**0**	**0.83**	**1.3**	**2**	**VTGADGKP**

5.170 2 169 cAMP-dependent protein kinase regulatory subunit [Crassostrea gigas]; 125 511 cAMP-dependent protein kinase regulatory subunit [Aplysia californica] gi

Protein Accession	CL24422.Contig2$_H$1 CL24422.Contig1$_H$1
Mean Expression Ratio	1.14
Median Expression Ratio	1.14
Credible Interval	(0.779, 1.69)
Associated Peptides	1
Associated Spectra	1
Coverage	NaN

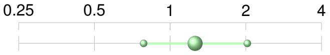

A	B	2.5	50	97.5	Sequence
**0**	**1**	**0.78**	**1.3**	**2**	**VLGPCSDILK**

5.171 118 1866 Lamin Dm0 [Crassostrea gigas]

Protein Accession	CL4079.Contig7$_H$1
Mean Expression Ratio	0.878
Median Expression Ratio	0.878
Credible Interval	(0.732, 1.05)
Associated Peptides	16
Associated Spectra	26
Coverage	NaN

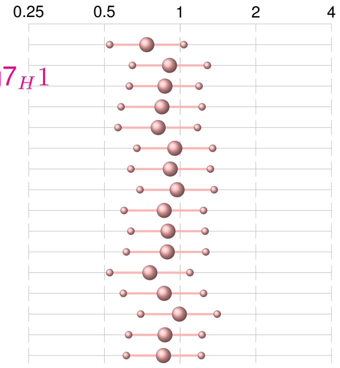

A	B	2.5	50	97.5	Sequence
1	1	0.53	0.74	1	ADDLSAELAK
1	1	0.65	0.91	1.3	GGIDIEEVDPEGK
2	1	0.63	0.87	1.2	KQLEEETLLR
1	0	0.58	0.85	1.2	LSVQVQSTEEVVK
1	0	0.57	0.82	1.2	RVETLEAQVAELTAK
1	1	0.68	0.95	1.3	WFVADEMK
1	0	0.64	0.91	1.3	SLVIKPGK
1	1	0.69	0.97	1.4	EDLHFK
1	0	0.6	0.86	1.2	YKTEYEDLLAK
1	1	0.64	0.89	1.3	EKHEFDLQTVR
0	1	0.61	0.89	1.3	LSEVEAPR
0	1	0.53	0.76	1.1	RLALKDDEIADLR
0	1	0.6	0.86	1.2	LLDETAKEK
0	2	0.7	0.99	1.4	IADLR
0	2	0.62	0.87	1.2	IRDLENQLAHER
0	2	0.61	0.86	1.2	LLEALR

5.172 9 887 minus strand hypothetical protein CGI_10006272 [Crassostrea gigas]

Protein Accession	CL46976.Contig1$_H$1
Mean Expression Ratio	0.877
Median Expression Ratio	0.878
Credible Interval	(0.614, 1.24)
Associated Peptides	1
Associated Spectra	3
Coverage	NaN

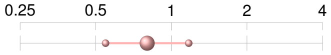

A	B	2.5	50	97.5	Sequence
2	1	0.55	0.8	1.2	MILPQINK

5.173 2156 3142 minus strand PREDICTED: hemicentin-1 [Xenopus (Silurana) tropicalis]

Protein Accession	CL2792.Contig4$_H$1
Mean Expression Ratio	1.14
Median Expression Ratio	1.14
Credible Interval	(0.902, 1.44)
Associated Peptides	7
Associated Spectra	15
Coverage	NaN

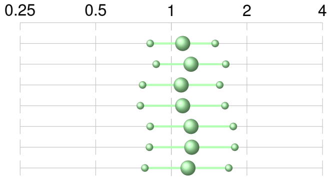

A	B	2.5	50	97.5	Sequence
4	1	0.82	1.1	1.5	GAPPPTVSWVK
2	2	0.87	1.2	1.6	NDVELYSK
1	1	0.77	1.1	1.6	SGVDAMKPYLR
1	0	0.75	1.1	1.6	LVEVIEEK
0	1	0.82	1.2	1.8	YECVAENLVHTR
0	1	0.82	1.2	1.8	NGLIIDTEKDPNLQVDPEGSLMFK
0	1	0.79	1.2	1.7	VDAMK

5.174 210 2201 minus strand Glycyl-tRNA synthetase [Crassostrea gigas]; 210 2201 minus strand Glycyl-tRNA synthetase [Crassostrea gigas]; 210 2201 minus strand Glycyl-tRNA synthetase [Crassostrea gigas]; 210 2201 minus strand Glycyl-tRNA synthetase [Crassostrea gigas]; 210 2201 minus strand Glycyl-tRNA synthetase [Crassostrea gigas]; 210 2201 minus strand Glycyl-tRNA synthetase [Crassostrea gigas]

Protein Accession	CL4073.Contig8$_{H}$1 CL4073.Contig7$_{H}$1 CL4073.Contig6$_{H}$1 CL4073.Contig5$_{H}$1 CL4073.Con
Mean Expression Ratio	1.14
Median Expression Ratio	1.14
Credible Interval	(0.793, 1.62)
Associated Peptides	2
Associated Spectra	2
Coverage	NaN

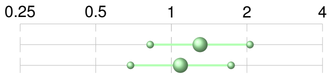

A	B	2.5	50	97.5	Sequence
0	1	0.82	1.3	2.1	ADHLIEATFEK
0	1	0.69	1.1	1.7	VLMPR

5.175 488 1039 Peptidyl-prolyl cis-trans isomerase B [Crassostrea gigas]; 488 1039 Peptidyl-prolyl cis-trans isomerase B [Crassostrea gigas]

Protein Accession	CL49248.Contig2$_{H}$1 CL49248.Contig1$_{H}$1
Mean Expression Ratio	1.14
Median Expression Ratio	1.14
Credible Interval	(0.879, 1.47)
Associated Peptides	4
Associated Spectra	19
Coverage	NaN

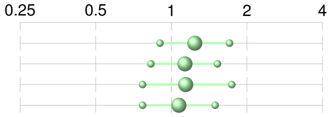

A	B	2.5	50	97.5	Sequence
3	3	0.9	1.2	1.7	ASLEFLDK
4	4	0.83	1.1	1.5	LVDCSATTLK
1	0	0.77	1.1	1.7	VTFSPK
3	1	0.77	1.1	1.5	INNVPVDK

5.176 6 650 minus strand glutathione S-transferase mu class protein [Crassostrea ariakensis]; 117 761 glutathione S-transferase mu class protein [Crassostrea ariakensis]; 122 766 glutathione S-transferase mu class protein [Crassostrea ariakensis]; 122 766 glutathione S-transferase mu class protein [Crassostrea ariakensis]

Protein Accession	Unigene56987$_{H}$1 CL6887.Contig3$_{H}$1 CL6887.Contig2$_{H}$1 CL6887.Contig1$_{H}$1
Mean Expression Ratio	1.13
Median Expression Ratio	1.13
Credible Interval	(0.89, 1.44)
Associated Peptides	6
Associated Spectra	13
Coverage	NaN

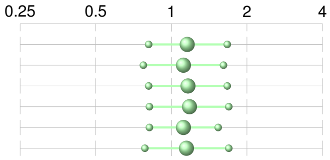

A	B	2.5	50	97.5	Sequence
2	0	0.81	1.2	1.7	ITQSNAILR
1	1	0.78	1.1	1.6	VKETLASYEK
1	1	0.81	1.2	1.7	PALGYWK
2	0	0.82	1.2	1.7	IMIPGILDDYPK
3	1	0.82	1.1	1.5	KHDLLGK
0	1	0.78	1.1	1.7	DAWFNVK

5.177 76 522 minus strand Troponin C [Crassostrea gigas]; 173 619 minus strand Troponin C [Crassostrea gigas]; 173 619 minus strand Troponin C [Crassostrea gigas]

Protein Accession	Unigene53263$_{H}1$ Unigene11674$_{H}1$ CL13001.Contig4$_{H}1$
Mean Expression Ratio	1.13
Median Expression Ratio	1.13
Credible Interval	(0.945, 1.37)
Associated Peptides	7
Associated Spectra	48
Coverage	NaN

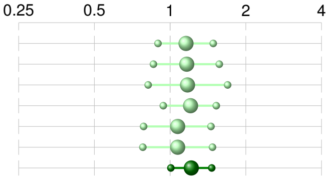

A	B	2.5	50	97.5	Sequence
4	4	0.9	1.2	1.5	GVIPVEDLR
3	1	0.86	1.2	1.6	QYNDANQTFNLFDK
1	0	0.82	1.2	1.7	QYNDANQTFNLFDKK
6	3	0.94	1.2	1.5	SLGLQVEDEK
2	2	0.79	1.1	1.4	VLDKGNK
2	1	0.78	1.1	1.5	SLGLQVEDEKLK
11	8	1	1.2	1.5	ELANVFK

5.178 3 347 beta-actin [Meretrix meretrix]; 3 215 Actin-2 OS=Onchocerca volvulus GN=act-2b PE=3 SV=1

Protein Accession	CL47466.Contig2$_{H}1$ CL47466.Contig1$_{H}1$
Mean Expression Ratio	1.13
Median Expression Ratio	1.13
Credible Interval	(0.96, 1.35)
Associated Peptides	11
Associated Spectra	307
Coverage	NaN

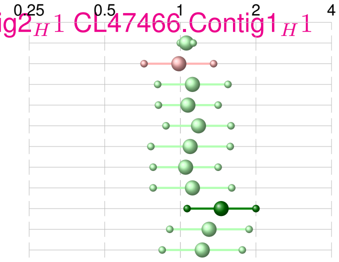

A	B	2.5	50	97.5	Sequence
147	133	1	1.1	1.1	EITALAPSTMK
2	1	0.72	0.99	1.4	EITALAPSTMKIK
3	0	0.81	1.1	1.5	KDLYANTVLSGGSTMYPGIADR
6	0	0.82	1.1	1.4	TVLSGGSTMYPGIADR
4	0	0.88	1.2	1.6	DLYANTVLSGGSTMYPGIADR
1	0	0.76	1.1	1.6	MQKEITALAPSTMK
0	4	0.78	1	1.4	LAPSTMK
0	1	0.78	1.1	1.6	ALAPSTMK
0	3	1.1	1.5	2	APSTMK
0	1	0.9	1.3	1.9	PSTMK
0	1	0.85	1.2	1.8	TALAPSTMK

5.179 30 536 putative mitochondrial ATP synthase [Haliotis discus discus]; 27 620 putative mitochondrial ATP synthase [Haliotis discus discus]

Protein Accession	CL13014.Contig4$_{H}1$ CL13014.Contig1$_{H}1$
Mean Expression Ratio	0.883
Median Expression Ratio	0.883
Credible Interval	(0.653, 1.18)
Associated Peptides	2
Associated Spectra	8
Coverage	NaN

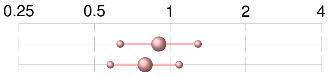

A	B	2.5	50	97.5	Sequence
2	1	0.63	0.9	1.3	LANPALVDQFQK
2	3	0.58	0.79	1.1	SAVDWVAFK

5.180 " 3475 4188 minus strand Heat shock 70 kDa protein cognate, putative [Pediculus humanus corporis] gi"

Protein Accession	CL5924.Contig14$_{H}$1
Mean Expression Ratio	0.884
Median Expression Ratio	0.884
Credible Interval	(0.625, 1.25)
Associated Peptides	2
Associated Spectra	3
Coverage	NaN

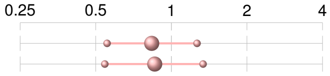

A	B	2.5	50	97.5	Sequence
2	0	0.56	0.83	1.3	STIEDEKLK
0	1	0.54	0.86	1.3	CNDVIR

5.181 99 3011 Eukaryotic translation initiation factor 3 subunit A [Crassostrea gigas]

Protein Accession	CL36771.Contig2$_{H}$1
Mean Expression Ratio	1.13
Median Expression Ratio	1.13
Credible Interval	(0.829, 1.54)
Associated Peptides	2
Associated Spectra	8
Coverage	NaN

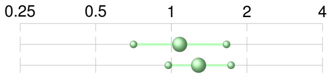

A	B	2.5	50	97.5	Sequence
1	0	0.71	1.1	1.7	NNSRVER
7	0	0.97	1.3	1.7	LFNLSREQRK

5.182 323 1192 cytosolic malate dehydrogenase [Lottia scabra]; 323 1192 cytosolic malate dehydrogenase [Lottia scabra]; 323 1192 cytosolic malate dehydrogenase [Lottia scabra]; 323 1192 cytosolic malate dehydrogenase [Lottia scabra]; 2474 3343 cytosolic malate dehydrogenase [Lottia scabra]; 116 1111 minus strand cytosolic malate dehydrogenase [Lottia scabra]

Protein Accession	CL280.Contig21$_{H}$1 CL280.Contig20$_{H}$1 CL280.Contig19$_{H}$1 CL280.Contig18$_{H}$1 CL280.Conti
Mean Expression Ratio	1.13
Median Expression Ratio	1.13
Credible Interval	(0.964, 1.33)
Associated Peptides	12
Associated Spectra	110
Coverage	NaN

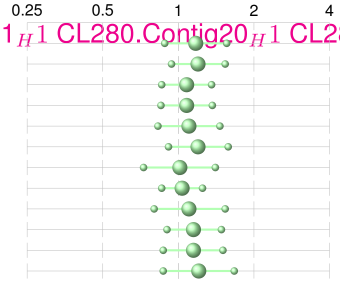

A	B	2.5	50	97.5	Sequence
3	3	0.88	1.2	1.6	EAVKDDNWINNEFISTVQK
6	5	0.94	1.2	1.5	ENFSCLTR
15	3	0.86	1.1	1.4	KLSSAMSAAK
6	6	0.85	1.1	1.4	LGIPNNAVSK
3	3	0.83	1.1	1.5	LSSAMSAAK
6	2	0.91	1.2	1.6	SQGANLDAVAK
2	1	0.73	1	1.4	YGIPEGVMYSYPVQIK
9	14	0.86	1	1.2	EMAEQILAA
2	1	0.8	1.1	1.5	ELLEER
6	4	0.9	1.2	1.5	DCDAAFLVGAMPR
4	3	0.87	1.1	1.5	DLLAANIK
2	1	0.87	1.2	1.7	YAPSIPK

5.183 " 158 355 Protein NAS-37, isoform b [Caenorhabditis elegans] gi; 15 227 Hemicentin-1 [Crassostrea gigas]"

Protein Accession	Unigene57180$_{H}$1 CL8016.Contig2$_{H}$1
Mean Expression Ratio	0.885
Median Expression Ratio	0.884
Credible Interval	(0.594, 1.31)
Associated Peptides	1
Associated Spectra	1
Coverage	NaN

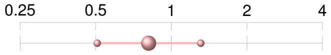

A	B	2.5	50	97.5	Sequence
**1**	**0**	**0.51**	**0.81**	**1.3**	**FKGSYGGVSR**

5.184 26 877 Ankyrin-2 [Crassostrea gigas]

Protein Accession	CL18051.Contig2$_{H}$1
Mean Expression Ratio	1.13
Median Expression Ratio	1.13
Credible Interval	(0.809, 1.57)
Associated Peptides	2
Associated Spectra	4
Coverage	NaN

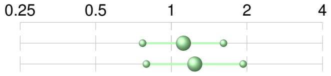

A	B	2.5	50	97.5	Sequence
**2**	**1**	**0.77**	**1.1**	**1.6**	**LPALHIAAK**
**0**	**1**	**0.8**	**1.2**	**1.9**	**DGLTPLHCAAR**

5.185 503 1090 minus strand myosin regulatory light chain [Fasciola hepatica]; 278 865 minus strand myosin regulatory light chain [Fasciola hepatica]

Protein Accession	CL43006.Contig6$_{H}$1 CL43006.Contig4$_{H}$1
Mean Expression Ratio	1.13
Median Expression Ratio	1.13
Credible Interval	(0.843, 1.51)
Associated Peptides	1
Associated Spectra	25
Coverage	NaN

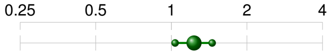

A	B	2.5	50	97.5	Sequence
**16**	**9**	**1**	**1.2**	**1.5**	**QAQVQEFK**

5.186 73 525 minus strand Actin-related protein 2/3 complex subunit 5-like protein [Salmo salar] gi; 73 525 Actin-related protein 2/3 complex subunit 5-like protein [Salmo salar] gi; 73 525 minus strand Actin-related protein 2/3 complex subunit 5-like protein [Salmo salar] gi

Protein Accession	CL14637.Contig6$_{H}1$ CL14637.Contig5$_{H}1$ CL14637.Contig4$_{H}1$
Mean Expression Ratio	0.883
Median Expression Ratio	0.886
Credible Interval	(0.593, 1.3)
Associated Peptides	1
Associated Spectra	1
Coverage	NaN

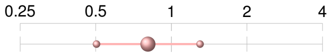

A	B	2.5	50	97.5	Sequence
**0**	**1**	**0.5**	**0.81**	**1.3**	**EVDILMK**

5.187 243 2165 minus strand Elongation factor 2 OS=Drosophila melanogaster GN=Ef2b PE=2 SV=4

Protein Accession	Unigene11527$_{H}1$
Mean Expression Ratio	0.885
Median Expression Ratio	0.886
Credible Interval	(0.612, 1.27)
Associated Peptides	2
Associated Spectra	2
Coverage	NaN

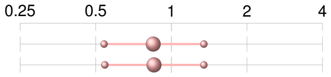

A	B	2.5	50	97.5	Sequence
**0**	**1**	**0.54**	**0.84**	**1.3**	**TQEDTLALADK**
**0**	**1**	**0.54**	**0.85**	**1.4**	**FNMEEVK**

5.188 167 2713 minus strand Major vault protein [Crassostrea gigas]

Protein Accession	CL18286.Contig3$_{H}1$
Mean Expression Ratio	1.13
Median Expression Ratio	1.13
Credible Interval	(0.847, 1.53)
Associated Peptides	5
Associated Spectra	6
Coverage	NaN

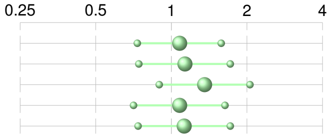

A	B	2.5	50	97.5	Sequence
**2**	**0**	**0.73**	**1.1**	**1.6**	**GAVAQVQFDDFHK**
**1**	**0**	**0.74**	**1.1**	**1.7**	**KEVVVEETIR**
**1**	**0**	**0.89**	**1.4**	**2.1**	**KPGDRWMIR**
**0**	**1**	**0.71**	**1.1**	**1.6**	**IEGEAAVEQAK**
**0**	**1**	**0.74**	**1.1**	**1.7**	**IVDEAEAEK**

5.189 231 902 Sodium- and chloride-dependent GABA transporter ine [Crassostrea gigas]

Protein Accession	CL11289.Contig3$_{H}1$
Mean Expression Ratio	0.887
Median Expression Ratio	0.887
Credible Interval	(0.68, 1.15)
Associated Peptides	3
Associated Spectra	12
Coverage	NaN

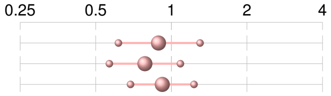

A	B	2.5	50	97.5	Sequence
**1**	**1**	**0.62**	**0.89**	**1.3**	**EAFELSFK**
**4**	**0**	**0.57**	**0.78**	**1.1**	**VPEIDAAAPFYGIPGADLADVSK**
**3**	**3**	**0.69**	**0.92**	**1.2**	**LVEFFQK**

5.190 74 1243 Actin-related protein 2 [Crassostrea gigas]

Protein Accession	CL8614.Contig4$_{H}1$
Mean Expression Ratio	1.13
Median Expression Ratio	1.13
Credible Interval	(0.818, 1.55)
Associated Peptides	2
Associated Spectra	6
Coverage	NaN

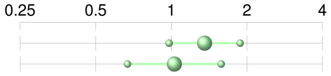

A	B	2.5	50	97.5	Sequence
**4**	**1**	**0.98**	**1.4**	**1.9**	**MVEVMFEK**
**0**	**1**	**0.67**	**1**	**1.6**	**DLMIGDEASQLR**

5.191 " 57 752 PREDICTED: adenylate kinase 2, mitochondrial-like isoform 2 [Callithrix jacchus]"

Protein Accession	CL4149.Contig1$_{H}1$
Mean Expression Ratio	1.13
Median Expression Ratio	1.13
Credible Interval	(0.837, 1.52)
Associated Peptides	3
Associated Spectra	6
Coverage	NaN

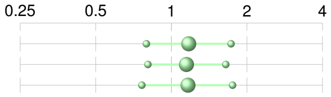

A	B	2.5	50	97.5	Sequence
**2**	**0**	**0.8**	**1.2**	**1.7**	**GIHTAVDASQPPHK**
**3**	**0**	**0.8**	**1.1**	**1.7**	**GFLLDGFPR**
**1**	**0**	**0.76**	**1.2**	**1.8**	**GTQAPLLAEK**

5.192 349 885 juvenile hormone diol kinase [Danaus plexippus]; 349 885 juvenile hormone diol kinase [Danaus plexippus]; 349 885 juvenile hormone diol kinase [Danaus plexippus]; 349 885 minus strand juvenile hormone diol kinase [Danaus plexippus]; 349 885 juvenile hormone diol kinase [Danaus plexippus]; 349 885 juvenile hormone diol kinase [Danaus plexippus]; 349 885 juvenile hormone diol kinase [Danaus plexippus]; 349 885 juvenile hormone diol kinase [Danaus plexippus]; 349 885 juvenile hormone diol kinase [Danaus plexippus]; 349 885 juvenile hormone diol kinase [Danaus plexippus]

Protein Accession	Unigene38920$_H$1 Unigene38919$_H$1 CL7590.Contig1$_H$1 CL7590.Contig16$_H$1 CL7590.Contig
Mean Expression Ratio	1.13
Median Expression Ratio	1.13
Credible Interval	(0.966, 1.32)
Associated Peptides	12
Associated Spectra	63
Coverage	NaN

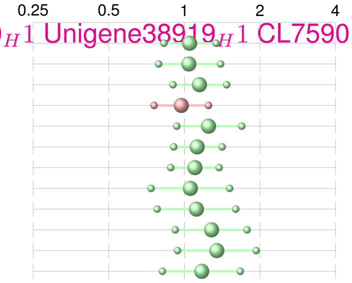

A	B	2.5	50	97.5	Sequence
5	4	0.83	1	1.3	DGIIDKNDFK
4	1	0.79	1	1.4	DILENVWEGLQK
6	2	0.9	1.2	1.5	FMFLANDTSGDGFVDR
4	4	0.76	0.98	1.2	IFGNSEADSNK
4	0	0.93	1.2	1.7	SVYQNLYDSNK
9	2	0.91	1.1	1.4	VPQWNEEYIK
9	3	0.88	1.1	1.4	GEFVTVQK
1	0	0.74	1.1	1.5	FSESGKVPQWNEEYIK
1	0	0.78	1.1	1.6	YIKFMFLANDTSGDGFVDR
0	2	0.92	1.3	1.8	DADINEDGIVTLDEWVK
0	1	0.94	1.3	1.9	DGIVTLDEWVK
0	1	0.82	1.2	1.7	AFDKLAQGAK

5.193 49 1038 Glyceraldehyde-3-phosphate dehydrogenase [Crotalus adamanteus]

Protein Accession	Unigene7744$_H$1
Mean Expression Ratio	1.13
Median Expression Ratio	1.13
Credible Interval	(0.829, 1.54)
Associated Peptides	1
Associated Spectra	12
Coverage	NaN

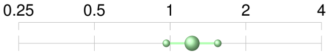

A	B	2.5	50	97.5	Sequence
12	0	0.97	1.2	1.5	SSIFDAK

5.194 7948 8541 Collagen alpha-3(VI) chain [Crassostrea gigas]

Protein Accession	CL47586.Contig9$_H$1
Mean Expression Ratio	1.13
Median Expression Ratio	1.13
Credible Interval	(0.822, 1.56)
Associated Peptides	2
Associated Spectra	5
Coverage	NaN

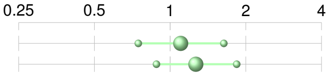

A	B	2.5	50	97.5	Sequence
2	0	0.74	1.1	1.6	ADIAFLLDSSGSVGYDNFR
3	0	0.88	1.3	1.8	DLQDAISAISYEYGNTNTAAGLK

5.195 313 2763 NCAM-related cell adhesion molecule precursor [Aplysia californica] gi

Protein Accession	Unigene11449$_{H}1$
Mean Expression Ratio	1.13
Median Expression Ratio	1.13
Credible Interval	(0.83, 1.53)
Associated Peptides	5
Associated Spectra	5
Coverage	NaN

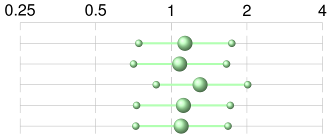

A	B	2.5	50	97.5	Sequence
1	0	0.74	1.1	1.7	EALEVDTSK
1	0	0.71	1.1	1.7	IGSSDPTVFVFK
1	0	0.87	1.3	2	NLAGSYDIEER
1	0	0.73	1.1	1.7	VPMTGGAEIANYVIEYR
0	1	0.72	1.1	1.7	AGAIASDETQLEVSIPPK

5.196 " 386 799 minus strand Myosin regulatory light chain LC-2, mantle muscle OS=Todarodes pacificus PE=1 SV=1; 656 1057 minus strand myosin regulatory light chain [Fasciola hepatica]"

Protein Accession	CL43006.Contig13$_{H}1$ CL43006.Contig11$_{H}1$
Mean Expression Ratio	1.13
Median Expression Ratio	1.13
Credible Interval	(0.868, 1.48)
Associated Peptides	2
Associated Spectra	27
Coverage	NaN

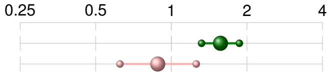

A	B	2.5	50	97.5	Sequence
15	9	1.3	1.6	1.9	DLLHNMGDNFTDDELK
0	3	0.62	0.88	1.3	DLLHNMGDNFTDDELKQTWK

5.197 71 523 minus strand hypothetical protein DAPPUDRAFT_309332 [Daphnia pulex]

Protein Accession	CL4487.Contig1$_{H}1$
Mean Expression Ratio	1.13
Median Expression Ratio	1.12
Credible Interval	(0.761, 1.67)
Associated Peptides	1
Associated Spectra	1
Coverage	NaN

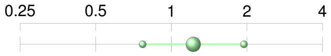

A	B	2.5	50	97.5	Sequence
0	1	0.77	1.2	2	IYHPNIDK

5.198 260 1597 isocitrate dehydrogenase [Mytilus galloprovincialis]; 260 1597 isocitrate dehydrogenase [Mytilus galloprovincialis]; 260 1597 isocitrate dehydrogenase [Mytilus galloprovincialis]

Protein Accession	CL10046.Contig8$_H$1 CL10046.Contig7$_H$1 CL10046.Contig6$_H$1
Mean Expression Ratio	0.889
Median Expression Ratio	0.89
Credible Interval	(0.666, 1.19)
Associated Peptides	5
Associated Spectra	6
Coverage	NaN

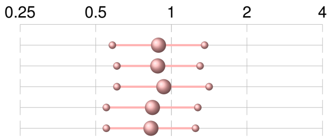

A	B	2.5	50	97.5	Sequence
1	0	0.58	0.89	1.4	DQTNDQVTIDSAEAIK
1	1	0.61	0.88	1.3	LTVFDFK
1	0	0.61	0.93	1.4	VKEFNLK
1	0	0.55	0.84	1.3	TSVLVCPDGK
0	1	0.55	0.83	1.2	SWPLYMSTK

5.199 " 276 659 Fatty acid-binding protein, adipocyte [Crassostrea gigas]; 426 809 Fatty acid-binding protein, adipocyte [Crassostrea gigas]; 277 660 Fatty acid-binding protein, adipocyte [Crassostrea gigas]; 283 666 Fatty acid-binding protein, adipocyte [Crassostrea gigas]; 270 653 Fatty acid-binding protein, adipocyte [Crassostrea gigas]; 283 666 Fatty acid-binding protein, adipocyte [Crassostrea gigas]; 258 641 Fatty acid-binding protein, adipocyte [Crassostrea gigas]; 427 810 Fatty acid-binding protein, adipocyte [Crassostrea gigas]; 277 660 Fatty acid-binding protein, adipocyte [Crassostrea gigas]"

Protein Accession	CL7666.Contig9$_H$1 CL7666.Contig8$_H$1 CL7666.Contig7$_H$1 CL7666.Contig6$_H$1 CL7666.Con
Mean Expression Ratio	1.12
Median Expression Ratio	1.12
Credible Interval	(0.887, 1.43)
Associated Peptides	5
Associated Spectra	16
Coverage	NaN

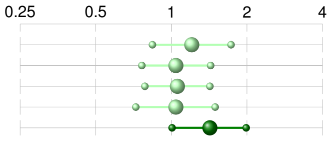

A	B	2.5	50	97.5	Sequence
1	1	0.84	1.2	1.7	AVLTEESGELVER
3	1	0.76	1	1.4	GVTATISFQK
4	1	0.78	1.1	1.4	LTTVAGDK
1	1	0.72	1	1.5	TVLAMER
1	2	1	1.4	2	AFVDGALK

5.200 3 239 minus strand alcohol dehydrogenase class 3 [Branchiostoma lanceolatum]

Protein Accession	Unigene58195$_H$1
Mean Expression Ratio	1.12
Median Expression Ratio	1.12
Credible Interval	(0.807, 1.56)
Associated Peptides	2
Associated Spectra	4
Coverage	NaN

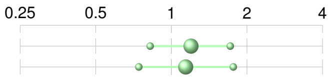

A	B	2.5	50	97.5	Sequence
3	0	0.82	1.2	1.7	GSAFGGWK
1	0	0.74	1.1	1.8	LVEAYMNGK

5.201 457 1842 Basement membrane-specific heparan sulfate proteoglycan core protein [Crassostrea gigas]; 457 1842 Basement membrane-specific heparan sulfate proteoglycan core protein [Crassostrea gigas]

Protein Accession	CL24300.Contig9$_{H}1$ CL24300.Contig10$_{H}1$
Mean Expression Ratio	1.12
Median Expression Ratio	1.12
Credible Interval	(0.81, 1.56)
Associated Peptides	2
Associated Spectra	4
Coverage	NaN

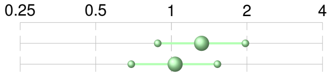

A	B	2.5	50	97.5	Sequence
1	1	0.88	1.3	2	KDGELPVEHK
1	1	0.69	1	1.5	IESSGALYIPR

5.202 101 1693 CALNUC [Spodoptera frugiperda]; 141 1325 Nucleobindin-2 [Crassostrea gigas]

Protein Accession	CL1888.Contig9$_{H}1$ CL1888.Contig6$_{H}1$
Mean Expression Ratio	0.891
Median Expression Ratio	0.892
Credible Interval	(0.623, 1.27)
Associated Peptides	2
Associated Spectra	3
Coverage	NaN

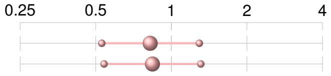

A	B	2.5	50	97.5	Sequence
1	1	0.53	0.82	1.3	LKELPEVER
1	0	0.54	0.84	1.3	REEFK

5.203 174 1649 minus strand Collagen alpha-1(XII) chain [Crassostrea gigas]; 135 1610 Collagen alpha-1(XII) chain [Crassostrea gigas]

Protein Accession	CL5340.Contig13$_{H}1$ CL5340.Contig11$_{H}1$
Mean Expression Ratio	1.12
Median Expression Ratio	1.12
Credible Interval	(0.909, 1.37)
Associated Peptides	10
Associated Spectra	27
Coverage	NaN

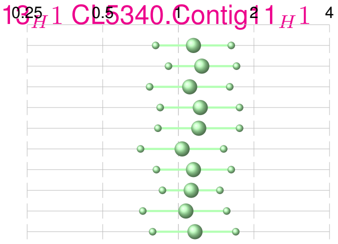

A	B	2.5	50	97.5	Sequence
2	0	0.81	1.1	1.6	ADVMFVTEFSQAGGR
4	0	0.92	1.2	1.7	ADVVFIVDSSR
1	1	0.77	1.1	1.6	DVMSVFHIAR
1	1	0.85	1.2	1.8	SIWMPDFK
1	0	0.83	1.2	1.8	VDGYSALDTIR
1	0	0.71	1	1.5	YWAEQLKK
2	0	0.82	1.2	1.6	KADVVFVLDK
6	5	0.87	1.1	1.5	DILAFR
1	0	0.72	1.1	1.6	TTELLK
1	0	0.79	1.2	1.7	SSSITAEHFQTQLR

5.204 85 1011 unnamed protein product [Mus musculus]

Protein Accession	CL42412.Contig1$_H$1
Mean Expression Ratio	1.12
Median Expression Ratio	1.12
Credible Interval	(0.799, 1.57)
Associated Peptides	3
Associated Spectra	3
Coverage	NaN

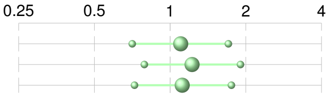

A	B	2.5	50	97.5	Sequence
1	0	0.71	1.1	1.7	NVIVEETVSEK
1	0	0.79	1.2	1.9	DDETMYVEAK
1	0	0.72	1.1	1.8	VFMQEFK

5.205 181 477 minus strand Zgc:56530 [Danio rerio]; 185 481 minus strand Zgc:56530 [Danio rerio]

Protein Accession	CL48470.Contig6$_H$1 CL27088.Contig2$_H$1
Mean Expression Ratio	1.12
Median Expression Ratio	1.12
Credible Interval	(0.818, 1.54)
Associated Peptides	2
Associated Spectra	6
Coverage	NaN

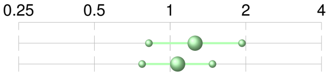

A	B	2.5	50	97.5	Sequence
1	0	0.82	1.3	1.9	TQVVAGTNYFIK
5	0	0.77	1.1	1.5	TFTDFK

5.206 116 1111 minus strand cytosolic malate dehydrogenase [Lottia scabra]

Protein Accession	CL280.Contig16$_H$1
Mean Expression Ratio	1.12
Median Expression Ratio	1.12
Credible Interval	(0.976, 1.28)
Associated Peptides	18
Associated Spectra	130
Coverage	NaN

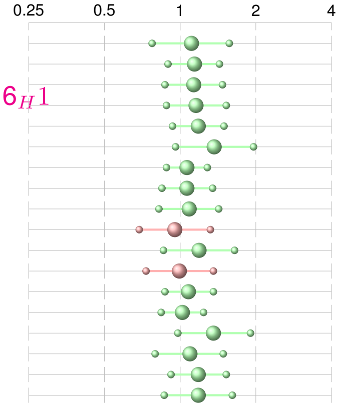

A	B	2.5	50	97.5	Sequence
1	0	0.77	1.1	1.6	ATADHMHDWWFGTEGDK
6	4	0.9	1.1	1.4	DCDAAFLVGAMPR
4	3	0.87	1.1	1.5	DLLAANIK
3	3	0.88	1.2	1.5	EAVKDDNWINNEFISTVQK
6	5	0.93	1.2	1.5	ENFSCLTR
1	0	0.96	1.4	2	EVIPTSDEK
15	3	0.88	1.1	1.3	KLSSAMSAAK
6	6	0.84	1.1	1.3	LGIPNNAVSK
3	3	0.82	1.1	1.4	LSSAMSAAK
2	0	0.69	0.95	1.3	QVTIVEGLSISDFAR
2	0	0.86	1.2	1.7	WVSMGVYSTGK
2	1	0.73	1	1.4	YGIPEGVMYSYPVQIK
12	0	0.87	1.1	1.4	KDLLAANIK
9	14	0.84	1	1.2	EMAEQILAA
2	0	0.98	1.4	1.9	SLLYSVAK
2	1	0.8	1.1	1.5	ELLEER
6	2	0.92	1.2	1.5	SQGANLDAVAK
2	1	0.87	1.2	1.6	YAPSIPK

5.207 811 1407 minus strand mitochondrial malate dehydrogenase 2 [Eospalax baileyi]

Protein Accession	CL10426.Contig13$_{H}1$
Mean Expression Ratio	0.897
Median Expression Ratio	0.896
Credible Interval	(0.681, 1.18)
Associated Peptides	3
Associated Spectra	11
Coverage	NaN

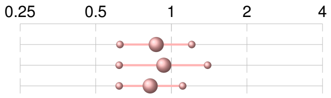

A	B	2.5	50	97.5	Sequence
2	2	0.63	0.87	1.2	IQNAGTEVVEAK
1	0	0.62	0.93	1.4	MQQLNSPR
3	3	0.62	0.83	1.1	AAMPELIANISK

5.208 2 475 unnamed protein product [Tetraodon nigroviridis]

Protein Accession	CL31420.Contig4$_{H}1$
Mean Expression Ratio	1.12
Median Expression Ratio	1.11
Credible Interval	(0.864, 1.43)
Associated Peptides	2
Associated Spectra	28
Coverage	NaN

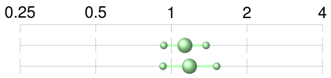

A	B	2.5	50	97.5	Sequence
18	0	0.93	1.1	1.4	DLYANTVLSGGTTMYPGIADR
10	0	0.93	1.2	1.5	KDLYANTVLSGGTTMYPGIADR

5.209 78 461 PREDICTED: hoi-polloi-like [Saccoglossus kowalevskii]; 103 492 NHP2-like protein 1 OS=Rattus norvegicus GN=Nhp2l1 PE=2 SV=4

Protein Accession	CL26515.Contig5$_{H}1$ CL26515.Contig4$_{H}1$
Mean Expression Ratio	0.898
Median Expression Ratio	0.897
Credible Interval	(0.602, 1.33)
Associated Peptides	1
Associated Spectra	1
Coverage	NaN

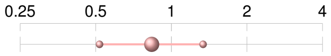

A	B	2.5	50	97.5	Sequence
1	0	0.52	0.84	1.3	NVPYVFVR

5.210 606 1730 UDP-glucuronosyltransferase 2C1 precursor [Oryctolagus cuniculus]

Protein Accession	CL29849.Contig5$_{H}1$
Mean Expression Ratio	1.12
Median Expression Ratio	1.11
Credible Interval	(0.778, 1.61)
Associated Peptides	2
Associated Spectra	3
Coverage	NaN

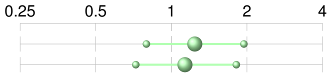

A	B	2.5	50	97.5	Sequence
1	1	0.79	1.2	1.9	LFVSHCGK
0	1	0.72	1.1	1.8	AAHAVEFVLK

5.211 85 633 hypothetical protein [Amblyomma maculatum]

Protein Accession	CL2221.Contig1$_H1$
Mean Expression Ratio	1.11
Median Expression Ratio	1.11
Credible Interval	(0.802, 1.54)
Associated Peptides	3
Associated Spectra	4
Coverage	NaN

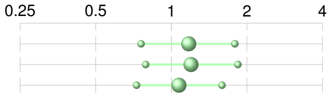

A	B	2.5	50	97.5	Sequence
1	0	0.76	1.2	1.8	DANDEWIR
1	0	0.79	1.2	1.8	EKLPDYDNK
0	2	0.72	1.1	1.6	IFYTEHLHADEEIR

5.212 151 1449 rab GDP-dissociation inhibitor [Branchiostoma belcheri]

Protein Accession	CL30719.Contig3$_H1$
Mean Expression Ratio	1.12
Median Expression Ratio	1.11
Credible Interval	(0.875, 1.43)
Associated Peptides	7
Associated Spectra	12
Coverage	NaN

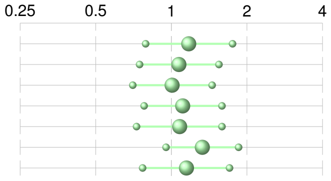

A	B	2.5	50	97.5	Sequence
1	0	0.79	1.2	1.7	EALGTSLMGMFEK
1	1	0.75	1.1	1.5	FLMANGQLVK
1	1	0.7	1	1.5	GVDCNGMTAAQLYEK
1	1	0.78	1.1	1.6	ITGEAFDFSK
1	0	0.73	1.1	1.6	IVYDETGVAVGVQDADGAVAK
2	1	0.95	1.3	1.9	LLVATGVTR
1	0	0.77	1.1	1.7	QWNVDLIPK

5.213 4 243 40S ribosomal protein S13 OS=Caenorhabditis elegans GN=rps-13 PE=2 SV=2; 4 243 40S ribosomal protein S13 OS=Caenorhabditis elegans GN=rps-13 PE=2 SV=2; 1 252 40S ribosomal protein S13 OS=Xenopus laevis GN=rps13 PE=3 SV=2; 1 213 40S ribosomal protein S13 OS=Xenopus laevis GN=rps13 PE=3 SV=2

Protein Accession	CL18049.Contig7$_H1$ CL18049.Contig4$_H1$ CL18049.Contig3$_H1$ CL18049.Contig2$_H1$
Mean Expression Ratio	0.896
Median Expression Ratio	0.898
Credible Interval	(0.625, 1.28)
Associated Peptides	2
Associated Spectra	2
Coverage	NaN

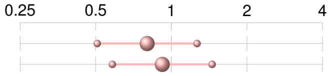

A	B	2.5	50	97.5	Sequence
1	0	0.51	0.8	1.3	GLAPDIPEDLYSLIK
1	0	0.58	0.92	1.5	SLIKK

5.214 252 848 sigma class glutathione-s-transferase 2 [Haliotis discus discus]

Protein Accession	CL19285.Contig21$_H1$
Mean Expression Ratio	0.9
Median Expression Ratio	0.9
Credible Interval	(0.67, 1.2)
Associated Peptides	4
Associated Spectra	7
Coverage	NaN

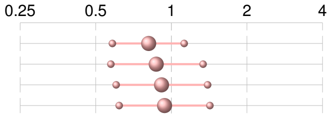

A	B	2.5	50	97.5	Sequence
2	2	0.58	0.81	1.1	LQDVDKDYPEVQK
1	0	0.58	0.87	1.3	LQAESLPK
1	0	0.6	0.92	1.4	LGNELTMA
0	1	0.62	0.94	1.4	KLRGR

5.215 " 28 1104 minus strand Pyruvate dehydrogenase E1 component subunit beta, mitochondrial [Crassostrea gigas]"

Protein Accession	CL18015.Contig14$_H1$
Mean Expression Ratio	1.11
Median Expression Ratio	1.11
Credible Interval	(0.771, 1.6)
Associated Peptides	2
Associated Spectra	2
Coverage	NaN

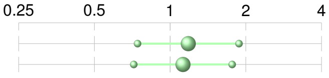

A	B	2.5	50	97.5	Sequence
**1**	**0**	**0.74**	**1.2**	**1.9**	**GIECEVINLR**
**1**	**0**	**0.72**	**1.1**	**1.8**	**SLRPVDDETIIK**

5.216 111 530 minus strand Nucleoside diphosphate kinase B OS=Homo sapiens GN=NME2 PE=1 SV=1

Protein Accession	CL22280.Contig3$_H1$
Mean Expression Ratio	1.11
Median Expression Ratio	1.11
Credible Interval	(0.845, 1.47)
Associated Peptides	4
Associated Spectra	9
Coverage	NaN

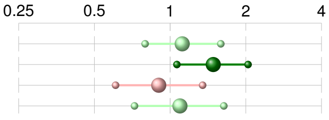

A	B	2.5	50	97.5	Sequence
**2**	**1**	**0.8**	**1.1**	**1.6**	**GDFCIEMGR**
**2**	**2**	**1.1**	**1.5**	**2**	**VMLGETNPLNSKPGTIR**
**1**	**0**	**0.61**	**0.9**	**1.3**	**LCRPGK**
**0**	**1**	**0.72**	**1.1**	**1.6**	**LVAAK**

5.217 " 1 1887 Pyruvate carboxylase, mitochondrial [Crassostrea gigas]"

Protein Accession	CL26073.Contig5$_H1$
Mean Expression Ratio	0.901
Median Expression Ratio	0.901
Credible Interval	(0.658, 1.24)
Associated Peptides	4
Associated Spectra	4
Coverage	NaN

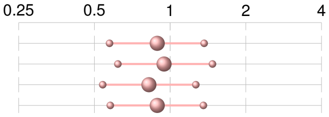

A	B	2.5	50	97.5	Sequence
**0**	**1**	**0.58**	**0.89**	**1.4**	**AGTHILGIK**
**0**	**1**	**0.62**	**0.95**	**1.5**	**HLEDVEENFKR**
**0**	**1**	**0.54**	**0.83**	**1.3**	**FLHECPWDR**
**0**	**1**	**0.58**	**0.89**	**1.4**	**IEVFR**

5.218 552 1499 minus strand uncharacterized protein LOC100158466 [Xenopus (Silurana) tropicalis] gi

Protein Accession	Unigene57645$_H1$
Mean Expression Ratio	0.902
Median Expression Ratio	0.901
Credible Interval	(0.633, 1.29)
Associated Peptides	2
Associated Spectra	3
Coverage	NaN

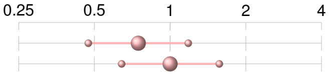

A	B	2.5	50	97.5	Sequence
**1**	**0**	**0.47**	**0.75**	**1.2**	**TAFSLLK**
**1**	**1**	**0.64**	**1**	**1.6**	**TGEPVEVDGELATLLAR**

5.219 721 1242 Lactoylglutathione lyase [Crassostrea gigas]; 882 1334 Lactoylglutathione lyase OS=Rattus norvegicus GN=Glo1 PE=1 SV=3; 113 634 Lactoylglutathione lyase [Crassostrea gigas]

Protein Accession	Unigene26921$_{H}1$ Unigene26919$_{H}1$ CL10946.Contig1$_{H}1$
Mean Expression Ratio	0.9
Median Expression Ratio	0.902
Credible Interval	(0.607, 1.34)
Associated Peptides	1
Associated Spectra	1
Coverage	NaN

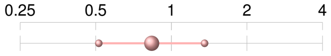

A	B	2.5	50	97.5	Sequence
**1**	**0**	**0.51**	**0.84**	**1.4**	**FESLGVEFIK**

5.220 2 256 Polyubiquitin (Fragment) OS=Xenopus laevis PE=1 SV=2; 20 388 large subunit ribosomal protein L40e [Clonorchis sinensis]

Protein Accession	CL47507.Contig2$_{H}1$ CL39010.Contig1$_{H}1$
Mean Expression Ratio	1.11
Median Expression Ratio	1.11
Credible Interval	(0.856, 1.43)
Associated Peptides	5
Associated Spectra	18
Coverage	NaN

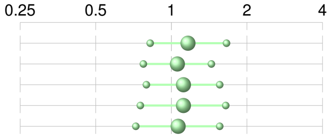

A	B	2.5	50	97.5	Sequence
**2**	**1**	**0.82**	**1.2**	**1.7**	**TITLEVEPSDTIENVK**
**4**	**4**	**0.77**	**1.1**	**1.4**	**TLSDYNIQK**
**3**	**0**	**0.79**	**1.1**	**1.6**	**LIFAGK**
**1**	**1**	**0.76**	**1.1**	**1.6**	**ESTLHLVLR**
**1**	**1**	**0.72**	**1.1**	**1.6**	**MQIFVK**

5.221 1050 1307 calreticulin precursor [Aplysia californica] gi

Protein Accession	CL4813.Contig1$_{H}1$
Mean Expression Ratio	0.9
Median Expression Ratio	0.902
Credible Interval	(0.607, 1.32)
Associated Peptides	1
Associated Spectra	1
Coverage	NaN

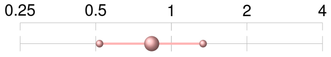

A	B	2.5	50	97.5	Sequence
**0**	**1**	**0.52**	**0.84**	**1.3**	**EFGDSTWGK**

5.222 108 593 hypothetical protein CGI_10025497 [Crassostrea gigas]

Protein Accession	CL36597.Contig1$_{H}1$
Mean Expression Ratio	1.11
Median Expression Ratio	1.11
Credible Interval	(0.789, 1.55)
Associated Peptides	1
Associated Spectra	5
Coverage	NaN

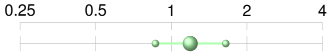

A	B	2.5	50	97.5	Sequence
**4**	**1**	**0.86**	**1.2**	**1.6**	**SFELADFHK**

5.223 161 301 Heat shock protein beta-6 OS=Mus musculus GN=Hspb6 PE=1 SV=1; 167 310 Heat shock protein beta-1 OS=Canis familiaris GN=HSPB1 PE=2 SV=1

Protein Accession	CL46983.Contig2$_{H}1$ CL46983.Contig1$_{H}1$
Mean Expression Ratio	0.903
Median Expression Ratio	0.903
Credible Interval	(0.664, 1.22)
Associated Peptides	2
Associated Spectra	9
Coverage	NaN

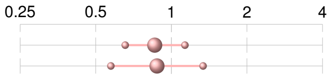

A	B	2.5	50	97.5	Sequence
6	2	0.66	0.86	1.1	VNMDVQHFKPEEIK
0	1	0.58	0.88	1.3	NMDVQHFKPEEIK

5.224 " 2041 3315 minus strand Myosin-10 OS=Mus musculus GN=Myh10 PE=1 SV=2; 2041 3345 minus strand Myosin heavy chain, non-muscle OS=Drosophila melanogaster GN=zip PE=1 SV=2; 2041 3351 minus strand Myosin-10 OS=Mus musculus GN=Myh10 PE=1 SV=2"

Protein Accession	Unigene62170$_{H}1$ Unigene62168$_{H}1$ Unigene62165$_{H}1$
Mean Expression Ratio	0.904
Median Expression Ratio	0.903
Credible Interval	(0.677, 1.21)
Associated Peptides	5
Associated Spectra	6
Coverage	NaN

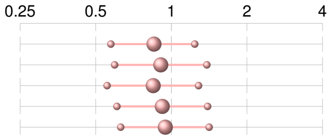

A	B	2.5	50	97.5	Sequence
0	2	0.57	0.85	1.2	AGVLAHLEEER
0	1	0.59	0.9	1.4	CIIPNHEK
0	1	0.55	0.85	1.3	IDSPLVLDQLR
0	1	0.61	0.92	1.4	ILFQEFR
0	1	0.63	0.94	1.4	LMATLR

5.225 697 954 minus strand Peptidyl-prolyl cis-trans isomerase B [Crassostrea gigas]; 614 877 Peptidyl-prolyl cis-trans isomerase B [Crassostrea gigas]

Protein Accession	CL49248.Contig4$_{H}1$ CL49248.Contig3$_{H}1$
Mean Expression Ratio	0.904
Median Expression Ratio	0.903
Credible Interval	(0.686, 1.19)
Associated Peptides	4
Associated Spectra	19
Coverage	NaN

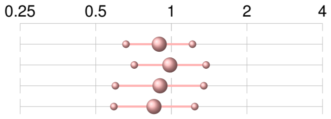

A	B	2.5	50	97.5	Sequence
4	4	0.66	0.9	1.2	LVDCSATTLK
3	3	0.71	0.98	1.4	ASLEFLDK
0	1	0.6	0.9	1.4	SMQNLDGR
3	1	0.59	0.85	1.2	INNVPVDK

5.226 507 1277 alpha tubulin [Notothenia coriiceps]

Protein Accession	Unigene56691$_{H}1$
Mean Expression Ratio	0.903
Median Expression Ratio	0.903
Credible Interval	(0.671, 1.21)
Associated Peptides	2
Associated Spectra	9
Coverage	NaN

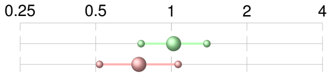

A	B	2.5	50	97.5	Sequence
5	1	0.76	1	1.4	LSVDYGK
2	1	0.52	0.74	1.1	LSVDYGKK

5.227 87 1454 hypothetical protein BRAFLDRAFT_287788 [Branchiostoma floridae] gi; 117 1469 hypothetical protein BRAFLDRAFT_287788 [Branchiostoma floridae] gi; 117 1469 hypothetical protein BRAFLDRAFT_287788 [Branchiostoma floridae] gi

Protein Accession	Unigene48935$_{H}1$ Unigene21344$_{H}1$ CL945.Contig6$_{H}1$
Mean Expression Ratio	0.903
Median Expression Ratio	0.904
Credible Interval	(0.709, 1.14)
Associated Peptides	6
Associated Spectra	17
Coverage	NaN

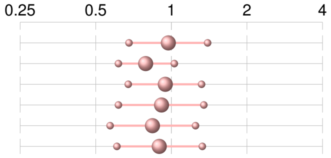

A	B	2.5	50	97.5	Sequence
1	1	0.68	0.97	1.4	AATLEQTILNVPETR
8	1	0.62	0.79	1	MVLAAAGGVNHGELVDLAK
2	1	0.68	0.94	1.3	RIPLPELEAR
1	0	0.61	0.92	1.3	QMLCYGR
1	0	0.57	0.84	1.2	PETRISSLDNGLR
0	1	0.61	0.9	1.3	ISSLDNGLR

5.228 " 1144 1956 minus strand Retinol dehydrogenase 12, partial [Crassostrea gigas]"

Protein Accession	CL3944.Contig7$_{H}1$
Mean Expression Ratio	1.11
Median Expression Ratio	1.11
Credible Interval	(0.807, 1.54)
Associated Peptides	3
Associated Spectra	4
Coverage	NaN

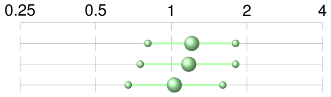

A	B	2.5	50	97.5	Sequence
2	0	0.81	1.2	1.8	LDFTDLALLK
1	0	0.75	1.2	1.8	LILGCR
0	1	0.67	1	1.6	LDLASLR

5.229 35 505 minus strand Basement membrane-specific heparan sulfate proteoglycan core protein [Crassostrea gigas]

Protein Accession	CL8101.Contig1$_H$1
Mean Expression Ratio	0.902
Median Expression Ratio	0.904
Credible Interval	(0.607, 1.34)
Associated Peptides	1
Associated Spectra	1
Coverage	NaN

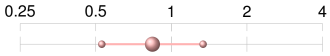

A	B	2.5	50	97.5	Sequence
**0**	**1**	**0.53**	**0.84**	**1.3**	**AAFAIR**

5.230 1118 1255 minus strand equistatin precursor [Actinia equina]

Protein Accession	CL27616.Contig7$_H$1
Mean Expression Ratio	0.905
Median Expression Ratio	0.905
Credible Interval	(0.612, 1.33)
Associated Peptides	1
Associated Spectra	1
Coverage	NaN

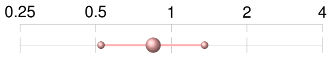

A	B	2.5	50	97.5	Sequence
**1**	**0**	**0.53**	**0.84**	**1.4**	**HLLGSYIPR**

5.231 718 1155 Annexin A7 [Crassostrea gigas]

Protein Accession	CL15539.Contig2$_H$1
Mean Expression Ratio	1.1
Median Expression Ratio	1.1
Credible Interval	(0.855, 1.42)
Associated Peptides	5
Associated Spectra	12
Coverage	NaN

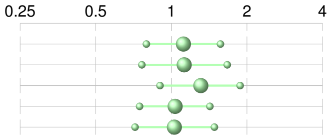

A	B	2.5	50	97.5	Sequence
**3**	**0**	**0.8**	**1.1**	**1.6**	**ATFDAYQQIAGK**
**1**	**0**	**0.76**	**1.1**	**1.7**	**DIESAIK**
**2**	**0**	**0.9**	**1.3**	**1.9**	**KLGTDEETFNR**
**4**	**0**	**0.75**	**1**	**1.4**	**NVPGYFAQR**
**2**	**0**	**0.72**	**1**	**1.5**	**SFPQLR**

5.232 4 399 Spectrin beta chain [Crassostrea gigas]

Protein Accession	Unigene17190$_H$1
Mean Expression Ratio	0.908
Median Expression Ratio	0.907
Credible Interval	(0.63, 1.31)
Associated Peptides	1
Associated Spectra	2
Coverage	NaN

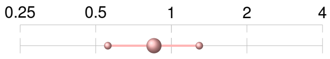

A	B	2.5	50	97.5	Sequence
**0**	**2**	**0.56**	**0.85**	**1.3**	**VHLENMGAHDIVDGSAR**

5.233 2 307 Laminin subunit gamma-1 [Crassostrea gigas]

Protein Accession	Unigene32982$_H$1
Mean Expression Ratio	1.1
Median Expression Ratio	1.1
Credible Interval	(0.751, 1.63)
Associated Peptides	1
Associated Spectra	1
Coverage	NaN

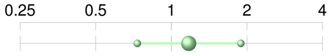

A	B	2.5	50	97.5	Sequence
0	1	0.73	1.2	1.9	AEISLR

5.234 203 1942 ezrin/radixin/moesin [Aplysia californica] gi; 243 1982 minus strand ezrin/radixin/moesin [Aplysia californica] gi

Protein Accession	CL13506.Contig5$_H$1 CL13506.Contig4$_H$1
Mean Expression Ratio	1.1
Median Expression Ratio	1.1
Credible Interval	(0.905, 1.34)
Associated Peptides	13
Associated Spectra	24
Coverage	NaN

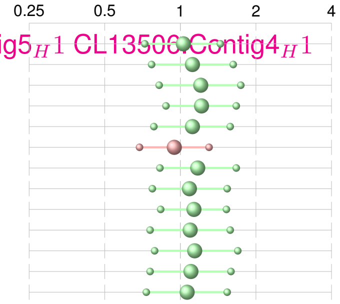

A	B	2.5	50	97.5	Sequence
1	1	0.72	1	1.4	DDAMMEYLK
1	0	0.77	1.1	1.6	FVIKPIDK
1	0	0.83	1.2	1.7	KAPDFVFYAPR
3	0	0.88	1.2	1.7	NISFNDKK
2	0	0.78	1.1	1.6	RKPDTIEVQQMK
2	1	0.69	0.95	1.3	KESPLQFK
1	1	0.83	1.2	1.7	QLFDQVVK
2	0	0.77	1.1	1.5	QLEMLK
4	0	0.83	1.1	1.5	SWWGEHK
1	0	0.76	1.1	1.6	LADELETAQR
0	1	0.79	1.1	1.7	MAELLALR
0	1	0.76	1.1	1.6	IGFPWSEIR
0	1	0.73	1.1	1.5	ESPLQFK

5.235 87 485 Sex-regulated protein janus-A [Crassostrea gigas]; 92 310 PREDICTED: hypothetical protein [Saccoglossus kowalevskii]; 92 310 PREDICTED: hypothetical protein [Saccoglossus kowalevskii]

Protein Accession	CL13131.Contig3$_H$1 CL13131.Contig2$_H$1 CL13131.Contig1$_H$1
Mean Expression Ratio	1.1
Median Expression Ratio	1.1
Credible Interval	(0.747, 1.63)
Associated Peptides	1
Associated Spectra	1
Coverage	NaN

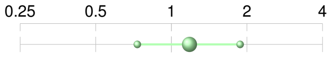

A	B	2.5	50	97.5	Sequence
1	0	0.73	1.2	1.9	VVGEIESK

5.236 816 1211 H2 [Haliotis tuberculata]

Protein Accession	CL11519.Contig5$_{H}1$
Mean Expression Ratio	0.913
Median Expression Ratio	0.91
Credible Interval	(0.638, 1.31)
Associated Peptides	2
Associated Spectra	4
Coverage	NaN

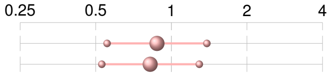

A	B	2.5	50	97.5	Sequence
**0**	**1**	**0.56**	**0.87**	**1.4**	**FDISDAVR**
**2**	**1**	**0.53**	**0.82**	**1.3**	**SLILAMR**

5.237 104 445 minus strand macrophage migration inhibitory factor [Ascaris suum]

Protein Accession	CL34536.Contig3$_{H}1$
Mean Expression Ratio	1.1
Median Expression Ratio	1.1
Credible Interval	(0.817, 1.47)
Associated Peptides	4
Associated Spectra	6
Coverage	NaN

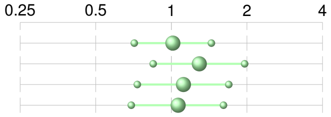

A	B	2.5	50	97.5	Sequence
**2**	**1**	**0.71**	**1**	**1.4**	**HIEEALGIPQDR**
**1**	**0**	**0.85**	**1.3**	**2**	**PVFILNTNLPADK**
**1**	**0**	**0.73**	**1.1**	**1.7**	**QLGKPESYVCVR**
**0**	**1**	**0.69**	**1.1**	**1.6**	**LVVPFMK**

5.238 54 278 ATP-citrate synthase [Crassostrea gigas]; 61 465 ATP-citrate synthase [Crassostrea gigas]

Protein Accession	CL35133.Contig2$_{H}1$ CL35133.Contig1$_{H}1$
Mean Expression Ratio	1.1
Median Expression Ratio	1.1
Credible Interval	(0.748, 1.63)
Associated Peptides	1
Associated Spectra	1
Coverage	NaN

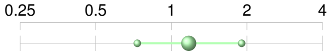

A	B	2.5	50	97.5	Sequence
**1**	**0**	**0.73**	**1.2**	**1.9**	**TIAIIAEGIPENLTR**

5.239 527 1321 minus strand troponin T [Mizuhopecten yessoensis]; 305 1099 minus strand troponin T [Mizuhopecten yessoensis]; 305 1099 minus strand troponin T [Mizuhopecten yessoensis]

Protein Accession	Unigene65787$_{H}1$ CL2214.Contig4$_{H}1$ CL2214.Contig3$_{H}1$
Mean Expression Ratio	0.911
Median Expression Ratio	0.911
Credible Interval	(0.783, 1.07)
Associated Peptides	10
Associated Spectra	104
Coverage	NaN

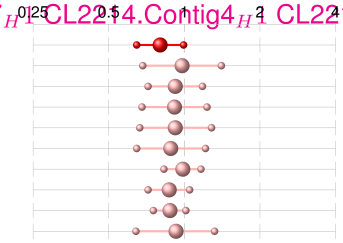

A	B	2.5	50	97.5	Sequence
2	10	0.65	0.8	0.99	LDSVMSKEELQK
1	0	0.69	0.98	1.4	SKEQLEDEK
5	3	0.72	0.92	1.2	SKEQLEDEKR
2	2	0.68	0.91	1.2	GPNFVITK
2	0	0.66	0.92	1.3	KGPNFVIT
3	0	0.65	0.88	1.2	AILSQR
17	6	0.83	0.98	1.2	KGPNFVITK
15	3	0.72	0.87	1.1	LDSVMSK
17	15	0.75	0.88	1	LTELR
0	1	0.64	0.93	1.3	EAREAEER

5.240 211 990 PREDICTED: gamma-glutamyl hydrolase-like [Xenopus (Silurana) tropicalis]; 223 1080 PREDICTED: gamma-glutamyl hydrolase [Gallus gallus]

Protein Accession	CL20208.Contig9$_{H}1$ CL20208.Contig3$_{H}1$
Mean Expression Ratio	0.909
Median Expression Ratio	0.912
Credible Interval	(0.626, 1.31)
Associated Peptides	1
Associated Spectra	2
Coverage	NaN

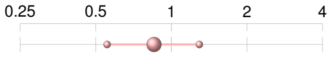

A	B	2.5	50	97.5	Sequence
2	0	0.56	0.85	1.3	YLWQAGAR

5.241 156 1106 ELAV 2-like protein [Aplysia californica] gi

Protein Accession	CL1500.Contig1$_{H}1$
Mean Expression Ratio	0.91
Median Expression Ratio	0.912
Credible Interval	(0.644, 1.28)
Associated Peptides	2
Associated Spectra	3
Coverage	NaN

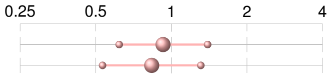

A	B	2.5	50	97.5	Sequence
1	1	0.62	0.93	1.4	NLPIPWTENELNK
0	1	0.53	0.83	1.3	VLVDLQTGVSK

5.242 448 1626 matrilin [Biomphalaria glabrata]

Protein Accession	CL7510.Contig3$_H$1
Mean Expression Ratio	1.1
Median Expression Ratio	1.1
Credible Interval	(0.938, 1.28)
Associated Peptides	13
Associated Spectra	63
Coverage	NaN

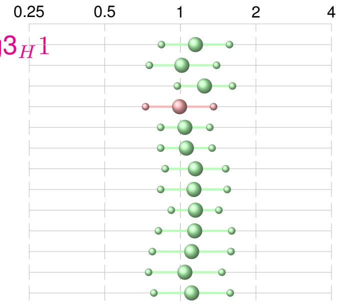

A	B	2.5	50	97.5	Sequence
2	1	0.84	1.2	1.6	EFLSMYDIGPAR
1	2	0.75	1	1.4	FAVVLTDGNSQESR
6	1	0.97	1.2	1.6	IDETSLNSAIDELVK
2	1	0.73	0.99	1.4	KTALVGFSDTR
5	6	0.83	1	1.3	TKEEVLEHVANMR
7	3	0.83	1.1	1.3	VFVVTTDSAAK
3	2	0.87	1.1	1.5	VIQVSSYDDLQK
3	0	0.84	1.1	1.5	VSNTELLNIAGDQER
6	6	0.92	1.1	1.4	DSLPGLVR
2	0	0.82	1.1	1.6	TALVGFSDTR
1	0	0.78	1.1	1.6	HEYGTFTDTGPGIDYMR
0	2	0.75	1	1.5	HKIDETSLNSAIDELVK
0	1	0.78	1.1	1.6	VSAIK

5.243 " 223 423 ADPATP carrier protein; K05863 solute carrier family 25 (mitochondrial adenine nucleotide translocator), member 4/5/6/31"

Protein Accession	CL27196.Contig3$_H$1
Mean Expression Ratio	1.09
Median Expression Ratio	1.1
Credible Interval	(0.789, 1.52)
Associated Peptides	2
Associated Spectra	4
Coverage	NaN

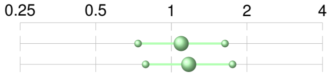

A	B	2.5	50	97.5	Sequence
2	0	0.74	1.1	1.6	LLVQNQNEMIK
0	2	0.79	1.2	1.7	GVIDCTIR

5.244 165 635 hypothetical protein CGI_10007639 [Crassostrea gigas]

Protein Accession	CL36715.Contig1$_H$1
Mean Expression Ratio	1.1
Median Expression Ratio	1.1
Credible Interval	(0.762, 1.58)
Associated Peptides	2
Associated Spectra	2
Coverage	NaN

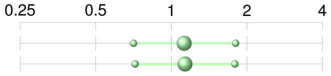

A	B	2.5	50	97.5	Sequence
1	0	0.71	1.1	1.8	AAIVAQVAK
1	0	0.72	1.1	1.8	MPLHASCK

5.245 " 249 1271 N(G),N(G)-dimethylarginine dimethylaminohydrolase 1 [Crassostrea gigas]"

Protein Accession	Unigene64051$_{H}1$
Mean Expression Ratio	1.1
Median Expression Ratio	1.1
Credible Interval	(0.765, 1.58)
Associated Peptides	2
Associated Spectra	3
Coverage	NaN

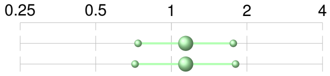

A	B	2.5	50	97.5	Sequence
**1**	**1**	**0.74**	**1.1**	**1.8**	**LGEVGEIR**
**1**	**0**	**0.72**	**1.1**	**1.8**	**EIFVGIGK**

5.246 340 615 putative Low-density lipoprotein receptor repeat class B [Trichinella spiralis] gi

Protein Accession	Unigene31531$_{H}1$
Mean Expression Ratio	1.1
Median Expression Ratio	1.1
Credible Interval	(0.748, 1.63)
Associated Peptides	1
Associated Spectra	1
Coverage	NaN

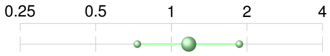

A	B	2.5	50	97.5	Sequence
**0**	**1**	**0.73**	**1.2**	**1.9**	**VLPFGLK**

5.247 201 632 USP-like protein isoform 1 [Crassostrea ariakensis] gi; 189 620 minus strand USP-like protein isoform 1 [Crassostrea ariakensis] gi

Protein Accession	Unigene39588$_{H}1$ Unigene20286$_{H}1$
Mean Expression Ratio	1.1
Median Expression Ratio	1.1
Credible Interval	(0.743, 1.63)
Associated Peptides	1
Associated Spectra	1
Coverage	NaN

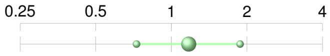

A	B	2.5	50	97.5	Sequence
**1**	**0**	**0.72**	**1.2**	**1.9**	**DPWHAIVDAAK**

5.248 290 2458 Putative beta-D-xylosidase 5 [Crassostrea gigas]; 299 2428 Putative beta-D-xylosidase 2 [Crassostrea gigas]

Protein Accession	CL49577.Contig2$_{H}1$ CL1594.Contig5$_{H}1$
Mean Expression Ratio	1.09
Median Expression Ratio	1.1
Credible Interval	(0.758, 1.56)
Associated Peptides	2
Associated Spectra	3
Coverage	NaN

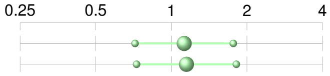

A	B	2.5	50	97.5	Sequence
**1**	**1**	**0.71**	**1.1**	**1.8**	**VAEAAGVEVR**
**1**	**0**	**0.73**	**1.1**	**1.8**	**VTPLFYTR**

5.249 7073 7339 minus strand Collagen alpha-3(VI) chain [Crassostrea gigas]

Protein Accession	Unigene26547$_H1$
Mean Expression Ratio	1.09
Median Expression Ratio	1.1
Credible Interval	(0.852, 1.4)
Associated Peptides	3
Associated Spectra	18
Coverage	NaN

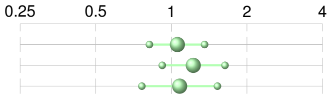

A	B	2.5	50	97.5	Sequence
6	3	0.82	1.1	1.4	ALVEELSGSGQNHR
6	0	0.92	1.2	1.6	YTPGSTNTAGGLR
2	1	0.76	1.1	1.5	YDTPQQVLEAIATTR

5.250 356 619 PREDICTED: hypothetical protein LOC100698726 [Oreochromis niloticus]

Protein Accession	CL3567.Contig6$_H1$
Mean Expression Ratio	1.1
Median Expression Ratio	1.09
Credible Interval	(0.76, 1.57)
Associated Peptides	2
Associated Spectra	2
Coverage	NaN

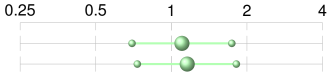

A	B	2.5	50	97.5	Sequence
1	0	0.7	1.1	1.7	LETEVNDLR
1	0	0.73	1.2	1.8	GELQKEEAFSAK

5.251 230 334 protein disulfide isomerase [Conus virgo]

Protein Accession	Unigene67026$_H1$
Mean Expression Ratio	1.09
Median Expression Ratio	1.09
Credible Interval	(0.794, 1.5)
Associated Peptides	1
Associated Spectra	8
Coverage	NaN

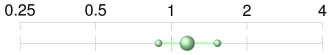

A	B	2.5	50	97.5	Sequence
4	4	0.89	1.2	1.5	VDATVENDLASK

5.252 628 1140 paramyosin [Haliotis discus discus]

Protein Accession	CL43455.Contig3$_H1$
Mean Expression Ratio	0.914
Median Expression Ratio	0.915
Credible Interval	(0.69, 1.2)
Associated Peptides	2
Associated Spectra	43
Coverage	NaN

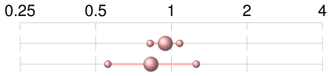

A	B	2.5	50	97.5	Sequence
23	19	0.82	0.94	1.1	LEEAEAFAVK
0	1	0.56	0.83	1.3	LEEAEAF

5.253 462 671 enhancer protein [Homo sapiens]

Protein Accession	CL3160.Contig12$_{H}1$
Mean Expression Ratio	1.09
Median Expression Ratio	1.09
Credible Interval	(0.825, 1.45)
Associated Peptides	3
Associated Spectra	11
Coverage	NaN

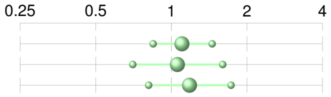

A	B	2.5	50	97.5	Sequence
**6**	**2**	**0.84**	**1.1**	**1.5**	**GLFIIDDK**
**0**	**1**	**0.7**	**1.1**	**1.6**	**PLLEDK**
**0**	**2**	**0.81**	**1.2**	**1.7**	**KIGGLGNMNIPLLEDK**

5.254 9 161 PREDICTED: tubulin alpha-1C chain-like [Acyrthosiphon pisum]; 9 161 Tubulin alpha-3 chain (Fragments) OS=Mesocricetus auratus PE=1 SV=1

Protein Accession	Unigene74551$_{H}1$ Unigene70816$_{H}1$
Mean Expression Ratio	1.09
Median Expression Ratio	1.09
Credible Interval	(0.734, 1.61)
Associated Peptides	1
Associated Spectra	1
Coverage	NaN

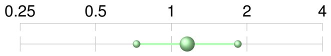

A	B	2.5	50	97.5	Sequence
**0**	**1**	**0.73**	**1.2**	**1.8**	**IHFPLATY**

5.255 " 431 880 Myosin essential light chain, striated adductor muscle OS=Aequipecten irradians PE=1 SV=2"

Protein Accession	CL36.Contig2$_{H}1$
Mean Expression Ratio	1.09
Median Expression Ratio	1.09
Credible Interval	(0.891, 1.33)
Associated Peptides	3
Associated Spectra	176
Coverage	NaN

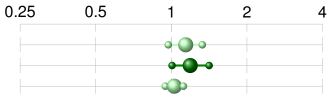

A	B	2.5	50	97.5	Sequence
**12**	**17**	**0.97**	**1.1**	**1.3**	**LSDEEVDQIMK**
**15**	**9**	**1**	**1.2**	**1.4**	**LTDITIDLEGNLK**
**46**	**77**	**0.94**	**1**	**1.1**	**VGDLLR**

5.256 247 726 Annexin A7 [Crassostrea gigas]; 286 675 Annexin A7 [Crassostrea gigas]

Protein Accession	CL15539.Contig3$_{H}1$ CL15539.Contig1$_{H}1$
Mean Expression Ratio	1.09
Median Expression Ratio	1.09
Credible Interval	(0.795, 1.5)
Associated Peptides	3
Associated Spectra	5
Coverage	NaN

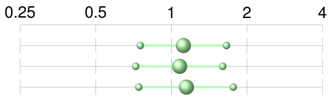

A	B	2.5	50	97.5	Sequence
**2**	**0**	**0.75**	**1.1**	**1.7**	**GLGTDEAAVIR**
**1**	**1**	**0.72**	**1.1**	**1.6**	**DLVSDLK**
**1**	**0**	**0.74**	**1.2**	**1.8**	**DIVSDTSGHFR**

5.257 680 991 minus strand PDZ and LIM domain protein 1 [Crassostrea gigas]; 553 864 minus strand PDZ and LIM domain protein 1 [Crassostrea gigas]

Protein Accession	Unigene7664$_{H}1$ Unigene7663$_{H}1$
Mean Expression Ratio	1.09
Median Expression Ratio	1.09
Credible Interval	(0.885, 1.35)
Associated Peptides	6
Associated Spectra	23
Coverage	NaN

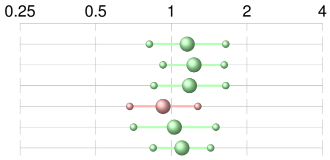

A	B	2.5	50	97.5	Sequence
2	0	0.82	1.2	1.6	AMGQPWGFR
3	3	0.93	1.2	1.6	DYGQALSVK
3	0	0.85	1.2	1.6	GPSTDFSAIKPK
3	1	0.68	0.93	1.3	SAGTQLQLTVLR
1	0	0.71	1	1.5	KVQPGTPSAATLRPGDVIQR
3	4	0.85	1.1	1.4	QLTHMQAHQLIK

5.258 8 271 minus strand Laminin subunit gamma-1 [Crassostrea gigas]

Protein Accession	Unigene36706$_{H}1$
Mean Expression Ratio	0.916
Median Expression Ratio	0.916
Credible Interval	(0.667, 1.26)
Associated Peptides	3
Associated Spectra	4
Coverage	NaN

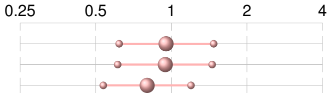

A	B	2.5	50	97.5	Sequence
1	0	0.62	0.95	1.5	EFTDASELYDR
0	1	0.61	0.95	1.5	LAEETLR
0	2	0.54	0.8	1.2	ALDLYR

5.259 213 881 PREDICTED: hypothetical protein [Saccoglossus kowalevskii]; 260 928 PREDICTED: hypothetical protein [Saccoglossus kowalevskii]; 294 962 PREDICTED: hypothetical protein [Saccoglossus kowalevskii]; 287 955 PREDICTED: hypothetical protein [Saccoglossus kowalevskii]; 316 984 PREDICTED: hypothetical protein [Saccoglossus kowalevskii]

Protein Accession	CL23253.Contig8$_{H}1$ CL23253.Contig7$_{H}1$ CL23253.Contig3$_{H}1$ CL23253.Contig2$_{H}1$ CL2325
Mean Expression Ratio	0.916
Median Expression Ratio	0.917
Credible Interval	(0.616, 1.35)
Associated Peptides	1
Associated Spectra	1
Coverage	NaN

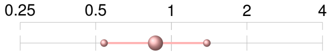

A	B	2.5	50	97.5	Sequence
0	1	0.54	0.86	1.4	VDLPGEFR

5.260 96 497 AAEL009772-PB [Aedes aegypti]

Protein Accession	CL27854.Contig2$_{H}1$
Mean Expression Ratio	1.09
Median Expression Ratio	1.09
Credible Interval	(0.754, 1.57)
Associated Peptides	1
Associated Spectra	3
Coverage	NaN

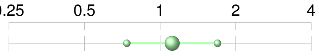

A	B	2.5	50	97.5	Sequence
2	1	0.74	1.1	1.7	LVEEFPMGNIK

5.261 3591 4526 minus strand myosin heavy chain cardiac muscle specific isoform 1 [Argopecten irradians]

Protein Accession	CL49594.Contig2$_{H}1$
Mean Expression Ratio	1.09
Median Expression Ratio	1.09
Credible Interval	(0.979, 1.21)
Associated Peptides	23
Associated Spectra	297
Coverage	NaN

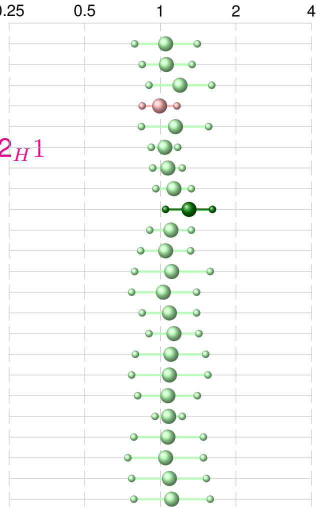

A	B	2.5	50	97.5	Sequence
3	1	0.79	1	1.4	AKLEQTLDELEDNLER
7	3	0.84	1.1	1.3	ATQANVEDLER
3	1	0.9	1.2	1.6	LEQTLDELEDNLER
22	7	0.85	1	1.2	LQQETANLTR
2	1	0.84	1.1	1.6	MSKQVEAQLAELSAK
34	20	0.92	1	1.2	QVEAQLAELSAK
22	19	0.94	1.1	1.2	SEVDDLQMQLQNVGK
18	9	0.96	1.1	1.3	TINDLQTQK
11	1	1	1.3	1.6	TMEDLQAEEDKVNHLNK
16	1	0.91	1.1	1.3	KLEQDLK
9	1	0.84	1.1	1.3	AATSRSRTLQHEAQVSALR
1	0	0.79	1.1	1.6	SEVDDLQMQLQNVGKNK
2	1	0.77	1	1.4	SASRSCSSCAATSRSRTLQHEAQVSALR
6	2	0.85	1.1	1.4	QQDAANEMADQIDQLLQKVK
10	0	0.9	1.1	1.4	ATSRSRTLQHEAQVSALR
1	1	0.8	1.1	1.5	ELEENVR
1	0	0.77	1.1	1.5	KKQQDAANEMADQIDQ
2	3	0.81	1.1	1.4	KTMEDLQAEEDKVNHLNK
25	26	0.96	1.1	1.2	QLEEAEHK
2	0	0.79	1.1	1.5	VGALTK
0	1	0.74	1.1	1.5	RKLEQDLK
0	1	0.77	1.1	1.5	LEENVR
0	1	0.78	1.1	1.6	QDAANEMADQIDQLLQK

5.262 " 3195 3968 Spectrin alpha chain, putative [Pediculus humanus corporis] gi"

Protein Accession	CL2195.Contig1$_{H}1$
Mean Expression Ratio	0.916
Median Expression Ratio	0.918
Credible Interval	(0.723, 1.16)
Associated Peptides	6
Associated Spectra	16
Coverage	NaN

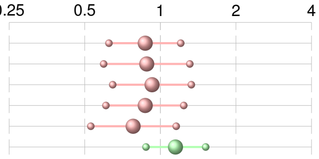

A	B	2.5	50	97.5	Sequence
2	1	0.62	0.87	1.2	DLSSVQTLLTK
1	0	0.59	0.88	1.3	LIDEGNHNADGIVER
1	1	0.65	0.92	1.3	LLLLQEQYR
1	1	0.61	0.87	1.2	LSELEAAASQR
1	0	0.53	0.78	1.2	QIEDLYLTFAK
4	3	0.87	1.2	1.5	YNDVIAR

5.263 484 1104 Calponin-2 [Crassostrea gigas]; 309 938 Calponin-2 [Crassostrea gigas]

Protein Accession	CL30203.Contig3$_H1$ CL21394.Contig2$_H1$
Mean Expression Ratio	0.919
Median Expression Ratio	0.919
Credible Interval	(0.798, 1.06)
Associated Peptides	11
Associated Spectra	138
Coverage	NaN

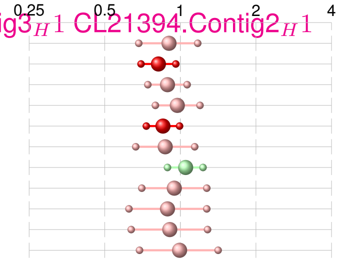

A	B	2.5	50	97.5	Sequence
5	1	0.69	0.9	1.2	AGQGIIGLQAGTNK
21	6	0.7	0.82	0.96	AVYDGTPNLPPAAK
10	10	0.74	0.89	1.1	MNTLAAPFK
11	3	0.79	0.97	1.2	QLTGEDIGSGAVQVEK
20	11	0.73	0.85	0.99	QMENIEVFLK
4	2	0.67	0.87	1.1	YDTGAEYEVR
16	11	0.89	1	1.2	NGQILCR
2	2	0.7	0.94	1.3	NLRNGQILCR
0	1	0.62	0.89	1.3	IIGLQAGTNK
0	1	0.64	0.91	1.3	PNLPPAAK
0	1	0.69	0.99	1.4	PTTLPR

5.264 156 1163 cathepsin B [Crassostrea ariakensis]

Protein Accession	CL26836.Contig9$_H1$
Mean Expression Ratio	0.919
Median Expression Ratio	0.919
Credible Interval	(0.631, 1.33)
Associated Peptides	1
Associated Spectra	2
Coverage	NaN

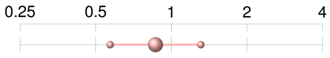

A	B	2.5	50	97.5	Sequence
1	1	0.57	0.87	1.3	DLPDSFDPR

5.265 118 513 Protein CAL-5 [Caenorhabditis elegans] gi

Protein Accession	Unigene47320$_H1$
Mean Expression Ratio	1.09
Median Expression Ratio	1.09
Credible Interval	(0.758, 1.56)
Associated Peptides	2
Associated Spectra	3
Coverage	NaN

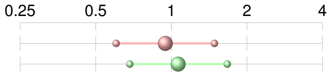

A	B	2.5	50	97.5	Sequence
1	1	0.6	0.95	1.5	EADANGDGYLSIQELK
1	0	0.68	1.1	1.7	TLLAHSGK

5.266 136 531 hypothetical protein CGI_10017533 [Crassostrea gigas]

Protein Accession	CL35548.Contig3$_H1$
Mean Expression Ratio	1.09
Median Expression Ratio	1.09
Credible Interval	(0.739, 1.61)
Associated Peptides	1
Associated Spectra	1
Coverage	NaN

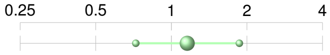

A	B	2.5	50	97.5	Sequence
0	1	0.72	1.2	1.9	DPVFQHPAIR

5.267 " 103 981 PREDICTED: ATP synthase, H+ transporting, mitochondrial F1 complex, gamma subunit isoform H (heart)-like [Saccoglossus kowalevskii]"

Protein Accession	CL25149.Contig1$_H$1
Mean Expression Ratio	0.922
Median Expression Ratio	0.92
Credible Interval	(0.658, 1.3)
Associated Peptides	2
Associated Spectra	3
Coverage	NaN

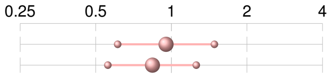

A	B	2.5	50	97.5	Sequence
1	0	0.61	0.95	1.5	ELKPARPYGAGAK
1	1	0.56	0.84	1.3	LTLTFNR

5.268 " 134 2047 PREDICTED: succinate dehydrogenase [ubiquinone] flavoprotein subunit, mitochondrial-like isoform 3, partial [Strongylocentrotus purpuratus]; 134 2047 PREDICTED: succinate dehydrogenase [ubiquinone] flavoprotein subunit, mitochondrial-like isoform 3, partial [Strongylocentrotus purpuratus]; 134 2047 PREDICTED: succinate dehydrogenase [ubiquinone] flavoprotein subunit, mitochondrial-like isoform 3, partial [Strongylocentrotus purpuratus]"

Protein Accession	CL47322.Contig4$_H$1 CL47322.Contig3$_H$1 CL47322.Contig2$_H$1
Mean Expression Ratio	0.92
Median Expression Ratio	0.92
Credible Interval	(0.774, 1.09)
Associated Peptides	14
Associated Spectra	42
Coverage	NaN

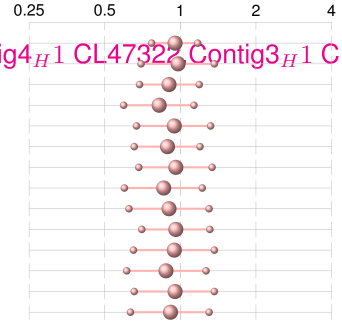

A	B	2.5	50	97.5	Sequence
10	4	0.77	0.95	1.2	AAFGLAEEGFK
1	1	0.7	0.98	1.4	ACANTIIEENKPGDR
3	3	0.69	0.9	1.2	AITIEIR
1	2	0.6	0.82	1.1	IYQQMEDLK
2	0	0.67	0.94	1.3	KHTLAYTNVDTGK
4	0	0.65	0.89	1.2	NTILATGGYGR
1	1	0.68	0.96	1.3	QDAGEASVGNLDK
1	0	0.6	0.86	1.2	SHTVAAQGGINAALGHMEEDDWR
1	0	0.62	0.9	1.3	AVIELENYGMPF
3	0	0.7	0.96	1.3	AFGGQSLK
1	0	0.65	0.94	1.4	GGINAAL
0	1	0.61	0.88	1.3	AVIELENYGMPFSR
0	1	0.66	0.95	1.4	LPPIR
0	1	0.63	0.91	1.3	WHFYDTVK

5.269 900 1616 minus strand hemocyanin isoform 1 [Haliotis diversicolor]

Protein Accession	Unigene30321$_H$1
Mean Expression Ratio	0.919
Median Expression Ratio	0.92
Credible Interval	(0.641, 1.31)
Associated Peptides	1
Associated Spectra	3
Coverage	NaN

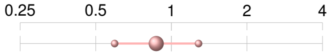

A	B	2.5	50	97.5	Sequence
2	1	0.59	0.87	1.3	VEITDDLK

5.270 496 2151 NADP-dependent malic enzyme [Crassostrea gigas]

Protein Accession	CL26274.Contig1$_H$1
Mean Expression Ratio	0.919
Median Expression Ratio	0.92
Credible Interval	(0.766, 1.1)
Associated Peptides	11
Associated Spectra	40
Coverage	NaN

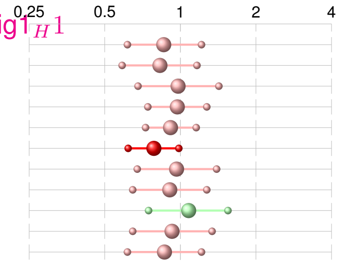

A	B	2.5	50	97.5	Sequence
1	1	0.61	0.86	1.2	AECTAEEAYTLTEGR
2	0	0.59	0.83	1.2	AIVVTDGER
1	0	0.68	0.98	1.4	GLFISIKDK
4	2	0.74	0.97	1.3	IWMVDSK
8	3	0.73	0.92	1.2	VLVNFR
7	4	0.62	0.78	0.99	GIDILR
1	0	0.67	0.96	1.4	GTAFSLK
1	1	0.64	0.91	1.3	GLLVK
1	0	0.75	1.1	1.6	GDLGVYGMGIP
0	1	0.65	0.93	1.3	QGTAAVAAAGVFAAVR
0	2	0.62	0.86	1.2	INERPIIFALSNPTSK

5.271 " 6 1274 talin, putative [Ixodes scapularis] gi"

Protein Accession	CL39200.Contig1$_H$1
Mean Expression Ratio	1.09
Median Expression Ratio	1.09
Credible Interval	(0.759, 1.56)
Associated Peptides	2
Associated Spectra	2
Coverage	NaN

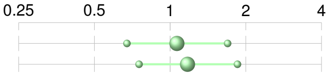

A	B	2.5	50	97.5	Sequence
0	1	0.67	1.1	1.7	SIAAATAALVK
0	1	0.75	1.2	1.9	LEMIR

5.272 2 1384 minus strand twitchin [Mytilus galloprovincialis]; 2 1384 minus strand twitchin [Mytilus galloprovincialis]; 2 1384 minus strand twitchin [Mytilus galloprovincialis]; 2 1384 minus strand twitchin [Mytilus galloprovincialis]; 2 1384 minus strand twitchin [Mytilus galloprovincialis]; 2 1384 minus strand twitchin [Mytilus galloprovincialis]

Protein Accession	Unigene62659$_H$1 Unigene62658$_H$1 Unigene62657$_H$1 Unigene62656$_H$1 Unigene62655$_H$1 U
Mean Expression Ratio	1.09
Median Expression Ratio	1.09
Credible Interval	(0.738, 1.61)
Associated Peptides	1
Associated Spectra	1
Coverage	NaN

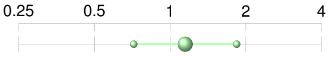

A	B	2.5	50	97.5	Sequence
1	0	0.72	1.2	1.8	DGGGADLYTAVLQIQDPKPEDGGTYK

5.273 633 806 Peroxiredoxin-1 [Crassostrea gigas]; 648 821 Peroxiredoxin-1 [Crassostrea gigas]; 666 839 Peroxiredoxin-1 [Crassostrea gigas]; 625 798 Peroxiredoxin-1 [Crassostrea gigas]; 633 824 Peroxiredoxin-1 [Crassostrea gigas]

Protein Accession	CL3160.Contig18$_{H}1$ CL3160.Contig17$_{H}1$ CL3160.Contig16$_{H}1$ CL3160.Contig15$_{H}1$ CL3160
Mean Expression Ratio	0.921
Median Expression Ratio	0.921
Credible Interval	(0.672, 1.26)
Associated Peptides	1
Associated Spectra	12
Coverage	NaN

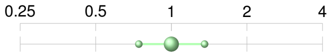

A	B	2.5	50	97.5	Sequence
6	6	0.74	1	1.4	LVQAFQFTDK

5.274 2 2542 kinesin heavy chain 1 [Aplysia californica] gi; 2 2542 kinesin heavy chain 1 [Aplysia californica] gi

Protein Accession	CL4142.Contig6$_{H}1$ CL4142.Contig5$_{H}1$
Mean Expression Ratio	0.919
Median Expression Ratio	0.921
Credible Interval	(0.681, 1.25)
Associated Peptides	3
Associated Spectra	6
Coverage	NaN

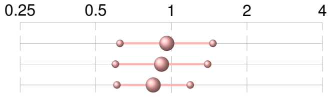

A	B	2.5	50	97.5	Sequence
1	0	0.62	0.96	1.5	QQLEAEK
1	0	0.6	0.91	1.4	ELQTLHNLR
4	0	0.61	0.85	1.2	LSELSIQMSRREQAK

5.275 266 934 minus strand Kyphoscoliosis peptidase [Crassostrea gigas]

Protein Accession	CL7490.Contig16$_{H}1$
Mean Expression Ratio	1.08
Median Expression Ratio	1.08
Credible Interval	(0.927, 1.27)
Associated Peptides	12
Associated Spectra	64
Coverage	NaN

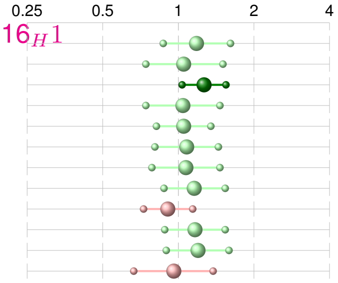

A	B	2.5	50	97.5	Sequence
3	0	0.87	1.2	1.6	ASLNANYGPDESK
1	0	0.74	1.1	1.5	DGCYLWEPLNLR
8	7	1	1.3	1.6	DLDNVHFK
2	0	0.74	1	1.5	DTVSLVVTLPDSGYYK
5	3	0.82	1	1.3	ELVFTQTER
3	1	0.81	1.1	1.4	LQIYALPSSDDSK
2	1	0.78	1.1	1.5	VNIPGAK
4	1	0.88	1.2	1.5	VTSNLIACGTEQEYK
8	3	0.72	0.91	1.1	YSSLLEYDL
5	1	0.89	1.2	1.5	QYAQWK
4	1	0.9	1.2	1.6	SRPELPHGYLGAQSK
0	1	0.67	0.96	1.4	GLKDLDNVHFK

5.276 " 65 1576 minus strand Aldehyde dehydrogenase, mitochondrial [Crassostrea gigas]"

Protein Accession	CL5835.Contig1$_{H}1$
Mean Expression Ratio	0.92
Median Expression Ratio	0.922
Credible Interval	(0.691, 1.23)
Associated Peptides	5
Associated Spectra	6
Coverage	NaN

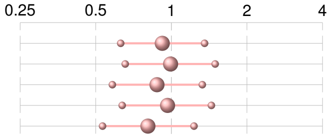

A	B	2.5	50	97.5	Sequence
1	1	0.63	0.92	1.4	LADLLER
1	0	0.66	0.99	1.5	ELGEYGLEAYTEVK
1	0	0.58	0.88	1.3	IFVEEK
1	0	0.64	0.96	1.4	VAEGDK
0	1	0.53	0.81	1.2	LIPVMAAK

5.277 64 393 Tubulin beta chain OS=Paracentrotus lividus PE=2 SV=1; 109 438 Tubulin beta chain OS=Paracentrotus lividus PE=2 SV=1; 160 489 Tubulin beta chain OS=Paracentrotus lividus PE=2 SV=1

Protein Accession	Unigene62663$_{H}1$ Unigene62661$_{H}1$ Unigene62660$_{H}1$
Mean Expression Ratio	1.08
Median Expression Ratio	1.08
Credible Interval	(0.77, 1.52)
Associated Peptides	1
Associated Spectra	6
Coverage	NaN

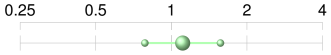

A	B	2.5	50	97.5	Sequence
4	2	0.78	1.1	1.6	INVYFNEATGGK

5.278 718 957 minus strand Calponin-1 OS=Gallus gallus GN=CNN1 PE=1 SV=2

Protein Accession	CL21394.Contig4$_{H}1$
Mean Expression Ratio	1.08
Median Expression Ratio	1.08
Credible Interval	(0.793, 1.47)
Associated Peptides	3
Associated Spectra	5
Coverage	NaN

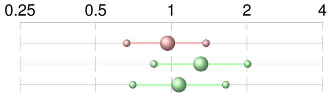

A	B	2.5	50	97.5	Sequence
3	0	0.66	0.96	1.4	EGQTVIGLQAGSNK
1	0	0.85	1.3	2	GASQAGMSMGAVR
1	0	0.7	1.1	1.6	IGLQAGSNK

5.279 511 1062 unknown [Dendroctonus ponderosae]; 511 1062 unknown [Dendroctonus ponderosae]

Protein Accession	CL18443.Contig3$_{H}1$ CL18443.Contig1$_{H}1$
Mean Expression Ratio	0.922
Median Expression Ratio	0.922
Credible Interval	(0.66, 1.29)
Associated Peptides	2
Associated Spectra	3
Coverage	NaN

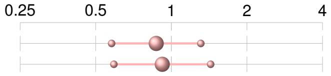

A	B	2.5	50	97.5	Sequence
1	1	0.58	0.87	1.3	KHNFLIDGFPR
0	1	0.59	0.92	1.4	FKDEIEMHLK

5.280 1 252 Aquaporin-4 [Crassostrea gigas]

Protein Accession	Unigene16706$_{H}1$
Mean Expression Ratio	1.08
Median Expression Ratio	1.08
Credible Interval	(0.757, 1.56)
Associated Peptides	2
Associated Spectra	2
Coverage	NaN

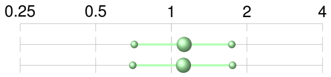

A	B	2.5	50	97.5	Sequence
1	0	0.71	1.1	1.7	APLDDAAAAEVIEIDETK
1	0	0.7	1.1	1.7	KFSPSQAAAAPQEK

5.281 316 1512 Alpha-N-acetylgalactosaminidase [Crassostrea gigas]; 282 1478 Alpha-N-acetylgalactosaminidase [Crassostrea gigas]

Protein Accession	CL2428.Contig4$_{H}1$ CL2428.Contig1$_{H}1$
Mean Expression Ratio	0.924
Median Expression Ratio	0.922
Credible Interval	(0.649, 1.32)
Associated Peptides	2
Associated Spectra	5
Coverage	NaN

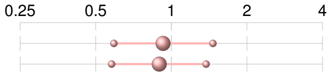

A	B	2.5	50	97.5	Sequence
1	0	0.59	0.93	1.5	LGIYEDFGSK
1	3	0.58	0.89	1.4	LVELGLR

5.282 871 1062 Adenylate kinase isoenzyme 5 [Crassostrea gigas]

Protein Accession	Unigene7895$_{H}1$
Mean Expression Ratio	1.08
Median Expression Ratio	1.08
Credible Interval	(0.819, 1.42)
Associated Peptides	2
Associated Spectra	13
Coverage	NaN

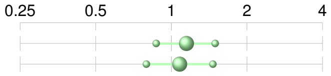

A	B	2.5	50	97.5	Sequence
6	2	0.87	1.1	1.5	VVFVVGGPGSGK
3	2	0.8	1.1	1.5	ALNEIMQK

5.283 1292 1471 minus strand hemocyanin isoform 1 [Haliotis diversicolor]

Protein Accession	Unigene65233$_{H}1$
Mean Expression Ratio	0.921
Median Expression Ratio	0.922
Credible Interval	(0.631, 1.33)
Associated Peptides	1
Associated Spectra	2
Coverage	NaN

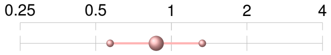

A	B	2.5	50	97.5	Sequence
1	1	0.57	0.87	1.3	LYTVQFEDALR

5.284 141 1325 Nucleobindin-2 [Crassostrea gigas]; 141 1208 minus strand Nucleobindin-2 [Crassostrea gigas]

Protein Accession	CL1888.Contig6$_H$1 CL1888.Contig2$_H$1
Mean Expression Ratio	0.923
Median Expression Ratio	0.923
Credible Interval	(0.624, 1.36)
Associated Peptides	1
Associated Spectra	2
Coverage	NaN

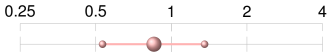

A	B	2.5	50	97.5	Sequence
**1**	**1**	**0.53**	**0.85**	**1.4**	**LKELPEVER**

5.285 28 642 minus strand Transmembrane emp24 domain-containing protein 9 [Crassostrea gigas]; 236 709 minus strand Transmembrane emp24 domain-containing protein 9 OS=Rattus norvegicus GN=Tmed9 PE=1 SV=1; 239 742 minus strand Transmembrane emp24 domain-containing protein eca OS=Drosophila erecta GN=eca PE=3 SV=1; 236 709 minus strand Transmembrane emp24 domain-containing protein 9 OS=Rattus norvegicus GN=Tmed9 PE=1 SV=1; 236 709 minus strand Transmembrane emp24 domain-containing protein 9 OS=Rattus norvegicus GN=Tmed9 PE=1 SV=1; 236 709 minus strand Transmembrane emp24 domain-containing protein 9 OS=Rattus norvegicus GN=Tmed9 PE=1 SV=1; 44 658 minus strand Transmembrane emp24 domain-containing protein 9 [Crassostrea gigas]

Protein Accession	CL2503.Contig8$_H$1 CL2503.Contig7$_H$1 CL2503.Contig5$_H$1 CL2503.Contig4$_H$1 CL2503.Con
Mean Expression Ratio	1.08
Median Expression Ratio	1.08
Credible Interval	(0.729, 1.6)
Associated Peptides	1
Associated Spectra	1
Coverage	NaN

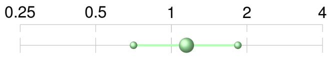

A	B	2.5	50	97.5	Sequence
**1**	**0**	**0.71**	**1.1**	**1.8**	**QLLDQVEQITK**

5.286 587 811 hemocyanin 2 [Megathura crenulata]

Protein Accession	Unigene4334$_H$1
Mean Expression Ratio	1.08
Median Expression Ratio	1.08
Credible Interval	(0.755, 1.57)
Associated Peptides	2
Associated Spectra	2
Coverage	NaN

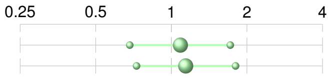

A	B	2.5	50	97.5	Sequence
**1**	**0**	**0.68**	**1.1**	**1.7**	**SVEDLTAGEAESLK**
**0**	**1**	**0.73**	**1.1**	**1.8**	**AIDDGTFR**

5.287 642 1415 minus strand cytochrome c oxidase subunit II [Oncomelania hupensis hupensis]

Protein Accession	CL6764.Contig3$_{H}1$
Mean Expression Ratio	0.923
Median Expression Ratio	0.924
Credible Interval	(0.625, 1.36)
Associated Peptides	1
Associated Spectra	1
Coverage	NaN

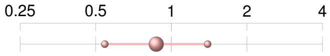

A	B	2.5	50	97.5	Sequence
0	1	0.54	0.87	1.4	IVLPTHTDIR

5.288 2939 3208 minus strand beta-thymosin domain repeat protein CSP24KDa_v1 [Hermissenda crassicornis] gi

Protein Accession	CL9548.Contig3$_{H}1$
Mean Expression Ratio	0.924
Median Expression Ratio	0.924
Credible Interval	(0.702, 1.22)
Associated Peptides	4
Associated Spectra	8
Coverage	NaN

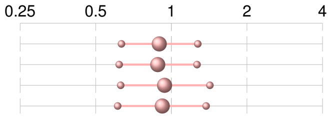

A	B	2.5	50	97.5	Sequence
2	1	0.63	0.9	1.3	NPLPSTDAIAAEK
2	1	0.62	0.88	1.3	AETEEKNPLPTK
1	0	0.63	0.94	1.4	KAETEEKNPLPTK
0	1	0.61	0.92	1.4	SIGEFDK

5.289 " 242 1504 Glutamate receptor, ionotropic kainate 3 [Crassostrea gigas]; 239 1501 Glutamate receptor, ionotropic kainate 3 [Crassostrea gigas]; 238 1500 Glutamate receptor, ionotropic kainate 3 [Crassostrea gigas]; 661 1923 minus strand Glutamate receptor, ionotropic kainate 3 [Crassostrea gigas]"

Protein Accession	CL12478.Contig7$_{H}1$ CL12478.Contig6$_{H}1$ CL12478.Contig5$_{H}1$ CL12478.Contig3$_{H}1$
Mean Expression Ratio	1.08
Median Expression Ratio	1.08
Credible Interval	(0.768, 1.52)
Associated Peptides	2
Associated Spectra	3
Coverage	NaN

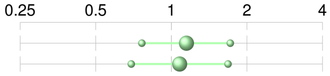

A	B	2.5	50	97.5	Sequence
2	0	0.76	1.1	1.7	RPTFAQPSFQER
1	0	0.69	1.1	1.7	LAILR

5.290 79 606 minus strand PREDICTED: similar to Ras-like protein 3 precursor (Protein roughened) [Tribolium castaneum] gi

Protein Accession	Unigene4246$_{H}$1
Mean Expression Ratio	1.08
Median Expression Ratio	1.08
Credible Interval	(0.822, 1.42)
Associated Peptides	4
Associated Spectra	9
Coverage	NaN

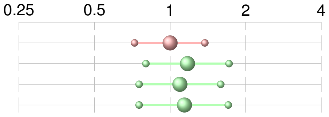

A	B	2.5	50	97.5	Sequence
2	2	0.72	1	1.4	LVVLGSGGVGK
2	0	0.8	1.2	1.7	VKDTDDVPMLLVGNK
1	1	0.75	1.1	1.6	CDLEVDR
0	1	0.75	1.1	1.7	DLYMK

5.291 18 647 minus strand ATP synthase-like protein [Biomphalaria glabrata]

Protein Accession	CL42054.Contig4$_{H}$1
Mean Expression Ratio	1.08
Median Expression Ratio	1.08
Credible Interval	(0.845, 1.37)
Associated Peptides	6
Associated Spectra	16
Coverage	NaN

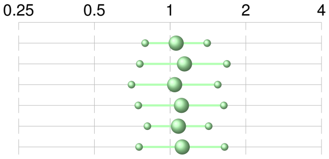

A	B	2.5	50	97.5	Sequence
2	4	0.79	1.1	1.4	LAEFVANPSLQR
1	0	0.76	1.1	1.7	KAVLEQALK
1	0	0.7	1	1.6	NFQNLLK
1	0	0.75	1.1	1.6	KLDEVEK
3	3	0.81	1.1	1.4	GALQGFLK
1	0	0.75	1.1	1.7	NYTNLIK

5.292 185 1258 minus strand Severin [Crassostrea gigas]

Protein Accession	CL7486.Contig1$_{H}$1
Mean Expression Ratio	1.08
Median Expression Ratio	1.08
Credible Interval	(0.829, 1.41)
Associated Peptides	7
Associated Spectra	9
Coverage	NaN

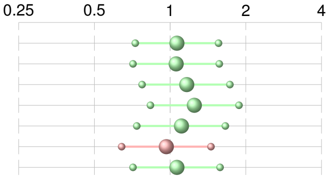

A	B	2.5	50	97.5	Sequence
2	0	0.73	1.1	1.6	EVQGHESDLFR
1	1	0.71	1.1	1.6	TDHPLVPITCLTEGTK
1	0	0.78	1.2	1.7	TDTVDEGSGTEEFDR
1	0	0.83	1.2	1.9	TVELDTFLNDVPVQHR
1	0	0.74	1.1	1.7	VTEWPVGDYGK
1	0	0.64	0.97	1.5	SDFISEDVFILDTK
1	0	0.71	1.1	1.6	TAGQKPGLQIWR

5.293 3 239 minus strand p38 MAPK [Littorina littorea]

Protein Accession	CL49144.Contig2$_{H}$1
Mean Expression Ratio	1.08
Median Expression Ratio	1.08
Credible Interval	(0.731, 1.61)
Associated Peptides	1
Associated Spectra	1
Coverage	NaN

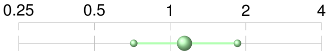

A	B	2.5	50	97.5	Sequence
0	1	0.71	1.1	1.9	MLDLDTDHR

5.294 729 1541 troponin T [Mizuhopecten yessoensis]

Protein Accession	Unigene34289$_{H}1$
Mean Expression Ratio	0.926
Median Expression Ratio	0.925
Credible Interval	(0.691, 1.25)
Associated Peptides	2
Associated Spectra	8
Coverage	NaN

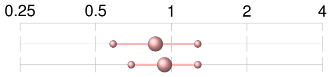

A	B	2.5	50	97.5	Sequence
1	1	0.59	0.86	1.3	RQQYDMIELAER
2	4	0.69	0.94	1.3	QQYDMIELAER

5.295 " 116 352 minus strand troponin I 4 [Clonorchis sinensis]; 491 706 minus strand troponin-I, TnI {C-terminal} [Chlamys nipponensis=Akazara scallop, akazara, striated adductor muscle, Peptide Partial, 135 aa]"

Protein Accession	Unigene63816$_{H}1$ Unigene63815$_{H}1$
Mean Expression Ratio	0.926
Median Expression Ratio	0.926
Credible Interval	(0.706, 1.21)
Associated Peptides	2
Associated Spectra	21
Coverage	NaN

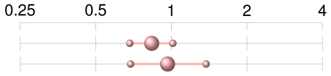

A	B	2.5	50	97.5	Sequence
12	6	0.68	0.83	1	FIKPVLR
2	1	0.69	0.97	1.4	FALKEEDESSK

5.296 247 762 Myophilin [Crassostrea gigas]; 244 759 Myophilin [Crassostrea gigas]; 119 634 Myophilin [Crassostrea gigas]

Protein Accession	CL16175.Contig6$_{H}1$ CL16175.Contig5$_{H}1$ CL16175.Contig4$_{H}1$
Mean Expression Ratio	1.08
Median Expression Ratio	1.08
Credible Interval	(0.797, 1.45)
Associated Peptides	3
Associated Spectra	6
Coverage	NaN

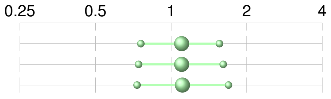

A	B	2.5	50	97.5	Sequence
2	1	0.76	1.1	1.6	GGGFALMQNIER
1	1	0.74	1.1	1.6	LMPGCIK
1	0	0.73	1.1	1.7	DGIVLCK

5.297 1200 1547 protein disulfide isomerase [Conus eburneus]

Protein Accession	CL3770.Contig1$_{H}1$
Mean Expression Ratio	1.08
Median Expression Ratio	1.08
Credible Interval	(0.886, 1.33)
Associated Peptides	8
Associated Spectra	24
Coverage	NaN

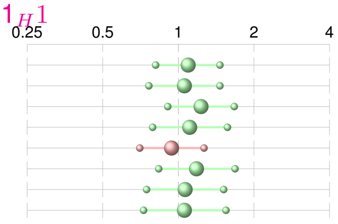

A	B	2.5	50	97.5	Sequence
2	3	0.81	1.1	1.5	IDSTANDVENIR
3	0	0.76	1.1	1.5	QLAPIWDELAEK
3	1	0.91	1.2	1.7	VQSFPTLK
1	1	0.79	1.1	1.6	YKDSNDIVIAK
1	4	0.7	0.94	1.3	NFNDVAFDK
1	1	0.83	1.2	1.7	VVDYNGER
1	1	0.75	1.1	1.5	VLVGK
0	1	0.73	1.1	1.5	TLSAFVK

5.298 64 435 peptidoglycan recognition protein S1L [Pomacea canaliculata]

Protein Accession	CL1297.Contig6$_{H}1$
Mean Expression Ratio	1.08
Median Expression Ratio	1.08
Credible Interval	(0.739, 1.61)
Associated Peptides	1
Associated Spectra	1
Coverage	NaN

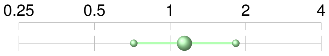

A	B	2.5	50	97.5	Sequence
1	0	0.72	1.1	1.8	MYSVCHGGR

5.299 51 1295 PREDICTED: phosphoglycerate kinase-like [Nasonia vitripennis]; 97 1341 PREDICTED: phosphoglycerate kinase-like [Nasonia vitripennis]

Protein Accession	CL14417.Contig13$_{H}1$ CL14417.Contig12$_{H}1$
Mean Expression Ratio	0.927
Median Expression Ratio	0.927
Credible Interval	(0.819, 1.05)
Associated Peptides	24
Associated Spectra	81
Coverage	NaN

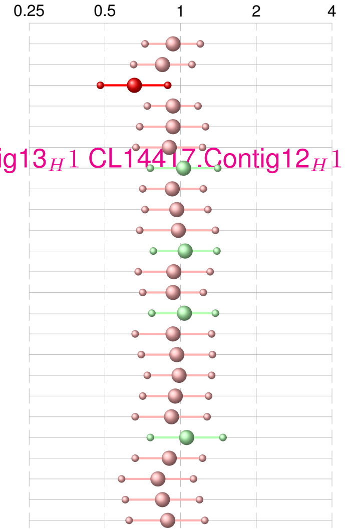

A	B	2.5	50	97.5	Sequence
4	3	0.72	0.93	1.2	ALMDAVVEATK
4	2	0.65	0.85	1.1	ATQEDIDKFR
2	1	0.48	0.65	0.89	FHVEEEGKGVAPDGTK
6	4	0.74	0.93	1.2	IVQGLMDK
2	1	0.69	0.93	1.3	LGDIYVNDAFGTAHR
1	2	0.66	0.9	1.2	LSIDKVDVK
3	0	0.76	1	1.4	SGIPADWMGLDIGPK
3	2	0.71	0.93	1.2	SVVLMSHLGRPDGTK
2	2	0.72	0.96	1.3	VDFNVPLKDGK
1	0	0.69	0.98	1.4	VLNNMEIGK
2	2	0.78	1	1.4	VLPGVAALTDA
2	0	0.68	0.94	1.3	VSMVSTGGGASLELLEGK
2	3	0.71	0.93	1.2	YSMKPVADELK
4	0	0.77	1	1.4	KELMYFAK
1	0	0.66	0.93	1.3	FTDAVK
2	0	0.7	0.97	1.3	VDFNVPLK
4	0	0.74	0.99	1.3	ASGFLLK
2	1	0.71	0.95	1.3	ELMYFAK
1	1	0.66	0.92	1.3	AHSSMVGCQLPIR
2	0	0.76	1.1	1.5	KNNVQIHLPEDFVTGDKFDEK
1	2	0.66	0.9	1.2	SLFDEEGAK
1	1	0.58	0.81	1.1	FHVEEEGK
0	1	0.6	0.85	1.2	LENLR
0	1	0.63	0.89	1.3	IVAALPTIK

5.300 " 125 628 minus strand collectin sub-family member 12, partial [Oncorhynchus mykiss]"

Protein Accession	CL46744.Contig5$_{H}1$
Mean Expression Ratio	1.08
Median Expression Ratio	1.08
Credible Interval	(0.738, 1.59)
Associated Peptides	1
Associated Spectra	1
Coverage	NaN

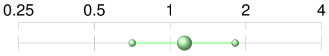

A	B	2.5	50	97.5	Sequence
1	0	0.71	1.1	1.8	WVQGDNRPLAYTR

5.301 176 670 hypothetical protein BRAFLDRAFT_122621 [Branchiostoma floridae] gi

Protein Accession	Unigene180$_{H}1$
Mean Expression Ratio	1.08
Median Expression Ratio	1.08
Credible Interval	(0.899, 1.3)
Associated Peptides	7
Associated Spectra	64
Coverage	NaN

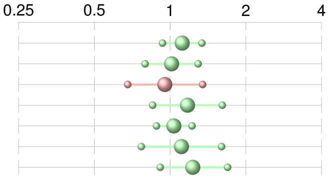

A	B	2.5	50	97.5	Sequence
11	9	0.93	1.1	1.3	FADENFLLK
6	3	0.8	1	1.3	VFFDITADGQPLGR
1	1	0.68	0.96	1.3	VIPNFMCQGGDFTNHNGTGGR
3	0	0.85	1.2	1.6	VLCTGEK
12	13	0.88	1	1.2	IVMELR
1	0	0.77	1.1	1.6	ADVVPK
2	2	0.91	1.2	1.7	HVVFGQVVEGMDVVR

5.302 228 1553 hypothetical protein CGI_10024545 [Crassostrea gigas]; 228 1553 hypothetical protein CGI_10024545 [Crassostrea gigas]

Protein Accession	CL14644.Contig9$_{H}1$ CL14644.Contig8$_{H}1$
Mean Expression Ratio	1.08
Median Expression Ratio	1.08
Credible Interval	(0.753, 1.55)
Associated Peptides	2
Associated Spectra	2
Coverage	NaN

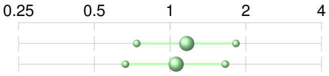

A	B	2.5	50	97.5	Sequence
0	1	0.73	1.2	1.8	LMEQPQSMLL
0	1	0.66	1.1	1.7	AEDILR

5.303 27 515 heat shock protein 70 [Capsaspora owczarzaki ATCC 30864]; 30 464 minus strand hypothetical protein [Trichinella spiralis]; 27 491 minus strand heat shock protein 70 [Capsaspora owczarzaki ATCC 30864]

Protein Accession	CL14449.Contig4$_{H}1$ CL14449.Contig2$_{H}1$ CL14449.Contig1$_{H}1$
Mean Expression Ratio	0.928
Median Expression Ratio	0.927
Credible Interval	(0.688, 1.26)
Associated Peptides	4
Associated Spectra	5
Coverage	NaN

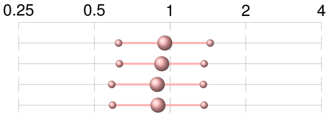

A	B	2.5	50	97.5	Sequence
1	0	0.63	0.95	1.4	RFDDPSVQSDMK
2	0	0.63	0.93	1.4	VEIIANDQGNR
1	0	0.58	0.89	1.4	NQVAMNPENTVFDAK
1	0	0.59	0.9	1.4	EVAEGYLGK

5.304 98 850 minus strand 14-3-3 protein epsilon [Schistocerca gregaria]

Protein Accession	CL27652.Contig3$_{H}1$
Mean Expression Ratio	1.08
Median Expression Ratio	1.08
Credible Interval	(0.862, 1.34)
Associated Peptides	8
Associated Spectra	18
Coverage	NaN

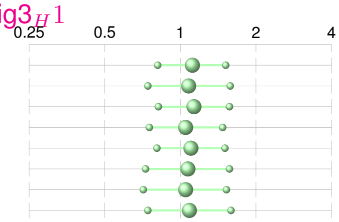

A	B	2.5	50	97.5	Sequence
3	1	0.81	1.1	1.5	AASDISMTELPPTHPIR
1	0	0.74	1.1	1.6	EAAENSLVAYK
3	0	0.82	1.1	1.6	HLIPNAGGGESK
1	2	0.75	1.1	1.5	IISSMEQK
3	1	0.81	1.1	1.5	YDEMVEAMK
1	0	0.73	1.1	1.6	VFYYK
0	1	0.71	1.1	1.5	YLAEFATGNDR
0	1	0.74	1.1	1.6	TELPPTHPIR

5.305 1497 1889 UDP-glucuronosyltransferase 2C1 (Fragment) OS=Oryctolagus cuniculus GN=UGT2C1 PE=2 SV=1

Protein Accession	CL29849.Contig1$_{H}1$
Mean Expression Ratio	1.08
Median Expression Ratio	1.08
Credible Interval	(0.738, 1.57)
Associated Peptides	1
Associated Spectra	2
Coverage	NaN

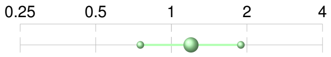

A	B	2.5	50	97.5	Sequence
**1**	**1**	**0.75**	**1.2**	**1.9**	**LFVSHCGK**

5.306 426 896 PREDICTED: calmodulin-like [Metaseiulus occidentalis]

Protein Accession	Unigene667$_{H}1$
Mean Expression Ratio	1.08
Median Expression Ratio	1.08
Credible Interval	(0.779, 1.48)
Associated Peptides	2
Associated Spectra	8
Coverage	NaN

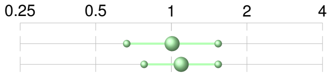

A	B	2.5	50	97.5	Sequence
**1**	**1**	**0.67**	**1**	**1.5**	**ADQLTEEQIAEFK**
**2**	**4**	**0.78**	**1.1**	**1.5**	**ELGTVMR**

5.307 182 1846 minus strand hypothetical protein DAPPUDRAFT_302108 [Daphnia pulex]; 110 1774 minus strand hypothetical protein DAPPUDRAFT_302108 [Daphnia pulex]

Protein Accession	CL11561.Contig6$_{H}1$ CL11561.Contig1$_{H}1$
Mean Expression Ratio	1.08
Median Expression Ratio	1.08
Credible Interval	(0.77, 1.53)
Associated Peptides	3
Associated Spectra	3
Coverage	NaN

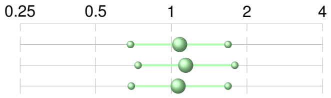

A	B	2.5	50	97.5	Sequence
**1**	**0**	**0.69**	**1.1**	**1.7**	**LRDAIRK**
**0**	**1**	**0.74**	**1.1**	**1.8**	**LFLPGGLK**
**0**	**1**	**0.69**	**1.1**	**1.7**	**LIIGQR**

5.308 98 1012 minus strand RecName: Full=Substrate-specific endoprotease Tex31; Flags: Precursor gi|32187775|emb|CAD36507.1| substrate-specific endoprotease [Conus textile]

Protein Accession	CL13681.Contig1$_{H}$1
Mean Expression Ratio	0.929
Median Expression Ratio	0.928
Credible Interval	(0.69, 1.25)
Associated Peptides	3
Associated Spectra	7
Coverage	NaN

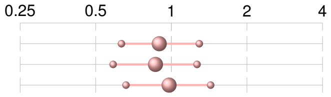

A	B	2.5	50	97.5	Sequence
2	1	0.63	0.89	1.3	NIPLSASDISAIVER
2	0	0.59	0.86	1.3	VPGAVFSK
1	1	0.66	0.98	1.4	ALDPQHTMCLADK

5.309 337 474 minus strand heat shock protein 22 isoform 1 [Ruditapes philippinarum]

Protein Accession	CL30681.Contig1$_{H}$1
Mean Expression Ratio	0.927
Median Expression Ratio	0.928
Credible Interval	(0.656, 1.3)
Associated Peptides	2
Associated Spectra	3
Coverage	NaN

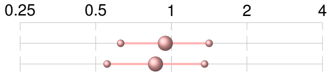

A	B	2.5	50	97.5	Sequence
1	1	0.63	0.94	1.4	EDWNFFDR
0	1	0.55	0.87	1.4	REDWNFFDR

5.310 2 2248 PREDICTED: heat shock 70 kDa protein 4-like [Oreochromis niloticus]; 2 2227 PREDICTED: heat shock 70 kDa protein 4-like [Oreochromis niloticus]

Protein Accession	CL12749.Contig5$_{H}$1 CL12749.Contig4$_{H}$1
Mean Expression Ratio	0.932
Median Expression Ratio	0.928
Credible Interval	(0.668, 1.31)
Associated Peptides	3
Associated Spectra	3
Coverage	NaN

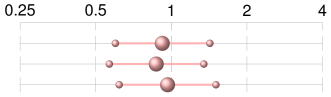

A	B	2.5	50	97.5	Sequence
1	0	0.6	0.92	1.4	EMLNLLIEK
1	0	0.57	0.87	1.3	TAWWTPK
0	1	0.62	0.96	1.5	HENPVIFVSQVMSEK

5.311 " 166 1248 PREDICTED: probable pyruvate dehydrogenase E1 component subunit alpha, mitochondrial-like [Metaseiulus occidentalis]; 166 1248 PREDICTED: probable pyruvate dehydrogenase E1 component subunit alpha, mitochondrial-like [Metaseiulus occidentalis]; 166 1248 PREDICTED: probable pyruvate dehydrogenase E1 component subunit alpha, mitochondrial-like [Metaseiulus occidentalis]; 166 1248 PREDICTED: probable pyruvate dehydrogenase E1 component subunit alpha, mitochondrial-like [Metaseiulus occidentalis]; 166 1248 PREDICTED: probable pyruvate dehydrogenase E1 component subunit alpha, mitochondrial-like [Metaseiulus occidentalis]; 166 1248 minus strand PREDICTED: probable pyruvate dehydrogenase E1 component subunit alpha, mitochondrial-like [Metaseiulus occidentalis]; 166 1248 minus strand PREDICTED: probable pyruvate dehydrogenase E1 component subunit alpha, mitochondrial-like [Metaseiulus occidentalis]; 166 1248 PREDICTED: probable pyruvate dehydrogenase E1 component subunit alpha, mitochondrial-like [Metaseiulus occidentalis]; 166 1248 PREDICTED: probable pyruvate dehydrogenase E1 component subunit alpha, mitochondrial-like [Metaseiulus occidentalis]"

Protein Accession	Unigene34082$_{H}1$ Unigene34081$_{H}1$ Unigene34079$_{H}1$ Unigene34077$_{H}1$ Unigene34075$_{H}1$ U
Mean Expression Ratio	0.926
Median Expression Ratio	0.929
Credible Interval	(0.67, 1.28)
Associated Peptides	3
Associated Spectra	4
Coverage	NaN

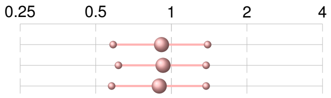

A	B	2.5	50	97.5	Sequence
1	0	0.59	0.92	1.4	TGLATEEELK
1	1	0.62	0.92	1.4	TGLATEEELKK
0	1	0.58	0.9	1.4	KIDGEVRSEVDAAAER

5.312 " 656 787 collagen alpha-1(XII) chain-like; K08132 collagen, type XII, alpha"

Protein Accession	Unigene26545$_{H}1$
Mean Expression Ratio	1.08
Median Expression Ratio	1.08
Credible Interval	(0.782, 1.51)
Associated Peptides	2
Associated Spectra	4
Coverage	NaN

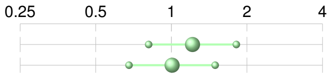

A	B	2.5	50	97.5	Sequence
1	1	0.81	1.2	1.8	AEVFDAVDNIPYR
1	1	0.68	1	1.5	TKAEVFDAVDNIPYR

5.313 2302 2547 minus strand hemocyanin isoform 1 [Haliotis diversicolor]; 816 1211 H2 [Haliotis tuberculata]

Protein Accession	CL11519.Contig6$_{H}1$ CL11519.Contig5$_{H}1$
Mean Expression Ratio	0.931
Median Expression Ratio	0.929
Credible Interval	(0.662, 1.31)
Associated Peptides	2
Associated Spectra	4
Coverage	NaN

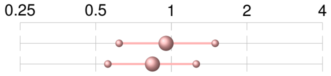

A	B	2.5	50	97.5	Sequence
**1**	**0**	**0.62**	**0.95**	**1.5**	**KNVLSLTPAER**
**2**	**1**	**0.56**	**0.84**	**1.3**	**SLILAMR**

5.314 172 678 Myosin regulatory light chain sqh [Crassostrea gigas]

Protein Accession	CL23773.Contig1$_{H}1$
Mean Expression Ratio	0.929
Median Expression Ratio	0.93
Credible Interval	(0.657, 1.31)
Associated Peptides	2
Associated Spectra	3
Coverage	NaN

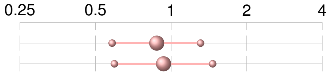

A	B	2.5	50	97.5	Sequence
**1**	**1**	**0.58**	**0.87**	**1.3**	**LTDEEVDEMFR**
**1**	**0**	**0.59**	**0.94**	**1.5**	**ELLTTMGDR**

5.315 238 1038 PREDICTED: hypothetical protein [Saccoglossus kowalevskii]; 294 992 PREDICTED: hypothetical protein [Saccoglossus kowalevskii]

Protein Accession	CL6273.Contig7$_{H}1$ CL6273.Contig6$_{H}1$
Mean Expression Ratio	0.929
Median Expression Ratio	0.93
Credible Interval	(0.626, 1.36)
Associated Peptides	1
Associated Spectra	1
Coverage	NaN

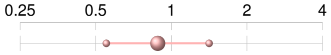

A	B	2.5	50	97.5	Sequence
**0**	**1**	**0.55**	**0.88**	**1.4**	**DVVLLHQLGR**

5.316 1 576 minus strand Tubulin beta chain [Crassostrea gigas]

Protein Accession	Unigene6463$_{H}1$
Mean Expression Ratio	1.08
Median Expression Ratio	1.08
Credible Interval	(0.731, 1.59)
Associated Peptides	1
Associated Spectra	4
Coverage	NaN

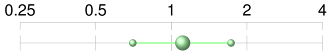

A	B	2.5	50	97.5	Sequence
**3**	**1**	**0.7**	**1.1**	**1.7**	**EVDEQMLNVQNK**

5.317 311 928 minus strand peroxiredoxin 3 [Clonorchis sinensis] gi; 288 905 minus strand peroxiredoxin 3 [Clonorchis sinensis] gi

Protein Accession	CL2043.Contig4$_H$1 CL2043.Contig1$_H$1
Mean Expression Ratio	0.931
Median Expression Ratio	0.93
Credible Interval	(0.712, 1.21)
Associated Peptides	3
Associated Spectra	13
Coverage	NaN

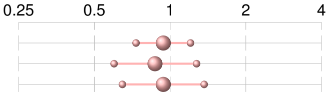

A	B	2.5	50	97.5	Sequence
5	4	0.73	0.94	1.2	QITINDLPVGR
1	1	0.6	0.87	1.3	SVDEILR
0	2	0.65	0.94	1.4	GLFIIDPK

5.318 764 1015 minus strand heat shock protein beta-1 [Trichinella spiralis] gi

Protein Accession	CL46983.Contig3$_H$1
Mean Expression Ratio	0.93
Median Expression Ratio	0.93
Credible Interval	(0.7, 1.23)
Associated Peptides	2
Associated Spectra	13
Coverage	NaN

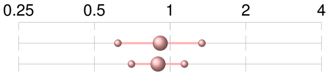

A	B	2.5	50	97.5	Sequence
2	0	0.62	0.91	1.3	SYVLPK
8	3	0.7	0.9	1.1	TVTSSLSQNGVLTLK

5.319 58 732 glutathione-s-transferase [Haliotis discus discus]

Protein Accession	CL4861.Contig1$_H$1
Mean Expression Ratio	0.931
Median Expression Ratio	0.931
Credible Interval	(0.688, 1.26)
Associated Peptides	4
Associated Spectra	5
Coverage	NaN

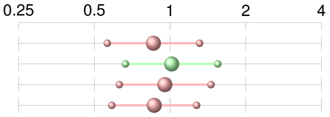

A	B	2.5	50	97.5	Sequence
0	1	0.56	0.86	1.3	IDKEYLEVK
0	1	0.66	1	1.5	GQVPTFR
0	1	0.63	0.95	1.5	WEEYLR
0	2	0.59	0.87	1.3	LISFSANEHK

5.320 613 2421 PREDICTED: lysosomal alpha-glucosidase [Danio rerio]

Protein Accession	CL5792.Contig1$_H$1
Mean Expression Ratio	1.07
Median Expression Ratio	1.07
Credible Interval	(0.726, 1.59)
Associated Peptides	1
Associated Spectra	1
Coverage	NaN

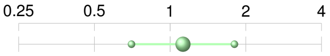

A	B	2.5	50	97.5	Sequence
1	0	0.7	1.1	1.8	ATMGGLQK

5.321 1714 2121 Phosphoenolpyruvate carboxykinase [GTP] [Crassostrea gigas]

Protein Accession: CL25948.Contig1$_{H}1$
Mean Expression Ratio: 0.931
Median Expression Ratio: 0.931
Credible Interval: (0.68, 1.26)
Associated Peptides: 1
Associated Spectra: 10
Coverage: NaN

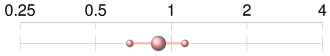

A	B	2.5	50	97.5	Sequence
**7**	**3**	**0.68**	**0.89**	**1.1**	**SEATAAAEFK**

5.322 387 1064 minus strand PREDICTED: hypothetical protein [Hydra magnipapillata]; 275 952 minus strand PREDICTED: hypothetical protein [Hydra magnipapillata]; 172 849 minus strand PREDICTED: hypothetical protein [Hydra magnipapillata]

Protein Accession: CL20613.Contig3$_{H}1$ CL20613.Contig2$_{H}1$ CL20613.Contig1$_{H}1$
Mean Expression Ratio: 0.93
Median Expression Ratio: 0.931
Credible Interval: (0.685, 1.25)
Associated Peptides: 3
Associated Spectra: 6
Coverage: NaN

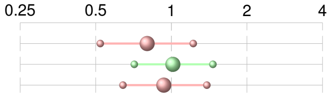

A	B	2.5	50	97.5	Sequence
**1**	**0**	**0.52**	**0.8**	**1.2**	**ELGATFEAADNFKPNVVVSGR**
**3**	**0**	**0.71**	**1**	**1.5**	**NAGYTVHFVSPK**
**1**	**1**	**0.64**	**0.93**	**1.4**	**GFLADQEVQK**

5.323 145 459 fk506-binding protein 2 [Papilio xuthus]; 237 551 fk506-binding protein 2 [Papilio xuthus]

Protein Accession: Unigene7883$_{H}1$ CL4621.Contig4$_{H}1$
Mean Expression Ratio: 1.07
Median Expression Ratio: 1.07
Credible Interval: (0.822, 1.4)
Associated Peptides: 4
Associated Spectra: 11
Coverage: NaN

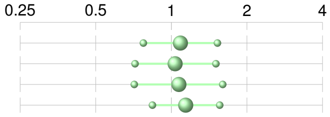

A	B	2.5	50	97.5	Sequence
**2**	**1**	**0.77**	**1.1**	**1.5**	**GVDVETISEGDKK**
**1**	**1**	**0.72**	**1**	**1.5**	**GWDEGVAQMSK**
**1**	**0**	**0.71**	**1.1**	**1.6**	**IGKGEVIK**
**4**	**1**	**0.84**	**1.1**	**1.6**	**NKPFQFK**

5.324 138 713 Rac [Aplysia californica] gi; 101 676 Rac [Aplysia californica] gi

Protein Accession: CL11009.Contig9$_{H}1$ CL11009.Contig1$_{H}1$
Mean Expression Ratio: 1.08
Median Expression Ratio: 1.07
Credible Interval: (0.729, 1.59)
Associated Peptides: 1
Associated Spectra: 1
Coverage: NaN

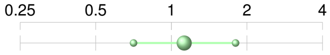

A	B	2.5	50	97.5	Sequence
**1**	**0**	**0.71**	**1.1**	**1.8**	**CVVVGDGAVGK**

5.325 " 3799 4371 papilin, partial [Rhipicephalus microplus]"

Protein Accession	CL19397.Contig5$_{H}$1
Mean Expression Ratio	1.07
Median Expression Ratio	1.07
Credible Interval	(0.741, 1.54)
Associated Peptides	1
Associated Spectra	2
Coverage	NaN

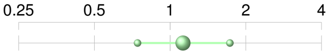

A	B	2.5	50	97.5	Sequence
**1**	**1**	**0.74**	**1.1**	**1.7**	**TVTLVLAK**

5.326 166 1563 arylsulfatase [Haliotis discus discus]

Protein Accession	CL49030.Contig16$_{H}$1
Mean Expression Ratio	1.08
Median Expression Ratio	1.07
Credible Interval	(0.729, 1.61)
Associated Peptides	1
Associated Spectra	1
Coverage	NaN

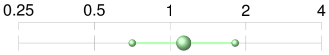

A	B	2.5	50	97.5	Sequence
**0**	**1**	**0.7**	**1.1**	**1.8**	**VPLVLHSK**

5.327 2 811 voltage-dependent anion channel 2-like protein [Haliotis diversicolor]; 2 811 voltage-dependent anion channel 2-like protein [Haliotis diversicolor]; 2 811 voltage-dependent anion channel 2-like protein [Haliotis diversicolor]

Protein Accession	CL2477.Contig3$_{H}$1 CL2477.Contig2$_{H}$1 CL2477.Contig1$_{H}$1
Mean Expression Ratio	0.933
Median Expression Ratio	0.933
Credible Interval	(0.758, 1.15)
Associated Peptides	9
Associated Spectra	22
Coverage	NaN

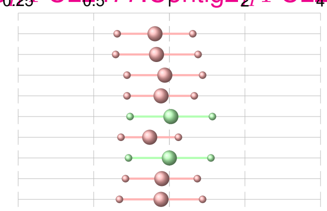

A	B	2.5	50	97.5	Sequence
**2**	**0**	**0.62**	**0.88**	**1.2**	**LAFDTSFAPQTGK**
**1**	**0**	**0.61**	**0.89**	**1.3**	**VSGSLETK**
**1**	**1**	**0.68**	**0.96**	**1.4**	**WSEYGLTFTEK**
**4**	**0**	**0.68**	**0.92**	**1.3**	**YTLDSSASVSAK**
**1**	**0**	**0.7**	**1**	**1.5**	**GYNYGFFK**
**2**	**5**	**0.64**	**0.83**	**1.1**	**VGLGLDVEA**
**1**	**0**	**0.69**	**1**	**1.5**	**FALGTK**
**1**	**2**	**0.67**	**0.93**	**1.3**	**LTLSALIEGK**
**0**	**1**	**0.63**	**0.92**	**1.4**	**DIFSK**

5.328 410 619 minus strand Guanine nucleotide-binding protein subunit gamma-1 [Crassostrea gigas]

Protein Accession	CL36920.Contig1$_{H}$1
Mean Expression Ratio	1.07
Median Expression Ratio	1.07
Credible Interval	(0.788, 1.46)
Associated Peptides	2
Associated Spectra	6
Coverage	NaN

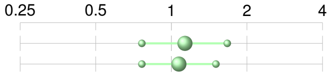

A	B	2.5	50	97.5	Sequence
**1**	**1**	**0.76**	**1.1**	**1.7**	**CEAQITR**
**2**	**2**	**0.76**	**1.1**	**1.5**	**LIEQLR**

5.329 6043 6174 myosin heavy chain [Mytilus galloprovincialis]; 5869 6000 myosin heavy chain [Mytilus galloprovincialis]

Protein Accession	Unigene19025$_{H}$1 CL49594.Contig1$_{H}$1
Mean Expression Ratio	0.933
Median Expression Ratio	0.933
Credible Interval	(0.691, 1.26)
Associated Peptides	1
Associated Spectra	12
Coverage	NaN

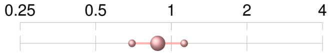

A	B	2.5	50	97.5	Sequence
**12**	**0**	**0.7**	**0.88**	**1.1**	**KLEADIQAMQTDLDEMNGELR**

5.330 87 485 Sex-regulated protein janus-A [Crassostrea gigas]

Protein Accession	CL13131.Contig3$_{H}$1
Mean Expression Ratio	0.931
Median Expression Ratio	0.934
Credible Interval	(0.632, 1.38)
Associated Peptides	1
Associated Spectra	1
Coverage	NaN

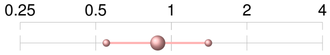

A	B	2.5	50	97.5	Sequence
**0**	**1**	**0.55**	**0.88**	**1.4**	**ADHSITQTLLLR**

5.331 75 803 hypothetical protein DAPPUDRAFT_231350 [Daphnia pulex]

Protein Accession	CL10537.Contig4$_{H}$1
Mean Expression Ratio	1.07
Median Expression Ratio	1.07
Credible Interval	(0.806, 1.43)
Associated Peptides	4
Associated Spectra	7
Coverage	NaN

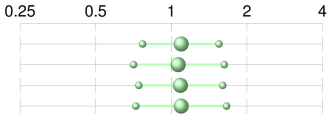

A	B	2.5	50	97.5	Sequence
**2**	**1**	**0.77**	**1.1**	**1.5**	**LDYQLEIQSAK**
**1**	**0**	**0.71**	**1.1**	**1.6**	**SITPQQEAESIK**
**1**	**1**	**0.74**	**1.1**	**1.6**	**VCIAQLK**
**1**	**0**	**0.72**	**1.1**	**1.7**	**ENVALQLEASYR**

5.332 95 271 minus strand hypothetical protein EAI_01530 [Harpegnathos saltator]

Protein Accession	CL9369.Contig9$_{H}1$
Mean Expression Ratio	0.932
Median Expression Ratio	0.934
Credible Interval	(0.648, 1.34)
Associated Peptides	1
Associated Spectra	2
Coverage	NaN

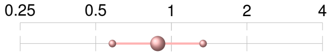

A	B	2.5	50	97.5	Sequence
**0**	**2**	**0.58**	**0.88**	**1.3**	**VAQFPFR**

5.333 2764 3420 PREDICTED: filamin-C-like [Nasonia vitripennis]

Protein Accession	CL4145.Contig1$_{H}1$
Mean Expression Ratio	1.07
Median Expression Ratio	1.07
Credible Interval	(0.763, 1.53)
Associated Peptides	1
Associated Spectra	3
Coverage	NaN

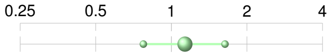

A	B	2.5	50	97.5	Sequence
**2**	**1**	**0.77**	**1.1**	**1.6**	**GVDVPVNTIDNK**

5.334 236 733 Spectrin alpha chain [Crassostrea gigas]

Protein Accession	Unigene61767$_{H}1$
Mean Expression Ratio	1.07
Median Expression Ratio	1.07
Credible Interval	(0.776, 1.48)
Associated Peptides	3
Associated Spectra	4
Coverage	NaN

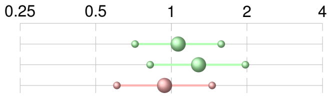

A	B	2.5	50	97.5	Sequence
**1**	**1**	**0.72**	**1.1**	**1.6**	**DIDDEEAWIK**
**1**	**0**	**0.83**	**1.3**	**2**	**DLTGVQNLR**
**0**	**1**	**0.61**	**0.94**	**1.5**	**LNEANTMHQFLR**

5.335 3 434 minus strand myosin essential light chain [Haliotis discus discus]

Protein Accession	CL17177.Contig8$_{H}1$
Mean Expression Ratio	1.07
Median Expression Ratio	1.07
Credible Interval	(0.916, 1.25)
Associated Peptides	11
Associated Spectra	69
Coverage	NaN

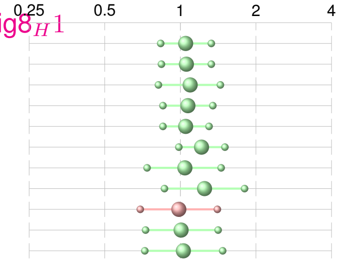

A	B	2.5	50	97.5	Sequence
**6**	**4**	**0.83**	**1.1**	**1.3**	**EGQGFMSAAELR**
**5**	**6**	**0.84**	**1.1**	**1.3**	**FTDTREDLEGNVK**
**4**	**1**	**0.82**	**1.1**	**1.4**	**HVLTGLGEK**
**5**	**5**	**0.85**	**1.1**	**1.3**	**LTDQQVTDVIK**
**6**	**7**	**0.85**	**1.1**	**1.3**	**YADFIEK**
**7**	**6**	**0.98**	**1.2**	**1.5**	**DGDVDAFK**
**1**	**1**	**0.74**	**1**	**1.5**	**VGDMCF**
**1**	**0**	**0.87**	**1.3**	**1.8**	**EDLEGNVK**
**1**	**0**	**0.69**	**0.99**	**1.4**	**LSDREGQGFMSAAELR**
**0**	**2**	**0.73**	**1**	**1.4**	**FTDTREDLEGN**
**0**	**1**	**0.72**	**1**	**1.5**	**HVLTGLG**

5.336 7825 8754 minus strand H2 [Haliotis tuberculata]

Protein Accession	CL7906.Contig3$_{H}1$
Mean Expression Ratio	0.934
Median Expression Ratio	0.935
Credible Interval	(0.629, 1.38)
Associated Peptides	1
Associated Spectra	1
Coverage	NaN

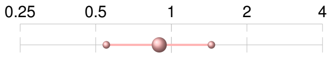

A	B	2.5	50	97.5	Sequence
1	0	0.55	0.89	1.4	ALPSLFTDPYDSNPFK

5.337 " 112 1320 Aspartate aminotransferase, cytoplasmic [Crassostrea gigas]"

Protein Accession	CL44136.Contig1$_{H}1$
Mean Expression Ratio	0.936
Median Expression Ratio	0.936
Credible Interval	(0.769, 1.14)
Associated Peptides	9
Associated Spectra	35
Coverage	NaN

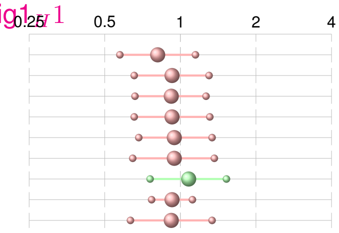

A	B	2.5	50	97.5	Sequence
1	1	0.57	0.81	1.1	ASIFADVK
1	1	0.65	0.93	1.3	IADVMEAK
2	1	0.66	0.92	1.3	SQMEIIVR
2	0	0.66	0.92	1.3	TPSVIPQIR
2	0	0.68	0.95	1.3	VNLGVGAYR
1	0	0.64	0.94	1.4	ISMAGVTNK
2	0	0.76	1.1	1.5	LAAEFLK
13	7	0.77	0.93	1.1	VEGIQVQGGTGGIR
0	1	0.63	0.92	1.3	TLANALILGK

5.338 205 2490 minus strand glucose-regulated protein 94 [Crassostrea gigas]

Protein Accession	CL5089.Contig1$_{H}1$
Mean Expression Ratio	0.935
Median Expression Ratio	0.936
Credible Interval	(0.687, 1.26)
Associated Peptides	5
Associated Spectra	5
Coverage	NaN

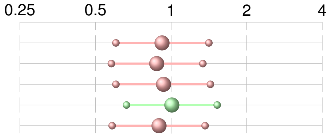

A	B	2.5	50	97.5	Sequence
1	0	0.6	0.92	1.4	ADKFAFQAEVNR
1	0	0.58	0.88	1.3	NLGTIAR
1	0	0.6	0.93	1.4	GTTVSLHLK
1	0	0.66	1	1.5	NDMFNNYGK
0	1	0.58	0.9	1.4	GVVDSDDLPLNVSR

5.339 89 838 Putative phosphoglycerate mutase [Crassostrea gigas]; 89 838 Putative phosphoglycerate mutase [Crassostrea gigas]; 89 835 Putative phosphoglycerate mutase [Crassostrea gigas]; 89 838 Putative phosphoglycerate mutase [Crassostrea gigas]

Protein Accession	CL6793.Contig6$_{H}1$ CL6793.Contig5$_{H}1$ CL6793.Contig4$_{H}1$ CL6793.Contig3$_{H}1$
Mean Expression Ratio	1.07
Median Expression Ratio	1.07
Credible Interval	(0.854, 1.34)
Associated Peptides	5
Associated Spectra	22
Coverage	NaN

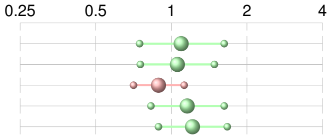

A	B	2.5	50	97.5	Sequence
1	0	0.75	1.1	1.6	DIPACECLK
2	1	0.75	1.1	1.5	HYYLATEEEVK
6	5	0.71	0.89	1.1	VLIAAHGNSLR
2	1	0.83	1.2	1.6	VLIAAHGN
3	1	0.89	1.2	1.7	EAQYAGELIK

5.340 106 1701 T-complex protein 1 subunit beta [Crassostrea gigas]

Protein Accession	CL8176.Contig1$_{H}1$
Mean Expression Ratio	0.937
Median Expression Ratio	0.937
Credible Interval	(0.65, 1.36)
Associated Peptides	2
Associated Spectra	2
Coverage	NaN

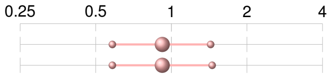

A	B	2.5	50	97.5	Sequence
1	0	0.58	0.92	1.4	GSGNLDAIQIIK
1	0	0.58	0.92	1.5	CTIVLR

5.341 472 1551 minus strand hypothetical protein [Amblyomma maculatum]

Protein Accession	Unigene11523$_{H}1$
Mean Expression Ratio	1.07
Median Expression Ratio	1.07
Credible Interval	(0.876, 1.3)
Associated Peptides	7
Associated Spectra	30
Coverage	NaN

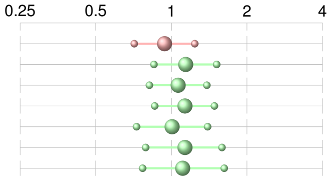

A	B	2.5	50	97.5	Sequence
6	0	0.71	0.94	1.2	LFQNAQLSR
5	0	0.85	1.1	1.5	MVTGPVPGNLAVSK
7	0	0.82	1.1	1.4	QSLTDILR
4	2	0.86	1.1	1.5	VANGLYYDDTAVTLAEAFK
1	2	0.73	1	1.4	VLELPYGTDGR
2	0	0.79	1.1	1.6	SKQSLTDILR
0	1	0.77	1.1	1.6	FMVSLPK

5.342 22 429 AGAP006937-PA [Anopheles gambiae str. PEST] gi; 1 474 Ubiquitin-conjugating enzyme E2 variant 2 OS=Rattus norvegicus GN=Ube2v2 PE=1 SV=3

Protein Accession	CL27581.Contig3$_{H}1$ CL27581.Contig2$_{H}1$
Mean Expression Ratio	0.936
Median Expression Ratio	0.939
Credible Interval	(0.635, 1.38)
Associated Peptides	1
Associated Spectra	1
Coverage	NaN

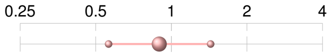

A	B	2.5	50	97.5	Sequence
0	1	0.56	0.89	1.4	LLDELELGEK

5.343 124 396 minus strand 40S ribosomal protein S10 OS=Lumbricus rubellus GN=RPS10 PE=2 SV=1

Protein Accession	Unigene42157$_{H}1$
Mean Expression Ratio	0.939
Median Expression Ratio	0.939
Credible Interval	(0.682, 1.29)
Associated Peptides	2
Associated Spectra	6
Coverage	NaN

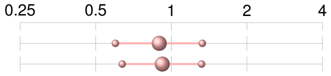

A	B	2.5	50	97.5	Sequence
2	1	0.6	0.89	1.3	KDFFSPK
3	0	0.64	0.92	1.3	YLRER

5.344 552 1886 minus strand intermediate filament protein [Biomphalaria glabrata]

Protein Accession	Unigene26795$_{H}$1
Mean Expression Ratio	1.06
Median Expression Ratio	1.07
Credible Interval	(0.951, 1.19)
Associated Peptides	22
Associated Spectra	158
Coverage	NaN

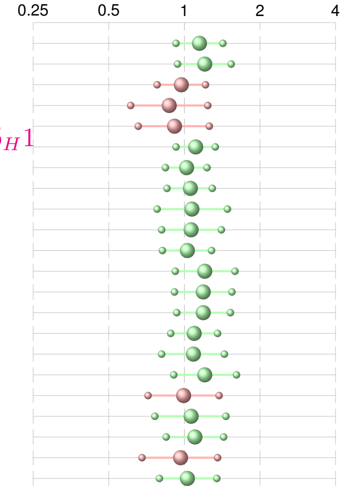

A	B	2.5	50	97.5	Sequence
10	2	0.93	1.1	1.4	ADLDAETAAHIENEVLAQTK
5	3	0.94	1.2	1.5	CGSLEDELAK
5	6	0.78	0.97	1.2	DMYEGELAQAR
1	0	0.61	0.87	1.2	DSYLVQLNELQLEFER
2	0	0.66	0.91	1.3	IAELEAALAAMK
12	7	0.93	1.1	1.3	KQEDWKPIR
14	4	0.84	1	1.2	KVITEFSTQR
11	3	0.85	1.1	1.3	LATYIEK
1	1	0.78	1.1	1.5	LEAELEALR
3	2	0.81	1.1	1.4	LLEGEENR
6	4	0.82	1	1.3	LRDQLAEK
4	1	0.92	1.2	1.6	SATGISEFR
4	2	0.91	1.2	1.5	VITEFSTQR
6	2	0.93	1.2	1.5	YAAQMDQLR
8	4	0.88	1.1	1.4	KLLEGEENR
4	0	0.81	1.1	1.4	MSATGISEFR
3	1	0.91	1.2	1.6	ELMDHK
1	1	0.72	0.99	1.4	EMQDCNER
1	1	0.76	1.1	1.5	IANLQDQIQALK
4	2	0.85	1.1	1.4	WLEAQNK
0	1	0.68	0.97	1.4	ALVDKLK
0	6	0.79	1	1.3	KLEAELEALR

5.345 605 1153 Peptidyl-prolyl cis-trans isomerase B [Crassostrea gigas]; 1046 1630 Peptidyl-prolyl cis-trans isomerase B [Crassostrea gigas]; 589 1173 Peptidyl-prolyl cis-trans isomerase B [Crassostrea gigas]; 883 1467 Peptidyl-prolyl cis-trans isomerase B [Crassostrea gigas]

Protein Accession	CL24747.Contig5$_{H}$1 CL24747.Contig4$_{H}$1 CL24747.Contig3$_{H}$1 CL24747.Contig1$_{H}$1
Mean Expression Ratio	0.938
Median Expression Ratio	0.939
Credible Interval	(0.678, 1.3)
Associated Peptides	3
Associated Spectra	7
Coverage	NaN

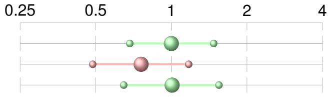

A	B	2.5	50	97.5	Sequence
2	3	0.69	1	1.5	IASSGVVGIASK
1	0	0.49	0.76	1.2	KLGELETSIIGRPK
1	0	0.65	1	1.5	SGMSIYGETFKDENFK

5.346 280 726 minus strand RecName: Full=Calmodulin; Short=CaM gi|14588595|dbj|BAB61794.1| calmodulin [Metridium senile] gi

Protein Accession	Unigene4486$_{H}$1
Mean Expression Ratio	0.94
Median Expression Ratio	0.939
Credible Interval	(0.711, 1.24)
Associated Peptides	5
Associated Spectra	13
Coverage	NaN

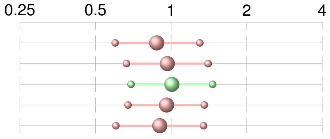

A	B	2.5	50	97.5	Sequence
1	1	0.6	0.88	1.3	ADQLTEEQIAEFK
2	0	0.67	0.96	1.4	EAFSLFDK
2	0	0.69	1	1.5	EAFSLFDKDGDGTITTK
2	4	0.67	0.96	1.4	ELGTVMR
1	0	0.6	0.9	1.3	HVMTNLGEKLTDEEVDEMIR

5.347 158 862 X-box binding protein [Haliotis discus discus]

Protein Accession	CL6396.Contig3$_{H}1$
Mean Expression Ratio	0.939
Median Expression Ratio	0.939
Credible Interval	(0.744, 1.19)
Associated Peptides	6
Associated Spectra	18
Coverage	NaN

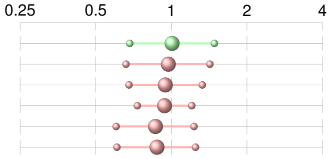

A	B	2.5	50	97.5	Sequence
1	0	0.68	1	1.5	GSISDPSVFNVPEACQK
1	0	0.66	0.97	1.4	VFPDFYFGAGDDK
3	0	0.68	0.95	1.3	ACIPDK
5	4	0.73	0.94	1.2	LYIVDLAK
1	1	0.6	0.87	1.2	SYFILK
2	0	0.61	0.88	1.2	SYYIGAR

5.348 212 1336 6-phosphofructokinase [Crassostrea gigas]

Protein Accession	CL48438.Contig4$_{H}1$
Mean Expression Ratio	0.941
Median Expression Ratio	0.939
Credible Interval	(0.668, 1.33)
Associated Peptides	2
Associated Spectra	3
Coverage	NaN

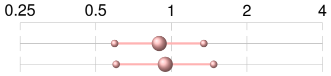

A	B	2.5	50	97.5	Sequence
2	0	0.59	0.89	1.3	LGLTQGYR
1	0	0.6	0.94	1.5	SVQWFK

5.349 177 791 Ras-related protein Rab-1A [Haliotis discus discus]

Protein Accession	CL21389.Contig6$_{H}1$
Mean Expression Ratio	1.06
Median Expression Ratio	1.06
Credible Interval	(0.811, 1.4)
Associated Peptides	5
Associated Spectra	8
Coverage	NaN

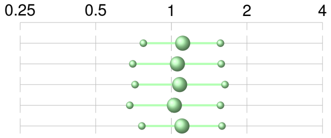

A	B	2.5	50	97.5	Sequence
3	0	0.77	1.1	1.6	MGPVTSASENKPSVK
1	0	0.7	1.1	1.6	QWLQEIDR
1	0	0.72	1.1	1.6	KVVDYTTAK
1	0	0.68	1	1.6	SCLLLR
0	2	0.76	1.1	1.6	LLLIGDSGVGK

5.350 11 244 minus strand Tubulin alpha chain (Fragment) OS=Lytechinus pictus PE=3 SV=1

Protein Accession	CL3047.Contig6$_{H}1$
Mean Expression Ratio	0.937
Median Expression Ratio	0.939
Credible Interval	(0.648, 1.35)
Associated Peptides	2
Associated Spectra	2
Coverage	NaN

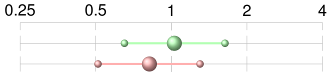

A	B	2.5	50	97.5	Sequence
0	1	0.65	1	1.6	EDLAALEK
0	1	0.51	0.82	1.3	FDLMYAK

5.351 274 666 Calmodulin-4 [Heterocephalus glaber]

Protein Accession	Unigene19050$_{H}1$
Mean Expression Ratio	1.06
Median Expression Ratio	1.06
Credible Interval	(0.834, 1.35)
Associated Peptides	4
Associated Spectra	17
Coverage	NaN

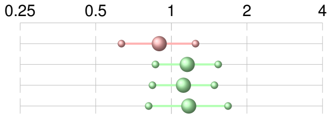

A	B	2.5	50	97.5	Sequence
2	1	0.64	0.89	1.3	GLDPENAVLDMDINK
3	3	0.87	1.2	1.5	SGFITLDELK
5	1	0.84	1.1	1.5	VLLSVGR
2	0	0.81	1.2	1.7	GTLSILELHK

5.352 290 2458 Putative beta-D-xylosidase 5 [Crassostrea gigas]

Protein Accession	CL49577.Contig2$_{H}1$
Mean Expression Ratio	1.06
Median Expression Ratio	1.06
Credible Interval	(0.761, 1.5)
Associated Peptides	3
Associated Spectra	4
Coverage	NaN

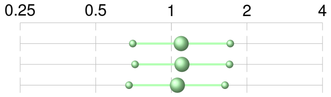

A	B	2.5	50	97.5	Sequence
0	1	0.7	1.1	1.7	QLLTDILR
1	1	0.72	1.1	1.7	VAEAAGVEVR
0	1	0.68	1.1	1.6	LEALEQGK

5.353 77 502 hypothetical protein CGI_10008263 [Crassostrea gigas]

Protein Accession	CL38960.Contig1$_{H}1$
Mean Expression Ratio	1.06
Median Expression Ratio	1.06
Credible Interval	(0.752, 1.49)
Associated Peptides	2
Associated Spectra	3
Coverage	NaN

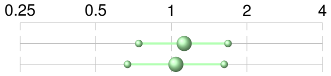

A	B	2.5	50	97.5	Sequence
2	0	0.74	1.1	1.7	IFVAIDGSK
1	0	0.67	1	1.6	KIFVAIDGSK

5.354 " 199 417 GG13156 [Drosophila erecta] gi; 253 501 minus strand NADH-ubiquinone oxidoreductase MLRQ subunit-like protein, partial [Lymnaea stagnalis]; 294 515 GG13156 [Drosophila erecta] gi"

Protein Accession	CL49605.Contig4$_{H}1$ CL49605.Contig2$_{H}1$ CL49605.Contig1$_{H}1$
Mean Expression Ratio	1.06
Median Expression Ratio	1.06
Credible Interval	(0.723, 1.58)
Associated Peptides	1
Associated Spectra	1
Coverage	NaN

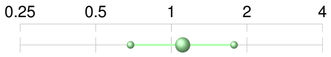

A	B	2.5	50	97.5	Sequence
1	0	0.69	1.1	1.8	GLTLSSLK

5.355 " 134 1567 minus strand 6-phosphogluconate dehydrogenase, decarboxylating [Crassostrea gigas]; 134 1573 minus strand 6-phosphogluconate dehydrogenase, decarboxylating [Crassostrea gigas]"

Protein Accession	CL5140.Contig5$_H$1 CL5140.Contig4$_H$1
Mean Expression Ratio	0.94
Median Expression Ratio	0.941
Credible Interval	(0.733, 1.21)
Associated Peptides	7
Associated Spectra	12
Coverage	NaN

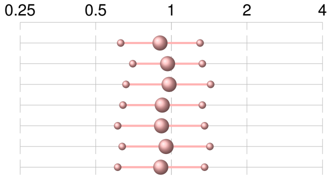

A	B	2.5	50	97.5	Sequence
1	1	0.63	0.9	1.3	AGQAVDDFIDK
2	2	0.7	0.96	1.3	SLEDMVDKLK
1	0	0.66	0.98	1.4	SVFLGNIK
1	1	0.64	0.92	1.3	LVPLLEK
1	0	0.61	0.91	1.4	YGPSLMPGGAEEAWPAIK
0	1	0.64	0.95	1.4	NIFQSISAK
0	1	0.61	0.91	1.4	AFVEDIKR

5.356 14 1642 minus strand chaperonin containing tcp1 [Haliotis discus discus]

Protein Accession	CL16298.Contig4$_H$1
Mean Expression Ratio	1.06
Median Expression Ratio	1.06
Credible Interval	(0.761, 1.48)
Associated Peptides	3
Associated Spectra	3
Coverage	NaN

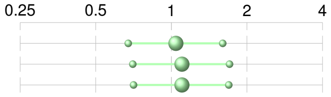

A	B	2.5	50	97.5	Sequence
1	0	0.67	1	1.6	ELEVQHPAAK
1	0	0.7	1.1	1.7	MVINHLEK
1	0	0.71	1.1	1.7	AIGATALPK

5.357 11 394 regucalcin [Salmo salar] gi

Protein Accession	CL42615.Contig1$_H$1
Mean Expression Ratio	0.941
Median Expression Ratio	0.941
Credible Interval	(0.638, 1.4)
Associated Peptides	1
Associated Spectra	1
Coverage	NaN

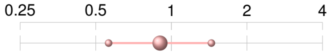

A	B	2.5	50	97.5	Sequence
1	0	0.56	0.9	1.4	NQPLAGSLFK

5.358 161 3001 minus strand E3 ubiquitin-protein ligase MIB2 [Crassostrea gigas]

Protein Accession	CL49497.Contig7$_H$1
Mean Expression Ratio	1.06
Median Expression Ratio	1.06
Credible Interval	(0.718, 1.56)
Associated Peptides	1
Associated Spectra	1
Coverage	NaN

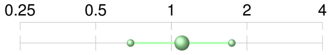

A	B	2.5	50	97.5	Sequence
0	1	0.69	1.1	1.7	GDAHAVEGILQR

5.359 392 808 Whirlin [Crassostrea gigas]

Protein Accession	CL22731.Contig1$_{H}1$
Mean Expression Ratio	0.941
Median Expression Ratio	0.942
Credible Interval	(0.669, 1.33)
Associated Peptides	1
Associated Spectra	4
Coverage	NaN

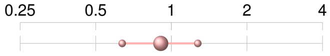

A	B	2.5	50	97.5	Sequence
**2**	**2**	**0.64**	**0.91**	**1.3**	**AGNDLHLLVQR**

5.360 165 377 Cysteine and glycine-rich protein 3 [Crassostrea gigas]; 135 344 Cysteine and glycine-rich protein 3 [Crassostrea gigas]

Protein Accession	CL38347.Contig2$_{H}1$ CL38347.Contig1$_{H}1$
Mean Expression Ratio	1.06
Median Expression Ratio	1.06
Credible Interval	(0.735, 1.54)
Associated Peptides	1
Associated Spectra	2
Coverage	NaN

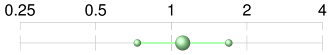

A	B	2.5	50	97.5	Sequence
**1**	**1**	**0.73**	**1.1**	**1.7**	**VEAGNIPFHK**

5.361 144 800 minus strand PREDICTED: protein F37C4.5-like [Amphimedon queenslandica]

Protein Accession	CL7246.Contig3$_{H}1$
Mean Expression Ratio	1.06
Median Expression Ratio	1.06
Credible Interval	(0.803, 1.42)
Associated Peptides	4
Associated Spectra	7
Coverage	NaN

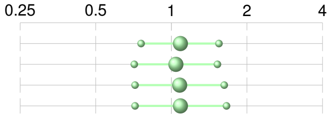

A	B	2.5	50	97.5	Sequence
**2**	**1**	**0.76**	**1.1**	**1.5**	**VTVGLVDIKPK**
**1**	**1**	**0.71**	**1**	**1.5**	**VSLLEPQLDLK**
**1**	**0**	**0.72**	**1.1**	**1.6**	**NKDKDVTLNK**
**1**	**0**	**0.72**	**1.1**	**1.7**	**VISNTHEQVIEVK**

5.362 113 1402 minus strand S-adenosylhomocysteine hydrolase [Crassostrea ariakensis]; 113 1402 minus strand S-adenosylhomocysteine hydrolase [Crassostrea ariakensis]

Protein Accession	CL7405.Contig2$_{H}1$ CL7405.Contig1$_{H}1$
Mean Expression Ratio	0.946
Median Expression Ratio	0.942
Credible Interval	(0.664, 1.36)
Associated Peptides	2
Associated Spectra	2
Coverage	NaN

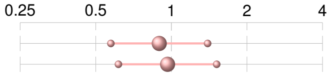

A	B	2.5	50	97.5	Sequence
**1**	**0**	**0.57**	**0.9**	**1.4**	**GLSEETTTGVHNLYR**
**1**	**0**	**0.62**	**0.96**	**1.5**	**LKVPAINVNDSVTK**

5.363 288 1487 minus strand putative procollagen type VI alpha 4 [Haliotis diversicolor]

Protein Accession CL6899.Contig2$_H$1
Mean Expression Ratio 1.06
Median Expression Ratio 1.06
Credible Interval (0.948, 1.19)
Associated Peptides 20
Associated Spectra 186
Coverage NaN

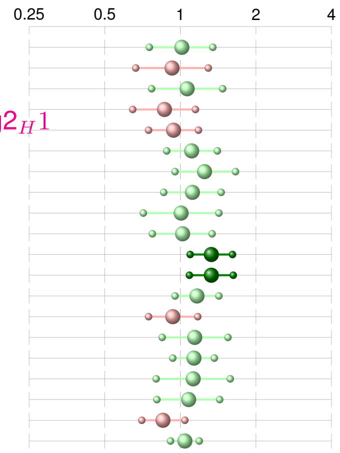

A	B	2.5	50	97.5	Sequence
4	0	0.75	1	1.3	AEVLAAIDQVR
2	0	0.67	0.93	1.3	ELTNQVAVLFLDDQALR
1	1	0.77	1.1	1.5	ENLYYIQSVVDR
1	3	0.64	0.86	1.1	MGFDVDQFR
5	5	0.75	0.94	1.2	TKAEVLAAIDQVR
8	2	0.88	1.1	1.4	VMVVITDGSSR
4	1	0.95	1.3	1.7	FDISVDDTR
3	3	0.86	1.1	1.4	FVVVDLGVR
1	0	0.71	1	1.4	LGNVVPQDENTYNNLLR
4	1	0.77	1	1.3	PDPTIGSCR
8	8	1.1	1.3	1.6	ALETLR
8	7	1.1	1.3	1.6	DLLPMR
9	5	0.95	1.2	1.4	FRDDITK
6	4	0.75	0.94	1.2	QVLQFVSQVITR
2	1	0.85	1.1	1.5	LRENLK
9	7	0.93	1.1	1.4	IGALTFSDDVK
1	0	0.8	1.1	1.6	FRALETLR
2	2	0.81	1.1	1.4	ADIINFASQSLAR
10	5	0.7	0.85	1	GANGVTLAQQVR
34	9	0.92	1	1.2	TGVTNTHLAIR

5.364 " 3 587 elongation factor 1 alpha, partial [Phorbas sp. nov. SB-2012]"

Protein Accession Unigene20048$_H$1
Mean Expression Ratio 0.944
Median Expression Ratio 0.943
Credible Interval (0.708, 1.26)
Associated Peptides 2
Associated Spectra 12
Coverage NaN

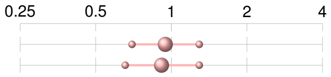

A	B	2.5	50	97.5	Sequence
5	1	0.7	0.95	1.3	IGGIGTVPVGR
3	3	0.65	0.92	1.3	LPLQDVYK

5.365 66 263 minus strand twitchin [Mytilus galloprovincialis]; 66 263 minus strand twitchin [Mytilus galloprovincialis]; 66 263 minus strand twitchin [Mytilus galloprovincialis]; 66 263 minus strand twitchin [Mytilus galloprovincialis]; 66 263 minus strand twitchin [Mytilus galloprovincialis]

Protein Accession Unigene64526$_H$1 Unigene64525$_H$1 Unigene64524$_H$1 Unigene64522$_H$1 Unigene64521$_H$1
Mean Expression Ratio 1.06
Median Expression Ratio 1.06
Credible Interval (0.745, 1.51)
Associated Peptides 1
Associated Spectra 3
Coverage NaN

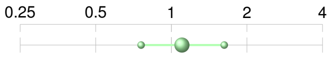

A	B	2.5	50	97.5	Sequence
2	1	0.76	1.1	1.6	FTETLSPVEVMQGDK

5.366 114 2633 Filamin-A [Crassostrea gigas]; 135 2654 Filamin-A [Crassostrea gigas]

Protein Accession	CL393.Contig21$_H1$ CL393.Contig16$_H1$
Mean Expression Ratio	1.06
Median Expression Ratio	1.06
Credible Interval	(0.953, 1.18)
Associated Peptides	35
Associated Spectra	168
Coverage	NaN

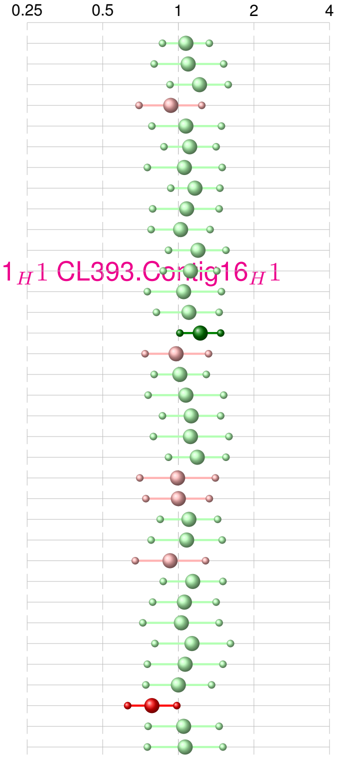

A	B	2.5	50	97.5	Sequence
9	3	0.86	1.1	1.3	AVANKPNEIVIDTSK
2	0	0.8	1.1	1.5	DAGEALLSIGMIAPSGHPEAELGYK
5	1	0.93	1.2	1.6	DNNDGTIVVEYQPK
4	0	0.7	0.93	1.2	FADEHVPGSPFLLNVGGEASGR
2	0	0.78	1.1	1.5	FDDVHIPGSPFIVNIAPDSGTAR
5	4	0.88	1.1	1.4	IGDSELGHAAGCK
1	0	0.75	1.1	1.5	KMGDGEIGIASFNPR
8	3	0.93	1.2	1.5	KPSPIQEQSSMVVETVEK
3	0	0.79	1.1	1.5	KVTANSYTVSYK
3	3	0.78	1	1.3	MGDGEIGIASFNPR
4	2	0.92	1.2	1.5	QMEAAEAAVPNK
8	0	0.87	1.1	1.4	SQLSLGNSAEYSLK
1	0	0.75	1.1	1.5	TNVSDETGCYSK
3	1	0.82	1.1	1.5	TPSGGHEDCFIQEMDK
12	9	1	1.2	1.5	VINFQIETK
3	1	0.74	0.98	1.3	VPKPGSYGLHIK
5	4	0.8	1	1.3	VQVGGPGLEK
1	0	0.76	1.1	1.5	VRDNNDGTIVVEYQPK
4	2	0.86	1.1	1.5	VTGALDK
1	0	0.8	1.1	1.6	WGDEDIPGSPFVIST
4	2	0.91	1.2	1.5	YGNIPIAGSPYK
1	0	0.7	0.99	1.4	YIPKENGVHYVDVK
3	1	0.74	1	1.3	YKGNPIK
5	1	0.85	1.1	1.4	EAGPGTLTVAVEGPGK
1	1	0.78	1.1	1.5	AALSCK
1	1	0.67	0.93	1.3	PGSYGLHIK
4	1	0.87	1.1	1.5	HINNSPFK
3	1	0.79	1.1	1.4	IMFTPK
1	0	0.72	1	1.4	FRGDASK
1	0	0.81	1.1	1.6	AQIFLEERPNGFLGVQYK
1	0	0.75	1.1	1.5	CPGDYLITIK
2	1	0.74	1	1.4	ENGVHYVDVK
6	6	0.63	0.79	0.99	MNGIHTLSVR
2	0	0.76	1.1	1.4	SDIECYK
1	0	0.75	1.1	1.5	VTANSYTVSYK

5.367 19 1218 Dynactin subunit 2 [Crassostrea gigas]; 48 830 minus strand Dynactin subunit 2 [Crassostrea gigas]

Protein Accession	CL5827.Contig5$_H1$ CL5827.Contig1$_H1$
Mean Expression Ratio	0.943
Median Expression Ratio	0.943
Credible Interval	(0.641, 1.39)
Associated Peptides	1
Associated Spectra	1
Coverage	NaN

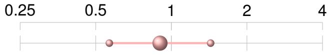

A	B	2.5	50	97.5	Sequence
1	0	0.57	0.9	1.4	VNELYEVMK

5.368 54 791 GMP reductase 2 [Crassostrea gigas]

Protein Accession CL26888.Contig1$_H$1
Mean Expression Ratio 1.06
Median Expression Ratio 1.06
Credible Interval (0.718, 1.58)
Associated Peptides 1
Associated Spectra 1
Coverage NaN

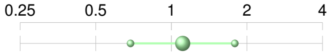

A	B	2.5	50	97.5	Sequence
**1**	**0**	**0.69**	**1.1**	**1.8**	**LFTTIHK**

5.369 4 675 Laminin subunit gamma-1 [Crassostrea gigas]

Protein Accession CL35712.Contig1$_H$1
Mean Expression Ratio 1.06
Median Expression Ratio 1.06
Credible Interval (0.79, 1.41)
Associated Peptides 6
Associated Spectra 6
Coverage NaN

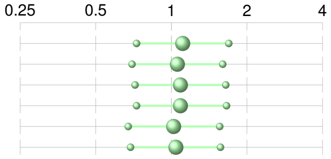

A	B	2.5	50	97.5	Sequence
**1**	**0**	**0.73**	**1.1**	**1.7**	**AAQLTASETASK**
**1**	**0**	**0.7**	**1.1**	**1.6**	**LAQLAEDTAIQASAEAR**
**1**	**0**	**0.71**	**1.1**	**1.6**	**TLQTLLEFDR**
**1**	**0**	**0.73**	**1.1**	**1.7**	**VPEIEK**
**1**	**0**	**0.67**	**1**	**1.6**	**TLSQEVNDAEGR**
**0**	**1**	**0.69**	**1**	**1.6**	**ANIEAEIK**

5.370 43 525 Reticulon-4 [Crassostrea gigas]; 143 757 Reticulon-4 [Crassostrea gigas]; 43 564 Reticulon-4 [Crassostrea gigas]

Protein Accession CL29775.Contig4$_H$1 CL29775.Contig3$_H$1 CL29775.Contig2$_H$1
Mean Expression Ratio 0.945
Median Expression Ratio 0.944
Credible Interval (0.655, 1.37)
Associated Peptides 1
Associated Spectra 3
Coverage NaN

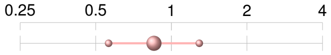

A	B	2.5	50	97.5	Sequence
**2**	**1**	**0.56**	**0.85**	**1.3**	**VTQTILQHVNR**

5.371 " 926 1189 minus strand Myosin heavy chain, striated muscle [Crassostrea gigas]"

Protein Accession Unigene11948$_H$1
Mean Expression Ratio 1.06
Median Expression Ratio 1.06
Credible Interval (0.837, 1.34)
Associated Peptides 3
Associated Spectra 28
Coverage NaN

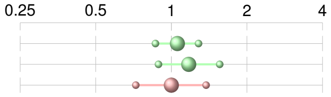

A	B	2.5	50	97.5	Sequence
**9**	**9**	**0.87**	**1.1**	**1.3**	**LNGNHLGK**
**6**	**0**	**0.89**	**1.2**	**1.6**	**NKDPLNETVVELLSHSK**
**1**	**3**	**0.72**	**1**	**1.4**	**TFLDKLNGNHLGK**

5.372 68 667 minus strand hypothetical protein BRAFLDRAFT_272416 [Branchiostoma floridae] gi; 68 667 minus strand hypothetical protein BRAFLDRAFT_272416 [Branchiostoma floridae] gi

Protein Accession	CL37759.Contig4$_H1$ CL37759.Contig1$_H1$
Mean Expression Ratio	0.945
Median Expression Ratio	0.944
Credible Interval	(0.659, 1.36)
Associated Peptides	2
Associated Spectra	2
Coverage	NaN

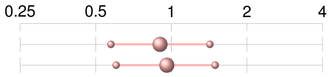

A	B	2.5	50	97.5	Sequence
**0**	**1**	**0.57**	**0.9**	**1.4**	**TLEEDEDVLLQLR**
**0**	**1**	**0.6**	**0.96**	**1.5**	**VLLQLR**

5.373 160 588 hypothetical protein CGI_10001640 [Crassostrea gigas]

Protein Accession	CL2347.Contig8$_H1$
Mean Expression Ratio	1.06
Median Expression Ratio	1.06
Credible Interval	(0.799, 1.4)
Associated Peptides	2
Associated Spectra	13
Coverage	NaN

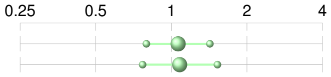

A	B	2.5	50	97.5	Sequence
**6**	**3**	**0.8**	**1.1**	**1.4**	**AFLEQLGSK**
**3**	**1**	**0.77**	**1.1**	**1.5**	**ANLIVIGSR**

5.374 16 288 SJCHGC01950 protein [Schistosoma japonicum] gi

Protein Accession	Unigene16338$_H1$
Mean Expression Ratio	0.945
Median Expression Ratio	0.944
Credible Interval	(0.684, 1.31)
Associated Peptides	1
Associated Spectra	7
Coverage	NaN

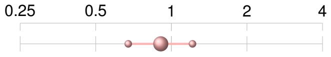

A	B	2.5	50	97.5	Sequence
**6**	**1**	**0.68**	**0.91**	**1.2**	**DIAAFIK**

5.375 132 590 minus strand Non-neuronal cytoplasmic intermediate filament protein OS=Helix aspersa PE=3 SV=2

Protein Accession	Unigene7770$_H1$
Mean Expression Ratio	1.06
Median Expression Ratio	1.06
Credible Interval	(0.741, 1.5)
Associated Peptides	1
Associated Spectra	3
Coverage	NaN

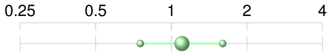

A	B	2.5	50	97.5	Sequence
**3**	**0**	**0.75**	**1.1**	**1.6**	**TMAEVPLGEK**

5.376 108 1259 minus strand PREDICTED: Na(+)/H(+) exchange regulatory cofactor NHE-RF3-like [Metaseiulus occidentalis]; 108 1190 minus strand PREDICTED: Na(+)/H(+) exchange regulatory cofactor NHE-RF3-like [Metaseiulus occidentalis]

Protein Accession	CL26023.Contig7$_{H}1$ CL26023.Contig6$_{H}1$
Mean Expression Ratio	0.945
Median Expression Ratio	0.945
Credible Interval	(0.64, 1.39)
Associated Peptides	1
Associated Spectra	1
Coverage	NaN

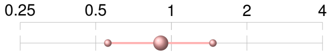

A	B	2.5	50	97.5	Sequence
**0**	**1**	**0.56**	**0.91**	**1.5**	**GDLPEVLHLTAVR**

5.377 145 972 LIM and SH3 domain protein F42H10.3 OS=Caenorhabditis elegans GN=F42H10.3 PE=2 SV=3

Protein Accession	CL49642.Contig8$_{H}1$
Mean Expression Ratio	1.06
Median Expression Ratio	1.06
Credible Interval	(0.729, 1.53)
Associated Peptides	1
Associated Spectra	2
Coverage	NaN

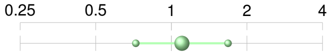

A	B	2.5	50	97.5	Sequence
**1**	**1**	**0.72**	**1.1**	**1.7**	**TVYPTEELK**

5.378 1592 1768 minus strand beta-actin [Diabolocatantops pinguis]

Protein Accession	CL22741.Contig2$_{H}1$
Mean Expression Ratio	1.06
Median Expression Ratio	1.06
Credible Interval	(0.808, 1.39)
Associated Peptides	2
Associated Spectra	17
Coverage	NaN

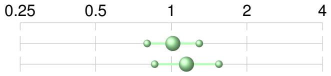

A	B	2.5	50	97.5	Sequence
**1**	**10**	**0.8**	**1**	**1.3**	**EISALAPPTMK**
**3**	**3**	**0.86**	**1.2**	**1.5**	**VIAPPER**

5.379 121 1452 minus strand Hexokinase type 2 [Crassostrea gigas]; 245 1576 minus strand Hexokinase type 2 [Crassostrea gigas]; 121 1452 minus strand Hexokinase type 2 [Crassostrea gigas]; 121 1452 minus strand Hexokinase type 2 [Crassostrea gigas]; 161 1513 minus strand Hexokinase type 2 [Crassostrea gigas]; 113 1471 minus strand Hexokinase type 2 [Crassostrea gigas]; 225 1577 minus strand Hexokinase type 2 [Crassostrea gigas]; 121 1452 minus strand Hexokinase type 2 [Crassostrea gigas]

Protein Accession	CL350.Contig9$_{H}1$ CL350.Contig8$_{H}1$ CL350.Contig6$_{H}1$ CL350.Contig5$_{H}1$ CL350.Contig3$_{H}1$
Mean Expression Ratio	0.944
Median Expression Ratio	0.946
Credible Interval	(0.64, 1.38)
Associated Peptides	1
Associated Spectra	1
Coverage	NaN

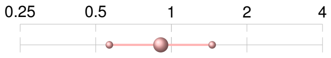

A	B	2.5	50	97.5	Sequence
**0**	**1**	**0.57**	**0.91**	**1.5**	**VLAPGIEFK**

5.380 56 937 Chloride intracellular channel exc-4 [Crassostrea gigas]

Protein Accession	CL34620.Contig3$_{H}1$
Mean Expression Ratio	1.06
Median Expression Ratio	1.06
Credible Interval	(0.749, 1.49)
Associated Peptides	2
Associated Spectra	3
Coverage	NaN

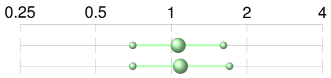

A	B	2.5	50	97.5	Sequence
**2**	**0**	**0.7**	**1.1**	**1.6**	**KVQGPLVK**
**1**	**0**	**0.7**	**1.1**	**1.7**	**KLLAELK**

5.381 16 438 hypothetical protein KGM_06034 [Danaus plexippus]; 16 438 hypothetical protein KGM_06034 [Danaus plexippus]

Protein Accession	CL18196.Contig2$_{H}1$ CL18196.Contig1$_{H}1$
Mean Expression Ratio	0.947
Median Expression Ratio	0.946
Credible Interval	(0.658, 1.36)
Associated Peptides	2
Associated Spectra	2
Coverage	NaN

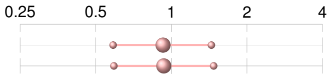

A	B	2.5	50	97.5	Sequence
**1**	**0**	**0.59**	**0.93**	**1.4**	**VHCGPNYPDEPPTVQFK**
**1**	**0**	**0.59**	**0.93**	**1.5**	**LLEELEDGQK**

5.382 185 742 Transmembrane protein Tmp21 [Crassostrea gigas]; 1 444 minus strand Transmembrane protein Tmp21 [Crassostrea gigas]

Protein Accession	CL3732.Contig3$_H1$ CL3732.Contig1$_H1$
Mean Expression Ratio	0.947
Median Expression Ratio	0.946
Credible Interval	(0.665, 1.37)
Associated Peptides	2
Associated Spectra	2
Coverage	NaN

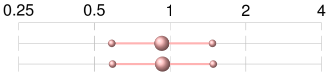

A	B	2.5	50	97.5	Sequence
**1**	**0**	**0.59**	**0.93**	**1.5**	**LKPLEVELR**
**1**	**0**	**0.59**	**0.93**	**1.5**	**EVHLNLK**

5.383 813 1244 Probable protein disulfide-isomerase A6 OS=Caenorhabditis elegans GN=tag-320 PE=2 SV=1

Protein Accession	CL39540.Contig4$_H1$
Mean Expression Ratio	0.945
Median Expression Ratio	0.946
Credible Interval	(0.641, 1.38)
Associated Peptides	1
Associated Spectra	2
Coverage	NaN

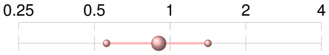

A	B	2.5	50	97.5	Sequence
**1**	**1**	**0.56**	**0.9**	**1.4**	**TASDIINWATEK**

5.384 280 1239 minus strand PREDICTED: smoothelin isoform 2 [Oryctolagus cuniculus]; 280 1239 minus strand PREDICTED: smoothelin isoform 2 [Oryctolagus cuniculus]; 280 1239 PREDICTED: smoothelin isoform 2 [Oryctolagus cuniculus]; 280 1239 minus strand PREDICTED: smoothelin isoform 2 [Oryctolagus cuniculus]; 280 1239 PREDICTED: smoothelin isoform 2 [Oryctolagus cuniculus]; 280 1239 minus strand PREDICTED: smoothelin isoform 2 [Oryctolagus cuniculus]; 280 1239 minus strand PREDICTED: smoothelin isoform 2 [Oryctolagus cuniculus]

Protein Accession	CL2374.Contig7$_H1$ CL2374.Contig6$_H1$ CL2374.Contig5$_H1$ CL2374.Contig4$_H1$ CL2374.Con
Mean Expression Ratio	1.06
Median Expression Ratio	1.06
Credible Interval	(0.774, 1.44)
Associated Peptides	3
Associated Spectra	5
Coverage	NaN

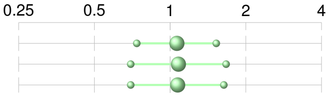

A	B	2.5	50	97.5	Sequence
**2**	**1**	**0.74**	**1.1**	**1.5**	**HNFQLAFDTAEK**
**1**	**0**	**0.7**	**1.1**	**1.7**	**LMNATDSFDER**
**0**	**1**	**0.7**	**1.1**	**1.6**	**PLLDVEDMVK**

5.385 585 944 minus strand Protein disulfide-isomerase A6 [Crassostrea gigas]

Protein Accession	CL39540.Contig7$_{H}1$
Mean Expression Ratio	0.945
Median Expression Ratio	0.947
Credible Interval	(0.658, 1.36)
Associated Peptides	2
Associated Spectra	3
Coverage	NaN

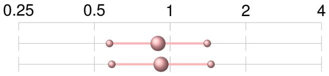

A	B	2.5	50	97.5	Sequence
**1**	**1**	**0.58**	**0.9**	**1.4**	**TASDIINWATEK**
**1**	**0**	**0.59**	**0.92**	**1.5**	**GFPTIK**

5.386 31 735 hypothetical protein BRAFLDRAFT_78603 [Branchiostoma floridae] gi; 55 735 hypothetical protein BRAFLDRAFT_78603 [Branchiostoma floridae] gi; 319 948 hypothetical protein BRAFLDRAFT_78603 [Branchiostoma floridae] gi; 353 1039 hypothetical protein BRAFLDRAFT_78603 [Branchiostoma floridae] gi; 207 893 hypothetical protein BRAFLDRAFT_78603 [Branchiostoma floridae] gi

Protein Accession	CL877.Contig7$_{H}1$ CL877.Contig6$_{H}1$ CL877.Contig5$_{H}1$ CL877.Contig4$_{H}1$ CL877.Contig3$_{H}1$
Mean Expression Ratio	0.946
Median Expression Ratio	0.948
Credible Interval	(0.658, 1.35)
Associated Peptides	2
Associated Spectra	5
Coverage	NaN

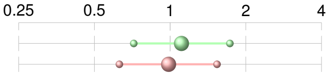

A	B	2.5	50	97.5	Sequence
**2**	**1**	**0.72**	**1.1**	**1.7**	**DDQTLVTMTGEVAGR**
**1**	**1**	**0.63**	**0.99**	**1.5**	**LKLPLDSSNR**

5.387 91 1392 minus strand hypothetical protein AND_03103 [Anopheles darlingi]

Protein Accession	CL10598.Contig5$_{H}1$
Mean Expression Ratio	0.947
Median Expression Ratio	0.948
Credible Interval	(0.663, 1.37)
Associated Peptides	2
Associated Spectra	2
Coverage	NaN

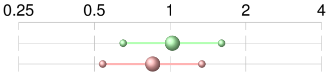

A	B	2.5	50	97.5	Sequence
**1**	**0**	**0.65**	**1**	**1.6**	**LPPFNEHALVQEK**
**1**	**0**	**0.54**	**0.85**	**1.3**	**LDEVDDLLTENR**

5.388 37 885 glyceraldehyde 3-phosphate dehydrogenase [Dugesia japonica]

Protein Accession	CL47424.Contig2$_H$1
Mean Expression Ratio	1.05
Median Expression Ratio	1.06
Credible Interval	(0.774, 1.44)
Associated Peptides	1
Associated Spectra	12
Coverage	NaN

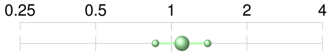

A	B	2.5	50	97.5	Sequence
10	2	0.86	1.1	1.4	GAAQNIIPASTGAAK

5.389 1971 3368 minus strand Kyphoscoliosis peptidase [Crassostrea gigas]; 1767 3158 minus strand hillarin [Hirudo medicinalis]; 1977 3368 minus strand hillarin [Hirudo medicinalis]

Protein Accession	CL3923.Contig9$_H$1 CL3923.Contig8$_H$1 CL3923.Contig10$_H$1
Mean Expression Ratio	1.06
Median Expression Ratio	1.05
Credible Interval	(0.887, 1.25)
Associated Peptides	13
Associated Spectra	40
Coverage	NaN

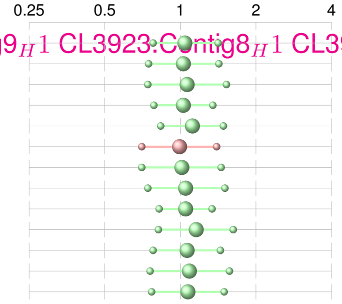

A	B	2.5	50	97.5	Sequence
2	2	0.78	1	1.4	EKGEAPPPVPATGPQQKPTLEAIDR
1	2	0.75	1	1.4	EMNFDKVEK
1	0	0.74	1.1	1.5	GEAPPPVPATGPQQKPTLEAIDR
4	2	0.78	1	1.3	GSPEEVLMGLK
4	1	0.84	1.1	1.5	NITNEIEK
1	1	0.7	0.99	1.4	TAVIYSR
1	0	0.7	1	1.4	TVGNTAYFNVTAPGR
1	0	0.74	1.1	1.5	IISGYAK
5	4	0.82	1	1.3	WQLLDR
2	0	0.82	1.2	1.6	MKPQFFK
2	1	0.78	1.1	1.4	NVLQSVDDHAIQVSK
0	1	0.76	1.1	1.6	WLATK
0	2	0.77	1.1	1.5	QLLDR

5.390 613 846 cytochrome c oxidase subunit Va [Antheraea yamamai] gi

Protein Accession	Unigene3832$_H$1
Mean Expression Ratio	1.05
Median Expression Ratio	1.05
Credible Interval	(0.712, 1.56)
Associated Peptides	1
Associated Spectra	1
Coverage	NaN

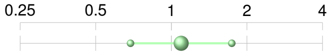

A	B	2.5	50	97.5	Sequence
1	0	0.69	1.1	1.7	AMNDLQGEDLVPEPK

5.391 52 1599 minus strand ATP synthase beta subunit [Pinctada fucata]

Protein Accession	CL34251.Contig5$_H1$
Mean Expression Ratio	1.05
Median Expression Ratio	1.05
Credible Interval	(0.876, 1.26)
Associated Peptides	11
Associated Spectra	34
Coverage	NaN

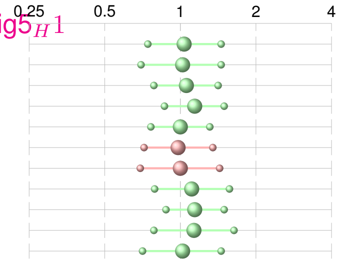

A	B	2.5	50	97.5	Sequence
1	1	0.74	1	1.5	AHGGYSVFAGVGER
1	0	0.7	1	1.5	EAAKPAAAGNLAVGR
2	2	0.78	1.1	1.4	IINVIGEPIDER
4	2	0.87	1.1	1.5	ILDPNVVGMEHYK
3	3	0.76	1	1.3	TIAMDGTEGLVR
2	1	0.72	0.98	1.3	VISLTDDTSK
1	0	0.69	1	1.4	VSLVYGQMNEPPGAR
2	0	0.79	1.1	1.6	VVDMLAPYAR
6	1	0.88	1.1	1.5	ILQDFK
0	1	0.79	1.1	1.6	FTQAGSEVSALLGR
0	1	0.71	1	1.5	IGLFGGAGVGK

5.392 142 588 AAEL009772-PB [Aedes aegypti]; 96 497 AAEL009772-PB [Aedes aegypti]

Protein Accession	Unigene73475$_H1$ CL27854.Contig2$_H1$
Mean Expression Ratio	0.95
Median Expression Ratio	0.949
Credible Interval	(0.643, 1.4)
Associated Peptides	1
Associated Spectra	3
Coverage	NaN

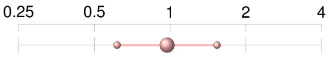

A	B	2.5	50	97.5	Sequence
2	1	0.62	0.97	1.5	LVEEFPMGNIK

5.393 187 1323 minus strand gelsolin [Aplysia californica] gi

Protein Accession	CL10220.Contig1$_H1$
Mean Expression Ratio	1.06
Median Expression Ratio	1.05
Credible Interval	(0.722, 1.55)
Associated Peptides	1
Associated Spectra	1
Coverage	NaN

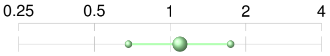

A	B	2.5	50	97.5	Sequence
1	0	0.68	1.1	1.7	LSDASGELDFK

5.394 61 2865 AP-2 complex subunit beta [Crassostrea gigas]

Protein Accession	CL6957.Contig1$_H1$
Mean Expression Ratio	0.947
Median Expression Ratio	0.95
Credible Interval	(0.638, 1.4)
Associated Peptides	1
Associated Spectra	1
Coverage	NaN

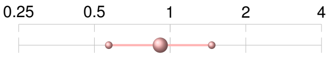

A	B	2.5	50	97.5	Sequence
1	0	0.57	0.91	1.5	NSNIFTIAK

5.395 21 239 minus strand calponin-like protein [Taenia asiatica]

Protein Accession	CL26318.Contig1$_H$1
Mean Expression Ratio	0.952
Median Expression Ratio	0.95
Credible Interval	(0.64, 1.41)
Associated Peptides	1
Associated Spectra	1
Coverage	NaN

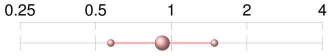

A	B	2.5	50	97.5	Sequence
**1**	**0**	**0.57**	**0.92**	**1.5**	**HIADIK**

5.396 211 1413 Alpha-N-acetylgalactosaminidase [Crassostrea gigas]

Protein Accession	CL5989.Contig1$_H$1
Mean Expression Ratio	0.951
Median Expression Ratio	0.95
Credible Interval	(0.681, 1.32)
Associated Peptides	2
Associated Spectra	4
Coverage	NaN

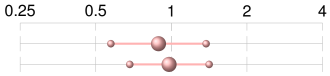

A	B	2.5	50	97.5	Sequence
**1**	**0**	**0.57**	**0.89**	**1.4**	**ALADYMHDR**
**3**	**0**	**0.68**	**0.98**	**1.4**	**ALLLNR**

5.397 338 661 Spectrin alpha chain [Crassostrea gigas]

Protein Accession	CL26928.Contig1$_H$1
Mean Expression Ratio	0.95
Median Expression Ratio	0.95
Credible Interval	(0.673, 1.33)
Associated Peptides	2
Associated Spectra	3
Coverage	NaN

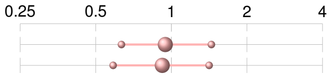

A	B	2.5	50	97.5	Sequence
**1**	**1**	**0.63**	**0.95**	**1.4**	**DVAGAEALLER**
**0**	**1**	**0.58**	**0.92**	**1.4**	**AIISADDLAK**

5.398 1213 3234 Protein-glutamine gamma-glutamyltransferase K [Crassostrea gigas]; 478 2499 Protein-glutamine gamma-glutamyltransferase K [Crassostrea gigas]

Protein Accession	CL29134.Contig6$_H$1 CL29134.Contig5$_H$1
Mean Expression Ratio	1.05
Median Expression Ratio	1.05
Credible Interval	(0.71, 1.54)
Associated Peptides	1
Associated Spectra	1
Coverage	NaN

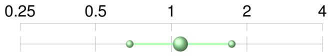

A	B	2.5	50	97.5	Sequence
**0**	**1**	**0.68**	**1.1**	**1.7**	**FLFAEAHGER**

5.399 546 995 Hypothetical protein CBG12002 [Caenorhabditis briggsae]; 543 995 minus strand PREDICTED: tubulin polymerization-promoting protein family member 2-like [Anolis carolinensis]; 546 995 Hypothetical protein CBG12002 [Caenorhabditis briggsae]; 546 995 minus strand Hypothetical protein CBG12002 [Caenorhabditis briggsae]

Protein Accession	CL6849.Contig7$_{H}$1 CL6849.Contig6$_{H}$1 CL6849.Contig2$_{H}$1 CL6849.Contig1$_{H}$1
Mean Expression Ratio	1.05
Median Expression Ratio	1.05
Credible Interval	(0.735, 1.53)
Associated Peptides	1
Associated Spectra	2
Coverage	NaN

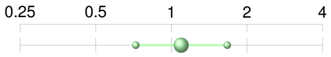

A	B	2.5	50	97.5	Sequence
0	2	0.72	1.1	1.7	LLPQLAADK

5.400 397 510 PREDICTED: actin-5C-like [Megachile rotundata]

Protein Accession	CL48442.Contig2$_{H}$1
Mean Expression Ratio	0.951
Median Expression Ratio	0.951
Credible Interval	(0.743, 1.22)
Associated Peptides	3
Associated Spectra	29
Coverage	NaN

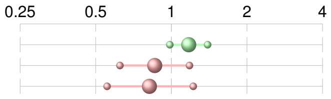

A	B	2.5	50	97.5	Sequence
15	9	0.98	1.2	1.4	DLTDYLMK
2	2	0.62	0.86	1.2	GRDLTDYLMK
0	1	0.55	0.82	1.2	LDLAGRDLTDY

5.401 58 1203 minus strand Obg-like ATPase 1 [Crassostrea gigas]

Protein Accession	CL33105.Contig1$_{H}$1
Mean Expression Ratio	0.951
Median Expression Ratio	0.951
Credible Interval	(0.641, 1.4)
Associated Peptides	1
Associated Spectra	1
Coverage	NaN

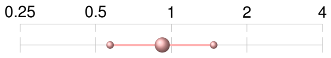

A	B	2.5	50	97.5	Sequence
1	0	0.57	0.92	1.5	IHTDFEK

5.402 15 428 PREDICTED: histone H2A.V-like [Apis florea]

Protein Accession	CL14228.Contig5$_{H}$1
Mean Expression Ratio	0.951
Median Expression Ratio	0.951
Credible Interval	(0.738, 1.23)
Associated Peptides	4
Associated Spectra	22
Coverage	NaN

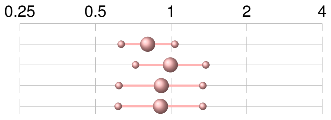

A	B	2.5	50	97.5	Sequence
9	3	0.63	0.81	1	AGLQFPVGR
3	3	0.72	0.99	1.4	ATIAGGGVIPHIHK
1	1	0.62	0.92	1.3	GDEELDSLIK
1	1	0.62	0.91	1.3	HLQLAIR

5.403 84 425 minus strand Transcription elongation factor B polypeptide 2 [Crassostrea gigas]; 84 425 minus strand Transcription elongation factor B polypeptide 2 [Crassostrea gigas]; 84 425 minus strand Transcription elongation factor B polypeptide 2 [Crassostrea gigas]; 101 442 minus strand Transcription elongation factor B polypeptide 2 [Crassostrea gigas]; 101 442 minus strand Transcription elongation factor B polypeptide 2 [Crassostrea gigas]; 84 425 minus strand Transcription elongation factor B polypeptide 2 [Crassostrea gigas]

Protein Accession	CL19448.Contig9$_{H}1$ CL19448.Contig8$_{H}1$ CL19448.Contig7$_{H}1$ CL19448.Contig1$_{H}1$ CL1944
Mean Expression Ratio	1.05
Median Expression Ratio	1.05
Credible Interval	(0.71, 1.54)
Associated Peptides	1
Associated Spectra	1
Coverage	NaN

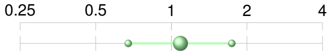

A	B	2.5	50	97.5	Sequence
**1**	**0**	**0.68**	**1.1**	**1.7**	**AQAPATLGLVYK**

5.404 1 852 beta actin-like 1 protein [Macrostomum lignano]

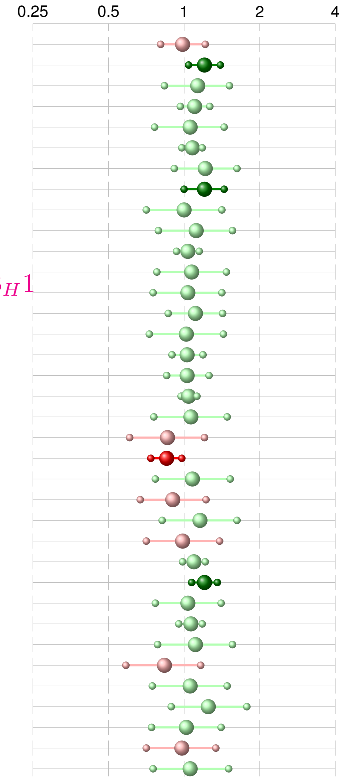

A	B	2.5	50	97.5	Sequence
9	5	0.81	0.99	1.2	AGFAGDDAPR
27	7	1	1.2	1.4	AVFPSIVGR
2	1	0.83	1.1	1.5	AVFPSIVGRPR
19	21	0.96	1.1	1.3	DSYVGDEAQSK
1	1	0.76	1.1	1.4	GVMVGMGQK
80	17	0.98	1.1	1.2	GYSFTTTAER
2	2	0.91	1.2	1.6	HQGVMVGMGQ
11	8	1	1.2	1.4	IWHHTFYNELR
1	0	0.71	1	1.4	MTQIMFETF
1	0	0.79	1.1	1.6	SYELPDGQVITIGN
63	15	0.93	1	1.1	SYELPDGQVITIGNER
1	1	0.78	1.1	1.5	TTGIVLDSGDGVTH
2	0	0.75	1	1.4	TTGIVLDSGDGVTHTVPIYEGY
7	0	0.87	1.1	1.4	TTGIVLDSGDGVTHTVPIYEGYALPH
1	0	0.73	1	1.4	VALDFEQEMATAAASSSLEK
18	17	0.9	1	1.2	VAPEEHPVLLTEA
10	6	0.85	1	1.3	VAPEEHPVLLTEAPLN
94	64	0.97	1	1.1	VAPEEHPVLLTEAPLNPK
1	0	0.76	1.1	1.5	VAPEEHPVLLTEAPLNPKANR
1	0	0.61	0.86	1.2	TEAPLNPK
23	13	0.74	0.85	0.98	RGILTLK
1	0	0.77	1.1	1.5	VAPEEHPVLLTEAPLNPKANREKMTQI
2	1	0.67	0.9	1.2	WDDMEK
1	0	0.82	1.2	1.6	HQGVMVGMGQKDSYVGDEAQSK
1	0	0.71	0.99	1.4	MTQIMFETFN
37	36	0.98	1.1	1.2	GILTLK
44	16	1.1	1.2	1.4	HQGVMVGMGQK
1	2	0.77	1	1.4	TTGIVLDSGDGVTHTVPIYEGYALPHAILR
50	19	0.95	1.1	1.2	YPIEHGIVTNWDDMEK
0	1	0.78	1.1	1.6	GIVTNWDDMEK
0	1	0.59	0.83	1.2	EIVRDIKEK
0	1	0.75	1.1	1.5	VMVGMGQK
0	1	0.89	1.2	1.8	GDEAQSK
0	2	0.74	1	1.4	VAPEEHPVLLTEAPL
0	2	0.71	0.98	1.3	VAPEEHPVLLTEAP
0	1	0.75	1.1	1.5	YPIEHGIVTNWDDME

Protein Accession	Unigene31438$_{H}1$
Mean Expression Ratio	1.05
Median Expression Ratio	1.05
Credible Interval	(0.961, 1.15)
Associated Peptides	36
Associated Spectra	772
Coverage	NaN

5.405 22 870 minus strand PREDICTED: alcohol dehydrogenase class-3 [Sarcophilus harrisii]

Protein Accession	Unigene27690$_{H}1$
Mean Expression Ratio	0.952
Median Expression Ratio	0.951
Credible Interval	(0.745, 1.22)
Associated Peptides	6
Associated Spectra	14
Coverage	NaN

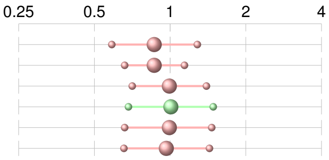

A	B	2.5	50	97.5	Sequence
1	0	0.58	0.87	1.3	AALEACHK
5	2	0.66	0.87	1.1	AAVAWEAK
1	2	0.71	0.99	1.4	IIGIDVNQDK
1	0	0.68	1	1.5	KPLVIEDIEVAPPAAGEVR
1	0	0.66	0.99	1.5	TNLCQK
0	1	0.65	0.97	1.4	FAMAK

5.406 3 1670 Spectrin alpha chain [Crassostrea gigas]

Protein Accession	CL2195.Contig7$_{H}1$
Mean Expression Ratio	1.05
Median Expression Ratio	1.05
Credible Interval	(0.795, 1.39)
Associated Peptides	6
Associated Spectra	7
Coverage	NaN

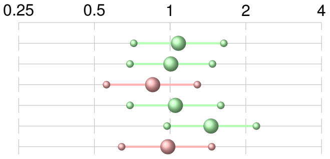

A	B	2.5	50	97.5	Sequence
1	0	0.72	1.1	1.6	NNLMDEFTISVR
1	1	0.69	1	1.5	VAALYDYTEK
1	0	0.56	0.85	1.3	HQALQAEIAGHEPR
1	0	0.69	1.1	1.6	KLDPSLSASR
1	0	0.97	1.5	2.2	ADLEAYRSV
0	1	0.64	0.98	1.5	LTAMGHTEAAEK

5.407 4 444 Vinculin [Crassostrea gigas]

Protein Accession	Unigene21732$_{H}1$
Mean Expression Ratio	1.05
Median Expression Ratio	1.05
Credible Interval	(0.797, 1.41)
Associated Peptides	4
Associated Spectra	9
Coverage	NaN

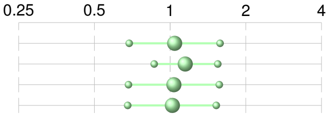

A	B	2.5	50	97.5	Sequence
1	0	0.69	1	1.6	AIAEASEEVTR
4	2	0.86	1.2	1.6	IPTIGTQLK
1	0	0.68	1	1.6	TNLLNVCER
1	0	0.68	1	1.5	DNEIISAAK

5.408 145 1020 minus strand PREDICTED: inorganic pyrophosphatase-like [Saccoglossus kowalevskii]

Protein Accession	CL12968.Contig14$_{H}1$
Mean Expression Ratio	1.05
Median Expression Ratio	1.05
Credible Interval	(0.814, 1.36)
Associated Peptides	6
Associated Spectra	10
Coverage	NaN

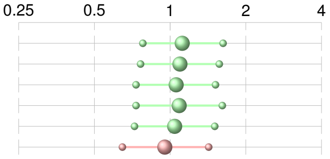

A	B	2.5	50	97.5	Sequence
1	1	0.78	1.1	1.6	AEVIVQESK
1	1	0.76	1.1	1.6	IIAIDVTDPLASK
1	1	0.73	1.1	1.5	LNDIDDVHR
1	0	0.73	1.1	1.6	VLGTMLLVDEGETDWK
1	1	0.72	1	1.5	EMPGFLR
0	1	0.65	0.95	1.4	VFFR

5.409 57 2009 hypothetical protein BRAFLDRAFT_285279 [Branchiostoma floridae] gi; 3 1973 hypothetical protein BRAFLDRAFT_285279 [Branchiostoma floridae] gi

Protein Accession	CL24444.Contig3$_{H}1$ CL24444.Contig1$_{H}1$
Mean Expression Ratio	0.951
Median Expression Ratio	0.952
Credible Interval	(0.666, 1.36)
Associated Peptides	2
Associated Spectra	2
Coverage	NaN

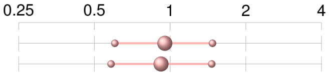

A	B	2.5	50	97.5	Sequence
1	0	0.6	0.95	1.5	INQIYEQ
0	1	0.58	0.92	1.5	KLTLK

5.410 2 211 minus strand sulfatase 1A precursor [Haliotis discus discus]

Protein Accession	Unigene22814$_H 1$
Mean Expression Ratio	1.05
Median Expression Ratio	1.05
Credible Interval	(0.727, 1.51)
Associated Peptides	1
Associated Spectra	2
Coverage	NaN

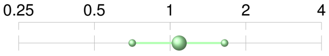

A	B	2.5	50	97.5	Sequence
**2**	**0**	**0.71**	**1.1**	**1.6**	**CGMVSAMDESIGK**

5.411 " 451 1026 PREDICTED: GTP-binding protein SAR1B, putative-like [Saccoglossus kowalevskii]"

Protein Accession	Unigene4124$_H 1$
Mean Expression Ratio	1.05
Median Expression Ratio	1.05
Credible Interval	(0.707, 1.55)
Associated Peptides	1
Associated Spectra	1
Coverage	NaN

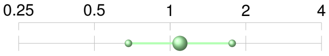

A	B	2.5	50	97.5	Sequence
**1**	**0**	**0.68**	**1.1**	**1.8**	**AELDSLLTDEQVANAPILVLGNK**

5.412 246 452 minus strand mitochondrial malate dehydrogenase precursor [Nucella lapillus]; 106 312 minus strand mitochondrial malate dehydrogenase precursor [Nucella lapillus]

Protein Accession	CL10426.Contig4$_H 1$ CL10426.Contig3$_H 1$
Mean Expression Ratio	0.953
Median Expression Ratio	0.952
Credible Interval	(0.675, 1.35)
Associated Peptides	1
Associated Spectra	4
Coverage	NaN

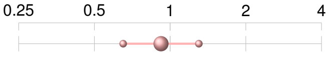

A	B	2.5	50	97.5	Sequence
**2**	**2**	**0.65**	**0.92**	**1.3**	**DDLFNTNAGIVR**

5.413 REVERSED 68 358 endonuclease-reverse transcriptase [Danaus plexippus]

Protein Accession	RRRRRUnigene39307$_H 1$
Mean Expression Ratio	0.953
Median Expression Ratio	0.952
Credible Interval	(0.663, 1.37)
Associated Peptides	2
Associated Spectra	2
Coverage	NaN

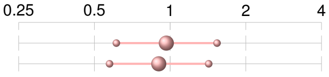

A	B	2.5	50	97.5	Sequence
**0**	**1**	**0.61**	**0.97**	**1.5**	**TIITALDK**
**0**	**1**	**0.57**	**0.9**	**1.4**	**CQSLLDENSIK**

5.414 365 2530 minus strand calpain B [Gecarcinus lateralis]

Protein Accession	CL34074.Contig5$_{H}1$
Mean Expression Ratio	1.05
Median Expression Ratio	1.05
Credible Interval	(0.834, 1.33)
Associated Peptides	7
Associated Spectra	14
Coverage	NaN

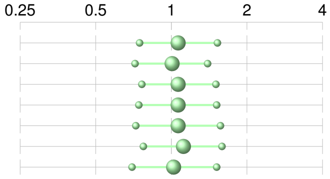

A	B	2.5	50	97.5	Sequence
1	1	0.75	1.1	1.5	LGYDEFK
2	1	0.72	1	1.4	SLQSLVMR
2	1	0.76	1.1	1.5	SMVAMHDGDLSGK
1	1	0.74	1.1	1.5	TLWEDLR
1	0	0.72	1.1	1.6	MQGQIPMVR
1	1	0.77	1.1	1.6	TMLTSFR
0	1	0.7	1	1.5	TLWEDLRR

5.415 44 1465 minus strand Adenylyl cyclase-associated protein 1 [Crassostrea gigas]; 52 1473 minus strand Adenylyl cyclase-associated protein 1 [Crassostrea gigas]; 48 1469 Adenylyl cyclase-associated protein 1 [Crassostrea gigas]

Protein Accession	CL19470.Contig9$_{H}1$ CL19470.Contig8$_{H}1$ CL19470.Contig1$_{H}1$
Mean Expression Ratio	1.05
Median Expression Ratio	1.05
Credible Interval	(0.865, 1.27)
Associated Peptides	13
Associated Spectra	24
Coverage	NaN

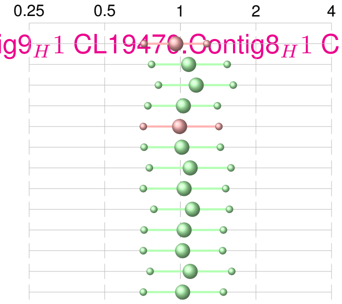

A	B	2.5	50	97.5	Sequence
2	3	0.72	0.96	1.3	AQLDIVNAAFK
1	1	0.77	1.1	1.5	DSLDTEIVTAK
1	1	0.82	1.2	1.6	EFALPEQFK
2	1	0.74	1	1.4	IQAVQEFR
1	1	0.71	1	1.4	NIVLDNVELK
1	1	0.72	1	1.4	QFLEVASK
1	0	0.75	1.1	1.6	SKQPPQADLPK
1	0	0.71	1	1.5	VNSIILDQCK
2	0	0.78	1.1	1.6	VPTISIDK
1	0	0.72	1	1.5	QAVYIYK
1	0	0.71	1	1.5	LETLALR
1	0	0.76	1.1	1.6	DTALQNAVAR
0	1	0.71	1	1.5	LKDFK

5.416 2 517 minus strand Anhydro-N-acetylmuramic acid kinase [Crassostrea gigas]

Protein Accession	Unigene23377$_{H}1$
Mean Expression Ratio	0.954
Median Expression Ratio	0.953
Credible Interval	(0.677, 1.34)
Associated Peptides	2
Associated Spectra	3
Coverage	NaN

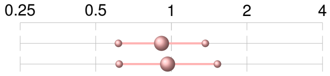

A	B	2.5	50	97.5	Sequence
1	1	0.61	0.91	1.4	KYGLQDVAR
0	1	0.62	0.97	1.5	KLVDAGLAVDK

5.417 1017 1499 minus strand PREDICTED: leukocyte elastase inhibitor-like [Oreochromis niloticus]

Protein Accession	CL39031.Contig4$_{H}1$
Mean Expression Ratio	0.953
Median Expression Ratio	0.953
Credible Interval	(0.657, 1.37)
Associated Peptides	1
Associated Spectra	2
Coverage	NaN

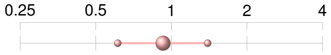

A	B	2.5	50	97.5	Sequence
**0**	**2**	**0.61**	**0.92**	**1.4**	**VLELPYGTDDR**

5.418 64 393 Tubulin beta chain OS=Paracentrotus lividus PE=2 SV=1; 111 398 Tubulin beta chain OS=Paracentrotus lividus PE=2 SV=1; 109 438 Tubulin beta chain OS=Paracentrotus lividus PE=2 SV=1; 160 489 Tubulin beta chain OS=Paracentrotus lividus PE=2 SV=1

Protein Accession	Unigene62663$_{H}1$ Unigene62662$_{H}1$ Unigene62661$_{H}1$ Unigene62660$_{H}1$
Mean Expression Ratio	0.952
Median Expression Ratio	0.953
Credible Interval	(0.654, 1.36)
Associated Peptides	1
Associated Spectra	6
Coverage	NaN

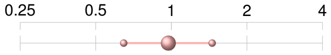

A	B	2.5	50	97.5	Sequence
**4**	**2**	**0.65**	**0.97**	**1.5**	**INVYFNEATGGK**

5.419 " 519 3182 minus strand Alpha-actinin, sarcomeric [Crassostrea gigas]"

Protein Accession	CL28960.Contig2$_{H}$1
Mean Expression Ratio	1.05
Median Expression Ratio	1.05
Credible Interval	(0.94, 1.17)
Associated Peptides	41
Associated Spectra	116
Coverage	NaN

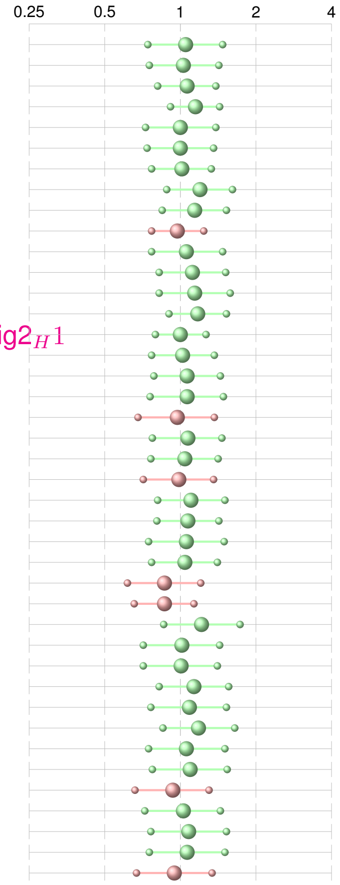

A	B	2.5	50	97.5	Sequence
1	0	0.74	1	1.5	AAPFNNWMDGAK
1	1	0.75	1	1.4	AGTGIENIEEDFR
5	1	0.81	1.1	1.4	AIIQYKPHIDELER
8	3	0.92	1.1	1.4	ALDFIASK
1	1	0.73	1	1.4	DNPIENLNTAFDIAEK
2	1	0.74	1	1.4	EALEEAER
3	2	0.77	1	1.3	ETADTDTAEQVMQSFK
2	1	0.88	1.2	1.6	FAIQDITVEELTAK
2	2	0.85	1.1	1.5	GISEDQMNEFR
6	3	0.77	0.97	1.2	IDQLHLEFAK
1	1	0.77	1.1	1.5	KAGTGIENIEEDFR
2	1	0.82	1.1	1.5	LSNRPAYLPTEGK
2	0	0.82	1.1	1.6	LVSIGAEEIVDGNVK
3	3	0.9	1.2	1.5	MSFNHFDK
4	5	0.79	1	1.3	MVSDIANAWK
2	2	0.77	1	1.4	NINEVENQILTR
2	1	0.78	1.1	1.4	RDQTLQQEMMR
1	0	0.76	1.1	1.5	GSLEEQLKK
1	0	0.68	0.97	1.4	HKPPKLEDK
2	0	0.77	1.1	1.5	MLDPEDMVNSAKPDER
2	1	0.77	1	1.4	LMEEYER
2	0	0.71	0.98	1.4	TRPWLENR
3	0	0.81	1.1	1.5	LQAFDK
2	2	0.8	1.1	1.4	KLDEFR
1	0	0.74	1.1	1.5	EDMLQSADFK
2	1	0.77	1	1.4	LGQLTVK
1	0	0.61	0.86	1.2	PHIDELER
2	3	0.65	0.86	1.1	LDEFR
1	0	0.86	1.2	1.7	HLDIPR
1	0	0.71	1	1.4	PGALDYMSFSTAL
1	0	0.71	1	1.4	DGLAFCALIHR
2	0	0.82	1.1	1.6	HRPELIDYHNLSK
1	0	0.77	1.1	1.5	KLQAFDK
2	0	0.85	1.2	1.6	LDHLAQK
1	0	0.75	1.1	1.5	YTQYTMETLR
0	1	0.77	1.1	1.5	EGLLDPAWEK
0	1	0.66	0.93	1.3	LENSFNTLQTR
0	1	0.72	1	1.4	KHKPPKLEDK
0	1	0.76	1.1	1.5	ELVPR
0	1	0.75	1.1	1.5	DQLHLEFAK
0	1	0.67	0.95	1.3	SIGVQMR

5.420 85 546 minus strand RecName: Full=Eukaryotic translation initiation factor 5A; Short=eIF-5A gi|51702279|sp|P62925.1|IF5A_SPOFR RecName: Full=Eukaryotic translation initiation factor 5A; Short=eIF-5A gi|6469515|gb|AAF13315.1|AF109730_1 translation initiation factor 5A [Spodoptera exigua] gi

Protein Accession	CL40725.Contig2$_{H}$1
Mean Expression Ratio	0.953
Median Expression Ratio	0.953
Credible Interval	(0.72, 1.26)
Associated Peptides	3
Associated Spectra	9
Coverage	NaN

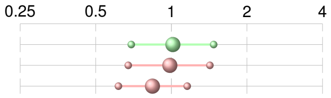

A	B	2.5	50	97.5	Sequence
1	1	0.69	1	1.5	AMGEECVVAVK
1	1	0.67	0.98	1.4	EDSLAAEIQK
2	3	0.62	0.84	1.2	IVEMSTSK

5.421 " 703 1173 60S acidic ribosomal protein P0, putative [Ixodes scapularis] gi"

Protein Accession	Unigene34489$_{H}$1
Mean Expression Ratio	0.956
Median Expression Ratio	0.954
Credible Interval	(0.647, 1.42)
Associated Peptides	1
Associated Spectra	1
Coverage	NaN

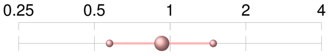

A	B	2.5	50	97.5	Sequence
0	1	0.58	0.92	1.5	KSFLEGVR

5.422 913 1560 minus strand Calcium-dependent protein kinase isoform 2 [Crassostrea gigas]

Protein Accession	CL5866.Contig1$_{H}$1
Mean Expression Ratio	1.05
Median Expression Ratio	1.05
Credible Interval	(0.774, 1.43)
Associated Peptides	4
Associated Spectra	5
Coverage	NaN

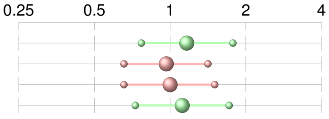

A	B	2.5	50	97.5	Sequence
0	1	0.77	1.2	1.8	DEFSVAVDK
0	2	0.65	0.96	1.4	FSFDEADKDK
0	1	0.65	1	1.5	IHYDEFVK
0	1	0.73	1.1	1.7	LLDINDDQK

5.423 1265 1831 minus strand Adenosine deaminase CECR1 [Crassostrea gigas]

Protein Accession	CL20850.Contig11$_{H}$1
Mean Expression Ratio	0.953
Median Expression Ratio	0.954
Credible Interval	(0.645, 1.42)
Associated Peptides	1
Associated Spectra	1
Coverage	NaN

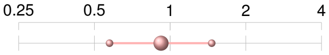

A	B	2.5	50	97.5	Sequence
0	1	0.57	0.92	1.5	GIAVEVNPISNQVLK

5.424 210 473 Abdominal ganglion neuropeptides L5-67 OS=Aplysia californica PE=1 SV=1; 879 1124 abdominal ganglion neuropeptides L5-67 precursor [Aplysia californica] gi; 210 482 minus strand abdominal ganglion neuropeptides L5-67 precursor [Aplysia californica] gi

Protein Accession	Unigene58290$_{H}$1 Unigene39670$_{H}$1 Unigene206$_{H}$1
Mean Expression Ratio	0.954
Median Expression Ratio	0.954
Credible Interval	(0.66, 1.37)
Associated Peptides	1
Associated Spectra	2
Coverage	NaN

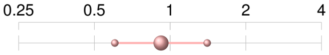

A	B	2.5	50	97.5	Sequence
0	2	0.6	0.92	1.4	VNGEIELPVEVFGAR

5.425 " 193 333 minus strand RecName: Full=Globin; AltName: Full=Myoglobin gi|299274|gb|AAB25743.1| myoglobin [Nassa mutabilis=marine gastropods, radular muscles, Peptide, 147 aa] gi; 193 333 minus strand RecName: Full=Globin; AltName: Full=Myoglobin gi|299274|gb|AAB25743.1| myoglobin [Nassa mutabilis=marine gastropods, radular muscles, Peptide, 147 aa] gi"

Protein Accession	CL8458.Contig5$_{H}1$ CL8458.Contig1$_{H}1$
Mean Expression Ratio	1.05
Median Expression Ratio	1.05
Credible Interval	(0.726, 1.53)
Associated Peptides	1
Associated Spectra	2
Coverage	NaN

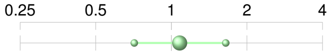

A	B	2.5	50	97.5	Sequence
2	0	0.71	1.1	1.7	AAINSTWPK

5.426 14 223 minus strand Endothelin-converting enzyme 2 OS=Homo sapiens GN=ECE2 PE=1 SV=4

Protein Accession	Unigene41869$_{H}1$
Mean Expression Ratio	0.953
Median Expression Ratio	0.955
Credible Interval	(0.657, 1.37)
Associated Peptides	1
Associated Spectra	2
Coverage	NaN

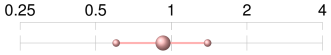

A	B	2.5	50	97.5	Sequence
2	0	0.6	0.92	1.4	KLLEAQNK

5.427 " 3341 4240 minus strand nonmuscle myosin II, partial [Aplysia californica]"

Protein Accession	CL19183.Contig2$_{H}1$
Mean Expression Ratio	1.05
Median Expression Ratio	1.05
Credible Interval	(0.813, 1.35)
Associated Peptides	7
Associated Spectra	11
Coverage	NaN

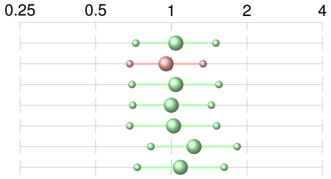

A	B	2.5	50	97.5	Sequence
1	1	0.72	1	1.5	RLETELQDLR
2	1	0.68	0.96	1.3	REEEVQIALQK
1	0	0.7	1	1.6	TATMLEEK
1	1	0.7	1	1.4	YAQMIEEK
1	0	0.68	1	1.5	QKQDVEQLEAR
1	0	0.83	1.2	1.8	QELEK
0	1	0.73	1.1	1.6	ELQDLR

5.428 195 2201 minus strand hypothetical protein DAPPUDRAFT_315656 [Daphnia pulex]

Protein Accession	Unigene38538$_H1$
Mean Expression Ratio	1.05
Median Expression Ratio	1.05
Credible Interval	(0.809, 1.36)
Associated Peptides	6
Associated Spectra	9
Coverage	NaN

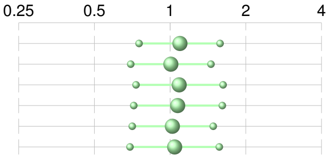

A	B	2.5	50	97.5	Sequence
1	1	0.75	1.1	1.6	FFTGQITAAGK
1	1	0.7	1	1.5	LLLSFGER
1	0	0.73	1.1	1.6	VALSPAATQALVK
1	0	0.72	1.1	1.6	VQIEAGAGEEAK
1	1	0.71	1	1.5	GPQFEEIGK
0	1	0.69	1	1.6	GSIVLHDGK

5.429 2 244 Ras homolog gene family member A [Clonorchis sinensis]

Protein Accession	Unigene32424$_H1$
Mean Expression Ratio	1.05
Median Expression Ratio	1.05
Credible Interval	(0.712, 1.55)
Associated Peptides	1
Associated Spectra	2
Coverage	NaN

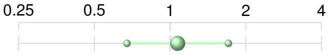

A	B	2.5	50	97.5	Sequence
1	1	0.67	1.1	1.7	EVFETATR

5.430 3 572 Receptor-type tyrosine-protein phosphatase N2 [Crassostrea gigas]; 3 542 minus strand Receptor-type tyrosine-protein phosphatase N2 [Crassostrea gigas]

Protein Accession	CL8283.Contig5$_H1$ CL8283.Contig2$_H1$
Mean Expression Ratio	0.953
Median Expression Ratio	0.956
Credible Interval	(0.637, 1.39)
Associated Peptides	1
Associated Spectra	1
Coverage	NaN

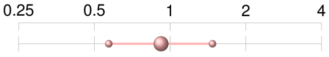

A	B	2.5	50	97.5	Sequence
0	1	0.57	0.92	1.5	EIDMAATLEHIR

5.431 252 1289 minus strand PDZ and LIM domain protein 3 OS=Gallus gallus GN=PDLIM3 PE=1 SV=1

Protein Accession	CL15373.Contig10$_H1$
Mean Expression Ratio	1.05
Median Expression Ratio	1.05
Credible Interval	(0.783, 1.4)
Associated Peptides	5
Associated Spectra	6
Coverage	NaN

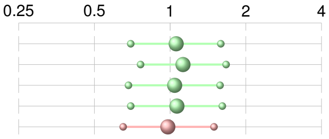

A	B	2.5	50	97.5	Sequence
1	0	0.7	1.1	1.6	SGDAILSINGVNTDHLTHEQAK
1	1	0.76	1.1	1.7	GFFVVEGK
1	0	0.68	1	1.6	IWKPQVTPLSE
1	0	0.7	1.1	1.6	CGGCDMLATGVIVK
0	1	0.65	0.98	1.5	AGCDIDFLIQR

5.432 11 1462 coronin [Schistosoma mansoni]

Protein Accession	CL11779.Contig2$_{H}1$
Mean Expression Ratio	1.05
Median Expression Ratio	1.05
Credible Interval	(0.799, 1.37)
Associated Peptides	4
Associated Spectra	10
Coverage	NaN

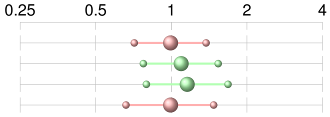

A	B	2.5	50	97.5	Sequence
2	2	0.71	0.99	1.4	DANPILVSLK
2	1	0.77	1.1	1.5	HVFGKPEK
2	0	0.8	1.2	1.7	VPMDAPVVAGHK
0	1	0.66	0.99	1.5	DAPVVAGHK

5.433 " 116 1168 minus strand hypothetical protein CGI_10014553, partial [Crassostrea gigas]"

Protein Accession	CL19215.Contig5$_{H}1$
Mean Expression Ratio	0.956
Median Expression Ratio	0.957
Credible Interval	(0.701, 1.31)
Associated Peptides	4
Associated Spectra	5
Coverage	NaN

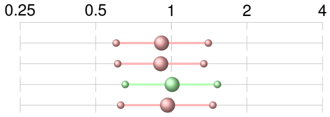

A	B	2.5	50	97.5	Sequence
1	0	0.6	0.92	1.4	SSFNDAVHSLFDK
1	1	0.61	0.91	1.3	SVIALGEGAGANIVAR
0	1	0.66	1	1.5	AAFIHVDVPGQEDDAK
0	1	0.63	0.97	1.5	VIENFQLSLR

5.434 42 296 minus strand twitchin [Mytilus galloprovincialis]

Protein Accession	Unigene19029$_{H}1$
Mean Expression Ratio	0.959
Median Expression Ratio	0.958
Credible Interval	(0.742, 1.25)
Associated Peptides	4
Associated Spectra	12
Coverage	NaN

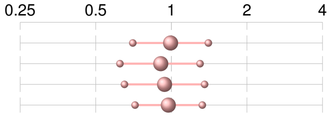

A	B	2.5	50	97.5	Sequence
2	1	0.7	1	1.4	APHFTQKPTIK
1	1	0.63	0.91	1.3	VTLADAGEYK
2	0	0.65	0.94	1.4	KPQLPDGK
3	2	0.72	0.97	1.3	LNFDSAK

5.435 " 27 974 PREDICTED: succinyl-CoA ligase [GDP-forming] subunit alpha, mitochondrial-like [Oreochromis niloticus]; 27 974 minus strand PREDICTED: succinyl-CoA ligase [GDP-forming] subunit alpha, mitochondrial-like [Oreochromis niloticus]"

Protein Accession	CL10674.Contig2$_{H}1$ CL10674.Contig1$_{H}1$
Mean Expression Ratio	0.958
Median Expression Ratio	0.958
Credible Interval	(0.71, 1.3)
Associated Peptides	3
Associated Spectra	6
Coverage	NaN

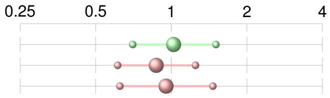

A	B	2.5	50	97.5	Sequence
2	0	0.7	1	1.5	MGHAGAIIAGGK
1	2	0.61	0.87	1.2	IYMATR
0	1	0.63	0.95	1.5	LICQGFTGK

5.436 187 696 minus strand putative ribosomal protein S10 [Sipunculus nudus]

Protein Accession	Unigene1233$_H$1
Mean Expression Ratio	0.959
Median Expression Ratio	0.958
Credible Interval	(0.655, 1.41)
Associated Peptides	1
Associated Spectra	3
Coverage	NaN

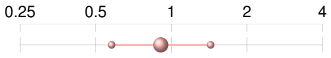

A	B	2.5	50	97.5	Sequence
**2**	**1**	**0.58**	**0.91**	**1.4**	**KDFFSPK**

5.437 229 699 hypothetical protein CGI_10017744 [Crassostrea gigas]; 199 669 hypothetical protein CGI_10017744 [Crassostrea gigas]; 202 672 hypothetical protein CGI_10017744 [Crassostrea gigas]

Protein Accession	CL5082.Contig3$_H$1 CL5082.Contig2$_H$1 CL5082.Contig1$_H$1
Mean Expression Ratio	1.04
Median Expression Ratio	1.04
Credible Interval	(0.789, 1.38)
Associated Peptides	3
Associated Spectra	9
Coverage	NaN

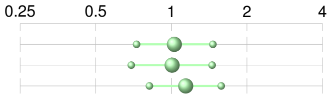

A	B	2.5	50	97.5	Sequence
**2**	**1**	**0.73**	**1**	**1.5**	**EVLELFDR**
**2**	**0**	**0.69**	**1**	**1.5**	**EGLHSLK**
**3**	**1**	**0.82**	**1.1**	**1.6**	**HLNKPDAIK**

5.438 630 1199 neuronal calcium sensor-1 [Lymnaea stagnalis]

Protein Accession	CL14574.Contig3$_H$1
Mean Expression Ratio	1.04
Median Expression Ratio	1.04
Credible Interval	(0.726, 1.5)
Associated Peptides	2
Associated Spectra	2
Coverage	NaN

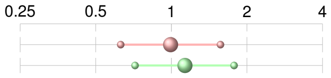

A	B	2.5	50	97.5	Sequence
**1**	**0**	**0.63**	**1**	**1.6**	**IFSVMDK**
**1**	**0**	**0.71**	**1.1**	**1.8**	**LYDLDNDGFITK**

5.439 316 1512 Alpha-N-acetylgalactosaminidase [Crassostrea gigas]; 282 1568 Alpha-N-acetylgalactosaminidase [Crassostrea gigas]; 282 1478 Alpha-N-acetylgalactosaminidase [Crassostrea gigas]

Protein Accession	CL2428.Contig4$_H$1 CL2428.Contig2$_H$1 CL2428.Contig1$_H$1
Mean Expression Ratio	1.04
Median Expression Ratio	1.04
Credible Interval	(0.753, 1.44)
Associated Peptides	2
Associated Spectra	5
Coverage	NaN

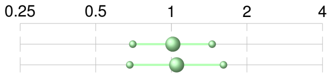

A	B	2.5	50	97.5	Sequence
**1**	**3**	**0.7**	**1**	**1.5**	**LVELGLR**
**0**	**1**	**0.68**	**1.1**	**1.6**	**ELFAGK**

5.440 116 1882 Actin-interacting protein 1 [Crassostrea gigas]

Protein Accession	CL12416.Contig1$_{H}1$
Mean Expression Ratio	0.96
Median Expression Ratio	0.959
Credible Interval	(0.768, 1.2)
Associated Peptides	9
Associated Spectra	17
Coverage	NaN

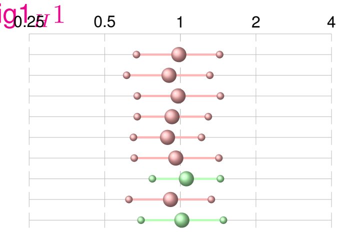

A	B	2.5	50	97.5	Sequence
1	0	0.67	0.99	1.4	FSPDGEVAVSGGADGK
1	0	0.61	0.9	1.3	ILTASEDNSVGFHEGPPFK
1	0	0.68	0.98	1.4	LVSIGVDDVVR
1	2	0.67	0.93	1.3	QFLDGEVK
3	1	0.65	0.89	1.2	TLDELDNKEIGAPGAHK
1	0	0.66	0.96	1.4	VFDVQSGSLVQEFK
4	0	0.77	1.1	1.4	TNEVTDMVIEGDR
1	0	0.62	0.91	1.3	KLESQPK
0	1	0.69	1	1.5	QILTASGDASAK

5.441 43 591 minus strand cyclophilin type peptidyl-prolyl cis-trans isomerase [Glossina morsitans morsitans]

Protein Accession	CL39270.Contig1$_{H}1$
Mean Expression Ratio	1.04
Median Expression Ratio	1.04
Credible Interval	(0.848, 1.29)
Associated Peptides	5
Associated Spectra	35
Coverage	NaN

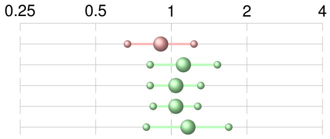

A	B	2.5	50	97.5	Sequence
1	3	0.67	0.91	1.2	VFFDMTADGVPLGR
4	0	0.82	1.1	1.5	KIESYGSKNGATSQK
7	4	0.83	1	1.3	FADENFTLK
7	8	0.85	1	1.3	ITMELR
0	1	0.79	1.2	1.7	VIPQFMCQGGDFTR

5.442 " 42 905 PREDICTED: enoyl-CoA hydratase, mitochondrial-like isoform 1 [Oreochromis niloticus]"

Protein Accession	CL6477.Contig7$_{H}1$
Mean Expression Ratio	0.961
Median Expression Ratio	0.96
Credible Interval	(0.697, 1.32)
Associated Peptides	2
Associated Spectra	5
Coverage	NaN

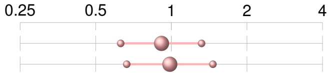

A	B	2.5	50	97.5	Sequence
1	2	0.63	0.91	1.3	AFAAGADIK
1	1	0.66	0.99	1.5	SVVLQASR

5.443 362 553 putative RNA-binding protein [Haliotis diversicolor]

Protein Accession	CL41007.Contig2$_{H}1$
Mean Expression Ratio	0.96
Median Expression Ratio	0.96
Credible Interval	(0.725, 1.27)
Associated Peptides	2
Associated Spectra	11
Coverage	NaN

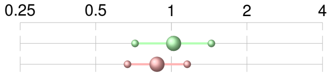

A	B	2.5	50	97.5	Sequence
2	1	0.72	1	1.4	ELQDETGCR
4	4	0.67	0.88	1.2	EVMHVDSSFVGR

5.444 194 775 minus strand Collagen alpha-1(XV) chain [Harpegnathos saltator]

Protein Accession	Unigene19084$_H1$
Mean Expression Ratio	1.04
Median Expression Ratio	1.04
Credible Interval	(0.73, 1.49)
Associated Peptides	2
Associated Spectra	2
Coverage	NaN

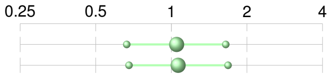

A	B	2.5	50	97.5	Sequence
1	0	0.67	1	1.7	GADYECHK
1	0	0.68	1.1	1.7	IIWHGSSK

5.445 227 3073 minus strand Lysosomal alpha-mannosidase [Crassostrea gigas]

Protein Accession	CL2930.Contig2$_H1$
Mean Expression Ratio	1.04
Median Expression Ratio	1.04
Credible Interval	(0.742, 1.47)
Associated Peptides	2
Associated Spectra	4
Coverage	NaN

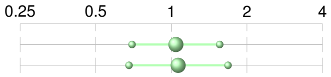

A	B	2.5	50	97.5	Sequence
2	1	0.7	1	1.6	VASELIPITPDTFR
1	0	0.68	1.1	1.7	ASLTWSGVR

5.446 77 1561 retinal dehydrogenase [Lymnaea stagnalis]; 69 1553 retinal dehydrogenase [Lymnaea stagnalis]; 308 1747 retinal dehydrogenase [Lymnaea stagnalis]

Protein Accession	CL13284.Contig7$_H1$ CL13284.Contig2$_H1$ CL13284.Contig1$_H1$
Mean Expression Ratio	0.962
Median Expression Ratio	0.96
Credible Interval	(0.799, 1.17)
Associated Peptides	15
Associated Spectra	23
Coverage	NaN

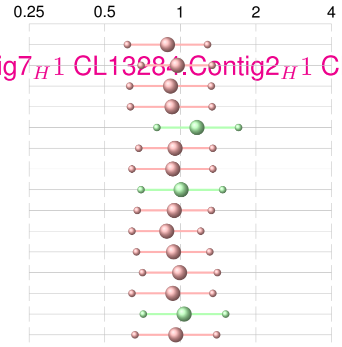

A	B	2.5	50	97.5	Sequence
1	0	0.62	0.89	1.3	ALMFVNGVR
2	1	0.7	0.97	1.3	AVEVAQDAFK
1	0	0.63	0.91	1.3	FSTVEEAIER
1	0	0.63	0.93	1.3	IYDEFVR
1	0	0.81	1.2	1.7	NIAYLASLETLDNGKPFK
1	1	0.68	0.95	1.3	TFPTINPATGK
1	0	0.64	0.94	1.4	VAFTGSTEVGQIIMK
1	0	0.7	1	1.5	KTAELANKR
2	0	0.67	0.95	1.3	KTAELANK
3	0	0.64	0.88	1.2	RGALLYK
1	1	0.67	0.94	1.3	LGSAWR
1	1	0.71	0.99	1.4	AIQIIR
1	0	0.64	0.93	1.3	EEIFGPVQQLLK
0	1	0.71	1	1.5	KIVDVQEADK
0	1	0.66	0.96	1.4	KIVDVQEADKADVDK

5.447 2 244 minus strand hypothetical protein KGM_08771 [Danaus plexippus]

Protein Accession	CL49466.Contig2$_{H}1$
Mean Expression Ratio	0.961
Median Expression Ratio	0.961
Credible Interval	(0.706, 1.31)
Associated Peptides	2
Associated Spectra	6
Coverage	NaN

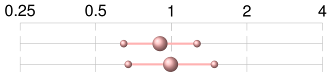

A	B	2.5	50	97.5	Sequence
**3**	**1**	**0.65**	**0.9**	**1.3**	**AVFIDLEPTVVDEVR**
**1**	**1**	**0.68**	**1**	**1.5**	**TVGGGDDSFNTFFSETGAGK**

5.448 154 621 minus strand Stress response protein nhaX [Crassostrea gigas]

Protein Accession	CL4265.Contig1$_{H}1$
Mean Expression Ratio	1.04
Median Expression Ratio	1.04
Credible Interval	(0.767, 1.4)
Associated Peptides	5
Associated Spectra	5
Coverage	NaN

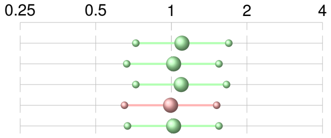

A	B	2.5	50	97.5	Sequence
**0**	**1**	**0.72**	**1.1**	**1.7**	**IQHDLEK**
**0**	**1**	**0.66**	**1**	**1.6**	**SVAAAKPGEGILK**
**0**	**1**	**0.72**	**1.1**	**1.7**	**EVLEAMLK**
**0**	**1**	**0.65**	**1**	**1.5**	**PGEGILK**
**0**	**1**	**0.67**	**1**	**1.5**	**EGILK**

5.449 636 1232 sigma class glutathione-s-transferase 3 [Haliotis discus discus]; 963 1559 sigma class glutathione-s-transferase 3 [Haliotis discus discus]; 907 1503 sigma class glutathione-s-transferase 3 [Haliotis discus discus]; 673 1269 sigma class glutathione-s-transferase 3 [Haliotis discus discus]; 177 773 sigma class glutathione-s-transferase 3 [Haliotis discus discus]; 208 804 sigma class glutathione-s-transferase 3 [Haliotis discus discus]; 327 923 sigma class glutathione-s-transferase 3 [Haliotis discus discus]

Protein Accession	Unigene22829$_{H}1$ CL19285.Contig31$_{H}1$ CL19285.Contig30$_{H}1$ CL19285.Contig27$_{H}1$ CL192
Mean Expression Ratio	1.04
Median Expression Ratio	1.04
Credible Interval	(0.724, 1.49)
Associated Peptides	2
Associated Spectra	2
Coverage	NaN

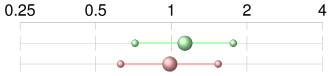

A	B	2.5	50	97.5	Sequence
**1**	**0**	**0.72**	**1.1**	**1.8**	**LLLAASGKDWEDVR**
**0**	**1**	**0.63**	**0.98**	**1.5**	**YMNIFEK**

5.450 54 638 minus strand ubiquitin carboxyl-terminal hydrolase [Aplysia californica] gi

Protein Accession	CL20666.Contig3$_H1$
Mean Expression Ratio	1.04
Median Expression Ratio	1.04
Credible Interval	(0.708, 1.54)
Associated Peptides	1
Associated Spectra	1
Coverage	NaN

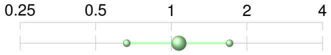

A	B	2.5	50	97.5	Sequence
**1**	**0**	**0.67**	**1.1**	**1.7**	**THADTLLEDTVK**

5.451 40 1752 minus strand heat shock protein 60 [Biomphalaria glabrata]; 40 1752 minus strand heat shock protein 60 [Biomphalaria glabrata]

Protein Accession	CL5583.Contig6$_H1$ CL5583.Contig5$_H1$
Mean Expression Ratio	1.04
Median Expression Ratio	1.04
Credible Interval	(0.793, 1.37)
Associated Peptides	5
Associated Spectra	8
Coverage	NaN

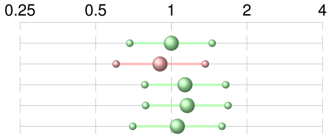

A	B	2.5	50	97.5	Sequence
**2**	**0**	**0.68**	**1**	**1.5**	**AVGDLISSAMK**
**1**	**0**	**0.6**	**0.9**	**1.4**	**TLRDELEVIEGMK**
**1**	**1**	**0.78**	**1.1**	**1.6**	**VGLQVVAVK**
**2**	**0**	**0.79**	**1.2**	**1.7**	**LPNLLR**
**0**	**1**	**0.7**	**1.1**	**1.6**	**DDTLLMR**

5.452 210 779 GH20056 [Drosophila grimshawi] gi; 216 785 minus strand GH20056 [Drosophila grimshawi] gi; 210 779 GH20056 [Drosophila grimshawi] gi; 216 785 GH20056 [Drosophila grimshawi] gi

Protein Accession	CL34497.Contig4$_H1$ CL34497.Contig3$_H1$ CL34497.Contig2$_H1$ CL34497.Contig1$_H1$
Mean Expression Ratio	0.96
Median Expression Ratio	0.962
Credible Interval	(0.707, 1.29)
Associated Peptides	3
Associated Spectra	9
Coverage	NaN

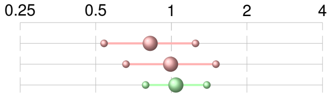

A	B	2.5	50	97.5	Sequence
**1**	**0**	**0.54**	**0.83**	**1.2**	**KQPFEFQIGVGQVIK**
**1**	**0**	**0.66**	**1**	**1.5**	**QLSQEEVSNFLK**
**5**	**2**	**0.79**	**1**	**1.4**	**GLLDMCAGEK**

5.453 1155 1649 78 kDa glucose-regulated protein OS=Aplysia californica PE=1 SV=1

Protein Accession	CL41594.Contig1$_{H}1$
Mean Expression Ratio	0.96
Median Expression Ratio	0.962
Credible Interval	(0.646, 1.41)
Associated Peptides	1
Associated Spectra	1
Coverage	NaN

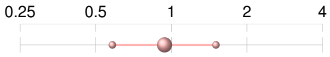

A	B	2.5	50	97.5	Sequence
**1**	**0**	**0.58**	**0.94**	**1.5**	**VEVESLFDGEDFSEQLTR**

5.454 241 561 minus strand thioredoxin [Crassostrea ariakensis]

Protein Accession	CL33159.Contig3$_{H}1$
Mean Expression Ratio	0.963
Median Expression Ratio	0.963
Credible Interval	(0.718, 1.29)
Associated Peptides	3
Associated Spectra	7
Coverage	NaN

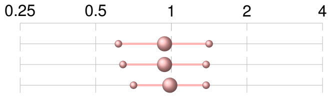

A	B	2.5	50	97.5	Sequence
**1**	**0**	**0.62**	**0.94**	**1.4**	**EVGEVVGASEEEIK**
**1**	**1**	**0.64**	**0.94**	**1.4**	**EVGEVVGASEEEIKK**
**2**	**2**	**0.71**	**0.99**	**1.4**	**IETQEDFEK**

5.455 96 536 minus strand hypothetical protein BRAFLDRAFT_285353 [Branchiostoma floridae] gi

Protein Accession	CL14282.Contig5$_{H}1$
Mean Expression Ratio	1.04
Median Expression Ratio	1.04
Credible Interval	(0.709, 1.53)
Associated Peptides	1
Associated Spectra	1
Coverage	NaN

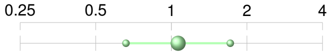

A	B	2.5	50	97.5	Sequence
**0**	**1**	**0.66**	**1.1**	**1.7**	**SQWSPALTISK**

5.456 192 380 minus strand RAB protein [Haliotis discus discus]

Protein Accession	Unigene35065$_{H}1$
Mean Expression Ratio	0.964
Median Expression Ratio	0.963
Credible Interval	(0.652, 1.44)
Associated Peptides	1
Associated Spectra	1
Coverage	NaN

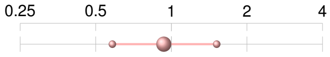

A	B	2.5	50	97.5	Sequence
**1**	**0**	**0.58**	**0.94**	**1.5**	**NVEEMFLAITK**

5.457 1 219 minus strand PREDICTED: nucleoside diphosphate kinase 3-like [Pan troglodytes]

Protein Accession	Unigene5384$_H$1
Mean Expression Ratio	0.963
Median Expression Ratio	0.963
Credible Interval	(0.721, 1.28)
Associated Peptides	2
Associated Spectra	11
Coverage	NaN

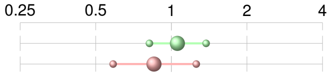

A	B	2.5	50	97.5	Sequence
6	3	0.82	1.1	1.4	GLVGEIIK
0	2	0.59	0.85	1.3	GLVGEIIKR

5.458 77 2278 Far upstream element-binding protein 3 [Crassostrea gigas]

Protein Accession	CL5030.Contig3$_H$1
Mean Expression Ratio	0.962
Median Expression Ratio	0.963
Credible Interval	(0.667, 1.39)
Associated Peptides	2
Associated Spectra	2
Coverage	NaN

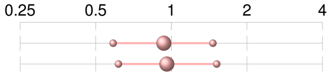

A	B	2.5	50	97.5	Sequence
1	0	0.59	0.94	1.5	EMVLELLAEK
1	0	0.62	0.96	1.5	LFNIR

5.459 299 7507 minus strand filamin [Clonorchis sinensis]

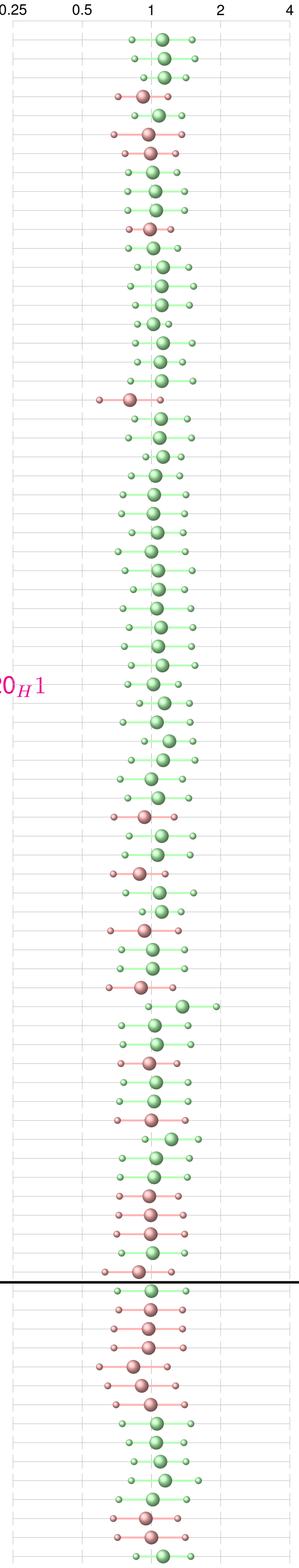

A	B	2.5	50	97.5	Sequence
3	0	0.82	1.1	1.5	AYGPGLEPK
2	1	0.85	1.1	1.5	EAGLEEDEEMTTTER
11	2	0.93	1.1	1.4	EETKPGSLGVTVEGPK
1	6	0.72	0.92	1.2	ELADDAQWK
3	6	0.85	1.1	1.4	EPVEVVFNNDR
1	0	0.69	0.97	1.4	EVNKSPFEVTVEGAAGDASK
4	3	0.77	0.99	1.3	FLEEDEGIR
5	3	0.8	1	1.3	FTVETFSAGK
4	0	0.79	1	1.4	GAGEGEVAVQIIGPGGSEEK
4	0	0.79	1	1.4	GAGNGSVDVVILDPHGR
8	5	0.8	0.99	1.2	GTLEVTLLNPK
3	4	0.79	1	1.3	IEASIDVSGIK
6	0	0.87	1.1	1.5	INVTHADKPIPGSPFK
1	1	0.81	1.1	1.5	IVNIDSTDIVDK
4	1	0.85	1.1	1.5	KDTVRPSIQPVVGK
12	18	0.87	1	1.2	LENVTMVLK
2	2	0.85	1.1	1.5	NFTTDWNDGR
4	6	0.87	1.1	1.4	QIADLESDFSDGLR
2	0	0.81	1.1	1.5	SPFEVTVEGAAGDASK
1	2	0.6	0.81	1.1	TQKLENVTMVLK
4	2	0.85	1.1	1.4	TTNVPVQDLVDKK
1	1	0.79	1.1	1.5	VDAVPDTGIPK
14	6	0.94	1.1	1.3	VEVGPFWK
7	1	0.82	1	1.3	VPDLPIK
2	0	0.75	1	1.4	VYVSEPTNPAK
1	1	0.74	1	1.4	TWFEVYTK
4	2	0.82	1.1	1.4	ACFATGR
1	0	0.72	1	1.4	IEASIDVSGIKVEGLEPK
1	0	0.77	1.1	1.5	YMPTKNGK
5	1	0.83	1.1	1.4	MAITSPSGK
1	0	0.75	1.1	1.5	VEGLEPK
2	0	0.8	1.1	1.5	AFGPGLTGGEALKPAFFTIDAR
1	0	0.76	1.1	1.5	GLEEDEEMTTTER
1	1	0.82	1.1	1.6	KTWFEVYTK
4	3	0.79	1	1.3	QVEVQVK
5	2	0.89	1.1	1.5	VDDLSMMTYLSQFPNAK
1	0	0.75	1.1	1.5	VKDSADFYVHTK
3	5	0.93	1.2	1.5	WANEHLK
2	0	0.82	1.1	1.6	WLDVPQLIKPDEMTNPK
0	2	0.73	1	1.4	AAADVTHVGSECELSLK
0	3	0.79	1.1	1.5	AEIDFQDR
0	3	0.69	0.93	1.3	CYGPGLDSK
0	2	0.8	1.1	1.5	EEPCLLK
0	2	0.77	1.1	1.5	ETFITVDAAEAGNGK
0	6	0.68	0.89	1.2	FNDEHIPDSPYR
0	1	0.77	1.1	1.5	GEVQQPCDFNIYTR
0	15	0.91	1.1	1.3	GGLGLGIEGPVEAK
0	1	0.66	0.93	1.3	IMVGESELGNASK
0	2	0.74	1	1.4	ISISALQDK
0	2	0.73	1	1.4	LANGHLGITFTPR
0	2	0.65	0.9	1.2	PAAFVVNFNGAK
0	1	0.97	1.4	1.9	RLANGHLGITFTPR
0	1	0.74	1	1.4	SPTGSDIDIDIIDR
0	1	0.75	1.1	1.5	SPVDELTVK
0	4	0.73	0.98	1.3	VKPAALDVSGVK
0	2	0.76	1	1.4	VQNPVDASK
0	1	0.73	1	1.4	VQVSPSIGDAR
0	1	0.71	1	1.4	VSEELITR
0	5	0.94	1.2	1.6	VTETDITDLTASIR
0	1	0.75	1	1.5	VTGPGVQGTTPASLPAVFK
0	1	0.73	1	1.4	YAGVNIAGSPFK
0	3	0.73	0.98	1.3	HIANSPFK
0	2	0.72	0.99	1.4	KISISALQDK
0	1	0.71	0.99	1.4	APGDYLISIR
0	2	0.74	1	1.4	EMGIHTVSVK
0	1	0.63	0.88	1.2	FADQHVPGSPFTVK
0	1	0.71	1	1.4	IMVGK
0	2	0.72	0.99	1.4	VKPAALDVSG
0	1	0.69	0.97	1.4	IPGTSPFDMTASVK
0	1	0.69	0.97	1.4	SVTLNGEGTVQAILTTPTGR
0	1	0.6	0.83	1.2	FADQHVPGSPF
0	1	0.65	0.91	1.3	VTYRPTEPGTYIINIK
0	1	0.7	0.99	1.4	QIADLESDFSDGL
0	1	0.75	1.1	1.5	EVPNSPFQMR
0	5	0.8	1	1.4	DVPGAPFPVK
0	6	0.84	1.1	1.4	ETGEHLVNVFR
0	1	0.82	1.1	1.6	GLQVGKPAAFVVNFNGAK
0	1	0.72	1	1.4	INVTHADKPIPGS
0	2	0.68	0.94	1.3	NIMGSPFTAK
0	1	0.71	1	1.4	PGVDASK
0	5	0.86	1.1	1.5	VLPGVDASK

Protein Accession	CL4145.Contig20 $_{H}1$
Mean Expression Ratio	1.04
Median Expression Ratio	1.04
Credible Interval	(0.965, 1.12)
Associated Peptides	81
Associated Spectra	324
Coverage	NaN

5.460 58 198 minus strand Protein DJ-1 [Crassostrea gigas]

Protein Accession	CL16588.Contig4$_{H}1$
Mean Expression Ratio	0.964
Median Expression Ratio	0.964
Credible Interval	(0.694, 1.33)
Associated Peptides	1
Associated Spectra	6
Coverage	NaN

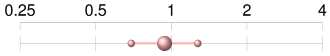

A	B	2.5	50	97.5	Sequence
**3**	**3**	**0.69**	**0.94**	**1.3**	**ADSLVAPMLVK**

5.461 " 1240 1911 PREDICTED: ATP synthase subunit alpha, mitochondrial-like [Anolis carolinensis]"

Protein Accession	Unigene30533$_{H}1$
Mean Expression Ratio	0.963
Median Expression Ratio	0.964
Credible Interval	(0.704, 1.33)
Associated Peptides	2
Associated Spectra	5
Coverage	NaN

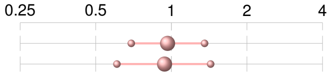

A	B	2.5	50	97.5	Sequence
**1**	**3**	**0.69**	**0.97**	**1.4**	**LELAQYR**
**0**	**1**	**0.61**	**0.94**	**1.4**	**LTELLK**

5.462 3 218 beta-tubulin [Fasciola hepatica] gi

Protein Accession	Unigene7955$_{H}1$
Mean Expression Ratio	0.963
Median Expression Ratio	0.965
Credible Interval	(0.732, 1.26)
Associated Peptides	3
Associated Spectra	12
Coverage	NaN

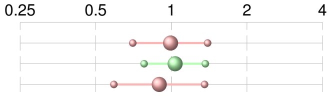

A	B	2.5	50	97.5	Sequence
**3**	**1**	**0.7**	**0.99**	**1.4**	**EVDEQMLNVQNK**
**7**	**0**	**0.78**	**1**	**1.4**	**TAVCDIPPR**
**1**	**0**	**0.59**	**0.89**	**1.4**	**VSEQFTAMFR**

5.463 160 501 hypothetical protein CGI_10001640 [Crassostrea gigas]; 160 588 hypothetical protein CGI_10001640 [Crassostrea gigas]; 160 501 hypothetical protein CGI_10001640 [Crassostrea gigas]; 160 552 hypothetical protein CGI_10001640 [Crassostrea gigas]; 160 501 hypothetical protein CGI_10001640 [Crassostrea gigas]; 160 501 hypothetical protein CGI_10001640 [Crassostrea gigas]

Protein Accession	Unigene62839$_{H}1$ CL2347.Contig8$_{H}1$ CL2347.Contig15$_{H}1$ CL2347.Contig14$_{H}1$ CL2347.Co
Mean Expression Ratio	0.967
Median Expression Ratio	0.965
Credible Interval	(0.704, 1.34)
Associated Peptides	2
Associated Spectra	13
Coverage	NaN

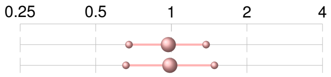

A	B	2.5	50	97.5	Sequence
6	3	0.68	0.97	1.4	AFLEQLGSK
3	1	0.66	0.99	1.5	ANLIVIGSR

5.464 977 1852 PREDICTED: similar to prohibitin [Tribolium castaneum] gi

Protein Accession	CL8219.Contig4$_{H}1$
Mean Expression Ratio	0.965
Median Expression Ratio	0.965
Credible Interval	(0.695, 1.33)
Associated Peptides	3
Associated Spectra	4
Coverage	NaN

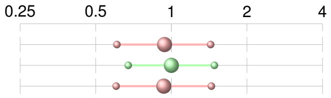

A	B	2.5	50	97.5	Sequence
1	0	0.61	0.94	1.4	VVQAEGEAAAAK
2	0	0.67	1	1.5	KLGDLAGR
1	0	0.6	0.93	1.4	QQVSLLIR

5.465 128 532 minus strand mitochondrial ATPase inhibitor [Ornithodoros parkeri]

Protein Accession	Unigene1376$_{H}1$
Mean Expression Ratio	1.04
Median Expression Ratio	1.04
Credible Interval	(0.707, 1.54)
Associated Peptides	1
Associated Spectra	1
Coverage	NaN

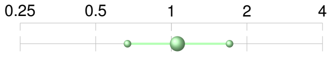

A	B	2.5	50	97.5	Sequence
1	0	0.67	1.1	1.7	MEAAHEELYFK

5.466 155 3214 sarco/endoplasmic reticulum calcium ATPase isoform B [Pinctada fucata]; 132 3110 sarco/endoplasmic reticulum calcium ATPase isoform C [Pinctada fucata]; 132 3110 minus strand sarco/endoplasmic reticulum calcium ATPase isoform C [Pinctada fucata]; 155 3133 sarco/endoplasmic reticulum calcium ATPase isoform C [Pinctada fucata]; 155 3133 sarco/endoplasmic reticulum calcium ATPase isoform C [Pinctada fucata]; 155 3214 minus strand sarco/endoplasmic reticulum calcium ATPase isoform B [Pinctada fucata]

Protein Accession	CL1323.Contig9$_H$1 CL1323.Contig8$_H$1 CL1323.Contig6$_H$1 CL1323.Contig21$_H$1 CL1323.Co
Mean Expression Ratio	1.04
Median Expression Ratio	1.04
Credible Interval	(0.897, 1.19)
Associated Peptides	25
Associated Spectra	74
Coverage	NaN

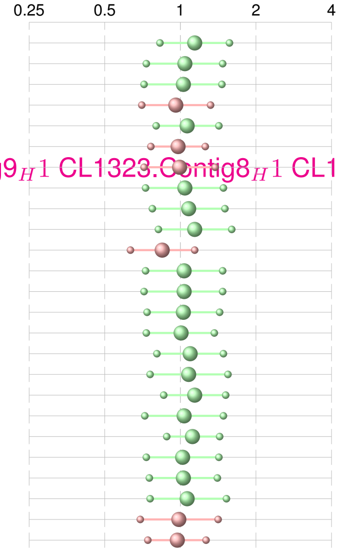

A	B	2.5	50	97.5	Sequence
1	2	0.83	1.1	1.6	ATAEAICR
1	0	0.73	1	1.5	DLVPGDIVEVSVGDK
1	0	0.71	1	1.5	DLVPGDIVEVSVGDKVPADIR
2	1	0.7	0.96	1.3	GAPEGLLDR
3	2	0.8	1.1	1.4	GIVVGTGLATEIGK
7	1	0.76	0.98	1.3	IGHNKVPMSPAIK
2	0	0.72	1	1.4	IGIFTENESTEGMSFTGR
1	0	0.73	1	1.5	IRNEMMDTEQEK
1	1	0.77	1.1	1.5	KEFTLEFSR
1	1	0.82	1.1	1.6	NAESAIEALK
2	3	0.63	0.85	1.1	NMLFSGTNIAAGK
1	0	0.73	1	1.5	SMSVYCVPNKPTR
1	0	0.72	1	1.5	TGTLTTNQMSVCK
2	0	0.74	1	1.4	VDQAILTGESVSVMK
3	0	0.73	1	1.4	VGEATETALTVLVEK
2	2	0.81	1.1	1.5	VIVITGDNK
1	0	0.76	1.1	1.6	VPMSPAIK
3	2	0.86	1.1	1.5	EFDELPPEDQR
1	0	0.72	1	1.5	NAESAIEALKEYEPEVAK
7	2	0.89	1.1	1.4	KAEIGIAMGSGTAVAK
1	1	0.73	1	1.4	LNFYNTDK
2	1	0.75	1	1.4	MDQAFTK
1	0	0.76	1.1	1.5	KNFEK
1	0	0.69	0.99	1.4	KNMLFSGTNIAAGK
3	4	0.74	0.97	1.3	CLALATIDNPPR

5.467 " 4146 6107 minus strand nonmuscle myosin II, partial [Aplysia californica]"

Protein Accession	Unigene30363$_{H}1$
Mean Expression Ratio	1.04
Median Expression Ratio	1.04
Credible Interval	(0.88, 1.23)
Associated Peptides	20
Associated Spectra	32
Coverage	NaN

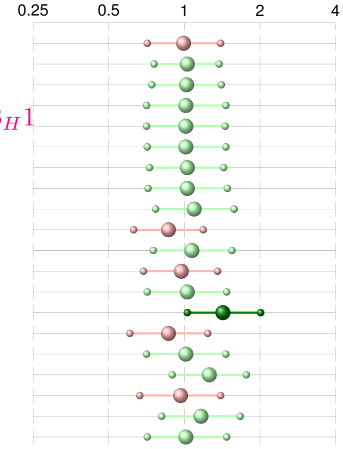

A	B	2.5	50	97.5	Sequence
2	0	0.71	1	1.4	ALEATVQEQR
2	2	0.76	1	1.4	ATIAALEGK
2	1	0.74	1	1.4	DLEQVSTQLEQVETK
1	0	0.71	1	1.5	ELDDQLEQNDVLVR
1	0	0.71	1	1.5	ELQIQAEDER
1	0	0.71	1	1.5	LAELEGQSR
1	1	0.73	1	1.4	LQEDLAAAER
1	0	0.72	1	1.5	QVDEAEEEVAR
1	0	0.77	1.1	1.6	VSSLEAQLADSQELMEEETR
2	1	0.63	0.87	1.2	VQIEELEDELQAAEDAK
1	0	0.75	1.1	1.5	EMEAELEEER
1	1	0.69	0.97	1.4	NLEAELVK
1	0	0.71	1	1.5	QSLLDEK
2	0	1	1.4	2	LKEDAIK
1	0	0.61	0.87	1.2	LSREEAYAQAK
1	0	0.71	1	1.5	LQAEVEDLTITLDNQR
1	1	0.9	1.3	1.8	AALARTNR
0	1	0.66	0.97	1.4	LKELQIQAEDERR
0	1	0.81	1.2	1.7	EMEQLHAQLEEARANADR
0	1	0.71	1	1.5	IEQLER

5.468 59 379 hypothetical protein DAPPUDRAFT_230756 [Daphnia pulex]; 117 443 minus strand hypothetical protein DAPPUDRAFT_230756 [Daphnia pulex]

Protein Accession	CL2137.Contig6$_{H}1$ CL2137.Contig5$_{H}1$
Mean Expression Ratio	1.04
Median Expression Ratio	1.04
Credible Interval	(0.705, 1.54)
Associated Peptides	1
Associated Spectra	1
Coverage	NaN

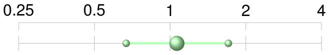

A	B	2.5	50	97.5	Sequence
1	0	0.67	1.1	1.7	YAQPRLK

5.469 94 519 minus strand hypothetical protein BRAFLDRAFT_115029 [Branchiostoma floridae] gi

Protein Accession	Unigene52953$_{H}1$
Mean Expression Ratio	0.966
Median Expression Ratio	0.966
Credible Interval	(0.713, 1.31)
Associated Peptides	2
Associated Spectra	7
Coverage	NaN

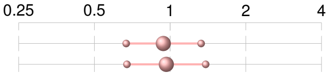

A	B	2.5	50	97.5	Sequence
2	2	0.67	0.94	1.3	SLDTDGDGTVSAAELR
2	1	0.68	0.97	1.4	FINKK

5.470 120 635 PREDICTED: similar to collagen type VI alpha 6 [Ciona intestinalis]; 120 635 minus strand PREDICTED: similar to collagen type VI alpha 6 [Ciona intestinalis]

Protein Accession	Unigene46785$_{H1}$ Unigene26613$_{H1}$
Mean Expression Ratio	0.967
Median Expression Ratio	0.967
Credible Interval	(0.671, 1.39)
Associated Peptides	2
Associated Spectra	2
Coverage	NaN

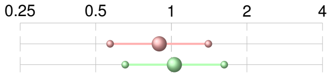

A	B	2.5	50	97.5	Sequence
**1**	**0**	**0.57**	**0.89**	**1.4**	**HGNTNTGDGIMYMLER**
**1**	**0**	**0.65**	**1**	**1.6**	**VSLVTYSDQANPESAIDFDSYSSK**

5.471 148 609 minus strand Titin [Crassostrea gigas]

Protein Accession	CL5825.Contig1$_{H1}$
Mean Expression Ratio	1.03
Median Expression Ratio	1.03
Credible Interval	(0.759, 1.41)
Associated Peptides	2
Associated Spectra	6
Coverage	NaN

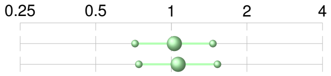

A	B	2.5	50	97.5	Sequence
**3**	**0**	**0.72**	**1**	**1.5**	**FTQKPALR**
**3**	**0**	**0.74**	**1.1**	**1.5**	**QQEGVAPNFIQKPVTR**

5.472 3 470 minus strand Spectrin beta chain [Crassostrea gigas]

Protein Accession	CL19757.Contig2$_{H1}$
Mean Expression Ratio	0.967
Median Expression Ratio	0.967
Credible Interval	(0.666, 1.4)
Associated Peptides	1
Associated Spectra	2
Coverage	NaN

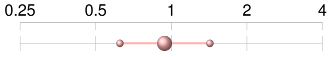

A	B	2.5	50	97.5	Sequence
**2**	**0**	**0.63**	**0.94**	**1.4**	**HSLLEADIHVVGER**

5.473 880 1434 minus strand Prohormone-4 [Crassostrea gigas]

Protein Accession	CL27941.Contig2$_{H1}$
Mean Expression Ratio	0.966
Median Expression Ratio	0.967
Credible Interval	(0.654, 1.43)
Associated Peptides	1
Associated Spectra	1
Coverage	NaN

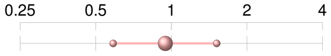

A	B	2.5	50	97.5	Sequence
**1**	**0**	**0.58**	**0.94**	**1.5**	**AALQGAQAGDERPLLR**

5.474 296 493 hypothetical protein BRAFLDRAFT_66287 [Branchiostoma floridae] gi

Protein Accession	Unigene414$_{H}1$
Mean Expression Ratio	1.04
Median Expression Ratio	1.03
Credible Interval	(0.719, 1.5)
Associated Peptides	1
Associated Spectra	2
Coverage	NaN

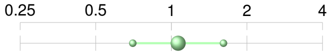

A	B	2.5	50	97.5	Sequence
**1**	**1**	**0.7**	**1.1**	**1.6**	**SLDDFLQAWK**

5.475 122 1195 minus strand Severin [Crassostrea gigas]

Protein Accession	CL7486.Contig12$_{H}1$
Mean Expression Ratio	1.03
Median Expression Ratio	1.03
Credible Interval	(0.702, 1.52)
Associated Peptides	1
Associated Spectra	2
Coverage	NaN

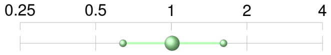

A	B	2.5	50	97.5	Sequence
**1**	**1**	**0.64**	**1**	**1.6**	**TDHPLVPITCLTEGTK**

5.476 " 757 1443 minus strand nonmuscle myosin II, partial [Aplysia californica]"

Protein Accession	CL43455.Contig2$_{H}1$
Mean Expression Ratio	1.03
Median Expression Ratio	1.03
Credible Interval	(0.695, 1.52)
Associated Peptides	1
Associated Spectra	1
Coverage	NaN

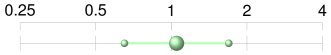

A	B	2.5	50	97.5	Sequence
**1**	**0**	**0.65**	**1.1**	**1.7**	**LVNEIK**

5.477 36 1310 minus strand elongation factor-1 gamma [Diguetia canities]

Protein Accession	CL14269.Contig5$_{H}1$
Mean Expression Ratio	1.03
Median Expression Ratio	1.03
Credible Interval	(0.756, 1.41)
Associated Peptides	3
Associated Spectra	5
Coverage	NaN

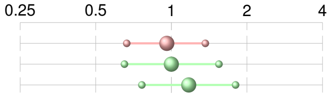

A	B	2.5	50	97.5	Sequence
**2**	**1**	**0.66**	**0.96**	**1.4**	**LAETMAQFDAK**
**1**	**0**	**0.65**	**1**	**1.5**	**SADFLAK**
**0**	**1**	**0.76**	**1.2**	**1.8**	**LDPDDPETK**

5.478 " 285 437 Propionyl-CoA carboxylase alpha chain, mitochondrial [Crassostrea gigas]"

Protein Accession	CL45581.Contig2$_{H}1$
Mean Expression Ratio	0.97
Median Expression Ratio	0.969
Credible Interval	(0.674, 1.4)
Associated Peptides	1
Associated Spectra	2
Coverage	NaN

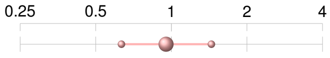

A	B	2.5	50	97.5	Sequence
2	0	0.63	0.95	1.4	GVTHNIPLLR

5.479 135 2630 Filamin-A [Crassostrea gigas]; 118 2613 Filamin-A [Crassostrea gigas]; 157 2652 Filamin-A [Crassostrea gigas]

Protein Accession	CL393.Contig15$_{H}1$ CL393.Contig13$_{H}1$ CL393.Contig12$_{H}1$
Mean Expression Ratio	0.97
Median Expression Ratio	0.969
Credible Interval	(0.839, 1.12)
Associated Peptides	23
Associated Spectra	141
Coverage	NaN

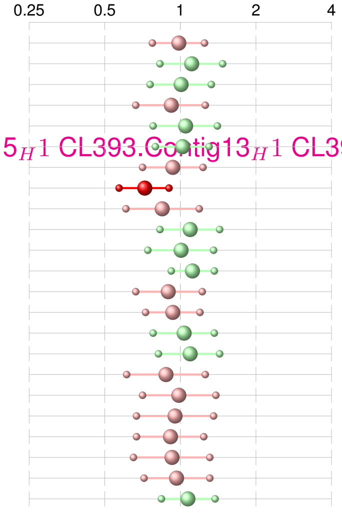

A	B	2.5	50	97.5	Sequence
9	3	0.77	0.98	1.2	AVANKPNEIVIDTSK
5	1	0.83	1.1	1.5	DNNDGTIVVEYQPK
5	1	0.76	1	1.3	EAGPGTLTVAVEGPGK
2	1	0.67	0.92	1.3	ENGVHYVDVK
4	1	0.78	1	1.4	HINNSPFK
5	4	0.79	1	1.3	IGDSELGHAAGCK
3	3	0.71	0.93	1.2	MGDGEIGIASFNPR
6	6	0.57	0.72	0.9	MNGIHTLSVR
1	1	0.61	0.85	1.2	PGSYGLHIK
4	2	0.83	1.1	1.4	QMEAAEAAVPNK
3	1	0.74	1	1.4	TPSGGHEDCFIQEMDK
12	9	0.92	1.1	1.4	VINFQIETK
3	1	0.66	0.9	1.2	VPKPGSYGLHIK
5	4	0.73	0.93	1.2	VQVGGPGLEK
4	2	0.78	1	1.4	VTGALDK
4	2	0.82	1.1	1.4	YGNIPIAGSPYK
0	1	0.61	0.88	1.3	GDSELGHAAGCK
1	1	0.71	0.99	1.4	AALSCK
0	1	0.67	0.95	1.4	QVTVHSLR
3	1	0.67	0.91	1.2	YKGNPIK
0	1	0.65	0.93	1.3	PKPGSYGLHIK
3	1	0.71	0.97	1.3	IMFTPK
8	3	0.84	1.1	1.4	KPSPIQEQSSMVVETVEK

5.480 " 343 1179 ATP synthase subunit alpha, mitochondrial OS=Pongo abelii GN=ATP5A1 PE=2 SV=1"

Protein Accession	Unigene30534$_{H}1$
Mean Expression Ratio	0.97
Median Expression Ratio	0.97
Credible Interval	(0.803, 1.17)
Associated Peptides	7
Associated Spectra	33
Coverage	NaN

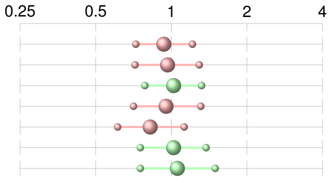

A	B	2.5	50	97.5	Sequence
3	4	0.72	0.94	1.2	TAVAIDTIINQK
4	0	0.72	0.97	1.3	TSVKEPMQTGIK
5	3	0.79	1	1.3	VLSIGDGIAR
1	3	0.71	0.95	1.3	VVDALGTPIDGK
2	2	0.61	0.83	1.1	APGIIPR
3	1	0.76	1	1.4	STVAQLVK
0	2	0.75	1.1	1.5	LSDADAMK

5.481 2 1795 minus strand Plectin-1 [Crassostrea gigas]

Protein Accession	CL32322.Contig2$_{H}$1
Mean Expression Ratio	1.03
Median Expression Ratio	1.03
Credible Interval	(0.716, 1.48)
Associated Peptides	2
Associated Spectra	2
Coverage	NaN

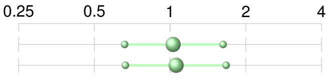

A	B	2.5	50	97.5	Sequence
1	0	0.66	1	1.6	TKDGDVIPIHDAIMTK
1	0	0.67	1.1	1.7	YITTAHFVR

5.482 965 1687 Arylsulfatase B [Crassostrea gigas]

Protein Accession	Unigene30559$_{H}$1
Mean Expression Ratio	1.03
Median Expression Ratio	1.03
Credible Interval	(0.696, 1.53)
Associated Peptides	1
Associated Spectra	1
Coverage	NaN

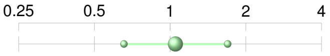

A	B	2.5	50	97.5	Sequence
0	1	0.66	1.1	1.7	WGLYDIEADPEER

5.483 45 944 minus strand unnamed protein product [Mus musculus] gi; 5 334 minus strand receptor of activated protein kinase C [Platynereis dumerilii]

Protein Accession	CL20494.Contig3$_{H}$1 CL20494.Contig1$_{H}$1
Mean Expression Ratio	1.03
Median Expression Ratio	1.03
Credible Interval	(0.718, 1.47)
Associated Peptides	2
Associated Spectra	2
Coverage	NaN

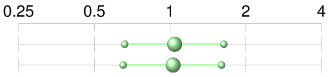

A	B	2.5	50	97.5	Sequence
1	0	0.66	1	1.6	IWDLEGK
1	0	0.65	1	1.6	AMCFSPNR

5.484 587 1081 hypothetical protein CGI_10026502 [Crassostrea gigas]

Protein Accession	Unigene22889$_{H}$1
Mean Expression Ratio	0.972
Median Expression Ratio	0.97
Credible Interval	(0.652, 1.44)
Associated Peptides	1
Associated Spectra	1
Coverage	NaN

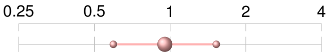

A	B	2.5	50	97.5	Sequence
0	1	0.59	0.95	1.5	LNGDAGHAIVK

5.485 5 1018 minus strand Glutaredoxin-3 [Crassostrea gigas]; 5 1018 Glutaredoxin-3 [Crassostrea gigas]

Protein Accession	CL7734.Contig9$_{H}1$ CL7734.Contig10$_{H}1$
Mean Expression Ratio	0.973
Median Expression Ratio	0.97
Credible Interval	(0.675, 1.41)
Associated Peptides	1
Associated Spectra	2
Coverage	NaN

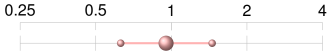

A	B	2.5	50	97.5	Sequence
**1**	**1**	**0.63**	**0.95**	**1.5**	**VAAEDLAEVSHR**

5.486 143 247 minus strand dynein light chain [Aplysia californica] gi

Protein Accession	CL13886.Contig1$_{H}1$
Mean Expression Ratio	0.97
Median Expression Ratio	0.971
Credible Interval	(0.672, 1.39)
Associated Peptides	1
Associated Spectra	2
Coverage	NaN

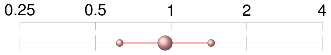

A	B	2.5	50	97.5	Sequence
**2**	**0**	**0.62**	**0.95**	**1.4**	**NADMAEDMQQDAVDCATQALEK**

5.487 160 393 minus strand PREDICTED: cysteine-rich protein 1-like [Megachile rotundata]; 148 381 PREDICTED: cysteine-rich protein 1-like [Megachile rotundata]; 148 381 PREDICTED: cysteine-rich protein 1-like [Megachile rotundata]; 148 381 PREDICTED: cysteine-rich protein 1-like [Megachile rotundata]; 160 393 minus strand PREDICTED: cysteine-rich protein 1-like [Megachile rotundata]; 160 393 minus strand PREDICTED: cysteine-rich protein 1-like [Megachile rotundata]

Protein Accession	CL11328.Contig9$_{H}1$ CL11328.Contig8$_{H}1$ CL11328.Contig7$_{H}1$ CL11328.Contig6$_{H}1$ CL1132
Mean Expression Ratio	1.03
Median Expression Ratio	1.03
Credible Interval	(0.737, 1.43)
Associated Peptides	2
Associated Spectra	4
Coverage	NaN

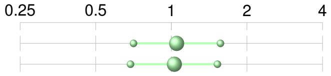

A	B	2.5	50	97.5	Sequence
**1**	**1**	**0.7**	**1.1**	**1.6**	**DWHAACLK**
**1**	**1**	**0.69**	**1**	**1.5**	**EVYFAER**

5.488 5 841 minus strand Spectrin alpha chain [Crassostrea gigas]

Protein Accession	CL44897.Contig1$_H$1
Mean Expression Ratio	0.972
Median Expression Ratio	0.971
Credible Interval	(0.692, 1.37)
Associated Peptides	2
Associated Spectra	3
Coverage	NaN

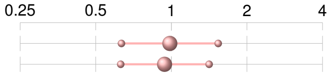

A	B	2.5	50	97.5	Sequence
**1**	**0**	**0.63**	**0.98**	**1.5**	**DMEDEEDWIR**
**1**	**1**	**0.63**	**0.94**	**1.4**	**HQNFEAEIEANR**

5.489 " 154 591 microsomal GST1, partial [Reishia clavigera]; 154 531 microsomal GST1, partial [Reishia clavigera]"

Protein Accession	CL3934.Contig2$_H$1 CL3934.Contig1$_H$1
Mean Expression Ratio	1.03
Median Expression Ratio	1.03
Credible Interval	(0.702, 1.53)
Associated Peptides	1
Associated Spectra	1
Coverage	NaN

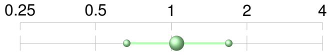

A	B	2.5	50	97.5	Sequence
**1**	**0**	**0.66**	**1**	**1.7**	**FFCPGTAVNTNDALVER**

5.490 35 271 PREDICTED: SH3 domain-binding glutamic acid-rich-like protein 3-like [Anolis carolinensis]; 35 271 PREDICTED: SH3 domain-binding glutamic acid-rich-like protein 3-like [Anolis carolinensis]; 245 523 PREDICTED: SH3 domain-binding glutamic acid-rich-like protein 3-like [Anolis carolinensis]; 192 470 PREDICTED: SH3 domain-binding glutamic acid-rich-like protein 3-like [Anolis carolinensis]; 192 470 PREDICTED: SH3 domain-binding glutamic acid-rich-like protein 3-like [Anolis carolinensis]; 192 470 PREDICTED: SH3 domain-binding glutamic acid-rich-like protein 3-like [Anolis carolinensis]; 111 389 PREDICTED: SH3 domain-binding glutamic acid-rich-like protein 3-like [Anolis carolinensis]; 144 380 PREDICTED: SH3 domain-binding glutamic acid-rich-like protein 3-like [Anolis carolinensis]; 111 389 PREDICTED: SH3 domain-binding glutamic acid-rich-like protein 3-like [Anolis carolinensis]; 35 271 PREDICTED: SH3 domain-binding glutamic acid-rich-like protein 3-like [Anolis carolinensis]

Protein Accession	Unigene58804$_{H}1$ Unigene57834$_{H}1$ Unigene53929$_{H}1$ Unigene46648$_{H}1$ Unigene46647$_{H}1$ U
Mean Expression Ratio	1.03
Median Expression Ratio	1.03
Credible Interval	(0.734, 1.46)
Associated Peptides	2
Associated Spectra	3
Coverage	NaN

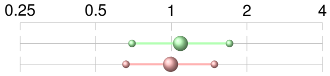

A	B	2.5	50	97.5	Sequence
1	0	0.7	1.1	1.7	SFQQAVEEEK
2	0	0.66	1	1.5	ILEYLR

5.491 155 3214 sarco/endoplasmic reticulum calcium ATPase isoform B [Pinctada fucata]; 155 3214 minus strand sarco/endoplasmic reticulum calcium ATPase isoform B [Pinctada fucata]

Protein Accession	CL1323.Contig9$_{H}1$ CL1323.Contig1$_{H}1$
Mean Expression Ratio	0.971
Median Expression Ratio	0.972
Credible Interval	(0.805, 1.17)
Associated Peptides	14
Associated Spectra	59
Coverage	NaN

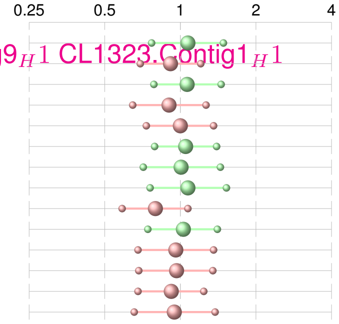

A	B	2.5	50	97.5	Sequence
1	2	0.77	1.1	1.5	ATAEAICR
3	4	0.69	0.91	1.2	CLALATIDNPPR
3	2	0.78	1.1	1.5	EFDELPPEDQR
2	1	0.65	0.9	1.3	GAPEGLLDR
3	2	0.73	1	1.4	GIVVGTGLATEIGK
7	2	0.79	1.1	1.4	KAEIGIAMGSGTAVAK
1	1	0.71	1	1.4	KEFTLEFSR
1	1	0.76	1.1	1.5	NAESAIEALK
2	3	0.59	0.8	1.1	NMLFSGTNIAAGK
2	2	0.74	1	1.4	VIVITGDNK
1	1	0.68	0.96	1.4	LNFYNTDK
2	1	0.68	0.96	1.3	MDQAFTK
7	1	0.68	0.92	1.2	IGHNKVPMSPAIK
0	1	0.65	0.95	1.4	KAEIGIAMGSGTA

5.492 49 1107 Eukaryotic translation initiation factor 3 subunit B [Crassostrea gigas]

Protein Accession	CL19479.Contig1$_H$1
Mean Expression Ratio	1.03
Median Expression Ratio	1.03
Credible Interval	(0.701, 1.52)
Associated Peptides	1
Associated Spectra	1
Coverage	NaN

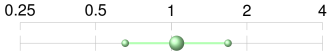

A	B	2.5	50	97.5	Sequence
**0**	**1**	**0.65**	**1**	**1.7**	**LMEEFSNLR**

5.493 1139 1378 Titin [Crassostrea gigas]; 1116 1355 Titin [Crassostrea gigas]; 1139 1378 Titin [Crassostrea gigas]; 1139 1378 Titin [Crassostrea gigas]; 1139 1378 Titin [Crassostrea gigas]; 1139 1378 Titin [Crassostrea gigas]; 1139 1378 Titin [Crassostrea gigas]; 1170 1409 Titin [Crassostrea gigas]

Protein Accession	CL181.Contig9$_H$1 CL181.Contig8$_H$1 CL181.Contig7$_H$1 CL181.Contig6$_H$1 CL181.Contig13$_H$
Mean Expression Ratio	0.973
Median Expression Ratio	0.972
Credible Interval	(0.704, 1.35)
Associated Peptides	2
Associated Spectra	4
Coverage	NaN

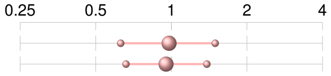

A	B	2.5	50	97.5	Sequence
**1**	**0**	**0.63**	**0.98**	**1.5**	**ITGYQIER**
**3**	**0**	**0.66**	**0.95**	**1.4**	**LSWKPPK**

5.494 249 845 sigma class glutathione-s-transferase 2 [Haliotis discus discus]; 252 848 sigma class glutathione-s-transferase 2 [Haliotis discus discus]

Protein Accession	CL19285.Contig32$_H$1 CL19285.Contig20$_H$1
Mean Expression Ratio	1.03
Median Expression Ratio	1.03
Credible Interval	(0.69, 1.53)
Associated Peptides	1
Associated Spectra	1
Coverage	NaN

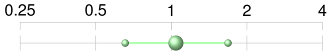

A	B	2.5	50	97.5	Sequence
**1**	**0**	**0.66**	**1**	**1.7**	**APFGQAPFMEYEGK**

5.495 102 1475 minus strand Cytosolic non-specific dipeptidase [Crassostrea gigas]

Protein Accession	CL4706.Contig1$_{H}1$
Mean Expression Ratio	0.971
Median Expression Ratio	0.972
Credible Interval	(0.657, 1.43)
Associated Peptides	1
Associated Spectra	1
Coverage	NaN

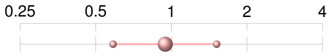

A	B	2.5	50	97.5	Sequence
**1**	**0**	**0.59**	**0.95**	**1.5**	**LVPDQEPHVIESLVR**

5.496 293 1795 Amidase [Crassostrea gigas]

Protein Accession	CL5248.Contig6$_{H}1$
Mean Expression Ratio	1.03
Median Expression Ratio	1.03
Credible Interval	(0.712, 1.48)
Associated Peptides	1
Associated Spectra	2
Coverage	NaN

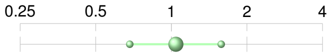

A	B	2.5	50	97.5	Sequence
**2**	**0**	**0.69**	**1**	**1.6**	**LSTIGVTHFDR**

5.497 206 1513 hypothetical protein CGI_10019003 [Crassostrea gigas]

Protein Accession	CL6385.Contig8$_{H}1$
Mean Expression Ratio	1.03
Median Expression Ratio	1.03
Credible Interval	(0.695, 1.52)
Associated Peptides	1
Associated Spectra	1
Coverage	NaN

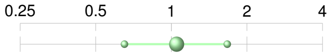

A	B	2.5	50	97.5	Sequence
**1**	**0**	**0.65**	**1.1**	**1.7**	**AALEDLLR**

5.498 " 220 627 Fatty acid-binding protein, brain [Crassostrea gigas]"

Protein Accession	CL9041.Contig2$_{H}1$
Mean Expression Ratio	0.973
Median Expression Ratio	0.973
Credible Interval	(0.753, 1.26)
Associated Peptides	5
Associated Spectra	13
Coverage	NaN

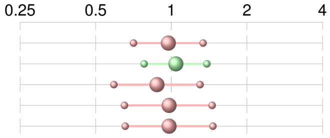

A	B	2.5	50	97.5	Sequence
**3**	**1**	**0.71**	**0.98**	**1.3**	**EVDGGVMTSTTTFDGK**
**3**	**3**	**0.78**	**1**	**1.4**	**LTITVEGGK**
**1**	**0**	**0.59**	**0.88**	**1.3**	**VDLASTTGLEEFGTAIGFSEEK**
**1**	**0**	**0.65**	**0.98**	**1.5**	**IVENYKLEDK**
**0**	**1**	**0.65**	**0.98**	**1.5**	**QNYSFK**

5.499 93 1034 Annexin A7 [Crassostrea gigas]; 73 1014 Annexin A7 [Crassostrea gigas]

Protein Accession	CL718.Contig3$_{H}1$ CL718.Contig1$_{H}1$
Mean Expression Ratio	1.03
Median Expression Ratio	1.03
Credible Interval	(0.8, 1.32)
Associated Peptides	6
Associated Spectra	12
Coverage	NaN

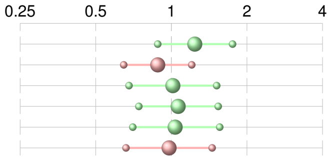

A	B	2.5	50	97.5	Sequence
2	1	0.88	1.2	1.8	ESDEWEAGDDAK
2	2	0.65	0.88	1.2	LEVAQAFK
1	0	0.68	1	1.5	KYEQLTGR
1	1	0.74	1.1	1.5	SEIDMAEIKK
0	1	0.7	1	1.6	TGTVFPADHFDAGK
0	1	0.66	0.98	1.5	PADHFDAGK

5.500 209 781 minus strand high mobility group protein 1 [Biomphalaria glabrata]; 277 771 high mobility group protein 1 [Biomphalaria glabrata]; 277 867 minus strand high mobility group protein 1 [Biomphalaria glabrata]; 277 870 minus strand high mobility group protein 1 [Biomphalaria glabrata]

Protein Accession	CL25076.Contig4$_{H}1$ CL25076.Contig3$_{H}1$ CL25076.Contig2$_{H}1$ CL25076.Contig1$_{H}1$
Mean Expression Ratio	1.03
Median Expression Ratio	1.03
Credible Interval	(0.715, 1.48)
Associated Peptides	2
Associated Spectra	2
Coverage	NaN

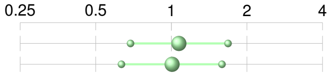

A	B	2.5	50	97.5	Sequence
1	0	0.69	1.1	1.7	LAGEEWR
1	0	0.63	1	1.6	KYEAEMAEYR

5.501 65 490 PREDICTED: cofilin-2 [Otolemur garnettii]

Protein Accession	CL15471.Contig3$_{H}1$
Mean Expression Ratio	1.03
Median Expression Ratio	1.03
Credible Interval	(0.786, 1.35)
Associated Peptides	4
Associated Spectra	11
Coverage	NaN

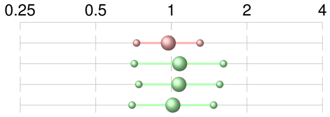

A	B	2.5	50	97.5	Sequence
4	2	0.73	0.97	1.3	IVMENEFAK
1	0	0.71	1.1	1.6	SQEDEYNSIIMTLPK
2	0	0.74	1.1	1.6	MLYSSSSSAVK
1	1	0.7	1	1.5	ALVFK

5.502 3 254 Calpain-5 [Crassostrea gigas]; 3 254 minus strand Calpain-5 [Crassostrea gigas]

Protein Accession	CL41869.Contig2$_H$1 CL41869.Contig1$_H$1
Mean Expression Ratio	1.03
Median Expression Ratio	1.03
Credible Interval	(0.711, 1.49)
Associated Peptides	1
Associated Spectra	2
Coverage	NaN

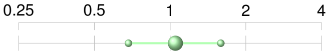

A	B	2.5	50	97.5	Sequence
1	1	0.69	1	1.6	EVDLFGHK

5.503 346 702 minus strand PREDICTED: galectin-4-like [Anolis carolinensis]

Protein Accession	Unigene42203$_H$1
Mean Expression Ratio	0.973
Median Expression Ratio	0.973
Credible Interval	(0.752, 1.26)
Associated Peptides	5
Associated Spectra	11
Coverage	NaN

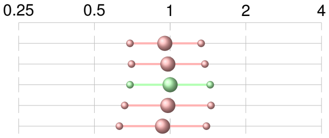

A	B	2.5	50	97.5	Sequence
3	1	0.69	0.96	1.3	AEATLEVR
2	1	0.7	0.98	1.4	SVPHGLVK
1	1	0.69	1	1.4	GKDFDIVIK
1	0	0.66	0.98	1.5	VKDSGFK
0	1	0.63	0.93	1.4	KSVPHGLVK

5.504 116 337 minus strand Golgi-associated plant pathogenesis-related protein 1 [Crassostrea gigas]; 538 759 minus strand Golgi-associated plant pathogenesis-related protein 1 [Crassostrea gigas]; 116 337 minus strand Golgi-associated plant pathogenesis-related protein 1 [Crassostrea gigas]; 261 482 minus strand Golgi-associated plant pathogenesis-related protein 1 [Crassostrea gigas]; 116 337 minus strand Golgi-associated plant pathogenesis-related protein 1 [Crassostrea gigas]; 116 337 minus strand Golgi-associated plant pathogenesis-related protein 1 [Crassostrea gigas]

Protein Accession	CL15263.Contig9$_H$1 CL15263.Contig8$_H$1 CL15263.Contig7$_H$1 CL15263.Contig5$_H$1 CL1526
Mean Expression Ratio	0.973
Median Expression Ratio	0.973
Credible Interval	(0.661, 1.43)
Associated Peptides	1
Associated Spectra	1
Coverage	NaN

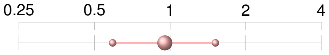

A	B	2.5	50	97.5	Sequence
1	0	0.59	0.95	1.5	KEHQVQPLVINK

5.505 410 709 calmodulin-like; K02183 calmodulin

Protein Accession	CL30882.Contig6$_{H}$1
Mean Expression Ratio	0.975
Median Expression Ratio	0.973
Credible Interval	(0.679, 1.4)
Associated Peptides	2
Associated Spectra	2
Coverage	NaN

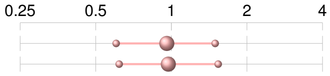

A	B	2.5	50	97.5	Sequence
**1**	**0**	**0.6**	**0.96**	**1.5**	**ELNDYVTEGGQSAAQAVLEK**
**0**	**1**	**0.62**	**0.97**	**1.5**	**EALRDFIEK**

5.506 333 770 USP-like protein isoform 2 [Crassostrea ariakensis]

Protein Accession	Unigene7710$_{H}$1
Mean Expression Ratio	0.972
Median Expression Ratio	0.974
Credible Interval	(0.658, 1.43)
Associated Peptides	1
Associated Spectra	1
Coverage	NaN

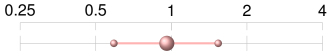

A	B	2.5	50	97.5	Sequence
**1**	**0**	**0.59**	**0.96**	**1.5**	**KIVIAMDGSK**

5.507 4 849 minus strand elongation factor 1-alpha [Cryptosporidium muris RN66]

Protein Accession	CL35888.Contig1$_{H}$1
Mean Expression Ratio	1.03
Median Expression Ratio	1.03
Credible Interval	(0.739, 1.42)
Associated Peptides	2
Associated Spectra	12
Coverage	NaN

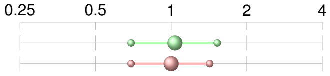

A	B	2.5	50	97.5	Sequence
**5**	**1**	**0.69**	**1**	**1.5**	**IGGIGTVPVGR**
**3**	**3**	**0.69**	**1**	**1.4**	**LPLQDVYK**

5.508 140 349 copper chaperone Atox1 [Crassostrea ariakensis]

Protein Accession	CL8999.Contig5$_{H}$1
Mean Expression Ratio	1.03
Median Expression Ratio	1.03
Credible Interval	(0.714, 1.48)
Associated Peptides	1
Associated Spectra	2
Coverage	NaN

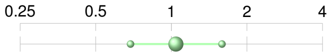

A	B	2.5	50	97.5	Sequence
**1**	**1**	**0.69**	**1**	**1.6**	**EELMATLQK**

5.509 138 713 Rac [Aplysia californica] gi; 125 691 Rac [Aplysia californica] gi; 149 871 minus strand Rac [Aplysia californica] gi; 101 676 Rac [Aplysia californica] gi

Protein Accession	CL11009.Contig9$_H1$ CL11009.Contig8$_H1$ CL11009.Contig4$_H1$ CL11009.Contig1$_H1$
Mean Expression Ratio	1.03
Median Expression Ratio	1.03
Credible Interval	(0.716, 1.47)
Associated Peptides	2
Associated Spectra	2
Coverage	NaN

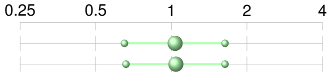

A	B	2.5	50	97.5	Sequence
0	1	0.65	1	1.6	NVFDEAIR
0	1	0.66	1	1.6	AVLCPR

5.510 3 230 minus strand RH21460p [Drosophila melanogaster]; 3 230 minus strand RH21460p [Drosophila melanogaster]

Protein Accession	Unigene57704$_H1$ CL18123.Contig3$_H1$
Mean Expression Ratio	0.976
Median Expression Ratio	0.975
Credible Interval	(0.664, 1.43)
Associated Peptides	1
Associated Spectra	1
Coverage	NaN

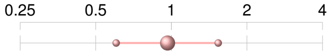

A	B	2.5	50	97.5	Sequence
0	1	0.6	0.96	1.5	EVDEDEAAFK

5.511 273 1304 Guanine nucleotide-binding protein subunit beta [Crassostrea gigas]

Protein Accession	CL15682.Contig1$_H1$
Mean Expression Ratio	1.03
Median Expression Ratio	1.03
Credible Interval	(0.829, 1.27)
Associated Peptides	6
Associated Spectra	23
Coverage	NaN

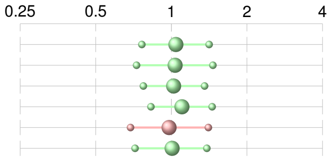

A	B	2.5	50	97.5	Sequence
3	1	0.77	1	1.4	IYAMHWASDSR
2	0	0.73	1	1.5	KAAGDTTLAQATAQCEPVGR
3	3	0.77	1	1.4	TFVSGACDASAK
3	3	0.83	1.1	1.4	LWDIR
0	2	0.69	0.98	1.4	LIVWDGYTTNK
0	3	0.72	1	1.4	LFDIR

5.512 126 704 minus strand ribosomal protein L12 [Ixodes scapularis]

Protein Accession	CL24053.Contig3$_H1$
Mean Expression Ratio	0.972
Median Expression Ratio	0.975
Credible Interval	(0.694, 1.36)
Associated Peptides	1
Associated Spectra	4
Coverage	NaN

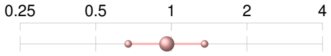

A	B	2.5	50	97.5	Sequence
2	2	0.67	0.96	1.4	IGPLGLSPK

5.513 130 2145 minus strand Glucosamine–fructose-6-phosphate aminotransferase [isomerizing] 1 [Crassostrea gigas]; 130 2121 minus strand Glucosamine–fructose-6-phosphate aminotransferase [isomerizing] 1 [Crassostrea gigas]

Protein Accession	CL2589.Contig3$_H$1 CL2589.Contig2$_H$1
Mean Expression Ratio	1.03
Median Expression Ratio	1.03
Credible Interval	(0.735, 1.43)
Associated Peptides	3
Associated Spectra	3
Coverage	NaN

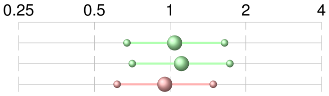

A	B	2.5	50	97.5	Sequence
1	0	0.68	1	1.6	KLPDQIKDVLK
0	1	0.71	1.1	1.7	HGPLALVDK
0	1	0.61	0.95	1.5	VANLEAQIK

5.514 835 1083 minus strand Calmodulin [Crassostrea gigas]

Protein Accession	CL49568.Contig2$_H$1
Mean Expression Ratio	1.02
Median Expression Ratio	1.03
Credible Interval	(0.848, 1.23)
Associated Peptides	7
Associated Spectra	48
Coverage	NaN

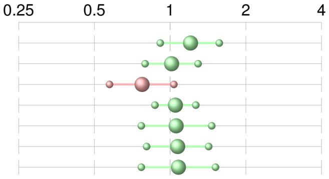

A	B	2.5	50	97.5	Sequence
6	0	0.91	1.2	1.6	HLLTDKDEETCYK
7	2	0.79	1	1.3	KGFIANEEMK
4	0	0.57	0.77	1	QQVQMTEQEVDEVIDYIDK
9	10	0.87	1	1.3	VDFTEFYK
2	1	0.77	1.1	1.5	GFIANEEMK
5	0	0.81	1.1	1.4	LLLNK
1	1	0.77	1.1	1.5	ILDDQR

5.515 126 287 minus strand Cytochrome b-c1 complex subunit 10 [Cricetulus griseus]; 126 287 minus strand Cytochrome b-c1 complex subunit 10 [Cricetulus griseus]; 126 287 minus strand Cytochrome b-c1 complex subunit 10 [Cricetulus griseus]; 126 287 minus strand Cytochrome b-c1 complex subunit 10 [Cricetulus griseus]

Protein Accession	CL46231.Contig6$_H$1 CL46231.Contig5$_H$1 CL46231.Contig4$_H$1 CL46231.Contig3$_H$1
Mean Expression Ratio	0.973
Median Expression Ratio	0.975
Credible Interval	(0.659, 1.43)
Associated Peptides	1
Associated Spectra	1
Coverage	NaN

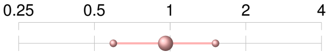

A	B	2.5	50	97.5	Sequence
0	1	0.6	0.96	1.5	FIPFYGSK

5.516 36 770 minus strand Endoplasmic reticulum protein ERp29 [Crassostrea gigas]

Protein Accession	CL37188.Contig1$_H$1
Mean Expression Ratio	0.974
Median Expression Ratio	0.975
Credible Interval	(0.677, 1.41)
Associated Peptides	2
Associated Spectra	2
Coverage	NaN

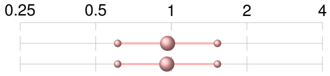

A	B	2.5	50	97.5	Sequence
1	0	0.61	0.97	1.5	KAQTAAAALTK
1	0	0.61	0.96	1.5	KPDFPVYK

5.517 113 808 minus strand omega class glutathione-s-transferase [Chlamys farreri]; 335 1045 minus strand omega class glutathione-s-transferase [Chlamys farreri]

Protein Accession	CL8096.Contig3$_H$1 CL8096.Contig2$_H$1
Mean Expression Ratio	0.977
Median Expression Ratio	0.976
Credible Interval	(0.679, 1.42)
Associated Peptides	1
Associated Spectra	2
Coverage	NaN

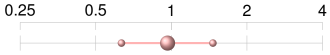

A	B	2.5	50	97.5	Sequence
2	0	0.63	0.96	1.5	HPLGLVPTLEQDSK

5.518 " 64 1332 Flotillin-1, putative [Pediculus humanus corporis] gi"

Protein Accession	CL33952.Contig1$_H$1
Mean Expression Ratio	1.02
Median Expression Ratio	1.02
Credible Interval	(0.792, 1.32)
Associated Peptides	4
Associated Spectra	16
Coverage	NaN

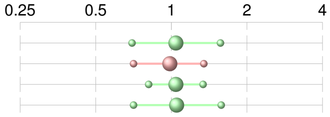

A	B	2.5	50	97.5	Sequence
1	0	0.7	1	1.6	AAYDMEVMTTK
3	1	0.71	0.98	1.3	VKGEAEAFAIEAK
6	4	0.81	1	1.3	LPSIVETL
0	1	0.71	1.1	1.6	FLNDIEIAK

5.519 54 662 minus strand Protein DJ-1 [Crassostrea gigas]

Protein Accession	CL16588.Contig3$_H$1
Mean Expression Ratio	0.976
Median Expression Ratio	0.976
Credible Interval	(0.663, 1.44)
Associated Peptides	1
Associated Spectra	1
Coverage	NaN

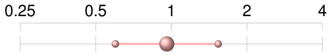

A	B	2.5	50	97.5	Sequence
1	0	0.6	0.96	1.5	NVNIVPDASMEEALK

5.520 170 460 Spectrin beta chain [Crassostrea gigas]

Protein Accession	CL38255.Contig1$_{H}1$
Mean Expression Ratio	1.02
Median Expression Ratio	1.02
Credible Interval	(0.718, 1.48)
Associated Peptides	2
Associated Spectra	2
Coverage	NaN

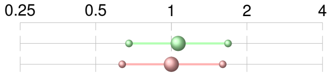

A	B	2.5	50	97.5	Sequence
1	0	0.68	1.1	1.7	LLEVLSGER
1	0	0.64	1	1.6	WVNSHLIR

5.521 2831 4900 minus strand Collagen alpha-3(VI) chain [Crassostrea gigas]

Protein Accession	Unigene26542$_{H}1$
Mean Expression Ratio	1.02
Median Expression Ratio	1.02
Credible Interval	(0.723, 1.47)
Associated Peptides	1
Associated Spectra	3
Coverage	NaN

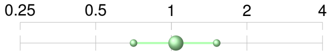

A	B	2.5	50	97.5	Sequence
3	0	0.71	1	1.5	TEMFTPANGDRPDVPNVAIVITDGVSNINSR

5.522 104 418 Tubulin-specific chaperone A [Crassostrea gigas]; 104 418 minus strand Tubulin-specific chaperone A [Crassostrea gigas]; 104 418 Tubulin-specific chaperone A [Crassostrea gigas]

Protein Accession	Unigene53595$_{H}1$ Unigene32457$_{H}1$ CL7190.Contig3$_{H}1$
Mean Expression Ratio	0.977
Median Expression Ratio	0.976
Credible Interval	(0.657, 1.45)
Associated Peptides	1
Associated Spectra	2
Coverage	NaN

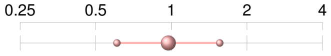

A	B	2.5	50	97.5	Sequence
1	1	0.61	0.98	1.6	ADLGETEELK

5.523 114 1001 Vinculin [Crassostrea gigas]

Protein Accession	CL18499.Contig1$_{H}1$
Mean Expression Ratio	1.02
Median Expression Ratio	1.02
Credible Interval	(0.8, 1.31)
Associated Peptides	8
Associated Spectra	11
Coverage	NaN

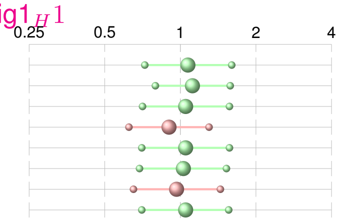

A	B	2.5	50	97.5	Sequence
1	0	0.72	1.1	1.6	MEDLVTFVK
2	1	0.8	1.1	1.6	TIESILEPVAQQVSR
1	0	0.71	1	1.6	NLSPVLTR
1	1	0.62	0.9	1.3	KMSDEIHEIIR
0	1	0.7	1	1.6	AAVDNLVR
0	1	0.69	1	1.5	MSDEIHEIIR
0	1	0.65	0.97	1.4	NLTPVLISGIK
0	1	0.7	1	1.6	IFITAK

5.524 324 1982 minus strand Pikachurin [Crassostrea gigas]

Protein Accession	CL4889.Contig10$_H$1
Mean Expression Ratio	1.02
Median Expression Ratio	1.02
Credible Interval	(0.727, 1.43)
Associated Peptides	2
Associated Spectra	3
Coverage	NaN

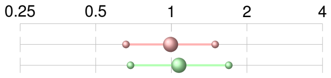

A	B	2.5	50	97.5	Sequence
2	0	0.66	0.99	1.5	VTAPIITEECGK
1	0	0.69	1.1	1.7	KGVLMR

5.525 180 560 calreticulin precursor [Aplysia californica] gi; 180 545 calreticulin precursor [Aplysia californica] gi

Protein Accession	CL4813.Contig4$_H$1 CL4813.Contig2$_H$1
Mean Expression Ratio	0.977
Median Expression Ratio	0.977
Credible Interval	(0.729, 1.31)
Associated Peptides	3
Associated Spectra	10
Coverage	NaN

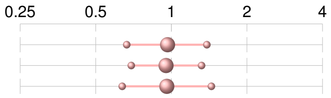

A	B	2.5	50	97.5	Sequence
2	1	0.66	0.97	1.4	FVWSAGK
4	1	0.69	0.96	1.3	WIPSVHK
1	1	0.64	0.96	1.4	FYGISAK

5.526 17 1681 minus strand Glucose-6-phosphate isomerase [Crassostrea gigas]

Protein Accession	CL34850.Contig1$_H$1
Mean Expression Ratio	1.02
Median Expression Ratio	1.02
Credible Interval	(0.802, 1.3)
Associated Peptides	7
Associated Spectra	13
Coverage	NaN

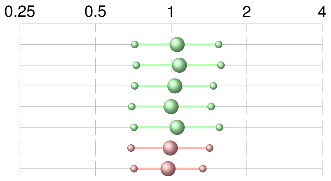

A	B	2.5	50	97.5	Sequence
1	0	0.72	1.1	1.5	EFTEQVISGAWK
1	0	0.72	1.1	1.6	KQLTSEPVWR
1	1	0.72	1	1.5	CKEFGIDEK
1	1	0.7	1	1.4	VHFISNVDGTHLAK
0	1	0.71	1.1	1.6	EAAEDELR
0	2	0.69	1	1.4	INFTEDR
0	4	0.71	0.97	1.3	INMAQMFK

5.527 151 1110 minus strand hypothetical protein DAPPUDRAFT_303085 [Daphnia pulex]

Protein Accession	CL12652.Contig2$_H$1
Mean Expression Ratio	1.02
Median Expression Ratio	1.02
Credible Interval	(0.715, 1.45)
Associated Peptides	1
Associated Spectra	3
Coverage	NaN

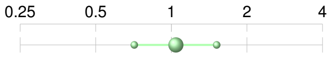

A	B	2.5	50	97.5	Sequence
2	1	0.71	1	1.5	TVIITGANTGIGK

5.528 42 434 cytochrome b5 [Haliotis discus discus]; 42 434 cytochrome b5 [Haliotis discus discus]

Protein Accession	CL6985.Contig9$_{H}1$ CL6985.Contig5$_{H}1$
Mean Expression Ratio	0.977
Median Expression Ratio	0.977
Credible Interval	(0.7, 1.38)
Associated Peptides	3
Associated Spectra	3
Coverage	NaN

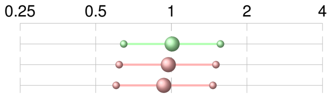

A	B	2.5	50	97.5	Sequence
**1**	**0**	**0.65**	**1**	**1.6**	**KTFETNTGPVGR**
**1**	**0**	**0.62**	**0.97**	**1.5**	**VFRSEEVK**
**0**	**1**	**0.6**	**0.93**	**1.5**	**ELMKDYLIGK**

5.529 179 2140 minus strand PREDICTED: hypothetical protein [Saccoglossus kowalevskii]

Protein Accession	CL13749.Contig6$_{H}1$
Mean Expression Ratio	1.02
Median Expression Ratio	1.02
Credible Interval	(0.696, 1.51)
Associated Peptides	1
Associated Spectra	1
Coverage	NaN

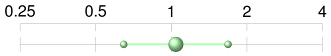

A	B	2.5	50	97.5	Sequence
**1**	**0**	**0.64**	**1**	**1.7**	**KFDGIGGLSGGGATSK**

5.530 112 930 calcineurin A [Haliotis discus discus]

Protein Accession	CL3137.Contig1$_{H}1$
Mean Expression Ratio	1.02
Median Expression Ratio	1.02
Credible Interval	(0.73, 1.44)
Associated Peptides	2
Associated Spectra	3
Coverage	NaN

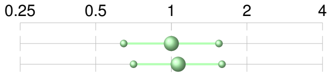

A	B	2.5	50	97.5	Sequence
**0**	**1**	**0.64**	**1**	**1.6**	**VTEDVALR**
**0**	**2**	**0.71**	**1.1**	**1.6**	**SEAFDAK**

5.531 " 241 1110 minus strand N(G),N(G)-dimethylarginine dimethylaminohydrolase 1 [Crassostrea gigas]; 241 1110 minus strand N(G),N(G)-dimethylarginine dimethylaminohydrolase 1 [Crassostrea gigas]; 241 1110 minus strand N(G),N(G)-dimethylarginine dimethylaminohydrolase 1 [Crassostrea gigas]"

Protein Accession	Unigene30451$_H$1 CL7107.Contig3$_H$1 CL7107.Contig1$_H$1
Mean Expression Ratio	1.02
Median Expression Ratio	1.02
Credible Interval	(0.688, 1.5)
Associated Peptides	1
Associated Spectra	2
Coverage	NaN

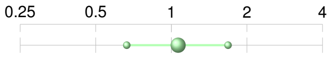

A	B	2.5	50	97.5	Sequence
1	1	0.67	1.1	1.7	LGEVGEIR

5.532 147 272 Spectrin alpha chain [Crassostrea gigas]

Protein Accession	CL2014.Contig2$_H$1
Mean Expression Ratio	0.978
Median Expression Ratio	0.978
Credible Interval	(0.659, 1.46)
Associated Peptides	1
Associated Spectra	1
Coverage	NaN

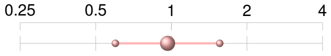

A	B	2.5	50	97.5	Sequence
0	1	0.6	0.96	1.6	VLETAEDIQQR

5.533 261 1361 tandem-repeat galectin [Pinctada fucata]; 261 1361 tandem-repeat galectin [Pinctada fucata]

Protein Accession	CL474.Contig2$_H$1 CL474.Contig1$_H$1
Mean Expression Ratio	0.978
Median Expression Ratio	0.978
Credible Interval	(0.673, 1.41)
Associated Peptides	1
Associated Spectra	2
Coverage	NaN

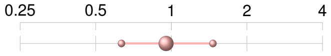

A	B	2.5	50	97.5	Sequence
0	2	0.63	0.96	1.5	LENNVLLHANQR

5.534 102 887 hypothetical protein DAPPUDRAFT_304712 [Daphnia pulex]

Protein Accession	CL6869.Contig1$_H$1
Mean Expression Ratio	0.978
Median Expression Ratio	0.978
Credible Interval	(0.739, 1.29)
Associated Peptides	4
Associated Spectra	8
Coverage	NaN

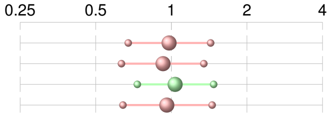

A	B	2.5	50	97.5	Sequence
2	0	0.67	0.98	1.4	GKPLFVR
1	1	0.63	0.93	1.3	LADIPEGK
2	1	0.73	1	1.5	HPEADAER
0	1	0.64	0.96	1.5	VDLSQLR

5.535 22 369 Protein farnesyltransferase/geranylgeranyltransferase type-1 subunit alpha [Crassostrea gigas]

Protein Accession	CL38456.Contig1$_H$1
Mean Expression Ratio	0.977
Median Expression Ratio	0.978
Credible Interval	(0.678, 1.41)
Associated Peptides	2
Associated Spectra	2
Coverage	NaN

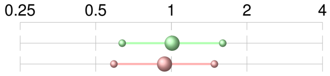

A	B	2.5	50	97.5	Sequence
1	0	0.64	1	1.6	YISAVIEDHPK
1	0	0.59	0.94	1.5	AIVASEEK

5.536 227 2920 minus strand Lysosomal alpha-mannosidase [Crassostrea gigas]; 227 3073 minus strand Lysosomal alpha-mannosidase [Crassostrea gigas]; 193 2886 minus strand Lysosomal alpha-mannosidase [Crassostrea gigas]

Protein Accession	CL2930.Contig3$_H$1 CL2930.Contig2$_H$1 CL2930.Contig1$_H$1
Mean Expression Ratio	0.979
Median Expression Ratio	0.978
Credible Interval	(0.734, 1.31)
Associated Peptides	5
Associated Spectra	8
Coverage	NaN

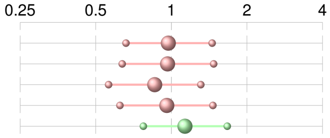

A	B	2.5	50	97.5	Sequence
2	1	0.66	0.97	1.5	VASELIPITPDTFR
0	1	0.64	0.97	1.5	PDNVHLLTLEQFR
0	1	0.56	0.86	1.3	ALPDNVHLLTLEQFR
0	1	0.62	0.96	1.5	QLNHR
0	2	0.77	1.1	1.7	AGASLTWSGVR

5.537 " 698 1225 minus strand actin related protein 2/3 complex, 21 kDa subunit [Crassostrea gigas]"

Protein Accession	CL17389.Contig2$_H$1
Mean Expression Ratio	0.978
Median Expression Ratio	0.978
Credible Interval	(0.718, 1.32)
Associated Peptides	3
Associated Spectra	7
Coverage	NaN

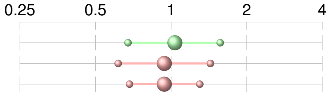

A	B	2.5	50	97.5	Sequence
**0**	**1**	**0.67**	**1**	**1.6**	**AYLQQLR**
**0**	**1**	**0.62**	**0.94**	**1.4**	**ANIFFR**
**0**	**5**	**0.68**	**0.94**	**1.3**	**LLPLK**

5.538 385 906 Soma ferritin OS=Lymnaea stagnalis PE=2 SV=2

Protein Accession	CL2484.Contig1$_H$1
Mean Expression Ratio	1.02
Median Expression Ratio	1.02
Credible Interval	(0.693, 1.51)
Associated Peptides	1
Associated Spectra	1
Coverage	NaN

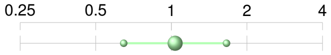

A	B	2.5	50	97.5	Sequence
**1**	**0**	**0.65**	**1**	**1.7**	**ELADHITNLK**

5.539 91 2658 minus strand puromycin-sensitive aminopeptidase [Saccoglossus kowalevskii]

Protein Accession	CL6708.Contig2$_H$1
Mean Expression Ratio	1.02
Median Expression Ratio	1.02
Credible Interval	(0.708, 1.45)
Associated Peptides	2
Associated Spectra	2
Coverage	NaN

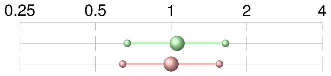

A	B	2.5	50	97.5	Sequence
**1**	**0**	**0.67**	**1.1**	**1.7**	**GMNLYLNR**
**1**	**0**	**0.64**	**1**	**1.6**	**LPKDVIPSNYNIR**

5.540 188 4135 Vigilin [Crassostrea gigas]

Protein Accession	CL846.Contig3$_H$1
Mean Expression Ratio	0.98
Median Expression Ratio	0.978
Credible Interval	(0.664, 1.45)
Associated Peptides	1
Associated Spectra	1
Coverage	NaN

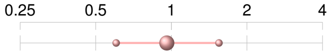

A	B	2.5	50	97.5	Sequence
**1**	**0**	**0.6**	**0.96**	**1.5**	**EVSIDIPHR**

5.541 " 196 1053 minus strand Cytochrome b-c1 complex subunit 2, mitochondrial OS=Homo sapiens GN=UQCRC2 PE=1 SV=3"

Protein Accession	CL7847.Contig5$_H1$
Mean Expression Ratio	1.02
Median Expression Ratio	1.02
Credible Interval	(0.707, 1.48)
Associated Peptides	1
Associated Spectra	2
Coverage	NaN

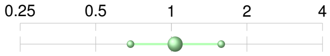

A	B	2.5	50	97.5	Sequence
**1**	**1**	**0.69**	**1**	**1.6**	**LDLAILK**

5.542 2 511 hypothetical protein CGI_10006272 [Crassostrea gigas]

Protein Accession	CL10577.Contig2$_H1$
Mean Expression Ratio	1.02
Median Expression Ratio	1.02
Credible Interval	(0.69, 1.52)
Associated Peptides	1
Associated Spectra	1
Coverage	NaN

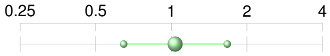

A	B	2.5	50	97.5	Sequence
**1**	**0**	**0.64**	**1**	**1.7**	**LLEGAVPK**

5.543 " 1 681 40S ribosomal protein S4, X isoform [Crassostrea gigas]"

Protein Accession	CL2560.Contig4$_H1$
Mean Expression Ratio	0.98
Median Expression Ratio	0.979
Credible Interval	(0.665, 1.45)
Associated Peptides	1
Associated Spectra	1
Coverage	NaN

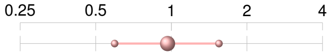

A	B	2.5	50	97.5	Sequence
**1**	**0**	**0.6**	**0.96**	**1.6**	**GVPYIVTHDGR**

5.544 225 839 minus strand Metallo-beta-lactamase domain-containing protein 1 [Crassostrea gigas]; 201 851 minus strand Metallo-beta-lactamase domain-containing protein 1 [Crassostrea gigas]

Protein Accession	CL35672.Contig2$_H1$ CL35672.Contig1$_H1$
Mean Expression Ratio	1.02
Median Expression Ratio	1.02
Credible Interval	(0.712, 1.47)
Associated Peptides	1
Associated Spectra	2
Coverage	NaN

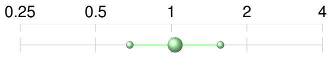

A	B	2.5	50	97.5	Sequence
**1**	**1**	**0.68**	**1**	**1.6**	**EADFIVPGHGPMFR**

5.545 362 874 minus strand Transgelin-2 [Crassostrea gigas]

Protein Accession	CL445.Contig3$_H$1
Mean Expression Ratio	1.02
Median Expression Ratio	1.02
Credible Interval	(0.893, 1.17)
Associated Peptides	14
Associated Spectra	146
Coverage	NaN

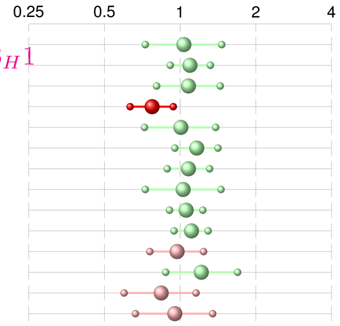

A	B	2.5	50	97.5	Sequence
1	0	0.73	1	1.5	AGLGAEIEKK
11	9	0.92	1.1	1.3	EFLVQSADLWEGR
3	1	0.8	1.1	1.4	EFLVQSADLWEGRK
16	0	0.63	0.77	0.94	GFQPSYTGSQSR
2	0	0.72	1	1.4	KAGLGAEIEKK
12	3	0.95	1.2	1.4	LLESEGKPK
8	8	0.89	1.1	1.3	LINFD
1	0	0.73	1	1.5	LGAEIEK
20	10	0.91	1.1	1.2	AGLGAEIEK
20	8	0.94	1.1	1.3	DGVMLCK
4	4	0.76	0.97	1.2	KAGLGAEIEK
1	1	0.88	1.2	1.7	RDGVMLCKLINK
0	2	0.6	0.84	1.2	LINKLLESEGKPK
0	1	0.67	0.95	1.3	QSADLWEGR

5.546 131 541 minus strand nervous system adducin [Aplysia californica] gi

Protein Accession	CL27337.Contig1$_H$1
Mean Expression Ratio	1.02
Median Expression Ratio	1.02
Credible Interval	(0.749, 1.39)
Associated Peptides	3
Associated Spectra	5
Coverage	NaN

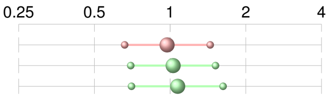

A	B	2.5	50	97.5	Sequence
1	1	0.66	0.97	1.4	FIDTIDPDDPEYIR
1	1	0.7	1	1.5	GIGASFGR
1	0	0.7	1.1	1.6	LAALYR

5.547 160 387 small nuclear ribonucleoprotein polypeptide G protein [Crassostrea ariakensis]; 180 407 small nuclear ribonucleoprotein polypeptide G protein [Crassostrea ariakensis]

Protein Accession	CL1272.Contig8$_H$1 CL1272.Contig7$_H$1
Mean Expression Ratio	1.02
Median Expression Ratio	1.02
Credible Interval	(0.728, 1.44)
Associated Peptides	2
Associated Spectra	3
Coverage	NaN

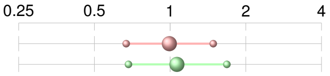

A	B	2.5	50	97.5	Sequence
2	0	0.67	0.99	1.5	TITGILR
1	0	0.68	1.1	1.7	AHPPELK

5.548 43 525 Reticulon-4 [Crassostrea gigas]; 143 757 Reticulon-4 [Crassostrea gigas]; 43 564 Reticulon-4 [Crassostrea gigas]; 302 583 Reticulon-4 OS=Rattus norvegicus GN=Rtn4 PE=1 SV=1

Protein Accession	CL29775.Contig4$_{H}1$ CL29775.Contig3$_{H}1$ CL29775.Contig2$_{H}1$ CL29775.Contig1$_{H}1$
Mean Expression Ratio	0.981
Median Expression Ratio	0.981
Credible Interval	(0.702, 1.38)
Associated Peptides	2
Associated Spectra	6
Coverage	NaN

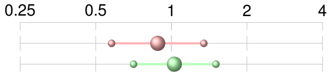

A	B	2.5	50	97.5	Sequence
2	1	0.58	0.89	1.4	VTQTILQHVNR
0	3	0.71	1	1.5	SLLELR

5.549 147 383 minus strand Tubulin-specific chaperone A OS=Rattus norvegicus GN=Tbca PE=1 SV=1; 104 418 Tubulin-specific chaperone A [Crassostrea gigas]; 104 418 minus strand Tubulin-specific chaperone A [Crassostrea gigas]; 104 418 Tubulin-specific chaperone A [Crassostrea gigas]

Protein Accession	Unigene66350$_{H}1$ Unigene53595$_{H}1$ Unigene32457$_{H}1$ CL7190.Contig3$_{H}1$
Mean Expression Ratio	1.02
Median Expression Ratio	1.02
Credible Interval	(0.687, 1.52)
Associated Peptides	1
Associated Spectra	2
Coverage	NaN

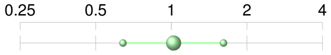

A	B	2.5	50	97.5	Sequence
1	1	0.64	1	1.6	ADLGETEELK

5.550 2 328 cytoskeletal actin 2 [Molgula oculata]

Protein Accession	Unigene2743$_{H}1$
Mean Expression Ratio	1.02
Median Expression Ratio	1.02
Credible Interval	(0.845, 1.23)
Associated Peptides	7
Associated Spectra	137
Coverage	NaN

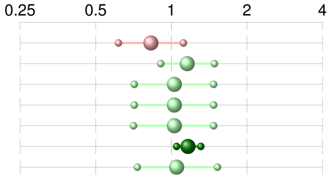

A	B	2.5	50	97.5	Sequence
3	2	0.62	0.83	1.1	IIAPPERK
7	2	0.9	1.2	1.5	IKIIAPPER
1	0	0.71	1	1.5	KIIAPPER
1	0	0.71	1	1.5	DITALAPPTMK
1	0	0.71	1	1.5	DLYANVVLSGGSTMYPGIADR
63	56	1	1.2	1.3	IIAPPER
1	0	0.73	1	1.5	KDLYANVVLSGGSTMYPGIADR

5.551 188 1021 hypothetical protein BRAFLDRAFT_214346 [Branchiostoma floridae] gi

Protein Accession	CL38498.Contig2$_H1$
Mean Expression Ratio	0.981
Median Expression Ratio	0.981
Credible Interval	(0.695, 1.38)
Associated Peptides	3
Associated Spectra	3
Coverage	NaN

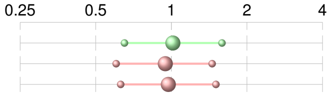

A	B	2.5	50	97.5	Sequence
1	0	0.65	1	1.6	EGLELHPPK
0	1	0.6	0.95	1.5	NLVGAIMTEEEAKK
0	1	0.63	0.97	1.5	DICTFLR

5.552 146 841 minus strand hypothetical protein CGI_10006609 [Crassostrea gigas]; 146 841 minus strand hypothetical protein CGI_10006609 [Crassostrea gigas]; 146 841 minus strand hypothetical protein CGI_10006609 [Crassostrea gigas]

Protein Accession	CL6681.Contig3$_H1$ CL6681.Contig2$_H1$ CL6681.Contig1$_H1$
Mean Expression Ratio	0.979
Median Expression Ratio	0.981
Credible Interval	(0.663, 1.44)
Associated Peptides	1
Associated Spectra	1
Coverage	NaN

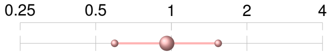

A	B	2.5	50	97.5	Sequence
0	1	0.59	0.96	1.5	GHVEGLEVLPAR

5.553 184 438 PREDICTED: similar to CG14235 CG14235-PA [Tribolium castaneum] gi

Protein Accession	Unigene638$_H1$
Mean Expression Ratio	1.02
Median Expression Ratio	1.02
Credible Interval	(0.739, 1.42)
Associated Peptides	2
Associated Spectra	5
Coverage	NaN

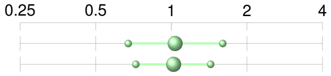

A	B	2.5	50	97.5	Sequence
1	0	0.67	1	1.6	SLCPNEWVAK
3	1	0.72	1	1.4	WDDQVANGTFAGK

5.554 1573 2385 minus strand Spectrin beta chain [Crassostrea gigas]

Protein Accession	CL5904.Contig4$_{H}1$
Mean Expression Ratio	0.983
Median Expression Ratio	0.982
Credible Interval	(0.665, 1.45)
Associated Peptides	1
Associated Spectra	1
Coverage	NaN

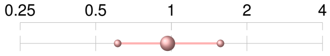

A	B	2.5	50	97.5	Sequence
**0**	**1**	**0.61**	**0.97**	**1.6**	**RLIQEEHPESDQIAVR**

5.555 366 935 minus strand 40S ribosomal protein S2 OS=Urechis caupo GN=RPS2 PE=2 SV=1

Protein Accession	CL2084.Contig7$_{H}1$
Mean Expression Ratio	1.02
Median Expression Ratio	1.02
Credible Interval	(0.69, 1.5)
Associated Peptides	1
Associated Spectra	1
Coverage	NaN

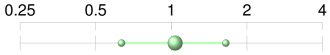

A	B	2.5	50	97.5	Sequence
**0**	**1**	**0.63**	**1**	**1.6**	**TYSFLTPDLWK**

5.556 140 1564 selenium-binding protein [Haliotis discus hannai]

Protein Accession	CL3868.Contig7$_{H}1$
Mean Expression Ratio	1.02
Median Expression Ratio	1.02
Credible Interval	(0.694, 1.51)
Associated Peptides	1
Associated Spectra	1
Coverage	NaN

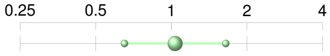

A	B	2.5	50	97.5	Sequence
**1**	**0**	**0.65**	**1**	**1.6**	**VVEAVDIHR**

5.557 " 40 813 minus strand NADH dehydrogenase [ubiquinone] iron-sulfur protein 3, mitochondrial [Crassostrea gigas]"

Protein Accession	CL8338.Contig9$_{H}1$
Mean Expression Ratio	0.98
Median Expression Ratio	0.982
Credible Interval	(0.682, 1.42)
Associated Peptides	2
Associated Spectra	2
Coverage	NaN

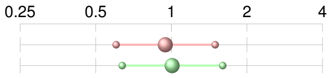

A	B	2.5	50	97.5	Sequence
**0**	**1**	**0.6**	**0.95**	**1.5**	**VVIEPIELQQEFR**
**0**	**1**	**0.64**	**1**	**1.6**	**ILTDYGFEGHPFR**

5.558 219 554 minus strand ribosomal protein P1 [Biomphalaria glabrata]

Protein Accession	CL45629.Contig2$_H$1
Mean Expression Ratio	1.02
Median Expression Ratio	1.02
Credible Interval	(0.687, 1.5)
Associated Peptides	1
Associated Spectra	1
Coverage	NaN

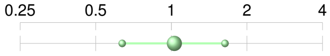

A	B	2.5	50	97.5	Sequence
1	0	0.64	1	1.6	ALEGVNIK

5.559 594 1253 peroxiredoxin 6 [Eriocheir sinensis]

Protein Accession	CL21894.Contig2$_H$1
Mean Expression Ratio	0.981
Median Expression Ratio	0.982
Credible Interval	(0.764, 1.27)
Associated Peptides	5
Associated Spectra	13
Coverage	NaN

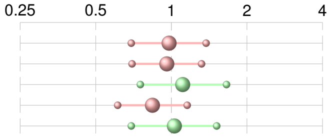

A	B	2.5	50	97.5	Sequence
2	1	0.69	0.98	1.4	VNLGDVFPDFK
4	0	0.7	0.96	1.3	AVFIIGPDK
1	0	0.75	1.1	1.7	AVFIIGPDKK
2	2	0.61	0.84	1.2	NFDEILR
1	0	0.69	1	1.5	QLTATQK

5.560 " 169 792 minus strand rho GDP dissociation inhibitor, putative [Ixodes scapularis] gi"

Protein Accession	CL39798.Contig1$_H$1
Mean Expression Ratio	0.981
Median Expression Ratio	0.982
Credible Interval	(0.718, 1.34)
Associated Peptides	3
Associated Spectra	5
Coverage	NaN

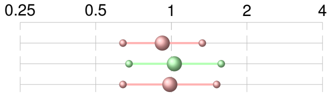

A	B	2.5	50	97.5	Sequence
2	1	0.64	0.92	1.3	IIIDDSNPLNVIVK
1	0	0.68	1	1.6	LAISVEGRPDAELDLTKPK
0	1	0.64	0.99	1.5	DIVAGLR

5.561 390 2267 neurofilament protein NF70 [Helix aspersa]; 318 2195 neurofilament protein NF70 [Helix aspersa]

Protein Accession	CL1353.Contig5$_H$1 CL1353.Contig4$_H$1
Mean Expression Ratio	0.981
Median Expression Ratio	0.982
Credible Interval	(0.766, 1.25)
Associated Peptides	7
Associated Spectra	13
Coverage	NaN

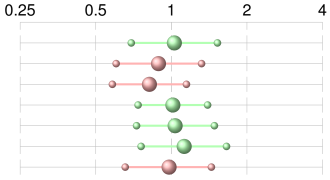

A	B	2.5	50	97.5	Sequence
1	0	0.69	1	1.5	GEMETFYNLK
1	0	0.6	0.89	1.3	LNQQISDLEAEIR
2	1	0.58	0.82	1.2	VGQLEELVDDLQR
4	0	0.74	1	1.4	FANYIEK
1	1	0.73	1	1.5	MADLEAR
0	1	0.76	1.1	1.7	ELQQEMDVK
0	1	0.66	0.98	1.4	LEFLK

5.562 10 1902 Mitochondrial inner membrane protein [Crassostrea gigas]; 10 1902 Mitochondrial inner membrane protein [Crassostrea gigas]; 10 1902 Mitochondrial inner membrane protein [Crassostrea gigas]; 10 1902 Mitochondrial inner membrane protein [Crassostrea gigas]; 10 1902 Mitochondrial inner membrane protein [Crassostrea gigas]; 10 1902 Mitochondrial inner membrane protein [Crassostrea gigas]

Protein Accession	CL6414.Contig8$_{H}1$ CL6414.Contig7$_{H}1$ CL6414.Contig6$_{H}1$ CL6414.Contig5$_{H}1$ CL6414.Con
Mean Expression Ratio	0.984
Median Expression Ratio	0.983
Credible Interval	(0.667, 1.47)
Associated Peptides	1
Associated Spectra	1
Coverage	NaN

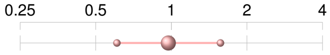

A	B	2.5	50	97.5	Sequence
**1**	**0**	**0.61**	**0.97**	**1.6**	**EIHTQLLQER**

5.563 582 2135 sulfatase 1A precursor [Haliotis discus discus]

Protein Accession	CL1700.Contig8$_{H}1$
Mean Expression Ratio	1.02
Median Expression Ratio	1.02
Credible Interval	(0.757, 1.37)
Associated Peptides	4
Associated Spectra	6
Coverage	NaN

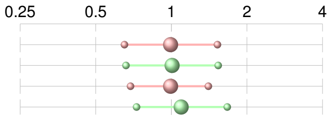

A	B	2.5	50	97.5	Sequence
**1**	**0**	**0.65**	**0.99**	**1.5**	**HNEDVFYSAK**
**1**	**0**	**0.66**	**1**	**1.5**	**GKYPDVFK**
**2**	**1**	**0.69**	**0.99**	**1.4**	**AVSFLHSPK**
**0**	**1**	**0.73**	**1.1**	**1.7**	**EGHMYTPTLDGLAR**

5.564 351 1826 Virulence metalloprotease [Crassostrea gigas]

Protein Accession	CL7226.Contig18$_{H}1$
Mean Expression Ratio	0.984
Median Expression Ratio	0.983
Credible Interval	(0.67, 1.44)
Associated Peptides	1
Associated Spectra	1
Coverage	NaN

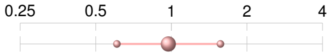

A	B	2.5	50	97.5	Sequence
**1**	**0**	**0.61**	**0.97**	**1.6**	**EGDVCYLENK**

5.565 " 2057 2497 Phosphate carrier protein, mitochondrial [Crassostrea gigas]"

Protein Accession	CL2712.Contig11$_{H}1$
Mean Expression Ratio	0.986
Median Expression Ratio	0.984
Credible Interval	(0.665, 1.45)
Associated Peptides	1
Associated Spectra	1
Coverage	NaN

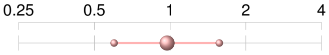

A	B	2.5	50	97.5	Sequence
**1**	**0**	**0.6**	**0.97**	**1.6**	**YVVPKPR**

5.566 " 45 506 minus strand PREDICTED: dihydrolipoyl dehydrogenase, mitochondrial [Monodelphis domestica]"

Protein Accession	CL24292.Contig3$_{H}1$
Mean Expression Ratio	1.02
Median Expression Ratio	1.02
Credible Interval	(0.703, 1.46)
Associated Peptides	2
Associated Spectra	2
Coverage	NaN

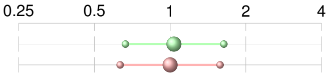

A	B	2.5	50	97.5	Sequence
**0**	**1**	**0.66**	**1**	**1.6**	**ALTGGIAHLFK**
**0**	**1**	**0.63**	**1**	**1.6**	**GIEIDGLR**

5.567 8 1636 minus strand V-type proton ATPase catalytic subunit A [Crassostrea gigas]; 8 1636 minus strand V-type proton ATPase catalytic subunit A [Crassostrea gigas]; 8 1636 minus strand V-type proton ATPase catalytic subunit A [Crassostrea gigas]

Protein Accession	CL33671.Contig3$_{H}1$ CL33671.Contig2$_{H}1$ CL33671.Contig1$_{H}1$
Mean Expression Ratio	0.984
Median Expression Ratio	0.984
Credible Interval	(0.682, 1.42)
Associated Peptides	1
Associated Spectra	2
Coverage	NaN

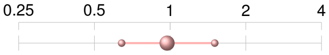

A	B	2.5	50	97.5	Sequence
**2**	**0**	**0.64**	**0.98**	**1.5**	**LAANHPLLTGQR**

5.568 248 2053 PDZ and LIM domain protein 5 [Crassostrea gigas]

Protein Accession	CL2142.Contig4$_{H}1$
Mean Expression Ratio	0.985
Median Expression Ratio	0.984
Credible Interval	(0.806, 1.2)
Associated Peptides	8
Associated Spectra	32
Coverage	NaN

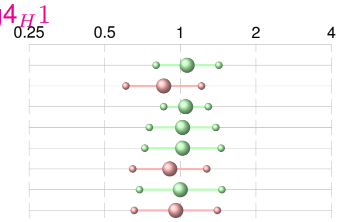

A	B	2.5	50	97.5	Sequence
**2**	**3**	**0.8**	**1.1**	**1.4**	**CGLQTGDVILK**
**1**	**1**	**0.61**	**0.86**	**1.2**	**IGNTDTSALR**
**4**	**11**	**0.86**	**1.1**	**1.3**	**LDLLLQR**
**3**	**1**	**0.75**	**1**	**1.4**	**VNPGSLAAK**
**1**	**1**	**0.72**	**1**	**1.5**	**YLAPHCK**
**0**	**2**	**0.65**	**0.91**	**1.3**	**MDSTTPWGFR**
**0**	**1**	**0.69**	**1**	**1.5**	**SVLTLEAK**
**0**	**1**	**0.66**	**0.96**	**1.4**	**LQTGDVILK**

5.569 87 1997 Transketolase-like protein 2 [Crassostrea gigas]; 87 1997 Transketolase-like protein 2 [Crassostrea gigas]; 87 1997 Transketolase-like protein 2 [Crassostrea gigas]; 87 1997 Transketolase-like protein 2 [Crassostrea gigas]; 87 1997 Transketolase-like protein 2 [Crassostrea gigas]; 87 1997 Transketolase-like protein 2 [Crassostrea gigas]; 87 1997 Transketolase-like protein 2 [Crassostrea gigas]

Protein Accession	CL1127.Contig6$_{H}1$ CL1127.Contig5$_{H}1$ CL1127.Contig4$_{H}1$ CL1127.Contig3$_{H}1$ CL1127.Con
Mean Expression Ratio	1.02
Median Expression Ratio	1.02
Credible Interval	(0.746, 1.39)
Associated Peptides	3
Associated Spectra	5
Coverage	NaN

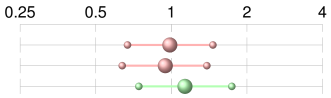

A	B	2.5	50	97.5	Sequence
1	1	0.67	0.99	1.5	VISLDGDVK
1	1	0.64	0.94	1.4	GMCFIR
1	0	0.74	1.1	1.7	NARETGGR

5.570 900 1142 minus strand heat shock protein 20 [Cyclina sinensis]

Protein Accession	CL19759.Contig1$_{H}1$
Mean Expression Ratio	0.983
Median Expression Ratio	0.985
Credible Interval	(0.742, 1.29)
Associated Peptides	3
Associated Spectra	11
Coverage	NaN

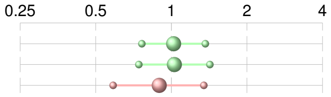

A	B	2.5	50	97.5	Sequence
4	2	0.76	1	1.4	QFKPEEITVK
3	1	0.74	1	1.4	QYILPEK
1	0	0.59	0.9	1.3	VEHPIVMDHQGNK

5.571 253 765 cytochrome c oxidase subunit IV [Urechis caupo]

Protein Accession	CL38636.Contig3$_{H}1$
Mean Expression Ratio	1.01
Median Expression Ratio	1.02
Credible Interval	(0.721, 1.44)
Associated Peptides	2
Associated Spectra	3
Coverage	NaN

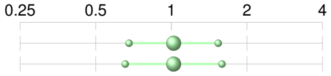

A	B	2.5	50	97.5	Sequence
2	0	0.68	1	1.5	FKENTPDVMALR
1	0	0.65	1	1.6	SLTLEDKK

5.572 783 1463 minus strand twitchin [Mytilus galloprovincialis]; 783 1463 minus strand twitchin [Mytilus galloprovincialis]; 752 1432 twitchin [Mytilus galloprovincialis]

Protein Accession	CL181.Contig5$_H$1 CL181.Contig3$_H$1 CL181.Contig1$_H$1
Mean Expression Ratio	0.985
Median Expression Ratio	0.985
Credible Interval	(0.772, 1.25)
Associated Peptides	7
Associated Spectra	13
Coverage	NaN

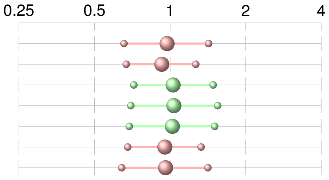

A	B	2.5	50	97.5	Sequence
1	0	0.66	0.97	1.4	DGKPVNSSDIYSITFDK
2	2	0.67	0.93	1.3	LNPQVVNDGDAVDLVVK
2	0	0.71	1	1.5	NSGGEVTSTATVNVDALKPK
1	0	0.7	1	1.6	VTGTEPFDITWAK
1	0	0.69	1	1.5	NQWGTASSMASLQVK
0	3	0.68	0.95	1.3	LEGDTIIIQQDITSK
0	1	0.64	0.96	1.4	SKPDLLWYR

5.573 264 1505 Ribosome-binding protein 1 [Crassostrea gigas]; 260 1501 Ribosome-binding protein 1 [Crassostrea gigas]; 256 1497 Ribosome-binding protein 1 [Crassostrea gigas]

Protein Accession	CL8282.Contig7$_H$1 CL8282.Contig4$_H$1 CL8282.Contig1$_H$1
Mean Expression Ratio	1.02
Median Expression Ratio	1.02
Credible Interval	(0.684, 1.51)
Associated Peptides	1
Associated Spectra	1
Coverage	NaN

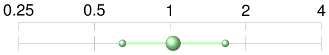

A	B	2.5	50	97.5	Sequence
1	0	0.64	1	1.7	LKEDKETLEAR

5.574 1363 1527 Protein disulfide-isomerase A3 [Crassostrea gigas]

Protein Accession	CL28563.Contig1$_H$1
Mean Expression Ratio	0.988
Median Expression Ratio	0.985
Credible Interval	(0.691, 1.43)
Associated Peptides	2
Associated Spectra	2
Coverage	NaN

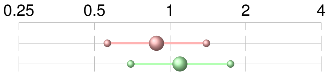

A	B	2.5	50	97.5	Sequence
0	1	0.56	0.89	1.4	LKDEPEITIAK
0	1	0.7	1.1	1.7	FEELAEK

5.575 " 154 534 minus strand Muscle M-line assembly protein unc-89, partial [Crassostrea gigas]"

Protein Accession	Unigene64523$_H1$
Mean Expression Ratio	1.01
Median Expression Ratio	1.02
Credible Interval	(0.687, 1.5)
Associated Peptides	1
Associated Spectra	2
Coverage	NaN

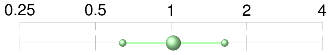

A	B	2.5	50	97.5	Sequence
1	1	0.64	1	1.6	LEIGEVVVDDEGEYICK

5.576 " 31 2061 minus strand 1,4-alpha-glucan-branching enzyme [Crassostrea gigas]"

Protein Accession	CL12555.Contig7$_H1$
Mean Expression Ratio	0.984
Median Expression Ratio	0.985
Credible Interval	(0.682, 1.42)
Associated Peptides	1
Associated Spectra	2
Coverage	NaN

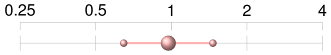

A	B	2.5	50	97.5	Sequence
0	2	0.65	0.98	1.5	LDGYLR

5.577 689 1984 hypothetical protein BRAFLDRAFT_78257 [Branchiostoma floridae] gi

Protein Accession	Unigene34076$_H1$
Mean Expression Ratio	1.02
Median Expression Ratio	1.01
Credible Interval	(0.805, 1.29)
Associated Peptides	8
Associated Spectra	14
Coverage	NaN

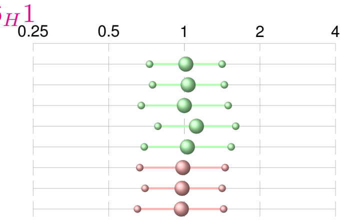

A	B	2.5	50	97.5	Sequence
2	1	0.73	1	1.4	TVGGFQLR
3	0	0.75	1	1.4	VQQKPEPSPAEGEVLIR
1	0	0.67	1	1.5	YLLFGSSNIVTGETK
1	1	0.79	1.1	1.6	YVSEIR
1	0	0.69	1	1.5	ILLDPSKAPTPK
0	1	0.66	0.98	1.4	SLVLTGFGSMK
0	2	0.7	0.98	1.4	GISILKPMGK
0	1	0.65	0.97	1.4	SFFSFAK

5.578 2295 2510 beta-thymosin domain repeat protein CSP24KDa_v1 [Hermissenda crassicornis] gi

Protein Accession	CL9548.Contig1$_H1$
Mean Expression Ratio	1.02
Median Expression Ratio	1.01
Credible Interval	(0.688, 1.51)
Associated Peptides	1
Associated Spectra	1
Coverage	NaN

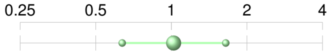

A	B	2.5	50	97.5	Sequence
1	0	0.64	1	1.6	ESVAAFDK

5.579 302 1273 minus strand hypothetical protein CGI_10007007 [Crassostrea gigas]

Protein Accession	CL18045.Contig10$_{H}1$
Mean Expression Ratio	0.985
Median Expression Ratio	0.985
Credible Interval	(0.667, 1.46)
Associated Peptides	1
Associated Spectra	3
Coverage	NaN

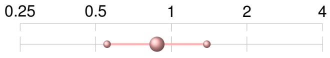

A	B	2.5	50	97.5	Sequence
2	1	0.56	0.88	1.4	IEAEFPGANIILHK

5.580 25 405 minus strand Elongation factor 1-delta [Anoplopoma fimbria]

Protein Accession	CL10502.Contig1$_{H}1$
Mean Expression Ratio	0.987
Median Expression Ratio	0.986
Credible Interval	(0.729, 1.36)
Associated Peptides	4
Associated Spectra	5
Coverage	NaN

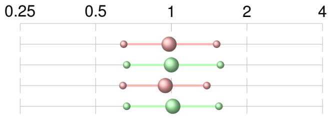

A	B	2.5	50	97.5	Sequence
1	0	0.64	0.98	1.5	KPVLIAK
1	0	0.66	1	1.6	VQTDGLVWGASK
1	1	0.64	0.95	1.4	LVPVGYGINK
0	1	0.66	1	1.6	RVQTDGLVWGASK

5.581 1 222 cAMP-dependent protein kinase regulatory subunit [Aplysia californica] gi

Protein Accession	Unigene13629$_{H}1$
Mean Expression Ratio	0.986
Median Expression Ratio	0.986
Credible Interval	(0.695, 1.4)
Associated Peptides	2
Associated Spectra	3
Coverage	NaN

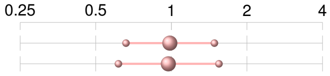

A	B	2.5	50	97.5	Sequence
1	1	0.66	0.99	1.5	NVLFSHLDDNER
0	1	0.62	0.98	1.5	SDIFDAMFPVHR

5.582 768 980 muscle LIM protein isoform 1 [Danaus plexippus]

Protein Accession	CL29824.Contig1$_{H}1$
Mean Expression Ratio	1.01
Median Expression Ratio	1.01
Credible Interval	(0.798, 1.28)
Associated Peptides	4
Associated Spectra	31
Coverage	NaN

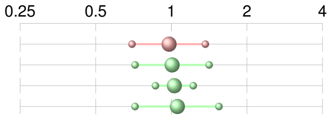

A	B	2.5	50	97.5	Sequence
3	0	0.7	0.98	1.4	SLDSTTMAMHETEVYCK
2	1	0.72	1	1.4	GVNTPWHK
18	6	0.87	1	1.2	AIGVNTPWHK
1	0	0.72	1.1	1.5	NFGPSGYGFGQGAGT

5.583 71 1321 minus strand Vinculin [Crassostrea gigas]

Protein Accession	Unigene23324$_H1$
Mean Expression Ratio	1.01
Median Expression Ratio	1.01
Credible Interval	(0.825, 1.25)
Associated Peptides	12
Associated Spectra	20
Coverage	NaN

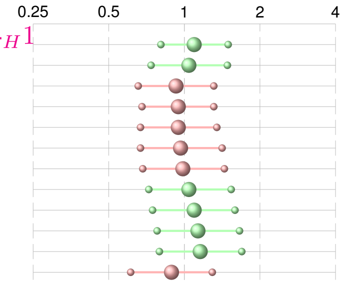

A	B	2.5	50	97.5	Sequence
3	1	0.81	1.1	1.5	DTITPMVQNAK
1	1	0.74	1	1.5	GLVDEAVDTAALIR
2	0	0.65	0.92	1.3	KPAPTVDGK
2	1	0.68	0.95	1.3	LINAVGGVR
2	0	0.67	0.94	1.4	NVGDIESMANALSELR
1	0	0.67	0.97	1.4	QWLANPGLDDK
1	0	0.68	0.99	1.4	VLQVAEMEAQNSEDPLFVDR
1	0	0.72	1	1.5	ASADLR
0	1	0.75	1.1	1.6	AEEEAILHDTDR
0	1	0.78	1.1	1.7	VNEASADLR
0	1	0.8	1.2	1.7	DREAILK
0	1	0.61	0.89	1.3	AIARNLSEKL

5.584 305 937 Clathrin light chain A [Crassostrea gigas]

Protein Accession	CL831.Contig4$_H1$
Mean Expression Ratio	1.01
Median Expression Ratio	1.01
Credible Interval	(0.689, 1.51)
Associated Peptides	1
Associated Spectra	1
Coverage	NaN

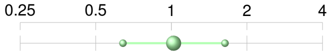

A	B	2.5	50	97.5	Sequence
0	1	0.64	1	1.6	AAEEAFIK

5.585 123 314 PREDICTED: ubiquinol-cytochrome c reductase hinge protein-like [Saccoglossus kowalevskii]; 279 530 PREDICTED: ubiquinol-cytochrome c reductase hinge protein-like [Saccoglossus kowalevskii]

Protein Accession	CL4684.Contig5$_H1$ CL4684.Contig4$_H1$
Mean Expression Ratio	1.01
Median Expression Ratio	1.01
Credible Interval	(0.701, 1.46)
Associated Peptides	1
Associated Spectra	2
Coverage	NaN

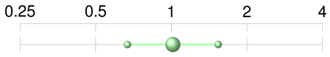

A	B	2.5	50	97.5	Sequence
2	0	0.67	1	1.5	CLFDELK

5.586 895 1410 Galectin-3 OS=Rattus norvegicus GN=Lgals3 PE=1 SV=4; 1067 1405 Galectin-9 [Crassostrea gigas]; 1068 1421 tandem-repeat galectin [Pinctada fucata]; 1041 1394 tandem-repeat galectin [Pinctada fucata]; 1046 1399 tandem-repeat galectin [Pinctada fucata]; 1068 1421 tandem-repeat galectin [Pinctada fucata]; 1067 1420 tandem-repeat galectin [Pinctada fucata]

Protein Accession	CL474.Contig9$_H$1 CL474.Contig8$_H$1 CL474.Contig7$_H$1 CL474.Contig6$_H$1 CL474.Contig5$_H$1
Mean Expression Ratio	1.01
Median Expression Ratio	1.01
Credible Interval	(0.702, 1.45)
Associated Peptides	1
Associated Spectra	2
Coverage	NaN

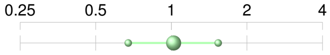

A	B	2.5	50	97.5	Sequence
2	0	0.67	1	1.5	VAVNNQHLLDFR

5.587 3 263 minus strand beta-actin [Urechis unicinctus]

Protein Accession	CL31420.Contig5$_H$1
Mean Expression Ratio	0.988
Median Expression Ratio	0.987
Credible Interval	(0.809, 1.21)
Associated Peptides	4
Associated Spectra	134
Coverage	NaN

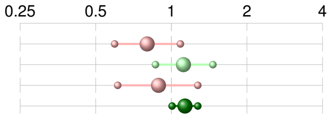

A	B	2.5	50	97.5	Sequence
3	2	0.59	0.8	1.1	IIAPPERK
7	2	0.86	1.1	1.5	IKIIAPPER
0	1	0.61	0.89	1.3	EITALAPPTMK
63	56	1	1.1	1.3	IIAPPER

5.588 530 679 minus strand GJ11201 [Drosophila virilis] gi

Protein Accession	CL45804.Contig2$_H$1
Mean Expression Ratio	0.986
Median Expression Ratio	0.988
Credible Interval	(0.677, 1.42)
Associated Peptides	1
Associated Spectra	2
Coverage	NaN

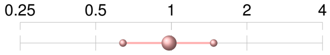

A	B	2.5	50	97.5	Sequence
2	0	0.64	0.98	1.5	QSQYALTLGPR

5.589 76 822 14-3-3 protein [Heterodera glycines]; 76 822 minus strand 14-3-3 protein [Heterodera glycines]

Protein Accession	Unigene34590$_{H}1$ Unigene28010$_{H}1$
Mean Expression Ratio	0.987
Median Expression Ratio	0.988
Credible Interval	(0.726, 1.34)
Associated Peptides	2
Associated Spectra	8
Coverage	NaN

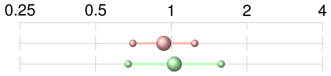

A	B	2.5	50	97.5	Sequence
**4**	**3**	**0.7**	**0.93**	**1.2**	**VVSSIEQK**
**1**	**0**	**0.67**	**1**	**1.6**	**KLEMAK**

5.590 227 1201 PREDICTED: quinone oxidoreductase-like isoform 1 [Ornithorhynchus anatinus]; 109 1083 PREDICTED: quinone oxidoreductase-like isoform 1 [Ornithorhynchus anatinus]; 164 1138 PREDICTED: quinone oxidoreductase-like isoform 1 [Ornithorhynchus anatinus]

Protein Accession	CL11899.Contig2$_{H}1$ CL11899.Contig1$_{H}1$ CL11899.Contig13$_{H}1$
Mean Expression Ratio	0.991
Median Expression Ratio	0.988
Credible Interval	(0.666, 1.48)
Associated Peptides	1
Associated Spectra	1
Coverage	NaN

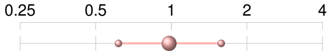

A	B	2.5	50	97.5	Sequence
**1**	**0**	**0.62**	**0.98**	**1.6**	**VHASGVNPVDTYIR**

5.591 4 219 hypothetical protein BRAFLDRAFT_66351 [Branchiostoma floridae] gi; 3 239 hypothetical protein BRAFLDRAFT_66351 [Branchiostoma floridae] gi

Protein Accession	CL31114.Contig2$_{H}1$ CL31114.Contig1$_{H}1$
Mean Expression Ratio	1.01
Median Expression Ratio	1.01
Credible Interval	(0.683, 1.5)
Associated Peptides	1
Associated Spectra	1
Coverage	NaN

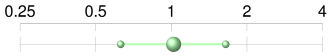

A	B	2.5	50	97.5	Sequence
**0**	**1**	**0.63**	**1**	**1.6**	**KLEEVSGELR**

5.592 68 481 minus strand netrin receptor UNC5A [Danio rerio] gi

Protein Accession	Unigene38845$_{H}1$
Mean Expression Ratio	0.986
Median Expression Ratio	0.989
Credible Interval	(0.662, 1.46)
Associated Peptides	1
Associated Spectra	1
Coverage	NaN

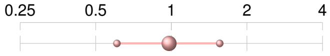

A	B	2.5	50	97.5	Sequence
1	0	0.61	0.98	1.6	ELMECQK

5.593 20 511 Neurexin-2-alpha [Crassostrea gigas]; 20 511 Neurexin-2-alpha [Crassostrea gigas]

Protein Accession	CL36998.Contig2$_{H}1$ CL36998.Contig1$_{H}1$
Mean Expression Ratio	0.987
Median Expression Ratio	0.989
Credible Interval	(0.668, 1.45)
Associated Peptides	1
Associated Spectra	1
Coverage	NaN

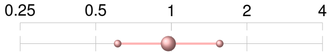

A	B	2.5	50	97.5	Sequence
0	1	0.61	0.98	1.6	AVFLDGR

5.594 63 443 PREDICTED: RAS protein activator like 1-like [Saccoglossus kowalevskii]

Protein Accession	CL19305.Contig2$_{H}1$
Mean Expression Ratio	0.99
Median Expression Ratio	0.989
Credible Interval	(0.793, 1.24)
Associated Peptides	4
Associated Spectra	40
Coverage	NaN

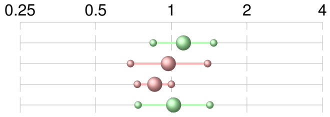

A	B	2.5	50	97.5	Sequence
2	4	0.84	1.1	1.5	DYAQAADGALASVLAR
1	1	0.69	0.97	1.4	YLEDWIK
16	13	0.73	0.86	1	LASVLAR
2	1	0.74	1	1.4	ALNIQLTK

5.595 3 386 fem-1-like protein C [Crassostrea gigas]

Protein Accession	Unigene19937$_{H}1$
Mean Expression Ratio	1.01
Median Expression Ratio	1.01
Credible Interval	(0.708, 1.43)
Associated Peptides	1
Associated Spectra	3
Coverage	NaN

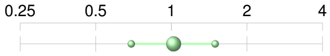

A	B	2.5	50	97.5	Sequence
3	0	0.69	1	1.5	MQSLLIR

5.596 429 1013 PDZ domain protein [Ixodes scapularis] gi

Protein Accession	CL8129.Contig12$_H$1
Mean Expression Ratio	0.99
Median Expression Ratio	0.99
Credible Interval	(0.796, 1.23)
Associated Peptides	6
Associated Spectra	20
Coverage	NaN

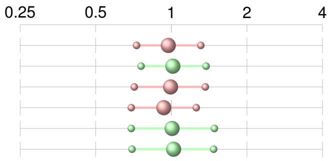

A	B	2.5	50	97.5	Sequence
4	1	0.73	0.97	1.3	DLGQPLSIQR
4	1	0.76	1	1.4	HGGGQIQLVIQR
1	2	0.71	0.99	1.4	VFSNSPAEGELQR
3	2	0.69	0.93	1.3	QALDLIK
1	0	0.69	1	1.5	DSFNTPWGFR
0	1	0.69	1	1.5	GDLILSINNR

5.597 6 317 myosin heavy chain cardiac muscle specific isoform 2 [Argopecten irradians]

Protein Accession	Unigene19023$_H$1
Mean Expression Ratio	0.99
Median Expression Ratio	0.99
Credible Interval	(0.879, 1.11)
Associated Peptides	16
Associated Spectra	245
Coverage	NaN

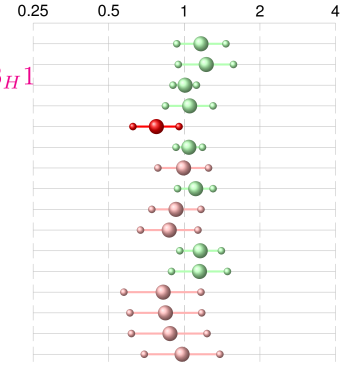

A	B	2.5	50	97.5	Sequence
6	5	0.93	1.2	1.5	ELAFQSDEDR
4	3	0.95	1.2	1.6	ELAFQSDEDRK
37	34	0.9	1	1.1	ELEMELDSEQR
11	1	0.84	1.1	1.3	KALETQLK
6	7	0.62	0.77	0.95	LADELRQEQEHSLQVEK
22	29	0.92	1	1.2	LDEAEAQALK
6	3	0.78	0.99	1.2	LKELAFQSDEDR
18	8	0.94	1.1	1.3	ALETQLK
5	5	0.74	0.93	1.2	LKELAFQSDEDRK
1	5	0.67	0.87	1.1	KAMADAAR
3	14	0.96	1.2	1.4	LADELR
7	0	0.89	1.1	1.5	QEQEHSLQVEK
0	1	0.57	0.82	1.2	ELEMELDSEQRR
0	2	0.61	0.84	1.2	LDEAEAQALKGGK
0	1	0.61	0.87	1.2	VRELEMELDSEQR
0	1	0.69	0.98	1.4	AFQSDEDRK

5.598 212 1405 minus strand unnamed protein product [Tetraodon nigroviridis]

Protein Accession	CL37546.Contig3$_H$1
Mean Expression Ratio	0.991
Median Expression Ratio	0.99
Credible Interval	(0.669, 1.46)
Associated Peptides	1
Associated Spectra	1
Coverage	NaN

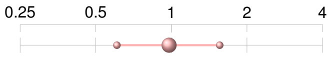

A	B	2.5	50	97.5	Sequence
0	1	0.61	0.98	1.6	TIAELGEK

5.599 552 1499 minus strand uncharacterized protein LOC100158466 [Xenopus (Silurana) tropicalis] gi; 552 1499 minus strand uncharacterized protein LOC100158466 [Xenopus (Silurana) tropicalis] gi; 552 1499 minus strand uncharacterized protein LOC100158466 [Xenopus (Silurana) tropicalis] gi

Protein Accession	Unigene57645$_{H}1$ Unigene52825$_{H}1$ Unigene48504$_{H}1$
Mean Expression Ratio	0.992
Median Expression Ratio	0.991
Credible Interval	(0.671, 1.47)
Associated Peptides	1
Associated Spectra	2
Coverage	NaN

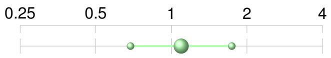

A	B	2.5	50	97.5	Sequence
**1**	**1**	**0.69**	**1.1**	**1.7**	**TGEPVEVDGELATLLAR**

5.600 181 744 unknown [Dendroctonus ponderosae]

Protein Accession	CL25948.Contig3$_{H}1$
Mean Expression Ratio	0.99
Median Expression Ratio	0.991
Credible Interval	(0.803, 1.22)
Associated Peptides	7
Associated Spectra	23
Coverage	NaN

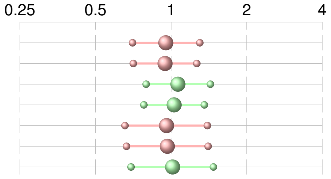

A	B	2.5	50	97.5	Sequence
**3**	**1**	**0.7**	**0.95**	**1.3**	**GTLTQLTK**
**4**	**1**	**0.71**	**0.95**	**1.3**	**SMGPIGSPMSK**
**4**	**1**	**0.8**	**1.1**	**1.4**	**TFICTPDR**
**4**	**2**	**0.78**	**1**	**1.4**	**VGHVPVAK**
**1**	**0**	**0.65**	**0.96**	**1.4**	**YKNNYLCR**
**1**	**0**	**0.66**	**0.97**	**1.4**	**GIYICDGSDEEAEEIIHK**
**1**	**0**	**0.69**	**1**	**1.5**	**NNYLCR**

5.601 242 589 Ribonucleoprotein RB97D OS=Drosophila melanogaster GN=Rb97D PE=2 SV=1

Protein Accession	CL6198.Contig1$_{H}1$
Mean Expression Ratio	1.01
Median Expression Ratio	1.01
Credible Interval	(0.681, 1.48)
Associated Peptides	1
Associated Spectra	1
Coverage	NaN

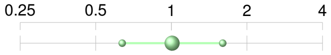

A	B	2.5	50	97.5	Sequence
**1**	**0**	**0.64**	**1**	**1.6**	**LFIGGLSYDTDEK**

5.602 " 1 510 Glycerol-3-phosphate dehydrogenase [NAD+], cytoplasmic [Crassostrea gigas]"

Protein Accession	Unigene16246$_{H}1$
Mean Expression Ratio	1.01
Median Expression Ratio	1.01
Credible Interval	(0.718, 1.42)
Associated Peptides	1
Associated Spectra	4
Coverage	NaN

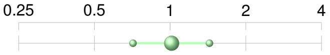

A	B	2.5	50	97.5	Sequence
2	2	0.71	1	1.4	EIFGMLEK

5.603 95 2506 Transitional endoplasmic reticulum ATPase [Crassostrea gigas]

Protein Accession	CL15973.Contig2$_{H}1$
Mean Expression Ratio	1.01
Median Expression Ratio	1.01
Credible Interval	(0.787, 1.29)
Associated Peptides	9
Associated Spectra	11
Coverage	NaN

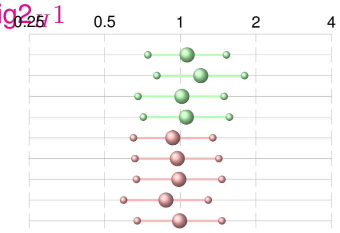

A	B	2.5	50	97.5	Sequence
1	1	0.74	1.1	1.5	EMVELPLR
1	0	0.8	1.2	1.8	LDQLIYIPLPDEK
1	0	0.68	1	1.5	MAIEAEIR
1	0	0.71	1.1	1.6	MDELQLFR
1	1	0.65	0.93	1.3	EVDIGIPDATGR
0	1	0.66	0.97	1.4	DIDLDYLAK
0	1	0.67	0.99	1.5	GILLYGPPGTGK
0	1	0.6	0.87	1.3	AHFEEAMK
0	1	0.67	1	1.5	GDIFLVR

5.604 970 1854 fasciclin-like protein precursor [Aplysia californica] gi

Protein Accession	CL9799.Contig1$_{H}1$
Mean Expression Ratio	1.01
Median Expression Ratio	1.01
Credible Interval	(0.713, 1.41)
Associated Peptides	3
Associated Spectra	3
Coverage	NaN

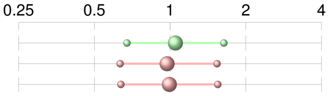

A	B	2.5	50	97.5	Sequence
1	0	0.67	1.1	1.6	VLIPDDALDLVTMAK
0	1	0.63	0.98	1.5	ANLVSMLK
0	1	0.64	0.99	1.5	ELQTVLLGHVLPEAR

5.605 134 385 minus strand CDGSH iron sulfur domain-containing protein 2 [Crassostrea gigas]; 11 511 minus strand CDGSH iron sulfur domain-containing protein 2 [Crassostrea gigas]

Protein Accession	CL36832.Contig5$_{H}1$ CL36832.Contig4$_{H}1$
Mean Expression Ratio	1.01
Median Expression Ratio	1.01
Credible Interval	(0.688, 1.49)
Associated Peptides	1
Associated Spectra	1
Coverage	NaN

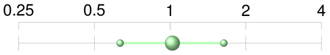

A	B	2.5	50	97.5	Sequence
1	0	0.63	1	1.6	VVNMVDIEDLGDK

5.606 205 1341 collagen precursor [Bombyx mori]

Protein Accession	CL38816.Contig2$_H$1
Mean Expression Ratio	1.01
Median Expression Ratio	1.01
Credible Interval	(0.685, 1.49)
Associated Peptides	1
Associated Spectra	1
Coverage	NaN

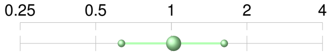

A	B	2.5	50	97.5	Sequence
0	1	0.63	1	1.6	GQLIPHIQK

5.607 34 1122 Fructose-bisphosphate aldolase [Crassostrea gigas]

Protein Accession	CL24228.Contig1$_H$1
Mean Expression Ratio	1.01
Median Expression Ratio	1.01
Credible Interval	(0.873, 1.17)
Associated Peptides	18
Associated Spectra	62
Coverage	NaN

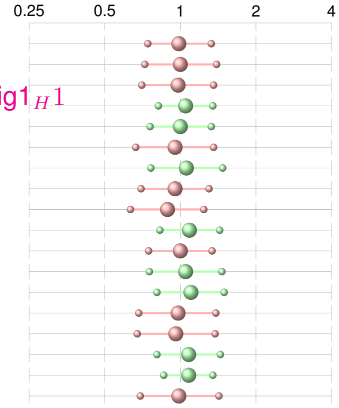

A	B	2.5	50	97.5	Sequence
3	1	0.74	0.99	1.3	DSQGTPFVK
1	1	0.72	1	1.4	GAQDELLK
1	1	0.7	0.98	1.4	GAQDELLKR
5	3	0.82	1	1.3	GIIPGIK
3	2	0.76	1	1.3	GILAADESVGSMGK
1	0	0.67	0.95	1.4	GVVPLAGSNNECTTQGLDGLAER
2	0	0.76	1.1	1.5	ISDNTPSYQAMIENANVLAR
3	0	0.69	0.95	1.3	KDGAQFAK
1	1	0.63	0.89	1.2	LEGIGLENTEENR
4	2	0.83	1.1	1.4	QIANSIVAPGK
3	1	0.74	1	1.3	RLEGIGLENTEENR
1	1	0.75	1.1	1.5	RLEGIGLENTEENRR
3	0	0.81	1.1	1.5	YASICQQNGLVPIVEPEVLPDGDHDLK
1	0	0.68	0.98	1.4	VKGAQDELLKR
1	0	0.67	0.96	1.4	ANSEAALGK
2	2	0.8	1.1	1.4	PVIAQYLTK
5	6	0.86	1.1	1.3	VTEQVLAFTYK
0	1	0.69	0.98	1.4	RLEGIGLENTEENRRRYR

5.608 260 892 minus strand calponin [Haliotis diversicolor]; 260 892 minus strand calponin [Haliotis diversicolor]

Protein Accession	CL48702.Contig2$_H$1 CL48702.Contig1$_H$1
Mean Expression Ratio	1.01
Median Expression Ratio	1.01
Credible Interval	(0.859, 1.19)
Associated Peptides	12
Associated Spectra	107
Coverage	NaN

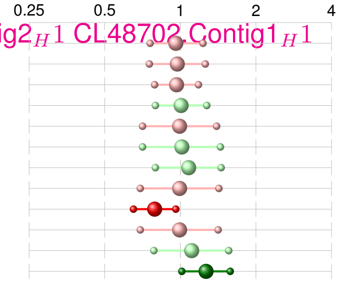

A	B	2.5	50	97.5	Sequence
7	4	0.76	0.96	1.2	AATGQQFPIEESNER
7	3	0.75	0.97	1.3	NFSEDKLR
18	8	0.79	0.97	1.2	TFQPASNLAFEAAR
7	5	0.79	1	1.3	VESSFDPEDAKK
1	1	0.71	0.99	1.4	YKVMENFYK
0	1	0.71	1	1.4	AFGIEVESHPSYTGQR
1	3	0.8	1.1	1.5	VESSFDPEDAK
0	1	0.69	1	1.4	FPIEESNER
7	13	0.65	0.79	0.96	IELFIQK
0	1	0.69	0.99	1.4	PASNLAFEAAR
1	1	0.78	1.1	1.6	VLACIR
12	5	1	1.3	1.6	VMENFYK

5.609 " 1 321 Dynein heavy chain, cytoplasmic [Crassostrea gigas]"

Protein Accession	Unigene35195$_{H}1$
Mean Expression Ratio	1.01
Median Expression Ratio	1.01
Credible Interval	(0.738, 1.38)
Associated Peptides	1
Associated Spectra	11
Coverage	NaN

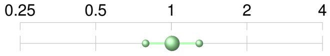

A	B	2.5	50	97.5	Sequence
**6**	**5**	**0.79**	**1**	**1.3**	**EILAVIQK**

5.610 " 154 534 minus strand Muscle M-line assembly protein unc-89, partial [Crassostrea gigas]; 259 690 minus strand Myosin light chain kinase, smooth muscle OS=Gallus gallus PE=1 SV=2"

Protein Accession	Unigene64523$_{H}1$ Unigene64520$_{H}1$
Mean Expression Ratio	0.995
Median Expression Ratio	0.993
Credible Interval	(0.673, 1.47)
Associated Peptides	1
Associated Spectra	2
Coverage	NaN

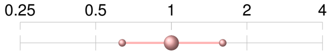

A	B	2.5	50	97.5	Sequence
**1**	**1**	**0.64**	**1**	**1.6**	**LEIGEVVVDDEGEYICK**

5.611 771 1505 O-methyltransferase family 3 [Dicentrarchus labrax]

Protein Accession	Unigene19132$_{H}1$
Mean Expression Ratio	1.01
Median Expression Ratio	1.01
Credible Interval	(0.715, 1.41)
Associated Peptides	3
Associated Spectra	3
Coverage	NaN

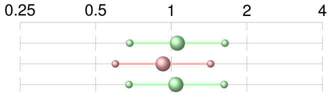

A	B	2.5	50	97.5	Sequence
**1**	**0**	**0.68**	**1.1**	**1.6**	**FGASYLPR**
**0**	**1**	**0.6**	**0.92**	**1.4**	**IAETTLTHPWR**
**0**	**1**	**0.68**	**1**	**1.6**	**VLIPVR**

5.612 116 1321 PREDICTED: similar to aspartate aminotransferase [Tribolium castaneum] gi

Protein Accession	CL11692.Contig6$_{H}1$
Mean Expression Ratio	0.994
Median Expression Ratio	0.993
Credible Interval	(0.772, 1.28)
Associated Peptides	8
Associated Spectra	10
Coverage	NaN

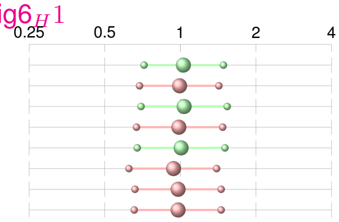

A	B	2.5	50	97.5	Sequence
**2**	**0**	**0.71**	**1**	**1.5**	**DGHQVALAQSFAK**
**1**	**1**	**0.69**	**0.99**	**1.4**	**ILSTPALR**
**1**	**0**	**0.7**	**1**	**1.5**	**INLGVGAYR**
**1**	**0**	**0.67**	**0.99**	**1.5**	**TQWLGEVK**
**1**	**0**	**0.67**	**1**	**1.5**	**ISVAGLSSK**
**0**	**1**	**0.63**	**0.94**	**1.4**	**ILSTPALRTQ**
**0**	**1**	**0.66**	**0.98**	**1.5**	**DAFALR**
**0**	**1**	**0.66**	**0.98**	**1.5**	**IIAMR**

5.613 145 1524 PREDICTED: aspartyl aminopeptidase-like [Oreochromis niloticus]

Protein Accession	CL19117.Contig2$_H$1
Mean Expression Ratio	0.992
Median Expression Ratio	0.993
Credible Interval	(0.691, 1.43)
Associated Peptides	2
Associated Spectra	2
Coverage	NaN

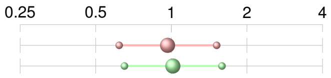

A	B	2.5	50	97.5	Sequence
1	0	0.62	0.96	1.5	ITVSPSNPVAFEEAIGK
1	0	0.65	1	1.6	KLVAHGFK

5.614 112 1050 Enolase-phosphatase E1 [Crassostrea gigas]; 112 1050 Enolase-phosphatase E1 [Crassostrea gigas]

Protein Accession	CL25175.Contig3$_H$1 CL25175.Contig2$_H$1
Mean Expression Ratio	0.994
Median Expression Ratio	0.993
Credible Interval	(0.728, 1.36)
Associated Peptides	3
Associated Spectra	5
Coverage	NaN

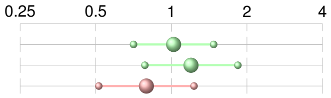

A	B	2.5	50	97.5	Sequence
1	2	0.71	1	1.5	SAAEAESLLVGVK
1	0	0.78	1.2	1.8	AIIFDVEGTTTPISFVK
0	1	0.51	0.8	1.2	QLQGHIWR

5.615 622 2733 PREDICTED: protein-glutamine gamma-glutamyltransferase K isoform 1 [Danio rerio]; 423 2498 PREDICTED: protein-glutamine gamma-glutamyltransferase K isoform 1 [Danio rerio]; 423 2534 PREDICTED: protein-glutamine gamma-glutamyltransferase K isoform 1 [Danio rerio]

Protein Accession	Unigene4042$_H$1 CL5652.Contig2$_H$1 CL5652.Contig1$_H$1
Mean Expression Ratio	0.994
Median Expression Ratio	0.994
Credible Interval	(0.775, 1.28)
Associated Peptides	6
Associated Spectra	12
Coverage	NaN

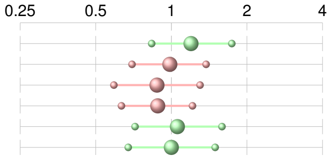

A	B	2.5	50	97.5	Sequence
1	1	0.84	1.2	1.7	LVFGIGEDPK
2	1	0.7	0.98	1.4	ILDMSGLK
1	0	0.59	0.88	1.3	GTQVVFILSAPDEK
2	2	0.63	0.88	1.2	KPHLQIK
1	0	0.72	1.1	1.6	GNPIPVVR
0	1	0.67	1	1.5	DVVFELR

5.616 2 1126 Prolyl endopeptidase [Crassostrea gigas]

Protein Accession	CL47692.Contig1$_{H}$1
Mean Expression Ratio	0.995
Median Expression Ratio	0.994
Credible Interval	(0.673, 1.49)
Associated Peptides	1
Associated Spectra	1
Coverage	NaN

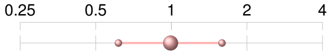

A	B	2.5	50	97.5	Sequence
**1**	**0**	**0.62**	**0.99**	**1.6**	**VVPLHSLK**

5.617 10 300 minus strand unnamed protein product [Tetraodon nigroviridis]

Protein Accession	CL14559.Contig2$_{H}$1
Mean Expression Ratio	0.993
Median Expression Ratio	0.994
Credible Interval	(0.705, 1.38)
Associated Peptides	1
Associated Spectra	4
Coverage	NaN

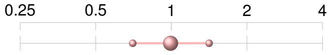

A	B	2.5	50	97.5	Sequence
**3**	**1**	**0.7**	**0.99**	**1.4**	**ILVQEGIHDR**

5.618 58 2268 hypothetical protein BRAFLDRAFT_267717 [Branchiostoma floridae] gi; 62 2245 hypothetical protein BRAFLDRAFT_267717 [Branchiostoma floridae] gi

Protein Accession	CL26176.Contig18$_{H}$1 CL26176.Contig17$_{H}$1
Mean Expression Ratio	0.994
Median Expression Ratio	0.994
Credible Interval	(0.671, 1.46)
Associated Peptides	1
Associated Spectra	1
Coverage	NaN

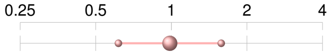

A	B	2.5	50	97.5	Sequence
**1**	**0**	**0.61**	**0.99**	**1.6**	**ALITIANK**

5.619 362 1084 Ectoine hydroxylase [Crassostrea gigas]; 362 1084 Ectoine hydroxylase [Crassostrea gigas]; 366 1220 Ectoine hydroxylase [Crassostrea gigas]

Protein Accession	CL2342.Contig4$_{H}$1 CL2342.Contig3$_{H}$1 CL2342.Contig2$_{H}$1
Mean Expression Ratio	0.996
Median Expression Ratio	0.994
Credible Interval	(0.673, 1.48)
Associated Peptides	1
Associated Spectra	1
Coverage	NaN

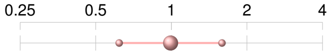

A	B	2.5	50	97.5	Sequence
**1**	**0**	**0.62**	**0.99**	**1.6**	**NFVLTDEMK**

5.620 981 1379 minus strand Multimerin-1 [Crassostrea gigas]; 998 1396 Multimerin-1 [Crassostrea gigas]; 975 1373 Multimerin-1 [Crassostrea gigas]; 960 1358 Multimerin-1 [Crassostrea gigas]; 810 1208 Multimerin-1 [Crassostrea gigas]

Protein Accession	CL22447.Contig9$_H$1 CL22447.Contig7$_H$1 CL22447.Contig6$_H$1 CL22447.Contig5$_H$1 CL2244
Mean Expression Ratio	0.994
Median Expression Ratio	0.994
Credible Interval	(0.742, 1.34)
Associated Peptides	3
Associated Spectra	7
Coverage	NaN

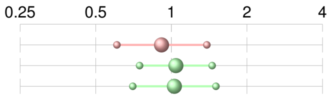

A	B	2.5	50	97.5	Sequence
1	0	0.61	0.92	1.4	TNTEFHTGPIVFDTTLSNK
2	2	0.75	1	1.5	VDAVLVADGQDVAR
0	2	0.7	1	1.5	VSFSAILK

5.621 5 514 hypothetical protein KGM_05418 [Danaus plexippus]

Protein Accession	CL11785.Contig1$_H$1
Mean Expression Ratio	0.993
Median Expression Ratio	0.994
Credible Interval	(0.717, 1.37)
Associated Peptides	3
Associated Spectra	4
Coverage	NaN

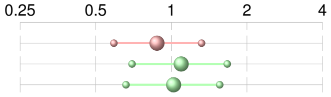

A	B	2.5	50	97.5	Sequence
1	1	0.59	0.88	1.3	ALLDLSENIDELNR
1	0	0.7	1.1	1.7	KLTNQSLK
1	0	0.66	1	1.6	NNLDIGK

5.622 4365 4553 minus strand Basement membrane-specific heparan sulfate proteoglycan core protein [Crassostrea gigas]; 2502 2696 minus strand hypothetical protein DAPPUDRAFT_186952 [Daphnia pulex]

Protein Accession	CL24300.Contig6$_H$1 CL24300.Contig2$_H$1
Mean Expression Ratio	0.995
Median Expression Ratio	0.995
Credible Interval	(0.71, 1.4)
Associated Peptides	2
Associated Spectra	3
Coverage	NaN

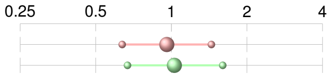

A	B	2.5	50	97.5	Sequence
1	1	0.64	0.96	1.4	DGSIIVDGQEVK
0	1	0.67	1	1.6	VDDGFR

5.623 72 521 minus strand GF14593 [Drosophila ananassae] gi

Protein Accession	CL38325.Contig2$_{H}$1
Mean Expression Ratio	0.995
Median Expression Ratio	0.995
Credible Interval	(0.683, 1.47)
Associated Peptides	1
Associated Spectra	1
Coverage	NaN

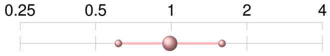

A	B	2.5	50	97.5	Sequence
1	0	0.61	0.99	1.6	LTADVHGK

5.624 1 264 Spectrin beta chain [Crassostrea gigas]

Protein Accession	Unigene36334$_{H}$1
Mean Expression Ratio	1.01
Median Expression Ratio	1.01
Credible Interval	(0.702, 1.45)
Associated Peptides	2
Associated Spectra	2
Coverage	NaN

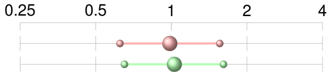

A	B	2.5	50	97.5	Sequence
1	0	0.63	0.99	1.6	QLLQVEHPNAAEVVQR
1	0	0.65	1	1.6	VAVVNQLAR

5.625 91 408 minus strand hypothetical protein [Amblyomma maculatum]

Protein Accession	CL19180.Contig1$_{H}$1
Mean Expression Ratio	1
Median Expression Ratio	1.01
Credible Interval	(0.671, 1.48)
Associated Peptides	1
Associated Spectra	1
Coverage	NaN

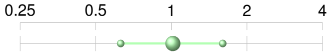

A	B	2.5	50	97.5	Sequence
1	0	0.63	1	1.6	EVGWTIVDGR

5.626 69 779 PREDICTED: 15-hydroxyprostaglandin dehydrogenase [NAD+] [Gallus gallus]

Protein Accession	CL44148.Contig4$_{H}$1
Mean Expression Ratio	0.995
Median Expression Ratio	0.995
Credible Interval	(0.694, 1.41)
Associated Peptides	2
Associated Spectra	2
Coverage	NaN

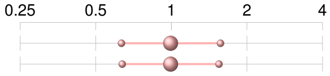

A	B	2.5	50	97.5	Sequence
1	0	0.63	0.99	1.6	PLVENPPSPTVIGEAVIK
1	0	0.64	0.99	1.6	PPSPTVIGEAVIK

5.627 152 1930 hypothetical protein BRAFLDRAFT_223365 [Branchiostoma floridae] gi

Protein Accession	CL49005.Contig4$_H$1
Mean Expression Ratio	0.995
Median Expression Ratio	0.995
Credible Interval	(0.671, 1.48)
Associated Peptides	1
Associated Spectra	1
Coverage	NaN

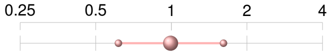

A	B	2.5	50	97.5	Sequence
**1**	**0**	**0.61**	**0.99**	**1.6**	**AQGYQLIR**

5.628 191 919 PREDICTED: 15-hydroxyprostaglandin dehydrogenase [NAD+] [Gallus gallus]

Protein Accession	CL7033.Contig5$_H$1
Mean Expression Ratio	1
Median Expression Ratio	1
Credible Interval	(0.679, 1.48)
Associated Peptides	1
Associated Spectra	1
Coverage	NaN

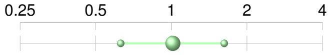

A	B	2.5	50	97.5	Sequence
**1**	**0**	**0.63**	**1**	**1.6**	**AMVEALLDK**

5.629 297 734 minus strand PREDICTED: calmodulin [Canis lupus familiaris]; 297 734 PREDICTED: calmodulin [Canis lupus familiaris]; 143 508 minus strand Calmodulin [Crassostrea gigas]

Protein Accession	Unigene26719$_H$1 Unigene17836$_H$1 Unigene10331$_H$1
Mean Expression Ratio	1.01
Median Expression Ratio	1
Credible Interval	(0.684, 1.48)
Associated Peptides	1
Associated Spectra	1
Coverage	NaN

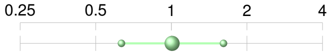

A	B	2.5	50	97.5	Sequence
**1**	**0**	**0.63**	**1**	**1.6**	**TLTFQEVDAIVTK**

5.630 998 1705 minus strand Basement membrane-specific heparan sulfate proteoglycan core protein [Crassostrea gigas]

Protein Accession	CL22963.Contig1$_{H}1$
Mean Expression Ratio	0.996
Median Expression Ratio	0.996
Credible Interval	(0.671, 1.48)
Associated Peptides	1
Associated Spectra	1
Coverage	NaN

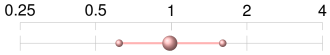

A	B	2.5	50	97.5	Sequence
**1**	**0**	**0.62**	**0.99**	**1.6**	**ITAYGGSLR**

5.631 27 722 Spectrin beta chain OS=Drosophila melanogaster GN=beta-Spec PE=1 SV=2; 27 722 Spectrin beta chain OS=Drosophila melanogaster GN=beta-Spec PE=1 SV=2

Protein Accession	CL5904.Contig3$_{H}1$ CL5904.Contig1$_{H}1$
Mean Expression Ratio	0.995
Median Expression Ratio	0.996
Credible Interval	(0.716, 1.38)
Associated Peptides	2
Associated Spectra	4
Coverage	NaN

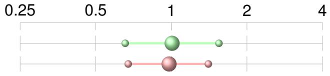

A	B	2.5	50	97.5	Sequence
**1**	**0**	**0.65**	**1**	**1.5**	**EQIEVMLADLDK**
**3**	**0**	**0.67**	**0.98**	**1.4**	**HQAFEAEIGHYAR**

5.632 616 1740 5'-AMP-activated protein kinase subunit beta-2 [Crassostrea gigas]

Protein Accession	Unigene52845$_{H}1$
Mean Expression Ratio	1.01
Median Expression Ratio	1
Credible Interval	(0.675, 1.49)
Associated Peptides	1
Associated Spectra	1
Coverage	NaN

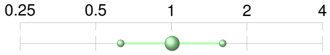

A	B	2.5	50	97.5	Sequence
**1**	**0**	**0.63**	**1**	**1.6**	**FIVDEQWVVSK**

5.633 " 492 3473 PREDICTED: 2-oxoglutarate dehydrogenase, mitochondrial isoform 1 [Sarcophilus harrisii]; 492 3458 PREDICTED: 2-oxoglutarate dehydrogenase, mitochondrial isoform 1 [Sarcophilus harrisii]"

Protein Accession	CL2200.Contig1$_{H}$1 CL2200.Contig16$_{H}$1
Mean Expression Ratio	1
Median Expression Ratio	1
Credible Interval	(0.699, 1.44)
Associated Peptides	2
Associated Spectra	2
Coverage	NaN

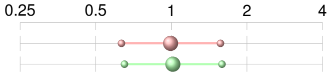

A	B	2.5	50	97.5	Sequence
**1**	**0**	**0.63**	**1**	**1.6**	**FSEVPEGFVVHGGLK**
**1**	**0**	**0.65**	**1**	**1.6**	**KPLVVATPK**

5.634 128 559 minus strand predicted protein [Nematostella vectensis] gi

Protein Accession	CL19343.Contig1$_{H}$1
Mean Expression Ratio	0.997
Median Expression Ratio	0.996
Credible Interval	(0.717, 1.39)
Associated Peptides	1
Associated Spectra	6
Coverage	NaN

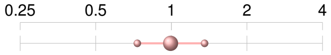

A	B	2.5	50	97.5	Sequence
**3**	**3**	**0.73**	**0.99**	**1.4**	**TVILGDLTGSK**

5.635 2019 2723 minus strand Kyphoscoliosis peptidase [Crassostrea gigas]; 2060 2764 minus strand Kyphoscoliosis peptidase [Crassostrea gigas]; 1074 1778 minus strand Kyphoscoliosis peptidase [Crassostrea gigas]; 2063 2767 minus strand Kyphoscoliosis peptidase [Crassostrea gigas]; 2044 2748 minus strand Kyphoscoliosis peptidase [Crassostrea gigas]; 2044 2748 minus strand Kyphoscoliosis peptidase [Crassostrea gigas]

Protein Accession	CL3923.Contig7$_{H}$1 CL3923.Contig6$_{H}$1 CL3923.Contig5$_{H}$1 CL3923.Contig4$_{H}$1 CL3923.Con
Mean Expression Ratio	1
Median Expression Ratio	1
Credible Interval	(0.7, 1.42)
Associated Peptides	1
Associated Spectra	3
Coverage	NaN

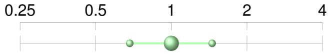

A	B	2.5	50	97.5	Sequence
**3**	**0**	**0.69**	**1**	**1.5**	**TMGQAPPTVPK**

5.636 150 1511 Tensin OS=Gallus gallus GN=TNS PE=1 SV=2

Protein Accession	CL2743.Contig2$_{H}1$
Mean Expression Ratio	1
Median Expression Ratio	1
Credible Interval	(0.725, 1.4)
Associated Peptides	3
Associated Spectra	4
Coverage	NaN

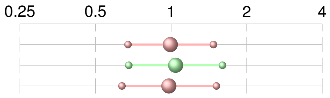

A	B	2.5	50	97.5	Sequence
1	1	0.67	0.99	1.5	DDAIVMLR
1	0	0.68	1	1.6	DKPPGTFVIR
1	0	0.64	0.98	1.5	GTFVIR

5.637 67 825 unnamed protein product [Oikopleura dioica]

Protein Accession	Unigene71508$_{H}1$
Mean Expression Ratio	1.01
Median Expression Ratio	1
Credible Interval	(0.698, 1.46)
Associated Peptides	1
Associated Spectra	2
Coverage	NaN

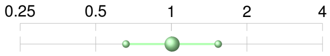

A	B	2.5	50	97.5	Sequence
2	0	0.66	1	1.5	TAANVEEAFINTAK

5.638 " 1 1563 Putative methylmalonate-semialdehyde dehydrogenase [acylating], mitochondrial [Crassostrea gigas]"

Protein Accession	Unigene56839$_{H}1$
Mean Expression Ratio	0.998
Median Expression Ratio	0.998
Credible Interval	(0.763, 1.31)
Associated Peptides	7
Associated Spectra	8
Coverage	NaN

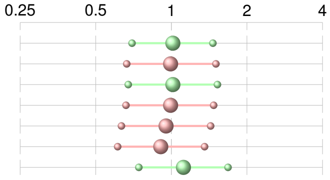

A	B	2.5	50	97.5	Sequence
1	1	0.7	1	1.5	AEMDAAVAAAK
1	0	0.66	1	1.5	GNFVGPTILHK
1	0	0.67	1	1.5	LFINNEFVESK
1	0	0.66	0.99	1.5	WIDVHNPATNEVVTR
1	0	0.63	0.95	1.4	TVTQMWR
0	1	0.61	0.91	1.4	TLVDAEGDVLR
0	1	0.74	1.1	1.7	PPGVVNVIHGR

5.639 1 111 glycogen synthase [Crassostrea gigas]

Protein Accession	Unigene78448$_{H}1$
Mean Expression Ratio	1
Median Expression Ratio	0.998
Credible Interval	(0.689, 1.46)
Associated Peptides	1
Associated Spectra	1
Coverage	NaN

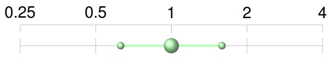

A	B	2.5	50	97.5	Sequence
0	1	0.63	1	1.6	NVEPDLDDDYLDR

5.640 285 1637 PREDICTED: talin-2-like [Cricetulus griseus]

Protein Accession	CL18079.Contig3$_{H}1$
Mean Expression Ratio	0.999
Median Expression Ratio	0.998
Credible Interval	(0.678, 1.47)
Associated Peptides	1
Associated Spectra	1
Coverage	NaN

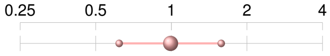

A	B	2.5	50	97.5	Sequence
0	1	0.62	0.99	1.6	ISIVGQNVIK

5.641 2881 3411 myosin heavy chain [Todarodes pacificus]; 2915 3445 myosin heavy chain [Todarodes pacificus]

Protein Accession	Unigene19024$_{H}1$ Unigene19021$_{H}1$
Mean Expression Ratio	1
Median Expression Ratio	1
Credible Interval	(0.873, 1.15)
Associated Peptides	13
Associated Spectra	135
Coverage	NaN

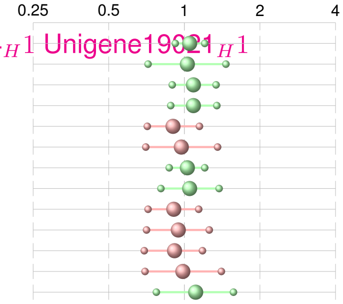

A	B	2.5	50	97.5	Sequence
28	13	0.92	1.1	1.2	ALDSMQASLEAEAK
1	0	0.72	1	1.5	ALTGEVEELR
9	6	0.89	1.1	1.3	CTALTGEVEELR
9	4	0.88	1.1	1.3	IQEKEEEFENTR
5	4	0.71	0.9	1.1	IQEKEEEFENTRR
2	0	0.7	0.98	1.4	KLEQDINELEIALDQANR
25	1	0.87	1	1.2	KLQQQVIETQQHVEEEAR
6	0	0.8	1.1	1.4	LQQQVIETQQHVEEEAR
4	6	0.72	0.91	1.1	RCTALTGEVEELR
1	3	0.71	0.95	1.3	RIQEKEEEFENTR
3	3	0.69	0.91	1.2	TALTGEVEELR
0	1	0.7	0.98	1.4	IQEKEEEFEN
0	1	0.77	1.1	1.6	ELEIALDQANR

5.642 49 747 PREDICTED: elongation factor 1-delta-like [Strongylocentrotus purpuratus]; 49 819 PREDICTED: elongation factor 1-delta-like isoform 2 [Strongylocentrotus purpuratus]

Protein Accession	CL10790.Contig13$_{H}1$ CL10790.Contig12$_{H}1$
Mean Expression Ratio	1
Median Expression Ratio	1
Credible Interval	(0.699, 1.44)
Associated Peptides	2
Associated Spectra	5
Coverage	NaN

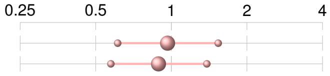

A	B	2.5	50	97.5	Sequence
1	0	0.61	0.97	1.5	VAALEVVGTGGASPK
1	3	0.57	0.89	1.4	LVDLAYGIK

5.643 373 579 myosin heavy chain [Argopecten irradians] gi

Protein Accession: Unigene64289$_{H}$1
Mean Expression Ratio: 1
Median Expression Ratio: 1
Credible Interval: (0.841, 1.19)
Associated Peptides: 9
Associated Spectra: 62
Coverage: NaN

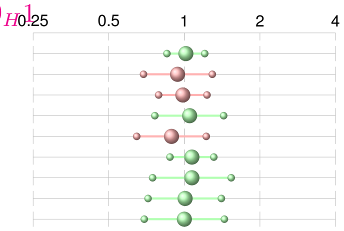

A	B	2.5	50	97.5	Sequence
15	7	0.85	1	1.2	QVEEAEEIAAVNLAK
2	1	0.69	0.94	1.3	RQVEEAEEIAAVNLAK
8	4	0.79	0.99	1.2	VQNELEDAEER
2	1	0.76	1	1.4	ELEDAEER
2	1	0.65	0.89	1.2	ADSAEGTLSK
11	4	0.88	1.1	1.3	KVQNELEDAEER
1	0	0.75	1.1	1.5	QELVDKLQAK
1	1	0.72	1	1.4	VDKLQAK
0	1	0.69	1	1.4	VSVSRR

5.644 3 293 Titin [Crassostrea gigas]

Protein Accession: Unigene9241$_{H}$1
Mean Expression Ratio: 0.995
Median Expression Ratio: 0.998
Credible Interval: (0.672, 1.47)
Associated Peptides: 1
Associated Spectra: 1
Coverage: NaN

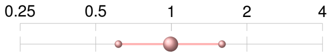

A	B	2.5	50	97.5	Sequence
1	0	0.62	1	1.6	TAINTVESSLSELGQR

5.645 605 1153 Peptidyl-prolyl cis-trans isomerase B [Crassostrea gigas]; 1046 1630 Peptidyl-prolyl cis-trans isomerase B [Crassostrea gigas]; 589 1173 Peptidyl-prolyl cis-trans isomerase B [Crassostrea gigas]; 938 1414 Peptidyl-prolyl cis-trans isomerase B [Crassostrea gigas]; 883 1467 Peptidyl-prolyl cis-trans isomerase B [Crassostrea gigas]

Protein Accession: CL24747.Contig5$_{H}$1 CL24747.Contig4$_{H}$1 CL24747.Contig3$_{H}$1 CL24747.Contig2$_{H}$1 CL2474
Mean Expression Ratio: 0.997
Median Expression Ratio: 0.998
Credible Interval: (0.718, 1.38)
Associated Peptides: 2
Associated Spectra: 6
Coverage: NaN

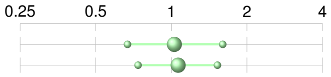

A	B	2.5	50	97.5	Sequence
0	1	0.67	1	1.6	DFVIQMGDVTGK
2	3	0.74	1.1	1.5	IASSGVVGIASK

5.646 113 445 ribosomal protein rps19 [Lineus viridis]

Protein Accession	CL8932.Contig6$_H1$
Mean Expression Ratio	0.997
Median Expression Ratio	0.998
Credible Interval	(0.673, 1.47)
Associated Peptides	1
Associated Spectra	1
Coverage	NaN

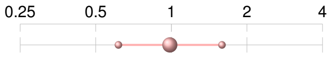

A	B	2.5	50	97.5	Sequence
**0**	**1**	**0.62**	**0.99**	**1.6**	**LEEIR**

5.647 125 754 minus strand peptidyl prolyl cis-trans isomerase B [Conus novaehollandiae]

Protein Accession	Unigene19558$_H1$
Mean Expression Ratio	0.998
Median Expression Ratio	0.998
Credible Interval	(0.731, 1.37)
Associated Peptides	4
Associated Spectra	5
Coverage	NaN

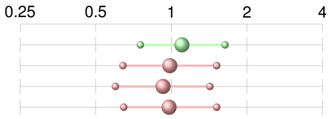

A	B	2.5	50	97.5	Sequence
**2**	**0**	**0.75**	**1.1**	**1.6**	**HLALGDKEDGGVK**
**1**	**0**	**0.64**	**0.99**	**1.5**	**KTQWLDGR**
**1**	**0**	**0.6**	**0.93**	**1.4**	**DTNGSQFFITTK**
**1**	**0**	**0.65**	**0.98**	**1.5**	**FPDENFK**

5.648 1070 1888 minus strand galectin [Pinctada fucata]; 1104 1922 minus strand galectin [Pinctada fucata]

Protein Accession	CL7245.Contig4$_H1$ CL7245.Contig2$_H1$
Mean Expression Ratio	1
Median Expression Ratio	1
Credible Interval	(0.67, 1.49)
Associated Peptides	1
Associated Spectra	1
Coverage	NaN

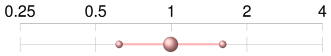

A	B	2.5	50	97.5	Sequence
**1**	**0**	**0.62**	**1**	**1.6**	**FNEGVTVR**

5.649 63 1865 Dolichyl-diphosphooligosaccharide–protein glycosyltransferase subunit 1 [Crassostrea gigas]; 63 1865 Dolichyl-diphosphooligosaccharide–protein glycosyltransferase subunit 1 [Crassostrea gigas]

Protein Accession	CL41703.Contig2$_{H}$1 CL41703.Contig1$_{H}$1
Mean Expression Ratio	1
Median Expression Ratio	1
Credible Interval	(0.737, 1.37)
Associated Peptides	4
Associated Spectra	5
Coverage	NaN

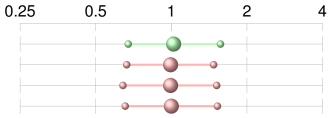

A	B	2.5	50	97.5	Sequence
1	0	0.67	1	1.6	KVDIATHIVK
1	1	0.66	0.99	1.5	VASLVEEVQSAQDKR
1	0	0.64	0.99	1.5	DLNAFQVAR
0	1	0.65	1	1.5	ELDDSVDIELRPR

5.650 125 550 minus strand ribosomal protein S20 [Chlamys farreri]; 125 550 minus strand ribosomal protein S20 [Chlamys farreri]; 125 550 minus strand ribosomal protein S20 [Chlamys farreri]

Protein Accession	CL6833.Contig3$_{H}$1 CL6833.Contig2$_{H}$1 CL6833.Contig1$_{H}$1
Mean Expression Ratio	0.999
Median Expression Ratio	1
Credible Interval	(0.706, 1.4)
Associated Peptides	2
Associated Spectra	3
Coverage	NaN

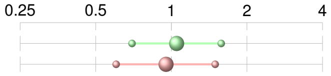

A	B	2.5	50	97.5	Sequence
2	0	0.7	1	1.6	IIDLHSPSEIVK
1	0	0.6	0.95	1.5	KTPCGEGSK

5.651 112 819 pyruvate kinase [Crassostrea gigas]; 132 932 pyruvate kinase [Crassostrea gigas]

Protein Accession	CL31920.Contig3$_{H}$1 CL31920.Contig1$_{H}$1
Mean Expression Ratio	0.998
Median Expression Ratio	0.999
Credible Interval	(0.674, 1.48)
Associated Peptides	1
Associated Spectra	1
Coverage	NaN

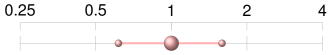

A	B	2.5	50	97.5	Sequence
1	0	0.61	1	1.6	ITIDDSYKEK

5.652 120 833 B-cell receptor-associated protein 31 [Crassostrea gigas]; 238 951 B-cell receptor-associated protein 31 [Crassostrea gigas]; 88 801 B-cell receptor-associated protein 31 [Crassostrea gigas]; 88 801 B-cell receptor-associated protein 31 [Crassostrea gigas]

Protein Accession	CL9442.Contig7$_{H}$1 CL9442.Contig5$_{H}$1 CL9442.Contig4$_{H}$1 CL9442.Contig3$_{H}$1
Mean Expression Ratio	1
Median Expression Ratio	1
Credible Interval	(0.68, 1.48)
Associated Peptides	1
Associated Spectra	1
Coverage	NaN

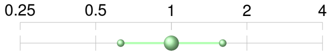

A	B	2.5	50	97.5	Sequence
1	0	0.63	1	1.6	LLEEHAQLQK

5.653 303 1793 PREDICTED: matrix metalloproteinase 19 isoform rasi-1 preproprotein-like [Saccoglossus kowalevskii]

Protein Accession	CL7632.Contig1$_{H}$1
Mean Expression Ratio	0.999
Median Expression Ratio	0.999
Credible Interval	(0.697, 1.43)
Associated Peptides	2
Associated Spectra	2
Coverage	NaN

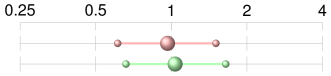

A	B	2.5	50	97.5	Sequence
1	0	0.61	0.96	1.5	FDAIDR
1	0	0.66	1	1.6	LGRVAR

5.654 549 821 minus strand Calponin-2 [Crassostrea gigas]

Protein Accession	CL21394.Contig10$_{H}$1
Mean Expression Ratio	0.998
Median Expression Ratio	1
Credible Interval	(0.758, 1.31)
Associated Peptides	3
Associated Spectra	10
Coverage	NaN

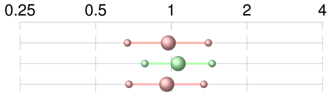

A	B	2.5	50	97.5	Sequence
2	0	0.67	0.97	1.4	QQGRASQAGMSMGAVR
3	2	0.78	1.1	1.5	SLFPTADLFEGR
3	0	0.68	0.96	1.3	NSLFPTADLFEGR

5.655 835 1116 tropomyosin [Ilyanassa obsoleta]

Protein Accession	CL2925.Contig8$_{H}$1
Mean Expression Ratio	1
Median Expression Ratio	1
Credible Interval	(0.782, 1.28)
Associated Peptides	4
Associated Spectra	15
Coverage	NaN

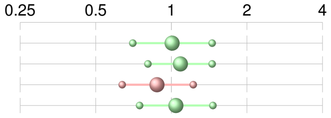

A	B	2.5	50	97.5	Sequence
1	1	0.7	1	1.5	ELEEQSHALTNSLK
2	4	0.81	1.1	1.5	LAVMEAELEK
2	2	0.64	0.87	1.2	QLELYEEK
0	3	0.75	1	1.5	LEDVETELR

5.656 242 1834 Regulator of differentiation 1 [Crassostrea gigas]; 458 2050 minus strand Regulator of differentiation 1 [Crassostrea gigas]; 503 2095 Regulator of differentiation 1 [Crassostrea gigas]; 274 1866 Regulator of differentiation 1 [Crassostrea gigas]; 434 1942 Regulator of differentiation 1 [Crassostrea gigas]

Protein Accession	CL11169.Contig7$_H1$ CL11169.Contig5$_H1$ CL11169.Contig4$_H1$ CL11169.Contig3$_H1$ CL1116
Mean Expression Ratio	0.999
Median Expression Ratio	1
Credible Interval	(0.691, 1.43)
Associated Peptides	2
Associated Spectra	2
Coverage	NaN

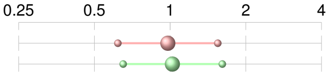

A	B	2.5	50	97.5	Sequence
**1**	**0**	**0.62**	**0.98**	**1.5**	**SLPHEVAETDIVQLGMPFGK**
**0**	**1**	**0.65**	**1**	**1.6**	**IITFTK**

iTRAQ Data Analysis Report

Javier, Head 7d versus control

April 7, 2014

1 Introduction

This document summarizes an analysis of relative protein expression using iTRAQ. The reporter ion peak area measurements supplied by the ABI software are used to estimate treatment-dependent peptide and protein relative expression. Estimation is accomplished using a Bayesian approach with the model given below. The document includes a protein relative expression summary and a per-protein detailed analysis. The document is internally hyperlinked and linked externally to NCBI.

2 Experiment and Model Description

2.1 Experiment Design

The report summarizes data from one or more iTRAQ experiments addressing a common comparison. The experiment design, used in this analysis, is given in the table below.

	Experiment	Treatment	Channel	Sample
1	A	control	115	s1
2	A	test	117	s1
3	B	control	115	s2
4	B	test	117	s2

2.2 Input Files

Data for this analysis was extracted from the following tandem mass spectra (MSMS) summary files.

Experiment	MSMS Summary File
A	head2.txt
B	head3.txt

2.3 Statistical Model

The following statistical model was used to estimate the treatment-dependent effects.

LogIntensity ˜ Channel + Spectrum + Protein + Peptide + Protein:Treatment + Peptide:Treatment

3 Data Summary

The data supplied in the MSMS summary is filtered to remove unidentified proteins, contaminants, and peptides containing selected modifications. The following table sumarizes the data provided and used in the analysis.

	A	B	Combined
Supplied Spectra	21342	14837	36179
Unidentified Spectra	8154	6352	14506
Disallowed Modifications	363	183	546
Spectra from Contaminants	0	0	0
Missing Data	3157	2212	5369
Low Confidence Spectra	8154	6352	14506
Degenerate Peptides	3594	2230	5824
Remaining Spectra			9934
Unique Proteins			657
Unique Peptides			2364
Model R^2			0.939

4 Protein Summary

Each protein identified in one or more of the MSMS summaries is listed below in decreasing order of expression change magnitude. The median and estimated credible interval for each protein is given to the left in the table. Proteins identified by a single peptide are listed in a separate table.

4.1 Identified Proteins

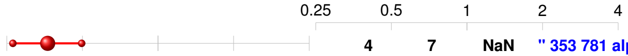

Peptides	Spectra	Coverage	Protein
4	7	NaN	**" 353 781 alpha-B-crystallin, putative [Ixodes scapularis] gi"**

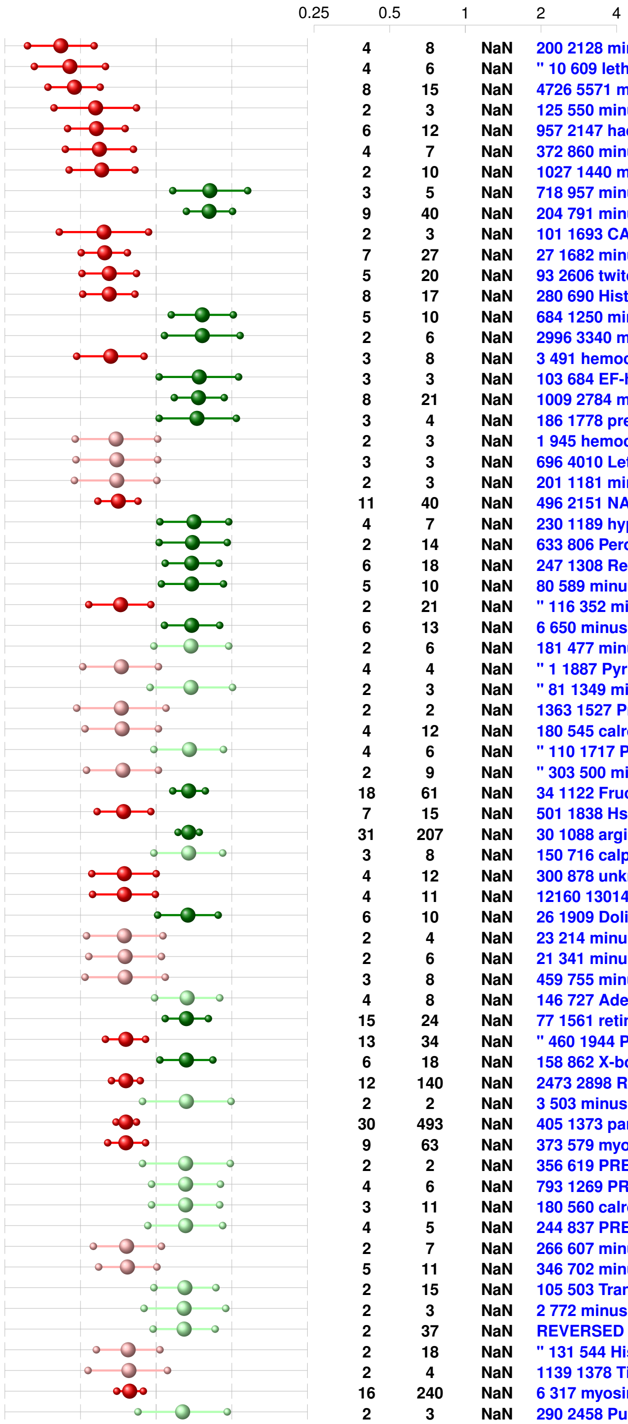

Peptides	Spectra	Coverage	Protein
4	8	NaN	200 2128 minus strand heat shock protein 70 [Cristaria plicata]
4	6	NaN	" 10 609 lethal(2)essential for life protein, l2efl [Aedes aegypti] gi"
8	15	NaN	4726 5571 minus strand twitchin [Mytilus galloprovincialis]
2	3	NaN	125 550 minus strand ribosomal protein S20 [Chlamys farreri]; 125 550 minus strand ribosomal protein S20 [Chlamy
6	12	NaN	957 2147 haemocyanin [Aplysia californica]
4	7	NaN	372 860 minus strand small heat shock protein [Tegillarca granosa]; 651 998 minus strand small heat shock protein
2	10	NaN	1027 1440 minus strand hemocyanin isoform 1 [Haliotis diversicolor]
3	5	NaN	718 957 minus strand Calponin-1 OS=Gallus gallus GN=CNN1 PE=1 SV=2
9	40	NaN	204 791 minus strand PREDICTED: 15-hydroxyprostaglandin dehydrogenase [NAD+] [Gallus gallus]
2	3	NaN	101 1693 CALNUC [Spodoptera frugiperda]; 141 1325 Nucleobindin-2 [Crassostrea gigas]
7	27	NaN	27 1682 minus strand PREDICTED: nucleolar protein 56 [Oreochromis niloticus]
5	20	NaN	93 2606 twitchin [Mytilus galloprovincialis]
8	17	NaN	280 690 Histone H3.3 [Harpegnathos saltator]
5	10	NaN	684 1250 minus strand Collagen alpha-5(VI) chain [Crassostrea gigas]
2	6	NaN	2996 3340 minus strand Collagen alpha-5(VI) chain [Crassostrea gigas]
3	8	NaN	3 491 hemocyanin [Haliotis tuberculata]
3	3	NaN	103 684 EF-hand domain-containing protein D2 [Crassostrea gigas]
8	21	NaN	1009 2784 minus strand Collagen alpha-3(VI) chain [Crassostrea gigas]
3	4	NaN	186 1778 predicted protein [Nematostella vectensis] gi
2	3	NaN	1 945 hemocyanin isoform 1 [Haliotis diversicolor]
3	3	NaN	696 4010 Lethal(2) giant larvae-like protein 1 [Crassostrea gigas]; 657 3971 minus strand Lethal(2) giant larvae-like p
2	3	NaN	201 1181 minus strand Serine/threonine-protein phosphatase PP1-alpha catalytic subunit [Crassostrea gigas]
11	40	NaN	496 2151 NADP-dependent malic enzyme [Crassostrea gigas]
4	7	NaN	230 1189 hypothetical protein [Amblyomma maculatum]
2	14	NaN	633 806 Peroxiredoxin-1 [Crassostrea gigas]; 648 821 Peroxiredoxin-1 [Crassostrea gigas]; 666 839 Peroxiredoxin-1
6	18	NaN	247 1308 RecName: Full=Guanine nucleotide-binding protein G(o) subunit alpha gi|9633|emb|CAA78806.1| GTP-bin
5	10	NaN	80 589 minus strand PREDICTED: adenine phosphoribosyltransferase-like [Strongylocentrotus purpuratus]
2	21	NaN	" 116 352 minus strand troponin I 4 [Clonorchis sinensis]; 491 706 minus strand troponin-I, TnI {C-terminal} [Chlamy
6	13	NaN	6 650 minus strand glutathione S-transferase mu class protein [Crassostrea ariakensis]; 117 761 glutathione S-trans
2	6	NaN	181 477 minus strand Zgc:56530 [Danio rerio]; 185 481 minus strand Zgc:56530 [Danio rerio]
4	4	NaN	" 1 1887 Pyruvate carboxylase, mitochondrial [Crassostrea gigas]"
2	3	NaN	" 81 1349 minus strand PREDICTED: LOW QUALITY PROTEIN: dolichyl-diphosphooligosaccharide–protein glycosyl
2	2	NaN	1363 1527 Protein disulfide-isomerase A3 [Crassostrea gigas]
4	12	NaN	180 545 calreticulin precursor [Aplysia californica] gi
4	6	NaN	" 110 1717 PREDICTED: glutamate dehydrogenase, mitochondrial-like [Oreochromis niloticus]; 110 1717 PREDICTE
2	9	NaN	" 303 500 minus strand protein lethal, putative [Pediculus humanus corporis] gi"
18	61	NaN	34 1122 Fructose-bisphosphate aldolase [Crassostrea gigas]
7	15	NaN	501 1838 Hsp90A [Haliotis asinina]
31	207	NaN	30 1088 arginine kinase [Conus novaehollandiae]
3	8	NaN	150 716 calponin/transgelin [Papilio xuthus]
4	12	NaN	300 878 unknown [Schistosoma japonicum]; 567 1145 unknown [Schistosoma japonicum]; 1110 1688 unknown [Sch
4	11	NaN	12160 13014 twitchin [Mytilus galloprovincialis]
6	10	NaN	26 1909 Dolichyl-diphosphooligosaccharide–protein glycosyltransferase subunit 2 [Crassostrea gigas]
2	4	NaN	23 214 minus strand hemocyanin isoform 1 [Nucula nucleus]
2	6	NaN	21 341 minus strand hemocyanin isoform 1 [Nucula nucleus]
3	8	NaN	459 755 minus strand heat shock protein beta-1 [Trichinella spiralis] gi; 464 763 minus strand heat shock protein bet
4	8	NaN	146 727 Adenylate kinase isoenzyme 1 [Crassostrea gigas]
15	24	NaN	77 1561 retinal dehydrogenase [Lymnaea stagnalis]; 69 1553 retinal dehydrogenase [Lymnaea stagnalis]; 308 1747 r
13	34	NaN	" 460 1944 PREDICTED: succinyl-CoA:3-ketoacid-coenzyme A transferase 1, mitochondrial [Canis lupus familiaris]"
6	18	NaN	158 862 X-box binding protein [Haliotis discus discus]
12	140	NaN	2473 2898 RecName: Full=Paramyosin gi|397603|emb|CAA79849.1| paramyosin [Echinococcus granulosus]
2	2	NaN	3 503 minus strand elongation factor-1 alpha [Pomacea bridgesi]
30	493	NaN	405 1373 paramyosin [Haliotis discus discus]
9	63	NaN	373 579 myosin heavy chain [Argopecten irradians] gi
2	2	NaN	356 619 PREDICTED: hypothetical protein LOC100698726 [Oreochromis niloticus]
4	6	NaN	793 1269 PREDICTED: calmodulin-like [Saccoglossus kowalevskii]
3	11	NaN	180 560 calreticulin precursor [Aplysia californica] gi; 180 545 calreticulin precursor [Aplysia californica] gi
4	5	NaN	244 837 PREDICTED: ras-like GTP-binding protein Rho1-like [Megachile rotundata]
2	7	NaN	266 607 minus strand H2 [Haliotis tuberculata]
5	11	NaN	346 702 minus strand PREDICTED: galectin-4-like [Anolis carolinensis]
2	15	NaN	105 503 Transgelin-2 [Crassostrea gigas]
2	3	NaN	2 772 minus strand arylsulfatase [Haliotis discus discus]
2	37	NaN	REVERSED 6 395 PREDICTED: sister chromatid cohesion protein PDS5 homolog B-A isoform 1 [Nasonia vitripennis
2	18	NaN	" 131 544 Histone H2B, gonadal OS=Patella granatina PE=1 SV=2"
2	4	NaN	1139 1378 Titin [Crassostrea gigas]; 1116 1355 Titin [Crassostrea gigas]; 1139 1378 Titin [Crassostrea gigas]; 1139 1
16	240	NaN	6 317 myosin heavy chain cardiac muscle specific isoform 2 [Argopecten irradians]
2	3	NaN	290 2458 Putative beta-D-xylosidase 5 [Crassostrea gigas]; 299 2428 Putative beta-D-xylosidase 2 [Crassostrea giga

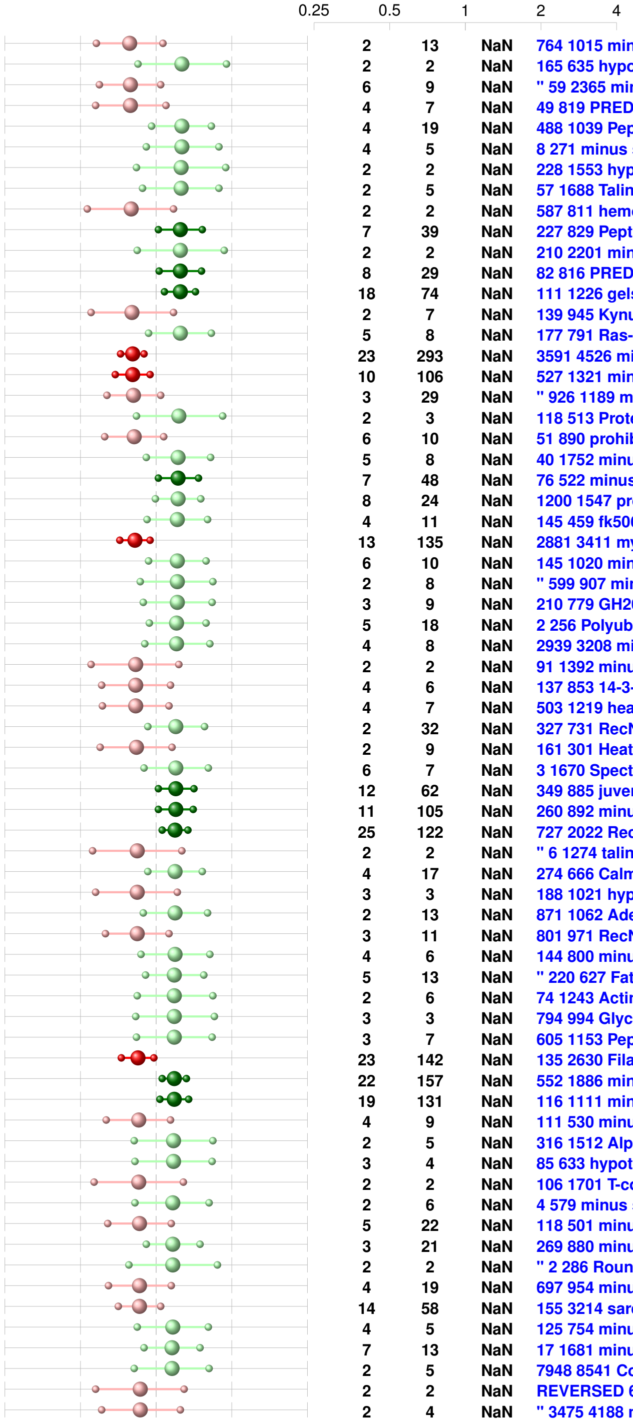

Peptides	Spectra	Coverage	Protein
2	13	NaN	764 1015 minus strand heat shock protein beta-1 [Trichinella spiralis] gi
2	2	NaN	165 635 hypothetical protein CGI_10007639 [Crassostrea gigas]
6	9	NaN	" 59 2365 minus strand Aconitate hydratase, mitochondrial [Crassostrea gigas]"
4	7	NaN	49 819 PREDICTED: elongation factor 1-delta-like isoform 2 [Strongylocentrotus purpuratus]
4	19	NaN	488 1039 Peptidyl-prolyl cis-trans isomerase B [Crassostrea gigas]; 488 1039 Peptidyl-prolyl cis-trans isomerase B [
4	5	NaN	8 271 minus strand Laminin subunit gamma-1 [Crassostrea gigas]
2	2	NaN	228 1553 hypothetical protein CGI_10024545 [Crassostrea gigas]; 228 1553 hypothetical protein CGI_10024545 [Cras
2	5	NaN	57 1688 Talin-1 [Camponotus floridanus]; 57 1865 Talin-1 [Camponotus floridanus]; 57 1835 Talin-1 [Camponotus flo
2	2	NaN	587 811 hemocyanin 2 [Megathura crenulata]
7	39	NaN	227 829 Peptidyl-prolyl cis-trans isomerase B [Crassostrea gigas]
2	2	NaN	210 2201 minus strand Glycyl-tRNA synthetase [Crassostrea gigas]; 210 2201 minus strand Glycyl-tRNA synthetase
8	29	NaN	82 816 PREDICTED: triosephosphate isomerase B [Oreochromis niloticus]
18	74	NaN	111 1226 gelsolin [Aplysia californica] gi; 42 1139 minus strand gelsolin [Aplysia californica] gi
2	7	NaN	139 945 Kynurenine formamidase [Crassostrea gigas]; 145 951 Kynurenine formamidase [Crassostrea gigas]; 220 1
5	8	NaN	177 791 Ras-related protein Rab-1A [Haliotis discus discus]
23	293	NaN	3591 4526 minus strand myosin heavy chain cardiac muscle specific isoform 1 [Argopecten irradians]
10	106	NaN	527 1321 minus strand troponin T [Mizuhopecten yessoensis]; 305 1099 minus strand troponin T [Mizuhopecten yes
3	29	NaN	" 926 1189 minus strand Myosin heavy chain, striated muscle [Crassostrea gigas]"
2	3	NaN	118 513 Protein CAL-5 [Caenorhabditis elegans] gi
6	10	NaN	51 890 prohibitin [Octopus tankahkeei]
5	8	NaN	40 1752 minus strand heat shock protein 60 [Biomphalaria glabrata]; 40 1752 minus strand heat shock protein 60 [B
7	48	NaN	76 522 minus strand Troponin C [Crassostrea gigas]; 173 619 minus strand Troponin C [Crassostrea gigas]; 173 619
8	24	NaN	1200 1547 protein disulfide isomerase [Conus eburneus]
4	11	NaN	145 459 fk506-binding protein 2 [Papilio xuthus]; 237 551 fk506-binding protein 2 [Papilio xuthus]
13	135	NaN	2881 3411 myosin heavy chain [Todarodes pacificus]; 2915 3445 myosin heavy chain [Todarodes pacificus]
6	10	NaN	145 1020 minus strand PREDICTED: inorganic pyrophosphatase-like [Saccoglossus kowalevskii]
2	8	NaN	" 599 907 minus strand Tryptophanyl-tRNA synthetase, cytoplasmic [Heterocephalus glaber]"
3	9	NaN	210 779 GH20056 [Drosophila grimshawi] gi; 216 785 minus strand GH20056 [Drosophila grimshawi] gi; 210 779 GH2
5	18	NaN	2 256 Polyubiquitin (Fragment) OS=Xenopus laevis PE=1 SV=2; 20 388 large subunit ribosomal protein L40e [Clonor
4	8	NaN	2939 3208 minus strand beta-thymosin domain repeat protein CSP24KDa_v1 [Hermissenda crassicornis] gi
2	2	NaN	91 1392 minus strand hypothetical protein AND_03103 [Anopheles darlingi]
4	6	NaN	137 853 14-3-3 protein [Heterodera glycines]
4	7	NaN	503 1219 heat shock cognate 71 [Mytilus galloprovincialis]
2	32	NaN	327 731 RecName: Full=Arginine kinase; Short=AK gi|439719|dbj|BAA05100.1| arginine kinase [Haliotis madaka]
2	9	NaN	161 301 Heat shock protein beta-6 OS=Mus musculus GN=Hspb6 PE=1 SV=1; 167 310 Heat shock protein beta-1 OS
6	7	NaN	3 1670 Spectrin alpha chain [Crassostrea gigas]
12	62	NaN	349 885 juvenile hormone diol kinase [Danaus plexippus]; 349 885 juvenile hormone diol kinase [Danaus plexippus]
11	105	NaN	260 892 minus strand calponin [Haliotis diversicolor]
25	122	NaN	727 2022 RecName: Full=Enolase; AltName: Full=2-phospho-D-glycerate hydro-lyase; AltName: Full=2-phosphoglyc
2	2	NaN	" 6 1274 talin, putative [Ixodes scapularis] gi"
4	17	NaN	274 666 Calmodulin-4 [Heterocephalus glaber]
3	3	NaN	188 1021 hypothetical protein BRAFLDRAFT_214346 [Branchiostoma floridae] gi
2	13	NaN	871 1062 Adenylate kinase isoenzyme 5 [Crassostrea gigas]
3	11	NaN	801 971 RecName: Full=Tropomyosin-2; AltName: Full=Tropomyosin II; Short=BgTMII; Short=TMII gi|155940|gb|AA
4	6	NaN	144 800 minus strand PREDICTED: protein F37C4.5-like [Amphimedon queenslandica]
5	13	NaN	" 220 627 Fatty acid-binding protein, brain [Crassostrea gigas]"
2	6	NaN	74 1243 Actin-related protein 2 [Crassostrea gigas]
3	3	NaN	794 994 Glycogenin-1 [Crassostrea gigas]; 1425 1739 minus strand Glycogenin-1 [Crassostrea gigas]
3	7	NaN	605 1153 Peptidyl-prolyl cis-trans isomerase B [Crassostrea gigas]; 1046 1630 Peptidyl-prolyl cis-trans isomerase B
23	142	NaN	135 2630 Filamin-A [Crassostrea gigas]; 118 2613 Filamin-A [Crassostrea gigas]; 157 2652 Filamin-A [Crassostrea g
22	157	NaN	552 1886 minus strand intermediate filament protein [Biomphalaria glabrata]
19	131	NaN	116 1111 minus strand cytosolic malate dehydrogenase [Lottia scabra]
4	9	NaN	111 530 minus strand Nucleoside diphosphate kinase B OS=Homo sapiens GN=NME2 PE=1 SV=1
2	5	NaN	316 1512 Alpha-N-acetylgalactosaminidase [Crassostrea gigas]; 282 1568 Alpha-N-acetylgalactosaminidase [Crasso
3	4	NaN	85 633 hypothetical protein [Amblyomma maculatum]
2	2	NaN	106 1701 T-complex protein 1 subunit beta [Crassostrea gigas]
2	6	NaN	4 579 minus strand Collagen alpha-3(VI) chain [Crassostrea gigas]
5	22	NaN	118 501 minus strand histone 2A.Z [Aplysia californica]
3	21	NaN	269 880 minus strand myosin regulatory light chain [Fasciola hepatica]
2	2	NaN	" 2 286 Roundabout 2, partial [Ascaris suum]"
4	19	NaN	697 954 minus strand Peptidyl-prolyl cis-trans isomerase B [Crassostrea gigas]; 614 877 Peptidyl-prolyl cis-trans iso
14	58	NaN	155 3214 sarco/endoplasmic reticulum calcium ATPase isoform B [Pinctada fucata]; 155 3214 minus strand sarco/er
4	5	NaN	125 754 minus strand peptidyl prolyl cis-trans isomerase B [Conus novaehollandiae]
7	13	NaN	17 1681 minus strand Glucose-6-phosphate isomerase [Crassostrea gigas]
2	5	NaN	7948 8541 Collagen alpha-3(VI) chain [Crassostrea gigas]
2	2	NaN	REVERSED 68 358 endonuclease-reverse transcriptase [Danaus plexippus]
2	4	NaN	" 3475 4188 minus strand Heat shock 70 kDa protein cognate, putative [Pediculus humanus corporis] gi"

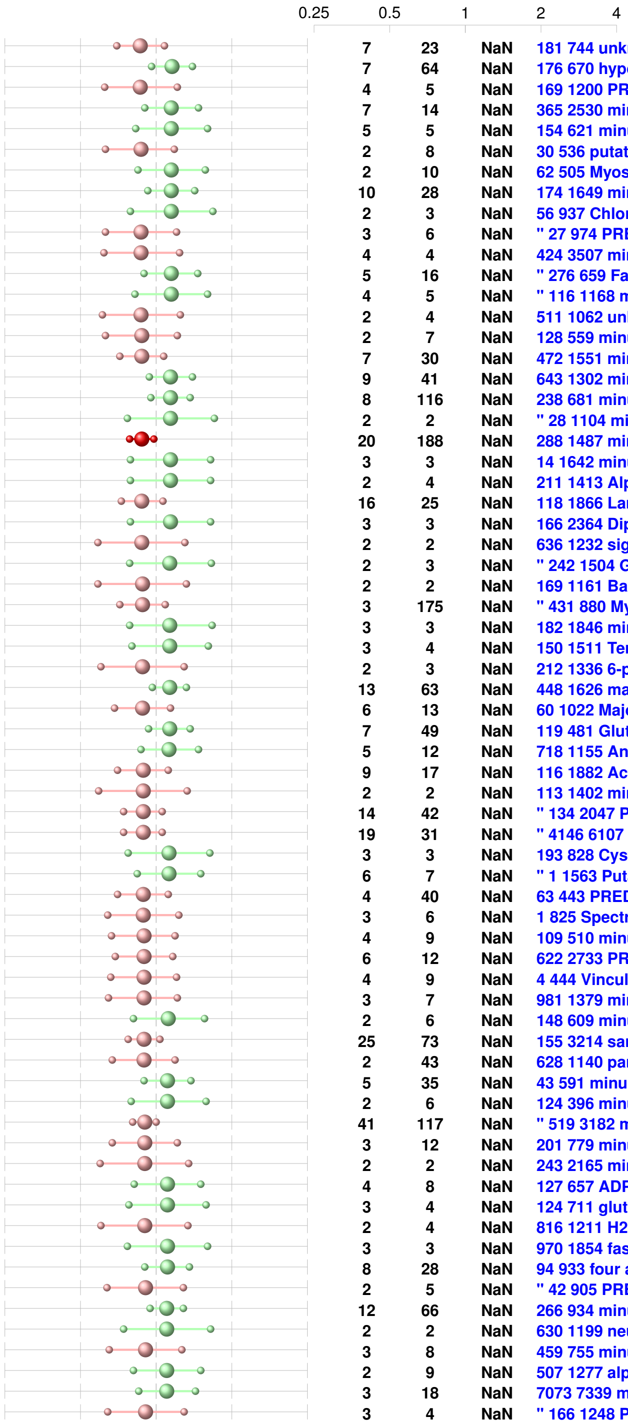

Peptides	Spectra	Coverage	Protein
7	23	NaN	181 744 unknown [Dendroctonus ponderosae]
7	64	NaN	176 670 hypothetical protein BRAFLDRAFT_122621 [Branchiostoma floridae] gi
4	5	NaN	169 1200 PREDICTED: nidogen-2-like isoform 1 [Strongylocentrotus purpuratus]
7	14	NaN	365 2530 minus strand calpain B [Gecarcinus lateralis]
5	5	NaN	154 621 minus strand Stress response protein nhaX [Crassostrea gigas]
2	8	NaN	30 536 putative mitochondrial ATP synthase [Haliotis discus discus]; 27 620 putative mitochondrial ATP synthase [H
2	10	NaN	62 505 Myosin-2 essential light chain [Crassostrea gigas]
10	28	NaN	174 1649 minus strand Collagen alpha-1(XII) chain [Crassostrea gigas]; 135 1610 Collagen alpha-1(XII) chain [Crasso
2	3	NaN	56 937 Chloride intracellular channel exc-4 [Crassostrea gigas]
3	6	NaN	" 27 974 PREDICTED: succinyl-CoA ligase [GDP-forming] subunit alpha, mitochondrial-like [Oreochromis niloticus];
4	4	NaN	424 3507 minus strand putative Na+/K+-ATPase alpha subunit [Paroctopus digueti]
5	16	NaN	" 276 659 Fatty acid-binding protein, adipocyte [Crassostrea gigas]; 426 809 Fatty acid-binding protein, adipocyte [C
4	5	NaN	" 116 1168 minus strand hypothetical protein CGI_10014553, partial [Crassostrea gigas]"
2	4	NaN	511 1062 unknown [Dendroctonus ponderosae]; 511 1062 unknown [Dendroctonus ponderosae]
2	7	NaN	128 559 minus strand predicted protein [Nematostella vectensis] gi
7	30	NaN	472 1551 minus strand hypothetical protein [Amblyomma maculatum]
9	41	NaN	643 1302 minus strand Kyphoscoliosis peptidase [Crassostrea gigas]; 634 1293 minus strand Kyphoscoliosis peptic
8	116	NaN	238 681 minus strand Globin OS=Cerithidea rhizophorarum PE=1 SV=1
2	2	NaN	" 28 1104 minus strand Pyruvate dehydrogenase E1 component subunit beta, mitochondrial [Crassostrea gigas]"
20	188	NaN	288 1487 minus strand putative procollagen type VI alpha 4 [Haliotis diversicolor]
3	3	NaN	14 1642 minus strand chaperonin containing tcp1 [Haliotis discus discus]
2	4	NaN	211 1413 Alpha-N-acetylgalactosaminidase [Crassostrea gigas]
16	25	NaN	118 1866 Lamin Dm0 [Crassostrea gigas]
3	3	NaN	166 2364 Dipeptidyl-peptidase 3 [Crassostrea gigas]
2	2	NaN	636 1232 sigma class glutathione-s-transferase 3 [Haliotis discus discus]; 963 1559 sigma class glutathione-s-transf
2	3	NaN	" 242 1504 Glutamate receptor, ionotropic kainate 3 [Crassostrea gigas]; 239 1501 Glutamate receptor, ionotropic ka
2	2	NaN	169 1161 Basement membrane-specific heparan sulfate proteoglycan core protein OS=Homo sapiens GN=HSPG2 PI
3	175	NaN	" 431 880 Myosin essential light chain, striated adductor muscle OS=Aequipecten irradians PE=1 SV=2"
3	3	NaN	182 1846 minus strand hypothetical protein DAPPUDRAFT_302108 [Daphnia pulex]; 110 1774 minus strand hypothe
3	4	NaN	150 1511 Tensin OS=Gallus gallus GN=TNS PE=1 SV=2
2	3	NaN	212 1336 6-phosphofructokinase [Crassostrea gigas]
13	63	NaN	448 1626 matrilin [Biomphalaria glabrata]
6	13	NaN	60 1022 Major egg antigen [Crassostrea gigas]; 60 1022 Major egg antigen [Crassostrea gigas]
7	49	NaN	119 481 Glutamate receptor U1 [Crassostrea gigas]
5	12	NaN	718 1155 Annexin A7 [Crassostrea gigas]
9	17	NaN	116 1882 Actin-interacting protein 1 [Crassostrea gigas]
2	2	NaN	113 1402 minus strand S-adenosylhomocysteine hydrolase [Crassostrea ariakensis]; 113 1402 minus strand S-aden
14	42	NaN	" 134 2047 PREDICTED: succinate dehydrogenase [ubiquinone] flavoprotein subunit, mitochondrial-like isoform 3, p
19	31	NaN	" 4146 6107 minus strand nonmuscle myosin II, partial [Aplysia californica]"
3	3	NaN	193 828 Cysteine-rich secretory protein LCCL domain-containing 2 [Crassostrea gigas]
6	7	NaN	" 1 1563 Putative methylmalonate-semialdehyde dehydrogenase [acylating], mitochondrial [Crassostrea gigas]"
4	40	NaN	63 443 PREDICTED: RAS protein activator like 1-like [Saccoglossus kowalevskii]
3	6	NaN	1 825 Spectrin alpha chain [Crassostrea gigas]
4	9	NaN	109 510 minus strand hypothetical protein DAPPUDRAFT_231281 [Daphnia pulex]
6	12	NaN	622 2733 PREDICTED: protein-glutamine gamma-glutamyltransferase K isoform 1 [Danio rerio]; 423 2498 PREDICTE
4	9	NaN	4 444 Vinculin [Crassostrea gigas]
3	7	NaN	981 1379 minus strand Multimerin-1 [Crassostrea gigas]; 998 1396 Multimerin-1 [Crassostrea gigas]; 975 1373 Multir
2	6	NaN	148 609 minus strand Titin [Crassostrea gigas]
25	73	NaN	155 3214 sarco/endoplasmic reticulum calcium ATPase isoform B [Pinctada fucata]; 132 3110 sarco/endoplasmic ret
2	43	NaN	628 1140 paramyosin [Haliotis discus discus]
5	35	NaN	43 591 minus strand cyclophilin type peptidyl-prolyl cis-trans isomerase [Glossina morsitans morsitans]
2	6	NaN	124 396 minus strand 40S ribosomal protein S10 OS=Lumbricus rubellus GN=RPS10 PE=2 SV=1
41	117	NaN	" 519 3182 minus strand Alpha-actinin, sarcomeric [Crassostrea gigas]"
3	12	NaN	201 779 minus strand sigma class glutathione-s-transferase 2 [Haliotis discus discus]
2	2	NaN	243 2165 minus strand Elongation factor 2 OS=Drosophila melanogaster GN=Ef2b PE=2 SV=4
4	8	NaN	127 657 ADP ribosylation factor 4 [Marsupenaeus japonicus]
3	4	NaN	124 711 glutathione S-transferase sigma 3 [Mytilus galloprovincialis]; 124 711 glutathione S-transferase sigma 3 [My
2	4	NaN	816 1211 H2 [Haliotis tuberculata]
3	3	NaN	970 1854 fasciclin-like protein precursor [Aplysia californica] gi
8	28	NaN	94 933 four and A half lim domains [Schistosoma mansoni]
2	5	NaN	" 42 905 PREDICTED: enoyl-CoA hydratase, mitochondrial-like isoform 1 [Oreochromis niloticus]"
12	66	NaN	266 934 minus strand Kyphoscoliosis peptidase [Crassostrea gigas]
2	2	NaN	630 1199 neuronal calcium sensor-1 [Lymnaea stagnalis]
3	8	NaN	459 755 minus strand heat shock protein beta-1 [Trichinella spiralis] gi
2	9	NaN	507 1277 alpha tubulin [Notothenia coriiceps]
3	18	NaN	7073 7339 minus strand Collagen alpha-3(VI) chain [Crassostrea gigas]
3	4	NaN	" 166 1248 PREDICTED: probable pyruvate dehydrogenase E1 component subunit alpha, mitochondrial-like [Metase

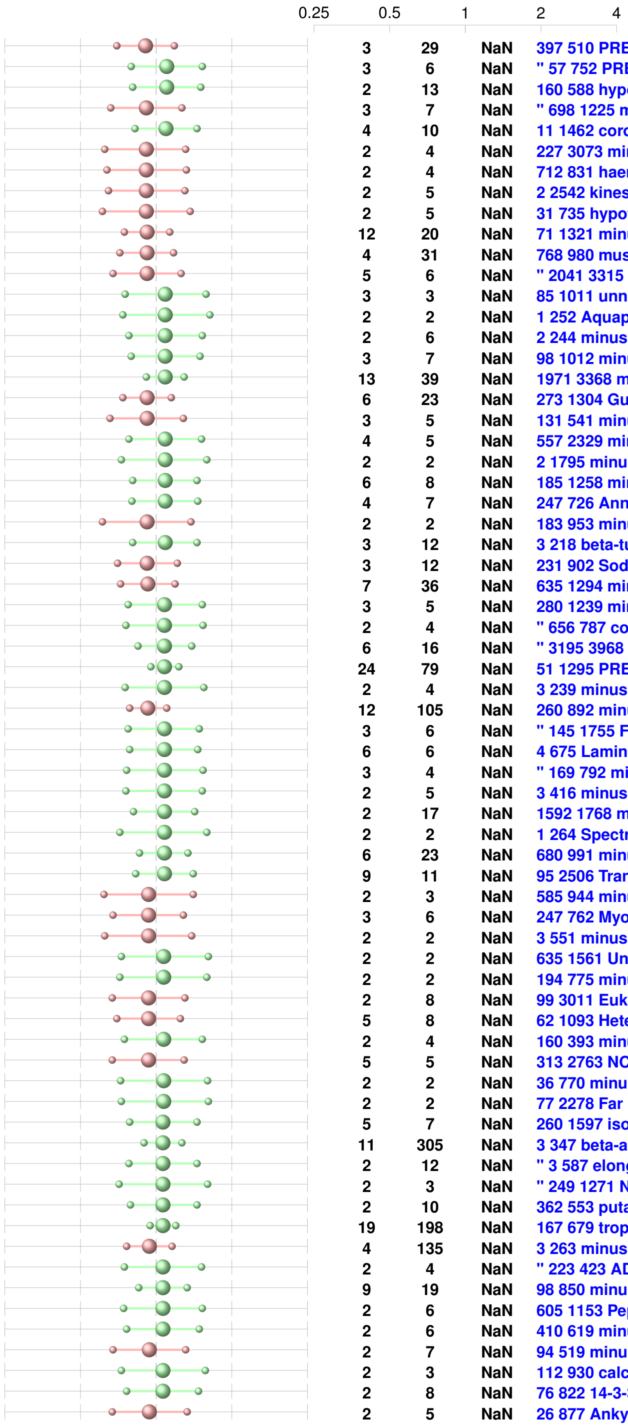

Peptides	Spectra	Coverage	Protein
3	29	NaN	397 510 PREDICTED: actin-5C-like [Megachile rotundata]
3	6	NaN	" 57 752 PREDICTED: adenylate kinase 2, mitochondrial-like isoform 2 [Callithrix jacchus]"
2	13	NaN	160 588 hypothetical protein CGI_10001640 [Crassostrea gigas]
3	7	NaN	" 698 1225 minus strand actin related protein 2/3 complex, 21 kDa subunit [Crassostrea gigas]"
4	10	NaN	11 1462 coronin [Schistosoma mansoni]
2	4	NaN	227 3073 minus strand Lysosomal alpha-mannosidase [Crassostrea gigas]
2	4	NaN	712 831 haemocyanin [Aplysia californica]
2	5	NaN	2 2542 kinesin heavy chain 1 [Aplysia californica] gi; 2 2542 kinesin heavy chain 1 [Aplysia californica] gi
2	5	NaN	31 735 hypothetical protein BRAFLDRAFT_78603 [Branchiostoma floridae] gi; 55 735 hypothetical protein BRAFLDF
12	20	NaN	71 1321 minus strand Vinculin [Crassostrea gigas]
4	31	NaN	768 980 muscle LIM protein isoform 1 [Danaus plexippus]
5	6	NaN	" 2041 3315 minus strand Myosin-10 OS=Mus musculus GN=Myh10 PE=1 SV=2; 2041 3345 minus strand Myosin hea
3	3	NaN	85 1011 unnamed protein product [Mus musculus]
2	2	NaN	1 252 Aquaporin-4 [Crassostrea gigas]
2	6	NaN	2 244 minus strand hypothetical protein KGM_08771 [Danaus plexippus]
3	7	NaN	98 1012 minus strand RecName: Full=Substrate-specific endoprotease Tex31; Flags: Precursor gi|32187775|emb|CA
13	39	NaN	1971 3368 minus strand Kyphoscoliosis peptidase [Crassostrea gigas]; 1767 3158 minus strand hillarin [Hirudo med
6	23	NaN	273 1304 Guanine nucleotide-binding protein subunit beta [Crassostrea gigas]
3	5	NaN	131 541 minus strand nervous system adducin [Aplysia californica] gi
4	5	NaN	557 2329 minus strand Collagen alpha-3(VI) chain [Crassostrea gigas]
2	2	NaN	2 1795 minus strand Plectin-1 [Crassostrea gigas]
6	8	NaN	185 1258 minus strand Severin [Crassostrea gigas]
4	7	NaN	247 726 Annexin A7 [Crassostrea gigas]; 286 675 Annexin A7 [Crassostrea gigas]
2	2	NaN	183 953 minus strand glutathione-S-transferase [Haliotis diversicolor supertexta]; 275 1045 minus strand glutathion
3	12	NaN	3 218 beta-tubulin [Fasciola hepatica] gi
3	12	NaN	231 902 Sodium- and chloride-dependent GABA transporter ine [Crassostrea gigas]
7	36	NaN	635 1294 minus strand Kyphoscoliosis peptidase [Crassostrea gigas]; 643 1302 minus strand Kyphoscoliosis peptic
3	5	NaN	280 1239 minus strand PREDICTED: smoothelin isoform 2 [Oryctolagus cuniculus]; 280 1239 minus strand PREDICT
2	4	NaN	" 656 787 collagen alpha-1(XII) chain-like; K08132 collagen, type XII, alpha"
6	16	NaN	" 3195 3968 Spectrin alpha chain, putative [Pediculus humanus corporis] gi"
24	79	NaN	51 1295 PREDICTED: phosphoglycerate kinase-like [Nasonia vitripennis]; 97 1341 PREDICTED: phosphoglycerate k
2	4	NaN	3 239 minus strand alcohol dehydrogenase class 3 [Branchiostoma lanceolatum]
12	105	NaN	260 892 minus strand calponin [Haliotis diversicolor]; 260 892 minus strand calponin [Haliotis diversicolor]
3	6	NaN	" 145 1755 Fumarate hydratase class I, aerobic [Crassostrea gigas]"
6	6	NaN	4 675 Laminin subunit gamma-1 [Crassostrea gigas]
3	4	NaN	" 169 792 minus strand rho GDP dissociation inhibitor, putative [Ixodes scapularis] gi"
2	5	NaN	3 416 minus strand myosin essential light chain [Crassostrea gigas]
2	17	NaN	1592 1768 minus strand beta-actin [Diabolocatantops pinguis]
2	2	NaN	1 264 Spectrin beta chain [Crassostrea gigas]
6	23	NaN	680 991 minus strand PDZ and LIM domain protein 1 [Crassostrea gigas]; 553 864 minus strand PDZ and LIM domai
9	11	NaN	95 2506 Transitional endoplasmic reticulum ATPase [Crassostrea gigas]
2	3	NaN	585 944 minus strand Protein disulfide-isomerase A6 [Crassostrea gigas]
3	6	NaN	247 762 Myophilin [Crassostrea gigas]; 244 759 Myophilin [Crassostrea gigas]; 119 634 Myophilin [Crassostrea giga
2	2	NaN	3 551 minus strand Spectrin beta chain [Crassostrea gigas]
2	2	NaN	635 1561 Uncharacterized protein yfeX [Crassostrea gigas]
2	2	NaN	194 775 minus strand Collagen alpha-1(XV) chain [Harpegnathos saltator]
2	8	NaN	99 3011 Eukaryotic translation initiation factor 3 subunit A [Crassostrea gigas]
5	8	NaN	62 1093 Heterogeneous nuclear ribonucleoprotein A2-like protein 1 [Crassostrea gigas]
2	4	NaN	160 393 minus strand PREDICTED: cysteine-rich protein 1-like [Megachile rotundata]; 148 381 PREDICTED: cysteine
5	5	NaN	313 2763 NCAM-related cell adhesion molecule precursor [Aplysia californica] gi
2	2	NaN	36 770 minus strand Endoplasmic reticulum protein ERp29 [Crassostrea gigas]
2	2	NaN	77 2278 Far upstream element-binding protein 3 [Crassostrea gigas]
5	7	NaN	260 1597 isocitrate dehydrogenase [Mytilus galloprovincialis]; 260 1597 isocitrate dehydrogenase [Mytilus galloprov
11	305	NaN	3 347 beta-actin [Meretrix meretrix]; 3 215 Actin-2 OS=Onchocerca volvulus GN=act-2b PE=3 SV=1
2	12	NaN	" 3 587 elongation factor 1 alpha, partial [Phorbas sp. nov. SB-2012]"
2	3	NaN	" 249 1271 N(G),N(G)-dimethylarginine dimethylaminohydrolase 1 [Crassostrea gigas]"
2	10	NaN	362 553 putative RNA-binding protein [Haliotis diversicolor]
19	198	NaN	167 679 tropomyosin [Haliotis discus discus]
4	135	NaN	3 263 minus strand beta-actin [Urechis unicinctus]
2	4	NaN	" 223 423 ADPATP carrier protein; K05863 solute carrier family 25 (mitochondrial adenine nucleotide translocator), m
9	19	NaN	98 850 minus strand 14-3-3 protein epsilon [Schistocerca gregaria]
2	6	NaN	605 1153 Peptidyl-prolyl cis-trans isomerase B [Crassostrea gigas]; 1046 1630 Peptidyl-prolyl cis-trans isomerase B
2	6	NaN	410 619 minus strand Guanine nucleotide-binding protein subunit gamma-1 [Crassostrea gigas]
2	7	NaN	94 519 minus strand hypothetical protein BRAFLDRAFT_115029 [Branchiostoma floridae] gi
2	3	NaN	112 930 calcineurin A [Haliotis discus discus]
2	8	NaN	76 822 14-3-3 protein [Heterodera glycines]; 76 822 minus strand 14-3-3 protein [Heterodera glycines]
2	5	NaN	26 877 Ankyrin-2 [Crassostrea gigas]

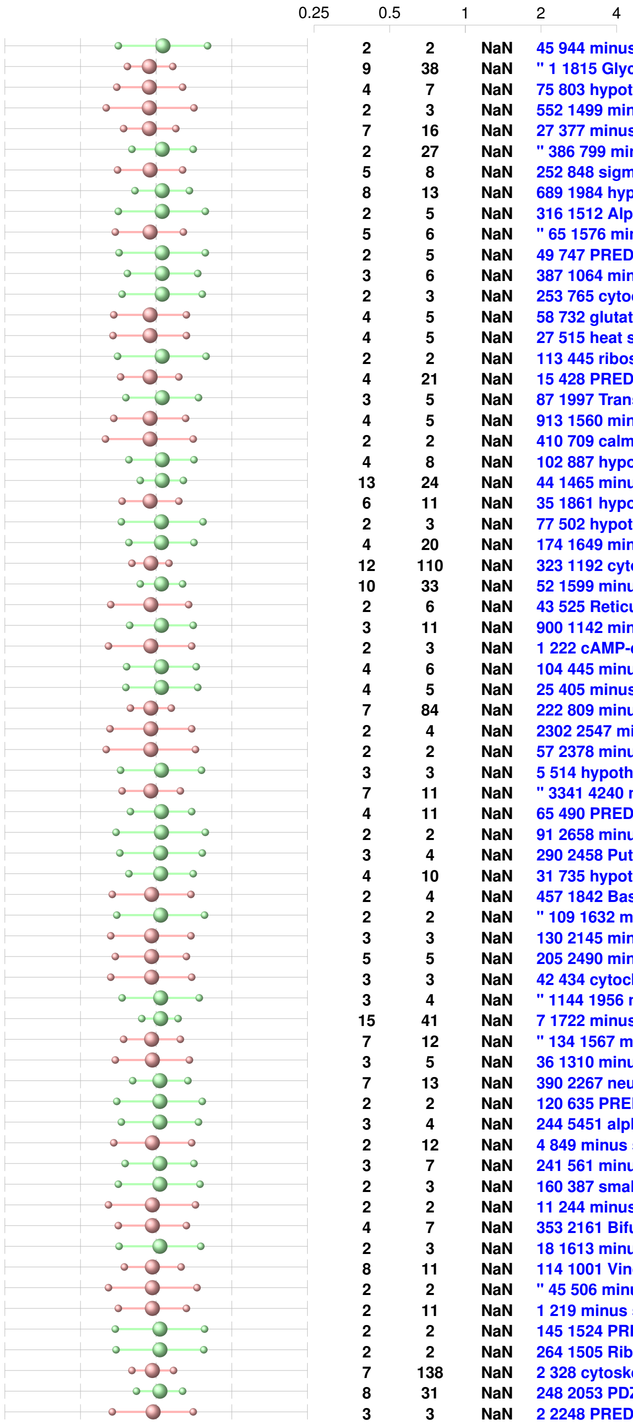

Peptides	Spectra	Coverage	Protein
2	2	NaN	45 944 minus strand unnamed protein product [Mus musculus] gi; 5 334 minus strand receptor of activated protein k
9	38	NaN	" 1 1815 Glycogen phosphorylase, muscle form [Crassostrea gigas]"
4	7	NaN	75 803 hypothetical protein DAPPUDRAFT_231350 [Daphnia pulex]
2	3	NaN	552 1499 minus strand uncharacterized protein LOC100158466 [Xenopus (Silurana) tropicalis] gi
7	16	NaN	27 377 minus strand ribosomal protein LP2 [Papilio xuthus] gi; 27 377 minus strand ribosomal protein LP2 [Papilio x
2	27	NaN	" 386 799 minus strand Myosin regulatory light chain LC-2, mantle muscle OS=Todarodes pacificus PE=1 SV=1; 656
5	8	NaN	252 848 sigma class glutathione-s-transferase 2 [Haliotis discus discus]
8	13	NaN	689 1984 hypothetical protein BRAFLDRAFT_78257 [Branchiostoma floridae] gi
2	5	NaN	316 1512 Alpha-N-acetylgalactosaminidase [Crassostrea gigas]; 282 1478 Alpha-N-acetylgalactosaminidase [Crasso
5	6	NaN	" 65 1576 minus strand Aldehyde dehydrogenase, mitochondrial [Crassostrea gigas]"
2	5	NaN	49 747 PREDICTED: elongation factor 1-delta-like [Strongylocentrotus purpuratus]; 49 819 PREDICTED: elongation f
3	6	NaN	387 1064 minus strand PREDICTED: hypothetical protein [Hydra magnipapillata]; 275 952 minus strand PREDICTED:
2	3	NaN	253 765 cytochrome c oxidase subunit IV [Urechis caupo]
4	5	NaN	58 732 glutathione-s-transferase [Haliotis discus discus]
4	5	NaN	27 515 heat shock protein 70 [Capsaspora owczarzaki ATCC 30864]; 30 464 minus strand hypothetical protein [Trich
2	2	NaN	113 445 ribosomal protein rps19 [Lineus viridis]
4	21	NaN	15 428 PREDICTED: histone H2A.V-like [Apis florea]
3	5	NaN	87 1997 Transketolase-like protein 2 [Crassostrea gigas]; 87 1997 Transketolase-like protein 2 [Crassostrea gigas]; 8
4	5	NaN	913 1560 minus strand Calcium-dependent protein kinase isoform 2 [Crassostrea gigas]
2	2	NaN	410 709 calmodulin-like; K02183 calmodulin
4	8	NaN	102 887 hypothetical protein DAPPUDRAFT_304712 [Daphnia pulex]
13	24	NaN	44 1465 minus strand Adenylyl cyclase-associated protein 1 [Crassostrea gigas]; 52 1473 minus strand Adenylyl cyc
6	11	NaN	35 1861 hypothetical protein DAPPUDRAFT_309082 [Daphnia pulex]
2	3	NaN	77 502 hypothetical protein CGI_10008263 [Crassostrea gigas]
4	20	NaN	174 1649 minus strand Collagen alpha-1(XII) chain [Crassostrea gigas]; 123 1490 minus strand Collagen alpha-1(XII)
12	110	NaN	323 1192 cytosolic malate dehydrogenase [Lottia scabra]; 323 1192 cytosolic malate dehydrogenase [Lottia scabra];
10	33	NaN	52 1599 minus strand ATP synthase beta subunit [Pinctada fucata]
2	6	NaN	43 525 Reticulon-4 [Crassostrea gigas]; 143 757 Reticulon-4 [Crassostrea gigas]; 43 564 Reticulon-4 [Crassostrea gi
3	11	NaN	900 1142 minus strand heat shock protein 20 [Cyclina sinensis]
2	3	NaN	1 222 cAMP-dependent protein kinase regulatory subunit [Aplysia californica] gi
4	6	NaN	104 445 minus strand macrophage migration inhibitory factor [Ascaris suum]
4	5	NaN	25 405 minus strand Elongation factor 1-delta [Anoplopoma fimbria]
7	84	NaN	222 809 minus strand PREDICTED: 15-hydroxyprostaglandin dehydrogenase [NAD+] [Gallus gallus]
2	4	NaN	2302 2547 minus strand hemocyanin isoform 1 [Haliotis diversicolor]; 816 1211 H2 [Haliotis tuberculata]
2	2	NaN	57 2378 minus strand Heterogeneous nuclear ribonucleoprotein U-like protein 1 [Crassostrea gigas]
3	3	NaN	5 514 hypothetical protein KGM_05418 [Danaus plexippus]
7	11	NaN	" 3341 4240 minus strand nonmuscle myosin II, partial [Aplysia californica]"
4	11	NaN	65 490 PREDICTED: cofilin-2 [Otolemur garnettii]
2	2	NaN	91 2658 minus strand puromycin-sensitive aminopeptidase [Saccoglossus kowalevskii]
3	4	NaN	290 2458 Putative beta-D-xylosidase 5 [Crassostrea gigas]
4	10	NaN	31 735 hypothetical protein BRAFLDRAFT_78603 [Branchiostoma floridae] gi; 55 735 hypothetical protein BRAFLDF
2	4	NaN	457 1842 Basement membrane-specific heparan sulfate proteoglycan core protein [Crassostrea gigas]; 457 1842 Ba
2	2	NaN	" 109 1632 minus strand Seryl-tRNA synthetase, cytoplasmic [Crassostrea gigas]; 109 1614 minus strand Seryl-tRNA
3	3	NaN	130 2145 minus strand Glucosamine--fructose-6-phosphate aminotransferase [isomerizing] 1 [Crassostrea gigas]; 1
5	5	NaN	205 2490 minus strand glucose-regulated protein 94 [Crassostrea gigas]
3	3	NaN	42 434 cytochrome b5 [Haliotis discus discus]; 42 434 cytochrome b5 [Haliotis discus discus]
3	4	NaN	" 1144 1956 minus strand Retinol dehydrogenase 12, partial [Crassostrea gigas]"
15	41	NaN	7 1722 minus strand Membrane metallo-endopeptidase-like 1 OS=Mus musculus GN=Mmel1 PE=1 SV=1
7	12	NaN	" 134 1567 minus strand 6-phosphogluconate dehydrogenase, decarboxylating [Crassostrea gigas]; 134 1573 minus
3	5	NaN	36 1310 minus strand elongation factor-1 gamma [Diguetia canities]
7	13	NaN	390 2267 neurofilament protein NF70 [Helix aspersa]; 318 2195 neurofilament protein NF70 [Helix aspersa]
2	2	NaN	120 635 PREDICTED: similar to collagen type VI alpha 6 [Ciona intestinalis]; 120 635 minus strand PREDICTED: simi
3	4	NaN	244 5451 alpha macroglobulin [Chlamys farreri]; 244 5493 alpha macroglobulin [Chlamys farreri]
2	12	NaN	4 849 minus strand elongation factor 1-alpha [Cryptosporidium muris RN66]
3	7	NaN	241 561 minus strand thioredoxin [Crassostrea ariakensis]
2	3	NaN	160 387 small nuclear ribonucleoprotein polypeptide G protein [Crassostrea ariakensis]; 180 407 small nuclear ribor
2	2	NaN	11 244 minus strand Tubulin alpha chain (Fragment) OS=Lytechinus pictus PE=3 SV=1
4	7	NaN	353 2161 Bifunctional 3'-phosphoadenosine 5'-phosphosulfate synthetase [Crassostrea gigas]; 141 19
2	3	NaN	18 1613 minus strand chaperonin containing T-complex polypeptide subunit zeta [Haliotis discus hannai]; 527 2089
8	11	NaN	114 1001 Vinculin [Crassostrea gigas]
2	2	NaN	" 45 506 minus strand PREDICTED: dihydrolipoyl dehydrogenase, mitochondrial [Monodelphis domestica]"
2	11	NaN	1 219 minus strand PREDICTED: nucleoside diphosphate kinase 3-like [Pan troglodytes]
2	2	NaN	145 1524 PREDICTED: aspartyl aminopeptidase-like [Oreochromis niloticus]
2	2	NaN	264 1505 Ribosome-binding protein 1 [Crassostrea gigas]; 260 1501 Ribosome-binding protein 1 [Crassostrea gigas
7	138	NaN	2 328 cytoskeletal actin 2 [Molgula oculata]
8	31	NaN	248 2053 PDZ and LIM domain protein 5 [Crassostrea gigas]
3	3	NaN	2 2248 PREDICTED: heat shock 70 kDa protein 4-like [Oreochromis niloticus]; 2 2227 PREDICTED: heat shock 70 kD

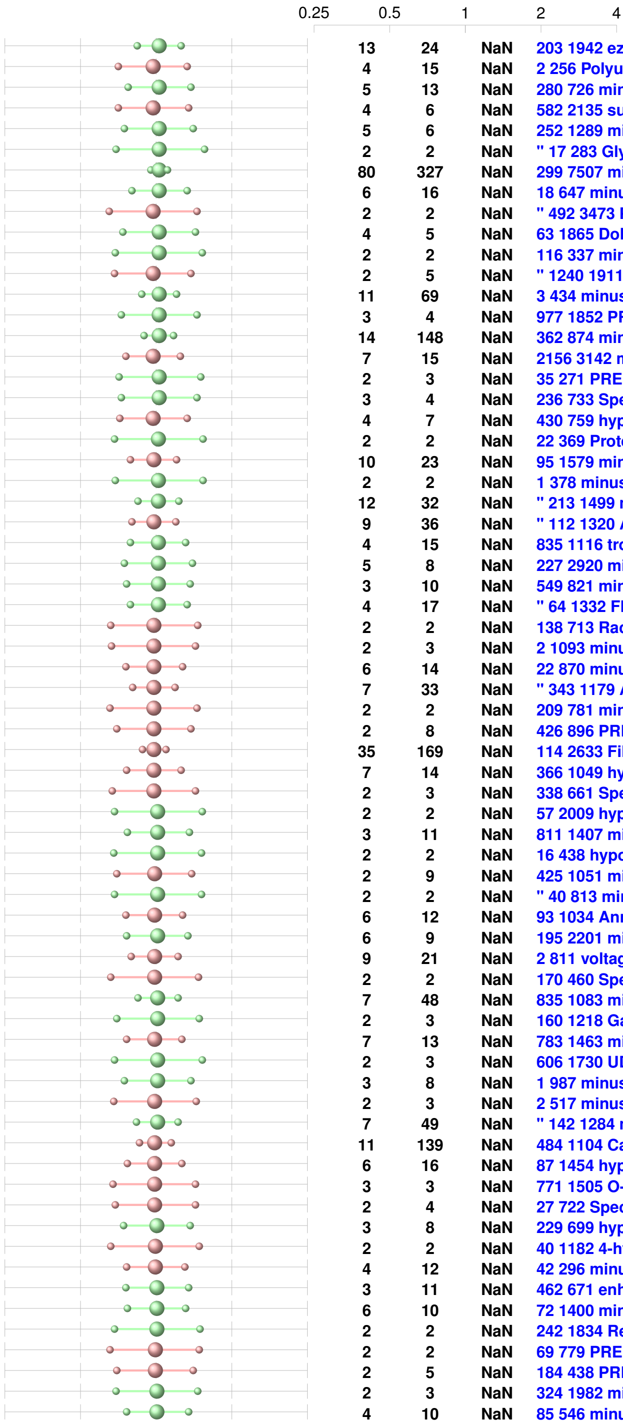

Peptides	Spectra	Coverage	Protein
13	24	NaN	203 1942 ezrin/radixin/moesin [Aplysia californica] gi; 243 1982 minus strand ezrin/radixin/moesin [Aplysia californic
4	15	NaN	2 256 Polyubiquitin (Fragment) OS=Xenopus laevis PE=1 SV=2; 20 472 minus strand large subunit ribosomal protein
5	13	NaN	280 726 minus strand RecName: Full=Calmodulin; Short=CaM gi|14588595|dbj|BAB61794.1| calmodulin [Metridium
4	6	NaN	582 2135 sulfatase 1A precursor [Haliotis discus discus]
5	6	NaN	252 1289 minus strand PDZ and LIM domain protein 3 OS=Gallus gallus GN=PDLIM3 PE=1 SV=1
2	2	NaN	" 17 283 Glycerol-3-phosphate dehydrogenase [NAD+], cytoplasmic [Crassostrea gigas]"
80	327	NaN	299 7507 minus strand filamin [Clonorchis sinensis]
6	16	NaN	18 647 minus strand ATP synthase-like protein [Biomphalaria glabrata]
2	2	NaN	" 492 3473 PREDICTED: 2-oxoglutarate dehydrogenase, mitochondrial isoform 1 [Sarcophilus harrisii]; 492 3458 PR
4	5	NaN	63 1865 Dolichyl-diphosphooligosaccharide–protein glycosyltransferase subunit 1 [Crassostrea gigas]; 63 1865 Dol
2	2	NaN	116 337 minus strand Golgi-associated plant pathogenesis-related protein 1 [Crassostrea gigas]; 538 759 minus stra
2	5	NaN	" 1240 1911 PREDICTED: ATP synthase subunit alpha, mitochondrial-like [Anolis carolinensis]"
11	69	NaN	3 434 minus strand myosin essential light chain [Haliotis discus discus]
3	4	NaN	977 1852 PREDICTED: similar to prohibitin [Tribolium castaneum] gi
14	148	NaN	362 874 minus strand Transgelin-2 [Crassostrea gigas]
7	15	NaN	2156 3142 minus strand PREDICTED: hemicentin-1 [Xenopus (Silurana) tropicalis]
2	3	NaN	35 271 PREDICTED: SH3 domain-binding glutamic acid-rich-like protein 3-like [Anolis carolinensis]; 35 271 PREDICT
3	4	NaN	236 733 Spectrin alpha chain [Crassostrea gigas]
4	7	NaN	430 759 hypothetical protein [Amblyomma maculatum]; 336 665 hypothetical protein [Amblyomma maculatum]
2	2	NaN	22 369 Protein farnesyltransferase/geranylgeranyltransferase type-1 subunit alpha [Crassostrea gigas]
10	23	NaN	95 1579 minus strand hypothetical protein BRAFLDRAFT_113974 [Branchiostoma floridae] gi
2	2	NaN	1 378 minus strand PREDICTED: CG10098-like [Saccoglossus kowalevskii]
12	32	NaN	" 213 1499 minus strand Citrate synthase, mitochondrial [Crassostrea gigas]"
9	36	NaN	" 112 1320 Aspartate aminotransferase, cytoplasmic [Crassostrea gigas]"
4	15	NaN	835 1116 tropomyosin [Ilyanassa obsoleta]
5	8	NaN	227 2920 minus strand Lysosomal alpha-mannosidase [Crassostrea gigas]; 227 3073 minus strand Lysosomal alpha
3	10	NaN	549 821 minus strand Calponin-2 [Crassostrea gigas]
4	17	NaN	" 64 1332 Flotillin-1, putative [Pediculus humanus corporis] gi"
2	2	NaN	138 713 Rac [Aplysia californica] gi; 125 691 Rac [Aplysia californica] gi; 149 871 minus strand Rac [Aplysia californ
2	3	NaN	2 1093 minus strand Neurexin-1-alpha [Crassostrea gigas]
6	14	NaN	22 870 minus strand PREDICTED: alcohol dehydrogenase class-3 [Sarcophilus harrisii]
7	33	NaN	" 343 1179 ATP synthase subunit alpha, mitochondrial OS=Pongo abelii GN=ATP5A1 PE=2 SV=1"
2	2	NaN	209 781 minus strand high mobility group protein 1 [Biomphalaria glabrata]; 277 771 high mobility group protein 1 [
2	8	NaN	426 896 PREDICTED: calmodulin-like [Metaseiulus occidentalis]
35	169	NaN	114 2633 Filamin-A [Crassostrea gigas]; 135 2654 Filamin-A [Crassostrea gigas]
7	14	NaN	366 1049 hypothetical protein CGI_10004855 [Crassostrea gigas]
2	3	NaN	338 661 Spectrin alpha chain [Crassostrea gigas]
2	2	NaN	57 2009 hypothetical protein BRAFLDRAFT_285279 [Branchiostoma floridae] gi; 3 1973 hypothetical protein BRAFL
3	11	NaN	811 1407 minus strand mitochondrial malate dehydrogenase 2 [Eospalax baileyi]
2	2	NaN	16 438 hypothetical protein KGM_06034 [Danaus plexippus]; 16 438 hypothetical protein KGM_06034 [Danaus plexip
2	9	NaN	425 1051 minus strand unknown [Schistosoma japonicum]; 300 878 unknown [Schistosoma japonicum]; 567 1145 u
2	2	NaN	" 40 813 minus strand NADH dehydrogenase [ubiquinone] iron-sulfur protein 3, mitochondrial [Crassostrea gigas]"
6	12	NaN	93 1034 Annexin A7 [Crassostrea gigas]; 73 1014 Annexin A7 [Crassostrea gigas]
6	9	NaN	195 2201 minus strand hypothetical protein DAPPUDRAFT_315656 [Daphnia pulex]
9	21	NaN	2 811 voltage-dependent anion channel 2-like protein [Haliotis diversicolor]; 2 811 voltage-dependent anion channel
2	2	NaN	170 460 Spectrin beta chain [Crassostrea gigas]
7	48	NaN	835 1083 minus strand Calmodulin [Crassostrea gigas]
2	3	NaN	160 1218 Galectin-9 [Crassostrea gigas]
7	13	NaN	783 1463 minus strand twitchin [Mytilus galloprovincialis]; 783 1463 minus strand twitchin [Mytilus galloprovincialis
2	3	NaN	606 1730 UDP-glucuronosyltransferase 2C1 precursor [Oryctolagus cuniculus]
3	8	NaN	1 987 minus strand RecName: Full=Tubulin beta chain; AltName: Full=Beta-tubulin
2	3	NaN	2 517 minus strand Anhydro-N-acetylmuramic acid kinase [Crassostrea gigas]
7	49	NaN	" 142 1284 minus strand Chain O, Crsytal Structure Of Human Liver Gapdh gi|67464044|pdb|1ZNQ|P Chain P, Crsyta
11	139	NaN	484 1104 Calponin-2 [Crassostrea gigas]; 309 938 Calponin-2 [Crassostrea gigas]
6	16	NaN	87 1454 hypothetical protein BRAFLDRAFT_287788 [Branchiostoma floridae] gi; 117 1469 hypothetical protein BRAF
3	3	NaN	771 1505 O-methyltransferase family 3 [Dicentrarchus labrax]
2	4	NaN	27 722 Spectrin beta chain OS=Drosophila melanogaster GN=beta-Spec PE=1 SV=2; 27 722 Spectrin beta chain OS=
3	8	NaN	229 699 hypothetical protein CGI_10017744 [Crassostrea gigas]; 199 669 hypothetical protein CGI_10017744 [Crasso
2	2	NaN	40 1182 4-hydroxyphenylpyruvate dioxygenase [Crassostrea gigas]
4	12	NaN	42 296 minus strand twitchin [Mytilus galloprovincialis]
3	11	NaN	462 671 enhancer protein [Homo sapiens]
6	10	NaN	72 1400 minus strand hypothetical protein RCJMB04_22i13 [Gallus gallus]
2	2	NaN	242 1834 Regulator of differentiation 1 [Crassostrea gigas]; 458 2050 minus strand Regulator of differentiation 1 [Cra
2	2	NaN	69 779 PREDICTED: 15-hydroxyprostaglandin dehydrogenase [NAD+] [Gallus gallus]
2	5	NaN	184 438 PREDICTED: similar to CG14235 CG14235-PA [Tribolium castaneum] gi
2	3	NaN	324 1982 minus strand Pikachurin [Crassostrea gigas]
4	10	NaN	85 546 minus strand RecName: Full=Eukaryotic translation initiation factor 5A; Short=eIF-5A gi|51702279|sp|P6292

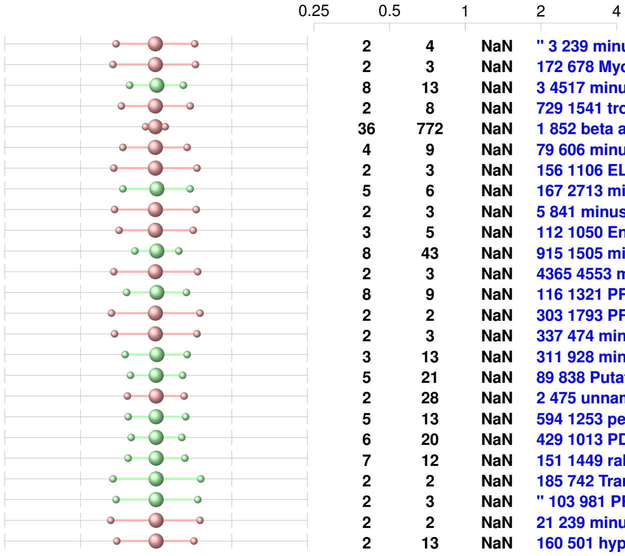

Peptides	Spectra	Coverage	Protein
2	4	NaN	" 3 239 minus strand laminin beta 1 chain, putative [Ixodes scapularis] gi"
2	3	NaN	172 678 Myosin regulatory light chain sqh [Crassostrea gigas]
8	13	NaN	3 4517 minus strand Clathrin heavy chain 1 [Crassostrea gigas]
2	8	NaN	729 1541 troponin T [Mizuhopecten yessoensis]
36	772	NaN	1 852 beta actin-like 1 protein [Macrostomum lignano]
4	9	NaN	79 606 minus strand PREDICTED: similar to Ras-like protein 3 precursor (Protein roughened) [Tribolium castaneum]
2	3	NaN	156 1106 ELAV 2-like protein [Aplysia californica] gi
5	6	NaN	167 2713 minus strand Major vault protein [Crassostrea gigas]
2	3	NaN	5 841 minus strand Spectrin alpha chain [Crassostrea gigas]
3	5	NaN	112 1050 Enolase-phosphatase E1 [Crassostrea gigas]; 112 1050 Enolase-phosphatase E1 [Crassostrea gigas]
8	43	NaN	915 1505 minus strand Collagen alpha-5(VI) chain [Crassostrea gigas]
2	3	NaN	4365 4553 minus strand Basement membrane-specific heparan sulfate proteoglycan core protein [Crassostrea gigas
8	9	NaN	116 1321 PREDICTED: similar to aspartate aminotransferase [Tribolium castaneum] gi
2	2	NaN	303 1793 PREDICTED: matrix metalloproteinase 19 isoform rasi-1 preproprotein-like [Saccoglossus kowalevskii]
2	3	NaN	337 474 minus strand heat shock protein 22 isoform 1 [Ruditapes philippinarum]
3	13	NaN	311 928 minus strand peroxiredoxin 3 [Clonorchis sinensis] gi; 288 905 minus strand peroxiredoxin 3 [Clonorchis si
5	21	NaN	89 838 Putative phosphoglycerate mutase [Crassostrea gigas]; 89 838 Putative phosphoglycerate mutase [Crassost
2	28	NaN	2 475 unnamed protein product [Tetraodon nigroviridis]
5	13	NaN	594 1253 peroxiredoxin 6 [Eriocheir sinensis]
6	20	NaN	429 1013 PDZ domain protein [Ixodes scapularis] gi
7	12	NaN	151 1449 rab GDP-dissociation inhibitor [Branchiostoma belcheri]
2	2	NaN	185 742 Transmembrane protein Tmp21 [Crassostrea gigas]; 1 444 minus strand Transmembrane protein Tmp21 [Cr
2	3	NaN	" 103 981 PREDICTED: ATP synthase, H+ transporting, mitochondrial F1 complex, gamma subunit isoform H (heart)
2	2	NaN	21 239 minus strand calponin-like protein [Taenia asiatica]
2	13	NaN	160 501 hypothetical protein CGI_10001640 [Crassostrea gigas]; 160 588 hypothetical protein CGI_10001640 [Crasso

4.2 Proteins Identified by a Single Peptide

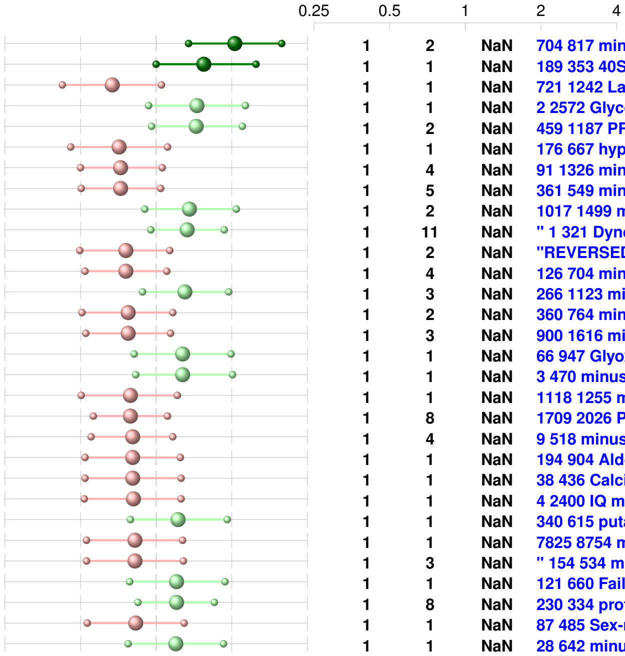

Peptides	Spectra	Coverage	Protein
1	2	NaN	704 817 minus strand expressed hypothetical protein [Trichoplax adhaerens] gi; 688 801 minus strand Lactoylglutat
1	1	NaN	189 353 40S ribosomal protein S12 [Crassostrea gigas]
1	1	NaN	721 1242 Lactoylglutathione lyase [Crassostrea gigas]; 882 1334 Lactoylglutathione lyase OS=Rattus norvegicus GN
1	1	NaN	2 2572 Glycogen debranching enzyme [Crassostrea gigas]
1	2	NaN	459 1187 PREDICTED: protein-L-isoaspartate(D-aspartate) O-methyltransferase-like isoform 2 [Anolis carolinensis]
1	1	NaN	176 667 hypothetical protein CGI_10018452 [Crassostrea gigas]; 176 634 hypothetical protein CGI_10018452 [Crasso
1	4	NaN	91 1326 minus strand Adipocyte plasma membrane-associated protein [Crassostrea gigas]; 107 1273 minus strand
1	5	NaN	361 549 minus strand 32 kDa beta-galactoside-binding lectin [Ascaris suum]
1	2	NaN	1017 1499 minus strand PREDICTED: leukocyte elastase inhibitor-like [Oreochromis niloticus]
1	11	NaN	" 1 321 Dynein heavy chain, cytoplasmic [Crassostrea gigas]"
1	2	NaN	"REVERSED 2 1810 minus strand Glutaminyl-tRNA synthetase, partial [Crassostrea gigas]"
1	4	NaN	126 704 minus strand ribosomal protein L12 [Ixodes scapularis]
1	3	NaN	266 1123 minus strand mitochondrial malate dehydrogenase precursor [Nucella lapillus]
1	2	NaN	360 764 minus strand Peptidyl-prolyl cis-trans isomerase A OS=Dictyostelium discoideum GN=ppiA PE=1 SV=1
1	3	NaN	900 1616 minus strand hemocyanin isoform 1 [Haliotis diversicolor]
1	1	NaN	66 947 Glyoxalase domain-containing protein 4 [Crassostrea gigas]
1	1	NaN	3 470 minus strand Spectrin beta chain [Crassostrea gigas]; 5 451 minus strand Spectrin beta chain [Crassostrea gi
1	1	NaN	1118 1255 minus strand equistatin precursor [Actinia equina]
1	8	NaN	1709 2026 Phosphoenolpyruvate carboxykinase [GTP] [Crassostrea gigas]
1	4	NaN	9 518 minus strand hemocyanin isoform 1 [Nucula nucleus]
1	1	NaN	194 904 Aldo-keto reductase family 1 member B10 [Crassostrea gigas]
1	1	NaN	38 436 Calcium-dependent protein kinase isoform 2 [Crassostrea gigas]
1	1	NaN	4 2400 IQ motif containing GTPase activating protein 2 [Xenopus laevis] gi; 4 2169 IQ motif containing GTPase activ
1	1	NaN	340 615 putative Low-density lipoprotein receptor repeat class B [Trichinella spiralis] gi
1	1	NaN	7825 8754 minus strand H2 [Haliotis tuberculata]
1	3	NaN	" 154 534 minus strand Muscle M-line assembly protein unc-89, partial [Crassostrea gigas]"
1	1	NaN	121 660 Failed axon connections homolog OS=Rattus norvegicus GN=Faxc PE=3 SV=1
1	8	NaN	230 334 protein disulfide isomerase [Conus virgo]
1	1	NaN	87 485 Sex-regulated protein janus-A [Crassostrea gigas]
1	1	NaN	28 642 minus strand Transmembrane emp24 domain-containing protein 9 [Crassostrea gigas]; 236 709 minus strand

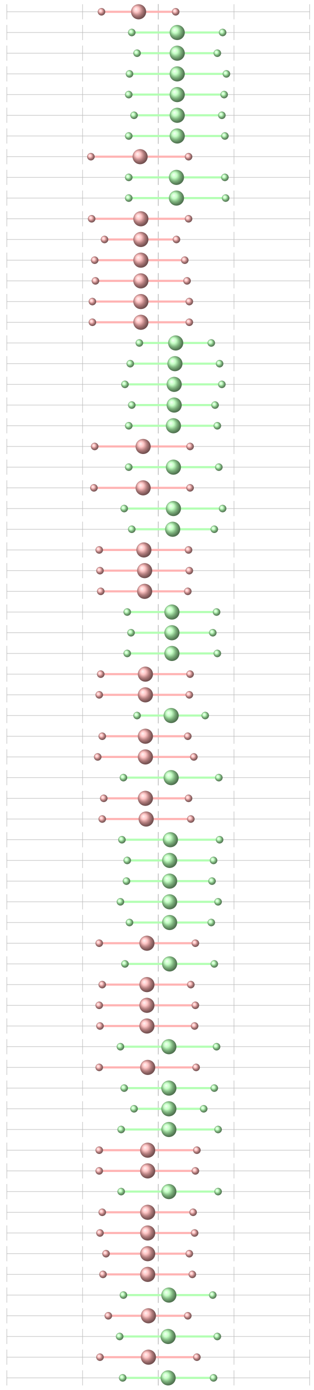

0.25 0.5 1 2 4

Peptides	Spectra	Coverage	Protein
1	10	NaN	1714 2121 Phosphoenolpyruvate carboxykinase [GTP] [Crassostrea gigas]
1	2	NaN	" 196 1053 minus strand Cytochrome b-c1 complex subunit 2, mitochondrial OS=Homo sapiens GN=UQCRC2 PE=1
1	6	NaN	64 393 Tubulin beta chain OS=Paracentrotus lividus PE=2 SV=1; 109 438 Tubulin beta chain OS=Paracentrotus lividu
1	1	NaN	" 125 628 minus strand collectin sub-family member 12, partial [Oncorhynchus mykiss]"
1	1	NaN	401 1174 minus strand Mechanosensory protein 2 [Crassostrea gigas]
1	2	NaN	143 247 minus strand dynein light chain [Aplysia californica] gi
1	1	NaN	" 158 355 Protein NAS-37, isoform b [Caenorhabditis elegans] gi; 15 227 Hemicentin-1 [Crassostrea gigas]"
1	1	NaN	625 1902 minus strand hypothetical protein CGI_10009847 [Crassostrea gigas]
1	1	NaN	140 1564 selenium-binding protein [Haliotis discus hannai]
1	1	NaN	258 629 Glycogenin-1 OS=Oryctolagus cuniculus GN=GYG1 PE=1 SV=3
1	2	NaN	118 513 Protein CAL-5 [Caenorhabditis elegans] gi
1	12	NaN	6043 6174 myosin heavy chain [Mytilus galloprovincialis]; 5869 6000 myosin heavy chain [Mytilus galloprovincialis]
1	2	NaN	261 1361 tandem-repeat galectin [Pinctada fucata]; 261 1361 tandem-repeat galectin [Pinctada fucata]
1	2	NaN	416 1717 minus strand T-complex protein 1 subunit delta OS=Aedes triseriatus PE=2 SV=1
1	1	NaN	213 881 PREDICTED: hypothetical protein [Saccoglossus kowalevskii]; 260 928 PREDICTED: hypothetical protein [S
1	1	NaN	3 572 Receptor-type tyrosine-protein phosphatase N2 [Crassostrea gigas]; 3 542 minus strand Receptor-type tyrosin
1	12	NaN	49 1038 Glyceraldehyde-3-phosphate dehydrogenase [Crotalus adamanteus]
1	2	NaN	140 349 copper chaperone Atox1 [Crassostrea ariakensis]
1	1	NaN	54 638 minus strand ubiquitin carboxyl-terminal hydrolase [Aplysia californica] gi
1	4	NaN	246 452 minus strand mitochondrial malate dehydrogenase precursor [Nucella lapillus]; 106 312 minus strand mitoc
1	3	NaN	96 497 AAEL009772-PB [Aedes aegypti]
1	1	NaN	192 380 minus strand RAB protein [Haliotis discus discus]
1	2	NaN	546 995 Hypothetical protein CBG12002 [Caenorhabditis briggsae]; 543 995 minus strand PREDICTED: tubulin polyr
1	2	NaN	552 1499 minus strand uncharacterized protein LOC100158466 [Xenopus (Silurana) tropicalis] gi; 552 1499 minus st
1	1	NaN	613 2421 PREDICTED: lysosomal alpha-glucosidase [Danio rerio]
1	4	NaN	10 300 minus strand unnamed protein product [Tetraodon nigroviridis]
1	2	NaN	62 1174 Tyrosine-protein kinase CSK [Crassostrea gigas]; 62 1174 minus strand Tyrosine-protein kinase CSK [Crass
1	2	NaN	" 34 1656 Chaperonin containing TCP1, subunit 7 (eta) [Danio rerio] gi; 34 1656 Chaperonin containing TCP1, subun
1	3	NaN	9 887 minus strand hypothetical protein CGI_10006272 [Crassostrea gigas]
1	2	NaN	" 193 333 minus strand RecName: Full=Globin; AltName: Full=Myoglobin gi|299274|gb|AAB25743.1| myoglobin [Na
1	4	NaN	219 428 Peptidyl-prolyl cis-trans isomerase OS=Hemicentrotus pulcherrimus PE=2 SV=1
1	3	NaN	" 154 534 minus strand Muscle M-line assembly protein unc-89, partial [Crassostrea gigas]; 259 690 minus strand M
1	2	NaN	1292 1471 minus strand hemocyanin isoform 1 [Haliotis diversicolor]
1	2	NaN	225 839 minus strand Metallo-beta-lactamase domain-containing protein 1 [Crassostrea gigas]; 201 851 minus stran
1	25	NaN	503 1090 minus strand myosin regulatory light chain [Fasciola hepatica]; 278 865 minus strand myosin regulatory li
1	3	NaN	2831 4900 minus strand Collagen alpha-3(VI) chain [Crassostrea gigas]
1	1	NaN	68 667 minus strand hypothetical protein BRAFLDRAFT_272416 [Branchiostoma floridae] gi; 68 667 minus strand h
1	1	NaN	2 169 cAMP-dependent protein kinase regulatory subunit [Crassostrea gigas]; 125 511 cAMP-dependent protein kin
1	3	NaN	66 263 minus strand twitchin [Mytilus galloprovincialis]; 66 263 minus strand twitchin [Mytilus galloprovincialis]; 66
1	2	NaN	523 1086 minus strand juvenile hormone diol kinase [Danaus plexippus]
1	1	NaN	201 632 USP-like protein isoform 1 [Crassostrea ariakensis] gi; 189 620 minus strand USP-like protein isoform 1 [Cr
1	3	NaN	132 590 minus strand Non-neuronal cytoplasmic intermediate filament protein OS=Helix aspersa PE=3 SV=2
1	3	NaN	2764 3420 PREDICTED: filamin-C-like [Nasonia vitripennis]
1	1	NaN	59 379 hypothetical protein DAPPUDRAFT_230756 [Daphnia pulex]; 117 443 minus strand hypothetical protein DAPP
1	4	NaN	" 1 510 Glycerol-3-phosphate dehydrogenase [NAD+], cytoplasmic [Crassostrea gigas]"
1	1	NaN	366 935 minus strand 40S ribosomal protein S2 OS=Urechis caupo GN=RPS2 PE=2 SV=1
1	2	NaN	895 1410 Galectin-3 OS=Rattus norvegicus GN=Lgals3 PE=1 SV=4; 1067 1405 Galectin-9 [Crassostrea gigas]; 1068 1
1	2	NaN	14 223 minus strand Endothelin-converting enzyme 2 OS=Homo sapiens GN=ECE2 PE=1 SV=4
1	1	NaN	78 461 PREDICTED: hoi-polloi-like [Saccoglossus kowalevskii]; 103 492 NHP2-like protein 1 OS=Rattus norvegicus (
1	1	NaN	1050 1307 calreticulin precursor [Aplysia californica] gi
1	1	NaN	297 734 minus strand PREDICTED: calmodulin [Canis lupus familiaris]; 297 734 PREDICTED: calmodulin [Canis lupu
1	1	NaN	642 1415 minus strand cytochrome c oxidase subunit II [Oncomelania hupensis hupensis]
1	2	NaN	" 148 1989 Acyl-CoA synthetase family member 2, mitochondrial [Crassostrea gigas]; 146 1987 minus strand Acyl-C
1	24	NaN	187 642 minus strand PREDICTED: similar to pol-like protein [Tribolium castaneum]
1	1	NaN	176 517 PREDICTED: elongin C-like [Saccoglossus kowalevskii]; 115 456 PREDICTED: elongin C-like [Saccoglossus
1	1	NaN	58 1203 minus strand Obg-like ATPase 1 [Crassostrea gigas]
1	1	NaN	54 278 ATP-citrate synthase [Crassostrea gigas]; 61 465 ATP-citrate synthase [Crassostrea gigas]
1	1	NaN	179 2140 minus strand PREDICTED: hypothetical protein [Saccoglossus kowalevskii]
1	2	NaN	67 825 unnamed protein product [Oikopleura dioica]
1	1	NaN	227 1201 PREDICTED: quinone oxidoreductase-like isoform 1 [Ornithorhynchus anatinus]; 109 1083 PREDICTED: qu
1	4	NaN	4 399 Spectrin beta chain [Crassostrea gigas]
1	2	NaN	325 642 minus strand Protein phosphatase 1B [Crassostrea gigas]
1	2	NaN	113 808 minus strand omega class glutathione-s-transferase [Chlamys farreri]; 335 1045 minus strand omega class
1	5	NaN	392 808 Whirlin [Crassostrea gigas]
1	1	NaN	613 846 cytochrome c oxidase subunit Va [Antheraea yamamai] gi
1	1	NaN	1265 1831 minus strand Adenosine deaminase CECR1 [Crassostrea gigas]
1	2	NaN	211 990 PREDICTED: gamma-glutamyl hydrolase-like [Xenopus (Silurana) tropicalis]; 223 1080 PREDICTED: gamma

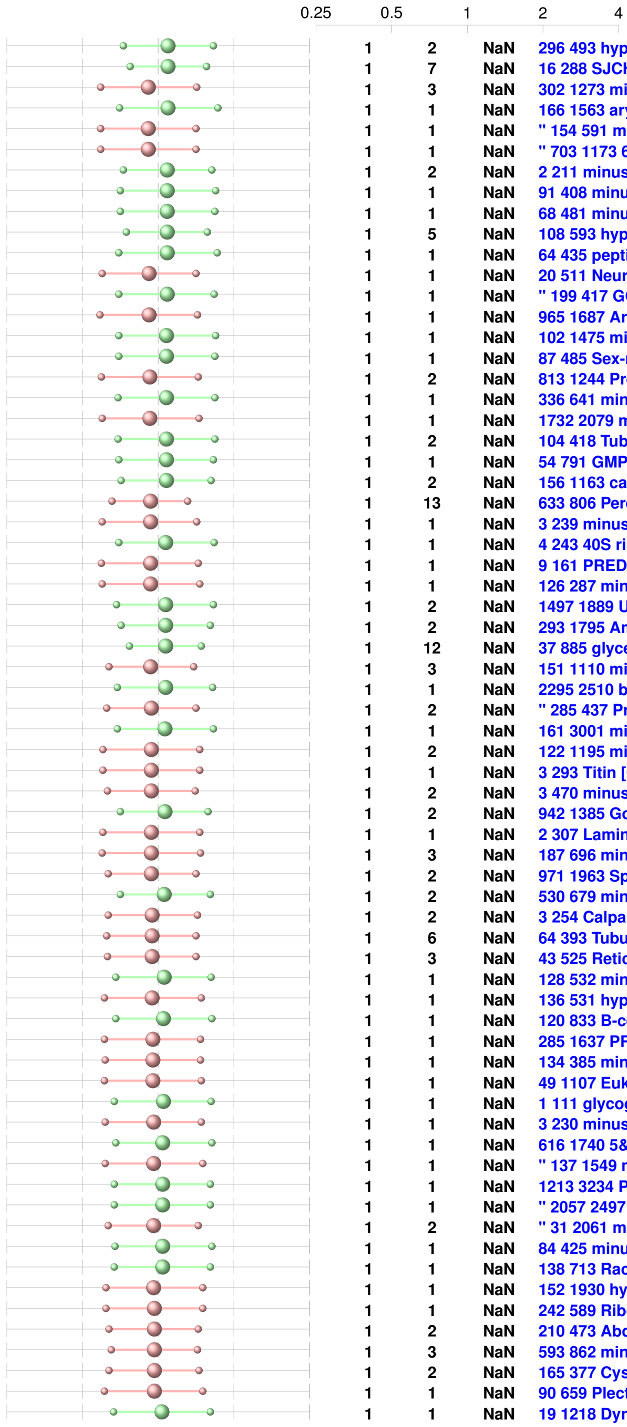

Peptides	Spectra	Coverage	Protein
1	2	NaN	296 493 hypothetical protein BRAFLDRAFT_66287 [Branchiostoma floridae] gi
1	7	NaN	16 288 SJCHGC01950 protein [Schistosoma japonicum] gi
1	3	NaN	302 1273 minus strand hypothetical protein CGI_10007007 [Crassostrea gigas]
1	1	NaN	166 1563 arylsulfatase [Haliotis discus discus]
1	1	NaN	" 154 591 microsomal GST1, partial [Reishia clavigera]; 154 531 microsomal GST1, partial [Reishia clavigera]"
1	1	NaN	" 703 1173 60S acidic ribosomal protein P0, putative [Ixodes scapularis] gi"
1	2	NaN	2 211 minus strand sulfatase 1A precursor [Haliotis discus discus]
1	1	NaN	91 408 minus strand hypothetical protein [Amblyomma maculatum]
1	1	NaN	68 481 minus strand netrin receptor UNC5A [Danio rerio] gi
1	5	NaN	108 593 hypothetical protein CGI_10025497 [Crassostrea gigas]
1	1	NaN	64 435 peptidoglycan recognition protein S1L [Pomacea canaliculata]
1	1	NaN	20 511 Neurexin-2-alpha [Crassostrea gigas]; 20 511 Neurexin-2-alpha [Crassostrea gigas]
1	1	NaN	" 199 417 GG13156 [Drosophila erecta] gi; 253 501 minus strand NADH-ubiquinone oxidoreductase MLRQ subunit-li
1	1	NaN	965 1687 Arylsulfatase B [Crassostrea gigas]
1	1	NaN	102 1475 minus strand Cytosolic non-specific dipeptidase [Crassostrea gigas]
1	1	NaN	87 485 Sex-regulated protein janus-A [Crassostrea gigas]; 92 310 PREDICTED: hypothetical protein [Saccoglossus k
1	2	NaN	813 1244 Probable protein disulfide-isomerase A6 OS=Caenorhabditis elegans GN=tag-320 PE=2 SV=1
1	1	NaN	336 641 minus strand malate dehydrogenase [Schistosoma japonicum]
1	1	NaN	1732 2079 minus strand PREDICTED: annexin A7-like [Ornithorhynchus anatinus]
1	2	NaN	104 418 Tubulin-specific chaperone A [Crassostrea gigas]; 104 418 minus strand Tubulin-specific chaperone A [Cras
1	1	NaN	54 791 GMP reductase 2 [Crassostrea gigas]
1	2	NaN	156 1163 cathepsin B [Crassostrea ariakensis]
1	13	NaN	633 806 Peroxiredoxin-1 [Crassostrea gigas]; 648 821 Peroxiredoxin-1 [Crassostrea gigas]; 666 839 Peroxiredoxin-1 [
1	1	NaN	3 239 minus strand p38 MAPK [Littorina littorea]
1	1	NaN	4 243 40S ribosomal protein S13 OS=Caenorhabditis elegans GN=rps-13 PE=2 SV=2; 4 243 40S ribosomal protein S1
1	1	NaN	9 161 PREDICTED: tubulin alpha-1C chain-like [Acyrthosiphon pisum]; 9 161 Tubulin alpha-3 chain (Fragments) OS=
1	1	NaN	126 287 minus strand Cytochrome b-c1 complex subunit 10 [Cricetulus griseus]; 126 287 minus strand Cytochrome
1	2	NaN	1497 1889 UDP-glucuronosyltransferase 2C1 (Fragment) OS=Oryctolagus cuniculus GN=UGT2C1 PE=2 SV=1
1	2	NaN	293 1795 Amidase [Crassostrea gigas]
1	12	NaN	37 885 glyceraldehyde 3-phosphate dehydrogenase [Dugesia japonica]
1	3	NaN	151 1110 minus strand hypothetical protein DAPPUDRAFT_303085 [Daphnia pulex]
1	1	NaN	2295 2510 beta-thymosin domain repeat protein CSP24KDa_v1 [Hermissenda crassicornis] gi
1	2	NaN	" 285 437 Propionyl-CoA carboxylase alpha chain, mitochondrial [Crassostrea gigas]"
1	1	NaN	161 3001 minus strand E3 ubiquitin-protein ligase MIB2 [Crassostrea gigas]
1	2	NaN	122 1195 minus strand Severin [Crassostrea gigas]
1	1	NaN	3 293 Titin [Crassostrea gigas]
1	2	NaN	3 470 minus strand Spectrin beta chain [Crassostrea gigas]
1	2	NaN	942 1385 Golgi-associated plant pathogenesis-related protein 1 [Crassostrea gigas]; 338 817 Golgi-associated plant
1	1	NaN	2 307 Laminin subunit gamma-1 [Crassostrea gigas]
1	3	NaN	187 696 minus strand putative ribosomal protein S10 [Sipunculus nudus]
1	2	NaN	971 1963 Spectrin alpha chain [Crassostrea gigas]; 972 1979 Spectrin alpha chain [Crassostrea gigas]
1	2	NaN	530 679 minus strand GJ11201 [Drosophila virilis] gi
1	2	NaN	3 254 Calpain-5 [Crassostrea gigas]; 3 254 minus strand Calpain-5 [Crassostrea gigas]
1	6	NaN	64 393 Tubulin beta chain OS=Paracentrotus lividus PE=2 SV=1; 111 398 Tubulin beta chain OS=Paracentrotus lividu
1	3	NaN	43 525 Reticulon-4 [Crassostrea gigas]; 143 757 Reticulon-4 [Crassostrea gigas]; 43 564 Reticulon-4 [Crassostrea gi
1	1	NaN	128 532 minus strand mitochondrial ATPase inhibitor [Ornithodoros parkeri]
1	1	NaN	136 531 hypothetical protein CGI_10017533 [Crassostrea gigas]
1	1	NaN	120 833 B-cell receptor-associated protein 31 [Crassostrea gigas]; 238 951 B-cell receptor-associated protein 31 [Cr
1	1	NaN	285 1637 PREDICTED: talin-2-like [Cricetulus griseus]
1	1	NaN	134 385 minus strand CDGSH iron sulfur domain-containing protein 2 [Crassostrea gigas]; 11 511 minus strand CDG
1	1	NaN	49 1107 Eukaryotic translation initiation factor 3 subunit B [Crassostrea gigas]
1	1	NaN	1 111 glycogen synthase [Crassostrea gigas]
1	1	NaN	3 230 minus strand RH21460p [Drosophila melanogaster]; 3 230 minus strand RH21460p [Drosophila melanogaster]
1	1	NaN	616 1740 5'-AMP-activated protein kinase subunit beta-2 [Crassostrea gigas]
1	1	NaN	" 137 1549 minus strand Serine hydroxymethyltransferase, mitochondrial [Crassostrea gigas]; 205 1608 minus stran
1	1	NaN	1213 3234 Protein-glutamine gamma-glutamyltransferase K [Crassostrea gigas]; 478 2499 Protein-glutamine gamma
1	1	NaN	" 2057 2497 Phosphate carrier protein, mitochondrial [Crassostrea gigas]"
1	2	NaN	" 31 2061 minus strand 1,4-alpha-glucan-branching enzyme [Crassostrea gigas]"
1	1	NaN	84 425 minus strand Transcription elongation factor B polypeptide 2 [Crassostrea gigas]; 84 425 minus strand Trans
1	1	NaN	138 713 Rac [Aplysia californica] gi; 101 676 Rac [Aplysia californica] gi
1	1	NaN	152 1930 hypothetical protein BRAFLDRAFT_223365 [Branchiostoma floridae] gi
1	1	NaN	242 589 Ribonucleoprotein RB97D OS=Drosophila melanogaster GN=Rb97D PE=2 SV=1
1	2	NaN	210 473 Abdominal ganglion neuropeptides L5-67 OS=Aplysia californica PE=1 SV=1; 879 1124 abdominal ganglion
1	3	NaN	593 862 minus strand mitochondrial ATP synthase coupling factor 6 precursor [Ochlerotatus taeniorhynchus]
1	2	NaN	165 377 Cysteine and glycine-rich protein 3 [Crassostrea gigas]; 135 344 Cysteine and glycine-rich protein 3 [Crasso
1	1	NaN	90 659 Plectin-1 [Crassostrea gigas]; 327 881 Plectin-1 [Crassostrea gigas]; 646 1290 minus strand Plectin-1 [Crasso
1	1	NaN	19 1218 Dynactin subunit 2 [Crassostrea gigas]; 48 830 minus strand Dynactin subunit 2 [Crassostrea gigas]

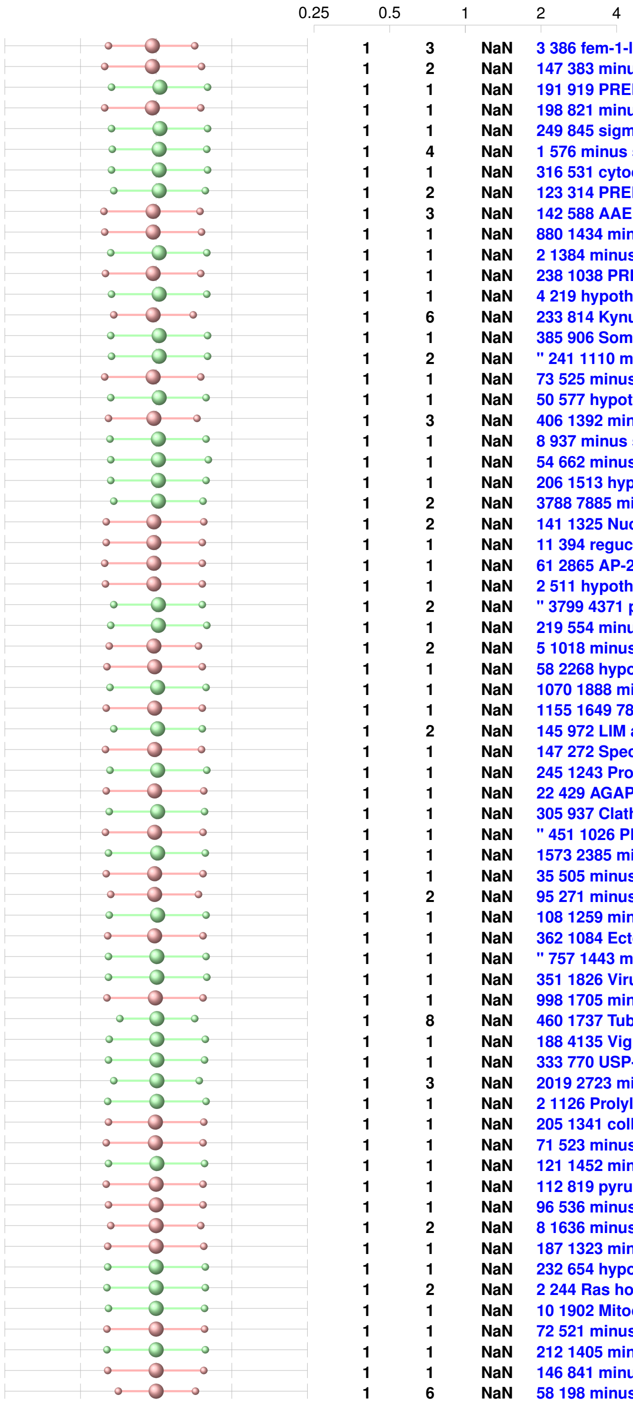

Peptides	Spectra	Coverage	Protein
1	3	NaN	3 386 fem-1-like protein C [Crassostrea gigas]
1	2	NaN	147 383 minus strand Tubulin-specific chaperone A OS=Rattus norvegicus GN=Tbca PE=1 SV=1; 104 418 Tubulin-sp
1	1	NaN	191 919 PREDICTED: 15-hydroxyprostaglandin dehydrogenase [NAD+] [Gallus gallus]
1	1	NaN	198 821 minus strand sigma class glutathione-s-transferase 2 [Haliotis discus discus]
1	1	NaN	249 845 sigma class glutathione-s-transferase 2 [Haliotis discus discus]; 252 848 sigma class glutathione-s-transfer
1	4	NaN	1 576 minus strand Tubulin beta chain [Crassostrea gigas]
1	1	NaN	316 531 cytochrome c oxidase subunit VIIa polypeptide 2 like [Xenopus (Silurana) tropicalis]
1	2	NaN	123 314 PREDICTED: ubiquinol-cytochrome c reductase hinge protein-like [Saccoglossus kowalevskii]; 279 530 PRE
1	3	NaN	142 588 AAEL009772-PB [Aedes aegypti]; 96 497 AAEL009772-PB [Aedes aegypti]
1	1	NaN	880 1434 minus strand Prohormone-4 [Crassostrea gigas]
1	1	NaN	2 1384 minus strand twitchin [Mytilus galloprovincialis]; 2 1384 minus strand twitchin [Mytilus galloprovincialis]; 2 1
1	1	NaN	238 1038 PREDICTED: hypothetical protein [Saccoglossus kowalevskii]; 294 992 PREDICTED: hypothetical protein [
1	1	NaN	4 219 hypothetical protein BRAFLDRAFT_66351 [Branchiostoma floridae] gi; 3 239 hypothetical protein BRAFLDRA
1	6	NaN	233 814 Kynurenine formamidase [Crassostrea gigas]; 233 979 Kynurenine formamidase [Crassostrea gigas]; 139 94
1	1	NaN	385 906 Soma ferritin OS=Lymnaea stagnalis PE=2 SV=2
1	2	NaN	" 241 1110 minus strand N(G),N(G)-dimethylarginine dimethylaminohydrolase 1 [Crassostrea gigas]; 241 1110 minus
1	1	NaN	73 525 minus strand Actin-related protein 2/3 complex subunit 5-like protein [Salmo salar] gi; 73 525 Actin-related pr
1	1	NaN	50 577 hypothetical protein BRAFLDRAFT_119799 [Branchiostoma floridae] gi; 50 577 hypothetical protein BRAFLD
1	3	NaN	406 1392 minus strand hypothetical protein CGI_10007007 [Crassostrea gigas]
1	1	NaN	8 937 minus strand galectin [Crassostrea virginica]
1	1	NaN	54 662 minus strand Protein DJ-1 [Crassostrea gigas]
1	1	NaN	206 1513 hypothetical protein CGI_10019003 [Crassostrea gigas]
1	2	NaN	3788 7885 minus strand Filamin-C [Crassostrea gigas]
1	2	NaN	141 1325 Nucleobindin-2 [Crassostrea gigas]; 141 1208 minus strand Nucleobindin-2 [Crassostrea gigas]
1	1	NaN	11 394 regucalcin [Salmo salar] gi
1	1	NaN	61 2865 AP-2 complex subunit beta [Crassostrea gigas]
1	1	NaN	2 511 hypothetical protein CGI_10006272 [Crassostrea gigas]
1	2	NaN	" 3799 4371 papilin, partial [Rhipicephalus microplus]"
1	1	NaN	219 554 minus strand ribosomal protein P1 [Biomphalaria glabrata]
1	2	NaN	5 1018 minus strand Glutaredoxin-3 [Crassostrea gigas]; 5 1018 Glutaredoxin-3 [Crassostrea gigas]
1	1	NaN	58 2268 hypothetical protein BRAFLDRAFT_267717 [Branchiostoma floridae] gi; 62 2245 hypothetical protein BRAFI
1	1	NaN	1070 1888 minus strand galectin [Pinctada fucata]; 1104 1922 minus strand galectin [Pinctada fucata]
1	1	NaN	1155 1649 78 kDa glucose-regulated protein OS=Aplysia californica PE=1 SV=1
1	2	NaN	145 972 LIM and SH3 domain protein F42H10.3 OS=Caenorhabditis elegans GN=F42H10.3 PE=2 SV=3
1	1	NaN	147 272 Spectrin alpha chain [Crassostrea gigas]
1	1	NaN	245 1243 Prostaglandin reductase 1 [Crassostrea gigas]
1	1	NaN	22 429 AGAP006937-PA [Anopheles gambiae str. PEST] gi; 1 474 Ubiquitin-conjugating enzyme E2 variant 2 OS=Rat
1	1	NaN	305 937 Clathrin light chain A [Crassostrea gigas]
1	1	NaN	" 451 1026 PREDICTED: GTP-binding protein SAR1B, putative-like [Saccoglossus kowalevskii]"
1	1	NaN	1573 2385 minus strand Spectrin beta chain [Crassostrea gigas]
1	1	NaN	35 505 minus strand Basement membrane-specific heparan sulfate proteoglycan core protein [Crassostrea gigas]
1	2	NaN	95 271 minus strand hypothetical protein EAI_01530 [Harpegnathos saltator]
1	1	NaN	108 1259 minus strand PREDICTED: Na(+)/H(+) exchange regulatory cofactor NHE-RF3-like [Metaseiulus occidentali
1	1	NaN	362 1084 Ectoine hydroxylase [Crassostrea gigas]; 362 1084 Ectoine hydroxylase [Crassostrea gigas]; 366 1220 Ecto
1	1	NaN	" 757 1443 minus strand nonmuscle myosin II, partial [Aplysia californica]"
1	1	NaN	351 1826 Virulence metalloprotease [Crassostrea gigas]
1	1	NaN	998 1705 minus strand Basement membrane-specific heparan sulfate proteoglycan core protein [Crassostrea gigas]
1	8	NaN	460 1737 Tubulin beta-4B chain OS=Mus musculus GN=Tubb4b PE=1 SV=1
1	1	NaN	188 4135 Vigilin [Crassostrea gigas]
1	1	NaN	333 770 USP-like protein isoform 2 [Crassostrea ariakensis]
1	3	NaN	2019 2723 minus strand Kyphoscoliosis peptidase [Crassostrea gigas]; 2060 2764 minus strand Kyphoscoliosis pep
1	1	NaN	2 1126 Prolyl endopeptidase [Crassostrea gigas]
1	1	NaN	205 1341 collagen precursor [Bombyx mori]
1	1	NaN	71 523 minus strand hypothetical protein DAPPUDRAFT_309332 [Daphnia pulex]
1	1	NaN	121 1452 minus strand Hexokinase type 2 [Crassostrea gigas]; 245 1576 minus strand Hexokinase type 2 [Crassostr
1	1	NaN	112 819 pyruvate kinase [Crassostrea gigas]; 132 932 pyruvate kinase [Crassostrea gigas]
1	1	NaN	96 536 minus strand hypothetical protein BRAFLDRAFT_285353 [Branchiostoma floridae] gi
1	2	NaN	8 1636 minus strand V-type proton ATPase catalytic subunit A [Crassostrea gigas]; 8 1636 minus strand V-type proto
1	1	NaN	187 1323 minus strand gelsolin [Aplysia californica] gi
1	1	NaN	232 654 hypothetical protein BRAFLDRAFT_87758 [Branchiostoma floridae] gi
1	2	NaN	2 244 Ras homolog gene family member A [Clonorchis sinensis]
1	1	NaN	10 1902 Mitochondrial inner membrane protein [Crassostrea gigas]; 10 1902 Mitochondrial inner membrane protein
1	1	NaN	72 521 minus strand GF14593 [Drosophila ananassae] gi
1	1	NaN	212 1405 minus strand unnamed protein product [Tetraodon nigroviridis]
1	1	NaN	146 841 minus strand hypothetical protein CGI_10006609 [Crassostrea gigas]; 146 841 minus strand hypothetical pr
1	6	NaN	58 198 minus strand Protein DJ-1 [Crassostrea gigas]

5 Protein Details

A detailed summary of each protein is given below. These sections include peptide relative expression estimates in addition to protein-level estimates.

5.1 " 353 781 alpha-B-crystallin, putative [Ixodes scapularis] gi"

Protein Accession	CL37349.Contig1$_H1$
Mean Expression Ratio	0.362
Median Expression Ratio	0.363
Credible Interval	(0.265, 0.497)
Associated Peptides	4
Associated Spectra	7
Coverage	NaN

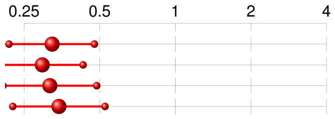

A	B	2.5	50	97.5	Sequence
2	0	0.22	0.32	0.48	LQESGTSEVVNNDKEFR
2	1	0.2	0.3	0.43	VAMDVQHFKPEELK
0	1	0.21	0.32	0.48	IIINAK
0	1	0.22	0.34	0.53	TVTSSLSPSGVLTLK

5.2 200 2128 minus strand heat shock protein 70 [Cristaria plicata]

Protein Accession	CL8787.Contig2$_H1$
Mean Expression Ratio	0.418
Median Expression Ratio	0.417
Credible Interval	(0.308, 0.566)
Associated Peptides	4
Associated Spectra	8
Coverage	NaN

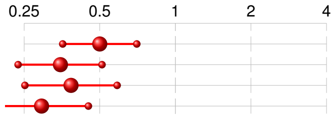

A	B	2.5	50	97.5	Sequence
1	3	0.36	0.5	0.7	IINEPTAAALAYGLDK
1	1	0.24	0.35	0.51	NGQVEIIANDQGQR
1	0	0.25	0.38	0.59	TLAPEEISSMVLVK
0	1	0.19	0.29	0.45	LSKEEIDR

5.3 " 10 609 lethal(2)essential for life protein, l2efl [Aedes aegypti] gi"

Protein Accession	Unigene67975$_H1$
Mean Expression Ratio	0.453
Median Expression Ratio	0.453
Credible Interval	(0.327, 0.628)
Associated Peptides	4
Associated Spectra	6
Coverage	NaN

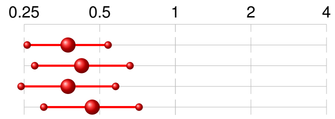

A	B	2.5	50	97.5	Sequence
1	2	0.26	0.37	0.54	ALEAPGETVIPITFEGK
0	1	0.28	0.42	0.66	SEVVNDDKEFR
0	1	0.24	0.37	0.58	KALEAPGETVIPITFEGKGDGK
0	1	0.3	0.47	0.72	KALEAPGETVIPITFEGK

5.4 4726 5571 minus strand twitchin [Mytilus galloprovincialis]

Protein Accession	CL181.Contig4$_{H}1$
Mean Expression Ratio	0.472
Median Expression Ratio	0.472
Credible Interval	(0.371, 0.599)
Associated Peptides	8
Associated Spectra	15
Coverage	NaN

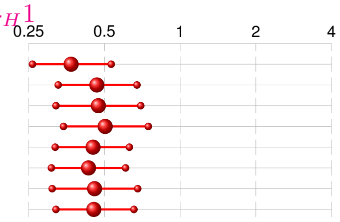

A	B	2.5	50	97.5	Sequence
1	1	0.26	0.37	0.53	EDGGAPITHYIVEK
1	1	0.33	0.47	0.67	GTVEGLQEGKEYEFR
1	0	0.32	0.47	0.7	VCDTDDLEIDVSDLTPGHK
1	0	0.34	0.5	0.75	CALLWDKPK
1	2	0.32	0.45	0.63	NGTPLNEANLDTETVPK
2	1	0.31	0.43	0.61	GLQEGKEYEFR
0	1	0.31	0.46	0.68	EIFVK
0	2	0.32	0.45	0.65	IPDVQR

5.5 704 817 minus strand expressed hypothetical protein [Trichoplax adhaerens] gi; 688 801 minus strand Lactoylglutathione lyase [Crassostrea gigas]; 735 848 minus strand expressed hypothetical protein [Trichoplax adhaerens] gi

Protein Accession	Unigene64106$_{H}1$ Unigene64104$_{H}1$ Unigene64103$_{H}1$
Mean Expression Ratio	2.06
Median Expression Ratio	2.06
Credible Interval	(1.35, 3.17)
Associated Peptides	1
Associated Spectra	2
Coverage	NaN

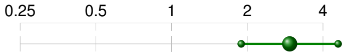

A	B	2.5	50	97.5	Sequence
1	1	1.9	3	4.6	FESLGVELVK

5.6 125 550 minus strand ribosomal protein S20 [Chlamys farreri]; 125 550 minus strand ribosomal protein S20 [Chlamys farreri]; 125 550 minus strand ribosomal protein S20 [Chlamys farreri]

Protein Accession	CL6833.Contig3$_{H}1$ CL6833.Contig2$_{H}1$ CL6833.Contig1$_{H}1$
Mean Expression Ratio	0.574
Median Expression Ratio	0.573
Credible Interval	(0.393, 0.837)
Associated Peptides	2
Associated Spectra	3
Coverage	NaN

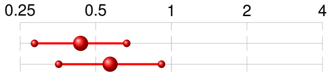

A	B	2.5	50	97.5	Sequence
2	0	0.28	0.44	0.66	IIDLHSPSEIVK
1	0	0.36	0.57	0.91	KTPCGEGSK

5.7 957 2147 haemocyanin [Aplysia californica]

Protein Accession	Unigene30320$_{H}1$
Mean Expression Ratio	0.58
Median Expression Ratio	0.579
Credible Interval	(0.444, 0.755)
Associated Peptides	6
Associated Spectra	12
Coverage	NaN

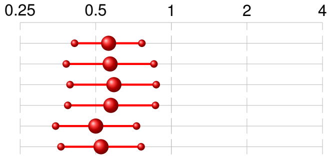

A	B	2.5	50	97.5	Sequence
3	2	0.41	0.56	0.77	LLTVQFER
1	0	0.38	0.57	0.85	YEVSELSLEQVSNLR
1	0	0.4	0.59	0.87	TFHISGVNQDITR
1	0	0.39	0.57	0.86	LLTVQFERAL
0	2	0.35	0.5	0.73	KAGQNIGDTLK
0	2	0.36	0.52	0.76	IDITDAVK

5.8 372 860 minus strand small heat shock protein [Tegillarca granosa]; 651 998 minus strand small heat shock protein [Tegillarca granosa]

Protein Accession	CL18190.Contig4$_{H}$1 CL18190.Contig1$_{H}$1
Mean Expression Ratio	0.593
Median Expression Ratio	0.593
Credible Interval	(0.436, 0.81)
Associated Peptides	4
Associated Spectra	7
Coverage	NaN

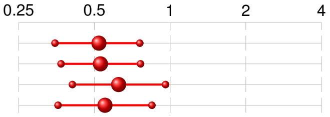

A	B	2.5	50	97.5	Sequence
1	1	0.35	0.52	0.76	EFLIPEEIDAETLGSR
2	1	0.37	0.53	0.76	LTEDGHLIISAR
1	0	0.41	0.63	0.96	IDKDGLSIK
0	1	0.36	0.55	0.84	TIAIESDK

5.9 1027 1440 minus strand hemocyanin isoform 1 [Haliotis diversicolor]

Protein Accession	CL40281.Contig1$_{H}$1
Mean Expression Ratio	0.608
Median Expression Ratio	0.607
Credible Interval	(0.45, 0.825)
Associated Peptides	2
Associated Spectra	10
Coverage	NaN

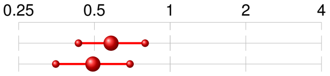

A	B	2.5	50	97.5	Sequence
3	3	0.43	0.58	0.79	LFVVQVEDELK
0	4	0.35	0.49	0.69	HNPFYDADIAFLQK

5.10 718 957 minus strand Calponin-1 OS=Gallus gallus GN=CNN1 PE=1 SV=2

Protein Accession	CL21394.Contig4$_{H}$1
Mean Expression Ratio	1.64
Median Expression Ratio	1.64
Credible Interval	(1.17, 2.31)
Associated Peptides	3
Associated Spectra	5
Coverage	NaN

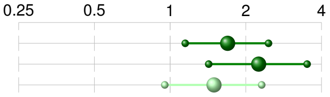

A	B	2.5	50	97.5	Sequence
3	0	1.2	1.7	2.5	EGQTVIGLQAGSNK
1	0	1.4	2.3	3.5	GASQAGMSMGAVR
1	0	0.95	1.5	2.3	IGLQAGSNK

5.11 204 791 minus strand PREDICTED: 15-hydroxyprostaglandin dehydrogenase [NAD+] [Gallus gallus]

Protein Accession	Unigene3818$_{H}$1
Mean Expression Ratio	1.63
Median Expression Ratio	1.63
Credible Interval	(1.32, 2.01)
Associated Peptides	9
Associated Spectra	40
Coverage	NaN

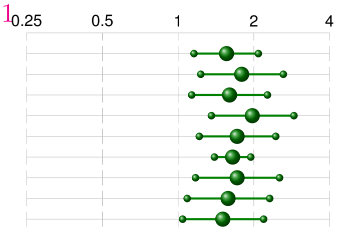

A	B	2.5	50	97.5	Sequence
4	1	1.2	1.6	2.1	AITMWGK
1	0	1.2	1.8	2.6	MGTTTDKELK
2	0	1.1	1.6	2.3	VDIMVNNAEIFSETDMEK
1	0	1.4	2	2.9	NAEIFSETDMEK
1	1	1.2	1.7	2.4	MDITDK
15	11	1.4	1.6	1.9	AVTEALLAK
1	0	1.2	1.7	2.5	IAFVSGGAMGTGR
0	1	1.1	1.6	2.3	TMTVNVTALMHGTK
0	1	1	1.5	2.2	HFISAFEK

5.12 101 1693 CALNUC [Spodoptera frugiperda]; 141 1325 Nucleobindin-2 [Crassostrea gigas]

Protein Accession	CL1888.Contig9$_H1$ CL1888.Contig6$_H1$
Mean Expression Ratio	0.62
Median Expression Ratio	0.621
Credible Interval	(0.413, 0.936)
Associated Peptides	2
Associated Spectra	3
Coverage	NaN

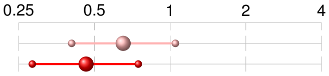

A	B	2.5	50	97.5	Sequence
1	1	0.41	0.65	1.1	LKELPEVER
1	0	0.28	0.46	0.75	REEFK

5.13 27 1682 minus strand PREDICTED: nucleolar protein 56 [Oreochromis niloticus]

Protein Accession	Unigene26699$_H1$
Mean Expression Ratio	0.623
Median Expression Ratio	0.623
Credible Interval	(0.503, 0.771)
Associated Peptides	7
Associated Spectra	27
Coverage	NaN

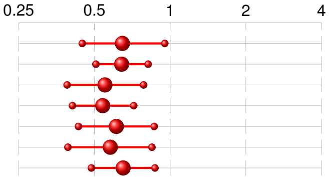

A	B	2.5	50	97.5	Sequence
1	0	0.45	0.65	0.95	KGLGGLSTEKK
7	3	0.51	0.64	0.82	LDGLGQAELQK
2	0	0.39	0.55	0.79	VATLEEEVYDWEIK
2	4	0.41	0.54	0.71	VTPLKLDGLGQAELQK
1	1	0.43	0.61	0.86	LIMQK
1	0	0.39	0.58	0.85	AAEDLRNEAK
4	1	0.49	0.65	0.87	KLIMQK

5.14 189 353 40S ribosomal protein S12 [Crassostrea gigas]

Protein Accession	Unigene385$_H1$
Mean Expression Ratio	1.56
Median Expression Ratio	1.55
Credible Interval	(1, 2.49)
Associated Peptides	1
Associated Spectra	1
Coverage	NaN

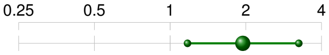

A	B	2.5	50	97.5	Sequence
1	0	1.2	1.9	3.3	TALIHDGLAR

5.15 93 2606 twitchin [Mytilus galloprovincialis]

Protein Accession	Unigene71883$_H1$
Mean Expression Ratio	0.651
Median Expression Ratio	0.65
Credible Interval	(0.508, 0.835)
Associated Peptides	5
Associated Spectra	20
Coverage	NaN

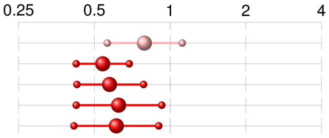

A	B	2.5	50	97.5	Sequence
2	1	0.56	0.79	1.1	FAFHNITGLSPNK
6	4	0.42	0.54	0.69	IIDFGLATR
2	3	0.43	0.58	0.78	FVPQPVEIK
1	0	0.42	0.62	0.93	VIAENFYGR
0	1	0.42	0.61	0.9	LNPDEIVK

5.16 280 690 Histone H3.3 [Harpegnathos saltator]

Protein Accession	Unigene26607$_H$1
Mean Expression Ratio	0.65
Median Expression Ratio	0.651
Credible Interval	(0.51, 0.823)
Associated Peptides	8
Associated Spectra	17
Coverage	NaN

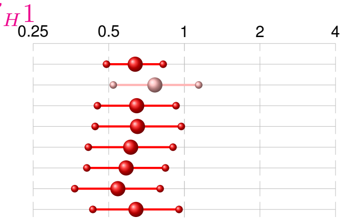

A	B	2.5	50	97.5	Sequence
4	4	0.49	0.64	0.82	STELLIR
1	0	0.52	0.76	1.1	KQLATK
1	1	0.45	0.64	0.93	RVTIMPK
1	0	0.44	0.65	0.97	RTKQTARK
1	0	0.41	0.61	0.9	DIQLAR
2	0	0.41	0.59	0.84	MARTKQTARK
0	1	0.37	0.54	0.8	EIAQDFK
0	1	0.43	0.64	0.95	KLPFQR

5.17 684 1250 minus strand Collagen alpha-5(VI) chain [Crassostrea gigas]

Protein Accession	CL284.Contig4$_H$1
Mean Expression Ratio	1.53
Median Expression Ratio	1.53
Credible Interval	(1.15, 2.02)
Associated Peptides	5
Associated Spectra	10
Coverage	NaN

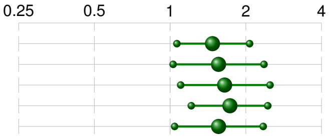

A	B	2.5	50	97.5	Sequence
1	3	1.1	1.5	2.1	HVMDALSAIDHHQGDR
1	0	1	1.6	2.4	VCIVITDGQSQEIQQTADEAR
1	0	1.1	1.6	2.5	EAGITMFAVGVGK
1	2	1.2	1.7	2.4	VAAITFGK
0	1	1	1.6	2.4	EAGITMF

5.18 2996 3340 minus strand Collagen alpha-5(VI) chain [Crassostrea gigas]

Protein Accession	CL47586.Contig8$_H$1
Mean Expression Ratio	1.52
Median Expression Ratio	1.52
Credible Interval	(1.08, 2.16)
Associated Peptides	2
Associated Spectra	6
Coverage	NaN

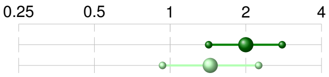

A	B	2.5	50	97.5	Sequence
2	3	1.4	2	2.8	ASIFDAIDEIPYR
1	0	0.94	1.4	2.2	YGSTNTADGIK

5.19 3 491 hemocyanin [Haliotis tuberculata]

Protein Accession	CL40281.Contig3$_H$1
Mean Expression Ratio	0.66
Median Expression Ratio	0.66
Credible Interval	(0.484, 0.893)
Associated Peptides	3
Associated Spectra	8
Coverage	NaN

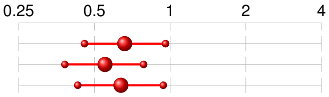

A	B	2.5	50	97.5	Sequence
2	1	0.46	0.66	0.96	DINPFALTK
1	2	0.38	0.55	0.78	TLEPFDRDINPFALTK
1	1	0.43	0.64	0.94	TLEPFDR

5.20 721 1242 Lactoylglutathione lyase [Crassostrea gigas]; 882 1334 Lactoylglutathione lyase OS=Rattus norvegicus GN=Glo1 PE=1 SV=3; 113 634 Lactoylglutathione lyase [Crassostrea gigas]

Protein Accession	Unigene26921$_H$1 Unigene26919$_H$1 CL10946.Contig1$_H$1
Mean Expression Ratio	0.669
Median Expression Ratio	0.671
Credible Interval	(0.424, 1.05)
Associated Peptides	1
Associated Spectra	1
Coverage	NaN

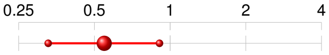

A	B	2.5	50	97.5	Sequence
1	0	0.33	0.55	0.91	FESLGVEFIK

5.21 103 684 EF-hand domain-containing protein D2 [Crassostrea gigas]

Protein Accession	CL20544.Contig1$_H$1
Mean Expression Ratio	1.48
Median Expression Ratio	1.48
Credible Interval	(1.03, 2.13)
Associated Peptides	3
Associated Spectra	3
Coverage	NaN

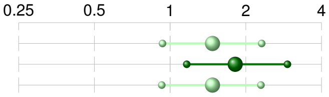

A	B	2.5	50	97.5	Sequence
1	0	0.93	1.5	2.3	VPQTHLALK
1	0	1.2	1.8	2.9	KFEEEIR
1	0	0.93	1.5	2.3	FIDFNELK

5.22 1009 2784 minus strand Collagen alpha-3(VI) chain [Crassostrea gigas]

Protein Accession	Unigene46961$_H$1
Mean Expression Ratio	1.48
Median Expression Ratio	1.48
Credible Interval	(1.18, 1.86)
Associated Peptides	8
Associated Spectra	21
Coverage	NaN

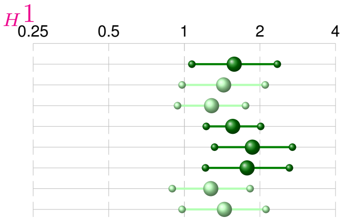

A	B	2.5	50	97.5	Sequence
1	0	1.1	1.6	2.3	FRKSPTLMFNLNEHK
1	0	0.98	1.4	2.1	IITVGIGEANRK
2	2	0.94	1.3	1.8	KSPTLMFNLNEHK
5	4	1.2	1.6	2	VAMSNFR
1	1	1.3	1.9	2.7	YADIDGGNVR
1	0	1.2	1.8	2.6	KAFNK
1	1	0.9	1.3	1.8	MFNLNEHK
0	1	0.98	1.4	2.1	FNLNEHK

5.23 2 2572 Glycogen debranching enzyme [Crassostrea gigas]

Protein Accession	CL45503.Contig1$_H$1
Mean Expression Ratio	1.45
Median Expression Ratio	1.45
Credible Interval	(0.934, 2.27)
Associated Peptides	1
Associated Spectra	1
Coverage	NaN

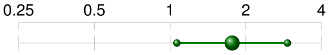

A	B	2.5	50	97.5	Sequence
0	1	1.1	1.8	2.9	IMAVNGWVMGDDPLR

5.24 186 1778 predicted protein [Nematostella vectensis] gi

Protein Accession	CL20488.Contig9$_H1$
Mean Expression Ratio	1.45
Median Expression Ratio	1.45
Credible Interval	(1.03, 2.08)
Associated Peptides	3
Associated Spectra	4
Coverage	NaN

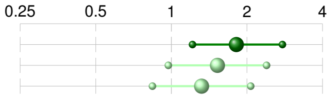

A	B	2.5	50	97.5	Sequence
1	1	1.2	1.8	2.8	EGTENVEMHFR
0	1	0.97	1.5	2.4	DGTTHVIWK
0	1	0.84	1.3	2.1	TRQLLK

5.25 459 1187 PREDICTED: protein-L-isoaspartate(D-aspartate) O-methyltransferase-like isoform 2 [Anolis carolinensis]

Protein Accession	CL3223.Contig3$_H1$
Mean Expression Ratio	1.45
Median Expression Ratio	1.45
Credible Interval	(0.962, 2.2)
Associated Peptides	1
Associated Spectra	2
Coverage	NaN

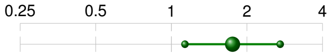

A	B	2.5	50	97.5	Sequence
2	0	1.1	1.8	2.7	AVGIEHIEELVK

5.26 1 945 hemocyanin isoform 1 [Haliotis diversicolor]

Protein Accession	Unigene30318$_H1$
Mean Expression Ratio	0.695
Median Expression Ratio	0.694
Credible Interval	(0.477, 1.02)
Associated Peptides	2
Associated Spectra	3
Coverage	NaN

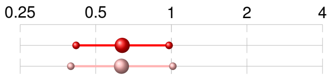

A	B	2.5	50	97.5	Sequence
1	1	0.42	0.64	0.98	VEITDVLK
0	1	0.4	0.63	1	LFVVEVEDELR

5.27 696 4010 Lethal(2) giant larvae-like protein 1 [Crassostrea gigas]; 657 3971 minus strand Lethal(2) giant larvae-like protein 1 [Crassostrea gigas]; 156 3470 Lethal(2) giant larvae-like protein 1 [Crassostrea gigas]

Protein Accession	CL3717.Contig4$_H1$ CL3717.Contig3$_H1$ CL3717.Contig16$_H1$
Mean Expression Ratio	0.696
Median Expression Ratio	0.696
Credible Interval	(0.478, 1.01)
Associated Peptides	3
Associated Spectra	3
Coverage	NaN

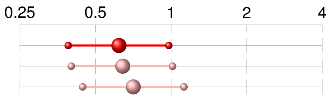

A	B	2.5	50	97.5	Sequence
0	1	0.39	0.62	0.98	LDTTADITIDSVR
0	1	0.4	0.64	1	VLPESLEVIHER
0	1	0.44	0.71	1.1	ASMIR

5.28 201 1181 minus strand Serine/threonine-protein phosphatase PP1-alpha catalytic subunit [Crassostrea gigas]

Protein Accession	CL28655.Contig4$_{H}1$
Mean Expression Ratio	0.696
Median Expression Ratio	0.698
Credible Interval	(0.473, 1.01)
Associated Peptides	2
Associated Spectra	3
Coverage	NaN

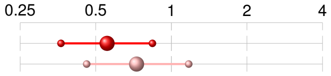

A	B	2.5	50	97.5	Sequence
1	1	0.36	0.55	0.84	ADTDKLNIDSIIAR
0	1	0.46	0.73	1.2	AHQVVEDGYEFFAK

5.29 496 2151 NADP-dependent malic enzyme [Crassostrea gigas]

Protein Accession	CL26274.Contig1$_{H}1$
Mean Expression Ratio	0.706
Median Expression Ratio	0.707
Credible Interval	(0.587, 0.845)
Associated Peptides	11
Associated Spectra	40
Coverage	NaN

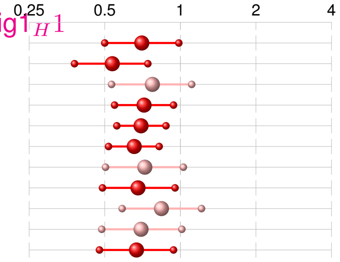

A	B	2.5	50	97.5	Sequence
1	1	0.5	0.7	0.99	AECTAEEAYTLTEGR
2	0	0.38	0.54	0.74	AIVVTDGER
1	0	0.53	0.77	1.1	GLFISIKDK
4	2	0.55	0.72	0.94	IWMVDSK
8	3	0.56	0.7	0.88	VLVNFR
7	4	0.52	0.66	0.82	GIDILR
1	0	0.5	0.72	1	GTAFSLK
1	1	0.49	0.68	0.95	GLLVK
1	0	0.59	0.84	1.2	GDLGVYGMGIP
0	1	0.49	0.7	1	QGTAAVAAAGVFAAVR
0	2	0.48	0.67	0.94	INERPIIFALSNPTSK

5.30 230 1189 hypothetical protein [Amblyomma maculatum]

Protein Accession	CL11202.Contig4$_{H}1$
Mean Expression Ratio	1.41
Median Expression Ratio	1.41
Credible Interval	(1.04, 1.95)
Associated Peptides	4
Associated Spectra	7
Coverage	NaN

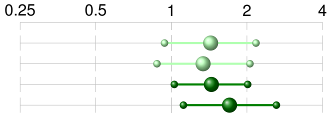

A	B	2.5	50	97.5	Sequence
1	0	0.94	1.4	2.2	KFAVDAVK
1	0	0.88	1.3	2.1	LSFDKEGMVK
0	4	1	1.4	2	ILEIIPGR
0	1	1.1	1.7	2.6	SFEPLEDPGVR

5.31 176 667 hypothetical protein CGI_10018452 [Crassostrea gigas]; 176 634 hypothetical protein CGI_10018452 [Crassostrea gigas]

Protein Accession	CL35173.Contig3$_{H}1$ CL35173.Contig1$_{H}1$
Mean Expression Ratio	0.713
Median Expression Ratio	0.714
Credible Interval	(0.458, 1.11)
Associated Peptides	1
Associated Spectra	1
Coverage	NaN

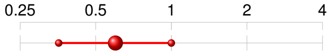

A	B	2.5	50	97.5	Sequence
0	1	0.36	0.6	1	NFLELTELK

5.32 633 806 Peroxiredoxin-1 [Crassostrea gigas]; 648 821 Peroxiredoxin-1 [Crassostrea gigas]; 666 839 Peroxiredoxin-1 [Crassostrea gigas]; 625 798 Peroxiredoxin-1 [Crassostrea gigas]

Protein Accession	CL3160.Contig18$_H$1 CL3160.Contig17$_H$1 CL3160.Contig16$_H$1 CL3160.Contig15$_H$1
Mean Expression Ratio	1.4
Median Expression Ratio	1.4
Credible Interval	(1.03, 1.92)
Associated Peptides	2
Associated Spectra	14
Coverage	NaN

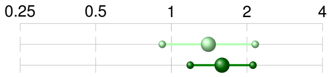

A	B	2.5	50	97.5	Sequence
1	0	0.92	1.4	2.2	EYFSK
7	6	1.2	1.6	2.1	LVQAFQFTDK

5.33 247 1308 RecName: Full=Guanine nucleotide-binding protein G(o) subunit alpha gi|9633|emb|CAA78806.1| GTP-binding protein alpha-o subunit [Lymnaea stagnalis]

Protein Accession	CL26602.Contig4$_H$1
Mean Expression Ratio	1.39
Median Expression Ratio	1.39
Credible Interval	(1.09, 1.77)
Associated Peptides	6
Associated Spectra	18
Coverage	NaN

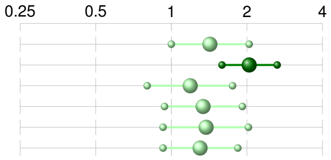

A	B	2.5	50	97.5	Sequence
1	1	1	1.4	2	IIHEGGFTSEDNK
4	5	1.6	2	2.6	LLLLGAGESGK
1	0	0.8	1.2	1.8	QTYEEAAAYIQAQFEAK
1	1	0.94	1.3	1.9	YFLDNLDR
1	0	0.92	1.4	2	KDLFEEK
0	3	0.93	1.3	1.8	MVLDVIAR

5.34 80 589 minus strand PREDICTED: adenine phosphoribosyltransferase-like [Strongylocentrotus purpuratus]

Protein Accession	CL8682.Contig6$_H$1
Mean Expression Ratio	1.39
Median Expression Ratio	1.39
Credible Interval	(1.05, 1.85)
Associated Peptides	5
Associated Spectra	10
Coverage	NaN

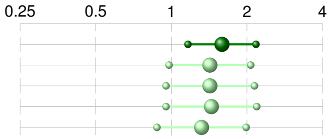

A	B	2.5	50	97.5	Sequence
2	3	1.2	1.6	2.2	LTVPVESLIK
1	1	0.98	1.4	2.1	AAVELVEK
0	1	0.95	1.4	2.1	GFLFGPMIAEK
0	1	0.95	1.4	2.2	NAPDADVIIGLESR
0	1	0.88	1.3	2	DIFPILR

5.35 " 116 352 minus strand troponin I 4 [Clonorchis sinensis]; 491 706 minus strand troponin-I, TnI {C-terminal} [Chlamys nipponensis=Akazara scallop, akazara, striated adductor muscle, Peptide Partial, 135 aa]"

Protein Accession	Unigene63816$_{H}1$ Unigene63815$_{H}1$
Mean Expression Ratio	0.721
Median Expression Ratio	0.721
Credible Interval	(0.539, 0.954)
Associated Peptides	2
Associated Spectra	21
Coverage	NaN

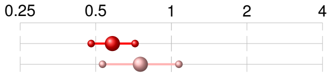

A	B	2.5	50	97.5	Sequence
12	6	0.48	0.58	0.72	FIKPVLR
2	1	0.53	0.75	1.1	FALKEEDESSK

5.36 6 650 minus strand glutathione S-transferase mu class protein [Crassostrea ariakensis]; 117 761 glutathione S-transferase mu class protein [Crassostrea ariakensis]; 122 766 glutathione S-transferase mu class protein [Crassostrea ariakensis]; 122 766 glutathione S-transferase mu class protein [Crassostrea ariakensis]

Protein Accession	Unigene56987$_{H}1$ CL6887.Contig3$_{H}1$ CL6887.Contig2$_{H}1$ CL6887.Contig1$_{H}1$
Mean Expression Ratio	1.39
Median Expression Ratio	1.39
Credible Interval	(1.08, 1.79)
Associated Peptides	6
Associated Spectra	13
Coverage	NaN

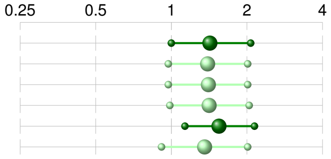

A	B	2.5	50	97.5	Sequence
2	0	1	1.4	2.1	ITQSNAILR
1	1	0.97	1.4	2	VKETLASYEK
1	1	0.97	1.4	2	PALGYWK
2	0	0.99	1.4	2	IMIPGILDDYPK
3	1	1.1	1.6	2.1	KHDLLGK
0	1	0.91	1.4	2	DAWFNVK

5.37 91 1326 minus strand Adipocyte plasma membrane-associated protein [Crassostrea gigas]; 107 1273 minus strand Adipocyte plasma membrane-associated protein [Crassostrea gigas]

Protein Accession	CL11239.Contig2$_{H}1$ CL11239.Contig1$_{H}1$
Mean Expression Ratio	0.725
Median Expression Ratio	0.723
Credible Interval	(0.499, 1.06)
Associated Peptides	1
Associated Spectra	4
Coverage	NaN

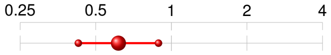

A	B	2.5	50	97.5	Sequence
0	4	0.43	0.61	0.89	YGLIAELDKEGK

5.38 361 549 minus strand 32 kDa beta-galactoside-binding lectin [Ascaris suum]

Protein Accession	CL5658.Contig3$_{H}1$
Mean Expression Ratio	0.725
Median Expression Ratio	0.724
Credible Interval	(0.502, 1.05)
Associated Peptides	1
Associated Spectra	5
Coverage	NaN

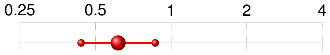

A	B	2.5	50	97.5	Sequence
3	2	0.44	0.62	0.86	TIVLNHMQDGR

5.39 181 477 minus strand Zgc:56530 [Danio rerio]; 185 481 minus strand Zgc:56530 [Danio rerio]

Protein Accession	CL48470.Contig6$_{H}1$ CL27088.Contig2$_{H}1$
Mean Expression Ratio	1.38
Median Expression Ratio	1.38
Credible Interval	(0.979, 1.94)
Associated Peptides	2
Associated Spectra	6
Coverage	NaN

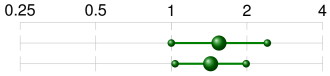

A	B	2.5	50	97.5	Sequence
1	0	1	1.6	2.4	TQVVAGTNYFIK
5	0	1	1.4	2	TFTDFK

5.40 " 1 1887 Pyruvate carboxylase, mitochondrial [Crassostrea gigas]"

Protein Accession	CL26073.Contig5$_{H}1$
Mean Expression Ratio	0.727
Median Expression Ratio	0.728
Credible Interval	(0.512, 1.02)
Associated Peptides	4
Associated Spectra	4
Coverage	NaN

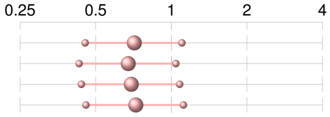

A	B	2.5	50	97.5	Sequence
0	1	0.45	0.71	1.1	AGTHILGIK
0	1	0.43	0.67	1	HLEDVEENFKR
0	1	0.44	0.69	1.1	FLHECPWDR
0	1	0.46	0.72	1.1	IEVFR

5.41 " 81 1349 minus strand PREDICTED: LOW QUALITY PROTEIN: dolichyl-diphosphooligosaccharide–protein glycosyltransferase 48 kDa subunit-like, partial [Cavia porcellus]; 81 1349 minus strand PREDICTED: LOW QUALITY PROTEIN: dolichyl-diphosphooligosaccharide–protein glycosyltransferase 48 kDa subunit-like, partial [Cavia porcellus]"

Protein Accession	CL49524.Contig2$_{H}1$ CL49524.Contig1$_{H}1$
Mean Expression Ratio	1.37
Median Expression Ratio	1.37
Credible Interval	(0.944, 2.02)
Associated Peptides	2
Associated Spectra	3
Coverage	NaN

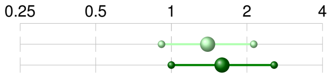

A	B	2.5	50	97.5	Sequence
1	1	0.91	1.4	2.1	DLGFDLTFK
1	0	1	1.6	2.6	FPQSGNLELAK

5.42 1363 1527 Protein disulfide-isomerase A3 [Crassostrea gigas]

Protein Accession	CL28563.Contig1$_{H}1$
Mean Expression Ratio	0.728
Median Expression Ratio	0.729
Credible Interval	(0.483, 1.09)
Associated Peptides	2
Associated Spectra	2
Coverage	NaN

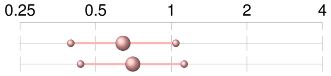

A	B	2.5	50	97.5	Sequence
0	1	0.4	0.64	1	LKDEPEITIAK
0	1	0.44	0.7	1.1	FEELAEK

5.43 180 545 calreticulin precursor [Aplysia californica] gi

Protein Accession	CL4813.Contig2$_{H}1$
Mean Expression Ratio	0.732
Median Expression Ratio	0.734
Credible Interval	(0.523, 1.02)
Associated Peptides	4
Associated Spectra	12
Coverage	NaN

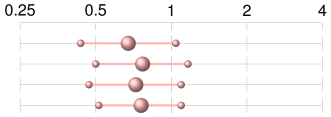

A	B	2.5	50	97.5	Sequence
0	1	0.44	0.68	1	EEFDANWEDR
1	1	0.5	0.77	1.2	FYGISAK
2	1	0.47	0.72	1.1	FVWSAGK
4	2	0.52	0.76	1.1	WIPSVHK

5.44 1017 1499 minus strand PREDICTED: leukocyte elastase inhibitor-like [Oreochromis niloticus]

Protein Accession	CL39031.Contig4$_H$1
Mean Expression Ratio	1.36
Median Expression Ratio	1.36
Credible Interval	(0.903, 2.09)
Associated Peptides	1
Associated Spectra	2
Coverage	NaN

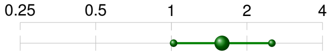

A	B	2.5	50	97.5	Sequence
**0**	**2**	**1**	**1.6**	**2.5**	**VLELPYGTDDR**

5.45 " 110 1717 PREDICTED: glutamate dehydrogenase, mitochondrial-like [Oreochromis niloticus]; 110 1717 PREDICTED: glutamate dehydrogenase, mitochondrial-like [Oreochromis niloticus]; 110 1717 PREDICTED: glutamate dehydrogenase, mitochondrial-like [Oreochromis niloticus]"

Protein Accession	CL16377.Contig9$_H$1 CL16377.Contig8$_H$1 CL16377.Contig10$_H$1
Mean Expression Ratio	1.36
Median Expression Ratio	1.36
Credible Interval	(0.981, 1.86)
Associated Peptides	4
Associated Spectra	6
Coverage	NaN

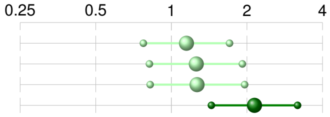

A	B	2.5	50	97.5	Sequence
**1**	**1**	**0.77**	**1.1**	**1.7**	**IIAEGANGPTTAGADEIFLK**
**1**	**0**	**0.82**	**1.3**	**1.9**	**DGSIYNPVDGIHPR**
**0**	**1**	**0.82**	**1.3**	**2**	**TAAYIAAIEK**
**0**	**2**	**1.4**	**2.1**	**3.2**	**EAGLTFT**

5.46 " 303 500 minus strand protein lethal, putative [Pediculus humanus corporis] gi"

Protein Accession	CL15038.Contig1$_H$1
Mean Expression Ratio	0.738
Median Expression Ratio	0.738
Credible Interval	(0.53, 1.02)
Associated Peptides	2
Associated Spectra	9
Coverage	NaN

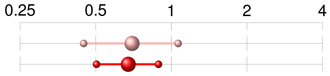

A	B	2.5	50	97.5	Sequence
**1**	**0**	**0.45**	**0.69**	**1.1**	**QDMLATGR**
**6**	**2**	**0.5**	**0.67**	**0.89**	**QMPMLGR**

5.47 34 1122 Fructose-bisphosphate aldolase [Crassostrea gigas]

Protein Accession	CL24228.Contig1$_H$1
Mean Expression Ratio	1.35
Median Expression Ratio	1.35
Credible Interval	(1.16, 1.57)
Associated Peptides	18
Associated Spectra	61
Coverage	NaN

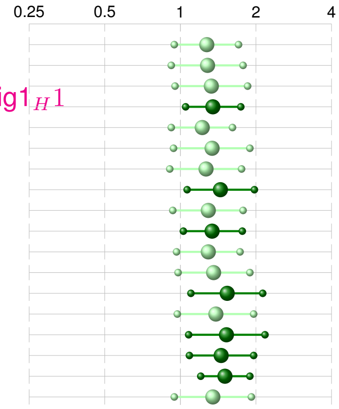

A	B	2.5	50	97.5	Sequence
3	1	0.94	1.3	1.7	DSQGTPFVK
1	1	0.92	1.3	1.8	GAQDELLK
1	1	0.96	1.3	1.9	GAQDELLKR
5	3	1.1	1.3	1.7	GIIPGIK
3	2	0.92	1.2	1.6	GILAADESVGSMGK
1	0	0.94	1.3	1.9	GVVPLAGSNNECTTQGLDGLAER
2	0	0.91	1.3	1.7	ISDNTPSYQAMIENANVLAR
3	0	1.1	1.4	2	KDGAQFAK
1	1	0.94	1.3	1.8	LEGIGLENTEENR
4	2	1	1.3	1.8	QIANSIVAPGK
3	1	0.96	1.3	1.7	RLEGIGLENTEENR
1	1	0.98	1.4	1.9	RLEGIGLENTEENRR
2	0	1.1	1.5	2.1	YASICQQNGLVPIVEPEVLPDGDHDLK
1	0	0.98	1.4	2	VKGAQDELLKR
1	0	1.1	1.5	2.2	ANSEAALGK
2	2	1.1	1.5	2	PVIAQYLTK
5	6	1.2	1.5	1.9	VTEQVLAFTYK
0	1	0.94	1.3	1.9	RLEGIGLENTEENRRRYR

5.48 501 1838 Hsp90A [Haliotis asinina]

Protein Accession	CL5184.Contig2$_H$1
Mean Expression Ratio	0.744
Median Expression Ratio	0.743
Credible Interval	(0.582, 0.955)
Associated Peptides	7
Associated Spectra	15
Coverage	NaN

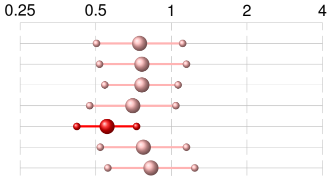

A	B	2.5	50	97.5	Sequence
1	0	0.5	0.75	1.1	AEFEGLCK
1	0	0.52	0.76	1.1	AIYYITGESLDAVK
1	2	0.54	0.76	1.1	GVVDSEDLPLNISR
1	0	0.47	0.7	1	NPDDITPEEYAEFYK
3	4	0.42	0.55	0.73	LAEFLR
1	0	0.52	0.78	1.1	RAPFDLFENK
1	0	0.56	0.83	1.2	VERVK

5.49 30 1088 arginine kinase [Conus novaehollandiae]

Protein Accession	CL5308.Contig5$_{H}1$
Mean Expression Ratio	1.35
Median Expression Ratio	1.34
Credible Interval	(1.22, 1.49)
Associated Peptides	31
Associated Spectra	207
Coverage	NaN

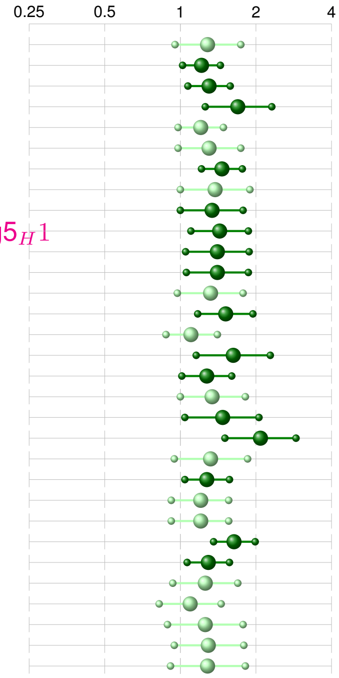

A	B	2.5	50	97.5	Sequence
2	1	0.95	1.3	1.7	AAGGYDDWPVGR
12	10	1	1.2	1.4	ATVEALNTFEGELK
8	8	1.1	1.3	1.6	FNFPDLDPSGK
2	1	1.3	1.7	2.3	GIHGEHTESVGGVYDISNK
9	5	0.98	1.2	1.5	GTYYPLLGMDK
2	2	0.98	1.3	1.7	KLIDDHFLFKDDDR
11	7	1.2	1.5	1.8	LAALPEFK
1	1	1	1.4	1.9	LGLSEFDAIMEMK
2	2	1	1.3	1.8	LIDDHFLFKDDDR
5	1	1.1	1.4	1.9	RFNFPDLDPSGK
3	1	1	1.4	1.9	SHASYGFPPVLSK
3	1	1.1	1.4	1.9	TFLVWVNEEDHIR
2	1	0.97	1.3	1.8	NGVEEILKK
7	0	1.2	1.5	1.9	KLTFAR
5	4	0.88	1.1	1.4	NGVEEILK
1	0	1.2	1.6	2.3	KLIDDHFLFK
6	4	1	1.3	1.6	LTFAR
2	1	1	1.3	1.8	EFCDK
1	0	1	1.5	2.1	TFEGELK
2	0	1.5	2.1	2.9	GTYYPLLGMDKATEK
1	0	0.95	1.3	1.9	APDFGEK
14	0	1	1.3	1.6	GGNLTQVYQR
1	5	0.92	1.2	1.6	GIFHNPDK
5	1	0.92	1.2	1.6	HPAPDFGEK
10	7	1.4	1.6	2	LVSGIHALEK
7	9	1.1	1.3	1.6	YIVSTR
0	3	0.93	1.3	1.7	LIDDHFLFK
0	4	0.82	1.1	1.5	IISMQK
0	1	0.89	1.3	1.8	VAEVK
0	2	0.95	1.3	1.8	IDDHFLFK
0	1	0.91	1.3	1.8	ATVEALNTFEGE

5.50 150 716 calponin/transgelin [Papilio xuthus]

Protein Accession	CL11747.Contig2$_{H}1$
Mean Expression Ratio	1.34
Median Expression Ratio	1.34
Credible Interval	(0.983, 1.84)
Associated Peptides	3
Associated Spectra	8
Coverage	NaN

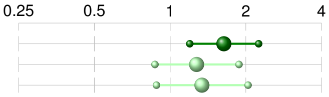

A	B	2.5	50	97.5	Sequence
4	1	1.2	1.6	2.3	GPPLNTPEHSVFATPDLYEK
1	1	0.87	1.3	1.9	MAFQQMEAIEK
1	0	0.88	1.3	2	FTTAIK

5.51 300 878 unknown [Schistosoma japonicum]; 567 1145 unknown [Schistosoma japonicum]; 1110 1688 unknown [Schistosoma japonicum]; 94 672 unknown [Schistosoma japonicum]; 599 1177 unknown [Schistosoma japonicum]; 336 914 unknown [Schistosoma japonicum]

Protein Accession: CL11292.Contig5$_{H}1$ CL11292.Contig3$_{H}1$ CL11292.Contig2$_{H}1$ CL11292.Contig1$_{H}1$ CL1129
Mean Expression Ratio: 0.746
Median Expression Ratio: 0.746
Credible Interval: (0.553, 1)
Associated Peptides: 4
Associated Spectra: 12
Coverage: NaN

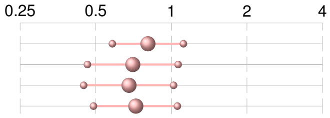

A	B	2.5	50	97.5	Sequence
4	4	0.58	0.81	1.1	AGNDVDLVVQR
1	0	0.46	0.7	1.1	GVVNTQSLPQEPEGEKR
1	0	0.45	0.68	1	NGDAILQICGESTAAMTHAQAK
2	0	0.49	0.72	1.1	VQLRRYDTSQPWGFR

5.52 12160 13014 twitchin [Mytilus galloprovincialis]

Protein Accession: CL181.Contig2$_{H}1$
Mean Expression Ratio: 0.747
Median Expression Ratio: 0.747
Credible Interval: (0.56, 0.995)
Associated Peptides: 4
Associated Spectra: 11
Coverage: NaN

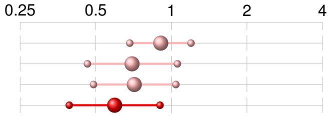

A	B	2.5	50	97.5	Sequence
1	6	0.68	0.91	1.2	ATLTVEPGVEGLR
1	0	0.46	0.7	1.1	AVNSLGEEETSCK
1	1	0.49	0.71	1	AFYKPK
1	0	0.39	0.59	0.9	NQLSAEGHTAK

5.53 26 1909 Dolichyl-diphosphooligosaccharide–protein glycosyltransferase subunit 2 [Crassostrea gigas]

Protein Accession: CL6515.Contig1$_{H}1$
Mean Expression Ratio: 1.34
Median Expression Ratio: 1.34
Credible Interval: (1.01, 1.76)
Associated Peptides: 6
Associated Spectra: 10
Coverage: NaN

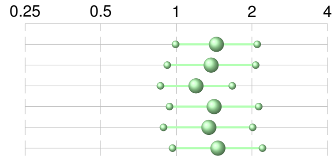

A	B	2.5	50	97.5	Sequence
1	1	0.99	1.4	2.1	AVSSALLDALK
1	0	0.92	1.4	2.1	KQFTASASDK
2	2	0.86	1.2	1.7	NLGLAVDEK
1	0	0.94	1.4	2.1	TLYELNFMEAK
1	0	0.89	1.3	2	LLTDAISAK
1	0	0.97	1.5	2.2	VSVTNLMGGDLGQLTVTADSAR

5.54 23 214 minus strand hemocyanin isoform 1 [Nucula nucleus]

Protein Accession: Unigene58881$_{H}1$
Mean Expression Ratio: 0.751
Median Expression Ratio: 0.749
Credible Interval: (0.53, 1.07)
Associated Peptides: 2
Associated Spectra: 4
Coverage: NaN

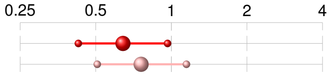

A	B	2.5	50	97.5	Sequence
2	0	0.43	0.64	0.97	GKIPNLNIYTSR
2	0	0.51	0.76	1.1	QEINPFFR

5.55 " 1 321 Dynein heavy chain, cytoplasmic [Crassostrea gigas]"

Protein Accession	Unigene35195$_{H}1$
Mean Expression Ratio	1.33
Median Expression Ratio	1.33
Credible Interval	(0.953, 1.86)
Associated Peptides	1
Associated Spectra	11
Coverage	NaN

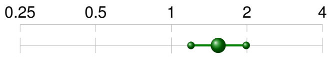

A	B	2.5	50	97.5	Sequence
6	5	1.2	1.5	2	EILAVIQK

5.56 21 341 minus strand hemocyanin isoform 1 [Nucula nucleus]

Protein Accession	CL40281.Contig2$_{H}1$
Mean Expression Ratio	0.753
Median Expression Ratio	0.751
Credible Interval	(0.539, 1.05)
Associated Peptides	2
Associated Spectra	6
Coverage	NaN

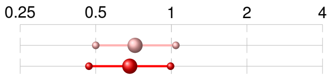

A	B	2.5	50	97.5	Sequence
1	2	0.5	0.72	1	GDELFSYLK
2	1	0.47	0.68	0.99	KPLEPFNR

5.57 459 755 minus strand heat shock protein beta-1 [Trichinella spiralis] gi; 464 763 minus strand heat shock protein beta-1 [Trichinella spiralis] gi; 437 736 minus strand heat shock protein beta-1 [Trichinella spiralis] gi; 437 736 minus strand heat shock protein beta-1 [Trichinella spiralis] gi; 437 736 minus strand heat shock protein beta-1 [Trichinella spiralis] gi

Protein Accession	CL17234.Contig5$_{H}1$ CL17234.Contig4$_{H}1$ CL17234.Contig3$_{H}1$ CL17234.Contig2$_{H}1$ CL1723
Mean Expression Ratio	0.753
Median Expression Ratio	0.752
Credible Interval	(0.52, 1.09)
Associated Peptides	3
Associated Spectra	8
Coverage	NaN

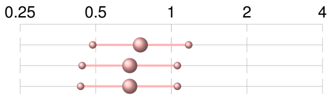

A	B	2.5	50	97.5	Sequence
2	1	0.49	0.75	1.2	DVEPTTVTSSLNADGVLTLK
2	1	0.44	0.68	1.1	IVVEGCHEEK
1	1	0.44	0.68	1.1	IVVEGCHEEKQDEHGFITR

5.58 146 727 Adenylate kinase isoenzyme 1 [Crassostrea gigas]

Protein Accession	CL42551.Contig1$_{H}1$
Mean Expression Ratio	1.33
Median Expression Ratio	1.33
Credible Interval	(0.985, 1.79)
Associated Peptides	4
Associated Spectra	8
Coverage	NaN

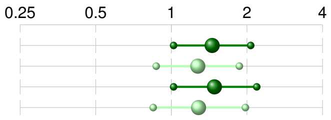

A	B	2.5	50	97.5	Sequence
3	0	1	1.5	2.1	DAMIQNITTTK
1	1	0.87	1.3	1.9	ELEQGLR
0	2	1	1.5	2.2	VVFVIGGPGSGK
0	1	0.85	1.3	2	LNAIMEK

5.59 "REVERSED 2 1810 minus strand Glutaminyl-tRNA synthetase, partial [Crassostrea gigas]"

Protein Accession	RRRRRCL16949.Contig1$_H$1
Mean Expression Ratio	0.753
Median Expression Ratio	0.756
Credible Interval	(0.498, 1.13)
Associated Peptides	1
Associated Spectra	2
Coverage	NaN

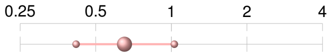

A	B	2.5	50	97.5	Sequence
2	0	0.42	0.65	1	GEELTVK

5.60 77 1561 retinal dehydrogenase [Lymnaea stagnalis]; 69 1553 retinal dehydrogenase [Lymnaea stagnalis]; 308 1747 retinal dehydrogenase [Lymnaea stagnalis]

Protein Accession	CL13284.Contig7$_H$1 CL13284.Contig2$_H$1 CL13284.Contig1$_H$1
Mean Expression Ratio	1.32
Median Expression Ratio	1.32
Credible Interval	(1.09, 1.61)
Associated Peptides	15
Associated Spectra	24
Coverage	NaN

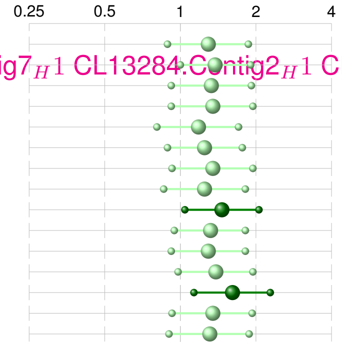

A	B	2.5	50	97.5	Sequence
1	0	0.89	1.3	1.9	ALMFVNGVR
2	1	1	1.4	1.9	AVEVAQDAFK
1	0	0.92	1.3	1.9	FSTVEEAIER
1	0	0.92	1.3	1.9	IYDEFVR
1	0	0.81	1.2	1.7	NIAYLASLETLDNGKPFK
1	1	0.89	1.3	1.8	TFPTINPATGK
1	0	0.93	1.3	1.9	VAFTGSTEVGQIIMK
1	0	0.86	1.3	1.8	KTAELANKR
2	0	1	1.5	2.1	KTAELANK
3	0	0.95	1.3	1.8	RGALLYK
1	1	0.92	1.3	1.8	LGSAWR
1	1	0.98	1.4	1.9	AIQIIR
2	0	1.1	1.6	2.3	EEIFGPVQQLLK
0	1	0.92	1.3	1.9	KIVDVQEADK
0	1	0.9	1.3	1.9	KIVDVQEADKADVDK

5.61 " 460 1944 PREDICTED: succinyl-CoA:3-ketoacid-coenzyme A transferase 1, mitochondrial [Canis lupus familiaris]"

Protein Accession	Unigene4332$_H$1
Mean Expression Ratio	0.756
Median Expression Ratio	0.757
Credible Interval	(0.629, 0.905)
Associated Peptides	13
Associated Spectra	34
Coverage	NaN

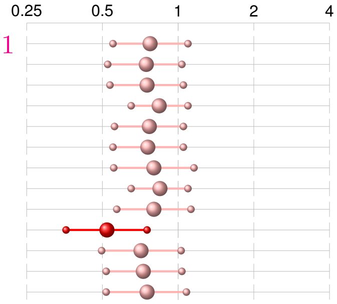

A	B	2.5	50	97.5	Sequence
1	1	0.55	0.78	1.1	AVFDVDNK
1	1	0.53	0.75	1	ECVSSYIGENK
1	1	0.54	0.75	1.1	EVAEGETAESIAK
2	5	0.65	0.84	1.1	EVVNMIVTEK
2	1	0.56	0.77	1	FNPDGTILHGSAGR
2	1	0.55	0.76	1	LGNLTFR
1	0	0.55	0.8	1.2	VMGMGGAMDLVSADK
3	4	0.65	0.85	1.1	ITGVEFR
2	0	0.57	0.8	1.1	VTLPK
0	1	0.36	0.52	0.75	AVFDVDNKEGLLLR
0	1	0.5	0.71	1	KGEEDADLINAGK
0	2	0.52	0.73	1	IFNGR
0	1	0.52	0.75	1.1	VFLGK

5.62 158 862 X-box binding protein [Haliotis discus discus]

Protein Accession	CL6396.Contig3$_{H}$1
Mean Expression Ratio	1.32
Median Expression Ratio	1.32
Credible Interval	(1.04, 1.68)
Associated Peptides	6
Associated Spectra	18
Coverage	NaN

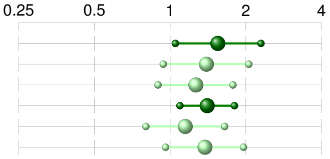

A	B	2.5	50	97.5	Sequence
1	0	1	1.5	2.3	GSISDPSVFNVPEACQK
1	0	0.94	1.4	2.1	VFPDFYFGAGDDK
3	0	0.89	1.3	1.8	ACIPDK
5	4	1.1	1.4	1.8	LYIVDLAK
1	1	0.8	1.1	1.6	SYFILK
2	0	0.96	1.4	2	SYYIGAR

5.63 2473 2898 RecName: Full=Paramyosin gi|397603|emb|CAA79849.1| paramyosin [Echinococcus granulosus]

Protein Accession	CL43455.Contig1$_{H}$1
Mean Expression Ratio	0.759
Median Expression Ratio	0.759
Credible Interval	(0.662, 0.867)
Associated Peptides	12
Associated Spectra	140
Coverage	NaN

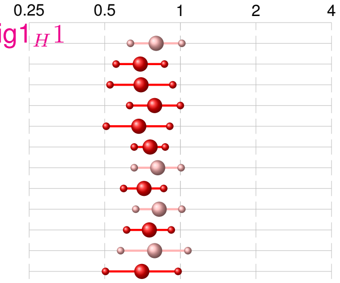

A	B	2.5	50	97.5	Sequence
5	4	0.63	0.8	1	AQTLIEEAENR
7	4	0.55	0.69	0.87	EILAQTEEER
2	2	0.52	0.7	0.93	EILAQTEEERR
6	4	0.63	0.79	1	INKEILAQTEEER
3	1	0.51	0.68	0.91	INKEILAQTEEERR
26	11	0.65	0.76	0.87	KAQTLIEEAENR
6	6	0.65	0.81	1	KQLEIEIR
14	6	0.6	0.72	0.86	QLAEFASLNEQLNLR
8	5	0.66	0.82	1	QLEEAEDVANPTMNK
9	6	0.61	0.75	0.92	QLEIEIR
1	2	0.58	0.79	1.1	RQLAEFASLNEQLNLR
1	1	0.5	0.7	0.98	RQLEEAEDVANPTMNK

5.64 126 704 minus strand ribosomal protein L12 [Ixodes scapularis]

Protein Accession	CL24053.Contig3$_{H}$1
Mean Expression Ratio	0.76
Median Expression Ratio	0.759
Credible Interval	(0.522, 1.11)
Associated Peptides	1
Associated Spectra	4
Coverage	NaN

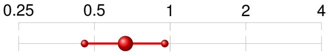

A	B	2.5	50	97.5	Sequence
2	2	0.46	0.66	0.95	IGPLGLSPK

5.65 3 503 minus strand elongation factor-1 alpha [Pomacea bridgesi]

Protein Accession	Unigene72143$_{H}$1
Mean Expression Ratio	1.32
Median Expression Ratio	1.32
Credible Interval	(0.885, 1.99)
Associated Peptides	2
Associated Spectra	2
Coverage	NaN

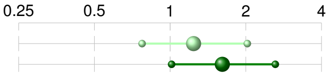

A	B	2.5	50	97.5	Sequence
0	1	0.77	1.2	2	QLIICANK
0	1	1	1.6	2.6	LVSPR

5.66 405 1373 paramyosin [Haliotis discus discus]

Protein Accession	CL43455.Contig4$_H$1
Mean Expression Ratio	0.76
Median Expression Ratio	0.76
Credible Interval	(0.692, 0.836)
Associated Peptides	30
Associated Spectra	493
Coverage	NaN

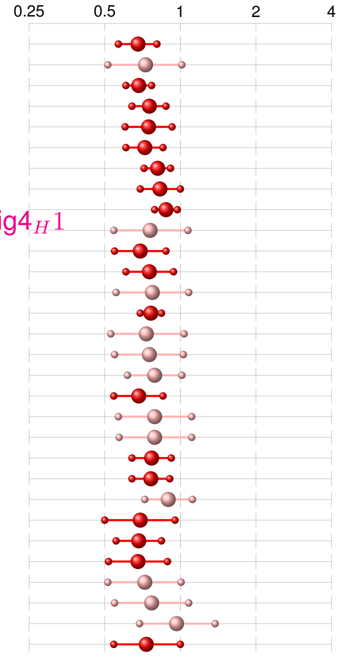

A	B	2.5	50	97.5	Sequence
14	7	0.57	0.68	0.81	DLELATAQFEAAEASLR
1	0	0.52	0.72	1	EKQTLVIELDGLSGQLDSITK
44	11	0.61	0.68	0.77	ELEDALDAER
23	5	0.64	0.75	0.88	HTEAVNDLSDQVDYLTK
5	7	0.6	0.75	0.92	IRELEDALDAER
14	10	0.61	0.72	0.85	LDEEAETSSNLR
33	19	0.72	0.81	0.92	LKGQVDDLTR
14	4	0.69	0.83	1	LTQENFDLQHQVQELDSSNAALAK
39	35	0.79	0.88	0.98	QLNDLTALK
1	0	0.54	0.76	1.1	QLNDLTALKAR
8	1	0.55	0.69	0.87	QNLQVNLSALQSDYDSLNAR
9	2	0.61	0.75	0.94	QTLVIELDGLSGQLDSITK
1	0	0.55	0.77	1.1	RHTEAVNDLSDQVDYLTK
46	38	0.69	0.76	0.84	TVAELSFR
1	0	0.53	0.73	1	LDQVQDSLDEQGSSTVAQIELNKK
1	1	0.55	0.75	1	ELEDALDAERDGR
4	3	0.62	0.79	1	KDLELATAQFEAAEASLR
9	1	0.54	0.68	0.85	DEQGSSTVAQIELNK
1	0	0.57	0.79	1.1	DEQGSSTVAQIELNKK
1	0	0.57	0.79	1.1	ESEIYK
20	0	0.64	0.77	0.92	LDQVQDSLDEQGSSTVAQIELNK
11	12	0.64	0.76	0.91	LSLESR
0	11	0.72	0.9	1.1	ITELEDSCEQLR
0	2	0.5	0.69	0.95	TRFDKELAAK
0	13	0.55	0.68	0.84	VSADYAALK
0	5	0.52	0.68	0.89	FDKELAAK
0	1	0.51	0.72	1	ITELEDSCEQLRV
0	1	0.55	0.77	1.1	ARLDEEAETSSNLR
0	1	0.69	0.97	1.4	LEDALDAER
0	3	0.54	0.73	1	TEELEETR

5.67 373 579 myosin heavy chain [Argopecten irradians] gi

Protein Accession	Unigene64289$_H$1
Mean Expression Ratio	0.761
Median Expression Ratio	0.76
Credible Interval	(0.64, 0.909)
Associated Peptides	9
Associated Spectra	63
Coverage	NaN

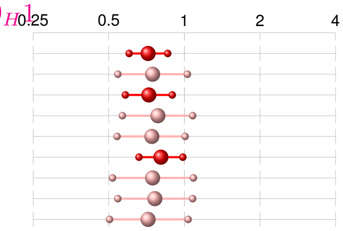

A	B	2.5	50	97.5	Sequence
15	7	0.6	0.72	0.86	QVEEAEEIAAVNLAK
2	1	0.54	0.75	1	RQVEEAEEIAAVNLAK
8	5	0.58	0.72	0.9	VQNELEDAEER
2	1	0.57	0.78	1.1	ELEDAEER
2	1	0.54	0.74	1	ADSAEGTLSK
11	4	0.66	0.81	0.99	KVQNELEDAEER
1	0	0.52	0.75	1.1	QELVDKLQAK
1	1	0.54	0.76	1.1	VDKLQAK
0	1	0.5	0.72	1	VSVSRR

5.68 356 619 PREDICTED: hypothetical protein LOC100698726 [Oreochromis niloticus]

Protein Accession	CL3567.Contig6$_{H}1$
Mean Expression Ratio	1.32
Median Expression Ratio	1.31
Credible Interval	(0.88, 1.97)
Associated Peptides	2
Associated Spectra	2
Coverage	NaN

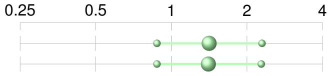

A	B	2.5	50	97.5	Sequence
1	0	0.88	1.4	2.3	LETEVNDLR
1	0	0.88	1.4	2.3	GELQKEEAFSAK

5.69 793 1269 PREDICTED: calmodulin-like [Saccoglossus kowalevskii]

Protein Accession	CL42648.Contig1$_{H}1$
Mean Expression Ratio	1.31
Median Expression Ratio	1.31
Credible Interval	(0.956, 1.8)
Associated Peptides	4
Associated Spectra	6
Coverage	NaN

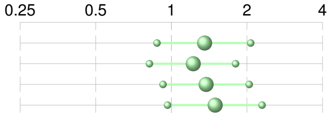

A	B	2.5	50	97.5	Sequence
1	0	0.88	1.4	2.1	EADKDGNGLSIQELASLIR
1	1	0.82	1.2	1.8	KVSLVDITDEEIIK
2	0	0.93	1.4	2	KIQESELK
0	1	0.97	1.5	2.3	IFLDLDDNCDR

5.70 180 560 calreticulin precursor [Aplysia californica] gi; 180 545 calreticulin precursor [Aplysia californica] gi

Protein Accession	CL4813.Contig4$_{H}1$ CL4813.Contig2$_{H}1$
Mean Expression Ratio	1.31
Median Expression Ratio	1.31
Credible Interval	(0.962, 1.79)
Associated Peptides	3
Associated Spectra	11
Coverage	NaN

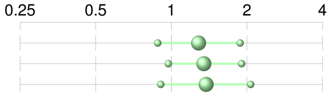

A	B	2.5	50	97.5	Sequence
2	1	0.88	1.3	1.9	FVWSAGK
4	2	0.97	1.4	1.9	WIPSVHK
1	1	0.91	1.4	2.1	FYGISAK

5.71 244 837 PREDICTED: ras-like GTP-binding protein Rho1-like [Megachile rotundata]

Protein Accession	Unigene11792$_{H}1$
Mean Expression Ratio	1.31
Median Expression Ratio	1.31
Credible Interval	(0.93, 1.84)
Associated Peptides	4
Associated Spectra	5
Coverage	NaN

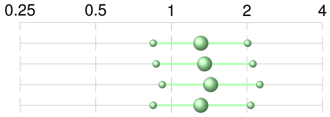

A	B	2.5	50	97.5	Sequence
1	1	0.85	1.3	2	EVFETATR
1	0	0.87	1.4	2.1	KLVIVGDGACGK
1	0	0.92	1.4	2.2	QVELALWDTAGQEDYDR
1	0	0.85	1.3	2.1	LMATVR

5.72 266 607 minus strand H2 [Haliotis tuberculata]

Protein Accession	Unigene62350$_{H}1$
Mean Expression Ratio	0.766
Median Expression Ratio	0.766
Credible Interval	(0.561, 1.05)
Associated Peptides	2
Associated Spectra	7
Coverage	NaN

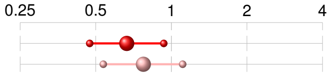

A	B	2.5	50	97.5	Sequence
**3**	**1**	**0.47**	**0.67**	**0.93**	**YAVAGIGVR**
**2**	**1**	**0.54**	**0.77**	**1.1**	**KDINTLTDEEIK**

5.73 266 1123 minus strand mitochondrial malate dehydrogenase precursor [Nucella lapillus]

Protein Accession	CL10426.Contig11$_{H}1$
Mean Expression Ratio	1.3
Median Expression Ratio	1.31
Credible Interval	(0.883, 1.95)
Associated Peptides	1
Associated Spectra	3
Coverage	NaN

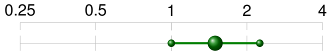

A	B	2.5	50	97.5	Sequence
**1**	**2**	**1**	**1.5**	**2.3**	**SLIEALNGMEGR**

5.74 346 702 minus strand PREDICTED: galectin-4-like [Anolis carolinensis]

Protein Accession	Unigene42203$_{H}1$
Mean Expression Ratio	0.769
Median Expression Ratio	0.768
Credible Interval	(0.59, 1.01)
Associated Peptides	5
Associated Spectra	11
Coverage	NaN

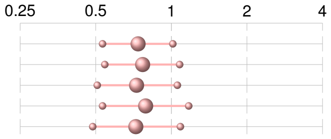

A	B	2.5	50	97.5	Sequence
**3**	**1**	**0.53**	**0.74**	**1**	**AEATLEVR**
**2**	**1**	**0.54**	**0.77**	**1.1**	**SVPHGLVK**
**1**	**1**	**0.51**	**0.73**	**1.1**	**GKDFDIVIK**
**1**	**0**	**0.53**	**0.79**	**1.2**	**VKDSGFK**
**0**	**1**	**0.48**	**0.72**	**1.1**	**KSVPHGLVK**

5.75 105 503 Transgelin-2 [Crassostrea gigas]

Protein Accession	CL445.Contig2$_{H}1$
Mean Expression Ratio	1.3
Median Expression Ratio	1.3
Credible Interval	(0.979, 1.73)
Associated Peptides	2
Associated Spectra	15
Coverage	NaN

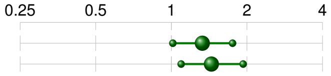

A	B	2.5	50	97.5	Sequence
**4**	**4**	**1**	**1.3**	**1.7**	**SGLGLEIDK**
**5**	**2**	**1.1**	**1.4**	**1.9**	**SGLGLEIDKK**

5.76 360 764 minus strand Peptidyl-prolyl cis-trans isomerase A OS=Dictyostelium discoideum GN=ppiA PE=1 SV=1

Protein Accession	Unigene64429$_H$1
Mean Expression Ratio	0.77
Median Expression Ratio	0.771
Credible Interval	(0.507, 1.17)
Associated Peptides	1
Associated Spectra	2
Coverage	NaN

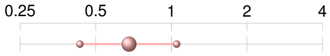

A	B	2.5	50	97.5	Sequence
**0**	**2**	**0.43**	**0.68**	**1.1**	**HVVFGQVVEGLDVVR**

5.77 2 772 minus strand arylsulfatase [Haliotis discus discus]

Protein Accession	CL48181.Contig1$_H$1
Mean Expression Ratio	1.3
Median Expression Ratio	1.3
Credible Interval	(0.893, 1.89)
Associated Peptides	2
Associated Spectra	3
Coverage	NaN

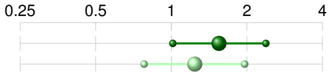

A	B	2.5	50	97.5	Sequence
**1**	**1**	**1**	**1.5**	**2.4**	**LLPQLK**
**0**	**1**	**0.78**	**1.2**	**2**	**GVGMVVDALK**

5.78 REVERSED 6 395 PREDICTED: sister chromatid cohesion protein PDS5 homolog B-A isoform 1 [Nasonia vitripennis]

Protein Accession	RRRRRUnigene51506$_H$1
Mean Expression Ratio	1.3
Median Expression Ratio	1.3
Credible Interval	(0.975, 1.72)
Associated Peptides	2
Associated Spectra	37
Coverage	NaN

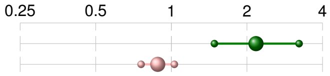

A	B	2.5	50	97.5	Sequence
**2**	**0**	**1.5**	**2.2**	**3.2**	**IGICIDNFR**
**25**	**10**	**0.76**	**0.88**	**1**	**LQELVDK**

5.79 " 131 544 Histone H2B, gonadal OS=Patella granatina PE=1 SV=2"

Protein Accession	CL29794.Contig2$_H$1
Mean Expression Ratio	0.775
Median Expression Ratio	0.776
Credible Interval	(0.578, 1.04)
Associated Peptides	2
Associated Spectra	18
Coverage	NaN

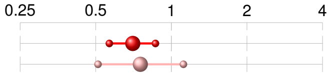

A	B	2.5	50	97.5	Sequence
**9**	**7**	**0.57**	**0.7**	**0.86**	**LLLPGELAK**
**0**	**2**	**0.51**	**0.75**	**1.1**	**PGELAK**

5.80 900 1616 minus strand hemocyanin isoform 1 [Haliotis diversicolor]

Protein Accession	Unigene30321$_{H}1$
Mean Expression Ratio	0.775
Median Expression Ratio	0.776
Credible Interval	(0.523, 1.14)
Associated Peptides	1
Associated Spectra	3
Coverage	NaN

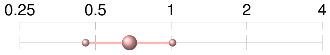

A	B	2.5	50	97.5	Sequence
**2**	**1**	**0.46**	**0.68**	**1**	**VEITDDLK**

5.81 1139 1378 Titin [Crassostrea gigas]; 1116 1355 Titin [Crassostrea gigas]; 1139 1378 Titin [Crassostrea gigas]; 1139 1378 Titin [Crassostrea gigas]; 1139 1378 Titin [Crassostrea gigas]; 1139 1378 Titin [Crassostrea gigas]; 1139 1378 Titin [Crassostrea gigas]; 1170 1409 Titin [Crassostrea gigas]

Protein Accession	CL181.Contig9$_{H}1$ CL181.Contig8$_{H}1$ CL181.Contig7$_{H}1$ CL181.Contig6$_{H}1$ CL181.Contig13$_{H}$
Mean Expression Ratio	0.777
Median Expression Ratio	0.777
Credible Interval	(0.537, 1.11)
Associated Peptides	2
Associated Spectra	4
Coverage	NaN

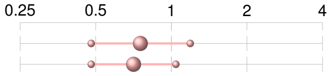

A	B	2.5	50	97.5	Sequence
**1**	**0**	**0.48**	**0.75**	**1.2**	**ITGYQIER**
**3**	**0**	**0.48**	**0.71**	**1**	**LSWKPPK**

5.82 66 947 Glyoxalase domain-containing protein 4 [Crassostrea gigas]

Protein Accession	CL23175.Contig2$_{H}1$
Mean Expression Ratio	1.28
Median Expression Ratio	1.28
Credible Interval	(0.816, 1.99)
Associated Peptides	1
Associated Spectra	1
Coverage	NaN

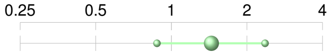

A	B	2.5	50	97.5	Sequence
**0**	**1**	**0.87**	**1.4**	**2.4**	**LELVEIGQK**

5.83 3 470 minus strand Spectrin beta chain [Crassostrea gigas]; 5 451 minus strand Spectrin beta chain [Crassostrea gigas]

Protein Accession	CL19757.Contig2$_{H}1$ CL19757.Contig1$_{H}1$
Mean Expression Ratio	1.28
Median Expression Ratio	1.28
Credible Interval	(0.831, 2.02)
Associated Peptides	1
Associated Spectra	1
Coverage	NaN

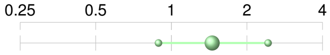

A	B	2.5	50	97.5	Sequence
**0**	**1**	**0.89**	**1.5**	**2.4**	**HLMGVEDLLQK**

5.84 6 317 myosin heavy chain cardiac muscle specific isoform 2 [Argopecten irradians]

Protein Accession Unigene19023$_{H}1$
Mean Expression Ratio 0.786
Median Expression Ratio 0.785
Credible Interval (0.696, 0.886)
Associated Peptides 16
Associated Spectra 240
Coverage NaN

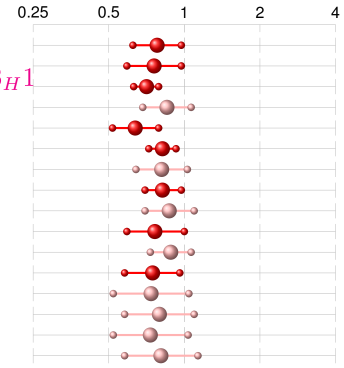

A	B	2.5	50	97.5	Sequence
6	5	0.62	0.78	0.97	ELAFQSDEDR
4	3	0.59	0.76	0.98	ELAFQSDEDRK
33	33	0.63	0.71	0.79	ELEMELDSEQR
11	1	0.68	0.85	1.1	KALETQLK
6	7	0.52	0.64	0.79	LADELRQEQEHSLQVEK
23	29	0.72	0.82	0.92	LDEAEAQALK
6	3	0.64	0.81	1	LKELAFQSDEDR
17	8	0.7	0.82	0.97	ALETQLK
5	5	0.7	0.87	1.1	LKELAFQSDEDRK
1	5	0.59	0.77	1	KAMADAAR
3	14	0.73	0.88	1.1	LADELR
7	0	0.58	0.75	0.96	QEQEHSLQVEK
0	1	0.52	0.74	1	ELEMELDSEQRR
0	2	0.58	0.79	1.1	LDEAEAQALKGGK
0	1	0.52	0.73	1	VRELEMELDSEQR
0	1	0.58	0.81	1.1	AFQSDEDRK

5.85 290 2458 Putative beta-D-xylosidase 5 [Crassostrea gigas]; 299 2428 Putative beta-D-xylosidase 2 [Crassostrea gigas]

Protein Accession CL49577.Contig2$_{H}1$ CL1594.Contig5$_{H}1$
Mean Expression Ratio 1.27
Median Expression Ratio 1.27
Credible Interval (0.847, 1.92)
Associated Peptides 2
Associated Spectra 3
Coverage NaN

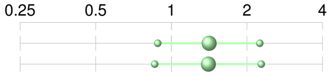

A	B	2.5	50	97.5	Sequence
1	1	0.88	1.4	2.3	VAEAAGVEVR
1	0	0.86	1.4	2.3	VTPLFYTR

5.86 764 1015 minus strand heat shock protein beta-1 [Trichinella spiralis] gi

Protein Accession CL46983.Contig3$_{H}1$
Mean Expression Ratio 0.786
Median Expression Ratio 0.786
Credible Interval (0.579, 1.06)
Associated Peptides 2
Associated Spectra 13
Coverage NaN

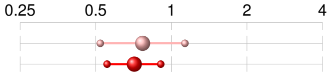

A	B	2.5	50	97.5	Sequence
2	0	0.52	0.77	1.1	SYVLPK
8	3	0.55	0.71	0.91	TVTSSLSQNGVLTLK

5.87 165 635 hypothetical protein CGI_10007639 [Crassostrea gigas]

Protein Accession	CL36715.Contig1$_H$1
Mean Expression Ratio	1.27
Median Expression Ratio	1.27
Credible Interval	(0.847, 1.91)
Associated Peptides	2
Associated Spectra	2
Coverage	NaN

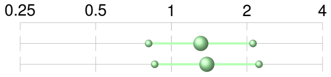

A	B	2.5	50	97.5	Sequence
1	0	0.81	1.3	2.1	AAIVAQVAK
1	0	0.86	1.4	2.2	MPLHASCK

5.88 " 59 2365 minus strand Aconitate hydratase, mitochondrial [Crassostrea gigas]"

Protein Accession	CL13509.Contig1$_H$1
Mean Expression Ratio	0.788
Median Expression Ratio	0.789
Credible Interval	(0.596, 1.04)
Associated Peptides	6
Associated Spectra	9
Coverage	NaN

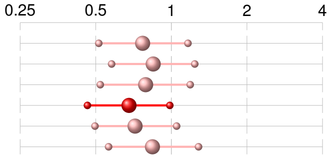

A	B	2.5	50	97.5	Sequence
1	0	0.51	0.77	1.2	HTMNEGQIEWFR
1	1	0.58	0.85	1.2	KNGWPLNIR
1	0	0.52	0.79	1.2	DLKPGAPVVCEIK
1	1	0.46	0.68	0.98	IHETNLK
0	2	0.5	0.72	1.1	VAGILTVK
0	1	0.56	0.84	1.3	LQLLQPFEK

5.89 49 819 PREDICTED: elongation factor 1-delta-like isoform 2 [Strongylocentrotus purpuratus]

Protein Accession	CL10790.Contig12$_H$1
Mean Expression Ratio	0.79
Median Expression Ratio	0.79
Credible Interval	(0.573, 1.09)
Associated Peptides	4
Associated Spectra	7
Coverage	NaN

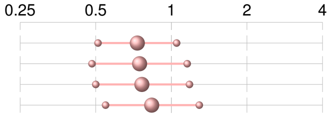

A	B	2.5	50	97.5	Sequence
1	3	0.51	0.73	1.1	LVDLAYGIK
0	1	0.48	0.75	1.2	LLEEYAAK
0	1	0.5	0.76	1.2	RLEEIEK
0	1	0.55	0.84	1.3	EISLAR

5.90 488 1039 Peptidyl-prolyl cis-trans isomerase B [Crassostrea gigas]; 488 1039 Peptidyl-prolyl cis-trans isomerase B [Crassostrea gigas]

Protein Accession	CL49248.Contig2$_H$1 CL49248.Contig1$_H$1
Mean Expression Ratio	1.26
Median Expression Ratio	1.26
Credible Interval	(0.961, 1.66)
Associated Peptides	4
Associated Spectra	19
Coverage	NaN

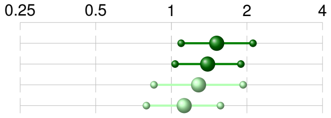

A	B	2.5	50	97.5	Sequence
3	3	1.1	1.5	2.1	ASLEFLDK
4	4	1	1.4	1.9	LVDCSATTLK
1	0	0.85	1.3	1.9	VTFSPK
3	1	0.8	1.1	1.6	INNVPVDK

5.91 1118 1255 minus strand equistatin precursor [Actinia equina]

Protein Accession	CL27616.Contig7$_H1$
Mean Expression Ratio	0.79
Median Expression Ratio	0.792
Credible Interval	(0.504, 1.21)
Associated Peptides	1
Associated Spectra	1
Coverage	NaN

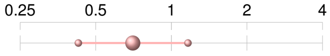

A	B	2.5	50	97.5	Sequence
1	0	0.43	0.7	1.2	HLLGSYIPR

5.92 8 271 minus strand Laminin subunit gamma-1 [Crassostrea gigas]

Protein Accession	Unigene36706$_H1$
Mean Expression Ratio	1.26
Median Expression Ratio	1.26
Credible Interval	(0.912, 1.78)
Associated Peptides	4
Associated Spectra	5
Coverage	NaN

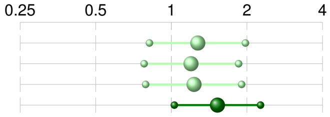

A	B	2.5	50	97.5	Sequence
1	0	0.82	1.3	2	EFTDASELYDR
0	1	0.78	1.2	1.8	HMQEAAQIDNIAR
0	1	0.79	1.2	1.9	LAEETLR
0	2	1	1.5	2.3	ALDLYR

5.93 1709 2026 Phosphoenolpyruvate carboxykinase [GTP] [Crassostrea gigas]

Protein Accession	CL25948.Contig2$_H1$
Mean Expression Ratio	0.791
Median Expression Ratio	0.792
Credible Interval	(0.562, 1.11)
Associated Peptides	1
Associated Spectra	8
Coverage	NaN

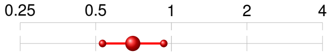

A	B	2.5	50	97.5	Sequence
5	3	0.53	0.7	0.93	GVPLVYEAK

5.94 228 1553 hypothetical protein CGI_10024545 [Crassostrea gigas]; 228 1553 hypothetical protein CGI_10024545 [Crassostrea gigas]

Protein Accession	CL14644.Contig9$_H1$ CL14644.Contig8$_H1$
Mean Expression Ratio	1.26
Median Expression Ratio	1.26
Credible Interval	(0.835, 1.89)
Associated Peptides	2
Associated Spectra	2
Coverage	NaN

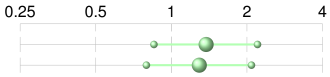

A	B	2.5	50	97.5	Sequence
0	1	0.85	1.4	2.2	LMEQPQSMLL
0	1	0.8	1.3	2.1	AEDILR

5.95 57 1688 Talin-1 [Camponotus floridanus]; 57 1865 Talin-1 [Camponotus floridanus]; 57 1835 Talin-1 [Camponotus floridanus]

Protein Accession	CL49468.Contig2$_H1$ CL45858.Contig2$_H1$ CL45858.Contig1$_H1$
Mean Expression Ratio	1.25
Median Expression Ratio	1.25
Credible Interval	(0.883, 1.77)
Associated Peptides	2
Associated Spectra	5
Coverage	NaN

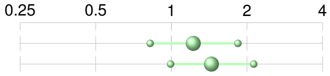

A	B	2.5	50	97.5	Sequence
2	0	0.82	1.2	1.8	KAQEPIVYAGK
3	0	0.99	1.4	2.1	ISIQAR

5.96 587 811 hemocyanin 2 [Megathura crenulata]

Protein Accession	Unigene4334$_H1$
Mean Expression Ratio	0.797
Median Expression Ratio	0.798
Credible Interval	(0.534, 1.18)
Associated Peptides	2
Associated Spectra	2
Coverage	NaN

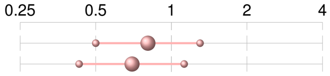

A	B	2.5	50	97.5	Sequence
1	0	0.5	0.81	1.3	SVEDLTAGEAESLK
0	1	0.43	0.7	1.1	AIDDGTFR

5.97 227 829 Peptidyl-prolyl cis-trans isomerase B [Crassostrea gigas]

Protein Accession	CL16665.Contig2$_H1$
Mean Expression Ratio	1.25
Median Expression Ratio	1.25
Credible Interval	(1.02, 1.53)
Associated Peptides	7
Associated Spectra	39
Coverage	NaN

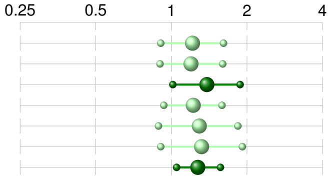

A	B	2.5	50	97.5	Sequence
4	1	0.9	1.2	1.6	DTNGSQFFFTLGPSR
3	2	0.9	1.2	1.6	MGPGADFQSPK
3	1	1	1.4	1.9	NYNGQGDDISGK
3	4	0.93	1.2	1.6	TLCDGWVR
1	0	0.89	1.3	1.8	FLDEK
1	0	0.91	1.3	1.9	NFTVTHEATLEIEVK
10	6	1	1.3	1.6	GFNFLR

5.98 210 2201 minus strand Glycyl-tRNA synthetase [Crassostrea gigas]; 210 2201 minus strand Glycyl-tRNA synthetase [Crassostrea gigas]; 210 2201 minus strand Glycyl-tRNA synthetase [Crassostrea gigas]; 210 2201 minus strand Glycyl-tRNA synthetase [Crassostrea gigas]; 210 2201 minus strand Glycyl-tRNA synthetase [Crassostrea gigas]; 210 2201 minus strand Glycyl-tRNA synthetase [Crassostrea gigas]

Protein Accession	CL4073.Contig8$_{H}1$ CL4073.Contig7$_{H}1$ CL4073.Contig6$_{H}1$ CL4073.Contig5$_{H}1$ CL4073.Con
Mean Expression Ratio	1.25
Median Expression Ratio	1.25
Credible Interval	(0.839, 1.87)
Associated Peptides	2
Associated Spectra	2
Coverage	NaN

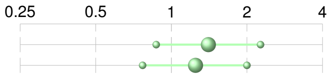

A	B	2.5	50	97.5	Sequence
0	1	0.87	1.4	2.3	ADHLIEATFEK
0	1	0.77	1.3	2	VLMPR

5.99 82 816 PREDICTED: triosephosphate isomerase B [Oreochromis niloticus]

Protein Accession	CL26247.Contig4$_{H}1$
Mean Expression Ratio	1.25
Median Expression Ratio	1.25
Credible Interval	(1.02, 1.52)
Associated Peptides	8
Associated Spectra	29
Coverage	NaN

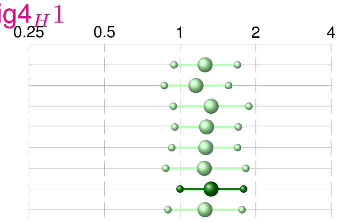

A	B	2.5	50	97.5	Sequence
3	2	0.94	1.3	1.7	AIADNVNDWK
3	2	0.87	1.2	1.6	GAFTGEISPAMIK
2	0	0.94	1.3	1.9	HVFGESDELIGQK
3	2	0.95	1.3	1.7	QFFVGGNWK
4	0	0.93	1.3	1.7	ELATKPDVDGFLVGGASLK
1	0	0.88	1.3	1.8	IGVAAQNCYKEPK
3	2	1	1.3	1.8	TEFITIINSK
0	2	0.9	1.3	1.8	SVDEIIK

5.100 111 1226 gelsolin [Aplysia californica] gi; 42 1139 minus strand gelsolin [Aplysia californica] gi

Protein Accession	Unigene39723$_{H}1$ Unigene32782$_{H}1$
Mean Expression Ratio	1.25
Median Expression Ratio	1.25
Credible Interval	(1.08, 1.44)
Associated Peptides	18
Associated Spectra	74
Coverage	NaN

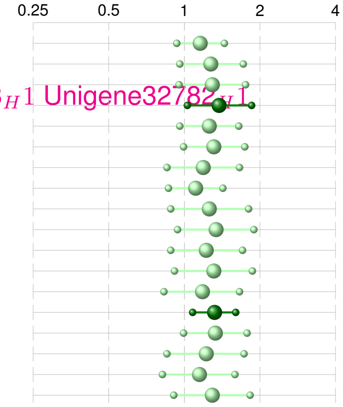

A	B	2.5	50	97.5	Sequence
4	8	0.93	1.2	1.4	AMQFIGEIK
3	1	0.96	1.3	1.7	DLVTSDDVYILDTGLK
3	0	0.95	1.3	1.8	ESGNPWRPVTVIK
4	0	1	1.4	1.8	EVFVWVGSNASPDEK
5	1	0.96	1.3	1.7	EVQGHESDLFK
3	2	0.99	1.3	1.7	KNGLPLAHNYLR
1	1	0.85	1.2	1.7	QGIVTMEGGAASGFR
4	4	0.87	1.1	1.4	SDDVFIFDSGK
1	0	0.88	1.3	1.8	TVELDTK
1	0	0.94	1.3	1.9	SVPAEEYK
1	1	0.88	1.2	1.7	QFDTALAA
1	0	0.91	1.3	1.9	KESADSEPAWDGAGQKPGLEIWR
1	0	0.83	1.2	1.7	NSTQDEYGTAAYK
13	3	1.1	1.3	1.6	LFHFAK
3	1	0.99	1.3	1.8	VVPWPK
0	1	0.85	1.2	1.7	DSNLALFGSDTEK
0	2	0.82	1.1	1.6	LNDFK
0	1	0.9	1.3	1.8	NGLPLAHNYLR

5.101 139 945 Kynurenine formamidase [Crassostrea gigas]; 145 951 Kynurenine formamidase [Crassostrea gigas]; 220 1026 Kynurenine formamidase [Crassostrea gigas]; 39 743 Kynurenine formamidase [Crassostrea gigas]; 123 929 Kynurenine formamidase [Crassostrea gigas]; 39 743 Kynurenine formamidase [Crassostrea gigas]

Protein Accession	CL8132.Contig9$_{H}1$ CL8132.Contig8$_{H}1$ CL8132.Contig7$_{H}1$ CL8132.Contig3$_{H}1$ CL8132.Con
Mean Expression Ratio	0.803
Median Expression Ratio	0.803
Credible Interval	(0.551, 1.17)
Associated Peptides	2
Associated Spectra	7
Coverage	NaN

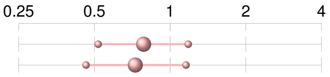

A	B	2.5	50	97.5	Sequence
**4**	**2**	**0.52**	**0.78**	**1.2**	**LVGPGVVIDVR**
**0**	**1**	**0.46**	**0.73**	**1.2**	**WLTTNR**

5.102 177 791 Ras-related protein Rab-1A [Haliotis discus discus]

Protein Accession	CL21389.Contig6$_{H}1$
Mean Expression Ratio	1.24
Median Expression Ratio	1.24
Credible Interval	(0.933, 1.66)
Associated Peptides	5
Associated Spectra	8
Coverage	NaN

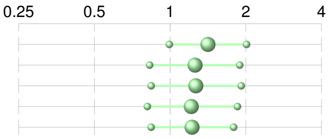

A	B	2.5	50	97.5	Sequence
**3**	**0**	**0.99**	**1.4**	**2**	**MGPVTSASENKPSVK**
**1**	**0**	**0.83**	**1.3**	**1.9**	**QWLQEIDR**
**1**	**0**	**0.84**	**1.3**	**1.9**	**KVVDYTTAK**
**1**	**0**	**0.81**	**1.2**	**1.8**	**SCLLLR**
**0**	**2**	**0.84**	**1.2**	**1.8**	**LLLIGDSGVGK**

5.103 9 518 minus strand hemocyanin isoform 1 [Nucula nucleus]

Protein Accession	Unigene71735$_{H}1$
Mean Expression Ratio	0.803
Median Expression Ratio	0.804
Credible Interval	(0.55, 1.17)
Associated Peptides	1
Associated Spectra	4
Coverage	NaN

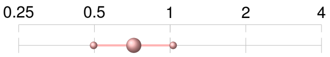

A	B	2.5	50	97.5	Sequence
**2**	**2**	**0.5**	**0.72**	**1**	**IDFEHADVAR**

5.104 3591 4526 minus strand myosin heavy chain cardiac muscle specific isoform 1 [Argopecten irradians]

Protein Accession	CL49594.Contig2$_H$1
Mean Expression Ratio	0.805
Median Expression Ratio	0.805
Credible Interval	(0.721, 0.897)
Associated Peptides	23
Associated Spectra	293
Coverage	NaN

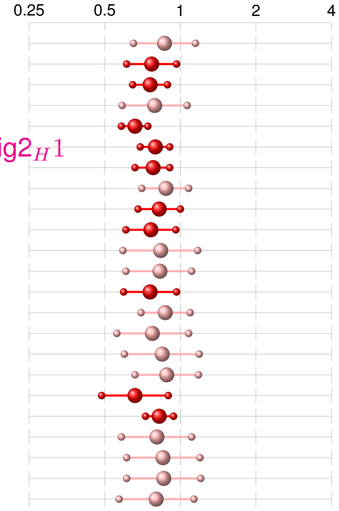

A	B	2.5	50	97.5	Sequence
3	1	0.65	0.87	1.2	AKLEQTLDELEDNLER
7	3	0.61	0.77	0.96	ATQANVEDLER
22	7	0.65	0.76	0.89	LQQETANLTR
2	1	0.59	0.79	1.1	MSKQVEAQLAELSAK
34	20	0.58	0.66	0.74	QVEAQLAELSAK
22	18	0.69	0.79	0.91	SEVDDLQMQLQNVGK
17	10	0.66	0.78	0.91	TINDLQTQK
11	1	0.7	0.87	1.1	TMEDLQAEEDKVNHLNK
16	1	0.68	0.82	1	KLEQDLK
9	1	0.61	0.76	0.96	AATSRSRTLQHEAQVSALR
1	0	0.59	0.84	1.2	SEVDDLQMQLQNVGKNK
2	1	0.61	0.83	1.1	SASRSCSSCAATSRSRTLQHEAQVSALR
6	2	0.59	0.76	0.96	QQDAANEMADQIDQLLQKVK
10	0	0.7	0.87	1.1	ATSRSRTLQHEAQVSALR
1	1	0.56	0.78	1.1	ELEENVR
1	0	0.6	0.84	1.2	KKQQDAANEMADQIDQ
2	2	0.66	0.88	1.2	KTMEDLQAEEDKVNHLNK
2	1	0.49	0.66	0.89	LEQTLDELEDNLER
25	25	0.73	0.83	0.94	QLEEAEHK
2	0	0.58	0.8	1.1	VGALTK
0	1	0.61	0.85	1.2	RKLEQDLK
0	1	0.61	0.86	1.2	LEENVR
0	1	0.57	0.8	1.1	QDAANEMADQIDQLLQK

5.105 194 904 Aldo-keto reductase family 1 member B10 [Crassostrea gigas]

Protein Accession	CL3558.Contig4$_H$1
Mean Expression Ratio	0.804
Median Expression Ratio	0.805
Credible Interval	(0.521, 1.25)
Associated Peptides	1
Associated Spectra	1
Coverage	NaN

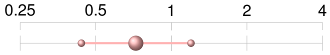

A	B	2.5	50	97.5	Sequence
0	1	0.44	0.72	1.2	ALEVLVSEGK

5.106 527 1321 minus strand troponin T [Mizuhopecten yessoensis]; 305 1099 minus strand troponin T [Mizuhopecten yessoensis]; 305 1099 minus strand troponin T [Mizuhopecten yessoensis]

Protein Accession	Unigene65787$_H$1 CL2214.Contig4$_H$1 CL2214.Contig3$_H$1
Mean Expression Ratio	0.806
Median Expression Ratio	0.806
Credible Interval	(0.69, 0.944)
Associated Peptides	10
Associated Spectra	106
Coverage	NaN

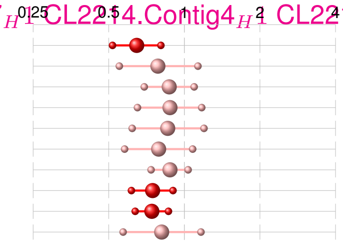

A	B	2.5	50	97.5	Sequence
2	10	0.52	0.65	0.81	LDSVMSKEELQK
1	0	0.55	0.79	1.1	SKEQLEDEK
5	5	0.69	0.87	1.1	SKEQLEDEKR
2	2	0.65	0.87	1.2	GPNFVITK
2	0	0.62	0.86	1.2	KGPNFVIT
3	0	0.58	0.79	1.1	AILSQR
17	6	0.74	0.88	1	KGPNFVITK
15	3	0.62	0.75	0.9	LDSVMSK
17	15	0.64	0.74	0.87	LTELR
0	1	0.57	0.81	1.2	EAREAEER

5.107 38 436 Calcium-dependent protein kinase isoform 2 [Crassostrea gigas]

Protein Accession	CL5866.Contig5$_{H}$1
Mean Expression Ratio	0.809
Median Expression Ratio	0.809
Credible Interval	(0.52, 1.26)
Associated Peptides	1
Associated Spectra	1
Coverage	NaN

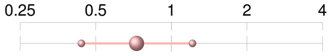

A	B	2.5	50	97.5	Sequence
1	0	0.44	0.73	1.2	SHAGLDVEADK

5.108 4 2400 IQ motif containing GTPase activating protein 2 [Xenopus laevis] gi; 4 2169 IQ motif containing GTPase activating protein 2 [Xenopus laevis] gi

Protein Accession	CL875.Contig2$_{H}$1 CL875.Contig1$_{H}$1
Mean Expression Ratio	0.809
Median Expression Ratio	0.81
Credible Interval	(0.519, 1.26)
Associated Peptides	1
Associated Spectra	1
Coverage	NaN

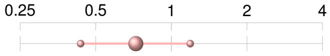

A	B	2.5	50	97.5	Sequence
0	1	0.43	0.72	1.2	ITLQDVVTHNR

5.109 " 926 1189 minus strand Myosin heavy chain, striated muscle [Crassostrea gigas]"

Protein Accession	Unigene11948$_{H}$1
Mean Expression Ratio	0.812
Median Expression Ratio	0.811
Credible Interval	(0.637, 1.04)
Associated Peptides	3
Associated Spectra	29
Coverage	NaN

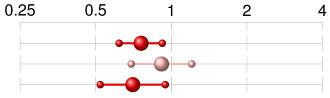

A	B	2.5	50	97.5	Sequence
10	8	0.62	0.76	0.92	LNGNHLGK
6	0	0.69	0.91	1.2	NKDPLNETVVELLSHSK
1	4	0.52	0.7	0.95	TFLDKLNGNHLGK

5.110 118 513 Protein CAL-5 [Caenorhabditis elegans] gi

Protein Accession	Unigene47320$_{H}$1
Mean Expression Ratio	1.23
Median Expression Ratio	1.23
Credible Interval	(0.836, 1.84)
Associated Peptides	2
Associated Spectra	3
Coverage	NaN

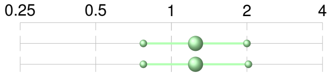

A	B	2.5	50	97.5	Sequence
1	1	0.77	1.2	2	EADANGDGYLSIQELK
1	0	0.77	1.2	2	TLLAHSGK

5.111 51 890 prohibitin [Octopus tankahkeei]

Protein Accession	CL26635.Contig2$_{H}$1
Mean Expression Ratio	0.817
Median Expression Ratio	0.816
Credible Interval	(0.623, 1.07)
Associated Peptides	6
Associated Spectra	10
Coverage	NaN

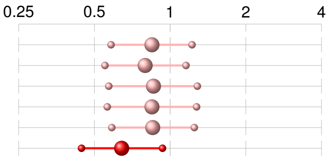

A	B	2.5	50	97.5	Sequence
1	1	0.58	0.85	1.2	AFTEAGEGLVELR
1	1	0.55	0.8	1.2	FLVEKEEQK
1	0	0.57	0.86	1.3	NVPVITGSK
1	0	0.56	0.85	1.3	IEAAEDIA
0	2	0.59	0.85	1.2	AVIFDR
0	2	0.44	0.64	0.93	QVAQQDAERAR

5.112 40 1752 minus strand heat shock protein 60 [Biomphalaria glabrata]; 40 1752 minus strand heat shock protein 60 [Biomphalaria glabrata]

Protein Accession	CL5583.Contig6$_{H}$1 CL5583.Contig5$_{H}$1
Mean Expression Ratio	1.23
Median Expression Ratio	1.23
Credible Interval	(0.915, 1.65)
Associated Peptides	5
Associated Spectra	8
Coverage	NaN

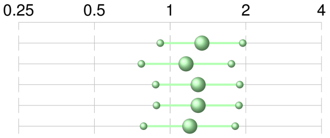

A	B	2.5	50	97.5	Sequence
2	0	0.91	1.3	1.9	AVGDLISSAMK
1	0	0.77	1.2	1.8	TLRDELEVIEGMK
1	1	0.87	1.3	1.9	VGLQVVAVK
2	0	0.88	1.3	1.9	LPNLLR
0	1	0.78	1.2	1.8	DDTLLMR

5.113 76 522 minus strand Troponin C [Crassostrea gigas]; 173 619 minus strand Troponin C [Crassostrea gigas]; 173 619 minus strand Troponin C [Crassostrea gigas]

Protein Accession	Unigene53263$_{H}$1 Unigene11674$_{H}$1 CL13001.Contig4$_{H}$1
Mean Expression Ratio	1.22
Median Expression Ratio	1.23
Credible Interval	(1.02, 1.48)
Associated Peptides	7
Associated Spectra	48
Coverage	NaN

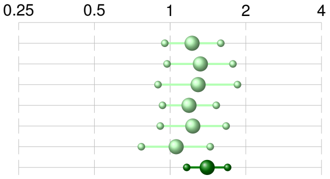

A	B	2.5	50	97.5	Sequence
4	4	0.95	1.2	1.6	GVIPVEDLR
3	1	0.97	1.3	1.8	QYNDANQTFNLFDK
1	0	0.9	1.3	1.9	QYNDANQTFNLFDKK
6	3	0.93	1.2	1.5	SLGLQVEDEK
2	2	0.91	1.2	1.7	VLDKGNK
2	1	0.77	1.1	1.4	SLGLQVEDEKLK
11	8	1.2	1.4	1.7	ELANVFK

5.114 1200 1547 protein disulfide isomerase [Conus eburneus]

Protein Accession	CL3770.Contig1$_{H}$1
Mean Expression Ratio	1.22
Median Expression Ratio	1.22
Credible Interval	(0.995, 1.5)
Associated Peptides	8
Associated Spectra	24
Coverage	NaN

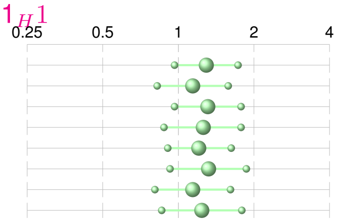

A	B	2.5	50	97.5	Sequence
2	3	0.96	1.3	1.7	IDSTANDVENIR
3	0	0.82	1.1	1.6	QLAPIWDELAEK
3	1	0.97	1.3	1.8	VQSFPTLK
1	1	0.88	1.3	1.8	YKDSNDIVIAK
1	4	0.91	1.2	1.6	NFNDVAFDK
1	1	0.92	1.3	1.9	VVDYNGER
1	1	0.81	1.1	1.6	VLVGK
0	1	0.86	1.2	1.8	TLSAFVK

5.115 340 615 putative Low-density lipoprotein receptor repeat class B [Trichinella spiralis] gi

Protein Accession	Unigene31531$_H$1
Mean Expression Ratio	1.22
Median Expression Ratio	1.22
Credible Interval	(0.791, 1.92)
Associated Peptides	1
Associated Spectra	1
Coverage	NaN

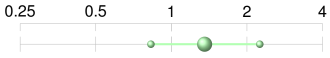

A	B	2.5	50	97.5	Sequence
0	1	0.83	1.4	2.3	VLPFGLK

5.116 145 459 fk506-binding protein 2 [Papilio xuthus]; 237 551 fk506-binding protein 2 [Papilio xuthus]

Protein Accession	Unigene7883$_H$1 CL4621.Contig4$_H$1
Mean Expression Ratio	1.22
Median Expression Ratio	1.22
Credible Interval	(0.922, 1.6)
Associated Peptides	4
Associated Spectra	11
Coverage	NaN

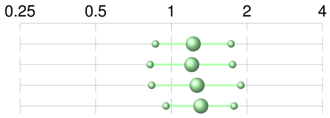

A	B	2.5	50	97.5	Sequence
2	1	0.87	1.2	1.7	GVDVETISEGDKK
1	1	0.82	1.2	1.8	GWDEGVAQMSK
1	0	0.84	1.3	1.9	IGKGEVIK
4	1	0.95	1.3	1.8	NKPFQFK

5.117 2881 3411 myosin heavy chain [Todarodes pacificus]; 2915 3445 myosin heavy chain [Todarodes pacificus]

Protein Accession	Unigene19024$_H$1 Unigene19021$_H$1
Mean Expression Ratio	0.823
Median Expression Ratio	0.823
Credible Interval	(0.715, 0.945)
Associated Peptides	13
Associated Spectra	135
Coverage	NaN

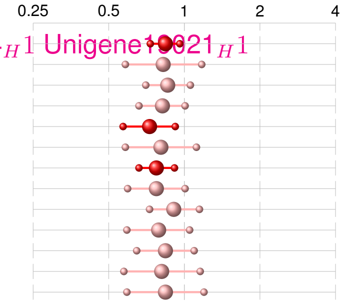

A	B	2.5	50	97.5	Sequence
28	13	0.73	0.84	0.96	ALDSMQASLEAEAK
1	0	0.58	0.82	1.2	ALTGEVEELR
9	6	0.7	0.86	1.1	CTALTGEVEELR
9	4	0.66	0.82	1	IQEKEEEFENTR
5	4	0.57	0.73	0.92	IQEKEEEFENTRR
2	0	0.58	0.81	1.1	KLEQDINELEIALDQANR
25	1	0.66	0.78	0.91	KLQQQVIETQQHVEEEAR
6	0	0.6	0.78	1	LQQQVIETQQHVEEEAR
4	6	0.73	0.91	1.1	RCTALTGEVEELR
1	3	0.59	0.79	1.1	RIQEKEEEFENTR
3	3	0.65	0.84	1.1	TALTGEVEELR
0	1	0.57	0.81	1.2	IQEKEEEFEN
0	1	0.59	0.84	1.2	ELEIALDQANR

5.118 145 1020 minus strand PREDICTED: inorganic pyrophosphatase-like [Saccoglossus kowalevskii]

Protein Accession	CL12968.Contig14$_H1$
Mean Expression Ratio	1.21
Median Expression Ratio	1.22
Credible Interval	(0.93, 1.58)
Associated Peptides	6
Associated Spectra	10
Coverage	NaN

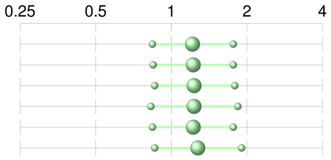

A	B	2.5	50	97.5	Sequence
1	1	0.84	1.2	1.8	AEVIVQESK
1	1	0.85	1.2	1.8	IIAIDVTDPLASK
1	1	0.86	1.2	1.8	LNDIDDVHR
1	0	0.83	1.2	1.8	VLGTMLLVDEGETDWK
1	1	0.84	1.2	1.8	EMPGFLR
0	1	0.86	1.3	1.9	VFFR

5.119 7825 8754 minus strand H2 [Haliotis tuberculata]

Protein Accession	CL7906.Contig3$_H1$
Mean Expression Ratio	0.823
Median Expression Ratio	0.823
Credible Interval	(0.53, 1.27)
Associated Peptides	1
Associated Spectra	1
Coverage	NaN

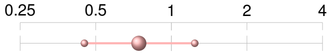

A	B	2.5	50	97.5	Sequence
1	0	0.45	0.74	1.2	ALPSLFTDPYDSNPFK

5.120 " 599 907 minus strand Tryptophanyl-tRNA synthetase, cytoplasmic [Heterocephalus glaber]"

Protein Accession	Unigene26755$_H1$
Mean Expression Ratio	1.21
Median Expression Ratio	1.21
Credible Interval	(0.865, 1.69)
Associated Peptides	2
Associated Spectra	8
Coverage	NaN

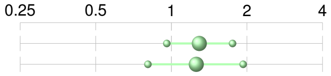

A	B	2.5	50	97.5	Sequence
3	4	0.96	1.3	1.8	DMDPLLDLYEK
1	0	0.8	1.3	1.9	KTPFYLYTGR

5.121 210 779 GH20056 [Drosophila grimshawi] gi; 216 785 minus strand GH20056 [Drosophila grimshawi] gi; 210 779 GH20056 [Drosophila grimshawi] gi; 216 785 GH20056 [Drosophila grimshawi] gi

Protein Accession	CL34497.Contig4$_H1$ CL34497.Contig3$_H1$ CL34497.Contig2$_H1$ CL34497.Contig1$_H1$
Mean Expression Ratio	1.21
Median Expression Ratio	1.21
Credible Interval	(0.886, 1.67)
Associated Peptides	3
Associated Spectra	9
Coverage	NaN

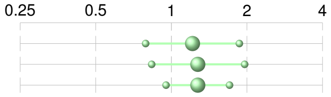

A	B	2.5	50	97.5	Sequence
1	0	0.79	1.2	1.9	KQPFEFQIGVGQVIK
1	0	0.83	1.3	2	QLSQEEVSNFLK
5	2	0.95	1.3	1.7	GLLDMCAGEK

5.122 " 154 534 minus strand Muscle M-line assembly protein unc-89, partial [Crassostrea gigas]"

Protein Accession	Unigene64523$_{H}1$
Mean Expression Ratio	0.825
Median Expression Ratio	0.825
Credible Interval	(0.528, 1.28)
Associated Peptides	1
Associated Spectra	3
Coverage	NaN

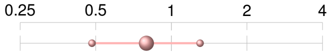

A	B	2.5	50	97.5	Sequence
2	1	0.48	0.8	1.3	LEIGEVVVDDEGEYICK

5.123 121 660 Failed axon connections homolog OS=Rattus norvegicus GN=Faxc PE=3 SV=1

Protein Accession	Unigene63215$_{H}1$
Mean Expression Ratio	1.21
Median Expression Ratio	1.21
Credible Interval	(0.786, 1.88)
Associated Peptides	1
Associated Spectra	1
Coverage	NaN

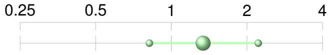

A	B	2.5	50	97.5	Sequence
0	1	0.82	1.3	2.2	LLVLR

5.124 2 256 Polyubiquitin (Fragment) OS=Xenopus laevis PE=1 SV=2; 20 388 large subunit ribosomal protein L40e [Clonorchis sinensis]

Protein Accession	CL47507.Contig2$_{H}1$ CL39010.Contig1$_{H}1$
Mean Expression Ratio	1.21
Median Expression Ratio	1.21
Credible Interval	(0.937, 1.58)
Associated Peptides	5
Associated Spectra	18
Coverage	NaN

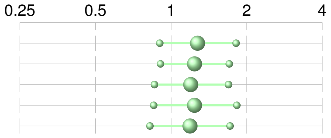

A	B	2.5	50	97.5	Sequence
2	1	0.9	1.3	1.8	TITLEVEPSDTIENVK
4	3	0.91	1.2	1.7	TLSDYNIQK
3	0	0.86	1.2	1.7	LIFAGK
1	1	0.85	1.2	1.8	ESTLHLVLR
2	1	0.83	1.2	1.7	MQIFVK

5.125 2939 3208 minus strand beta-thymosin domain repeat protein CSP24KDa_v1 [Hermissenda crassicornis] gi

Protein Accession	CL9548.Contig3$_{H}1$
Mean Expression Ratio	1.21
Median Expression Ratio	1.21
Credible Interval	(0.903, 1.64)
Associated Peptides	4
Associated Spectra	8
Coverage	NaN

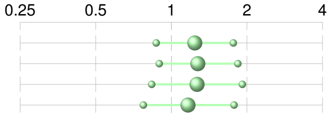

A	B	2.5	50	97.5	Sequence
2	1	0.87	1.2	1.8	NPLPSTDAIAAEK
2	1	0.9	1.3	1.8	AETEEKNPLPTK
1	0	0.84	1.3	1.9	KAETEEKNPLPTK
0	1	0.77	1.2	1.8	SIGEFDK

5.126 91 1392 minus strand hypothetical protein AND_03103 [Anopheles darlingi]

Protein Accession	CL10598.Contig5$_{H}1$
Mean Expression Ratio	0.828
Median Expression Ratio	0.829
Credible Interval	(0.552, 1.23)
Associated Peptides	2
Associated Spectra	2
Coverage	NaN

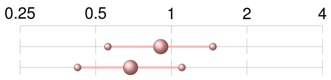

A	B	2.5	50	97.5	Sequence
1	0	0.56	0.91	1.5	LPPFNEHALVQEK
1	0	0.42	0.69	1.1	LDEVDDLLTENR

5.127 137 853 14-3-3 protein [Heterodera glycines]

Protein Accession	CL9040.Contig4$_{H}1$
Mean Expression Ratio	0.831
Median Expression Ratio	0.829
Credible Interval	(0.606, 1.14)
Associated Peptides	4
Associated Spectra	6
Coverage	NaN

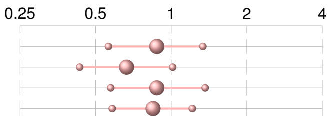

A	B	2.5	50	97.5	Sequence
1	0	0.56	0.88	1.3	AIECANQAYQK
1	0	0.43	0.67	1	EAFDEAISELDSLQGEDCK
1	0	0.57	0.88	1.4	NASNEEENVFYLK
3	0	0.58	0.84	1.2	VICMIEQK

5.128 503 1219 heat shock cognate 71 [Mytilus galloprovincialis]

Protein Accession	CL23109.Contig5$_{H}1$
Mean Expression Ratio	0.83
Median Expression Ratio	0.83
Credible Interval	(0.61, 1.12)
Associated Peptides	4
Associated Spectra	7
Coverage	NaN

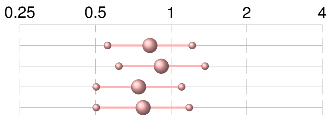

A	B	2.5	50	97.5	Sequence
1	1	0.56	0.83	1.2	FEELNADLFR
2	0	0.62	0.92	1.4	LLQDFFNGK
2	0	0.5	0.74	1.1	GTLEPVEK
0	1	0.51	0.77	1.2	SAIHEIVLVGGSTR

5.129 230 334 protein disulfide isomerase [Conus virgo]

Protein Accession	Unigene67026$_{H}1$
Mean Expression Ratio	1.2
Median Expression Ratio	1.2
Credible Interval	(0.848, 1.7)
Associated Peptides	1
Associated Spectra	8
Coverage	NaN

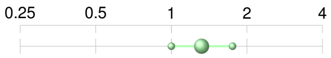

A	B	2.5	50	97.5	Sequence
4	4	1	1.3	1.7	VDATVENDLASK

5.130 87 485 Sex-regulated protein janus-A [Crassostrea gigas]

Protein Accession	CL13131.Contig3$_{H}1$
Mean Expression Ratio	0.833
Median Expression Ratio	0.832
Credible Interval	(0.532, 1.29)
Associated Peptides	1
Associated Spectra	1
Coverage	NaN

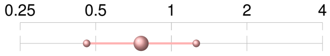

A	B	2.5	50	97.5	Sequence
**0**	**1**	**0.46**	**0.76**	**1.3**	**ADHSITQTLLLR**

5.131 28 642 minus strand Transmembrane emp24 domain-containing protein 9 [Crassostrea gigas]; 236 709 minus strand Transmembrane emp24 domain-containing protein 9 OS=Rattus norvegicus GN=Tmed9 PE=1 SV=1; 239 742 minus strand Transmembrane emp24 domain-containing protein eca OS=Drosophila erecta GN=eca PE=3 SV=1; 236 709 minus strand Transmembrane emp24 domain-containing protein 9 OS=Rattus norvegicus GN=Tmed9 PE=1 SV=1; 236 709 minus strand Transmembrane emp24 domain-containing protein 9 OS=Rattus norvegicus GN=Tmed9 PE=1 SV=1; 236 709 minus strand Transmembrane emp24 domain-containing protein 9 OS=Rattus norvegicus GN=Tmed9 PE=1 SV=1; 44 658 minus strand Transmembrane emp24 domain-containing protein 9 [Crassostrea gigas]

Protein Accession	CL2503.Contig8$_{H}1$ CL2503.Contig7$_{H}1$ CL2503.Contig5$_{H}1$ CL2503.Contig4$_{H}1$ CL2503.Con
Mean Expression Ratio	1.2
Median Expression Ratio	1.2
Credible Interval	(0.772, 1.85)
Associated Peptides	1
Associated Spectra	1
Coverage	NaN

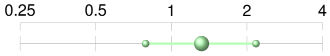

A	B	2.5	50	97.5	Sequence
**1**	**0**	**0.79**	**1.3**	**2.2**	**QLLDQVEQITK**

5.132 327 731 RecName: Full=Arginine kinase; Short=AK gi|439719|dbj|BAA05100.1| arginine kinase [Haliotis madaka]

Protein Accession	Unigene63526$_{H}1$
Mean Expression Ratio	1.2
Median Expression Ratio	1.2
Credible Interval	(0.927, 1.55)
Associated Peptides	2
Associated Spectra	32
Coverage	NaN

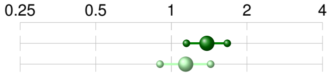

A	B	2.5	50	97.5	Sequence
**10**	**10**	**1.2**	**1.4**	**1.7**	**DLYEQLK**
**7**	**5**	**0.9**	**1.1**	**1.4**	**FGGTLAECIR**

5.133 161 301 Heat shock protein beta-6 OS=Mus musculus GN=Hspb6 PE=1 SV=1; 167 310 Heat shock protein beta-1 OS=Canis familiaris GN=HSPB1 PE=2 SV=1

Protein Accession	CL46983.Contig2$_H$1 CL46983.Contig1$_H$1
Mean Expression Ratio	0.833
Median Expression Ratio	0.834
Credible Interval	(0.599, 1.15)
Associated Peptides	2
Associated Spectra	9
Coverage	NaN

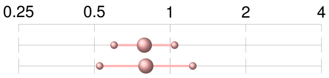

A	B	2.5	50	97.5	Sequence
6	2	0.6	0.79	1	VNMDVQHFKPEEIK
0	1	0.52	0.8	1.2	NMDVQHFKPEEIK

5.134 1714 2121 Phosphoenolpyruvate carboxykinase [GTP] [Crassostrea gigas]

Protein Accession	CL25948.Contig1$_H$1
Mean Expression Ratio	0.836
Median Expression Ratio	0.836
Credible Interval	(0.595, 1.17)
Associated Peptides	1
Associated Spectra	10
Coverage	NaN

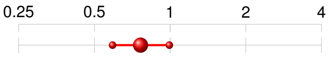

A	B	2.5	50	97.5	Sequence
7	3	0.59	0.76	0.99	SEATAAAEFK

5.135 3 1670 Spectrin alpha chain [Crassostrea gigas]

Protein Accession	CL2195.Contig7$_H$1
Mean Expression Ratio	1.2
Median Expression Ratio	1.2
Credible Interval	(0.893, 1.61)
Associated Peptides	6
Associated Spectra	7
Coverage	NaN

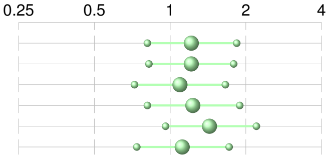

A	B	2.5	50	97.5	Sequence
1	0	0.81	1.2	1.8	NNLMDEFTISVR
1	1	0.82	1.2	1.8	VAALYDYTEK
1	0	0.72	1.1	1.7	HQALQAEIAGHEPR
1	0	0.81	1.2	1.9	KLDPSLSASR
1	0	0.96	1.4	2.2	ADLEAYRSV
0	1	0.74	1.1	1.7	LTAMGHTEAAEK

5.136 349 885 juvenile hormone diol kinase [Danaus plexippus]; 349 885 juvenile hormone diol kinase [Danaus plexippus]; 349 885 juvenile hormone diol kinase [Danaus plexippus]; 349 885 minus strand juvenile hormone diol kinase [Danaus plexippus]; 349 885 juvenile hormone diol kinase [Danaus plexippus]; 349 885 juvenile hormone diol kinase [Danaus plexippus]; 349 885 juvenile hormone diol kinase [Danaus plexippus]; 349 885 juvenile hormone diol kinase [Danaus plexippus]; 349 885 juvenile hormone diol kinase [Danaus plexippus]; 349 885 juvenile hormone diol kinase [Danaus plexippus]

Protein Accession	Unigene38920$_H$1 Unigene38919$_H$1 CL7590.Contig1$_H$1 CL7590.Contig16$_H$1 CL7590.Contig
Mean Expression Ratio	1.2
Median Expression Ratio	1.2
Credible Interval	(1.02, 1.41)
Associated Peptides	12
Associated Spectra	62
Coverage	NaN

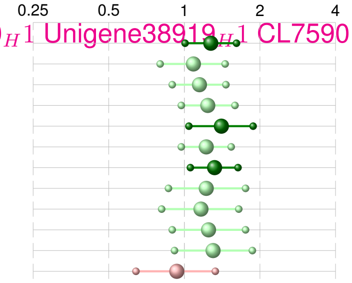

A	B	2.5	50	97.5	Sequence
5	4	1	1.3	1.6	DGIIDKNDFK
3	1	0.8	1.1	1.5	DILENVWEGLQK
6	2	0.89	1.1	1.5	FMFLANDTSGDGFVDR
4	4	0.97	1.2	1.6	IFGNSEADSNK
4	0	1	1.4	1.9	SVYQNLYDSNK
9	2	0.98	1.2	1.5	VPQWNEEYIK
9	3	1.1	1.3	1.6	GEFVTVQK
1	0	0.86	1.2	1.8	FSESGKVPQWNEEYIK
1	0	0.81	1.2	1.7	YIKFMFLANDTSGDGFVDR
0	2	0.9	1.2	1.8	DADINEDGIVTLDEWVK
0	1	0.92	1.3	1.9	DGIVTLDEWVK
0	1	0.64	0.93	1.3	AFDKLAQGAK

5.137 260 892 minus strand calponin [Haliotis diversicolor]

Protein Accession	CL48702.Contig1$_H$1
Mean Expression Ratio	1.19
Median Expression Ratio	1.2
Credible Interval	(1.02, 1.4)
Associated Peptides	11
Associated Spectra	105
Coverage	NaN

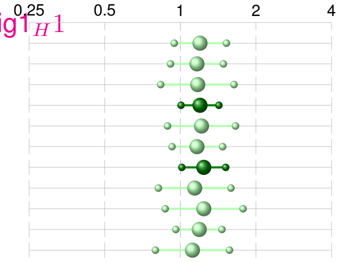

A	B	2.5	50	97.5	Sequence
7	4	0.94	1.2	1.5	AATGQQFPIEESNER
7	3	0.92	1.2	1.5	NFSEDKLR
2	0	0.83	1.2	1.6	TFQPASNLAF
18	8	1	1.2	1.4	TFQPASNLAFEAAR
1	3	0.89	1.2	1.7	VESSFDPEDAK
7	5	0.93	1.2	1.5	VESSFDPEDAKK
12	5	1	1.2	1.5	VMENFYK
1	1	0.82	1.1	1.6	YKVMENFYK
1	0	0.87	1.2	1.8	AKVESSFDPEDAKK
7	11	0.96	1.2	1.5	IELFIQK
1	1	0.8	1.1	1.6	VLACIR

5.138 " 196 1053 minus strand Cytochrome b-c1 complex subunit 2, mitochondrial OS=Homo sapiens GN=UQCRC2 PE=1 SV=3"

Protein Accession	CL7847.Contig5$_H$1
Mean Expression Ratio	1.19
Median Expression Ratio	1.19
Credible Interval	(0.782, 1.8)
Associated Peptides	1
Associated Spectra	2
Coverage	NaN

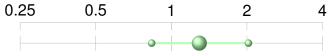

A	B	2.5	50	97.5	Sequence
1	1	0.84	1.3	2	LDLAILK

5.139 64 393 Tubulin beta chain OS=Paracentrotus lividus PE=2 SV=1; 109 438 Tubulin beta chain OS=Paracentrotus lividus PE=2 SV=1; 160 489 Tubulin beta chain OS=Paracentrotus lividus PE=2 SV=1

Protein Accession	Unigene62663$_H$1 Unigene62661$_H$1 Unigene62660$_H$1
Mean Expression Ratio	1.19
Median Expression Ratio	1.19
Credible Interval	(0.822, 1.72)
Associated Peptides	1
Associated Spectra	6
Coverage	NaN

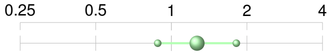

A	B	2.5	50	97.5	Sequence
4	2	0.89	1.3	1.8	INVYFNEATGGK

5.140 727 2022 RecName: Full=Enolase; AltName: Full=2-phospho-D-glycerate hydro-lyase; AltName: Full=2-phosphoglycerate dehydratase gi|1911573|gb|AAB50731.1| enolase [Doryteuthis pealeii]; 690 1985 RecName: Full=Enolase; AltName: Full=2-phospho-D-glycerate hydro-lyase; AltName: Full=2-phosphoglycerate dehydratase gi|1911573|gb|AAB50731.1| enolase [Doryteuthis pealeii]; 213 1508 RecName: Full=Enolase; AltName: Full=2-phospho-D-glycerate hydro-lyase; AltName: Full=2-phosphoglycerate dehydratase gi|1911573|gb|AAB50731.1| enolase [Doryteuthis pealeii]; 246 1541 RecName: Full=Enolase; AltName: Full=2-phospho-D-glycerate hydro-lyase; AltName: Full=2-phosphoglycerate dehydratase gi|1911573|gb|AAB50731.1| enolase [Doryteuthis pealeii]

Protein Accession	CL15687.Contig6$_H$1 CL15687.Contig5$_H$1 CL15687.Contig3$_H$1 CL15687.Contig2$_H$1
Mean Expression Ratio	1.19
Median Expression Ratio	1.19
Credible Interval	(1.06, 1.34)
Associated Peptides	25
Associated Spectra	122
Coverage	NaN

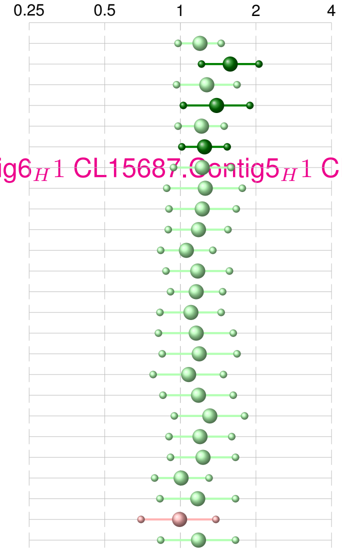

A	B	2.5	50	97.5	Sequence
11	4	0.98	1.2	1.5	AAGWGVMVSHR
3	3	1.2	1.6	2.1	AAVPSGASTGIYEALELR
3	2	0.96	1.3	1.7	DGKYDLDFK
2	1	1	1.4	1.9	FQIVGDDLLVTNPK
12	1	0.98	1.2	1.5	GVPLYR
9	4	1	1.2	1.5	IAPAFVGK
4	2	0.94	1.2	1.6	IEEELGVDAFYAGK
1	0	0.89	1.3	1.8	IEIGMDVAASEFCK
2	1	0.9	1.2	1.7	IMLDLDGTPNK
4	1	0.9	1.2	1.6	IMLDLDGTPNKEK
3	6	0.84	1.1	1.3	TLDLADQTAMDK
3	1	0.88	1.2	1.6	TSQQMIDMYHDFVK
4	4	0.91	1.2	1.5	VQMGIDR
1	4	0.83	1.1	1.5	QIFDSR
1	0	0.82	1.2	1.6	SYPIVTIEDPFDQDDWDAYTK
1	0	0.85	1.2	1.7	HIADLCGNKR
1	1	0.78	1.1	1.5	MGSETYHHLK
1	1	0.85	1.2	1.6	YDLDFK
1	1	0.95	1.3	1.8	LAMQEF
2	2	0.9	1.2	1.6	ACNALLLK
2	1	0.91	1.2	1.7	VNQIGSVSESIQAFK
5	3	0.79	1	1.3	YNQILR
0	1	0.83	1.2	1.7	HIADLCGNK
0	1	0.7	0.99	1.4	EGLELLK
0	1	0.83	1.2	1.7	ADLVVGLSTGQIK

5.141 " 125 628 minus strand collectin sub-family member 12, partial [Oncorhynchus mykiss]"

Protein Accession	CL46744.Contig5$_H$1
Mean Expression Ratio	1.19
Median Expression Ratio	1.19
Credible Interval	(0.769, 1.87)
Associated Peptides	1
Associated Spectra	1
Coverage	NaN

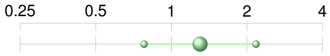

A	B	2.5	50	97.5	Sequence
1	0	0.78	1.3	2.2	WVQGDNRPLAYTR

5.142 " 6 1274 talin, putative [Ixodes scapularis] gi"

Protein Accession	CL39200.Contig1$_H$1
Mean Expression Ratio	0.842
Median Expression Ratio	0.842
Credible Interval	(0.559, 1.27)
Associated Peptides	2
Associated Spectra	2
Coverage	NaN

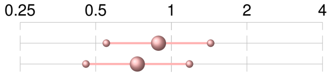

A	B	2.5	50	97.5	Sequence
0	1	0.55	0.89	1.4	SIAAATAALVK
0	1	0.46	0.73	1.2	LEMIR

5.143 274 666 Calmodulin-4 [Heterocephalus glaber]

Protein Accession	Unigene19050$_H$1
Mean Expression Ratio	1.19
Median Expression Ratio	1.19
Credible Interval	(0.924, 1.53)
Associated Peptides	4
Associated Spectra	17
Coverage	NaN

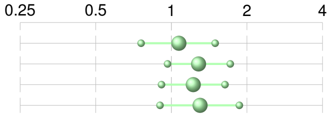

A	B	2.5	50	97.5	Sequence
2	1	0.76	1.1	1.5	GLDPENAVLDMDINK
3	3	0.96	1.3	1.7	SGFITLDELK
5	1	0.92	1.2	1.6	VLLSVGR
2	0	0.9	1.3	1.9	GTLSILELHK

5.144 188 1021 hypothetical protein BRAFLDRAFT_214346 [Branchiostoma floridae] gi

Protein Accession	CL38498.Contig2$_H$1
Mean Expression Ratio	0.839
Median Expression Ratio	0.842
Credible Interval	(0.575, 1.21)
Associated Peptides	3
Associated Spectra	3
Coverage	NaN

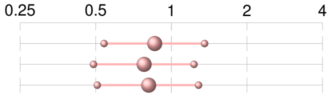

A	B	2.5	50	97.5	Sequence
1	0	0.54	0.86	1.4	EGLELHPPK
0	1	0.49	0.78	1.2	NLVGAIMTEEEAKK
0	1	0.51	0.81	1.3	DICTFLR

5.145 871 1062 Adenylate kinase isoenzyme 5 [Crassostrea gigas]

Protein Accession	Unigene7895$_H$1
Mean Expression Ratio	1.19
Median Expression Ratio	1.19
Credible Interval	(0.889, 1.6)
Associated Peptides	2
Associated Spectra	13
Coverage	NaN

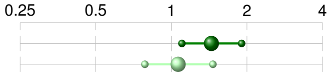

A	B	2.5	50	97.5	Sequence
6	2	1.1	1.4	1.9	VVFVVGGPGSGK
3	2	0.79	1.1	1.5	ALNEIMQK

5.146 401 1174 minus strand Mechanosensory protein 2 [Crassostrea gigas]

Protein Accession	Unigene7703$_H$1
Mean Expression Ratio	1.19
Median Expression Ratio	1.19
Credible Interval	(0.763, 1.83)
Associated Peptides	1
Associated Spectra	1
Coverage	NaN

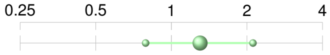

A	B	2.5	50	97.5	Sequence
1	0	0.79	1.3	2.1	VIAAEGEQK

5.147 143 247 minus strand dynein light chain [Aplysia californica] gi

Protein Accession	CL13886.Contig1$_H$1
Mean Expression Ratio	1.19
Median Expression Ratio	1.19
Credible Interval	(0.8, 1.79)
Associated Peptides	1
Associated Spectra	2
Coverage	NaN

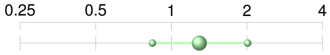

A	B	2.5	50	97.5	Sequence
2	0	0.84	1.3	2	NADMAEDMQQDAVDCATQALEK

5.148 801 971 RecName: Full=Tropomyosin-2; AltName: Full=Tropomyosin II; Short=BgTMII; Short=TMII gi|155940|gb|AAA27816.1| tropomyosin [Biomphalaria glabrata]

Protein Accession	CL2925.Contig4$_H$1
Mean Expression Ratio	0.843
Median Expression Ratio	0.843
Credible Interval	(0.629, 1.13)
Associated Peptides	3
Associated Spectra	11
Coverage	NaN

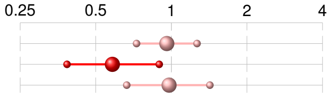

A	B	2.5	50	97.5	Sequence
7	1	0.73	0.96	1.3	EDSYEETIR
0	1	0.38	0.58	0.89	ISEQEASQR
0	2	0.66	0.98	1.4	QQYEIFGISEQEASQR

5.149 " 158 355 Protein NAS-37, isoform b [Caenorhabditis elegans] gi; 15 227 Hemicentin-1 [Crassostrea gigas]"

Protein Accession Unigene57180$_{H}1$ CL8016.Contig2$_{H}1$
Mean Expression Ratio 1.19
Median Expression Ratio 1.19
Credible Interval (0.766, 1.84)
Associated Peptides 1
Associated Spectra 1
Coverage NaN

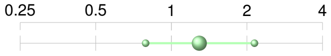

A	B	2.5	50	97.5	Sequence
1	0	0.79	1.3	2.1	FKGSYGGVSR

5.150 144 800 minus strand PREDICTED: protein F37C4.5-like [Amphimedon queenslandica]

Protein Accession CL7246.Contig3$_{H}1$
Mean Expression Ratio 1.19
Median Expression Ratio 1.19
Credible Interval (0.868, 1.64)
Associated Peptides 4
Associated Spectra 6
Coverage NaN

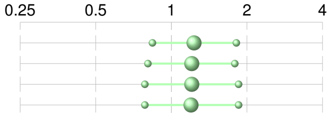

A	B	2.5	50	97.5	Sequence
2	0	0.84	1.2	1.8	VTVGLVDIKPK
1	1	0.81	1.2	1.8	VSLLEPQLDLK
1	0	0.79	1.2	1.8	NKDKDVTLNK
1	0	0.78	1.2	1.8	VISNTHEQVIEVK

5.151 " 220 627 Fatty acid-binding protein, brain [Crassostrea gigas]"

Protein Accession CL9041.Contig2$_{H}1$
Mean Expression Ratio 1.19
Median Expression Ratio 1.19
Credible Interval (0.908, 1.55)
Associated Peptides 5
Associated Spectra 13
Coverage NaN

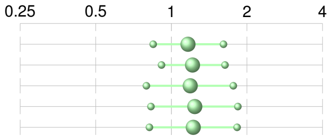

A	B	2.5	50	97.5	Sequence
3	1	0.85	1.2	1.6	EVDGGVMTSTTTFDGK
3	3	0.91	1.2	1.6	LTITVEGGK
1	0	0.79	1.2	1.8	VDLASTTGLEEFGTAIGFSEEK
1	0	0.83	1.2	1.8	IVENYKLEDK
0	1	0.82	1.2	1.8	QNYSFK

5.152 74 1243 Actin-related protein 2 [Crassostrea gigas]

Protein Accession CL8614.Contig4$_{H}1$
Mean Expression Ratio 1.19
Median Expression Ratio 1.18
Credible Interval (0.842, 1.68)
Associated Peptides 2
Associated Spectra 6
Coverage NaN

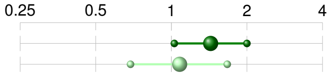

A	B	2.5	50	97.5	Sequence
4	1	1	1.4	2	MVEVMFEK
0	1	0.69	1.1	1.7	DLMIGDEASQLR

5.153 625 1902 minus strand hypothetical protein CGI_10009847 [Crassostrea gigas]

Protein Accession	Unigene8159$_{H}$1
Mean Expression Ratio	0.846
Median Expression Ratio	0.845
Credible Interval	(0.54, 1.32)
Associated Peptides	1
Associated Spectra	1
Coverage	NaN

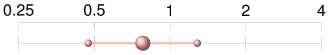

A	B	2.5	50	97.5	Sequence
0	1	0.47	0.78	1.3	LEHVPVTTFR

5.154 794 994 Glycogenin-1 [Crassostrea gigas]; 1425 1739 minus strand Glycogenin-1 [Crassostrea gigas]

Protein Accession	CL18608.Contig3$_{H}$1 CL18608.Contig2$_{H}$1
Mean Expression Ratio	1.19
Median Expression Ratio	1.18
Credible Interval	(0.827, 1.71)
Associated Peptides	3
Associated Spectra	3
Coverage	NaN

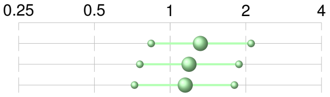

A	B	2.5	50	97.5	Sequence
1	0	0.84	1.3	2.1	VGGGESADYLILDDR
1	0	0.76	1.2	1.9	VGGGESADYLILDDRER
0	1	0.72	1.1	1.8	IAAPVLSFK

5.155 605 1153 Peptidyl-prolyl cis-trans isomerase B [Crassostrea gigas]; 1046 1630 Peptidyl-prolyl cis-trans isomerase B [Crassostrea gigas]; 589 1173 Peptidyl-prolyl cis-trans isomerase B [Crassostrea gigas]; 883 1467 Peptidyl-prolyl cis-trans isomerase B [Crassostrea gigas]

Protein Accession	CL24747.Contig5$_{H}$1 CL24747.Contig4$_{H}$1 CL24747.Contig3$_{H}$1 CL24747.Contig1$_{H}$1
Mean Expression Ratio	1.18
Median Expression Ratio	1.18
Credible Interval	(0.836, 1.67)
Associated Peptides	3
Associated Spectra	7
Coverage	NaN

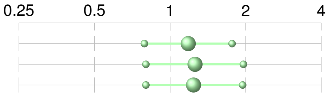

A	B	2.5	50	97.5	Sequence
2	3	0.79	1.2	1.8	IASSGVVGIASK
1	0	0.8	1.3	2	KLGELETSIIGRPK
1	0	0.8	1.2	1.9	SGMSIYGETFKDENFK

5.156 135 2630 Filamin-A [Crassostrea gigas]; 118 2613 Filamin-A [Crassostrea gigas]; 157 2652 Filamin-A [Crassostrea gigas]

Protein Accession	CL393.Contig15$_{H}1$ CL393.Contig13$_{H}1$ CL393.Contig12$_{H}1$
Mean Expression Ratio	0.845
Median Expression Ratio	0.845
Credible Interval	(0.728, 0.976)
Associated Peptides	23
Associated Spectra	142
Coverage	NaN

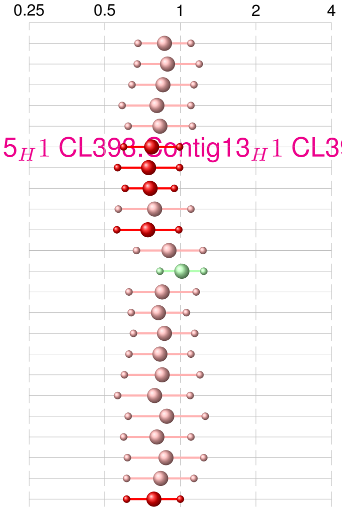

A	B	2.5	50	97.5	Sequence
9	3	0.68	0.86	1.1	AVANKPNEIVIDTSK
5	1	0.67	0.89	1.2	DNNDGTIVVEYQPK
5	1	0.64	0.85	1.1	EAGPGTLTVAVEGPGK
2	1	0.59	0.8	1.1	ENGVHYVDVK
4	1	0.62	0.83	1.1	HINNSPFK
5	4	0.6	0.77	0.99	IGDSELGHAAGCK
3	2	0.56	0.75	1	MGDGEIGIASFNPR
9	5	0.6	0.76	0.95	MNGIHTLSVR
1	1	0.57	0.79	1.1	PGSYGLHIK
4	2	0.56	0.74	0.98	QMEAAAEAAVPNK
3	1	0.67	0.9	1.2	TPSGGHEDCFIQEMDK
12	9	0.83	1	1.2	VINFQIETK
3	1	0.62	0.85	1.2	VPKPGSYGLHIK
5	4	0.64	0.82	1.1	VQVGGPGLEK
4	2	0.65	0.86	1.1	VTGALDK
4	2	0.63	0.83	1.1	YGNIPIAGSPYK
0	1	0.6	0.85	1.2	GDSELGHAAGCK
1	1	0.56	0.79	1.1	AALSCK
0	1	0.62	0.88	1.3	QVTVHSLR
3	1	0.6	0.81	1.1	YKGNPIK
0	1	0.62	0.88	1.2	PKPGSYGLHIK
3	1	0.61	0.83	1.1	IMFTPK
8	3	0.61	0.78	1	KPSPIQEQSSMVVETVEK

5.157 552 1886 minus strand intermediate filament protein [Biomphalaria glabrata]

Protein Accession	Unigene26795$_{H}1$
Mean Expression Ratio	1.18
Median Expression Ratio	1.18
Credible Interval	(1.06, 1.32)
Associated Peptides	22
Associated Spectra	157
Coverage	NaN

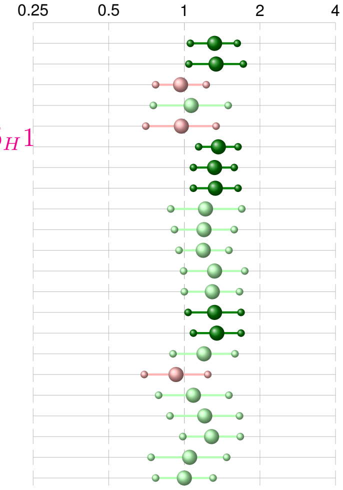

A	B	2.5	50	97.5	Sequence
10	2	1.1	1.3	1.6	ADLDAETAAHIENEVLAQTK
5	3	1	1.3	1.7	CGSLEDELAK
5	5	0.77	0.97	1.2	DMYEGELAQAR
1	0	0.75	1.1	1.5	DSYLVQLNELQLEFER
2	0	0.7	0.97	1.3	IAELEAALAAMK
12	7	1.1	1.4	1.6	KQEDWKPIR
14	4	1.1	1.3	1.6	KVITEFSTQR
11	3	1.1	1.3	1.6	LATYIEK
1	1	0.88	1.2	1.7	LEAELEALR
3	2	0.91	1.2	1.6	LLEGEENR
6	4	0.95	1.2	1.5	LRDQLAEK
4	1	1	1.3	1.7	SATGISEFR
4	2	1	1.3	1.7	VITEFSTQR
6	2	1	1.3	1.7	YAAQMDQLR
8	4	1.1	1.3	1.7	KLLEGEENR
4	0	0.9	1.2	1.6	MSATGISEFR
3	1	0.69	0.93	1.2	ELMDHK
1	1	0.79	1.1	1.5	EMQDCNER
1	1	0.88	1.2	1.7	IANLQDQIQALK
4	2	0.99	1.3	1.7	WLEAQNK
0	1	0.74	1	1.5	ALVDKLK
0	6	0.77	1	1.3	KLEAELEALR

5.158 140 1564 selenium-binding protein [Haliotis discus hannai]

Protein Accession	CL3868.Contig7$_H$1
Mean Expression Ratio	1.18
Median Expression Ratio	1.18
Credible Interval	(0.764, 1.84)
Associated Peptides	1
Associated Spectra	1
Coverage	NaN

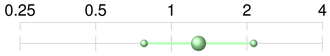

A	B	2.5	50	97.5	Sequence
**1**	**0**	**0.78**	**1.3**	**2.1**	**VVEAVDIHR**

5.159 258 629 Glycogenin-1 OS=Oryctolagus cuniculus GN=GYG1 PE=1 SV=3

Protein Accession	Unigene77101$_H$1
Mean Expression Ratio	1.18
Median Expression Ratio	1.18
Credible Interval	(0.763, 1.86)
Associated Peptides	1
Associated Spectra	1
Coverage	NaN

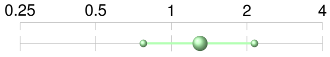

A	B	2.5	50	97.5	Sequence
**1**	**0**	**0.77**	**1.3**	**2.1**	**IHCWELTQYEK**

5.160 116 1111 minus strand cytosolic malate dehydrogenase [Lottia scabra]

Protein Accession	CL280.Contig16$_H$1
Mean Expression Ratio	1.18
Median Expression Ratio	1.18
Credible Interval	(1.03, 1.34)
Associated Peptides	19
Associated Spectra	131
Coverage	NaN

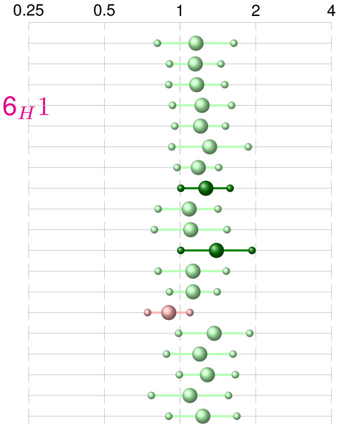

A	B	2.5	50	97.5	Sequence
**1**	**0**	**0.81**	**1.2**	**1.6**	**ATADHMHDWWFGTEGDK**
**6**	**4**	**0.91**	**1.2**	**1.5**	**DCDAAFLVGAMPR**
**4**	**3**	**0.9**	**1.2**	**1.5**	**DLLAANIK**
**3**	**3**	**0.94**	**1.2**	**1.6**	**EAVKDDNWINNEFISTVQK**
**6**	**5**	**0.95**	**1.2**	**1.5**	**ENFSCLTR**
**1**	**0**	**0.93**	**1.3**	**1.9**	**EVIPTSDEK**
**15**	**4**	**0.97**	**1.2**	**1.4**	**KLSSAMSAAK**
**6**	**6**	**1**	**1.3**	**1.6**	**LGIPNNAVSK**
**3**	**3**	**0.82**	**1.1**	**1.4**	**LSSAMSAAK**
**2**	**0**	**0.79**	**1.1**	**1.5**	**QVTIVEGLSISDFAR**
**2**	**0**	**1**	**1.4**	**1.9**	**WVSMGVYSTGK**
**2**	**1**	**0.82**	**1.1**	**1.5**	**YGIPEGVMYSYPVQIK**
**12**	**0**	**0.9**	**1.1**	**1.4**	**KDLLAANIK**
**9**	**14**	**0.74**	**0.9**	**1.1**	**EMAEQILAA**
**2**	**0**	**0.99**	**1.4**	**1.9**	**SLLYSVAK**
**2**	**1**	**0.88**	**1.2**	**1.6**	**ELLEER**
**6**	**1**	**0.99**	**1.3**	**1.7**	**SQGANLDAVAK**
**1**	**0**	**0.77**	**1.1**	**1.6**	**VVIVGNPANTNALICAK**
**2**	**1**	**0.9**	**1.2**	**1.7**	**YAPSIPK**

5.161 118 513 Protein CAL-5 [Caenorhabditis elegans] gi

Protein Accession	CL18668.Contig1$_{H}1$
Mean Expression Ratio	0.851
Median Expression Ratio	0.85
Credible Interval	(0.545, 1.32)
Associated Peptides	1
Associated Spectra	2
Coverage	NaN

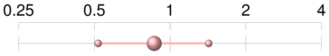

A	B	2.5	50	97.5	Sequence
**1**	**1**	**0.52**	**0.86**	**1.4**	**EADANGDGYLSIQELK**

5.162 6043 6174 myosin heavy chain [Mytilus galloprovincialis]; 5869 6000 myosin heavy chain [Mytilus galloprovincialis]

Protein Accession	Unigene19025$_{H}1$ CL49594.Contig1$_{H}1$
Mean Expression Ratio	0.85
Median Expression Ratio	0.85
Credible Interval	(0.613, 1.18)
Associated Peptides	1
Associated Spectra	12
Coverage	NaN

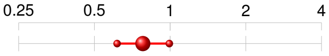

A	B	2.5	50	97.5	Sequence
**12**	**0**	**0.62**	**0.78**	**0.99**	**KLEADIQAMQTDLDEMNGELR**

5.163 111 530 minus strand Nucleoside diphosphate kinase B OS=Homo sapiens GN=NME2 PE=1 SV=1

Protein Accession	CL22280.Contig3$_{H}1$
Mean Expression Ratio	0.85
Median Expression Ratio	0.851
Credible Interval	(0.633, 1.14)
Associated Peptides	4
Associated Spectra	9
Coverage	NaN

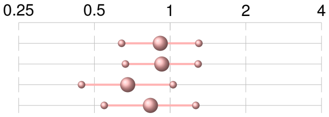

A	B	2.5	50	97.5	Sequence
**2**	**1**	**0.64**	**0.91**	**1.3**	**GDFCIEMGR**
**2**	**2**	**0.66**	**0.92**	**1.3**	**VMLGETNPLNSKPGTIR**
**1**	**0**	**0.44**	**0.68**	**1**	**LCRPGK**
**0**	**1**	**0.55**	**0.84**	**1.3**	**LVAAK**

5.164 261 1361 tandem-repeat galectin [Pinctada fucata]; 261 1361 tandem-repeat galectin [Pinctada fucata]

Protein Accession	CL474.Contig2$_{H}1$ CL474.Contig1$_{H}1$
Mean Expression Ratio	0.849
Median Expression Ratio	0.852
Credible Interval	(0.56, 1.28)
Associated Peptides	1
Associated Spectra	2
Coverage	NaN

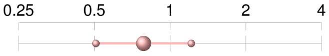

A	B	2.5	50	97.5	Sequence
**0**	**2**	**0.51**	**0.78**	**1.2**	**LENNVLLHANQR**

5.165 316 1512 Alpha-N-acetylgalactosaminidase [Crassostrea gigas]; 282 1568 Alpha-N-acetylgalactosaminidase [Crassostrea gigas]; 282 1478 Alpha-N-acetylgalactosaminidase [Crassostrea gigas]

Protein Accession	CL2428.Contig4$_H$1 CL2428.Contig2$_H$1 CL2428.Contig1$_H$1
Mean Expression Ratio	1.17
Median Expression Ratio	1.17
Credible Interval	(0.817, 1.69)
Associated Peptides	2
Associated Spectra	5
Coverage	NaN

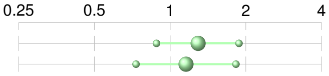

A	B	2.5	50	97.5	Sequence
**1**	**3**	**0.88**	**1.3**	**1.9**	**LVELGLR**
**0**	**1**	**0.73**	**1.2**	**1.8**	**ELFAGK**

5.166 85 633 hypothetical protein [Amblyomma maculatum]

Protein Accession	CL2221.Contig1$_H$1
Mean Expression Ratio	1.17
Median Expression Ratio	1.17
Credible Interval	(0.826, 1.67)
Associated Peptides	3
Associated Spectra	4
Coverage	NaN

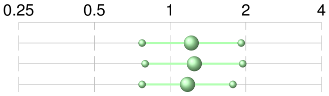

A	B	2.5	50	97.5	Sequence
**1**	**0**	**0.77**	**1.2**	**1.9**	**DANDEWIR**
**1**	**0**	**0.8**	**1.2**	**1.9**	**EKLPDYDNK**
**0**	**2**	**0.77**	**1.2**	**1.8**	**IFYTEHLHADEEIR**

5.167 416 1717 minus strand T-complex protein 1 subunit delta OS=Aedes triseriatus PE=2 SV=1

Protein Accession	CL8566.Contig1$_H$1
Mean Expression Ratio	0.853
Median Expression Ratio	0.853
Credible Interval	(0.563, 1.3)
Associated Peptides	1
Associated Spectra	2
Coverage	NaN

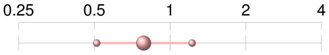

A	B	2.5	50	97.5	Sequence
**1**	**1**	**0.51**	**0.79**	**1.2**	**HAAGELTAGINVR**

5.168 106 1701 T-complex protein 1 subunit beta [Crassostrea gigas]

Protein Accession	CL8176.Contig1$_H$1
Mean Expression Ratio	0.854
Median Expression Ratio	0.853
Credible Interval	(0.567, 1.28)
Associated Peptides	2
Associated Spectra	2
Coverage	NaN

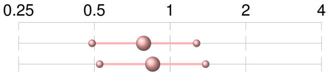

A	B	2.5	50	97.5	Sequence
**1**	**0**	**0.49**	**0.79**	**1.3**	**GSGNLDAIQIIK**
**1**	**0**	**0.52**	**0.85**	**1.4**	**CTIVLR**

5.169 213 881 PREDICTED: hypothetical protein [Saccoglossus kowalevskii]; 260 928 PREDICTED: hypothetical protein [Saccoglossus kowalevskii]; 294 962 PREDICTED: hypothetical protein [Saccoglossus kowalevskii]; 287 955 PREDICTED: hypothetical protein [Saccoglossus kowalevskii]; 316 984 PREDICTED: hypothetical protein [Saccoglossus kowalevskii]

Protein Accession	CL23253.Contig8$_{H}1$ CL23253.Contig7$_{H}1$ CL23253.Contig3$_{H}1$ CL23253.Contig2$_{H}1$ CL2325
Mean Expression Ratio	0.853
Median Expression Ratio	0.854
Credible Interval	(0.547, 1.33)
Associated Peptides	1
Associated Spectra	1
Coverage	NaN

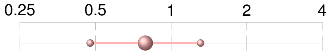

A	B	2.5	50	97.5	Sequence
**0**	**1**	**0.48**	**0.79**	**1.3**	**VDLPGEFR**

5.170 3 572 Receptor-type tyrosine-protein phosphatase N2 [Crassostrea gigas]; 3 542 minus strand Receptor-type tyrosine-protein phosphatase N2 [Crassostrea gigas]

Protein Accession	CL8283.Contig5$_{H}1$ CL8283.Contig2$_{H}1$
Mean Expression Ratio	0.855
Median Expression Ratio	0.855
Credible Interval	(0.547, 1.33)
Associated Peptides	1
Associated Spectra	1
Coverage	NaN

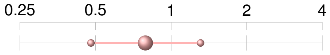

A	B	2.5	50	97.5	Sequence
**0**	**1**	**0.48**	**0.79**	**1.3**	**EIDMAATLEHIR**

5.171 49 1038 Glyceraldehyde-3-phosphate dehydrogenase [Crotalus adamanteus]

Protein Accession	Unigene7744$_{H}1$
Mean Expression Ratio	1.17
Median Expression Ratio	1.17
Credible Interval	(0.842, 1.63)
Associated Peptides	1
Associated Spectra	12
Coverage	NaN

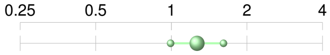

A	B	2.5	50	97.5	Sequence
**12**	**0**	**0.99**	**1.3**	**1.6**	**SSIFDAK**

5.172 4 579 minus strand Collagen alpha-3(VI) chain [Crassostrea gigas]

Protein Accession	CL46502.Contig3$_H1$
Mean Expression Ratio	1.17
Median Expression Ratio	1.17
Credible Interval	(0.823, 1.63)
Associated Peptides	2
Associated Spectra	6
Coverage	NaN

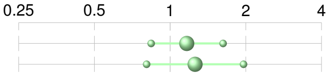

A	B	2.5	50	97.5	Sequence
**5**	**0**	**0.84**	**1.2**	**1.6**	**FAVQEFSELR**
**1**	**0**	**0.81**	**1.3**	**2**	**LIKDFVK**

5.173 118 501 minus strand histone 2A.Z [Aplysia californica]

Protein Accession	CL14228.Contig6$_H1$
Mean Expression Ratio	0.858
Median Expression Ratio	0.857
Credible Interval	(0.642, 1.15)
Associated Peptides	5
Associated Spectra	22
Coverage	NaN

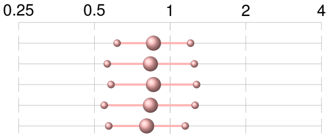

A	B	2.5	50	97.5	Sequence
**3**	**3**	**0.62**	**0.86**	**1.2**	**ATIAGGGVIPHIHK**
**1**	**1**	**0.56**	**0.84**	**1.2**	**GDEELDSLIK**
**1**	**1**	**0.58**	**0.86**	**1.3**	**HLQLAIR**
**0**	**1**	**0.55**	**0.83**	**1.3**	**GGGVIPHIHK**
**9**	**2**	**0.57**	**0.81**	**1.1**	**AGLQFPVGR**

5.174 269 880 minus strand myosin regulatory light chain [Fasciola hepatica]

Protein Accession	CL43006.Contig5$_H1$
Mean Expression Ratio	1.17
Median Expression Ratio	1.17
Credible Interval	(0.912, 1.5)
Associated Peptides	3
Associated Spectra	21
Coverage	NaN

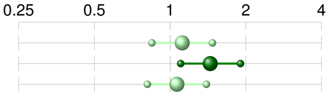

A	B	2.5	50	97.5	Sequence
**5**	**2**	**0.85**	**1.1**	**1.5**	**ATSNVFAK**
**7**	**0**	**1.1**	**1.4**	**1.9**	**KLMQEMK**
**4**	**3**	**0.81**	**1.1**	**1.4**	**LMQEMK**

5.175 " 2 286 Roundabout 2, partial [Ascaris suum]"

Protein Accession	CL8589.Contig2$_H1$
Mean Expression Ratio	1.17
Median Expression Ratio	1.17
Credible Interval	(0.78, 1.75)
Associated Peptides	2
Associated Spectra	2
Coverage	NaN

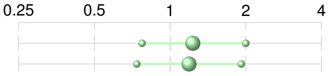

A	B	2.5	50	97.5	Sequence
**1**	**0**	**0.77**	**1.2**	**2**	**GEPTPEITWLK**
**1**	**0**	**0.74**	**1.2**	**1.9**	**KSQTAVLDVIR**

5.176 697 954 minus strand Peptidyl-prolyl cis-trans isomerase B [Crassostrea gigas]; 614 877 Peptidyl-prolyl cis-trans isomerase B [Crassostrea gigas]

Protein Accession	CL49248.Contig4$_{H}1$ CL49248.Contig3$_{H}1$
Mean Expression Ratio	0.861
Median Expression Ratio	0.859
Credible Interval	(0.646, 1.15)
Associated Peptides	4
Associated Spectra	19
Coverage	NaN

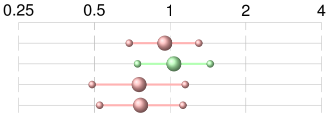

A	B	2.5	50	97.5	Sequence
4	4	0.69	0.95	1.3	LVDCSATTLK
3	3	0.74	1	1.4	ASLEFLDK
0	1	0.49	0.75	1.2	SMQNLDGR
3	1	0.53	0.76	1.1	INNVPVDK

5.177 140 349 copper chaperone Atox1 [Crassostrea ariakensis]

Protein Accession	CL8999.Contig5$_{H}1$
Mean Expression Ratio	1.16
Median Expression Ratio	1.16
Credible Interval	(0.773, 1.75)
Associated Peptides	1
Associated Spectra	2
Coverage	NaN

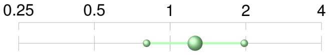

A	B	2.5	50	97.5	Sequence
1	1	0.81	1.3	2	EELMATLQK

5.178 155 3214 sarco/endoplasmic reticulum calcium ATPase isoform B [Pinctada fucata]; 155 3214 minus strand sarco/endoplasmic reticulum calcium ATPase isoform B [Pinctada fucata]

Protein Accession	CL1323.Contig9$_{H}1$ CL1323.Contig1$_{H}1$
Mean Expression Ratio	0.86
Median Expression Ratio	0.86
Credible Interval	(0.705, 1.04)
Associated Peptides	14
Associated Spectra	58
Coverage	NaN

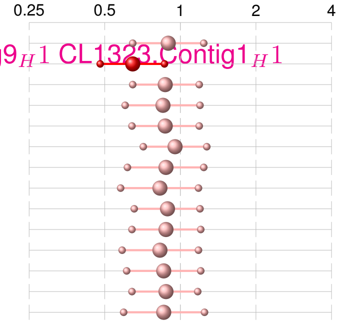

A	B	2.5	50	97.5	Sequence
1	2	0.64	0.89	1.2	ATAEAICR
3	3	0.48	0.64	0.87	CLALATIDNPPR
3	2	0.65	0.87	1.2	EFDELPPEDQR
2	1	0.6	0.85	1.2	GAPEGLLDR
3	2	0.64	0.87	1.2	GIVVGTGLATEIGK
7	2	0.71	0.95	1.3	KAEIGIAMGSGTAVAK
1	1	0.61	0.87	1.2	KEFTLEFSR
1	1	0.58	0.83	1.2	NAESAIEALK
2	3	0.65	0.89	1.2	NMLFSGTNIAAGK
2	2	0.64	0.88	1.2	VIVITGDNK
1	1	0.59	0.83	1.2	LNFYNTDK
2	1	0.61	0.86	1.2	MDQAFTK
7	1	0.64	0.88	1.2	IGHNKVPMSPAIK
0	1	0.59	0.86	1.2	KAEIGIAMGSGTA

5.179 125 754 minus strand peptidyl prolyl cis-trans isomerase B [Conus novaehollandiae]

Protein Accession	Unigene19558$_{H}1$
Mean Expression Ratio	1.16
Median Expression Ratio	1.16
Credible Interval	(0.841, 1.61)
Associated Peptides	4
Associated Spectra	5
Coverage	NaN

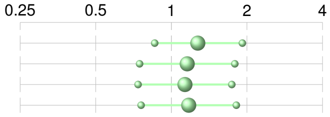

A	B	2.5	50	97.5	Sequence
2	0	0.86	1.3	1.9	HLALGDKEDGGVK
1	0	0.75	1.2	1.8	KTQWLDGR
1	0	0.74	1.1	1.7	DTNGSQFFITTK
1	0	0.76	1.2	1.8	FPDENFK

5.180 17 1681 minus strand Glucose-6-phosphate isomerase [Crassostrea gigas]

Protein Accession	CL34850.Contig1$_{H}1$
Mean Expression Ratio	1.16
Median Expression Ratio	1.16
Credible Interval	(0.898, 1.49)
Associated Peptides	7
Associated Spectra	13
Coverage	NaN

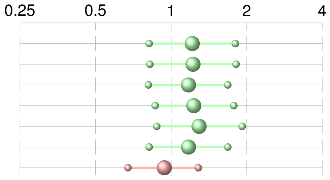

A	B	2.5	50	97.5	Sequence
1	0	0.82	1.2	1.8	EFTEQVISGAWK
1	0	0.82	1.2	1.8	KQLTSEPVWR
1	1	0.81	1.2	1.7	CKEFGIDEK
1	1	0.87	1.2	1.8	VHFISNVDGTHLAK
0	1	0.87	1.3	1.9	EAAEDELR
0	2	0.82	1.2	1.7	INFTEDR
0	4	0.67	0.94	1.3	INMAQMFK

5.181 7948 8541 Collagen alpha-3(VI) chain [Crassostrea gigas]

Protein Accession	CL47586.Contig9$_{H}1$
Mean Expression Ratio	1.16
Median Expression Ratio	1.16
Credible Interval	(0.819, 1.63)
Associated Peptides	2
Associated Spectra	5
Coverage	NaN

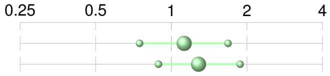

A	B	2.5	50	97.5	Sequence
2	0	0.75	1.1	1.7	ADIAFLLDSSGSVGYDNFR
3	0	0.89	1.3	1.9	DLQDAISAISYEYGNTNTAAGLK

5.182 REVERSED 68 358 endonuclease-reverse transcriptase [Danaus plexippus]

Protein Accession	RRRRRUnigene39307$_{H}1$
Mean Expression Ratio	0.863
Median Expression Ratio	0.863
Credible Interval	(0.576, 1.29)
Associated Peptides	2
Associated Spectra	2
Coverage	NaN

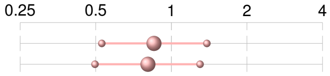

A	B	2.5	50	97.5	Sequence
0	1	0.53	0.85	1.4	TIITALDK
0	1	0.5	0.81	1.3	CQSLLDENSIK

5.183 54 638 minus strand ubiquitin carboxyl-terminal hydrolase [Aplysia californica] gi

Protein Accession	CL20666.Contig3$_{H1}$
Mean Expression Ratio	1.15
Median Expression Ratio	1.16
Credible Interval	(0.737, 1.79)
Associated Peptides	1
Associated Spectra	1
Coverage	NaN

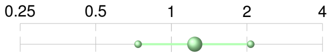

A	B	2.5	50	97.5	Sequence
**1**	**0**	**0.74**	**1.2**	**2.1**	**THADTLLEDTVK**

5.184 " 3475 4188 minus strand Heat shock 70 kDa protein cognate, putative [Pediculus humanus corporis] gi"

Protein Accession	CL5924.Contig14$_{H1}$
Mean Expression Ratio	0.868
Median Expression Ratio	0.866
Credible Interval	(0.606, 1.25)
Associated Peptides	2
Associated Spectra	4
Coverage	NaN

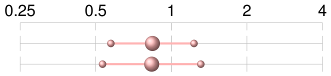

A	B	2.5	50	97.5	Sequence
**2**	**1**	**0.58**	**0.84**	**1.2**	**STIEDEKLK**
**0**	**1**	**0.53**	**0.84**	**1.3**	**CNDVIR**

5.185 181 744 unknown [Dendroctonus ponderosae]

Protein Accession	CL25948.Contig3$_{H1}$
Mean Expression Ratio	0.866
Median Expression Ratio	0.867
Credible Interval	(0.696, 1.08)
Associated Peptides	7
Associated Spectra	23
Coverage	NaN

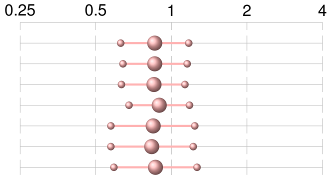

A	B	2.5	50	97.5	Sequence
**3**	**1**	**0.63**	**0.86**	**1.2**	**GTLTQLTK**
**4**	**1**	**0.64**	**0.86**	**1.2**	**SMGPIGSPMSK**
**4**	**1**	**0.63**	**0.85**	**1.1**	**TFICTPDR**
**4**	**2**	**0.68**	**0.9**	**1.2**	**VGHVPVAK**
**1**	**0**	**0.58**	**0.85**	**1.2**	**YKNNYLCR**
**1**	**0**	**0.58**	**0.83**	**1.2**	**GIYICDGSDEEAEEIIHK**
**1**	**0**	**0.59**	**0.87**	**1.3**	**NNYLCR**

5.186 246 452 minus strand mitochondrial malate dehydrogenase precursor [Nucella lapillus]; 106 312 minus strand mitochondrial malate dehydrogenase precursor [Nucella lapillus]

Protein Accession	CL10426.Contig4$_{H1}$ CL10426.Contig3$_{H1}$
Mean Expression Ratio	1.15
Median Expression Ratio	1.15
Credible Interval	(0.783, 1.68)
Associated Peptides	1
Associated Spectra	4
Coverage	NaN

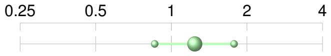

A	B	2.5	50	97.5	Sequence
**2**	**2**	**0.86**	**1.2**	**1.8**	**DDLFNTNAGIVR**

5.187 176 670 hypothetical protein BRAFLDRAFT_122621 [Branchiostoma floridae] gi

Protein Accession	Unigene180$_{H}1$
Mean Expression Ratio	1.15
Median Expression Ratio	1.15
Credible Interval	(0.956, 1.39)
Associated Peptides	7
Associated Spectra	64
Coverage	NaN

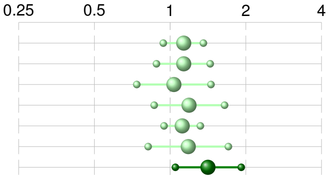

A	B	2.5	50	97.5	Sequence
11	9	0.94	1.1	1.4	FADENFLLK
6	3	0.88	1.1	1.4	VFFDITADGQPLGR
1	1	0.73	1	1.5	VIPNFMCQGGDFTNHNGTGGR
3	0	0.87	1.2	1.6	VLCTGEK
12	13	0.95	1.1	1.3	IVMELR
1	0	0.82	1.2	1.7	ADVVPK
2	2	1	1.4	1.9	HVVFGQVVEGMDVVR

5.188 169 1200 PREDICTED: nidogen-2-like isoform 1 [Strongylocentrotus purpuratus]

Protein Accession	CL478.Contig2$_{H}1$
Mean Expression Ratio	0.869
Median Expression Ratio	0.867
Credible Interval	(0.626, 1.21)
Associated Peptides	4
Associated Spectra	5
Coverage	NaN

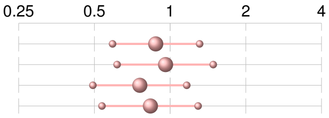

A	B	2.5	50	97.5	Sequence
0	2	0.59	0.88	1.3	LDIVATAR
0	1	0.62	0.96	1.5	LPNGLTFDQVSR
0	1	0.49	0.76	1.2	IECVHR
0	1	0.53	0.83	1.3	VFTVPALSR

5.189 365 2530 minus strand calpain B [Gecarcinus lateralis]

Protein Accession	CL34074.Contig5$_{H}1$
Mean Expression Ratio	1.15
Median Expression Ratio	1.15
Credible Interval	(0.901, 1.47)
Associated Peptides	7
Associated Spectra	14
Coverage	NaN

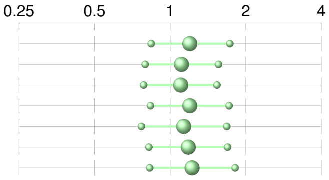

A	B	2.5	50	97.5	Sequence
1	1	0.84	1.2	1.7	LGYDEFK
2	1	0.8	1.1	1.6	SLQSLVMR
2	1	0.78	1.1	1.5	SMVAMHDGDLSGK
1	1	0.83	1.2	1.7	TLWEDLR
1	0	0.77	1.1	1.7	MQGQIPMVR
1	1	0.82	1.2	1.7	TMLTSFR
0	1	0.83	1.2	1.8	TLWEDLRR

5.190 96 497 AAEL009772-PB [Aedes aegypti]

Protein Accession	CL27854.Contig2$_{H}1$
Mean Expression Ratio	1.15
Median Expression Ratio	1.15
Credible Interval	(0.761, 1.72)
Associated Peptides	1
Associated Spectra	3
Coverage	NaN

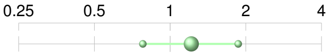

A	B	2.5	50	97.5	Sequence
2	1	0.78	1.2	1.9	LVEEFPMGNIK

5.191 154 621 minus strand Stress response protein nhaX [Crassostrea gigas]

Protein Accession	CL4265.Contig1$_{H}$1
Mean Expression Ratio	1.15
Median Expression Ratio	1.15
Credible Interval	(0.83, 1.6)
Associated Peptides	5
Associated Spectra	5
Coverage	NaN

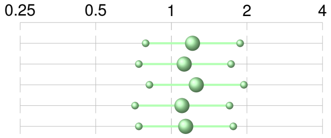

A	B	2.5	50	97.5	Sequence
**0**	**1**	**0.79**	**1.2**	**1.9**	**IQHDLEK**
**0**	**1**	**0.74**	**1.1**	**1.7**	**SVAAAKPGEGILK**
**0**	**1**	**0.82**	**1.3**	**1.9**	**EVLEAMLK**
**0**	**1**	**0.72**	**1.1**	**1.7**	**PGEGILK**
**0**	**1**	**0.74**	**1.1**	**1.8**	**EGILK**

5.192 30 536 putative mitochondrial ATP synthase [Haliotis discus discus]; 27 620 putative mitochondrial ATP synthase [Haliotis discus discus]

Protein Accession	CL13014.Contig4$_{H}$1 CL13014.Contig1$_{H}$1
Mean Expression Ratio	0.867
Median Expression Ratio	0.869
Credible Interval	(0.63, 1.18)
Associated Peptides	2
Associated Spectra	8
Coverage	NaN

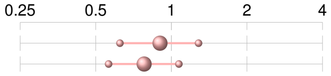

A	B	2.5	50	97.5	Sequence
**2**	**1**	**0.63**	**0.9**	**1.3**	**LANPALVDQFQK**
**2**	**3**	**0.56**	**0.78**	**1.1**	**SAVDWVAFK**

5.193 192 380 minus strand RAB protein [Haliotis discus discus]

Protein Accession	Unigene35065$_{H}$1
Mean Expression Ratio	0.867
Median Expression Ratio	0.869
Credible Interval	(0.558, 1.34)
Associated Peptides	1
Associated Spectra	1
Coverage	NaN

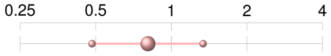

A	B	2.5	50	97.5	Sequence
**1**	**0**	**0.48**	**0.81**	**1.3**	**NVEEMFLAITK**

5.194 546 995 Hypothetical protein CBG12002 [Caenorhabditis briggsae]; 543 995 minus strand PREDICTED: tubulin polymerization-promoting protein family member 2-like [Anolis carolinensis]; 546 995 Hypothetical protein CBG12002 [Caenorhabditis briggsae]; 546 995 minus strand Hypothetical protein CBG12002 [Caenorhabditis briggsae]

Protein Accession	CL6849.Contig7$_H$1 CL6849.Contig6$_H$1 CL6849.Contig2$_H$1 CL6849.Contig1$_H$1
Mean Expression Ratio	1.15
Median Expression Ratio	1.15
Credible Interval	(0.764, 1.74)
Associated Peptides	1
Associated Spectra	2
Coverage	NaN

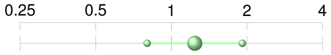

A	B	2.5	50	97.5	Sequence
**0**	**2**	**0.8**	**1.2**	**1.9**	**LLPQLAADK**

5.195 62 505 Myosin-2 essential light chain [Crassostrea gigas]

Protein Accession	CL47077.Contig1$_H$1
Mean Expression Ratio	1.15
Median Expression Ratio	1.15
Credible Interval	(0.849, 1.57)
Associated Peptides	2
Associated Spectra	10
Coverage	NaN

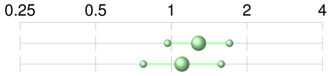

A	B	2.5	50	97.5	Sequence
**5**	**2**	**0.97**	**1.3**	**1.7**	**IAASQLGDVLR**
**2**	**1**	**0.77**	**1.1**	**1.6**	**HILTSLGEK**

5.196 174 1649 minus strand Collagen alpha-1(XII) chain [Crassostrea gigas]; 135 1610 Collagen alpha-1(XII) chain [Crassostrea gigas]

Protein Accession	CL5340.Contig13$_H$1 CL5340.Contig11$_H$1
Mean Expression Ratio	1.15
Median Expression Ratio	1.15
Credible Interval	(0.928, 1.42)
Associated Peptides	10
Associated Spectra	28
Coverage	NaN

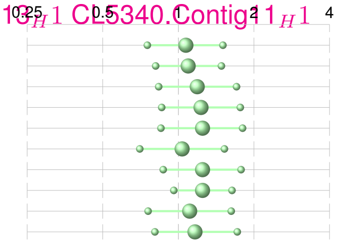

A	B	2.5	50	97.5	Sequence
**2**	**0**	**0.75**	**1.1**	**1.5**	**ADVMFVTEFSQAGGR**
**4**	**1**	**0.81**	**1.1**	**1.5**	**ADVVFIVDSSR**
**1**	**1**	**0.83**	**1.2**	**1.7**	**DVMSVFHIAR**
**1**	**1**	**0.86**	**1.2**	**1.8**	**SIWMPDFK**
**1**	**0**	**0.85**	**1.2**	**1.8**	**VDGYSALDTIR**
**1**	**0**	**0.7**	**1**	**1.5**	**YWAEQLKK**
**2**	**0**	**0.87**	**1.2**	**1.8**	**KADVVFVLDK**
**6**	**5**	**0.96**	**1.2**	**1.6**	**DILAFR**
**1**	**0**	**0.76**	**1.1**	**1.6**	**TTELLK**
**1**	**0**	**0.81**	**1.2**	**1.7**	**SSSITAEHFQTQLR**

5.197 56 937 Chloride intracellular channel exc-4 [Crassostrea gigas]

Protein Accession	CL34620.Contig3$_H$1
Mean Expression Ratio	1.15
Median Expression Ratio	1.15
Credible Interval	(0.793, 1.68)
Associated Peptides	2
Associated Spectra	3
Coverage	NaN

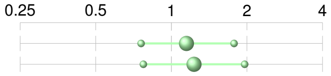

A	B	2.5	50	97.5	Sequence
2	0	0.76	1.2	1.8	KVQGPLVK
1	0	0.78	1.2	2	KLLAELK

5.198 " 27 974 PREDICTED: succinyl-CoA ligase [GDP-forming] subunit alpha, mitochondrial-like [Oreochromis niloticus]; 27 974 minus strand PREDICTED: succinyl-CoA ligase [GDP-forming] subunit alpha, mitochondrial-like [Oreochromis niloticus]"

Protein Accession	CL10674.Contig2$_H$1 CL10674.Contig1$_H$1
Mean Expression Ratio	0.871
Median Expression Ratio	0.872
Credible Interval	(0.628, 1.21)
Associated Peptides	3
Associated Spectra	6
Coverage	NaN

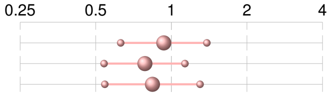

A	B	2.5	50	97.5	Sequence
2	0	0.63	0.93	1.4	MGHAGAIIAGGK
1	2	0.54	0.79	1.1	IYMATR
0	1	0.54	0.84	1.3	LICQGFTGK

5.199 424 3507 minus strand putative Na+/K+-ATPase alpha subunit [Paroctopus digueti]

Protein Accession	Unigene11494$_H$1
Mean Expression Ratio	0.872
Median Expression Ratio	0.872
Credible Interval	(0.621, 1.24)
Associated Peptides	4
Associated Spectra	4
Coverage	NaN

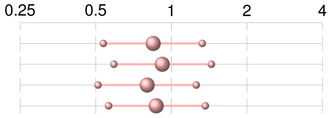

A	B	2.5	50	97.5	Sequence
1	0	0.54	0.85	1.3	SAEFTHENPLETR
0	1	0.59	0.92	1.4	DMTPAQIDEILR
0	1	0.51	0.8	1.3	MTVAHMWFDGR
0	1	0.56	0.87	1.4	VIVAHGFK

5.200 552 1499 minus strand uncharacterized protein LOC100158466 [Xenopus (Silurana) tropicalis] gi; 552 1499 minus strand uncharacterized protein LOC100158466 [Xenopus (Silurana) tropicalis] gi; 552 1499 minus strand uncharacterized protein LOC100158466 [Xenopus (Silurana) tropicalis] gi

Protein Accession	Unigene57645$_{H}1$ Unigene52825$_{H}1$ Unigene48504$_{H}1$
Mean Expression Ratio	0.868
Median Expression Ratio	0.872
Credible Interval	(0.553, 1.34)
Associated Peptides	1
Associated Spectra	2
Coverage	NaN

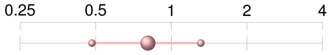

A	B	2.5	50	97.5	Sequence
1	1	0.48	0.8	1.3	TGEPVEVDGELATLLAR

5.201 " 276 659 Fatty acid-binding protein, adipocyte [Crassostrea gigas]; 426 809 Fatty acid-binding protein, adipocyte [Crassostrea gigas]; 277 660 Fatty acid-binding protein, adipocyte [Crassostrea gigas]; 283 666 Fatty acid-binding protein, adipocyte [Crassostrea gigas]; 270 653 Fatty acid-binding protein, adipocyte [Crassostrea gigas]; 283 666 Fatty acid-binding protein, adipocyte [Crassostrea gigas]; 258 641 Fatty acid-binding protein, adipocyte [Crassostrea gigas]; 427 810 Fatty acid-binding protein, adipocyte [Crassostrea gigas]; 277 660 Fatty acid-binding protein, adipocyte [Crassostrea gigas]"

Protein Accession	CL7666.Contig9$_{H}1$ CL7666.Contig8$_{H}1$ CL7666.Contig7$_{H}1$ CL7666.Contig6$_{H}1$ CL7666.Con
Mean Expression Ratio	1.15
Median Expression Ratio	1.15
Credible Interval	(0.897, 1.46)
Associated Peptides	5
Associated Spectra	16
Coverage	NaN

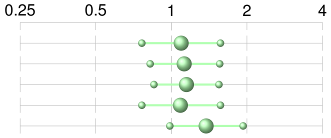

A	B	2.5	50	97.5	Sequence
1	1	0.76	1.1	1.6	AVLTEESGELVER
3	1	0.82	1.1	1.6	GVTATISFQK
4	1	0.85	1.1	1.5	LTTVAGDK
1	1	0.76	1.1	1.6	TVLAMER
1	2	0.98	1.4	1.9	AFVDGALK

5.202 " 116 1168 minus strand hypothetical protein CGI_10014553, partial [Crassostrea gigas]"

Protein Accession	CL19215.Contig5$_{H}1$
Mean Expression Ratio	1.15
Median Expression Ratio	1.15
Credible Interval	(0.824, 1.61)
Associated Peptides	4
Associated Spectra	5
Coverage	NaN

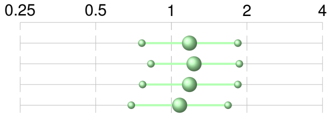

A	B	2.5	50	97.5	Sequence
1	0	0.76	1.2	1.8	SSFNDAVHSLFDK
1	1	0.83	1.2	1.9	SVIALGEGAGANIVAR
0	1	0.77	1.2	1.8	AAFIHVDVPGQEDDAK
0	1	0.69	1.1	1.7	VIENFQLSLR

5.203 613 2421 PREDICTED: lysosomal alpha-glucosidase [Danio rerio]

Protein Accession	CL5792.Contig1$_{H}$1
Mean Expression Ratio	1.15
Median Expression Ratio	1.15
Credible Interval	(0.732, 1.8)
Associated Peptides	1
Associated Spectra	1
Coverage	NaN

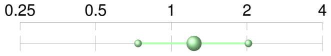

A	B	2.5	50	97.5	Sequence
**1**	**0**	**0.74**	**1.2**	**2**	**ATMGGLQK**

5.204 511 1062 unknown [Dendroctonus ponderosae]; 511 1062 unknown [Dendroctonus ponderosae]

Protein Accession	CL18443.Contig3$_{H}$1 CL18443.Contig1$_{H}$1
Mean Expression Ratio	0.873
Median Expression Ratio	0.873
Credible Interval	(0.609, 1.25)
Associated Peptides	2
Associated Spectra	4
Coverage	NaN

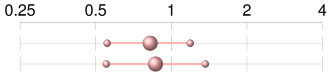

A	B	2.5	50	97.5	Sequence
**1**	**2**	**0.56**	**0.82**	**1.2**	**KHNFLIDGFPR**
**0**	**1**	**0.55**	**0.87**	**1.4**	**FKDEIEMHLK**

5.205 128 559 minus strand predicted protein [Nematostella vectensis] gi

Protein Accession	CL19343.Contig1$_{H}$1
Mean Expression Ratio	0.875
Median Expression Ratio	0.874
Credible Interval	(0.629, 1.22)
Associated Peptides	2
Associated Spectra	7
Coverage	NaN

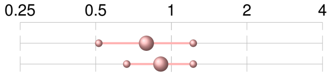

A	B	2.5	50	97.5	Sequence
**1**	**0**	**0.51**	**0.79**	**1.2**	**GIPCLILLNGEDASEISR**
**3**	**3**	**0.66**	**0.91**	**1.2**	**TVILGDLTGSK**

5.206 472 1551 minus strand hypothetical protein [Amblyomma maculatum]

Protein Accession	Unigene11523$_{H}$1
Mean Expression Ratio	0.873
Median Expression Ratio	0.874
Credible Interval	(0.715, 1.07)
Associated Peptides	7
Associated Spectra	30
Coverage	NaN

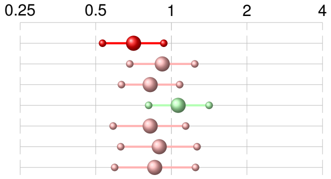

A	B	2.5	50	97.5	Sequence
**6**	**0**	**0.53**	**0.71**	**0.93**	**LFQNAQLSR**
**5**	**0**	**0.68**	**0.92**	**1.2**	**MVTGPVPGNLAVSK**
**7**	**0**	**0.63**	**0.83**	**1.1**	**QSLTDILR**
**4**	**2**	**0.81**	**1.1**	**1.4**	**VANGLYYDDTAVTLAEAFK**
**1**	**2**	**0.59**	**0.82**	**1.1**	**VLELPYGTDGR**
**2**	**0**	**0.63**	**0.89**	**1.3**	**SKQSLTDILR**
**0**	**1**	**0.59**	**0.86**	**1.2**	**FMVSLPK**

5.207 10 300 minus strand unnamed protein product [Tetraodon nigroviridis]

Protein Accession	CL14559.Contig2$_{H}1$
Mean Expression Ratio	1.14
Median Expression Ratio	1.14
Credible Interval	(0.783, 1.67)
Associated Peptides	1
Associated Spectra	4
Coverage	NaN

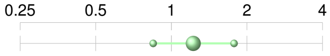

A	B	2.5	50	97.5	Sequence
**3**	**1**	**0.85**	**1.2**	**1.8**	**ILVQEGIHDR**

5.208 643 1302 minus strand Kyphoscoliosis peptidase [Crassostrea gigas]; 634 1293 minus strand Kyphoscoliosis peptidase [Crassostrea gigas]; 634 1293 minus strand Kyphoscoliosis peptidase [Crassostrea gigas]; 634 1293 minus strand Kyphoscoliosis peptidase [Crassostrea gigas]; 639 1298 minus strand Kyphoscoliosis peptidase [Crassostrea gigas]

Protein Accession	CL7772.Contig1$_{H}1$ CL7772.Contig16$_{H}1$ CL7772.Contig15$_{H}1$ CL7772.Contig14$_{H}1$ CL7772.C
Mean Expression Ratio	1.14
Median Expression Ratio	1.14
Credible Interval	(0.942, 1.4)
Associated Peptides	9
Associated Spectra	41
Coverage	NaN

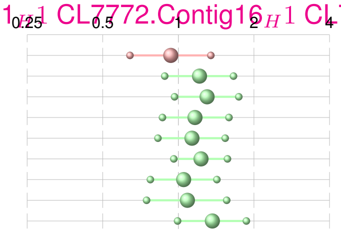

A	B	2.5	50	97.5	Sequence
**1**	**0**	**0.64**	**0.93**	**1.3**	**DGCYLYSPLVLNSSSR**
**2**	**2**	**0.88**	**1.2**	**1.7**	**FQIFALPTSDDSK**
**4**	**1**	**0.97**	**1.3**	**1.7**	**GAGEVWEGTAALDK**
**4**	**0**	**0.87**	**1.2**	**1.6**	**STTNLIQCR**
**3**	**1**	**0.83**	**1.1**	**1.5**	**VTLNACFGEDESK**
**7**	**6**	**0.96**	**1.2**	**1.6**	**LSNVTFK**
**3**	**2**	**0.77**	**1.1**	**1.4**	**QYAPWR**
**1**	**0**	**0.75**	**1.1**	**1.6**	**LELVSHPQAEFTLAQDNEVEIK**
**3**	**1**	**0.99**	**1.4**	**1.9**	**AVYPFPK**

5.209 238 681 minus strand Globin OS=Cerithidea rhizophorarum PE=1 SV=1

Protein Accession	CL8458.Contig2$_{H}1$
Mean Expression Ratio	1.14
Median Expression Ratio	1.14
Credible Interval	(0.952, 1.37)
Associated Peptides	8
Associated Spectra	116
Coverage	NaN

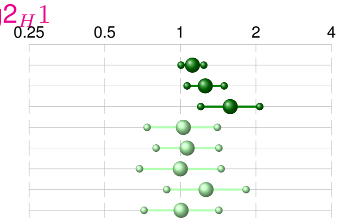

A	B	2.5	50	97.5	Sequence
**42**	**32**	**1**	**1.1**	**1.2**	**AAITSTWSK**
**15**	**9**	**1.1**	**1.3**	**1.5**	**AYFPFLPGASEGDIK**
**4**	**2**	**1.2**	**1.6**	**2.1**	**ILAMDDADCLDGLGTK**
**0**	**3**	**0.74**	**1**	**1.4**	**PFLPGASEGDIK**
**0**	**5**	**0.8**	**1.1**	**1.4**	**PGASEGDIK**
**0**	**1**	**0.69**	**1**	**1.5**	**ATMENNGAK**
**0**	**1**	**0.88**	**1.3**	**1.8**	**EVFGSFLEATL**
**0**	**2**	**0.71**	**1**	**1.4**	**EVFGSFLEATLGGAATPDVK**

5.210 " 28 1104 minus strand Pyruvate dehydrogenase E1 component subunit beta, mitochondrial [Crassostrea gigas]"

Protein Accession	CL18015.Contig14$_{H}1$
Mean Expression Ratio	1.14
Median Expression Ratio	1.14
Credible Interval	(0.767, 1.71)
Associated Peptides	2
Associated Spectra	2
Coverage	NaN

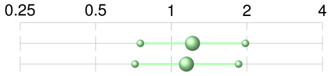

A	B	2.5	50	97.5	Sequence
1	0	0.75	1.2	2	GIECEVINLR
1	0	0.72	1.2	1.9	SLRPVDDETIIK

5.211 288 1487 minus strand putative procollagen type VI alpha 4 [Haliotis diversicolor]

Protein Accession	CL6899.Contig2$_{H}1$
Mean Expression Ratio	0.876
Median Expression Ratio	0.876
Credible Interval	(0.782, 0.982)
Associated Peptides	20
Associated Spectra	188
Coverage	NaN

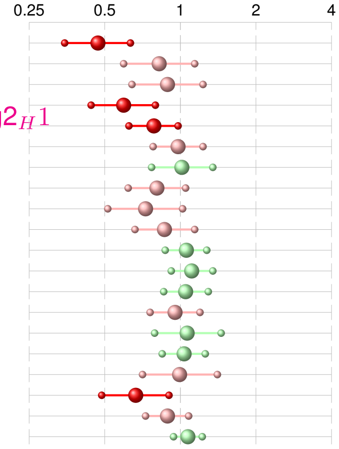

A	B	2.5	50	97.5	Sequence
4	0	0.35	0.47	0.63	AEVLAAIDQVR
2	0	0.6	0.82	1.1	ELTNQVAVLFLDDQALR
1	1	0.64	0.89	1.2	ENLYYIQSVVDR
1	3	0.44	0.59	0.79	MGFDVDQFR
5	5	0.62	0.78	0.98	TKAEVLAAIDQVR
8	2	0.78	0.98	1.2	VMVVITDGSSR
4	1	0.77	1	1.3	FDISVDDTR
3	3	0.62	0.81	1.1	FVVVDLGVR
1	0	0.51	0.73	1	LGNVVPQDENTYNNLLR
4	1	0.66	0.87	1.1	PDPTIGSCR
8	9	0.87	1.1	1.3	ALETLR
8	8	0.92	1.1	1.4	DLLPMR
9	5	0.86	1.1	1.3	FRDDITK
6	4	0.76	0.95	1.2	QVLQFVSQVITR
2	1	0.79	1.1	1.5	LRENLK
9	7	0.85	1	1.3	IGALTFSDDVK
1	0	0.71	0.99	1.4	FRALETLR
1	2	0.49	0.66	0.9	ADIINFASQSLAR
10	5	0.73	0.89	1.1	GANGVTLAQQVR
35	9	0.94	1.1	1.2	TGVTNTHLAIR

5.212 14 1642 minus strand chaperonin containing tcp1 [Haliotis discus discus]

Protein Accession	CL16298.Contig4$_{H}1$
Mean Expression Ratio	1.14
Median Expression Ratio	1.14
Credible Interval	(0.79, 1.65)
Associated Peptides	3
Associated Spectra	3
Coverage	NaN

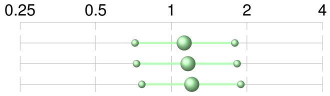

A	B	2.5	50	97.5	Sequence
1	0	0.71	1.1	1.8	ELEVQHPAAK
1	0	0.73	1.2	1.8	MVINHLEK
1	0	0.77	1.2	1.9	AIGATALPK

5.213 62 1174 Tyrosine-protein kinase CSK [Crassostrea gigas]; 62 1174 minus strand Tyrosine-protein kinase CSK [Crassostrea gigas]

Protein Accession	CL22249.Contig5$_H$1 CL22249.Contig3$_H$1
Mean Expression Ratio	0.877
Median Expression Ratio	0.878
Credible Interval	(0.581, 1.32)
Associated Peptides	1
Associated Spectra	2
Coverage	NaN

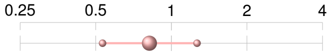

A	B	2.5	50	97.5	Sequence
0	2	0.53	0.82	1.3	IPLTDVVMHVER

5.214 211 1413 Alpha-N-acetylgalactosaminidase [Crassostrea gigas]

Protein Accession	CL5989.Contig1$_H$1
Mean Expression Ratio	1.14
Median Expression Ratio	1.14
Credible Interval	(0.791, 1.65)
Associated Peptides	2
Associated Spectra	4
Coverage	NaN

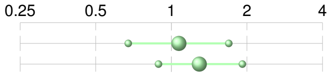

A	B	2.5	50	97.5	Sequence
1	0	0.68	1.1	1.7	ALADYMHDR
3	0	0.89	1.3	1.9	ALLLNR

5.215 118 1866 Lamin Dm0 [Crassostrea gigas]

Protein Accession	CL4079.Contig7$_H$1
Mean Expression Ratio	0.879
Median Expression Ratio	0.879
Credible Interval	(0.729, 1.06)
Associated Peptides	16
Associated Spectra	25
Coverage	NaN

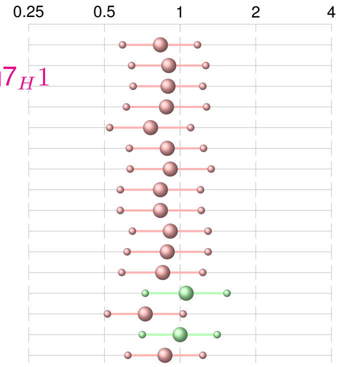

A	B	2.5	50	97.5	Sequence
1	1	0.59	0.83	1.2	ADDLSAELAK
1	1	0.64	0.9	1.3	GGIDIEEVDPEGK
2	1	0.65	0.89	1.2	KQLEEETLLR
1	0	0.61	0.88	1.3	LSVQVQSTEEVVK
1	0	0.52	0.76	1.1	RVETLEAQVAELTAK
1	1	0.63	0.89	1.2	WFVADEMK
1	0	0.63	0.92	1.3	SLVIKPGK
1	0	0.58	0.84	1.2	EDLHFK
1	0	0.58	0.84	1.2	YKTEYEDLLAK
1	1	0.65	0.91	1.3	EKHEFDLQTVR
0	1	0.62	0.89	1.3	LSEVEAPR
0	1	0.59	0.85	1.2	RLALKDDEIADLR
0	1	0.73	1.1	1.5	LLDETAKEK
0	2	0.51	0.73	1	IADLR
0	2	0.71	1	1.4	IRDLENQLAHER
0	2	0.62	0.87	1.2	LLEALR

5.216 166 2364 Dipeptidyl-peptidase 3 [Crassostrea gigas]

Protein Accession	CL9071.Contig2$_{H}$1
Mean Expression Ratio	1.14
Median Expression Ratio	1.14
Credible Interval	(0.79, 1.65)
Associated Peptides	3
Associated Spectra	3
Coverage	NaN

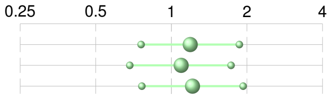

A	B	2.5	50	97.5	Sequence
1	0	0.76	1.2	1.9	SFETGSIPAHK
1	0	0.68	1.1	1.7	YVILR
1	0	0.76	1.2	1.9	VTGADGKP

5.217 636 1232 sigma class glutathione-s-transferase 3 [Haliotis discus discus]; 963 1559 sigma class glutathione-s-transferase 3 [Haliotis discus discus]; 907 1503 sigma class glutathione-s-transferase 3 [Haliotis discus discus]; 673 1269 sigma class glutathione-s-transferase 3 [Haliotis discus discus]; 177 773 sigma class glutathione-s-transferase 3 [Haliotis discus discus]; 208 804 sigma class glutathione-s-transferase 3 [Haliotis discus discus]; 327 923 sigma class glutathione-s-transferase 3 [Haliotis discus discus]

Protein Accession	Unigene22829$_{H}$1 CL19285.Contig31$_{H}$1 CL19285.Contig30$_{H}$1 CL19285.Contig27$_{H}$1 CL192
Mean Expression Ratio	0.879
Median Expression Ratio	0.879
Credible Interval	(0.588, 1.3)
Associated Peptides	2
Associated Spectra	2
Coverage	NaN

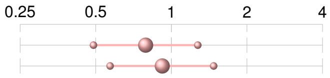

A	B	2.5	50	97.5	Sequence
1	0	0.49	0.79	1.3	LLLAASGKDWEDVR
0	1	0.57	0.92	1.5	YMNIFEK

5.218 " 242 1504 Glutamate receptor, ionotropic kainate 3 [Crassostrea gigas]; 239 1501 Glutamate receptor, ionotropic kainate 3 [Crassostrea gigas]; 238 1500 Glutamate receptor, ionotropic kainate 3 [Crassostrea gigas]; 661 1923 minus strand Glutamate receptor, ionotropic kainate 3 [Crassostrea gigas]"

Protein Accession	CL12478.Contig7$_{H}$1 CL12478.Contig6$_{H}$1 CL12478.Contig5$_{H}$1 CL12478.Contig3$_{H}$1
Mean Expression Ratio	1.14
Median Expression Ratio	1.14
Credible Interval	(0.785, 1.66)
Associated Peptides	2
Associated Spectra	3
Coverage	NaN

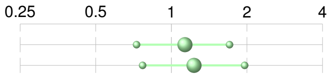

A	B	2.5	50	97.5	Sequence
2	0	0.73	1.1	1.7	RPTFAQPSFQER
1	0	0.77	1.2	2	LAILR

5.219 169 1161 Basement membrane-specific heparan sulfate proteoglycan core protein OS=Homo sapiens GN=HSPG2 PE=1 SV=4

Protein Accession	CL5208.Contig1$_{H}1$
Mean Expression Ratio	0.88
Median Expression Ratio	0.88
Credible Interval	(0.588, 1.32)
Associated Peptides	2
Associated Spectra	2
Coverage	NaN

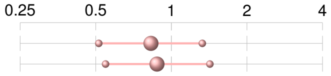

A	B	2.5	50	97.5	Sequence
1	0	0.51	0.83	1.3	GGDLTANHVVQGDVLR
1	0	0.55	0.88	1.4	PTITWSR

5.220 " 431 880 Myosin essential light chain, striated adductor muscle OS=Aequipecten irradians PE=1 SV=2"

Protein Accession	CL36.Contig2$_{H}1$
Mean Expression Ratio	0.881
Median Expression Ratio	0.88
Credible Interval	(0.719, 1.09)
Associated Peptides	3
Associated Spectra	175
Coverage	NaN

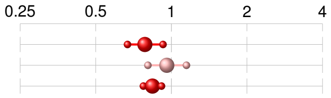

A	B	2.5	50	97.5	Sequence
12	16	0.67	0.79	0.93	LSDEEVDQIMK
15	9	0.81	0.96	1.1	LTDITIDLEGNLK
45	78	0.77	0.84	0.92	VGDLLR

5.221 " 34 1656 Chaperonin containing TCP1, subunit 7 (eta) [Danio rerio] gi; 34 1656 Chaperonin containing TCP1, subunit 7 (eta) [Danio rerio] gi"

Protein Accession	CL24061.Contig2$_{H}1$ CL24061.Contig1$_{H}1$
Mean Expression Ratio	0.883
Median Expression Ratio	0.881
Credible Interval	(0.588, 1.33)
Associated Peptides	1
Associated Spectra	2
Coverage	NaN

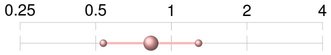

A	B	2.5	50	97.5	Sequence
2	0	0.53	0.83	1.3	SIAVSIKK

5.222 182 1846 minus strand hypothetical protein DAPPUDRAFT_302108 [Daphnia pulex]; 110 1774 minus strand hypothetical protein DAPPUDRAFT_302108 [Daphnia pulex]

Protein Accession	CL11561.Contig6$_{H}1$ CL11561.Contig1$_{H}1$
Mean Expression Ratio	1.14
Median Expression Ratio	1.14
Credible Interval	(0.785, 1.67)
Associated Peptides	3
Associated Spectra	3
Coverage	NaN

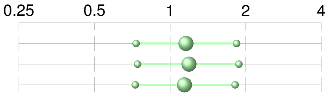

A	B	2.5	50	97.5	Sequence
**1**	**0**	**0.73**	**1.2**	**1.8**	**LRDAIRK**
**0**	**1**	**0.74**	**1.2**	**1.9**	**LFLPGGLK**
**0**	**1**	**0.73**	**1.1**	**1.8**	**LIIGQR**

5.223 9 887 minus strand hypothetical protein CGI_10006272 [Crassostrea gigas]

Protein Accession	CL46976.Contig1$_{H}1$
Mean Expression Ratio	0.88
Median Expression Ratio	0.881
Credible Interval	(0.592, 1.31)
Associated Peptides	1
Associated Spectra	3
Coverage	NaN

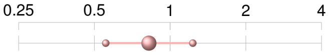

A	B	2.5	50	97.5	Sequence
**2**	**1**	**0.55**	**0.82**	**1.2**	**MILPQINK**

5.224 150 1511 Tensin OS=Gallus gallus GN=TNS PE=1 SV=2

Protein Accession	CL2743.Contig2$_{H}1$
Mean Expression Ratio	1.13
Median Expression Ratio	1.14
Credible Interval	(0.801, 1.61)
Associated Peptides	3
Associated Spectra	4
Coverage	NaN

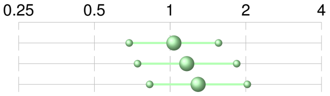

A	B	2.5	50	97.5	Sequence
**1**	**1**	**0.69**	**1**	**1.6**	**DDAIVMLR**
**1**	**0**	**0.74**	**1.2**	**1.8**	**DKPPGTFVIR**
**1**	**0**	**0.83**	**1.3**	**2**	**GTFVIR**

5.225 " 193 333 minus strand RecName: Full=Globin; AltName: Full=Myoglobin gi|299274|gb|AAB25743.1| myoglobin [Nassa mutabilis=marine gastropods, radular muscles, Peptide, 147 aa] gi; 193 333 minus strand RecName: Full=Globin; AltName: Full=Myoglobin gi|299274|gb|AAB25743.1| myoglobin [Nassa mutabilis=marine gastropods, radular muscles, Peptide, 147 aa] gi"

Protein Accession	CL8458.Contig5$_H$1 CL8458.Contig1$_H$1
Mean Expression Ratio	1.14
Median Expression Ratio	1.13
Credible Interval	(0.752, 1.7)
Associated Peptides	1
Associated Spectra	2
Coverage	NaN

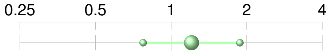

A	B	2.5	50	97.5	Sequence
**2**	**0**	**0.78**	**1.2**	**1.9**	**AAINSTWPK**

5.226 212 1336 6-phosphofructokinase [Crassostrea gigas]

Protein Accession	CL48438.Contig4$_H$1
Mean Expression Ratio	0.883
Median Expression Ratio	0.883
Credible Interval	(0.604, 1.29)
Associated Peptides	2
Associated Spectra	3
Coverage	NaN

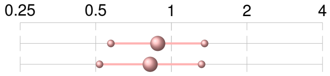

A	B	2.5	50	97.5	Sequence
**2**	**0**	**0.58**	**0.88**	**1.4**	**LGLTQGYR**
**1**	**0**	**0.52**	**0.83**	**1.3**	**SVQWFK**

5.227 219 428 Peptidyl-prolyl cis-trans isomerase OS=Hemicentrotus pulcherrimus PE=2 SV=1

Protein Accession	CL39270.Contig2$_H$1
Mean Expression Ratio	1.13
Median Expression Ratio	1.13
Credible Interval	(0.78, 1.64)
Associated Peptides	1
Associated Spectra	4
Coverage	NaN

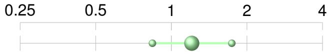

A	B	2.5	50	97.5	Sequence
**2**	**2**	**0.84**	**1.2**	**1.7**	**IIIANSGQV**

5.228 448 1626 matrilin [Biomphalaria glabrata]

Protein Accession	CL7510.Contig3$_H1$
Mean Expression Ratio	1.13
Median Expression Ratio	1.13
Credible Interval	(0.968, 1.32)
Associated Peptides	13
Associated Spectra	63
Coverage	NaN

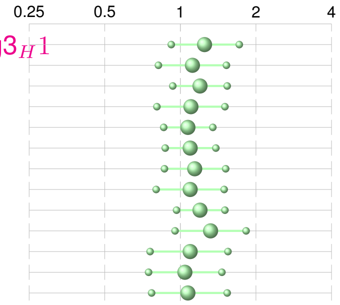

A	B	2.5	50	97.5	Sequence
2	1	0.92	1.3	1.7	EFLSMYDIGPAR
1	2	0.82	1.1	1.5	FAVVLTDGNSQESR
6	1	0.93	1.2	1.5	IDETSLNSAIDELVK
2	1	0.81	1.1	1.5	KTALVGFSDTR
5	6	0.86	1.1	1.3	TKEEVLEHVANMR
7	3	0.87	1.1	1.4	VFVVTTDSAAK
3	2	0.87	1.1	1.5	VIQVSSYDDLQK
3	0	0.8	1.1	1.5	VSNTELLNIAGDQER
6	6	0.97	1.2	1.5	DSLPGLVR
2	0	0.96	1.3	1.8	TALVGFSDTR
1	0	0.76	1.1	1.5	HEYGTFTDTGPGIDYMR
0	2	0.75	1	1.5	HKIDETSLNSAIDELVK
0	1	0.77	1.1	1.5	VSAIK

5.229 60 1022 Major egg antigen [Crassostrea gigas]; 60 1022 Major egg antigen [Crassostrea gigas]

Protein Accession	CL18744.Contig3$_H1$ CL18744.Contig2$_H1$
Mean Expression Ratio	0.885
Median Expression Ratio	0.885
Credible Interval	(0.684, 1.14)
Associated Peptides	6
Associated Spectra	13
Coverage	NaN

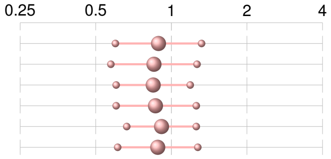

A	B	2.5	50	97.5	Sequence
1	0	0.6	0.89	1.3	QLTIEAPLKPATVQQR
1	0	0.57	0.85	1.3	TAIEVDSEGR
1	2	0.6	0.85	1.2	SEVHIPVQK
2	0	0.6	0.86	1.3	FDVSEFKPEEVSVK
1	3	0.67	0.91	1.3	ETFLPVR
0	2	0.61	0.88	1.3	LTVDIGEFSPDEIVVK

5.230 " 154 534 minus strand Muscle M-line assembly protein unc-89, partial [Crassostrea gigas]; 259 690 minus strand Myosin light chain kinase, smooth muscle OS=Gallus gallus PE=1 SV=2"

Protein Accession	Unigene64523$_H1$ Unigene64520$_H1$
Mean Expression Ratio	1.13
Median Expression Ratio	1.13
Credible Interval	(0.751, 1.71)
Associated Peptides	1
Associated Spectra	3
Coverage	NaN

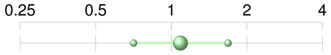

A	B	2.5	50	97.5	Sequence
2	1	0.71	1.1	1.7	LEIGEVVVDDEGEYICK

5.231 119 481 Glutamate receptor U1 [Crassostrea gigas]

Protein Accession	CL570.Contig5$_H$1
Mean Expression Ratio	1.13
Median Expression Ratio	1.13
Credible Interval	(0.933, 1.37)
Associated Peptides	7
Associated Spectra	49
Coverage	NaN

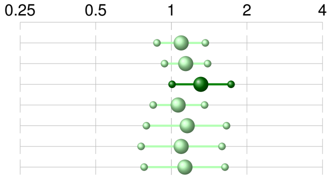

A	B	2.5	50	97.5	Sequence
9	3	0.88	1.1	1.4	GTANIGAGALTVTSER
10	7	0.94	1.1	1.4	RTPDGWSGIIGDVVK
6	1	1	1.3	1.7	TPDGWSGIIGDVVK
7	3	0.84	1.1	1.4	FQYTFR
0	1	0.8	1.2	1.7	AEVVK
0	1	0.76	1.1	1.6	GAGALTVTSER
0	1	0.78	1.1	1.6	GTANIGAGALTVT

5.232 718 1155 Annexin A7 [Crassostrea gigas]

Protein Accession	CL15539.Contig2$_H$1
Mean Expression Ratio	1.13
Median Expression Ratio	1.13
Credible Interval	(0.871, 1.47)
Associated Peptides	5
Associated Spectra	12
Coverage	NaN

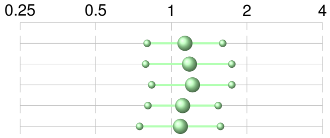

A	B	2.5	50	97.5	Sequence
3	0	0.8	1.1	1.6	ATFDAYQQIAGK
1	0	0.79	1.2	1.7	DIESAIK
2	0	0.84	1.2	1.7	KLGTDEETFNR
4	0	0.81	1.1	1.5	NVPGYFAQR
2	0	0.75	1.1	1.6	SFPQLR

5.233 116 1882 Actin-interacting protein 1 [Crassostrea gigas]

Protein Accession	CL12416.Contig1$_H$1
Mean Expression Ratio	0.887
Median Expression Ratio	0.886
Credible Interval	(0.703, 1.11)
Associated Peptides	9
Associated Spectra	17
Coverage	NaN

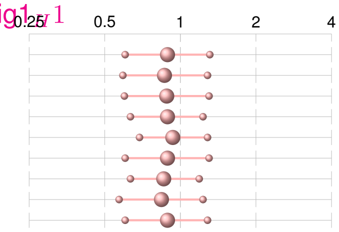

A	B	2.5	50	97.5	Sequence
1	0	0.6	0.89	1.3	FSPDGEVAVSGGADGK
1	0	0.59	0.87	1.3	ILTASEDNSVGFHEGPPFK
1	0	0.6	0.88	1.3	LVSIGVDDVVR
1	2	0.63	0.89	1.2	QFLDGEVK
3	1	0.69	0.93	1.3	TLDELDNKEIGAPGAHK
1	0	0.6	0.89	1.3	VFDVQSGSLVQEFK
4	0	0.63	0.86	1.2	TNEVTDMVIEGDR
1	0	0.57	0.84	1.2	KLESQPK
0	1	0.6	0.89	1.3	QILTASGDASAK

5.234 1292 1471 minus strand hemocyanin isoform 1 [Haliotis diversicolor]

Protein Accession	Unigene65233$_H$1
Mean Expression Ratio	0.885
Median Expression Ratio	0.886
Credible Interval	(0.591, 1.34)
Associated Peptides	1
Associated Spectra	2
Coverage	NaN

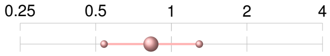

A	B	2.5	50	97.5	Sequence
1	1	0.54	0.83	1.3	LYTVQFEDALR

5.235 225 839 minus strand Metallo-beta-lactamase domain-containing protein 1 [Crassostrea gigas]; 201 851 minus strand Metallo-beta-lactamase domain-containing protein 1 [Crassostrea gigas]

Protein Accession	CL35672.Contig2$_H$1 CL35672.Contig1$_H$1
Mean Expression Ratio	0.883
Median Expression Ratio	0.886
Credible Interval	(0.583, 1.33)
Associated Peptides	1
Associated Spectra	2
Coverage	NaN

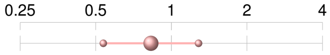

A	B	2.5	50	97.5	Sequence
1	1	0.53	0.83	1.3	EADFIVPGHGPMFR

5.236 113 1402 minus strand S-adenosylhomocysteine hydrolase [Crassostrea ariakensis]; 113 1402 minus strand S-adenosylhomocysteine hydrolase [Crassostrea ariakensis]

Protein Accession	CL7405.Contig2$_H$1 CL7405.Contig1$_H$1
Mean Expression Ratio	0.886
Median Expression Ratio	0.887
Credible Interval	(0.592, 1.33)
Associated Peptides	2
Associated Spectra	2
Coverage	NaN

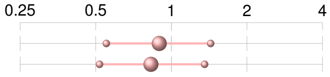

A	B	2.5	50	97.5	Sequence
1	0	0.55	0.89	1.4	GLSEETTTGVHNLYR
1	0	0.52	0.83	1.4	LKVPAINVNDSVTK

5.237 " 134 2047 PREDICTED: succinate dehydrogenase [ubiquinone] flavoprotein subunit, mitochondrial-like isoform 3, partial [Strongylocentrotus purpuratus]; 134 2047 PREDICTED: succinate dehydrogenase [ubiquinone] flavoprotein subunit, mitochondrial-like isoform 3, partial [Strongylocentrotus purpuratus]; 134 2047 PREDICTED: succinate dehydrogenase [ubiquinone] flavoprotein subunit, mitochondrial-like isoform 3, partial [Strongylocentrotus purpuratus]"

Protein Accession	CL47322.Contig4$_H$1 CL47322.Contig3$_H$1 CL47322.Contig2$_H$1
Mean Expression Ratio	0.887
Median Expression Ratio	0.888
Credible Interval	(0.744, 1.06)
Associated Peptides	14
Associated Spectra	42
Coverage	NaN

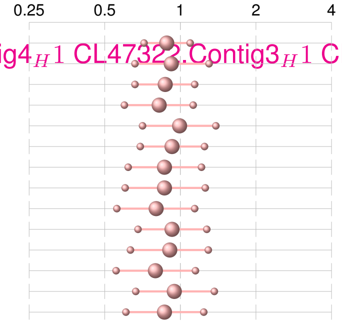

A	B	2.5	50	97.5	Sequence
10	4	0.72	0.89	1.1	AAFGLAEEGFK
1	1	0.66	0.92	1.3	ACANTIIEENKPGDR
3	3	0.66	0.87	1.1	AITIEIR
1	2	0.6	0.82	1.1	IYQQMEDLK
2	0	0.71	0.99	1.4	KHTLAYTNVDTGK
4	0	0.69	0.93	1.3	NTILATGGYGR
1	1	0.62	0.87	1.2	QDAGEASVGNLDK
1	0	0.6	0.87	1.3	SHTVAAQGGINAALGHMEEDDWR
1	0	0.56	0.8	1.1	AVIELENYGMPF
3	0	0.68	0.93	1.3	AFGGQSLK
1	0	0.63	0.91	1.3	GGINAAL
0	1	0.56	0.8	1.1	AVIELENYGMPFSR
0	1	0.67	0.95	1.4	LPPIR
0	1	0.61	0.86	1.2	WHFYDTVK

5.238 " 4146 6107 minus strand nonmuscle myosin II, partial [Aplysia californica]"

Protein Accession	Unigene30363$_{H}1$
Mean Expression Ratio	0.887
Median Expression Ratio	0.888
Credible Interval	(0.743, 1.06)
Associated Peptides	19
Associated Spectra	31
Coverage	NaN

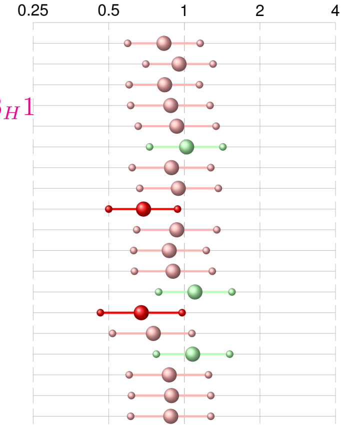

A	B	2.5	50	97.5	Sequence
2	0	0.59	0.83	1.2	ALEATVQEQR
2	2	0.7	0.95	1.3	ATIAALEGK
2	1	0.6	0.83	1.1	DLEQVSTQLEQVETK
1	0	0.61	0.88	1.3	ELDDQLEQNDVLVR
1	0	0.65	0.93	1.3	LAELEGQSR
1	1	0.73	1	1.4	LQEDLAAAER
1	0	0.62	0.89	1.3	QVDEAEEEVAR
1	0	0.66	0.95	1.4	VSSLEAQLADSQELMEEETR
2	1	0.5	0.69	0.94	VQIEELEDELQAAEDAK
1	0	0.64	0.93	1.3	EMEAELEEER
1	1	0.63	0.87	1.2	NLEAELVK
1	0	0.63	0.9	1.3	QSLLDEK
2	0	0.79	1.1	1.5	LKEDAIK
1	0	0.46	0.68	0.98	LSREEAYAQAK
1	0	0.52	0.75	1.1	LQAEVEDLTITLDNQR
1	1	0.77	1.1	1.5	AALARTNR
0	1	0.6	0.87	1.3	LKELQIQAEDERR
0	1	0.62	0.89	1.3	EMEQLHAQLEEARANADR
0	1	0.61	0.88	1.3	IEQLER

5.239 193 828 Cysteine-rich secretory protein LCCL domain-containing 2 [Crassostrea gigas]

Protein Accession	CL40761.Contig2$_{H}1$
Mean Expression Ratio	1.13
Median Expression Ratio	1.13
Credible Interval	(0.774, 1.64)
Associated Peptides	3
Associated Spectra	3
Coverage	NaN

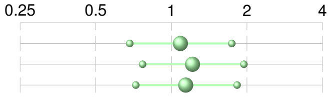

A	B	2.5	50	97.5	Sequence
1	0	0.68	1.1	1.7	LEWDAQMESEATSWAAR
1	0	0.77	1.2	1.9	YSPEGNWVNEKPYEK
1	0	0.72	1.1	1.8	ICQPLR

5.240 503 1090 minus strand myosin regulatory light chain [Fasciola hepatica]; 278 865 minus strand myosin regulatory light chain [Fasciola hepatica]

Protein Accession	CL43006.Contig6$_{H}1$ CL43006.Contig4$_{H}1$
Mean Expression Ratio	1.13
Median Expression Ratio	1.13
Credible Interval	(0.823, 1.54)
Associated Peptides	1
Associated Spectra	25
Coverage	NaN

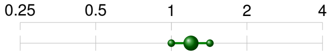

A	B	2.5	50	97.5	Sequence
16	9	1	1.2	1.4	QAQVQEFK

5.241 2831 4900 minus strand Collagen alpha-3(VI) chain [Crassostrea gigas]

Protein Accession	Unigene26542$_{H1}$
Mean Expression Ratio	0.888
Median Expression Ratio	0.889
Credible Interval	(0.601, 1.31)
Associated Peptides	1
Associated Spectra	3
Coverage	NaN

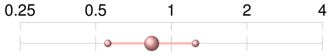

A	B	2.5	50	97.5	Sequence
3	0	0.56	0.83	1.3	TEMFTPANGDRPDVPNVAIVITDGVSNINSR

5.242 " 1 1563 Putative methylmalonate-semialdehyde dehydrogenase [acylating], mitochondrial [Crassostrea gigas]"

Protein Accession	Unigene56839$_{H1}$
Mean Expression Ratio	1.13
Median Expression Ratio	1.13
Credible Interval	(0.839, 1.51)
Associated Peptides	6
Associated Spectra	7
Coverage	NaN

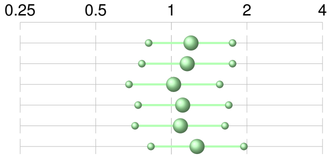

A	B	2.5	50	97.5	Sequence
1	1	0.81	1.2	1.8	AEMDAAVAAAK
1	0	0.76	1.2	1.7	GNFVGPTILHK
1	0	0.68	1	1.6	LFINNEFVESK
1	0	0.74	1.1	1.7	WIDVHNPATNEVVTR
1	0	0.72	1.1	1.6	TVTQMWR
0	1	0.83	1.3	1.9	TLVDAEGDVLR

5.243 63 443 PREDICTED: RAS protein activator like 1-like [Saccoglossus kowalevskii]

Protein Accession	CL19305.Contig2$_{H1}$
Mean Expression Ratio	0.889
Median Expression Ratio	0.889
Credible Interval	(0.704, 1.12)
Associated Peptides	4
Associated Spectra	40
Coverage	NaN

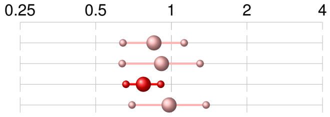

A	B	2.5	50	97.5	Sequence
2	4	0.64	0.85	1.1	DYAQAADGALASVLAR
1	1	0.64	0.91	1.3	YLEDWIK
16	13	0.66	0.78	0.91	LASVLAR
2	1	0.7	0.98	1.4	ALNIQLTK

5.244 1 825 Spectrin alpha chain [Crassostrea gigas]

Protein Accession	CL10282.Contig3$_{H1}$
Mean Expression Ratio	0.889
Median Expression Ratio	0.89
Credible Interval	(0.643, 1.23)
Associated Peptides	3
Associated Spectra	6
Coverage	NaN

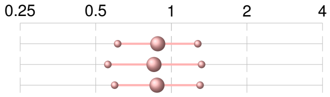

A	B	2.5	50	97.5	Sequence
2	1	0.61	0.88	1.3	MQHNLEQQIEAR
1	0	0.56	0.85	1.3	IEDLGAAMEER
0	2	0.6	0.88	1.3	LILDNR

5.245 68 667 minus strand hypothetical protein BRAFLDRAFT_272416 [Branchiostoma floridae] gi; 68 667 minus strand hypothetical protein BRAFLDRAFT_272416 [Branchiostoma floridae] gi

Protein Accession	CL37759.Contig4$_{H}1$ CL37759.Contig1$_{H}1$
Mean Expression Ratio	0.892
Median Expression Ratio	0.891
Credible Interval	(0.576, 1.39)
Associated Peptides	1
Associated Spectra	1
Coverage	NaN

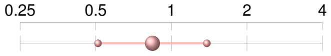

A	B	2.5	50	97.5	Sequence
**0**	**1**	**0.51**	**0.84**	**1.4**	**VLLQLR**

5.246 2 169 cAMP-dependent protein kinase regulatory subunit [Crassostrea gigas]; 125 511 cAMP-dependent protein kinase regulatory subunit [Aplysia californica] gi

Protein Accession	CL24422.Contig2$_{H}1$ CL24422.Contig1$_{H}1$
Mean Expression Ratio	1.12
Median Expression Ratio	1.12
Credible Interval	(0.729, 1.74)
Associated Peptides	1
Associated Spectra	1
Coverage	NaN

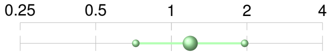

A	B	2.5	50	97.5	Sequence
**0**	**1**	**0.72**	**1.2**	**2**	**VLGPCSDILK**

5.247 66 263 minus strand twitchin [Mytilus galloprovincialis]; 66 263 minus strand twitchin [Mytilus galloprovincialis]; 66 263 minus strand twitchin [Mytilus galloprovincialis]; 66 263 minus strand twitchin [Mytilus galloprovincialis]; 66 263 minus strand twitchin [Mytilus galloprovincialis]

Protein Accession	Unigene64526$_{H}1$ Unigene64525$_{H}1$ Unigene64524$_{H}1$ Unigene64522$_{H}1$ Unigene64521$_{H}1$
Mean Expression Ratio	0.891
Median Expression Ratio	0.892
Credible Interval	(0.606, 1.32)
Associated Peptides	1
Associated Spectra	3
Coverage	NaN

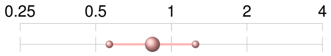

A	B	2.5	50	97.5	Sequence
**2**	**1**	**0.56**	**0.84**	**1.2**	**FTETLSPVEVMQGDK**

5.248 109 510 minus strand hypothetical protein DAPPUDRAFT_231281 [Daphnia pulex]

Protein Accession	Unigene4945$_{H}$1
Mean Expression Ratio	0.892
Median Expression Ratio	0.892
Credible Interval	(0.666, 1.19)
Associated Peptides	4
Associated Spectra	9
Coverage	NaN

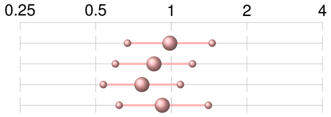

A	B	2.5	50	97.5	Sequence
1	1	0.67	0.99	1.4	LAGIEDPFEMNVK
2	1	0.6	0.85	1.2	VSMPDTLGHAVGIER
2	1	0.54	0.76	1.1	YELLAK
0	1	0.62	0.92	1.4	PDTLGHAVGIER

5.249 622 2733 PREDICTED: protein-glutamine gamma-glutamyltransferase K isoform 1 [Danio rerio]; 423 2498 PREDICTED: protein-glutamine gamma-glutamyltransferase K isoform 1 [Danio rerio]; 423 2534 PREDICTED: protein-glutamine gamma-glutamyltransferase K isoform 1 [Danio rerio]

Protein Accession	Unigene4042$_{H}$1 CL5652.Contig2$_{H}$1 CL5652.Contig1$_{H}$1
Mean Expression Ratio	0.894
Median Expression Ratio	0.893
Credible Interval	(0.688, 1.16)
Associated Peptides	6
Associated Spectra	12
Coverage	NaN

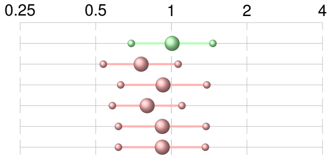

A	B	2.5	50	97.5	Sequence
1	1	0.69	1	1.5	LVFGIGEDPK
2	1	0.54	0.76	1.1	ILDMSGLK
1	0	0.63	0.93	1.4	GTQVVFILSAPDEK
2	2	0.58	0.8	1.1	KPHLQIK
1	0	0.62	0.92	1.4	GNPIPVVR
0	1	0.62	0.92	1.4	DVVFELR

5.250 523 1086 minus strand juvenile hormone diol kinase [Danaus plexippus]

Protein Accession	CL7590.Contig5$_{H}$1
Mean Expression Ratio	0.893
Median Expression Ratio	0.893
Credible Interval	(0.599, 1.35)
Associated Peptides	1
Associated Spectra	2
Coverage	NaN

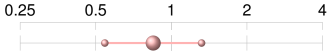

A	B	2.5	50	97.5	Sequence
2	0	0.54	0.84	1.3	GEFVTVQTIFGNSVEDSHK

5.251 4 444 Vinculin [Crassostrea gigas]

Protein Accession	Unigene21732$_{H}1$
Mean Expression Ratio	0.894
Median Expression Ratio	0.893
Credible Interval	(0.659, 1.2)
Associated Peptides	4
Associated Spectra	9
Coverage	NaN

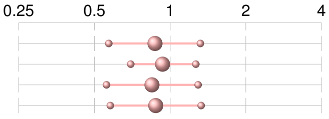

A	B	2.5	50	97.5	Sequence
**1**	**0**	**0.57**	**0.87**	**1.3**	**AIAEASEEVTR**
**4**	**2**	**0.7**	**0.93**	**1.3**	**IPTIGTQLK**
**1**	**0**	**0.56**	**0.85**	**1.3**	**TNLLNVCER**
**1**	**0**	**0.58**	**0.87**	**1.3**	**DNEIISAAK**

5.252 981 1379 minus strand Multimerin-1 [Crassostrea gigas]; 998 1396 Multimerin-1 [Crassostrea gigas]; 975 1373 Multimerin-1 [Crassostrea gigas]; 960 1358 Multimerin-1 [Crassostrea gigas]; 810 1208 Multimerin-1 [Crassostrea gigas]

Protein Accession	CL22447.Contig9$_{H}1$ CL22447.Contig7$_{H}1$ CL22447.Contig6$_{H}1$ CL22447.Contig5$_{H}1$ CL2244
Mean Expression Ratio	0.893
Median Expression Ratio	0.893
Credible Interval	(0.648, 1.22)
Associated Peptides	3
Associated Spectra	7
Coverage	NaN

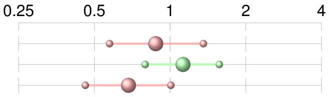

A	B	2.5	50	97.5	Sequence
**1**	**0**	**0.57**	**0.87**	**1.4**	**TNTEFHTGPIVFDTTLSNK**
**2**	**2**	**0.8**	**1.1**	**1.6**	**VDAVLVADGQDVAR**
**0**	**2**	**0.46**	**0.68**	**1**	**VSFSAILK**

5.253 148 609 minus strand Titin [Crassostrea gigas]

Protein Accession	CL5825.Contig1$_{H}1$
Mean Expression Ratio	1.12
Median Expression Ratio	1.12
Credible Interval	(0.81, 1.56)
Associated Peptides	2
Associated Spectra	6
Coverage	NaN

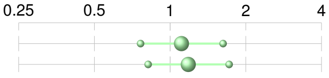

A	B	2.5	50	97.5	Sequence
**3**	**0**	**0.76**	**1.1**	**1.6**	**FTQKPALR**
**3**	**0**	**0.82**	**1.2**	**1.7**	**QQEGVAPNFIQKPVTR**

5.254 155 3214 sarco/endoplasmic reticulum calcium ATPase isoform B [Pinctada fucata]; 132 3110 sarco/endoplasmic reticulum calcium ATPase isoform C [Pinctada fucata]; 132 3110 minus strand sarco/endoplasmic reticulum calcium ATPase isoform C [Pinctada fucata]; 155 3133 sarco/endoplasmic reticulum calcium ATPase isoform C [Pinctada fucata]; 155 3133 sarco/endoplasmic reticulum calcium ATPase isoform C [Pinctada fucata]; 155 3214 minus strand sarco/endoplasmic reticulum calcium ATPase isoform B [Pinctada fucata]

Protein Accession	CL1323.Contig9$_H$1 CL1323.Contig8$_H$1 CL1323.Contig6$_H$1 CL1323.Contig21$_H$1 CL1323.Co
Mean Expression Ratio	0.895
Median Expression Ratio	0.895
Credible Interval	(0.771, 1.03)
Associated Peptides	25
Associated Spectra	73
Coverage	NaN

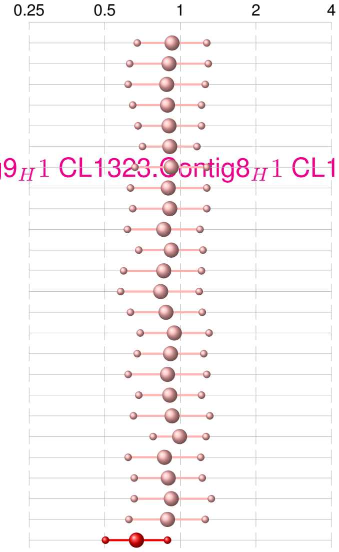

A	B	2.5	50	97.5	Sequence
1	2	0.68	0.93	1.3	ATAEAICR
1	0	0.63	0.9	1.3	DLVPGDIVEVSVGDK
1	0	0.62	0.88	1.3	DLVPGDIVEVSVGDKVPADIR
2	1	0.64	0.89	1.2	GAPEGLLDR
3	2	0.68	0.9	1.2	GIVVGTGLATEIGK
7	1	0.71	0.91	1.2	IGHNKVPMSPAIK
2	0	0.66	0.92	1.3	IGIFTENESTEGMSFTGR
1	0	0.63	0.9	1.3	IRNEMMDTEQEK
1	1	0.65	0.91	1.3	KEFTLEFSR
1	1	0.61	0.86	1.2	NAESAIEALK
2	3	0.68	0.92	1.2	NMLFSGTNIAAGK
1	0	0.6	0.86	1.2	SMSVYCVPNKPTR
1	0	0.58	0.83	1.2	TGTLTTNQMSVCK
2	0	0.63	0.87	1.2	VDQAILTGESVSVMK
3	0	0.69	0.95	1.3	VGEATETALTVLVEK
2	2	0.67	0.92	1.2	VIVITGDNK
1	0	0.62	0.89	1.3	VPMSPAIK
3	2	0.68	0.9	1.2	EFDELPPEDQR
1	0	0.65	0.92	1.3	NAESAIEALKEYEPEVAK
7	2	0.78	0.99	1.3	KAEIGIAMGSGTAVAK
1	1	0.62	0.86	1.2	LNFYNTDK
2	1	0.66	0.89	1.2	MDQAFTK
1	0	0.65	0.92	1.3	KNFEK
1	0	0.63	0.89	1.3	KNMLFSGTNIAAGK
3	3	0.5	0.67	0.89	CLALATIDNPPR

5.255 201 632 USP-like protein isoform 1 [Crassostrea ariakensis] gi; 189 620 minus strand USP-like protein isoform 1 [Crassostrea ariakensis] gi

Protein Accession	Unigene39588$_H$1 Unigene20286$_H$1
Mean Expression Ratio	1.12
Median Expression Ratio	1.12
Credible Interval	(0.717, 1.75)
Associated Peptides	1
Associated Spectra	1
Coverage	NaN

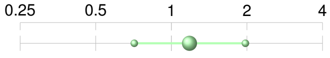

A	B	2.5	50	97.5	Sequence
1	0	0.71	1.2	2	DPWHAIVDAAK

5.256 628 1140 paramyosin [Haliotis discus discus]

Protein Accession	CL43455.Contig3$_H$1
Mean Expression Ratio	0.897
Median Expression Ratio	0.897
Credible Interval	(0.671, 1.19)
Associated Peptides	2
Associated Spectra	43
Coverage	NaN

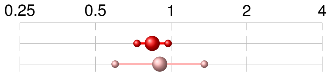

A	B	2.5	50	97.5	Sequence
**22**	**20**	**0.73**	**0.84**	**0.97**	**LEEAEAFAVK**
**0**	**1**	**0.6**	**0.9**	**1.4**	**LEEAEAF**

5.257 43 591 minus strand cyclophilin type peptidyl-prolyl cis-trans isomerase [Glossina morsitans morsitans]

Protein Accession	CL39270.Contig1$_H$1
Mean Expression Ratio	1.11
Median Expression Ratio	1.11
Credible Interval	(0.894, 1.38)
Associated Peptides	5
Associated Spectra	35
Coverage	NaN

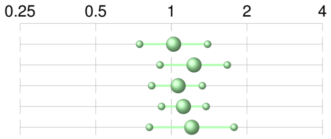

A	B	2.5	50	97.5	Sequence
**1**	**3**	**0.75**	**1**	**1.4**	**VFFDMTADGVPLGR**
**4**	**0**	**0.9**	**1.2**	**1.7**	**KIESYGSKNGATSQK**
**7**	**4**	**0.84**	**1.1**	**1.3**	**FADENFTLK**
**7**	**8**	**0.91**	**1.1**	**1.4**	**ITMELR**
**0**	**1**	**0.82**	**1.2**	**1.8**	**VIPQFMCQGGDFTR**

5.258 132 590 minus strand Non-neuronal cytoplasmic intermediate filament protein OS=Helix aspersa PE=3 SV=2

Protein Accession	Unigene7770$_H$1
Mean Expression Ratio	1.11
Median Expression Ratio	1.11
Credible Interval	(0.752, 1.65)
Associated Peptides	1
Associated Spectra	3
Coverage	NaN

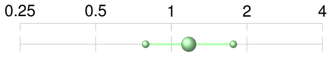

A	B	2.5	50	97.5	Sequence
**3**	**0**	**0.79**	**1.2**	**1.8**	**TMAEVPLGEK**

5.259 124 396 minus strand 40S ribosomal protein S10 OS=Lumbricus rubellus GN=RPS10 PE=2 SV=1

Protein Accession	Unigene42157$_H$1
Mean Expression Ratio	1.12
Median Expression Ratio	1.11
Credible Interval	(0.794, 1.58)
Associated Peptides	2
Associated Spectra	6
Coverage	NaN

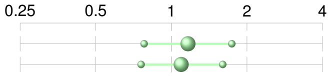

A	B	2.5	50	97.5	Sequence
**2**	**1**	**0.78**	**1.2**	**1.7**	**KDFFSPK**
**3**	**0**	**0.76**	**1.1**	**1.6**	**YLRER**

5.260 2764 3420 PREDICTED: filamin-C-like [Nasonia vitripennis]

Protein Accession	CL4145.Contig1$_H$1
Mean Expression Ratio	1.11
Median Expression Ratio	1.11
Credible Interval	(0.75, 1.64)
Associated Peptides	1
Associated Spectra	3
Coverage	NaN

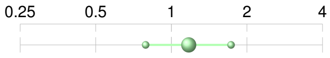

A	B	2.5	50	97.5	Sequence
2	1	0.79	1.2	1.7	GVDVPVNTIDNK

5.261 " 519 3182 minus strand Alpha-actinin, sarcomeric [Crassostrea gigas]"

Protein Accession	CL28960.Contig2$_H$1
Mean Expression Ratio	0.899
Median Expression Ratio	0.899
Credible Interval	(0.806, 1)
Associated Peptides	41
Associated Spectra	117
Coverage	NaN

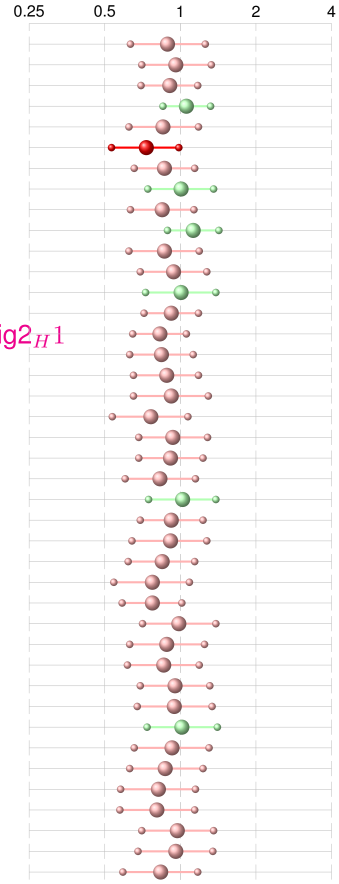

A	B	2.5	50	97.5	Sequence
1	0	0.63	0.89	1.3	AAPFNNWMDGAK
1	1	0.7	0.96	1.3	AGTGIENIEEDFR
5	1	0.7	0.91	1.2	AIIQYKPHIDELER
8	3	0.85	1.1	1.3	ALDFIASK
1	1	0.62	0.85	1.2	DNPIENLNTAFDIAEK
2	1	0.53	0.73	0.99	EALEEAER
3	2	0.65	0.87	1.1	ETADTDTAEQVMQSFK
2	1	0.74	1	1.4	FAIQDITVEELTAK
2	2	0.63	0.85	1.1	GISEDQMNEFR
6	3	0.89	1.1	1.4	IDQLHLEFAK
1	1	0.62	0.86	1.2	KAGTGIENIEEDFR
2	1	0.69	0.94	1.3	LSNRPAYLPTEGK
2	0	0.73	1	1.4	LVSIGAEEIVDGNVK
3	4	0.72	0.92	1.2	MSFNHFDK
3	5	0.65	0.83	1.1	MVSDIANAWK
2	2	0.63	0.84	1.1	NINEVENQILTR
2	1	0.65	0.88	1.2	RDQTLQQEMMR
1	0	0.65	0.92	1.3	GSLEEQLKK
1	0	0.54	0.76	1.1	HKPPKLEDK
2	0	0.68	0.94	1.3	MLDPEDMVNSAKPDER
2	1	0.68	0.91	1.2	LMEEYER
2	0	0.6	0.83	1.1	TRPWLENR
3	0	0.75	1	1.4	LQAFDK
2	2	0.69	0.92	1.2	KLDEFR
1	0	0.64	0.91	1.3	EDMLQSADFK
2	1	0.62	0.85	1.1	LGGQLTVK
1	0	0.54	0.77	1.1	PHIDELER
2	3	0.59	0.77	1	LDEFR
1	0	0.7	0.99	1.4	HLDIPR
1	0	0.63	0.89	1.2	PGALDYMSFSTAL
1	0	0.61	0.86	1.2	DGLAFCALIHR
2	0	0.69	0.95	1.3	HRPELIDYHNLSK
1	0	0.68	0.95	1.3	KLQAFDK
2	0	0.74	1	1.4	LDHLAQK
1	0	0.65	0.92	1.3	YTQYTMETLR
0	1	0.63	0.87	1.2	EGLLDPAWEK
0	1	0.58	0.82	1.1	LENSFNTLQTR
0	1	0.57	0.81	1.1	KHKPPKLEDK
0	2	0.7	0.98	1.4	ELVPR
0	1	0.68	0.96	1.3	DQLHLEFAK
0	1	0.59	0.83	1.2	SIGVQMR

5.262 201 779 minus strand sigma class glutathione-s-transferase 2 [Haliotis discus discus]

Protein Accession	Unigene22998$_H1$
Mean Expression Ratio	0.901
Median Expression Ratio	0.9
Credible Interval	(0.667, 1.21)
Associated Peptides	3
Associated Spectra	12
Coverage	NaN

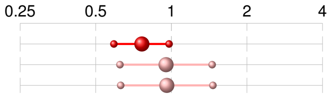

A	B	2.5	50	97.5	Sequence
**4**	**6**	**0.59**	**0.76**	**0.98**	**LVLALADQK**
**1**	**0**	**0.62**	**0.95**	**1.5**	**KLEEEEFPK**
**0**	**1**	**0.63**	**0.96**	**1.5**	**YFGMFQK**

5.263 243 2165 minus strand Elongation factor 2 OS=Drosophila melanogaster GN=Ef2b PE=2 SV=4

Protein Accession	Unigene11527$_H1$
Mean Expression Ratio	0.898
Median Expression Ratio	0.9
Credible Interval	(0.599, 1.34)
Associated Peptides	2
Associated Spectra	2
Coverage	NaN

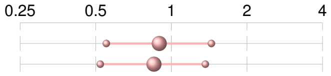

A	B	2.5	50	97.5	Sequence
**0**	**1**	**0.55**	**0.9**	**1.4**	**TQEDTLALADK**
**0**	**1**	**0.52**	**0.85**	**1.4**	**FNMEEVK**

5.264 59 379 hypothetical protein DAPPUDRAFT_230756 [Daphnia pulex]; 117 443 minus strand hypothetical protein DAPPUDRAFT_230756 [Daphnia pulex]

Protein Accession	CL2137.Contig6$_H1$ CL2137.Contig5$_H1$
Mean Expression Ratio	1.11
Median Expression Ratio	1.11
Credible Interval	(0.705, 1.73)
Associated Peptides	1
Associated Spectra	1
Coverage	NaN

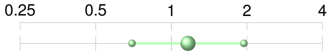

A	B	2.5	50	97.5	Sequence
**1**	**0**	**0.7**	**1.2**	**2**	**YAQPRLK**

5.265 127 657 ADP ribosylation factor 4 [Marsupenaeus japonicus]

Protein Accession	Unigene23129$_H1$
Mean Expression Ratio	1.11
Median Expression Ratio	1.11
Credible Interval	(0.821, 1.51)
Associated Peptides	4
Associated Spectra	8
Coverage	NaN

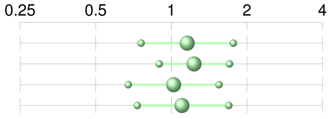

A	B	2.5	50	97.5	Sequence
**1**	**0**	**0.76**	**1.2**	**1.8**	**IEEAKDELQK**
**2**	**3**	**0.89**	**1.2**	**1.7**	**ILMVGLDAAGK**
**0**	**1**	**0.67**	**1**	**1.5**	**LLVFANK**
**0**	**1**	**0.73**	**1.1**	**1.7**	**LGLNQLR**

5.266 124 711 glutathione S-transferase sigma 3 [Mytilus galloprovincialis]; 124 711 glutathione S-transferase sigma 3 [Mytilus galloprovincialis]; 110 697 glutathione S-transferase sigma 3 [Mytilus galloprovincialis]

Protein Accession	CL27284.Contig5$_{H}1$ CL27284.Contig4$_{H}1$ CL27284.Contig3$_{H}1$
Mean Expression Ratio	1.11
Median Expression Ratio	1.11
Credible Interval	(0.781, 1.58)
Associated Peptides	3
Associated Spectra	4
Coverage	NaN

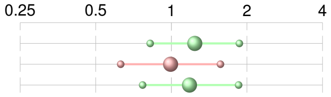

A	B	2.5	50	97.5	Sequence
1	1	0.82	1.2	1.9	VDEATQGVVDIR
1	0	0.63	1	1.6	TALYGYAFEKDEAR
1	0	0.77	1.2	1.8	LVPEAEK

5.267 " 1 510 Glycerol-3-phosphate dehydrogenase [NAD+], cytoplasmic [Crassostrea gigas]"

Protein Accession	Unigene16246$_{H}1$
Mean Expression Ratio	1.11
Median Expression Ratio	1.11
Credible Interval	(0.766, 1.62)
Associated Peptides	1
Associated Spectra	4
Coverage	NaN

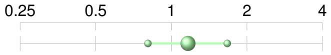

A	B	2.5	50	97.5	Sequence
2	2	0.8	1.2	1.7	EIFGMLEK

5.268 816 1211 H2 [Haliotis tuberculata]

Protein Accession	CL11519.Contig5$_{H}1$
Mean Expression Ratio	0.901
Median Expression Ratio	0.902
Credible Interval	(0.603, 1.34)
Associated Peptides	2
Associated Spectra	4
Coverage	NaN

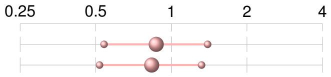

A	B	2.5	50	97.5	Sequence
0	1	0.54	0.87	1.4	FDISDAVR
2	1	0.52	0.84	1.3	SLILAMR

5.269 366 935 minus strand 40S ribosomal protein S2 OS=Urechis caupo GN=RPS2 PE=2 SV=1

Protein Accession	CL2084.Contig7$_{H}1$
Mean Expression Ratio	0.901
Median Expression Ratio	0.902
Credible Interval	(0.581, 1.4)
Associated Peptides	1
Associated Spectra	1
Coverage	NaN

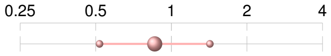

A	B	2.5	50	97.5	Sequence
0	1	0.52	0.86	1.4	TYSFLTPDLWK

5.270 895 1410 Galectin-3 OS=Rattus norvegicus GN=Lgals3 PE=1 SV=4; 1067 1405 Galectin-9 [Crassostrea gigas]; 1068 1421 tandem-repeat galectin [Pinctada fucata]; 1041 1394 tandem-repeat galectin [Pinctada fucata]; 1046 1399 tandem-repeat galectin [Pinctada fucata]; 1068 1421 tandem-repeat galectin [Pinctada fucata]; 1067 1420 tandem-repeat galectin [Pinctada fucata]

Protein Accession	CL474.Contig9$_H$1 CL474.Contig8$_H$1 CL474.Contig7$_H$1 CL474.Contig6$_H$1 CL474.Contig5$_H$1
Mean Expression Ratio	1.11
Median Expression Ratio	1.11
Credible Interval	(0.737, 1.67)
Associated Peptides	1
Associated Spectra	2
Coverage	NaN

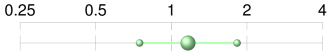

A	B	2.5	50	97.5	Sequence
2	0	0.75	1.2	1.8	VAVNNQHLLDFR

5.271 970 1854 fasciclin-like protein precursor [Aplysia californica] gi

Protein Accession	CL9799.Contig1$_H$1
Mean Expression Ratio	1.11
Median Expression Ratio	1.11
Credible Interval	(0.77, 1.61)
Associated Peptides	3
Associated Spectra	3
Coverage	NaN

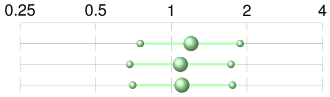

A	B	2.5	50	97.5	Sequence
1	0	0.75	1.2	1.9	VLIPDDALDLVTMAK
0	1	0.68	1.1	1.7	ANLVSMLK
0	1	0.7	1.1	1.8	ELQTVLLGHVLPEAR

5.272 94 933 four and A half lim domains [Schistosoma mansoni]

Protein Accession	CL7046.Contig6$_H$1
Mean Expression Ratio	1.11
Median Expression Ratio	1.11
Credible Interval	(0.904, 1.36)
Associated Peptides	8
Associated Spectra	28
Coverage	NaN

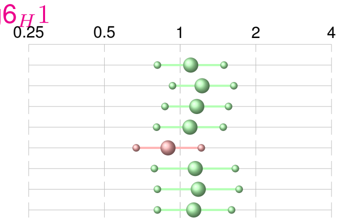

A	B	2.5	50	97.5	Sequence
3	1	0.81	1.1	1.5	CDASMVGR
4	2	0.94	1.2	1.6	CTKPITGIGGTK
3	2	0.87	1.2	1.6	FISFEDR
2	2	0.81	1.1	1.5	SLVDQPFAPK
3	2	0.67	0.89	1.2	SFIPR
1	0	0.79	1.1	1.7	CSGVINK
1	0	0.81	1.2	1.7	KFEYK
1	1	0.81	1.1	1.6	DDQPYCGDCFGELFAK

5.273 14 223 minus strand Endothelin-converting enzyme 2 OS=Homo sapiens GN=ECE2 PE=1 SV=4

Protein Accession	Unigene41869$_{H}1$
Mean Expression Ratio	0.902
Median Expression Ratio	0.903
Credible Interval	(0.601, 1.34)
Associated Peptides	1
Associated Spectra	2
Coverage	NaN

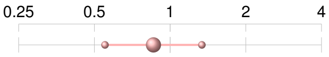

A	B	2.5	50	97.5	Sequence
**2**	**0**	**0.55**	**0.86**	**1.3**	**KLLEAQNK**

5.274 78 461 PREDICTED: hoi-polloi-like [Saccoglossus kowalevskii]; 103 492 NHP2-like protein 1 OS=Rattus norvegicus GN=Nhp2l1 PE=2 SV=4

Protein Accession	CL26515.Contig5$_{H}1$ CL26515.Contig4$_{H}1$
Mean Expression Ratio	0.902
Median Expression Ratio	0.903
Credible Interval	(0.582, 1.4)
Associated Peptides	1
Associated Spectra	1
Coverage	NaN

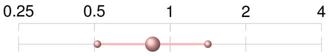

A	B	2.5	50	97.5	Sequence
**1**	**0**	**0.52**	**0.85**	**1.4**	**NVPYVFVR**

5.275 1050 1307 calreticulin precursor [Aplysia californica] gi

Protein Accession	CL4813.Contig1$_{H}1$
Mean Expression Ratio	0.904
Median Expression Ratio	0.904
Credible Interval	(0.586, 1.4)
Associated Peptides	1
Associated Spectra	1
Coverage	NaN

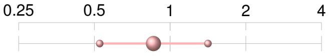

A	B	2.5	50	97.5	Sequence
**0**	**1**	**0.52**	**0.86**	**1.4**	**EFGDSTWGK**

5.276 297 734 minus strand PREDICTED: calmodulin [Canis lupus familiaris]; 297 734 PREDICTED: calmodulin [Canis lupus familiaris]; 143 508 minus strand Calmodulin [Crassostrea gigas]

Protein Accession	Unigene26719$_{H}1$ Unigene17836$_{H}1$ Unigene10331$_{H}1$
Mean Expression Ratio	1.1
Median Expression Ratio	1.11
Credible Interval	(0.706, 1.71)
Associated Peptides	1
Associated Spectra	1
Coverage	NaN

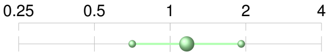

A	B	2.5	50	97.5	Sequence
**1**	**0**	**0.71**	**1.2**	**1.9**	**TLTFQEVDAIVTK**

5.277 " 42 905 PREDICTED: enoyl-CoA hydratase, mitochondrial-like isoform 1 [Oreochromis niloticus]"

Protein Accession	CL6477.Contig7$_{H}1$
Mean Expression Ratio	0.906
Median Expression Ratio	0.905
Credible Interval	(0.637, 1.29)
Associated Peptides	2
Associated Spectra	5
Coverage	NaN

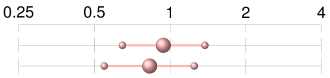

A	B	2.5	50	97.5	Sequence
1	2	0.65	0.94	1.4	AFAAGADIK
1	1	0.55	0.83	1.2	SVVLQASR

5.278 642 1415 minus strand cytochrome c oxidase subunit II [Oncomelania hupensis hupensis]

Protein Accession	CL6764.Contig3$_{H}1$
Mean Expression Ratio	0.905
Median Expression Ratio	0.905
Credible Interval	(0.582, 1.41)
Associated Peptides	1
Associated Spectra	1
Coverage	NaN

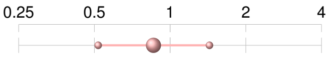

A	B	2.5	50	97.5	Sequence
0	1	0.52	0.86	1.4	IVLPTHTDIR

5.279 266 934 minus strand Kyphoscoliosis peptidase [Crassostrea gigas]

Protein Accession	CL7490.Contig16$_{H}1$
Mean Expression Ratio	1.1
Median Expression Ratio	1.11
Credible Interval	(0.949, 1.29)
Associated Peptides	12
Associated Spectra	66
Coverage	NaN

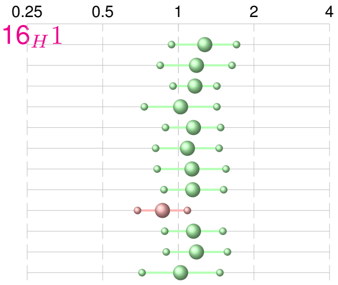

A	B	2.5	50	97.5	Sequence
3	1	0.94	1.3	1.7	ASLNANYGPDESK
2	0	0.85	1.2	1.6	DGCYLWEPLNLR
8	7	0.95	1.2	1.4	DLDNVHFK
2	0	0.73	1	1.4	DTVSLVVTLPDSGYYK
5	3	0.89	1.1	1.5	ELVFTQTER
3	1	0.81	1.1	1.5	LQIYALPSSDDSK
2	1	0.82	1.1	1.5	VNIPGAK
4	1	0.87	1.1	1.5	VTSNLIACGTEQEYK
8	3	0.69	0.87	1.1	YSSLLEYDL
5	1	0.88	1.2	1.5	QYAQWK
4	1	0.9	1.2	1.6	SRPELPHGYLGAQSK
0	1	0.72	1	1.5	GLKDLDNVHFK

5.280 " 148 1989 Acyl-CoA synthetase family member 2, mitochondrial [Crassostrea gigas]; 146 1987 minus strand Acyl-CoA synthetase family member 2, mitochondrial [Crassostrea gigas]"

Protein Accession	CL19799.Contig6$_{H}1$ CL19799.Contig5$_{H}1$
Mean Expression Ratio	1.1
Median Expression Ratio	1.1
Credible Interval	(0.732, 1.68)
Associated Peptides	1
Associated Spectra	2
Coverage	NaN

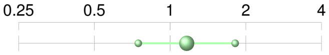

A	B	2.5	50	97.5	Sequence
**0**	**2**	**0.75**	**1.2**	**1.8**	**LAGPGELGEILVR**

5.281 630 1199 neuronal calcium sensor-1 [Lymnaea stagnalis]

Protein Accession	CL14574.Contig3$_{H}1$
Mean Expression Ratio	1.11
Median Expression Ratio	1.1
Credible Interval	(0.745, 1.64)
Associated Peptides	2
Associated Spectra	2
Coverage	NaN

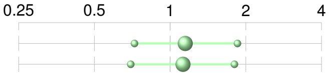

A	B	2.5	50	97.5	Sequence
**1**	**0**	**0.72**	**1.1**	**1.9**	**IFSVMDK**
**1**	**0**	**0.7**	**1.1**	**1.8**	**LYDLDNDGFITK**

5.282 187 642 minus strand PREDICTED: similar to pol-like protein [Tribolium castaneum]

Protein Accession	CL5644.Contig3$_{H}1$
Mean Expression Ratio	1.1
Median Expression Ratio	1.1
Credible Interval	(0.802, 1.51)
Associated Peptides	1
Associated Spectra	24
Coverage	NaN

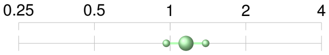

A	B	2.5	50	97.5	Sequence
**24**	**0**	**0.97**	**1.2**	**1.4**	**IILEQEELK**

5.283 459 755 minus strand heat shock protein beta-1 [Trichinella spiralis] gi

Protein Accession	CL17234.Contig5$_{H}1$
Mean Expression Ratio	0.905
Median Expression Ratio	0.906
Credible Interval	(0.649, 1.27)
Associated Peptides	3
Associated Spectra	8
Coverage	NaN

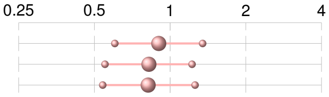

A	B	2.5	50	97.5	Sequence
**2**	**1**	**0.6**	**0.9**	**1.3**	**DVEPTTVTSSLNADGVLTLK**
**2**	**1**	**0.55**	**0.83**	**1.2**	**IVVEGCHEEK**
**1**	**1**	**0.54**	**0.82**	**1.3**	**IVVEGCHEEKQDEHGFITR**

5.284 507 1277 alpha tubulin [Notothenia coriiceps]

Protein Accession	Unigene56691$_H$1
Mean Expression Ratio	1.11
Median Expression Ratio	1.1
Credible Interval	(0.811, 1.51)
Associated Peptides	2
Associated Spectra	9
Coverage	NaN

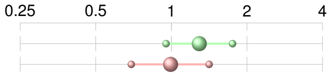

A	B	2.5	50	97.5	Sequence
**5**	**1**	**0.95**	**1.3**	**1.8**	**LSVDYGK**
**2**	**1**	**0.69**	**0.99**	**1.4**	**LSVDYGKK**

5.285 7073 7339 minus strand Collagen alpha-3(VI) chain [Crassostrea gigas]

Protein Accession	Unigene26547$_H$1
Mean Expression Ratio	1.1
Median Expression Ratio	1.1
Credible Interval	(0.856, 1.44)
Associated Peptides	3
Associated Spectra	18
Coverage	NaN

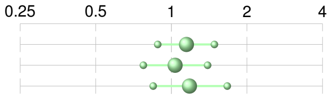

A	B	2.5	50	97.5	Sequence
**6**	**3**	**0.88**	**1.1**	**1.5**	**ALVEELSGSGQNHR**
**6**	**0**	**0.78**	**1**	**1.4**	**YTPGSTNTAGGLR**
**2**	**1**	**0.85**	**1.2**	**1.7**	**YDTPQQVLEAIATTR**

5.286 176 517 PREDICTED: elongin C-like [Saccoglossus kowalevskii]; 115 456 PREDICTED: elongin C-like [Saccoglossus kowalevskii]; 994 1350 PREDICTED: elongin C-like [Saccoglossus kowalevskii]; 126 482 PREDICTED: elongin C-like [Saccoglossus kowalevskii]; 30 371 PREDICTED: elongin C-like [Saccoglossus kowalevskii]

Protein Accession	Unigene38248$_H$1 CL27549.Contig5$_H$1 CL27549.Contig4$_H$1 CL27549.Contig3$_H$1 CL27549.C
Mean Expression Ratio	1.1
Median Expression Ratio	1.1
Credible Interval	(0.71, 1.73)
Associated Peptides	1
Associated Spectra	1
Coverage	NaN

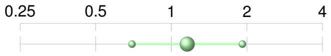

A	B	2.5	50	97.5	Sequence
**1**	**0**	**0.7**	**1.2**	**1.9**	**LISSDGHEFIVK**

5.287 58 1203 minus strand Obg-like ATPase 1 [Crassostrea gigas]

Protein Accession	CL33105.Contig1$_H$1
Mean Expression Ratio	0.906
Median Expression Ratio	0.907
Credible Interval	(0.583, 1.42)
Associated Peptides	1
Associated Spectra	1
Coverage	NaN

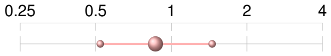

A	B	2.5	50	97.5	Sequence
**1**	**0**	**0.52**	**0.86**	**1.4**	**IHTDFEK**

5.288 54 278 ATP-citrate synthase [Crassostrea gigas]; 61 465 ATP-citrate synthase [Crassostrea gigas]

Protein Accession	CL35133.Contig2$_H$1 CL35133.Contig1$_H$1
Mean Expression Ratio	0.906
Median Expression Ratio	0.907
Credible Interval	(0.582, 1.41)
Associated Peptides	1
Associated Spectra	1
Coverage	NaN

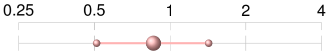

A	B	2.5	50	97.5	Sequence
**1**	**0**	**0.51**	**0.86**	**1.4**	**TIAIIAEGIPENLTR**

5.289 " 166 1248 PREDICTED: probable pyruvate dehydrogenase E1 component subunit alpha, mitochondrial-like [Metaseiulus occidentalis]; 166 1248 PREDICTED: probable pyruvate dehydrogenase E1 component subunit alpha, mitochondrial-like [Metaseiulus occidentalis]; 166 1248 PREDICTED: probable pyruvate dehydrogenase E1 component subunit alpha, mitochondrial-like [Metaseiulus occidentalis]; 166 1248 PREDICTED: probable pyruvate dehydrogenase E1 component subunit alpha, mitochondrial-like [Metaseiulus occidentalis]; 166 1248 PREDICTED: probable pyruvate dehydrogenase E1 component subunit alpha, mitochondrial-like [Metaseiulus occidentalis]; 166 1248 minus strand PREDICTED: probable pyruvate dehydrogenase E1 component subunit alpha, mitochondrial-like [Metaseiulus occidentalis]; 166 1248 minus strand PREDICTED: probable pyruvate dehydrogenase E1 component subunit alpha, mitochondrial-like [Metaseiulus occidentalis]; 166 1248 PREDICTED: probable pyruvate dehydrogenase E1 component subunit alpha, mitochondrial-like [Metaseiulus occidentalis]; 166 1248 PREDICTED: probable pyruvate dehydrogenase E1 component subunit alpha, mitochondrial-like [Metaseiulus occidentalis]"

Protein Accession	Unigene34082$_H$1 Unigene34081$_H$1 Unigene34079$_H$1 Unigene34077$_H$1 Unigene34075$_H$1 U
Mean Expression Ratio	0.908
Median Expression Ratio	0.907
Credible Interval	(0.644, 1.29)
Associated Peptides	3
Associated Spectra	4
Coverage	NaN

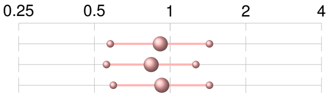

A	B	2.5	50	97.5	Sequence
**1**	**0**	**0.58**	**0.91**	**1.4**	**TGLATEEELK**
**1**	**1**	**0.56**	**0.84**	**1.3**	**TGLATEEELKK**
**0**	**1**	**0.6**	**0.93**	**1.4**	**KIDGEVRSEVDAAAER**

5.290 179 2140 minus strand PREDICTED: hypothetical protein [Saccoglossus kowalevskii]

Protein Accession	CL13749.Contig6$_{H}1$
Mean Expression Ratio	1.1
Median Expression Ratio	1.1
Credible Interval	(0.711, 1.73)
Associated Peptides	1
Associated Spectra	1
Coverage	NaN

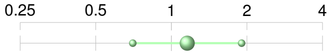

A	B	2.5	50	97.5	Sequence
**1**	**0**	**0.7**	**1.2**	**1.9**	**KFDGIGGLSGGGATSK**

5.291 67 825 unnamed protein product [Oikopleura dioica]

Protein Accession	Unigene71508$_{H}1$
Mean Expression Ratio	0.908
Median Expression Ratio	0.908
Credible Interval	(0.599, 1.38)
Associated Peptides	1
Associated Spectra	2
Coverage	NaN

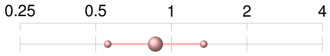

A	B	2.5	50	97.5	Sequence
**2**	**0**	**0.56**	**0.86**	**1.3**	**TAANVEEAFINTAK**

5.292 227 1201 PREDICTED: quinone oxidoreductase-like isoform 1 [Ornithorhynchus anatinus]; 109 1083 PREDICTED: quinone oxidoreductase-like isoform 1 [Ornithorhynchus anatinus]; 164 1138 PREDICTED: quinone oxidoreductase-like isoform 1 [Ornithorhynchus anatinus]

Protein Accession	CL11899.Contig2$_{H}1$ CL11899.Contig1$_{H}1$ CL11899.Contig13$_{H}1$
Mean Expression Ratio	0.904
Median Expression Ratio	0.908
Credible Interval	(0.585, 1.4)
Associated Peptides	1
Associated Spectra	1
Coverage	NaN

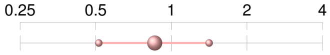

A	B	2.5	50	97.5	Sequence
**1**	**0**	**0.51**	**0.86**	**1.4**	**VHASGVNPVDTYIR**

5.293 4 399 Spectrin beta chain [Crassostrea gigas]

Protein Accession	Unigene17190$_H1$
Mean Expression Ratio	0.909
Median Expression Ratio	0.908
Credible Interval	(0.621, 1.33)
Associated Peptides	1
Associated Spectra	4
Coverage	NaN

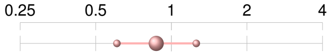

A	B	2.5	50	97.5	Sequence
**1**	**3**	**0.61**	**0.87**	**1.3**	**VHLENMGAHDIVDGSAR**

5.294 397 510 PREDICTED: actin-5C-like [Megachile rotundata]

Protein Accession	CL48442.Contig2$_H1$
Mean Expression Ratio	0.909
Median Expression Ratio	0.908
Credible Interval	(0.699, 1.18)
Associated Peptides	3
Associated Spectra	29
Coverage	NaN

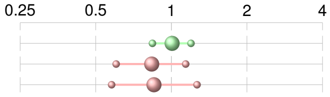

A	B	2.5	50	97.5	Sequence
**15**	**9**	**0.84**	**1**	**1.2**	**DLTDYLMK**
**2**	**2**	**0.6**	**0.83**	**1.1**	**GRDLTDYLMK**
**0**	**1**	**0.58**	**0.85**	**1.3**	**LDLAGRDLTDY**

5.295 " 57 752 PREDICTED: adenylate kinase 2, mitochondrial-like isoform 2 [Callithrix jacchus]"

Protein Accession	CL4149.Contig1$_H1$
Mean Expression Ratio	1.1
Median Expression Ratio	1.1
Credible Interval	(0.798, 1.53)
Associated Peptides	3
Associated Spectra	6
Coverage	NaN

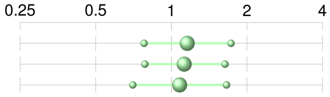

A	B	2.5	50	97.5	Sequence
**2**	**0**	**0.78**	**1.2**	**1.7**	**GIHTAVDASQPPHK**
**3**	**0**	**0.78**	**1.1**	**1.6**	**GFLLDGFPR**
**1**	**0**	**0.7**	**1.1**	**1.7**	**GTQAPLLAEK**

5.296 160 588 hypothetical protein CGI_10001640 [Crassostrea gigas]

Protein Accession	CL2347.Contig8$_H1$
Mean Expression Ratio	1.1
Median Expression Ratio	1.1
Credible Interval	(0.806, 1.5)
Associated Peptides	2
Associated Spectra	13
Coverage	NaN

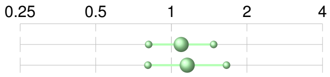

A	B	2.5	50	97.5	Sequence
**6**	**3**	**0.81**	**1.1**	**1.5**	**AFLEQLGSK**
**3**	**1**	**0.81**	**1.2**	**1.7**	**ANLIVIGSR**

5.297 325 642 minus strand Protein phosphatase 1B [Crassostrea gigas]

Protein Accession	Unigene268$_{H}1$
Mean Expression Ratio	0.907
Median Expression Ratio	0.91
Credible Interval	(0.604, 1.37)
Associated Peptides	1
Associated Spectra	2
Coverage	NaN

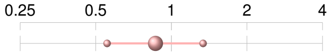

A	B	2.5	50	97.5	Sequence
**1**	**1**	**0.56**	**0.87**	**1.3**	**VEMEDAHTAVVSIPHAR**

5.298 113 808 minus strand omega class glutathione-s-transferase [Chlamys farreri]; 335 1045 minus strand omega class glutathione-s-transferase [Chlamys farreri]

Protein Accession	CL8096.Contig3$_{H}1$ CL8096.Contig2$_{H}1$
Mean Expression Ratio	1.1
Median Expression Ratio	1.1
Credible Interval	(0.725, 1.65)
Associated Peptides	1
Associated Spectra	2
Coverage	NaN

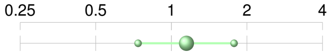

A	B	2.5	50	97.5	Sequence
**2**	**0**	**0.74**	**1.2**	**1.8**	**HPLGLVPTLEQDSK**

5.299 " 698 1225 minus strand actin related protein 2/3 complex, 21 kDa subunit [Crassostrea gigas]"

Protein Accession	CL17389.Contig2$_{H}1$
Mean Expression Ratio	0.911
Median Expression Ratio	0.911
Credible Interval	(0.66, 1.26)
Associated Peptides	3
Associated Spectra	7
Coverage	NaN

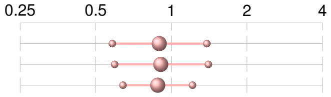

A	B	2.5	50	97.5	Sequence
**0**	**1**	**0.58**	**0.89**	**1.4**	**AYLQQLR**
**0**	**1**	**0.6**	**0.91**	**1.4**	**ANIFFR**
**0**	**5**	**0.64**	**0.88**	**1.2**	**LLPLK**

5.300 392 808 Whirlin [Crassostrea gigas]

Protein Accession	CL22731.Contig1$_{H}1$
Mean Expression Ratio	0.912
Median Expression Ratio	0.911
Credible Interval	(0.635, 1.31)
Associated Peptides	1
Associated Spectra	5
Coverage	NaN

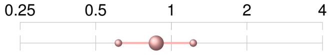

A	B	2.5	50	97.5	Sequence
**2**	**3**	**0.62**	**0.87**	**1.2**	**AGNDLHLLVQR**

5.301 613 846 cytochrome c oxidase subunit Va [Antheraea yamamai] gi

Protein Accession	Unigene3832$_H$1
Mean Expression Ratio	1.1
Median Expression Ratio	1.1
Credible Interval	(0.704, 1.72)
Associated Peptides	1
Associated Spectra	1
Coverage	NaN

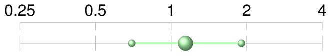

A	B	2.5	50	97.5	Sequence
1	0	0.7	1.1	1.9	AMNDLQGEDLVPEPK

5.302 11 1462 coronin [Schistosoma mansoni]

Protein Accession	CL11779.Contig2$_H$1
Mean Expression Ratio	1.1
Median Expression Ratio	1.1
Credible Interval	(0.826, 1.46)
Associated Peptides	4
Associated Spectra	10
Coverage	NaN

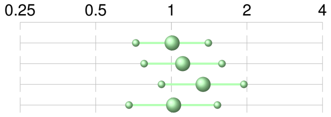

A	B	2.5	50	97.5	Sequence
2	2	0.72	1	1.4	DANPILVSLK
2	1	0.78	1.1	1.6	HVFGKPEK
2	0	0.91	1.3	2	VPMDAPVVAGHK
0	1	0.68	1	1.5	DAPVVAGHK

5.303 1265 1831 minus strand Adenosine deaminase CECR1 [Crassostrea gigas]

Protein Accession	CL20850.Contig11$_H$1
Mean Expression Ratio	0.912
Median Expression Ratio	0.912
Credible Interval	(0.586, 1.43)
Associated Peptides	1
Associated Spectra	1
Coverage	NaN

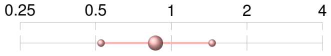

A	B	2.5	50	97.5	Sequence
0	1	0.52	0.87	1.4	GIAVEVNPISNQVLK

5.304 227 3073 minus strand Lysosomal alpha-mannosidase [Crassostrea gigas]

Protein Accession	CL2930.Contig2$_H$1
Mean Expression Ratio	0.911
Median Expression Ratio	0.913
Credible Interval	(0.625, 1.31)
Associated Peptides	2
Associated Spectra	4
Coverage	NaN

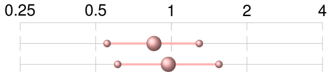

A	B	2.5	50	97.5	Sequence
2	1	0.56	0.85	1.3	VASELIPITPDTFR
1	0	0.61	0.98	1.5	ASLTWSGVR

5.305 211 990 PREDICTED: gamma-glutamyl hydrolase-like [Xenopus (Silurana) tropicalis]; 223 1080 PREDICTED: gamma-glutamyl hydrolase [Gallus gallus]

Protein Accession	CL20208.Contig9$_H$1 CL20208.Contig3$_H$1
Mean Expression Ratio	1.1
Median Expression Ratio	1.1
Credible Interval	(0.721, 1.66)
Associated Peptides	1
Associated Spectra	2
Coverage	NaN

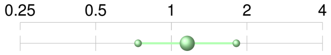

A	B	2.5	50	97.5	Sequence
**2**	**0**	**0.74**	**1.2**	**1.8**	**YLWQAGAR**

5.306 712 831 haemocyanin [Aplysia californica]

Protein Accession	Unigene65929$_H$1
Mean Expression Ratio	0.913
Median Expression Ratio	0.913
Credible Interval	(0.635, 1.32)
Associated Peptides	2
Associated Spectra	4
Coverage	NaN

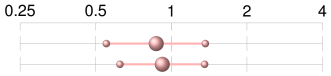

A	B	2.5	50	97.5	Sequence
**1**	**0**	**0.55**	**0.87**	**1.4**	**YDVTDVFNQMR**
**3**	**0**	**0.62**	**0.92**	**1.4**	**YHFNVK**

5.307 2 2542 kinesin heavy chain 1 [Aplysia californica] gi; 2 2542 kinesin heavy chain 1 [Aplysia californica] gi

Protein Accession	CL4142.Contig6$_H$1 CL4142.Contig5$_H$1
Mean Expression Ratio	0.913
Median Expression Ratio	0.913
Credible Interval	(0.646, 1.3)
Associated Peptides	2
Associated Spectra	5
Coverage	NaN

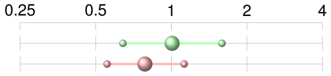

A	B	2.5	50	97.5	Sequence
**1**	**0**	**0.64**	**1**	**1.6**	**QQLEAEK**
**4**	**0**	**0.55**	**0.79**	**1.1**	**LSELSIQMSRREQAK**

5.308 31 735 hypothetical protein BRAFLDRAFT_78603 [Branchiostoma floridae] gi; 55 735 hypothetical protein BRAFLDRAFT_78603 [Branchiostoma floridae] gi; 319 948 hypothetical protein BRAFLDRAFT_78603 [Branchiostoma floridae] gi; 353 1039 hypothetical protein BRAFLDRAFT_78603 [Branchiostoma floridae] gi; 207 893 hypothetical protein BRAFLDRAFT_78603 [Branchiostoma floridae] gi

Protein Accession	CL877.Contig7$_{H1}$ CL877.Contig6$_{H1}$ CL877.Contig5$_{H1}$ CL877.Contig4$_{H1}$ CL877.Contig3$_{H1}$
Mean Expression Ratio	0.912
Median Expression Ratio	0.913
Credible Interval	(0.611, 1.37)
Associated Peptides	2
Associated Spectra	5
Coverage	NaN

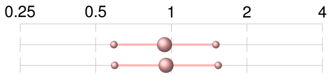

A	B	2.5	50	97.5	Sequence
**2**	**1**	**0.59**	**0.94**	**1.5**	**DDQTLVTMTGEVAGR**
**1**	**1**	**0.59**	**0.95**	**1.5**	**LKLPLDSSNR**

5.309 296 493 hypothetical protein BRAFLDRAFT_66287 [Branchiostoma floridae] gi

Protein Accession	Unigene414$_{H1}$
Mean Expression Ratio	1.1
Median Expression Ratio	1.09
Credible Interval	(0.729, 1.65)
Associated Peptides	1
Associated Spectra	2
Coverage	NaN

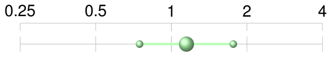

A	B	2.5	50	97.5	Sequence
**1**	**1**	**0.75**	**1.1**	**1.8**	**SLDDFLQAWK**

5.310 16 288 SJCHGC01950 protein [Schistosoma japonicum] gi

Protein Accession	Unigene16338$_{H1}$
Mean Expression Ratio	1.09
Median Expression Ratio	1.09
Credible Interval	(0.775, 1.56)
Associated Peptides	1
Associated Spectra	7
Coverage	NaN

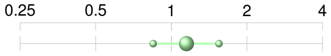

A	B	2.5	50	97.5	Sequence
**6**	**1**	**0.85**	**1.1**	**1.5**	**DIAAFIK**

5.311 302 1273 minus strand hypothetical protein CGI_10007007 [Crassostrea gigas]

Protein Accession	CL18045.Contig10$_H$1
Mean Expression Ratio	0.916
Median Expression Ratio	0.915
Credible Interval	(0.591, 1.43)
Associated Peptides	1
Associated Spectra	3
Coverage	NaN

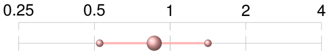

A	B	2.5	50	97.5	Sequence
**2**	**1**	**0.53**	**0.86**	**1.4**	**IEAEFPGANIILHK**

5.312 166 1563 arylsulfatase [Haliotis discus discus]

Protein Accession	CL49030.Contig16$_H$1
Mean Expression Ratio	1.1
Median Expression Ratio	1.09
Credible Interval	(0.703, 1.73)
Associated Peptides	1
Associated Spectra	1
Coverage	NaN

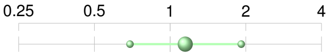

A	B	2.5	50	97.5	Sequence
**0**	**1**	**0.69**	**1.1**	**1.9**	**VPLVLHSK**

5.313 " 154 591 microsomal GST1, partial [Reishia clavigera]; 154 531 microsomal GST1, partial [Reishia clavigera]"

Protein Accession	CL3934.Contig2$_H$1 CL3934.Contig1$_H$1
Mean Expression Ratio	0.917
Median Expression Ratio	0.917
Credible Interval	(0.589, 1.42)
Associated Peptides	1
Associated Spectra	1
Coverage	NaN

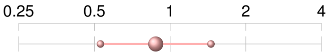

A	B	2.5	50	97.5	Sequence
**1**	**0**	**0.53**	**0.88**	**1.5**	**FFCPGTAVNTNDALVER**

5.314 " 703 1173 60S acidic ribosomal protein P0, putative [Ixodes scapularis] gi"

Protein Accession	Unigene34489$_H$1
Mean Expression Ratio	0.917
Median Expression Ratio	0.917
Credible Interval	(0.592, 1.42)
Associated Peptides	1
Associated Spectra	1
Coverage	NaN

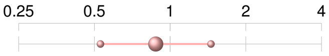

A	B	2.5	50	97.5	Sequence
**0**	**1**	**0.53**	**0.88**	**1.5**	**KSFLEGVR**

5.315 2 211 minus strand sulfatase 1A precursor [Haliotis discus discus]

Protein Accession	Unigene22814$_{H}1$
Mean Expression Ratio	1.09
Median Expression Ratio	1.09
Credible Interval	(0.725, 1.63)
Associated Peptides	1
Associated Spectra	2
Coverage	NaN

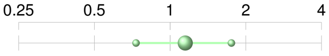

A	B	2.5	50	97.5	Sequence
2	0	0.73	1.1	1.8	CGMVSAMDESIGK

5.316 71 1321 minus strand Vinculin [Crassostrea gigas]

Protein Accession	Unigene23324$_{H}1$
Mean Expression Ratio	0.918
Median Expression Ratio	0.918
Credible Interval	(0.745, 1.13)
Associated Peptides	12
Associated Spectra	20
Coverage	NaN

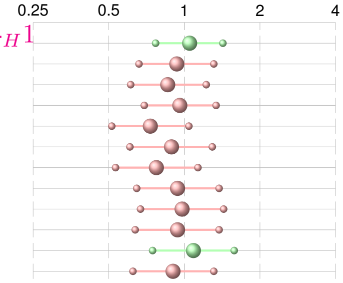

A	B	2.5	50	97.5	Sequence
3	1	0.77	1	1.4	DTITPMVQNAK
1	1	0.66	0.93	1.3	GLVDEAVDTAALIR
2	0	0.61	0.86	1.2	KPAPTVDGK
2	1	0.69	0.96	1.3	LINAVGGVR
2	0	0.51	0.73	1	NVGDIESMANALSELR
1	0	0.61	0.89	1.3	QWLANPGLDDK
1	0	0.53	0.77	1.1	VLQVAEMEAQNSEDPLFVDR
1	0	0.65	0.94	1.4	ASADLR
0	1	0.67	0.98	1.4	AEEEAILHDTDR
0	1	0.64	0.94	1.4	VNEASADLR
0	1	0.75	1.1	1.6	DREAILK
0	1	0.62	0.9	1.3	AIARNLSEKL

5.317 768 980 muscle LIM protein isoform 1 [Danaus plexippus]

Protein Accession	CL29824.Contig1$_{H}1$
Mean Expression Ratio	0.917
Median Expression Ratio	0.918
Credible Interval	(0.717, 1.18)
Associated Peptides	4
Associated Spectra	31
Coverage	NaN

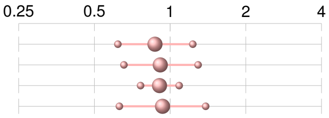

A	B	2.5	50	97.5	Sequence
3	0	0.62	0.87	1.2	SLDSTTMAMHETEVYCK
2	1	0.65	0.92	1.3	GVNTPWHK
18	6	0.77	0.91	1.1	AIGVNTPWHK
1	0	0.63	0.94	1.4	NFGPSGYGFGQGAGT

5.318 " 2041 3315 minus strand Myosin-10 OS=Mus musculus GN=Myh10 PE=1 SV=2; 2041 3345 minus strand Myosin heavy chain, non-muscle OS=Drosophila melanogaster GN=zip PE=1 SV=2; 2041 3351 minus strand Myosin-10 OS=Mus musculus GN=Myh10 PE=1 SV=2"

Protein Accession	Unigene62170$_{H}1$ Unigene62168$_{H}1$ Unigene62165$_{H}1$
Mean Expression Ratio	0.92
Median Expression Ratio	0.918
Credible Interval	(0.675, 1.26)
Associated Peptides	5
Associated Spectra	6
Coverage	NaN

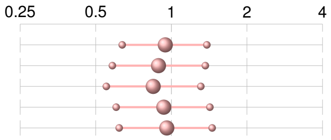

A	B	2.5	50	97.5	Sequence
0	2	0.64	0.95	1.4	AGVLAHLEEER
0	1	0.58	0.89	1.4	CIIPNHEK
0	1	0.55	0.84	1.3	IDSPLVLDQLR
0	1	0.6	0.93	1.4	ILFQEFR
0	1	0.62	0.96	1.5	LMATLR

5.319 91 408 minus strand hypothetical protein [Amblyomma maculatum]

Protein Accession	CL19180.Contig1$_{H}1$
Mean Expression Ratio	1.09
Median Expression Ratio	1.09
Credible Interval	(0.708, 1.69)
Associated Peptides	1
Associated Spectra	1
Coverage	NaN

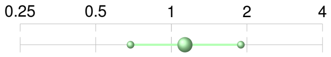

A	B	2.5	50	97.5	Sequence
1	0	0.69	1.1	1.9	EVGWTIVDGR

5.320 85 1011 unnamed protein product [Mus musculus]

Protein Accession	CL42412.Contig1$_{H}1$
Mean Expression Ratio	1.09
Median Expression Ratio	1.09
Credible Interval	(0.752, 1.58)
Associated Peptides	3
Associated Spectra	3
Coverage	NaN

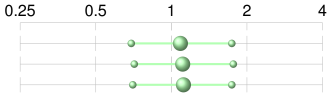

A	B	2.5	50	97.5	Sequence
1	0	0.69	1.1	1.7	NVIVEETVSEK
1	0	0.71	1.1	1.8	DDETMYVEAK
1	0	0.7	1.1	1.7	VFMQEFK

5.321 1 252 Aquaporin-4 [Crassostrea gigas]

Protein Accession	Unigene16706$_{H}1$
Mean Expression Ratio	1.09
Median Expression Ratio	1.09
Credible Interval	(0.735, 1.63)
Associated Peptides	2
Associated Spectra	2
Coverage	NaN

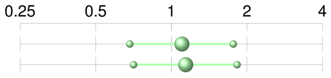

A	B	2.5	50	97.5	Sequence
1	0	0.69	1.1	1.8	APLDDAAAAEVIEIDETK
1	0	0.71	1.1	1.8	KFSPSQAAAAPQEK

5.322 68 481 minus strand netrin receptor UNC5A [Danio rerio] gi

Protein Accession	Unigene38845$_{H1}$
Mean Expression Ratio	1.09
Median Expression Ratio	1.09
Credible Interval	(0.707, 1.68)
Associated Peptides	1
Associated Spectra	1
Coverage	NaN

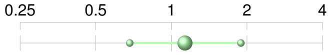

A	B	2.5	50	97.5	Sequence
**1**	**0**	**0.68**	**1.1**	**1.9**	**ELMECQK**

5.323 2 244 minus strand hypothetical protein KGM_08771 [Danaus plexippus]

Protein Accession	CL49466.Contig2$_{H1}$
Mean Expression Ratio	1.09
Median Expression Ratio	1.09
Credible Interval	(0.781, 1.52)
Associated Peptides	2
Associated Spectra	6
Coverage	NaN

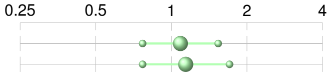

A	B	2.5	50	97.5	Sequence
**3**	**1**	**0.77**	**1.1**	**1.5**	**AVFIDLEPTVVDEVR**
**1**	**1**	**0.77**	**1.1**	**1.7**	**TVGGGDDSFNTFFSETGAGK**

5.324 108 593 hypothetical protein CGI_10025497 [Crassostrea gigas]

Protein Accession	CL36597.Contig1$_{H1}$
Mean Expression Ratio	1.09
Median Expression Ratio	1.09
Credible Interval	(0.749, 1.57)
Associated Peptides	1
Associated Spectra	5
Coverage	NaN

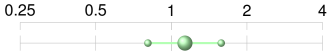

A	B	2.5	50	97.5	Sequence
**4**	**1**	**0.8**	**1.1**	**1.6**	**SFELADFHK**

5.325 98 1012 minus strand RecName: Full=Substrate-specific endoprotease Tex31; Flags: Precursor gi|32187775|emb|CAD36507.1| substrate-specific endoprotease [Conus textile]

Protein Accession	CL13681.Contig1$_{H1}$
Mean Expression Ratio	1.09
Median Expression Ratio	1.09
Credible Interval	(0.796, 1.49)
Associated Peptides	3
Associated Spectra	7
Coverage	NaN

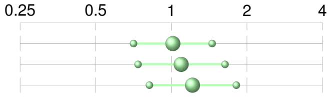

A	B	2.5	50	97.5	Sequence
**2**	**1**	**0.71**	**1**	**1.5**	**NIPLSASDISAIVER**
**2**	**0**	**0.74**	**1.1**	**1.6**	**VPGAVFSK**
**1**	**1**	**0.82**	**1.2**	**1.8**	**ALDPQHTMCLADK**

5.326 1971 3368 minus strand Kyphoscoliosis peptidase [Crassostrea gigas]; 1767 3158 minus strand hillarin [Hirudo medicinalis]; 1977 3368 minus strand hillarin [Hirudo medicinalis]

Protein Accession	CL3923.Contig9$_H1$ CL3923.Contig8$_H1$ CL3923.Contig10$_H1$
Mean Expression Ratio	1.09
Median Expression Ratio	1.09
Credible Interval	(0.913, 1.29)
Associated Peptides	13
Associated Spectra	39
Coverage	NaN

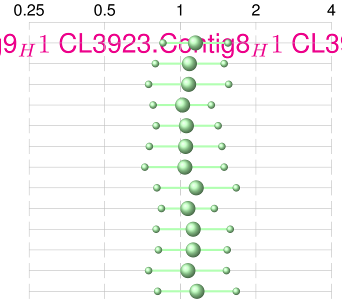

A	B	2.5	50	97.5	Sequence
2	2	0.85	1.1	1.5	EKGEAPPPVPATGPQQKPTLEAIDR
1	2	0.79	1.1	1.5	EMNFDKVEK
1	0	0.75	1.1	1.6	GEAPPPVPATGPQQKPTLEAIDR
4	2	0.78	1	1.3	GSPEEVLMGLK
4	1	0.8	1.1	1.4	NITNEIEK
1	1	0.75	1	1.5	TAVIYSR
1	0	0.72	1	1.5	TVGNTAYFNVTAPGR
1	0	0.81	1.2	1.7	IISGYAK
5	4	0.84	1.1	1.4	WQLLDR
2	0	0.8	1.1	1.6	MKPQFFK
2	1	0.82	1.1	1.5	NVLQSVDDHAIQVSK
0	1	0.75	1.1	1.5	WLATK
0	1	0.81	1.2	1.7	QLLDR

5.327 64 435 peptidoglycan recognition protein S1L [Pomacea canaliculata]

Protein Accession	CL1297.Contig6$_H1$
Mean Expression Ratio	1.09
Median Expression Ratio	1.09
Credible Interval	(0.699, 1.71)
Associated Peptides	1
Associated Spectra	1
Coverage	NaN

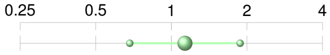

A	B	2.5	50	97.5	Sequence
1	0	0.68	1.1	1.9	MYSVCHGGR

5.328 20 511 Neurexin-2-alpha [Crassostrea gigas]; 20 511 Neurexin-2-alpha [Crassostrea gigas]

Protein Accession	CL36998.Contig2$_H1$ CL36998.Contig1$_H1$
Mean Expression Ratio	0.921
Median Expression Ratio	0.921
Credible Interval	(0.599, 1.41)
Associated Peptides	1
Associated Spectra	1
Coverage	NaN

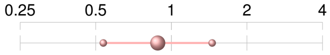

A	B	2.5	50	97.5	Sequence
0	1	0.53	0.88	1.5	AVFLDGR

5.329 273 1304 Guanine nucleotide-binding protein subunit beta [Crassostrea gigas]

Protein Accession	CL15682.Contig1$_H$1
Mean Expression Ratio	0.92
Median Expression Ratio	0.921
Credible Interval	(0.738, 1.15)
Associated Peptides	6
Associated Spectra	23
Coverage	NaN

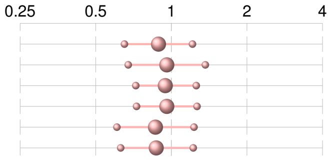

A	B	2.5	50	97.5	Sequence
3	1	0.65	0.89	1.2	IYAMHWASDSR
2	0	0.67	0.96	1.4	KAAGDTTLAQATAQCEPVGR
3	3	0.72	0.95	1.3	TFVSGACDASAK
3	3	0.72	0.96	1.3	LWDIR
0	2	0.61	0.87	1.2	LIVWDGYTTNK
0	3	0.63	0.87	1.2	LFDIR

5.330 131 541 minus strand nervous system adducin [Aplysia californica] gi

Protein Accession	CL27337.Contig1$_H$1
Mean Expression Ratio	0.922
Median Expression Ratio	0.921
Credible Interval	(0.656, 1.29)
Associated Peptides	3
Associated Spectra	5
Coverage	NaN

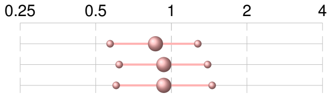

A	B	2.5	50	97.5	Sequence
1	1	0.57	0.86	1.3	FIDTIDPDDPEYIR
1	1	0.62	0.93	1.4	GIGASFGR
1	0	0.6	0.93	1.4	LAALYR

5.331 557 2329 minus strand Collagen alpha-3(VI) chain [Crassostrea gigas]

Protein Accession	CL47586.Contig7$_H$1
Mean Expression Ratio	1.09
Median Expression Ratio	1.09
Credible Interval	(0.778, 1.52)
Associated Peptides	4
Associated Spectra	5
Coverage	NaN

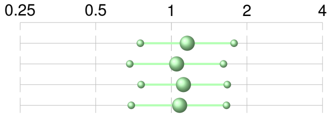

A	B	2.5	50	97.5	Sequence
1	0	0.75	1.2	1.8	KADYSAALGK
1	0	0.68	1	1.6	KLDVVVAFHASPR
0	2	0.76	1.1	1.7	VALGFFNK
0	1	0.69	1.1	1.7	VVAVGVGAVVK

5.332 2 1795 minus strand Plectin-1 [Crassostrea gigas]

Protein Accession	CL32322.Contig2$_H$1
Mean Expression Ratio	1.08
Median Expression Ratio	1.09
Credible Interval	(0.726, 1.6)
Associated Peptides	2
Associated Spectra	2
Coverage	NaN

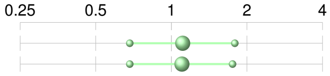

A	B	2.5	50	97.5	Sequence
1	0	0.68	1.1	1.8	TKDGDVIPIHDAIMTK
1	0	0.68	1.1	1.8	YITTAHFVR

5.333 185 1258 minus strand Severin [Crassostrea gigas]

Protein Accession	CL7486.Contig1$_H1$
Mean Expression Ratio	1.08
Median Expression Ratio	1.08
Credible Interval	(0.806, 1.46)
Associated Peptides	6
Associated Spectra	8
Coverage	NaN

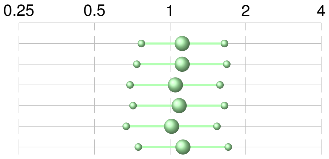

A	B	2.5	50	97.5	Sequence
2	0	0.77	1.1	1.6	EVQGHESDLFR
1	1	0.74	1.1	1.7	TDHPLVPITCLTEGTK
1	0	0.69	1.1	1.6	TVELDTFLNDVPVQHR
1	0	0.71	1.1	1.6	VTEWPVGDYGK
1	0	0.67	1	1.5	SDFISEDVFILDTK
1	0	0.75	1.1	1.7	TAGQKPGLQIWR

5.334 " 199 417 GG13156 [Drosophila erecta] gi; 253 501 minus strand NADH-ubiquinone oxidoreductase MLRQ subunit-like protein, partial [Lymnaea stagnalis]; 294 515 GG13156 [Drosophila erecta] gi"

Protein Accession	CL49605.Contig4$_H1$ CL49605.Contig2$_H1$ CL49605.Contig1$_H1$
Mean Expression Ratio	1.08
Median Expression Ratio	1.08
Credible Interval	(0.696, 1.67)
Associated Peptides	1
Associated Spectra	1
Coverage	NaN

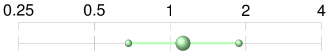

A	B	2.5	50	97.5	Sequence
1	0	0.68	1.1	1.9	GLTLSSLK

5.335 247 726 Annexin A7 [Crassostrea gigas]; 286 675 Annexin A7 [Crassostrea gigas]

Protein Accession	CL15539.Contig3$_H1$ CL15539.Contig1$_H1$
Mean Expression Ratio	1.08
Median Expression Ratio	1.08
Credible Interval	(0.803, 1.47)
Associated Peptides	4
Associated Spectra	7
Coverage	NaN

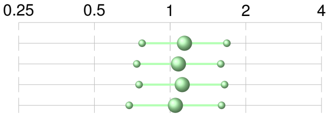

A	B	2.5	50	97.5	Sequence
2	0	0.77	1.1	1.7	GLGTDEAAVIR
1	1	0.74	1.1	1.6	DLVSDLK
1	1	0.75	1.1	1.6	DIVSDTSGHFR
1	0	0.69	1	1.6	ILTSHNNMQR

5.336 965 1687 Arylsulfatase B [Crassostrea gigas]

Protein Accession	Unigene30559$_H1$
Mean Expression Ratio	0.921
Median Expression Ratio	0.923
Credible Interval	(0.586, 1.43)
Associated Peptides	1
Associated Spectra	1
Coverage	NaN

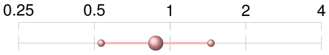

A	B	2.5	50	97.5	Sequence
0	1	0.53	0.88	1.5	WGLYDIEADPEER

5.337 183 953 minus strand glutathione-S-transferase [Haliotis diversicolor supertexta]; 275 1045 minus strand glutathione-S-transferase [Haliotis diversicolor supertexta]; 343 1113 minus strand glutathione-S-transferase [Haliotis diversicolor supertexta]

Protein Accession	Unigene40627$_H1$ CL6878.Contig3$_H1$ CL6878.Contig2$_H1$
Mean Expression Ratio	0.92
Median Expression Ratio	0.923
Credible Interval	(0.61, 1.37)
Associated Peptides	2
Associated Spectra	2
Coverage	NaN

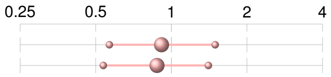

A	B	2.5	50	97.5	Sequence
0	1	0.57	0.92	1.5	LPHDPLPK
0	1	0.54	0.87	1.4	KIDPK

5.338 3 218 beta-tubulin [Fasciola hepatica] gi

Protein Accession	Unigene7955$_H1$
Mean Expression Ratio	1.08
Median Expression Ratio	1.08
Credible Interval	(0.806, 1.45)
Associated Peptides	3
Associated Spectra	12
Coverage	NaN

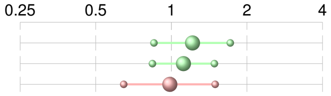

A	B	2.5	50	97.5	Sequence
3	1	0.85	1.2	1.7	EVDEQMLNVQNK
7	0	0.84	1.1	1.5	TAVCDIPPR
1	0	0.65	0.99	1.5	VSEQFTAMFR

5.339 231 902 Sodium- and chloride-dependent GABA transporter ine [Crassostrea gigas]

Protein Accession	CL11289.Contig3$_H1$
Mean Expression Ratio	0.923
Median Expression Ratio	0.923
Credible Interval	(0.7, 1.21)
Associated Peptides	3
Associated Spectra	12
Coverage	NaN

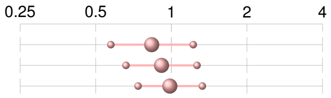

A	B	2.5	50	97.5	Sequence
1	1	0.58	0.84	1.2	EAFELSFK
4	0	0.66	0.91	1.3	VPEIDAAAPFYGIPGADLADVSK
3	3	0.74	0.98	1.3	LVEFFQK

5.340 635 1294 minus strand Kyphoscoliosis peptidase [Crassostrea gigas]; 643 1302 minus strand Kyphoscoliosis peptidase [Crassostrea gigas]; 635 1294 minus strand Kyphoscoliosis peptidase [Crassostrea gigas]; 634 1293 minus strand Kyphoscoliosis peptidase [Crassostrea gigas]; 634 1293 minus strand Kyphoscoliosis peptidase [Crassostrea gigas]; 634 1293 minus strand Kyphoscoliosis peptidase [Crassostrea gigas]; 639 1298 minus strand Kyphoscoliosis peptidase [Crassostrea gigas]

Protein Accession	CL7772.Contig20$_{H}$1 CL7772.Contig1$_{H}$1 CL7772.Contig19$_{H}$1 CL7772.Contig16$_{H}$1 CL7772.(
Mean Expression Ratio	0.926
Median Expression Ratio	0.924
Credible Interval	(0.723, 1.19)
Associated Peptides	7
Associated Spectra	36
Coverage	NaN

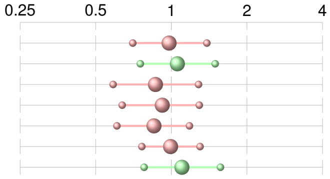

A	B	2.5	50	97.5	Sequence
2	2	0.7	0.98	1.4	FQIFALPTSDDSK
4	1	0.75	1.1	1.5	GAGEVWEGTAALDK
0	1	0.59	0.87	1.3	GAGEVWEGTAALDKHR
3	1	0.64	0.92	1.3	VTLNACFGEDESK
3	2	0.61	0.85	1.2	QYAPWR
7	6	0.76	0.99	1.3	LSNVTFK
3	1	0.78	1.1	1.6	AVYPFPK

5.341 280 1239 minus strand PREDICTED: smoothelin isoform 2 [Oryctolagus cuniculus]; 280 1239 minus strand PREDICTED: smoothelin isoform 2 [Oryctolagus cuniculus]; 280 1239 PREDICTED: smoothelin isoform 2 [Oryctolagus cuniculus]; 280 1239 minus strand PREDICTED: smoothelin isoform 2 [Oryctolagus cuniculus]; 280 1239 PREDICTED: smoothelin isoform 2 [Oryctolagus cuniculus]; 280 1239 minus strand PREDICTED: smoothelin isoform 2 [Oryctolagus cuniculus]; 280 1239 minus strand PREDICTED: smoothelin isoform 2 [Oryctolagus cuniculus]

Protein Accession	CL2374.Contig7$_{H}$1 CL2374.Contig6$_{H}$1 CL2374.Contig5$_{H}$1 CL2374.Contig4$_{H}$1 CL2374.Con
Mean Expression Ratio	1.08
Median Expression Ratio	1.08
Credible Interval	(0.773, 1.52)
Associated Peptides	3
Associated Spectra	5
Coverage	NaN

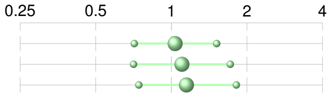

A	B	2.5	50	97.5	Sequence
2	1	0.71	1	1.5	HNFQLAFDTAEK
1	0	0.71	1.1	1.7	LMNATDSFDER
0	1	0.74	1.1	1.8	PLLDVEDMVK

5.342 " 656 787 collagen alpha-1(XII) chain-like; K08132 collagen, type XII, alpha"

Protein Accession	Unigene26545$_{H}$1
Mean Expression Ratio	1.08
Median Expression Ratio	1.08
Credible Interval	(0.757, 1.54)
Associated Peptides	2
Associated Spectra	4
Coverage	NaN

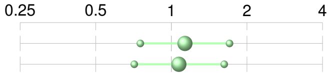

A	B	2.5	50	97.5	Sequence
1	1	0.75	1.1	1.7	AEVFDAVDNIPYR
1	1	0.71	1.1	1.6	TKAEVFDAVDNIPYR

5.343 102 1475 minus strand Cytosolic non-specific dipeptidase [Crassostrea gigas]

Protein Accession	CL4706.Contig1$_H1$
Mean Expression Ratio	1.08
Median Expression Ratio	1.08
Credible Interval	(0.695, 1.7)
Associated Peptides	1
Associated Spectra	1
Coverage	NaN

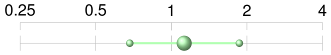

A	B	2.5	50	97.5	Sequence
**1**	**0**	**0.68**	**1.1**	**1.9**	**LVPDQEPHVIESLVR**

5.344 " 3195 3968 Spectrin alpha chain, putative [Pediculus humanus corporis] gi"

Protein Accession	CL2195.Contig1$_H1$
Mean Expression Ratio	1.08
Median Expression Ratio	1.08
Credible Interval	(0.846, 1.39)
Associated Peptides	6
Associated Spectra	16
Coverage	NaN

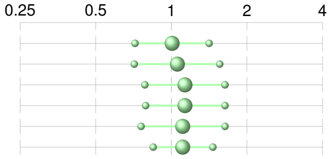

A	B	2.5	50	97.5	Sequence
**2**	**1**	**0.72**	**1**	**1.4**	**DLSSVQTLLTK**
**1**	**0**	**0.71**	**1.1**	**1.6**	**LIDEGNHNADGIVER**
**1**	**1**	**0.78**	**1.1**	**1.6**	**LLLLQEQYR**
**1**	**1**	**0.79**	**1.1**	**1.6**	**LSELEAAASQR**
**1**	**0**	**0.76**	**1.1**	**1.6**	**QIEDLYLTFAK**
**4**	**3**	**0.85**	**1.1**	**1.5**	**YNDVIAR**

5.345 87 485 Sex-regulated protein janus-A [Crassostrea gigas]; 92 310 PREDICTED: hypothetical protein [Saccoglossus kowalevskii]; 92 310 PREDICTED: hypothetical protein [Saccoglossus kowalevskii]

Protein Accession	CL13131.Contig3$_H1$ CL13131.Contig2$_H1$ CL13131.Contig1$_H1$
Mean Expression Ratio	1.08
Median Expression Ratio	1.08
Credible Interval	(0.698, 1.69)
Associated Peptides	1
Associated Spectra	1
Coverage	NaN

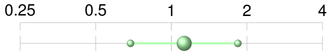

A	B	2.5	50	97.5	Sequence
**1**	**0**	**0.69**	**1.1**	**1.8**	**VVGEIESK**

5.346 813 1244 Probable protein disulfide-isomerase A6 OS=Caenorhabditis elegans GN=tag-320 PE=2 SV=1

Protein Accession	CL39540.Contig4$_{H}1$
Mean Expression Ratio	0.926
Median Expression Ratio	0.926
Credible Interval	(0.593, 1.44)
Associated Peptides	1
Associated Spectra	2
Coverage	NaN

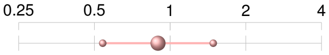

A	B	2.5	50	97.5	Sequence
1	1	0.54	0.89	1.5	TASDIINWATEK

5.347 51 1295 PREDICTED: phosphoglycerate kinase-like [Nasonia vitripennis]; 97 1341 PREDICTED: phosphoglycerate kinase-like [Nasonia vitripennis]

Protein Accession	CL14417.Contig13$_{H}1$ CL14417.Contig12$_{H}1$
Mean Expression Ratio	1.08
Median Expression Ratio	1.08
Credible Interval	(0.953, 1.23)
Associated Peptides	24
Associated Spectra	79
Coverage	NaN

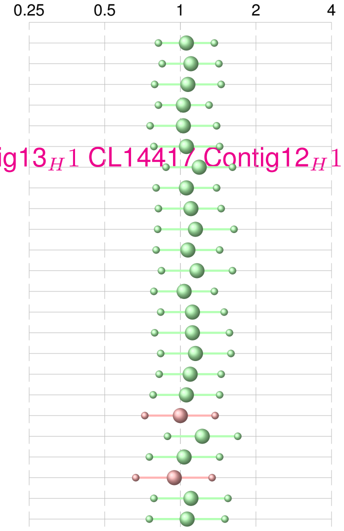

A	B	2.5	50	97.5	Sequence
4	3	0.82	1.1	1.4	ALMDAVVEATK
4	2	0.85	1.1	1.4	ATQEDIDKFR
2	1	0.79	1.1	1.5	FHVEEEGKGVAPDGTK
6	4	0.82	1	1.3	IVQGLMDK
2	1	0.76	1	1.4	LGDIYVNDAFGTAHR
1	2	0.78	1.1	1.4	LSIDKVDVK
3	0	0.88	1.2	1.6	SGIPADWMGLDIGPK
3	2	0.8	1.1	1.4	SVVLMSHLGRPDGTK
2	2	0.82	1.1	1.5	VDFNVPLKDGK
1	0	0.81	1.2	1.6	VLNNMEIGK
2	2	0.8	1.1	1.4	VLPGVAALTDA
2	0	0.84	1.2	1.6	VSMVSTGGGASLELLEGK
2	3	0.79	1	1.4	YSMKPVADELK
4	0	0.84	1.1	1.5	KELMYFAK
1	0	0.79	1.1	1.6	FTDAVK
2	0	0.83	1.1	1.6	VDFNVPLK
4	0	0.82	1.1	1.5	ASGFLLK
2	1	0.78	1.1	1.4	ELMYFAK
1	1	0.72	1	1.4	AHSSMVGCQLPIR
2	0	0.89	1.2	1.7	KNNVQIHLPEDFVTGDKFDEK
1	1	0.75	1	1.4	SLFDEEGAK
1	0	0.66	0.94	1.3	FHVEEEGK
0	1	0.78	1.1	1.6	LENLR
0	1	0.75	1.1	1.5	IVAALPTIK

5.348 3 239 minus strand alcohol dehydrogenase class 3 [Branchiostoma lanceolatum]

Protein Accession	Unigene58195$_{H}1$
Mean Expression Ratio	1.08
Median Expression Ratio	1.08
Credible Interval	(0.752, 1.55)
Associated Peptides	2
Associated Spectra	4
Coverage	NaN

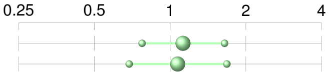

A	B	2.5	50	97.5	Sequence
3	0	0.77	1.1	1.6	GSAFGGWK
1	0	0.69	1.1	1.7	LVEAYMNGK

5.349 260 892 minus strand calponin [Haliotis diversicolor]; 260 892 minus strand calponin [Haliotis diversicolor]

Protein Accession	CL48702.Contig2$_H$1 CL48702.Contig1$_H$1
Mean Expression Ratio	0.928
Median Expression Ratio	0.927
Credible Interval	(0.786, 1.1)
Associated Peptides	12
Associated Spectra	105
Coverage	NaN

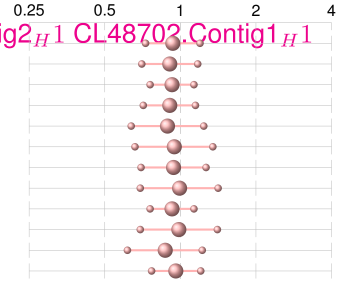

A	B	2.5	50	97.5	Sequence
7	4	0.73	0.93	1.2	AATGQQFPIEESNER
7	3	0.7	0.91	1.2	NFSEDKLR
18	8	0.76	0.93	1.1	TFQPASNLAFEAAR
7	5	0.71	0.91	1.2	VESSFDPEDAKK
1	1	0.64	0.89	1.2	YKVMENFYK
0	1	0.66	0.94	1.3	AFGIEVESHPSYTGQR
1	3	0.7	0.94	1.3	VESSFDPEDAK
0	1	0.69	0.99	1.4	FPIEESNER
7	11	0.76	0.92	1.1	IELFIQK
0	1	0.69	0.98	1.4	PASNLAFEAAR
1	1	0.62	0.87	1.2	VLACIR
12	5	0.77	0.96	1.2	VMENFYK

5.350 " 145 1755 Fumarate hydratase class I, aerobic [Crassostrea gigas]"

Protein Accession	CL32374.Contig1$_H$1
Mean Expression Ratio	1.08
Median Expression Ratio	1.08
Credible Interval	(0.775, 1.49)
Associated Peptides	3
Associated Spectra	6
Coverage	NaN

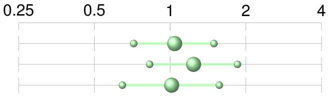

A	B	2.5	50	97.5	Sequence
2	1	0.72	1	1.5	IGIGAQFGGK
1	1	0.83	1.2	1.9	KVDTPWK
0	1	0.65	1	1.6	LEQLE

5.351 336 641 minus strand malate dehydrogenase [Schistosoma japonicum]

Protein Accession	CL11441.Contig9$_H$1
Mean Expression Ratio	1.08
Median Expression Ratio	1.08
Credible Interval	(0.694, 1.68)
Associated Peptides	1
Associated Spectra	1
Coverage	NaN

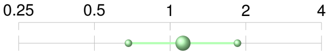

A	B	2.5	50	97.5	Sequence
0	1	0.68	1.1	1.9	ALADLLVAADHR

5.352 1732 2079 minus strand PREDICTED: annexin A7-like [Ornithorhynchus anatinus]

Protein Accession	CL9274.Contig2$_H$1
Mean Expression Ratio	0.927
Median Expression Ratio	0.928
Credible Interval	(0.599, 1.45)
Associated Peptides	1
Associated Spectra	1
Coverage	NaN

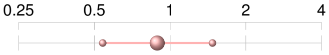

A	B	2.5	50	97.5	Sequence
1	0	0.54	0.89	1.5	DLLSAISSETSGDFETGLR

5.353 4 675 Laminin subunit gamma-1 [Crassostrea gigas]

Protein Accession	CL35712.Contig1$_{H}1$
Mean Expression Ratio	1.08
Median Expression Ratio	1.08
Credible Interval	(0.786, 1.46)
Associated Peptides	6
Associated Spectra	6
Coverage	NaN

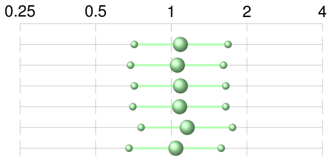

A	B	2.5	50	97.5	Sequence
1	0	0.71	1.1	1.7	AAQLTASETASK
1	0	0.69	1.1	1.6	LAQLAEDTAIQASAEAR
1	0	0.71	1.1	1.6	TLQTLLEFDR
1	0	0.7	1.1	1.6	VPEIEK
1	0	0.76	1.2	1.7	TLSQEVNDAEGR
0	1	0.68	1	1.6	ANIEAEIK

5.354 " 169 792 minus strand rho GDP dissociation inhibitor, putative [Ixodes scapularis] gi"

Protein Accession	CL39798.Contig1$_{H}1$
Mean Expression Ratio	1.08
Median Expression Ratio	1.08
Credible Interval	(0.761, 1.54)
Associated Peptides	3
Associated Spectra	4
Coverage	NaN

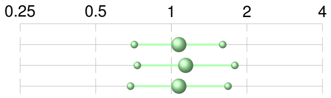

A	B	2.5	50	97.5	Sequence
2	0	0.71	1.1	1.6	IIIDDSNPLNVIVK
1	0	0.73	1.1	1.8	LAISVEGRPDAELDLTKPK
0	1	0.69	1.1	1.7	DIVAGLR

5.355 3 416 minus strand myosin essential light chain [Crassostrea gigas]

Protein Accession	Unigene47974$_{H}1$
Mean Expression Ratio	1.08
Median Expression Ratio	1.08
Credible Interval	(0.76, 1.53)
Associated Peptides	2
Associated Spectra	5
Coverage	NaN

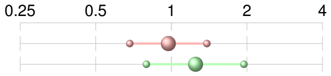

A	B	2.5	50	97.5	Sequence
0	4	0.68	0.97	1.4	CVGLNPTLATIK
0	1	0.79	1.2	2	NPTLATIK

5.356 104 418 Tubulin-specific chaperone A [Crassostrea gigas]; 104 418 minus strand Tubulin-specific chaperone A [Crassostrea gigas]; 104 418 Tubulin-specific chaperone A [Crassostrea gigas]

Protein Accession	Unigene53595$_{H}1$ Unigene32457$_{H}1$ CL7190.Contig3$_{H}1$
Mean Expression Ratio	1.08
Median Expression Ratio	1.08
Credible Interval	(0.692, 1.68)
Associated Peptides	1
Associated Spectra	2
Coverage	NaN

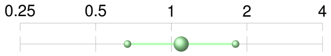

A	B	2.5	50	97.5	Sequence
1	1	0.67	1.1	1.8	ADLGETEELK

5.357 1592 1768 minus strand beta-actin [Diabolocatantops pinguis]

Protein Accession	CL22741.Contig2$_H$1
Mean Expression Ratio	1.08
Median Expression Ratio	1.08
Credible Interval	(0.812, 1.42)
Associated Peptides	2
Associated Spectra	17
Coverage	NaN

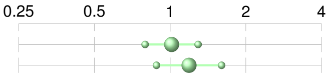

A	B	2.5	50	97.5	Sequence
**1**	**10**	**0.79**	**1**	**1.3**	**EISALAPPTMK**
**3**	**3**	**0.88**	**1.2**	**1.6**	**VIAPPER**

5.358 1 264 Spectrin beta chain [Crassostrea gigas]

Protein Accession	Unigene36334$_H$1
Mean Expression Ratio	1.07
Median Expression Ratio	1.08
Credible Interval	(0.715, 1.59)
Associated Peptides	2
Associated Spectra	2
Coverage	NaN

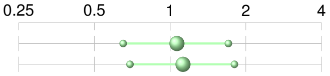

A	B	2.5	50	97.5	Sequence
**1**	**0**	**0.65**	**1.1**	**1.7**	**QLLQVEHPNAAEVVQR**
**1**	**0**	**0.69**	**1.1**	**1.8**	**VAVVNQLAR**

5.359 54 791 GMP reductase 2 [Crassostrea gigas]

Protein Accession	CL26888.Contig1$_H$1
Mean Expression Ratio	1.07
Median Expression Ratio	1.08
Credible Interval	(0.697, 1.66)
Associated Peptides	1
Associated Spectra	1
Coverage	NaN

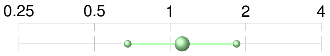

A	B	2.5	50	97.5	Sequence
**1**	**0**	**0.68**	**1.1**	**1.8**	**LFTTIHK**

5.360 156 1163 cathepsin B [Crassostrea ariakensis]

Protein Accession	CL26836.Contig9$_H$1
Mean Expression Ratio	1.07
Median Expression Ratio	1.08
Credible Interval	(0.714, 1.63)
Associated Peptides	1
Associated Spectra	2
Coverage	NaN

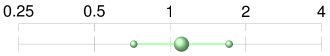

A	B	2.5	50	97.5	Sequence
**1**	**1**	**0.72**	**1.1**	**1.7**	**DLPDSFDPR**

5.361 680 991 minus strand PDZ and LIM domain protein 1 [Crassostrea gigas]; 553 864 minus strand PDZ and LIM domain protein 1 [Crassostrea gigas]

Protein Accession	Unigene7664$_H$1 Unigene7663$_H$1
Mean Expression Ratio	1.08
Median Expression Ratio	1.08
Credible Interval	(0.86, 1.34)
Associated Peptides	6
Associated Spectra	23
Coverage	NaN

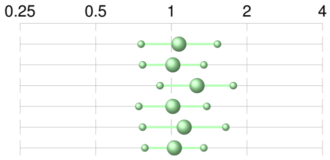

A	B	2.5	50	97.5	Sequence
2	0	0.76	1.1	1.5	AMGQPWGFR
3	3	0.77	1	1.3	DYGQALSVK
3	0	0.9	1.3	1.8	GPSTDFSAIKPK
3	1	0.74	1	1.4	SAGTQLQLTVLR
1	0	0.77	1.1	1.6	KVQPGTPSAATLRPGDVIQR
3	4	0.78	1	1.3	QLTHMQAHQLIK

5.362 95 2506 Transitional endoplasmic reticulum ATPase [Crassostrea gigas]

Protein Accession	CL15973.Contig2$_H$1
Mean Expression Ratio	1.08
Median Expression Ratio	1.08
Credible Interval	(0.831, 1.41)
Associated Peptides	9
Associated Spectra	11
Coverage	NaN

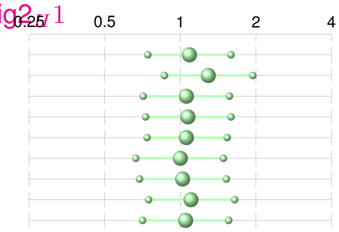

A	B	2.5	50	97.5	Sequence
1	1	0.74	1.1	1.6	EMVELPLR
1	0	0.86	1.3	1.9	LDQLIYIPLPDEK
1	0	0.71	1.1	1.6	MAIEAEIR
1	0	0.73	1.1	1.6	MDELQLFR
1	1	0.74	1.1	1.5	EVDIGIPDATGR
0	1	0.67	1	1.5	DIDLDYLAK
0	1	0.69	1	1.5	GILLYGPPGTGK
0	1	0.75	1.1	1.7	AHFEEAMK
0	1	0.71	1.1	1.6	GDIFLVR

5.363 585 944 minus strand Protein disulfide-isomerase A6 [Crassostrea gigas]

Protein Accession	CL39540.Contig7$_H$1
Mean Expression Ratio	0.928
Median Expression Ratio	0.93
Credible Interval	(0.618, 1.4)
Associated Peptides	2
Associated Spectra	3
Coverage	NaN

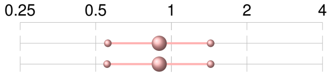

A	B	2.5	50	97.5	Sequence
1	1	0.56	0.89	1.4	TASDIINWATEK
1	0	0.56	0.89	1.4	GFPTIK

5.364 633 806 Peroxiredoxin-1 [Crassostrea gigas]; 648 821 Peroxiredoxin-1 [Crassostrea gigas]; 666 839 Peroxiredoxin-1 [Crassostrea gigas]; 625 798 Peroxiredoxin-1 [Crassostrea gigas]; 633 824 Peroxiredoxin-1 [Crassostrea gigas]

Protein Accession	CL3160.Contig18$_H$1 CL3160.Contig17$_H$1 CL3160.Contig16$_H$1 CL3160.Contig15$_H$1 CL3160
Mean Expression Ratio	0.93
Median Expression Ratio	0.93
Credible Interval	(0.657, 1.31)
Associated Peptides	1
Associated Spectra	13
Coverage	NaN

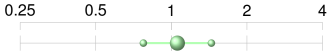

A	B	2.5	50	97.5	Sequence
7	6	0.77	1.1	1.4	LVQAFQFTDK

5.365 247 762 Myophilin [Crassostrea gigas]; 244 759 Myophilin [Crassostrea gigas]; 119 634 Myophilin [Crassostrea gigas]

Protein Accession	CL16175.Contig6$_H$1 CL16175.Contig5$_H$1 CL16175.Contig4$_H$1
Mean Expression Ratio	0.93
Median Expression Ratio	0.93
Credible Interval	(0.673, 1.28)
Associated Peptides	3
Associated Spectra	6
Coverage	NaN

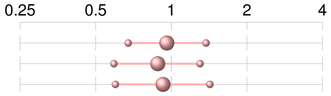

A	B	2.5	50	97.5	Sequence
2	1	0.67	0.96	1.4	GGGFALMQNIER
1	1	0.59	0.88	1.3	LMPGCIK
1	0	0.6	0.92	1.4	DGIVLCK

5.366 3 239 minus strand p38 MAPK [Littorina littorea]

Protein Accession	CL49144.Contig2$_H$1
Mean Expression Ratio	0.93
Median Expression Ratio	0.931
Credible Interval	(0.599, 1.43)
Associated Peptides	1
Associated Spectra	1
Coverage	NaN

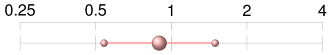

A	B	2.5	50	97.5	Sequence
0	1	0.54	0.9	1.5	MLDLDTDHR

5.367 3 551 minus strand Spectrin beta chain [Crassostrea gigas]

Protein Accession	Unigene35960$_H$1
Mean Expression Ratio	0.931
Median Expression Ratio	0.931
Credible Interval	(0.626, 1.39)
Associated Peptides	2
Associated Spectra	2
Coverage	NaN

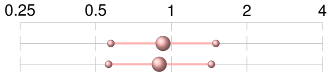

A	B	2.5	50	97.5	Sequence
0	1	0.57	0.93	1.5	ELALREELIR
0	1	0.56	0.9	1.4	IVEKPPKFDEK

5.368 635 1561 Uncharacterized protein yfeX [Crassostrea gigas]

Protein Accession	CL12046.Contig4$_{H}$1
Mean Expression Ratio	1.07
Median Expression Ratio	1.07
Credible Interval	(0.725, 1.61)
Associated Peptides	2
Associated Spectra	2
Coverage	NaN

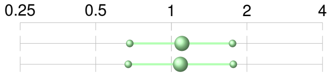

A	B	2.5	50	97.5	Sequence
**1**	**0**	**0.68**	**1.1**	**1.8**	**TLESWVGR**
**1**	**0**	**0.67**	**1.1**	**1.8**	**LFDLAQLVLK**

5.369 4 243 40S ribosomal protein S13 OS=Caenorhabditis elegans GN=rps-13 PE=2 SV=2; 4 243 40S ribosomal protein S13 OS=Caenorhabditis elegans GN=rps-13 PE=2 SV=2; 1 252 40S ribosomal protein S13 OS=Xenopus laevis GN=rps13 PE=3 SV=2; 1 213 40S ribosomal protein S13 OS=Xenopus laevis GN=rps13 PE=3 SV=2

Protein Accession	CL18049.Contig7$_{H}$1 CL18049.Contig4$_{H}$1 CL18049.Contig3$_{H}$1 CL18049.Contig2$_{H}$1
Mean Expression Ratio	1.07
Median Expression Ratio	1.07
Credible Interval	(0.698, 1.67)
Associated Peptides	1
Associated Spectra	1
Coverage	NaN

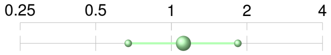

A	B	2.5	50	97.5	Sequence
**1**	**0**	**0.67**	**1.1**	**1.8**	**SLIKK**

5.370 194 775 minus strand Collagen alpha-1(XV) chain [Harpegnathos saltator]

Protein Accession	Unigene19084$_{H}$1
Mean Expression Ratio	1.07
Median Expression Ratio	1.07
Credible Interval	(0.719, 1.6)
Associated Peptides	2
Associated Spectra	2
Coverage	NaN

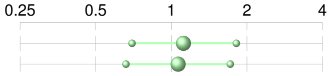

A	B	2.5	50	97.5	Sequence
**1**	**0**	**0.7**	**1.1**	**1.8**	**GADYECHK**
**1**	**0**	**0.66**	**1.1**	**1.7**	**IIWHGSSK**

5.371 99 3011 Eukaryotic translation initiation factor 3 subunit A [Crassostrea gigas]

Protein Accession	CL36771.Contig2$_{H}1$
Mean Expression Ratio	0.932
Median Expression Ratio	0.932
Credible Interval	(0.669, 1.3)
Associated Peptides	2
Associated Spectra	8
Coverage	NaN

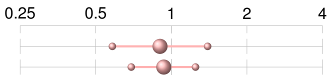

A	B	2.5	50	97.5	Sequence
**1**	**0**	**0.58**	**0.9**	**1.4**	**NNSRVER**
**7**	**0**	**0.69**	**0.93**	**1.2**	**LFNLSREQRK**

5.372 9 161 PREDICTED: tubulin alpha-1C chain-like [Acyrthosiphon pisum]; 9 161 Tubulin alpha-3 chain (Fragments) OS=Mesocricetus auratus PE=1 SV=1

Protein Accession	Unigene74551$_{H}1$ Unigene70816$_{H}1$
Mean Expression Ratio	0.933
Median Expression Ratio	0.932
Credible Interval	(0.595, 1.44)
Associated Peptides	1
Associated Spectra	1
Coverage	NaN

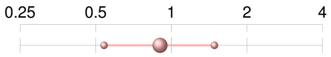

A	B	2.5	50	97.5	Sequence
**0**	**1**	**0.54**	**0.9**	**1.5**	**IHFPLATY**

5.373 126 287 minus strand Cytochrome b-c1 complex subunit 10 [Cricetulus griseus]; 126 287 minus strand Cytochrome b-c1 complex subunit 10 [Cricetulus griseus]; 126 287 minus strand Cytochrome b-c1 complex subunit 10 [Cricetulus griseus]; 126 287 minus strand Cytochrome b-c1 complex subunit 10 [Cricetulus griseus]

Protein Accession	CL46231.Contig6$_{H}1$ CL46231.Contig5$_{H}1$ CL46231.Contig4$_{H}1$ CL46231.Contig3$_{H}1$
Mean Expression Ratio	0.935
Median Expression Ratio	0.932
Credible Interval	(0.601, 1.46)
Associated Peptides	1
Associated Spectra	1
Coverage	NaN

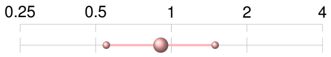

A	B	2.5	50	97.5	Sequence
**0**	**1**	**0.55**	**0.91**	**1.5**	**FIPFYGSK**

5.374 1497 1889 UDP-glucuronosyltransferase 2C1 (Fragment) OS=Oryctolagus cuniculus GN=UGT2C1 PE=2 SV=1

Protein Accession	CL29849.Contig1_H1
Mean Expression Ratio	1.07
Median Expression Ratio	1.07
Credible Interval	(0.683, 1.66)
Associated Peptides	1
Associated Spectra	2
Coverage	NaN

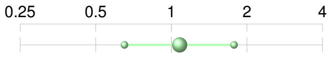

A	B	2.5	50	97.5	Sequence
1	1	0.65	1.1	1.8	LFVSHCGK

5.375 62 1093 Heterogeneous nuclear ribonucleoprotein A2-like protein 1 [Crassostrea gigas]

Protein Accession	CL47083.Contig4_H1
Mean Expression Ratio	0.932
Median Expression Ratio	0.933
Credible Interval	(0.696, 1.25)
Associated Peptides	5
Associated Spectra	8
Coverage	NaN

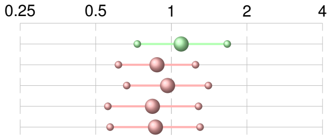

A	B	2.5	50	97.5	Sequence
1	0	0.73	1.1	1.7	ETFSPFGTISQIDLITDK
2	1	0.62	0.88	1.3	EDTTEDMIR
1	1	0.66	0.96	1.4	MFVGGIKEDTTEDMIR
0	1	0.56	0.84	1.3	GGSRGGR
0	1	0.57	0.86	1.3	GGGRGGSR

5.376 160 393 minus strand PREDICTED: cysteine-rich protein 1-like [Megachile rotundata]; 148 381 PREDICTED: cysteine-rich protein 1-like [Megachile rotundata]; 148 381 PREDICTED: cysteine-rich protein 1-like [Megachile rotundata]; 148 381 PREDICTED: cysteine-rich protein 1-like [Megachile rotundata]; 160 393 minus strand PREDICTED: cysteine-rich protein 1-like [Megachile rotundata]; 160 393 minus strand PREDICTED: cysteine-rich protein 1-like [Megachile rotundata]

Protein Accession	CL11328.Contig9_H1 CL11328.Contig8_H1 CL11328.Contig7_H1 CL11328.Contig6_H1 CL1132
Mean Expression Ratio	1.07
Median Expression Ratio	1.07
Credible Interval	(0.749, 1.53)
Associated Peptides	2
Associated Spectra	4
Coverage	NaN

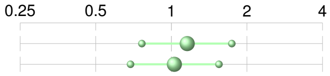

A	B	2.5	50	97.5	Sequence
1	1	0.76	1.2	1.7	DWHAACLK
1	1	0.69	1	1.5	EVYFAER

5.377 313 2763 NCAM-related cell adhesion molecule precursor [Aplysia californica] gi

Protein Accession	Unigene11449$_{H}1$
Mean Expression Ratio	0.934
Median Expression Ratio	0.933
Credible Interval	(0.671, 1.29)
Associated Peptides	5
Associated Spectra	5
Coverage	NaN

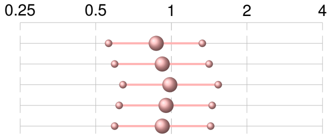

A	B	2.5	50	97.5	Sequence
1	0	0.56	0.87	1.3	EALEVDTSK
1	0	0.6	0.92	1.4	IGSSDPTVFVFK
1	0	0.64	0.98	1.5	NLAGSYDIEER
1	0	0.62	0.95	1.5	VPMTGGAEIANYVIEYR
0	1	0.59	0.92	1.4	AGAIASDETQLEVSIPPK

5.378 293 1795 Amidase [Crassostrea gigas]

Protein Accession	CL5248.Contig6$_{H}1$
Mean Expression Ratio	1.07
Median Expression Ratio	1.07
Credible Interval	(0.713, 1.62)
Associated Peptides	1
Associated Spectra	2
Coverage	NaN

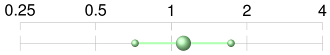

A	B	2.5	50	97.5	Sequence
2	0	0.72	1.1	1.7	LSTIGVTHFDR

5.379 36 770 minus strand Endoplasmic reticulum protein ERp29 [Crassostrea gigas]

Protein Accession	CL37188.Contig1$_{H}1$
Mean Expression Ratio	1.07
Median Expression Ratio	1.07
Credible Interval	(0.719, 1.61)
Associated Peptides	2
Associated Spectra	2
Coverage	NaN

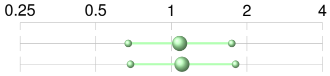

A	B	2.5	50	97.5	Sequence
1	0	0.67	1.1	1.7	KAQTAAAALTK
1	0	0.69	1.1	1.8	KPDFPVYK

5.380 37 885 glyceraldehyde 3-phosphate dehydrogenase [Dugesia japonica]

Protein Accession	CL47424.Contig2$_{H}1$
Mean Expression Ratio	1.07
Median Expression Ratio	1.07
Credible Interval	(0.767, 1.48)
Associated Peptides	1
Associated Spectra	12
Coverage	NaN

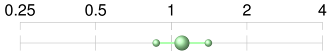

A	B	2.5	50	97.5	Sequence
10	2	0.87	1.1	1.4	GAAQNIIPASTGAAK

5.381 77 2278 Far upstream element-binding protein 3 [Crassostrea gigas]

Protein Accession	CL5030.Contig3$_H$1
Mean Expression Ratio	1.07
Median Expression Ratio	1.07
Credible Interval	(0.727, 1.61)
Associated Peptides	2
Associated Spectra	2
Coverage	NaN

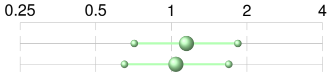

A	B	2.5	50	97.5	Sequence
**1**	**0**	**0.71**	**1.1**	**1.8**	**EMVLELLAEK**
**1**	**0**	**0.65**	**1**	**1.7**	**LFNIR**

5.382 151 1110 minus strand hypothetical protein DAPPUDRAFT_303085 [Daphnia pulex]

Protein Accession	CL12652.Contig2$_H$1
Mean Expression Ratio	0.937
Median Expression Ratio	0.935
Credible Interval	(0.637, 1.38)
Associated Peptides	1
Associated Spectra	3
Coverage	NaN

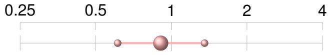

A	B	2.5	50	97.5	Sequence
**2**	**1**	**0.61**	**0.91**	**1.4**	**TVIITGANTGIGK**

5.383 2295 2510 beta-thymosin domain repeat protein CSP24KDa_v1 [Hermissenda crassicornis] gi

Protein Accession	CL9548.Contig1$_H$1
Mean Expression Ratio	1.07
Median Expression Ratio	1.07
Credible Interval	(0.686, 1.64)
Associated Peptides	1
Associated Spectra	1
Coverage	NaN

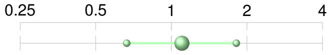

A	B	2.5	50	97.5	Sequence
**1**	**0**	**0.67**	**1.1**	**1.8**	**ESVAAFDK**

5.384 260 1597 isocitrate dehydrogenase [Mytilus galloprovincialis]; 260 1597 isocitrate dehydrogenase [Mytilus galloprovincialis]; 260 1597 isocitrate dehydrogenase [Mytilus galloprovincialis]

Protein Accession	CL10046.Contig8$_H$1 CL10046.Contig7$_H$1 CL10046.Contig6$_H$1
Mean Expression Ratio	1.07
Median Expression Ratio	1.07
Credible Interval	(0.786, 1.45)
Associated Peptides	5
Associated Spectra	7
Coverage	NaN

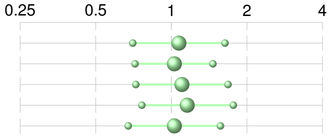

A	B	2.5	50	97.5	Sequence
**1**	**0**	**0.7**	**1.1**	**1.6**	**DQTNDQVTIDSAEAIK**
**1**	**2**	**0.72**	**1**	**1.5**	**LTVFDFK**
**1**	**0**	**0.72**	**1.1**	**1.7**	**VKEFNLK**
**1**	**0**	**0.76**	**1.2**	**1.8**	**TSVLVCPDGK**
**0**	**1**	**0.67**	**1**	**1.6**	**SWPLYMSTK**

5.385 3 347 beta-actin [Meretrix meretrix]; 3 215 Actin-2 OS=Onchocerca volvulus GN=act-2b PE=3 SV=1

Protein Accession	CL47466.Contig2$_{H}$1 CL47466.Contig1$_{H}$1
Mean Expression Ratio	1.07
Median Expression Ratio	1.07
Credible Interval	(0.898, 1.27)
Associated Peptides	11
Associated Spectra	305
Coverage	NaN

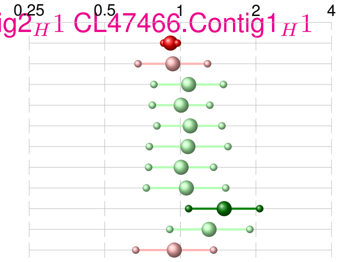

A	B	2.5	50	97.5	Sequence
148	129	0.86	0.91	0.97	EITALAPSTMK
2	1	0.68	0.93	1.3	EITALAPSTMKIK
3	0	0.79	1.1	1.5	KDLYANTVLSGGSTMYPGIADR
7	0	0.77	1	1.3	TVLSGGSTMYPGIADR
4	0	0.81	1.1	1.5	DLYANTVLSGGSTMYPGIADR
1	0	0.75	1.1	1.5	MQKEITALAPSTMK
0	4	0.75	1	1.4	LAPSTMK
0	1	0.73	1.1	1.5	ALAPSTMK
0	3	1.1	1.5	2.1	APSTMK
0	1	0.91	1.3	1.9	PSTMK
0	1	0.66	0.95	1.4	TALAPSTMK

5.386 " 3 587 elongation factor 1 alpha, partial [Phorbas sp. nov. SB-2012]"

Protein Accession	Unigene20048$_{H}$1
Mean Expression Ratio	1.07
Median Expression Ratio	1.07
Credible Interval	(0.781, 1.45)
Associated Peptides	2
Associated Spectra	12
Coverage	NaN

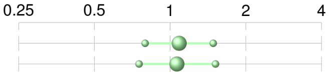

A	B	2.5	50	97.5	Sequence
5	1	0.79	1.1	1.5	IGGIGTVPVGR
3	3	0.75	1.1	1.5	LPLQDVYK

5.387 " 249 1271 N(G),N(G)-dimethylarginine dimethylaminohydrolase 1 [Crassostrea gigas]"

Protein Accession	Unigene64051$_{H}$1
Mean Expression Ratio	1.07
Median Expression Ratio	1.07
Credible Interval	(0.714, 1.61)
Associated Peptides	2
Associated Spectra	3
Coverage	NaN

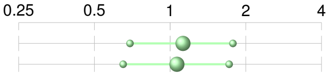

A	B	2.5	50	97.5	Sequence
1	1	0.69	1.1	1.8	LGEVGEIR
1	0	0.65	1.1	1.7	EIFVGIGK

5.388 362 553 putative RNA-binding protein [Haliotis diversicolor]

Protein Accession	CL41007.Contig2$_{H}$1
Mean Expression Ratio	1.07
Median Expression Ratio	1.07
Credible Interval	(0.788, 1.46)
Associated Peptides	2
Associated Spectra	10
Coverage	NaN

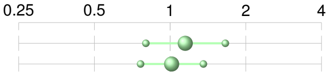

A	B	2.5	50	97.5	Sequence
2	1	0.8	1.2	1.7	ELQDETGCR
3	4	0.76	1	1.4	EVMHVDSSFVGR

5.389 167 679 tropomyosin [Haliotis discus discus]

Protein Accession	CL2925.Contig3$_H1$
Mean Expression Ratio	1.07
Median Expression Ratio	1.07
Credible Interval	(0.948, 1.2)
Associated Peptides	19
Associated Spectra	198
Coverage	NaN

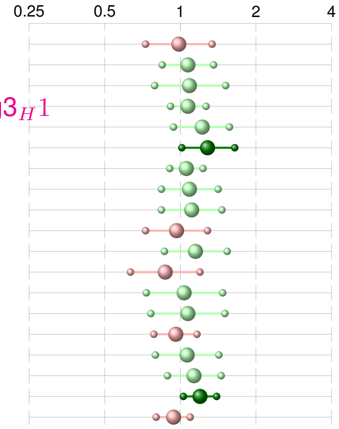

A	B	2.5	50	97.5	Sequence
2	1	0.73	0.99	1.3	AMDKAEQMEQK
6	3	0.85	1.1	1.4	ANEGLIEANTK
1	1	0.79	1.1	1.5	ENAMDKAEQMEQK
22	4	0.91	1.1	1.3	IKLLEEDLER
6	1	0.94	1.2	1.6	KYANLENEYDKANEGLIEANTK
8	0	1	1.3	1.7	MDKENAMDK
24	8	0.91	1.1	1.2	MDKENAMDKAEQMEQK
3	3	0.84	1.1	1.4	YANLENEYDK
5	0	0.84	1.1	1.5	YANLENEYDKANEGLIEANTK
2	2	0.73	0.97	1.3	KMLAMK
4	0	0.87	1.2	1.5	KYANLENEYDK
1	1	0.63	0.87	1.2	ANEGLIEAN
1	0	0.73	1	1.5	SKLEEDLN
1	0	0.76	1.1	1.5	LLEEDLER
3	13	0.79	0.96	1.2	RIKLLEEDLER
4	0	0.79	1.1	1.4	SKLEEDLNPLQK
7	1	0.89	1.1	1.5	LRDTEVQK
14	17	1	1.2	1.4	MDAIK
14	15	0.8	0.94	1.1	MLAMK

5.390 " 285 437 Propionyl-CoA carboxylase alpha chain, mitochondrial [Crassostrea gigas]"

Protein Accession	CL45581.Contig2$_H1$
Mean Expression Ratio	0.939
Median Expression Ratio	0.939
Credible Interval	(0.622, 1.42)
Associated Peptides	1
Associated Spectra	2
Coverage	NaN

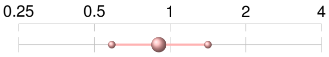

A	B	2.5	50	97.5	Sequence
2	0	0.59	0.9	1.4	GVTHNIPLLR

5.391 161 3001 minus strand E3 ubiquitin-protein ligase MIB2 [Crassostrea gigas]

Protein Accession	CL49497.Contig7$_H1$
Mean Expression Ratio	1.06
Median Expression Ratio	1.07
Credible Interval	(0.687, 1.66)
Associated Peptides	1
Associated Spectra	1
Coverage	NaN

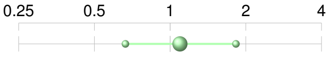

A	B	2.5	50	97.5	Sequence
0	1	0.66	1.1	1.8	GDAHAVEGILQR

5.392 3 263 minus strand beta-actin [Urechis unicinctus]

Protein Accession	CL31420.Contig5$_{H}1$
Mean Expression Ratio	0.94
Median Expression Ratio	0.939
Credible Interval	(0.764, 1.16)
Associated Peptides	4
Associated Spectra	135
Coverage	NaN

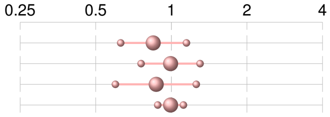

A	B	2.5	50	97.5	Sequence
3	2	0.63	0.85	1.1	IIAPPERK
7	2	0.76	0.99	1.3	IKIIAPPER
0	1	0.6	0.87	1.3	EITALAPPTMK
63	57	0.88	0.99	1.1	IIAPPER

5.393 " 223 423 ADPATP carrier protein; K05863 solute carrier family 25 (mitochondrial adenine nucleotide translocator), member 4/5/6/31"

Protein Accession	CL27196.Contig3$_{H}1$
Mean Expression Ratio	1.06
Median Expression Ratio	1.06
Credible Interval	(0.748, 1.52)
Associated Peptides	2
Associated Spectra	4
Coverage	NaN

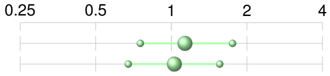

A	B	2.5	50	97.5	Sequence
2	0	0.75	1.1	1.8	LLVQNQNEMIK
0	2	0.67	1	1.6	GVIDCTIR

5.394 98 850 minus strand 14-3-3 protein epsilon [Schistocerca gregaria]

Protein Accession	CL27652.Contig3$_{H}1$
Mean Expression Ratio	1.06
Median Expression Ratio	1.06
Credible Interval	(0.851, 1.33)
Associated Peptides	9
Associated Spectra	19
Coverage	NaN

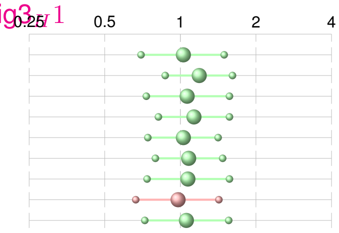

A	B	2.5	50	97.5	Sequence
1	0	0.7	1	1.5	AAFDDAIAELDTLSEESYK
3	1	0.87	1.2	1.6	AASDISMTELPPTHPIR
1	0	0.73	1.1	1.6	EAAENSLVAYK
3	0	0.82	1.1	1.6	HLIPNAGGGESK
1	2	0.74	1	1.4	IISSMEQK
3	1	0.8	1.1	1.5	YDEMVEAMK
1	0	0.73	1.1	1.6	VFYYK
0	1	0.67	0.98	1.4	YLAEFATGNDR
0	1	0.72	1.1	1.6	TELPPTHPIR

5.395 605 1153 Peptidyl-prolyl cis-trans isomerase B [Crassostrea gigas]; 1046 1630 Peptidyl-prolyl cis-trans isomerase B [Crassostrea gigas]; 589 1173 Peptidyl-prolyl cis-trans isomerase B [Crassostrea gigas]; 938 1414 Peptidyl-prolyl cis-trans isomerase B [Crassostrea gigas]; 883 1467 Peptidyl-prolyl cis-trans isomerase B [Crassostrea gigas]

Protein Accession	CL24747.Contig5$_H$1 CL24747.Contig4$_H$1 CL24747.Contig3$_H$1 CL24747.Contig2$_H$1 CL2474
Mean Expression Ratio	1.06
Median Expression Ratio	1.06
Credible Interval	(0.745, 1.53)
Associated Peptides	2
Associated Spectra	6
Coverage	NaN

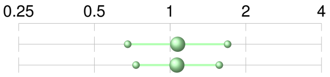

A	B	2.5	50	97.5	Sequence
0	1	0.68	1.1	1.7	DFVIQMGDVTGK
2	3	0.73	1.1	1.6	IASSGVVGIASK

5.396 410 619 minus strand Guanine nucleotide-binding protein subunit gamma-1 [Crassostrea gigas]

Protein Accession	CL36920.Contig1$_H$1
Mean Expression Ratio	1.06
Median Expression Ratio	1.06
Credible Interval	(0.762, 1.49)
Associated Peptides	2
Associated Spectra	6
Coverage	NaN

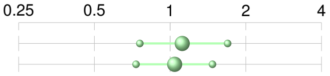

A	B	2.5	50	97.5	Sequence
1	1	0.76	1.1	1.7	CEAQITR
2	2	0.73	1	1.5	LIEQLR

5.397 94 519 minus strand hypothetical protein BRAFLDRAFT_115029 [Branchiostoma floridae] gi

Protein Accession	Unigene52953$_H$1
Mean Expression Ratio	0.94
Median Expression Ratio	0.94
Credible Interval	(0.675, 1.31)
Associated Peptides	2
Associated Spectra	7
Coverage	NaN

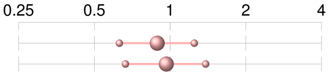

A	B	2.5	50	97.5	Sequence
2	2	0.63	0.89	1.3	SLDTDGDGTVSAAELR
2	1	0.66	0.97	1.4	FINKK

5.398 112 930 calcineurin A [Haliotis discus discus]

Protein Accession	CL3137.Contig1$_H$1
Mean Expression Ratio	1.06
Median Expression Ratio	1.06
Credible Interval	(0.727, 1.56)
Associated Peptides	2
Associated Spectra	3
Coverage	NaN

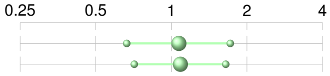

A	B	2.5	50	97.5	Sequence
**0**	**1**	**0.67**	**1.1**	**1.7**	**VTEDVALR**
**0**	**2**	**0.71**	**1.1**	**1.7**	**SEAFDAK**

5.399 76 822 14-3-3 protein [Heterodera glycines]; 76 822 minus strand 14-3-3 protein [Heterodera glycines]

Protein Accession	Unigene34590$_H$1 Unigene28010$_H$1
Mean Expression Ratio	1.06
Median Expression Ratio	1.06
Credible Interval	(0.763, 1.48)
Associated Peptides	2
Associated Spectra	8
Coverage	NaN

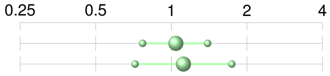

A	B	2.5	50	97.5	Sequence
**4**	**3**	**0.77**	**1**	**1.4**	**VVSSIEQK**
**1**	**0**	**0.72**	**1.1**	**1.7**	**KLEMAK**

5.400 122 1195 minus strand Severin [Crassostrea gigas]

Protein Accession	CL7486.Contig12$_H$1
Mean Expression Ratio	0.942
Median Expression Ratio	0.941
Credible Interval	(0.602, 1.46)
Associated Peptides	1
Associated Spectra	2
Coverage	NaN

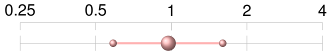

A	B	2.5	50	97.5	Sequence
**1**	**1**	**0.59**	**0.97**	**1.6**	**TDHPLVPITCLTEGTK**

5.401 3 293 Titin [Crassostrea gigas]

Protein Accession	Unigene9241$_H$1
Mean Expression Ratio	0.939
Median Expression Ratio	0.941
Credible Interval	(0.604, 1.46)
Associated Peptides	1
Associated Spectra	1
Coverage	NaN

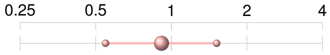

A	B	2.5	50	97.5	Sequence
**1**	**0**	**0.55**	**0.91**	**1.5**	**TAINTVESSLSELGQR**

5.402 3 470 minus strand Spectrin beta chain [Crassostrea gigas]

Protein Accession	CL19757.Contig2$_{H}1$
Mean Expression Ratio	0.94
Median Expression Ratio	0.941
Credible Interval	(0.627, 1.4)
Associated Peptides	1
Associated Spectra	2
Coverage	NaN

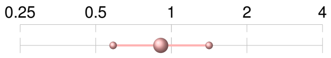

A	B	2.5	50	97.5	Sequence
**2**	**0**	**0.59**	**0.91**	**1.4**	**HSLLEADIHVVGER**

5.403 26 877 Ankyrin-2 [Crassostrea gigas]

Protein Accession	CL18051.Contig2$_{H}1$
Mean Expression Ratio	0.94
Median Expression Ratio	0.941
Credible Interval	(0.668, 1.33)
Associated Peptides	2
Associated Spectra	5
Coverage	NaN

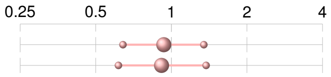

A	B	2.5	50	97.5	Sequence
**2**	**1**	**0.64**	**0.93**	**1.3**	**LPALHIAAK**
**1**	**1**	**0.61**	**0.91**	**1.4**	**DGLTPLHCAAR**

5.404 45 944 minus strand unnamed protein product [Mus musculus] gi; 5 334 minus strand receptor of activated protein kinase C [Platynereis dumerilii]

Protein Accession	CL20494.Contig3$_{H}1$ CL20494.Contig1$_{H}1$
Mean Expression Ratio	1.06
Median Expression Ratio	1.06
Credible Interval	(0.706, 1.6)
Associated Peptides	2
Associated Spectra	2
Coverage	NaN

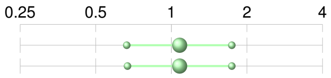

A	B	2.5	50	97.5	Sequence
**1**	**0**	**0.66**	**1.1**	**1.7**	**IWDLEGK**
**1**	**0**	**0.67**	**1.1**	**1.7**	**AMCFSPNR**

5.405 942 1385 Golgi-associated plant pathogenesis-related protein 1 [Crassostrea gigas]; 338 817 Golgi-associated plant pathogenesis-related protein 1 [Crassostrea gigas]; 354 833 Golgi-associated plant pathogenesis-related protein 1 [Crassostrea gigas]; 576 1055 Golgi-associated plant pathogenesis-related protein 1 [Crassostrea gigas]; 618 1097 Golgi-associated plant pathogenesis-related protein 1 [Crassostrea gigas]; 536 979 Golgi-associated plant pathogenesis-related protein 1 [Crassostrea gigas]; 740 1219 minus strand Golgi-associated plant pathogenesis-related protein 1 [Crassostrea gigas]

Protein Accession	Unigene391$_H$1 CL15263.Contig2$_H$1 CL15263.Contig1$_H$1 CL15263.Contig13$_H$1 CL15263.Co
Mean Expression Ratio	1.06
Median Expression Ratio	1.06
Credible Interval	(0.705, 1.58)
Associated Peptides	1
Associated Spectra	2
Coverage	NaN

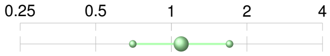

A	B	2.5	50	97.5	Sequence
1	1	0.7	1.1	1.7	IITEETIIK

5.406 " 1 1815 Glycogen phosphorylase, muscle form [Crassostrea gigas]"

Protein Accession	CL20630.Contig5$_H$1
Mean Expression Ratio	0.943
Median Expression Ratio	0.942
Credible Interval	(0.766, 1.16)
Associated Peptides	9
Associated Spectra	38
Coverage	NaN

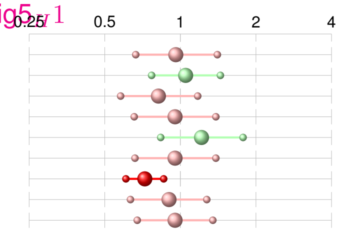

A	B	2.5	50	97.5	Sequence
1	0	0.66	0.96	1.4	VVFLENYR
2	2	0.77	1.1	1.4	VVNNDPIIGDR
1	1	0.58	0.82	1.2	TSFDMFPEK
1	0	0.66	0.95	1.4	WPVSMLER
1	0	0.84	1.2	1.8	DFAQMFPER
1	0	0.66	0.96	1.4	FGNPWEK
16	8	0.61	0.72	0.86	IEELEEIEEDAGL
0	2	0.63	0.9	1.3	AWDLVVR
0	2	0.67	0.95	1.3	LLLCNSGLADVIAEK

5.407 2 307 Laminin subunit gamma-1 [Crassostrea gigas]

Protein Accession	Unigene32982$_H$1
Mean Expression Ratio	0.941
Median Expression Ratio	0.942
Credible Interval	(0.603, 1.46)
Associated Peptides	1
Associated Spectra	1
Coverage	NaN

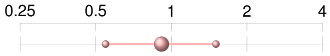

A	B	2.5	50	97.5	Sequence
0	1	0.55	0.91	1.5	AEISLR

5.408 187 696 minus strand putative ribosomal protein S10 [Sipunculus nudus]

Protein Accession Unigene1233$_H$1
Mean Expression Ratio 0.942
Median Expression Ratio 0.942
Credible Interval (0.599, 1.47)
Associated Peptides 1
Associated Spectra 3
Coverage NaN

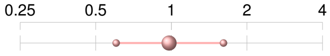

A	B	2.5	50	97.5	Sequence
2	1	0.6	0.98	1.6	KDFFSPK

5.409 75 803 hypothetical protein DAPPUDRAFT_231350 [Daphnia pulex]

Protein Accession CL10537.Contig4$_H$1
Mean Expression Ratio 0.942
Median Expression Ratio 0.942
Credible Interval (0.698, 1.27)
Associated Peptides 4
Associated Spectra 7
Coverage NaN

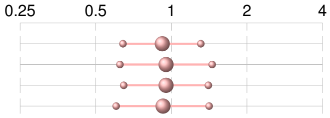

A	B	2.5	50	97.5	Sequence
2	1	0.64	0.92	1.3	LDYQLEIQSAK
1	0	0.62	0.95	1.5	SITPQQEAESIK
1	1	0.65	0.95	1.4	VCIAQLK
1	0	0.6	0.93	1.4	ENVALQLEASYR

5.410 552 1499 minus strand uncharacterized protein LOC100158466 [Xenopus (Silurana) tropicalis] gi

Protein Accession Unigene57645$_H$1
Mean Expression Ratio 0.942
Median Expression Ratio 0.942
Credible Interval (0.632, 1.41)
Associated Peptides 2
Associated Spectra 3
Coverage NaN

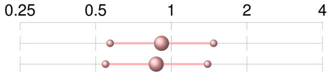

A	B	2.5	50	97.5	Sequence
1	0	0.57	0.92	1.5	TAFSLLK
1	1	0.55	0.87	1.4	TGEPVEVDGELATLLAR

5.411 27 377 minus strand ribosomal protein LP2 [Papilio xuthus] gi; 27 377 minus strand ribosomal protein LP2 [Papilio xuthus] gi; 27 377 minus strand ribosomal protein LP2 [Papilio xuthus] gi; 1538 1891 minus strand ribosomal protein LP2 [Papilio xuthus] gi

Protein Accession Unigene53017$_H$1 Unigene53016$_H$1 Unigene53015$_H$1 CL49012.Contig9$_H$1
Mean Expression Ratio 0.942
Median Expression Ratio 0.942
Credible Interval (0.74, 1.19)
Associated Peptides 7
Associated Spectra 16
Coverage NaN

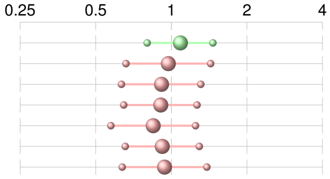

A	B	2.5	50	97.5	Sequence
4	1	0.8	1.1	1.5	KVVSQLSGK
1	0	0.66	0.98	1.4	SIDQLIAEGSR
2	0	0.63	0.91	1.3	VVSQLSGK
3	0	0.65	0.91	1.3	ILSSVGIEAEADK
1	0	0.57	0.85	1.3	AAAPAAEAK
1	2	0.66	0.92	1.3	ILSSVGIEAEADKVK
1	0	0.64	0.94	1.4	SGKEPTAADLEK

5.412 971 1963 Spectrin alpha chain [Crassostrea gigas]; 972 1979 Spectrin alpha chain [Crassostrea gigas]

Protein Accession	CL2195.Contig6$_H1$ CL2195.Contig4$_H1$
Mean Expression Ratio	0.944
Median Expression Ratio	0.943
Credible Interval	(0.631, 1.41)
Associated Peptides	1
Associated Spectra	2
Coverage	NaN

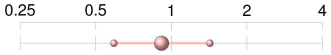

A	B	2.5	50	97.5	Sequence
1	1	0.59	0.92	1.4	EAFLAGDTVDGDNVETLIK

5.413 " 386 799 minus strand Myosin regulatory light chain LC-2, mantle muscle OS=Todarodes pacificus PE=1 SV=1; 656 1057 minus strand myosin regulatory light chain [Fasciola hepatica]"

Protein Accession	CL43006.Contig13$_H1$ CL43006.Contig11$_H1$
Mean Expression Ratio	1.06
Median Expression Ratio	1.06
Credible Interval	(0.799, 1.41)
Associated Peptides	2
Associated Spectra	27
Coverage	NaN

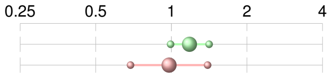

A	B	2.5	50	97.5	Sequence
15	9	0.99	1.2	1.4	DLLHNMGDNFTDDELK
0	3	0.69	0.98	1.4	DLLHNMGDNFTDDELKQTWK

5.414 252 848 sigma class glutathione-s-transferase 2 [Haliotis discus discus]

Protein Accession	CL19285.Contig21$_H1$
Mean Expression Ratio	0.943
Median Expression Ratio	0.943
Credible Interval	(0.701, 1.27)
Associated Peptides	5
Associated Spectra	8
Coverage	NaN

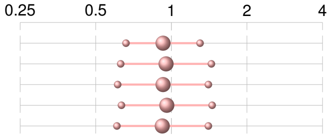

A	B	2.5	50	97.5	Sequence
2	2	0.66	0.92	1.3	LQDVDKDYPEVQK
1	0	0.63	0.95	1.4	LQAESLPK
1	0	0.61	0.92	1.4	LGNELTMA
0	1	0.63	0.96	1.5	KLRGR
0	1	0.61	0.92	1.4	EWEDVR

5.415 689 1984 hypothetical protein BRAFLDRAFT_78257 [Branchiostoma floridae] gi

Protein Accession	Unigene34076$_H1$
Mean Expression Ratio	1.06
Median Expression Ratio	1.06
Credible Interval	(0.825, 1.36)
Associated Peptides	8
Associated Spectra	13
Coverage	NaN

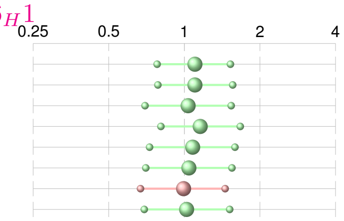

A	B	2.5	50	97.5	Sequence
2	1	0.78	1.1	1.5	TVGGFQLR
3	0	0.79	1.1	1.6	VQQKPEPSPAEGEVLIR
1	0	0.7	1	1.5	YLLFGSSNIVTGETK
1	1	0.8	1.2	1.7	YVSEIR
1	0	0.73	1.1	1.6	ILLDPSKAPTPK
0	1	0.7	1	1.5	SLVLTGFGSMK
0	1	0.67	0.99	1.5	SFFSFAK
0	1	0.69	1	1.5	GISILKPMGK

5.416 316 1512 Alpha-N-acetylgalactosaminidase [Crassostrea gigas]; 282 1478 Alpha-N-acetylgalactosaminidase [Crassostrea gigas]

Protein Accession	CL2428.Contig4$_H$1 CL2428.Contig1$_H$1
Mean Expression Ratio	1.06
Median Expression Ratio	1.06
Credible Interval	(0.709, 1.57)
Associated Peptides	2
Associated Spectra	5
Coverage	NaN

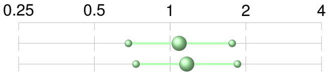

A	B	2.5	50	97.5	Sequence
**1**	**0**	**0.68**	**1.1**	**1.8**	**LGIYEDFGSK**
**1**	**3**	**0.73**	**1.2**	**1.9**	**LVELGLR**

5.417 530 679 minus strand GJ11201 [Drosophila virilis] gi

Protein Accession	CL45804.Contig2$_H$1
Mean Expression Ratio	1.06
Median Expression Ratio	1.06
Credible Interval	(0.709, 1.61)
Associated Peptides	1
Associated Spectra	2
Coverage	NaN

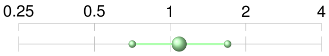

A	B	2.5	50	97.5	Sequence
**2**	**0**	**0.71**	**1.1**	**1.7**	**QSQYALTLGPR**

5.418 " 65 1576 minus strand Aldehyde dehydrogenase, mitochondrial [Crassostrea gigas]"

Protein Accession	CL5835.Contig1$_H$1
Mean Expression Ratio	0.943
Median Expression Ratio	0.944
Credible Interval	(0.69, 1.28)
Associated Peptides	5
Associated Spectra	6
Coverage	NaN

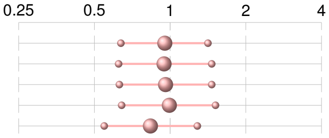

A	B	2.5	50	97.5	Sequence
**1**	**1**	**0.64**	**0.95**	**1.4**	**LADLLER**
**1**	**0**	**0.63**	**0.95**	**1.5**	**ELGEYGLEAYTEVK**
**1**	**0**	**0.63**	**0.96**	**1.5**	**IFVEEK**
**1**	**0**	**0.64**	**0.99**	**1.5**	**VAEGDK**
**0**	**1**	**0.55**	**0.83**	**1.3**	**LIPVMAAK**

5.419 3 254 Calpain-5 [Crassostrea gigas]; 3 254 minus strand Calpain-5 [Crassostrea gigas]

Protein Accession	CL41869.Contig2$_H$1 CL41869.Contig1$_H$1
Mean Expression Ratio	0.945
Median Expression Ratio	0.944
Credible Interval	(0.633, 1.44)
Associated Peptides	1
Associated Spectra	2
Coverage	NaN

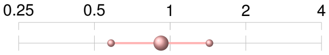

A	B	2.5	50	97.5	Sequence
**1**	**1**	**0.58**	**0.92**	**1.4**	**EVDLFGHK**

5.420 49 747 PREDICTED: elongation factor 1-delta-like [Strongylocentrotus purpuratus]; 49 819 PREDICTED: elongation factor 1-delta-like isoform 2 [Strongylocentrotus purpuratus]

Protein Accession	CL10790.Contig13$_H$1 CL10790.Contig12$_H$1
Mean Expression Ratio	1.06
Median Expression Ratio	1.06
Credible Interval	(0.71, 1.57)
Associated Peptides	2
Associated Spectra	5
Coverage	NaN

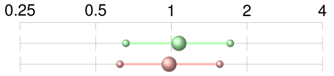

A	B	2.5	50	97.5	Sequence
1	0	0.66	1.1	1.7	VAALEVVGTGGASPK
1	3	0.62	0.98	1.6	LVDLAYGIK

5.421 387 1064 minus strand PREDICTED: hypothetical protein [Hydra magnipapillata]; 275 952 minus strand PREDICTED: hypothetical protein [Hydra magnipapillata]; 172 849 minus strand PREDICTED: hypothetical protein [Hydra magnipapillata]

Protein Accession	CL20613.Contig3$_H$1 CL20613.Contig2$_H$1 CL20613.Contig1$_H$1
Mean Expression Ratio	1.06
Median Expression Ratio	1.06
Credible Interval	(0.768, 1.47)
Associated Peptides	3
Associated Spectra	6
Coverage	NaN

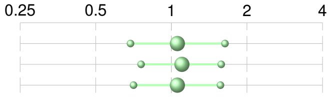

A	B	2.5	50	97.5	Sequence
1	0	0.69	1.1	1.6	ELGATFEAADNFKPNVVVSGR
3	0	0.76	1.1	1.6	NAGYTVHFVSPK
1	1	0.71	1.1	1.6	GFLADQEVQK

5.422 253 765 cytochrome c oxidase subunit IV [Urechis caupo]

Protein Accession	CL38636.Contig3$_H$1
Mean Expression Ratio	1.06
Median Expression Ratio	1.06
Credible Interval	(0.731, 1.53)
Associated Peptides	2
Associated Spectra	3
Coverage	NaN

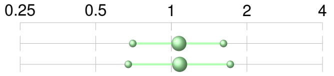

A	B	2.5	50	97.5	Sequence
2	0	0.7	1.1	1.6	FKENTPDVMALR
1	0	0.68	1.1	1.7	SLTLEDKK

5.423 64 393 Tubulin beta chain OS=Paracentrotus lividus PE=2 SV=1; 111 398 Tubulin beta chain OS=Paracentrotus lividus PE=2 SV=1; 109 438 Tubulin beta chain OS=Paracentrotus lividus PE=2 SV=1; 160 489 Tubulin beta chain OS=Paracentrotus lividus PE=2 SV=1

Protein Accession	Unigene62663$_H$1 Unigene62662$_H$1 Unigene62661$_H$1 Unigene62660$_H$1
Mean Expression Ratio	0.944
Median Expression Ratio	0.945
Credible Interval	(0.625, 1.41)
Associated Peptides	1
Associated Spectra	6
Coverage	NaN

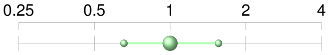

A	B	2.5	50	97.5	Sequence
4	2	0.65	1	1.6	INVYFNEATGGK

5.424 58 732 glutathione-s-transferase [Haliotis discus discus]

Protein Accession	CL4861.Contig1$_H$1
Mean Expression Ratio	0.946
Median Expression Ratio	0.945
Credible Interval	(0.68, 1.32)
Associated Peptides	4
Associated Spectra	5
Coverage	NaN

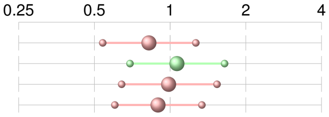

A	B	2.5	50	97.5	Sequence
0	1	0.54	0.83	1.3	IDKEYLEVK
0	1	0.69	1.1	1.6	GQVPTFR
0	1	0.64	0.99	1.5	WEEYLR
0	2	0.6	0.9	1.3	LISFSANEHK

5.425 27 515 heat shock protein 70 [Capsaspora owczarzaki ATCC 30864]; 30 464 minus strand hypothetical protein [Trichinella spiralis]; 27 491 minus strand heat shock protein 70 [Capsaspora owczarzaki ATCC 30864]

Protein Accession	CL14449.Contig4$_H$1 CL14449.Contig2$_H$1 CL14449.Contig1$_H$1
Mean Expression Ratio	0.944
Median Expression Ratio	0.945
Credible Interval	(0.675, 1.32)
Associated Peptides	4
Associated Spectra	5
Coverage	NaN

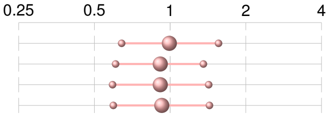

A	B	2.5	50	97.5	Sequence
1	0	0.64	0.99	1.6	RFDDPSVQSDMK
2	0	0.61	0.91	1.4	VEIIANDQGNR
1	0	0.59	0.92	1.4	NQVAMNPENTVFDAK
1	0	0.59	0.93	1.4	EVAEGYLGK

5.426 43 525 Reticulon-4 [Crassostrea gigas]; 143 757 Reticulon-4 [Crassostrea gigas]; 43 564 Reticulon-4 [Crassostrea gigas]

Protein Accession	CL29775.Contig4$_{H}1$ CL29775.Contig3$_{H}1$ CL29775.Contig2$_{H}1$
Mean Expression Ratio	0.945
Median Expression Ratio	0.945
Credible Interval	(0.627, 1.41)
Associated Peptides	1
Associated Spectra	3
Coverage	NaN

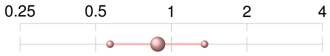

A	B	2.5	50	97.5	Sequence
2	1	0.57	0.88	1.4	VTQTILQHVNR

5.427 113 445 ribosomal protein rps19 [Lineus viridis]

Protein Accession	CL8932.Contig6$_{H}1$
Mean Expression Ratio	1.06
Median Expression Ratio	1.06
Credible Interval	(0.702, 1.58)
Associated Peptides	2
Associated Spectra	2
Coverage	NaN

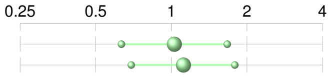

A	B	2.5	50	97.5	Sequence
1	0	0.63	1	1.7	KVLQSLEGLK
0	1	0.69	1.1	1.8	LEEIR

5.428 15 428 PREDICTED: histone H2A.V-like [Apis florea]

Protein Accession	CL14228.Contig5$_{H}1$
Mean Expression Ratio	0.946
Median Expression Ratio	0.946
Credible Interval	(0.724, 1.23)
Associated Peptides	4
Associated Spectra	21
Coverage	NaN

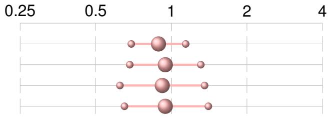

A	B	2.5	50	97.5	Sequence
9	2	0.69	0.89	1.1	AGLQFPVGR
3	3	0.68	0.95	1.3	ATIAGGGVIPHIHK
1	1	0.62	0.92	1.4	GDEELDSLIK
1	1	0.65	0.94	1.4	HLQLAIR

5.429 87 1997 Transketolase-like protein 2 [Crassostrea gigas]; 87 1997 Transketolase-like protein 2 [Crassostrea gigas]; 87 1997 Transketolase-like protein 2 [Crassostrea gigas]; 87 1997 Transketolase-like protein 2 [Crassostrea gigas]; 87 1997 Transketolase-like protein 2 [Crassostrea gigas]; 87 1997 Transketolase-like protein 2 [Crassostrea gigas]; 87 1997 Transketolase-like protein 2 [Crassostrea gigas]

Protein Accession	CL1127.Contig6$_H1$ CL1127.Contig5$_H1$ CL1127.Contig4$_H1$ CL1127.Contig3$_H1$ CL1127.Con
Mean Expression Ratio	1.06
Median Expression Ratio	1.06
Credible Interval	(0.758, 1.48)
Associated Peptides	3
Associated Spectra	5
Coverage	NaN

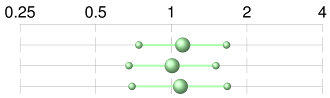

A	B	2.5	50	97.5	Sequence
1	1	0.74	1.1	1.7	VISLDGDVK
1	1	0.68	1	1.5	GMCFIR
1	0	0.7	1.1	1.7	NARETGGR

5.430 128 532 minus strand mitochondrial ATPase inhibitor [Ornithodoros parkeri]

Protein Accession	Unigene1376$_H1$
Mean Expression Ratio	1.06
Median Expression Ratio	1.06
Credible Interval	(0.676, 1.62)
Associated Peptides	1
Associated Spectra	1
Coverage	NaN

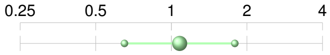

A	B	2.5	50	97.5	Sequence
1	0	0.65	1.1	1.8	MEAAHEELYFK

5.431 913 1560 minus strand Calcium-dependent protein kinase isoform 2 [Crassostrea gigas]

Protein Accession	CL5866.Contig1$_H1$
Mean Expression Ratio	0.945
Median Expression Ratio	0.948
Credible Interval	(0.677, 1.31)
Associated Peptides	4
Associated Spectra	5
Coverage	NaN

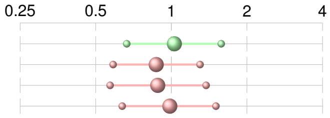

A	B	2.5	50	97.5	Sequence
0	1	0.67	1	1.6	DEFSVAVDK
0	2	0.59	0.87	1.3	FSFDEADKDK
0	1	0.57	0.88	1.4	IHYDEFVK
0	1	0.64	0.99	1.5	LLDINDDQK

5.432 410 709 calmodulin-like; K02183 calmodulin

Protein Accession	CL30882.Contig6$_{H}1$
Mean Expression Ratio	0.945
Median Expression Ratio	0.948
Credible Interval	(0.627, 1.4)
Associated Peptides	2
Associated Spectra	2
Coverage	NaN

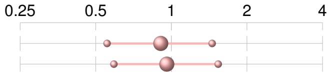

A	B	2.5	50	97.5	Sequence
**1**	**0**	**0.56**	**0.91**	**1.5**	**ELNDYVTEGGQSAAQAVLEK**
**0**	**1**	**0.59**	**0.96**	**1.5**	**EALRDFIEK**

5.433 136 531 hypothetical protein CGI_10017533 [Crassostrea gigas]

Protein Accession	CL35548.Contig3$_{H}1$
Mean Expression Ratio	0.945
Median Expression Ratio	0.948
Credible Interval	(0.609, 1.49)
Associated Peptides	1
Associated Spectra	1
Coverage	NaN

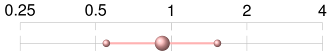

A	B	2.5	50	97.5	Sequence
**0**	**1**	**0.55**	**0.92**	**1.5**	**DPVFQHPAIR**

5.434 102 887 hypothetical protein DAPPUDRAFT_304712 [Daphnia pulex]

Protein Accession	CL6869.Contig1$_{H}1$
Mean Expression Ratio	1.05
Median Expression Ratio	1.05
Credible Interval	(0.778, 1.41)
Associated Peptides	4
Associated Spectra	8
Coverage	NaN

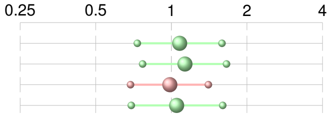

A	B	2.5	50	97.5	Sequence
**2**	**0**	**0.73**	**1.1**	**1.6**	**GKPLFVR**
**1**	**1**	**0.77**	**1.1**	**1.7**	**LADIPEGK**
**2**	**1**	**0.69**	**0.98**	**1.4**	**HPEADAER**
**0**	**1**	**0.69**	**1.1**	**1.6**	**VDLSQLR**

5.435 44 1465 minus strand Adenylyl cyclase-associated protein 1 [Crassostrea gigas]; 52 1473 minus strand Adenylyl cyclase-associated protein 1 [Crassostrea gigas]; 48 1469 Adenylyl cyclase-associated protein 1 [Crassostrea gigas]

Protein Accession	CL19470.Contig9$_H$1 CL19470.Contig8$_H$1 CL19470.Contig1$_H$1
Mean Expression Ratio	1.05
Median Expression Ratio	1.05
Credible Interval	(0.863, 1.28)
Associated Peptides	13
Associated Spectra	24
Coverage	NaN

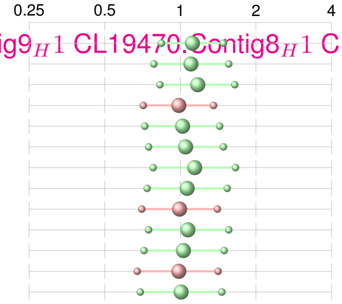

A	B	2.5	50	97.5	Sequence
2	3	0.84	1.1	1.5	AQLDIVNAAFK
1	1	0.78	1.1	1.6	DSLDTEIVTAK
1	1	0.83	1.2	1.7	EFALPEQFK
2	1	0.71	0.98	1.4	IQAVQEFR
1	1	0.72	1	1.4	NIVLDNVELK
1	1	0.75	1.1	1.5	QFLEVASK
1	0	0.78	1.1	1.7	SKQPPQADLPK
1	0	0.74	1.1	1.5	VNSIILDQCK
2	0	0.7	0.99	1.4	VPTISIDK
1	0	0.74	1.1	1.6	QAVYIYK
1	0	0.72	1	1.5	LETLALR
1	0	0.67	0.98	1.4	DTALQNAVAR
0	1	0.69	1	1.5	LKDFK

5.436 35 1861 hypothetical protein DAPPUDRAFT_309082 [Daphnia pulex]

Protein Accession	CL49314.Contig5$_H$1
Mean Expression Ratio	0.947
Median Expression Ratio	0.949
Credible Interval	(0.732, 1.23)
Associated Peptides	6
Associated Spectra	11
Coverage	NaN

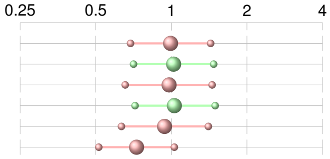

A	B	2.5	50	97.5	Sequence
2	0	0.69	0.99	1.4	ELDVALTAVGLK
1	1	0.71	1	1.5	LINHSVPDTIDER
1	0	0.66	0.98	1.5	WVNYHLAR
1	1	0.72	1	1.5	YAIGLAR
1	0	0.63	0.94	1.4	DIGHTFK
0	3	0.51	0.73	1	MADGILLCK

5.437 77 502 hypothetical protein CGI_10008263 [Crassostrea gigas]

Protein Accession	CL38960.Contig1$_H$1
Mean Expression Ratio	1.05
Median Expression Ratio	1.05
Credible Interval	(0.728, 1.54)
Associated Peptides	2
Associated Spectra	3
Coverage	NaN

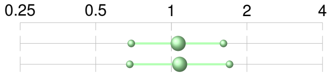

A	B	2.5	50	97.5	Sequence
2	0	0.69	1.1	1.6	IFVAIDGSK
1	0	0.68	1.1	1.7	KIFVAIDGSK

5.438 120 833 B-cell receptor-associated protein 31 [Crassostrea gigas]; 238 951 B-cell receptor-associated protein 31 [Crassostrea gigas]; 88 801 B-cell receptor-associated protein 31 [Crassostrea gigas]; 88 801 B-cell receptor-associated protein 31 [Crassostrea gigas]

Protein Accession	CL9442.Contig7$_{H}1$ CL9442.Contig5$_{H}1$ CL9442.Contig4$_{H}1$ CL9442.Contig3$_{H}1$
Mean Expression Ratio	1.05
Median Expression Ratio	1.05
Credible Interval	(0.679, 1.64)
Associated Peptides	1
Associated Spectra	1
Coverage	NaN

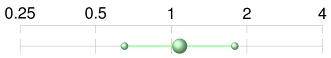

A	B	2.5	50	97.5	Sequence
**1**	**0**	**0.65**	**1.1**	**1.8**	**LLEEHAQLQK**

5.439 174 1649 minus strand Collagen alpha-1(XII) chain [Crassostrea gigas]; 123 1490 minus strand Collagen alpha-1(XII) chain [Crassostrea gigas]; 135 1610 Collagen alpha-1(XII) chain [Crassostrea gigas]

Protein Accession	CL5340.Contig13$_{H}1$ CL5340.Contig12$_{H}1$ CL5340.Contig11$_{H}1$
Mean Expression Ratio	1.05
Median Expression Ratio	1.05
Credible Interval	(0.781, 1.41)
Associated Peptides	4
Associated Spectra	20
Coverage	NaN

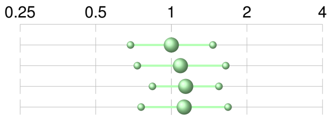

A	B	2.5	50	97.5	Sequence
**4**	**1**	**0.69**	**1**	**1.5**	**ADVVFIVDSSR**
**1**	**1**	**0.73**	**1.1**	**1.6**	**DVMSVFHIAR**
**6**	**5**	**0.84**	**1.1**	**1.5**	**DILAFR**
**1**	**1**	**0.76**	**1.1**	**1.7**	**SIWMPDFK**

5.440 285 1637 PREDICTED: talin-2-like [Cricetulus griseus]

Protein Accession	CL18079.Contig3$_{H}1$
Mean Expression Ratio	0.95
Median Expression Ratio	0.95
Credible Interval	(0.61, 1.47)
Associated Peptides	1
Associated Spectra	1
Coverage	NaN

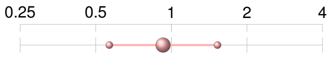

A	B	2.5	50	97.5	Sequence
**0**	**1**	**0.56**	**0.92**	**1.5**	**ISIVGQNVIK**

5.441 323 1192 cytosolic malate dehydrogenase [Lottia scabra]; 323 1192 cytosolic malate dehydrogenase [Lottia scabra]; 323 1192 cytosolic malate dehydrogenase [Lottia scabra]; 323 1192 cytosolic malate dehydrogenase [Lottia scabra]; 2474 3343 cytosolic malate dehydrogenase [Lottia scabra]; 116 1111 minus strand cytosolic malate dehydrogenase [Lottia scabra]

Protein Accession	CL280.Contig21$_H$1 CL280.Contig20$_H$1 CL280.Contig19$_H$1 CL280.Contig18$_H$1 CL280.Conti
Mean Expression Ratio	0.951
Median Expression Ratio	0.95
Credible Interval	(0.804, 1.12)
Associated Peptides	12
Associated Spectra	110
Coverage	NaN

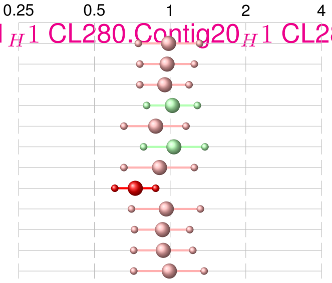

A	B	2.5	50	97.5	Sequence
3	3	0.75	0.99	1.3	EAVKDDNWINNEFISTVQK
6	5	0.76	0.97	1.2	ENFSCLTR
15	4	0.76	0.95	1.2	KLSSAMSAAK
6	6	0.81	1	1.3	LGIPNNAVSK
3	3	0.66	0.87	1.2	LSSAMSAAK
6	1	0.78	1	1.4	SQGANLDAVAK
2	1	0.66	0.91	1.2	YGIPEGVMYSYPVQIK
9	14	0.6	0.73	0.88	EMAEQILAA
2	1	0.7	0.96	1.3	ELLEER
6	4	0.72	0.93	1.2	DCDAAFLVGAMPR
4	3	0.72	0.94	1.2	DLLAANIK
2	1	0.72	0.99	1.4	YAPSIPK

5.442 52 1599 minus strand ATP synthase beta subunit [Pinctada fucata]

Protein Accession	CL34251.Contig5$_H$1
Mean Expression Ratio	1.05
Median Expression Ratio	1.05
Credible Interval	(0.866, 1.27)
Associated Peptides	10
Associated Spectra	33
Coverage	NaN

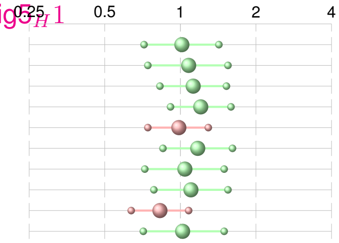

A	B	2.5	50	97.5	Sequence
1	1	0.72	1	1.4	AHGGYSVFAGVGER
1	0	0.74	1.1	1.6	EAAKPAAAGNLAVGR
2	2	0.83	1.1	1.5	IINVIGEPIDER
4	2	0.92	1.2	1.6	ILDPNVVGMEHYK
3	3	0.74	0.98	1.3	TIAMDGTEGLVR
2	1	0.85	1.2	1.6	VISLTDDTSK
1	0	0.72	1	1.5	VSLVYGQMNEPPGAR
2	0	0.79	1.1	1.5	VVDMLAPYAR
6	1	0.64	0.83	1.1	ILQDFK
0	1	0.71	1	1.5	IGLFGGAGVGK

5.443 43 525 Reticulon-4 [Crassostrea gigas]; 143 757 Reticulon-4 [Crassostrea gigas]; 43 564 Reticulon-4 [Crassostrea gigas]; 302 583 Reticulon-4 OS=Rattus norvegicus GN=Rtn4 PE=1 SV=1

Protein Accession	CL29775.Contig4$_H$1 CL29775.Contig3$_H$1 CL29775.Contig2$_H$1 CL29775.Contig1$_H$1
Mean Expression Ratio	0.95
Median Expression Ratio	0.951
Credible Interval	(0.661, 1.35)
Associated Peptides	2
Associated Spectra	6
Coverage	NaN

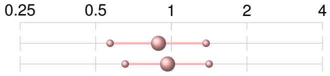

A	B	2.5	50	97.5	Sequence
2	1	0.57	0.89	1.4	VTQTILQHVNR
0	3	0.66	0.97	1.4	SLLELR

5.444 900 1142 minus strand heat shock protein 20 [Cyclina sinensis]

Protein Accession	CL19759.Contig1$_H1$
Mean Expression Ratio	1.05
Median Expression Ratio	1.05
Credible Interval	(0.787, 1.4)
Associated Peptides	3
Associated Spectra	11
Coverage	NaN

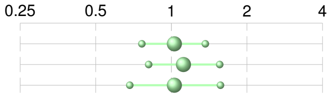

A	B	2.5	50	97.5	Sequence
4	2	0.76	1	1.4	QFKPEEITVK
3	1	0.81	1.1	1.6	QYILPEK
1	0	0.68	1	1.6	VEHPIVMDHQGNK

5.445 1 222 cAMP-dependent protein kinase regulatory subunit [Aplysia californica] gi

Protein Accession	Unigene13629$_H1$
Mean Expression Ratio	0.95
Median Expression Ratio	0.951
Credible Interval	(0.648, 1.39)
Associated Peptides	2
Associated Spectra	3
Coverage	NaN

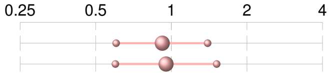

A	B	2.5	50	97.5	Sequence
1	1	0.6	0.92	1.4	NVLFSHLDDNER
0	1	0.6	0.95	1.5	SDIFDAMFPVHR

5.446 104 445 minus strand macrophage migration inhibitory factor [Ascaris suum]

Protein Accession	CL34536.Contig3$_H1$
Mean Expression Ratio	1.05
Median Expression Ratio	1.05
Credible Interval	(0.764, 1.44)
Associated Peptides	4
Associated Spectra	6
Coverage	NaN

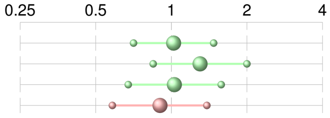

A	B	2.5	50	97.5	Sequence
2	1	0.71	1	1.5	HIEEALGIPQDR
1	0	0.85	1.3	2	PVFILNTNLPADK
1	0	0.67	1	1.6	QLGKPESYVCVR
0	1	0.58	0.9	1.4	LVVPFMK

5.447 25 405 minus strand Elongation factor 1-delta [Anoplopoma fimbria]

Protein Accession	CL10502.Contig1$_H1$
Mean Expression Ratio	1.05
Median Expression Ratio	1.05
Credible Interval	(0.756, 1.46)
Associated Peptides	4
Associated Spectra	5
Coverage	NaN

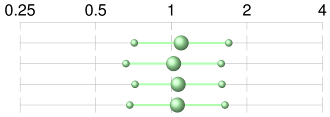

A	B	2.5	50	97.5	Sequence
1	0	0.71	1.1	1.7	KPVLIAK
1	0	0.66	1	1.6	VQTDGLVWGASK
1	1	0.72	1.1	1.6	LVPVGYGINK
0	1	0.68	1.1	1.6	RVQTDGLVWGASK

5.448 222 809 minus strand PREDICTED: 15-hydroxyprostaglandin dehydrogenase [NAD+] [Gallus gallus]

Protein Accession	CL44148.Contig2$_H$1
Mean Expression Ratio	0.954
Median Expression Ratio	0.953
Credible Interval	(0.792, 1.15)
Associated Peptides	7
Associated Spectra	84
Coverage	NaN

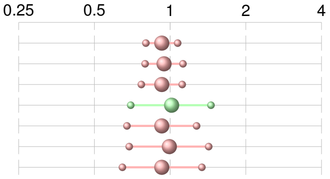

A	B	2.5	50	97.5	Sequence
20	16	0.8	0.93	1.1	ATVEAFLTK
17	6	0.79	0.95	1.1	VFLTGGAHGIGK
13	6	0.77	0.93	1.1	VMFCDINDK
1	0	0.7	1	1.5	RVFLTGGAHGIGK
0	3	0.67	0.92	1.3	TGGAHGIGK
0	1	0.69	0.99	1.4	LTGGAHGIGK
0	1	0.64	0.93	1.3	GGAHGIGK

5.449 134 385 minus strand CDGSH iron sulfur domain-containing protein 2 [Crassostrea gigas]; 11 511 minus strand CDGSH iron sulfur domain-containing protein 2 [Crassostrea gigas]

Protein Accession	CL36832.Contig5$_H$1 CL36832.Contig4$_H$1
Mean Expression Ratio	0.951
Median Expression Ratio	0.953
Credible Interval	(0.616, 1.47)
Associated Peptides	1
Associated Spectra	1
Coverage	NaN

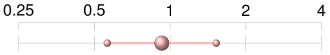

A	B	2.5	50	97.5	Sequence
1	0	0.56	0.93	1.5	VVNMVDIEDLGDK

5.450 2302 2547 minus strand hemocyanin isoform 1 [Haliotis diversicolor]; 816 1211 H2 [Haliotis tuberculata]

Protein Accession	CL11519.Contig6$_H$1 CL11519.Contig5$_H$1
Mean Expression Ratio	0.952
Median Expression Ratio	0.953
Credible Interval	(0.654, 1.38)
Associated Peptides	2
Associated Spectra	4
Coverage	NaN

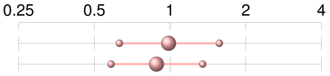

A	B	2.5	50	97.5	Sequence
1	0	0.63	0.98	1.6	KNVLSLTPAER
2	1	0.58	0.88	1.3	SLILAMR

5.451 57 2378 minus strand Heterogeneous nuclear ribonucleoprotein U-like protein 1 [Crassostrea gigas]

Protein Accession	CL15560.Contig5$_H$1
Mean Expression Ratio	0.954
Median Expression Ratio	0.954
Credible Interval	(0.633, 1.44)
Associated Peptides	2
Associated Spectra	2
Coverage	NaN

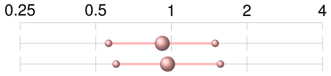

A	B	2.5	50	97.5	Sequence
**0**	**1**	**0.56**	**0.92**	**1.5**	**ALFPHILTK**
**0**	**1**	**0.6**	**0.97**	**1.6**	**VTELRDELK**

5.452 5 514 hypothetical protein KGM_05418 [Danaus plexippus]

Protein Accession	CL11785.Contig1$_H$1
Mean Expression Ratio	1.05
Median Expression Ratio	1.05
Credible Interval	(0.722, 1.51)
Associated Peptides	3
Associated Spectra	3
Coverage	NaN

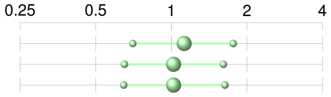

A	B	2.5	50	97.5	Sequence
**1**	**0**	**0.7**	**1.1**	**1.8**	**KLTNQSLK**
**1**	**0**	**0.65**	**1**	**1.6**	**NNLDIGK**
**0**	**1**	**0.65**	**1**	**1.6**	**ALLDLSENIDELNR**

5.453 49 1107 Eukaryotic translation initiation factor 3 subunit B [Crassostrea gigas]

Protein Accession	CL19479.Contig1$_H$1
Mean Expression Ratio	0.955
Median Expression Ratio	0.955
Credible Interval	(0.612, 1.48)
Associated Peptides	1
Associated Spectra	1
Coverage	NaN

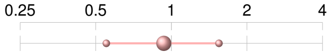

A	B	2.5	50	97.5	Sequence
**0**	**1**	**0.55**	**0.93**	**1.6**	**LMEEFSNLR**

5.454 " 3341 4240 minus strand nonmuscle myosin II, partial [Aplysia californica]"

Protein Accession	CL19183.Contig2$_H$1
Mean Expression Ratio	0.955
Median Expression Ratio	0.955
Credible Interval	(0.731, 1.24)
Associated Peptides	7
Associated Spectra	11
Coverage	NaN

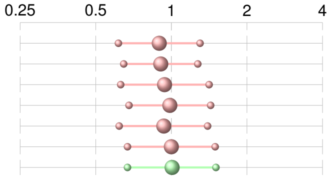

A	B	2.5	50	97.5	Sequence
**1**	**1**	**0.62**	**0.89**	**1.3**	**RLETELQDLR**
**2**	**1**	**0.64**	**0.91**	**1.3**	**REEEVQIALQK**
**1**	**0**	**0.63**	**0.94**	**1.4**	**TATMLEEK**
**1**	**1**	**0.68**	**0.99**	**1.4**	**YAQMIEEK**
**1**	**0**	**0.62**	**0.93**	**1.4**	**QKQDVEQLEAR**
**1**	**0**	**0.67**	**1**	**1.5**	**QELEK**
**0**	**1**	**0.67**	**1**	**1.5**	**ELQDLR**

5.455 1 111 glycogen synthase [Crassostrea gigas]

Protein Accession	Unigene78448$_{H}1$
Mean Expression Ratio	1.05
Median Expression Ratio	1.05
Credible Interval	(0.669, 1.63)
Associated Peptides	1
Associated Spectra	1
Coverage	NaN

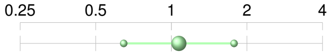

A	B	2.5	50	97.5	Sequence
0	1	0.65	1.1	1.8	NVEPDLDDDYLDR

5.456 65 490 PREDICTED: cofilin-2 [Otolemur garnettii]

Protein Accession	CL15471.Contig3$_{H}1$
Mean Expression Ratio	1.05
Median Expression Ratio	1.05
Credible Interval	(0.788, 1.39)
Associated Peptides	4
Associated Spectra	11
Coverage	NaN

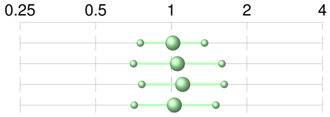

A	B	2.5	50	97.5	Sequence
4	2	0.75	1	1.4	IVMENEFAK
1	0	0.71	1.1	1.6	SQEDEYNSIIMTLPK
2	0	0.76	1.1	1.6	MLYSSSSSAVK
1	1	0.71	1	1.5	ALVFK

5.457 91 2658 minus strand puromycin-sensitive aminopeptidase [Saccoglossus kowalevskii]

Protein Accession	CL6708.Contig2$_{H}1$
Mean Expression Ratio	1.05
Median Expression Ratio	1.05
Credible Interval	(0.695, 1.57)
Associated Peptides	2
Associated Spectra	2
Coverage	NaN

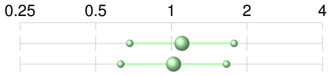

A	B	2.5	50	97.5	Sequence
1	0	0.68	1.1	1.8	GMNLYLNR
1	0	0.63	1	1.7	LPKDVIPSNYNIR

5.458 290 2458 Putative beta-D-xylosidase 5 [Crassostrea gigas]

Protein Accession	CL49577.Contig2$_{H}1$
Mean Expression Ratio	1.05
Median Expression Ratio	1.05
Credible Interval	(0.718, 1.52)
Associated Peptides	3
Associated Spectra	4
Coverage	NaN

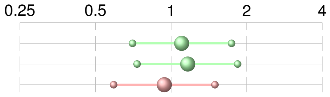

A	B	2.5	50	97.5	Sequence
0	1	0.7	1.1	1.7	QLLTDILR
1	1	0.73	1.2	1.8	VAEAAGVEVR
0	1	0.59	0.94	1.5	LEALEQGK

5.459 31 735 hypothetical protein BRAFLDRAFT_78603 [Branchiostoma floridae] gi; 55 735 hypothetical protein BRAFLDRAFT_78603 [Branchiostoma floridae] gi; 353 1039 hypothetical protein BRAFLDRAFT_78603 [Branchiostoma floridae] gi; 207 893 hypothetical protein BRAFLDRAFT_78603 [Branchiostoma floridae] gi

Protein Accession	CL877.Contig7$_{H}1$ CL877.Contig6$_{H}1$ CL877.Contig4$_{H}1$ CL877.Contig3$_{H}1$
Mean Expression Ratio	1.05
Median Expression Ratio	1.05
Credible Interval	(0.781, 1.41)
Associated Peptides	4
Associated Spectra	10
Coverage	NaN

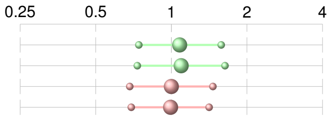

A	B	2.5	50	97.5	Sequence
2	1	0.74	1.1	1.6	DDQTLVTMTGEVAGR
1	1	0.73	1.1	1.6	LKLPLDSSNR
2	0	0.68	1	1.5	KVIHSIVGPHFAEGR
3	0	0.69	0.99	1.4	VALVPK

5.460 3 230 minus strand RH21460p [Drosophila melanogaster]; 3 230 minus strand RH21460p [Drosophila melanogaster]

Protein Accession	Unigene57704$_{H}1$ CL18123.Contig3$_{H}1$
Mean Expression Ratio	0.955
Median Expression Ratio	0.957
Credible Interval	(0.615, 1.48)
Associated Peptides	1
Associated Spectra	1
Coverage	NaN

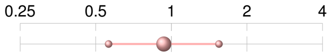

A	B	2.5	50	97.5	Sequence
0	1	0.56	0.93	1.5	EVDEDEAAFK

5.461 457 1842 Basement membrane-specific heparan sulfate proteoglycan core protein [Crassostrea gigas]; 457 1842 Basement membrane-specific heparan sulfate proteoglycan core protein [Crassostrea gigas]

Protein Accession	CL24300.Contig9$_{H}1$ CL24300.Contig10$_{H}1$
Mean Expression Ratio	0.956
Median Expression Ratio	0.957
Credible Interval	(0.669, 1.37)
Associated Peptides	2
Associated Spectra	4
Coverage	NaN

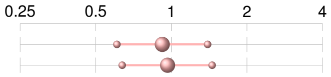

A	B	2.5	50	97.5	Sequence
1	1	0.61	0.92	1.4	KDGELPVEHK
1	1	0.64	0.96	1.5	IESSGALYIPR

5.462 " 109 1632 minus strand Seryl-tRNA synthetase, cytoplasmic [Crassostrea gigas]; 109 1614 minus strand Seryl-tRNA synthetase, cytoplasmic [Crassostrea gigas]"

Protein Accession	CL11056.Contig9$_H1$ CL11056.Contig1$_H1$
Mean Expression Ratio	1.04
Median Expression Ratio	1.04
Credible Interval	(0.696, 1.56)
Associated Peptides	2
Associated Spectra	2
Coverage	NaN

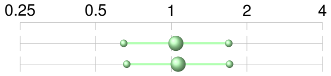

A	B	2.5	50	97.5	Sequence
0	1	0.65	1	1.7	LVDTVVDQDAAWR
0	1	0.66	1.1	1.7	SLGIPYR

5.463 130 2145 minus strand Glucosamine–fructose-6-phosphate aminotransferase [isomerizing] 1 [Crassostrea gigas]; 130 2121 minus strand Glucosamine–fructose-6-phosphate aminotransferase [isomerizing] 1 [Crassostrea gigas]

Protein Accession	CL2589.Contig3$_H1$ CL2589.Contig2$_H1$
Mean Expression Ratio	0.955
Median Expression Ratio	0.958
Credible Interval	(0.66, 1.38)
Associated Peptides	3
Associated Spectra	3
Coverage	NaN

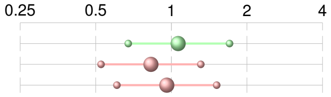

A	B	2.5	50	97.5	Sequence
1	0	0.67	1.1	1.7	KLPDQIKDVLK
0	1	0.52	0.83	1.3	HGPLALVDK
0	1	0.61	0.96	1.5	VANLEAQIK

5.464 616 1740 5'-AMP-activated protein kinase subunit beta-2 [Crassostrea gigas]

Protein Accession	Unigene52845$_H1$
Mean Expression Ratio	1.05
Median Expression Ratio	1.04
Credible Interval	(0.676, 1.63)
Associated Peptides	1
Associated Spectra	1
Coverage	NaN

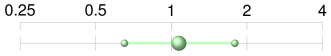

A	B	2.5	50	97.5	Sequence
1	0	0.65	1.1	1.8	FIVDEQWVVSK

5.465 205 2490 minus strand glucose-regulated protein 94 [Crassostrea gigas]

Protein Accession	CL5089.Contig1$_H$1
Mean Expression Ratio	0.956
Median Expression Ratio	0.958
Credible Interval	(0.689, 1.32)
Associated Peptides	5
Associated Spectra	5
Coverage	NaN

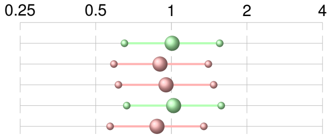

A	B	2.5	50	97.5	Sequence
1	0	0.65	1	1.6	ADKFAFQAEVNR
1	0	0.59	0.9	1.4	NLGTIAR
1	0	0.61	0.95	1.5	GTTVSLHLK
1	0	0.66	1	1.6	NDMFNNYGK
0	1	0.57	0.88	1.3	GVVDSDDLPLNVSR

5.466 42 434 cytochrome b5 [Haliotis discus discus]; 42 434 cytochrome b5 [Haliotis discus discus]

Protein Accession	CL6985.Contig9$_H$1 CL6985.Contig5$_H$1
Mean Expression Ratio	0.959
Median Expression Ratio	0.958
Credible Interval	(0.66, 1.38)
Associated Peptides	3
Associated Spectra	3
Coverage	NaN

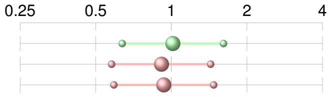

A	B	2.5	50	97.5	Sequence
1	0	0.64	1	1.6	KTFETNTGPVGR
1	0	0.58	0.91	1.4	VFRSEEVK
0	1	0.59	0.94	1.5	ELMKDYLIGK

5.467 " 1144 1956 minus strand Retinol dehydrogenase 12, partial [Crassostrea gigas]"

Protein Accession	CL3944.Contig7$_H$1
Mean Expression Ratio	1.04
Median Expression Ratio	1.04
Credible Interval	(0.733, 1.48)
Associated Peptides	3
Associated Spectra	4
Coverage	NaN

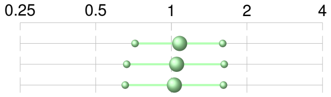

A	B	2.5	50	97.5	Sequence
2	0	0.72	1.1	1.6	LDFTDLALLK
1	0	0.66	1	1.6	LILGCR
0	1	0.65	1	1.6	LDLASLR

5.468 " 137 1549 minus strand Serine hydroxymethyltransferase, mitochondrial [Crassostrea gigas]; 205 1608 minus strand Serine hydroxymethyltransferase, mitochondrial [Crassostrea gigas]; 161 1573 minus strand Serine hydroxymethyltransferase, mitochondrial [Crassostrea gigas]; 205 1386 minus strand Serine hydroxymethyltransferase, mitochondrial [Crassostrea gigas]"

Protein Accession	CL22722.Contig6$_{H}1$ CL22722.Contig5$_{H}1$ CL22722.Contig4$_{H}1$ CL22722.Contig1$_{H}1$
Mean Expression Ratio	0.959
Median Expression Ratio	0.959
Credible Interval	(0.614, 1.5)
Associated Peptides	1
Associated Spectra	1
Coverage	NaN

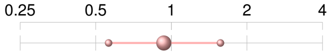

A	B	2.5	50	97.5	Sequence
**1**	**0**	**0.56**	**0.94**	**1.6**	**IIFYR**

5.469 1213 3234 Protein-glutamine gamma-glutamyltransferase K [Crassostrea gigas]; 478 2499 Protein-glutamine gamma-glutamyltransferase K [Crassostrea gigas]

Protein Accession	CL29134.Contig6$_{H}1$ CL29134.Contig5$_{H}1$
Mean Expression Ratio	1.04
Median Expression Ratio	1.04
Credible Interval	(0.671, 1.62)
Associated Peptides	1
Associated Spectra	1
Coverage	NaN

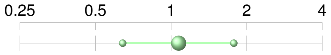

A	B	2.5	50	97.5	Sequence
**0**	**1**	**0.64**	**1.1**	**1.8**	**FLFAEAHGER**

5.470 " 2057 2497 Phosphate carrier protein, mitochondrial [Crassostrea gigas]"

Protein Accession	CL2712.Contig11$_{H}1$
Mean Expression Ratio	1.04
Median Expression Ratio	1.04
Credible Interval	(0.667, 1.62)
Associated Peptides	1
Associated Spectra	1
Coverage	NaN

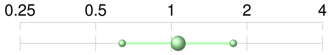

A	B	2.5	50	97.5	Sequence
**1**	**0**	**0.64**	**1.1**	**1.8**	**YVVPKPR**

5.471 " 31 2061 minus strand 1,4-alpha-glucan-branching enzyme [Crassostrea gigas]"

Protein Accession	CL12555.Contig7$_H$1
Mean Expression Ratio	0.958
Median Expression Ratio	0.961
Credible Interval	(0.632, 1.44)
Associated Peptides	1
Associated Spectra	2
Coverage	NaN

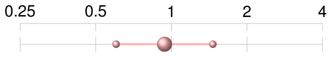

A	B	2.5	50	97.5	Sequence
0	2	0.6	0.94	1.5	LDGYLR

5.472 7 1722 minus strand Membrane metallo-endopeptidase-like 1 OS=Mus musculus GN=Mmel1 PE=1 SV=1

Protein Accession	CL19557.Contig1$_H$1
Mean Expression Ratio	1.04
Median Expression Ratio	1.04
Credible Interval	(0.879, 1.22)
Associated Peptides	15
Associated Spectra	41
Coverage	NaN

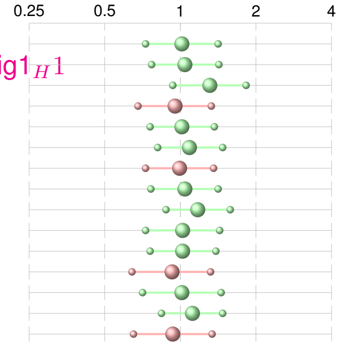

A	B	2.5	50	97.5	Sequence
1	1	0.73	1	1.4	AVLIEAIR
3	0	0.77	1	1.4	HQVENPNLHEVTSLADLEK
1	1	0.94	1.3	1.8	IGYPDYIMDDLR
1	1	0.68	0.95	1.3	LFVLNSFDQNQR
3	1	0.76	1	1.4	QDAQDVADFK
3	1	0.81	1.1	1.5	TLVENIADNAGLK
2	1	0.72	0.99	1.4	WPVSAAAVK
2	1	0.76	1	1.4	WLTDASK
4	0	0.87	1.2	1.6	LFPEVTGR
2	0	0.73	1	1.4	GLELFR
2	2	0.76	1	1.4	MNIDGYR
1	0	0.64	0.92	1.3	AQALNAK
1	0	0.71	1	1.4	QTALDK
2	3	0.84	1.1	1.5	LQLAEISLPK
0	1	0.65	0.94	1.3	LTDASKQTALDK

5.473 84 425 minus strand Transcription elongation factor B polypeptide 2 [Crassostrea gigas]; 84 425 minus strand Transcription elongation factor B polypeptide 2 [Crassostrea gigas]; 84 425 minus strand Transcription elongation factor B polypeptide 2 [Crassostrea gigas]; 101 442 minus strand Transcription elongation factor B polypeptide 2 [Crassostrea gigas]; 101 442 minus strand Transcription elongation factor B polypeptide 2 [Crassostrea gigas]; 84 425 minus strand Transcription elongation factor B polypeptide 2 [Crassostrea gigas]

Protein Accession	CL19448.Contig9$_H$1 CL19448.Contig8$_H$1 CL19448.Contig7$_H$1 CL19448.Contig1$_H$1 CL1944
Mean Expression Ratio	1.04
Median Expression Ratio	1.04
Credible Interval	(0.675, 1.63)
Associated Peptides	1
Associated Spectra	1
Coverage	NaN

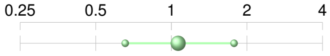

A	B	2.5	50	97.5	Sequence
1	0	0.66	1.1	1.8	AQAPATLGLVYK

5.474 138 713 Rac [Aplysia californica] gi; 101 676 Rac [Aplysia californica] gi

Protein Accession	CL11009.Contig9$_{H}1$ CL11009.Contig1$_{H}1$
Mean Expression Ratio	1.04
Median Expression Ratio	1.04
Credible Interval	(0.67, 1.61)
Associated Peptides	1
Associated Spectra	1
Coverage	NaN

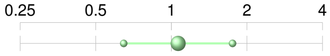

A	B	2.5	50	97.5	Sequence
1	0	0.65	1.1	1.8	CVVVGDGAVGK

5.475 " 134 1567 minus strand 6-phosphogluconate dehydrogenase, decarboxylating [Crassostrea gigas]; 134 1573 minus strand 6-phosphogluconate dehydrogenase, decarboxylating [Crassostrea gigas]"

Protein Accession	CL5140.Contig5$_{H}1$ CL5140.Contig4$_{H}1$
Mean Expression Ratio	0.961
Median Expression Ratio	0.962
Credible Interval	(0.745, 1.24)
Associated Peptides	7
Associated Spectra	12
Coverage	NaN

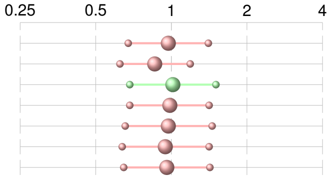

A	B	2.5	50	97.5	Sequence
1	1	0.67	0.97	1.4	AGQAVDDFIDK
2	2	0.62	0.86	1.2	SLEDMVDKLK
1	0	0.68	1	1.5	SVFLGNIK
1	1	0.68	0.99	1.4	LVPLLEK
1	0	0.66	0.98	1.5	YGPSLMPGGAEEAWPAIK
0	1	0.64	0.95	1.4	NIFQSISAK
0	1	0.65	0.96	1.4	AFVEDIKR

5.476 152 1930 hypothetical protein BRAFLDRAFT_223365 [Branchiostoma floridae] gi

Protein Accession	CL49005.Contig4$_{H}1$
Mean Expression Ratio	0.964
Median Expression Ratio	0.962
Credible Interval	(0.622, 1.5)
Associated Peptides	1
Associated Spectra	1
Coverage	NaN

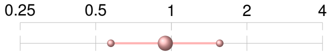

A	B	2.5	50	97.5	Sequence
1	0	0.57	0.95	1.6	AQGYQLIR

5.477 36 1310 minus strand elongation factor-1 gamma [Diguetia canities]

Protein Accession	CL14269.Contig5$_{H}1$
Mean Expression Ratio	0.964
Median Expression Ratio	0.963
Credible Interval	(0.69, 1.36)
Associated Peptides	3
Associated Spectra	5
Coverage	NaN

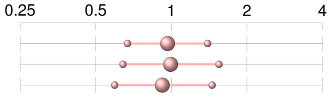

A	B	2.5	50	97.5	Sequence
2	1	0.67	0.96	1.4	LAETMAQFDAK
1	0	0.64	1	1.5	SADFLAK
0	1	0.59	0.92	1.5	LDPDDPETK

5.478 390 2267 neurofilament protein NF70 [Helix aspersa]; 318 2195 neurofilament protein NF70 [Helix aspersa]

Protein Accession	CL1353.Contig5$_H$1 CL1353.Contig4$_H$1
Mean Expression Ratio	1.04
Median Expression Ratio	1.04
Credible Interval	(0.805, 1.34)
Associated Peptides	7
Associated Spectra	13
Coverage	NaN

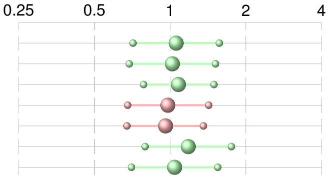

A	B	2.5	50	97.5	Sequence
1	0	0.71	1.1	1.6	GEMETFYNLK
1	0	0.69	1	1.5	LNQQISDLEAEIR
4	0	0.78	1.1	1.5	FANYIEK
1	1	0.68	0.98	1.4	MADLEAR
1	2	0.67	0.96	1.4	VGQLEELVDDLQR
0	1	0.79	1.2	1.8	ELQQEMDVK
0	1	0.7	1	1.5	LEFLK

5.479 242 589 Ribonucleoprotein RB97D OS=Drosophila melanogaster GN=Rb97D PE=2 SV=1

Protein Accession	CL6198.Contig1$_H$1
Mean Expression Ratio	0.963
Median Expression Ratio	0.963
Credible Interval	(0.62, 1.5)
Associated Peptides	1
Associated Spectra	1
Coverage	NaN

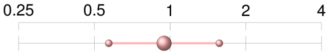

A	B	2.5	50	97.5	Sequence
1	0	0.57	0.95	1.6	LFIGGLSYDTDEK

5.480 120 635 PREDICTED: similar to collagen type VI alpha 6 [Ciona intestinalis]; 120 635 minus strand PREDICTED: similar to collagen type VI alpha 6 [Ciona intestinalis]

Protein Accession	Unigene46785$_H$1 Unigene26613$_H$1
Mean Expression Ratio	1.04
Median Expression Ratio	1.04
Credible Interval	(0.696, 1.52)
Associated Peptides	2
Associated Spectra	2
Coverage	NaN

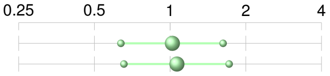

A	B	2.5	50	97.5	Sequence
1	0	0.64	1	1.6	HGNTNTGDGIMYMLER
1	0	0.66	1.1	1.7	VSLVTYSDQANPESAIDFDSYSSK

5.481 244 5451 alpha macroglobulin [Chlamys farreri]; 244 5493 alpha macroglobulin [Chlamys farreri]

Protein Accession	CL20671.Contig5$_{H}1$ CL20671.Contig4$_{H}1$
Mean Expression Ratio	1.04
Median Expression Ratio	1.04
Credible Interval	(0.725, 1.48)
Associated Peptides	3
Associated Spectra	4
Coverage	NaN

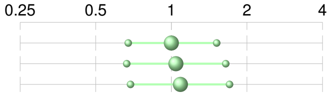

A	B	2.5	50	97.5	Sequence
1	1	0.67	1	1.5	HLALDVEQLNR
0	1	0.67	1	1.6	SLKIK
0	1	0.69	1.1	1.7	VAVNGIECINR

5.482 210 473 Abdominal ganglion neuropeptides L5-67 OS=Aplysia californica PE=1 SV=1; 879 1124 abdominal ganglion neuropeptides L5-67 precursor [Aplysia californica] gi; 210 482 minus strand abdominal ganglion neuropeptides L5-67 precursor [Aplysia californica] gi

Protein Accession	Unigene58290$_{H}1$ Unigene39670$_{H}1$ Unigene206$_{H}1$
Mean Expression Ratio	0.964
Median Expression Ratio	0.964
Credible Interval	(0.636, 1.45)
Associated Peptides	1
Associated Spectra	2
Coverage	NaN

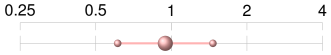

A	B	2.5	50	97.5	Sequence
0	2	0.61	0.94	1.5	VNGEIELPVEVFGAR

5.483 593 862 minus strand mitochondrial ATP synthase coupling factor 6 precursor [Ochlerotatus taeniorhynchus]

Protein Accession	Unigene4216$_{H}1$
Mean Expression Ratio	0.964
Median Expression Ratio	0.965
Credible Interval	(0.651, 1.43)
Associated Peptides	1
Associated Spectra	3
Coverage	NaN

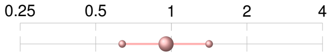

A	B	2.5	50	97.5	Sequence
0	3	0.64	0.95	1.4	LVDASPTVQAALKEELEK

5.484 4 849 minus strand elongation factor 1-alpha [Cryptosporidium muris RN66]

Protein Accession CL35888.Contig1$_H$1
Mean Expression Ratio 0.967
Median Expression Ratio 0.965
Credible Interval (0.68, 1.38)
Associated Peptides 2
Associated Spectra 12
Coverage NaN

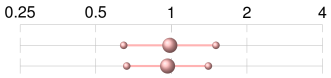

A	B	2.5	50	97.5	Sequence
**5**	**1**	**0.65**	**0.98**	**1.5**	**IGGIGTVPVGR**
**3**	**3**	**0.66**	**0.97**	**1.4**	**LPLQDVYK**

5.485 165 377 Cysteine and glycine-rich protein 3 [Crassostrea gigas]; 135 344 Cysteine and glycine-rich protein 3 [Crassostrea gigas]

Protein Accession CL38347.Contig2$_H$1 CL38347.Contig1$_H$1
Mean Expression Ratio 0.965
Median Expression Ratio 0.965
Credible Interval (0.638, 1.46)
Associated Peptides 1
Associated Spectra 2
Coverage NaN

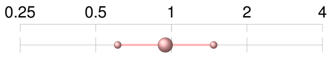

A	B	2.5	50	97.5	Sequence
**1**	**1**	**0.61**	**0.95**	**1.5**	**VEAGNIPFHK**

5.486 241 561 minus strand thioredoxin [Crassostrea ariakensis]

Protein Accession CL33159.Contig3$_H$1
Mean Expression Ratio 1.04
Median Expression Ratio 1.04
Credible Interval (0.753, 1.42)
Associated Peptides 3
Associated Spectra 7
Coverage NaN

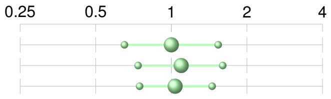

A	B	2.5	50	97.5	Sequence
**1**	**0**	**0.65**	**1**	**1.5**	**EVGEVVGASEEEIK**
**1**	**1**	**0.74**	**1.1**	**1.6**	**EVGEVVGASEEEIKK**
**2**	**2**	**0.75**	**1**	**1.4**	**IETQEDFEK**

5.487 160 387 small nuclear ribonucleoprotein polypeptide G protein [Crassostrea ariakensis]; 180 407 small nuclear ribonucleoprotein polypeptide G protein [Crassostrea ariakensis]

Protein Accession CL1272.Contig8$_H$1 CL1272.Contig7$_H$1
Mean Expression Ratio 1.03
Median Expression Ratio 1.04
Credible Interval (0.709, 1.5)
Associated Peptides 2
Associated Spectra 3
Coverage NaN

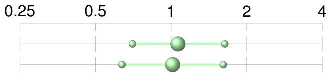

A	B	2.5	50	97.5	Sequence
**2**	**0**	**0.7**	**1.1**	**1.6**	**TITGILR**
**1**	**0**	**0.64**	**1**	**1.6**	**AHPPELK**

5.488 11 244 minus strand Tubulin alpha chain (Fragment) OS=Lytechinus pictus PE=3 SV=1

Protein Accession	CL3047.Contig6$_{H}$1
Mean Expression Ratio	0.968
Median Expression Ratio	0.966
Credible Interval	(0.646, 1.44)
Associated Peptides	2
Associated Spectra	2
Coverage	NaN

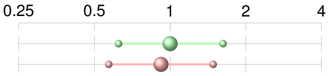

A	B	2.5	50	97.5	Sequence
0	1	0.62	1	1.6	EDLAALEK
0	1	0.57	0.92	1.5	FDLMYAK

5.489 353 2161 Bifunctional 3'-phosphoadenosine 5'-phosphosulfate synthetase [Crassostrea gigas]; 141 1943 Bifunctional 3'-phosphoadenosine 5'-phosphosulfate synthetase [Crassostrea gigas]

Protein Accession	CL8992.Contig2$_{H}$1 CL8992.Contig1$_{H}$1
Mean Expression Ratio	0.966
Median Expression Ratio	0.966
Credible Interval	(0.707, 1.32)
Associated Peptides	4
Associated Spectra	7
Coverage	NaN

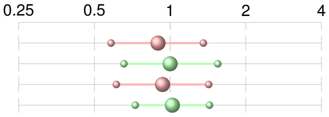

A	B	2.5	50	97.5	Sequence
1	0	0.58	0.9	1.4	QHAAILEEK
1	0	0.66	1	1.5	QFGTSNLGHPYVK
1	0	0.61	0.93	1.4	AGPTEVQWHAK
2	2	0.73	1	1.4	LDGSAAFALK

5.490 90 659 Plectin-1 [Crassostrea gigas]; 327 881 Plectin-1 [Crassostrea gigas]; 646 1290 minus strand Plectin-1 [Crassostrea gigas]

Protein Accession	CL7983.Contig3$_{H}$1 CL7983.Contig2$_{H}$1 CL7983.Contig1$_{H}$1
Mean Expression Ratio	0.965
Median Expression Ratio	0.966
Credible Interval	(0.613, 1.51)
Associated Peptides	1
Associated Spectra	1
Coverage	NaN

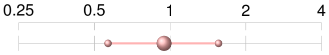

A	B	2.5	50	97.5	Sequence
0	1	0.57	0.95	1.6	AQHLVNVEEAR

5.491 19 1218 Dynactin subunit 2 [Crassostrea gigas]; 48 830 minus strand Dynactin subunit 2 [Crassostrea gigas]

Protein Accession	CL5827.Contig5$_{H}1$ CL5827.Contig1$_{H}1$
Mean Expression Ratio	1.03
Median Expression Ratio	1.04
Credible Interval	(0.662, 1.61)
Associated Peptides	1
Associated Spectra	1
Coverage	NaN

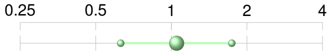

A	B	2.5	50	97.5	Sequence
1	0	0.63	1.1	1.7	VNELYEVMK

5.492 18 1613 minus strand chaperonin containing T-complex polypeptide subunit zeta [Haliotis discus hannai]; 527 2089 minus strand chaperonin containing T-complex polypeptide subunit zeta [Haliotis discus hannai]

Protein Accession	CL23722.Contig7$_{H}1$ CL23722.Contig5$_{H}1$
Mean Expression Ratio	1.03
Median Expression Ratio	1.03
Credible Interval	(0.711, 1.51)
Associated Peptides	2
Associated Spectra	3
Coverage	NaN

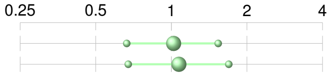

A	B	2.5	50	97.5	Sequence
2	0	0.66	1	1.5	LITEGFELAR
1	0	0.67	1.1	1.7	AAQALAVNISAA

5.493 114 1001 Vinculin [Crassostrea gigas]

Protein Accession	CL18499.Contig1$_{H}1$
Mean Expression Ratio	0.967
Median Expression Ratio	0.967
Credible Interval	(0.748, 1.26)
Associated Peptides	8
Associated Spectra	11
Coverage	NaN

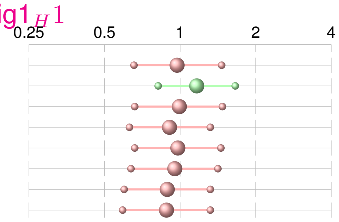

A	B	2.5	50	97.5	Sequence
1	0	0.65	0.97	1.5	MEDLVTFVK
2	1	0.82	1.2	1.7	TIESILEPVAQQVSR
1	0	0.66	0.99	1.5	NLSPVLTR
1	1	0.63	0.91	1.3	KMSDEIHEIIR
0	1	0.66	0.98	1.5	AAVDNLVR
0	1	0.64	0.95	1.4	MSDEIHEIIR
0	1	0.6	0.89	1.3	NLTPVLISGIK
0	1	0.59	0.88	1.3	IFITAK

5.494 " 45 506 minus strand PREDICTED: dihydrolipoyl dehydrogenase, mitochondrial [Monodelphis domestica]"

Protein Accession	CL24292.Contig3$_{H}1$
Mean Expression Ratio	0.968
Median Expression Ratio	0.967
Credible Interval	(0.645, 1.45)
Associated Peptides	2
Associated Spectra	2
Coverage	NaN

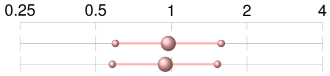

A	B	2.5	50	97.5	Sequence
0	1	0.6	0.98	1.6	ALTGGIAHLFK
0	1	0.58	0.95	1.5	GIEIDGLR

5.495 1 219 minus strand PREDICTED: nucleoside diphosphate kinase 3-like [Pan troglodytes]

Protein Accession	Unigene5384$_{H1}$
Mean Expression Ratio	0.967
Median Expression Ratio	0.967
Credible Interval	(0.709, 1.32)
Associated Peptides	2
Associated Spectra	11
Coverage	NaN

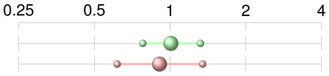

A	B	2.5	50	97.5	Sequence
6	3	0.78	1	1.3	GLVGEIIK
0	2	0.62	0.91	1.3	GLVGEIIKR

5.496 145 1524 PREDICTED: aspartyl aminopeptidase-like [Oreochromis niloticus]

Protein Accession	CL19117.Contig2$_{H1}$
Mean Expression Ratio	1.03
Median Expression Ratio	1.03
Credible Interval	(0.688, 1.55)
Associated Peptides	2
Associated Spectra	2
Coverage	NaN

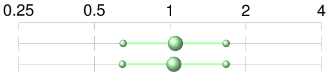

A	B	2.5	50	97.5	Sequence
1	0	0.65	1.1	1.7	ITVSPSNPVAFEEAIGK
1	0	0.65	1	1.7	KLVAHGFK

5.497 264 1505 Ribosome-binding protein 1 [Crassostrea gigas]; 260 1501 Ribosome-binding protein 1 [Crassostrea gigas]; 256 1497 Ribosome-binding protein 1 [Crassostrea gigas]

Protein Accession	CL8282.Contig7$_{H1}$ CL8282.Contig4$_{H1}$ CL8282.Contig1$_{H1}$
Mean Expression Ratio	1.03
Median Expression Ratio	1.03
Credible Interval	(0.691, 1.55)
Associated Peptides	2
Associated Spectra	2
Coverage	NaN

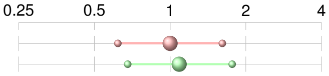

A	B	2.5	50	97.5	Sequence
1	0	0.62	1	1.6	LVQQLQEQLSEK
1	0	0.68	1.1	1.8	LKEDKETLEAR

5.498 2 328 cytoskeletal actin 2 [Molgula oculata]

Protein Accession	Unigene2743$_{H1}$
Mean Expression Ratio	0.968
Median Expression Ratio	0.968
Credible Interval	(0.799, 1.18)
Associated Peptides	7
Associated Spectra	138
Coverage	NaN

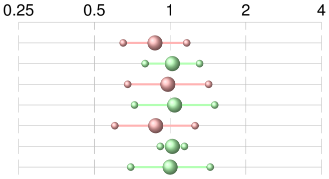

A	B	2.5	50	97.5	Sequence
3	2	0.65	0.87	1.2	IIAPPERK
7	2	0.8	1	1.3	IKIIAPPER
1	0	0.68	0.98	1.4	KIIAPPER
1	0	0.72	1	1.5	DITALAPPTMK
1	0	0.6	0.88	1.3	DLYANVVLSGGSTMYPGIADR
63	57	0.91	1	1.1	IIAPPER
1	0	0.7	1	1.4	KDLYANVVLSGGSTMYPGIADR

5.499 3 386 fem-1-like protein C [Crassostrea gigas]

Protein Accession	Unigene19937$_{H}$1
Mean Expression Ratio	0.966
Median Expression Ratio	0.968
Credible Interval	(0.646, 1.42)
Associated Peptides	1
Associated Spectra	3
Coverage	NaN

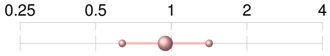

A	B	2.5	50	97.5	Sequence
**3**	**0**	**0.64**	**0.95**	**1.4**	**MQSLLIR**

5.500 147 383 minus strand Tubulin-specific chaperone A OS=Rattus norvegicus GN=Tbca PE=1 SV=1; 104 418 Tubulin-specific chaperone A [Crassostrea gigas]; 104 418 minus strand Tubulin-specific chaperone A [Crassostrea gigas]; 104 418 Tubulin-specific chaperone A [Crassostrea gigas]

Protein Accession	Unigene66350$_{H}$1 Unigene53595$_{H}$1 Unigene32457$_{H}$1 CL7190.Contig3$_{H}$1
Mean Expression Ratio	0.968
Median Expression Ratio	0.968
Credible Interval	(0.625, 1.51)
Associated Peptides	1
Associated Spectra	2
Coverage	NaN

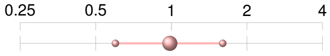

A	B	2.5	50	97.5	Sequence
**1**	**1**	**0.6**	**0.99**	**1.6**	**ADLGETEELK**

5.501 191 919 PREDICTED: 15-hydroxyprostaglandin dehydrogenase [NAD+] [Gallus gallus]

Protein Accession	CL7033.Contig5$_{H}$1
Mean Expression Ratio	1.03
Median Expression Ratio	1.03
Credible Interval	(0.667, 1.6)
Associated Peptides	1
Associated Spectra	1
Coverage	NaN

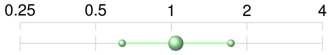

A	B	2.5	50	97.5	Sequence
**1**	**0**	**0.64**	**1**	**1.7**	**AMVEALLDK**

5.502 248 2053 PDZ and LIM domain protein 5 [Crassostrea gigas]

Protein Accession	CL2142.Contig4$_{H}$1
Mean Expression Ratio	1.03
Median Expression Ratio	1.03
Credible Interval	(0.836, 1.27)
Associated Peptides	8
Associated Spectra	31
Coverage	NaN

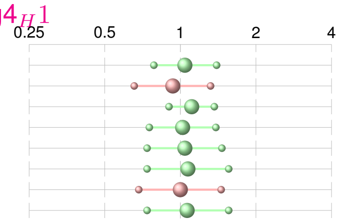

A	B	2.5	50	97.5	Sequence
**2**	**3**	**0.78**	**1**	**1.4**	**CGLQTGDVILK**
**1**	**1**	**0.66**	**0.93**	**1.3**	**IGNTDTSALR**
**4**	**11**	**0.9**	**1.1**	**1.4**	**LDLLLQR**
**3**	**1**	**0.75**	**1**	**1.4**	**VNPGSLAAK**
**1**	**1**	**0.73**	**1**	**1.5**	**YLAPHCK**
**0**	**1**	**0.74**	**1.1**	**1.6**	**SVLTLEAK**
**0**	**1**	**0.68**	**1**	**1.5**	**LQTGDVILK**
**0**	**1**	**0.74**	**1.1**	**1.6**	**MDSTTPWGFR**

5.503 198 821 minus strand sigma class glutathione-s-transferase 2 [Haliotis discus discus]

Protein Accession: CL1352.Contig4$_{H}$1
Mean Expression Ratio: 0.968
Median Expression Ratio: 0.969
Credible Interval: (0.625, 1.51)
Associated Peptides: 1
Associated Spectra: 1
Coverage: NaN

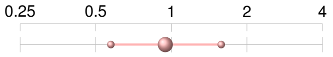

A	B	2.5	50	97.5	Sequence
**0**	**1**	**0.57**	**0.95**	**1.6**	**AAGQPFDDNVVDR**

5.504 249 845 sigma class glutathione-s-transferase 2 [Haliotis discus discus]; 252 848 sigma class glutathione-s-transferase 2 [Haliotis discus discus]

Protein Accession: CL19285.Contig32$_{H}$1 CL19285.Contig20$_{H}$1
Mean Expression Ratio: 1.03
Median Expression Ratio: 1.03
Credible Interval: (0.663, 1.6)
Associated Peptides: 1
Associated Spectra: 1
Coverage: NaN

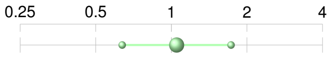

A	B	2.5	50	97.5	Sequence
**1**	**0**	**0.64**	**1.1**	**1.7**	**APFGQAPFMEYEGK**

5.505 1 576 minus strand Tubulin beta chain [Crassostrea gigas]

Protein Accession: Unigene6463$_{H}$1
Mean Expression Ratio: 1.03
Median Expression Ratio: 1.03
Credible Interval: (0.671, 1.59)
Associated Peptides: 1
Associated Spectra: 4
Coverage: NaN

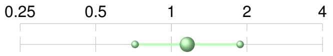

A	B	2.5	50	97.5	Sequence
**3**	**1**	**0.72**	**1.2**	**1.9**	**EVDEQMLNVQNK**

5.506 316 531 cytochrome c oxidase subunit VIIa polypeptide 2 like [Xenopus (Silurana) tropicalis]

Protein Accession: CL17601.Contig5$_{H}$1
Mean Expression Ratio: 1.03
Median Expression Ratio: 1.03
Credible Interval: (0.666, 1.61)
Associated Peptides: 1
Associated Spectra: 1
Coverage: NaN

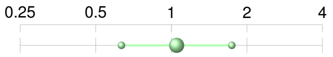

A	B	2.5	50	97.5	Sequence
**0**	**1**	**0.63**	**1**	**1.7**	**QDGLLVWQK**

5.507 123 314 PREDICTED: ubiquinol-cytochrome c reductase hinge protein-like [Saccoglossus kowalevskii]; 279 530 PREDICTED: ubiquinol-cytochrome c reductase hinge protein-like [Saccoglossus kowalevskii]

Protein Accession	CL4684.Contig5$_{H}$1 CL4684.Contig4$_{H}$1
Mean Expression Ratio	1.03
Median Expression Ratio	1.03
Credible Interval	(0.68, 1.57)
Associated Peptides	1
Associated Spectra	2
Coverage	NaN

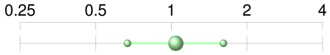

A	B	2.5	50	97.5	Sequence
**2**	**0**	**0.67**	**1**	**1.6**	**CLFDELK**

5.508 142 588 AAEL009772-PB [Aedes aegypti]; 96 497 AAEL009772-PB [Aedes aegypti]

Protein Accession	Unigene73475$_{H}$1 CL27854.Contig2$_{H}$1
Mean Expression Ratio	0.966
Median Expression Ratio	0.97
Credible Interval	(0.618, 1.5)
Associated Peptides	1
Associated Spectra	3
Coverage	NaN

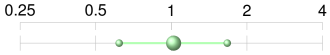

A	B	2.5	50	97.5	Sequence
**2**	**1**	**0.62**	**1**	**1.7**	**LVEEFPMGNIK**

5.509 2 2248 PREDICTED: heat shock 70 kDa protein 4-like [Oreochromis niloticus]; 2 2227 PREDICTED: heat shock 70 kDa protein 4-like [Oreochromis niloticus]

Protein Accession	CL12749.Contig5$_{H}$1 CL12749.Contig4$_{H}$1
Mean Expression Ratio	0.968
Median Expression Ratio	0.97
Credible Interval	(0.668, 1.4)
Associated Peptides	3
Associated Spectra	3
Coverage	NaN

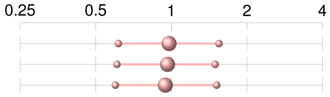

A	B	2.5	50	97.5	Sequence
**1**	**0**	**0.62**	**0.98**	**1.6**	**EMLNLLIEK**
**1**	**0**	**0.61**	**0.96**	**1.5**	**TAWWTPK**
**0**	**1**	**0.6**	**0.95**	**1.5**	**HENPVIFVSQVMSEK**

5.510 203 1942 ezrin/radixin/moesin [Aplysia californica] gi; 243 1982 minus strand ezrin/radixin/moesin [Aplysia californica] gi

Protein Accession	CL13506.Contig5$_H$1 CL13503.Contig4$_H$1
Mean Expression Ratio	1.03
Median Expression Ratio	1.03
Credible Interval	(0.841, 1.26)
Associated Peptides	13
Associated Spectra	24
Coverage	NaN

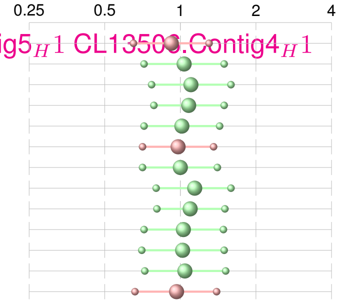

A	B	2.5	50	97.5	Sequence
1	1	0.65	0.92	1.3	DDAMMEYLK
1	0	0.72	1	1.5	FVIKPIDK
1	0	0.77	1.1	1.6	KAPDFVFYAPR
3	0	0.78	1.1	1.5	NISFNDKK
2	0	0.72	1	1.4	RKPDTIEVQQMK
2	1	0.71	0.98	1.4	KESPLQFK
1	1	0.71	1	1.4	QLFDQVVK
2	0	0.8	1.1	1.6	QLEMLK
4	0	0.81	1.1	1.5	SWWGEHK
1	0	0.71	1	1.5	LADELETAQR
0	1	0.7	1	1.5	MAELLALR
0	1	0.72	1	1.5	IGFPWSEIR
0	1	0.66	0.97	1.4	ESPLQFK

5.511 2 256 Polyubiquitin (Fragment) OS=Xenopus laevis PE=1 SV=2; 20 472 minus strand large subunit ribosomal protein L40e [Clonorchis sinensis]; 20 388 large subunit ribosomal protein L40e [Clonorchis sinensis]

Protein Accession	CL47507.Contig2$_H$1 CL39010.Contig2$_H$1 CL39010.Contig1$_H$1
Mean Expression Ratio	0.969
Median Expression Ratio	0.97
Credible Interval	(0.708, 1.32)
Associated Peptides	4
Associated Spectra	15
Coverage	NaN

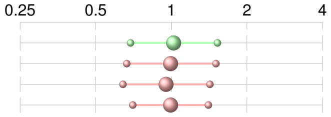

A	B	2.5	50	97.5	Sequence
2	1	0.69	1	1.5	TITLEVEPSDTIENVK
1	1	0.66	1	1.5	ESTLHLVLR
2	1	0.64	0.95	1.4	MQIFVK
4	3	0.7	0.99	1.4	TLSDYNIQK

5.512 880 1434 minus strand Prohormone-4 [Crassostrea gigas]

Protein Accession	CL27941.Contig2$_H$1
Mean Expression Ratio	0.97
Median Expression Ratio	0.97
Credible Interval	(0.623, 1.51)
Associated Peptides	1
Associated Spectra	1
Coverage	NaN

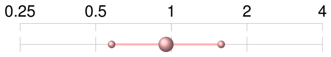

A	B	2.5	50	97.5	Sequence
1	0	0.58	0.95	1.6	AALQGAQAGDERPLLR

5.513 2 1384 minus strand twitchin [Mytilus galloprovincialis]; 2 1384 minus strand twitchin [Mytilus galloprovincialis]; 2 1384 minus strand twitchin [Mytilus galloprovincialis]; 2 1384 minus strand twitchin [Mytilus galloprovincialis]; 2 1384 minus strand twitchin [Mytilus galloprovincialis]; 2 1384 minus strand twitchin [Mytilus galloprovincialis]

Protein Accession	Unigene62659$_{H}1$ Unigene62658$_{H}1$ Unigene62657$_{H}1$ Unigene62656$_{H}1$ Unigene62655$_{H}1$ U
Mean Expression Ratio	1.03
Median Expression Ratio	1.03
Credible Interval	(0.659, 1.59)
Associated Peptides	1
Associated Spectra	1
Coverage	NaN

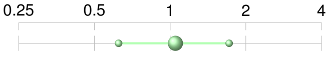

A	B	2.5	50	97.5	Sequence
1	0	0.63	1	1.7	DGGGADLYTAVLQIQDPKPEDGGTYK

5.514 280 726 minus strand RecName: Full=Calmodulin; Short=CaM gi|14588595|dbj|BAB61794.1| calmodulin [Metridium senile] gi

Protein Accession	Unigene4486$_{H}1$
Mean Expression Ratio	1.03
Median Expression Ratio	1.03
Credible Interval	(0.772, 1.37)
Associated Peptides	5
Associated Spectra	13
Coverage	NaN

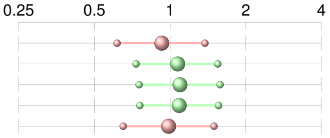

A	B	2.5	50	97.5	Sequence
1	1	0.62	0.93	1.4	ADQLTEEQIAEFK
2	0	0.73	1.1	1.5	EAFSLFDK
2	0	0.75	1.1	1.6	EAFSLFDKDGDGTITTK
2	4	0.76	1.1	1.6	ELGTVMR
1	0	0.65	0.99	1.5	HVMTNLGEKLTDEEVDEMIR

5.515 582 2135 sulfatase 1A precursor [Haliotis discus discus]

Protein Accession	CL1700.Contig8$_{H}1$
Mean Expression Ratio	0.972
Median Expression Ratio	0.971
Credible Interval	(0.708, 1.34)
Associated Peptides	4
Associated Spectra	6
Coverage	NaN

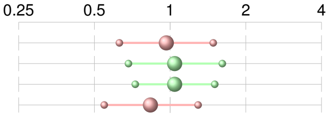

A	B	2.5	50	97.5	Sequence
1	0	0.63	0.97	1.5	HNEDVFYSAK
1	0	0.68	1	1.6	GKYPDVFK
2	1	0.72	1	1.5	AVSFLHSPK
0	1	0.55	0.84	1.3	EGHMYTPTLDGLAR

5.516 252 1289 minus strand PDZ and LIM domain protein 3 OS=Gallus gallus GN=PDLIM3 PE=1 SV=1

Protein Accession	CL15373.Contig10$_{H}1$
Mean Expression Ratio	1.03
Median Expression Ratio	1.03
Credible Interval	(0.75, 1.41)
Associated Peptides	5
Associated Spectra	6
Coverage	NaN

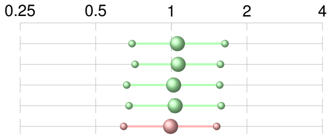

A	B	2.5	50	97.5	Sequence
1	0	0.7	1.1	1.6	SGDAILSINGVNTDHLTHEQAK
1	1	0.72	1.1	1.6	GFFVVEGK
1	0	0.67	1	1.6	IWKPQVTPLSE
1	0	0.68	1	1.6	CGGCDMLATGVIVK
0	1	0.64	0.99	1.5	AGCDIDFLIQR

5.517 " 17 283 Glycerol-3-phosphate dehydrogenase [NAD+], cytoplasmic [Crassostrea gigas]"

Protein Accession	Unigene9883$_{H}1$
Mean Expression Ratio	1.03
Median Expression Ratio	1.03
Credible Interval	(0.69, 1.56)
Associated Peptides	2
Associated Spectra	2
Coverage	NaN

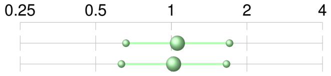

A	B	2.5	50	97.5	Sequence
0	1	0.66	1.1	1.7	KLTEIVNTEHENVK
0	1	0.63	1	1.7	LTEIVNTEHENVK

5.518 299 7507 minus strand filamin [Clonorchis sinensis]

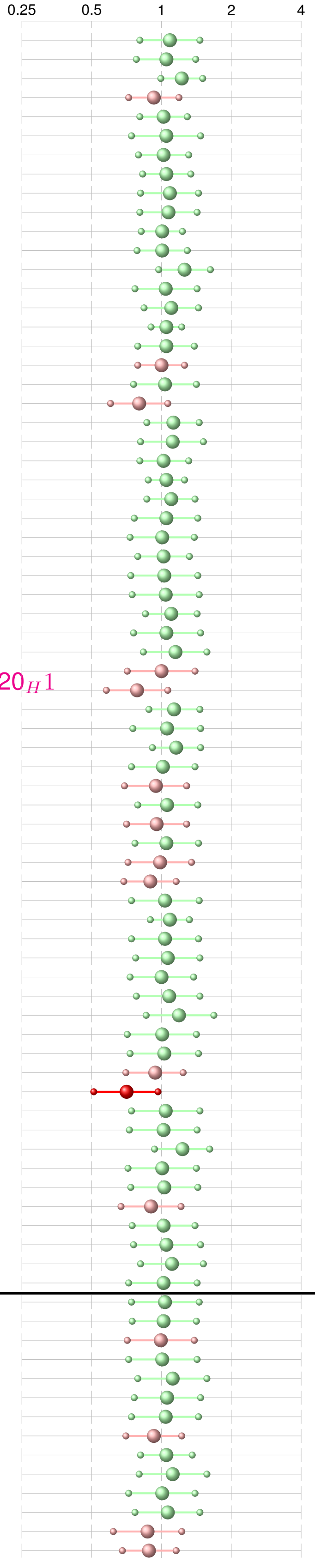

Protein Accession	CL4145.Contig20$_{H}$1
Mean Expression Ratio	1.03
Median Expression Ratio	1.03
Credible Interval	(0.954, 1.11)
Associated Peptides	80
Associated Spectra	327
Coverage	NaN

A	B	2.5	50	97.5	Sequence
3	0	0.81	1.1	1.5	AYGPGLEPK
2	1	0.78	1	1.4	EAGLEEDEEMTTTER
11	2	1	1.2	1.5	EETKPGSLGVTVEGPK
1	6	0.72	0.93	1.2	ELADDAQWK
3	6	0.81	1	1.3	EPVEVVFNNDR
1	0	0.74	1	1.5	EVNKSPFEVTVEGAAGDASK
4	3	0.8	1	1.3	FLEEDEGIR
5	3	0.83	1.1	1.3	FTVETFSAGK
4	0	0.81	1.1	1.4	GAGEGEVAVQIIGPGGSEEK
4	0	0.8	1.1	1.4	GAGNGSVDVVILDPHGR
8	5	0.82	1	1.2	GTLEVTLLNPK
3	4	0.78	1	1.3	IEASIDVSGIK
6	0	0.97	1.3	1.6	INVTHADKPIPGSPFK
1	1	0.77	1	1.4	IVNIDSTDIVDK
4	1	0.84	1.1	1.4	KDTVRPSIQPVVGK
13	18	0.9	1	1.2	LENVTMVLK
2	2	0.79	1	1.4	NFTTDWNDGR
4	6	0.79	1	1.3	QIADLESDFSDGLR
2	0	0.76	1	1.4	SPFEVTVEGAAGDASK
2	2	0.6	0.8	1.1	TQKLENVTMVLK
4	2	0.87	1.1	1.5	TTNVPVQDLVDKK
1	1	0.81	1.1	1.5	VDAVPDTGIPK
6	2	0.81	1	1.3	VDDLSMMTYLSQFPNAK
14	6	0.88	1	1.3	VEVGPFWK
7	1	0.87	1.1	1.4	VPDLPIK
2	0	0.76	1.1	1.4	VYVSEPTNPAK
1	1	0.73	1	1.4	TWFEVYTK
4	2	0.79	1	1.3	ACFATGR
1	0	0.74	1	1.4	IEASIDVSGIKVEGLEPK
1	0	0.75	1	1.5	YMPTKNGK
5	1	0.85	1.1	1.4	MAITSPSGK
1	0	0.76	1.1	1.5	VEGLEPK
2	0	0.84	1.1	1.6	AFGPGLTGGEALKPAFFTIDAR
1	0	0.71	1	1.4	GLEEDEEMTTTER
2	1	0.58	0.78	1.1	KTWFEVYTK
4	3	0.88	1.1	1.5	QVEVQVK
1	0	0.75	1.1	1.5	VKDSADFYVHTK
3	5	0.92	1.2	1.5	WANEHLK
2	0	0.74	1	1.4	WLDVPQLIKPDEMTNPK
0	2	0.69	0.94	1.3	AAADVTHVGSECELSLK
0	3	0.79	1.1	1.4	AEIDFQDR
0	3	0.71	0.95	1.3	CYGPGLDSK
0	2	0.77	1	1.4	EEPCLLK
0	2	0.72	0.98	1.4	ETFITVDAAEAGNGK
0	6	0.69	0.89	1.2	FNDEHIPDSPYR
0	1	0.74	1	1.5	GEVQQPCDFNIYTR
0	15	0.89	1.1	1.3	GGLGLGIEGPVEAK
0	1	0.74	1	1.4	IMVGESELGNASK
0	2	0.77	1.1	1.5	ISISALQDK
0	2	0.73	1	1.4	LANGHLGITFTPR
0	2	0.78	1.1	1.5	PAAFVVNFNGAK
0	1	0.86	1.2	1.7	RLANGHLGITFTPR
0	1	0.71	1	1.4	SPTGSDIDIDIIDR
0	1	0.73	1	1.4	SPVDELTVK
0	4	0.7	0.94	1.2	VKPAALDVSGVK
0	2	0.51	0.71	0.97	VQNPVDASK
0	1	0.74	1	1.5	VQVSPSIGDAR
0	1	0.73	1	1.4	VSEELITR
0	5	0.94	1.2	1.6	VTETDITDLTASIR
0	1	0.72	1	1.4	VTGPGVQGTTPASLPAVFK
0	1	0.73	1	1.4	YAGVNIAGSPFK
0	3	0.67	0.9	1.2	HIANSPFK
0	2	0.75	1	1.4	KISISALQDK
0	1	0.76	1	1.5	APGDYLISIR
0	2	0.81	1.1	1.5	EMGIHTVSVK
0	1	0.72	1	1.4	FADQHVPGSPFTVK
0	1	0.74	1	1.5	IMVGK
0	2	0.75	1	1.4	VKPAALDVSG
0	1	0.71	0.99	1.4	IPGTSPFDMTASVK
0	1	0.72	1	1.4	SVTLNGEGTVQAILTTPTGR
0	1	0.79	1.1	1.6	FADQHVPGSPF
0	1	0.76	1.1	1.5	QIADLESDFSDGL
0	1	0.74	1	1.4	EVPNSPFQMR
0	5	0.7	0.93	1.2	DVPGAPFPVK
0	6	0.81	1	1.4	ETGEHLVNVFR
0	1	0.8	1.1	1.6	GLQVGKPAAFVVNFNGAK
0	1	0.72	1	1.4	INVTHADKPIPGS
0	2	0.77	1.1	1.5	NIMGSPFTAK
0	1	0.62	0.87	1.2	PGVDASK
0	5	0.68	0.89	1.2	VLPGVDASK

5.519 18 647 minus strand ATP synthase-like protein [Biomphalaria glabrata]

Protein Accession	CL42054.Contig4$_{H}1$
Mean Expression Ratio	1.03
Median Expression Ratio	1.03
Credible Interval	(0.798, 1.33)
Associated Peptides	6
Associated Spectra	16
Coverage	NaN

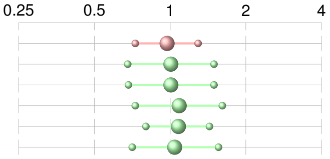

A	B	2.5	50	97.5	Sequence
2	4	0.73	0.97	1.3	LAEFVANPSLQR
1	0	0.68	1	1.5	KAVLEQALK
1	0	0.68	1	1.5	NFQNLLK
1	0	0.72	1.1	1.6	KLDEVEK
3	3	0.8	1.1	1.4	GALQGFLK
1	0	0.71	1	1.6	NYTNLIK

5.520 238 1038 PREDICTED: hypothetical protein [Saccoglossus kowalevskii]; 294 992 PREDICTED: hypothetical protein [Saccoglossus kowalevskii]

Protein Accession	CL6273.Contig7$_{H}1$ CL6273.Contig6$_{H}1$
Mean Expression Ratio	0.974
Median Expression Ratio	0.972
Credible Interval	(0.63, 1.51)
Associated Peptides	1
Associated Spectra	1
Coverage	NaN

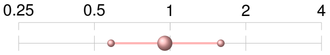

A	B	2.5	50	97.5	Sequence
0	1	0.58	0.95	1.6	DVVLLHQLGR

5.521 " 492 3473 PREDICTED: 2-oxoglutarate dehydrogenase, mitochondrial isoform 1 [Sarcophilus harrisii]; 492 3458 PREDICTED: 2-oxoglutarate dehydrogenase, mitochondrial isoform 1 [Sarcophilus harrisii]"

Protein Accession	CL2200.Contig1$_{H}1$ CL2200.Contig16$_{H}1$
Mean Expression Ratio	0.974
Median Expression Ratio	0.973
Credible Interval	(0.653, 1.45)
Associated Peptides	2
Associated Spectra	2
Coverage	NaN

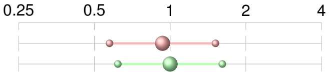

A	B	2.5	50	97.5	Sequence
1	0	0.57	0.94	1.5	FSEVPEGFVVHGGLK
1	0	0.62	1	1.6	KPLVVATPK

5.522 63 1865 Dolichyl-diphosphooligosaccharide–protein glycosyltransferase subunit 1 [Crassostrea gigas]; 63 1865 Dolichyl-diphosphooligosaccharide–protein glycosyltransferase subunit 1 [Crassostrea gigas]

Protein Accession	CL41703.Contig2$_{H}1$ CL41703.Contig1$_{H}1$
Mean Expression Ratio	1.03
Median Expression Ratio	1.03
Credible Interval	(0.736, 1.44)
Associated Peptides	4
Associated Spectra	5
Coverage	NaN

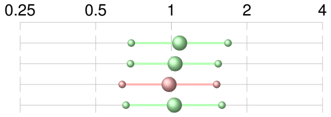

A	B	2.5	50	97.5	Sequence
1	0	0.69	1.1	1.7	KVDIATHIVK
1	1	0.69	1	1.5	VASLVEEVQSAQDKR
1	0	0.64	0.98	1.5	DLNAFQVAR
0	1	0.66	1	1.6	ELDDSVDIELRPR

5.523 4 219 hypothetical protein BRAFLDRAFT_66351 [Branchiostoma floridae] gi; 3 239 hypothetical protein BRAFLDRAFT_66351 [Branchiostoma floridae] gi

Protein Accession	CL31114.Contig2$_{H}1$ CL31114.Contig1$_{H}1$
Mean Expression Ratio	1.03
Median Expression Ratio	1.03
Credible Interval	(0.665, 1.59)
Associated Peptides	1
Associated Spectra	1
Coverage	NaN

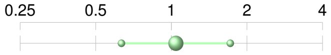

A	B	2.5	50	97.5	Sequence
0	1	0.63	1	1.7	KLEEVSGELR

5.524 116 337 minus strand Golgi-associated plant pathogenesis-related protein 1 [Crassostrea gigas]; 538 759 minus strand Golgi-associated plant pathogenesis-related protein 1 [Crassostrea gigas]; 116 337 minus strand Golgi-associated plant pathogenesis-related protein 1 [Crassostrea gigas]; 261 482 minus strand Golgi-associated plant pathogenesis-related protein 1 [Crassostrea gigas]; 116 337 minus strand Golgi-associated plant pathogenesis-related protein 1 [Crassostrea gigas]; 116 337 minus strand Golgi-associated plant pathogenesis-related protein 1 [Crassostrea gigas]

Protein Accession	CL15263.Contig9$_{H}1$ CL15263.Contig8$_{H}1$ CL15263.Contig7$_{H}1$ CL15263.Contig5$_{H}1$ CL1526
Mean Expression Ratio	1.03
Median Expression Ratio	1.03
Credible Interval	(0.69, 1.52)
Associated Peptides	2
Associated Spectra	2
Coverage	NaN

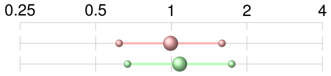

A	B	2.5	50	97.5	Sequence
1	0	0.62	0.99	1.6	FIDEMLEAHNK
1	0	0.67	1.1	1.7	KEHQVQPLVINK

5.525 " 1240 1911 PREDICTED: ATP synthase subunit alpha, mitochondrial-like [Anolis carolinensis]"

Protein Accession	Unigene30533$_{H}1$
Mean Expression Ratio	0.973
Median Expression Ratio	0.973
Credible Interval	(0.685, 1.38)
Associated Peptides	2
Associated Spectra	5
Coverage	NaN

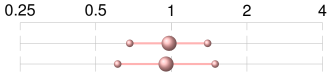

A	B	2.5	50	97.5	Sequence
1	3	0.68	0.98	1.4	LELAQYR
0	1	0.61	0.95	1.5	LTELLK

5.526 233 814 Kynurenine formamidase [Crassostrea gigas]; 233 979 Kynurenine formamidase [Crassostrea gigas]; 139 945 Kynurenine formamidase [Crassostrea gigas]; 145 951 Kynurenine formamidase [Crassostrea gigas]; 220 1026 Kynurenine formamidase [Crassostrea gigas]; 39 725 Kynurenine formamidase [Crassostrea gigas]; 198 986 Kynurenine formamidase [Crassostrea gigas]; 39 743 Kynurenine formamidase [Crassostrea gigas]; 123 929 Kynurenine formamidase [Crassostrea gigas]; 39 743 Kynurenine formamidase [Crassostrea gigas]

Protein Accession	Unigene7804$_{H}1$ Unigene42149$_{H}1$ CL8132.Contig9$_{H}1$ CL8132.Contig8$_{H}1$ CL8132.Contig7$_{H}$
Mean Expression Ratio	0.974
Median Expression Ratio	0.974
Credible Interval	(0.676, 1.41)
Associated Peptides	1
Associated Spectra	6
Coverage	NaN

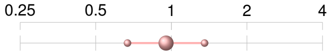

A	B	2.5	50	97.5	Sequence
4	2	0.67	0.95	1.4	LVGPGVVIDVR

5.527 3 434 minus strand myosin essential light chain [Haliotis discus discus]

Protein Accession	CL17177.Contig8$_{H}1$
Mean Expression Ratio	1.03
Median Expression Ratio	1.03
Credible Interval	(0.875, 1.21)
Associated Peptides	11
Associated Spectra	69
Coverage	NaN

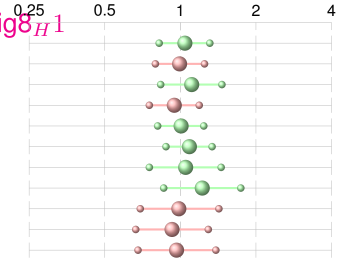

A	B	2.5	50	97.5	Sequence
6	4	0.82	1	1.3	EGQGFMSAAELR
5	6	0.79	0.99	1.2	FTDTREDLEGNVK
4	1	0.84	1.1	1.5	HVLTGLGEK
5	5	0.75	0.95	1.2	LTDQQVTDVIK
6	7	0.81	1	1.2	YADFIEK
7	6	0.88	1.1	1.3	DGDVDAFK
1	1	0.75	1	1.5	VGDMCF
1	0	0.86	1.2	1.7	EDLEGNVK
1	0	0.69	0.99	1.4	LSDREGQGFMSAAELR
0	2	0.67	0.93	1.3	FTDTREDLEGN
0	1	0.68	0.97	1.4	HVLTGLG

5.528 977 1852 PREDICTED: similar to prohibitin [Tribolium castaneum] gi

Protein Accession	CL8219.Contig4$_{H}1$
Mean Expression Ratio	1.03
Median Expression Ratio	1.03
Credible Interval	(0.727, 1.46)
Associated Peptides	3
Associated Spectra	4
Coverage	NaN

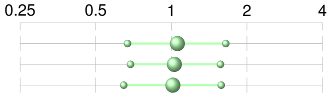

A	B	2.5	50	97.5	Sequence
1	0	0.67	1.1	1.7	VVQAEGEAAAAK
2	0	0.69	1	1.6	KLGDLAGR
1	0	0.65	1	1.6	QQVSLLIR

5.529 385 906 Soma ferritin OS=Lymnaea stagnalis PE=2 SV=2

Protein Accession	CL2484.Contig1$_{H}1$
Mean Expression Ratio	1.03
Median Expression Ratio	1.03
Credible Interval	(0.661, 1.6)
Associated Peptides	1
Associated Spectra	1
Coverage	NaN

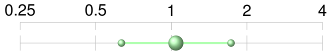

A	B	2.5	50	97.5	Sequence
1	0	0.63	1	1.7	ELADHITNLK

5.530 362 874 minus strand Transgelin-2 [Crassostrea gigas]

Protein Accession	CL445.Contig3$_{H}1$
Mean Expression Ratio	1.03
Median Expression Ratio	1.03
Credible Interval	(0.897, 1.17)
Associated Peptides	14
Associated Spectra	148
Coverage	NaN

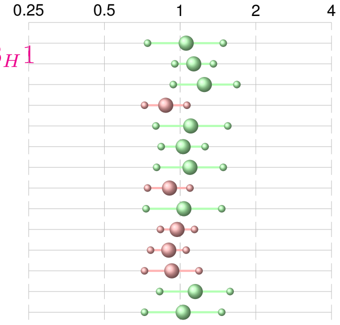

A	B	2.5	50	97.5	Sequence
1	0	0.74	1.1	1.5	AGLGAEIEKK
12	9	0.95	1.1	1.4	EFLVQSADLWEGR
3	1	0.94	1.3	1.7	EFLVQSADLWEGRK
17	0	0.72	0.88	1.1	GFQPSYTGSQSR
2	0	0.8	1.1	1.5	KAGLGAEIEKK
12	3	0.84	1	1.3	LLESEGKPK
1	2	0.81	1.1	1.5	LINKLLESEGKPK
8	8	0.74	0.91	1.1	LINFD
1	0	0.73	1	1.5	LGAEIEK
20	9	0.83	0.98	1.1	AGLGAEIEK
20	8	0.77	0.9	1.1	DGVMLCK
4	4	0.72	0.93	1.2	KAGLGAEIEK
1	1	0.83	1.1	1.6	RDGVMLCKLINK
0	1	0.72	1	1.5	QSADLWEGR

5.531 " 241 1110 minus strand N(G),N(G)-dimethylarginine dimethylaminohydrolase 1 [Crassostrea gigas]; 241 1110 minus strand N(G),N(G)-dimethylarginine dimethylaminohydrolase 1 [Crassostrea gigas]; 241 1110 minus strand N(G),N(G)-dimethylarginine dimethylaminohydrolase 1 [Crassostrea gigas]"

Protein Accession	Unigene30451$_{H}1$ CL7107.Contig3$_{H}1$ CL7107.Contig1$_{H}1$
Mean Expression Ratio	1.03
Median Expression Ratio	1.03
Credible Interval	(0.665, 1.6)
Associated Peptides	1
Associated Spectra	2
Coverage	NaN

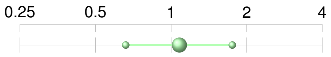

A	B	2.5	50	97.5	Sequence
1	1	0.66	1.1	1.8	LGEVGEIR

5.532 2156 3142 minus strand PREDICTED: hemicentin-1 [Xenopus (Silurana) tropicalis]

Protein Accession	CL2792.Contig4$_{H}1$
Mean Expression Ratio	0.975
Median Expression Ratio	0.975
Credible Interval	(0.76, 1.25)
Associated Peptides	7
Associated Spectra	15
Coverage	NaN

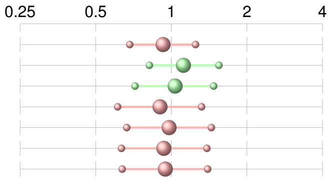

A	B	2.5	50	97.5	Sequence
4	1	0.68	0.93	1.2	GAPPPTVSWVK
2	2	0.82	1.1	1.5	NDVELYSK
1	1	0.72	1	1.5	SGVDAMKPYLR
1	0	0.61	0.9	1.3	LVEVIEEK
0	1	0.66	0.98	1.4	YECVAENLVHTR
0	1	0.63	0.93	1.4	NGLIIDTEKDPNLQVDPEGSLMFK
0	1	0.64	0.94	1.4	VDAMK

5.533 35 271 PREDICTED: SH3 domain-binding glutamic acid-rich-like protein 3-like [Anolis carolinensis]; 35 271 PREDICTED: SH3 domain-binding glutamic acid-rich-like protein 3-like [Anolis carolinensis]; 245 523 PREDICTED: SH3 domain-binding glutamic acid-rich-like protein 3-like [Anolis carolinensis]; 192 470 PREDICTED: SH3 domain-binding glutamic acid-rich-like protein 3-like [Anolis carolinensis]; 192 470 PREDICTED: SH3 domain-binding glutamic acid-rich-like protein 3-like [Anolis carolinensis]; 192 470 PREDICTED: SH3 domain-binding glutamic acid-rich-like protein 3-like [Anolis carolinensis]; 111 389 PREDICTED: SH3 domain-binding glutamic acid-rich-like protein 3-like [Anolis carolinensis]; 144 380 PREDICTED: SH3 domain-binding glutamic acid-rich-like protein 3-like [Anolis carolinensis]; 111 389 PREDICTED: SH3 domain-binding glutamic acid-rich-like protein 3-like [Anolis carolinensis]; 35 271 PREDICTED: SH3 domain-binding glutamic acid-rich-like protein 3-like [Anolis carolinensis]

Protein Accession	Unigene58804$_H1$ Unigene57834$_H1$ Unigene53929$_H1$ Unigene46648$_H1$ Unigene46647$_H1$ U
Mean Expression Ratio	1.03
Median Expression Ratio	1.03
Credible Interval	(0.71, 1.5)
Associated Peptides	2
Associated Spectra	3
Coverage	NaN

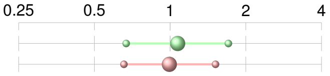

A	B	2.5	50	97.5	Sequence
**1**	**0**	**0.67**	**1.1**	**1.7**	**SFQQAVEEEK**
**2**	**0**	**0.65**	**0.99**	**1.5**	**ILEYLR**

5.534 73 525 minus strand Actin-related protein 2/3 complex subunit 5-like protein [Salmo salar] gi; 73 525 Actin-related protein 2/3 complex subunit 5-like protein [Salmo salar] gi; 73 525 minus strand Actin-related protein 2/3 complex subunit 5-like protein [Salmo salar] gi

Protein Accession	CL14637.Contig6$_H1$ CL14637.Contig5$_H1$ CL14637.Contig4$_H1$
Mean Expression Ratio	0.975
Median Expression Ratio	0.975
Credible Interval	(0.624, 1.51)
Associated Peptides	1
Associated Spectra	1
Coverage	NaN

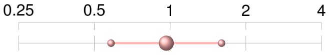

A	B	2.5	50	97.5	Sequence
**0**	**1**	**0.58**	**0.97**	**1.6**	**EVDILMK**

5.535 236 733 Spectrin alpha chain [Crassostrea gigas]

Protein Accession	Unigene61767$_H1$
Mean Expression Ratio	1.03
Median Expression Ratio	1.03
Credible Interval	(0.725, 1.46)
Associated Peptides	3
Associated Spectra	4
Coverage	NaN

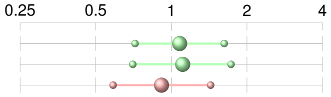

A	B	2.5	50	97.5	Sequence
1	1	0.72	1.1	1.6	DIDDEEAWIK
1	0	0.7	1.1	1.7	DLTGVQNLR
0	1	0.59	0.91	1.4	LNEANTMHQFLR

5.536 50 577 hypothetical protein BRAFLDRAFT_119799 [Branchiostoma floridae] gi; 50 577 hypothetical protein BRAFLDRAFT_119799 [Branchiostoma floridae] gi

Protein Accession	CL5795.Contig4$_H1$ CL5795.Contig3$_H1$
Mean Expression Ratio	1.02
Median Expression Ratio	1.02
Credible Interval	(0.658, 1.58)
Associated Peptides	1
Associated Spectra	1
Coverage	NaN

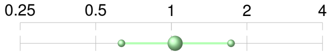

A	B	2.5	50	97.5	Sequence
0	1	0.63	1	1.7	ADLGIDLSSVLGNVR

5.537 430 759 hypothetical protein [Amblyomma maculatum]; 336 665 hypothetical protein [Amblyomma maculatum]

Protein Accession	CL4062.Contig3$_H1$ CL4062.Contig2$_H1$
Mean Expression Ratio	0.976
Median Expression Ratio	0.976
Credible Interval	(0.719, 1.33)
Associated Peptides	4
Associated Spectra	7
Coverage	NaN

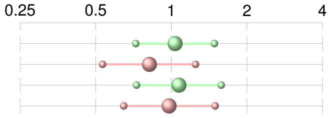

A	B	2.5	50	97.5	Sequence
2	1	0.72	1	1.5	ETLFEYLADPK
1	0	0.53	0.82	1.2	KTGQSAGFSYTDANK
1	1	0.72	1.1	1.6	KYIPGTK
0	1	0.65	0.98	1.5	MVFAGIK

5.538 22 369 Protein farnesyltransferase/geranylgeranyltransferase type-1 subunit alpha [Crassostrea gigas]

Protein Accession	CL38456.Contig1$_H1$
Mean Expression Ratio	1.03
Median Expression Ratio	1.02
Credible Interval	(0.685, 1.54)
Associated Peptides	2
Associated Spectra	2
Coverage	NaN

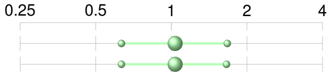

A	B	2.5	50	97.5	Sequence
1	0	0.63	1	1.7	YISAVIEDHPK
1	0	0.63	1	1.7	AIVASEEK

5.539 406 1392 minus strand hypothetical protein CGI_10007007 [Crassostrea gigas]

Protein Accession	CL18045.Contig1$_{H}1$
Mean Expression Ratio	0.975
Median Expression Ratio	0.976
Credible Interval	(0.647, 1.45)
Associated Peptides	1
Associated Spectra	3
Coverage	NaN

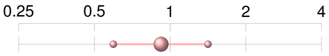

A	B	2.5	50	97.5	Sequence
**2**	**1**	**0.6**	**0.92**	**1.4**	**IEAEFPGANIILHK**

5.540 8 937 minus strand galectin [Crassostrea virginica]

Protein Accession	CL7245.Contig6$_{H}1$
Mean Expression Ratio	1.02
Median Expression Ratio	1.02
Credible Interval	(0.657, 1.58)
Associated Peptides	1
Associated Spectra	1
Coverage	NaN

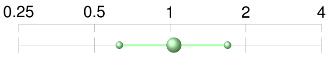

A	B	2.5	50	97.5	Sequence
**1**	**0**	**0.63**	**1**	**1.7**	**QDQGDVVLNAK**

5.541 95 1579 minus strand hypothetical protein BRAFLDRAFT_113974 [Branchiostoma floridae] gi

Protein Accession	CL19753.Contig5$_{H}1$
Mean Expression Ratio	0.977
Median Expression Ratio	0.976
Credible Interval	(0.79, 1.2)
Associated Peptides	10
Associated Spectra	23
Coverage	NaN

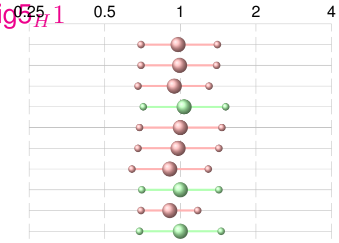

A	B	2.5	50	97.5	Sequence
**1**	**1**	**0.7**	**0.98**	**1.4**	**ELGEYALK**
**1**	**1**	**0.7**	**0.99**	**1.4**	**FKDINEVIER**
**2**	**1**	**0.68**	**0.94**	**1.3**	**LIMQAAGR**
**1**	**0**	**0.71**	**1**	**1.5**	**TFPVVNPATEK**
**1**	**0**	**0.69**	**1**	**1.5**	**TSYGLAAGVITK**
**1**	**0**	**0.68**	**0.98**	**1.4**	**TVGDPFDAVTMQGPQVDQEQFEK**
**1**	**1**	**0.64**	**0.91**	**1.3**	**ADLRPDPNIK**
**1**	**1**	**0.7**	**1**	**1.4**	**DEIFGPVQSIIK**
**3**	**5**	**0.7**	**0.91**	**1.2**	**FADLVIR**
**0**	**1**	**0.69**	**1**	**1.5**	**VICEVQEADK**

5.542 1 378 minus strand PREDICTED: CG10098-like [Saccoglossus kowalevskii]

Protein Accession	Unigene15933$_{H}1$
Mean Expression Ratio	1.02
Median Expression Ratio	1.02
Credible Interval	(0.686, 1.54)
Associated Peptides	2
Associated Spectra	2
Coverage	NaN

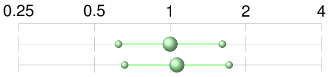

A	B	2.5	50	97.5	Sequence
**1**	**0**	**0.62**	**1**	**1.6**	**AFNASFEGLSLSEK**
**1**	**0**	**0.66**	**1.1**	**1.7**	**ALMAEK**

5.543 54 662 minus strand Protein DJ-1 [Crassostrea gigas]

Protein Accession	CL16588.Contig3$_{H}$1
Mean Expression Ratio	1.03
Median Expression Ratio	1.02
Credible Interval	(0.658, 1.61)
Associated Peptides	1
Associated Spectra	1
Coverage	NaN

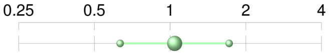

A	B	2.5	50	97.5	Sequence
1	0	0.63	1	1.7	NVNIVPDASMEEALK

5.544 " 213 1499 minus strand Citrate synthase, mitochondrial [Crassostrea gigas]"

Protein Accession	CL992.Contig2$_{H}$1
Mean Expression Ratio	1.02
Median Expression Ratio	1.02
Credible Interval	(0.849, 1.23)
Associated Peptides	12
Associated Spectra	32
Coverage	NaN

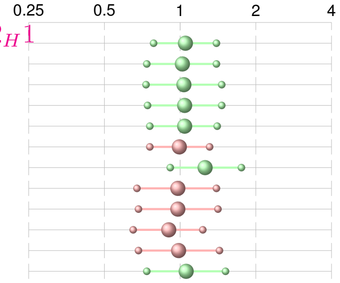

A	B	2.5	50	97.5	Sequence
4	1	0.79	1	1.4	ALGLPIERPK
3	0	0.74	1	1.4	ALVTETSVLDPEEGIR
1	1	0.73	1	1.5	DVLSQIIPEK
2	0	0.74	1	1.5	LMESLGGEVSDEQLR
3	1	0.76	1	1.4	SGQVVPGYGHAVLR
4	2	0.76	0.99	1.3	VVPDILIEQGK
3	0	0.91	1.3	1.8	SFDTPSLR
1	0	0.67	0.98	1.4	VGEVTVDMMYGGMR
1	0	0.68	0.98	1.4	EVSEFR
2	1	0.65	0.9	1.2	HLPNDPMFK
0	1	0.69	0.99	1.4	NFIWNTLK
0	1	0.74	1.1	1.5	LISQLYK

5.545 206 1513 hypothetical protein CGI_10019003 [Crassostrea gigas]

Protein Accession	CL6385.Contig8$_{H}$1
Mean Expression Ratio	1.02
Median Expression Ratio	1.02
Credible Interval	(0.658, 1.58)
Associated Peptides	1
Associated Spectra	1
Coverage	NaN

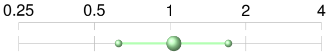

A	B	2.5	50	97.5	Sequence
1	0	0.62	1	1.7	AALEDLLR

5.546 3788 7885 minus strand Filamin-C [Crassostrea gigas]

Protein Accession	CL4145.Contig29$_{H}$1
Mean Expression Ratio	1.02
Median Expression Ratio	1.02
Credible Interval	(0.679, 1.54)
Associated Peptides	1
Associated Spectra	2
Coverage	NaN

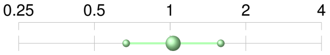

A	B	2.5	50	97.5	Sequence
2	0	0.67	1	1.6	WGEEDIPGSPFAVEVI

5.547 141 1325 Nucleobindin-2 [Crassostrea gigas]; 141 1208 minus strand Nucleobindin-2 [Crassostrea gigas]

Protein Accession	CL1888.Contig6$_H$1 CL1888.Contig2$_H$1
Mean Expression Ratio	0.979
Median Expression Ratio	0.978
Credible Interval	(0.634, 1.54)
Associated Peptides	1
Associated Spectra	2
Coverage	NaN

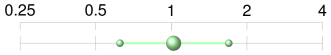

A	B	2.5	50	97.5	Sequence
1	1	0.62	1	1.7	LKELPEVER

5.548 " 112 1320 Aspartate aminotransferase, cytoplasmic [Crassostrea gigas]"

Protein Accession	CL44136.Contig1$_H$1
Mean Expression Ratio	0.978
Median Expression Ratio	0.978
Credible Interval	(0.798, 1.2)
Associated Peptides	9
Associated Spectra	36
Coverage	NaN

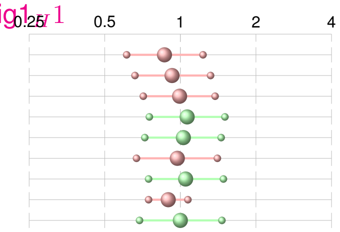

A	B	2.5	50	97.5	Sequence
1	1	0.61	0.87	1.2	ASIFADVK
1	1	0.66	0.93	1.3	IADVMEAK
2	1	0.71	0.99	1.4	SQMEIIVR
2	0	0.75	1.1	1.5	TPSVIPQIR
2	0	0.72	1	1.5	VNLGVGAYR
1	0	0.67	0.98	1.4	ISMAGVTNK
2	0	0.75	1.1	1.5	LAAEFLK
12	9	0.75	0.89	1.1	VEGIQVQGGTGGIR
0	1	0.69	1	1.5	TLANALILGK

5.549 835 1116 tropomyosin [Ilyanassa obsoleta]

Protein Accession	CL2925.Contig8$_H$1
Mean Expression Ratio	1.02
Median Expression Ratio	1.02
Credible Interval	(0.791, 1.31)
Associated Peptides	4
Associated Spectra	15
Coverage	NaN

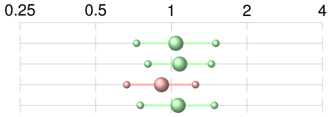

A	B	2.5	50	97.5	Sequence
1	1	0.73	1	1.5	ELEEQSHALTNSLK
2	4	0.81	1.1	1.4	LAVMEAELEK
2	2	0.66	0.91	1.3	QLELYEEK
0	3	0.75	1.1	1.5	LEDVETELR

5.550 11 394 regucalcin [Salmo salar] gi

Protein Accession	CL42615.Contig1$_H$1
Mean Expression Ratio	0.979
Median Expression Ratio	0.979
Credible Interval	(0.633, 1.53)
Associated Peptides	1
Associated Spectra	1
Coverage	NaN

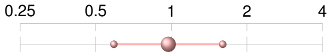

A	B	2.5	50	97.5	Sequence
1	0	0.59	0.98	1.6	NQPLAGSLFK

5.551 227 2920 minus strand Lysosomal alpha-mannosidase [Crassostrea gigas]; 227 3073 minus strand Lysosomal alpha-mannosidase [Crassostrea gigas]; 193 2886 minus strand Lysosomal alpha-mannosidase [Crassostrea gigas]

Protein Accession	CL2930.Contig3$_{H}1$ CL2930.Contig2$_{H}1$ CL2930.Contig1$_{H}1$
Mean Expression Ratio	1.02
Median Expression Ratio	1.02
Credible Interval	(0.749, 1.39)
Associated Peptides	5
Associated Spectra	8
Coverage	NaN

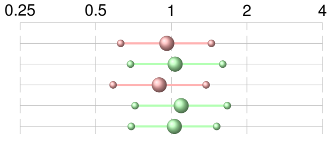

A	B	2.5	50	97.5	Sequence
2	1	0.63	0.96	1.4	VASELIPITPDTFR
0	1	0.69	1	1.6	PDNVHLLTLEQFR
0	1	0.58	0.9	1.4	ALPDNVHLLTLEQFR
0	1	0.71	1.1	1.7	QLNHR
0	2	0.69	1	1.5	AGASLTWSGVR

5.552 549 821 minus strand Calponin-2 [Crassostrea gigas]

Protein Accession	CL21394.Contig10$_{H}1$
Mean Expression Ratio	1.02
Median Expression Ratio	1.02
Credible Interval	(0.764, 1.36)
Associated Peptides	3
Associated Spectra	10
Coverage	NaN

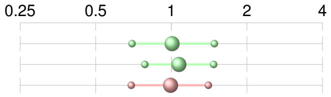

A	B	2.5	50	97.5	Sequence
2	0	0.7	1	1.5	QQGRASQAGMSMGAVR
3	2	0.78	1.1	1.5	SLFPTADLFEGR
3	0	0.69	1	1.4	NSLFPTADLFEGR

5.553 " 64 1332 Flotillin-1, putative [Pediculus humanus corporis] gi"

Protein Accession	CL33952.Contig1$_{H}1$
Mean Expression Ratio	1.02
Median Expression Ratio	1.02
Credible Interval	(0.789, 1.33)
Associated Peptides	4
Associated Spectra	17
Coverage	NaN

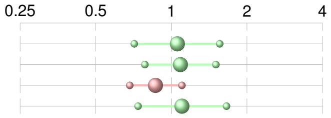

A	B	2.5	50	97.5	Sequence
1	0	0.71	1.1	1.6	AAYDMEVMTTK
3	1	0.78	1.1	1.5	VKGEAEAFAIEAK
6	5	0.68	0.86	1.1	LPSIVETL
0	1	0.74	1.1	1.7	FLNDIEIAK

5.554 138 713 Rac [Aplysia californica] gi; 125 691 Rac [Aplysia californica] gi; 149 871 minus strand Rac [Aplysia californica] gi; 101 676 Rac [Aplysia californica] gi

Protein Accession	CL11009.Contig9$_H$1 CL11009.Contig8$_H$1 CL11009.Contig4$_H$1 CL11009.Contig1$_H$1
Mean Expression Ratio	0.981
Median Expression Ratio	0.98
Credible Interval	(0.659, 1.47)
Associated Peptides	2
Associated Spectra	2
Coverage	NaN

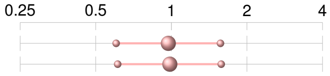

A	B	2.5	50	97.5	Sequence
0	1	0.6	0.97	1.6	NVFDEAIR
0	1	0.61	0.99	1.6	AVLCPR

5.555 2 1093 minus strand Neurexin-1-alpha [Crassostrea gigas]

Protein Accession	Unigene11591$_H$1
Mean Expression Ratio	0.979
Median Expression Ratio	0.98
Credible Interval	(0.664, 1.43)
Associated Peptides	2
Associated Spectra	3
Coverage	NaN

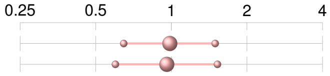

A	B	2.5	50	97.5	Sequence
1	1	0.65	0.98	1.5	IYVGGILDKEEK
0	1	0.6	0.96	1.5	IGDVVEAERPFSLIK

5.556 22 870 minus strand PREDICTED: alcohol dehydrogenase class-3 [Sarcophilus harrisii]

Protein Accession	Unigene27690$_H$1
Mean Expression Ratio	0.981
Median Expression Ratio	0.981
Credible Interval	(0.759, 1.27)
Associated Peptides	6
Associated Spectra	14
Coverage	NaN

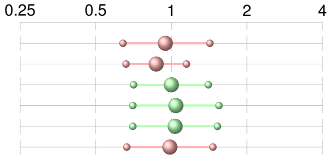

A	B	2.5	50	97.5	Sequence
1	0	0.64	0.95	1.4	AALEACHK
5	2	0.66	0.87	1.1	AAVAWEAK
1	2	0.71	1	1.4	IIGIDVNQDK
1	0	0.7	1	1.6	KPLVIEDIEVAPPAAGEVR
1	0	0.7	1	1.5	TNLCQK
0	1	0.66	0.98	1.5	FAMAK

5.557 " 343 1179 ATP synthase subunit alpha, mitochondrial OS=Pongo abelii GN=ATP5A1 PE=2 SV=1"

Protein Accession	Unigene30534$_H$1
Mean Expression Ratio	0.981
Median Expression Ratio	0.981
Credible Interval	(0.807, 1.19)
Associated Peptides	7
Associated Spectra	33
Coverage	NaN

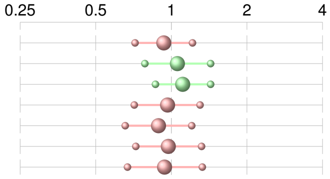

A	B	2.5	50	97.5	Sequence
3	4	0.72	0.93	1.2	TAVAIDTIINQK
4	0	0.78	1.1	1.4	TSVKEPMQTGIK
5	3	0.86	1.1	1.4	VLSIGDGIAR
1	3	0.71	0.96	1.3	VVDALGTPIDGK
2	2	0.66	0.89	1.2	APGIIPR
3	1	0.72	0.97	1.3	STVAQLVK
0	2	0.67	0.94	1.3	LSDADAMK

5.558 61 2865 AP-2 complex subunit beta [Crassostrea gigas]

Protein Accession	CL6957.Contig1$_{H}1$
Mean Expression Ratio	0.98
Median Expression Ratio	0.981
Credible Interval	(0.625, 1.52)
Associated Peptides	1
Associated Spectra	1
Coverage	NaN

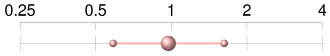

A	B	2.5	50	97.5	Sequence
**1**	**0**	**0.59**	**0.97**	**1.6**	**NSNIFTIAK**

5.559 2 511 hypothetical protein CGI_10006272 [Crassostrea gigas]

Protein Accession	CL10577.Contig2$_{H}1$
Mean Expression Ratio	0.98
Median Expression Ratio	0.981
Credible Interval	(0.631, 1.52)
Associated Peptides	1
Associated Spectra	1
Coverage	NaN

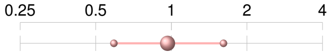

A	B	2.5	50	97.5	Sequence
**1**	**0**	**0.59**	**0.97**	**1.6**	**LLEGAVPK**

5.560 " 3799 4371 papilin, partial [Rhipicephalus microplus]"

Protein Accession	CL19397.Contig5$_{H}1$
Mean Expression Ratio	1.02
Median Expression Ratio	1.02
Credible Interval	(0.677, 1.53)
Associated Peptides	1
Associated Spectra	2
Coverage	NaN

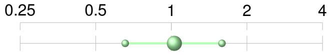

A	B	2.5	50	97.5	Sequence
**1**	**1**	**0.66**	**1**	**1.6**	**TVTLVLAK**

5.561 209 781 minus strand high mobility group protein 1 [Biomphalaria glabrata]; 277 771 high mobility group protein 1 [Biomphalaria glabrata]; 277 867 minus strand high mobility group protein 1 [Biomphalaria glabrata]; 277 870 minus strand high mobility group protein 1 [Biomphalaria glabrata]

Protein Accession	CL25076.Contig4$_{H}1$ CL25076.Contig3$_{H}1$ CL25076.Contig2$_{H}1$ CL25076.Contig1$_{H}1$
Mean Expression Ratio	0.98
Median Expression Ratio	0.981
Credible Interval	(0.653, 1.46)
Associated Peptides	2
Associated Spectra	2
Coverage	NaN

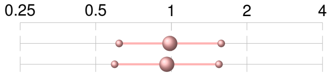

A	B	2.5	50	97.5	Sequence
**1**	**0**	**0.62**	**0.99**	**1.6**	**LAGEEWR**
**1**	**0**	**0.59**	**0.96**	**1.5**	**KYEAEMAEYR**

5.562 219 554 minus strand ribosomal protein P1 [Biomphalaria glabrata]

Protein Accession	CL45629.Contig2$_H$1
Mean Expression Ratio	1.02
Median Expression Ratio	1.02
Credible Interval	(0.658, 1.59)
Associated Peptides	1
Associated Spectra	1
Coverage	NaN

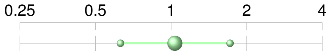

A	B	2.5	50	97.5	Sequence
**1**	**0**	**0.63**	**1**	**1.7**	**ALEGVNIK**

5.563 426 896 PREDICTED: calmodulin-like [Metaseiulus occidentalis]

Protein Accession	Unigene667$_H$1
Mean Expression Ratio	0.982
Median Expression Ratio	0.981
Credible Interval	(0.696, 1.38)
Associated Peptides	2
Associated Spectra	8
Coverage	NaN

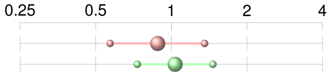

A	B	2.5	50	97.5	Sequence
**1**	**1**	**0.57**	**0.88**	**1.4**	**ADQLTEEQIAEFK**
**2**	**4**	**0.73**	**1**	**1.5**	**ELGTVMR**

5.564 114 2633 Filamin-A [Crassostrea gigas]; 135 2654 Filamin-A [Crassostrea gigas]

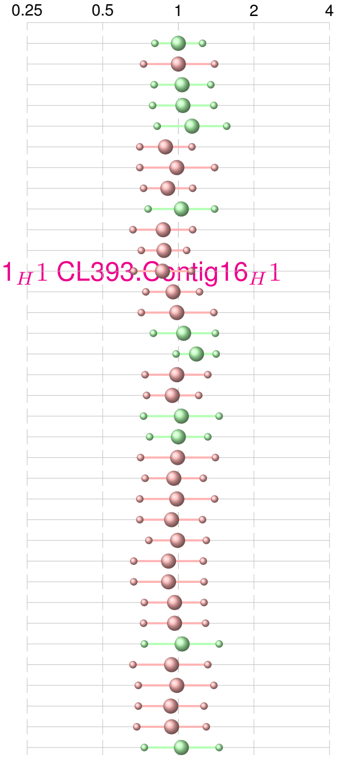

A	B	2.5	50	97.5	Sequence
9	3	0.81	1	1.2	AVANKPNEIVIDTSK
2	0	0.73	1	1.4	DAGEALLSIGMIAPSGHPEAELGYK
5	1	0.8	1	1.3	DNNDGTIVVEYQPK
4	0	0.79	1	1.4	FADEHVPGSPFLLNVGGEASGR
2	0	0.83	1.1	1.6	FDDVHIPGSPFIVNIAPDSGTAR
5	4	0.7	0.89	1.1	IGDSELGHAAGCK
1	0	0.7	0.99	1.4	KMGDGEIGIASFNPR
8	3	0.73	0.91	1.1	KPSPIQEQSSMVVETVEK
3	0	0.76	1	1.4	KVTANSYTVSYK
3	2	0.66	0.87	1.1	MGDGEIGIASFNPR
9	5	0.71	0.88	1.1	MNGIHTLSVR
4	2	0.66	0.87	1.1	QMEAAEAAVPNK
8	0	0.74	0.95	1.2	SQLSLGNSAEYSLK
1	0	0.71	0.99	1.4	TNVSDETGCYSK
3	1	0.79	1	1.4	TPSGGHEDCFIQEMDK
12	9	0.98	1.2	1.4	VINFQIETK
3	1	0.74	0.99	1.3	VPKPGSYGLHIK
5	4	0.75	0.95	1.2	VQVGGPGLEK
1	0	0.73	1	1.5	VRDNNDGTIVVEYQPK
4	2	0.77	1	1.3	VTGALDK
1	0	0.71	0.99	1.4	WGDEDIPGSPFVIST
4	2	0.74	0.96	1.3	YGNIPIAGSPYK
1	0	0.7	0.99	1.4	YIPKENGVHYVDVK
3	1	0.7	0.94	1.3	YKGNPIK
5	1	0.76	0.99	1.3	EAGPGTLTVAVEGPGK
1	1	0.66	0.92	1.3	AALSCK
1	1	0.67	0.92	1.3	PGSYGLHIK
4	1	0.73	0.96	1.3	HINNSPFK
3	1	0.72	0.97	1.3	IMFTPK
1	0	0.73	1	1.5	FRGDASK
1	0	0.66	0.94	1.3	AQIFLEERPNGFLGVQYK
1	0	0.69	0.98	1.4	CPGDYLITIK
2	1	0.69	0.94	1.3	ENGVHYVDVK
2	0	0.68	0.94	1.3	SDIECYK
1	0	0.73	1	1.5	VTANSYTVSYK

Protein Accession	CL393.Contig21$_H$1 CL393.Contig16$_H$1
Mean Expression Ratio	0.982
Median Expression Ratio	0.981
Credible Interval	(0.881, 1.09)
Associated Peptides	35
Associated Spectra	169
Coverage	NaN

5.565 366 1049 hypothetical protein CGI_10004855 [Crassostrea gigas]

Protein Accession	CL43590.Contig5$_H$1
Mean Expression Ratio	0.982
Median Expression Ratio	0.982
Credible Interval	(0.764, 1.26)
Associated Peptides	7
Associated Spectra	14
Coverage	NaN

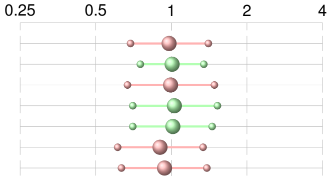

A	B	2.5	50	97.5	Sequence
1	1	0.69	0.98	1.4	DEWTHLTAK
2	4	0.75	1	1.3	GGDLWQGEVSLEQHR
1	0	0.67	1	1.5	ADSDRPSVSAGIGQKPQFQDLK
1	0	0.7	1	1.5	VKVPGAK
2	0	0.7	1	1.5	KVFPYPK
0	1	0.61	0.9	1.3	TTVQLHK
0	1	0.63	0.94	1.4	KVEIK

5.566 5 1018 minus strand Glutaredoxin-3 [Crassostrea gigas]; 5 1018 Glutaredoxin-3 [Crassostrea gigas]

Protein Accession	CL7734.Contig9$_{H}$1 CL7734.Contig10$_{H}$1
Mean Expression Ratio	0.982
Median Expression Ratio	0.982
Credible Interval	(0.649, 1.48)
Associated Peptides	1
Associated Spectra	2
Coverage	NaN

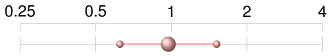

A	B	2.5	50	97.5	Sequence
**1**	**1**	**0.63**	**0.97**	**1.5**	**VAAEDLAEVSHR**

5.567 338 661 Spectrin alpha chain [Crassostrea gigas]

Protein Accession	CL26928.Contig1$_{H}$1
Mean Expression Ratio	0.983
Median Expression Ratio	0.983
Credible Interval	(0.67, 1.44)
Associated Peptides	2
Associated Spectra	3
Coverage	NaN

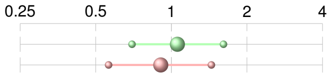

A	B	2.5	50	97.5	Sequence
**1**	**1**	**0.7**	**1.1**	**1.6**	**DVAGAEALLER**
**0**	**1**	**0.56**	**0.91**	**1.4**	**AIISADDLAK**

5.568 57 2009 hypothetical protein BRAFLDRAFT_285279 [Branchiostoma floridae] gi; 3 1973 hypothetical protein BRAFLDRAFT_285279 [Branchiostoma floridae] gi

Protein Accession	CL24444.Contig3$_{H}$1 CL24444.Contig1$_{H}$1
Mean Expression Ratio	1.02
Median Expression Ratio	1.02
Credible Interval	(0.684, 1.52)
Associated Peptides	2
Associated Spectra	2
Coverage	NaN

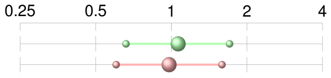

A	B	2.5	50	97.5	Sequence
**1**	**0**	**0.66**	**1.1**	**1.7**	**INQIYEQ**
**0**	**1**	**0.6**	**0.98**	**1.6**	**KLTLK**

5.569 811 1407 minus strand mitochondrial malate dehydrogenase 2 [Eospalax baileyi]

Protein Accession	CL10426.Contig13$_{H}$1
Mean Expression Ratio	1.02
Median Expression Ratio	1.02
Credible Interval	(0.767, 1.36)
Associated Peptides	3
Associated Spectra	11
Coverage	NaN

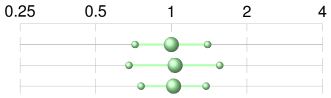

A	B	2.5	50	97.5	Sequence
**2**	**2**	**0.72**	**1**	**1.4**	**IQNAGTEVVEAK**
**1**	**0**	**0.68**	**1**	**1.6**	**MQQLNSPR**
**3**	**3**	**0.76**	**1**	**1.4**	**AAMPELIANISK**

5.570 58 2268 hypothetical protein BRAFLDRAFT_267717 [Branchiostoma floridae] gi; 62 2245 hypothetical protein BRAFLDRAFT_267717 [Branchiostoma floridae] gi

Protein Accession	CL26176.Contig18$_{H}$1 CL26176.Contig17$_{H}$1
Mean Expression Ratio	0.983
Median Expression Ratio	0.983
Credible Interval	(0.636, 1.52)
Associated Peptides	1
Associated Spectra	1
Coverage	NaN

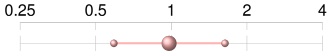

A	B	2.5	50	97.5	Sequence
**1**	**0**	**0.59**	**0.98**	**1.6**	**ALITIANK**

5.571 16 438 hypothetical protein KGM_06034 [Danaus plexippus]; 16 438 hypothetical protein KGM_06034 [Danaus plexippus]

Protein Accession	CL18196.Contig2$_{H}$1 CL18196.Contig1$_{H}$1
Mean Expression Ratio	1.02
Median Expression Ratio	1.02
Credible Interval	(0.68, 1.51)
Associated Peptides	2
Associated Spectra	2
Coverage	NaN

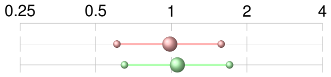

A	B	2.5	50	97.5	Sequence
**1**	**0**	**0.61**	**0.98**	**1.6**	**VHCGPNYPDEPPTVQFK**
**1**	**0**	**0.65**	**1.1**	**1.7**	**LLEELEDGQK**

5.572 1070 1888 minus strand galectin [Pinctada fucata]; 1104 1922 minus strand galectin [Pinctada fucata]

Protein Accession	CL7245.Contig4$_{H}$1 CL7245.Contig2$_{H}$1
Mean Expression Ratio	1.02
Median Expression Ratio	1.02
Credible Interval	(0.657, 1.58)
Associated Peptides	1
Associated Spectra	1
Coverage	NaN

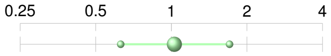

A	B	2.5	50	97.5	Sequence
**1**	**0**	**0.63**	**1**	**1.7**	**FNEGVTVR**

5.573 1155 1649 78 kDa glucose-regulated protein OS=Aplysia californica PE=1 SV=1

Protein Accession	CL41594.Contig1$_H$1
Mean Expression Ratio	0.982
Median Expression Ratio	0.984
Credible Interval	(0.631, 1.52)
Associated Peptides	1
Associated Spectra	1
Coverage	NaN

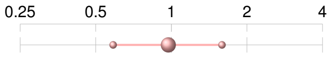

A	B	2.5	50	97.5	Sequence
**1**	**0**	**0.58**	**0.97**	**1.6**	**VEVESLFDGEDFSEQLTR**

5.574 425 1051 minus strand unknown [Schistosoma japonicum]; 300 878 unknown [Schistosoma japonicum]; 567 1145 unknown [Schistosoma japonicum]; 1110 1688 unknown [Schistosoma japonicum]; 94 672 unknown [Schistosoma japonicum]; 599 1177 unknown [Schistosoma japonicum]; 336 914 unknown [Schistosoma japonicum]

Protein Accession	CL11292.Contig9$_H$1 CL11292.Contig5$_H$1 CL11292.Contig3$_H$1 CL11292.Contig2$_H$1 CL1129
Mean Expression Ratio	0.987
Median Expression Ratio	0.984
Credible Interval	(0.699, 1.38)
Associated Peptides	2
Associated Spectra	9
Coverage	NaN

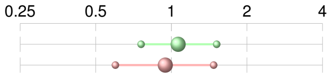

A	B	2.5	50	97.5	Sequence
**4**	**4**	**0.76**	**1.1**	**1.5**	**AGNDVDLVVQR**
**0**	**1**	**0.6**	**0.95**	**1.5**	**RYDTSQPWGFR**

5.575 145 972 LIM and SH3 domain protein F42H10.3 OS=Caenorhabditis elegans GN=F42H10.3 PE=2 SV=3

Protein Accession	CL49642.Contig8$_H$1
Mean Expression Ratio	1.02
Median Expression Ratio	1.02
Credible Interval	(0.677, 1.53)
Associated Peptides	1
Associated Spectra	2
Coverage	NaN

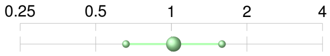

A	B	2.5	50	97.5	Sequence
**1**	**1**	**0.66**	**1**	**1.6**	**TVYPTEELK**

5.576 " 40 813 minus strand NADH dehydrogenase [ubiquinone] iron-sulfur protein 3, mitochondrial [Crassostrea gigas]"

Protein Accession	CL8338.Contig9$_{H}1$
Mean Expression Ratio	1.01
Median Expression Ratio	1.02
Credible Interval	(0.684, 1.51)
Associated Peptides	2
Associated Spectra	2
Coverage	NaN

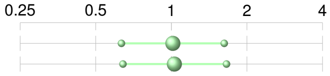

A	B	2.5	50	97.5	Sequence
0	1	0.63	1	1.6	VVIEPIELQQEFR
0	1	0.64	1	1.7	ILTDYGFEGHPFR

5.577 93 1034 Annexin A7 [Crassostrea gigas]; 73 1014 Annexin A7 [Crassostrea gigas]

Protein Accession	CL718.Contig3$_{H}1$ CL718.Contig1$_{H}1$
Mean Expression Ratio	0.984
Median Expression Ratio	0.984
Credible Interval	(0.758, 1.27)
Associated Peptides	6
Associated Spectra	12
Coverage	NaN

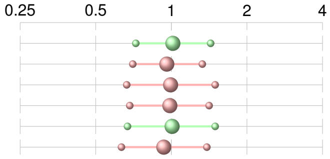

A	B	2.5	50	97.5	Sequence
2	1	0.72	1	1.4	ESDEWEAGDDAK
2	2	0.7	0.96	1.3	LEVAQAFK
1	0	0.67	0.99	1.5	KYEQLTGR
1	1	0.68	0.99	1.4	SEIDMAEIKK
0	1	0.67	1	1.5	TGTVFPADHFDAGK
0	1	0.63	0.93	1.4	PADHFDAGK

5.578 195 2201 minus strand hypothetical protein DAPPUDRAFT_315656 [Daphnia pulex]

Protein Accession	Unigene38538$_{H}1$
Mean Expression Ratio	1.01
Median Expression Ratio	1.02
Credible Interval	(0.766, 1.34)
Associated Peptides	6
Associated Spectra	9
Coverage	NaN

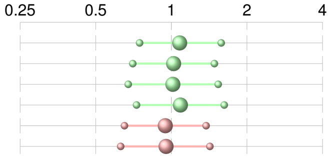

A	B	2.5	50	97.5	Sequence
1	1	0.75	1.1	1.6	FFTGQITAAGK
1	1	0.7	1	1.5	LLLSFGER
1	0	0.67	1	1.5	VALSPAATQALVK
1	0	0.73	1.1	1.6	VQIEAGAGEEAK
1	1	0.65	0.95	1.4	GPQFEEIGK
0	1	0.63	0.95	1.4	GSIVLHDGK

5.579 2 811 voltage-dependent anion channel 2-like protein [Haliotis diversicolor]; 2 811 voltage-dependent anion channel 2-like protein [Haliotis diversicolor]; 2 811 voltage-dependent anion channel 2-like protein [Haliotis diversicolor]

Protein Accession	CL2477.Contig3$_H$1 CL2477.Contig2$_H$1 CL2477.Contig1$_H$1
Mean Expression Ratio	0.986
Median Expression Ratio	0.985
Credible Interval	(0.794, 1.22)
Associated Peptides	9
Associated Spectra	21
Coverage	NaN

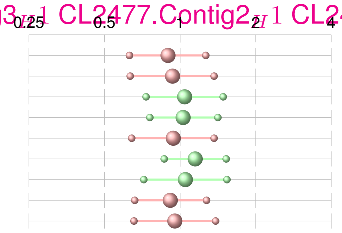

A	B	2.5	50	97.5	Sequence
2	0	0.63	0.9	1.3	LAFDTSFAPQTGK
1	0	0.63	0.93	1.4	VSGSLETK
1	1	0.73	1	1.5	WSEYGLTFTEK
4	0	0.76	1	1.4	YTLDSSASVSAK
1	0	0.64	0.94	1.4	GYNYGFFK
2	4	0.86	1.1	1.5	VGLGLDVEA
1	0	0.72	1	1.5	FALGTK
1	2	0.66	0.92	1.3	LTLSALIEGK
0	1	0.66	0.95	1.4	DIFSK

5.580 147 272 Spectrin alpha chain [Crassostrea gigas]

Protein Accession	CL2014.Contig2$_H$1
Mean Expression Ratio	0.983
Median Expression Ratio	0.985
Credible Interval	(0.63, 1.52)
Associated Peptides	1
Associated Spectra	1
Coverage	NaN

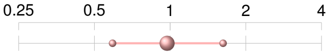

A	B	2.5	50	97.5	Sequence
0	1	0.59	0.98	1.6	VLETAEDIQQR

5.581 245 1243 Prostaglandin reductase 1 [Crassostrea gigas]

Protein Accession	CL37890.Contig1$_H$1
Mean Expression Ratio	1.02
Median Expression Ratio	1.02
Credible Interval	(0.656, 1.59)
Associated Peptides	1
Associated Spectra	1
Coverage	NaN

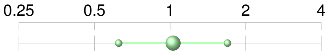

A	B	2.5	50	97.5	Sequence
1	0	0.62	1	1.7	TLAVLHPDMPR

5.582 170 460 Spectrin beta chain [Crassostrea gigas]

Protein Accession	CL38255.Contig1$_H$1
Mean Expression Ratio	0.983
Median Expression Ratio	0.985
Credible Interval	(0.659, 1.47)
Associated Peptides	2
Associated Spectra	2
Coverage	NaN

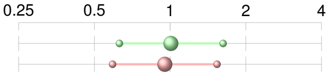

A	B	2.5	50	97.5	Sequence
1	0	0.63	1	1.6	LLEVLSGER
1	0	0.59	0.95	1.5	WVNSHLIR

5.583 835 1083 minus strand Calmodulin [Crassostrea gigas]

Protein Accession	CL49568.Contig2$_{H}1$
Mean Expression Ratio	1.02
Median Expression Ratio	1.01
Credible Interval	(0.847, 1.23)
Associated Peptides	7
Associated Spectra	48
Coverage	NaN

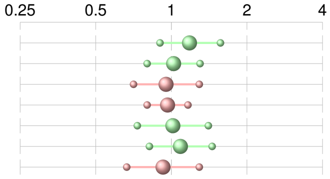

A	B	2.5	50	97.5	Sequence
6	0	0.9	1.2	1.6	HLLTDKDEETCYK
7	2	0.8	1	1.3	KGFIANEEMK
4	0	0.71	0.95	1.3	QQVQMTEQEVDEVIDYIDK
9	10	0.8	0.97	1.2	VDFTEFYK
2	1	0.73	1	1.4	GFIANEEMK
5	0	0.82	1.1	1.5	LLLNK
1	1	0.66	0.92	1.3	ILDDQR

5.584 22 429 AGAP006937-PA [Anopheles gambiae str. PEST] gi; 1 474 Ubiquitin-conjugating enzyme E2 variant 2 OS=Rattus norvegicus GN=Ube2v2 PE=1 SV=3

Protein Accession	CL27581.Contig3$_{H}1$ CL27581.Contig2$_{H}1$
Mean Expression Ratio	0.985
Median Expression Ratio	0.986
Credible Interval	(0.634, 1.55)
Associated Peptides	1
Associated Spectra	1
Coverage	NaN

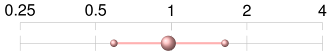

A	B	2.5	50	97.5	Sequence
0	1	0.59	0.98	1.6	LLDELELGEK

5.585 305 937 Clathrin light chain A [Crassostrea gigas]

Protein Accession	CL831.Contig4$_{H}1$
Mean Expression Ratio	1.01
Median Expression Ratio	1.01
Credible Interval	(0.652, 1.56)
Associated Peptides	1
Associated Spectra	1
Coverage	NaN

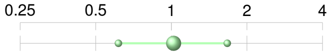

A	B	2.5	50	97.5	Sequence
0	1	0.62	1	1.7	AAEEAFIK

5.586 160 1218 Galectin-9 [Crassostrea gigas]

Protein Accession	Unigene165$_{H}1$
Mean Expression Ratio	1.01
Median Expression Ratio	1.01
Credible Interval	(0.697, 1.49)
Associated Peptides	2
Associated Spectra	3
Coverage	NaN

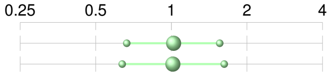

A	B	2.5	50	97.5	Sequence
1	1	0.66	1	1.6	IAVNNQHLVEFR
1	0	0.64	1	1.6	SGIPGGLAPGR

5.587 783 1463 minus strand twitchin [Mytilus galloprovincialis]; 783 1463 minus strand twitchin [Mytilus galloprovincialis]; 752 1432 twitchin [Mytilus galloprovincialis]

Protein Accession	CL181.Contig5$_H$1 CL181.Contig3$_H$1 CL181.Contig1$_H$1
Mean Expression Ratio	0.985
Median Expression Ratio	0.986
Credible Interval	(0.763, 1.27)
Associated Peptides	7
Associated Spectra	13
Coverage	NaN

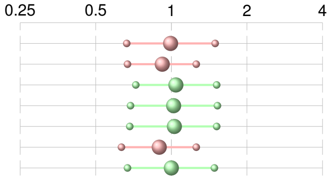

A	B	2.5	50	97.5	Sequence
1	0	0.67	1	1.5	DGKPVNSSDIYSITFDK
2	2	0.67	0.92	1.3	LNPQVVNDGDAVDLVVK
2	0	0.72	1	1.5	NSGGEVTSTATVNVDALKPK
1	0	0.69	1	1.5	VTGTEPFDITWAK
1	0	0.68	1	1.5	NQWGTASSMASLQVK
0	3	0.63	0.9	1.3	LEGDTIIIQQDITSK
0	1	0.67	1	1.5	SKPDLLWYR

5.588 " 451 1026 PREDICTED: GTP-binding protein SAR1B, putative-like [Saccoglossus kowalevskii]"

Protein Accession	Unigene4124$_H$1
Mean Expression Ratio	0.985
Median Expression Ratio	0.987
Credible Interval	(0.627, 1.53)
Associated Peptides	1
Associated Spectra	1
Coverage	NaN

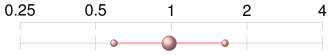

A	B	2.5	50	97.5	Sequence
1	0	0.59	0.98	1.6	AELDSLLTDEQVANAPILVLGNK

5.589 606 1730 UDP-glucuronosyltransferase 2C1 precursor [Oryctolagus cuniculus]

Protein Accession	CL29849.Contig5$_H$1
Mean Expression Ratio	1.01
Median Expression Ratio	1.01
Credible Interval	(0.683, 1.53)
Associated Peptides	2
Associated Spectra	3
Coverage	NaN

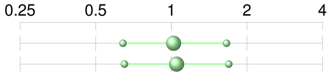

A	B	2.5	50	97.5	Sequence
1	1	0.64	1	1.7	LFVSHCGK
0	1	0.65	1	1.7	AAHAVEFVLK

5.590 1 987 minus strand RecName: Full=Tubulin beta chain; AltName: Full=Beta-tubulin

Protein Accession	Unigene7954$_{H}1$
Mean Expression Ratio	1.01
Median Expression Ratio	1.01
Credible Interval	(0.746, 1.38)
Associated Peptides	3
Associated Spectra	8
Coverage	NaN

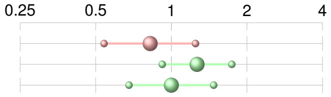

A	B	2.5	50	97.5	Sequence
1	0	0.54	0.83	1.2	AILVDLEPGTMDSVR
5	0	0.92	1.3	1.7	ALTVPELTQQMFDAK
2	0	0.68	1	1.5	PELTQQMFDAK

5.591 2 517 minus strand Anhydro-N-acetylmuramic acid kinase [Crassostrea gigas]

Protein Accession	Unigene23377$_{H}1$
Mean Expression Ratio	0.989
Median Expression Ratio	0.988
Credible Interval	(0.679, 1.44)
Associated Peptides	2
Associated Spectra	3
Coverage	NaN

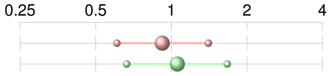

A	B	2.5	50	97.5	Sequence
1	1	0.61	0.92	1.4	KYGLQDVAR
0	1	0.66	1.1	1.7	KLVDAGLAVDK

5.592 " 142 1284 minus strand Chain O, Crsytal Structure Of Human Liver Gapdh gi|67464044|pdb|1ZNQ|P Chain P, Crsytal Structure Of Human Liver Gapdh gi|67464045|pdb|1ZNQ|R Chain R, Crsytal Structure Of Human Liver Gapdh gi|67464046|pdb|1ZNQ|Q Chain Q, Crsytal Structure Of Human Liver Gapdh"

Protein Accession	CL34318.Contig1$_{H}1$
Mean Expression Ratio	1.01
Median Expression Ratio	1.01
Credible Interval	(0.838, 1.22)
Associated Peptides	7
Associated Spectra	49
Coverage	NaN

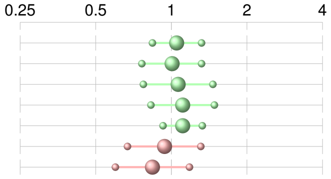

A	B	2.5	50	97.5	Sequence
5	6	0.84	1.1	1.3	DPTAIPWR
4	2	0.76	1	1.3	INVYQEKDPTAIPWR
2	1	0.77	1.1	1.5	VIPELNGK
4	1	0.83	1.1	1.5	INVYQEK
0	20	0.92	1.1	1.3	IGINGFGR
0	2	0.67	0.94	1.3	VIPELNGKLTGMAFR
0	2	0.6	0.84	1.2	CLAPLAK

5.593 1573 2385 minus strand Spectrin beta chain [Crassostrea gigas]

Protein Accession	CL5904.Contig4$_{H}1$
Mean Expression Ratio	1.01
Median Expression Ratio	1.01
Credible Interval	(0.647, 1.57)
Associated Peptides	1
Associated Spectra	1
Coverage	NaN

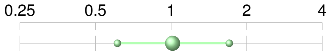

A	B	2.5	50	97.5	Sequence
**0**	**1**	**0.61**	**1**	**1.7**	**RLIQEEHPESDQIAVR**

5.594 35 505 minus strand Basement membrane-specific heparan sulfate proteoglycan core protein [Crassostrea gigas]

Protein Accession	CL8101.Contig1$_{H}1$
Mean Expression Ratio	0.988
Median Expression Ratio	0.988
Credible Interval	(0.635, 1.53)
Associated Peptides	1
Associated Spectra	1
Coverage	NaN

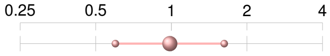

A	B	2.5	50	97.5	Sequence
**0**	**1**	**0.6**	**0.99**	**1.6**	**AAFAIR**

5.595 484 1104 Calponin-2 [Crassostrea gigas]; 309 938 Calponin-2 [Crassostrea gigas]

Protein Accession	CL30203.Contig3$_{H}1$ CL21394.Contig2$_{H}1$
Mean Expression Ratio	0.989
Median Expression Ratio	0.988
Credible Interval	(0.856, 1.15)
Associated Peptides	11
Associated Spectra	139
Coverage	NaN

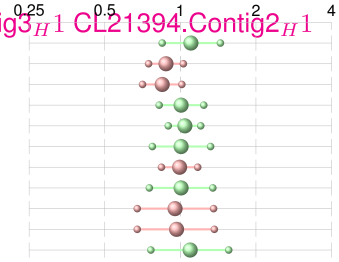

A	B	2.5	50	97.5	Sequence
**5**	**1**	**0.85**	**1.1**	**1.4**	**AGQGIIGLQAGTNK**
**21**	**6**	**0.75**	**0.88**	**1**	**AVYDGTPNLPPAAK**
**10**	**11**	**0.71**	**0.85**	**1**	**MNTLAAPFK**
**11**	**3**	**0.82**	**1**	**1.2**	**QLTGEDIGSGAVQVEK**
**21**	**11**	**0.89**	**1**	**1.2**	**QMENIEVFLK**
**4**	**2**	**0.77**	**1**	**1.3**	**YDTGAEYEVR**
**15**	**11**	**0.84**	**0.99**	**1.2**	**NGQILCR**
**2**	**2**	**0.75**	**1**	**1.3**	**NLRNGQILCR**
**0**	**1**	**0.67**	**0.96**	**1.4**	**IIGLQAGTNK**
**0**	**1**	**0.67**	**0.97**	**1.4**	**PNLPPAAK**
**0**	**1**	**0.76**	**1.1**	**1.6**	**PTTLPR**

5.596 87 1454 hypothetical protein BRAFLDRAFT_287788 [Branchiostoma floridae] gi; 117 1469 hypothetical protein BRAFLDRAFT_287788 [Branchiostoma floridae] gi; 117 1469 hypothetical protein BRAFLDRAFT_287788 [Branchiostoma floridae] gi

Protein Accession	Unigene48935$_H$1 Unigene21344$_H$1 CL945.Contig6$_H$1
Mean Expression Ratio	0.989
Median Expression Ratio	0.988
Credible Interval	(0.769, 1.26)
Associated Peptides	6
Associated Spectra	16
Coverage	NaN

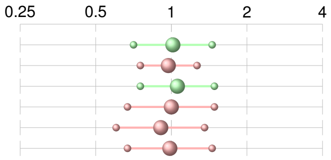

A	B	2.5	50	97.5	Sequence
1	1	0.71	1	1.5	AATLEQTILNVPETR
7	1	0.75	0.97	1.3	MVLAAAGGVNHGELVDLAK
2	1	0.75	1.1	1.5	RIPLPELEAR
1	0	0.67	1	1.5	QMLCYGR
1	0	0.6	0.91	1.4	PETRISSLDNGLR
0	1	0.67	0.99	1.5	ISSLDNGLR

5.597 95 271 minus strand hypothetical protein EAI_01530 [Harpegnathos saltator]

Protein Accession	CL9369.Contig9$_H$1
Mean Expression Ratio	0.988
Median Expression Ratio	0.988
Credible Interval	(0.66, 1.48)
Associated Peptides	1
Associated Spectra	2
Coverage	NaN

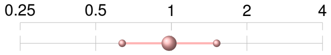

A	B	2.5	50	97.5	Sequence
0	2	0.64	0.98	1.5	VAQFPFR

5.598 108 1259 minus strand PREDICTED: Na(+)/H(+) exchange regulatory cofactor NHE-RF3-like [Metaseiulus occidentalis]; 108 1190 minus strand PREDICTED: Na(+)/H(+) exchange regulatory cofactor NHE-RF3-like [Metaseiulus occidentalis]

Protein Accession	CL26023.Contig7$_H$1 CL26023.Contig6$_H$1
Mean Expression Ratio	1.01
Median Expression Ratio	1.01
Credible Interval	(0.65, 1.58)
Associated Peptides	1
Associated Spectra	1
Coverage	NaN

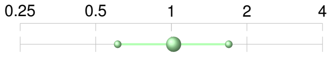

A	B	2.5	50	97.5	Sequence
0	1	0.61	1	1.7	GDLPEVLHLTAVR

5.599 771 1505 O-methyltransferase family 3 [Dicentrarchus labrax]

Protein Accession	Unigene19132$_{H1}$
Mean Expression Ratio	0.986
Median Expression Ratio	0.989
Credible Interval	(0.675, 1.44)
Associated Peptides	3
Associated Spectra	3
Coverage	NaN

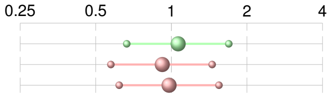

A	B	2.5	50	97.5	Sequence
**1**	**0**	**0.67**	**1.1**	**1.7**	**FGASYLPR**
**0**	**1**	**0.58**	**0.92**	**1.4**	**IAETTLTHPWR**
**0**	**1**	**0.62**	**0.98**	**1.6**	**VLIPVR**

5.600 362 1084 Ectoine hydroxylase [Crassostrea gigas]; 362 1084 Ectoine hydroxylase [Crassostrea gigas]; 366 1220 Ectoine hydroxylase [Crassostrea gigas]

Protein Accession	CL2342.Contig4$_{H1}$ CL2342.Contig3$_{H1}$ CL2342.Contig2$_{H1}$
Mean Expression Ratio	0.99
Median Expression Ratio	0.989
Credible Interval	(0.64, 1.54)
Associated Peptides	1
Associated Spectra	1
Coverage	NaN

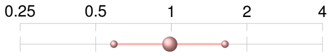

A	B	2.5	50	97.5	Sequence
**1**	**0**	**0.59**	**0.99**	**1.6**	**NFVLTDEMK**

5.601 27 722 Spectrin beta chain OS=Drosophila melanogaster GN=beta-Spec PE=1 SV=2; 27 722 Spectrin beta chain OS=Drosophila melanogaster GN=beta-Spec PE=1 SV=2

Protein Accession	CL5904.Contig3$_{H1}$ CL5904.Contig1$_{H1}$
Mean Expression Ratio	0.991
Median Expression Ratio	0.989
Credible Interval	(0.688, 1.43)
Associated Peptides	2
Associated Spectra	4
Coverage	NaN

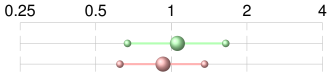

A	B	2.5	50	97.5	Sequence
**1**	**0**	**0.67**	**1.1**	**1.6**	**EQIEVMLADLDK**
**3**	**0**	**0.63**	**0.93**	**1.4**	**HQAFEAEIGHYAR**

5.602 " 757 1443 minus strand nonmuscle myosin II, partial [Aplysia californica]"

Protein Accession	CL43455.Contig2$_{H1}$
Mean Expression Ratio	1.01
Median Expression Ratio	1.01
Credible Interval	(0.645, 1.58)
Associated Peptides	1
Associated Spectra	1
Coverage	NaN

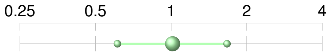

A	B	2.5	50	97.5	Sequence
**1**	**0**	**0.61**	**1**	**1.7**	**LVNEIK**

5.603 229 699 hypothetical protein CGI_10017744 [Crassostrea gigas]; 199 669 hypothetical protein CGI_10017744 [Crassostrea gigas]; 202 672 hypothetical protein CGI_10017744 [Crassostrea gigas]

Protein Accession	CL5082.Contig3$_H$1 CL5082.Contig2$_H$1 CL5082.Contig1$_H$1
Mean Expression Ratio	1.01
Median Expression Ratio	1.01
Credible Interval	(0.741, 1.37)
Associated Peptides	3
Associated Spectra	8
Coverage	NaN

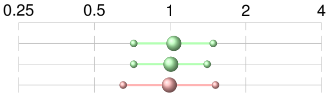

A	B	2.5	50	97.5	Sequence
**2**	**1**	**0.72**	**1**	**1.5**	**EVLELFDR**
**3**	**1**	**0.72**	**1**	**1.4**	**HLNKPDAIK**
**1**	**0**	**0.65**	**0.99**	**1.5**	**EGLHSLK**

5.604 40 1182 4-hydroxyphenylpyruvate dioxygenase [Crassostrea gigas]

Protein Accession	CL7585.Contig2$_H$1
Mean Expression Ratio	0.989
Median Expression Ratio	0.99
Credible Interval	(0.66, 1.48)
Associated Peptides	2
Associated Spectra	2
Coverage	NaN

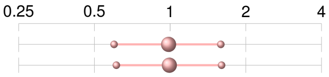

A	B	2.5	50	97.5	Sequence
**1**	**0**	**0.6**	**0.98**	**1.6**	**DVAFEVEDLDAIFK**
**0**	**1**	**0.61**	**0.99**	**1.6**	**SLFEAIELDQAER**

5.605 42 296 minus strand twitchin [Mytilus galloprovincialis]

Protein Accession	Unigene19029$_H$1
Mean Expression Ratio	0.992
Median Expression Ratio	0.991
Credible Interval	(0.761, 1.3)
Associated Peptides	4
Associated Spectra	12
Coverage	NaN

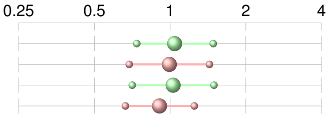

A	B	2.5	50	97.5	Sequence
**2**	**1**	**0.74**	**1**	**1.5**	**APHFTQKPTIK**
**1**	**1**	**0.69**	**1**	**1.4**	**VTLADAGEYK**
**2**	**0**	**0.71**	**1**	**1.5**	**KPQLPDGK**
**3**	**2**	**0.66**	**0.91**	**1.2**	**LNFDSAK**

5.606 462 671 enhancer protein [Homo sapiens]

Protein Accession	CL3160.Contig12$_H$1
Mean Expression Ratio	1.01
Median Expression Ratio	1.01
Credible Interval	(0.756, 1.35)
Associated Peptides	3
Associated Spectra	11
Coverage	NaN

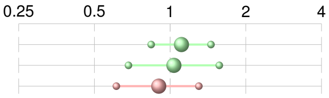

A	B	2.5	50	97.5	Sequence
**6**	**2**	**0.84**	**1.1**	**1.5**	**GLFIIDDK**
**0**	**1**	**0.68**	**1**	**1.6**	**PLLEDK**
**0**	**2**	**0.61**	**0.9**	**1.3**	**KIGGLGNMNIPLLEDK**

5.607 351 1826 Virulence metalloprotease [Crassostrea gigas]

Protein Accession	CL7226.Contig18$_{H}1$
Mean Expression Ratio	1.01
Median Expression Ratio	1.01
Credible Interval	(0.644, 1.59)
Associated Peptides	1
Associated Spectra	1
Coverage	NaN

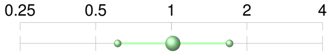

A	B	2.5	50	97.5	Sequence
**1**	**0**	**0.61**	**1**	**1.7**	**EGDVCYLENK**

5.608 998 1705 minus strand Basement membrane-specific heparan sulfate proteoglycan core protein [Crassostrea gigas]

Protein Accession	CL22963.Contig1$_{H}1$
Mean Expression Ratio	0.992
Median Expression Ratio	0.991
Credible Interval	(0.637, 1.54)
Associated Peptides	1
Associated Spectra	1
Coverage	NaN

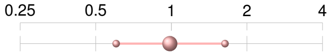

A	B	2.5	50	97.5	Sequence
**1**	**0**	**0.6**	**0.99**	**1.6**	**ITAYGGSLR**

5.609 72 1400 minus strand hypothetical protein RCJMB04_22i13 [Gallus gallus]

Protein Accession	CL44755.Contig5$_{H}1$
Mean Expression Ratio	1.01
Median Expression Ratio	1.01
Credible Interval	(0.769, 1.31)
Associated Peptides	6
Associated Spectra	10
Coverage	NaN

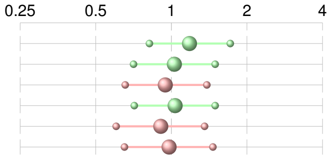

A	B	2.5	50	97.5	Sequence
**2**	**0**	**0.82**	**1.2**	**1.7**	**FLVASTPDEALQHAAELGK**
**1**	**1**	**0.71**	**1**	**1.5**	**LVFSADEAK**
**1**	**1**	**0.66**	**0.95**	**1.4**	**AVFQMR**
**1**	**1**	**0.71**	**1**	**1.5**	**LSGIPDVFVK**
**0**	**1**	**0.6**	**0.91**	**1.4**	**AIIAASSMK**
**0**	**1**	**0.65**	**0.98**	**1.5**	**LSTIIDLAR**

5.610 242 1834 Regulator of differentiation 1 [Crassostrea gigas]; 458 2050 minus strand Regulator of differentiation 1 [Crassostrea gigas]; 503 2095 Regulator of differentiation 1 [Crassostrea gigas]; 274 1866 Regulator of differentiation 1 [Crassostrea gigas]; 434 1942 Regulator of differentiation 1 [Crassostrea gigas]

Protein Accession	CL11169.Contig7$_{H}1$ CL11169.Contig5$_{H}1$ CL11169.Contig4$_{H}1$ CL11169.Contig3$_{H}1$ CL1116
Mean Expression Ratio	1.01
Median Expression Ratio	1.01
Credible Interval	(0.682, 1.49)
Associated Peptides	2
Associated Spectra	2
Coverage	NaN

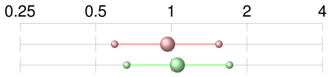

A	B	2.5	50	97.5	Sequence
1	0	0.59	0.97	1.5	SLPHEVAETDIVQLGMPFGK
0	1	0.66	1.1	1.7	IITFTK

5.611 460 1737 Tubulin beta-4B chain OS=Mus musculus GN=Tubb4b PE=1 SV=1

Protein Accession	Unigene34147$_{H}1$
Mean Expression Ratio	1.01
Median Expression Ratio	1.01
Credible Interval	(0.715, 1.42)
Associated Peptides	1
Associated Spectra	8
Coverage	NaN

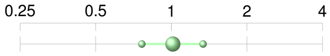

A	B	2.5	50	97.5	Sequence
3	5	0.76	1	1.3	AVLVDLEPGTMDSVR

5.612 188 4135 Vigilin [Crassostrea gigas]

Protein Accession	CL846.Contig3$_{H}1$
Mean Expression Ratio	1.01
Median Expression Ratio	1.01
Credible Interval	(0.649, 1.57)
Associated Peptides	1
Associated Spectra	1
Coverage	NaN

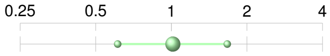

A	B	2.5	50	97.5	Sequence
1	0	0.61	1	1.7	EVSIDIPHR

5.613 69 779 PREDICTED: 15-hydroxyprostaglandin dehydrogenase [NAD+] [Gallus gallus]

Protein Accession	CL44148.Contig4$_{H}1$
Mean Expression Ratio	0.991
Median Expression Ratio	0.992
Credible Interval	(0.657, 1.48)
Associated Peptides	2
Associated Spectra	2
Coverage	NaN

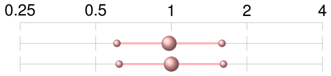

A	B	2.5	50	97.5	Sequence
**1**	**0**	**0.61**	**0.98**	**1.6**	**PLVENPPSPTVIGEAVIK**
**1**	**0**	**0.62**	**1**	**1.6**	**PPSPTVIGEAVIK**

5.614 184 438 PREDICTED: similar to CG14235 CG14235-PA [Tribolium castaneum] gi

Protein Accession	Unigene638$_{H}1$
Mean Expression Ratio	0.993
Median Expression Ratio	0.992
Credible Interval	(0.699, 1.4)
Associated Peptides	2
Associated Spectra	5
Coverage	NaN

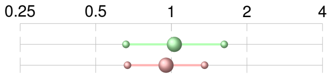

A	B	2.5	50	97.5	Sequence
**1**	**0**	**0.66**	**1**	**1.6**	**SLCPNEWVAK**
**3**	**1**	**0.67**	**0.95**	**1.4**	**WDDQVANGTFAGK**

5.615 324 1982 minus strand Pikachurin [Crassostrea gigas]

Protein Accession	CL4889.Contig10$_{H}1$
Mean Expression Ratio	1.01
Median Expression Ratio	1.01
Credible Interval	(0.692, 1.46)
Associated Peptides	2
Associated Spectra	3
Coverage	NaN

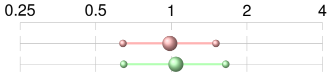

A	B	2.5	50	97.5	Sequence
**2**	**0**	**0.64**	**0.98**	**1.5**	**VTAPIITEECGK**
**1**	**0**	**0.65**	**1**	**1.7**	**KGVLMR**

5.616 85 546 minus strand RecName: Full=Eukaryotic translation initiation factor 5A; Short=eIF-5A gi|51702279|sp|P62925.1|IF5A_SPOFR RecName: Full=Eukaryotic translation initiation factor 5A; Short=eIF-5A gi|6469515|gb|AAF13315.1|AF109730_1 translation initiation factor 5A [Spodoptera exigua] gi

Protein Accession	CL40725.Contig2$_H$1
Mean Expression Ratio	1.01
Median Expression Ratio	1.01
Credible Interval	(0.761, 1.35)
Associated Peptides	4
Associated Spectra	10
Coverage	NaN

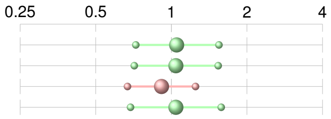

A	B	2.5	50	97.5	Sequence
1	1	0.72	1.1	1.5	AMGEECVVAVK
1	1	0.71	1	1.5	EDSLAAEIQK
2	3	0.67	0.91	1.3	IVEMSTSK
0	1	0.69	1	1.6	KNEFVVIK

5.617 333 770 USP-like protein isoform 2 [Crassostrea ariakensis]

Protein Accession	Unigene7710$_H$1
Mean Expression Ratio	1.01
Median Expression Ratio	1.01
Credible Interval	(0.645, 1.56)
Associated Peptides	1
Associated Spectra	1
Coverage	NaN

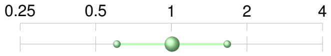

A	B	2.5	50	97.5	Sequence
1	0	0.61	1	1.7	KIVIAMDGSK

5.618 " 3 239 minus strand laminin beta 1 chain, putative [Ixodes scapularis] gi"

Protein Accession	CL48498.Contig2$_H$1
Mean Expression Ratio	0.994
Median Expression Ratio	0.993
Credible Interval	(0.693, 1.42)
Associated Peptides	2
Associated Spectra	4
Coverage	NaN

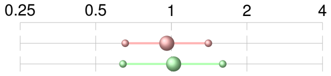

A	B	2.5	50	97.5	Sequence
2	1	0.66	0.96	1.4	LAQQAEQLLTTK
1	0	0.64	1	1.6	ARNTSETVR

5.619 172 678 Myosin regulatory light chain sqh [Crassostrea gigas]

Protein Accession	CL23773.Contig1$_H$1
Mean Expression Ratio	0.991
Median Expression Ratio	0.993
Credible Interval	(0.673, 1.44)
Associated Peptides	2
Associated Spectra	3
Coverage	NaN

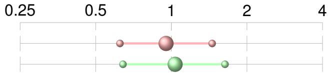

A	B	2.5	50	97.5	Sequence
1	1	0.62	0.95	1.5	LTDEEVDEMFR
1	0	0.64	1	1.6	ELLTTMGDR

5.620 3 4517 minus strand Clathrin heavy chain 1 [Crassostrea gigas]

Protein Accession	CL16446.Contig1$_{H}1$
Mean Expression Ratio	1.01
Median Expression Ratio	1.01
Credible Interval	(0.785, 1.29)
Associated Peptides	8
Associated Spectra	13
Coverage	NaN

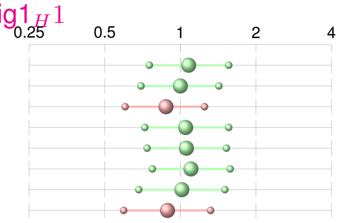

A	B	2.5	50	97.5	Sequence
2	0	0.75	1.1	1.6	KFEVNTSAIK
2	0	0.7	1	1.4	AQLNQGMVK
1	1	0.6	0.87	1.3	AGLLQR
1	0	0.72	1.1	1.6	KFNTLFQSGQYQEAAK
1	1	0.74	1.1	1.5	FLQMAR
2	0	0.77	1.1	1.6	VDGAR
1	0	0.68	1	1.5	NFLKEAK
0	1	0.6	0.89	1.3	AVNFFIK

5.621 2019 2723 minus strand Kyphoscoliosis peptidase [Crassostrea gigas]; 2060 2764 minus strand Kyphoscoliosis peptidase [Crassostrea gigas]; 1074 1778 minus strand Kyphoscoliosis peptidase [Crassostrea gigas]; 2063 2767 minus strand Kyphoscoliosis peptidase [Crassostrea gigas]; 2044 2748 minus strand Kyphoscoliosis peptidase [Crassostrea gigas]; 2044 2748 minus strand Kyphoscoliosis peptidase [Crassostrea gigas]

Protein Accession	CL3923.Contig7$_{H}1$ CL3923.Contig6$_{H}1$ CL3923.Contig5$_{H}1$ CL3923.Contig4$_{H}1$ CL3923.Con
Mean Expression Ratio	1.01
Median Expression Ratio	1.01
Credible Interval	(0.678, 1.48)
Associated Peptides	1
Associated Spectra	3
Coverage	NaN

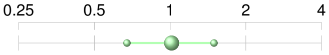

A	B	2.5	50	97.5	Sequence
3	0	0.67	1	1.5	TMGQAPPTVPK

5.622 729 1541 troponin T [Mizuhopecten yessoensis]

Protein Accession	Unigene34289$_{H}1$
Mean Expression Ratio	0.994
Median Expression Ratio	0.994
Credible Interval	(0.727, 1.36)
Associated Peptides	2
Associated Spectra	8
Coverage	NaN

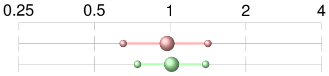

A	B	2.5	50	97.5	Sequence
1	1	0.65	0.97	1.4	RQQYDMIELAER
2	4	0.74	1	1.4	QQYDMIELAER

5.623 2 1126 Prolyl endopeptidase [Crassostrea gigas]

Protein Accession	CL47692.Contig1$_{H1}$
Mean Expression Ratio	1.01
Median Expression Ratio	1.01
Credible Interval	(0.643, 1.58)
Associated Peptides	1
Associated Spectra	1
Coverage	NaN

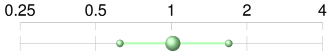

A	B	2.5	50	97.5	Sequence
1	0	0.62	1	1.7	VVPLHSLK

5.624 1 852 beta actin-like 1 protein [Macrostomum lignano]

Protein Accession	Unigene31438$_{H1}$
Mean Expression Ratio	0.994
Median Expression Ratio	0.994
Credible Interval	(0.907, 1.09)
Associated Peptides	36
Associated Spectra	772
Coverage	NaN

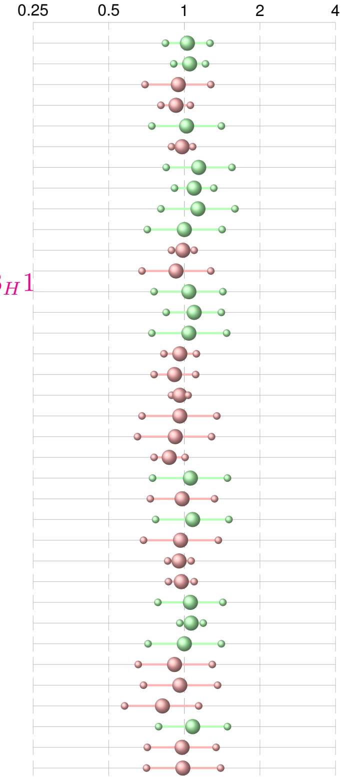

A	B	2.5	50	97.5	Sequence
9	5	0.84	1	1.3	AGFAGDDAPR
27	7	0.91	1.1	1.2	AVFPSIVGR
2	1	0.7	0.94	1.3	AVFPSIVGRPR
21	21	0.81	0.92	1.1	DSYVGDEAQSK
1	1	0.74	1	1.4	GVMVGMGQK
80	17	0.89	0.98	1.1	GYSFTTTAER
2	1	0.85	1.1	1.6	HQGVMVGMGQ
12	8	0.91	1.1	1.3	IWHHTFYNELR
1	0	0.8	1.1	1.6	MTQIMFETF
1	0	0.71	1	1.4	SYELPDGQVITIGN
61	15	0.89	0.98	1.1	SYELPDGQVITIGNER
1	1	0.68	0.93	1.3	TTGIVLDSGDGVTH
2	0	0.76	1	1.4	TTGIVLDSGDGVTHTVPIYEGY
7	0	0.85	1.1	1.4	TTGIVLDSGDGVTHTVPIYEGYALPH
1	0	0.74	1	1.5	VALDFEQEMATAAASSSLEK
18	17	0.83	0.96	1.1	VAPEEHPVLLTEA
10	6	0.76	0.92	1.1	VAPEEHPVLLTEAPLN
96	65	0.89	0.96	1	VAPEEHPVLLTEAPLNPK
1	0	0.68	0.96	1.3	VAPEEHPVLLTEAPLNPKANR
1	0	0.65	0.92	1.3	TEAPLNPK
23	13	0.76	0.87	1	RGILTLK
1	0	0.75	1.1	1.5	VAPEEHPVLLTEAPLNPKANREKMTQI
2	1	0.73	0.98	1.3	WDDMEK
1	0	0.77	1.1	1.5	HQGVMVGMGQKDSYVGDEAQSK
1	0	0.69	0.96	1.4	MTQIMFETFN
36	36	0.86	0.96	1.1	GILTLK
43	16	0.86	0.97	1.1	HQGVMVGMGQK
1	2	0.78	1.1	1.4	TTGIVLDSGDGVTHTVPIYEGYALPHAILR
50	18	0.96	1.1	1.2	YPIEHGIVTNWDDMEK
0	1	0.72	1	1.4	GIVTNWDDMEK
0	1	0.65	0.91	1.3	EIVRDIKEK
0	1	0.69	0.96	1.4	VMVGMGQK
0	1	0.58	0.82	1.1	GDEAQSK
0	2	0.79	1.1	1.5	VAPEEHPVLLTEAPL
0	2	0.71	0.98	1.3	VAPEEHPVLLTEAP
0	1	0.7	0.99	1.4	YPIEHGIVTNWDDME

5.625 79 606 minus strand PREDICTED: similar to Ras-like protein 3 precursor (Protein roughened) [Tribolium castaneum] gi

Protein Accession	Unigene4246$_{H1}$
Mean Expression Ratio	0.994
Median Expression Ratio	0.995
Credible Interval	(0.739, 1.32)
Associated Peptides	4
Associated Spectra	9
Coverage	NaN

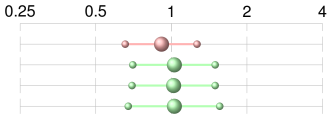

A	B	2.5	50	97.5	Sequence
2	2	0.66	0.92	1.3	LVVLGSGGVGK
2	0	0.7	1	1.5	VKDTDDVPMLLVGNK
1	1	0.7	1	1.5	CDLEVDR
0	1	0.67	1	1.6	DLYMK

5.626 156 1106 ELAV 2-like protein [Aplysia californica] gi

Protein Accession	CL1500.Contig1$_{H1}$
Mean Expression Ratio	0.993
Median Expression Ratio	0.995
Credible Interval	(0.677, 1.45)
Associated Peptides	2
Associated Spectra	3
Coverage	NaN

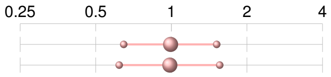

A	B	2.5	50	97.5	Sequence
1	1	0.65	1	1.5	NLPIPWTENELNK
0	1	0.62	0.98	1.6	VLVDLQTGVSK

5.627 167 2713 minus strand Major vault protein [Crassostrea gigas]

Protein Accession	CL18286.Contig3$_{H1}$
Mean Expression Ratio	1.01
Median Expression Ratio	1.01
Credible Interval	(0.737, 1.37)
Associated Peptides	5
Associated Spectra	6
Coverage	NaN

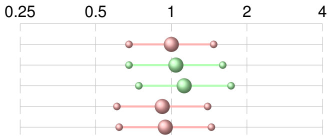

A	B	2.5	50	97.5	Sequence
2	0	0.68	1	1.5	GAVAQVQFDDFHK
1	0	0.68	1	1.6	KEVVVEETIR
1	0	0.74	1.1	1.7	KPGDRWMIR
0	1	0.61	0.92	1.4	IEGEAAVEQAK
0	1	0.62	0.95	1.4	IVDEAEAEK

5.628 205 1341 collagen precursor [Bombyx mori]

Protein Accession	CL38816.Contig2$_{H1}$
Mean Expression Ratio	0.995
Median Expression Ratio	0.995
Credible Interval	(0.646, 1.55)
Associated Peptides	1
Associated Spectra	1
Coverage	NaN

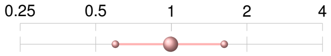

A	B	2.5	50	97.5	Sequence
0	1	0.6	0.99	1.6	GQLIPHIQK

5.629 5 841 minus strand Spectrin alpha chain [Crassostrea gigas]

Protein Accession	CL44897.Contig1$_{H}1$
Mean Expression Ratio	0.996
Median Expression Ratio	0.995
Credible Interval	(0.682, 1.45)
Associated Peptides	2
Associated Spectra	3
Coverage	NaN

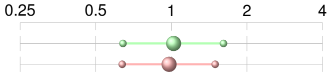

A	B	2.5	50	97.5	Sequence
1	0	0.64	1	1.6	DMEDEEDWIR
1	1	0.64	0.98	1.5	HQNFEAEIEANR

5.630 112 1050 Enolase-phosphatase E1 [Crassostrea gigas]; 112 1050 Enolase-phosphatase E1 [Crassostrea gigas]

Protein Accession	CL25175.Contig3$_{H}1$ CL25175.Contig2$_{H}1$
Mean Expression Ratio	0.996
Median Expression Ratio	0.995
Credible Interval	(0.712, 1.4)
Associated Peptides	3
Associated Spectra	5
Coverage	NaN

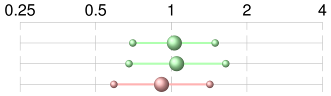

A	B	2.5	50	97.5	Sequence
1	2	0.7	1	1.5	SAAEAESLLVGVK
1	0	0.68	1.1	1.6	AIIFDVEGTTTPISFVK
0	1	0.59	0.91	1.4	QLQGHIWR

5.631 915 1505 minus strand Collagen alpha-5(VI) chain [Crassostrea gigas]

Protein Accession	CL40183.Contig3$_{H}1$
Mean Expression Ratio	1.01
Median Expression Ratio	1
Credible Interval	(0.821, 1.23)
Associated Peptides	8
Associated Spectra	43
Coverage	NaN

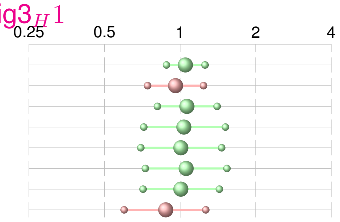

A	B	2.5	50	97.5	Sequence
13	10	0.88	1.1	1.3	LEAIPWR
4	4	0.74	0.96	1.2	VGEQLDAEELLNIAGDPK
2	4	0.81	1.1	1.4	DFVNAFQINPTAVR
1	0	0.72	1	1.5	HGLATNTGAGIK
1	0	0.7	1	1.5	HGLATNTGAGIKYMR
1	0	0.73	1.1	1.5	VGEQLDAEELL
0	2	0.71	1	1.4	RVVTAENYAHLNLVK
0	1	0.6	0.87	1.3	VVTAENYAHLNLVK

5.632 4365 4553 minus strand Basement membrane-specific heparan sulfate proteoglycan core protein [Crassostrea gigas]; 2502 2696 minus strand hypothetical protein DAPPUDRAFT_186952 [Daphnia pulex]

Protein Accession	CL24300.Contig6$_{H}1$ CL24300.Contig2$_{H}1$
Mean Expression Ratio	0.994
Median Expression Ratio	0.995
Credible Interval	(0.677, 1.46)
Associated Peptides	2
Associated Spectra	3
Coverage	NaN

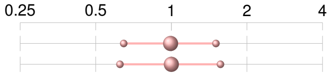

A	B	2.5	50	97.5	Sequence
1	1	0.65	0.99	1.5	DGSIIVDGQEVK
0	1	0.63	1	1.6	VDDGFR

5.633 71 523 minus strand hypothetical protein DAPPUDRAFT_309332 [Daphnia pulex]

Protein Accession	CL4487.Contig1$_H$1
Mean Expression Ratio	0.994
Median Expression Ratio	0.996
Credible Interval	(0.634, 1.54)
Associated Peptides	1
Associated Spectra	1
Coverage	NaN

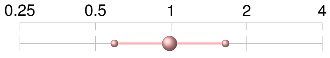

A	B	2.5	50	97.5	Sequence
**0**	**1**	**0.6**	**0.98**	**1.6**	**IYHPNIDK**

5.634 121 1452 minus strand Hexokinase type 2 [Crassostrea gigas]; 245 1576 minus strand Hexokinase type 2 [Crassostrea gigas]; 121 1452 minus strand Hexokinase type 2 [Crassostrea gigas]; 121 1452 minus strand Hexokinase type 2 [Crassostrea gigas]; 161 1513 minus strand Hexokinase type 2 [Crassostrea gigas]; 113 1471 minus strand Hexokinase type 2 [Crassostrea gigas]; 225 1577 minus strand Hexokinase type 2 [Crassostrea gigas]; 121 1452 minus strand Hexokinase type 2 [Crassostrea gigas]

Protein Accession	CL350.Contig9$_H$1 CL350.Contig8$_H$1 CL350.Contig6$_H$1 CL350.Contig5$_H$1 CL350.Contig3$_H$1
Mean Expression Ratio	1
Median Expression Ratio	1
Credible Interval	(0.644, 1.55)
Associated Peptides	1
Associated Spectra	1
Coverage	NaN

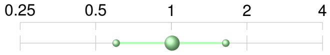

A	B	2.5	50	97.5	Sequence
**0**	**1**	**0.6**	**1**	**1.6**	**VLAPGIEFK**

5.635 116 1321 PREDICTED: similar to aspartate aminotransferase [Tribolium castaneum] gi

Protein Accession	CL11692.Contig6$_H$1
Mean Expression Ratio	1
Median Expression Ratio	1
Credible Interval	(0.765, 1.32)
Associated Peptides	8
Associated Spectra	9
Coverage	NaN

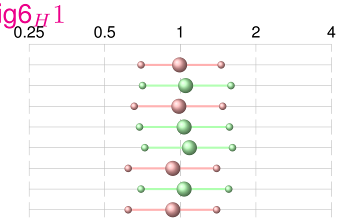

A	B	2.5	50	97.5	Sequence
**1**	**1**	**0.7**	**1**	**1.5**	**ILSTPALR**
**1**	**0**	**0.71**	**1**	**1.6**	**INLGVGAYR**
**1**	**0**	**0.65**	**0.98**	**1.5**	**TQWLGEVK**
**1**	**0**	**0.69**	**1**	**1.6**	**ISVAGLSSK**
**1**	**0**	**0.72**	**1.1**	**1.6**	**DGHQVALAQSFAK**
**0**	**1**	**0.62**	**0.93**	**1.4**	**ILSTPALRTQ**
**0**	**1**	**0.7**	**1**	**1.6**	**DAFALR**
**0**	**1**	**0.62**	**0.93**	**1.4**	**IIAMR**

5.636 303 1793 PREDICTED: matrix metalloproteinase 19 isoform rasi-1 preproprotein-like [Saccoglossus kowalevskii]

Protein Accession	CL7632.Contig1$_{H}1$
Mean Expression Ratio	0.995
Median Expression Ratio	0.996
Credible Interval	(0.663, 1.49)
Associated Peptides	2
Associated Spectra	2
Coverage	NaN

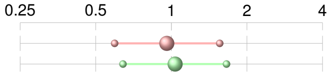

A	B	2.5	50	97.5	Sequence
1	0	0.59	0.96	1.6	FDAIDR
1	0	0.64	1	1.7	LGRVAR

5.637 337 474 minus strand heat shock protein 22 isoform 1 [Ruditapes philippinarum]

Protein Accession	CL30681.Contig1$_{H}1$
Mean Expression Ratio	0.994
Median Expression Ratio	0.996
Credible Interval	(0.683, 1.45)
Associated Peptides	2
Associated Spectra	3
Coverage	NaN

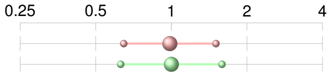

A	B	2.5	50	97.5	Sequence
1	1	0.65	0.98	1.5	EDWNFFDR
0	1	0.63	1	1.6	REDWNFFDR

5.638 311 928 minus strand peroxiredoxin 3 [Clonorchis sinensis] gi; 288 905 minus strand peroxiredoxin 3 [Clonorchis sinensis] gi

Protein Accession	CL2043.Contig4$_{H}1$ CL2043.Contig1$_{H}1$
Mean Expression Ratio	1
Median Expression Ratio	1
Credible Interval	(0.754, 1.33)
Associated Peptides	3
Associated Spectra	13
Coverage	NaN

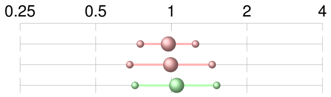

A	B	2.5	50	97.5	Sequence
5	4	0.75	0.97	1.2	QITINDLPVGR
1	1	0.68	1	1.5	SVDEILR
0	2	0.71	1	1.5	GLFIIDPK

5.639 89 838 Putative phosphoglycerate mutase [Crassostrea gigas]; 89 838 Putative phosphoglycerate mutase [Crassostrea gigas]; 89 835 Putative phosphoglycerate mutase [Crassostrea gigas]; 89 838 Putative phosphoglycerate mutase [Crassostrea gigas]

Protein Accession	CL6793.Contig6$_{H}1$ CL6793.Contig5$_{H}1$ CL6793.Contig4$_{H}1$ CL6793.Contig3$_{H}1$
Mean Expression Ratio	1
Median Expression Ratio	1
Credible Interval	(0.79, 1.28)
Associated Peptides	5
Associated Spectra	21
Coverage	NaN

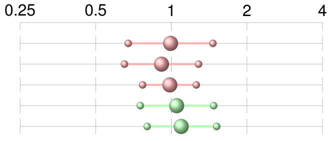

A	B	2.5	50	97.5	Sequence
1	0	0.67	0.99	1.5	DIPACECLK
2	1	0.65	0.92	1.3	HYYLATEEEVK
6	4	0.77	0.98	1.3	VLIAAHGNSLR
2	1	0.75	1	1.5	VLIAAHGN
3	1	0.8	1.1	1.5	EAQYAGELIK

5.640 2 475 unnamed protein product [Tetraodon nigroviridis]

Protein Accession	CL31420.Contig4$_{H}1$
Mean Expression Ratio	0.997
Median Expression Ratio	0.997
Credible Interval	(0.769, 1.29)
Associated Peptides	2
Associated Spectra	28
Coverage	NaN

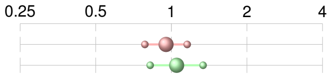

A	B	2.5	50	97.5	Sequence
18	0	0.78	0.95	1.2	DLYANTVLSGGTTMYPGIADR
10	0	0.82	1	1.3	KDLYANTVLSGGTTMYPGIADR

5.641 112 819 pyruvate kinase [Crassostrea gigas]; 132 932 pyruvate kinase [Crassostrea gigas]

Protein Accession	CL31920.Contig3$_{H}1$ CL31920.Contig1$_{H}1$
Mean Expression Ratio	0.995
Median Expression Ratio	0.997
Credible Interval	(0.632, 1.54)
Associated Peptides	1
Associated Spectra	1
Coverage	NaN

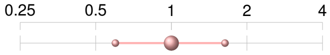

A	B	2.5	50	97.5	Sequence
1	0	0.6	1	1.6	ITIDDSYKEK

5.642 594 1253 peroxiredoxin 6 [Eriocheir sinensis]

Protein Accession	CL21894.Contig2$_{H}1$
Mean Expression Ratio	1
Median Expression Ratio	1
Credible Interval	(0.774, 1.31)
Associated Peptides	5
Associated Spectra	13
Coverage	NaN

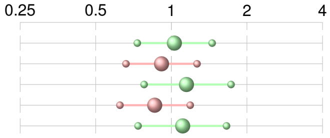

A	B	2.5	50	97.5	Sequence
2	1	0.73	1	1.5	VNLGDVFPDFK
4	0	0.66	0.91	1.3	AVFIIGPDK
1	0	0.78	1.1	1.7	AVFIIGPDKK
2	2	0.62	0.86	1.2	NFDEILR
1	0	0.74	1.1	1.7	QLTATQK

5.643 96 536 minus strand hypothetical protein BRAFLDRAFT_285353 [Branchiostoma floridae] gi

Protein Accession	CL14282.Contig5$_{H}1$
Mean Expression Ratio	0.997
Median Expression Ratio	0.998
Credible Interval	(0.648, 1.54)
Associated Peptides	1
Associated Spectra	1
Coverage	NaN

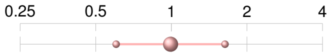

A	B	2.5	50	97.5	Sequence
**0**	**1**	**0.6**	**1**	**1.6**	**SQWSPALTISK**

5.644 8 1636 minus strand V-type proton ATPase catalytic subunit A [Crassostrea gigas]; 8 1636 minus strand V-type proton ATPase catalytic subunit A [Crassostrea gigas]; 8 1636 minus strand V-type proton ATPase catalytic subunit A [Crassostrea gigas]

Protein Accession	CL33671.Contig3$_{H}1$ CL33671.Contig2$_{H}1$ CL33671.Contig1$_{H}1$
Mean Expression Ratio	0.997
Median Expression Ratio	0.998
Credible Interval	(0.661, 1.5)
Associated Peptides	1
Associated Spectra	2
Coverage	NaN

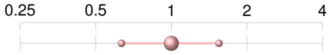

A	B	2.5	50	97.5	Sequence
**2**	**0**	**0.63**	**1**	**1.5**	**LAANHPLLTGQR**

5.645 187 1323 minus strand gelsolin [Aplysia californica] gi

Protein Accession	CL10220.Contig1$_{H}1$
Mean Expression Ratio	0.996
Median Expression Ratio	0.998
Credible Interval	(0.643, 1.54)
Associated Peptides	1
Associated Spectra	1
Coverage	NaN

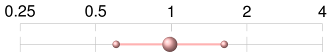

A	B	2.5	50	97.5	Sequence
**1**	**0**	**0.6**	**0.99**	**1.6**	**LSDASGELDFK**

5.646 232 654 hypothetical protein BRAFLDRAFT_87758 [Branchiostoma floridae] gi

Protein Accession	CL34711.Contig8$_{H}$1
Mean Expression Ratio	1
Median Expression Ratio	1
Credible Interval	(0.642, 1.56)
Associated Peptides	1
Associated Spectra	1
Coverage	NaN

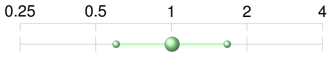

A	B	2.5	50	97.5	Sequence
1	0	0.6	1	1.7	CVATGQGGYR

5.647 429 1013 PDZ domain protein [Ixodes scapularis] gi

Protein Accession	CL8129.Contig12$_{H}$1
Mean Expression Ratio	1
Median Expression Ratio	1
Credible Interval	(0.796, 1.27)
Associated Peptides	6
Associated Spectra	20
Coverage	NaN

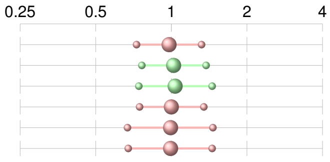

A	B	2.5	50	97.5	Sequence
4	1	0.72	0.98	1.3	DLGQPLSIQR
4	1	0.76	1	1.4	HGGGQIQLVIQR
1	2	0.74	1	1.5	VFSNSPAEGELQR
3	2	0.75	1	1.3	QALDLIK
1	0	0.67	0.99	1.5	DSFNTPWGFR
0	1	0.68	0.99	1.5	GDLILSINNR

5.648 151 1449 rab GDP-dissociation inhibitor [Branchiostoma belcheri]

Protein Accession	CL30719.Contig3$_{H}$1
Mean Expression Ratio	1
Median Expression Ratio	1
Credible Interval	(0.776, 1.3)
Associated Peptides	7
Associated Spectra	12
Coverage	NaN

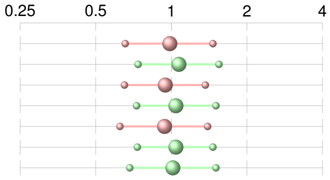

A	B	2.5	50	97.5	Sequence
1	0	0.65	0.99	1.5	EALGTSLMGMFEK
1	1	0.74	1.1	1.5	FLMANGQLVK
1	1	0.65	0.95	1.4	GVDCNGMTAAQLYEK
1	1	0.73	1	1.5	ITGEAFDFSK
1	0	0.63	0.94	1.4	IVYDETGVAVGVQDADGAVAK
2	1	0.73	1	1.5	LLVATGVTR
1	0	0.68	1	1.5	QWNVDLIPK

5.649 2 244 Ras homolog gene family member A [Clonorchis sinensis]

Protein Accession	Unigene32424$_{H}$1
Mean Expression Ratio	1
Median Expression Ratio	1
Credible Interval	(0.639, 1.57)
Associated Peptides	1
Associated Spectra	2
Coverage	NaN

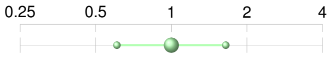

A	B	2.5	50	97.5	Sequence
1	1	0.61	1	1.6	EVFETATR

5.650 10 1902 Mitochondrial inner membrane protein [Crassostrea gigas]; 10 1902 Mitochondrial inner membrane protein [Crassostrea gigas]; 10 1902 Mitochondrial inner membrane protein [Crassostrea gigas]; 10 1902 Mitochondrial inner membrane protein [Crassostrea gigas]; 10 1902 Mitochondrial inner membrane protein [Crassostrea gigas]; 10 1902 Mitochondrial inner membrane protein [Crassostrea gigas]

Protein Accession	CL6414.Contig8$_{H}1$ CL6414.Contig7$_{H}1$ CL6414.Contig6$_{H}1$ CL6414.Contig5$_{H}1$ CL6414.Con
Mean Expression Ratio	1
Median Expression Ratio	1
Credible Interval	(0.647, 1.56)
Associated Peptides	1
Associated Spectra	1
Coverage	NaN

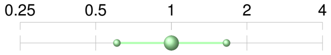

A	B	2.5	50	97.5	Sequence
**1**	**0**	**0.61**	**1**	**1.7**	**EIHTQLLQER**

5.651 185 742 Transmembrane protein Tmp21 [Crassostrea gigas]; 1 444 minus strand Transmembrane protein Tmp21 [Crassostrea gigas]

Protein Accession	CL3732.Contig3$_{H}1$ CL3732.Contig1$_{H}1$
Mean Expression Ratio	1
Median Expression Ratio	1
Credible Interval	(0.674, 1.5)
Associated Peptides	2
Associated Spectra	2
Coverage	NaN

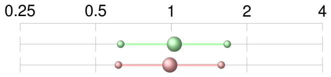

A	B	2.5	50	97.5	Sequence
**1**	**0**	**0.63**	**1**	**1.7**	**LKPLEVELR**
**1**	**0**	**0.62**	**0.99**	**1.6**	**EVHLNLK**

5.652 72 521 minus strand GF14593 [Drosophila ananassae] gi

Protein Accession	CL38325.Contig2$_{H}1$
Mean Expression Ratio	0.999
Median Expression Ratio	0.999
Credible Interval	(0.639, 1.56)
Associated Peptides	1
Associated Spectra	1
Coverage	NaN

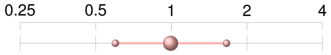

A	B	2.5	50	97.5	Sequence
**1**	**0**	**0.6**	**1**	**1.7**	**LTADVHGK**

5.653 " 103 981 PREDICTED: ATP synthase, H+ transporting, mitochondrial F1 complex, gamma subunit isoform H (heart)-like [Saccoglossus kowalevskii]"

Protein Accession	CL25149.Contig1$_{H}1$
Mean Expression Ratio	1
Median Expression Ratio	1
Credible Interval	(0.692, 1.46)
Associated Peptides	2
Associated Spectra	3
Coverage	NaN

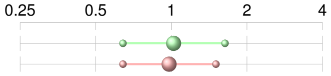

A	B	2.5	50	97.5	Sequence
**1**	**0**	**0.64**	**1**	**1.6**	**ELKPARPYGAGAK**
**1**	**1**	**0.64**	**0.98**	**1.5**	**LTLTFNR**

5.654 21 239 minus strand calponin-like protein [Taenia asiatica]

Protein Accession	CL26318.Contig1$_{H}1$
Mean Expression Ratio	0.998
Median Expression Ratio	0.999
Credible Interval	(0.662, 1.5)
Associated Peptides	2
Associated Spectra	2
Coverage	NaN

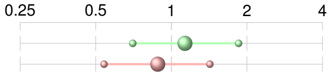

A	B	2.5	50	97.5	Sequence
**1**	**0**	**0.7**	**1.1**	**1.9**	**HIADIK**
**1**	**0**	**0.54**	**0.88**	**1.4**	**VDDMSAASK**

5.655 212 1405 minus strand unnamed protein product [Tetraodon nigroviridis]

Protein Accession	CL37546.Contig3$_{H}1$
Mean Expression Ratio	1
Median Expression Ratio	1
Credible Interval	(0.638, 1.57)
Associated Peptides	1
Associated Spectra	1
Coverage	NaN

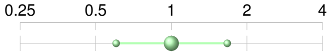

A	B	2.5	50	97.5	Sequence
**0**	**1**	**0.6**	**1**	**1.7**	**TIAELGEK**

5.656 146 841 minus strand hypothetical protein CGI_10006609 [Crassostrea gigas]; 146 841 minus strand hypothetical protein CGI_10006609 [Crassostrea gigas]; 146 841 minus strand hypothetical protein CGI_10006609 [Crassostrea gigas]

Protein Accession	CL6681.Contig3$_{H}1$ CL6681.Contig2$_{H}1$ CL6681.Contig1$_{H}1$
Mean Expression Ratio	0.998
Median Expression Ratio	0.999
Credible Interval	(0.641, 1.55)
Associated Peptides	1
Associated Spectra	1
Coverage	NaN

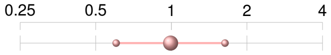

A	B	2.5	50	97.5	Sequence
**0**	**1**	**0.6**	**1**	**1.6**	**GHVEGLEVLPAR**

5.657 58 198 minus strand Protein DJ-1 [Crassostrea gigas]

Protein Accession	CL16588.Contig4$_{H}1$
Mean Expression Ratio	1
Median Expression Ratio	1
Credible Interval	(0.706, 1.43)
Associated Peptides	1
Associated Spectra	6
Coverage	NaN

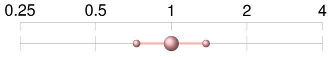

A	B	2.5	50	97.5	Sequence
**3**	**3**	**0.72**	**1**	**1.4**	**ADSLVAPMLVK**

5.658 160 501 hypothetical protein CGI_10001640 [Crassostrea gigas]; 160 588 hypothetical protein CGI_10001640 [Crassostrea gigas]; 160 501 hypothetical protein CGI_10001640 [Crassostrea gigas]; 160 552 hypothetical protein CGI_10001640 [Crassostrea gigas]; 160 501 hypothetical protein CGI_10001640 [Crassostrea gigas]; 160 501 hypothetical protein CGI_10001640 [Crassostrea gigas]

Protein Accession	Unigene62839$_{H}1$ CL2347.Contig8$_{H}1$ CL2347.Contig15$_{H}1$ CL2347.Contig14$_{H}1$ CL2347.Co
Mean Expression Ratio	0.997
Median Expression Ratio	1
Credible Interval	(0.697, 1.42)
Associated Peptides	2
Associated Spectra	13
Coverage	NaN

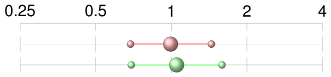

A	B	2.5	50	97.5	Sequence
**6**	**3**	**0.69**	**0.99**	**1.4**	**AFLEQLGSK**
**3**	**1**	**0.69**	**1**	**1.6**	**ANLIVIGSR**

iTRAQ Data Analysis Report

Javier, Head 14d versus control

April 7, 2014

1 Introduction

This document summarizes an analysis of relative protein expression using iTRAQ. The reporter ion peak area measurements supplied by the ABI software are used to estimate treatment-dependent peptide and protein relative expression. Estimation is accomplished using a Bayesian approach with the model given below. The document includes a protein relative expression summary and a per-protein detailed analysis. The document is internally hyperlinked and linked externally to NCBI.

2 Experiment and Model Description

2.1 Experiment Design

The report summarizes data from one or more iTRAQ experiments addressing a common comparison. The experiment design, used in this analysis, is given in the table below.

	Experiment	Treatment	Channel	Sample
1	A	control	115	s1
2	A	test	118	s1
3	B	control	115	s2
4	B	test	118	s2

2.2 Input Files

Data for this analysis was extracted from the following tandem mass spectra (MSMS) summary files.

Experiment	MSMS Summary File
A	head2.txt
B	head3.txt

2.3 Statistical Model

The following statistical model was used to estimate the treatment-dependent effects.

LogIntensity ˜ Channel + Spectrum + Protein + Peptide + Protein:Treatment + Peptide:Treatment

3 Data Summary

The data supplied in the MSMS summary is filtered to remove unidentified proteins, contaminants, and peptides containing selected modifications. The following table sumarizes the data provided and used in the analysis.

	A	B	Combined
Supplied Spectra	21342	14837	36179
Unidentified Spectra	8154	6352	14506
Disallowed Modifications	363	186	549
Spectra from Contaminants	0	0	0
Missing Data	3160	2214	5374
Low Confidence Spectra	8154	6352	14506
Degenerate Peptides	3602	2236	5838
Remaining Spectra			9912
Unique Proteins			656
Unique Peptides			2366
Model R^2			0.938

4 Protein Summary

Each protein identified in one or more of the MSMS summaries is listed below in decreasing order of expression change magnitude. The median and estimated credible interval for each protein is given to the left in the table. Proteins identified by a single peptide are listed in a separate table.

4.1 Identified Proteins

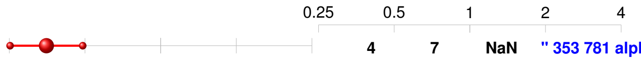

Peptides	Spectra	Coverage	Protein
4	7	NaN	**" 353 781 alpha-B-crystallin, putative [Ixodes scapularis] gi"**

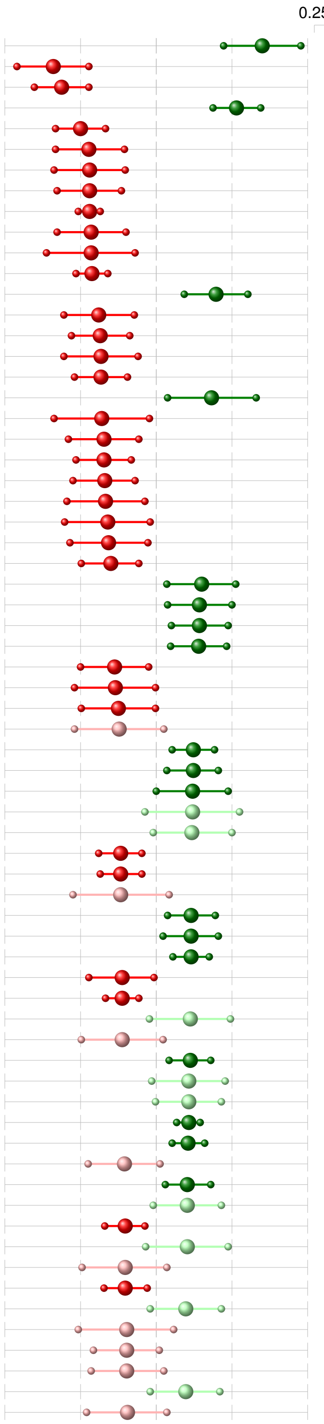

Peptides	Spectra	Coverage	Protein
3	6	NaN	718 957 minus strand Calponin-1 OS=Gallus gallus GN=CNN1 PE=1 SV=2
4	8	NaN	200 2128 minus strand heat shock protein 70 [Cristaria plicata]
8	17	NaN	280 690 Histone H3.3 [Harpegnathos saltator]
9	41	NaN	204 791 minus strand PREDICTED: 15-hydroxyprostaglandin dehydrogenase [NAD+] [Gallus gallus]
7	27	NaN	27 1682 minus strand PREDICTED: nucleolar protein 56 [Oreochromis niloticus]
2	18	NaN	" 131 544 Histone H2B, gonadal OS=Patella granatina PE=1 SV=2"
5	7	NaN	" 10 609 lethal(2)essential for life protein, l2efl [Aedes aegypti] gi"
5	23	NaN	118 501 minus strand histone 2A.Z [Aplysia californica]
30	480	NaN	405 1373 paramyosin [Haliotis discus discus]
2	43	NaN	628 1140 paramyosin [Haliotis discus discus]
2	3	NaN	1 945 hemocyanin isoform 1 [Haliotis diversicolor]
12	138	NaN	2473 2898 RecName: Full=Paramyosin gi|397603|emb|CAA79849.1| paramyosin [Echinococcus granulosus]
3	28	NaN	397 510 PREDICTED: actin-5C-like [Megachile rotundata]
3	8	NaN	3 491 hemocyanin [Haliotis tuberculata]
7	13	NaN	957 2147 haemocyanin [Aplysia californica]
3	7	NaN	372 860 minus strand small heat shock protein [Tegillarca granosa]; 651 998 minus strand small heat shock protein
8	15	NaN	4726 5571 minus strand twitchin [Mytilus galloprovincialis]
3	3	NaN	103 684 EF-hand domain-containing protein D2 [Crassostrea gigas]
2	2	NaN	1363 1527 Protein disulfide-isomerase A3 [Crassostrea gigas]
2	10	NaN	1027 1440 minus strand hemocyanin isoform 1 [Haliotis diversicolor]
5	21	NaN	93 2606 twitchin [Mytilus galloprovincialis]
5	11	NaN	346 702 minus strand PREDICTED: galectin-4-like [Anolis carolinensis]
2	8	NaN	" 599 907 minus strand Tryptophanyl-tRNA synthetase, cytoplasmic [Heterocephalus glaber]"
2	4	NaN	23 214 minus strand hemocyanin isoform 1 [Nucula nucleus]
2	6	NaN	21 341 minus strand hemocyanin isoform 1 [Nucula nucleus]
7	15	NaN	501 1838 Hsp90A [Haliotis asinina]
4	20	NaN	174 1649 minus strand Collagen alpha-1(XII) chain [Crassostrea gigas]; 123 1490 minus strand Collagen alpha-1(XII)
5	10	NaN	80 589 minus strand PREDICTED: adenine phosphoribosyltransferase-like [Strongylocentrotus purpuratus]
3	21	NaN	269 880 minus strand myosin regulatory light chain [Fasciola hepatica]
5	16	NaN	" 276 659 Fatty acid-binding protein, adipocyte [Crassostrea gigas]; 426 809 Fatty acid-binding protein, adipocyte [C
2	21	NaN	" 116 352 minus strand troponin I 4 [Clonorchis sinensis]; 491 706 minus strand troponin-I, TnI {C-terminal} [Chlamy
4	4	NaN	" 1 1887 Pyruvate carboxylase, mitochondrial [Crassostrea gigas]"
2	7	NaN	266 607 minus strand H2 [Haliotis tuberculata]
2	3	NaN	511 1062 unknown [Dendroctonus ponderosae]; 511 1062 unknown [Dendroctonus ponderosae]
7	48	NaN	76 522 minus strand Troponin C [Crassostrea gigas]; 173 619 minus strand Troponin C [Crassostrea gigas]; 173 619
8	21	NaN	1009 2784 minus strand Collagen alpha-3(VI) chain [Crassostrea gigas]
3	8	NaN	150 716 calponin/transgelin [Papilio xuthus]
2	3	NaN	552 1499 minus strand uncharacterized protein LOC100158466 [Xenopus (Silurana) tropicalis] gi
2	6	NaN	181 477 minus strand Zgc:56530 [Danio rerio]; 185 481 minus strand Zgc:56530 [Danio rerio]
14	58	NaN	155 3214 sarco/endoplasmic reticulum calcium ATPase isoform B [Pinctada fucata]; 155 3214 minus strand sarco/er
13	34	NaN	" 460 1944 PREDICTED: succinyl-CoA:3-ketoacid-coenzyme A transferase 1, mitochondrial [Canis lupus familiaris]"
2	2	NaN	169 1161 Basement membrane-specific heparan sulfate proteoglycan core protein OS=Homo sapiens GN=HSPG2 P
8	24	NaN	1200 1547 protein disulfide isomerase [Conus eburneus]
6	18	NaN	247 1308 RecName: Full=Guanine nucleotide-binding protein G(o) subunit alpha gi|9633|emb|CAA78806.1| GTP-bin
11	102	NaN	260 892 minus strand calponin [Haliotis diversicolor]
4	11	NaN	12160 13014 twitchin [Mytilus galloprovincialis]
25	73	NaN	155 3214 sarco/endoplasmic reticulum calcium ATPase isoform B [Pinctada fucata]; 132 3110 sarco/endoplasmic ret
4	5	NaN	244 837 PREDICTED: ras-like GTP-binding protein Rho1-like [Megachile rotundata]
2	5	NaN	2 2542 kinesin heavy chain 1 [Aplysia californica] gi; 2 2542 kinesin heavy chain 1 [Aplysia californica] gi
8	119	NaN	238 681 minus strand Globin OS=Cerithidea rhizophorarum PE=1 SV=1
4	6	NaN	" 110 1717 PREDICTED: glutamate dehydrogenase, mitochondrial-like [Oreochromis niloticus]; 110 1717 PREDICTE
2	15	NaN	105 503 Transgelin-2 [Crassostrea gigas]
31	204	NaN	30 1088 arginine kinase [Conus novaehollandiae]
18	76	NaN	111 1226 gelsolin [Aplysia californica] gi; 42 1139 minus strand gelsolin [Aplysia californica] gi
6	6	NaN	4 675 Laminin subunit gamma-1 [Crassostrea gigas]
8	29	NaN	82 816 PREDICTED: triosephosphate isomerase B [Oreochromis niloticus]
4	8	NaN	2939 3208 minus strand beta-thymosin domain repeat protein CSP24KDa_v1 [Hermissenda crassicornis] gi
9	63	NaN	373 579 myosin heavy chain [Argopecten irradians] gi
3	4	NaN	186 1778 predicted protein [Nematostella vectensis] gi
2	4	NaN	457 1842 Basement membrane-specific heparan sulfate proteoglycan core protein [Crassostrea gigas]; 457 1842 Ba
11	40	NaN	496 2151 NADP-dependent malic enzyme [Crassostrea gigas]
2	10	NaN	62 505 Myosin-2 essential light chain [Crassostrea gigas]
2	2	NaN	185 742 Transmembrane protein Tmp21 [Crassostrea gigas]; 1 444 minus strand Transmembrane protein Tmp21 [Cr
4	19	NaN	697 954 minus strand Peptidyl-prolyl cis-trans isomerase B [Crassostrea gigas]; 614 877 Peptidyl-prolyl cis-trans iso
4	7	NaN	49 819 PREDICTED: elongation factor 1-delta-like isoform 2 [Strongylocentrotus purpuratus]
3	11	NaN	801 971 RecName: Full=Tropomyosin-2; AltName: Full=Tropomyosin II; Short=BgTMII; Short=TMII gi|155940|gb|AA
2	9	NaN	425 1051 minus strand unknown [Schistosoma japonicum]; 300 878 unknown [Schistosoma japonicum]; 567 1145 u

0.25 0.5 1 2 4

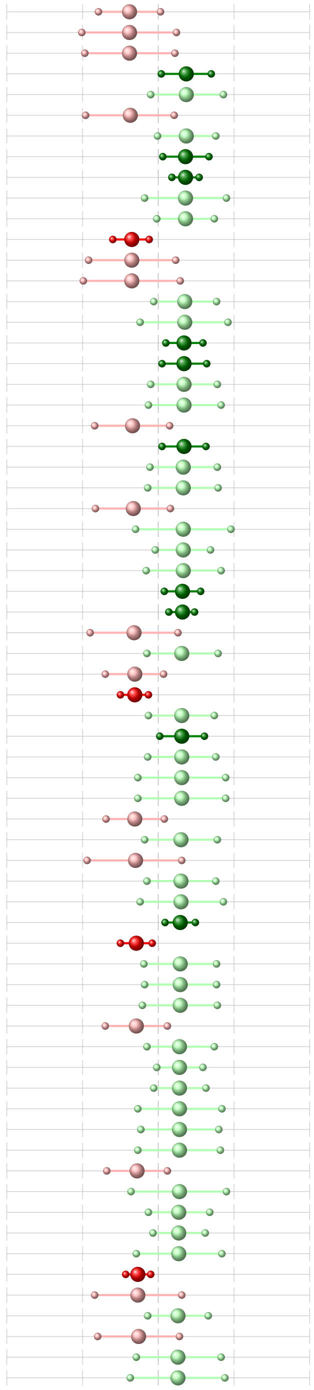

Peptides	Spectra	Coverage	Protein
4	22	NaN	15 428 PREDICTED: histone H2A.V-like [Apis florea]
2	2	NaN	243 2165 minus strand Elongation factor 2 OS=Drosophila melanogaster GN=Ef2b PE=2 SV=4
2	3	NaN	201 1181 minus strand Serine/threonine-protein phosphatase PP1-alpha catalytic subunit [Crassostrea gigas]
10	28	NaN	174 1649 minus strand Collagen alpha-1(XII) chain [Crassostrea gigas]; 135 1610 Collagen alpha-1(XII) chain [Crasso
4	7	NaN	230 1189 hypothetical protein [Amblyomma maculatum]
2	3	NaN	160 387 small nuclear ribonucleoprotein polypeptide G protein [Crassostrea ariakensis]; 180 407 small nuclear ribor
9	11	NaN	95 2506 Transitional endoplasmic reticulum ATPase [Crassostrea gigas]
8	43	NaN	915 1505 minus strand Collagen alpha-5(VI) chain [Crassostrea gigas]
19	202	NaN	167 679 tropomyosin [Haliotis discus discus]
2	5	NaN	57 1688 Talin-1 [Camponotus floridanus]; 57 1865 Talin-1 [Camponotus floridanus]; 57 1835 Talin-1 [Camponotus flo
6	13	NaN	6 650 minus strand glutathione S-transferase mu class protein [Crassostrea ariakensis]; 117 761 glutathione S-trans
10	105	NaN	527 1321 minus strand troponin T [Mizuhopecten yessoensis]; 305 1099 minus strand troponin T [Mizuhopecten yes
3	8	NaN	459 755 minus strand heat shock protein beta-1 [Trichinella spiralis] gi; 464 763 minus strand heat shock protein bet
2	2	NaN	3 551 minus strand Spectrin beta chain [Crassostrea gigas]
4	19	NaN	488 1039 Peptidyl-prolyl cis-trans isomerase B [Crassostrea gigas]; 488 1039 Peptidyl-prolyl cis-trans isomerase B [
2	3	NaN	" 81 1349 minus strand PREDICTED: LOW QUALITY PROTEIN: dolichyl-diphosphooligosaccharide–protein glycosylt
11	70	NaN	3 434 minus strand myosin essential light chain [Haliotis discus discus]
9	41	NaN	643 1302 minus strand Kyphoscoliosis peptidase [Crassostrea gigas]; 634 1293 minus strand Kyphoscoliosis peptic
2	37	NaN	REVERSED 6 395 PREDICTED: sister chromatid cohesion protein PDS5 homolog B-A isoform 1 [Nasonia vitripennis
3	11	NaN	180 560 calreticulin precursor [Aplysia californica] gi; 180 545 calreticulin precursor [Aplysia californica] gi
3	7	NaN	" 698 1225 minus strand actin related protein 2/3 complex, 21 kDa subunit [Crassostrea gigas]"
7	49	NaN	119 481 Glutamate receptor U1 [Crassostrea gigas]
2	13	NaN	871 1062 Adenylate kinase isoenzyme 5 [Crassostrea gigas]
2	13	NaN	160 588 hypothetical protein CGI_10001640 [Crassostrea gigas]
2	8	NaN	729 1541 troponin T [Mizuhopecten yessoensis]
2	3	NaN	290 2458 Putative beta-D-xylosidase 5 [Crassostrea gigas]; 299 2428 Putative beta-D-xylosidase 2 [Crassostrea giga
7	15	NaN	2156 3142 minus strand PREDICTED: hemicentin-1 [Xenopus (Silurana) tropicalis]
2	14	NaN	633 806 Peroxiredoxin-1 [Crassostrea gigas]; 648 821 Peroxiredoxin-1 [Crassostrea gigas]; 666 839 Peroxiredoxin-1 [
12	62	NaN	349 885 juvenile hormone diol kinase [Danaus plexippus]; 349 885 juvenile hormone diol kinase [Danaus plexippus]
22	156	NaN	552 1886 minus strand intermediate filament protein [Biomphalaria glabrata]
2	3	NaN	125 550 minus strand ribosomal protein S20 [Chlamys farreri]; 125 550 minus strand ribosomal protein S20 [Chlamy
4	8	NaN	127 657 ADP ribosylation factor 4 [Marsupenaeus japonicus]
7	13	NaN	783 1463 minus strand twitchin [Mytilus galloprovincialis]; 783 1463 minus strand twitchin [Mytilus galloprovincialis
16	242	NaN	6 317 myosin heavy chain cardiac muscle specific isoform 2 [Argopecten irradians]
2	27	NaN	" 386 799 minus strand Myosin regulatory light chain LC-2, mantle muscle OS=Todarodes pacificus PE=1 SV=1; 656
15	24	NaN	77 1561 retinal dehydrogenase [Lymnaea stagnalis]; 69 1553 retinal dehydrogenase [Lymnaea stagnalis]; 308 1747 r
6	7	NaN	3 1670 Spectrin alpha chain [Crassostrea gigas]
3	3	NaN	182 1846 minus strand hypothetical protein DAPPUDRAFT_302108 [Daphnia pulex]; 110 1774 minus strand hypothet
2	3	NaN	2 772 minus strand arylsulfatase [Haliotis discus discus]
7	14	NaN	366 1049 hypothetical protein CGI_10004855 [Crassostrea gigas]
2	9	NaN	507 1277 alpha tubulin [Notothenia coriiceps]
2	2	NaN	1 378 minus strand PREDICTED: CG10098-like [Saccoglossus kowalevskii]
4	8	NaN	146 727 Adenylate kinase isoenzyme 1 [Crassostrea gigas]
3	4	NaN	150 1511 Tensin OS=Gallus gallus GN=TNS PE=1 SV=2
19	130	NaN	116 1111 minus strand cytosolic malate dehydrogenase [Lottia scabra]
13	136	NaN	2881 3411 myosin heavy chain [Todarodes pacificus]; 2915 3445 myosin heavy chain [Todarodes pacificus]
4	6	NaN	137 853 14-3-3 protein [Heterodera glycines]
5	6	NaN	167 2713 minus strand Major vault protein [Crassostrea gigas]
3	6	NaN	" 145 1755 Fumarate hydratase class I, aerobic [Crassostrea gigas]"
6	10	NaN	51 890 prohibitin [Octopus tankahkeei]
4	10	NaN	31 735 hypothetical protein BRAFLDRAFT_78603 [Branchiostoma floridae] gi; 55 735 hypothetical protein BRAFLDF
13	23	NaN	203 1942 ezrin/radixin/moesin [Aplysia californica] gi; 243 1982 minus strand ezrin/radixin/moesin [Aplysia californic
6	20	NaN	429 1013 PDZ domain protein [Ixodes scapularis] gi
2	4	NaN	2 1093 minus strand Neurexin-1-alpha [Crassostrea gigas]
2	6	NaN	3 416 minus strand myosin essential light chain [Crassostrea gigas]
3	4	NaN	8 271 minus strand Laminin subunit gamma-1 [Crassostrea gigas]
7	11	NaN	" 3341 4240 minus strand nonmuscle myosin II, partial [Aplysia californica]"
2	2	NaN	" 2 286 Roundabout 2, partial [Ascaris suum]"
5	12	NaN	718 1155 Annexin A7 [Crassostrea gigas]
6	21	NaN	680 991 minus strand PDZ and LIM domain protein 1 [Crassostrea gigas]; 553 864 minus strand PDZ and LIM domair
3	3	NaN	85 1011 unnamed protein product [Mus musculus]
23	291	NaN	3591 4526 minus strand myosin heavy chain cardiac muscle specific isoform 1 [Argopecten irradians]
3	3	NaN	40 1182 4-hydroxyphenylpyruvate dioxygenase [Crassostrea gigas]
3	18	NaN	7073 7339 minus strand Collagen alpha-3(VI) chain [Crassostrea gigas]
3	4	NaN	" 166 1248 PREDICTED: probable pyruvate dehydrogenase E1 component subunit alpha, mitochondrial-like [Metase
2	4	NaN	2996 3340 minus strand Collagen alpha-5(VI) chain [Crassostrea gigas]
2	2	NaN	587 811 hemocyanin 2 [Megathura crenulata]

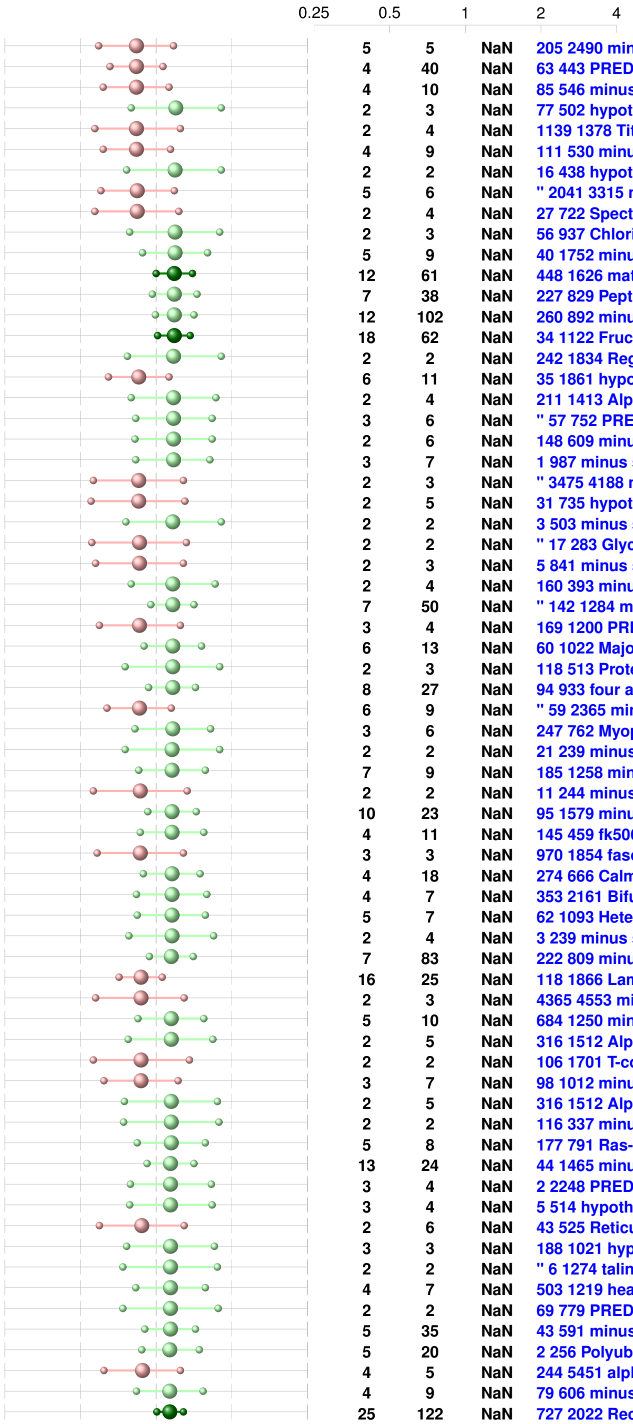

Peptides	Spectra	Coverage	Protein
5	5	NaN	205 2490 minus strand glucose-regulated protein 94 [Crassostrea gigas]
4	40	NaN	63 443 PREDICTED: RAS protein activator like 1-like [Saccoglossus kowalevskii]
4	10	NaN	85 546 minus strand RecName: Full=Eukaryotic translation initiation factor 5A; Short=eIF-5A gi|51702279|sp|P6292
2	3	NaN	77 502 hypothetical protein CGI_10008263 [Crassostrea gigas]
2	4	NaN	1139 1378 Titin [Crassostrea gigas]; 1116 1355 Titin [Crassostrea gigas]; 1139 1378 Titin [Crassostrea gigas]; 1139 1
4	9	NaN	111 530 minus strand Nucleoside diphosphate kinase B OS=Homo sapiens GN=NME2 PE=1 SV=1
2	2	NaN	16 438 hypothetical protein KGM_06034 [Danaus plexippus]; 16 438 hypothetical protein KGM_06034 [Danaus plexip
5	6	NaN	" 2041 3315 minus strand Myosin-10 OS=Mus musculus GN=Myh10 PE=1 SV=2; 2041 3345 minus strand Myosin hea
2	4	NaN	27 722 Spectrin beta chain OS=Drosophila melanogaster GN=beta-Spec PE=1 SV=2; 27 722 Spectrin beta chain OS=
2	3	NaN	56 937 Chloride intracellular channel exc-4 [Crassostrea gigas]
5	9	NaN	40 1752 minus strand heat shock protein 60 [Biomphalaria glabrata]; 40 1752 minus strand heat shock protein 60 [B
12	61	NaN	448 1626 matrilin [Biomphalaria glabrata]
7	38	NaN	227 829 Peptidyl-prolyl cis-trans isomerase B [Crassostrea gigas]
12	102	NaN	260 892 minus strand calponin [Haliotis diversicolor]; 260 892 minus strand calponin [Haliotis diversicolor]
18	62	NaN	34 1122 Fructose-bisphosphate aldolase [Crassostrea gigas]
2	2	NaN	242 1834 Regulator of differentiation 1 [Crassostrea gigas]; 458 2050 minus strand Regulator of differentiation 1 [Cr
6	11	NaN	35 1861 hypothetical protein DAPPUDRAFT_309082 [Daphnia pulex]
2	4	NaN	211 1413 Alpha-N-acetylgalactosaminidase [Crassostrea gigas]
3	6	NaN	" 57 752 PREDICTED: adenylate kinase 2, mitochondrial-like isoform 2 [Callithrix jacchus]"
2	6	NaN	148 609 minus strand Titin [Crassostrea gigas]
3	7	NaN	1 987 minus strand RecName: Full=Tubulin beta chain; AltName: Full=Beta-tubulin
2	3	NaN	" 3475 4188 minus strand Heat shock 70 kDa protein cognate, putative [Pediculus humanus corporis] gi"
2	5	NaN	31 735 hypothetical protein BRAFLDRAFT_78603 [Branchiostoma floridae] gi; 55 735 hypothetical protein BRAFLDR
2	2	NaN	3 503 minus strand elongation factor-1 alpha [Pomacea bridgesi]
2	2	NaN	" 17 283 Glycerol-3-phosphate dehydrogenase [NAD+], cytoplasmic [Crassostrea gigas]"
2	3	NaN	5 841 minus strand Spectrin alpha chain [Crassostrea gigas]
2	4	NaN	160 393 minus strand PREDICTED: cysteine-rich protein 1-like [Megachile rotundata]; 148 381 PREDICTED: cysteine
7	50	NaN	" 142 1284 minus strand Chain O, Crsytal Structure Of Human Liver Gapdh gi|67464044|pdb|1ZNQ|P Chain P, Crsyta
3	4	NaN	169 1200 PREDICTED: nidogen-2-like isoform 1 [Strongylocentrotus purpuratus]
6	13	NaN	60 1022 Major egg antigen [Crassostrea gigas]; 60 1022 Major egg antigen [Crassostrea gigas]
2	3	NaN	118 513 Protein CAL-5 [Caenorhabditis elegans] gi
8	27	NaN	94 933 four and A half lim domains [Schistosoma mansoni]
6	9	NaN	" 59 2365 minus strand Aconitate hydratase, mitochondrial [Crassostrea gigas]"
3	6	NaN	247 762 Myophilin [Crassostrea gigas]; 244 759 Myophilin [Crassostrea gigas]; 119 634 Myophilin [Crassostrea giga
2	2	NaN	21 239 minus strand calponin-like protein [Taenia asiatica]
7	9	NaN	185 1258 minus strand Severin [Crassostrea gigas]
2	2	NaN	11 244 minus strand Tubulin alpha chain (Fragment) OS=Lytechinus pictus PE=3 SV=1
10	23	NaN	95 1579 minus strand hypothetical protein BRAFLDRAFT_113974 [Branchiostoma floridae] gi
4	11	NaN	145 459 fk506-binding protein 2 [Papilio xuthus]; 237 551 fk506-binding protein 2 [Papilio xuthus]
3	3	NaN	970 1854 fasciclin-like protein precursor [Aplysia californica] gi
4	18	NaN	274 666 Calmodulin-4 [Heterocephalus glaber]
4	7	NaN	353 2161 Bifunctional 3'-phosphoadenosine 5'-phosphosulfate synthetase [Crassostrea gigas]; 141 19
5	7	NaN	62 1093 Heterogeneous nuclear ribonucleoprotein A2-like protein 1 [Crassostrea gigas]
2	4	NaN	3 239 minus strand alcohol dehydrogenase class 3 [Branchiostoma lanceolatum]
7	83	NaN	222 809 minus strand PREDICTED: 15-hydroxyprostaglandin dehydrogenase [NAD+] [Gallus gallus]
16	25	NaN	118 1866 Lamin Dm0 [Crassostrea gigas]
2	3	NaN	4365 4553 minus strand Basement membrane-specific heparan sulfate proteoglycan core protein [Crassostrea gigas
5	10	NaN	684 1250 minus strand Collagen alpha-5(VI) chain [Crassostrea gigas]
2	5	NaN	316 1512 Alpha-N-acetylgalactosaminidase [Crassostrea gigas]; 282 1568 Alpha-N-acetylgalactosaminidase [Crasso
2	2	NaN	106 1701 T-complex protein 1 subunit beta [Crassostrea gigas]
3	7	NaN	98 1012 minus strand RecName: Full=Substrate-specific endoprotease Tex31; Flags: Precursor gi|32187775|emb|C
2	5	NaN	316 1512 Alpha-N-acetylgalactosaminidase [Crassostrea gigas]; 282 1478 Alpha-N-acetylgalactosaminidase [Crasso
2	2	NaN	116 337 minus strand Golgi-associated plant pathogenesis-related protein 1 [Crassostrea gigas]; 538 759 minus stra
5	8	NaN	177 791 Ras-related protein Rab-1A [Haliotis discus discus]
13	24	NaN	44 1465 minus strand Adenylyl cyclase-associated protein 1 [Crassostrea gigas]; 52 1473 minus strand Adenylyl cyc
3	4	NaN	2 2248 PREDICTED: heat shock 70 kDa protein 4-like [Oreochromis niloticus]; 2 2227 PREDICTED: heat shock 70 kD
3	4	NaN	5 514 hypothetical protein KGM_05418 [Danaus plexippus]
2	6	NaN	43 525 Reticulon-4 [Crassostrea gigas]; 143 757 Reticulon-4 [Crassostrea gigas]; 43 564 Reticulon-4 [Crassostrea gi
3	3	NaN	188 1021 hypothetical protein BRAFLDRAFT_214346 [Branchiostoma floridae] gi
2	2	NaN	" 6 1274 talin, putative [Ixodes scapularis] gi"
4	7	NaN	503 1219 heat shock cognate 71 [Mytilus galloprovincialis]
2	2	NaN	69 779 PREDICTED: 15-hydroxyprostaglandin dehydrogenase [NAD+] [Gallus gallus]
5	35	NaN	43 591 minus strand cyclophilin type peptidyl-prolyl cis-trans isomerase [Glossina morsitans morsitans]
5	20	NaN	2 256 Polyubiquitin (Fragment) OS=Xenopus laevis PE=1 SV=2; 20 388 large subunit ribosomal protein L40e [Clonor
4	5	NaN	244 5451 alpha macroglobulin [Chlamys farreri]; 244 5493 alpha macroglobulin [Chlamys farreri]
4	9	NaN	79 606 minus strand PREDICTED: similar to Ras-like protein 3 precursor (Protein roughened) [Tribolium castaneum]
25	122	NaN	727 2022 RecName: Full=Enolase; AltName: Full=2-phospho-D-glycerate hydro-lyase; AltName: Full=2-phosphoglyc

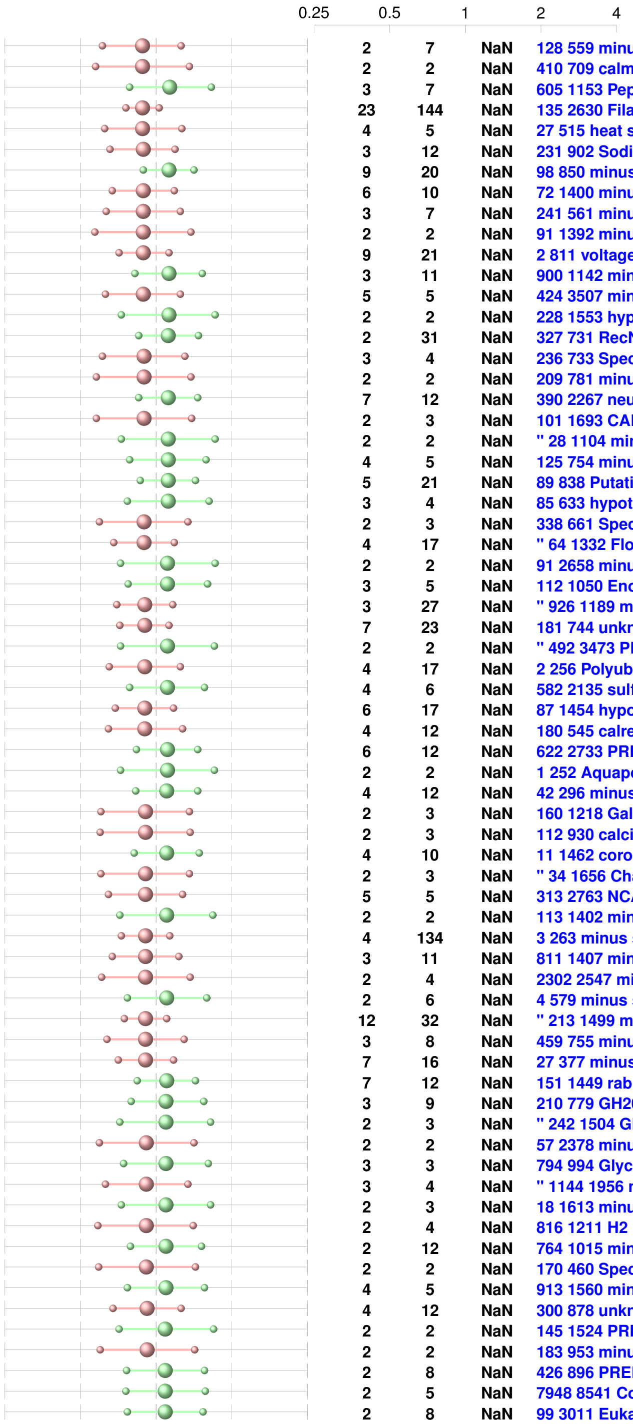

Peptides	Spectra	Coverage	Protein
2	7	NaN	128 559 minus strand predicted protein [Nematostella vectensis] gi
2	2	NaN	410 709 calmodulin-like; K02183 calmodulin
3	7	NaN	605 1153 Peptidyl-prolyl cis-trans isomerase B [Crassostrea gigas]; 1046 1630 Peptidyl-prolyl cis-trans isomerase B
23	144	NaN	135 2630 Filamin-A [Crassostrea gigas]; 118 2613 Filamin-A [Crassostrea gigas]; 157 2652 Filamin-A [Crassostrea g
4	5	NaN	27 515 heat shock protein 70 [Capsaspora owczarzaki ATCC 30864]; 30 464 minus strand hypothetical protein [Trich
3	12	NaN	231 902 Sodium- and chloride-dependent GABA transporter ine [Crassostrea gigas]
9	20	NaN	98 850 minus strand 14-3-3 protein epsilon [Schistocerca gregaria]
6	10	NaN	72 1400 minus strand hypothetical protein RCJMB04_22i13 [Gallus gallus]
3	7	NaN	241 561 minus strand thioredoxin [Crassostrea ariakensis]
2	2	NaN	91 1392 minus strand hypothetical protein AND_03103 [Anopheles darlingi]
9	21	NaN	2 811 voltage-dependent anion channel 2-like protein [Haliotis diversicolor]; 2 811 voltage-dependent anion channel
3	11	NaN	900 1142 minus strand heat shock protein 20 [Cyclina sinensis]
5	5	NaN	424 3507 minus strand putative Na+/K+-ATPase alpha subunit [Paroctopus digueti]
2	2	NaN	228 1553 hypothetical protein CGI_10024545 [Crassostrea gigas]; 228 1553 hypothetical protein CGI_10024545 [Cras
2	31	NaN	327 731 RecName: Full=Arginine kinase; Short=AK gi|439719|dbj|BAA05100.1| arginine kinase [Haliotis madaka]
3	4	NaN	236 733 Spectrin alpha chain [Crassostrea gigas]
2	2	NaN	209 781 minus strand high mobility group protein 1 [Biomphalaria glabrata]; 277 771 high mobility group protein 1 [
7	12	NaN	390 2267 neurofilament protein NF70 [Helix aspersa]; 318 2195 neurofilament protein NF70 [Helix aspersa]
2	3	NaN	101 1693 CALNUC [Spodoptera frugiperda]; 141 1325 Nucleobindin-2 [Crassostrea gigas]
2	2	NaN	" 28 1104 minus strand Pyruvate dehydrogenase E1 component subunit beta, mitochondrial [Crassostrea gigas]"
4	5	NaN	125 754 minus strand peptidyl prolyl cis-trans isomerase B [Conus novaehollandiae]
5	21	NaN	89 838 Putative phosphoglycerate mutase [Crassostrea gigas]; 89 838 Putative phosphoglycerate mutase [Crassost
3	4	NaN	85 633 hypothetical protein [Amblyomma maculatum]
2	3	NaN	338 661 Spectrin alpha chain [Crassostrea gigas]
4	17	NaN	" 64 1332 Flotillin-1, putative [Pediculus humanus corporis] gi"
2	2	NaN	91 2658 minus strand puromycin-sensitive aminopeptidase [Saccoglossus kowalevskii]
3	5	NaN	112 1050 Enolase-phosphatase E1 [Crassostrea gigas]; 112 1050 Enolase-phosphatase E1 [Crassostrea gigas]
3	27	NaN	" 926 1189 minus strand Myosin heavy chain, striated muscle [Crassostrea gigas]"
7	23	NaN	181 744 unknown [Dendroctonus ponderosae]
2	2	NaN	" 492 3473 PREDICTED: 2-oxoglutarate dehydrogenase, mitochondrial isoform 1 [Sarcophilus harrisii]; 492 3458 PR
4	17	NaN	2 256 Polyubiquitin (Fragment) OS=Xenopus laevis PE=1 SV=2; 20 472 minus strand large subunit ribosomal protein
4	6	NaN	582 2135 sulfatase 1A precursor [Haliotis discus discus]
6	17	NaN	87 1454 hypothetical protein BRAFLDRAFT_287788 [Branchiostoma floridae] gi; 117 1469 hypothetical protein BRAI
4	12	NaN	180 545 calreticulin precursor [Aplysia californica] gi
6	12	NaN	622 2733 PREDICTED: protein-glutamine gamma-glutamyltransferase K isoform 1 [Danio rerio]; 423 2498 PREDICTE
2	2	NaN	1 252 Aquaporin-4 [Crassostrea gigas]
4	12	NaN	42 296 minus strand twitchin [Mytilus galloprovincialis]
2	3	NaN	160 1218 Galectin-9 [Crassostrea gigas]
2	3	NaN	112 930 calcineurin A [Haliotis discus discus]
4	10	NaN	11 1462 coronin [Schistosoma mansoni]
2	3	NaN	" 34 1656 Chaperonin containing TCP1, subunit 7 (eta) [Danio rerio] gi; 34 1656 Chaperonin containing TCP1, subun
5	5	NaN	313 2763 NCAM-related cell adhesion molecule precursor [Aplysia californica] gi
2	2	NaN	113 1402 minus strand S-adenosylhomocysteine hydrolase [Crassostrea ariakensis]; 113 1402 minus strand S-aden
4	134	NaN	3 263 minus strand beta-actin [Urechis unicinctus]
3	11	NaN	811 1407 minus strand mitochondrial malate dehydrogenase 2 [Eospalax baileyi]
2	4	NaN	2302 2547 minus strand hemocyanin isoform 1 [Haliotis diversicolor]; 816 1211 H2 [Haliotis tuberculata]
2	6	NaN	4 579 minus strand Collagen alpha-3(VI) chain [Crassostrea gigas]
12	32	NaN	" 213 1499 minus strand Citrate synthase, mitochondrial [Crassostrea gigas]"
3	8	NaN	459 755 minus strand heat shock protein beta-1 [Trichinella spiralis] gi
7	16	NaN	27 377 minus strand ribosomal protein LP2 [Papilio xuthus] gi; 27 377 minus strand ribosomal protein LP2 [Papilio x
7	12	NaN	151 1449 rab GDP-dissociation inhibitor [Branchiostoma belcheri]
3	9	NaN	210 779 GH20056 [Drosophila grimshawi] gi; 216 785 minus strand GH20056 [Drosophila grimshawi] gi; 210 779 GH2
2	3	NaN	" 242 1504 Glutamate receptor, ionotropic kainate 3 [Crassostrea gigas]; 239 1501 Glutamate receptor, ionotropic ka
2	2	NaN	57 2378 minus strand Heterogeneous nuclear ribonucleoprotein U-like protein 1 [Crassostrea gigas]
3	3	NaN	794 994 Glycogenin-1 [Crassostrea gigas]; 1425 1739 minus strand Glycogenin-1 [Crassostrea gigas]
3	4	NaN	" 1144 1956 minus strand Retinol dehydrogenase 12, partial [Crassostrea gigas]"
2	3	NaN	18 1613 minus strand chaperonin containing T-complex polypeptide subunit zeta [Haliotis discus hannai]; 527 2089
2	4	NaN	816 1211 H2 [Haliotis tuberculata]
2	12	NaN	764 1015 minus strand heat shock protein beta-1 [Trichinella spiralis] gi
2	2	NaN	170 460 Spectrin beta chain [Crassostrea gigas]
4	5	NaN	913 1560 minus strand Calcium-dependent protein kinase isoform 2 [Crassostrea gigas]
4	12	NaN	300 878 unknown [Schistosoma japonicum]; 567 1145 unknown [Schistosoma japonicum]; 1110 1688 unknown [Sch
2	2	NaN	145 1524 PREDICTED: aspartyl aminopeptidase-like [Oreochromis niloticus]
2	2	NaN	183 953 minus strand glutathione-S-transferase [Haliotis diversicolor supertexta]; 275 1045 minus strand glutathion
2	8	NaN	426 896 PREDICTED: calmodulin-like [Metaseiulus occidentalis]
2	5	NaN	7948 8541 Collagen alpha-3(VI) chain [Crassostrea gigas]
2	8	NaN	99 3011 Eukaryotic translation initiation factor 3 subunit A [Crassostrea gigas]

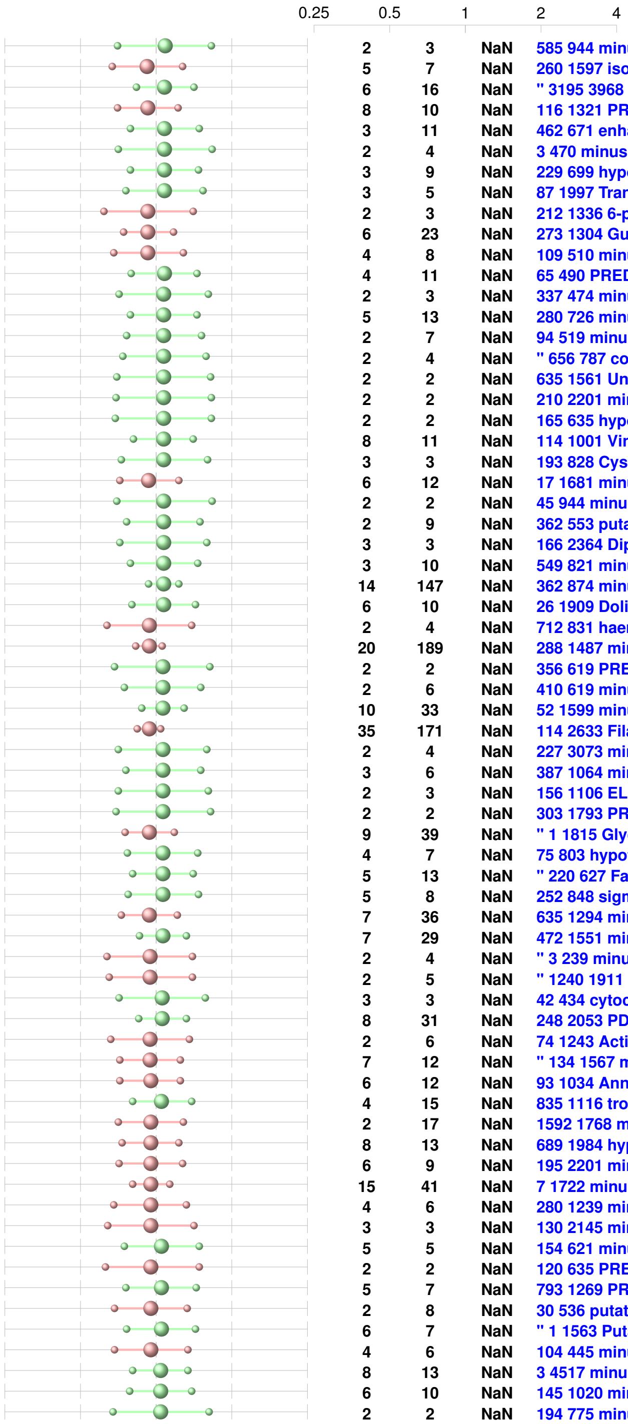

Peptides	Spectra	Coverage	Protein
2	3	NaN	585 944 minus strand Protein disulfide-isomerase A6 [Crassostrea gigas]
5	7	NaN	260 1597 isocitrate dehydrogenase [Mytilus galloprovincialis]; 260 1597 isocitrate dehydrogenase [Mytilus galloprov
6	16	NaN	" 3195 3968 Spectrin alpha chain, putative [Pediculus humanus corporis] gi"
8	10	NaN	116 1321 PREDICTED: similar to aspartate aminotransferase [Tribolium castaneum] gi
3	11	NaN	462 671 enhancer protein [Homo sapiens]
2	4	NaN	3 470 minus strand Spectrin beta chain [Crassostrea gigas]; 5 451 minus strand Spectrin beta chain [Crassostrea gi
3	9	NaN	229 699 hypothetical protein CGI_10017744 [Crassostrea gigas]; 199 669 hypothetical protein CGI_10017744 [Crasso
3	5	NaN	87 1997 Transketolase-like protein 2 [Crassostrea gigas]; 87 1997 Transketolase-like protein 2 [Crassostrea gigas]; 8
2	3	NaN	212 1336 6-phosphofructokinase [Crassostrea gigas]
6	23	NaN	273 1304 Guanine nucleotide-binding protein subunit beta [Crassostrea gigas]
4	8	NaN	109 510 minus strand hypothetical protein DAPPUDRAFT_231281 [Daphnia pulex]
4	11	NaN	65 490 PREDICTED: cofilin-2 [Otolemur garnettii]
2	3	NaN	337 474 minus strand heat shock protein 22 isoform 1 [Ruditapes philippinarum]
5	13	NaN	280 726 minus strand RecName: Full=Calmodulin; Short=CaM gi|14588595|dbj|BAB61794.1| calmodulin [Metridium
2	7	NaN	94 519 minus strand hypothetical protein BRAFLDRAFT_115029 [Branchiostoma floridae] gi
2	4	NaN	" 656 787 collagen alpha-1(XII) chain-like; K08132 collagen, type XII, alpha"
2	2	NaN	635 1561 Uncharacterized protein yfeX [Crassostrea gigas]
2	2	NaN	210 2201 minus strand Glycyl-tRNA synthetase [Crassostrea gigas]; 210 2201 minus strand Glycyl-tRNA synthetase
2	2	NaN	165 635 hypothetical protein CGI_10007639 [Crassostrea gigas]
8	11	NaN	114 1001 Vinculin [Crassostrea gigas]
3	3	NaN	193 828 Cysteine-rich secretory protein LCCL domain-containing 2 [Crassostrea gigas]
6	12	NaN	17 1681 minus strand Glucose-6-phosphate isomerase [Crassostrea gigas]
2	2	NaN	45 944 minus strand unnamed protein product [Mus musculus] gi; 5 334 minus strand receptor of activated protein k
2	9	NaN	362 553 putative RNA-binding protein [Haliotis diversicolor]
3	3	NaN	166 2364 Dipeptidyl-peptidase 3 [Crassostrea gigas]
3	10	NaN	549 821 minus strand Calponin-2 [Crassostrea gigas]
14	147	NaN	362 874 minus strand Transgelin-2 [Crassostrea gigas]
6	10	NaN	26 1909 Dolichyl-diphosphooligosaccharide–protein glycosyltransferase subunit 2 [Crassostrea gigas]
2	4	NaN	712 831 haemocyanin [Aplysia californica]
20	189	NaN	288 1487 minus strand putative procollagen type VI alpha 4 [Haliotis diversicolor]
2	2	NaN	356 619 PREDICTED: hypothetical protein LOC100698726 [Oreochromis niloticus]
2	6	NaN	410 619 minus strand Guanine nucleotide-binding protein subunit gamma-1 [Crassostrea gigas]
10	33	NaN	52 1599 minus strand ATP synthase beta subunit [Pinctada fucata]
35	171	NaN	114 2633 Filamin-A [Crassostrea gigas]; 135 2654 Filamin-A [Crassostrea gigas]
2	4	NaN	227 3073 minus strand Lysosomal alpha-mannosidase [Crassostrea gigas]
3	6	NaN	387 1064 minus strand PREDICTED: hypothetical protein [Hydra magnipapillata]; 275 952 minus strand PREDICTED
2	3	NaN	156 1106 ELAV 2-like protein [Aplysia californica] gi
2	2	NaN	303 1793 PREDICTED: matrix metalloproteinase 19 isoform rasi-1 preproprotein-like [Saccoglossus kowalevskii]
9	39	NaN	" 1 1815 Glycogen phosphorylase, muscle form [Crassostrea gigas]"
4	7	NaN	75 803 hypothetical protein DAPPUDRAFT_231350 [Daphnia pulex]
5	13	NaN	" 220 627 Fatty acid-binding protein, brain [Crassostrea gigas]"
5	8	NaN	252 848 sigma class glutathione-s-transferase 2 [Haliotis discus discus]
7	36	NaN	635 1294 minus strand Kyphoscoliosis peptidase [Crassostrea gigas]; 643 1302 minus strand Kyphoscoliosis peptic
7	29	NaN	472 1551 minus strand hypothetical protein [Amblyomma maculatum]
2	4	NaN	" 3 239 minus strand laminin beta 1 chain, putative [Ixodes scapularis] gi"
2	5	NaN	" 1240 1911 PREDICTED: ATP synthase subunit alpha, mitochondrial-like [Anolis carolinensis]"
3	3	NaN	42 434 cytochrome b5 [Haliotis discus discus]; 42 434 cytochrome b5 [Haliotis discus discus]
8	31	NaN	248 2053 PDZ and LIM domain protein 5 [Crassostrea gigas]
2	6	NaN	74 1243 Actin-related protein 2 [Crassostrea gigas]
7	12	NaN	" 134 1567 minus strand 6-phosphogluconate dehydrogenase, decarboxylating [Crassostrea gigas]; 134 1573 minus
6	12	NaN	93 1034 Annexin A7 [Crassostrea gigas]; 73 1014 Annexin A7 [Crassostrea gigas]
4	15	NaN	835 1116 tropomyosin [Ilyanassa obsoleta]
2	17	NaN	1592 1768 minus strand beta-actin [Diabolocatantops pinguis]
8	13	NaN	689 1984 hypothetical protein BRAFLDRAFT_78257 [Branchiostoma floridae] gi
6	9	NaN	195 2201 minus strand hypothetical protein DAPPUDRAFT_315656 [Daphnia pulex]
15	41	NaN	7 1722 minus strand Membrane metallo-endopeptidase-like 1 OS=Mus musculus GN=Mmel1 PE=1 SV=1
4	6	NaN	280 1239 minus strand PREDICTED: smoothelin isoform 2 [Oryctolagus cuniculus]; 280 1239 minus strand PREDICT
3	3	NaN	130 2145 minus strand Glucosamine–fructose-6-phosphate aminotransferase [isomerizing] 1 [Crassostrea gigas]; 1
5	5	NaN	154 621 minus strand Stress response protein nhaX [Crassostrea gigas]
2	2	NaN	120 635 PREDICTED: similar to collagen type VI alpha 6 [Ciona intestinalis]; 120 635 minus strand PREDICTED: simi
5	7	NaN	793 1269 PREDICTED: calmodulin-like [Saccoglossus kowalevskii]
2	8	NaN	30 536 putative mitochondrial ATP synthase [Haliotis discus discus]; 27 620 putative mitochondrial ATP synthase [H
6	7	NaN	" 1 1563 Putative methylmalonate-semialdehyde dehydrogenase [acylating], mitochondrial [Crassostrea gigas]"
4	6	NaN	104 445 minus strand macrophage migration inhibitory factor [Ascaris suum]
8	13	NaN	3 4517 minus strand Clathrin heavy chain 1 [Crassostrea gigas]
6	10	NaN	145 1020 minus strand PREDICTED: inorganic pyrophosphatase-like [Saccoglossus kowalevskii]
2	2	NaN	194 775 minus strand Collagen alpha-1(XV) chain [Harpegnathos saltator]

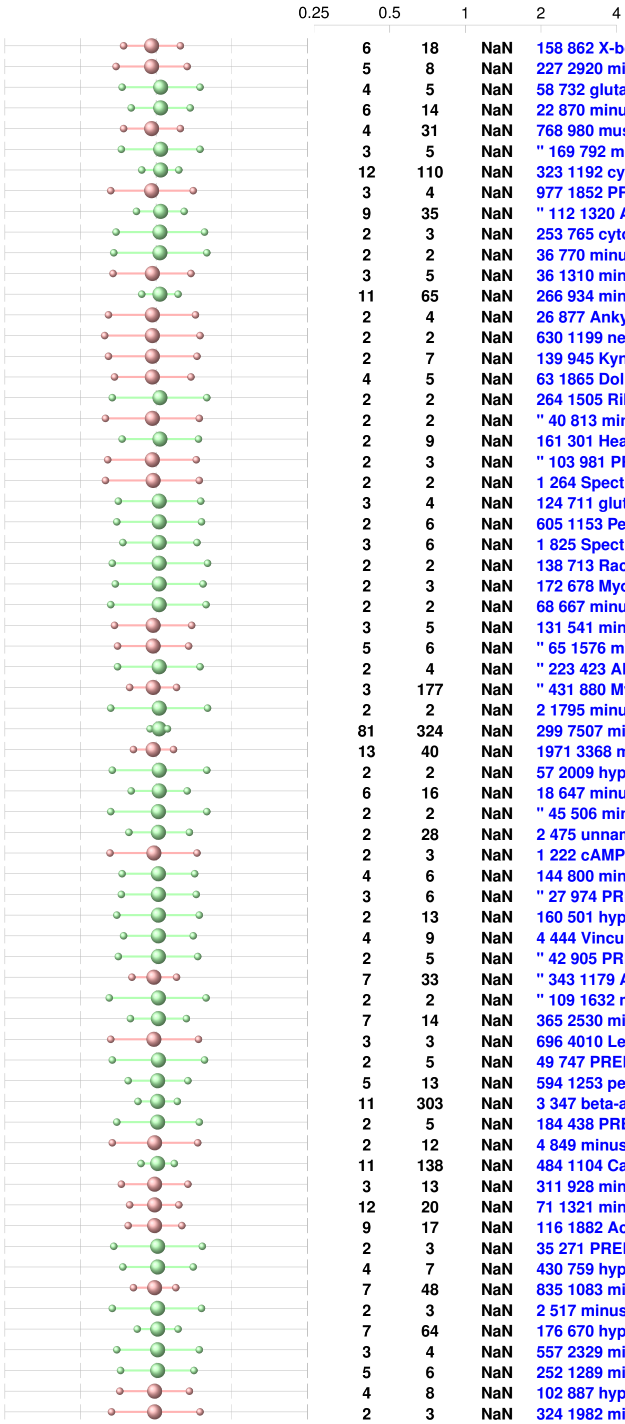

Peptides	Spectra	Coverage	Protein
6	18	NaN	158 862 X-box binding protein [Haliotis discus discus]
5	8	NaN	227 2920 minus strand Lysosomal alpha-mannosidase [Crassostrea gigas]; 227 3073 minus strand Lysosomal alpha
4	5	NaN	58 732 glutathione-s-transferase [Haliotis discus discus]
6	14	NaN	22 870 minus strand PREDICTED: alcohol dehydrogenase class-3 [Sarcophilus harrisii]
4	31	NaN	768 980 muscle LIM protein isoform 1 [Danaus plexippus]
3	5	NaN	" 169 792 minus strand rho GDP dissociation inhibitor, putative [Ixodes scapularis] gi"
12	110	NaN	323 1192 cytosolic malate dehydrogenase [Lottia scabra]; 323 1192 cytosolic malate dehydrogenase [Lottia scabra];
3	4	NaN	977 1852 PREDICTED: similar to prohibitin [Tribolium castaneum] gi
9	35	NaN	" 112 1320 Aspartate aminotransferase, cytoplasmic [Crassostrea gigas]"
2	3	NaN	253 765 cytochrome c oxidase subunit IV [Urechis caupo]
2	2	NaN	36 770 minus strand Endoplasmic reticulum protein ERp29 [Crassostrea gigas]
3	5	NaN	36 1310 minus strand elongation factor-1 gamma [Diguetia canities]
11	65	NaN	266 934 minus strand Kyphoscoliosis peptidase [Crassostrea gigas]
2	4	NaN	26 877 Ankyrin-2 [Crassostrea gigas]
2	2	NaN	630 1199 neuronal calcium sensor-1 [Lymnaea stagnalis]
2	7	NaN	139 945 Kynurenine formamidase [Crassostrea gigas]; 145 951 Kynurenine formamidase [Crassostrea gigas]; 220 10
4	5	NaN	63 1865 Dolichyl-diphosphooligosaccharide–protein glycosyltransferase subunit 1 [Crassostrea gigas]; 63 1865 Dol
2	2	NaN	264 1505 Ribosome-binding protein 1 [Crassostrea gigas]; 260 1501 Ribosome-binding protein 1 [Crassostrea gigas
2	2	NaN	" 40 813 minus strand NADH dehydrogenase [ubiquinone] iron-sulfur protein 3, mitochondrial [Crassostrea gigas]"
2	9	NaN	161 301 Heat shock protein beta-6 OS=Mus musculus GN=Hspb6 PE=1 SV=1; 167 310 Heat shock protein beta-1 OS=
2	3	NaN	" 103 981 PREDICTED: ATP synthase, H+ transporting, mitochondrial F1 complex, gamma subunit isoform H (heart)
2	2	NaN	1 264 Spectrin beta chain [Crassostrea gigas]
3	4	NaN	124 711 glutathione S-transferase sigma 3 [Mytilus galloprovincialis]; 124 711 glutathione S-transferase sigma 3 [My
2	6	NaN	605 1153 Peptidyl-prolyl cis-trans isomerase B [Crassostrea gigas]; 1046 1630 Peptidyl-prolyl cis-trans isomerase B
3	6	NaN	1 825 Spectrin alpha chain [Crassostrea gigas]
2	2	NaN	138 713 Rac [Aplysia californica] gi; 125 691 Rac [Aplysia californica] gi; 149 871 minus strand Rac [Aplysia californ
2	3	NaN	172 678 Myosin regulatory light chain sqh [Crassostrea gigas]
2	2	NaN	68 667 minus strand hypothetical protein BRAFLDRAFT_272416 [Branchiostoma floridae] gi; 68 667 minus strand hy
3	5	NaN	131 541 minus strand nervous system adducin [Aplysia californica] gi
5	6	NaN	" 65 1576 minus strand Aldehyde dehydrogenase, mitochondrial [Crassostrea gigas]"
2	4	NaN	" 223 423 ADPATP carrier protein; K05863 solute carrier family 25 (mitochondrial adenine nucleotide translocator), m
3	177	NaN	" 431 880 Myosin essential light chain, striated adductor muscle OS=Aequipecten irradians PE=1 SV=2"
2	2	NaN	2 1795 minus strand Plectin-1 [Crassostrea gigas]
81	324	NaN	299 7507 minus strand filamin [Clonorchis sinensis]
13	40	NaN	1971 3368 minus strand Kyphoscoliosis peptidase [Crassostrea gigas]; 1767 3158 minus strand hillarin [Hirudo med
2	2	NaN	57 2009 hypothetical protein BRAFLDRAFT_285279 [Branchiostoma floridae] gi; 3 1973 hypothetical protein BRAFL
6	16	NaN	18 647 minus strand ATP synthase-like protein [Biomphalaria glabrata]
2	2	NaN	" 45 506 minus strand PREDICTED: dihydrolipoyl dehydrogenase, mitochondrial [Monodelphis domestica]"
2	28	NaN	2 475 unnamed protein product [Tetraodon nigroviridis]
2	3	NaN	1 222 cAMP-dependent protein kinase regulatory subunit [Aplysia californica] gi
4	6	NaN	144 800 minus strand PREDICTED: protein F37C4.5-like [Amphimedon queenslandica]
3	6	NaN	" 27 974 PREDICTED: succinyl-CoA ligase [GDP-forming] subunit alpha, mitochondrial-like [Oreochromis niloticus];
2	13	NaN	160 501 hypothetical protein CGI_10001640 [Crassostrea gigas]; 160 588 hypothetical protein CGI_10001640 [Crasso
4	9	NaN	4 444 Vinculin [Crassostrea gigas]
2	5	NaN	" 42 905 PREDICTED: enoyl-CoA hydratase, mitochondrial-like isoform 1 [Oreochromis niloticus]"
7	33	NaN	" 343 1179 ATP synthase subunit alpha, mitochondrial OS=Pongo abelii GN=ATP5A1 PE=2 SV=1"
2	2	NaN	" 109 1632 minus strand Seryl-tRNA synthetase, cytoplasmic [Crassostrea gigas]; 109 1614 minus strand Seryl-tRNA
7	14	NaN	365 2530 minus strand calpain B [Gecarcinus lateralis]
3	3	NaN	696 4010 Lethal(2) giant larvae-like protein 1 [Crassostrea gigas]; 657 3971 minus strand Lethal(2) giant larvae-like p
2	5	NaN	49 747 PREDICTED: elongation factor 1-delta-like [Strongylocentrotus purpuratus]; 49 819 PREDICTED: elongation f
5	13	NaN	594 1253 peroxiredoxin 6 [Eriocheir sinensis]
11	303	NaN	3 347 beta-actin [Meretrix meretrix]; 3 215 Actin-2 OS=Onchocerca volvulus GN=act-2b PE=3 SV=1
2	5	NaN	184 438 PREDICTED: similar to CG14235 CG14235-PA [Tribolium castaneum] gi
2	12	NaN	4 849 minus strand elongation factor 1-alpha [Cryptosporidium muris RN66]
11	138	NaN	484 1104 Calponin-2 [Crassostrea gigas]; 309 938 Calponin-2 [Crassostrea gigas]
3	13	NaN	311 928 minus strand peroxiredoxin 3 [Clonorchis sinensis] gi; 288 905 minus strand peroxiredoxin 3 [Clonorchis sin
12	20	NaN	71 1321 minus strand Vinculin [Crassostrea gigas]
9	17	NaN	116 1882 Actin-interacting protein 1 [Crassostrea gigas]
2	3	NaN	35 271 PREDICTED: SH3 domain-binding glutamic acid-rich-like protein 3-like [Anolis carolinensis]; 35 271 PREDICT
4	7	NaN	430 759 hypothetical protein [Amblyomma maculatum]; 336 665 hypothetical protein [Amblyomma maculatum]
7	48	NaN	835 1083 minus strand Calmodulin [Crassostrea gigas]
2	3	NaN	2 517 minus strand Anhydro-N-acetylmuramic acid kinase [Crassostrea gigas]
7	64	NaN	176 670 hypothetical protein BRAFLDRAFT_122621 [Branchiostoma floridae] gi
3	4	NaN	557 2329 minus strand Collagen alpha-3(VI) chain [Crassostrea gigas]
5	6	NaN	252 1289 minus strand PDZ and LIM domain protein 3 OS=Gallus gallus GN=PDLIM3 PE=1 SV=1
4	8	NaN	102 887 hypothetical protein DAPPUDRAFT_304712 [Daphnia pulex]
2	3	NaN	324 1982 minus strand Pikachurin [Crassostrea gigas]

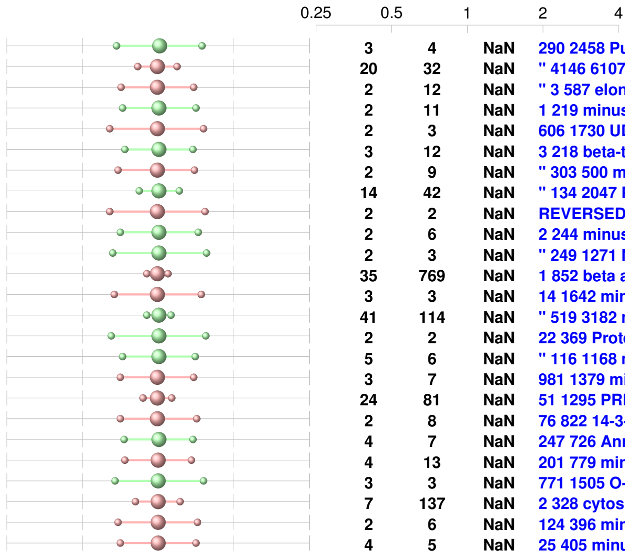

Peptides	Spectra	Coverage	Protein
3	4	NaN	290 2458 Putative beta-D-xylosidase 5 [Crassostrea gigas]
20	32	NaN	" 4146 6107 minus strand nonmuscle myosin II, partial [Aplysia californica]"
2	12	NaN	" 3 587 elongation factor 1 alpha, partial [Phorbas sp. nov. SB-2012]"
2	11	NaN	1 219 minus strand PREDICTED: nucleoside diphosphate kinase 3-like [Pan troglodytes]
2	3	NaN	606 1730 UDP-glucuronosyltransferase 2C1 precursor [Oryctolagus cuniculus]
3	12	NaN	3 218 beta-tubulin [Fasciola hepatica] gi
2	9	NaN	" 303 500 minus strand protein lethal, putative [Pediculus humanus corporis] gi"
14	42	NaN	" 134 2047 PREDICTED: succinate dehydrogenase [ubiquinone] flavoprotein subunit, mitochondrial-like isoform 3, p
2	2	NaN	REVERSED 68 358 endonuclease-reverse transcriptase [Danaus plexippus]
2	6	NaN	2 244 minus strand hypothetical protein KGM_08771 [Danaus plexippus]
2	3	NaN	" 249 1271 N(G),N(G)-dimethylarginine dimethylaminohydrolase 1 [Crassostrea gigas]"
35	769	NaN	1 852 beta actin-like 1 protein [Macrostomum lignano]
3	3	NaN	14 1642 minus strand chaperonin containing tcp1 [Haliotis discus discus]
41	114	NaN	" 519 3182 minus strand Alpha-actinin, sarcomeric [Crassostrea gigas]"
2	2	NaN	22 369 Protein farnesyltransferase/geranylgeranyltransferase type-1 subunit alpha [Crassostrea gigas]
5	6	NaN	" 116 1168 minus strand hypothetical protein CGI_10014553, partial [Crassostrea gigas]"
3	7	NaN	981 1379 minus strand Multimerin-1 [Crassostrea gigas]; 998 1396 Multimerin-1 [Crassostrea gigas]; 975 1373 Multir
24	81	NaN	51 1295 PREDICTED: phosphoglycerate kinase-like [Nasonia vitripennis]; 97 1341 PREDICTED: phosphoglycerate k
2	8	NaN	76 822 14-3-3 protein [Heterodera glycines]; 76 822 minus strand 14-3-3 protein [Heterodera glycines]
4	7	NaN	247 726 Annexin A7 [Crassostrea gigas]; 286 675 Annexin A7 [Crassostrea gigas]
4	13	NaN	201 779 minus strand sigma class glutathione-s-transferase 2 [Haliotis discus discus]
3	3	NaN	771 1505 O-methyltransferase family 3 [Dicentrarchus labrax]
7	137	NaN	2 328 cytoskeletal actin 2 [Molgula oculata]
2	6	NaN	124 396 minus strand 40S ribosomal protein S10 OS=Lumbricus rubellus GN=RPS10 PE=2 SV=1
4	5	NaN	25 405 minus strand Elongation factor 1-delta [Anoplopoma fimbria]

4.2 Proteins Identified by a Single Peptide

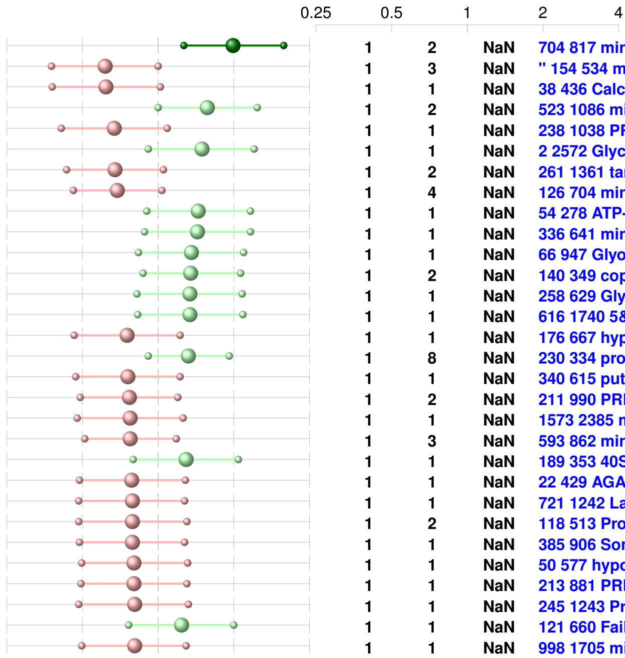

Peptides	Spectra	Coverage	Protein
1	2	NaN	704 817 minus strand expressed hypothetical protein [Trichoplax adhaerens] gi; 688 801 minus strand Lactoylglutat
1	3	NaN	" 154 534 minus strand Muscle M-line assembly protein unc-89, partial [Crassostrea gigas]"
1	1	NaN	38 436 Calcium-dependent protein kinase isoform 2 [Crassostrea gigas]
1	2	NaN	523 1086 minus strand juvenile hormone diol kinase [Danaus plexippus]
1	1	NaN	238 1038 PREDICTED: hypothetical protein [Saccoglossus kowalevskii]; 294 992 PREDICTED: hypothetical protein [
1	1	NaN	2 2572 Glycogen debranching enzyme [Crassostrea gigas]
1	2	NaN	261 1361 tandem-repeat galectin [Pinctada fucata]; 261 1361 tandem-repeat galectin [Pinctada fucata]
1	4	NaN	126 704 minus strand ribosomal protein L12 [Ixodes scapularis]
1	1	NaN	54 278 ATP-citrate synthase [Crassostrea gigas]; 61 465 ATP-citrate synthase [Crassostrea gigas]
1	1	NaN	336 641 minus strand malate dehydrogenase [Schistosoma japonicum]
1	1	NaN	66 947 Glyoxalase domain-containing protein 4 [Crassostrea gigas]
1	2	NaN	140 349 copper chaperone Atox1 [Crassostrea ariakensis]
1	1	NaN	258 629 Glycogenin-1 OS=Oryctolagus cuniculus GN=GYG1 PE=1 SV=3
1	1	NaN	616 1740 5'-AMP-activated protein kinase subunit beta-2 [Crassostrea gigas]
1	1	NaN	176 667 hypothetical protein CGI_10018452 [Crassostrea gigas]; 176 634 hypothetical protein CGI_10018452 [Crasso
1	8	NaN	230 334 protein disulfide isomerase [Conus virgo]
1	1	NaN	340 615 putative Low-density lipoprotein receptor repeat class B [Trichinella spiralis] gi
1	2	NaN	211 990 PREDICTED: gamma-glutamyl hydrolase-like [Xenopus (Silurana) tropicalis]; 223 1080 PREDICTED: gamma
1	1	NaN	1573 2385 minus strand Spectrin beta chain [Crassostrea gigas]
1	3	NaN	593 862 minus strand mitochondrial ATP synthase coupling factor 6 precursor [Ochlerotatus taeniorhynchus]
1	1	NaN	189 353 40S ribosomal protein S12 [Crassostrea gigas]
1	1	NaN	22 429 AGAP006937-PA [Anopheles gambiae str. PEST] gi; 1 474 Ubiquitin-conjugating enzyme E2 variant 2 OS=Rat
1	1	NaN	721 1242 Lactoylglutathione lyase [Crassostrea gigas]; 882 1334 Lactoylglutathione lyase OS=Rattus norvegicus GN
1	2	NaN	118 513 Protein CAL-5 [Caenorhabditis elegans] gi
1	1	NaN	385 906 Soma ferritin OS=Lymnaea stagnalis PE=2 SV=2
1	1	NaN	50 577 hypothetical protein BRAFLDRAFT_119799 [Branchiostoma floridae] gi; 50 577 hypothetical protein BRAFLD
1	1	NaN	213 881 PREDICTED: hypothetical protein [Saccoglossus kowalevskii]; 260 928 PREDICTED: hypothetical protein [S
1	1	NaN	245 1243 Prostaglandin reductase 1 [Crassostrea gigas]
1	1	NaN	121 660 Failed axon connections homolog OS=Rattus norvegicus GN=Faxc PE=3 SV=1
1	1	NaN	998 1705 minus strand Basement membrane-specific heparan sulfate proteoglycan core protein [Crassostrea gigas]

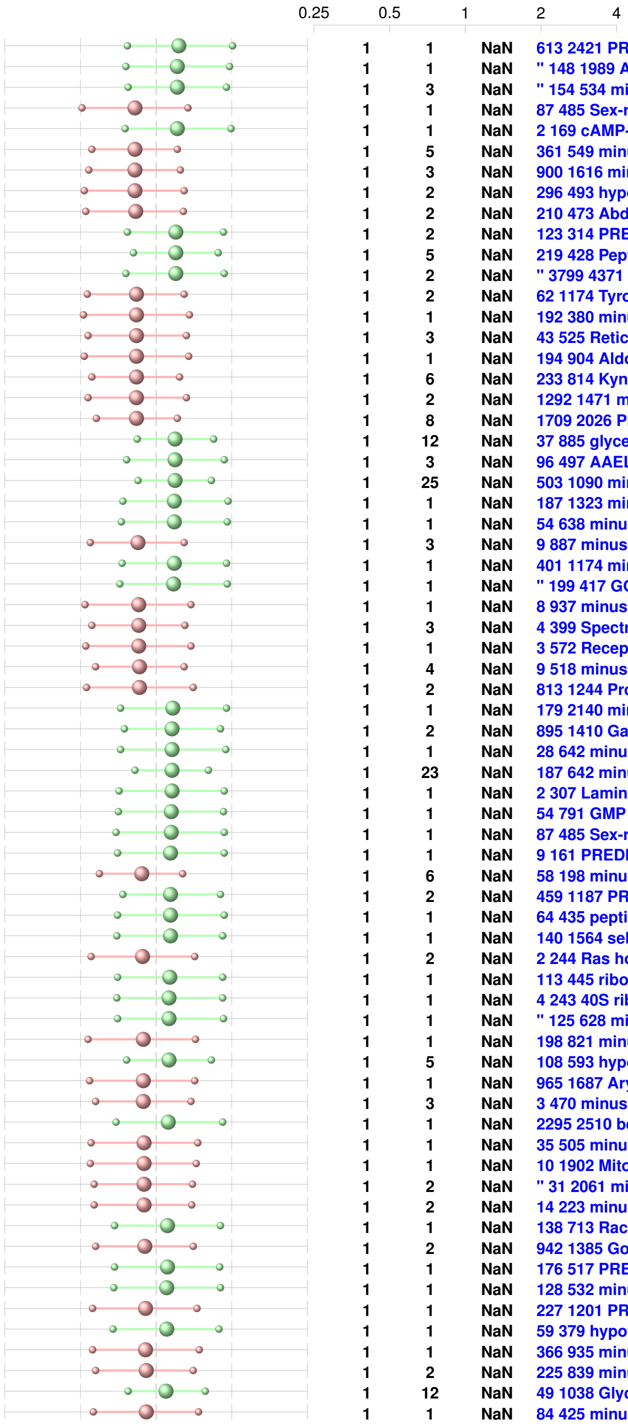

Peptides	Spectra	Coverage	Protein
1	1	NaN	613 2421 PREDICTED: lysosomal alpha-glucosidase [Danio rerio]
1	1	NaN	" 148 1989 Acyl-CoA synthetase family member 2, mitochondrial [Crassostrea gigas]; 146 1987 minus strand Acyl-C
1	3	NaN	" 154 534 minus strand Muscle M-line assembly protein unc-89, partial [Crassostrea gigas]; 259 690 minus strand M
1	1	NaN	87 485 Sex-regulated protein janus-A [Crassostrea gigas]
1	1	NaN	2 169 cAMP-dependent protein kinase regulatory subunit [Crassostrea gigas]; 125 511 cAMP-dependent protein kina
1	5	NaN	361 549 minus strand 32 kDa beta-galactoside-binding lectin [Ascaris suum]
1	3	NaN	900 1616 minus strand hemocyanin isoform 1 [Haliotis diversicolor]
1	2	NaN	296 493 hypothetical protein BRAFLDRAFT_66287 [Branchiostoma floridae] gi
1	2	NaN	210 473 Abdominal ganglion neuropeptides L5-67 OS=Aplysia californica PE=1 SV=1; 879 1124 abdominal ganglion
1	2	NaN	123 314 PREDICTED: ubiquinol-cytochrome c reductase hinge protein-like [Saccoglossus kowalevskii]; 279 530 PRE
1	5	NaN	219 428 Peptidyl-prolyl cis-trans isomerase OS=Hemicentrotus pulcherrimus PE=2 SV=1
1	2	NaN	" 3799 4371 papilin, partial [Rhipicephalus microplus]"
1	2	NaN	62 1174 Tyrosine-protein kinase CSK [Crassostrea gigas]; 62 1174 minus strand Tyrosine-protein kinase CSK [Crass
1	1	NaN	192 380 minus strand RAB protein [Haliotis discus discus]
1	3	NaN	43 525 Reticulon-4 [Crassostrea gigas]; 143 757 Reticulon-4 [Crassostrea gigas]; 43 564 Reticulon-4 [Crassostrea gi
1	1	NaN	194 904 Aldo-keto reductase family 1 member B10 [Crassostrea gigas]
1	6	NaN	233 814 Kynurenine formamidase [Crassostrea gigas]; 233 979 Kynurenine formamidase [Crassostrea gigas]; 139 9
1	2	NaN	1292 1471 minus strand hemocyanin isoform 1 [Haliotis diversicolor]
1	8	NaN	1709 2026 Phosphoenolpyruvate carboxykinase [GTP] [Crassostrea gigas]
1	12	NaN	37 885 glyceraldehyde 3-phosphate dehydrogenase [Dugesia japonica]
1	3	NaN	96 497 AAEL009772-PB [Aedes aegypti]
1	25	NaN	503 1090 minus strand myosin regulatory light chain [Fasciola hepatica]; 278 865 minus strand myosin regulatory lig
1	1	NaN	187 1323 minus strand gelsolin [Aplysia californica] gi
1	1	NaN	54 638 minus strand ubiquitin carboxyl-terminal hydrolase [Aplysia californica] gi
1	3	NaN	9 887 minus strand hypothetical protein CGI_10006272 [Crassostrea gigas]
1	1	NaN	401 1174 minus strand Mechanosensory protein 2 [Crassostrea gigas]
1	1	NaN	" 199 417 GG13156 [Drosophila erecta] gi; 253 501 minus strand NADH-ubiquinone oxidoreductase MLRQ subunit-li
1	1	NaN	8 937 minus strand galectin [Crassostrea virginica]
1	3	NaN	4 399 Spectrin beta chain [Crassostrea gigas]
1	1	NaN	3 572 Receptor-type tyrosine-protein phosphatase N2 [Crassostrea gigas]; 3 542 minus strand Receptor-type tyrosin
1	4	NaN	9 518 minus strand hemocyanin isoform 1 [Nucula nucleus]
1	2	NaN	813 1244 Probable protein disulfide-isomerase A6 OS=Caenorhabditis elegans GN=tag-320 PE=2 SV=1
1	1	NaN	179 2140 minus strand PREDICTED: hypothetical protein [Saccoglossus kowalevskii]
1	2	NaN	895 1410 Galectin-3 OS=Rattus norvegicus GN=Lgals3 PE=1 SV=4; 1067 1405 Galectin-9 [Crassostrea gigas]; 1068 1
1	1	NaN	28 642 minus strand Transmembrane emp24 domain-containing protein 9 [Crassostrea gigas]; 236 709 minus strand
1	23	NaN	187 642 minus strand PREDICTED: similar to pol-like protein [Tribolium castaneum]
1	1	NaN	2 307 Laminin subunit gamma-1 [Crassostrea gigas]
1	1	NaN	54 791 GMP reductase 2 [Crassostrea gigas]
1	1	NaN	87 485 Sex-regulated protein janus-A [Crassostrea gigas]; 92 310 PREDICTED: hypothetical protein [Saccoglossus k
1	1	NaN	9 161 PREDICTED: tubulin alpha-1C chain-like [Acyrthosiphon pisum]; 9 161 Tubulin alpha-3 chain (Fragments) OS=
1	6	NaN	58 198 minus strand Protein DJ-1 [Crassostrea gigas]
1	2	NaN	459 1187 PREDICTED: protein-L-isoaspartate(D-aspartate) O-methyltransferase-like isoform 2 [Anolis carolinensis]
1	1	NaN	64 435 peptidoglycan recognition protein S1L [Pomacea canaliculata]
1	1	NaN	140 1564 selenium-binding protein [Haliotis discus hannai]
1	2	NaN	2 244 Ras homolog gene family member A [Clonorchis sinensis]
1	1	NaN	113 445 ribosomal protein rps19 [Lineus viridis]
1	1	NaN	4 243 40S ribosomal protein S13 OS=Caenorhabditis elegans GN=rps-13 PE=2 SV=2; 4 243 40S ribosomal protein S1
1	1	NaN	" 125 628 minus strand collectin sub-family member 12, partial [Oncorhynchus mykiss]"
1	1	NaN	198 821 minus strand sigma class glutathione-s-transferase 2 [Haliotis discus discus]
1	5	NaN	108 593 hypothetical protein CGI_10025497 [Crassostrea gigas]
1	1	NaN	965 1687 Arylsulfatase B [Crassostrea gigas]
1	3	NaN	3 470 minus strand Spectrin beta chain [Crassostrea gigas]
1	1	NaN	2295 2510 beta-thymosin domain repeat protein CSP24KDa_v1 [Hermissenda crassicornis] gi
1	1	NaN	35 505 minus strand Basement membrane-specific heparan sulfate proteoglycan core protein [Crassostrea gigas]
1	1	NaN	10 1902 Mitochondrial inner membrane protein [Crassostrea gigas]; 10 1902 Mitochondrial inner membrane protein
1	2	NaN	" 31 2061 minus strand 1,4-alpha-glucan-branching enzyme [Crassostrea gigas]"
1	2	NaN	14 223 minus strand Endothelin-converting enzyme 2 OS=Homo sapiens GN=ECE2 PE=1 SV=4
1	1	NaN	138 713 Rac [Aplysia californica] gi; 101 676 Rac [Aplysia californica] gi
1	2	NaN	942 1385 Golgi-associated plant pathogenesis-related protein 1 [Crassostrea gigas]; 338 817 Golgi-associated plant
1	1	NaN	176 517 PREDICTED: elongin C-like [Saccoglossus kowalevskii]; 115 456 PREDICTED: elongin C-like [Saccoglossus
1	1	NaN	128 532 minus strand mitochondrial ATPase inhibitor [Ornithodoros parkeri]
1	1	NaN	227 1201 PREDICTED: quinone oxidoreductase-like isoform 1 [Ornithorhynchus anatinus]; 109 1083 PREDICTED: qu
1	1	NaN	59 379 hypothetical protein DAPPUDRAFT_230756 [Daphnia pulex]; 117 443 minus strand hypothetical protein DAPP
1	1	NaN	366 935 minus strand 40S ribosomal protein S2 OS=Urechis caupo GN=RPS2 PE=2 SV=1
1	2	NaN	225 839 minus strand Metallo-beta-lactamase domain-containing protein 1 [Crassostrea gigas]; 201 851 minus stran
1	12	NaN	49 1038 Glyceraldehyde-3-phosphate dehydrogenase [Crotalus adamanteus]
1	1	NaN	84 425 minus strand Transcription elongation factor B polypeptide 2 [Crassostrea gigas]; 84 425 minus strand Trans

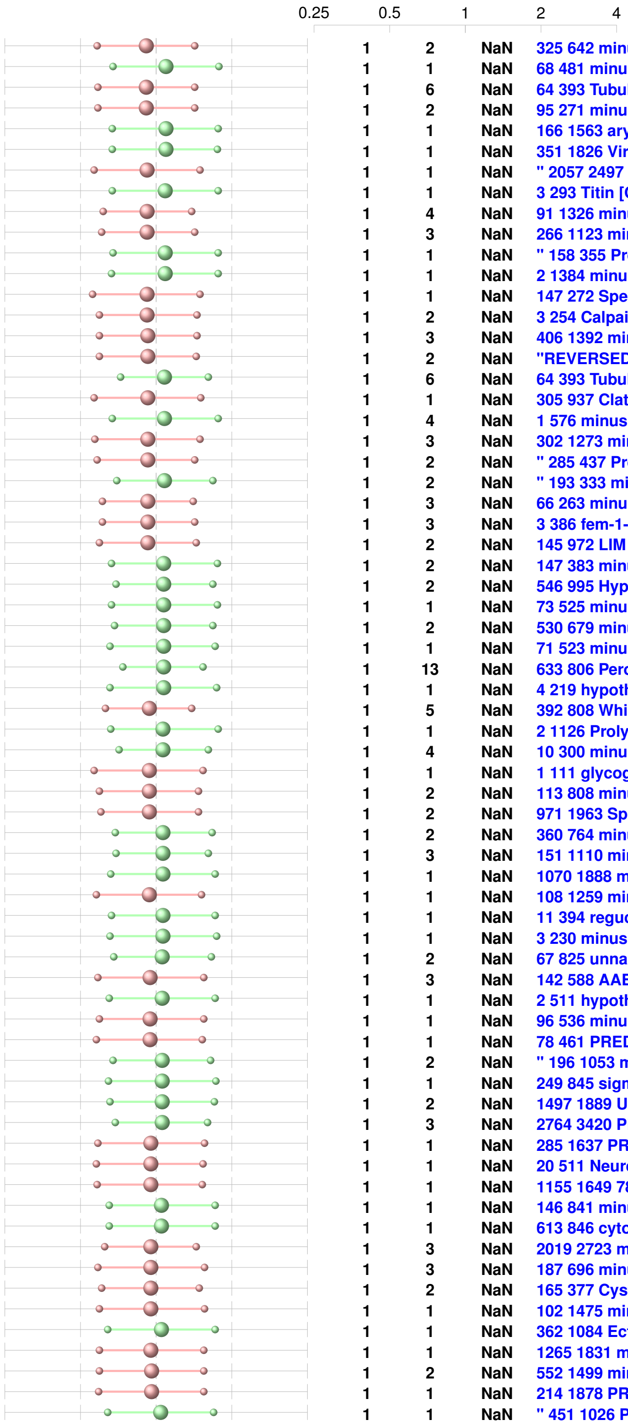

Peptides	Spectra	Coverage	Protein
1	2	NaN	325 642 minus strand Protein phosphatase 1B [Crassostrea gigas]
1	1	NaN	68 481 minus strand netrin receptor UNC5A [Danio rerio] gi
1	6	NaN	64 393 Tubulin beta chain OS=Paracentrotus lividus PE=2 SV=1; 111 398 Tubulin beta chain OS=Paracentrotus lividu
1	2	NaN	95 271 minus strand hypothetical protein EAI_01530 [Harpegnathos saltator]
1	1	NaN	166 1563 arylsulfatase [Haliotis discus discus]
1	1	NaN	351 1826 Virulence metalloprotease [Crassostrea gigas]
1	1	NaN	" 2057 2497 Phosphate carrier protein, mitochondrial [Crassostrea gigas]"
1	1	NaN	3 293 Titin [Crassostrea gigas]
1	4	NaN	91 1326 minus strand Adipocyte plasma membrane-associated protein [Crassostrea gigas]; 107 1273 minus strand A
1	3	NaN	266 1123 minus strand mitochondrial malate dehydrogenase precursor [Nucella lapillus]
1	1	NaN	" 158 355 Protein NAS-37, isoform b [Caenorhabditis elegans] gi; 15 227 Hemicentin-1 [Crassostrea gigas]"
1	1	NaN	2 1384 minus strand twitchin [Mytilus galloprovincialis]; 2 1384 minus strand twitchin [Mytilus galloprovincialis]; 2 1
1	1	NaN	147 272 Spectrin alpha chain [Crassostrea gigas]
1	2	NaN	3 254 Calpain-5 [Crassostrea gigas]; 3 254 minus strand Calpain-5 [Crassostrea gigas]
1	3	NaN	406 1392 minus strand hypothetical protein CGI_10007007 [Crassostrea gigas]
1	2	NaN	"REVERSED 2 1810 minus strand Glutaminyl-tRNA synthetase, partial [Crassostrea gigas]"
1	6	NaN	64 393 Tubulin beta chain OS=Paracentrotus lividus PE=2 SV=1; 109 438 Tubulin beta chain OS=Paracentrotus lividu
1	1	NaN	305 937 Clathrin light chain A [Crassostrea gigas]
1	4	NaN	1 576 minus strand Tubulin beta chain [Crassostrea gigas]
1	3	NaN	302 1273 minus strand hypothetical protein CGI_10007007 [Crassostrea gigas]
1	2	NaN	" 285 437 Propionyl-CoA carboxylase alpha chain, mitochondrial [Crassostrea gigas]"
1	2	NaN	" 193 333 minus strand RecName: Full=Globin; AltName: Full=Myoglobin gi|299274|gb|AAB25743.1| myoglobin [Na
1	3	NaN	66 263 minus strand twitchin [Mytilus galloprovincialis]; 66 263 minus strand twitchin [Mytilus galloprovincialis]; 66
1	3	NaN	3 386 fem-1-like protein C [Crassostrea gigas]
1	2	NaN	145 972 LIM and SH3 domain protein F42H10.3 OS=Caenorhabditis elegans GN=F42H10.3 PE=2 SV=3
1	2	NaN	147 383 minus strand Tubulin-specific chaperone A OS=Rattus norvegicus GN=Tbca PE=1 SV=1; 104 418 Tubulin-sp
1	2	NaN	546 995 Hypothetical protein CBG12002 [Caenorhabditis briggsae]; 543 995 minus strand PREDICTED: tubulin polyr
1	1	NaN	73 525 minus strand Actin-related protein 2/3 complex subunit 5-like protein [Salmo salar] gi; 73 525 Actin-related pr
1	2	NaN	530 679 minus strand GJ11201 [Drosophila virilis] gi
1	1	NaN	71 523 minus strand hypothetical protein DAPPUDRAFT_309332 [Daphnia pulex]
1	13	NaN	633 806 Peroxiredoxin-1 [Crassostrea gigas]; 648 821 Peroxiredoxin-1 [Crassostrea gigas]; 666 839 Peroxiredoxin-1
1	1	NaN	4 219 hypothetical protein BRAFLDRAFT_66351 [Branchiostoma floridae] gi; 3 239 hypothetical protein BRAFLDRAI
1	5	NaN	392 808 Whirlin [Crassostrea gigas]
1	1	NaN	2 1126 Prolyl endopeptidase [Crassostrea gigas]
1	4	NaN	10 300 minus strand unnamed protein product [Tetraodon nigroviridis]
1	1	NaN	1 111 glycogen synthase [Crassostrea gigas]
1	2	NaN	113 808 minus strand omega class glutathione-s-transferase [Chlamys farreri]; 335 1045 minus strand omega class
1	2	NaN	971 1963 Spectrin alpha chain [Crassostrea gigas]; 972 1979 Spectrin alpha chain [Crassostrea gigas]
1	2	NaN	360 764 minus strand Peptidyl-prolyl cis-trans isomerase A OS=Dictyostelium discoideum GN=ppiA PE=1 SV=1
1	3	NaN	151 1110 minus strand hypothetical protein DAPPUDRAFT_303085 [Daphnia pulex]
1	1	NaN	1070 1888 minus strand galectin [Pinctada fucata]; 1104 1922 minus strand galectin [Pinctada fucata]
1	1	NaN	108 1259 minus strand PREDICTED: Na(+)/H(+) exchange regulatory cofactor NHE-RF3-like [Metaseiulus occidentali
1	1	NaN	11 394 regucalcin [Salmo salar] gi
1	1	NaN	3 230 minus strand RH21460p [Drosophila melanogaster]; 3 230 minus strand RH21460p [Drosophila melanogaster]
1	2	NaN	67 825 unnamed protein product [Oikopleura dioica]
1	3	NaN	142 588 AAEL009772-PB [Aedes aegypti]; 96 497 AAEL009772-PB [Aedes aegypti]
1	1	NaN	2 511 hypothetical protein CGI_10006272 [Crassostrea gigas]
1	1	NaN	96 536 minus strand hypothetical protein BRAFLDRAFT_285353 [Branchiostoma floridae] gi
1	1	NaN	78 461 PREDICTED: hoi-polloi-like [Saccoglossus kowalevskii]; 103 492 NHP2-like protein 1 OS=Rattus norvegicus (
1	2	NaN	" 196 1053 minus strand Cytochrome b-c1 complex subunit 2, mitochondrial OS=Homo sapiens GN=UQCRC2 PE=1
1	1	NaN	249 845 sigma class glutathione-s-transferase 2 [Haliotis discus discus]; 252 848 sigma class glutathione-s-transfer
1	2	NaN	1497 1889 UDP-glucuronosyltransferase 2C1 (Fragment) OS=Oryctolagus cuniculus GN=UGT2C1 PE=2 SV=1
1	3	NaN	2764 3420 PREDICTED: filamin-C-like [Nasonia vitripennis]
1	1	NaN	285 1637 PREDICTED: talin-2-like [Cricetulus griseus]
1	1	NaN	20 511 Neurexin-2-alpha [Crassostrea gigas]; 20 511 Neurexin-2-alpha [Crassostrea gigas]
1	1	NaN	1155 1649 78 kDa glucose-regulated protein OS=Aplysia californica PE=1 SV=1
1	1	NaN	146 841 minus strand hypothetical protein CGI_10006609 [Crassostrea gigas]; 146 841 minus strand hypothetical pr
1	1	NaN	613 846 cytochrome c oxidase subunit Va [Antheraea yamamai] gi
1	3	NaN	2019 2723 minus strand Kyphoscoliosis peptidase [Crassostrea gigas]; 2060 2764 minus strand Kyphoscoliosis pep
1	3	NaN	187 696 minus strand putative ribosomal protein S10 [Sipunculus nudus]
1	2	NaN	165 377 Cysteine and glycine-rich protein 3 [Crassostrea gigas]; 135 344 Cysteine and glycine-rich protein 3 [Crasso
1	1	NaN	102 1475 minus strand Cytosolic non-specific dipeptidase [Crassostrea gigas]
1	1	NaN	362 1084 Ectoine hydroxylase [Crassostrea gigas]; 362 1084 Ectoine hydroxylase [Crassostrea gigas]; 366 1220 Ecto
1	1	NaN	1265 1831 minus strand Adenosine deaminase CECR1 [Crassostrea gigas]
1	2	NaN	552 1499 minus strand uncharacterized protein LOC100158466 [Xenopus (Silurana) tropicalis] gi; 552 1499 minus st
1	1	NaN	214 1878 PREDICTED: lysyl-tRNA synthetase-like [Oreochromis niloticus]
1	1	NaN	" 451 1026 PREDICTED: GTP-binding protein SAR1B, putative-like [Saccoglossus kowalevskii]"

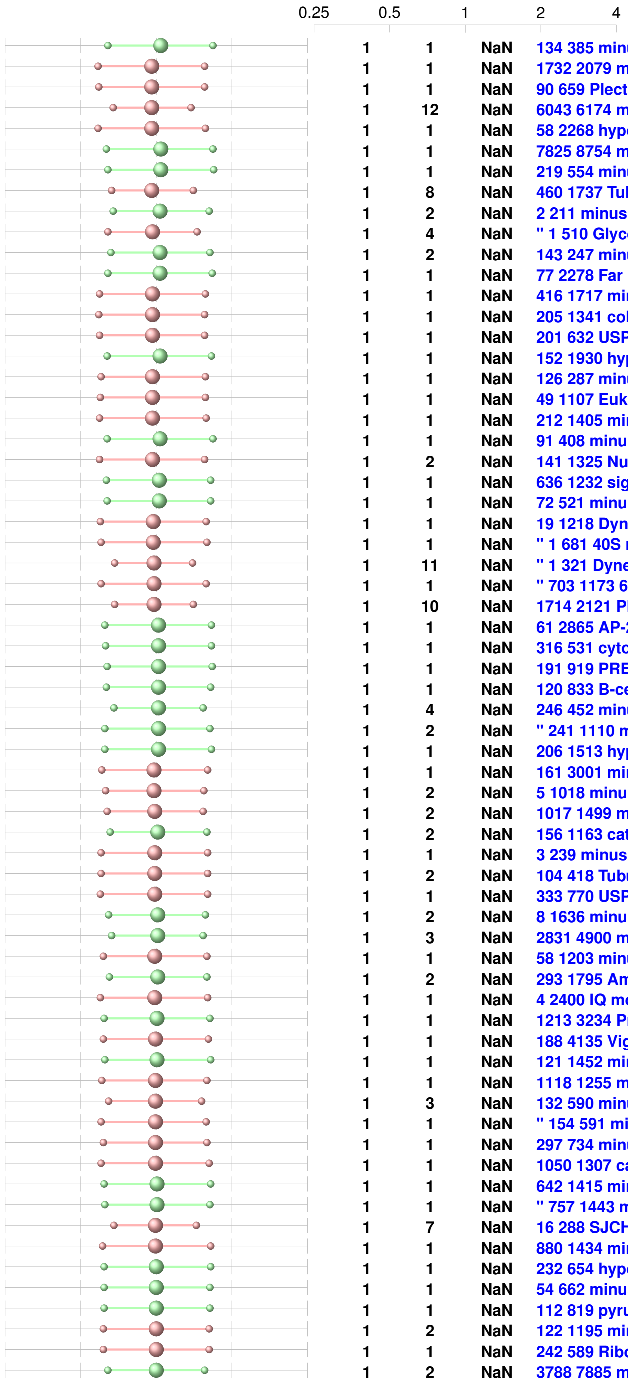

Peptides	Spectra	Coverage	Protein
1	1	NaN	134 385 minus strand CDGSH iron sulfur domain-containing protein 2 [Crassostrea gigas]; 11 511 minus strand CDC
1	1	NaN	1732 2079 minus strand PREDICTED: annexin A7-like [Ornithorhynchus anatinus]
1	1	NaN	90 659 Plectin-1 [Crassostrea gigas]; 327 881 Plectin-1 [Crassostrea gigas]; 646 1290 minus strand Plectin-1 [Crasso
1	12	NaN	6043 6174 myosin heavy chain [Mytilus galloprovincialis]; 5869 6000 myosin heavy chain [Mytilus galloprovincialis]
1	1	NaN	58 2268 hypothetical protein BRAFLDRAFT_267717 [Branchiostoma floridae] gi; 62 2245 hypothetical protein BRAFL
1	1	NaN	7825 8754 minus strand H2 [Haliotis tuberculata]
1	1	NaN	219 554 minus strand ribosomal protein P1 [Biomphalaria glabrata]
1	8	NaN	460 1737 Tubulin beta-4B chain OS=Mus musculus GN=Tubb4b PE=1 SV=1
1	2	NaN	2 211 minus strand sulfatase 1A precursor [Haliotis discus discus]
1	4	NaN	" 1 510 Glycerol-3-phosphate dehydrogenase [NAD+], cytoplasmic [Crassostrea gigas]"
1	2	NaN	143 247 minus strand dynein light chain [Aplysia californica] gi
1	1	NaN	77 2278 Far upstream element-binding protein 3 [Crassostrea gigas]
1	1	NaN	416 1717 minus strand T-complex protein 1 subunit delta OS=Aedes triseriatus PE=2 SV=1
1	1	NaN	205 1341 collagen precursor [Bombyx mori]
1	1	NaN	201 632 USP-like protein isoform 1 [Crassostrea ariakensis] gi; 189 620 minus strand USP-like protein isoform 1 [Cra
1	1	NaN	152 1930 hypothetical protein BRAFLDRAFT_223365 [Branchiostoma floridae] gi
1	1	NaN	126 287 minus strand Cytochrome b-c1 complex subunit 10 [Cricetulus griseus]; 126 287 minus strand Cytochrome
1	1	NaN	49 1107 Eukaryotic translation initiation factor 3 subunit B [Crassostrea gigas]
1	1	NaN	212 1405 minus strand unnamed protein product [Tetraodon nigroviridis]
1	1	NaN	91 408 minus strand hypothetical protein [Amblyomma maculatum]
1	2	NaN	141 1325 Nucleobindin-2 [Crassostrea gigas]; 141 1208 minus strand Nucleobindin-2 [Crassostrea gigas]
1	1	NaN	636 1232 sigma class glutathione-s-transferase 3 [Haliotis discus discus]; 963 1559 sigma class glutathione-s-transf
1	1	NaN	72 521 minus strand GF14593 [Drosophila ananassae] gi
1	1	NaN	19 1218 Dynactin subunit 2 [Crassostrea gigas]; 48 830 minus strand Dynactin subunit 2 [Crassostrea gigas]
1	1	NaN	" 1 681 40S ribosomal protein S4, X isoform [Crassostrea gigas]"
1	11	NaN	" 1 321 Dynein heavy chain, cytoplasmic [Crassostrea gigas]"
1	1	NaN	" 703 1173 60S acidic ribosomal protein P0, putative [Ixodes scapularis] gi"
1	10	NaN	1714 2121 Phosphoenolpyruvate carboxykinase [GTP] [Crassostrea gigas]
1	1	NaN	61 2865 AP-2 complex subunit beta [Crassostrea gigas]
1	1	NaN	316 531 cytochrome c oxidase subunit VIIa polypeptide 2 like [Xenopus (Silurana) tropicalis]
1	1	NaN	191 919 PREDICTED: 15-hydroxyprostaglandin dehydrogenase [NAD+] [Gallus gallus]
1	1	NaN	120 833 B-cell receptor-associated protein 31 [Crassostrea gigas]; 238 951 B-cell receptor-associated protein 31 [Cr
1	4	NaN	246 452 minus strand mitochondrial malate dehydrogenase precursor [Nucella lapillus]; 106 312 minus strand mitoc
1	2	NaN	" 241 1110 minus strand N(G),N(G)-dimethylarginine dimethylaminohydrolase 1 [Crassostrea gigas]; 241 1110 minus
1	1	NaN	206 1513 hypothetical protein CGI_10019003 [Crassostrea gigas]
1	1	NaN	161 3001 minus strand E3 ubiquitin-protein ligase MIB2 [Crassostrea gigas]
1	2	NaN	5 1018 minus strand Glutaredoxin-3 [Crassostrea gigas]; 5 1018 Glutaredoxin-3 [Crassostrea gigas]
1	2	NaN	1017 1499 minus strand PREDICTED: leukocyte elastase inhibitor-like [Oreochromis niloticus]
1	2	NaN	156 1163 cathepsin B [Crassostrea ariakensis]
1	1	NaN	3 239 minus strand p38 MAPK [Littorina littorea]
1	2	NaN	104 418 Tubulin-specific chaperone A [Crassostrea gigas]; 104 418 minus strand Tubulin-specific chaperone A [Cras
1	1	NaN	333 770 USP-like protein isoform 2 [Crassostrea ariakensis]
1	2	NaN	8 1636 minus strand V-type proton ATPase catalytic subunit A [Crassostrea gigas]; 8 1636 minus strand V-type proto
1	3	NaN	2831 4900 minus strand Collagen alpha-3(VI) chain [Crassostrea gigas]
1	1	NaN	58 1203 minus strand Obg-like ATPase 1 [Crassostrea gigas]
1	2	NaN	293 1795 Amidase [Crassostrea gigas]
1	1	NaN	4 2400 IQ motif containing GTPase activating protein 2 [Xenopus laevis] gi; 4 2169 IQ motif containing GTPase activ
1	1	NaN	1213 3234 Protein-glutamine gamma-glutamyltransferase K [Crassostrea gigas]; 478 2499 Protein-glutamine gamma
1	1	NaN	188 4135 Vigilin [Crassostrea gigas]
1	1	NaN	121 1452 minus strand Hexokinase type 2 [Crassostrea gigas]; 245 1576 minus strand Hexokinase type 2 [Crassostr
1	1	NaN	1118 1255 minus strand equistatin precursor [Actinia equina]
1	3	NaN	132 590 minus strand Non-neuronal cytoplasmic intermediate filament protein OS=Helix aspersa PE=3 SV=2
1	1	NaN	" 154 591 microsomal GST1, partial [Reishia clavigera]; 154 531 microsomal GST1, partial [Reishia clavigera]"
1	1	NaN	297 734 minus strand PREDICTED: calmodulin [Canis lupus familiaris]; 297 734 PREDICTED: calmodulin [Canis lupu
1	1	NaN	1050 1307 calreticulin precursor [Aplysia californica] gi
1	1	NaN	642 1415 minus strand cytochrome c oxidase subunit II [Oncomelania hupensis hupensis]
1	1	NaN	" 757 1443 minus strand nonmuscle myosin II, partial [Aplysia californica]"
1	7	NaN	16 288 SJCHGC01950 protein [Schistosoma japonicum] gi
1	1	NaN	880 1434 minus strand Prohormone-4 [Crassostrea gigas]
1	1	NaN	232 654 hypothetical protein BRAFLDRAFT_87758 [Branchiostoma floridae] gi
1	1	NaN	54 662 minus strand Protein DJ-1 [Crassostrea gigas]
1	1	NaN	112 819 pyruvate kinase [Crassostrea gigas]; 132 932 pyruvate kinase [Crassostrea gigas]
1	2	NaN	122 1195 minus strand Severin [Crassostrea gigas]
1	1	NaN	242 589 Ribonucleoprotein RB97D OS=Drosophila melanogaster GN=Rb97D PE=2 SV=1
1	2	NaN	3788 7885 minus strand Filamin-C [Crassostrea gigas]

5 Protein Details

A detailed summary of each protein is given below. These sections include peptide relative expression estimates in addition to protein-level estimates.

5.1 " 353 781 alpha-B-crystallin, putative [Ixodes scapularis] gi"

Protein Accession	CL37349.Contig1$_{H}1$
Mean Expression Ratio	0.351
Median Expression Ratio	0.35
Credible Interval	(0.252, 0.488)
Associated Peptides	4
Associated Spectra	7
Coverage	NaN

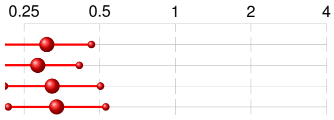

A	B	2.5	50	97.5	Sequence
2	0	0.2	0.31	0.46	LQESGTSEVVNNDKEFR
2	1	0.2	0.28	0.41	VAMDVQHFKPEELK
0	1	0.21	0.32	0.5	IIINAK
0	1	0.22	0.34	0.53	TVTSSLSPSGVLTLK

5.2 718 957 minus strand Calponin-1 OS=Gallus gallus GN=CNN1 PE=1 SV=2

Protein Accession	CL21394.Contig4$_{H}1$
Mean Expression Ratio	2.64
Median Expression Ratio	2.64
Credible Interval	(1.86, 3.77)
Associated Peptides	3
Associated Spectra	6
Coverage	NaN

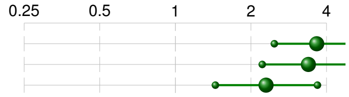

A	B	2.5	50	97.5	Sequence
3	0	2.5	3.7	5.4	EGQTVIGLQAGSNK
2	0	2.2	3.4	5.2	GASQAGMSMGAVR
1	0	1.4	2.3	3.7	IGLQAGSNK

5.3 200 2128 minus strand heat shock protein 70 [Cristaria plicata]

Protein Accession	CL8787.Contig2$_{H}1$
Mean Expression Ratio	0.389
Median Expression Ratio	0.39
Credible Interval	(0.28, 0.538)
Associated Peptides	4
Associated Spectra	8
Coverage	NaN

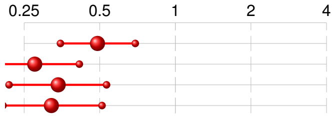

A	B	2.5	50	97.5	Sequence
1	3	0.35	0.49	0.69	IINEPTAAALAYGLDK
1	1	0.18	0.28	0.41	NGQVEIIANDQGQR
1	0	0.22	0.34	0.53	TLAPEEISSMVLVK
0	1	0.21	0.32	0.51	LSKEEIDR

5.4 280 690 Histone H3.3 [Harpegnathos saltator]

Protein Accession	Unigene26607$_{H}1$
Mean Expression Ratio	0.419
Median Expression Ratio	0.42
Credible Interval	(0.327, 0.539)
Associated Peptides	8
Associated Spectra	17
Coverage	NaN

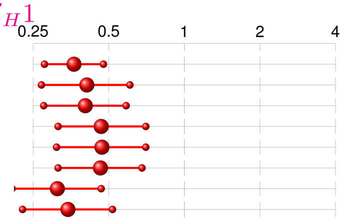

A	B	2.5	50	97.5	Sequence
4	4	0.28	0.36	0.48	STELLIR
1	0	0.27	0.41	0.61	KQLATK
1	1	0.28	0.4	0.59	RVTIMPK
1	0	0.31	0.47	0.7	RTKQTARK
1	0	0.31	0.47	0.7	DIQLAR
2	0	0.31	0.46	0.68	MARTKQTARK
0	1	0.21	0.31	0.47	EIAQDFK
0	1	0.23	0.34	0.52	KLPFQR

5.5 204 791 minus strand PREDICTED: 15-hydroxyprostaglandin dehydrogenase [NAD+] [Gallus gallus]

Protein Accession	Unigene3818$_{H}1$
Mean Expression Ratio	2.09
Median Expression Ratio	2.08
Credible Interval	(1.68, 2.6)
Associated Peptides	9
Associated Spectra	41
Coverage	NaN

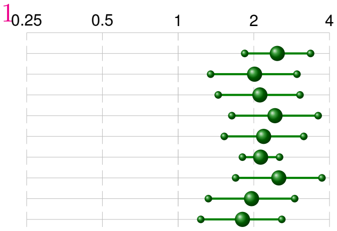

A	B	2.5	50	97.5	Sequence
4	1	1.8	2.5	3.4	AITMWGK
1	0	1.3	2	3	MGTTTDKELK
2	0	1.4	2.1	3.1	VDIMVNNAEIFSETDMEK
1	0	1.6	2.4	3.6	NAEIFSETDMEK
1	1	1.5	2.2	3.2	MDITDK
15	11	1.8	2.1	2.5	AVTEALLAK
1	0	1.7	2.5	3.7	IAFVSGGAMGTGR
0	1	1.3	2	2.9	TMTVNVTALMHGTK
0	2	1.2	1.8	2.6	HFISAFEK

5.6 27 1682 minus strand PREDICTED: nucleolar protein 56 [Oreochromis niloticus]

Protein Accession	Unigene26699$_{H}1$
Mean Expression Ratio	0.5
Median Expression Ratio	0.5
Credible Interval	(0.398, 0.626)
Associated Peptides	7
Associated Spectra	27
Coverage	NaN

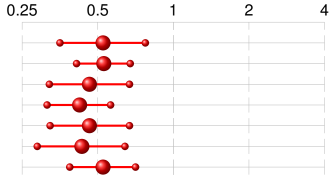

A	B	2.5	50	97.5	Sequence
1	0	0.35	0.52	0.77	KGLGGLSTEKK
7	3	0.41	0.53	0.67	LDGLGQAELQK
2	0	0.32	0.46	0.67	VATLEEEVYDWEIK
2	4	0.32	0.42	0.56	VTPLKLDGLGQAELQK
1	1	0.32	0.46	0.67	LIMQK
1	0	0.29	0.43	0.64	AAEDLRNEAK
4	1	0.39	0.52	0.71	KLIMQK

5.7 704 817 minus strand expressed hypothetical protein [Trichoplax adhaerens] gi; 688 801 minus strand Lactoylglutathione lyase [Crassostrea gigas]; 735 848 minus strand expressed hypothetical protein [Trichoplax adhaerens] gi

Protein Accession	Unigene64106$_{H}1$ Unigene64104$_{H}1$ Unigene64103$_{H}1$
Mean Expression Ratio	1.99
Median Expression Ratio	1.99
Credible Interval	(1.27, 3.16)
Associated Peptides	1
Associated Spectra	2
Coverage	NaN

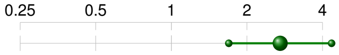

A	B	2.5	50	97.5	Sequence
1	1	1.7	2.7	4.3	FESLGVELVK

5.8 " 131 544 Histone H2B, gonadal OS=Patella granatina PE=1 SV=2"

Protein Accession	CL29794.Contig2$_{H}1$
Mean Expression Ratio	0.542
Median Expression Ratio	0.541
Credible Interval	(0.397, 0.749)
Associated Peptides	2
Associated Spectra	18
Coverage	NaN

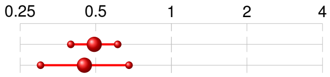

A	B	2.5	50	97.5	Sequence
9	7	0.4	0.49	0.61	LLLPGELAK
0	2	0.3	0.45	0.68	PGELAK

5.9 " 10 609 lethal(2)essential for life protein, l2efl [Aedes aegypti] gi"

Protein Accession	Unigene67975$_{H}1$
Mean Expression Ratio	0.542
Median Expression Ratio	0.542
Credible Interval	(0.393, 0.751)
Associated Peptides	5
Associated Spectra	7
Coverage	NaN

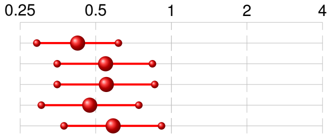

A	B	2.5	50	97.5	Sequence
1	2	0.29	0.42	0.61	ALEAPGETVIPITFEGK
1	0	0.35	0.55	0.84	MQMGETGK
0	1	0.35	0.55	0.86	SEVVNDDKEFR
0	1	0.3	0.47	0.74	KALEAPGETVIPITFEGKGDGK
0	1	0.37	0.59	0.91	KALEAPGETVIPITFEGK

5.10 118 501 minus strand histone 2A.Z [Aplysia californica]

Protein Accession	CL14228.Contig6$_{H}1$
Mean Expression Ratio	0.542
Median Expression Ratio	0.542
Credible Interval	(0.403, 0.727)
Associated Peptides	5
Associated Spectra	23
Coverage	NaN

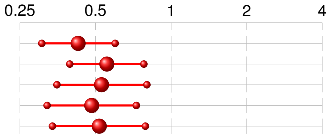

A	B	2.5	50	97.5	Sequence
9	3	0.31	0.43	0.6	AGLQFPVGR
3	3	0.39	0.55	0.78	ATIAGGGVIPHIHK
1	1	0.35	0.53	0.8	GDEELDSLIK
1	1	0.32	0.48	0.73	HLQLAIR
0	1	0.34	0.52	0.79	GGGVIPHIHK

5.11 405 1373 paramyosin [Haliotis discus discus]

Protein Accession	CL43455.Contig4$_H1$
Mean Expression Ratio	0.543
Median Expression Ratio	0.543
Credible Interval	(0.49, 0.6)
Associated Peptides	30
Associated Spectra	480
Coverage	NaN

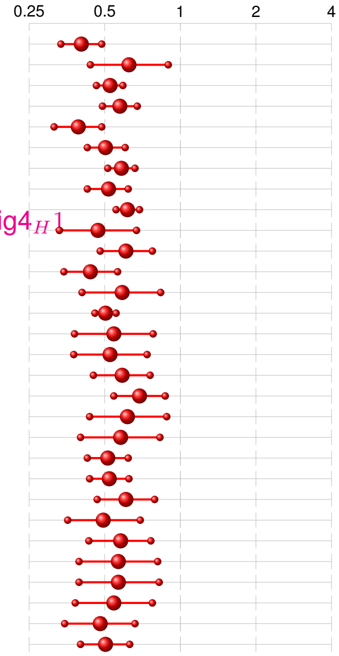

A	B	2.5	50	97.5	Sequence
12	7	0.34	0.4	0.49	DLELATAQFEAAEASLR
1	0	0.44	0.63	0.9	EKQTLVIELDGLSGQLDSITK
44	11	0.46	0.52	0.59	ELEDALDAER
23	5	0.49	0.58	0.68	HTEAVNDLSDQVDYLTK
5	7	0.31	0.39	0.49	IRELEDALDAER
14	10	0.43	0.5	0.6	LDEEAETSSNLR
33	19	0.51	0.58	0.66	LKGQVDDLTR
14	4	0.43	0.52	0.62	LTQENFDLQHQVQELDSSNAALAK
39	36	0.55	0.62	0.69	QLNDLTALK
1	0	0.33	0.47	0.67	QLNDLTALKAR
8	1	0.48	0.61	0.77	QNLQVNLSALQSDYDSLNAR
8	1	0.34	0.44	0.56	QTLVIELDGLSGQLDSITK
1	0	0.41	0.59	0.83	RHTEAVNDLSDQVDYLTK
46	36	0.46	0.5	0.56	TVAELSFR
1	0	0.38	0.54	0.78	LDQVQDSLDEQGSSTVAQIELNKK
1	1	0.38	0.53	0.74	ELEDALDAERDGR
4	3	0.45	0.59	0.76	KDLELATAQFEAAEASLR
9	1	0.54	0.69	0.87	DEQGSSTVAQIELNK
1	0	0.44	0.61	0.88	DEQGSSTVAQIELNKK
1	0	0.4	0.58	0.83	ESEIYK
20	0	0.43	0.51	0.62	LDQVQDSLDEQGSSTVAQIELNK
10	11	0.43	0.52	0.62	LSLESR
0	7	0.47	0.61	0.79	ITELEDSCEQLR
0	2	0.36	0.49	0.69	TRFDKELAAK
0	5	0.43	0.58	0.77	FDKELAAK
0	1	0.39	0.57	0.81	ITELEDSCEQLRV
0	1	0.4	0.57	0.82	ARLDEEAETSSNLR
0	1	0.38	0.54	0.77	LEDALDAER
0	3	0.35	0.48	0.66	TEELEETR
0	11	0.4	0.5	0.63	VSADYAALK

5.12 628 1140 paramyosin [Haliotis discus discus]

Protein Accession	CL43455.Contig3$_H1$
Mean Expression Ratio	0.552
Median Expression Ratio	0.551
Credible Interval	(0.404, 0.757)
Associated Peptides	2
Associated Spectra	43
Coverage	NaN

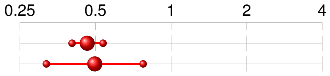

A	B	2.5	50	97.5	Sequence
22	20	0.4	0.46	0.53	LEEAEAFAVK
0	1	0.32	0.5	0.78	LEEAEAF

5.13 1 945 hemocyanin isoform 1 [Haliotis diversicolor]

Protein Accession	Unigene30318$_H1$
Mean Expression Ratio	0.552
Median Expression Ratio	0.552
Credible Interval	(0.367, 0.825)
Associated Peptides	2
Associated Spectra	3
Coverage	NaN

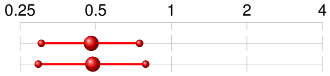

A	B	2.5	50	97.5	Sequence
1	1	0.3	0.48	0.75	VEITDVLK
0	1	0.3	0.49	0.79	LFVVEVEDELR

5.14 2473 2898 RecName: Full=Paramyosin gi|397603|emb|CAA79849.1| paramyosin [Echinococcus granulosus]

Protein Accession	CL43455.Contig1$_H$1
Mean Expression Ratio	0.554
Median Expression Ratio	0.554
Credible Interval	(0.481, 0.64)
Associated Peptides	12
Associated Spectra	138
Coverage	NaN

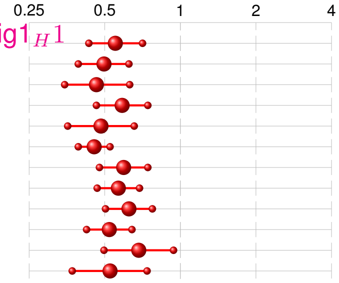

A	B	2.5	50	97.5	Sequence
5	4	0.43	0.55	0.71	AQTLIEEAENR
7	4	0.39	0.49	0.62	EILAQTEEER
2	2	0.35	0.46	0.63	EILAQTEEERR
6	4	0.46	0.59	0.74	INKEILAQTEEER
3	1	0.35	0.48	0.65	INKEILAQTEEERR
26	10	0.39	0.45	0.53	KAQTLIEEAENR
6	6	0.48	0.59	0.74	KQLEIEIR
13	6	0.47	0.57	0.69	QLAEFASLNEQLNLR
8	5	0.5	0.63	0.77	QLEEAEDVANPTMNK
9	6	0.42	0.52	0.64	QLEIEIR
1	2	0.5	0.68	0.94	RQLAEFASLNEQLNLR
1	1	0.37	0.52	0.74	RQLEEAEDVANPTMNK

5.15 397 510 PREDICTED: actin-5C-like [Megachile rotundata]

Protein Accession	CL48442.Contig2$_H$1
Mean Expression Ratio	1.73
Median Expression Ratio	1.73
Credible Interval	(1.3, 2.31)
Associated Peptides	3
Associated Spectra	28
Coverage	NaN

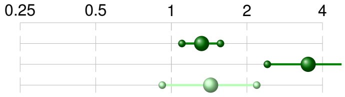

A	B	2.5	50	97.5	Sequence
15	9	1.1	1.3	1.6	DLTDYLMK
1	2	2.4	3.5	5.1	GRDLTDYLMK
0	1	0.92	1.4	2.2	LDLAGRDLTDY

5.16 3 491 hemocyanin [Haliotis tuberculata]

Protein Accession	CL40281.Contig3$_H$1
Mean Expression Ratio	0.592
Median Expression Ratio	0.592
Credible Interval	(0.428, 0.82)
Associated Peptides	3
Associated Spectra	8
Coverage	NaN

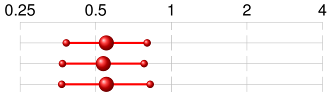

A	B	2.5	50	97.5	Sequence
2	1	0.38	0.55	0.8	DINPFALTK
1	2	0.37	0.54	0.78	TLEPFDRDINPFALTK
1	1	0.37	0.55	0.82	TLEPFDR

5.17 957 2147 haemocyanin [Aplysia californica]

Protein Accession	Unigene30320$_H$1
Mean Expression Ratio	0.601
Median Expression Ratio	0.6
Credible Interval	(0.459, 0.786)
Associated Peptides	7
Associated Spectra	13
Coverage	NaN

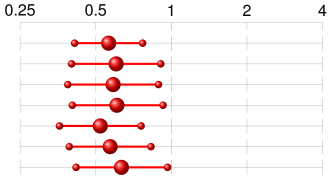

A	B	2.5	50	97.5	Sequence
3	2	0.41	0.56	0.77	LLTVQFER
1	0	0.4	0.6	0.91	YEVSELSLEQVSNLR
1	0	0.39	0.58	0.89	TFHISGVNQDITR
1	0	0.4	0.61	0.93	LLTVQFERAL
0	2	0.36	0.52	0.76	KAGQNIGDTLK
0	2	0.39	0.57	0.83	IDITDAVK
0	1	0.42	0.63	0.97	ANALPVQVFDTEK

5.18 372 860 minus strand small heat shock protein [Tegillarca granosa]; 651 998 minus strand small heat shock protein [Tegillarca granosa]

Protein Accession	CL18190.Contig4$_{H}1$ CL18190.Contig1$_{H}1$
Mean Expression Ratio	0.602
Median Expression Ratio	0.601
Credible Interval	(0.43, 0.845)
Associated Peptides	3
Associated Spectra	7
Coverage	NaN

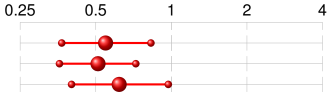

A	B	2.5	50	97.5	Sequence
1	1	0.37	0.55	0.83	EFLIPEEIDAETLGSR
2	2	0.36	0.51	0.72	LTEDGHLIISAR
1	0	0.4	0.62	0.97	IDKDGLSIK

5.19 4726 5571 minus strand twitchin [Mytilus galloprovincialis]

Protein Accession	CL181.Contig4$_{H}1$
Mean Expression Ratio	0.603
Median Expression Ratio	0.602
Credible Interval	(0.472, 0.771)
Associated Peptides	8
Associated Spectra	15
Coverage	NaN

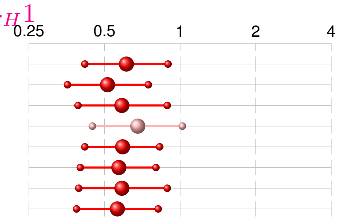

A	B	2.5	50	97.5	Sequence
1	1	0.42	0.61	0.89	EDGGAPITHYIVEK
1	1	0.36	0.52	0.75	GTVEGLQEGKEYEFR
1	0	0.39	0.59	0.89	VCDTDDLEIDVSDLTPGHK
1	0	0.45	0.68	1	CALLWDKPK
1	2	0.42	0.59	0.83	NGTPLNEANLDTETVPK
2	1	0.4	0.57	0.8	GLQEGKEYEFR
0	1	0.4	0.59	0.89	EIFVK
0	2	0.39	0.56	0.82	IPDVQR

5.20 103 684 EF-hand domain-containing protein D2 [Crassostrea gigas]

Protein Accession	CL20544.Contig1$_{H}1$
Mean Expression Ratio	1.66
Median Expression Ratio	1.66
Credible Interval	(1.11, 2.49)
Associated Peptides	3
Associated Spectra	3
Coverage	NaN

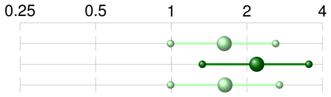

A	B	2.5	50	97.5	Sequence
1	0	0.99	1.6	2.6	VPQTHLALK
1	0	1.3	2.2	3.5	KFEEEIR
1	0	0.99	1.6	2.7	FIDFNELK

5.21 1363 1527 Protein disulfide-isomerase A3 [Crassostrea gigas]

Protein Accession	CL28563.Contig1$_{H}1$
Mean Expression Ratio	0.607
Median Expression Ratio	0.607
Credible Interval	(0.392, 0.942)
Associated Peptides	2
Associated Spectra	2
Coverage	NaN

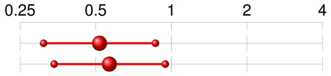

A	B	2.5	50	97.5	Sequence
0	1	0.31	0.52	0.86	LKDEPEITIAK
0	1	0.34	0.57	0.94	FEELAEK

5.22 " 154 534 minus strand Muscle M-line assembly protein unc-89, partial [Crassostrea gigas]"

Protein Accession	Unigene64523$_H1$
Mean Expression Ratio	0.614
Median Expression Ratio	0.614
Credible Interval	(0.375, 1)
Associated Peptides	1
Associated Spectra	3
Coverage	NaN

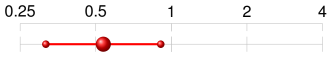

A	B	2.5	50	97.5	Sequence
**2**	**1**	**0.32**	**0.54**	**0.91**	**LEIGEVVVDDEGEYICK**

5.23 1027 1440 minus strand hemocyanin isoform 1 [Haliotis diversicolor]

Protein Accession	CL40281.Contig1$_H1$
Mean Expression Ratio	0.618
Median Expression Ratio	0.618
Credible Interval	(0.447, 0.855)
Associated Peptides	2
Associated Spectra	10
Coverage	NaN

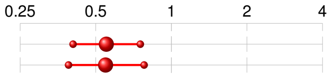

A	B	2.5	50	97.5	Sequence
**3**	**3**	**0.41**	**0.55**	**0.75**	**LFVVQVEDELK**
**0**	**4**	**0.39**	**0.55**	**0.78**	**HNPFYDADIAFLQK**

5.24 93 2606 twitchin [Mytilus galloprovincialis]

Protein Accession	Unigene71883$_H1$
Mean Expression Ratio	0.618
Median Expression Ratio	0.618
Credible Interval	(0.478, 0.797)
Associated Peptides	5
Associated Spectra	21
Coverage	NaN

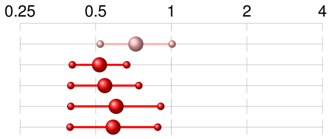

A	B	2.5	50	97.5	Sequence
**3**	**1**	**0.52**	**0.72**	**1**	**FAFHNITGLSPNK**
**6**	**4**	**0.4**	**0.52**	**0.67**	**IIDFGLATR**
**2**	**3**	**0.4**	**0.54**	**0.74**	**FVPQPVEIK**
**1**	**0**	**0.4**	**0.6**	**0.91**	**VIAENFYGR**
**0**	**1**	**0.39**	**0.59**	**0.88**	**LNPDEIVK**

5.25 38 436 Calcium-dependent protein kinase isoform 2 [Crassostrea gigas]

Protein Accession	CL5866.Contig5$_H1$
Mean Expression Ratio	0.622
Median Expression Ratio	0.62
Credible Interval	(0.38, 1.02)
Associated Peptides	1
Associated Spectra	1
Coverage	NaN

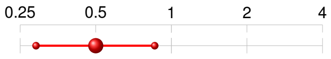

A	B	2.5	50	97.5	Sequence
**1**	**0**	**0.29**	**0.5**	**0.86**	**SHAGLDVEADK**

5.26 346 702 minus strand PREDICTED: galectin-4-like [Anolis carolinensis]

Protein Accession	Unigene42203$_{H}$1
Mean Expression Ratio	0.622
Median Expression Ratio	0.623
Credible Interval	(0.468, 0.826)
Associated Peptides	5
Associated Spectra	11
Coverage	NaN

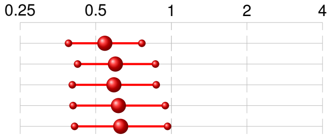

A	B	2.5	50	97.5	Sequence
3	1	0.39	0.54	0.77	AEATLEVR
2	1	0.42	0.6	0.86	SVPHGLVK
1	1	0.4	0.59	0.87	GKDFDIVIK
1	0	0.41	0.62	0.94	VKDSGFK
0	1	0.41	0.63	0.97	KSVPHGLVK

5.27 " 599 907 minus strand Tryptophanyl-tRNA synthetase, cytoplasmic [Heterocephalus glaber]"

Protein Accession	Unigene26755$_{H}$1
Mean Expression Ratio	0.628
Median Expression Ratio	0.627
Credible Interval	(0.44, 0.903)
Associated Peptides	2
Associated Spectra	8
Coverage	NaN

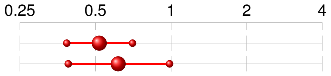

A	B	2.5	50	97.5	Sequence
3	4	0.38	0.52	0.7	DMDPLLDLYEK
1	0	0.39	0.62	0.99	KTPFYLYTGR

5.28 523 1086 minus strand juvenile hormone diol kinase [Danaus plexippus]

Protein Accession	CL7590.Contig5$_{H}$1
Mean Expression Ratio	1.57
Median Expression Ratio	1.57
Credible Interval	(0.998, 2.47)
Associated Peptides	1
Associated Spectra	2
Coverage	NaN

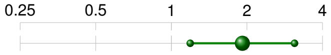

A	B	2.5	50	97.5	Sequence
2	0	1.2	1.9	3.1	GEFVTVQTIFGNSVEDSHK

5.29 23 214 minus strand hemocyanin isoform 1 [Nucula nucleus]

Protein Accession	Unigene58881$_{H}$1
Mean Expression Ratio	0.641
Median Expression Ratio	0.642
Credible Interval	(0.434, 0.944)
Associated Peptides	2
Associated Spectra	4
Coverage	NaN

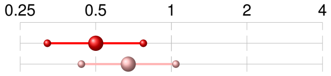

A	B	2.5	50	97.5	Sequence
2	0	0.32	0.5	0.77	GKIPNLNIYTSR
2	0	0.44	0.67	1	QEINPFFR

5.30 21 341 minus strand hemocyanin isoform 1 [Nucula nucleus]

Protein Accession	CL40281.Contig2$_{H}1$
Mean Expression Ratio	0.646
Median Expression Ratio	0.646
Credible Interval	(0.454, 0.924)
Associated Peptides	2
Associated Spectra	6
Coverage	NaN

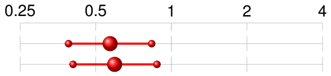

A	B	2.5	50	97.5	Sequence
**1**	**2**	**0.39**	**0.57**	**0.84**	**GDELFSYLK**
**2**	**1**	**0.41**	**0.6**	**0.88**	**KPLEPFNR**

5.31 501 1838 Hsp90A [Haliotis asinina]

Protein Accession	CL5184.Contig2$_{H}1$
Mean Expression Ratio	0.657
Median Expression Ratio	0.657
Credible Interval	(0.503, 0.855)
Associated Peptides	7
Associated Spectra	15
Coverage	NaN

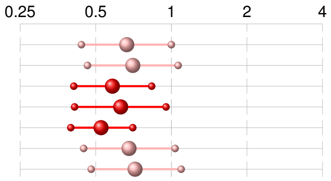

A	B	2.5	50	97.5	Sequence
**1**	**0**	**0.44**	**0.66**	**1**	**AEFEGLCK**
**1**	**0**	**0.46**	**0.7**	**1.1**	**AIYYITGESLDAVK**
**1**	**2**	**0.41**	**0.58**	**0.83**	**GVVDSEDLPLNISR**
**1**	**0**	**0.41**	**0.63**	**0.95**	**NPDDITPEEYAEFYK**
**3**	**4**	**0.4**	**0.53**	**0.7**	**LAEFLR**
**1**	**0**	**0.45**	**0.68**	**1**	**RAPFDLFENK**
**1**	**0**	**0.48**	**0.72**	**1.1**	**VERVK**

5.32 174 1649 minus strand Collagen alpha-1(XII) chain [Crassostrea gigas]; 123 1490 minus strand Collagen alpha-1(XII) chain [Crassostrea gigas]; 135 1610 Collagen alpha-1(XII) chain [Crassostrea gigas]

Protein Accession	CL5340.Contig13$_{H}1$ CL5340.Contig12$_{H}1$ CL5340.Contig11$_{H}1$
Mean Expression Ratio	1.51
Median Expression Ratio	1.51
Credible Interval	(1.1, 2.08)
Associated Peptides	4
Associated Spectra	20
Coverage	NaN

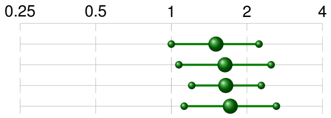

A	B	2.5	50	97.5	Sequence
**4**	**1**	**1**	**1.5**	**2.2**	**ADVVFIVDSSR**
**1**	**1**	**1.1**	**1.6**	**2.5**	**DVMSVFHIAR**
**6**	**5**	**1.2**	**1.7**	**2.3**	**DILAFR**
**1**	**1**	**1.1**	**1.7**	**2.6**	**SIWMPDFK**

5.33 238 1038 PREDICTED: hypothetical protein [Saccoglossus kowalevskii]; 294 992 PREDICTED: hypothetical protein [Saccoglossus kowalevskii]

Protein Accession	CL6273.Contig7$_{H}1$ CL6273.Contig6$_{H}1$
Mean Expression Ratio	0.668
Median Expression Ratio	0.667
Credible Interval	(0.412, 1.08)
Associated Peptides	1
Associated Spectra	1
Coverage	NaN

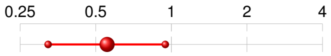

A	B	2.5	50	97.5	Sequence
**0**	**1**	**0.32**	**0.56**	**0.94**	**DVVLLHQLGR**

5.34 2 2572 Glycogen debranching enzyme [Crassostrea gigas]

Protein Accession	CL45503.Contig1$_H$1
Mean Expression Ratio	1.49
Median Expression Ratio	1.5
Credible Interval	(0.911, 2.41)
Associated Peptides	1
Associated Spectra	1
Coverage	NaN

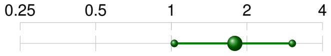

A	B	2.5	50	97.5	Sequence
**0**	**1**	**1**	**1.8**	**3**	**IMAVNGWVMGDDPLR**

5.35 80 589 minus strand PREDICTED: adenine phosphoribosyltransferase-like [Strongylocentrotus purpuratus]

Protein Accession	CL8682.Contig6$_H$1
Mean Expression Ratio	1.49
Median Expression Ratio	1.49
Credible Interval	(1.11, 2)
Associated Peptides	5
Associated Spectra	10
Coverage	NaN

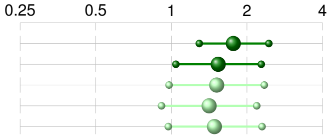

A	B	2.5	50	97.5	Sequence
**2**	**3**	**1.3**	**1.8**	**2.4**	**LTVPVESLIK**
**1**	**1**	**1**	**1.5**	**2.3**	**AAVELVEK**
**0**	**1**	**0.98**	**1.5**	**2.3**	**GFLFGPMIAEK**
**0**	**1**	**0.91**	**1.4**	**2.2**	**NAPDADVIIGLESR**
**0**	**1**	**0.98**	**1.5**	**2.3**	**DIFPILR**

5.36 269 880 minus strand myosin regulatory light chain [Fasciola hepatica]

Protein Accession	CL43006.Contig5$_H$1
Mean Expression Ratio	1.49
Median Expression Ratio	1.48
Credible Interval	(1.15, 1.93)
Associated Peptides	3
Associated Spectra	21
Coverage	NaN

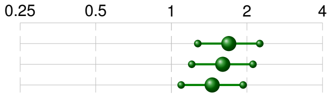

A	B	2.5	50	97.5	Sequence
**5**	**2**	**1.3**	**1.7**	**2.2**	**ATSNVFAK**
**7**	**0**	**1.2**	**1.6**	**2.1**	**KLMQEMK**
**4**	**3**	**1.1**	**1.5**	**1.9**	**LMQEMK**

5.37 261 1361 tandem-repeat galectin [Pinctada fucata]; 261 1361 tandem-repeat galectin [Pinctada fucata]

Protein Accession	CL474.Contig2$_H$1 CL474.Contig1$_H$1
Mean Expression Ratio	0.674
Median Expression Ratio	0.674
Credible Interval	(0.432, 1.05)
Associated Peptides	1
Associated Spectra	2
Coverage	NaN

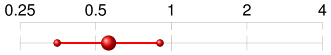

A	B	2.5	50	97.5	Sequence
**0**	**2**	**0.35**	**0.56**	**0.9**	**LENNVLLHANQR**

5.38 " 276 659 Fatty acid-binding protein, adipocyte [Crassostrea gigas]; 426 809 Fatty acid-binding protein, adipocyte [Crassostrea gigas]; 277 660 Fatty acid-binding protein, adipocyte [Crassostrea gigas]; 283 666 Fatty acid-binding protein, adipocyte [Crassostrea gigas]; 270 653 Fatty acid-binding protein, adipocyte [Crassostrea gigas]; 283 666 Fatty acid-binding protein, adipocyte [Crassostrea gigas]; 258 641 Fatty acid-binding protein, adipocyte [Crassostrea gigas]; 427 810 Fatty acid-binding protein, adipocyte [Crassostrea gigas]; 277 660 Fatty acid-binding protein, adipocyte [Crassostrea gigas]"

Protein Accession	CL7666.Contig9$_H$1 CL7666.Contig8$_H$1 CL7666.Contig7$_H$1 CL7666.Contig6$_H$1 CL7666.Con
Mean Expression Ratio	1.47
Median Expression Ratio	1.47
Credible Interval	(1.14, 1.91)
Associated Peptides	5
Associated Spectra	16
Coverage	NaN

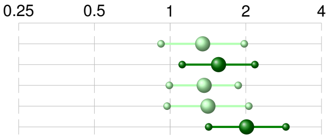

A	B	2.5	50	97.5	Sequence
1	1	0.92	1.4	2	AVLTEESGELVER
3	1	1.1	1.6	2.2	GVTATISFQK
4	1	1	1.4	1.9	LTTVAGDK
1	1	0.97	1.4	2.1	TVLAMER
1	2	1.4	2	2.9	AFVDGALK

5.39 " 116 352 minus strand troponin I 4 [Clonorchis sinensis]; 491 706 minus strand troponin-I, TnI {C-terminal} [Chlamys nipponensis=Akazara scallop, akazara, striated adductor muscle, Peptide Partial, 135 aa]"

Protein Accession	Unigene63816$_H$1 Unigene63815$_H$1
Mean Expression Ratio	0.682
Median Expression Ratio	0.683
Credible Interval	(0.498, 0.934)
Associated Peptides	2
Associated Spectra	21
Coverage	NaN

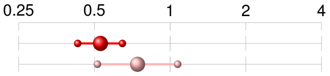

A	B	2.5	50	97.5	Sequence
12	6	0.43	0.53	0.65	FIKPVLR
2	1	0.51	0.74	1.1	FALKEEDESSK

5.40 " 1 1887 Pyruvate carboxylase, mitochondrial [Crassostrea gigas]"

Protein Accession	CL26073.Contig5$_H$1
Mean Expression Ratio	0.684
Median Expression Ratio	0.686
Credible Interval	(0.472, 0.993)
Associated Peptides	4
Associated Spectra	4
Coverage	NaN

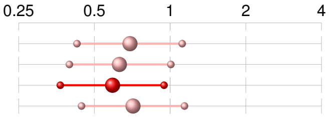

A	B	2.5	50	97.5	Sequence
0	1	0.43	0.69	1.1	AGTHILGIK
0	1	0.4	0.63	1	HLEDVEENFKR
0	1	0.37	0.59	0.94	FLHECPWDR
0	1	0.44	0.71	1.1	IEVFR

5.41 126 704 minus strand ribosomal protein L12 [Ixodes scapularis]

Protein Accession	CL24053.Contig3$_{H}1$
Mean Expression Ratio	0.69
Median Expression Ratio	0.69
Credible Interval	(0.46, 1.04)
Associated Peptides	1
Associated Spectra	4
Coverage	NaN

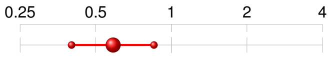

A	B	2.5	50	97.5	Sequence
**2**	**2**	**0.4**	**0.58**	**0.85**	**IGPLGLSPK**

5.42 54 278 ATP-citrate synthase [Crassostrea gigas]; 61 465 ATP-citrate synthase [Crassostrea gigas]

Protein Accession	CL35133.Contig2$_{H}1$ CL35133.Contig1$_{H}1$
Mean Expression Ratio	1.44
Median Expression Ratio	1.44
Credible Interval	(0.9, 2.33)
Associated Peptides	1
Associated Spectra	1
Coverage	NaN

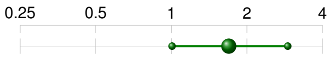

A	B	2.5	50	97.5	Sequence
**1**	**0**	**1**	**1.7**	**2.9**	**TIAIIAEGIPENLTR**

5.43 336 641 minus strand malate dehydrogenase [Schistosoma japonicum]

Protein Accession	CL11441.Contig9$_{H}1$
Mean Expression Ratio	1.43
Median Expression Ratio	1.44
Credible Interval	(0.884, 2.34)
Associated Peptides	1
Associated Spectra	1
Coverage	NaN

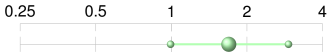

A	B	2.5	50	97.5	Sequence
**0**	**1**	**1**	**1.7**	**2.9**	**ALADLLVAADHR**

5.44 266 607 minus strand H2 [Haliotis tuberculata]

Protein Accession	Unigene62350$_{H}1$
Mean Expression Ratio	0.707
Median Expression Ratio	0.706
Credible Interval	(0.502, 0.996)
Associated Peptides	2
Associated Spectra	7
Coverage	NaN

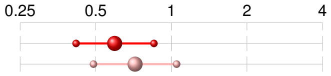

A	B	2.5	50	97.5	Sequence
**3**	**1**	**0.42**	**0.59**	**0.86**	**YAVAGIGVR**
**2**	**1**	**0.49**	**0.72**	**1.1**	**KDINTLTDEEIK**

5.45 511 1062 unknown [Dendroctonus ponderosae]; 511 1062 unknown [Dendroctonus ponderosae]

Protein Accession	CL18443.Contig3$_H$1 CL18443.Contig1$_H$1
Mean Expression Ratio	0.711
Median Expression Ratio	0.712
Credible Interval	(0.473, 1.07)
Associated Peptides	2
Associated Spectra	3
Coverage	NaN

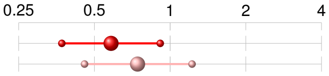

A	B	2.5	50	97.5	Sequence
1	1	0.37	0.58	0.91	KHNFLIDGFPR
0	1	0.46	0.74	1.2	FKDEIEMHLK

5.46 76 522 minus strand Troponin C [Crassostrea gigas]; 173 619 minus strand Troponin C [Crassostrea gigas]; 173 619 minus strand Troponin C [Crassostrea gigas]

Protein Accession	Unigene53263$_H$1 Unigene11674$_H$1 CL13001.Contig4$_H$1
Mean Expression Ratio	1.4
Median Expression Ratio	1.41
Credible Interval	(1.15, 1.71)
Associated Peptides	7
Associated Spectra	48
Coverage	NaN

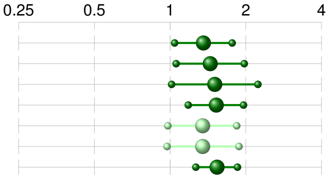

A	B	2.5	50	97.5	Sequence
4	4	1	1.4	1.8	GVIPVEDLR
3	1	1.1	1.4	2	QYNDANQTFNLFDK
1	0	1	1.5	2.2	QYNDANQTFNLFDKK
6	3	1.2	1.5	2	SLGLQVEDEK
2	2	0.98	1.3	1.8	VLDKGNK
2	1	0.97	1.3	1.9	SLGLQVEDEKLK
11	8	1.3	1.5	1.9	ELANVFK

5.47 1009 2784 minus strand Collagen alpha-3(VI) chain [Crassostrea gigas]

Protein Accession	Unigene46961$_H$1
Mean Expression Ratio	1.4
Median Expression Ratio	1.4
Credible Interval	(1.1, 1.76)
Associated Peptides	8
Associated Spectra	21
Coverage	NaN

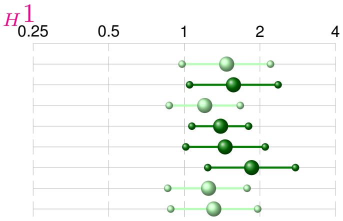

A	B	2.5	50	97.5	Sequence
1	0	0.98	1.5	2.2	FRKSPTLMFNLNEHK
1	0	1	1.6	2.4	IITVGIGEANRK
2	2	0.87	1.2	1.7	KSPTLMFNLNEHK
5	4	1.1	1.4	1.8	VAMSNFR
1	1	1	1.5	2.1	YADIDGGNVR
1	0	1.2	1.8	2.8	KAFNK
1	1	0.86	1.2	1.8	MFNLNEHK
0	1	0.88	1.3	2	FNLNEHK

5.48 150 716 calponin/transgelin [Papilio xuthus]

Protein Accession	CL11747.Contig2$_H$1
Mean Expression Ratio	1.4
Median Expression Ratio	1.4
Credible Interval	(1, 1.93)
Associated Peptides	3
Associated Spectra	8
Coverage	NaN

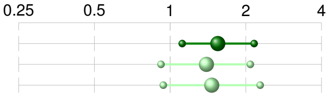

A	B	2.5	50	97.5	Sequence
4	1	1.1	1.6	2.2	GPPLNTPEHSVFATPDLYEK
1	1	0.92	1.4	2.1	MAFQQMEAIEK
1	0	0.94	1.5	2.3	FTTAIK

5.49 552 1499 minus strand uncharacterized protein LOC100158466 [Xenopus (Silurana) tropicalis] gi

Protein Accession	Unigene57645$_H$1
Mean Expression Ratio	1.39
Median Expression Ratio	1.39
Credible Interval	(0.903, 2.14)
Associated Peptides	2
Associated Spectra	3
Coverage	NaN

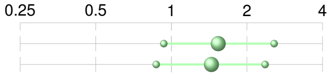

A	B	2.5	50	97.5	Sequence
1	0	0.93	1.5	2.6	TAFSLLK
1	1	0.87	1.4	2.4	TGEPVEVDGELATLLAR

5.50 181 477 minus strand Zgc:56530 [Danio rerio]; 185 481 minus strand Zgc:56530 [Danio rerio]

Protein Accession	CL48470.Contig6$_H$1 CL27088.Contig2$_H$1
Mean Expression Ratio	1.39
Median Expression Ratio	1.39
Credible Interval	(0.97, 2)
Associated Peptides	2
Associated Spectra	6
Coverage	NaN

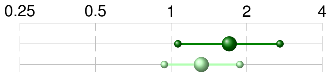

A	B	2.5	50	97.5	Sequence
1	0	1.1	1.7	2.7	TQVVAGTNYFIK
5	0	0.94	1.3	1.9	TFTDFK

5.51 155 3214 sarco/endoplasmic reticulum calcium ATPase isoform B [Pinctada fucata]; 155 3214 minus strand sarco/endoplasmic reticulum calcium ATPase isoform B [Pinctada fucata]

Protein Accession	CL1323.Contig9$_H$1 CL1323.Contig1$_H$1
Mean Expression Ratio	0.722
Median Expression Ratio	0.722
Credible Interval	(0.591, 0.878)
Associated Peptides	14
Associated Spectra	58
Coverage	NaN

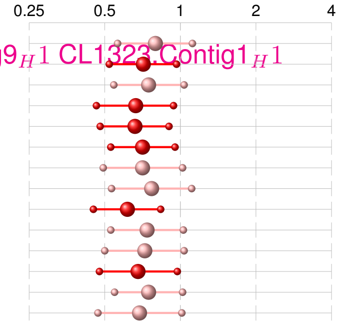

A	B	2.5	50	97.5	Sequence
1	2	0.56	0.79	1.1	ATAEAICR
3	3	0.52	0.71	0.96	CLALATIDNPPR
3	2	0.54	0.75	1	EFDELPPEDQR
2	1	0.46	0.66	0.94	GAPEGLLDR
3	2	0.48	0.66	0.9	GIVVGTGLATEIGK
7	2	0.53	0.71	0.95	KAEIGIAMGSGTAVAK
1	1	0.49	0.71	1	KEFTLEFSR
1	1	0.53	0.77	1.1	NAESAIEALK
2	3	0.45	0.61	0.84	NMLFSGTNIAAGK
2	2	0.53	0.74	1	VIVITGDNK
1	1	0.5	0.72	1	LNFYNTDK
2	1	0.48	0.68	0.97	MDQAFTK
7	1	0.55	0.75	1	IGHNKVPMSPAIK
0	1	0.47	0.69	1	KAEIGIAMGSGTA

5.52 " 460 1944 PREDICTED: succinyl-CoA:3-ketoacid-coenzyme A transferase 1, mitochondrial [Canis lupus familiaris]"

Protein Accession	Unigene4332$_{H}$1
Mean Expression Ratio	0.723
Median Expression Ratio	0.722
Credible Interval	(0.599, 0.874)
Associated Peptides	13
Associated Spectra	34
Coverage	NaN

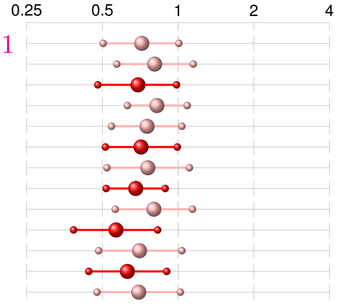

A	B	2.5	50	97.5	Sequence
1	1	0.5	0.72	1	AVFDVDNK
1	1	0.57	0.81	1.2	ECVSSYIGENK
1	1	0.48	0.69	0.98	EVAEGETAESIAK
2	5	0.63	0.83	1.1	EVVNMIVTEK
2	1	0.54	0.75	1	FNPDGTILHGSAGR
2	1	0.51	0.71	0.99	LGNLTFR
1	0	0.52	0.76	1.1	VMGMGGAMDLVSADK
3	4	0.52	0.68	0.89	ITGVEFR
2	0	0.56	0.8	1.1	VTLPK
0	1	0.38	0.57	0.83	AVFDVDNKEGLLLR
0	1	0.48	0.7	1	KGEEDADLINAGK
0	2	0.44	0.63	0.9	IFNGR
0	1	0.48	0.7	1	VFLGK

5.53 169 1161 Basement membrane-specific heparan sulfate proteoglycan core protein OS=Homo sapiens GN=HSPG2 PE=1 SV=4

Protein Accession	CL5208.Contig1$_{H}$1
Mean Expression Ratio	0.724
Median Expression Ratio	0.723
Credible Interval	(0.466, 1.13)
Associated Peptides	2
Associated Spectra	2
Coverage	NaN

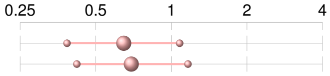

A	B	2.5	50	97.5	Sequence
1	0	0.38	0.65	1.1	GGDLTANHVVQGDVLR
1	0	0.42	0.69	1.2	PTITWSR

5.54 1200 1547 protein disulfide isomerase [Conus eburneus]

Protein Accession	CL3770.Contig1$_{H}$1
Mean Expression Ratio	1.38
Median Expression Ratio	1.38
Credible Interval	(1.11, 1.72)
Associated Peptides	8
Associated Spectra	24
Coverage	NaN

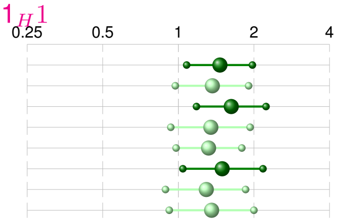

A	B	2.5	50	97.5	Sequence
2	3	1.1	1.5	2	IDSTANDVENIR
3	0	0.97	1.4	1.9	QLAPIWDELAEK
3	1	1.2	1.6	2.2	VQSFPTLK
1	1	0.94	1.3	1.9	YKDSNDIVIAK
1	4	0.98	1.3	1.8	NFNDVAFDK
1	1	1	1.5	2.2	VVDYNGER
1	1	0.89	1.3	1.8	VLVGK
0	1	0.92	1.4	2	TLSAFVK

5.55 247 1308 RecName: Full=Guanine nucleotide-binding protein G(o) subunit alpha gi|9633|emb|CAA78806.1| GTP-binding protein alpha-o subunit [Lymnaea stagnalis]

Protein Accession	CL26602.Contig4$_{H}1$
Mean Expression Ratio	1.38
Median Expression Ratio	1.37
Credible Interval	(1.07, 1.77)
Associated Peptides	6
Associated Spectra	18
Coverage	NaN

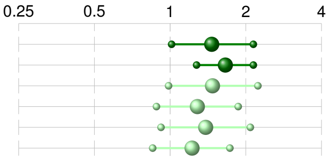

A	B	2.5	50	97.5	Sequence
1	1	1	1.5	2.1	IIHEGGFTSEDNK
4	5	1.3	1.7	2.1	LLLLGAGESGK
1	0	0.99	1.5	2.2	QTYEEAAAYIQAQFEAK
1	1	0.88	1.3	1.9	YFLDNLDR
1	0	0.92	1.4	2.1	KDLFEEK
0	3	0.85	1.2	1.7	MVLDVIAR

5.56 260 892 minus strand calponin [Haliotis diversicolor]

Protein Accession	CL48702.Contig1$_{H}1$
Mean Expression Ratio	1.37
Median Expression Ratio	1.37
Credible Interval	(1.17, 1.63)
Associated Peptides	11
Associated Spectra	102
Coverage	NaN

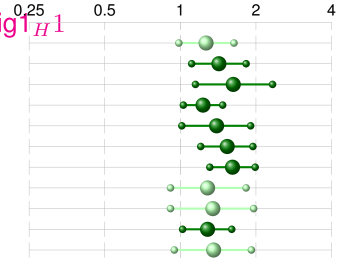

A	B	2.5	50	97.5	Sequence
6	4	0.99	1.3	1.6	AATGQQFPIEESNER
7	3	1.1	1.4	1.8	NFSEDKLR
2	0	1.2	1.6	2.3	TFQPASNLAF
18	8	1	1.2	1.5	TFQPASNLAFEAAR
1	3	1	1.4	1.9	VESSFDPEDAK
7	5	1.2	1.5	1.9	VESSFDPEDAKK
12	5	1.3	1.6	2	VMENFYK
1	1	0.91	1.3	1.8	YKVMENFYK
1	0	0.92	1.3	2	AKVESSFDPEDAKK
7	9	1	1.3	1.6	IELFIQK
1	1	0.95	1.4	1.9	VLACIR

5.57 12160 13014 twitchin [Mytilus galloprovincialis]

Protein Accession	CL181.Contig2$_{H}1$
Mean Expression Ratio	0.73
Median Expression Ratio	0.73
Credible Interval	(0.539, 0.981)
Associated Peptides	4
Associated Spectra	11
Coverage	NaN

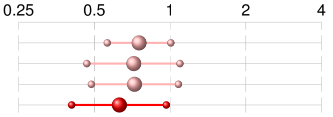

A	B	2.5	50	97.5	Sequence
1	6	0.56	0.75	1	ATLTVEPGVEGLR
1	0	0.47	0.72	1.1	AVNSLGEEETSCK
1	1	0.49	0.72	1.1	AFYKPK
1	0	0.41	0.63	0.97	NQLSAEGHTAK

5.58 155 3214 sarco/endoplasmic reticulum calcium ATPase isoform B [Pinctada fucata]; 132 3110 sarco/endoplasmic reticulum calcium ATPase isoform C [Pinctada fucata]; 132 3110 minus strand sarco/endoplasmic reticulum calcium ATPase isoform C [Pinctada fucata]; 155 3133 sarco/endoplasmic reticulum calcium ATPase isoform C [Pinctada fucata]; 155 3133 sarco/endoplasmic reticulum calcium ATPase isoform C [Pinctada fucata]; 155 3214 minus strand sarco/endoplasmic reticulum calcium ATPase isoform B [Pinctada fucata]

Protein Accession	CL1323.Contig9$_H$1 CL1323.Contig8$_H$1 CL1323.Contig6$_H$1 CL1323.Contig21$_H$1 CL1323.Co
Mean Expression Ratio	0.73
Median Expression Ratio	0.73
Credible Interval	(0.629, 0.85)
Associated Peptides	25
Associated Spectra	73
Coverage	NaN

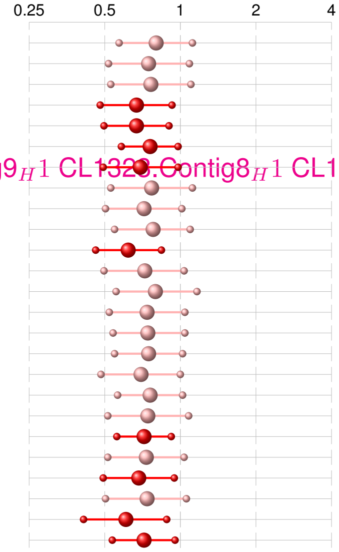

A	B	2.5	50	97.5	Sequence
1	2	0.57	0.8	1.1	ATAEAICR
1	0	0.52	0.75	1.1	DLVPGDIVEVSVGDK
1	0	0.53	0.76	1.1	DLVPGDIVEVSVGDKVPADIR
2	1	0.48	0.67	0.92	GAPEGLLDR
3	2	0.49	0.67	0.9	GIVVGTGLATEIGK
7	1	0.58	0.76	0.98	IGHNKVPMSPAIK
2	0	0.49	0.69	0.98	IGIFTENESTEGMSFTGR
1	0	0.53	0.77	1.1	IRNEMMDTEQEK
1	1	0.5	0.72	1	KEFTLEFSR
1	1	0.55	0.78	1.1	NAESAIEALK
2	3	0.46	0.62	0.84	NMLFSGTNIAAGK
1	0	0.5	0.72	1	SMSVYCVPNKPTR
1	0	0.55	0.8	1.2	TGTLTTNQMSVCK
2	0	0.52	0.74	1	VDQAILTGESVSVMK
3	0	0.54	0.74	1	VGEATETALTVLVEK
2	2	0.55	0.74	1	VIVITGDNK
1	0	0.48	0.7	1	VPMSPAIK
3	2	0.56	0.76	1	EFDELPPEDQR
1	0	0.51	0.74	1.1	NAESAIEALKEYEPEVAK
7	2	0.56	0.72	0.92	KAEIGIAMGSGTAVAK
1	1	0.51	0.73	1	LNFYNTDK
2	1	0.49	0.68	0.95	MDQAFTK
1	0	0.5	0.73	1.1	KNFEK
1	0	0.41	0.61	0.88	KNMLFSGTNIAAGK
3	3	0.54	0.72	0.96	CLALATIDNPPR

5.59 244 837 PREDICTED: ras-like GTP-binding protein Rho1-like [Megachile rotundata]

Protein Accession	Unigene11792$_H$1
Mean Expression Ratio	1.36
Median Expression Ratio	1.37
Credible Interval	(0.939, 1.97)
Associated Peptides	4
Associated Spectra	5
Coverage	NaN

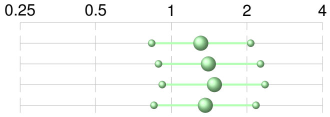

A	B	2.5	50	97.5	Sequence
1	1	0.83	1.3	2.1	EVFETATR
1	0	0.89	1.4	2.3	KLVIVGDGACGK
1	0	0.92	1.5	2.4	QVELALWDTAGQEDYDR
1	0	0.85	1.4	2.2	LMATVR

5.60 2 2542 kinesin heavy chain 1 [Aplysia californica] gi; 2 2542 kinesin heavy chain 1 [Aplysia californica] gi

Protein Accession	CL4142.Contig6$_{H}1$ CL4142.Contig5$_{H}1$
Mean Expression Ratio	0.734
Median Expression Ratio	0.734
Credible Interval	(0.504, 1.06)
Associated Peptides	2
Associated Spectra	5
Coverage	NaN

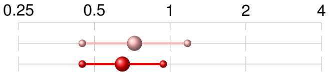

A	B	2.5	50	97.5	Sequence
1	0	0.45	0.72	1.2	QQLEAEK
4	0	0.45	0.65	0.94	LSELSIQMSRREQAK

5.61 238 681 minus strand Globin OS=Cerithidea rhizophorarum PE=1 SV=1

Protein Accession	CL8458.Contig2$_{H}1$
Mean Expression Ratio	1.36
Median Expression Ratio	1.36
Credible Interval	(1.12, 1.64)
Associated Peptides	8
Associated Spectra	119
Coverage	NaN

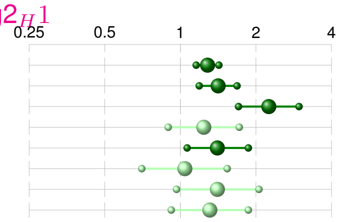

A	B	2.5	50	97.5	Sequence
43	33	1.2	1.3	1.4	AAITSTWSK
15	9	1.2	1.4	1.7	AYFPFLPGASEGDIK
4	2	1.7	2.2	3	ILAMDDADCLDGLGTK
0	3	0.89	1.2	1.7	PFLPGASEGDIK
0	6	1.1	1.4	1.9	PGASEGDIK
0	1	0.7	1	1.5	ATMENNGAK
0	1	0.96	1.4	2.1	EVFGSFLEATL
0	2	0.92	1.3	1.9	EVFGSFLEATLGGAATPDVK

5.62 66 947 Glyoxalase domain-containing protein 4 [Crassostrea gigas]

Protein Accession	CL23175.Contig2$_{H}1$
Mean Expression Ratio	1.36
Median Expression Ratio	1.36
Credible Interval	(0.836, 2.19)
Associated Peptides	1
Associated Spectra	1
Coverage	NaN

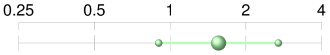

A	B	2.5	50	97.5	Sequence
0	1	0.9	1.6	2.7	LELVEIGQK

5.63 " 110 1717 PREDICTED: glutamate dehydrogenase, mitochondrial-like [Oreochromis niloticus]; 110 1717 PREDICTED: glutamate dehydrogenase, mitochondrial-like [Oreochromis niloticus]; 110 1717 PREDICTED: glutamate dehydrogenase, mitochondrial-like [Oreochromis niloticus]"

Protein Accession	CL16377.Contig9$_{H}1$ CL16377.Contig8$_{H}1$ CL16377.Contig10$_{H}1$
Mean Expression Ratio	1.35
Median Expression Ratio	1.35
Credible Interval	(0.959, 1.88)
Associated Peptides	4
Associated Spectra	6
Coverage	NaN

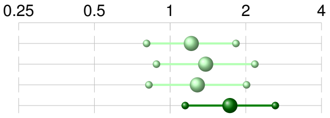

A	B	2.5	50	97.5	Sequence
1	1	0.8	1.2	1.8	IIAEGANGPTTAGADEIFLK
1	0	0.88	1.4	2.2	DGSIYNPVDGIHPR
0	1	0.82	1.3	2	TAAYIAAIEK
0	2	1.2	1.7	2.6	EAGLTFT

5.64 140 349 copper chaperone Atox1 [Crassostrea ariakensis]

Protein Accession	CL8999.Contig5$_{H}1$
Mean Expression Ratio	1.35
Median Expression Ratio	1.34
Credible Interval	(0.871, 2.13)
Associated Peptides	1
Associated Spectra	2
Coverage	NaN

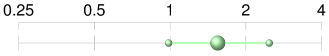

A	B	2.5	50	97.5	Sequence
1	1	0.99	1.5	2.5	EELMATLQK

5.65 105 503 Transgelin-2 [Crassostrea gigas]

Protein Accession	CL445.Contig2$_{H}1$
Mean Expression Ratio	1.34
Median Expression Ratio	1.34
Credible Interval	(0.993, 1.82)
Associated Peptides	2
Associated Spectra	15
Coverage	NaN

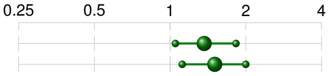

A	B	2.5	50	97.5	Sequence
4	4	1	1.4	1.8	SGLGLEIDK
5	2	1.1	1.5	2	SGLGLEIDKK

5.66 30 1088 arginine kinase [Conus novaehollandiae]

Protein Accession	CL5308.Contig5$_{H}1$
Mean Expression Ratio	1.34
Median Expression Ratio	1.34
Credible Interval	(1.21, 1.49)
Associated Peptides	31
Associated Spectra	204
Coverage	NaN

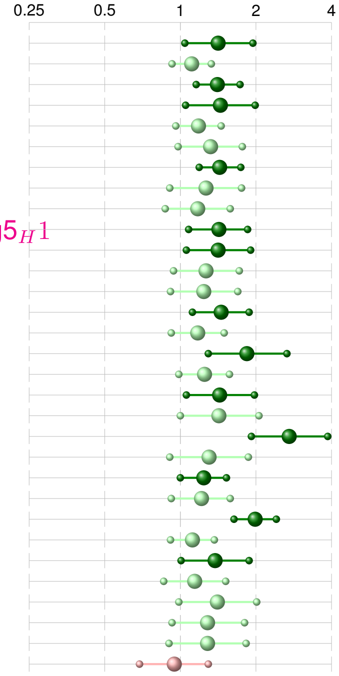

A	B	2.5	50	97.5	Sequence
2	1	1	1.4	2	AAGGYDDWPVGR
12	10	0.93	1.1	1.3	ATVEALNTFEGELK
8	8	1.2	1.4	1.7	FNFPDLDPSGK
2	1	1.1	1.4	2	GIHGEHTESVGGVYDISNK
9	5	0.96	1.2	1.5	GTYYPLLGMDK
2	2	0.98	1.3	1.8	KLIDDHFLFKDDDR
11	7	1.2	1.4	1.7	LAALPEFK
1	1	0.9	1.3	1.8	LGLSEFDAIMEMK
2	2	0.87	1.2	1.6	LIDDHFLFKDDDR
5	1	1.1	1.4	1.8	RFNFPDLDPSGK
3	1	1.1	1.4	1.9	SHASYGFPPVLSK
3	1	0.94	1.3	1.7	TFLVWVNEEDHIR
2	1	0.91	1.2	1.7	NGVEEILKK
7	0	1.1	1.4	1.9	KLTFAR
5	4	0.92	1.2	1.5	NGVEEILK
1	0	1.3	1.8	2.7	KLIDDHFLFK
6	4	0.99	1.2	1.6	LTFAR
2	1	1.1	1.4	2	EFCDK
1	0	1	1.4	2.1	TFEGELK
2	0	1.9	2.7	3.9	GTYYPLLGMDKATEK
1	0	0.91	1.3	1.9	APDFGEK
13	0	1	1.2	1.5	GGNLTQVYQR
5	1	0.92	1.2	1.6	HPAPDFGEK
10	7	1.6	2	2.4	LVSGIHALEK
7	9	0.91	1.1	1.4	YIVSTR
0	3	1	1.4	1.9	LIDDHFLFK
0	5	0.86	1.1	1.5	IISMQK
0	1	0.99	1.4	2	VAEVK
0	2	0.92	1.3	1.8	IDDHFLFK
0	1	0.9	1.3	1.8	ATVEALNTFEGE
0	3	0.69	0.94	1.3	GIFHNPDK

5.67 111 1226 gelsolin [Aplysia californica] gi; 42 1139 minus strand gelsolin [Aplysia californica] gi

Protein Accession	Unigene39723$_{H}1$ Unigene32782$_{H}1$
Mean Expression Ratio	1.34
Median Expression Ratio	1.34
Credible Interval	(1.16, 1.56)
Associated Peptides	18
Associated Spectra	76
Coverage	NaN

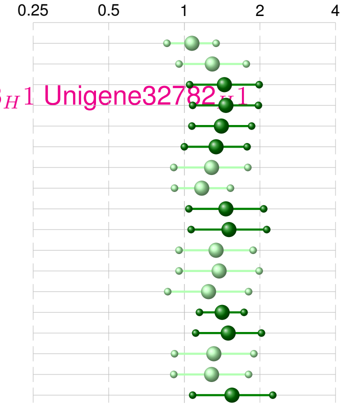

A	B	2.5	50	97.5	Sequence
4	8	0.85	1.1	1.3	AMQFIGEIK
3	1	0.96	1.3	1.8	DLVTSDDVYILDTGLK
3	0	1	1.4	2	ESGNPWRPVTVIK
4	0	1.1	1.5	2	EVFVWVGSNASPDEK
5	1	1.1	1.4	1.9	EVQGHESDLFK
3	2	1	1.3	1.8	KNGLPLAHNYLR
1	1	0.91	1.3	1.8	QGIVTMEGGAASGFR
4	4	0.91	1.2	1.5	SDDVFIFDSGK
1	1	1	1.5	2.1	TVELDTK
2	0	1.1	1.5	2.1	SVPAEEYK
1	1	0.95	1.3	1.9	QFDTALAA
1	0	0.95	1.4	2	KESADSEPAWDGAGQKPGLEIWR
1	0	0.86	1.2	1.8	NSTQDEYGTAAYK
13	3	1.2	1.4	1.7	LFHFAK
3	1	1.1	1.5	2	VVPWPK
0	1	0.91	1.3	1.9	DSNLALFGSDTEK
0	2	0.91	1.3	1.8	LNDFK
0	1	1.1	1.5	2.2	NGLPLAHNYLR

5.68 4 675 Laminin subunit gamma-1 [Crassostrea gigas]

Protein Accession	CL35712.Contig1$_{H}1$
Mean Expression Ratio	0.745
Median Expression Ratio	0.746
Credible Interval	(0.534, 1.03)
Associated Peptides	6
Associated Spectra	6
Coverage	NaN

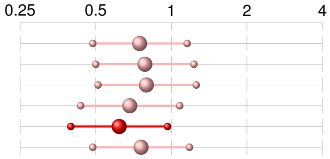

A	B	2.5	50	97.5	Sequence
1	0	0.49	0.75	1.2	AAQLTASETASK
1	0	0.5	0.78	1.2	LAQLAEDTAIQASAEAR
1	0	0.51	0.8	1.3	TLQTLLEFDR
1	0	0.44	0.68	1.1	VPEIEK
1	0	0.4	0.62	0.97	TLSQEVNDAEGR
0	1	0.49	0.76	1.2	ANIEAEIK

5.69 258 629 Glycogenin-1 OS=Oryctolagus cuniculus GN=GYG1 PE=1 SV=3

Protein Accession	Unigene77101$_{H}1$
Mean Expression Ratio	1.33
Median Expression Ratio	1.34
Credible Interval	(0.822, 2.16)
Associated Peptides	1
Associated Spectra	1
Coverage	NaN

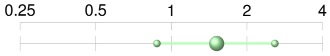

A	B	2.5	50	97.5	Sequence
1	0	0.88	1.5	2.6	IHCWELTQYEK

5.70 616 1740 5'-AMP-activated protein kinase subunit beta-2 [Crassostrea gigas]

Protein Accession	Unigene52845$_{H}1$
Mean Expression Ratio	1.34
Median Expression Ratio	1.33
Credible Interval	(0.828, 2.17)
Associated Peptides	1
Associated Spectra	1
Coverage	NaN

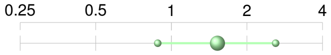

A	B	2.5	50	97.5	Sequence
1	0	0.88	1.5	2.6	FIVDEQWVVSK

5.71 82 816 PREDICTED: triosephosphate isomerase B [Oreochromis niloticus]

Protein Accession	CL26247.Contig4$_{H}1$
Mean Expression Ratio	1.33
Median Expression Ratio	1.33
Credible Interval	(1.09, 1.65)
Associated Peptides	8
Associated Spectra	29
Coverage	NaN

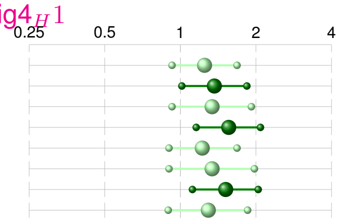

A	B	2.5	50	97.5	Sequence
3	2	0.92	1.2	1.7	AIADNVNDWK
3	2	1	1.4	1.8	GAFTGEISPAMIK
2	0	0.93	1.3	1.9	HVFGESDELIGQK
3	2	1.2	1.6	2.1	QFFVGGNWK
4	0	0.9	1.2	1.7	ELATKPDVDGFLVGGASLK
1	0	0.9	1.3	2	IGVAAQNCYKEPK
3	2	1.1	1.5	2	TEFITIINSK
0	2	0.9	1.3	1.9	SVDEIIK

5.72 2939 3208 minus strand beta-thymosin domain repeat protein CSP24KDa_v1 [Hermissenda crassicornis] gi

Protein Accession	CL9548.Contig3$_{H}1$
Mean Expression Ratio	1.33
Median Expression Ratio	1.33
Credible Interval	(0.974, 1.82)
Associated Peptides	4
Associated Spectra	8
Coverage	NaN

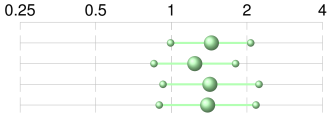

A	B	2.5	50	97.5	Sequence
2	1	0.99	1.4	2.1	NPLPSTDAIAAEK
2	1	0.85	1.2	1.8	AETEEKNPLPTK
1	0	0.92	1.4	2.2	KAETEEKNPLPTK
0	1	0.9	1.4	2.2	SIGEFDK

5.73 373 579 myosin heavy chain [Argopecten irradians] gi

Protein Accession	Unigene64289$_{H}1$
Mean Expression Ratio	0.751
Median Expression Ratio	0.752
Credible Interval	(0.625, 0.902)
Associated Peptides	9
Associated Spectra	63
Coverage	NaN

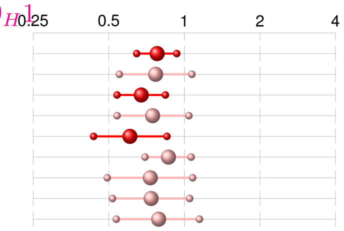

A	B	2.5	50	97.5	Sequence
15	7	0.65	0.78	0.93	QVEEAEEIAAVNLAK
2	1	0.55	0.77	1.1	RQVEEAEEIAAVNLAK
8	5	0.54	0.68	0.84	VQNELEDAEER
2	1	0.54	0.75	1	ELEDAEER
2	1	0.43	0.61	0.85	ADSAEGTLSK
11	4	0.7	0.86	1.1	KVQNELEDAEER
1	0	0.49	0.73	1.1	QELVDKLQAK
1	1	0.52	0.74	1.1	VDKLQAK
0	1	0.54	0.79	1.2	VSVSRR

5.74 186 1778 predicted protein [Nematostella vectensis] gi

Protein Accession	CL20488.Contig9$_{H}1$
Mean Expression Ratio	1.33
Median Expression Ratio	1.33
Credible Interval	(0.908, 1.93)
Associated Peptides	3
Associated Spectra	4
Coverage	NaN

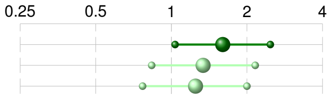

A	B	2.5	50	97.5	Sequence
**1**	**1**	**1**	**1.6**	**2.5**	**EGTENVEMHFR**
**0**	**1**	**0.83**	**1.3**	**2.2**	**DGTTHVIWK**
**0**	**1**	**0.77**	**1.2**	**2**	**TRQLLK**

5.75 457 1842 Basement membrane-specific heparan sulfate proteoglycan core protein [Crassostrea gigas]; 457 1842 Basement membrane-specific heparan sulfate proteoglycan core protein [Crassostrea gigas]

Protein Accession	CL24300.Contig9$_{H}1$ CL24300.Contig10$_{H}1$
Mean Expression Ratio	0.753
Median Expression Ratio	0.753
Credible Interval	(0.508, 1.1)
Associated Peptides	2
Associated Spectra	4
Coverage	NaN

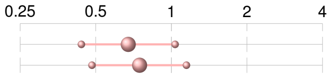

A	B	2.5	50	97.5	Sequence
**1**	**1**	**0.44**	**0.67**	**1**	**KDGELPVEHK**
**1**	**1**	**0.48**	**0.75**	**1.1**	**IESSGALYIPR**

5.76 176 667 hypothetical protein CGI_10018452 [Crassostrea gigas]; 176 634 hypothetical protein CGI_10018452 [Crassostrea gigas]

Protein Accession	CL35173.Contig3$_{H}1$ CL35173.Contig1$_{H}1$
Mean Expression Ratio	0.755
Median Expression Ratio	0.754
Credible Interval	(0.462, 1.22)
Associated Peptides	1
Associated Spectra	1
Coverage	NaN

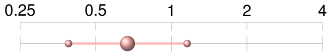

A	B	2.5	50	97.5	Sequence
**0**	**1**	**0.39**	**0.67**	**1.2**	**NFLELTELK**

5.77 496 2151 NADP-dependent malic enzyme [Crassostrea gigas]

Protein Accession	CL26274.Contig1$_H1$
Mean Expression Ratio	0.755
Median Expression Ratio	0.755
Credible Interval	(0.621, 0.92)
Associated Peptides	11
Associated Spectra	40
Coverage	NaN

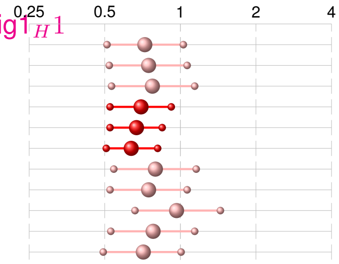

A	B	2.5	50	97.5	Sequence
1	1	0.51	0.72	1	AECTAEEAYTLTEGR
2	0	0.52	0.75	1.1	AIVVTDGER
1	0	0.53	0.78	1.1	GLFISIKDK
4	2	0.52	0.7	0.92	IWMVDSK
8	3	0.53	0.67	0.85	VLVNFR
7	4	0.51	0.64	0.81	GIDILR
1	0	0.54	0.8	1.2	GTAFSLK
1	1	0.53	0.75	1.1	GLLVK
1	0	0.66	0.97	1.4	GDLGVYGMGIP
0	1	0.53	0.78	1.1	QGTAAVAAAGVFAAVR
0	2	0.49	0.71	1	INERPIIFALSNPTSK

5.78 230 334 protein disulfide isomerase [Conus virgo]

Protein Accession	Unigene67026$_H1$
Mean Expression Ratio	1.32
Median Expression Ratio	1.32
Credible Interval	(0.912, 1.92)
Associated Peptides	1
Associated Spectra	8
Coverage	NaN

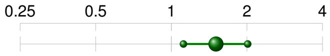

A	B	2.5	50	97.5	Sequence
4	4	1.1	1.5	2	VDATVENDLASK

5.79 340 615 putative Low-density lipoprotein receptor repeat class B [Trichinella spiralis] gi

Protein Accession	Unigene31531$_H1$
Mean Expression Ratio	0.758
Median Expression Ratio	0.758
Credible Interval	(0.468, 1.22)
Associated Peptides	1
Associated Spectra	1
Coverage	NaN

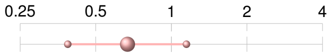

A	B	2.5	50	97.5	Sequence
0	1	0.39	0.67	1.2	VLPFGLK

5.80 62 505 Myosin-2 essential light chain [Crassostrea gigas]

Protein Accession	CL47077.Contig1$_H1$
Mean Expression Ratio	1.31
Median Expression Ratio	1.31
Credible Interval	(0.946, 1.82)
Associated Peptides	2
Associated Spectra	10
Coverage	NaN

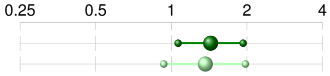

A	B	2.5	50	97.5	Sequence
5	2	1.1	1.4	1.9	IAASQLGDVLR
2	1	0.93	1.4	2	HILTSLGEK

5.81 185 742 Transmembrane protein Tmp21 [Crassostrea gigas]; 1 444 minus strand Transmembrane protein Tmp21 [Crassostrea gigas]

Protein Accession	CL3732.Contig3$_H$1 CL3732.Contig1$_H$1
Mean Expression Ratio	0.761
Median Expression Ratio	0.763
Credible Interval	(0.49, 1.17)
Associated Peptides	2
Associated Spectra	2
Coverage	NaN

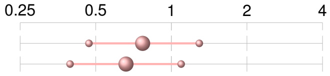

A	B	2.5	50	97.5	Sequence
**1**	**0**	**0.47**	**0.77**	**1.3**	**LKPLEVELR**
**1**	**0**	**0.4**	**0.66**	**1.1**	**EVHLNLK**

5.82 697 954 minus strand Peptidyl-prolyl cis-trans isomerase B [Crassostrea gigas]; 614 877 Peptidyl-prolyl cis-trans isomerase B [Crassostrea gigas]

Protein Accession	CL49248.Contig4$_H$1 CL49248.Contig3$_H$1
Mean Expression Ratio	0.762
Median Expression Ratio	0.763
Credible Interval	(0.562, 1.03)
Associated Peptides	4
Associated Spectra	19
Coverage	NaN

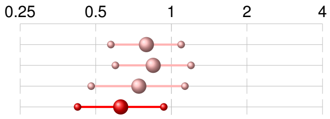

A	B	2.5	50	97.5	Sequence
**4**	**4**	**0.58**	**0.79**	**1.1**	**LVDCSATTLK**
**3**	**3**	**0.6**	**0.85**	**1.2**	**ASLEFLDK**
**0**	**1**	**0.48**	**0.74**	**1.1**	**SMQNLDGR**
**3**	**1**	**0.42**	**0.63**	**0.93**	**INNVPVDK**

5.83 49 819 PREDICTED: elongation factor 1-delta-like isoform 2 [Strongylocentrotus purpuratus]

Protein Accession	CL10790.Contig12$_H$1
Mean Expression Ratio	0.764
Median Expression Ratio	0.763
Credible Interval	(0.552, 1.07)
Associated Peptides	4
Associated Spectra	7
Coverage	NaN

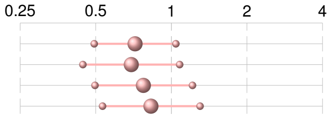

A	B	2.5	50	97.5	Sequence
**1**	**3**	**0.49**	**0.72**	**1**	**LVDLAYGIK**
**0**	**1**	**0.45**	**0.69**	**1.1**	**LLEEYAAK**
**0**	**1**	**0.49**	**0.77**	**1.2**	**RLEEIEK**
**0**	**1**	**0.53**	**0.83**	**1.3**	**EISLAR**

5.84 801 971 RecName: Full=Tropomyosin-2; AltName: Full=Tropomyosin II; Short=BgTMII; Short=TMII gi|155940|gb|AAA27816.1| tropomyosin [Biomphalaria glabrata]

Protein Accession	CL2925.Contig4$_{H}1$
Mean Expression Ratio	1.31
Median Expression Ratio	1.31
Credible Interval	(0.947, 1.79)
Associated Peptides	3
Associated Spectra	11
Coverage	NaN

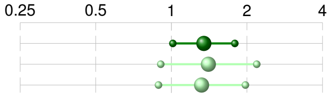

A	B	2.5	50	97.5	Sequence
7	1	1	1.3	1.8	EDSYEETIR
0	1	0.91	1.4	2.2	ISEQEASQR
0	2	0.89	1.3	2	QQYEIFGISEQEASQR

5.85 425 1051 minus strand unknown [Schistosoma japonicum]; 300 878 unknown [Schistosoma japonicum]; 567 1145 unknown [Schistosoma japonicum]; 1110 1688 unknown [Schistosoma japonicum]; 94 672 unknown [Schistosoma japonicum]; 599 1177 unknown [Schistosoma japonicum]; 336 914 unknown [Schistosoma japonicum]

Protein Accession	CL11292.Contig9$_{H}1$ CL11292.Contig5$_{H}1$ CL11292.Contig3$_{H}1$ CL11292.Contig2$_{H}1$ CL1129
Mean Expression Ratio	0.767
Median Expression Ratio	0.767
Credible Interval	(0.53, 1.1)
Associated Peptides	2
Associated Spectra	9
Coverage	NaN

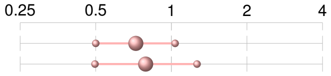

A	B	2.5	50	97.5	Sequence
4	4	0.5	0.72	1	AGNDVDLVVQR
0	1	0.5	0.79	1.3	RYDTSQPWGFR

5.86 15 428 PREDICTED: histone H2A.V-like [Apis florea]

Protein Accession	CL14228.Contig5$_{H}1$
Mean Expression Ratio	0.768
Median Expression Ratio	0.768
Credible Interval	(0.577, 1.02)
Associated Peptides	4
Associated Spectra	22
Coverage	NaN

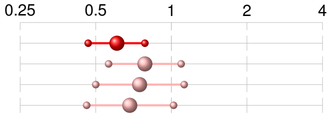

A	B	2.5	50	97.5	Sequence
9	3	0.47	0.61	0.78	AGLQFPVGR
3	3	0.56	0.79	1.1	ATIAGGGVIPHIHK
1	1	0.5	0.75	1.1	GDEELDSLIK
1	1	0.46	0.68	1	HLQLAIR

5.87 243 2165 minus strand Elongation factor 2 OS=Drosophila melanogaster GN=Ef2b PE=2 SV=4

Protein Accession	Unigene11527$_{H}$1
Mean Expression Ratio	0.769
Median Expression Ratio	0.769
Credible Interval	(0.497, 1.18)
Associated Peptides	2
Associated Spectra	2
Coverage	NaN

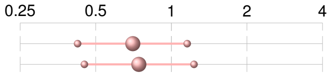

A	B	2.5	50	97.5	Sequence
0	1	0.42	0.7	1.2	TQEDTLALADK
0	1	0.45	0.74	1.2	FNMEEVK

5.88 211 990 PREDICTED: gamma-glutamyl hydrolase-like [Xenopus (Silurana) tropicalis]; 223 1080 PREDICTED: gamma-glutamyl hydrolase [Gallus gallus]

Protein Accession	CL20208.Contig9$_{H}$1 CL20208.Contig3$_{H}$1
Mean Expression Ratio	0.769
Median Expression Ratio	0.77
Credible Interval	(0.491, 1.2)
Associated Peptides	1
Associated Spectra	2
Coverage	NaN

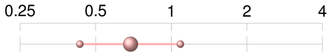

A	B	2.5	50	97.5	Sequence
2	0	0.43	0.69	1.1	YLWQAGAR

5.89 201 1181 minus strand Serine/threonine-protein phosphatase PP1-alpha catalytic subunit [Crassostrea gigas]

Protein Accession	CL28655.Contig4$_{H}$1
Mean Expression Ratio	0.77
Median Expression Ratio	0.771
Credible Interval	(0.509, 1.17)
Associated Peptides	2
Associated Spectra	3
Coverage	NaN

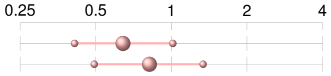

A	B	2.5	50	97.5	Sequence
1	1	0.41	0.64	1	ADTDKLNIDSIIAR
0	1	0.49	0.82	1.3	AHQVVEDGYEFFAK

5.90 174 1649 minus strand Collagen alpha-1(XII) chain [Crassostrea gigas]; 135 1610 Collagen alpha-1(XII) chain [Crassostrea gigas]

Protein Accession	CL5340.Contig13$_{H}1$ CL5340.Contig11$_{H}1$
Mean Expression Ratio	1.29
Median Expression Ratio	1.3
Credible Interval	(1.03, 1.62)
Associated Peptides	10
Associated Spectra	28
Coverage	NaN

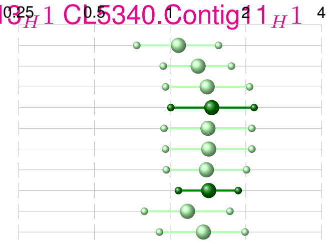

A	B	2.5	50	97.5	Sequence
2	0	0.74	1.1	1.6	ADVMFVTEFSQAGGR
4	1	0.94	1.3	1.8	ADVVFIVDSSR
1	1	0.96	1.4	2.1	DVMSVFHIAR
1	1	1	1.5	2.2	SIWMPDFK
1	0	0.95	1.4	2.1	VDGYSALDTIR
1	0	0.96	1.4	2.1	YWAEQLKK
2	0	0.96	1.4	2	KADVVFVLDK
6	5	1.1	1.4	1.9	DILAFR
1	0	0.79	1.2	1.7	TTELLK
1	0	0.91	1.4	2	SSSITAEHFQTQLR

5.91 230 1189 hypothetical protein [Amblyomma maculatum]

Protein Accession	CL11202.Contig4$_{H}1$
Mean Expression Ratio	1.3
Median Expression Ratio	1.29
Credible Interval	(0.93, 1.82)
Associated Peptides	4
Associated Spectra	7
Coverage	NaN

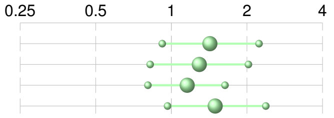

A	B	2.5	50	97.5	Sequence
1	0	0.92	1.4	2.2	KFAVDAVK
1	0	0.82	1.3	2	LSFDKEGMVK
0	4	0.81	1.2	1.6	ILEIIPGR
0	1	0.97	1.5	2.4	SFEPLEDPGVR

5.92 1573 2385 minus strand Spectrin beta chain [Crassostrea gigas]

Protein Accession	CL5904.Contig4$_{H}1$
Mean Expression Ratio	0.772
Median Expression Ratio	0.772
Credible Interval	(0.477, 1.26)
Associated Peptides	1
Associated Spectra	1
Coverage	NaN

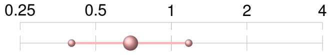

A	B	2.5	50	97.5	Sequence
0	1	0.4	0.69	1.2	RLIQEEHPESDQIAVR

5.93 160 387 small nuclear ribonucleoprotein polypeptide G protein [Crassostrea ariakensis]; 180 407 small nuclear ribonucleoprotein polypeptide G protein [Crassostrea ariakensis]

Protein Accession	CL1272.Contig8$_{H}1$ CL1272.Contig7$_{H}1$
Mean Expression Ratio	0.771
Median Expression Ratio	0.773
Credible Interval	(0.514, 1.16)
Associated Peptides	2
Associated Spectra	3
Coverage	NaN

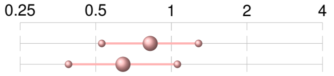

A	B	2.5	50	97.5	Sequence
2	0	0.53	0.82	1.3	TITGILR
1	0	0.39	0.64	1.1	AHPPELK

5.94 95 2506 Transitional endoplasmic reticulum ATPase [Crassostrea gigas]

Protein Accession	CL15973.Contig2$_{H}1$
Mean Expression Ratio	1.29
Median Expression Ratio	1.29
Credible Interval	(0.994, 1.69)
Associated Peptides	9
Associated Spectra	11
Coverage	NaN

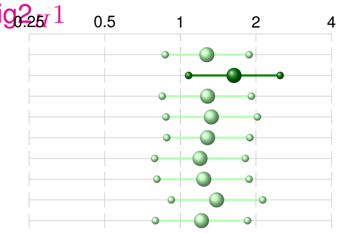

A	B	2.5	50	97.5	Sequence
1	1	0.87	1.3	1.9	EMVELPLR
1	0	1.1	1.6	2.5	LDQLIYIPLPDEK
1	0	0.84	1.3	1.9	MAIEAEIR
1	0	0.87	1.3	2	MDELQLFR
1	1	0.88	1.3	1.9	EVDIGIPDATGR
0	1	0.79	1.2	1.8	DIDLDYLAK
0	1	0.81	1.2	1.9	GILLYGPPGTGK
0	1	0.92	1.4	2.1	AHFEEAMK
0	1	0.8	1.2	1.8	GDIFLVR

5.95 593 862 minus strand mitochondrial ATP synthase coupling factor 6 precursor [Ochlerotatus taeniorhynchus]

Protein Accession	Unigene4216$_{H}1$
Mean Expression Ratio	0.777
Median Expression Ratio	0.776
Credible Interval	(0.511, 1.18)
Associated Peptides	1
Associated Spectra	3
Coverage	NaN

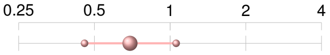

A	B	2.5	50	97.5	Sequence
0	3	0.46	0.69	1.1	LVDASPTVQAALKEELEK

5.96 189 353 40S ribosomal protein S12 [Crassostrea gigas]

Protein Accession	Unigene385$_{H}1$
Mean Expression Ratio	1.29
Median Expression Ratio	1.29
Credible Interval	(0.793, 2.09)
Associated Peptides	1
Associated Spectra	1
Coverage	NaN

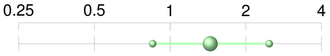

A	B	2.5	50	97.5	Sequence
1	0	0.85	1.4	2.5	TALIHDGLAR

5.97 915 1505 minus strand Collagen alpha-5(VI) chain [Crassostrea gigas]

Protein Accession	CL40183.Contig3$_{H}1$
Mean Expression Ratio	1.29
Median Expression Ratio	1.29
Credible Interval	(1.04, 1.59)
Associated Peptides	8
Associated Spectra	43
Coverage	NaN

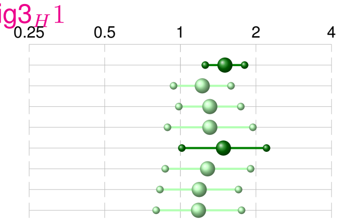

A	B	2.5	50	97.5	Sequence
13	10	1.3	1.5	1.8	LEAIPWR
4	4	0.94	1.2	1.6	VGEQLDAEELLNIAGDPK
2	4	0.98	1.3	1.7	DFVNAFQINPTAVR
1	0	0.89	1.3	1.9	HGLATNTGAGIK
1	0	1	1.5	2.2	HGLATNTGAGIKYMR
1	0	0.87	1.3	1.9	VGEQLDAEELL
0	2	0.83	1.2	1.7	RVVTAENYAHLNLVK
0	1	0.8	1.2	1.8	VVTAENYAHLNLVK

5.98 167 679 tropomyosin [Haliotis discus discus]

Protein Accession	CL2925.Contig3$_{H}$1
Mean Expression Ratio	1.28
Median Expression Ratio	1.28
Credible Interval	(1.14, 1.45)
Associated Peptides	19
Associated Spectra	202
Coverage	NaN

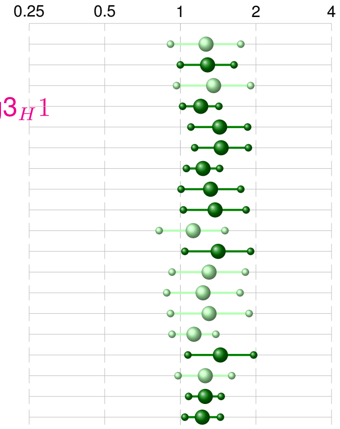

A	B	2.5	50	97.5	Sequence
2	1	0.91	1.3	1.7	AMDKAEQMEQK
6	3	1	1.3	1.6	ANEGLIEANTK
1	1	0.96	1.4	1.9	ENAMDKAEQMEQK
23	3	1	1.2	1.4	IKLLEEDLER
6	1	1.1	1.4	1.9	KYANLENEYDKANEGLIEANTK
8	0	1.1	1.5	1.9	MDKENAMDK
24	8	1.1	1.2	1.4	MDKENAMDKAEQMEQK
3	3	1	1.3	1.7	YANLENEYDK
5	0	1	1.4	1.8	YANLENEYDKANEGLIEANTK
2	2	0.83	1.1	1.5	KMLAMK
4	0	1	1.4	1.9	KYANLENEYDK
1	1	0.92	1.3	1.8	ANEGLIEAN
1	1	0.88	1.2	1.7	SKLEEDLN
1	0	0.91	1.3	1.9	LLEEDLER
3	13	0.93	1.1	1.4	RIKLLEEDLER
4	0	1.1	1.4	2	SKLEEDLNPLQK
8	1	0.98	1.3	1.6	LRDTEVQK
14	19	1.1	1.3	1.5	MDAIK
14	15	1	1.2	1.4	MLAMK

5.99 57 1688 Talin-1 [Camponotus floridanus]; 57 1865 Talin-1 [Camponotus floridanus]; 57 1835 Talin-1 [Camponotus floridanus]

Protein Accession	CL49468.Contig2$_{H}$1 CL45858.Contig2$_{H}$1 CL45858.Contig1$_{H}$1
Mean Expression Ratio	1.28
Median Expression Ratio	1.28
Credible Interval	(0.881, 1.86)
Associated Peptides	2
Associated Spectra	5
Coverage	NaN

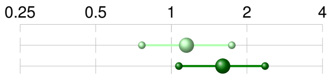

A	B	2.5	50	97.5	Sequence
2	0	0.76	1.1	1.7	KAQEPIVYAGK
3	0	1.1	1.6	2.4	ISIQAR

5.100 6 650 minus strand glutathione S-transferase mu class protein [Crassostrea ariakensis]; 117 761 glutathione S-transferase mu class protein [Crassostrea ariakensis]; 122 766 glutathione S-transferase mu class protein [Crassostrea ariakensis]; 122 766 glutathione S-transferase mu class protein [Crassostrea ariakensis]

Protein Accession	Unigene56987$_{H}$1 CL6887.Contig3$_{H}$1 CL6887.Contig2$_{H}$1 CL6887.Contig1$_{H}$1
Mean Expression Ratio	1.28
Median Expression Ratio	1.28
Credible Interval	(0.983, 1.67)
Associated Peptides	6
Associated Spectra	13
Coverage	NaN

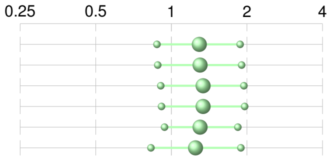

A	B	2.5	50	97.5	Sequence
2	0	0.87	1.3	1.9	ITQSNAILR
1	1	0.88	1.3	1.9	VKETLASYEK
1	1	0.91	1.3	2	PALGYWK
2	0	0.91	1.3	2	IMIPGILDDYPK
3	1	0.94	1.3	1.8	KHDLLGK
0	1	0.83	1.2	1.9	DAWFNVK

5.101 527 1321 minus strand troponin T [Mizuhopecten yessoensis]; 305 1099 minus strand troponin T [Mizuhopecten yessoensis]; 305 1099 minus strand troponin T [Mizuhopecten yessoensis]

Protein Accession	Unigene65787$_H$1 CL2214.Contig4$_H$1 CL2214.Contig3$_H$1
Mean Expression Ratio	0.782
Median Expression Ratio	0.782
Credible Interval	(0.661, 0.922)
Associated Peptides	10
Associated Spectra	105
Coverage	NaN

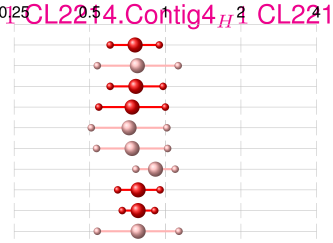

A	B	2.5	50	97.5	Sequence
2	10	0.6	0.76	0.95	LDSVMSKEELQK
1	0	0.54	0.78	1.1	SKEQLEDEK
5	5	0.6	0.76	0.98	SKEQLEDEKR
2	2	0.54	0.74	1	GPNFVITK
2	0	0.51	0.72	1	KGPNFVIT
3	0	0.53	0.74	1	AILSQR
16	6	0.77	0.92	1.1	KGPNFVITK
15	3	0.65	0.78	0.95	LDSVMSK
17	15	0.67	0.78	0.91	LTELR
0	1	0.54	0.78	1.1	EAREAEER

5.102 459 755 minus strand heat shock protein beta-1 [Trichinella spiralis] gi; 464 763 minus strand heat shock protein beta-1 [Trichinella spiralis] gi; 437 736 minus strand heat shock protein beta-1 [Trichinella spiralis] gi; 437 736 minus strand heat shock protein beta-1 [Trichinella spiralis] gi; 437 736 minus strand heat shock protein beta-1 [Trichinella spiralis] gi

Protein Accession	CL17234.Contig5$_H$1 CL17234.Contig4$_H$1 CL17234.Contig3$_H$1 CL17234.Contig2$_H$1 CL1723
Mean Expression Ratio	0.786
Median Expression Ratio	0.784
Credible Interval	(0.53, 1.17)
Associated Peptides	3
Associated Spectra	8
Coverage	NaN

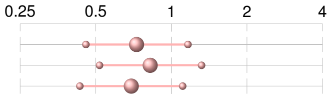

A	B	2.5	50	97.5	Sequence
2	1	0.46	0.73	1.2	DVEPTTVTSSLNADGVLTLK
2	1	0.52	0.82	1.3	IVVEGCHEEK
1	1	0.43	0.69	1.1	IVVEGCHEEKQDEHGFITR

5.103 3 551 minus strand Spectrin beta chain [Crassostrea gigas]

Protein Accession	Unigene35960$_H$1
Mean Expression Ratio	0.785
Median Expression Ratio	0.784
Credible Interval	(0.504, 1.22)
Associated Peptides	2
Associated Spectra	2
Coverage	NaN

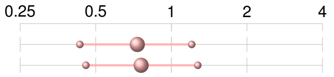

A	B	2.5	50	97.5	Sequence
0	1	0.43	0.73	1.2	ELALREELIR
0	1	0.46	0.76	1.3	IVEKPPKFDEK

5.104 488 1039 Peptidyl-prolyl cis-trans isomerase B [Crassostrea gigas]; 488 1039 Peptidyl-prolyl cis-trans isomerase B [Crassostrea gigas]

Protein Accession	CL49248.Contig2$_{H1}$ CL49248.Contig1$_{H1}$
Mean Expression Ratio	1.28
Median Expression Ratio	1.27
Credible Interval	(0.958, 1.7)
Associated Peptides	4
Associated Spectra	19
Coverage	NaN

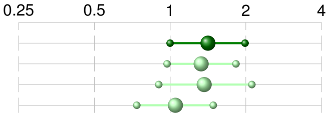

A	B	2.5	50	97.5	Sequence
3	3	1	1.4	2	ASLEFLDK
4	4	0.97	1.3	1.8	LVDCSATTLK
1	0	0.9	1.4	2.1	VTFSPK
3	1	0.74	1	1.5	INNVPVDK

5.105 22 429 AGAP006937-PA [Anopheles gambiae str. PEST] gi; 1 474 Ubiquitin-conjugating enzyme E2 variant 2 OS=Rattus norvegicus GN=Ube2v2 PE=1 SV=3

Protein Accession	CL27581.Contig3$_{H1}$ CL27581.Contig2$_{H1}$
Mean Expression Ratio	0.785
Median Expression Ratio	0.785
Credible Interval	(0.485, 1.29)
Associated Peptides	1
Associated Spectra	1
Coverage	NaN

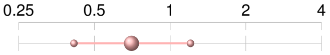

A	B	2.5	50	97.5	Sequence
0	1	0.41	0.7	1.2	LLDELELGEK

5.106 " 81 1349 minus strand PREDICTED: LOW QUALITY PROTEIN: dolichyl-diphosphooligosaccharide–protein glycosyltransferase 48 kDa subunit-like, partial [Cavia porcellus]; 81 1349 minus strand PREDICTED: LOW QUALITY PROTEIN: dolichyl-diphosphooligosaccharide–protein glycosyltransferase 48 kDa subunit-like, partial [Cavia porcellus]"

Protein Accession	CL49524.Contig2$_{H1}$ CL49524.Contig1$_{H1}$
Mean Expression Ratio	1.27
Median Expression Ratio	1.27
Credible Interval	(0.845, 1.9)
Associated Peptides	2
Associated Spectra	3
Coverage	NaN

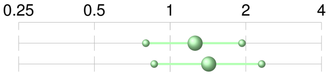

A	B	2.5	50	97.5	Sequence
1	1	0.8	1.3	1.9	DLGFDLTFK
1	0	0.86	1.4	2.3	FPQSGNLELAK

5.107 3 434 minus strand myosin essential light chain [Haliotis discus discus]

Protein Accession	CL17177.Contig8$_{H}1$
Mean Expression Ratio	1.27
Median Expression Ratio	1.27
Credible Interval	(1.07, 1.5)
Associated Peptides	11
Associated Spectra	70
Coverage	NaN

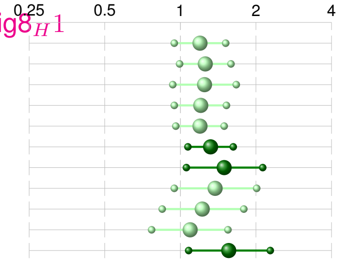

A	B	2.5	50	97.5	Sequence
6	4	0.95	1.2	1.5	EGQGFMSAAELR
5	6	0.99	1.3	1.6	FTDTREDLEGNVK
4	1	0.93	1.2	1.7	HVLTGLGEK
5	5	0.94	1.2	1.5	LTDQQVTDVIK
6	7	0.96	1.2	1.5	YADFIEK
7	7	1.1	1.3	1.6	DGDVDAFK
1	1	1.1	1.5	2.1	VGDMCF
1	0	0.95	1.4	2	EDLEGNVK
1	0	0.85	1.2	1.8	LSDREGQGFMSAAELR
0	2	0.77	1.1	1.6	FTDTREDLEGN
0	1	1.1	1.6	2.3	HVLTGLG

5.108 643 1302 minus strand Kyphoscoliosis peptidase [Crassostrea gigas]; 634 1293 minus strand Kyphoscoliosis peptidase [Crassostrea gigas]; 634 1293 minus strand Kyphoscoliosis peptidase [Crassostrea gigas]; 634 1293 minus strand Kyphoscoliosis peptidase [Crassostrea gigas]; 639 1298 minus strand Kyphoscoliosis peptidase [Crassostrea gigas]

Protein Accession	CL7772.Contig1$_{H}1$ CL7772.Contig16$_{H}1$ CL7772.Contig15$_{H}1$ CL7772.Contig14$_{H}1$ CL7772.C
Mean Expression Ratio	1.27
Median Expression Ratio	1.27
Credible Interval	(1.03, 1.56)
Associated Peptides	9
Associated Spectra	41
Coverage	NaN

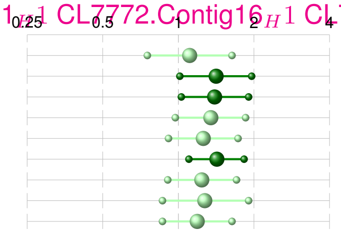

A	B	2.5	50	97.5	Sequence
1	0	0.76	1.1	1.6	DGCYLYSPLVLNSSSR
2	2	1	1.4	2	FQIFALPTSDDSK
4	1	1	1.4	1.9	GAGEVWEGTAALDK
4	0	0.98	1.3	1.9	STTNLIQCR
3	1	0.91	1.3	1.7	VTLNACFGEDESK
7	6	1.1	1.4	1.8	LSNVTFK
3	2	0.91	1.2	1.7	QYAPWR
1	0	0.87	1.3	1.9	LELVSHPQAEFTLAQDNEVEIK
3	1	0.86	1.2	1.6	AVYPFPK

5.109 REVERSED 6 395 PREDICTED: sister chromatid cohesion protein PDS5 homolog B-A isoform 1 [Nasonia vitripennis]

Protein Accession	RRRRRUnigene51506$_{H}1$
Mean Expression Ratio	1.27
Median Expression Ratio	1.27
Credible Interval	(0.932, 1.72)
Associated Peptides	2
Associated Spectra	37
Coverage	NaN

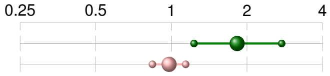

A	B	2.5	50	97.5	Sequence
2	0	1.2	1.8	2.8	IGICIDNFR
25	10	0.84	0.98	1.1	LQELVDK

5.110 180 560 calreticulin precursor [Aplysia californica] gi; 180 545 calreticulin precursor [Aplysia californica] gi

Protein Accession	CL4813.Contig4$_H$1 CL4813.Contig2$_H$1
Mean Expression Ratio	1.27
Median Expression Ratio	1.27
Credible Interval	(0.913, 1.77)
Associated Peptides	3
Associated Spectra	11
Coverage	NaN

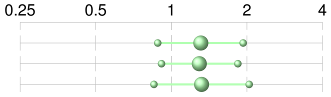

A	B	2.5	50	97.5	Sequence
**2**	**1**	**0.88**	**1.3**	**1.9**	**FVWSAGK**
**4**	**2**	**0.92**	**1.3**	**1.8**	**WIPSVHK**
**1**	**1**	**0.86**	**1.3**	**2**	**FYGISAK**

5.111 " 698 1225 minus strand actin related protein 2/3 complex, 21 kDa subunit [Crassostrea gigas]"

Protein Accession	CL17389.Contig2$_H$1
Mean Expression Ratio	0.789
Median Expression Ratio	0.79
Credible Interval	(0.558, 1.11)
Associated Peptides	3
Associated Spectra	7
Coverage	NaN

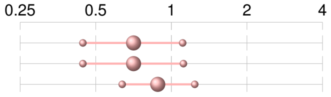

A	B	2.5	50	97.5	Sequence
**0**	**1**	**0.44**	**0.71**	**1.1**	**AYLQQLR**
**0**	**1**	**0.44**	**0.71**	**1.1**	**ANIFFR**
**0**	**5**	**0.64**	**0.88**	**1.2**	**LLPLK**

5.112 721 1242 Lactoylglutathione lyase [Crassostrea gigas]; 882 1334 Lactoylglutathione lyase OS=Rattus norvegicus GN=Glo1 PE=1 SV=3; 113 634 Lactoylglutathione lyase [Crassostrea gigas]

Protein Accession	Unigene26921$_H$1 Unigene26919$_H$1 CL10946.Contig1$_H$1
Mean Expression Ratio	0.789
Median Expression Ratio	0.79
Credible Interval	(0.483, 1.27)
Associated Peptides	1
Associated Spectra	1
Coverage	NaN

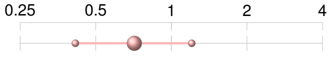

A	B	2.5	50	97.5	Sequence
**1**	**0**	**0.41**	**0.71**	**1.2**	**FESLGVEFIK**

5.113 118 513 Protein CAL-5 [Caenorhabditis elegans] gi

Protein Accession	CL18668.Contig1$_H$1
Mean Expression Ratio	0.792
Median Expression Ratio	0.791
Credible Interval	(0.483, 1.3)
Associated Peptides	1
Associated Spectra	2
Coverage	NaN

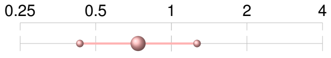

A	B	2.5	50	97.5	Sequence
**1**	**1**	**0.43**	**0.74**	**1.3**	**EADANGDGYLSIQELK**

5.114 119 481 Glutamate receptor U1 [Crassostrea gigas]

Protein Accession	CL570.Contig5$_H$1
Mean Expression Ratio	1.26
Median Expression Ratio	1.26
Credible Interval	(1.03, 1.55)
Associated Peptides	7
Associated Spectra	49
Coverage	NaN

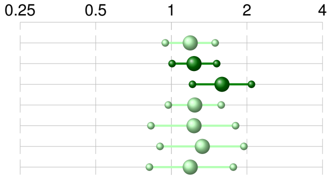

A	B	2.5	50	97.5	Sequence
9	3	0.95	1.2	1.5	GTANIGAGALTVTSER
10	7	1	1.2	1.5	RTPDGWSGIIGDVVK
6	1	1.2	1.6	2.1	TPDGWSGIIGDVVK
7	3	0.97	1.2	1.6	FQYTFR
0	1	0.83	1.2	1.8	AEVVK
0	1	0.9	1.3	1.9	GAGALTVTSER
0	1	0.82	1.2	1.8	GTANIGAGALTVT

5.115 385 906 Soma ferritin OS=Lymnaea stagnalis PE=2 SV=2

Protein Accession	CL2484.Contig1$_H$1
Mean Expression Ratio	0.792
Median Expression Ratio	0.793
Credible Interval	(0.486, 1.28)
Associated Peptides	1
Associated Spectra	1
Coverage	NaN

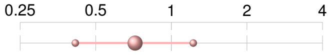

A	B	2.5	50	97.5	Sequence
1	0	0.42	0.71	1.2	ELADHITNLK

5.116 871 1062 Adenylate kinase isoenzyme 5 [Crassostrea gigas]

Protein Accession	Unigene7895$_H$1
Mean Expression Ratio	1.26
Median Expression Ratio	1.26
Credible Interval	(0.924, 1.72)
Associated Peptides	2
Associated Spectra	13
Coverage	NaN

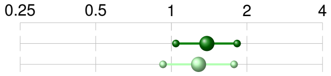

A	B	2.5	50	97.5	Sequence
6	2	1	1.4	1.8	VVFVVGGPGSGK
3	2	0.93	1.3	1.8	ALNEIMQK

5.117 160 588 hypothetical protein CGI_10001640 [Crassostrea gigas]

Protein Accession	CL2347.Contig8$_H$1
Mean Expression Ratio	1.26
Median Expression Ratio	1.26
Credible Interval	(0.908, 1.73)
Associated Peptides	2
Associated Spectra	13
Coverage	NaN

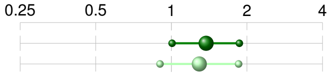

A	B	2.5	50	97.5	Sequence
6	3	1	1.4	1.9	AFLEQLGSK
3	1	0.9	1.3	1.9	ANLIVIGSR

5.118 729 1541 troponin T [Mizuhopecten yessoensis]

Protein Accession	Unigene34289$_H1$
Mean Expression Ratio	0.796
Median Expression Ratio	0.795
Credible Interval	(0.564, 1.12)
Associated Peptides	2
Associated Spectra	8
Coverage	NaN

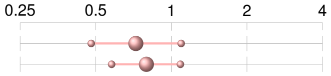

A	B	2.5	50	97.5	Sequence
1	1	0.48	0.72	1.1	RQQYDMIELAER
2	4	0.58	0.79	1.1	QQYDMIELAER

5.119 290 2458 Putative beta-D-xylosidase 5 [Crassostrea gigas]; 299 2428 Putative beta-D-xylosidase 2 [Crassostrea gigas]

Protein Accession	CL49577.Contig2$_H1$ CL1594.Contig5$_H1$
Mean Expression Ratio	1.26
Median Expression Ratio	1.26
Credible Interval	(0.813, 1.95)
Associated Peptides	2
Associated Spectra	3
Coverage	NaN

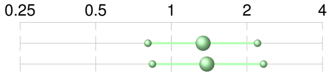

A	B	2.5	50	97.5	Sequence
1	1	0.81	1.3	2.2	VAEAAGVEVR
1	0	0.84	1.4	2.3	VTPLFYTR

5.120 2156 3142 minus strand PREDICTED: hemicentin-1 [Xenopus (Silurana) tropicalis]

Protein Accession	CL2792.Contig4$_H1$
Mean Expression Ratio	1.25
Median Expression Ratio	1.26
Credible Interval	(0.974, 1.62)
Associated Peptides	7
Associated Spectra	15
Coverage	NaN

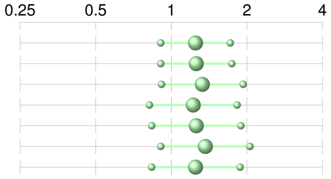

A	B	2.5	50	97.5	Sequence
4	1	0.91	1.3	1.7	GAPPPTVSWVK
2	2	0.91	1.3	1.7	NDVELYSK
1	1	0.91	1.3	1.9	SGVDAMKPYLR
1	0	0.82	1.2	1.8	LVEVIEEK
0	1	0.83	1.3	1.9	YECVAENLVHTR
0	1	0.91	1.4	2.1	NGLIIDTEKDPNLQVDPEGSLMFK
0	1	0.83	1.2	1.9	VDAMK

5.121 633 806 Peroxiredoxin-1 [Crassostrea gigas]; 648 821 Peroxiredoxin-1 [Crassostrea gigas]; 666 839 Peroxiredoxin-1 [Crassostrea gigas]; 625 798 Peroxiredoxin-1 [Crassostrea gigas]

Protein Accession	CL3160.Contig18$_H1$ CL3160.Contig17$_H1$ CL3160.Contig16$_H1$ CL3160.Contig15$_H1$
Mean Expression Ratio	1.26
Median Expression Ratio	1.26
Credible Interval	(0.895, 1.77)
Associated Peptides	2
Associated Spectra	14
Coverage	NaN

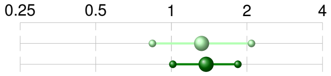

A	B	2.5	50	97.5	Sequence
1	0	0.84	1.3	2.1	EYFSK
7	6	1	1.4	1.8	LVQAFQFTDK

5.122 349 885 juvenile hormone diol kinase [Danaus plexippus]; 349 885 juvenile hormone diol kinase [Danaus plexippus]; 349 885 juvenile hormone diol kinase [Danaus plexippus]; 349 885 minus strand juvenile hormone diol kinase [Danaus plexippus]; 349 885 juvenile hormone diol kinase [Danaus plexippus]; 349 885 juvenile hormone diol kinase [Danaus plexippus]; 349 885 juvenile hormone diol kinase [Danaus plexippus]; 349 885 juvenile hormone diol kinase [Danaus plexippus]; 349 885 juvenile hormone diol kinase [Danaus plexippus]; 349 885 juvenile hormone diol kinase [Danaus plexippus]

Protein Accession	Unigene38920 $_H1$ Unigene38919 $_H1$ CL7590.Contig1 $_H1$ CL7590.Contig16 $_H1$ CL7590.Contig
Mean Expression Ratio	1.25
Median Expression Ratio	1.25
Credible Interval	(1.05, 1.47)
Associated Peptides	12
Associated Spectra	62
Coverage	NaN

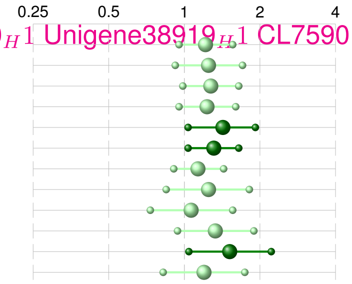

A	B	2.5	50	97.5	Sequence
5	4	0.95	1.2	1.6	DGIIDKNDFK
3	1	0.92	1.3	1.7	DILENVWEGLQK
6	2	0.99	1.3	1.6	FMFLANDTSGDGFVDR
4	4	0.95	1.2	1.6	IFGNSEADSNK
4	0	1	1.4	1.9	SVYQNLYDSNK
9	2	1	1.3	1.7	VPQWNEEYIK
9	3	0.91	1.1	1.4	GEFVTVQK
1	0	0.85	1.2	1.8	FSESGKVPQWNEEYIK
1	0	0.73	1.1	1.6	YIKFMFLANDTSGDGFVDR
0	2	0.94	1.3	1.9	DADINEDGIVTLDEWVK
0	1	1	1.5	2.2	DGIVTLDEWVK
0	1	0.82	1.2	1.7	AFDKLAQGAK

5.123 552 1886 minus strand intermediate filament protein [Biomphalaria glabrata]

Protein Accession	Unigene26795 $_H1$
Mean Expression Ratio	1.24
Median Expression Ratio	1.25
Credible Interval	(1.11, 1.4)
Associated Peptides	22
Associated Spectra	156
Coverage	NaN

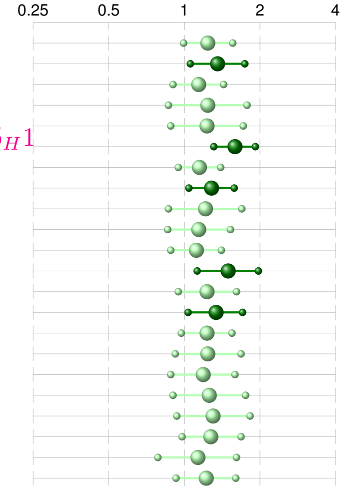

A	B	2.5	50	97.5	Sequence
10	2	0.99	1.2	1.6	ADLDAETAAHIENEVLAQTK
5	3	1.1	1.4	1.7	CGSLEDELAK
5	5	0.9	1.1	1.4	DMYEGELAQAR
1	0	0.87	1.2	1.8	DSYLVQLNELQLEFER
2	0	0.88	1.2	1.7	IAELEAALAAMK
12	7	1.3	1.6	1.9	KQEDWKPIR
14	4	0.94	1.1	1.4	KVITEFSTQR
11	3	1	1.3	1.6	LATYIEK
1	1	0.86	1.2	1.7	LEAELEALR
3	2	0.86	1.1	1.5	LLEGEENR
6	4	0.88	1.1	1.4	LRDQLAEK
4	1	1.1	1.5	2	SATGISEFR
4	2	0.94	1.2	1.6	VITEFSTQR
6	2	1	1.3	1.7	YAAQMDQLR
7	4	0.97	1.2	1.5	KLLEGEENR
4	0	0.92	1.2	1.7	MSATGISEFR
3	1	0.88	1.2	1.6	ELMDHK
1	1	0.9	1.3	1.8	EMQDCNER
1	1	0.93	1.3	1.8	IANLQDQIQALK
4	2	0.98	1.3	1.7	WLEAQNK
0	1	0.78	1.1	1.6	ALVDKLK
0	6	0.93	1.2	1.6	KLEAELEALR

5.124 50 577 hypothetical protein BRAFLDRAFT_119799 [Branchiostoma floridae] gi; 50 577 hypothetical protein BRAFLDRAFT_119799 [Branchiostoma floridae] gi

Protein Accession	CL5795.Contig4$_{H}$1 CL5795.Contig3$_{H}$1
Mean Expression Ratio	0.804
Median Expression Ratio	0.803
Credible Interval	(0.496, 1.31)
Associated Peptides	1
Associated Spectra	1
Coverage	NaN

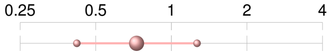

A	B	2.5	50	97.5	Sequence
**0**	**1**	**0.42**	**0.73**	**1.3**	**ADLGIDLSSVLGNVR**

5.125 125 550 minus strand ribosomal protein S20 [Chlamys farreri]; 125 550 minus strand ribosomal protein S20 [Chlamys farreri]; 125 550 minus strand ribosomal protein S20 [Chlamys farreri]

Protein Accession	CL6833.Contig3$_{H}$1 CL6833.Contig2$_{H}$1 CL6833.Contig1$_{H}$1
Mean Expression Ratio	0.803
Median Expression Ratio	0.804
Credible Interval	(0.536, 1.2)
Associated Peptides	2
Associated Spectra	3
Coverage	NaN

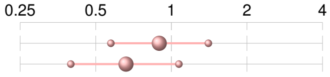

A	B	2.5	50	97.5	Sequence
**2**	**0**	**0.57**	**0.89**	**1.4**	**IIDLHSPSEIVK**
**1**	**0**	**0.4**	**0.66**	**1.1**	**KTPCGEGSK**

5.126 213 881 PREDICTED: hypothetical protein [Saccoglossus kowalevskii]; 260 928 PREDICTED: hypothetical protein [Saccoglossus kowalevskii]; 294 962 PREDICTED: hypothetical protein [Saccoglossus kowalevskii]; 287 955 PREDICTED: hypothetical protein [Saccoglossus kowalevskii]; 316 984 PREDICTED: hypothetical protein [Saccoglossus kowalevskii]

Protein Accession	CL23253.Contig8$_{H}$1 CL23253.Contig7$_{H}$1 CL23253.Contig3$_{H}$1 CL23253.Contig2$_{H}$1 CL2325
Mean Expression Ratio	0.802
Median Expression Ratio	0.804
Credible Interval	(0.493, 1.3)
Associated Peptides	1
Associated Spectra	1
Coverage	NaN

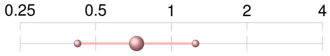

A	B	2.5	50	97.5	Sequence
**0**	**1**	**0.42**	**0.73**	**1.3**	**VDLPGEFR**

5.127 127 657 ADP ribosylation factor 4 [Marsupenaeus japonicus]

Protein Accession	Unigene23129$_H1$
Mean Expression Ratio	1.24
Median Expression Ratio	1.24
Credible Interval	(0.902, 1.73)
Associated Peptides	4
Associated Spectra	8
Coverage	NaN

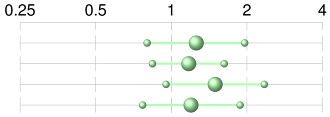

A	B	2.5	50	97.5	Sequence
1	0	0.8	1.3	2	IEEAKDELQK
2	3	0.84	1.2	1.6	ILMVGLDAAGK
0	1	0.95	1.5	2.4	LLVFANK
0	1	0.77	1.2	1.9	LGLNQLR

5.128 245 1243 Prostaglandin reductase 1 [Crassostrea gigas]

Protein Accession	CL37890.Contig1$_H1$
Mean Expression Ratio	0.804
Median Expression Ratio	0.805
Credible Interval	(0.484, 1.32)
Associated Peptides	1
Associated Spectra	1
Coverage	NaN

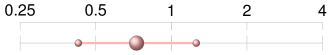

A	B	2.5	50	97.5	Sequence
1	0	0.43	0.73	1.3	TLAVLHPDMPR

5.129 783 1463 minus strand twitchin [Mytilus galloprovincialis]; 783 1463 minus strand twitchin [Mytilus galloprovincialis]; 752 1432 twitchin [Mytilus galloprovincialis]

Protein Accession	CL181.Contig5$_H1$ CL181.Contig3$_H1$ CL181.Contig1$_H1$
Mean Expression Ratio	0.805
Median Expression Ratio	0.805
Credible Interval	(0.617, 1.05)
Associated Peptides	7
Associated Spectra	13
Coverage	NaN

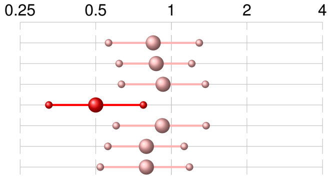

A	B	2.5	50	97.5	Sequence
1	0	0.56	0.85	1.3	DGKPVNSSDIYSITFDK
2	2	0.62	0.87	1.2	LNPQVVNDGDAVDLVVK
2	0	0.63	0.93	1.4	NSGGEVTSTATVNVDALKPK
1	0	0.32	0.5	0.77	VTGTEPFDITWAK
1	0	0.6	0.92	1.4	NQWGTASSMASLQVK
0	3	0.56	0.8	1.1	LEGDTIIIQQDITSK
0	1	0.52	0.79	1.2	SKPDLLWYR

5.130 6 317 myosin heavy chain cardiac muscle specific isoform 2 [Argopecten irradians]

Protein Accession	Unigene19023$_{H}1$
Mean Expression Ratio	0.805
Median Expression Ratio	0.805
Credible Interval	(0.708, 0.915)
Associated Peptides	16
Associated Spectra	242
Coverage	NaN

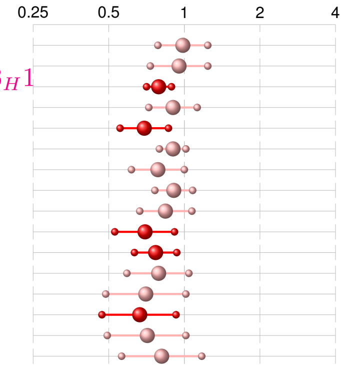

A	B	2.5	50	97.5	Sequence
6	5	0.79	0.98	1.2	ELAFQSDEDR
4	3	0.73	0.95	1.2	ELAFQSDEDRK
37	32	0.71	0.79	0.89	ELEMELDSEQR
11	1	0.72	0.9	1.1	KALETQLK
6	7	0.56	0.69	0.86	LADELRQEQEHSLQVEK
24	30	0.79	0.9	1	LDEAEAQALK
6	3	0.62	0.78	1	LKELAFQSDEDR
17	7	0.76	0.91	1.1	ALETQLK
5	5	0.66	0.84	1.1	LKELAFQSDEDRK
1	5	0.53	0.7	0.92	KAMADAAR
3	14	0.63	0.77	0.93	LADELR
5	0	0.59	0.79	1	QEQEHSLQVEK
0	1	0.49	0.7	1	ELEMELDSEQRR
0	2	0.47	0.66	0.92	LDEAEAQALKGGK
0	1	0.49	0.71	1	VRELEMELDSEQR
0	1	0.56	0.81	1.2	AFQSDEDRK

5.131 " 386 799 minus strand Myosin regulatory light chain LC-2, mantle muscle OS=Todarodes pacificus PE=1 SV=1; 656 1057 minus strand myosin regulatory light chain [Fasciola hepatica]"

Protein Accession	CL43006.Contig13$_{H}1$ CL43006.Contig11$_{H}1$
Mean Expression Ratio	1.24
Median Expression Ratio	1.24
Credible Interval	(0.913, 1.67)
Associated Peptides	2
Associated Spectra	27
Coverage	NaN

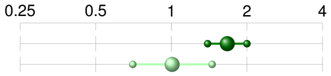

A	B	2.5	50	97.5	Sequence
15	9	1.4	1.7	2	DLLHNMGDNFTDDELK
0	3	0.7	1	1.5	DLLHNMGDNFTDDELKQTWK

5.132 121 660 Failed axon connections homolog OS=Rattus norvegicus GN=Faxc PE=3 SV=1

Protein Accession	Unigene63215$_{H}1$
Mean Expression Ratio	1.24
Median Expression Ratio	1.24
Credible Interval	(0.762, 2)
Associated Peptides	1
Associated Spectra	1
Coverage	NaN

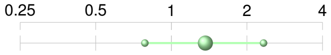

A	B	2.5	50	97.5	Sequence
0	1	0.78	1.4	2.3	LLVLR

5.133 77 1561 retinal dehydrogenase [Lymnaea stagnalis]; 69 1553 retinal dehydrogenase [Lymnaea stagnalis]; 308 1747 retinal dehydrogenase [Lymnaea stagnalis]

Protein Accession	CL13284.Contig7$_{H}$1 CL13284.Contig2$_{H}$1 CL13284.Contig1$_{H}$1
Mean Expression Ratio	1.24
Median Expression Ratio	1.24
Credible Interval	(1.01, 1.52)
Associated Peptides	15
Associated Spectra	24
Coverage	NaN

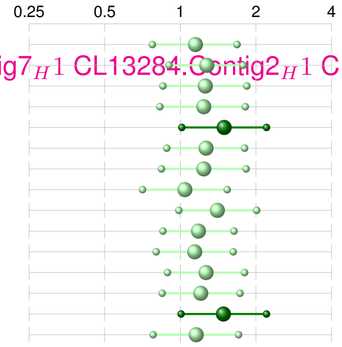

A	B	2.5	50	97.5	Sequence
1	0	0.77	1.1	1.7	ALMFVNGVR
2	1	0.9	1.3	1.8	AVEVAQDAFK
1	0	0.85	1.3	1.8	FSTVEEAIER
1	0	0.83	1.2	1.8	IYDEFVR
1	0	1	1.5	2.2	NIAYLASLETLDNGKPFK
1	1	0.88	1.3	1.8	TFPTINPATGK
1	0	0.84	1.2	1.8	VAFTGSTEVGQIIMK
1	0	0.71	1	1.5	KTAELANKR
2	0	0.98	1.4	2	KTAELANK
3	0	0.85	1.2	1.6	RGALLYK
1	1	0.8	1.1	1.6	LGSAWR
1	1	0.89	1.3	1.8	AIQIIR
2	0	0.84	1.2	1.7	EEIFGPVQQLLK
0	1	1	1.5	2.2	KIVDVQEADK
0	1	0.78	1.2	1.7	KIVDVQEADKADVDK

5.134 3 1670 Spectrin alpha chain [Crassostrea gigas]

Protein Accession	CL2195.Contig7$_{H}$1
Mean Expression Ratio	1.24
Median Expression Ratio	1.24
Credible Interval	(0.904, 1.69)
Associated Peptides	6
Associated Spectra	7
Coverage	NaN

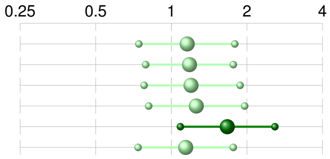

A	B	2.5	50	97.5	Sequence
1	0	0.74	1.2	1.8	NNLMDEFTISVR
1	1	0.79	1.2	1.8	VAALYDYTEK
1	0	0.78	1.2	1.9	HQALQAEIAGHEPR
1	0	0.81	1.3	2	KLDPSLSASR
1	0	1.1	1.7	2.6	ADLEAYRSV
0	1	0.74	1.1	1.8	LTAMGHTEAAEK

5.135 182 1846 minus strand hypothetical protein DAPPUDRAFT_302108 [Daphnia pulex]; 110 1774 minus strand hypothetical protein DAPPUDRAFT_302108 [Daphnia pulex]

Protein Accession	CL11561.Contig6$_{H}$1 CL11561.Contig1$_{H}$1
Mean Expression Ratio	1.24
Median Expression Ratio	1.24
Credible Interval	(0.828, 1.85)
Associated Peptides	3
Associated Spectra	3
Coverage	NaN

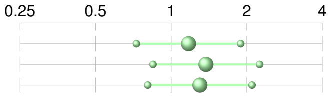

A	B	2.5	50	97.5	Sequence
1	0	0.72	1.2	1.9	LRDAIRK
0	1	0.85	1.4	2.2	LFLPGGLK
0	1	0.8	1.3	2.1	LIIGQR

5.136 2 772 minus strand arylsulfatase [Haliotis discus discus]

Protein Accession	CL48181.Contig1$_{H}1$
Mean Expression Ratio	1.24
Median Expression Ratio	1.24
Credible Interval	(0.83, 1.86)
Associated Peptides	2
Associated Spectra	3
Coverage	NaN

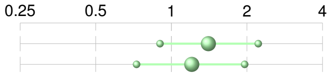

A	B	2.5	50	97.5	Sequence
**1**	**1**	**0.9**	**1.4**	**2.2**	**LLPQLK**
**0**	**1**	**0.73**	**1.2**	**2**	**GVGMVVDALK**

5.137 366 1049 hypothetical protein CGI_10004855 [Crassostrea gigas]

Protein Accession	CL43590.Contig5$_{H}1$
Mean Expression Ratio	0.809
Median Expression Ratio	0.809
Credible Interval	(0.619, 1.06)
Associated Peptides	7
Associated Spectra	14
Coverage	NaN

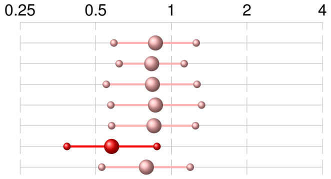

A	B	2.5	50	97.5	Sequence
**1**	**1**	**0.59**	**0.86**	**1.3**	**DEWTHLTAK**
**2**	**4**	**0.62**	**0.84**	**1.1**	**GGDLWQGEVSLEQHR**
**1**	**0**	**0.55**	**0.84**	**1.3**	**ADSDRPSVSAGIGQKPQFQDLK**
**1**	**0**	**0.58**	**0.87**	**1.3**	**VKVPGAK**
**2**	**0**	**0.58**	**0.85**	**1.2**	**KVFPYPK**
**0**	**1**	**0.38**	**0.58**	**0.87**	**TTVQLHK**
**0**	**1**	**0.53**	**0.79**	**1.2**	**KVEIK**

5.138 998 1705 minus strand Basement membrane-specific heparan sulfate proteoglycan core protein [Crassostrea gigas]

Protein Accession	CL22963.Contig1$_{H}1$
Mean Expression Ratio	0.807
Median Expression Ratio	0.809
Credible Interval	(0.497, 1.3)
Associated Peptides	1
Associated Spectra	1
Coverage	NaN

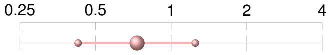

A	B	2.5	50	97.5	Sequence
**1**	**0**	**0.43**	**0.73**	**1.2**	**ITAYGGSLR**

5.139 613 2421 PREDICTED: lysosomal alpha-glucosidase [Danio rerio]

Protein Accession	CL5792.Contig1$_{H}1$
Mean Expression Ratio	1.24
Median Expression Ratio	1.23
Credible Interval	(0.767, 2.01)
Associated Peptides	1
Associated Spectra	1
Coverage	NaN

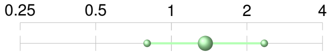

A	B	2.5	50	97.5	Sequence
**1**	**0**	**0.8**	**1.4**	**2.3**	**ATMGGLQK**

5.140 507 1277 alpha tubulin [Notothenia coriiceps]

Protein Accession	Unigene56691$_{H1}$
Mean Expression Ratio	1.23
Median Expression Ratio	1.23
Credible Interval	(0.885, 1.71)
Associated Peptides	2
Associated Spectra	9
Coverage	NaN

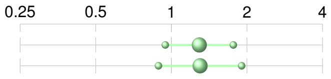

A	B	2.5	50	97.5	Sequence
5	1	0.95	1.3	1.8	LSVDYGK
2	1	0.89	1.3	1.9	LSVDYGKK

5.141 1 378 minus strand PREDICTED: CG10098-like [Saccoglossus kowalevskii]

Protein Accession	Unigene15933$_{H1}$
Mean Expression Ratio	0.81
Median Expression Ratio	0.81
Credible Interval	(0.522, 1.24)
Associated Peptides	2
Associated Spectra	2
Coverage	NaN

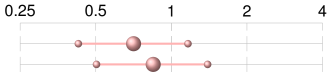

A	B	2.5	50	97.5	Sequence
1	0	0.43	0.71	1.2	AFNASFEGLSLSEK
1	0	0.5	0.85	1.4	ALMAEK

5.142 146 727 Adenylate kinase isoenzyme 1 [Crassostrea gigas]

Protein Accession	CL42551.Contig1$_{H1}$
Mean Expression Ratio	1.23
Median Expression Ratio	1.23
Credible Interval	(0.901, 1.7)
Associated Peptides	4
Associated Spectra	8
Coverage	NaN

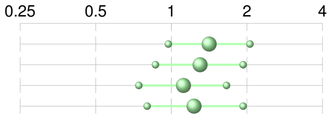

A	B	2.5	50	97.5	Sequence
3	0	0.97	1.4	2.1	DAMIQNITTTK
1	1	0.87	1.3	1.9	ELEQGLR
0	2	0.74	1.1	1.7	VVFVIGGPGSGK
0	1	0.8	1.2	1.9	LNAIMEK

5.143 150 1511 Tensin OS=Gallus gallus GN=TNS PE=1 SV=2

Protein Accession	CL2743.Contig2$_{H1}$
Mean Expression Ratio	1.23
Median Expression Ratio	1.23
Credible Interval	(0.847, 1.81)
Associated Peptides	3
Associated Spectra	4
Coverage	NaN

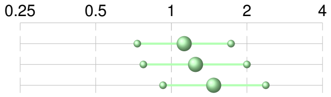

A	B	2.5	50	97.5	Sequence
1	1	0.73	1.1	1.7	DDAIVMLR
1	0	0.77	1.2	2	DKPPGTFVIR
1	0	0.93	1.5	2.4	GTFVIR

5.144 116 1111 minus strand cytosolic malate dehydrogenase [Lottia scabra]

Protein Accession	CL280.Contig16$_H$1
Mean Expression Ratio	1.22
Median Expression Ratio	1.22
Credible Interval	(1.06, 1.41)
Associated Peptides	19
Associated Spectra	130
Coverage	NaN

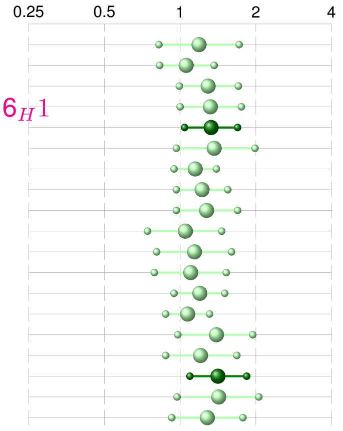

A	B	2.5	50	97.5	Sequence
1	0	0.82	1.2	1.7	ATADHMHDWWFGTEGDK
5	4	0.83	1.1	1.4	DCDAAFLVGAMPR
4	3	0.99	1.3	1.7	DLLAANIK
3	3	1	1.3	1.8	EAVKDDNWINNEFISTVQK
6	5	1	1.3	1.7	ENFSCLTR
1	0	0.97	1.4	2	EVIPTSDEK
15	4	0.95	1.2	1.4	KLSSAMSAAK
6	6	0.97	1.2	1.6	LGIPNNAVSK
3	3	0.96	1.3	1.7	LSSAMSAAK
2	0	0.74	1.1	1.5	QVTIVEGLSISDFAR
2	0	0.81	1.1	1.6	WVSMGVYSTGK
2	1	0.79	1.1	1.5	YGIPEGVMYSYPVQIK
11	0	0.95	1.2	1.5	KDLLAANIK
9	14	0.88	1.1	1.3	EMAEQILAA
2	0	0.98	1.4	1.9	SLLYSVAK
2	1	0.88	1.2	1.7	ELLEER
6	2	1.1	1.4	1.8	SQGANLDAVAK
1	0	0.98	1.4	2.1	VVIVGNPANTNALICAK
2	1	0.93	1.3	1.8	YAPSIPK

5.145 2881 3411 myosin heavy chain [Todarodes pacificus]; 2915 3445 myosin heavy chain [Todarodes pacificus]

Protein Accession	Unigene19024$_H$1 Unigene19021$_H$1
Mean Expression Ratio	0.818
Median Expression Ratio	0.818
Credible Interval	(0.708, 0.948)
Associated Peptides	13
Associated Spectra	136
Coverage	NaN

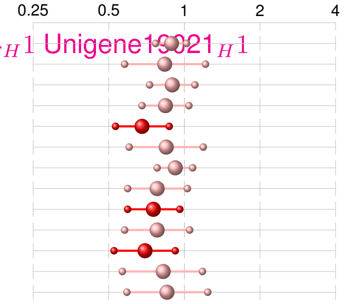

A	B	2.5	50	97.5	Sequence
28	13	0.77	0.89	1	ALDSMQASLEAEAK
1	0	0.58	0.84	1.2	ALTGEVEELR
9	6	0.73	0.89	1.1	CTALTGEVEELR
9	4	0.68	0.84	1	IQEKEEEFENTR
5	4	0.53	0.68	0.87	IQEKEEEFENTRR
2	0	0.6	0.85	1.2	KLEQDINELEIALDQANR
26	1	0.78	0.92	1.1	KLQQQVIETQQHVEEEAR
6	0	0.59	0.78	1	LQQQVIETQQHVEEEAR
4	6	0.6	0.75	0.96	RCTALTGEVEELR
1	3	0.58	0.78	1.1	RIQEKEEEFENTR
3	3	0.53	0.7	0.92	TALTGEVEELR
0	1	0.57	0.83	1.2	IQEKEEEFEN
0	1	0.59	0.85	1.2	ELEIALDQANR

5.146 137 853 14-3-3 protein [Heterodera glycines]

Protein Accession	CL9040.Contig4$_H$1
Mean Expression Ratio	1.22
Median Expression Ratio	1.22
Credible Interval	(0.875, 1.71)
Associated Peptides	4
Associated Spectra	6
Coverage	NaN

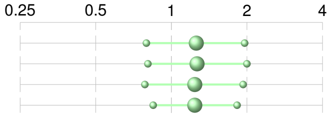

A	B	2.5	50	97.5	Sequence
1	0	0.79	1.3	2	AIECANQAYQK
1	0	0.81	1.3	2	EAFDEAISELDSLQGEDCK
1	0	0.79	1.2	1.9	NASNEEENVFYLK
3	0	0.85	1.2	1.8	VICMIEQK

5.147 167 2713 minus strand Major vault protein [Crassostrea gigas]

Protein Accession	CL18286.Contig3$_{H}1$
Mean Expression Ratio	1.22
Median Expression Ratio	1.22
Credible Interval	(0.881, 1.7)
Associated Peptides	5
Associated Spectra	6
Coverage	NaN

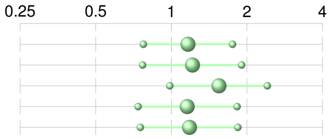

A	B	2.5	50	97.5	Sequence
2	0	0.77	1.2	1.8	GAVAQVQFDDFHK
1	0	0.77	1.2	1.9	KEVVVEETIR
1	0	0.99	1.6	2.4	KPGDRWMIR
0	1	0.74	1.2	1.8	IEGEAAVEQAK
0	1	0.75	1.2	1.8	IVDEAEAEK

5.148 " 145 1755 Fumarate hydratase class I, aerobic [Crassostrea gigas]"

Protein Accession	CL32374.Contig1$_{H}1$
Mean Expression Ratio	1.22
Median Expression Ratio	1.22
Credible Interval	(0.862, 1.72)
Associated Peptides	3
Associated Spectra	6
Coverage	NaN

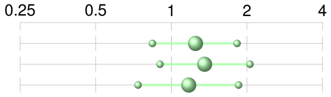

A	B	2.5	50	97.5	Sequence
2	1	0.84	1.2	1.8	IGIGAQFGGK
1	1	0.9	1.4	2.1	KVDTPWK
0	1	0.74	1.2	1.9	LEQLE

5.149 51 890 prohibitin [Octopus tankahkeei]

Protein Accession	CL26635.Contig2$_{H}1$
Mean Expression Ratio	0.82
Median Expression Ratio	0.82
Credible Interval	(0.616, 1.09)
Associated Peptides	6
Associated Spectra	10
Coverage	NaN

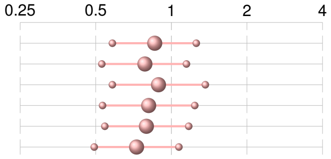

A	B	2.5	50	97.5	Sequence
1	1	0.58	0.86	1.3	AFTEAGEGLVELR
1	1	0.53	0.78	1.1	FLVEKEEQK
1	0	0.58	0.89	1.4	NVPVITGSK
1	0	0.53	0.81	1.2	IEAAEDIA
0	2	0.54	0.79	1.2	AVIFDR
0	2	0.49	0.73	1.1	QVAQQDAERAR

5.150 31 735 hypothetical protein BRAFLDRAFT_78603 [Branchiostoma floridae] gi; 55 735 hypothetical protein BRAFLDRAFT_78603 [Branchiostoma floridae] gi; 353 1039 hypothetical protein BRAFLDRAFT_78603 [Branchiostoma floridae] gi; 207 893 hypothetical protein BRAFLDRAFT_78603 [Branchiostoma floridae] gi

Protein Accession	CL877.Contig7$_{H}1$ CL877.Contig6$_{H}1$ CL877.Contig4$_{H}1$ CL877.Contig3$_{H}1$
Mean Expression Ratio	1.22
Median Expression Ratio	1.22
Credible Interval	(0.9, 1.67)
Associated Peptides	4
Associated Spectra	10
Coverage	NaN

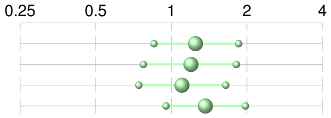

A	B	2.5	50	97.5	Sequence
2	1	0.85	1.3	1.9	DDQTLVTMTGEVAGR
1	1	0.78	1.2	1.8	LKLPLDSSNR
2	0	0.74	1.1	1.6	KVIHSIVGPHFAEGR
3	0	0.95	1.4	2	VALVPK

5.151 203 1942 ezrin/radixin/moesin [Aplysia californica] gi; 243 1982 minus strand ezrin/radixin/moesin [Aplysia californica] gi

Protein Accession	CL13506.Contig5$_{H}1$ CL13506.Contig4$_{H}1$
Mean Expression Ratio	1.22
Median Expression Ratio	1.22
Credible Interval	(0.988, 1.5)
Associated Peptides	13
Associated Spectra	23
Coverage	NaN

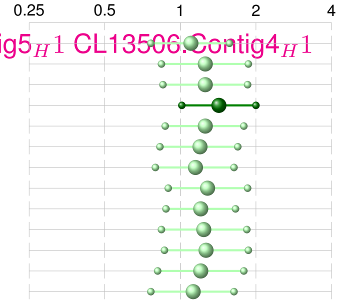

A	B	2.5	50	97.5	Sequence
1	1	0.76	1.1	1.6	DDAMMEYLK
1	0	0.84	1.3	1.9	FVIKPIDK
1	0	0.85	1.3	1.9	KAPDFVFYAPR
3	0	1	1.4	2	NISFNDKK
2	0	0.87	1.3	1.8	RKPDTIEVQQMK
2	0	0.83	1.2	1.7	KESPLQFK
1	1	0.8	1.2	1.6	QLFDQVVK
2	0	0.89	1.3	1.9	QLEMLK
4	0	0.88	1.2	1.7	SWWGEHK
1	0	0.84	1.2	1.8	LADELETAQR
0	1	0.86	1.3	1.9	MAELLALR
0	1	0.81	1.2	1.8	IGFPWSEIR
0	1	0.76	1.1	1.6	ESPLQFK

5.152 " 148 1989 Acyl-CoA synthetase family member 2, mitochondrial [Crassostrea gigas]; 146 1987 minus strand Acyl-CoA synthetase family member 2, mitochondrial [Crassostrea gigas]"

Protein Accession	CL19799.Contig6$_{H}1$ CL19799.Contig5$_{H}1$
Mean Expression Ratio	1.22
Median Expression Ratio	1.22
Credible Interval	(0.757, 1.96)
Associated Peptides	1
Associated Spectra	1
Coverage	NaN

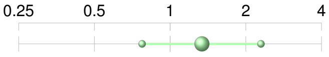

A	B	2.5	50	97.5	Sequence
0	1	0.77	1.3	2.3	LAGPGELGEILVR

5.153 " 154 534 minus strand Muscle M-line assembly protein unc-89, partial [Crassostrea gigas]; 259 690 minus strand Myosin light chain kinase, smooth muscle OS=Gallus gallus PE=1 SV=2"

Protein Accession	Unigene64523$_{H}1$ Unigene64520$_{H}1$
Mean Expression Ratio	1.22
Median Expression Ratio	1.22
Credible Interval	(0.774, 1.9)
Associated Peptides	1
Associated Spectra	3
Coverage	NaN

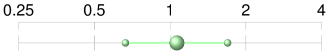

A	B	2.5	50	97.5	Sequence
2	1	0.67	1.1	1.7	LEIGEVVVDDEGEYICK

5.154 87 485 Sex-regulated protein janus-A [Crassostrea gigas]

Protein Accession	CL13131.Contig3$_H$1
Mean Expression Ratio	0.823
Median Expression Ratio	0.822
Credible Interval	(0.506, 1.34)
Associated Peptides	1
Associated Spectra	1
Coverage	NaN

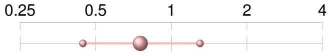

A	B	2.5	50	97.5	Sequence
0	1	0.44	0.75	1.3	ADHSITQTLLLR

5.155 2 169 cAMP-dependent protein kinase regulatory subunit [Crassostrea gigas]; 125 511 cAMP-dependent protein kinase regulatory subunit [Aplysia californica] gi

Protein Accession	CL24422.Contig2$_H$1 CL24422.Contig1$_H$1
Mean Expression Ratio	1.22
Median Expression Ratio	1.22
Credible Interval	(0.754, 1.99)
Associated Peptides	1
Associated Spectra	1
Coverage	NaN

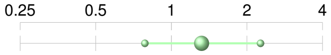

A	B	2.5	50	97.5	Sequence
0	1	0.78	1.3	2.3	VLGPCSDILK

5.156 429 1013 PDZ domain protein [Ixodes scapularis] gi

Protein Accession	CL8129.Contig12$_H$1
Mean Expression Ratio	1.22
Median Expression Ratio	1.22
Credible Interval	(0.96, 1.54)
Associated Peptides	6
Associated Spectra	20
Coverage	NaN

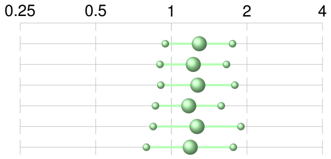

A	B	2.5	50	97.5	Sequence
4	1	0.94	1.3	1.8	DLGQPLSIQR
4	1	0.9	1.2	1.7	HGGGQIQLVIQR
1	2	0.91	1.3	1.8	VFSNSPAEGELQR
3	2	0.86	1.2	1.6	QALDLIK
1	0	0.85	1.3	1.9	DSFNTPWGFR
0	1	0.8	1.2	1.8	GDLILSINNR

5.157 2 1093 minus strand Neurexin-1-alpha [Crassostrea gigas]

Protein Accession	Unigene11591$_H$1
Mean Expression Ratio	1.22
Median Expression Ratio	1.21
Credible Interval	(0.83, 1.79)
Associated Peptides	2
Associated Spectra	4
Coverage	NaN

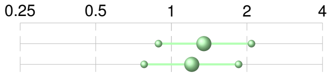

A	B	2.5	50	97.5	Sequence
1	1	0.89	1.3	2.1	IGDVVEAERPFSLIK
1	1	0.78	1.2	1.8	IYVGGILDKEEK

5.158 3 416 minus strand myosin essential light chain [Crassostrea gigas]

Protein Accession	Unigene47974$_{H1}$
Mean Expression Ratio	1.22
Median Expression Ratio	1.21
Credible Interval	(0.85, 1.75)
Associated Peptides	2
Associated Spectra	6
Coverage	NaN

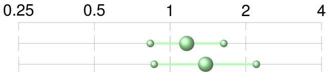

A	B	2.5	50	97.5	Sequence
0	5	0.83	1.2	1.6	CVGLNPTLATIK
0	1	0.86	1.4	2.2	NPTLATIK

5.159 361 549 minus strand 32 kDa beta-galactoside-binding lectin [Ascaris suum]

Protein Accession	CL5658.Contig3$_{H1}$
Mean Expression Ratio	0.822
Median Expression Ratio	0.824
Credible Interval	(0.554, 1.21)
Associated Peptides	1
Associated Spectra	5
Coverage	NaN

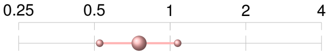

A	B	2.5	50	97.5	Sequence
3	2	0.52	0.75	1.1	TIVLNHMQDGR

5.160 8 271 minus strand Laminin subunit gamma-1 [Crassostrea gigas]

Protein Accession	Unigene36706$_{H1}$
Mean Expression Ratio	1.21
Median Expression Ratio	1.21
Credible Interval	(0.832, 1.76)
Associated Peptides	3
Associated Spectra	4
Coverage	NaN

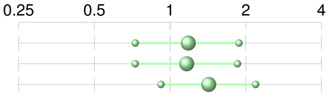

A	B	2.5	50	97.5	Sequence
1	0	0.73	1.2	1.9	EFTDASELYDR
0	1	0.72	1.2	1.9	LAEETLR
0	2	0.92	1.4	2.2	ALDLYR

5.161 900 1616 minus strand hemocyanin isoform 1 [Haliotis diversicolor]

Protein Accession	Unigene30321$_{H1}$
Mean Expression Ratio	0.823
Median Expression Ratio	0.825
Credible Interval	(0.538, 1.25)
Associated Peptides	1
Associated Spectra	3
Coverage	NaN

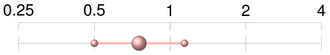

A	B	2.5	50	97.5	Sequence
2	1	0.5	0.75	1.1	VEITDDLK

5.162 296 493 hypothetical protein BRAFLDRAFT_66287 [Branchiostoma floridae] gi

Protein Accession	Unigene414$_{H}1$
Mean Expression Ratio	0.824
Median Expression Ratio	0.825
Credible Interval	(0.519, 1.29)
Associated Peptides	1
Associated Spectra	2
Coverage	NaN

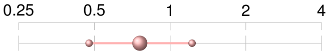

A	B	2.5	50	97.5	Sequence
1	1	0.48	0.76	1.2	SLDDFLQAWK

5.163 " 3341 4240 minus strand nonmuscle myosin II, partial [Aplysia californica]"

Protein Accession	CL19183.Contig2$_{H}1$
Mean Expression Ratio	0.826
Median Expression Ratio	0.825
Credible Interval	(0.626, 1.09)
Associated Peptides	7
Associated Spectra	11
Coverage	NaN

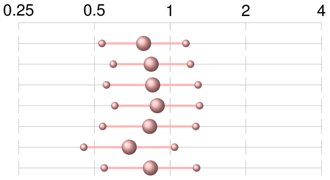

A	B	2.5	50	97.5	Sequence
1	1	0.54	0.79	1.2	RLETELQDLR
2	1	0.59	0.84	1.2	REEEVQIALQK
1	0	0.56	0.85	1.3	TATMLEEK
1	1	0.6	0.89	1.3	YAQMIEEK
1	0	0.54	0.83	1.3	QKQDVEQLEAR
1	0	0.45	0.69	1	QELEK
0	1	0.55	0.84	1.3	ELQDLR

5.164 " 2 286 Roundabout 2, partial [Ascaris suum]"

Protein Accession	CL8589.Contig2$_{H}1$
Mean Expression Ratio	1.21
Median Expression Ratio	1.21
Credible Interval	(0.779, 1.86)
Associated Peptides	2
Associated Spectra	2
Coverage	NaN

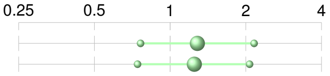

A	B	2.5	50	97.5	Sequence
1	0	0.77	1.3	2.2	GEPTPEITWLK
1	0	0.74	1.2	2.1	KSQTAVLDVIR

5.165 718 1155 Annexin A7 [Crassostrea gigas]

Protein Accession	CL15539.Contig2$_{H}1$
Mean Expression Ratio	1.21
Median Expression Ratio	1.21
Credible Interval	(0.913, 1.6)
Associated Peptides	5
Associated Spectra	12
Coverage	NaN

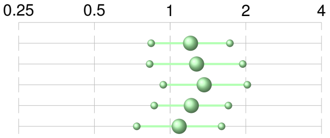

A	B	2.5	50	97.5	Sequence
3	0	0.84	1.2	1.7	ATFDAYQQIAGK
1	0	0.83	1.3	1.9	DIESAIK
2	0	0.94	1.4	2	KLGTDEETFNR
4	0	0.87	1.2	1.7	NVPGYFAQR
2	0	0.74	1.1	1.6	SFPQLR

5.166 680 991 minus strand PDZ and LIM domain protein 1 [Crassostrea gigas]; 553 864 minus strand PDZ and LIM domain protein 1 [Crassostrea gigas]

Protein Accession	Unigene7664$_{H}1$ Unigene7663$_{H}1$
Mean Expression Ratio	1.21
Median Expression Ratio	1.21
Credible Interval	(0.95, 1.53)
Associated Peptides	6
Associated Spectra	21
Coverage	NaN

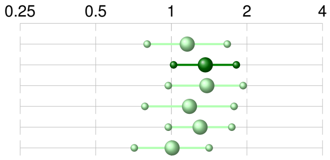

A	B	2.5	50	97.5	Sequence
2	0	0.8	1.2	1.7	AMGQPWGFR
3	3	1	1.4	1.8	DYGQALSVK
3	0	0.97	1.4	1.9	GPSTDFSAIKPK
1	0	0.79	1.2	1.8	KVQPGTPSAATLRPGDVIQR
3	3	0.97	1.3	1.7	QLTHMQAHQLIK
2	1	0.71	1	1.4	SAGTQLQLTVLR

5.167 85 1011 unnamed protein product [Mus musculus]

Protein Accession	CL42412.Contig1$_{H}1$
Mean Expression Ratio	1.21
Median Expression Ratio	1.21
Credible Interval	(0.818, 1.79)
Associated Peptides	3
Associated Spectra	3
Coverage	NaN

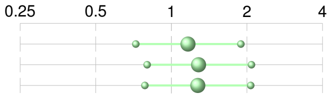

A	B	2.5	50	97.5	Sequence
1	0	0.72	1.2	1.9	NVIVEETVSEK
1	0	0.8	1.3	2.1	DDETMYVEAK
1	0	0.79	1.3	2.1	VFMQEFK

5.168 210 473 Abdominal ganglion neuropeptides L5-67 OS=Aplysia californica PE=1 SV=1; 879 1124 abdominal ganglion neuropeptides L5-67 precursor [Aplysia californica] gi; 210 482 minus strand abdominal ganglion neuropeptides L5-67 precursor [Aplysia californica] gi

Protein Accession	Unigene58290$_{H}1$ Unigene39670$_{H}1$ Unigene206$_{H}1$
Mean Expression Ratio	0.828
Median Expression Ratio	0.83
Credible Interval	(0.526, 1.29)
Associated Peptides	1
Associated Spectra	2
Coverage	NaN

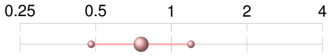

A	B	2.5	50	97.5	Sequence
0	2	0.48	0.76	1.2	VNGEIELPVEVFGAR

5.169 3591 4526 minus strand myosin heavy chain cardiac muscle specific isoform 1 [Argopecten irradians]

Protein Accession	CL49594.Contig2$_{H}1$
Mean Expression Ratio	0.832
Median Expression Ratio	0.831
Credible Interval	(0.742, 0.935)
Associated Peptides	23
Associated Spectra	291
Coverage	NaN

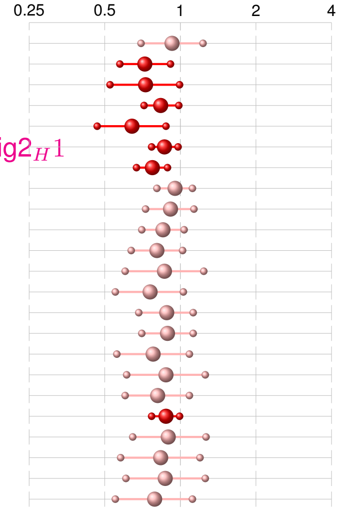

A	B	2.5	50	97.5	Sequence
3	2	0.7	0.93	1.2	AKLEQTLDELEDNLER
7	3	0.57	0.72	0.92	ATQANVEDLER
2	1	0.53	0.73	0.99	LEQTLDELEDNLER
22	7	0.71	0.84	0.98	LQQETANLTR
2	1	0.47	0.64	0.88	MSKQVEAQLAELSAK
34	19	0.77	0.87	0.98	QVEAQLAELSAK
22	17	0.67	0.77	0.89	SEVDDLQMQLQNVGK
18	9	0.81	0.95	1.1	TINDLQTQK
11	1	0.73	0.91	1.1	TMEDLQAEEDKVNHLNK
16	1	0.7	0.85	1	KLEQDLK
9	1	0.64	0.81	1	AATSRSRTLQHEAQVSALR
1	0	0.6	0.87	1.2	SEVDDLQMQLQNVGKNK
2	1	0.55	0.76	1	SASRSCSSCAATSRSRTLQHEAQVSALR
6	2	0.68	0.88	1.1	QQDAANEMADQIDQLLQKVK
10	0	0.7	0.89	1.1	ATSRSRTLQHEAQVSALR
1	1	0.56	0.78	1.1	ELEENVR
1	0	0.61	0.88	1.3	KKQQDAANEMADQIDQ
2	2	0.6	0.81	1.1	KTMEDLQAEEDKVNHLNK
24	25	0.77	0.87	1	QLEEAEHK
2	0	0.65	0.9	1.3	VGALTK
0	1	0.58	0.83	1.2	RKLEQDLK
0	1	0.61	0.87	1.3	LEENVR
0	1	0.55	0.79	1.1	QDAANEMADQIDQLLQK

5.170 40 1182 4-hydroxyphenylpyruvate dioxygenase [Crassostrea gigas]

Protein Accession	CL7585.Contig2$_{H}1$
Mean Expression Ratio	0.834
Median Expression Ratio	0.832
Credible Interval	(0.56, 1.24)
Associated Peptides	3
Associated Spectra	3
Coverage	NaN

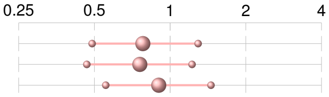

A	B	2.5	50	97.5	Sequence
1	0	0.49	0.78	1.3	DVAFEVEDLDAIFK
1	0	0.47	0.76	1.2	TYGDTTHTLIDR
0	1	0.55	0.9	1.4	SLFEAIELDQAER

5.171 7073 7339 minus strand Collagen alpha-3(VI) chain [Crassostrea gigas]

Protein Accession	Unigene26547$_{H}1$
Mean Expression Ratio	1.2
Median Expression Ratio	1.2
Credible Interval	(0.91, 1.59)
Associated Peptides	3
Associated Spectra	18
Coverage	NaN

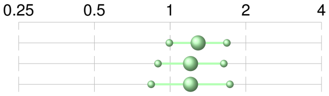

A	B	2.5	50	97.5	Sequence
6	3	0.99	1.3	1.7	ALVEELSGSGQNHR
6	0	0.9	1.2	1.6	YTPGSTNTAGGLR
2	1	0.84	1.2	1.7	YDTPQQVLEAIATTR

5.172 " 166 1248 PREDICTED: probable pyruvate dehydrogenase E1 component subunit alpha, mitochondrial-like [Metaseiulus occidentalis]; 166 1248 PREDICTED: probable pyruvate dehydrogenase E1 component subunit alpha, mitochondrial-like [Metaseiulus occidentalis]; 166 1248 PREDICTED: probable pyruvate dehydrogenase E1 component subunit alpha, mitochondrial-like [Metaseiulus occidentalis]; 166 1248 PREDICTED: probable pyruvate dehydrogenase E1 component subunit alpha, mitochondrial-like [Metaseiulus occidentalis]; 166 1248 PREDICTED: probable pyruvate dehydrogenase E1 component subunit alpha, mitochondrial-like [Metaseiulus occidentalis]; 166 1248 minus strand PREDICTED: probable pyruvate dehydrogenase E1 component subunit alpha, mitochondrial-like [Metaseiulus occidentalis]; 166 1248 minus strand PREDICTED: probable pyruvate dehydrogenase E1 component subunit alpha, mitochondrial-like [Metaseiulus occidentalis]; 166 1248 PREDICTED: probable pyruvate dehydrogenase E1 component subunit alpha, mitochondrial-like [Metaseiulus occidentalis]; 166 1248 PREDICTED: probable pyruvate dehydrogenase E1 component subunit alpha, mitochondrial-like [Metaseiulus occidentalis]"

Protein Accession	Unigene34082$_H$1 Unigene34081$_H$1 Unigene34079$_H$1 Unigene34077$_H$1 Unigene34075$_H$1 U
Mean Expression Ratio	0.833
Median Expression Ratio	0.832
Credible Interval	(0.574, 1.21)
Associated Peptides	3
Associated Spectra	4
Coverage	NaN

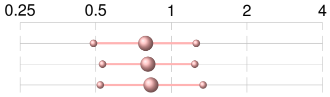

A	B	2.5	50	97.5	Sequence
1	0	0.49	0.79	1.3	TGLATEEELK
1	1	0.53	0.81	1.2	TGLATEEELKK
0	1	0.52	0.83	1.3	KIDGEVRSEVDAAAER

5.173 123 314 PREDICTED: ubiquinol-cytochrome c reductase hinge protein-like [Saccoglossus kowalevskii]; 279 530 PREDICTED: ubiquinol-cytochrome c reductase hinge protein-like [Saccoglossus kowalevskii]

Protein Accession	CL4684.Contig5$_H$1 CL4684.Contig4$_H$1
Mean Expression Ratio	1.2
Median Expression Ratio	1.2
Credible Interval	(0.77, 1.86)
Associated Peptides	1
Associated Spectra	2
Coverage	NaN

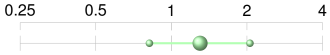

A	B	2.5	50	97.5	Sequence
2	0	0.82	1.3	2.1	CLFDELK

5.174 219 428 Peptidyl-prolyl cis-trans isomerase OS=Hemicentrotus pulcherrimus PE=2 SV=1

Protein Accession	CL39270.Contig2$_{H}1$
Mean Expression Ratio	1.2
Median Expression Ratio	1.2
Credible Interval	(0.811, 1.77)
Associated Peptides	1
Associated Spectra	5
Coverage	NaN

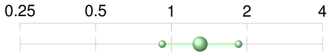

A	B	2.5	50	97.5	Sequence
**2**	**3**	**0.92**	**1.3**	**1.9**	**IIIANSGQV**

5.175 2996 3340 minus strand Collagen alpha-5(VI) chain [Crassostrea gigas]

Protein Accession	CL47586.Contig8$_{H}1$
Mean Expression Ratio	1.2
Median Expression Ratio	1.2
Credible Interval	(0.817, 1.78)
Associated Peptides	2
Associated Spectra	4
Coverage	NaN

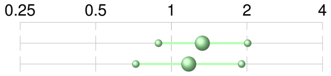

A	B	2.5	50	97.5	Sequence
**2**	**1**	**0.89**	**1.3**	**2**	**ASIFDAIDEIPYR**
**1**	**0**	**0.72**	**1.2**	**1.9**	**YGSTNTADGIK**

5.176 " 3799 4371 papilin, partial [Rhipicephalus microplus]"

Protein Accession	CL19397.Contig5$_{H}1$
Mean Expression Ratio	1.2
Median Expression Ratio	1.2
Credible Interval	(0.76, 1.87)
Associated Peptides	1
Associated Spectra	2
Coverage	NaN

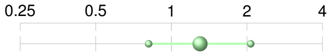

A	B	2.5	50	97.5	Sequence
**1**	**1**	**0.81**	**1.3**	**2.1**	**TVTLVLAK**

5.177 62 1174 Tyrosine-protein kinase CSK [Crassostrea gigas]; 62 1174 minus strand Tyrosine-protein kinase CSK [Crassostrea gigas]

Protein Accession	CL22249.Contig5$_{H}1$ CL22249.Contig3$_{H}1$
Mean Expression Ratio	0.831
Median Expression Ratio	0.833
Credible Interval	(0.532, 1.29)
Associated Peptides	1
Associated Spectra	2
Coverage	NaN

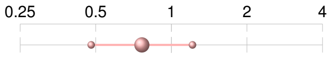

A	B	2.5	50	97.5	Sequence
**0**	**2**	**0.48**	**0.77**	**1.2**	**IPLTDVVMHVER**

5.178 192 380 minus strand RAB protein [Haliotis discus discus]

Protein Accession	Unigene35065$_{H}1$
Mean Expression Ratio	0.834
Median Expression Ratio	0.834
Credible Interval	(0.513, 1.35)
Associated Peptides	1
Associated Spectra	1
Coverage	NaN

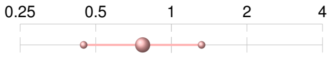

A	B	2.5	50	97.5	Sequence
**1**	**0**	**0.45**	**0.77**	**1.3**	**NVEEMFLAITK**

5.179 587 811 hemocyanin 2 [Megathura crenulata]

Protein Accession	Unigene4334$_{H}1$
Mean Expression Ratio	1.2
Median Expression Ratio	1.2
Credible Interval	(0.775, 1.84)
Associated Peptides	2
Associated Spectra	2
Coverage	NaN

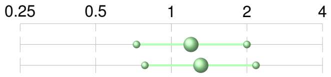

A	B	2.5	50	97.5	Sequence
**1**	**0**	**0.73**	**1.2**	**2**	**SVEDLTAGEAESLK**
**0**	**1**	**0.78**	**1.3**	**2.2**	**AIDDGTFR**

5.180 205 2490 minus strand glucose-regulated protein 94 [Crassostrea gigas]

Protein Accession	CL5089.Contig1$_{H}1$
Mean Expression Ratio	0.834
Median Expression Ratio	0.836
Credible Interval	(0.59, 1.17)
Associated Peptides	5
Associated Spectra	5
Coverage	NaN

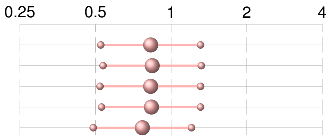

A	B	2.5	50	97.5	Sequence
**1**	**0**	**0.53**	**0.83**	**1.3**	**ADKFAFQAEVNR**
**1**	**0**	**0.53**	**0.84**	**1.3**	**NLGTIAR**
**1**	**0**	**0.52**	**0.83**	**1.3**	**GTTVSLHLK**
**1**	**0**	**0.53**	**0.84**	**1.3**	**NDMFNNYGK**
**0**	**1**	**0.49**	**0.77**	**1.2**	**GVVDSDDLPLNVSR**

5.181 63 443 PREDICTED: RAS protein activator like 1-like [Saccoglossus kowalevskii]

Protein Accession	CL19305.Contig2$_{H}1$
Mean Expression Ratio	0.836
Median Expression Ratio	0.836
Credible Interval	(0.655, 1.07)
Associated Peptides	4
Associated Spectra	40
Coverage	NaN

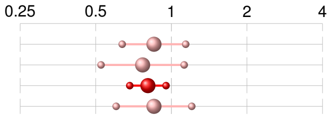

A	B	2.5	50	97.5	Sequence
**2**	**4**	**0.64**	**0.85**	**1.1**	**DYAQAADGALASVLAR**
**1**	**1**	**0.52**	**0.77**	**1.1**	**YLEDWIK**
**16**	**13**	**0.68**	**0.81**	**0.95**	**LASVLAR**
**2**	**1**	**0.6**	**0.85**	**1.2**	**ALNIQLTK**

5.182 43 525 Reticulon-4 [Crassostrea gigas]; 143 757 Reticulon-4 [Crassostrea gigas]; 43 564 Reticulon-4 [Crassostrea gigas]

Protein Accession	CL29775.Contig4$_H$1 CL29775.Contig3$_H$1 CL29775.Contig2$_H$1
Mean Expression Ratio	0.837
Median Expression Ratio	0.836
Credible Interval	(0.535, 1.32)
Associated Peptides	1
Associated Spectra	3
Coverage	NaN

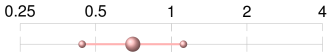

A	B	2.5	50	97.5	Sequence
**2**	**1**	**0.44**	**0.7**	**1.1**	**VTQTILQHVNR**

5.183 194 904 Aldo-keto reductase family 1 member B10 [Crassostrea gigas]

Protein Accession	CL3558.Contig4$_H$1
Mean Expression Ratio	0.837
Median Expression Ratio	0.836
Credible Interval	(0.516, 1.35)
Associated Peptides	1
Associated Spectra	1
Coverage	NaN

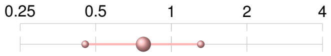

A	B	2.5	50	97.5	Sequence
**0**	**1**	**0.45**	**0.77**	**1.3**	**ALEVLVSEGK**

5.184 233 814 Kynurenine formamidase [Crassostrea gigas]; 233 979 Kynurenine formamidase [Crassostrea gigas]; 139 945 Kynurenine formamidase [Crassostrea gigas]; 145 951 Kynurenine formamidase [Crassostrea gigas]; 220 1026 Kynurenine formamidase [Crassostrea gigas]; 39 725 Kynurenine formamidase [Crassostrea gigas]; 198 986 Kynurenine formamidase [Crassostrea gigas]; 39 743 Kynurenine formamidase [Crassostrea gigas]; 123 929 Kynurenine formamidase [Crassostrea gigas]; 39 743 Kynurenine formamidase [Crassostrea gigas]

Protein Accession	Unigene7804$_H$1 Unigene42149$_H$1 CL8132.Contig9$_H$1 CL8132.Contig8$_H$1 CL8132.Contig7$_H$
Mean Expression Ratio	0.835
Median Expression Ratio	0.837
Credible Interval	(0.557, 1.24)
Associated Peptides	1
Associated Spectra	6
Coverage	NaN

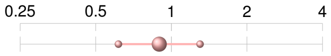

A	B	2.5	50	97.5	Sequence
**4**	**2**	**0.62**	**0.89**	**1.3**	**LVGPGVVIDVR**

5.185 85 546 minus strand RecName: Full=Eukaryotic translation initiation factor 5A; Short=eIF-5A gi|51702279|sp|P62925.1|IF5A_SPOFR RecName: Full=Eukaryotic translation initiation factor 5A; Short=eIF-5A gi|6469515|gb|AAF13315.1|AF109730_1 translation initiation factor 5A [Spodoptera exigua] gi

Protein Accession	CL40725.Contig2$_H$1
Mean Expression Ratio	0.835
Median Expression Ratio	0.837
Credible Interval	(0.618, 1.13)
Associated Peptides	4
Associated Spectra	10
Coverage	NaN

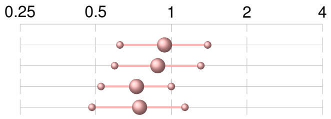

A	B	2.5	50	97.5	Sequence
1	1	0.62	0.94	1.4	AMGEECVVAVK
1	1	0.59	0.88	1.3	EDSLAAEIQK
2	3	0.52	0.73	1	IVEMSTSK
0	1	0.48	0.75	1.1	KNEFVVIK

5.186 1292 1471 minus strand hemocyanin isoform 1 [Haliotis diversicolor]

Protein Accession	Unigene65233$_H$1
Mean Expression Ratio	0.838
Median Expression Ratio	0.837
Credible Interval	(0.537, 1.32)
Associated Peptides	1
Associated Spectra	2
Coverage	NaN

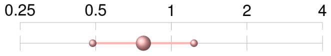

A	B	2.5	50	97.5	Sequence
1	1	0.49	0.77	1.2	LYTVQFEDALR

5.187 1709 2026 Phosphoenolpyruvate carboxykinase [GTP] [Crassostrea gigas]

Protein Accession	CL25948.Contig2$_H$1
Mean Expression Ratio	0.838
Median Expression Ratio	0.837
Credible Interval	(0.576, 1.21)
Associated Peptides	1
Associated Spectra	8
Coverage	NaN

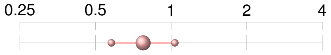

A	B	2.5	50	97.5	Sequence
5	3	0.58	0.77	1	GVPLVYEAK

5.188 77 502 hypothetical protein CGI_10008263 [Crassostrea gigas]

Protein Accession	CL38960.Contig1$_H$1
Mean Expression Ratio	1.2
Median Expression Ratio	1.19
Credible Interval	(0.794, 1.81)
Associated Peptides	2
Associated Spectra	3
Coverage	NaN

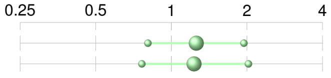

A	B	2.5	50	97.5	Sequence
2	0	0.81	1.3	1.9	IFVAIDGSK
1	0	0.76	1.2	2	KIFVAIDGSK

5.189 1139 1378 Titin [Crassostrea gigas]; 1116 1355 Titin [Crassostrea gigas]; 1139 1378 Titin [Crassostrea gigas]; 1139 1378 Titin [Crassostrea gigas]; 1139 1378 Titin [Crassostrea gigas]; 1139 1378 Titin [Crassostrea gigas]; 1139 1378 Titin [Crassostrea gigas]; 1170 1409 Titin [Crassostrea gigas]

Protein Accession	CL181.Contig9$_{H}1$ CL181.Contig8$_{H}1$ CL181.Contig7$_{H}1$ CL181.Contig6$_{H}1$ CL181.Contig13$_{H}$
Mean Expression Ratio	0.838
Median Expression Ratio	0.837
Credible Interval	(0.569, 1.25)
Associated Peptides	2
Associated Spectra	4
Coverage	NaN

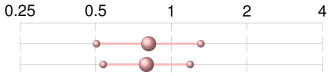

A	B	2.5	50	97.5	Sequence
1	0	0.5	0.81	1.3	ITGYQIER
3	0	0.53	0.8	1.2	LSWKPPK

5.190 111 530 minus strand Nucleoside diphosphate kinase B OS=Homo sapiens GN=NME2 PE=1 SV=1

Protein Accession	CL22280.Contig3$_{H}1$
Mean Expression Ratio	0.837
Median Expression Ratio	0.838
Credible Interval	(0.616, 1.14)
Associated Peptides	4
Associated Spectra	9
Coverage	NaN

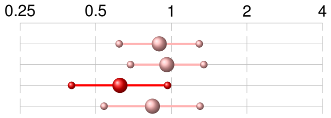

A	B	2.5	50	97.5	Sequence
2	1	0.62	0.9	1.3	GDFCIEMGR
2	2	0.69	0.96	1.4	VMLGETNPLNSKPGTIR
1	0	0.4	0.62	0.96	LCRPGK
0	1	0.54	0.84	1.3	LVAAK

5.191 37 885 glyceraldehyde 3-phosphate dehydrogenase [Dugesia japonica]

Protein Accession	CL47424.Contig2$_{H}1$
Mean Expression Ratio	1.19
Median Expression Ratio	1.19
Credible Interval	(0.842, 1.7)
Associated Peptides	1
Associated Spectra	12
Coverage	NaN

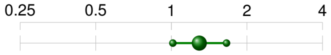

A	B	2.5	50	97.5	Sequence
10	2	1	1.3	1.7	GAAQNIIPASTGAAK

5.192 96 497 AAEL009772-PB [Aedes aegypti]

Protein Accession	CL27854.Contig2$_{H}1$
Mean Expression Ratio	1.19
Median Expression Ratio	1.19
Credible Interval	(0.764, 1.87)
Associated Peptides	1
Associated Spectra	3
Coverage	NaN

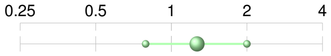

A	B	2.5	50	97.5	Sequence
2	1	0.79	1.3	2	LVEEFPMGNIK

5.193 16 438 hypothetical protein KGM_06034 [Danaus plexippus]; 16 438 hypothetical protein KGM_06034 [Danaus plexippus]

Protein Accession	CL18196.Contig2$_H$1 CL18196.Contig1$_H$1
Mean Expression Ratio	1.19
Median Expression Ratio	1.19
Credible Interval	(0.765, 1.82)
Associated Peptides	2
Associated Spectra	2
Coverage	NaN

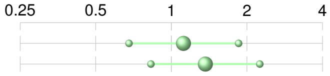

A	B	2.5	50	97.5	Sequence
**1**	**0**	**0.68**	**1.1**	**1.8**	**VHCGPNYPDEPPTVQFK**
**1**	**0**	**0.83**	**1.4**	**2.2**	**LLEELEDGQK**

5.194 " 2041 3315 minus strand Myosin-10 OS=Mus musculus GN=Myh10 PE=1 SV=2; 2041 3345 minus strand Myosin heavy chain, non-muscle OS=Drosophila melanogaster GN=zip PE=1 SV=2; 2041 3351 minus strand Myosin-10 OS=Mus musculus GN=Myh10 PE=1 SV=2"

Protein Accession	Unigene62170$_H$1 Unigene62168$_H$1 Unigene62165$_H$1
Mean Expression Ratio	0.842
Median Expression Ratio	0.842
Credible Interval	(0.603, 1.18)
Associated Peptides	5
Associated Spectra	6
Coverage	NaN

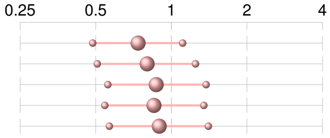

A	B	2.5	50	97.5	Sequence
**0**	**2**	**0.49**	**0.73**	**1.1**	**AGVLAHLEEER**
**0**	**1**	**0.51**	**0.8**	**1.2**	**CIIPNHEK**
**0**	**1**	**0.56**	**0.87**	**1.4**	**IDSPLVLDQLR**
**0**	**1**	**0.54**	**0.85**	**1.3**	**ILFQEFR**
**0**	**1**	**0.57**	**0.9**	**1.4**	**LMATLR**

5.195 27 722 Spectrin beta chain OS=Drosophila melanogaster GN=beta-Spec PE=1 SV=2; 27 722 Spectrin beta chain OS=Drosophila melanogaster GN=beta-Spec PE=1 SV=2

Protein Accession	CL5904.Contig3$_H$1 CL5904.Contig1$_H$1
Mean Expression Ratio	0.84
Median Expression Ratio	0.842
Credible Interval	(0.569, 1.23)
Associated Peptides	2
Associated Spectra	4
Coverage	NaN

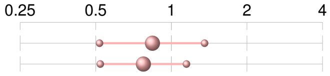

A	B	2.5	50	97.5	Sequence
**1**	**0**	**0.52**	**0.84**	**1.4**	**EQIEVMLADLDK**
**3**	**0**	**0.52**	**0.78**	**1.1**	**HQAFEAEIGHYAR**

5.196 56 937 Chloride intracellular channel exc-4 [Crassostrea gigas]

Protein Accession	CL34620.Contig3$_{H}$1
Mean Expression Ratio	1.19
Median Expression Ratio	1.19
Credible Interval	(0.786, 1.79)
Associated Peptides	2
Associated Spectra	3
Coverage	NaN

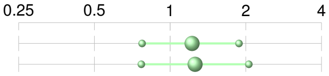

A	B	2.5	50	97.5	Sequence
2	0	0.78	1.2	1.9	KVQGPLVK
1	0	0.77	1.3	2.1	KLLAELK

5.197 503 1090 minus strand myosin regulatory light chain [Fasciola hepatica]; 278 865 minus strand myosin regulatory light chain [Fasciola hepatica]

Protein Accession	CL43006.Contig6$_{H}$1 CL43006.Contig4$_{H}$1
Mean Expression Ratio	1.19
Median Expression Ratio	1.19
Credible Interval	(0.847, 1.66)
Associated Peptides	1
Associated Spectra	25
Coverage	NaN

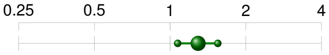

A	B	2.5	50	97.5	Sequence
16	9	1.1	1.3	1.5	QAQVQEFK

5.198 40 1752 minus strand heat shock protein 60 [Biomphalaria glabrata]; 40 1752 minus strand heat shock protein 60 [Biomphalaria glabrata]

Protein Accession	CL5583.Contig6$_{H}$1 CL5583.Contig5$_{H}$1
Mean Expression Ratio	1.19
Median Expression Ratio	1.19
Credible Interval	(0.883, 1.6)
Associated Peptides	5
Associated Spectra	9
Coverage	NaN

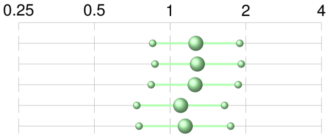

A	B	2.5	50	97.5	Sequence
2	0	0.85	1.3	1.9	AVGDLISSAMK
1	1	0.87	1.3	1.9	TLRDELEVIEGMK
1	1	0.84	1.3	1.9	VGLQVVAVK
2	0	0.74	1.1	1.6	LPNLLR
0	1	0.75	1.2	1.7	DDTLLMR

5.199 448 1626 matrilin [Biomphalaria glabrata]

Protein Accession	CL7510.Contig3$_{H}$1
Mean Expression Ratio	1.18
Median Expression Ratio	1.18
Credible Interval	(1, 1.4)
Associated Peptides	12
Associated Spectra	61
Coverage	NaN

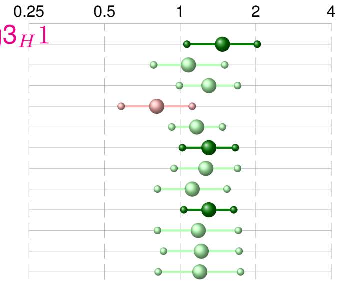

A	B	2.5	50	97.5	Sequence
2	1	1.1	1.5	2	EFLSMYDIGPAR
1	2	0.78	1.1	1.5	FAVVLTDGNSQESR
6	1	0.99	1.3	1.7	IDETSLNSAIDELVK
2	1	0.58	0.81	1.1	KTALVGFSDTR
5	6	0.92	1.2	1.5	TKEEVLEHVANMR
7	3	1	1.3	1.7	VFVVTTDSAAK
3	2	0.94	1.3	1.7	VIQVSSYDDLQK
3	0	0.81	1.1	1.5	VSNTELLNIAGDQER
6	6	1	1.3	1.6	DSLPGLVR
1	0	0.81	1.2	1.7	HEYGTFTDTGPGIDYMR
0	2	0.86	1.2	1.7	HKIDETSLNSAIDELVK
0	1	0.82	1.2	1.7	VSAIK

5.200 227 829 Peptidyl-prolyl cis-trans isomerase B [Crassostrea gigas]

Protein Accession	CL16665.Contig2$_H$1
Mean Expression Ratio	1.18
Median Expression Ratio	1.18
Credible Interval	(0.964, 1.46)
Associated Peptides	7
Associated Spectra	38
Coverage	NaN

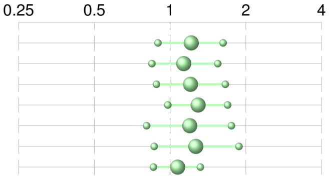

A	B	2.5	50	97.5	Sequence
4	1	0.9	1.2	1.6	DTNGSQFFFTLGPSR
3	2	0.84	1.1	1.5	MGPGADFQSPK
3	1	0.88	1.2	1.7	NYNGQGDDISGK
3	4	0.98	1.3	1.7	TLCDGWVR
1	0	0.8	1.2	1.7	FLDEK
1	0	0.86	1.3	1.9	NFTVTHEATLEIEVK
10	5	0.86	1.1	1.3	GFNFLR

5.201 187 1323 minus strand gelsolin [Aplysia californica] gi

Protein Accession	CL10220.Contig1$_H$1
Mean Expression Ratio	1.18
Median Expression Ratio	1.18
Credible Interval	(0.736, 1.93)
Associated Peptides	1
Associated Spectra	1
Coverage	NaN

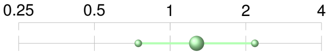

A	B	2.5	50	97.5	Sequence
1	0	0.75	1.3	2.2	LSDASGELDFK

5.202 260 892 minus strand calponin [Haliotis diversicolor]; 260 892 minus strand calponin [Haliotis diversicolor]

Protein Accession	CL48702.Contig2$_H$1 CL48702.Contig1$_H$1
Mean Expression Ratio	1.18
Median Expression Ratio	1.18
Credible Interval	(0.991, 1.41)
Associated Peptides	12
Associated Spectra	102
Coverage	NaN

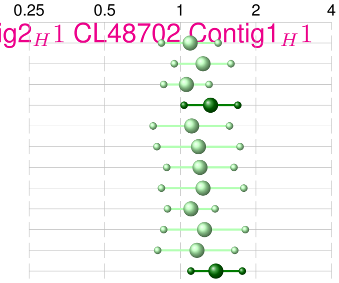

A	B	2.5	50	97.5	Sequence
6	4	0.84	1.1	1.4	AATGQQFPIEESNER
7	3	0.94	1.2	1.6	NFSEDKLR
18	8	0.86	1.1	1.3	TFQPASNLAFEAAR
7	5	1	1.3	1.7	VESSFDPEDAKK
1	1	0.78	1.1	1.6	YKVMENFYK
0	1	0.81	1.2	1.7	AFGIEVESHPSYTGQR
1	3	0.88	1.2	1.6	VESSFDPEDAK
0	1	0.84	1.2	1.8	FPIEESNER
7	9	0.89	1.1	1.4	IELFIQK
0	1	0.86	1.2	1.8	PASNLAFEAAR
1	1	0.81	1.2	1.7	VLACIR
12	5	1.1	1.4	1.8	VMENFYK

5.203 54 638 minus strand ubiquitin carboxyl-terminal hydrolase [Aplysia californica] gi

Protein Accession	CL20666.Contig3$_H$1
Mean Expression Ratio	1.18
Median Expression Ratio	1.18
Credible Interval	(0.728, 1.91)
Associated Peptides	1
Associated Spectra	1
Coverage	NaN

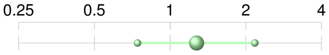

A	B	2.5	50	97.5	Sequence
1	0	0.74	1.3	2.2	THADTLLEDTVK

5.204 9 887 minus strand hypothetical protein CGI_10006272 [Crassostrea gigas]

Protein Accession	CL46976.Contig1$_{H}$1
Mean Expression Ratio	0.845
Median Expression Ratio	0.847
Credible Interval	(0.547, 1.29)
Associated Peptides	1
Associated Spectra	3
Coverage	NaN

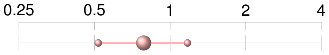

A	B	2.5	50	97.5	Sequence
2	1	0.52	0.78	1.2	MILPQINK

5.205 401 1174 minus strand Mechanosensory protein 2 [Crassostrea gigas]

Protein Accession	Unigene7703$_{H}$1
Mean Expression Ratio	1.18
Median Expression Ratio	1.18
Credible Interval	(0.73, 1.91)
Associated Peptides	1
Associated Spectra	1
Coverage	NaN

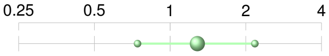

A	B	2.5	50	97.5	Sequence
1	0	0.74	1.3	2.2	VIAAEGEQK

5.206 34 1122 Fructose-bisphosphate aldolase [Crassostrea gigas]

Protein Accession	CL24228.Contig1$_{H}$1
Mean Expression Ratio	1.18
Median Expression Ratio	1.18
Credible Interval	(1.02, 1.37)
Associated Peptides	18
Associated Spectra	62
Coverage	NaN

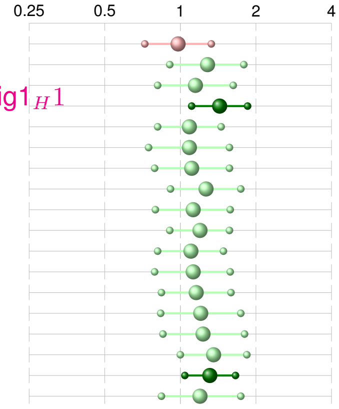

A	B	2.5	50	97.5	Sequence
3	1	0.72	0.98	1.3	DSQGTPFVK
1	1	0.91	1.3	1.8	GAQDELLK
1	1	0.81	1.2	1.6	GAQDELLKR
5	3	1.1	1.4	1.9	GIIPGIK
3	2	0.81	1.1	1.5	GILAADESVGSMGK
1	0	0.75	1.1	1.6	GVVPLAGSNNECTTQGLDGLAER
2	0	0.79	1.1	1.6	ISDNTPSYQAMIENANVLAR
3	0	0.92	1.3	1.7	KDGAQFAK
1	1	0.8	1.1	1.6	LEGIGLENTEENR
4	2	0.91	1.2	1.6	QIANSIVAPGK
3	1	0.81	1.1	1.5	RLEGIGLENTEENR
1	1	0.79	1.1	1.6	RLEGIGLENTEENRR
3	0	0.84	1.2	1.6	YASICQQNGLVPIVEPEVLPDGDHDLK
1	0	0.83	1.2	1.7	VKGAQDELLKR
1	0	0.85	1.2	1.8	ANSEAALGK
2	2	1	1.4	1.8	PVIAQYLTK
5	6	1	1.3	1.7	VTEQVLAFTYK
0	1	0.84	1.2	1.7	RLEGIGLENTEENRRRYR

5.207 242 1834 Regulator of differentiation 1 [Crassostrea gigas]; 458 2050 minus strand Regulator of differentiation 1 [Crassostrea gigas]; 503 2095 Regulator of differentiation 1 [Crassostrea gigas]; 274 1866 Regulator of differentiation 1 [Crassostrea gigas]; 434 1942 Regulator of differentiation 1 [Crassostrea gigas]

Protein Accession	CL11169.Contig7$_H$1 CL11169.Contig5$_H$1 CL11169.Contig4$_H$1 CL11169.Contig3$_H$1 CL1116
Mean Expression Ratio	1.18
Median Expression Ratio	1.18
Credible Interval	(0.768, 1.82)
Associated Peptides	2
Associated Spectra	2
Coverage	NaN

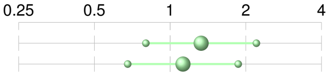

A	B	2.5	50	97.5	Sequence
1	0	0.8	1.3	2.2	SLPHEVAETDIVQLGMPFGK
0	1	0.68	1.1	1.9	IITFTK

5.208 35 1861 hypothetical protein DAPPUDRAFT_309082 [Daphnia pulex]

Protein Accession	CL49314.Contig5$_H$1
Mean Expression Ratio	0.849
Median Expression Ratio	0.851
Credible Interval	(0.645, 1.13)
Associated Peptides	6
Associated Spectra	11
Coverage	NaN

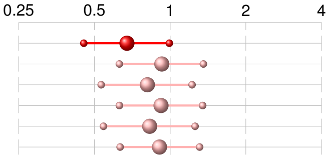

A	B	2.5	50	97.5	Sequence
2	0	0.45	0.67	0.99	ELDVALTAVGLK
1	1	0.63	0.92	1.4	LINHSVPDTIDER
1	0	0.53	0.81	1.2	WVNYHLAR
1	1	0.63	0.92	1.3	YAIGLAR
1	0	0.54	0.83	1.3	DIGHTFK
0	3	0.63	0.91	1.3	MADGILLCK

5.209 " 199 417 GG13156 [Drosophila erecta] gi; 253 501 minus strand NADH-ubiquinone oxidoreductase MLRQ subunit-like protein, partial [Lymnaea stagnalis]; 294 515 GG13156 [Drosophila erecta] gi"

Protein Accession	CL49605.Contig4$_H$1 CL49605.Contig2$_H$1 CL49605.Contig1$_H$1
Mean Expression Ratio	1.18
Median Expression Ratio	1.18
Credible Interval	(0.717, 1.92)
Associated Peptides	1
Associated Spectra	1
Coverage	NaN

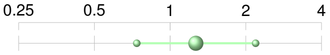

A	B	2.5	50	97.5	Sequence
1	0	0.74	1.3	2.2	GLTLSSLK

5.210 211 1413 Alpha-N-acetylgalactosaminidase [Crassostrea gigas]

Protein Accession	CL5989.Contig1$_{H}$1
Mean Expression Ratio	1.18
Median Expression Ratio	1.18
Credible Interval	(0.794, 1.73)
Associated Peptides	2
Associated Spectra	4
Coverage	NaN

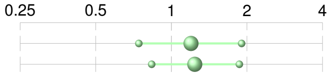

A	B	2.5	50	97.5	Sequence
1	0	0.74	1.2	1.9	ALADYMHDR
3	0	0.83	1.2	1.9	ALLLNR

5.211 8 937 minus strand galectin [Crassostrea virginica]

Protein Accession	CL7245.Contig6$_{H}$1
Mean Expression Ratio	0.852
Median Expression Ratio	0.852
Credible Interval	(0.52, 1.38)
Associated Peptides	1
Associated Spectra	1
Coverage	NaN

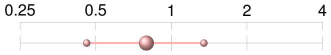

A	B	2.5	50	97.5	Sequence
1	0	0.46	0.79	1.4	QDQGDVVLNAK

5.212 " 57 752 PREDICTED: adenylate kinase 2, mitochondrial-like isoform 2 [Callithrix jacchus]"

Protein Accession	CL4149.Contig1$_{H}$1
Mean Expression Ratio	1.18
Median Expression Ratio	1.17
Credible Interval	(0.828, 1.67)
Associated Peptides	3
Associated Spectra	6
Coverage	NaN

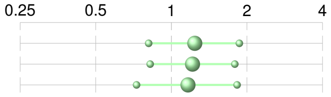

A	B	2.5	50	97.5	Sequence
2	0	0.81	1.2	1.9	GIHTAVDASQPPHK
3	0	0.82	1.2	1.8	GFLLDGFPR
1	0	0.73	1.2	1.8	GTQAPLLAEK

5.213 4 399 Spectrin beta chain [Crassostrea gigas]

Protein Accession	Unigene17190$_{H}$1
Mean Expression Ratio	0.853
Median Expression Ratio	0.853
Credible Interval	(0.557, 1.3)
Associated Peptides	1
Associated Spectra	3
Coverage	NaN

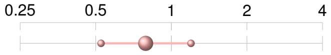

A	B	2.5	50	97.5	Sequence
1	2	0.52	0.79	1.2	VHLENMGAHDIVDGSAR

5.214 148 609 minus strand Titin [Crassostrea gigas]

Protein Accession	CL5825.Contig1$_{H}$1
Mean Expression Ratio	1.17
Median Expression Ratio	1.17
Credible Interval	(0.821, 1.67)
Associated Peptides	2
Associated Spectra	6
Coverage	NaN

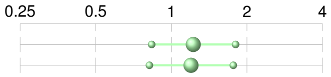

A	B	2.5	50	97.5	Sequence
3	0	0.83	1.2	1.8	FTQKPALR
3	0	0.82	1.2	1.8	QQEGVAPNFIQKPVTR

5.215 1 987 minus strand RecName: Full=Tubulin beta chain; AltName: Full=Beta-tubulin

Protein Accession	Unigene7954$_{H}$1
Mean Expression Ratio	1.17
Median Expression Ratio	1.17
Credible Interval	(0.828, 1.64)
Associated Peptides	3
Associated Spectra	7
Coverage	NaN

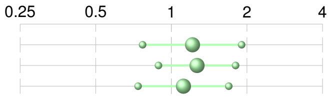

A	B	2.5	50	97.5	Sequence
1	0	0.77	1.2	1.9	AILVDLEPGTMDSVR
4	0	0.89	1.3	1.8	ALTVPELTQQMFDAK
2	0	0.74	1.1	1.7	PELTQQMFDAK

5.216 " 3475 4188 minus strand Heat shock 70 kDa protein cognate, putative [Pediculus humanus corporis] gi"

Protein Accession	CL5924.Contig14$_{H}$1
Mean Expression Ratio	0.853
Median Expression Ratio	0.855
Credible Interval	(0.564, 1.28)
Associated Peptides	2
Associated Spectra	3
Coverage	NaN

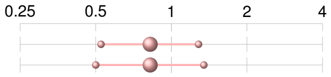

A	B	2.5	50	97.5	Sequence
2	0	0.53	0.82	1.3	STIEDEKLK
0	1	0.5	0.82	1.3	CNDVIR

5.217 3 572 Receptor-type tyrosine-protein phosphatase N2 [Crassostrea gigas]; 3 542 minus strand Receptor-type tyrosine-protein phosphatase N2 [Crassostrea gigas]

Protein Accession	CL8283.Contig5$_{H}$1 CL8283.Contig2$_{H}$1
Mean Expression Ratio	0.854
Median Expression Ratio	0.855
Credible Interval	(0.526, 1.38)
Associated Peptides	1
Associated Spectra	1
Coverage	NaN

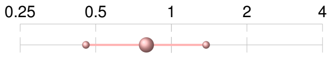

A	B	2.5	50	97.5	Sequence
0	1	0.46	0.79	1.4	EIDMAATLEHIR

5.218 31 735 hypothetical protein BRAFLDRAFT_78603 [Branchiostoma floridae] gi; 55 735 hypothetical protein BRAFLDRAFT_78603 [Branchiostoma floridae] gi; 319 948 hypothetical protein BRAFLDRAFT_78603 [Branchiostoma floridae] gi; 353 1039 hypothetical protein BRAFLDRAFT_78603 [Branchiostoma floridae] gi; 207 893 hypothetical protein BRAFLDRAFT_78603 [Branchiostoma floridae] gi

Protein Accession	CL877.Contig7$_{H}1$ CL877.Contig6$_{H}1$ CL877.Contig5$_{H}1$ CL877.Contig4$_{H}1$ CL877.Contig3$_{H}1$
Mean Expression Ratio	0.852
Median Expression Ratio	0.855
Credible Interval	(0.552, 1.3)
Associated Peptides	2
Associated Spectra	5
Coverage	NaN

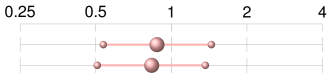

A	B	2.5	50	97.5	Sequence
**2**	**1**	**0.53**	**0.88**	**1.4**	**DDQTLVTMTGEVAGR**
**1**	**1**	**0.51**	**0.83**	**1.4**	**LKLPLDSSNR**

5.219 3 503 minus strand elongation factor-1 alpha [Pomacea bridgesi]

Protein Accession	Unigene72143$_{H}1$
Mean Expression Ratio	1.17
Median Expression Ratio	1.17
Credible Interval	(0.758, 1.81)
Associated Peptides	2
Associated Spectra	2
Coverage	NaN

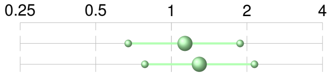

A	B	2.5	50	97.5	Sequence
**0**	**1**	**0.68**	**1.1**	**1.9**	**QLIICANK**
**0**	**1**	**0.78**	**1.3**	**2.1**	**LVSPR**

5.220 " 17 283 Glycerol-3-phosphate dehydrogenase [NAD+], cytoplasmic [Crassostrea gigas]"

Protein Accession	Unigene9883$_{H}1$
Mean Expression Ratio	0.857
Median Expression Ratio	0.857
Credible Interval	(0.555, 1.32)
Associated Peptides	2
Associated Spectra	2
Coverage	NaN

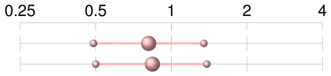

A	B	2.5	50	97.5	Sequence
**0**	**1**	**0.49**	**0.81**	**1.3**	**KLTEIVNTEHENVK**
**0**	**1**	**0.5**	**0.84**	**1.4**	**LTEIVNTEHENVK**

5.221 9 518 minus strand hemocyanin isoform 1 [Nucula nucleus]

Protein Accession	Unigene71735$_{H}1$
Mean Expression Ratio	0.86
Median Expression Ratio	0.857
Credible Interval	(0.574, 1.29)
Associated Peptides	1
Associated Spectra	4
Coverage	NaN

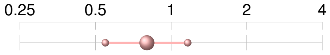

A	B	2.5	50	97.5	Sequence
**2**	**2**	**0.55**	**0.8**	**1.2**	**IDFEHADVAR**

5.222 5 841 minus strand Spectrin alpha chain [Crassostrea gigas]

Protein Accession	CL44897.Contig1$_{H}1$
Mean Expression Ratio	0.859
Median Expression Ratio	0.858
Credible Interval	(0.574, 1.28)
Associated Peptides	2
Associated Spectra	3
Coverage	NaN

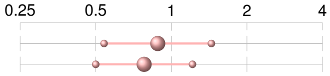

A	B	2.5	50	97.5	Sequence
**1**	**0**	**0.54**	**0.88**	**1.4**	**DMEDEEDWIR**
**1**	**1**	**0.5**	**0.78**	**1.2**	**HQNFEAEIEANR**

5.223 813 1244 Probable protein disulfide-isomerase A6 OS=Caenorhabditis elegans GN=tag-320 PE=2 SV=1

Protein Accession	CL39540.Contig4$_{H}1$
Mean Expression Ratio	0.86
Median Expression Ratio	0.858
Credible Interval	(0.53, 1.4)
Associated Peptides	1
Associated Spectra	2
Coverage	NaN

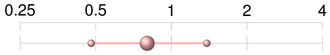

A	B	2.5	50	97.5	Sequence
**1**	**1**	**0.48**	**0.8**	**1.4**	**TASDIINWATEK**

5.224 160 393 minus strand PREDICTED: cysteine-rich protein 1-like [Megachile rotundata]; 148 381 PREDICTED: cysteine-rich protein 1-like [Megachile rotundata]; 148 381 PREDICTED: cysteine-rich protein 1-like [Megachile rotundata]; 148 381 PREDICTED: cysteine-rich protein 1-like [Megachile rotundata]; 160 393 minus strand PREDICTED: cysteine-rich protein 1-like [Megachile rotundata]; 160 393 minus strand PREDICTED: cysteine-rich protein 1-like [Megachile rotundata]

Protein Accession	CL11328.Contig9$_{H}1$ CL11328.Contig8$_{H}1$ CL11328.Contig7$_{H}1$ CL11328.Contig6$_{H}1$ CL1132
Mean Expression Ratio	1.16
Median Expression Ratio	1.16
Credible Interval	(0.797, 1.72)
Associated Peptides	2
Associated Spectra	4
Coverage	NaN

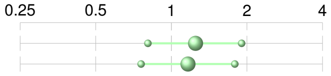

A	B	2.5	50	97.5	Sequence
1	1	0.8	1.2	1.9	DWHAACLK
1	1	0.76	1.2	1.8	EVYFAER

5.225 179 2140 minus strand PREDICTED: hypothetical protein [Saccoglossus kowalevskii]

Protein Accession	CL13749.Contig6$_{H}1$
Mean Expression Ratio	1.16
Median Expression Ratio	1.16
Credible Interval	(0.72, 1.9)
Associated Peptides	1
Associated Spectra	1
Coverage	NaN

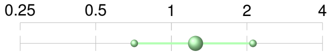

A	B	2.5	50	97.5	Sequence
1	0	0.71	1.2	2.1	KFDGIGGLSGGGATSK

5.226 " 142 1284 minus strand Chain O, Crsytal Structure Of Human Liver Gapdh gi|67464044|pdb|1ZNQ|P Chain P, Crsytal Structure Of Human Liver Gapdh gi|67464045|pdb|1ZNQ|R Chain R, Crsytal Structure Of Human Liver Gapdh gi|67464046|pdb|1ZNQ|Q Chain Q, Crsytal Structure Of Human Liver Gapdh"

Protein Accession	CL34318.Contig1$_{H}1$
Mean Expression Ratio	1.16
Median Expression Ratio	1.16
Credible Interval	(0.955, 1.41)
Associated Peptides	7
Associated Spectra	50
Coverage	NaN

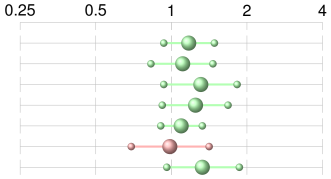

A	B	2.5	50	97.5	Sequence
5	6	0.93	1.2	1.5	DPTAIPWR
4	2	0.83	1.1	1.5	INVYQEKDPTAIPWR
2	1	0.93	1.3	1.8	VIPELNGK
4	1	0.92	1.2	1.7	INVYQEK
0	20	0.91	1.1	1.3	IGINGFGR
0	2	0.69	0.99	1.4	VIPELNGKLTGMAFR
0	3	0.96	1.3	1.9	CLAPLAK

5.227 169 1200 PREDICTED: nidogen-2-like isoform 1 [Strongylocentrotus purpuratus]

Protein Accession	CL478.Contig2$_H$1
Mean Expression Ratio	0.861
Median Expression Ratio	0.86
Credible Interval	(0.593, 1.25)
Associated Peptides	3
Associated Spectra	4
Coverage	NaN

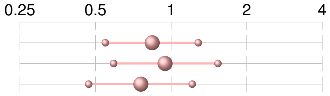

A	B	2.5	50	97.5	Sequence
0	2	0.55	0.84	1.3	LDIVATAR
0	1	0.59	0.95	1.5	LPNGLTFDQVSR
0	1	0.47	0.76	1.2	VFTVPALSR

5.228 60 1022 Major egg antigen [Crassostrea gigas]; 60 1022 Major egg antigen [Crassostrea gigas]

Protein Accession	CL18744.Contig3$_H$1 CL18744.Contig2$_H$1
Mean Expression Ratio	1.16
Median Expression Ratio	1.16
Credible Interval	(0.892, 1.52)
Associated Peptides	6
Associated Spectra	13
Coverage	NaN

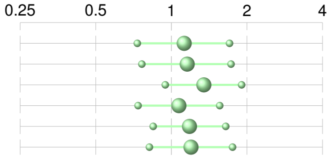

A	B	2.5	50	97.5	Sequence
1	0	0.73	1.1	1.7	QLTIEAPLKPATVQQR
1	0	0.76	1.2	1.7	TAIEVDSEGR
1	2	0.95	1.3	1.9	SEVHIPVQK
2	0	0.74	1.1	1.6	FDVSEFKPEEVSVK
1	3	0.85	1.2	1.6	ETFLPVR
0	2	0.82	1.2	1.7	LTVDIGEFSPDEIVVK

5.229 118 513 Protein CAL-5 [Caenorhabditis elegans] gi

Protein Accession	Unigene47320$_H$1
Mean Expression Ratio	1.16
Median Expression Ratio	1.16
Credible Interval	(0.755, 1.79)
Associated Peptides	2
Associated Spectra	3
Coverage	NaN

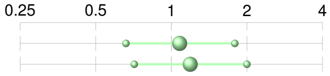

A	B	2.5	50	97.5	Sequence
1	1	0.66	1.1	1.8	EADANGDGYLSIQELK
1	0	0.71	1.2	2	TLLAHSGK

5.230 94 933 four and A half lim domains [Schistosoma mansoni]

Protein Accession	CL7046.Contig6$_H$1
Mean Expression Ratio	1.16
Median Expression Ratio	1.16
Credible Interval	(0.935, 1.44)
Associated Peptides	8
Associated Spectra	27
Coverage	NaN

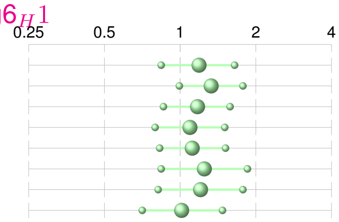

A	B	2.5	50	97.5	Sequence
2	1	0.84	1.2	1.7	CDASMVGR
4	2	0.99	1.3	1.8	CTKPITGIGGTK
3	2	0.86	1.2	1.6	FISFEDR
2	2	0.8	1.1	1.5	SLVDQPFAPK
3	2	0.83	1.1	1.5	SFIPR
1	0	0.84	1.3	1.9	CSGVINK
1	0	0.82	1.2	1.8	KFEYK
1	1	0.71	1	1.5	DDQPYCGDCFGELFAK

5.231 " 59 2365 minus strand Aconitate hydratase, mitochondrial [Crassostrea gigas]"

Protein Accession	CL13509.Contig1$_H$1
Mean Expression Ratio	0.859
Median Expression Ratio	0.861
Credible Interval	(0.637, 1.15)
Associated Peptides	6
Associated Spectra	9
Coverage	NaN

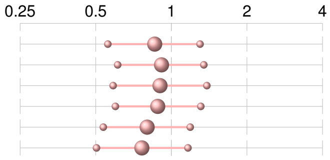

A	B	2.5	50	97.5	Sequence
1	0	0.56	0.86	1.3	HTMNEGQIEWFR
1	1	0.61	0.91	1.4	KNGWPLNIR
1	0	0.59	0.9	1.4	DLKPGAPVVCEIK
1	1	0.6	0.88	1.3	IHETNLK
0	2	0.54	0.8	1.2	VAGILTVK
0	1	0.5	0.76	1.2	LQLLQPFEK

5.232 247 762 Myophilin [Crassostrea gigas]; 244 759 Myophilin [Crassostrea gigas]; 119 634 Myophilin [Crassostrea gigas]

Protein Accession	CL16175.Contig6$_H$1 CL16175.Contig5$_H$1 CL16175.Contig4$_H$1
Mean Expression Ratio	1.16
Median Expression Ratio	1.16
Credible Interval	(0.825, 1.65)
Associated Peptides	3
Associated Spectra	6
Coverage	NaN

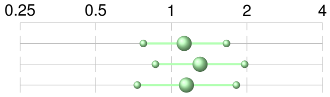

A	B	2.5	50	97.5	Sequence
2	1	0.77	1.1	1.7	GGGFALMQNIER
1	1	0.86	1.3	2	LMPGCIK
1	0	0.73	1.1	1.8	DGIVLCK

5.233 21 239 minus strand calponin-like protein [Taenia asiatica]

Protein Accession	CL26318.Contig1$_H$1
Mean Expression Ratio	1.16
Median Expression Ratio	1.16
Credible Interval	(0.753, 1.78)
Associated Peptides	2
Associated Spectra	2
Coverage	NaN

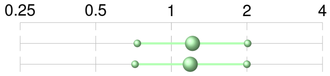

A	B	2.5	50	97.5	Sequence
1	0	0.73	1.2	2	HIADIK
1	0	0.72	1.2	2	VDDMSAASK

5.234 185 1258 minus strand Severin [Crassostrea gigas]

Protein Accession	CL7486.Contig1$_H$1
Mean Expression Ratio	1.16
Median Expression Ratio	1.16
Credible Interval	(0.855, 1.57)
Associated Peptides	7
Associated Spectra	9
Coverage	NaN

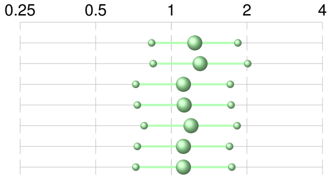

A	B	2.5	50	97.5	Sequence
2	0	0.84	1.2	1.8	EVQGHESDLFR
1	1	0.85	1.3	2	TDHPLVPITCLTEGTK
1	0	0.72	1.1	1.7	TDTVDEGSGTEEFDR
1	0	0.73	1.1	1.7	TVELDTFLNDVPVQHR
1	0	0.78	1.2	1.8	VTEWPVGDYGK
1	0	0.73	1.1	1.7	SDFISEDVFILDTK
1	0	0.72	1.1	1.7	TAGQKPGLQIWR

5.235 11 244 minus strand Tubulin alpha chain (Fragment) OS=Lytechinus pictus PE=3 SV=1

Protein Accession	CL3047.Contig6$_H1$
Mean Expression Ratio	0.863
Median Expression Ratio	0.862
Credible Interval	(0.562, 1.33)
Associated Peptides	2
Associated Spectra	2
Coverage	NaN

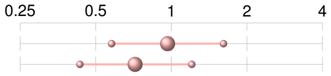

A	B	2.5	50	97.5	Sequence
0	1	0.58	0.96	1.6	EDLAALEK
0	1	0.43	0.72	1.2	FDLMYAK

5.236 95 1579 minus strand hypothetical protein BRAFLDRAFT_113974 [Branchiostoma floridae] gi

Protein Accession	CL19753.Contig3$_H1$
Mean Expression Ratio	1.16
Median Expression Ratio	1.16
Credible Interval	(0.929, 1.44)
Associated Peptides	10
Associated Spectra	23
Coverage	NaN

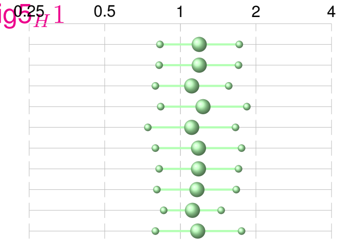

A	B	2.5	50	97.5	Sequence
1	1	0.83	1.2	1.7	ELGEYALK
1	1	0.82	1.2	1.7	FKDINEVIER
2	1	0.79	1.1	1.6	LIMQAAGR
1	0	0.84	1.2	1.8	TFPVVNPATEK
1	0	0.74	1.1	1.7	TSYGLAAGVITK
1	0	0.79	1.2	1.7	TVGDPFDAVTMQGPQVDQEQFEK
1	1	0.82	1.2	1.7	ADLRPDPNIK
1	1	0.81	1.2	1.7	DEIFGPVQSIIK
3	5	0.86	1.1	1.5	FADLVIR
0	1	0.79	1.2	1.8	VICEVQEADK

5.237 145 459 fk506-binding protein 2 [Papilio xuthus]; 237 551 fk506-binding protein 2 [Papilio xuthus]

Protein Accession	Unigene7883$_H1$ CL4621.Contig4$_H1$
Mean Expression Ratio	1.16
Median Expression Ratio	1.16
Credible Interval	(0.867, 1.55)
Associated Peptides	4
Associated Spectra	11
Coverage	NaN

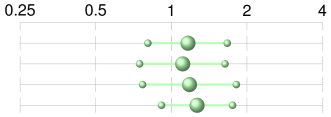

A	B	2.5	50	97.5	Sequence
2	1	0.81	1.2	1.7	GVDVETISEGDKK
1	1	0.75	1.1	1.6	GWDEGVAQMSK
1	0	0.77	1.2	1.8	IGKGEVIK
4	1	0.91	1.3	1.8	NKPFQFK

5.238 895 1410 Galectin-3 OS=Rattus norvegicus GN=Lgals3 PE=1 SV=4; 1067 1405 Galectin-9 [Crassostrea gigas]; 1068 1421 tandem-repeat galectin [Pinctada fucata]; 1041 1394 tandem-repeat galectin [Pinctada fucata]; 1046 1399 tandem-repeat galectin [Pinctada fucata]; 1068 1421 tandem-repeat galectin [Pinctada fucata]; 1067 1420 tandem-repeat galectin [Pinctada fucata]

Protein Accession	CL474.Contig9$_{H}1$ CL474.Contig8$_{H}1$ CL474.Contig7$_{H}1$ CL474.Contig6$_{H}1$ CL474.Contig5$_{H}1$
Mean Expression Ratio	1.16
Median Expression Ratio	1.16
Credible Interval	(0.745, 1.8)
Associated Peptides	1
Associated Spectra	2
Coverage	NaN

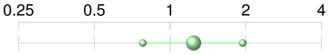

A	B	2.5	50	97.5	Sequence
**2**	**0**	**0.78**	**1.2**	**1.9**	**VAVNNQHLLDFR**

5.239 970 1854 fasciclin-like protein precursor [Aplysia californica] gi

Protein Accession	CL9799.Contig1$_{H}1$
Mean Expression Ratio	0.863
Median Expression Ratio	0.864
Credible Interval	(0.581, 1.28)
Associated Peptides	3
Associated Spectra	3
Coverage	NaN

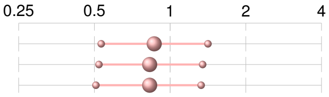

A	B	2.5	50	97.5	Sequence
**1**	**0**	**0.53**	**0.87**	**1.4**	**VLIPDDALDLVTMAK**
**0**	**1**	**0.52**	**0.83**	**1.3**	**ANLVSMLK**
**0**	**1**	**0.51**	**0.83**	**1.3**	**ELQTVLLGHVLPEAR**

5.240 28 642 minus strand Transmembrane emp24 domain-containing protein 9 [Crassostrea gigas]; 236 709 minus strand Transmembrane emp24 domain-containing protein 9 OS=Rattus norvegicus GN=Tmed9 PE=1 SV=1; 239 742 minus strand Transmembrane emp24 domain-containing protein eca OS=Drosophila erecta GN=eca PE=3 SV=1; 236 709 minus strand Transmembrane emp24 domain-containing protein 9 OS=Rattus norvegicus GN=Tmed9 PE=1 SV=1; 236 709 minus strand Transmembrane emp24 domain-containing protein 9 OS=Rattus norvegicus GN=Tmed9 PE=1 SV=1; 236 709 minus strand Transmembrane emp24 domain-containing protein 9 OS=Rattus norvegicus GN=Tmed9 PE=1 SV=1; 44 658 minus strand Transmembrane emp24 domain-containing protein 9 [Crassostrea gigas]

Protein Accession	CL2503.Contig8$_{H}$1 CL2503.Contig7$_{H}$1 CL2503.Contig5$_{H}$1 CL2503.Contig4$_{H}$1 CL2503.Con
Mean Expression Ratio	1.16
Median Expression Ratio	1.16
Credible Interval	(0.72, 1.89)
Associated Peptides	1
Associated Spectra	1
Coverage	NaN

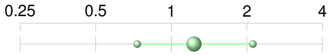

A	B	2.5	50	97.5	Sequence
1	0	0.73	1.2	2.1	QLLDQVEQITK

5.241 187 642 minus strand PREDICTED: similar to pol-like protein [Tribolium castaneum]

Protein Accession	CL5644.Contig3$_{H}$1
Mean Expression Ratio	1.16
Median Expression Ratio	1.16
Credible Interval	(0.825, 1.62)
Associated Peptides	1
Associated Spectra	23
Coverage	NaN

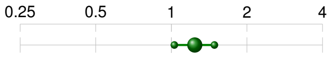

A	B	2.5	50	97.5	Sequence
23	0	1	1.2	1.5	IILEQEELK

5.242 274 666 Calmodulin-4 [Heterocephalus glaber]

Protein Accession	Unigene19050$_{H}$1
Mean Expression Ratio	1.16
Median Expression Ratio	1.16
Credible Interval	(0.891, 1.49)
Associated Peptides	4
Associated Spectra	18
Coverage	NaN

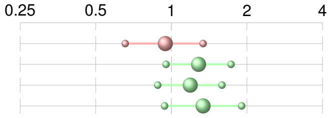

A	B	2.5	50	97.5	Sequence
2	1	0.66	0.94	1.3	GLDPENAVLDMDINK
3	3	0.95	1.3	1.7	SGFITLDELK
5	1	0.88	1.2	1.6	VLLSVGR
2	1	0.94	1.3	1.9	GTLSILELHK

5.243 2 307 Laminin subunit gamma-1 [Crassostrea gigas]

Protein Accession	Unigene32982$_{H1}$
Mean Expression Ratio	1.16
Median Expression Ratio	1.16
Credible Interval	(0.713, 1.87)
Associated Peptides	1
Associated Spectra	1
Coverage	NaN

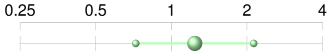

A	B	2.5	50	97.5	Sequence
**0**	**1**	**0.72**	**1.2**	**2.1**	**AEISLR**

5.244 353 2161 Bifunctional 3'-phosphoadenosine 5'-phosphosulfate synthetase [Crassostrea gigas]; 141 1943 Bifunctional 3'-phosphoadenosine 5'-phosphosulfate synthetase [Crassostrea gigas]

Protein Accession	CL8992.Contig2$_{H1}$ CL8992.Contig1$_{H1}$
Mean Expression Ratio	1.15
Median Expression Ratio	1.15
Credible Interval	(0.832, 1.6)
Associated Peptides	4
Associated Spectra	7
Coverage	NaN

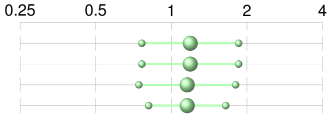

A	B	2.5	50	97.5	Sequence
**1**	**0**	**0.76**	**1.2**	**1.9**	**QHAAILEEK**
**1**	**0**	**0.76**	**1.2**	**1.9**	**QFGTSNLGHPYVK**
**1**	**0**	**0.74**	**1.2**	**1.8**	**AGPTEVQWHAK**
**2**	**2**	**0.81**	**1.2**	**1.7**	**LDGSAAFALK**

5.245 62 1093 Heterogeneous nuclear ribonucleoprotein A2-like protein 1 [Crassostrea gigas]

Protein Accession	CL47083.Contig4$_{H1}$
Mean Expression Ratio	1.15
Median Expression Ratio	1.15
Credible Interval	(0.84, 1.57)
Associated Peptides	5
Associated Spectra	7
Coverage	NaN

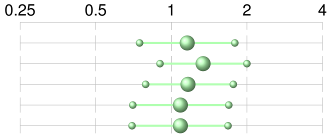

A	B	2.5	50	97.5	Sequence
**1**	**0**	**0.75**	**1.2**	**1.8**	**ETFSPFGTISQIDLITDK**
**2**	**0**	**0.9**	**1.3**	**2**	**EDTTEDMIR**
**1**	**1**	**0.79**	**1.2**	**1.8**	**MFVGGIKEDTTEDMIR**
**0**	**1**	**0.7**	**1.1**	**1.7**	**GGSRGGR**
**0**	**1**	**0.7**	**1.1**	**1.7**	**GGGRGGSR**

5.246 3 239 minus strand alcohol dehydrogenase class 3 [Branchiostoma lanceolatum]

Protein Accession	Unigene58195$_H1$
Mean Expression Ratio	1.15
Median Expression Ratio	1.15
Credible Interval	(0.778, 1.69)
Associated Peptides	2
Associated Spectra	4
Coverage	NaN

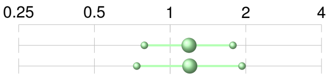

A	B	2.5	50	97.5	Sequence
3	0	0.79	1.2	1.8	GSAFGGWK
1	0	0.74	1.2	1.9	LVEAYMNGK

5.247 222 809 minus strand PREDICTED: 15-hydroxyprostaglandin dehydrogenase [NAD+] [Gallus gallus]

Protein Accession	CL44148.Contig2$_H1$
Mean Expression Ratio	1.15
Median Expression Ratio	1.15
Credible Interval	(0.938, 1.4)
Associated Peptides	7
Associated Spectra	83
Coverage	NaN

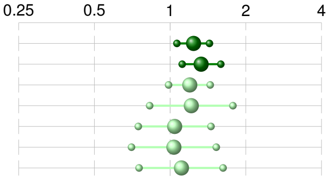

A	B	2.5	50	97.5	Sequence
19	16	1.1	1.2	1.4	ATVEAFLTK
17	6	1.1	1.3	1.6	VFLTGGAHGIGK
13	6	0.99	1.2	1.4	VMFCDINDK
1	0	0.83	1.2	1.8	RVFLTGGAHGIGK
0	3	0.75	1	1.5	TGGAHGIGK
0	1	0.7	1	1.5	LTGGAHGIGK
0	1	0.75	1.1	1.6	GGAHGIGK

5.248 118 1866 Lamin Dm0 [Crassostrea gigas]

Protein Accession	CL4079.Contig7$_H1$
Mean Expression Ratio	0.868
Median Expression Ratio	0.869
Credible Interval	(0.714, 1.06)
Associated Peptides	16
Associated Spectra	25
Coverage	NaN

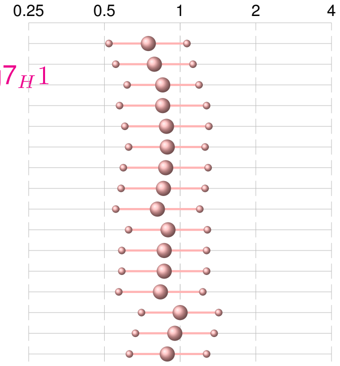

A	B	2.5	50	97.5	Sequence
1	1	0.52	0.74	1.1	ADDLSAELAK
1	1	0.55	0.79	1.1	GGIDIEEVDPEGK
2	1	0.62	0.85	1.2	KQLEEETLLR
1	0	0.57	0.85	1.3	LSVQVQSTEEVVK
1	0	0.6	0.88	1.3	RVETLEAQVAELTAK
1	1	0.62	0.89	1.3	WFVADEMK
1	0	0.59	0.88	1.3	SLVIKPGK
1	0	0.58	0.86	1.3	EDLHFK
1	0	0.56	0.81	1.2	YKTEYEDLLAK
1	1	0.63	0.89	1.3	EKHEFDLQTVR
1	0	0.58	0.86	1.3	LTIDEQMAEYSDLLDIK
0	1	0.59	0.87	1.3	LSEVEAPR
0	1	0.57	0.84	1.2	RLALKDDEIADLR
0	2	0.7	1	1.4	IADLR
0	2	0.67	0.95	1.4	IRDLENQLAHER
0	2	0.63	0.89	1.3	LLEALR

5.249 54 791 GMP reductase 2 [Crassostrea gigas]

Protein Accession	CL26888.Contig1$_H$1
Mean Expression Ratio	1.15
Median Expression Ratio	1.15
Credible Interval	(0.707, 1.85)
Associated Peptides	1
Associated Spectra	1
Coverage	NaN

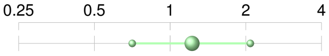

A	B	2.5	50	97.5	Sequence
**1**	**0**	**0.71**	**1.2**	**2.1**	**LFTTIHK**

5.250 87 485 Sex-regulated protein janus-A [Crassostrea gigas]; 92 310 PREDICTED: hypothetical protein [Saccoglossus kowalevskii]; 92 310 PREDICTED: hypothetical protein [Saccoglossus kowalevskii]

Protein Accession	CL13131.Contig3$_H$1 CL13131.Contig2$_H$1 CL13131.Contig1$_H$1
Mean Expression Ratio	1.15
Median Expression Ratio	1.15
Credible Interval	(0.694, 1.86)
Associated Peptides	1
Associated Spectra	1
Coverage	NaN

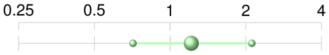

A	B	2.5	50	97.5	Sequence
**1**	**0**	**0.71**	**1.2**	**2.1**	**VVGEIESK**

5.251 4365 4553 minus strand Basement membrane-specific heparan sulfate proteoglycan core protein [Crassostrea gigas]; 2502 2696 minus strand hypothetical protein DAPPUDRAFT_186952 [Daphnia pulex]

Protein Accession	CL24300.Contig6$_H$1 CL24300.Contig2$_H$1
Mean Expression Ratio	0.867
Median Expression Ratio	0.871
Credible Interval	(0.575, 1.29)
Associated Peptides	2
Associated Spectra	3
Coverage	NaN

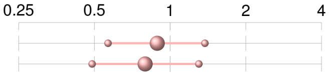

A	B	2.5	50	97.5	Sequence
**1**	**1**	**0.57**	**0.89**	**1.4**	**DGSIIVDGQEVK**
**0**	**1**	**0.49**	**0.79**	**1.3**	**VDDGFR**

5.252 684 1250 minus strand Collagen alpha-5(VI) chain [Crassostrea gigas]

Protein Accession	CL284.Contig4$_H$1
Mean Expression Ratio	1.15
Median Expression Ratio	1.15
Credible Interval	(0.848, 1.55)
Associated Peptides	5
Associated Spectra	10
Coverage	NaN

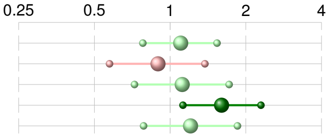

A	B	2.5	50	97.5	Sequence
**1**	**3**	**0.78**	**1.1**	**1.5**	**HVMDALSAIDHHQGDR**
**1**	**0**	**0.58**	**0.9**	**1.4**	**VCIVITDGQSQEIQQTADEAR**
**1**	**0**	**0.72**	**1.1**	**1.7**	**EAGITMFAVGVGK**
**1**	**2**	**1.1**	**1.6**	**2.3**	**VAAITFGK**
**0**	**1**	**0.79**	**1.2**	**1.9**	**EAGITMF**

5.253 316 1512 Alpha-N-acetylgalactosaminidase [Crassostrea gigas]; 282 1568 Alpha-N-acetylgalactosaminidase [Crassostrea gigas]; 282 1478 Alpha-N-acetylgalactosaminidase [Crassostrea gigas]

Protein Accession	CL2428.Contig4$_H$1 CL2428.Contig2$_H$1 CL2428.Contig1$_H$1
Mean Expression Ratio	1.15
Median Expression Ratio	1.15
Credible Interval	(0.775, 1.69)
Associated Peptides	2
Associated Spectra	5
Coverage	NaN

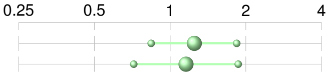

A	B	2.5	50	97.5	Sequence
1	3	0.84	1.2	1.8	LVELGLR
0	1	0.72	1.2	1.9	ELFAGK

5.254 106 1701 T-complex protein 1 subunit beta [Crassostrea gigas]

Protein Accession	CL8176.Contig1$_H$1
Mean Expression Ratio	0.872
Median Expression Ratio	0.872
Credible Interval	(0.563, 1.36)
Associated Peptides	2
Associated Spectra	2
Coverage	NaN

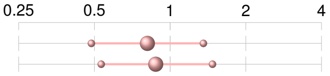

A	B	2.5	50	97.5	Sequence
1	0	0.49	0.81	1.4	GSGNLDAIQIIK
1	0	0.53	0.87	1.5	CTIVLR

5.255 98 1012 minus strand RecName: Full=Substrate-specific endoprotease Tex31; Flags: Precursor gi|32187775|emb|CAD36507.1| substrate-specific endoprotease [Conus textile]

Protein Accession	CL13681.Contig1$_H$1
Mean Expression Ratio	0.873
Median Expression Ratio	0.872
Credible Interval	(0.621, 1.22)
Associated Peptides	3
Associated Spectra	7
Coverage	NaN

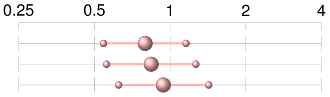

A	B	2.5	50	97.5	Sequence
2	1	0.54	0.79	1.2	NIPLSASDISAIVER
2	0	0.56	0.84	1.3	VPGAVFSK
1	1	0.63	0.94	1.4	ALDPQHTMCLADK

5.256 316 1512 Alpha-N-acetylgalactosaminidase [Crassostrea gigas]; 282 1478 Alpha-N-acetylgalactosaminidase [Crassostrea gigas]

Protein Accession	CL2428.Contig4$_{H}$1 CL2428.Contig1$_{H}$1
Mean Expression Ratio	1.15
Median Expression Ratio	1.15
Credible Interval	(0.747, 1.76)
Associated Peptides	2
Associated Spectra	5
Coverage	NaN

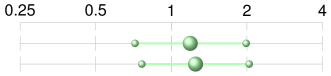

A	B	2.5	50	97.5	Sequence
1	0	0.72	1.2	2	LGIYEDFGSK
1	3	0.76	1.2	2	LVELGLR

5.257 116 337 minus strand Golgi-associated plant pathogenesis-related protein 1 [Crassostrea gigas]; 538 759 minus strand Golgi-associated plant pathogenesis-related protein 1 [Crassostrea gigas]; 116 337 minus strand Golgi-associated plant pathogenesis-related protein 1 [Crassostrea gigas]; 261 482 minus strand Golgi-associated plant pathogenesis-related protein 1 [Crassostrea gigas]; 116 337 minus strand Golgi-associated plant pathogenesis-related protein 1 [Crassostrea gigas]; 116 337 minus strand Golgi-associated plant pathogenesis-related protein 1 [Crassostrea gigas]

Protein Accession	CL15263.Contig9$_{H}$1 CL15263.Contig8$_{H}$1 CL15263.Contig7$_{H}$1 CL15263.Contig5$_{H}$1 CL1526[illegible]
Mean Expression Ratio	1.14
Median Expression Ratio	1.15
Credible Interval	(0.74, 1.78)
Associated Peptides	2
Associated Spectra	2
Coverage	NaN

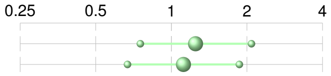

A	B	2.5	50	97.5	Sequence
1	0	0.75	1.2	2.1	FIDEMLEAHNK
1	0	0.67	1.1	1.9	KEHQVQPLVINK

5.258 177 791 Ras-related protein Rab-1A [Haliotis discus discus]

Protein Accession	CL21389.Contig6$_{H}$1
Mean Expression Ratio	1.14
Median Expression Ratio	1.14
Credible Interval	(0.839, 1.57)
Associated Peptides	5
Associated Spectra	8
Coverage	NaN

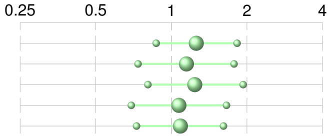

A	B	2.5	50	97.5	Sequence
3	0	0.87	1.3	1.8	MGPVTSASENKPSVK
1	0	0.74	1.2	1.8	QWLQEIDR
1	0	0.81	1.2	1.9	KVVDYTTAK
1	0	0.69	1.1	1.7	SCLLLR
0	2	0.73	1.1	1.6	LLLIGDSGVGK

5.259 9 161 PREDICTED: tubulin alpha-1C chain-like [Acyrthosiphon pisum]; 9 161 Tubulin alpha-3 chain (Fragments) OS=Mesocricetus auratus PE=1 SV=1

Protein Accession	Unigene74551$_H1$ Unigene70816$_H1$
Mean Expression Ratio	1.14
Median Expression Ratio	1.14
Credible Interval	(0.705, 1.87)
Associated Peptides	1
Associated Spectra	1
Coverage	NaN

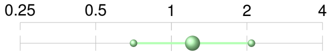

A	B	2.5	50	97.5	Sequence
**0**	**1**	**0.71**	**1.2**	**2.1**	**IHFPLATY**

5.260 58 198 minus strand Protein DJ-1 [Crassostrea gigas]

Protein Accession	CL16588.Contig4$_H1$
Mean Expression Ratio	0.875
Median Expression Ratio	0.875
Credible Interval	(0.596, 1.28)
Associated Peptides	1
Associated Spectra	6
Coverage	NaN

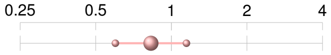

A	B	2.5	50	97.5	Sequence
**3**	**3**	**0.6**	**0.83**	**1.1**	**ADSLVAPMLVK**

5.261 459 1187 PREDICTED: protein-L-isoaspartate(D-aspartate) O-methyltransferase-like isoform 2 [Anolis carolinensis]

Protein Accession	CL3223.Contig3$_H1$
Mean Expression Ratio	1.15
Median Expression Ratio	1.14
Credible Interval	(0.738, 1.8)
Associated Peptides	1
Associated Spectra	2
Coverage	NaN

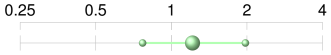

A	B	2.5	50	97.5	Sequence
**2**	**0**	**0.77**	**1.2**	**2**	**AVGIEHIEELVK**

5.262 44 1465 minus strand Adenylyl cyclase-associated protein 1 [Crassostrea gigas]; 52 1473 minus strand Adenylyl cyclase-associated protein 1 [Crassostrea gigas]; 48 1469 Adenylyl cyclase-associated protein 1 [Crassostrea gigas]

Protein Accession	CL19470.Contig9$_{H}$1 CL19470.Contig8$_{H}$1 CL19470.Contig1$_{H}$1
Mean Expression Ratio	1.14
Median Expression Ratio	1.14
Credible Interval	(0.923, 1.41)
Associated Peptides	13
Associated Spectra	24
Coverage	NaN

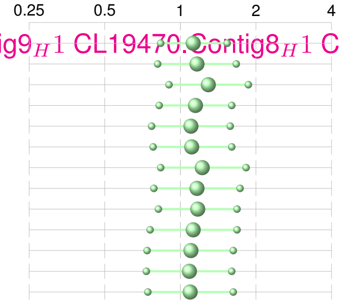

A	B	2.5	50	97.5	Sequence
2	3	0.83	1.1	1.5	AQLDIVNAAFK
1	1	0.81	1.2	1.7	DSLDTEIVTAK
1	1	0.9	1.3	1.9	EFALPEQFK
2	1	0.82	1.1	1.6	IQAVQEFR
1	1	0.77	1.1	1.6	NIVLDNVELK
1	1	0.78	1.1	1.6	QFLEVASK
1	0	0.83	1.2	1.8	SKQPPQADLPK
1	0	0.78	1.2	1.7	VNSIILDQCK
2	0	0.82	1.2	1.7	VPTISIDK
1	0	0.76	1.1	1.7	QAVYIYK
1	0	0.74	1.1	1.6	LETLALR
1	0	0.73	1.1	1.6	DTALQNAVAR
0	1	0.74	1.1	1.6	LKDFK

5.263 2 2248 PREDICTED: heat shock 70 kDa protein 4-like [Oreochromis niloticus]; 2 2227 PREDICTED: heat shock 70 kDa protein 4-like [Oreochromis niloticus]

Protein Accession	CL12749.Contig5$_{H}$1 CL12749.Contig4$_{H}$1
Mean Expression Ratio	1.14
Median Expression Ratio	1.14
Credible Interval	(0.789, 1.66)
Associated Peptides	3
Associated Spectra	4
Coverage	NaN

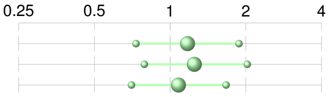

A	B	2.5	50	97.5	Sequence
1	0	0.73	1.2	1.9	EMLNLLIEK
1	0	0.79	1.2	2	TAWWTPK
0	2	0.7	1.1	1.7	HENPVIFVSQVMSEK

5.264 5 514 hypothetical protein KGM_05418 [Danaus plexippus]

Protein Accession	CL11785.Contig1$_{H}$1
Mean Expression Ratio	1.14
Median Expression Ratio	1.14
Credible Interval	(0.782, 1.67)
Associated Peptides	3
Associated Spectra	4
Coverage	NaN

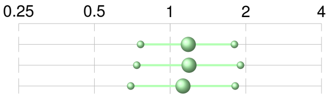

A	B	2.5	50	97.5	Sequence
1	1	0.76	1.2	1.8	ALLDLSENIDELNR
1	0	0.74	1.2	1.9	KLTNQSLK
1	0	0.7	1.1	1.8	NNLDIGK

5.265 43 525 Reticulon-4 [Crassostrea gigas]; 143 757 Reticulon-4 [Crassostrea gigas]; 43 564 Reticulon-4 [Crassostrea gigas]; 302 583 Reticulon-4 OS=Rattus norvegicus GN=Rtn4 PE=1 SV=1

Protein Accession	CL29775.Contig4$_{H}$1 CL29775.Contig3$_{H}$1 CL29775.Contig2$_{H}$1 CL29775.Contig1$_{H}$1
Mean Expression Ratio	0.877
Median Expression Ratio	0.878
Credible Interval	(0.595, 1.29)
Associated Peptides	2
Associated Spectra	6
Coverage	NaN

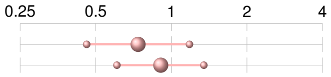

A	B	2.5	50	97.5	Sequence
**2**	**1**	**0.46**	**0.74**	**1.2**	**VTQTILQHVNR**
**0**	**3**	**0.61**	**0.91**	**1.3**	**SLLELR**

5.266 188 1021 hypothetical protein BRAFLDRAFT_214346 [Branchiostoma floridae] gi

Protein Accession	CL38498.Contig2$_{H}$1
Mean Expression Ratio	1.14
Median Expression Ratio	1.14
Credible Interval	(0.762, 1.7)
Associated Peptides	3
Associated Spectra	3
Coverage	NaN

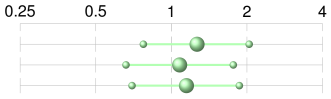

A	B	2.5	50	97.5	Sequence
**1**	**0**	**0.78**	**1.3**	**2**	**EGLELHPPK**
**0**	**1**	**0.66**	**1.1**	**1.8**	**NLVGAIMTEEEAKK**
**0**	**1**	**0.7**	**1.1**	**1.9**	**DICTFLR**

5.267 " 6 1274 talin, putative [Ixodes scapularis] gi"

Protein Accession	CL39200.Contig1$_{H}$1
Mean Expression Ratio	1.14
Median Expression Ratio	1.14
Credible Interval	(0.737, 1.76)
Associated Peptides	2
Associated Spectra	2
Coverage	NaN

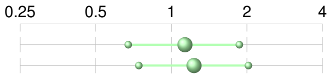

A	B	2.5	50	97.5	Sequence
**0**	**1**	**0.67**	**1.1**	**1.9**	**SIAAATAALVK**
**0**	**1**	**0.74**	**1.2**	**2**	**LEMIR**

5.268 64 435 peptidoglycan recognition protein S1L [Pomacea canaliculata]

Protein Accession	CL1297.Contig6$_{H}$1
Mean Expression Ratio	1.14
Median Expression Ratio	1.14
Credible Interval	(0.7, 1.86)
Associated Peptides	1
Associated Spectra	1
Coverage	NaN

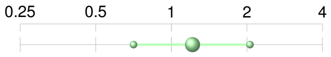

A	B	2.5	50	97.5	Sequence
**1**	**0**	**0.71**	**1.2**	**2.1**	**MYSVCHGGR**

5.269 503 1219 heat shock cognate 71 [Mytilus galloprovincialis]

Protein Accession	CL23109.Contig5$_{H}1$
Mean Expression Ratio	1.14
Median Expression Ratio	1.14
Credible Interval	(0.827, 1.57)
Associated Peptides	4
Associated Spectra	7
Coverage	NaN

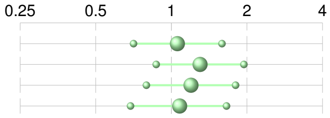

A	B	2.5	50	97.5	Sequence
1	1	0.71	1.1	1.6	FEELNADLFR
2	0	0.87	1.3	1.9	LLQDFFNGK
2	0	0.8	1.2	1.8	GTLEPVEK
0	1	0.69	1.1	1.7	SAIHEIVLVGGSTR

5.270 69 779 PREDICTED: 15-hydroxyprostaglandin dehydrogenase [NAD+] [Gallus gallus]

Protein Accession	CL44148.Contig4$_{H}1$
Mean Expression Ratio	1.14
Median Expression Ratio	1.14
Credible Interval	(0.739, 1.77)
Associated Peptides	2
Associated Spectra	2
Coverage	NaN

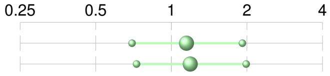

A	B	2.5	50	97.5	Sequence
1	0	0.7	1.2	1.9	PLVENPPSPTVIGEAVIK
1	0	0.73	1.2	2	PPSPTVIGEAVIK

5.271 43 591 minus strand cyclophilin type peptidyl-prolyl cis-trans isomerase [Glossina morsitans morsitans]

Protein Accession	CL39270.Contig1$_{H}1$
Mean Expression Ratio	1.14
Median Expression Ratio	1.14
Credible Interval	(0.904, 1.43)
Associated Peptides	5
Associated Spectra	35
Coverage	NaN

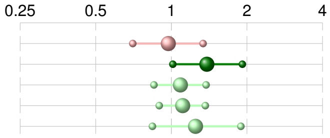

A	B	2.5	50	97.5	Sequence
1	3	0.7	0.97	1.3	VFFDMTADGVPLGR
4	0	1	1.4	1.9	KIESYGSKNGATSQK
7	4	0.85	1.1	1.4	FADENFTLK
7	8	0.9	1.1	1.4	ITMELR
0	1	0.84	1.2	1.9	VIPQFMCQGGDFTR

5.272 140 1564 selenium-binding protein [Haliotis discus hannai]

Protein Accession	CL3868.Contig7$_{H}1$
Mean Expression Ratio	1.14
Median Expression Ratio	1.14
Credible Interval	(0.695, 1.84)
Associated Peptides	1
Associated Spectra	1
Coverage	NaN

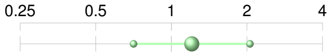

A	B	2.5	50	97.5	Sequence
1	0	0.71	1.2	2.1	VVEAVDIHR

5.273 2 256 Polyubiquitin (Fragment) OS=Xenopus laevis PE=1 SV=2; 20 388 large subunit ribosomal protein L40e [Clonorchis sinensis]

Protein Accession	CL47507.Contig2$_H$1 CL39010.Contig1$_H$1
Mean Expression Ratio	1.14
Median Expression Ratio	1.14
Credible Interval	(0.876, 1.49)
Associated Peptides	5
Associated Spectra	20
Coverage	NaN

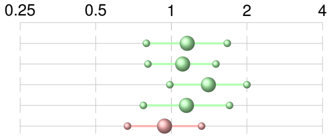

A	B	2.5	50	97.5	Sequence
2	1	0.8	1.2	1.7	TITLEVEPSDTIENVK
4	4	0.81	1.1	1.5	TLSDYNIQK
3	0	0.98	1.4	2	LIFAGK
1	1	0.77	1.2	1.7	ESTLHLVLR
3	1	0.67	0.94	1.3	MQIFVK

5.274 244 5451 alpha macroglobulin [Chlamys farreri]; 244 5493 alpha macroglobulin [Chlamys farreri]

Protein Accession	CL20671.Contig5$_H$1 CL20671.Contig4$_H$1
Mean Expression Ratio	0.88
Median Expression Ratio	0.88
Credible Interval	(0.618, 1.25)
Associated Peptides	4
Associated Spectra	5
Coverage	NaN

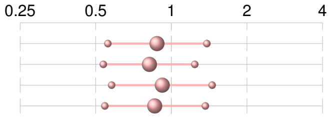

A	B	2.5	50	97.5	Sequence
1	0	0.56	0.88	1.4	DSFSLPLPLDMVPDSGR
1	1	0.53	0.82	1.2	HLALDVEQLNR
0	1	0.58	0.92	1.5	SLKIK
0	1	0.54	0.86	1.4	VAVNGIECINR

5.275 79 606 minus strand PREDICTED: similar to Ras-like protein 3 precursor (Protein roughened) [Tribolium castaneum] gi

Protein Accession	Unigene4246$_H$1
Mean Expression Ratio	1.13
Median Expression Ratio	1.14
Credible Interval	(0.837, 1.54)
Associated Peptides	4
Associated Spectra	9
Coverage	NaN

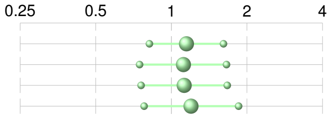

A	B	2.5	50	97.5	Sequence
2	2	0.82	1.2	1.6	LVVLGSGGVGK
2	0	0.75	1.1	1.7	VKDTDDVPMLLVGNK
1	1	0.76	1.1	1.7	CDLEVDR
0	1	0.78	1.2	1.9	DLYMK

5.276 727 2022 RecName: Full=Enolase; AltName: Full=2-phospho-D-glycerate hydro-lyase; AltName: Full=2-phosphoglycerate dehydratase gi|1911573|gb|AAB50731.1| enolase [Doryteuthis pealeii]; 690 1985 RecName: Full=Enolase; AltName: Full=2-phospho-D-glycerate hydro-lyase; AltName: Full=2-phosphoglycerate dehydratase gi|1911573|gb|AAB50731.1| enolase [Doryteuthis pealeii]; 213 1508 RecName: Full=Enolase; AltName: Full=2-phospho-D-glycerate hydro-lyase; AltName: Full=2-phosphoglycerate dehydratase gi|1911573|gb|AAB50731.1| enolase [Doryteuthis pealeii]; 246 1541 RecName: Full=Enolase; AltName: Full=2-phospho-D-glycerate hydro-lyase; AltName: Full=2-phosphoglycerate dehydratase gi|1911573|gb|AAB50731.1| enolase [Doryteuthis pealeii]

Protein Accession	CL15687.Contig6$_{H}1$ CL15687.Contig5$_{H}1$ CL15687.Contig3$_{H}1$ CL15687.Contig2$_{H}1$
Mean Expression Ratio	1.14
Median Expression Ratio	1.14
Credible Interval	(1.01, 1.28)
Associated Peptides	25
Associated Spectra	122
Coverage	NaN

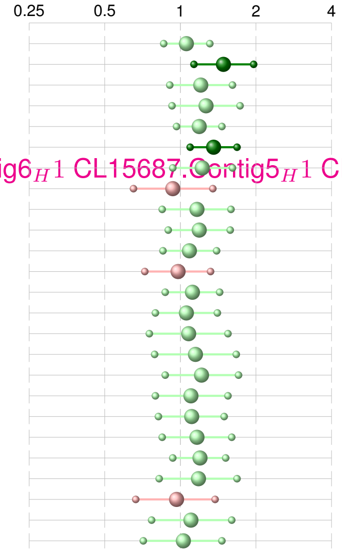

A	B	2.5	50	97.5	Sequence
11	3	0.86	1.1	1.3	AAGWGVMVSHR
3	3	1.1	1.5	2	AAVPSGASTGIYEALELR
3	2	0.91	1.2	1.6	DGKYDLDFK
2	1	0.92	1.3	1.7	FQIVGDDLLVTNPK
12	2	0.97	1.2	1.5	GVPLYR
9	4	1.1	1.4	1.7	IAPAFVGK
4	2	0.93	1.2	1.6	IEEELGVDAFYAGK
1	0	0.65	0.94	1.3	IEIGMDVAASEFCK
2	1	0.85	1.2	1.6	IMLDLDGTPNK
4	1	0.89	1.2	1.6	IMLDLDGTPNKEK
3	6	0.85	1.1	1.4	TLDLADQTAMDK
3	1	0.72	0.98	1.3	TSQQMIDMYHDFVK
4	4	0.87	1.1	1.4	VQMGIDR
1	4	0.8	1.1	1.4	QIFDSR
1	0	0.75	1.1	1.5	SYPIVTIEDPFDQDDWDAYTK
1	0	0.79	1.1	1.7	HIADLCGNKR
1	1	0.87	1.2	1.7	YDLDFK
1	1	0.79	1.1	1.5	LAMQEF
2	2	0.82	1.1	1.5	ACNALLLK
2	1	0.84	1.2	1.6	VNQIGSVSESIQAFK
5	4	0.93	1.2	1.5	YNQILR
0	1	0.83	1.2	1.7	HIADLCGNK
0	1	0.67	0.96	1.4	EGLELLK
0	1	0.77	1.1	1.6	MGSETYHHLK
0	1	0.71	1	1.5	ADLVVGLSTGQIK

5.277 128 559 minus strand predicted protein [Nematostella vectensis] gi

Protein Accession	CL19343.Contig1$_{H}1$
Mean Expression Ratio	0.879
Median Expression Ratio	0.881
Credible Interval	(0.613, 1.26)
Associated Peptides	2
Associated Spectra	7
Coverage	NaN

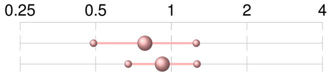

A	B	2.5	50	97.5	Sequence
1	0	0.49	0.78	1.3	GIPCLILLNGEDASEISR
3	3	0.67	0.92	1.3	TVILGDLTGSK

5.278 410 709 calmodulin-like; K02183 calmodulin

Protein Accession	CL30882.Contig6$_H$1
Mean Expression Ratio	0.881
Median Expression Ratio	0.881
Credible Interval	(0.573, 1.35)
Associated Peptides	2
Associated Spectra	2
Coverage	NaN

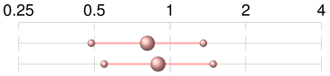

A	B	2.5	50	97.5	Sequence
**1**	**0**	**0.49**	**0.81**	**1.4**	**ELNDYVTEGGQSAAQAVLEK**
**0**	**1**	**0.55**	**0.89**	**1.5**	**EALRDFIEK**

5.279 2 244 Ras homolog gene family member A [Clonorchis sinensis]

Protein Accession	Unigene32424$_H$1
Mean Expression Ratio	0.884
Median Expression Ratio	0.881
Credible Interval	(0.551, 1.42)
Associated Peptides	1
Associated Spectra	2
Coverage	NaN

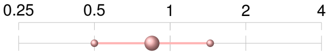

A	B	2.5	50	97.5	Sequence
**1**	**1**	**0.5**	**0.85**	**1.4**	**EVFETATR**

5.280 113 445 ribosomal protein rps19 [Lineus viridis]

Protein Accession	CL8932.Contig6$_H$1
Mean Expression Ratio	1.13
Median Expression Ratio	1.13
Credible Interval	(0.701, 1.84)
Associated Peptides	1
Associated Spectra	1
Coverage	NaN

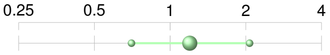

A	B	2.5	50	97.5	Sequence
**0**	**1**	**0.7**	**1.2**	**2.1**	**LEEIR**

5.281 605 1153 Peptidyl-prolyl cis-trans isomerase B [Crassostrea gigas]; 1046 1630 Peptidyl-prolyl cis-trans isomerase B [Crassostrea gigas]; 589 1173 Peptidyl-prolyl cis-trans isomerase B [Crassostrea gigas]; 883 1467 Peptidyl-prolyl cis-trans isomerase B [Crassostrea gigas]

Protein Accession	CL24747.Contig5$_H$1 CL24747.Contig4$_H$1 CL24747.Contig3$_H$1 CL24747.Contig1$_H$1
Mean Expression Ratio	1.13
Median Expression Ratio	1.13
Credible Interval	(0.782, 1.66)
Associated Peptides	3
Associated Spectra	7
Coverage	NaN

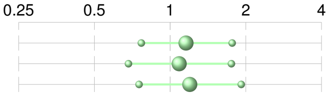

A	B	2.5	50	97.5	Sequence
**2**	**3**	**0.77**	**1.2**	**1.8**	**IASSGVVGIASK**
**1**	**0**	**0.68**	**1.1**	**1.8**	**KLGELETSIIGRPK**
**1**	**0**	**0.75**	**1.2**	**1.9**	**SGMSIYGETFKDENFK**

5.282 135 2630 Filamin-A [Crassostrea gigas]; 118 2613 Filamin-A [Crassostrea gigas]; 157 2652 Filamin-A [Crassostrea gigas]

Protein Accession	CL393.Contig15$_H$1 CL393.Contig13$_H$1 CL393.Contig12$_H$1
Mean Expression Ratio	0.884
Median Expression Ratio	0.884
Credible Interval	(0.76, 1.03)
Associated Peptides	23
Associated Spectra	144
Coverage	NaN

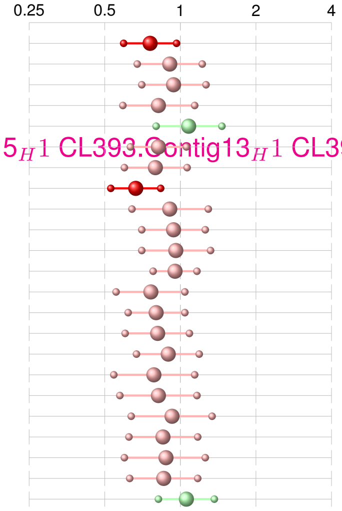

A	B	2.5	50	97.5	Sequence
9	3	0.59	0.76	0.97	AVANKPNEIVIDTSK
5	1	0.68	0.9	1.2	DNNDGTIVVEYQPK
5	1	0.7	0.94	1.3	EAGPGTLTVAVEGPGK
2	1	0.59	0.82	1.1	ENGVHYVDVK
4	1	0.8	1.1	1.5	HINNSPFK
6	4	0.63	0.82	1.1	IGDSELGHAAGCK
3	3	0.6	0.8	1.1	MGDGEIGIASFNPR
7	7	0.53	0.67	0.83	MNGIHTLSVR
1	1	0.64	0.91	1.3	PGSYGLHIK
4	2	0.7	0.94	1.3	QMEAAEAAVPNK
3	1	0.7	0.96	1.3	TPSGGHEDCFIQEMDK
12	9	0.78	0.95	1.2	VINFQIETK
3	1	0.55	0.76	1	VPKPGSYGLHIK
5	4	0.62	0.8	1	VQVGGPGLEK
4	2	0.6	0.81	1.1	VTGALDK
4	2	0.67	0.89	1.2	YGNIPIAGSPYK
0	1	0.54	0.79	1.1	GDSELGHAAGCK
1	1	0.58	0.82	1.2	AALSCK
0	1	0.64	0.92	1.3	QVTVHSLR
3	1	0.62	0.85	1.2	YKGNPIK
0	1	0.6	0.88	1.3	PKPGSYGLHIK
3	1	0.63	0.86	1.2	IMFTPK
8	3	0.82	1.1	1.4	KPSPIQEQSSMVVETVEK

5.283 27 515 heat shock protein 70 [Capsaspora owczarzaki ATCC 30864]; 30 464 minus strand hypothetical protein [Trichinella spiralis]; 27 491 minus strand heat shock protein 70 [Capsaspora owczarzaki ATCC 30864]

Protein Accession	CL14449.Contig4$_H$1 CL14449.Contig2$_H$1 CL14449.Contig1$_H$1
Mean Expression Ratio	0.885
Median Expression Ratio	0.885
Credible Interval	(0.625, 1.26)
Associated Peptides	4
Associated Spectra	5
Coverage	NaN

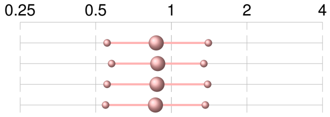

A	B	2.5	50	97.5	Sequence
1	0	0.55	0.87	1.4	RFDDPSVQSDMK
2	0	0.58	0.88	1.3	VEIIANDQGNR
1	0	0.55	0.88	1.4	NQVAMNPENTVFDAK
1	0	0.55	0.86	1.4	EVAEGYLGK

5.284 4 243 40S ribosomal protein S13 OS=Caenorhabditis elegans GN=rps-13 PE=2 SV=2; 4 243 40S ribosomal protein S13 OS=Caenorhabditis elegans GN=rps-13 PE=2 SV=2; 1 252 40S ribosomal protein S13 OS=Xenopus laevis GN=rps13 PE=3 SV=2; 1 213 40S ribosomal protein S13 OS=Xenopus laevis GN=rps13 PE=3 SV=2

Protein Accession	CL18049.Contig7$_{H}1$ CL18049.Contig4$_{H}1$ CL18049.Contig3$_{H}1$ CL18049.Contig2$_{H}1$
Mean Expression Ratio	1.13
Median Expression Ratio	1.13
Credible Interval	(0.698, 1.84)
Associated Peptides	1
Associated Spectra	1
Coverage	NaN

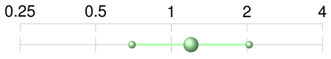

A	B	2.5	50	97.5	Sequence
**1**	**0**	**0.7**	**1.2**	**2**	**SLIKK**

5.285 " 125 628 minus strand collectin sub-family member 12, partial [Oncorhynchus mykiss]"

Protein Accession	CL46744.Contig5$_{H}1$
Mean Expression Ratio	1.13
Median Expression Ratio	1.13
Credible Interval	(0.701, 1.85)
Associated Peptides	1
Associated Spectra	1
Coverage	NaN

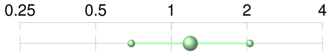

A	B	2.5	50	97.5	Sequence
**1**	**0**	**0.69**	**1.2**	**2.1**	**WVQGDNRPLAYTR**

5.286 231 902 Sodium- and chloride-dependent GABA transporter ine [Crassostrea gigas]

Protein Accession	CL11289.Contig3$_{H}1$
Mean Expression Ratio	0.887
Median Expression Ratio	0.887
Credible Interval	(0.656, 1.19)
Associated Peptides	3
Associated Spectra	12
Coverage	NaN

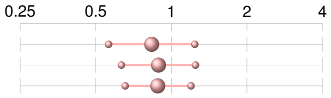

A	B	2.5	50	97.5	Sequence
**1**	**1**	**0.56**	**0.84**	**1.2**	**EAFELSFK**
**4**	**0**	**0.63**	**0.89**	**1.3**	**VPEIDAAAPFYGIPGADLADVSK**
**3**	**3**	**0.65**	**0.88**	**1.2**	**LVEFFQK**

5.287 198 821 minus strand sigma class glutathione-s-transferase 2 [Haliotis discus discus]

Protein Accession	CL1352.Contig4$_{H}1$
Mean Expression Ratio	0.883
Median Expression Ratio	0.888
Credible Interval	(0.537, 1.43)
Associated Peptides	1
Associated Spectra	1
Coverage	NaN

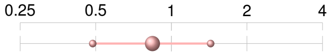

A	B	2.5	50	97.5	Sequence
0	1	0.49	0.84	1.4	AAGQPFDDNVVDR

5.288 98 850 minus strand 14-3-3 protein epsilon [Schistocerca gregaria]

Protein Accession	CL27652.Contig3$_{H}1$
Mean Expression Ratio	1.12
Median Expression Ratio	1.13
Credible Interval	(0.888, 1.41)
Associated Peptides	9
Associated Spectra	20
Coverage	NaN

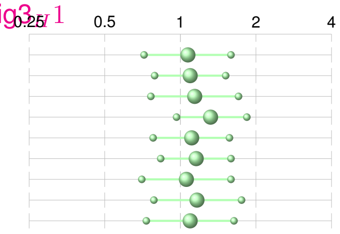

A	B	2.5	50	97.5	Sequence
1	0	0.71	1.1	1.6	AAFDDAIAELDTLSEESYK
3	1	0.79	1.1	1.5	AASDISMTELPPTHPIR
1	0	0.76	1.1	1.7	EAAENSLVAYK
4	0	0.96	1.3	1.8	HLIPNAGGGESK
1	2	0.78	1.1	1.6	IISSMEQK
3	1	0.83	1.2	1.6	YDEMVEAMK
1	0	0.7	1.1	1.6	VFYYK
0	1	0.78	1.2	1.8	YLAEFATGNDR
0	1	0.73	1.1	1.6	TELPPTHPIR

5.289 72 1400 minus strand hypothetical protein RCJMB04_22i13 [Gallus gallus]

Protein Accession	CL44755.Contig5$_{H}1$
Mean Expression Ratio	0.888
Median Expression Ratio	0.888
Credible Interval	(0.667, 1.19)
Associated Peptides	6
Associated Spectra	10
Coverage	NaN

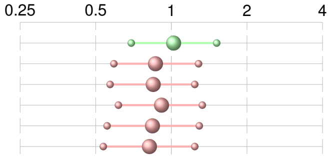

A	B	2.5	50	97.5	Sequence
2	0	0.69	1	1.5	FLVASTPDEALQHAAELGK
1	1	0.59	0.86	1.3	LVFSADEAK
1	1	0.57	0.84	1.2	AVFQMR
1	1	0.62	0.91	1.3	LSGIPDVFVK
0	1	0.56	0.84	1.3	AIIAASSMK
0	1	0.54	0.82	1.2	LSTIIDLAR

5.290 108 593 hypothetical protein CGI_10025497 [Crassostrea gigas]

Protein Accession	CL36597.Contig1$_{H}1$
Mean Expression Ratio	1.13
Median Expression Ratio	1.13
Credible Interval	(0.762, 1.66)
Associated Peptides	1
Associated Spectra	5
Coverage	NaN

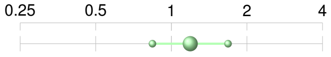

A	B	2.5	50	97.5	Sequence
4	1	0.84	1.2	1.7	SFELADFHK

5.291 241 561 minus strand thioredoxin [Crassostrea ariakensis]

Protein Accession	CL33159.Contig3$_H$1
Mean Expression Ratio	0.888
Median Expression Ratio	0.889
Credible Interval	(0.632, 1.25)
Associated Peptides	3
Associated Spectra	7
Coverage	NaN

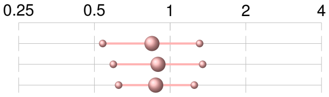

A	B	2.5	50	97.5	Sequence
1	0	0.54	0.84	1.3	EVGEVVGASEEEIK
1	1	0.6	0.89	1.3	EVGEVVGASEEEIKK
2	2	0.62	0.88	1.2	IETQEDFEK

5.292 91 1392 minus strand hypothetical protein AND_03103 [Anopheles darlingi]

Protein Accession	CL10598.Contig5$_H$1
Mean Expression Ratio	0.887
Median Expression Ratio	0.889
Credible Interval	(0.569, 1.37)
Associated Peptides	2
Associated Spectra	2
Coverage	NaN

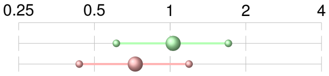

A	B	2.5	50	97.5	Sequence
1	0	0.61	1	1.7	LPPFNEHALVQEK
1	0	0.43	0.73	1.2	LDEVDDLLTENR

5.293 2 811 voltage-dependent anion channel 2-like protein [Haliotis diversicolor]; 2 811 voltage-dependent anion channel 2-like protein [Haliotis diversicolor]; 2 811 voltage-dependent anion channel 2-like protein [Haliotis diversicolor]

Protein Accession	CL2477.Contig3$_H$1 CL2477.Contig2$_H$1 CL2477.Contig1$_H$1
Mean Expression Ratio	0.889
Median Expression Ratio	0.889
Credible Interval	(0.713, 1.12)
Associated Peptides	9
Associated Spectra	21
Coverage	NaN

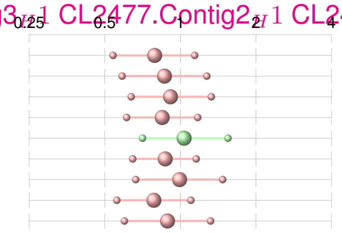

A	B	2.5	50	97.5	Sequence
2	0	0.54	0.79	1.1	LAFDTSFAPQTGK
1	0	0.59	0.87	1.3	VSGSLETK
1	1	0.64	0.91	1.3	WSEYGLTFTEK
4	0	0.61	0.85	1.2	YTLDSSASVSAK
1	0	0.71	1	1.5	GYNYGFFK
2	4	0.65	0.87	1.2	VGLGLDVEA
1	0	0.66	0.99	1.5	FALGTK
1	2	0.56	0.78	1.1	LTLSALIEGK
0	1	0.6	0.89	1.3	DIFSK

5.294 900 1142 minus strand heat shock protein 20 [Cyclina sinensis]

Protein Accession	CL19759.Contig1$_{H}1$
Mean Expression Ratio	1.12
Median Expression Ratio	1.12
Credible Interval	(0.826, 1.52)
Associated Peptides	3
Associated Spectra	11
Coverage	NaN

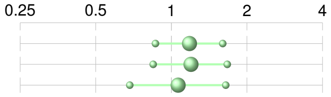

A	B	2.5	50	97.5	Sequence
4	2	0.87	1.2	1.6	QFKPEEITVK
3	1	0.85	1.2	1.7	QYILPEK
1	0	0.69	1.1	1.6	VEHPIVMDHQGNK

5.295 424 3507 minus strand putative Na+/K+-ATPase alpha subunit [Paroctopus digueti]

Protein Accession	Unigene11494$_{H}1$
Mean Expression Ratio	0.889
Median Expression Ratio	0.89
Credible Interval	(0.63, 1.25)
Associated Peptides	5
Associated Spectra	5
Coverage	NaN

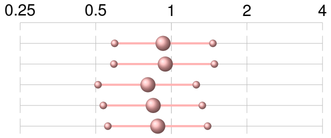

A	B	2.5	50	97.5	Sequence
1	0	0.6	0.93	1.5	SAEFTHENPLETR
0	1	0.59	0.94	1.5	DMTPAQIDEILR
0	1	0.51	0.81	1.3	MTVAHMWFDGR
0	1	0.53	0.85	1.3	VIMVTGDHPITAK
0	1	0.56	0.88	1.4	VIVAHGFK

5.296 965 1687 Arylsulfatase B [Crassostrea gigas]

Protein Accession	Unigene30559$_{H}1$
Mean Expression Ratio	0.889
Median Expression Ratio	0.89
Credible Interval	(0.545, 1.42)
Associated Peptides	1
Associated Spectra	1
Coverage	NaN

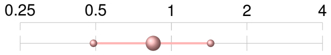

A	B	2.5	50	97.5	Sequence
0	1	0.49	0.85	1.4	WGLYDIEADPEER

5.297 3 470 minus strand Spectrin beta chain [Crassostrea gigas]

Protein Accession	CL19757.Contig2$_{H}1$
Mean Expression Ratio	0.893
Median Expression Ratio	0.891
Credible Interval	(0.575, 1.38)
Associated Peptides	1
Associated Spectra	3
Coverage	NaN

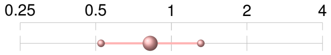

A	B	2.5	50	97.5	Sequence
2	1	0.52	0.83	1.3	HSLLEADIHVVGER

5.298 228 1553 hypothetical protein CGI_10024545 [Crassostrea gigas]; 228 1553 hypothetical protein CGI_10024545 [Crassostrea gigas]

Protein Accession	CL14644.Contig9$_{H}1$ CL14644.Contig8$_{H}1$
Mean Expression Ratio	1.12
Median Expression Ratio	1.12
Credible Interval	(0.727, 1.72)
Associated Peptides	2
Associated Spectra	2
Coverage	NaN

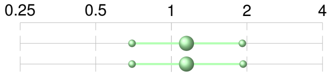

A	B	2.5	50	97.5	Sequence
**0**	**1**	**0.7**	**1.2**	**1.9**	**LMEQPQSMLL**
**0**	**1**	**0.7**	**1.2**	**1.9**	**AEDILR**

5.299 327 731 RecName: Full=Arginine kinase; Short=AK gi|439719|dbj|BAA05100.1| arginine kinase [Haliotis madaka]

Protein Accession	Unigene63526$_{H}1$
Mean Expression Ratio	1.12
Median Expression Ratio	1.12
Credible Interval	(0.851, 1.47)
Associated Peptides	2
Associated Spectra	31
Coverage	NaN

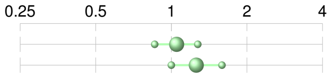

A	B	2.5	50	97.5	Sequence
**10**	**9**	**0.86**	**1**	**1.3**	**DLYEQLK**
**7**	**5**	**1**	**1.3**	**1.6**	**FGGTLAECIR**

5.300 2295 2510 beta-thymosin domain repeat protein CSP24KDa_v1 [Hermissenda crassicornis] gi

Protein Accession	CL9548.Contig1$_{H}1$
Mean Expression Ratio	1.12
Median Expression Ratio	1.12
Credible Interval	(0.691, 1.84)
Associated Peptides	1
Associated Spectra	1
Coverage	NaN

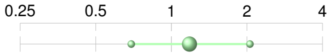

A	B	2.5	50	97.5	Sequence
**1**	**0**	**0.69**	**1.2**	**2.1**	**ESVAAFDK**

5.301 236 733 Spectrin alpha chain [Crassostrea gigas]

Protein Accession	Unigene61767$_{H}1$
Mean Expression Ratio	0.893
Median Expression Ratio	0.893
Credible Interval	(0.612, 1.3)
Associated Peptides	3
Associated Spectra	4
Coverage	NaN

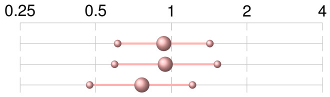

A	B	2.5	50	97.5	Sequence
**1**	**1**	**0.61**	**0.93**	**1.4**	**DIDDEEAWIK**
**1**	**0**	**0.6**	**0.95**	**1.5**	**DLTGVQNLR**
**0**	**1**	**0.47**	**0.76**	**1.2**	**LNEANTMHQFLR**

5.302 209 781 minus strand high mobility group protein 1 [Biomphalaria glabrata]; 277 771 high mobility group protein 1 [Biomphalaria glabrata]; 277 867 minus strand high mobility group protein 1 [Biomphalaria glabrata]; 277 870 minus strand high mobility group protein 1 [Biomphalaria glabrata]

Protein Accession	CL25076.Contig4$_H$1 CL25076.Contig3$_H$1 CL25076.Contig2$_H$1 CL25076.Contig1$_H$1
Mean Expression Ratio	0.895
Median Expression Ratio	0.894
Credible Interval	(0.579, 1.37)
Associated Peptides	2
Associated Spectra	2
Coverage	NaN

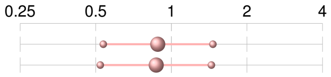

A	B	2.5	50	97.5	Sequence
**1**	**0**	**0.54**	**0.88**	**1.5**	**LAGEEWR**
**1**	**0**	**0.52**	**0.87**	**1.4**	**KYEAEMAEYR**

5.303 390 2267 neurofilament protein NF70 [Helix aspersa]; 318 2195 neurofilament protein NF70 [Helix aspersa]

Protein Accession	CL1353.Contig5$_H$1 CL1353.Contig4$_H$1
Mean Expression Ratio	1.12
Median Expression Ratio	1.12
Credible Interval	(0.853, 1.46)
Associated Peptides	7
Associated Spectra	12
Coverage	NaN

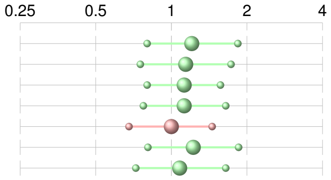

A	B	2.5	50	97.5	Sequence
**1**	**0**	**0.8**	**1.2**	**1.8**	**GEMETFYNLK**
**1**	**0**	**0.75**	**1.1**	**1.7**	**LNQQISDLEAEIR**
**4**	**0**	**0.8**	**1.1**	**1.6**	**FANYIEK**
**1**	**1**	**0.77**	**1.1**	**1.6**	**MADLEAR**
**1**	**1**	**0.68**	**1**	**1.5**	**VGQLEELVDDLQR**
**0**	**1**	**0.81**	**1.2**	**1.9**	**ELQQEMDVK**
**0**	**1**	**0.72**	**1.1**	**1.7**	**LEFLK**

5.304 101 1693 CALNUC [Spodoptera frugiperda]; 141 1325 Nucleobindin-2 [Crassostrea gigas]

Protein Accession	CL1888.Contig9$_H$1 CL1888.Contig6$_H$1
Mean Expression Ratio	0.895
Median Expression Ratio	0.895
Credible Interval	(0.576, 1.39)
Associated Peptides	2
Associated Spectra	3
Coverage	NaN

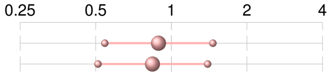

A	B	2.5	50	97.5	Sequence
**1**	**1**	**0.54**	**0.89**	**1.5**	**LKELPEVER**
**1**	**0**	**0.51**	**0.84**	**1.4**	**REEFK**

5.305 " 28 1104 minus strand Pyruvate dehydrogenase E1 component subunit beta, mitochondrial [Crassostrea gigas]"

Protein Accession	CL18015.Contig14$_{H}1$
Mean Expression Ratio	1.12
Median Expression Ratio	1.12
Credible Interval	(0.729, 1.72)
Associated Peptides	2
Associated Spectra	2
Coverage	NaN

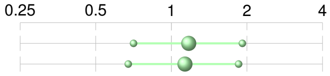

A	B	2.5	50	97.5	Sequence
1	0	0.71	1.2	1.9	GIECEVINLR
1	0	0.67	1.1	1.9	SLRPVDDETIIK

5.306 125 754 minus strand peptidyl prolyl cis-trans isomerase B [Conus novaehollandiae]

Protein Accession	Unigene19558$_{H}1$
Mean Expression Ratio	1.12
Median Expression Ratio	1.12
Credible Interval	(0.784, 1.58)
Associated Peptides	4
Associated Spectra	5
Coverage	NaN

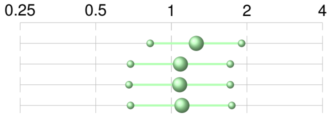

A	B	2.5	50	97.5	Sequence
2	0	0.82	1.3	1.9	HLALGDKEDGGVK
1	0	0.69	1.1	1.7	KTQWLDGR
1	0	0.68	1.1	1.7	DTNGSQFFITTK
1	0	0.69	1.1	1.7	FPDENFK

5.307 89 838 Putative phosphoglycerate mutase [Crassostrea gigas]; 89 838 Putative phosphoglycerate mutase [Crassostrea gigas]; 89 835 Putative phosphoglycerate mutase [Crassostrea gigas]; 89 838 Putative phosphoglycerate mutase [Crassostrea gigas]

Protein Accession	CL6793.Contig6$_{H}1$ CL6793.Contig5$_{H}1$ CL6793.Contig4$_{H}1$ CL6793.Contig3$_{H}1$
Mean Expression Ratio	1.12
Median Expression Ratio	1.12
Credible Interval	(0.865, 1.43)
Associated Peptides	5
Associated Spectra	21
Coverage	NaN

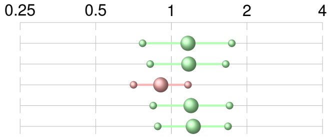

A	B	2.5	50	97.5	Sequence
1	0	0.77	1.2	1.7	DIPACECLK
2	1	0.83	1.2	1.6	HYYLATEEEVK
6	4	0.71	0.91	1.2	VLIAAHGNSLR
2	1	0.84	1.2	1.7	VLIAAHGN
3	1	0.88	1.2	1.7	EAQYAGELIK

5.308 35 505 minus strand Basement membrane-specific heparan sulfate proteoglycan core protein [Crassostrea gigas]

Protein Accession	CL8101.Contig1$_{H}1$
Mean Expression Ratio	0.895
Median Expression Ratio	0.896
Credible Interval	(0.553, 1.46)
Associated Peptides	1
Associated Spectra	1
Coverage	NaN

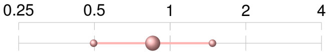

A	B	2.5	50	97.5	Sequence
**0**	**1**	**0.5**	**0.85**	**1.5**	**AAFAIR**

5.309 10 1902 Mitochondrial inner membrane protein [Crassostrea gigas]; 10 1902 Mitochondrial inner membrane protein [Crassostrea gigas]; 10 1902 Mitochondrial inner membrane protein [Crassostrea gigas]; 10 1902 Mitochondrial inner membrane protein [Crassostrea gigas]; 10 1902 Mitochondrial inner membrane protein [Crassostrea gigas]; 10 1902 Mitochondrial inner membrane protein [Crassostrea gigas]

Protein Accession	CL6414.Contig8$_{H}1$ CL6414.Contig7$_{H}1$ CL6414.Contig6$_{H}1$ CL6414.Contig5$_{H}1$ CL6414.Con
Mean Expression Ratio	0.898
Median Expression Ratio	0.896
Credible Interval	(0.548, 1.45)
Associated Peptides	1
Associated Spectra	1
Coverage	NaN

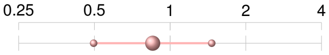

A	B	2.5	50	97.5	Sequence
**1**	**0**	**0.5**	**0.85**	**1.5**	**EIHTQLLQER**

5.310 85 633 hypothetical protein [Amblyomma maculatum]

Protein Accession	CL2221.Contig1$_{H}1$
Mean Expression Ratio	1.12
Median Expression Ratio	1.12
Credible Interval	(0.768, 1.63)
Associated Peptides	3
Associated Spectra	4
Coverage	NaN

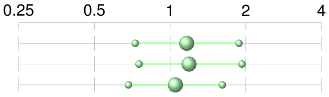

A	B	2.5	50	97.5	Sequence
**1**	**0**	**0.73**	**1.2**	**1.9**	**DANDEWIR**
**1**	**0**	**0.75**	**1.2**	**1.9**	**EKLPDYDNK**
**0**	**2**	**0.68**	**1**	**1.6**	**IFYTEHLHADEEIR**

5.311 " 31 2061 minus strand 1,4-alpha-glucan-branching enzyme [Crassostrea gigas]"

Protein Accession	CL12555.Contig7$_{H}$1
Mean Expression Ratio	0.895
Median Expression Ratio	0.896
Credible Interval	(0.565, 1.4)
Associated Peptides	1
Associated Spectra	2
Coverage	NaN

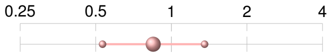

A	B	2.5	50	97.5	Sequence
0	2	0.53	0.85	1.4	LDGYLR

5.312 338 661 Spectrin alpha chain [Crassostrea gigas]

Protein Accession	CL26928.Contig1$_{H}$1
Mean Expression Ratio	0.895
Median Expression Ratio	0.896
Credible Interval	(0.593, 1.34)
Associated Peptides	2
Associated Spectra	3
Coverage	NaN

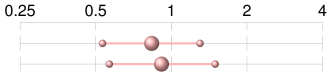

A	B	2.5	50	97.5	Sequence
1	1	0.53	0.83	1.3	DVAGAEALLER
0	1	0.57	0.91	1.5	AIISADDLAK

5.313 " 64 1332 Flotillin-1, putative [Pediculus humanus corporis] gi"

Protein Accession	CL33952.Contig1$_{H}$1
Mean Expression Ratio	0.898
Median Expression Ratio	0.897
Credible Interval	(0.677, 1.18)
Associated Peptides	4
Associated Spectra	17
Coverage	NaN

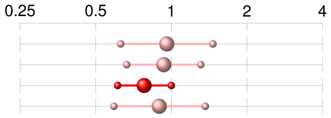

A	B	2.5	50	97.5	Sequence
1	0	0.63	0.96	1.5	AAYDMEVMTTK
3	1	0.67	0.93	1.3	VKGEAEAFAIEAK
6	5	0.61	0.78	1	LPSIVETL
0	1	0.59	0.89	1.4	FLNDIEIAK

5.314 14 223 minus strand Endothelin-converting enzyme 2 OS=Homo sapiens GN=ECE2 PE=1 SV=4

Protein Accession	Unigene41869$_{H}$1
Mean Expression Ratio	0.896
Median Expression Ratio	0.898
Credible Interval	(0.567, 1.39)
Associated Peptides	1
Associated Spectra	2
Coverage	NaN

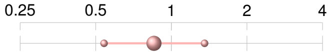

A	B	2.5	50	97.5	Sequence
2	0	0.54	0.85	1.4	KLLEAQNK

5.315 91 2658 minus strand puromycin-sensitive aminopeptidase [Saccoglossus kowalevskii]

Protein Accession	CL6708.Contig2$_{H}1$
Mean Expression Ratio	1.11
Median Expression Ratio	1.11
Credible Interval	(0.723, 1.71)
Associated Peptides	2
Associated Spectra	2
Coverage	NaN

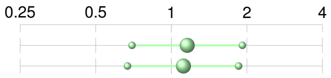

A	B	2.5	50	97.5	Sequence
1	0	0.7	1.2	1.9	GMNLYLNR
1	0	0.67	1.1	1.9	LPKDVIPSNYNIR

5.316 112 1050 Enolase-phosphatase E1 [Crassostrea gigas]; 112 1050 Enolase-phosphatase E1 [Crassostrea gigas]

Protein Accession	CL25175.Contig3$_{H}1$ CL25175.Contig2$_{H}1$
Mean Expression Ratio	1.11
Median Expression Ratio	1.11
Credible Interval	(0.772, 1.61)
Associated Peptides	3
Associated Spectra	5
Coverage	NaN

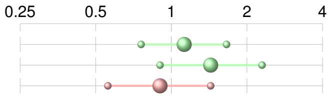

A	B	2.5	50	97.5	Sequence
1	2	0.76	1.1	1.7	SAAEAESLLVGVK
1	0	0.9	1.4	2.3	AIIFDVEGTTTPISFVK
0	1	0.56	0.9	1.4	QLQGHIWR

5.317 " 926 1189 minus strand Myosin heavy chain, striated muscle [Crassostrea gigas]"

Protein Accession	Unigene11948$_{H}1$
Mean Expression Ratio	0.899
Median Expression Ratio	0.899
Credible Interval	(0.698, 1.16)
Associated Peptides	3
Associated Spectra	27
Coverage	NaN

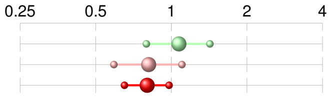

A	B	2.5	50	97.5	Sequence
6	0	0.79	1.1	1.4	NKDPLNETVVELLSHSK
1	4	0.59	0.81	1.1	TFLDKLNGNHLGK
10	6	0.65	0.8	0.98	LNGNHLGK

5.318 181 744 unknown [Dendroctonus ponderosae]

Protein Accession	CL25948.Contig3$_{H}1$
Mean Expression Ratio	0.899
Median Expression Ratio	0.899
Credible Interval	(0.717, 1.13)
Associated Peptides	7
Associated Spectra	23
Coverage	NaN

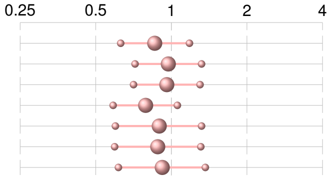

A	B	2.5	50	97.5	Sequence
3	1	0.63	0.86	1.2	GTLTQLTK
4	1	0.72	0.97	1.3	SMGPIGSPMSK
4	1	0.71	0.96	1.3	TFICTPDR
4	2	0.59	0.79	1.1	VGHVPVAK
1	0	0.6	0.89	1.3	YKNNYLCR
1	0	0.59	0.88	1.3	GIYICDGSDEEAEEIIHK
1	0	0.62	0.92	1.4	NNYLCR

5.319 " 492 3473 PREDICTED: 2-oxoglutarate dehydrogenase, mitochondrial isoform 1 [Sarcophilus harrisii]; 492 3458 PREDICTED: 2-oxoglutarate dehydrogenase, mitochondrial isoform 1 [Sarcophilus harrisii]"

Protein Accession	CL2200.Contig1$_H1$ CL2200.Contig16$_H1$
Mean Expression Ratio	1.11
Median Expression Ratio	1.11
Credible Interval	(0.722, 1.71)
Associated Peptides	2
Associated Spectra	2
Coverage	NaN

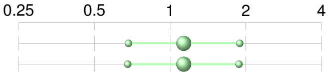

A	B	2.5	50	97.5	Sequence
1	0	0.68	1.1	1.9	FSEVPEGFVVHGGLK
1	0	0.68	1.1	1.9	KPLVVATPK

5.320 2 256 Polyubiquitin (Fragment) OS=Xenopus laevis PE=1 SV=2; 20 472 minus strand large subunit ribosomal protein L40e [Clonorchis sinensis]; 20 388 large subunit ribosomal protein L40e [Clonorchis sinensis]

Protein Accession	CL47507.Contig2$_H1$ CL39010.Contig2$_H1$ CL39010.Contig1$_H1$
Mean Expression Ratio	0.899
Median Expression Ratio	0.901
Credible Interval	(0.65, 1.24)
Associated Peptides	4
Associated Spectra	17
Coverage	NaN

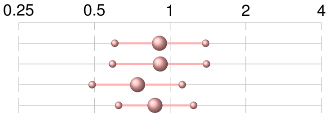

A	B	2.5	50	97.5	Sequence
2	1	0.6	0.91	1.4	TITLEVEPSDTIENVK
1	1	0.59	0.91	1.4	ESTLHLVLR
3	1	0.49	0.74	1.1	MQIFVK
4	4	0.62	0.87	1.2	TLSDYNIQK

5.321 582 2135 sulfatase 1A precursor [Haliotis discus discus]

Protein Accession	CL1700.Contig8$_H1$
Mean Expression Ratio	1.11
Median Expression Ratio	1.11
Credible Interval	(0.787, 1.56)
Associated Peptides	4
Associated Spectra	6
Coverage	NaN

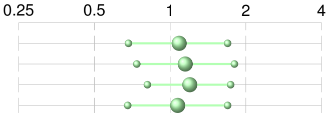

A	B	2.5	50	97.5	Sequence
1	0	0.68	1.1	1.7	HNEDVFYSAK
1	0	0.74	1.2	1.8	GKYPDVFK
2	1	0.81	1.2	1.7	AVSFLHSPK
0	1	0.68	1.1	1.7	EGHMYTPTLDGLAR

5.322 87 1454 hypothetical protein BRAFLDRAFT_287788 [Branchiostoma floridae] gi; 117 1469 hypothetical protein BRAFLDRAFT_287788 [Branchiostoma floridae] gi; 117 1469 hypothetical protein BRAFLDRAFT_287788 [Branchiostoma floridae] gi

Protein Accession	Unigene48935$_{H}1$ Unigene21344$_{H}1$ CL945.Contig6$_{H}1$
Mean Expression Ratio	0.901
Median Expression Ratio	0.901
Credible Interval	(0.69, 1.18)
Associated Peptides	6
Associated Spectra	17
Coverage	NaN

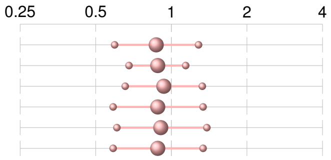

A	B	2.5	50	97.5	Sequence
1	1	0.59	0.87	1.3	AATLEQTILNVPETR
8	1	0.68	0.88	1.1	MVLAAAGGVNHGELVDLAK
2	1	0.65	0.93	1.3	RIPLPELEAR
1	0	0.59	0.89	1.3	QMLCYGR
1	0	0.61	0.91	1.4	PETRISSLDNGLR
0	1	0.59	0.88	1.3	ISSLDNGLR

5.323 138 713 Rac [Aplysia californica] gi; 101 676 Rac [Aplysia californica] gi

Protein Accession	CL11009.Contig9$_{H}1$ CL11009.Contig1$_{H}1$
Mean Expression Ratio	1.11
Median Expression Ratio	1.11
Credible Interval	(0.682, 1.81)
Associated Peptides	1
Associated Spectra	1
Coverage	NaN

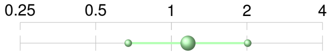

A	B	2.5	50	97.5	Sequence
1	0	0.67	1.2	2	CVVVGDGAVGK

5.324 942 1385 Golgi-associated plant pathogenesis-related protein 1 [Crassostrea gigas]; 338 817 Golgi-associated plant pathogenesis-related protein 1 [Crassostrea gigas]; 354 833 Golgi-associated plant pathogenesis-related protein 1 [Crassostrea gigas]; 576 1055 Golgi-associated plant pathogenesis-related protein 1 [Crassostrea gigas]; 618 1097 Golgi-associated plant pathogenesis-related protein 1 [Crassostrea gigas]; 536 979 Golgi-associated plant pathogenesis-related protein 1 [Crassostrea gigas]; 740 1219 minus strand Golgi-associated plant pathogenesis-related protein 1 [Crassostrea gigas]

Protein Accession	Unigene391$_{H}1$ CL15263.Contig2$_{H}1$ CL15263.Contig1$_{H}1$ CL15263.Contig13$_{H}1$ CL15263.Co
Mean Expression Ratio	0.901
Median Expression Ratio	0.902
Credible Interval	(0.575, 1.4)
Associated Peptides	1
Associated Spectra	2
Coverage	NaN

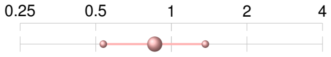

A	B	2.5	50	97.5	Sequence
1	1	0.54	0.86	1.4	IITEETIIK

5.325 180 545 calreticulin precursor [Aplysia californica] gi

Protein Accession	CL4813.Contig2$_H$1
Mean Expression Ratio	0.901
Median Expression Ratio	0.902
Credible Interval	(0.644, 1.27)
Associated Peptides	4
Associated Spectra	12
Coverage	NaN

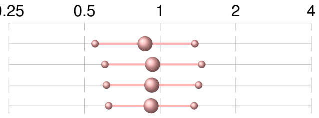

A	B	2.5	50	97.5	Sequence
0	1	0.55	0.87	1.4	EEFDANWEDR
1	1	0.6	0.93	1.5	FYGISAK
2	1	0.61	0.93	1.4	FVWSAGK
4	2	0.63	0.92	1.4	WIPSVHK

5.326 176 517 PREDICTED: elongin C-like [Saccoglossus kowalevskii]; 115 456 PREDICTED: elongin C-like [Saccoglossus kowalevskii]; 994 1350 PREDICTED: elongin C-like [Saccoglossus kowalevskii]; 126 482 PREDICTED: elongin C-like [Saccoglossus kowalevskii]; 30 371 PREDICTED: elongin C-like [Saccoglossus kowalevskii]

Protein Accession	Unigene38248$_H$1 CL27549.Contig5$_H$1 CL27549.Contig4$_H$1 CL27549.Contig3$_H$1 CL27549.C
Mean Expression Ratio	1.11
Median Expression Ratio	1.11
Credible Interval	(0.681, 1.79)
Associated Peptides	1
Associated Spectra	1
Coverage	NaN

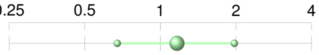

A	B	2.5	50	97.5	Sequence
1	0	0.67	1.2	2	LISSDGHEFIVK

5.327 622 2733 PREDICTED: protein-glutamine gamma-glutamyltransferase K isoform 1 [Danio rerio]; 423 2498 PREDICTED: protein-glutamine gamma-glutamyltransferase K isoform 1 [Danio rerio]; 423 2534 PREDICTED: protein-glutamine gamma-glutamyltransferase K isoform 1 [Danio rerio]

Protein Accession	Unigene4042$_H$1 CL5652.Contig2$_H$1 CL5652.Contig1$_H$1
Mean Expression Ratio	1.11
Median Expression Ratio	1.11
Credible Interval	(0.835, 1.46)
Associated Peptides	6
Associated Spectra	12
Coverage	NaN

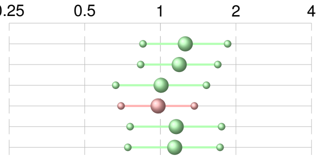

A	B	2.5	50	97.5	Sequence
1	1	0.85	1.3	1.9	LVFGIGEDPK
2	1	0.84	1.2	1.7	ILDMSGLK
1	0	0.66	1	1.5	GTQVVFILSAPDEK
2	2	0.7	0.98	1.4	KPHLQIK
1	0	0.76	1.2	1.8	GNPIPVVR
0	1	0.74	1.1	1.7	DVVFELR

5.328 1 252 Aquaporin-4 [Crassostrea gigas]

Protein Accession	Unigene16706$_{H}1$
Mean Expression Ratio	1.11
Median Expression Ratio	1.11
Credible Interval	(0.72, 1.71)
Associated Peptides	2
Associated Spectra	2
Coverage	NaN

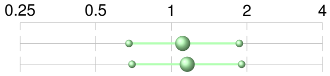

A	B	2.5	50	97.5	Sequence
1	0	0.68	1.1	1.9	APLDDAAAAEVIEIDETK
1	0	0.7	1.2	1.9	KFSPSQAAAAPQEK

5.329 42 296 minus strand twitchin [Mytilus galloprovincialis]

Protein Accession	Unigene19029$_{H}1$
Mean Expression Ratio	1.11
Median Expression Ratio	1.11
Credible Interval	(0.832, 1.47)
Associated Peptides	4
Associated Spectra	12
Coverage	NaN

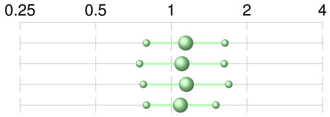

A	B	2.5	50	97.5	Sequence
2	1	0.8	1.1	1.6	APHFTQKPTIK
1	1	0.75	1.1	1.6	VTLADAGEYK
2	0	0.78	1.1	1.7	KPQLPDGK
3	2	0.79	1.1	1.5	LNFDSAK

5.330 160 1218 Galectin-9 [Crassostrea gigas]

Protein Accession	Unigene165$_{H}1$
Mean Expression Ratio	0.906
Median Expression Ratio	0.905
Credible Interval	(0.601, 1.36)
Associated Peptides	2
Associated Spectra	3
Coverage	NaN

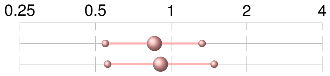

A	B	2.5	50	97.5	Sequence
1	1	0.55	0.86	1.3	IAVNNQHLVEFR
1	0	0.56	0.91	1.5	SGIPGGLAPGR

5.331 112 930 calcineurin A [Haliotis discus discus]

Protein Accession	CL3137.Contig1$_{H}1$
Mean Expression Ratio	0.905
Median Expression Ratio	0.906
Credible Interval	(0.599, 1.36)
Associated Peptides	2
Associated Spectra	3
Coverage	NaN

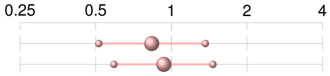

A	B	2.5	50	97.5	Sequence
0	1	0.51	0.84	1.4	VTEDVALR
0	2	0.59	0.93	1.5	SEAFDAK

5.332 11 1462 coronin [Schistosoma mansoni]

Protein Accession	CL11779.Contig2$_{H}1$
Mean Expression Ratio	1.1
Median Expression Ratio	1.1
Credible Interval	(0.817, 1.48)
Associated Peptides	4
Associated Spectra	10
Coverage	NaN

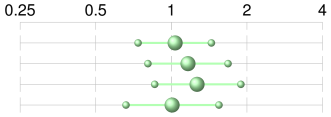

A	B	2.5	50	97.5	Sequence
2	2	0.74	1	1.4	DANPILVSLK
2	1	0.81	1.2	1.7	HVFGKPEK
2	0	0.86	1.3	1.9	VPMDAPVVAGHK
0	1	0.66	1	1.5	DAPVVAGHK

5.333 " 34 1656 Chaperonin containing TCP1, subunit 7 (eta) [Danio rerio] gi; 34 1656 Chaperonin containing TCP1, subunit 7 (eta) [Danio rerio] gi"

Protein Accession	CL24061.Contig2$_{H}1$ CL24061.Contig1$_{H}1$
Mean Expression Ratio	0.908
Median Expression Ratio	0.907
Credible Interval	(0.603, 1.36)
Associated Peptides	2
Associated Spectra	3
Coverage	NaN

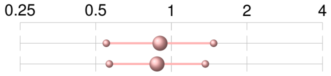

A	B	2.5	50	97.5	Sequence
1	0	0.55	0.9	1.5	TLDIVHPAAK
2	0	0.57	0.88	1.4	SIAVSIKK

5.334 313 2763 NCAM-related cell adhesion molecule precursor [Aplysia californica] gi

Protein Accession	Unigene11449$_{H}1$
Mean Expression Ratio	0.906
Median Expression Ratio	0.907
Credible Interval	(0.644, 1.27)
Associated Peptides	5
Associated Spectra	5
Coverage	NaN

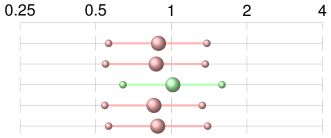

A	B	2.5	50	97.5	Sequence
1	0	0.56	0.89	1.4	EALEVDTSK
1	0	0.55	0.87	1.4	IGSSDPTVFVFK
1	0	0.64	1	1.6	NLAGSYDIEER
1	0	0.54	0.85	1.3	VPMTGGAEIANYVIEYR
0	1	0.56	0.88	1.4	AGAIASDETQLEVSIPPK

5.335 113 1402 minus strand S-adenosylhomocysteine hydrolase [Crassostrea ariakensis]; 113 1402 minus strand S-adenosylhomocysteine hydrolase [Crassostrea ariakensis]

Protein Accession	CL7405.Contig2$_{H}1$ CL7405.Contig1$_{H}1$
Mean Expression Ratio	1.1
Median Expression Ratio	1.1
Credible Interval	(0.717, 1.69)
Associated Peptides	2
Associated Spectra	2
Coverage	NaN

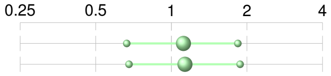

A	B	2.5	50	97.5	Sequence
1	0	0.67	1.1	1.8	GLSEETTTGVHNLYR
1	0	0.68	1.1	1.9	LKVPAINVNDSVTK

5.336 128 532 minus strand mitochondrial ATPase inhibitor [Ornithodoros parkeri]

Protein Accession	Unigene1376$_{H}1$
Mean Expression Ratio	1.1
Median Expression Ratio	1.1
Credible Interval	(0.68, 1.8)
Associated Peptides	1
Associated Spectra	1
Coverage	NaN

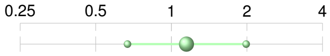

A	B	2.5	50	97.5	Sequence
1	0	0.67	1.2	2	MEAAHEELYFK

5.337 3 263 minus strand beta-actin [Urechis unicinctus]

Protein Accession	CL31420.Contig5$_{H}1$
Mean Expression Ratio	0.908
Median Expression Ratio	0.908
Credible Interval	(0.728, 1.13)
Associated Peptides	4
Associated Spectra	134
Coverage	NaN

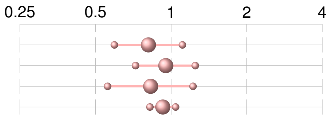

A	B	2.5	50	97.5	Sequence
3	2	0.59	0.81	1.1	IIAPPERK
7	2	0.72	0.95	1.3	IKIIAPPER
0	1	0.56	0.83	1.2	EITALAPPTMK
63	56	0.82	0.93	1	IIAPPER

5.338 811 1407 minus strand mitochondrial malate dehydrogenase 2 [Eospalax baileyi]

Protein Accession	CL10426.Contig13$_{H}1$
Mean Expression Ratio	0.909
Median Expression Ratio	0.908
Credible Interval	(0.671, 1.23)
Associated Peptides	3
Associated Spectra	11
Coverage	NaN

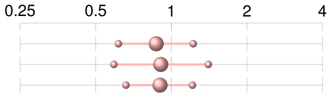

A	B	2.5	50	97.5	Sequence
2	2	0.62	0.87	1.2	IQNAGTEVVEAK
1	0	0.59	0.91	1.4	MQQLNSPR
3	3	0.66	0.9	1.2	AAMPELIANISK

5.339 227 1201 PREDICTED: quinone oxidoreductase-like isoform 1 [Ornithorhynchus anatinus]; 109 1083 PREDICTED: quinone oxidoreductase-like isoform 1 [Ornithorhynchus anatinus]; 164 1138 PREDICTED: quinone oxidoreductase-like isoform 1 [Ornithorhynchus anatinus]

Protein Accession	CL11899.Contig2$_H$1 CL11899.Contig1$_H$1 CL11899.Contig13$_H$1
Mean Expression Ratio	0.91
Median Expression Ratio	0.908
Credible Interval	(0.56, 1.45)
Associated Peptides	1
Associated Spectra	1
Coverage	NaN

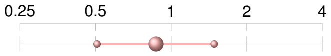

A	B	2.5	50	97.5	Sequence
1	0	0.51	0.87	1.5	VHASGVNPVDTYIR

5.340 2302 2547 minus strand hemocyanin isoform 1 [Haliotis diversicolor]; 816 1211 H2 [Haliotis tuberculata]

Protein Accession	CL11519.Contig6$_H$1 CL11519.Contig5$_H$1
Mean Expression Ratio	0.908
Median Expression Ratio	0.909
Credible Interval	(0.606, 1.36)
Associated Peptides	2
Associated Spectra	4
Coverage	NaN

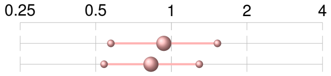

A	B	2.5	50	97.5	Sequence
1	0	0.58	0.93	1.5	KNVLSLTPAER
2	1	0.54	0.83	1.3	SLILAMR

5.341 4 579 minus strand Collagen alpha-3(VI) chain [Crassostrea gigas]

Protein Accession	CL46502.Contig3$_H$1
Mean Expression Ratio	1.1
Median Expression Ratio	1.1
Credible Interval	(0.768, 1.59)
Associated Peptides	2
Associated Spectra	6
Coverage	NaN

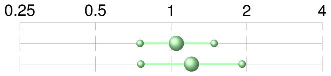

A	B	2.5	50	97.5	Sequence
5	0	0.75	1.1	1.5	FAVQEFSELR
1	0	0.76	1.2	1.9	LIKDFVK

5.342 " 213 1499 minus strand Citrate synthase, mitochondrial [Crassostrea gigas]"

Protein Accession	CL992.Contig2$_H$1
Mean Expression Ratio	0.908
Median Expression Ratio	0.909
Credible Interval	(0.749, 1.1)
Associated Peptides	12
Associated Spectra	32
Coverage	NaN

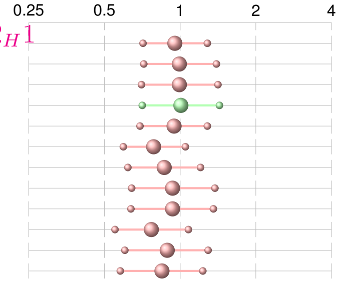

A	B	2.5	50	97.5	Sequence
4	1	0.71	0.96	1.3	ALGLPIERPK
3	0	0.71	1	1.4	ALVTETSVLDPEEGIR
1	1	0.7	0.99	1.4	DVLSQIIPEK
2	0	0.71	1	1.4	LMESLGGEVSDEQLR
3	1	0.69	0.95	1.3	SGQVVPGYGHAVLR
4	2	0.59	0.78	1.1	VVPDILIEQGK
3	0	0.62	0.86	1.2	SFDTPSLR
1	0	0.64	0.94	1.4	VGEVTVDMMYGGMR
1	0	0.64	0.93	1.4	EVSEFR
2	1	0.55	0.77	1.1	HLPNDPMFK
0	1	0.6	0.89	1.3	NFIWNTLK
0	1	0.58	0.84	1.2	LISQLYK

5.343 459 755 minus strand heat shock protein beta-1 [Trichinella spiralis] gi

Protein Accession	CL17234.Contig5$_H$1
Mean Expression Ratio	0.91
Median Expression Ratio	0.909
Credible Interval	(0.639, 1.29)
Associated Peptides	3
Associated Spectra	8
Coverage	NaN

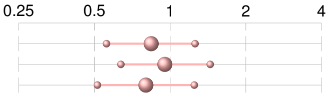

A	B	2.5	50	97.5	Sequence
2	1	0.56	0.84	1.3	DVEPTTVTSSLNADGVLTLK
2	1	0.64	0.96	1.4	IVVEGCHEEK
1	1	0.52	0.8	1.3	IVVEGCHEEKQDEHGFITR

5.344 27 377 minus strand ribosomal protein LP2 [Papilio xuthus] gi; 27 377 minus strand ribosomal protein LP2 [Papilio xuthus] gi; 27 377 minus strand ribosomal protein LP2 [Papilio xuthus] gi; 1538 1891 minus strand ribosomal protein LP2 [Papilio xuthus] gi

Protein Accession	Unigene53017$_H$1 Unigene53016$_H$1 Unigene53015$_H$1 CL49012.Contig9$_H$1
Mean Expression Ratio	0.91
Median Expression Ratio	0.91
Credible Interval	(0.707, 1.17)
Associated Peptides	7
Associated Spectra	16
Coverage	NaN

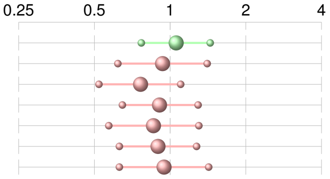

A	B	2.5	50	97.5	Sequence
4	1	0.77	1.1	1.4	KVVSQLSGK
1	0	0.62	0.93	1.4	SIDQLIAEGSR
2	0	0.52	0.76	1.1	VVSQLSGK
3	0	0.65	0.91	1.3	ILSSVGIEAEADK
1	0	0.57	0.86	1.3	AAAPAAEAK
1	2	0.63	0.89	1.3	ILSSVGIEAEADKVK
1	0	0.63	0.94	1.4	SGKEPTAADLEK

5.345 151 1449 rab GDP-dissociation inhibitor [Branchiostoma belcheri]

Protein Accession	CL30719.Contig3$_H1$
Mean Expression Ratio	1.1
Median Expression Ratio	1.1
Credible Interval	(0.839, 1.43)
Associated Peptides	7
Associated Spectra	12
Coverage	NaN

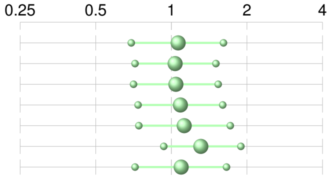

A	B	2.5	50	97.5	Sequence
1	0	0.69	1.1	1.6	EALGTSLMGMFEK
1	1	0.72	1	1.5	FLMANGQLVK
1	1	0.71	1	1.5	GVDCNGMTAAQLYEK
1	1	0.74	1.1	1.6	ITGEAFDFSK
1	0	0.74	1.1	1.7	IVYDETGVAVGVQDADGAVAK
2	1	0.93	1.3	1.9	LLVATGVTR
1	0	0.72	1.1	1.7	QWNVDLIPK

5.346 59 379 hypothetical protein DAPPUDRAFT_230756 [Daphnia pulex]; 117 443 minus strand hypothetical protein DAPPUDRAFT_230756 [Daphnia pulex]

Protein Accession	CL2137.Contig6$_H1$ CL2137.Contig5$_H1$
Mean Expression Ratio	1.1
Median Expression Ratio	1.1
Credible Interval	(0.675, 1.78)
Associated Peptides	1
Associated Spectra	1
Coverage	NaN

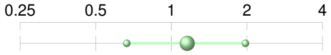

A	B	2.5	50	97.5	Sequence
1	0	0.67	1.2	2	YAQPRLK

5.347 366 935 minus strand 40S ribosomal protein S2 OS=Urechis caupo GN=RPS2 PE=2 SV=1

Protein Accession	CL2084.Contig7$_H1$
Mean Expression Ratio	0.911
Median Expression Ratio	0.91
Credible Interval	(0.558, 1.49)
Associated Peptides	1
Associated Spectra	1
Coverage	NaN

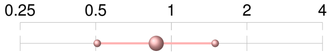

A	B	2.5	50	97.5	Sequence
0	1	0.51	0.87	1.5	TYSFLTPDLWK

5.348 225 839 minus strand Metallo-beta-lactamase domain-containing protein 1 [Crassostrea gigas]; 201 851 minus strand Metallo-beta-lactamase domain-containing protein 1 [Crassostrea gigas]

Protein Accession	CL35672.Contig2$_{H}1$ CL35672.Contig1$_{H}1$
Mean Expression Ratio	0.908
Median Expression Ratio	0.911
Credible Interval	(0.575, 1.41)
Associated Peptides	1
Associated Spectra	2
Coverage	NaN

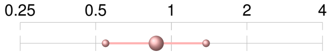

A	B	2.5	50	97.5	Sequence
**1**	**1**	**0.55**	**0.87**	**1.4**	**EADFIVPGHGPMFR**

5.349 210 779 GH20056 [Drosophila grimshawi] gi; 216 785 minus strand GH20056 [Drosophila grimshawi] gi; 210 779 GH20056 [Drosophila grimshawi] gi; 216 785 GH20056 [Drosophila grimshawi] gi

Protein Accession	CL34497.Contig4$_{H}1$ CL34497.Contig3$_{H}1$ CL34497.Contig2$_{H}1$ CL34497.Contig1$_{H}1$
Mean Expression Ratio	1.1
Median Expression Ratio	1.1
Credible Interval	(0.796, 1.54)
Associated Peptides	3
Associated Spectra	9
Coverage	NaN

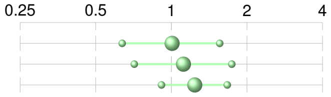

A	B	2.5	50	97.5	Sequence
**1**	**0**	**0.64**	**1**	**1.6**	**KQPFEFQIGVGQVIK**
**1**	**0**	**0.71**	**1.1**	**1.7**	**QLSQEEVSNFLK**
**5**	**2**	**0.91**	**1.2**	**1.7**	**GLLDMCAGEK**

5.350 49 1038 Glyceraldehyde-3-phosphate dehydrogenase [Crotalus adamanteus]

Protein Accession	Unigene7744$_{H}1$
Mean Expression Ratio	1.1
Median Expression Ratio	1.1
Credible Interval	(0.772, 1.57)
Associated Peptides	1
Associated Spectra	12
Coverage	NaN

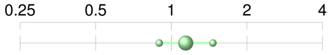

A	B	2.5	50	97.5	Sequence
**12**	**0**	**0.9**	**1.1**	**1.5**	**SSIFDAK**

5.351 84 425 minus strand Transcription elongation factor B polypeptide 2 [Crassostrea gigas]; 84 425 minus strand Transcription elongation factor B polypeptide 2 [Crassostrea gigas]; 84 425 minus strand Transcription elongation factor B polypeptide 2 [Crassostrea gigas]; 101 442 minus strand Transcription elongation factor B polypeptide 2 [Crassostrea gigas]; 101 442 minus strand Transcription elongation factor B polypeptide 2 [Crassostrea gigas]; 84 425 minus strand Transcription elongation factor B polypeptide 2 [Crassostrea gigas]

Protein Accession	CL19448.Contig9$_{H}1$ CL19448.Contig8$_{H}1$ CL19448.Contig7$_{H}1$ CL19448.Contig1$_{H}1$ CL1944
Mean Expression Ratio	0.912
Median Expression Ratio	0.912
Credible Interval	(0.564, 1.48)
Associated Peptides	1
Associated Spectra	1
Coverage	NaN

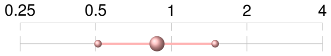

A	B	2.5	50	97.5	Sequence
**1**	**0**	**0.51**	**0.88**	**1.5**	**AQAPATLGLVYK**

5.352 " 242 1504 Glutamate receptor, ionotropic kainate 3 [Crassostrea gigas]; 239 1501 Glutamate receptor, ionotropic kainate 3 [Crassostrea gigas]; 238 1500 Glutamate receptor, ionotropic kainate 3 [Crassostrea gigas]; 661 1923 minus strand Glutamate receptor, ionotropic kainate 3 [Crassostrea gigas]"

Protein Accession	CL12478.Contig7$_{H}1$ CL12478.Contig6$_{H}1$ CL12478.Contig5$_{H}1$ CL12478.Contig3$_{H}1$
Mean Expression Ratio	1.09
Median Expression Ratio	1.1
Credible Interval	(0.719, 1.65)
Associated Peptides	2
Associated Spectra	3
Coverage	NaN

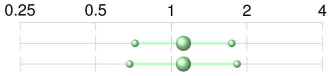

A	B	2.5	50	97.5	Sequence
**2**	**0**	**0.72**	**1.1**	**1.7**	**RPTFAQPSFQER**
**1**	**0**	**0.68**	**1.1**	**1.8**	**LAILR**

5.353 325 642 minus strand Protein phosphatase 1B [Crassostrea gigas]

Protein Accession	Unigene268$_{H}1$
Mean Expression Ratio	0.913
Median Expression Ratio	0.913
Credible Interval	(0.582, 1.42)
Associated Peptides	1
Associated Spectra	2
Coverage	NaN

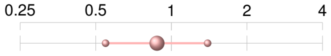

A	B	2.5	50	97.5	Sequence
**1**	**1**	**0.55**	**0.88**	**1.4**	**VEMEDAHTAVVSIPHAR**

5.354 68 481 minus strand netrin receptor UNC5A [Danio rerio] gi

Protein Accession	Unigene38845$_{H}1$
Mean Expression Ratio	1.09
Median Expression Ratio	1.1
Credible Interval	(0.671, 1.78)
Associated Peptides	1
Associated Spectra	1
Coverage	NaN

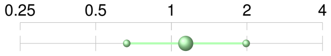

A	B	2.5	50	97.5	Sequence
**1**	**0**	**0.66**	**1.1**	**2**	**ELMECQK**

5.355 64 393 Tubulin beta chain OS=Paracentrotus lividus PE=2 SV=1; 111 398 Tubulin beta chain OS=Paracentrotus lividus PE=2 SV=1; 109 438 Tubulin beta chain OS=Paracentrotus lividus PE=2 SV=1; 160 489 Tubulin beta chain OS=Paracentrotus lividus PE=2 SV=1

Protein Accession	Unigene62663$_{H}1$ Unigene62662$_{H}1$ Unigene62661$_{H}1$ Unigene62660$_{H}1$
Mean Expression Ratio	0.915
Median Expression Ratio	0.914
Credible Interval	(0.585, 1.43)
Associated Peptides	1
Associated Spectra	6
Coverage	NaN

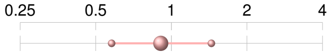

A	B	2.5	50	97.5	Sequence
**4**	**2**	**0.58**	**0.91**	**1.4**	**INVYFNEATGGK**

5.356 57 2378 minus strand Heterogeneous nuclear ribonucleoprotein U-like protein 1 [Crassostrea gigas]

Protein Accession	CL15560.Contig5$_{H}1$
Mean Expression Ratio	0.914
Median Expression Ratio	0.914
Credible Interval	(0.593, 1.41)
Associated Peptides	2
Associated Spectra	2
Coverage	NaN

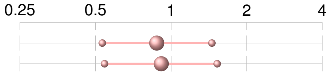

A	B	2.5	50	97.5	Sequence
**0**	**1**	**0.53**	**0.88**	**1.5**	**ALFPHILTK**
**0**	**1**	**0.55**	**0.91**	**1.5**	**VTELRDELK**

5.357 794 994 Glycogenin-1 [Crassostrea gigas]; 1425 1739 minus strand Glycogenin-1 [Crassostrea gigas]

Protein Accession	CL18608.Contig3$_H1$ CL18608.Contig2$_H1$
Mean Expression Ratio	1.09
Median Expression Ratio	1.09
Credible Interval	(0.74, 1.61)
Associated Peptides	3
Associated Spectra	3
Coverage	NaN

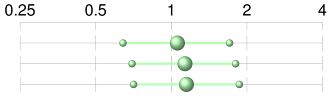

A	B	2.5	50	97.5	Sequence
1	0	0.64	1.1	1.7	VGGGESADYLILDDR
1	0	0.7	1.1	1.8	VGGGESADYLILDDRER
0	1	0.71	1.2	1.9	IAAPVLSFK

5.358 " 1144 1956 minus strand Retinol dehydrogenase 12, partial [Crassostrea gigas]"

Protein Accession	CL3944.Contig7$_H1$
Mean Expression Ratio	0.914
Median Expression Ratio	0.915
Credible Interval	(0.628, 1.33)
Associated Peptides	3
Associated Spectra	4
Coverage	NaN

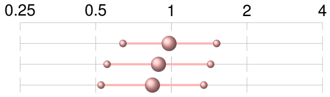

A	B	2.5	50	97.5	Sequence
2	0	0.64	0.98	1.5	LDFTDLALLK
1	0	0.55	0.89	1.4	LILGCR
0	1	0.52	0.84	1.3	LDLASLR

5.359 18 1613 minus strand chaperonin containing T-complex polypeptide subunit zeta [Haliotis discus hannai]; 527 2089 minus strand chaperonin containing T-complex polypeptide subunit zeta [Haliotis discus hannai]

Protein Accession	CL23722.Contig7$_H1$ CL23722.Contig5$_H1$
Mean Expression Ratio	1.09
Median Expression Ratio	1.09
Credible Interval	(0.728, 1.64)
Associated Peptides	2
Associated Spectra	3
Coverage	NaN

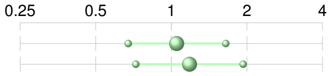

A	B	2.5	50	97.5	Sequence
2	0	0.67	1.1	1.6	LITEGFELAR
1	0	0.72	1.2	1.9	AAQALAVNISAA

5.360 95 271 minus strand hypothetical protein EAI_01530 [Harpegnathos saltator]

Protein Accession	CL9369.Contig9$_{H}1$
Mean Expression Ratio	0.915
Median Expression Ratio	0.915
Credible Interval	(0.586, 1.43)
Associated Peptides	1
Associated Spectra	2
Coverage	NaN

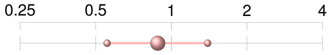

A	B	2.5	50	97.5	Sequence
**0**	**2**	**0.55**	**0.88**	**1.4**	**VAQFPFR**

5.361 816 1211 H2 [Haliotis tuberculata]

Protein Accession	CL11519.Contig5$_{H}1$
Mean Expression Ratio	0.914
Median Expression Ratio	0.915
Credible Interval	(0.587, 1.41)
Associated Peptides	2
Associated Spectra	4
Coverage	NaN

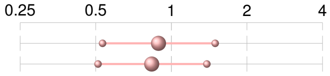

A	B	2.5	50	97.5	Sequence
**0**	**1**	**0.53**	**0.89**	**1.5**	**FDISDAVR**
**2**	**1**	**0.51**	**0.83**	**1.4**	**SLILAMR**

5.362 764 1015 minus strand heat shock protein beta-1 [Trichinella spiralis] gi

Protein Accession	CL46983.Contig3$_{H}1$
Mean Expression Ratio	1.09
Median Expression Ratio	1.09
Credible Interval	(0.789, 1.51)
Associated Peptides	2
Associated Spectra	12
Coverage	NaN

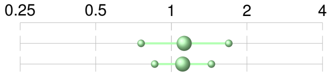

A	B	2.5	50	97.5	Sequence
**2**	**0**	**0.76**	**1.1**	**1.7**	**SYVLPK**
**8**	**2**	**0.86**	**1.1**	**1.4**	**TVTSSLSQNGVLTLK**

5.363 170 460 Spectrin beta chain [Crassostrea gigas]

Protein Accession	CL38255.Contig1$_{H}1$
Mean Expression Ratio	0.917
Median Expression Ratio	0.916
Credible Interval	(0.591, 1.43)
Associated Peptides	2
Associated Spectra	2
Coverage	NaN

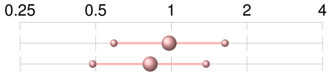

A	B	2.5	50	97.5	Sequence
**1**	**0**	**0.59**	**0.98**	**1.6**	**LLEVLSGER**
**1**	**0**	**0.49**	**0.83**	**1.4**	**WVNSHLIR**

5.364 913 1560 minus strand Calcium-dependent protein kinase isoform 2 [Crassostrea gigas]

Protein Accession	CL5866.Contig1$_{H}1$
Mean Expression Ratio	1.09
Median Expression Ratio	1.09
Credible Interval	(0.769, 1.55)
Associated Peptides	4
Associated Spectra	5
Coverage	NaN

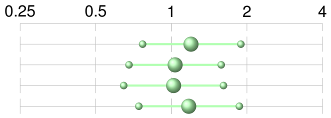

A	B	2.5	50	97.5	Sequence
0	1	0.77	1.2	1.9	DEFSVAVDK
0	2	0.68	1	1.6	FSFDEADKDK
0	1	0.65	1	1.6	IHYDEFVK
0	1	0.74	1.2	1.9	LLDINDDQK

5.365 166 1563 arylsulfatase [Haliotis discus discus]

Protein Accession	CL49030.Contig16$_{H}1$
Mean Expression Ratio	1.09
Median Expression Ratio	1.09
Credible Interval	(0.67, 1.76)
Associated Peptides	1
Associated Spectra	1
Coverage	NaN

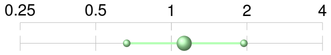

A	B	2.5	50	97.5	Sequence
0	1	0.66	1.1	2	VPLVLHSK

5.366 351 1826 Virulence metalloprotease [Crassostrea gigas]

Protein Accession	CL7226.Contig18$_{H}1$
Mean Expression Ratio	1.09
Median Expression Ratio	1.09
Credible Interval	(0.67, 1.76)
Associated Peptides	1
Associated Spectra	1
Coverage	NaN

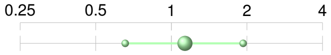

A	B	2.5	50	97.5	Sequence
1	0	0.65	1.1	1.9	EGDVCYLENK

5.367 300 878 unknown [Schistosoma japonicum]; 567 1145 unknown [Schistosoma japonicum]; 1110 1688 unknown [Schistosoma japonicum]; 94 672 unknown [Schistosoma japonicum]; 599 1177 unknown [Schistosoma japonicum]; 336 914 unknown [Schistosoma japonicum]

Protein Accession	CL11292.Contig5$_{H}1$ CL11292.Contig3$_{H}1$ CL11292.Contig2$_{H}1$ CL11292.Contig1$_{H}1$ CL1129
Mean Expression Ratio	0.918
Median Expression Ratio	0.918
Credible Interval	(0.672, 1.26)
Associated Peptides	4
Associated Spectra	12
Coverage	NaN

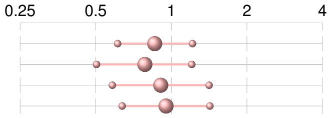

A	B	2.5	50	97.5	Sequence
4	4	0.61	0.86	1.2	AGNDVDLVVQR
1	0	0.5	0.78	1.2	GVVNTQSLPQEPEGEKR
1	0	0.58	0.91	1.4	NGDAILQICGESTAAMTHAQAK
2	0	0.64	0.95	1.4	VQLRRYDTSQPWGFR

5.368 " 2057 2497 Phosphate carrier protein, mitochondrial [Crassostrea gigas]"

Protein Accession	CL2712.Contig11$_{H}1$
Mean Expression Ratio	0.922
Median Expression Ratio	0.918
Credible Interval	(0.568, 1.5)
Associated Peptides	1
Associated Spectra	1
Coverage	NaN

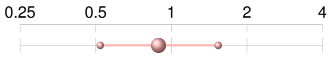

A	B	2.5	50	97.5	Sequence
**1**	**0**	**0.52**	**0.89**	**1.5**	**YVVPKPR**

5.369 145 1524 PREDICTED: aspartyl aminopeptidase-like [Oreochromis niloticus]

Protein Accession	CL19117.Contig2$_{H}1$
Mean Expression Ratio	1.09
Median Expression Ratio	1.09
Credible Interval	(0.71, 1.7)
Associated Peptides	2
Associated Spectra	2
Coverage	NaN

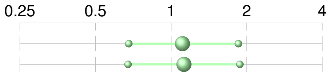

A	B	2.5	50	97.5	Sequence
**1**	**0**	**0.68**	**1.1**	**1.9**	**ITVSPSNPVAFEEAIGK**
**1**	**0**	**0.67**	**1.1**	**1.9**	**KLVAHGFK**

5.370 183 953 minus strand glutathione-S-transferase [Haliotis diversicolor supertexta]; 275 1045 minus strand glutathione-S-transferase [Haliotis diversicolor supertexta]; 343 1113 minus strand glutathione-S-transferase [Haliotis diversicolor supertexta]

Protein Accession	Unigene40627$_{H}1$ CL6878.Contig3$_{H}1$ CL6878.Contig2$_{H}1$
Mean Expression Ratio	0.92
Median Expression Ratio	0.918
Credible Interval	(0.597, 1.43)
Associated Peptides	2
Associated Spectra	2
Coverage	NaN

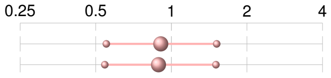

A	B	2.5	50	97.5	Sequence
**0**	**1**	**0.55**	**0.91**	**1.5**	**LPHDPLPK**
**0**	**1**	**0.55**	**0.89**	**1.5**	**KIDPK**

5.371 426 896 PREDICTED: calmodulin-like [Metaseiulus occidentalis]

Protein Accession	Unigene667$_{H}1$
Mean Expression Ratio	1.09
Median Expression Ratio	1.09
Credible Interval	(0.764, 1.56)
Associated Peptides	2
Associated Spectra	8
Coverage	NaN

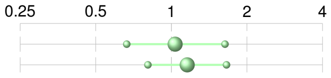

A	B	2.5	50	97.5	Sequence
**1**	**1**	**0.66**	**1**	**1.6**	**ADQLTEEQIAEFK**
**2**	**4**	**0.8**	**1.2**	**1.7**	**ELGTVMR**

5.372 3 293 Titin [Crassostrea gigas]

Protein Accession	Unigene9241$_H1$
Mean Expression Ratio	1.09
Median Expression Ratio	1.09
Credible Interval	(0.667, 1.77)
Associated Peptides	1
Associated Spectra	1
Coverage	NaN

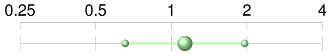

A	B	2.5	50	97.5	Sequence
**1**	**0**	**0.66**	**1.1**	**2**	**TAINTVESSLSELGQR**

5.373 91 1326 minus strand Adipocyte plasma membrane-associated protein [Crassostrea gigas]; 107 1273 minus strand Adipocyte plasma membrane-associated protein [Crassostrea gigas]

Protein Accession	CL11239.Contig2$_H1$ CL11239.Contig1$_H1$
Mean Expression Ratio	0.92
Median Expression Ratio	0.919
Credible Interval	(0.616, 1.38)
Associated Peptides	1
Associated Spectra	4
Coverage	NaN

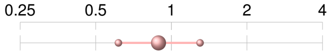

A	B	2.5	50	97.5	Sequence
**0**	**4**	**0.61**	**0.89**	**1.3**	**YGLIAELDKEGK**

5.374 7948 8541 Collagen alpha-3(VI) chain [Crassostrea gigas]

Protein Accession	CL47586.Contig9$_H1$
Mean Expression Ratio	1.09
Median Expression Ratio	1.09
Credible Interval	(0.757, 1.57)
Associated Peptides	2
Associated Spectra	5
Coverage	NaN

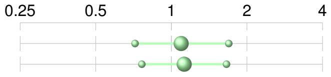

A	B	2.5	50	97.5	Sequence
**2**	**0**	**0.72**	**1.1**	**1.7**	**ADIAFLLDSSGSVGYDNFR**
**3**	**0**	**0.76**	**1.1**	**1.7**	**DLQDAISAISYEYGNTNTAAGLK**

5.375 266 1123 minus strand mitochondrial malate dehydrogenase precursor [Nucella lapillus]

Protein Accession	CL10426.Contig11$_H1$
Mean Expression Ratio	0.92
Median Expression Ratio	0.919
Credible Interval	(0.606, 1.42)
Associated Peptides	1
Associated Spectra	3
Coverage	NaN

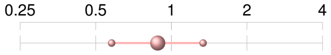

A	B	2.5	50	97.5	Sequence
**1**	**2**	**0.58**	**0.88**	**1.3**	**SLIEALNGMEGR**

5.376 99 3011 Eukaryotic translation initiation factor 3 subunit A [Crassostrea gigas]

Protein Accession	CL36771.Contig2$_{H}1$
Mean Expression Ratio	1.09
Median Expression Ratio	1.09
Credible Interval	(0.769, 1.53)
Associated Peptides	2
Associated Spectra	8
Coverage	NaN

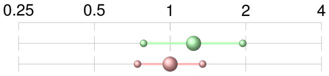

A	B	2.5	50	97.5	Sequence
**1**	**0**	**0.78**	**1.2**	**1.9**	**NNSRVER**
**7**	**0**	**0.74**	**1**	**1.3**	**LFNLSREQRK**

5.377 " 158 355 Protein NAS-37, isoform b [Caenorhabditis elegans] gi; 15 227 Hemicentin-1 [Crassostrea gigas]"

Protein Accession	Unigene57180$_{H}1$ CL8016.Contig2$_{H}1$
Mean Expression Ratio	1.09
Median Expression Ratio	1.09
Credible Interval	(0.675, 1.76)
Associated Peptides	1
Associated Spectra	1
Coverage	NaN

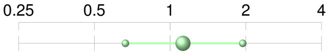

A	B	2.5	50	97.5	Sequence
**1**	**0**	**0.66**	**1.1**	**1.9**	**FKGSYGGVSR**

5.378 2 1384 minus strand twitchin [Mytilus galloprovincialis]; 2 1384 minus strand twitchin [Mytilus galloprovincialis]; 2 1384 minus strand twitchin [Mytilus galloprovincialis]; 2 1384 minus strand twitchin [Mytilus galloprovincialis]; 2 1384 minus strand twitchin [Mytilus galloprovincialis]; 2 1384 minus strand twitchin [Mytilus galloprovincialis]

Protein Accession	Unigene62659$_{H}1$ Unigene62658$_{H}1$ Unigene62657$_{H}1$ Unigene62656$_{H}1$ Unigene62655$_{H}1$ U
Mean Expression Ratio	1.09
Median Expression Ratio	1.09
Credible Interval	(0.666, 1.77)
Associated Peptides	1
Associated Spectra	1
Coverage	NaN

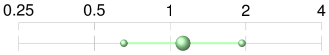

A	B	2.5	50	97.5	Sequence
**1**	**0**	**0.66**	**1.1**	**1.9**	**DGGGADLYTAVLQIQDPKPEDGGTYK**

5.379 585 944 minus strand Protein disulfide-isomerase A6 [Crassostrea gigas]

Protein Accession	CL39540.Contig7$_{H}$1
Mean Expression Ratio	1.09
Median Expression Ratio	1.08
Credible Interval	(0.701, 1.66)
Associated Peptides	2
Associated Spectra	3
Coverage	NaN

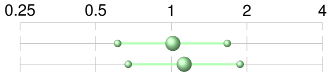

A	B	2.5	50	97.5	Sequence
1	1	0.61	1	1.7	TASDIINWATEK
1	0	0.67	1.1	1.9	GFPTIK

5.380 260 1597 isocitrate dehydrogenase [Mytilus galloprovincialis]; 260 1597 isocitrate dehydrogenase [Mytilus galloprovincialis]; 260 1597 isocitrate dehydrogenase [Mytilus galloprovincialis]

Protein Accession	CL10046.Contig8$_{H}$1 CL10046.Contig7$_{H}$1 CL10046.Contig6$_{H}$1
Mean Expression Ratio	0.924
Median Expression Ratio	0.923
Credible Interval	(0.668, 1.27)
Associated Peptides	5
Associated Spectra	7
Coverage	NaN

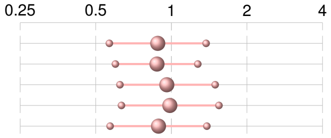

A	B	2.5	50	97.5	Sequence
1	0	0.57	0.88	1.4	DQTNDQVTIDSAEAIK
1	2	0.6	0.87	1.3	LTVFDFK
1	0	0.62	0.96	1.5	VKEFNLK
1	0	0.64	0.99	1.5	TSVLVCPDGK
0	1	0.57	0.89	1.4	SWPLYMSTK

5.381 147 272 Spectrin alpha chain [Crassostrea gigas]

Protein Accession	CL2014.Contig2$_{H}$1
Mean Expression Ratio	0.919
Median Expression Ratio	0.923
Credible Interval	(0.56, 1.49)
Associated Peptides	1
Associated Spectra	1
Coverage	NaN

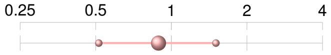

A	B	2.5	50	97.5	Sequence
0	1	0.51	0.89	1.5	VLETAEDIQQR

5.382 3 254 Calpain-5 [Crassostrea gigas]; 3 254 minus strand Calpain-5 [Crassostrea gigas]

Protein Accession	CL41869.Contig2$_{H}$1 CL41869.Contig1$_{H}$1
Mean Expression Ratio	0.925
Median Expression Ratio	0.923
Credible Interval	(0.595, 1.45)
Associated Peptides	1
Associated Spectra	2
Coverage	NaN

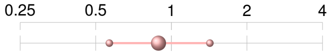

A	B	2.5	50	97.5	Sequence
1	1	0.56	0.89	1.4	EVDLFGHK

5.383 " 3195 3968 Spectrin alpha chain, putative [Pediculus humanus corporis] gi"

Protein Accession	CL2195.Contig1$_{H}$1
Mean Expression Ratio	1.08
Median Expression Ratio	1.08
Credible Interval	(0.833, 1.41)
Associated Peptides	6
Associated Spectra	16
Coverage	NaN

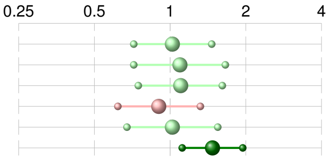

A	B	2.5	50	97.5	Sequence
2	1	0.72	1	1.5	DLSSVQTLLTK
1	0	0.72	1.1	1.7	LIDEGNHNADGIVER
1	1	0.75	1.1	1.6	LLLLQEQYR
1	1	0.62	0.9	1.3	LSELEAAASQR
1	0	0.67	1	1.5	QIEDLYLTFAK
4	3	1.1	1.5	1.9	YNDVIAR

5.384 116 1321 PREDICTED: similar to aspartate aminotransferase [Tribolium castaneum] gi

Protein Accession	CL11692.Contig6$_{H}$1
Mean Expression Ratio	0.922
Median Expression Ratio	0.924
Credible Interval	(0.7, 1.22)
Associated Peptides	8
Associated Spectra	10
Coverage	NaN

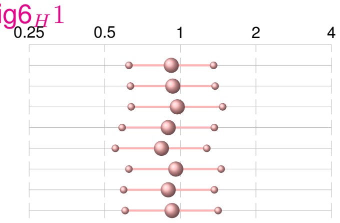

A	B	2.5	50	97.5	Sequence
2	0	0.63	0.92	1.4	DGHQVALAQSFAK
1	1	0.63	0.93	1.4	ILSTPALR
1	0	0.64	0.97	1.5	INLGVGAYR
1	0	0.59	0.9	1.4	TQWLGEVK
1	0	0.55	0.84	1.3	ISVAGLSSK
0	1	0.62	0.96	1.5	ILSTPALRTQ
0	1	0.59	0.9	1.4	DAFALR
0	1	0.6	0.92	1.4	IIAMR

5.385 406 1392 minus strand hypothetical protein CGI_10007007 [Crassostrea gigas]

Protein Accession	CL18045.Contig1$_{H}$1
Mean Expression Ratio	0.924
Median Expression Ratio	0.924
Credible Interval	(0.594, 1.45)
Associated Peptides	1
Associated Spectra	3
Coverage	NaN

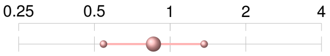

A	B	2.5	50	97.5	Sequence
2	1	0.54	0.86	1.4	IEAEFPGANIILHK

5.386 "REVERSED 2 1810 minus strand Glutaminyl-tRNA synthetase, partial [Crassostrea gigas]"

Protein Accession	RRRRRCL16949.Contig1$_{H}$1
Mean Expression Ratio	0.925
Median Expression Ratio	0.924
Credible Interval	(0.593, 1.44)
Associated Peptides	1
Associated Spectra	2
Coverage	NaN

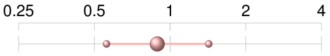

A	B	2.5	50	97.5	Sequence
2	0	0.56	0.89	1.4	GEELTVK

5.387 462 671 enhancer protein [Homo sapiens]

Protein Accession	CL3160.Contig12$_{H}1$
Mean Expression Ratio	1.08
Median Expression Ratio	1.08
Credible Interval	(0.788, 1.49)
Associated Peptides	3
Associated Spectra	11
Coverage	NaN

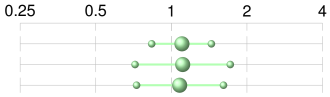

A	B	2.5	50	97.5	Sequence
**6**	**2**	**0.83**	**1.1**	**1.4**	**GLFIIDDK**
**0**	**1**	**0.72**	**1.1**	**1.7**	**PLLEDK**
**0**	**2**	**0.73**	**1.1**	**1.6**	**KIGGLGNMNIPLLEDK**

5.388 64 393 Tubulin beta chain OS=Paracentrotus lividus PE=2 SV=1; 109 438 Tubulin beta chain OS=Paracentrotus lividus PE=2 SV=1; 160 489 Tubulin beta chain OS=Paracentrotus lividus PE=2 SV=1

Protein Accession	Unigene62663$_{H}1$ Unigene62661$_{H}1$ Unigene62660$_{H}1$
Mean Expression Ratio	1.08
Median Expression Ratio	1.08
Credible Interval	(0.721, 1.61)
Associated Peptides	1
Associated Spectra	6
Coverage	NaN

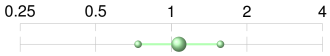

A	B	2.5	50	97.5	Sequence
**4**	**2**	**0.74**	**1.1**	**1.6**	**INVYFNEATGGK**

5.389 305 937 Clathrin light chain A [Crassostrea gigas]

Protein Accession	CL831.Contig4$_{H}1$
Mean Expression Ratio	0.926
Median Expression Ratio	0.924
Credible Interval	(0.566, 1.5)
Associated Peptides	1
Associated Spectra	1
Coverage	NaN

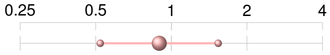

A	B	2.5	50	97.5	Sequence
**0**	**1**	**0.52**	**0.89**	**1.5**	**AAEEAFIK**

5.390 1 576 minus strand Tubulin beta chain [Crassostrea gigas]

Protein Accession	Unigene6463$_{H}1$
Mean Expression Ratio	1.08
Median Expression Ratio	1.08
Credible Interval	(0.667, 1.76)
Associated Peptides	1
Associated Spectra	4
Coverage	NaN

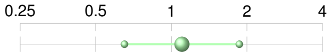

A	B	2.5	50	97.5	Sequence
**3**	**1**	**0.65**	**1.1**	**1.9**	**EVDEQMLNVQNK**

5.391 302 1273 minus strand hypothetical protein CGI_10007007 [Crassostrea gigas]

Protein Accession	CL18045.Contig10$_{H}1$
Mean Expression Ratio	0.923
Median Expression Ratio	0.925
Credible Interval	(0.572, 1.49)
Associated Peptides	1
Associated Spectra	3
Coverage	NaN

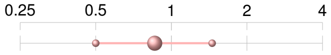

A	B	2.5	50	97.5	Sequence
**2**	**1**	**0.5**	**0.86**	**1.5**	**IEAEFPGANIILHK**

5.392 3 470 minus strand Spectrin beta chain [Crassostrea gigas]; 5 451 minus strand Spectrin beta chain [Crassostrea gigas]

Protein Accession	CL19757.Contig2$_{H}1$ CL19757.Contig1$_{H}1$
Mean Expression Ratio	1.08
Median Expression Ratio	1.08
Credible Interval	(0.705, 1.67)
Associated Peptides	2
Associated Spectra	4
Coverage	NaN

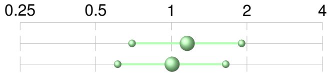

A	B	2.5	50	97.5	Sequence
**0**	**1**	**0.7**	**1.2**	**1.9**	**HLMGVEDLLQK**
**2**	**1**	**0.61**	**1**	**1.7**	**HSLLEADIHVVGER**

5.393 " 285 437 Propionyl-CoA carboxylase alpha chain, mitochondrial [Crassostrea gigas]"

Protein Accession	CL45581.Contig2$_{H}1$
Mean Expression Ratio	0.924
Median Expression Ratio	0.925
Credible Interval	(0.582, 1.43)
Associated Peptides	1
Associated Spectra	2
Coverage	NaN

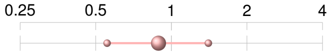

A	B	2.5	50	97.5	Sequence
**2**	**0**	**0.56**	**0.89**	**1.4**	**GVTHNIPLLR**

5.394 229 699 hypothetical protein CGI_10017744 [Crassostrea gigas]; 199 669 hypothetical protein CGI_10017744 [Crassostrea gigas]; 202 672 hypothetical protein CGI_10017744 [Crassostrea gigas]

Protein Accession	CL5082.Contig3$_{H}1$ CL5082.Contig2$_{H}1$ CL5082.Contig1$_{H}1$
Mean Expression Ratio	1.08
Median Expression Ratio	1.08
Credible Interval	(0.791, 1.48)
Associated Peptides	3
Associated Spectra	9
Coverage	NaN

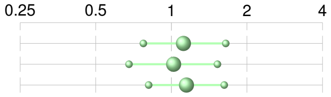

A	B	2.5	50	97.5	Sequence
**2**	**1**	**0.77**	**1.1**	**1.6**	**EVLELFDR**
**2**	**0**	**0.68**	**1**	**1.5**	**EGLHSLK**
**3**	**1**	**0.81**	**1.2**	**1.6**	**HLNKPDAIK**

5.395 " 193 333 minus strand RecName: Full=Globin; AltName: Full=Myoglobin gi|299274|gb|AAB25743.1| myoglobin [Nassa mutabilis=marine gastropods, radular muscles, Peptide, 147 aa] gi; 193 333 minus strand RecName: Full=Globin; AltName: Full=Myoglobin gi|299274|gb|AAB25743.1| myoglobin [Nassa mutabilis=marine gastropods, radular muscles, Peptide, 147 aa] gi"

Protein Accession	CL8458.Contig5$_{H}1$ CL8458.Contig1$_{H}1$
Mean Expression Ratio	1.08
Median Expression Ratio	1.08
Credible Interval	(0.695, 1.68)
Associated Peptides	1
Associated Spectra	2
Coverage	NaN

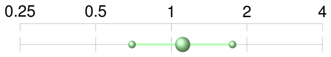

A	B	2.5	50	97.5	Sequence
**2**	**0**	**0.7**	**1.1**	**1.8**	**AAINSTWPK**

5.396 87 1997 Transketolase-like protein 2 [Crassostrea gigas]; 87 1997 Transketolase-like protein 2 [Crassostrea gigas]; 87 1997 Transketolase-like protein 2 [Crassostrea gigas]; 87 1997 Transketolase-like protein 2 [Crassostrea gigas]; 87 1997 Transketolase-like protein 2 [Crassostrea gigas]; 87 1997 Transketolase-like protein 2 [Crassostrea gigas]; 87 1997 Transketolase-like protein 2 [Crassostrea gigas]

Protein Accession	CL1127.Contig6$_{H}1$ CL1127.Contig5$_{H}1$ CL1127.Contig4$_{H}1$ CL1127.Contig3$_{H}1$ CL1127.Con
Mean Expression Ratio	1.08
Median Expression Ratio	1.08
Credible Interval	(0.756, 1.54)
Associated Peptides	3
Associated Spectra	5
Coverage	NaN

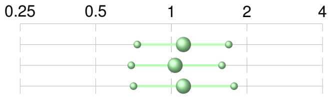

A	B	2.5	50	97.5	Sequence
**1**	**1**	**0.73**	**1.1**	**1.7**	**VISLDGDVK**
**1**	**1**	**0.69**	**1**	**1.6**	**GMCFIR**
**1**	**0**	**0.71**	**1.1**	**1.8**	**NARETGGR**

5.397 212 1336 6-phosphofructokinase [Crassostrea gigas]

Protein Accession	CL48438.Contig4$_H$1
Mean Expression Ratio	0.928
Median Expression Ratio	0.928
Credible Interval	(0.62, 1.4)
Associated Peptides	2
Associated Spectra	3
Coverage	NaN

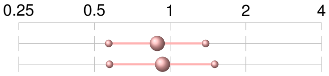

A	B	2.5	50	97.5	Sequence
**2**	**0**	**0.57**	**0.89**	**1.4**	**LGLTQGYR**
**1**	**0**	**0.58**	**0.93**	**1.5**	**SVQWFK**

5.398 66 263 minus strand twitchin [Mytilus galloprovincialis]; 66 263 minus strand twitchin [Mytilus galloprovincialis]; 66 263 minus strand twitchin [Mytilus galloprovincialis]; 66 263 minus strand twitchin [Mytilus galloprovincialis]; 66 263 minus strand twitchin [Mytilus galloprovincialis]

Protein Accession	Unigene64526$_H$1 Unigene64525$_H$1 Unigene64524$_H$1 Unigene64522$_H$1 Unigene64521$_H$1
Mean Expression Ratio	0.93
Median Expression Ratio	0.929
Credible Interval	(0.612, 1.41)
Associated Peptides	1
Associated Spectra	3
Coverage	NaN

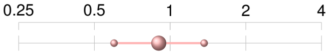

A	B	2.5	50	97.5	Sequence
**2**	**1**	**0.6**	**0.9**	**1.4**	**FTETLSPVEVMQGDK**

5.399 273 1304 Guanine nucleotide-binding protein subunit beta [Crassostrea gigas]

Protein Accession	CL15682.Contig1$_H$1
Mean Expression Ratio	0.93
Median Expression Ratio	0.929
Credible Interval	(0.743, 1.17)
Associated Peptides	6
Associated Spectra	23
Coverage	NaN

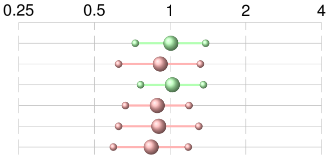

A	B	2.5	50	97.5	Sequence
**3**	**1**	**0.73**	**1**	**1.4**	**IYAMHWASDSR**
**2**	**0**	**0.63**	**0.92**	**1.3**	**KAAGDTTLAQATAQCEPVGR**
**3**	**3**	**0.76**	**1**	**1.4**	**TFVSGACDASAK**
**3**	**3**	**0.66**	**0.89**	**1.2**	**LWDIR**
**0**	**2**	**0.62**	**0.9**	**1.3**	**LIVWDGYTTNK**
**0**	**3**	**0.59**	**0.84**	**1.2**	**LFDIR**

5.400 109 510 minus strand hypothetical protein DAPPUDRAFT_231281 [Daphnia pulex]

Protein Accession	Unigene4945$_H$1
Mean Expression Ratio	0.933
Median Expression Ratio	0.929
Credible Interval	(0.68, 1.28)
Associated Peptides	4
Associated Spectra	8
Coverage	NaN

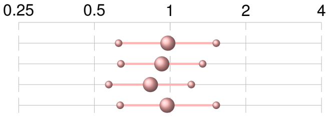

A	B	2.5	50	97.5	Sequence
1	0	0.63	0.98	1.5	LAGIEDPFEMNVK
2	1	0.64	0.93	1.3	VSMPDTLGHAVGIER
2	1	0.57	0.83	1.2	YELLAK
0	1	0.63	0.97	1.5	PDTLGHAVGIER

5.401 3 386 fem-1-like protein C [Crassostrea gigas]

Protein Accession	Unigene19937$_H$1
Mean Expression Ratio	0.93
Median Expression Ratio	0.929
Credible Interval	(0.612, 1.42)
Associated Peptides	1
Associated Spectra	3
Coverage	NaN

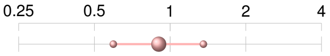

A	B	2.5	50	97.5	Sequence
3	0	0.59	0.9	1.4	MQSLLIR

5.402 65 490 PREDICTED: cofilin-2 [Otolemur garnettii]

Protein Accession	CL15471.Contig3$_H$1
Mean Expression Ratio	1.08
Median Expression Ratio	1.08
Credible Interval	(0.793, 1.45)
Associated Peptides	4
Associated Spectra	11
Coverage	NaN

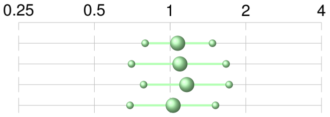

A	B	2.5	50	97.5	Sequence
4	2	0.79	1.1	1.5	IVMENEFAK
1	0	0.7	1.1	1.7	SQEDEYNSIIMTLPK
2	0	0.79	1.2	1.7	MLYSSSSSAVK
1	1	0.69	1	1.5	ALVFK

5.403 145 972 LIM and SH3 domain protein F42H10.3 OS=Caenorhabditis elegans GN=F42H10.3 PE=2 SV=3

Protein Accession	CL49642.Contig8$_H$1
Mean Expression Ratio	0.93
Median Expression Ratio	0.93
Credible Interval	(0.593, 1.45)
Associated Peptides	1
Associated Spectra	2
Coverage	NaN

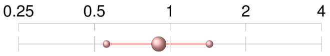

A	B	2.5	50	97.5	Sequence
1	1	0.56	0.9	1.4	TVYPTEELK

5.404 147 383 minus strand Tubulin-specific chaperone A OS=Rattus norvegicus GN=Tbca PE=1 SV=1; 104 418 Tubulin-specific chaperone A [Crassostrea gigas]; 104 418 minus strand Tubulin-specific chaperone A [Crassostrea gigas]; 104 418 Tubulin-specific chaperone A [Crassostrea gigas]

Protein Accession	Unigene66350$_{H}1$ Unigene53595$_{H}1$ Unigene32457$_{H}1$ CL7190.Contig3$_{H}1$
Mean Expression Ratio	1.08
Median Expression Ratio	1.08
Credible Interval	(0.664, 1.75)
Associated Peptides	1
Associated Spectra	2
Coverage	NaN

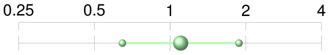

A	B	2.5	50	97.5	Sequence
**1**	**1**	**0.64**	**1.1**	**1.9**	**ADLGETEELK**

5.405 546 995 Hypothetical protein CBG12002 [Caenorhabditis briggsae]; 543 995 minus strand PREDICTED: tubulin polymerization-promoting protein family member 2-like [Anolis carolinensis]; 546 995 Hypothetical protein CBG12002 [Caenorhabditis briggsae]; 546 995 minus strand Hypothetical protein CBG12002 [Caenorhabditis briggsae]

Protein Accession	CL6849.Contig7$_{H}1$ CL6849.Contig6$_{H}1$ CL6849.Contig2$_{H}1$ CL6849.Contig1$_{H}1$
Mean Expression Ratio	1.08
Median Expression Ratio	1.08
Credible Interval	(0.692, 1.69)
Associated Peptides	1
Associated Spectra	2
Coverage	NaN

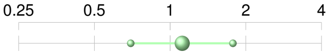

A	B	2.5	50	97.5	Sequence
**0**	**2**	**0.7**	**1.1**	**1.8**	**LLPQLAADK**

5.406 337 474 minus strand heat shock protein 22 isoform 1 [Ruditapes philippinarum]

Protein Accession	CL30681.Contig1$_{H}1$
Mean Expression Ratio	1.07
Median Expression Ratio	1.07
Credible Interval	(0.713, 1.61)
Associated Peptides	2
Associated Spectra	3
Coverage	NaN

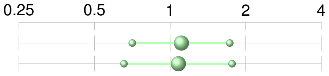

A	B	2.5	50	97.5	Sequence
**1**	**1**	**0.71**	**1.1**	**1.7**	**EDWNFFDR**
**0**	**1**	**0.66**	**1.1**	**1.8**	**REDWNFFDR**

5.407 280 726 minus strand RecName: Full=Calmodulin; Short=CaM gi|14588595|dbj|BAB61794.1| calmodulin [Metridium senile] gi

Protein Accession	Unigene4486$_H$1
Mean Expression Ratio	1.07
Median Expression Ratio	1.07
Credible Interval	(0.791, 1.45)
Associated Peptides	5
Associated Spectra	13
Coverage	NaN

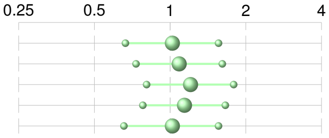

A	B	2.5	50	97.5	Sequence
1	1	0.67	1	1.6	ADQLTEEQIAEFK
2	0	0.73	1.1	1.6	EAFSLFDK
2	0	0.8	1.2	1.8	EAFSLFDKDGDGTITTK
2	4	0.78	1.1	1.7	ELGTVMR
1	0	0.66	1	1.6	HVMTNLGEKLTDEEVDEMIR

5.408 94 519 minus strand hypothetical protein BRAFLDRAFT_115029 [Branchiostoma floridae] gi

Protein Accession	Unigene52953$_H$1
Mean Expression Ratio	1.07
Median Expression Ratio	1.07
Credible Interval	(0.761, 1.51)
Associated Peptides	2
Associated Spectra	7
Coverage	NaN

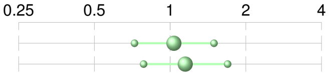

A	B	2.5	50	97.5	Sequence
2	2	0.72	1	1.5	SLDTDGDGTVSAAELR
2	1	0.79	1.2	1.7	FINKK

5.409 73 525 minus strand Actin-related protein 2/3 complex subunit 5-like protein [Salmo salar] gi; 73 525 Actin-related protein 2/3 complex subunit 5-like protein [Salmo salar] gi; 73 525 minus strand Actin-related protein 2/3 complex subunit 5-like protein [Salmo salar] gi

Protein Accession	CL14637.Contig6$_H$1 CL14637.Contig5$_H$1 CL14637.Contig4$_H$1
Mean Expression Ratio	1.08
Median Expression Ratio	1.07
Credible Interval	(0.665, 1.75)
Associated Peptides	1
Associated Spectra	1
Coverage	NaN

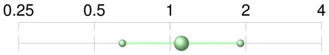

A	B	2.5	50	97.5	Sequence
0	1	0.64	1.1	1.9	EVDILMK

5.410 " 656 787 collagen alpha-1(XII) chain-like; K08132 collagen, type XII, alpha"

Protein Accession	Unigene26545$_{H}1$
Mean Expression Ratio	1.07
Median Expression Ratio	1.07
Credible Interval	(0.738, 1.58)
Associated Peptides	2
Associated Spectra	4
Coverage	NaN

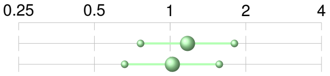

A	B	2.5	50	97.5	Sequence
1	1	0.76	1.2	1.8	AEVFDAVDNIPYR
1	1	0.66	1	1.6	TKAEVFDAVDNIPYR

5.411 635 1561 Uncharacterized protein yfeX [Crassostrea gigas]

Protein Accession	CL12046.Contig4$_{H}1$
Mean Expression Ratio	1.07
Median Expression Ratio	1.07
Credible Interval	(0.699, 1.65)
Associated Peptides	2
Associated Spectra	2
Coverage	NaN

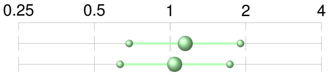

A	B	2.5	50	97.5	Sequence
1	0	0.69	1.2	1.9	TLESWVGR
1	0	0.63	1	1.7	LFDLAQLVLK

5.412 210 2201 minus strand Glycyl-tRNA synthetase [Crassostrea gigas]; 210 2201 minus strand Glycyl-tRNA synthetase [Crassostrea gigas]; 210 2201 minus strand Glycyl-tRNA synthetase [Crassostrea gigas]; 210 2201 minus strand Glycyl-tRNA synthetase [Crassostrea gigas]; 210 2201 minus strand Glycyl-tRNA synthetase [Crassostrea gigas]; 210 2201 minus strand Glycyl-tRNA synthetase [Crassostrea gigas]

Protein Accession	CL4073.Contig8$_{H}1$ CL4073.Contig7$_{H}1$ CL4073.Contig6$_{H}1$ CL4073.Contig5$_{H}1$ CL4073.Con
Mean Expression Ratio	1.07
Median Expression Ratio	1.07
Credible Interval	(0.694, 1.66)
Associated Peptides	2
Associated Spectra	2
Coverage	NaN

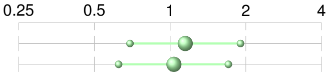

A	B	2.5	50	97.5	Sequence
0	1	0.69	1.1	1.9	ADHLIEATFEK
0	1	0.62	1	1.7	VLMPR

5.413 165 635 hypothetical protein CGI_10007639 [Crassostrea gigas]

Protein Accession	CL36715.Contig1$_{H}1$
Mean Expression Ratio	1.07
Median Expression Ratio	1.07
Credible Interval	(0.69, 1.65)
Associated Peptides	2
Associated Spectra	2
Coverage	NaN

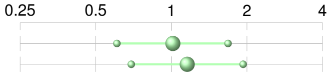

A	B	2.5	50	97.5	Sequence
1	0	0.61	1	1.7	AAIVAQVAK
1	0	0.69	1.2	1.9	MPLHASCK

5.414 114 1001 Vinculin [Crassostrea gigas]

Protein Accession	CL18499.Contig1$_{H}1$
Mean Expression Ratio	1.07
Median Expression Ratio	1.07
Credible Interval	(0.81, 1.4)
Associated Peptides	8
Associated Spectra	11
Coverage	NaN

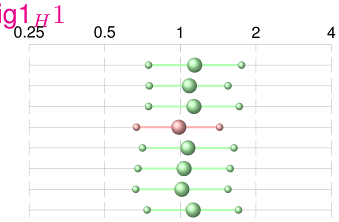

A	B	2.5	50	97.5	Sequence
1	0	0.75	1.1	1.8	MEDLVTFVK
2	1	0.75	1.1	1.5	TIESILEPVAQQVSR
1	0	0.75	1.1	1.7	NLSPVLTR
1	1	0.67	0.98	1.4	KMSDEIHEIIR
0	1	0.71	1.1	1.6	AAVDNLVR
0	1	0.68	1	1.6	MSDEIHEIIR
0	1	0.66	1	1.5	NLTPVLISGIK
0	1	0.74	1.1	1.7	IFITAK

5.415 193 828 Cysteine-rich secretory protein LCCL domain-containing 2 [Crassostrea gigas]

Protein Accession	CL40761.Contig2$_{H}1$
Mean Expression Ratio	1.07
Median Expression Ratio	1.07
Credible Interval	(0.725, 1.6)
Associated Peptides	3
Associated Spectra	3
Coverage	NaN

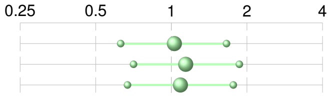

A	B	2.5	50	97.5	Sequence
1	0	0.63	1	1.7	LEWDAQMESEATSWAAR
1	0	0.71	1.1	1.9	YSPEGNWVNEKPYEK
1	0	0.67	1.1	1.8	ICQPLR

5.416 530 679 minus strand GJ11201 [Drosophila virilis] gi

Protein Accession	CL45804.Contig2$_{H}1$
Mean Expression Ratio	1.07
Median Expression Ratio	1.07
Credible Interval	(0.684, 1.69)
Associated Peptides	1
Associated Spectra	2
Coverage	NaN

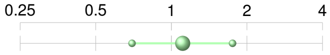

A	B	2.5	50	97.5	Sequence
2	0	0.7	1.1	1.8	QSQYALTLGPR

5.417 17 1681 minus strand Glucose-6-phosphate isomerase [Crassostrea gigas]

Protein Accession	CL34850.Contig1$_{H1}$
Mean Expression Ratio	0.935
Median Expression Ratio	0.935
Credible Interval	(0.715, 1.23)
Associated Peptides	6
Associated Spectra	12
Coverage	NaN

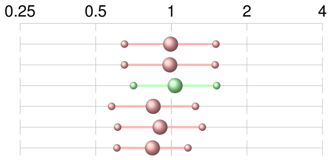

A	B	2.5	50	97.5	Sequence
1	0	0.65	0.99	1.5	EFTEQVISGAWK
1	0	0.65	0.99	1.5	KQLTSEPVWR
1	1	0.71	1	1.5	CKEFGIDEK
1	1	0.58	0.85	1.3	VHFISNVDGTHLAK
0	2	0.61	0.9	1.3	INFTEDR
0	4	0.61	0.84	1.2	INMAQMFK

5.418 45 944 minus strand unnamed protein product [Mus musculus] gi; 5 334 minus strand receptor of activated protein kinase C [Platynereis dumerilii]

Protein Accession	CL20494.Contig3$_{H1}$ CL20494.Contig1$_{H1}$
Mean Expression Ratio	1.07
Median Expression Ratio	1.07
Credible Interval	(0.698, 1.67)
Associated Peptides	2
Associated Spectra	2
Coverage	NaN

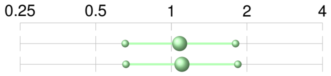

A	B	2.5	50	97.5	Sequence
1	0	0.66	1.1	1.8	IWDLEGK
1	0	0.66	1.1	1.8	AMCFSPNR

5.419 71 523 minus strand hypothetical protein DAPPUDRAFT_309332 [Daphnia pulex]

Protein Accession	CL4487.Contig1$_{H1}$
Mean Expression Ratio	1.07
Median Expression Ratio	1.07
Credible Interval	(0.656, 1.72)
Associated Peptides	1
Associated Spectra	1
Coverage	NaN

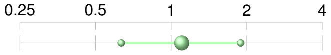

A	B	2.5	50	97.5	Sequence
0	1	0.63	1.1	1.9	IYHPNIDK

5.420 633 806 Peroxiredoxin-1 [Crassostrea gigas]; 648 821 Peroxiredoxin-1 [Crassostrea gigas]; 666 839 Peroxiredoxin-1 [Crassostrea gigas]; 625 798 Peroxiredoxin-1 [Crassostrea gigas]; 633 824 Peroxiredoxin-1 [Crassostrea gigas]

Protein Accession	CL3160.Contig18$_{H1}$ CL3160.Contig17$_{H1}$ CL3160.Contig16$_{H1}$ CL3160.Contig15$_{H1}$ CL3160
Mean Expression Ratio	1.07
Median Expression Ratio	1.07
Credible Interval	(0.739, 1.54)
Associated Peptides	1
Associated Spectra	13
Coverage	NaN

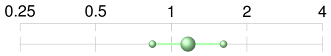

A	B	2.5	50	97.5	Sequence
**7**	**6**	**0.84**	**1.2**	**1.6**	**LVQAFQFTDK**

5.421 4 219 hypothetical protein BRAFLDRAFT_66351 [Branchiostoma floridae] gi; 3 239 hypothetical protein BRAFLDRAFT_66351 [Branchiostoma floridae] gi

Protein Accession	CL31114.Contig2$_{H1}$ CL31114.Contig1$_{H1}$
Mean Expression Ratio	1.07
Median Expression Ratio	1.07
Credible Interval	(0.653, 1.75)
Associated Peptides	1
Associated Spectra	1
Coverage	NaN

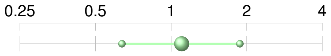

A	B	2.5	50	97.5	Sequence
**0**	**1**	**0.64**	**1.1**	**1.9**	**KLEEVSGELR**

5.422 362 553 putative RNA-binding protein [Haliotis diversicolor]

Protein Accession	CL41007.Contig2$_{H1}$
Mean Expression Ratio	1.07
Median Expression Ratio	1.07
Credible Interval	(0.765, 1.49)
Associated Peptides	2
Associated Spectra	9
Coverage	NaN

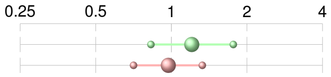

A	B	2.5	50	97.5	Sequence
**2**	**1**	**0.83**	**1.2**	**1.8**	**ELQDETGCR**
**3**	**3**	**0.71**	**0.97**	**1.3**	**EVMHVDSSFVGR**

5.423 166 2364 Dipeptidyl-peptidase 3 [Crassostrea gigas]

Protein Accession	CL9071.Contig2$_{H}$1
Mean Expression Ratio	1.07
Median Expression Ratio	1.07
Credible Interval	(0.715, 1.59)
Associated Peptides	3
Associated Spectra	3
Coverage	NaN

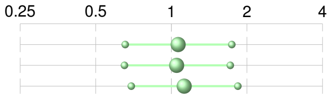

A	B	2.5	50	97.5	Sequence
1	0	0.65	1.1	1.7	SFETGSIPAHK
1	0	0.65	1.1	1.7	YVILR
1	0	0.69	1.1	1.8	VTGADGKP

5.424 549 821 minus strand Calponin-2 [Crassostrea gigas]

Protein Accession	CL21394.Contig10$_{H}$1
Mean Expression Ratio	1.07
Median Expression Ratio	1.07
Credible Interval	(0.789, 1.46)
Associated Peptides	3
Associated Spectra	10
Coverage	NaN

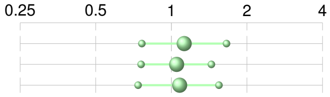

A	B	2.5	50	97.5	Sequence
2	0	0.76	1.1	1.7	QQGRASQAGMSMGAVR
3	2	0.76	1	1.4	SLFPTADLFEGR
3	0	0.74	1.1	1.6	NSLFPTADLFEGR

5.425 362 874 minus strand Transgelin-2 [Crassostrea gigas]

Protein Accession	CL445.Contig3$_{H}$1
Mean Expression Ratio	1.07
Median Expression Ratio	1.07
Credible Interval	(0.93, 1.23)
Associated Peptides	14
Associated Spectra	147
Coverage	NaN

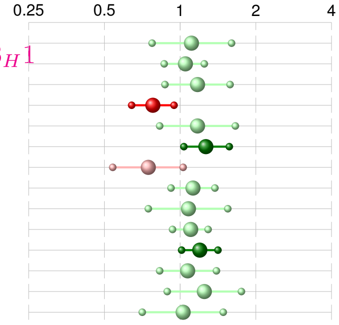

A	B	2.5	50	97.5	Sequence
1	0	0.78	1.1	1.6	AGLGAEIEKK
11	9	0.87	1	1.3	EFLVQSADLWEGR
3	1	0.87	1.2	1.6	EFLVQSADLWEGRK
17	0	0.64	0.78	0.95	GFQPSYTGSQSR
2	0	0.83	1.2	1.7	KAGLGAEIEKK
12	3	1	1.3	1.6	LLESEGKPK
1	2	0.54	0.75	1	LINKLLESEGKPK
8	8	0.92	1.1	1.4	LINFD
1	0	0.75	1.1	1.6	LGAEIEK
20	9	0.93	1.1	1.3	AGLGAEIEK
20	8	1	1.2	1.4	DGVMLCK
4	4	0.83	1.1	1.4	KAGLGAEIEK
1	1	0.89	1.2	1.8	RDGVMLCKLINK
0	1	0.71	1	1.5	QSADLWEGR

5.426 26 1909 Dolichyl-diphosphooligosaccharide–protein glycosyltransferase subunit 2 [Crassostrea gigas]

Protein Accession	CL6515.Contig1$_{H}1$
Mean Expression Ratio	1.07
Median Expression Ratio	1.07
Credible Interval	(0.801, 1.43)
Associated Peptides	6
Associated Spectra	10
Coverage	NaN

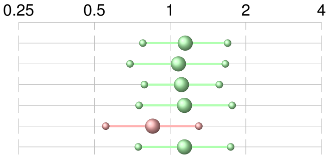

A	B	2.5	50	97.5	Sequence
1	1	0.78	1.2	1.7	AVSSALLDALK
1	0	0.69	1.1	1.7	KQFTASASDK
2	2	0.79	1.1	1.6	NLGLAVDEK
1	0	0.75	1.1	1.8	TLYELNFMEAK
1	0	0.56	0.85	1.3	LLTDAISAK
1	0	0.75	1.1	1.7	VSVTNLMGGDLGQLTVTADSAR

5.427 712 831 haemocyanin [Aplysia californica]

Protein Accession	Unigene65929$_{H}1$
Mean Expression Ratio	0.934
Median Expression Ratio	0.936
Credible Interval	(0.635, 1.39)
Associated Peptides	2
Associated Spectra	4
Coverage	NaN

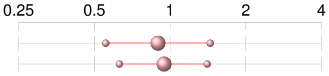

A	B	2.5	50	97.5	Sequence
1	0	0.56	0.89	1.4	YDVTDVFNQMR
3	0	0.63	0.95	1.4	YHFNVK

5.428 288 1487 minus strand putative procollagen type VI alpha 4 [Haliotis diversicolor]

Protein Accession	CL6899.Contig2$_{H}1$
Mean Expression Ratio	0.937
Median Expression Ratio	0.936
Credible Interval	(0.831, 1.06)
Associated Peptides	20
Associated Spectra	189
Coverage	NaN

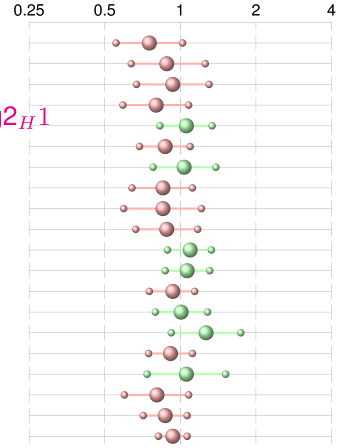

A	B	2.5	50	97.5	Sequence
4	0	0.56	0.75	1	AEVLAAIDQVR
2	0	0.64	0.89	1.3	ELTNQVAVLFLDDQALR
1	1	0.67	0.94	1.3	ENLYYIQSVVDR
1	3	0.59	0.8	1.1	MGFDVDQFR
5	5	0.83	1.1	1.3	TKAEVLAAIDQVR
9	2	0.69	0.87	1.1	VMVVITDGSSR
4	1	0.78	1	1.4	FDISVDDTR
3	3	0.64	0.85	1.1	FVVVDLGVR
1	0	0.59	0.85	1.2	LGNVVPQDENTYNNLLR
4	1	0.66	0.88	1.2	PDPTIGSCR
8	8	0.89	1.1	1.3	ALETLR
8	7	0.87	1.1	1.3	DLLPMR
9	5	0.75	0.93	1.1	FRDDITK
6	4	0.8	1	1.3	QVLQFVSQVITR
2	1	0.92	1.3	1.7	LRENLK
9	7	0.75	0.91	1.1	IGALTFSDDVK
1	0	0.74	1.1	1.5	FRALETLR
2	2	0.6	0.81	1.1	ADIINFASQSLAR
10	5	0.71	0.87	1.1	GANGVTLAQQVR
36	9	0.82	0.94	1.1	TGVTNTHLAIR

5.429 392 808 Whirlin [Crassostrea gigas]

Protein Accession	CL22731.Contig1$_{H}1$
Mean Expression Ratio	0.935
Median Expression Ratio	0.936
Credible Interval	(0.629, 1.39)
Associated Peptides	1
Associated Spectra	5
Coverage	NaN

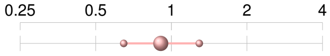

A	B	2.5	50	97.5	Sequence
**2**	**3**	**0.65**	**0.91**	**1.3**	**AGNDLHLLVQR**

5.430 2 1126 Prolyl endopeptidase [Crassostrea gigas]

Protein Accession	CL47692.Contig1$_{H}1$
Mean Expression Ratio	1.07
Median Expression Ratio	1.07
Credible Interval	(0.66, 1.76)
Associated Peptides	1
Associated Spectra	1
Coverage	NaN

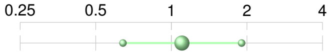

A	B	2.5	50	97.5	Sequence
**1**	**0**	**0.64**	**1.1**	**1.9**	**VVPLHSLK**

5.431 356 619 PREDICTED: hypothetical protein LOC100698726 [Oreochromis niloticus]

Protein Accession	CL3567.Contig6$_{H}1$
Mean Expression Ratio	1.06
Median Expression Ratio	1.07
Credible Interval	(0.682, 1.64)
Associated Peptides	2
Associated Spectra	2
Coverage	NaN

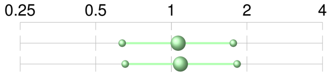

A	B	2.5	50	97.5	Sequence
**1**	**0**	**0.64**	**1.1**	**1.8**	**LETEVNDLR**
**1**	**0**	**0.65**	**1.1**	**1.8**	**GELQKEEAFSAK**

5.432 10 300 minus strand unnamed protein product [Tetraodon nigroviridis]

Protein Accession	CL14559.Contig2$_{H}1$
Mean Expression Ratio	1.06
Median Expression Ratio	1.07
Credible Interval	(0.71, 1.61)
Associated Peptides	1
Associated Spectra	4
Coverage	NaN

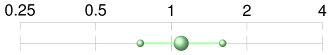

A	B	2.5	50	97.5	Sequence
**3**	**1**	**0.75**	**1.1**	**1.6**	**ILVQEGIHDR**

5.433 410 619 minus strand Guanine nucleotide-binding protein subunit gamma-1 [Crassostrea gigas]

Protein Accession	CL36920.Contig1$_{H}1$
Mean Expression Ratio	1.07
Median Expression Ratio	1.07
Credible Interval	(0.747, 1.51)
Associated Peptides	2
Associated Spectra	6
Coverage	NaN

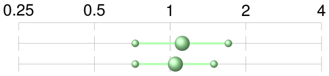

A	B	2.5	50	97.5	Sequence
1	1	0.73	1.1	1.7	CEAQITR
2	2	0.73	1	1.5	LIEQLR

5.434 52 1599 minus strand ATP synthase beta subunit [Pinctada fucata]

Protein Accession	CL34251.Contig5$_{H}1$
Mean Expression Ratio	1.07
Median Expression Ratio	1.07
Credible Interval	(0.876, 1.3)
Associated Peptides	10
Associated Spectra	33
Coverage	NaN

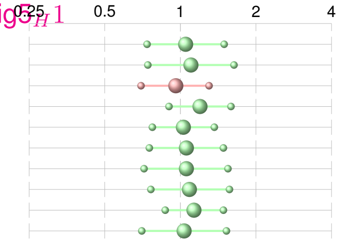

A	B	2.5	50	97.5	Sequence
1	1	0.74	1	1.5	AHGGYSVFAGVGER
1	0	0.74	1.1	1.6	EAAKPAAAGNLAVGR
2	2	0.7	0.96	1.3	IINVIGEPIDER
4	2	0.9	1.2	1.6	ILDPNVVGMEHYK
3	3	0.77	1	1.4	TIAMDGTEGLVR
2	1	0.76	1.1	1.5	VISLTDDTSK
1	0	0.72	1.1	1.5	VSLVYGQMNEPPGAR
2	0	0.77	1.1	1.6	VVDMLAPYAR
6	1	0.87	1.1	1.5	ILQDFK
0	1	0.7	1	1.5	IGLFGGAGVGK

5.435 1 111 glycogen synthase [Crassostrea gigas]

Protein Accession	Unigene78448$_{H}1$
Mean Expression Ratio	0.939
Median Expression Ratio	0.938
Credible Interval	(0.568, 1.54)
Associated Peptides	1
Associated Spectra	1
Coverage	NaN

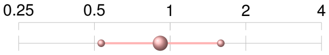

A	B	2.5	50	97.5	Sequence
0	1	0.53	0.91	1.6	NVEPDLDDDYLDR

5.436 113 808 minus strand omega class glutathione-s-transferase [Chlamys farreri]; 335 1045 minus strand omega class glutathione-s-transferase [Chlamys farreri]

Protein Accession	CL8096.Contig3$_{H}1$ CL8096.Contig2$_{H}1$
Mean Expression Ratio	0.936
Median Expression Ratio	0.939
Credible Interval	(0.597, 1.47)
Associated Peptides	1
Associated Spectra	2
Coverage	NaN

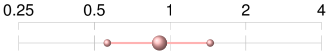

A	B	2.5	50	97.5	Sequence
2	0	0.56	0.91	1.4	HPLGLVPTLEQDSK

5.437 114 2633 Filamin-A [Crassostrea gigas]; 135 2654 Filamin-A [Crassostrea gigas]

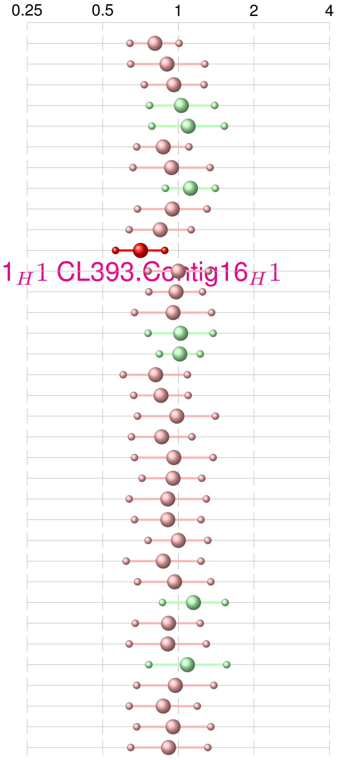

A	B	2.5	50	97.5	Sequence
9	3	0.64	0.81	1	AVANKPNEIVIDTSK
2	0	0.65	0.9	1.3	DAGEALLSIGMIAPSGHPEAELGYK
5	1	0.73	0.96	1.3	DNNDGTIVVEYQPK
4	0	0.77	1	1.4	FADEHVPGSPFLLNVGGEASGR
2	0	0.78	1.1	1.5	FDDVHIPGSPFIVNIAPDSGTAR
6	4	0.68	0.87	1.1	IGDSELGHAAGCK
1	0	0.66	0.94	1.3	KMGDGEIGIASFNPR
8	3	0.89	1.1	1.4	KPSPIQEQSSMVVETVEK
3	0	0.69	0.94	1.3	KVTANSYTVSYK
3	3	0.64	0.85	1.1	MGDGEIGIASFNPR
7	7	0.56	0.71	0.88	MNGIHTLSVR
4	2	0.76	1	1.3	QMEAAEAAVPNK
8	0	0.76	0.98	1.2	SQLSLGNSAEYSLK
1	0	0.67	0.95	1.4	TNVSDETGCYSK
3	1	0.76	1	1.4	TPSGGHEDCFIQEMDK
12	9	0.84	1	1.2	VINFQIETK
3	1	0.6	0.81	1.1	VPKPGSYGLHIK
5	4	0.67	0.85	1.1	VQVGGPGLEK
1	0	0.69	0.99	1.4	VRDNNDGTIVVEYQPK
4	2	0.65	0.86	1.1	VTGALDK
1	0	0.67	0.96	1.4	WGDEDIPGSPFVIST
4	2	0.72	0.95	1.2	YGNIPIAGSPYK
1	0	0.64	0.91	1.3	YIPKENGVHYVDVK
3	1	0.67	0.91	1.2	YKGNPIK
5	1	0.76	1	1.3	EAGPGTLTVAVEGPGK
1	1	0.62	0.87	1.2	AALSCK
1	1	0.69	0.97	1.3	PGSYGLHIK
4	1	0.86	1.1	1.5	HINNSPFK
3	1	0.68	0.91	1.2	IMFTPK
1	0	0.64	0.91	1.3	FRGDASK
1	0	0.76	1.1	1.6	AQIFLEERPNGFLGVQYK
1	0	0.68	0.97	1.4	CPGDYLITIK
2	1	0.64	0.87	1.2	ENGVHYVDVK
2	0	0.68	0.95	1.3	SDIECYK
1	0	0.64	0.92	1.3	VTANSYTVSYK

Protein Accession	CL393.Contig21$_H$1 CL393.Contig16$_H$1
Mean Expression Ratio	0.939
Median Expression Ratio	0.939
Credible Interval	(0.841, 1.05)
Associated Peptides	35
Associated Spectra	171
Coverage	NaN

5.438 227 3073 minus strand Lysosomal alpha-mannosidase [Crassostrea gigas]

Protein Accession	CL2930.Contig2$_H$1
Mean Expression Ratio	1.06
Median Expression Ratio	1.07
Credible Interval	(0.709, 1.59)
Associated Peptides	2
Associated Spectra	4
Coverage	NaN

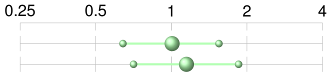

A	B	2.5	50	97.5	Sequence
2	1	0.64	1	1.5	VASELIPITPDTFR
1	0	0.71	1.2	1.9	ASLTWSGVR

5.439 387 1064 minus strand PREDICTED: hypothetical protein [Hydra magnipapillata]; 275 952 minus strand PREDICTED: hypothetical protein [Hydra magnipapillata]; 172 849 minus strand PREDICTED: hypothetical protein [Hydra magnipapillata]

Protein Accession	CL20613.Contig3$_{H}1$ CL20613.Contig2$_{H}1$ CL20613.Contig1$_{H}1$
Mean Expression Ratio	1.07
Median Expression Ratio	1.06
Credible Interval	(0.756, 1.51)
Associated Peptides	3
Associated Spectra	6
Coverage	NaN

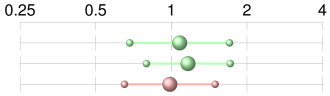

A	B	2.5	50	97.5	Sequence
1	0	0.68	1.1	1.7	ELGATFEAADNFKPNVVVSGR
3	0	0.8	1.2	1.7	NAGYTVHFVSPK
1	1	0.65	0.99	1.5	GFLADQEVQK

5.440 971 1963 Spectrin alpha chain [Crassostrea gigas]; 972 1979 Spectrin alpha chain [Crassostrea gigas]

Protein Accession	CL2195.Contig6$_{H}1$ CL2195.Contig4$_{H}1$
Mean Expression Ratio	0.942
Median Expression Ratio	0.939
Credible Interval	(0.604, 1.48)
Associated Peptides	1
Associated Spectra	2
Coverage	NaN

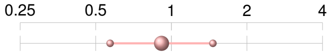

A	B	2.5	50	97.5	Sequence
1	1	0.57	0.91	1.5	EAFLAGDTVDGDNVETLIK

5.441 156 1106 ELAV 2-like protein [Aplysia californica] gi

Protein Accession	CL1500.Contig1$_{H}1$
Mean Expression Ratio	1.07
Median Expression Ratio	1.06
Credible Interval	(0.706, 1.61)
Associated Peptides	2
Associated Spectra	3
Coverage	NaN

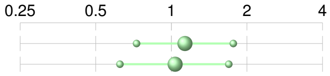

A	B	2.5	50	97.5	Sequence
1	1	0.73	1.1	1.8	NLPIPWTENELNK
0	1	0.62	1	1.7	VLVDLQTGVSK

5.442 303 1793 PREDICTED: matrix metalloproteinase 19 isoform rasi-1 preproprotein-like [Saccoglossus kowalevskii]

Protein Accession	CL7632.Contig1$_{H}1$
Mean Expression Ratio	1.06
Median Expression Ratio	1.06
Credible Interval	(0.693, 1.65)
Associated Peptides	2
Associated Spectra	2
Coverage	NaN

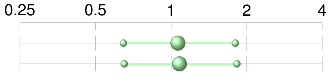

A	B	2.5	50	97.5	Sequence
1	0	0.65	1.1	1.8	FDAIDR
1	0	0.65	1.1	1.8	LGRVAR

5.443 " 1 1815 Glycogen phosphorylase, muscle form [Crassostrea gigas]"

Protein Accession	CL20630.Contig5$_{H}1$
Mean Expression Ratio	0.941
Median Expression Ratio	0.94
Credible Interval	(0.753, 1.18)
Associated Peptides	9
Associated Spectra	39
Coverage	NaN

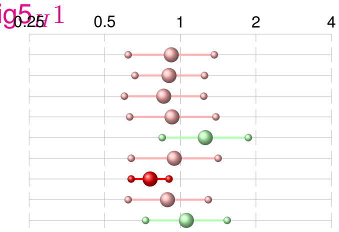

A	B	2.5	50	97.5	Sequence
1	0	0.62	0.92	1.4	VVFLENYR
2	2	0.66	0.9	1.2	VVNNDPIIGDR
1	1	0.6	0.86	1.2	TSFDMFPEK
1	0	0.63	0.93	1.4	WPVSMLER
1	0	0.84	1.3	1.9	DFAQMFPER
1	0	0.64	0.95	1.4	FGNPWEK
16	9	0.64	0.76	0.9	IEELEEIEEDAGL
0	2	0.62	0.89	1.3	AWDLVVR
0	2	0.73	1.1	1.5	LLLCNSGLADVIAEK

5.444 360 764 minus strand Peptidyl-prolyl cis-trans isomerase A OS=Dictyostelium discoideum GN=ppiA PE=1 SV=1

Protein Accession	Unigene64429$_{H}1$
Mean Expression Ratio	1.06
Median Expression Ratio	1.06
Credible Interval	(0.687, 1.66)
Associated Peptides	1
Associated Spectra	2
Coverage	NaN

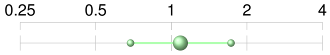

A	B	2.5	50	97.5	Sequence
0	2	0.69	1.1	1.7	HVVFGQVVEGLDVVR

5.445 75 803 hypothetical protein DAPPUDRAFT_231350 [Daphnia pulex]

Protein Accession	CL10537.Contig4$_{H}1$
Mean Expression Ratio	1.06
Median Expression Ratio	1.06
Credible Interval	(0.768, 1.46)
Associated Peptides	4
Associated Spectra	7
Coverage	NaN

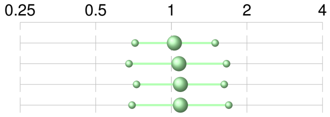

A	B	2.5	50	97.5	Sequence
2	1	0.71	1	1.5	LDYQLEIQSAK
1	0	0.68	1.1	1.7	SITPQQEAESIK
1	1	0.73	1.1	1.6	VCIAQLK
1	0	0.7	1.1	1.7	ENVALQLEASYR

5.446 151 1110 minus strand hypothetical protein DAPPUDRAFT_303085 [Daphnia pulex]

Protein Accession	CL12652.Contig2$_H$1
Mean Expression Ratio	1.06
Median Expression Ratio	1.06
Credible Interval	(0.694, 1.61)
Associated Peptides	1
Associated Spectra	3
Coverage	NaN

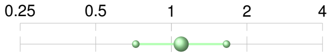

A	B	2.5	50	97.5	Sequence
2	1	0.72	1.1	1.7	TVIITGANTGIGK

5.447 " 220 627 Fatty acid-binding protein, brain [Crassostrea gigas]"

Protein Accession	CL9041.Contig2$_H$1
Mean Expression Ratio	1.06
Median Expression Ratio	1.06
Credible Interval	(0.806, 1.41)
Associated Peptides	5
Associated Spectra	13
Coverage	NaN

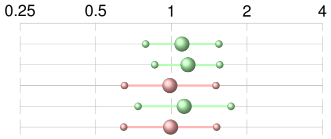

A	B	2.5	50	97.5	Sequence
3	1	0.79	1.1	1.5	EVDGGVMTSTTTFDGK
3	3	0.86	1.2	1.6	LTITVEGGK
1	0	0.65	0.98	1.5	VDLASTTGLEEFGTAIGFSEEK
1	0	0.74	1.1	1.7	IVENYKLEDK
0	1	0.65	0.99	1.5	QNYSFK

5.448 1070 1888 minus strand galectin [Pinctada fucata]; 1104 1922 minus strand galectin [Pinctada fucata]

Protein Accession	CL7245.Contig4$_H$1 CL7245.Contig2$_H$1
Mean Expression Ratio	1.06
Median Expression Ratio	1.06
Credible Interval	(0.658, 1.72)
Associated Peptides	1
Associated Spectra	1
Coverage	NaN

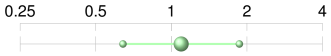

A	B	2.5	50	97.5	Sequence
1	0	0.64	1.1	1.9	FNEGVTVR

5.449 108 1259 minus strand PREDICTED: Na(+)/H(+) exchange regulatory cofactor NHE-RF3-like [Metaseiulus occidentalis]; 108 1190 minus strand PREDICTED: Na(+)/H(+) exchange regulatory cofactor NHE-RF3-like [Metaseiulus occidentalis]

Protein Accession	CL26023.Contig7$_{H}1$ CL26023.Contig6$_{H}1$
Mean Expression Ratio	0.943
Median Expression Ratio	0.941
Credible Interval	(0.577, 1.51)
Associated Peptides	1
Associated Spectra	1
Coverage	NaN

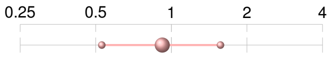

A	B	2.5	50	97.5	Sequence
0	1	0.53	0.92	1.6	GDLPEVLHLTAVR

5.450 252 848 sigma class glutathione-s-transferase 2 [Haliotis discus discus]

Protein Accession	CL19285.Contig21$_{H}1$
Mean Expression Ratio	1.06
Median Expression Ratio	1.06
Credible Interval	(0.776, 1.47)
Associated Peptides	5
Associated Spectra	8
Coverage	NaN

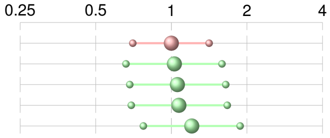

A	B	2.5	50	97.5	Sequence
2	2	0.7	1	1.4	LQDVDKDYPEVQK
1	0	0.66	1	1.6	LQAESLPK
1	0	0.68	1.1	1.7	LGNELTMA
0	1	0.69	1.1	1.7	KLRGR
0	1	0.78	1.2	1.9	EWEDVR

5.451 635 1294 minus strand Kyphoscoliosis peptidase [Crassostrea gigas]; 643 1302 minus strand Kyphoscoliosis peptidase [Crassostrea gigas]; 635 1294 minus strand Kyphoscoliosis peptidase [Crassostrea gigas]; 634 1293 minus strand Kyphoscoliosis peptidase [Crassostrea gigas]; 634 1293 minus strand Kyphoscoliosis peptidase [Crassostrea gigas]; 634 1293 minus strand Kyphoscoliosis peptidase [Crassostrea gigas]; 639 1298 minus strand Kyphoscoliosis peptidase [Crassostrea gigas]

Protein Accession	CL7772.Contig20$_{H}1$ CL7772.Contig1$_{H}1$ CL7772.Contig19$_{H}1$ CL7772.Contig16$_{H}1$ CL7772.[illegible]
Mean Expression Ratio	0.942
Median Expression Ratio	0.942
Credible Interval	(0.725, 1.22)
Associated Peptides	7
Associated Spectra	36
Coverage	NaN

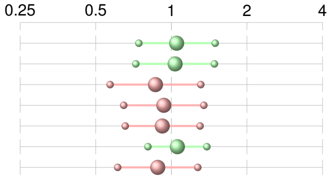

A	B	2.5	50	97.5	Sequence
2	2	0.74	1.1	1.5	FQIFALPTSDDSK
4	1	0.72	1	1.5	GAGEVWEGTAALDK
0	1	0.57	0.87	1.3	GAGEVWEGTAALDKHR
3	1	0.65	0.93	1.3	VTLNACFGEDESK
3	2	0.65	0.92	1.3	QYAPWR
7	6	0.8	1.1	1.4	LSNVTFK
3	1	0.61	0.88	1.3	AVYPFPK

5.452 11 394 regucalcin [Salmo salar] gi

Protein Accession	CL42615.Contig1$_{H}1$
Mean Expression Ratio	1.06
Median Expression Ratio	1.06
Credible Interval	(0.663, 1.72)
Associated Peptides	1
Associated Spectra	1
Coverage	NaN

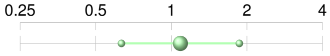

A	B	2.5	50	97.5	Sequence
**1**	**0**	**0.63**	**1.1**	**1.9**	**NQPLAGSLFK**

5.453 3 230 minus strand RH21460p [Drosophila melanogaster]; 3 230 minus strand RH21460p [Drosophila melanogaster]

Protein Accession	Unigene57704$_{H}1$ CL18123.Contig3$_{H}1$
Mean Expression Ratio	1.06
Median Expression Ratio	1.06
Credible Interval	(0.655, 1.74)
Associated Peptides	1
Associated Spectra	1
Coverage	NaN

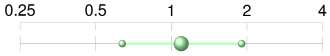

A	B	2.5	50	97.5	Sequence
**0**	**1**	**0.64**	**1.1**	**1.9**	**EVDEDEAAFK**

5.454 472 1551 minus strand hypothetical protein [Amblyomma maculatum]

Protein Accession	Unigene11523$_{H}1$
Mean Expression Ratio	1.06
Median Expression Ratio	1.06
Credible Interval	(0.858, 1.32)
Associated Peptides	7
Associated Spectra	29
Coverage	NaN

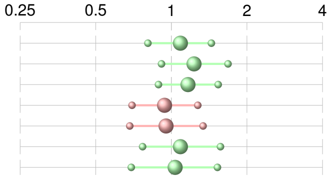

A	B	2.5	50	97.5	Sequence
**6**	**0**	**0.81**	**1.1**	**1.4**	**LFQNAQLSR**
**5**	**0**	**0.91**	**1.2**	**1.7**	**MVTGPVPGNLAVSK**
**7**	**0**	**0.89**	**1.2**	**1.5**	**QSLTDILR**
**3**	**2**	**0.7**	**0.94**	**1.3**	**VANGLYYDDTAVTLAEAFK**
**1**	**2**	**0.68**	**0.95**	**1.3**	**VLELPYGTDGR**
**2**	**0**	**0.77**	**1.1**	**1.6**	**SKQSLTDILR**
**0**	**1**	**0.69**	**1**	**1.5**	**FMVSLPK**

5.455 " 3 239 minus strand laminin beta 1 chain, putative [Ixodes scapularis] gi"

Protein Accession	CL48498.Contig2$_{H}1$
Mean Expression Ratio	0.944
Median Expression Ratio	0.944
Credible Interval	(0.638, 1.39)
Associated Peptides	2
Associated Spectra	4
Coverage	NaN

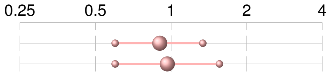

A	B	2.5	50	97.5	Sequence
**2**	**1**	**0.6**	**0.9**	**1.3**	**LAQQAEQLLTTK**
**1**	**0**	**0.6**	**0.96**	**1.6**	**ARNTSETVR**

5.456 67 825 unnamed protein product [Oikopleura dioica]

Protein Accession	Unigene71508$_H1$
Mean Expression Ratio	1.06
Median Expression Ratio	1.06
Credible Interval	(0.678, 1.65)
Associated Peptides	1
Associated Spectra	2
Coverage	NaN

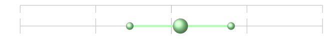

A	B	2.5	50	97.5	Sequence
2	0	0.69	1.1	1.7	TAANVEEAFINTAK

5.457 " 1240 1911 PREDICTED: ATP synthase subunit alpha, mitochondrial-like [Anolis carolinensis]"

Protein Accession	Unigene30533$_H1$
Mean Expression Ratio	0.947
Median Expression Ratio	0.945
Credible Interval	(0.649, 1.39)
Associated Peptides	2
Associated Spectra	5
Coverage	NaN

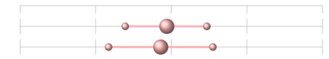

A	B	2.5	50	97.5	Sequence
1	3	0.66	0.96	1.4	LELAQYR
0	1	0.56	0.91	1.5	LTELLK

5.458 42 434 cytochrome b5 [Haliotis discus discus]; 42 434 cytochrome b5 [Haliotis discus discus]

Protein Accession	CL6985.Contig9$_H1$ CL6985.Contig5$_H1$
Mean Expression Ratio	1.06
Median Expression Ratio	1.06
Credible Interval	(0.713, 1.57)
Associated Peptides	3
Associated Spectra	3
Coverage	NaN

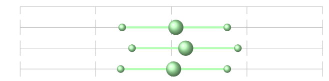

A	B	2.5	50	97.5	Sequence
1	0	0.64	1	1.7	KTFETNTGPVGR
1	0	0.7	1.1	1.8	VFRSEEVK
0	1	0.63	1	1.7	ELMKDYLIGK

5.459 248 2053 PDZ and LIM domain protein 5 [Crassostrea gigas]

Protein Accession	CL2142.Contig4$_H1$
Mean Expression Ratio	1.06
Median Expression Ratio	1.06
Credible Interval	(0.85, 1.32)
Associated Peptides	8
Associated Spectra	31
Coverage	NaN

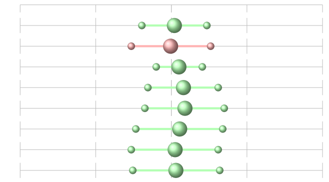

A	B	2.5	50	97.5	Sequence
2	3	0.76	1	1.4	CGLQTGDVILK
1	1	0.69	0.99	1.4	IGNTDTSALR
4	11	0.87	1.1	1.3	LDLLLQR
3	1	0.81	1.1	1.5	VNPGSLAAK
1	1	0.78	1.1	1.6	YLAPHCK
0	1	0.72	1.1	1.6	SVLTLEAK
0	1	0.69	1	1.5	LQTGDVILK
0	1	0.7	1	1.6	MDSTTPWGFR

5.460 142 588 AAEL009772-PB [Aedes aegypti]; 96 497 AAEL009772-PB [Aedes aegypti]

Protein Accession	Unigene73475$_H$1 CL27854.Contig2$_H$1
Mean Expression Ratio	0.948
Median Expression Ratio	0.946
Credible Interval	(0.588, 1.55)
Associated Peptides	1
Associated Spectra	3
Coverage	NaN

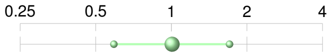

A	B	2.5	50	97.5	Sequence
**2**	**1**	**0.59**	**1**	**1.7**	**LVEEFPMGNIK**

5.461 2 511 hypothetical protein CGI_10006272 [Crassostrea gigas]

Protein Accession	CL10577.Contig2$_H$1
Mean Expression Ratio	1.06
Median Expression Ratio	1.06
Credible Interval	(0.65, 1.72)
Associated Peptides	1
Associated Spectra	1
Coverage	NaN

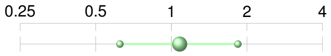

A	B	2.5	50	97.5	Sequence
**1**	**0**	**0.62**	**1.1**	**1.8**	**LLEGAVPK**

5.462 96 536 minus strand hypothetical protein BRAFLDRAFT_285353 [Branchiostoma floridae] gi

Protein Accession	CL14282.Contig5$_H$1
Mean Expression Ratio	0.949
Median Expression Ratio	0.946
Credible Interval	(0.594, 1.54)
Associated Peptides	1
Associated Spectra	1
Coverage	NaN

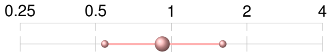

A	B	2.5	50	97.5	Sequence
**0**	**1**	**0.54**	**0.92**	**1.6**	**SQWSPALTISK**

5.463 78 461 PREDICTED: hoi-polloi-like [Saccoglossus kowalevskii]; 103 492 NHP2-like protein 1 OS=Rattus norvegicus GN=Nhp2l1 PE=2 SV=4

Protein Accession	CL26515.Contig5$_H$1 CL26515.Contig4$_H$1
Mean Expression Ratio	0.946
Median Expression Ratio	0.946
Credible Interval	(0.579, 1.53)
Associated Peptides	1
Associated Spectra	1
Coverage	NaN

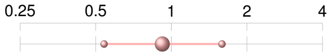

A	B	2.5	50	97.5	Sequence
**1**	**0**	**0.54**	**0.92**	**1.6**	**NVPYVFVR**

5.464 74 1243 Actin-related protein 2 [Crassostrea gigas]

Protein Accession	CL8614.Contig4$_{H}1$
Mean Expression Ratio	0.946
Median Expression Ratio	0.946
Credible Interval	(0.659, 1.36)
Associated Peptides	2
Associated Spectra	6
Coverage	NaN

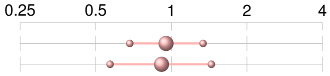

A	B	2.5	50	97.5	Sequence
**4**	**1**	**0.68**	**0.95**	**1.3**	**MVEVMFEK**
**0**	**1**	**0.57**	**0.91**	**1.4**	**DLMIGDEASQLR**

5.465 " 196 1053 minus strand Cytochrome b-c1 complex subunit 2, mitochondrial OS=Homo sapiens GN=UQCRC2 PE=1 SV=3"

Protein Accession	CL7847.Contig5$_{H}1$
Mean Expression Ratio	1.06
Median Expression Ratio	1.06
Credible Interval	(0.673, 1.64)
Associated Peptides	1
Associated Spectra	2
Coverage	NaN

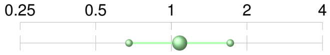

A	B	2.5	50	97.5	Sequence
**1**	**1**	**0.68**	**1.1**	**1.7**	**LDLAILK**

5.466 249 845 sigma class glutathione-s-transferase 2 [Haliotis discus discus]; 252 848 sigma class glutathione-s-transferase 2 [Haliotis discus discus]

Protein Accession	CL19285.Contig32$_{H}1$ CL19285.Contig20$_{H}1$
Mean Expression Ratio	1.06
Median Expression Ratio	1.06
Credible Interval	(0.646, 1.73)
Associated Peptides	1
Associated Spectra	1
Coverage	NaN

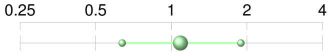

A	B	2.5	50	97.5	Sequence
**1**	**0**	**0.64**	**1.1**	**1.9**	**APFGQAPFMEYEGK**

5.467 1497 1889 UDP-glucuronosyltransferase 2C1 (Fragment) OS=Oryctolagus cuniculus GN=UGT2C1 PE=2 SV=1

Protein Accession	CL29849.Contig1$_{H}1$
Mean Expression Ratio	1.05
Median Expression Ratio	1.06
Credible Interval	(0.656, 1.7)
Associated Peptides	1
Associated Spectra	2
Coverage	NaN

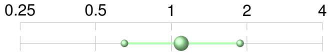

A	B	2.5	50	97.5	Sequence
**1**	**1**	**0.65**	**1.1**	**1.9**	**LFVSHCGK**

5.468 2764 3420 PREDICTED: filamin-C-like [Nasonia vitripennis]

Protein Accession	CL4145.Contig1$_{H}$1
Mean Expression Ratio	1.05
Median Expression Ratio	1.05
Credible Interval	(0.688, 1.61)
Associated Peptides	1
Associated Spectra	3
Coverage	NaN

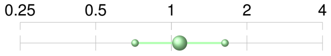

A	B	2.5	50	97.5	Sequence
**2**	**1**	**0.72**	**1.1**	**1.6**	**GVDVPVNTIDNK**

5.469 " 134 1567 minus strand 6-phosphogluconate dehydrogenase, decarboxylating [Crassostrea gigas]; 134 1573 minus strand 6-phosphogluconate dehydrogenase, decarboxylating [Crassostrea gigas]"

Protein Accession	CL5140.Contig5$_{H}$1 CL5140.Contig4$_{H}$1
Mean Expression Ratio	0.948
Median Expression Ratio	0.949
Credible Interval	(0.718, 1.25)
Associated Peptides	7
Associated Spectra	12
Coverage	NaN

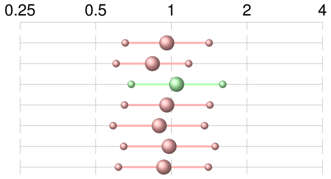

A	B	2.5	50	97.5	Sequence
**1**	**1**	**0.66**	**0.96**	**1.4**	**AGQAVDDFIDK**
**2**	**2**	**0.6**	**0.84**	**1.2**	**SLEDMVDKLK**
**1**	**0**	**0.69**	**1.1**	**1.6**	**SVFLGNIK**
**1**	**1**	**0.65**	**0.96**	**1.4**	**LVPLLEK**
**1**	**0**	**0.58**	**0.89**	**1.4**	**YGPSLMPGGAEEAWPAIK**
**0**	**1**	**0.65**	**0.98**	**1.5**	**NIFQSISAK**
**0**	**1**	**0.61**	**0.93**	**1.4**	**AFVEDIKR**

5.470 285 1637 PREDICTED: talin-2-like [Cricetulus griseus]

Protein Accession	CL18079.Contig3$_{H}$1
Mean Expression Ratio	0.951
Median Expression Ratio	0.95
Credible Interval	(0.586, 1.56)
Associated Peptides	1
Associated Spectra	1
Coverage	NaN

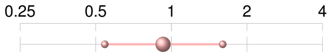

A	B	2.5	50	97.5	Sequence
**0**	**1**	**0.55**	**0.93**	**1.6**	**ISIVGQNVIK**

5.471 20 511 Neurexin-2-alpha [Crassostrea gigas]; 20 511 Neurexin-2-alpha [Crassostrea gigas]

Protein Accession	CL36998.Contig2$_{H}$1 CL36998.Contig1$_{H}$1
Mean Expression Ratio	0.948
Median Expression Ratio	0.95
Credible Interval	(0.579, 1.54)
Associated Peptides	1
Associated Spectra	1
Coverage	NaN

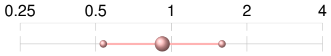

A	B	2.5	50	97.5	Sequence
**0**	**1**	**0.53**	**0.92**	**1.6**	**AVFLDGR**

5.472 1155 1649 78 kDa glucose-regulated protein OS=Aplysia californica PE=1 SV=1

Protein Accession	CL41594.Contig1$_H$1
Mean Expression Ratio	0.949
Median Expression Ratio	0.95
Credible Interval	(0.583, 1.53)
Associated Peptides	1
Associated Spectra	1
Coverage	NaN

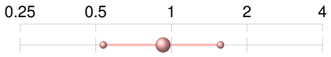

A	B	2.5	50	97.5	Sequence
**1**	**0**	**0.54**	**0.93**	**1.6**	**VEVESLFDGEDFSEQLTR**

5.473 93 1034 Annexin A7 [Crassostrea gigas]; 73 1014 Annexin A7 [Crassostrea gigas]

Protein Accession	CL718.Contig3$_H$1 CL718.Contig1$_H$1
Mean Expression Ratio	0.949
Median Expression Ratio	0.95
Credible Interval	(0.716, 1.25)
Associated Peptides	6
Associated Spectra	12
Coverage	NaN

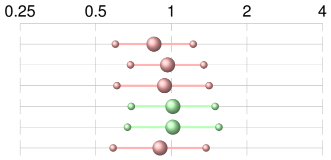

A	B	2.5	50	97.5	Sequence
**2**	**1**	**0.6**	**0.85**	**1.2**	**ESDEWEAGDDAK**
**2**	**2**	**0.69**	**0.96**	**1.4**	**LEVAQAFK**
**1**	**0**	**0.61**	**0.94**	**1.4**	**KYEQLTGR**
**1**	**1**	**0.69**	**1**	**1.5**	**SEIDMAEIKK**
**0**	**1**	**0.67**	**1**	**1.5**	**TGTVFPADHFDAGK**
**0**	**1**	**0.59**	**0.9**	**1.4**	**PADHFDAGK**

5.474 146 841 minus strand hypothetical protein CGI_10006609 [Crassostrea gigas]; 146 841 minus strand hypothetical protein CGI_10006609 [Crassostrea gigas]; 146 841 minus strand hypothetical protein CGI_10006609 [Crassostrea gigas]

Protein Accession	CL6681.Contig3$_H$1 CL6681.Contig2$_H$1 CL6681.Contig1$_H$1
Mean Expression Ratio	1.06
Median Expression Ratio	1.05
Credible Interval	(0.652, 1.72)
Associated Peptides	1
Associated Spectra	1
Coverage	NaN

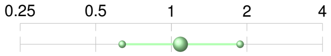

A	B	2.5	50	97.5	Sequence
**0**	**1**	**0.64**	**1.1**	**1.9**	**GHVEGLEVLPAR**

5.475 835 1116 tropomyosin [Ilyanassa obsoleta]

Protein Accession	CL2925.Contig8$_H$1
Mean Expression Ratio	1.05
Median Expression Ratio	1.05
Credible Interval	(0.805, 1.38)
Associated Peptides	4
Associated Spectra	15
Coverage	NaN

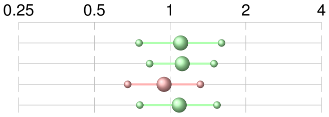

A	B	2.5	50	97.5	Sequence
**1**	**1**	**0.75**	**1.1**	**1.6**	**ELEEQSHALTNSLK**
**2**	**4**	**0.83**	**1.1**	**1.5**	**LAVMEAELEK**
**2**	**2**	**0.68**	**0.95**	**1.3**	**QLELYEEK**
**0**	**3**	**0.76**	**1.1**	**1.5**	**LEDVETELR**

5.476 1592 1768 minus strand beta-actin [Diabolocatantops pinguis]

Protein Accession	CL22741.Contig2$_H$1
Mean Expression Ratio	0.951
Median Expression Ratio	0.951
Credible Interval	(0.706, 1.28)
Associated Peptides	2
Associated Spectra	17
Coverage	NaN

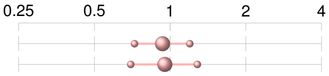

A	B	2.5	50	97.5	Sequence
**1**	**10**	**0.72**	**0.93**	**1.2**	**EISALAPPTMK**
**3**	**3**	**0.7**	**0.95**	**1.3**	**VIAPPER**

5.477 613 846 cytochrome c oxidase subunit Va [Antheraea yamamai] gi

Protein Accession	Unigene3832$_H$1
Mean Expression Ratio	1.05
Median Expression Ratio	1.05
Credible Interval	(0.652, 1.71)
Associated Peptides	1
Associated Spectra	1
Coverage	NaN

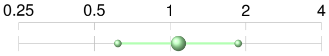

A	B	2.5	50	97.5	Sequence
**1**	**0**	**0.62**	**1.1**	**1.9**	**AMNDLQGEDLVPEPK**

5.478 2019 2723 minus strand Kyphoscoliosis peptidase [Crassostrea gigas]; 2060 2764 minus strand Kyphoscoliosis peptidase [Crassostrea gigas]; 1074 1778 minus strand Kyphoscoliosis peptidase [Crassostrea gigas]; 2063 2767 minus strand Kyphoscoliosis peptidase [Crassostrea gigas]; 2044 2748 minus strand Kyphoscoliosis peptidase [Crassostrea gigas]; 2044 2748 minus strand Kyphoscoliosis peptidase [Crassostrea gigas]

Protein Accession	CL3923.Contig7$_{H}1$ CL3923.Contig6$_{H}1$ CL3923.Contig5$_{H}1$ CL3923.Contig4$_{H}1$ CL3923.Con
Mean Expression Ratio	0.95
Median Expression Ratio	0.951
Credible Interval	(0.623, 1.45)
Associated Peptides	1
Associated Spectra	3
Coverage	NaN

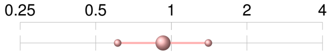

A	B	2.5	50	97.5	Sequence
3	0	0.61	0.93	1.4	TMGQAPPTVPK

5.479 689 1984 hypothetical protein BRAFLDRAFT_78257 [Branchiostoma floridae] gi

Protein Accession	Unigene34076$_{H}1$
Mean Expression Ratio	0.951
Median Expression Ratio	0.952
Credible Interval	(0.73, 1.23)
Associated Peptides	8
Associated Spectra	13
Coverage	NaN

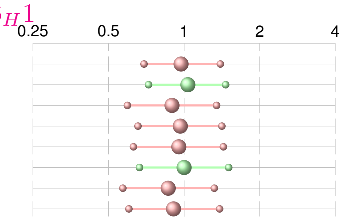

A	B	2.5	50	97.5	Sequence
2	1	0.69	0.97	1.4	TVGGFQLR
3	0	0.72	1	1.5	VQQKPEPSPAEGEVLIR
1	0	0.59	0.9	1.3	YLLFGSSNIVTGETK
1	1	0.66	0.97	1.4	YVSEIR
1	0	0.63	0.95	1.4	ILLDPSKAPTPK
0	1	0.66	1	1.5	SLVLTGFGSMK
0	1	0.57	0.86	1.3	GISILKPMGK
0	1	0.6	0.91	1.4	SFFSFAK

5.480 195 2201 minus strand hypothetical protein DAPPUDRAFT_315656 [Daphnia pulex]

Protein Accession	Unigene38538$_{H}1$
Mean Expression Ratio	0.951
Median Expression Ratio	0.952
Credible Interval	(0.712, 1.27)
Associated Peptides	6
Associated Spectra	9
Coverage	NaN

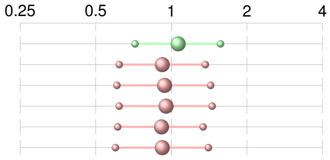

A	B	2.5	50	97.5	Sequence
1	1	0.72	1.1	1.6	FFTGQITAAGK
1	1	0.62	0.92	1.4	LLLSFGER
1	0	0.61	0.94	1.4	VALSPAATQALVK
1	0	0.62	0.95	1.5	VQIEAGAGEEAK
1	1	0.61	0.91	1.3	GPQFEEIGK
0	1	0.6	0.92	1.4	GSIVLHDGK

5.481 7 1722 minus strand Membrane metallo-endopeptidase-like 1 OS=Mus musculus GN=Mmel1 PE=1 SV=1

Protein Accession	CL19557.Contig1$_{H}1$
Mean Expression Ratio	0.953
Median Expression Ratio	0.952
Credible Interval	(0.804, 1.13)
Associated Peptides	15
Associated Spectra	41
Coverage	NaN

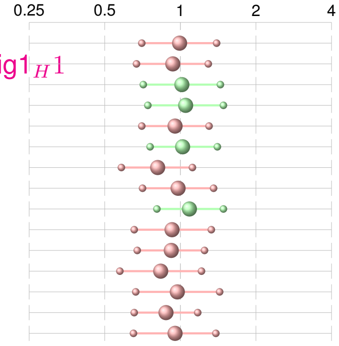

A	B	2.5	50	97.5	Sequence
1	1	0.7	0.99	1.4	AVLIEAIR
3	0	0.67	0.93	1.3	HQVENPNLHEVTSLADLEK
1	1	0.71	1	1.4	IGYPDYIMDDLR
1	1	0.74	1	1.5	LFVLNSFDQNQR
3	1	0.7	0.95	1.3	QDAQDVADFK
3	1	0.76	1	1.4	TLVENIADNAGLK
2	1	0.58	0.81	1.1	WPVSAAAVK
2	1	0.71	0.98	1.4	WLTDASK
4	0	0.81	1.1	1.5	LFPEVTGR
2	0	0.65	0.93	1.3	GLELFR
2	2	0.68	0.92	1.2	MNIDGYR
1	0	0.57	0.84	1.2	AQALNAK
1	0	0.67	0.97	1.4	QTALDK
2	3	0.65	0.87	1.2	LQLAEISLPK
0	1	0.65	0.96	1.4	LTDASKQTALDK

5.482 280 1239 minus strand PREDICTED: smoothelin isoform 2 [Oryctolagus cuniculus]; 280 1239 minus strand PREDICTED: smoothelin isoform 2 [Oryctolagus cuniculus]; 280 1239 PREDICTED: smoothelin isoform 2 [Oryctolagus cuniculus]; 280 1239 minus strand PREDICTED: smoothelin isoform 2 [Oryctolagus cuniculus]; 280 1239 PREDICTED: smoothelin isoform 2 [Oryctolagus cuniculus]; 280 1239 minus strand PREDICTED: smoothelin isoform 2 [Oryctolagus cuniculus]; 280 1239 minus strand PREDICTED: smoothelin isoform 2 [Oryctolagus cuniculus]

Protein Accession	CL2374.Contig7$_{H}1$ CL2374.Contig6$_{H}1$ CL2374.Contig5$_{H}1$ CL2374.Contig4$_{H}1$ CL2374.Con
Mean Expression Ratio	0.951
Median Expression Ratio	0.953
Credible Interval	(0.677, 1.32)
Associated Peptides	4
Associated Spectra	6
Coverage	NaN

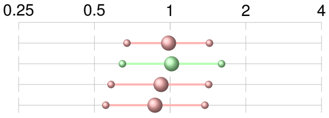

A	B	2.5	50	97.5	Sequence
2	1	0.68	0.98	1.4	HNFQLAFDTAEK
1	0	0.65	1	1.6	LMNATDSFDER
1	0	0.58	0.92	1.4	EKETEDLVR
0	1	0.55	0.87	1.4	PLLDVEDMVK

5.483 187 696 minus strand putative ribosomal protein S10 [Sipunculus nudus]

Protein Accession	Unigene1233$_{H}1$
Mean Expression Ratio	0.955
Median Expression Ratio	0.953
Credible Interval	(0.585, 1.55)
Associated Peptides	1
Associated Spectra	3
Coverage	NaN

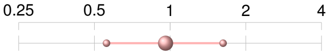

A	B	2.5	50	97.5	Sequence
2	1	0.56	0.96	1.6	KDFFSPK

5.484 165 377 Cysteine and glycine-rich protein 3 [Crassostrea gigas]; 135 344 Cysteine and glycine-rich protein 3 [Crassostrea gigas]

Protein Accession	CL38347.Contig2$_H$1 CL38347.Contig1$_H$1
Mean Expression Ratio	0.951
Median Expression Ratio	0.953
Credible Interval	(0.605, 1.48)
Associated Peptides	1
Associated Spectra	2
Coverage	NaN

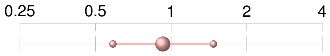

A	B	2.5	50	97.5	Sequence
**1**	**1**	**0.59**	**0.93**	**1.5**	**VEAGNIPFHK**

5.485 130 2145 minus strand Glucosamine–fructose-6-phosphate aminotransferase [isomerizing] 1 [Crassostrea gigas]; 130 2121 minus strand Glucosamine–fructose-6-phosphate aminotransferase [isomerizing] 1 [Crassostrea gigas]

Protein Accession	CL2589.Contig3$_H$1 CL2589.Contig2$_H$1
Mean Expression Ratio	0.952
Median Expression Ratio	0.954
Credible Interval	(0.643, 1.42)
Associated Peptides	3
Associated Spectra	3
Coverage	NaN

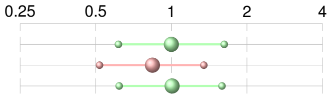

A	B	2.5	50	97.5	Sequence
**1**	**0**	**0.62**	**1**	**1.6**	**KLPDQIKDVLK**
**0**	**1**	**0.52**	**0.84**	**1.4**	**HGPLALVDK**
**0**	**1**	**0.62**	**1**	**1.6**	**VANLEAQIK**

5.486 154 621 minus strand Stress response protein nhaX [Crassostrea gigas]

Protein Accession	CL4265.Contig1$_H$1
Mean Expression Ratio	1.05
Median Expression Ratio	1.05
Credible Interval	(0.746, 1.49)
Associated Peptides	5
Associated Spectra	5
Coverage	NaN

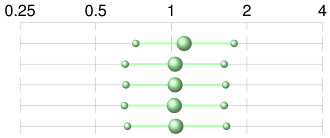

A	B	2.5	50	97.5	Sequence
**0**	**1**	**0.72**	**1.1**	**1.8**	**IQHDLEK**
**0**	**1**	**0.66**	**1**	**1.6**	**SVAAAKPGEGILK**
**0**	**1**	**0.66**	**1**	**1.6**	**EVLEAMLK**
**0**	**1**	**0.65**	**1**	**1.6**	**PGEGILK**
**0**	**1**	**0.67**	**1**	**1.7**	**EGILK**

5.487 102 1475 minus strand Cytosolic non-specific dipeptidase [Crassostrea gigas]

Protein Accession	CL4706.Contig1$_H$1
Mean Expression Ratio	0.954
Median Expression Ratio	0.954
Credible Interval	(0.594, 1.56)
Associated Peptides	1
Associated Spectra	1
Coverage	NaN

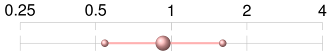

A	B	2.5	50	97.5	Sequence
**1**	**0**	**0.54**	**0.93**	**1.6**	**LVPDQEPHVIESLVR**

5.488 120 635 PREDICTED: similar to collagen type VI alpha 6 [Ciona intestinalis]; 120 635 minus strand PREDICTED: similar to collagen type VI alpha 6 [Ciona intestinalis]

Protein Accession	Unigene46785$_H$1 Unigene26613$_H$1
Mean Expression Ratio	0.958
Median Expression Ratio	0.955
Credible Interval	(0.627, 1.49)
Associated Peptides	2
Associated Spectra	2
Coverage	NaN

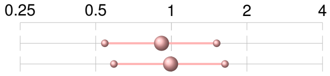

A	B	2.5	50	97.5	Sequence
**1**	**0**	**0.54**	**0.91**	**1.5**	**HGNTNTGDGIMYMLER**
**1**	**0**	**0.59**	**0.99**	**1.6**	**VSLVTYSDQANPESAIDFDSYSSK**

5.489 362 1084 Ectoine hydroxylase [Crassostrea gigas]; 362 1084 Ectoine hydroxylase [Crassostrea gigas]; 366 1220 Ectoine hydroxylase [Crassostrea gigas]

Protein Accession	CL2342.Contig4$_H$1 CL2342.Contig3$_H$1 CL2342.Contig2$_H$1
Mean Expression Ratio	1.05
Median Expression Ratio	1.05
Credible Interval	(0.643, 1.72)
Associated Peptides	1
Associated Spectra	1
Coverage	NaN

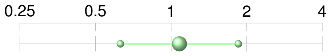

A	B	2.5	50	97.5	Sequence
**1**	**0**	**0.63**	**1.1**	**1.9**	**NFVLTDEMK**

5.490 793 1269 PREDICTED: calmodulin-like [Saccoglossus kowalevskii]

Protein Accession	CL42648.Contig1$_{H}$1
Mean Expression Ratio	1.05
Median Expression Ratio	1.05
Credible Interval	(0.76, 1.44)
Associated Peptides	5
Associated Spectra	7
Coverage	NaN

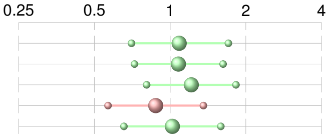

A	B	2.5	50	97.5	Sequence
1	0	0.7	1.1	1.7	EADKDGNGLSIQELASLIR
1	1	0.72	1.1	1.6	KVSLVDITDEEIIK
2	0	0.81	1.2	1.8	KIQESELK
0	1	0.57	0.88	1.4	LAEADVDR
0	1	0.65	1	1.6	IFLDLDDNCDR

5.491 30 536 putative mitochondrial ATP synthase [Haliotis discus discus]; 27 620 putative mitochondrial ATP synthase [Haliotis discus discus]

Protein Accession	CL13014.Contig4$_{H}$1 CL13014.Contig1$_{H}$1
Mean Expression Ratio	0.953
Median Expression Ratio	0.955
Credible Interval	(0.684, 1.33)
Associated Peptides	2
Associated Spectra	8
Coverage	NaN

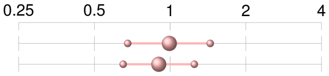

A	B	2.5	50	97.5	Sequence
2	1	0.68	0.99	1.4	LANPALVDQFQK
2	3	0.65	0.9	1.3	SAVDWVAFK

5.492 " 1 1563 Putative methylmalonate-semialdehyde dehydrogenase [acylating], mitochondrial [Crassostrea gigas]"

Protein Accession	Unigene56839$_{H}$1
Mean Expression Ratio	1.05
Median Expression Ratio	1.05
Credible Interval	(0.763, 1.44)
Associated Peptides	6
Associated Spectra	7
Coverage	NaN

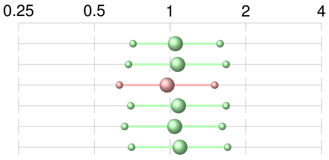

A	B	2.5	50	97.5	Sequence
1	1	0.71	1.1	1.6	AEMDAAVAAAK
1	0	0.68	1.1	1.7	GNFVGPTILHK
1	0	0.63	0.98	1.5	LFINNEFVESK
1	0	0.7	1.1	1.7	WIDVHNPATNEVVTR
1	0	0.66	1	1.6	TVTQMWR
0	1	0.7	1.1	1.7	TLVDAEGDVLR

5.493 104 445 minus strand macrophage migration inhibitory factor [Ascaris suum]

Protein Accession	CL34536.Contig3$_{H}$1
Mean Expression Ratio	0.956
Median Expression Ratio	0.956
Credible Interval	(0.684, 1.34)
Associated Peptides	4
Associated Spectra	6
Coverage	NaN

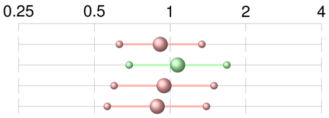

A	B	2.5	50	97.5	Sequence
2	1	0.63	0.91	1.3	HIEEALGIPQDR
1	0	0.69	1.1	1.7	PVFILNTNLPADK
1	0	0.6	0.95	1.5	QLGKPESYVCVR
0	1	0.56	0.89	1.4	LVVPFMK

5.494 1265 1831 minus strand Adenosine deaminase CECR1 [Crassostrea gigas]

Protein Accession	CL20850.Contig11$_{H}1$
Mean Expression Ratio	0.957
Median Expression Ratio	0.956
Credible Interval	(0.593, 1.55)
Associated Peptides	1
Associated Spectra	1
Coverage	NaN

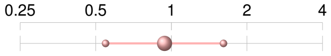

A	B	2.5	50	97.5	Sequence
0	1	0.55	0.94	1.6	GIAVEVNPISNQVLK

5.495 552 1499 minus strand uncharacterized protein LOC100158466 [Xenopus (Silurana) tropicalis] gi; 552 1499 minus strand uncharacterized protein LOC100158466 [Xenopus (Silurana) tropicalis] gi; 552 1499 minus strand uncharacterized protein LOC100158466 [Xenopus (Silurana) tropicalis] gi

Protein Accession	Unigene57645$_{H}1$ Unigene52825$_{H}1$ Unigene48504$_{H}1$
Mean Expression Ratio	0.959
Median Expression Ratio	0.957
Credible Interval	(0.595, 1.55)
Associated Peptides	1
Associated Spectra	2
Coverage	NaN

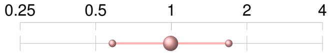

A	B	2.5	50	97.5	Sequence
1	1	0.58	0.99	1.7	TGEPVEVDGELATLLAR

5.496 214 1878 PREDICTED: lysyl-tRNA synthetase-like [Oreochromis niloticus]

Protein Accession	CL25588.Contig2$_{H}1$
Mean Expression Ratio	0.957
Median Expression Ratio	0.957
Credible Interval	(0.591, 1.55)
Associated Peptides	1
Associated Spectra	1
Coverage	NaN

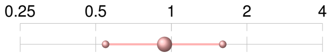

A	B	2.5	50	97.5	Sequence
0	1	0.55	0.94	1.6	ISLISDLEK

5.497 " 451 1026 PREDICTED: GTP-binding protein SAR1B, putative-like [Saccoglossus kowalevskii]"

Protein Accession	Unigene4124$_{H}1$
Mean Expression Ratio	1.04
Median Expression Ratio	1.05
Credible Interval	(0.642, 1.7)
Associated Peptides	1
Associated Spectra	1
Coverage	NaN

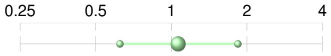

A	B	2.5	50	97.5	Sequence
1	0	0.62	1.1	1.8	AELDSLLTDEQVANAPILVLGNK

5.498 3 4517 minus strand Clathrin heavy chain 1 [Crassostrea gigas]

Protein Accession	CL16446.Contig1$_H$1
Mean Expression Ratio	1.04
Median Expression Ratio	1.04
Credible Interval	(0.806, 1.34)
Associated Peptides	8
Associated Spectra	13
Coverage	NaN

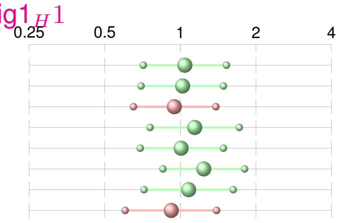

A	B	2.5	50	97.5	Sequence
2	0	0.71	1	1.5	KFEVNTSAIK
2	0	0.7	1	1.5	AQLNQGMVK
1	1	0.65	0.95	1.4	AGLLQR
1	0	0.76	1.1	1.7	KFNTLFQSGQYQEAAK
1	1	0.69	1	1.5	FLQMAR
2	0	0.85	1.2	1.8	VDGAR
1	0	0.72	1.1	1.6	NFLKEAK
0	1	0.6	0.92	1.4	AVNFFIK

5.499 134 385 minus strand CDGSH iron sulfur domain-containing protein 2 [Crassostrea gigas]; 11 511 minus strand CDGSH iron sulfur domain-containing protein 2 [Crassostrea gigas]

Protein Accession	CL36832.Contig5$_H$1 CL36832.Contig4$_H$1
Mean Expression Ratio	1.04
Median Expression Ratio	1.04
Credible Interval	(0.641, 1.68)
Associated Peptides	1
Associated Spectra	1
Coverage	NaN

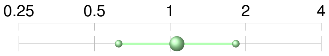

A	B	2.5	50	97.5	Sequence
1	0	0.63	1.1	1.8	VVNMVDIEDLGDK

5.500 145 1020 minus strand PREDICTED: inorganic pyrophosphatase-like [Saccoglossus kowalevskii]

Protein Accession	CL12968.Contig14$_H$1
Mean Expression Ratio	1.04
Median Expression Ratio	1.04
Credible Interval	(0.785, 1.39)
Associated Peptides	6
Associated Spectra	10
Coverage	NaN

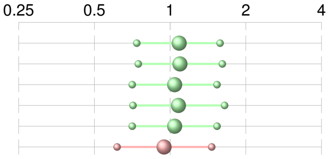

A	B	2.5	50	97.5	Sequence
1	1	0.74	1.1	1.6	AEVIVQESK
1	1	0.75	1.1	1.6	IIAIDVTDPLASK
1	1	0.71	1	1.5	LNDIDDVHR
1	0	0.71	1.1	1.6	VLGTMLLVDEGETDWK
1	1	0.7	1	1.5	EMPGFLR
0	1	0.62	0.94	1.5	VFFR

5.501 1732 2079 minus strand PREDICTED: annexin A7-like [Ornithorhynchus anatinus]

Protein Accession	CL9274.Contig2$_H$1
Mean Expression Ratio	0.958
Median Expression Ratio	0.958
Credible Interval	(0.586, 1.56)
Associated Peptides	1
Associated Spectra	1
Coverage	NaN

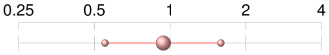

A	B	2.5	50	97.5	Sequence
1	0	0.55	0.94	1.6	DLLSAISSETSGDFETGLR

5.502 194 775 minus strand Collagen alpha-1(XV) chain [Harpegnathos saltator]

Protein Accession: Unigene19084$_{H}1$
Mean Expression Ratio: 1.04
Median Expression Ratio: 1.04
Credible Interval: (0.676, 1.62)
Associated Peptides: 2
Associated Spectra: 2
Coverage: NaN

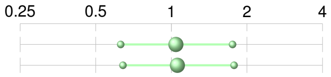

A	B	2.5	50	97.5	Sequence
1	0	0.63	1	1.8	GADYECHK
1	0	0.64	1.1	1.8	IIWHGSSK

5.503 90 659 Plectin-1 [Crassostrea gigas]; 327 881 Plectin-1 [Crassostrea gigas]; 646 1290 minus strand Plectin-1 [Crassostrea gigas]

Protein Accession: CL7983.Contig3$_{H}1$ CL7983.Contig2$_{H}1$ CL7983.Contig1$_{H}1$
Mean Expression Ratio: 0.957
Median Expression Ratio: 0.958
Credible Interval: (0.59, 1.55)
Associated Peptides: 1
Associated Spectra: 1
Coverage: NaN

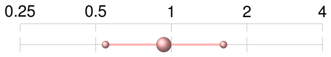

A	B	2.5	50	97.5	Sequence
0	1	0.55	0.94	1.6	AQHLVNVEEAR

5.504 158 862 X-box binding protein [Haliotis discus discus]

Protein Accession: CL6396.Contig3$_{H}1$
Mean Expression Ratio: 0.959
Median Expression Ratio: 0.958
Credible Interval: (0.745, 1.25)
Associated Peptides: 6
Associated Spectra: 18
Coverage: NaN

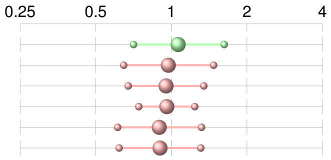

A	B	2.5	50	97.5	Sequence
1	0	0.71	1.1	1.6	GSISDPSVFNVPEACQK
1	0	0.65	0.98	1.5	VFPDFYFGAGDDK
3	0	0.68	0.95	1.3	ACIPDK
5	4	0.74	0.96	1.2	LYIVDLAK
1	1	0.61	0.89	1.3	SYFILK
2	0	0.62	0.9	1.3	SYYIGAR

5.505 6043 6174 myosin heavy chain [Mytilus galloprovincialis]; 5869 6000 myosin heavy chain [Mytilus galloprovincialis]

Protein Accession: Unigene19025$_{H}1$ CL49594.Contig1$_{H}1$
Mean Expression Ratio: 0.96
Median Expression Ratio: 0.958
Credible Interval: (0.676, 1.37)
Associated Peptides: 1
Associated Spectra: 12
Coverage: NaN

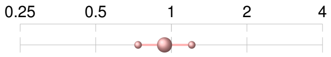

A	B	2.5	50	97.5	Sequence
12	0	0.74	0.94	1.2	KLEADIQAMQTDLDEMNGELR

5.506 227 2920 minus strand Lysosomal alpha-mannosidase [Crassostrea gigas]; 227 3073 minus strand Lysosomal alpha-mannosidase [Crassostrea gigas]; 193 2886 minus strand Lysosomal alpha-mannosidase [Crassostrea gigas]

Protein Accession	CL2930.Contig3$_{H}1$ CL2930.Contig2$_{H}1$ CL2930.Contig1$_{H}1$
Mean Expression Ratio	0.96
Median Expression Ratio	0.958
Credible Interval	(0.693, 1.33)
Associated Peptides	5
Associated Spectra	8
Coverage	NaN

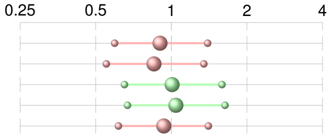

A	B	2.5	50	97.5	Sequence
2	1	0.59	0.9	1.4	VASELIPITPDTFR
0	1	0.55	0.86	1.3	PDNVHLLTLEQFR
0	1	0.65	1	1.6	ALPDNVHLLTLEQFR
0	1	0.67	1	1.6	QLNHR
0	2	0.62	0.93	1.4	AGASLTWSGVR

5.507 58 732 glutathione-s-transferase [Haliotis discus discus]

Protein Accession	CL4861.Contig1$_{H}1$
Mean Expression Ratio	1.04
Median Expression Ratio	1.04
Credible Interval	(0.732, 1.49)
Associated Peptides	4
Associated Spectra	5
Coverage	NaN

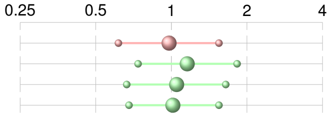

A	B	2.5	50	97.5	Sequence
0	1	0.61	0.98	1.5	IDKEYLEVK
0	1	0.74	1.2	1.8	GQVPTFR
0	1	0.66	1	1.7	WEEYLR
0	2	0.68	1	1.6	LISFSANEHK

5.508 22 870 minus strand PREDICTED: alcohol dehydrogenase class-3 [Sarcophilus harrisii]

Protein Accession	Unigene27690$_{H}1$
Mean Expression Ratio	1.04
Median Expression Ratio	1.04
Credible Interval	(0.795, 1.37)
Associated Peptides	6
Associated Spectra	14
Coverage	NaN

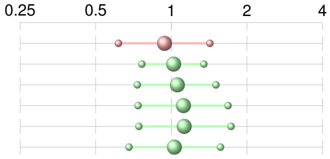

A	B	2.5	50	97.5	Sequence
1	0	0.61	0.94	1.4	AALEACHK
5	2	0.76	1	1.3	AAVAWEAK
1	2	0.73	1.1	1.5	IIGIDVNQDK
1	0	0.74	1.1	1.7	KPLVIEDIEVAPPAAGEVR
1	0	0.74	1.1	1.7	TNLCQK
0	1	0.68	1	1.6	FAMAK

5.509 768 980 muscle LIM protein isoform 1 [Danaus plexippus]

Protein Accession	CL29824.Contig1$_{H}1$
Mean Expression Ratio	0.959
Median Expression Ratio	0.959
Credible Interval	(0.742, 1.25)
Associated Peptides	4
Associated Spectra	31
Coverage	NaN

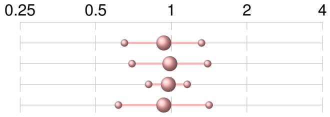

A	B	2.5	50	97.5	Sequence
3	0	0.65	0.93	1.3	SLDSTTMAMHETEVYCK
2	1	0.7	0.99	1.4	GVNTPWHK
18	6	0.81	0.97	1.2	AIGVNTPWHK
1	0	0.62	0.93	1.4	NFGPSGYGFGQGAGT

5.510 " 169 792 minus strand rho GDP dissociation inhibitor, putative [Ixodes scapularis] gi"

Protein Accession	CL39798.Contig1$_H$1
Mean Expression Ratio	1.04
Median Expression Ratio	1.04
Credible Interval	(0.727, 1.49)
Associated Peptides	3
Associated Spectra	5
Coverage	NaN

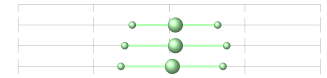

A	B	2.5	50	97.5	Sequence
2	1	0.71	1.1	1.6	IIIDDSNPLNVIVK
1	0	0.66	1.1	1.7	LAISVEGRPDAELDLTKPK
0	1	0.64	1	1.6	DIVAGLR

5.511 58 2268 hypothetical protein BRAFLDRAFT_267717 [Branchiostoma floridae] gi; 62 2245 hypothetical protein BRAFLDRAFT_267717 [Branchiostoma floridae] gi

Protein Accession	CL26176.Contig18$_H$1 CL26176.Contig17$_H$1
Mean Expression Ratio	0.958
Median Expression Ratio	0.959
Credible Interval	(0.588, 1.57)
Associated Peptides	1
Associated Spectra	1
Coverage	NaN

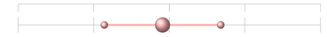

A	B	2.5	50	97.5	Sequence
1	0	0.55	0.94	1.6	ALITIANK

5.512 323 1192 cytosolic malate dehydrogenase [Lottia scabra]; 323 1192 cytosolic malate dehydrogenase [Lottia scabra]; 323 1192 cytosolic malate dehydrogenase [Lottia scabra]; 323 1192 cytosolic malate dehydrogenase [Lottia scabra]; 2474 3343 cytosolic malate dehydrogenase [Lottia scabra]; 116 1111 minus strand cytosolic malate dehydrogenase [Lottia scabra]

Protein Accession	CL280.Contig21$_H$1 CL280.Contig20$_H$1 CL280.Contig19$_H$1 CL280.Contig18$_H$1 CL280.Conti
Mean Expression Ratio	1.04
Median Expression Ratio	1.04
Credible Interval	(0.878, 1.23)
Associated Peptides	12
Associated Spectra	110
Coverage	NaN

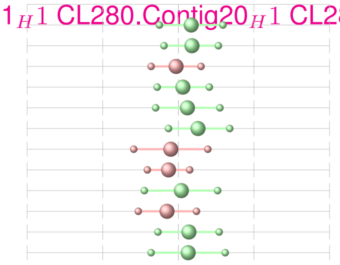

A	B	2.5	50	97.5	Sequence
3	3	0.84	1.1	1.5	EAVKDDNWINNEFISTVQK
6	5	0.88	1.1	1.4	ENFSCLTR
15	4	0.78	0.98	1.2	KLSSAMSAAK
6	6	0.82	1	1.3	LGIPNNAVSK
3	3	0.81	1.1	1.5	LSSAMSAAK
6	2	0.91	1.2	1.6	SQGANLDAVAK
2	1	0.66	0.94	1.3	YGIPEGVMYSYPVQIK
9	14	0.75	0.91	1.1	EMAEQILAA
2	1	0.73	1	1.4	ELLEER
5	4	0.69	0.9	1.2	DCDAAFLVGAMPR
4	3	0.83	1.1	1.5	DLLAANIK
2	1	0.78	1.1	1.5	YAPSIPK

5.513 977 1852 PREDICTED: similar to prohibitin [Tribolium castaneum] gi

Protein Accession	CL8219.Contig4$_{H}1$
Mean Expression Ratio	0.962
Median Expression Ratio	0.961
Credible Interval	(0.661, 1.4)
Associated Peptides	3
Associated Spectra	4
Coverage	NaN

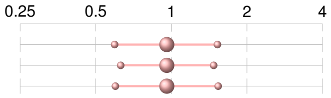

A	B	2.5	50	97.5	Sequence
1	0	0.59	0.96	1.5	VVQAEGEAAAAK
2	0	0.63	0.96	1.5	KLGDLAGR
1	0	0.6	0.96	1.5	QQVSLLIR

5.514 7825 8754 minus strand H2 [Haliotis tuberculata]

Protein Accession	CL7906.Contig3$_{H}1$
Mean Expression Ratio	1.04
Median Expression Ratio	1.04
Credible Interval	(0.632, 1.68)
Associated Peptides	1
Associated Spectra	1
Coverage	NaN

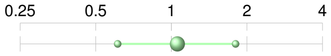

A	B	2.5	50	97.5	Sequence
1	0	0.61	1.1	1.8	ALPSLFTDPYDSNPFK

5.515 " 112 1320 Aspartate aminotransferase, cytoplasmic [Crassostrea gigas]"

Protein Accession	CL44136.Contig1$_{H}1$
Mean Expression Ratio	1.04
Median Expression Ratio	1.04
Credible Interval	(0.837, 1.29)
Associated Peptides	9
Associated Spectra	35
Coverage	NaN

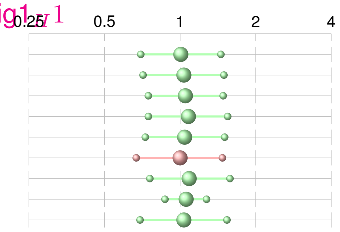

A	B	2.5	50	97.5	Sequence
1	1	0.7	1	1.5	ASIFADVK
1	1	0.71	1	1.5	IADVMEAK
2	1	0.75	1.1	1.5	SQMEIIVR
2	0	0.75	1.1	1.6	TPSVIPQIR
2	0	0.73	1	1.5	VNLGVGAYR
1	0	0.67	1	1.5	ISMAGVTNK
2	0	0.76	1.1	1.6	LAAEFLK
13	7	0.87	1.1	1.3	VEGIQVQGGTGGIR
0	1	0.69	1	1.5	TLANALILGK

5.516 219 554 minus strand ribosomal protein P1 [Biomphalaria glabrata]

Protein Accession	CL45629.Contig2$_{H}1$
Mean Expression Ratio	1.04
Median Expression Ratio	1.04
Credible Interval	(0.643, 1.7)
Associated Peptides	1
Associated Spectra	1
Coverage	NaN

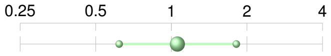

A	B	2.5	50	97.5	Sequence
1	0	0.62	1.1	1.8	ALEGVNIK

5.517 460 1737 Tubulin beta-4B chain OS=Mus musculus GN=Tubb4b PE=1 SV=1

Protein Accession	Unigene34147$_{H1}$
Mean Expression Ratio	0.963
Median Expression Ratio	0.963
Credible Interval	(0.665, 1.41)
Associated Peptides	1
Associated Spectra	8
Coverage	NaN

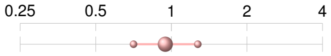

A	B	2.5	50	97.5	Sequence
**3**	**5**	**0.71**	**0.95**	**1.3**	**AVLVDLEPGTMDSVR**

5.518 2 211 minus strand sulfatase 1A precursor [Haliotis discus discus]

Protein Accession	Unigene22814$_{H1}$
Mean Expression Ratio	1.04
Median Expression Ratio	1.04
Credible Interval	(0.673, 1.62)
Associated Peptides	1
Associated Spectra	2
Coverage	NaN

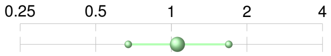

A	B	2.5	50	97.5	Sequence
**2**	**0**	**0.67**	**1.1**	**1.7**	**CGMVSAMDESIGK**

5.519 " 1 510 Glycerol-3-phosphate dehydrogenase [NAD+], cytoplasmic [Crassostrea gigas]"

Protein Accession	Unigene16246$_{H1}$
Mean Expression Ratio	0.964
Median Expression Ratio	0.963
Credible Interval	(0.641, 1.45)
Associated Peptides	1
Associated Spectra	4
Coverage	NaN

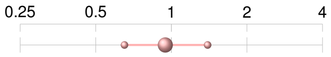

A	B	2.5	50	97.5	Sequence
**2**	**2**	**0.65**	**0.95**	**1.4**	**EIFGMLEK**

5.520 253 765 cytochrome c oxidase subunit IV [Urechis caupo]

Protein Accession	CL38636.Contig3$_{H1}$
Mean Expression Ratio	1.04
Median Expression Ratio	1.04
Credible Interval	(0.694, 1.56)
Associated Peptides	2
Associated Spectra	3
Coverage	NaN

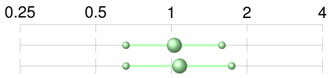

A	B	2.5	50	97.5	Sequence
**2**	**0**	**0.66**	**1**	**1.6**	**FKENTPDVMALR**
**1**	**0**	**0.66**	**1.1**	**1.7**	**SLTLEDKK**

5.521 36 770 minus strand Endoplasmic reticulum protein ERp29 [Crassostrea gigas]

Protein Accession	CL37188.Contig1$_H$1
Mean Expression Ratio	1.04
Median Expression Ratio	1.04
Credible Interval	(0.676, 1.59)
Associated Peptides	2
Associated Spectra	2
Coverage	NaN

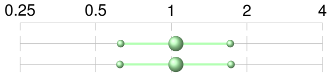

A	B	2.5	50	97.5	Sequence
**1**	**0**	**0.63**	**1**	**1.7**	**KAQTAAAALTK**
**1**	**0**	**0.62**	**1**	**1.7**	**KPDFPVYK**

5.522 143 247 minus strand dynein light chain [Aplysia californica] gi

Protein Accession	CL13886.Contig1$_H$1
Mean Expression Ratio	1.04
Median Expression Ratio	1.04
Credible Interval	(0.661, 1.63)
Associated Peptides	1
Associated Spectra	2
Coverage	NaN

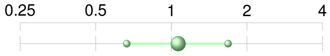

A	B	2.5	50	97.5	Sequence
**2**	**0**	**0.66**	**1.1**	**1.7**	**NADMAEDMQQDAVDCATQALEK**

5.523 36 1310 minus strand elongation factor-1 gamma [Diguetia canities]

Protein Accession	CL14269.Contig5$_H$1
Mean Expression Ratio	0.962
Median Expression Ratio	0.963
Credible Interval	(0.673, 1.38)
Associated Peptides	3
Associated Spectra	5
Coverage	NaN

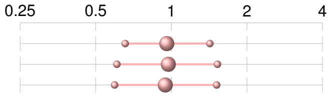

A	B	2.5	50	97.5	Sequence
**2**	**1**	**0.65**	**0.96**	**1.4**	**LAETMAQFDAK**
**1**	**0**	**0.61**	**0.97**	**1.5**	**SADFLAK**
**0**	**1**	**0.6**	**0.94**	**1.5**	**LDPDDPETK**

5.524 77 2278 Far upstream element-binding protein 3 [Crassostrea gigas]

Protein Accession	CL5030.Contig3$_H$1
Mean Expression Ratio	1.04
Median Expression Ratio	1.04
Credible Interval	(0.64, 1.67)
Associated Peptides	1
Associated Spectra	1
Coverage	NaN

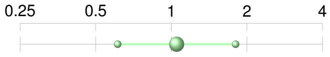

A	B	2.5	50	97.5	Sequence
**1**	**0**	**0.61**	**1.1**	**1.8**	**LFNIR**

5.525 416 1717 minus strand T-complex protein 1 subunit delta OS=Aedes triseriatus PE=2 SV=1

Protein Accession CL8566.Contig1$_{H}1$
Mean Expression Ratio 0.965
Median Expression Ratio 0.964
Credible Interval (0.593, 1.57)
Associated Peptides 1
Associated Spectra 1
Coverage NaN

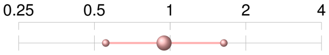

A	B	2.5	50	97.5	Sequence
0	1	0.55	0.95	1.6	HAAGELTAGINVR

5.526 266 934 minus strand Kyphoscoliosis peptidase [Crassostrea gigas]

Protein Accession CL7490.Contig16$_{H}1$
Mean Expression Ratio 1.04
Median Expression Ratio 1.04
Credible Interval (0.879, 1.22)
Associated Peptides 11
Associated Spectra 65
Coverage NaN

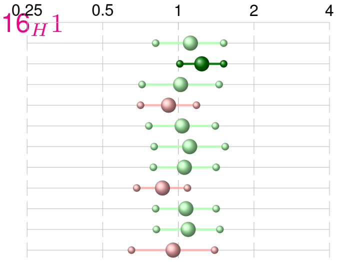

A	B	2.5	50	97.5	Sequence
3	1	0.81	1.1	1.5	ASLNANYGPDESK
8	8	1	1.2	1.5	DLDNVHFK
2	0	0.72	1	1.5	DTVSLVVTLPDSGYYK
5	3	0.71	0.91	1.2	ELVFTQTER
3	1	0.76	1	1.4	LQIYALPSSDDSK
2	1	0.8	1.1	1.5	VNIPGAK
4	1	0.79	1.1	1.4	VTSNLIACGTEQEYK
8	3	0.68	0.86	1.1	YSSLLEYDL
5	1	0.81	1.1	1.4	QYAQWK
4	1	0.82	1.1	1.5	SRPELPHGYLGAQSK
0	1	0.65	0.95	1.4	GLKDLDNVHFK

5.527 205 1341 collagen precursor [Bombyx mori]

Protein Accession CL38816.Contig2$_{H}1$
Mean Expression Ratio 0.962
Median Expression Ratio 0.964
Credible Interval (0.589, 1.56)
Associated Peptides 1
Associated Spectra 1
Coverage NaN

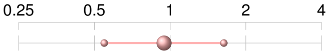

A	B	2.5	50	97.5	Sequence
0	1	0.55	0.95	1.6	GQLIPHIQK

5.528 26 877 Ankyrin-2 [Crassostrea gigas]

Protein Accession CL18051.Contig2$_{H}1$
Mean Expression Ratio 0.965
Median Expression Ratio 0.964
Credible Interval (0.645, 1.43)
Associated Peptides 2
Associated Spectra 4
Coverage NaN

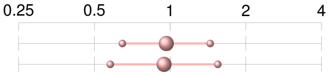

A	B	2.5	50	97.5	Sequence
2	1	0.64	0.97	1.4	LPALHIAAK
0	1	0.58	0.94	1.5	DGLTPLHCAAR

5.529 201 632 USP-like protein isoform 1 [Crassostrea ariakensis] gi; 189 620 minus strand USP-like protein isoform 1 [Crassostrea ariakensis] gi

Protein Accession	Unigene39588$_{H}$1 Unigene20286$_{H}$1
Mean Expression Ratio	0.963
Median Expression Ratio	0.965
Credible Interval	(0.594, 1.56)
Associated Peptides	1
Associated Spectra	1
Coverage	NaN

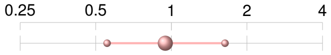

A	B	2.5	50	97.5	Sequence
**1**	**0**	**0.55**	**0.94**	**1.6**	**DPWHAIVDAAK**

5.530 152 1930 hypothetical protein BRAFLDRAFT_223365 [Branchiostoma floridae] gi

Protein Accession	CL49005.Contig4$_{H}$1
Mean Expression Ratio	1.03
Median Expression Ratio	1.04
Credible Interval	(0.638, 1.66)
Associated Peptides	1
Associated Spectra	1
Coverage	NaN

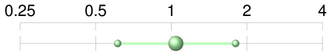

A	B	2.5	50	97.5	Sequence
**1**	**0**	**0.61**	**1**	**1.8**	**AQGYQLIR**

5.531 126 287 minus strand Cytochrome b-c1 complex subunit 10 [Cricetulus griseus]; 126 287 minus strand Cytochrome b-c1 complex subunit 10 [Cricetulus griseus]; 126 287 minus strand Cytochrome b-c1 complex subunit 10 [Cricetulus griseus]; 126 287 minus strand Cytochrome b-c1 complex subunit 10 [Cricetulus griseus]

Protein Accession	CL46231.Contig6$_{H}$1 CL46231.Contig5$_{H}$1 CL46231.Contig4$_{H}$1 CL46231.Contig3$_{H}$1
Mean Expression Ratio	0.966
Median Expression Ratio	0.966
Credible Interval	(0.602, 1.57)
Associated Peptides	1
Associated Spectra	1
Coverage	NaN

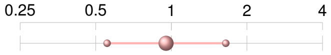

A	B	2.5	50	97.5	Sequence
**0**	**1**	**0.55**	**0.95**	**1.6**	**FIPFYGSK**

5.532 630 1199 neuronal calcium sensor-1 [Lymnaea stagnalis]

Protein Accession	CL14574.Contig3$_{H}1$
Mean Expression Ratio	0.966
Median Expression Ratio	0.966
Credible Interval	(0.623, 1.5)
Associated Peptides	2
Associated Spectra	2
Coverage	NaN

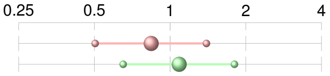

A	B	2.5	50	97.5	Sequence
**1**	**0**	**0.5**	**0.84**	**1.4**	**IFSVMDK**
**1**	**0**	**0.65**	**1.1**	**1.8**	**LYDLDNDGFITK**

5.533 139 945 Kynurenine formamidase [Crassostrea gigas]; 145 951 Kynurenine formamidase [Crassostrea gigas]; 220 1026 Kynurenine formamidase [Crassostrea gigas]; 39 743 Kynurenine formamidase [Crassostrea gigas]; 123 929 Kynurenine formamidase [Crassostrea gigas]; 39 743 Kynurenine formamidase [Crassostrea gigas]

Protein Accession	CL8132.Contig9$_{H}1$ CL8132.Contig8$_{H}1$ CL8132.Contig7$_{H}1$ CL8132.Contig3$_{H}1$ CL8132.Con
Mean Expression Ratio	0.966
Median Expression Ratio	0.967
Credible Interval	(0.644, 1.45)
Associated Peptides	2
Associated Spectra	7
Coverage	NaN

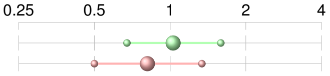

A	B	2.5	50	97.5	Sequence
**4**	**2**	**0.67**	**1**	**1.6**	**LVGPGVVIDVR**
**0**	**1**	**0.5**	**0.81**	**1.3**	**WLTTNR**

5.534 63 1865 Dolichyl-diphosphooligosaccharide–protein glycosyltransferase subunit 1 [Crassostrea gigas]; 63 1865 Dolichyl-diphosphooligosaccharide–protein glycosyltransferase subunit 1 [Crassostrea gigas]

Protein Accession	CL41703.Contig2$_{H}1$ CL41703.Contig1$_{H}1$
Mean Expression Ratio	0.968
Median Expression Ratio	0.967
Credible Interval	(0.683, 1.38)
Associated Peptides	4
Associated Spectra	5
Coverage	NaN

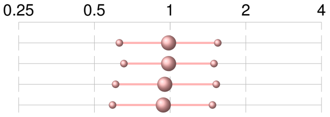

A	B	2.5	50	97.5	Sequence
**1**	**0**	**0.63**	**0.98**	**1.5**	**KVDIATHIVK**
**1**	**1**	**0.66**	**0.99**	**1.5**	**VASLVEEVQSAQDKR**
**1**	**0**	**0.61**	**0.95**	**1.5**	**DLNAFQVAR**
**0**	**1**	**0.59**	**0.94**	**1.5**	**ELDDSVDIELRPR**

5.535 264 1505 Ribosome-binding protein 1 [Crassostrea gigas]; 260 1501 Ribosome-binding protein 1 [Crassostrea gigas]; 256 1497 Ribosome-binding protein 1 [Crassostrea gigas]

Protein Accession	CL8282.Contig7$_{H}$1 CL8282.Contig4$_{H}$1 CL8282.Contig1$_{H}$1
Mean Expression Ratio	1.03
Median Expression Ratio	1.03
Credible Interval	(0.67, 1.59)
Associated Peptides	2
Associated Spectra	2
Coverage	NaN

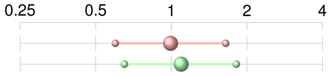

A	B	2.5	50	97.5	Sequence
**1**	**0**	**0.6**	**0.99**	**1.6**	**LVQQLQEQLSEK**
**1**	**0**	**0.65**	**1.1**	**1.8**	**LKEDKETLEAR**

5.536 49 1107 Eukaryotic translation initiation factor 3 subunit B [Crassostrea gigas]

Protein Accession	CL19479.Contig1$_{H}$1
Mean Expression Ratio	0.967
Median Expression Ratio	0.967
Credible Interval	(0.597, 1.57)
Associated Peptides	1
Associated Spectra	1
Coverage	NaN

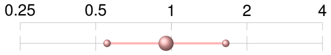

A	B	2.5	50	97.5	Sequence
**0**	**1**	**0.55**	**0.95**	**1.6**	**LMEEFSNLR**

5.537 212 1405 minus strand unnamed protein product [Tetraodon nigroviridis]

Protein Accession	CL37546.Contig3$_{H}$1
Mean Expression Ratio	0.971
Median Expression Ratio	0.967
Credible Interval	(0.596, 1.58)
Associated Peptides	1
Associated Spectra	1
Coverage	NaN

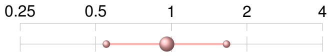

A	B	2.5	50	97.5	Sequence
**0**	**1**	**0.55**	**0.96**	**1.7**	**TIAELGEK**

5.538 " 40 813 minus strand NADH dehydrogenase [ubiquinone] iron-sulfur protein 3, mitochondrial [Crassostrea gigas]"

Protein Accession	CL8338.Contig9$_{H}$1
Mean Expression Ratio	0.966
Median Expression Ratio	0.968
Credible Interval	(0.627, 1.49)
Associated Peptides	2
Associated Spectra	2
Coverage	NaN

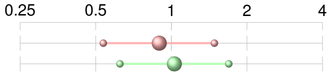

A	B	2.5	50	97.5	Sequence
**0**	**1**	**0.54**	**0.9**	**1.5**	**VVIEPIELQQEFR**
**0**	**1**	**0.62**	**1**	**1.7**	**ILTDYGFEGHPFR**

5.539 91 408 minus strand hypothetical protein [Amblyomma maculatum]

Protein Accession	CL19180.Contig1$_H$1
Mean Expression Ratio	1.03
Median Expression Ratio	1.03
Credible Interval	(0.638, 1.68)
Associated Peptides	1
Associated Spectra	1
Coverage	NaN

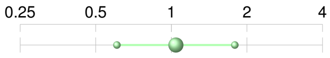

A	B	2.5	50	97.5	Sequence
**1**	**0**	**0.61**	**1**	**1.8**	**EVGWTIVDGR**

5.540 161 301 Heat shock protein beta-6 OS=Mus musculus GN=Hspb6 PE=1 SV=1; 167 310 Heat shock protein beta-1 OS=Canis familiaris GN=HSPB1 PE=2 SV=1

Protein Accession	CL46983.Contig2$_H$1 CL46983.Contig1$_H$1
Mean Expression Ratio	1.03
Median Expression Ratio	1.03
Credible Interval	(0.73, 1.47)
Associated Peptides	2
Associated Spectra	9
Coverage	NaN

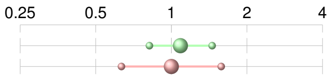

A	B	2.5	50	97.5	Sequence
**6**	**2**	**0.82**	**1.1**	**1.5**	**VNMDVQHFKPEEIK**
**0**	**1**	**0.63**	**1**	**1.6**	**NMDVQHFKPEEIK**

5.541 " 103 981 PREDICTED: ATP synthase, H+ transporting, mitochondrial F1 complex, gamma subunit isoform H (heart)-like [Saccoglossus kowalevskii]"

Protein Accession	CL25149.Contig1$_H$1
Mean Expression Ratio	0.968
Median Expression Ratio	0.969
Credible Interval	(0.642, 1.44)
Associated Peptides	2
Associated Spectra	3
Coverage	NaN

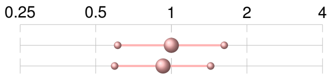

A	B	2.5	50	97.5	Sequence
**1**	**0**	**0.61**	**1**	**1.6**	**ELKPARPYGAGAK**
**1**	**1**	**0.6**	**0.93**	**1.4**	**LTLTFNR**

5.542 141 1325 Nucleobindin-2 [Crassostrea gigas]; 141 1208 minus strand Nucleobindin-2 [Crassostrea gigas]

Protein Accession	CL1888.Contig6$_{H}1$ CL1888.Contig2$_{H}1$
Mean Expression Ratio	0.964
Median Expression Ratio	0.969
Credible Interval	(0.596, 1.56)
Associated Peptides	1
Associated Spectra	2
Coverage	NaN

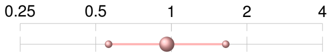

A	B	2.5	50	97.5	Sequence
**1**	**1**	**0.56**	**0.96**	**1.6**	**LKELPEVER**

5.543 1 264 Spectrin beta chain [Crassostrea gigas]

Protein Accession	Unigene36334$_{H}1$
Mean Expression Ratio	0.969
Median Expression Ratio	0.97
Credible Interval	(0.628, 1.48)
Associated Peptides	2
Associated Spectra	2
Coverage	NaN

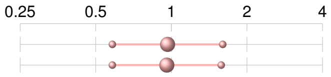

A	B	2.5	50	97.5	Sequence
**1**	**0**	**0.58**	**0.97**	**1.6**	**QLLQVEHPNAAEVVQR**
**1**	**0**	**0.58**	**0.96**	**1.6**	**VAVVNQLAR**

5.544 124 711 glutathione S-transferase sigma 3 [Mytilus galloprovincialis]; 124 711 glutathione S-transferase sigma 3 [Mytilus galloprovincialis]; 110 697 glutathione S-transferase sigma 3 [Mytilus galloprovincialis]

Protein Accession	CL27284.Contig5$_{H}1$ CL27284.Contig4$_{H}1$ CL27284.Contig3$_{H}1$
Mean Expression Ratio	1.03
Median Expression Ratio	1.03
Credible Interval	(0.709, 1.51)
Associated Peptides	3
Associated Spectra	4
Coverage	NaN

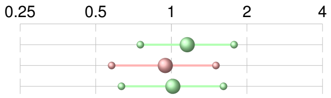

A	B	2.5	50	97.5	Sequence
**1**	**1**	**0.75**	**1.2**	**1.8**	**VDEATQGVVDIR**
**1**	**0**	**0.58**	**0.94**	**1.5**	**TALYGYAFEKDEAR**
**1**	**0**	**0.63**	**1**	**1.6**	**LVPEAEK**

5.545 605 1153 Peptidyl-prolyl cis-trans isomerase B [Crassostrea gigas]; 1046 1630 Peptidyl-prolyl cis-trans isomerase B [Crassostrea gigas]; 589 1173 Peptidyl-prolyl cis-trans isomerase B [Crassostrea gigas]; 938 1414 Peptidyl-prolyl cis-trans isomerase B [Crassostrea gigas]; 883 1467 Peptidyl-prolyl cis-trans isomerase B [Crassostrea gigas]

Protein Accession	CL24747.Contig5$_H$1 CL24747.Contig4$_H$1 CL24747.Contig3$_H$1 CL24747.Contig2$_H$1 CL2474
Mean Expression Ratio	1.03
Median Expression Ratio	1.03
Credible Interval	(0.698, 1.51)
Associated Peptides	2
Associated Spectra	6
Coverage	NaN

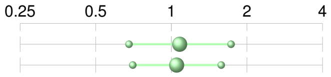

A	B	2.5	50	97.5	Sequence
0	1	0.68	1.1	1.7	DFVIQMGDVTGK
2	3	0.7	1.1	1.6	IASSGVVGIASK

5.546 1 825 Spectrin alpha chain [Crassostrea gigas]

Protein Accession	CL10282.Contig3$_H$1
Mean Expression Ratio	1.03
Median Expression Ratio	1.03
Credible Interval	(0.735, 1.45)
Associated Peptides	3
Associated Spectra	6
Coverage	NaN

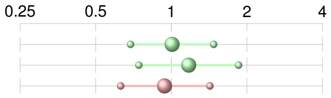

A	B	2.5	50	97.5	Sequence
2	1	0.69	1	1.5	MQHNLEQQIEAR
1	0	0.74	1.2	1.8	IEDLGAAMEER
0	2	0.63	0.94	1.4	LILDNR

5.547 636 1232 sigma class glutathione-s-transferase 3 [Haliotis discus discus]; 963 1559 sigma class glutathione-s-transferase 3 [Haliotis discus discus]; 907 1503 sigma class glutathione-s-transferase 3 [Haliotis discus discus]; 673 1269 sigma class glutathione-s-transferase 3 [Haliotis discus discus]; 177 773 sigma class glutathione-s-transferase 3 [Haliotis discus discus]; 208 804 sigma class glutathione-s-transferase 3 [Haliotis discus discus]; 327 923 sigma class glutathione-s-transferase 3 [Haliotis discus discus]

Protein Accession	Unigene22829$_H$1 CL19285.Contig31$_H$1 CL19285.Contig30$_H$1 CL19285.Contig27$_H$1 CL192
Mean Expression Ratio	1.03
Median Expression Ratio	1.03
Credible Interval	(0.631, 1.65)
Associated Peptides	1
Associated Spectra	1
Coverage	NaN

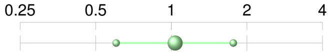

A	B	2.5	50	97.5	Sequence
0	1	0.6	1	1.8	YMNIFEK

5.548 138 713 Rac [Aplysia californica] gi; 125 691 Rac [Aplysia californica] gi; 149 871 minus strand Rac [Aplysia californica] gi; 101 676 Rac [Aplysia californica] gi

Protein Accession	CL11009.Contig9$_{H}$1 CL11009.Contig8$_{H}$1 CL11009.Contig4$_{H}$1 CL11009.Contig1$_{H}$1
Mean Expression Ratio	1.03
Median Expression Ratio	1.03
Credible Interval	(0.668, 1.6)
Associated Peptides	2
Associated Spectra	2
Coverage	NaN

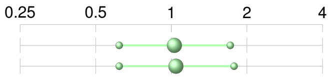

A	B	2.5	50	97.5	Sequence
0	1	0.62	1	1.7	NVFDEAIR
0	1	0.62	1	1.8	AVLCPR

5.549 72 521 minus strand GF14593 [Drosophila ananassae] gi

Protein Accession	CL38325.Contig2$_{H}$1
Mean Expression Ratio	1.03
Median Expression Ratio	1.03
Credible Interval	(0.639, 1.65)
Associated Peptides	1
Associated Spectra	1
Coverage	NaN

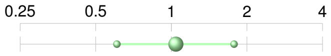

A	B	2.5	50	97.5	Sequence
1	0	0.61	1	1.8	LTADVHGK

5.550 172 678 Myosin regulatory light chain sqh [Crassostrea gigas]

Protein Accession	CL23773.Contig1$_{H}$1
Mean Expression Ratio	1.03
Median Expression Ratio	1.03
Credible Interval	(0.687, 1.54)
Associated Peptides	2
Associated Spectra	3
Coverage	NaN

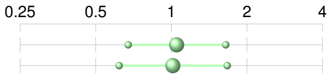

A	B	2.5	50	97.5	Sequence
1	1	0.67	1.1	1.6	LTDEEVDEMFR
1	0	0.62	1	1.7	ELLTTMGDR

5.551 68 667 minus strand hypothetical protein BRAFLDRAFT_272416 [Branchiostoma floridae] gi; 68 667 minus strand hypothetical protein BRAFLDRAFT_272416 [Branchiostoma floridae] gi

Protein Accession	CL37759.Contig4$_{H}$1 CL37759.Contig1$_{H}$1
Mean Expression Ratio	1.03
Median Expression Ratio	1.03
Credible Interval	(0.661, 1.58)
Associated Peptides	2
Associated Spectra	2
Coverage	NaN

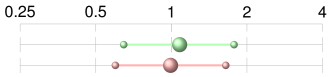

A	B	2.5	50	97.5	Sequence
0	1	0.65	1.1	1.8	TLEEDEDVLLQLR
0	1	0.6	0.99	1.6	VLLQLR

5.552 131 541 minus strand nervous system adducin [Aplysia californica] gi

Protein Accession	CL27337.Contig1$_H$1
Mean Expression Ratio	0.974
Median Expression Ratio	0.974
Credible Interval	(0.683, 1.38)
Associated Peptides	3
Associated Spectra	5
Coverage	NaN

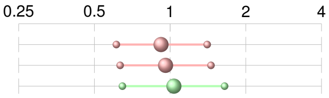

A	B	2.5	50	97.5	Sequence
1	1	0.61	0.92	1.4	FIDTIDPDDPEYIR
1	1	0.63	0.96	1.5	GIGASFGR
1	0	0.65	1	1.6	LAALYR

5.553 " 65 1576 minus strand Aldehyde dehydrogenase, mitochondrial [Crassostrea gigas]"

Protein Accession	CL5835.Contig1$_H$1
Mean Expression Ratio	0.974
Median Expression Ratio	0.974
Credible Interval	(0.703, 1.35)
Associated Peptides	5
Associated Spectra	6
Coverage	NaN

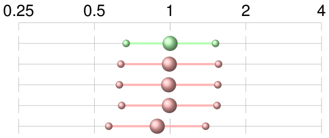

A	B	2.5	50	97.5	Sequence
1	1	0.67	1	1.5	LADLLER
1	0	0.64	0.99	1.6	ELGEYGLEAYTEVK
1	0	0.63	0.99	1.6	IFVEEK
1	0	0.64	0.99	1.5	VAEGDK
0	1	0.57	0.89	1.4	LIPVMAAK

5.554 " 223 423 ADPATP carrier protein; K05863 solute carrier family 25 (mitochondrial adenine nucleotide translocator), member 4/5/6/31"

Protein Accession	CL27196.Contig3$_H$1
Mean Expression Ratio	1.03
Median Expression Ratio	1.03
Credible Interval	(0.701, 1.5)
Associated Peptides	2
Associated Spectra	4
Coverage	NaN

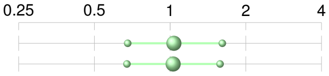

A	B	2.5	50	97.5	Sequence
2	0	0.68	1	1.6	LLVQNQNEMIK
0	2	0.67	1	1.6	GVIDCTIR

5.555 " 431 880 Myosin essential light chain, striated adductor muscle OS=Aequipecten irradians PE=1 SV=2"

Protein Accession	CL36.Contig2$_H$1
Mean Expression Ratio	0.974
Median Expression Ratio	0.974
Credible Interval	(0.787, 1.21)
Associated Peptides	3
Associated Spectra	177
Coverage	NaN

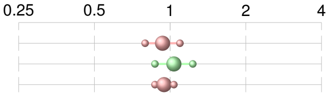

A	B	2.5	50	97.5	Sequence
12	17	0.79	0.93	1.1	LSDEEVDQIMK
15	9	0.87	1	1.2	LTDITIDLEGNLK
46	78	0.87	0.95	1	VGDLLR

5.556 2 1795 minus strand Plectin-1 [Crassostrea gigas]

Protein Accession	CL32322.Contig2$_{H}1$
Mean Expression Ratio	1.03
Median Expression Ratio	1.03
Credible Interval	(0.658, 1.6)
Associated Peptides	2
Associated Spectra	2
Coverage	NaN

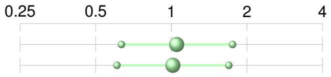

A	B	2.5	50	97.5	Sequence
**1**	**0**	**0.63**	**1.1**	**1.7**	**TKDGDVIPIHDAIMTK**
**1**	**0**	**0.61**	**1**	**1.7**	**YITTAHFVR**

5.557 299 7507 minus strand filamin [Clonorchis sinensis]

Protein Accession	CL4145.Contig20$_{H}$1
Mean Expression Ratio	1.03
Median Expression Ratio	1.03
Credible Interval	(0.949, 1.11)
Associated Peptides	81
Associated Spectra	324
Coverage	NaN

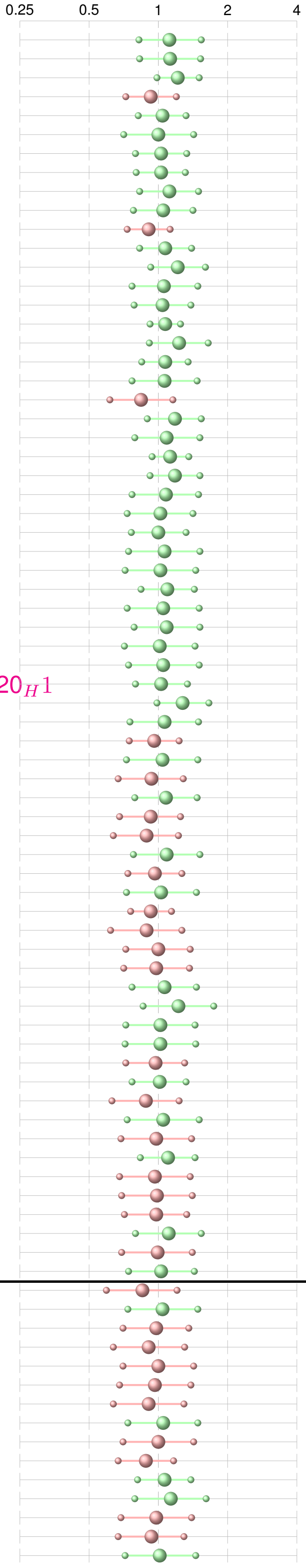

A	B	2.5	50	97.5	Sequence
3	0	0.82	1.1	1.5	AYGPGLEPK
2	1	0.83	1.1	1.5	EAGLEEDEEMTTTER
11	2	0.98	1.2	1.5	EETKPGSLGVTVEGPK
1	6	0.72	0.93	1.2	ELADDAQWK
3	6	0.82	1	1.3	EPVEVVFNNDR
1	0	0.71	1	1.4	EVNKSPFEVTVEGAAGDASK
4	3	0.79	1	1.3	FLEEDEGIR
5	3	0.8	1	1.3	FTVETFSAGK
4	0	0.83	1.1	1.5	GAGEGEVAVQIIGPGGSEEK
4	0	0.78	1	1.4	GAGNGSVDVVILDPHGR
8	5	0.73	0.91	1.1	GTLEVTLLNPK
3	4	0.83	1.1	1.4	IEASIDVSGIK
6	0	0.93	1.2	1.6	INVTHADKPIPGSPFK
1	1	0.77	1.1	1.5	IVNIDSTDIVDK
4	1	0.78	1	1.4	KDTVRPSIQPVVGK
13	18	0.92	1.1	1.3	LENVTMVLK
2	2	0.92	1.2	1.7	NFTTDWNDGR
4	6	0.85	1.1	1.4	QIADLESDFSDGLR
2	0	0.77	1.1	1.5	SPFEVTVEGAAGDASK
1	2	0.62	0.84	1.2	TQKLENVTMVLK
4	2	0.9	1.2	1.5	TTNVPVQDLVDKK
1	1	0.79	1.1	1.5	VDAVPDTGIPK
14	6	0.94	1.1	1.4	VEVGPFWK
7	1	0.92	1.2	1.5	VPDLPIK
2	0	0.77	1.1	1.5	VYVSEPTNPAK
1	1	0.73	1	1.4	TWFEVYTK
4	2	0.77	1	1.3	ACFATGR
1	0	0.74	1.1	1.5	IEASIDVSGIKVEGLEPK
1	0	0.72	1	1.5	YMPTKNGK
5	1	0.84	1.1	1.4	MAITSPSGK
1	0	0.73	1	1.5	VEGLEPK
2	0	0.79	1.1	1.5	AFGPGLTGGEALKPAFFTIDAR
1	0	0.71	1	1.4	GLEEDEEMTTTER
1	0	0.74	1.1	1.5	KTWFEVYTK
4	3	0.8	1	1.3	QVEVQVK
4	3	0.99	1.3	1.7	VDDLSMMTYLSQFPNAK
1	0	0.75	1.1	1.5	VKDSADFYVHTK
3	5	0.75	0.96	1.2	WANEHLK
1	0	0.73	1	1.5	WLDVPQLIKPDEMTNPK
0	2	0.67	0.93	1.3	AAADVTHVGSECELSLK
0	3	0.79	1.1	1.5	AEIDFQDR
0	3	0.68	0.92	1.3	CYGPGLDSK
0	2	0.64	0.89	1.2	EEPCLLK
0	2	0.78	1.1	1.5	ETFITVDAAEAGNGK
0	6	0.74	0.96	1.3	FNDEHIPDSPYR
0	1	0.72	1	1.5	GEVQQPCDFNIYTR
0	15	0.76	0.93	1.1	GGLGLGIEGPVEAK
0	1	0.62	0.89	1.3	IMVGESELGNASK
0	2	0.72	1	1.4	ISISALQDK
0	2	0.71	0.98	1.4	LANGHLGITFTPR
0	2	0.77	1.1	1.5	PAAFVVNFNGAK
0	1	0.86	1.2	1.7	RLANGHLGITFTPR
0	1	0.72	1	1.4	SPTGSDIDIDIIDR
0	1	0.72	1	1.5	SPVDELTVK
0	4	0.72	0.97	1.3	VKPAALDVSGVK
0	6	0.77	1	1.3	VLPGVDASK
0	2	0.63	0.88	1.2	VQNPVDASK
0	1	0.73	1.1	1.5	VQVSPSIGDAR
0	1	0.69	0.98	1.4	VSEELITR
0	5	0.83	1.1	1.4	VTETDITDLTASIR
0	1	0.68	0.96	1.4	VTGPGVQGTTPASLPAVFK
0	1	0.69	0.98	1.4	YAGVNIAGSPFK
0	3	0.71	0.98	1.3	HIANSPFK
0	2	0.79	1.1	1.5	KISISALQDK
0	1	0.69	0.99	1.4	APGDYLISIR
0	2	0.74	1	1.4	EMGIHTVSVK
0	1	0.59	0.85	1.2	FADQHVPGSPFTVK
0	1	0.74	1	1.5	IMVGK
0	2	0.7	0.98	1.4	VKPAALDVSG
0	1	0.64	0.91	1.3	IPGTSPFDMTASVK
0	1	0.7	1	1.4	SVTLNGEGTVQAILTTPTGR
0	1	0.68	0.97	1.4	FADQHVPGSPF
0	1	0.64	0.9	1.3	VTYRPTEPGTYIINIK
0	1	0.74	1	1.5	QIADLESDFSDGL
0	1	0.7	1	1.4	EVPNSPFQMR
0	5	0.67	0.88	1.2	DVPGAPFPVK
0	6	0.81	1.1	1.4	ETGEHLVNVFR
0	1	0.79	1.1	1.6	GLQVGKPAAFVVNFNGAK
0	1	0.69	0.98	1.4	INVTHADKPIPGS
0	2	0.67	0.93	1.3	NIMGSPFTAK
0	1	0.72	1	1.5	PGVDASK

5.558 19 1218 Dynactin subunit 2 [Crassostrea gigas]; 48 830 minus strand Dynactin subunit 2 [Crassostrea gigas]

Protein Accession	CL5827.Contig5$_{H}1$ CL5827.Contig1$_{H}1$
Mean Expression Ratio	0.973
Median Expression Ratio	0.974
Credible Interval	(0.597, 1.58)
Associated Peptides	1
Associated Spectra	1
Coverage	NaN

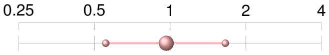

A	B	2.5	50	97.5	Sequence
1	0	0.56	0.97	1.7	VNELYEVMK

5.559 1971 3368 minus strand Kyphoscoliosis peptidase [Crassostrea gigas]; 1767 3158 minus strand hillarin [Hirudo medicinalis]; 1977 3368 minus strand hillarin [Hirudo medicinalis]

Protein Accession	CL3923.Contig9$_{H}1$ CL3923.Contig8$_{H}1$ CL3923.Contig10$_{H}1$
Mean Expression Ratio	0.974
Median Expression Ratio	0.974
Credible Interval	(0.814, 1.17)
Associated Peptides	13
Associated Spectra	40
Coverage	NaN

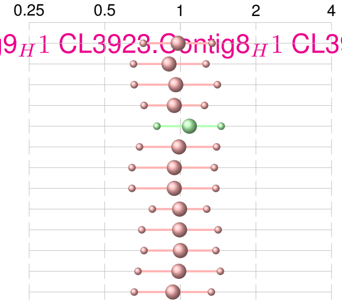

A	B	2.5	50	97.5	Sequence
2	2	0.71	0.98	1.3	EKGEAPPPVPATGPQQKPTLEAIDR
1	2	0.65	0.9	1.3	EMNFDKVEK
1	0	0.65	0.96	1.4	GEAPPPVPATGPQQKPTLEAIDR
4	2	0.72	0.95	1.3	GSPEEVLMGLK
4	1	0.81	1.1	1.5	NITNEIEK
1	1	0.69	0.98	1.4	TAVIYSR
1	0	0.64	0.95	1.4	TVGNTAYFNVTAPGR
1	0	0.64	0.95	1.4	IISGYAK
5	4	0.77	0.99	1.3	WQLLDR
2	0	0.7	0.99	1.4	MKPQFFK
2	1	0.72	1	1.4	NVLQSVDDHAIQVSK
0	1	0.68	0.99	1.4	WLATK
0	2	0.65	0.94	1.3	QLLDR

5.560 " 1 681 40S ribosomal protein S4, X isoform [Crassostrea gigas]"

Protein Accession	CL2560.Contig4$_{H}1$
Mean Expression Ratio	0.977
Median Expression Ratio	0.975
Credible Interval	(0.601, 1.59)
Associated Peptides	1
Associated Spectra	1
Coverage	NaN

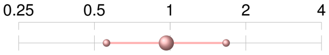

A	B	2.5	50	97.5	Sequence
1	0	0.56	0.97	1.7	GVPYIVTHDGR

5.561 57 2009 hypothetical protein BRAFLDRAFT_285279 [Branchiostoma floridae] gi; 3 1973 hypothetical protein BRAFLDRAFT_285279 [Branchiostoma floridae] gi

Protein Accession	CL24444.Contig3$_H1$ CL24444.Contig1$_H1$
Mean Expression Ratio	1.03
Median Expression Ratio	1.03
Credible Interval	(0.668, 1.59)
Associated Peptides	2
Associated Spectra	2
Coverage	NaN

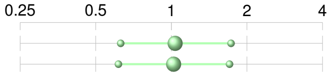

A	B	2.5	50	97.5	Sequence
1	0	0.63	1	1.7	INQIYEQ
0	1	0.62	1	1.7	KLTLK

5.562 18 647 minus strand ATP synthase-like protein [Biomphalaria glabrata]

Protein Accession	CL42054.Contig4$_H1$
Mean Expression Ratio	1.03
Median Expression Ratio	1.03
Credible Interval	(0.797, 1.33)
Associated Peptides	6
Associated Spectra	16
Coverage	NaN

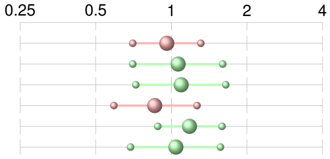

A	B	2.5	50	97.5	Sequence
1	4	0.7	0.96	1.3	LAEFVANPSLQR
1	0	0.7	1.1	1.6	KAVLEQALK
1	0	0.72	1.1	1.6	NFQNLLK
2	0	0.59	0.86	1.3	KLDEVEK
3	3	0.88	1.2	1.6	GALQGFLK
1	0	0.69	1	1.6	NYTNLIK

5.563 " 45 506 minus strand PREDICTED: dihydrolipoyl dehydrogenase, mitochondrial [Monodelphis domestica]"

Protein Accession	CL24292.Contig3$_H1$
Mean Expression Ratio	1.02
Median Expression Ratio	1.02
Credible Interval	(0.66, 1.59)
Associated Peptides	2
Associated Spectra	2
Coverage	NaN

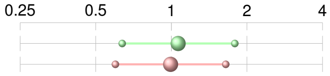

A	B	2.5	50	97.5	Sequence
0	1	0.64	1.1	1.8	ALTGGIAHLFK
0	1	0.6	0.99	1.6	GIEIDGLR

5.564 2 475 unnamed protein product [Tetraodon nigroviridis]

Protein Accession	CL31420.Contig4$_H1$
Mean Expression Ratio	1.02
Median Expression Ratio	1.02
Credible Interval	(0.777, 1.36)
Associated Peptides	2
Associated Spectra	28
Coverage	NaN

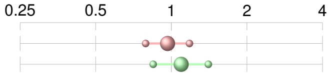

A	B	2.5	50	97.5	Sequence
18	0	0.79	0.97	1.2	DLYANTVLSGGTTMYPGIADR
10	0	0.84	1.1	1.4	KDLYANTVLSGGTTMYPGIADR

5.565 " 1 321 Dynein heavy chain, cytoplasmic [Crassostrea gigas]"

Protein Accession	Unigene35195$_{H}1$
Mean Expression Ratio	0.977
Median Expression Ratio	0.977
Credible Interval	(0.682, 1.4)
Associated Peptides	1
Associated Spectra	11
Coverage	NaN

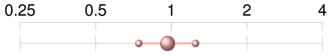

A	B	2.5	50	97.5	Sequence
6	5	0.74	0.97	1.2	EILAVIQK

5.566 1 222 cAMP-dependent protein kinase regulatory subunit [Aplysia californica] gi

Protein Accession	Unigene13629$_{H}1$
Mean Expression Ratio	0.976
Median Expression Ratio	0.977
Credible Interval	(0.655, 1.46)
Associated Peptides	2
Associated Spectra	3
Coverage	NaN

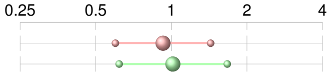

A	B	2.5	50	97.5	Sequence
1	1	0.6	0.93	1.4	NVLFSHLDDNER
0	1	0.62	1	1.7	SDIFDAMFPVHR

5.567 144 800 minus strand PREDICTED: protein F37C4.5-like [Amphimedon queenslandica]

Protein Accession	CL7246.Contig3$_{H}1$
Mean Expression Ratio	1.02
Median Expression Ratio	1.02
Credible Interval	(0.733, 1.42)
Associated Peptides	4
Associated Spectra	6
Coverage	NaN

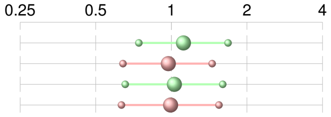

A	B	2.5	50	97.5	Sequence
2	0	0.74	1.1	1.7	VTVGLVDIKPK
1	1	0.64	0.97	1.5	VSLLEPQLDLK
1	0	0.66	1	1.6	NKDKDVTLNK
1	0	0.63	1	1.6	VISNTHEQVIEVK

5.568 " 703 1173 60S acidic ribosomal protein P0, putative [Ixodes scapularis] gi"

Protein Accession	Unigene34489$_{H}1$
Mean Expression Ratio	0.975
Median Expression Ratio	0.978
Credible Interval	(0.602, 1.58)
Associated Peptides	1
Associated Spectra	1
Coverage	NaN

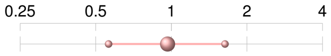

A	B	2.5	50	97.5	Sequence
0	1	0.56	0.97	1.6	KSFLEGVR

5.569 1714 2121 Phosphoenolpyruvate carboxykinase [GTP] [Crassostrea gigas]

Protein Accession	CL25948.Contig1$_{H}1$
Mean Expression Ratio	0.977
Median Expression Ratio	0.978
Credible Interval	(0.684, 1.4)
Associated Peptides	1
Associated Spectra	10
Coverage	NaN

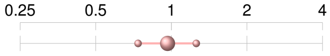

A	B	2.5	50	97.5	Sequence
**7**	**3**	**0.74**	**0.97**	**1.3**	**SEATAAAEFK**

5.570 61 2865 AP-2 complex subunit beta [Crassostrea gigas]

Protein Accession	CL6957.Contig1$_{H}1$
Mean Expression Ratio	1.02
Median Expression Ratio	1.02
Credible Interval	(0.626, 1.66)
Associated Peptides	1
Associated Spectra	1
Coverage	NaN

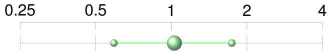

A	B	2.5	50	97.5	Sequence
**1**	**0**	**0.59**	**1**	**1.7**	**NSNIFTIAK**

5.571 " 27 974 PREDICTED: succinyl-CoA ligase [GDP-forming] subunit alpha, mitochondrial-like [Oreochromis niloticus]; 27 974 minus strand PREDICTED: succinyl-CoA ligase [GDP-forming] subunit alpha, mitochondrial-like [Oreochromis niloticus]"

Protein Accession	CL10674.Contig2$_{H}1$ CL10674.Contig1$_{H}1$
Mean Expression Ratio	1.02
Median Expression Ratio	1.02
Credible Interval	(0.725, 1.45)
Associated Peptides	3
Associated Spectra	6
Coverage	NaN

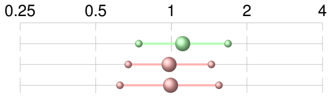

A	B	2.5	50	97.5	Sequence
**2**	**0**	**0.74**	**1.1**	**1.7**	**MGHAGAIIAGGK**
**1**	**2**	**0.67**	**0.98**	**1.4**	**IYMATR**
**0**	**1**	**0.62**	**0.99**	**1.5**	**LICQGFTGK**

5.572 160 501 hypothetical protein CGI_10001640 [Crassostrea gigas]; 160 588 hypothetical protein CGI_10001640 [Crassostrea gigas]; 160 501 hypothetical protein CGI_10001640 [Crassostrea gigas]; 160 552 hypothetical protein CGI_10001640 [Crassostrea gigas]; 160 501 hypothetical protein CGI_10001640 [Crassostrea gigas]; 160 501 hypothetical protein CGI_10001640 [Crassostrea gigas]

Protein Accession	Unigene62839$_{H}1$ CL2347.Contig8$_{H}1$ CL2347.Contig15$_{H}1$ CL2347.Contig14$_{H}1$ CL2347.Co
Mean Expression Ratio	1.02
Median Expression Ratio	1.02
Credible Interval	(0.696, 1.49)
Associated Peptides	2
Associated Spectra	13
Coverage	NaN

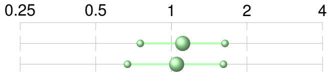

A	B	2.5	50	97.5	Sequence
6	3	0.75	1.1	1.6	AFLEQLGSK
3	1	0.67	1	1.6	ANLIVIGSR

5.573 316 531 cytochrome c oxidase subunit VIIa polypeptide 2 like [Xenopus (Silurana) tropicalis]

Protein Accession	CL17601.Contig5$_{H}1$
Mean Expression Ratio	1.02
Median Expression Ratio	1.02
Credible Interval	(0.628, 1.66)
Associated Peptides	1
Associated Spectra	1
Coverage	NaN

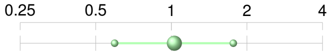

A	B	2.5	50	97.5	Sequence
0	1	0.6	1	1.8	QDGLLVWQK

5.574 191 919 PREDICTED: 15-hydroxyprostaglandin dehydrogenase [NAD+] [Gallus gallus]

Protein Accession	CL7033.Contig5$_{H}1$
Mean Expression Ratio	1.02
Median Expression Ratio	1.02
Credible Interval	(0.635, 1.67)
Associated Peptides	1
Associated Spectra	1
Coverage	NaN

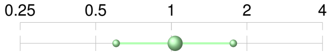

A	B	2.5	50	97.5	Sequence
1	0	0.6	1	1.8	AMVEALLDK

5.575 4 444 Vinculin [Crassostrea gigas]

Protein Accession	Unigene21732$_{H}1$
Mean Expression Ratio	1.02
Median Expression Ratio	1.02
Credible Interval	(0.742, 1.41)
Associated Peptides	4
Associated Spectra	9
Coverage	NaN

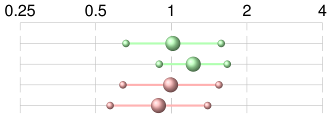

A	B	2.5	50	97.5	Sequence
**1**	**0**	**0.66**	**1**	**1.6**	**AIAEASEEVTR**
**4**	**2**	**0.9**	**1.2**	**1.7**	**IPTIGTQLK**
**1**	**0**	**0.64**	**0.99**	**1.5**	**TNLLNVCER**
**1**	**0**	**0.57**	**0.89**	**1.4**	**DNEIISAAK**

5.576 " 42 905 PREDICTED: enoyl-CoA hydratase, mitochondrial-like isoform 1 [Oreochromis niloticus]"

Protein Accession	CL6477.Contig7$_{H}1$
Mean Expression Ratio	1.02
Median Expression Ratio	1.02
Credible Interval	(0.708, 1.47)
Associated Peptides	2
Associated Spectra	5
Coverage	NaN

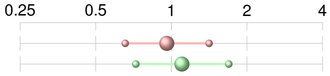

A	B	2.5	50	97.5	Sequence
**1**	**2**	**0.65**	**0.96**	**1.4**	**AFAAGADIK**
**1**	**1**	**0.72**	**1.1**	**1.7**	**SVVLQASR**

5.577 120 833 B-cell receptor-associated protein 31 [Crassostrea gigas]; 238 951 B-cell receptor-associated protein 31 [Crassostrea gigas]; 88 801 B-cell receptor-associated protein 31 [Crassostrea gigas]; 88 801 B-cell receptor-associated protein 31 [Crassostrea gigas]

Protein Accession	CL9442.Contig7$_{H}1$ CL9442.Contig5$_{H}1$ CL9442.Contig4$_{H}1$ CL9442.Contig3$_{H}1$
Mean Expression Ratio	1.02
Median Expression Ratio	1.02
Credible Interval	(0.632, 1.65)
Associated Peptides	1
Associated Spectra	1
Coverage	NaN

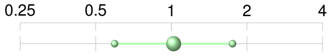

A	B	2.5	50	97.5	Sequence
**1**	**0**	**0.6**	**1**	**1.7**	**LLEEHAQLQK**

5.578 " 343 1179 ATP synthase subunit alpha, mitochondrial OS=Pongo abelii GN=ATP5A1 PE=2 SV=1"

Protein Accession	Unigene30534$_{H}1$
Mean Expression Ratio	0.981
Median Expression Ratio	0.98
Credible Interval	(0.801, 1.2)
Associated Peptides	7
Associated Spectra	33
Coverage	NaN

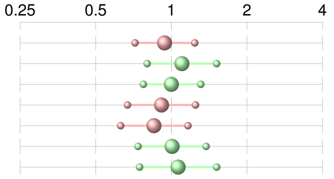

A	B	2.5	50	97.5	Sequence
3	4	0.72	0.94	1.2	TAVAIDTIINQK
4	0	0.8	1.1	1.5	TSVKEPMQTGIK
5	3	0.77	1	1.3	VLSIGDGIAR
1	3	0.67	0.91	1.2	VVDALGTPIDGK
2	2	0.63	0.85	1.2	APGIIPR
3	1	0.74	1	1.4	STVAQLVK
0	2	0.75	1.1	1.5	LSDADAMK

5.579 246 452 minus strand mitochondrial malate dehydrogenase precursor [Nucella lapillus]; 106 312 minus strand mitochondrial malate dehydrogenase precursor [Nucella lapillus]

Protein Accession	CL10426.Contig4$_{H}1$ CL10426.Contig3$_{H}1$
Mean Expression Ratio	1.02
Median Expression Ratio	1.02
Credible Interval	(0.68, 1.54)
Associated Peptides	1
Associated Spectra	4
Coverage	NaN

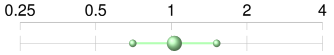

A	B	2.5	50	97.5	Sequence
2	2	0.7	1	1.5	DDLFNTNAGIVR

5.580 " 109 1632 minus strand Seryl-tRNA synthetase, cytoplasmic [Crassostrea gigas]; 109 1614 minus strand Seryl-tRNA synthetase, cytoplasmic [Crassostrea gigas]"

Protein Accession	CL11056.Contig9$_{H}1$ CL11056.Contig1$_{H}1$
Mean Expression Ratio	1.02
Median Expression Ratio	1.02
Credible Interval	(0.652, 1.58)
Associated Peptides	2
Associated Spectra	2
Coverage	NaN

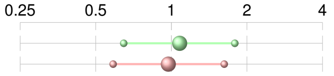

A	B	2.5	50	97.5	Sequence
0	1	0.65	1.1	1.8	LVDTVVDQDAAWR
0	1	0.58	0.97	1.6	SLGIPYR

5.581 " 241 1110 minus strand N(G),N(G)-dimethylarginine dimethylaminohydrolase 1 [Crassostrea gigas]; 241 1110 minus strand N(G),N(G)-dimethylarginine dimethylaminohydrolase 1 [Crassostrea gigas]; 241 1110 minus strand N(G),N(G)-dimethylarginine dimethylaminohydrolase 1 [Crassostrea gigas]"

Protein Accession	Unigene30451$_{H}1$ CL7107.Contig3$_{H}1$ CL7107.Contig1$_{H}1$
Mean Expression Ratio	1.02
Median Expression Ratio	1.02
Credible Interval	(0.625, 1.64)
Associated Peptides	1
Associated Spectra	2
Coverage	NaN

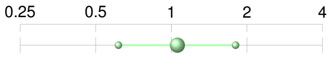

A	B	2.5	50	97.5	Sequence
**1**	**1**	**0.62**	**1.1**	**1.8**	**LGEVGEIR**

5.582 206 1513 hypothetical protein CGI_10019003 [Crassostrea gigas]

Protein Accession	CL6385.Contig8$_{H}1$
Mean Expression Ratio	1.02
Median Expression Ratio	1.02
Credible Interval	(0.625, 1.66)
Associated Peptides	1
Associated Spectra	1
Coverage	NaN

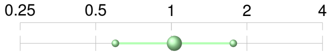

A	B	2.5	50	97.5	Sequence
**1**	**0**	**0.6**	**1**	**1.8**	**AALEDLLR**

5.583 161 3001 minus strand E3 ubiquitin-protein ligase MIB2 [Crassostrea gigas]

Protein Accession	CL49497.Contig7$_{H}1$
Mean Expression Ratio	0.985
Median Expression Ratio	0.982
Credible Interval	(0.608, 1.6)
Associated Peptides	1
Associated Spectra	1
Coverage	NaN

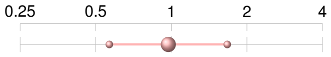

A	B	2.5	50	97.5	Sequence
**0**	**1**	**0.57**	**0.97**	**1.7**	**GDAHAVEGILQR**

5.584 365 2530 minus strand calpain B [Gecarcinus lateralis]

Protein Accession	CL34074.Contig5$_{H}1$
Mean Expression Ratio	1.02
Median Expression Ratio	1.02
Credible Interval	(0.791, 1.32)
Associated Peptides	7
Associated Spectra	14
Coverage	NaN

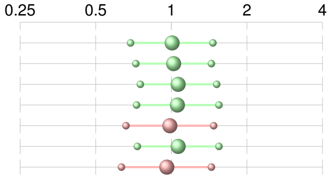

A	B	2.5	50	97.5	Sequence
1	1	0.69	1	1.5	LGYDEFK
2	1	0.72	1	1.4	SLQSLVMR
2	1	0.75	1.1	1.5	SMVAMHDGDLSGK
1	1	0.73	1.1	1.5	TLWEDLR
1	0	0.66	0.99	1.5	MQGQIPMVR
1	1	0.73	1.1	1.5	TMLTSFR
0	1	0.63	0.96	1.4	TLWEDLRR

5.585 5 1018 minus strand Glutaredoxin-3 [Crassostrea gigas]; 5 1018 Glutaredoxin-3 [Crassostrea gigas]

Protein Accession	CL7734.Contig9$_{H}1$ CL7734.Contig10$_{H}1$
Mean Expression Ratio	0.983
Median Expression Ratio	0.982
Credible Interval	(0.628, 1.55)
Associated Peptides	1
Associated Spectra	2
Coverage	NaN

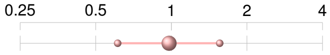

A	B	2.5	50	97.5	Sequence
1	1	0.61	0.98	1.6	VAAEDLAEVSHR

5.586 696 4010 Lethal(2) giant larvae-like protein 1 [Crassostrea gigas]; 657 3971 minus strand Lethal(2) giant larvae-like protein 1 [Crassostrea gigas]; 156 3470 Lethal(2) giant larvae-like protein 1 [Crassostrea gigas]

Protein Accession	CL3717.Contig4$_{H}1$ CL3717.Contig3$_{H}1$ CL3717.Contig16$_{H}1$
Mean Expression Ratio	0.98
Median Expression Ratio	0.982
Credible Interval	(0.661, 1.47)
Associated Peptides	3
Associated Spectra	3
Coverage	NaN

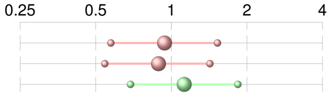

A	B	2.5	50	97.5	Sequence
0	1	0.57	0.94	1.5	LDTTADITIDSVR
0	1	0.54	0.89	1.4	VLPESLEVIHER
0	1	0.69	1.1	1.8	ASMIR

5.587 49 747 PREDICTED: elongation factor 1-delta-like [Strongylocentrotus purpuratus]; 49 819 PREDICTED: elongation factor 1-delta-like isoform 2 [Strongylocentrotus purpuratus]

Protein Accession	CL10790.Contig13$_H1$ CL10790.Contig12$_H1$
Mean Expression Ratio	1.02
Median Expression Ratio	1.02
Credible Interval	(0.667, 1.56)
Associated Peptides	2
Associated Spectra	5
Coverage	NaN

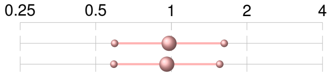

A	B	2.5	50	97.5	Sequence
1	0	0.59	0.98	1.6	VAALEVVGTGGASPK
1	3	0.59	0.96	1.6	LVDLAYGIK

5.588 594 1253 peroxiredoxin 6 [Eriocheir sinensis]

Protein Accession	CL21894.Contig2$_H1$
Mean Expression Ratio	1.02
Median Expression Ratio	1.02
Credible Interval	(0.774, 1.34)
Associated Peptides	5
Associated Spectra	13
Coverage	NaN

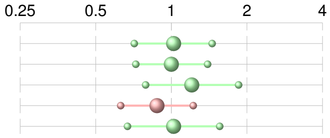

A	B	2.5	50	97.5	Sequence
2	1	0.71	1	1.5	VNLGDVFPDFK
4	0	0.72	1	1.4	AVFIIGPDK
1	0	0.79	1.2	1.8	AVFIIGPDKK
2	2	0.63	0.88	1.2	NFDEILR
1	0	0.67	1	1.6	QLTATQK

5.589 3 347 beta-actin [Meretrix meretrix]; 3 215 Actin-2 OS=Onchocerca volvulus GN=act-2b PE=3 SV=1

Protein Accession	CL47466.Contig2$_H1$ CL47466.Contig1$_H1$
Mean Expression Ratio	1.02
Median Expression Ratio	1.02
Credible Interval	(0.847, 1.21)
Associated Peptides	11
Associated Spectra	303
Coverage	NaN

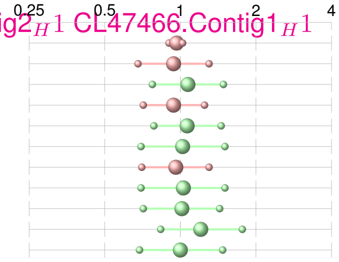

A	B	2.5	50	97.5	Sequence
146	131	0.9	0.96	1	EITALAPSTMK
2	1	0.68	0.94	1.3	EITALAPSTMKIK
3	0	0.77	1.1	1.5	KDLYANTVLSGGSTMYPGIADR
6	0	0.71	0.94	1.2	TVLSGGSTMYPGIADR
4	0	0.79	1.1	1.5	DLYANTVLSGGSTMYPGIADR
1	0	0.7	1	1.5	MQKEITALAPSTMK
0	4	0.7	0.96	1.3	LAPSTMK
0	1	0.7	1	1.5	ALAPSTMK
0	2	0.71	1	1.4	APSTMK
0	1	0.83	1.2	1.8	PSTMK
0	1	0.69	1	1.5	TALAPSTMK

5.590 184 438 PREDICTED: similar to CG14235 CG14235-PA [Tribolium castaneum] gi

Protein Accession	Unigene638$_{H}1$
Mean Expression Ratio	1.02
Median Expression Ratio	1.02
Credible Interval	(0.697, 1.48)
Associated Peptides	2
Associated Spectra	5
Coverage	NaN

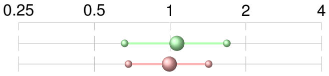

A	B	2.5	50	97.5	Sequence
1	0	0.66	1.1	1.7	SLCPNEWVAK
3	1	0.68	0.99	1.4	WDDQVANGTFAGK

5.591 4 849 minus strand elongation factor 1-alpha [Cryptosporidium muris RN66]

Protein Accession	CL35888.Contig1$_{H}1$
Mean Expression Ratio	0.985
Median Expression Ratio	0.983
Credible Interval	(0.671, 1.46)
Associated Peptides	2
Associated Spectra	12
Coverage	NaN

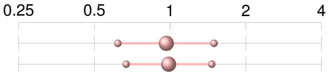

A	B	2.5	50	97.5	Sequence
5	1	0.62	0.96	1.5	IGGIGTVPVGR
3	3	0.67	0.99	1.5	LPLQDVYK

5.592 484 1104 Calponin-2 [Crassostrea gigas]; 309 938 Calponin-2 [Crassostrea gigas]

Protein Accession	CL30203.Contig3$_{H}1$ CL21394.Contig2$_{H}1$
Mean Expression Ratio	1.02
Median Expression Ratio	1.02
Credible Interval	(0.871, 1.18)
Associated Peptides	11
Associated Spectra	138
Coverage	NaN

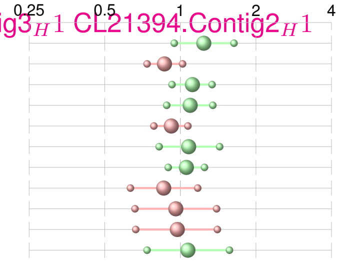

A	B	2.5	50	97.5	Sequence
5	1	0.94	1.2	1.6	AGQGIIGLQAGTNK
21	6	0.74	0.87	1	AVYDGTPNLPPAAK
10	11	0.93	1.1	1.3	MNTLAAPFK
11	3	0.88	1.1	1.3	QLTGEDIGSGAVQVEK
20	11	0.78	0.92	1.1	QMENIEVFLK
4	2	0.82	1.1	1.4	YDTGAEYEVR
15	11	0.89	1.1	1.3	NGQILCR
2	2	0.63	0.86	1.2	NLRNGQILCR
0	1	0.66	0.96	1.4	IIGLQAGTNK
0	1	0.66	0.97	1.4	PNLPPAAK
0	1	0.74	1.1	1.6	PTTLPR

5.593 1017 1499 minus strand PREDICTED: leukocyte elastase inhibitor-like [Oreochromis niloticus]

Protein Accession	CL39031.Contig4$_{H}$1
Mean Expression Ratio	0.984
Median Expression Ratio	0.984
Credible Interval	(0.638, 1.53)
Associated Peptides	1
Associated Spectra	2
Coverage	NaN

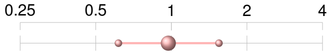

A	B	2.5	50	97.5	Sequence
0	2	0.62	0.97	1.5	VLELPYGTDDR

5.594 156 1163 cathepsin B [Crassostrea ariakensis]

Protein Accession	CL26836.Contig9$_{H}$1
Mean Expression Ratio	1.02
Median Expression Ratio	1.02
Credible Interval	(0.653, 1.59)
Associated Peptides	1
Associated Spectra	2
Coverage	NaN

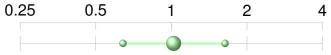

A	B	2.5	50	97.5	Sequence
1	1	0.64	1	1.6	DLPDSFDPR

5.595 311 928 minus strand peroxiredoxin 3 [Clonorchis sinensis] gi; 288 905 minus strand peroxiredoxin 3 [Clonorchis sinensis] gi

Protein Accession	CL2043.Contig4$_{H}$1 CL2043.Contig1$_{H}$1
Mean Expression Ratio	0.985
Median Expression Ratio	0.984
Credible Interval	(0.729, 1.33)
Associated Peptides	3
Associated Spectra	13
Coverage	NaN

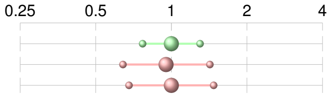

A	B	2.5	50	97.5	Sequence
5	4	0.77	1	1.3	QITINDLPVGR
1	1	0.64	0.96	1.4	SVDEILR
0	2	0.68	1	1.5	GLFIIDPK

5.596 71 1321 minus strand Vinculin [Crassostrea gigas]

Protein Accession	Unigene23324$_{H}$1
Mean Expression Ratio	0.985
Median Expression Ratio	0.985
Credible Interval	(0.786, 1.23)
Associated Peptides	12
Associated Spectra	20
Coverage	NaN

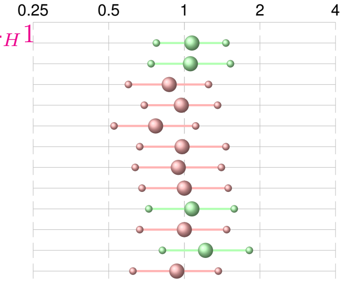

A	B	2.5	50	97.5	Sequence
3	1	0.77	1.1	1.5	DTITPMVQNAK
1	1	0.74	1.1	1.5	GLVDEAVDTAALIR
2	0	0.6	0.87	1.3	KPAPTVDGK
2	1	0.69	0.97	1.4	LINAVGGVR
2	0	0.52	0.77	1.1	NVGDIESMANALSELR
1	0	0.66	0.98	1.5	QWLANPGLDDK
1	0	0.64	0.95	1.4	VLQVAEMEAQNSEDPLFVDR
1	0	0.68	1	1.5	ASADLR
0	1	0.72	1.1	1.6	AEEEAILHDTDR
0	1	0.67	1	1.5	VNEASADLR
0	1	0.82	1.2	1.8	DREAILK
0	1	0.62	0.93	1.4	AIARNLSEKL

5.597 3 239 minus strand p38 MAPK [Littorina littorea]

Protein Accession	CL49144.Contig2$_{H}$1
Mean Expression Ratio	0.984
Median Expression Ratio	0.985
Credible Interval	(0.602, 1.6)
Associated Peptides	1
Associated Spectra	1
Coverage	NaN

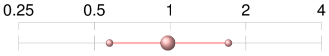

A	B	2.5	50	97.5	Sequence
**0**	**1**	**0.57**	**0.98**	**1.7**	**MLDLDTDHR**

5.598 104 418 Tubulin-specific chaperone A [Crassostrea gigas]; 104 418 minus strand Tubulin-specific chaperone A [Crassostrea gigas]; 104 418 Tubulin-specific chaperone A [Crassostrea gigas]

Protein Accession	Unigene53595$_{H}$1 Unigene32457$_{H}$1 CL7190.Contig3$_{H}$1
Mean Expression Ratio	0.984
Median Expression Ratio	0.985
Credible Interval	(0.604, 1.6)
Associated Peptides	1
Associated Spectra	2
Coverage	NaN

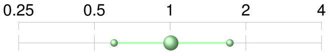

A	B	2.5	50	97.5	Sequence
**1**	**1**	**0.6**	**1**	**1.7**	**ADLGETEELK**

5.599 333 770 USP-like protein isoform 2 [Crassostrea ariakensis]

Protein Accession	Unigene7710$_{H}$1
Mean Expression Ratio	0.984
Median Expression Ratio	0.986
Credible Interval	(0.6, 1.6)
Associated Peptides	1
Associated Spectra	1
Coverage	NaN

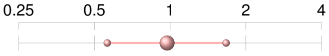

A	B	2.5	50	97.5	Sequence
**1**	**0**	**0.56**	**0.97**	**1.7**	**KIVIAMDGSK**

5.600 116 1882 Actin-interacting protein 1 [Crassostrea gigas]

Protein Accession	CL12416.Contig1$_{H}$1
Mean Expression Ratio	0.987
Median Expression Ratio	0.987
Credible Interval	(0.775, 1.26)
Associated Peptides	9
Associated Spectra	17
Coverage	NaN

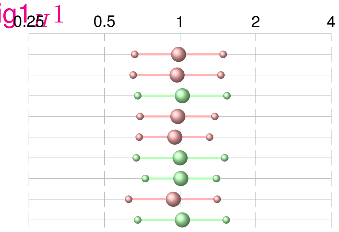

A	B	2.5	50	97.5	Sequence
**1**	**0**	**0.66**	**0.98**	**1.5**	**FSPDGEVAVSGGADGK**
**1**	**0**	**0.65**	**0.98**	**1.5**	**ILTASEDNSVGFHEGPPFK**
**1**	**0**	**0.68**	**1**	**1.5**	**LVSIGVDDVVR**
**1**	**2**	**0.69**	**0.98**	**1.4**	**QFLDGEVK**
**3**	**1**	**0.69**	**0.95**	**1.3**	**TLDELDNKEIGAPGAHK**
**1**	**0**	**0.67**	**1**	**1.5**	**VFDVQSGSLVQEFK**
**4**	**0**	**0.73**	**1**	**1.4**	**TNEVTDMVIEGDR**
**1**	**0**	**0.62**	**0.94**	**1.4**	**KLESQPK**
**0**	**1**	**0.68**	**1**	**1.5**	**QILTASGDASAK**

5.601 35 271 PREDICTED: SH3 domain-binding glutamic acid-rich-like protein 3-like [Anolis carolinensis]; 35 271 PREDICTED: SH3 domain-binding glutamic acid-rich-like protein 3-like [Anolis carolinensis]; 245 523 PREDICTED: SH3 domain-binding glutamic acid-rich-like protein 3-like [Anolis carolinensis]; 192 470 PREDICTED: SH3 domain-binding glutamic acid-rich-like protein 3-like [Anolis carolinensis]; 192 470 PREDICTED: SH3 domain-binding glutamic acid-rich-like protein 3-like [Anolis carolinensis]; 192 470 PREDICTED: SH3 domain-binding glutamic acid-rich-like protein 3-like [Anolis carolinensis]; 111 389 PREDICTED: SH3 domain-binding glutamic acid-rich-like protein 3-like [Anolis carolinensis]; 144 380 PREDICTED: SH3 domain-binding glutamic acid-rich-like protein 3-like [Anolis carolinensis]; 111 389 PREDICTED: SH3 domain-binding glutamic acid-rich-like protein 3-like [Anolis carolinensis]; 35 271 PREDICTED: SH3 domain-binding glutamic acid-rich-like protein 3-like [Anolis carolinensis]

Protein Accession	Unigene58804$_{H}1$ Unigene57834$_{H}1$ Unigene53929$_{H}1$ Unigene46648$_{H}1$ Unigene46647$_{H}1$ U
Mean Expression Ratio	1.01
Median Expression Ratio	1.01
Credible Interval	(0.678, 1.52)
Associated Peptides	2
Associated Spectra	3
Coverage	NaN

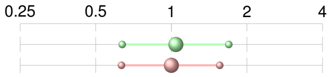

A	B	2.5	50	97.5	Sequence
1	0	0.64	1	1.7	SFQQAVEEEK
2	0	0.63	1	1.6	ILEYLR

5.602 430 759 hypothetical protein [Amblyomma maculatum]; 336 665 hypothetical protein [Amblyomma maculatum]

Protein Accession	CL4062.Contig3$_{H}1$ CL4062.Contig2$_{H}1$
Mean Expression Ratio	1.02
Median Expression Ratio	1.01
Credible Interval	(0.736, 1.4)
Associated Peptides	4
Associated Spectra	7
Coverage	NaN

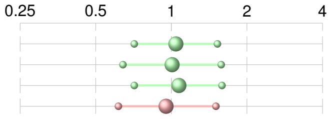

A	B	2.5	50	97.5	Sequence
2	1	0.71	1	1.5	ETLFEYLADPK
1	0	0.64	1	1.6	KTGQSAGFSYTDANK
1	1	0.71	1.1	1.6	KYIPGTK
0	1	0.62	0.95	1.5	MVFAGIK

5.603 8 1636 minus strand V-type proton ATPase catalytic subunit A [Crassostrea gigas]; 8 1636 minus strand V-type proton ATPase catalytic subunit A [Crassostrea gigas]; 8 1636 minus strand V-type proton ATPase catalytic subunit A [Crassostrea gigas]

Protein Accession	CL33671.Contig3$_{H}1$ CL33671.Contig2$_{H}1$ CL33671.Contig1$_{H}1$
Mean Expression Ratio	1.01
Median Expression Ratio	1.01
Credible Interval	(0.647, 1.57)
Associated Peptides	1
Associated Spectra	2
Coverage	NaN

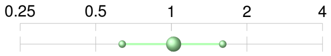

A	B	2.5	50	97.5	Sequence
**2**	**0**	**0.64**	**1**	**1.6**	**LAANHPLLTGQR**

5.604 2831 4900 minus strand Collagen alpha-3(VI) chain [Crassostrea gigas]

Protein Accession	Unigene26542$_{H}1$
Mean Expression Ratio	1.01
Median Expression Ratio	1.01
Credible Interval	(0.662, 1.54)
Associated Peptides	1
Associated Spectra	3
Coverage	NaN

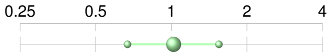

A	B	2.5	50	97.5	Sequence
**3**	**0**	**0.67**	**1**	**1.5**	**TEMFTPANGDRPDVPNVAIVITDGVSNINSR**

5.605 835 1083 minus strand Calmodulin [Crassostrea gigas]

Protein Accession	CL49568.Contig2$_{H}1$
Mean Expression Ratio	0.988
Median Expression Ratio	0.987
Credible Interval	(0.814, 1.2)
Associated Peptides	7
Associated Spectra	48
Coverage	NaN

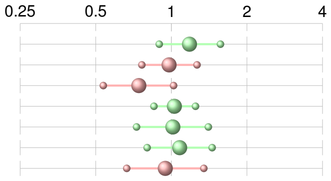

A	B	2.5	50	97.5	Sequence
**6**	**0**	**0.89**	**1.2**	**1.6**	**HLLTDKDEETCYK**
**7**	**2**	**0.76**	**0.98**	**1.3**	**KGFIANEEMK**
**4**	**0**	**0.54**	**0.74**	**1**	**QQVQMTEQEVDEVIDYIDK**
**9**	**10**	**0.85**	**1**	**1.3**	**VDFTEFYK**
**2**	**1**	**0.73**	**1**	**1.4**	**GFIANEEMK**
**5**	**0**	**0.8**	**1.1**	**1.5**	**LLLNK**
**1**	**1**	**0.67**	**0.95**	**1.3**	**ILDDQR**

5.606 58 1203 minus strand Obg-like ATPase 1 [Crassostrea gigas]

Protein Accession	CL33105.Contig1$_{H}1$
Mean Expression Ratio	0.989
Median Expression Ratio	0.987
Credible Interval	(0.615, 1.59)
Associated Peptides	1
Associated Spectra	1
Coverage	NaN

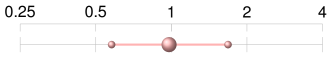

A	B	2.5	50	97.5	Sequence
**1**	**0**	**0.58**	**0.98**	**1.7**	**IHTDFEK**

5.607 2 517 minus strand Anhydro-N-acetylmuramic acid kinase [Crassostrea gigas]

Protein Accession	Unigene23377$_{H}1$
Mean Expression Ratio	1.01
Median Expression Ratio	1.01
Credible Interval	(0.67, 1.52)
Associated Peptides	2
Associated Spectra	3
Coverage	NaN

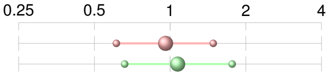

A	B	2.5	50	97.5	Sequence
1	1	0.61	0.96	1.5	KYGLQDVAR
0	1	0.66	1.1	1.8	KLVDAGLAVDK

5.608 176 670 hypothetical protein BRAFLDRAFT_122621 [Branchiostoma floridae] gi

Protein Accession	Unigene180$_{H}1$
Mean Expression Ratio	1.01
Median Expression Ratio	1.01
Credible Interval	(0.838, 1.22)
Associated Peptides	7
Associated Spectra	64
Coverage	NaN

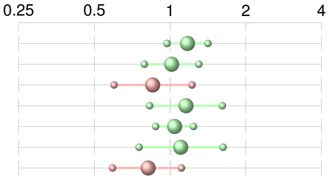

A	B	2.5	50	97.5	Sequence
11	9	0.97	1.2	1.4	FADENFLLK
6	3	0.79	1	1.3	VFFDITADGQPLGR
1	1	0.6	0.86	1.2	VIPNFMCQGGDFTNHNGTGGR
3	0	0.83	1.2	1.6	VLCTGEK
12	13	0.88	1	1.2	IVMELR
1	0	0.75	1.1	1.6	ADVVPK
2	2	0.59	0.82	1.1	HVVFGQVVEGMDVVR

5.609 293 1795 Amidase [Crassostrea gigas]

Protein Accession	CL5248.Contig6$_{H}1$
Mean Expression Ratio	1.01
Median Expression Ratio	1.01
Credible Interval	(0.649, 1.59)
Associated Peptides	1
Associated Spectra	2
Coverage	NaN

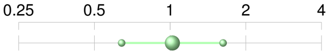

A	B	2.5	50	97.5	Sequence
2	0	0.64	1	1.6	LSTIGVTHFDR

5.610 557 2329 minus strand Collagen alpha-3(VI) chain [Crassostrea gigas]

Protein Accession	CL47586.Contig7$_{H}1$
Mean Expression Ratio	1.01
Median Expression Ratio	1.01
Credible Interval	(0.698, 1.48)
Associated Peptides	3
Associated Spectra	4
Coverage	NaN

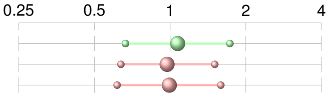

A	B	2.5	50	97.5	Sequence
1	0	0.66	1.1	1.7	KADYSAALGK
0	2	0.64	0.97	1.5	VALGFFNK
0	1	0.62	1	1.6	VVAVGVGAVVK

5.611 252 1289 minus strand PDZ and LIM domain protein 3 OS=Gallus gallus GN=PDLIM3 PE=1 SV=1

Protein Accession	CL15373.Contig10$_H$1
Mean Expression Ratio	1.01
Median Expression Ratio	1.01
Credible Interval	(0.721, 1.41)
Associated Peptides	5
Associated Spectra	6
Coverage	NaN

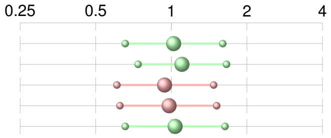

A	B	2.5	50	97.5	Sequence
1	0	0.65	1	1.6	SGDAILSINGVNTDHLTHEQAK
1	1	0.74	1.1	1.7	GFFVVEGK
1	0	0.61	0.94	1.5	IWKPQVTPLSE
1	0	0.62	0.98	1.5	CGGCDMLATGVIVK
0	1	0.66	1	1.6	AGCDIDFLIQR

5.612 102 887 hypothetical protein DAPPUDRAFT_304712 [Daphnia pulex]

Protein Accession	CL6869.Contig1$_H$1
Mean Expression Ratio	0.988
Median Expression Ratio	0.989
Credible Interval	(0.715, 1.35)
Associated Peptides	4
Associated Spectra	8
Coverage	NaN

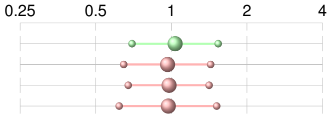

A	B	2.5	50	97.5	Sequence
2	0	0.7	1	1.5	GKPLFVR
1	1	0.65	0.97	1.4	LADIPEGK
2	1	0.68	0.98	1.4	HPEADAER
0	1	0.62	0.97	1.5	VDLSQLR

5.613 4 2400 IQ motif containing GTPase activating protein 2 [Xenopus laevis] gi; 4 2169 IQ motif containing GTPase activating protein 2 [Xenopus laevis] gi

Protein Accession	CL875.Contig2$_H$1 CL875.Contig1$_H$1
Mean Expression Ratio	0.989
Median Expression Ratio	0.989
Credible Interval	(0.6, 1.6)
Associated Peptides	1
Associated Spectra	1
Coverage	NaN

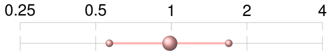

A	B	2.5	50	97.5	Sequence
0	1	0.57	0.99	1.7	ITLQDVVTHNR

5.614 324 1982 minus strand Pikachurin [Crassostrea gigas]

Protein Accession	CL4889.Contig10$_H$1
Mean Expression Ratio	0.991
Median Expression Ratio	0.989
Credible Interval	(0.664, 1.49)
Associated Peptides	2
Associated Spectra	3
Coverage	NaN

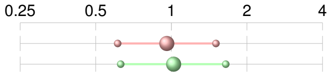

A	B	2.5	50	97.5	Sequence
2	0	0.61	0.96	1.5	VTAPIITEECGK
1	0	0.63	1	1.6	KGVLMR

5.615 290 2458 Putative beta-D-xylosidase 5 [Crassostrea gigas]

Protein Accession	CL49577.Contig2$_{H}1$
Mean Expression Ratio	1.01
Median Expression Ratio	1.01
Credible Interval	(0.683, 1.5)
Associated Peptides	3
Associated Spectra	4
Coverage	NaN

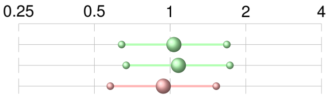

A	B	2.5	50	97.5	Sequence
0	1	0.64	1	1.7	QLLTDILR
1	1	0.67	1.1	1.7	VAEAAGVEVR
0	1	0.58	0.94	1.5	LEALEQGK

5.616 " 4146 6107 minus strand nonmuscle myosin II, partial [Aplysia californica]"

Protein Accession	Unigene30363$_{H}1$
Mean Expression Ratio	0.99
Median Expression Ratio	0.99
Credible Interval	(0.831, 1.19)
Associated Peptides	20
Associated Spectra	32
Coverage	NaN

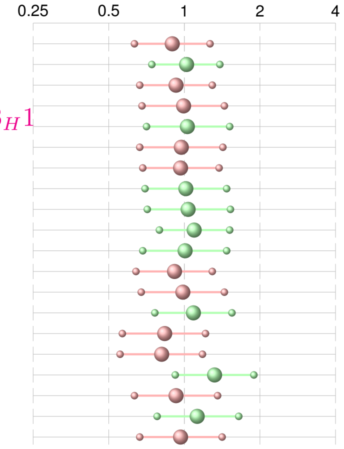

A	B	2.5	50	97.5	Sequence
2	0	0.63	0.89	1.3	ALEATVQEQR
2	2	0.74	1	1.4	ATIAALEGK
2	1	0.67	0.92	1.3	DLEQVSTQLEQVETK
1	0	0.68	0.99	1.4	ELDDQLEQNDVLVR
1	0	0.71	1	1.5	ELQIQAEDER
1	0	0.67	0.97	1.4	LAELEGQSR
1	1	0.68	0.97	1.4	LQEDLAAAER
1	0	0.7	1	1.5	QVDEAEEEVAR
1	0	0.71	1	1.5	VSSLEAQLADSQELMEEETR
2	1	0.79	1.1	1.5	VQIEELEDELQAAEDAK
1	0	0.68	1	1.5	EMEAELEEER
1	1	0.64	0.91	1.3	NLEAELVK
1	0	0.67	0.99	1.4	QSLLDEK
2	0	0.76	1.1	1.5	LKEDAIK
1	0	0.57	0.83	1.2	LSREEAYAQAK
1	0	0.56	0.81	1.2	LQAEVEDLTITLDNQR
1	1	0.92	1.3	1.9	AALARTNR
0	1	0.63	0.93	1.4	LKELQIQAEDERR
0	1	0.78	1.1	1.6	EMEQLHAQLEEARANADR
0	1	0.66	0.96	1.4	IEQLER

5.617 " 3 587 elongation factor 1 alpha, partial [Phorbas sp. nov. SB-2012]"

Protein Accession	Unigene20048$_{H}1$
Mean Expression Ratio	0.99
Median Expression Ratio	0.99
Credible Interval	(0.714, 1.39)
Associated Peptides	2
Associated Spectra	12
Coverage	NaN

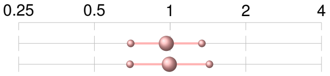

A	B	2.5	50	97.5	Sequence
5	1	0.7	0.97	1.3	IGGIGTVPVGR
3	3	0.69	1	1.4	LPLQDVYK

5.618 1 219 minus strand PREDICTED: nucleoside diphosphate kinase 3-like [Pan troglodytes]

Protein Accession	Unigene5384$_H$1
Mean Expression Ratio	1.01
Median Expression Ratio	1.01
Credible Interval	(0.724, 1.41)
Associated Peptides	2
Associated Spectra	11
Coverage	NaN

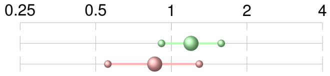

A	B	2.5	50	97.5	Sequence
6	3	0.91	1.2	1.6	GLVGEIIK
0	2	0.56	0.86	1.3	GLVGEIIKR

5.619 1213 3234 Protein-glutamine gamma-glutamyltransferase K [Crassostrea gigas]; 478 2499 Protein-glutamine gamma-glutamyltransferase K [Crassostrea gigas]

Protein Accession	CL29134.Contig6$_H$1 CL29134.Contig5$_H$1
Mean Expression Ratio	1.01
Median Expression Ratio	1.01
Credible Interval	(0.621, 1.64)
Associated Peptides	1
Associated Spectra	1
Coverage	NaN

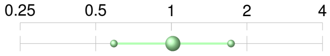

A	B	2.5	50	97.5	Sequence
0	1	0.59	1	1.7	FLFAEAHGER

5.620 606 1730 UDP-glucuronosyltransferase 2C1 precursor [Oryctolagus cuniculus]

Protein Accession	CL29849.Contig5$_H$1
Mean Expression Ratio	0.989
Median Expression Ratio	0.991
Credible Interval	(0.641, 1.52)
Associated Peptides	2
Associated Spectra	3
Coverage	NaN

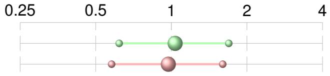

A	B	2.5	50	97.5	Sequence
1	1	0.62	1	1.7	LFVSHCGK
0	1	0.58	0.97	1.6	AAHAVEFVLK

5.621 3 218 beta-tubulin [Fasciola hepatica] gi

Protein Accession	Unigene7955$_H$1
Mean Expression Ratio	1.01
Median Expression Ratio	1.01
Credible Interval	(0.738, 1.38)
Associated Peptides	3
Associated Spectra	12
Coverage	NaN

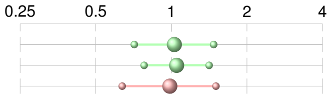

A	B	2.5	50	97.5	Sequence
3	1	0.71	1	1.5	EVDEQMLNVQNK
7	0	0.78	1.1	1.4	TAVCDIPPR
1	0	0.64	0.98	1.5	VSEQFTAMFR

5.622 " 303 500 minus strand protein lethal, putative [Pediculus humanus corporis] gi"

Protein Accession	CL15038.Contig1$_{H}1$
Mean Expression Ratio	0.989
Median Expression Ratio	0.991
Credible Interval	(0.69, 1.4)
Associated Peptides	2
Associated Spectra	9
Coverage	NaN

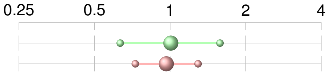

A	B	2.5	50	97.5	Sequence
1	0	0.63	1	1.6	QDMLATGR
6	2	0.73	0.97	1.3	QMPMLGR

5.623 " 134 2047 PREDICTED: succinate dehydrogenase [ubiquinone] flavoprotein subunit, mitochondrial-like isoform 3, partial [Strongylocentrotus purpuratus]; 134 2047 PREDICTED: succinate dehydrogenase [ubiquinone] flavoprotein subunit, mitochondrial-like isoform 3, partial [Strongylocentrotus purpuratus]; 134 2047 PREDICTED: succinate dehydrogenase [ubiquinone] flavoprotein subunit, mitochondrial-like isoform 3, partial [Strongylocentrotus purpuratus]"

Protein Accession	CL47322.Contig4$_{H}1$ CL47322.Contig3$_{H}1$ CL47322.Contig2$_{H}1$
Mean Expression Ratio	1.01
Median Expression Ratio	1.01
Credible Interval	(0.843, 1.21)
Associated Peptides	14
Associated Spectra	42
Coverage	NaN

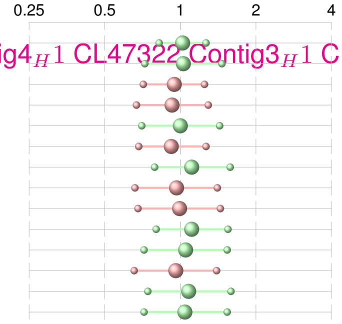

A	B	2.5	50	97.5	Sequence
10	4	0.83	1	1.3	AAFGLAEEGFK
1	1	0.72	1	1.5	ACANTIIEENKPGDR
3	3	0.71	0.95	1.3	AITIEIR
1	2	0.67	0.93	1.3	IYQQMEDLK
2	0	0.7	1	1.4	KHTLAYTNVDTGK
4	0	0.68	0.92	1.3	NTILATGGYGR
1	1	0.79	1.1	1.6	QDAGEASVGNLDK
1	0	0.66	0.97	1.4	SHTVAAQGGINAALGHMEEDDWR
1	0	0.68	0.99	1.4	AVIELENYGMPF
3	0	0.8	1.1	1.6	AFGGQSLK
1	0	0.72	1	1.5	GGINAAL
0	1	0.66	0.96	1.4	AVIELENYGMPFSR
0	1	0.74	1.1	1.6	LPPIR
0	1	0.71	1	1.5	WHFYDTVK

5.624 REVERSED 68 358 endonuclease-reverse transcriptase [Danaus plexippus]

Protein Accession	RRRRRUnigene39307$_{H}1$
Mean Expression Ratio	0.99
Median Expression Ratio	0.991
Credible Interval	(0.642, 1.54)
Associated Peptides	2
Associated Spectra	2
Coverage	NaN

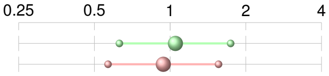

A	B	2.5	50	97.5	Sequence
0	1	0.63	1	1.7	TIITALDK
0	1	0.57	0.94	1.6	CQSLLDENSIK

5.625 188 4135 Vigilin [Crassostrea gigas]

Protein Accession	CL846.Contig3$_H$1
Mean Expression Ratio	0.99
Median Expression Ratio	0.991
Credible Interval	(0.614, 1.61)
Associated Peptides	1
Associated Spectra	1
Coverage	NaN

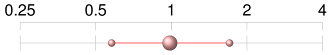

A	B	2.5	50	97.5	Sequence
**1**	**0**	**0.58**	**0.98**	**1.7**	**EVSIDIPHR**

5.626 2 244 minus strand hypothetical protein KGM_08771 [Danaus plexippus]

Protein Accession	CL49466.Contig2$_H$1
Mean Expression Ratio	1.01
Median Expression Ratio	1.01
Credible Interval	(0.705, 1.45)
Associated Peptides	2
Associated Spectra	6
Coverage	NaN

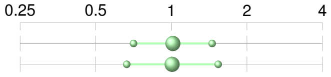

A	B	2.5	50	97.5	Sequence
**3**	**1**	**0.71**	**1**	**1.5**	**AVFIDLEPTVVDEVR**
**1**	**1**	**0.67**	**1**	**1.5**	**TVGGGDDSFNTFFSETGAGK**

5.627 121 1452 minus strand Hexokinase type 2 [Crassostrea gigas]; 245 1576 minus strand Hexokinase type 2 [Crassostrea gigas]; 121 1452 minus strand Hexokinase type 2 [Crassostrea gigas]; 121 1452 minus strand Hexokinase type 2 [Crassostrea gigas]; 161 1513 minus strand Hexokinase type 2 [Crassostrea gigas]; 113 1471 minus strand Hexokinase type 2 [Crassostrea gigas]; 225 1577 minus strand Hexokinase type 2 [Crassostrea gigas]; 121 1452 minus strand Hexokinase type 2 [Crassostrea gigas]

Protein Accession	CL350.Contig9$_H$1 CL350.Contig8$_H$1 CL350.Contig6$_H$1 CL350.Contig5$_H$1 CL350.Contig3$_H$1
Mean Expression Ratio	1.01
Median Expression Ratio	1.01
Credible Interval	(0.623, 1.65)
Associated Peptides	1
Associated Spectra	1
Coverage	NaN

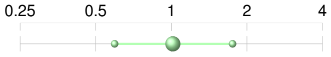

A	B	2.5	50	97.5	Sequence
**0**	**1**	**0.6**	**1**	**1.8**	**VLAPGIEFK**

5.628 " 249 1271 N(G),N(G)-dimethylarginine dimethylaminohydrolase 1 [Crassostrea gigas]"

Protein Accession	Unigene64051$_{H1}$
Mean Expression Ratio	1.01
Median Expression Ratio	1.01
Credible Interval	(0.658, 1.55)
Associated Peptides	2
Associated Spectra	3
Coverage	NaN

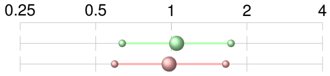

A	B	2.5	50	97.5	Sequence
1	1	0.64	1.1	1.7	LGEVGEIR
1	0	0.59	0.98	1.6	EIFVGIGK

5.629 1 852 beta actin-like 1 protein [Macrostomum lignano]

Protein Accession	Unigene31438$_{H1}$
Mean Expression Ratio	0.991
Median Expression Ratio	0.992
Credible Interval	(0.901, 1.09)
Associated Peptides	35
Associated Spectra	769
Coverage	NaN

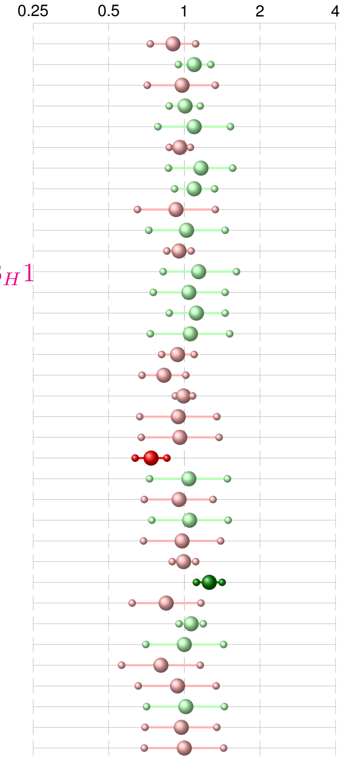

A	B	2.5	50	97.5	Sequence
9	5	0.73	0.9	1.1	AGFAGDDAPR
27	7	0.94	1.1	1.3	AVFPSIVGR
2	1	0.71	0.98	1.3	AVFPSIVGRPR
19	21	0.87	1	1.2	DSYVGDEAQSK
1	1	0.79	1.1	1.5	GVMVGMGQK
80	17	0.87	0.96	1.1	GYSFTTTAER
2	2	0.86	1.2	1.6	HQGVMVGMGQ
12	8	0.91	1.1	1.3	IWHHTFYNELR
1	0	0.65	0.93	1.3	MTQIMFETF
1	0	0.72	1	1.5	SYELPDGQVITIGN
60	15	0.85	0.95	1.1	SYELPDGQVITIGNER
1	1	0.82	1.1	1.6	TTGIVLDSGDGVTH
2	0	0.75	1	1.5	TTGIVLDSGDGVTHTVPIYEGY
7	0	0.87	1.1	1.5	TTGIVLDSGDGVTHTVPIYEGYALPH
1	0	0.73	1.1	1.5	VALDFEQEMATAAASSSLEK
18	17	0.81	0.94	1.1	VAPEEHPVLLTEA
10	6	0.68	0.83	1	VAPEEHPVLLTEAPLN
95	65	0.92	0.99	1.1	VAPEEHPVLLTEAPLNPK
1	0	0.67	0.95	1.3	VAPEEHPVLLTEAPLNPKANR
1	0	0.67	0.96	1.4	TEAPLNPK
23	13	0.64	0.74	0.85	RGILTLK
1	0	0.73	1	1.5	VAPEEHPVLLTEAPLNPKANREKMTQI
2	1	0.69	0.95	1.3	WDDMEK
1	0	0.74	1	1.5	HQGVMVGMGQKDSYVGDEAQSK
1	0	0.69	0.98	1.4	MTQIMFETFN
37	36	0.89	0.99	1.1	GILTLK
44	15	1.1	1.3	1.4	HQGVMVGMGQK
1	2	0.62	0.85	1.2	TTGIVLDSGDGVTHTVPIYEGYALPHAILR
50	19	0.95	1.1	1.2	YPIEHGIVTNWDDMEK
0	1	0.7	1	1.4	GIVTNWDDMEK
0	1	0.56	0.81	1.2	EIVRDIKEK
0	1	0.65	0.94	1.3	VMVGMGQK
0	1	0.71	1	1.4	VAPEEHPVLLTEAPL
0	2	0.7	0.97	1.4	VAPEEHPVLLTEAP
0	1	0.69	1	1.4	YPIEHGIVTNWDDME

5.630 1118 1255 minus strand equistatin precursor [Actinia equina]

Protein Accession	CL27616.Contig7$_{H}1$
Mean Expression Ratio	0.991
Median Expression Ratio	0.992
Credible Interval	(0.606, 1.59)
Associated Peptides	1
Associated Spectra	1
Coverage	NaN

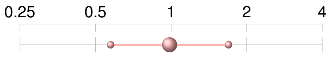

A	B	2.5	50	97.5	Sequence
**1**	**0**	**0.57**	**0.99**	**1.7**	**HLLGSYIPR**

5.631 132 590 minus strand Non-neuronal cytoplasmic intermediate filament protein OS=Helix aspersa PE=3 SV=2

Protein Accession	Unigene7770$_{H}1$
Mean Expression Ratio	0.991
Median Expression Ratio	0.992
Credible Interval	(0.646, 1.51)
Associated Peptides	1
Associated Spectra	3
Coverage	NaN

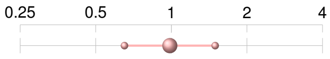

A	B	2.5	50	97.5	Sequence
**3**	**0**	**0.65**	**0.99**	**1.5**	**TMAEVPLGEK**

5.632 14 1642 minus strand chaperonin containing tcp1 [Haliotis discus discus]

Protein Accession	CL16298.Contig4$_{H}1$
Mean Expression Ratio	0.995
Median Expression Ratio	0.993
Credible Interval	(0.67, 1.49)
Associated Peptides	3
Associated Spectra	3
Coverage	NaN

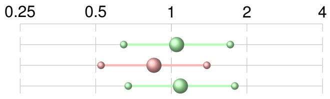

A	B	2.5	50	97.5	Sequence
**1**	**0**	**0.65**	**1.1**	**1.7**	**ELEVQHPAAK**
**1**	**0**	**0.52**	**0.85**	**1.4**	**MVINHLEK**
**1**	**0**	**0.67**	**1.1**	**1.8**	**AIGATALPK**

5.633 " 519 3182 minus strand Alpha-actinin, sarcomeric [Crassostrea gigas]"

Protein Accession	CL28960.Contig2$_H$1
Mean Expression Ratio	1.01
Median Expression Ratio	1.01
Credible Interval	(0.899, 1.13)
Associated Peptides	41
Associated Spectra	114
Coverage	NaN

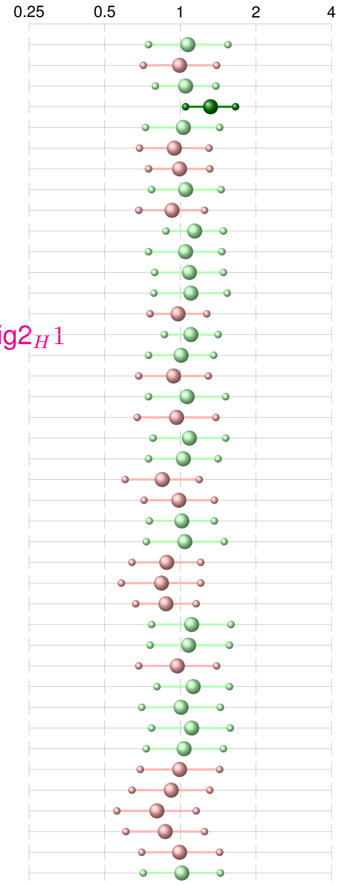

A	B	2.5	50	97.5	Sequence
1	0	0.75	1.1	1.5	AAPFNNWMDGAK
1	1	0.71	1	1.4	AGTGIENIEEDFR
5	1	0.79	1	1.4	AIIQYKPHIDELER
8	3	1	1.3	1.7	ALDFIASK
1	1	0.73	1	1.4	DNPIENLNTAFDIAEK
2	1	0.69	0.94	1.3	EALEEAER
3	2	0.75	0.99	1.3	ETADTDTAEQVMQSFK
2	1	0.77	1.1	1.5	FAIQDITVEELTAK
2	2	0.68	0.93	1.2	GISEDQMNEFR
6	1	0.88	1.1	1.5	IDQLHLEFAK
1	1	0.75	1	1.5	KAGTGIENIEEDFR
2	1	0.79	1.1	1.5	LSNRPAYLPTEGK
2	0	0.79	1.1	1.5	LVSIGAEEIVDGNVK
3	4	0.76	0.98	1.3	MSFNHFDK
4	5	0.87	1.1	1.4	MVSDIANAWK
2	2	0.75	1	1.4	NINEVENQILTR
2	1	0.68	0.94	1.3	RDQTLQQEMMR
1	0	0.75	1.1	1.5	GSLEEQLKK
1	0	0.67	0.96	1.4	HKPPKLEDK
2	0	0.78	1.1	1.5	MLDPEDMVNSAKPDER
2	1	0.75	1	1.4	LMEEYER
2	0	0.6	0.85	1.2	TRPWLENR
3	0	0.71	0.99	1.4	LQAFDK
2	2	0.75	1	1.4	KLDEFR
1	0	0.73	1	1.5	EDMLQSADFK
2	1	0.64	0.88	1.2	LGQLTVK
1	0	0.58	0.84	1.2	PHIDELER
2	3	0.66	0.87	1.2	LDEFR
1	0	0.77	1.1	1.6	HLDIPR
1	0	0.76	1.1	1.6	PGALDYMSFSTAL
1	0	0.68	0.97	1.4	DGLAFCALIHR
2	0	0.81	1.1	1.6	HRPELIDYHNLSK
1	0	0.7	1	1.4	KLQAFDK
1	0	0.77	1.1	1.6	LDHLAQK
1	0	0.73	1	1.5	YTQYTMETLR
0	1	0.69	0.99	1.4	EGLLDPAWEK
0	1	0.64	0.92	1.3	LENSFNTLQTR
0	1	0.56	0.8	1.2	KHKPPKLEDK
0	1	0.61	0.87	1.2	ELVPR
0	1	0.7	1	1.4	DQLHLEFAK
0	1	0.71	1	1.4	SIGVQMR

5.634 22 369 Protein farnesyltransferase/geranylgeranyltransferase type-1 subunit alpha [Crassostrea gigas]

Protein Accession	CL38456.Contig1$_H$1
Mean Expression Ratio	1.01
Median Expression Ratio	1.01
Credible Interval	(0.652, 1.55)
Associated Peptides	2
Associated Spectra	2
Coverage	NaN

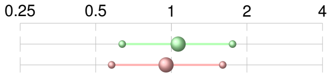

A	B	2.5	50	97.5	Sequence
1	0	0.64	1.1	1.7	YISAVIEDHPK
1	0	0.58	0.95	1.6	AIVASEEK

5.635 " 154 591 microsomal GST1, partial [Reishia clavigera]; 154 531 microsomal GST1, partial [Reishia clavigera]"

Protein Accession	CL3934.Contig2$_{H}1$ CL3934.Contig1$_{H}1$
Mean Expression Ratio	0.987
Median Expression Ratio	0.993
Credible Interval	(0.604, 1.58)
Associated Peptides	1
Associated Spectra	1
Coverage	NaN

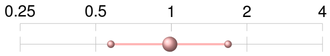

A	B	2.5	50	97.5	Sequence
1	0	0.57	0.98	1.7	FFCPGTAVNTNDALVER

5.636 " 116 1168 minus strand hypothetical protein CGI_10014553, partial [Crassostrea gigas]"

Protein Accession	CL19215.Contig5$_{H}1$
Mean Expression Ratio	1.01
Median Expression Ratio	1.01
Credible Interval	(0.722, 1.4)
Associated Peptides	5
Associated Spectra	6
Coverage	NaN

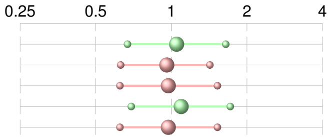

A	B	2.5	50	97.5	Sequence
1	0	0.67	1	1.6	SSFNDAVHSLFDK
1	1	0.63	0.96	1.4	SVIALGEGAGANIVAR
1	0	0.63	0.97	1.5	AQNKDQLQK
0	1	0.69	1.1	1.7	AAFIHVDVPGQEDDAK
0	1	0.62	0.97	1.5	VIENFQLSLR

5.637 981 1379 minus strand Multimerin-1 [Crassostrea gigas]; 998 1396 Multimerin-1 [Crassostrea gigas]; 975 1373 Multimerin-1 [Crassostrea gigas]; 960 1358 Multimerin-1 [Crassostrea gigas]; 810 1208 Multimerin-1 [Crassostrea gigas]

Protein Accession	CL22447.Contig9$_{H}1$ CL22447.Contig7$_{H}1$ CL22447.Contig6$_{H}1$ CL22447.Contig5$_{H}1$ CL2244
Mean Expression Ratio	0.995
Median Expression Ratio	0.993
Credible Interval	(0.708, 1.39)
Associated Peptides	3
Associated Spectra	7
Coverage	NaN

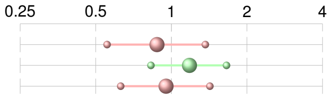

A	B	2.5	50	97.5	Sequence
1	0	0.55	0.88	1.4	TNTEFHTGPIVFDTTLSNK
2	2	0.83	1.2	1.7	VDAVLVADGQDVAR
0	2	0.63	0.95	1.4	VSFSAILK

5.638 51 1295 PREDICTED: phosphoglycerate kinase-like [Nasonia vitripennis]; 97 1341 PREDICTED: phosphoglycerate kinase-like [Nasonia vitripennis]

Protein Accession	CL14417.Contig13$_H$1 CL14417.Contig12$_H$1
Mean Expression Ratio	0.994
Median Expression Ratio	0.994
Credible Interval	(0.872, 1.13)
Associated Peptides	24
Associated Spectra	81
Coverage	NaN

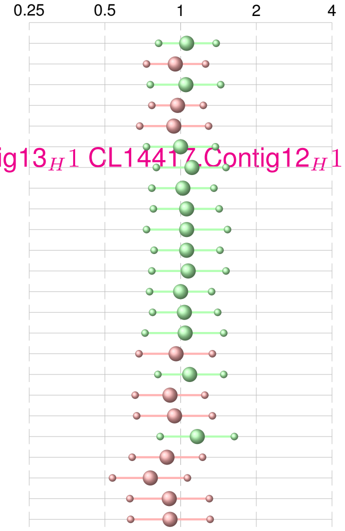

A	B	2.5	50	97.5	Sequence
4	3	0.82	1.1	1.4	ALMDAVVEATK
4	2	0.73	0.95	1.3	ATQEDIDKFR
2	1	0.76	1	1.4	FHVEEEGKGVAPDGTK
6	4	0.77	0.97	1.2	IVQGLMDK
2	1	0.69	0.94	1.3	LGDIYVNDAFGTAHR
1	2	0.73	1	1.4	LSIDKVDVK
3	0	0.8	1.1	1.5	SGIPADWMGLDIGPK
3	2	0.77	1	1.4	SVVLMSHLGRPDGTK
2	2	0.78	1.1	1.4	VDFNVPLKDGK
1	0	0.73	1.1	1.5	VLNNMEIGK
2	2	0.79	1.1	1.4	VLPGVAALTDA
2	0	0.77	1.1	1.5	VSMVSTGGGASLELLEGK
2	3	0.75	1	1.3	YSMKPVADELK
4	0	0.77	1	1.4	KELMYFAK
1	0	0.72	1	1.5	FTDAVK
2	0	0.68	0.96	1.3	VDFNVPLK
4	0	0.81	1.1	1.5	ASGFLLK
2	1	0.66	0.91	1.2	ELMYFAK
1	1	0.67	0.95	1.3	AHSSMVGCQLPIR
2	0	0.83	1.2	1.6	KNNVQIHLPEDFVTGDKFDEK
1	2	0.64	0.89	1.2	SLFDEEGAK
1	1	0.54	0.76	1.1	FHVEEEGK
0	1	0.63	0.9	1.3	LENLR
0	1	0.63	0.91	1.3	IVAALPTIK

5.639 297 734 minus strand PREDICTED: calmodulin [Canis lupus familiaris]; 297 734 PREDICTED: calmodulin [Canis lupus familiaris]; 143 508 minus strand Calmodulin [Crassostrea gigas]

Protein Accession	Unigene26719$_H$1 Unigene17836$_H$1 Unigene10331$_H$1
Mean Expression Ratio	0.992
Median Expression Ratio	0.994
Credible Interval	(0.614, 1.6)
Associated Peptides	1
Associated Spectra	1
Coverage	NaN

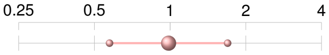

A	B	2.5	50	97.5	Sequence
1	0	0.57	0.99	1.7	TLTFQEVDAIVTK

5.640 1050 1307 calreticulin precursor [Aplysia californica] gi

Protein Accession	CL4813.Contig1$_H$1
Mean Expression Ratio	0.992
Median Expression Ratio	0.994
Credible Interval	(0.605, 1.62)
Associated Peptides	1
Associated Spectra	1
Coverage	NaN

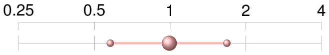

A	B	2.5	50	97.5	Sequence
0	1	0.58	0.99	1.7	EFGDSTWGK

5.641 76 822 14-3-3 protein [Heterodera glycines]; 76 822 minus strand 14-3-3 protein [Heterodera glycines]

Protein Accession	Unigene34590$_{H}1$ Unigene28010$_{H}1$
Mean Expression Ratio	0.997
Median Expression Ratio	0.995
Credible Interval	(0.705, 1.42)
Associated Peptides	2
Associated Spectra	8
Coverage	NaN

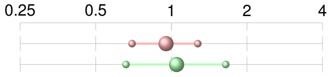

A	B	2.5	50	97.5	Sequence
**4**	**3**	**0.7**	**0.95**	**1.3**	**VVSSIEQK**
**1**	**0**	**0.66**	**1.1**	**1.6**	**KLEMAK**

5.642 247 726 Annexin A7 [Crassostrea gigas]; 286 675 Annexin A7 [Crassostrea gigas]

Protein Accession	CL15539.Contig3$_{H}1$ CL15539.Contig1$_{H}1$
Mean Expression Ratio	1
Median Expression Ratio	1
Credible Interval	(0.731, 1.38)
Associated Peptides	4
Associated Spectra	7
Coverage	NaN

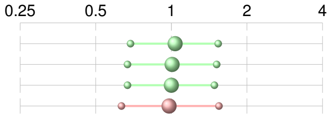

A	B	2.5	50	97.5	Sequence
**2**	**0**	**0.69**	**1**	**1.5**	**GLGTDEAAVIR**
**1**	**1**	**0.67**	**1**	**1.5**	**DLVSDLK**
**1**	**1**	**0.67**	**1**	**1.5**	**DIVSDTSGHFR**
**1**	**0**	**0.63**	**0.98**	**1.5**	**ILTSHNNMQR**

5.643 642 1415 minus strand cytochrome c oxidase subunit II [Oncomelania hupensis hupensis]

Protein Accession	CL6764.Contig3$_{H}1$
Mean Expression Ratio	1.01
Median Expression Ratio	1
Credible Interval	(0.622, 1.64)
Associated Peptides	1
Associated Spectra	1
Coverage	NaN

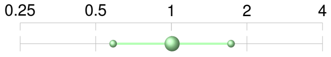

A	B	2.5	50	97.5	Sequence
**0**	**1**	**0.59**	**1**	**1.7**	**IVLPTHTDIR**

5.644 " 757 1443 minus strand nonmuscle myosin II, partial [Aplysia californica]"

Protein Accession	CL43455.Contig2$_{H}1$
Mean Expression Ratio	1
Median Expression Ratio	1
Credible Interval	(0.623, 1.63)
Associated Peptides	1
Associated Spectra	1
Coverage	NaN

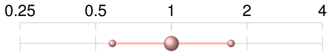

A	B	2.5	50	97.5	Sequence
**1**	**0**	**0.58**	**1**	**1.7**	**LVNEIK**

5.645 16 288 SJCHGC01950 protein [Schistosoma japonicum] gi

Protein Accession	Unigene16338$_{H}1$
Mean Expression Ratio	0.995
Median Expression Ratio	0.996
Credible Interval	(0.68, 1.44)
Associated Peptides	1
Associated Spectra	7
Coverage	NaN

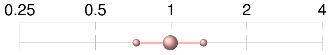

A	B	2.5	50	97.5	Sequence
**6**	**1**	**0.73**	**0.99**	**1.3**	**DIAAFIK**

5.646 880 1434 minus strand Prohormone-4 [Crassostrea gigas]

Protein Accession	CL27941.Contig2$_{H}1$
Mean Expression Ratio	0.997
Median Expression Ratio	0.996
Credible Interval	(0.611, 1.65)
Associated Peptides	1
Associated Spectra	1
Coverage	NaN

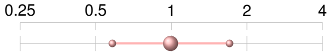

A	B	2.5	50	97.5	Sequence
**1**	**0**	**0.58**	**0.99**	**1.7**	**AALQGAQAGDERPLLR**

5.647 201 779 minus strand sigma class glutathione-s-transferase 2 [Haliotis discus discus]

Protein Accession	Unigene22998$_{H}1$
Mean Expression Ratio	0.997
Median Expression Ratio	0.997
Credible Interval	(0.737, 1.36)
Associated Peptides	4
Associated Spectra	13
Coverage	NaN

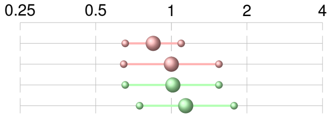

A	B	2.5	50	97.5	Sequence
**4**	**6**	**0.66**	**0.85**	**1.1**	**LVLALADQK**
**1**	**0**	**0.64**	**1**	**1.5**	**KLEEEEFPK**
**0**	**1**	**0.66**	**1**	**1.5**	**YFGMFQK**
**0**	**1**	**0.75**	**1.1**	**1.8**	**CFLAR**

5.648 232 654 hypothetical protein BRAFLDRAFT_87758 [Branchiostoma floridae] gi

Protein Accession	CL34711.Contig8$_{H}1$
Mean Expression Ratio	1.01
Median Expression Ratio	1
Credible Interval	(0.618, 1.64)
Associated Peptides	1
Associated Spectra	1
Coverage	NaN

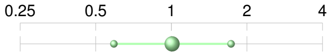

A	B	2.5	50	97.5	Sequence
**1**	**0**	**0.59**	**1**	**1.7**	**CVATGQGGYR**

5.649 771 1505 O-methyltransferase family 3 [Dicentrarchus labrax]

Protein Accession	Unigene19132$_{H}1$
Mean Expression Ratio	1.01
Median Expression Ratio	1
Credible Interval	(0.672, 1.51)
Associated Peptides	3
Associated Spectra	3
Coverage	NaN

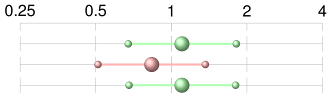

A	B	2.5	50	97.5	Sequence
1	0	0.67	1.1	1.8	FGASYLPR
0	1	0.51	0.83	1.4	IAETTLTHPWR
0	1	0.68	1.1	1.8	VLIPVR

5.650 54 662 minus strand Protein DJ-1 [Crassostrea gigas]

Protein Accession	CL16588.Contig3$_{H}1$
Mean Expression Ratio	1
Median Expression Ratio	1
Credible Interval	(0.62, 1.64)
Associated Peptides	1
Associated Spectra	1
Coverage	NaN

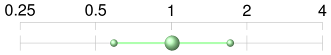

A	B	2.5	50	97.5	Sequence
1	0	0.59	1	1.7	NVNIVPDASMEEALK

5.651 112 819 pyruvate kinase [Crassostrea gigas]; 132 932 pyruvate kinase [Crassostrea gigas]

Protein Accession	CL31920.Contig3$_{H}1$ CL31920.Contig1$_{H}1$
Mean Expression Ratio	1
Median Expression Ratio	1
Credible Interval	(0.619, 1.64)
Associated Peptides	1
Associated Spectra	1
Coverage	NaN

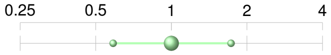

A	B	2.5	50	97.5	Sequence
1	0	0.59	1	1.7	ITIDDSYKEK

5.652 2 328 cytoskeletal actin 2 [Molgula oculata]

Protein Accession	Unigene2743$_{H}1$
Mean Expression Ratio	0.996
Median Expression Ratio	0.998
Credible Interval	(0.811, 1.22)
Associated Peptides	7
Associated Spectra	137
Coverage	NaN

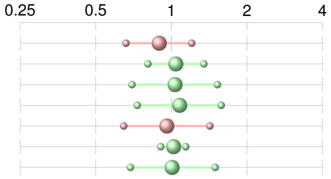

A	B	2.5	50	97.5	Sequence
3	2	0.66	0.89	1.2	IIAPPERK
7	2	0.81	1	1.3	IKIIAPPER
1	0	0.7	1	1.5	KIIAPPER
1	0	0.73	1.1	1.6	DITALAPPTMK
1	0	0.64	0.96	1.4	DLYANVVLSGGSTMYPGIADR
63	56	0.91	1	1.1	IIAPPER
1	0	0.69	1	1.5	KDLYANVVLSGGSTMYPGIADR

5.653 122 1195 minus strand Severin [Crassostrea gigas]

Protein Accession	CL7486.Contig12$_H1$
Mean Expression Ratio	0.996
Median Expression Ratio	0.998
Credible Interval	(0.614, 1.63)
Associated Peptides	1
Associated Spectra	2
Coverage	NaN

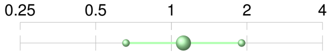

A	B	2.5	50	97.5	Sequence
1	1	0.66	1.1	1.9	TDHPLVPITCLTEGTK

5.654 242 589 Ribonucleoprotein RB97D OS=Drosophila melanogaster GN=Rb97D PE=2 SV=1

Protein Accession	CL6198.Contig1$_H1$
Mean Expression Ratio	0.998
Median Expression Ratio	0.998
Credible Interval	(0.613, 1.62)
Associated Peptides	1
Associated Spectra	1
Coverage	NaN

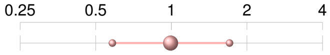

A	B	2.5	50	97.5	Sequence
1	0	0.58	0.99	1.7	LFIGGLSYDTDEK

5.655 124 396 minus strand 40S ribosomal protein S10 OS=Lumbricus rubellus GN=RPS10 PE=2 SV=1

Protein Accession	Unigene42157$_H1$
Mean Expression Ratio	0.999
Median Expression Ratio	0.999
Credible Interval	(0.693, 1.44)
Associated Peptides	2
Associated Spectra	6
Coverage	NaN

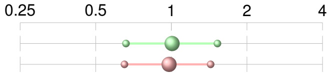

A	B	2.5	50	97.5	Sequence
2	1	0.66	1	1.5	KDFFSPK
3	0	0.65	0.98	1.4	YLRER

5.656 25 405 minus strand Elongation factor 1-delta [Anoplopoma fimbria]

Protein Accession	CL10502.Contig1$_H1$
Mean Expression Ratio	0.998
Median Expression Ratio	0.999
Credible Interval	(0.705, 1.41)
Associated Peptides	4
Associated Spectra	5
Coverage	NaN

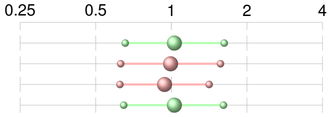

A	B	2.5	50	97.5	Sequence
1	0	0.66	1	1.6	KPVLIAK
1	0	0.63	1	1.6	VQTDGLVWGASK
1	1	0.62	0.94	1.4	LVPVGYGINK
0	1	0.65	1	1.6	RVQTDGLVWGASK

5.657 3788 7885 minus strand Filamin-C [Crassostrea gigas]

Protein Accession	CL4145.Contig29$_{H}1$
Mean Expression Ratio	0.999
Median Expression Ratio	1
Credible Interval	(0.644, 1.56)
Associated Peptides	1
Associated Spectra	2
Coverage	NaN

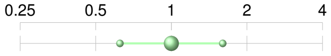

A	B	2.5	50	97.5	Sequence
**2**	**0**	**0.63**	**1**	**1.6**	**WGEEDIPGSPFAVEVI**

iTRAQ Data Analysis Report

Javier, Head 28d versus control

April 7, 2014

1 Introduction

This document summarizes an analysis of relative protein expression using iTRAQ. The reporter ion peak area measurements supplied by the ABI software are used to estimate treatment-dependent peptide and protein relative expression. Estimation is accomplished using a Bayesian approach with the model given below. The document includes a protein relative expression summary and a per-protein detailed analysis. The document is internally hyperlinked and linked externally to NCBI.

2 Experiment and Model Description

2.1 Experiment Design

The report summarizes data from one or more iTRAQ experiments addressing a common comparison. The experiment design, used in this analysis, is given in the table below.

	Experiment	Treatment	Channel	Sample
1	A	control	115	s1
2	A	test	119	s1
3	B	control	115	s2
4	B	test	119	s2

2.2 Input Files

Data for this analysis was extracted from the following tandem mass spectra (MSMS) summary files.

Experiment	MSMS Summary File
A	head2.txt
B	head3.txt

2.3 Statistical Model

The following statistical model was used to estimate the treatment-dependent effects.

LogIntensity ˜ Channel + Spectrum + Protein + Peptide + Protein:Treatment + Peptide:Treatment

3 Data Summary

The data supplied in the MSMS summary is filtered to remove unidentified proteins, contaminants, and peptides containing selected modifications. The following table sumarizes the data provided and used in the analysis.

	A	B	Combined
Supplied Spectra	21342	14837	36179
Unidentified Spectra	8154	6352	14506
Disallowed Modifications	361	186	547
Spectra from Contaminants	0	0	0
Missing Data	3154	2172	5326
Low Confidence Spectra	8154	6352	14506
Degenerate Peptides	3605	2243	5848
Remaining Spectra			9952
Unique Proteins			657
Unique Peptides			2370
Model R^2			0.943

4 Protein Summary

Each protein identified in one or more of the MSMS summaries is listed below in decreasing order of expression change magnitude. The median and estimated credible interval for each protein is given to the left in the table. Proteins identified by a single peptide are listed in a separate table.

4.1 Identified Proteins

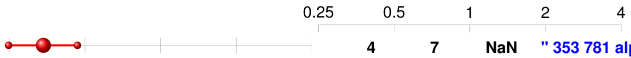

Peptides	Spectra	Coverage	Protein
4	7	NaN	" 353 781 alpha-B-crystallin, putative [Ixodes scapularis] gi"

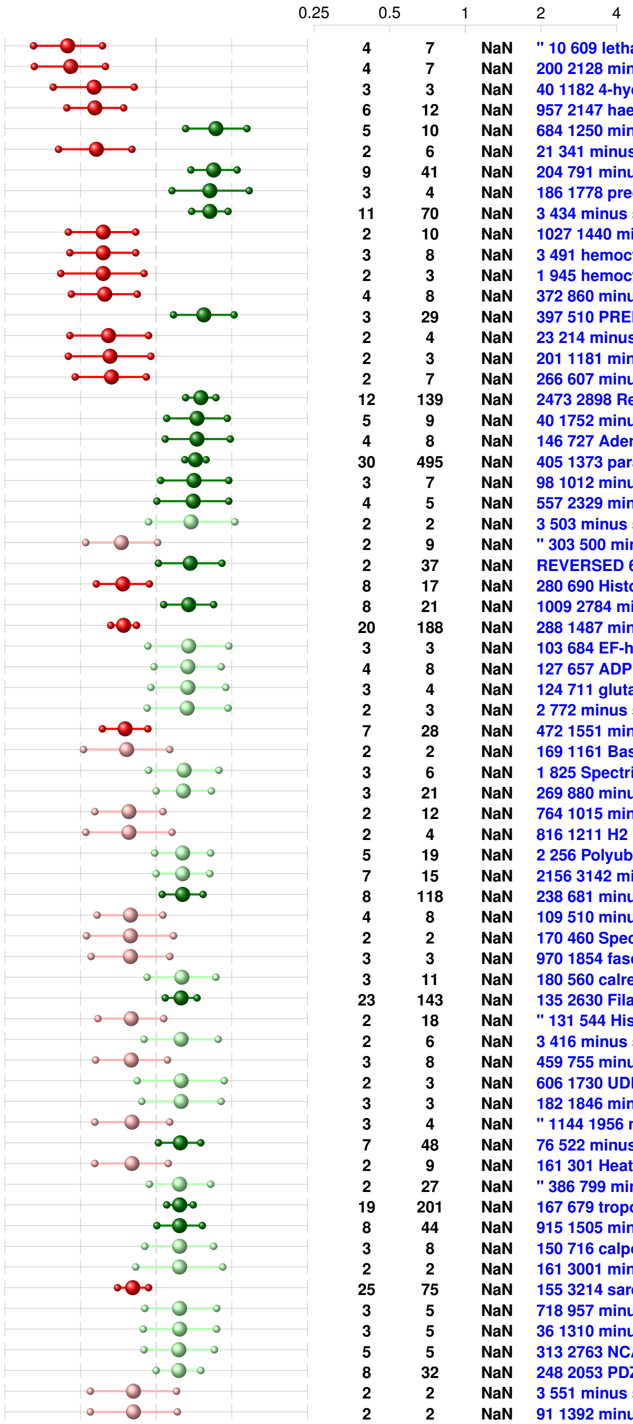

Peptides	Spectra	Coverage	Protein
4	7	NaN	" 10 609 lethal(2)essential for life protein, l2efl [Aedes aegypti] gi"
4	7	NaN	200 2128 minus strand heat shock protein 70 [Cristaria plicata]
3	3	NaN	40 1182 4-hydroxyphenylpyruvate dioxygenase [Crassostrea gigas]
6	12	NaN	957 2147 haemocyanin [Aplysia californica]
5	10	NaN	684 1250 minus strand Collagen alpha-5(VI) chain [Crassostrea gigas]
2	6	NaN	21 341 minus strand hemocyanin isoform 1 [Nucula nucleus]
9	41	NaN	204 791 minus strand PREDICTED: 15-hydroxyprostaglandin dehydrogenase [NAD+] [Gallus gallus]
3	4	NaN	186 1778 predicted protein [Nematostella vectensis] gi
11	70	NaN	3 434 minus strand myosin essential light chain [Haliotis discus discus]
2	10	NaN	1027 1440 minus strand hemocyanin isoform 1 [Haliotis diversicolor]
3	8	NaN	3 491 hemocyanin [Haliotis tuberculata]
2	3	NaN	1 945 hemocyanin isoform 1 [Haliotis diversicolor]
4	8	NaN	372 860 minus strand small heat shock protein [Tegillarca granosa]; 651 998 minus strand small heat shock protein
3	29	NaN	397 510 PREDICTED: actin-5C-like [Megachile rotundata]
2	4	NaN	23 214 minus strand hemocyanin isoform 1 [Nucula nucleus]
2	3	NaN	201 1181 minus strand Serine/threonine-protein phosphatase PP1-alpha catalytic subunit [Crassostrea gigas]
2	7	NaN	266 607 minus strand H2 [Haliotis tuberculata]
12	139	NaN	2473 2898 RecName: Full=Paramyosin gi|397603|emb|CAA79849.1| paramyosin [Echinococcus granulosus]
5	9	NaN	40 1752 minus strand heat shock protein 60 [Biomphalaria glabrata]; 40 1752 minus strand heat shock protein 60 [B
4	8	NaN	146 727 Adenylate kinase isoenzyme 1 [Crassostrea gigas]
30	495	NaN	405 1373 paramyosin [Haliotis discus discus]
3	7	NaN	98 1012 minus strand RecName: Full=Substrate-specific endoprotease Tex31; Flags: Precursor gi|32187775|emb|C
4	5	NaN	557 2329 minus strand Collagen alpha-3(VI) chain [Crassostrea gigas]
2	2	NaN	3 503 minus strand elongation factor-1 alpha [Pomacea bridgesi]
2	9	NaN	" 303 500 minus strand protein lethal, putative [Pediculus humanus corporis] gi"
2	37	NaN	REVERSED 6 395 PREDICTED: sister chromatid cohesion protein PDS5 homolog B-A isoform 1 [Nasonia vitripennis
8	17	NaN	280 690 Histone H3.3 [Harpegnathos saltator]
8	21	NaN	1009 2784 minus strand Collagen alpha-3(VI) chain [Crassostrea gigas]
20	188	NaN	288 1487 minus strand putative procollagen type VI alpha 4 [Haliotis diversicolor]
3	3	NaN	103 684 EF-hand domain-containing protein D2 [Crassostrea gigas]
4	8	NaN	127 657 ADP ribosylation factor 4 [Marsupenaeus japonicus]
3	4	NaN	124 711 glutathione S-transferase sigma 3 [Mytilus galloprovincialis]; 124 711 glutathione S-transferase sigma 3 [My
2	3	NaN	2 772 minus strand arylsulfatase [Haliotis discus discus]
7	28	NaN	472 1551 minus strand hypothetical protein [Amblyomma maculatum]
2	2	NaN	169 1161 Basement membrane-specific heparan sulfate proteoglycan core protein OS=Homo sapiens GN=HSPG2 P
3	6	NaN	1 825 Spectrin alpha chain [Crassostrea gigas]
3	21	NaN	269 880 minus strand myosin regulatory light chain [Fasciola hepatica]
2	12	NaN	764 1015 minus strand heat shock protein beta-1 [Trichinella spiralis] gi
2	4	NaN	816 1211 H2 [Haliotis tuberculata]
5	19	NaN	2 256 Polyubiquitin (Fragment) OS=Xenopus laevis PE=1 SV=2; 20 388 large subunit ribosomal protein L40e [Clonor
7	15	NaN	2156 3142 minus strand PREDICTED: hemicentin-1 [Xenopus (Silurana) tropicalis]
8	118	NaN	238 681 minus strand Globin OS=Cerithidea rhizophorarum PE=1 SV=1
4	8	NaN	109 510 minus strand hypothetical protein DAPPUDRAFT_231281 [Daphnia pulex]
2	2	NaN	170 460 Spectrin beta chain [Crassostrea gigas]
3	3	NaN	970 1854 fasciclin-like protein precursor [Aplysia californica] gi
3	11	NaN	180 560 calreticulin precursor [Aplysia californica] gi; 180 545 calreticulin precursor [Aplysia californica] gi
23	143	NaN	135 2630 Filamin-A [Crassostrea gigas]; 118 2613 Filamin-A [Crassostrea gigas]; 157 2652 Filamin-A [Crassostrea g
2	18	NaN	" 131 544 Histone H2B, gonadal OS=Patella granatina PE=1 SV=2"
2	6	NaN	3 416 minus strand myosin essential light chain [Crassostrea gigas]
3	8	NaN	459 755 minus strand heat shock protein beta-1 [Trichinella spiralis] gi
2	3	NaN	606 1730 UDP-glucuronosyltransferase 2C1 precursor [Oryctolagus cuniculus]
3	3	NaN	182 1846 minus strand hypothetical protein DAPPUDRAFT_302108 [Daphnia pulex]; 110 1774 minus strand hypothe
3	4	NaN	" 1144 1956 minus strand Retinol dehydrogenase 12, partial [Crassostrea gigas]"
7	48	NaN	76 522 minus strand Troponin C [Crassostrea gigas]; 173 619 minus strand Troponin C [Crassostrea gigas]; 173 619
2	9	NaN	161 301 Heat shock protein beta-6 OS=Mus musculus GN=Hspb6 PE=1 SV=1; 167 310 Heat shock protein beta-1 OS
2	27	NaN	" 386 799 minus strand Myosin regulatory light chain LC-2, mantle muscle OS=Todarodes pacificus PE=1 SV=1; 656
19	201	NaN	167 679 tropomyosin [Haliotis discus discus]
8	44	NaN	915 1505 minus strand Collagen alpha-5(VI) chain [Crassostrea gigas]
3	8	NaN	150 716 calponin/transgelin [Papilio xuthus]
2	2	NaN	161 3001 minus strand E3 ubiquitin-protein ligase MIB2 [Crassostrea gigas]
25	75	NaN	155 3214 sarco/endoplasmic reticulum calcium ATPase isoform B [Pinctada fucata]; 132 3110 sarco/endoplasmic re
3	5	NaN	718 957 minus strand Calponin-1 OS=Gallus gallus GN=CNN1 PE=1 SV=2
3	5	NaN	36 1310 minus strand elongation factor-1 gamma [Diguetia canities]
5	5	NaN	313 2763 NCAM-related cell adhesion molecule precursor [Aplysia californica] gi
8	32	NaN	248 2053 PDZ and LIM domain protein 5 [Crassostrea gigas]
2	2	NaN	3 551 minus strand Spectrin beta chain [Crassostrea gigas]
2	2	NaN	91 1392 minus strand hypothetical protein AND_03103 [Anopheles darlingi]

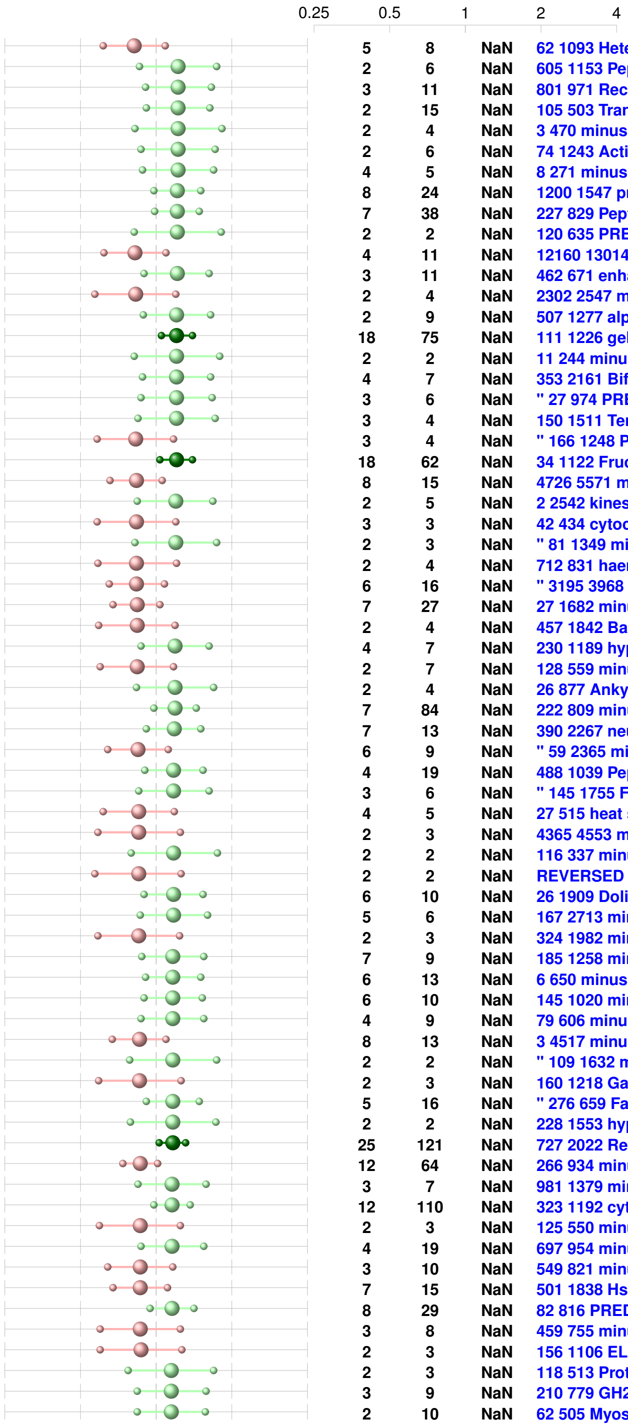

Peptides	Spectra	Coverage	Protein
5	8	NaN	62 1093 Heterogeneous nuclear ribonucleoprotein A2-like protein 1 [Crassostrea gigas]
2	6	NaN	605 1153 Peptidyl-prolyl cis-trans isomerase B [Crassostrea gigas]; 1046 1630 Peptidyl-prolyl cis-trans isomerase B
3	11	NaN	801 971 RecName: Full=Tropomyosin-2; AltName: Full=Tropomyosin II; Short=BgTMII; Short=TMII gi|155940|gb|AA
2	15	NaN	105 503 Transgelin-2 [Crassostrea gigas]
2	4	NaN	3 470 minus strand Spectrin beta chain [Crassostrea gigas]; 5 451 minus strand Spectrin beta chain [Crassostrea gi
2	6	NaN	74 1243 Actin-related protein 2 [Crassostrea gigas]
4	5	NaN	8 271 minus strand Laminin subunit gamma-1 [Crassostrea gigas]
8	24	NaN	1200 1547 protein disulfide isomerase [Conus eburneus]
7	38	NaN	227 829 Peptidyl-prolyl cis-trans isomerase B [Crassostrea gigas]
2	2	NaN	120 635 PREDICTED: similar to collagen type VI alpha 6 [Ciona intestinalis]; 120 635 minus strand PREDICTED: simi
4	11	NaN	12160 13014 twitchin [Mytilus galloprovincialis]
3	11	NaN	462 671 enhancer protein [Homo sapiens]
2	4	NaN	2302 2547 minus strand hemocyanin isoform 1 [Haliotis diversicolor]; 816 1211 H2 [Haliotis tuberculata]
2	9	NaN	507 1277 alpha tubulin [Notothenia coriiceps]
18	75	NaN	111 1226 gelsolin [Aplysia californica] gi; 42 1139 minus strand gelsolin [Aplysia californica] gi
2	2	NaN	11 244 minus strand Tubulin alpha chain (Fragment) OS=Lytechinus pictus PE=3 SV=1
4	7	NaN	353 2161 Bifunctional 3'-phosphoadenosine 5'-phosphosulfate synthetase [Crassostrea gigas]; 141 19
3	6	NaN	" 27 974 PREDICTED: succinyl-CoA ligase [GDP-forming] subunit alpha, mitochondrial-like [Oreochromis niloticus];
3	4	NaN	150 1511 Tensin OS=Gallus gallus GN=TNS PE=1 SV=2
3	4	NaN	" 166 1248 PREDICTED: probable pyruvate dehydrogenase E1 component subunit alpha, mitochondrial-like [Metase
18	62	NaN	34 1122 Fructose-bisphosphate aldolase [Crassostrea gigas]
8	15	NaN	4726 5571 minus strand twitchin [Mytilus galloprovincialis]
2	5	NaN	2 2542 kinesin heavy chain 1 [Aplysia californica] gi; 2 2542 kinesin heavy chain 1 [Aplysia californica] gi
3	3	NaN	42 434 cytochrome b5 [Haliotis discus discus]; 42 434 cytochrome b5 [Haliotis discus discus]
2	3	NaN	" 81 1349 minus strand PREDICTED: LOW QUALITY PROTEIN: dolichyl-diphosphooligosaccharide–protein glycosyl
2	4	NaN	712 831 haemocyanin [Aplysia californica]
6	16	NaN	" 3195 3968 Spectrin alpha chain, putative [Pediculus humanus corporis] gi"
7	27	NaN	27 1682 minus strand PREDICTED: nucleolar protein 56 [Oreochromis niloticus]
2	4	NaN	457 1842 Basement membrane-specific heparan sulfate proteoglycan core protein [Crassostrea gigas]; 457 1842 Ba
4	7	NaN	230 1189 hypothetical protein [Amblyomma maculatum]
2	7	NaN	128 559 minus strand predicted protein [Nematostella vectensis] gi
2	4	NaN	26 877 Ankyrin-2 [Crassostrea gigas]
7	84	NaN	222 809 minus strand PREDICTED: 15-hydroxyprostaglandin dehydrogenase [NAD+] [Gallus gallus]
7	13	NaN	390 2267 neurofilament protein NF70 [Helix aspersa]; 318 2195 neurofilament protein NF70 [Helix aspersa]
6	9	NaN	" 59 2365 minus strand Aconitate hydratase, mitochondrial [Crassostrea gigas]"
4	19	NaN	488 1039 Peptidyl-prolyl cis-trans isomerase B [Crassostrea gigas]; 488 1039 Peptidyl-prolyl cis-trans isomerase B [
3	6	NaN	" 145 1755 Fumarate hydratase class I, aerobic [Crassostrea gigas]"
4	5	NaN	27 515 heat shock protein 70 [Capsaspora owczarzaki ATCC 30864]; 30 464 minus strand hypothetical protein [Trich
2	3	NaN	4365 4553 minus strand Basement membrane-specific heparan sulfate proteoglycan core protein [Crassostrea gigas
2	2	NaN	116 337 minus strand Golgi-associated plant pathogenesis-related protein 1 [Crassostrea gigas]; 538 759 minus stra
2	2	NaN	REVERSED 68 358 endonuclease-reverse transcriptase [Danaus plexippus]
6	10	NaN	26 1909 Dolichyl-diphosphooligosaccharide–protein glycosyltransferase subunit 2 [Crassostrea gigas]
5	6	NaN	167 2713 minus strand Major vault protein [Crassostrea gigas]
2	3	NaN	324 1982 minus strand Pikachurin [Crassostrea gigas]
7	9	NaN	185 1258 minus strand Severin [Crassostrea gigas]
6	13	NaN	6 650 minus strand glutathione S-transferase mu class protein [Crassostrea ariakensis]; 117 761 glutathione S-trans
6	10	NaN	145 1020 minus strand PREDICTED: inorganic pyrophosphatase-like [Saccoglossus kowalevskii]
4	9	NaN	79 606 minus strand PREDICTED: similar to Ras-like protein 3 precursor (Protein roughened) [Tribolium castaneum]
8	13	NaN	3 4517 minus strand Clathrin heavy chain 1 [Crassostrea gigas]
2	2	NaN	" 109 1632 minus strand Seryl-tRNA synthetase, cytoplasmic [Crassostrea gigas]; 109 1614 minus strand Seryl-tRN
2	3	NaN	160 1218 Galectin-9 [Crassostrea gigas]
5	16	NaN	" 276 659 Fatty acid-binding protein, adipocyte [Crassostrea gigas]; 426 809 Fatty acid-binding protein, adipocyte [C
2	2	NaN	228 1553 hypothetical protein CGI_10024545 [Crassostrea gigas]; 228 1553 hypothetical protein CGI_10024545 [Cras
25	121	NaN	727 2022 RecName: Full=Enolase; AltName: Full=2-phospho-D-glycerate hydro-lyase; AltName: Full=2-phosphoglyc
12	64	NaN	266 934 minus strand Kyphoscoliosis peptidase [Crassostrea gigas]
3	7	NaN	981 1379 minus strand Multimerin-1 [Crassostrea gigas]; 998 1396 Multimerin-1 [Crassostrea gigas]; 975 1373 Multin
12	110	NaN	323 1192 cytosolic malate dehydrogenase [Lottia scabra]; 323 1192 cytosolic malate dehydrogenase [Lottia scabra];
2	3	NaN	125 550 minus strand ribosomal protein S20 [Chlamys farreri]; 125 550 minus strand ribosomal protein S20 [Chlamy
4	19	NaN	697 954 minus strand Peptidyl-prolyl cis-trans isomerase B [Crassostrea gigas]; 614 877 Peptidyl-prolyl cis-trans iso
3	10	NaN	549 821 minus strand Calponin-2 [Crassostrea gigas]
7	15	NaN	501 1838 Hsp90A [Haliotis asinina]
8	29	NaN	82 816 PREDICTED: triosephosphate isomerase B [Oreochromis niloticus]
3	8	NaN	459 755 minus strand heat shock protein beta-1 [Trichinella spiralis] gi; 464 763 minus strand heat shock protein bet
2	3	NaN	156 1106 ELAV 2-like protein [Aplysia californica] gi
2	3	NaN	118 513 Protein CAL-5 [Caenorhabditis elegans] gi
3	9	NaN	210 779 GH20056 [Drosophila grimshawi] gi; 216 785 minus strand GH20056 [Drosophila grimshawi] gi; 210 779 GH2
2	10	NaN	62 505 Myosin-2 essential light chain [Crassostrea gigas]

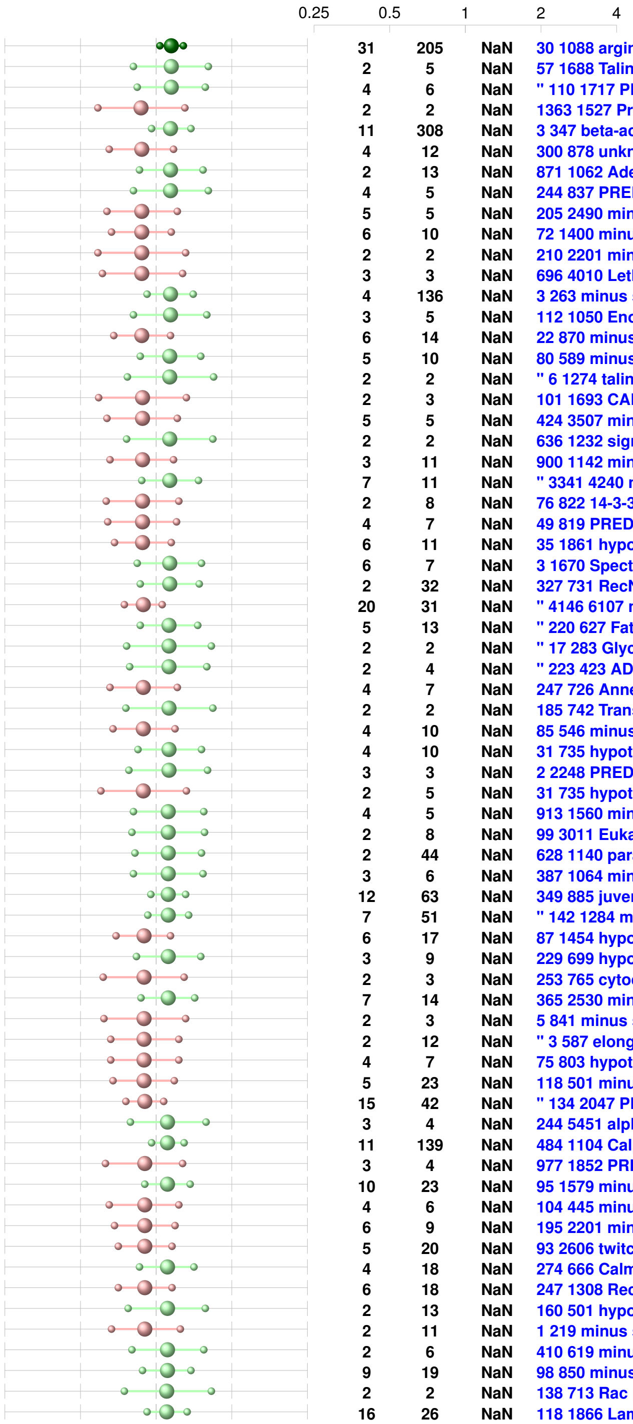

Peptides	Spectra	Coverage	Protein
31	205	NaN	30 1088 arginine kinase [Conus novaehollandiae]
2	5	NaN	57 1688 Talin-1 [Camponotus floridanus]; 57 1865 Talin-1 [Camponotus floridanus]; 57 1835 Talin-1 [Camponotus flo
4	6	NaN	" 110 1717 PREDICTED: glutamate dehydrogenase, mitochondrial-like [Oreochromis niloticus]; 110 1717 PREDICTE
2	2	NaN	1363 1527 Protein disulfide-isomerase A3 [Crassostrea gigas]
11	308	NaN	3 347 beta-actin [Meretrix meretrix]; 3 215 Actin-2 OS=Onchocerca volvulus GN=act-2b PE=3 SV=1
4	12	NaN	300 878 unknown [Schistosoma japonicum]; 567 1145 unknown [Schistosoma japonicum]; 1110 1688 unknown [Sch
2	13	NaN	871 1062 Adenylate kinase isoenzyme 5 [Crassostrea gigas]
4	5	NaN	244 837 PREDICTED: ras-like GTP-binding protein Rho1-like [Megachile rotundata]
5	5	NaN	205 2490 minus strand glucose-regulated protein 94 [Crassostrea gigas]
6	10	NaN	72 1400 minus strand hypothetical protein RCJMB04_22i13 [Gallus gallus]
2	2	NaN	210 2201 minus strand Glycyl-tRNA synthetase [Crassostrea gigas]; 210 2201 minus strand Glycyl-tRNA synthetase
3	3	NaN	696 4010 Lethal(2) giant larvae-like protein 1 [Crassostrea gigas]; 657 3971 minus strand Lethal(2) giant larvae-like p
4	136	NaN	3 263 minus strand beta-actin [Urechis unicinctus]
3	5	NaN	112 1050 Enolase-phosphatase E1 [Crassostrea gigas]; 112 1050 Enolase-phosphatase E1 [Crassostrea gigas]
6	14	NaN	22 870 minus strand PREDICTED: alcohol dehydrogenase class-3 [Sarcophilus harrisii]
5	10	NaN	80 589 minus strand PREDICTED: adenine phosphoribosyltransferase-like [Strongylocentrotus purpuratus]
2	2	NaN	" 6 1274 talin, putative [Ixodes scapularis] gi"
2	3	NaN	101 1693 CALNUC [Spodoptera frugiperda]; 141 1325 Nucleobindin-2 [Crassostrea gigas]
5	5	NaN	424 3507 minus strand putative Na+/K+-ATPase alpha subunit [Paroctopus digueti]
2	2	NaN	636 1232 sigma class glutathione-s-transferase 3 [Haliotis discus discus]; 963 1559 sigma class glutathione-s-transf
3	11	NaN	900 1142 minus strand heat shock protein 20 [Cyclina sinensis]
7	11	NaN	" 3341 4240 minus strand nonmuscle myosin II, partial [Aplysia californica]"
2	8	NaN	76 822 14-3-3 protein [Heterodera glycines]; 76 822 minus strand 14-3-3 protein [Heterodera glycines]
4	7	NaN	49 819 PREDICTED: elongation factor 1-delta-like isoform 2 [Strongylocentrotus purpuratus]
6	11	NaN	35 1861 hypothetical protein DAPPUDRAFT_309082 [Daphnia pulex]
6	7	NaN	3 1670 Spectrin alpha chain [Crassostrea gigas]
2	32	NaN	327 731 RecName: Full=Arginine kinase; Short=AK gi|439719|dbj|BAA05100.1| arginine kinase [Haliotis madaka]
20	31	NaN	" 4146 6107 minus strand nonmuscle myosin II, partial [Aplysia californica]"
5	13	NaN	" 220 627 Fatty acid-binding protein, brain [Crassostrea gigas]"
2	2	NaN	" 17 283 Glycerol-3-phosphate dehydrogenase [NAD+], cytoplasmic [Crassostrea gigas]"
2	4	NaN	" 223 423 ADPATP carrier protein; K05863 solute carrier family 25 (mitochondrial adenine nucleotide translocator), m
4	7	NaN	247 726 Annexin A7 [Crassostrea gigas]; 286 675 Annexin A7 [Crassostrea gigas]
2	2	NaN	185 742 Transmembrane protein Tmp21 [Crassostrea gigas]; 1 444 minus strand Transmembrane protein Tmp21 [Cr
4	10	NaN	85 546 minus strand RecName: Full=Eukaryotic translation initiation factor 5A; Short=eIF-5A gi|51702279|sp|P6292
4	10	NaN	31 735 hypothetical protein BRAFLDRAFT_78603 [Branchiostoma floridae] gi; 55 735 hypothetical protein BRAFLDF
3	3	NaN	2 2248 PREDICTED: heat shock 70 kDa protein 4-like [Oreochromis niloticus]; 2 2227 PREDICTED: heat shock 70 kD
2	5	NaN	31 735 hypothetical protein BRAFLDRAFT_78603 [Branchiostoma floridae] gi; 55 735 hypothetical protein BRAFLDF
4	5	NaN	913 1560 minus strand Calcium-dependent protein kinase isoform 2 [Crassostrea gigas]
2	8	NaN	99 3011 Eukaryotic translation initiation factor 3 subunit A [Crassostrea gigas]
2	44	NaN	628 1140 paramyosin [Haliotis discus discus]
3	6	NaN	387 1064 minus strand PREDICTED: hypothetical protein [Hydra magnipapillata]; 275 952 minus strand PREDICTED
12	63	NaN	349 885 juvenile hormone diol kinase [Danaus plexippus]; 349 885 juvenile hormone diol kinase [Danaus plexippus]
7	51	NaN	" 142 1284 minus strand Chain O, Crsytal Structure Of Human Liver Gapdh gi|67464044|pdb|1ZNQ|P Chain P, Crsyta
6	17	NaN	87 1454 hypothetical protein BRAFLDRAFT_287788 [Branchiostoma floridae] gi; 117 1469 hypothetical protein BRAF
3	9	NaN	229 699 hypothetical protein CGI_10017744 [Crassostrea gigas]; 199 669 hypothetical protein CGI_10017744 [Crasso
2	3	NaN	253 765 cytochrome c oxidase subunit IV [Urechis caupo]
7	14	NaN	365 2530 minus strand calpain B [Gecarcinus lateralis]
2	3	NaN	5 841 minus strand Spectrin alpha chain [Crassostrea gigas]
2	12	NaN	" 3 587 elongation factor 1 alpha, partial [Phorbas sp. nov. SB-2012]"
4	7	NaN	75 803 hypothetical protein DAPPUDRAFT_231350 [Daphnia pulex]
5	23	NaN	118 501 minus strand histone 2A.Z [Aplysia californica]
15	42	NaN	" 134 2047 PREDICTED: succinate dehydrogenase [ubiquinone] flavoprotein subunit, mitochondrial-like isoform 3, p
3	4	NaN	244 5451 alpha macroglobulin [Chlamys farreri]; 244 5493 alpha macroglobulin [Chlamys farreri]
11	139	NaN	484 1104 Calponin-2 [Crassostrea gigas]; 309 938 Calponin-2 [Crassostrea gigas]
3	4	NaN	977 1852 PREDICTED: similar to prohibitin [Tribolium castaneum] gi
10	23	NaN	95 1579 minus strand hypothetical protein BRAFLDRAFT_113974 [Branchiostoma floridae] gi
4	6	NaN	104 445 minus strand macrophage migration inhibitory factor [Ascaris suum]
6	9	NaN	195 2201 minus strand hypothetical protein DAPPUDRAFT_315656 [Daphnia pulex]
5	20	NaN	93 2606 twitchin [Mytilus galloprovincialis]
4	18	NaN	274 666 Calmodulin-4 [Heterocephalus glaber]
6	18	NaN	247 1308 RecName: Full=Guanine nucleotide-binding protein G(o) subunit alpha gi|9633|emb|CAA78806.1| GTP-bin
2	13	NaN	160 501 hypothetical protein CGI_10001640 [Crassostrea gigas]; 160 588 hypothetical protein CGI_10001640 [Crasso
2	11	NaN	1 219 minus strand PREDICTED: nucleoside diphosphate kinase 3-like [Pan troglodytes]
2	6	NaN	410 619 minus strand Guanine nucleotide-binding protein subunit gamma-1 [Crassostrea gigas]
9	19	NaN	98 850 minus strand 14-3-3 protein epsilon [Schistocerca gregaria]
2	2	NaN	138 713 Rac [Aplysia californica] gi; 125 691 Rac [Aplysia californica] gi; 149 871 minus strand Rac [Aplysia californ
16	26	NaN	118 1866 Lamin Dm0 [Crassostrea gigas]

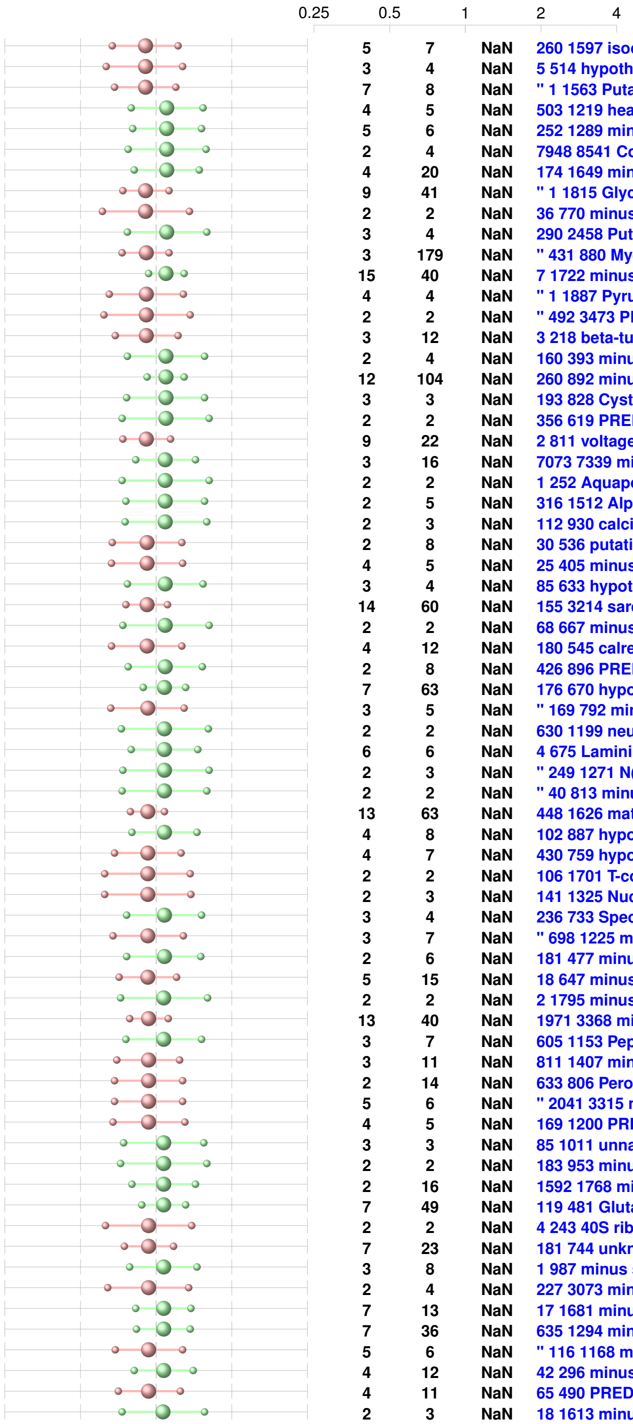

Peptides	Spectra	Coverage	Protein
5	7	NaN	260 1597 isocitrate dehydrogenase [Mytilus galloprovincialis]; 260 1597 isocitrate dehydrogenase [Mytilus galloprov
3	4	NaN	5 514 hypothetical protein KGM_05418 [Danaus plexippus]
7	8	NaN	" 1 1563 Putative methylmalonate-semialdehyde dehydrogenase [acylating], mitochondrial [Crassostrea gigas]"
4	5	NaN	503 1219 heat shock cognate 71 [Mytilus galloprovincialis]
5	6	NaN	252 1289 minus strand PDZ and LIM domain protein 3 OS=Gallus gallus GN=PDLIM3 PE=1 SV=1
2	4	NaN	7948 8541 Collagen alpha-3(VI) chain [Crassostrea gigas]
4	20	NaN	174 1649 minus strand Collagen alpha-1(XII) chain [Crassostrea gigas]; 123 1490 minus strand Collagen alpha-1(XII)
9	41	NaN	" 1 1815 Glycogen phosphorylase, muscle form [Crassostrea gigas]"
2	2	NaN	36 770 minus strand Endoplasmic reticulum protein ERp29 [Crassostrea gigas]
3	4	NaN	290 2458 Putative beta-D-xylosidase 5 [Crassostrea gigas]
3	179	NaN	" 431 880 Myosin essential light chain, striated adductor muscle OS=Aequipecten irradians PE=1 SV=2"
15	40	NaN	7 1722 minus strand Membrane metallo-endopeptidase-like 1 OS=Mus musculus GN=Mmel1 PE=1 SV=1
4	4	NaN	" 1 1887 Pyruvate carboxylase, mitochondrial [Crassostrea gigas]"
2	2	NaN	" 492 3473 PREDICTED: 2-oxoglutarate dehydrogenase, mitochondrial isoform 1 [Sarcophilus harrisii]; 492 3458 PR
3	12	NaN	3 218 beta-tubulin [Fasciola hepatica] gi
2	4	NaN	160 393 minus strand PREDICTED: cysteine-rich protein 1-like [Megachile rotundata]; 148 381 PREDICTED: cysteine
12	104	NaN	260 892 minus strand calponin [Haliotis diversicolor]; 260 892 minus strand calponin [Haliotis diversicolor]
3	3	NaN	193 828 Cysteine-rich secretory protein LCCL domain-containing 2 [Crassostrea gigas]
2	2	NaN	356 619 PREDICTED: hypothetical protein LOC100698726 [Oreochromis niloticus]
9	22	NaN	2 811 voltage-dependent anion channel 2-like protein [Haliotis diversicolor]; 2 811 voltage-dependent anion channel
3	16	NaN	7073 7339 minus strand Collagen alpha-3(VI) chain [Crassostrea gigas]
2	2	NaN	1 252 Aquaporin-4 [Crassostrea gigas]
2	5	NaN	316 1512 Alpha-N-acetylgalactosaminidase [Crassostrea gigas]; 282 1568 Alpha-N-acetylgalactosaminidase [Crasso
2	3	NaN	112 930 calcineurin A [Haliotis discus discus]
2	8	NaN	30 536 putative mitochondrial ATP synthase [Haliotis discus discus]; 27 620 putative mitochondrial ATP synthase [H
4	5	NaN	25 405 minus strand Elongation factor 1-delta [Anoplopoma fimbria]
3	4	NaN	85 633 hypothetical protein [Amblyomma maculatum]
14	60	NaN	155 3214 sarco/endoplasmic reticulum calcium ATPase isoform B [Pinctada fucata]; 155 3214 minus strand sarco/er
2	2	NaN	68 667 minus strand hypothetical protein BRAFLDRAFT_272416 [Branchiostoma floridae] gi; 68 667 minus strand hy
4	12	NaN	180 545 calreticulin precursor [Aplysia californica] gi
2	8	NaN	426 896 PREDICTED: calmodulin-like [Metaseiulus occidentalis]
7	63	NaN	176 670 hypothetical protein BRAFLDRAFT_122621 [Branchiostoma floridae] gi
3	5	NaN	" 169 792 minus strand rho GDP dissociation inhibitor, putative [Ixodes scapularis] gi"
2	2	NaN	630 1199 neuronal calcium sensor-1 [Lymnaea stagnalis]
6	6	NaN	4 675 Laminin subunit gamma-1 [Crassostrea gigas]
2	3	NaN	" 249 1271 N(G),N(G)-dimethylarginine dimethylaminohydrolase 1 [Crassostrea gigas]"
2	2	NaN	" 40 813 minus strand NADH dehydrogenase [ubiquinone] iron-sulfur protein 3, mitochondrial [Crassostrea gigas]"
13	63	NaN	448 1626 matrilin [Biomphalaria glabrata]
4	8	NaN	102 887 hypothetical protein DAPPUDRAFT_304712 [Daphnia pulex]
4	7	NaN	430 759 hypothetical protein [Amblyomma maculatum]; 336 665 hypothetical protein [Amblyomma maculatum]
2	2	NaN	106 1701 T-complex protein 1 subunit beta [Crassostrea gigas]
2	3	NaN	141 1325 Nucleobindin-2 [Crassostrea gigas]; 141 1208 minus strand Nucleobindin-2 [Crassostrea gigas]
3	4	NaN	236 733 Spectrin alpha chain [Crassostrea gigas]
3	7	NaN	" 698 1225 minus strand actin related protein 2/3 complex, 21 kDa subunit [Crassostrea gigas]"
2	6	NaN	181 477 minus strand Zgc:56530 [Danio rerio]; 185 481 minus strand Zgc:56530 [Danio rerio]
5	15	NaN	18 647 minus strand ATP synthase-like protein [Biomphalaria glabrata]
2	2	NaN	2 1795 minus strand Plectin-1 [Crassostrea gigas]
13	40	NaN	1971 3368 minus strand Kyphoscoliosis peptidase [Crassostrea gigas]; 1767 3158 minus strand hillarin [Hirudo med
3	7	NaN	605 1153 Peptidyl-prolyl cis-trans isomerase B [Crassostrea gigas]; 1046 1630 Peptidyl-prolyl cis-trans isomerase B
3	11	NaN	811 1407 minus strand mitochondrial malate dehydrogenase 2 [Eospalax baileyi]
2	14	NaN	633 806 Peroxiredoxin-1 [Crassostrea gigas]; 648 821 Peroxiredoxin-1 [Crassostrea gigas]; 666 839 Peroxiredoxin-1 [
5	6	NaN	" 2041 3315 minus strand Myosin-10 OS=Mus musculus GN=Myh10 PE=1 SV=2; 2041 3345 minus strand Myosin hea
4	5	NaN	169 1200 PREDICTED: nidogen-2-like isoform 1 [Strongylocentrotus purpuratus]
3	3	NaN	85 1011 unnamed protein product [Mus musculus]
2	2	NaN	183 953 minus strand glutathione-S-transferase [Haliotis diversicolor supertexta]; 275 1045 minus strand glutathion
2	16	NaN	1592 1768 minus strand beta-actin [Diabolocatantops pinguis]
7	49	NaN	119 481 Glutamate receptor U1 [Crassostrea gigas]
2	2	NaN	4 243 40S ribosomal protein S13 OS=Caenorhabditis elegans GN=rps-13 PE=2 SV=2; 4 243 40S ribosomal protein S1
7	23	NaN	181 744 unknown [Dendroctonus ponderosae]
3	8	NaN	1 987 minus strand RecName: Full=Tubulin beta chain; AltName: Full=Beta-tubulin
2	4	NaN	227 3073 minus strand Lysosomal alpha-mannosidase [Crassostrea gigas]
7	13	NaN	17 1681 minus strand Glucose-6-phosphate isomerase [Crassostrea gigas]
7	36	NaN	635 1294 minus strand Kyphoscoliosis peptidase [Crassostrea gigas]; 643 1302 minus strand Kyphoscoliosis peptid
5	6	NaN	" 116 1168 minus strand hypothetical protein CGI_10014553, partial [Crassostrea gigas]"
4	12	NaN	42 296 minus strand twitchin [Mytilus galloprovincialis]
4	11	NaN	65 490 PREDICTED: cofilin-2 [Otolemur garnettii]
2	3	NaN	18 1613 minus strand chaperonin containing T-complex polypeptide subunit zeta [Haliotis discus hannai]; 527 2089

0.25 0.5 1 2 4

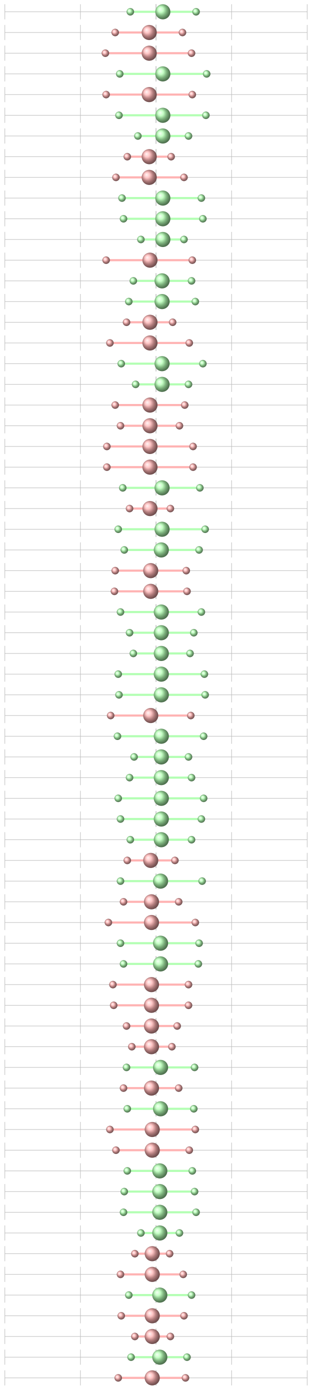

Peptides	Spectra	Coverage	Protein
2	13	NaN	160 588 hypothetical protein CGI_10001640 [Crassostrea gigas]
5	6	NaN	" 65 1576 minus strand Aldehyde dehydrogenase, mitochondrial [Crassostrea gigas]"
2	2	NaN	145 1524 PREDICTED: aspartyl aminopeptidase-like [Oreochromis niloticus]
2	5	NaN	49 747 PREDICTED: elongation factor 1-delta-like [Strongylocentrotus purpuratus]; 49 819 PREDICTED: elongation f
2	2	NaN	243 2165 minus strand Elongation factor 2 OS=Drosophila melanogaster GN=Ef2b PE=2 SV=4
2	2	NaN	91 2658 minus strand puromycin-sensitive aminopeptidase [Saccoglossus kowalevskii]
6	20	NaN	680 991 minus strand PDZ and LIM domain protein 1 [Crassostrea gigas]; 553 864 minus strand PDZ and LIM domai
7	32	NaN	" 343 1179 ATP synthase subunit alpha, mitochondrial OS=Pongo abelii GN=ATP5A1 PE=2 SV=1"
4	6	NaN	280 1239 minus strand PREDICTED: smoothelin isoform 2 [Oryctolagus cuniculus]; 280 1239 minus strand PREDICT
3	3	NaN	166 2364 Dipeptidyl-peptidase 3 [Crassostrea gigas]
3	3	NaN	794 994 Glycogenin-1 [Crassostrea gigas]; 1425 1739 minus strand Glycogenin-1 [Crassostrea gigas]
13	23	NaN	203 1942 ezrin/radixin/moesin [Aplysia californica] gi; 243 1982 minus strand ezrin/radixin/moesin [Aplysia californi
2	5	NaN	316 1512 Alpha-N-acetylgalactosaminidase [Crassostrea gigas]; 282 1478 Alpha-N-acetylgalactosaminidase [Crasso
6	11	NaN	93 1034 Annexin A7 [Crassostrea gigas]; 73 1014 Annexin A7 [Crassostrea gigas]
4	7	NaN	144 800 minus strand PREDICTED: protein F37C4.5-like [Amphimedon queenslandica]
9	35	NaN	" 112 1320 Aspartate aminotransferase, cytoplasmic [Crassostrea gigas]"
2	7	NaN	139 945 Kynurenine formamidase [Crassostrea gigas]; 145 951 Kynurenine formamidase [Crassostrea gigas]; 220 10
2	3	NaN	338 661 Spectrin alpha chain [Crassostrea gigas]
8	13	NaN	151 1449 rab GDP-dissociation inhibitor [Branchiostoma belcheri]
4	6	NaN	137 853 14-3-3 protein [Heterodera glycines]
4	22	NaN	15 428 PREDICTED: histone H2A.V-like [Apis florea]
2	2	NaN	194 775 minus strand Collagen alpha-1(XV) chain [Harpegnathos saltator]
2	2	NaN	69 779 PREDICTED: 15-hydroxyprostaglandin dehydrogenase [NAD+] [Gallus gallus]
2	12	NaN	4 849 minus strand elongation factor 1-alpha [Cryptosporidium muris RN66]
12	32	NaN	" 213 1499 minus strand Citrate synthase, mitochondrial [Crassostrea gigas]"
2	2	NaN	1 378 minus strand PREDICTED: CG10098-like [Saccoglossus kowalevskii]
2	6	NaN	124 396 minus strand 40S ribosomal protein S10 OS=Lumbricus rubellus GN=RPS10 PE=2 SV=1
4	5	NaN	58 732 glutathione-s-transferase [Haliotis discus discus]
3	5	NaN	131 541 minus strand nervous system adducin [Aplysia californica] gi
2	3	NaN	35 271 PREDICTED: SH3 domain-binding glutamic acid-rich-like protein 3-like [Anolis carolinensis]; 35 271 PREDICT
4	8	NaN	2939 3208 minus strand beta-thymosin domain repeat protein CSP24KDa_v1 [Hermissenda crassicornis] gi
4	15	NaN	835 1116 tropomyosin [Ilyanassa obsoleta]
2	2	NaN	" 45 506 minus strand PREDICTED: dihydrolipoyl dehydrogenase, mitochondrial [Monodelphis domestica]"
2	3	NaN	290 2458 Putative beta-D-xylosidase 5 [Crassostrea gigas]; 299 2428 Putative beta-D-xylosidase 2 [Crassostrea giga
3	3	NaN	771 1505 O-methyltransferase family 3 [Dicentrarchus labrax]
2	2	NaN	209 781 minus strand high mobility group protein 1 [Biomphalaria glabrata]; 277 771 high mobility group protein 1 [
3	32	NaN	" 926 1189 minus strand Myosin heavy chain, striated muscle [Crassostrea gigas]"
5	8	NaN	793 1269 PREDICTED: calmodulin-like [Saccoglossus kowalevskii]
2	2	NaN	587 811 hemocyanin 2 [Megathura crenulata]
2	3	NaN	511 1062 unknown [Dendroctonus ponderosae]; 511 1062 unknown [Dendroctonus ponderosae]
4	11	NaN	145 459 fk506-binding protein 2 [Papilio xuthus]; 237 551 fk506-binding protein 2 [Papilio xuthus]
5	35	NaN	43 591 minus strand cyclophilin type peptidyl-prolyl cis-trans isomerase [Glossina morsitans morsitans]
2	3	NaN	" 242 1504 Glutamate receptor, ionotropic kainate 3 [Crassostrea gigas]; 239 1501 Glutamate receptor, ionotropic ka
7	12	NaN	" 134 1567 minus strand 6-phosphogluconate dehydrogenase, decarboxylating [Crassostrea gigas]; 134 1573 minus
2	2	NaN	57 2009 hypothetical protein BRAFLDRAFT_285279 [Branchiostoma floridae] gi; 3 1973 hypothetical protein BRAFL
3	3	NaN	14 1642 minus strand chaperonin containing tcp1 [Haliotis discus discus]
2	5	NaN	" 42 905 PREDICTED: enoyl-CoA hydratase, mitochondrial-like isoform 1 [Oreochromis niloticus]"
2	5	NaN	184 438 PREDICTED: similar to CG14235 CG14235-PA [Tribolium castaneum] gi
2	5	NaN	" 1240 1911 PREDICTED: ATP synthase subunit alpha, mitochondrial-like [Anolis carolinensis]"
9	17	NaN	116 1882 Actin-interacting protein 1 [Crassostrea gigas]
11	40	NaN	496 2151 NADP-dependent malic enzyme [Crassostrea gigas]
4	6	NaN	582 2135 sulfatase 1A precursor [Haliotis discus discus]
4	31	NaN	768 980 muscle LIM protein isoform 1 [Danaus plexippus]
4	9	NaN	4 444 Vinculin [Crassostrea gigas]
2	3	NaN	552 1499 minus strand uncharacterized protein LOC100158466 [Xenopus (Silurana) tropicalis] gi
2	9	NaN	425 1051 minus strand unknown [Schistosoma japonicum]; 300 878 unknown [Schistosoma japonicum]; 567 1145 u
5	8	NaN	252 848 sigma class glutathione-s-transferase 2 [Haliotis discus discus]
2	8	NaN	729 1541 troponin T [Mizuhopecten yessoensis]
3	5	NaN	87 1997 Transketolase-like protein 2 [Crassostrea gigas]; 87 1997 Transketolase-like protein 2 [Crassostrea gigas]; 8
13	34	NaN	" 460 1944 PREDICTED: succinyl-CoA:3-ketoacid-coenzyme A transferase 1, mitochondrial [Canis lupus familiaris]"
11	104	NaN	260 892 minus strand calponin [Haliotis diversicolor]
3	13	NaN	311 928 minus strand peroxiredoxin 3 [Clonorchis sinensis] gi; 288 905 minus strand peroxiredoxin 3 [Clonorchis si
5	8	NaN	177 791 Ras-related protein Rab-1A [Haliotis discus discus]
3	11	NaN	231 902 Sodium- and chloride-dependent GABA transporter ine [Crassostrea gigas]
10	105	NaN	527 1321 minus strand troponin T [Mizuhopecten yessoensis]; 305 1099 minus strand troponin T [Mizuhopecten yes
5	14	NaN	594 1253 peroxiredoxin 6 [Eriocheir sinensis]
3	12	NaN	201 779 minus strand sigma class glutathione-s-transferase 2 [Haliotis discus discus]

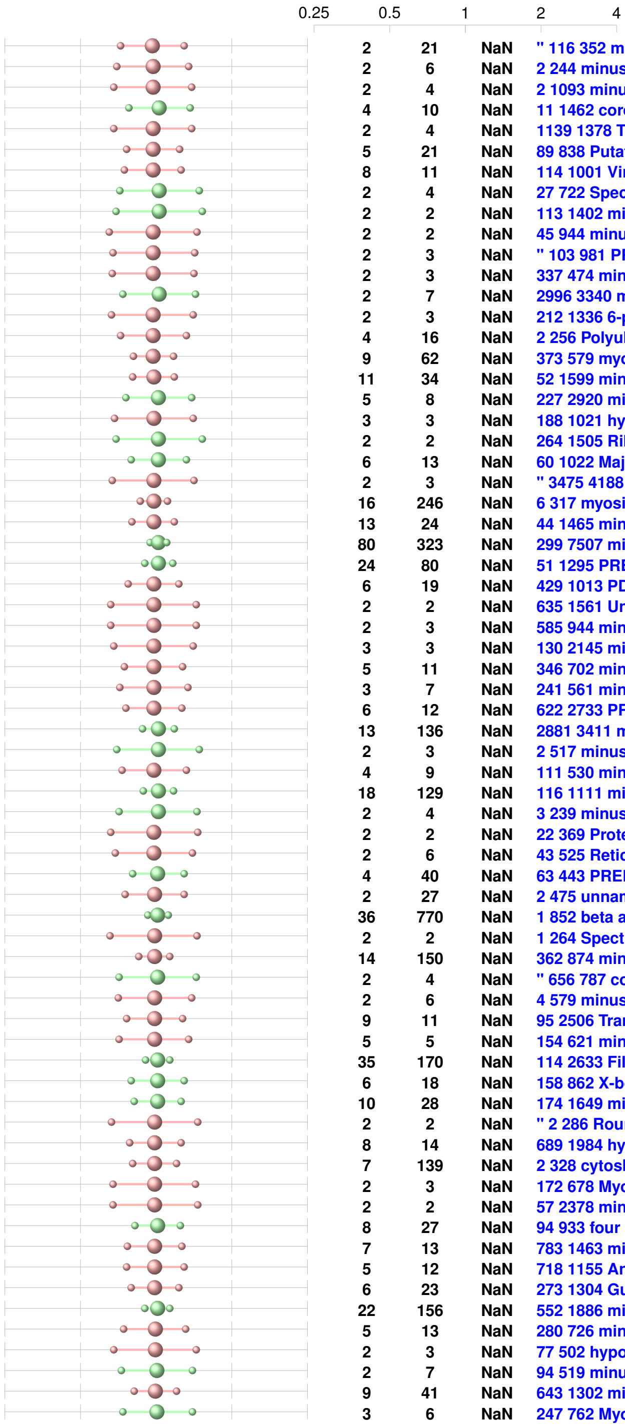

Peptides	Spectra	Coverage	Protein
2	21	NaN	" 116 352 minus strand troponin I 4 [Clonorchis sinensis]; 491 706 minus strand troponin-I, TnI {C-terminal} [Chlamy
2	6	NaN	2 244 minus strand hypothetical protein KGM_08771 [Danaus plexippus]
2	4	NaN	2 1093 minus strand Neurexin-1-alpha [Crassostrea gigas]
4	10	NaN	11 1462 coronin [Schistosoma mansoni]
2	4	NaN	1139 1378 Titin [Crassostrea gigas]; 1116 1355 Titin [Crassostrea gigas]; 1139 1378 Titin [Crassostrea gigas]; 1139 1
5	21	NaN	89 838 Putative phosphoglycerate mutase [Crassostrea gigas]; 89 838 Putative phosphoglycerate mutase [Crassost
8	11	NaN	114 1001 Vinculin [Crassostrea gigas]
2	4	NaN	27 722 Spectrin beta chain OS=Drosophila melanogaster GN=beta-Spec PE=1 SV=2; 27 722 Spectrin beta chain OS=
2	2	NaN	113 1402 minus strand S-adenosylhomocysteine hydrolase [Crassostrea ariakensis]; 113 1402 minus strand S-aden
2	2	NaN	45 944 minus strand unnamed protein product [Mus musculus] gi; 5 334 minus strand receptor of activated protein I
2	3	NaN	" 103 981 PREDICTED: ATP synthase, H+ transporting, mitochondrial F1 complex, gamma subunit isoform H (heart)
2	3	NaN	337 474 minus strand heat shock protein 22 isoform 1 [Ruditapes philippinarum]
2	7	NaN	2996 3340 minus strand Collagen alpha-5(VI) chain [Crassostrea gigas]
2	3	NaN	212 1336 6-phosphofructokinase [Crassostrea gigas]
4	16	NaN	2 256 Polyubiquitin (Fragment) OS=Xenopus laevis PE=1 SV=2; 20 472 minus strand large subunit ribosomal protein
9	62	NaN	373 579 myosin heavy chain [Argopecten irradians] gi
11	34	NaN	52 1599 minus strand ATP synthase beta subunit [Pinctada fucata]
5	8	NaN	227 2920 minus strand Lysosomal alpha-mannosidase [Crassostrea gigas]; 227 3073 minus strand Lysosomal alpha
3	3	NaN	188 1021 hypothetical protein BRAFLDRAFT_214346 [Branchiostoma floridae] gi
2	2	NaN	264 1505 Ribosome-binding protein 1 [Crassostrea gigas]; 260 1501 Ribosome-binding protein 1 [Crassostrea gigas
6	13	NaN	60 1022 Major egg antigen [Crassostrea gigas]; 60 1022 Major egg antigen [Crassostrea gigas]
2	3	NaN	" 3475 4188 minus strand Heat shock 70 kDa protein cognate, putative [Pediculus humanus corporis] gi"
16	246	NaN	6 317 myosin heavy chain cardiac muscle specific isoform 2 [Argopecten irradians]
13	24	NaN	44 1465 minus strand Adenylyl cyclase-associated protein 1 [Crassostrea gigas]; 52 1473 minus strand Adenylyl cyc
80	323	NaN	299 7507 minus strand filamin [Clonorchis sinensis]
24	80	NaN	51 1295 PREDICTED: phosphoglycerate kinase-like [Nasonia vitripennis]; 97 1341 PREDICTED: phosphoglycerate k
6	19	NaN	429 1013 PDZ domain protein [Ixodes scapularis] gi
2	2	NaN	635 1561 Uncharacterized protein yfeX [Crassostrea gigas]
2	3	NaN	585 944 minus strand Protein disulfide-isomerase A6 [Crassostrea gigas]
3	3	NaN	130 2145 minus strand Glucosamine--fructose-6-phosphate aminotransferase [isomerizing] 1 [Crassostrea gigas]; 1
5	11	NaN	346 702 minus strand PREDICTED: galectin-4-like [Anolis carolinensis]
3	7	NaN	241 561 minus strand thioredoxin [Crassostrea ariakensis]
6	12	NaN	622 2733 PREDICTED: protein-glutamine gamma-glutamyltransferase K isoform 1 [Danio rerio]; 423 2498 PREDICTE
13	136	NaN	2881 3411 myosin heavy chain [Todarodes pacificus]; 2915 3445 myosin heavy chain [Todarodes pacificus]
2	3	NaN	2 517 minus strand Anhydro-N-acetylmuramic acid kinase [Crassostrea gigas]
4	9	NaN	111 530 minus strand Nucleoside diphosphate kinase B OS=Homo sapiens GN=NME2 PE=1 SV=1
18	129	NaN	116 1111 minus strand cytosolic malate dehydrogenase [Lottia scabra]
2	4	NaN	3 239 minus strand alcohol dehydrogenase class 3 [Branchiostoma lanceolatum]
2	2	NaN	22 369 Protein farnesyltransferase/geranylgeranyltransferase type-1 subunit alpha [Crassostrea gigas]
2	6	NaN	43 525 Reticulon-4 [Crassostrea gigas]; 143 757 Reticulon-4 [Crassostrea gigas]; 43 564 Reticulon-4 [Crassostrea gi
4	40	NaN	63 443 PREDICTED: RAS protein activator like 1-like [Saccoglossus kowalevskii]
2	27	NaN	2 475 unnamed protein product [Tetraodon nigroviridis]
36	770	NaN	1 852 beta actin-like 1 protein [Macrostomum lignano]
2	2	NaN	1 264 Spectrin beta chain [Crassostrea gigas]
14	150	NaN	362 874 minus strand Transgelin-2 [Crassostrea gigas]
2	4	NaN	" 656 787 collagen alpha-1(XII) chain-like; K08132 collagen, type XII, alpha"
2	6	NaN	4 579 minus strand Collagen alpha-3(VI) chain [Crassostrea gigas]
9	11	NaN	95 2506 Transitional endoplasmic reticulum ATPase [Crassostrea gigas]
5	5	NaN	154 621 minus strand Stress response protein nhaX [Crassostrea gigas]
35	170	NaN	114 2633 Filamin-A [Crassostrea gigas]; 135 2654 Filamin-A [Crassostrea gigas]
6	18	NaN	158 862 X-box binding protein [Haliotis discus discus]
10	28	NaN	174 1649 minus strand Collagen alpha-1(XII) chain [Crassostrea gigas]; 135 1610 Collagen alpha-1(XII) chain [Crasso
2	2	NaN	" 2 286 Roundabout 2, partial [Ascaris suum]"
8	14	NaN	689 1984 hypothetical protein BRAFLDRAFT_78257 [Branchiostoma floridae] gi
7	139	NaN	2 328 cytoskeletal actin 2 [Molgula oculata]
2	3	NaN	172 678 Myosin regulatory light chain sqh [Crassostrea gigas]
2	2	NaN	57 2378 minus strand Heterogeneous nuclear ribonucleoprotein U-like protein 1 [Crassostrea gigas]
8	27	NaN	94 933 four and A half lim domains [Schistosoma mansoni]
7	13	NaN	783 1463 minus strand twitchin [Mytilus galloprovincialis]; 783 1463 minus strand twitchin [Mytilus galloprovincialis
5	12	NaN	718 1155 Annexin A7 [Crassostrea gigas]
6	23	NaN	273 1304 Guanine nucleotide-binding protein subunit beta [Crassostrea gigas]
22	156	NaN	552 1886 minus strand intermediate filament protein [Biomphalaria glabrata]
5	13	NaN	280 726 minus strand RecName: Full=Calmodulin; Short=CaM gi|14588595|dbj|BAB61794.1| calmodulin [Metridium
2	3	NaN	77 502 hypothetical protein CGI_10008263 [Crassostrea gigas]
2	7	NaN	94 519 minus strand hypothetical protein BRAFLDRAFT_115029 [Branchiostoma floridae] gi
9	41	NaN	643 1302 minus strand Kyphoscoliosis peptidase [Crassostrea gigas]; 634 1293 minus strand Kyphoscoliosis peptid
3	6	NaN	247 762 Myophilin [Crassostrea gigas]; 244 759 Myophilin [Crassostrea gigas]; 119 634 Myophilin [Crassostrea giga

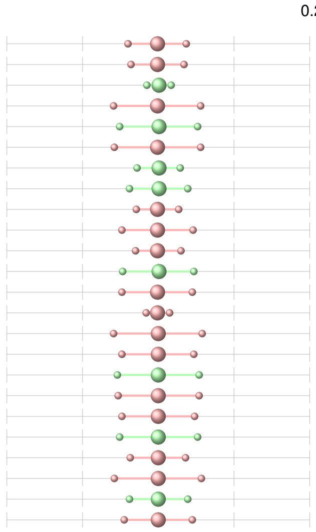

Peptides	Spectra	Coverage	Protein
4	17	NaN	" 64 1332 Flotillin-1, putative [Pediculus humanus corporis] gi"
7	16	NaN	27 377 minus strand ribosomal protein LP2 [Papilio xuthus] gi; 27 377 minus strand ribosomal protein LP2 [Papilio x
23	294	NaN	3591 4526 minus strand myosin heavy chain cardiac muscle specific isoform 1 [Argopecten irradians]
2	2	NaN	410 709 calmodulin-like; K02183 calmodulin
2	4	NaN	211 1413 Alpha-N-acetylgalactosaminidase [Crassostrea gigas]
2	2	NaN	165 635 hypothetical protein CGI_10007639 [Crassostrea gigas]
14	23	NaN	77 1561 retinal dehydrogenase [Lymnaea stagnalis]; 69 1553 retinal dehydrogenase [Lymnaea stagnalis]; 308 1747 r
8	10	NaN	116 1321 PREDICTED: similar to aspartate aminotransferase [Tribolium castaneum] gi
7	48	NaN	835 1083 minus strand Calmodulin [Crassostrea gigas]
4	5	NaN	125 754 minus strand peptidyl prolyl cis-trans isomerase B [Conus novaehollandiae]
12	20	NaN	71 1321 minus strand Vinculin [Crassostrea gigas]
3	6	NaN	" 57 752 PREDICTED: adenylate kinase 2, mitochondrial-like isoform 2 [Callithrix jacchus]"
4	5	NaN	63 1865 Dolichyl-diphosphooligosaccharide–protein glycosyltransferase subunit 1 [Crassostrea gigas]; 63 1865 Dol
41	115	NaN	" 519 3182 minus strand Alpha-actinin, sarcomeric [Crassostrea gigas]"
2	2	NaN	" 28 1104 minus strand Pyruvate dehydrogenase E1 component subunit beta, mitochondrial [Crassostrea gigas]"
2	8	NaN	" 599 907 minus strand Tryptophanyl-tRNA synthetase, cytoplasmic [Heterocephalus glaber]"
2	3	NaN	1 222 cAMP-dependent protein kinase regulatory subunit [Aplysia californica] gi
2	3	NaN	56 937 Chloride intracellular channel exc-4 [Crassostrea gigas]
2	6	NaN	148 609 minus strand Titin [Crassostrea gigas]
2	4	NaN	" 3 239 minus strand laminin beta 1 chain, putative [Ixodes scapularis] gi"
7	14	NaN	366 1049 hypothetical protein CGI_10004855 [Crassostrea gigas]
2	2	NaN	303 1793 PREDICTED: matrix metalloproteinase 19 isoform rasi-1 preproprotein-like [Saccoglossus kowalevskii]
6	10	NaN	51 890 prohibitin [Octopus tankahkeei]
2	10	NaN	362 553 putative RNA-binding protein [Haliotis diversicolor]

4.2 Proteins Identified by a Single Peptide

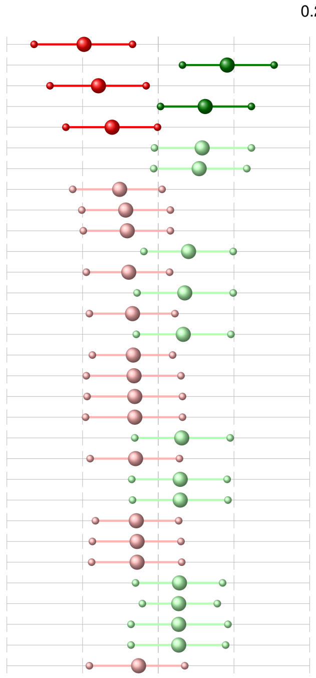

Peptides	Spectra	Coverage	Protein
1	1	NaN	721 1242 Lactoylglutathione lyase [Crassostrea gigas]; 882 1334 Lactoylglutathione lyase OS=Rattus norvegicus GN
1	2	NaN	704 817 minus strand expressed hypothetical protein [Trichoplax adhaerens] gi; 688 801 minus strand Lactoylglutat
1	1	NaN	38 436 Calcium-dependent protein kinase isoform 2 [Crassostrea gigas]
1	2	NaN	523 1086 minus strand juvenile hormone diol kinase [Danaus plexippus]
1	2	NaN	360 764 minus strand Peptidyl-prolyl cis-trans isomerase A OS=Dictyostelium discoideum GN=ppiA PE=1 SV=1
1	1	NaN	66 947 Glyoxalase domain-containing protein 4 [Crassostrea gigas]
1	1	NaN	2 2572 Glycogen debranching enzyme [Crassostrea gigas]
1	2	NaN	971 1963 Spectrin alpha chain [Crassostrea gigas]; 972 1979 Spectrin alpha chain [Crassostrea gigas]
1	2	NaN	" 285 437 Propionyl-CoA carboxylase alpha chain, mitochondrial [Crassostrea gigas]"
1	3	NaN	900 1616 minus strand hemocyanin isoform 1 [Haliotis diversicolor]
1	2	NaN	" 196 1053 minus strand Cytochrome b-c1 complex subunit 2, mitochondrial OS=Homo sapiens GN=UQCRC2 PE=1
1	3	NaN	361 549 minus strand 32 kDa beta-galactoside-binding lectin [Ascaris suum]
1	1	NaN	" 158 355 Protein NAS-37, isoform b [Caenorhabditis elegans] gi; 15 227 Hemicentin-1 [Crassostrea gigas]"
1	3	NaN	91 1326 minus strand Adipocyte plasma membrane-associated protein [Crassostrea gigas]; 107 1273 minus strand A
1	1	NaN	" 125 628 minus strand collectin sub-family member 12, partial [Oncorhynchus mykiss]"
1	6	NaN	233 814 Kynurenine formamidase [Crassostrea gigas]; 233 979 Kynurenine formamidase [Crassostrea gigas]; 139 94
1	1	NaN	8 937 minus strand galectin [Crassostrea virginica]
1	1	NaN	401 1174 minus strand Mechanosensory protein 2 [Crassostrea gigas]
1	1	NaN	19 1218 Dynactin subunit 2 [Crassostrea gigas]; 48 830 minus strand Dynactin subunit 2 [Crassostrea gigas]
1	1	NaN	232 654 hypothetical protein BRAFLDRAFT_87758 [Branchiostoma floridae] gi
1	2	NaN	" 34 1656 Chaperonin containing TCP1, subunit 7 (eta) [Danio rerio] gi; 34 1656 Chaperonin containing TCP1, subun
1	1	NaN	336 641 minus strand malate dehydrogenase [Schistosoma japonicum]
1	1	NaN	59 379 hypothetical protein DAPPUDRAFT_230756 [Daphnia pulex]; 117 443 minus strand hypothetical protein DAPP
1	4	NaN	9 518 minus strand hemocyanin isoform 1 [Nucula nucleus]
1	2	NaN	1292 1471 minus strand hemocyanin isoform 1 [Haliotis diversicolor]
1	2	NaN	296 493 hypothetical protein BRAFLDRAFT_66287 [Branchiostoma floridae] gi
1	3	NaN	96 497 AAEL009772-PB [Aedes aegypti]
1	8	NaN	230 334 protein disulfide isomerase [Conus virgo]
1	1	NaN	21 239 minus strand calponin-like protein [Taenia asiatica]
1	1	NaN	71 523 minus strand hypothetical protein DAPPUDRAFT_309332 [Daphnia pulex]
1	1	NaN	16 438 hypothetical protein KGM_06034 [Danaus plexippus]; 16 438 hypothetical protein KGM_06034 [Danaus plexip

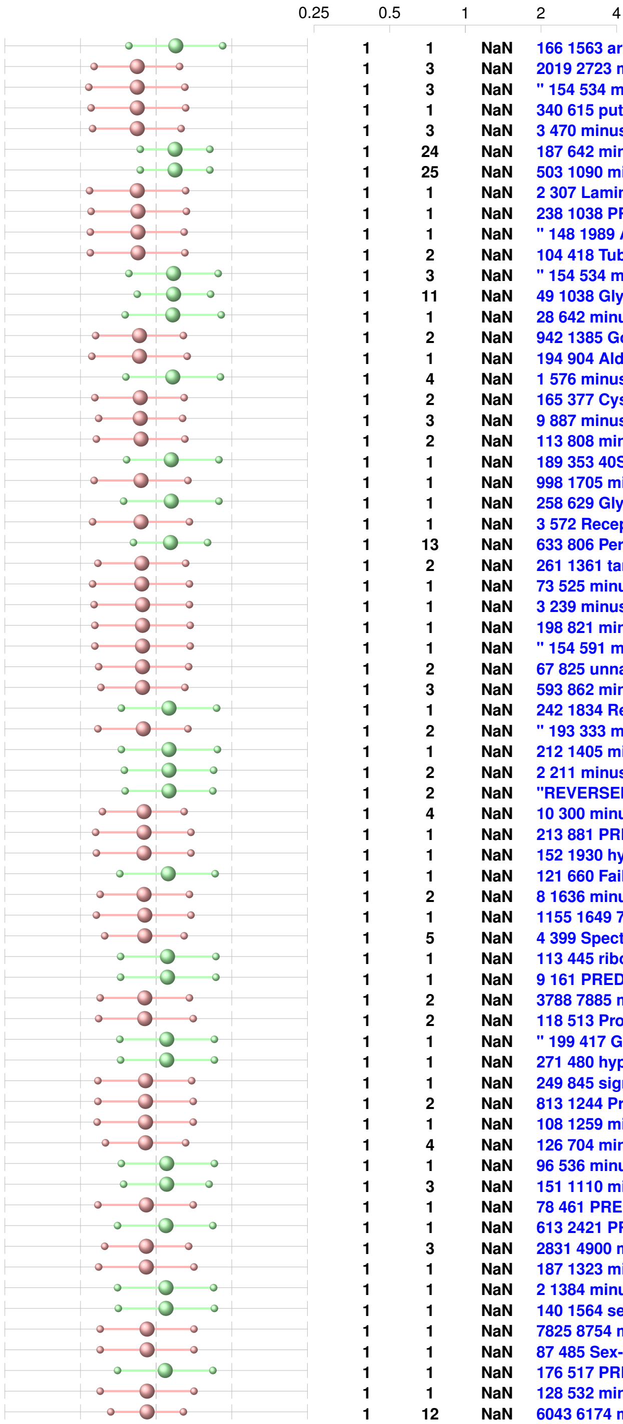

Peptides	Spectra	Coverage	Protein
1	1	NaN	166 1563 arylsulfatase [Haliotis discus discus]
1	3	NaN	2019 2723 minus strand Kyphoscoliosis peptidase [Crassostrea gigas]; 2060 2764 minus strand Kyphoscoliosis pep
1	3	NaN	" 154 534 minus strand Muscle M-line assembly protein unc-89, partial [Crassostrea gigas]"
1	1	NaN	340 615 putative Low-density lipoprotein receptor repeat class B [Trichinella spiralis] gi
1	3	NaN	3 470 minus strand Spectrin beta chain [Crassostrea gigas]
1	24	NaN	187 642 minus strand PREDICTED: similar to pol-like protein [Tribolium castaneum]
1	25	NaN	503 1090 minus strand myosin regulatory light chain [Fasciola hepatica]; 278 865 minus strand myosin regulatory li
1	1	NaN	2 307 Laminin subunit gamma-1 [Crassostrea gigas]
1	1	NaN	238 1038 PREDICTED: hypothetical protein [Saccoglossus kowalevskii]; 294 992 PREDICTED: hypothetical protein [
1	1	NaN	" 148 1989 Acyl-CoA synthetase family member 2, mitochondrial [Crassostrea gigas]; 146 1987 minus strand Acyl-C
1	2	NaN	104 418 Tubulin-specific chaperone A [Crassostrea gigas]; 104 418 minus strand Tubulin-specific chaperone A [Cras
1	3	NaN	" 154 534 minus strand Muscle M-line assembly protein unc-89, partial [Crassostrea gigas]; 259 690 minus strand M
1	11	NaN	49 1038 Glyceraldehyde-3-phosphate dehydrogenase [Crotalus adamanteus]
1	1	NaN	28 642 minus strand Transmembrane emp24 domain-containing protein 9 [Crassostrea gigas]; 236 709 minus strand
1	2	NaN	942 1385 Golgi-associated plant pathogenesis-related protein 1 [Crassostrea gigas]; 338 817 Golgi-associated plant
1	1	NaN	194 904 Aldo-keto reductase family 1 member B10 [Crassostrea gigas]
1	4	NaN	1 576 minus strand Tubulin beta chain [Crassostrea gigas]
1	2	NaN	165 377 Cysteine and glycine-rich protein 3 [Crassostrea gigas]; 135 344 Cysteine and glycine-rich protein 3 [Crasso
1	3	NaN	9 887 minus strand hypothetical protein CGI_10006272 [Crassostrea gigas]
1	2	NaN	113 808 minus strand omega class glutathione-s-transferase [Chlamys farreri]; 335 1045 minus strand omega class
1	1	NaN	189 353 40S ribosomal protein S12 [Crassostrea gigas]
1	1	NaN	998 1705 minus strand Basement membrane-specific heparan sulfate proteoglycan core protein [Crassostrea gigas]
1	1	NaN	258 629 Glycogenin-1 OS=Oryctolagus cuniculus GN=GYG1 PE=1 SV=3
1	1	NaN	3 572 Receptor-type tyrosine-protein phosphatase N2 [Crassostrea gigas]; 3 542 minus strand Receptor-type tyrosin
1	13	NaN	633 806 Peroxiredoxin-1 [Crassostrea gigas]; 648 821 Peroxiredoxin-1 [Crassostrea gigas]; 666 839 Peroxiredoxin-1
1	2	NaN	261 1361 tandem-repeat galectin [Pinctada fucata]; 261 1361 tandem-repeat galectin [Pinctada fucata]
1	1	NaN	73 525 minus strand Actin-related protein 2/3 complex subunit 5-like protein [Salmo salar] gi; 73 525 Actin-related pr
1	1	NaN	3 239 minus strand p38 MAPK [Littorina littorea]
1	1	NaN	198 821 minus strand sigma class glutathione-s-transferase 2 [Haliotis discus discus]
1	1	NaN	" 154 591 microsomal GST1, partial [Reishia clavigera]; 154 531 microsomal GST1, partial [Reishia clavigera]"
1	2	NaN	67 825 unnamed protein product [Oikopleura dioica]
1	3	NaN	593 862 minus strand mitochondrial ATP synthase coupling factor 6 precursor [Ochlerotatus taeniorhynchus]
1	1	NaN	242 1834 Regulator of differentiation 1 [Crassostrea gigas]; 458 2050 minus strand Regulator of differentiation 1 [Cr
1	2	NaN	" 193 333 minus strand RecName: Full=Globin; AltName: Full=Myoglobin gi|299274|gb|AAB25743.1| myoglobin [Na
1	1	NaN	212 1405 minus strand unnamed protein product [Tetraodon nigroviridis]
1	2	NaN	2 211 minus strand sulfatase 1A precursor [Haliotis discus discus]
1	2	NaN	"REVERSED 2 1810 minus strand Glutaminyl-tRNA synthetase, partial [Crassostrea gigas]"
1	4	NaN	10 300 minus strand unnamed protein product [Tetraodon nigroviridis]
1	1	NaN	213 881 PREDICTED: hypothetical protein [Saccoglossus kowalevskii]; 260 928 PREDICTED: hypothetical protein [S
1	1	NaN	152 1930 hypothetical protein BRAFLDRAFT_223365 [Branchiostoma floridae] gi
1	1	NaN	121 660 Failed axon connections homolog OS=Rattus norvegicus GN=Faxc PE=3 SV=1
1	2	NaN	8 1636 minus strand V-type proton ATPase catalytic subunit A [Crassostrea gigas]; 8 1636 minus strand V-type proto
1	1	NaN	1155 1649 78 kDa glucose-regulated protein OS=Aplysia californica PE=1 SV=1
1	5	NaN	4 399 Spectrin beta chain [Crassostrea gigas]
1	1	NaN	113 445 ribosomal protein rps19 [Lineus viridis]
1	1	NaN	9 161 PREDICTED: tubulin alpha-1C chain-like [Acyrthosiphon pisum]; 9 161 Tubulin alpha-3 chain (Fragments) OS=
1	2	NaN	3788 7885 minus strand Filamin-C [Crassostrea gigas]
1	2	NaN	118 513 Protein CAL-5 [Caenorhabditis elegans] gi
1	1	NaN	" 199 417 GG13156 [Drosophila erecta] gi; 253 501 minus strand NADH-ubiquinone oxidoreductase MLRQ subunit-li
1	1	NaN	271 480 hypothetical protein BRAFLDRAFT_75518 [Branchiostoma floridae] gi
1	1	NaN	249 845 sigma class glutathione-s-transferase 2 [Haliotis discus discus]; 252 848 sigma class glutathione-s-transfer
1	2	NaN	813 1244 Probable protein disulfide-isomerase A6 OS=Caenorhabditis elegans GN=tag-320 PE=2 SV=1
1	1	NaN	108 1259 minus strand PREDICTED: Na(+)/H(+) exchange regulatory cofactor NHE-RF3-like [Metaseiulus occidentali
1	4	NaN	126 704 minus strand ribosomal protein L12 [Ixodes scapularis]
1	1	NaN	96 536 minus strand hypothetical protein BRAFLDRAFT_285353 [Branchiostoma floridae] gi
1	3	NaN	151 1110 minus strand hypothetical protein DAPPUDRAFT_303085 [Daphnia pulex]
1	1	NaN	78 461 PREDICTED: hoi-polloi-like [Saccoglossus kowalevskii]; 103 492 NHP2-like protein 1 OS=Rattus norvegicus (
1	1	NaN	613 2421 PREDICTED: lysosomal alpha-glucosidase [Danio rerio]
1	3	NaN	2831 4900 minus strand Collagen alpha-3(VI) chain [Crassostrea gigas]
1	1	NaN	187 1323 minus strand gelsolin [Aplysia californica] gi
1	1	NaN	2 1384 minus strand twitchin [Mytilus galloprovincialis]; 2 1384 minus strand twitchin [Mytilus galloprovincialis]; 2 1
1	1	NaN	140 1564 selenium-binding protein [Haliotis discus hannai]
1	1	NaN	7825 8754 minus strand H2 [Haliotis tuberculata]
1	1	NaN	87 485 Sex-regulated protein janus-A [Crassostrea gigas]
1	1	NaN	176 517 PREDICTED: elongin C-like [Saccoglossus kowalevskii]; 115 456 PREDICTED: elongin C-like [Saccoglossus
1	1	NaN	128 532 minus strand mitochondrial ATPase inhibitor [Ornithodoros parkeri]
1	12	NaN	6043 6174 myosin heavy chain [Mytilus galloprovincialis]; 5869 6000 myosin heavy chain [Mytilus galloprovincialis]

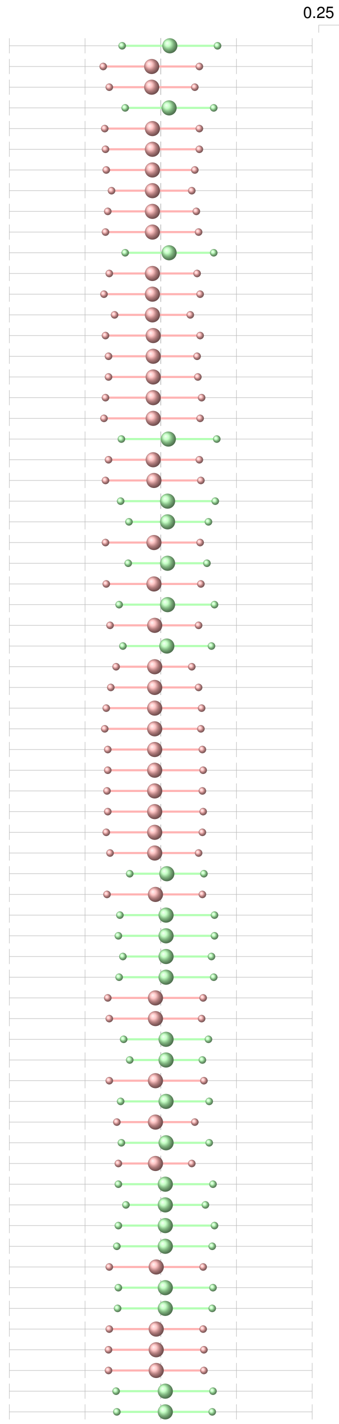

Peptides	Spectra	Coverage	Protein
1	2	NaN	147 383 minus strand Tubulin-specific chaperone A OS=Rattus norvegicus GN=Tbca PE=1 SV=1; 104 418 Tubulin-sp
1	1	NaN	121 1452 minus strand Hexokinase type 2 [Crassostrea gigas]; 245 1576 minus strand Hexokinase type 2 [Crassostr
1	3	NaN	3 386 fem-1-like protein C [Crassostrea gigas]
1	2	NaN	266 1123 minus strand mitochondrial malate dehydrogenase precursor [Nucella lapillus]
1	1	NaN	50 577 hypothetical protein BRAFLDRAFT_119799 [Branchiostoma floridae] gi; 50 577 hypothetical protein BRAFLD
1	1	NaN	120 833 B-cell receptor-associated protein 31 [Crassostrea gigas]; 238 951 B-cell receptor-associated protein 31 [Cr
1	2	NaN	" 31 2061 minus strand 1,4-alpha-glucan-branching enzyme [Crassostrea gigas]"
1	4	NaN	246 452 minus strand mitochondrial malate dehydrogenase precursor [Nucella lapillus]; 106 312 minus strand mitoc
1	2	NaN	293 1795 Amidase [Crassostrea gigas]
1	1	NaN	2 511 hypothetical protein CGI_10006272 [Crassostrea gigas]
1	2	NaN	546 995 Hypothetical protein CBG12002 [Caenorhabditis briggsae]; 543 995 minus strand PREDICTED: tubulin polyr
1	2	NaN	160 387 small nuclear ribonucleoprotein polypeptide G protein [Crassostrea ariakensis]; 180 407 small nuclear ribor
1	1	NaN	333 770 USP-like protein isoform 2 [Crassostrea ariakensis]
1	8	NaN	460 1737 Tubulin beta-4B chain OS=Mus musculus GN=Tubb4b PE=1 SV=1
1	1	NaN	72 521 minus strand GF14593 [Drosophila ananassae] gi
1	2	NaN	156 1163 cathepsin B [Crassostrea ariakensis]
1	2	NaN	325 642 minus strand Protein phosphatase 1B [Crassostrea gigas]
1	2	NaN	552 1499 minus strand uncharacterized protein LOC100158466 [Xenopus (Silurana) tropicalis] gi; 552 1499 minus st
1	1	NaN	227 1201 PREDICTED: quinone oxidoreductase-like isoform 1 [Ornithorhynchus anatinus]; 109 1083 PREDICTED: qu
1	1	NaN	54 791 GMP reductase 2 [Crassostrea gigas]
1	2	NaN	123 314 PREDICTED: ubiquinol-cytochrome c reductase hinge protein-like [Saccoglossus kowalevskii]; 279 530 PRE
1	1	NaN	3 230 minus strand RH21460p [Drosophila melanogaster]; 3 230 minus strand RH21460p [Drosophila melanogaster]
1	3	NaN	187 696 minus strand putative ribosomal protein S10 [Sipunculus nudus]
1	5	NaN	108 593 hypothetical protein CGI_10025497 [Crassostrea gigas]
1	1	NaN	176 667 hypothetical protein CGI_10018452 [Crassostrea gigas]; 176 634 hypothetical protein CGI_10018452 [Crasso
1	5	NaN	219 428 Peptidyl-prolyl cis-trans isomerase OS=Hemicentrotus pulcherrimus PE=2 SV=1
1	1	NaN	" 2057 2497 Phosphate carrier protein, mitochondrial [Crassostrea gigas]"
1	1	NaN	87 485 Sex-regulated protein janus-A [Crassostrea gigas]; 92 310 PREDICTED: hypothetical protein [Saccoglossus k
1	2	NaN	3 254 Calpain-5 [Crassostrea gigas]; 3 254 minus strand Calpain-5 [Crassostrea gigas]
1	2	NaN	5 1018 minus strand Glutaredoxin-3 [Crassostrea gigas]; 5 1018 Glutaredoxin-3 [Crassostrea gigas]
1	8	NaN	1709 2026 Phosphoenolpyruvate carboxykinase [GTP] [Crassostrea gigas]
1	2	NaN	1017 1499 minus strand PREDICTED: leukocyte elastase inhibitor-like [Oreochromis niloticus]
1	1	NaN	1050 1307 calreticulin precursor [Aplysia californica] gi
1	1	NaN	54 662 minus strand Protein DJ-1 [Crassostrea gigas]
1	1	NaN	84 425 minus strand Transcription elongation factor B polypeptide 2 [Crassostrea gigas]; 84 425 minus strand Trans
1	1	NaN	245 1243 Prostaglandin reductase 1 [Crassostrea gigas]
1	1	NaN	134 385 minus strand CDGSH iron sulfur domain-containing protein 2 [Crassostrea gigas]; 11 511 minus strand CDG
1	1	NaN	112 819 pyruvate kinase [Crassostrea gigas]; 132 932 pyruvate kinase [Crassostrea gigas]
1	1	NaN	1118 1255 minus strand equistatin precursor [Actinia equina]
1	2	NaN	14 223 minus strand Endothelin-converting enzyme 2 OS=Homo sapiens GN=ECE2 PE=1 SV=4
1	11	NaN	" 1 321 Dynein heavy chain, cytoplasmic [Crassostrea gigas]"
1	1	NaN	642 1415 minus strand cytochrome c oxidase subunit II [Oncomelania hupensis hupensis]
1	1	NaN	206 1513 hypothetical protein CGI_10019003 [Crassostrea gigas]
1	1	NaN	2 169 cAMP-dependent protein kinase regulatory subunit [Crassostrea gigas]; 125 511 cAMP-dependent protein kina
1	2	NaN	95 271 minus strand hypothetical protein EAI_01530 [Harpegnathos saltator]
1	1	NaN	625 1902 minus strand hypothetical protein CGI_10009847 [Crassostrea gigas]
1	1	NaN	54 278 ATP-citrate synthase [Crassostrea gigas]; 61 465 ATP-citrate synthase [Crassostrea gigas]
1	1	NaN	242 589 Ribonucleoprotein RB97D OS=Drosophila melanogaster GN=Rb97D PE=2 SV=1
1	3	NaN	132 590 minus strand Non-neuronal cytoplasmic intermediate filament protein OS=Helix aspersa PE=3 SV=2
1	12	NaN	37 885 glyceraldehyde 3-phosphate dehydrogenase [Dugesia japonica]
1	1	NaN	10 1902 Mitochondrial inner membrane protein [Crassostrea gigas]; 10 1902 Mitochondrial inner membrane protein
1	2	NaN	" 3799 4371 papilin, partial [Rhipicephalus microplus]"
1	7	NaN	16 288 SJCHGC01950 protein [Schistosoma japonicum] gi
1	2	NaN	459 1187 PREDICTED: protein-L-isoaspartate(D-aspartate) O-methyltransferase-like isoform 2 [Anolis carolinensis]
1	10	NaN	1714 2121 Phosphoenolpyruvate carboxykinase [GTP] [Crassostrea gigas]
1	1	NaN	138 713 Rac [Aplysia californica] gi; 101 676 Rac [Aplysia californica] gi
1	5	NaN	392 808 Whirlin [Crassostrea gigas]
1	1	NaN	54 638 minus strand ubiquitin carboxyl-terminal hydrolase [Aplysia californica] gi
1	1	NaN	4 219 hypothetical protein BRAFLDRAFT_66351 [Branchiostoma floridae] gi; 3 239 hypothetical protein BRAFLDRAF
1	1	NaN	880 1434 minus strand Prohormone-4 [Crassostrea gigas]
1	2	NaN	" 241 1110 minus strand N(G),N(G)-dimethylarginine dimethylaminohydrolase 1 [Crassostrea gigas]; 241 1110 minus
1	1	NaN	61 2865 AP-2 complex subunit beta [Crassostrea gigas]
1	1	NaN	91 408 minus strand hypothetical protein [Amblyomma maculatum]
1	1	NaN	58 1203 minus strand Obg-like ATPase 1 [Crassostrea gigas]
1	1	NaN	90 659 Plectin-1 [Crassostrea gigas]; 327 881 Plectin-1 [Crassostrea gigas]; 646 1290 minus strand Plectin-1 [Crasso
1	1	NaN	192 380 minus strand RAB protein [Haliotis discus discus]
1	1	NaN	616 1740 5'-AMP-activated protein kinase subunit beta-2 [Crassostrea gigas]

Javier, Head 28d versus control

0.25 0.5 1 2 4

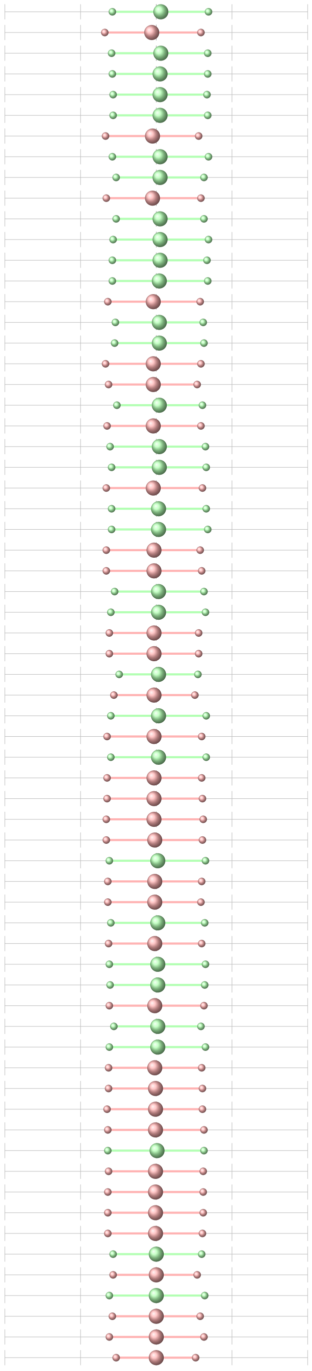

	Peptides	Spectra	Coverage	Protein
	1	1	NaN	22 429 AGAP006937-PA [Anopheles gambiae str. PEST] gi; 1 474 Ubiquitin-conjugating enzyme E2 variant 2 OS=Rat
	1	1	NaN	214 1878 PREDICTED: lysyl-tRNA synthetase-like [Oreochromis niloticus]
	1	1	NaN	" 757 1443 minus strand nonmuscle myosin II, partial [Aplysia californica]"
	1	1	NaN	2 1126 Prolyl endopeptidase [Crassostrea gigas]
	1	1	NaN	351 1826 Virulence metalloprotease [Crassostrea gigas]
	1	1	NaN	613 846 cytochrome c oxidase subunit Va [Antheraea yamamai] gi
	1	1	NaN	416 1717 minus strand T-complex protein 1 subunit delta OS=Aedes triseriatus PE=2 SV=1
	1	1	NaN	64 435 peptidoglycan recognition protein S1L [Pomacea canaliculata]
	1	2	NaN	140 349 copper chaperone Atox1 [Crassostrea ariakensis]
	1	1	NaN	362 1084 Ectoine hydroxylase [Crassostrea gigas]; 362 1084 Ectoine hydroxylase [Crassostrea gigas]; 366 1220 Ect
	1	2	NaN	225 839 minus strand Metallo-beta-lactamase domain-containing protein 1 [Crassostrea gigas]; 201 851 minus stran
	1	2	NaN	122 1195 minus strand Severin [Crassostrea gigas]
	1	1	NaN	2295 2510 beta-thymosin domain repeat protein CSP24KDa_v1 [Hermissenda crassicornis] gi
	1	1	NaN	205 1341 collagen precursor [Bombyx mori]
	1	2	NaN	406 1392 minus strand hypothetical protein CGI_10007007 [Crassostrea gigas]
	1	3	NaN	43 525 Reticulon-4 [Crassostrea gigas]; 143 757 Reticulon-4 [Crassostrea gigas]; 43 564 Reticulon-4 [Crassostrea gi
	1	2	NaN	530 679 minus strand GJ11201 [Drosophila virilis] gi
	1	1	NaN	20 511 Neurexin-2-alpha [Crassostrea gigas]; 20 511 Neurexin-2-alpha [Crassostrea gigas]
	1	2	NaN	62 1174 Tyrosine-protein kinase CSK [Crassostrea gigas]; 62 1174 minus strand Tyrosine-protein kinase CSK [Crass
	1	3	NaN	2764 3420 PREDICTED: filamin-C-like [Nasonia vitripennis]
	1	1	NaN	77 2278 Far upstream element-binding protein 3 [Crassostrea gigas]
	1	1	NaN	147 272 Spectrin alpha chain [Crassostrea gigas]
	1	1	NaN	" 451 1026 PREDICTED: GTP-binding protein SAR1B, putative-like [Saccoglossus kowalevskii]"
	1	1	NaN	49 1107 Eukaryotic translation initiation factor 3 subunit B [Crassostrea gigas]
	1	2	NaN	2 244 Ras homolog gene family member A [Clonorchis sinensis]
	1	1	NaN	285 1637 PREDICTED: talin-2-like [Cricetulus griseus]
	1	1	NaN	385 906 Soma ferritin OS=Lymnaea stagnalis PE=2 SV=2
	1	1	NaN	316 531 cytochrome c oxidase subunit VIIa polypeptide 2 like [Xenopus (Silurana) tropicalis]
	1	2	NaN	145 972 LIM and SH3 domain protein F42H10.3 OS=Caenorhabditis elegans GN=F42H10.3 PE=2 SV=3
	1	2	NaN	1497 1889 UDP-glucuronosyltransferase 2C1 (Fragment) OS=Oryctolagus cuniculus GN=UGT2C1 PE=2 SV=1
	1	2	NaN	143 247 minus strand dynein light chain [Aplysia californica] gi
	1	2	NaN	211 990 PREDICTED: gamma-glutamyl hydrolase-like [Xenopus (Silurana) tropicalis]; 223 1080 PREDICTED: gamma
	1	6	NaN	58 198 minus strand Protein DJ-1 [Crassostrea gigas]
	1	4	NaN	" 1 510 Glycerol-3-phosphate dehydrogenase [NAD+], cytoplasmic [Crassostrea gigas]"
	1	1	NaN	58 2268 hypothetical protein BRAFLDRAFT_267717 [Branchiostoma floridae] gi; 62 2245 hypothetical protein BRAF
	1	1	NaN	366 935 minus strand 40S ribosomal protein S2 OS=Urechis caupo GN=RPS2 PE=2 SV=1
	1	1	NaN	219 554 minus strand ribosomal protein P1 [Biomphalaria glabrata]
	1	1	NaN	1573 2385 minus strand Spectrin beta chain [Crassostrea gigas]
	1	1	NaN	297 734 minus strand PREDICTED: calmodulin [Canis lupus familiaris]; 297 734 PREDICTED: calmodulin [Canis lupu
	1	2	NaN	302 1273 minus strand hypothetical protein CGI_10007007 [Crassostrea gigas]
	1	1	NaN	1213 3234 Protein-glutamine gamma-glutamyltransferase K [Crassostrea gigas]; 478 2499 Protein-glutamine gamma
	1	1	NaN	179 2140 minus strand PREDICTED: hypothetical protein [Saccoglossus kowalevskii]
	1	1	NaN	11 394 regucalcin [Salmo salar] gi
	1	1	NaN	" 137 1549 minus strand Serine hydroxymethyltransferase, mitochondrial [Crassostrea gigas]; 205 1608 minus stran
	1	1	NaN	68 481 minus strand netrin receptor UNC5A [Danio rerio] gi
	1	3	NaN	142 588 AAEL009772-PB [Aedes aegypti]; 96 497 AAEL009772-PB [Aedes aegypti]
	1	1	NaN	126 287 minus strand Cytochrome b-c1 complex subunit 10 [Cricetulus griseus]; 126 287 minus strand Cytochrome
	1	1	NaN	3 293 Titin [Crassostrea gigas]
	1	1	NaN	" 703 1173 60S acidic ribosomal protein P0, putative [Ixodes scapularis] gi"
	1	6	NaN	64 393 Tubulin beta chain OS=Paracentrotus lividus PE=2 SV=1; 111 398 Tubulin beta chain OS=Paracentrotus lividu
	1	1	NaN	4 2400 IQ motif containing GTPase activating protein 2 [Xenopus laevis] gi; 4 2169 IQ motif containing GTPase activ
	1	1	NaN	191 919 PREDICTED: 15-hydroxyprostaglandin dehydrogenase [NAD+] [Gallus gallus]
	1	1	NaN	188 4135 Vigilin [Crassostrea gigas]
	1	1	NaN	1265 1831 minus strand Adenosine deaminase CECR1 [Crassostrea gigas]
	1	1	NaN	35 505 minus strand Basement membrane-specific heparan sulfate proteoglycan core protein [Crassostrea gigas]
	1	1	NaN	1732 2079 minus strand PREDICTED: annexin A7-like [Ornithorhynchus anatinus]
	1	1	NaN	305 937 Clathrin light chain A [Crassostrea gigas]
	1	1	NaN	1 111 glycogen synthase [Crassostrea gigas]
	1	1	NaN	102 1475 minus strand Cytosolic non-specific dipeptidase [Crassostrea gigas]
	1	1	NaN	146 841 minus strand hypothetical protein CGI_10006609 [Crassostrea gigas]; 146 841 minus strand hypothetical pr
	1	2	NaN	895 1410 Galectin-3 OS=Rattus norvegicus GN=Lgals3 PE=1 SV=4; 1067 1405 Galectin-9 [Crassostrea gigas]; 1068 1
	1	3	NaN	66 263 minus strand twitchin [Mytilus galloprovincialis]; 66 263 minus strand twitchin [Mytilus galloprovincialis]; 66
	1	1	NaN	1070 1888 minus strand galectin [Pinctada fucata]; 1104 1922 minus strand galectin [Pinctada fucata]
	1	2	NaN	210 473 Abdominal ganglion neuropeptides L5-67 OS=Aplysia californica PE=1 SV=1; 879 1124 abdominal ganglion
	1	1	NaN	965 1687 Arylsulfatase B [Crassostrea gigas]
	1	6	NaN	64 393 Tubulin beta chain OS=Paracentrotus lividus PE=2 SV=1; 109 438 Tubulin beta chain OS=Paracentrotus lividu

5 Protein Details

A detailed summary of each protein is given below. These sections include peptide relative expression estimates in addition to protein-level estimates.

5.1 " 353 781 alpha-B-crystallin, putative [Ixodes scapularis] gi"

Protein Accession	CL37349.Contig1$_H$1
Mean Expression Ratio	0.341
Median Expression Ratio	0.341
Credible Interval	(0.248, 0.468)
Associated Peptides	4
Associated Spectra	7
Coverage	NaN

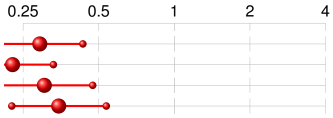

A	B	2.5	50	97.5	Sequence
2	0	0.2	0.29	0.43	LQESGTSEVVNNDKEFR
2	1	0.16	0.23	0.33	VAMDVQHFKPEELK
0	1	0.2	0.3	0.47	IIINAK
0	1	0.22	0.35	0.54	TVTSSLSPSGVLTLK

5.2 " 10 609 lethal(2)essential for life protein, l2efl [Aedes aegypti] gi"

Protein Accession	Unigene67975$_H$1
Mean Expression Ratio	0.444
Median Expression Ratio	0.443
Credible Interval	(0.325, 0.609)
Associated Peptides	4
Associated Spectra	7
Coverage	NaN

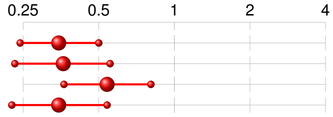

A	B	2.5	50	97.5	Sequence
1	2	0.24	0.35	0.5	ALEAPGETVIPITFEGK
1	0	0.23	0.36	0.56	MQMGETGK
0	2	0.36	0.54	0.81	SEVVNDDKEFR
0	1	0.22	0.35	0.54	KALEAPGETVIPITFEGKGDGK

5.3 200 2128 minus strand heat shock protein 70 [Cristaria plicata]

Protein Accession	CL8787.Contig2$_H$1
Mean Expression Ratio	0.457
Median Expression Ratio	0.458
Credible Interval	(0.328, 0.63)
Associated Peptides	4
Associated Spectra	7
Coverage	NaN

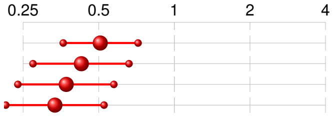

A	B	2.5	50	97.5	Sequence
1	3	0.36	0.51	0.72	IINEPTAAALAYGLDK
1	0	0.27	0.42	0.66	TLAPEEISSMVLVK
0	1	0.24	0.37	0.57	NGQVEIIANDQGQR
0	1	0.21	0.33	0.52	LSKEEIDR

5.4 721 1242 Lactoylglutathione lyase [Crassostrea gigas]; 882 1334 Lactoylglutathione lyase OS=Rattus norvegicus GN=Glo1 PE=1 SV=3; 113 634 Lactoylglutathione lyase [Crassostrea gigas]

Protein Accession	Unigene26921$_{H}1$ Unigene26919$_{H}1$ CL10946.Contig1$_{H}1$
Mean Expression Ratio	0.505
Median Expression Ratio	0.506
Credible Interval	(0.321, 0.788)
Associated Peptides	1
Associated Spectra	1
Coverage	NaN

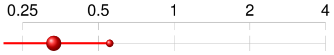

A	B	2.5	50	97.5	Sequence
**1**	**0**	**0.2**	**0.33**	**0.56**	**FESLGVEFIK**

5.5 704 817 minus strand expressed hypothetical protein [Trichoplax adhaerens] gi; 688 801 minus strand Lactoylglutathione lyase [Crassostrea gigas]; 735 848 minus strand expressed hypothetical protein [Trichoplax adhaerens] gi

Protein Accession	Unigene64106$_{H}1$ Unigene64104$_{H}1$ Unigene64103$_{H}1$
Mean Expression Ratio	1.89
Median Expression Ratio	1.88
Credible Interval	(1.25, 2.88)
Associated Peptides	1
Associated Spectra	2
Coverage	NaN

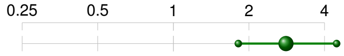

A	B	2.5	50	97.5	Sequence
**1**	**1**	**1.8**	**2.8**	**4.5**	**FESLGVELVK**

5.6 40 1182 4-hydroxyphenylpyruvate dioxygenase [Crassostrea gigas]

Protein Accession	CL7585.Contig2$_{H}1$
Mean Expression Ratio	0.566
Median Expression Ratio	0.566
Credible Interval	(0.389, 0.817)
Associated Peptides	3
Associated Spectra	3
Coverage	NaN

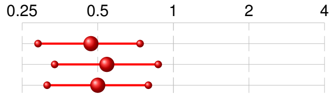

A	B	2.5	50	97.5	Sequence
**1**	**0**	**0.29**	**0.47**	**0.74**	**DVAFEVEDLDAIFK**
**1**	**0**	**0.34**	**0.54**	**0.87**	**TYGDTTHTLIDR**
**0**	**1**	**0.31**	**0.5**	**0.79**	**SLFEAIELDQAER**

5.7 957 2147 haemocyanin [Aplysia californica]

Protein Accession	Unigene30320$_{H}1$
Mean Expression Ratio	0.573
Median Expression Ratio	0.572
Credible Interval	(0.441, 0.745)
Associated Peptides	6
Associated Spectra	12
Coverage	NaN

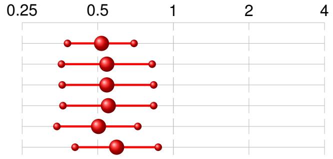

A	B	2.5	50	97.5	Sequence
**3**	**2**	**0.38**	**0.52**	**0.7**	**LLTVQFER**
**1**	**0**	**0.36**	**0.54**	**0.82**	**YEVSELSLEQVSNLR**
**1**	**0**	**0.36**	**0.54**	**0.83**	**TFHISGVNQDITR**
**1**	**0**	**0.36**	**0.55**	**0.83**	**LLTVQFERAL**
**0**	**2**	**0.34**	**0.5**	**0.72**	**KAGQNIGDTLK**
**0**	**2**	**0.41**	**0.59**	**0.87**	**IDITDAVK**

5.8 38 436 Calcium-dependent protein kinase isoform 2 [Crassostrea gigas]

Protein Accession	CL5866.Contig5$_{H}1$
Mean Expression Ratio	0.577
Median Expression Ratio	0.576
Credible Interval	(0.37, 0.894)
Associated Peptides	1
Associated Spectra	1
Coverage	NaN

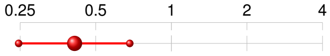

A	B	2.5	50	97.5	Sequence
1	0	0.25	0.41	0.68	SHAGLDVEADK

5.9 684 1250 minus strand Collagen alpha-5(VI) chain [Crassostrea gigas]

Protein Accession	CL284.Contig4$_{H}1$
Mean Expression Ratio	1.73
Median Expression Ratio	1.73
Credible Interval	(1.31, 2.29)
Associated Peptides	5
Associated Spectra	10
Coverage	NaN

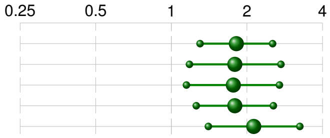

A	B	2.5	50	97.5	Sequence
1	3	1.3	1.8	2.5	HVMDALSAIDHHQGDR
1	0	1.2	1.8	2.7	VCIVITDGQSQEIQQTADEAR
1	0	1.2	1.8	2.7	EAGITMFAVGVGK
1	2	1.3	1.8	2.6	VAAITFGK
0	1	1.4	2.1	3.3	EAGITMF

5.10 21 341 minus strand hemocyanin isoform 1 [Nucula nucleus]

Protein Accession	CL40281.Contig2$_{H}1$
Mean Expression Ratio	0.576
Median Expression Ratio	0.577
Credible Interval	(0.41, 0.802)
Associated Peptides	2
Associated Spectra	6
Coverage	NaN

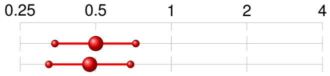

A	B	2.5	50	97.5	Sequence
1	2	0.34	0.5	0.72	GDELFSYLK
2	1	0.33	0.47	0.69	KPLEPFNR

5.11 204 791 minus strand PREDICTED: 15-hydroxyprostaglandin dehydrogenase [NAD+] [Gallus gallus]

Protein Accession	Unigene3818$_{H}1$
Mean Expression Ratio	1.69
Median Expression Ratio	1.69
Credible Interval	(1.38, 2.1)
Associated Peptides	9
Associated Spectra	41
Coverage	NaN

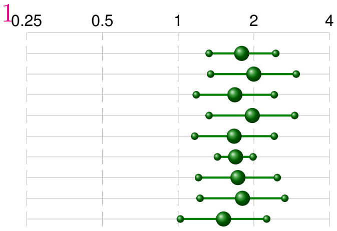

A	B	2.5	50	97.5	Sequence
4	1	1.3	1.8	2.5	AITMWGK
1	0	1.3	2	2.9	MGTTTDKELK
2	0	1.2	1.7	2.4	VDIMVNNAEIFSETDMEK
1	0	1.3	2	2.9	NAEIFSETDMEK
1	1	1.2	1.7	2.4	MDITDK
15	11	1.4	1.7	2	AVTEALLAK
1	1	1.2	1.7	2.5	IAFVSGGAMGTGR
0	1	1.2	1.8	2.7	TMTVNVTALMHGTK
0	1	1	1.5	2.2	HFISAFEK

5.12 186 1778 predicted protein [Nematostella vectensis] gi

Protein Accession	CL20488.Contig9$_H$1
Mean Expression Ratio	1.64
Median Expression Ratio	1.64
Credible Interval	(1.16, 2.34)
Associated Peptides	3
Associated Spectra	4
Coverage	NaN

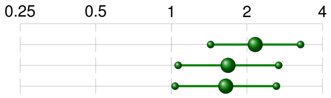

A	B	2.5	50	97.5	Sequence
1	1	1.4	2.2	3.3	EGTENVEMHFR
0	1	1.1	1.7	2.7	DGTTHVIWK
0	1	1	1.6	2.6	TRQLLK

5.13 3 434 minus strand myosin essential light chain [Haliotis discus discus]

Protein Accession	CL17177.Contig8$_H$1
Mean Expression Ratio	1.64
Median Expression Ratio	1.64
Credible Interval	(1.39, 1.93)
Associated Peptides	11
Associated Spectra	70
Coverage	NaN

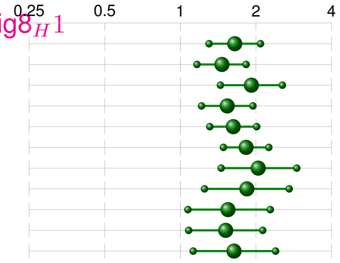

A	B	2.5	50	97.5	Sequence
6	4	1.3	1.7	2.1	EGQGFMSAAELR
5	6	1.2	1.5	1.8	FTDTREDLEGNVK
4	1	1.4	1.9	2.6	HVLTGLGEK
5	5	1.2	1.5	1.9	LTDQQVTDVIK
6	7	1.3	1.6	2	YADFIEK
7	7	1.5	1.8	2.3	DGDVDAFK
1	1	1.5	2	2.9	VGDMCF
1	0	1.2	1.8	2.7	EDLEGNVK
1	0	1.1	1.5	2.3	LSDREGQGFMSAAELR
0	2	1.1	1.5	2.1	FTDTREDLEGN
0	1	1.1	1.6	2.4	HVLTGLG

5.14 1027 1440 minus strand hemocyanin isoform 1 [Haliotis diversicolor]

Protein Accession	CL40281.Contig1$_H$1
Mean Expression Ratio	0.613
Median Expression Ratio	0.614
Credible Interval	(0.449, 0.832)
Associated Peptides	2
Associated Spectra	10
Coverage	NaN

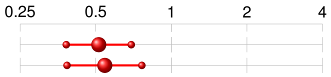

A	B	2.5	50	97.5	Sequence
3	3	0.38	0.51	0.69	LFVVQVEDELK
0	4	0.39	0.54	0.76	HNPFYDADIAFLQK

5.15 3 491 hemocyanin [Haliotis tuberculata]

Protein Accession	CL40281.Contig3$_H$1
Mean Expression Ratio	0.614
Median Expression Ratio	0.614
Credible Interval	(0.453, 0.831)
Associated Peptides	3
Associated Spectra	8
Coverage	NaN

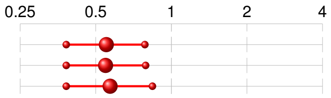

A	B	2.5	50	97.5	Sequence
2	1	0.38	0.55	0.79	DINPFALTK
1	2	0.38	0.55	0.79	TLEPFDRDINPFALTK
1	1	0.38	0.57	0.84	TLEPFDR

5.16 1 945 hemocyanin isoform 1 [Haliotis diversicolor]

Protein Accession	Unigene30318$_{H}1$
Mean Expression Ratio	0.616
Median Expression Ratio	0.616
Credible Interval	(0.419, 0.895)
Associated Peptides	2
Associated Spectra	3
Coverage	NaN

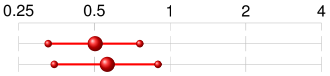

A	B	2.5	50	97.5	Sequence
1	1	0.33	0.5	0.76	VEITDVLK
0	1	0.35	0.56	0.9	LFVVEVEDELR

5.17 372 860 minus strand small heat shock protein [Tegillarca granosa]; 651 998 minus strand small heat shock protein [Tegillarca granosa]

Protein Accession	CL18190.Contig4$_{H}1$ CL18190.Contig1$_{H}1$
Mean Expression Ratio	0.624
Median Expression Ratio	0.624
Credible Interval	(0.459, 0.842)
Associated Peptides	4
Associated Spectra	8
Coverage	NaN

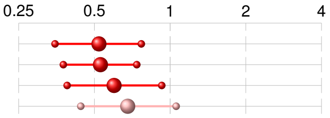

A	B	2.5	50	97.5	Sequence
1	1	0.35	0.52	0.77	EFLIPEEIDAETLGSR
2	2	0.38	0.53	0.74	LTEDGHLIISAR
1	0	0.39	0.6	0.93	IDKDGLSIK
0	1	0.44	0.68	1.1	TIAIESDK

5.18 397 510 PREDICTED: actin-5C-like [Megachile rotundata]

Protein Accession	CL48442.Contig2$_{H}1$
Mean Expression Ratio	1.55
Median Expression Ratio	1.55
Credible Interval	(1.17, 2.04)
Associated Peptides	3
Associated Spectra	29
Coverage	NaN

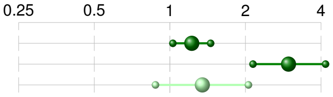

A	B	2.5	50	97.5	Sequence
15	9	1	1.2	1.5	DLTDYLMK
2	2	2.1	3	4.2	GRDLTDYLMK
0	1	0.88	1.4	2.1	LDLAGRDLTDY

5.19 23 214 minus strand hemocyanin isoform 1 [Nucula nucleus]

Protein Accession	Unigene58881$_{H}1$
Mean Expression Ratio	0.649
Median Expression Ratio	0.648
Credible Interval	(0.453, 0.932)
Associated Peptides	2
Associated Spectra	4
Coverage	NaN

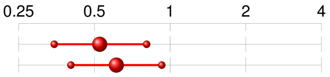

A	B	2.5	50	97.5	Sequence
2	0	0.35	0.53	0.8	GKIPNLNIYTSR
2	0	0.4	0.61	0.93	QEINPFFR

5.20 523 1086 minus strand juvenile hormone diol kinase [Danaus plexippus]

Protein Accession	CL7590.Contig5$_H 1$
Mean Expression Ratio	1.55
Median Expression Ratio	1.54
Credible Interval	(1.02, 2.34)
Associated Peptides	1
Associated Spectra	2
Coverage	NaN

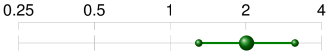

A	B	2.5	50	97.5	Sequence
2	0	1.3	2	3.1	GEFVTVQTIFGNSVEDSHK

5.21 360 764 minus strand Peptidyl-prolyl cis-trans isomerase A OS=Dictyostelium discoideum GN=ppiA PE=1 SV=1

Protein Accession	Unigene64429$_H 1$
Mean Expression Ratio	0.656
Median Expression Ratio	0.656
Credible Interval	(0.429, 0.99)
Associated Peptides	1
Associated Spectra	2
Coverage	NaN

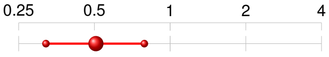

A	B	2.5	50	97.5	Sequence
0	2	0.32	0.51	0.79	HVVFGQVVEGLDVVR

5.22 201 1181 minus strand Serine/threonine-protein phosphatase PP1-alpha catalytic subunit [Crassostrea gigas]

Protein Accession	CL28655.Contig4$_H 1$
Mean Expression Ratio	0.655
Median Expression Ratio	0.657
Credible Interval	(0.447, 0.952)
Associated Peptides	2
Associated Spectra	3
Coverage	NaN

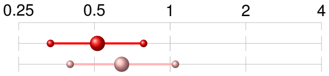

A	B	2.5	50	97.5	Sequence
1	1	0.34	0.51	0.79	ADTDKLNIDSIIAR
0	1	0.4	0.64	1	AHQVVEDGYEFFAK

5.23 266 607 minus strand H2 [Haliotis tuberculata]

Protein Accession	Unigene62350$_H 1$
Mean Expression Ratio	0.662
Median Expression Ratio	0.663
Credible Interval	(0.477, 0.912)
Associated Peptides	2
Associated Spectra	7
Coverage	NaN

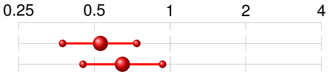

A	B	2.5	50	97.5	Sequence
3	1	0.37	0.53	0.74	YAVAGIGVR
2	1	0.45	0.64	0.93	KDINTLTDEEIK

5.24 2473 2898 RecName: Full=Paramyosin gi|397603|emb|CAA79849.1| paramyosin [Echinococcus granulosus]

Protein Accession	CL43455.Contig1$_{H}$1
Mean Expression Ratio	1.51
Median Expression Ratio	1.51
Credible Interval	(1.31, 1.73)
Associated Peptides	12
Associated Spectra	139
Coverage	NaN

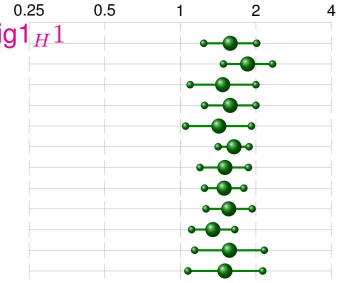

A	B	2.5	50	97.5	Sequence
5	4	1.2	1.6	2	AQTLIEEAENR
7	4	1.5	1.9	2.3	EILAQTEEER
2	2	1.1	1.5	2	EILAQTEEERR
6	4	1.2	1.6	2	INKEILAQTEEER
3	1	1	1.4	1.9	INKEILAQTEEERR
25	11	1.4	1.6	1.9	KAQTLIEEAENR
6	6	1.2	1.5	1.9	KQLEIEIR
14	6	1.2	1.5	1.8	QLAEFASLNEQLNLR
8	5	1.3	1.6	1.9	QLEEAEDVANPTMNK
9	6	1.1	1.4	1.7	QLEIEIR
1	2	1.1	1.6	2.2	RQLAEFASLNEQLNLR
1	1	1.1	1.5	2.1	RQLEEAEDVANPTMNK

5.25 66 947 Glyoxalase domain-containing protein 4 [Crassostrea gigas]

Protein Accession	CL23175.Contig2$_{H}$1
Mean Expression Ratio	1.5
Median Expression Ratio	1.5
Credible Interval	(0.963, 2.34)
Associated Peptides	1
Associated Spectra	1
Coverage	NaN

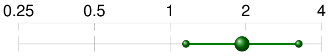

A	B	2.5	50	97.5	Sequence
0	1	1.2	1.9	3.2	LELVEIGQK

5.26 2 2572 Glycogen debranching enzyme [Crassostrea gigas]

Protein Accession	CL45503.Contig1$_{H}$1
Mean Expression Ratio	1.46
Median Expression Ratio	1.46
Credible Interval	(0.96, 2.26)
Associated Peptides	1
Associated Spectra	1
Coverage	NaN

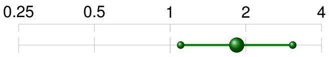

A	B	2.5	50	97.5	Sequence
0	1	1.1	1.8	3.1	IMAVNGWVMGDDPLR

5.27 40 1752 minus strand heat shock protein 60 [Biomphalaria glabrata]; 40 1752 minus strand heat shock protein 60 [Biomphalaria glabrata]

Protein Accession	CL5583.Contig6$_{H}$1 CL5583.Contig5$_{H}$1
Mean Expression Ratio	1.45
Median Expression Ratio	1.45
Credible Interval	(1.1, 1.91)
Associated Peptides	5
Associated Spectra	9
Coverage	NaN

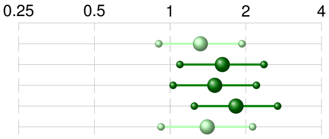

A	B	2.5	50	97.5	Sequence
2	0	0.9	1.3	1.9	AVGDLISSAMK
1	1	1.1	1.6	2.4	TLRDELEVIEGMK
1	1	1	1.5	2.2	VGLQVVAVK
2	0	1.3	1.8	2.7	LPNLLR
0	1	0.92	1.4	2.1	DDTLLMR

5.28 146 727 Adenylate kinase isoenzyme 1 [Crassostrea gigas]

Protein Accession	CL42551.Contig1$_H$1
Mean Expression Ratio	1.45
Median Expression Ratio	1.45
Credible Interval	(1.08, 1.97)
Associated Peptides	4
Associated Spectra	8
Coverage	NaN

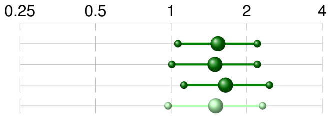

A	B	2.5	50	97.5	Sequence
3	0	1.1	1.5	2.2	DAMIQNITTTK
1	1	1	1.5	2.2	ELEQGLR
0	2	1.1	1.6	2.5	VVFVIGGPGSGK
0	1	0.97	1.5	2.3	LNAIMEK

5.29 405 1373 paramyosin [Haliotis discus discus]

Protein Accession	CL43455.Contig4$_H$1
Mean Expression Ratio	1.44
Median Expression Ratio	1.44
Credible Interval	(1.3, 1.58)
Associated Peptides	30
Associated Spectra	495
Coverage	NaN

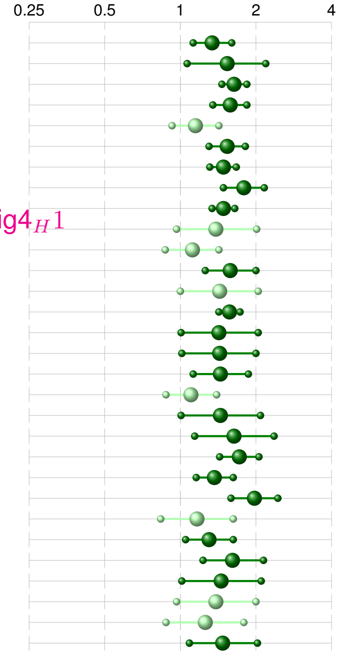

A	B	2.5	50	97.5	Sequence
14	7	1.1	1.3	1.6	DLELATAQFEAAEASLR
1	0	1.1	1.5	2.2	EKQTLVIELDGLSGQLDSITK
45	12	1.5	1.6	1.8	ELEDALDAER
23	5	1.4	1.6	1.8	HTEAVNDLSDQVDYLTK
5	7	0.93	1.2	1.4	IRELEDALDAER
14	10	1.3	1.5	1.8	LDEEAETSSNLR
33	19	1.3	1.5	1.7	LKGQVDDLTR
14	4	1.5	1.8	2.2	LTQENFDLQHQVQELDSSNAALAK
39	36	1.3	1.5	1.6	QLNDLTALK
1	0	0.96	1.4	2	QLNDLTALKAR
8	1	0.87	1.1	1.4	QNLQVNLSALQSDYDSLNAR
9	1	1.3	1.6	2	QTLVIELDGLSGQLDSITK
1	0	1	1.4	2	RHTEAVNDLSDQVDYLTK
46	38	1.4	1.6	1.7	TVAELSFR
1	0	1	1.4	2	LDQVQDSLDEQGGSSTVAQIELNKK
1	1	1	1.4	2	ELEDALDAERDGR
4	3	1.1	1.4	1.9	KDLELATAQFEAAEASLR
9	1	0.88	1.1	1.4	DEQGSSTVAQIELNK
1	0	1	1.4	2.1	DEQGSSTVAQIELNKK
1	0	1.1	1.6	2.4	ESEIYK
20	0	1.4	1.7	2.1	LDQVQDSLDEQGGSSTVAQIELNK
11	12	1.2	1.4	1.6	LSLESR
0	12	1.6	2	2.4	ITELEDSCEQLR
0	2	0.83	1.2	1.6	TRFDKELAAK
0	12	1.1	1.3	1.6	VSADYAALK
0	5	1.2	1.6	2.1	FDKELAAK
0	1	1	1.5	2.1	ITELEDSCEQLRV
0	1	0.97	1.4	2	ARLDEEAETSSNLR
0	1	0.88	1.3	1.8	LEDALDAER
0	3	1.1	1.5	2	TEELEETR

5.30 971 1963 Spectrin alpha chain [Crassostrea gigas]; 972 1979 Spectrin alpha chain [Crassostrea gigas]

Protein Accession	CL2195.Contig6$_H$1 CL2195.Contig4$_H$1
Mean Expression Ratio	0.697
Median Expression Ratio	0.7
Credible Interval	(0.458, 1.03)
Associated Peptides	1
Associated Spectra	2
Coverage	NaN

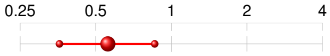

A	B	2.5	50	97.5	Sequence
1	1	0.36	0.56	0.86	EAFLAGDTVDGDNVETLIK

5.31 98 1012 minus strand RecName: Full=Substrate-specific endoprotease Tex31; Flags: Precursor gi|32187775|emb|CAD36507.1| substrate-specific endoprotease [Conus textile]

Protein Accession	CL13681.Contig1$_H$1
Mean Expression Ratio	1.42
Median Expression Ratio	1.42
Credible Interval	(1.04, 1.94)
Associated Peptides	3
Associated Spectra	7
Coverage	NaN

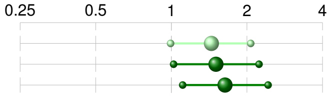

A	B	2.5	50	97.5	Sequence
2	1	1	1.4	2.1	NIPLSASDISAIVER
2	0	1	1.5	2.2	VPGAVFSK
1	1	1.1	1.6	2.4	ALDPQHTMCLADK

5.32 557 2329 minus strand Collagen alpha-3(VI) chain [Crassostrea gigas]

Protein Accession	CL47586.Contig7$_H$1
Mean Expression Ratio	1.4
Median Expression Ratio	1.4
Credible Interval	(1.01, 1.94)
Associated Peptides	4
Associated Spectra	5
Coverage	NaN

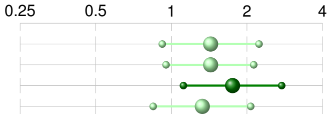

A	B	2.5	50	97.5	Sequence
1	0	0.92	1.4	2.2	KADYSAALGK
1	1	0.95	1.4	2.1	VVAVGVGAVVK
1	0	1.1	1.7	2.7	KLDVVVAFHASPR
0	1	0.84	1.3	2.1	VALGFFNK

5.33 3 503 minus strand elongation factor-1 alpha [Pomacea bridgesi]

Protein Accession	Unigene72143$_H$1
Mean Expression Ratio	1.38
Median Expression Ratio	1.38
Credible Interval	(0.935, 2.06)
Associated Peptides	2
Associated Spectra	2
Coverage	NaN

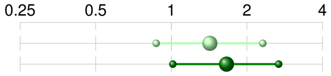

A	B	2.5	50	97.5	Sequence
0	1	0.87	1.4	2.3	QLIICANK
0	1	1	1.7	2.7	LVSPR

5.34 " 303 500 minus strand protein lethal, putative [Pediculus humanus corporis] gi"

Protein Accession	CL15038.Contig1$_H$1
Mean Expression Ratio	0.73
Median Expression Ratio	0.729
Credible Interval	(0.525, 1.01)
Associated Peptides	2
Associated Spectra	9
Coverage	NaN

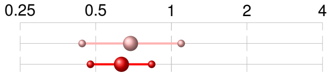

A	B	2.5	50	97.5	Sequence
1	0	0.44	0.69	1.1	QDMLATGR
6	2	0.48	0.63	0.83	QMPMLGR

5.35 REVERSED 6 395 PREDICTED: sister chromatid cohesion protein PDS5 homolog B-A isoform 1 [Nasonia vitripennis]

Protein Accession	RRRRRUnigene51506$_H$1
Mean Expression Ratio	1.36
Median Expression Ratio	1.36
Credible Interval	(1.02, 1.82)
Associated Peptides	2
Associated Spectra	37
Coverage	NaN

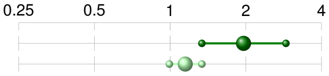

A	B	2.5	50	97.5	Sequence
2	0	1.3	2	2.9	IGICIDNFR
25	10	0.99	1.2	1.3	LQELVDK

5.36 280 690 Histone H3.3 [Harpegnathos saltator]

Protein Accession	Unigene26607$_H$1
Mean Expression Ratio	0.739
Median Expression Ratio	0.74
Credible Interval	(0.579, 0.937)
Associated Peptides	8
Associated Spectra	17
Coverage	NaN

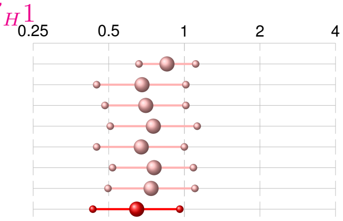

A	B	2.5	50	97.5	Sequence
4	4	0.66	0.85	1.1	STELLIR
1	0	0.45	0.68	1	KQLATK
1	1	0.48	0.7	1	RVTIMPK
1	0	0.51	0.75	1.1	RTKQTARK
1	0	0.45	0.67	1	DIQLAR
2	0	0.52	0.76	1.1	MARTKQTARK
0	1	0.5	0.74	1.1	EIAQDFK
0	1	0.43	0.65	0.96	KLPFQR

5.37 1009 2784 minus strand Collagen alpha-3(VI) chain [Crassostrea gigas]

Protein Accession	Unigene46961$_H$1
Mean Expression Ratio	1.35
Median Expression Ratio	1.35
Credible Interval	(1.08, 1.69)
Associated Peptides	8
Associated Spectra	21
Coverage	NaN

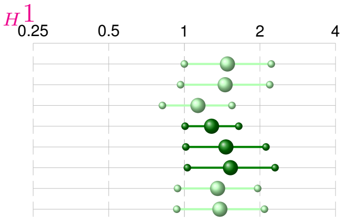

A	B	2.5	50	97.5	Sequence
1	0	1	1.5	2.2	FRKSPTLMFNLNEHK
1	0	0.97	1.5	2.2	IITVGIGEANRK
2	2	0.82	1.1	1.5	KSPTLMFNLNEHK
5	4	1	1.3	1.7	VAMSNFR
1	1	1	1.5	2.1	YADIDGGNVR
1	0	1	1.5	2.3	KAFNK
1	1	0.94	1.4	2	MFNLNEHK
0	1	0.93	1.4	2.1	FNLNEHK

5.38 288 1487 minus strand putative procollagen type VI alpha 4 [Haliotis diversicolor]

Protein Accession	CL6899.Contig2$_{H}$1
Mean Expression Ratio	0.742
Median Expression Ratio	0.742
Credible Interval	(0.661, 0.835)
Associated Peptides	20
Associated Spectra	188
Coverage	NaN

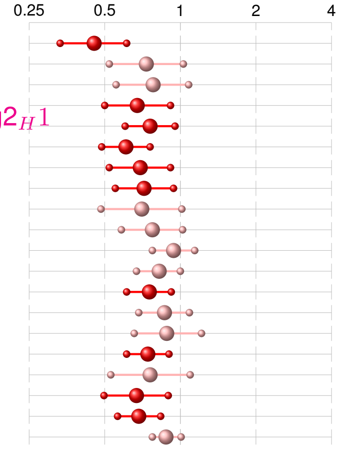

A	B	2.5	50	97.5	Sequence
4	0	0.33	0.45	0.61	AEVLAAIDQVR
2	0	0.52	0.73	1	ELTNQVAVLFLDDQALR
1	1	0.55	0.78	1.1	ENLYYIQSVVDR
1	3	0.5	0.67	0.91	MGFDVDQFR
5	5	0.6	0.76	0.95	TKAEVLAAIDQVR
9	2	0.49	0.61	0.76	VMVVITDGSSR
4	1	0.52	0.69	0.91	FDISVDDTR
3	3	0.55	0.72	0.94	FVVVDLGVR
1	0	0.48	0.7	1	LGNVVPQDENTYNNLLR
4	1	0.58	0.77	1	PDPTIGSCR
8	9	0.78	0.94	1.1	ALETLR
8	7	0.67	0.82	1	DLLPMR
9	5	0.61	0.75	0.92	FRDDITK
6	4	0.68	0.86	1.1	QVLQFVSQVITR
2	1	0.65	0.88	1.2	LRENLK
9	7	0.61	0.74	0.9	IGALTFSDDVK
1	0	0.53	0.76	1.1	FRALETLR
2	2	0.5	0.67	0.89	ADIINFASQSLAR
10	5	0.56	0.68	0.84	GANGVTLAQQVR
34	9	0.77	0.88	1	TGVTNTHLAIR

5.39 103 684 EF-hand domain-containing protein D2 [Crassostrea gigas]

Protein Accession	CL20544.Contig1$_{H}$1
Mean Expression Ratio	1.34
Median Expression Ratio	1.34
Credible Interval	(0.929, 1.94)
Associated Peptides	3
Associated Spectra	3
Coverage	NaN

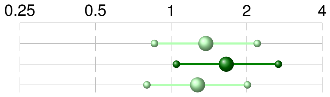

A	B	2.5	50	97.5	Sequence
1	0	0.86	1.4	2.2	VPQTHLALK
1	0	1	1.7	2.7	KFEEEIR
1	0	0.8	1.3	2	FIDFNELK

5.40 " 285 437 Propionyl-CoA carboxylase alpha chain, mitochondrial [Crassostrea gigas]"

Protein Accession	CL45581.Contig2$_{H}$1
Mean Expression Ratio	0.745
Median Expression Ratio	0.745
Credible Interval	(0.498, 1.11)
Associated Peptides	1
Associated Spectra	2
Coverage	NaN

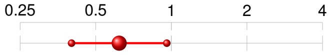

A	B	2.5	50	97.5	Sequence
2	0	0.4	0.62	0.96	GVTHNIPLLR

5.41 127 657 ADP ribosylation factor 4 [Marsupenaeus japonicus]

Protein Accession	Unigene23129$_{H}1$
Mean Expression Ratio	1.34
Median Expression Ratio	1.34
Credible Interval	(0.98, 1.81)
Associated Peptides	4
Associated Spectra	8
Coverage	NaN

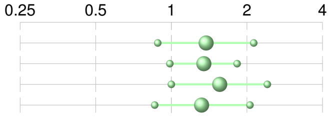

A	B	2.5	50	97.5	Sequence
1	0	0.88	1.4	2.1	IEEAKDELQK
2	3	0.99	1.3	1.8	ILMVGLDAAGK
0	1	1	1.6	2.4	LLVFANK
0	1	0.86	1.3	2.1	LGLNQLR

5.42 124 711 glutathione S-transferase sigma 3 [Mytilus galloprovincialis]; 124 711 glutathione S-transferase sigma 3 [Mytilus galloprovincialis]; 110 697 glutathione S-transferase sigma 3 [Mytilus galloprovincialis]

Protein Accession	CL27284.Contig5$_{H}1$ CL27284.Contig4$_{H}1$ CL27284.Contig3$_{H}1$
Mean Expression Ratio	1.34
Median Expression Ratio	1.34
Credible Interval	(0.956, 1.89)
Associated Peptides	3
Associated Spectra	4
Coverage	NaN

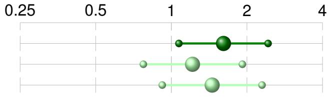

A	B	2.5	50	97.5	Sequence
1	1	1.1	1.6	2.4	VDEATQGVVDIR
1	0	0.77	1.2	1.9	TALYGYAFEKDEAR
1	0	0.92	1.5	2.3	LVPEAEK

5.43 2 772 minus strand arylsulfatase [Haliotis discus discus]

Protein Accession	CL48181.Contig1$_{H}1$
Mean Expression Ratio	1.33
Median Expression Ratio	1.33
Credible Interval	(0.923, 1.93)
Associated Peptides	2
Associated Spectra	3
Coverage	NaN

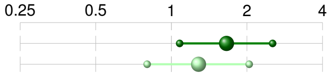

A	B	2.5	50	97.5	Sequence
1	1	1.1	1.7	2.5	LLPQLK
0	1	0.8	1.3	2	GVGMVVDALK

5.44 472 1551 minus strand hypothetical protein [Amblyomma maculatum]

Protein Accession	Unigene11523$_{H}1$
Mean Expression Ratio	0.753
Median Expression Ratio	0.752
Credible Interval	(0.612, 0.927)
Associated Peptides	7
Associated Spectra	28
Coverage	NaN

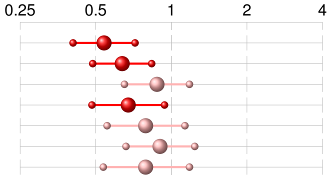

A	B	2.5	50	97.5	Sequence
6	0	0.41	0.54	0.71	LFQNAQLSR
7	0	0.49	0.64	0.83	QSLTDILR
3	2	0.65	0.88	1.2	VANGLYYDDTAVTLAEAFK
1	2	0.48	0.67	0.94	VLELPYGTDGR
2	0	0.56	0.79	1.1	SKQSLTDILR
4	0	0.66	0.9	1.2	MVTGPVPGNLAVSK
0	1	0.53	0.79	1.2	FMVSLPK

5.45 900 1616 minus strand hemocyanin isoform 1 [Haliotis diversicolor]

Protein Accession	Unigene30321$_{H}1$
Mean Expression Ratio	0.753
Median Expression Ratio	0.754
Credible Interval	(0.503, 1.12)
Associated Peptides	1
Associated Spectra	3
Coverage	NaN

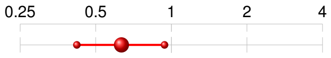

A	B	2.5	50	97.5	Sequence
**2**	**1**	**0.42**	**0.63**	**0.94**	**VEITDDLK**

5.46 " 196 1053 minus strand Cytochrome b-c1 complex subunit 2, mitochondrial OS=Homo sapiens GN=UQCRC2 PE=1 SV=3"

Protein Accession	CL7847.Contig5$_{H}1$
Mean Expression Ratio	1.31
Median Expression Ratio	1.32
Credible Interval	(0.875, 1.98)
Associated Peptides	1
Associated Spectra	2
Coverage	NaN

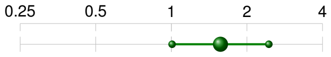

A	B	2.5	50	97.5	Sequence
**1**	**1**	**1**	**1.6**	**2.4**	**LDLAILK**

5.47 169 1161 Basement membrane-specific heparan sulfate proteoglycan core protein OS=Homo sapiens GN=HSPG2 PE=1 SV=4

Protein Accession	CL5208.Contig1$_{H}1$
Mean Expression Ratio	0.761
Median Expression Ratio	0.761
Credible Interval	(0.515, 1.13)
Associated Peptides	2
Associated Spectra	2
Coverage	NaN

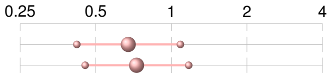

A	B	2.5	50	97.5	Sequence
**1**	**0**	**0.42**	**0.67**	**1.1**	**GGDLTANHVVQGDVLR**
**1**	**0**	**0.45**	**0.73**	**1.2**	**PTITWSR**

5.48 361 549 minus strand 32 kDa beta-galactoside-binding lectin [Ascaris suum]

Protein Accession	CL5658.Contig3$_{H}1$
Mean Expression Ratio	0.76
Median Expression Ratio	0.761
Credible Interval	(0.517, 1.11)
Associated Peptides	1
Associated Spectra	3
Coverage	NaN

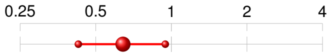

A	B	2.5	50	97.5	Sequence
**1**	**2**	**0.43**	**0.64**	**0.94**	**TIVLNHMQDGR**

5.49 1 825 Spectrin alpha chain [Crassostrea gigas]

Protein Accession	CL10282.Contig3$_{H}1$
Mean Expression Ratio	1.29
Median Expression Ratio	1.29
Credible Interval	(0.936, 1.78)
Associated Peptides	3
Associated Spectra	6
Coverage	NaN

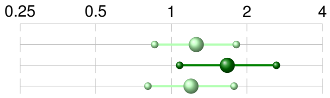

A	B	2.5	50	97.5	Sequence
2	1	0.86	1.3	1.8	MQHNLEQQIEAR
1	0	1.1	1.7	2.6	IEDLGAAMEER
0	2	0.81	1.2	1.8	LILDNR

5.50 269 880 minus strand myosin regulatory light chain [Fasciola hepatica]

Protein Accession	CL43006.Contig5$_{H}1$
Mean Expression Ratio	1.28
Median Expression Ratio	1.28
Credible Interval	(1, 1.66)
Associated Peptides	3
Associated Spectra	21
Coverage	NaN

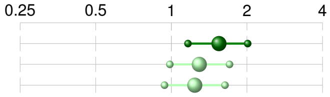

A	B	2.5	50	97.5	Sequence
5	2	1.2	1.5	2	ATSNVFAK
7	0	0.99	1.3	1.7	KLMQEMK
4	3	0.94	1.2	1.6	LMQEMK

5.51 764 1015 minus strand heat shock protein beta-1 [Trichinella spiralis] gi

Protein Accession	CL46983.Contig3$_{H}1$
Mean Expression Ratio	0.78
Median Expression Ratio	0.78
Credible Interval	(0.571, 1.06)
Associated Peptides	2
Associated Spectra	12
Coverage	NaN

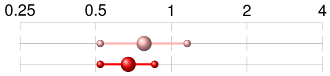

A	B	2.5	50	97.5	Sequence
2	0	0.52	0.78	1.2	SYVLPK
8	2	0.52	0.67	0.86	TVTSSLSQNGVLTLK

5.52 816 1211 H2 [Haliotis tuberculata]

Protein Accession	CL11519.Contig5$_{H}1$
Mean Expression Ratio	0.779
Median Expression Ratio	0.781
Credible Interval	(0.525, 1.16)
Associated Peptides	2
Associated Spectra	4
Coverage	NaN

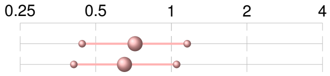

A	B	2.5	50	97.5	Sequence
0	1	0.44	0.72	1.2	FDISDAVR
2	1	0.41	0.65	1.1	SLILAMR

5.53 2 256 Polyubiquitin (Fragment) OS=Xenopus laevis PE=1 SV=2; 20 388 large subunit ribosomal protein L40e [Clonorchis sinensis]

Protein Accession	CL47507.Contig2$_{H}$1 CL39010.Contig1$_{H}$1
Mean Expression Ratio	1.28
Median Expression Ratio	1.28
Credible Interval	(0.983, 1.65)
Associated Peptides	5
Associated Spectra	19
Coverage	NaN

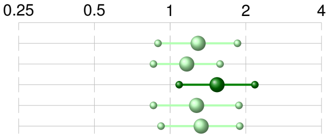

A	B	2.5	50	97.5	Sequence
2	1	0.9	1.3	1.9	TITLEVEPSDTIENVK
4	4	0.86	1.2	1.6	TLSDYNIQK
3	0	1.1	1.5	2.2	LIFAGK
1	1	0.86	1.3	1.9	ESTLHLVLR
2	1	0.92	1.3	1.9	MQIFVK

5.54 2156 3142 minus strand PREDICTED: hemicentin-1 [Xenopus (Silurana) tropicalis]

Protein Accession	CL2792.Contig4$_{H}$1
Mean Expression Ratio	1.28
Median Expression Ratio	1.28
Credible Interval	(1, 1.64)
Associated Peptides	7
Associated Spectra	15
Coverage	NaN

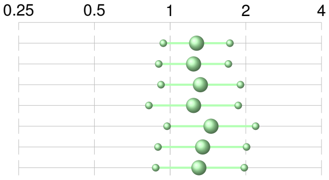

A	B	2.5	50	97.5	Sequence
4	1	0.94	1.3	1.7	GAPPPTVSWVK
2	2	0.9	1.2	1.7	NDVELYSK
1	1	0.92	1.3	1.9	SGVDAMKPYLR
1	0	0.83	1.2	1.9	LVEVIEEK
0	1	0.97	1.5	2.2	YECVAENLVHTR
0	1	0.9	1.3	2	NGLIIDTEKDPNLQVDPEGSLMFK
0	1	0.87	1.3	2	VDAMK

5.55 238 681 minus strand Globin OS=Cerithidea rhizophorarum PE=1 SV=1

Protein Accession	CL8458.Contig2$_{H}$1
Mean Expression Ratio	1.28
Median Expression Ratio	1.28
Credible Interval	(1.06, 1.54)
Associated Peptides	8
Associated Spectra	118
Coverage	NaN

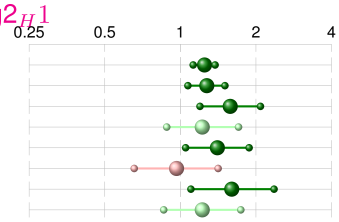

A	B	2.5	50	97.5	Sequence
43	33	1.1	1.2	1.4	AAITSTWSK
15	9	1.1	1.3	1.5	AYFPFLPGASEGDIK
4	2	1.2	1.6	2.1	ILAMDDADCLDGLGTK
0	3	0.88	1.2	1.7	PFLPGASEGDIK
0	5	1	1.4	1.9	PGASEGDIK
0	1	0.65	0.96	1.4	ATMENNGAK
0	1	1.1	1.6	2.4	EVFGSFLEATL
0	2	0.86	1.2	1.7	EVFGSFLEATLGGAATPDVK

5.56 " 158 355 Protein NAS-37, isoform b [Caenorhabditis elegans] gi; 15 227 Hemicentin-1 [Crassostrea gigas]"

Protein Accession	Unigene57180$_{H}$1 CL8016.Contig2$_{H}$1
Mean Expression Ratio	1.27
Median Expression Ratio	1.27
Credible Interval	(0.821, 1.99)
Associated Peptides	1
Associated Spectra	1
Coverage	NaN

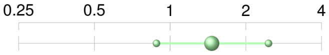

A	B	2.5	50	97.5	Sequence
1	0	0.88	1.5	2.5	FKGSYGGVSR

5.57 109 510 minus strand hypothetical protein DAPPUDRAFT_231281 [Daphnia pulex]

Protein Accession	Unigene4945$_{H}1$
Mean Expression Ratio	0.787
Median Expression Ratio	0.788
Credible Interval	(0.583, 1.07)
Associated Peptides	4
Associated Spectra	8
Coverage	NaN

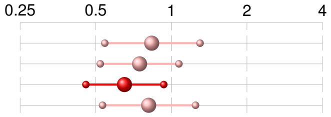

A	B	2.5	50	97.5	Sequence
1	0	0.54	0.84	1.3	LAGIEDPFEMNVK
2	1	0.52	0.75	1.1	VSMPDTLGHAVGIER
2	1	0.46	0.65	0.93	YELLAK
0	1	0.53	0.81	1.2	PDTLGHAVGIER

5.58 170 460 Spectrin beta chain [Crassostrea gigas]

Protein Accession	CL38255.Contig1$_{H}1$
Mean Expression Ratio	0.787
Median Expression Ratio	0.788
Credible Interval	(0.529, 1.17)
Associated Peptides	2
Associated Spectra	2
Coverage	NaN

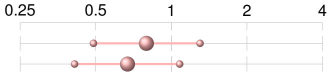

A	B	2.5	50	97.5	Sequence
1	0	0.49	0.79	1.3	LLEVLSGER
1	0	0.41	0.67	1.1	WVNSHLIR

5.59 91 1326 minus strand Adipocyte plasma membrane-associated protein [Crassostrea gigas]; 107 1273 minus strand Adipocyte plasma membrane-associated protein [Crassostrea gigas]

Protein Accession	CL11239.Contig2$_{H}1$ CL11239.Contig1$_{H}1$
Mean Expression Ratio	0.789
Median Expression Ratio	0.789
Credible Interval	(0.534, 1.17)
Associated Peptides	1
Associated Spectra	3
Coverage	NaN

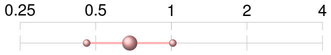

A	B	2.5	50	97.5	Sequence
0	3	0.46	0.68	1	YGLIAELDKEGK

5.60 970 1854 fasciclin-like protein precursor [Aplysia californica] gi

Protein Accession	CL9799.Contig1$_{H}1$
Mean Expression Ratio	0.792
Median Expression Ratio	0.791
Credible Interval	(0.551, 1.13)
Associated Peptides	3
Associated Spectra	3
Coverage	NaN

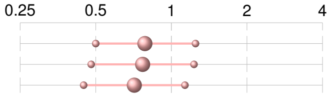

A	B	2.5	50	97.5	Sequence
1	0	0.5	0.79	1.2	VLIPDDALDLVTMAK
0	1	0.48	0.77	1.2	ANLVSMLK
0	1	0.45	0.71	1.1	ELQTVLLGHVLPEAR

5.61 180 560 calreticulin precursor [Aplysia californica] gi; 180 545 calreticulin precursor [Aplysia californica] gi

Protein Accession	CL4813.Contig4$_{H}$1 CL4813.Contig2$_{H}$1
Mean Expression Ratio	1.26
Median Expression Ratio	1.26
Credible Interval	(0.922, 1.73)
Associated Peptides	3
Associated Spectra	11
Coverage	NaN

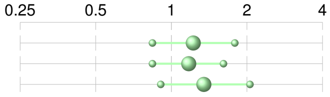

A	B	2.5	50	97.5	Sequence
2	1	0.84	1.2	1.8	FVWSAGK
4	2	0.84	1.2	1.6	WIPSVHK
1	1	0.9	1.3	2.1	FYGISAK

5.62 135 2630 Filamin-A [Crassostrea gigas]; 118 2613 Filamin-A [Crassostrea gigas]; 157 2652 Filamin-A [Crassostrea gigas]

Protein Accession	CL393.Contig15$_{H}$1 CL393.Contig13$_{H}$1 CL393.Contig12$_{H}$1
Mean Expression Ratio	1.26
Median Expression Ratio	1.26
Credible Interval	(1.09, 1.46)
Associated Peptides	23
Associated Spectra	143
Coverage	NaN

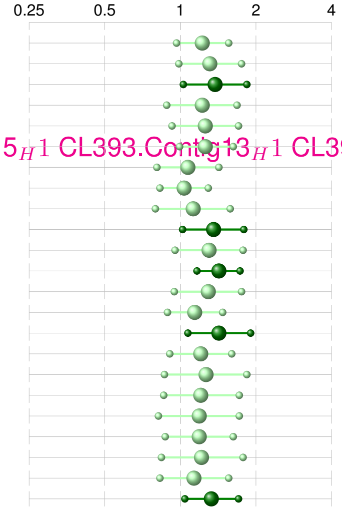

A	B	2.5	50	97.5	Sequence
9	3	0.96	1.2	1.6	AVANKPNEIVIDTSK
5	1	0.98	1.3	1.8	DNNDGTIVVEYQPK
5	1	1	1.4	1.8	EAGPGTLTVAVEGPGK
2	1	0.88	1.2	1.7	ENGVHYVDVK
4	1	0.93	1.3	1.7	HINNSPFK
6	4	0.99	1.3	1.6	IGDSELGHAAGCK
3	3	0.8	1.1	1.4	MGDGEIGIASFNPR
7	6	0.83	1	1.3	MNGIHTLSVR
1	1	0.79	1.1	1.6	PGSYGLHIK
4	2	1	1.4	1.8	QMEAAEAAVPNK
3	1	0.95	1.3	1.8	TPSGGHEDCFIQEMDK
12	9	1.2	1.4	1.7	VINFQIETK
3	1	0.95	1.3	1.8	VPKPGSYGLHIK
5	4	0.89	1.1	1.5	VQVGGPGLEK
4	2	1.1	1.4	1.9	VTGALDK
4	2	0.91	1.2	1.6	YGNIPIAGSPYK
0	1	0.87	1.3	1.8	GDSELGHAAGCK
1	1	0.86	1.2	1.7	AALSCK
0	1	0.82	1.2	1.7	QVTVHSLR
3	1	0.87	1.2	1.6	YKGNPIK
0	1	0.84	1.2	1.8	PKPGSYGLHIK
3	1	0.83	1.1	1.6	IMFTPK
8	3	1	1.3	1.7	KPSPIQEQSSMVVETVEK

5.63 " 131 544 Histone H2B, gonadal OS=Patella granatina PE=1 SV=2"

Protein Accession	CL29794.Contig2$_{H}$1
Mean Expression Ratio	0.795
Median Expression Ratio	0.794
Credible Interval	(0.587, 1.07)
Associated Peptides	2
Associated Spectra	18
Coverage	NaN

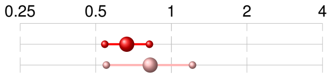

A	B	2.5	50	97.5	Sequence
9	7	0.54	0.67	0.82	LLLPGELAK
0	2	0.55	0.82	1.2	PGELAK

5.64 3 416 minus strand myosin essential light chain [Crassostrea gigas]

Protein Accession	Unigene47974$_{H}1$
Mean Expression Ratio	1.26
Median Expression Ratio	1.26
Credible Interval	(0.894, 1.76)
Associated Peptides	2
Associated Spectra	6
Coverage	NaN

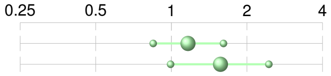

A	B	2.5	50	97.5	Sequence
**0**	**5**	**0.84**	**1.2**	**1.6**	**CVGLNPTLATIK**
**0**	**1**	**0.99**	**1.6**	**2.4**	**NPTLATIK**

5.65 459 755 minus strand heat shock protein beta-1 [Trichinella spiralis] gi

Protein Accession	CL17234.Contig5$_{H}1$
Mean Expression Ratio	0.796
Median Expression Ratio	0.796
Credible Interval	(0.574, 1.11)
Associated Peptides	3
Associated Spectra	8
Coverage	NaN

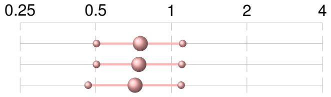

A	B	2.5	50	97.5	Sequence
**2**	**1**	**0.5**	**0.75**	**1.1**	**DVEPTTVTSSLNADGVLTLK**
**2**	**1**	**0.5**	**0.74**	**1.1**	**IVVEGCHEEK**
**1**	**1**	**0.47**	**0.72**	**1.1**	**IVVEGCHEEKQDEHGFITR**

5.66 " 125 628 minus strand collectin sub-family member 12, partial [Oncorhynchus mykiss]"

Protein Accession	CL46744.Contig5$_{H}1$
Mean Expression Ratio	1.26
Median Expression Ratio	1.26
Credible Interval	(0.816, 1.95)
Associated Peptides	1
Associated Spectra	1
Coverage	NaN

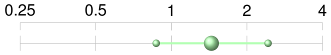

A	B	2.5	50	97.5	Sequence
**1**	**0**	**0.87**	**1.4**	**2.4**	**WVQGDNRPLAYTR**

5.67 606 1730 UDP-glucuronosyltransferase 2C1 precursor [Oryctolagus cuniculus]

Protein Accession	CL29849.Contig5$_{H}1$
Mean Expression Ratio	1.25
Median Expression Ratio	1.25
Credible Interval	(0.842, 1.87)
Associated Peptides	2
Associated Spectra	3
Coverage	NaN

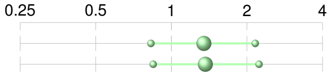

A	B	2.5	50	97.5	Sequence
**1**	**1**	**0.83**	**1.3**	**2.2**	**LFVSHCGK**
**0**	**1**	**0.85**	**1.4**	**2.2**	**AAHAVEFVLK**

5.68 182 1846 minus strand hypothetical protein DAPPUDRAFT_302108 [Daphnia pulex]; 110 1774 minus strand hypothetical protein DAPPUDRAFT_302108 [Daphnia pulex]

Protein Accession	CL11561.Contig6$_{H}1$ CL11561.Contig1$_{H}1$
Mean Expression Ratio	1.26
Median Expression Ratio	1.25
Credible Interval	(0.879, 1.82)
Associated Peptides	3
Associated Spectra	3
Coverage	NaN

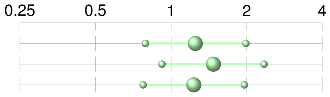

A	B	2.5	50	97.5	Sequence
**1**	**0**	**0.79**	**1.2**	**2**	**LRDAIRK**
**0**	**1**	**0.92**	**1.5**	**2.3**	**LFLPGGLK**
**0**	**1**	**0.77**	**1.2**	**2**	**LIIGQR**

5.69 233 814 Kynurenine formamidase [Crassostrea gigas]; 233 979 Kynurenine formamidase [Crassostrea gigas]; 139 945 Kynurenine formamidase [Crassostrea gigas]; 145 951 Kynurenine formamidase [Crassostrea gigas]; 220 1026 Kynurenine formamidase [Crassostrea gigas]; 39 725 Kynurenine formamidase [Crassostrea gigas]; 198 986 Kynurenine formamidase [Crassostrea gigas]; 39 743 Kynurenine formamidase [Crassostrea gigas]; 123 929 Kynurenine formamidase [Crassostrea gigas]; 39 743 Kynurenine formamidase [Crassostrea gigas]

Protein Accession	Unigene7804$_{H}1$ Unigene42149$_{H}1$ CL8132.Contig9$_{H}1$ CL8132.Contig8$_{H}1$ CL8132.Contig7$_{H}$
Mean Expression Ratio	0.797
Median Expression Ratio	0.798
Credible Interval	(0.548, 1.14)
Associated Peptides	1
Associated Spectra	6
Coverage	NaN

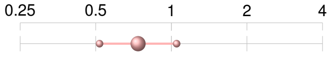

A	B	2.5	50	97.5	Sequence
**4**	**2**	**0.52**	**0.74**	**1**	**LVGPGVVIDVR**

5.70 " 1144 1956 minus strand Retinol dehydrogenase 12, partial [Crassostrea gigas]"

Protein Accession	CL3944.Contig7$_{H}1$
Mean Expression Ratio	0.8
Median Expression Ratio	0.799
Credible Interval	(0.569, 1.13)
Associated Peptides	3
Associated Spectra	4
Coverage	NaN

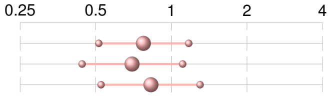

A	B	2.5	50	97.5	Sequence
**2**	**0**	**0.51**	**0.77**	**1.2**	**LDFTDLALLK**
**1**	**0**	**0.44**	**0.7**	**1.1**	**LILGCR**
**0**	**1**	**0.52**	**0.83**	**1.3**	**LDLASLR**

5.71 8 937 minus strand galectin [Crassostrea virginica]

Protein Accession	CL7245.Contig6$_{H}1$
Mean Expression Ratio	0.803
Median Expression Ratio	0.802
Credible Interval	(0.518, 1.23)
Associated Peptides	1
Associated Spectra	1
Coverage	NaN

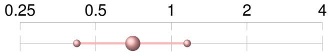

A	B	2.5	50	97.5	Sequence
1	0	0.42	0.7	1.2	QDQGDVVLNAK

5.72 76 522 minus strand Troponin C [Crassostrea gigas]; 173 619 minus strand Troponin C [Crassostrea gigas]; 173 619 minus strand Troponin C [Crassostrea gigas]

Protein Accession	Unigene53263$_{H}1$ Unigene11674$_{H}1$ CL13001.Contig4$_{H}1$
Mean Expression Ratio	1.24
Median Expression Ratio	1.25
Credible Interval	(1.02, 1.5)
Associated Peptides	7
Associated Spectra	48
Coverage	NaN

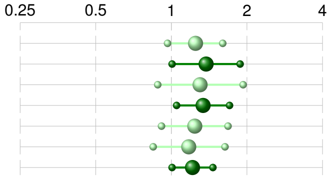

A	B	2.5	50	97.5	Sequence
4	4	0.96	1.2	1.6	GVIPVEDLR
3	1	1	1.4	1.9	QYNDANQTFNLFDK
1	0	0.88	1.3	1.9	QYNDANQTFNLFDKK
6	3	1	1.3	1.7	SLGLQVEDEK
2	2	0.91	1.2	1.7	VLDKGNK
2	1	0.84	1.2	1.6	SLGLQVEDEKLK
11	8	1	1.2	1.5	ELANVFK

5.73 161 301 Heat shock protein beta-6 OS=Mus musculus GN=Hspb6 PE=1 SV=1; 167 310 Heat shock protein beta-1 OS=Canis familiaris GN=HSPB1 PE=2 SV=1

Protein Accession	CL46983.Contig2$_{H}1$ CL46983.Contig1$_{H}1$
Mean Expression Ratio	0.802
Median Expression Ratio	0.804
Credible Interval	(0.572, 1.11)
Associated Peptides	2
Associated Spectra	9
Coverage	NaN

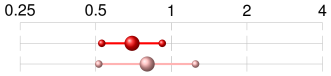

A	B	2.5	50	97.5	Sequence
6	2	0.53	0.7	0.92	VNMDVQHFKPEEIK
0	1	0.52	0.8	1.2	NMDVQHFKPEEIK

5.74 " 386 799 minus strand Myosin regulatory light chain LC-2, mantle muscle OS=Todarodes pacificus PE=1 SV=1; 656 1057 minus strand myosin regulatory light chain [Fasciola hepatica]"

Protein Accession	CL43006.Contig13$_H$1 CL43006.Contig11$_H$1
Mean Expression Ratio	1.24
Median Expression Ratio	1.24
Credible Interval	(0.937, 1.65)
Associated Peptides	2
Associated Spectra	27
Coverage	NaN

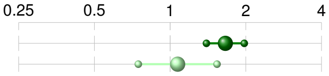

A	B	2.5	50	97.5	Sequence
15	9	1.4	1.7	2	DLLHNMGDNFTDDELK
0	3	0.75	1.1	1.5	DLLHNMGDNFTDDELKQTWK

5.75 167 679 tropomyosin [Haliotis discus discus]

Protein Accession	CL2925.Contig3$_H$1
Mean Expression Ratio	1.24
Median Expression Ratio	1.24
Credible Interval	(1.1, 1.4)
Associated Peptides	19
Associated Spectra	201
Coverage	NaN

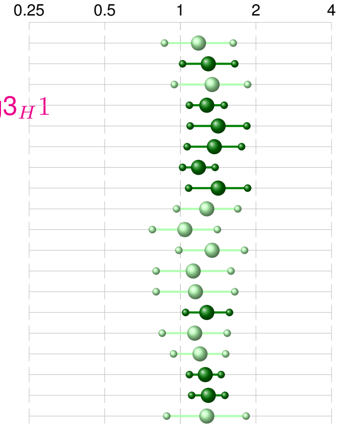

A	B	2.5	50	97.5	Sequence
2	1	0.87	1.2	1.6	AMDKAEQMEQK
6	3	1	1.3	1.6	ANEGLIEANTK
1	1	0.95	1.3	1.9	ENAMDKAEQMEQK
23	3	1.1	1.3	1.5	IKLLEEDLER
6	1	1.1	1.4	1.8	KYANLENEYDKANEGLIEANTK
8	0	1.1	1.4	1.7	MDKENAMDK
24	8	1	1.2	1.4	MDKENAMDKAEQMEQK
3	3	1.1	1.4	1.9	YANLENEYDK
5	0	0.97	1.3	1.7	YANLENEYDKANEGLIEANTK
2	2	0.77	1	1.4	KMLAMK
4	0	0.99	1.3	1.8	KYANLENEYDK
1	1	0.8	1.1	1.6	SKLEEDLN
1	0	0.8	1.2	1.7	LLEEDLER
3	13	1.1	1.3	1.6	RIKLLEEDLER
4	0	0.85	1.1	1.5	SKLEEDLNPLQK
8	1	0.94	1.2	1.5	LRDTEVQK
14	19	1.1	1.3	1.5	MDAIK
14	15	1.1	1.3	1.5	MLAMK
0	1	0.88	1.3	1.8	ANEGLIEAN

5.76 401 1174 minus strand Mechanosensory protein 2 [Crassostrea gigas]

Protein Accession	Unigene7703$_H$1
Mean Expression Ratio	0.805
Median Expression Ratio	0.805
Credible Interval	(0.52, 1.25)
Associated Peptides	1
Associated Spectra	1
Coverage	NaN

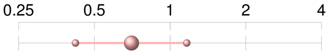

A	B	2.5	50	97.5	Sequence
1	0	0.42	0.7	1.2	VIAAEGEQK

5.77 19 1218 Dynactin subunit 2 [Crassostrea gigas]; 48 830 minus strand Dynactin subunit 2 [Crassostrea gigas]

Protein Accession	CL5827.Contig5$_{H}1$ CL5827.Contig1$_{H}1$
Mean Expression Ratio	0.803
Median Expression Ratio	0.805
Credible Interval	(0.516, 1.24)
Associated Peptides	1
Associated Spectra	1
Coverage	NaN

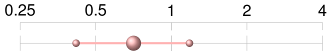

A	B	2.5	50	97.5	Sequence
1	0	0.42	0.71	1.2	VNELYEVMK

5.78 915 1505 minus strand Collagen alpha-5(VI) chain [Crassostrea gigas]

Protein Accession	CL40183.Contig3$_{H}1$
Mean Expression Ratio	1.24
Median Expression Ratio	1.24
Credible Interval	(1.01, 1.53)
Associated Peptides	8
Associated Spectra	44
Coverage	NaN

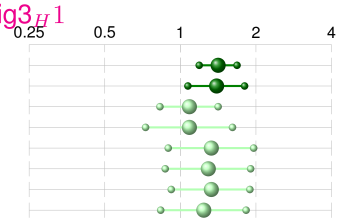

A	B	2.5	50	97.5	Sequence
13	10	1.2	1.4	1.7	LEAIPWR
4	4	1.1	1.4	1.8	VGEQLDAEELLNIAGDPK
3	4	0.83	1.1	1.4	DFVNAFQINPTAVR
1	0	0.73	1.1	1.6	HGLATNTGAGIK
1	0	0.89	1.3	2	HGLATNTGAGIKYMR
1	0	0.87	1.3	1.9	VGEQLDAEELL
0	2	0.92	1.3	1.9	RVVTAENYAHLNLVK
0	1	0.83	1.2	1.8	VVTAENYAHLNLVK

5.79 150 716 calponin/transgelin [Papilio xuthus]

Protein Accession	CL11747.Contig2$_{H}1$
Mean Expression Ratio	1.24
Median Expression Ratio	1.24
Credible Interval	(0.903, 1.69)
Associated Peptides	3
Associated Spectra	8
Coverage	NaN

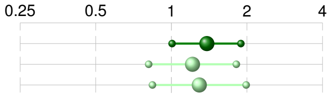

A	B	2.5	50	97.5	Sequence
4	1	1	1.4	1.9	GPPLNTPEHSVFATPDLYEK
1	1	0.81	1.2	1.8	MAFQQMEAIEK
1	0	0.84	1.3	2	FTTAIK

5.80 161 3001 minus strand E3 ubiquitin-protein ligase MIB2 [Crassostrea gigas]

Protein Accession	CL49497.Contig7$_{H}1$
Mean Expression Ratio	1.24
Median Expression Ratio	1.24
Credible Interval	(0.83, 1.83)
Associated Peptides	2
Associated Spectra	2
Coverage	NaN

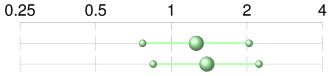

A	B	2.5	50	97.5	Sequence
0	1	0.77	1.3	2	GDAHAVEGILQR
0	1	0.85	1.4	2.2	EQVALYLLEK

5.81 155 3214 sarco/endoplasmic reticulum calcium ATPase isoform B [Pinctada fucata]; 132 3110 sarco/endoplasmic reticulum calcium ATPase isoform C [Pinctada fucata]; 132 3110 minus strand sarco/endoplasmic reticulum calcium ATPase isoform C [Pinctada fucata]; 155 3133 sarco/endoplasmic reticulum calcium ATPase isoform C [Pinctada fucata]; 155 3133 sarco/endoplasmic reticulum calcium ATPase isoform C [Pinctada fucata]; 155 3214 minus strand sarco/endoplasmic reticulum calcium ATPase isoform B [Pinctada fucata]

Protein Accession	CL1323.Contig9$_H1$ CL1323.Contig8$_H1$ CL1323.Contig6$_H1$ CL1323.Contig21$_H1$ CL1323.Co
Mean Expression Ratio	0.808
Median Expression Ratio	0.808
Credible Interval	(0.7, 0.934)
Associated Peptides	25
Associated Spectra	75
Coverage	NaN

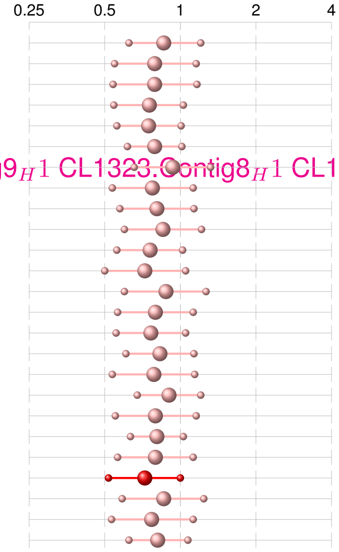

A	B	2.5	50	97.5	Sequence
1	2	0.62	0.86	1.2	ATAEAICR
1	0	0.55	0.79	1.2	DLVPGDIVEVSVGDK
1	0	0.54	0.79	1.2	DLVPGDIVEVSVGDKVPADIR
2	1	0.54	0.75	1	GAPEGLLDR
3	2	0.56	0.75	1	GIVVGTGLATEIGK
7	1	0.62	0.79	1	IGHNKVPMSPAIK
2	0	0.66	0.93	1.3	IGIFTENESTEGMSFTGR
1	0	0.53	0.77	1.1	IRNEMMDTEQEK
1	1	0.58	0.81	1.1	KEFTLEFSR
1	1	0.6	0.85	1.2	NAESAIEALK
2	3	0.56	0.76	1	NMLFSGTNIAAGK
1	0	0.5	0.72	1	SMSVYCVPNKPTR
1	0	0.6	0.88	1.3	TGTLTTNQMSVCK
2	0	0.56	0.79	1.1	VDQAILTGESVSVMK
3	0	0.55	0.76	1.1	VGEATETALTVLVEK
2	2	0.61	0.83	1.1	VIVITGDNK
1	0	0.54	0.78	1.1	VPMSPAIK
3	2	0.67	0.9	1.2	EFDELPPEDQR
1	0	0.55	0.8	1.2	NAESAIEALKEYEPEVAK
7	2	0.63	0.81	1	KAEIGIAMGSGTAVAK
1	1	0.56	0.79	1.1	LNFYNTDK
2	1	0.52	0.72	1	MDQAFTK
1	0	0.58	0.86	1.2	KNFEK
1	0	0.53	0.77	1.1	KNMLFSGTNIAAGK
3	5	0.62	0.81	1.1	CLALATIDNPPR

5.82 718 957 minus strand Calponin-1 OS=Gallus gallus GN=CNN1 PE=1 SV=2

Protein Accession	CL21394.Contig4$_H1$
Mean Expression Ratio	1.24
Median Expression Ratio	1.24
Credible Interval	(0.899, 1.74)
Associated Peptides	3
Associated Spectra	5
Coverage	NaN

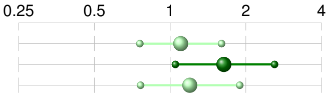

A	B	2.5	50	97.5	Sequence
3	0	0.76	1.1	1.6	EGQTVIGLQAGSNK
1	0	1.1	1.6	2.6	GASQAGMSMGAVR
1	0	0.76	1.2	1.9	IGLQAGSNK

5.83 36 1310 minus strand elongation factor-1 gamma [Diguetia canities]

Protein Accession	CL14269.Contig5$_H1$
Mean Expression Ratio	1.24
Median Expression Ratio	1.24
Credible Interval	(0.887, 1.74)
Associated Peptides	3
Associated Spectra	5
Coverage	NaN

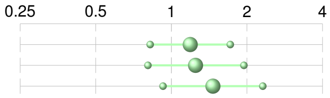

A	B	2.5	50	97.5	Sequence
**2**	**1**	**0.83**	**1.2**	**1.7**	**LAETMAQFDAK**
**1**	**0**	**0.8**	**1.3**	**1.9**	**SADFLAK**
**0**	**1**	**0.93**	**1.5**	**2.3**	**LDPDDPETK**

5.84 232 654 hypothetical protein BRAFLDRAFT_87758 [Branchiostoma floridae] gi

Protein Accession	CL34711.Contig8$_H1$
Mean Expression Ratio	1.24
Median Expression Ratio	1.24
Credible Interval	(0.807, 1.93)
Associated Peptides	1
Associated Spectra	1
Coverage	NaN

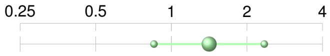

A	B	2.5	50	97.5	Sequence
**1**	**0**	**0.85**	**1.4**	**2.3**	**CVATGQGGYR**

5.85 313 2763 NCAM-related cell adhesion molecule precursor [Aplysia californica] gi

Protein Accession	Unigene11449$_H1$
Mean Expression Ratio	1.23
Median Expression Ratio	1.23
Credible Interval	(0.897, 1.71)
Associated Peptides	5
Associated Spectra	5
Coverage	NaN

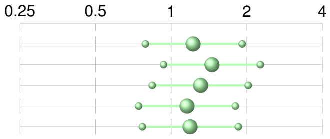

A	B	2.5	50	97.5	Sequence
**1**	**0**	**0.79**	**1.2**	**1.9**	**EALEVDTSK**
**1**	**0**	**0.93**	**1.5**	**2.3**	**IGSSDPTVFVFK**
**1**	**0**	**0.84**	**1.3**	**2**	**NLAGSYDIEER**
**1**	**0**	**0.74**	**1.2**	**1.8**	**VPMTGGAEIANYVIEYR**
**0**	**1**	**0.77**	**1.2**	**1.9**	**AGAIASDETQLEVSIPPK**

5.86 " 34 1656 Chaperonin containing TCP1, subunit 7 (eta) [Danio rerio] gi; 34 1656 Chaperonin containing TCP1, subunit 7 (eta) [Danio rerio] gi"

Protein Accession	CL24061.Contig2$_H1$ CL24061.Contig1$_H1$
Mean Expression Ratio	0.809
Median Expression Ratio	0.813
Credible Interval	(0.535, 1.21)
Associated Peptides	1
Associated Spectra	2
Coverage	NaN

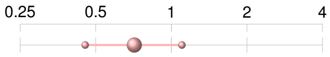

A	B	2.5	50	97.5	Sequence
**2**	**0**	**0.45**	**0.71**	**1.1**	**SIAVSIKK**

5.87 248 2053 PDZ and LIM domain protein 5 [Crassostrea gigas]

Protein Accession	CL2142.Contig4$_{H}1$
Mean Expression Ratio	1.23
Median Expression Ratio	1.23
Credible Interval	(0.999, 1.51)
Associated Peptides	8
Associated Spectra	32
Coverage	NaN

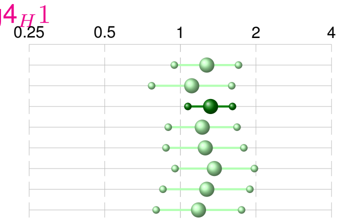

A	B	2.5	50	97.5	Sequence
2	3	0.95	1.3	1.7	CGLQTGDVILK
1	1	0.77	1.1	1.6	IGNTDTSALR
4	11	1.1	1.3	1.6	LDLLLQR
3	1	0.89	1.2	1.7	VNPGSLAAK
1	1	0.88	1.3	1.8	YLAPHCK
0	2	0.96	1.4	2	MDSTTPWGFR
0	1	0.86	1.3	1.9	SVLTLEAK
0	1	0.8	1.2	1.8	LQTGDVILK

5.88 3 551 minus strand Spectrin beta chain [Crassostrea gigas]

Protein Accession	Unigene35960$_{H}1$
Mean Expression Ratio	0.813
Median Expression Ratio	0.814
Credible Interval	(0.548, 1.21)
Associated Peptides	2
Associated Spectra	2
Coverage	NaN

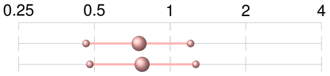

A	B	2.5	50	97.5	Sequence
0	1	0.46	0.75	1.2	ELALREELIR
0	1	0.48	0.77	1.3	IVEKPPKFDEK

5.89 91 1392 minus strand hypothetical protein AND_03103 [Anopheles darlingi]

Protein Accession	CL10598.Contig5$_{H}1$
Mean Expression Ratio	0.815
Median Expression Ratio	0.815
Credible Interval	(0.546, 1.21)
Associated Peptides	2
Associated Spectra	2
Coverage	NaN

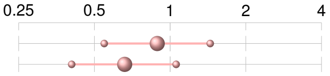

A	B	2.5	50	97.5	Sequence
1	0	0.55	0.89	1.4	LPPFNEHALVQEK
1	0	0.41	0.66	1.1	LDEVDDLLTENR

5.90 62 1093 Heterogeneous nuclear ribonucleoprotein A2-like protein 1 [Crassostrea gigas]

Protein Accession	CL47083.Contig4$_{H}1$
Mean Expression Ratio	0.815
Median Expression Ratio	0.816
Credible Interval	(0.614, 1.09)
Associated Peptides	5
Associated Spectra	8
Coverage	NaN

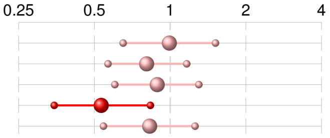

A	B	2.5	50	97.5	Sequence
1	0	0.65	0.99	1.5	ETFSPFGTISQIDLITDK
2	1	0.57	0.81	1.2	EDTTEDMIR
1	1	0.6	0.89	1.3	MFVGGIKEDTTEDMIR
0	1	0.35	0.53	0.83	GGSRGGR
0	1	0.54	0.83	1.3	GGGRGGSR

5.91 605 1153 Peptidyl-prolyl cis-trans isomerase B [Crassostrea gigas]; 1046 1630 Peptidyl-prolyl cis-trans isomerase B [Crassostrea gigas]; 589 1173 Peptidyl-prolyl cis-trans isomerase B [Crassostrea gigas]; 938 1414 Peptidyl-prolyl cis-trans isomerase B [Crassostrea gigas]; 883 1467 Peptidyl-prolyl cis-trans isomerase B [Crassostrea gigas]

Protein Accession	CL24747.Contig5$_{H}1$ CL24747.Contig4$_{H}1$ CL24747.Contig3$_{H}1$ CL24747.Contig2$_{H}1$ CL2474
Mean Expression Ratio	1.22
Median Expression Ratio	1.23
Credible Interval	(0.856, 1.74)
Associated Peptides	2
Associated Spectra	6
Coverage	NaN

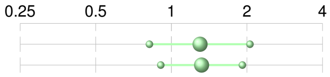

A	B	2.5	50	97.5	Sequence
**0**	**1**	**0.82**	**1.3**	**2.1**	**DFVIQMGDVTGK**
**2**	**3**	**0.9**	**1.3**	**1.9**	**IASSGVVGIASK**

5.92 801 971 RecName: Full=Tropomyosin-2; AltName: Full=Tropomyosin II; Short=BgTMII; Short=TMII gi|155940|gb|AAA27816.1| tropomyosin [Biomphalaria glabrata]

Protein Accession	CL2925.Contig4$_{H}1$
Mean Expression Ratio	1.23
Median Expression Ratio	1.22
Credible Interval	(0.909, 1.66)
Associated Peptides	3
Associated Spectra	11
Coverage	NaN

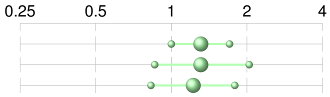

A	B	2.5	50	97.5	Sequence
**7**	**1**	**1**	**1.3**	**1.7**	**EDSYEETIR**
**0**	**1**	**0.86**	**1.3**	**2**	**ISEQEASQR**
**0**	**2**	**0.83**	**1.2**	**1.8**	**QQYEIFGISEQEASQR**

5.93 336 641 minus strand malate dehydrogenase [Schistosoma japonicum]

Protein Accession	CL11441.Contig9$_{H}1$
Mean Expression Ratio	1.22
Median Expression Ratio	1.22
Credible Interval	(0.785, 1.88)
Associated Peptides	1
Associated Spectra	1
Coverage	NaN

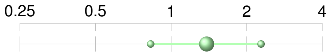

A	B	2.5	50	97.5	Sequence
**0**	**1**	**0.83**	**1.4**	**2.3**	**ALADLLVAADHR**

5.94 105 503 Transgelin-2 [Crassostrea gigas]

Protein Accession	CL445.Contig2$_{H}1$
Mean Expression Ratio	1.22
Median Expression Ratio	1.22
Credible Interval	(0.916, 1.63)
Associated Peptides	2
Associated Spectra	15
Coverage	NaN

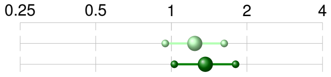

A	B	2.5	50	97.5	Sequence
4	4	0.95	1.2	1.6	SGLGLEIDK
5	2	1	1.4	1.8	SGLGLEIDKK

5.95 59 379 hypothetical protein DAPPUDRAFT_230756 [Daphnia pulex]; 117 443 minus strand hypothetical protein DAPPUDRAFT_230756 [Daphnia pulex]

Protein Accession	CL2137.Contig6$_{H}1$ CL2137.Contig5$_{H}1$
Mean Expression Ratio	1.22
Median Expression Ratio	1.22
Credible Interval	(0.788, 1.89)
Associated Peptides	1
Associated Spectra	1
Coverage	NaN

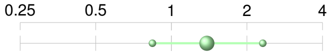

A	B	2.5	50	97.5	Sequence
1	0	0.84	1.4	2.3	YAQPRLK

5.96 3 470 minus strand Spectrin beta chain [Crassostrea gigas]; 5 451 minus strand Spectrin beta chain [Crassostrea gigas]

Protein Accession	CL19757.Contig2$_{H}1$ CL19757.Contig1$_{H}1$
Mean Expression Ratio	1.22
Median Expression Ratio	1.22
Credible Interval	(0.825, 1.82)
Associated Peptides	2
Associated Spectra	4
Coverage	NaN

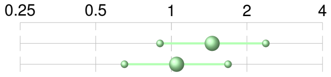

A	B	2.5	50	97.5	Sequence
0	1	0.9	1.5	2.4	HLMGVEDLLQK
2	1	0.65	1	1.7	HSLLEADIHVVGER

5.97 74 1243 Actin-related protein 2 [Crassostrea gigas]

Protein Accession	CL8614.Contig4$_{H}1$
Mean Expression Ratio	1.22
Median Expression Ratio	1.22
Credible Interval	(0.869, 1.72)
Associated Peptides	2
Associated Spectra	6
Coverage	NaN

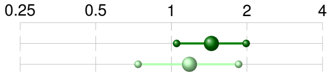

A	B	2.5	50	97.5	Sequence
4	1	1	1.4	2	MVEVMFEK
0	1	0.74	1.2	1.9	DLMIGDEASQLR

5.98 9 518 minus strand hemocyanin isoform 1 [Nucula nucleus]

Protein Accession	Unigene71735$_{H}1$
Mean Expression Ratio	0.82
Median Expression Ratio	0.82
Credible Interval	(0.561, 1.2)
Associated Peptides	1
Associated Spectra	4
Coverage	NaN

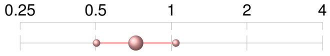

A	B	2.5	50	97.5	Sequence
2	2	0.5	0.72	1	IDFEHADVAR

5.99 8 271 minus strand Laminin subunit gamma-1 [Crassostrea gigas]

Protein Accession	Unigene36706$_{H}1$
Mean Expression Ratio	1.22
Median Expression Ratio	1.22
Credible Interval	(0.885, 1.7)
Associated Peptides	4
Associated Spectra	5
Coverage	NaN

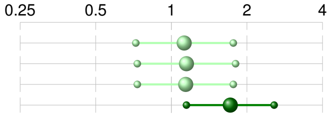

A	B	2.5	50	97.5	Sequence
1	0	0.72	1.1	1.8	EFTDASELYDR
0	1	0.73	1.1	1.8	HMQEAAQIDNIAR
0	1	0.73	1.1	1.8	LAEETLR
0	2	1.2	1.7	2.6	ALDLYR

5.100 1292 1471 minus strand hemocyanin isoform 1 [Haliotis diversicolor]

Protein Accession	Unigene65233$_{H}1$
Mean Expression Ratio	0.823
Median Expression Ratio	0.822
Credible Interval	(0.546, 1.23)
Associated Peptides	1
Associated Spectra	2
Coverage	NaN

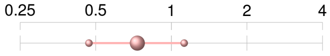

A	B	2.5	50	97.5	Sequence
1	1	0.47	0.73	1.1	LYTVQFEDALR

5.101 1200 1547 protein disulfide isomerase [Conus eburneus]

Protein Accession	CL3770.Contig1$_{H}1$
Mean Expression Ratio	1.22
Median Expression Ratio	1.22
Credible Interval	(0.982, 1.51)
Associated Peptides	8
Associated Spectra	24
Coverage	NaN

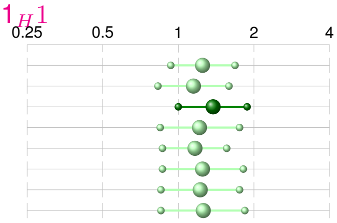

A	B	2.5	50	97.5	Sequence
2	3	0.93	1.2	1.7	IDSTANDVENIR
3	0	0.83	1.1	1.6	QLAPIWDELAEK
3	1	1	1.4	1.9	VQSFPTLK
1	1	0.85	1.2	1.8	YKDSNDIVIAK
1	4	0.87	1.2	1.6	NFNDVAFDK
1	1	0.87	1.2	1.8	VVDYNGER
1	1	0.85	1.2	1.8	VLVGK
0	1	0.85	1.3	1.8	TLSAFVK

5.102 296 493 hypothetical protein BRAFLDRAFT_66287 [Branchiostoma floridae] gi

Protein Accession	Unigene414$_H$1
Mean Expression Ratio	0.823
Median Expression Ratio	0.825
Credible Interval	(0.544, 1.24)
Associated Peptides	1
Associated Spectra	2
Coverage	NaN

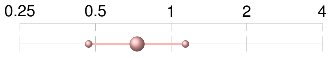

A	B	2.5	50	97.5	Sequence
1	1	0.47	0.73	1.1	SLDDFLQAWK

5.103 227 829 Peptidyl-prolyl cis-trans isomerase B [Crassostrea gigas]

Protein Accession	CL16665.Contig2$_H$1
Mean Expression Ratio	1.21
Median Expression Ratio	1.21
Credible Interval	(0.987, 1.49)
Associated Peptides	7
Associated Spectra	38
Coverage	NaN

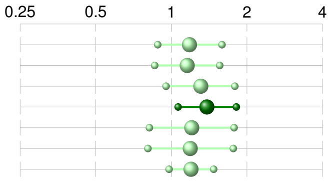

A	B	2.5	50	97.5	Sequence
4	1	0.88	1.2	1.6	DTNGSQFFFTLGPSR
3	2	0.86	1.2	1.6	MGPGADFQSPK
3	1	0.96	1.3	1.8	NYNGQGDDISGK
3	4	1.1	1.4	1.8	TLCDGWVR
1	0	0.82	1.2	1.8	FLDEK
1	0	0.81	1.2	1.8	NFTVTHEATLEIEVK
10	5	0.98	1.2	1.5	GFNFLR

5.104 120 635 PREDICTED: similar to collagen type VI alpha 6 [Ciona intestinalis]; 120 635 minus strand PREDICTED: similar to collagen type VI alpha 6 [Ciona intestinalis]

Protein Accession	Unigene46785$_H$1 Unigene26613$_H$1
Mean Expression Ratio	1.21
Median Expression Ratio	1.21
Credible Interval	(0.821, 1.81)
Associated Peptides	2
Associated Spectra	2
Coverage	NaN

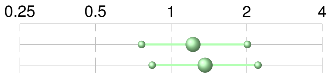

A	B	2.5	50	97.5	Sequence
1	0	0.76	1.2	2	HGNTNTGDGIMYMLER
1	0	0.84	1.4	2.2	VSLVTYSDQANPESAIDFDSYSSK

5.105 12160 13014 twitchin [Mytilus galloprovincialis]

Protein Accession	CL181.Contig2$_H$1
Mean Expression Ratio	0.825
Median Expression Ratio	0.826
Credible Interval	(0.619, 1.1)
Associated Peptides	4
Associated Spectra	11
Coverage	NaN

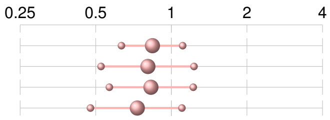

A	B	2.5	50	97.5	Sequence
1	6	0.63	0.84	1.1	ATLTVEPGVEGLR
1	0	0.52	0.81	1.2	AVNSLGEEETSCK
1	1	0.57	0.83	1.2	AFYKPK
1	0	0.48	0.73	1.1	NQLSAEGHTAK

5.106 462 671 enhancer protein [Homo sapiens]

Protein Accession	CL3160.Contig12$_{H}1$
Mean Expression Ratio	1.21
Median Expression Ratio	1.21
Credible Interval	(0.896, 1.62)
Associated Peptides	3
Associated Spectra	11
Coverage	NaN

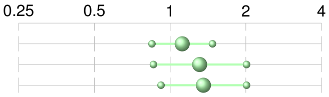

A	B	2.5	50	97.5	Sequence
**6**	**2**	**0.85**	**1.1**	**1.5**	**GLFIIDDK**
**0**	**1**	**0.86**	**1.3**	**2**	**PLLEDK**
**0**	**2**	**0.92**	**1.4**	**2**	**KIGGLGNMNIPLLEDK**

5.107 96 497 AAEL009772-PB [Aedes aegypti]

Protein Accession	CL27854.Contig2$_{H}1$
Mean Expression Ratio	1.21
Median Expression Ratio	1.21
Credible Interval	(0.81, 1.81)
Associated Peptides	1
Associated Spectra	3
Coverage	NaN

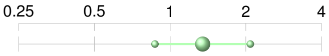

A	B	2.5	50	97.5	Sequence
**2**	**1**	**0.87**	**1.3**	**2.1**	**LVEEFPMGNIK**

5.108 2302 2547 minus strand hemocyanin isoform 1 [Haliotis diversicolor]; 816 1211 H2 [Haliotis tuberculata]

Protein Accession	CL11519.Contig6$_{H}1$ CL11519.Contig5$_{H}1$
Mean Expression Ratio	0.826
Median Expression Ratio	0.827
Credible Interval	(0.572, 1.2)
Associated Peptides	2
Associated Spectra	4
Coverage	NaN

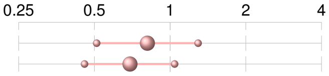

A	B	2.5	50	97.5	Sequence
**1**	**0**	**0.51**	**0.81**	**1.3**	**KNVLSLTPAER**
**2**	**1**	**0.46**	**0.69**	**1**	**SLILAMR**

5.109 230 334 protein disulfide isomerase [Conus virgo]

Protein Accession	Unigene67026$_{H}1$
Mean Expression Ratio	1.21
Median Expression Ratio	1.21
Credible Interval	(0.864, 1.71)
Associated Peptides	1
Associated Spectra	8
Coverage	NaN

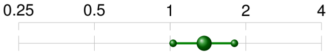

A	B	2.5	50	97.5	Sequence
**4**	**4**	**1**	**1.4**	**1.8**	**VDATVENDLASK**

5.110 507 1277 alpha tubulin [Notothenia coriiceps]

Protein Accession	Unigene56691$_{H}1$
Mean Expression Ratio	1.21
Median Expression Ratio	1.21
Credible Interval	(0.886, 1.65)
Associated Peptides	2
Associated Spectra	9
Coverage	NaN

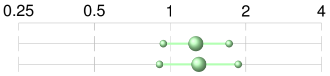

A	B	2.5	50	97.5	Sequence
5	1	0.94	1.3	1.7	LSVDYGK
2	1	0.91	1.3	1.9	LSVDYGKK

5.111 111 1226 gelsolin [Aplysia californica] gi; 42 1139 minus strand gelsolin [Aplysia californica] gi

Protein Accession	Unigene39723$_{H}1$ Unigene32782$_{H}1$
Mean Expression Ratio	1.21
Median Expression Ratio	1.21
Credible Interval	(1.05, 1.4)
Associated Peptides	18
Associated Spectra	75
Coverage	NaN

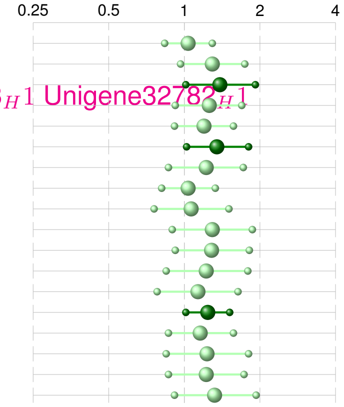

A	B	2.5	50	97.5	Sequence
4	8	0.83	1	1.3	AMQFIGEIK
3	1	0.97	1.3	1.7	DLVTSDDVYILDTGLK
3	0	1	1.4	1.9	ESGNPWRPVTVIK
4	0	0.92	1.3	1.7	EVFVWVGSNASPDEK
5	1	0.91	1.2	1.6	EVQGHESDLFK
3	2	1	1.3	1.8	KNGLPLAHNYLR
1	1	0.87	1.2	1.7	QGIVTMEGGAASGFR
4	4	0.81	1	1.3	SDDVFIFDSGK
1	1	0.76	1.1	1.5	TVELDTK
1	0	0.89	1.3	1.9	SVPAEEYK
1	1	0.92	1.3	1.8	QFDTALAA
1	0	0.85	1.2	1.8	KESADSEPAWDGAGQKPGLEIWR
1	0	0.78	1.1	1.6	NSTQDEYGTAAYK
13	3	1	1.2	1.5	LFHFAK
3	1	0.87	1.2	1.6	VVPWPK
0	1	0.85	1.2	1.8	DSNLALFGSDTEK
0	2	0.87	1.2	1.7	LNDFK
0	1	0.91	1.3	1.9	NGLPLAHNYLR

5.112 11 244 minus strand Tubulin alpha chain (Fragment) OS=Lytechinus pictus PE=3 SV=1

Protein Accession	CL3047.Contig6$_{H}1$
Mean Expression Ratio	1.21
Median Expression Ratio	1.21
Credible Interval	(0.819, 1.8)
Associated Peptides	2
Associated Spectra	2
Coverage	NaN

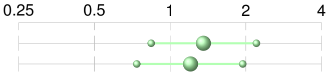

A	B	2.5	50	97.5	Sequence
0	1	0.84	1.4	2.2	EDLAALEK
0	1	0.74	1.2	1.9	FDLMYAK

5.113 353 2161 Bifunctional 3'-phosphoadenosine 5'-phosphosulfate synthetase [Crassostrea gigas]; 141 1943 Bifunctional 3'-phosphoadenosine 5'-phosphosulfate synthetase [Crassostrea gigas]

Protein Accession	CL8992.Contig2$_{H}$1 CL8992.Contig1$_{H}$1
Mean Expression Ratio	1.21
Median Expression Ratio	1.21
Credible Interval	(0.884, 1.65)
Associated Peptides	4
Associated Spectra	7
Coverage	NaN

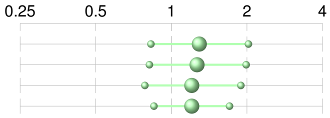

A	B	2.5	50	97.5	Sequence
1	0	0.83	1.3	2	QHAAILEEK
1	0	0.82	1.3	2	QFGTSNLGHPYVK
1	0	0.78	1.2	1.9	AGPTEVQWHAK
2	2	0.85	1.2	1.7	LDGSAAFALK

5.114 " 27 974 PREDICTED: succinyl-CoA ligase [GDP-forming] subunit alpha, mitochondrial-like [Oreochromis niloticus]; 27 974 minus strand PREDICTED: succinyl-CoA ligase [GDP-forming] subunit alpha, mitochondrial-like [Oreochromis niloticus]"

Protein Accession	CL10674.Contig2$_{H}$1 CL10674.Contig1$_{H}$1
Mean Expression Ratio	1.21
Median Expression Ratio	1.21
Credible Interval	(0.873, 1.67)
Associated Peptides	3
Associated Spectra	6
Coverage	NaN

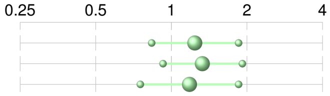

A	B	2.5	50	97.5	Sequence
2	0	0.83	1.2	1.9	MGHAGAIIAGGK
1	2	0.93	1.3	1.9	IYMATR
0	1	0.75	1.2	1.9	LICQGFTGK

5.115 21 239 minus strand calponin-like protein [Taenia asiatica]

Protein Accession	CL26318.Contig1$_{H}$1
Mean Expression Ratio	1.21
Median Expression Ratio	1.2
Credible Interval	(0.78, 1.89)
Associated Peptides	1
Associated Spectra	1
Coverage	NaN

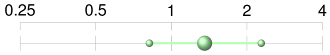

A	B	2.5	50	97.5	Sequence
1	0	0.82	1.4	2.3	HIADIK

5.116 150 1511 Tensin OS=Gallus gallus GN=TNS PE=1 SV=2

Protein Accession	CL2743.Contig2$_{H}1$
Mean Expression Ratio	1.2
Median Expression Ratio	1.2
Credible Interval	(0.849, 1.71)
Associated Peptides	3
Associated Spectra	4
Coverage	NaN

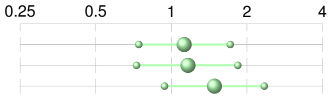

A	B	2.5	50	97.5	Sequence
1	1	0.74	1.1	1.7	DDAIVMLR
1	0	0.73	1.2	1.8	DKPPGTFVIR
1	0	0.94	1.5	2.3	GTFVIR

5.117 " 166 1248 PREDICTED: probable pyruvate dehydrogenase E1 component subunit alpha, mitochondrial-like [Metaseiulus occidentalis]; 166 1248 PREDICTED: probable pyruvate dehydrogenase E1 component subunit alpha, mitochondrial-like [Metaseiulus occidentalis]; 166 1248 PREDICTED: probable pyruvate dehydrogenase E1 component subunit alpha, mitochondrial-like [Metaseiulus occidentalis]; 166 1248 PREDICTED: probable pyruvate dehydrogenase E1 component subunit alpha, mitochondrial-like [Metaseiulus occidentalis]; 166 1248 PREDICTED: probable pyruvate dehydrogenase E1 component subunit alpha, mitochondrial-like [Metaseiulus occidentalis]; 166 1248 minus strand PREDICTED: probable pyruvate dehydrogenase E1 component subunit alpha, mitochondrial-like [Metaseiulus occidentalis]; 166 1248 minus strand PREDICTED: probable pyruvate dehydrogenase E1 component subunit alpha, mitochondrial-like [Metaseiulus occidentalis]; 166 1248 PREDICTED: probable pyruvate dehydrogenase E1 component subunit alpha, mitochondrial-like [Metaseiulus occidentalis]; 166 1248 PREDICTED: probable pyruvate dehydrogenase E1 component subunit alpha, mitochondrial-like [Metaseiulus occidentalis]"

Protein Accession	Unigene34082$_{H}1$ Unigene34081$_{H}1$ Unigene34079$_{H}1$ Unigene34077$_{H}1$ Unigene34075$_{H}1$ U
Mean Expression Ratio	0.829
Median Expression Ratio	0.831
Credible Interval	(0.584, 1.17)
Associated Peptides	3
Associated Spectra	4
Coverage	NaN

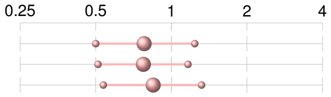

A	B	2.5	50	97.5	Sequence
1	0	0.5	0.78	1.2	TGLATEEELK
1	1	0.51	0.77	1.2	TGLATEEELKK
0	1	0.53	0.85	1.3	KIDGEVRSEVDAAAER

5.118 34 1122 Fructose-bisphosphate aldolase [Crassostrea gigas]

Protein Accession	CL24228.Contig1$_H$1
Mean Expression Ratio	1.2
Median Expression Ratio	1.2
Credible Interval	(1.03, 1.4)
Associated Peptides	18
Associated Spectra	62
Coverage	NaN

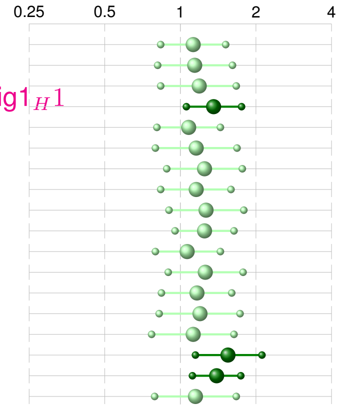

A	B	2.5	50	97.5	Sequence
3	1	0.83	1.1	1.5	DSQGTPFVK
1	1	0.81	1.1	1.6	GAQDELLK
1	1	0.84	1.2	1.7	GAQDELLKR
5	3	1.1	1.4	1.7	GIIPGIK
3	2	0.81	1.1	1.4	GILAADESVGSMGK
1	0	0.79	1.2	1.7	GVVPLAGSNNECTTQGLDGLAER
2	0	0.88	1.2	1.8	ISDNTPSYQAMIENANVLAR
3	0	0.83	1.2	1.6	KDGAQFAK
1	1	0.9	1.3	1.8	LEGIGLENTEENR
4	2	0.95	1.3	1.6	QIANSIVAPGK
3	1	0.79	1.1	1.4	RLEGIGLENTEENR
1	1	0.9	1.3	1.8	RLEGIGLENTEENRR
3	0	0.84	1.2	1.6	YASICQQNGLVPIVEPEVLPDGDHDLK
1	0	0.82	1.2	1.7	VKGAQDELLKR
1	0	0.77	1.1	1.6	ANSEAALGK
2	2	1.1	1.5	2.1	PVIAQYLTK
5	6	1.1	1.4	1.7	VTEQVLAFTYK
0	1	0.79	1.1	1.7	RLEGIGLENTEENRRRYR

5.119 71 523 minus strand hypothetical protein DAPPUDRAFT_309332 [Daphnia pulex]

Protein Accession	CL4487.Contig1$_H$1
Mean Expression Ratio	1.2
Median Expression Ratio	1.2
Credible Interval	(0.781, 1.85)
Associated Peptides	1
Associated Spectra	1
Coverage	NaN

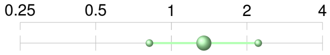

A	B	2.5	50	97.5	Sequence
0	1	0.82	1.3	2.2	IYHPNIDK

5.120 16 438 hypothetical protein KGM_06034 [Danaus plexippus]; 16 438 hypothetical protein KGM_06034 [Danaus plexippus]

Protein Accession	CL18196.Contig2$_H$1 CL18196.Contig1$_H$1
Mean Expression Ratio	0.832
Median Expression Ratio	0.833
Credible Interval	(0.532, 1.27)
Associated Peptides	1
Associated Spectra	1
Coverage	NaN

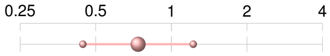

A	B	2.5	50	97.5	Sequence
1	0	0.45	0.74	1.2	VHCGPNYPDEPPTVQFK

5.121 4726 5571 minus strand twitchin [Mytilus galloprovincialis]

Protein Accession	CL181.Contig4$_H$1
Mean Expression Ratio	0.832
Median Expression Ratio	0.833
Credible Interval	(0.655, 1.05)
Associated Peptides	8
Associated Spectra	15
Coverage	NaN

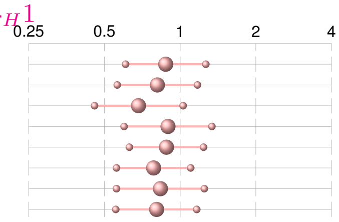

A	B	2.5	50	97.5	Sequence
1	1	0.61	0.88	1.3	EDGGAPITHYIVEK
1	1	0.56	0.81	1.2	GTVEGLQEGKEYEFR
1	0	0.46	0.68	1	VCDTDDLEIDVSDLTPGHK
1	0	0.6	0.9	1.3	CALLWDKPK
1	2	0.63	0.88	1.2	NGTPLNEANLDTETVPK
2	1	0.56	0.78	1.1	GLQEGKEYEFR
0	1	0.56	0.84	1.2	EIFVK
0	2	0.55	0.8	1.2	IPDVQR

5.122 166 1563 arylsulfatase [Haliotis discus discus]

Protein Accession	CL49030.Contig16$_H$1
Mean Expression Ratio	1.2
Median Expression Ratio	1.2
Credible Interval	(0.779, 1.84)
Associated Peptides	1
Associated Spectra	1
Coverage	NaN

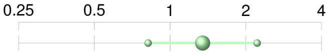

A	B	2.5	50	97.5	Sequence
0	1	0.82	1.3	2.2	VPLVLHSK

5.123 2 2542 kinesin heavy chain 1 [Aplysia californica] gi; 2 2542 kinesin heavy chain 1 [Aplysia californica] gi

Protein Accession	CL4142.Contig6$_H$1 CL4142.Contig5$_H$1
Mean Expression Ratio	1.2
Median Expression Ratio	1.2
Credible Interval	(0.842, 1.69)
Associated Peptides	2
Associated Spectra	5
Coverage	NaN

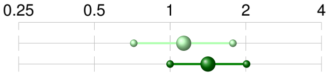

A	B	2.5	50	97.5	Sequence
1	0	0.71	1.1	1.8	QQLEAEK
4	0	1	1.4	2	LSELSIQMSRREQAK

5.124 42 434 cytochrome b5 [Haliotis discus discus]; 42 434 cytochrome b5 [Haliotis discus discus]

Protein Accession	CL6985.Contig9$_H$1 CL6985.Contig5$_H$1
Mean Expression Ratio	0.835
Median Expression Ratio	0.837
Credible Interval	(0.581, 1.2)
Associated Peptides	3
Associated Spectra	3
Coverage	NaN

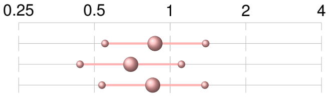

A	B	2.5	50	97.5	Sequence
1	0	0.55	0.87	1.4	KTFETNTGPVGR
1	0	0.44	0.69	1.1	VFRSEEVK
0	1	0.54	0.85	1.4	ELMKDYLIGK

5.125 " 81 1349 minus strand PREDICTED: LOW QUALITY PROTEIN: dolichyl-diphosphooligosaccharide–protein glycosyltransferase 48 kDa subunit-like, partial [Cavia porcellus]; 81 1349 minus strand PREDICTED: LOW QUALITY PROTEIN: dolichyl-diphosphooligosaccharide–protein glycosyltransferase 48 kDa subunit-like, partial [Cavia porcellus]"

Protein Accession	CL49524.Contig2$_{H}1$ CL49524.Contig1$_{H}1$
Mean Expression Ratio	1.19
Median Expression Ratio	1.2
Credible Interval	(0.825, 1.74)
Associated Peptides	2
Associated Spectra	3
Coverage	NaN

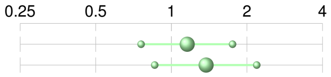

A	B	2.5	50	97.5	Sequence
1	1	0.76	1.2	1.8	DLGFDLTFK
1	0	0.86	1.4	2.2	FPQSGNLELAK

5.126 712 831 haemocyanin [Aplysia californica]

Protein Accession	Unigene65929$_{H}1$
Mean Expression Ratio	0.836
Median Expression Ratio	0.837
Credible Interval	(0.585, 1.21)
Associated Peptides	2
Associated Spectra	4
Coverage	NaN

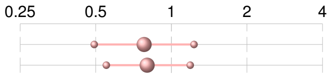

A	B	2.5	50	97.5	Sequence
1	0	0.49	0.78	1.2	YDVTDVFNQMR
3	0	0.55	0.8	1.2	YHFNVK

5.127 " 3195 3968 Spectrin alpha chain, putative [Pediculus humanus corporis] gi"

Protein Accession	CL2195.Contig1$_{H}1$
Mean Expression Ratio	0.838
Median Expression Ratio	0.838
Credible Interval	(0.652, 1.08)
Associated Peptides	6
Associated Spectra	16
Coverage	NaN

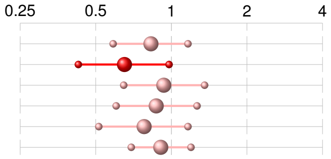

A	B	2.5	50	97.5	Sequence
2	1	0.59	0.83	1.2	DLSSVQTLLTK
1	0	0.43	0.65	0.98	LIDEGNHNADGIVER
1	1	0.64	0.94	1.4	LLLLQEQYR
1	1	0.6	0.87	1.3	LSELEAAASQR
1	0	0.51	0.78	1.2	QIEDLYLTFAK
4	3	0.69	0.91	1.2	YNDVIAR

5.128 2019 2723 minus strand Kyphoscoliosis peptidase [Crassostrea gigas]; 2060 2764 minus strand Kyphoscoliosis peptidase [Crassostrea gigas]; 1074 1778 minus strand Kyphoscoliosis peptidase [Crassostrea gigas]; 2063 2767 minus strand Kyphoscoliosis peptidase [Crassostrea gigas]; 2044 2748 minus strand Kyphoscoliosis peptidase [Crassostrea gigas]; 2044 2748 minus strand Kyphoscoliosis peptidase [Crassostrea gigas]

Protein Accession	CL3923.Contig7$_{H}1$ CL3923.Contig6$_{H}1$ CL3923.Contig5$_{H}1$ CL3923.Contig4$_{H}1$ CL3923.Con
Mean Expression Ratio	0.838
Median Expression Ratio	0.839
Credible Interval	(0.566, 1.24)
Associated Peptides	1
Associated Spectra	3
Coverage	NaN

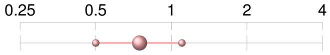

A	B	2.5	50	97.5	Sequence
**3**	**0**	**0.5**	**0.75**	**1.1**	**TMGQAPPTVPK**

5.129 " 154 534 minus strand Muscle M-line assembly protein unc-89, partial [Crassostrea gigas]"

Protein Accession	Unigene64523$_{H}1$
Mean Expression Ratio	0.841
Median Expression Ratio	0.839
Credible Interval	(0.541, 1.3)
Associated Peptides	1
Associated Spectra	3
Coverage	NaN

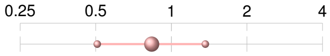

A	B	2.5	50	97.5	Sequence
**2**	**1**	**0.51**	**0.84**	**1.4**	**LEIGEVVVDDEGEYICK**

5.130 340 615 putative Low-density lipoprotein receptor repeat class B [Trichinella spiralis] gi

Protein Accession	Unigene31531$_{H}1$
Mean Expression Ratio	0.84
Median Expression Ratio	0.839
Credible Interval	(0.549, 1.31)
Associated Peptides	1
Associated Spectra	1
Coverage	NaN

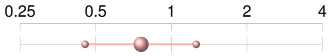

A	B	2.5	50	97.5	Sequence
**0**	**1**	**0.45**	**0.76**	**1.3**	**VLPFGLK**

5.131 27 1682 minus strand PREDICTED: nucleolar protein 56 [Oreochromis niloticus]

Protein Accession	Unigene26699$_H$1
Mean Expression Ratio	0.839
Median Expression Ratio	0.84
Credible Interval	(0.674, 1.04)
Associated Peptides	7
Associated Spectra	27
Coverage	NaN

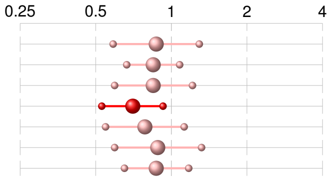

A	B	2.5	50	97.5	Sequence
1	0	0.59	0.87	1.3	KGLGGLSTEKK
7	3	0.67	0.84	1.1	LDGLGQAELQK
2	0	0.6	0.85	1.2	VATLEEEVYDWEIK
2	4	0.53	0.7	0.93	VTPLKLDGLGQAELQK
1	1	0.55	0.78	1.1	LIMQK
1	0	0.6	0.88	1.3	AAEDLRNEAK
4	1	0.65	0.87	1.2	KLIMQK

5.132 457 1842 Basement membrane-specific heparan sulfate proteoglycan core protein [Crassostrea gigas]; 457 1842 Basement membrane-specific heparan sulfate proteoglycan core protein [Crassostrea gigas]

Protein Accession	CL24300.Contig9$_H$1 CL24300.Contig10$_H$1
Mean Expression Ratio	0.84
Median Expression Ratio	0.84
Credible Interval	(0.589, 1.19)
Associated Peptides	2
Associated Spectra	4
Coverage	NaN

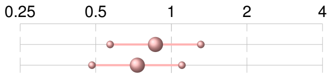

A	B	2.5	50	97.5	Sequence
1	1	0.57	0.87	1.3	KDGELPVEHK
1	1	0.48	0.73	1.1	IESSGALYIPR

5.133 230 1189 hypothetical protein [Amblyomma maculatum]

Protein Accession	CL11202.Contig4$_H$1
Mean Expression Ratio	1.19
Median Expression Ratio	1.19
Credible Interval	(0.873, 1.63)
Associated Peptides	4
Associated Spectra	7
Coverage	NaN

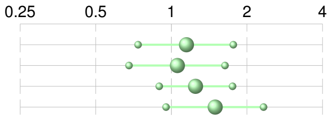

A	B	2.5	50	97.5	Sequence
1	0	0.74	1.1	1.8	KFAVDAVK
1	0	0.68	1.1	1.6	LSFDKEGMVK
0	4	0.89	1.3	1.8	ILEIIPGR
0	1	0.95	1.5	2.3	SFEPLEDPGVR

5.134 3 470 minus strand Spectrin beta chain [Crassostrea gigas]

Protein Accession	CL19757.Contig2$_H$1
Mean Expression Ratio	0.84
Median Expression Ratio	0.841
Credible Interval	(0.558, 1.26)
Associated Peptides	1
Associated Spectra	3
Coverage	NaN

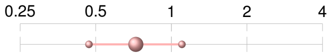

A	B	2.5	50	97.5	Sequence
2	1	0.47	0.72	1.1	HSLLEADIHVVGER

5.135 128 559 minus strand predicted protein [Nematostella vectensis] gi

Protein Accession	CL19343.Contig1$_H1$
Mean Expression Ratio	0.842
Median Expression Ratio	0.842
Credible Interval	(0.598, 1.17)
Associated Peptides	2
Associated Spectra	7
Coverage	NaN

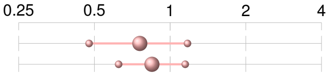

A	B	2.5	50	97.5	Sequence
1	0	0.48	0.76	1.2	GIPCLILLNGEDASEISR
3	3	0.62	0.85	1.1	TVILGDLTGSK

5.136 26 877 Ankyrin-2 [Crassostrea gigas]

Protein Accession	CL18051.Contig2$_H1$
Mean Expression Ratio	1.19
Median Expression Ratio	1.19
Credible Interval	(0.833, 1.69)
Associated Peptides	2
Associated Spectra	4
Coverage	NaN

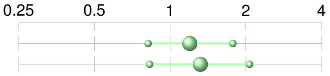

A	B	2.5	50	97.5	Sequence
2	1	0.82	1.2	1.8	LPALHIAAK
0	1	0.83	1.3	2.1	DGLTPLHCAAR

5.137 222 809 minus strand PREDICTED: 15-hydroxyprostaglandin dehydrogenase [NAD+] [Gallus gallus]

Protein Accession	CL44148.Contig2$_H1$
Mean Expression Ratio	1.19
Median Expression Ratio	1.19
Credible Interval	(0.977, 1.45)
Associated Peptides	7
Associated Spectra	84
Coverage	NaN

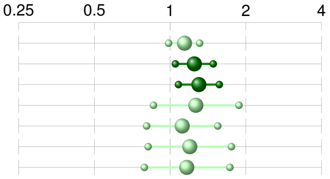

A	B	2.5	50	97.5	Sequence
20	16	0.99	1.1	1.3	ATVEAFLTK
17	6	1	1.2	1.5	VFLTGGAHGIGK
13	6	1.1	1.3	1.6	VMFCDINDK
1	0	0.86	1.3	1.9	RVFLTGGAHGIGK
0	3	0.8	1.1	1.5	TGGAHGIGK
0	1	0.82	1.2	1.8	LTGGAHGIGK
0	1	0.79	1.2	1.7	GGAHGIGK

5.138 187 642 minus strand PREDICTED: similar to pol-like protein [Tribolium castaneum]

Protein Accession	CL5644.Contig3$_H1$
Mean Expression Ratio	1.19
Median Expression Ratio	1.19
Credible Interval	(0.864, 1.64)
Associated Peptides	1
Associated Spectra	24
Coverage	NaN

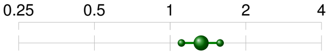

A	B	2.5	50	97.5	Sequence
24	0	1.1	1.3	1.6	IILEQEELK

5.139 503 1090 minus strand myosin regulatory light chain [Fasciola hepatica]; 278 865 minus strand myosin regulatory light chain [Fasciola hepatica]

Protein Accession CL43006.Contig6$_H1$ CL43006.Contig4$_H1$
Mean Expression Ratio 1.19
Median Expression Ratio 1.19
Credible Interval (0.865, 1.63)
Associated Peptides 1
Associated Spectra 25
Coverage NaN

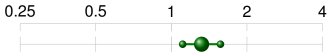

A	B	2.5	50	97.5	Sequence
**16**	**9**	**1.1**	**1.3**	**1.6**	**QAQVQEFK**

5.140 2 307 Laminin subunit gamma-1 [Crassostrea gigas]

Protein Accession Unigene32982$_H1$
Mean Expression Ratio 0.844
Median Expression Ratio 0.844
Credible Interval (0.542, 1.31)
Associated Peptides 1
Associated Spectra 1
Coverage NaN

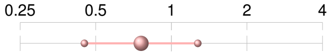

A	B	2.5	50	97.5	Sequence
**0**	**1**	**0.45**	**0.76**	**1.3**	**AEISLR**

5.141 238 1038 PREDICTED: hypothetical protein [Saccoglossus kowalevskii]; 294 992 PREDICTED: hypothetical protein [Saccoglossus kowalevskii]

Protein Accession CL6273.Contig7$_H1$ CL6273.Contig6$_H1$
Mean Expression Ratio 0.848
Median Expression Ratio 0.844
Credible Interval (0.551, 1.32)
Associated Peptides 1
Associated Spectra 1
Coverage NaN

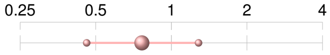

A	B	2.5	50	97.5	Sequence
**0**	**1**	**0.46**	**0.76**	**1.3**	**DVVLLHQLGR**

5.142 " 148 1989 Acyl-CoA synthetase family member 2, mitochondrial [Crassostrea gigas]; 146 1987 minus strand Acyl-CoA synthetase family member 2, mitochondrial [Crassostrea gigas]"

Protein Accession CL19799.Contig6$_H1$ CL19799.Contig5$_H1$
Mean Expression Ratio 0.842
Median Expression Ratio 0.844
Credible Interval (0.546, 1.29)
Associated Peptides 1
Associated Spectra 1
Coverage NaN

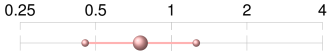

A	B	2.5	50	97.5	Sequence
**0**	**1**	**0.45**	**0.75**	**1.3**	**LAGPGELGEILVR**

5.143 104 418 Tubulin-specific chaperone A [Crassostrea gigas]; 104 418 minus strand Tubulin-specific chaperone A [Crassostrea gigas]; 104 418 Tubulin-specific chaperone A [Crassostrea gigas]

Protein Accession	Unigene53595$_H1$ Unigene32457$_H1$ CL7190.Contig3$_H1$
Mean Expression Ratio	0.845
Median Expression Ratio	0.844
Credible Interval	(0.546, 1.3)
Associated Peptides	1
Associated Spectra	2
Coverage	NaN

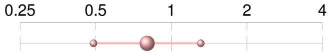

A	B	2.5	50	97.5	Sequence
**1**	**1**	**0.49**	**0.8**	**1.3**	**ADLGETEELK**

5.144 390 2267 neurofilament protein NF70 [Helix aspersa]; 318 2195 neurofilament protein NF70 [Helix aspersa]

Protein Accession	CL1353.Contig5$_H1$ CL1353.Contig4$_H1$
Mean Expression Ratio	1.18
Median Expression Ratio	1.18
Credible Interval	(0.916, 1.51)
Associated Peptides	7
Associated Spectra	13
Coverage	NaN

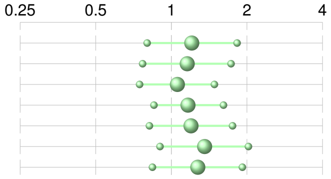

A	B	2.5	50	97.5	Sequence
**1**	**0**	**0.8**	**1.2**	**1.8**	**GEMETFYNLK**
**1**	**0**	**0.77**	**1.2**	**1.7**	**LNQQISDLEAEIR**
**2**	**1**	**0.75**	**1.1**	**1.5**	**VGQLEELVDDLQR**
**4**	**0**	**0.85**	**1.2**	**1.6**	**FANYIEK**
**1**	**1**	**0.82**	**1.2**	**1.8**	**MADLEAR**
**0**	**1**	**0.9**	**1.4**	**2**	**ELQQEMDVK**
**0**	**1**	**0.84**	**1.3**	**1.9**	**LEFLK**

5.145 " 59 2365 minus strand Aconitate hydratase, mitochondrial [Crassostrea gigas]"

Protein Accession	CL13509.Contig1$_H1$
Mean Expression Ratio	0.85
Median Expression Ratio	0.849
Credible Interval	(0.642, 1.12)
Associated Peptides	6
Associated Spectra	9
Coverage	NaN

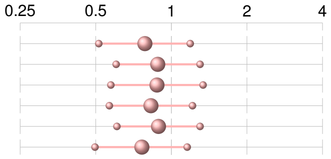

A	B	2.5	50	97.5	Sequence
**1**	**0**	**0.51**	**0.78**	**1.2**	**HTMNEGQIEWFR**
**1**	**1**	**0.6**	**0.88**	**1.3**	**KNGWPLNIR**
**1**	**0**	**0.57**	**0.88**	**1.3**	**DLKPGAPVVCEIK**
**1**	**1**	**0.57**	**0.83**	**1.2**	**IHETNLK**
**0**	**2**	**0.61**	**0.89**	**1.3**	**VAGILTVK**
**0**	**1**	**0.5**	**0.76**	**1.2**	**LQLLQPFEK**

5.146 488 1039 Peptidyl-prolyl cis-trans isomerase B [Crassostrea gigas]; 488 1039 Peptidyl-prolyl cis-trans isomerase B [Crassostrea gigas]

Protein Accession	CL49248.Contig2$_{H}1$ CL49248.Contig1$_{H}1$
Mean Expression Ratio	1.18
Median Expression Ratio	1.18
Credible Interval	(0.9, 1.54)
Associated Peptides	4
Associated Spectra	19
Coverage	NaN

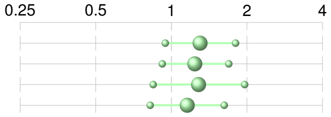

A	B	2.5	50	97.5	Sequence
3	3	0.94	1.3	1.8	ASLEFLDK
4	4	0.92	1.2	1.7	LVDCSATTLK
1	0	0.85	1.3	2	VTFSPK
3	1	0.82	1.2	1.6	INNVPVDK

5.147 " 145 1755 Fumarate hydratase class I, aerobic [Crassostrea gigas]"

Protein Accession	CL32374.Contig1$_{H}1$
Mean Expression Ratio	1.18
Median Expression Ratio	1.18
Credible Interval	(0.851, 1.62)
Associated Peptides	3
Associated Spectra	6
Coverage	NaN

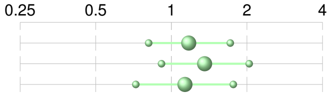

A	B	2.5	50	97.5	Sequence
2	1	0.81	1.2	1.7	IGIGAQFGGK
1	1	0.92	1.4	2	KVDTPWK
0	1	0.72	1.1	1.8	LEQLE

5.148 27 515 heat shock protein 70 [Capsaspora owczarzaki ATCC 30864]; 30 464 minus strand hypothetical protein [Trichinella spiralis]; 27 491 minus strand heat shock protein 70 [Capsaspora owczarzaki ATCC 30864]

Protein Accession	CL14449.Contig4$_{H}1$ CL14449.Contig2$_{H}1$ CL14449.Contig1$_{H}1$
Mean Expression Ratio	0.852
Median Expression Ratio	0.851
Credible Interval	(0.618, 1.18)
Associated Peptides	4
Associated Spectra	5
Coverage	NaN

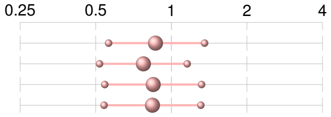

A	B	2.5	50	97.5	Sequence
1	0	0.56	0.87	1.4	RFDDPSVQSDMK
2	0	0.52	0.78	1.2	VEIIANDQGNR
1	0	0.54	0.85	1.3	NQVAMNPENTVFDAK
1	0	0.54	0.84	1.3	EVAEGYLGK

5.149 4365 4553 minus strand Basement membrane-specific heparan sulfate proteoglycan core protein [Crassostrea gigas]; 2502 2696 minus strand hypothetical protein DAPPUDRAFT_186952 [Daphnia pulex]

Protein Accession	CL24300.Contig6$_{H}1$ CL24300.Contig2$_{H}1$
Mean Expression Ratio	0.854
Median Expression Ratio	0.852
Credible Interval	(0.588, 1.25)
Associated Peptides	2
Associated Spectra	3
Coverage	NaN

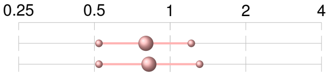

A	B	2.5	50	97.5	Sequence
**1**	**1**	**0.52**	**0.8**	**1.2**	**DGSIIVDGQEVK**
**0**	**1**	**0.52**	**0.83**	**1.3**	**VDDGFR**

5.150 116 337 minus strand Golgi-associated plant pathogenesis-related protein 1 [Crassostrea gigas]; 538 759 minus strand Golgi-associated plant pathogenesis-related protein 1 [Crassostrea gigas]; 116 337 minus strand Golgi-associated plant pathogenesis-related protein 1 [Crassostrea gigas]; 261 482 minus strand Golgi-associated plant pathogenesis-related protein 1 [Crassostrea gigas]; 116 337 minus strand Golgi-associated plant pathogenesis-related protein 1 [Crassostrea gigas]; 116 337 minus strand Golgi-associated plant pathogenesis-related protein 1 [Crassostrea gigas]

Protein Accession	CL15263.Contig9$_{H}1$ CL15263.Contig8$_{H}1$ CL15263.Contig7$_{H}1$ CL15263.Contig5$_{H}1$ CL1526
Mean Expression Ratio	1.18
Median Expression Ratio	1.17
Credible Interval	(0.797, 1.75)
Associated Peptides	2
Associated Spectra	2
Coverage	NaN

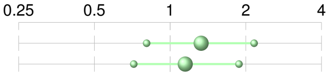

A	B	2.5	50	97.5	Sequence
**1**	**0**	**0.81**	**1.3**	**2.2**	**FIDEMLEAHNK**
**1**	**0**	**0.72**	**1.1**	**1.9**	**KEHQVQPLVINK**

5.151 REVERSED 68 358 endonuclease-reverse transcriptase [Danaus plexippus]

Protein Accession	RRRRRUnigene39307$_{H}1$
Mean Expression Ratio	0.85
Median Expression Ratio	0.852
Credible Interval	(0.572, 1.26)
Associated Peptides	2
Associated Spectra	2
Coverage	NaN

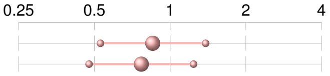

A	B	2.5	50	97.5	Sequence
**0**	**1**	**0.53**	**0.85**	**1.4**	**TIITALDK**
**0**	**1**	**0.48**	**0.77**	**1.2**	**CQSLLDENSIK**

5.152 26 1909 Dolichyl-diphosphooligosaccharide–protein glycosyltransferase subunit 2 [Crassostrea gigas]

Protein Accession	CL6515.Contig1$_{H}1$
Mean Expression Ratio	1.17
Median Expression Ratio	1.17
Credible Interval	(0.892, 1.54)
Associated Peptides	6
Associated Spectra	10
Coverage	NaN

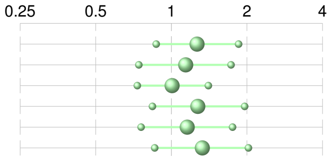

A	B	2.5	50	97.5	Sequence
1	1	0.87	1.3	1.9	AVSSALLDALK
1	0	0.74	1.1	1.7	KQFTASASDK
2	2	0.73	1	1.4	NLGLAVDEK
1	0	0.84	1.3	2	TLYELNFMEAK
1	0	0.76	1.2	1.8	LLTDAISAK
1	0	0.86	1.3	2	VSVTNLMGGDLGQLTVTADSAR

5.153 " 154 534 minus strand Muscle M-line assembly protein unc-89, partial [Crassostrea gigas]; 259 690 minus strand Myosin light chain kinase, smooth muscle OS=Gallus gallus PE=1 SV=2"

Protein Accession	Unigene64523$_{H}1$ Unigene64520$_{H}1$
Mean Expression Ratio	1.17
Median Expression Ratio	1.17
Credible Interval	(0.78, 1.76)
Associated Peptides	1
Associated Spectra	3
Coverage	NaN

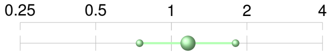

A	B	2.5	50	97.5	Sequence
2	1	0.75	1.2	1.8	LEIGEVVVDDEGEYICK

5.154 167 2713 minus strand Major vault protein [Crassostrea gigas]

Protein Accession	CL18286.Contig3$_{H}1$
Mean Expression Ratio	1.18
Median Expression Ratio	1.17
Credible Interval	(0.865, 1.61)
Associated Peptides	5
Associated Spectra	6
Coverage	NaN

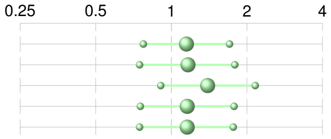

A	B	2.5	50	97.5	Sequence
2	0	0.78	1.2	1.7	GAVAQVQFDDFHK
1	0	0.75	1.2	1.8	KEVVVEETIR
1	0	0.91	1.4	2.2	KPGDRWMIR
0	1	0.75	1.2	1.8	IEGEAAVEQAK
0	1	0.75	1.2	1.8	IVDEAEAEK

5.155 49 1038 Glyceraldehyde-3-phosphate dehydrogenase [Crotalus adamanteus]

Protein Accession	Unigene7744$_{H}1$
Mean Expression Ratio	1.17
Median Expression Ratio	1.17
Credible Interval	(0.842, 1.65)
Associated Peptides	1
Associated Spectra	11
Coverage	NaN

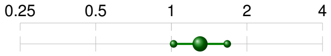

A	B	2.5	50	97.5	Sequence
11	0	1	1.3	1.7	SSIFDAK

5.156 324 1982 minus strand Pikachurin [Crassostrea gigas]

Protein Accession	CL4889.Contig10$_H1$
Mean Expression Ratio	0.854
Median Expression Ratio	0.855
Credible Interval	(0.587, 1.24)
Associated Peptides	2
Associated Spectra	3
Coverage	NaN

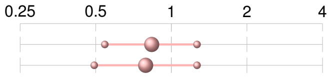

A	B	2.5	50	97.5	Sequence
2	0	0.54	0.84	1.3	VTAPIITEECGK
1	0	0.49	0.79	1.3	KGVLMR

5.157 185 1258 minus strand Severin [Crassostrea gigas]

Protein Accession	CL7486.Contig1$_H1$
Mean Expression Ratio	1.17
Median Expression Ratio	1.17
Credible Interval	(0.878, 1.55)
Associated Peptides	7
Associated Spectra	9
Coverage	NaN

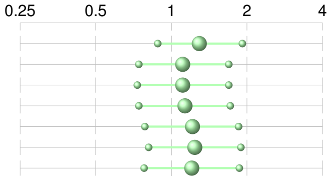

A	B	2.5	50	97.5	Sequence
2	0	0.88	1.3	1.9	EVQGHESDLFR
1	1	0.74	1.1	1.7	TDHPLVPITCLTEGTK
1	0	0.73	1.1	1.7	TDTVDEGSGTEEFDR
1	0	0.74	1.1	1.7	TVELDTFLNDVPVQHR
1	0	0.79	1.2	1.9	VTEWPVGDYGK
1	0	0.81	1.2	1.9	SDFISEDVFILDTK
1	0	0.78	1.2	1.9	TAGQKPGLQIWR

5.158 6 650 minus strand glutathione S-transferase mu class protein [Crassostrea ariakensis]; 117 761 glutathione S-transferase mu class protein [Crassostrea ariakensis]; 122 766 glutathione S-transferase mu class protein [Crassostrea ariakensis]; 122 766 glutathione S-transferase mu class protein [Crassostrea ariakensis]

Protein Accession	Unigene56987$_H1$ CL6887.Contig3$_H1$ CL6887.Contig2$_H1$ CL6887.Contig1$_H1$
Mean Expression Ratio	1.17
Median Expression Ratio	1.17
Credible Interval	(0.906, 1.51)
Associated Peptides	6
Associated Spectra	13
Coverage	NaN

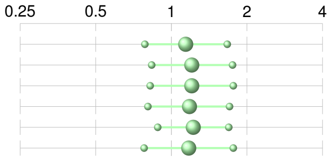

A	B	2.5	50	97.5	Sequence
2	0	0.78	1.1	1.7	ITQSNAILR
1	1	0.83	1.2	1.8	VKETLASYEK
1	1	0.83	1.2	1.8	PALGYWK
2	0	0.81	1.2	1.7	IMIPGILDDYPK
3	1	0.88	1.2	1.7	KHDLLGK
0	1	0.78	1.2	1.8	DAWFNVK

5.159 145 1020 minus strand PREDICTED: inorganic pyrophosphatase-like [Saccoglossus kowalevskii]

Protein Accession	CL12968.Contig14$_{H}1$
Mean Expression Ratio	1.17
Median Expression Ratio	1.17
Credible Interval	(0.893, 1.53)
Associated Peptides	6
Associated Spectra	10
Coverage	NaN

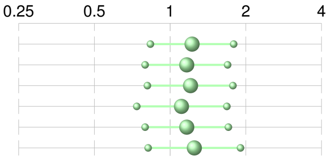

A	B	2.5	50	97.5	Sequence
1	1	0.84	1.2	1.8	AEVIVQESK
1	1	0.8	1.2	1.7	IIAIDVTDPLASK
1	1	0.81	1.2	1.8	LNDIDDVHR
1	0	0.74	1.1	1.7	VLGTMLLVDEGETDWK
1	1	0.8	1.2	1.7	EMPGFLR
0	1	0.82	1.3	1.9	VFFR

5.160 79 606 minus strand PREDICTED: similar to Ras-like protein 3 precursor (Protein roughened) [Tribolium castaneum] gi

Protein Accession	Unigene4246$_{H}1$
Mean Expression Ratio	1.17
Median Expression Ratio	1.17
Credible Interval	(0.869, 1.54)
Associated Peptides	4
Associated Spectra	9
Coverage	NaN

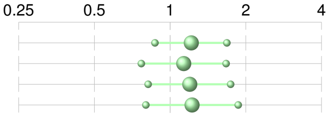

A	B	2.5	50	97.5	Sequence
2	2	0.87	1.2	1.7	LVVLGSGGVGK
2	0	0.77	1.1	1.7	VKDTDDVPMLLVGNK
1	1	0.82	1.2	1.7	CDLEVDR
0	1	0.8	1.2	1.9	DLYMK

5.161 3 4517 minus strand Clathrin heavy chain 1 [Crassostrea gigas]

Protein Accession	CL16446.Contig1$_{H}1$
Mean Expression Ratio	0.858
Median Expression Ratio	0.857
Credible Interval	(0.67, 1.1)
Associated Peptides	8
Associated Spectra	13
Coverage	NaN

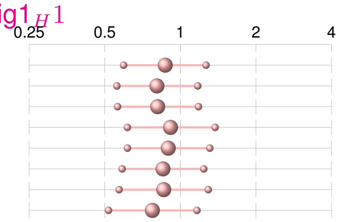

A	B	2.5	50	97.5	Sequence
2	0	0.6	0.87	1.3	KFEVNTSAIK
2	0	0.56	0.81	1.2	AQLNQGMVK
1	1	0.56	0.81	1.2	AGLLQR
1	0	0.61	0.91	1.4	KFNTLFQSGQYQEAAK
1	1	0.61	0.9	1.3	FLQMAR
2	0	0.59	0.86	1.2	VDGAR
1	0	0.57	0.86	1.3	NFLKEAK
0	1	0.52	0.77	1.2	AVNFFIK

5.162 28 642 minus strand Transmembrane emp24 domain-containing protein 9 [Crassostrea gigas]; 236 709 minus strand Transmembrane emp24 domain-containing protein 9 OS=Rattus norvegicus GN=Tmed9 PE=1 SV=1; 239 742 minus strand Transmembrane emp24 domain-containing protein eca OS=Drosophila erecta GN=eca PE=3 SV=1; 236 709 minus strand Transmembrane emp24 domain-containing protein 9 OS=Rattus norvegicus GN=Tmed9 PE=1 SV=1; 236 709 minus strand Transmembrane emp24 domain-containing protein 9 OS=Rattus norvegicus GN=Tmed9 PE=1 SV=1; 236 709 minus strand Transmembrane emp24 domain-containing protein 9 OS=Rattus norvegicus GN=Tmed9 PE=1 SV=1; 44 658 minus strand Transmembrane emp24 domain-containing protein 9 [Crassostrea gigas]

Protein Accession	CL2503.Contig8$_{H}$1 CL2503.Contig7$_{H}$1 CL2503.Contig5$_{H}$1 CL2503.Contig4$_{H}$1 CL2503.Con
Mean Expression Ratio	1.17
Median Expression Ratio	1.17
Credible Interval	(0.754, 1.81)
Associated Peptides	1
Associated Spectra	1
Coverage	NaN

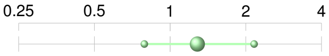

A	B	2.5	50	97.5	Sequence
1	0	0.79	1.3	2.2	QLLDQVEQITK

5.163 942 1385 Golgi-associated plant pathogenesis-related protein 1 [Crassostrea gigas]; 338 817 Golgi-associated plant pathogenesis-related protein 1 [Crassostrea gigas]; 354 833 Golgi-associated plant pathogenesis-related protein 1 [Crassostrea gigas]; 576 1055 Golgi-associated plant pathogenesis-related protein 1 [Crassostrea gigas]; 618 1097 Golgi-associated plant pathogenesis-related protein 1 [Crassostrea gigas]; 536 979 Golgi-associated plant pathogenesis-related protein 1 [Crassostrea gigas]; 740 1219 minus strand Golgi-associated plant pathogenesis-related protein 1 [Crassostrea gigas]

Protein Accession	Unigene391$_{H}$1 CL15263.Contig2$_{H}$1 CL15263.Contig1$_{H}$1 CL15263.Contig13$_{H}$1 CL15263.Co
Mean Expression Ratio	0.858
Median Expression Ratio	0.858
Credible Interval	(0.573, 1.28)
Associated Peptides	1
Associated Spectra	2
Coverage	NaN

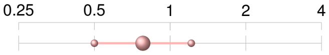

A	B	2.5	50	97.5	Sequence
1	1	0.5	0.78	1.2	IITEETIIK

5.164 194 904 Aldo-keto reductase family 1 member B10 [Crassostrea gigas]

Protein Accession	CL3558.Contig4$_{H}1$
Mean Expression Ratio	0.858
Median Expression Ratio	0.858
Credible Interval	(0.556, 1.33)
Associated Peptides	1
Associated Spectra	1
Coverage	NaN

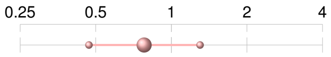

A	B	2.5	50	97.5	Sequence
**0**	**1**	**0.47**	**0.78**	**1.3**	**ALEVLVSEGK**

5.165 " 109 1632 minus strand Seryl-tRNA synthetase, cytoplasmic [Crassostrea gigas]; 109 1614 minus strand Seryl-tRNA synthetase, cytoplasmic [Crassostrea gigas]"

Protein Accession	CL11056.Contig9$_{H}1$ CL11056.Contig1$_{H}1$
Mean Expression Ratio	1.16
Median Expression Ratio	1.16
Credible Interval	(0.785, 1.74)
Associated Peptides	2
Associated Spectra	2
Coverage	NaN

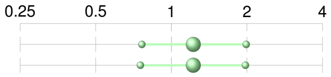

A	B	2.5	50	97.5	Sequence
**0**	**1**	**0.76**	**1.2**	**2**	**LVDTVVDQDAAWR**
**0**	**1**	**0.75**	**1.2**	**2**	**SLGIPYR**

5.166 160 1218 Galectin-9 [Crassostrea gigas]

Protein Accession	Unigene165$_{H}1$
Mean Expression Ratio	0.861
Median Expression Ratio	0.859
Credible Interval	(0.591, 1.25)
Associated Peptides	2
Associated Spectra	3
Coverage	NaN

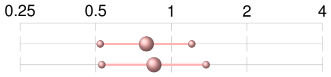

A	B	2.5	50	97.5	Sequence
**1**	**1**	**0.52**	**0.79**	**1.2**	**IAVNNQHLVEFR**
**1**	**0**	**0.53**	**0.85**	**1.4**	**SGIPGGLAPGR**

5.167 " 276 659 Fatty acid-binding protein, adipocyte [Crassostrea gigas]; 426 809 Fatty acid-binding protein, adipocyte [Crassostrea gigas]; 277 660 Fatty acid-binding protein, adipocyte [Crassostrea gigas]; 283 666 Fatty acid-binding protein, adipocyte [Crassostrea gigas]; 270 653 Fatty acid-binding protein, adipocyte [Crassostrea gigas]; 283 666 Fatty acid-binding protein, adipocyte [Crassostrea gigas]; 258 641 Fatty acid-binding protein, adipocyte [Crassostrea gigas]; 427 810 Fatty acid-binding protein, adipocyte [Crassostrea gigas]; 277 660 Fatty acid-binding protein, adipocyte [Crassostrea gigas]"

Protein Accession	CL7666.Contig9$_{H}$1 CL7666.Contig8$_{H}$1 CL7666.Contig7$_{H}$1 CL7666.Contig6$_{H}$1 CL7666.Con
Mean Expression Ratio	1.16
Median Expression Ratio	1.16
Credible Interval	(0.916, 1.49)
Associated Peptides	5
Associated Spectra	16
Coverage	NaN

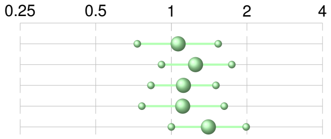

A	B	2.5	50	97.5	Sequence
1	1	0.73	1.1	1.5	AVLTEESGELVER
3	1	0.91	1.3	1.7	GVTATISFQK
4	1	0.83	1.1	1.5	LTTVAGDK
1	1	0.77	1.1	1.6	TVLAMER
1	2	1	1.4	2	AFVDGALK

5.168 228 1553 hypothetical protein CGI_10024545 [Crassostrea gigas]; 228 1553 hypothetical protein CGI_10024545 [Crassostrea gigas]

Protein Accession	CL14644.Contig9$_{H}$1 CL14644.Contig8$_{H}$1
Mean Expression Ratio	1.17
Median Expression Ratio	1.16
Credible Interval	(0.789, 1.73)
Associated Peptides	2
Associated Spectra	2
Coverage	NaN

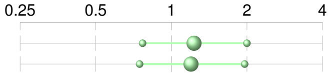

A	B	2.5	50	97.5	Sequence
0	1	0.77	1.2	2	LMEQPQSMLL
0	1	0.75	1.2	2	AEDILR

5.169 1 576 minus strand Tubulin beta chain [Crassostrea gigas]

Protein Accession	Unigene6463$_{H}$1
Mean Expression Ratio	1.16
Median Expression Ratio	1.16
Credible Interval	(0.759, 1.8)
Associated Peptides	1
Associated Spectra	4
Coverage	NaN

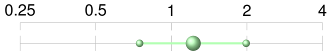

A	B	2.5	50	97.5	Sequence
3	1	0.75	1.2	2	EVDEQMLNVQNK

5.170 727 2022 RecName: Full=Enolase; AltName: Full=2-phospho-D-glycerate hydro-lyase; AltName: Full=2-phosphoglycerate dehydratase gi|1911573|gb|AAB50731.1| enolase [Doryteuthis pealeii]; 690 1985 RecName: Full=Enolase; AltName: Full=2-phospho-D-glycerate hydro-lyase; AltName: Full=2-phosphoglycerate dehydratase gi|1911573|gb|AAB50731.1| enolase [Doryteuthis pealeii]; 213 1508 RecName: Full=Enolase; AltName: Full=2-phospho-D-glycerate hydro-lyase; AltName: Full=2-phosphoglycerate dehydratase gi|1911573|gb|AAB50731.1| enolase [Doryteuthis pealeii]; 246 1541 RecName: Full=Enolase; AltName: Full=2-phospho-D-glycerate hydro-lyase; AltName: Full=2-phosphoglycerate dehydratase gi|1911573|gb|AAB50731.1| enolase [Doryteuthis pealeii]

Protein Accession	CL15687.Contig6$_{H}1$ CL15687.Contig5$_{H}1$ CL15687.Contig3$_{H}1$ CL15687.Contig2$_{H}1$
Mean Expression Ratio	1.16
Median Expression Ratio	1.16
Credible Interval	(1.03, 1.31)
Associated Peptides	25
Associated Spectra	121
Coverage	NaN

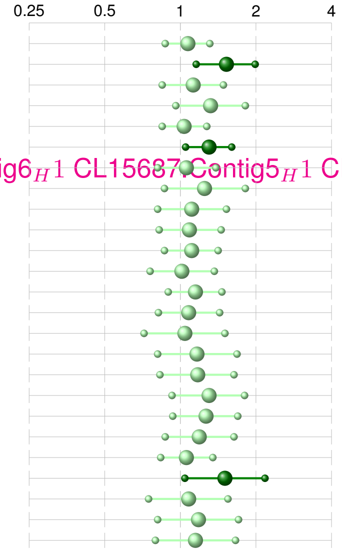

A	B	2.5	50	97.5	Sequence
11	3	0.87	1.1	1.3	AAGWGVMVSHR
3	3	1.2	1.5	2	AAVPSGASTGIYEALELR
3	2	0.85	1.1	1.5	DGKYDLDFK
2	1	0.96	1.3	1.8	FQIVGDDLLVTNPK
12	2	0.85	1	1.3	GVPLYR
9	4	1	1.3	1.6	IAPAFVGK
4	2	0.81	1.1	1.4	IEEELGVDAFYAGK
1	0	0.86	1.2	1.8	IEIGMDVAASEFCK
2	1	0.81	1.1	1.5	IMLDLDGTPNK
4	1	0.82	1.1	1.5	IMLDLDGTPNKEK
3	5	0.87	1.1	1.4	TLDLADQTAMDK
3	1	0.76	1	1.4	TSQQMIDMYHDFVK
4	4	0.89	1.1	1.5	VQMGIDR
1	4	0.82	1.1	1.4	QIFDSR
1	0	0.71	1	1.5	SYPIVTIEDPFDQDDWDAYTK
1	0	0.81	1.2	1.7	HIADLCGNKR
1	1	0.83	1.2	1.6	YDLDFK
1	1	0.93	1.3	1.8	LAMQEF
2	2	0.94	1.3	1.7	ACNALLLK
2	1	0.87	1.2	1.6	VNQIGSVSESIQAFK
5	4	0.84	1.1	1.3	YNQILR
0	1	1	1.5	2.2	HIADLCGNK
0	1	0.75	1.1	1.6	EGLELLK
0	1	0.81	1.2	1.7	MGSETYHHLK
0	1	0.8	1.1	1.7	ADLVVGLSTGQIK

5.171 266 934 minus strand Kyphoscoliosis peptidase [Crassostrea gigas]

Protein Accession	CL7490.Contig16$_H$1
Mean Expression Ratio	0.863
Median Expression Ratio	0.862
Credible Interval	(0.736, 1.01)
Associated Peptides	12
Associated Spectra	64
Coverage	NaN

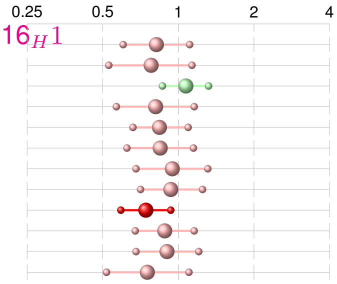

A	B	2.5	50	97.5	Sequence
3	1	0.6	0.82	1.1	ASLNANYGPDESK
1	0	0.53	0.78	1.1	DGCYLWEPLNLR
8	6	0.86	1.1	1.3	DLDNVHFK
2	0	0.57	0.81	1.2	DTVSLVVTLPDSGYYK
5	3	0.66	0.84	1.1	ELVFTQTER
3	1	0.62	0.85	1.1	LQIYALPSSDDSK
2	1	0.68	0.94	1.3	VNIPGAK
4	1	0.71	0.93	1.2	VTSNLIACGTEQEYK
8	3	0.59	0.74	0.93	YSSLLEYDL
5	1	0.67	0.88	1.2	QYAQWK
4	1	0.68	0.9	1.2	SRPELPHGYLGAQSK
0	1	0.52	0.75	1.1	GLKDLDNVHFK

5.172 981 1379 minus strand Multimerin-1 [Crassostrea gigas]; 998 1396 Multimerin-1 [Crassostrea gigas]; 975 1373 Multimerin-1 [Crassostrea gigas]; 960 1358 Multimerin-1 [Crassostrea gigas]; 810 1208 Multimerin-1 [Crassostrea gigas]

Protein Accession	CL22447.Contig9$_H$1 CL22447.Contig7$_H$1 CL22447.Contig6$_H$1 CL22447.Contig5$_H$1 CL2244
Mean Expression Ratio	1.16
Median Expression Ratio	1.16
Credible Interval	(0.844, 1.58)
Associated Peptides	3
Associated Spectra	7
Coverage	NaN

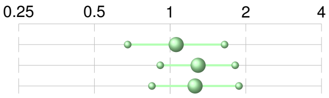

A	B	2.5	50	97.5	Sequence
1	0	0.68	1.1	1.6	TNTEFHTGPIVFDTTLSNK
2	2	0.91	1.3	1.8	VDAVLVADGQDVAR
0	2	0.84	1.3	1.9	VSFSAILK

5.173 323 1192 cytosolic malate dehydrogenase [Lottia scabra]; 323 1192 cytosolic malate dehydrogenase [Lottia scabra]; 323 1192 cytosolic malate dehydrogenase [Lottia scabra]; 323 1192 cytosolic malate dehydrogenase [Lottia scabra]; 2474 3343 cytosolic malate dehydrogenase [Lottia scabra]; 116 1111 minus strand cytosolic malate dehydrogenase [Lottia scabra]

Protein Accession	CL280.Contig21$_H$1 CL280.Contig20$_H$1 CL280.Contig19$_H$1 CL280.Contig18$_H$1 CL280.Conti
Mean Expression Ratio	1.16
Median Expression Ratio	1.16
Credible Interval	(0.981, 1.37)
Associated Peptides	12
Associated Spectra	110
Coverage	NaN

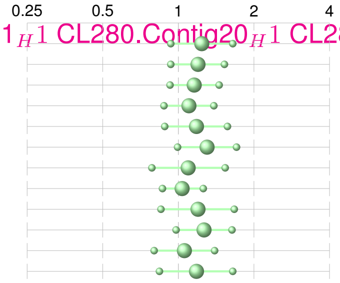

A	B	2.5	50	97.5	Sequence
3	3	0.93	1.2	1.7	EAVKDDNWINNEFISTVQK
6	5	0.93	1.2	1.5	ENFSCLTR
15	4	0.92	1.2	1.5	KLSSAMSAAK
6	6	0.88	1.1	1.4	LGIPNNAVSK
3	3	0.88	1.2	1.6	LSSAMSAAK
6	2	0.99	1.3	1.7	SQGANLDAVAK
2	1	0.79	1.1	1.5	YGIPEGVMYSYPVQIK
9	14	0.87	1	1.3	EMAEQILAA
2	1	0.85	1.2	1.7	ELLEER
5	4	0.98	1.3	1.6	DCDAAFLVGAMPR
4	3	0.8	1.1	1.4	DLLAANIK
2	1	0.84	1.2	1.6	YAPSIPK

5.174 165 377 Cysteine and glycine-rich protein 3 [Crassostrea gigas]; 135 344 Cysteine and glycine-rich protein 3 [Crassostrea gigas]

Protein Accession	CL38347.Contig2$_{H}$1 CL38347.Contig1$_{H}$1
Mean Expression Ratio	0.862
Median Expression Ratio	0.863
Credible Interval	(0.57, 1.29)
Associated Peptides	1
Associated Spectra	2
Coverage	NaN

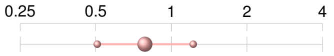

A	B	2.5	50	97.5	Sequence
**1**	**1**	**0.51**	**0.79**	**1.2**	**VEAGNIPFHK**

5.175 125 550 minus strand ribosomal protein S20 [Chlamys farreri]; 125 550 minus strand ribosomal protein S20 [Chlamys farreri]; 125 550 minus strand ribosomal protein S20 [Chlamys farreri]

Protein Accession	CL6833.Contig3$_{H}$1 CL6833.Contig2$_{H}$1 CL6833.Contig1$_{H}$1
Mean Expression Ratio	0.864
Median Expression Ratio	0.864
Credible Interval	(0.593, 1.25)
Associated Peptides	2
Associated Spectra	3
Coverage	NaN

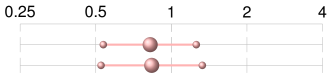

A	B	2.5	50	97.5	Sequence
**2**	**0**	**0.53**	**0.82**	**1.3**	**IIDLHSPSEIVK**
**1**	**0**	**0.52**	**0.83**	**1.3**	**KTPCGEGSK**

5.176 697 954 minus strand Peptidyl-prolyl cis-trans isomerase B [Crassostrea gigas]; 614 877 Peptidyl-prolyl cis-trans isomerase B [Crassostrea gigas]

Protein Accession	CL49248.Contig4$_{H}$1 CL49248.Contig3$_{H}$1
Mean Expression Ratio	1.16
Median Expression Ratio	1.16
Credible Interval	(0.873, 1.55)
Associated Peptides	4
Associated Spectra	19
Coverage	NaN

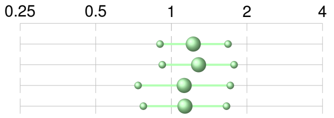

A	B	2.5	50	97.5	Sequence
**4**	**4**	**0.9**	**1.2**	**1.7**	**LVDCSATTLK**
**3**	**3**	**0.92**	**1.3**	**1.8**	**ASLEFLDK**
**0**	**1**	**0.74**	**1.1**	**1.7**	**SMQNLDGR**
**3**	**1**	**0.78**	**1.1**	**1.7**	**INNVPVDK**

5.177 549 821 minus strand Calponin-2 [Crassostrea gigas]

Protein Accession	CL21394.Contig10$_{H}$1
Mean Expression Ratio	0.864
Median Expression Ratio	0.864
Credible Interval	(0.642, 1.16)
Associated Peptides	3
Associated Spectra	10
Coverage	NaN

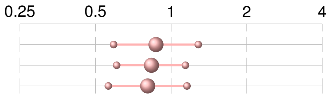

A	B	2.5	50	97.5	Sequence
**2**	**0**	**0.59**	**0.87**	**1.3**	**QQGRASQAGMSMGAVR**
**3**	**2**	**0.61**	**0.83**	**1.1**	**SLFPTADLFEGR**
**3**	**0**	**0.56**	**0.81**	**1.2**	**NSLFPTADLFEGR**

5.178 501 1838 Hsp90A [Haliotis asinina]

Protein Accession	CL5184.Contig2$_{H}$1
Mean Expression Ratio	0.864
Median Expression Ratio	0.864
Credible Interval	(0.672, 1.11)
Associated Peptides	7
Associated Spectra	15
Coverage	NaN

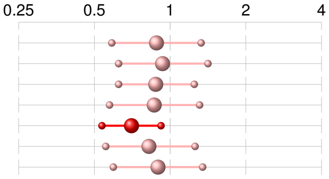

A	B	2.5	50	97.5	Sequence
1	0	0.58	0.88	1.3	AEFEGLCK
1	0	0.62	0.93	1.4	AIYYITGESLDAVK
1	2	0.63	0.88	1.2	GVVDSEDLPLNISR
1	0	0.57	0.87	1.3	NPDDITPEEYAEFYK
3	4	0.54	0.7	0.92	LAEFLR
1	0	0.55	0.83	1.3	RAPFDLFENK
1	0	0.6	0.9	1.3	VERVK

5.179 82 816 PREDICTED: triosephosphate isomerase B [Oreochromis niloticus]

Protein Accession	CL26247.Contig4$_{H}$1
Mean Expression Ratio	1.16
Median Expression Ratio	1.15
Credible Interval	(0.946, 1.42)
Associated Peptides	8
Associated Spectra	29
Coverage	NaN

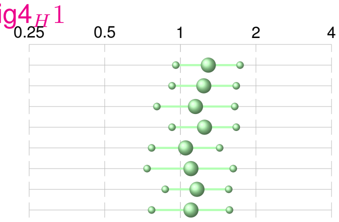

A	B	2.5	50	97.5	Sequence
3	2	0.96	1.3	1.7	AIADNVNDWK
3	2	0.93	1.2	1.7	GAFTGEISPAMIK
2	0	0.81	1.2	1.7	HVFGESDELIGQK
3	2	0.92	1.2	1.7	QFFVGGNWK
4	0	0.77	1.1	1.4	ELATKPDVDGFLVGGASLK
1	0	0.74	1.1	1.6	IGVAAQNCYKEPK
3	2	0.87	1.2	1.6	TEFITIINSK
0	2	0.77	1.1	1.6	SVDEIIK

5.180 459 755 minus strand heat shock protein beta-1 [Trichinella spiralis] gi; 464 763 minus strand heat shock protein beta-1 [Trichinella spiralis] gi; 437 736 minus strand heat shock protein beta-1 [Trichinella spiralis] gi; 437 736 minus strand heat shock protein beta-1 [Trichinella spiralis] gi; 437 736 minus strand heat shock protein beta-1 [Trichinella spiralis] gi

Protein Accession	CL17234.Contig5$_{H}$1 CL17234.Contig4$_{H}$1 CL17234.Contig3$_{H}$1 CL17234.Contig2$_{H}$1 CL1723
Mean Expression Ratio	0.867
Median Expression Ratio	0.866
Credible Interval	(0.601, 1.25)
Associated Peptides	3
Associated Spectra	8
Coverage	NaN

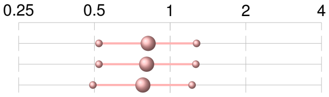

A	B	2.5	50	97.5	Sequence
2	1	0.52	0.82	1.3	DVEPTTVTSSLNADGVLTLK
2	1	0.52	0.81	1.3	IVVEGCHEEK
1	1	0.49	0.78	1.2	IVVEGCHEEKQDEHGFITR

5.181 9 887 minus strand hypothetical protein CGI_10006272 [Crassostrea gigas]

Protein Accession	CL46976.Contig1$_H$1
Mean Expression Ratio	0.866
Median Expression Ratio	0.867
Credible Interval	(0.589, 1.27)
Associated Peptides	1
Associated Spectra	3
Coverage	NaN

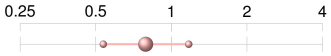

A	B	2.5	50	97.5	Sequence
**2**	**1**	**0.53**	**0.79**	**1.2**	**MILPQINK**

5.182 156 1106 ELAV 2-like protein [Aplysia californica] gi

Protein Accession	CL1500.Contig1$_H$1
Mean Expression Ratio	0.869
Median Expression Ratio	0.868
Credible Interval	(0.598, 1.26)
Associated Peptides	2
Associated Spectra	3
Coverage	NaN

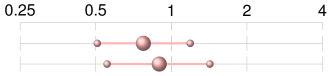

A	B	2.5	50	97.5	Sequence
**1**	**1**	**0.51**	**0.77**	**1.2**	**NLPIPWTENELNK**
**0**	**1**	**0.55**	**0.89**	**1.4**	**VLVDLQTGVSK**

5.183 113 808 minus strand omega class glutathione-s-transferase [Chlamys farreri]; 335 1045 minus strand omega class glutathione-s-transferase [Chlamys farreri]

Protein Accession	CL8096.Contig3$_H$1 CL8096.Contig2$_H$1
Mean Expression Ratio	0.867
Median Expression Ratio	0.868
Credible Interval	(0.577, 1.3)
Associated Peptides	1
Associated Spectra	2
Coverage	NaN

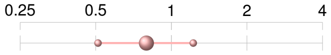

A	B	2.5	50	97.5	Sequence
**2**	**0**	**0.51**	**0.8**	**1.2**	**HPLGLVPTLEQDSK**

5.184 118 513 Protein CAL-5 [Caenorhabditis elegans] gi

Protein Accession	Unigene47320$_H$1
Mean Expression Ratio	1.15
Median Expression Ratio	1.15
Credible Interval	(0.773, 1.69)
Associated Peptides	2
Associated Spectra	3
Coverage	NaN

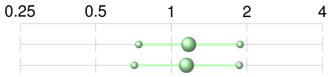

A	B	2.5	50	97.5	Sequence
**1**	**1**	**0.74**	**1.2**	**1.9**	**EADANGDGYLSIQELK**
**1**	**0**	**0.71**	**1.2**	**1.9**	**TLLAHSGK**

5.185 189 353 40S ribosomal protein S12 [Crassostrea gigas]

Protein Accession	Unigene385$_{H1}$
Mean Expression Ratio	1.15
Median Expression Ratio	1.15
Credible Interval	(0.761, 1.78)
Associated Peptides	1
Associated Spectra	1
Coverage	NaN

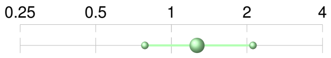

A	B	2.5	50	97.5	Sequence
**1**	**0**	**0.78**	**1.3**	**2.1**	**TALIHDGLAR**

5.186 210 779 GH20056 [Drosophila grimshawi] gi; 216 785 minus strand GH20056 [Drosophila grimshawi] gi; 210 779 GH20056 [Drosophila grimshawi] gi; 216 785 GH20056 [Drosophila grimshawi] gi

Protein Accession	CL34497.Contig4$_{H1}$ CL34497.Contig3$_{H1}$ CL34497.Contig2$_{H1}$ CL34497.Contig1$_{H1}$
Mean Expression Ratio	1.15
Median Expression Ratio	1.15
Credible Interval	(0.842, 1.58)
Associated Peptides	3
Associated Spectra	9
Coverage	NaN

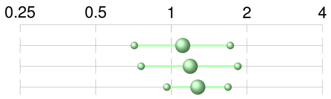

A	B	2.5	50	97.5	Sequence
**1**	**0**	**0.71**	**1.1**	**1.7**	**KQPFEFQIGVGQVIK**
**1**	**0**	**0.76**	**1.2**	**1.8**	**QLSQEEVSNFLK**
**5**	**2**	**0.96**	**1.3**	**1.7**	**GLLDMCAGEK**

5.187 62 505 Myosin-2 essential light chain [Crassostrea gigas]

Protein Accession	CL47077.Contig1$_{H1}$
Mean Expression Ratio	1.15
Median Expression Ratio	1.15
Credible Interval	(0.843, 1.57)
Associated Peptides	2
Associated Spectra	10
Coverage	NaN

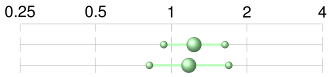

A	B	2.5	50	97.5	Sequence
**5**	**2**	**0.93**	**1.2**	**1.6**	**IAASQLGDVLR**
**2**	**1**	**0.82**	**1.2**	**1.7**	**HILTSLGEK**

5.188 30 1088 arginine kinase [Conus novaehollandiae]

Protein Accession	CL5308.Contig5$_H$1
Mean Expression Ratio	1.15
Median Expression Ratio	1.15
Credible Interval	(1.04, 1.28)
Associated Peptides	31
Associated Spectra	205
Coverage	NaN

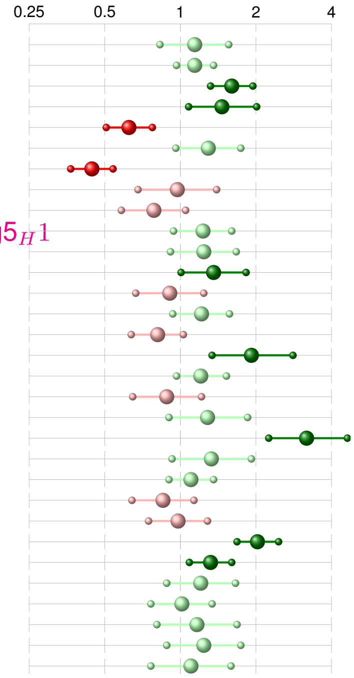

A	B	2.5	50	97.5	Sequence
2	1	0.83	1.1	1.6	AAGGYDDWPVGR
11	11	0.96	1.1	1.4	ATVEALNTFEGELK
8	8	1.3	1.6	1.9	FNFPDLDPSGK
2	1	1.1	1.5	2	GIHGEHTESVGGVYDISNK
9	5	0.51	0.62	0.77	GTYYPLLGMDK
2	2	0.96	1.3	1.7	KLIDDHFLFKDDDR
10	7	0.37	0.44	0.54	LAALPEFK
1	0	0.68	0.97	1.4	LGLSEFDAIMEMK
2	2	0.58	0.78	1.1	LIDDHFLFKDDDR
5	1	0.94	1.2	1.6	RFNFPDLDPSGK
3	1	0.92	1.2	1.7	SHASYGFPPVLSK
3	1	1	1.4	1.8	TFLVWVNEEDHIR
2	1	0.66	0.91	1.2	NGVEEILKK
7	0	0.93	1.2	1.6	KLTFAR
5	4	0.64	0.81	1	NGVEEILK
1	0	1.3	1.9	2.8	KLIDDHFLFK
6	4	0.96	1.2	1.5	LTFAR
2	1	0.64	0.89	1.2	EFCDK
1	0	0.9	1.3	1.8	TFEGELK
2	0	2.3	3.2	4.6	GTYYPLLGMDKATEK
1	0	0.93	1.3	1.9	APDFGEK
14	0	0.9	1.1	1.4	GGNLTQVYQR
1	4	0.64	0.85	1.1	GIFHNPDK
5	1	0.75	0.98	1.3	HPAPDFGEK
10	7	1.7	2	2.5	LVSGIHALEK
7	9	1.1	1.3	1.6	YIVSTR
0	3	0.88	1.2	1.7	LIDDHFLFK
0	5	0.76	1	1.3	IISMQK
0	1	0.81	1.2	1.7	VAEVK
0	2	0.88	1.2	1.7	IDDHFLFK
0	1	0.76	1.1	1.6	ATVEALNTFEGE

5.189 57 1688 Talin-1 [Camponotus floridanus]; 57 1865 Talin-1 [Camponotus floridanus]; 57 1835 Talin-1 [Camponotus floridanus]

Protein Accession	CL49468.Contig2$_H$1 CL45858.Contig2$_H$1 CL45858.Contig1$_H$1
Mean Expression Ratio	1.15
Median Expression Ratio	1.15
Credible Interval	(0.814, 1.61)
Associated Peptides	2
Associated Spectra	5
Coverage	NaN

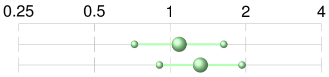

A	B	2.5	50	97.5	Sequence
2	0	0.72	1.1	1.6	KAQEPIVYAGK
3	0	0.91	1.3	1.9	ISIQAR

5.190 " 110 1717 PREDICTED: glutamate dehydrogenase, mitochondrial-like [Oreochromis niloticus]; 110 1717 PREDICTED: glutamate dehydrogenase, mitochondrial-like [Oreochromis niloticus]; 110 1717 PREDICTED: glutamate dehydrogenase, mitochondrial-like [Oreochromis niloticus]"

Protein Accession	CL16377.Contig9$_H1$ CL16377.Contig8$_H1$ CL16377.Contig10$_H1$
Mean Expression Ratio	1.15
Median Expression Ratio	1.15
Credible Interval	(0.839, 1.57)
Associated Peptides	4
Associated Spectra	6
Coverage	NaN

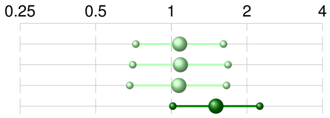

A	B	2.5	50	97.5	Sequence
**1**	**1**	**0.72**	**1.1**	**1.6**	**IIAEGANGPTTAGADEIFLK**
**1**	**0**	**0.7**	**1.1**	**1.7**	**DGSIYNPVDGIHPR**
**0**	**1**	**0.68**	**1.1**	**1.7**	**TAAYIAAIEK**
**0**	**2**	**1**	**1.5**	**2.2**	**EAGLTFT**

5.191 998 1705 minus strand Basement membrane-specific heparan sulfate proteoglycan core protein [Crassostrea gigas]

Protein Accession	CL22963.Contig1$_H1$
Mean Expression Ratio	0.871
Median Expression Ratio	0.872
Credible Interval	(0.567, 1.34)
Associated Peptides	1
Associated Spectra	1
Coverage	NaN

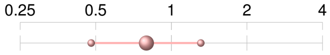

A	B	2.5	50	97.5	Sequence
**1**	**0**	**0.48**	**0.8**	**1.3**	**ITAYGGSLR**

5.192 258 629 Glycogenin-1 OS=Oryctolagus cuniculus GN=GYG1 PE=1 SV=3

Protein Accession	Unigene77101$_H1$
Mean Expression Ratio	1.15
Median Expression Ratio	1.15
Credible Interval	(0.741, 1.78)
Associated Peptides	1
Associated Spectra	1
Coverage	NaN

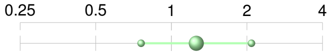

A	B	2.5	50	97.5	Sequence
**1**	**0**	**0.76**	**1.3**	**2.1**	**IHCWELTQYEK**

5.193 1363 1527 Protein disulfide-isomerase A3 [Crassostrea gigas]

Protein Accession	CL28563.Contig1$_H1$
Mean Expression Ratio	0.874
Median Expression Ratio	0.872
Credible Interval	(0.588, 1.3)
Associated Peptides	2
Associated Spectra	2
Coverage	NaN

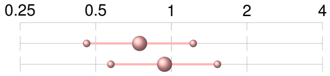

A	B	2.5	50	97.5	Sequence
**0**	**1**	**0.46**	**0.75**	**1.2**	**LKDEPEITIAK**
**0**	**1**	**0.57**	**0.94**	**1.5**	**FEELAEK**

5.194 3 572 Receptor-type tyrosine-protein phosphatase N2 [Crassostrea gigas]; 3 542 minus strand Receptor-type tyrosine-protein phosphatase N2 [Crassostrea gigas]

Protein Accession	CL8283.Contig5$_{H}1$ CL8283.Contig2$_{H}1$
Mean Expression Ratio	0.872
Median Expression Ratio	0.873
Credible Interval	(0.559, 1.35)
Associated Peptides	1
Associated Spectra	1
Coverage	NaN

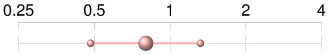

A	B	2.5	50	97.5	Sequence
0	1	0.48	0.8	1.3	EIDMAATLEHIR

5.195 3 347 beta-actin [Meretrix meretrix]; 3 215 Actin-2 OS=Onchocerca volvulus GN=act-2b PE=3 SV=1

Protein Accession	CL47466.Contig2$_{H}1$ CL47466.Contig1$_{H}1$
Mean Expression Ratio	1.15
Median Expression Ratio	1.14
Credible Interval	(0.959, 1.37)
Associated Peptides	11
Associated Spectra	308
Coverage	NaN

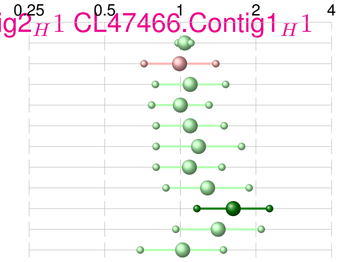

A	B	2.5	50	97.5	Sequence
151	129	0.98	1	1.1	EITALAPSTMK
2	1	0.72	0.99	1.4	EITALAPSTMKIK
3	0	0.79	1.1	1.5	KDLYANTVLSGGSTMYPGIADR
7	0	0.77	1	1.3	TVLSGGSTMYPGIADR
4	0	0.8	1.1	1.5	DLYANTVLSGGSTMYPGIADR
1	0	0.8	1.2	1.8	MQKEITALAPSTMK
0	4	0.8	1.1	1.5	LAPSTMK
0	1	0.88	1.3	1.9	ALAPSTMK
0	3	1.2	1.6	2.3	APSTMK
0	1	0.96	1.4	2.1	PSTMK
0	1	0.69	1	1.5	TALAPSTMK

5.196 300 878 unknown [Schistosoma japonicum]; 567 1145 unknown [Schistosoma japonicum]; 1110 1688 unknown [Schistosoma japonicum]; 94 672 unknown [Schistosoma japonicum]; 599 1177 unknown [Schistosoma japonicum]; 336 914 unknown [Schistosoma japonicum]

Protein Accession	CL11292.Contig5$_{H}1$ CL11292.Contig3$_{H}1$ CL11292.Contig2$_{H}1$ CL11292.Contig1$_{H}1$ CL1129
Mean Expression Ratio	0.874
Median Expression Ratio	0.875
Credible Interval	(0.65, 1.17)
Associated Peptides	4
Associated Spectra	12
Coverage	NaN

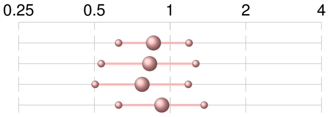

A	B	2.5	50	97.5	Sequence
4	4	0.62	0.86	1.2	AGNDVDLVVQR
1	0	0.53	0.83	1.3	GVVNTQSLPQEPEGEKR
1	0	0.5	0.78	1.2	NGDAILQICGESTAAMTHAQAK
2	0	0.62	0.92	1.4	VQLRRYDTSQPWGFR

5.197 871 1062 Adenylate kinase isoenzyme 5 [Crassostrea gigas]

Protein Accession	Unigene7895$_{H}1$
Mean Expression Ratio	1.15
Median Expression Ratio	1.14
Credible Interval	(0.861, 1.53)
Associated Peptides	2
Associated Spectra	13
Coverage	NaN

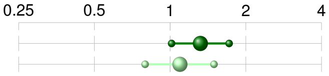

A	B	2.5	50	97.5	Sequence
6	2	1	1.3	1.7	VVFVVGGPGSGK
3	2	0.8	1.1	1.5	ALNEIMQK

5.198 244 837 PREDICTED: ras-like GTP-binding protein Rho1-like [Megachile rotundata]

Protein Accession	Unigene11792$_{H}1$
Mean Expression Ratio	1.14
Median Expression Ratio	1.14
Credible Interval	(0.812, 1.61)
Associated Peptides	4
Associated Spectra	5
Coverage	NaN

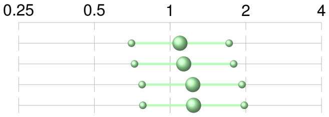

A	B	2.5	50	97.5	Sequence
1	1	0.7	1.1	1.7	EVFETATR
1	0	0.72	1.1	1.8	KLVIVGDGACGK
1	0	0.77	1.2	1.9	QVELALWDTAGQEDYDR
1	0	0.78	1.2	2	LMATVR

5.199 205 2490 minus strand glucose-regulated protein 94 [Crassostrea gigas]

Protein Accession	CL5089.Contig1$_{H}1$
Mean Expression Ratio	0.878
Median Expression Ratio	0.875
Credible Interval	(0.639, 1.21)
Associated Peptides	5
Associated Spectra	5
Coverage	NaN

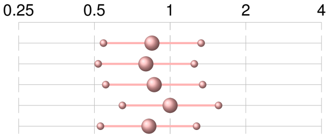

A	B	2.5	50	97.5	Sequence
1	0	0.55	0.85	1.3	ADKFAFQAEVNR
1	0	0.52	0.8	1.3	NLGTIAR
1	0	0.55	0.87	1.3	GTTVSLHLK
1	0	0.65	1	1.6	NDMFNNYGK
0	1	0.53	0.82	1.3	GVVDSDDLPLNVSR

5.200 633 806 Peroxiredoxin-1 [Crassostrea gigas]; 648 821 Peroxiredoxin-1 [Crassostrea gigas]; 666 839 Peroxiredoxin-1 [Crassostrea gigas]; 625 798 Peroxiredoxin-1 [Crassostrea gigas]; 633 824 Peroxiredoxin-1 [Crassostrea gigas]

Protein Accession	CL3160.Contig18$_{H}1$ CL3160.Contig17$_{H}1$ CL3160.Contig16$_{H}1$ CL3160.Contig15$_{H}1$ CL3160
Mean Expression Ratio	1.14
Median Expression Ratio	1.14
Credible Interval	(0.81, 1.6)
Associated Peptides	1
Associated Spectra	13
Coverage	NaN

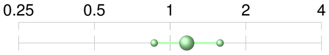

A	B	2.5	50	97.5	Sequence
7	6	0.86	1.2	1.6	LVQAFQFTDK

5.201 72 1400 minus strand hypothetical protein RCJMB04_22i13 [Gallus gallus]

Protein Accession	CL44755.Contig5$_H1$
Mean Expression Ratio	0.876
Median Expression Ratio	0.876
Credible Interval	(0.666, 1.15)
Associated Peptides	6
Associated Spectra	10
Coverage	NaN

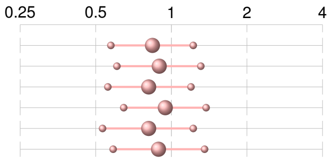

A	B	2.5	50	97.5	Sequence
2	0	0.57	0.84	1.2	FLVASTPDEALQHAAELGK
1	1	0.61	0.89	1.3	LVFSADEAK
1	1	0.56	0.81	1.2	AVFQMR
1	1	0.65	0.95	1.4	LSGIPDVFVK
0	1	0.53	0.81	1.2	AIIAASSMK
0	1	0.59	0.89	1.4	LSTIIDLAR

5.202 261 1361 tandem-repeat galectin [Pinctada fucata]; 261 1361 tandem-repeat galectin [Pinctada fucata]

Protein Accession	CL474.Contig2$_H1$ CL474.Contig1$_H1$
Mean Expression Ratio	0.877
Median Expression Ratio	0.877
Credible Interval	(0.585, 1.31)
Associated Peptides	1
Associated Spectra	2
Coverage	NaN

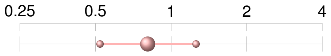

A	B	2.5	50	97.5	Sequence
0	2	0.52	0.81	1.3	LENNVLLHANQR

5.203 210 2201 minus strand Glycyl-tRNA synthetase [Crassostrea gigas]; 210 2201 minus strand Glycyl-tRNA synthetase [Crassostrea gigas]; 210 2201 minus strand Glycyl-tRNA synthetase [Crassostrea gigas]; 210 2201 minus strand Glycyl-tRNA synthetase [Crassostrea gigas]; 210 2201 minus strand Glycyl-tRNA synthetase [Crassostrea gigas]; 210 2201 minus strand Glycyl-tRNA synthetase [Crassostrea gigas]

Protein Accession	CL4073.Contig8$_H1$ CL4073.Contig7$_H1$ CL4073.Contig6$_H1$ CL4073.Contig5$_H1$ CL4073.Con
Mean Expression Ratio	0.876
Median Expression Ratio	0.877
Credible Interval	(0.588, 1.31)
Associated Peptides	2
Associated Spectra	2
Coverage	NaN

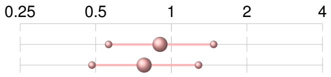

A	B	2.5	50	97.5	Sequence
0	1	0.56	0.9	1.5	ADHLIEATFEK
0	1	0.48	0.78	1.3	VLMPR

5.204 73 525 minus strand Actin-related protein 2/3 complex subunit 5-like protein [Salmo salar] gi; 73 525 Actin-related protein 2/3 complex subunit 5-like protein [Salmo salar] gi; 73 525 minus strand Actin-related protein 2/3 complex subunit 5-like protein [Salmo salar] gi

Protein Accession	CL14637.Contig6$_H$1 CL14637.Contig5$_H$1 CL14637.Contig4$_H$1
Mean Expression Ratio	0.877
Median Expression Ratio	0.878
Credible Interval	(0.557, 1.35)
Associated Peptides	1
Associated Spectra	1
Coverage	NaN

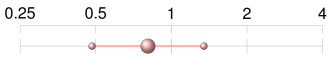

A	B	2.5	50	97.5	Sequence
0	1	0.48	0.81	1.3	EVDILMK

5.205 696 4010 Lethal(2) giant larvae-like protein 1 [Crassostrea gigas]; 657 3971 minus strand Lethal(2) giant larvae-like protein 1 [Crassostrea gigas]; 156 3470 Lethal(2) giant larvae-like protein 1 [Crassostrea gigas]

Protein Accession	CL3717.Contig4$_H$1 CL3717.Contig3$_H$1 CL3717.Contig16$_H$1
Mean Expression Ratio	0.879
Median Expression Ratio	0.878
Credible Interval	(0.609, 1.27)
Associated Peptides	3
Associated Spectra	3
Coverage	NaN

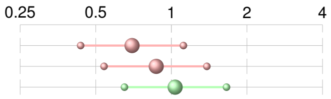

A	B	2.5	50	97.5	Sequence
0	1	0.44	0.7	1.1	LDTTADITIDSVR
0	1	0.54	0.87	1.4	VLPESLEVIHER
0	1	0.65	1	1.7	ASMIR

5.206 3 263 minus strand beta-actin [Urechis unicinctus]

Protein Accession	CL31420.Contig5$_H$1
Mean Expression Ratio	1.14
Median Expression Ratio	1.14
Credible Interval	(0.92, 1.4)
Associated Peptides	4
Associated Spectra	136
Coverage	NaN

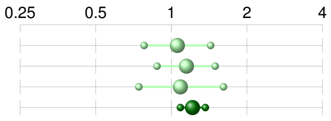

A	B	2.5	50	97.5	Sequence
3	2	0.78	1.1	1.4	IIAPPERK
7	2	0.87	1.1	1.5	IKIIAPPER
0	1	0.74	1.1	1.6	EITALAPPTMK
63	58	1.1	1.2	1.4	IIAPPER

5.207 112 1050 Enolase-phosphatase E1 [Crassostrea gigas]; 112 1050 Enolase-phosphatase E1 [Crassostrea gigas]

Protein Accession	CL25175.Contig3$_{H}1$ CL25175.Contig2$_{H}1$
Mean Expression Ratio	1.14
Median Expression Ratio	1.14
Credible Interval	(0.812, 1.59)
Associated Peptides	3
Associated Spectra	5
Coverage	NaN

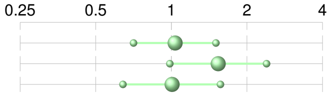

A	B	2.5	50	97.5	Sequence
1	2	0.71	1	1.5	SAAEAESLLVGVK
1	0	0.98	1.5	2.4	AIIFDVEGTTTPISFVK
0	1	0.64	1	1.6	QLQGHIWR

5.208 22 870 minus strand PREDICTED: alcohol dehydrogenase class-3 [Sarcophilus harrisii]

Protein Accession	Unigene27690$_{H}1$
Mean Expression Ratio	0.878
Median Expression Ratio	0.879
Credible Interval	(0.679, 1.14)
Associated Peptides	6
Associated Spectra	14
Coverage	NaN

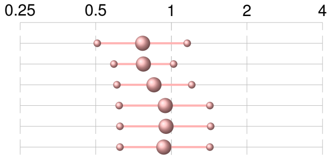

A	B	2.5	50	97.5	Sequence
1	0	0.51	0.77	1.2	AALEACHK
5	2	0.59	0.78	1	AAVAWEAK
1	2	0.61	0.85	1.2	IIGIDVNQDK
1	0	0.62	0.95	1.4	KPLVIEDIEVAPPAAGEVR
1	0	0.62	0.95	1.4	TNLCQK
0	1	0.62	0.93	1.4	FAMAK

5.209 80 589 minus strand PREDICTED: adenine phosphoribosyltransferase-like [Strongylocentrotus purpuratus]

Protein Accession	CL8682.Contig6$_{H}1$
Mean Expression Ratio	1.14
Median Expression Ratio	1.14
Credible Interval	(0.865, 1.51)
Associated Peptides	5
Associated Spectra	10
Coverage	NaN

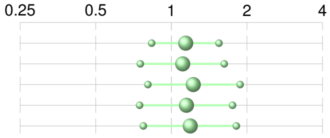

A	B	2.5	50	97.5	Sequence
2	3	0.84	1.1	1.5	LTVPVESLIK
1	1	0.75	1.1	1.6	AAVELVEK
0	1	0.81	1.2	1.9	GFLFGPMIAEK
0	1	0.75	1.2	1.8	NAPDADVIIGLESR
0	1	0.77	1.2	1.8	DIFPILR

5.210 " 6 1274 talin, putative [Ixodes scapularis] gi"

Protein Accession	CL39200.Contig1$_{H}1$
Mean Expression Ratio	1.14
Median Expression Ratio	1.14
Credible Interval	(0.77, 1.69)
Associated Peptides	2
Associated Spectra	2
Coverage	NaN

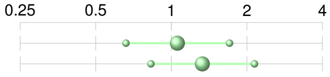

A	B	2.5	50	97.5	Sequence
0	1	0.66	1.1	1.7	SIAAATAALVK
0	1	0.83	1.3	2.1	LEMIR

5.211 101 1693 CALNUC [Spodoptera frugiperda]; 141 1325 Nucleobindin-2 [Crassostrea gigas]

Protein Accession	CL1888.Contig9$_{H}1$ CL1888.Contig6$_{H}1$
Mean Expression Ratio	0.881
Median Expression Ratio	0.88
Credible Interval	(0.591, 1.32)
Associated Peptides	2
Associated Spectra	3
Coverage	NaN

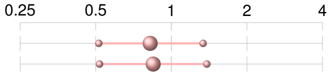

A	B	2.5	50	97.5	Sequence
1	1	0.51	0.82	1.3	LKELPEVER
1	0	0.52	0.85	1.4	REEFK

5.212 3 239 minus strand p38 MAPK [Littorina littorea]

Protein Accession	CL49144.Contig2$_{H}1$
Mean Expression Ratio	0.878
Median Expression Ratio	0.88
Credible Interval	(0.568, 1.36)
Associated Peptides	1
Associated Spectra	1
Coverage	NaN

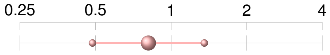

A	B	2.5	50	97.5	Sequence
0	1	0.49	0.81	1.4	MLDLDTDHR

5.213 424 3507 minus strand putative Na+/K+-ATPase alpha subunit [Paroctopus digueti]

Protein Accession	Unigene11494$_{H}1$
Mean Expression Ratio	0.879
Median Expression Ratio	0.881
Credible Interval	(0.639, 1.21)
Associated Peptides	5
Associated Spectra	5
Coverage	NaN

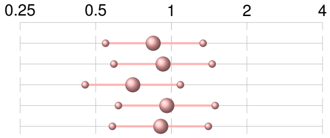

A	B	2.5	50	97.5	Sequence
1	0	0.55	0.85	1.3	SAEFTHENPLETR
0	1	0.59	0.93	1.4	DMTPAQIDEILR
0	1	0.45	0.7	1.1	MTVAHMWFDGR
0	1	0.61	0.96	1.5	VIMVTGDHPITAK
0	1	0.58	0.91	1.4	VIVAHGFK

5.214 636 1232 sigma class glutathione-s-transferase 3 [Haliotis discus discus]; 963 1559 sigma class glutathione-s-transferase 3 [Haliotis discus discus]; 907 1503 sigma class glutathione-s-transferase 3 [Haliotis discus discus]; 673 1269 sigma class glutathione-s-transferase 3 [Haliotis discus discus]; 177 773 sigma class glutathione-s-transferase 3 [Haliotis discus discus]; 208 804 sigma class glutathione-s-transferase 3 [Haliotis discus discus]; 327 923 sigma class glutathione-s-transferase 3 [Haliotis discus discus]

Protein Accession	Unigene22829$_H$1 CL19285.Contig31$_H$1 CL19285.Contig30$_H$1 CL19285.Contig27$_H$1 CL192
Mean Expression Ratio	1.13
Median Expression Ratio	1.14
Credible Interval	(0.763, 1.69)
Associated Peptides	2
Associated Spectra	2
Coverage	NaN

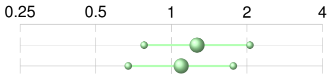

A	B	2.5	50	97.5	Sequence
1	0	0.78	1.3	2.1	LLLAASGKDWEDVR
0	1	0.68	1.1	1.8	YMNIFEK

5.215 900 1142 minus strand heat shock protein 20 [Cyclina sinensis]

Protein Accession	CL19759.Contig1$_H$1
Mean Expression Ratio	0.88
Median Expression Ratio	0.881
Credible Interval	(0.655, 1.18)
Associated Peptides	3
Associated Spectra	11
Coverage	NaN

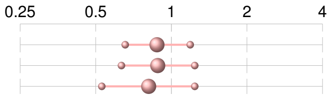

A	B	2.5	50	97.5	Sequence
4	2	0.65	0.88	1.2	QFKPEEITVK
3	1	0.63	0.88	1.2	QYILPEK
1	0	0.53	0.81	1.2	VEHPIVMDHQGNK

5.216 " 3341 4240 minus strand nonmuscle myosin II, partial [Aplysia californica]"

Protein Accession	CL19183.Contig2$_H$1
Mean Expression Ratio	1.14
Median Expression Ratio	1.13
Credible Interval	(0.879, 1.47)
Associated Peptides	7
Associated Spectra	11
Coverage	NaN

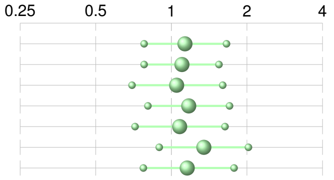

A	B	2.5	50	97.5	Sequence
1	1	0.78	1.1	1.7	RLETELQDLR
2	1	0.78	1.1	1.5	REEEVQIALQK
1	0	0.7	1.1	1.6	TATMLEEK
1	1	0.81	1.2	1.7	YAQMIEEK
1	0	0.72	1.1	1.6	QKQDVEQLEAR
1	0	0.89	1.4	2	QELEK
0	1	0.77	1.2	1.8	ELQDLR

5.217 76 822 14-3-3 protein [Heterodera glycines]; 76 822 minus strand 14-3-3 protein [Heterodera glycines]

Protein Accession	Unigene34590$_{H}1$ Unigene28010$_{H}1$
Mean Expression Ratio	0.882
Median Expression Ratio	0.882
Credible Interval	(0.635, 1.23)
Associated Peptides	2
Associated Spectra	8
Coverage	NaN

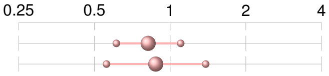

A	B	2.5	50	97.5	Sequence
4	3	0.61	0.82	1.1	VVSSIEQK
1	0	0.56	0.88	1.4	KLEMAK

5.218 49 819 PREDICTED: elongation factor 1-delta-like isoform 2 [Strongylocentrotus purpuratus]

Protein Accession	CL10790.Contig12$_{H}1$
Mean Expression Ratio	0.883
Median Expression Ratio	0.882
Credible Interval	(0.642, 1.2)
Associated Peptides	4
Associated Spectra	7
Coverage	NaN

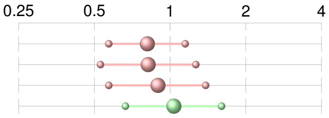

A	B	2.5	50	97.5	Sequence
1	3	0.57	0.81	1.2	LVDLAYGIK
0	1	0.53	0.82	1.3	LLEEYAAK
0	1	0.57	0.89	1.4	RLEEIEK
0	1	0.66	1	1.6	EISLAR

5.219 35 1861 hypothetical protein DAPPUDRAFT_309082 [Daphnia pulex]

Protein Accession	CL49314.Contig5$_{H}1$
Mean Expression Ratio	0.883
Median Expression Ratio	0.883
Credible Interval	(0.681, 1.15)
Associated Peptides	6
Associated Spectra	11
Coverage	NaN

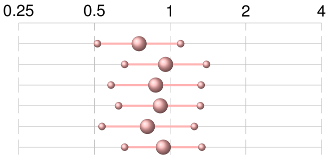

A	B	2.5	50	97.5	Sequence
2	0	0.51	0.75	1.1	ELDVALTAVGLK
1	1	0.66	0.96	1.4	LINHSVPDTIDER
1	0	0.58	0.88	1.3	WVNYHLAR
1	1	0.62	0.91	1.3	YAIGLAR
1	0	0.54	0.81	1.2	DIGHTFK
0	3	0.66	0.94	1.3	MADGILLCK

5.220 198 821 minus strand sigma class glutathione-s-transferase 2 [Haliotis discus discus]

Protein Accession	CL1352.Contig4$_{H}1$
Mean Expression Ratio	0.884
Median Expression Ratio	0.883
Credible Interval	(0.571, 1.36)
Associated Peptides	1
Associated Spectra	1
Coverage	NaN

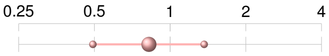

A	B	2.5	50	97.5	Sequence
0	1	0.49	0.82	1.4	AAGQPFDDNVVDR

5.221 " 154 591 microsomal GST1, partial [Reishia clavigera]; 154 531 microsomal GST1, partial [Reishia clavigera]"

Protein Accession CL3934.Contig2$_{H}1$ CL3934.Contig1$_{H}1$
Mean Expression Ratio 0.882
Median Expression Ratio 0.884
Credible Interval (0.571, 1.37)
Associated Peptides 1
Associated Spectra 1
Coverage NaN

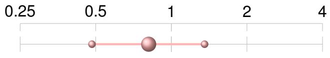

A	B	2.5	50	97.5	Sequence
1	0	0.48	0.81	1.4	FFCPGTAVNTNDALVER

5.222 3 1670 Spectrin alpha chain [Crassostrea gigas]

Protein Accession CL2195.Contig7$_{H}1$
Mean Expression Ratio 1.13
Median Expression Ratio 1.13
Credible Interval (0.842, 1.51)
Associated Peptides 6
Associated Spectra 7
Coverage NaN

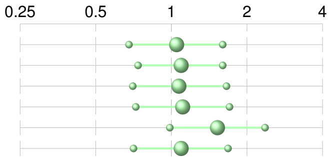

A	B	2.5	50	97.5	Sequence
1	0	0.68	1	1.6	NNLMDEFTISVR
1	1	0.74	1.1	1.6	VAALYDYTEK
1	0	0.7	1.1	1.7	HQALQAEIAGHEPR
1	0	0.72	1.1	1.7	KLDPSLSASR
1	0	0.99	1.5	2.4	ADLEAYRSV
0	1	0.71	1.1	1.7	LTAMGHTEAAEK

5.223 67 825 unnamed protein product [Oikopleura dioica]

Protein Accession Unigene71508$_{H}1$
Mean Expression Ratio 0.886
Median Expression Ratio 0.884
Credible Interval (0.59, 1.35)
Associated Peptides 1
Associated Spectra 2
Coverage NaN

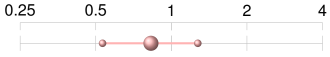

A	B	2.5	50	97.5	Sequence
2	0	0.53	0.83	1.3	TAANVEEAFINTAK

5.224 327 731 RecName: Full=Arginine kinase; Short=AK gi|439719|dbj|BAA05100.1| arginine kinase [Haliotis madaka]

Protein Accession Unigene63526$_{H}1$
Mean Expression Ratio 1.13
Median Expression Ratio 1.13
Credible Interval (0.863, 1.48)
Associated Peptides 2
Associated Spectra 32
Coverage NaN

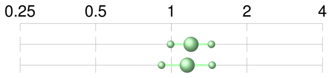

A	B	2.5	50	97.5	Sequence
10	10	1	1.2	1.4	DLYEQLK
7	5	0.92	1.2	1.5	FGGTLAECIR

5.225 593 862 minus strand mitochondrial ATP synthase coupling factor 6 precursor [Ochlerotatus taeniorhynchus]

Protein Accession	Unigene4216$_H$1
Mean Expression Ratio	0.886
Median Expression Ratio	0.885
Credible Interval	(0.604, 1.3)
Associated Peptides	1
Associated Spectra	3
Coverage	NaN

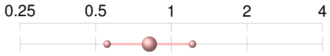

A	B	2.5	50	97.5	Sequence
0	3	0.55	0.82	1.2	LVDASPTVQAALKEELEK

5.226 " 4146 6107 minus strand nonmuscle myosin II, partial [Aplysia californica]"

Protein Accession	Unigene30363$_H$1
Mean Expression Ratio	0.885
Median Expression Ratio	0.886
Credible Interval	(0.745, 1.06)
Associated Peptides	20
Associated Spectra	31
Coverage	NaN

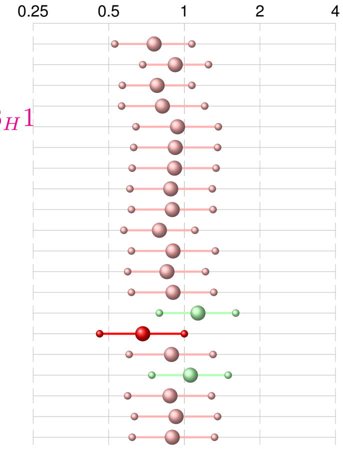

A	B	2.5	50	97.5	Sequence
2	0	0.53	0.76	1.1	ALEATVQEQR
2	2	0.68	0.92	1.3	ATIAALEGK
2	1	0.57	0.78	1.1	DLEQVSTQLEQVETK
1	0	0.56	0.82	1.2	ELDDQLEQNDVLVR
1	0	0.64	0.94	1.4	ELQIQAEDER
1	0	0.63	0.92	1.4	LAELEGQSR
1	0	0.62	0.91	1.3	LQEDLAAAER
1	0	0.61	0.89	1.3	QVDEAEEEVAR
1	0	0.62	0.9	1.3	VSSLEAQLADSQELMEEETR
2	1	0.57	0.8	1.1	VQIEELEDELQAAEDAK
1	0	0.62	0.9	1.3	EMEAELEEER
1	1	0.59	0.85	1.2	NLEAELVK
1	0	0.62	0.9	1.3	QSLLDEK
2	0	0.8	1.1	1.6	LKEDAIK
1	0	0.46	0.68	1	LSREEAYAQAK
1	0	0.6	0.89	1.3	LQAEVEDLTITLDNQR
1	1	0.74	1.1	1.5	AALARTNR
0	1	0.6	0.88	1.3	LKELQIQAEDERR
0	1	0.63	0.92	1.4	EMEQLHAQLEEARANADR
0	1	0.62	0.9	1.3	IEQLER

5.227 " 220 627 Fatty acid-binding protein, brain [Crassostrea gigas]"

Protein Accession	CL9041.Contig2$_H$1
Mean Expression Ratio	1.13
Median Expression Ratio	1.13
Credible Interval	(0.864, 1.46)
Associated Peptides	5
Associated Spectra	13
Coverage	NaN

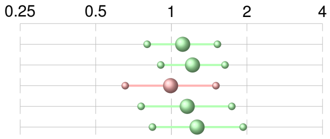

A	B	2.5	50	97.5	Sequence
3	1	0.8	1.1	1.5	EVDGGVMTSTTTFDGK
3	3	0.91	1.2	1.6	LTITVEGGK
1	0	0.66	0.99	1.5	VDLASTTGLEEFGTAIGFSEEK
1	0	0.76	1.2	1.7	IVENYKLEDK
0	1	0.84	1.3	1.9	QNYSFK

5.228 " 17 283 Glycerol-3-phosphate dehydrogenase [NAD+], cytoplasmic [Crassostrea gigas]"

Protein Accession	Unigene9883$_{H}$1
Mean Expression Ratio	1.13
Median Expression Ratio	1.13
Credible Interval	(0.763, 1.66)
Associated Peptides	2
Associated Spectra	2
Coverage	NaN

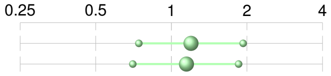

A	B	2.5	50	97.5	Sequence
0	1	0.74	1.2	1.9	KLTEIVNTEHENVK
0	1	0.7	1.1	1.8	LTEIVNTEHENVK

5.229 " 223 423 ADPATP carrier protein; K05863 solute carrier family 25 (mitochondrial adenine nucleotide translocator), member 4/5/6/31"

Protein Accession	CL27196.Contig3$_{H}$1
Mean Expression Ratio	1.12
Median Expression Ratio	1.13
Credible Interval	(0.785, 1.6)
Associated Peptides	2
Associated Spectra	4
Coverage	NaN

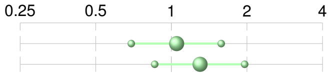

A	B	2.5	50	97.5	Sequence
2	0	0.69	1	1.6	LLVQNQNEMIK
0	2	0.86	1.3	2	GVIDCTIR

5.230 242 1834 Regulator of differentiation 1 [Crassostrea gigas]; 458 2050 minus strand Regulator of differentiation 1 [Crassostrea gigas]; 503 2095 Regulator of differentiation 1 [Crassostrea gigas]; 274 1866 Regulator of differentiation 1 [Crassostrea gigas]; 434 1942 Regulator of differentiation 1 [Crassostrea gigas]

Protein Accession	CL11169.Contig7$_{H}$1 CL11169.Contig5$_{H}$1 CL11169.Contig4$_{H}$1 CL11169.Contig3$_{H}$1 CL1116
Mean Expression Ratio	1.13
Median Expression Ratio	1.13
Credible Interval	(0.728, 1.74)
Associated Peptides	1
Associated Spectra	1
Coverage	NaN

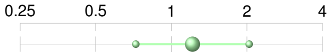

A	B	2.5	50	97.5	Sequence
0	1	0.72	1.2	2	IITFTK

5.231 " 193 333 minus strand RecName: Full=Globin; AltName: Full=Myoglobin gi|299274|gb|AAB25743.1| myoglobin [Nassa mutabilis=marine gastropods, radular muscles, Peptide, 147 aa] gi; 193 333 minus strand RecName: Full=Globin; AltName: Full=Myoglobin gi|299274|gb|AAB25743.1| myoglobin [Nassa mutabilis=marine gastropods, radular muscles, Peptide, 147 aa] gi"

Protein Accession	CL8458.Contig5$_{H}1$ CL8458.Contig1$_{H}1$
Mean Expression Ratio	0.889
Median Expression Ratio	0.889
Credible Interval	(0.585, 1.34)
Associated Peptides	1
Associated Spectra	2
Coverage	NaN

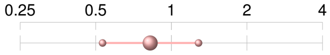

A	B	2.5	50	97.5	Sequence
**2**	**0**	**0.53**	**0.82**	**1.3**	**AAINSTWPK**

5.232 247 726 Annexin A7 [Crassostrea gigas]; 286 675 Annexin A7 [Crassostrea gigas]

Protein Accession	CL15539.Contig3$_{H}1$ CL15539.Contig1$_{H}1$
Mean Expression Ratio	0.889
Median Expression Ratio	0.889
Credible Interval	(0.654, 1.21)
Associated Peptides	4
Associated Spectra	7
Coverage	NaN

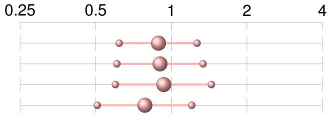

A	B	2.5	50	97.5	Sequence
**2**	**1**	**0.62**	**0.89**	**1.3**	**GLGTDEAAVIR**
**1**	**1**	**0.61**	**0.9**	**1.3**	**DLVSDLK**
**1**	**0**	**0.6**	**0.93**	**1.4**	**DIVSDTSGHFR**
**1**	**0**	**0.51**	**0.78**	**1.2**	**ILTSHNNMQR**

5.233 212 1405 minus strand unnamed protein product [Tetraodon nigroviridis]

Protein Accession	CL37546.Contig3$_{H}1$
Mean Expression Ratio	1.12
Median Expression Ratio	1.12
Credible Interval	(0.729, 1.75)
Associated Peptides	1
Associated Spectra	1
Coverage	NaN

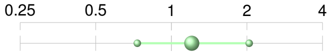

A	B	2.5	50	97.5	Sequence
**0**	**1**	**0.73**	**1.2**	**2**	**TIAELGEK**

5.234 185 742 Transmembrane protein Tmp21 [Crassostrea gigas]; 1 444 minus strand Transmembrane protein Tmp21 [Crassostrea gigas]

Protein Accession	CL3732.Contig3$_{H}1$ CL3732.Contig1$_{H}1$
Mean Expression Ratio	1.12
Median Expression Ratio	1.12
Credible Interval	(0.756, 1.68)
Associated Peptides	2
Associated Spectra	2
Coverage	NaN

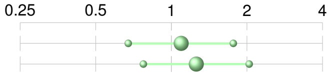

A	B	2.5	50	97.5	Sequence
1	0	0.67	1.1	1.8	LKPLEVELR
1	0	0.77	1.3	2	EVHLNLK

5.235 85 546 minus strand RecName: Full=Eukaryotic translation initiation factor 5A; Short=eIF-5A gi|51702279|sp|P62925.1|IF5A_SPOFR RecName: Full=Eukaryotic translation initiation factor 5A; Short=eIF-5A gi|6469515|gb|AAF13315.1|AF109730_1 translation initiation factor 5A [Spodoptera exigua] gi

Protein Accession	CL40725.Contig2$_{H}1$
Mean Expression Ratio	0.892
Median Expression Ratio	0.89
Credible Interval	(0.674, 1.19)
Associated Peptides	4
Associated Spectra	10
Coverage	NaN

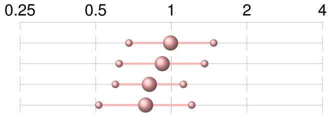

A	B	2.5	50	97.5	Sequence
1	1	0.68	1	1.5	AMGEECVVAVK
1	1	0.62	0.92	1.4	EDSLAAEIQK
2	3	0.6	0.82	1.1	IVEMSTSK
0	1	0.52	0.79	1.2	KNEFVVIK

5.236 31 735 hypothetical protein BRAFLDRAFT_78603 [Branchiostoma floridae] gi; 55 735 hypothetical protein BRAFLDRAFT_78603 [Branchiostoma floridae] gi; 353 1039 hypothetical protein BRAFLDRAFT_78603 [Branchiostoma floridae] gi; 207 893 hypothetical protein BRAFLDRAFT_78603 [Branchiostoma floridae] gi

Protein Accession	CL877.Contig7$_{H}1$ CL877.Contig6$_{H}1$ CL877.Contig4$_{H}1$ CL877.Contig3$_{H}1$
Mean Expression Ratio	1.12
Median Expression Ratio	1.12
Credible Interval	(0.847, 1.52)
Associated Peptides	4
Associated Spectra	10
Coverage	NaN

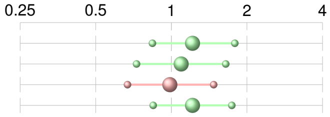

A	B	2.5	50	97.5	Sequence
2	1	0.84	1.2	1.8	DDQTLVTMTGEVAGR
1	1	0.72	1.1	1.6	LKLPLDSSNR
2	0	0.67	0.99	1.5	KVIHSIVGPHFAEGR
3	0	0.85	1.2	1.7	VALVPK

5.237 2 2248 PREDICTED: heat shock 70 kDa protein 4-like [Oreochromis niloticus]; 2 2227 PREDICTED: heat shock 70 kDa protein 4-like [Oreochromis niloticus]

Protein Accession	CL12749.Contig5$_{H}1$ CL12749.Contig4$_{H}1$
Mean Expression Ratio	1.12
Median Expression Ratio	1.12
Credible Interval	(0.778, 1.61)
Associated Peptides	3
Associated Spectra	3
Coverage	NaN

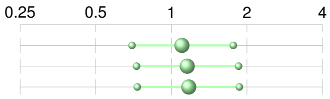

A	B	2.5	50	97.5	Sequence
**1**	**0**	**0.7**	**1.1**	**1.8**	**EMLNLLIEK**
**1**	**0**	**0.73**	**1.2**	**1.8**	**TAWWTPK**
**0**	**1**	**0.73**	**1.2**	**1.9**	**HENPVIFVSQVMSEK**

5.238 2 211 minus strand sulfatase 1A precursor [Haliotis discus discus]

Protein Accession	Unigene22814$_{H}1$
Mean Expression Ratio	1.12
Median Expression Ratio	1.12
Credible Interval	(0.749, 1.69)
Associated Peptides	1
Associated Spectra	2
Coverage	NaN

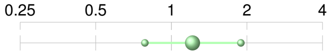

A	B	2.5	50	97.5	Sequence
**2**	**0**	**0.78**	**1.2**	**1.9**	**CGMVSAMDESIGK**

5.239 "REVERSED 2 1810 minus strand Glutaminyl-tRNA synthetase, partial [Crassostrea gigas]"

Protein Accession	RRRRRCL16949.Contig1$_{H}1$
Mean Expression Ratio	1.12
Median Expression Ratio	1.12
Credible Interval	(0.753, 1.69)
Associated Peptides	1
Associated Spectra	2
Coverage	NaN

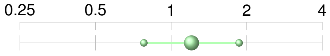

A	B	2.5	50	97.5	Sequence
**2**	**0**	**0.78**	**1.2**	**1.9**	**GEELTVK**

5.240 31 735 hypothetical protein BRAFLDRAFT_78603 [Branchiostoma floridae] gi; 55 735 hypothetical protein BRAFLDRAFT_78603 [Branchiostoma floridae] gi; 319 948 hypothetical protein BRAFLDRAFT_78603 [Branchiostoma floridae] gi; 353 1039 hypothetical protein BRAFLDRAFT_78603 [Branchiostoma floridae] gi; 207 893 hypothetical protein BRAFLDRAFT_78603 [Branchiostoma floridae] gi

Protein Accession	CL877.Contig7$_{H}$1 CL877.Contig6$_{H}$1 CL877.Contig5$_{H}$1 CL877.Contig4$_{H}$1 CL877.Contig3$_{H}$1
Mean Expression Ratio	0.891
Median Expression Ratio	0.892
Credible Interval	(0.603, 1.32)
Associated Peptides	2
Associated Spectra	5
Coverage	NaN

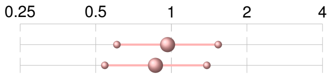

A	B	2.5	50	97.5	Sequence
2	1	0.61	0.97	1.5	DDQTLVTMTGEVAGR
1	1	0.54	0.87	1.4	LKLPLDSSNR

5.241 913 1560 minus strand Calcium-dependent protein kinase isoform 2 [Crassostrea gigas]

Protein Accession	CL5866.Contig1$_{H}$1
Mean Expression Ratio	1.12
Median Expression Ratio	1.12
Credible Interval	(0.811, 1.55)
Associated Peptides	4
Associated Spectra	5
Coverage	NaN

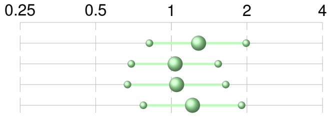

A	B	2.5	50	97.5	Sequence
0	1	0.82	1.3	2	DEFSVAVDK
0	2	0.69	1	1.5	FSFDEADKDK
0	1	0.67	1	1.6	IHYDEFVK
0	1	0.77	1.2	1.9	LLDINDDQK

5.242 10 300 minus strand unnamed protein product [Tetraodon nigroviridis]

Protein Accession	CL14559.Contig2$_{H}$1
Mean Expression Ratio	0.891
Median Expression Ratio	0.892
Credible Interval	(0.613, 1.29)
Associated Peptides	1
Associated Spectra	4
Coverage	NaN

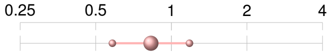

A	B	2.5	50	97.5	Sequence
3	1	0.58	0.83	1.2	ILVQEGIHDR

5.243 99 3011 Eukaryotic translation initiation factor 3 subunit A [Crassostrea gigas]

Protein Accession	CL36771.Contig2$_{H}$1
Mean Expression Ratio	1.12
Median Expression Ratio	1.12
Credible Interval	(0.799, 1.56)
Associated Peptides	2
Associated Spectra	8
Coverage	NaN

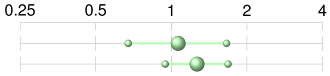

A	B	2.5	50	97.5	Sequence
**1**	**0**	**0.68**	**1.1**	**1.7**	**NNSRVER**
**7**	**0**	**0.95**	**1.3**	**1.7**	**LFNLSREQRK**

5.244 628 1140 paramyosin [Haliotis discus discus]

Protein Accession	CL43455.Contig3$_{H}$1
Mean Expression Ratio	1.12
Median Expression Ratio	1.12
Credible Interval	(0.826, 1.51)
Associated Peptides	2
Associated Spectra	44
Coverage	NaN

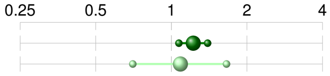

A	B	2.5	50	97.5	Sequence
**23**	**20**	**1.1**	**1.2**	**1.4**	**LEEAEAFAVK**
**0**	**1**	**0.7**	**1.1**	**1.7**	**LEEAEAF**

5.245 387 1064 minus strand PREDICTED: hypothetical protein [Hydra magnipapillata]; 275 952 minus strand PREDICTED: hypothetical protein [Hydra magnipapillata]; 172 849 minus strand PREDICTED: hypothetical protein [Hydra magnipapillata]

Protein Accession	CL20613.Contig3$_{H}$1 CL20613.Contig2$_{H}$1 CL20613.Contig1$_{H}$1
Mean Expression Ratio	1.12
Median Expression Ratio	1.12
Credible Interval	(0.814, 1.54)
Associated Peptides	3
Associated Spectra	6
Coverage	NaN

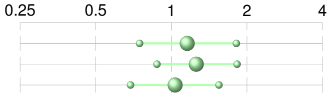

A	B	2.5	50	97.5	Sequence
**1**	**0**	**0.75**	**1.2**	**1.8**	**ELGATFEAADNFKPNVVVSGR**
**3**	**0**	**0.87**	**1.3**	**1.8**	**NAGYTVHFVSPK**
**1**	**1**	**0.69**	**1**	**1.5**	**GFLADQEVQK**

5.246 349 885 juvenile hormone diol kinase [Danaus plexippus]; 349 885 juvenile hormone diol kinase [Danaus plexippus]; 349 885 juvenile hormone diol kinase [Danaus plexippus]; 349 885 minus strand juvenile hormone diol kinase [Danaus plexippus]; 349 885 juvenile hormone diol kinase [Danaus plexippus]; 349 885 juvenile hormone diol kinase [Danaus plexippus]; 349 885 juvenile hormone diol kinase [Danaus plexippus]; 349 885 juvenile hormone diol kinase [Danaus plexippus]; 349 885 juvenile hormone diol kinase [Danaus plexippus]; 349 885 juvenile hormone diol kinase [Danaus plexippus]

Protein Accession Unigene38920$_H1$ Unigene38919$_H1$ CL7590.Contig1$_H1$ CL7590.Contig16$_H1$ CL7590.Contig

Mean Expression Ratio 1.12

Median Expression Ratio 1.12

Credible Interval (0.95, 1.31)

Associated Peptides 12

Associated Spectra 63

Coverage NaN

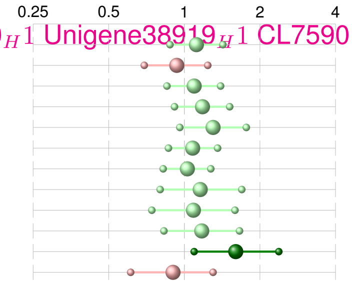

A	B	2.5	50	97.5	Sequence
5	4	0.88	1.1	1.4	DGIIDKNDFK
4	1	0.69	0.93	1.2	DILENVWEGLQK
6	2	0.85	1.1	1.4	FMFLANDTSGDGFVDR
4	4	0.92	1.2	1.5	IFGNSEADSNK
4	0	0.96	1.3	1.8	SVYQNLYDSNK
9	2	0.86	1.1	1.4	VPQWNEEYIK
9	3	0.82	1	1.3	GEFVTVQK
1	0	0.8	1.2	1.7	FSESGKVPQWNEEYIK
1	0	0.74	1.1	1.6	YIKFMFLANDTSGDGFVDR
0	2	0.83	1.2	1.7	DADINEDGIVTLDEWVK
0	1	1.1	1.6	2.4	DGIVTLDEWVK
0	1	0.61	0.9	1.3	AFDKLAQGAK

5.247 " 142 1284 minus strand Chain O, Crsytal Structure Of Human Liver Gapdh gi|67464044|pdb|1ZNQ|P Chain P, Crsytal Structure Of Human Liver Gapdh gi|67464045|pdb|1ZNQ|R Chain R, Crsytal Structure Of Human Liver Gapdh gi|67464046|pdb|1ZNQ|Q Chain Q, Crsytal Structure Of Human Liver Gapdh"

Protein Accession CL34318.Contig1$_H1$

Mean Expression Ratio 1.12

Median Expression Ratio 1.12

Credible Interval (0.926, 1.35)

Associated Peptides 7

Associated Spectra 51

Coverage NaN

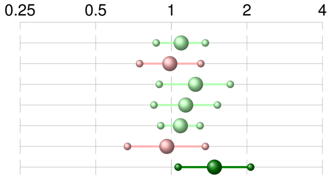

A	B	2.5	50	97.5	Sequence
5	6	0.87	1.1	1.4	DPTAIPWR
4	2	0.75	0.99	1.3	INVYQEKDPTAIPWR
2	1	0.9	1.3	1.7	VIPELNGK
4	1	0.85	1.1	1.5	INVYQEK
0	21	0.91	1.1	1.3	IGINGFGR
0	2	0.67	0.96	1.4	VIPELNGKLTGMAFR
0	3	1.1	1.5	2.1	CLAPLAK

5.248 213 881 PREDICTED: hypothetical protein [Saccoglossus kowalevskii]; 260 928 PREDICTED: hypothetical protein [Saccoglossus kowalevskii]; 294 962 PREDICTED: hypothetical protein [Saccoglossus kowalevskii]; 287 955 PREDICTED: hypothetical protein [Saccoglossus kowalevskii]; 316 984 PREDICTED: hypothetical protein [Saccoglossus kowalevskii]

Protein Accession	CL23253.Contig8$_H$1 CL23253.Contig7$_H$1 CL23253.Contig3$_H$1 CL23253.Contig2$_H$1 CL2325
Mean Expression Ratio	0.892
Median Expression Ratio	0.895
Credible Interval	(0.576, 1.38)
Associated Peptides	1
Associated Spectra	1
Coverage	NaN

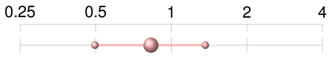

A	B	2.5	50	97.5	Sequence
0	1	0.5	0.83	1.4	VDLPGEFR

5.249 87 1454 hypothetical protein BRAFLDRAFT_287788 [Branchiostoma floridae] gi; 117 1469 hypothetical protein BRAFLDRAFT_287788 [Branchiostoma floridae] gi; 117 1469 hypothetical protein BRAFLDRAFT_287788 [Branchiostoma floridae] gi

Protein Accession	Unigene48935$_H$1 Unigene21344$_H$1 CL945.Contig6$_H$1
Mean Expression Ratio	0.895
Median Expression Ratio	0.896
Credible Interval	(0.695, 1.14)
Associated Peptides	6
Associated Spectra	17
Coverage	NaN

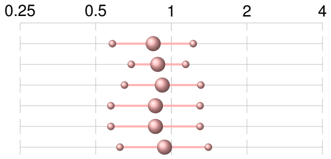

A	B	2.5	50	97.5	Sequence
1	1	0.58	0.85	1.2	AATLEQTILNVPETR
8	1	0.69	0.88	1.1	MVLAAAGGVNHGELVDLAK
2	1	0.65	0.92	1.3	RIPLPELEAR
1	0	0.57	0.86	1.3	QMLCYGR
1	0	0.57	0.87	1.3	PETRISSLDNGLR
0	1	0.62	0.94	1.4	ISSLDNGLR

5.250 229 699 hypothetical protein CGI_10017744 [Crassostrea gigas]; 199 669 hypothetical protein CGI_10017744 [Crassostrea gigas]; 202 672 hypothetical protein CGI_10017744 [Crassostrea gigas]

Protein Accession	CL5082.Contig3$_H$1 CL5082.Contig2$_H$1 CL5082.Contig1$_H$1
Mean Expression Ratio	1.12
Median Expression Ratio	1.12
Credible Interval	(0.838, 1.5)
Associated Peptides	3
Associated Spectra	9
Coverage	NaN

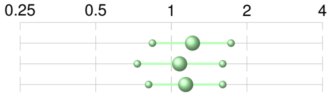

A	B	2.5	50	97.5	Sequence
2	1	0.84	1.2	1.7	EVLELFDR
2	0	0.73	1.1	1.6	EGLHSLK
3	1	0.81	1.1	1.6	HLNKPDAIK

5.251 253 765 cytochrome c oxidase subunit IV [Urechis caupo]

Protein Accession	CL38636.Contig3$_{H}1$
Mean Expression Ratio	0.894
Median Expression Ratio	0.896
Credible Interval	(0.614, 1.3)
Associated Peptides	2
Associated Spectra	3
Coverage	NaN

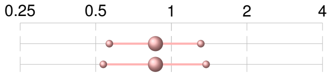

A	B	2.5	50	97.5	Sequence
2	0	0.57	0.87	1.3	FKENTPDVMALR
1	0	0.54	0.86	1.4	SLTLEDKK

5.252 365 2530 minus strand calpain B [Gecarcinus lateralis]

Protein Accession	CL34074.Contig5$_{H}1$
Mean Expression Ratio	1.12
Median Expression Ratio	1.12
Credible Interval	(0.873, 1.42)
Associated Peptides	7
Associated Spectra	14
Coverage	NaN

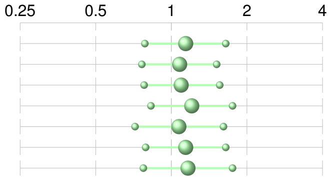

A	B	2.5	50	97.5	Sequence
1	1	0.78	1.1	1.6	LGYDEFK
2	1	0.76	1.1	1.5	SLQSLVMR
2	1	0.78	1.1	1.6	SMVAMHDGDLSGK
1	1	0.83	1.2	1.8	TLWEDLR
1	0	0.72	1.1	1.6	MQGQIPMVR
1	1	0.79	1.1	1.6	TMLTSFR
0	1	0.78	1.2	1.7	TLWEDLRR

5.253 5 841 minus strand Spectrin alpha chain [Crassostrea gigas]

Protein Accession	CL44897.Contig1$_{H}1$
Mean Expression Ratio	0.897
Median Expression Ratio	0.896
Credible Interval	(0.618, 1.31)
Associated Peptides	2
Associated Spectra	3
Coverage	NaN

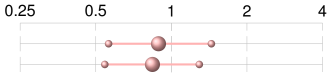

A	B	2.5	50	97.5	Sequence
1	0	0.56	0.89	1.4	DMEDEEDWIR
1	1	0.54	0.84	1.3	HQNFEAEIEANR

5.254 152 1930 hypothetical protein BRAFLDRAFT_223365 [Branchiostoma floridae] gi

Protein Accession	CL49005.Contig4$_{H}1$
Mean Expression Ratio	0.894
Median Expression Ratio	0.897
Credible Interval	(0.58, 1.37)
Associated Peptides	1
Associated Spectra	1
Coverage	NaN

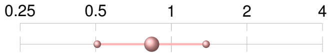

A	B	2.5	50	97.5	Sequence
1	0	0.51	0.83	1.4	AQGYQLIR

5.255 " 3 587 elongation factor 1 alpha, partial [Phorbas sp. nov. SB-2012]"

Protein Accession	Unigene20048$_{H}1$
Mean Expression Ratio	0.897
Median Expression Ratio	0.897
Credible Interval	(0.658, 1.23)
Associated Peptides	2
Associated Spectra	12
Coverage	NaN

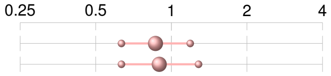

A	B	2.5	50	97.5	Sequence
5	1	0.63	0.86	1.2	IGGIGTVPVGR
3	3	0.63	0.89	1.3	LPLQDVYK

5.256 121 660 Failed axon connections homolog OS=Rattus norvegicus GN=Faxc PE=3 SV=1

Protein Accession	Unigene63215$_{H}1$
Mean Expression Ratio	1.11
Median Expression Ratio	1.11
Credible Interval	(0.716, 1.72)
Associated Peptides	1
Associated Spectra	1
Coverage	NaN

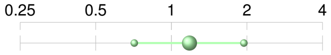

A	B	2.5	50	97.5	Sequence
0	1	0.71	1.2	2	LLVLR

5.257 75 803 hypothetical protein DAPPUDRAFT_231350 [Daphnia pulex]

Protein Accession	CL10537.Contig4$_{H}1$
Mean Expression Ratio	0.898
Median Expression Ratio	0.897
Credible Interval	(0.661, 1.23)
Associated Peptides	4
Associated Spectra	7
Coverage	NaN

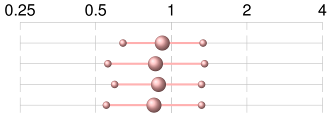

A	B	2.5	50	97.5	Sequence
2	1	0.64	0.92	1.3	LDYQLEIQSAK
1	0	0.56	0.87	1.4	SITPQQEAESIK
1	1	0.59	0.89	1.3	VCIAQLK
1	0	0.55	0.85	1.3	ENVALQLEASYR

5.258 118 501 minus strand histone 2A.Z [Aplysia californica]

Protein Accession	CL14228.Contig6$_{H}1$
Mean Expression Ratio	0.898
Median Expression Ratio	0.898
Credible Interval	(0.674, 1.18)
Associated Peptides	5
Associated Spectra	23
Coverage	NaN

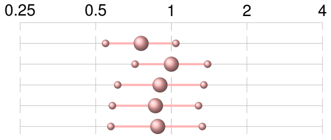

A	B	2.5	50	97.5	Sequence
9	3	0.55	0.76	1	AGLQFPVGR
3	3	0.72	1	1.4	ATIAGGGVIPHIHK
1	1	0.61	0.9	1.3	GDEELDSLIK
1	1	0.58	0.87	1.3	HLQLAIR
0	1	0.57	0.88	1.3	GGGVIPHIHK

5.259 8 1636 minus strand V-type proton ATPase catalytic subunit A [Crassostrea gigas]; 8 1636 minus strand V-type proton ATPase catalytic subunit A [Crassostrea gigas]; 8 1636 minus strand V-type proton ATPase catalytic subunit A [Crassostrea gigas]

Protein Accession	CL33671.Contig3$_H1$ CL33671.Contig2$_H1$ CL33671.Contig1$_H1$
Mean Expression Ratio	0.898
Median Expression Ratio	0.898
Credible Interval	(0.6, 1.36)
Associated Peptides	1
Associated Spectra	2
Coverage	NaN

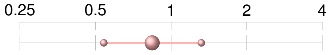

A	B	2.5	50	97.5	Sequence
**2**	**0**	**0.54**	**0.84**	**1.3**	**LAANHPLLTGQR**

5.260 1155 1649 78 kDa glucose-regulated protein OS=Aplysia californica PE=1 SV=1

Protein Accession	CL41594.Contig1$_H1$
Mean Expression Ratio	0.895
Median Expression Ratio	0.898
Credible Interval	(0.579, 1.38)
Associated Peptides	1
Associated Spectra	1
Coverage	NaN

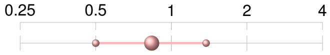

A	B	2.5	50	97.5	Sequence
**1**	**0**	**0.5**	**0.83**	**1.4**	**VEVESLFDGEDFSEQLTR**

5.261 4 399 Spectrin beta chain [Crassostrea gigas]

Protein Accession	Unigene17190$_H1$
Mean Expression Ratio	0.898
Median Expression Ratio	0.898
Credible Interval	(0.626, 1.29)
Associated Peptides	1
Associated Spectra	5
Coverage	NaN

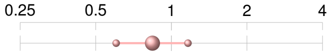

A	B	2.5	50	97.5	Sequence
**2**	**3**	**0.6**	**0.84**	**1.2**	**VHLENMGAHDIVDGSAR**

5.262 " 134 2047 PREDICTED: succinate dehydrogenase [ubiquinone] flavoprotein subunit, mitochondrial-like isoform 3, partial [Strongylocentrotus purpuratus]; 134 2047 PREDICTED: succinate dehydrogenase [ubiquinone] flavoprotein subunit, mitochondrial-like isoform 3, partial [Strongylocentrotus purpuratus]; 134 2047 PREDICTED: succinate dehydrogenase [ubiquinone] flavoprotein subunit, mitochondrial-like isoform 3, partial [Strongylocentrotus purpuratus]"

Protein Accession	CL47322.Contig4$_H$1 CL47322.Contig3$_H$1 CL47322.Contig2$_H$1
Mean Expression Ratio	0.899
Median Expression Ratio	0.898
Credible Interval	(0.756, 1.07)
Associated Peptides	15
Associated Spectra	42
Coverage	NaN

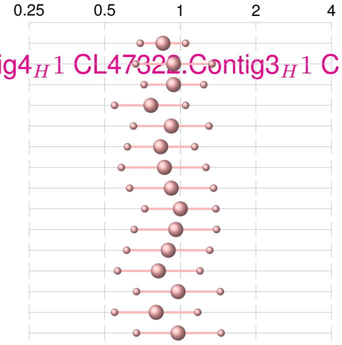

A	B	2.5	50	97.5	Sequence
10	4	0.69	0.85	1	AAFGLAEEGFK
1	1	0.66	0.94	1.3	ACANTIIEENKPGDR
3	3	0.71	0.94	1.2	AITIEIR
1	2	0.55	0.76	1.1	IYQQMEDLK
2	0	0.65	0.92	1.3	KHTLAYTNVDTGK
4	0	0.62	0.83	1.1	NTILATGGYGR
1	0	0.58	0.87	1.3	SHTVAAQGGINAALGHMEEDDWR
1	0	0.63	0.92	1.4	AVIELENYGMPF
3	0	0.72	1	1.4	AFGGQSLK
1	0	0.65	0.96	1.4	GGINAAL
0	1	0.61	0.89	1.3	AITIEIREGR
0	1	0.56	0.82	1.2	AVIELENYGMPFSR
0	1	0.67	0.98	1.4	LPPIR
0	1	0.55	0.8	1.2	QDAGEASVGNLDK
0	1	0.67	0.98	1.5	WHFYDTVK

5.263 113 445 ribosomal protein rps19 [Lineus viridis]

Protein Accession	CL8932.Contig6$_H$1
Mean Expression Ratio	1.11
Median Expression Ratio	1.11
Credible Interval	(0.723, 1.73)
Associated Peptides	1
Associated Spectra	1
Coverage	NaN

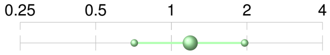

A	B	2.5	50	97.5	Sequence
0	1	0.71	1.2	2	LEEIR

5.264 9 161 PREDICTED: tubulin alpha-1C chain-like [Acyrthosiphon pisum]; 9 161 Tubulin alpha-3 chain (Fragments) OS=Mesocricetus auratus PE=1 SV=1

Protein Accession	Unigene74551$_H$1 Unigene70816$_H$1
Mean Expression Ratio	1.11
Median Expression Ratio	1.11
Credible Interval	(0.72, 1.73)
Associated Peptides	1
Associated Spectra	1
Coverage	NaN

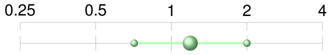

A	B	2.5	50	97.5	Sequence
0	1	0.71	1.2	2	IHFPLATY

5.265 244 5451 alpha macroglobulin [Chlamys farreri]; 244 5493 alpha macroglobulin [Chlamys farreri]

Protein Accession	CL20671.Contig5$_H$1 CL20671.Contig4$_H$1
Mean Expression Ratio	1.11
Median Expression Ratio	1.11
Credible Interval	(0.788, 1.58)
Associated Peptides	3
Associated Spectra	4
Coverage	NaN

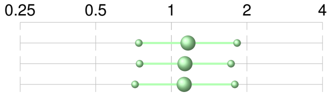

A	B	2.5	50	97.5	Sequence
1	0	0.74	1.2	1.8	DSFSLPLPLDMVPDSGR
1	1	0.75	1.1	1.7	HLALDVEQLNR
0	1	0.72	1.1	1.8	SLKIK

5.266 3788 7885 minus strand Filamin-C [Crassostrea gigas]

Protein Accession	CL4145.Contig29$_H$1
Mean Expression Ratio	0.899
Median Expression Ratio	0.9
Credible Interval	(0.597, 1.35)
Associated Peptides	1
Associated Spectra	2
Coverage	NaN

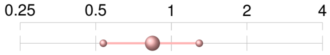

A	B	2.5	50	97.5	Sequence
2	0	0.53	0.84	1.3	WGEEDIPGSPFAVEVI

5.267 484 1104 Calponin-2 [Crassostrea gigas]; 309 938 Calponin-2 [Crassostrea gigas]

Protein Accession	CL30203.Contig3$_H$1 CL21394.Contig2$_H$1
Mean Expression Ratio	1.11
Median Expression Ratio	1.11
Credible Interval	(0.956, 1.29)
Associated Peptides	11
Associated Spectra	139
Coverage	NaN

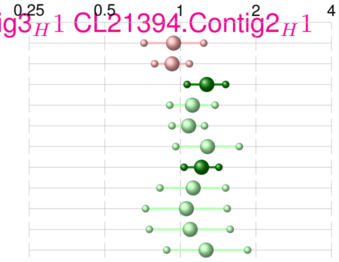

A	B	2.5	50	97.5	Sequence
5	1	0.72	0.94	1.2	AGQGIIGLQAGTNK
21	6	0.79	0.93	1.1	AVYDGTPNLPPAAK
10	11	1.1	1.3	1.5	MNTLAAPFK
11	3	0.91	1.1	1.4	QLTGEDIGSGAVQVEK
21	11	0.93	1.1	1.2	QMENIEVFLK
4	1	0.96	1.3	1.7	YDTGAEYEVR
16	11	1	1.2	1.4	NGQILCR
2	2	0.83	1.1	1.5	NLRNGQILCR
0	1	0.73	1.1	1.5	IIGLQAGTNK
0	1	0.75	1.1	1.6	PNLPPAAK
0	1	0.88	1.3	1.9	PTTLPR

5.268 977 1852 PREDICTED: similar to prohibitin [Tribolium castaneum] gi

Protein Accession	CL8219.Contig4$_H$1
Mean Expression Ratio	0.899
Median Expression Ratio	0.901
Credible Interval	(0.629, 1.27)
Associated Peptides	3
Associated Spectra	4
Coverage	NaN

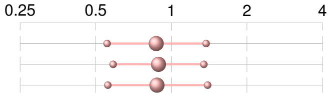

A	B	2.5	50	97.5	Sequence
1	0	0.55	0.87	1.4	VVQAEGEAAAAK
2	0	0.59	0.89	1.3	KLGDLAGR
1	0	0.56	0.87	1.4	QQVSLLIR

5.269 95 1579 minus strand hypothetical protein BRAFLDRAFT_113974 [Branchiostoma floridae] gi

Protein Accession	CL19753.Contig3$_H$1
Mean Expression Ratio	1.11
Median Expression Ratio	1.11
Credible Interval	(0.899, 1.37)
Associated Peptides	10
Associated Spectra	23
Coverage	NaN

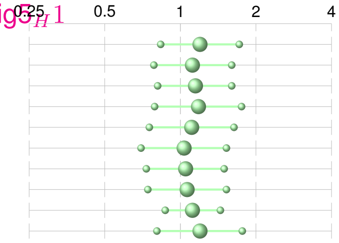

A	B	2.5	50	97.5	Sequence
1	1	0.83	1.2	1.7	ELGEYALK
1	1	0.78	1.1	1.6	FKDINEVIER
2	1	0.81	1.1	1.6	LIMQAAGR
1	0	0.79	1.2	1.7	TFPVVNPATEK
1	0	0.75	1.1	1.6	TSYGLAAGVITK
1	0	0.7	1	1.5	TVGDPFDAVTMQGPQVDQEQFEK
1	1	0.73	1	1.5	ADLRPDPNIK
1	1	0.74	1.1	1.5	DEIFGPVQSIIK
3	5	0.87	1.1	1.4	FADLVIR
0	1	0.8	1.2	1.8	VICEVQEADK

5.270 104 445 minus strand macrophage migration inhibitory factor [Ascaris suum]

Protein Accession	CL34536.Contig3$_H$1
Mean Expression Ratio	0.901
Median Expression Ratio	0.902
Credible Interval	(0.651, 1.23)
Associated Peptides	4
Associated Spectra	6
Coverage	NaN

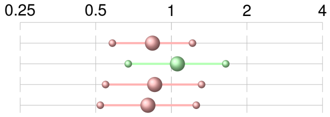

A	B	2.5	50	97.5	Sequence
2	1	0.58	0.84	1.2	HIEEALGIPQDR
1	0	0.67	1.1	1.6	PVFILNTNLPADK
1	0	0.55	0.86	1.3	QLGKPESYVCVR
0	1	0.52	0.81	1.3	LVVPFMK

5.271 195 2201 minus strand hypothetical protein DAPPUDRAFT_315656 [Daphnia pulex]

Protein Accession	Unigene38538$_H$1
Mean Expression Ratio	0.902
Median Expression Ratio	0.902
Credible Interval	(0.682, 1.19)
Associated Peptides	6
Associated Spectra	9
Coverage	NaN

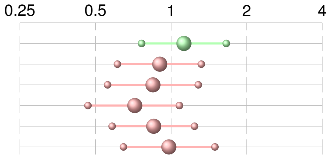

A	B	2.5	50	97.5	Sequence
1	1	0.77	1.1	1.7	FFTGQITAAGK
1	1	0.61	0.9	1.3	LLLSFGER
1	0	0.56	0.85	1.3	VALSPAATQALVK
1	0	0.47	0.72	1.1	VQIEAGAGEEAK
1	1	0.58	0.85	1.2	GPQFEEIGK
0	1	0.64	0.98	1.5	GSIVLHDGK

5.272 93 2606 twitchin [Mytilus galloprovincialis]

Protein Accession	Unigene71883$_H$1
Mean Expression Ratio	0.902
Median Expression Ratio	0.902
Credible Interval	(0.708, 1.16)
Associated Peptides	5
Associated Spectra	20
Coverage	NaN

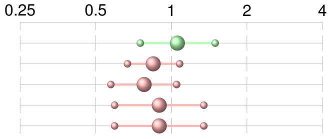

A	B	2.5	50	97.5	Sequence
2	1	0.75	1.1	1.5	FAFHNITGLSPNK
6	4	0.67	0.85	1.1	IIDFGLATR
2	3	0.58	0.78	1.1	FVPQPVEIK
1	0	0.59	0.89	1.4	VIAENFYGR
0	1	0.59	0.89	1.3	LNPDEIVK

5.273 118 513 Protein CAL-5 [Caenorhabditis elegans] gi

Protein Accession	CL18668.Contig1$_H$1
Mean Expression Ratio	0.905
Median Expression Ratio	0.902
Credible Interval	(0.589, 1.4)
Associated Peptides	1
Associated Spectra	2
Coverage	NaN

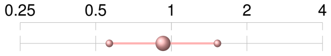

A	B	2.5	50	97.5	Sequence
1	1	0.57	0.93	1.5	EADANGDGYLSIQELK

5.274 274 666 Calmodulin-4 [Heterocephalus glaber]

Protein Accession	Unigene19050$_H$1
Mean Expression Ratio	1.11
Median Expression Ratio	1.11
Credible Interval	(0.861, 1.41)
Associated Peptides	4
Associated Spectra	18
Coverage	NaN

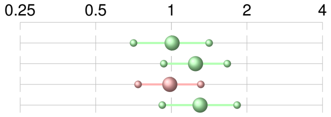

A	B	2.5	50	97.5	Sequence
2	1	0.71	1	1.4	GLDPENAVLDMDINK
3	3	0.94	1.2	1.7	SGFITLDELK
5	1	0.74	0.98	1.3	VLLSVGR
2	1	0.92	1.3	1.8	GTLSILELHK

5.275 247 1308 RecName: Full=Guanine nucleotide-binding protein G(o) subunit alpha gi|9633|emb|CAA78806.1| GTP-binding protein alpha-o subunit [Lymnaea stagnalis]

Protein Accession	CL26602.Contig4$_H$1
Mean Expression Ratio	0.905
Median Expression Ratio	0.904
Credible Interval	(0.709, 1.15)
Associated Peptides	6
Associated Spectra	18
Coverage	NaN

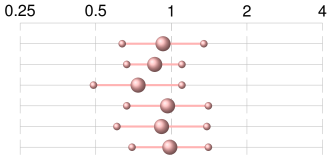

A	B	2.5	50	97.5	Sequence
1	1	0.64	0.92	1.3	IIHEGGFTSEDNK
4	5	0.67	0.86	1.1	LLLLGAGESGK
1	0	0.49	0.74	1.1	QTYEEAAAYIQAQFEAK
1	1	0.67	0.97	1.4	YFLDNLDR
1	0	0.61	0.92	1.4	KDLFEEK
0	3	0.7	0.99	1.4	MVLDVIAR

5.276 160 501 hypothetical protein CGI_10001640 [Crassostrea gigas]; 160 588 hypothetical protein CGI_10001640 [Crassostrea gigas]; 160 501 hypothetical protein CGI_10001640 [Crassostrea gigas]; 160 552 hypothetical protein CGI_10001640 [Crassostrea gigas]; 160 501 hypothetical protein CGI_10001640 [Crassostrea gigas]; 160 501 hypothetical protein CGI_10001640 [Crassostrea gigas]

Protein Accession	Unigene62839$_{H}1$ CL2347.Contig8$_{H}1$ CL2347.Contig15$_{H}1$ CL2347.Contig14$_{H}1$ CL2347.Co
Mean Expression Ratio	1.1
Median Expression Ratio	1.11
Credible Interval	(0.776, 1.57)
Associated Peptides	2
Associated Spectra	13
Coverage	NaN

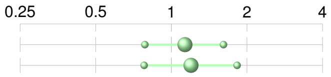

A	B	2.5	50	97.5	Sequence
**6**	**3**	**0.78**	**1.1**	**1.6**	**AFLEQLGSK**
**3**	**1**	**0.78**	**1.2**	**1.8**	**ANLIVIGSR**

5.277 1 219 minus strand PREDICTED: nucleoside diphosphate kinase 3-like [Pan troglodytes]

Protein Accession	Unigene5384$_{H}1$
Mean Expression Ratio	0.906
Median Expression Ratio	0.904
Credible Interval	(0.664, 1.25)
Associated Peptides	2
Associated Spectra	11
Coverage	NaN

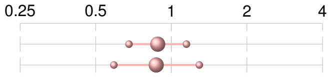

A	B	2.5	50	97.5	Sequence
**6**	**3**	**0.68**	**0.88**	**1.1**	**GLVGEIIK**
**0**	**2**	**0.59**	**0.87**	**1.3**	**GLVGEIIKR**

5.278 410 619 minus strand Guanine nucleotide-binding protein subunit gamma-1 [Crassostrea gigas]

Protein Accession	CL36920.Contig1$_{H}1$
Mean Expression Ratio	1.11
Median Expression Ratio	1.11
Credible Interval	(0.8, 1.55)
Associated Peptides	2
Associated Spectra	6
Coverage	NaN

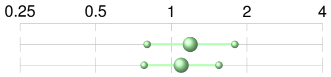

A	B	2.5	50	97.5	Sequence
**1**	**1**	**0.8**	**1.2**	**1.8**	**CEAQITR**
**2**	**2**	**0.78**	**1.1**	**1.5**	**LIEQLR**

5.279 98 850 minus strand 14-3-3 protein epsilon [Schistocerca gregaria]

Protein Accession	CL27652.Contig3$_H$1
Mean Expression Ratio	1.11
Median Expression Ratio	1.11
Credible Interval	(0.885, 1.38)
Associated Peptides	9
Associated Spectra	19
Coverage	NaN

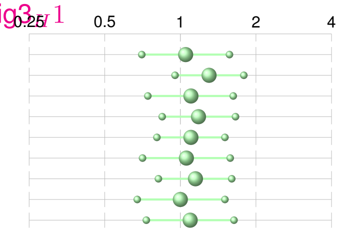

A	B	2.5	50	97.5	Sequence
1	0	0.7	1.1	1.6	AAFDDAIAELDTLSEESYK
3	1	0.95	1.3	1.8	AASDISMTELPPTHPIR
1	0	0.74	1.1	1.6	EAAENSLVAYK
1	2	0.85	1.2	1.7	IISSMEQK
3	1	0.81	1.1	1.5	YDEMVEAMK
1	0	0.71	1.1	1.6	VFYYK
3	0	0.82	1.1	1.6	HLIPNAGGGESK
0	1	0.68	1	1.5	YLAEFATGNDR
0	1	0.73	1.1	1.6	TELPPTHPIR

5.280 138 713 Rac [Aplysia californica] gi; 125 691 Rac [Aplysia californica] gi; 149 871 minus strand Rac [Aplysia californica] gi; 101 676 Rac [Aplysia californica] gi

Protein Accession	CL11009.Contig9$_H$1 CL11009.Contig8$_H$1 CL11009.Contig4$_H$1 CL11009.Contig1$_H$1
Mean Expression Ratio	1.11
Median Expression Ratio	1.11
Credible Interval	(0.747, 1.66)
Associated Peptides	2
Associated Spectra	2
Coverage	NaN

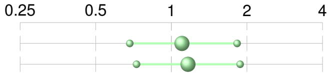

A	B	2.5	50	97.5	Sequence
0	1	0.68	1.1	1.8	NVFDEAIR
0	1	0.73	1.2	1.9	AVLCPR

5.281 " 199 417 GG13156 [Drosophila erecta] gi; 253 501 minus strand NADH-ubiquinone oxidoreductase MLRQ subunit-like protein, partial [Lymnaea stagnalis]; 294 515 GG13156 [Drosophila erecta] gi"

Protein Accession	CL49605.Contig4$_H$1 CL49605.Contig2$_H$1 CL49605.Contig1$_H$1
Mean Expression Ratio	1.11
Median Expression Ratio	1.11
Credible Interval	(0.719, 1.71)
Associated Peptides	1
Associated Spectra	1
Coverage	NaN

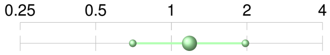

A	B	2.5	50	97.5	Sequence
1	0	0.7	1.2	2	GLTLSSLK

5.282 118 1866 Lamin Dm0 [Crassostrea gigas]

Protein Accession	CL4079.Contig7$_{H}$1
Mean Expression Ratio	1.1
Median Expression Ratio	1.1
Credible Interval	(0.918, 1.33)
Associated Peptides	16
Associated Spectra	26
Coverage	NaN

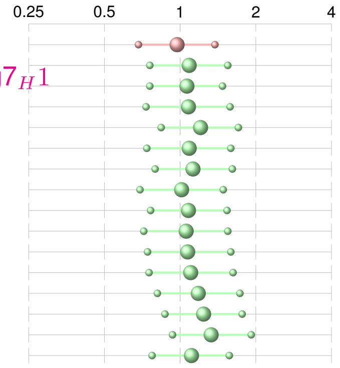

A	B	2.5	50	97.5	Sequence
1	1	0.68	0.97	1.4	ADDLSAELAK
1	1	0.76	1.1	1.5	GGIDIEEVDPEGK
2	1	0.76	1.1	1.5	KQLEEETLLR
1	0	0.73	1.1	1.6	LSVQVQSTEEVVK
1	1	0.84	1.2	1.7	WFVADEMK
1	0	0.74	1.1	1.6	SLVIKPGK
1	1	0.8	1.1	1.6	EDLHFK
1	0	0.69	1	1.5	YKTEYEDLLAK
1	1	0.76	1.1	1.5	EKHEFDLQTVR
1	0	0.72	1.1	1.5	LTIDEQMAEYSDLLDIK
0	1	0.74	1.1	1.6	LSEVEAPR
0	1	0.75	1.1	1.6	RLALKDDEIADLR
0	1	0.81	1.2	1.7	LLDETAKEK
0	2	0.87	1.2	1.8	IADLR
0	2	0.94	1.3	1.9	IRDLENQLAHER
0	2	0.77	1.1	1.6	LLEALR

5.283 271 480 hypothetical protein BRAFLDRAFT_75518 [Branchiostoma floridae] gi

Protein Accession	Unigene73452$_{H}$1
Mean Expression Ratio	1.11
Median Expression Ratio	1.1
Credible Interval	(0.72, 1.71)
Associated Peptides	1
Associated Spectra	1
Coverage	NaN

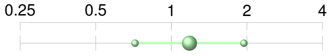

A	B	2.5	50	97.5	Sequence
1	0	0.71	1.2	2	GTHLDAPAHFAQGK

5.284 249 845 sigma class glutathione-s-transferase 2 [Haliotis discus discus]; 252 848 sigma class glutathione-s-transferase 2 [Haliotis discus discus]

Protein Accession	CL19285.Contig32$_{H}$1 CL19285.Contig20$_{H}$1
Mean Expression Ratio	0.904
Median Expression Ratio	0.906
Credible Interval	(0.587, 1.38)
Associated Peptides	1
Associated Spectra	1
Coverage	NaN

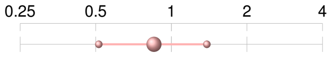

A	B	2.5	50	97.5	Sequence
1	0	0.51	0.85	1.4	APFGQAPFMEYEGK

5.285 260 1597 isocitrate dehydrogenase [Mytilus galloprovincialis]; 260 1597 isocitrate dehydrogenase [Mytilus galloprovincialis]; 260 1597 isocitrate dehydrogenase [Mytilus galloprovincialis]

Protein Accession	CL10046.Contig8$_{H}1$ CL10046.Contig7$_{H}1$ CL10046.Contig6$_{H}1$
Mean Expression Ratio	0.907
Median Expression Ratio	0.906
Credible Interval	(0.671, 1.23)
Associated Peptides	5
Associated Spectra	7
Coverage	NaN

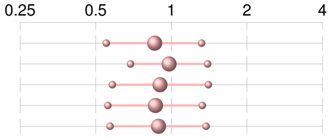

A	B	2.5	50	97.5	Sequence
1	0	0.55	0.86	1.3	DQTNDQVTIDSAEAIK
1	2	0.69	0.98	1.4	LTVFDFK
1	0	0.58	0.9	1.4	VKEFNLK
1	0	0.56	0.87	1.3	TSVLVCPDGK
0	1	0.57	0.89	1.4	SWPLYMSTK

5.286 5 514 hypothetical protein KGM_05418 [Danaus plexippus]

Protein Accession	CL11785.Contig1$_{H}1$
Mean Expression Ratio	0.904
Median Expression Ratio	0.906
Credible Interval	(0.635, 1.27)
Associated Peptides	3
Associated Spectra	4
Coverage	NaN

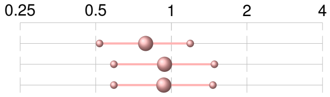

A	B	2.5	50	97.5	Sequence
1	1	0.52	0.79	1.2	ALLDLSENIDELNR
1	0	0.59	0.94	1.5	KLTNQSLK
1	0	0.59	0.93	1.5	NNLDIGK

5.287 813 1244 Probable protein disulfide-isomerase A6 OS=Caenorhabditis elegans GN=tag-320 PE=2 SV=1

Protein Accession	CL39540.Contig4$_{H}1$
Mean Expression Ratio	0.908
Median Expression Ratio	0.906
Credible Interval	(0.586, 1.4)
Associated Peptides	1
Associated Spectra	2
Coverage	NaN

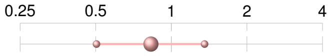

A	B	2.5	50	97.5	Sequence
1	1	0.5	0.83	1.4	TASDIINWATEK

5.288 108 1259 minus strand PREDICTED: Na(+)/H(+) exchange regulatory cofactor NHE-RF3-like [Metaseiulus occidentalis]; 108 1190 minus strand PREDICTED: Na(+)/H(+) exchange regulatory cofactor NHE-RF3-like [Metaseiulus occidentalis]

Protein Accession	CL26023.Contig7$_H1$ CL26023.Contig6$_H1$
Mean Expression Ratio	0.905
Median Expression Ratio	0.906
Credible Interval	(0.583, 1.4)
Associated Peptides	1
Associated Spectra	1
Coverage	NaN

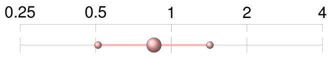

A	B	2.5	50	97.5	Sequence
0	1	0.51	0.85	1.4	GDLPEVLHLTAVR

5.289 " 1 1563 Putative methylmalonate-semialdehyde dehydrogenase [acylating], mitochondrial [Crassostrea gigas]"

Protein Accession	Unigene56839$_H1$
Mean Expression Ratio	0.906
Median Expression Ratio	0.906
Credible Interval	(0.685, 1.2)
Associated Peptides	7
Associated Spectra	8
Coverage	NaN

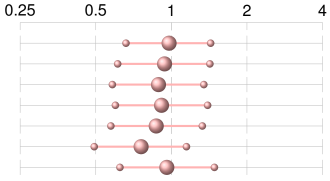

A	B	2.5	50	97.5	Sequence
1	1	0.66	0.98	1.4	AEMDAAVAAAK
1	0	0.61	0.94	1.4	GNFVGPTILHK
1	0	0.58	0.89	1.3	LFINNEFVESK
1	0	0.6	0.91	1.4	WIDVHNPATNEVVTR
1	0	0.57	0.87	1.3	TVTQMWR
0	1	0.49	0.76	1.1	TLVDAEGDVLR
0	1	0.63	0.96	1.5	PPGVVNVIHGR

5.290 503 1219 heat shock cognate 71 [Mytilus galloprovincialis]

Protein Accession	CL23109.Contig5$_H1$
Mean Expression Ratio	1.1
Median Expression Ratio	1.1
Credible Interval	(0.798, 1.54)
Associated Peptides	4
Associated Spectra	5
Coverage	NaN

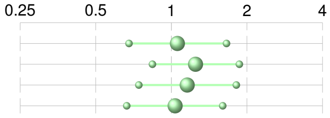

A	B	2.5	50	97.5	Sequence
1	0	0.68	1.1	1.7	FEELNADLFR
2	0	0.84	1.3	1.9	LLQDFFNGK
1	0	0.74	1.2	1.8	GTLEPVEK
0	1	0.66	1	1.6	SAIHEIVLVGGSTR

5.291 252 1289 minus strand PDZ and LIM domain protein 3 OS=Gallus gallus GN=PDLIM3 PE=1 SV=1

Protein Accession	CL15373.Contig10$_{H}1$
Mean Expression Ratio	1.1
Median Expression Ratio	1.1
Credible Interval	(0.808, 1.51)
Associated Peptides	5
Associated Spectra	6
Coverage	NaN

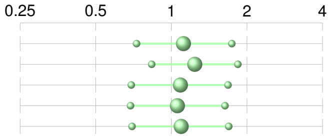

A	B	2.5	50	97.5	Sequence
1	0	0.73	1.1	1.7	SGDAILSINGVNTDHLTHEQAK
1	1	0.84	1.2	1.8	GFFVVEGK
1	0	0.69	1.1	1.7	IWKPQVTPLSE
1	0	0.69	1.1	1.6	CGGCDMLATGVIVK
0	1	0.7	1.1	1.7	AGCDIDFLIQR

5.292 126 704 minus strand ribosomal protein L12 [Ixodes scapularis]

Protein Accession	CL24053.Contig3$_{H}1$
Mean Expression Ratio	0.907
Median Expression Ratio	0.907
Credible Interval	(0.627, 1.31)
Associated Peptides	1
Associated Spectra	4
Coverage	NaN

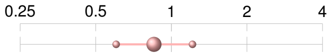

A	B	2.5	50	97.5	Sequence
2	2	0.6	0.85	1.2	IGPLGLSPK

5.293 7948 8541 Collagen alpha-3(VI) chain [Crassostrea gigas]

Protein Accession	CL47586.Contig9$_{H}1$
Mean Expression Ratio	1.1
Median Expression Ratio	1.1
Credible Interval	(0.773, 1.58)
Associated Peptides	2
Associated Spectra	4
Coverage	NaN

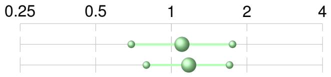

A	B	2.5	50	97.5	Sequence
1	0	0.69	1.1	1.7	ADIAFLLDSSGSVGYDNFR
3	0	0.8	1.2	1.7	DLQDAISAISYEYGNTNTAAGLK

5.294 174 1649 minus strand Collagen alpha-1(XII) chain [Crassostrea gigas]; 123 1490 minus strand Collagen alpha-1(XII) chain [Crassostrea gigas]; 135 1610 Collagen alpha-1(XII) chain [Crassostrea gigas]

Protein Accession	CL5340.Contig13$_{H}1$ CL5340.Contig12$_{H}1$ CL5340.Contig11$_{H}1$
Mean Expression Ratio	1.1
Median Expression Ratio	1.1
Credible Interval	(0.817, 1.48)
Associated Peptides	4
Associated Spectra	20
Coverage	NaN

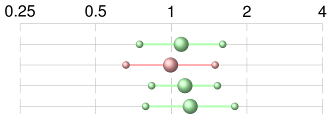

A	B	2.5	50	97.5	Sequence
4	1	0.75	1.1	1.6	ADVVFIVDSSR
1	1	0.66	0.99	1.5	DVMSVFHIAR
6	5	0.84	1.1	1.5	DILAFR
1	1	0.79	1.2	1.8	SIWMPDFK

5.295 96 536 minus strand hypothetical protein BRAFLDRAFT_285353 [Branchiostoma floridae] gi

Protein Accession	CL14282.Contig5$_H$1
Mean Expression Ratio	1.1
Median Expression Ratio	1.1
Credible Interval	(0.727, 1.71)
Associated Peptides	1
Associated Spectra	1
Coverage	NaN

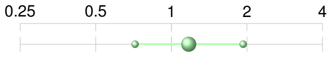

A	B	2.5	50	97.5	Sequence
**0**	**1**	**0.72**	**1.2**	**1.9**	**SQWSPALTISK**

5.296 " 1 1815 Glycogen phosphorylase, muscle form [Crassostrea gigas]"

Protein Accession	CL20630.Contig5$_H$1
Mean Expression Ratio	0.91
Median Expression Ratio	0.909
Credible Interval	(0.738, 1.12)
Associated Peptides	9
Associated Spectra	41
Coverage	NaN

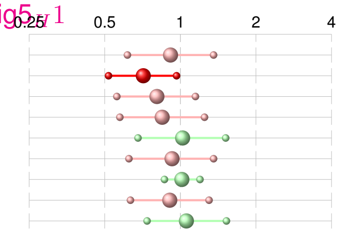

A	B	2.5	50	97.5	Sequence
**1**	**0**	**0.61**	**0.91**	**1.4**	**VVFLENYR**
**2**	**2**	**0.52**	**0.71**	**0.96**	**VVNNDPIIGDR**
**1**	**1**	**0.56**	**0.8**	**1.1**	**TSFDMFPEK**
**1**	**0**	**0.57**	**0.85**	**1.3**	**WPVSMLER**
**1**	**0**	**0.68**	**1**	**1.5**	**DFAQMFPER**
**1**	**0**	**0.62**	**0.92**	**1.4**	**FGNPWEK**
**17**	**10**	**0.87**	**1**	**1.2**	**IEELEEIEEDAGL**
**0**	**2**	**0.63**	**0.91**	**1.3**	**AWDLVVR**
**0**	**2**	**0.74**	**1.1**	**1.5**	**LLLCNSGLADVIAEK**

5.297 151 1110 minus strand hypothetical protein DAPPUDRAFT_303085 [Daphnia pulex]

Protein Accession	CL12652.Contig2$_H$1
Mean Expression Ratio	1.1
Median Expression Ratio	1.1
Credible Interval	(0.744, 1.62)
Associated Peptides	1
Associated Spectra	3
Coverage	NaN

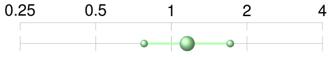

A	B	2.5	50	97.5	Sequence
**2**	**1**	**0.78**	**1.2**	**1.7**	**TVIITGANTGIGK**

5.298 36 770 minus strand Endoplasmic reticulum protein ERp29 [Crassostrea gigas]

Protein Accession	CL37188.Contig1$_H$1
Mean Expression Ratio	0.908
Median Expression Ratio	0.91
Credible Interval	(0.61, 1.35)
Associated Peptides	2
Associated Spectra	2
Coverage	NaN

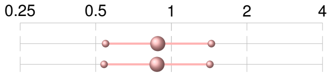

A	B	2.5	50	97.5	Sequence
**1**	**0**	**0.55**	**0.88**	**1.4**	**KAQTAAAALTK**
**1**	**0**	**0.54**	**0.88**	**1.4**	**KPDFPVYK**

5.299 290 2458 Putative beta-D-xylosidase 5 [Crassostrea gigas]

Protein Accession	CL49577.Contig2$_{H}1$
Mean Expression Ratio	1.1
Median Expression Ratio	1.1
Credible Interval	(0.766, 1.59)
Associated Peptides	3
Associated Spectra	4
Coverage	NaN

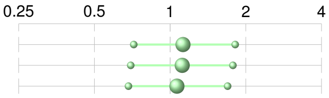

A	B	2.5	50	97.5	Sequence
0	1	0.72	1.1	1.8	QLLTDILR
1	1	0.7	1.1	1.8	VAEAAGVEVR
0	1	0.68	1.1	1.7	LEALEQGK

5.300 " 431 880 Myosin essential light chain, striated adductor muscle OS=Aequipecten irradians PE=1 SV=2"

Protein Accession	CL36.Contig2$_{H}1$
Mean Expression Ratio	0.909
Median Expression Ratio	0.911
Credible Interval	(0.73, 1.12)
Associated Peptides	3
Associated Spectra	179
Coverage	NaN

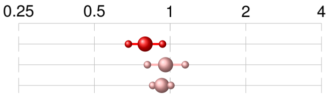

A	B	2.5	50	97.5	Sequence
12	17	0.68	0.8	0.93	LSDEEVDQIMK
15	9	0.81	0.96	1.1	LTDITIDLEGNLK
47	79	0.86	0.93	1	VGDLLR

5.301 7 1722 minus strand Membrane metallo-endopeptidase-like 1 OS=Mus musculus GN=Mmel1 PE=1 SV=1

Protein Accession	CL19557.Contig1$_{H}1$
Mean Expression Ratio	1.1
Median Expression Ratio	1.1
Credible Interval	(0.93, 1.29)
Associated Peptides	15
Associated Spectra	40
Coverage	NaN

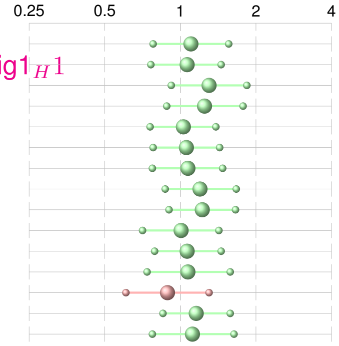

A	B	2.5	50	97.5	Sequence
1	1	0.78	1.1	1.6	AVLIEAIR
3	0	0.76	1.1	1.5	HQVENPNLHEVTSLADLEK
1	1	0.92	1.3	1.8	IGYPDYIMDDLR
1	1	0.89	1.3	1.8	LFVLNSFDQNQR
3	1	0.76	1	1.4	QDAQDVADFK
3	1	0.78	1.1	1.4	TLVENIADNAGLK
2	1	0.78	1.1	1.5	WPVSAAAVK
2	1	0.87	1.2	1.7	WLTDASK
4	0	0.9	1.2	1.7	LFPEVTGR
2	0	0.71	1	1.4	GLELFR
2	2	0.79	1.1	1.5	MNIDGYR
1	0	0.74	1.1	1.6	AQALNAK
1	0	0.61	0.89	1.3	QTALDK
1	3	0.85	1.2	1.6	LQLAEISLPK
0	1	0.77	1.1	1.6	LTDASKQTALDK

5.302 78 461 PREDICTED: hoi-polloi-like [Saccoglossus kowalevskii]; 103 492 NHP2-like protein 1 OS=Rattus norvegicus GN=Nhp2l1 PE=2 SV=4

Protein Accession	CL26515.Contig5$_{H}1$ CL26515.Contig4$_{H}1$
Mean Expression Ratio	0.91
Median Expression Ratio	0.912
Credible Interval	(0.585, 1.4)
Associated Peptides	1
Associated Spectra	1
Coverage	NaN

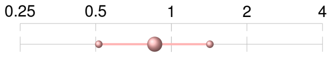

A	B	2.5	50	97.5	Sequence
1	0	0.51	0.86	1.4	NVPYVFVR

5.303 " 1 1887 Pyruvate carboxylase, mitochondrial [Crassostrea gigas]"

Protein Accession	CL26073.Contig5$_{H}1$
Mean Expression Ratio	0.912
Median Expression Ratio	0.913
Credible Interval	(0.649, 1.28)
Associated Peptides	4
Associated Spectra	4
Coverage	NaN

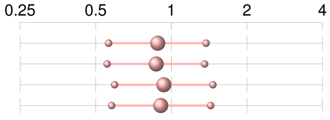

A	B	2.5	50	97.5	Sequence
0	1	0.56	0.88	1.4	AGTHILGIK
0	1	0.55	0.87	1.4	HLEDVEENFKR
0	1	0.59	0.93	1.5	FLHECPWDR
0	1	0.58	0.91	1.4	IEVFR

5.304 " 492 3473 PREDICTED: 2-oxoglutarate dehydrogenase, mitochondrial isoform 1 [Sarcophilus harrisii]; 492 3458 PREDICTED: 2-oxoglutarate dehydrogenase, mitochondrial isoform 1 [Sarcophilus harrisii]"

Protein Accession	CL2200.Contig1$_{H}1$ CL2200.Contig16$_{H}1$
Mean Expression Ratio	0.915
Median Expression Ratio	0.913
Credible Interval	(0.621, 1.37)
Associated Peptides	2
Associated Spectra	2
Coverage	NaN

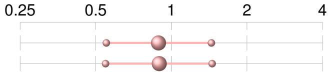

A	B	2.5	50	97.5	Sequence
1	0	0.55	0.89	1.4	FSEVPEGFVVHGGLK
1	0	0.55	0.9	1.5	KPLVVATPK

5.305 3 218 beta-tubulin [Fasciola hepatica] gi

Protein Accession	Unigene7955$_{H}1$
Mean Expression Ratio	0.915
Median Expression Ratio	0.914
Credible Interval	(0.686, 1.22)
Associated Peptides	3
Associated Spectra	12
Coverage	NaN

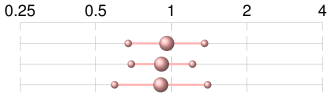

A	B	2.5	50	97.5	Sequence
3	1	0.67	0.96	1.4	EVDEQMLNVQNK
7	0	0.69	0.91	1.2	TAVCDIPPR
1	0	0.59	0.91	1.4	VSEQFTAMFR

5.306 160 393 minus strand PREDICTED: cysteine-rich protein 1-like [Megachile rotundata]; 148 381 PREDICTED: cysteine-rich protein 1-like [Megachile rotundata]; 148 381 PREDICTED: cysteine-rich protein 1-like [Megachile rotundata]; 148 381 PREDICTED: cysteine-rich protein 1-like [Megachile rotundata]; 160 393 minus strand PREDICTED: cysteine-rich protein 1-like [Megachile rotundata]; 160 393 minus strand PREDICTED: cysteine-rich protein 1-like [Megachile rotundata]

Protein Accession	CL11328.Contig9$_{H}1$ CL11328.Contig8$_{H}1$ CL11328.Contig7$_{H}1$ CL11328.Contig6$_{H}1$ CL1132
Mean Expression Ratio	1.09
Median Expression Ratio	1.09
Credible Interval	(0.77, 1.56)
Associated Peptides	2
Associated Spectra	4
Coverage	NaN

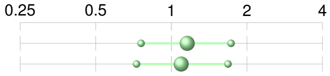

A	B	2.5	50	97.5	Sequence
**1**	**1**	**0.76**	**1.2**	**1.7**	**DWHAACLK**
**1**	**1**	**0.73**	**1.1**	**1.7**	**EVYFAER**

5.307 613 2421 PREDICTED: lysosomal alpha-glucosidase [Danio rerio]

Protein Accession	CL5792.Contig1$_{H}1$
Mean Expression Ratio	1.09
Median Expression Ratio	1.09
Credible Interval	(0.704, 1.68)
Associated Peptides	1
Associated Spectra	1
Coverage	NaN

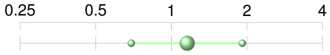

A	B	2.5	50	97.5	Sequence
**1**	**0**	**0.69**	**1.2**	**1.9**	**ATMGGLQK**

5.308 2831 4900 minus strand Collagen alpha-3(VI) chain [Crassostrea gigas]

Protein Accession	Unigene26542$_{H}1$
Mean Expression Ratio	0.916
Median Expression Ratio	0.914
Credible Interval	(0.625, 1.35)
Associated Peptides	1
Associated Spectra	3
Coverage	NaN

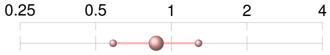

A	B	2.5	50	97.5	Sequence
**3**	**0**	**0.59**	**0.87**	**1.3**	**TEMFTPANGDRPDVPNVAIVITDGVSNINSR**

5.309 187 1323 minus strand gelsolin [Aplysia californica] gi

Protein Accession	CL10220.Contig1$_H$1
Mean Expression Ratio	0.915
Median Expression Ratio	0.915
Credible Interval	(0.589, 1.41)
Associated Peptides	1
Associated Spectra	1
Coverage	NaN

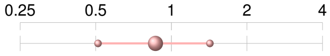

A	B	2.5	50	97.5	Sequence
1	0	0.51	0.86	1.4	LSDASGELDFK

5.310 2 1384 minus strand twitchin [Mytilus galloprovincialis]; 2 1384 minus strand twitchin [Mytilus galloprovincialis]; 2 1384 minus strand twitchin [Mytilus galloprovincialis]; 2 1384 minus strand twitchin [Mytilus galloprovincialis]; 2 1384 minus strand twitchin [Mytilus galloprovincialis]; 2 1384 minus strand twitchin [Mytilus galloprovincialis]

Protein Accession	Unigene62659$_H$1 Unigene62658$_H$1 Unigene62657$_H$1 Unigene62656$_H$1 Unigene62655$_H$1 U
Mean Expression Ratio	1.09
Median Expression Ratio	1.09
Credible Interval	(0.704, 1.69)
Associated Peptides	1
Associated Spectra	1
Coverage	NaN

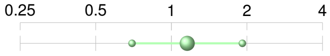

A	B	2.5	50	97.5	Sequence
1	0	0.7	1.2	1.9	DGGGADLYTAVLQIQDPKPEDGGTYK

5.311 260 892 minus strand calponin [Haliotis diversicolor]; 260 892 minus strand calponin [Haliotis diversicolor]

Protein Accession	CL48702.Contig2$_H$1 CL48702.Contig1$_H$1
Mean Expression Ratio	1.09
Median Expression Ratio	1.09
Credible Interval	(0.922, 1.29)
Associated Peptides	12
Associated Spectra	104
Coverage	NaN

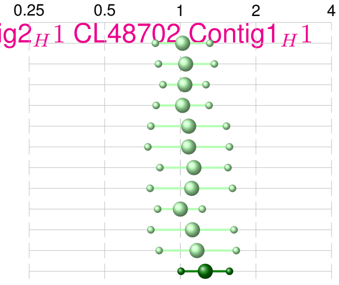

A	B	2.5	50	97.5	Sequence
7	4	0.79	1	1.3	AATGQQFPIEESNER
7	3	0.82	1.1	1.4	NFSEDKLR
17	8	0.85	1	1.3	TFQPASNLAFEAAR
7	5	0.8	1	1.3	VESSFDPEDAKK
1	1	0.76	1.1	1.5	YKVMENFYK
0	1	0.74	1.1	1.6	AFGIEVESHPSYTGQR
1	3	0.83	1.1	1.5	VESSFDPEDAK
0	1	0.76	1.1	1.6	FPIEESNER
7	11	0.81	1	1.2	IELFIQK
0	1	0.76	1.1	1.6	PASNLAFEAAR
1	1	0.82	1.2	1.7	VLACIR
12	5	1	1.3	1.6	VMENFYK

5.312 140 1564 selenium-binding protein [Haliotis discus hannai]

Protein Accession	CL3868.Contig7$_{H}1$
Mean Expression Ratio	1.09
Median Expression Ratio	1.09
Credible Interval	(0.709, 1.7)
Associated Peptides	1
Associated Spectra	1
Coverage	NaN

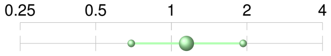

A	B	2.5	50	97.5	Sequence
**1**	**0**	**0.69**	**1.2**	**1.9**	**VVEAVDIHR**

5.313 193 828 Cysteine-rich secretory protein LCCL domain-containing 2 [Crassostrea gigas]

Protein Accession	CL40761.Contig2$_{H}1$
Mean Expression Ratio	1.09
Median Expression Ratio	1.09
Credible Interval	(0.761, 1.56)
Associated Peptides	3
Associated Spectra	3
Coverage	NaN

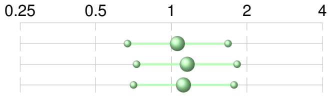

A	B	2.5	50	97.5	Sequence
**1**	**0**	**0.67**	**1.1**	**1.7**	**LEWDAQMESEATSWAAR**
**1**	**0**	**0.73**	**1.2**	**1.8**	**YSPEGNWVNEKPYEK**
**1**	**0**	**0.71**	**1.1**	**1.8**	**ICQPLR**

5.314 356 619 PREDICTED: hypothetical protein LOC100698726 [Oreochromis niloticus]

Protein Accession	CL3567.Contig6$_{H}1$
Mean Expression Ratio	1.09
Median Expression Ratio	1.09
Credible Interval	(0.73, 1.62)
Associated Peptides	2
Associated Spectra	2
Coverage	NaN

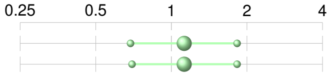

A	B	2.5	50	97.5	Sequence
**1**	**0**	**0.69**	**1.1**	**1.8**	**LETEVNDLR**
**1**	**0**	**0.7**	**1.1**	**1.8**	**GELQKEEAFSAK**

5.315 2 811 voltage-dependent anion channel 2-like protein [Haliotis diversicolor]; 2 811 voltage-dependent anion channel 2-like protein [Haliotis diversicolor]; 2 811 voltage-dependent anion channel 2-like protein [Haliotis diversicolor]

Protein Accession	CL2477.Contig3$_{H}$1 CL2477.Contig2$_{H}$1 CL2477.Contig1$_{H}$1
Mean Expression Ratio	0.917
Median Expression Ratio	0.917
Credible Interval	(0.738, 1.14)
Associated Peptides	9
Associated Spectra	22
Coverage	NaN

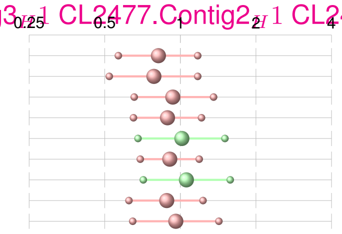

A	B	2.5	50	97.5	Sequence
2	0	0.57	0.82	1.2	LAFDTSFAPQTGK
1	0	0.52	0.79	1.2	VSGSLETK
1	1	0.66	0.94	1.4	WSEYGLTFTEK
4	0	0.65	0.89	1.2	YTLDSSASVSAK
1	0	0.68	1	1.5	GYNYGFFK
2	5	0.69	0.91	1.2	VGLGLDVEA
1	0	0.71	1.1	1.6	FALGTK
1	2	0.63	0.88	1.2	LTLSALIEGK
0	1	0.64	0.96	1.4	DIFSK

5.316 7825 8754 minus strand H2 [Haliotis tuberculata]

Protein Accession	CL7906.Contig3$_{H}$1
Mean Expression Ratio	0.92
Median Expression Ratio	0.917
Credible Interval	(0.598, 1.41)
Associated Peptides	1
Associated Spectra	1
Coverage	NaN

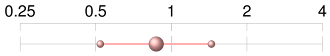

A	B	2.5	50	97.5	Sequence
1	0	0.52	0.87	1.4	ALPSLFTDPYDSNPFK

5.317 87 485 Sex-regulated protein janus-A [Crassostrea gigas]

Protein Accession	CL13131.Contig3$_{H}$1
Mean Expression Ratio	0.92
Median Expression Ratio	0.918
Credible Interval	(0.597, 1.42)
Associated Peptides	1
Associated Spectra	1
Coverage	NaN

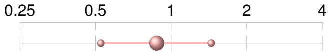

A	B	2.5	50	97.5	Sequence
0	1	0.52	0.88	1.4	ADHSITQTLLLR

5.318 7073 7339 minus strand Collagen alpha-3(VI) chain [Crassostrea gigas]

Protein Accession	Unigene26547$_{H}$1
Mean Expression Ratio	1.09
Median Expression Ratio	1.09
Credible Interval	(0.83, 1.43)
Associated Peptides	3
Associated Spectra	16
Coverage	NaN

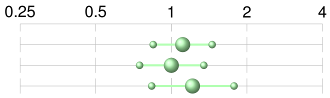

A	B	2.5	50	97.5	Sequence
5	3	0.85	1.1	1.5	ALVEELSGSGQNHR
6	0	0.75	1	1.3	YTPGSTNTAGGLR
1	1	0.83	1.2	1.8	YDTPQQVLEAIATTR

5.319 1 252 Aquaporin-4 [Crassostrea gigas]

Protein Accession	Unigene16706$_{H}1$
Mean Expression Ratio	1.09
Median Expression Ratio	1.09
Credible Interval	(0.732, 1.63)
Associated Peptides	2
Associated Spectra	2
Coverage	NaN

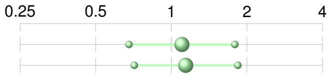

A	B	2.5	50	97.5	Sequence
**1**	**0**	**0.68**	**1.1**	**1.8**	**APLDDAAAAEVIEIDETK**
**1**	**0**	**0.71**	**1.1**	**1.8**	**KFSPSQAAAAPQEK**

5.320 316 1512 Alpha-N-acetylgalactosaminidase [Crassostrea gigas]; 282 1568 Alpha-N-acetylgalactosaminidase [Crassostrea gigas]; 282 1478 Alpha-N-acetylgalactosaminidase [Crassostrea gigas]

Protein Accession	CL2428.Contig4$_{H}1$ CL2428.Contig2$_{H}1$ CL2428.Contig1$_{H}1$
Mean Expression Ratio	1.09
Median Expression Ratio	1.09
Credible Interval	(0.759, 1.56)
Associated Peptides	2
Associated Spectra	5
Coverage	NaN

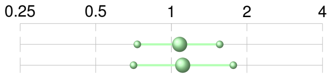

A	B	2.5	50	97.5	Sequence
**1**	**3**	**0.73**	**1.1**	**1.6**	**LVELGLR**
**0**	**1**	**0.71**	**1.1**	**1.8**	**ELFAGK**

5.321 176 517 PREDICTED: elongin C-like [Saccoglossus kowalevskii]; 115 456 PREDICTED: elongin C-like [Saccoglossus kowalevskii]; 994 1350 PREDICTED: elongin C-like [Saccoglossus kowalevskii]; 126 482 PREDICTED: elongin C-like [Saccoglossus kowalevskii]; 30 371 PREDICTED: elongin C-like [Saccoglossus kowalevskii]

Protein Accession	Unigene38248$_{H}1$ CL27549.Contig5$_{H}1$ CL27549.Contig4$_{H}1$ CL27549.Contig3$_{H}1$ CL27549.C
Mean Expression Ratio	1.09
Median Expression Ratio	1.09
Credible Interval	(0.701, 1.69)
Associated Peptides	1
Associated Spectra	1
Coverage	NaN

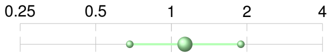

A	B	2.5	50	97.5	Sequence
**1**	**0**	**0.68**	**1.1**	**1.9**	**LISSDGHEFIVK**

5.322 112 930 calcineurin A [Haliotis discus discus]

Protein Accession	CL3137.Contig1$_{H}1$
Mean Expression Ratio	1.09
Median Expression Ratio	1.09
Credible Interval	(0.753, 1.59)
Associated Peptides	2
Associated Spectra	3
Coverage	NaN

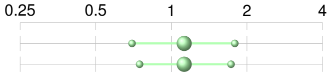

A	B	2.5	50	97.5	Sequence
**0**	**1**	**0.7**	**1.1**	**1.8**	**VTEDVALR**
**0**	**2**	**0.75**	**1.1**	**1.7**	**SEAFDAK**

5.323 30 536 putative mitochondrial ATP synthase [Haliotis discus discus]; 27 620 putative mitochondrial ATP synthase [Haliotis discus discus]

Protein Accession	CL13014.Contig4$_{H}1$ CL13014.Contig1$_{H}1$
Mean Expression Ratio	0.919
Median Expression Ratio	0.919
Credible Interval	(0.668, 1.26)
Associated Peptides	2
Associated Spectra	8
Coverage	NaN

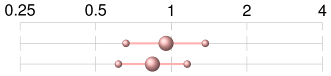

A	B	2.5	50	97.5	Sequence
**2**	**1**	**0.66**	**0.95**	**1.4**	**LANPALVDQFQK**
**2**	**3**	**0.62**	**0.84**	**1.2**	**SAVDWVAFK**

5.324 25 405 minus strand Elongation factor 1-delta [Anoplopoma fimbria]

Protein Accession	CL10502.Contig1$_{H}1$
Mean Expression Ratio	0.921
Median Expression Ratio	0.92
Credible Interval	(0.663, 1.27)
Associated Peptides	4
Associated Spectra	5
Coverage	NaN

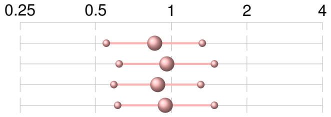

A	B	2.5	50	97.5	Sequence
**1**	**0**	**0.55**	**0.86**	**1.3**	**KPVLIAK**
**1**	**0**	**0.62**	**0.96**	**1.5**	**VQTDGLVWGASK**
**1**	**1**	**0.59**	**0.88**	**1.3**	**LVPVGYGINK**
**0**	**1**	**0.61**	**0.95**	**1.5**	**RVQTDGLVWGASK**

5.325 85 633 hypothetical protein [Amblyomma maculatum]

Protein Accession	CL2221.Contig1$_{H}1$
Mean Expression Ratio	1.09
Median Expression Ratio	1.09
Credible Interval	(0.766, 1.54)
Associated Peptides	3
Associated Spectra	4
Coverage	NaN

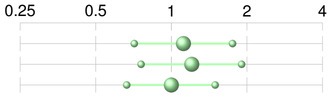

A	B	2.5	50	97.5	Sequence
**1**	**0**	**0.71**	**1.1**	**1.7**	**DANDEWIR**
**1**	**0**	**0.76**	**1.2**	**1.9**	**EKLPDYDNK**
**0**	**2**	**0.66**	**1**	**1.5**	**IFYTEHLHADEEIR**

5.326 155 3214 sarco/endoplasmic reticulum calcium ATPase isoform B [Pinctada fucata]; 155 3214 minus strand sarco/endoplasmic reticulum calcium ATPase isoform B [Pinctada fucata]

Protein Accession	CL1323.Contig9$_{H}$1 CL1323.Contig1$_{H}$1
Mean Expression Ratio	0.92
Median Expression Ratio	0.921
Credible Interval	(0.759, 1.11)
Associated Peptides	14
Associated Spectra	60
Coverage	NaN

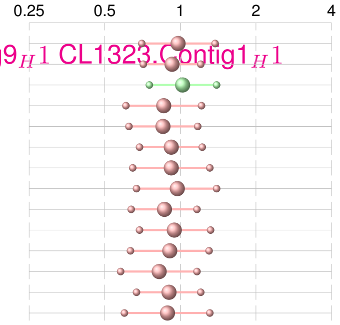

A	B	2.5	50	97.5	Sequence
1	2	0.7	0.98	1.4	ATAEAICR
3	5	0.71	0.93	1.2	CLALATIDNPPR
3	2	0.75	1	1.4	EFDELPPEDQR
2	1	0.61	0.86	1.2	GAPEGLLDR
3	2	0.62	0.85	1.2	GIVVGTGLATEIGK
7	2	0.69	0.92	1.2	KAEIGIAMGSGTAVAK
1	1	0.65	0.92	1.3	KEFTLEFSR
1	1	0.67	0.97	1.4	NAESAIEALK
2	3	0.64	0.86	1.2	NMLFSGTNIAAGK
2	2	0.69	0.95	1.3	VIVITGDNK
1	1	0.64	0.91	1.3	LNFYNTDK
2	1	0.58	0.82	1.2	MDQAFTK
7	1	0.67	0.9	1.2	IGHNKVPMSPAIK
0	1	0.6	0.89	1.3	KAEIGIAMGSGTA

5.327 128 532 minus strand mitochondrial ATPase inhibitor [Ornithodoros parkeri]

Protein Accession	Unigene1376$_{H}$1
Mean Expression Ratio	0.919
Median Expression Ratio	0.921
Credible Interval	(0.598, 1.41)
Associated Peptides	1
Associated Spectra	1
Coverage	NaN

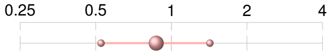

A	B	2.5	50	97.5	Sequence
1	0	0.52	0.87	1.4	MEAAHEELYFK

5.328 6043 6174 myosin heavy chain [Mytilus galloprovincialis]; 5869 6000 myosin heavy chain [Mytilus galloprovincialis]

Protein Accession	Unigene19025$_{H}$1 CL49594.Contig1$_{H}$1
Mean Expression Ratio	0.922
Median Expression Ratio	0.922
Credible Interval	(0.657, 1.29)
Associated Peptides	1
Associated Spectra	12
Coverage	NaN

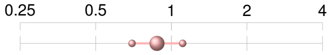

A	B	2.5	50	97.5	Sequence
12	0	0.7	0.88	1.1	KLEADIQAMQTDLDEMNGELR

5.329 68 667 minus strand hypothetical protein BRAFLDRAFT_272416 [Branchiostoma floridae] gi; 68 667 minus strand hypothetical protein BRAFLDRAFT_272416 [Branchiostoma floridae] gi

Protein Accession	CL37759.Contig4$_H$1 CL37759.Contig1$_H$1
Mean Expression Ratio	1.09
Median Expression Ratio	1.08
Credible Interval	(0.739, 1.62)
Associated Peptides	2
Associated Spectra	2
Coverage	NaN

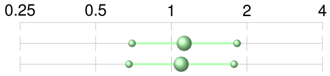

A	B	2.5	50	97.5	Sequence
**0**	**1**	**0.7**	**1.1**	**1.8**	**TLEEDEDVLLQLR**
**0**	**1**	**0.68**	**1.1**	**1.8**	**VLLQLR**

5.330 180 545 calreticulin precursor [Aplysia californica] gi

Protein Accession	CL4813.Contig2$_H$1
Mean Expression Ratio	0.921
Median Expression Ratio	0.922
Credible Interval	(0.662, 1.27)
Associated Peptides	4
Associated Spectra	12
Coverage	NaN

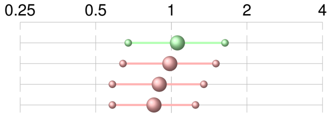

A	B	2.5	50	97.5	Sequence
**0**	**1**	**0.67**	**1.1**	**1.6**	**EEFDANWEDR**
**1**	**1**	**0.64**	**0.99**	**1.5**	**FYGISAK**
**2**	**1**	**0.58**	**0.89**	**1.3**	**FVWSAGK**
**4**	**2**	**0.58**	**0.85**	**1.2**	**WIPSVHK**

5.331 147 383 minus strand Tubulin-specific chaperone A OS=Rattus norvegicus GN=Tbca PE=1 SV=1; 104 418 Tubulin-specific chaperone A [Crassostrea gigas]; 104 418 minus strand Tubulin-specific chaperone A [Crassostrea gigas]; 104 418 Tubulin-specific chaperone A [Crassostrea gigas]

Protein Accession	Unigene66350$_H$1 Unigene53595$_H$1 Unigene32457$_H$1 CL7190.Contig3$_H$1
Mean Expression Ratio	1.08
Median Expression Ratio	1.08
Credible Interval	(0.703, 1.68)
Associated Peptides	1
Associated Spectra	2
Coverage	NaN

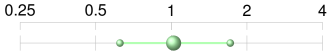

A	B	2.5	50	97.5	Sequence
**1**	**1**	**0.62**	**1**	**1.7**	**ADLGETEELK**

5.332 121 1452 minus strand Hexokinase type 2 [Crassostrea gigas]; 245 1576 minus strand Hexokinase type 2 [Crassostrea gigas]; 121 1452 minus strand Hexokinase type 2 [Crassostrea gigas]; 121 1452 minus strand Hexokinase type 2 [Crassostrea gigas]; 161 1513 minus strand Hexokinase type 2 [Crassostrea gigas]; 113 1471 minus strand Hexokinase type 2 [Crassostrea gigas]; 225 1577 minus strand Hexokinase type 2 [Crassostrea gigas]; 121 1452 minus strand Hexokinase type 2 [Crassostrea gigas]

Protein Accession	CL350.Contig9$_H1$ CL350.Contig8$_H1$ CL350.Contig6$_H1$ CL350.Contig5$_H1$ CL350.Contig3$_H1$
Mean Expression Ratio	0.92
Median Expression Ratio	0.923
Credible Interval	(0.588, 1.42)
Associated Peptides	1
Associated Spectra	1
Coverage	NaN

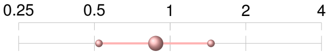

A	B	2.5	50	97.5	Sequence
**0**	**1**	**0.52**	**0.88**	**1.5**	**VLAPGIEFK**

5.333 426 896 PREDICTED: calmodulin-like [Metaseiulus occidentalis]

Protein Accession	Unigene667$_H1$
Mean Expression Ratio	1.08
Median Expression Ratio	1.08
Credible Interval	(0.772, 1.52)
Associated Peptides	2
Associated Spectra	8
Coverage	NaN

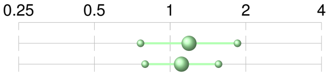

A	B	2.5	50	97.5	Sequence
**1**	**1**	**0.76**	**1.2**	**1.8**	**ADQLTEEQIAEFK**
**2**	**4**	**0.79**	**1.1**	**1.6**	**ELGTVMR**

5.334 3 386 fem-1-like protein C [Crassostrea gigas]

Protein Accession	Unigene19937$_H1$
Mean Expression Ratio	0.924
Median Expression Ratio	0.923
Credible Interval	(0.626, 1.37)
Associated Peptides	1
Associated Spectra	3
Coverage	NaN

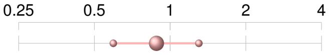

A	B	2.5	50	97.5	Sequence
**3**	**0**	**0.6**	**0.88**	**1.3**	**MQSLLIR**

5.335 266 1123 minus strand mitochondrial malate dehydrogenase precursor [Nucella lapillus]

Protein Accession	CL10426.Contig11$_{H}1$
Mean Expression Ratio	1.08
Median Expression Ratio	1.08
Credible Interval	(0.723, 1.62)
Associated Peptides	1
Associated Spectra	2
Coverage	NaN

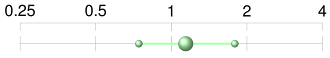

A	B	2.5	50	97.5	Sequence
**1**	**1**	**0.74**	**1.1**	**1.8**	**SLIEALNGMEGR**

5.336 50 577 hypothetical protein BRAFLDRAFT_119799 [Branchiostoma floridae] gi; 50 577 hypothetical protein BRAFLDRAFT_119799 [Branchiostoma floridae] gi

Protein Accession	CL5795.Contig4$_{H}1$ CL5795.Contig3$_{H}1$
Mean Expression Ratio	0.923
Median Expression Ratio	0.924
Credible Interval	(0.597, 1.43)
Associated Peptides	1
Associated Spectra	1
Coverage	NaN

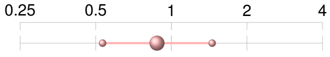

A	B	2.5	50	97.5	Sequence
**0**	**1**	**0.53**	**0.87**	**1.5**	**ADLGIDLSSVLGNVR**

5.337 176 670 hypothetical protein BRAFLDRAFT_122621 [Branchiostoma floridae] gi

Protein Accession	Unigene180$_{H}1$
Mean Expression Ratio	1.08
Median Expression Ratio	1.08
Credible Interval	(0.892, 1.31)
Associated Peptides	7
Associated Spectra	63
Coverage	NaN

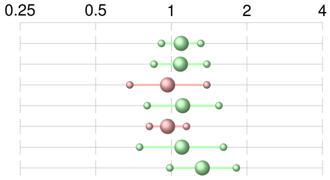

A	B	2.5	50	97.5	Sequence
**11**	**9**	**0.91**	**1.1**	**1.3**	**FADENFLLK**
**6**	**3**	**0.85**	**1.1**	**1.4**	**VFFDITADGQPLGR**
**1**	**1**	**0.68**	**0.97**	**1.4**	**VIPNFMCQGGDFTNHNGTGGR**
**3**	**0**	**0.8**	**1.1**	**1.5**	**VLCTGEK**
**12**	**12**	**0.82**	**0.97**	**1.1**	**IVMELR**
**1**	**0**	**0.75**	**1.1**	**1.6**	**ADVVPK**
**2**	**2**	**0.98**	**1.3**	**1.8**	**HVVFGQVVEGMDVVR**

5.338 " 169 792 minus strand rho GDP dissociation inhibitor, putative [Ixodes scapularis] gi"

Protein Accession	CL39798.Contig1$_{H}1$
Mean Expression Ratio	0.924
Median Expression Ratio	0.925
Credible Interval	(0.658, 1.29)
Associated Peptides	3
Associated Spectra	5
Coverage	NaN

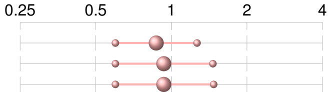

A	B	2.5	50	97.5	Sequence
2	1	0.6	0.87	1.3	IIIDDSNPLNVIVK
1	0	0.6	0.93	1.5	LAISVEGRPDAELDLTKPK
0	1	0.6	0.93	1.5	DIVAGLR

5.339 630 1199 neuronal calcium sensor-1 [Lymnaea stagnalis]

Protein Accession	CL14574.Contig3$_{H}1$
Mean Expression Ratio	1.08
Median Expression Ratio	1.08
Credible Interval	(0.729, 1.61)
Associated Peptides	2
Associated Spectra	2
Coverage	NaN

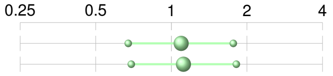

A	B	2.5	50	97.5	Sequence
1	0	0.68	1.1	1.8	IFSVMDK
1	0	0.69	1.1	1.8	LYDLDNDGFITK

5.340 120 833 B-cell receptor-associated protein 31 [Crassostrea gigas]; 238 951 B-cell receptor-associated protein 31 [Crassostrea gigas]; 88 801 B-cell receptor-associated protein 31 [Crassostrea gigas]; 88 801 B-cell receptor-associated protein 31 [Crassostrea gigas]

Protein Accession	CL9442.Contig7$_{H}1$ CL9442.Contig5$_{H}1$ CL9442.Contig4$_{H}1$ CL9442.Contig3$_{H}1$
Mean Expression Ratio	0.925
Median Expression Ratio	0.925
Credible Interval	(0.601, 1.43)
Associated Peptides	1
Associated Spectra	1
Coverage	NaN

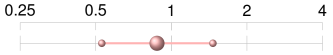

A	B	2.5	50	97.5	Sequence
1	0	0.53	0.87	1.5	LLEEHAQLQK

5.341 4 675 Laminin subunit gamma-1 [Crassostrea gigas]

Protein Accession	CL35712.Contig1$_{H}1$
Mean Expression Ratio	1.08
Median Expression Ratio	1.08
Credible Interval	(0.798, 1.46)
Associated Peptides	6
Associated Spectra	6
Coverage	NaN

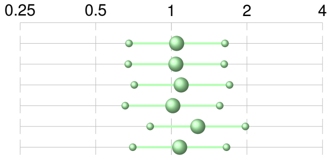

A	B	2.5	50	97.5	Sequence
1	0	0.68	1.1	1.6	AAQLTASETASK
1	0	0.67	1	1.6	LAQLAEDTAIQASAEAR
1	0	0.71	1.1	1.7	TLQTLLEFDR
1	0	0.65	1	1.6	VPEIEK
1	0	0.83	1.3	2	TLSQEVNDAEGR
0	1	0.7	1.1	1.7	ANIEAEIK

5.342 " 31 2061 minus strand 1,4-alpha-glucan-branching enzyme [Crassostrea gigas]"

Protein Accession	CL12555.Contig7$_H1$
Mean Expression Ratio	0.922
Median Expression Ratio	0.925
Credible Interval	(0.609, 1.37)
Associated Peptides	1
Associated Spectra	2
Coverage	NaN

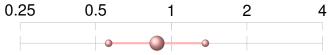

A	B	2.5	50	97.5	Sequence
**0**	**2**	**0.56**	**0.88**	**1.4**	**LDGYLR**

5.343 " 249 1271 N(G),N(G)-dimethylarginine dimethylaminohydrolase 1 [Crassostrea gigas]"

Protein Accession	Unigene64051$_H1$
Mean Expression Ratio	1.08
Median Expression Ratio	1.08
Credible Interval	(0.735, 1.62)
Associated Peptides	2
Associated Spectra	3
Coverage	NaN

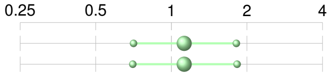

A	B	2.5	50	97.5	Sequence
**1**	**1**	**0.71**	**1.1**	**1.8**	**LGEVGEIR**
**1**	**0**	**0.7**	**1.1**	**1.8**	**EIFVGIGK**

5.344 " 40 813 minus strand NADH dehydrogenase [ubiquinone] iron-sulfur protein 3, mitochondrial [Crassostrea gigas]"

Protein Accession	CL8338.Contig9$_H1$
Mean Expression Ratio	1.08
Median Expression Ratio	1.08
Credible Interval	(0.732, 1.6)
Associated Peptides	2
Associated Spectra	2
Coverage	NaN

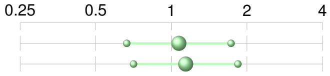

A	B	2.5	50	97.5	Sequence
**0**	**1**	**0.66**	**1.1**	**1.7**	**VVIEPIELQQEFR**
**0**	**1**	**0.71**	**1.1**	**1.8**	**ILTDYGFEGHPFR**

5.345 246 452 minus strand mitochondrial malate dehydrogenase precursor [Nucella lapillus]; 106 312 minus strand mitochondrial malate dehydrogenase precursor [Nucella lapillus]

Protein Accession	CL10426.Contig4$_H$1 CL10426.Contig3$_H$1
Mean Expression Ratio	0.924
Median Expression Ratio	0.925
Credible Interval	(0.635, 1.33)
Associated Peptides	1
Associated Spectra	4
Coverage	NaN

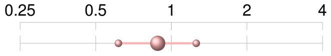

A	B	2.5	50	97.5	Sequence
2	2	0.61	0.88	1.3	DDLFNTNAGIVR

5.346 448 1626 matrilin [Biomphalaria glabrata]

Protein Accession	CL7510.Contig3$_H$1
Mean Expression Ratio	0.924
Median Expression Ratio	0.925
Credible Interval	(0.789, 1.08)
Associated Peptides	13
Associated Spectra	63
Coverage	NaN

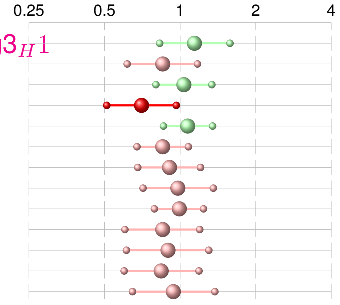

A	B	2.5	50	97.5	Sequence
2	1	0.83	1.1	1.6	EFLSMYDIGPAR
1	2	0.62	0.85	1.2	FAVVLTDGNSQESR
6	1	0.8	1	1.3	IDETSLNSAIDELVK
2	1	0.51	0.7	0.97	KTALVGFSDTR
5	6	0.86	1.1	1.3	TKEEVLEHVANMR
7	3	0.67	0.85	1.1	VFVVTTDSAAK
3	2	0.68	0.91	1.2	VIQVSSYDDLQK
3	0	0.71	0.98	1.4	VSNTELLNIAGDQER
6	6	0.79	0.99	1.2	DSLPGLVR
2	0	0.6	0.85	1.2	TALVGFSDTR
1	0	0.61	0.9	1.3	HEYGTFTDTGPGIDYMR
0	2	0.6	0.84	1.2	HKIDETSLNSAIDELVK
0	1	0.65	0.94	1.4	VSAIK

5.347 102 887 hypothetical protein DAPPUDRAFT_304712 [Daphnia pulex]

Protein Accession	CL6869.Contig1$_H$1
Mean Expression Ratio	1.08
Median Expression Ratio	1.08
Credible Interval	(0.801, 1.45)
Associated Peptides	4
Associated Spectra	8
Coverage	NaN

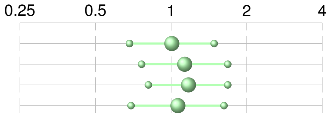

A	B	2.5	50	97.5	Sequence
2	0	0.68	1	1.5	GKPLFVR
1	1	0.76	1.1	1.7	LADIPEGK
2	1	0.81	1.2	1.7	HPEADAER
0	1	0.69	1.1	1.6	VDLSQLR

5.348 430 759 hypothetical protein [Amblyomma maculatum]; 336 665 hypothetical protein [Amblyomma maculatum]

Protein Accession	CL4062.Contig3$_{H}$1 CL4062.Contig2$_{H}$1
Mean Expression Ratio	0.925
Median Expression Ratio	0.926
Credible Interval	(0.682, 1.25)
Associated Peptides	4
Associated Spectra	7
Coverage	NaN

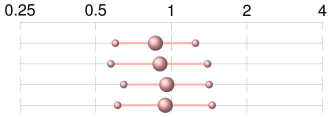

A	B	2.5	50	97.5	Sequence
2	1	0.6	0.86	1.2	ETLFEYLADPK
1	0	0.57	0.9	1.4	KTGQSAGFSYTDANK
1	1	0.64	0.96	1.4	KYIPGTK
0	1	0.61	0.94	1.5	MVFAGIK

5.349 106 1701 T-complex protein 1 subunit beta [Crassostrea gigas]

Protein Accession	CL8176.Contig1$_{H}$1
Mean Expression Ratio	0.926
Median Expression Ratio	0.926
Credible Interval	(0.625, 1.37)
Associated Peptides	2
Associated Spectra	2
Coverage	NaN

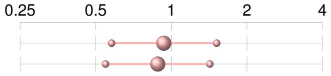

A	B	2.5	50	97.5	Sequence
1	0	0.58	0.93	1.5	GSGNLDAIQIIK
1	0	0.55	0.88	1.4	CTIVLR

5.350 293 1795 Amidase [Crassostrea gigas]

Protein Accession	CL5248.Contig6$_{H}$1
Mean Expression Ratio	0.927
Median Expression Ratio	0.926
Credible Interval	(0.617, 1.39)
Associated Peptides	1
Associated Spectra	2
Coverage	NaN

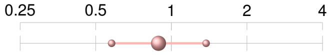

A	B	2.5	50	97.5	Sequence
2	0	0.58	0.89	1.4	LSTIGVTHFDR

5.351 2 511 hypothetical protein CGI_10006272 [Crassostrea gigas]

Protein Accession	CL10577.Contig2$_{H}$1
Mean Expression Ratio	0.926
Median Expression Ratio	0.926
Credible Interval	(0.604, 1.41)
Associated Peptides	1
Associated Spectra	1
Coverage	NaN

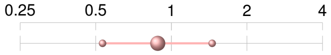

A	B	2.5	50	97.5	Sequence
1	0	0.53	0.88	1.5	LLEGAVPK

5.352 141 1325 Nucleobindin-2 [Crassostrea gigas]; 141 1208 minus strand Nucleobindin-2 [Crassostrea gigas]

Protein Accession	CL1888.Contig6$_{H}1$ CL1888.Contig2$_{H}1$
Mean Expression Ratio	0.927
Median Expression Ratio	0.927
Credible Interval	(0.623, 1.38)
Associated Peptides	2
Associated Spectra	3
Coverage	NaN

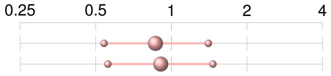

A	B	2.5	50	97.5	Sequence
1	1	0.54	0.87	1.4	LKELPEVER
0	1	0.56	0.91	1.5	LQELAR

5.353 236 733 Spectrin alpha chain [Crassostrea gigas]

Protein Accession	Unigene61767$_{H}1$
Mean Expression Ratio	1.08
Median Expression Ratio	1.08
Credible Interval	(0.765, 1.52)
Associated Peptides	3
Associated Spectra	4
Coverage	NaN

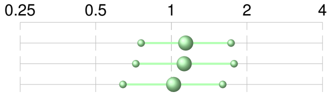

A	B	2.5	50	97.5	Sequence
1	1	0.76	1.1	1.7	DIDDEEAWIK
1	0	0.72	1.1	1.8	DLTGVQNLR
0	1	0.64	1	1.6	LNEANTMHQFLR

5.354 546 995 Hypothetical protein CBG12002 [Caenorhabditis briggsae]; 543 995 minus strand PREDICTED: tubulin polymerization-promoting protein family member 2-like [Anolis carolinensis]; 546 995 Hypothetical protein CBG12002 [Caenorhabditis briggsae]; 546 995 minus strand Hypothetical protein CBG12002 [Caenorhabditis briggsae]

Protein Accession	CL6849.Contig7$_{H}1$ CL6849.Contig6$_{H}1$ CL6849.Contig2$_{H}1$ CL6849.Contig1$_{H}1$
Mean Expression Ratio	1.08
Median Expression Ratio	1.08
Credible Interval	(0.724, 1.62)
Associated Peptides	1
Associated Spectra	2
Coverage	NaN

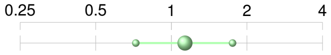

A	B	2.5	50	97.5	Sequence
0	2	0.72	1.1	1.8	LLPQLAADK

5.355 160 387 small nuclear ribonucleoprotein polypeptide G protein [Crassostrea ariakensis]; 180 407 small nuclear ribonucleoprotein polypeptide G protein [Crassostrea ariakensis]

Protein Accession	CL1272.Contig8$_{H}$1 CL1272.Contig7$_{H}$1
Mean Expression Ratio	0.926
Median Expression Ratio	0.928
Credible Interval	(0.623, 1.39)
Associated Peptides	1
Associated Spectra	2
Coverage	NaN

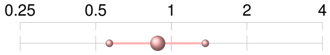

A	B	2.5	50	97.5	Sequence
2	0	0.57	0.89	1.4	TITGILR

5.356 " 698 1225 minus strand actin related protein 2/3 complex, 21 kDa subunit [Crassostrea gigas]"

Protein Accession	CL17389.Contig2$_{H}$1
Mean Expression Ratio	0.927
Median Expression Ratio	0.928
Credible Interval	(0.674, 1.28)
Associated Peptides	3
Associated Spectra	7
Coverage	NaN

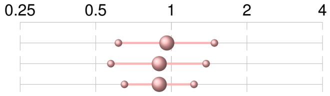

A	B	2.5	50	97.5	Sequence
0	1	0.62	0.96	1.5	AYLQQLR
0	1	0.57	0.9	1.4	ANIFFR
0	5	0.65	0.89	1.2	LLPLK

5.357 181 477 minus strand Zgc:56530 [Danio rerio]; 185 481 minus strand Zgc:56530 [Danio rerio]

Protein Accession	CL48470.Contig6$_{H}$1 CL27088.Contig2$_{H}$1
Mean Expression Ratio	1.08
Median Expression Ratio	1.08
Credible Interval	(0.762, 1.51)
Associated Peptides	2
Associated Spectra	6
Coverage	NaN

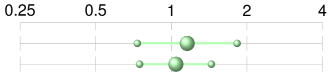

A	B	2.5	50	97.5	Sequence
1	0	0.73	1.2	1.8	TQVVAGTNYFIK
5	0	0.75	1	1.4	TFTDFK

5.358 333 770 USP-like protein isoform 2 [Crassostrea ariakensis]

Protein Accession	Unigene7710$_{H}$1
Mean Expression Ratio	0.929
Median Expression Ratio	0.929
Credible Interval	(0.596, 1.44)
Associated Peptides	1
Associated Spectra	1
Coverage	NaN

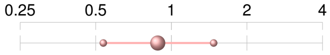

A	B	2.5	50	97.5	Sequence
1	0	0.53	0.88	1.5	KIVIAMDGSK

5.359 18 647 minus strand ATP synthase-like protein [Biomphalaria glabrata]

Protein Accession	CL42054.Contig4$_{H}1$
Mean Expression Ratio	0.928
Median Expression Ratio	0.929
Credible Interval	(0.71, 1.21)
Associated Peptides	5
Associated Spectra	15
Coverage	NaN

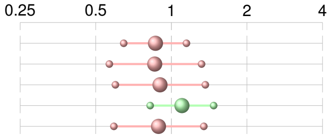

A	B	2.5	50	97.5	Sequence
**2**	**4**	**0.65**	**0.86**	**1.1**	**LAEFVANPSLQR**
**1**	**0**	**0.57**	**0.86**	**1.3**	**KAVLEQALK**
**1**	**0**	**0.6**	**0.9**	**1.4**	**NFQNLLK**
**3**	**3**	**0.83**	**1.1**	**1.5**	**GALQGFLK**
**1**	**0**	**0.59**	**0.89**	**1.4**	**NYTNLIK**

5.360 460 1737 Tubulin beta-4B chain OS=Mus musculus GN=Tubb4b PE=1 SV=1

Protein Accession	Unigene34147$_{H}1$
Mean Expression Ratio	0.927
Median Expression Ratio	0.929
Credible Interval	(0.655, 1.31)
Associated Peptides	1
Associated Spectra	8
Coverage	NaN

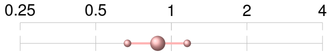

A	B	2.5	50	97.5	Sequence
**3**	**5**	**0.67**	**0.88**	**1.2**	**AVLVDLEPGTMDSVR**

5.361 72 521 minus strand GF14593 [Drosophila ananassae] gi

Protein Accession	CL38325.Contig2$_{H}1$
Mean Expression Ratio	0.93
Median Expression Ratio	0.93
Credible Interval	(0.602, 1.44)
Associated Peptides	1
Associated Spectra	1
Coverage	NaN

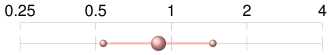

A	B	2.5	50	97.5	Sequence
**1**	**0**	**0.54**	**0.89**	**1.5**	**LTADVHGK**

5.362 156 1163 cathepsin B [Crassostrea ariakensis]

Protein Accession	CL26836.Contig9$_{H}1$
Mean Expression Ratio	0.928
Median Expression Ratio	0.93
Credible Interval	(0.619, 1.39)
Associated Peptides	1
Associated Spectra	2
Coverage	NaN

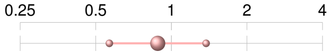

A	B	2.5	50	97.5	Sequence
**1**	**1**	**0.57**	**0.88**	**1.4**	**DLPDSFDPR**

5.363 325 642 minus strand Protein phosphatase 1B [Crassostrea gigas]

Protein Accession	Unigene268$_H$1
Mean Expression Ratio	0.93
Median Expression Ratio	0.93
Credible Interval	(0.618, 1.4)
Associated Peptides	1
Associated Spectra	2
Coverage	NaN

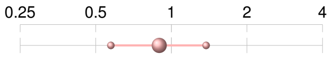

A	B	2.5	50	97.5	Sequence
**1**	**1**	**0.57**	**0.89**	**1.4**	**VEMEDAHTAVVSIPHAR**

5.364 2 1795 minus strand Plectin-1 [Crassostrea gigas]

Protein Accession	CL32322.Contig2$_H$1
Mean Expression Ratio	1.08
Median Expression Ratio	1.07
Credible Interval	(0.721, 1.6)
Associated Peptides	2
Associated Spectra	2
Coverage	NaN

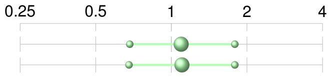

A	B	2.5	50	97.5	Sequence
**1**	**0**	**0.68**	**1.1**	**1.8**	**TKDGDVIPIHDAIMTK**
**1**	**0**	**0.68**	**1.1**	**1.8**	**YITTAHFVR**

5.365 552 1499 minus strand uncharacterized protein LOC100158466 [Xenopus (Silurana) tropicalis] gi; 552 1499 minus strand uncharacterized protein LOC100158466 [Xenopus (Silurana) tropicalis] gi; 552 1499 minus strand uncharacterized protein LOC100158466 [Xenopus (Silurana) tropicalis] gi

Protein Accession	Unigene57645$_H$1 Unigene52825$_H$1 Unigene48504$_H$1
Mean Expression Ratio	0.931
Median Expression Ratio	0.931
Credible Interval	(0.602, 1.45)
Associated Peptides	1
Associated Spectra	2
Coverage	NaN

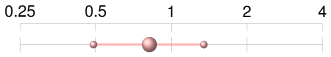

A	B	2.5	50	97.5	Sequence
**1**	**1**	**0.49**	**0.82**	**1.3**	**TGEPVEVDGELATLLAR**

5.366 1971 3368 minus strand Kyphoscoliosis peptidase [Crassostrea gigas]; 1767 3158 minus strand hillarin [Hirudo medicinalis]; 1977 3368 minus strand hillarin [Hirudo medicinalis]

Protein Accession	CL3923.Contig9$_H$1 CL3923.Contig8$_H$1 CL3923.Contig10$_H$1
Mean Expression Ratio	0.932
Median Expression Ratio	0.931
Credible Interval	(0.785, 1.11)
Associated Peptides	13
Associated Spectra	40
Coverage	NaN

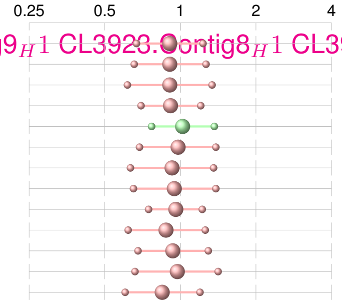

A	B	2.5	50	97.5	Sequence
2	2	0.67	0.91	1.2	EKGEAPPPVPATGPQQKPTLEAIDR
1	2	0.65	0.91	1.3	EMNFDKVEK
1	0	0.62	0.91	1.3	GEAPPPVPATGPQQKPTLEAIDR
4	2	0.7	0.91	1.2	GSPEEVLMGLK
4	1	0.77	1	1.4	NITNEIEK
1	1	0.69	0.98	1.4	TAVIYSR
1	0	0.63	0.93	1.4	TVGNTAYFNVTAPGR
1	0	0.65	0.95	1.4	IISGYAK
5	4	0.75	0.96	1.2	WQLLDR
2	0	0.62	0.88	1.3	MKPQFFK
2	1	0.68	0.93	1.3	NVLQSVDDHAIQVSK
0	1	0.66	0.97	1.4	WLATK
0	2	0.6	0.85	1.2	QLLDR

5.367 605 1153 Peptidyl-prolyl cis-trans isomerase B [Crassostrea gigas]; 1046 1630 Peptidyl-prolyl cis-trans isomerase B [Crassostrea gigas]; 589 1173 Peptidyl-prolyl cis-trans isomerase B [Crassostrea gigas]; 883 1467 Peptidyl-prolyl cis-trans isomerase B [Crassostrea gigas]

Protein Accession	CL24747.Contig5$_H$1 CL24747.Contig4$_H$1 CL24747.Contig3$_H$1 CL24747.Contig1$_H$1
Mean Expression Ratio	1.07
Median Expression Ratio	1.07
Credible Interval	(0.757, 1.51)
Associated Peptides	3
Associated Spectra	7
Coverage	NaN

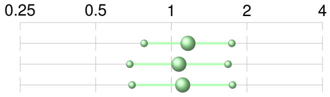

A	B	2.5	50	97.5	Sequence
2	3	0.78	1.2	1.7	IASSGVVGIASK
1	0	0.68	1.1	1.7	KLGELETSIIGRPK
1	0	0.7	1.1	1.8	SGMSIYGETFKDENFK

5.368 227 1201 PREDICTED: quinone oxidoreductase-like isoform 1 [Ornithorhynchus anatinus]; 109 1083 PREDICTED: quinone oxidoreductase-like isoform 1 [Ornithorhynchus anatinus]; 164 1138 PREDICTED: quinone oxidoreductase-like isoform 1 [Ornithorhynchus anatinus]

Protein Accession	CL11899.Contig2$_H$1 CL11899.Contig1$_H$1 CL11899.Contig13$_H$1
Mean Expression Ratio	0.93
Median Expression Ratio	0.932
Credible Interval	(0.596, 1.44)
Associated Peptides	1
Associated Spectra	1
Coverage	NaN

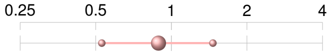

A	B	2.5	50	97.5	Sequence
1	0	0.53	0.89	1.5	VHASGVNPVDTYIR

5.369 811 1407 minus strand mitochondrial malate dehydrogenase 2 [Eospalax baileyi]

Protein Accession	CL10426.Contig13$_{H}1$
Mean Expression Ratio	0.934
Median Expression Ratio	0.932
Credible Interval	(0.7, 1.24)
Associated Peptides	3
Associated Spectra	11
Coverage	NaN

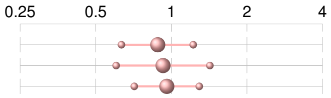

A	B	2.5	50	97.5	Sequence
2	2	0.63	0.88	1.2	IQNAGTEVVEAK
1	0	0.6	0.93	1.4	MQQLNSPR
3	3	0.71	0.96	1.3	AAMPELIANISK

5.370 633 806 Peroxiredoxin-1 [Crassostrea gigas]; 648 821 Peroxiredoxin-1 [Crassostrea gigas]; 666 839 Peroxiredoxin-1 [Crassostrea gigas]; 625 798 Peroxiredoxin-1 [Crassostrea gigas]

Protein Accession	CL3160.Contig18$_{H}1$ CL3160.Contig17$_{H}1$ CL3160.Contig16$_{H}1$ CL3160.Contig15$_{H}1$
Mean Expression Ratio	0.934
Median Expression Ratio	0.932
Credible Interval	(0.685, 1.28)
Associated Peptides	2
Associated Spectra	14
Coverage	NaN

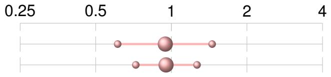

A	B	2.5	50	97.5	Sequence
1	0	0.61	0.95	1.5	EYFSK
7	6	0.72	0.96	1.3	LVQAFQFTDK

5.371 54 791 GMP reductase 2 [Crassostrea gigas]

Protein Accession	CL26888.Contig1$_{H}1$
Mean Expression Ratio	1.07
Median Expression Ratio	1.07
Credible Interval	(0.699, 1.67)
Associated Peptides	1
Associated Spectra	1
Coverage	NaN

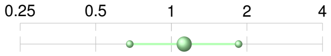

A	B	2.5	50	97.5	Sequence
1	0	0.68	1.1	1.9	LFTTIHK

5.372 " 2041 3315 minus strand Myosin-10 OS=Mus musculus GN=Myh10 PE=1 SV=2; 2041 3345 minus strand Myosin heavy chain, non-muscle OS=Drosophila melanogaster GN=zip PE=1 SV=2; 2041 3351 minus strand Myosin-10 OS=Mus musculus GN=Myh10 PE=1 SV=2"

Protein Accession	Unigene62170$_H$1 Unigene62168$_H$1 Unigene62165$_H$1
Mean Expression Ratio	0.932
Median Expression Ratio	0.933
Credible Interval	(0.685, 1.27)
Associated Peptides	5
Associated Spectra	6
Coverage	NaN

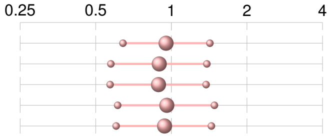

A	B	2.5	50	97.5	Sequence
0	2	0.64	0.96	1.4	AGVLAHLEEER
0	1	0.57	0.9	1.4	CIIPNHEK
0	1	0.57	0.89	1.4	IDSPLVLDQLR
0	1	0.61	0.96	1.5	ILFQEFR
0	1	0.6	0.94	1.4	LMATLR

5.373 169 1200 PREDICTED: nidogen-2-like isoform 1 [Strongylocentrotus purpuratus]

Protein Accession	CL478.Contig2$_H$1
Mean Expression Ratio	0.932
Median Expression Ratio	0.933
Credible Interval	(0.672, 1.3)
Associated Peptides	4
Associated Spectra	5
Coverage	NaN

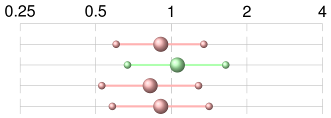

A	B	2.5	50	97.5	Sequence
0	2	0.6	0.91	1.3	LDIVATAR
0	1	0.67	1.1	1.6	LPNGLTFDQVSR
0	1	0.53	0.83	1.3	IECVHR
0	1	0.58	0.91	1.4	VFTVPALSR

5.374 85 1011 unnamed protein product [Mus musculus]

Protein Accession	CL42412.Contig1$_H$1
Mean Expression Ratio	1.07
Median Expression Ratio	1.07
Credible Interval	(0.743, 1.55)
Associated Peptides	3
Associated Spectra	3
Coverage	NaN

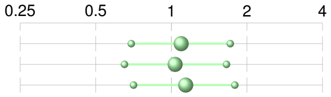

A	B	2.5	50	97.5	Sequence
1	0	0.69	1.1	1.7	NVIVEETVSEK
1	0	0.65	1	1.7	DDETMYVEAK
1	0	0.71	1.1	1.8	VFMQEFK

5.375 183 953 minus strand glutathione-S-transferase [Haliotis diversicolor supertexta]; 275 1045 minus strand glutathione-S-transferase [Haliotis diversicolor supertexta]; 343 1113 minus strand glutathione-S-transferase [Haliotis diversicolor supertexta]

Protein Accession	Unigene40627$_{H}1$ CL6878.Contig3$_{H}1$ CL6878.Contig2$_{H}1$
Mean Expression Ratio	1.07
Median Expression Ratio	1.07
Credible Interval	(0.72, 1.59)
Associated Peptides	2
Associated Spectra	2
Coverage	NaN

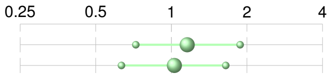

A	B	2.5	50	97.5	Sequence
0	1	0.72	1.2	1.9	LPHDPLPK
0	1	0.63	1	1.7	KIDPK

5.376 1592 1768 minus strand beta-actin [Diabolocatantops pinguis]

Protein Accession	CL22741.Contig2$_{H}1$
Mean Expression Ratio	1.07
Median Expression Ratio	1.07
Credible Interval	(0.798, 1.43)
Associated Peptides	2
Associated Spectra	16
Coverage	NaN

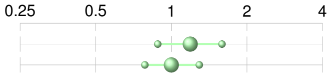

A	B	2.5	50	97.5	Sequence
3	3	0.88	1.2	1.6	VIAPPER
0	10	0.78	1	1.3	EISALAPPTMK

5.377 119 481 Glutamate receptor U1 [Crassostrea gigas]

Protein Accession	CL570.Contig5$_{H}1$
Mean Expression Ratio	1.07
Median Expression Ratio	1.07
Credible Interval	(0.877, 1.31)
Associated Peptides	7
Associated Spectra	49
Coverage	NaN

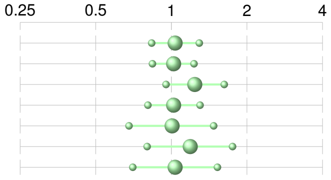

A	B	2.5	50	97.5	Sequence
9	3	0.83	1	1.3	GTANIGAGALTVTSER
10	7	0.84	1	1.2	RTPDGWSGIIGDVVK
6	1	0.95	1.2	1.6	TPDGWSGIIGDVVK
7	3	0.8	1	1.3	FQYTFR
0	1	0.68	1	1.5	AEVVK
0	1	0.8	1.2	1.8	GAGALTVTSER
0	1	0.7	1	1.5	GTANIGAGALTVT

5.378 4 243 40S ribosomal protein S13 OS=Caenorhabditis elegans GN=rps-13 PE=2 SV=2; 4 243 40S ribosomal protein S13 OS=Caenorhabditis elegans GN=rps-13 PE=2 SV=2; 1 252 40S ribosomal protein S13 OS=Xenopus laevis GN=rps13 PE=3 SV=2; 1 213 40S ribosomal protein S13 OS=Xenopus laevis GN=rps13 PE=3 SV=2

Protein Accession	CL18049.Contig7$_{H}$1 CL18049.Contig4$_{H}$1 CL18049.Contig3$_{H}$1 CL18049.Contig2$_{H}$1
Mean Expression Ratio	0.934
Median Expression Ratio	0.934
Credible Interval	(0.627, 1.38)
Associated Peptides	2
Associated Spectra	2
Coverage	NaN

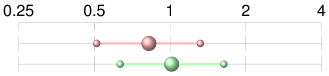

A	B	2.5	50	97.5	Sequence
1	0	0.51	0.82	1.3	GLAPDIPEDLYSLIK
1	0	0.63	1	1.6	SLIKK

5.379 181 744 unknown [Dendroctonus ponderosae]

Protein Accession	CL25948.Contig3$_{H}$1
Mean Expression Ratio	0.933
Median Expression Ratio	0.934
Credible Interval	(0.746, 1.17)
Associated Peptides	7
Associated Spectra	23
Coverage	NaN

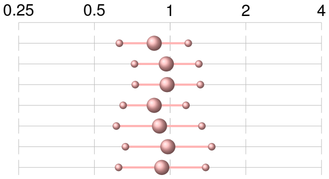

A	B	2.5	50	97.5	Sequence
3	1	0.63	0.86	1.2	GTLTQLTK
4	1	0.72	0.97	1.3	SMGPIGSPMSK
4	1	0.73	0.97	1.3	TFICTPDR
4	2	0.65	0.87	1.2	VGHVPVAK
1	0	0.61	0.91	1.3	YKNNYLCR
1	0	0.66	0.98	1.5	GIYICDGSDEEAEEIIHK
1	0	0.63	0.93	1.4	NNYLCR

5.380 1 987 minus strand RecName: Full=Tubulin beta chain; AltName: Full=Beta-tubulin

Protein Accession	Unigene7954$_{H}$1
Mean Expression Ratio	1.07
Median Expression Ratio	1.07
Credible Interval	(0.785, 1.45)
Associated Peptides	3
Associated Spectra	8
Coverage	NaN

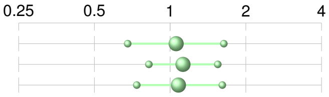

A	B	2.5	50	97.5	Sequence
1	0	0.68	1.1	1.6	AILVDLEPGTMDSVR
5	0	0.82	1.1	1.5	ALTVPELTQQMFDAK
2	0	0.73	1.1	1.6	PELTQQMFDAK

5.381 227 3073 minus strand Lysosomal alpha-mannosidase [Crassostrea gigas]

Protein Accession	CL2930.Contig2$_{H}1$
Mean Expression Ratio	0.935
Median Expression Ratio	0.935
Credible Interval	(0.641, 1.35)
Associated Peptides	2
Associated Spectra	4
Coverage	NaN

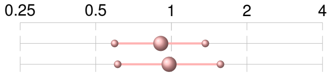

A	B	2.5	50	97.5	Sequence
**2**	**1**	**0.59**	**0.91**	**1.4**	**VASELIPITPDTFR**
**1**	**0**	**0.61**	**0.98**	**1.6**	**ASLTWSGVR**

5.382 17 1681 minus strand Glucose-6-phosphate isomerase [Crassostrea gigas]

Protein Accession	CL34850.Contig1$_{H}1$
Mean Expression Ratio	1.07
Median Expression Ratio	1.07
Credible Interval	(0.832, 1.38)
Associated Peptides	7
Associated Spectra	13
Coverage	NaN

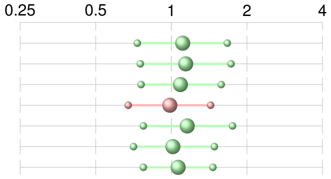

A	B	2.5	50	97.5	Sequence
**1**	**0**	**0.73**	**1.1**	**1.7**	**EFTEQVISGAWK**
**1**	**0**	**0.75**	**1.1**	**1.7**	**KQLTSEPVWR**
**1**	**1**	**0.76**	**1.1**	**1.6**	**CKEFGIDEK**
**1**	**1**	**0.68**	**0.99**	**1.4**	**VHFISNVDGTHLAK**
**0**	**1**	**0.77**	**1.2**	**1.8**	**EAAEDELR**
**0**	**2**	**0.71**	**1**	**1.5**	**INFTEDR**
**0**	**4**	**0.77**	**1.1**	**1.5**	**INMAQMFK**

5.383 123 314 PREDICTED: ubiquinol-cytochrome c reductase hinge protein-like [Saccoglossus kowalevskii]; 279 530 PREDICTED: ubiquinol-cytochrome c reductase hinge protein-like [Saccoglossus kowalevskii]

Protein Accession	CL4684.Contig5$_{H}1$ CL4684.Contig4$_{H}1$
Mean Expression Ratio	0.937
Median Expression Ratio	0.935
Credible Interval	(0.621, 1.42)
Associated Peptides	1
Associated Spectra	2
Coverage	NaN

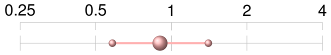

A	B	2.5	50	97.5	Sequence
**2**	**0**	**0.58**	**0.9**	**1.4**	**CLFDELK**

5.384 635 1294 minus strand Kyphoscoliosis peptidase [Crassostrea gigas]; 643 1302 minus strand Kyphoscoliosis peptidase [Crassostrea gigas]; 635 1294 minus strand Kyphoscoliosis peptidase [Crassostrea gigas]; 634 1293 minus strand Kyphoscoliosis peptidase [Crassostrea gigas]; 634 1293 minus strand Kyphoscoliosis peptidase [Crassostrea gigas]; 634 1293 minus strand Kyphoscoliosis peptidase [Crassostrea gigas]; 639 1298 minus strand Kyphoscoliosis peptidase [Crassostrea gigas]

Protein Accession	CL7772.Contig20$_H$1 CL7772.Contig1$_H$1 CL7772.Contig19$_H$1 CL7772.Contig16$_H$1 CL7772.(
Mean Expression Ratio	1.07
Median Expression Ratio	1.07
Credible Interval	(0.833, 1.37)
Associated Peptides	7
Associated Spectra	36
Coverage	NaN

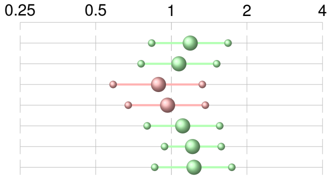

A	B	2.5	50	97.5	Sequence
2	2	0.84	1.2	1.7	FQIFALPTSDDSK
4	1	0.76	1.1	1.5	GAGEVWEGTAALDK
0	1	0.59	0.89	1.3	GAGEVWEGTAALDKHR
3	1	0.68	0.96	1.4	VTLNACFGEDESK
3	2	0.8	1.1	1.6	QYAPWR
7	6	0.94	1.2	1.6	LSNVTFK
3	1	0.86	1.2	1.7	AVYPFPK

5.385 " 116 1168 minus strand hypothetical protein CGI_10014553, partial [Crassostrea gigas]"

Protein Accession	CL19215.Contig5$_H$1
Mean Expression Ratio	0.936
Median Expression Ratio	0.936
Credible Interval	(0.69, 1.27)
Associated Peptides	5
Associated Spectra	6
Coverage	NaN

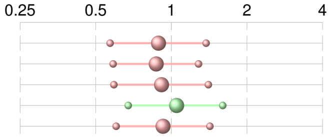

A	B	2.5	50	97.5	Sequence
1	0	0.57	0.89	1.4	SSFNDAVHSLFDK
1	1	0.59	0.87	1.3	SVIALGEGAGANIVAR
1	0	0.59	0.91	1.4	AQNKDQLQK
0	1	0.67	1	1.6	AAFIHVDVPGQEDDAK
0	1	0.6	0.93	1.4	VIENFQLSLR

5.386 42 296 minus strand twitchin [Mytilus galloprovincialis]

Protein Accession	Unigene19029$_H$1
Mean Expression Ratio	1.07
Median Expression Ratio	1.07
Credible Interval	(0.818, 1.41)
Associated Peptides	4
Associated Spectra	12
Coverage	NaN

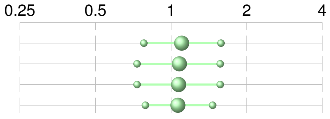

A	B	2.5	50	97.5	Sequence
2	1	0.78	1.1	1.6	APHFTQKPTIK
1	1	0.73	1.1	1.6	VTLADAGEYK
2	0	0.73	1.1	1.6	KPQLPDGK
3	2	0.79	1.1	1.5	LNFDSAK

5.387 65 490 PREDICTED: cofilin-2 [Otolemur garnettii]

Protein Accession	CL15471.Contig3$_H$1
Mean Expression Ratio	0.937
Median Expression Ratio	0.936
Credible Interval	(0.706, 1.25)
Associated Peptides	4
Associated Spectra	11
Coverage	NaN

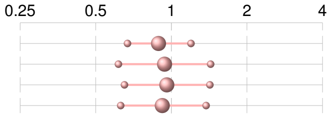

A	B	2.5	50	97.5	Sequence
**4**	**2**	**0.67**	**0.89**	**1.2**	**IVMENEFAK**
**1**	**0**	**0.62**	**0.94**	**1.4**	**SQEDEYNSIIMTLPK**
**2**	**0**	**0.65**	**0.96**	**1.4**	**MLYSSSSSAVK**
**1**	**1**	**0.63**	**0.92**	**1.4**	**ALVFK**

5.388 3 230 minus strand RH21460p [Drosophila melanogaster]; 3 230 minus strand RH21460p [Drosophila melanogaster]

Protein Accession	Unigene57704$_H$1 CL18123.Contig3$_H$1
Mean Expression Ratio	0.934
Median Expression Ratio	0.936
Credible Interval	(0.601, 1.44)
Associated Peptides	1
Associated Spectra	1
Coverage	NaN

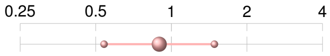

A	B	2.5	50	97.5	Sequence
**0**	**1**	**0.54**	**0.89**	**1.5**	**EVDEDEAAFK**

5.389 187 696 minus strand putative ribosomal protein S10 [Sipunculus nudus]

Protein Accession	Unigene1233$_H$1
Mean Expression Ratio	1.07
Median Expression Ratio	1.07
Credible Interval	(0.695, 1.65)
Associated Peptides	1
Associated Spectra	3
Coverage	NaN

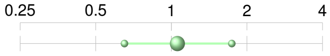

A	B	2.5	50	97.5	Sequence
**2**	**1**	**0.65**	**1.1**	**1.7**	**KDFFSPK**

5.390 18 1613 minus strand chaperonin containing T-complex polypeptide subunit zeta [Haliotis discus hannai]; 527 2089 minus strand chaperonin containing T-complex polypeptide subunit zeta [Haliotis discus hannai]

Protein Accession	CL23722.Contig7$_H$1 CL23722.Contig5$_H$1
Mean Expression Ratio	1.07
Median Expression Ratio	1.07
Credible Interval	(0.734, 1.55)
Associated Peptides	2
Associated Spectra	3
Coverage	NaN

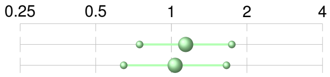

A	B	2.5	50	97.5	Sequence
**2**	**0**	**0.75**	**1.1**	**1.7**	**LITEGFELAR**
**1**	**0**	**0.65**	**1**	**1.7**	**AAQALAVNISAA**

5.391 160 588 hypothetical protein CGI_10001640 [Crassostrea gigas]

Protein Accession CL2347.Contig8$_H1$
Mean Expression Ratio 1.07
Median Expression Ratio 1.07
Credible Interval (0.788, 1.44)
Associated Peptides 2
Associated Spectra 13
Coverage NaN

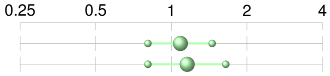

A	B	2.5	50	97.5	Sequence
6	3	0.81	1.1	1.5	AFLEQLGSK
3	1	0.81	1.2	1.6	ANLIVIGSR

5.392 " 65 1576 minus strand Aldehyde dehydrogenase, mitochondrial [Crassostrea gigas]"

Protein Accession CL5835.Contig1$_H1$
Mean Expression Ratio 0.937
Median Expression Ratio 0.937
Credible Interval (0.687, 1.28)
Associated Peptides 5
Associated Spectra 6
Coverage NaN

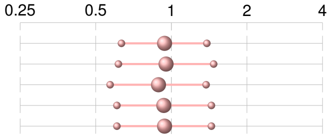

A	B	2.5	50	97.5	Sequence
1	1	0.63	0.94	1.4	LADLLER
1	0	0.61	0.96	1.5	ELGEYGLEAYTEVK
1	0	0.57	0.89	1.4	IFVEEK
1	0	0.61	0.93	1.4	VAEGDK
0	1	0.61	0.94	1.4	LIPVMAAK

5.393 108 593 hypothetical protein CGI_10025497 [Crassostrea gigas]

Protein Accession CL36597.Contig1$_H1$
Mean Expression Ratio 1.07
Median Expression Ratio 1.07
Credible Interval (0.746, 1.55)
Associated Peptides 1
Associated Spectra 5
Coverage NaN

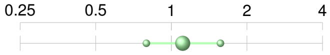

A	B	2.5	50	97.5	Sequence
4	1	0.8	1.1	1.6	SFELADFHK

5.394 176 667 hypothetical protein CGI_10018452 [Crassostrea gigas]; 176 634 hypothetical protein CGI_10018452 [Crassostrea gigas]

Protein Accession CL35173.Contig3$_H1$ CL35173.Contig1$_H1$
Mean Expression Ratio 0.934
Median Expression Ratio 0.938
Credible Interval (0.602, 1.44)
Associated Peptides 1
Associated Spectra 1
Coverage NaN

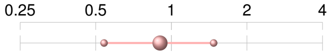

A	B	2.5	50	97.5	Sequence
0	1	0.54	0.9	1.5	NFLELTELK

5.395 145 1524 PREDICTED: aspartyl aminopeptidase-like [Oreochromis niloticus]

Protein Accession	CL19117.Contig2$_{H}1$
Mean Expression Ratio	0.938
Median Expression Ratio	0.939
Credible Interval	(0.63, 1.39)
Associated Peptides	2
Associated Spectra	2
Coverage	NaN

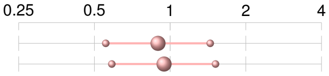

A	B	2.5	50	97.5	Sequence
**1**	**0**	**0.55**	**0.9**	**1.4**	**ITVSPSNPVAFEEAIGK**
**1**	**0**	**0.59**	**0.95**	**1.5**	**KLVAHGFK**

5.396 49 747 PREDICTED: elongation factor 1-delta-like [Strongylocentrotus purpuratus]; 49 819 PREDICTED: elongation factor 1-delta-like isoform 2 [Strongylocentrotus purpuratus]

Protein Accession	CL10790.Contig13$_{H}1$ CL10790.Contig12$_{H}1$
Mean Expression Ratio	1.07
Median Expression Ratio	1.06
Credible Interval	(0.719, 1.59)
Associated Peptides	2
Associated Spectra	5
Coverage	NaN

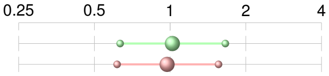

A	B	2.5	50	97.5	Sequence
**1**	**0**	**0.63**	**1**	**1.7**	**VAALEVVGTGGASPK**
**1**	**3**	**0.62**	**0.97**	**1.6**	**LVDLAYGIK**

5.397 243 2165 minus strand Elongation factor 2 OS=Drosophila melanogaster GN=Ef2b PE=2 SV=4

Protein Accession	Unigene11527$_{H}1$
Mean Expression Ratio	0.939
Median Expression Ratio	0.94
Credible Interval	(0.633, 1.39)
Associated Peptides	2
Associated Spectra	2
Coverage	NaN

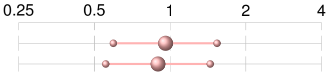

A	B	2.5	50	97.5	Sequence
**0**	**1**	**0.59**	**0.96**	**1.5**	**TQEDTLALADK**
**0**	**1**	**0.55**	**0.9**	**1.4**	**FNMEEVK**

5.398 91 2658 minus strand puromycin-sensitive aminopeptidase [Saccoglossus kowalevskii]

Protein Accession	CL6708.Contig2$_H$1
Mean Expression Ratio	1.06
Median Expression Ratio	1.06
Credible Interval	(0.714, 1.58)
Associated Peptides	2
Associated Spectra	2
Coverage	NaN

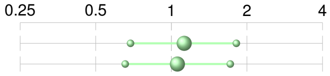

A	B	2.5	50	97.5	Sequence
**1**	**0**	**0.69**	**1.1**	**1.8**	**GMNLYLNR**
**1**	**0**	**0.66**	**1.1**	**1.7**	**LPKDVIPSNYNIR**

5.399 219 428 Peptidyl-prolyl cis-trans isomerase OS=Hemicentrotus pulcherrimus PE=2 SV=1

Protein Accession	CL39270.Contig2$_H$1
Mean Expression Ratio	1.06
Median Expression Ratio	1.06
Credible Interval	(0.742, 1.53)
Associated Peptides	1
Associated Spectra	5
Coverage	NaN

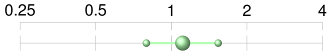

A	B	2.5	50	97.5	Sequence
**2**	**3**	**0.8**	**1.1**	**1.5**	**IIIANSGQV**

5.400 " 2057 2497 Phosphate carrier protein, mitochondrial [Crassostrea gigas]"

Protein Accession	CL2712.Contig11$_H$1
Mean Expression Ratio	0.94
Median Expression Ratio	0.941
Credible Interval	(0.607, 1.44)
Associated Peptides	1
Associated Spectra	1
Coverage	NaN

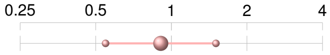

A	B	2.5	50	97.5	Sequence
**1**	**0**	**0.55**	**0.9**	**1.5**	**YVVPKPR**

5.401 87 485 Sex-regulated protein janus-A [Crassostrea gigas]; 92 310 PREDICTED: hypothetical protein [Saccoglossus kowalevskii]; 92 310 PREDICTED: hypothetical protein [Saccoglossus kowalevskii]

Protein Accession	CL13131.Contig3$_H$1 CL13131.Contig2$_H$1 CL13131.Contig1$_H$1
Mean Expression Ratio	1.06
Median Expression Ratio	1.06
Credible Interval	(0.682, 1.64)
Associated Peptides	1
Associated Spectra	1
Coverage	NaN

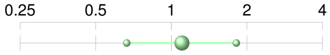

A	B	2.5	50	97.5	Sequence
**1**	**0**	**0.66**	**1.1**	**1.8**	**VVGEIESK**

5.402 680 991 minus strand PDZ and LIM domain protein 1 [Crassostrea gigas]; 553 864 minus strand PDZ and LIM domain protein 1 [Crassostrea gigas]

Protein Accession	Unigene7664$_{H}$1 Unigene7663$_{H}$1
Mean Expression Ratio	1.06
Median Expression Ratio	1.06
Credible Interval	(0.846, 1.35)
Associated Peptides	6
Associated Spectra	20
Coverage	NaN

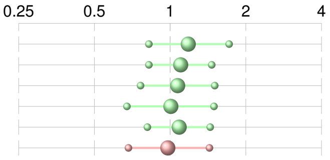

A	B	2.5	50	97.5	Sequence
2	0	0.82	1.2	1.7	AMGQPWGFR
3	3	0.83	1.1	1.5	DYGQALSVK
3	0	0.76	1.1	1.5	GPSTDFSAIKPK
1	0	0.67	1	1.5	KVQPGTPSAATLRPGDVIQR
3	3	0.82	1.1	1.4	QLTHMQAHQLIK
1	1	0.68	0.98	1.4	SAGTQLQLTVLR

5.403 " 343 1179 ATP synthase subunit alpha, mitochondrial OS=Pongo abelii GN=ATP5A1 PE=2 SV=1"

Protein Accession	Unigene30534$_{H}$1
Mean Expression Ratio	0.942
Median Expression Ratio	0.941
Credible Interval	(0.77, 1.15)
Associated Peptides	7
Associated Spectra	32
Coverage	NaN

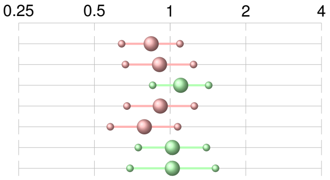

A	B	2.5	50	97.5	Sequence
3	4	0.64	0.84	1.1	TAVAIDTIINQK
4	0	0.66	0.91	1.2	TSVKEPMQTGIK
5	3	0.85	1.1	1.4	VLSIGDGIAR
1	3	0.67	0.91	1.2	VVDALGTPIDGK
2	2	0.58	0.79	1.1	APGIIPR
3	1	0.75	1	1.4	STVAQLVK
0	1	0.69	1	1.5	LSDADAMK

5.404 280 1239 minus strand PREDICTED: smoothelin isoform 2 [Oryctolagus cuniculus]; 280 1239 minus strand PREDICTED: smoothelin isoform 2 [Oryctolagus cuniculus]; 280 1239 PREDICTED: smoothelin isoform 2 [Oryctolagus cuniculus]; 280 1239 minus strand PREDICTED: smoothelin isoform 2 [Oryctolagus cuniculus]; 280 1239 PREDICTED: smoothelin isoform 2 [Oryctolagus cuniculus]; 280 1239 minus strand PREDICTED: smoothelin isoform 2 [Oryctolagus cuniculus]; 280 1239 minus strand PREDICTED: smoothelin isoform 2 [Oryctolagus cuniculus]

Protein Accession	CL2374.Contig7$_{H}$1 CL2374.Contig6$_{H}$1 CL2374.Contig5$_{H}$1 CL2374.Contig4$_{H}$1 CL2374.Con
Mean Expression Ratio	0.944
Median Expression Ratio	0.941
Credible Interval	(0.69, 1.3)
Associated Peptides	4
Associated Spectra	6
Coverage	NaN

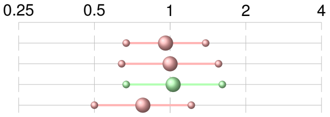

A	B	2.5	50	97.5	Sequence
2	1	0.67	0.96	1.4	HNFQLAFDTAEK
1	0	0.64	1	1.6	LMNATDSFDER
1	0	0.67	1	1.6	EKETEDLVR
0	1	0.5	0.78	1.2	PLLDVEDMVK

5.405 166 2364 Dipeptidyl-peptidase 3 [Crassostrea gigas]

Protein Accession	CL9071.Contig2$_{H}1$
Mean Expression Ratio	1.06
Median Expression Ratio	1.06
Credible Interval	(0.734, 1.52)
Associated Peptides	3
Associated Spectra	3
Coverage	NaN

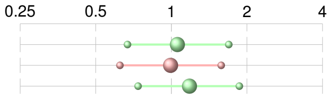

A	B	2.5	50	97.5	Sequence
1	0	0.67	1.1	1.7	SFETGSIPAHK
1	0	0.62	0.99	1.6	YVILR
1	0	0.74	1.2	1.9	VTGADGKP

5.406 794 994 Glycogenin-1 [Crassostrea gigas]; 1425 1739 minus strand Glycogenin-1 [Crassostrea gigas]

Protein Accession	CL18608.Contig3$_{H}1$ CL18608.Contig2$_{H}1$
Mean Expression Ratio	1.06
Median Expression Ratio	1.06
Credible Interval	(0.745, 1.53)
Associated Peptides	3
Associated Spectra	3
Coverage	NaN

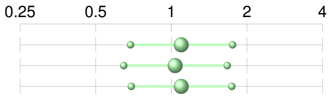

A	B	2.5	50	97.5	Sequence
1	0	0.69	1.1	1.7	VGGGESADYLILDDR
1	0	0.64	1	1.7	VGGGESADYLILDDRER
0	1	0.69	1.1	1.7	IAAPVLSFK

5.407 203 1942 ezrin/radixin/moesin [Aplysia californica] gi; 243 1982 minus strand ezrin/radixin/moesin [Aplysia californica] gi

Protein Accession	CL13506.Contig5$_{H}1$ CL13506.Contig4$_{H}1$
Mean Expression Ratio	1.06
Median Expression Ratio	1.06
Credible Interval	(0.872, 1.29)
Associated Peptides	13
Associated Spectra	23
Coverage	NaN

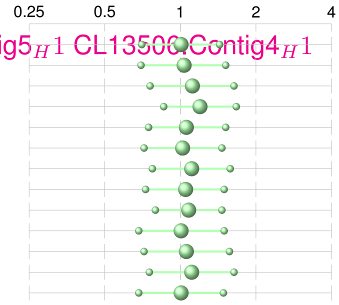

A	B	2.5	50	97.5	Sequence
1	1	0.7	1	1.4	DDAMMEYLK
1	0	0.7	1	1.5	FVIKPIDK
1	0	0.76	1.1	1.6	KAPDFVFYAPR
3	0	0.86	1.2	1.7	NISFNDKK
2	0	0.75	1.1	1.5	RKPDTIEVQQMK
2	0	0.72	1	1.5	KESPLQFK
1	1	0.77	1.1	1.6	QLFDQVVK
2	0	0.73	1	1.5	QLEMLK
4	0	0.79	1.1	1.5	SWWGEHK
1	0	0.68	1	1.5	LADELETAQR
0	1	0.72	1.1	1.6	MAELLALR
0	1	0.75	1.1	1.6	IGFPWSEIR
0	1	0.68	1	1.5	ESPLQFK

5.408 3 254 Calpain-5 [Crassostrea gigas]; 3 254 minus strand Calpain-5 [Crassostrea gigas]

Protein Accession	CL41869.Contig2$_{H}1$ CL41869.Contig1$_{H}1$
Mean Expression Ratio	0.943
Median Expression Ratio	0.943
Credible Interval	(0.63, 1.41)
Associated Peptides	1
Associated Spectra	2
Coverage	NaN

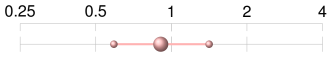

A	B	2.5	50	97.5	Sequence
**1**	**1**	**0.59**	**0.91**	**1.4**	**EVDLFGHK**

5.409 316 1512 Alpha-N-acetylgalactosaminidase [Crassostrea gigas]; 282 1478 Alpha-N-acetylgalactosaminidase [Crassostrea gigas]

Protein Accession	CL2428.Contig4$_{H}1$ CL2428.Contig1$_{H}1$
Mean Expression Ratio	0.944
Median Expression Ratio	0.943
Credible Interval	(0.634, 1.4)
Associated Peptides	2
Associated Spectra	5
Coverage	NaN

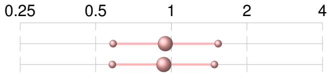

A	B	2.5	50	97.5	Sequence
**1**	**0**	**0.59**	**0.95**	**1.5**	**LGIYEDFGSK**
**1**	**3**	**0.58**	**0.93**	**1.5**	**LVELGLR**

5.410 5 1018 minus strand Glutaredoxin-3 [Crassostrea gigas]; 5 1018 Glutaredoxin-3 [Crassostrea gigas]

Protein Accession	CL7734.Contig9$_{H}1$ CL7734.Contig10$_{H}1$
Mean Expression Ratio	1.06
Median Expression Ratio	1.06
Credible Interval	(0.706, 1.59)
Associated Peptides	1
Associated Spectra	2
Coverage	NaN

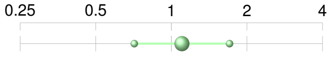

A	B	2.5	50	97.5	Sequence
**1**	**1**	**0.71**	**1.1**	**1.7**	**VAAEDLAEVSHR**

5.411 1709 2026 Phosphoenolpyruvate carboxykinase [GTP] [Crassostrea gigas]

Protein Accession	CL25948.Contig2$_{H}1$
Mean Expression Ratio	0.943
Median Expression Ratio	0.944
Credible Interval	(0.664, 1.33)
Associated Peptides	1
Associated Spectra	8
Coverage	NaN

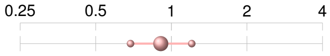

A	B	2.5	50	97.5	Sequence
**5**	**3**	**0.69**	**0.91**	**1.2**	**GVPLVYEAK**

5.412 1017 1499 minus strand PREDICTED: leukocyte elastase inhibitor-like [Oreochromis niloticus]

Protein Accession	CL39031.Contig4$_H1$
Mean Expression Ratio	0.945
Median Expression Ratio	0.944
Credible Interval	(0.632, 1.42)
Associated Peptides	1
Associated Spectra	2
Coverage	NaN

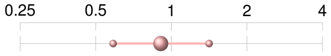

A	B	2.5	50	97.5	Sequence
**0**	**2**	**0.59**	**0.91**	**1.4**	**VLELPYGTDDR**

5.413 93 1034 Annexin A7 [Crassostrea gigas]; 73 1014 Annexin A7 [Crassostrea gigas]

Protein Accession	CL718.Contig3$_H1$ CL718.Contig1$_H1$
Mean Expression Ratio	1.06
Median Expression Ratio	1.06
Credible Interval	(0.814, 1.39)
Associated Peptides	6
Associated Spectra	11
Coverage	NaN

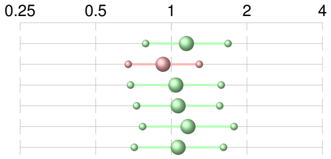

A	B	2.5	50	97.5	Sequence
**2**	**0**	**0.79**	**1.2**	**1.7**	**ESDEWEAGDDAK**
**2**	**2**	**0.67**	**0.93**	**1.3**	**LEVAQAFK**
**1**	**0**	**0.69**	**1**	**1.6**	**KYEQLTGR**
**1**	**1**	**0.73**	**1.1**	**1.6**	**SEIDMAEIKK**
**0**	**1**	**0.77**	**1.2**	**1.8**	**TGTVFPADHFDAGK**
**0**	**1**	**0.71**	**1.1**	**1.6**	**PADHFDAGK**

5.414 1050 1307 calreticulin precursor [Aplysia californica] gi

Protein Accession	CL4813.Contig1$_H1$
Mean Expression Ratio	0.945
Median Expression Ratio	0.944
Credible Interval	(0.609, 1.45)
Associated Peptides	1
Associated Spectra	1
Coverage	NaN

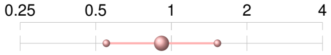

A	B	2.5	50	97.5	Sequence
**0**	**1**	**0.55**	**0.91**	**1.5**	**EFGDSTWGK**

5.415 144 800 minus strand PREDICTED: protein F37C4.5-like [Amphimedon queenslandica]

Protein Accession	CL7246.Contig3$_H1$
Mean Expression Ratio	1.06
Median Expression Ratio	1.06
Credible Interval	(0.779, 1.44)
Associated Peptides	4
Associated Spectra	7
Coverage	NaN

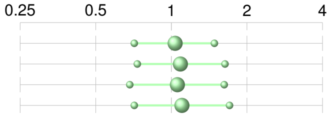

A	B	2.5	50	97.5	Sequence
**2**	**1**	**0.71**	**1**	**1.5**	**VTVGLVDIKPK**
**1**	**1**	**0.73**	**1.1**	**1.6**	**VSLLEPQLDLK**
**1**	**0**	**0.68**	**1.1**	**1.6**	**NKDKDVTLNK**
**1**	**0**	**0.71**	**1.1**	**1.7**	**VISNTHEQVIEVK**

5.416 " 112 1320 Aspartate aminotransferase, cytoplasmic [Crassostrea gigas]"

Protein Accession	CL44136.Contig1$_{H}1$
Mean Expression Ratio	0.944
Median Expression Ratio	0.945
Credible Interval	(0.765, 1.16)
Associated Peptides	9
Associated Spectra	35
Coverage	NaN

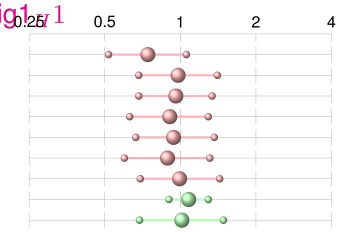

A	B	2.5	50	97.5	Sequence
1	1	0.52	0.74	1.1	ASIFADVK
1	1	0.68	0.98	1.4	IADVMEAK
2	1	0.68	0.96	1.3	SQMEIIVR
2	0	0.63	0.91	1.3	TPSVIPQIR
2	0	0.67	0.94	1.4	VNLGVGAYR
1	0	0.6	0.89	1.3	ISMAGVTNK
2	0	0.69	0.99	1.4	LAAEFLK
12	8	0.9	1.1	1.3	VEGIQVQGGTGGIR
0	1	0.69	1	1.5	TLANALILGK

5.417 54 662 minus strand Protein DJ-1 [Crassostrea gigas]

Protein Accession	CL16588.Contig3$_{H}1$
Mean Expression Ratio	0.939
Median Expression Ratio	0.945
Credible Interval	(0.601, 1.45)
Associated Peptides	1
Associated Spectra	1
Coverage	NaN

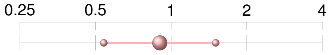

A	B	2.5	50	97.5	Sequence
1	0	0.54	0.9	1.5	NVNIVPDASMEEALK

5.418 84 425 minus strand Transcription elongation factor B polypeptide 2 [Crassostrea gigas]; 84 425 minus strand Transcription elongation factor B polypeptide 2 [Crassostrea gigas]; 84 425 minus strand Transcription elongation factor B polypeptide 2 [Crassostrea gigas]; 101 442 minus strand Transcription elongation factor B polypeptide 2 [Crassostrea gigas]; 101 442 minus strand Transcription elongation factor B polypeptide 2 [Crassostrea gigas]; 84 425 minus strand Transcription elongation factor B polypeptide 2 [Crassostrea gigas]

Protein Accession	CL19448.Contig9$_{H}1$ CL19448.Contig8$_{H}1$ CL19448.Contig7$_{H}1$ CL19448.Contig1$_{H}1$ CL1944
Mean Expression Ratio	0.948
Median Expression Ratio	0.945
Credible Interval	(0.615, 1.46)
Associated Peptides	1
Associated Spectra	1
Coverage	NaN

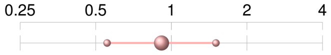

A	B	2.5	50	97.5	Sequence
1	0	0.55	0.91	1.5	AQAPATLGLVYK

5.419 245 1243 Prostaglandin reductase 1 [Crassostrea gigas]

Protein Accession	CL37890.Contig1$_H$1
Mean Expression Ratio	0.946
Median Expression Ratio	0.945
Credible Interval	(0.616, 1.47)
Associated Peptides	1
Associated Spectra	1
Coverage	NaN

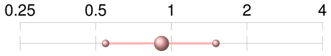

A	B	2.5	50	97.5	Sequence
1	0	0.55	0.91	1.5	TLAVLHPDMPR

5.420 139 945 Kynurenine formamidase [Crassostrea gigas]; 145 951 Kynurenine formamidase [Crassostrea gigas]; 220 1026 Kynurenine formamidase [Crassostrea gigas]; 39 743 Kynurenine formamidase [Crassostrea gigas]; 123 929 Kynurenine formamidase [Crassostrea gigas]; 39 743 Kynurenine formamidase [Crassostrea gigas]

Protein Accession	CL8132.Contig9$_H$1 CL8132.Contig8$_H$1 CL8132.Contig7$_H$1 CL8132.Contig3$_H$1 CL8132.Con
Mean Expression Ratio	0.943
Median Expression Ratio	0.945
Credible Interval	(0.656, 1.36)
Associated Peptides	2
Associated Spectra	7
Coverage	NaN

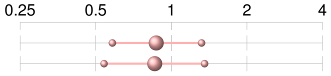

A	B	2.5	50	97.5	Sequence
4	2	0.58	0.87	1.3	LVGPGVVIDVR
0	1	0.54	0.86	1.4	WLTTNR

5.421 134 385 minus strand CDGSH iron sulfur domain-containing protein 2 [Crassostrea gigas]; 11 511 minus strand CDGSH iron sulfur domain-containing protein 2 [Crassostrea gigas]

Protein Accession	CL36832.Contig5$_H$1 CL36832.Contig4$_H$1
Mean Expression Ratio	0.945
Median Expression Ratio	0.945
Credible Interval	(0.61, 1.46)
Associated Peptides	1
Associated Spectra	1
Coverage	NaN

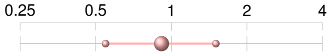

A	B	2.5	50	97.5	Sequence
1	0	0.55	0.91	1.5	VVNMVDIEDLGDK

5.422 112 819 pyruvate kinase [Crassostrea gigas]; 132 932 pyruvate kinase [Crassostrea gigas]

Protein Accession	CL31920.Contig3$_H$1 CL31920.Contig1$_H$1
Mean Expression Ratio	0.948
Median Expression Ratio	0.946
Credible Interval	(0.616, 1.48)
Associated Peptides	1
Associated Spectra	1
Coverage	NaN

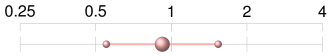

A	B	2.5	50	97.5	Sequence
1	0	0.55	0.92	1.5	ITIDDSYKEK

5.423 1118 1255 minus strand equistatin precursor [Actinia equina]

Protein Accession	CL27616.Contig7$_H$1
Mean Expression Ratio	0.944
Median Expression Ratio	0.946
Credible Interval	(0.608, 1.47)
Associated Peptides	1
Associated Spectra	1
Coverage	NaN

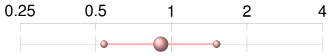

A	B	2.5	50	97.5	Sequence
1	0	0.54	0.91	1.5	HLLGSYIPR

5.424 338 661 Spectrin alpha chain [Crassostrea gigas]

Protein Accession	CL26928.Contig1$_H$1
Mean Expression Ratio	1.06
Median Expression Ratio	1.06
Credible Interval	(0.729, 1.54)
Associated Peptides	2
Associated Spectra	3
Coverage	NaN

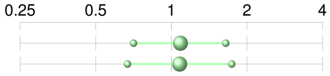

A	B	2.5	50	97.5	Sequence
1	1	0.71	1.1	1.7	DVAGAEALLER
0	1	0.67	1.1	1.7	AIISADDLAK

5.425 151 1449 rab GDP-dissociation inhibitor [Branchiostoma belcheri]

Protein Accession	CL30719.Contig3$_H$1
Mean Expression Ratio	1.06
Median Expression Ratio	1.06
Credible Interval	(0.828, 1.35)
Associated Peptides	8
Associated Spectra	13
Coverage	NaN

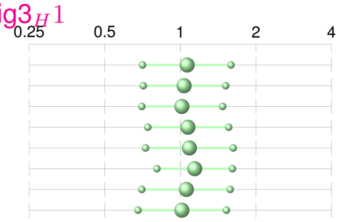

A	B	2.5	50	97.5	Sequence
1	0	0.71	1.1	1.6	EALGTSLMGMFEK
1	1	0.71	1	1.5	FLMANGQLVK
1	1	0.7	1	1.5	GVDCNGMTAAQLYEK
1	1	0.74	1.1	1.6	ITGEAFDFSK
1	0	0.73	1.1	1.6	IVYDETGVAVGVQDADGAVAK
2	1	0.81	1.1	1.6	LLVATGVTR
1	0	0.7	1.1	1.6	QWNVDLIPK
0	1	0.68	1	1.5	DLIAR

5.426 14 223 minus strand Endothelin-converting enzyme 2 OS=Homo sapiens GN=ECE2 PE=1 SV=4

Protein Accession	Unigene41869$_{H}1$
Mean Expression Ratio	0.948
Median Expression Ratio	0.947
Credible Interval	(0.63, 1.42)
Associated Peptides	1
Associated Spectra	2
Coverage	NaN

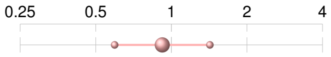

A	B	2.5	50	97.5	Sequence
2	0	0.59	0.92	1.4	KLLEAQNK

5.427 137 853 14-3-3 protein [Heterodera glycines]

Protein Accession	CL9040.Contig4$_{H}1$
Mean Expression Ratio	0.947
Median Expression Ratio	0.947
Credible Interval	(0.687, 1.3)
Associated Peptides	4
Associated Spectra	6
Coverage	NaN

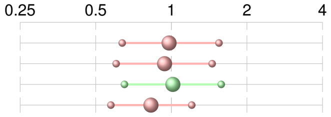

A	B	2.5	50	97.5	Sequence
1	0	0.64	0.98	1.6	AIECANQAYQK
1	0	0.6	0.94	1.5	EAFDEAISELDSLQGEDCK
1	0	0.65	1	1.6	NASNEEENVFYLK
3	0	0.57	0.83	1.2	VICMIEQK

5.428 15 428 PREDICTED: histone H2A.V-like [Apis florea]

Protein Accession	CL14228.Contig5$_{H}1$
Mean Expression Ratio	0.948
Median Expression Ratio	0.947
Credible Interval	(0.723, 1.24)
Associated Peptides	4
Associated Spectra	22
Coverage	NaN

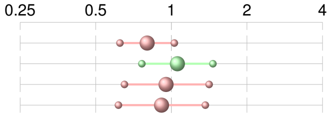

A	B	2.5	50	97.5	Sequence
9	3	0.62	0.8	1	AGLQFPVGR
3	3	0.76	1.1	1.5	ATIAGGGVIPHIHK
1	1	0.65	0.95	1.4	GDEELDSLIK
1	1	0.61	0.91	1.4	HLQLAIR

5.429 194 775 minus strand Collagen alpha-1(XV) chain [Harpegnathos saltator]

Protein Accession	Unigene19084$_{H}1$
Mean Expression Ratio	0.949
Median Expression Ratio	0.947
Credible Interval	(0.636, 1.41)
Associated Peptides	2
Associated Spectra	2
Coverage	NaN

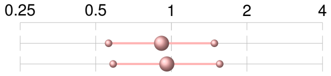

A	B	2.5	50	97.5	Sequence
1	0	0.56	0.91	1.5	GADYECHK
1	0	0.59	0.96	1.6	IIWHGSSK

5.430 69 779 PREDICTED: 15-hydroxyprostaglandin dehydrogenase [NAD+] [Gallus gallus]

Protein Accession	CL44148.Contig4$_{H1}$
Mean Expression Ratio	0.949
Median Expression Ratio	0.947
Credible Interval	(0.638, 1.4)
Associated Peptides	2
Associated Spectra	2
Coverage	NaN

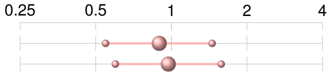

A	B	2.5	50	97.5	Sequence
**1**	**0**	**0.55**	**0.89**	**1.5**	**PLVENPPSPTVIGEAVIK**
**1**	**0**	**0.6**	**0.97**	**1.6**	**PPSPTVIGEAVIK**

5.431 " 1 321 Dynein heavy chain, cytoplasmic [Crassostrea gigas]"

Protein Accession	Unigene35195$_{H1}$
Mean Expression Ratio	1.06
Median Expression Ratio	1.06
Credible Interval	(0.75, 1.49)
Associated Peptides	1
Associated Spectra	11
Coverage	NaN

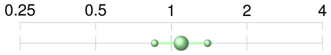

A	B	2.5	50	97.5	Sequence
**6**	**5**	**0.86**	**1.1**	**1.4**	**EILAVIQK**

5.432 4 849 minus strand elongation factor 1-alpha [Cryptosporidium muris RN66]

Protein Accession	CL35888.Contig1$_{H1}$
Mean Expression Ratio	1.05
Median Expression Ratio	1.05
Credible Interval	(0.735, 1.49)
Associated Peptides	2
Associated Spectra	12
Coverage	NaN

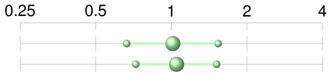

A	B	2.5	50	97.5	Sequence
**5**	**1**	**0.66**	**1**	**1.5**	**IGGIGTVPVGR**
**3**	**3**	**0.72**	**1**	**1.5**	**LPLQDVYK**

5.433 " 213 1499 minus strand Citrate synthase, mitochondrial [Crassostrea gigas]"

Protein Accession	CL992.Contig2$_H$1
Mean Expression Ratio	0.948
Median Expression Ratio	0.949
Credible Interval	(0.784, 1.14)
Associated Peptides	12
Associated Spectra	32
Coverage	NaN

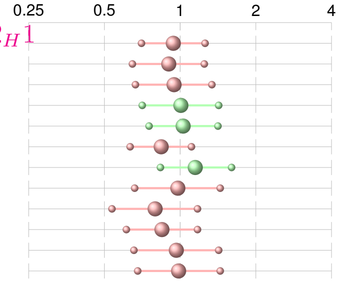

A	B	2.5	50	97.5	Sequence
4	1	0.7	0.94	1.3	ALGLPIERPK
3	0	0.65	0.9	1.3	ALVTETSVLDPEEGIR
1	1	0.66	0.95	1.3	DVLSQIIPEK
2	0	0.71	1	1.4	LMESLGGEVSDEQLR
3	1	0.75	1	1.4	SGQVVPGYGHAVLR
4	2	0.63	0.84	1.1	VVPDILIEQGK
3	0	0.84	1.2	1.6	SFDTPSLR
1	0	0.66	0.98	1.4	VGEVTVDMMYGGMR
1	0	0.53	0.8	1.2	EVSEFR
2	1	0.61	0.85	1.2	HLPNDPMFK
0	1	0.65	0.97	1.4	NFIWNTLK
0	1	0.68	0.99	1.4	LISQLYK

5.434 1 378 minus strand PREDICTED: CG10098-like [Saccoglossus kowalevskii]

Protein Accession	Unigene15933$_H$1
Mean Expression Ratio	1.05
Median Expression Ratio	1.05
Credible Interval	(0.706, 1.57)
Associated Peptides	2
Associated Spectra	2
Coverage	NaN

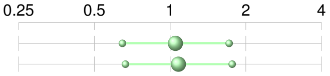

A	B	2.5	50	97.5	Sequence
1	0	0.65	1.1	1.7	AFNASFEGLSLSEK
1	0	0.66	1.1	1.8	ALMAEK

5.435 124 396 minus strand 40S ribosomal protein S10 OS=Lumbricus rubellus GN=RPS10 PE=2 SV=1

Protein Accession	Unigene42157$_H$1
Mean Expression Ratio	1.05
Median Expression Ratio	1.05
Credible Interval	(0.747, 1.48)
Associated Peptides	2
Associated Spectra	6
Coverage	NaN

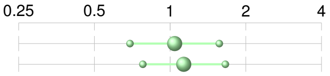

A	B	2.5	50	97.5	Sequence
2	1	0.69	1	1.6	KDFFSPK
3	0	0.78	1.1	1.7	YLRER

5.436 642 1415 minus strand cytochrome c oxidase subunit II [Oncomelania hupensis hupensis]

Protein Accession	CL6764.Contig3$_H1$
Mean Expression Ratio	0.948
Median Expression Ratio	0.95
Credible Interval	(0.611, 1.47)
Associated Peptides	1
Associated Spectra	1
Coverage	NaN

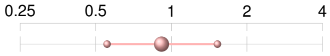

A	B	2.5	50	97.5	Sequence
**0**	**1**	**0.55**	**0.92**	**1.5**	**IVLPTHTDIR**

5.437 206 1513 hypothetical protein CGI_10019003 [Crassostrea gigas]

Protein Accession	CL6385.Contig8$_H1$
Mean Expression Ratio	1.06
Median Expression Ratio	1.05
Credible Interval	(0.685, 1.64)
Associated Peptides	1
Associated Spectra	1
Coverage	NaN

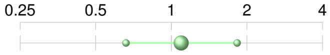

A	B	2.5	50	97.5	Sequence
**1**	**0**	**0.66**	**1.1**	**1.8**	**AALEDLLR**

5.438 2 169 cAMP-dependent protein kinase regulatory subunit [Crassostrea gigas]; 125 511 cAMP-dependent protein kinase regulatory subunit [Aplysia californica] gi

Protein Accession	CL24422.Contig2$_H1$ CL24422.Contig1$_H1$
Mean Expression Ratio	1.05
Median Expression Ratio	1.05
Credible Interval	(0.68, 1.63)
Associated Peptides	1
Associated Spectra	1
Coverage	NaN

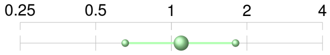

A	B	2.5	50	97.5	Sequence
**0**	**1**	**0.65**	**1.1**	**1.8**	**VLGPCSDILK**

5.439 95 271 minus strand hypothetical protein EAI_01530 [Harpegnathos saltator]

Protein Accession	CL9369.Contig9$_H1$
Mean Expression Ratio	1.05
Median Expression Ratio	1.05
Credible Interval	(0.705, 1.59)
Associated Peptides	1
Associated Spectra	2
Coverage	NaN

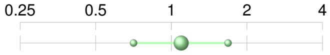

A	B	2.5	50	97.5	Sequence
**0**	**2**	**0.71**	**1.1**	**1.7**	**VAQFPFR**

5.440 58 732 glutathione-s-transferase [Haliotis discus discus]

Protein Accession CL4861.Contig1$_H$1
Mean Expression Ratio 0.949
Median Expression Ratio 0.95
Credible Interval (0.687, 1.32)
Associated Peptides 4
Associated Spectra 5
Coverage NaN

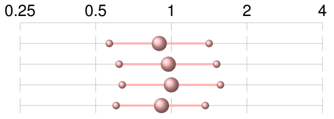

A	B	2.5	50	97.5	Sequence
0	1	0.57	0.89	1.4	IDKEYLEVK
0	1	0.62	0.97	1.5	GQVPTFR
0	1	0.64	1	1.6	WEEYLR
0	2	0.6	0.91	1.4	LISFSANEHK

5.441 131 541 minus strand nervous system adducin [Aplysia californica] gi

Protein Accession CL27337.Contig1$_H$1
Mean Expression Ratio 0.952
Median Expression Ratio 0.95
Credible Interval (0.683, 1.32)
Associated Peptides 3
Associated Spectra 5
Coverage NaN

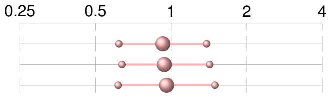

A	B	2.5	50	97.5	Sequence
1	1	0.62	0.92	1.4	FIDTIDPDDPEYIR
1	1	0.64	0.94	1.4	GIGASFGR
1	0	0.62	0.96	1.5	LAALYR

5.442 35 271 PREDICTED: SH3 domain-binding glutamic acid-rich-like protein 3-like [Anolis carolinensis]; 35 271 PREDICTED: SH3 domain-binding glutamic acid-rich-like protein 3-like [Anolis carolinensis]; 245 523 PREDICTED: SH3 domain-binding glutamic acid-rich-like protein 3-like [Anolis carolinensis]; 192 470 PREDICTED: SH3 domain-binding glutamic acid-rich-like protein 3-like [Anolis carolinensis]; 192 470 PREDICTED: SH3 domain-binding glutamic acid-rich-like protein 3-like [Anolis carolinensis]; 192 470 PREDICTED: SH3 domain-binding glutamic acid-rich-like protein 3-like [Anolis carolinensis]; 111 389 PREDICTED: SH3 domain-binding glutamic acid-rich-like protein 3-like [Anolis carolinensis]; 144 380 PREDICTED: SH3 domain-binding glutamic acid-rich-like protein 3-like [Anolis carolinensis]; 111 389 PREDICTED: SH3 domain-binding glutamic acid-rich-like protein 3-like [Anolis carolinensis]; 35 271 PREDICTED: SH3 domain-binding glutamic acid-rich-like protein 3-like [Anolis carolinensis]

Protein Accession Unigene58804$_H$1 Unigene57834$_H$1 Unigene53929$_H$1 Unigene46648$_H$1 Unigene46647$_H$1 U
Mean Expression Ratio 1.05
Median Expression Ratio 1.05
Credible Interval (0.721, 1.52)
Associated Peptides 2
Associated Spectra 3
Coverage NaN

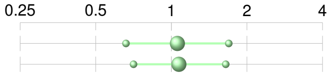

A	B	2.5	50	97.5	Sequence
1	0	0.66	1.1	1.7	SFQQAVEEEK
2	0	0.71	1.1	1.6	ILEYLR

5.443 625 1902 minus strand hypothetical protein CGI_10009847 [Crassostrea gigas]

Protein Accession	Unigene8159$_{H}1$
Mean Expression Ratio	1.05
Median Expression Ratio	1.05
Credible Interval	(0.683, 1.63)
Associated Peptides	1
Associated Spectra	1
Coverage	NaN

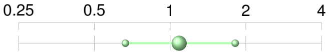

A	B	2.5	50	97.5	Sequence
**0**	**1**	**0.66**	**1.1**	**1.8**	**LEHVPVTTFR**

5.444 54 278 ATP-citrate synthase [Crassostrea gigas]; 61 465 ATP-citrate synthase [Crassostrea gigas]

Protein Accession	CL35133.Contig2$_{H}1$ CL35133.Contig1$_{H}1$
Mean Expression Ratio	0.952
Median Expression Ratio	0.951
Credible Interval	(0.614, 1.47)
Associated Peptides	1
Associated Spectra	1
Coverage	NaN

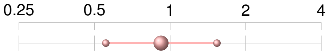

A	B	2.5	50	97.5	Sequence
**1**	**0**	**0.56**	**0.92**	**1.5**	**TIAIIAEGIPENLTR**

5.445 2939 3208 minus strand beta-thymosin domain repeat protein CSP24KDa_v1 [Hermissenda crassicornis] gi

Protein Accession	CL9548.Contig3$_{H}1$
Mean Expression Ratio	1.05
Median Expression Ratio	1.05
Credible Interval	(0.784, 1.41)
Associated Peptides	4
Associated Spectra	8
Coverage	NaN

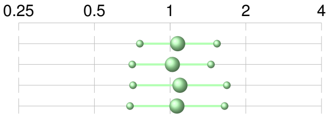

A	B	2.5	50	97.5	Sequence
**2**	**1**	**0.76**	**1.1**	**1.5**	**NPLPSTDAIAAEK**
**2**	**1**	**0.71**	**1**	**1.5**	**AETEEKNPLPTK**
**1**	**0**	**0.71**	**1.1**	**1.7**	**KAETEEKNPLPTK**
**0**	**1**	**0.69**	**1.1**	**1.6**	**SIGEFDK**

5.446 242 589 Ribonucleoprotein RB97D OS=Drosophila melanogaster GN=Rb97D PE=2 SV=1

Protein Accession	CL6198.Contig1$_{H}1$
Mean Expression Ratio	0.952
Median Expression Ratio	0.952
Credible Interval	(0.623, 1.46)
Associated Peptides	1
Associated Spectra	1
Coverage	NaN

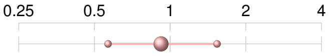

A	B	2.5	50	97.5	Sequence
**1**	**0**	**0.57**	**0.92**	**1.5**	**LFIGGLSYDTDEK**

5.447 835 1116 tropomyosin [Ilyanassa obsoleta]

Protein Accession	CL2925.Contig8$_{H}1$
Mean Expression Ratio	1.05
Median Expression Ratio	1.05
Credible Interval	(0.811, 1.36)
Associated Peptides	4
Associated Spectra	15
Coverage	NaN

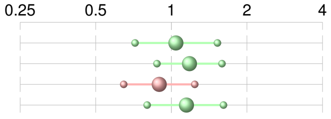

A	B	2.5	50	97.5	Sequence
1	1	0.72	1	1.5	ELEEQSHALTNSLK
2	4	0.88	1.2	1.6	LAVMEAELEK
2	2	0.65	0.89	1.2	QLELYEEK
0	3	0.8	1.1	1.6	LEDVETELR

5.448 " 45 506 minus strand PREDICTED: dihydrolipoyl dehydrogenase, mitochondrial [Monodelphis domestica]"

Protein Accession	CL24292.Contig3$_{H}1$
Mean Expression Ratio	1.05
Median Expression Ratio	1.05
Credible Interval	(0.707, 1.56)
Associated Peptides	2
Associated Spectra	2
Coverage	NaN

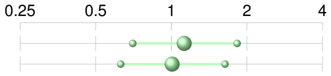

A	B	2.5	50	97.5	Sequence
0	1	0.7	1.1	1.8	ALTGGIAHLFK
0	1	0.63	1	1.6	GIEIDGLR

5.449 290 2458 Putative beta-D-xylosidase 5 [Crassostrea gigas]; 299 2428 Putative beta-D-xylosidase 2 [Crassostrea gigas]

Protein Accession	CL49577.Contig2$_{H}1$ CL1594.Contig5$_{H}1$
Mean Expression Ratio	1.05
Median Expression Ratio	1.05
Credible Interval	(0.711, 1.57)
Associated Peptides	2
Associated Spectra	3
Coverage	NaN

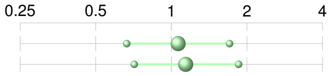

A	B	2.5	50	97.5	Sequence
1	1	0.66	1.1	1.7	VAEAAGVEVR
1	0	0.71	1.1	1.9	VTPLFYTR

5.450 771 1505 O-methyltransferase family 3 [Dicentrarchus labrax]

Protein Accession	Unigene19132$_{H}1$
Mean Expression Ratio	0.952
Median Expression Ratio	0.953
Credible Interval	(0.662, 1.37)
Associated Peptides	3
Associated Spectra	3
Coverage	NaN

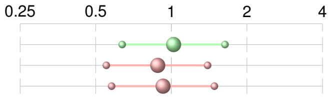

A	B	2.5	50	97.5	Sequence
1	0	0.64	1	1.6	FGASYLPR
0	1	0.55	0.88	1.4	IAETTLTHPWR
0	1	0.58	0.93	1.5	VLIPVR

5.451 209 781 minus strand high mobility group protein 1 [Biomphalaria glabrata]; 277 771 high mobility group protein 1 [Biomphalaria glabrata]; 277 867 minus strand high mobility group protein 1 [Biomphalaria glabrata]; 277 870 minus strand high mobility group protein 1 [Biomphalaria glabrata]

Protein Accession	CL25076.Contig4$_{H}1$ CL25076.Contig3$_{H}1$ CL25076.Contig2$_{H}1$ CL25076.Contig1$_{H}1$
Mean Expression Ratio	1.05
Median Expression Ratio	1.05
Credible Interval	(0.703, 1.55)
Associated Peptides	2
Associated Spectra	2
Coverage	NaN

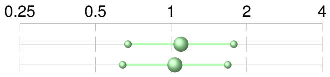

A	B	2.5	50	97.5	Sequence
1	0	0.67	1.1	1.8	LAGEEWR
1	0	0.64	1	1.7	KYEAEMAEYR

5.452 " 926 1189 minus strand Myosin heavy chain, striated muscle [Crassostrea gigas]"

Protein Accession	Unigene11948$_{H}1$
Mean Expression Ratio	1.05
Median Expression Ratio	1.05
Credible Interval	(0.82, 1.35)
Associated Peptides	3
Associated Spectra	32
Coverage	NaN

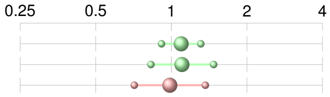

A	B	2.5	50	97.5	Sequence
11	11	0.91	1.1	1.3	LNGNHLGK
6	0	0.83	1.1	1.5	NKDPLNETVVELLSHSK
1	3	0.71	0.99	1.4	TFLDKLNGNHLGK

5.453 132 590 minus strand Non-neuronal cytoplasmic intermediate filament protein OS=Helix aspersa PE=3 SV=2

Protein Accession	Unigene7770$_{H}1$
Mean Expression Ratio	1.05
Median Expression Ratio	1.05
Credible Interval	(0.711, 1.54)
Associated Peptides	1
Associated Spectra	3
Coverage	NaN

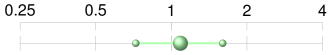

A	B	2.5	50	97.5	Sequence
3	0	0.72	1.1	1.6	TMAEVPLGEK

5.454 37 885 glyceraldehyde 3-phosphate dehydrogenase [Dugesia japonica]

Protein Accession	CL47424.Contig2$_{H}1$
Mean Expression Ratio	1.05
Median Expression Ratio	1.05
Credible Interval	(0.753, 1.46)
Associated Peptides	1
Associated Spectra	12
Coverage	NaN

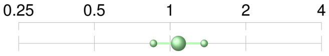

A	B	2.5	50	97.5	Sequence
**10**	**2**	**0.86**	**1.1**	**1.4**	**GAAQNIIPASTGAAK**

5.455 10 1902 Mitochondrial inner membrane protein [Crassostrea gigas]; 10 1902 Mitochondrial inner membrane protein [Crassostrea gigas]; 10 1902 Mitochondrial inner membrane protein [Crassostrea gigas]; 10 1902 Mitochondrial inner membrane protein [Crassostrea gigas]; 10 1902 Mitochondrial inner membrane protein [Crassostrea gigas]; 10 1902 Mitochondrial inner membrane protein [Crassostrea gigas]

Protein Accession	CL6414.Contig8$_{H}1$ CL6414.Contig7$_{H}1$ CL6414.Contig6$_{H}1$ CL6414.Contig5$_{H}1$ CL6414.Con
Mean Expression Ratio	0.96
Median Expression Ratio	0.954
Credible Interval	(0.623, 1.48)
Associated Peptides	1
Associated Spectra	1
Coverage	NaN

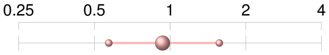

A	B	2.5	50	97.5	Sequence
**1**	**0**	**0.57**	**0.93**	**1.6**	**EIHTQLLQER**

5.456 793 1269 PREDICTED: calmodulin-like [Saccoglossus kowalevskii]

Protein Accession	CL42648.Contig1$_{H}1$
Mean Expression Ratio	1.05
Median Expression Ratio	1.05
Credible Interval	(0.784, 1.39)
Associated Peptides	5
Associated Spectra	8
Coverage	NaN

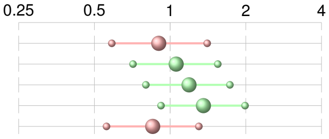

A	B	2.5	50	97.5	Sequence
**1**	**0**	**0.59**	**0.9**	**1.4**	**EADKDGNGLSIQELASLIR**
**1**	**1**	**0.71**	**1.1**	**1.5**	**KVSLVDITDEEIIK**
**2**	**0**	**0.8**	**1.2**	**1.7**	**KIQESELK**
**0**	**2**	**0.92**	**1.4**	**2**	**IFLDLDDNCDR**
**0**	**1**	**0.56**	**0.86**	**1.3**	**LAEADVDR**

5.457 " 3799 4371 papilin, partial [Rhipicephalus microplus]"

Protein Accession	CL19397.Contig5$_H$1
Mean Expression Ratio	1.05
Median Expression Ratio	1.05
Credible Interval	(0.695, 1.56)
Associated Peptides	1
Associated Spectra	2
Coverage	NaN

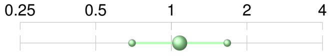

A	B	2.5	50	97.5	Sequence
**1**	**1**	**0.7**	**1.1**	**1.7**	**TVTLVLAK**

5.458 16 288 SJCHGC01950 protein [Schistosoma japonicum] gi

Protein Accession	Unigene16338$_H$1
Mean Expression Ratio	0.953
Median Expression Ratio	0.954
Credible Interval	(0.668, 1.36)
Associated Peptides	1
Associated Spectra	7
Coverage	NaN

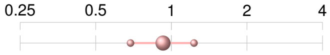

A	B	2.5	50	97.5	Sequence
**6**	**1**	**0.69**	**0.92**	**1.2**	**DIAAFIK**

5.459 587 811 hemocyanin 2 [Megathura crenulata]

Protein Accession	Unigene4334$_H$1
Mean Expression Ratio	1.05
Median Expression Ratio	1.05
Credible Interval	(0.709, 1.55)
Associated Peptides	2
Associated Spectra	2
Coverage	NaN

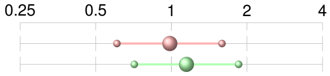

A	B	2.5	50	97.5	Sequence
**1**	**0**	**0.61**	**0.98**	**1.6**	**SVEDLTAGEAESLK**
**0**	**1**	**0.71**	**1.1**	**1.9**	**AIDDGTFR**

5.460 511 1062 unknown [Dendroctonus ponderosae]; 511 1062 unknown [Dendroctonus ponderosae]

Protein Accession	CL18443.Contig3$_H$1 CL18443.Contig1$_H$1
Mean Expression Ratio	1.05
Median Expression Ratio	1.05
Credible Interval	(0.72, 1.52)
Associated Peptides	2
Associated Spectra	3
Coverage	NaN

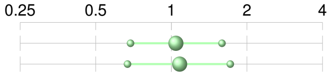

A	B	2.5	50	97.5	Sequence
**1**	**1**	**0.69**	**1**	**1.6**	**KHNFLIDGFPR**
**0**	**1**	**0.67**	**1.1**	**1.7**	**FKDEIEMHLK**

5.461 459 1187 PREDICTED: protein-L-isoaspartate(D-aspartate) O-methyltransferase-like isoform 2 [Anolis carolinensis]

Protein Accession	CL3223.Contig3$_{H}1$
Mean Expression Ratio	1.05
Median Expression Ratio	1.05
Credible Interval	(0.697, 1.56)
Associated Peptides	1
Associated Spectra	2
Coverage	NaN

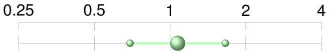

A	B	2.5	50	97.5	Sequence
**2**	**0**	**0.69**	**1.1**	**1.7**	**AVGIEHIEELVK**

5.462 1714 2121 Phosphoenolpyruvate carboxykinase [GTP] [Crassostrea gigas]

Protein Accession	CL25948.Contig1$_{H}1$
Mean Expression Ratio	0.954
Median Expression Ratio	0.956
Credible Interval	(0.679, 1.33)
Associated Peptides	1
Associated Spectra	10
Coverage	NaN

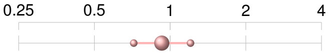

A	B	2.5	50	97.5	Sequence
**7**	**3**	**0.72**	**0.93**	**1.2**	**SEATAAAEFK**

5.463 145 459 fk506-binding protein 2 [Papilio xuthus]; 237 551 fk506-binding protein 2 [Papilio xuthus]

Protein Accession	Unigene7883$_{H}1$ CL4621.Contig4$_{H}1$
Mean Expression Ratio	1.04
Median Expression Ratio	1.05
Credible Interval	(0.792, 1.38)
Associated Peptides	4
Associated Spectra	11
Coverage	NaN

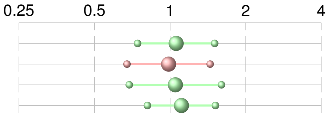

A	B	2.5	50	97.5	Sequence
**2**	**1**	**0.74**	**1.1**	**1.5**	**GVDVETISEGDKK**
**1**	**1**	**0.67**	**0.99**	**1.4**	**GWDEGVAQMSK**
**1**	**0**	**0.69**	**1.1**	**1.6**	**IGKGEVIK**
**4**	**1**	**0.81**	**1.1**	**1.5**	**NKPFQFK**

5.464 43 591 minus strand cyclophilin type peptidyl-prolyl cis-trans isomerase [Glossina morsitans morsitans]

Protein Accession	CL39270.Contig1$_{H}1$
Mean Expression Ratio	0.956
Median Expression Ratio	0.956
Credible Interval	(0.768, 1.19)
Associated Peptides	5
Associated Spectra	35
Coverage	NaN

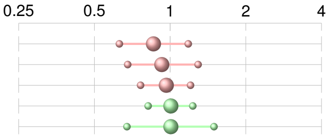

A	B	2.5	50	97.5	Sequence
**1**	**3**	**0.63**	**0.86**	**1.2**	**VFFDMTADGVPLGR**
**4**	**0**	**0.68**	**0.93**	**1.3**	**KIESYGSKNGATSQK**
**7**	**4**	**0.76**	**0.96**	**1.2**	**FADENFTLK**
**7**	**8**	**0.82**	**1**	**1.2**	**ITMELR**
**0**	**1**	**0.68**	**1**	**1.5**	**VIPQFMCQGGDFTR**

5.465 " 242 1504 Glutamate receptor, ionotropic kainate 3 [Crassostrea gigas]; 239 1501 Glutamate receptor, ionotropic kainate 3 [Crassostrea gigas]; 238 1500 Glutamate receptor, ionotropic kainate 3 [Crassostrea gigas]; 661 1923 minus strand Glutamate receptor, ionotropic kainate 3 [Crassostrea gigas]"

Protein Accession	CL12478.Contig7$_H1$ CL12478.Contig6$_H1$ CL12478.Contig5$_H1$ CL12478.Contig3$_H1$
Mean Expression Ratio	1.04
Median Expression Ratio	1.05
Credible Interval	(0.72, 1.52)
Associated Peptides	2
Associated Spectra	3
Coverage	NaN

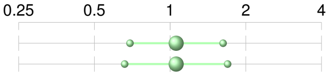

A	B	2.5	50	97.5	Sequence
**2**	**0**	**0.69**	**1.1**	**1.6**	**RPTFAQPSFQER**
**1**	**0**	**0.66**	**1.1**	**1.7**	**LAILR**

5.466 138 713 Rac [Aplysia californica] gi; 101 676 Rac [Aplysia californica] gi

Protein Accession	CL11009.Contig9$_H1$ CL11009.Contig1$_H1$
Mean Expression Ratio	1.05
Median Expression Ratio	1.05
Credible Interval	(0.679, 1.61)
Associated Peptides	1
Associated Spectra	1
Coverage	NaN

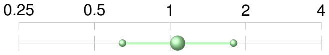

A	B	2.5	50	97.5	Sequence
**1**	**0**	**0.65**	**1.1**	**1.8**	**CVVVGDGAVGK**

5.467 392 808 Whirlin [Crassostrea gigas]

Protein Accession	CL22731.Contig1$_H1$
Mean Expression Ratio	1.05
Median Expression Ratio	1.05
Credible Interval	(0.729, 1.51)
Associated Peptides	1
Associated Spectra	5
Coverage	NaN

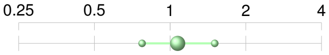

A	B	2.5	50	97.5	Sequence
**2**	**3**	**0.77**	**1.1**	**1.5**	**AGNDLHLLVQR**

5.468 54 638 minus strand ubiquitin carboxyl-terminal hydrolase [Aplysia californica] gi

Protein Accession	CL20666.Contig3$_{H1}$
Mean Expression Ratio	1.05
Median Expression Ratio	1.05
Credible Interval	(0.678, 1.64)
Associated Peptides	1
Associated Spectra	1
Coverage	NaN

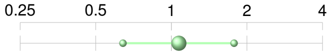

A	B	2.5	50	97.5	Sequence
1	0	0.64	1.1	1.8	THADTLLEDTVK

5.469 4 219 hypothetical protein BRAFLDRAFT_66351 [Branchiostoma floridae] gi; 3 239 hypothetical protein BRAFLDRAFT_66351 [Branchiostoma floridae] gi

Protein Accession	CL31114.Contig2$_{H1}$ CL31114.Contig1$_{H1}$
Mean Expression Ratio	1.04
Median Expression Ratio	1.05
Credible Interval	(0.671, 1.6)
Associated Peptides	1
Associated Spectra	1
Coverage	NaN

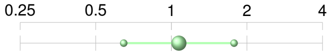

A	B	2.5	50	97.5	Sequence
0	1	0.64	1.1	1.8	KLEEVSGELR

5.470 " 134 1567 minus strand 6-phosphogluconate dehydrogenase, decarboxylating [Crassostrea gigas]; 134 1573 minus strand 6-phosphogluconate dehydrogenase, decarboxylating [Crassostrea gigas]"

Protein Accession	CL5140.Contig5$_{H1}$ CL5140.Contig4$_{H1}$
Mean Expression Ratio	0.957
Median Expression Ratio	0.957
Credible Interval	(0.741, 1.23)
Associated Peptides	7
Associated Spectra	12
Coverage	NaN

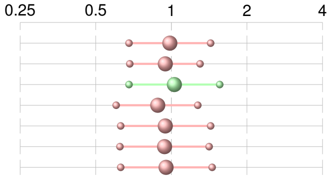

A	B	2.5	50	97.5	Sequence
1	1	0.68	0.99	1.4	AGQAVDDFIDK
2	2	0.68	0.94	1.3	SLEDMVDKLK
1	0	0.68	1	1.6	SVFLGNIK
1	1	0.6	0.88	1.3	LVPLLEK
1	0	0.63	0.95	1.4	YGPSLMPGGAEEAWPAIK
0	1	0.62	0.94	1.4	NIFQSISAK
0	1	0.63	0.95	1.5	AFVEDIKR

5.471 57 2009 hypothetical protein BRAFLDRAFT_285279 [Branchiostoma floridae] gi; 3 1973 hypothetical protein BRAFLDRAFT_285279 [Branchiostoma floridae] gi

Protein Accession	CL24444.Contig3$_H$1 CL24444.Contig1$_H$1
Mean Expression Ratio	0.962
Median Expression Ratio	0.958
Credible Interval	(0.647, 1.44)
Associated Peptides	2
Associated Spectra	2
Coverage	NaN

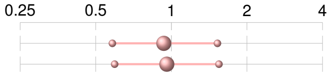

A	B	2.5	50	97.5	Sequence
1	0	0.58	0.93	1.5	INQIYEQ
0	1	0.6	0.96	1.6	KLTLK

5.472 880 1434 minus strand Prohormone-4 [Crassostrea gigas]

Protein Accession	CL27941.Contig2$_H$1
Mean Expression Ratio	0.96
Median Expression Ratio	0.958
Credible Interval	(0.625, 1.48)
Associated Peptides	1
Associated Spectra	1
Coverage	NaN

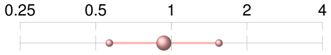

A	B	2.5	50	97.5	Sequence
1	0	0.57	0.93	1.6	AALQGAQAGDERPLLR

5.473 14 1642 minus strand chaperonin containing tcp1 [Haliotis discus discus]

Protein Accession	CL16298.Contig4$_H$1
Mean Expression Ratio	1.04
Median Expression Ratio	1.04
Credible Interval	(0.721, 1.49)
Associated Peptides	3
Associated Spectra	3
Coverage	NaN

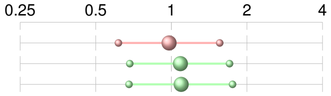

A	B	2.5	50	97.5	Sequence
1	0	0.61	0.98	1.6	ELEVQHPAAK
1	0	0.68	1.1	1.7	MVINHLEK
1	0	0.68	1.1	1.8	AIGATALPK

5.474 " 42 905 PREDICTED: enoyl-CoA hydratase, mitochondrial-like isoform 1 [Oreochromis niloticus]"

Protein Accession	CL6477.Contig7$_H$1
Mean Expression Ratio	1.04
Median Expression Ratio	1.04
Credible Interval	(0.74, 1.47)
Associated Peptides	2
Associated Spectra	5
Coverage	NaN

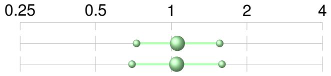

A	B	2.5	50	97.5	Sequence
1	2	0.72	1.1	1.6	AFAAGADIK
1	1	0.7	1.1	1.6	SVVLQASR

5.475 " 241 1110 minus strand N(G),N(G)-dimethylarginine dimethylaminohydrolase 1 [Crassostrea gigas]; 241 1110 minus strand N(G),N(G)-dimethylarginine dimethylaminohydrolase 1 [Crassostrea gigas]; 241 1110 minus strand N(G),N(G)-dimethylarginine dimethylaminohydrolase 1 [Crassostrea gigas]"

Protein Accession	Unigene30451$_{H}1$ CL7107.Contig3$_{H}1$ CL7107.Contig1$_{H}1$
Mean Expression Ratio	1.04
Median Expression Ratio	1.04
Credible Interval	(0.677, 1.62)
Associated Peptides	1
Associated Spectra	2
Coverage	NaN

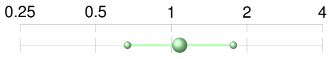

A	B	2.5	50	97.5	Sequence
**1**	**1**	**0.67**	**1.1**	**1.8**	**LGEVGEIR**

5.476 61 2865 AP-2 complex subunit beta [Crassostrea gigas]

Protein Accession	CL6957.Contig1$_{H}1$
Mean Expression Ratio	1.04
Median Expression Ratio	1.04
Credible Interval	(0.675, 1.6)
Associated Peptides	1
Associated Spectra	1
Coverage	NaN

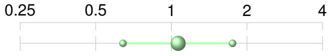

A	B	2.5	50	97.5	Sequence
**1**	**0**	**0.64**	**1.1**	**1.8**	**NSNIFTIAK**

5.477 184 438 PREDICTED: similar to CG14235 CG14235-PA [Tribolium castaneum] gi

Protein Accession	Unigene638$_{H}1$
Mean Expression Ratio	0.957
Median Expression Ratio	0.96
Credible Interval	(0.674, 1.35)
Associated Peptides	2
Associated Spectra	5
Coverage	NaN

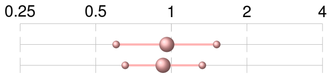

A	B	2.5	50	97.5	Sequence
**1**	**0**	**0.6**	**0.96**	**1.5**	**SLCPNEWVAK**
**3**	**1**	**0.66**	**0.93**	**1.3**	**WDDQVANGTFAGK**

5.478 " 1240 1911 PREDICTED: ATP synthase subunit alpha, mitochondrial-like [Anolis carolinensis]"

Protein Accession	Unigene30533$_{H}1$
Mean Expression Ratio	0.959
Median Expression Ratio	0.96
Credible Interval	(0.68, 1.35)
Associated Peptides	2
Associated Spectra	5
Coverage	NaN

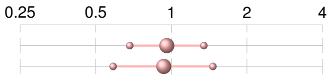

A	B	2.5	50	97.5	Sequence
**1**	**3**	**0.68**	**0.96**	**1.4**	**LELAQYR**
**0**	**1**	**0.59**	**0.93**	**1.5**	**LTELLK**

5.479 91 408 minus strand hypothetical protein [Amblyomma maculatum]

Protein Accession	CL19180.Contig1$_{H}1$
Mean Expression Ratio	0.961
Median Expression Ratio	0.96
Credible Interval	(0.622, 1.48)
Associated Peptides	1
Associated Spectra	1
Coverage	NaN

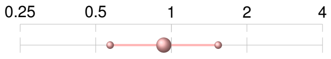

A	B	2.5	50	97.5	Sequence
**1**	**0**	**0.57**	**0.94**	**1.5**	**EVGWTIVDGR**

5.480 58 1203 minus strand Obg-like ATPase 1 [Crassostrea gigas]

Protein Accession	CL33105.Contig1$_{H}1$
Mean Expression Ratio	0.959
Median Expression Ratio	0.96
Credible Interval	(0.621, 1.49)
Associated Peptides	1
Associated Spectra	1
Coverage	NaN

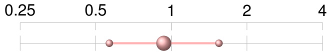

A	B	2.5	50	97.5	Sequence
**1**	**0**	**0.57**	**0.93**	**1.5**	**IHTDFEK**

5.481 116 1882 Actin-interacting protein 1 [Crassostrea gigas]

Protein Accession	CL12416.Contig1$_{H}1$
Mean Expression Ratio	0.96
Median Expression Ratio	0.96
Credible Interval	(0.764, 1.21)
Associated Peptides	9
Associated Spectra	17
Coverage	NaN

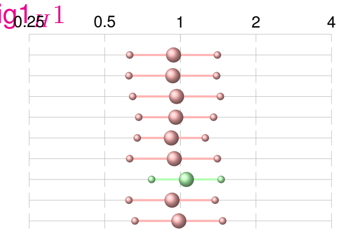

A	B	2.5	50	97.5	Sequence
**1**	**0**	**0.63**	**0.94**	**1.4**	**FSPDGEVAVSGGADGK**
**1**	**0**	**0.63**	**0.93**	**1.4**	**ILTASEDNSVGFHEGPPFK**
**1**	**0**	**0.64**	**0.96**	**1.4**	**LVSIGVDDVVR**
**1**	**2**	**0.68**	**0.96**	**1.4**	**QFLDGEVK**
**3**	**1**	**0.67**	**0.92**	**1.3**	**TLDELDNKEIGAPGAHK**
**1**	**0**	**0.63**	**0.94**	**1.4**	**VFDVQSGSLVQEFK**
**4**	**0**	**0.77**	**1.1**	**1.5**	**TNEVTDMVIEGDR**
**1**	**0**	**0.63**	**0.93**	**1.4**	**KLESQPK**
**0**	**1**	**0.66**	**0.99**	**1.5**	**QILTASGDASAK**

5.482 496 2151 NADP-dependent malic enzyme [Crassostrea gigas]

Protein Accession	CL26274.Contig1$_H$1
Mean Expression Ratio	0.961
Median Expression Ratio	0.96
Credible Interval	(0.8, 1.16)
Associated Peptides	11
Associated Spectra	40
Coverage	NaN

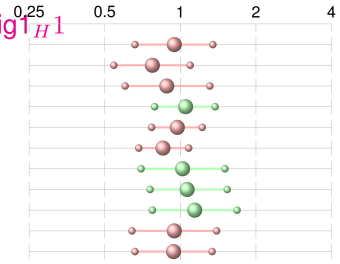

A	B	2.5	50	97.5	Sequence
1	1	0.66	0.94	1.3	AECTAEEAYTLTEGR
2	0	0.54	0.77	1.1	AIVVTDGER
1	0	0.6	0.88	1.3	GLFISIKDK
4	2	0.79	1	1.4	IWMVDSK
8	3	0.77	0.97	1.2	VLVNFR
7	4	0.68	0.85	1.1	GIDILR
1	0	0.7	1	1.5	GTAFSLK
1	1	0.76	1.1	1.5	GLLVK
1	0	0.77	1.1	1.7	GDLGVYGMGIP
0	1	0.64	0.95	1.4	QGTAAVAAAGVFAAVR
0	2	0.66	0.94	1.3	INERPIIFALSNPTSK

5.483 90 659 Plectin-1 [Crassostrea gigas]; 327 881 Plectin-1 [Crassostrea gigas]; 646 1290 minus strand Plectin-1 [Crassostrea gigas]

Protein Accession	CL7983.Contig3$_H$1 CL7983.Contig2$_H$1 CL7983.Contig1$_H$1
Mean Expression Ratio	0.961
Median Expression Ratio	0.961
Credible Interval	(0.621, 1.48)
Associated Peptides	1
Associated Spectra	1
Coverage	NaN

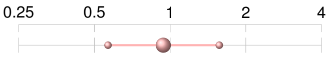

A	B	2.5	50	97.5	Sequence
0	1	0.57	0.94	1.6	AQHLVNVEEAR

5.484 582 2135 sulfatase 1A precursor [Haliotis discus discus]

Protein Accession	CL1700.Contig8$_H$1
Mean Expression Ratio	1.04
Median Expression Ratio	1.04
Credible Interval	(0.762, 1.43)
Associated Peptides	4
Associated Spectra	6
Coverage	NaN

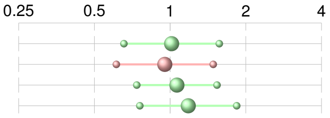

A	B	2.5	50	97.5	Sequence
1	0	0.65	1	1.6	HNEDVFYSAK
1	0	0.61	0.95	1.5	GKYPDVFK
2	1	0.74	1.1	1.5	AVSFLHSPK
0	1	0.76	1.2	1.8	EGHMYTPTLDGLAR

5.485 768 980 muscle LIM protein isoform 1 [Danaus plexippus]

Protein Accession	CL29824.Contig1$_H$1
Mean Expression Ratio	0.961
Median Expression Ratio	0.962
Credible Interval	(0.744, 1.23)
Associated Peptides	4
Associated Spectra	31
Coverage	NaN

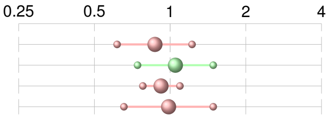

A	B	2.5	50	97.5	Sequence
3	0	0.62	0.87	1.2	SLDSTTMAMHETEVYCK
2	1	0.74	1	1.5	GVNTPWHK
18	6	0.78	0.92	1.1	AIGVNTPWHK
1	0	0.65	0.99	1.5	NFGPSGYGFGQGAGT

5.486 192 380 minus strand RAB protein [Haliotis discus discus]

Protein Accession	Unigene35065$_{H}1$
Mean Expression Ratio	1.04
Median Expression Ratio	1.04
Credible Interval	(0.667, 1.61)
Associated Peptides	1
Associated Spectra	1
Coverage	NaN

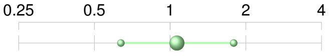

A	B	2.5	50	97.5	Sequence
**1**	**0**	**0.64**	**1.1**	**1.8**	**NVEEMFLAITK**

5.487 616 1740 5'-AMP-activated protein kinase subunit beta-2 [Crassostrea gigas]

Protein Accession	Unigene52845$_{H}1$
Mean Expression Ratio	1.04
Median Expression Ratio	1.04
Credible Interval	(0.67, 1.6)
Associated Peptides	1
Associated Spectra	1
Coverage	NaN

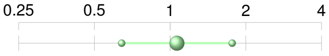

A	B	2.5	50	97.5	Sequence
**1**	**0**	**0.64**	**1.1**	**1.8**	**FIVDEQWVVSK**

5.488 22 429 AGAP006937-PA [Anopheles gambiae str. PEST] gi; 1 474 Ubiquitin-conjugating enzyme E2 variant 2 OS=Rattus norvegicus GN=Ube2v2 PE=1 SV=3

Protein Accession	CL27581.Contig3$_{H}1$ CL27581.Contig2$_{H}1$
Mean Expression Ratio	1.04
Median Expression Ratio	1.04
Credible Interval	(0.668, 1.61)
Associated Peptides	1
Associated Spectra	1
Coverage	NaN

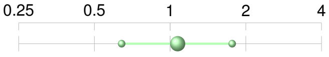

A	B	2.5	50	97.5	Sequence
**0**	**1**	**0.64**	**1.1**	**1.8**	**LLDELELGEK**

5.489 214 1878 PREDICTED: lysyl-tRNA synthetase-like [Oreochromis niloticus]

Protein Accession	CL25588.Contig2$_{H}1$
Mean Expression Ratio	0.963
Median Expression Ratio	0.962
Credible Interval	(0.624, 1.5)
Associated Peptides	1
Associated Spectra	1
Coverage	NaN

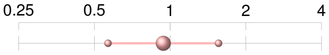

A	B	2.5	50	97.5	Sequence
**0**	**1**	**0.57**	**0.94**	**1.6**	**ISLISDLEK**

5.490 4 444 Vinculin [Crassostrea gigas]

Protein Accession	Unigene21732$_{H}1$
Mean Expression Ratio	1.04
Median Expression Ratio	1.04
Credible Interval	(0.767, 1.41)
Associated Peptides	4
Associated Spectra	9
Coverage	NaN

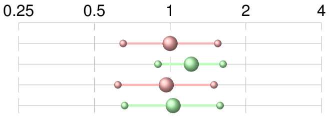

A	B	2.5	50	97.5	Sequence
**1**	**0**	**0.65**	**1**	**1.5**	**AIAEASEEVTR**
**4**	**2**	**0.9**	**1.2**	**1.6**	**IPTIGTQLK**
**1**	**0**	**0.62**	**0.96**	**1.5**	**TNLLNVCER**
**1**	**0**	**0.66**	**1**	**1.6**	**DNEIISAAK**

5.491 " 757 1443 minus strand nonmuscle myosin II, partial [Aplysia californica]"

Protein Accession	CL43455.Contig2$_{H}1$
Mean Expression Ratio	1.04
Median Expression Ratio	1.04
Credible Interval	(0.663, 1.6)
Associated Peptides	1
Associated Spectra	1
Coverage	NaN

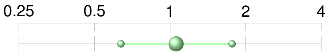

A	B	2.5	50	97.5	Sequence
**1**	**0**	**0.64**	**1.1**	**1.8**	**LVNEIK**

5.492 552 1499 minus strand uncharacterized protein LOC100158466 [Xenopus (Silurana) tropicalis] gi

Protein Accession	Unigene57645$_{H}1$
Mean Expression Ratio	0.963
Median Expression Ratio	0.963
Credible Interval	(0.653, 1.44)
Associated Peptides	2
Associated Spectra	3
Coverage	NaN

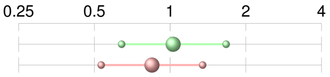

A	B	2.5	50	97.5	Sequence
**1**	**0**	**0.64**	**1**	**1.7**	**TAFSLLK**
**1**	**1**	**0.53**	**0.85**	**1.4**	**TGEPVEVDGELATLLAR**

5.493 425 1051 minus strand unknown [Schistosoma japonicum]; 300 878 unknown [Schistosoma japonicum]; 567 1145 unknown [Schistosoma japonicum]; 1110 1688 unknown [Schistosoma japonicum]; 94 672 unknown [Schistosoma japonicum]; 599 1177 unknown [Schistosoma japonicum]; 336 914 unknown [Schistosoma japonicum]

Protein Accession	CL11292.Contig9$_{H}1$ CL11292.Contig5$_{H}1$ CL11292.Contig3$_{H}1$ CL11292.Contig2$_{H}1$ CL1129
Mean Expression Ratio	0.965
Median Expression Ratio	0.963
Credible Interval	(0.691, 1.35)
Associated Peptides	2
Associated Spectra	9
Coverage	NaN

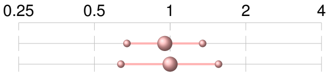

A	B	2.5	50	97.5	Sequence
4	4	0.67	0.95	1.3	AGNDVDLVVQR
0	1	0.64	1	1.6	RYDTSQPWGFR

5.494 2 1126 Prolyl endopeptidase [Crassostrea gigas]

Protein Accession	CL47692.Contig1$_{H}1$
Mean Expression Ratio	1.04
Median Expression Ratio	1.04
Credible Interval	(0.669, 1.6)
Associated Peptides	1
Associated Spectra	1
Coverage	NaN

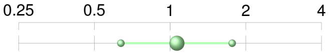

A	B	2.5	50	97.5	Sequence
1	0	0.64	1.1	1.8	VVPLHSLK

5.495 351 1826 Virulence metalloprotease [Crassostrea gigas]

Protein Accession	CL7226.Contig18$_{H}1$
Mean Expression Ratio	1.04
Median Expression Ratio	1.04
Credible Interval	(0.672, 1.59)
Associated Peptides	1
Associated Spectra	1
Coverage	NaN

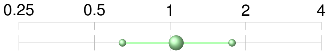

A	B	2.5	50	97.5	Sequence
1	0	0.65	1.1	1.8	EGDVCYLENK

5.496 252 848 sigma class glutathione-s-transferase 2 [Haliotis discus discus]

Protein Accession	CL19285.Contig21$_{H}1$
Mean Expression Ratio	1.04
Median Expression Ratio	1.04
Credible Interval	(0.771, 1.39)
Associated Peptides	5
Associated Spectra	8
Coverage	NaN

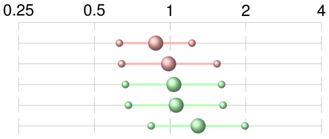

A	B	2.5	50	97.5	Sequence
2	2	0.63	0.88	1.2	LQDVDKDYPEVQK
1	0	0.64	0.98	1.5	LQAESLPK
1	0	0.67	1	1.6	LGNELTMA
0	1	0.68	1.1	1.6	KLRGR
0	1	0.84	1.3	2	EWEDVR

5.497 613 846 cytochrome c oxidase subunit Va [Antheraea yamamai] gi

Protein Accession	Unigene3832$_H$1
Mean Expression Ratio	1.04
Median Expression Ratio	1.04
Credible Interval	(0.672, 1.61)
Associated Peptides	1
Associated Spectra	1
Coverage	NaN

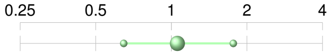

A	B	2.5	50	97.5	Sequence
**1**	**0**	**0.65**	**1.1**	**1.8**	**AMNDLQGEDLVPEPK**

5.498 729 1541 troponin T [Mizuhopecten yessoensis]

Protein Accession	Unigene34289$_H$1
Mean Expression Ratio	1.04
Median Expression Ratio	1.04
Credible Interval	(0.748, 1.43)
Associated Peptides	2
Associated Spectra	8
Coverage	NaN

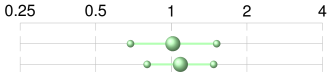

A	B	2.5	50	97.5	Sequence
**1**	**1**	**0.69**	**1**	**1.5**	**RQQYDMIELAER**
**2**	**4**	**0.8**	**1.1**	**1.5**	**QQYDMIELAER**

5.499 87 1997 Transketolase-like protein 2 [Crassostrea gigas]; 87 1997 Transketolase-like protein 2 [Crassostrea gigas]; 87 1997 Transketolase-like protein 2 [Crassostrea gigas]; 87 1997 Transketolase-like protein 2 [Crassostrea gigas]; 87 1997 Transketolase-like protein 2 [Crassostrea gigas]; 87 1997 Transketolase-like protein 2 [Crassostrea gigas]; 87 1997 Transketolase-like protein 2 [Crassostrea gigas]

Protein Accession	CL1127.Contig6$_H$1 CL1127.Contig5$_H$1 CL1127.Contig4$_H$1 CL1127.Contig3$_H$1 CL1127.Con
Mean Expression Ratio	1.04
Median Expression Ratio	1.04
Credible Interval	(0.741, 1.44)
Associated Peptides	3
Associated Spectra	5
Coverage	NaN

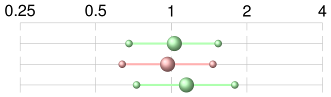

A	B	2.5	50	97.5	Sequence
**1**	**1**	**0.68**	**1**	**1.5**	**VISLDGDVK**
**1**	**1**	**0.64**	**0.97**	**1.5**	**GMCFIR**
**1**	**0**	**0.73**	**1.1**	**1.8**	**NARETGGR**

5.500 416 1717 minus strand T-complex protein 1 subunit delta OS=Aedes triseriatus PE=2 SV=1

Protein Accession	CL8566.Contig1$_H$1
Mean Expression Ratio	0.963
Median Expression Ratio	0.964
Credible Interval	(0.628, 1.48)
Associated Peptides	1
Associated Spectra	1
Coverage	NaN

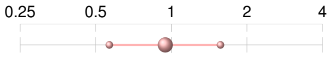

A	B	2.5	50	97.5	Sequence
1	0	0.56	0.94	1.6	HAAGELTAGINVR

5.501 " 460 1944 PREDICTED: succinyl-CoA:3-ketoacid-coenzyme A transferase 1, mitochondrial [Canis lupus familiaris]"

Protein Accession	Unigene4332$_H$1
Mean Expression Ratio	1.04
Median Expression Ratio	1.04
Credible Interval	(0.868, 1.24)
Associated Peptides	13
Associated Spectra	34
Coverage	NaN

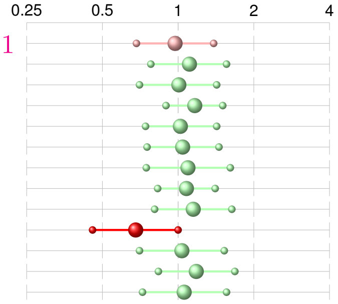

A	B	2.5	50	97.5	Sequence
1	1	0.68	0.97	1.4	AVFDVDNK
1	1	0.78	1.1	1.6	ECVSSYIGENK
1	1	0.7	1	1.4	EVAEGETAESIAK
2	5	0.9	1.2	1.5	EVVNMIVTEK
2	1	0.74	1	1.4	FNPDGTILHGSAGR
2	1	0.75	1	1.4	LGNLTFR
1	0	0.75	1.1	1.6	VMGMGGAMDLVSADK
3	4	0.83	1.1	1.4	ITGVEFR
2	0	0.81	1.1	1.6	VTLPK
0	1	0.46	0.68	1	AVFDVDNKEGLLLR
0	1	0.7	1	1.5	KGEEDADLINAGK
0	2	0.84	1.2	1.7	IFNGR
0	1	0.72	1.1	1.6	VFLGK

5.502 260 892 minus strand calponin [Haliotis diversicolor]

Protein Accession	CL48702.Contig1$_H$1
Mean Expression Ratio	0.967
Median Expression Ratio	0.965
Credible Interval	(0.823, 1.13)
Associated Peptides	11
Associated Spectra	104
Coverage	NaN

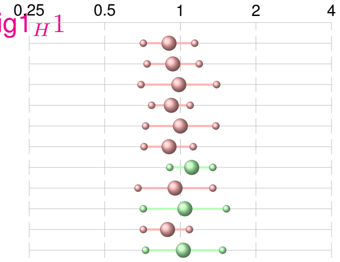

A	B	2.5	50	97.5	Sequence
7	4	0.71	0.9	1.1	AATGQQFPIEESNER
7	3	0.73	0.93	1.2	NFSEDKLR
2	0	0.7	0.99	1.4	TFQPASNLAF
17	8	0.77	0.92	1.1	TFQPASNLAFEAAR
1	3	0.73	1	1.4	VESSFDPEDAK
7	5	0.72	0.9	1.1	VESSFDPEDAKK
12	5	0.91	1.1	1.4	VMENFYK
1	1	0.68	0.95	1.3	YKVMENFYK
1	0	0.71	1	1.5	AKVESSFDPEDAKK
7	11	0.71	0.89	1.1	IELFIQK
1	1	0.73	1	1.5	VLACIR

5.503 311 928 minus strand peroxiredoxin 3 [Clonorchis sinensis] gi; 288 905 minus strand peroxiredoxin 3 [Clonorchis sinensis] gi

Protein Accession	CL2043.Contig4$_H$1 CL2043.Contig1$_H$1
Mean Expression Ratio	0.965
Median Expression Ratio	0.966
Credible Interval	(0.722, 1.28)
Associated Peptides	3
Associated Spectra	13
Coverage	NaN

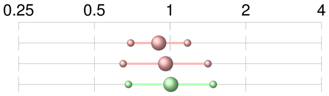

A	B	2.5	50	97.5	Sequence
5	4	0.7	0.9	1.2	QITINDLPVGR
1	1	0.65	0.96	1.4	SVDEILR
0	2	0.69	1	1.5	GLFIIDPK

5.504 64 435 peptidoglycan recognition protein S1L [Pomacea canaliculata]

Protein Accession	CL1297.Contig6$_H$1
Mean Expression Ratio	1.04
Median Expression Ratio	1.04
Credible Interval	(0.67, 1.61)
Associated Peptides	1
Associated Spectra	1
Coverage	NaN

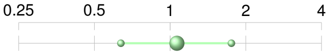

A	B	2.5	50	97.5	Sequence
1	0	0.64	1.1	1.8	MYSVCHGGR

5.505 140 349 copper chaperone Atox1 [Crassostrea ariakensis]

Protein Accession	CL8999.Contig5$_H$1
Mean Expression Ratio	1.04
Median Expression Ratio	1.04
Credible Interval	(0.694, 1.55)
Associated Peptides	1
Associated Spectra	2
Coverage	NaN

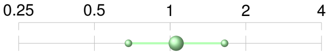

A	B	2.5	50	97.5	Sequence
1	1	0.68	1.1	1.6	EELMATLQK

5.506 177 791 Ras-related protein Rab-1A [Haliotis discus discus]

Protein Accession	CL21389.Contig6$_H$1
Mean Expression Ratio	1.04
Median Expression Ratio	1.03
Credible Interval	(0.781, 1.39)
Associated Peptides	5
Associated Spectra	8
Coverage	NaN

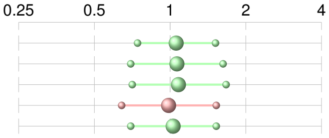

A	B	2.5	50	97.5	Sequence
3	0	0.74	1.1	1.5	MGPVTSASENKPSVK
1	0	0.7	1.1	1.6	QWLQEIDR
1	0	0.71	1.1	1.7	KVVDYTTAK
1	0	0.64	0.99	1.5	SCLLLR
0	2	0.7	1	1.5	LLLIGDSGVGK

5.507 362 1084 Ectoine hydroxylase [Crassostrea gigas]; 362 1084 Ectoine hydroxylase [Crassostrea gigas]; 366 1220 Ectoine hydroxylase [Crassostrea gigas]

Protein Accession	CL2342.Contig4$_{H}1$ CL2342.Contig3$_{H}1$ CL2342.Contig2$_{H}1$
Mean Expression Ratio	0.967
Median Expression Ratio	0.967
Credible Interval	(0.631, 1.5)
Associated Peptides	1
Associated Spectra	1
Coverage	NaN

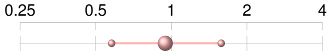

A	B	2.5	50	97.5	Sequence
**1**	**0**	**0.58**	**0.95**	**1.6**	**NFVLTDEMK**

5.508 231 902 Sodium- and chloride-dependent GABA transporter ine [Crassostrea gigas]

Protein Accession	CL11289.Contig3$_{H}1$
Mean Expression Ratio	0.966
Median Expression Ratio	0.967
Credible Interval	(0.728, 1.29)
Associated Peptides	3
Associated Spectra	11
Coverage	NaN

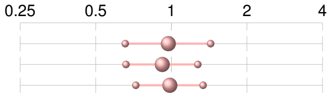

A	B	2.5	50	97.5	Sequence
**1**	**1**	**0.66**	**0.97**	**1.4**	**EAFELSFK**
**4**	**0**	**0.66**	**0.92**	**1.3**	**VPEIDAAAPFYGIPGADLADVSK**
**2**	**3**	**0.72**	**0.98**	**1.3**	**LVEFFQK**

5.509 225 839 minus strand Metallo-beta-lactamase domain-containing protein 1 [Crassostrea gigas]; 201 851 minus strand Metallo-beta-lactamase domain-containing protein 1 [Crassostrea gigas]

Protein Accession	CL35672.Contig2$_{H}1$ CL35672.Contig1$_{H}1$
Mean Expression Ratio	1.03
Median Expression Ratio	1.03
Credible Interval	(0.694, 1.55)
Associated Peptides	1
Associated Spectra	2
Coverage	NaN

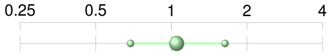

A	B	2.5	50	97.5	Sequence
**1**	**1**	**0.69**	**1.1**	**1.6**	**EADFIVPGHGPMFR**

5.510 122 1195 minus strand Severin [Crassostrea gigas]

Protein Accession	CL7486.Contig12$_H$1
Mean Expression Ratio	1.03
Median Expression Ratio	1.03
Credible Interval	(0.673, 1.61)
Associated Peptides	1
Associated Spectra	2
Coverage	NaN

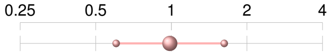

A	B	2.5	50	97.5	Sequence
1	1	0.6	0.99	1.6	TDHPLVPITCLTEGTK

5.511 527 1321 minus strand troponin T [Mizuhopecten yessoensis]; 305 1099 minus strand troponin T [Mizuhopecten yessoensis]; 305 1099 minus strand troponin T [Mizuhopecten yessoensis]

Protein Accession	Unigene65787$_H$1 CL2214.Contig4$_H$1 CL2214.Contig3$_H$1
Mean Expression Ratio	0.968
Median Expression Ratio	0.967
Credible Interval	(0.822, 1.14)
Associated Peptides	10
Associated Spectra	105
Coverage	NaN

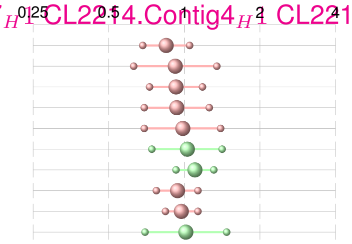

A	B	2.5	50	97.5	Sequence
2	10	0.68	0.85	1.1	LDSVMSKEELQK
1	0	0.63	0.92	1.4	SKEQLEDEK
5	4	0.73	0.93	1.2	SKEQLEDEKR
2	2	0.69	0.93	1.3	GPNFVITK
2	0	0.69	0.99	1.4	KGPNFVIT
3	0	0.74	1	1.4	AILSQR
17	6	0.93	1.1	1.3	KGPNFVITK
15	3	0.78	0.94	1.1	LDSVMSK
17	15	0.84	0.97	1.1	LTELR
0	1	0.7	1	1.5	EAREAEER

5.512 594 1253 peroxiredoxin 6 [Eriocheir sinensis]

Protein Accession	CL21894.Contig2$_H$1
Mean Expression Ratio	1.03
Median Expression Ratio	1.03
Credible Interval	(0.796, 1.33)
Associated Peptides	5
Associated Spectra	14
Coverage	NaN

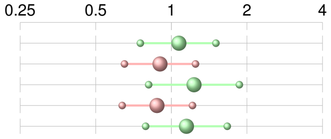

A	B	2.5	50	97.5	Sequence
2	1	0.75	1.1	1.5	VNLGDVFPDFK
4	0	0.65	0.9	1.2	AVFIIGPDK
1	0	0.81	1.2	1.9	AVFIIGPDKK
2	2	0.64	0.88	1.2	NFDEILR
1	1	0.79	1.1	1.7	QLTATQK

5.513 2295 2510 beta-thymosin domain repeat protein CSP24KDa_v1 [Hermissenda crassicornis] gi

Protein Accession	CL9548.Contig1$_H$1
Mean Expression Ratio	1.03
Median Expression Ratio	1.03
Credible Interval	(0.67, 1.59)
Associated Peptides	1
Associated Spectra	1
Coverage	NaN

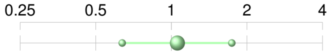

A	B	2.5	50	97.5	Sequence
1	0	0.64	1.1	1.7	ESVAAFDK

5.514 201 779 minus strand sigma class glutathione-s-transferase 2 [Haliotis discus discus]

Protein Accession	Unigene22998$_{H}1$
Mean Expression Ratio	0.967
Median Expression Ratio	0.967
Credible Interval	(0.708, 1.31)
Associated Peptides	3
Associated Spectra	12
Coverage	NaN

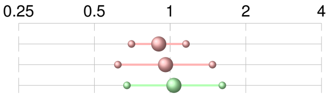

A	B	2.5	50	97.5	Sequence
4	6	0.7	0.9	1.2	LVLALADQK
1	0	0.62	0.96	1.5	KLEEEEFPK
0	1	0.67	1	1.6	YFGMFQK

5.515 " 116 352 minus strand troponin I 4 [Clonorchis sinensis]; 491 706 minus strand troponin-I, TnI {C-terminal} [Chlamys nipponensis=Akazara scallop, akazara, striated adductor muscle, Peptide Partial, 135 aa]"

Protein Accession	Unigene63816$_{H}1$ Unigene63815$_{H}1$
Mean Expression Ratio	0.969
Median Expression Ratio	0.968
Credible Interval	(0.724, 1.3)
Associated Peptides	2
Associated Spectra	21
Coverage	NaN

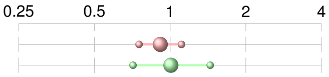

A	B	2.5	50	97.5	Sequence
12	6	0.75	0.91	1.1	FIKPVLR
2	1	0.71	1	1.4	FALKEEDESSK

5.516 2 244 minus strand hypothetical protein KGM_08771 [Danaus plexippus]

Protein Accession	CL49466.Contig2$_{H}1$
Mean Expression Ratio	0.969
Median Expression Ratio	0.969
Credible Interval	(0.697, 1.35)
Associated Peptides	2
Associated Spectra	6
Coverage	NaN

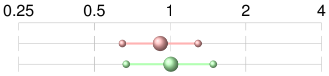

A	B	2.5	50	97.5	Sequence
3	1	0.65	0.92	1.3	AVFIDLEPTVVDEVR
1	1	0.67	1	1.5	TVGGGDDSFNTFFSETGAGK

5.517 2 1093 minus strand Neurexin-1-alpha [Crassostrea gigas]

Protein Accession	Unigene11591$_{H}1$
Mean Expression Ratio	0.969
Median Expression Ratio	0.97
Credible Interval	(0.676, 1.39)
Associated Peptides	2
Associated Spectra	4
Coverage	NaN

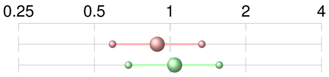

A	B	2.5	50	97.5	Sequence
1	1	0.59	0.89	1.3	IGDVVEAERPFSLIK
1	1	0.69	1	1.6	IYVGGILDKEEK

5.518 205 1341 collagen precursor [Bombyx mori]

Protein Accession	CL38816.Contig2$_{H}1$
Mean Expression Ratio	1.03
Median Expression Ratio	1.03
Credible Interval	(0.671, 1.6)
Associated Peptides	1
Associated Spectra	1
Coverage	NaN

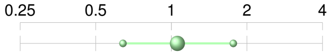

A	B	2.5	50	97.5	Sequence
**0**	**1**	**0.64**	**1.1**	**1.8**	**GQLIPHIQK**

5.519 406 1392 minus strand hypothetical protein CGI_10007007 [Crassostrea gigas]

Protein Accession	CL18045.Contig1$_{H}1$
Mean Expression Ratio	0.974
Median Expression Ratio	0.97
Credible Interval	(0.64, 1.49)
Associated Peptides	1
Associated Spectra	2
Coverage	NaN

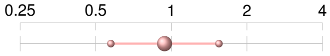

A	B	2.5	50	97.5	Sequence
**1**	**1**	**0.57**	**0.94**	**1.5**	**IEAEFPGANIILHK**

5.520 43 525 Reticulon-4 [Crassostrea gigas]; 143 757 Reticulon-4 [Crassostrea gigas]; 43 564 Reticulon-4 [Crassostrea gigas]

Protein Accession	CL29775.Contig4$_{H}1$ CL29775.Contig3$_{H}1$ CL29775.Contig2$_{H}1$
Mean Expression Ratio	1.03
Median Expression Ratio	1.03
Credible Interval	(0.69, 1.54)
Associated Peptides	1
Associated Spectra	3
Coverage	NaN

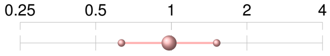

A	B	2.5	50	97.5	Sequence
**2**	**1**	**0.63**	**0.98**	**1.5**	**VTQTILQHVNR**

5.521 11 1462 coronin [Schistosoma mansoni]

Protein Accession	CL11779.Contig2$_{H}1$
Mean Expression Ratio	1.03
Median Expression Ratio	1.03
Credible Interval	(0.778, 1.37)
Associated Peptides	4
Associated Spectra	10
Coverage	NaN

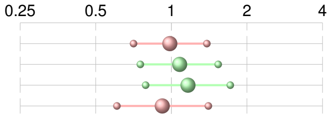

A	B	2.5	50	97.5	Sequence
**2**	**2**	**0.71**	**0.99**	**1.4**	**DANPILVSLK**
**2**	**1**	**0.75**	**1.1**	**1.5**	**HVFGKPEK**
**2**	**0**	**0.79**	**1.2**	**1.7**	**VPMDAPVVAGHK**
**0**	**1**	**0.61**	**0.92**	**1.4**	**DAPVVAGHK**

5.522 530 679 minus strand GJ11201 [Drosophila virilis] gi

Protein Accession	CL45804.Contig2$_{H}1$
Mean Expression Ratio	1.03
Median Expression Ratio	1.03
Credible Interval	(0.685, 1.55)
Associated Peptides	1
Associated Spectra	2
Coverage	NaN

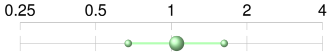

A	B	2.5	50	97.5	Sequence
2	0	0.67	1	1.6	QSQYALTLGPR

5.523 1139 1378 Titin [Crassostrea gigas]; 1116 1355 Titin [Crassostrea gigas]; 1139 1378 Titin [Crassostrea gigas]; 1139 1378 Titin [Crassostrea gigas]; 1139 1378 Titin [Crassostrea gigas]; 1139 1378 Titin [Crassostrea gigas]; 1139 1378 Titin [Crassostrea gigas]; 1170 1409 Titin [Crassostrea gigas]

Protein Accession	CL181.Contig9$_{H}1$ CL181.Contig8$_{H}1$ CL181.Contig7$_{H}1$ CL181.Contig6$_{H}1$ CL181.Contig13$_{H}$
Mean Expression Ratio	0.973
Median Expression Ratio	0.972
Credible Interval	(0.677, 1.39)
Associated Peptides	2
Associated Spectra	4
Coverage	NaN

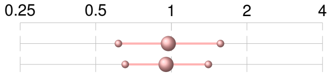

A	B	2.5	50	97.5	Sequence
1	0	0.61	0.97	1.6	ITGYQIER
3	0	0.65	0.95	1.4	LSWKPPK

5.524 89 838 Putative phosphoglycerate mutase [Crassostrea gigas]; 89 838 Putative phosphoglycerate mutase [Crassostrea gigas]; 89 835 Putative phosphoglycerate mutase [Crassostrea gigas]; 89 838 Putative phosphoglycerate mutase [Crassostrea gigas]

Protein Accession	CL6793.Contig6$_{H}1$ CL6793.Contig5$_{H}1$ CL6793.Contig4$_{H}1$ CL6793.Contig3$_{H}1$
Mean Expression Ratio	0.972
Median Expression Ratio	0.972
Credible Interval	(0.763, 1.24)
Associated Peptides	5
Associated Spectra	21
Coverage	NaN

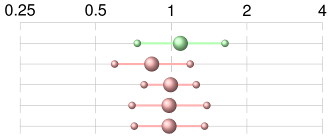

A	B	2.5	50	97.5	Sequence
1	0	0.73	1.1	1.6	DIPACECLK
2	1	0.59	0.83	1.2	HYYLATEEEVK
6	4	0.78	0.99	1.3	VLIAAHGNSLR
2	1	0.7	0.98	1.4	VLIAAHGN
3	1	0.71	0.98	1.4	EAQYAGELIK

5.525 20 511 Neurexin-2-alpha [Crassostrea gigas]; 20 511 Neurexin-2-alpha [Crassostrea gigas]

Protein Accession	CL36998.Contig2$_H$1 CL36998.Contig1$_H$1
Mean Expression Ratio	0.971
Median Expression Ratio	0.972
Credible Interval	(0.63, 1.51)
Associated Peptides	1
Associated Spectra	1
Coverage	NaN

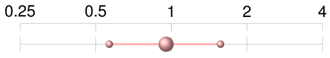

A	B	2.5	50	97.5	Sequence
0	1	0.57	0.95	1.6	AVFLDGR

5.526 62 1174 Tyrosine-protein kinase CSK [Crassostrea gigas]; 62 1174 minus strand Tyrosine-protein kinase CSK [Crassostrea gigas]

Protein Accession	CL22249.Contig5$_H$1 CL22249.Contig3$_H$1
Mean Expression Ratio	0.971
Median Expression Ratio	0.972
Credible Interval	(0.648, 1.45)
Associated Peptides	1
Associated Spectra	2
Coverage	NaN

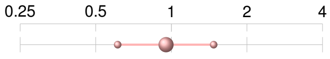

A	B	2.5	50	97.5	Sequence
0	2	0.61	0.95	1.5	IPLTDVVMHVER

5.527 114 1001 Vinculin [Crassostrea gigas]

Protein Accession	CL18499.Contig1$_H$1
Mean Expression Ratio	0.972
Median Expression Ratio	0.972
Credible Interval	(0.75, 1.26)
Associated Peptides	8
Associated Spectra	11
Coverage	NaN

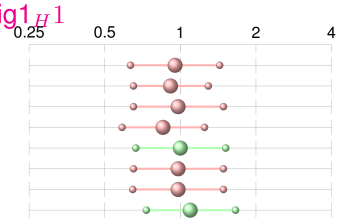

A	B	2.5	50	97.5	Sequence
1	0	0.63	0.95	1.4	MEDLVTFVK
2	1	0.65	0.91	1.3	TIESILEPVAQQVSR
1	0	0.65	0.98	1.5	NLSPVLTR
1	1	0.59	0.85	1.2	KMSDEIHEIIR
0	1	0.66	1	1.5	AAVDNLVR
0	1	0.65	0.98	1.5	MSDEIHEIIR
0	1	0.65	0.98	1.5	NLTPVLISGIK
0	1	0.73	1.1	1.7	IFITAK

5.528 27 722 Spectrin beta chain OS=Drosophila melanogaster GN=beta-Spec PE=1 SV=2; 27 722 Spectrin beta chain OS=Drosophila melanogaster GN=beta-Spec PE=1 SV=2

Protein Accession	CL5904.Contig3$_H$1 CL5904.Contig1$_H$1
Mean Expression Ratio	1.03
Median Expression Ratio	1.03
Credible Interval	(0.718, 1.49)
Associated Peptides	2
Associated Spectra	4
Coverage	NaN

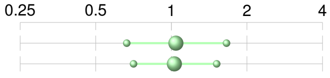

A	B	2.5	50	97.5	Sequence
1	0	0.66	1	1.7	EQIEVMLADLDK
3	0	0.71	1	1.5	HQAFEAEIGHYAR

5.529 113 1402 minus strand S-adenosylhomocysteine hydrolase [Crassostrea ariakensis]; 113 1402 minus strand S-adenosylhomocysteine hydrolase [Crassostrea ariakensis]

Protein Accession	CL7405.Contig2$_{H}1$ CL7405.Contig1$_{H}1$
Mean Expression Ratio	1.03
Median Expression Ratio	1.03
Credible Interval	(0.693, 1.53)
Associated Peptides	2
Associated Spectra	2
Coverage	NaN

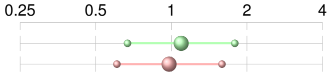

A	B	2.5	50	97.5	Sequence
**1**	**0**	**0.67**	**1.1**	**1.8**	**GLSEETTTGVHNLYR**
**1**	**0**	**0.61**	**0.98**	**1.6**	**LKVPAINVNDSVTK**

5.530 2764 3420 PREDICTED: filamin-C-like [Nasonia vitripennis]

Protein Accession	CL4145.Contig1$_{H}1$
Mean Expression Ratio	1.03
Median Expression Ratio	1.03
Credible Interval	(0.697, 1.52)
Associated Peptides	1
Associated Spectra	3
Coverage	NaN

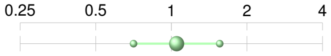

A	B	2.5	50	97.5	Sequence
**2**	**1**	**0.71**	**1**	**1.6**	**GVDVPVNTIDNK**

5.531 45 944 minus strand unnamed protein product [Mus musculus] gi; 5 334 minus strand receptor of activated protein kinase C [Platynereis dumerilii]

Protein Accession	CL20494.Contig3$_{H}1$ CL20494.Contig1$_{H}1$
Mean Expression Ratio	0.972
Median Expression Ratio	0.973
Credible Interval	(0.652, 1.45)
Associated Peptides	2
Associated Spectra	2
Coverage	NaN

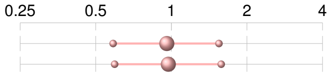

A	B	2.5	50	97.5	Sequence
**1**	**0**	**0.59**	**0.96**	**1.5**	**IWDLEGK**
**1**	**0**	**0.59**	**0.97**	**1.6**	**AMCFSPNR**

5.532 77 2278 Far upstream element-binding protein 3 [Crassostrea gigas]

Protein Accession	CL5030.Contig3$_{H}1$
Mean Expression Ratio	0.974
Median Expression Ratio	0.974
Credible Interval	(0.635, 1.5)
Associated Peptides	1
Associated Spectra	1
Coverage	NaN

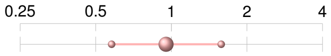

A	B	2.5	50	97.5	Sequence
**1**	**0**	**0.58**	**0.95**	**1.6**	**LFNIR**

5.533 " 103 981 PREDICTED: ATP synthase, H+ transporting, mitochondrial F1 complex, gamma subunit isoform H (heart)-like [Saccoglossus kowalevskii]"

Protein Accession	CL25149.Contig1$_{H}1$
Mean Expression Ratio	0.975
Median Expression Ratio	0.974
Credible Interval	(0.672, 1.42)
Associated Peptides	2
Associated Spectra	3
Coverage	NaN

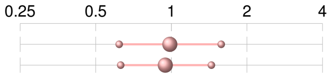

A	B	2.5	50	97.5	Sequence
**1**	**0**	**0.62**	**0.99**	**1.6**	**ELKPARPYGAGAK**
**1**	**1**	**0.63**	**0.95**	**1.4**	**LTLTFNR**

5.534 337 474 minus strand heat shock protein 22 isoform 1 [Ruditapes philippinarum]

Protein Accession	CL30681.Contig1$_{H}1$
Mean Expression Ratio	0.974
Median Expression Ratio	0.974
Credible Interval	(0.671, 1.41)
Associated Peptides	2
Associated Spectra	3
Coverage	NaN

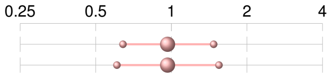

A	B	2.5	50	97.5	Sequence
**1**	**1**	**0.64**	**0.97**	**1.5**	**EDWNFFDR**
**0**	**1**	**0.61**	**0.97**	**1.5**	**REDWNFFDR**

5.535 2996 3340 minus strand Collagen alpha-5(VI) chain [Crassostrea gigas]

Protein Accession	CL47586.Contig8$_{H}1$
Mean Expression Ratio	1.03
Median Expression Ratio	1.03
Credible Interval	(0.74, 1.43)
Associated Peptides	2
Associated Spectra	7
Coverage	NaN

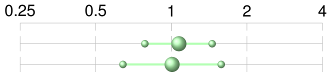

A	B	2.5	50	97.5	Sequence
**3**	**3**	**0.78**	**1.1**	**1.5**	**ASIFDAIDEIPYR**
**1**	**0**	**0.64**	**1**	**1.6**	**YGSTNTADGIK**

5.536 212 1336 6-phosphofructokinase [Crassostrea gigas]

Protein Accession	CL48438.Contig4$_{H}1$
Mean Expression Ratio	0.971
Median Expression Ratio	0.974
Credible Interval	(0.664, 1.4)
Associated Peptides	2
Associated Spectra	3
Coverage	NaN

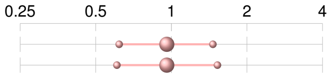

A	B	2.5	50	97.5	Sequence
**2**	**0**	**0.62**	**0.96**	**1.5**	**LGLTQGYR**
**1**	**0**	**0.61**	**0.96**	**1.5**	**SVQWFK**

5.537 2 256 Polyubiquitin (Fragment) OS=Xenopus laevis PE=1 SV=2; 20 472 minus strand large subunit ribosomal protein L40e [Clonorchis sinensis]; 20 388 large subunit ribosomal protein L40e [Clonorchis sinensis]

Protein Accession	CL47507.Contig2$_H$1 CL39010.Contig2$_H$1 CL39010.Contig1$_H$1
Mean Expression Ratio	0.976
Median Expression Ratio	0.974
Credible Interval	(0.721, 1.32)
Associated Peptides	4
Associated Spectra	16
Coverage	NaN

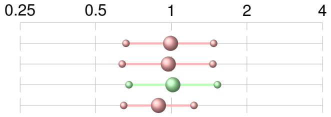

A	B	2.5	50	97.5	Sequence
2	1	0.66	0.99	1.5	TITLEVEPSDTIENVK
1	1	0.64	0.97	1.5	ESTLHLVLR
2	1	0.68	1	1.5	MQIFVK
4	4	0.65	0.89	1.2	TLSDYNIQK

5.538 147 272 Spectrin alpha chain [Crassostrea gigas]

Protein Accession	CL2014.Contig2$_H$1
Mean Expression Ratio	1.02
Median Expression Ratio	1.03
Credible Interval	(0.657, 1.57)
Associated Peptides	1
Associated Spectra	1
Coverage	NaN

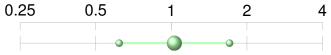

A	B	2.5	50	97.5	Sequence
0	1	0.62	1	1.7	VLETAEDIQQR

5.539 " 451 1026 PREDICTED: GTP-binding protein SAR1B, putative-like [Saccoglossus kowalevskii]"

Protein Accession	Unigene4124$_H$1
Mean Expression Ratio	1.03
Median Expression Ratio	1.03
Credible Interval	(0.666, 1.58)
Associated Peptides	1
Associated Spectra	1
Coverage	NaN

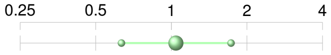

A	B	2.5	50	97.5	Sequence
1	0	0.63	1	1.7	AELDSLLTDEQVANAPILVLGNK

5.540 49 1107 Eukaryotic translation initiation factor 3 subunit B [Crassostrea gigas]

Protein Accession	CL19479.Contig1$_H$1
Mean Expression Ratio	0.979
Median Expression Ratio	0.975
Credible Interval	(0.632, 1.52)
Associated Peptides	1
Associated Spectra	1
Coverage	NaN

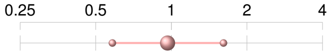

A	B	2.5	50	97.5	Sequence
0	1	0.58	0.96	1.6	LMEEFSNLR

5.541 373 579 myosin heavy chain [Argopecten irradians] gi

Protein Accession	Unigene64289$_{H}1$
Mean Expression Ratio	0.975
Median Expression Ratio	0.976
Credible Interval	(0.813, 1.17)
Associated Peptides	9
Associated Spectra	62
Coverage	NaN

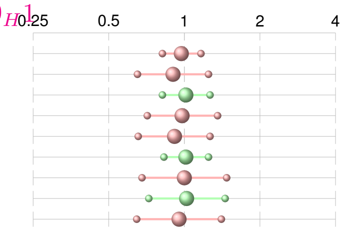

A	B	2.5	50	97.5	Sequence
15	7	0.82	0.97	1.2	QVEEAEEIAAVNLAK
2	1	0.65	0.9	1.2	RQVEEAEEIAAVNLAK
8	4	0.82	1	1.3	VQNELEDAEER
2	1	0.71	0.98	1.4	ELEDAEER
2	1	0.65	0.91	1.3	ADSAEGTLSK
11	4	0.83	1	1.2	KVQNELEDAEER
1	0	0.68	1	1.5	QELVDKLQAK
1	1	0.72	1	1.5	VDKLQAK
0	1	0.65	0.95	1.4	VSVSRR

5.542 52 1599 minus strand ATP synthase beta subunit [Pinctada fucata]

Protein Accession	CL34251.Contig5$_{H}1$
Mean Expression Ratio	0.976
Median Expression Ratio	0.976
Credible Interval	(0.809, 1.18)
Associated Peptides	11
Associated Spectra	34
Coverage	NaN

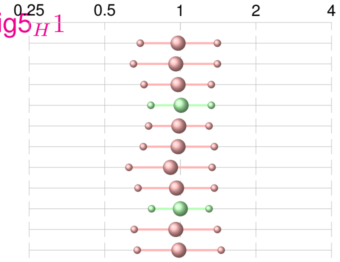

A	B	2.5	50	97.5	Sequence
1	1	0.69	0.98	1.4	AHGGYSVFAGVGER
1	0	0.65	0.96	1.4	EAAKPAAAGNLAVGR
2	2	0.72	0.98	1.3	IINVIGEPIDER
4	2	0.76	1	1.3	ILDPNVVGMEHYK
3	3	0.75	0.98	1.3	TIAMDGTEGLVR
2	1	0.71	0.98	1.4	VISLTDDTSK
1	0	0.62	0.91	1.3	VSLVYGQMNEPPGAR
2	0	0.68	0.96	1.4	VVDMLAPYAR
6	1	0.77	1	1.3	ILQDFK
0	1	0.66	0.96	1.4	FTQAGSEVSALLGR
0	1	0.67	0.99	1.4	IGLFGGAGVGK

5.543 227 2920 minus strand Lysosomal alpha-mannosidase [Crassostrea gigas]; 227 3073 minus strand Lysosomal alpha-mannosidase [Crassostrea gigas]; 193 2886 minus strand Lysosomal alpha-mannosidase [Crassostrea gigas]

Protein Accession	CL2930.Contig3$_{H}1$ CL2930.Contig2$_{H}1$ CL2930.Contig1$_{H}1$
Mean Expression Ratio	1.02
Median Expression Ratio	1.02
Credible Interval	(0.757, 1.39)
Associated Peptides	5
Associated Spectra	8
Coverage	NaN

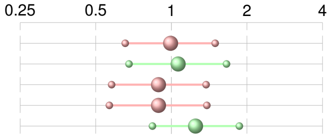

A	B	2.5	50	97.5	Sequence
2	1	0.66	0.99	1.5	VASELIPITPDTFR
0	1	0.68	1.1	1.7	PDNVHLLTLEQFR
0	1	0.58	0.89	1.4	ALPDNVHLLTLEQFR
0	1	0.57	0.89	1.4	QLNHR
0	2	0.84	1.2	1.9	AGASLTWSGVR

5.544 188 1021 hypothetical protein BRAFLDRAFT_214346 [Branchiostoma floridae] gi

Protein Accession	CL38498.Contig2$_H$1
Mean Expression Ratio	0.977
Median Expression Ratio	0.977
Credible Interval	(0.683, 1.4)
Associated Peptides	3
Associated Spectra	3
Coverage	NaN

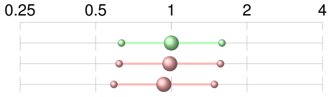

A	B	2.5	50	97.5	Sequence
1	0	0.63	1	1.6	EGLELHPPK
0	1	0.62	0.98	1.6	NLVGAIMTEEEAKK
0	1	0.59	0.93	1.5	DICTFLR

5.545 264 1505 Ribosome-binding protein 1 [Crassostrea gigas]; 260 1501 Ribosome-binding protein 1 [Crassostrea gigas]; 256 1497 Ribosome-binding protein 1 [Crassostrea gigas]

Protein Accession	CL8282.Contig7$_H$1 CL8282.Contig4$_H$1 CL8282.Contig1$_H$1
Mean Expression Ratio	1.03
Median Expression Ratio	1.02
Credible Interval	(0.692, 1.52)
Associated Peptides	2
Associated Spectra	2
Coverage	NaN

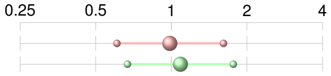

A	B	2.5	50	97.5	Sequence
1	0	0.61	0.99	1.6	LVQQLQEQLSEK
1	0	0.67	1.1	1.8	LKEDKETLEAR

5.546 2 244 Ras homolog gene family member A [Clonorchis sinensis]

Protein Accession	Unigene32424$_H$1
Mean Expression Ratio	1.03
Median Expression Ratio	1.02
Credible Interval	(0.664, 1.58)
Associated Peptides	1
Associated Spectra	2
Coverage	NaN

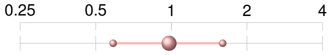

A	B	2.5	50	97.5	Sequence
1	1	0.59	0.98	1.6	EVFETATR

5.547 285 1637 PREDICTED: talin-2-like [Cricetulus griseus]

Protein Accession	CL18079.Contig3$_H$1
Mean Expression Ratio	1.02
Median Expression Ratio	1.02
Credible Interval	(0.662, 1.6)
Associated Peptides	1
Associated Spectra	1
Coverage	NaN

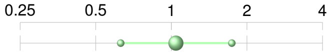

A	B	2.5	50	97.5	Sequence
0	1	0.63	1	1.7	ISIVGQNVIK

5.548 60 1022 Major egg antigen [Crassostrea gigas]; 60 1022 Major egg antigen [Crassostrea gigas]

Protein Accession	CL18744.Contig3$_H1$ CL18744.Contig2$_H1$
Mean Expression Ratio	1.02
Median Expression Ratio	1.02
Credible Interval	(0.795, 1.32)
Associated Peptides	6
Associated Spectra	13
Coverage	NaN

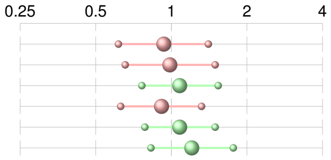

A	B	2.5	50	97.5	Sequence
1	0	0.61	0.93	1.4	QLTIEAPLKPATVQQR
1	0	0.65	0.99	1.5	TAIEVDSEGR
1	2	0.76	1.1	1.5	SEVHIPVQK
2	0	0.63	0.91	1.3	FDVSEFKPEEVSVK
1	3	0.78	1.1	1.5	ETFLPVR
0	2	0.83	1.2	1.8	LTVDIGEFSPDEIVVK

5.549 " 3475 4188 minus strand Heat shock 70 kDa protein cognate, putative [Pediculus humanus corporis] gi"

Protein Accession	CL5924.Contig14$_H1$
Mean Expression Ratio	0.975
Median Expression Ratio	0.977
Credible Interval	(0.67, 1.41)
Associated Peptides	2
Associated Spectra	3
Coverage	NaN

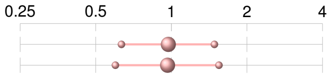

A	B	2.5	50	97.5	Sequence
2	0	0.63	0.97	1.5	STIEDEKLK
0	1	0.6	0.96	1.5	CNDVIR

5.550 6 317 myosin heavy chain cardiac muscle specific isoform 2 [Argopecten irradians]

Protein Accession	Unigene19023$_H1$
Mean Expression Ratio	0.978
Median Expression Ratio	0.977
Credible Interval	(0.864, 1.11)
Associated Peptides	16
Associated Spectra	246
Coverage	NaN

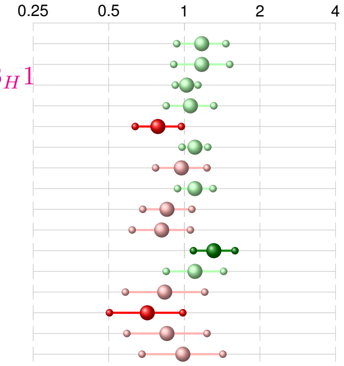

A	B	2.5	50	97.5	Sequence
6	5	0.94	1.2	1.5	ELAFQSDEDR
4	3	0.91	1.2	1.5	ELAFQSDEDRK
37	33	0.92	1	1.1	ELEMELDSEQR
11	1	0.85	1.1	1.3	KALETQLK
6	7	0.64	0.79	0.97	LADELRQEQEHSLQVEK
24	30	0.98	1.1	1.2	LDEAEAQALK
6	3	0.77	0.97	1.2	LKELAFQSDEDR
17	8	0.94	1.1	1.3	ALETQLK
5	5	0.68	0.85	1.1	LKELAFQSDEDRK
1	5	0.62	0.81	1.1	KAMADAAR
3	14	1.1	1.3	1.6	LADELR
7	0	0.85	1.1	1.4	QEQEHSLQVEK
0	1	0.58	0.83	1.2	ELEMELDSEQRR
0	2	0.5	0.71	0.99	LDEAEAQALKGGK
0	1	0.59	0.85	1.2	VRELEMELDSEQR
0	1	0.68	0.98	1.4	AFQSDEDRK

5.551 44 1465 minus strand Adenylyl cyclase-associated protein 1 [Crassostrea gigas]; 52 1473 minus strand Adenylyl cyclase-associated protein 1 [Crassostrea gigas]; 48 1469 Adenylyl cyclase-associated protein 1 [Crassostrea gigas]

Protein Accession	CL19470.Contig9$_{H}$1 CL19470.Contig8$_{H}$1 CL19470.Contig1$_{H}$1
Mean Expression Ratio	0.978
Median Expression Ratio	0.977
Credible Interval	(0.802, 1.18)
Associated Peptides	13
Associated Spectra	24
Coverage	NaN

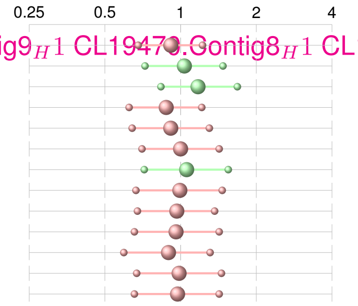

A	B	2.5	50	97.5	Sequence
2	3	0.68	0.91	1.2	AQLDIVNAAFK
1	1	0.72	1	1.5	DSLDTEIVTAK
1	1	0.84	1.2	1.7	EFALPEQFK
2	1	0.62	0.88	1.2	IQAVQEFR
1	1	0.64	0.92	1.3	NIVLDNVELK
1	1	0.7	1	1.4	QFLEVASK
1	0	0.72	1.1	1.5	SKQPPQADLPK
1	0	0.67	0.99	1.5	VNSIILDQCK
2	0	0.67	0.96	1.4	VPTISIDK
1	0	0.65	0.96	1.4	QAVYIYK
1	0	0.59	0.89	1.3	LETLALR
1	0	0.67	0.98	1.4	DTALQNAVAR
0	1	0.65	0.97	1.4	LKDFK

5.552 299 7507 minus strand filamin [Clonorchis sinensis]

Protein Accession	CL4145.Contig20$_H$1
Mean Expression Ratio	1.02
Median Expression Ratio	1.02
Credible Interval	(0.948, 1.1)
Associated Peptides	80
Associated Spectra	323
Coverage	NaN

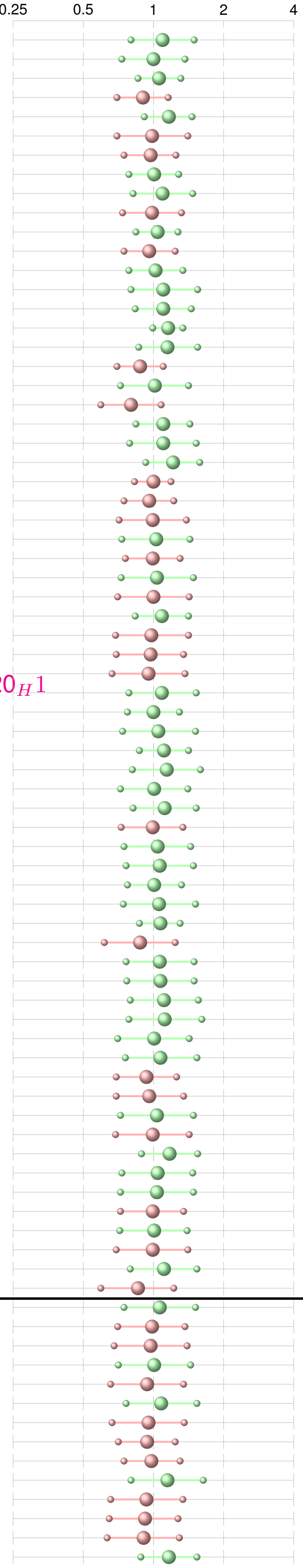

A	B	2.5	50	97.5	Sequence
3	0	0.8	1.1	1.5	AYGPGLEPK
2	1	0.73	1	1.4	EAGLEEDEEMTTTER
11	2	0.86	1.1	1.3	EETKPGSLGVTVEGPK
1	6	0.7	0.9	1.2	ELADDAQWK
3	6	0.92	1.2	1.5	EPVEVVFNNDR
1	0	0.7	0.99	1.4	EVNKSPFEVTVEGAAGDASK
4	3	0.75	0.97	1.2	FLEEDEGIR
5	3	0.78	1	1.3	FTVETFSAGK
4	0	0.82	1.1	1.5	GAGEGEVAVQIIGPGGSEEK
4	0	0.74	0.99	1.3	GAGNGSVDVVILDPHGR
8	5	0.84	1	1.3	GTLEVTLLNPK
3	4	0.74	0.96	1.2	IEASIDVSGIK
6	0	0.78	1	1.3	INVTHADKPIPGSPFK
1	1	0.8	1.1	1.5	IVNIDSTDIVDK
4	1	0.84	1.1	1.5	KDTVRPSIQPVVGK
12	19	0.99	1.2	1.3	LENVTMVLK
2	2	0.86	1.1	1.5	NFTTDWNDGR
4	6	0.7	0.88	1.1	QIADLESDFSDGLR
2	0	0.72	1	1.4	SPFEVTVEGAAGDASK
2	2	0.6	0.8	1.1	TQKLENVTMVLK
4	2	0.84	1.1	1.4	TTNVPVQDLVDKK
1	1	0.79	1.1	1.5	VDAVPDTGIPK
4	2	0.93	1.2	1.6	VDDLSMMTYLSQFPNAK
14	6	0.83	1	1.2	VEVGPFWK
7	1	0.75	0.96	1.2	VPDLPIK
2	0	0.71	1	1.4	VYVSEPTNPAK
1	1	0.73	1	1.4	TWFEVYTK
4	2	0.76	0.99	1.3	ACFATGR
1	0	0.73	1	1.5	IEASIDVSGIKVEGLEPK
1	0	0.7	1	1.4	YMPTKNGK
5	1	0.83	1.1	1.4	MAITSPSGK
1	0	0.69	0.98	1.4	VEGLEPK
2	0	0.69	0.97	1.3	AFGPGLTGGEALKPAFFTIDAR
1	0	0.66	0.95	1.4	GLEEDEEMTTTER
1	1	0.79	1.1	1.5	KTWFEVYTK
4	3	0.78	1	1.3	QVEVQVK
1	0	0.74	1.1	1.5	VKDSADFYVHTK
3	5	0.87	1.1	1.4	WANEHLK
2	0	0.81	1.1	1.6	WLDVPQLIKPDEMTNPK
0	2	0.72	1	1.4	AAADVTHVGSECELSLK
0	3	0.82	1.1	1.5	AEIDFQDR
0	3	0.73	0.99	1.3	CYGPGLDSK
0	2	0.75	1	1.4	EEPCLLK
0	2	0.76	1.1	1.5	ETFITVDAAEAGNGK
0	6	0.77	1	1.3	FNDEHIPDSPYR
0	1	0.74	1.1	1.5	GEVQQPCDFNIYTR
0	15	0.87	1.1	1.3	GGLGLGIEGPVEAK
0	1	0.61	0.88	1.2	IMVGESELGNASK
0	2	0.76	1.1	1.5	ISISALQDK
0	2	0.77	1.1	1.5	LANGHLGITFTPR
0	2	0.79	1.1	1.6	PAAFVVNFNGAK
0	1	0.79	1.1	1.6	RLANGHLGITFTPR
0	1	0.7	1	1.4	SPTGSDIDIDIIDR
0	1	0.76	1.1	1.5	SPVDELTVK
0	4	0.69	0.93	1.3	VKPAALDVSGVK
0	2	0.69	0.96	1.3	VQNPVDASK
0	1	0.72	1	1.5	VQVSPSIGDAR
0	1	0.69	0.99	1.4	VSEELITR
0	5	0.89	1.2	1.6	VTETDITDLTASIR
0	1	0.73	1	1.5	VTGPGVQGTTPASLPAVFK
0	1	0.72	1	1.5	YAGVNIAGSPFK
0	3	0.72	0.99	1.3	HIANSPFK
0	2	0.72	1	1.4	KISISALQDK
0	1	0.69	0.99	1.4	APGDYLISIR
0	2	0.79	1.1	1.5	EMGIHTVSVK
0	1	0.59	0.86	1.2	FADQHVPGSPFTVK
0	1	0.75	1.1	1.5	IMVGK
0	2	0.7	0.98	1.4	VKPAALDVSG
0	1	0.68	0.97	1.4	IPGTSPFDMTASVK
0	1	0.71	1	1.4	SVTLNGEGTVQAILTTPTGR
0	1	0.65	0.94	1.3	FADQHVPGSPF
0	1	0.76	1.1	1.5	QIADLESDFSDGL
0	1	0.67	0.95	1.4	EVPNSPFQMR
0	5	0.71	0.94	1.2	DVPGAPFPVK
0	5	0.75	0.98	1.3	ETGEHLVNVFR
0	1	0.8	1.1	1.6	GLQVGKPAAFVVNFNGAK
0	1	0.66	0.93	1.3	INVTHADKPIPGS
0	2	0.65	0.92	1.3	NIMGSPFTAK
0	1	0.63	0.91	1.3	PGVDASK
0	5	0.88	1.2	1.5	VLPGVDASK

5.553 51 1295 PREDICTED: phosphoglycerate kinase-like [Nasonia vitripennis]; 97 1341 PREDICTED: phosphoglycerate kinase-like [Nasonia vitripennis]

Protein Accession	CL14417.Contig13$_H$1 CL14417.Contig12$_H$1
Mean Expression Ratio	1.02
Median Expression Ratio	1.02
Credible Interval	(0.9, 1.16)
Associated Peptides	24
Associated Spectra	80
Coverage	NaN

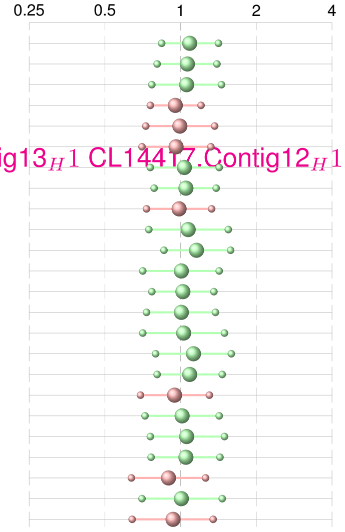

A	B	2.5	50	97.5	Sequence
4	3	0.84	1.1	1.4	ALMDAVVEATK
4	2	0.81	1.1	1.4	ATQEDIDKFR
2	1	0.77	1.1	1.5	FHVEEEGKGVAPDGTK
6	4	0.76	0.95	1.2	IVQGLMDK
2	1	0.73	1	1.4	LGDIYVNDAFGTAHR
1	2	0.7	0.96	1.3	LSIDKVDVK
3	0	0.76	1	1.4	SGIPADWMGLDIGPK
3	2	0.79	1	1.4	SVVLMSHLGRPDGTK
2	2	0.73	0.99	1.3	VDFNVPLKDGK
1	0	0.75	1.1	1.5	VLNNMEIGK
2	2	0.86	1.2	1.6	VLPGVAALTDA
2	0	0.71	1	1.4	VSMVSTGGGASLELLEGK
2	3	0.77	1	1.4	YSMKPVADELK
3	0	0.73	1	1.4	KELMYFAK
1	0	0.71	1	1.5	FTDAVK
2	0	0.8	1.1	1.6	VDFNVPLK
4	0	0.8	1.1	1.5	ASGFLLK
2	1	0.69	0.95	1.3	ELMYFAK
1	1	0.72	1	1.4	AHSSMVGCQLPIR
2	0	0.76	1.1	1.5	KNNVQIHLPEDFVTGDKFDEK
1	2	0.76	1	1.4	SLFDEEGAK
1	1	0.64	0.9	1.3	FHVEEEGK
0	1	0.7	1	1.5	LENLR
0	1	0.64	0.93	1.3	IVAALPTIK

5.554 429 1013 PDZ domain protein [Ixodes scapularis] gi

Protein Accession	CL8129.Contig12$_H$1
Mean Expression Ratio	0.978
Median Expression Ratio	0.978
Credible Interval	(0.775, 1.23)
Associated Peptides	6
Associated Spectra	19
Coverage	NaN

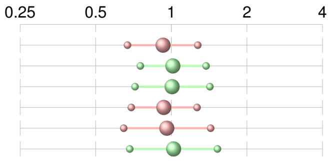

A	B	2.5	50	97.5	Sequence
4	0	0.67	0.93	1.3	DLGQPLSIQR
4	1	0.75	1	1.4	HGGGQIQLVIQR
1	2	0.72	1	1.4	VFSNSPAEGELQR
3	2	0.69	0.93	1.3	QALDLIK
1	0	0.65	0.96	1.4	DSFNTPWGFR
0	1	0.69	1	1.5	GDLILSINNR

5.555 385 906 Soma ferritin OS=Lymnaea stagnalis PE=2 SV=2

Protein Accession	CL2484.Contig1$_H$1
Mean Expression Ratio	0.977
Median Expression Ratio	0.978
Credible Interval	(0.633, 1.5)
Associated Peptides	1
Associated Spectra	1
Coverage	NaN

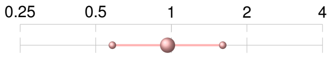

A	B	2.5	50	97.5	Sequence
1	0	0.58	0.97	1.6	ELADHITNLK

5.556 635 1561 Uncharacterized protein yfeX [Crassostrea gigas]

Protein Accession	CL12046.Contig4$_{H}$1
Mean Expression Ratio	0.978
Median Expression Ratio	0.978
Credible Interval	(0.659, 1.45)
Associated Peptides	2
Associated Spectra	2
Coverage	NaN

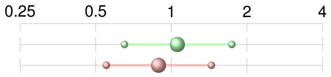

A	B	2.5	50	97.5	Sequence
**1**	**0**	**0.65**	**1.1**	**1.7**	**TLESWVGR**
**1**	**0**	**0.55**	**0.89**	**1.4**	**LFDLAQLVLK**

5.557 316 531 cytochrome c oxidase subunit VIIa polypeptide 2 like [Xenopus (Silurana) tropicalis]

Protein Accession	CL17601.Contig5$_{H}$1
Mean Expression Ratio	0.978
Median Expression Ratio	0.978
Credible Interval	(0.632, 1.51)
Associated Peptides	1
Associated Spectra	1
Coverage	NaN

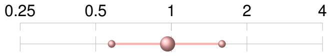

A	B	2.5	50	97.5	Sequence
**0**	**1**	**0.58**	**0.97**	**1.6**	**QDGLLVWQK**

5.558 145 972 LIM and SH3 domain protein F42H10.3 OS=Caenorhabditis elegans GN=F42H10.3 PE=2 SV=3

Protein Accession	CL49642.Contig8$_{H}$1
Mean Expression Ratio	1.02
Median Expression Ratio	1.02
Credible Interval	(0.685, 1.54)
Associated Peptides	1
Associated Spectra	2
Coverage	NaN

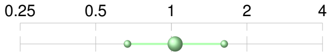

A	B	2.5	50	97.5	Sequence
**1**	**1**	**0.67**	**1**	**1.6**	**TVYPTEELK**

5.559 1497 1889 UDP-glucuronosyltransferase 2C1 (Fragment) OS=Oryctolagus cuniculus GN=UGT2C1 PE=2 SV=1

Protein Accession	CL29849.Contig1$_{H}$1
Mean Expression Ratio	1.02
Median Expression Ratio	1.02
Credible Interval	(0.658, 1.57)
Associated Peptides	1
Associated Spectra	2
Coverage	NaN

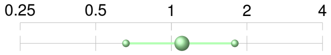

A	B	2.5	50	97.5	Sequence
**1**	**1**	**0.66**	**1.1**	**1.8**	**LFVSHCGK**

5.560 585 944 minus strand Protein disulfide-isomerase A6 [Crassostrea gigas]

Protein Accession	CL39540.Contig7$_H$1
Mean Expression Ratio	0.977
Median Expression Ratio	0.979
Credible Interval	(0.661, 1.44)
Associated Peptides	2
Associated Spectra	3
Coverage	NaN

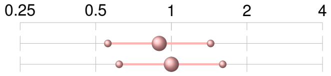

A	B	2.5	50	97.5	Sequence
**1**	**1**	**0.56**	**0.9**	**1.4**	**TASDIINWATEK**
**1**	**0**	**0.62**	**1**	**1.6**	**GFPTIK**

5.561 130 2145 minus strand Glucosamine–fructose-6-phosphate aminotransferase [isomerizing] 1 [Crassostrea gigas]; 130 2121 minus strand Glucosamine–fructose-6-phosphate aminotransferase [isomerizing] 1 [Crassostrea gigas]

Protein Accession	CL2589.Contig3$_H$1 CL2589.Contig2$_H$1
Mean Expression Ratio	0.98
Median Expression Ratio	0.979
Credible Interval	(0.679, 1.41)
Associated Peptides	3
Associated Spectra	3
Coverage	NaN

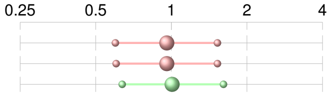

A	B	2.5	50	97.5	Sequence
**1**	**0**	**0.6**	**0.96**	**1.5**	**KLPDQIKDVLK**
**0**	**1**	**0.6**	**0.96**	**1.5**	**HGPLALVDK**
**0**	**1**	**0.64**	**1**	**1.6**	**VANLEAQIK**

5.562 143 247 minus strand dynein light chain [Aplysia californica] gi

Protein Accession	CL13886.Contig1$_H$1
Mean Expression Ratio	0.981
Median Expression Ratio	0.979
Credible Interval	(0.651, 1.47)
Associated Peptides	1
Associated Spectra	2
Coverage	NaN

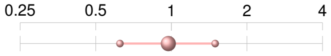

A	B	2.5	50	97.5	Sequence
**2**	**0**	**0.63**	**0.97**	**1.5**	**NADMAEDMQQDAVDCATQALEK**

5.563 211 990 PREDICTED: gamma-glutamyl hydrolase-like [Xenopus (Silurana) tropicalis]; 223 1080 PREDICTED: gamma-glutamyl hydrolase [Gallus gallus]

Protein Accession	CL20208.Contig9$_H$1 CL20208.Contig3$_H$1
Mean Expression Ratio	0.979
Median Expression Ratio	0.979
Credible Interval	(0.652, 1.47)
Associated Peptides	1
Associated Spectra	2
Coverage	NaN

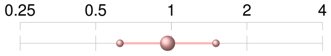

A	B	2.5	50	97.5	Sequence
2	0	0.62	0.96	1.5	YLWQAGAR

5.564 346 702 minus strand PREDICTED: galectin-4-like [Anolis carolinensis]

Protein Accession	Unigene42203$_H$1
Mean Expression Ratio	0.98
Median Expression Ratio	0.979
Credible Interval	(0.748, 1.28)
Associated Peptides	5
Associated Spectra	11
Coverage	NaN

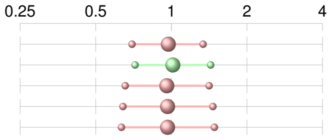

A	B	2.5	50	97.5	Sequence
3	1	0.7	0.97	1.3	AEATLEVR
2	1	0.72	1	1.4	SVPHGLVK
1	1	0.66	0.96	1.4	GKDFDIVIK
1	0	0.64	0.97	1.5	VKDSGFK
0	1	0.63	0.97	1.5	KSVPHGLVK

5.565 241 561 minus strand thioredoxin [Crassostrea ariakensis]

Protein Accession	CL33159.Contig3$_H$1
Mean Expression Ratio	0.98
Median Expression Ratio	0.98
Credible Interval	(0.716, 1.34)
Associated Peptides	3
Associated Spectra	7
Coverage	NaN

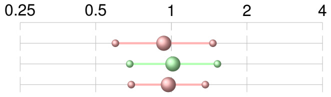

A	B	2.5	50	97.5	Sequence
1	0	0.6	0.93	1.5	EVGEVVGASEEEIK
1	1	0.68	1	1.5	EVGEVVGASEEEIKK
2	2	0.69	0.98	1.4	IETQEDFEK

5.566 58 198 minus strand Protein DJ-1 [Crassostrea gigas]

Protein Accession	CL16588.Contig4$_H$1
Mean Expression Ratio	1.02
Median Expression Ratio	1.02
Credible Interval	(0.713, 1.46)
Associated Peptides	1
Associated Spectra	6
Coverage	NaN

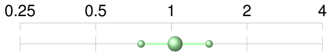

A	B	2.5	50	97.5	Sequence
3	3	0.76	1	1.4	ADSLVAPMLVK

5.567 " 1 510 Glycerol-3-phosphate dehydrogenase [NAD+], cytoplasmic [Crassostrea gigas]"

Protein Accession	Unigene16246$_{H}1$
Mean Expression Ratio	0.98
Median Expression Ratio	0.981
Credible Interval	(0.678, 1.42)
Associated Peptides	1
Associated Spectra	4
Coverage	NaN

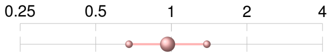

A	B	2.5	50	97.5	Sequence
2	2	0.68	0.96	1.4	EIFGMLEK

5.568 58 2268 hypothetical protein BRAFLDRAFT_267717 [Branchiostoma floridae] gi; 62 2245 hypothetical protein BRAFLDRAFT_267717 [Branchiostoma floridae] gi

Protein Accession	CL26176.Contig18$_{H}1$ CL26176.Contig17$_{H}1$
Mean Expression Ratio	1.02
Median Expression Ratio	1.02
Credible Interval	(0.659, 1.58)
Associated Peptides	1
Associated Spectra	1
Coverage	NaN

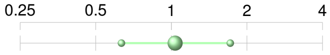

A	B	2.5	50	97.5	Sequence
1	0	0.63	1	1.7	ALITIANK

5.569 622 2733 PREDICTED: protein-glutamine gamma-glutamyltransferase K isoform 1 [Danio rerio]; 423 2498 PREDICTED: protein-glutamine gamma-glutamyltransferase K isoform 1 [Danio rerio]; 423 2534 PREDICTED: protein-glutamine gamma-glutamyltransferase K isoform 1 [Danio rerio]

Protein Accession	Unigene4042$_{H}1$ CL5652.Contig2$_{H}1$ CL5652.Contig1$_{H}1$
Mean Expression Ratio	0.981
Median Expression Ratio	0.981
Credible Interval	(0.756, 1.27)
Associated Peptides	6
Associated Spectra	12
Coverage	NaN

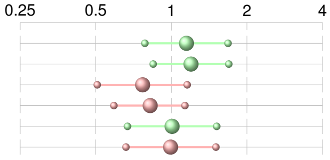

A	B	2.5	50	97.5	Sequence
1	1	0.79	1.1	1.7	LVFGIGEDPK
2	1	0.85	1.2	1.7	ILDMSGLK
1	0	0.51	0.77	1.2	GTQVVFILSAPDEK
2	2	0.59	0.82	1.1	KPHLQIK
1	0	0.67	1	1.5	GNPIPVVR
0	1	0.66	0.99	1.5	DVVFELR

5.570 2881 3411 myosin heavy chain [Todarodes pacificus]; 2915 3445 myosin heavy chain [Todarodes pacificus]

Protein Accession	Unigene19024$_H$1 Unigene19021$_H$1
Mean Expression Ratio	1.02
Median Expression Ratio	1.02
Credible Interval	(0.882, 1.18)
Associated Peptides	13
Associated Spectra	136
Coverage	NaN

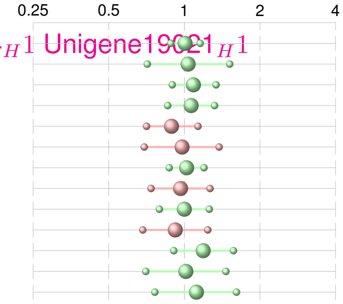

A	B	2.5	50	97.5	Sequence
28	13	0.88	1	1.2	ALDSMQASLEAEAK
1	0	0.71	1	1.5	ALTGEVEELR
9	6	0.89	1.1	1.3	CTALTGEVEELR
9	4	0.86	1.1	1.3	IQEKEEEFENTR
5	4	0.71	0.89	1.1	IQEKEEEFENTRR
2	0	0.69	0.98	1.4	KLEQDINELEIALDQANR
26	1	0.87	1	1.2	KLQQQVIETQQHVEEEAR
6	0	0.74	0.97	1.3	LQQQVIETQQHVEEEAR
4	6	0.8	1	1.3	RCTALTGEVEELR
1	3	0.68	0.92	1.2	RIQEKEEEFENTR
3	3	0.91	1.2	1.6	TALTGEVEELR
0	1	0.7	1	1.5	IQEKEEEFEN
0	1	0.76	1.1	1.6	ELEIALDQANR

5.571 2 517 minus strand Anhydro-N-acetylmuramic acid kinase [Crassostrea gigas]

Protein Accession	Unigene23377$_H$1
Mean Expression Ratio	1.02
Median Expression Ratio	1.02
Credible Interval	(0.699, 1.48)
Associated Peptides	2
Associated Spectra	3
Coverage	NaN

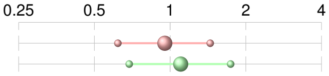

A	B	2.5	50	97.5	Sequence
1	1	0.62	0.96	1.4	KYGLQDVAR
0	1	0.69	1.1	1.7	KLVDAGLAVDK

5.572 366 935 minus strand 40S ribosomal protein S2 OS=Urechis caupo GN=RPS2 PE=2 SV=1

Protein Accession	CL2084.Contig7$_H$1
Mean Expression Ratio	0.982
Median Expression Ratio	0.982
Credible Interval	(0.637, 1.51)
Associated Peptides	1
Associated Spectra	1
Coverage	NaN

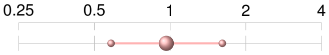

A	B	2.5	50	97.5	Sequence
0	1	0.58	0.97	1.6	TYSFLTPDLWK

5.573 219 554 minus strand ribosomal protein P1 [Biomphalaria glabrata]

Protein Accession	CL45629.Contig2$_{H}1$
Mean Expression Ratio	1.02
Median Expression Ratio	1.02
Credible Interval	(0.662, 1.58)
Associated Peptides	1
Associated Spectra	1
Coverage	NaN

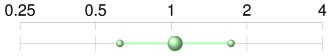

A	B	2.5	50	97.5	Sequence
**1**	**0**	**0.62**	**1**	**1.7**	**ALEGVNIK**

5.574 111 530 minus strand Nucleoside diphosphate kinase B OS=Homo sapiens GN=NME2 PE=1 SV=1

Protein Accession	CL22280.Contig3$_{H}1$
Mean Expression Ratio	0.981
Median Expression Ratio	0.982
Credible Interval	(0.733, 1.32)
Associated Peptides	4
Associated Spectra	9
Coverage	NaN

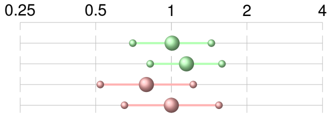

A	B	2.5	50	97.5	Sequence
**2**	**1**	**0.7**	**1**	**1.4**	**GDFCIEMGR**
**2**	**2**	**0.82**	**1.1**	**1.6**	**VMLGETNPLNSKPGTIR**
**1**	**0**	**0.52**	**0.8**	**1.2**	**LCRPGK**
**0**	**1**	**0.65**	**1**	**1.5**	**LVAAK**

5.575 1573 2385 minus strand Spectrin beta chain [Crassostrea gigas]

Protein Accession	CL5904.Contig4$_{H}1$
Mean Expression Ratio	0.982
Median Expression Ratio	0.982
Credible Interval	(0.637, 1.52)
Associated Peptides	1
Associated Spectra	1
Coverage	NaN

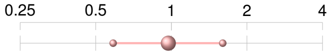

A	B	2.5	50	97.5	Sequence
**0**	**1**	**0.59**	**0.97**	**1.6**	**RLIQEEHPESDQIAVR**

5.576 297 734 minus strand PREDICTED: calmodulin [Canis lupus familiaris]; 297 734 PREDICTED: calmodulin [Canis lupus familiaris]; 143 508 minus strand Calmodulin [Crassostrea gigas]

Protein Accession	Unigene26719$_{H}1$ Unigene17836$_{H}1$ Unigene10331$_{H}1$
Mean Expression Ratio	0.982
Median Expression Ratio	0.982
Credible Interval	(0.639, 1.53)
Associated Peptides	1
Associated Spectra	1
Coverage	NaN

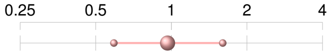

A	B	2.5	50	97.5	Sequence
**1**	**0**	**0.59**	**0.97**	**1.6**	**TLTFQEVDAIVTK**

5.577 116 1111 minus strand cytosolic malate dehydrogenase [Lottia scabra]

Protein Accession	CL280.Contig16$_H$1
Mean Expression Ratio	1.02
Median Expression Ratio	1.02
Credible Interval	(0.889, 1.17)
Associated Peptides	18
Associated Spectra	129
Coverage	NaN

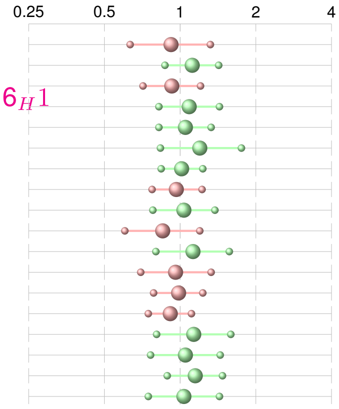

A	B	2.5	50	97.5	Sequence
1	0	0.63	0.92	1.3	ATADHMHDWWFGTEGDK
5	4	0.87	1.1	1.4	DCDAAFLVGAMPR
4	3	0.71	0.93	1.2	DLLAANIK
3	3	0.82	1.1	1.4	EAVKDDNWINNEFISTVQK
6	5	0.83	1.1	1.3	ENFSCLTR
1	0	0.84	1.2	1.8	EVIPTSDEK
15	4	0.84	1	1.2	KLSSAMSAAK
6	6	0.77	0.97	1.2	LGIPNNAVSK
3	3	0.78	1	1.4	LSSAMSAAK
2	0	0.6	0.85	1.2	QVTIVEGLSISDFAR
2	0	0.8	1.1	1.6	WVSMGVYSTGK
2	1	0.7	0.96	1.3	YGIPEGVMYSYPVQIK
11	0	0.78	0.98	1.2	KDLLAANIK
9	14	0.75	0.91	1.1	EMAEQILAA
2	0	0.8	1.1	1.6	SLLYSVAK
2	1	0.76	1	1.4	ELLEER
6	2	0.89	1.1	1.5	SQGANLDAVAK
2	1	0.75	1	1.4	YAPSIPK

5.578 3 239 minus strand alcohol dehydrogenase class 3 [Branchiostoma lanceolatum]

Protein Accession	Unigene58195$_H$1
Mean Expression Ratio	1.02
Median Expression Ratio	1.02
Credible Interval	(0.712, 1.45)
Associated Peptides	2
Associated Spectra	4
Coverage	NaN

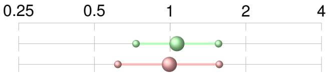

A	B	2.5	50	97.5	Sequence
3	0	0.73	1.1	1.6	GSAFGGWK
1	0	0.62	0.99	1.6	LVEAYMNGK

5.579 22 369 Protein farnesyltransferase/geranylgeranyltransferase type-1 subunit alpha [Crassostrea gigas]

Protein Accession	CL38456.Contig1$_H$1
Mean Expression Ratio	0.983
Median Expression Ratio	0.983
Credible Interval	(0.66, 1.46)
Associated Peptides	2
Associated Spectra	2
Coverage	NaN

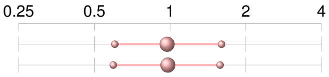

A	B	2.5	50	97.5	Sequence
1	0	0.6	0.98	1.6	YISAVIEDHPK
1	0	0.6	0.98	1.6	AIVASEEK

5.580 43 525 Reticulon-4 [Crassostrea gigas]; 143 757 Reticulon-4 [Crassostrea gigas]; 43 564 Reticulon-4 [Crassostrea gigas]; 302 583 Reticulon-4 OS=Rattus norvegicus GN=Rtn4 PE=1 SV=1

Protein Accession	CL29775.Contig4$_{H}1$ CL29775.Contig3$_{H}1$ CL29775.Contig2$_{H}1$ CL29775.Contig1$_{H}1$
Mean Expression Ratio	0.982
Median Expression Ratio	0.983
Credible Interval	(0.688, 1.4)
Associated Peptides	2
Associated Spectra	6
Coverage	NaN

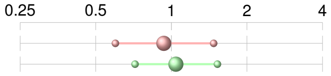

A	B	2.5	50	97.5	Sequence
**2**	**1**	**0.6**	**0.93**	**1.5**	**VTQTILQHVNR**
**0**	**3**	**0.72**	**1**	**1.5**	**SLLELR**

5.581 302 1273 minus strand hypothetical protein CGI_10007007 [Crassostrea gigas]

Protein Accession	CL18045.Contig10$_{H}1$
Mean Expression Ratio	0.986
Median Expression Ratio	0.983
Credible Interval	(0.632, 1.54)
Associated Peptides	1
Associated Spectra	2
Coverage	NaN

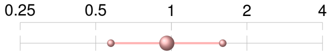

A	B	2.5	50	97.5	Sequence
**1**	**1**	**0.58**	**0.96**	**1.6**	**IEAEFPGANIILHK**

5.582 63 443 PREDICTED: RAS protein activator like 1-like [Saccoglossus kowalevskii]

Protein Accession	CL19305.Contig2$_{H}1$
Mean Expression Ratio	1.02
Median Expression Ratio	1.02
Credible Interval	(0.805, 1.29)
Associated Peptides	4
Associated Spectra	40
Coverage	NaN

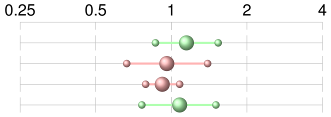

A	B	2.5	50	97.5	Sequence
**2**	**4**	**0.87**	**1.1**	**1.5**	**DYAQAADGALASVLAR**
**1**	**1**	**0.66**	**0.96**	**1.4**	**YLEDWIK**
**16**	**13**	**0.79**	**0.92**	**1.1**	**LASVLAR**
**2**	**1**	**0.76**	**1.1**	**1.5**	**ALNIQLTK**

5.583 2 475 unnamed protein product [Tetraodon nigroviridis]

Protein Accession	CL31420.Contig4$_{H}1$
Mean Expression Ratio	0.983
Median Expression Ratio	0.983
Credible Interval	(0.75, 1.29)
Associated Peptides	2
Associated Spectra	27
Coverage	NaN

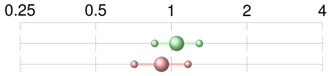

A	B	2.5	50	97.5	Sequence
**17**	**0**	**0.86**	**1.1**	**1.3**	**DLYANTVLSGGTTMYPGIADR**
**10**	**0**	**0.71**	**0.91**	**1.2**	**KDLYANTVLSGGTTMYPGIADR**

5.584 1 852 beta actin-like 1 protein [Macrostomum lignano]

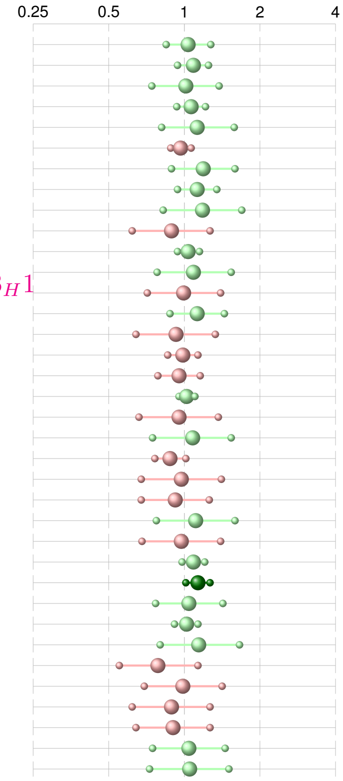

A	B	2.5	50	97.5	Sequence
9	5	0.85	1	1.3	AGFAGDDAPR
26	7	0.94	1.1	1.2	AVFPSIVGR
2	1	0.74	1	1.4	AVFPSIVGRPR
20	22	0.94	1.1	1.2	DSYVGDEAQSK
1	1	0.81	1.1	1.6	GVMVGMGQK
80	17	0.88	0.97	1.1	GYSFTTTAER
2	2	0.89	1.2	1.6	HQGVMVGMGQ
12	8	0.94	1.1	1.3	IWHHTFYNELR
1	0	0.82	1.2	1.7	MTQIMFETF
1	0	0.62	0.89	1.3	SYELPDGQVITIGN
60	15	0.94	1	1.1	SYELPDGQVITIGNER
1	1	0.78	1.1	1.5	TTGIVLDSGDGVTH
2	0	0.71	0.99	1.4	TTGIVLDSGDGVTHTVPIYEGY
7	0	0.87	1.1	1.4	TTGIVLDSGDGVTHTVPIYEGYALPH
1	0	0.64	0.93	1.3	VALDFEQEMATAAASSSLEK
18	17	0.86	0.99	1.1	VAPEEHPVLLTEA
10	6	0.79	0.95	1.2	VAPEEHPVLLTEAPLN
95	64	0.95	1	1.1	VAPEEHPVLLTEAPLNPK
1	0	0.66	0.95	1.4	VAPEEHPVLLTEAPLNPKANR
1	0	0.75	1.1	1.5	TEAPLNPK
23	13	0.76	0.88	1	RGILTLK
1	0	0.67	0.97	1.4	VAPEEHPVLLTEAPLNPKANREKMTQI
2	1	0.67	0.92	1.3	WDDMEK
1	0	0.77	1.1	1.6	HQGVMVGMGQKDSYVGDEAQSK
1	0	0.68	0.98	1.4	MTQIMFETFN
36	36	0.98	1.1	1.2	GILTLK
45	15	1	1.1	1.3	HQGVMVGMGQK
1	2	0.77	1	1.4	TTGIVLDSGDGVTHTVPIYEGYALPHAILR
49	19	0.92	1	1.1	YPIEHGIVTNWDDMEK
0	1	0.8	1.1	1.7	GIVTNWDDMEK
0	1	0.55	0.79	1.1	EIVRDIKEK
0	1	0.69	0.99	1.4	VMVGMGQK
0	1	0.62	0.89	1.3	GDEAQSK
0	2	0.64	0.9	1.3	VAPEEHPVLLTEAPL
0	2	0.75	1	1.5	VAPEEHPVLLTEAP
0	1	0.73	1.1	1.5	YPIEHGIVTNWDDME

Protein Accession	Unigene31438$_{H}1$
Mean Expression Ratio	1.02
Median Expression Ratio	1.02
Credible Interval	(0.925, 1.12)
Associated Peptides	36
Associated Spectra	770
Coverage	NaN

5.585 1 264 Spectrin beta chain [Crassostrea gigas]

Protein Accession	Unigene36334$_{H}1$
Mean Expression Ratio	0.983
Median Expression Ratio	0.984
Credible Interval	(0.657, 1.45)
Associated Peptides	2
Associated Spectra	2
Coverage	NaN

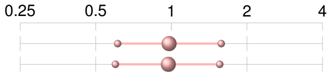

A	B	2.5	50	97.5	Sequence
1	0	0.61	0.98	1.6	QLLQVEHPNAAEVVQR
1	0	0.6	0.97	1.6	VAVVNQLAR

5.586 362 874 minus strand Transgelin-2 [Crassostrea gigas]

Protein Accession	CL445.Contig3$_{H}$1
Mean Expression Ratio	0.984
Median Expression Ratio	0.984
Credible Interval	(0.854, 1.13)
Associated Peptides	14
Associated Spectra	150
Coverage	NaN

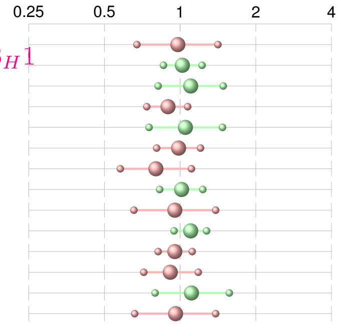

A	B	2.5	50	97.5	Sequence
1	0	0.67	0.98	1.4	AGLGAEIEKK
12	9	0.86	1	1.2	EFLVQSADLWEGR
3	1	0.82	1.1	1.5	EFLVQSADLWEGRK
17	0	0.74	0.89	1.1	GFQPSYTGSQSR
2	0	0.75	1.1	1.5	KAGLGAEIEKK
12	3	0.81	0.99	1.2	LLESEGKPK
1	2	0.58	0.8	1.1	LINKLLESEGKPK
8	8	0.83	1	1.2	LINFD
1	0	0.66	0.95	1.4	LGAEIEK
20	11	0.95	1.1	1.3	AGLGAEIEK
20	8	0.82	0.96	1.1	DGVMLCK
4	4	0.72	0.92	1.2	KAGLGAEIEK
1	1	0.79	1.1	1.6	RDGVMLCKLINK
0	1	0.66	0.96	1.4	QSADLWEGR

5.587 1213 3234 Protein-glutamine gamma-glutamyltransferase K [Crassostrea gigas]; 478 2499 Protein-glutamine gamma-glutamyltransferase K [Crassostrea gigas]

Protein Accession	CL29134.Contig6$_{H}$1 CL29134.Contig5$_{H}$1
Mean Expression Ratio	0.985
Median Expression Ratio	0.984
Credible Interval	(0.635, 1.53)
Associated Peptides	1
Associated Spectra	1
Coverage	NaN

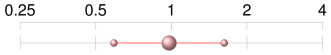

A	B	2.5	50	97.5	Sequence
0	1	0.59	0.98	1.6	FLFAEAHGER

5.588 179 2140 minus strand PREDICTED: hypothetical protein [Saccoglossus kowalevskii]

Protein Accession	CL13749.Contig6$_{H}$1
Mean Expression Ratio	1.02
Median Expression Ratio	1.02
Credible Interval	(0.652, 1.57)
Associated Peptides	1
Associated Spectra	1
Coverage	NaN

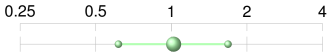

A	B	2.5	50	97.5	Sequence
1	0	0.62	1	1.7	KFDGIGGLSGGGATSK

5.589 11 394 regucalcin [Salmo salar] gi

Protein Accession	CL42615.Contig1$_{H}1$
Mean Expression Ratio	0.986
Median Expression Ratio	0.985
Credible Interval	(0.643, 1.52)
Associated Peptides	1
Associated Spectra	1
Coverage	NaN

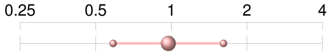

A	B	2.5	50	97.5	Sequence
1	0	0.58	0.98	1.6	NQPLAGSLFK

5.590 " 656 787 collagen alpha-1(XII) chain-like; K08132 collagen, type XII, alpha"

Protein Accession	Unigene26545$_{H}1$
Mean Expression Ratio	1.02
Median Expression Ratio	1.02
Credible Interval	(0.713, 1.45)
Associated Peptides	2
Associated Spectra	4
Coverage	NaN

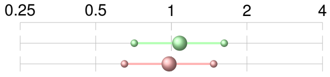

A	B	2.5	50	97.5	Sequence
1	1	0.71	1.1	1.6	AEVFDAVDNIPYR
1	1	0.65	0.98	1.5	TKAEVFDAVDNIPYR

5.591 4 579 minus strand Collagen alpha-3(VI) chain [Crassostrea gigas]

Protein Accession	CL46502.Contig3$_{H}1$
Mean Expression Ratio	0.985
Median Expression Ratio	0.985
Credible Interval	(0.705, 1.39)
Associated Peptides	2
Associated Spectra	6
Coverage	NaN

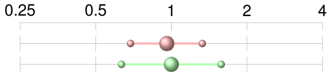

A	B	2.5	50	97.5	Sequence
5	0	0.69	0.96	1.3	FAVQEFSELR
1	0	0.63	1	1.6	LIKDFVK

5.592 95 2506 Transitional endoplasmic reticulum ATPase [Crassostrea gigas]

Protein Accession	CL15973.Contig2$_{H}1$
Mean Expression Ratio	0.986
Median Expression Ratio	0.985
Credible Interval	(0.766, 1.27)
Associated Peptides	9
Associated Spectra	11
Coverage	NaN

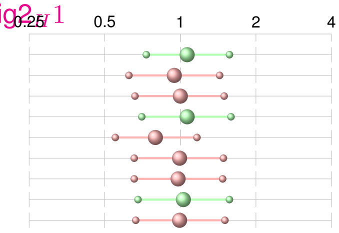

A	B	2.5	50	97.5	Sequence
1	1	0.73	1.1	1.6	EMVELPLR
1	0	0.62	0.95	1.4	LDQLIYIPLPDEK
1	0	0.66	1	1.5	MAIEAEIR
1	0	0.7	1.1	1.6	MDELQLFR
1	1	0.55	0.8	1.2	EVDIGIPDATGR
0	1	0.66	0.99	1.5	DIDLDYLAK
0	1	0.66	0.98	1.5	GILLYGPPGTGK
0	1	0.68	1	1.6	AHFEEAMK
0	1	0.66	0.99	1.5	GDIFLVR

5.593 154 621 minus strand Stress response protein nhaX [Crassostrea gigas]

Protein Accession	CL4265.Contig1$_H$1
Mean Expression Ratio	0.985
Median Expression Ratio	0.985
Credible Interval	(0.712, 1.35)
Associated Peptides	5
Associated Spectra	5
Coverage	NaN

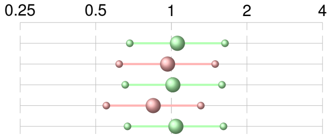

A	B	2.5	50	97.5	Sequence
0	1	0.69	1.1	1.6	IQHDLEK
0	1	0.62	0.96	1.5	SVAAAKPGEGILK
0	1	0.65	1	1.6	EVLEAMLK
0	1	0.55	0.85	1.3	PGEGILK
0	1	0.67	1	1.6	EGILK

5.594 " 137 1549 minus strand Serine hydroxymethyltransferase, mitochondrial [Crassostrea gigas]; 205 1608 minus strand Serine hydroxymethyltransferase, mitochondrial [Crassostrea gigas]; 161 1573 minus strand Serine hydroxymethyltransferase, mitochondrial [Crassostrea gigas]; 205 1386 minus strand Serine hydroxymethyltransferase, mitochondrial [Crassostrea gigas]"

Protein Accession	CL22722.Contig6$_H$1 CL22722.Contig5$_H$1 CL22722.Contig4$_H$1 CL22722.Contig1$_H$1
Mean Expression Ratio	0.984
Median Expression Ratio	0.985
Credible Interval	(0.641, 1.51)
Associated Peptides	1
Associated Spectra	1
Coverage	NaN

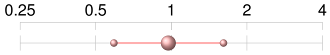

A	B	2.5	50	97.5	Sequence
1	0	0.59	0.97	1.6	IIFYR

5.595 68 481 minus strand netrin receptor UNC5A [Danio rerio] gi

Protein Accession	Unigene38845$_H$1
Mean Expression Ratio	1.01
Median Expression Ratio	1.01
Credible Interval	(0.66, 1.56)
Associated Peptides	1
Associated Spectra	1
Coverage	NaN

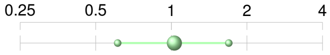

A	B	2.5	50	97.5	Sequence
1	0	0.61	1	1.7	ELMECQK

5.596 114 2633 Filamin-A [Crassostrea gigas]; 135 2654 Filamin-A [Crassostrea gigas]

Protein Accession	CL393.Contig21$_H$1 CL393.Contig16$_H$1
Mean Expression Ratio	1.01
Median Expression Ratio	1.01
Credible Interval	(0.909, 1.13)
Associated Peptides	35
Associated Spectra	170
Coverage	NaN

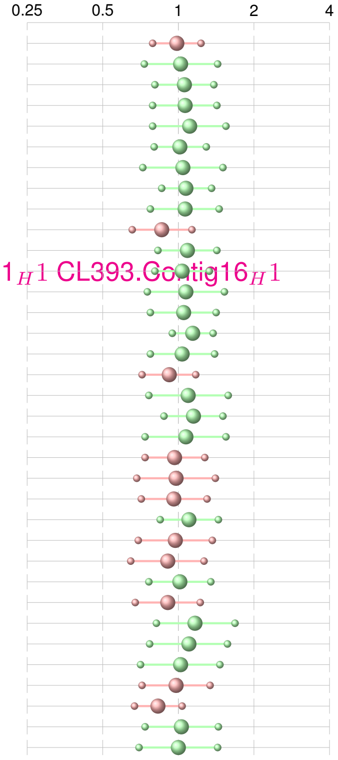

A	B	2.5	50	97.5	Sequence
9	3	0.79	0.99	1.2	AVANKPNEIVIDTSK
2	0	0.73	1	1.4	DAGEALLSIGMIAPSGHPEAELGYK
5	1	0.81	1.1	1.4	DNNDGTIVVEYQPK
4	0	0.79	1.1	1.4	FADEHVPGSPFLLNVGGEASGR
2	0	0.79	1.1	1.5	FDDVHIPGSPFIVNIAPDSGTAR
6	4	0.8	1	1.3	IGDSELGHAAGCK
1	0	0.72	1	1.5	KMGDGEIGIASFNPR
8	3	0.86	1.1	1.4	KPSPIQEQSSMVVETVEK
3	0	0.78	1.1	1.5	KVTANSYTVSYK
3	3	0.65	0.86	1.1	MGDGEIGIASFNPR
4	2	0.83	1.1	1.4	QMEAAEAAVPNK
8	0	0.81	1	1.3	SQLSLGNSAEYSLK
1	0	0.75	1.1	1.5	TNVSDETGCYSK
3	1	0.78	1	1.4	TPSGGHEDCFIQEMDK
12	9	0.94	1.1	1.4	VINFQIETK
3	1	0.77	1	1.4	VPKPGSYGLHIK
5	4	0.72	0.92	1.2	VQVGGPGLEK
1	0	0.77	1.1	1.6	VRDNNDGTIVVEYQPK
4	2	0.87	1.1	1.5	VTGALDK
1	0	0.74	1.1	1.5	WGDEDIPGSPFVIST
4	2	0.74	0.97	1.3	YGNIPIAGSPYK
1	0	0.68	0.98	1.4	YIPKENGVHYVDVK
3	1	0.71	0.96	1.3	YKGNPIK
5	1	0.85	1.1	1.4	EAGPGTLTVAVEGPGK
1	1	0.69	0.97	1.4	AALSCK
1	1	0.64	0.91	1.3	PGSYGLHIK
4	1	0.76	1	1.3	HINNSPFK
3	1	0.68	0.91	1.2	IMFTPK
1	0	0.82	1.2	1.7	FRGDASK
1	0	0.77	1.1	1.6	AQIFLEERPNGFLGVQYK
1	0	0.71	1	1.5	CPGDYLITIK
2	1	0.72	0.98	1.3	ENGVHYVDVK
7	6	0.67	0.83	1	MNGIHTLSVR
2	0	0.74	1	1.4	SDIECYK
1	0	0.7	1	1.4	VTANSYTVSYK

5.597 158 862 X-box binding protein [Haliotis discus discus]

Protein Accession	CL6396.Contig3$_H$1
Mean Expression Ratio	1.01
Median Expression Ratio	1.01
Credible Interval	(0.795, 1.29)
Associated Peptides	6
Associated Spectra	18
Coverage	NaN

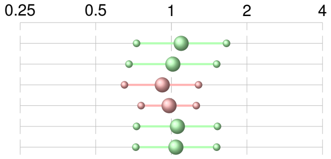

A	B	2.5	50	97.5	Sequence
1	0	0.73	1.1	1.7	GSISDPSVFNVPEACQK
1	0	0.68	1	1.5	VFPDFYFGAGDDK
3	0	0.65	0.92	1.3	ACIPDK
5	4	0.76	0.98	1.3	LYIVDLAK
1	1	0.73	1.1	1.5	SYFILK
2	0	0.72	1	1.5	SYYIGAR

5.598 174 1649 minus strand Collagen alpha-1(XII) chain [Crassostrea gigas]; 135 1610 Collagen alpha-1(XII) chain [Crassostrea gigas]

Protein Accession	CL5340.Contig13$_H$1 CL5340.Contig11$_H$1
Mean Expression Ratio	1.01
Median Expression Ratio	1.01
Credible Interval	(0.819, 1.26)
Associated Peptides	10
Associated Spectra	28
Coverage	NaN

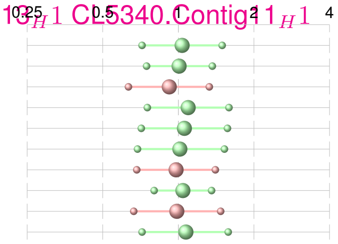

A	B	2.5	50	97.5	Sequence
2	0	0.72	1	1.5	ADVMFVTEFSQAGGR
4	1	0.75	1	1.4	ADVVFIVDSSR
1	1	0.63	0.92	1.3	DVMSVFHIAR
1	1	0.75	1.1	1.6	SIWMPDFK
1	0	0.71	1.1	1.6	VDGYSALDTIR
1	0	0.69	1	1.5	YWAEQLKK
2	0	0.68	0.98	1.4	KADVVFVLDK
6	5	0.8	1	1.4	DILAFR
1	0	0.67	0.99	1.5	TTELLK
1	0	0.72	1.1	1.6	SSSITAEHFQTQLR

5.599 142 588 AAEL009772-PB [Aedes aegypti]; 96 497 AAEL009772-PB [Aedes aegypti]

Protein Accession	Unigene73475$_H$1 CL27854.Contig2$_H$1
Mean Expression Ratio	0.987
Median Expression Ratio	0.986
Credible Interval	(0.644, 1.52)
Associated Peptides	1
Associated Spectra	3
Coverage	NaN

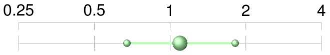

A	B	2.5	50	97.5	Sequence
2	1	0.67	1.1	1.8	LVEEFPMGNIK

5.600 " 2 286 Roundabout 2, partial [Ascaris suum]"

Protein Accession	CL8589.Contig2$_H$1
Mean Expression Ratio	0.985
Median Expression Ratio	0.987
Credible Interval	(0.663, 1.46)
Associated Peptides	2
Associated Spectra	2
Coverage	NaN

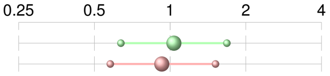

A	B	2.5	50	97.5	Sequence
1	0	0.64	1	1.7	GEPTPEITWLK
1	0	0.58	0.93	1.5	KSQTAVLDVIR

5.601 126 287 minus strand Cytochrome b-c1 complex subunit 10 [Cricetulus griseus]; 126 287 minus strand Cytochrome b-c1 complex subunit 10 [Cricetulus griseus]; 126 287 minus strand Cytochrome b-c1 complex subunit 10 [Cricetulus griseus]; 126 287 minus strand Cytochrome b-c1 complex subunit 10 [Cricetulus griseus]

Protein Accession	CL46231.Contig6$_{H}1$ CL46231.Contig5$_{H}1$ CL46231.Contig4$_{H}1$ CL46231.Contig3$_{H}1$
Mean Expression Ratio	1.01
Median Expression Ratio	1.01
Credible Interval	(0.651, 1.57)
Associated Peptides	1
Associated Spectra	1
Coverage	NaN

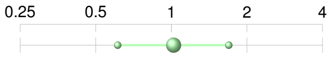

A	B	2.5	50	97.5	Sequence
0	1	0.61	1	1.7	FIPFYGSK

5.602 3 293 Titin [Crassostrea gigas]

Protein Accession	Unigene9241$_{H}1$
Mean Expression Ratio	1.02
Median Expression Ratio	1.01
Credible Interval	(0.656, 1.56)
Associated Peptides	1
Associated Spectra	1
Coverage	NaN

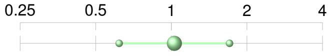

A	B	2.5	50	97.5	Sequence
1	0	0.62	1	1.7	TAINTVESSLSELGQR

5.603 689 1984 hypothetical protein BRAFLDRAFT_78257 [Branchiostoma floridae] gi

Protein Accession	Unigene34076$_{H}1$
Mean Expression Ratio	0.989
Median Expression Ratio	0.987
Credible Interval	(0.782, 1.25)
Associated Peptides	8
Associated Spectra	14
Coverage	NaN

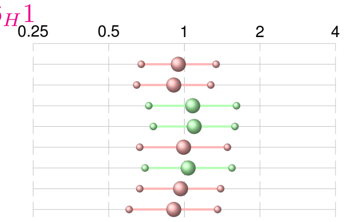

A	B	2.5	50	97.5	Sequence
2	1	0.67	0.94	1.3	TVGGFQLR
3	0	0.64	0.91	1.3	VQQKPEPSPAEGEVLIR
1	0	0.72	1.1	1.6	YLLFGSSNIVTGETK
1	1	0.75	1.1	1.6	YVSEIR
1	0	0.66	0.99	1.5	ILLDPSKAPTPK
0	1	0.7	1	1.5	SLVLTGFGSMK
0	2	0.66	0.97	1.4	GISILKPMGK
0	1	0.6	0.91	1.4	SFFSFAK

5.604 2 328 cytoskeletal actin 2 [Molgula oculata]

Protein Accession	Unigene2743$_{H}$1
Mean Expression Ratio	0.988
Median Expression Ratio	0.987
Credible Interval	(0.808, 1.2)
Associated Peptides	7
Associated Spectra	139
Coverage	NaN

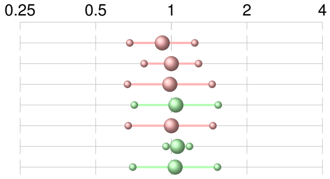

A	B	2.5	50	97.5	Sequence
3	2	0.68	0.92	1.2	IIAPPERK
7	2	0.78	1	1.3	IKIIAPPER
1	0	0.67	0.98	1.5	KIIAPPER
1	0	0.71	1	1.5	DITALAPPTMK
1	0	0.67	1	1.5	DLYANVVLSGGSTMYPGIADR
63	58	0.95	1.1	1.2	IIAPPER
1	0	0.7	1	1.5	KDLYANVVLSGGSTMYPGIADR

5.605 172 678 Myosin regulatory light chain sqh [Crassostrea gigas]

Protein Accession	CL23773.Contig1$_{H}$1
Mean Expression Ratio	0.987
Median Expression Ratio	0.988
Credible Interval	(0.676, 1.43)
Associated Peptides	2
Associated Spectra	3
Coverage	NaN

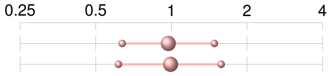

A	B	2.5	50	97.5	Sequence
1	1	0.64	0.97	1.5	LTDEEVDEMFR
1	0	0.62	0.99	1.6	ELLTTMGDR

5.606 " 703 1173 60S acidic ribosomal protein P0, putative [Ixodes scapularis] gi"

Protein Accession	Unigene34489$_{H}$1
Mean Expression Ratio	0.989
Median Expression Ratio	0.988
Credible Interval	(0.651, 1.54)
Associated Peptides	1
Associated Spectra	1
Coverage	NaN

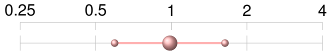

A	B	2.5	50	97.5	Sequence
0	1	0.6	0.98	1.6	KSFLEGVR

5.607 57 2378 minus strand Heterogeneous nuclear ribonucleoprotein U-like protein 1 [Crassostrea gigas]

Protein Accession	CL15560.Contig5$_{H}$1
Mean Expression Ratio	0.991
Median Expression Ratio	0.989
Credible Interval	(0.672, 1.46)
Associated Peptides	2
Associated Spectra	2
Coverage	NaN

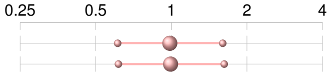

A	B	2.5	50	97.5	Sequence
0	1	0.61	0.98	1.6	ALFPHILTK
0	1	0.61	0.99	1.6	VTELRDELK

5.608 94 933 four and A half lim domains [Schistosoma mansoni]

Protein Accession	CL7046.Contig6$_H1$
Mean Expression Ratio	1.01
Median Expression Ratio	1.01
Credible Interval	(0.826, 1.25)
Associated Peptides	8
Associated Spectra	27
Coverage	NaN

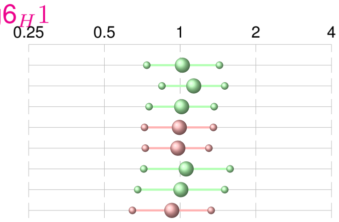

A	B	2.5	50	97.5	Sequence
2	1	0.74	1	1.4	CDASMVGR
4	2	0.85	1.1	1.5	CTKPITGIGGTK
3	2	0.75	1	1.4	FISFEDR
2	2	0.72	1	1.4	SLVDQPFAPK
3	2	0.73	0.98	1.3	SFIPR
1	0	0.72	1.1	1.6	CSGVINK
1	0	0.68	1	1.5	KFEYK
1	1	0.65	0.93	1.3	DDQPYCGDCFGELFAK

5.609 783 1463 minus strand twitchin [Mytilus galloprovincialis]; 783 1463 minus strand twitchin [Mytilus galloprovincialis]; 752 1432 twitchin [Mytilus galloprovincialis]

Protein Accession	CL181.Contig5$_H1$ CL181.Contig3$_H1$ CL181.Contig1$_H1$
Mean Expression Ratio	0.988
Median Expression Ratio	0.989
Credible Interval	(0.767, 1.27)
Associated Peptides	7
Associated Spectra	13
Coverage	NaN

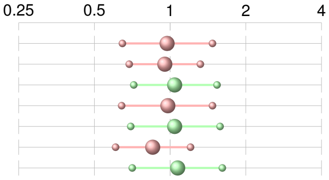

A	B	2.5	50	97.5	Sequence
1	0	0.65	0.97	1.5	DGKPVNSSDIYSITFDK
2	2	0.69	0.95	1.3	LNPQVVNDGDAVDLVVK
2	0	0.72	1	1.5	NSGGEVTSTATVNVDALKPK
1	0	0.64	0.98	1.5	VTGTEPFDITWAK
1	0	0.7	1	1.6	NQWGTASSMASLQVK
0	3	0.61	0.85	1.2	LEGDTIIIQQDITSK
0	1	0.71	1.1	1.6	SKPDLLWYR

5.610 718 1155 Annexin A7 [Crassostrea gigas]

Protein Accession	CL15539.Contig2$_H1$
Mean Expression Ratio	0.989
Median Expression Ratio	0.989
Credible Interval	(0.763, 1.29)
Associated Peptides	5
Associated Spectra	12
Coverage	NaN

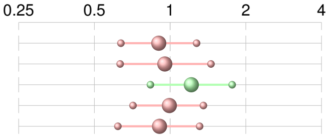

A	B	2.5	50	97.5	Sequence
3	0	0.64	0.9	1.3	ATFDAYQQIAGK
1	0	0.63	0.95	1.5	DIESAIK
2	0	0.83	1.2	1.8	KLGTDEETFNR
4	0	0.71	0.99	1.4	NVPGYFAQR
2	0	0.62	0.91	1.3	SFPQLR

5.611 64 393 Tubulin beta chain OS=Paracentrotus lividus PE=2 SV=1; 111 398 Tubulin beta chain OS=Paracentrotus lividus PE=2 SV=1; 109 438 Tubulin beta chain OS=Paracentrotus lividus PE=2 SV=1; 160 489 Tubulin beta chain OS=Paracentrotus lividus PE=2 SV=1

Protein Accession	Unigene62663$_H$1 Unigene62662$_H$1 Unigene62661$_H$1 Unigene62660$_H$1
Mean Expression Ratio	1.01
Median Expression Ratio	1.01
Credible Interval	(0.679, 1.5)
Associated Peptides	1
Associated Spectra	6
Coverage	NaN

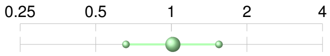

A	B	2.5	50	97.5	Sequence
4	2	0.66	1	1.5	INVYFNEATGGK

5.612 273 1304 Guanine nucleotide-binding protein subunit beta [Crassostrea gigas]

Protein Accession	CL15682.Contig1$_H$1
Mean Expression Ratio	0.988
Median Expression Ratio	0.989
Credible Interval	(0.794, 1.23)
Associated Peptides	6
Associated Spectra	23
Coverage	NaN

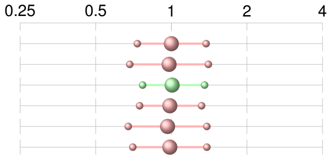

A	B	2.5	50	97.5	Sequence
3	1	0.73	1	1.4	IYAMHWASDSR
2	0	0.68	0.98	1.4	KAAGDTTLAQATAQCEPVGR
3	3	0.77	1	1.4	TFVSGACDASAK
3	3	0.75	0.99	1.3	LWDIR
0	2	0.67	0.97	1.4	LIVWDGYTTNK
0	3	0.7	0.98	1.4	LFDIR

5.613 552 1886 minus strand intermediate filament protein [Biomphalaria glabrata]

Protein Accession	Unigene26795$_H$1
Mean Expression Ratio	1.01
Median Expression Ratio	1.01
Credible Interval	(0.904, 1.14)
Associated Peptides	22
Associated Spectra	156
Coverage	NaN

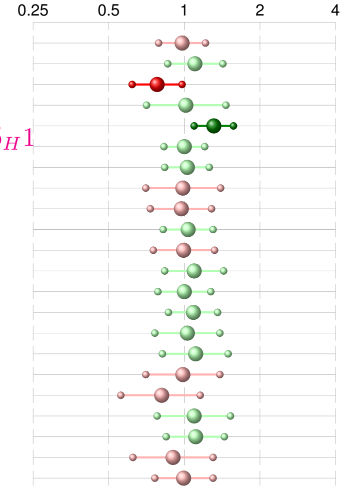

A	B	2.5	50	97.5	Sequence
10	2	0.79	0.98	1.2	ADLDAETAAHIENEVLAQTK
5	3	0.86	1.1	1.4	CGSLEDELAK
5	6	0.62	0.78	0.98	DMYEGELAQAR
1	0	0.7	1	1.5	DSYLVQLNELQLEFER
12	7	1.1	1.3	1.6	KQEDWKPIR
14	4	0.83	1	1.2	KVITEFSTQR
11	3	0.84	1	1.3	LATYIEK
1	1	0.7	0.99	1.4	LEAELEALR
3	2	0.73	0.97	1.3	LLEGEENR
6	4	0.82	1	1.3	LRDQLAEK
4	1	0.75	1	1.3	SATGISEFR
4	2	0.84	1.1	1.4	VITEFSTQR
6	2	0.78	1	1.3	YAAQMDQLR
8	3	0.87	1.1	1.4	KLLEGEENR
4	0	0.76	1	1.4	MSATGISEFR
3	1	0.82	1.1	1.5	ELMDHK
1	1	0.7	0.99	1.4	EMQDCNER
1	0	0.56	0.81	1.2	IAELEAALAAMK
1	1	0.78	1.1	1.5	IANLQDQIQALK
4	2	0.85	1.1	1.4	WLEAQNK
0	1	0.63	0.9	1.3	ALVDKLK
0	6	0.76	1	1.3	KLEAELEALR

5.614 4 2400 IQ motif containing GTPase activating protein 2 [Xenopus laevis] gi; 4 2169 IQ motif containing GTPase activating protein 2 [Xenopus laevis] gi

Protein Accession	CL875.Contig2$_H$1 CL875.Contig1$_H$1
Mean Expression Ratio	1.01
Median Expression Ratio	1.01
Credible Interval	(0.652, 1.57)
Associated Peptides	1
Associated Spectra	1
Coverage	NaN

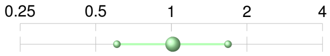

A	B	2.5	50	97.5	Sequence
**0**	**1**	**0.61**	**1**	**1.7**	**ITLQDVVTHNR**

5.615 191 919 PREDICTED: 15-hydroxyprostaglandin dehydrogenase [NAD+] [Gallus gallus]

Protein Accession	CL7033.Contig5$_H$1
Mean Expression Ratio	0.988
Median Expression Ratio	0.989
Credible Interval	(0.646, 1.51)
Associated Peptides	1
Associated Spectra	1
Coverage	NaN

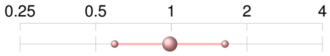

A	B	2.5	50	97.5	Sequence
**1**	**0**	**0.6**	**0.98**	**1.6**	**AMVEALLDK**

5.616 188 4135 Vigilin [Crassostrea gigas]

Protein Accession	CL846.Contig3$_H$1
Mean Expression Ratio	0.991
Median Expression Ratio	0.99
Credible Interval	(0.648, 1.52)
Associated Peptides	1
Associated Spectra	1
Coverage	NaN

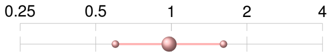

A	B	2.5	50	97.5	Sequence
**1**	**0**	**0.6**	**0.98**	**1.6**	**EVSIDIPHR**

5.617 280 726 minus strand RecName: Full=Calmodulin; Short=CaM gi|14588595|dbj|BAB61794.1| calmodulin [Metridium senile] gi

Protein Accession	Unigene4486$_H$1
Mean Expression Ratio	0.991
Median Expression Ratio	0.991
Credible Interval	(0.74, 1.31)
Associated Peptides	5
Associated Spectra	13
Coverage	NaN

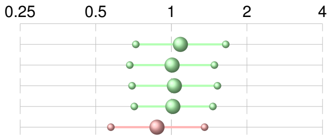

A	B	2.5	50	97.5	Sequence
**1**	**1**	**0.72**	**1.1**	**1.7**	**ADQLTEEQIAEFK**
**2**	**0**	**0.68**	**1**	**1.5**	**EAFSLFDK**
**2**	**0**	**0.7**	**1**	**1.5**	**EAFSLFDKDGDGTITTK**
**2**	**4**	**0.71**	**1**	**1.5**	**ELGTVMR**
**1**	**0**	**0.57**	**0.88**	**1.4**	**HVMTNLGEKLTDEEVDEMIR**

5.618 1265 1831 minus strand Adenosine deaminase CECR1 [Crassostrea gigas]

Protein Accession	CL20850.Contig11$_H1$
Mean Expression Ratio	0.991
Median Expression Ratio	0.991
Credible Interval	(0.638, 1.52)
Associated Peptides	1
Associated Spectra	1
Coverage	NaN

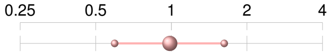

A	B	2.5	50	97.5	Sequence
0	1	0.59	0.99	1.6	GIAVEVNPISNQVLK

5.619 35 505 minus strand Basement membrane-specific heparan sulfate proteoglycan core protein [Crassostrea gigas]

Protein Accession	CL8101.Contig1$_H1$
Mean Expression Ratio	0.991
Median Expression Ratio	0.991
Credible Interval	(0.644, 1.54)
Associated Peptides	1
Associated Spectra	1
Coverage	NaN

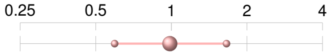

A	B	2.5	50	97.5	Sequence
0	1	0.6	0.99	1.7	AAFAIR

5.620 77 502 hypothetical protein CGI_10008263 [Crassostrea gigas]

Protein Accession	CL38960.Contig1$_H1$
Mean Expression Ratio	0.992
Median Expression Ratio	0.991
Credible Interval	(0.68, 1.45)
Associated Peptides	2
Associated Spectra	3
Coverage	NaN

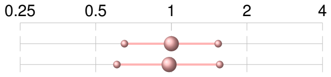

A	B	2.5	50	97.5	Sequence
2	0	0.65	1	1.5	IFVAIDGSK
1	0	0.61	0.98	1.6	KIFVAIDGSK

5.621 94 519 minus strand hypothetical protein BRAFLDRAFT_115029 [Branchiostoma floridae] gi

Protein Accession	Unigene52953$_H1$
Mean Expression Ratio	1.01
Median Expression Ratio	1.01
Credible Interval	(0.729, 1.4)
Associated Peptides	2
Associated Spectra	7
Coverage	NaN

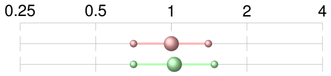

A	B	2.5	50	97.5	Sequence
2	2	0.71	1	1.4	SLDTDGDGTVSAAELR
2	1	0.71	1	1.5	FINKK

5.622 643 1302 minus strand Kyphoscoliosis peptidase [Crassostrea gigas]; 634 1293 minus strand Kyphoscoliosis peptidase [Crassostrea gigas]; 634 1293 minus strand Kyphoscoliosis peptidase [Crassostrea gigas]; 634 1293 minus strand Kyphoscoliosis peptidase [Crassostrea gigas]; 639 1298 minus strand Kyphoscoliosis peptidase [Crassostrea gigas]

Protein Accession	CL7772.Contig1$_H 1$ CL7772.Contig16$_H 1$ CL7772.Contig15$_H 1$ CL7772.Contig14$_H 1$ CL7772.C
Mean Expression Ratio	0.992
Median Expression Ratio	0.992
Credible Interval	(0.816, 1.21)
Associated Peptides	9
Associated Spectra	41
Coverage	NaN

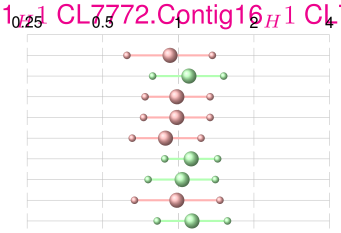

A	B	2.5	50	97.5	Sequence
1	0	0.62	0.92	1.4	DGCYLYSPLVLNSSSR
2	2	0.79	1.1	1.5	FQIFALPTSDDSK
4	1	0.74	0.99	1.3	GAGEVWEGTAALDK
4	0	0.73	0.99	1.3	STTNLIQCR
3	1	0.65	0.89	1.2	VTLNACFGEDESK
7	6	0.88	1.1	1.4	LSNVTFK
3	2	0.76	1	1.4	QYAPWR
1	0	0.67	0.99	1.5	LELVSHPQAEFTLAQDNEVEIK
3	1	0.83	1.1	1.6	AVYPFPK

5.623 247 762 Myophilin [Crassostrea gigas]; 244 759 Myophilin [Crassostrea gigas]; 119 634 Myophilin [Crassostrea gigas]

Protein Accession	CL16175.Contig6$_H 1$ CL16175.Contig5$_H 1$ CL16175.Contig4$_H 1$
Mean Expression Ratio	1.01
Median Expression Ratio	1.01
Credible Interval	(0.738, 1.39)
Associated Peptides	3
Associated Spectra	6
Coverage	NaN

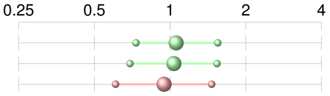

A	B	2.5	50	97.5	Sequence
2	1	0.73	1.1	1.5	GGGFALMQNIER
1	1	0.69	1	1.5	LMPGCIK
1	0	0.61	0.95	1.5	DGIVLCK

5.624 " 64 1332 Flotillin-1, putative [Pediculus humanus corporis] gi"

Protein Accession	CL33952.Contig1$_H 1$
Mean Expression Ratio	0.992
Median Expression Ratio	0.992
Credible Interval	(0.758, 1.3)
Associated Peptides	4
Associated Spectra	17
Coverage	NaN

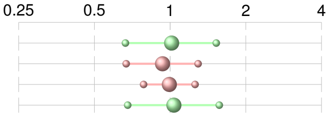

A	B	2.5	50	97.5	Sequence
1	0	0.66	1	1.5	AAYDMEVMTTK
3	1	0.67	0.93	1.3	VKGEAEAFAIEAK
6	5	0.78	0.99	1.3	LPSIVETL
0	1	0.68	1	1.6	FLNDIEIAK

5.625 1732 2079 minus strand PREDICTED: annexin A7-like [Ornithorhynchus anatinus]

Protein Accession	CL9274.Contig2$_{H1}$
Mean Expression Ratio	1.01
Median Expression Ratio	1.01
Credible Interval	(0.643, 1.55)
Associated Peptides	1
Associated Spectra	1
Coverage	NaN

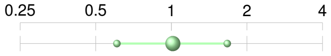

A	B	2.5	50	97.5	Sequence
1	0	0.61	1	1.7	DLLSAISSETSGDFETGLR

5.626 305 937 Clathrin light chain A [Crassostrea gigas]

Protein Accession	CL831.Contig4$_{H1}$
Mean Expression Ratio	0.991
Median Expression Ratio	0.992
Credible Interval	(0.648, 1.54)
Associated Peptides	1
Associated Spectra	1
Coverage	NaN

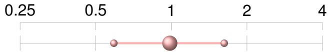

A	B	2.5	50	97.5	Sequence
0	1	0.59	0.99	1.6	AAEEAFIK

5.627 27 377 minus strand ribosomal protein LP2 [Papilio xuthus] gi; 27 377 minus strand ribosomal protein LP2 [Papilio xuthus] gi; 27 377 minus strand ribosomal protein LP2 [Papilio xuthus] gi; 1538 1891 minus strand ribosomal protein LP2 [Papilio xuthus] gi

Protein Accession	Unigene53017$_{H1}$ Unigene53016$_{H1}$ Unigene53015$_{H1}$ CL49012.Contig9$_{H1}$
Mean Expression Ratio	0.993
Median Expression Ratio	0.992
Credible Interval	(0.781, 1.26)
Associated Peptides	7
Associated Spectra	16
Coverage	NaN

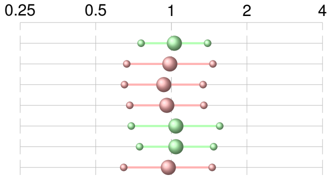

A	B	2.5	50	97.5	Sequence
4	1	0.76	1	1.4	KVVSQLSGK
1	0	0.66	0.99	1.5	SIDQLIAEGSR
2	0	0.65	0.93	1.3	VVSQLSGK
3	0	0.68	0.96	1.3	ILSSVGIEAEADK
1	0	0.69	1	1.6	AAAPAAEAK
1	2	0.75	1	1.5	ILSSVGIEAEADKVK
1	0	0.65	0.97	1.5	SGKEPTAADLEK

5.628 3591 4526 minus strand myosin heavy chain cardiac muscle specific isoform 1 [Argopecten irradians]

Protein Accession	CL49594.Contig2$_H$1
Mean Expression Ratio	1.01
Median Expression Ratio	1.01
Credible Interval	(0.9, 1.13)
Associated Peptides	23
Associated Spectra	294
Coverage	NaN

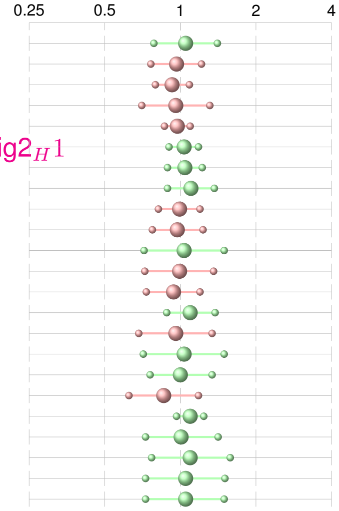

A	B	2.5	50	97.5	Sequence
3	1	0.79	1.1	1.4	AKLEQTLDELEDNLER
7	3	0.76	0.96	1.2	ATQANVEDLER
22	7	0.8	0.93	1.1	LQQETANLTR
2	1	0.7	0.96	1.3	MSKQVEAQLAELSAK
34	20	0.86	0.97	1.1	QVEAQLAELSAK
22	18	0.9	1	1.2	SEVDDLQMQLQNVGK
17	9	0.89	1	1.2	TINDLQTQK
11	1	0.89	1.1	1.4	TMEDLQAEEDKVNHLNK
16	1	0.82	0.99	1.2	KLEQDLK
9	1	0.77	0.97	1.2	AATSRSRTLQHEAQVSALR
1	0	0.72	1	1.5	SEVDDLQMQLQNVGKNK
2	1	0.72	0.99	1.4	SASRSCSSCAATSRSRTLQHEAQVSALR
6	2	0.73	0.94	1.2	QQDAANEMADQIDQLLQKVK
11	0	0.88	1.1	1.4	ATSRSRTLQHEAQVSALR
1	1	0.68	0.96	1.3	ELEENVR
1	0	0.71	1	1.5	KKQQDAANEMADQIDQ
2	3	0.76	1	1.3	KTMEDLQAEEDKVNHLNK
2	1	0.62	0.86	1.2	LEQTLDELEDNLER
24	26	0.97	1.1	1.2	QLEEAEHK
2	0	0.73	1	1.4	VGALTK
0	1	0.77	1.1	1.6	RKLEQDLK
0	1	0.73	1	1.5	LEENVR
0	1	0.73	1	1.5	QDAANEMADQIDQLLQK

5.629 410 709 calmodulin-like; K02183 calmodulin

Protein Accession	CL30882.Contig6$_H$1
Mean Expression Ratio	0.992
Median Expression Ratio	0.993
Credible Interval	(0.662, 1.48)
Associated Peptides	2
Associated Spectra	2
Coverage	NaN

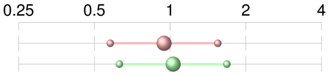

A	B	2.5	50	97.5	Sequence
1	0	0.58	0.95	1.5	ELNDYVTEGGQSAAQAVLEK
0	1	0.63	1	1.7	EALRDFIEK

5.630 211 1413 Alpha-N-acetylgalactosaminidase [Crassostrea gigas]

Protein Accession	CL5989.Contig1$_H$1
Mean Expression Ratio	1.01
Median Expression Ratio	1.01
Credible Interval	(0.704, 1.44)
Associated Peptides	2
Associated Spectra	4
Coverage	NaN

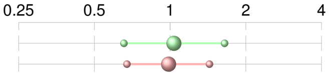

A	B	2.5	50	97.5	Sequence
1	0	0.65	1	1.7	ALADYMHDR
3	0	0.67	0.99	1.4	ALLLNR

5.631 165 635 hypothetical protein CGI_10007639 [Crassostrea gigas]

Protein Accession	CL36715.Contig1$_H$1
Mean Expression Ratio	0.994
Median Expression Ratio	0.993
Credible Interval	(0.671, 1.48)
Associated Peptides	2
Associated Spectra	2
Coverage	NaN

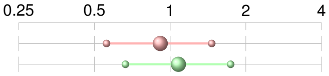

A	B	2.5	50	97.5	Sequence
1	0	0.56	0.91	1.5	AAIVAQVAK
1	0	0.66	1.1	1.7	MPLHASCK

5.632 77 1561 retinal dehydrogenase [Lymnaea stagnalis]; 69 1553 retinal dehydrogenase [Lymnaea stagnalis]; 308 1747 retinal dehydrogenase [Lymnaea stagnalis]

Protein Accession	CL13284.Contig7$_H$1 CL13284.Contig2$_H$1 CL13284.Contig1$_H$1
Mean Expression Ratio	1.01
Median Expression Ratio	1.01
Credible Interval	(0.826, 1.22)
Associated Peptides	14
Associated Spectra	23
Coverage	NaN

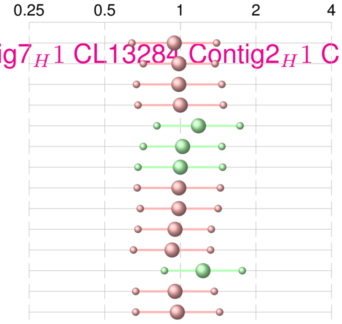

A	B	2.5	50	97.5	Sequence
1	0	0.64	0.95	1.4	ALMFVNGVR
2	1	0.71	0.99	1.4	AVEVAQDAFK
1	0	0.67	0.99	1.5	FSTVEEAIER
1	0	0.68	1	1.5	IYDEFVR
1	0	0.8	1.2	1.7	NIAYLASLETLDNGKPFK
1	1	0.71	1	1.5	TFPTINPATGK
1	0	0.68	1	1.5	VAFTGSTEVGQIIMK
1	0	0.68	0.99	1.4	KTAELANKR
2	0	0.69	0.99	1.4	KTAELANK
3	0	0.68	0.95	1.3	RGALLYK
1	1	0.65	0.93	1.3	LGSAWR
1	1	0.86	1.2	1.8	AIQIIR
2	0	0.67	0.95	1.4	EEIFGPVQQLLK
0	1	0.66	0.98	1.4	KIVDVQEADKADVDK

5.633 116 1321 PREDICTED: similar to aspartate aminotransferase [Tribolium castaneum] gi

Protein Accession	CL11692.Contig6$_H$1
Mean Expression Ratio	1.01
Median Expression Ratio	1.01
Credible Interval	(0.771, 1.31)
Associated Peptides	8
Associated Spectra	10
Coverage	NaN

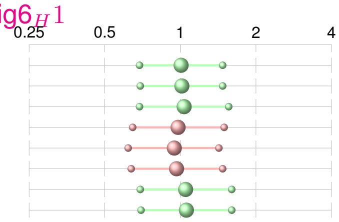

A	B	2.5	50	97.5	Sequence
2	0	0.69	1	1.5	DGHQVALAQSFAK
1	1	0.69	1	1.5	ILSTPALR
1	0	0.69	1	1.6	INLGVGAYR
1	0	0.65	0.98	1.5	TQWLGEVK
1	0	0.62	0.94	1.4	ISVAGLSSK
0	1	0.64	0.97	1.5	ILSTPALRTQ
0	1	0.69	1	1.6	DAFALR
0	1	0.7	1.1	1.6	IIAMR

5.634 835 1083 minus strand Calmodulin [Crassostrea gigas]

Protein Accession	CL49568.Contig2$_{H}1$
Mean Expression Ratio	0.994
Median Expression Ratio	0.995
Credible Interval	(0.82, 1.2)
Associated Peptides	7
Associated Spectra	48
Coverage	NaN

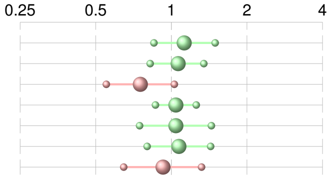

A	B	2.5	50	97.5	Sequence
6	0	0.85	1.1	1.5	HLLTDKDEETCYK
7	2	0.82	1.1	1.4	KGFIANEEMK
4	0	0.55	0.75	1	QQVQMTEQEVDEVIDYIDK
9	10	0.87	1	1.3	VDFTEFYK
2	1	0.75	1	1.4	GFIANEEMK
5	0	0.8	1.1	1.4	LLLNK
1	1	0.65	0.93	1.3	ILDDQR

5.635 1 111 glycogen synthase [Crassostrea gigas]

Protein Accession	Unigene78448$_{H}1$
Mean Expression Ratio	0.993
Median Expression Ratio	0.995
Credible Interval	(0.643, 1.53)
Associated Peptides	1
Associated Spectra	1
Coverage	NaN

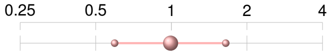

A	B	2.5	50	97.5	Sequence
0	1	0.59	0.99	1.6	NVEPDLDDDYLDR

5.636 125 754 minus strand peptidyl prolyl cis-trans isomerase B [Conus novaehollandiae]

Protein Accession	Unigene19558$_{H}1$
Mean Expression Ratio	0.993
Median Expression Ratio	0.995
Credible Interval	(0.719, 1.37)
Associated Peptides	4
Associated Spectra	5
Coverage	NaN

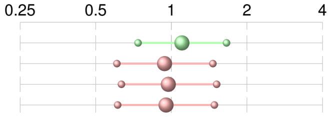

A	B	2.5	50	97.5	Sequence
2	0	0.74	1.1	1.7	HLALGDKEDGGVK
1	0	0.61	0.94	1.5	KTQWLDGR
1	0	0.63	0.97	1.5	DTNGSQFFITTK
1	0	0.61	0.95	1.5	FPDENFK

5.637 71 1321 minus strand Vinculin [Crassostrea gigas]

Protein Accession	Unigene23324$_{H}1$
Mean Expression Ratio	0.996
Median Expression Ratio	0.995
Credible Interval	(0.811, 1.23)
Associated Peptides	12
Associated Spectra	20
Coverage	NaN

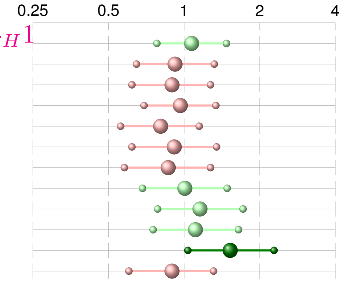

A	B	2.5	50	97.5	Sequence
3	1	0.78	1.1	1.5	DTITPMVQNAK
1	1	0.64	0.92	1.3	GLVDEAVDTAALIR
2	0	0.62	0.89	1.3	KPAPTVDGK
2	1	0.69	0.96	1.3	LINAVGGVR
2	0	0.56	0.81	1.2	NVGDIESMANALSELR
1	0	0.62	0.91	1.3	QWLANPGLDDK
1	0	0.58	0.86	1.3	VLQVAEMEAQNSEDPLFVDR
1	0	0.68	1	1.5	ASADLR
0	1	0.78	1.2	1.7	AEEEAILHDTDR
0	1	0.75	1.1	1.6	VNEASADLR
0	1	1	1.5	2.3	DREAILK
0	1	0.6	0.9	1.3	AIARNLSEKL

5.638 " 57 752 PREDICTED: adenylate kinase 2, mitochondrial-like isoform 2 [Callithrix jacchus]"

Protein Accession	CL4149.Contig1$_{H}1$
Mean Expression Ratio	1
Median Expression Ratio	1
Credible Interval	(0.724, 1.39)
Associated Peptides	3
Associated Spectra	6
Coverage	NaN

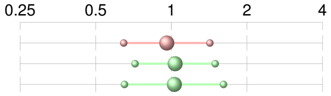

A	B	2.5	50	97.5	Sequence
2	0	0.65	0.96	1.4	GIHTAVDASQPPHK
3	0	0.72	1	1.5	GFLLDGFPR
1	0	0.65	1	1.6	GTQAPLLAEK

5.639 102 1475 minus strand Cytosolic non-specific dipeptidase [Crassostrea gigas]

Protein Accession	CL4706.Contig1$_{H}1$
Mean Expression Ratio	0.996
Median Expression Ratio	0.996
Credible Interval	(0.641, 1.53)
Associated Peptides	1
Associated Spectra	1
Coverage	NaN

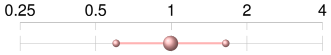

A	B	2.5	50	97.5	Sequence
1	0	0.6	1	1.7	LVPDQEPHVIESLVR

5.640 63 1865 Dolichyl-diphosphooligosaccharide–protein glycosyltransferase subunit 1 [Crassostrea gigas]; 63 1865 Dolichyl-diphosphooligosaccharide–protein glycosyltransferase subunit 1 [Crassostrea gigas]

Protein Accession	CL41703.Contig2$_{H}1$ CL41703.Contig1$_{H}1$
Mean Expression Ratio	0.994
Median Expression Ratio	0.996
Credible Interval	(0.719, 1.37)
Associated Peptides	4
Associated Spectra	5
Coverage	NaN

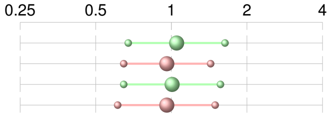

A	B	2.5	50	97.5	Sequence
1	0	0.67	1.1	1.6	KVDIATHIVK
1	1	0.65	0.96	1.4	VASLVEEVQSAQDKR
1	0	0.65	1	1.6	DLNAFQVAR
0	1	0.61	0.96	1.5	ELDDSVDIELRPR

5.641 " 519 3182 minus strand Alpha-actinin, sarcomeric [Crassostrea gigas]"

Protein Accession	CL28960.Contig2$_H1$
Mean Expression Ratio	0.996
Median Expression Ratio	0.996
Credible Interval	(0.894, 1.11)
Associated Peptides	41
Associated Spectra	115
Coverage	NaN

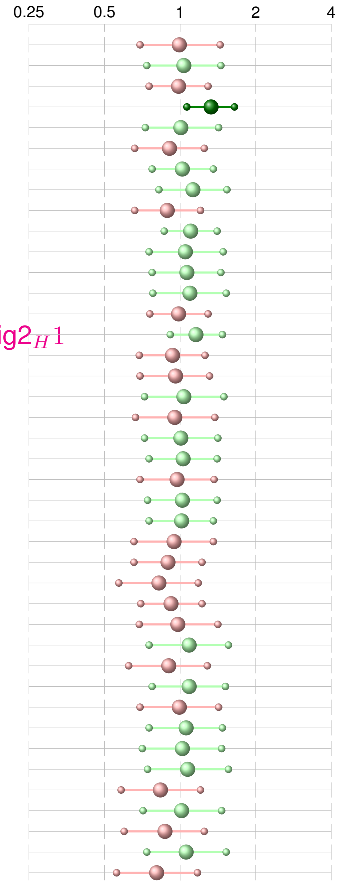

A	B	2.5	50	97.5	Sequence
1	0	0.69	0.99	1.4	AAPFNNWMDGAK
1	1	0.74	1	1.5	AGTGIENIEEDFR
5	1	0.75	0.99	1.3	AIIQYKPHIDELER
8	3	1.1	1.3	1.6	ALDFIASK
1	1	0.73	1	1.4	DNPIENLNTAFDIAEK
2	1	0.66	0.91	1.2	EALEEAER
3	2	0.77	1	1.4	ETADTDTAEQVMQSFK
2	1	0.83	1.1	1.5	FAIQDITVEELTAK
2	2	0.66	0.89	1.2	GISEDQMNEFR
6	2	0.86	1.1	1.4	IDQLHLEFAK
1	1	0.75	1.1	1.5	KAGTGIENIEEDFR
2	1	0.78	1.1	1.5	LSNRPAYLPTEGK
2	0	0.78	1.1	1.5	LVSIGAEEIVDGNVK
3	3	0.76	0.99	1.3	MSFNHFDK
4	5	0.91	1.2	1.5	MVSDIANAWK
2	2	0.69	0.93	1.3	NINEVENQILTR
2	1	0.69	0.96	1.3	RDQTLQQEMMR
1	0	0.72	1	1.5	GSLEEQLKK
1	0	0.67	0.95	1.4	HKPPKLEDK
2	0	0.72	1	1.4	MLDPEDMVNSAKPDER
2	1	0.75	1	1.4	LMEEYER
2	0	0.69	0.97	1.4	TRPWLENR
3	0	0.74	1	1.4	LQAFDK
2	2	0.75	1	1.4	KLDEFR
1	0	0.65	0.94	1.4	EDMLQSADFK
2	1	0.66	0.9	1.2	LGQLTVK
1	0	0.57	0.82	1.2	PHIDELER
2	3	0.7	0.92	1.2	LDEFR
1	0	0.69	0.98	1.4	HLDIPR
1	0	0.75	1.1	1.6	PGALDYMSFSTAL
1	0	0.63	0.9	1.3	DGLAFCALIHR
2	0	0.77	1.1	1.5	HRPELIDYHNLSK
1	0	0.69	1	1.4	KLQAFDK
2	0	0.75	1.1	1.5	LDHLAQK
1	0	0.71	1	1.5	YTQYTMETLR
0	1	0.74	1.1	1.6	EGLLDPAWEK
0	1	0.58	0.84	1.2	LENSFNTLQTR
0	1	0.71	1	1.5	KHKPPKLEDK
0	1	0.6	0.87	1.2	ELVPR
0	1	0.74	1.1	1.5	DQLHLEFAK
0	1	0.56	0.81	1.2	SIGVQMR

5.642 146 841 minus strand hypothetical protein CGI_10006609 [Crassostrea gigas]; 146 841 minus strand hypothetical protein CGI_10006609 [Crassostrea gigas]; 146 841 minus strand hypothetical protein CGI_10006609 [Crassostrea gigas]

Protein Accession	CL6681.Contig3$_H1$ CL6681.Contig2$_H1$ CL6681.Contig1$_H1$
Mean Expression Ratio	0.995
Median Expression Ratio	0.996
Credible Interval	(0.643, 1.53)
Associated Peptides	1
Associated Spectra	1
Coverage	NaN

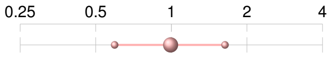

A	B	2.5	50	97.5	Sequence
0	1	0.6	0.99	1.6	GHVEGLEVLPAR

5.643 895 1410 Galectin-3 OS=Rattus norvegicus GN=Lgals3 PE=1 SV=4; 1067 1405 Galectin-9 [Crassostrea gigas]; 1068 1421 tandem-repeat galectin [Pinctada fucata]; 1041 1394 tandem-repeat galectin [Pinctada fucata]; 1046 1399 tandem-repeat galectin [Pinctada fucata]; 1068 1421 tandem-repeat galectin [Pinctada fucata]; 1067 1420 tandem-repeat galectin [Pinctada fucata]

Protein Accession	CL474.Contig9$_H$1 CL474.Contig8$_H$1 CL474.Contig7$_H$1 CL474.Contig6$_H$1 CL474.Contig5$_H$1
Mean Expression Ratio	1.01
Median Expression Ratio	1
Credible Interval	(0.672, 1.51)
Associated Peptides	1
Associated Spectra	2
Coverage	NaN

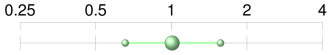

A	B	2.5	50	97.5	Sequence
**2**	**0**	**0.65**	**1**	**1.6**	**VAVNNQHLLDFR**

5.644 " 28 1104 minus strand Pyruvate dehydrogenase E1 component subunit beta, mitochondrial [Crassostrea gigas]"

Protein Accession	CL18015.Contig14$_H$1
Mean Expression Ratio	0.997
Median Expression Ratio	0.997
Credible Interval	(0.665, 1.49)
Associated Peptides	2
Associated Spectra	2
Coverage	NaN

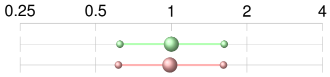

A	B	2.5	50	97.5	Sequence
**1**	**0**	**0.62**	**1**	**1.6**	**GIECEVINLR**
**1**	**0**	**0.62**	**0.99**	**1.6**	**SLRPVDDETIIK**

5.645 " 599 907 minus strand Tryptophanyl-tRNA synthetase, cytoplasmic [Heterocephalus glaber]"

Protein Accession	Unigene26755$_H$1
Mean Expression Ratio	0.997
Median Expression Ratio	0.997
Credible Interval	(0.717, 1.39)
Associated Peptides	2
Associated Spectra	8
Coverage	NaN

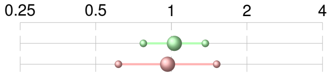

A	B	2.5	50	97.5	Sequence
**3**	**4**	**0.77**	**1**	**1.4**	**DMDPLLDLYEK**
**1**	**0**	**0.61**	**0.97**	**1.5**	**KTPFYLYTGR**

5.646 1 222 cAMP-dependent protein kinase regulatory subunit [Aplysia californica] gi

Protein Accession	Unigene13629$_H$1
Mean Expression Ratio	1
Median Expression Ratio	1
Credible Interval	(0.688, 1.46)
Associated Peptides	2
Associated Spectra	3
Coverage	NaN

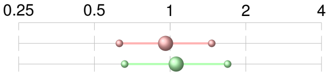

A	B	2.5	50	97.5	Sequence
**1**	**1**	**0.63**	**0.96**	**1.5**	**NVLFSHLDDNER**
**0**	**1**	**0.66**	**1.1**	**1.7**	**SDIFDAMFPVHR**

5.647 66 263 minus strand twitchin [Mytilus galloprovincialis]; 66 263 minus strand twitchin [Mytilus galloprovincialis]; 66 263 minus strand twitchin [Mytilus galloprovincialis]; 66 263 minus strand twitchin [Mytilus galloprovincialis]; 66 263 minus strand twitchin [Mytilus galloprovincialis]

Protein Accession	Unigene64526$_H$1 Unigene64525$_H$1 Unigene64524$_H$1 Unigene64522$_H$1 Unigene64521$_H$1
Mean Expression Ratio	0.995
Median Expression Ratio	0.997
Credible Interval	(0.671, 1.46)
Associated Peptides	1
Associated Spectra	3
Coverage	NaN

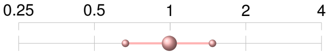

A	B	2.5	50	97.5	Sequence
**2**	**1**	**0.67**	**0.99**	**1.5**	**FTETLSPVEVMQGDK**

5.648 1070 1888 minus strand galectin [Pinctada fucata]; 1104 1922 minus strand galectin [Pinctada fucata]

Protein Accession	CL7245.Contig4$_H$1 CL7245.Contig2$_H$1
Mean Expression Ratio	1
Median Expression Ratio	1
Credible Interval	(0.652, 1.56)
Associated Peptides	1
Associated Spectra	1
Coverage	NaN

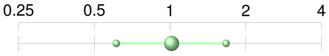

A	B	2.5	50	97.5	Sequence
**1**	**0**	**0.61**	**1**	**1.7**	**FNEGVTVR**

5.649 210 473 Abdominal ganglion neuropeptides L5-67 OS=Aplysia californica PE=1 SV=1; 879 1124 abdominal ganglion neuropeptides L5-67 precursor [Aplysia californica] gi; 210 482 minus strand abdominal ganglion neuropeptides L5-67 precursor [Aplysia californica] gi

Protein Accession	Unigene58290$_{H}1$ Unigene39670$_{H}1$ Unigene206$_{H}1$
Mean Expression Ratio	0.997
Median Expression Ratio	0.998
Credible Interval	(0.668, 1.5)
Associated Peptides	1
Associated Spectra	2
Coverage	NaN

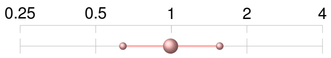

A	B	2.5	50	97.5	Sequence
**0**	**2**	**0.64**	**0.99**	**1.6**	**VNGEIELPVEVFGAR**

5.650 56 937 Chloride intracellular channel exc-4 [Crassostrea gigas]

Protein Accession	CL34620.Contig3$_{H}1$
Mean Expression Ratio	1
Median Expression Ratio	0.998
Credible Interval	(0.693, 1.46)
Associated Peptides	2
Associated Spectra	3
Coverage	NaN

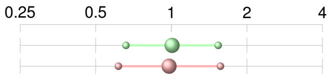

A	B	2.5	50	97.5	Sequence
**2**	**0**	**0.66**	**1**	**1.5**	**KVQGPLVK**
**1**	**0**	**0.62**	**0.98**	**1.6**	**KLLAELK**

5.651 965 1687 Arylsulfatase B [Crassostrea gigas]

Protein Accession	Unigene30559$_{H}1$
Mean Expression Ratio	0.998
Median Expression Ratio	0.998
Credible Interval	(0.649, 1.55)
Associated Peptides	1
Associated Spectra	1
Coverage	NaN

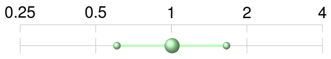

A	B	2.5	50	97.5	Sequence
**0**	**1**	**0.61**	**1**	**1.7**	**WGLYDIEADPEER**

5.652 148 609 minus strand Titin [Crassostrea gigas]

Protein Accession	CL5825.Contig1$_{H}1$
Mean Expression Ratio	0.999
Median Expression Ratio	0.998
Credible Interval	(0.718, 1.39)
Associated Peptides	2
Associated Spectra	6
Coverage	NaN

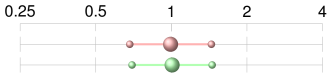

A	B	2.5	50	97.5	Sequence
**3**	**0**	**0.68**	**0.99**	**1.4**	**FTQKPALR**
**3**	**0**	**0.7**	**1**	**1.5**	**QQEGVAPNFIQKPVTR**

5.653 " 3 239 minus strand laminin beta 1 chain, putative [Ixodes scapularis] gi"

Protein Accession	CL48498.Contig2$_H$1
Mean Expression Ratio	1
Median Expression Ratio	1
Credible Interval	(0.704, 1.43)
Associated Peptides	2
Associated Spectra	4
Coverage	NaN

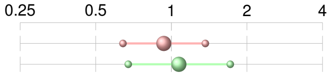

A	B	2.5	50	97.5	Sequence
2	1	0.64	0.93	1.4	LAQQAEQLLTTK
1	0	0.68	1.1	1.7	ARNTSETVR

5.654 366 1049 hypothetical protein CGI_10004855 [Crassostrea gigas]

Protein Accession	CL43590.Contig5$_H$1
Mean Expression Ratio	0.997
Median Expression Ratio	0.998
Credible Interval	(0.773, 1.28)
Associated Peptides	7
Associated Spectra	14
Coverage	NaN

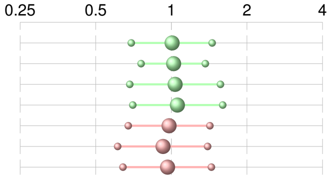

A	B	2.5	50	97.5	Sequence
1	1	0.69	1	1.5	DEWTHLTAK
2	4	0.76	1	1.4	GGDLWQGEVSLEQHR
1	0	0.68	1	1.6	ADSDRPSVSAGIGQKPQFQDLK
1	0	0.7	1.1	1.6	VKVPGAK
2	0	0.67	0.98	1.4	KVFPYPK
0	1	0.61	0.93	1.4	TTVQLHK
0	1	0.64	0.96	1.4	KVEIK

5.655 303 1793 PREDICTED: matrix metalloproteinase 19 isoform rasi-1 preproprotein-like [Saccoglossus kowalevskii]

Protein Accession	CL7632.Contig1$_H$1
Mean Expression Ratio	0.999
Median Expression Ratio	0.999
Credible Interval	(0.671, 1.48)
Associated Peptides	2
Associated Spectra	2
Coverage	NaN

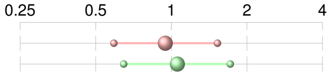

A	B	2.5	50	97.5	Sequence
1	0	0.59	0.95	1.5	FDAIDR
1	0	0.65	1.1	1.7	LGRVAR

5.656 51 890 prohibitin [Octopus tankahkeei]

Protein Accession	CL26635.Contig2$_H$1
Mean Expression Ratio	1
Median Expression Ratio	1
Credible Interval	(0.767, 1.31)
Associated Peptides	6
Associated Spectra	10
Coverage	NaN

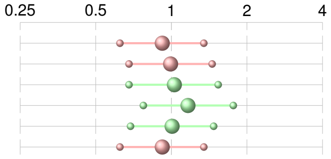

A	B	2.5	50	97.5	Sequence
1	1	0.62	0.92	1.3	AFTEAGEGLVELR
1	1	0.68	0.99	1.5	FLVEKEEQK
1	0	0.68	1	1.5	NVPVITGSK
1	0	0.77	1.2	1.8	IEAAEDIA
0	2	0.69	1	1.5	AVIFDR
0	2	0.63	0.92	1.3	QVAQQDAERAR

5.657 64 393 Tubulin beta chain OS=Paracentrotus lividus PE=2 SV=1; 109 438 Tubulin beta chain OS=Paracentrotus lividus PE=2 SV=1; 160 489 Tubulin beta chain OS=Paracentrotus lividus PE=2 SV=1

Protein Accession	Unigene62663$_H$1 Unigene62661$_H$1 Unigene62660$_H$1
Mean Expression Ratio	1
Median Expression Ratio	0.999
Credible Interval	(0.691, 1.44)
Associated Peptides	1
Associated Spectra	6
Coverage	NaN

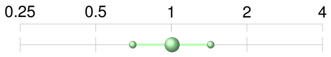

A	B	2.5	50	97.5	Sequence
**4**	**2**	**0.7**	**1**	**1.4**	**INVYFNEATGGK**

5.658 362 553 putative RNA-binding protein [Haliotis diversicolor]

Protein Accession	CL41007.Contig2$_H$1
Mean Expression Ratio	1
Median Expression Ratio	0.999
Credible Interval	(0.731, 1.37)
Associated Peptides	2
Associated Spectra	10
Coverage	NaN

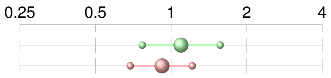

A	B	2.5	50	97.5	Sequence
**2**	**1**	**0.77**	**1.1**	**1.6**	**ELQDETGCR**
**3**	**4**	**0.69**	**0.92**	**1.2**	**EVMHVDSSFVGR**

iTRAQ Data Analysis Report

Javier, Head 56d versus control

April 7, 2014

1 Introduction

This document summarizes an analysis of relative protein expression using iTRAQ. The reporter ion peak area measurements supplied by the ABI software are used to estimate treatment-dependent peptide and protein relative expression. Estimation is accomplished using a Bayesian approach with the model given below. The document includes a protein relative expression summary and a per-protein detailed analysis. The document is internally hyperlinked and linked externally to NCBI.

2 Experiment and Model Description

2.1 Experiment Design

The report summarizes data from one or more iTRAQ experiments addressing a common comparison. The experiment design, used in this analysis, is given in the table below.

	Experiment	Treatment	Channel	Sample
1	A	control	115	s1
2	A	test	121	s1
3	B	control	115	s2
4	B	test	121	s2

2.2 Input Files

Data for this analysis was extracted from the following tandem mass spectra (MSMS) summary files.

Experiment	MSMS Summary File
A	head2.txt
B	head3.txt

2.3 Statistical Model

The following statistical model was used to estimate the treatment-dependent effects.

LogIntensity ˜ Channel + Spectrum + Protein + Peptide + Protein:Treatment + Peptide:Treatment

3 Data Summary

The data supplied in the MSMS summary is filtered to remove unidentified proteins, contaminants, and peptides containing selected modifications. The following table sumarizes the data provided and used in the analysis.

	A	B	Combined
Supplied Spectra	21342	14837	36179
Unidentified Spectra	8154	6352	14506
Disallowed Modifications	361	184	545
Spectra from Contaminants	0	0	0
Missing Data	3252	2211	5463
Low Confidence Spectra	8154	6352	14506
Degenerate Peptides	3567	2238	5805
Remaining Spectra			9860
Unique Proteins			653
Unique Peptides			2351
Model R^2			0.935

4 Protein Summary

Each protein identified in one or more of the MSMS summaries is listed below in decreasing order of expression change magnitude. The median and estimated credible interval for each protein is given to the left in the table. Proteins identified by a single peptide are listed in a separate table.

4.1 Identified Proteins

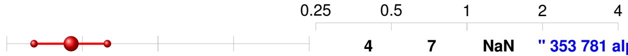

	Peptides	Spectra	Coverage	Protein
	4	7	NaN	**" 353 781 alpha-B-crystallin, putative [Ixodes scapularis] gi"**

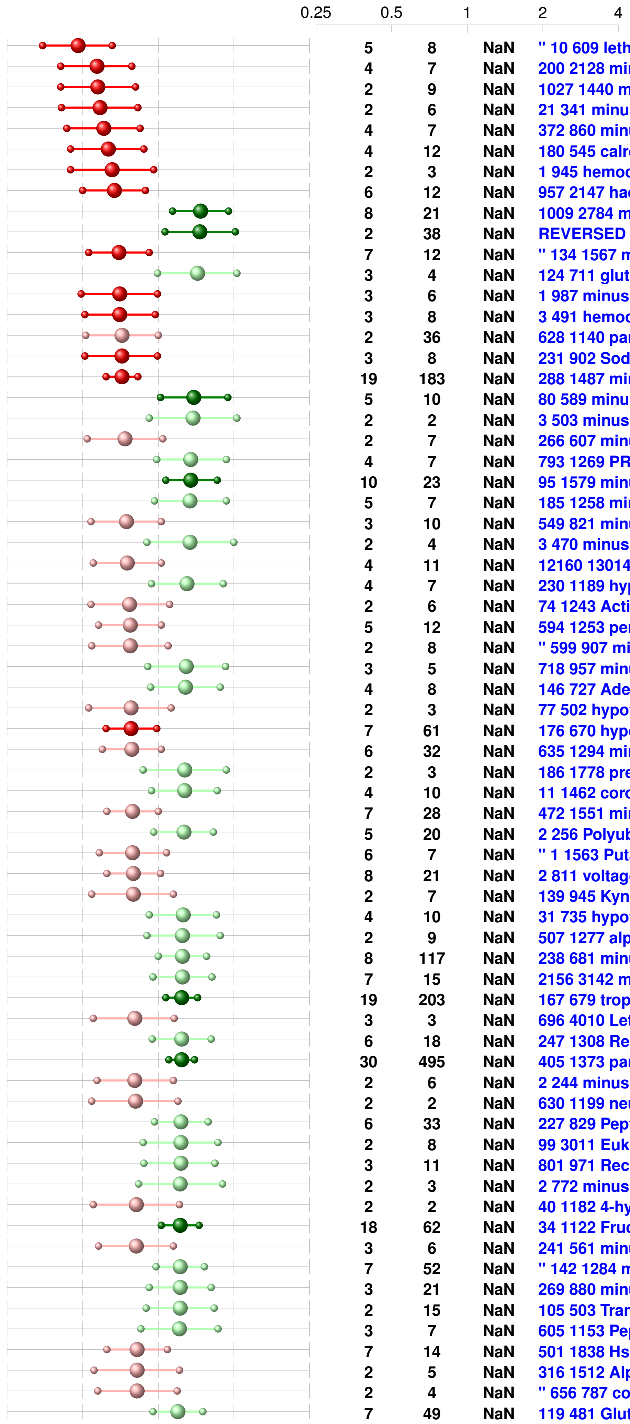

Peptides	Spectra	Coverage	Protein
5	8	NaN	" 10 609 lethal(2)essential for life protein, l2efl [Aedes aegypti] gi"
4	7	NaN	200 2128 minus strand heat shock protein 70 [Cristaria plicata]
2	9	NaN	1027 1440 minus strand hemocyanin isoform 1 [Haliotis diversicolor]
2	6	NaN	21 341 minus strand hemocyanin isoform 1 [Nucula nucleus]
4	7	NaN	372 860 minus strand small heat shock protein [Tegillarca granosa]; 651 998 minus strand small heat shock protein
4	12	NaN	180 545 calreticulin precursor [Aplysia californica] gi
2	3	NaN	1 945 hemocyanin isoform 1 [Haliotis diversicolor]
6	12	NaN	957 2147 haemocyanin [Aplysia californica]
8	21	NaN	1009 2784 minus strand Collagen alpha-3(VI) chain [Crassostrea gigas]
2	38	NaN	REVERSED 6 395 PREDICTED: sister chromatid cohesion protein PDS5 homolog B-A isoform 1 [Nasonia vitripennis
7	12	NaN	" 134 1567 minus strand 6-phosphogluconate dehydrogenase, decarboxylating [Crassostrea gigas]; 134 1573 minus
3	4	NaN	124 711 glutathione S-transferase sigma 3 [Mytilus galloprovincialis]; 124 711 glutathione S-transferase sigma 3 [My
3	6	NaN	1 987 minus strand RecName: Full=Tubulin beta chain; AltName: Full=Beta-tubulin
3	8	NaN	3 491 hemocyanin [Haliotis tuberculata]
2	36	NaN	628 1140 paramyosin [Haliotis discus discus]
3	8	NaN	231 902 Sodium- and chloride-dependent GABA transporter ine [Crassostrea gigas]
19	183	NaN	288 1487 minus strand putative procollagen type VI alpha 4 [Haliotis diversicolor]
5	10	NaN	80 589 minus strand PREDICTED: adenine phosphoribosyltransferase-like [Strongylocentrotus purpuratus]
2	2	NaN	3 503 minus strand elongation factor-1 alpha [Pomacea bridgesi]
2	7	NaN	266 607 minus strand H2 [Haliotis tuberculata]
4	7	NaN	793 1269 PREDICTED: calmodulin-like [Saccoglossus kowalevskii]
10	23	NaN	95 1579 minus strand hypothetical protein BRAFLDRAFT_113974 [Branchiostoma floridae] gi
5	7	NaN	185 1258 minus strand Severin [Crassostrea gigas]
3	10	NaN	549 821 minus strand Calponin-2 [Crassostrea gigas]
2	4	NaN	3 470 minus strand Spectrin beta chain [Crassostrea gigas]; 5 451 minus strand Spectrin beta chain [Crassostrea gi
4	11	NaN	12160 13014 twitchin [Mytilus galloprovincialis]
4	7	NaN	230 1189 hypothetical protein [Amblyomma maculatum]
2	6	NaN	74 1243 Actin-related protein 2 [Crassostrea gigas]
5	12	NaN	594 1253 peroxiredoxin 6 [Eriocheir sinensis]
2	8	NaN	" 599 907 minus strand Tryptophanyl-tRNA synthetase, cytoplasmic [Heterocephalus glaber]"
3	5	NaN	718 957 minus strand Calponin-1 OS=Gallus gallus GN=CNN1 PE=1 SV=2
4	8	NaN	146 727 Adenylate kinase isoenzyme 1 [Crassostrea gigas]
2	3	NaN	77 502 hypothetical protein CGI_10008263 [Crassostrea gigas]
7	61	NaN	176 670 hypothetical protein BRAFLDRAFT_122621 [Branchiostoma floridae] gi
6	32	NaN	635 1294 minus strand Kyphoscoliosis peptidase [Crassostrea gigas]; 643 1302 minus strand Kyphoscoliosis peptic
2	3	NaN	186 1778 predicted protein [Nematostella vectensis] gi
4	10	NaN	11 1462 coronin [Schistosoma mansoni]
7	28	NaN	472 1551 minus strand hypothetical protein [Amblyomma maculatum]
5	20	NaN	2 256 Polyubiquitin (Fragment) OS=Xenopus laevis PE=1 SV=2; 20 388 large subunit ribosomal protein L40e [Clonor
6	7	NaN	" 1 1563 Putative methylmalonate-semialdehyde dehydrogenase [acylating], mitochondrial [Crassostrea gigas]"
8	21	NaN	2 811 voltage-dependent anion channel 2-like protein [Haliotis diversicolor]; 2 811 voltage-dependent anion channel
2	7	NaN	139 945 Kynurenine formamidase [Crassostrea gigas]; 145 951 Kynurenine formamidase [Crassostrea gigas]; 220 10
4	10	NaN	31 735 hypothetical protein BRAFLDRAFT_78603 [Branchiostoma floridae] gi; 55 735 hypothetical protein BRAFLDF
2	9	NaN	507 1277 alpha tubulin [Notothenia coriiceps]
8	117	NaN	238 681 minus strand Globin OS=Cerithidea rhizophorarum PE=1 SV=1
7	15	NaN	2156 3142 minus strand PREDICTED: hemicentin-1 [Xenopus (Silurana) tropicalis]
19	203	NaN	167 679 tropomyosin [Haliotis discus discus]
3	3	NaN	696 4010 Lethal(2) giant larvae-like protein 1 [Crassostrea gigas]; 657 3971 minus strand Lethal(2) giant larvae-like p
6	18	NaN	247 1308 RecName: Full=Guanine nucleotide-binding protein G(o) subunit alpha gi|9633|emb|CAA78806.1| GTP-bin
30	495	NaN	405 1373 paramyosin [Haliotis discus discus]
2	6	NaN	2 244 minus strand hypothetical protein KGM_08771 [Danaus plexippus]
2	2	NaN	630 1199 neuronal calcium sensor-1 [Lymnaea stagnalis]
6	33	NaN	227 829 Peptidyl-prolyl cis-trans isomerase B [Crassostrea gigas]
2	8	NaN	99 3011 Eukaryotic translation initiation factor 3 subunit A [Crassostrea gigas]
3	11	NaN	801 971 RecName: Full=Tropomyosin-2; AltName: Full=Tropomyosin II; Short=BgTMII; Short=TMII gi|155940|gb|AAA
2	3	NaN	2 772 minus strand arylsulfatase [Haliotis discus discus]
2	2	NaN	40 1182 4-hydroxyphenylpyruvate dioxygenase [Crassostrea gigas]
18	62	NaN	34 1122 Fructose-bisphosphate aldolase [Crassostrea gigas]
3	6	NaN	241 561 minus strand thioredoxin [Crassostrea ariakensis]
7	52	NaN	" 142 1284 minus strand Chain O, Crsytal Structure Of Human Liver Gapdh gi|67464044|pdb|1ZNQ|P Chain P, Crsyta
3	21	NaN	269 880 minus strand myosin regulatory light chain [Fasciola hepatica]
2	15	NaN	105 503 Transgelin-2 [Crassostrea gigas]
3	7	NaN	605 1153 Peptidyl-prolyl cis-trans isomerase B [Crassostrea gigas]; 1046 1630 Peptidyl-prolyl cis-trans isomerase B
7	14	NaN	501 1838 Hsp90A [Haliotis asinina]
2	5	NaN	316 1512 Alpha-N-acetylgalactosaminidase [Crassostrea gigas]; 282 1478 Alpha-N-acetylgalactosaminidase [Crasso
2	4	NaN	" 656 787 collagen alpha-1(XII) chain-like; K08132 collagen, type XII, alpha"
7	49	NaN	119 481 Glutamate receptor U1 [Crassostrea gigas]

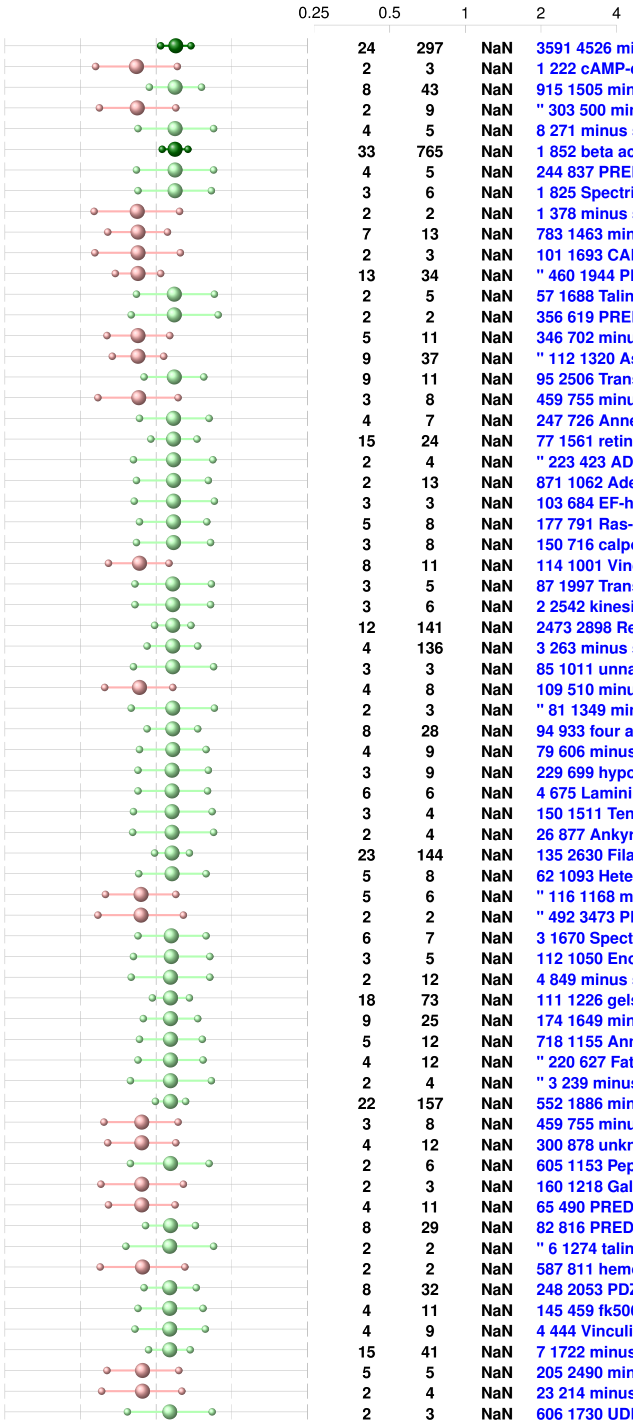

Peptides	Spectra	Coverage	Protein
24	297	NaN	3591 4526 minus strand myosin heavy chain cardiac muscle specific isoform 1 [Argopecten irradians]
2	3	NaN	1 222 cAMP-dependent protein kinase regulatory subunit [Aplysia californica] gi
8	43	NaN	915 1505 minus strand Collagen alpha-5(VI) chain [Crassostrea gigas]
2	9	NaN	" 303 500 minus strand protein lethal, putative [Pediculus humanus corporis] gi"
4	5	NaN	8 271 minus strand Laminin subunit gamma-1 [Crassostrea gigas]
33	765	NaN	1 852 beta actin-like 1 protein [Macrostomum lignano]
4	5	NaN	244 837 PREDICTED: ras-like GTP-binding protein Rho1-like [Megachile rotundata]
3	6	NaN	1 825 Spectrin alpha chain [Crassostrea gigas]
2	2	NaN	1 378 minus strand PREDICTED: CG10098-like [Saccoglossus kowalevskii]
7	13	NaN	783 1463 minus strand twitchin [Mytilus galloprovincialis]; 783 1463 minus strand twitchin [Mytilus galloprovincialis
2	3	NaN	101 1693 CALNUC [Spodoptera frugiperda]; 141 1325 Nucleobindin-2 [Crassostrea gigas]
13	34	NaN	" 460 1944 PREDICTED: succinyl-CoA:3-ketoacid-coenzyme A transferase 1, mitochondrial [Canis lupus familiaris]"
2	5	NaN	57 1688 Talin-1 [Camponotus floridanus]; 57 1865 Talin-1 [Camponotus floridanus]; 57 1835 Talin-1 [Camponotus flo
2	2	NaN	356 619 PREDICTED: hypothetical protein LOC100698726 [Oreochromis niloticus]
5	11	NaN	346 702 minus strand PREDICTED: galectin-4-like [Anolis carolinensis]
9	37	NaN	" 112 1320 Aspartate aminotransferase, cytoplasmic [Crassostrea gigas]"
9	11	NaN	95 2506 Transitional endoplasmic reticulum ATPase [Crassostrea gigas]
3	8	NaN	459 755 minus strand heat shock protein beta-1 [Trichinella spiralis] gi; 464 763 minus strand heat shock protein bet
4	7	NaN	247 726 Annexin A7 [Crassostrea gigas]; 286 675 Annexin A7 [Crassostrea gigas]
15	24	NaN	77 1561 retinal dehydrogenase [Lymnaea stagnalis]; 69 1553 retinal dehydrogenase [Lymnaea stagnalis]; 308 1747 r
2	4	NaN	" 223 423 ADPATP carrier protein; K05863 solute carrier family 25 (mitochondrial adenine nucleotide translocator), m
2	13	NaN	871 1062 Adenylate kinase isoenzyme 5 [Crassostrea gigas]
3	3	NaN	103 684 EF-hand domain-containing protein D2 [Crassostrea gigas]
5	8	NaN	177 791 Ras-related protein Rab-1A [Haliotis discus discus]
3	8	NaN	150 716 calponin/transgelin [Papilio xuthus]
8	11	NaN	114 1001 Vinculin [Crassostrea gigas]
3	5	NaN	87 1997 Transketolase-like protein 2 [Crassostrea gigas]; 87 1997 Transketolase-like protein 2 [Crassostrea gigas]; 8
3	6	NaN	2 2542 kinesin heavy chain 1 [Aplysia californica] gi; 2 2542 kinesin heavy chain 1 [Aplysia californica] gi
12	141	NaN	2473 2898 RecName: Full=Paramyosin gi|397603|emb|CAA79849.1| paramyosin [Echinococcus granulosus]
4	136	NaN	3 263 minus strand beta-actin [Urechis unicinctus]
3	3	NaN	85 1011 unnamed protein product [Mus musculus]
4	8	NaN	109 510 minus strand hypothetical protein DAPPUDRAFT_231281 [Daphnia pulex]
2	3	NaN	" 81 1349 minus strand PREDICTED: LOW QUALITY PROTEIN: dolichyl-diphosphooligosaccharide–protein glycosyl
8	28	NaN	94 933 four and A half lim domains [Schistosoma mansoni]
4	9	NaN	79 606 minus strand PREDICTED: similar to Ras-like protein 3 precursor (Protein roughened) [Tribolium castaneum]
3	9	NaN	229 699 hypothetical protein CGI_10017744 [Crassostrea gigas]; 199 669 hypothetical protein CGI_10017744 [Crasso
6	6	NaN	4 675 Laminin subunit gamma-1 [Crassostrea gigas]
3	4	NaN	150 1511 Tensin OS=Gallus gallus GN=TNS PE=1 SV=2
2	4	NaN	26 877 Ankyrin-2 [Crassostrea gigas]
23	144	NaN	135 2630 Filamin-A [Crassostrea gigas]; 118 2613 Filamin-A [Crassostrea gigas]; 157 2652 Filamin-A [Crassostrea g
5	8	NaN	62 1093 Heterogeneous nuclear ribonucleoprotein A2-like protein 1 [Crassostrea gigas]
5	6	NaN	" 116 1168 minus strand hypothetical protein CGI_10014553, partial [Crassostrea gigas]"
2	2	NaN	" 492 3473 PREDICTED: 2-oxoglutarate dehydrogenase, mitochondrial isoform 1 [Sarcophilus harrisii]; 492 3458 PR
6	7	NaN	3 1670 Spectrin alpha chain [Crassostrea gigas]
3	5	NaN	112 1050 Enolase-phosphatase E1 [Crassostrea gigas]; 112 1050 Enolase-phosphatase E1 [Crassostrea gigas]
2	12	NaN	4 849 minus strand elongation factor 1-alpha [Cryptosporidium muris RN66]
18	73	NaN	111 1226 gelsolin [Aplysia californica] gi; 42 1139 minus strand gelsolin [Aplysia californica] gi
9	25	NaN	174 1649 minus strand Collagen alpha-1(XII) chain [Crassostrea gigas]; 135 1610 Collagen alpha-1(XII) chain [Crasso
5	12	NaN	718 1155 Annexin A7 [Crassostrea gigas]
4	12	NaN	" 220 627 Fatty acid-binding protein, brain [Crassostrea gigas]"
2	4	NaN	" 3 239 minus strand laminin beta 1 chain, putative [Ixodes scapularis] gi"
22	157	NaN	552 1886 minus strand intermediate filament protein [Biomphalaria glabrata]
3	8	NaN	459 755 minus strand heat shock protein beta-1 [Trichinella spiralis] gi
4	12	NaN	300 878 unknown [Schistosoma japonicum]; 567 1145 unknown [Schistosoma japonicum]; 1110 1688 unknown [Sch
2	6	NaN	605 1153 Peptidyl-prolyl cis-trans isomerase B [Crassostrea gigas]; 1046 1630 Peptidyl-prolyl cis-trans isomerase B
2	3	NaN	160 1218 Galectin-9 [Crassostrea gigas]
4	11	NaN	65 490 PREDICTED: cofilin-2 [Otolemur garnettii]
8	29	NaN	82 816 PREDICTED: triosephosphate isomerase B [Oreochromis niloticus]
2	2	NaN	" 6 1274 talin, putative [Ixodes scapularis] gi"
2	2	NaN	587 811 hemocyanin 2 [Megathura crenulata]
8	32	NaN	248 2053 PDZ and LIM domain protein 5 [Crassostrea gigas]
4	11	NaN	145 459 fk506-binding protein 2 [Papilio xuthus]; 237 551 fk506-binding protein 2 [Papilio xuthus]
4	9	NaN	4 444 Vinculin [Crassostrea gigas]
15	41	NaN	7 1722 minus strand Membrane metallo-endopeptidase-like 1 OS=Mus musculus GN=Mmel1 PE=1 SV=1
5	5	NaN	205 2490 minus strand glucose-regulated protein 94 [Crassostrea gigas]
2	4	NaN	23 214 minus strand hemocyanin isoform 1 [Nucula nucleus]
2	3	NaN	606 1730 UDP-glucuronosyltransferase 2C1 precursor [Oryctolagus cuniculus]

0.25 0.5 1 2 4

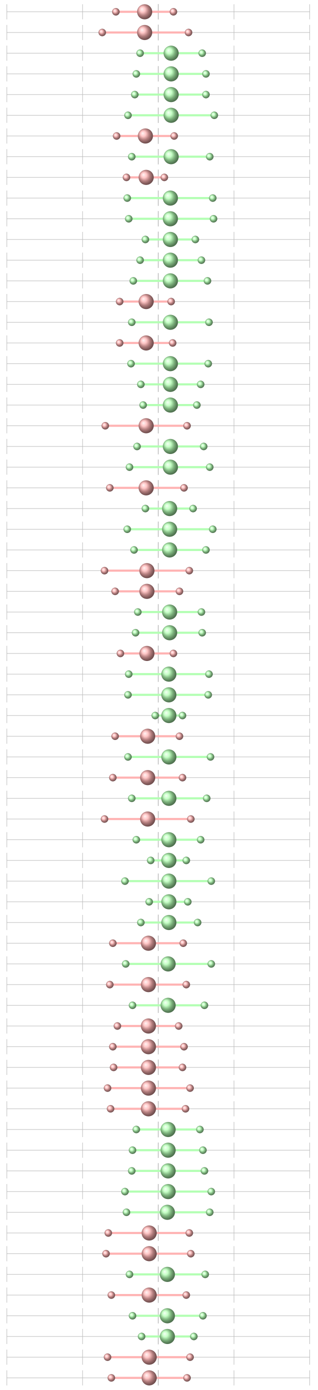

	Peptides	Spectra	Coverage	Protein
	7	14	NaN	4726 5571 minus strand twitchin [Mytilus galloprovincialis]
	2	2	NaN	16 438 hypothetical protein KGM_06034 [Danaus plexippus]; 16 438 hypothetical protein KGM_06034 [Danaus plexip
	4	18	NaN	274 666 Calmodulin-4 [Heterocephalus glaber]
	2	17	NaN	1592 1768 minus strand beta-actin [Diabolocatantops pinguis]
	3	11	NaN	180 560 calreticulin precursor [Aplysia californica] gi; 180 545 calreticulin precursor [Aplysia californica] gi
	2	2	NaN	1 252 Aquaporin-4 [Crassostrea gigas]
	8	17	NaN	280 690 Histone H3.3 [Harpegnathos saltator]
	2	6	NaN	4 579 minus strand Collagen alpha-3(VI) chain [Crassostrea gigas]
	14	135	NaN	362 874 minus strand Transgelin-2 [Crassostrea gigas]
	2	2	NaN	11 244 minus strand Tubulin alpha chain (Fragment) OS=Lytechinus pictus PE=3 SV=1
	2	3	NaN	118 513 Protein CAL-5 [Caenorhabditis elegans] gi
	7	47	NaN	76 522 minus strand Troponin C [Crassostrea gigas]; 173 619 minus strand Troponin C [Crassostrea gigas]; 173 619
	5	17	NaN	158 862 X-box binding protein [Haliotis discus discus]
	5	5	NaN	154 621 minus strand Stress response protein nhaX [Crassostrea gigas]
	8	24	NaN	1200 1547 protein disulfide isomerase [Conus eburneus]
	2	6	NaN	410 619 minus strand Guanine nucleotide-binding protein subunit gamma-1 [Crassostrea gigas]
	7	27	NaN	27 1682 minus strand PREDICTED: nucleolar protein 56 [Oreochromis niloticus]
	3	5	NaN	2 2248 PREDICTED: heat shock 70 kDa protein 4-like [Oreochromis niloticus]; 2 2227 PREDICTED: heat shock 70 kD
	7	13	NaN	390 2267 neurofilament protein NF70 [Helix aspersa]; 318 2195 neurofilament protein NF70 [Helix aspersa]
	6	23	NaN	273 1304 Guanine nucleotide-binding protein subunit beta [Crassostrea gigas]
	2	4	NaN	227 3073 minus strand Lysosomal alpha-mannosidase [Crassostrea gigas]
	3	11	NaN	900 1142 minus strand heat shock protein 20 [Cyclina sinensis]
	2	4	NaN	2 1093 minus strand Neurexin-1-alpha [Crassostrea gigas]
	4	5	NaN	913 1560 minus strand Calcium-dependent protein kinase isoform 2 [Crassostrea gigas]
	9	40	NaN	643 1302 minus strand Kyphoscoliosis peptidase [Crassostrea gigas]; 634 1293 minus strand Kyphoscoliosis peptic
	2	3	NaN	552 1499 minus strand uncharacterized protein LOC100158466 [Xenopus (Silurana) tropicalis] gi
	2	10	NaN	62 505 Myosin-2 essential light chain [Crassostrea gigas]
	2	2	NaN	113 1402 minus strand S-adenosylhomocysteine hydrolase [Crassostrea ariakensis]; 113 1402 minus strand S-aden
	6	9	NaN	" 59 2365 minus strand Aconitate hydratase, mitochondrial [Crassostrea gigas]"
	3	18	NaN	7073 7339 minus strand Collagen alpha-3(VI) chain [Crassostrea gigas]
	2	30	NaN	327 731 RecName: Full=Arginine kinase; Short=AK gi|439719|dbj|BAA05100.1| arginine kinase [Haliotis madaka]
	9	40	NaN	" 1 1815 Glycogen phosphorylase, muscle form [Crassostrea gigas]"
	3	3	NaN	193 828 Cysteine-rich secretory protein LCCL domain-containing 2 [Crassostrea gigas]
	3	3	NaN	188 1021 hypothetical protein BRAFLDRAFT_214346 [Branchiostoma floridae] gi
	35	171	NaN	114 2633 Filamin-A [Crassostrea gigas]; 135 2654 Filamin-A [Crassostrea gigas]
	5	23	NaN	118 501 minus strand histone 2A.Z [Aplysia californica]
	2	3	NaN	56 937 Chloride intracellular channel exc-4 [Crassostrea gigas]
	2	26	NaN	" 386 799 minus strand Myosin regulatory light chain LC-2, mantle muscle OS=Todarodes pacificus PE=1 SV=1; 656
	3	5	NaN	" 169 792 minus strand rho GDP dissociation inhibitor, putative [Ixodes scapularis] gi"
	2	2	NaN	REVERSED 68 358 endonuclease-reverse transcriptase [Danaus plexippus]
	6	9	NaN	195 2201 minus strand hypothetical protein DAPPUDRAFT_315656 [Daphnia pulex]
	19	126	NaN	116 1111 minus strand cytosolic malate dehydrogenase [Lottia scabra]
	2	2	NaN	185 742 Transmembrane protein Tmp21 [Crassostrea gigas]; 1 444 minus strand Transmembrane protein Tmp21 [Cr
	13	136	NaN	2881 3411 myosin heavy chain [Todarodes pacificus]; 2915 3445 myosin heavy chain [Todarodes pacificus]
	8	13	NaN	151 1449 rab GDP-dissociation inhibitor [Branchiostoma belcheri]
	5	7	NaN	260 1597 isocitrate dehydrogenase [Mytilus galloprovincialis]; 260 1597 isocitrate dehydrogenase [Mytilus galloprov
	2	2	NaN	242 1834 Regulator of differentiation 1 [Crassostrea gigas]; 458 2050 minus strand Regulator of differentiation 1 [Cr
	4	4	NaN	" 1 1887 Pyruvate carboxylase, mitochondrial [Crassostrea gigas]"
	5	8	NaN	227 2920 minus strand Lysosomal alpha-mannosidase [Crassostrea gigas]; 227 3073 minus strand Lysosomal alpha
	8	10	NaN	116 1321 PREDICTED: similar to aspartate aminotransferase [Tribolium castaneum] gi
	4	7	NaN	430 759 hypothetical protein [Amblyomma maculatum]; 336 665 hypothetical protein [Amblyomma maculatum]
	4	17	NaN	2 256 Polyubiquitin (Fragment) OS=Xenopus laevis PE=1 SV=2; 20 472 minus strand large subunit ribosomal protein
	2	3	NaN	125 550 minus strand ribosomal protein S20 [Chlamys farreri]; 125 550 minus strand ribosomal protein S20 [Chlamy
	3	5	NaN	36 1310 minus strand elongation factor-1 gamma [Diguetia canities]
	4	19	NaN	488 1039 Peptidyl-prolyl cis-trans isomerase B [Crassostrea gigas]; 488 1039 Peptidyl-prolyl cis-trans isomerase B [
	5	6	NaN	" 2041 3315 minus strand Myosin-10 OS=Mus musculus GN=Myh10 PE=1 SV=2; 2041 3345 minus strand Myosin hea
	3	6	NaN	" 57 752 PREDICTED: adenylate kinase 2, mitochondrial-like isoform 2 [Callithrix jacchus]"
	2	2	NaN	138 713 Rac [Aplysia californica] gi; 125 691 Rac [Aplysia californica] gi; 149 871 minus strand Rac [Aplysia californ
	2	3	NaN	18 1613 minus strand chaperonin containing T-complex polypeptide subunit zeta [Haliotis discus hannai]; 527 2089
	2	4	NaN	2302 2547 minus strand hemocyanin isoform 1 [Haliotis diversicolor]; 816 1211 H2 [Haliotis tuberculata]
	2	2	NaN	210 2201 minus strand Glycyl-tRNA synthetase [Crassostrea gigas]; 210 2201 minus strand Glycyl-tRNA synthetase
	3	5	NaN	503 1219 heat shock cognate 71 [Mytilus galloprovincialis]
	2	9	NaN	161 301 Heat shock protein beta-6 OS=Mus musculus GN=Hspb6 PE=1 SV=1; 167 310 Heat shock protein beta-1 OS=
	2	13	NaN	160 588 hypothetical protein CGI_10001640 [Crassostrea gigas]
	9	40	NaN	204 791 minus strand PREDICTED: 15-hydroxyprostaglandin dehydrogenase [NAD+] [Gallus gallus]
	2	3	NaN	172 678 Myosin regulatory light chain sqh [Crassostrea gigas]
	4	4	NaN	424 3507 minus strand putative Na+/K+-ATPase alpha subunit [Paroctopus digueti]

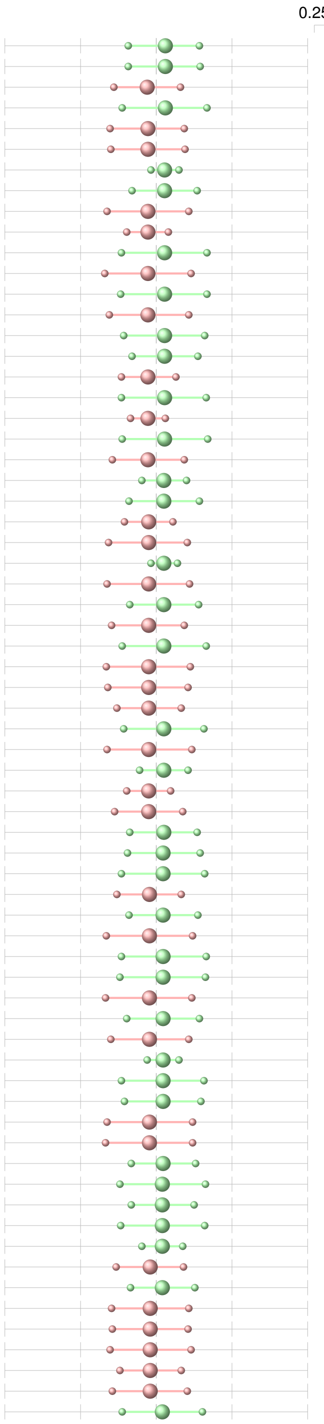

Peptides	Spectra	Coverage	Protein
4	6	NaN	104 445 minus strand macrophage migration inhibitory factor [Ascaris suum]
5	5	NaN	313 2763 NCAM-related cell adhesion molecule precursor [Aplysia californica] gi
6	9	NaN	252 848 sigma class glutathione-s-transferase 2 [Haliotis discus discus]
2	2	NaN	228 1553 hypothetical protein CGI_10024545 [Crassostrea gigas]; 228 1553 hypothetical protein CGI_10024545 [Cras
2	7	NaN	94 519 minus strand hypothetical protein BRAFLDRAFT_115029 [Branchiostoma floridae] gi
4	5	NaN	244 5451 alpha macroglobulin [Chlamys farreri]; 244 5493 alpha macroglobulin [Chlamys farreri]
40	115	NaN	" 519 3182 minus strand Alpha-actinin, sarcomeric [Crassostrea gigas]"
4	16	NaN	" 64 1332 Flotillin-1, putative [Pediculus humanus corporis] gi"
2	3	NaN	112 930 calcineurin A [Haliotis discus discus]
11	99	NaN	260 892 minus strand calponin [Haliotis diversicolor]
2	2	NaN	194 775 minus strand Collagen alpha-1(XV) chain [Harpegnathos saltator]
2	2	NaN	145 1524 PREDICTED: aspartyl aminopeptidase-like [Oreochromis niloticus]
2	2	NaN	1363 1527 Protein disulfide-isomerase A3 [Crassostrea gigas]
3	3	NaN	14 1642 minus strand chaperonin containing tcp1 [Haliotis discus discus]
3	3	NaN	130 2145 minus strand Glucosamine–fructose-6-phosphate aminotransferase [isomerizing] 1 [Crassostrea gigas]; 1
4	19	NaN	697 954 minus strand Peptidyl-prolyl cis-trans isomerase B [Crassostrea gigas]; 614 877 Peptidyl-prolyl cis-trans iso
5	34	NaN	43 591 minus strand cyclophilin type peptidyl-prolyl cis-trans isomerase [Glossina morsitans morsitans]
2	2	NaN	" 45 506 minus strand PREDICTED: dihydrolipoyl dehydrogenase, mitochondrial [Monodelphis domestica]"
25	75	NaN	155 3214 sarco/endoplasmic reticulum calcium ATPase isoform B [Pinctada fucata]; 132 3110 sarco/endoplasmic ret
2	2	NaN	" 2 286 Roundabout 2, partial [Ascaris suum]"
2	13	NaN	764 1015 minus strand heat shock protein beta-1 [Trichinella spiralis] gi
13	40	NaN	1971 3368 minus strand Kyphoscoliosis peptidase [Crassostrea gigas]; 1767 3158 minus strand hillarin [Hirudo med
4	7	NaN	353 2161 Bifunctional 3'-phosphoadenosine 5'-phosphosulfate synthetase [Crassostrea gigas]; 141 19
7	46	NaN	835 1083 minus strand Calmodulin [Crassostrea gigas]
3	4	NaN	977 1852 PREDICTED: similar to prohibitin [Tribolium castaneum] gi
31	207	NaN	30 1088 arginine kinase [Conus novaehollandiae]
2	3	NaN	337 474 minus strand heat shock protein 22 isoform 1 [Ruditapes philippinarum]
4	7	NaN	75 803 hypothetical protein DAPPUDRAFT_231350 [Daphnia pulex]
4	5	NaN	25 405 minus strand Elongation factor 1-delta [Anoplopoma fimbria]
2	3	NaN	338 661 Spectrin alpha chain [Crassostrea gigas]
2	2	NaN	68 667 minus strand hypothetical protein BRAFLDRAFT_272416 [Branchiostoma floridae] gi; 68 667 minus strand hy
3	3	NaN	166 2364 Dipeptidyl-peptidase 3 [Crassostrea gigas]
6	10	NaN	72 1400 minus strand hypothetical protein RCJMB04_22i13 [Gallus gallus]
2	4	NaN	" 3475 4188 minus strand Heat shock 70 kDa protein cognate, putative [Pediculus humanus corporis] gi"
2	5	NaN	31 735 hypothetical protein BRAFLDRAFT_78603 [Branchiostoma floridae] gi; 55 735 hypothetical protein BRAFLDR
13	24	NaN	44 1465 minus strand Adenylyl cyclase-associated protein 1 [Crassostrea gigas]; 52 1473 minus strand Adenylyl cyc
11	63	NaN	3 434 minus strand myosin essential light chain [Haliotis discus discus]
5	12	NaN	280 726 minus strand RecName: Full=Calmodulin; Short=CaM gi|14588595|dbj|BAB61794.1| calmodulin [Metridium
4	19	NaN	174 1649 minus strand Collagen alpha-1(XII) chain [Crassostrea gigas]; 123 1490 minus strand Collagen alpha-1(XII)
3	7	NaN	981 1379 minus strand Multimerin-1 [Crassostrea gigas]; 998 1396 Multimerin-1 [Crassostrea gigas]; 975 1373 Multin
2	3	NaN	" 103 981 PREDICTED: ATP synthase, H+ transporting, mitochondrial F1 complex, gamma subunit isoform H (heart)
4	12	NaN	42 296 minus strand twitchin [Mytilus galloprovincialis]
4	8	NaN	2939 3208 minus strand beta-thymosin domain repeat protein CSP24KDa_v1 [Hermissenda crassicornis] gi
2	2	NaN	165 635 hypothetical protein CGI_10007639 [Crassostrea gigas]
2	3	NaN	290 2458 Putative beta-D-xylosidase 5 [Crassostrea gigas]; 299 2428 Putative beta-D-xylosidase 2 [Crassostrea giga
2	2	NaN	45 944 minus strand unnamed protein product [Mus musculus] gi; 5 334 minus strand receptor of activated protein k
2	2	NaN	57 2378 minus strand Heterogeneous nuclear ribonucleoprotein U-like protein 1 [Crassostrea gigas]
4	6	NaN	137 853 14-3-3 protein [Heterodera glycines]
2	5	NaN	184 438 PREDICTED: similar to CG14235 CG14235-PA [Tribolium castaneum] gi
25	122	NaN	727 2022 RecName: Full=Enolase; AltName: Full=2-phospho-D-glycerate hydro-lyase; AltName: Full=2-phosphoglyc
3	4	NaN	290 2458 Putative beta-D-xylosidase 5 [Crassostrea gigas]
3	5	NaN	280 1239 minus strand PREDICTED: smoothelin isoform 2 [Oryctolagus cuniculus]; 280 1239 minus strand PREDICT
2	2	NaN	410 709 calmodulin-like; K02183 calmodulin
2	2	NaN	" 40 813 minus strand NADH dehydrogenase [ubiquinone] iron-sulfur protein 3, mitochondrial [Crassostrea gigas]"
5	10	NaN	684 1250 minus strand Collagen alpha-5(VI) chain [Crassostrea gigas]
2	2	NaN	170 460 Spectrin beta chain [Crassostrea gigas]
6	10	NaN	26 1909 Dolichyl-diphosphooligosaccharide–protein glycosyltransferase subunit 2 [Crassostrea gigas]
2	3	NaN	" 249 1271 N(G),N(G)-dimethylarginine dimethylaminohydrolase 1 [Crassostrea gigas]"
13	63	NaN	448 1626 matrilin [Biomphalaria glabrata]
3	13	NaN	311 928 minus strand peroxiredoxin 3 [Clonorchis sinensis] gi; 288 905 minus strand peroxiredoxin 3 [Clonorchis si
6	10	NaN	" 3341 4240 minus strand nonmuscle myosin II, partial [Aplysia californica]"
2	6	NaN	3 416 minus strand myosin essential light chain [Crassostrea gigas]
2	11	NaN	633 806 Peroxiredoxin-1 [Crassostrea gigas]; 648 821 Peroxiredoxin-1 [Crassostrea gigas]; 666 839 Peroxiredoxin-1 [
3	3	NaN	794 994 Glycogenin-1 [Crassostrea gigas]; 1425 1739 minus strand Glycogenin-1 [Crassostrea gigas]
5	21	NaN	93 2606 twitchin [Mytilus galloprovincialis]
4	5	NaN	557 2329 minus strand Collagen alpha-3(VI) chain [Crassostrea gigas]
3	3	NaN	771 1505 O-methyltransferase family 3 [Dicentrarchus labrax]

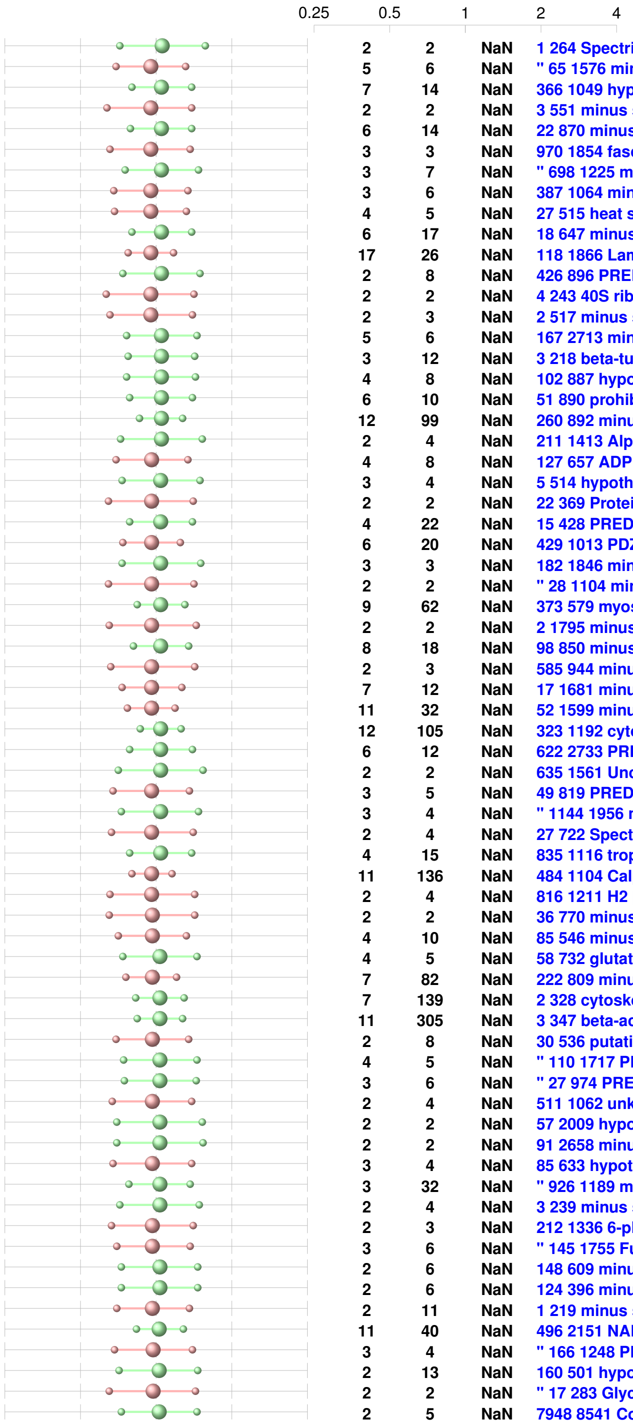

Peptides	Spectra	Coverage	Protein
2	2	NaN	1 264 Spectrin beta chain [Crassostrea gigas]
5	6	NaN	" 65 1576 minus strand Aldehyde dehydrogenase, mitochondrial [Crassostrea gigas]"
7	14	NaN	366 1049 hypothetical protein CGI_10004855 [Crassostrea gigas]
2	2	NaN	3 551 minus strand Spectrin beta chain [Crassostrea gigas]
6	14	NaN	22 870 minus strand PREDICTED: alcohol dehydrogenase class-3 [Sarcophilus harrisii]
3	3	NaN	970 1854 fasciclin-like protein precursor [Aplysia californica] gi
3	7	NaN	" 698 1225 minus strand actin related protein 2/3 complex, 21 kDa subunit [Crassostrea gigas]"
3	6	NaN	387 1064 minus strand PREDICTED: hypothetical protein [Hydra magnipapillata]; 275 952 minus strand PREDICTED
4	5	NaN	27 515 heat shock protein 70 [Capsaspora owczarzaki ATCC 30864]; 30 464 minus strand hypothetical protein [Trich
6	17	NaN	18 647 minus strand ATP synthase-like protein [Biomphalaria glabrata]
17	26	NaN	118 1866 Lamin Dm0 [Crassostrea gigas]
2	8	NaN	426 896 PREDICTED: calmodulin-like [Metaseiulus occidentalis]
2	2	NaN	4 243 40S ribosomal protein S13 OS=Caenorhabditis elegans GN=rps-13 PE=2 SV=2; 4 243 40S ribosomal protein S1
2	3	NaN	2 517 minus strand Anhydro-N-acetylmuramic acid kinase [Crassostrea gigas]
5	6	NaN	167 2713 minus strand Major vault protein [Crassostrea gigas]
3	12	NaN	3 218 beta-tubulin [Fasciola hepatica] gi
4	8	NaN	102 887 hypothetical protein DAPPUDRAFT_304712 [Daphnia pulex]
6	10	NaN	51 890 prohibitin [Octopus tankahkeei]
12	99	NaN	260 892 minus strand calponin [Haliotis diversicolor]; 260 892 minus strand calponin [Haliotis diversicolor]
2	4	NaN	211 1413 Alpha-N-acetylgalactosaminidase [Crassostrea gigas]
4	8	NaN	127 657 ADP ribosylation factor 4 [Marsupenaeus japonicus]
3	4	NaN	5 514 hypothetical protein KGM_05418 [Danaus plexippus]
2	2	NaN	22 369 Protein farnesyltransferase/geranylgeranyltransferase type-1 subunit alpha [Crassostrea gigas]
4	22	NaN	15 428 PREDICTED: histone H2A.V-like [Apis florea]
6	20	NaN	429 1013 PDZ domain protein [Ixodes scapularis] gi
3	3	NaN	182 1846 minus strand hypothetical protein DAPPUDRAFT_302108 [Daphnia pulex]; 110 1774 minus strand hypothe
2	2	NaN	" 28 1104 minus strand Pyruvate dehydrogenase E1 component subunit beta, mitochondrial [Crassostrea gigas]"
9	62	NaN	373 579 myosin heavy chain [Argopecten irradians] gi
2	2	NaN	2 1795 minus strand Plectin-1 [Crassostrea gigas]
8	18	NaN	98 850 minus strand 14-3-3 protein epsilon [Schistocerca gregaria]
2	3	NaN	585 944 minus strand Protein disulfide-isomerase A6 [Crassostrea gigas]
7	12	NaN	17 1681 minus strand Glucose-6-phosphate isomerase [Crassostrea gigas]
11	32	NaN	52 1599 minus strand ATP synthase beta subunit [Pinctada fucata]
12	105	NaN	323 1192 cytosolic malate dehydrogenase [Lottia scabra]; 323 1192 cytosolic malate dehydrogenase [Lottia scabra];
6	12	NaN	622 2733 PREDICTED: protein-glutamine gamma-glutamyltransferase K isoform 1 [Danio rerio]; 423 2498 PREDICTE
2	2	NaN	635 1561 Uncharacterized protein yfeX [Crassostrea gigas]
3	5	NaN	49 819 PREDICTED: elongation factor 1-delta-like isoform 2 [Strongylocentrotus purpuratus]
3	4	NaN	" 1144 1956 minus strand Retinol dehydrogenase 12, partial [Crassostrea gigas]"
2	4	NaN	27 722 Spectrin beta chain OS=Drosophila melanogaster GN=beta-Spec PE=1 SV=2; 27 722 Spectrin beta chain OS=
4	15	NaN	835 1116 tropomyosin [Ilyanassa obsoleta]
11	136	NaN	484 1104 Calponin-2 [Crassostrea gigas]; 309 938 Calponin-2 [Crassostrea gigas]
2	4	NaN	816 1211 H2 [Haliotis tuberculata]
2	2	NaN	36 770 minus strand Endoplasmic reticulum protein ERp29 [Crassostrea gigas]
4	10	NaN	85 546 minus strand RecName: Full=Eukaryotic translation initiation factor 5A; Short=eIF-5A gi|51702279|sp|P6292
4	5	NaN	58 732 glutathione-s-transferase [Haliotis discus discus]
7	82	NaN	222 809 minus strand PREDICTED: 15-hydroxyprostaglandin dehydrogenase [NAD+] [Gallus gallus]
7	139	NaN	2 328 cytoskeletal actin 2 [Molgula oculata]
11	305	NaN	3 347 beta-actin [Meretrix meretrix]; 3 215 Actin-2 OS=Onchocerca volvulus GN=act-2b PE=3 SV=1
2	8	NaN	30 536 putative mitochondrial ATP synthase [Haliotis discus discus]; 27 620 putative mitochondrial ATP synthase [H
4	5	NaN	" 110 1717 PREDICTED: glutamate dehydrogenase, mitochondrial-like [Oreochromis niloticus]; 110 1717 PREDICTE
3	6	NaN	" 27 974 PREDICTED: succinyl-CoA ligase [GDP-forming] subunit alpha, mitochondrial-like [Oreochromis niloticus];
2	4	NaN	511 1062 unknown [Dendroctonus ponderosae]; 511 1062 unknown [Dendroctonus ponderosae]
2	2	NaN	57 2009 hypothetical protein BRAFLDRAFT_285279 [Branchiostoma floridae] gi; 3 1973 hypothetical protein BRAFL
2	2	NaN	91 2658 minus strand puromycin-sensitive aminopeptidase [Saccoglossus kowalevskii]
3	4	NaN	85 633 hypothetical protein [Amblyomma maculatum]
3	32	NaN	" 926 1189 minus strand Myosin heavy chain, striated muscle [Crassostrea gigas]"
2	4	NaN	3 239 minus strand alcohol dehydrogenase class 3 [Branchiostoma lanceolatum]
2	3	NaN	212 1336 6-phosphofructokinase [Crassostrea gigas]
3	6	NaN	" 145 1755 Fumarate hydratase class I, aerobic [Crassostrea gigas]"
2	6	NaN	148 609 minus strand Titin [Crassostrea gigas]
2	6	NaN	124 396 minus strand 40S ribosomal protein S10 OS=Lumbricus rubellus GN=RPS10 PE=2 SV=1
2	11	NaN	1 219 minus strand PREDICTED: nucleoside diphosphate kinase 3-like [Pan troglodytes]
11	40	NaN	496 2151 NADP-dependent malic enzyme [Crassostrea gigas]
3	4	NaN	" 166 1248 PREDICTED: probable pyruvate dehydrogenase E1 component subunit alpha, mitochondrial-like [Metase
2	13	NaN	160 501 hypothetical protein CGI_10001640 [Crassostrea gigas]; 160 588 hypothetical protein CGI_10001640 [Crasso
2	2	NaN	" 17 283 Glycerol-3-phosphate dehydrogenase [NAD+], cytoplasmic [Crassostrea gigas]"
2	5	NaN	7948 8541 Collagen alpha-3(VI) chain [Crassostrea gigas]

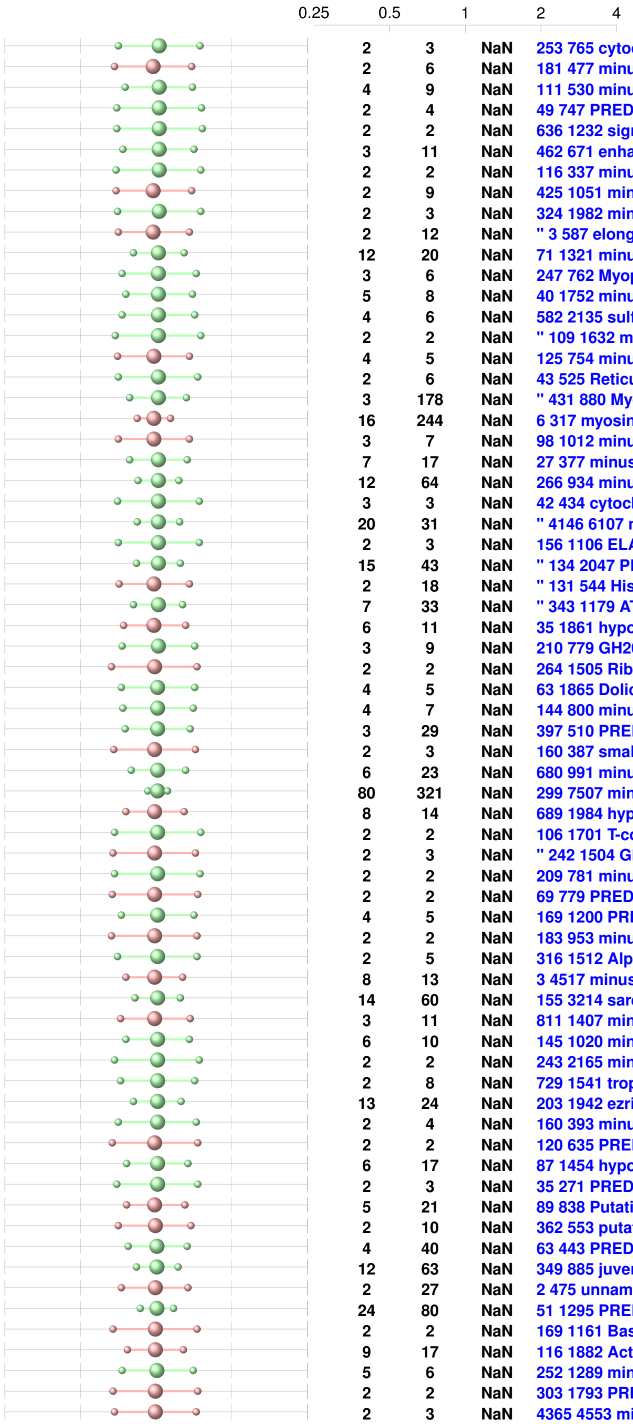

Peptides	Spectra	Coverage	Protein
2	3	NaN	253 765 cytochrome c oxidase subunit IV [Urechis caupo]
2	6	NaN	181 477 minus strand Zgc:56530 [Danio rerio]; 185 481 minus strand Zgc:56530 [Danio rerio]
4	9	NaN	111 530 minus strand Nucleoside diphosphate kinase B OS=Homo sapiens GN=NME2 PE=1 SV=1
2	4	NaN	49 747 PREDICTED: elongation factor 1-delta-like [Strongylocentrotus purpuratus]; 49 819 PREDICTED: elongation f
2	2	NaN	636 1232 sigma class glutathione-s-transferase 3 [Haliotis discus discus]; 963 1559 sigma class glutathione-s-transf
3	11	NaN	462 671 enhancer protein [Homo sapiens]
2	2	NaN	116 337 minus strand Golgi-associated plant pathogenesis-related protein 1 [Crassostrea gigas]; 538 759 minus stra
2	9	NaN	425 1051 minus strand unknown [Schistosoma japonicum]; 300 878 unknown [Schistosoma japonicum]; 567 1145 u
2	3	NaN	324 1982 minus strand Pikachurin [Crassostrea gigas]
2	12	NaN	" 3 587 elongation factor 1 alpha, partial [Phorbas sp. nov. SB-2012]"
12	20	NaN	71 1321 minus strand Vinculin [Crassostrea gigas]
3	6	NaN	247 762 Myophilin [Crassostrea gigas]; 244 759 Myophilin [Crassostrea gigas]; 119 634 Myophilin [Crassostrea giga
5	8	NaN	40 1752 minus strand heat shock protein 60 [Biomphalaria glabrata]; 40 1752 minus strand heat shock protein 60 [B
4	6	NaN	582 2135 sulfatase 1A precursor [Haliotis discus discus]
2	2	NaN	" 109 1632 minus strand Seryl-tRNA synthetase, cytoplasmic [Crassostrea gigas]; 109 1614 minus strand Seryl-tRNA
4	5	NaN	125 754 minus strand peptidyl prolyl cis-trans isomerase B [Conus novaehollandiae]
2	6	NaN	43 525 Reticulon-4 [Crassostrea gigas]; 143 757 Reticulon-4 [Crassostrea gigas]; 43 564 Reticulon-4 [Crassostrea gi
3	178	NaN	" 431 880 Myosin essential light chain, striated adductor muscle OS=Aequipecten irradians PE=1 SV=2"
16	244	NaN	6 317 myosin heavy chain cardiac muscle specific isoform 2 [Argopecten irradians]
3	7	NaN	98 1012 minus strand RecName: Full=Substrate-specific endoprotease Tex31; Flags: Precursor gi|32187775|emb|CA
7	17	NaN	27 377 minus strand ribosomal protein LP2 [Papilio xuthus] gi; 27 377 minus strand ribosomal protein LP2 [Papilio x
12	64	NaN	266 934 minus strand Kyphoscoliosis peptidase [Crassostrea gigas]
3	3	NaN	42 434 cytochrome b5 [Haliotis discus discus]; 42 434 cytochrome b5 [Haliotis discus discus]
20	31	NaN	" 4146 6107 minus strand nonmuscle myosin II, partial [Aplysia californica]"
2	3	NaN	156 1106 ELAV 2-like protein [Aplysia californica] gi
15	43	NaN	" 134 2047 PREDICTED: succinate dehydrogenase [ubiquinone] flavoprotein subunit, mitochondrial-like isoform 3, p
2	18	NaN	" 131 544 Histone H2B, gonadal OS=Patella granatina PE=1 SV=2"
7	33	NaN	" 343 1179 ATP synthase subunit alpha, mitochondrial OS=Pongo abelii GN=ATP5A1 PE=2 SV=1"
6	11	NaN	35 1861 hypothetical protein DAPPUDRAFT_309082 [Daphnia pulex]
3	9	NaN	210 779 GH20056 [Drosophila grimshawi] gi; 216 785 minus strand GH20056 [Drosophila grimshawi] gi; 210 779 GH2
2	2	NaN	264 1505 Ribosome-binding protein 1 [Crassostrea gigas]; 260 1501 Ribosome-binding protein 1 [Crassostrea gigas
4	5	NaN	63 1865 Dolichyl-diphosphooligosaccharide–protein glycosyltransferase subunit 1 [Crassostrea gigas]; 63 1865 Dol
4	7	NaN	144 800 minus strand PREDICTED: protein F37C4.5-like [Amphimedon queenslandica]
3	29	NaN	397 510 PREDICTED: actin-5C-like [Megachile rotundata]
2	3	NaN	160 387 small nuclear ribonucleoprotein polypeptide G protein [Crassostrea ariakensis]; 180 407 small nuclear ribon
6	23	NaN	680 991 minus strand PDZ and LIM domain protein 1 [Crassostrea gigas]; 553 864 minus strand PDZ and LIM domai
80	321	NaN	299 7507 minus strand filamin [Clonorchis sinensis]
8	14	NaN	689 1984 hypothetical protein BRAFLDRAFT_78257 [Branchiostoma floridae] gi
2	2	NaN	106 1701 T-complex protein 1 subunit beta [Crassostrea gigas]
2	3	NaN	" 242 1504 Glutamate receptor, ionotropic kainate 3 [Crassostrea gigas]; 239 1501 Glutamate receptor, ionotropic ka
2	2	NaN	209 781 minus strand high mobility group protein 1 [Biomphalaria glabrata]; 277 771 high mobility group protein 1 [B
2	2	NaN	69 779 PREDICTED: 15-hydroxyprostaglandin dehydrogenase [NAD+] [Gallus gallus]
4	5	NaN	169 1200 PREDICTED: nidogen-2-like isoform 1 [Strongylocentrotus purpuratus]
2	2	NaN	183 953 minus strand glutathione-S-transferase [Haliotis diversicolor supertexta]; 275 1045 minus strand glutathion
2	5	NaN	316 1512 Alpha-N-acetylgalactosaminidase [Crassostrea gigas]; 282 1568 Alpha-N-acetylgalactosaminidase [Crasso
8	13	NaN	3 4517 minus strand Clathrin heavy chain 1 [Crassostrea gigas]
14	60	NaN	155 3214 sarco/endoplasmic reticulum calcium ATPase isoform B [Pinctada fucata]; 155 3214 minus strand sarco/en
3	11	NaN	811 1407 minus strand mitochondrial malate dehydrogenase 2 [Eospalax baileyi]
6	10	NaN	145 1020 minus strand PREDICTED: inorganic pyrophosphatase-like [Saccoglossus kowalevskii]
2	2	NaN	243 2165 minus strand Elongation factor 2 OS=Drosophila melanogaster GN=Ef2b PE=2 SV=4
2	8	NaN	729 1541 troponin T [Mizuhopecten yessoensis]
13	24	NaN	203 1942 ezrin/radixin/moesin [Aplysia californica] gi; 243 1982 minus strand ezrin/radixin/moesin [Aplysia californi
2	4	NaN	160 393 minus strand PREDICTED: cysteine-rich protein 1-like [Megachile rotundata]; 148 381 PREDICTED: cysteine
2	2	NaN	120 635 PREDICTED: similar to collagen type VI alpha 6 [Ciona intestinalis]; 120 635 minus strand PREDICTED: simi
6	17	NaN	87 1454 hypothetical protein BRAFLDRAFT_287788 [Branchiostoma floridae] gi; 117 1469 hypothetical protein BRAF
2	3	NaN	35 271 PREDICTED: SH3 domain-binding glutamic acid-rich-like protein 3-like [Anolis carolinensis]; 35 271 PREDICT
5	21	NaN	89 838 Putative phosphoglycerate mutase [Crassostrea gigas]; 89 838 Putative phosphoglycerate mutase [Crassost
2	10	NaN	362 553 putative RNA-binding protein [Haliotis diversicolor]
4	40	NaN	63 443 PREDICTED: RAS protein activator like 1-like [Saccoglossus kowalevskii]
12	63	NaN	349 885 juvenile hormone diol kinase [Danaus plexippus]; 349 885 juvenile hormone diol kinase [Danaus plexippus]
2	27	NaN	2 475 unnamed protein product [Tetraodon nigroviridis]
24	80	NaN	51 1295 PREDICTED: phosphoglycerate kinase-like [Nasonia vitripennis]; 97 1341 PREDICTED: phosphoglycerate k
2	2	NaN	169 1161 Basement membrane-specific heparan sulfate proteoglycan core protein OS=Homo sapiens GN=HSPG2 PE
9	17	NaN	116 1882 Actin-interacting protein 1 [Crassostrea gigas]
5	6	NaN	252 1289 minus strand PDZ and LIM domain protein 3 OS=Gallus gallus GN=PDLIM3 PE=1 SV=1
2	2	NaN	303 1793 PREDICTED: matrix metalloproteinase 19 isoform rasi-1 preproprotein-like [Saccoglossus kowalevskii]
2	3	NaN	4365 4553 minus strand Basement membrane-specific heparan sulfate proteoglycan core protein [Crassostrea gigas

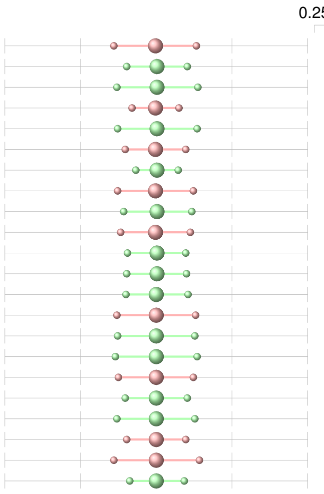

Peptides	Spectra	Coverage	Protein
2	3	NaN	2996 3340 minus strand Collagen alpha-5(VI) chain [Crassostrea gigas]
6	12	NaN	6 650 minus strand glutathione S-transferase mu class protein [Crassostrea ariakensis]; 117 761 glutathione S-trans
2	4	NaN	1139 1378 Titin [Crassostrea gigas]; 1116 1355 Titin [Crassostrea gigas]; 1139 1378 Titin [Crassostrea gigas]; 1139 1
12	31	NaN	" 213 1499 minus strand Citrate synthase, mitochondrial [Crassostrea gigas]"
2	5	NaN	" 1240 1911 PREDICTED: ATP synthase subunit alpha, mitochondrial-like [Anolis carolinensis]"
6	13	NaN	60 1022 Major egg antigen [Crassostrea gigas]; 60 1022 Major egg antigen [Crassostrea gigas]
10	105	NaN	527 1321 minus strand troponin T [Mizuhopecten yessoensis]; 305 1099 minus strand troponin T [Mizuhopecten yes
2	8	NaN	76 822 14-3-3 protein [Heterodera glycines]; 76 822 minus strand 14-3-3 protein [Heterodera glycines]
4	13	NaN	201 779 minus strand sigma class glutathione-s-transferase 2 [Haliotis discus discus]
2	21	NaN	" 116 352 minus strand troponin I 4 [Clonorchis sinensis]; 491 706 minus strand troponin-I, TnI {C-terminal} [Chlamy
7	14	NaN	365 2530 minus strand calpain B [Gecarcinus lateralis]
5	16	NaN	" 276 659 Fatty acid-binding protein, adipocyte [Crassostrea gigas]; 426 809 Fatty acid-binding protein, adipocyte [C
6	11	NaN	93 1034 Annexin A7 [Crassostrea gigas]; 73 1014 Annexin A7 [Crassostrea gigas]
2	4	NaN	457 1842 Basement membrane-specific heparan sulfate proteoglycan core protein [Crassostrea gigas]; 457 1842 Ba
3	4	NaN	236 733 Spectrin alpha chain [Crassostrea gigas]
2	3	NaN	5 841 minus strand Spectrin alpha chain [Crassostrea gigas]
3	5	NaN	131 541 minus strand nervous system adducin [Aplysia californica] gi
4	31	NaN	768 980 muscle LIM protein isoform 1 [Danaus plexippus]
2	5	NaN	" 42 905 PREDICTED: enoyl-CoA hydratase, mitochondrial-like isoform 1 [Oreochromis niloticus]"
6	16	NaN	" 3195 3968 Spectrin alpha chain, putative [Pediculus humanus corporis] gi"
2	2	NaN	91 1392 minus strand hypothetical protein AND_03103 [Anopheles darlingi]
7	23	NaN	181 744 unknown [Dendroctonus ponderosae]

4.2 Proteins Identified by a Single Peptide

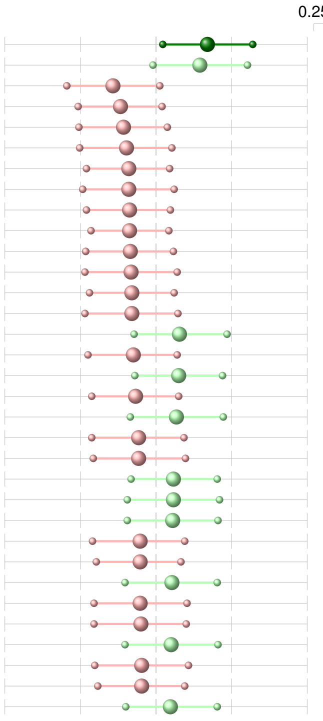

Peptides	Spectra	Coverage	Protein
1	2	NaN	704 817 minus strand expressed hypothetical protein [Trichoplax adhaerens] gi; 688 801 minus strand Lactoylglutat
1	1	NaN	66 947 Glyoxalase domain-containing protein 4 [Crassostrea gigas]
1	3	NaN	302 1273 minus strand hypothetical protein CGI_10007007 [Crassostrea gigas]
1	4	NaN	9 518 minus strand hemocyanin isoform 1 [Nucula nucleus]
1	2	NaN	360 764 minus strand Peptidyl-prolyl cis-trans isomerase A OS=Dictyostelium discoideum GN=ppiA PE=1 SV=1
1	1	NaN	245 1243 Prostaglandin reductase 1 [Crassostrea gigas]
1	5	NaN	361 549 minus strand 32 kDa beta-galactoside-binding lectin [Ascaris suum]
1	1	NaN	8 937 minus strand galectin [Crassostrea virginica]
1	4	NaN	91 1326 minus strand Adipocyte plasma membrane-associated protein [Crassostrea gigas]; 107 1273 minus strand
1	10	NaN	633 806 Peroxiredoxin-1 [Crassostrea gigas]; 648 821 Peroxiredoxin-1 [Crassostrea gigas]; 666 839 Peroxiredoxin-1
1	2	NaN	3788 7885 minus strand Filamin-C [Crassostrea gigas]
1	1	NaN	87 485 Sex-regulated protein janus-A [Crassostrea gigas]
1	3	NaN	900 1616 minus strand hemocyanin isoform 1 [Haliotis diversicolor]
1	1	NaN	3 239 minus strand p38 MAPK [Littorina littorea]
1	1	NaN	2 2572 Glycogen debranching enzyme [Crassostrea gigas]
1	2	NaN	201 1181 minus strand Serine/threonine-protein phosphatase PP1-alpha catalytic subunit [Crassostrea gigas]
1	2	NaN	" 196 1053 minus strand Cytochrome b-c1 complex subunit 2, mitochondrial OS=Homo sapiens GN=UQCRC2 PE=1
1	2	NaN	123 314 PREDICTED: ubiquinol-cytochrome c reductase hinge protein-like [Saccoglossus kowalevskii]; 279 530 PRE
1	1	NaN	401 1174 minus strand Mechanosensory protein 2 [Crassostrea gigas]
1	1	NaN	7825 8754 minus strand H2 [Haliotis tuberculata]
1	1	NaN	192 380 minus strand RAB protein [Haliotis discus discus]
1	3	NaN	406 1392 minus strand hypothetical protein CGI_10007007 [Crassostrea gigas]
1	1	NaN	258 629 Glycogenin-1 OS=Oryctolagus cuniculus GN=GYG1 PE=1 SV=3
1	1	NaN	" 137 1549 minus strand Serine hydroxymethyltransferase, mitochondrial [Crassostrea gigas]; 205 1608 minus stran
1	1	NaN	1155 1649 78 kDa glucose-regulated protein OS=Aplysia californica PE=1 SV=1
1	3	NaN	" 1 510 Glycerol-3-phosphate dehydrogenase [NAD+], cytoplasmic [Crassostrea gigas]"
1	1	NaN	21 239 minus strand calponin-like protein [Taenia asiatica]
1	1	NaN	" 451 1026 PREDICTED: GTP-binding protein SAR1B, putative-like [Saccoglossus kowalevskii]"
1	1	NaN	366 935 minus strand 40S ribosomal protein S2 OS=Urechis caupo GN=RPS2 PE=2 SV=1
1	1	NaN	121 660 Failed axon connections homolog OS=Rattus norvegicus GN=Faxc PE=3 SV=1
1	1	NaN	78 461 PREDICTED: hoi-polloi-like [Saccoglossus kowalevskii]; 103 492 NHP2-like protein 1 OS=Rattus norvegicus
1	2	NaN	1292 1471 minus strand hemocyanin isoform 1 [Haliotis diversicolor]
1	1	NaN	" 199 417 GG13156 [Drosophila erecta] gi; 253 501 minus strand NADH-ubiquinone oxidoreductase MLRQ subunit-li

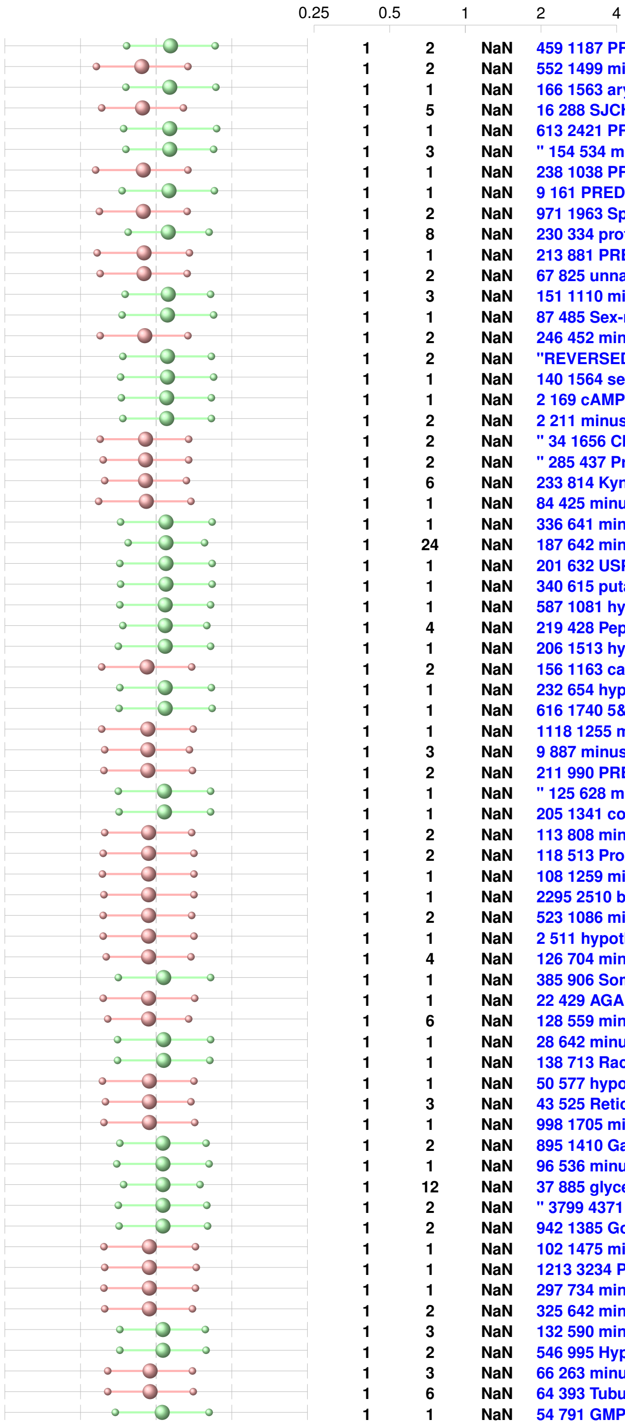

Peptides	Spectra	Coverage	Protein
1	2	NaN	459 1187 PREDICTED: protein-L-isoaspartate(D-aspartate) O-methyltransferase-like isoform 2 [Anolis carolinensis]
1	2	NaN	552 1499 minus strand uncharacterized protein LOC100158466 [Xenopus (Silurana) tropicalis] gi; 552 1499 minus st
1	1	NaN	166 1563 arylsulfatase [Haliotis discus discus]
1	5	NaN	16 288 SJCHGC01950 protein [Schistosoma japonicum] gi
1	1	NaN	613 2421 PREDICTED: lysosomal alpha-glucosidase [Danio rerio]
1	3	NaN	" 154 534 minus strand Muscle M-line assembly protein unc-89, partial [Crassostrea gigas]; 259 690 minus strand M
1	1	NaN	238 1038 PREDICTED: hypothetical protein [Saccoglossus kowalevskii]; 294 992 PREDICTED: hypothetical protein [
1	1	NaN	9 161 PREDICTED: tubulin alpha-1C chain-like [Acyrthosiphon pisum]; 9 161 Tubulin alpha-3 chain (Fragments) OS=
1	2	NaN	971 1963 Spectrin alpha chain [Crassostrea gigas]; 972 1979 Spectrin alpha chain [Crassostrea gigas]
1	8	NaN	230 334 protein disulfide isomerase [Conus virgo]
1	1	NaN	213 881 PREDICTED: hypothetical protein [Saccoglossus kowalevskii]; 260 928 PREDICTED: hypothetical protein [S
1	2	NaN	67 825 unnamed protein product [Oikopleura dioica]
1	3	NaN	151 1110 minus strand hypothetical protein DAPPUDRAFT_303085 [Daphnia pulex]
1	1	NaN	87 485 Sex-regulated protein janus-A [Crassostrea gigas]; 92 310 PREDICTED: hypothetical protein [Saccoglossus k
1	2	NaN	246 452 minus strand mitochondrial malate dehydrogenase precursor [Nucella lapillus]; 106 312 minus strand mitoc
1	2	NaN	"REVERSED 2 1810 minus strand Glutaminyl-tRNA synthetase, partial [Crassostrea gigas]"
1	1	NaN	140 1564 selenium-binding protein [Haliotis discus hannai]
1	1	NaN	2 169 cAMP-dependent protein kinase regulatory subunit [Crassostrea gigas]; 125 511 cAMP-dependent protein kina
1	2	NaN	2 211 minus strand sulfatase 1A precursor [Haliotis discus discus]
1	2	NaN	" 34 1656 Chaperonin containing TCP1, subunit 7 (eta) [Danio rerio] gi; 34 1656 Chaperonin containing TCP1, subun
1	2	NaN	" 285 437 Propionyl-CoA carboxylase alpha chain, mitochondrial [Crassostrea gigas]"
1	6	NaN	233 814 Kynurenine formamidase [Crassostrea gigas]; 233 979 Kynurenine formamidase [Crassostrea gigas]; 139 94
1	1	NaN	84 425 minus strand Transcription elongation factor B polypeptide 2 [Crassostrea gigas]; 84 425 minus strand Trans
1	1	NaN	336 641 minus strand malate dehydrogenase [Schistosoma japonicum]
1	24	NaN	187 642 minus strand PREDICTED: similar to pol-like protein [Tribolium castaneum]
1	1	NaN	201 632 USP-like protein isoform 1 [Crassostrea ariakensis] gi; 189 620 minus strand USP-like protein isoform 1 [Cra
1	1	NaN	340 615 putative Low-density lipoprotein receptor repeat class B [Trichinella spiralis] gi
1	1	NaN	587 1081 hypothetical protein CGI_10026502 [Crassostrea gigas]
1	4	NaN	219 428 Peptidyl-prolyl cis-trans isomerase OS=Hemicentrotus pulcherrimus PE=2 SV=1
1	1	NaN	206 1513 hypothetical protein CGI_10019003 [Crassostrea gigas]
1	2	NaN	156 1163 cathepsin B [Crassostrea ariakensis]
1	1	NaN	232 654 hypothetical protein BRAFLDRAFT_87758 [Branchiostoma floridae] gi
1	1	NaN	616 1740 5'-AMP-activated protein kinase subunit beta-2 [Crassostrea gigas]
1	1	NaN	1118 1255 minus strand equistatin precursor [Actinia equina]
1	3	NaN	9 887 minus strand hypothetical protein CGI_10006272 [Crassostrea gigas]
1	2	NaN	211 990 PREDICTED: gamma-glutamyl hydrolase-like [Xenopus (Silurana) tropicalis]; 223 1080 PREDICTED: gamma
1	1	NaN	" 125 628 minus strand collectin sub-family member 12, partial [Oncorhynchus mykiss]"
1	1	NaN	205 1341 collagen precursor [Bombyx mori]
1	2	NaN	113 808 minus strand omega class glutathione-s-transferase [Chlamys farreri]; 335 1045 minus strand omega class
1	2	NaN	118 513 Protein CAL-5 [Caenorhabditis elegans] gi
1	1	NaN	108 1259 minus strand PREDICTED: Na(+)/H(+) exchange regulatory cofactor NHE-RF3-like [Metaseiulus occidentali
1	1	NaN	2295 2510 beta-thymosin domain repeat protein CSP24KDa_v1 [Hermissenda crassicornis] gi
1	2	NaN	523 1086 minus strand juvenile hormone diol kinase [Danaus plexippus]
1	1	NaN	2 511 hypothetical protein CGI_10006272 [Crassostrea gigas]
1	4	NaN	126 704 minus strand ribosomal protein L12 [Ixodes scapularis]
1	1	NaN	385 906 Soma ferritin OS=Lymnaea stagnalis PE=2 SV=2
1	1	NaN	22 429 AGAP006937-PA [Anopheles gambiae str. PEST] gi; 1 474 Ubiquitin-conjugating enzyme E2 variant 2 OS=Rat
1	6	NaN	128 559 minus strand predicted protein [Nematostella vectensis] gi
1	1	NaN	28 642 minus strand Transmembrane emp24 domain-containing protein 9 [Crassostrea gigas]; 236 709 minus strand
1	1	NaN	138 713 Rac [Aplysia californica] gi; 101 676 Rac [Aplysia californica] gi
1	1	NaN	50 577 hypothetical protein BRAFLDRAFT_119799 [Branchiostoma floridae] gi; 50 577 hypothetical protein BRAFLD
1	3	NaN	43 525 Reticulon-4 [Crassostrea gigas]; 143 757 Reticulon-4 [Crassostrea gigas]; 43 564 Reticulon-4 [Crassostrea gi
1	1	NaN	998 1705 minus strand Basement membrane-specific heparan sulfate proteoglycan core protein [Crassostrea gigas]
1	2	NaN	895 1410 Galectin-3 OS=Rattus norvegicus GN=Lgals3 PE=1 SV=4; 1067 1405 Galectin-9 [Crassostrea gigas]; 1068 1
1	1	NaN	96 536 minus strand hypothetical protein BRAFLDRAFT_285353 [Branchiostoma floridae] gi
1	12	NaN	37 885 glyceraldehyde 3-phosphate dehydrogenase [Dugesia japonica]
1	2	NaN	" 3799 4371 papilin, partial [Rhipicephalus microplus]"
1	2	NaN	942 1385 Golgi-associated plant pathogenesis-related protein 1 [Crassostrea gigas]; 338 817 Golgi-associated plant
1	1	NaN	102 1475 minus strand Cytosolic non-specific dipeptidase [Crassostrea gigas]
1	1	NaN	1213 3234 Protein-glutamine gamma-glutamyltransferase K [Crassostrea gigas]; 478 2499 Protein-glutamine gamma
1	1	NaN	297 734 minus strand PREDICTED: calmodulin [Canis lupus familiaris]; 297 734 PREDICTED: calmodulin [Canis lupu
1	2	NaN	325 642 minus strand Protein phosphatase 1B [Crassostrea gigas]
1	3	NaN	132 590 minus strand Non-neuronal cytoplasmic intermediate filament protein OS=Helix aspersa PE=3 SV=2
1	2	NaN	546 995 Hypothetical protein CBG12002 [Caenorhabditis briggsae]; 543 995 minus strand PREDICTED: tubulin polyr
1	3	NaN	66 263 minus strand twitchin [Mytilus galloprovincialis]; 66 263 minus strand twitchin [Mytilus galloprovincialis]; 66
1	6	NaN	64 393 Tubulin beta chain OS=Paracentrotus lividus PE=2 SV=1; 111 398 Tubulin beta chain OS=Paracentrotus lividu
1	1	NaN	54 791 GMP reductase 2 [Crassostrea gigas]

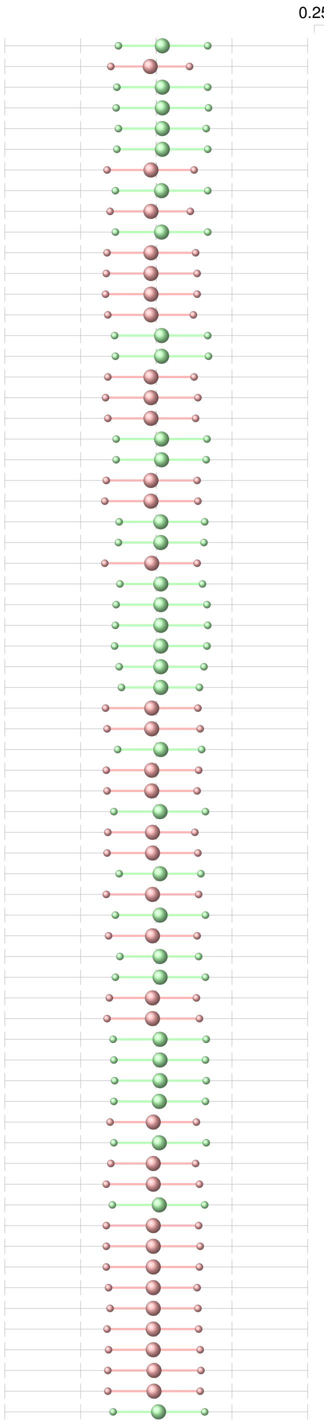

Peptides	Spectra	Coverage	Protein
1	2	NaN	530 679 minus strand GJ11201 [Drosophila virilis] gi
1	9	NaN	" 1 321 Dynein heavy chain, cytoplasmic [Crassostrea gigas]"
1	3	NaN	187 696 minus strand putative ribosomal protein S10 [Sipunculus nudus]
1	1	NaN	11 394 regucalcin [Salmo salar] gi
1	2	NaN	210 473 Abdominal ganglion neuropeptides L5-67 OS=Aplysia californica PE=1 SV=1; 879 1124 abdominal ganglion
1	1	NaN	176 517 PREDICTED: elongin C-like [Saccoglossus kowalevskii]; 115 456 PREDICTED: elongin C-like [Saccoglossus
1	2	NaN	14 223 minus strand Endothelin-converting enzyme 2 OS=Homo sapiens GN=ECE2 PE=1 SV=4
1	1	NaN	126 287 minus strand Cytochrome b-c1 complex subunit 10 [Cricetulus griseus]; 126 287 minus strand Cytochrome
1	10	NaN	1714 2121 Phosphoenolpyruvate carboxykinase [GTP] [Crassostrea gigas]
1	2	NaN	1497 1889 UDP-glucuronosyltransferase 2C1 (Fragment) OS=Oryctolagus cuniculus GN=UGT2C1 PE=2 SV=1
1	2	NaN	165 377 Cysteine and glycine-rich protein 3 [Crassostrea gigas]; 135 344 Cysteine and glycine-rich protein 3 [Crasso
1	1	NaN	" 148 1989 Acyl-CoA synthetase family member 2, mitochondrial [Crassostrea gigas]; 146 1987 minus strand Acyl-C
1	1	NaN	1050 1307 calreticulin precursor [Aplysia californica] gi
1	4	NaN	392 808 Whirlin [Crassostrea gigas]
1	1	NaN	" 757 1443 minus strand nonmuscle myosin II, partial [Aplysia californica]"
1	1	NaN	1070 1888 minus strand galectin [Pinctada fucata]; 1104 1922 minus strand galectin [Pinctada fucata]
1	3	NaN	2019 2723 minus strand Kyphoscoliosis peptidase [Crassostrea gigas]; 2060 2764 minus strand Kyphoscoliosis pep
1	1	NaN	187 1323 minus strand gelsolin [Aplysia californica] gi
1	2	NaN	5 1018 minus strand Glutaredoxin-3 [Crassostrea gigas]; 5 1018 Glutaredoxin-3 [Crassostrea gigas]
1	1	NaN	113 445 ribosomal protein rps19 [Lineus viridis]
1	1	NaN	4 219 hypothetical protein BRAFLDRAFT_66351 [Branchiostoma floridae] gi; 3 239 hypothetical protein BRAFLDRAI
1	2	NaN	141 1325 Nucleobindin-2 [Crassostrea gigas]; 141 1208 minus strand Nucleobindin-2 [Crassostrea gigas]
1	1	NaN	242 589 Ribonucleoprotein RB97D OS=Drosophila melanogaster GN=Rb97D PE=2 SV=1
1	3	NaN	2764 3420 PREDICTED: filamin-C-like [Nasonia vitripennis]
1	3	NaN	96 497 AAEL009772-PB [Aedes aegypti]
1	1	NaN	1573 2385 minus strand Spectrin beta chain [Crassostrea gigas]
1	5	NaN	108 593 hypothetical protein CGI_10025497 [Crassostrea gigas]
1	1	NaN	54 638 minus strand ubiquitin carboxyl-terminal hydrolase [Aplysia californica] gi
1	1	NaN	71 523 minus strand hypothetical protein DAPPUDRAFT_309332 [Daphnia pulex]
1	1	NaN	219 554 minus strand ribosomal protein P1 [Biomphalaria glabrata]
1	4	NaN	10 300 minus strand unnamed protein product [Tetraodon nigroviridis]
1	12	NaN	49 1038 Glyceraldehyde-3-phosphate dehydrogenase [Crotalus adamanteus]
1	1	NaN	" 154 591 microsomal GST1, partial [Reishia clavigera]; 154 531 microsomal GST1, partial [Reishia clavigera]"
1	1	NaN	194 904 Aldo-keto reductase family 1 member B10 [Crassostrea gigas]
1	3	NaN	3 386 fem-1-like protein C [Crassostrea gigas]
1	1	NaN	198 821 minus strand sigma class glutathione-s-transferase 2 [Haliotis discus discus]
1	1	NaN	227 1201 PREDICTED: quinone oxidoreductase-like isoform 1 [Ornithorhynchus anatinus]; 109 1083 PREDICTED: qu
1	1	NaN	179 2140 minus strand PREDICTED: hypothetical protein [Saccoglossus kowalevskii]
1	2	NaN	8 1636 minus strand V-type proton ATPase catalytic subunit A [Crassostrea gigas]; 8 1636 minus strand V-type proto
1	1	NaN	148 462 hypothetical protein DAPPUDRAFT_306954 [Daphnia pulex]; 148 462 hypothetical protein DAPPUDRAFT_30
1	6	NaN	58 198 minus strand Protein DJ-1 [Crassostrea gigas]
1	1	NaN	1265 1831 minus strand Adenosine deaminase CECR1 [Crassostrea gigas]
1	1	NaN	10 1902 Mitochondrial inner membrane protein [Crassostrea gigas]; 10 1902 Mitochondrial inner membrane protein
1	2	NaN	296 493 hypothetical protein BRAFLDRAFT_66287 [Branchiostoma floridae] gi
1	12	NaN	6043 6174 myosin heavy chain [Mytilus galloprovincialis]; 5869 6000 myosin heavy chain [Mytilus galloprovincialis]
1	1	NaN	191 919 PREDICTED: 15-hydroxyprostaglandin dehydrogenase [NAD+] [Gallus gallus]
1	2	NaN	261 1361 tandem-repeat galectin [Pinctada fucata]; 261 1361 tandem-repeat galectin [Pinctada fucata]
1	1	NaN	212 1405 minus strand unnamed protein product [Tetraodon nigroviridis]
1	2	NaN	" 241 1110 minus strand N(G),N(G)-dimethylarginine dimethylaminohydrolase 1 [Crassostrea gigas]; 241 1110 minus
1	1	NaN	1732 2079 minus strand PREDICTED: annexin A7-like [Ornithorhynchus anatinus]
1	1	NaN	316 531 cytochrome c oxidase subunit VIIa polypeptide 2 like [Xenopus (Silurana) tropicalis]
1	1	NaN	613 846 cytochrome c oxidase subunit Va [Antheraea yamamai] gi
1	3	NaN	2831 4900 minus strand Collagen alpha-3(VI) chain [Crassostrea gigas]
1	1	NaN	4 2400 IQ motif containing GTPase activating protein 2 [Xenopus laevis] gi; 4 2169 IQ motif containing GTPase activa
1	3	NaN	593 862 minus strand mitochondrial ATP synthase coupling factor 6 precursor [Ochlerotatus taeniorhynchus]
1	1	NaN	73 525 minus strand Actin-related protein 2/3 complex subunit 5-like protein [Salmo salar] gi; 73 525 Actin-related pr
1	1	NaN	642 1415 minus strand cytochrome c oxidase subunit II [Oncomelania hupensis hupensis]
1	1	NaN	90 659 Plectin-1 [Crassostrea gigas]; 327 881 Plectin-1 [Crassostrea gigas]; 646 1290 minus strand Plectin-1 [Crasso
1	1	NaN	3 230 minus strand RH21460p [Drosophila melanogaster]; 3 230 minus strand RH21460p [Drosophila melanogaster]
1	1	NaN	54 278 ATP-citrate synthase [Crassostrea gigas]; 61 465 ATP-citrate synthase [Crassostrea gigas]
1	2	NaN	3 254 Calpain-5 [Crassostrea gigas]; 3 254 minus strand Calpain-5 [Crassostrea gigas]
1	3	NaN	3 470 minus strand Spectrin beta chain [Crassostrea gigas]
1	1	NaN	35 505 minus strand Basement membrane-specific heparan sulfate proteoglycan core protein [Crassostrea gigas]
1	2	NaN	104 418 Tubulin-specific chaperone A [Crassostrea gigas]; 104 418 minus strand Tubulin-specific chaperone A [Cras
1	1	NaN	3 293 Titin [Crassostrea gigas]
1	1	NaN	2 1384 minus strand twitchin [Mytilus galloprovincialis]; 2 1384 minus strand twitchin [Mytilus galloprovincialis]; 2 1
1	1	NaN	58 1203 minus strand Obg-like ATPase 1 [Crassostrea gigas]

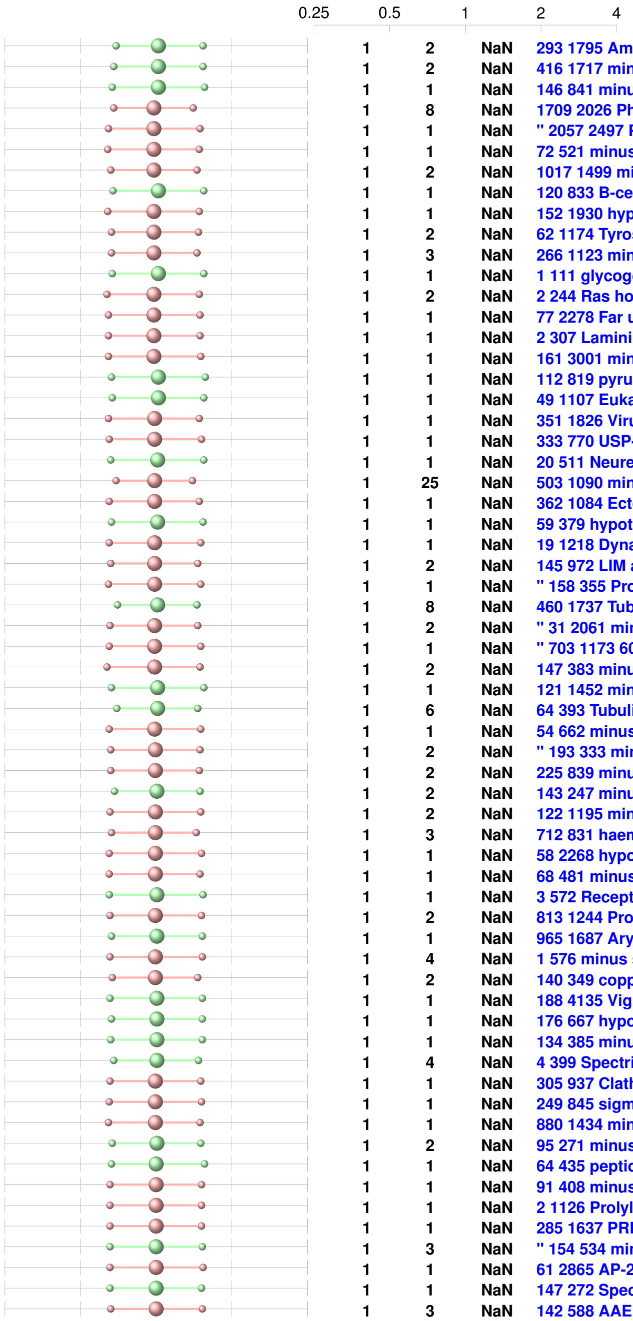

Peptides	Spectra	Coverage	Protein
1	2	NaN	293 1795 Amidase [Crassostrea gigas]
1	2	NaN	416 1717 minus strand T-complex protein 1 subunit delta OS=Aedes triseriatus PE=2 SV=1
1	1	NaN	146 841 minus strand hypothetical protein CGI_10006609 [Crassostrea gigas]; 146 841 minus strand hypothetical pr
1	8	NaN	1709 2026 Phosphoenolpyruvate carboxykinase [GTP] [Crassostrea gigas]
1	1	NaN	" 2057 2497 Phosphate carrier protein, mitochondrial [Crassostrea gigas]"
1	1	NaN	72 521 minus strand GF14593 [Drosophila ananassae] gi
1	2	NaN	1017 1499 minus strand PREDICTED: leukocyte elastase inhibitor-like [Oreochromis niloticus]
1	1	NaN	120 833 B-cell receptor-associated protein 31 [Crassostrea gigas]; 238 951 B-cell receptor-associated protein 31 [Cr
1	1	NaN	152 1930 hypothetical protein BRAFLDRAFT_223365 [Branchiostoma floridae] gi
1	2	NaN	62 1174 Tyrosine-protein kinase CSK [Crassostrea gigas]; 62 1174 minus strand Tyrosine-protein kinase CSK [Crass
1	3	NaN	266 1123 minus strand mitochondrial malate dehydrogenase precursor [Nucella lapillus]
1	1	NaN	1 111 glycogen synthase [Crassostrea gigas]
1	2	NaN	2 244 Ras homolog gene family member A [Clonorchis sinensis]
1	1	NaN	77 2278 Far upstream element-binding protein 3 [Crassostrea gigas]
1	1	NaN	2 307 Laminin subunit gamma-1 [Crassostrea gigas]
1	1	NaN	161 3001 minus strand E3 ubiquitin-protein ligase MIB2 [Crassostrea gigas]
1	1	NaN	112 819 pyruvate kinase [Crassostrea gigas]; 132 932 pyruvate kinase [Crassostrea gigas]
1	1	NaN	49 1107 Eukaryotic translation initiation factor 3 subunit B [Crassostrea gigas]
1	1	NaN	351 1826 Virulence metalloprotease [Crassostrea gigas]
1	1	NaN	333 770 USP-like protein isoform 2 [Crassostrea ariakensis]
1	1	NaN	20 511 Neurexin-2-alpha [Crassostrea gigas]; 20 511 Neurexin-2-alpha [Crassostrea gigas]
1	25	NaN	503 1090 minus strand myosin regulatory light chain [Fasciola hepatica]; 278 865 minus strand myosin regulatory li
1	1	NaN	362 1084 Ectoine hydroxylase [Crassostrea gigas]; 362 1084 Ectoine hydroxylase [Crassostrea gigas]; 366 1220 Ecto
1	1	NaN	59 379 hypothetical protein DAPPUDRAFT_230756 [Daphnia pulex]; 117 443 minus strand hypothetical protein DAPP
1	1	NaN	19 1218 Dynactin subunit 2 [Crassostrea gigas]; 48 830 minus strand Dynactin subunit 2 [Crassostrea gigas]
1	2	NaN	145 972 LIM and SH3 domain protein F42H10.3 OS=Caenorhabditis elegans GN=F42H10.3 PE=2 SV=3
1	1	NaN	" 158 355 Protein NAS-37, isoform b [Caenorhabditis elegans] gi; 15 227 Hemicentin-1 [Crassostrea gigas]"
1	8	NaN	460 1737 Tubulin beta-4B chain OS=Mus musculus GN=Tubb4b PE=1 SV=1
1	2	NaN	" 31 2061 minus strand 1,4-alpha-glucan-branching enzyme [Crassostrea gigas]"
1	1	NaN	" 703 1173 60S acidic ribosomal protein P0, putative [Ixodes scapularis] gi"
1	2	NaN	147 383 minus strand Tubulin-specific chaperone A OS=Rattus norvegicus GN=Tbca PE=1 SV=1; 104 418 Tubulin-sp
1	1	NaN	121 1452 minus strand Hexokinase type 2 [Crassostrea gigas]; 245 1576 minus strand Hexokinase type 2 [Crassostr
[truncated: 551,559 more chars]
